# The electronic tree of life (eToL): a net of long probes to characterize the microbiome from RNA-seq data

**DOI:** 10.1186/s12866-022-02671-2

**Published:** 2022-12-22

**Authors:** Xinyue Hu, Jürgen G. Haas, Richard Lathe

**Affiliations:** 1 Program in Bioinformatics, School of Biological Sciences, King’s Buildings, University of Edinburgh, Edinburgh, EH9 3FD UK; 2 Division of Infection Medicine, University of Edinburgh, Little France, Edinburgh, EH16 4SB UK

**Keywords:** Archaea, Bacteria, BLAST, brain, disease, Fungi, microbiome, RNA-seq, Tree of Life, virus

## Abstract

**Background:**

Microbiome analysis generally requires PCR-based or metagenomic shotgun sequencing, sophisticated programs, and large volumes of data. Alternative approaches based on widely available RNA-seq data are constrained because of sequence similarities between the transcriptomes of microbes/viruses and those of the host, compounded by the extreme abundance of host sequences in such libraries. Current approaches are also limited to specific microbial groups. There is a need for alternative methods of microbiome analysis that encompass the entire tree of life.

**Results:**

We report a method to specifically retrieve non-human sequences in human tissue RNA-seq data. For cellular microbes we used a bioinformatic 'net', based on filtered 64-mer sequences designed from small subunit ribosomal RNA (rRNA) sequences across the Tree of Life (the 'electronic tree of life', eToL), to comprehensively (98%) entrap all non-human rRNA sequences present in the target tissue. Using brain as a model, retrieval of matching reads, re-exclusion of human-related sequences, followed by contig building and species identification, is followed by confirmation of the abundance and identity of the corresponding species groups. We provide methods to automate this analysis. The method reduces the computation time versus metagenomics by a factor of >1000. A variant approach is necessary for viruses. Again, because of significant matches between viral and human sequences, a 'stripping' approach is essential. Contamination during workup is a potential problem, and we discuss strategies to circumvent this issue. To illustrate the versatility of the method we report the use of the eToL methodology to unambiguously identify exogenous microbial and viral sequences in human tissue RNA-seq data across the entire tree of life including Archaea, Bacteria, Chloroplastida, basal Eukaryota, Fungi, and Holozoa/Metazoa, and discuss the technical and bioinformatic challenges involved.

**Conclusions:**

This generic methodology is likely to find wide application in microbiome analysis including diagnostics.

**Supplementary Information:**

The online version contains supplementary material available at 10.1186/s12866-022-02671-2.

## Introduction

There is growing interest in the role that the microbiome plays in health and disease. Higher organisms including humans have evolved in the presence of diverse and abundant microbial species and viruses. Although some are evident pathogens, others are essential for normal physiological function. In plants there is a symbiotic relationship between the plant host and mycorrhizal microbes that are necessary for plant nutrition. In human, the gut microbiome is a key component of normal physiology where it contributes to nutrition, physiological homeostasis, and immunity development, and dysregulation of the gut microbiome (dysbiosis) is known to contribute to multiple disorders [1, 2]. As specific examples of the beneficial roles of the microbiome, the human microbiome is a major source of vitamins [3], and dysbiosis in experimental animals and human is implicated in immune system deficits [4] and neurocognitive disorders [5]. However, it is becoming increasingly clear that tissues other than the gut have their own microbiomes, most notably the skin, lung, and the oronasal cavities, as highlighted by the Human Microbiome Project [6, 7] (https://hmpdacc.org/). In addition, bacterial species are widely reported in blood of healthy individuals (reviewed in [8]), and the majority of cerebrospinal fluid (CSF) samples were found to be positive for one or more pathogens, confirmed in most cases by direct culture [9]; CSF of individuals with meningitis was found to contain diverse microbes [10], and multiple bacterial species were found in CSF of control children [11]. Diverse viruses are also present in CSF [12]. Furthermore, analysis of normal hamster liver revealed multiple bacterial species, confirmed by direct microbial culture [13]. The kidney also appears to have its own microbiome [14], and the human urinary microbiome (a proxy for the kidney) was found to comprise multiple bacterial species [15], suggesting that many solid tissues may harbor their own individual microbiomes, some detrimental whereas others may potentially be beneficial. However, the exact spectrum of microorganisms present in important tissues such as brain, heart, muscle, breast, and gonads has not been established, and future work will be necessary to address this issue.

Microbiome characterization is generally based on three key techniques: (i) ribosomal RNA (rRNA) analysis, generally assisted by PCR amplification, (ii) DNA-based metagenomics, and (iii) RNA-seq combined with informatic analysis (reviewed in [16–23]), although several hybrid methods have been used. In the first, rRNA (or rDNA) sequences are amplified using a suite of PCR primers, sequenced, and compared against the database. Because this depends on the use of short PCR primers, the method may lack specificity – risking amplification of non-RNA sequences in addition to missing any species whose rRNA sequence diverges from the primers. Furthermore, differences in abundance require quantitative PCR amplification of each sample followed by deep sequencing, and small differences in abundance (e.g., 2–4-fold changes) are difficult to detect.

The second method is based on metagenomic analysis through shotgun sequencing and genome assembly. This requires many-fold more data, often reaching terabyte (Tb) levels, and requires dedicated tools to remove human sequences and to assemble contigs. Moreover, because high sequencing depth is necessary, assembly-based methods are restricted to highly abundant members of the microbiome. Moreover, metagenomics does not easily address differential abundance. Both methods require extensive wet-lab work and can require machine-learning tools to unravel the true extent of the microbiome [24].

The third technique is generally based on analysis of RNA-seq (sometimes DNA-seq) data for exact matches to short *k*-mers (generally 31-mers) using techniques some as Kraken [25] and CLARK [26], but these methods require careful interpretation because the shortness of the sequences means that matches can be found by chance and, conversely, variants that differ by a single nucleotide can be missed.

Each of the above techniques has advantages and disadvantages. In addition to relatively high demands on data processing, and sometimes low selectivity, the different methodologies have often given conflicting results. We illustrate this through studies on the brain.

Like other tissues, the brain carries a burden of endogenous microbes and viruses [27], although these have not been well characterized, and some have questioned whether there is indeed a brain microbiome [28]. Bacterial sequences were reported in surgical epilepsy samples of human brain, and peptidoglycan-positive bodies consistent with bacteria were detected by immunohistochemistry and microscopy [29]; bacterial infection could be transmitted onwards by intracerebral inoculation of mice [29]. Roberts *et al.* reported diverse bacteria, identified by morphological criteria, upon high-resolution imaging of normal human brain [30], and brain of germfree mice (unlike that of conventionally reared mice) was reported to be devoid of microbes [30], although this awaits confirmation. In addition, an increasing body of evidence suggests that microbes readily enter the brain (e.g. [31]), can be detected by *in situ* immunohistochemistry of brain [32], and that brain infection may play a role in neurodegenerative disorders such as Alzheimer disease (AD) [33]. Nevertheless, there has been extensive debate about which microbes (and how many) are present. Chronic inflammation and infection caused by spirochetes have been suggested to contribute to the slow progression of AD [34]. *Chlamydia pneumoniae* shows associations with late-onset AD [35], and other bacteria such as Proteobacteria, Actinobacteria, and Firmicutes, as well as Fungi such as *Malassezia*, *Alternaria*, and *Candida* spp., have been reported [36]. An important PCR-based study revealed multiple bacterial species in AD brain [37]. Other work has focused on periodontal pathogens such as *Porphyromonas gingivalis* [38]. Nevertheless, in other studies very few microbes of this class (Bacteriodetes) were found, and other key species implicated such as spirochetes and *Chlamydia* (see above) were not well represented [36, 37]. Furthermore, beyond specific target groups, the relative abundances of these different species have not been established.

A major limitation is that the majority of studies to date have focused on select microbial groups such as bacteria or fungi. Several classes of cellular microbes across the known Tree of Life have not been widely studied to date, including Archaea, Amoebozoa, Chloroplastida, and Eukaryota. This relative dearth of such broader analysis extends more generally beyond our current target tissue (the brain) to tissues analyzed in the majority of endeavors.

Many viruses are also present in human tissues. Herpes simplex virus 1 (HSV-1) sequences were discovered in AD brain around 30 years ago [39]. Infections with viruses such as HSV-1 are widespread in the population; these generally remain in a silent (latent) form life-long, but may be reactivated because of stress, inflammation, or other factors, leading to proliferation and localized damage. Other viruses, notably human herpesvirus 6A (HHV-6A) and 7 (HHV-7), have also been suggested to be associated with AD. Readhead *et al.* used a modified ViromeScan workflow [40] and found that the abundance of these two viruses among 515 viral species was increased in the transcriptomes of AD brain in three of four cohorts compared to normal brain. For specific viruses, unique 31-mers were generated with Jellyfish [41], RNA-seq reads that are possible human or bacterial sequences were filtered, and then mapped to the filtered 31-mers to determine viral abundance [40]. However, Chorlton [42] challenged these results, suggesting that local alignment by Bowtie2 (http://bowtie-bio.sourceforge.net/bowtie2/index.shtml) required only a relatively low number of matching bases, and the Best Match Tagger (ftp://ftp.ncbi.nlm.nih.gov/pub/agarwala/bmtagger/) had a 2% false negative rate when filtering human-derived reads. Also, hepatitis C virus and eradicated variola virus were found in 100% and 97.5% of samples, respectively, using the modified ViromeScan. KrakenUniq [43], which performs efficient *k*-mer counts in metagenomics and can better identify false-positive reads, found few HHV-6A reads and no HHV-7 reads were detected [42]. Allnut *et al.* used digital droplet PCR (ddPCR) to amplify specific regions of HHV-6A and HHV-6B in 708 brain sections [44]. PathSeq [45], which has high specificity and sensitivity in distinguishing between human and non-human sequences, was also used as a complementary method with RNA-seq data, which contained part of the cohorts also used by Readhead *et al.*, to screen for pathogens from more than 25 000 microbes, containing 118 human viruses. Neither of these methods found associations between HHV-6 and AD [44], and the true contribution of herpes and other viruses to human brain disease remains unknown.

In addition to viruses, endogenous retroviruses and retroelements constitute a further class of replicative elements that might also contribute to human disease.

In the present work we have devised a different approach to microbial identification based on the use of long (64-mer) probes to identify sequence matches in RNA-seq data. The generation of RNA-seq datasets is increasingly rapid and cheap and, moreover, a large number of RNA-seq datasets generated by other laboratories for a variety of research purposes have been filed online at repositories such as the National Center for Biotechnology Information (NCBI) sequence read archive (SRA) database (which now contains over 25 million Terabases of open-access sequence information; https://www.ncbi.nlm.nih.gov/sra). The SRA repository can be searched for sequence matches either online or locally (see below) and is an increasingly important resource for microbiome studies.

Key objectives in the present report have been to devise methods that (i) extend to the entire tree of life, (ii) are applicable to widely available RNA-seq datasets such as those filed at the NCBI SRA repository, (iii) can determine both the identity and the absolute abundance of microbes including viruses and retroelements in human tissues, (iv) do not require sophisticated computer expertise or dedicated computer programs other than those that are widely available and/or freely downloadable, and (v) allow pictorial representation of the microbiome that facilitates comparative interpretation of the results.

We report the development of two related methods. First, an electronic tree of life (eToL) approach based on a 'net' of 16S/18S rRNA sequences across all cellular lifeforms to comprehensively retrieve all non-human sequences. Second, a 'stripping' method based on viral genomes to unambiguously detect key viral species. In this methodology paper we focus on technical and bioinformatic issues – case studies are presented that illustrate the versatility of the method; application of the eToL methodology to the human microbiome in select tissues will be presented elsewhere. The potential utility of the methodology in diagnostic applications is also discussed.

## Methodology development

The methodology we have developed is centrally based on Basic Local Alignment Search Tool (BLAST) screening of publicly available RNA-seq libraries (~50 SRAs were analyzed in this work) using a suite of probes (64-mers for microbial rRNA and whole-genome viral sequences) that are filtered to remove sequences with matches to human genomic/transcriptomic datasets. We present here the detailed rationale behind this approach and discuss relevant technical issues.

### Cellular microbes: the electronic Tree of Life (eToL) approach

**Fig. 1:**
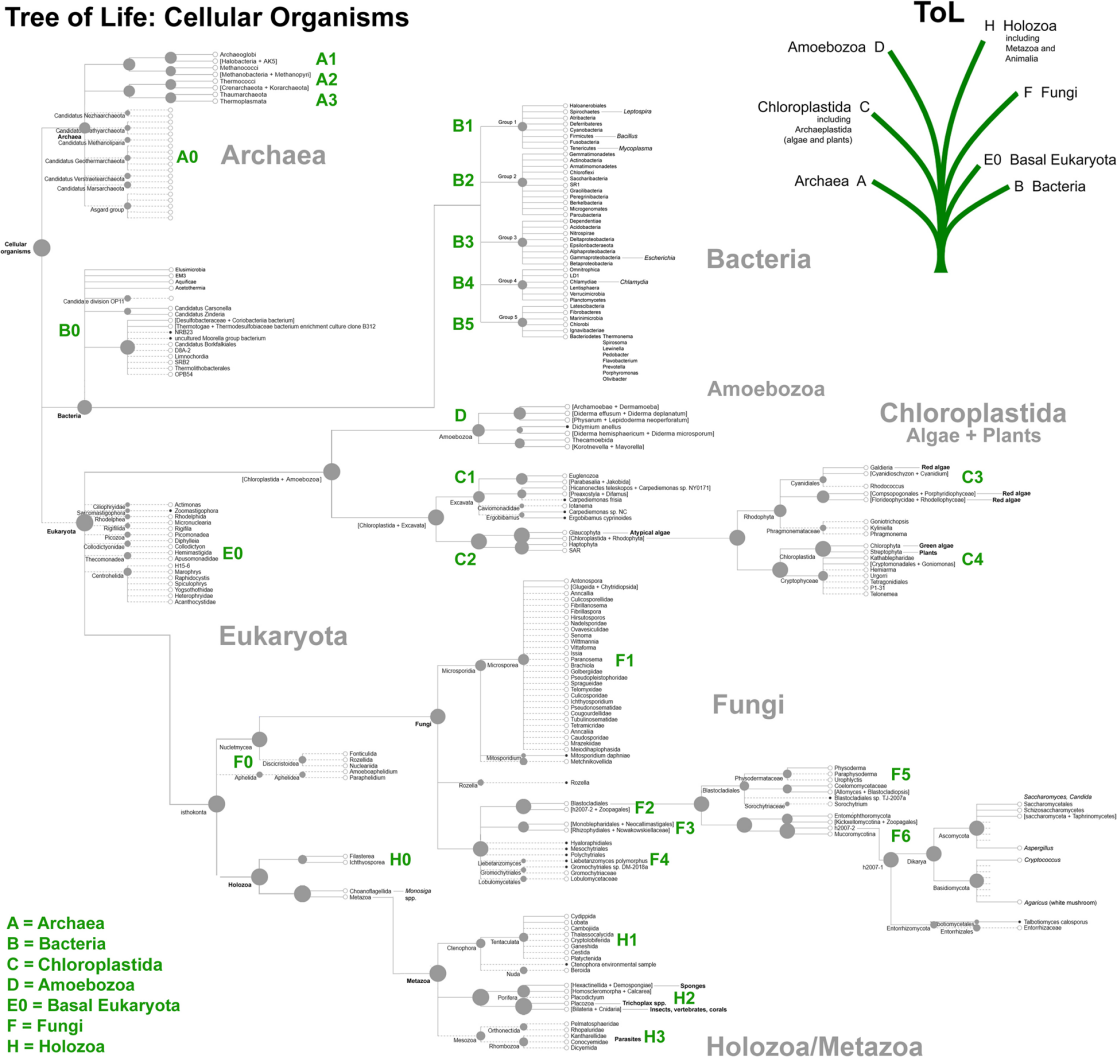
The phylogenetic tree for cellular microbes. The phylogenetic tree was obtained from Open Tree of Life project (modified from https://tree.opentreeoflife.org/) extended to include a recent bacterial consensus tree (main text for details)

According to the three-domain system, cellular life can be classified into Archaea, Bacteria, and Eukaryota [46]. To address the full diversity of cellular lifeforms we consulted the Open Tree of Life (OToL; https://tree.opentreeoflife.org/), a National Science Foundation (NSF) collaborative effort across 10 institutions that synthesizes phylogenetic trees based on sequence and taxonomic data [47–49]. For completeness, alternative phylograms include the LifeMap NCBI Version (http://lifemap-ncbi.univ-lyon1.fr/), and we refer to this where appropriate. Because OToL is not definitive on the placement of bacteria, we followed Schulz *et al.* [50] for a recent re-evaluation of the evolutionary phylogeny of bacteria, and synthesized a compromise ToL that includes all taxonomic groups, extending from Archaea and Bacteria to include Amoebozoa, Chloroplastida, Fungi, basal Eukaryota, and Holozoa/Metazoa (Fig. 1).

**Fig. 2:**
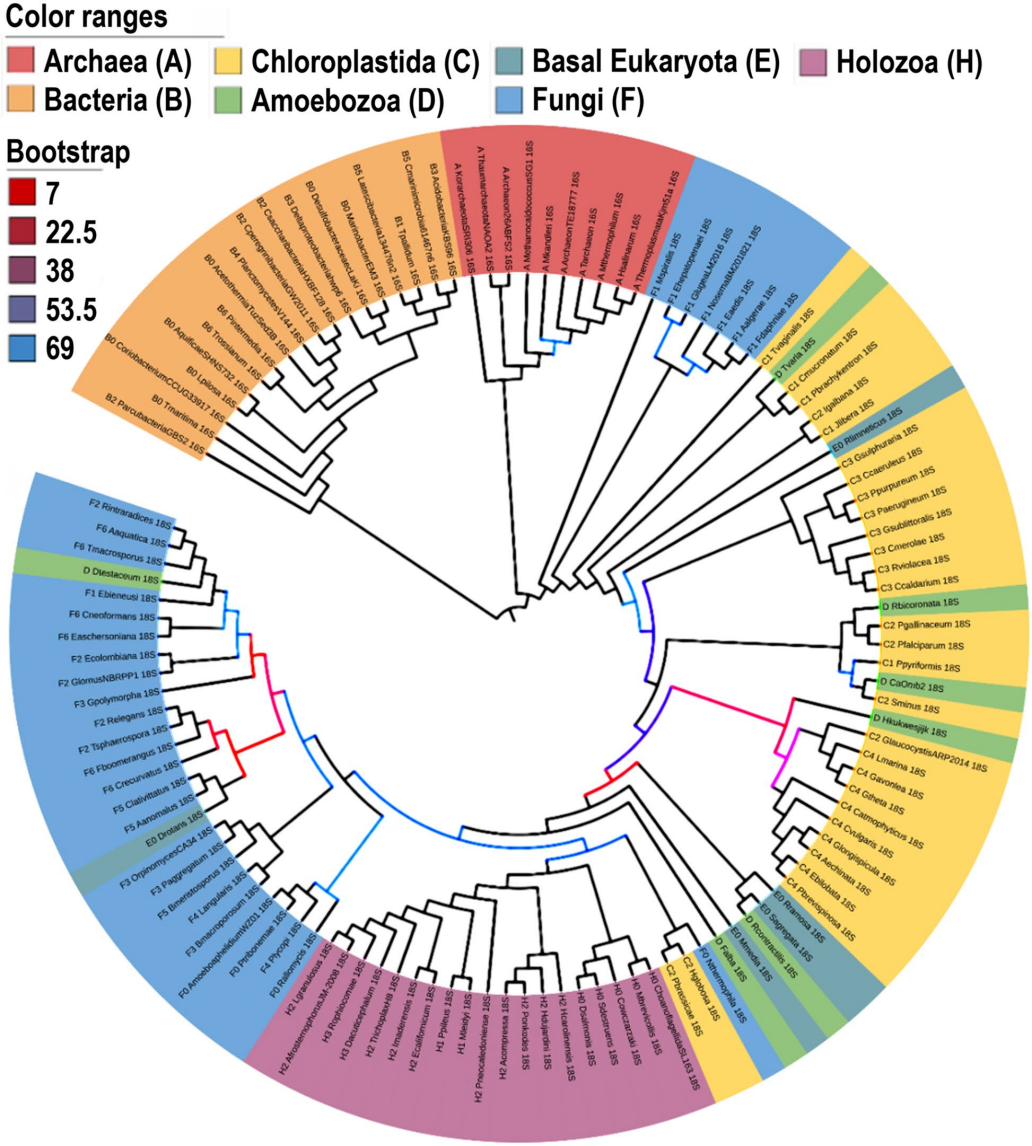
Phylogenetic tree built with rRNA sequences for the key 120 organisms. 16S rRNA sequences for Archaea (red) and Bacteria (orange), and 18S rRNA sequences for Chloroplastida (yellow), Amoebozoa (green), basal Eukaryota (grey blue), Fungi (blue) and Holozoa (purple). Bootstrap values of most of the nodes are over 70 (black), indicating strong phylogenetic support. Nodes with bootstrap values lower than 70 have weaker phylogenetic support and are shown in a color gradient from red (low value) to blue (high value. Note that the placements of Fungi, Chloroplastida, and Amoebozoa are intermingled, whereas the other groups are tightly clustered

From this tree 126 species were selected that were judged to be equally divergent from each other as estimated by their spacing/position on the tree, taking into account the relative diversity of Fungi in particular. Full-length 16S/18S sequences were downloaded from NCBI. To address the diversity of these sequences, and potential duplications within this dataset, we built a phylogenetic tree using Clustal Omega (https://www.ebi.ac.uk/Tools/msa/clustalo/). After exclusion of very close relatives (near-duplicates), we recompiled a list of 120 rRNA sequences that span the full diversity of cellular lifeforms (Table S1 in the supplementary data online). These sequences were then aligned using MUSCLE (v3.8.31) [51], and the IQTree tool (v1.6.6) was used to build a maximum likelihood tree with the aligned sequences [52]. To find the best-fit model and estimate the phylogenetic support of the nodes, the ModelFinder (MFP) option [53], and -bb option [54] with 1000 bootstrap replicates were chosen, respectively. The phylogenetic tree was modified and rooted with the outgroup bacteria using the iTOL tool (v6) [55], as shown in Fig. 2.

As will be noted from the figure, it is not entirely consistent with current trees for cellular organisms, and some phylogenetically distinct classes showed intermingling in their precise 16S/18S rRNA sequences. However, the objective of this work was not to address the phylogenetic relationships between the diverse species, but instead to devise a means to detect/extract, as far as possible, a comprehensive compendium of non-human sequences in human tissue. We therefore proceeded to use this collection of 120 rRNA sequences as a route towards evaluating the true complexity of cellular lifeforms.

### Databases

The RNA-seq datasets (*n* = ~50) used in this work are listed in Table S2.

### Probe length and specificity

Probing for microbes in RNA-seq data has generally exploited the fact that a typical cell contains many thousands of ribosomes (Box 1), whereas housekeeping transcripts are less well represented, often by a factor of 100 or more. However, a central problem in using rRNA-based probes to detect specific microbial matches in human RNA-seq data is that many, if not the majority, of full-length sequence probes detect significant (according to conventional criteria regarding the likelihood of detection by chance) matches, albeit often partial, in human rRNA. The problem is accentuated with long probes (bacterial 16S rRNA is ~1.5 kb in length, eukaryotic 18S rRNA is ~1.9 kb in length; for comparison, bacterial 23S large subunit rRNA is ~3 kb, and eukaryotic 28S rRNA is ~5 kb in length). As probe length increases, so too does the likelihood of finding matches in human rRNA sequences as well finding chance matches in non-rRNA sequences [56]. Conversely, using short sequences (in the range of 20–30 nucleotides) for PCR or *k*-mer analysis risks losing specificity, even though (at high stringency) these should be unique in the human genome/transcriptome, but this does not take into account the diversity of polymorphisms in the human population, *de novo* mutations, and sequencing errors. Given that *in silico* analysis by BLAST is formally equivalent to wet-lab probing by nucleic acid hybridization, we based our design on previous calculations that a minimum probe length of 62 nt is required, at a biologically plausible/significant level of 85% identity, to detect a unique sequence in a random collection of nucleotides of the size of the human genome (ca 3 × 10e9 nt) with a likelihood of 0.1 of encountering a similar sequence by chance [56]. For simplicity, we adopted a probe length of 64 nt.

Box 1. Transcripts per microbial cell/infected host cell **Cellular microbes: ribosomal copy number depends on cell type and growth rate**

Readcounts of rRNA provide an indicator of how many microbes are present, but there is no direct one-to-one relationship between rRNA readcounts and microbial cell number. The absolute abundance of ribosomes in each cell (ribosomes per cell, RBPC) depends on both growth rate and cell type/size. For bacteria such as *Escherichia coli*, RBPC values can be up to 70 000 during periods of rapid growth, but as low as a few thousand in poor growth conditions (http://book.bionumbers.org/how-many-ribosomes-are-in-a-cell/). In another bacterium, *Sphingomonas*, RBPC can be as low as 200 [57] under poor growth conditions. We assume that bacteria in human solid tissues grow very slowly, and a compromise estimate of 2000 RBPC has been adopted. The same value has been assumed for Archaea because they resemble bacteria in terms of size.

Eukaryotic cells are generally much larger, contain more ribosomes, and for yeast under fast-growing conditions 200 000 RBPC have been reported [58, 59]. However, as in *E. coli*, ribosome content depends on growth rate [60]. We therefore applied a similar reduction for what we expect to be very slow-growing cells, giving an estimate of 10000 RBPC (fivefold greater than in bacteria), noting that the exact RBPC values will depend on the species. Although this value could apply to other eukaryotes, it may be an underestimate because, for example, some large mammalian cells can have over a million ribosomes [61]; however, this extreme high value is unlikely to be representative of microbes more generally.

**Virus-infected cells**

The problem in estimating how many genomes are present per host cell becomes more acute for viruses because we must distinguish between latent/quiescent infection, where viral transcript counts are expected to be similar to those of endogenous housekeeping genes, and virulent replication, where there can be thousands of genomes/transcripts per cell. For this reason detection of viral sequences in RNA-seq data is likely to detect highly replicative viruses, but could potentially miss low-level latency-related transcripts from quiescent viruses. This point is discussed in more detail in Box 2.

### rRNA hypervariable or constant regions

rRNA genes of bacteria and fungi, in particular, are known to contain hypervariable regions [62, 63] as well as conserved regions. Although the use of hypervariable regions has the advantage that it can identify exact species (or groups of species), the drawback is that probes based exclusively on hypervariable regions are likely to miss other species that have a slightly different sequence, arguing against pre-selection. By contrast, probes based entirely on conserved regions may detect species irrespective of their class. As a further argument against pre-selection of regions, rRNAs contain a high degree of secondary structure that can (unpredictably) impede reverse transcriptase-mediated copying into cDNA, and some pre-designed probes may find few matches in RNA-seq archives. To illustrate, the number of RNA-seq reads matching different regions of *E. coli* 16S rRNA in a test dataset differed by a factor of 100 (not presented). As a working compromise, we generated probe sequences without any pre-selection based on knowledge of the target sequence (e.g., of variable versus conserved regions). Probe redundancy (a potential outcome of random design) is addressed in the sections below.

### Probe generation and nomenclature

We generated 64-mer probes from the 120 rRNA sequences (~10 per kb, noting that not all available sequences are complete). The non-overlapping 64-mer sequences were devised as probes by semi-random selection using probe.py script. The version of python was v3.6.3 [64]. This generated a list of 1323 probes. In naming the probes we aimed to devise a simple, but easily remembered, nomenclature. Probes were therefore prefixed by a single letter for each of the major domains as follows: A, Archaea; B, Bacteria; C, Chloroplastida (algae and plants), D, Amoebozoa; E0, basal Eukaryota (that may constitute a clade of their own); F, Fungi; and H, Holozoa/Metazoa. Group G was not allocated, and may be retained should any new branches of lifeforms to be discovered (if there are any). We use the term Chloroplastida to denote all organisms containing chloroplast-related organelles in preference to the term Viridiplantae because the latter implies that algae are plants [65]*.* Probes were thus named >X (code of ToL domain and number)_abridged species name_rRNA (16S or 18S)_probe number, for example, A_Hsalinarum_16S1.

### BLAST analysis

Analysis is based on the basic local sequence alignment search tool, BLAST/BLAST+ [66, 67], that is now widely accepted as the gold standard for detecting significant sequence similarities. Sequence searching employed a series of publicly available SRA databases (Table S2), and nucleotide BLAST (BLASTn) was run either online at NCBI (https://blast.ncbi.nlm.nih.gov/Blast.cgi?PROGRAM=blastn&BLAST_SPEC=GeoBlast&PAGE_TYPE=BlastSearch) or locally at the Edinburgh Compute and Data Facility (ECDF) Linux Compute Cluster (http://www.ecdf.ed.ac.uk/) ('EDDIE') at the University of Edinburgh using BLAST+ downloaded from NCBI (https://www.ncbi.nlm.nih.gov/guide/howto/run-blast-local/). In this work we carefully explored different settings for similarity detection, including wordsize (default = 28), match/mismatch parameters (default = 1,−2), and gap costs (linear), and in no case did this improve selectivity. We acknowledge that the default settings for evaluating sequence similarity have been carefully optimized by NCBI staff and their advisers, and we identified no obvious improvements. The basic settings (optimize for highly similar sequences) are that BLAST detects 64-mer sequences with 4 mismatches (94% identity), but also detects 28-mer sequences with no mismatches and 38-mer sequences with a single mismatch (which could be as low as 50% identity over the full length of the probe; values correct at time of writing, October 2021; these settings may evolve with updates of the NCBI website), although these shorter matches have lower overall scores. Nevertheless, coverage (length of the region of homology) for the majority of matches detected (80–90%) were generally in the range 70–100% and, because a second screening step is applied, the default settings were retained. Cut-off scores are discussed in the sections below.

### Removal of probes matching human sequences

Although 64-mer probes based on rRNA are less likely (because of their length) than full-length rRNA sequences to find matches in human databases by chance, rRNA is relatively conserved across species, and many of the probes found matches in human sequences. The 64-mers were therefore screened for probes that were significantly similar to human sequences (default megablast task and -entrez_query "Homo sapiens {Organism}") by BLASTn v2.11.0+ with nt database [68]. About 300 probes found matches in human sequence databases (default parameters), and these probes were discarded. The final list of probes (*N* = 1017) without matches in the human database was generated in Fasta format and is given in Table S3. An important aspect of our analysis has been to determine whether this list is sufficiently comprehensive (discussed further below).

### Determining the number of matching reads in SRAs

A script was devised to count the number of matches in NCBI RNA-seq SRA datasets to each probe according to our selection criteria (see also below). Readcounts after filtering can also be determined by accessing the hit table csv file and using the COUNTIF function of Microsoft Excel in the format =COUNTIF(A1:A5000,"probename"), a similar function is available in Apache Open Office (https://www.openoffice.org/welcome/credits.html) and other widely accessible spreadsheet programs.

### Refiltering is necessary: sequence similarity does not follow the transitive law

A central issue encountered in this work is that sequence similarity, as assessed by BLAST, does not follow the transitive law of mathematics and logic. The law dictates that, if A = B, and B = C, then A = C; also that if A > B, and B > C, then A > C. This rule does not apply to similarity between nucleotide (or protein) sequences. If sequence A is significantly similar (σ) to sequence B, i.e., A σ B, and B σ C, one may not conclude that A σ C. Conversely, if A is not similar (/σ) to B, and B σ C, one may not conclude that A /σ C. This is because, in three sequences A–C, sequence A (e.g., the probe) may not be similar to B (e.g., human sequence), but there may be an intermediate sequence C that is significantly similar to both A and B. For this reason, in searching human RNA-seq data with filtered probes, although most human sequences have been removed, many BLAST matches retrieved from human tissue are still of human origin. Second-round filtering of all matches detected is therefore necessary. In addition, representative human genome(s) and transcriptomes at NCBI do not encompass the full diversity of polymorphic variants that are present across the human population, and variant sequences may be encountered that achieve a threshold significant match with a filtered (i.e., no matches in human) probe despite being of human origin.

All matches retrieved from human RNA-seq libraries were therefore revalidated to exclude human sequences. This involves (i) retrieving the matching sequences, (ii) searching again for homologies to the downloaded human nucleotide database (ftp://ftp.ncbi.nlm.nih.gov/blast/db/; accessed 20 May 2021). Because the sequences retrieved are endogenous to the SRA that harbors them (and are therefore generally longer than the 64 nt probe length), they automatically generate higher similarity scores for a given extent of homology, and the threshold score for significance must therefore be adjusted according to the mean readsize in each dataset. The adjusted cutoffs were as follows. If the bitscore is >160 (MSBB) or >100 (Miami) or >126 (Rockefeller), the sequence was considered to be human in origin, and was discarded. For other datasets (liver, skin, and 17 brain samples a cutoff of >150 was applied; cutoffs for other databases were >160 for the Mount Sinai Brain Bank and >150 for the Edinburgh Brain Bank, not presented). Three scripts were generated for this step, EDDIE_ToL.sh, Abundance_ToL.py and Abundance_count.py. Finally, duplicate reads detected with different probes (if any) were filtered to allocate reads to a single probe showing the highest sequence similarity, and the number of confirmed non-human reads were counted for each probe (Abundance_count.py).

### Computational demands of different methods: eToL is fast

BLAST is the gold-standard method for comparing two nucleotide sequences. However, next-generation sequencing (NGS) of tissue samples for microbiome analysis, including both RNA-seq and DNA-based metagenomic sequencing, generates very large amounts of data. An obligatory first step is to remove all human sequences, and this requires that the sequence library is compared to the human genomic/transcriptomic databases. Nevertheless, BLAST is too slow for routine NGS analysis, and 'end-to-end processing times, even on multicore computational servers, can take several days to weeks' [69]. Faster algorithms such as SNAP [70] and SeqAlto [71] and workflows such as SURPI [69] are a little faster, but still require hours or more of computation time. eToL significantly reduces the computation time required; a brief analysis is presented below.

BLASTn and related methods generally employ iterative comparisons. The basic algorithm is to start with local identities and determine whether these can be extended across the two sequences that are being compared. To simplify mathematical analysis of the number of comparisons required by these methods, we first define a sequence 'unit' as 100 nt, and we consider that a probe in the eToL method (64 nt) and a single read generated by short-read RNA-seq (100 to 150 nt) are approximately the same size (i.e., 1 unit). Second, we introduce the concept of a 'block' (BL) that roughly represents the number of comparisons required to determine whether a probe sequence (1 unit in size) is related to a target sequence of the same size. In other words, 1 BL is the amount of computation required for a single 100 nt x 100 nt comparison. Comparing a typical RNA-seq library (a little over 10e10 nt = 10e8 units) to the human genome (10e9 nt, or a transcriptome of a similar size = 10e7 units) using BLASTn thus involves 10e8 × 10e7 BL, i.e., 10e15 BL or 10e6 GBL. This approximates the number of computations necessary to identify non-human reads in an NGS library using BLASTn.

The eToL method differs from the above because the probes are prefiltered to remove human sequences. BLASTn using 1000 probes, and screening a typical RNA-seq library, involves 10e3 × 10e8 BL, i.e., 10e11 BL, or 10e2 GBL, in the first instance. This is a very considerable saving. However, refiltering is necessary because some of the sequence matches detected will be human. In a typical eToL analysis ~10% of probes (100 units) find matches, and each 'positive' probe finds in the order of 100 matches, generating 10e4 units of sequence. These must now be compared against the human genome or transcriptome (10e7 units), in other words 10e4 x 10e7 = 10e11 BL, 10e5 MBL, or 10e2 GBL. Although these are approximate 'rule of thumb' calculations, the entire eToL analysis requires 2 x 10e2 GBL of comparisons, whereas 10e6 GBL of comparisons are necessary for direct NGS versus human comparison, in other words a computation reduction by a factor of 5000, thus requiring 5000-fold less time. In support, eToL analysis of a single RNA-seq library (online at NCBI) takes only 1–5 minutes to retrieve all non-human microbial sequences.

### The issue of rRNA depletion in RNA-seq libraries

Ribosomal RNA represents ~80% of the total RNA in each cell. Because most SRA studies in the data repositories have focused on endogenous human transcripts rather than on exogenous microbes, it is commonplace to use protocols to deplete rRNA. Precipitation with LiCl and/or selection of poly(A)^+^ RNA using immobilized oligo(dT) are widely used, but rarely remove more than 50–80% of rRNA. To address this we probed different datasets based on raw RNA-seq versus selection for poly(A)^+^ RNA before sequencing. As shown in Fig. 5A, there was no clear difference in the abundance of microbial rRNA transcripts between raw and poly(A)^+^ SRA datasets. Because poly(A) selection is often not performed, we could only analyze a small number of SRAs; however, we report this result because it suggests that poly(A)^+^ selection is not necessarily fatal for microbiome analysis, and this is likely to reflect (i) inefficiency of rRNA removal, (ii) different kits are likely to have different efficiencies of removal, and (iii) the extent of variation is comparable to that seen when the same tissue is worked up using alternative protocols in different laboratories. Although poly(A)^+^ datasets represent a increasingly small fraction of SRAs, we recommend careful checks on poly(A)^+^ datasets to determine the abundance of non-poly(A)^+^ RNA species before microbiome analysis. In addition, poly(A)^+^ datasets may be problematic for internal normalization to determine absolute abundances.

An alternative approach is to specifically deplete host rRNA in a sample through hybridization to specific complementary oligonucleotides [72]. This method can achieve >90% removal of host (e.g., human) rRNA but does not remove other rRNAs such as those of exogenous microbes. No adjustment for rRNA removal was therefore performed.

### Normalization: housekeeping transcripts

Because different tissue samples contain different amounts of RNA, sometimes partially degraded, and different RNA-seq protocols have different efficiencies of conversion to cDNA for sequencing, we normalized the number of readcounts obtained to the amount of biological material in the SRA. For this purpose we relied on three different housekeeping genes: phosphoglycerate kinase (*PGK1*), hydroxymethyl-CoA reductase (*HMGCR*), and neuron-specific enolase (*NSE*). An earlier study accurately determined the number of transcripts per cell of *PGK1*-driven GFP is 46–64 transcripts per cell [73] (Box 2). We found no significant differences between *PGK1* and *NSE* levels in these datasets, whereas *HMGCR* expression was lower. Importantly, different target tissues will require their own panel of housekeeping gene probes. Of note, the expression levels of *PGK1* and *NSE1* in human brain appear to be constant as a function of age (https://hbatlas.org/pages/development).

As before, 64-mer probes were devised, and BLAST searching was employed to determine the number of host cells that each SRA corresponds to. To minimize statistical fluctuation, the mean of *PGK* (two probes) and *NSE1* (two probes) was adopted for normalization. The number of host cells thus equals the mean readcounts of *PGK1* and *NSE* probes divided by 50, and in each case the raw readcount was normalized to the number of microbe transcripts per host cell by dividing the by the estimated number of host cells. Other tissue types will require dedicated housekeeping gene probes (discussed in Box 2).

Box 2. Normalization to endogenous transcripts and detection limits A key question concerns the extent of coverage of each sequence read archive (SRA). To address this we used *PGK1* probes to normalize RNA-seq data. *PGK1* stands out because Kempe *et al.* [73] carefully measured the level of expression of *PGK1*-driven GFP and accurately measured 46–64 transcripts per cell. In support, it is estimated that ~12 000 genes are expressed per typical cell, and between 360 000 and 10^6^ mRNAs are present: the average number of transcripts, per gene, is thus in the range 30–83, strictly comparable to the estimate of ca 50 *PGK1* transcripts per cell determined by Kempe *et al.*

Screening of RNA-seq libraries revealed that *PGK1* match numbers are in the general range of 10 to over 250 per library, with a median of 152, arguing that each sequence library roughly equates to the sequence content of ~3 cells, although more recent RNA-seq datasets appear to be a little larger (10 cells). This conclusion is substantiated by considerations of SRA file sizes (3–10 gigabytes, GB). Very approximately, an uncompressed file containing 1 megabyte (MB) of information contains 1 megabase of nucleotides, and a file containing 1 GB of information contains ~1 gigabase of nucleotides. The entire transcriptome of a cell, at 600 000 mRNAs per cell, and average mRNA length = 2.2 kb, equates to 1 320 000 000 nucleotides (1.3 GB of data). However, rRNA removal is usually far from complete and, in addition, 1/3 of the information in a typical SRA file is not sequence data (each entry contains details of the specific read in question). To cover the (non-rRNA) transcriptome of a single cell therefore requires a minimum of 2.5 GB of information in FASTA format. Although they may be partly compressed in some formats, consideration of SRA file size (3–10 GB) is consistent with the interpretation that a single SRA equates to the transcriptome of a small number of cells. A legitimate concern is therefore that poorly abundant (but biologically relevant) microbes/viruses may not be detected (see below).

Another consideration is that rRNAs tend to contain regions of secondary structure that reverse transcriptase (RT) may find difficult to copy into cDNA (Methodology Development). However, this is unpredictable, and we have made no explicit allowance for this factor.

Average read length is a further consideration. Using 64-mer probes, if the mean read length is 150 nt, then (if mRNAs are randomly sampled) some 40% of reads will not sufficiently overlap with the probe, but this falls to ~30% for 250 nt reads. However, this factor is identical for housekeeping genes and for microbial transcripts, and does not affect normalization.

Although our normalization is based on *PGK1* transcripts at 50 per host cell, even using two independent probes for *PGK1* we observed some statistical fluctuation. To dampen this effect we considered two other genes. The first, hydroxymethyl glutaryl-CoA reductase (*HMGCR*) is another housekeeping gene, but transcript levels were on average fivefold lower, making it unsuitable. Instead we turned to a neuron-specific housekeeping gene, neuron-specific enolase (*NSE*, also known as *ENO2*). It can legitimately be argued that, because *NSE* is neuron-specific, it is not entirely representative of our target tissue (brain) where neurons only constitute a little over one half of all host cells. However, we saw excellent agreement between *NSE* and *PGK1* transcripts, and for simplicity our results are presented following normalization to the mean number of *PGK1* and *NSE* transcripts per cell (or per 100 host cells), assuming that 1 host cell = (mean {*PGK1* and *NSE1*})/50. Although this probe combination is probably useful for normalizing RNA-seq data from brain, in other tissues it will be necessary to carefully choose the most appropriate panel of housekeeping genes for designing normalization probes.

**Calculated detection sensitivity**

If current sequence libraries represent circa 10 cells (above), and cellular microbes contain 2000 ribosomes (Box 1), then detection of a single rRNA transcript (detection limit) means that the corresponding microbe is present at a level of 0.0005 microbial cells per 10 host cells, which is very sensitive. For lytic viruses, where 1000+ transcripts per infected cell may be present, a similar level of detection (0.0005 infected cells per 10 host cells) is likely to apply. The exception is for latent viruses. For example, herpes viruses express latency transcripts of various types (LAT in the case of HSV-1), and the abundance of such transcripts is assumed to be low, possibly comparable to the level of housekeeping gene expression (50 transcripts per cell). If the RNA-seq dataset represents the transcriptome of at most 10 cells, then the detection of a single LAT transcript would represent 0.02 infected cells per 10 host cells, and this is likely to constitute the lower level of detection. However, multiple viruses are present in human tissues at low levels, where they persist throughout a lifetime [27], and it remains to be determined whether latent/quiescent infection such viruses compromises cell function; the lower sensitivity may therefore not be a drawback in the analysis of human physiology in health and disease.

### Visualization and display of readcounts: heatmapping

An objective of this work was to generate a pictorial description of the distribution of (filtered) sequence matches in a given sample. Two methods were used, both based on the sequential order of probes in the taxonomic groups A (Archaea) to H (Holozoa). In the first, Morpheus software at the Broad Institute of MIT and Harvard (Cambridge, MA, USA; https://software.broadinstitute.org/morpheus/) was used for the display either with or without conversion to log2. All matches below a cutoff value (specified in the figures) are shown in blue, and all values between cutoff and a maximum value (dictated by the experiment in hand) are shown on a white to red scale on a blue background where white = cutoff, red = maximum (or above), and blue = below cutoff. This presentation is outlined in Fig. 4. In the second, the normalized data for each probe (reads per host cell) were summed for each organism, converted to log2 scale, and heatmaps plotted using the pheatmap package (v1.0.12) of R. Because of the wider range of abundances for retroelements and viruses, these can require different displays (these are specified in all cases).

### Redundancy check

Because all our probes for cellular microbes are based on 16S/18S rRNA, we expected that some probes in the collection would be similar to others, perhaps because they identify highly conserved regions. A concern was therefore that the patterns we observe might to some extent reflect the degree of conservation across different species, as reflected by the number of probes in the collection with similar sequences, rather than the abundance of a target species. To address this, we catenated the entire probe collection into a single file and queried it by BLAST with the individual probes. Of the 1017 probes, 634 (62.3%) were unique within the collection, 100 (9.8%) found 2 matches (i.e., detected one similar sequence), and 122 (12.0%) gave 3–5 matches, and the remainder gave >5 matches (15.9%) (Fig. 6A). We refer to the number of matches identified by each probe as 'redundancy'. Nevertheless, inspection revealed that the 'next best' matches were on average 48.8 nt in coverage, with 2.5 mismatches, falling to homology stretches as short as 31-mers with one mismatch, arguing that the extent of sequence overlap/duplication within the collection is relatively low.

However, to formally address whether the signals detected in human tissues might reflect the number of probe/probe similarities, we plotted the extent of 'redundancy' against total readcounts across the cohort. As shown in Fig. 6B, there was a very slight correlation between redundancy and total readcount (trendline). However, the signals with the highest readcounts were at the lower end of the redundancy scale. Figure 6C–E shows the outcome of normalizing the readcount for each probe to the redundancy of the probe, indicating that normalization does not have a major effect on the overall pattern. In Fig. 6F we examined the effect of sequentially deleting readcounts for probes with a high redundancy (20 or above, 20+; 10 or above, 10+; 5 or above, 5+) and then deleting values for probes with redundancies of 2–5, leaving only signals generated by unique probes within the collection. As shown, progressive removal of redundant probes led to some degradation of the signal, but (i) many highly abundant signals remained despite restriction to unique probes, and (ii) the remaining signals continued to encompass the entire tree of life. However, coverage was slightly compromised, and probing the collection of known human commensals/pathogens (*N* = 104) with the 634 'unique' probes (not presented) failed to detect 6 species (5.8%; compared to only 2% missed with the complete probe list, next section).

One potential way to avoid this issue would be to generate a far larger collection of random probes, and then to discard probes with partial overlaps/duplications in the probe collection, and this could be done in the future. Overall, our observations argue that the overall pattern of microbe signals detected is not dependent on probe redundancy. Because our primary intention was to retrieve the maximum number of non-human sequences, we elected not to remove partially overlapping sequences from the probelist because this could compromise retrieval. A drawback is that different probes might potentially retrieve the same sequences. However, this ambiguity is resolved by separate confirmation with 23S/28S sequences and contig building for identification (see below).

### The probe collection is largely comprehensive

To address whether the filtered probe collection is sufficiently comprehensive, we used it to screen a manually assembled list of rRNAs for known human-associated species. A list of 104 organisms known to be present in human samples was assembled according to Archaea found in human gut [74], bacteria (listed in Wikipedia: https://en.wikipedia.org/wiki/Pathogenic_bacteria), fungi (https://en.wikipedia.org/wiki/Pathogenic_fungus), helminths [75], and a selection of rarer species assembled manually. Where two or more closely related species were listed, a single representative species was selected, noting that some species with similar names may in fact show significant divergence. Where the corresponding rRNA sequence was not available, the sequence from a closely related species was selected. In each case 16S/18S rRNA sequences were downloaded from nucleotide database of NCBI. This collection was named the PATHLIST (Table S4). Probing PATHLIST using the probe collection found matches in 98% of cases, and only missed two species (Discussion), indicating that the eToL v1.0 collection is largely sufficient for our purposes. Subsequent versions of eToL will include these 'missing species'.

### Specificity and cross-matching

To determine the extent of cross-matching between probes designed from the different taxonomic groups (A–H), the 1000+ probes were used to screen the PATHLIST dataset of human-associated microbes. Because very few known human-associated microbes fall into the groups C (Chloroplastida), D (Amoebozoa), and E0 (basal Eukaryota), these were not included. As shown in Fig. 5B, other than some overlaps between Fungi and Holozoa (as expected, given their relatedness), there was little cross-matching between groups, attesting to the group-specificity of the probes in the collection.

### Species identification, contig generation, and diversity

The eToL net collects a compendium of non-human sequences (confirmed by second-round filtering) in a target tissue. For identification, filtered matching reads for key signals of interest were downloaded, contigs were generated using one of several online tools (e.g., CAP3 assembly at the Rhone-Alpes Bioinformatics Pole PRABI-Doua; http://doua.prabi.fr/cgi-bin/run_cap3; also EG assembler https://www.genome.jp/tools-bin/eassembler4.cgi?status=seqclean&pmode=all [76]; unfortunately, EGassembler ended on July 4th 2022; a summary of available tools is given at https://onlinetoolweb.com/contig-assembly-online-tool/), and their abundances determined by BLAST of each contig generated from human tissue against the collection of sequence matches, retaining only sequences with 100% (or near-100%) identity to the probe. Where necessary key contigs are used as probes to retrieve additional sequences from the same libraries. For sequence identification, BLAST at NCBI allows retrieval of the closest homologs. However, an average of 4000 matches were retrieved across 20 SRA datasets, but this can be as high as 16000. Computation time for contig building using EGassembler is estimated at 30 minutes for 4000 matches, rising to 5 h for 16000 matches [76] (computation time is approximately proportional to the square of the number of sequences). By contrast, contig assembly by phylogenetic group (A–H, where the mean number of matches for each bacteria and fungi was ~1000) is significantly faster and this number of sequences can be assembled into contigs in ~5 minutes [76]. Nevertheless, because the microbial sequences retrieved are not monophyletic, and show divergence within a single individual sample and between different samples, as illustrated in Box 3; high-stringency contig building risks excluding important contributors to the microbiome and, as in all such microbiome analyses, caution is necessary in interpretation.

Box 3. Case study: signals detected are not monophyletic To illustrate the complexity of computer searching, we report the case of searching the first liver SRA with a 64-mer probe, B3_AcidobacteriaKBS96_16S_8. This found 44 matches, of which eight were 100% identical, whereas the major class (36) contained one or more mismatches. Contig building generated six contigs, of which two were highly abundant, the others less so. Sequence comparisons and tree building (Clustal Omega) revealed that they were divergent in sequence (Fig. A). To extend the two most abundant contigs, these were used to reprobe the SRA, and matches were used to generate contigs again. This gave two contigs of 496 and 336 nt. The former was 97% identical to an uncultured Betaproteobacteria clone, whereas the latter was 100% identical to a Gammaproteobacteria species, *Acinetobacter*.

Using the major contigs to search a second liver SRA revealed that the major contig from liver 1 found closely related, but not 100% identical, sequences in liver 2, whereas the second most abundant contig in liver 1 found no close relatives in liver 2. This illustrates two points: (i) the exact species present in a tissue sample are not monophyletic, but represent a spectrum of related microbes; and (ii) what is true of one tissue sample may not be true of a near-identical sample from a different individual. The best approach may be to address multiple samples from different individuals, and identify the commonalities.

**Fig. A:**
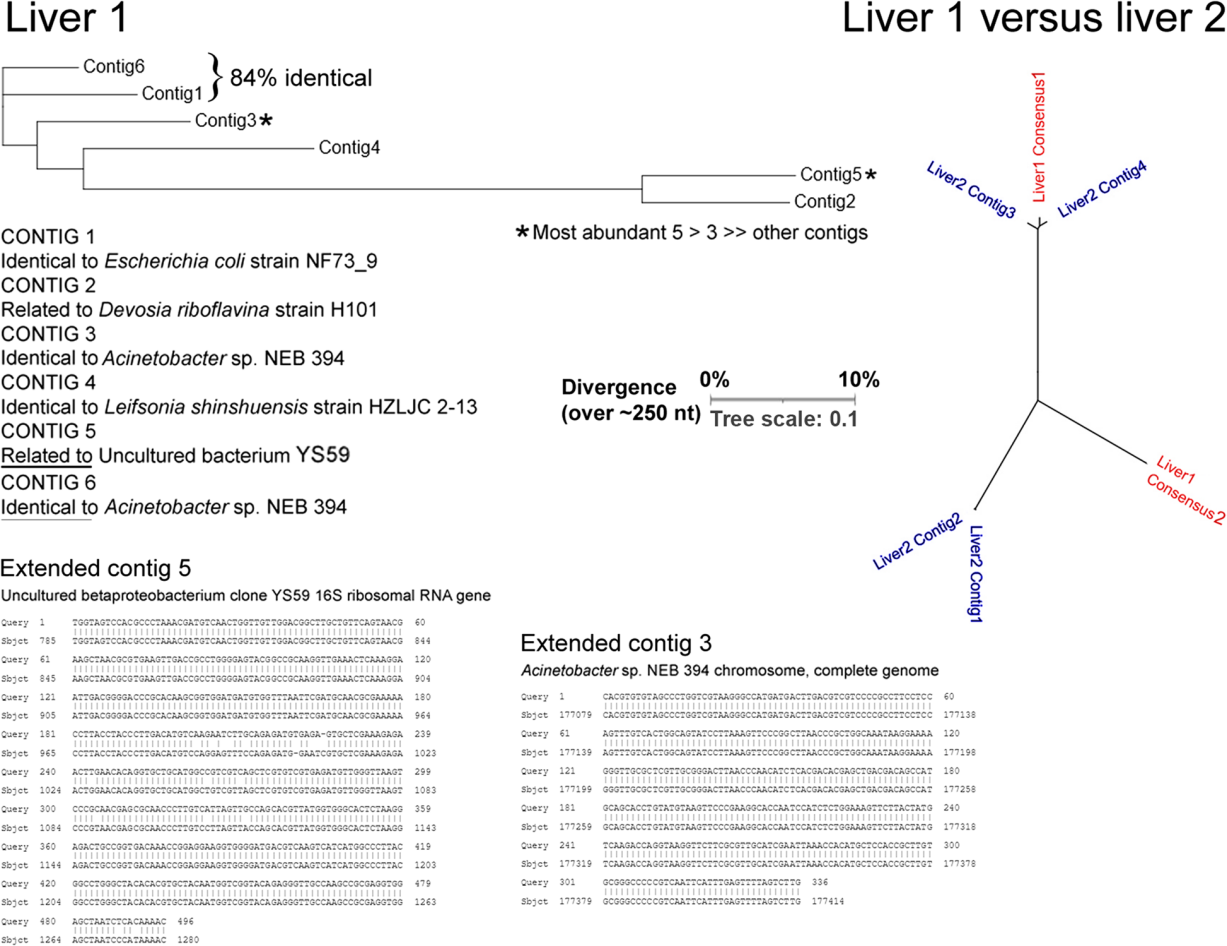
Matches obtained with a single probe highlight the most abundant species (*contigs 3 and 5) in the first liver sample, contig building (Consensus 1 and Consensus 2, respectively) and divergence in a second sample.

### Validation with 23S/28S and mitochondrial DNA

Because this approach employs a large number of probes (>1000), any observed high or low abundance in a particular tissue SRA could occur by chance (Bonferroni correction). To circumvent this issue the same protocols were used to devise fresh probes from 23S/28S rRNA for the same (or the closest available) key species signal of interest, and these were then used to reprobe the SRAs for matches. Again, all matches were filtered against human sequences, contigs assembled, followed by species identification.

An alternative approach that is valid for eukaryotic microbes including Fungi, but not for Bacteria or Archaea, is to employ mitochondrial DNA (mtDNA) sequences that have been 'stripped' (below) against human sequences.

### Viruses: whole-genome 'stripping'

Because viruses have no ribosomes, we turned to whole-genome sequences. For viruses where detailed transcriptomes are available, both in latency and in productive infection, we recommend the use of 64-mer probes based on abundant transcripts. However, transcriptome data are only available for a minority of virus types, and for general applications we based our methodology on intact viral genomes. Nevertheless, these also contain internal homologies with human sequences (see below). For methodology development we based our analysis on the report in Neuron by Readhead et al. [40] where they used a *k*-mer method to screen human brain SRAs for 515 viruses. Although this method is prone to false positives (Introduction) it appears to be immune to false negatives. For methodology development we therefore selected the top 20 viruses in terms of readcounts, representing >99.9% of all matches found in human brain (B. Readhead, pers. comm.).

To identify matching sequences in the human genome/transcriptome, complete genomes for these 20 key viruses were used to search human sequences in the NCBI databases (BLAST/nucleotide collection (nr/nt), organism name = *Homo sapiens*). This revealed many matches, some relatively close (Table S5). To refine this approach we applied a 'stripping' method, as follows. All regions of sequence similarity between the viral genomes and human sequences identified by BLAST were deleted from the viral genome. In addition, low-complexity regions were removed. The first-round stripped genomes were then searched again against *H. sapiens*, revealing further matches. Four rounds of stripping were necessary to remove all homologies with the human genome/transcriptome. Because some viruses are present as integrated copies in around 1% of the human population (in particular with homology to HHV-6A, HHV-6B, and potentially HHV-7) [77], these are present in the NCBI databases of 'human' sequences, and these were manually curated to remove key matching sequences (notably human telomeric repeats that are present in the viral genomes). The stripped genomes are given in Table S6.

### Retroelements

The principal retroelements in the human genome are long and short interspersed nuclear elements (LINEs and SINEs, respectively). LINE activation has been reported in neurological disease [78]. LINEs belong to several subfamilies, and representative 64-mer probes were devised for each of these. SINEs are generally relatively short highly conserved sequences related to human 7SL RNA, and probes were included to cover SINE elements. The human genome also includes several classes of human endogenous retroviruses (HERVs) that have been implicated in diverse disorders including neurodegenerative disease [79], and we included probes to assess their abundance. Following the same nomenclature scheme as before, probes for retroelements including HERVs were allocated domain code R (Table S2C).

### Recognizing and excluding contamination

Microbiome identification through rRNA sequencing and metagenomics is prone to various types of contamination that can obscure true signals [80–82]. Contamination may be classified into two principal categories (Table 1A). Type 1 contamination includes contamination of the sample and reagents used to examine it, whereas type II concerns *in vivo* biocontamination, as discussed below.

(i) **Reagent contamination: type 1A**

**Table 1:** Types of contamination and strategies to exclude them

**A Types of contamination**		
**Class**	**Type**	**Comments**
1A	Molecular biology reagents	If contaminated, the same signal will be present in all samples
1B	Sample contamination during dissection	Expect environmental contaminants such as spores, pollens, and skin microbes (caution, skin microbes have been implicated in several human diseases)
2A	Lifelong *in vivo* biocontamination from blood and the environment	Environmental contaminants such as microparticles of the same size as spores and pollens have been demonstrated to enter tissues; microbes of a similar size rapidly enter human tissues
2B	*In vivo* biocontamination: perimortem	When analyzing a tissue in relation to human disease, contamination may arise from *in vivo* dissemination of microbes (e.g., respiratory disease) unrelated to the primary disorder; invasion of diseased tissue may be a consequence rather than a cause of tissue degeneration
**BStrategies to exclude contaminants**		
**Method**	**Strategy**	**Comments**
Negative controls	Exclude all signals present in blank workups	Negative controls alone are not sufficient to detect all contaminating species. In addition, RNA-seq data rarely have blank controls because these are rejected as errors by the sequencing instrument
Common contaminants	Consider excluding common contaminant species such as those listed in Salter *et al.* [80] and Sanabria *et al.* [83]	Caution is urged because common contaminant microbes may themselves be the cause of disease
Within-batch consistency	Exclude signals that are present at similar levels in all samples	Caution is urged because, if applied to gut or lung, this would exclude many of the major species that are known to be present
Between-batch consistency	Only include signals that are present (and at different levels) in independent datasets from the same tissue	A major caveat is that, in different individuals with the same pathology, diverse organisms can be the cause of that pathology (e.g., viruses, bacteria, and fungi can all cause inflammatory lung disease).
Differential signals	Only include differential signals between, for example, disease samples versus controls	Consistent differential signals point unambiguously to species that are not contaminants
Microheterogeneity	High-resolution strain/substrain mapping. Contaminants introduced during sample work-up are likely to be of the same specific genotypes in different samples, whereas true signals are most likely heterogeneous in their exact sequences	Download sequences from different samples of the target tissue and prepare phylogenetic trees

Molecular biology reagents used to work up samples for sequence analysis are often contaminated with microorganisms. Salter *et al.* reported that sample dilution by a factor of 10^3^ to 10^4^ was necessary before contaminants represent 50% of the signal [80]. In terms of RNA-seq from PCR amplified material, this means that 0.1% of the signals could originate from contaminating material. In a series of 1000 signals, one or more may therefore arise from reagent contamination. Common bacterial contaminant species are given in Table 1, Table S1, and Table S2 of [80], and Fig. 5 of [83]. In addition, organisms commonly encountered in tap water may be consulted **(**Table S2). Although duplicate and/or blank samples worked up independently have been recommended [82], this is not possible with pre-existing RNA-seq data. In addition, workup and sequencing of blank samples generally fails because instrument settings reject very low numbers of sequence reads (Azenta Life Sciences/GENEWIZ, authorized personal communication).

(ii) **Sample contamination: type 1B**

This is an important issue because, if samples are not dissected under fully sterile conditions, they may become contaminated by exogenous organisms, for example airborne spores and microbes from human skin. These latter are likely to contaminate some samples, and analysis is complicated by the fact that the skin microbiome is very diverse and differs according to body site, individual, and geographical location [84–86]. One possible way to tackle this issue is to exclude known human skin microbes. In the present work we subtracted brain datasets against a series of RNA-seq datasets for human skin (Results). Caution is necessary because, for example, a common skin fungus, *Malassezia*, has been directly implicated in human diseases such as psoriasis and has been reliably been detected in other human tissues [87].

(iii) ***In vivo* biocontamination: types 2A and 2B**

This falls into two subtypes. Type 2A concerns contamination *in vivo* through life-long exposure to environmental agents. For example, we have observed signals in RNA-seq data corresponding to barley (*Hordeum vulgare*, not presented). Although airborne contamination of samples is not excluded, we suspect that *in vivo* contamination may take place. For example, inhaled ultrafine manganese oxide particles readily enter the central nervous system [88]. In mice exposed to microparticles (5 μm) and macroparticles (20 μm) in drinking water, both types of particle entered body tissues [89], and environmental exposure to both small (<2.5 μm) and large (2.5–10 μm) particles has been associated with cognitive decline in human [90]. Barley pollen (25 μm) is in the same broad size range; over the course of a lifetime it is possible that these might also enter the circulation including brain vasculature, and from there into the brain itself. A similar route of infection could apply to microbial spores.

Type 2B contamination concerns contamination of the target tissue *in vivo* before sampling. For example, in studying a body tissue obtained postmortem, the cause of death should be taken into consideration. To illustrate, in many elderly patients (the principal source of postmortem tissues) death is often precipitated by severe infection, often pulmonary, and it is possible if not likely that microbes enter the circulation and are thus present in diverse body tissues, independently of any disease process under investigation (Table 1A).

(iv) **Strategies to exclude contamination**

Key recommendations are summarized in Table 1B. However, there is likely to be substantial overlap between microbes that are representative of the natural microbiome in human tissues and common contaminants of human origin such as from the skin.

(v) **Viruses and contamination**

The problem of virus contamination is less severe because analysis is based on RNA-seq data, and contamination with mammalian cells expressing virus transcripts is thought to be unlikely. By contrast, contamination with viral genomes is possible, but these (particularly for DNA viruses) may be recognized because genomic reads are unlikely to correspond to the viral transcriptome. Viral reads were therefore mapped to the viral transcriptome to determine their authenticity (not presented).

### Microbes and viruses: how many cells/genomes are being detected?

This analysis is based, for cellular organisms, on the number of copies of rRNA transcripts in each sample. The question therefore arises of how many cells are present, which in turn depends on the number of ribosomes (or viral transcripts) per cell. Because this parameter introduces a further complexity, further discussion is provided in Boxes 1 and 2.

### Scripts

The scripts developed in this study are available at github (https://github.com/xinyuehu12/ToL).

## Results

### Tree of life

To generate the probe collection for cellular microbes, 120 key organisms were selected from Open Tree of Life Project and other sources to represent, as far as possible, the full spectrum of cellular organisms. The key organisms cover the domains of Archaea (A), Bacteria (B0–6), Chloroplastida (C1–4), Amoebozoa (D), basal Eukaryota (E0), fungi (F0–6), and Holozoa/Metazoa (H0–3) (Fig. 1). To address the distribution of these organisms across the Tree of Life, the 16S/18S sequences corresponding to these 120 organisms were downloaded and used to build a phylogenetic tree (Fig. 2). Because Archaea and Eukaryota are more related, the Bacteria group was used as outgroup [91]. This tree has relatively good statistical support because the bootstrap values for most of nodes are over 70. The nodes that have weak support (bootstrap value <70) are shown in gradient colors, ranging from red (7) to blue (69). The 16S rRNA sequences form two monophyletic groups, Archaea and Bacteria, whereas only one monophyletic group, Holozoa, was observed within the 18S rRNA sequences of eukaryotes. The species, especially from Chloroplastida and Amoebozoa, were not clustered well (Fig. 2), and their nodes show weak support.

**Fig. 3:**
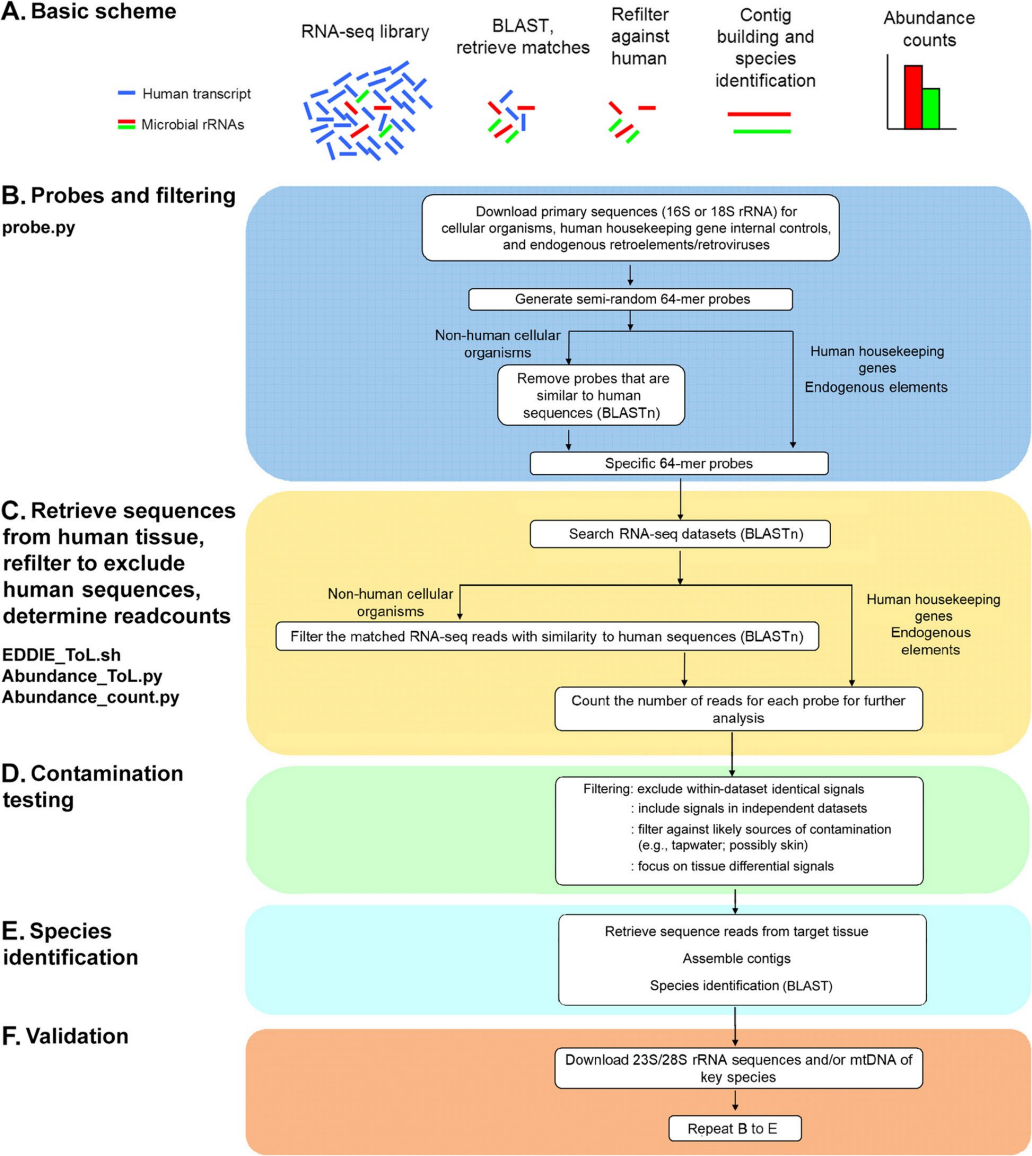
eToL workflow pipeline. The probe.py was used to download primary sequences and devise probes. The probes were aligned to RNA-seq datasets, and the matches (reads) were filtered and counted by EDDIE_ToL.sh, Abundance_ToL.py and Abundance_count.py

We then devised 64-mer probes for these species (the rationale is presented in the Methodology section), as shown in the pipeline (Fig. 3), and filtered them against human sequences. To validate this approach, we checked whether the probe collection detects known human pathogens/commensals. With few exceptions, all organisms in the PATHLIST were detected in the probe collection, generally with multiple matches. The mean number of matches between the probe collection and each sequence in the PATHLIST was 25.8, and the median was 15. Only two species were not detected, *Leishmania donovani* and *Ascaris lumbricoides*. These data suggest that the probe collection covers around 98% of species for which sequences are available (future editions of eToL will be revised to include any missing lineages).

**Fig. 4:**
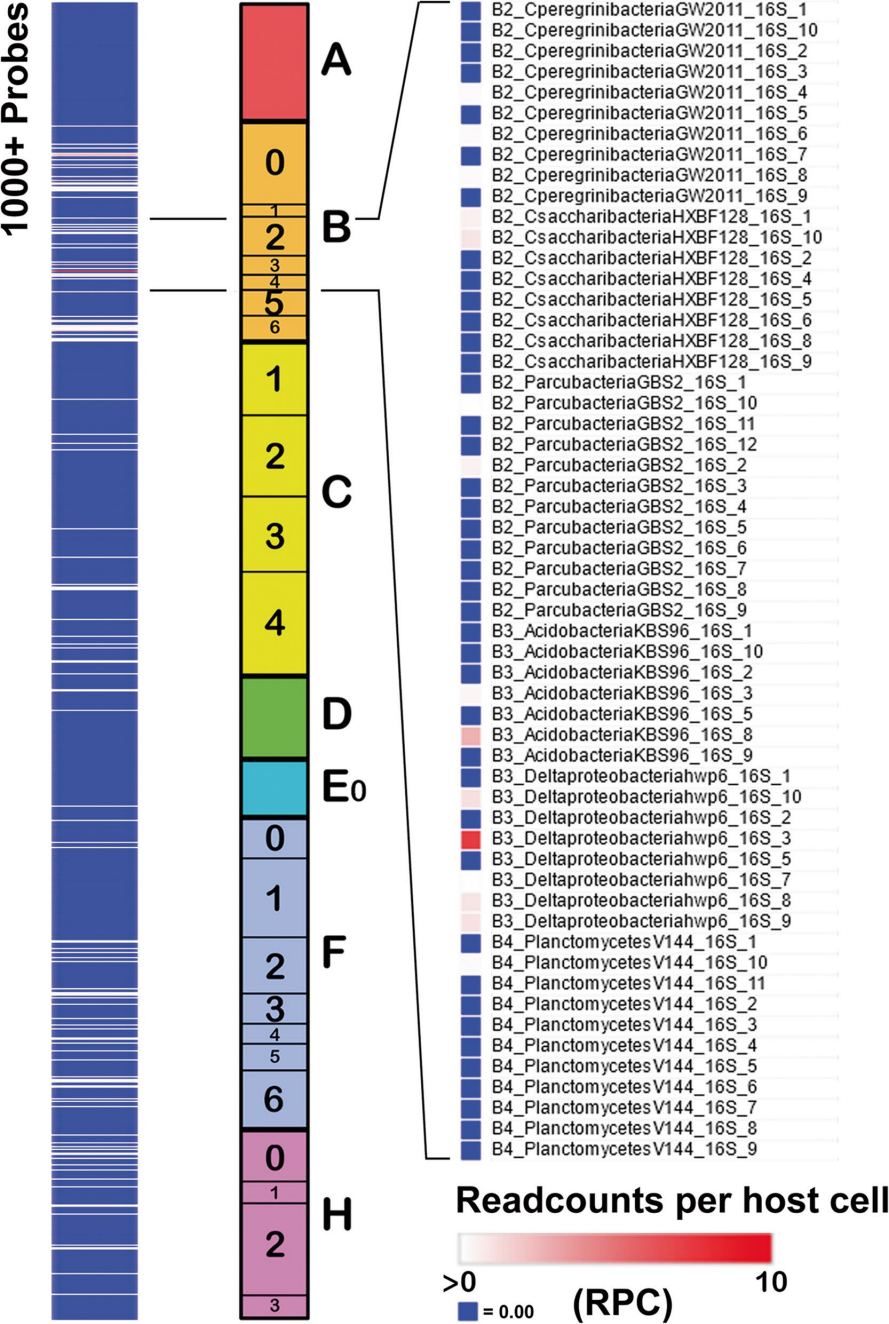
Visualization of the cellular microbiome profile. (Left) Standard display (first brain sequence read archive listed in the supplementary material) generated with Morpheus where the number of matches for the 1000+ probes in a constant order A–H (Center) are colored such that blue = 0, white = lower cutoff (generally any value >0) and red ≥ upper bound (differs between experiments). All values are normalized to the number of host cells determined by readcounts for two housekeeping genes. (Right) Magnification of the B2–B4 region showing matches for individual probes

We also developed a uniform method of display based on heat-mapping, as illustrated in Fig. 4.

**Fig. 5:**
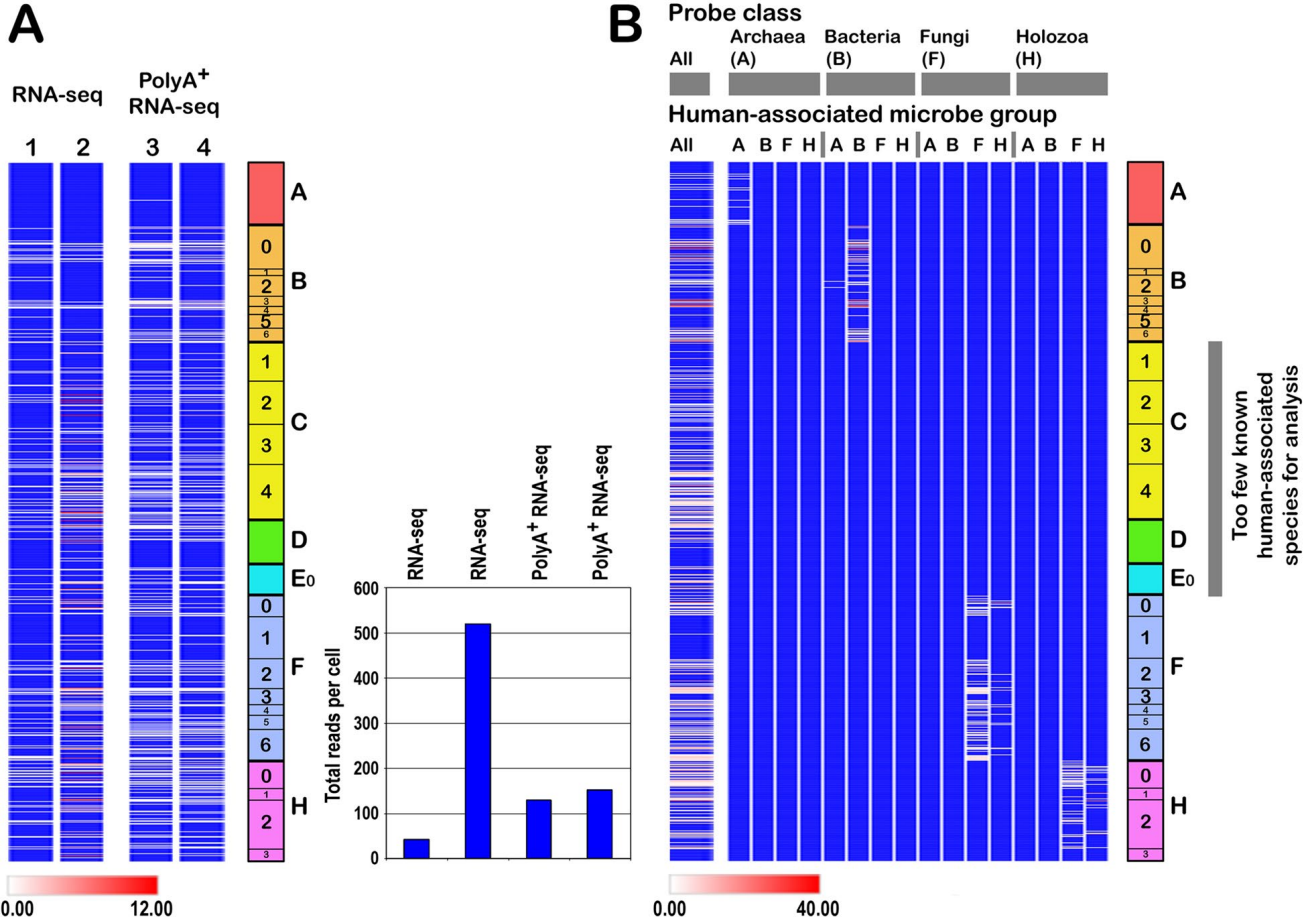
Analysis of RNA-seq of total RNA versus polyA^+^ RNA, and class specificity of the different probes. (A) Two brain (cortex) polyA^+^ RNA datasets were available for study, these were matched with two (cortex) total RNA datasets. Although the total number of microbial matches spanned a 10-fold range (inset), as expected given independent sample preparation and sequencing methods, the figure shows that polyA^+^ RNA selection does not remove all rRNA, and the number of overall matches (inset) for the two polyA^+^ datasets was intermediate between the two matched total RNA datasets. (B) (Left) The whole probe collection was used to probe the list of 100+ human-associated microbes (PATHLIST). (Right) Probes for classes A (Archaea), B (Bacteria), F (Fungi), and H (Holozoa) were then separately used to probe PATHLIST subclassified into the same groups. Classes C (Chloroplastida), D (Amoebozoa), and E0 (basal Eukaryota) were not studied because too few human-associated species are known. The figure demonstrates that each probe class principally detects species of the same class, although some cross-matching was observed between Fungi and Holozoa as expected because of evolutionary relationships. Class C–E0 probes failed to find matches in classes A, B, F, and H (panel B), but some crossmatching is expected because these probes found matches in the complete PATHLIST (left)

**Fig. 6:**
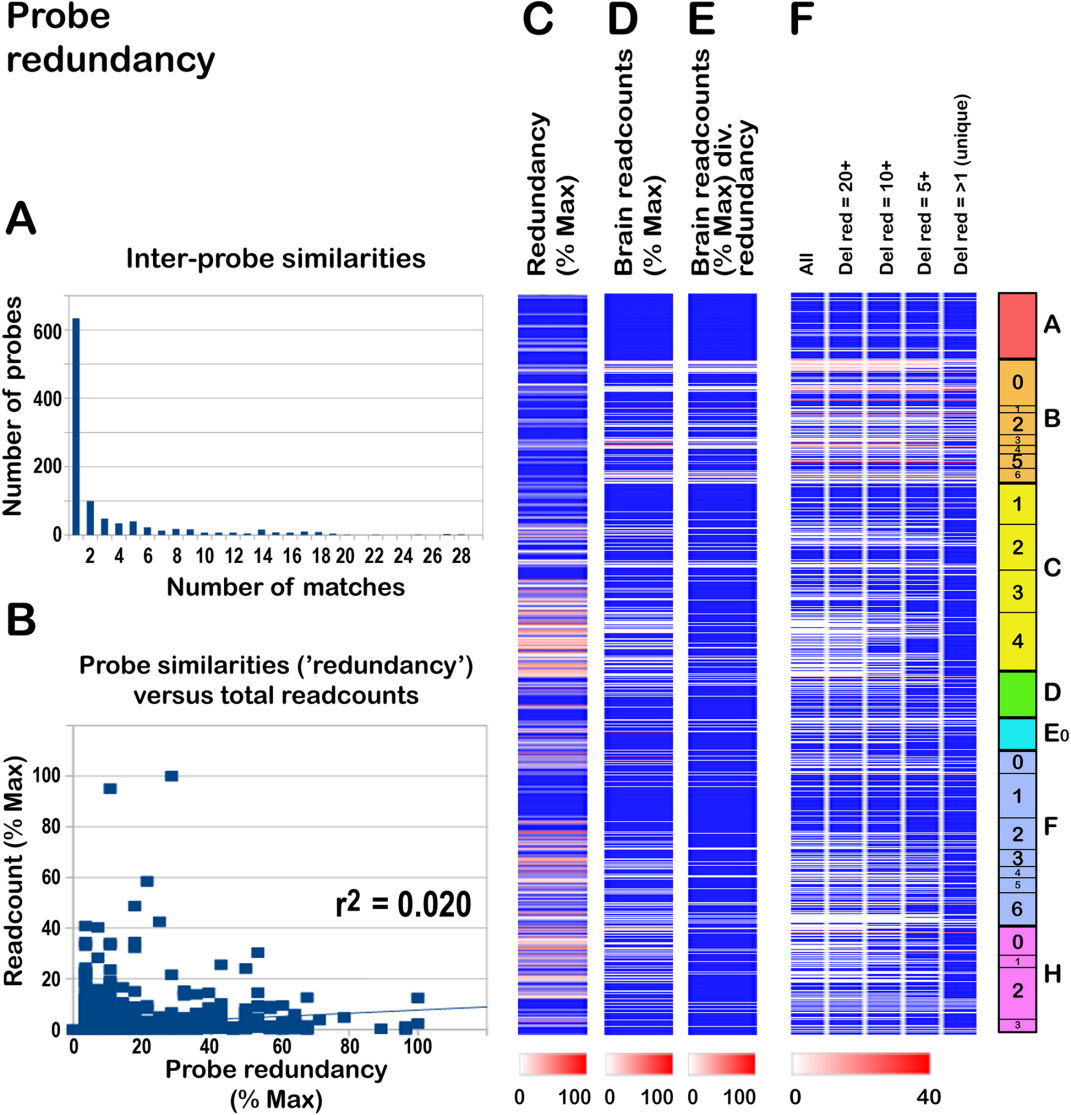
Overlaps within the probe collection ('redundancy'). (A) The collection was compared to itself, revealing that over 60% were unique. (B) To determine how probe overlaps might affect sequence detection, we compared the total readcounts (brain) to the extent of probe redundancy (number of overlaps in the probe collection), demonstrating that high readcounts do not correlate with redundancy. The r^2^ of the trendline (Microsoft Excel) was 0.02, showing that only 2% of the observed variation in numbers of matches can be ascribed to probe redundancy. (C,D) Side-by-side comparison of probe redundancy (C) with brain readcounts (D), demonstrating no obvious correlation. (E) As in (D), but the readcount scores have been divided by the redundancy of each probe, showing some changes, but conservation of the overall pattern. (F) Effect of removing probe signals with different levels of redundancy (≥20, ≥10, ≥5, and >1). Although the overall profile was retained, this degraded many signals, indicating that the complete probe list is preferable for comprehensive retrieval

A concern is that rRNA depletion {e.g., through poly(A) RNA selection} in RNA-seq libraries might compromise detection. However, this was not found to be a systematic problem, and robust microbe signals were detected in both poly(A)^+^ and unselected RNA-seq datasets (Fig. 5), although only a small number of poly(A)^+^ datasets were examined (section on Methodology development). A further concern is whether the probe collection we have developed contains probes that significantly overlap with each other, and thus constitute partial duplicates. As described in Methodology, this was carefully addressed (Fig. 6). The whole probe collection detected 98% of a test list of human pathogens and commensals, whereas selection of the unique probes detected only 92%. We will address this issue in further refinements, but the current analysis was continued with the complete list of probes.

### Excluding contamination

Contamination is increasingly recognized to be a problem in microbiome analysis (reviewed by de Goffau *et al.* 2018). We therefore adapted our protocols to address this issue. A signature of contamination is that different samples processed in parallel have consistent signals in all samples. Multi-positive signals were deleted from both the Miami and Rockefeller datasets. As shown in Figure S1, many of the signals in the Miami dataset might be contaminants, whereas the same issue was not encountered with the Rockefeller dataset (Figure S1). However, deleting multi-positive signals comes at the risk of removing true signals (Table 1B).

### Mutiple independent datasets: the brain has its own microbiome

**Fig. 7:**
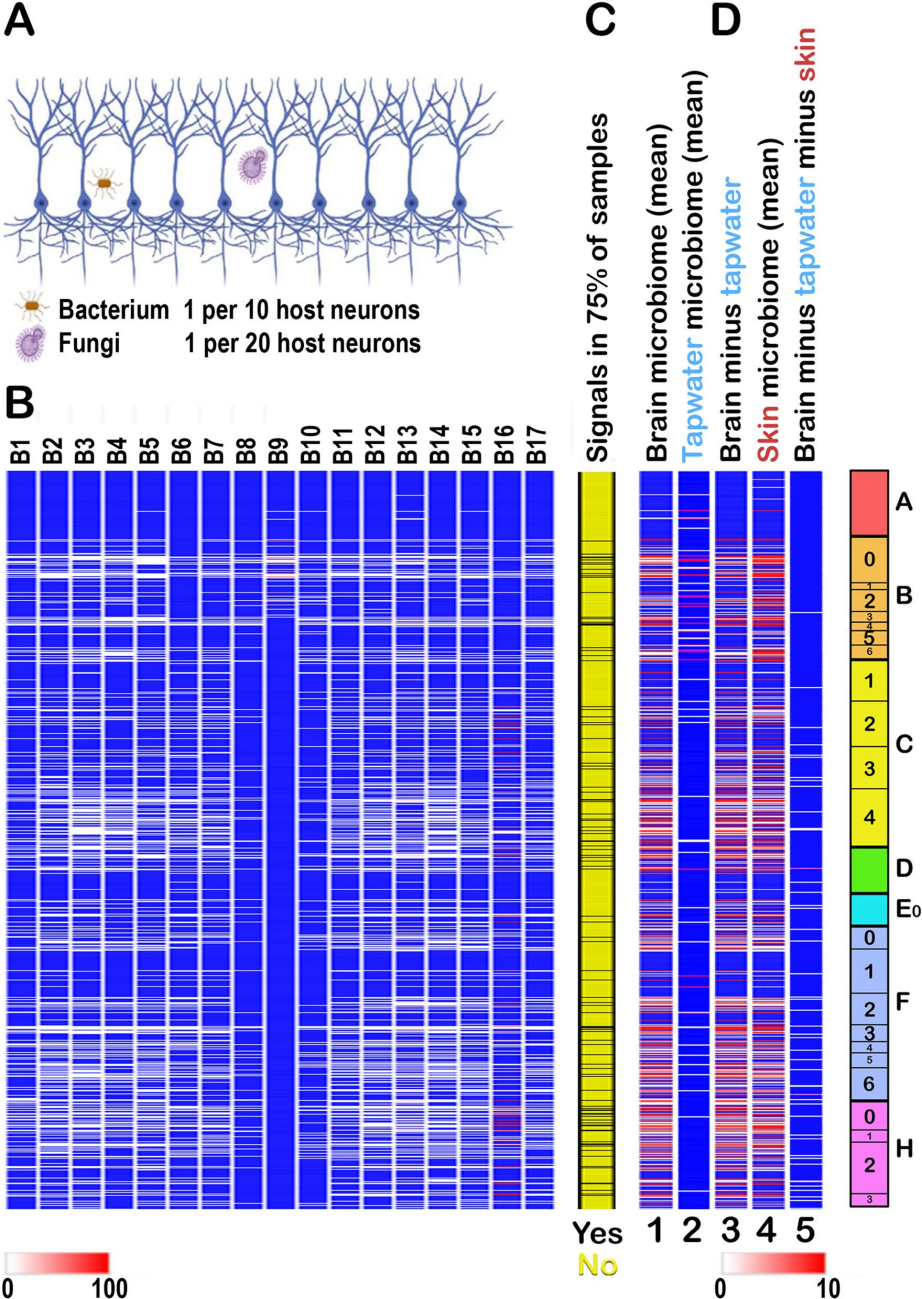
The profile of the cellular microbiome in human brain. (A) Schematic of the relative representation of microbes in normal brain (cortex): overall readcounts from 17 independent brain samples (below) indicate that bacteria and fungi are the major species in brain. (B) Profiles of readcounts in 17 independent brain samples showing different patterns in different brain RNA-seq datasets (where B9 and B16 have distinctive patters) but overall conservation of the profile. Estimated abundances from the calculated numbers of rRNA per cell in the different organisms are as follows Archaea (10^-5^ microbes per host cell), Bacteria (0.14), Chloroplastida (0.06, but type 2 contamination has not yet been excluded, Table 1), Amoebozoa (0.01), basal Eukaryota (0.01), Fungi (0.05), Holozoa (0.05, possibly because of cross-matching with Fungi, Figure 5B). Bacteria and Fungi represent 41% and 13% of the total burden (together >50%). (C) Signals that are present in >75% of all samples. (D) The brain has its own microbiome. (Lane 1) The mean brain microbiome profile from 17 independent samples. (Lane 2) The mean profile in tapwater. (Lane 3) Brain profile where all signals also detected in tapwater have been removed. (Lane 4) Mean skin microbiome profile. (Lane 5) Brain profile where all signals also detected in tapwater and skin have been removed, showing degradation of the signal. Although type 1 contamination cannot be formally excluded here, there may be overlaps between the brain and skin microbiomes (Discussion). However, despite attenuation of the signal, the subtraction demonstrates that there are microbial signals in brain that do not occur in skin. Panel (A) was created at Biorender.com

The second strategy was to only consider signals that are present in independent datasets from the same tissue. We therefore screened 17 independent RNA-seq datasets from human brain. As shown in Fig. 7A, although there were differences in the exact signals in each dataset, the patterns were substantially conserved despite entirely independent workup.

To rigorously confirm that the microbial signals detected in brain do not arise through contamination, we sequentially subtracted the signals from the 17 independent brain samples against tapwater (which, because of its chlorine content, has its own specific microbiome; details in the source references for the tapwater SRAs), and then against human skin. Specifically, if any signal appeared in any sample of tapwater or skin, all brain values were set to zero. Both procedures markedly depleted the brain microbial signal. However, multiple signals remained (Fig. 7B). This argues that brain has its own microbiome that differs from that of skin, and is unlikely to represent either type 1 or type 2 contamination.

### Tissue differences: liver versus brain

Previous studies highlighted likely overlaps between the brain and skin microbiomes (e.g., [36, 37]), which may be unsurprising because both tissues have an epithelial developmental origin. Subtraction of brain against skin signals could conceal species that are both (i) present in skin, and are (ii) opportunistic invaders of the CNS. We therefore focused on a different tissue, liver. As shown in Figure S2, the microbiome profiles for brain and liver display many similarities, but also some evident differences. The identities of key differentials were established by contig building and probing of NCBI datasets (Figure S3). In the samples analyzed, *Malassezia* spp. were found to be brain-specific, whereas *Staphylococcus aureus* appeared to be liver-specific (Table S7). The differential presence of key species was confirmed by second-round reprobing using 23S/28S-based probes (not presented).

### Heterogeneity

We addressed whether individual probes are generally detecting specific organisms, or clusters of organisms. We observe that, even using these highly selective probes, we identify clusters of organisms that are not monophyletic. We illustrate this in Box 3 through a case study. It is important to note that each probe detects a cluster of related species rather than a unique species (Box 3), and the exact identity of each sequence group retrieved from human tissue must be revalidated by 23S/28S analysis.

### Viruses and retroelements

**Fig. 8:**
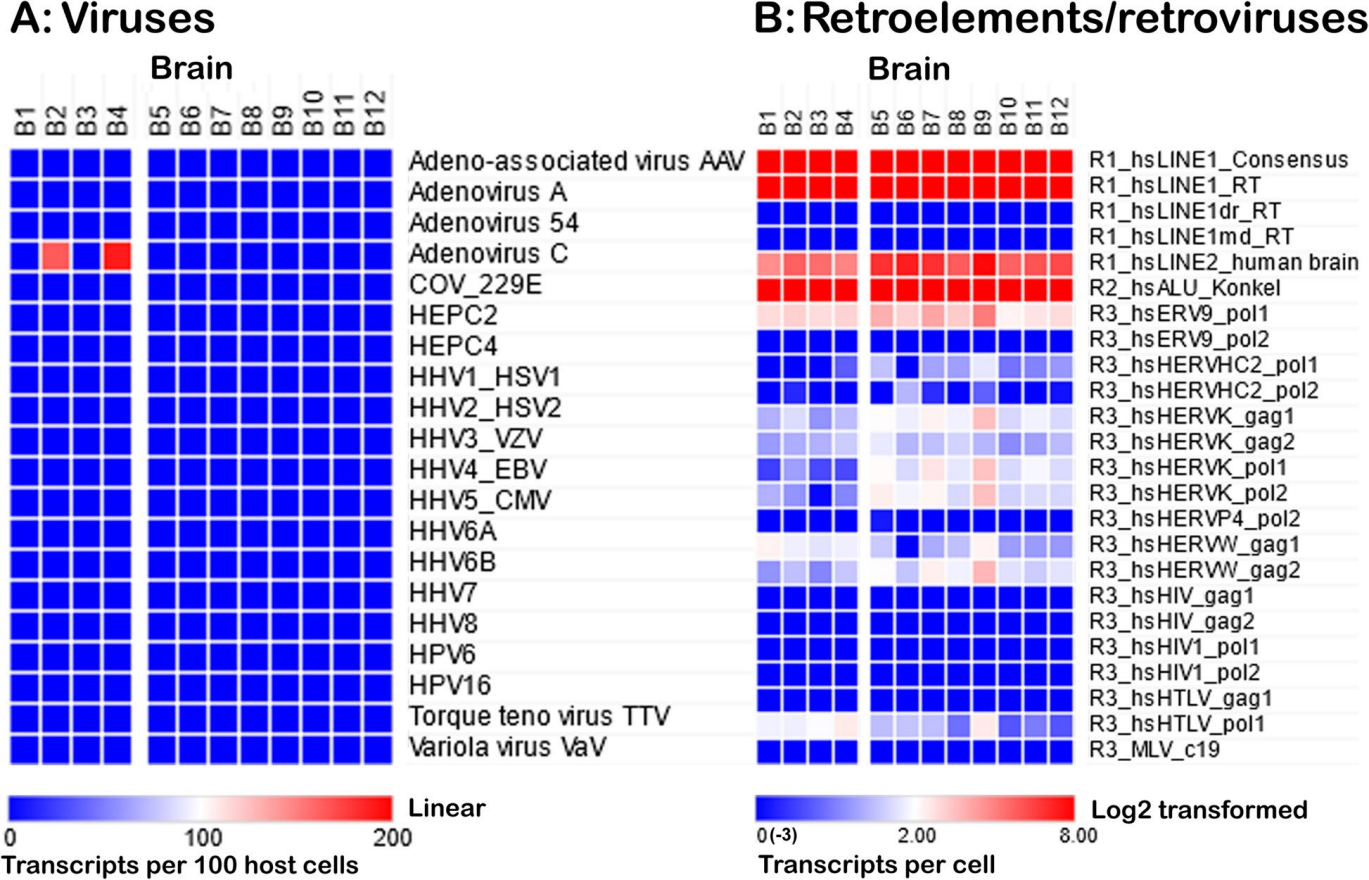
Viruses and retroelements in brain. (A) Screening of 12 normal brain samples (Miami and Rockefeller datasets) with the stripped (to remove all sequences similar to human) genomes corresponding to the top 20 viruses (>99% of brain matches in Readhead et al. 2018; text for details) revealed matches only for adenovirus type C. (B) Screening for retroelements and endogenous retroviruses showing that transcripts for LINE and SINE elements are highly abundant, whereas endogenous retrovirus transcripts are much less abundant (8–128-fold). All samples were HIV1-negative

In terms of readcounts, viruses were less abundant than the other microbes (Fig. 8A). Adenovirus C was the most abundant in these samples. Other viruses such as HSV-1, CMV, HHV-6A and TTV were also present in some individuals (not presented), but their overall abundance was low (comprehensive analysis using this methodology is in preparation).

We also screened, using 64-mer probes, the abundance of select retroelements and endogenous retroviruses in tissue samples. As shown in Fig. 8B, these are very well represented, although at present their potential contributions to health and disease remain unknown.

### How many microbes are there in brain?

Readcounts, normalized on a per cell basis, do not immediately indicate whether microbes in brain are (relative to host cells) rare or abundant. We therefore considered further normalization factors including the number of rRNA copies that are present in typical microbial cells of different types (Box 1) to calculate how many cells/genomes are present in normal human brain; we elaborate on this point in the Discussion section and in Box 2.

## Discussion

We report a new method to comprehensively analyze the entire microbiome of human tissue samples from transcriptomic (RNA-seq) data. The method comprises a 'net' of >1000 probes that covers all organisms from the known spectrum of lifeforms – the electronic Tree of Life (eToL). The method reported here is not intended to replace other established methodologies, but to provide an alternative that does not require any dedicated computer programs beyond those that are already widely available to the community (e.g., BLAST and BLAST+). Primarily based on 16S/18S sequences from cellular organisms, the approach has been extended to cover viruses and retroelements. Moreover, for the first time it addresses the entire ToL rather than selected subgroups of microbes. The most significant advantage of this method is that it requires around 5000-fold less computation than rival NGS techniques (e.g., metagenomics) and avoids the problems of non-selectivity encountered in some earlier studies.

This study is based on a spectrum of cellular organisms (*N* = 120) that span the entire ToL (Figs. 1 and 2), 20 viruses that are reported to be particularly abundant in our target tissue (brain), and 11 types of retroelements. For cellular species, the maximum likelihood phylogenetic tree shows the relationships between the rRNA sequences of the different cellular organisms, but species from domains such as Chloroplastida, Amoebozoa, and basal Eukaryota were not well clustered. Some nodes have weak statistical support although the overall statistical support is good. This may be because only partial rRNA sequences were available for some species. In addition, the branch of *Enterocytozoon bieneusi* (F1_Ebieneusi_18S) is long (not presented), which means it has high divergence compared to the other species, emphasizing the exceptional diversity of the fungi. However, the robustness of multiple alignment tools plays an important role in the accuracy of the phylogenetic tree. Although Multiple Alignment Using Fast Fourier Transform (MAFFT) has been reported to have good alignment accuracy, the MUSCLE tool was found to have better performance in reconstructing trees in our study. Other aligners may also be used to align such distant sequences [92]. To improve the accuracy of the alignments, rRNA secondary structures may also need to be considered [93].

Earlier studies of microbe analysis encountered problems including non-specificity and crossreactivity with human sequences. The *K* value (the frequency of probes aligning to target sequences by chance) can be used to calculate the probe length that is necessary for accurate target detection. By considering the background of incomplete but above-threshold matches (*K*_b_ value), the minimum probe length for finding unique matches in a typical mammalian genomic library was calculated to be 62 nt at 85% sequence identity and a *K*_b_ of 0.1 [56]. Therefore, our eToL approach was based on 64-mer (or longer) probes, that have been carefully filtered to remove any sequences matching human sequences. The 64-mers were semi-randomly selected because, if we used a sliding window method to produce overlapping 64-mers, the computation time required to process the many thousands of probes generated would become prohibitive.

The collection of probes (eToL) is designed as a 'net' to entrap all non-human microbial sequences. For this reason the identity of each probe does not indicate the exact species present, and retrieval of sequences from human tissue is necessary for species identification. However, the matches to our probes discovered in RNA-seq data unambiguously confirmed the presence of microorganisms because all matches were double-checked to exclude human sequences. Therefore, the false positive rate of detecting human sequences was reduced to near-zero.

The 64-mer probe collection for cellular organisms is believed to be largely comprehensive because 98% of known human pathogens and commensals were detected by the probe collection. In future editions of the ToL probe list will include probes for missing species such as *Leishmania donovani* and *Ascaris lumbricoides*, as well for species that are less well represented in the probe list.

We took strict precautions to recognize and exclude potential contaminants of either type 1 or type 2 (Table 1). This revealed that some RNA-seq datasets are potentially diluted by contaminant species inadvertently introduced during sample preparation and workup. Subtraction of matches against likely sources of contamination including human skin and tap water reduced but did not eliminate the microbiome signals, arguing that key species are indeed present in human brain (in preparation). To confirm the identity of these brain-resident species, the matches were retrieved from brain, the exact species identified, and then further validated by 23S/28S rRNA analysis, and for eukaryotic species, analysis of mitochondrial DNA (in preparation).

For viruses, we report that the false positives in the study of Readhead *et al.* may be explained, in part, by homologies between viral and human sequences. For example, HHV-6A, 6B, and 7 (that were asserted to be increased in AD) have pronounced matches with human telomeric DNA repeats, HHV-3 and HHV-8 contain sequences similar to human thymidylate synthase (*TYMS*), the HHV-4 genome has matches to human interleukin 10 receptor variant (*IL10RV*), and the genome of variola virus (the agent of smallpox, also detected in human brain by Readhead *et al.* contains homologies to human ribonucleotide reductase subunits (*RRM1* and *RRM2B*) (Table S5). The major virus type in human brain was identified to be adenovirus type C (transcript mapping will be reported elsewhere).

Overall, this work reveals that a remarkable diversity of microbes are present in brain (and liver) samples. All major taxonomic groups are represented. In addition to bacteria and fungi, as previously reported, we report that microbes ranging from Archaea to Amoebozoa, Chloroplastida, basal Eukaryota, and Holozoa/Metazoa are present in human brain (to be presented elsewhere). Few viruses were encountered, the majority being of the adenovirus C class.

The eToL method goes some way towards answering the question of whether there is indeed a brain microbiome [28]. Because this is a Methodology paper, we do not report systematically on the identities of the target organisms or experimental confirmation (in preparation) except in a specific examplar study (brain versus liver, Figure S2). However, in terms of microbes/genomes per cell, we estimate that Archaea are present at 10^−5^ microbes per host cell, Bacteria (0.14), Amoebozoa (0.01), basal Eukaryota (0.01), and Fungi (0.05), of which Bacteria and Fungi constitute >50% of the total microbial burden (Fig. 7). Although this number of microbes might appear to be high, brain neurons are extremely large, and we calculate that, in terms of volume, the total microbial cell volume amounts to no more than 1/10 000th of the neuronal volume.

We have considered whether the eToL method can be extended to medical diagnostic applications. The method correctly identified 98% of human-associated microbial sequences, and simple modifications would allow this to be raised to 99%+. A potential drawback is that short small subunit rRNA sequences do not allow precise identification of microbial species. For example, in one study *Bacillus cereus* was misidentified as *B. thurigenesis* because the 16S rRNA sequences of these two species are 99.7% identical [94]. However, simple refinement of the analysis by reference to 23S/28S sequences allows rapid confirmation that a given organism is indeed present. In addition, our analyses indicate that few if any signals detected in human samples are monophyletic – we observe multiple closely related species/sequences that probably reflect lifetime exposure to constantly evolving external and internal microbiomes. For this reason the eToL method has a significant advantage over standard metagenomic and/or PCR analyses that are based on the reference sequences of standard microbial types. The principle utility of the method is that it rapidly identifies and quantifies the principal organism groups that are present in a biological sample. 'Syndromic classifiers' (discussed in [95]) are of enormous utility in medical analysis because they permit patients to be classified into infectious versus non-infectious syndromes, or can distinguish between viral versus fungal versus bacterial infections, with 'important implications for clinical management' [95].

The approach presented here, unlike other strategies, also has the advantage that all cellular organisms across the ToL can be addressed in a single screen. For viruses, the genome-stripping method offers a route to ensure that only viral sequences are detected. In addition, the method has advantages over other strategies in terms of specificity, and employs widely available analytical tools (BLAST and BLAST+). It is also rapid (around 5000-fold faster than conventional NGS metagenomic methods). Moreover, unlike most PCR-based and metagenomic analyses, the method allows quantification of the absolute number of microbes present in a given sample. Simple modifications to the protocol make it applicable to other species such as non-human primates and rodents where whole-genome sequence data are available for stripping.

In sum, the methodologies presented here may find broad application in the analysis of microbes and viruses in widely available RNA-seq data for human tissues, and thereby enhance our understanding of the role of the microbome in human physiology in health and disease. In addition, given that RNA-seq data can now be obtained in a few days for under $400, and that the sequence analysis can be performed very quickly (a few minutes), the method is likely to lend itself to medical diagnostic applications in the analysis of oral, nasal, and pulmonary samples, as well as of blood, urine, and cerebrospinal fluid.

## Supplementary Information

**Additional file 1.**

## Data Availability

All relevant data and materials, including data repositories and sequence read archive identification numbers, are provided in the manuscript and/or in the supplementary material online.
