# Supplementary material for: The electronic tree of life (eToL): a net of long probes to characterize the microbiome from RNA-seq data
Source: BMC Microbiol. 2022 Dec 22;22:317. doi: 10.1186/s12866-022-02671-2 (PMC9773549; doi:10.1186/s12866-022-02671-2)
Supplement: Supplementary file 1 — Additional file 1. [file 12866_2022_2671_MOESM1_ESM.zip › SUPPLEMENTARY MATERIAL ONLINE.pdf]

Table S1. List of species accessions used to compile the Tree of Life.

Table S2. List of sequence read archive datasets.

Table S3. List of 64-mer probes. (A) Probes covering the microbial Tree of Life. (B) Housekeeping gene probes. (C) Probes for retroelements and endogenous retroviruses.

Table S4. List of human-associated species (PATHLIST).

Table S5. Matches between key viruses and the human genome.

Table S6. Stripped viral genomes. This Table is 350 pages in length and is supplied separately.

Table S7. Identification of liver- and brain (cortex)-specific signals.

Figure S1. Some brain microbiome profiles are suggestive of contamination.

Figure S2. Microbiome profiles of liver versus cortex.

Table S1. List of species accessions used to compile the Tree of Life.

Domain	Code	Kingdom/Phylum	Subtaxon	Species	Systematic name	Accession
Archaea	A	Halobacteria		*Halobacterium salinarum*	A_Hsalinarum_16S	NR_025555.1
		Methanococci		*Methanocaldococcus* SG1	A_MethanocaldococcusSG1_16S	MK602649.1
		Methanopyri		*Methanopyrus kandleri*	A_Mkandleri_16S	NR_074539.1
		Thermococci		Thermococci archaeon NE31B05cA	A_Tarchaeon_16S	DQ423947.1
		Korarchaeota		Korarchaeota SRI-306	A_KorarchaeotaSRI306_16S	AF255604.1
		Thermoplasmata		Thermoplasmata archaeon Kjm51a	A_ThermoplasmataKjm51a_16S	AB749767.1
		Thaumarchaeota		Thaumarchaeota archaeon NAOA2	A_ThaumarchaeotaNAOA2_16S	KT380501.1
		Archaeoglobi		Archaeon TE1-87-T7	A_ArchaeonTE187T7_16S	KX098446.1
		Methanoliparaceae		*Methanoliparum thermophilum*	A_Mthermophilum_16S	RXIF01000002.1
		Asgard		Archaeon 26ABF_S2	A_Archaeon26ABFS2_16S	MN444134.1
Bacteria	B0	Desulfobacteraceae		Desulfobacteraceae cLaKi	B0_DesulfobacteraceaecLaKi_16S	AJ582710.1
	B0	Coriobacteriia		*Coriobacterium* CCUG_33917	B0_CoriobacteriumCCUG33917_16S	AJ131149.1
	B0	Thermotogae		*Thermotoga maritima*	B0_Tmaritima_16S	NR_102775.2
	B0	Limnochordia		*Limnochorda pilosa*	B0_Lpilosa_16S	AP014924.1
	B0	EM3		*Marinobacter* EM3	B0_MarinobacterEM3_16S	HG004173.1
	B0	Aquificae		Aquificae SHNS732	B0_AquificaeSHNS732_16S	HM110206.1
	B0	Acetothermia		Acetothermia TuzSed3B	B0_AcetothermiaTuzSed3B_16S	MT523014.1

	B1	Spirochaetes	*Treponema pallidum* supbsp. *pertenue*	B1_Tpallidum_16S	AF426102.1
	B2	Saccharibacteria	Candidatus Saccharibacteria HXBF1-28	B2_CsaccharibacteriaHXBF128_16S	KJ540650.1
	B2	Peregrinibacteria	Candidatus Peregrinibacteria GW2011	B2_CperegrinibacteriaGW2011_16S	KX123616.1
	B2	Parcubacteria	Parcubacteria GBS-2	B2_ParcubacteriaGBS2_16S	KT033776.1
	B3	Acidobacteria	Acidobacteria KBS96	B3_AcidobacteriaKBS96_16S	FJ870384.1
	B3	Deltaproteobacteria	*Deltaproteobacteria* hwp6	B3_Deltaproteobacteriahwp6_16S	MT084045.1
	B4	Planctomycetes	*Planctomycetes* V144	B4_PlanctomycetesV144_16S	MK554519.1
	B5	Latescibacteria	Latescibacteria 134476n2	B5_Latescibacteria134476n2_16S	MK048603.1
	B5	Marinimicrobia	*Candidatus Marinimicrobia* 61467n6	B5_Cmarinimicrobia61467n6_16S	MK048793.1
	B6	Bacteriodetes	*Thermonema rossianum*	B6_Trossianum_16S	NR_026367.1
	B6	Bacteriodetes	*Prevotella intermedi*a	B6_Pintermedia_16S	LT707625.1
Chloroplastida	C1	Euglenozoa	*Phacus brachykentron*	C1_Pbrachykentron_18S	AJ532481.1
		Euglenozoa	*Colacium mucronatum*	C1_Cmucronatum_18S	AJ532440.1
		Parabasalia	*Trichomonas vaginalis*	C1_Tvaginalis_18S	KM282377.1
		Jakobida	*Jakoba libera*	C1_Jlibera_18S	AF411288.1
		Preaxostyla	*Paratrimastix pyriformis*	C1_Ppyriformis_18S	KT388052.1
	C2	Glaucophyta	*Glaucocystis* ARP-2014	C2_GlaucocystisARP2014_18S	KF631388.1
		Haptophyta	*Isochrysis galbana*	C2_Igalbana_18S	KX980524.1

	SAR/Ciliophora	*Spirostomum minus*	C2_Sminus_18S	MK929559.1
	SAR/Apicomplexa	*Plasmodium falciparum*	C2_Pfalciparum_18S	XR_002966654.1
	SAR/Apicomplexa	*Plasmodium gallinaceum*	C2_Pgallinaceum_18S	XR_003699198.1
	SAR/Stramenopiles	*Heteromita globosa*	C2_Hglobosa_18S	U42447.1
	SAR/Rhizaria	*Plasmodiophora brassicae*	C2_Pbrassicae_18S	AB094976.1
C3	Galdieria	*Galdieria sulphuraria*	C3_Gsulphuraria_18S	LC504058.1
	Cyanidioschyzon	*Cyanidioschyzon merolae*	C3_Cmerolae_18S	XR_002461616.1
	Cyanadinium	*Cyanadinium caldarium*	C3_Ccaldarium_18S	AB091231.1
	Compsopogonales	*Compsopogon caeruleus*	C3_Ccaeruleus_18S	KC596285.1
	Porphyridiophyceae	*Porphyridium aerugineum*	C3_Paerugineum_18S	AJ421145.1
	Floridophycidea	*Porphyridium purpureum*	C3_Ppurpureum_18S	KY054989.1
	Rhodellophyceae	*Rhodella violacea*	C3_Rviolacea_18S	AB045580.1
	Goniotrichopsis	*Goniotrichiopsis sublittoralis*	C3_Gsublittoralis_18S	AF168629.1
C4	Chlorophyta	*Atractomorpha echinata*	C4_Aechinata_18S	U73470.1
	Chlorophyta	*Golenkinia longispicula*	C4_Glongispicula_18S	AF499923.1
	Chlorophyta	*Pleurastrosarcina brevispinosa*	C4_Pbrevispinosa_18S	KM020187.1
	Chlorophyta	*Elliptochloris bilobata*	C4_Ebilobata_18S	AM422984.1
	Streptophyta	*Chlorokybus atmophyticus*	C4_Catmophyticus_18S	AF408244.1
	Streptophyta	*Chara vulgaris*	C4_Cvulgaris_18S	AF032747.1
	Kathablepharidae	*Leucocryptos marina*	C4_Lmarina_18S	KY980342.1
	Cryptomodadales	*Guillardia theta*	C4_Gtheta_18S	X57162.1

		Goniomonas	*Goniomonas avonlea*	C4_Gavonlea_18S	JQ434475.1
Amoebozoa	D	Amoebozoa	*Diderma testaceum*	D_Dtestaceum_18S	HM101142.1
		Archamoebae	*Rhizomastix bicoronata*	D_Rbicoronata_18S	KP343638.1
		Ciliophryidea	*Ciliophora* aOmb2	D_CaOmb2_18S	LN869948.1
		Hemimastigida	*Hemimastix kukwesjijk*	D_Hkukwesjijk_18S	MF682191.1
		Centrohelida	*Raphidocystis contractilis*	D_Rcontractilis_18S	AB196984.1
		Nucletmycea	*Fonticula alba*	D_Falba_18S	FJ816018.1
		Physarum	*Trichia varia*	D_Tvaria_18S	KM495055.1
Basal Eukaryota	E0	Rhodelphida	*Rhodelphis limneticus*	E0_Rlimneticus_18S	MK966713.1
		Micronuclearia	*Rigifila ramosa*	E0_Rramosa_18S	AB686266.1
		Diphylleia	*Diphylleia rotans*	E0_Drotans_18S	MF039354.1
		Apusomonadida	*Multimonas media*	E0_Mmedia_18S	KP996852.1
		Centrohelida	*Spiculophrys agregata*	E0_Sagregata_18S	KU178913.1
Fungi	F0	Paraphelidium	*Paraphelidium tribonemae*	F0_Ptribonemae_18S	KX576681.1
		Nucleariida	*Nuclearia thermophila*	F0_Nthermophila_18S	HG530253.1
		Rozellida	*Rozella allomycis*	F0_Rallomycis_18S	KX354828.1
		Amoebaphelidium	*Amoeboaphelidium* WZ01	F0_AmoeboaphelidiumWZ01_18S	KU983765.1
	F1	Antonospora/Chytridiopsidae	*Enterocytozoon bieneusi*	F1_Ebieneusi_18S	ABGB01001533.1
		Glugeida	*Glugea* LM2016	F1_GlugeaLM2016_18S	KU577431.1
		Chytridiopsida	*Enterocytozoon hepatopenaei*	F1_Ehepatopenaei_18S	KY643648.1
		Fibrillaspora	*Fibrillaspora daphniae*	F1_Fdaphniae_18S	MF278272.1
		Nosema	*Nosema* BM201821	F1_NosemaBM201821_18S	LC467329.1

	Culicosporidae	*Edhazardia aedis*	F1_Eaedis_18S	AF027684.1
	Anncaliia	*Anncaliia algerae*	F1_Aalgerae_18S	HM216911.1
	Metchnikovellida	*Metchnikovella spiralis*	F1_Mspiralis_18S	MW344837.1
	h2007-2/Diversisporales	*Entrophospora colombiana*	F2_Ecolombiana_18S	AB220170.1
	h2007-2/Glomerales	*Rhizophagus intraradices*	F2_Rintraradices_18S	MF401586.1
	h2007-2/Glomerales	*Glomus* NBRPP1	F2_GlomusNBRPP1_18S	EF136912.1
	Zoopagales	*Rhopalomyces elegans*	F2_Relegans_18S	AY635834.1
	Zoopagales	*Thamnocephalis sphaerospora*	F2_Tsphaerospora_18S	AB016013.1
F3	Monoblepharidales	*Gonapodya polymorpha*	F3_Gpolymorpha_18S	AY349028.1
	Neocallimastigales	*Orpinomyces* CA34	F3_OrpinomycesCA34_18S	KC922183.1
	Rhizophydiales	*Boothiomyces macroporosum*	F3_Bmacroporosum_18S	NG_017171.1
	Polychytrium	*Polychytrium aggregatum*	F3_Paggregatum_18S	MT409103.1
F4	Lobulomycetaceae	*Lobulomyces angularis*	F4_Langularis_18S	NG_061011.1
	Physoderma	*Physoderma lycopi*	F4_Plycopi_18S	HQ888716.1
F5	Coelomymycetaceae	*Coelomomyces lativittatus*	F5_Clativittatus_18S	HQ888713.1
	Allomyces	*Allomyces anomalus*	F5_Aanomalus_18S	JN941234.1
	Entomophthoromycota	*Basidiobolus meristosporus*	F5_Bmeristosporus_18S	JX242609.1
F6	Kickxellomycotina	*Furculomyces boomerangus*	F6_Fboomerangus_18S	AF277013.1
	Zoopageles	*Atractospora aquatica*	F6_Aaquatica_18S	NG_070122.1

		Mucoromycotina		*Cokeromyces recurvatus*	F6_Crecurvatus_18S	AF113416.1
		Cryptococcus		*Cryptococcus neoformans*	F6_Cneoformans_18S	NG_064879.1
		Eurotialis		*Talaromyces macrosporus*	F6_Tmacrosporus_18S	M83262.1
		Entorrhizaceae		*Entorrhiza aschersoniana*	F6_Easchersoniana_18S	DQ363318.1
Holozoa/Metazoa	H0	Filasterea		*Capsaspora owczarzaki*	H0_Cowczarzaki_18S	XR_889848.1
		Ichthysporea		*Dermocystidium salmonis*	H0_Dsalmonis_18S	U21337.1
		Ichthysporea		*Sphaerothecum destruens*	H0_Sdestruens_18S	FN996945.1
		Choanoflagellida		*Monosiga brevicollis*	H0_Mbrevicollis_18S	AF100940.1
		Choanoflagellida		*Choanoflagellida* SL163	H0_ChoanoflagellidaSL163_18S	EF432541.1
	H1	Ctenophora	Cydippida	*Pleurobrachia pileus*	H1_Ppileus_18S	MF599313.1
		Ctenophora	Lobata	*Mnemiopsis leidyi*	H1_Mleidyi_18S	KJ754158.1
	H2	Porifora	Hexactinellida	*Hexactinella carolinensis*	H2_Hcarolinensis_18S	AM886408.1
		Porifora	Desmospongiae	*Amphimedon compressa*	H2_Acompressa_18S	KC902400.1
		Porifora	Desmospongiae	*Halisarca dujardini*	H2_Hdujardini_18S	EU702418.1
		Porifora	Homoscleromorpha	*Plakinastrella onkodes*	H2_Ponkodes_18S	HM118548.1
		Porifora	Calcarea	*Plectroninia neocaledoniense*	H2_Pneocaledoniense_18S	AM180979.1
		Placozoa	Trichoplacidae	*Trichoplax* H8	H2_TrichoplaxH8_18S	AY652581.1
		Bilateria	Ecdysozoa	*Afrosternophorus* sp. JM-2008	H2_AfrosternophorusJM-2008_18S	EU559360.1

	Bilateria	Nematoda	*Leptonchus granulosus*	H2_Lgranulosus_18S	KR184128.1
	Cnidaria	Anthozoa	*Isarachnanthus maderensis*	H2_Imaderensis_18S	AB859825.1
	Cnidaria	Anthozoa	*Eudendrium californicum*	H2_Ecalifornicum_18S	EU305492.1
H3	Orthonectida	Rhopaluridae	*Rhophalura ophiocomae*	H3_Rophiocomae_18S	U58369.1
	Rhombozoa	Dicyema	*Dicyema acuticephalum*	H3_Dacuticephalum_18S	D26530.1

Table S2. List of sequence read archive datasets.

Sequence read archive	Dataset[b]	Tissue
Brain (first series)		
SRX1250555	Rockfeller, USA	DLPFC
SRX1250556	Rockfeller, USA	DLPFC
SRX1250557	Rockfeller, USA	DLPFC
SRX1250558	Rockfeller, USA	DLPFC
SRX1250559	Rockfeller, USA	DLPFC
SRX1250560	Rockfeller, USA	DLPFC
SRX1250561	Rockfeller, USA	DLPFC
SRX1250562	Rockfeller, USA	DLPFC
SRX970051	Miami, USA	HPC
SRX970052	Miami, USA	HPC
SRX970053	Miami, USA	HPC
SRX970054	Miami, USA	HPC
Brain (second series)		
SRX10561059	University of Sydney, Australia	Cortex[c]
SRX11187945	Johns Hopkins, USA	DLPFC
SRX12960438	TRON Mainz, Germany	Brain
SRX9350010	Boston University, USA	PFC
SRX5541734	Skolkovo, Russia	Cortex (polyA+ selection)[c]
SRX5527594	CRI Seattle, USA	Cortex[c]
SRX10869684	University of Pittsburgh, USA	DLPFC
SRX10859671	University of California Irvine, USA	PFC
SRX2983651	NINS, Japan	DPFC
SRX3119985	Icahn School of Medicine, USA	DLPFC

SRX3098239	Lieber Institute, USA	DLPFC
SRX3009308	Columbia University, USA	DLPFC
SRX2497777	PICB, Shanghai, China	PFC
SRX834731	Boston University, USA	PFC
SRX390440	NIMH, NIH, USA	DLPFC
SRX272967	University of Pennsylvania, USA	DLPFC
SRX081982	University of Lausanne, Switzerland	Cortex (polyA+ selection)[c]
Liver		
SRX10498444	Changsha, China	Liver
SRX10498445	Changsha, China	Liver
SRX10498446	Changsha, China	Liver
SRX10498447	Changsha, China	Liver
SRX10498448	Changsha, China	Liver
SRX10498449	Changsha, China	Liver
SRX10498450	Changsha, China	Liver
SRX10498451	Changsha, China	Liver
Skin		
SRX6457388	LSB, NIAMS, NIH, USA	Skin (diabetic foot)
SRX6457389	LSB, NIAMS, NIH, USA	Skin (diabetic foot)
SRX6457390	LSB, NIAMS, NIH, USA	Skin (diabetic foot)
SRX6457391	LSB, NIAMS, NIH, USA	Skin (diabetic foot)
SRX6457392	LSB, NIAMS, NIH, USA	Skin (diabetic foot)
SRX6457393	LSB, NIAMS, NIH, USA	Skin (diabetic foot)
SRX6457394	LSB, NIAMS, NIH, USA	Skin (diabetic foot)
SRX6457395	LSB, NIAMS, NIH, USA	Skin (diabetic foot)
Tapwater		

SRX831467	Peking University	Water ('pure')
SRX831468	Peking University	Tapwater 0.04 mg/l FC
SRX831470	Peking University	Tapwater 0.56 mg/l FC
SRX831472	Peking University	Tapwater 1.76 mg/l FC

[a]Abbreviations: CRI, Cancer Research Institute (USA) DPFC, dorsal prefrontal cortex; DLPFC, dorsolateral prefrontal cortex; FC, free chlorine; HPC, hippocampus; LSB, Laboratory for Skin Biology (USA); NIAMS, National Institute of Arthritis and Musculoskeletal and Skin Diseases; NIH, National Institutes of Health (USA); NIMH, National Institute of Mental Health (USA); NINS, National Institutes of Natural Sciences (Japan); PFC, prefrontal cortex; PICB, Partner Institute for Computational Biology (China).
[b]The key RNA-seq data used in this work were from Magistri *et al.* (Magistri *et al.* 2015) (Miami), Scheckel *et al.* (Scheckel *et al.* 2016) (Rockefeller), Sawaya et al. [Sawaya Stone Brooks 2020) (LSB), Wu *et al.* (Wu *et al.* 2021) (Changsha, China), and Peking University Bioproject PRJNA267517; 'Dynamics of microbial communities in tap water networks'. Full details for all datasets may be accessed at https://trace.ncbi.nlm.nih.gov/Traces/sra/sra.cgi?.
[c]Datasets used for polyA+ versus total RNA comparison.

Table S3 (A). List of 64-mer probes covering the Tree of Life.

>A_Hsalinarum_16S_1
GGCCATTGCTATCGGAGTCCGATTTAGCCATGCTAGTTGTGCGGGTTTAGACCCGCAGCGGAAA
>A_Hsalinarum_16S_2
TTGCTCCTGGAAGGGGCAAAGCCGGAAACGCTCCGGCGCCACAGGATGCGGCTGCGGTCGATTA
>A_Hsalinarum_16S_3
TGAGACAAGATTCCGGGCCCTACGGGGCGCAGCAGGCGCGAAACCTTTACACTGTACGAAAGTG
>A_Hsalinarum_16S_4
GGGCAAGACCGGTGCCAGCCGCCGCGGTAATACCGGCAGTCCGAGTGATGGCCGATCTTATTGG
>A_Hsalinarum_16S_5
CTGTTCAGCTTGGGACCGGAAGACCTGAGGGGTACGTCTGGGGTAGGAGTGAAATCCTGTAATC
>A_Hsalinarum_16S_6
CCGGATTAGATACCCGGGTAGTCCTAGCTGTAAACGATGTCCGCTAGGTGTGGCGCAGGCTACG
>A_Hsalinarum_16S_7
ATTGGCGGGGGAGCACTACAACCGGAGGAGCCTGCGGTTTAATTGGACTCAACGCCGGACATCT
>A_Hsalinarum_16S_8
CGTCAGCTCGTACCGTGAGGCGTCCTGTTAAGTCAGGCAACGAGCGAGACCCGCACTCCTAATT
>A_Hsalinarum_16S_9
GTAGGTCAGTATGCCCCGAATGGGCTGGGCAACACGCGGGCTACAATGGTCGAGACAATGGGAA
>A_Hsalinarum_16S_10
GCTGGATTCGGTAGTAATCGCGTGTCAGCAGCGCGCGGTGAATACGTCCCTGCTCCTTGCACAC
>A_MethanocaldococcusSG1_16S_1
GGGGCTCCCTTCGGGGAGCACCGGCGCACGGCTCAGTAACACGTGGCTAACCTACCCTCGGGTG
>A_MethanocaldococcusSG1_16S_2
GCGGCCGCCCGAGGATGGGGCTGCGGCGGATTAGGTAGTTGGTGGGGTAACGGCCCACCAAGCC
>A_MethanocaldococcusSG1_16S_3
GCAGGCGCGAAACCTCCGCAATGCGCGAAAGCGCGACGGGGGGACCCCGAGTGCCCACGCTCCG
>A_MethanocaldococcusSG1_16S_4
CGGCGGCCCGAGTGGTGGCCACTGTTATTGGGCCTAAAGCGTCCGTAGCCGGCCCGGTAAGTCT
>A_MethanocaldococcusSG1_16S_5
CCCCAGGGGTAGCGGTGAAATGCGTTGATCCCTGGGGGACCACCTGTGGCGAAGGCGCCCGGCT
>A_MethanocaldococcusSG1_16S_6
CTCTGCGGACTAGGTGTCGCGTCGGCTTCGGGCCGGCGCGGTGCCGAAGGGAAGCCGTTAAGTC
>A_MethanocaldococcusSG1_16S_7
CGGTTTAATTGGATTCAACGCCGGGCATCTTACCAGGGGCGACGGCAGGATGAAGGCCAGGTTG
>A_MethanocaldococcusSG1_16S_8
GTAACGAGCGAGACCCGTGCCCCATGTTGCTATCCCCTCCTCCGGGAGGGGGGCACTCATGGGG
>A_MethanocaldococcusSG1_16S_9
GGCTACAATGGCCGGGACAATGGGATGCGACCCCGAAAGGGGGAGCAAATCCCCTAAACCCGGT
>A_MethanocaldococcusSG1_16S_10
TGAATGCGTCCCTGCTCCTTGCACACACCGCCCGTCACGCCACCCGAGTTGGGCCCAAGTGAGG
>A_Mkandleri_16S_1
ACTCCGGTTGATCCTGCCGGAGGCCACCGCTATCGGGGTCCGACTAAGCCATGCAAGTCGAGGG
>A_Mkandleri_16S_2
CGGCGAAAGTGGGGCTAATCCCCGATAGGCGGGGCGGCCTGGAACGGTCCTCCGCCGAAAGGGC
>A_Mkandleri_16S_3
AAGCCGATAATCGGTACGGGCGGTGAGAGCCGGAGCCCGGAGACGGGGACTGAGACAAGGCCCC
>A_Mkandleri_16S_4
GGGCAAAGCCCCGGCGGCTGTACCGGGGTGTAAAAAGCCCCGGGTAGAAAGCGGCGGGCAAGAC

>A_Mkandleri_16S_5
TCCCGTGGGTCCCCGCCGAAAGCCCGCGGCTTAACCGCGGGAGTCGGCGGGGAAACTGCGGGAC
>A_Mkandleri_16S_6
AAGGCGTCCGGCTGGAACGGGTCCGACGGTGAGGGCCGAAAGCCGGGGGAGCAAACCGGATTAG
>A_Mkandleri_16S_7
AAGCCGTTAAGTCCGCCGCCTGGGGAGTACGGCCGCAAGGCTGAAACTTAAAGGAATTGGCGGG
>A_Mkandleri_16S_8
GAAGGCCAGGTTGACGACCTTGCCGGACGAGCTGAGAGGAGGTGCATGGCCGCCGTCAGCTCGT
>A_Mkandleri_16S_9
CCGGGCACTCTGCGGGGATCGCCGCCGTTAAGGCGGATGAAAGTGGGGGCGACGGCAGGTCCGT
>A_Mkandleri_16S_10
ATCCCCTAAACCCCGTCGTAGTTCGGATTGCGGGCTGCAACTCGCCCGCATGAAGGTGGAATCG
>A_Mkandleri_16S_11
CCCCCCGGGGGCAAGCCCCCGGTCCGCAAGGGCTGGGGGCGAGCCCCCGGGGGGTGAGGGGGGC
>A_Tarchaeon_16S_1
GTAGCCGGTATGGTAAGTTCCTGGTAAAATTGGGCAGCTTAACTGTCCTTGTGCTAGGGATACT
>A_Tarchaeon_16S_2
AGTGGCGAAAGCGTCAGACTGGAACGCGCCTGACGGTGAGGGACGAAAGCCAGGGGAGCGAACC
>A_Tarchaeon_16S_3
CGTAGAGAAGTTGATAAGCGTGCCGCCTGGGAAGTACCAGCGCAAGCTGGAAACTTAAAGGAAT
>A_Tarchaeon_16S_4
GCAGGATGAAGGCCAAGCTGACGACTTTGCTGGACGAGCCGAGAGGAGGTGCATGGCCGTCGTC
>A_Tarchaeon_16S_5
GGGTGCTGGACAAACTAAGGGGACCGCTTGCGATAGCAAGAGGAAGGAGCGGGCGACGATAGGT
>A_Tarchaeon_16S_6
GGAAATCCCCAAACCTAACCCAAGTTCGGATTGTGGGCTGTAACTCGCCCACATGAAGCTGGAA
>A_KorarchaeotaSRI306_16S_1
GATTCTTGCCGGAGGGAACCCCTATCGGGTTCAGACTAAGCCATGCGAGTCGGCTGGGGGCACT
>A_KorarchaeotaSRI306_16S_2
CTAATCCCGGATAGGTGTGGGGTGCTGAAATGCCCTCACACCGAAAGTAGGCGGGAAATGGACC
>A_KorarchaeotaSRI306_16S_3
GGGCCCTGAGAGGGGGAGCCCGGAGATGGGCACTGAGACAAGGGTCTAGGCCCTAAGGGGCGCA
>A_KorarchaeotaSRI306_16S_4
CCCTGTGTAAAAAGCAGGGGGCAGGAAGGGGAGGGCAAGGCTGGTGGCAGCCGCCGCGGTAAAA
>A_KorarchaeotaSRI306_16S_5
CGCCTGTAGACAGGCGGGTTGCTGCAAATACTGCAGGGCTAGGGAGCGGGAGGAGCCGGGGGTA
>A_KorarchaeotaSRI306_16S_6
ACGGTGAGGGACGAAAGCTGGGGGAGCAAACCGGATTAGATACCCGGGTAGTCCCAGCCGTAAA
>A_KorarchaeotaSRI306_16S_7
GAGTACGGCCGCAAGGCTGAAACTTGAAGGAATTGACGGGGGGGCACCACAAGGGGTGAATGCC
>A_KorarchaeotaSRI306_16S_8
CAGACGCGCTGAGGGGTGGTGCATGGCCGTCGCCAGCTCGTGCCGTGAGGTGTCCTGTTAAGTC
>A_KorarchaeotaSRI306_16S_9
CTCCGAAGAGGAGGAGGAAGGTGGGGGCTACGGCAGGTCAGTATGCCCCTAATCCCCCGGGCCG
>A_KorarchaeotaSRI306_16S_10
TGGGATCGAAGGGCTGCAACTCGCCCTCGTGAACCCGGAATCCCTAGTAACCGCGGTTCTCCAT
>A_ThermoplasmataKjm51a_16S_1
CGGCCACCGCTATAGGAATTCGATTAAGACATGCGAGTCGAGAGTCGTAATGGACTCGGCGGAC
>A_ThermoplasmataKjm51a_16S_2
ATGGCATCTGGAATGAGCTATGGTTCAAAGTTCCGGCGCTTAAGGATCGGTCTGCGGCCTATCA

>A_ThermoplasmataKjm51a_16S_3
TGAGACACGAATCCAGGCCCTACGGGGCGCAGCAGTCGCGAAAACTTCACACTGGGGGCAACCC
>A_ThermoplasmataKjm51a_16S_4
GGGTAAGACGGGTGCCAGCCGCCGCGGTAATACCTGCAGCCCAAGTGGTGGCCGATAGTATTGA
>A_ThermoplasmataKjm51a_16S_5
TGTCAGACTTGGGACCGGGAGAGGCTAGAGGTACTTCTGGGGTAGGGGTAAAATCCTGTAATCC
>A_ThermoplasmataKjm51a_16S_6
GGGATTAGATACCCCGGTAGTCCAGGGTGTAAACGCTGCAGACTTGGTGTTGGAAATCCTTCGA
>A_ThermoplasmataKjm51a_16S_7
TTGGCGGGGGAGCACCGCAACGGGAGGAGCGTGCGGTTTAATTGGATTCAACACCGGAAAACTC
>A_ThermoplasmataKjm51a_16S_8
CAGTTCGTACCGTAAGGCGTTCTCTTAAGTGAGATAACGAACGAGACCCTCACCAATAATTGCA
>A_ThermoplasmataKjm51a_16S_9
TCAGTATGCCCCGAATCTCCTGGGCTACACGCGCGCTACAAAGGGCGGGACAATGGGCTCCGAC
>A_ThaumarchaeotaNAOA2_16S_1
AACATTATGCCTGGAATGGTTTATGTTCCAAATGATTTATCGCCGTAGGATGGGACTGCGTCCT
>A_ThaumarchaeotaNAOA2_16S_2
GTACTGAGACACGGACCCAGGCCCTATGGGGCGCAGCAGGCGAGAAAACTTTGCAATGTGCGAA
>A_ThaumarchaeotaNAOA2_16S_3
GTGGGCAAGTTCTGGTGTCAGCCGCCGCGGTAAAACCAGCACCTCAAGTTGTCAGGATGATTAT
>A_ThaumarchaeotaNAOA2_16S_4
TACTACAGAGCTAGGGAGTGGGAGAGGTAGACGGTACTCGGTAGGAAGGGGTAAAATCCTTTGA
>A_ThaumarchaeotaNAOA2_16S_5
AACCGGATTAGATACCCGGGTAGTCCCAGCTGTAAACCATGCAAACTCAGTGATGCATTGGCTT
>A_ThaumarchaeotaNAOA2_16S_6
GAATTGGCGGGGGAGCACCACAAGGGGTGAAGCCTGCGGTTCAATTGGAGTCAACGCCAGAAAT
>A_ThaumarchaeotaNAOA2_16S_7
CGCCAGCTCGTGCCGTGAGATGTCCTGTTAAGTCAGGTAACGAGCGAGATCCCTGCCTCTAGTT
>A_ThaumarchaeotaNAOA2_16S_8
GGCAGGTCAGTATGCCCCGAAACTCTGGGGCCACACGCGGGCTGCAATGGTAACGACAATGGGT
>A_ArchaeonTE187T7_16S_1
CGGCTGCTCAGTAACACGTGGACAACCTGCCCTCGGGTGGGGGATAACCCCGGGAAACTGGGGC
>A_ArchaeonTE187T7_16S_2
GATTAGGTAGTTGGTGGGGTAACGGCCCACCAAGCCTAAGATCCGTACGGGCTGTGGGAGCAGG
>A_ArchaeonTE187T7_16S_3
AACCGCGACGGGGTTAGCCGGAGTGCCCGTGCATTGCACGGGCTGTCGGGGTGCCTAAACAGCA
>A_ArchaeonTE187T7_16S_4
TTGGGCCTAAAGCGTCCGTAGCCGGCCCAGTAAGTCCCCCGGGAAATCGGGTCGCTTAACGATC
>A_ArchaeonTE187T7_16S_5
AATCCCGGGAGGACCACCTGTGGCGAAGGCGCTCGGCTGGAACGGGTCCGACGGTGAGGGACGA
>A_ArchaeonTE187T7_16S_6
TACGAGCTCCGGTGGTGCCGGAGGGAAGCCGTTAAGCCCGCCGCCTGGGAAGTACGGCCGCAAG
>A_Mthermophilum_16S_1
GCAATTCCGGTTGATCCTGCCGGAGGCCACTGCTATGGGAATTCGACTAAGCCATGCAAGTTGA
>A_Mthermophilum_16S_2
GGAAACTGGGAATAATACCCAATAGATCATTGGCACTGGAATGTCCTTTGATCCAAATGCTTTT
>A_Mthermophilum_16S_3
GGTCGTGAGAGCGATCGCCCGGAGATTGGATCTGAGATATGATCCTAGGCCCTACGGGGTGCAG
>A_Mthermophilum_16S_4
GGTGCGTAAAAAACACCTAATAGAAAGGGCCGGGTAAGACCGGTGCCAGCCGCCGCGGTAATAC

>A_Mthermophilum_16S_5
GGTGGCCTAACCATTGGGCGGCCAGGTGATACTATCAGGCTTGGGACTAGGAGAGACCAGAGGT
>A_Mthermophilum_16S_6
GACGGTGATGGACGAAGGCTGGGGGCGCAAACCGGATTAGATACCCGGGTAGTCCCAGCAGTAA
>A_Mthermophilum_16S_7
AAGTACGGCCGCAAGGCTGAAACTTAAAGGAATTGGCGGGGGAGTACCACAACCGGTGGAGCCT
>A_Mthermophilum_16S_8
AGATCAGCTGAGAGGAGGTGCATGGCCGTCGCCAGTTCGTACCGTGAGGCATCCTGTTTAGTCA
>A_Mthermophilum_16S_9
CGCTAAGCTGAAGGAAGGAGCGGGCTACGGTAGGTCAGCATGCCCTGAATCCTCCGGGATACAC
>A_Mthermophilum_16S_10
GATCGAAGGCTGCAACTCGCCTTCGTGAAGATGGAATCGGTAGTAATCGTGACTCAAAATGTCA
>A_Mthermophilum_16S_11
CAGAGGAAGATCGAATCTGGATTCTGCAAGGGGGGTTAAGTCGTAACAAGGTAGCCGTAGGGGA
>A_Archaeon26ABFS2_16S_1
AGGATTAAGTCATGCAAGTCAAGGCGGGCTTGTCTCGCCTGGCGAACTGCTCCGTAACACGTAG
>A_Archaeon26ABFS2_16S_2
TACACATCCAAAGGAAACGCCACAGGATGAGCCTGCGTCCGATTATGCTTGTTGGCGGTGTCTT
>A_Archaeon26ABFS2_16S_3
CCTATGGGGTGCAGCAGGCGCGAAAACTGGCCAATGCGCGAAAGCGTGTGCCGGCTAATCCGAG
>A_Archaeon26ABFS2_16S_4
CCGCGGTAAAACCAGCTCTTAGAGTGGTGGGCGTGTTTATTTGGCTTAAAGTGTGCGTAGCAGG
>A_Archaeon26ABFS2_16S_5
ATAGGTATGATGTACGCGGTGGGTAGGGGTGAAATCCGATAATCCATCGCGGACAACCGGTGGC
>A_Archaeon26ABFS2_16S_6
GTCCTATGCTGTAAACGATGCGCACTAAGTGTTAGGCAATGCACGACATTGTCTAGTGCCGAAG
>A_Archaeon26ABFS2_16S_7
AAGAAGTGGAGCCTGCGGTTTAATTGGACTCAACTCCGGGAAGCTCACCTGCGCCGTAACGTGA
>A_Archaeon26ABFS2_16S_8
TCCTGTTAAGTCAGGCAACGAACGAGATCCCTACCGCTAATTGCCAGCGAGACCCCCGGGTCGT
>A_Archaeon26ABFS2_16S_9
GCGCAGGGCTACACGCGGGCTACAATGGCTGGTACAGAGGGTCTCCAACACCGAAAGGTGACGG
>A_Archaeon26ABFS2_16S_10
GGGTCAGCAGCCCGTGCTGAATACGTCCCTGGGCCTTGCACACACCGCCCGTCGCAGCACGCGA
>B0_DesulfobacteraceaecLaKi_16S_1
ATAGGGGAAACCCTGACGCAGCAACGCCGCGTGAGTGAAGAAGGCCCTCGGGTCGTAAAGCTCT
>B0_DesulfobacteraceaecLaKi_16S_2
CGTGCCAGCAGCCGCGGTAATACGGAGGGTGCAAGCGTTGTTCGGAATTACTGGGCGTAAAGGG
>B0_DesulfobacteraceaecLaKi_16S_3
GAGTATGGGAGAGGAGAGTGGAATTCCCAGTGTAGAGGTGAAATTCGTAGATATTGGGAGGAAC
>B0_DesulfobacteraceaecLaKi_16S_4
ACCCTGGTAGTCCACGCTGTAAACGATGAGCACTAGGTGTAGCGGGTATTGACCCCTGCTGTGC
>B0_DesulfobacteraceaecLaKi_16S_5
GCACAAGCGGTGGAGCATGTGGTTCAATTCGACGCAACGCGAAGAACCTTACCTGGATTTGACA
>B0_DesulfobacteraceaecLaKi_16S_6
CTCGTGTCGTGAGATGTTGGGTTAAGTCCCGCAACGAGCGCAACCCCTCTCTCTAGTTGCCCGT
>B0_DesulfobacteraceaecLaKi_16S_7
TTTATATCCAGGGCTACACACGTGCTACAATGGGCGGTACAAAGGGATGCTAGTCCGTGAGGAG
>B0_DesulfobacteraceaecLaKi_16S_8
ATCGTGGATCAGCATGCCACGGTGAATACGTTCCCGGGCCTTGTACACACCGCCCGTCACACCA

>B0_CoriobacteriumCCUG33917_16S_1
GATGAACGCTGGCGGCGCGCCTAACACATGCAAGTCGAACGGCACCCACCTTCGGGTGGAAGCG
>B0_CoriobacteriumCCUG33917_16S_2
CGGATACCCCCGGGCGCCGCATGGCGCCCGGGCTAAAGCCCCGACGGGAGGGGATGGCTCCGCG
>B0_CoriobacteriumCCUG33917_16S_5
TCAACCCCCCGAAGCCCCCGGAACCTCCGCGGCTTGGGTCCGGTAGGGGAGGGTGGAACACCCG
>B0_CoriobacteriumCCUG33917_16S_6
AGGCGCGAAAGCTGGGGGAGCGAACAGGATTAGATACCCTGGTAGTCCCAGCCGTAAACGATGG
>B0_CoriobacteriumCCUG33917_16S_7
AGGCTAAAACTCAAAGGAATTGACGGGGGCCCGCACAAGCAGCGGAGCATGTGGCTTAATTCGA
>B0_CoriobacteriumCCUG33917_16S_8
CAGGTGGTGCATGGCTGTCGTCAGCTCGTGTCGTGAGATGTTGGGTTAAGTCCCGCAACGAGCG
>B0_CoriobacteriumCCUG33917_16S_9
GGGGACGACGTCAAGTCATCATGCCCCTTATGCCCTGGGCTGCACACGTGCTACAATGGCCGGT
>B0_CoriobacteriumCCUG33917_16S_10
CCCCCATGAAGTCGGAGTTGCTAGTAATCGCGGATCAGCATGCCGCGGTGAATGCGTTCCCGGG
>B0_Tmaritima_16S_1
TATATGGAGGGTTTGATCCTGGCTCAGGGTGAACGCTGGCGGCGTGCCTAACACATGCAAGTCG
>B0_Tmaritima_16S_2
AGGGGGATAACCAGGGGAAACCCTGGTTAATACCCCATACGCTCCATCAACGCAAGTTGGTGGA
>B0_Tmaritima_16S_3
CGACGACGGGTAGCCGGCCTGAGAGGGTGGTCGGCCACAGGGGCACTGAGACACGGGCCCCACT
>B0_Tmaritima_16S_4
CTTCGGGGTGTAAACCGCTGTGGCGGGGGAAGAATAAGGTAGGGAGGAAATGCCCTACCGATGA
>B0_Tmaritima_16S_5
ATTTACTGGGCGTAAAGGGGGCGTAGGCGGCCTGGTGTGTCGGATGTGAAATCCCACGGCTCAA
>B0_Tmaritima_16S_6
CGTAGATATCGGCAGGAACGCCGGTGGGGAAGCCGGTCTCCTGGGCCGACCCCGACGCTGAGGC
>B0_Tmaritima_16S_7
GGTAATCCCTCCGTGCTGAAGCTAACGCGTTAAGTGGGCCGCCTGGGGAGTACGCCCGCAAGGG
>B0_Tmaritima_16S_8
TTACCAGGGCTTGACATGCCGGTGGTACCTCCCCGAAAGGGGTAGGGACCCAGTCCTTCGGGAC
>B0_Tmaritima_16S_9
CAACCCCTGCCCCTAGTTGCCAGCGGTTCGGCCGGGCACTCTAGGGGGACTGCCGGCGACGAGC
>B0_Tmaritima_16S_10
CAATGGGTTGCGACCCCGCGAGGGGGAGCCAATCCCCAAAGCCGCCCTCAGTTCGGATCGCAGG
>B0_Tmaritima_16S_11
CTTGTACACACCGCCCGTCACGCCACCCGAGTCGGGGGCTCCCGAAGACACCTACCCCAACCCG
>B0_Lpilosa_16S_1
GACGAACGCTGGCGGCGTGCCTCATACATGCAAGTCGAGCGGACCCTTGGTGCGGAAGCCTTCG
>B0_Lpilosa_16S_2
CCGAAAGGCGGGCTAATACCGCATGAGTTCCTTCCGGGGCATCTCGGAAGGAGCAAAGGCCTTC
>B0_Lpilosa_16S_4
AACCCCTGTCCCAGGGGACGAGAAGGGACGGTACCCTGGGAGGAAGCCCCGGCTAACTACGTGC
>B0_Lpilosa_16S_5
AGGTCCTGGGTGAAAGGCCCCGGCTCAACCGGGGAGGGCCCCAGGAAACCGGCGGGCTAGAGGG
>B0_Lpilosa_16S_6
CGGCCTGGACAGAACCTGACGCTGAGGCACGAAAGCTGGGGGAGCGAACAGGATTAGATACCCT
>B0_Lpilosa_16S_7
CACTCCGCCTGGGGAGTACGGCCGCAAGGCTGAAACTCAAAGGAATTGACGGGGGCCCGCACAA

>B0_Lpilosa_16S_9
GAGACTGCCGGCGACAAGCCGGAGGAAGGCGGGGATGAGGTCAAATCATCATGCCCCTTATGCC
>B0_Lpilosa_16S_10
GGTCCCAGTTCGGATCGCAGGCTGCAACCCGCCTGCGTGAAGGCGGAATCGCTAGTAATCGCGG
>B0_MarinobacterEM3_16S_1
GCATGCGGTAGGCTTAACTGCAAGTCGAGCGGTAACAGGGGGAGCTTGCTCCCCGCTGACGAGC
>B0_MarinobacterEM3_16S_2
TACGCCCTTTGGGGGAAAGCAGGGGATCTTCGGACCTTGCGCTATTGGATGTGCCTATGTCGGA
>B0_MarinobacterEM3_16S_3
GACTGAGACACGGCCCGAACTCCTACGGGAGGCAGCAGTGGGGAATATTGGACAATGGGGGCAA
>B0_MarinobacterEM3_16S_4
CGACTAATACTCGTGAGGCTTGACGTTACTCACAGAAGAAGCACCGGCTAACTCCGTGCCAGCA
>B0_MarinobacterEM3_16S_5
AGATGTGAAAGCCCCGGGCTCAACCTGGGAACGGCATTTCGAACTGTCAGGCTAGAGTATGGTA
>B0_MarinobacterEM3_16S_7
CGCCTGGGGAGTACGGCCGCAAGGTTAAAACTCAAATGAATTGACGGGGGCCCGCACAAGCGGT
>B0_MarinobacterEM3_16S_8
ATTGGTGCCTTCGGGAACTCTGACACAGGTGCTGCATGGCCGTCGTCAGCTCGTGTCGTGAGAT
>B0_MarinobacterEM3_16S_9
GCTGGTGACAAACCGGAGGAAGGTGGGGATGACGTCAGGTCATCATGGCCCTTCCGGCCAGGGC
>B0_MarinobacterEM3_16S_10
GTCCGGATCGGAGTCTGCAACTCGACTCCGTGAAGTCGGAATCGCTAGTAATCGCGAATCAGAA
>B0_MarinobacterEM3_16S_11
TAGTCTAACCTTCGGGAGGACGATCACCCCGGTGTGGTTCCTGACTGGGGTAAAGTCGTAACAA
>B0_AquificaeSHNS732_16S_1
GCGAACGCTGGCGGCATGCCTAACACATGCAAGTCGTACGGAGAGTGGGGCAACTCACTCTCAG
>B0_AquificaeSHNS732_16S_2
TATATTGCCTTTTGTCACTAAGACAAAAGGTGAAAGCGAGGCTGTCAAAGGCTTCGGCTCAAAG
>B0_AquificaeSHNS732_16S_3
TGATCAGCCACAGTGGGACTGAGACACGGCCCGCACCCCTACGGGGGGCAGCAGTGGGGAATCG
>B0_AquificaeSHNS732_16S_4
TGGGAAGATGGGACTAAGAGCTAATACCTCCTGGTCTTGACGGTACCATCAGAGGAAGGGACGG
>B0_AquificaeSHNS732_16S_5
AGCTGGTTTCGTAAGCGGATTGTCAAAGCCCGAAGCTCAACTTCGGCAAGGCATTCCGAACTGC
>B0_AquificaeSHNS732_16S_6
TGGCGAAGGCGGCCTACTGGGACGGTACTGACGGTCATGGACGAAAGCTGGGGGAGCAAACCGG
>B0_AquificaeSHNS732_16S_7
GCGTTAAACATCCCGCCTGGGGAGTACGGGCGCAAGCCTGAAACTCAAAGGAATTGGCGGGGGC
>B0_AquificaeSHNS732_16S_8
GGCTGTCGAAAGATAGCCGTGTTTCCCTTTTGGGAGAAATCCTACCACAGGTGGTGCATGGCCG
>B0_AquificaeSHNS732_16S_9
TGAGGTGCTCTATAAGGAGACTGCCGGCGACAAGCCGGAGGAAGGAGAGGACGACGTCAGGTCA
>B0_AquificaeSHNS732_16S_10
TAATCTCACAAACCTTGTCGTGGTGCGAATTGAAGGTTGAAACTCACCTTCATGAAGCCGGAAT
>B0_AcetothermiaTuzSed3B_16S_1
ATGGCTCAGGATGAACGCTAGCGGCGCGCTTAACACATGCAAGTCGCGCGATCGGCTCCTTCCA
>B0_AcetothermiaTuzSed3B_16S_2
GAGATAGCATCGGGAAACCGTTGTTAATATCCCATAACACCAGAAGAACCCAGGTTCTTCTGGT
>B0_AcetothermiaTuzSed3B_16S_3
CCAAGATAGGTAGGGGGCCTGAGAGGGCGACCCCCACACTGGGACTGAGACACGGCCCAGACT

>B0_AcetothermiaTuzSed3B_16S_4
GCCTTCGGGTTGTAAACTCCTTTTCCGGGGGACGAATAAGGTAAGTAGGCAATGGCTTACCGAT
>B0_AcetothermiaTuzSed3B_16S_5
GGAATTACTGGGCGTAAAGGGCGTCTAGGCGGTCGGTCAAGTCAGTCGTGAAAGTCCTCGGCTA
>B0_AcetothermiaTuzSed3B_16S_6
TGCGTAGAGACCGAGAGGTACCCCGATGGTGAAGACAGCTTCCTGGGCGTTTCCTGACGCTGAA
>B0_AcetothermiaTuzSed3B_16S_7
CCCCCTACAGAGGGTCGTTGCCGGAGCTAACGTGTTAAGCCCACCGCCTGGGGATTACGTCCGC
>B0_AcetothermiaTuzSed3B_16S_8
GAACCTTACCAGGGCTTGACCTAGCGGCAGTAGGACCCCGAAAGGGGAACGACCTCAAGCTTTG
>B0_AcetothermiaTuzSed3B_16S_9
GCGCAACCCCTGCCCTTAATTGCCAGCGGGTAGTGCCGGGTACTATAAGGGGACTGCCGGAGAA
>B0_AcetothermiaTuzSed3B_16S_10
GCTACAATGAGGAGCAAGTCCGCAAGGACGAGCAAATCTCAAAAAGGTGCCCCCAGTGCGGATT
>B0_AcetothermiaTuzSed3B_16S_11
TGGGCCTTGTACACACCGCCCGTCACACCAATCGAGTAGGCGACACCCGAAGTCGGCCCTTCGG
>B1_Tpallidum_16S_1
TTTGATCCTGGCTCAGAACGAACGCTGGCGGTGCGTTTTAAGCATGCAAGTCGAACGGCAAGGA
>B1_Tpallidum_16S_2
TCTAGAAATAGGGGGTAATACCGAATACGCTCTTTTGGACGTAGGTCTTTGAGAGGAAAGGGGG
>B1_Tpallidum_16S_4
TTGTAAAGTTCTTTTGCCGACGAAGAATGAGGACGGGAGGGAATGCCCGTTTGATGACGGTAGT
>B1_Tpallidum_16S_5
TGGGCGTAAAGGGCATGCAGGCGGACTGGTAAGCCTGGTGTGAAATCCCCGAGCTCAACTTGGG
>B1_Tpallidum_16S_6
TATTTGGAAGAACACCGGTGGCGAAGGCGGGTTTCTGGCCGATGATTGACGCTGAGGTGCGAAG
>B1_Tpallidum_16S_7
TCTCGGCGCCGACGCGAACGCATTAAGTGTACCGCCTGGGGAGTATGCTCGCAAGAGTGAAACT
>B1_Tpallidum_16S_8
GGTTTGACATCAAGAGGAGCGCCGTAGAAATGCGGTGGCGTAGCGATACGCCTCTTGACAGGTG
>B1_Tpallidum_16S_9
GCCAGCAAGTGGTGTTGGGGACTCTGGCGGAACTGCCGGTGACAAACCGGAGGAAGGTGGGGAT
>B1_Tpallidum_16S_10
GTGAAGTGGAGCAAACCGCAAAAAGGCAATCGTAGTCCGGATTGAAGTCTGAACTCGACTTCAT
>B1_Tpallidum_16S_11
CACACCATCCGAGTTGGAGATACCCGAAGTCACTAGCCTAACCCGCAAGGGAGGGCGGTGCCGA
>B2_CsaccharibacteriaHXBF128_16S_1
GATGAGCGCTGGCGGCGTGCCTAATACATGCAAGTCGAGCGGCAGCACGGTCTTCGGACTGGTG
>B2_CsaccharibacteriaHXBF128_16S_2
ACCGCATGTGATCTACGGATTAAAGCTTTATGCGCTTTGGGAACGGCCTGCGTCTGATTAGCTT
>B2_CsaccharibacteriaHXBF128_16S_4
TTGAATAAGGGTCTGCTAACTACGTGCCAGCAGCCGCGGTCATACGTAGGACCCAAGCGTTATC
>B2_CsaccharibacteriaHXBF128_16S_5
CCATTATTTGAACTGCTAAGCTAGAAGGCGAGAGAGGTAGATGGAATTCCTGATGTAGGGGTAA
>B2_CsaccharibacteriaHXBF128_16S_6
GTGGGGAGCAAACGGGATTAGATACCCCGGTAGTCCCCGCCGTAAACTATGGATGCTAGCTGTA
>B2_CsaccharibacteriaHXBF128_16S_8
GATGCATGGCCGTCGTCAGCTCGTGTCGTGAGATGTTAGGTTAAGTCCTTCAACGAGCGCAACC
>B2_CsaccharibacteriaHXBF128_16S_9
TTTCCCTTACGTCTGGGGCTACAAACACGCTACAATGGCCGGTACAAAGGGCAGCCAAGTCGCG

>B2_CsaccharibacteriaHXBF128_16S_10
CTAGTAACGGTAAGTCAGCACATTACCGTGAATACGTTCCCGGGTCTTGTACACACCGCCCGTC
>B2_CperegrinibacteriaGW2011_16S_1
TGGAGAGTTTGATCCTGGCTCAGGGTGAACGCTGGCGGTGTGTTTAATACATGCAAGTCGAGCG
>B2_CperegrinibacteriaGW2011_16S_2
AGTGGGTTAATACCGGATGGTCCCGAAAGGGTAAAGATTTATCGGTCTGAGAGGTGCCTGCGTC
>B2_CperegrinibacteriaGW2011_16S_3
TGGGACTGAGACACGGCCCACACTCCTACGGGAGGCAGCAGTAAAGAATCTTCCGCAATGGGCG
>B2_CperegrinibacteriaGW2011_16S_4
GACGGTACCCTAGGAATAAGCACCGGCTAACTTCATGCCAGCAGCCGCGGTAATACGAAGGGTG
>B2_CperegrinibacteriaGW2011_16S_5
AACTGAGAATTAGTACCCGAAACTGGTATGCTAGAGACTTGGAGAGGTTAGTGGAATTCGGTGT
>B2_CperegrinibacteriaGW2011_16S_6
GGACGAAAGCGTGGGGAGCAAACGGGATTAGATACCCCGGTAGTCCACGCCGTAAACGATGGAT
>B2_CperegrinibacteriaGW2011_16S_7
GCAAGACTAAAACTCAAAGGAATAGACGGGGACCCACACAAGCGGTGGAGCATCTGGTTTAATT
>B2_CperegrinibacteriaGW2011_16S_8
CCTAAGACAGGTGCTGCATGGTTGTCGTCAGCTCGTGCCTTGAGGTGTTCGGTTAAGTCCGTAA
>B2_CperegrinibacteriaGW2011_16S_9
CGTCAAATCAGCATGGCCCTTATGCCTGGGGCGACACAGGTGCTATAATGGCCGGTACAACGGG
>B2_CperegrinibacteriaGW2011_16S_10
AAGCTGGAATCGCTAGTAAACGCGCATCAGCCATGGCGCGTTGAATATGTTCCTGGGTCTTGTA
>B2_ParcubacteriaGBS2_16S_1
GCTAAAAGTGGTGCGGGGGATGTCATGCGGGACTGCCGGTTTGAGCCGGAGGAAGGTGGGGACG
>B2_ParcubacteriaGBS2_16S_2
TAAGGCGGAGCTAATCCCTTAAAAGCCGCCCCAGTTCGGATCGGGGTCTGCAACTCGACCCCGT
>B2_ParcubacteriaGBS2_16S_3
TCACGCCAAGCGAGCCGGTAACAGGCGAAACCCAGCGGAAGCTGGACTAGCCTGGGACCGGTGA
>B2_ParcubacteriaGBS2_16S_4
CCTGGTCCAGGCTGAACGCTGGCGGCGTGGATAAGGCATGCAAGTCAGAGGGCCAAGACAGTAT
>B2_ParcubacteriaGBS2_16S_5
TCTCTCGAGCTACGGTGGGTCCTCCAAAGCCCGTAAGGGCGACGGAGGAGAGACGGGGACAATC
>B2_ParcubacteriaGBS2_16S_6
CCGCTCTGGGAAGGGCCCGCGGCCTATCAGGTAGTTGGTGGGGTAATGGCCCACCAAGCCTATG
>B2_ParcubacteriaGBS2_16S_7
CCGAGAATATTGGGCAATGGGGGAAACCCTGACCCAGCGACGCCGCGTGGGGGAAGAAGGCCTT
>B2_ParcubacteriaGBS2_16S_8
TGGCTGCCAATCACGTGCCAGCAGCAGCGGTAATACGTGAGCCACAAGCGTTAGCCGGATTTAC
>B2_ParcubacteriaGBS2_16S_9
GATACTGTTTGACTAGAGGGCGGTAGAGGCCGGTGGAACTGCCGGTGTAGGGGTGAAATCCGTT
>B2_ParcubacteriaGBS2_16S_10
CAAAGGGGATTAGATACCCCTGTAGTCCACGCCCTAAACGATGGGCACTAGCTGCCTGCAGTGT
>B2_ParcubacteriaGBS2_16S_11
AATAGACGGGGGCTCGCACAAGCGGTGGTCCATGTGGCTCAATCCGACGACAACCGTGGAACCT
>B2_ParcubacteriaGBS2_16S_12
GGCCGTCGTCAGCAGGTACCGTGAGGCGCAAGCTTAGATGCCAAAACCTGCGCAACCCTCGCCG
>B3_AcidobacteriaKBS96_16S_1
AACGCTGGCGGCGCGCTTAACACATGCAAGTCGCACGAGAAAGGGGAGCAATCCCTGAGTACAG
>B3_AcidobacteriaKBS96_16S_2
CATAAGCCTGAGAAGGGAAAGCAGCAATGCGCTGAAGGAGGAGCTCGCGGCCGATTAGCTAGTT

>B3_AcidobacteriaKBS96_16S_3
CGGGCCAGACTCCTACGGGAGGCAGCAGTGGGGAATCTTGCACAATGGGGGAAACCCTGATGCA
>B3_AcidobacteriaKBS96_16S_5
GCCGGAAACTGGAGTGCTGGAGCGCGGGAGAGGAAAGCGGAATTCCTGGTGTAGCGGTGAAATG
>B3_AcidobacteriaKBS96_16S_8
GCATGGCTGTCGTCAGCTCGTGTCGTGAGATGTTGGGTTAAGTCCCGCAACGAGCGCAACCCTC
>B3_AcidobacteriaKBS96_16S_9
CGTCAAGTCATCATGGCCTTTATGTCCAGGGCTACACACGTGCTACAATGGACGGTACAAAGCG
>B3_AcidobacteriaKBS96_16S_10
AAGCTGGAATCGCTAGTAATGGCATATCAGAACGATGCCGTGAATACGTTCCCGGGCCTTGTAC
>B3_Deltaproteobacteriahwp6_16S_1
ACGAACGCTGGCGGCATGCCTAACACATGCAAGTCGAACGAGAAAGTCACTTCGGTGGCGATTA
>B3_Deltaproteobacteriahwp6_16S_2
CCGGATAACAATGCATGACACAAGTCATATATTTGAAAGCTTTATGTGCTGAAGGAGGGGTCTG
>B3_Deltaproteobacteriahwp6_16S_3
ACACTGGAACTGAGACACGGTCCAGACTCCTACGGGAGGCAGCAGTGGGGAATATTGCACAATG
>B3_Deltaproteobacteriahwp6_16S_5
CTCAACCCCGGAGGGTCTTTCGAAACTGCTAATCTAGAGAGGGTCAGGGGCCGGCAGAATTCCT
>B3_Deltaproteobacteriahwp6_16S_7
GTCGCAAGACTAAAACTCAAAGGAATTGACGGGGGCCCGCACAAGCGGTGGAACATGTGGTTTA
>B3_Deltaproteobacteriahwp6_16S_8
GGCCGAGTGACAGGTGCTGCATGGCTGTCGTCAGCTCGTGTCGTGAGATGTTGGGTTAAGTCCC
>B3_Deltaproteobacteriahwp6_16S_9
GGAGGAGGGTGGGGATGACGTCAAGTCCTCATGGCCTTTATGACCAGGGCTACACACGTGTTAC
>B3_Deltaproteobacteriahwp6_16S_10
CTGCAACTCGACTGCGTGAAGCTGGAATCGCTAGTAATCGCGGATCAGCACGCCGCGGTGAATA
>B4_PlanctomycetesV144_16S_1
TTCAAGGGTTTGATCCTGGCTCAGAATGAACGTTGGCGGCGTGGATTAGGCATGCAAGTCGGAC
>B4_PlanctomycetesV144_16S_2
AGCCACGGGAAACCGTGATTAATACCGGATAACCTTTTCGAGTATGGTGCTTGAAAAGCAAAGG
>B4_PlanctomycetesV144_16S_3
TAGGGGGTGTGAGAGCATGGCCCCCACCACTGGGACTGAGACACTGCCCAGACACCTACGGGTG
>B4_PlanctomycetesV144_16S_4
TGTAAACCGCTGTCAGAGGGGATGAAATGCAGAAGGGTTCTCCCTTTTGTTTGACAGAGCCTCA
>B4_PlanctomycetesV144_16S_5
TTAAAGGGTGCGTAGGCGGTTTAATAAGTAGGGTGTGAAATGCCAGGGCTCAACCTTGGCACGG
>B4_PlanctomycetesV144_16S_6
GAAGGAACGCCGGTGGCGAAAGCGGTACACTGGGTCTTAACTGACGCTGAGGCACGAAAGCTAG
>B4_PlanctomycetesV144_16S_7
TCCGGACGTAGCGAAAGCATTAAGTACTCCGCCTGGGGAGTATGGTCGCAAGGCTGAAACTCAA
>B4_PlanctomycetesV144_16S_8
TTTGACATGCTTGTATTAGCTCTGTGAAAGCAGAGTGACGCCTTCGGGTGGAACTTGCACAGGT
>B4_PlanctomycetesV144_16S_9
TGCCAGCACGTTATGGTGGGGACTCTAAGGAGACTGCCGGTGTCAAACCGGAGGAAGGTGGGGA
>B4_PlanctomycetesV144_16S_10
CGCGAGATCAAGCAAATCCCAAAAAGCGCCGCTCAGTTCGGATTGCAGGCTGCAACTCGCCTGC
>B4_PlanctomycetesV144_16S_11
CGTCAAGCCACGAAAGCGGGGGGCGTCCAAAGTCGCTAAGCTAACCTTCGGGAGGCAGGCGCCT
>B5_Latescibacteria134476n2_16S_1
AACGAACGTTGGCGGCGTGGATTAGGCATGCAAGTCGAACGAGAAAACTTCCTTCGGGAAGCGG

>B5_Latescibacteria134476n2_16S_2
TACCGCATGACTCGGCAAGTCGCATGGTTTGCCGGCAAAGGTGGCCTCTCCATGGAAGCTGCCG
>B5_Latescibacteria134476n2_16S_4
CTGTCAGTAGGGAATAAAGCCGGGGAACGAACAATTCCCCGGTCTGAAGGTACCTACAGAGGAA
>B5_Latescibacteria134476n2_16S_5
GGTGCGTAGGCGGCCTTGTCCGTCAGAGGTGAAATCTACCGGCTCACCCGGTCAGACTGCCTCT
>B5_Latescibacteria134476n2_16S_6
AACACCGGTGGCGAAGGCGGCCATCTGGGACGGTACTGACGCTGAGGCACGAAAGCTAGGGTAT
>B5_Latescibacteria134476n2_16S_7
GCCGCAGCTAACGCATTAAGTGGACCACCTGGGGAGTACGCTCGCAAGGGTGAAACTCAAAGGA
>B5_Latescibacteria134476n2_16S_8
CATCCAGGGGACCGGTGTAGAGATACACCTTCTCTTTGAGTCGCTGGACAGGTGCTGCATGGCT
>B5_Latescibacteria134476n2_16S_9
CATGGTGGGAACTCTAAGGGGACTGCCGGTGATAAACCGGAGGAAGGTGGGGATGACGTCAAGT
>B5_Latescibacteria134476n2_16S_10
GCCAATCCCAAAAAGCCGGCCTCAGTTCGGATTGCAGTCTGTAACTCGACTGCATGAAGTCGGA
>B5_Latescibacteria134476n2_16S_11
CGAATTGGCTGCACCCGAAGTCGTTCTGCCAACCCTTTTGGGAGGCATGCGCCGAAGGTGTGGT
>B5_Cmarinimicrobia61467n6_16S_1
GACGAACGCTGGCGGCGTGCTTAACACATGCAAGTCAAGGAGAAAGTACCTTTCGGGTGCGAGT
>B5_Cmarinimicrobia61467n6_16S_2
ACCGAATAATGCAGCGGACCCTTCGGGGTATGTTGTTAAAGCGGTCTCGATTTATCGGGTACGC
>B5_Cmarinimicrobia61467n6_16S_4
CTGTCGTGAGGGAAGAACAACTCGGATTCGAATAGGGTTCGAGCCTGACGGTACCTCACAAGAA
>B5_Cmarinimicrobia61467n6_16S_5
GGGTCCGTAGGCGTCTTGGAAAGTTGTTCGTTAAATCCACCGGCTCAACCGGTAACCTGCGAAC
>B5_Cmarinimicrobia61467n6_16S_6
AACACCGATGGCGAAGGCAGCTTTCTGGCCCAATACTGACGCTGAGGGACGAAAGCGTGGGGAG
>B5_Cmarinimicrobia61467n6_16S_7
TGTCGGAGCTAACGCATTAAGTACTCCGCCTGGGGACTACGACCGCAAGGTTGAAACTCAAAGG
>B5_Cmarinimicrobia61467n6_16S_8
ACATGTCAGTGAAAGTCCTGTGAAAGCAGGACCCTCTGCGAGCTTGCTCAAAGACACTGTCACA
>B5_Cmarinimicrobia61467n6_16S_9
AGTTACCAGCACATCACGGTGGGCACTCTAAGGAGACTGCCTGGGATAACCAGGAGGAAGGTGG
>B5_Cmarinimicrobia61467n6_16S_10
ACCTGCGAAGGGGAGCTAATCCCAAAAAACCGGTCCCAGTTCAGATTGGAGTCTGCAACTCGAC
>B5_Cmarinimicrobia61467n6_16S_11
CCCGTCAAGCCATGGAAGTCAGCAGTACCCGAAGTCAGTGGCCTAACCCCGATTCGTCGGGGAG
>B6_Trossianum_16S_1
TAGCGGCAGGCCTAATACATGCAAGTCGAGGGGCAGCGGGTCTCCTTTCGGGGAGATGCCGGCG
>B6_Trossianum_16S_2
CCCATATGCTCATCGAGGGGCATCCCTTGTTGAGGAAAGCTCCGGCGGTGCAGGATGGGCATGC
>B6_Trossianum_16S_3
CACGGACACTGAGACACGGGTCCGACTCCTACGGGAGGCAGCAGTAGGGAATATTGGGCAATGG
>B6_Trossianum_16S_4
AGAGCGCCTGCGGGCGTGTGTTTGACGGTACCTGACGAATAAGCACCGGCTAACTCCGTGCCAG
>B6_Trossianum_16S_5
TCCGTGTTGAAAGGCTACGGCTTAACCGTAGTAAGGCATTGGATACTGCGAGGCTTGAGTACAG
>B6_Trossianum_16S_7
CCTGGGGAGTACGCCGGCAACGGTGAAACTCAAAGGAATTGACGGGGGTCCGCACAAGCGGTGG

>B6_Trossianum_16S_8
CTTCCCTTCGGGGCAGAGTGCAAGGTGCTGCATGGCTGTCGTCAGCTCGTGTCGTGAGATGTTG
>B6_Trossianum_16S_9
TGCGCAAGCAGAGAGGAAGGTGGGGATGACGTCAAGTCATCACGGCCCTTACGCCCAGGGCGAC
>B6_Trossianum_16S_10
CGGATTGGAGTCTGCAACTCGACTCCATGAAGCTGGAATCGCTAGTAATCGCGCATCAGCCATG
>B6_Trossianum_16S_11
GCCGCTGAGGAGCCGTTTAGGGCGAAACCGATGACTGGGGCTAAGTCGTAACAAGGTAGCCGTA
>B6_Pintermedia_16S_1
CTATGTGCTTGCACATTTTGGACGTCGACCGGCGCACGGGTGAGTATCGCGTATCCAACCTTCC
>B6_Pintermedia_16S_2
AGATTCATCGGTGGAGGATGGGGATGCGTCTGATTAGCTTGTTGGTGCGGGTAACGGCCCACCA
>B6_Pintermedia_16S_3
GGCAGCAGTGAGGAATATTGGTCAATGGACGGAAGTCTGAACCAGCCAAGTAGCGTGCAGGATT
>B6_Pintermedia_16S_4
CAATAAGGACCGGCTAATTCCGTGCCAGCAGCCGCGGTAATACGGAAGGTCCAGGCGTTATCCG
>B6_Pintermedia_16S_5
CAGCGCGAACTGGCGGACCTGAGTGCACGCAACGTATGCGGAATTCATGGTGTAGCGGTGAAAT
>B6_Pintermedia_16S_6
GGTATCGAACAGGATTAGATACCCTGGTAGTCCGCACGGTAAACGATGGATGCCCGCTGTTAGC
>B6_Pintermedia_16S_7
AATTGACGGGGGCCCGCACAAGCGGAGGAACATGTGGTTTAATTCGATGATACGCGAGGAACCT
>B6_Pintermedia_16S_8
TTGTCGTCAGCTCGTGCCGTGAGGTGTCGGCTTAAGTGCCATAACGAGCGCAACCCCTTTCCTT
>B6_Pintermedia_16S_9
ATCAGCACGGCCCTTACGTCCGGGGCTACACACGTGTTACAATGGCCGGTACAGAGGGACGGTG
>C1_Pbrachykentron_18S_1
ATGCATGTCTCAGAGTACATGGAATTCCACAGTGGGTCTGTGAATGGCTCCTTACATCAGCAGT
>C1_Pbrachykentron_18S_2
TACATGGATGGAACAGTCTGCTGCATCATGGATGTATCGGTGGTTTACCATTGGTACTTCCAGC
>C1_Pbrachykentron_18S_3
CCTATTGGACACCTGTGCTCGCAGAGTGGAATCTTCATTGGTTCAGCATTGCTTGATCACACGA
>C1_Pbrachykentron_18S_4
ATTCCGGAGAGGGAGCCTGAGAGATGGCTACTACTACCAAGGTGGGCAGCAGGCACGCAAATTA
>C1_Pbrachykentron_18S_5
TAATGGATGCAATCCAAACACAGTGATGAGTATCAACTGGAGGGCAAGTCTGGTGCCAGCAGCT
>C1_Pbrachykentron_18S_6
TCTGTTGGATGGCTAGTGGAGGACGAGTTGCTTGCTATGAGTTGGTGTCTTCGGGCATTGCTTG
>C1_Pbrachykentron_18S_7
CTGTTCTGGCTCCAAGTCTCCTTCAGTGGAGATCGATGCGGTTTCATTGCTTGACACATGTGTG
>C1_Pbrachykentron_18S_8
TGGAAAACTCAGTGTGCTTCAAGCATCCTTACTGGAATTGTATGCCCGTCCATGGAATGACAAA
>C1_Pbrachykentron_18S_9
GTTACACAGAGATAGTACTGTTTGAGTGTCAAAGCTCAGACACCCTGCTACTCTCTGCTTGTCT
>C1_Pbrachykentron_18S_10
CTTAGACCGCTGTATGATCAACTGCAGCGAAGGCGCTCCGCAAGTGCTTGTTCGTCGATCAAGA
>C1_Pbrachykentron_18S_11
GATGCAAGTTCAACGTACTTCATCAGCACTCATCCCCATGTCACGAGAAATCCAAGCCTATGGG
>C1_Pbrachykentron_18S_12
GCTTAATTTGACTCAACACGGGGAATGTTACCAGGTCAGGACGTTGCTGGGATTGACAGATTGA

>C1_Pbrachykentron_18S_13
ATAACGAGTGAGATATCTACCTTCCACTAGCTACAGTCTCGCAATCTGTAGAGCTGGATGCTTG
>C1_Pbrachykentron_18S_14
AGGTTCTGTGTACGCTACCAAGTACAAGCATGCTAGAGCCAACAGCAGGTCTGTGATGCTCCCA
>C1_Pbrachykentron_18S_15
TGAGTGGGGAACCATTGAAACCTGTGAACGTACTGGGGATAGATGCTTGCAACTGTCTGCCTTG
>C1_Pbrachykentron_18S_16
GTTGCTACCGATGACAGTAGGATAGAGCCATCAGGAGACTTGATCACTTCCGTGGTTGAGTCGA
>C1_Cmucronatum_18S_1
ATGCATGTCTCAGCGCAAACGGTTCAACAGTGGGTCTGTGAATGGCTCCTTACATCAGCAGTCA
>C1_Cmucronatum_18S_2
GGAAGATCAGGTCTGCCACGTCACGTTTGTCGGCATTCATGCAGCCTAACGGCGGTGGATTGCG
>C1_Cmucronatum_18S_3
CGCGCTGGCAGAGTGAAGGCTTCGTGCCTACATTGCTTGACCACAGGAACTTCTGACCTATCAG
>C1_Cmucronatum_18S_4
CTGAGAGACGGCTACCACTACCAAGGTGGGCAGCAGGCGCGCAAATTGCCCAATGCAAAGACAT
>C1_Cmucronatum_18S_6
TGCATGGCCCTCGGGCTGTGTGCTGGATACAACCCTCAGCAGTATGTCCTGGTTCCAATCTCAT
>C1_Cmucronatum_18S_7
ACAACATGAGGACACAGTCTAGTGCCGTGCAACAGCCTCAGTCCACCACCGGAGTCCACCATTG
>C1_Cmucronatum_18S_8
GACCAAGTGTGGCCATGGAGTTGTGCTGGCCTTGGAGCTCACTCTGGAGAATGTGGTGCTGCAC
>C1_Cmucronatum_18S_9
AGATATTGCAACGCTAGAGGTGAAATTCTTAGACCGTTGCAAGATCAACGGCAGCGAAGGCGTT
>C1_Cmucronatum_18S_10
TGTAAACTATGCCAGCTCGGCCTCAGTGGAGCAAGTTCACGGACTCCATTGGTGCCATGCCCTC
>C1_Cmucronatum_18S_11
TGGCACCACAAGGCGTGGAGTATGCGGCTTAATTTGACTCAACACGGGGAATGTTACCAGGTCA
>C1_Cmucronatum_18S_12
TGGAGTGATTTGTCTGGTTGATTCCGATAACGAGTGAGACATCTGCCTCCCAATAGCCTTCATC
>C1_Cmucronatum_18S_13
GCTTCTCTGAGGTGCTGTGTTCGCTTCCAAGAGCACGCATGCTAGAGCCAACAGCAGGTCTGTG
>C1_Cmucronatum_18S_14
TGCTCAGACGTGAGTGGGAAACCCTGCAAAACTGTGCTGTACTGGGGATAGATGATTGCAACTG
>C1_Cmucronatum_18S_15
CCGCCCGTCGTTGCTACCGATGGTGGCAGGATAGAGCCATCAGGAGACCACGGAACCTTCGGGT
>C1_Tvaginalis_18S_1
GTCATAGATTAAGCCATGCAAGTGTTAGTTCAGGTAACGAAACTGCGAATAGCTCATTAATACG
>C1_Tvaginalis_18S_2
GTTTCTCCAGATGTGAATTATGGAGGAAAAGTTGACCTCATCAGAGGCACGCCATTCGACTGAG
>C1_Tvaginalis_18S_3
GGAGAAGGCGCCTGAGAGATAGCGACTATATCCACGGGTAGCAGCAGGCGCGAAACTTTCCCAC
>C1_Tvaginalis_18S_4
CGTACCGAAACCTAGCAGAGGGCCAGTCTGGTGCCAGCAGCTGCGGTAATTCCAGCTCTGCGAG
>C1_Tvaginalis_18S_5
ACTGTGAACAAATCAGGACGCTTAGAGTATGGCCACATGAATGACTCAGCGCAGTATGAAGTCT
>C1_Tvaginalis_18S_6
GACTCATGAGAGAGAAGCTGAGGCGAAGGCGTCTACCTAGAGGGTTTCTGTCGATCAAGGGCGA
>C1_Tvaginalis_18S_7
TTAATGGCAGAATCTTTGGAGAATCATAGTTCTTGGGCTCTGGGGGAACTACGACCGCAAGCTG

>C1_Jlibera_18S_1
AACCTGGTTGATCCTGCCAGTAGTCATATGCTTGTCTCAAGGATTAAGCCATGCATGTCTAAGT
>C1_Jlibera_18S_2
TACATGGATTACCGTAGTAACTCCAGAGCTAATACATGCAAAAAACTCCCGCGTTAGAGGGATG
>C1_Jlibera_18S_3
CGGCGATGAACCAATCAAGTTTCTGCCCTATCAGCTTTCGTCGGTAGGGTATTGGCCTACCGAG
>C1_Jlibera_18S_4
GCAGCAGGCGCGCAAATTACCCCATCTCAATTCGAGGAGGTAGCGACAAGAAATAACTGTTGAC
>C1_Jlibera_18S_6
CCTCCTGGACCCCACCCGATCAATGCGAGCCGCGCTGCTTGCAGCGCGCGCCTCGTACGGGTAC
>C1_Jlibera_18S_7
TCCGGTCGCGCTTTGTTGGTGCGGGCGCATCGGAGGACATGGTTAATAGGGATAGTTGGGGGCA
>C1_Jlibera_18S_8
TTCCCATTGATCAAGAACGAAAGTTAGGGGATCGAAGACGATCAGACACCGTCCTAGTCCTAAC
>C1_Jlibera_18S_10
AGATTGATAGTTCTTTCTTGATTCTGTGGGTGGTGGTGCATGCCCGTTCTTAGTTGGTGGAGTG
>C1_Jlibera_18S_11
GTATACTACTGCTTCTTAGAGAGATTATGGACGACAAGTCCATTGAAACGTTCGGCAAAAACAG
>C1_Jlibera_18S_12
AGGTCCCGGGGAATCTTGTCAAAGCTCACCGTGATTGGGATAGTCTATTGCAATTTTTAGACTT
>C1_Jlibera_18S_13
CGCTCCTACCGATTGGATGATGCGGTGAAGACTCTGGACCGTGCCGGGCTTGCGTGGCGGCTAG
>C1_Ppyriformis_18S_1
GTGAAACTGCGAATGGCTCATTATATCAGTAATAGTTTATTTGATAGTACCTTACTACATGGAT
>C1_Ppyriformis_18S_2
AACCAATCGCGGCCTCGCAAGGGGACGTGTCCTTTGGTGATTCATAATAACTGATCGAATCGAA
>C1_Ppyriformis_18S_4
TGATTACGTCATTCTGTAATTGGAATGGGCCGAACTTAAATAATTCGGCGAGTATCAATTAGAG
>C1_Ppyriformis_18S_5
GTAGTTGAACCTTGGAGGAGTTATGCTGCGGTCATTGGGTTTCGGCTCGGTGACGGGTGTTTCT
>C1_Ppyriformis_18S_6
AATTGGAGTGTTCAAAGCAGGCTTGATGCAATTGAACATTTAAGCATGGAATAATAAAATAGGA
>C1_Ppyriformis_18S_7
GTCAGAGGTGAAATTCTTGGATTGACGTAAGACGAACTACTGCGAAAGCATTTGCCAAGGATGT
>C1_Ppyriformis_18S_8
GACTCAGTATTGGAGGGGCTTCCAAACGACCCTTTCAGCACTGCTAGAGAAATCAAAGTCTCTG
>C1_Ppyriformis_18S_9
CGGCTTAATTTGACTCAACACGGGGAAACTTACCAGGTCCAGACACAGGAAGGATTGACAGATT
>C1_Ppyriformis_18S_10
CGATAACGAACGAGACCTCAGCCGGCTAAATAGCCACACTCGCCCTTCGGGGCGGGTGCCTATT
>C1_Ppyriformis_18S_11
ATGTTCTGGGCCGCACGCGCGCTACACTGACGCCGTCAACAAGCTCATCCTGGGCCGAAAGGTT
>C2_GlaucocystisARP2014_18S_2
ACGGTGGGTTCACGCCCTCCGATCCCTTGGTGATTCATAATAACTTCTCGAATCGCATGGCTTC
>C2_GlaucocystisARP2014_18S_3
GGGTAACGGAGAATTAGGGTTCGATTCCGGAGAGGGCGCCTGAGAGATGGCGACCACATCCAAG
>C2_GlaucocystisARP2014_18S_4
AAGTCGTGTAATTGGAATGAGAACAATTTAAATCCCTTATCGAGGATCAATTGGAGGGAAAGTC
>C2_GlaucocystisARP2014_18S_5
ATTTCGGGGCGGGGCGGCTGGTCTGCTCTATGAGTGTGTACTAGTCGTACCCGTCCTTCCTTCC

>C2_GlaucocystisARP2014_18S_6
CAGGCCTTGCGCTCTGAACATTATTAGCATGGAATAATAAAATAGGACCTTGGTTCTATTTTGT
>C2_GlaucocystisARP2014_18S_7
TGGATTTACGGAAGATGAACTACTGCGAAAGCATTTGCCAAGGATGTTTTCATTAATCAAGAAC
>C2_GlaucocystisARP2014_18S_8
TGTTTTTTTCACGACTCCTTTGGCACGCTATGAGAAATCAAAGTTTTTGGGTTCCGGGGGGAGT
>C2_GlaucocystisARP2014_18S_9
CAACACGGGGAAACTTACCAGGTCCGGACATAGTAAGGATTGACAGATTGAGAGCTCTTTCTTG
>C2_GlaucocystisARP2014_18S_10
CCTTAACCTACTAAATAGTTACGCGAACATTTTTGTTCACGGTCAACTTCTTAGAGGGACTATT
>C2_GlaucocystisARP2014_18S_11
CACTGATGAATTCAACGAGCTCTTCCTTGACCGATAGGTCTGGGTAATCTTTTGAAATTTCATC
>C2_Igalbana_18S_2
GGATAACCGTAGTAATTCTAGAGCTAATACATGCAGGAGTTCCCGACTTCGGAAGGGATGTATT
>C2_Igalbana_18S_3
TGGCGATGGTTCATTCAAATTTCTGCCCTATCAGCTTTCGATGGTAGGATAGAGGCCTACCATG
>C2_Igalbana_18S_4
GCAGCAGGCGCGTAAATTGCCCGAATCCTGACACAGGGAGGTAGTGACAAGAAATAACAATACA
>C2_Igalbana_18S_6
CGGCCGCTACTCTTAACTGAGCGGTGGTCGGAGACGGGATGTTTACTTTGAAAAAATCAGAGTG
>C2_Igalbana_18S_7
CGAGCACCGGAGTAATGATTAACAGGGACAGTCAGGGGCACTCGTATTCCGCCGAGAGAGGTGA
>C2_Igalbana_18S_8
TAGGGGATCGAAGACGATCAGATACCGTCGTAGTGTTAACCATAAACCATGCCGACTAGGGATT
>C2_Igalbana_18S_12
GGGATAGATTATTGCAACTATTAATCTTCAACGAGGAATTCCTAGTAAGCGTGTGTCATCAGCG
>C2_Igalbana_18S_13
GACTGCGGCGCCGCCGCTGGTTCTCCAGCGCTGGCGTCGCGGGAAGCTGTCCGAACCTTATCAT
>C2_Sminus_18S_1
ATTAAAACAGTTATAGTTTCTTTGCTGAACGTGTATATGGATAACCGTAGCAATTCTAGAGCTA
>C2_Sminus_18S_2
ATAGTAACTTGTCGGACTTTTCGAAGTGAAGCATTCAAGTTTCTGCCCTATCAGCTTTCGATGG
>C2_Sminus_18S_5
CTGAGGAGCTCGCTCTCATTCACTTGTGGGCGAGTGTTCAGGTACTTTACCTTGAGAAAATTAG
>C2_Sminus_18S_6
TGGCGCTGAGTAATGATTAACAGGGATAGTTGGGGGCATTCGTATTTAATTGTCAGAGGTGAAA
>C2_Sminus_18S_10
AAGTTTGAGGCAATAACAGGTCCGTGATGCCCTTAGATGTCCTGGGCCGCACGCGCGCTACACT
>C2_Sminus_18S_11
AATTATTGGTCATCAACGAGGAATTCCTAGTAAACGCAAGTCATCAGCTTGCATTGATTACGTC
>C2_Pfalciparum_18S_1
AACCTGGTTGATCTTGCCAGTAGTCATATGCTTGTCTCACAGATTAAGCCATGCAAGTGAAAGT
>C2_Pfalciparum_18S_2
GGATAACTACGGAAAATCTGTAGCTAATACTTGTGAAAATACCTTTTGATATATACATATGTAT
>C2_Pfalciparum_18S_3
AAAATATGTGTATTATCAATCGAGTATCTGACCTATCAGCTTTTGATGTTAGGGTATTGACCTA
>C2_Pfalciparum_18S_4
GGAAGGCAGCAGGCGCGTAAATTACCCAATTCTAAAAAAGAGAGGTAGTGACAAGAAATAACAA
>C2_Pfalciparum_18S_6
TTGCTTTGTTCAAAATAAGGTTTTCTAATAAATTATGTTTTTATCAGATATGACAGAATCTTTT

>C2_Pfalciparum_18S_7
ACGGGTAGTCATGATTGAGTTCATTGTGTTTGAATACTACAGCATGGAATAACAAATATGAATA
>C2_Pfalciparum_18S_8
GGGGCATTCGTATTCAGATGTCAGAGGTGAAATTCTAAGATTTTCTGGAGACGGACTACTGCGA
>C2_Pfalciparum_18S_9
CTTAACCATAAACTATACCGACTAGGTGTTGGATGAATATAAAAAATATATAAATATGTAGCAT
>C2_Pfalciparum_18S_10
GGGCGAGTATTCGCGCAAGCGAGAAAGTTAAAAGAATTGACGGAAGGGCACCACCAGGCGTGGA
>C2_Pfalciparum_18S_11
CTTTCTTGATTTCTTGGATGGTGATGCATGGCCGTTTTTAGTTCGTGAATATGATTTGTCTGGT
>C2_Pfalciparum_18S_12
AGGTAATTATACATGTTTATTCAGTGTTCAAATTAGGATATTTTTTTATTAAAATATTCTTTTC
>C2_Pfalciparum_18S_13
AAAGCTTCTTAGAGGAACAGTGTGTATCTAACACAAGGAAGTTTAAGGCAACAACAGGTCTGTG
>C2_Pfalciparum_18S_14
AAGTGTGTACAGTTTTTCCTGTACTGAAAAGTATAGGTAATCTTTATCAGTATATATCGTAATT
>C2_Pgallinaceum_18S_1
TTTTCCATTATTTATAGAATAAGTATTAATTTAATAATATATATAGTAACCTGGTTGATCTTGC
>C2_Pgallinaceum_18S_2
AACGGCTCATTAAAACAGTTATAATCTACTTGACATTTTTTTATAAGGATAACTACGGAAAAGC
>C2_Pgallinaceum_18S_3
GAAAAAAGTTACTAATTTAAGGAATTATAACAAAGAAGCAACACATAATAAAACTCTGTTTTAT
>C2_Pgallinaceum_18S_5
GTTTTGCAATTGGAATGATAGGAATTTAAAAACTTCCTAAAGTAACAATTGGAGGGCAAGTCTG
>C2_Pgallinaceum_18S_6
TTCAAAGAATCAATTTTTAAAAGATGCTTTATTAGATGCATGTTAAATGACACTACGGTGTATA
>C2_Pgallinaceum_18S_7
GTGATGAGAATTTTTGTTACTTTGAGTAAATTAGAGTGTTCATAGCAAACAGTTTACAACAGAC
>C2_Pgallinaceum_18S_8
CTTAGTTACGATTAATAGGAGTAGTATGGGGGCATTCGTATTTAGATGTTAGAGGTGAAATTCT
>C2_Pgallinaceum_18S_9
AGTGAAGACGATCAGATACCGTCGTAATCTTAATCATAAACTATACCGACTAGGTGTTGGATGA
>C2_Pgallinaceum_18S_10
GAAATCAAAGTCTTTGGGTTCTGGGGCGAGTATTCGCGCAAGCGAGAAAGTTAAAAGAATTGAC
>C2_Pgallinaceum_18S_11
GTAGGATTGACAGATTGATAGCTCTTTCTTGATTTCTTGGATGGTGATGCATGGCCGTTTTTAG
>C2_Pgallinaceum_18S_12
TATTCTTAAACAAAGAAGAATATAGATAAAAATTACAGATAAGTGAAAATATTAGGATATTTTT
>C2_Pgallinaceum_18S_13
TGATTGTAAAGCTTCTTAGAGGAACATTGTGTGTCTAACACAAGGAAGTTTAAGGCAACAACAG
>C2_Hglobosa_18S_2
TGGATACCCGTAGTAATTCTAGAGCTAATACATGCGTAAAGTCCCGACTTTTTGGAAGGGATGT
>C2_Hglobosa_18S_3
CACGCTGGCGACAAGTTCATTCAAATTTCTGCCCTATCAGCTTTCGACGGTACTGTAGGTGGAC
>C2_Hglobosa_18S_6
CGCCATCCTTCCAATGGCTATTCATCCTTCTTCATTGAAGGTGTGAGTGGTATTGGATCTTTTA
>C2_Hglobosa_18S_7
GGTTCTATTTTGGTGGTTTCTAGGACTGAAGTAATGATTGATAGGGATAGTTGGGGGTRCTAGT
>C2_Hglobosa_18S_8
TTTAATCAAAGAACGAAAGTTGGGGGATCGAAGACGATCAGATACCGTCGTAGTCTCAACCATA

>C2_Hglobosa_18S_11
GACTTCTTAGAGGGACTATCGGTGTGTAGCCGACGGAAGTTCGAGGCAATAACAGGTCTGTGAT
>C2_Hglobosa_18S_12
GGTAATCTTTGCAACATGCATCGTGCTGGGGATAGATTATTGTAATTATTAATCTTCAACGAGG
>C2_Hglobosa_18S_13
CCGATTGAATGGATTAGTGAGCTTCACGGATTGCTGCGTGTGTGGCTTCGCCACTTACGTAATG
>C2_Pbrassicae_18S_1
CAATGAAATGCGGATGGCTCATTAAAACAGTTTGAATTTATTTGATGGATGTACGCGAGTACTA
>C2_Pbrassicae_18S_2
ATACAAAAACCAAACCTGGCAACAGGTTTCGTTTGTTGATTCACAATAACTGATCGGACCGTTG
>C2_Pbrassicae_18S_3
ACGGGTAACGGAGAATCGGGGTTCGATTCCGGAGAGGGAGCTTGAGAATTGGCTACCACATCTA
>C2_Pbrassicae_18S_4
GTTAGGACTGGTAATTGGAATGAGAACAAGTTAAACCTATTATCGAGGATCCATTGGAGGGCAA
>C2_Pbrassicae_18S_5
TGGACTTGTGTGCCTGCGCGTGTTCAGCGGTCTGCGTTCAAAAGAGCGTTACGACATGCGGCGC
>C2_Pbrassicae_18S_6
TGTGAGAAAAGTAGAGTGTTCAAGGCAGGCATTATTGCAATTGAATATGTTAGCATGGAATAAT
>C3_Gsulphuraria_18S_2
TTTTTTGGATTCTCTTTTTTTTACGAGGGAATTGGAAAAAGCGACCCATCTTGAAACGAGTTTC
>C3_Gsulphuraria_18S_3
TGTGAAAGGATAGCTTAGCATGGTATAATTGTAATATAGGATAGAATTACGTTTGTAGAATGGT
>C3_Gsulphuraria_18S_4
TTTGGAAGACGAACGGATGCGAAAGCGTTTGGCAAGGATATACCCATTGATCAAGGACGAAAGT
>C3_Gsulphuraria_18S_5
ATTAAGCATCGTACATCCTTTTTTTCCGGGAAACCAAAGATTTTGGGTTCTGGGGGGAGTATGG
>C3_Gsulphuraria_18S_6
ACGGGGCAACTTACCAGGTCCGGACATTTTTATGATTGACAGATTGATAGCTCTTTCATGATTG
>C3_Gsulphuraria_18S_7
TTCCTGCTAACTAGTTGGTATCCTTTTTTTTGAAGAGAGGATGCATTGAACTTCTTAGAGGTAC
>C3_Gsulphuraria_18S_8
GCTACACTGATGCATTCACTGAGTTTTTTTGAAAACCTATTCTGAAAGGAATATGGGTAATCTT
>C3_Cmerolae_18S_2
ACCGTAGGAATTCTACAGCTAATACATGCCCACACACCCGACTTTGGAAGGGTGGGATTTATCA
>C3_Cmerolae_18S_3
GACAGATCATATAAATTTCTGCCCTATCAACTTTCGATGGTAGGATAGAGGCCTACCATGGTGG
>C3_Cmerolae_18S_4
CAGGCGCGCAAATTACCCAATCCTGACTCAGGGAGGTAGTGACAAGAAATAACGAGACCGGGCT
>C3_Cmerolae_18S_6
GGGGTCCTCGCGGGCTCCGCGCGGCGAGCCAGCCCGTTTACTGTGAACAAATTAGAGTGCTCCA
>C3_Cmerolae_18S_7
GCCGAAGTAATGATGAATAGGGACAGTCGGGGGCATTCGTATTCCATTGTCAGAGGTGAAATTC
>C3_Cmerolae_18S_12
GGAATTATGGGTCTTCAACGAGGAATTCCTTGTAAGCGCGAGTCATCAGCTCGCGCTGAATACG
>C3_Cmerolae_18S_13
CCGGCCGGTTCGCCGGCGCGGGCGCGCCCGAAAGCTCAACAAACCTTATCATTTAGAGGAAGGA
>C3_Ccaldarium_18S_2
AATACGTGCGAGCACACCCGACTTTGGGAGGGTGGTCTTTATTAGATACAAAACCGATCTGTCC
>C3_Ccaldarium_18S_3
TGCCCTATCAACTTTCGATGGTAGGATCGGGGCCTACCATGGTGTCAACGGGTGACGGGGAATG

>C3_Ccaldarium_18S_4
ATCCTGACTCAGGGAGGTAGTGACAAGAAATACCGGTGCGCGGCTCTTCGAGTTGCGCTTCGGA
>C3_Ccaldarium_18S_6
TCCAGGACGTATACTGTGAAAAAATTAGAGTGTTTCAAGCAGGCGATTGCCGTGAATACATTAG
>C3_Ccaldarium_18S_7
GGGGCATTCGGATTCCGTTGTCAGAGGTGAAATTCTTAGATTAGCGGAAGACGAACATCCGCGA
>C3_Ccaldarium_18S_8
CTTAACCATAAACGATGCCGACTCGGGATCGGTGGATGTTTTTGCGACGCCATCGGCACCGCGC
>C3_Ccaldarium_18S_10
GATTTGTCTGGTTAATTCCGTTAACGAGCGAGACCTTGACCTGCTAACTAGCGGCGCGGAGCGC
>C3_Ccaldarium_18S_11
ATGCCCTTAGATGTTCTGGGCCGCACGCGCGCTACACTGATGCATGCAACGAGTGTCGGATGCT
>C3_Ccaeruleus_18S_2
GCTAATACATGCCTACAGACCCGACTTTGGAAGGGTGGTATTTATTAGAATCAAAACCCCTCGG
>C3_Ccaeruleus_18S_3
TTTCTGCCCTATCAACTTTCGATGGTAGGGTAGTGGCCTACCATGGTGGTAACGGGTGACGGAG
>C3_Ccaeruleus_18S_4
CCCAATCCTGACACAGGGAGGTAGTGACAATAAATAACAGTAGAGGGCACTTTTGTGTTTTCTT
>C3_Ccaeruleus_18S_6
AGTGTGCTCTGGAGTTTGCGCGTTTACTGTGAACAAATTAGAGTGTTTAAAGCAGGCGGTTGCC
>C3_Ccaeruleus_18S_7
AATAGGGACAGTTGGGGGCATTCGTATTTCGTTGTCAGAGGTGAAATTCTTGGATTTACGAAAG
>C3_Ccaeruleus_18S_12
TTAATCTTCAACGAGGAATTCCTTGTAAGCGCGAGTCATCAGCTCGCGTTGAATACGTCCCTGC
>C3_Ccaeruleus_18S_13
TTCGCCGGCTGGCGGCCGCGGGAAGTTCATTAAACCTTATCGTTTAGAGGAAGGAGAAGTCGTA
>C3_Paerugineum_18S_2
CTAATACATGCGCCAACATCCGACTTTCGAAGGATGGTATTTATTAGCCGCAACCAGCCGGGCT
>C3_Paerugineum_18S_3
CTTTCGACGGTAGGGTAGTGGCCTACCGTGGTGTTTACGGGTAACGGAGAATTAGGGTTCGATT
>C3_Paerugineum_18S_4
GGGAGGTAGTGACAAGAAATAACAATGGGAGACCTTTTGGTTGACCACTTGGAATGAGTACATT
>C3_Paerugineum_18S_7
CGTATTTCATTGTCAGAGGTGAAATTCTTGGATTTTTGAAAGACGAACCGCTGCGAAAGCGTTT
>C3_Paerugineum_18S_8
TAAACTATGCCGACTGGGGATTGGTGGACGTTCCAATCTGACTCCATCAGCACCCTATGAGAAA
>C3_Paerugineum_18S_9
GCGTGGAGCCTGCGNCTTAATTTGACTCAACACGGGGAAACTTACCAGGTCCAGACGAAGTTAG
>C3_Paerugineum_18S_10
TCTGGTTGATTCCGTTAACGAACGAGACCTTAACCTGCTAACTAGTGGATTGAATCTTCGGATT
>C3_Paerugineum_18S_11
GATGTTCTGGGCCGCACGCGCGCTACACTGATGCATGCAGCGAGTGTACCTTGGTCGGAAGGCC
>C3_Paerugineum_18S_13
TAAACCTTATCATCTAGAGGAAGGAGAAGTCGTAACAAGGTCTCCGTAGGTGAACCTGCGGAGG
>C3_Ppurpureum_18S_2
CCCTGGTAATTCTAGAGCTAATACATGCGCACACATCCGACTCTGGGAGGATGGTATTTATTGG
>C3_Ppurpureum_18S_3
ATTCAAATTTCTGCCCTATCAACTTTCGACGGTAGGGTAGTGGCCTACCGTGGTGTTAACGGGT
>C3_Ppurpureum_18S_4
CAAATTACCCAATCCTGACTCAGGGAGGTAGTGACAAGAAATATCGATAGGCGAGTCATTTGGC

>C3_Ppurpureum_18S_7
GTCAAGAGGAACGGTCGGGGGCACTCGTATTTCATTGTCAGAGGTGAAATTCTTGGATTTTTGA
>C3_Ppurpureum_18S_12
TATTAATCTTCAACGAGGAATGCCTTGTAAGCGCAAGTCATCAGCTTGCGTTGAATACGTCCCT
>C3_Ppurpureum_18S_13
CAACCCTGCGCGCGCGAGAAGCTCACTGAACCTTATCATCTAGAGGAAGGAGAAGTCGTAACAA
>C3_Rviolacea_18S_2
AGCTAATACGTGCCTCAACGCCCAACTCACGAAGGGTGGTATTTATTAGTTTTAAACCAATCGG
>C3_Rviolacea_18S_3
CCCTATCAACTTTGGATGGTAAGGTAGTGGCTTACCATGGTGGTAACGGGTAACGGAGAATTAG
>C3_Rviolacea_18S_4
CCTGACTCAGGGAGGTAGTGACAAAAAATAACGGTAGAGCGCCCTCGTGGTGTCTCTAATTGGA
>C3_Rviolacea_18S_6
TCCAGGACGTTTACTGTGAAAAAATTAGAGTGTTTAAAGCAGGCGATTGCCGTGAATACATTAG
>C3_Rviolacea_18S_7
GGGGCATTCGGATTCCGTTGTCAGAGGTGAAATTCTTAGATTAGCGGAAGACGAACAGCTGCGA
>C3_Rviolacea_18S_10
TGATTTGTCTGGTTAATTCCGTTAACGAACGAGACCTTGACCTNCTAACTAGCTGTGGGAATTT
>C3_Rviolacea_18S_11
GCCCTTAGATGTTCTGGGCCGCACGCGCGCTACACTGATGCATTCAACGAGTTGCACCTTGGCT
>C3_Gsublittoralis_18S_2
AGTAACCTAGAGCTAATACATGCCTAAACGCCCGACTCACGAAGGGTGGTATTTATTAGATACA
>C3_Gsublittoralis_18S_3
ATTCAAATTTCTGCCCTATCAACTTTCGATGGTAGGGTAGTGGCCTACCATGGTGTTCACGGGT
>C3_Gsublittoralis_18S_4
CAAATTACCCAATCCTGACTCAGGGAGGTAGTGACAAAAAATAACGAACGGAGGCTTTTCAAGT
>C3_Gsublittoralis_18S_7
TGATTAATAGGGATAGTTGGGGGCATTCGTATTTCATTGTCAGAGGTGAAATTCTTGGATTTAT
>C3_Gsublittoralis_18S_10
NCCGNNCTTANTTGGNGGANTGATTTGTCTGGTTAATTCCGTTAACGAACGAGACCTTAACCTG
>C3_Gsublittoralis_18S_12
ATTGGAATTATTAATCTTGAACGAGGAATTCCTTGTAAGCGTGAGTCATCAGCTCGCGTTGAAT
>C3_Gsublittoralis_18S_13
GAGCCTGCTGGTTTTACTGGCGATTCGTCCCGAGAAGTTCATTAAACCTTATCATTTAGAGGAA
>C4_Aechinata_18S_1
AAAGATTAAGCCATGCATGTCTAAGTATAAACTGCTTATACTGTGAAACTGCGAATGGCTCATT
>C4_Aechinata_18S_2
GGTGACTTCGGAAGCCTTGCACACATTAGACCAAAGGCCGACCCGGCTCTGCCGGTTTCTCGGT
>C4_Aechinata_18S_3
GATGGTAGGATAGAGGCCTACCATGGTGGTAACGGGTGACGGAGGATTAGGGTTCGATTCCGGA
>C4_Aechinata_18S_4
GTAGTGACAAGAAATAACAAAGGAGGCCTTACGGCTTTCCTATTGGAATGAGTACAATCTAAAT
>C4_Aechinata_18S_5
AGTTGTTGCAGTTAAAAAGCTCGTAGTCGGATTTCGGGCAGGTGTCAGCGGTCCGCCCTTTGGG
>C4_Aechinata_18S_6
GTTACTGTGAGTAAATTAGAGTGTTCAAAGCAGGCCTACGCTCTGAATACGTTAGCATGGAATA
>C4_Aechinata_18S_7
GTATTTCATTGTCAGAGGTGAAATTCTTGGATTTATGAAAGACGAACTACTGCGAAAGCATTTG
>C4_Aechinata_18S_8
AAACGATGCCGACTAGGGATTGCAGGGTGTTTTTCGATGACCTCTGCAGCACCTTATGAGAAA

>C4_Aechinata_18S_10
TCAGGTTGATTCCGGTAACGAACGAGACCTCAGCCTGCTAAATAGTCGCGCTTGCCTCTGGCAG
>C4_Aechinata_18S_11
GATGTTCTGGGCCGCACGCGCGCTACACTGATGCATTCAACAAGCCTATCCTTGGCCGAAAGGC
>C4_Glongispicula_18S_2
GTAATTCTAGAGCTAATACGTGCGTAAATCCCGACTCTTGGAAGGGACGTATTTATTAGATAAA
>C4_Glongispicula_18S_3
TTTCATTCAAATTTCTGCCCTATCAACTTTCGATGGTAGGATAGAGGCCTACCATGGTGGTAAC
>C4_Glongispicula_18S_4
CGCGCAAATTACCCAATCCTGATACGGGGAGGTAGTGACAATAAATAACAATACCGGGCATTAC
>C4_Glongispicula_18S_6
GGGCTTCACTGTCCGGGACCCGGAGTCGGCGAGGTTACTTTGAGTAAATTAGAGTGTTCAAAGC
>C4_Glongispicula_18S_7
AGTAATGATTAAGAGGGACAGTCGGGGGCATTCGTATTGCGCTGTCAGAGGTGAAATTCTTGGA
>C4_Glongispicula_18S_8
AAGACGATTAGATACCGTCGTAGTCTCAACCATAAACGATGCCGACTAGGGATTGGTGGGAGTT
>C4_Glongispicula_18S_10
TGCATGGCCGTTCTTAGTTGGTGGGTTGCCTTGTCAGGTTGATTCCGGTAACGAACGAGACCTC
>C4_Glongispicula_18S_12
ATTGCAATTATTAGTCTTCAACGAGGAATGCCTAGTAAGCGCGAGTCATCAGCTCGCGTTGATT
>C4_Glongispicula_18S_13
GGTGGTGGTCTTCACTGCCCTTGCTGAGAAGTTCATTAAACCCTCCCGCCTAGAGGAAGGAGAA
>C4_Pbrevispinosa_18S_2
AGCTAATACGTGCGTAAATCCCGACTTCTGGAAGGGACGTATTTATTAGATAAAAGGCCGACCG
>C4_Pbrevispinosa_18S_3
TTTCTGCCCTATCAACTTTCGATGGTAGGATAGAGGCCTACCATGGTGGTAACGGGTGACGGAG
>C4_Pbrevispinosa_18S_4
CCCAATCCTGACACAGGGAGGTAGTGACAATAAATAACAATACCGGGCTTTTCAAGTCTGGTAA
>C4_Pbrevispinosa_18S_6
GTCCGGGACTCGGAGTCGGCGAGGTTACTTTGAGTAAATTAGAGTGTTCAAAGCAGGCCTACGC
>C4_Pbrevispinosa_18S_7
AAGAGGGACAGTCGGGGGCATTCGTATTTCATTGTCAGAGGTGAAATTCTTGGATTTATGAAAG
>C4_Pbrevispinosa_18S_8
GATACCGTCCTAGTCTCAACCATAAACGATGCCGACTAGGGATTGGCGGATGTTTCTTCGATGA
>C4_Pbrevispinosa_18S_10
TTCTTAGTTGGTGGGTTGCCTTGTCAGGTTGATTCCGGTAACGAACGAGACCTCAGCCTGCTAA
>C4_Pbrevispinosa_18S_12
TAATCTTCAACGAGGAATGCCTAGTAAGCGCGAGTCATCAGCTCGCGTTGATTACGTCCCTGCC
>C4_Pbrevispinosa_18S_13
CGCCGCCGGCTGCGGCCGAAAAGTTCGTTGAACCCTCCCACMTAGAGGAAGGAGAAGTCGTAAC
>C4_Ebilobata_18S_1
GTTTAAGTTAACCCTGCATGTCTAAGTATAAACTGCTTTATACTGTGAAACTGCGAATGGCTCA
>C4_Ebilobata_18S_2
ACATCCCGACTCCTGGAAGGGACGTATTTATTAGATAAAAGGCCGACCGGACCTAGTCCGACTC
>C4_Ebilobata_18S_3
CTTTCGACGGTAAGGTATTGGCTTACCGTGGTGGTAACGGGTGACGGAGGATTAGGGTTCGATT
>C4_Ebilobata_18S_4
GGGAGGTAGTGACAATAAATAACAATACCGGGGTTTTTCAACTCTGGTAATTGGAATGAGTACA
>C4_Ebilobata_18S_6
TCGGCGAGGTTACTTTGAGTAAATTAGAGTGTTCAAAGCAGGCCTACGCTCTGAATACATTAGC

>C4_Ebilobata_18S_7
GGGCATTCGTATTTCATTGTCAGAGGTGAAATTCTTGGATTTATGAAAGACGAACTACTGCGAA
>C4_Ebilobata_18S_8
TCAACCATAAACGATGCCGACTAGGGATCGGCGGGTGTTCCTTTGATGACCCCGCCGGCACCTT
>C4_Ebilobata_18S_10
TTGCCTTGTCAGGTTGATTCCGGTAACGAACGAGACCTCAGCCTGCTAAATAGCCACGCCTGGT
>C4_Ebilobata_18S_11
ATGCCCTTAGATGTTCTGGGCCGCACGCGCGCTACACTGATGCGATCAACGAGCCCAGCCTTGG
>C4_Ebilobata_18S_12
ATGCCTAGTAAGCGCGAGTCATCAGCTCGCGTTGATTACGTCCCTGCCCTTTGTACACACCGCC
>C4_Catmophyticus_18S_1
AAAGATTAAGCCATGCATGTCTAAGTATAAACTGTTATACTGTGAAACTGCGAATGGCTCATTA
>C4_Catmophyticus_18S_2
GTCCCGACTCTTGGAAGGGATGTATTTATTAGATAAAAAACCAATACGGGTTTCGGCCCGGTAT
>C4_Catmophyticus_18S_3
AACTTTCGATGGTAGGATAGAGGCCTACCATGGTGGTAACGGGTGACGGAGAATTAGGGTTCGA
>C4_Catmophyticus_18S_4
CAGGGAGGTAGTGACAATAAATAACAATACCGGGCTTTCTAAGTCTGGTAATTGGAATGAGTAC
>C4_Catmophyticus_18S_7
TGGGGGCATTCGTATTTCGTTGTCAGAGGTGAAATTCTTGGATTTACGAAAGACGAACTTCTGC
>C4_Catmophyticus_18S_8
GTCTCTACCATAAACGATGCCGACTAGGGATTGGCAGATGTTACTTAGATGACTCTGCCAGCAC
>C4_Catmophyticus_18S_10
GGAGTGATTTGTCTGGTTAATTCCGTTAACGAACGAGACCTCAGCCTGCTAAATAGTTACGCGA
>C4_Catmophyticus_18S_11
TGTGATGCCCTTAGATGTTCTGGGCCGCACGCGCGCTACACTGATGGATTCAACGAGTATATAA
>C4_Catmophyticus_18S_12
ACGAGGAATTCCTAGTAAGCGCGAGTCATCAGCTCGCGTTGATTACGTCCCTGCCCTTTGTACA
>C4_Cvulgaris_18S_1
GTCTCAAAGACTAAGCCATGCACGTCTAAGTGTGAACACTTTGCACTGTGAAACTGCGAATGGC
>C4_Cvulgaris_18S_2
GCAGCATATCCCGACTTTTGGAAGGGATGTATTTATTGGATAAAAGGCCGATCCGGGCTTGCCC
>C4_Cvulgaris_18S_3
CCTATCAACTTTCGATGGTAGGATAGAGGCCTACCATGGTGGTAACGGGTGACGGAGAATTAGG
>C4_Cvulgaris_18S_4
CTGACATAGGGAGGTAGTGACAATAAATAACAATACTGGGCTCTTTCGAGTCCGGTAATTGGAA
>C4_Cvulgaris_18S_7
TAGAGACGGTTGGGGGCATTCGTATTCCATTGTCAGAGGTGAAATTCTTGGATTTATGGATGAC
>C4_Cvulgaris_18S_8
TACCGTCCTAGTCTCAACCATAAACGATGCCGACTAGGGATTGGCGGATGTCTATTGGATGACT
>C4_Cvulgaris_18S_12
ATCGATCTTGAACGAGGAATGCCTAGTAAGCGTGAGTCATCAGCTCGCGCTGATTACGTCCCTG
>C4_Cvulgaris_18S_13
GTTTGCCGCTGGGGGTGCTGTAGAAGTTCATTGAACCTTATCATTTAGAGGAAGGAGAAGTCGT
>C4_Lmarina_18S_2
ATGCGCTTCGGCGGTGATTGGTGATTCATAATAACTTTGCGAATCGTATGGCCTTGCGCTGACG
>C4_Lmarina_18S_4
TAATTGGAATGAGAACAATTTAAATCCCTTATCGAGGATCCATTAGAGGGCAAGTCTGGTGCCA
>C4_Lmarina_18S_5
GTTTGACAAATGGTCTGCCTTCGGGTTTGCACTGTTTGTTGGCACTTCTTTTCTGGGGAACTCA

>C4_Lmarina_18S_6
ATTGAATACATTAGCATGGAATAATGGAATAGGACTTTGGTCTTATTTTGTTGGTTTCTAAGGC
>C4_Lmarina_18S_7
GATAAACTACTGCGAAAGCATTCGCCAAGGATGTTTTCATTAATCAAGAACGAAAGTTAGGGGA
>C4_Lmarina_18S_8
CTCTGTTGGCACCTTGTGAGAAATCAAAGTTTTTGGGTTCCGGGGGGAGTATGGTCGCAAGGCT
>C4_Lmarina_18S_9
TTACCAGGTCCAGACATGAGAAGGATTGACAGATTGAGAGCTCTTTCTTGATTCTATGGGTGGT
>C4_Lmarina_18S_10
ATAGTTACCTTCACAATTTGTGGGGGGCCAACTTCTTAGAGGGACTATTCGTGCCTAGCGAGTG
>C4_Lmarina_18S_11
CAAGTCTATAACCTTGGTCGAAAGGCCTGGGTAATCTTGCAAATTTCATCGTGATGGGGATAGA
>C4_Lmarina_18S_12
CCTTTGTACACACCGCCCGTCGCTCCTACCGATTGAATGGTCCGGTGAATTCTTCGGATTGTGC
>C4_Gtheta_18S_2
ATAACCGTAGTAATTCTAGAGCTAATACATGCACCAAGGCCCGACTCACGGAGGGTTGTATTTA
>C4_Gtheta_18S_3
ATTCAAATTTCTGCCCTATCAACTTTCGATGGTAGGATAGAGGCCTACCATGGTTTTAACGGGT
>C4_Gtheta_18S_4
CAAATTACCCAATCCCGACTCGGGGAGGTAGTGACAATAAATAACAATACAGGGCCAACGGTCT
>C4_Gtheta_18S_7
AAAAGGGACAGTTGGGGCCGTTTATATTTCGTTGTCAGAGGTGAAATTCTTGGATTTACGAAAG
>C4_Gtheta_18S_11
AACAGGTCTGTGATGCCCTTAGATGTTCTGGGCCGCACGCGCGCTACACTGATGAACGCAACGA
>C4_Gtheta_18S_12
ATCTTCAACGAGGAATTCCTAGTAAGCGCGAGTCATCAGCTCGCGTTGATTACGTCCCTGCCCT
>C4_Gtheta_18S_13
GTGAGTGGTTGCGAGAAGTTGATTGAACCTTATCATTTAGAGGAAGGAGAAGTCGTAACAAGGT
>C4_Gavonlea_18S_2
GCATCAAGCCCCGACTCACGAAGGGGTGTATTTATTAGATTCAAAGCCAACCCCTGGCAACAGG
>C4_Gavonlea_18S_3
TATCAACTTTCGATGGTAGGATAGAGGCCTACCATGGTTTTAACGGGTGACGGAGAATTAGGGT
>C4_Gavonlea_18S_4
GATACGGGGAGGTAGTGACAATAAATAACAATACAGGGCTCTTCGAGTCTTGTAATTGGAATGA
>C4_Gavonlea_18S_7
GGGGCCGTTTATATTCCGTTGTCAGAGGTGAAATTCTTGGATTTACGGAAGATAAACTTCTGCG
>C4_Gavonlea_18S_10
AGTGATTTGTCTGGTTAATTCCGTTAACGAACGAGACCTCAGCCTACTAAATAGTCACACGAAC
>C4_Gavonlea_18S_11
TGATGCCCTTAGATGTTCTGGGCCGCACGCGCGCTACACTGATGAATTCAACGAGCTCACAACC
>C4_Gavonlea_18S_12
AGGAATTCCTAGTAAGCGCGATTCATCAGATCGCGTTGATTACGTCCCTGCCCTTTGTACACAC
>D_Dtestaceum_18S_2
CCGGAAGGGATGTATTTATTAGATAAAAAACCAATGCCTTCGGGCTCCTTGGTGATTCATGATA
>D_Dtestaceum_18S_3
AGTGGCCTACCATGGTTTCTACGGGTAACGGGGAATTAGGGTTCGATTCCGGAGAGGGAGCCTG
>D_Dtestaceum_18S_4
AAATACTGATACAGGGCTCTTTTGGGTCTTGTAATTGGAATGAGTACAATTTAAATCCCTTAAC
>D_Dtestaceum_18S_5
GCAGTTAAAAAGCTCGTAGTTGAACCTTGGGTTTGGCTGCTCGGTCCGCCTAACCGCGTGAACT

>D_Dtestaceum_18S_7
TTGTCAGAGGTGAAATTCTTGGATTTATTGAAGACTAACTACTGCGAAAGCATTTGCCAAGGAT
>D_Dtestaceum_18S_8
GCCGACTAGGGATCGGGCGGTGTTCAACTTATGACCCGCTCGGCACCTTACGAGAAATCAAAGT
>D_Rbicoronata_18S_1
TTTTGTATGTTTGTGTGAAAGATTAAGCCATGCATGTGTAAGTATAAATGGTTCCCAGTGAAAC
>D_Rbicoronata_18S_2
GGCTAATACGAGCAAAGAAGATTATTTTTTGTAAAGAAAATAATTTGACTTGATATTCAAATAA
>D_Rbicoronata_18S_3
TATCGTTGGAGTAAATCTAATGATAACCCAATTTGATTTCTGCCCTATCAACTTGTGATGGTAC
>D_Rbicoronata_18S_5
AGAAACCATTGGAGGACAAGTCTGGTGCCAGCACCCGCGGTAATTCCAGCTCCAATAATGTATA
>D_Rbicoronata_18S_6
TTATCTTAAGAATAATATTTTAAAAATTTATTCAATTAATTTATTTATAATGAAATCTTTATAT
>D_Rbicoronata_18S_7
TTTCGTTGTTTTATTACTTTGAAGAAAATAAAATGTTTAAAATAAATAATTGTGTTTTGAATAT
>D_Rbicoronata_18S_8
GTTGATTAATAGGGCTAATTGGGGTGATTTATATTTAATTGTTAGAGGTGAAATTCTTGGATTT
>D_Rbicoronata_18S_9
ACGATCAGATACCGTCGTAGTCTCAACCATAAACTATATCAACCAGAGATTGGATAAATGTTAT
>D_Rbicoronata_18S_10
TCGGGGGGAGTATGGTCGCAAGGCTGAAACTTAAAGGAATTGATGGAAGGGGAAACAATGAGTG
>D_Rbicoronata_18S_12
TTACTTAAAAGAACTACTTTATGTTTTTAATTAATGGAAGTTTGAGGCAATAACAGGTCTGTGA
>D_Rbicoronata_18S_13
ATTAGCAATAATGAATTTTTAATTTTTATTTTATTATCGAAAGTGAGAACTTGAGAAAATCTTT
>D_Rbicoronata_18S_14
TCAGCTTTTGTTGATTATGTCCCTTCCCTTTGTACACACCGCCCGTCGCTCCTACCGATTGAGT
>D_CaOmb2_18S_1
TAGTCATATGCTTGTCTTAAAGATTAAGCCATGCATGTCTAAGTATAAATAGTATACAGTGAAA
>D_CaOmb2_18S_2
GCTAATACATGCTGCAAGACCTGACTTTTTGGAAAGGTTGTATTTATTAGATCTCAAACCAATA
>D_CaOmb2_18S_3
TGCCCTATCAGTTTTCGTTGGTAGTGTATTGGACTACCAAGACAGTCACGGGTAACGGGGAATT
>D_CaOmb2_18S_4
ATCCTGATTCAGGGAGGTAGTGACAAGAAATAACAACTCGGGGGCTCACGCCTTACGAGATTGA
>D_CaOmb2_18S_7
GGCATTAGTATTTAATTGTCAGAGGTGAAATTCTTGGATTTATTAAAGACTAACTTATGCGAAA
>D_CaOmb2_18S_8
TAACTATAAACTATACCGACTAGGGATTGGCTGGGTTTTCCAAGCTCAGTCAGCACCTTATGAG
>D_CaOmb2_18S_9
CAGGAGTGGATTTTGCGGCTTAATTTGACTCAACACGGGGAAACTTACCAGGTCAAGACATGGG
>D_Hkukwesjijk_18S_2
GCGGCGCCTGGCAACAGAGCGTGCAGCAGGTGGTGTAGTGGCCACTGGGTGGTAACAATCTAAG
>D_Hkukwesjijk_18S_3
TCTGGGTTGCTTAAGATATAGTCGGCCGGCCGCAGAAATGCGGTTCTCTAAGAGGAATCTTCAC
>D_Hkukwesjijk_18S_4
TCAGCGGAGAAATGGCTACCACATCCAAGGATGGCAGCAGGCGCGCAAATTACCCAATCCTGAC
>D_Hkukwesjijk_18S_6
TCTGCGGTTGTCGGCTTCTCTTACCTGTCTGGCATTGCCCTCGTTGGTGGTGCTAGGCTCGTTT

>D_Hkukwesjijk_18S_7
GGCCCTATTTTGTTGGTTTCTAGGACCGAAGTAATGATTAATAGGGACAGTTGGGGGCATTCGT
>D_Rcontractilis_18S_2
CACATGGACTAGTTGGACAACCGTAGTAATTCTAGAGCTAGTACATGCGAAAACAAGCCCTGAA
>D_Rcontractilis_18S_3
GATAAAAGCCCAGCCGATGGTGAGTCAAGATAACTTTGCGAATCGGGGCAAAGGAGGGTGTTTA
>D_Rcontractilis_18S_4
TATTTGCCTACCATGGCGATCACGGGTAACGGGGAATTGGGGTTCGATTCCGGAGAGGGAGCTT
>D_Rcontractilis_18S_5
TACATAACAATGCTCATTTGCGGTTTACGCCGCAGGAGCAATTGGAATGAGAGATGGACAAACG
>D_Rcontractilis_18S_6
TCCAATAGCATATATTAACATTGTTGCAGTTAAAAAGCTCGTAGTCTGAGTTGATAAGGGGAGG
>D_Rcontractilis_18S_7
GGGGGTGTTTTTAATTTTTAAAAACACAGCTCGCGATGGTTTACTTTGAGAAAAATAGAGCGTT
>D_Rcontractilis_18S_8
AGCATGGGATAATGGAATAGGACATTGGCCTTGTGTTTTCATCGCACGCCGACTTTTATTTAAA
>D_Rcontractilis_18S_9
GGTGAAATTCTTGGATTCATGGAAGATGAACCAGTGCGAAAGCATTTGTCAAAGATGTTTTCAT
>D_Rcontractilis_18S_10
GGATTGGGGGGCGCTACGTTGATGAATGATGTGGTCAAACATGTCGTTCATTGATATTCAGCCC
>D_Rcontractilis_18S_13
CACAAAACTCCTTTCTTCTGTCATTTGATTTATCAAATAGCAAACAACTTCTTAGAGGGACAAT
>D_Rcontractilis_18S_14
TACACTGACGAACGCAACGAGCATTGTTGGGGGGCGTTGATTTTGATTCATCAAATTGTTGACA
>D_Rcontractilis_18S_15
TTGTCTTGAACGAGGAATTCCTAGTAAGTGTGAGTCATCAGGTTGCGCTGATTACGTCCCTGCC
>D_Rcontractilis_18S_16
TTGACTATTGTTTCGGCAATGGTTGATGCCCAGCCCAGAAGTAGTTCAAATCTTACTATTTAGA
>D_Falba_18S_2
CGGATACCTGTGGTAATTCTAGAKCTAATACGTGCGCCTTGTGCGGGGGCAACCCCGCAGGCAT
>D_Falba_18S_3
CGTGCCGATCCAATTTCTGCCCTATCAACTTTCGATGGTAGGATCGGGGCCTACCATGGTTGCG
>D_Falba_18S_4
GGCGCGCAAATTACCCAATCCTGACACAGGGAGGTAGTGACAAGAAATAACGACGCGGGGCCGA
>D_Falba_18S_6
GGGGCGTGTCGTTCATTCGGCGCGCCTCGTGCTCCCCCGCATTTTACTTTGAAGAAATTAGAGT
>D_Falba_18S_7
CTCTAGAGCCGGAGTAATGATTAATAGGGATAGTTGGGGGCATTAGTATTTAATTGTCAGAGGT
>D_Falba_18S_8
GTTAGGGGATCGAAGACGATTAGATACCCGTCGTAGTCTTAACCATCAACGATGCCGACTCGGG
>D_Falba_18S_12
CCGTGATGGGGATTGAGCATTGCAATTGTTGCTCATGAACGAGGAATTCCTAGTAAGCGCACGT
>D_Falba_18S_13
GGTCTTTGGACGCTGTTGCGCTGCGGGGCGACCTGCGGCACTTTGGCGGAAGCTGCCCAAACTT
>D_Tvaria_18S_1
ACGTATAAGAGGAAGCGATCCATAAATCTGCGAACGGCTCCGTATATCTGTTATCACCCCCGGC
>D_Tvaria_18S_2
GTCCCCCACCGGGGGGGGCGCCGGGGCTAGCCGATTGATGCCGGGGGGCCCCGCAAGGGGTCCA
>D_Tvaria_18S_3
ATTAACTAGATGTCCGGCCAAGGCCCGGCCACGGTGATAACGGGTACAGAGGATTAGGGTTCGA

>D_Tvaria_18S_4
CCCTGAGGACGTGAGGGGATATAGCAATGCCCCGCCCATAAAGCGGGGCAATTGCAATGGGGGT
>D_Tvaria_18S_5
CATCTAATTAAGTTGTTGCAGTTAAAACGCTCGTAGTCCAAGCCAGTGCGCCCTCTGGCCGCCC
>D_Tvaria_18S_6
CCGGGGAAGGCGGATTCGGGGGGCCACTACCTTAGCCTGGGGGCTACGGGGGCGGGGAACTTCA
>D_Tvaria_18S_7
CCCGCAGCAAAGAATAACAGAACAGGGGCTCCCTGCCTCCTCTGGCCAGGATGGGTCAATGATT
>D_Tvaria_18S_8
GAAAGCAGCCCACAAGTACCCTTTCTTAGATCAAGAACGAAAGTCTGGGGATCGAAGATGATCA
>D_Tvaria_18S_9
TGTGAGAAATCATAAGCGTTTGGGCTCCGGGGGGAGTATGGTCGCAAGACTGAAACTTAAAGGA
>D_Tvaria_18S_10
GGGGGGTAGGATGGACAGCCCAGCAGGGCTTTCCTGATTCCTCCAGTGGTGGTGCATGGTCGTT
>D_Tvaria_18S_11
GCACGCCGCCGTGGCCTGCTCGGCGTCTCACAAGTGGCGCTGGGCGGGCTGTGGTGGCTGAGAA
>D_Tvaria_18S_12
CCTTAGATGCTCTAGGCTGCACGCGCGCTACAATGGGGCGAACACCGGGCTCTCCTGTGCCTAA
>D_Tvaria_18S_13
CTAGTAGTCGCCCATCACCAGTGGGCGACGATTCTGTCCCTGCTCTTTGTACACACCGCCCGTC
>E0_Rlimneticus_18S_1
GGGCTTAACTAGNNCTGCAGATGGCTCATTAAATCAGTTATAGTTTATTTGATTGTGCTTGCTA
>E0_Rlimneticus_18S_2
TACAAAACCAACTCGGGGCAACCCGGTTTTTGGTGATTCATAATAACTTCGCGAATCGCATGGC
>E0_Rlimneticus_18S_4
TTCAAGTCTTGTAATTGGAATGAGTACAATTTAAACCCCTTAACGAGGATCAACTGGAGGGCAA
>E0_Rlimneticus_18S_5
CGGATTTCGGAACGGGCCGGAAGGTCTGCCGATTGGTGTGTACTTTCTGGCCTGTTCTTCCTTC
>E0_Rlimneticus_18S_6
GCAGGCCATTTGCTCTGAATACATTAGCATGGAATAATCGAATAGGACTCTGGTTCTATTTTGT
>E0_Rlimneticus_18S_7
TGGATTTATGAAAGACGAACTTCTGCGAAAGCATTCGTCAGGGATGTTTTCATTGATCAAGAAC
>E0_Rlimneticus_18S_8
TGTTTTCTTTATGACCCCTTCAGCACCTTATGAGAAATCAAAGTCTTTGGGTTCCGGGGGGAGT
>E0_Rlimneticus_18S_9
CAACACGGGGCAACTTACCAGGTCAGGACATAAGAAGGATTGACAGATTGAGAGCTCTTTCTTG
>E0_Rlimneticus_18S_10
CCTTAACCTGCTAAATAGTCACACTTACCTTCGGGTAGGTGGCCGACTTCTTAGAGGGACGATG
>E0_Rlimneticus_18S_11
CACTGATGGATGCAACGAGTATATAACCTTGACCGAGAGGTCTGGGTAATCTTTTTAAACTCCA
>E0_Rramosa_18S_1
TGCATGTCTAAGTATAAATGACTTTATACAGTGAAACTGCGAATGGCTCATTACATCAGTTATA
>E0_Rramosa_18S_2
GGAAGGGATGCATTTGTTAGATACAAAACCAATTTCGTTCTTCGGGACGGATTTTGTTGATGAT
>E0_Rramosa_18S_3
CGTCGGTAAGATAGAGGCTTACCGAGGTTTCAACGGGTAACGGAGAATTAGGGTTCGATTCCGG
>E0_Rramosa_18S_4
GGTAGTGACAAGAAATAACAATACAGGGCCTTTTGGTCTTGTAATTGGAATGAGAACAATCTAA
>E0_Rramosa_18S_6
ATAACATATCTTTCTGTGGAAAGCCGCCAACTGCTCTTTACTGAGTGGGTGGAGTTACGGAGTT

>E0_Rramosa_18S_7
ACAGGACTTTGGTCATGTTTTGTTGGTTTTTGAGGACCGAAGTAATGATTAATAGGGATAGTTG
>E0_Rramosa_18S_8
AGGATGTTTTCATTAATCAAGAACGAAAGTTAGGGGATCGAAGACGATCAGATACCGTCGTAGT
>E0_Rramosa_18S_10
GAAGGATTGACAGATTGAGAGCTCTTTCTTGATTCTATGGGTGGTGGTGCATGGCCGTTCTTAG
>E0_Rramosa_18S_11
CAGATCCGTTTGCGTAACAGCTTCTTAGAGGGACTATCGGTGACTAACCGACGGAAGTTTGAGG
>E0_Rramosa_18S_12
ACAACGGGAGTGGCCGTCAAAAAGCTGCTCTTATTCCTTGGCCGAGAGGTCTGGGTAATCCTTT
>E0_Drotans_18S_1
AAGTGGAGTCATAATAACTTTTGCGAATCGCATGATTTCGAATCGGCGATGAATCATTCAAATT
>E0_Drotans_18S_4
TGCTTAGGAATTGATTGGAGCAATCTGGTCTTTTTCTGAGTAATTAGAATGGAAAACCTTTTTC
>E0_Drotans_18S_5
AGAGTGTTCAAAGCAGACATTTTTTGTCTTGAATACATTAGCATGGAATAATGAAATAGGACTC
>E0_Drotans_18S_6
TTGTCAGAGGTGAAATTCTTGGATTTATGAAAGATAAACTAATGCGAAAGCATCTGCCAAGGAT
>E0_Drotans_18S_7
CCGACTAGGGATCGGTGGGTGTTGTAATTTCTAATGAGTAAAAGTAGCAATACTTTTACTTTTA
>E0_Drotans_18S_10
TCTTGTAATGGAATTTAAAAAGTTGGAACTCTTCAAAAGATGGATATGCTTCTTAGAGGGACTA
>E0_Drotans_18S_11
TACACTGACCAAAGCAACGAGTTATTACATTTTATGGGTTTTGGAAACAAAATTTATAAAATTA
>E0_Mmedia_18S_1
TCTCAAGGATTAAGCCATGCATGTCTAAGTATAAGCAATTTATACAGCGAAACTGCGAGTGGCT
>E0_Mmedia_18S_2
TCTAGAGCTAATACGTGCGCAAAAGAAGGAGGAGAACTCAAAAGCTCCTCCTGTATTTATTAGA
>E0_Mmedia_18S_3
TGGCAACAGCCGGCGATGATTCATTCAAATTTCTGCCCTATCAATTGTCGACGGTAAGATAGAG
>E0_Mmedia_18S_6
GTGCCGGCCGCCGGGCCCTTTCGTCTGGAGGCCGGGCGCAACGACTTGATTGTCGTGCGATTCC
>E0_Mmedia_18S_7
GGAATAACCACTTAGGACTTTGGTTCTATTTTGTTGGTTTTAAGGACTAAAGTAATGATTAACA
>E0_Mmedia_18S_8
GCACTTGGCAAGGAGGTTTTCATTAATCAAGAACGAAAGTTAGGGGATCGAAGACGATCAGATA
>E0_Mmedia_18S_10
TAATAAGGATTGACAGATTGAGAGCTCTTTCTTGATTCTACGGGTGGTGGTGCATGGCCGTTCT
>E0_Mmedia_18S_11
TCCTGATCGTGGCGAACTTCTTAGAGGGACTTTTGCGTGTTAAACCAAAGGAAGTTTGGGGCAA
>E0_Mmedia_18S_12
AGACCGTTAGGTCCGGGTAATCTTTGAAAGTTTATCGTGATGGGGATCGTTGATTGTAATTTTT
>E0_Mmedia_18S_13
GCCCGTCGCTCCTACCGATTGAATGGTCCGGTGAGGTTCCAGGACTGGCCTTTGTTGATTGGTA
>E0_Sagregata_18S_2
GCTAATACATGCGCCAAGCCCCGACTACGCATTCGTGCGTGGAAGGGGTGCATTTATTAGATTC
>E0_Sagregata_18S_3
CATTCAAATTTCTGCCCTATCAGCTTTCGATGGTAGGATCTTGGCCTACCATGGCTATAACGGG
>E0_Sagregata_18S_4
GCAAATTACCCAATCCCGACACGGGGAGGTAGTGACCAAAAATAACAATGCTGGACTATTACTA

>E0_Sagregata_18S_6
GCGCCTTGGAAGTCAAACTCCAGGCGTAACTTTCGGAGGCCCATGGCTCACGCCATGGAATTCC
>E0_Sagregata_18S_7
AGTGGAATAGGACGTTGGTTCTATTTTGGTGGTGTTAAGCACCGACGTAATGATTAATAGGGAC
>E0_Sagregata_18S_8
TGTCAAGGATGTTTTCATTGATCAAGAACGAAAGTTAGGGGAGCAAAGACGATCAGATACCGTC
>E0_Sagregata_18S_10
CACCAGGTCCAGACATAGTAAGGACTGACAGATTGAGAGCTCTTTCTTGATTCTATGAAAGGTG
>E0_Sagregata_18S_11
TAGTTGTGTCTACTTTCGGGTAGGCATTCTGCGGGGTCAAACCCGCAAACTTCTTAGAGGGACA
>E0_Sagregata_18S_12
CTACACTGACTGGCTCAACGAGTTTTTTCGGGTCGTGCCCTCGGGTGCGTCCCGTGCCACCTTG
>F0_Ptribonemae_18S_1
TCACTTTCGATGGTAGGATAGAGGCCTACCATGGTTGTAACGGGTAACGGGGAATTAGGGTTCG
>F0_Ptribonemae_18S_2
ACAGGGAGGTAGTGACAATAAATAACAATACAGGGCTCTTTGAGTCTTGTAATTGGAATGAGTA
>F0_Ptribonemae_18S_5
GGGGGCATTAGTATTTCCGGGCTAGAGGTGAAATTCTTGGATTCCGGAAAGACTAACTACTGCG
>F0_Ptribonemae_18S_6
TCTTAACCATAAACTATGCCGACTCAGGATCAGTGAATGTTCGTTTTGACTTCATTGGCACTGT
>F0_Ptribonemae_18S_8
ATGGGGATAGACCATTGCAATTATTGGTCTTCAACGAGGAATTCCTAGTAAGCGCGAGTCATCA
>F0_Nthermophila_18S_2
ACTTCTGGAAGGGATGTATTTATTAGATAAAAAACCAACCGGGGCAACCCGTCTTTTGCTTGGT
>F0_Nthermophila_18S_3
CGATGGTAATGTAGTGGATTACCATGGTTGTAACGGGTAACGGAGAATTAGGGTTCGGTTCCGG
>F0_Nthermophila_18S_4
GGTAGTGACAAGAAATAACAATACAGGGTCCTAAACGGTCTTGTAATTGGAATGAGTACAATTT
>F0_Nthermophila_18S_6
GCTTGCCTCTCTCTACCTCCTTCTAAAGACCCAGGGTGCTCTTCACCGGGCGTCCTGGGGATTT
>F0_Nthermophila_18S_7
AAATAGGACTTTGGTTCTATTTTGTTGGCTTCTAGGACCGAAGTAATGATTAATAGGGATAGTT
>F0_Nthermophila_18S_8
AAGGATGTTTTCATTAATCAAGAACGAAAGTTAGGGGATCGAAGATGATCAGATACCGTCGTAG
>F0_Nthermophila_18S_11
GGGGAAGGGGTGGGGGGGTTTCGGCCTCCCCTCCTCGCTTCCCAGGGCGGTTCTGCGGTTTTCA
>F0_Nthermophila_18S_12
GTCCTGGGCTGCACGCGCGCTACACTGATGAAGTCAGCGAGTTTTATCACACCTTGTTCGGAAG
>F0_Nthermophila_18S_13
AATTCCTAGTAAGCGCGAGTCATCAGCTCGCGTTGATTACGTCCCTGCCCTTTGTACACACCGC
>F0_Rallomycis_18S_1
TAAATAACAATGCAGAACCTTTTAGGTTTTGCAATTGGAATGAGTACAATTTAAATCCCTTAAC
>F0_Rallomycis_18S_2
GCAGTTAAAAAGCTCGTAGTTGAACTTCGGGTTGAGCATTTAGTCCACTCCGGTGGTACTATAA
>F0_Rallomycis_18S_3
AAATTAGAGTGTTTAAAGCAGGCTTAGGCTTGAATACATTAGCATGGAATAATAGAATAGGACT
>F0_Rallomycis_18S_4
TAGAGGTGAAATTCTTGGATTTGCTCAAGGCTAACTACTGCGAAAGCATTTGCCAAGGATGTTT
>F0_Rallomycis_18S_5
CTAGGGATCGGTGGGCTTCATTTTGAACCCATCGGCACCTTATGAGAAATCAAAGTGTTTGGGT

>F0_Rallomycis_18S_6
CTTAATTTGACTCAACACGGGGAAACTCACCAGGTCCAGACATAGTAAGGATTGACAGATTGAG
>F0_Rallomycis_18S_7
TAACGAACGAGACCTTAACCTGCTAAATAGTTTCATGAACTTTGGTTCATTGTAACTTCTTAGA
>F0_Rallomycis_18S_8
CGCGCGCTACACTGATGAAACCAGCGAGTTTATAACCTTGGCCGAAAGGTCTGGGTAATCTTGT
>F0_AmoeboaphelidiumWZ01_18S_1
GGCTATCAGATTAGCCATGCAGTGCCAGTACAAATGACTCTGTACAGTGAAACTGCGAATGGCT
>F0_AmoeboaphelidiumWZ01_18S_2
ATAAATGCCCGACTTCTGGAAGGGCTGTTGTTATTAGATTAAAACCAATGGTGGCAACACCTCA
>F0_AmoeboaphelidiumWZ01_18S_4
GAGGTAGTGACAATACATAACGATACAGGGCCTTCGTGGTCCTGTAATTGGAATGAGTACAATC
>F0_AmoeboaphelidiumWZ01_18S_5
TATTAAAGTTGTTGCGTTAGAAACGCGCGTAGTTGAATTTGGGCACAGACTGGCTGTCCGCTTA
>F0_AmoeboaphelidiumWZ01_18S_6
GACTTTTACTTTGAAAAAATTAGAGTGTTTAAAGCAGGCGTTCGCTTGAATACATTAGCATGGA
>F0_AmoeboaphelidiumWZ01_18S_7
ATTAGTATTTCAGAGTCAGAGGTGAAATTCTTGGATTTCTGAAAGACTAACTACTGCGAAAGCA
>F0_AmoeboaphelidiumWZ01_18S_8
CCATAAACTATGCCGACTCGGGATTGGTGGAACGTAATTATACGCGTCCATCAGCACCGTACGA
>F0_AmoeboaphelidiumWZ01_18S_9
CAAGGCGTGGAATATGCGGCTTAATTTGACTCAACACGGGGAAACTTACCAGGTCCAGACTTAA
>F0_AmoeboaphelidiumWZ01_18S_10
TTTGTCTGCTTAATTGCGATAACGAACGAGACCTTTTCCTGCTAAATAGACTCACTCAGCCTTG
>F1_Ebieneusi_18S_1
GATGCTAGTCGGGAGTACATGAGATATCTCATAATTAGAGCATTCCGTGAGGACTTTTCGCATT
>F1_Ebieneusi_18S_2
CCCTACTGCCGCTCGCACCGCAACCTCCGATTTTCCTCACCCATACACACATCATTCCCCCATC
>F1_Ebieneusi_18S_3
AGATACACTCTCACGCCTGCACACACCACCGCACCGCTGGTCCCCTCGGCATCCCCCATACACA
>F1_Ebieneusi_18S_4
AAACCTTGTTACGACTTGTACTTTATCTAGCCATAGATATTCAGAGCCGAAGCTCTTCATCCCT
>F1_Ebieneusi_18S_5
TACTAGGAATTCCTTATTCACTACGTATTTACAAACGTAGATCCTACTCATCTCAAATCACTTT
>F1_Ebieneusi_18S_6
CCTCCTGTGTTACAGAACACCTGTCAAGGAGGTCTCACTGGTTTAAGCATTTAAGCTAAAGGTC
>F1_GlugeaLM2016_18S_1
CACCAGGTTGGATCTGCCTGACGTGGATGCTAGTCTCCATAAGTAAGCACATGCATGTGCAGCG
>F1_GlugeaLM2016_18S_2
GAGGGCAAAACACAAGATGAGCGATTGACGAGGTCGTTCGTTTAACGAATAGTGTAGGAGAGTA
>F1_GlugeaLM2016_18S_3
TTCCGGAGAGGGAGCCTGAGAGACGGCTACCAGGTCCAAGGACAGCAGCAGGCGCGAAAATTAC
>F1_GlugeaLM2016_18S_4
ACAAGACTGGTGCCAGCACCCGCGGTAATACCAGCTCCTGGAGTGTCTATGATGATTGCTGCAG
>F1_GlugeaLM2016_18S_5
ATGGAATAAGGAGCGTTTAGGGGCCAGGTTATTAAGCGACGAGGGGTGAAATCTGGTGACTCGC
>F1_GlugeaLM2016_18S_6
GATTAGAGACCGTTGTAGTTCTAGCAGTAAACGATGCCGATACCGTGGTGCGGATACGCGACGC
>F1_GlugeaLM2016_18S_7
CCAAGAGTGACTGTCGGCTTAATTTGACTCAACGCGGGACAGCTTACCAGGCCCGACGGCCGGA

>F1_GlugeaLM2016_18S_8
GGGAAGTAGTGAGACCCCTACCGCAAGGGACAGGTCCCGAAAGCACAGGAAGAAAGGGTCAAGA
>F1_Ehepatopenaei_18S_1
GGGAACGACGAACGGCTCAGTAATGTTGCGCTGATTTGCTCTATGCGGGAAGAATAACCACGGT
>F1_Ehepatopenaei_18S_2
ATGGGTAACGGGAAATCAGGGTTTGATTCCGGAGAGGGAGCCTGAGAGATGGCTCCCACGTCCA
>F1_Ehepatopenaei_18S_3
GTAAAAACCTTGACGTGAAGCAATTGGAGGGCAAGTTTTGGTGCCAGCAGCCGCGGTAATTCCA
>F1_Ehepatopenaei_18S_4
AGCCATTGAGTTTGTTGAGAGTAGCGGAACGGATAGGGAGCATGGTATAGGTGGGCAAAGAATG
>F1_Ehepatopenaei_18S_5
CTAGAGTATCGAAAGTGATTAGACACCGCTGTAGTTCTAGCAGTAAACTATGCCGACAATGCTG
>F1_Ehepatopenaei_18S_6
AAACTTAAAGCGAAATTGACGGAAGGACACTACCAGGAGTGGATTGTGCTGCTTAATTTAACTC
>F1_Ehepatopenaei_18S_7
TGGAAATTGATGGGGCGACTTTTAGCTTAAGTGCTGGAACCAGTGAGATCTTCTAGACAGGTGT
>F1_Ehepatopenaei_18S_8
ATATCTCTTGGAAAAGACAAAGCATTTTGAGATGAGTAGGATTAGCTTTTGTAAATAAGCTATG
>F1_Fdaphniae_18S_1
GTGCCAGCCGCCGCGGTAATTCCAGCTCCAGTAGTGCATATACATGCTGTAGTTAGAAAGTTTG
>F1_Fdaphniae_18S_2
AGCGGATGGAGGTAATTGTATTTGGCAGCGAGAGGTGAAAATTGAAGACCTGCCGAGGACAACC
>F1_Fdaphniae_18S_3
GTTGTAGTTCCGGCCGTAAACGATGCCTACTTATAGTGTCAGTGCGCTGATGCAATATAGAGAA
>F1_Fdaphniae_18S_4
AGGAGTGGAGCACGCGGTTCAATTTGACTCAACCCGGGACAACTTACCAGGGCCGGTGCACGCG
>F1_NosemaBM201821_18S_1
GTAGACGCTATACTCTAAGATTAACCCATGCATGTTTATTGAATATAAAGAAAAGACGAACAGC
>F1_NosemaBM201821_18S_2
TAAGATCTATCAGTTAGTTGTTAGGGTAATGGCTTAACAAGACTATGACGGATAACGGTATTAC
>F1_NosemaBM201821_18S_3
ATGATATTATATTGAGGCAGTTATGAGTAGTATTTTATAATTATTGTAGTATTGTAAGTACATA
>F1_NosemaBM201821_18S_4
GATGCAGTTAAAAAGTCTGTAGTTTATTTGTAATAAGCATTGTAAGGTATACTGTATGGTTAGG
>F1_NosemaBM201821_18S_5
GGACGTAAGCTAGAGGATCGAAGATGATTAGATACCATTGTAGTTCTAGCAGTAAACTATGTTG
>F1_NosemaBM201821_18S_6
AGATTGAAAATTAAAGAAATTGACGGAAGAATACCACAAGGAGTGGATTGTGCGGCTTAATTTG
>F1_NosemaBM201821_18S_7
TGGATGCTGTGAAGTAATGATTAATTTCAACAAGATGTGAGACCCTCATTTAGACAGATGTAGT
>F1_NosemaBM201821_18S_8
TATTTGATATTATAAGGGATAATATAATGTAAGATATATTTGAACATGGAATTGCTAGTAAATT
>F1_Eaedis_18S_1
CACCAGGTTGATTCTGCCTGACGTGGACGCTTGCCTCGAAGATTAAGCCATGCAAGTCTGTGAA
>F1_Eaedis_18S_2
TAATATAGTGGATGAGTGGGTGACCTATCAGCTTGACGGTACGGTAAGTGCGTACCGAGGCTAT
>F1_Eaedis_18S_3
AGGCGCGAAACTTACCCAATGAACGTTGAGGTAGTTACGAGGCGTAGTATTATGAAGTGTGTGT
>F1_Eaedis_18S_4
TTTATTGCTGCGGTTAAAACGTGCGTAGTCGGCTAGTTGTGTGAATAGTGATTCGGGATGGTAC

>F1_Eaedis_18S_5
GGTGCTTTTAATTTCAGGAACTTATAGCTTGAGACAGGGACGGGGTAATTTTATTTGGTAGCGA
>F1_Eaedis_18S_6
CGTGAGCAGGAGTATCGAAGAGGATTAGAGACCCACGTAGTTCCTTGCAGTCAACGATGCCAAC
>F1_Eaedis_18S_7
ACTTGAAGAAATTGACGGAAGGACACCACAAGGAGTGGAGTGTGCGGGTTAATTTGACTCAACG
>F1_Eaedis_18S_8
CTTAACACGTGGAGTGATCTGTCTGGTCAAATCTGATAACGCGTGAGAGGTGAGTGGTTTTTAA
>F1_Eaedis_18S_9
CGCGCACTACAATGAGTGGTGGTGCTATAAGTAGTAGCCAGTCGTAGTTGGGATTGACATATGT
>F1_Eaedis_18S_10
ACACCGCCCGTCGTTATCTAAGATGGAGGTGCGGGTGAAGATGTGAGTGTGTAGAGTTGGTGCG
>F1_Aalgerae_18S_1
CACCAGGTTGATTCTGCCTGGTATGTGTGCTAGCGTCAAAGATTTAGCCATGCATGCTTTTCGA
>F1_Aalgerae_18S_2
TAAGGCTAAGACTTAGCGTGACGCACTTTTGTGAAGAAAGGCGACTTGTGCAGCATTGGTTTCT
>F1_Aalgerae_18S_3
AGAGGAAGCCTTAGAGACAGCTTTCACGTCCAAGGATGGCAGCAGGCGCGAAACTTACCCAATT
>F1_Aalgerae_18S_5
TGAGTGTAGAAGCGATTGAAGGCGATTGTATTCACCAGCCAGAGGTAAAATTTGATGACCTGGT
>F1_Aalgerae_18S_6
ATTAGATACCGTTGTAGTTCCGGCCGTAAATTATGCCAACTTGTGCTTCTGCTTCTGCGGAGGC
>F1_Aalgerae_18S_7
GGACACCACAAGGAGTGGATTATGCGGCTTAATTTGACTCAACGCGGGACAACTCACCAGAGCC
>F1_Aalgerae_18S_8
AGGTGACTTGTCAGGTTTACTCCGGTAACGTGTGATGTGCTGTATGCAAGTATTTTTGTGAGAC
>F1_Aalgerae_18S_9
TACAGTGGGTGCTGTAGATATATATAGGTGGAAAAGGGCCCGAGACTGGGATCATGCTTTGTAA
>F1_Aalgerae_18S_10
ACCGCCCGTCGCTATCTGAGATGGATGTTTTTATGAAGATGCTGCTGTTAGAGGCATTTGAGTA
>F1_Mspiralis_18S_1
ATCACCAGGTTGATTCTGCCAGTGAGATACGCTAGTTTAAAGGACTAAGCCATGCAAGCGCGTT
>F1_Mspiralis_18S_2
CCACGGCTAAGCCGACAAGCGACAGCACGTAAGGGTAGTGTTGCAGAGTAGGAGTAGTGATTGA
>F1_Mspiralis_18S_3
GGGAGCCTGAGAGATAGCTACCAGGTCCACGGATCGCAGCAGGCGCGAAACTTGCCCAATGTGA
>F1_Mspiralis_18S_4
TCTGGTGCCAGCCGCCGCGGTAATTCCAGCTCAGGGAGCGTATAGCAGGTATGATGCGGTTAAG
>F1_Mspiralis_18S_5
GGGATGGAGGGAGTCCATGGGGAGGGGTGAAAACCGGAGATCCATGGAGGCCGAAGCGAGGCGA
>F1_Mspiralis_18S_6
CCCAGCAGCAAACAGCCCAGCGCCGTGGGGAATTATATCCACGACGAGACGAAAGAGAAGGGTT
>F1_Mspiralis_18S_7
TAATTTGACTCAACACGGGACAGCTTACCAAGCGAGGCCGTCAAGATGATCCAGTTTTGGAGAT
>F1_Mspiralis_18S_8
TTATTTTGTAGACGCCTCTTATGCAAGAGGGGAAGAAAAGGCAACAACAGGTCCGTGATGCCCC
>F1_Mspiralis_18S_9
GATTGTGGCATGTAATAGCCACATGAACGAGGAATTCCTAGTAGGGTTGTTTTATGAGAACGAA
>F2_Ecolombiana_18S_2
CTAGAGCTAATACATGCTAAAAATCCCGACTTCCGGAAGGGACGTATTTATTAGATAAAAAACC

>F2_Ecolombiana_18S_3
TCATTCAAATTTCTGCCCTATCAACTTTCGATGGTAGGATAGAGGCCTACCATGGTTTTAACGG
>F2_Ecolombiana_18S_4
CGCAAATTACCCAATCCCAACACGGGGAGGTAGTGACAATAAATAACAATATGGGGCCTTTACG
>F2_Ecolombiana_18S_6
TTGCCATTAATTTGGTGGGTCGGGGAAGCAGGACTGTTACCTTGAAAAAATTAGAGTGCTCAAA
>F2_Ecolombiana_18S_7
TCACCGTAATGATTAATAGGGATAGTTGGGGGCATTAGTATTCAATTGTCAGAGGTGAAATTCT
>F2_Rintraradices_18S_1
TTCAGCTTCTGTAGTCATATGCTTGTCTCAGTCGTACAAGGTTTCACTGTAATCATATGCTTGT
>F2_Rintraradices_18S_2
GAGGGGGGGGATTGTTCGATAAAAAAACAATGCCCTTCGGGTTTTCTTGGTGATGGAAGATAAC
>F2_Rintraradices_18S_3
GGCTGACCATGGATCCGACGGGCAAGGGGGAACAAAGGGACGGGGCATGAGGCTGAGCCTGAGA
>F2_Rintraradices_18S_4
TACTGAGACAGGGCTCTTTTGTGTATTGTCTCTGGAATGAGTACAATATAGACCTCATAACCAG
>F2_Rintraradices_18S_5
CTTTACAAGCTCGTAGTTCGAAACTTGGGCCTGGGTGGAAGGTCCACCGTCACCGCGGGTACTG
>F2_Rintraradices_18S_6
AAATCATTAGAGTTGGACGAAGCAAGACTGTGCTCAGAACGCTTAACTCGGAACCAAAGACATA
>F2_Rintraradices_18S_7
TCAGTTGGCGGAAGTTGAAATTCTTGTAATTACTGATAGAACTAACCTACGGTCAAAACATTAG
>F2_Rintraradices_18S_8
CCTATACCGTAACACTTGCGCGCACCTTCGGACCGGGCGGCAGCTACTATTGGCTCTACCGATC
>F2_Rintraradices_18S_9
ACTTACAGCAACTGGAAAGGGAAGCGTCATCGCAATGGGCTGCTGAACCGCGGCCGACTTTATC
>F2_GlomusNBRPP1_18S_2
TCTAGAGCTAATACATGCTAAAAATCCCGACTTCTGGAAGGGATGTATTTATTAGATAAAAAAC
>F2_GlomusNBRPP1_18S_3
ATCATTCAAATTTCTGCCCTATCAACTTTCGATGGTAGGATAGAGGCCTACCATGGTGGTAACG
>F2_GlomusNBRPP1_18S_4
GCGCAATTACCCAATCCCGACACGGGGAGGTAGTGACAATAAATAACAATACGGGGCTCTTTCG
>F2_GlomusNBRPP1_18S_6
CGATGCCCTTAATTGGGTGTCACGGGGAACCAGGACCTTTACTTTGAAAAAATTAGAGTGTTTA
>F2_GlomusNBRPP1_18S_7
GGATCACCGTAATGATTAATAGGGATAGTTGGGGGCATTAGTATTCAATTGTCAGAGGTGAAAT
>F2_GlomusNBRPP1_18S_12
CTGGGGATAGAGCATTGCAATTATCGCTCTTCAACGAGGAATCCCTAGTAAGCGTAAGTCATCA
>F2_GlomusNBRPP1_18S_13
TCGGATCGGCGATCGGCGAGTAGCAATATTCGCTGGTTGCTGAGAAGTTGATCAAACTTGGTCA
>F2_Relegans_18S_1
AGTTATATGCTTGTCTAAAAGATTAAGCCATGCATGTCTAAGTATAAACCAATTCATACGGTGA
>F2_Relegans_18S_2
AGCTAATACATGCAATTAAAGCGTTTTACGCTGTATTTATTAGATAAGCCAACAGCGGCCTCGG
>F2_Relegans_18S_3
CGATGGTAGGATAGAGGCCTACCATGGTCGTTACGGGTAACGGGGAATTAGGGTTCGATTCCGG
>F2_Relegans_18S_4
GGTAGTGACAATAAATAACAATGCAGGGCCCTTTGGGTCTTGCAATTGGAATGAGTACAATTTA
>F2_Relegans_18S_6
ACAGTCGCGGGCGGCCCATTACTGCTTGGGCAGGGTCGTCACTCCTAAACGGGTGGCAGCGCTG

>F2_Relegans_18S_7
TAATAGAATAAGACATGGTCTTGTTTTGTTGGTTTAAACCGGATCGACGTAATGATTAATAGGA
>F2_Relegans_18S_8
TTTGCCAAGTACATTTCCATTAATCAAGAACGAAAGTTAGGGGATCGAAGACGATTAGATACCG
>F2_Relegans_18S_9
GAAATCAAAGTTAGGCTCCGGGGGGAGTATGGTCGCAAGGCTGAAACTTGAAGGAATTGACGGA
>F2_Relegans_18S_11
GCAGAGTTGTACGGATTCTGCGGCGGCCTGGCGTTTCGTCAGTGCCCGTTGTGGGACACCGGCG
>F2_Relegans_18S_12
TGTGATGCCCTTAGATGTTCTGGGCCGCACGCGCGCTACACTGACAGAGCCAGCGAGTATTGGC
>F2_Relegans_18S_13
TTGGACTTGAACGAGGAATTCCTAGTAAGCGCGATTCATCAGATCGCGTTGATTACGTCCCTGC
>F2_Tsphaerospora_18S_2
CGAAATCCCGACTTTTTGGAAGGGATGTATTTATTAGATAAAAGCCAGCCCGTAGCTTGCTGCG
>F2_Tsphaerospora_18S_3
ATCAGTTCACGTTGGTAGGATAGAGGCCTACCAAGGTTTTGACGGGTAACGGGGAATTAGGGTT
>F2_Tsphaerospora_18S_4
ACACAGGGAGGTAGTGACAATAAATAACAATGCAGGGCCCTTACGGGTCTTGCAATTGGAATGA
>F2_Tsphaerospora_18S_6
TCTGCCGACGGTCGCGGATGGCTCACCTTTTCTTGGCGAGGCAGCGGTGCTCCTAACCGGGTGT
>F2_Tsphaerospora_18S_7
TTAGCATGGAATAATAGAATAGGACCTTGGTTCTATTTTGTTGGTTTTTGGGAACCACGGTAAT
>F2_Tsphaerospora_18S_8
TTGCGAAAGCATTTGCCAAGGATGTTTTCATTAATCAAGAACGAAAGTAGGGGGATCGAAGACG
>F2_Tsphaerospora_18S_9
CGGCACCTTACGAGAAATCAAAGTTTCTAGGCTCCGGGGGGAGTATGGTCGCAAGGCTGAAACT
>F2_Tsphaerospora_18S_10
GTCCAGACATAGTGAGGATTGACAGATTGAGAGCTCTTTCTTGATTCTATGGGTGGTGGTGCAT
>F2_Tsphaerospora_18S_11
CGGGGACACTTTCGGGCGGAGTAGTACGGACTGTGTGCGGCGGCGTTTCGGCGTTCGTTCGTGA
>F2_Tsphaerospora_18S_13
CCTTTGCAATTATCGGACTTGAACGAGGAATTCCTAGTAAGCGCGGTTCATCAGATCGCGTTGA
>F2_Tsphaerospora_18S_14
GGTGCGCAGCCGGCAACGGTTAGCCACAGGCCGAGAAGCTGGTCANATTAAGGCATTTAGAGGA
>F3_Gpolymorpha_18S_1
GAACGCGAAGGCCTATTAACTAGTATAGTTTAGTTGATAGTACCTTATCACNTGGATATCGTGG
>F3_Gpolymorpha_18S_2
CGGGCAACCGGTTCTTTGGTGATTCATAGTAAAAAATCGAATCGCAAGGCTTTAGCTAGCGATG
>F3_Gpolymorpha_18S_4
GAATGAGAACAATCTAAACCCCTTCACGAGGAACAATTGGAGGCAAGTCTGGTGCCAGCAGCCG
>F3_Gpolymorpha_18S_5
GAGAGGTCCGCCCTTGTGGTGAGTACTTTTCTGGCTGGGTCTTTCTTTCTGGTGAAGCATTGTG
>F3_Gpolymorpha_18S_6
GGATAGATTAGCATGAATAATAGAATAGGACTTTGGTTCTATTTTGTTGGTTTTTAGGCAGAGA
>F3_Gpolymorpha_18S_7
CGAAAGCATTTGCCAAGNATGTTTTCATTGATCAAGANCGAAGTTAGGGGATCGAAGACNATCA
>F3_OrpinomycesCA34_18S_1
GCAGGGTTTCCCAGACCAGGCGTTGCAAAAGTCGAAAAAGACATACGTACATGGCGGCCGGTCG
>F3_OrpinomycesCA34_18S_3
TCTAGAGCTAATACATGCATAAAAACCCGACTTCTGGAAGGGTTGTATTTATTAGATAAAAAAC

>F3_OrpinomycesCA34_18S_4
TCAAATTTCTGCCCTATCAACTTTCGATGGTAGGATAGAGGCCTACCATGGTTTTAACGGGTAA
>F3_OrpinomycesCA34_18S_5
AATTACCCAATCCTGACACAGGGAGGTAGTGACAATAAATAACGATACAGGGCTTTTTAGTCT
>F3_Bmacroporosum_18S_2
AGCTAATACATGCTAAAAACCCCGACTTCTGGAAGGGGTGTATTTATTAGATAAAAAACCAATC
>F3_Bmacroporosum_18S_3
TCTGCCCTATCAACTTTCGATGGTAGGATAGAGGCCTACCATGGTTTTAACGGGTAACGGAGAA
>F3_Bmacroporosum_18S_4
CAATCCTGACACAGGGAGGTAGTGACAATAAATAACAATACAGGGCTCTTTGAGTCTTGTAATT
>F3_Bmacroporosum_18S_6
AGAGATCCAGGACTTTTACTTTGAAAAAATTAGAGTGTTTAAAGCAGGCTTACGCTTGAATACA
>F3_Bmacroporosum_18S_7
AGTTGGGGGCATTAGTATTCAATCGTCAGAGGTGAAATTCTTGGATTGATTGAAGACTAACTAC
>F3_Bmacroporosum_18S_10
GTGGAGTGATTTGTCTGGTTAATTCCGTTAACGAACGAGACCTTAACCTGCTAAATAGTTACGA
>F3_Bmacroporosum_18S_11
TGTGATGCCCTTAGAAGTTCTGGGCCGCACGCGCGCTACACTGATGAAGGCAACAAGTAATTCA
>F3_Bmacroporosum_18S_12
ACGAGGAATTCCTAGTAAGCGTGAGTCATCAGCTCGCGTTGATTACGTCCCTGCCCTTTGTACA
>F3_Bmacroporosum_18S_13
GAGATGCTGAGAAGTTGGTCAAACTTGGTCATTTAGAGGAACTAAAAGTCGTAACAAGGTAACC
>F3_Paggregatum_18S_2
AAACCAATCCCAGCAATGGGTTTCCTGGTGATTCATAGTAACTTTTCGAATCGTATGACTTTAC
>F3_Paggregatum_18S_4
GTCTTGTAATTGGAATGAGTACAATTTAAATCCCTTAACGAGAACCAATTGGAGGGCAAGTCTG
>F3_Paggregatum_18S_5
TTTGGGCCTGGTTAGACGGTCTGCCGCAAGGCACGTACTGTTGGATCGGGTCTTTTACCTTCTG
>F3_Paggregatum_18S_6
AGGCATGATTGCTCGAATACATTAGCATGGAATAATAGAATAGGACTTTGGTTCTATTTTGTTG
>F3_Paggregatum_18S_7
GATTTATGAAAGACTAACTTCTGCGAAAGCATTTGCCAAGGATGTTTTCATTAATCAAGAACGA
>F4_Langularis_18S_2
CAGCTAATACATGCTTTAAACCCCGACTTCTGGAAGGGGCGTACTTATTAGATTTAAGCCAACC
>F4_Langularis_18S_3
TTCCCCATCAACTTTCGATGGTAGGATAGAGGCCTACCATGGTTTTAACGGGTAACGGAGAATT
>F4_Langularis_18S_4
ATCCTGACACAGGGAGGTAGTGACAATAAATAACAATCCAGGACCTTTTGGTCTTGGAATTGGA
>F4_Langularis_18S_5
TCTAATAGCGTATATTCAAGTTGTTGCAGTTAAAAAGCGCGTAGTTGAATTTCAGACTTGGTTA
>F4_Langularis_18S_6
GCGTTAGGGATCTAGGACTGTTACTTTGAGAAAATTAGAGTGTTTAAAACAGGCTTATGCTTGA
>F4_Langularis_18S_7
GGATAGTTGGGGGCATTAATATTTCATAGTCAGAGGTGAAATTCTTGGATTTATGAAAGATTAA
>F4_Langularis_18S_11
AGGTCTGTGATGCCCTTAGATGTTCTGGGCCGCACGCGCGCTACACTGATGAAGGCAACAAGTA
>F4_Langularis_18S_12
CTTCAACGAGGAATTCCTAGTAAGCGTTTGTCATCAGCAAGCGTTGATTACGTCCCTGCCCTTT
>F4_Langularis_18S_13
GAAGCCGTTTGTTAAGAAGTTGGTCAAACTTGGTCATTTAGAGGAAGTAAAAGTCGTAACAAGG

>F4_Plycopi_18S_2
TAAAAAACCAACCCGGGTAACCGGTTCTTTGGTGATTCATAATAACTTTTCGAATCGCATGGCT
>F4_Plycopi_18S_4
TTGAGTTTTGCAATTGGAATGAGTACAATTTAAATCCCTTAACGAGGAACAATTGGAGGGCAAG
>F4_Plycopi_18S_5
GAATTTTGGATCTGCTGCAGTGGTCAGCCTTCACGGTTTGTACTGCTTGCTGGTGGATCTTTTT
>F4_Plycopi_18S_6
TTCAAAGCAGGCATTTACGCTTGAATACATTAGCATGGAATAATAAAATAGGACTTTGGTTTTA
>F4_Plycopi_18S_7
AATTCTTGGATTTATGAAAGACTAACTTCTGCGAAAGCATTTGCCAAGGATGTTTTCATTAATC
>F5_Clativittatus_18S_2
TTTATGGAAGGGATGCAGTTATTAGATACAAAACCAACCCGGGCAACCGGTCCCTTGGTGATTC
>F5_Clativittatus_18S_3
GTAGGATAGAGGCCTACCGTGGTGATAACGGGTAACGGGGAATTAGGGTTCGATTCCGGAGAGG
>F5_Clativittatus_18S_4
TGACAATAAATAACGATGCGAGACCTTTTGGTTTTTGCAATTGGAATGAGTACAATTTAAATCC
>F5_Clativittatus_18S_7
ATAGGGATAGTTGGGGGCATTAGTATTTAATTGTCAGAGGTGAAATTCTTGGATTTAGGAAAGA
>F5_Clativittatus_18S_8
ATACCGTCGTAGTCTTAACCATAAACGATGCCGACCAGGGATCGGACGCTTGTAGTTTTTTTAA
>F5_Clativittatus_18S_12
GAAAGGTCTGGGTAATCTTTTGAAACTTCATCGTGATGGGGATTGACCTTTGTAATTATCGGTC
>F5_Clativittatus_18S_13
GTCGCTACTACCGATTGGATGGCTTAGTGAGATTTTTGGATTGCGAGGCAATGCAGCAATGTGA
>F5_Aanomalus_18S_2
GGGATGCAGTTATTAGATACAAAACCAACCCGGGCAACCGGTTTTTTGGTGATTCATGATAACT
>F5_Aanomalus_18S_4
TAACGATGCAGGACCTTATGGTCTTTGCAATTGGAATGAGTACAATTTAAATCCCTTAACGAGG
>F5_Aanomalus_18S_5
TTAAAAAGCTCGTAGTTGAATTTCAAGGGTTTTTTTCGTAGCTAGCCATTGTTCCCTTTGCGGT
>F5_Aanomalus_18S_7
ATAGTTGGGGGCATTAGTATTTAATTGTCAGAGGTGAAATTCTTGGATTTAGGAAAGACTAACT
>F5_Aanomalus_18S_8
TCGTAGTCTTAACCATAAACGATGCCGACCAGGGATCGGGCGCTTGTAGTTTTTCCAAAAAGAC
>F5_Bmeristosporus_18S_1
TTTGTACTGTGAAACTGCGAATGGCTCATGTATATCAGTTATAGTTTATTTGATAGTACCTTAC
>F5_Bmeristosporus_18S_2
ATAAAAAAACCAACGTGGGCAACCACTTTTTAGGTGATTCATAATAACTTTTCGAATCGTATGAC
>F5_Bmeristosporus_18S_4
TTTTGGGTCTTGTAATTGGAATGAGTACAATTTAAATCTCTTAACGAGGAACAATTGGAGGGCA
>F5_Bmeristosporus_18S_5
TTGAATTTTGGACCTCGGCCAGACGGTCTGCCTGTTTGGGTACGTACTGTCTTGGCTAGGTCTT
>F5_Bmeristosporus_18S_6
TTTAAAGCAGGCTTACGCTTGAATACATTAGCATGGAATAATAGAATAGGACTTTGGTTCTATT
>F5_Bmeristosporus_18S_7
TTCTTGGATTTATGAAAGACTAACTTCTGCGAAAGCATTTGCCAAGGATGTTTTCATTAATCAA
>F5_Bmeristosporus_18S_8
TCAATGTTATTTTATGACTTGATCGGCACCATATGAGAAATCAAAGTTTTTGGGTTCCGGGGGG
>F5_Bmeristosporus_18S_9
ACTCAACACGGGGAAACTCACCAGGTCCAGACATAGTAAGGATTGACAGATTGAGAGCTCTTTC

>F5_Bmeristosporus_18S_10
AGACCTTAACCTGCTAAATAGTTACGTTTACCTTTGTGTAGACGGTCAACTTCTTAGAGGGACT
>F5_Bmeristosporus_18S_11
TTACACTGATGAAATCAACGAGTTTTTCCTKGGCCGGAAGGTSWGGGTAATCTTGTAAAATTT
>F6_Fboomerangus_18S_2
AAAACCCCGACTTTTGGAAGGGGGGCATTTATTAGATACAGAACCAATGGGGGCAACCTCTTTA
>F6_Fboomerangus_18S_3
CTGTCGATGGTAGTATAGAGGACTACCATGGTGGTAACGGGTAACGGGGAATTAGGGTTCGATT
>F6_Fboomerangus_18S_4
GGGAGGTAGTGACAATAAATAACAATCCAGGGCTTTTTAAGTCTTGGAATTGTAATGAGTACAA
>F6_Fboomerangus_18S_6
TTTGACCAGCACTTTTACCTTGAAAAAATTAGAGTGTTCAAAGGCACCTAAASCCTGAATAMAT
>F6_Fboomerangus_18S_7
GTTGGGGGCATTTGTATGGCACTGTCAGTGGTGAAATACTTGAACCAGTGCCAGACAAACTGAT
>F6_Fboomerangus_18S_8
AGTCTTAACCATAAACTATGCCGACTCGGGATCAGGTAGTGTTAAAATTTGCTACTTGGCACCG
>F6_Fboomerangus_18S_9
CACCACCAGGAGTGGAGCCTGCGCCAATTCGACTCAACACGGGAAAACTCACCAGGTCCAGACA
>F6_Fboomerangus_18S_12
ACCATTGCAATTATTGGTCTTGAACGAGGAATTCCTAGTAAGCGCAAGTCATCAGCTTGCGTTG
>F6_Fboomerangus_18S_13
AACAACAACTTGGCAACAGGTTATTGATTGCTGGGAAGTTGGTCAAACTTGGTCATCTAGAGGA
>F6_Aaquatica_18S_1
TTAAATCAGTTATCAGTCTATTTGATAGTACCTTACTACTTGGATAACCGTGGTAATTCTAGAG
>F6_Aaquatica_18S_2
GGTGATTCATAATAACTTCTCGAATCGCATGGCCTTGTGCCGGCGATGGTTCATTCGAATTTCT
>F6_Aaquatica_18S_5
TCACCGCGAGCACTGGTTCGGTTGGGCCTTTTCCTCTAGGGAACCGCATGTTCTTTACTGGGCG
>F6_Aaquatica_18S_6
GAATAATAGAATAGGACGTGCGGTTCTATTTTGTTGGTTTCTAGGACCGCCGTAATGATTAAAA
>F6_Aaquatica_18S_7
GCATTTGCCAAGGATGTTTTCATTAATCAGGAACGAAAGTCAGGGGATCGAAGACGATCAGATA
>F6_Crecurvatus_18S_2
CAAAAGCCTGGCAACGGGTGCACTTATTAGGTACAAAACCAATATTGCGTCTTTGGCCCAATTT
>F6_Crecurvatus_18S_3
TGGTTGAGATTGTAAGGTAGTGGCTTACAATGCCTACAACGGGTAACGGGGAATTAGGGTTCGA
>F6_Crecurvatus_18S_4
CGGGGAGGTAGTGACAATAAATAACAATGCAGGGCCTTTAAGGTCTTGCAATTGGAATGAGTAC
>F6_Crecurvatus_18S_5
GTATATTAAAGTTGTTGCAGTTAAAACGTCCGTAGTCAAATTTTAGTCTTTTAGGTGATGCGGT
>F6_Crecurvatus_18S_6
AGTAGATAATTAAGCCATTACCATGAGCAAATCAGAGTGTTTAAACAGGCTTTTTAAGCTTGAT
>F6_Crecurvatus_18S_7
GAAACGGTTGGGGGCATTTGTATTTGGTCGCTAGAGGTGAAATTCTTGGATTGACCGAAGACAA
>F6_Crecurvatus_18S_8
ACCGTCGTAGTCTTAACCACAAACTATGCCGACTAGAGATTGGGCTTGTTTATTATGACTAGCT
>F6_Crecurvatus_18S_12
TTTGTAATTATCGCTCTTCAACGAGGAATTCCTAGTAAGCGCAAGTCATCAGCTTGCGTTGATT
>F6_Crecurvatus_18S_13
TTCGGACGACGCAAGTTGTCTTTAGCGGGACGAACTATGGCAAACTAAGCTATTTAGAGGAAGT

>F6_Cneoformans_18S_2
GAGCTAATACATGCTGAAAAGCCCCGACTTCTGGAAGGGGTGTATTTATTAGATAAAAAACCAA
>F6_Cneoformans_18S_3
ATATCTGCCCTATCAACTTTCGATGGTAGGATAGAGGCCTACCATGGTATCAACGGGTAACGGG
>F6_Cneoformans_18S_4
ACCCAATCCCGACACGGGGAGGTAGTGACAATAAATAACAATACAGGGCTCTTTTGGGCCTTGT
>F6_Cneoformans_18S_7
TTAATAGGGACGGTCGGGGGCATTGGTATTCCGTTGCTAGAGGTGAAATTCTTAGATTGACGGA
>F6_Cneoformans_18S_8
TAGATACCGTTGTAGTCTTAACAGTAAACGATGCCGACTAGGGATCGGCCCACGTCAATCTCTG
>F6_Cneoformans_18S_12
TTATTGCTCTTCAACGAGGAATACCTAGTAAGCGTGAGTCACCAGCTCGCGTTGATTACGTCCC
>F6_Tmacrosporus_18S_2
CGGAAGGGGTGTATTTATTAGATAAAAAACCAATGCCCTTCGGGGCTCCTTGGTGAATCATAAT
>F6_Tmacrosporus_18S_3
TAGTGGCCTACCATGGTGGCAACGGGTAACGGGGAATTAGGGTTCGATTCCGGAGAGGGAGCCT
>F6_Tmacrosporus_18S_4
TAAATACTGATACAGGGCTCTTTTGGGTCTTGTAATTGGAATGAGAACAATCTAAATCCCTTAA
>F6_Tmacrosporus_18S_5
TGCAGTTAAAAAGCTCGTAGTTGAACCTTGGGCCCGTCCTGCCGGTCCGCCTCACCGCGAGTAC
>F6_Tmacrosporus_18S_7
AGCTGTCAGAGGTGAAATTCTTGGATTTGCTGAAGACTAACTACTGCGAAAGCATTCGCCAAGG
>F6_Tmacrosporus_18S_8
ATGCCGACTAGGGATCGGGCGGGGTTTCTATGATGACCCGCTCGGCACCTTACGAGAAATCAAA
>F6_Tmacrosporus_18S_10
TTAATTGCGATAACGAACGAGACCTCGGCCCTTAAATAGCCCGGTCCGCGTTTGCGGCCGCTGG
>F6_Tmacrosporus_18S_11
GGGCCGCACGCGCGCTACACTGACAGGGCCAGCGAGTTCATCACCTTGGCCGAGAGGTCTGGGT
>F6_Easchersoniana_18S_1
ATTAAATCAGTTATAGTTTATTTGATGGTACCTTACTACTTGGATAACCGAGGTAATTCTAGAG
>F6_Easchersoniana_18S_2
TTGGTGAATCATAATAACTTTGCGAATCGTATGGCCTTGAGCCGACGATGCTTCATTCAAATAT
>F6_Easchersoniana_18S_5
CTCACGGTGTGTACTGGGTTGTGCTGGTCCTTTCCTTCTGGTAAACTGTTATGTCCTTTACTGG
>F6_Easchersoniana_18S_6
ATGGAATAATAGAATAGGATGTGTGGTTCTATTTTGTTGGTTTCTAGGATCACCATAATGATTA
>F6_Easchersoniana_18S_7
AAAGCATTTGCCAAGGATGTTTTCATTAATCAAGAACGAAAGTTAGGGGATCGAAAACGATCAG
>F6_Easchersoniana_18S_9
ACAAAGTAAGGATTGACAGATTGAGAGCTCTTTCTTGATTCTTTGGGTGGTGGTGCATGGCCGT
>F6_Easchersoniana_18S_10
CTTTTGCTGGTCGCTGTGCTTCTTAGAGGGACTATCGGCGTTTAGCCGATGGGAGTTTGAGGCA
>F6_Easchersoniana_18S_11
TGGCCGGAAGGTCTGGGTAATCTTGTTAAACTTCATCGTGCTGGGGATAGAGCATTGCAATTAT
>H0_Cowczarzaki_18S_2
GGATAACCGTAGTAATTCTAGAGCTAATACATGCAAAAAATCCCGACTTCTGGAAGGGATGTAT
>H0_Cowczarzaki_18S_3
GCTGGCGATGCATCATTCAAATTTCTGCCCTATCAACTTTCGATGGTAAGGTATTGGCTTACCA
>H0_Cowczarzaki_18S_4
AGGCAGCAGGCGCGCAAATTACCCAATCCTGACACAGGGAGGTAGTGACAATAAATAACAATCC

>H0_Cowczarzaki_18S_6
TAGACGTGCGCGTTCTTTACTGAGCGCGTATCGGATATAGGACTTTTACTGTGAAAAAATTAGA
>H0_Cowczarzaki_18S_7
TTCTAGGACCGAAGTAATGATTAATAGGGATAGTTGGGGCCGTTAGTATTTAATTGTCAGAGGT
>H0_Cowczarzaki_18S_8
GTTAGGGGATCGAAGACGATCAGATACCGTCGTAGTCTTAACCTTAAACGATGCCAACTAGGGA
>H0_Cowczarzaki_18S_11
CCAATGGAGGTTTGAGGCAATAACAGGTCTGTGATGCCCTTCGATGTCCCGGGCCGCACGCGCG
>H0_Cowczarzaki_18S_12
GATTGATCCTTGTAATTCTGGATCATCAACGAGGAATTCCTAGTACCCGCAAGTCATCAGCTTG
>H0_Cowczarzaki_18S_13
CTGACATTTTGCAGCTGGCAACAGCCGCGAGGTGACGAGAAGTTGCTCAAACTTGATCATTTAG
>H0_Dsalmonis_18S_1
CTGCCAGTAGTCATATGCTTGTCTCNNAGATTAAGCCATGCATGTCTAAGTATAAACAAATCTA
>H0_Dsalmonis_18S_2
TAATTCTAGAGCTAATACATGCTAAAAATCCCGACTTCTGGAAGGGATGTATTTATTAGATAAA
>H0_Dsalmonis_18S_3
ATGATTCATTCAAATTTCTGCCCTATCAACTTTCGATGGTAAGGTAGTGGCTTACCATGGTTAC
>H0_Dsalmonis_18S_6
GCGGCGTGTGCCCTTCACTGTGGTGTGCGTCCGTCTTATTTCGGGACTTTTACTGTGAAAAAAT
>H0_Dsalmonis_18S_7
GGTTTCTAGGACCAAAGTAATGATTAATAGGGATAGTTGGGGGCATTAGTATTTAATTGTCAGA
>H0_Dsalmonis_18S_8
AAAGTTAGGGGATCGAAGATGATCAGATACCGTCGTAGTCCTAACTATAAACTATGCCGACTAG
>H0_Dsalmonis_18S_12
GTGCTGGGGATAGATCTTTGCAATTTTCGATCTTAAACGAGGAATTCCTAGTAAGCGCAAGTCA
>H0_Dsalmonis_18S_13
TCTTCGGATTGGCGTTCTGCAGCTGGCGACAGCAGCGGAGTGCCGAAAAGTTGATCAAACTTGA
>H0_Sdestruens_18S_2
TAGAGCTAATACATGCTAAAAGTCCCGACTTTTCGGAAGGGATGTATTTATTAGATAAAAAACC
>H0_Sdestruens_18S_3
CATTCAAATTTCTGCCCTATCAACTTTCGATGGTAAGGTAGTGGCTTACCATGGTTGCAACGGG
>H0_Sdestruens_18S_6
GCTTAATTGAGTGTGCGTCGAGTTTGGGACTTTTACTGTGAAAAAATTAGAGTGTTCAAAGCAG
>H0_Sdestruens_18S_7
TAATGATTAATAGGGATAGTTGGGGGCATTAGTATTTAATTGTCAGAGGTGAAATTCTTGGATT
>H0_Sdestruens_18S_8
GATGATCAGATACCGTCGTAGTCCTAACCATAAACTATGCCGACTAGGGATTGGTGGATGTTAA
>H0_Sdestruens_18S_12
TGCAATTTTCGATCTTAAACGAGGAATTCCTAGTAAGCGCAAGTCATCAGCTTGCGTTGATTAC
>H0_Sdestruens_18S_13
ACCGCTGGCAACAGCAGTGAGGCTGCCGAAAAGTTGATCAAACTTGATCATTTAGAGGAAGTAA
>H0_Mbrevicollis_18S_2
GATACCCGTGGTAATTCTAGAGCTAATACATGCGACAAAACCCGACTTCTGGAAGGGTTGTATT
>H0_Mbrevicollis_18S_3
ATTCAAATTTCTGCCCTATCAACTTTCGATGGTAAGGTATTGGCTTACCATGGTTACAACGGGT
>H0_Mbrevicollis_18S_4
CAAATTACCCAATCCTGACACAGGGAGGTAGTGACAATAAATAACAATACAGAGCTCTTTGAGC
>H0_Mbrevicollis_18S_7
TGATTAATAGGGATAGTTGGGGGCATTAGTATTTAATTGTCAGAGGTGAAATTCTTGGATTTAT

>H0_Mbrevicollis_18S_11
GGCAACAACAGGTCTGTGATGCCCTTAGATGTTCTGGGCCGCACGCGCGCTACACTGACGGAGT
>H0_Mbrevicollis_18S_12
ATCGATCTTGAACGAGGAATTCCTAGTAAGCGTGATTCATCAGATCGCGTTGATTACGTCCCTG
>H0_Mbrevicollis_18S_13
AAGCCTGTTGGCGCCGGGAACTTGGTCAAACTTGATCATTTAGAGGAAGTAAAAGTCGTAACAA
>H0_ChoanoflagellidaSL163_18S_2
CTTCTGGGAGGGTTGTATTTATTAGATAAAAAACCAATCGCCTCGGCGTTTCCGGTGAATCATA
>H0_ChoanoflagellidaSL163_18S_3
GGTATTGGCTTACCATGGTTGCAACGGGTAACGGAGAATTAGGGTTCGATTCCGGAGAGGGAGC
>H0_ChoanoflagellidaSL163_18S_4
AATAAATAACAATACAGGGCCTTTTAGGTTTTGTAATTGGAATGAGTACAATTTAAATCTCTTA
>H0_ChoanoflagellidaSL163_18S_5
TTGCAGTTAAAAAGCTCGTAGTTGGATTTCTGGGCTGGCCGTGCGGTCCACCTCACGGTGAGAA
>H0_ChoanoflagellidaSL163_18S_7
ATTGTCAGAGGTGAAATTCTTGGATTTATGAAAGACTAACTACTGCGAAAGCATTTGCCAAGGA
>H0_ChoanoflagellidaSL163_18S_8
GCCGACTCGGGATCGGTGGGGTGATTATAGCCGCATCGGCACCGTATGAGAAATCAAAGTTTTT
>H0_ChoanoflagellidaSL163_18S_9
GCGGCTTAATTTGACTCAACACGGGGAAACTCACCAGGTCCAGACATAGTAAGGATTGACAGAT
>H0_ChoanoflagellidaSL163_18S_10
CCGTTAACGAACGAGACCTTAACCTGCTAAATAGTGACGCGATTCTCGAATCGCGGGTTACTTC
>H0_ChoanoflagellidaSL163_18S_11
CCGCACGCGCGCTACACTGACGAGGACAACGAGTTTATAACCTGCGCCGGAAGGTGTGGGTAAT
>H1_Ppileus_18S_2
TAACCGTAGTAATTCTAGAGCTAATACATGCGAAAAGTCCCGACTTCTGGAAGGGATGTATTTA
>H1_Ppileus_18S_3
GGCGATGTTTCATTCGAGTTTCTGCCCTATCAACTTTCGATGGTAAGGTATTGGCTTACCATGG
>H1_Ppileus_18S_4
CAGCAGGCGCGCAAATTACCCAATCCCGATTCGGGGAGGTAGTGACAATAAATAACGTTGCAGG
>H1_Ppileus_18S_6
ACTCCGTGTGCCCTTAACTGGGTGGGCGAAGGATTCGCGACGTTTACTTTGAAAAAATTAGAGT
>H1_Ppileus_18S_7
CGAGACCGAAGTAATGATTAATAGGGACAGTTGGGGGCATTCGTATTTCATTGTCAGAGGTGAA
>H1_Ppileus_18S_8
GGAGGCTCGAAGACGATCAGATACCGTCCTAGTTCCAACCATAAACGATGCCGTCTGCGGATCG
>H1_Ppileus_18S_12
GGATAGACCATTGCAATTTTTGGTCTTGAACGAGGAATTCCTAGTAAGCATGAGTCATCAACTC
>H1_Ppileus_18S_13
ATTGGAGACGTTGCGCCGCAAGGCACATCGTCCTCGAGAACTTGATCAAACTTGATCATTTAGA
>H1_Mleidyi_18S_2
TTCTGGAAGGGATGTATTTATTAGATTAAAAACCAATGCGTTTAACGACGCTTTTCGGTGATTC
>H1_Mleidyi_18S_3
GTAAGGTATTGGCTTACCATGGTGACAACGGGTAACGGAGAATTAGGGTTCGATTCCGGAGAGG
>H1_Mleidyi_18S_4
TGACAATAAATAACGTTGCAGGCGCCAACGGCTTCTGCAGTCGGAATGAGTACAATATAACACC
>H1_Mleidyi_18S_7
TATTTCATTGTCAGAGGTGAAATTCTTGGATTTATGAAAGACGAACTTCTGCGAAAGCATTTGC
>H1_Mleidyi_18S_8
AACGATGCCGTCTGCGGATCGGAGGTCGCTCAATTAAGGCTCCTTCGGCACGCTATGAGAAATC

>H1_Mleidyi_18S_9
GTGGAACCTGCGGTTTAATTTGACTCAACACGGGAAAACTCACCAGGTCCAGACATAGGAAGGA
>H1_Mleidyi_18S_10
TGGTTAATTCCGTTAACGAACGAGACCTTAACCTGCTAAATAGTGACACGGTTCTTTGAACCGT
>H1_Mleidyi_18S_11
ATGTTCTGGGCCACACGCGCGTTACACTGATGAAGCCAGCGAGTATATCGCCTTCACCGGAAGG
>H2_Hcarolinensis_18S_2
CGTAGTAACATAGAGCTAATACATGCTTGAAATCCTGACTGGTTGCTCTTTCGCGGGGGTGATC
>H2_Hcarolinensis_18S_3
CTTGGTGATTCATGGTAACTTATCGGATCGCATGGCCTTGCGCTGGCGATGCTTCATACAAGTT
>H2_Hcarolinensis_18S_6
GCCCTGTGTGGTGTGTACTGGCTTTGGTGTGCTAGTTGGGGTGCCTTGCCTTGAAAGGGGTGGG
>H2_Hcarolinensis_18S_8
ATGGAGTAATGATTGATAGGGACAGTTGGGGGCATTCGTATTTAATTGTCAGAGGTGAAATTCT
>H2_Acompressa_18S_2
TCTCGGAAGGGATGTATTTATTAGATCCAAAACCAATGCCGGGACTTCGGTCCCGGGAAGCTGG
>H2_Acompressa_18S_3
TCGATGGTAGGGTATTGGCCTACCATGGTCGCAACGGGTGACGGAGAATTAGGGTTCGATTCCG
>H2_Acompressa_18S_4
AGGTAGTGACAATAAATAACAATGCCGGCCTCTTGAGTAGGCCGGCAATTGGAATGAGTACAAT
>H2_Acompressa_18S_7
CATTCGTATTCAATTGTCAGAGGTGAAATTCTTGGATTTATGGAAGACGAACAACTGCGAAAGC
>H2_Acompressa_18S_8
ACCATAAACTATGCCGGCTAGGGATCGGCGGAGGTCGCAATCGCCTCCGTCGGCACCTTGAGAG
>H2_Acompressa_18S_10
TTGTCTGGTTAATTCCGTTAACGAACGAGACCTTAACCTGCTAACTAGCCGCGCCGTTCCCGAA
>H2_Acompressa_18S_11
GCCCTTAGATGTCCTGGGCCGCACGCGCGCTACACTGACGGAGCCAGCGAGCGTGTCCTTCGCC
>H2_Acompressa_18S_12
TTCCTAGTAAGCGCGAGTCACCAGCTCGCGTTGATTACGTCCCTGCCCTTTGTACACACCGCCC
>H2_Hdujardini_18S_2
TTCTAGAGCTAATACATGCTACAAGTCCCGACTTCTGGAAGGGATGTATTTATTAGATACAAAA
>H2_Hdujardini_18S_3
TTCATTCAAGTTTCTGCCCTATCAACTTTCGATGGTAGGGTATTGGCCTACCATGGTTACAACG
>H2_Hdujardini_18S_4
GCGCAAATTACCCAATCCCGACACGGGGAGGTAGTGACAATAAATACTGATACCGGGCTCTTTG
>H2_Hdujardini_18S_6
CTTCATTGCAGTGGACTGGGGACCTCGGGACGTTTACTGTGAAAAAATTAGAGTGTTCAAAGCA
>H2_Hdujardini_18S_7
GTAATGATCAATAGGGACAGTCGGGGGCATTCGTATTCTATTGTCAGAGGTGAAATTCTTGGAT
>H2_Hdujardini_18S_8
AGACGATCAGATACCGTCCTAGTCCTAACCGTAAACTATGCCGACTAGGGATCGGCGGGGTTCA
>H2_Hdujardini_18S_12
GCAATTATTGATCTTGAACGAGGAATTCCTAGTAAGCGCAAGTCATCAGCTTGCGTTGATTACG
>H2_Hdujardini_18S_13
TGGACTTTCTGTTCAACTAGGTAGTGGGAAGCTCAGCGAATGATATTTTTTCGAGGAAGTAAAA
>H2_Ponkodes_18S_2
CAAAACCAATGCGGGGCTCCGGTCCCGGTCTTTGGTGATTCATGGTAACTGTTCGGAGCGCACG
>H2_Ponkodes_18S_4
CTTTTTCAAGTCTTGTAATTGGAATGAGAACAATTTAAGTCCCTTAACGAGGAACAATTGGAGG

>H2_Ponkodes_18S_5
TAGTTGGATTTCGGCGGCGCGCTCTCGGTCCACCTGGCGGCGTGTCACTGGGAGCGCGCGGCTT
>H2_Ponkodes_18S_6
TCAAAGCAGGCCGTTGGGCTTGGATACATTAGCATGGAATAATGGAATAGGACGTCGGTCCTAT
>H2_Ponkodes_18S_7
ATTCTTGGATTTATGGAAGACGAACGCATGCGAAAGCATTTGCCAAGGATGTTTTCGTTAATCA
>H2_Ponkodes_18S_8
GAGGACGTTATATCACGACTCCTTCGGCACCTTTCGAGAAATCAAAGTTTCTGGGTTCCGGGGG
>H2_Ponkodes_18S_9
GACTCAACACGGGAAAACTCACCAGGTCCGGACACAGTAAGGATTGACAGATCGAGAGCTCTTT
>H2_Ponkodes_18S_10
GAGACCTTAACCTGCTAACTAGTCACGTGGCGTCGTCTTTCGGCGCGGCGGTCGACTTCTTAGA
>H2_Ponkodes_18S_11
CGCGCGCTACACTGACGGATGCAGCGAGTTGTGTCCTGCGTCAAGAGGCGCGGGTAATCTTGTC
>H2_Pneocaledoniense_18S_2
TTGGATAACCGTGGTAATTCTAGAGCTAATACATGCGTTAAAGTCCTGACCTCTCGGGGAAGGG
>H2_Pneocaledoniense_18S_3
CGCATGGTCTTGTGCCGGCGATGACTCATTCAAATTTCTGCCCTATCAACTTTCGATGGTAAGG
>H2_Pneocaledoniense_18S_6
GTTCTTCTTTCCGTGGAGCGTGTGTGCTCTTCACTGAGTGTGCACGTAACTCGGGACTTTTACT
>H2_Pneocaledoniense_18S_7
CTATTTTGTTGGTTTTCGGGACCAAGGTAATGACTAATAGGGACAGTTGGGGGCATTCGTATTC
>H2_Pneocaledoniense_18S_12
TTCATCGTGCTGGGGATAGACCATTGCAATTCTTGGTCTTCAACGAGGAATTCCTAGTAAGCGC
>H2_Pneocaledoniense_18S_13
GTGAGATCTTCGGATTTGTCGCCCGGCGACCTCGTGCCGCTGGGCCGTACGGAGAAGTTGATCA
>H2_TrichoplaxH8_18S_2
AACCGTGGTAATTCTAGAGCTAATACATGCGAAAAGTCCCGACTTTGCGGAAGGGATGTATTTA
>H2_TrichoplaxH8_18S_3
CCGGCGATGCTTCATTCAAGTTTCTGCCCTATCAACTTTCGATGGTAAGGTATTGGCTTACCAT
>H2_TrichoplaxH8_18S_4
GGCAGCAGGCGCGCAAATTACCCAATCCCGACACGGGGAGGTAGTGACAAGAAATAACGATACG
>H2_TrichoplaxH8_18S_6
TGCATATGCTCTTAATTGAGTGTATGTAGGACTTGAGACTTTTACTGTGAAAAAATTAGAGTGT
>H2_TrichoplaxH8_18S_7
GAACCGAAGTAATGATTAATAGGGACAGTTGGGGGCATTCGTACTTCATTGTCAGAGGTGAAAT
>H2_TrichoplaxH8_18S_12
TTGTAATTATTGGTCTTGAACGAGGAATTCCTAGTAAGCGCGAGTCATCAGCTCGCGTTGATTA
>H2_TrichoplaxH8_18S_13
GCGCGACTTCACGGTTGTGCCAATTATCGAGAAGTTGATCAAACTTGATCATCTAGAGGAAGTA
>H2_AfrosternophorusJM-2008_18S_2
TTTGGGAGAGGGTGCTTTTATTAGACCAAGACCAATATGGCTCCGGTCATATTTTGTGGTGACT
>H2_AfrosternophorusJM-2008_18S_3
TGGTAGGTTATGCGCCTACCATGGTTATTACGGGTAACGGGGAATCAGAGTTCGATTCCGGAGA
>H2_AfrosternophorusJM-2008_18S_4
AGTGACGAAAAATAACAATACGGGACTTTTTTAAGGCTCGTAATTGGAATGAGTACACTCTAAA
>H2_AfrosternophorusJM-2008_18S_5
AAAGTTGTTGCGGTTAAAAAGCTCGTAGTTGGATCTCAGTCGCGCTCCCAGCGATACGTTGCAA
>H2_AfrosternophorusJM-2008_18S_6
TTTACTTTGAAAAAATTAGGGTGCTCAAAGCAGGCTTGTGCCTGGATATTGGTGCATGGAATAA

>H2_AfrosternophorusJM-2008_18S_7
CGTATTGCGGCGTTAGAGGTGAAATTCTTGGATCGCTGCAAGACGAACTACTGCGAAAGCATTT
>H2_AfrosternophorusJM-2008_18S_8
TAAACGATGCCAACCAGCGATCCGCCTGAGTTCCTCAAACGACTCTGCGGGCAGCCTCCGGGAA
>H2_AfrosternophorusJM-2008_18S_11
TAGATGTCCGGGGCCGCACGCGCGCTACACTGAAGGAATCAACATGTGTCTACCCTAGTCCGAA
>H2_Lgranulosus_18S_1
CCTTAATACGGTGAAGCCGCGAATAGCTCATTACAACAGCCACCGTTTATTAGAATATCGTTAT
>H2_Lgranulosus_18S_2
TTAGAATAAAAACCAATCGGGCCTCGGCCCGTGATTTGGTGAATCTGAATAACTCCGCCGATCG
>H2_Lgranulosus_18S_4
AGTCCTCTTCGAGGTCTGTCATCGGAATGGGTACAATTTAAATCCTTTAACGAGGATCTATTGG
>H2_Lgranulosus_18S_5
TCGTAGTTGGATCTGCGGCCGAGCGGGGCGGTCCCCCGAAAGGGCGGTCACCGTACCCGCTAGC
>H2_Lgranulosus_18S_6
GAGTGCTTAAAGCAGGCGAAATAGCCTGAATAAGGTGCATGGAATAATGGAATAGGACCTCGGT
>H2_Lgranulosus_18S_8
CGATTAGTCGGCGTTAATCTACGACCCGACTAGCAGCTTCCGGGAAACCAAAGTTTTTCGTTTC
>H2_Lgranulosus_18S_9
TAATTTGACTCAACACGGGAAAACTCACCCGGCCCGGACACCGTAAGGATTGACAGACTGAGAG
>H2_Lgranulosus_18S_10
ACGAACGAGACTCTGGCCTATTAAATAGACGGCGTATCGAAAAGTACGCCGAGCTTCTTAGAGG
>H2_Lgranulosus_18S_11
CGCTACACTGAAAGAATCAGCGTGCGCGATTCGCCTGGTCCGGAAGGATCGGGTAACCCGAGTA
>H2_Imaderensis_18S_1
TGGCTCATTAAATCAGTTATCGTTTATTTGATTGTACCCTTACTACTTGGATAACCGTAGTAAT
>H2_Imaderensis_18S_2
TAGCTTCGGCCAGCCGGATCGTTGGTGATTCATAGTAACTGTTCGAATCGCAGGGCCCTTTGCG
>H2_Imaderensis_18S_5
TTCGGGAGGGGTCGGCCGGTCCGCCGCAAGGTGTGCTACTGGTCGGCCCGTCCTTCCTGGCGCA
>H2_Imaderensis_18S_6
GCTCGCCGGCTTGGATACATAAGCATGGAATAATGGAATAGGACTCTGGTTCTATTTTGTTGGT
>H2_Imaderensis_18S_9
CGGGAAAACTCACCAGGTCCGGACATGGTAAGGATTGAACAGATTGAGAGCTCTTTCTTGATTC
>H2_Imaderensis_18S_10
GACCTGCTAAATAGTCACGCCGTTCCCGAACGGTGGCTGACTTCTTAGAGGGACTGTTGGTGTC
>H2_Imaderensis_18S_11
GTATCAGCGAGTCCCTCCTTTGCCGTCAGGCATGGGTAATCTTGTGAAATATCATCGTGCTGGG
>H2_Ecalifornicum_18S_2
TTCTGGAAGGGATGTATTTATTAGATTAAAAACCAATGCGGGTCCTCGTGGCTCGTCTTCTTGG
>H2_Ecalifornicum_18S_3
CGATGGTAAGGTAGTGGCTTACCATGGTTGTAACGGGTGACGGAGAATTAGGGTTCGATTCCGG
>H2_Ecalifornicum_18S_4
GGTAGTGACAAGAAATAACGATACGGGGTCTTAATAGGTCTCGCAATTGGAATGAGTACAATTT
>H2_Ecalifornicum_18S_7
ATCCGTATTTCGTTGTCAGAGGTGAAATTCTTGGATTTACGAAAGACGAACAACTGCGAAAGCA
>H2_Ecalifornicum_18S_8
CCATAAACGATGTCGACTAGGGATCAGCGGGCGTTATTGTACGACCTCGTTGGCACCTTACGGG
>H2_Ecalifornicum_18S_10
TTGTCTGGTTAATTCCGTTAACGAACGAGACCTTAACCGGCTAAATAGTCACACGATTCTCGAA

>H2_Ecalifornicum_18S_11
AGATGTTCTGGGCCGCACGCGCGCTACACTGTCGGATTCAGCGAGTCTTAACCTTAACCGAAAG
>H3_Rophiocomae_18S_2
GATAACTGTGGCAANTCTAGAGCTAACACATGCAACAGAGCTTTCTTGGATCTTTCGGGATTCC
>H3_Rophiocomae_18S_3
TGCCGATCGTAGGGCCTAGAGCCGACGATAGATCCTTCAAGTGTCTGCCCTATCAACTTTCGAT
>H3_Rophiocomae_18S_4
ACAGCTCCTACTTCCAAGGAGGGCAGCAGGCGCGCAAACTACCCAATCCCGGCTCGGGGAGGTG
>H3_Rophiocomae_18S_6
AATCCTTATGTTAGTCGNTTTCAGTTCTCTTTATTGAGTGCTGTGAATGACTACAAATTTTACT
>H3_Rophiocomae_18S_7
TTCTATCTGTTGGTTGATGAACTTGAGGTAATGATTAAGAGGGACAGTCGGGGGCATTCATACT
>H3_Rophiocomae_18S_10
GATGGCTCTTTCTTGATTCAGTGATTGGTGGTGCATGCCCGTTCTTAGTTGGTGGAGCGATTTG
>H3_Rophiocomae_18S_11
TCAGCGGTCATTTATTCTTCTTAGAGGGACTGGTTGGTTCGGCTTAATATGTAGAAACACGAAA
>H3_Rophiocomae_18S_12
TAATTTTCCAAGCTCGAGAGAGTTCGGAAATCCGTGAAACTCCCTCGTGATAGGGATTGTGCGA
>H3_Rophiocomae_18S_13
TGTACACACCGCCCGTCGCTACTACTGATTGAATGGTTTAGTGAGGTTATTGGATCGAATTCTG
>H3_Dacuticephalum_18S_1
GTATAAGTTCATGCTCTCTAATGAGCGAGACCGCAACGGCTCATTAAATCGGACATAACTTACT
>H3_Dacuticephalum_18S_2
TATTAGATTAGTTGACTCTGGATAAATTTGCTGATCACGGGCTGTGCTGTGACGTATCAATTAG
>H3_Dacuticephalum_18S_3
ATTCCGGAGAGGGAGCATGAGAAATGGCTGCCAGATCTATGGATTGCAGCAGGTGCGAAAATTA
>H3_Dacuticephalum_18S_5
AAGTAGGCCTTTGCTGTAGTTGAGTGCTACGGCTTTACCTTGAACAAAATAGAGTGCTTAAGGC
>H3_Dacuticephalum_18S_6
GATTAACAGAGACAGACGGGGGCATTCGTATTGCACCGCTAGAGGTGAAATTCGTAGATCGGTG
>H3_Dacuticephalum_18S_7
TAGATCTCGTCGTAGTTCCAACTATAAACGATGCCAACTAGCACTCCGCTAATAAGATTTTGAC
>H3_Dacuticephalum_18S_8
TTGACGGAAGGGCACCACAGGAGTGAGCCTGCGCTAATTTGACTCAACACAGAAAAACTCACCC
>H3_Dacuticephalum_18S_10
TGCCTTAGACGTTCGGGGCTGAACGCGCGCTACAATGGAGAAAGCAGTGGGTGTCTTGCTTGGC

Table S3 (B). List of 64-mer probes for housekeeping genes

>Housekeeping_hPGK1_p1
AACTCAAATCTCTGCTGGGCAAGGATGTTCTGTTCTTGAAGGACTGTGTAGGCCCAGAAGTGGA
>Housekeeping_hPGK1_p3
GTACTTTCCTGCCTTTTAGTTCCTGTGCACAGCCCCTAAGTCAACTTAGCATTTTCTGCATCTC
>Housekeeping_hHMGCR_ex2
TGGCCTCTTTGTGGCCTCCCATCCCTGGGAAGTCATAGTGGGGACAGTGACACTGACCATCTGC
>Housekeeping_hHMGCR_ex3
ATAATTCTGACAATAACACGATGCATAGCCATCCTGTATATTTACTTCCAGTTCCAGAATTTAC
>Housekeeping_hNSE_ex2
GAGAAGATCTGGGCCCGGGAGATCCTGGACTCCCGCGGGAACCCCACAGTGGAGGTGGATCTCT

>Housekeeping_hNSE_ex3
TGCCCAGTGGAGCCTCTACGGGCATCTATGAGGCCCTGGAGCTGAGGGATGGAGACAAACAGCG

Table S3 (C). List of 64-mer probes for retroelements

>R1_LINE1_RT
TGGCAAACCAAATCCAGCAGCACATCAAAAAGCTTATCCACCATGATCAAGTGGGCTTCATCCC
>R1_LINE1md_RT
TCACATATAGTGTGGCAATGAACACTGTTGCTTGTTACATATACTTCAAGCATCCAGCAGCCAT
>R1_LINE1dr_RT
TGGAGTGGAATGTCAAGGAAGGACATCTGGACATTCAACCCCCTGAGAATCAGGTTCTTGATCC
>R1_CONSENSUS_hLINE1
AATACATCCCATCAATACCTAATTTATTGAGAGTTTTTAGCATGAAGGGTTGTTGAATTTTGTC
>R1_hLINE2_HUMAN_BRAIN
CACCACATAAACAGAATTAAAAACAAAAATCACATGATCATCTCACTAGATGCAGAAAAAGCAT
>R2_hALU_Konkel
GGCCGGGCGCGGTGGCTCACGCCTGTAATCCCAGCACTTTGGGAGGCCGAGGCGGGCGGATCAC
>R3_MLV_c19
AGGTCCCCATGGTTCACGACCCTGATATCCACCATTATGGGCCCTCTGATAGTACCTTAAAACC
>R3_hsHERVW_gag1
CAACTCACAATTATGTAAAAAGTGTGGTTTATGCCCTACAGGAAGCCCTCAGAGTCCACCTCCC
>R3_hsHERVW_gag2
CAGCCAGAGTGCCTGTACCTTTTTCTCTCTCAGACTTAAAGCAAATTAAAATAGACCTAGGTAA
>R3_hsHERVK_gag1
CCCAGAAAGTCAGTATGGATATCCAGGAATGCCCCCAGCACCACAGGGCAGGGCGCCATACCCT
>R3_hsHERVK_gag2
GGCAATTCCCAATAACGTTAGAACCGATGCCACCTGGAGAAGGAGCCCAAGAGGGAGAGCCTCC
>R3_hsHERVK_pol1
TCTCCGGCCATGATCCCAAAAGACTGGCCTTTAATTATAATTGATCTGAAAGATCGCTTTTTTA
>R3_hsHERVK_pol2
GGATTGTGAAAAATTTGCCTTTACTATACCAGCCATAAATAGTAAAGAACTAGCCACCAGGTTG
>R3_hsRTVL_pol1
TTCTCACCTTATTCAATATATTGATGACCTTCGTCTTTGTAGCCCCTCCTTTGAATCTTCTCAA
>R3_hsHERVP4_pol2
AAGAGGGACCTAATTTCCCACAAGAGAAGTGGCCAGATAGGATGAAGCTAACGGAAAGGAGTGC
>R3_hsHERVHC2_pol1
ATGCACTCCTTATGGGGCTGGGTTTTCACCTTTTGAAATCATGTATGGAAGGGCTCCGCCTATC
>R3_hsHERVHC2_pol2
GTGCAGTCTCTCCAACAGGTACAAGACATCATCCAGCCGCTTGTCTGGGGAGCTCATCCCAATC
>R3_hsERV9_pol1
TTCTGAAGTCCAGGCATTCTAGTCCTTCAGTATGTGGATGATTTACTTTTGGCTACCAGTTTGG
>R3_hsERV9_pol2
TATGCTGGCTTATCGGCACCCTAAGACATTAAAACAATTGTGGGGGTTCCTTGGAATCACTGGC
>R3_hsHIV_gag1
CAAATGTTAAAAGATACTATCAATGAGGAAGCTGCAGAATGGGACAGGGTACATCCAGTACATG
>R3_hsHIV_gag2
GATGAGAGAACCGAGGGGAAGTGACATAGCAGGAACTACCAGTACCCTTCAGGAACAAATAGGA
>R3_hsHIV1_pol1
TGACAAAAATTTTAGAGCCTTTTAGAAAACAAAACCCAGAAGTGGTTATCTATCAATACATGGA

>R3_hsHIV1_pol2
GTGCCCACACTAATGATGTAAAGCAATTAACAGAGGCAGTACAGAAAATAGCCACAGAAAGCAT
>R3_hsHTLV_gag1
TAGAAACGCCAGTCTGGATGTGTCCCATTAACTACTCCCTCCTAGCTAGTCTACTCCCAAAAGG

Table S4. List of human-associated species (PATHLIST).

Class	Species	Accession
A	*Methanobrevibacter massiliensis*	NR_115169.1
A	*Methanobrevibacter oralis*	LR590665.1
A	*Methanomassiliicoccus luminyensis*	HQ896499.1
A	*Methanomethylophilus alvus*	KC412010.1
A	*Methanosphaera stadtmanae*	NR_028236.1
B	*Actinomyces israelii*	AF479270.1
B	*Bacillus anthracis*	AB592486.1
B	*Bacteroides fragilis*	LR999640.1
B	*Bartonella henselae*	NR_074335.2
B	*Bordetella pertussis*	NR_025951.1
B	*Borrelia burgdorferi*	NR_044732.2
B	*Brucella abortus*	NR_042460.1
B	*Campylobacter jejuni*	NR_041834.1
B	*Chlamydophila psittaci*	NR_036864.2
B	*Clostridium botulinum*	NR_029157.1
B	*Corynebacterium diphtheria*	Y09044.1
B	*Ehrlichia canis*	NR_118741.1
B	*Enterococcus faecalis*	NR_040789.1
B	*Escherichia coli*	NR_024570.1
B	*Francisella tularensis*	NR_029362.1
B	*Haemophilus influenza*	NR_044682.2
B	*Helicobacter pylori*	NR_044761.1
B	*Klebsiella pneumoniae*	NR_036794.1
B	*Legionella pneumophila*	NR_041742.1
B	*Leptospira interrogans*	NR_029361.1
B	*Listeria monocytogenes*	NR_044823.1
B	*Mycobacterium leprae*	X53999.1
B	*Mycobacterium tuberculosis*	NR_102810.2
B	*Mycoplasma pneumoniae*	NR_041751.1
B	*Neisseria gonorrhoeae*	NR_026079.2
B	*Neisseria meningitidis*	NR_104946.1
B	*Nocardia asteroides*	NR_041856.1
B	*Porphyromonas gingivalis*	NR_040838.1
B	*Pseudomonas aeruginosa*	NR_026078.1
B	*Rickettsia rickettsii*	NR_028018.1
B	*Salmonella typhi*	NR_074799.1
B	*Shigella dysenteriae*	NR_026332.1
B	*Shigella sonnei*	NR_104826.1
B	*Staphylococcus aureus*	NR_118997.2
B	*Streptococcus agalactiae*	NR_040821.1
B	*Streptococcus pyogenes*	NR_028598.1
B	*Treponema pallidum*	M88726.1
B	*Ureaplasma urealyticum*	NR_041710.1
B	*Vibrio cholerae*	NR_044050.1
B	*Yersinia pestis*	NR_025160.1
C/D	*Cryptosporidium muris*	AB089284.1
C/D	*Toxoplasma gondii*	L24381.1

C/D	*Cyclospora cayetanensis*	FJ009129.1
E	*Microsporidium spp.*	AF151529.1
E	*Plasmodium falciparum*	XR_002966654.1
E	*Toxoplasma gondii*	XR_001974253.1
E	*Giardia intestinalis*	XR_005248693.1
E	*Leishmania donovani*	XR_002966730.1
E	*Naegleria fowleri*	KY062165.1
E	*Chlorella vulgaris*	MF686487.1
E	*Mallomonas papillosa*	HF549062.1
E	*Dientamoeba fragilis*	JQ677148.1
F	*Candida albicans*	NG_070791.1
F	*Candida tropicalis*	HG798647.1
F	*Aspergillus fumigatus*	AB008401.1
F	*Aspergillus flavus*	D63696.1
F	*Cryptococcus neoformans*	NG_064879.1
F	*Histoplasma capsulatum*	AF320009.1
F	*Pneumocystis jirovecii*	AB266392.1
F	*Stachybotrys chartaru*	DQ680066.1
F	*Blastomyces dermatitidis*	M63096.1
F	*Coccidioides immitis*	XR_001099882.1
F	*Paracaccidioides brasiliensis*	XR_001551862.1
F	*Talaromyces marneffei*	NG_074941.1
H	*Ancylostoma caninum*	AJ920347.2
H	*Anisakis pegreffi*	EF180082.1
H	*Ascaris lumbricoides*	X06225.1
H	*Blastocystis hominis*	AM275395.1
H	*Brachionus plicatilis*	U49911.1
H	*Brugia malayi*	KP760121.1
H	*Clonorchis sinensis*	JF314770.1
H	*Diphyllobothrium latum*	DQ925309.1
H	*Dipylidium caninum*	AB731643.1
H	*Dirofilaria immitis*	AF182647.1
H	*Echinococcus multilocularis*	AB731634.1
H	*Encentrum astridae*	DQ297695.1
H	*Endolimax nana*	LC230015.1
H	*Entamoeba histolytica*	KP233836.1
H	*Enterobius vermicularis*	JF934731.1
H	*Fasciola hepatica*	AJ004969.1
H	*Fasciolopsis buski*	L06668.1
H	*Giardia lamblia*	XR_005248690.1
H	*Leishmania braziliensis*	JX030192.1
H	*Loa loa*	XR_002251421.1
H	*Mansonella streptocerca*	KP760145.1
H	*Moniliformis moniliformis*	Z19562.1
H	*Necator americanus*	AY295811.1
H	*Onchocerca volvulus*	KT031393.1
H	*Paragonimus westermani*	AJ287556.1
H	*Plasmodium vivax*	XR_003001206.1
H	*Pneumocystis carinii*	AH001732.2
H	*Schistosoma japonicum*	AY157226.1

H	*Strongyloides stercoralis*	AB453316.1
H	*Taenia saginata*	AB731616.1
H	*Toxocara canis*	JN256977.1
H	*Trichinella spiralis*	MH289515.1
H	*Trichomonas vaginalis*	KX061409.1
H	*Wuchereria bancrofti*	AY843438.1

Table S5. Matches between key viruses and the human genome.

A Identities of human matches					
**Virus**	**Isolate; sequence identifier**	**Major matches**		**Partial matches**	
		**Gene symbol**	**Gene name**	**Gene symbol**	**Gene name**
Adeno-associated virus	Adeno-associated virus isolate MHH-05-2015; NC_040671.1	ND	NA	*CCNA2*	Cyclin A2
Adenovirus A	Human adenovirus A; NC_001460.1	ND	NA	Unknown	Chr.18
				IRF2BPL	Interferon regulatory factor 2 binding protein like
Adenovirus type C	Human mastadenovirus C K67-339; LC504573.1	Human integrated adenovirus 2 VA RNA	NA	*YWHAB*	Tyrosine/tryptophan monooxygenase activation protein beta
				TMEM9B	TMEM9 domain family member B
				NCBP3	Nuclear cap binding subunit 3
				PABPC1L2B	Poly(A) binding protein cytoplasmic 1 like 2B
Adenovirus 54	Human adenovirus 54; NC_012959.1	ND	NA	*CHIC1*	Cysteine rich hydrophobic domain 1
Coronavirus 229E	Human coronavirus 229E strain 229E/human/USA/933-40/1993; KF514433.1	ND	NA	*SPARC*	Secreted protein acidic and cysteine rich
				IL6ST	Interleukin 6 signal transducer
				PLC1B1	Phospholipase C beta1
				Unknown	Chr.11
HEPC2	Hepatitis C virus genotype 2; NC_009823.1	ND	NA	Unknown	Chr.11
HEPC4	Hepatitis C virus genotype; NC_009825.1	ND	NA		
HHV1/HSV1	Human herpesvirus 1 strain 17; LT576869.1	ND	NA	*MAPK3*	Mitogen-activated protein kinase 3

				CYT2H	Cytohesin 2
				PLPPR3	Phospholipid phosphatase-related 3
HHV2/HSV2	Human herpesvirus 2 strain HG52; NC_001798.2	ND	NA	*POLD1*	DNA polymerase delta1, catalytic subunit
				TAOK1	TAO kinase 1
				SOBP	Sine oculis binding protein homolog
				RPS6KB2	Ribosomal protein S6 kinase B2
				PGAP1	Inositol deacylase
				FGFR4	Fibroblast growth factor receptor 4
				CDH15	Cadherin 15
				MLLT10	Histone methyltransferase cofactor
				TBXAS1	Thromboxane A synthase 1
				RRAGC	Ras-related GTP binding C
				FBLL1	Fibrillarin-like 1
HHV3/VZV	Human alphaherpesvirus 3 strain VZVs/Pasadena.USA/48.12/Z{2}; KF811485.1	*TYMS/ENOSF1*	Thymidylate synthase		
HHV4/EBV	Human herpesvirus 4 strain YCCEL1; AP015016.1	*IL10*	Interleukin-10 variant	PFAS	Phosphoribosylformylglycinamidine synthase
				TAF4B	TATA-box binding protein-associated factor 4b
HHV5/CMV	Human herpesvirus 5 strain Merlin; NC_006273.2	ND	NA	ANKRD28	Ankyrin repeat domain 28
				SFPQ	Splicing factor factor proline and glutamine rich
				SMARCA2	SWI/SNF-related, matrix associated
				GNAO1	G protein subunit O1
				APP	Amyloid precursor protein
				RBFOX1	RNA-binding FOX1 homolog
				CDK16	Cyclin-dependent kinase 16
HHV6A	Human herpesvirus 6A isolate AJ; KP257584.1	Endogenous HHV6	NA	DCAF7	DDB1 and CUL4 associated factor 7
		*No matches to *Pan troglodytes*			
		**Gorilla gorilla*	Unknown		

		Human telomeric DNA repeats			
HHV6B	Human herpesvirus 6B; NC_000898.1	Endogenous HHV6	NA		
		*No matches to *Pan troglodytes*			
		**Gorilla gorilla* ribosomal RNA			
		Human telomeric DNA repeats			
HHV7	Human herpesvirus-7 (HHV7) JI; U43400.1	Endogenous HHV6	NA	DAPK1	
		*No matches to *Pan troglodytes*			
		*Five matches to *Gorilla gorilla*	Telomeric region and unknown		
		Human telomeric DNA repeats			
HHV8	Human herpesvirus 8 strain GK18; NC_009333.1	*TYMS/ENOSF 1*	Thymidylate synthase	GTF2A1	General transcription factor IIA subunit 1
				HMGB1	High mobility group box 1
				APLP2	Amyloid beta precursor like protein 2
				CENPB	Centromere protein B
				NCL	CLN8 transmembrane ER and ERGIC protein
				ARMH4	Armadillo like helical domain containing 4
HPV6	Human papillomavirus type 6 isolate HPV6-gw-0611; MK463909.1	ND	NA	Unknown	Chr.6
HPV16	Human papillomavirus type 16; K02718.1	NA	ND		
TTV	Torque teno virus; NC_015783.1	NA	ND	TRPM3	Transient receptor cation channel M3

VARIOLAV	Variola virus; NC_001611.1	*RRM1*	Ribonucleotide reductase		
		RRM2B	Ribonucleotide reductase		
		VACV		Unknown	Chr.1p32

Sequence matches were detected by two procedures with different cut-offs for similarity. (i) Direct BLASTn searching of human databases for matches with full-length viral genomes. (ii) BLASTn searching of SRA libraries with full-length viral genomes, retrieval of matches, and second-round searching of these human sequences in the human databases, then in virus databases, and then in the entire NCBI repository. In this second analysis a sequence was deemed to be of human origin if matches to the human database achieved higher scores (homology x coverage) than in any other search. *For HHV6A, HHV6B, and HHV7, searching was also performed against *Pan troglodytes* (chimpanzee) and *Gorilla gorilla* (gorilla) because their genomes/transcriptomes are similar to those of human but contain no integrated viruses of this class.

Abbreviations: NA, not applicable; ND, none detected

B Identity of viral genes and extent of homology

**Virus**	**Human gene**	**Virus gene name**	**Encoding**	**% Identity**	**Extent of homology**
HHV3	*TYMS/ENOSF1*	*ORF13/PTZ00 164*	Thymidylate synthase/bifunctional dihydrofolate reductase-thymidylate synthase	70%	797 nt
HHV4	*IL10*	*BCRF1*	Viral interleukin 10	77%	391 nt
HHV6A/6B	Telomeric repeats	Region 200–400 (HSV6A)	Left extremity of genome	81%	Multiple matches >100 nt
		Region 151500–151700 (HSV6A)	Close to DR1/US22, near to right extremity of genome	81%	Multiple matches >100 nt
HHV8	*TYMS/ENOSF1*	*ORF70/PTZ00 164*	Bifunctional dihydrofolate reductase-thymidylate synthase	69%	650 nt
Variola virus	*RRM1*	*PLN02437*	Ribonucleoside-diphosphate reductase large subunit	69%	2268 nt
Variola virus	*RRM2*	*PLN02437*	Ribonucleoside-diphosphate reductase large subunit	71%	933 nt

Table S6. Stripped viral genomes. Supplied separately (350 pages)

Table S7. Identification of liver- and brain (cortex)-specific signals

Probe	Species detected	Sequence similarity	
		Contig length	% Identity
**Liver-specific**			
C3_Rviolacea_18S_10	*Staphylococcus aureus*[a]	251	99.6
D_CaOmb2_18S_1	*Staphylococcus aureus*[a]	151	99.3
F1_Mspiralis_18S_4	*Cryptosporidium* sp.	159	99.4
F5_Clativittatus_18S_3	*Staphylococcus aureus*[a]	232	99.6
F6_Fboomerangus_18S_6	*Aspergillus flavis*	251	99.6
**Brain-specific**			
B6_Pintermedia_16S_8[b]	Uncultured Bacteriodetes	129	98.4
	Flavobacterium sp. (Bacteriodetes)	169	99.4
F2_GlomusNBRPP1_18S_6[b]	*Malassezia restricta*[c]	145	100
	Malassezia globosa[c]	200	100
F6_Aaquatica_18S_6	*Malassezia globosa*[c]	187	100
F6_Cneoformans_18S_2	*Malassezia restricta*[c]	180	100

[a]Insecure identification in view of similarities to human rRNA sequences.

[b]Two contigs of similar abundance were generated.

[c]The presence of *Malassezia* spp. in brain (but not liver) was also confirmed through mtDNA analysis.

Figure S1.

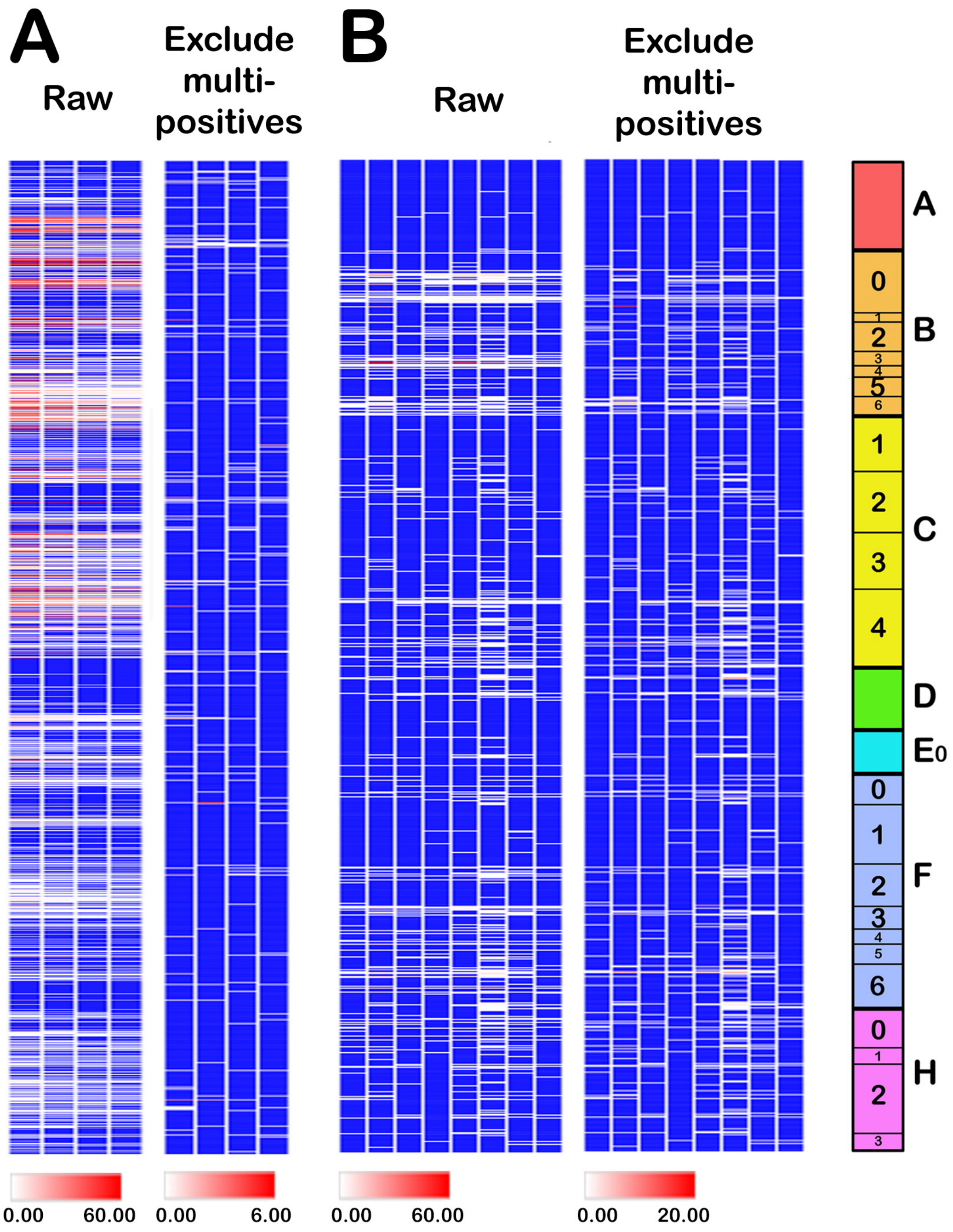

Figure S1. Profiles for four RNA-seq datasets from normal human hippocampus (Left, Miami dataset) and eight RNA-seq datasets from normal human dorsolateral prefrontal cortex (Right, Rockefeller dataset). (A) Exclusion of signals that were present in all samples led to a major reduction in the microbiome profile, suggestive of contamination, whereas in (B) the same exclusion techique led to minimal changes in the overall profile. Because (A) and (B) are RNA-seq from different brain regions, they are not directly comparable to each other.

Figure S2.

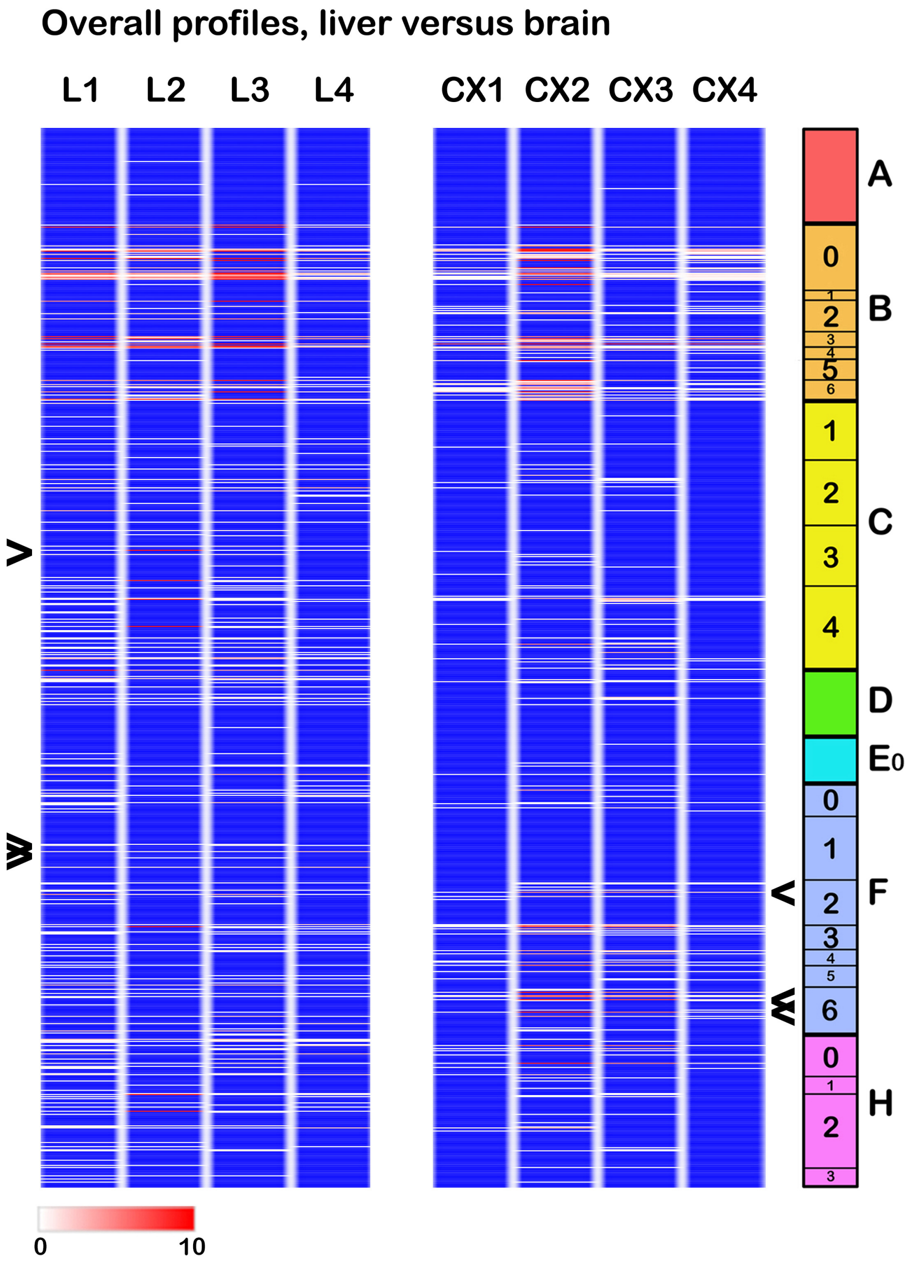

Figure S2. Similarities and differences between the overal microbiome profiles of liver (left) versus cortex (CX, right). Some differential signals are indicated (arrows).

Figure S3

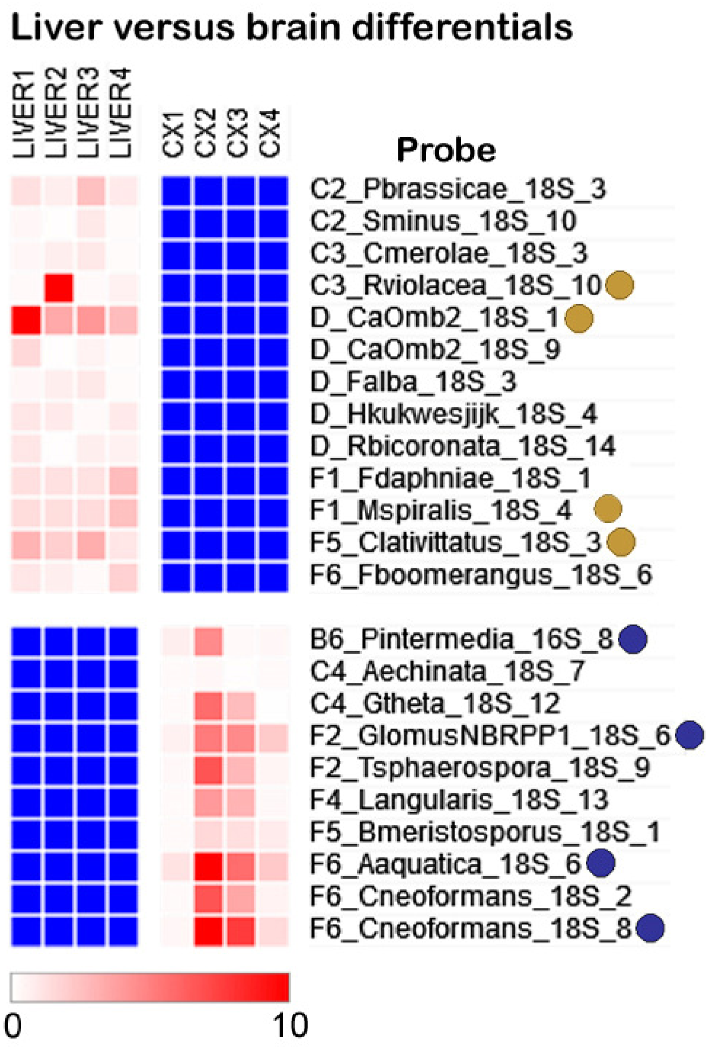

Figure S3. Microbiome signals that appear to be specific for liver (above) and brain (below). Differential signals were confirmed for the probes (marked in gold and blue) by retrieval of matching sequences from the original SRA datasets, contig assembly, and species identification (**Table S7**), as well as through 23S/28S analysis (not presented).
