# Supplementary material for: The electronic tree of life (eToL): a net of long probes to characterize the microbiome from RNA-seq data
Source: BMC Microbiol. 2022 Dec 22;22:317. doi: 10.1186/s12866-022-02671-2 (PMC9773549; doi:10.1186/s12866-022-02671-2)
Supplement: Supplementary file 1 — Additional file 1. [file 12866_2022_2671_MOESM1_ESM.zip › Hu et al. SUPPLEMENTARY TABLE S6_STRIPPED VIRUSES v5.doc]

**Table S6. Stripped viral genomes.**

>ADENOASSOCIATEDVIRUS_STRIPPED4

GTCATACTTTCTCACAATAAGCTTGTCCTCCTCCCCCCAATAACACGCTCGCTCGCTCGCTCGGGACAACCGGGCGAAGCCCGGTTGCAAGCGCCCTTCGGGCGCTTGTCCCGAGCGAGCGAGCGAGCGTGTTATTGGGGGGAGGAGGACAAGCTTATTGTGAGAAAGTATGACCAAAGTACGTAGGCCAGATACCGTAGGGGTTACACAACCAGGTTGTAACACTTGGGTGTCTGGGTTCGATTTCCAGTCTGGCCGTGTAGGGAGGGCGTGATTCTTCATCAAAGTGGTGGGTGGAGAATCTGACCCTTCTTTTTTCTCGCGAGACTTTGCGAGATTTCCAAATATCGCGAGACCTTAACAGTATAAAGTGAGCGCGAGCGAGCGACCGCGCCAGACGCCATCACGCCGTTGATCCCTACACACCAAGATGACGACACCTACGTACTACGAGCTGATTGTGGAGCTGCCGAGCGACATTGACACTCAACTCCCCCTGGTCAGCGACAGCTTTGTGCGATGGGTGACGAGCAAGACGTGGGAACCCCCCCTGGACAGCAAATGGGACATGGACCAAGTGGACCAAGTCCAGCTGACTCTCGGAGACAAGATTCAACGGGAAATTTTAAAACAGTGGAGGACCATTACCGGAGACCCTGATCCGAAATATTACGTCCAGCTGGAGCAAGGAGAGACGTACTTTCACCTGCACACGTTGCTGCAGTGCTGCAACATTAAGCCGTTGGTCCTCGGAAGATATGTCAAACAGATTGAGAAAAAGCTGGTGAGTACCGTCTACGGGGGGCACAATCCTCTGATCGACAATTGGCTCCGAATTACCAAGACAAAATCGATCGGGGGCTCCAATAAAATTCGCGCGCAAAGCTACATTCCCGCCTATCTCATTCCCAAAAAACAACCGGAAGTTCAGTGGGCGTGGACAAATATCGAGGAGTATATAAAGGCCGTTTTAAACTCTGAATTGCGTCATCAAATTGGAGAAGCGCATTTCCAGGAGCAAGGCCTCGCTCTGCGCGACAGCACCAACCTATCGAGAAACTCTGAGGGGGCTCCCATCATCGTCAGCAAGTGCACCAAGAAATACATGGAGCTCGTCGAGTGGCTGGTCGAGAAGGGCATCACGACCGAGAAGCAATGGCTCTTGGAAAACAAGGAGAGCTTTCGCTCCTTCCAGGCCTCGAGCAACTCGGCTCGCCAAATCAAGGCGGCCCTTCAGGGCGCCACTCAGGAAATGCTCCTCACCAAGACGGCGTCAGACTACCTGATTGGCAAAGACCCCATCGGAGACATGACCGACAACCGAATCTACAAAATTTTGGAAATGAACGGCTACGACCCTCTCTACGTGGCCAACCTGTTTGTAGGTTGGTGTCAGATGAAGTTTGGAAAACGAAACACAATCTGGCTGTTTGGACCTGCGACCACGGGCAAGACCAACATTGCGGAGGCTATTGCCCATGCTGTGCCCTTTTATGGATGCGTCAACTGGACCAACGAAAACTTTCCCTTCAACGACTGCCTGGAAAAGATGATCATTTGGTGGGAAGAGGGGAAAATGACGGCCAAGATTGTAGAGACGGCTAAAGCCATCCTCGGGGGATCCAAGGTTCGCGTCGACCAAAAGTGCAAGTCTTCGATGCAGCTGGAACCTACGCCGGTCATCATTACCAGCAACACCAACATGTGTTATGTCGTGGATGGAAACACGACCACCTTTGAGCACGCTCAACCCCTACAGGACCGAATGTTTAAGCTGGAACTCTTGAAGCGACTTCCCGACGACTTTGGAAAAGTGACCAAAAAGGAAGTCAGAGACTTTTTCGCGTGGGGGGCTAAACATACCGTAGAGGTCGATTCTTGCTTTTTAGTAAGAAAGGCGGAGTCTCGTAAAAGACACGCCCCGGAAGTGGCATCAGAGGATAAAAGCCCTCCCGCTAAGGCGGCTCGCACAGACGAGCTTCAGCATTTGAGCGGCGAGGAGGGAACCTCTGTCTCTGCCAGGTATGTTTTGAAATGCGCTAAACATTTGGGGATGGTAACCATGATGTGGCCATGTAGAGATTGTGAAAAGGCCAATTGTAATATAAATCAGTGCATTTTGCATAAAAGTTTGTCTTGTAAAGAGTGTTTTCCAGATTATGATTCCGATGTATCTGTTCAGGAAGGCGAGCCTTCCGGCAACCCCCCCTTGTCGAGCTCCGACGAGGACATTCCCTCTCACCAACCCCCCCTTGTCAAAGATTGTAAACCCTGGACTCCGTGTTCCTATCACCACCTGACCGGGGTAGCCAATAGAAATTGTAGCATGTGCAAATTGAGAAATGTGGATTTGGATGATTGTGACAGTGAGCAATAAAATGACTTAAACTAGACATGTCTGCTGCTGATTCTGTTCCAGATTGGTTGGAGAATTTTGTGCGCAAGCACATTGTCAATCCGGTTGCCGAATTTGCTCATTTGGAGGCTGGAGCCCCACAACCAAAGCCTAACCAGCAGCATCAAGATCGAGGCGGAACCAAGGACGATAGCCGAGGTCTTGTTTTACCTGGCTACAAGTATCTTGGTCCTTTTAACGGTCTTGACAAGGGTGAACCCGTCAACGCTGCTGACGCTGCTGCGCTCGAGCACGACAAGGCGTACGACCAGCAGCTGCAAGCGGGAGACAACCCCTATCTGAAATATAACCACGCAGACGCCGAATTTCAAGAGGCCCTAAAGGACGACACATCCTTTGGGGGAAATCTCGGTAAAGCGGTATTCCAGGCCAAAAAGAGGGTTCTCGAACCCTTTGGCTTGGCTGAAGACGGAAAGACGGCTCCTACCAACGAACGTCGAAAGGAGAATATAGACGACTACTATCCCAAGAGGAAAAAAGCCAAGGCGGGAGAAGAAAAGCCCCCTTCTACCGACGCAGTAGAAGGAGCTGGAGACGGAGAACCAAGCACATCTACCGGAGGGGAAACCCCCAGCGGTACTCAATCGTCTCGGGGCCTAACAACGCTGCCCAGATTCGTTACGACAAGGGTCGTAAGGAAGACATTGCCGGCCAATGCAGAAATTGGCTCCCCGGACCATTTTGGCAAAATCAGAGCGTGCGTCTGGACAATGCCAACAACAATCCCAAGTGGGATTACTGGGCAAACTCGAATCGAGTAAGGCTGGACGGCAAATTGTACTCCGTCAACCCTGGAATTCCTCAAGCCACAGAAGCTACGCAGAATCCCTACAACCAGTATCCGACGCAGTCGACTCTGGTGTTTGAAAAGAAGCCCGGAGGAAACCCCACCGGAGTAGATCCAAACAATCTCAACCTTACCAAGGATGAAGAAATTAGAACCACCAATCCCGTGGCCTATGCCGTGTCAGGAACAGTCACCGGAACGTCGGCCATCAATCAAAACAATGGAGCCAGTCAGACTCCTACGGCTTCCGACGTGGACATTTTGGGAGCCATGCCCGGAATGGTGTGGCAAAATAGAGACATTTATCTGCAAGGGCCCATTTGGGCCAAGATTCCCTCCACAGACAATCATTTTCATCCTTGTCCTCTCATGGGAGGATTCGGATTCAAGCATCCGCCTCCGCAGATTCTCATCAAAAACACACCGGTGCCGTCAGACCCTGCGGGATTTTCAGCGACCAAGTTCAACTCGTTCATCACTCAGTATTCCACCGGACAAGTCACCGTGGAAATCATGTGGGAACTACAAAAGGAAACGTCCAAGAGGTGGAATCCAGAAATTCAGTTTACGTCCAATTTCAACGCCACCCAAGAACTGCAGTTTGCACCCAACGTGTCTGGAGACTACGAAGAACCCAGAGCCATCGGCTCGAGATATCTCACCAAACCTCTGTAACT

CGTTTCAGTTTCAATTCGACTCGCGTCATACTTTCTCACAATAAGCTTGTCCTCCTCCCCCCAATAACACGCTCGCTCGCTCGCTCGGGACAAGCGCCCGAAGGGCGCTTGCAACCGGGCTTCGCCCGGTTGTCCCGAGCGAGCGAGCGAGCGTGTTATTGGGGGGAGGAGGACAAGCTTATTGTGAGAAAGTATGAC

>ADENOVIRUS_54_STRIPPED4

CATCATCAATAATATACCCCACAAAGTAAACAAAAGTTAATATGCAAATGAGCTTTTAAATTTTAACGGTTTTAGGGCAGGGCCAATGTTAATTGGTTAACAAGCGGTAATGCAGTTGACGTCAAGACGCACGGCCGGCGGTTGCCGCAGAGGCGTGGCCTAGCCCGGAAGCAAGTCGCAGGGCCGATGACGTATAAAAAAGCGGACTTTAAACCCGGAAACGGCCGATTTTCCCACGGCCACGCCCGGATATGAGTAAACTGTGCTCCCAAAGTTTAAGAAAAATGAGACACCTGCACCTCCTGTCTTCAACTGTGCCTATTGACATGGCCGCATTATTGCTGGAGGACTATGTAAATACAATATTGGAGGACGAACTGCATTTATCTCCGTTTGAGCTGGGACCCACACTTCAGGACCTATATGATTTGGAGGTAAATGCCCAGGAGAACGACCCAAACGAAGAGGCTGTGAATTTAATATTTCCAGAATCTATGATTCTTCAGGCTGACATAGCCAGCGAAGCTGTACCTACTTCAGTTTATACACCGACTCTGCCGCCTATACCTAAATTGGAAGAGGAGGATAAGCTAGACCTTCGGTGTTATGAGGAAGGTTTTCCTCCCAGCGATTCAGAGGACGAACGGGGTGAGCAGAGCATGGCTATAATCTCAGACTATGCGTGTGTGGTTGTGGAAAATCATTTTGTGTTGGACAATCCTGAGGTGCCAGGGCAAGGCTGTAAATCCTGTCAATATCACCGGGAACAGACCGGAGACCCAAATGCTTCCTGCGCTCTGTGTTACATGAAAATGAGCTTCAGCTTTATTTACAGTAAGTGGAGTAAATGTAAGAGAGGCTAAGTGCTTAAGACATTACTGTGCTTTGCTTGAACAGCTGTACTAAGTGTGGTTTATTTTTGTTACTAGGTCCGGTGTCAGAGGATGAGTCATCACCCTTAGAAGAAGACCACCCGTCTCCCCCTGATCTTACAAATGACACGCCCCTGCAAGTGCGCAAACCCACCCCGGTTAGACCGAGTGGCGAGAGGCGAGCGGCTGTTTACAAAATTGAGGATTTGTTGCAGGACGTGGGTGGGAATGAACCTTTGAACCTGAGCTTGAAACGCCCCAGGAACTAGGCGCAGCTGTGCTTAGTCATGTGTAAATAAAGTTGTACAATAAAAGTATGTGTGACGCATGCAAGGTGTGGTTTATGACTCATGGGCGGGGCTTAGTCCTATATAAGTGGTAACACCTGGGCACTTAGGCACAAACTTTAGGAAGTTCCTAATGGATGTGTGGAGTATTCTTGGGGAATTTAACAAGACACGCCGGCTTGTGGAGGATAGTTCAGACGGGTGCTCCGGGTTTTGGAGACACTGGTTTGGAACTCCTCTATCTCGCCTGGTGTACACAGTTAAGAAGGATTATAGCGAGGAATTTGAAAATCTTTTTGCCGACTGTTCTGGCCTGCTAGATTCTTTAAATTTTGGCCACCAGTCCCTTTTCCAGGAAAGGGTACTCCACAGCCTTGATTTTTCCAGCCCAGGGCGCACTACAGCCGGGGTTGCTTTTGTGGTTTTTTTGGTTGACAAATGGAGCCAGGACACCCAACTAAGCAGGGGCTACATTCTGGACTTTGCAGCCATGCACCTGTGGAGGGCCTGGATGAGGCAGCGGGGACAGAGAATCTTGAACTACTGGCTTTTACAGCCAGCAGCGTCGGGTCTTCTTCATCTACACAGACAAACATCCATGTTGGAGGAAGAAATGAGGGAGGCCATGGACGAGAACCCGAGGAGCGGCCTGGACCCTCCGTCGGAAGAGGAGCTGGATTAAATGAGGTATCCAGCCTGTACCCAGAGCTTAGCAAGGTGCTGACATCCATGGCCAGGGGAGTGAAGAGGGAGAGGAGCGATGGGGGCAATACCGGGCTGATGACCGAGCTAACTGCCAGCCTGATGAATCGCAAGCGCCCAGAGCGTATTACCTGGCACGAGCTACAGCAGGAGTGCAGGGATGAGATAGGCCTGATGCAGGATAAATATGGCCTGGAGCAAATAAAAACCCATTGGTTGAACCCAGACGAGGATTGGGAGGAGGCCATTAAGAAATATGCCAAGATAGCCCTGCGCCCAGATTGCAAGTACAGGGTGACTAAGACCGTGAATATTAAACATGCCTGCTACATCTCGGGGAACGGGGCAGAGGTGGACATCGATACTCTGGACAAGTCAGCCTTCAGGTGTTGCATGATGGGAATGAGAGTAGGAGTAATGAATATGAATTCCATGATCTTTATAAACATAAAGTTCAATGGAGAGAAGTTTAATGGGGTACTGTTTATGGCCAACAGCCACATGACCCTGCATGGTTGCAGTTTTTTTGGCTTTAACAATATGTGTGCAGAGGTCTGGGGTGCTGCTAAGATTAGGGGATGTAAGTTTTACGGCTGCTGGATGGGCGTGGTTGGAAGACCCAAGAGCGAGATGTCTGTAAAGCAGTGTGTGTTTGAAAAATGCTACCTGGGAGTCTGTACCGAGGGCAATGCTAGAGTAAGACACTGCTCTTCCCTAGAAACGGGCTGCTTTTGCCTGGTGAAGGGCACAGCCTCGATTAAGCATAATGTGGTAAAGGGCTGCACGGATGAGCGCATGTACAACATGCTGACCTGCGACTCGGGGGTCTGCCATATCCTGAAGAACATCCATGTGACCTCCCACCCCAGAAAAAAGTGGCCAGTGTTTGAGAATAACCTGCTGATTAAGTGCCATATGCACCTGGGTGCCAGAAGGGGCACCTTCCAGCCGTACCAGTGCAACTTTAGCCAGACCAAGCTGCTGTTGGAGAACGATGCCTTCTCCAGGGTGAACCTGAACGGCATCTTTGACATGGATGTCTCGGTGTACAAGATCCTGAGATACGATGAGACCAAGTCCAGGGTGCGCGCTTGCGAGTGCGGTGGCAGACACACCAGGATGCAGCCAGTGGCCCTGGATGTGACCGAGGAGCTGCGACCAGACCACCTGGTGATGGCCTGTACCGGGACCGAGTTCAGCTCCAGTGGGGAGGACACAGATTAGAGGTAGGTTTAAGTAGTGGGCGTGGCTAAGGTAAGTATAAAGGCGGGTGTCTTACAAGGGTCTTTTTGCTTTTCTGCAGACATCATGAACGGGACCGGCGGGGCCTTCGAAGGGGGGCTTTTTAGCCCTTATTTGACAACCCGCCTGCCGGGATGGGCCGGAGTTCGTCAGAATGTGATGGGATCAACGGTGGACGGGCGCCCAGTGCTTCCAGCAAATTCCTCGACCATGACCTACGCGACCGTGGGGACGAGCTCGTCGCTTGACAGCACCGCCGCAGCCGCGGCAGCCGCAGCCGCCATGACAGCGACGAGACTGGCCTCGAGCTACATGCCCAGCAGCGGTAGCAGCCCCTCCATCCCCAGTTCCATCATCGCCGAGGAGAAACTGCTGGCCCTGCTGGCTGAGCTGGAAGCCCTGAGCCGCCAGCTGACCGCCCTGACCCAGGAGGTGTCCGAGCTCCGCGAACAGCAGCAGCAGCAAAATAAATGATTTAATAAACACAAATTTTGATTCAAACAGCAAAGCATCTTTATTATTTATTTTTTCGCGCGCGGTAGGCCCTGGTCCACCTCTCCCGATCATTAAGAGTGCGGTGGATTTTTTCCAGGACCCGGTAGAGGTGGGATTGGATGTTGAGGTACATGGGCATGAGCCCGTCCCGGGGGTGGAGGTAGCACCACTGCATGGCCTCGTGTTCTGGGGTTGTGTTGTAGATGATCCAGTCATAGCAGGGGCGCTGGGCGTGGTGCTGGATGATGTCCTTAAGGAGGAGACTGATGGCCACGGGGAGCCCCTTGGTGTAGGTGTTGGCAAAGCGGTTGAGCTGGGAGGGATGCATGCGGGGGGAGATGATGTGTAGTTTGGCCTGGATCTTAAGGTTGGCGATGTTACCGCCCAGATCCCGCCGGGGGTTCATGTTGTGCAGGACCACCAGGACAGTGTAGCCCGTGCACTTGGGGAACTTGTCATGCAACTTGGAAGGGAATGCGTGGAAGAATTTGGAGACGCCCTTGTGCCCGCCCAGGTTTTCCATGCACTCATCCATGATGATGGCAATGGGCCCGTGGGCTGCGGCTTTGGCAAAGACGTTTCTGGGGTCAGAGACATCGTAATTATGCTCTTGGGTGAGATCATCATAAGACATTTTAATGAATTTGGGGCGGAGGGTGCCAGATTGTGGGACGATGGTTCCCTCGGGCCCCGGGGCGAAGTTCCCCTCGCAGATCTGCATCTCCCAGGCTTTCATCTCGGAGGGGGGGATCATGTCCACCTGCGGGGCGATGAAAAAAATGGTTTCCGGGGCGGGGGTGATGAGCTGCGAGGAGAGCAGGTTTCTTAACAGCTGGGACTTGCCGCACCCGGTTGGGCCGTAGATGACCCCGATGACGGGTTGCAGGTGGTAGTTCAAGGAGATGCAACTGCCGTCGTCCCGGAGGAGGGGGGCTACCTGGTTAAGCATGTCTCTGACTTGGAGGTTTTCCCGAACAAGCTCGCTGAGGAGGCGGTCCCCGCCCAGCGAGAGCAGTTCTTGCAGGGAAGCAAAGTTTTTTAGGGGCTTAAGCCCGTCGGCCATGGGCATCTTGGCGAGGGTCTGCGAGAGGAGCTCCAGGCGGTCCCAGAGCTCGGTGACGTGCTCTACGGCATCTGGATCCAGCAGACTTCCTCGTTTCGGGGGTTGGGACGACTGCGACTGTAGGGCACAAGACGATGGGCGTCCAGCGCGGCCAGCGTCATGTCCTTCCAGGGTCTCAGGGTCCGCGTGAGGGTGGTCTCCGTTACGGTGAATGGGTGGGCCCCGGGCTGGGCGCTTGCAAGTGTGCGCTTCAGACTCATCCTGCTGGTGCTGAAACGGGCACGGTCTTCGCCCTGCGCGTCGGCAAGATAGCAGTTAACCATGAGCTGGTAGTTCAGGGCCTTGGCGGCATGGCCCTTGGCGCGGAGCTTGCCCTTGGAAAAGCGCCCGCAGGCGGGACAGAGGAGGGATTGCAGGGCGTACAGTTTGGGCGCGAGAAAGACCGACTCGGGGGCAAAGGCGTCCGCTCCGCAGTGGGCGCAGACGGTCTCGCACTCGACAAGCCAGGTGAGCTCGGGCTGCTTGGGGTCAAAAACCAGTTTTCCCCCGTTCTTTTTGATGCGCTTCTTACCTCGCGTCTCCATAAGTCTGTGTCCGCGCTCGGTGACAAACAGGCTGTCGGTGTCCCCGTAGACGGACTTGATTGGCCTGTCCTGCAGGGGCGTCCCGCGGTCCTCCTCGTAAAGAAACTCGGACCACTCTGAGACGAAGGCGCGCGTCCACGCCAAGACAAAGGAGGCCACGTGCGAGGGGTAGCGGTCGTTTTCCACCAGGGGGTCCACCTTTTCCACCGTGTGCAGACACATGTCCCCCTCCTCCGCATCCAAGAAGGTGATTGGCTTGTAGGTGTAGGCCACGTGACCGGGGGTCCCCGACGGGGGGGTATAAAAGGGGGCGGGTCTGTGCTCGTCCTCACTCTCTTCCGCGTCGCTGTCCACGAGCGCCAGCTGTTGGGGTAGGTATTCCCTCTCAAGAGCGGGCATAACCTCGGCACTCAGGTTGTCAGTTTCTAGAAACGAGGAGGATTTGATGTTGGCCTGCCCTGCCGCAATGCTTTTTAGGAGACTTTCATCCATTTGGTCAGAAAAGACTATTTTTTTATTGTCAAGCTTGGTGGCAAAGGAGCCATAGAGGGCGTTGGAGAGAAGCTTGGCGATGGATCTCATGGTCTGATTTTTGTCACGGTCGGCGCGCTCCTTGGCCGCAATGTTGAGCTGGACATATTCGCGCGCAACGCACTTCCATTCGGGAAAAACAGTGGTGCGCTCGTCGGGCACAATCCTGACGCGCCAGCCTCGGTTATGCAGGGTGACCAGGTCCACGCTGGTGGCCACCTCGCCGCGCAGGGGCTCGTTAGTCCAGCAGAGGCGCCCGCCCTTGCGCGAGCAAAAAGGTGGTAACACATCAAGCAAATGCTCGTCAGGGGGGTCCGCATCGATGGTAAAGATGCCCGGACAGAGTTCCTTGTCAAAATAATCAATTTTTAAGGATGCATCATCCAAGGCCATTTGCCAGTTGCGGGCGGCCAGGGCTCGCTCGCAGGGGTTCAGGGGCGGACCCCAGGGCATGGGATGCGTAAGCGCGGAGGCGTACATGCCGCAGATGTCATACACATAGATGGGCTCCGAGAGGATGCCGATGTAGGTGGGATAACAGCGCCCCCCGCGGATGCTGGCGCGCACGTAGTCATACAACTCATGCGAGGGTGCCAAGAAGGCGGGGCCAAGATTGGTGCGCTGGGGCTGCTCGGCGCGAAAGACGATCTGGCGAAAGATGGCATGCGAGTTGGAGGAAATAGTGGGCCGTTGGAAGATGTTAAAGTGGGCGTGGGGCAGGCGGACCGAGTCGCGGATAAAGTGCGCGTAGGAGTCTTGCAGCTTGGCGACCAGCTCGGCAGTGACAAGGACGTCCATGGCGCAGTAGTCCAGTGTTTCGCGGATAATGTCATAACCCGCCTCTCCTTTTTTCTTCCACAGCTCGCGGTTAAGGGCGTACTCCTCGTCATCCTTCCAGTACTCCCGGAGCGGGAATCCTCGATCGTCCGCACGGTAAGAGCCCAGCATGTAGAAATGGTTAACGGCCTTGTAGGGACAGCAGCCCTTCTCCACGGGGAGGGCGTAAGCTTGAGCGGCCTTGCGGAGCGAGGTGTGCGTCAGGGCAAAGGTGTCCCTGACCATGACTTTCAAAAACTGGTACTTAAAGTCCGAGTCGTCGCAGCCGCCGTGCTCCCAGAGCTCAAAATCGGTGCGCTTCTTTGAGAGGGGGTTAGGCAGAGCGAAAGTGACGTCATTGAAGAGAATCTTGCCTGCTCGTGGCATAAAATTGCGGGTGATGCGGAAAGGGCCCGGAACGCAGGCTCGGTTGTTGATAACCTGGGCGGCTAGGACAATCTCGTCAAAGCCGTTGATGTTGTGCCCGACAATGTATAGTTCCATGAATCGCGGGCGGCCTTTGATGTGCGGCAGCTTTTTAAGCTCCTCGTAGGTGAGGTCCTCGGGGCATTGCAGGCCGTGCTGCTCCAGTGCCCACTCCTGAAGATGTGGGTTGGCTTGCATGAAGGAAGCCCAGAGCTCGCGGGCCATAAGGGTCTGGAGCTCGTCGCGAAAGAGGCGGAACTGCTGGCCCACGGCCATCTTTTCGGGGGTGATGCAGTAGAAGGTAAAGGGGTCCCGCTCCCAGCGATCCCAGCGTAAGCGCACGGCAAGATCGCGAGCAAGGGCGACCAGCTTGGGGTCCCCGGAGAATTTCATGACCAGCATGAAGGGGACAAGCTGCTTGCCGAAGGACCCCATCCAGGTGTAGGTTTCTACATCGTAGGTAACAAAGAGCCGCTCCGTGCGAGGATGAGAGCCGATTGGGAAGAACTGGATTTCCTGCCACCAGTTGGACGAGTGGCTGTTAATGTGATGAAAGTAGAAATCTCTCCGGCGAACCGAGCACTCGTGCTGATGCTTGTAAAAGCGTCCGCAGTACTCGCAGCGCTGCACGGGCTGTACCTCATCCACAAGATACACAGCGCGTCCCTTGAGGAGGAACTTCAGGAGTGGCGGCCCTGGCTGGTGGTTTTCATGTTCGCCTGCGTAGGACTTACCCTGGGGCTCCTCGAGGACGGAGAGGCTGACAAGCCCGCGCGGGAGCCAGGTCCAAATCTCGGCGCGGCGGGGGCGGAGAGCGAAGACGAGGGCGCGCAGTTGGGAGCTGTCCATGGTGTTGCGGAGATCCAGGTCCGGGGGCAGGGTTTTAAGGTTGACCTCGTAGAGGCGGGAGAGGGCGTGCTTAAGATGCAGATGGTACTTGATCTCCACGGGTGAGTTGGTGGCCGTGTCCACGCATTGCATGAGCCCGTAGCTGCGCGGGGCCACGACCGTGCCGCGGTGCGCTTTTAGAAGCGGTGTCGCGGACGCGCTCCTGGCGGCAGCGGCGGTTCCGGCCCCGTGGGCAGGGGCGGCAGAGGCACGTCGGCGTGGCGCTCGGGCAGGTCCCGGTGCTGCGCCCTGAGAGCGCTGGCGTGCGCGACGACGCGGCGGTTGACATCCTGGATCTGCCGCCTCTGCGTGAAGACCACGGGCCCCGTGACTTTGAACCTGAAAGACAGTTCAACAGAATCAATCTCGGCGTCATTGACGGCGGCCTGCCGCAGGATCTCTTGCACGTCGCCCGAGTTGTCCTGGTAGGCGATCTCGGACATGAACTGCTCGATCTCCTCCTCCTGGAGATCGCCGCGGCCCGCGCGCTCGACGGTGGCGGCAAGGTCATTTGAGATGCGACCCATAAGCTGCGAAAAGGCACCCAGGCCGCTCTCGTTCCAAACGCGGCTGTAAACCACGTCCCCGTCAACGTCGCGCGCGCGCATGACCACCTGCGCAAGGTTCAGCTCCACGTGCCGCGTAAAGACGGCGTAGTTGCGCAGGCGCTGAAAAAGGTAGTTAAGGGTGGTGGCGATGTGCTCGGTGACAAAGAAGTACATGATCCAGCGGCGCAGGGGCATTTCGCTGATGTCGCCAATGGCCTCCAGCCTTTCCATGGCCTCGTAAAAGTCCACGGCAAAGTTAAAAAACTGGGCGTTGCGGGCCGATACCGTGAGCTCGTCTTCCAGGAGCCGGATGAGCTCGGCAATGGTGGCGCGCACCTCGCGCTCAAAATCCCCGGGAGCCTCCTCTTGTTCCTCTTCCATGACGACCTCTTCTTCTATTTCCTCTGGGGGCGGTGGTGGTGGCGGGGCCCGACGACGACGGCGACGCACCGGGAGACGGTCGACGAAGCGCTCGATCATTTCCCCGCGGCGGCGACGCATGGTTTCGGTGACGGCGCGACCCTGTTCGCGAGGACGCAGCGTGAAGACGCCGCCGGTCATCTCCCGGTAATAGGGCGGGTCCCCGTTGGGTAGCGAAAGGGCGCTAACGATGCATCTTATCAATTGCGGCGTAGGGGACGTAAGCGCGTCAAGATCGACCGGATCGGAGAATCTTTGGAGGAAAGCGTCTAGCCAATCGCAGTCGCAAGGTAAGCTTAAACACGTAGCAGCCCTGTGGACGCTGTTAGAATTGCGATTGCTGATGATGTAATTGAAGTAGGCGTTTTTGAGGCGGCGGATGGTGGCGAGGAGGACCAGGTCCTTGGGTCCCGCTTGCTGGATGCGAAGCCGCTCGGCCATGCCCCAGGCCTGACCCTGACACCGGCTCAGGTTCTTGTAGTAGTCATGCATGAGCCTCTCAATGTCATCATTTGCGGCGGAGGCGGAGTCTTCCATGCGGGTAACCCCAACGCCCCTGAGCGGCTGCACGAGCGCCAGGTCGGCGACGACGCGCTCGGCAAGGATGGCCTGTTGCACGCGGGTGAGGGTGTCTTGGAAGTCGTCCATGTCAACAAAGCGGTGGTAGGCCCCGGTGTTAATGGTGTAGGTGCAGTTGGCCATAAGCGACCAGTTAACGGTCTGCAGGCCGGGCTGCACAACCTCGGAGTACCTAAGCCGCGAGAAGGCGCGCGAGTCAAAGACGTAGTCGTTGCAGGTGCGCACAAGGTACTGGTATCCAACTAGGAAGTGCGGCGGCGGCTGGCGGTACAGCGGCCAGCGCTGGGTGGCCGGCGCGCCCGGGGCCAGGTCCTCGAGCATGAGGCGGTGGTAGCCGTAGAGGTAGCGGGACATCCAGGTAATGCCGGCGGCGGTGGTGGAGGCGCGCGGGAACTCGCGGACGCGGTTCCAGATGTTGCGCAGCGGTAGGAAATAGTCCATGGTCGGCACGGTCTGGCCGGTAAGACGCGCGCAGTCATTGACGCTCTAGAGGCAAAAACGAAAGCGGTTGAGCAGGCTCTTCCTCCGTAGCCTGGCGGAACGCAAACGGGTTAGGCCGCGCGTGTACCCCGGTTCGAGTCCCCTCGAATCAGGCTAAAGCCGCGACTAACGTGGTATTGGCACTCCCGTCTCGACCCAAGCCCGATAGCCGCCAGGATACGGTGAAGAGCCTTTTTTGCCGGCCGAGAGGGGTCGCTAGATTTAAAAGCGGTCGAAAACCCCGCCGGGTAGTGGCTCGCGCCCGTAGTCTGGAGAAGCATTGCCAGGGTTACGTCGCGGCAGAACCCGGTTCGCAGACGGCCGCGGCGAGCGAGCCCCCTTTTTTCTTTTGCCAGATGCATCCCGTCCTGCGCCAAATGCGTCCCACCCCCCCGGCGACCACCGCAACCGCGGCCGTAGCAGGCGCCGGCGCTGTACAACAGCAGACAGAGATGGACTTGGAAGAGGGCGAAGGGCTGGCAAGACTGGGGGCACCGTCCCCGGAGCGACACCCCCGCGTGCAGCTGCAAAAGGACGTGCGCCCGGCGTACGTGCCTGCGCAGAACCTGTTCAGGGACCGCAGTGGGGAGGAGCCCGAGGAGATGCGCGACTGCCGGTTTCGGGCGGGCAGGGAGCTGCGCGAGGGCCTGGACCGCCAGCGCGTGCTGCGCGACGATGATTTCGAGCCGAACGAGCAGACGGGGATCAGCCCCGCGCGCGCGCACGTGGCGGCGGCCAACCTGGTGACGGCCTACGAGCAGACGGTGAAACAGGAGCGCAACTTCCAAAAGAGTTTCAACAACCACGTGCGCACCCTGATTGCGCGCGAGGAGGTGGCCCTGGGCCTGATGCACCTATGGGACCTGGCGGAGGCCATCGTGCAAAACCCGGACAGCAAGCCTCTGACGGCGCAGCTGTTCCTGGTGGTGCAGCACAGCAGGGACAACGAGGCGTTTAGGGAGGCGCTGCTAAACATTGCCGAGCCCGAGGGTCGCTGGCTGCTGGAGCTGATTAACATCTTGCAGAGCATCGTAGTGCAGGAGCGCAGCCTGAGCCTGGCCGAGAAGGTGGCGGCGATCAACTACTCAGTGCTGAGCCTGGGCAAGTTTTACGCGCGCAAGATTTACAAGACGCCGTACGTGCCCATAGACAAAGAGGTGAAGATAGACAGCTTTTACATGCGCATGGCGCTCAAGGTGCTGACGCTGAGCGACGACCTGGGCGTGTACCGCAACGACCGCATCCACAAGGCCGTGAGCACAAGCCGGCGGCGCGAGCTGAGCGACCGCGAGCTGATGCTGAGCCTGCGCCGGGCGCTGGTAGGGGGCGCCGCCAGCGGCGAAGAGTCCCACTTTGACATGGGGGCGGACCTGCATTGGCAGCCGAGCCGGCGCGCCTTGGAGGCCGCCTACGGTCCAGAGG

CACCCGCTGCGGGGTACTGACGCCTCCGTGATGTGTTTTTAGATGTCCCAGCAGCAAGCCCCGGACCCCGCCATAAGGGCGGCGCTGCAAAGCCAGCCGTCCGGTCTAGCATCGGACGACTGGGAGGCCGCGATGCAACGCATTATGGCCCTGACGACCCGCAACCCCGAGTCCTTTAGACAACAGCCGCAGGCCAACAGACTCTCGGCCATTCTGGAGGCGGTGGTCCCCTCTCGGACCAACCCCACGCACGAGAAGGTGCTGGCGATCGTGAACGCGCTGGCGGAGAACAAGGCCATCCGTCCCGACGAGGCCGGGCTGGTGTACAACGCCCTGCTGGAGCGCGTGGGCCGCTACAACAGCACAAACGTGCAGTCCAACCTGGACCGGCTGGTGACGGACGTGCGCGAGGCCGTGGCGCAGCGCGAGCGGTTCAAGAACGAGGGCCTGGGCTCGCTGGTGGCGCTTAACGCCTTTTTGGCAACGCAGCCGGCGAACGTGCCGCGCGGGCAGGACGATTACACCAATTTTATCAGCGCGCTGCGGCTGATGGTGACCGAGGTGCCCCAGAGCGAGGTGTACCAGTCGGGCCCGGACTACTTTTTCCAGACAAGCCGGCAGGGCCTGCAGACGGTGAACCTAAGCCAGGCTTTCAAGAACCTACGCGGGCTGTGGGGCGTGCAGGCGCCCGTGGGCGACCGGTCAACGGTGAGCAGCTTGTTGACGCCCAACTCGCGGCTGCTGCTGCTGCTGATCGCACCCTTCACCGACAGCGGCAGCGTAAACCGCAACTCGTACCTGGGCCACCTGCTAACGCTGTACCGCGAGGCCATAGGCCAGGCGCAGGTGGACGAGCAAACCTTCCAGGAGATCACAAGCGTAAGCCGTGCACTGGGGCAGAACGACACCGACAGTTTGAGGGCCACCCTAAACTTCCTGCTGACCAATAGACAGCAGAAAATTCCGCCGCAGTACGCACTGTCGGCCGAGGAAGAAAGGATTCTGAGATATGTGCAGCAGAGCGTAGGGCTGTTCCTGATGCAGGAGGGGGCCACCCCCAGCGCCGCGCTGGACATGACCGCGCGCAACATGGAACCTAGCATGTACGCCGCCAACCGGCCGTTTATTAATAAGTTGATGGACTACCTGCACCGCGCGGCGTCCATGAACTCGGACTACTTTACCAATGCCATCTTGAACCCGCATTGGCTCCCGCCGCCAGGGTTCTACACGGGCGAGTACGACATGCCCGACCCCAACGACGGGTTTCTGTGGGACGACGTGGACAGCGCGGTATTTTTGCCCGCCTTTCAAAAGAGACAGGAAGCAATGCGCACGCCTAGCGAGGGCGCTGTGGGAAGGAGCCCCTTTCCTAGCTTAGGAAGTTTGCATAGCCTACCTAACTCGGTAAACAGCGGCAGGGTGAGCCGGCCGCATTTGCTGGGCGAGGACGAGTACCTGAACGACTCGCTGCTGCGGCCGCCGCGGGCCAAGAACGCCATGGCCAATAACGGGATAGAAAGTCTGGTGGACAAACTAAACCGCTGGAAGACCTACGCTCAGGATCATAGGGACGCGCCCGCACCGCGGCGACAGCGCCACGACCGGCAGCGGGGCCTGGTGTGGGACGACGAGGACTCGGCCGACGATAGCAGCGTGTTGGACTTGGGCGGGAGCGGTGGTGGGGCCAACCCGTTCGCGCATCTGCAGCCCAGACTGGGGCAACGGATGTTTTGAATGCATAAAATAAAACTCACCAAGGCCATAGCGTGCGTTCTCTTCCTTGTTAGAGATGAGGCGCGCGGTGGTGTCCTCTCCTCCTCCCTCGTACGAGAGCGTGATGGCGCAGGCAACCCTGGAGGTTCCGTTTGTGCCTCCGCGGTATATGGCTCCTACGGAGGGCAGAAACAGCATTCGTTACTCGGAGCTGGCTCCGCAGTACGACACCACTCGCGTGTACTTGGTGGACAACAAGTCGGCGGACATCGCTTCCCTGAACTACCAAAACGACCACAGCAACTTCCTGACCACGGTGGTGCAGAACAACGATTTCACCACCGCCGAGGCCAGCACGCAGACGATAAATTTTGACGAGCGGTCCCGGTGGGGCGGTGATCTGAAGACCATTCTGCACACCAACATGCCCAATGTGAACGAGTACATGTTCACCAGCAAGTTTAAGGCGCGGGTGATGGTGGCTAGGAAGCACCCAGAAGGGGTAGACAACACAGATTTGAGTCAGGATAAGCTTGAATATCAGTGGTTTGAGTTTACCCTGCCCGAGGGCAATTTTTCCGAGACCATGACCATAGACCTGATGAACAACGCCATCTTGGAAAACTACTTGCAAGTGGGGCGTCAAAATGGCGTGCTGGAGAGCGATATTGGAGTCAAGTTTGACAGCAGAAATTTCAAGCTGGGCTGGGACCCGGTAACTAAGCTGGTGATGCCGGGGGTCTACACCTACGAGGCCTTCCACCCGGACGTGGTGCTGCTGCCGGGCTGCGGGGTGGACTTCACCGAGAGCCGCCTGAGCAACCTCCTGGGCATTCGCAAGAAGCAACCTTTCCAAGAGGGTTTCAGGATCATGTATGAGGATCTAGAAGGCGGCAACATCCCCGCACTTCTAAATGTAACCAAGTACTTGGAAAGCAAGAAGACGCTACAGAAGGCAGTGGAAAATGCTGCTAAAGTTAATGCTCCTGCAAGAGGAGATAGCAGTGTCCCAAGAGCTGTAGAAAAGGCTGCTGAGAAGGAGCTAGAGATTGTTCCCATTGAAAAGGATGACAGCAACAGAAGTTATAATATTATACCTGGAACCACGGACACCCTGTACCGCAGTTGGTACCTGTCCTATACCTACGGGGACCCCAAAAAGGGGGTACAGTCGTGGACGCTGCTCACCACCCCGGACGTTACCTGCGGCGCGGAGCAAGTTTACTGGTCGCTGCCGGACCTCATGCAAGACCCCGTCACCTTTCGCTCCACCCAGCAAGTCAGCAACTACCCCGTGGTTGGCGCCGAGCTCATGCCCTTCCGCGCCAAAAGCTTTTACAACGATCTCGCCGTCTACTCCCAGCTCATCCGCAGCTACACCTCCCTCACCCACGTCTTCAACCGCTTCCCCGACAACCAGATCCTGTGCCGCCCGCCCGCGCCCACCATCACCACCGTTAGTGAAAACGTGCCTGCTCTCACAGATCACGGGACGCTACCGCTGCGCAGCAGTATCCGCGGAGTCCAGCGAGTGACCGTTACTGACGCCCGTCGCCGCACCTGTCCCTACGTCTACAAGGCCCTGGGCATAGTCGCGCCGCGCGTGCTCTCCAGTCGCACCTTCTAGAAAATGTCCATTCTCATCTCGCCCAGCAATAACACCGGCTGGGGTCTTACTAGGCCCAGCACCATGTACGGAGGAGCCAAGAAGCGCTCCCAACAGCACCCCGTTCGCGTCCGCGGCCACTTTCGCGCTCCCTGGGGCGCTTACAAGCGCGGGCGGACCTCTGCCCCCGCCGCCGTGCGCACCACCGTCGACGACGTCATTGACTCCGTAGTCGCTGACGCGCGCAACTACACCCCCGCCCCCTCCACCGTGGACGCGGTCATTGACAGCGTGGTGGCTGACGCGCGCGACTATGCCAGACGCAAGAGCCGGCGGCGACGGATTGCCAGGCGCCACCGGAGCACGCCCGCCATGCGCGCCGCCCGGGCTCTGCTGCGCCGCGCCAGACGCACTGGCCGCCGGGCCATGATGCAAGCCGCGCGTCGCGCCGCCGCCGCACCCACCCCCACAGGCAGGACTCGCAGACGAGCGGCCGTTGCCGCCGCCGCGGCCATCTCTAGCATGACCAGACCCAGGCGCGGAAACGTGTACTGGGTGCGCGACTCCATCACGGGCGTGCGCGTGCCCGTGCGCACCCGTCCTCCTCGTCCCTGATCTAATGCTTGTGTCCTCCCCCGCAAGCGACGATGTCAAAGCGCAAAATCAAGGAGGAGATGCTCCAGGTCGTCGCCCCGGAGATTTACGGACCCCCGGCGGACCAGAAACCCCGCAAAATCAAACGGGTTAAAAAAAAGGATGAGGTGGGCGAGGGGGCAGTAGAGTTTGTGCGCGAGTTTGCTCCGCGGCGGCGCGTAAATTGGAAGGGGCGCAGGGTGCAGCGCGTGTTGCGGCCCGGCACGGCGGTGGTGTTTACGCCCGGCGAGCGGTCCTCGGTCAGGAGCAAGCGTAGCTATGACGAGGTGTACGGCGACAACGACATCCTGGACCAGGCGGCGGAGCGGGCGGGCGAGTTCGCCTACGGGAAGCGGTCGCGCGAAGAGGAGCTGATCTCGCTGCCGCTGGACGAGAGCAACCCCACGCCGAGCCTGAAGCCCGTGACCCTGCAGCAGGTGCTGCCCCAGGCGGTGCTGCTGCCGAGCCGCGGGGTCAAGCGCGAGGGCGAGAACATGTACCCGACCATGCAGATCATGGTGCCCAAGCGTCGGCGCGTGGAGGACGTGCTGGACACCGTGAAAATGGATGTGGAGCCCGAGGTTAAGGTGCGCCCCATTAAGCAGGTGGCGCCGGGCCTGGGCGTGCAGACCGTGGACATTCAGATCCCCACCGACATGGATGTCGACAAAAAACCCTCGACCAGCATCGAGGTGCAGACCGACCCCTGGCTCCCAGCCTCCACCGCCTTTACATCCACCGCCGCCACGGCCACCGAGCCTTCCAGGAGGCGAAGATGGGGCCCTGCCAACCGGCTGATGCCCAACTACGTGTTGCATCCTTCCATCATCCCGACGCCGGGCTACCGCGGCACCCGGTATTACGCCAGCCGCAGGCGCCCCGCAGGCAAGCGCCGCCGCCGCACCACCACCCGCCGCCGTCTGGCCCCCGCCCGCGTGCGCCGCGTAACTACGCGCCAGGGCCGCTCGTTTGTTCTGCCCACCGTGCGCTACCACCCCAGCATCCTTTAATCCGTGTGCTGTAATACTGTTGCAGAGAGATGGCTCTCACTTGCCGCCTGCGCATTCCCGTCCCGAATTACCGAGGAAGATCCCGCCGCAGGAGAGGCATGGCAGGCAGCGGCCTAAACCGCCGCCGGCGGCGGGCCATGCGCAGGCGCCTGAGTGGCGGGTTCCTGCCTGCGCTTATCCCCATAATCGCCGCGGCCATTGGCACGATCCCGGGCATAGCTTCCGTTGCGCTGCAAGCGTCGCAGCGCCGTTAATGTGCAAATAAAGCCTCTTTAGACTCTGACACACCTGGTCCTGTATATTTTTAGAATGGAAGACATCAATTTTGCGTCCCTGGCTCCGCGGCACGGCACGCGGCCGTTCATGGGCACCTGGAACGAGATCGGCACCAGCCAGCTGAACGGGGGCGCCTTCAATTGGAGCAGTGTCTGGAGCGGGCTTAAAAATTTTGGCTCAACGCTCCGGACCTATGGGAACAAGGCCTGGAATAGTAGCACAGGGCAAATGTTAAGGGAAAAGCTCGCAGACCAGAACTTCCAGCAAAAGGTGGTGGACGGCCTGGCCTCAGGCATTAACGGGGTAGTGGACATTGCAAACCAGGCCGTGCAGCGCGAGATAAACAGCCGCCTGAACCCGCGGCCGCCCACGGTGGTGGAGATGGAAGATGCAACTCCTCCGCCCAGGGGCGAGAAGCGGCCGCGGCCTGACGCGGAGGAGACGACCCTGCAGGTAGACGAGCCGCCCTCGTACGAGGAGGCCCTTAAGGCCGGCATGCCCACCACACGCATCATTGCGCCAATGGCCACGGGTGTCATGAAACCCGCCACCCTTGACCTGCCTTCACCACCCGTGCCCGCTCCACCAAAGGCAGCTCCGGTCGTGCAGGCCCCCCCGGTGGCAACCGCCGTGCGCCGCGTCCCCGCCCGCCGCCAGGCCCAGAACTGGCAGAGCACGCTGCACAGTATCGTGGGCCTGGGAGTGAAAAGTCTGAAGCGCCGCCGATGCTATTAAGAGAGGAAAGAGGACACTAAAGGGAGAGCTTAACTTGTATGTGCCTTACCGCCAGAGAACGCGCGAAGATGGCCACCCCCTCGATGATGCCGCAGTGGGCGTACATGCACATCGCCGGGCAGGACGCCTCGGAGTACCTGAGCCCGGGTCTGGTGCAGTTTGCCCGCGCCACCGACACGTACTTCAGCCTGGGCAACAAGTTTAGAAACCCCACGGTGGCTCCCACCCACGATGTGACCACGGACCGGTCCCAGCGTCTGACGCTGCGCTTTGTGCCCGTGGATCGCGAGGACACCACGTACTCGTACAAGGCGCGCTTCACTCTGGCCGTGGGCGACAACCGGGTGCTAGACATGGCCAGCACGTACTTTGACATCCGCGGCGTTTTGGACCGCGGTCCCAGCTTTAAACCCTACTCGGGCACGGCTTACAACAGTTTGGCCCCCAAGGGCGCCCCAAACTCCAGTCAGTGGATTACAAAACAAACTAATGCTGGAAACGAAACTACAAAAACTCACACATACGGTGTCGCTGCCATGGGAGGCGCTGACATAACAATTAAGGGTCTGCAAATTGGTGTTGACAAAACTGAAAACAAAAATGAGCCTATCTATGCAAACGAAATCTATCAGCCAGAACCTCAAGTAGGAGAAGAAAACCTGCAAGATGTTGAAAATTACTATGGAGGCAGAGCCCTTAAAAAGGAAACCAAAATGAAGCCTTGTTATGGCTCATTTGCTAGACCCACAAATGAAAAAGGAGGGCAGGCTGTATTTAAAACTGGAAATGATGGCCAGCCAACTACTGAGCATGACATAACAATGGCTTTCTTTGATACTCCTGGCGACACTAATGCTGAAGACACAGAACTTGAAGCAGACATTGTTATGTACACCGAAAATGTTAATCTTGAAACTCCAGATACTCATGTGGTGTACAAGCCAGGACCTTTAGAAGACAGTTCAGAAATTAATTTAACACAGCAGTCCATGCCAAACAGGCCAAACTACATTGGCTTCAGGGACAACTTTGTAGGCCTTATGTACTACAACAGCACTGGCAACATGGGTGTGCTAGCTGGTCAGGCCTCTCAGTTGAATGCTGTGGTTGACTTACAAGACAGAAACACGGAGCTGTCTTACCAGCTTTTGCTAGATTCTCTGGGTGACAGAACCAGATACTTTAGTATGTGGAACTCTGCGGTGGACAGTTACGATCCAGATGTCAGGATTATTGAGAATCACGGTGTGGAAGATGAACTTCCAAACTATTGCTTTCCATTGGACGGAGCTGGTACTAATGCTACATATCAGGGTGTTAAAGTTAAAAATGGACAGGATGGTGACAACAACGCAGAATGGGAGAAAGACAATGCAGTTGCGGATCGAAACCAAATTTGCAAGGGTAACATCGTCGCCATGGAAATTAACCTTCAGGCCAACCTGTGGAAAAGTTTTCTGTACTCGAACGTGGCCTTGTACCTGCCCGACTCCTTTAAGTACACGCCGGCCAACGTCACACTGCCCACCAACACCAACACCTACGAGTACATGAACGGGCGCGTGGTGGCCCCCTCGCTGGTGGACGCTTACGTTAACATTGGCGCCCGCTGGTCACTGGACCCCATGGACAACGTTAACCCCTTTAACCACCACCGCAACGCGGGCCTGCGCTATCGCTCCATGCTGCTGGGCAACGGGCGCTACGTGCCCTTTCACATCCAAGTGCCCCAAAAGTTCTTTGCCATTAAAAACCTGCTCCTGCTCCCGGGCTCCTACACCTACGAGTGGAACTTCCGCAAGGACGTCAACATGATCCTGCAGAGTTCCCTGGGCAACGACCTGCGCGTCGACGGCGCCTCCGTCCGCTTTGACAGTGTGAACCTCTACGCCACCTTCTTCCCCATGGCGCACAACACCGCCTCCACCCTGGAAGCCATGCTGCGCAACGACACCAACGACCAGTCCTTCAACGACTACCTCTCGGCCGCCAACATGCTTTACCCCATCCCGGCCAAGGCCACCAACGTGCCCATTTCCATCCCCTCGCGCAACTGGGCCGCCTTTCGCGGCTGGAGTTTTACCCGCCTAAAAACCAAGGAAACTCCCTCACTCGGCTCGGGTTTCGACCCCTACTTTGTCTACTCAGGCTCCATCCCCTACCTTGACGGGACCTTCTACCTCAACCACACCTTCAAGAAGGTCTCCATTATGTTCGACTCCTCGGTTAGCTGGCCCGGCAATGACCGGCTGCTTACGCCGAACGAGTTCGAGATTAAGCGCAGCGTCGACGGGGAGGGCTATAACGTGGCCCAATGCAACATGACCAAGGACTGGTTCCTTATCCAGATGCTTTCCCACTACAACATTGGCTACCAGGGCTTCCACGTGCCTGAGGGCTACAAGGACCGCATGTACTCCTTCTTCCGCAACTTCCAGCCCATGAGTAGGCAGGTGGTCGATGAGATTAACTACAAGGATTACAAGGCCGTTGCCCTGCCCTTCCAGCACAACAACTCGGGCTTCACCGGCTACCTCGCACCTACCATGCGCCAGGGGCAGCCCTACCCCGCCAACTTCCCCTACCCGCTTATTGGAGAGACCGCCGTGCCCTCCGTCACCCAAAAAAAGTTCCTCTGCGACAGGGTCATGTGGCGCATCCCATTCTCCAGCAACTTCATGTCTATGGGCGCCCTTACCGACCTGGGTCAAAACATGCTTTACGCCAACTCGGCGCACGCGCTCGACATGACCTTTGAGGTGGACCCCATGGATGAGCCCACCCTCCTCTATCTTCTCTTTGAAGTTTTCGACGTGGTCAGAGTGCACCAGCCGCACCGCGGCGTCATCGAGGCCGTCTACCTGCGCACGCCCTTCTCCGCCGGCAACGCCACCACCTAAGCATGAGCGGCTCCAGCGAACAAGAGCTCGCGGCCATTGTGCGCGACCTGGGCTGCGGGCCCTACTTTTTGGGCACCCACGACAAGCGCTTCCCGGGCTTCCTCGCCGGCAACAAGCTGGCATGCGCCATCGTCAACACGGCCGGCCGCGAGACCGGGGGCGTGCACTGGCTTGCCTTTGGCTGGAACCCGCGCTCGCGCACCTGCTACATGTTTGACCCCTTTGGGTTTTCGGACCGCCGGCTTAAGCAGATTTACAGCTTCGAGTACGAGGCCATGTTGCGTCGCAGCGCCCTGGCCTTATCGCCCGACCGCTGTCTCAGCCTCGAGCAGTCCACCCAGACCGTGCAGGGGCCCGACTCCGCCGCCTGCGGACTCTTCTGTTGCATGTTCTTGCATGCCTTCGTGCACTGGCCCGATCGACCCATGGACGGGAACCCCACCATGAACTTGCTAACGGGGGTGCCCAATGGCATGCTACAATCGCCACAGGTGCTGCCCACCCTCCGGCGCAACCAGGAGGAGCTCTACCGCTTCCTTGCGCACCACTCCCCCTACTTTCGCTCCCACCGCGCCGCCATTGAACACGCCACCGCTTTTGATAAAATGAAACAACTGCGTGTATGACTCAAATAAACAGCACTTTTATTTTACATGCACTGGAGTATATGCAAGTTATTTAAAAGTCAAAGGGGTTCTCGCGCTCGTCGTTGTGCGCCGCGCTGGGAAGGGCCACGTTGCGGTACTGGTACTTGGGCTGCCACTTAAACTCGGGGATCACCAGTTTGGGAACAGCAATCTCGGGGAAGGTCTCGCTCCACATGCGCCGGCTCATCTGCAGGGCGCCCAGCATGTCCGGGGCTGAAATTTTAAAATCGCAGTTGGGACCGGTGCTCTGCGCGCGCGAGTTGCGGTACACGGGGTTGCAGCACTGAAACACCATCAAACTGGGGTACTTTACGCTGGCCAGCACGCTCTTATCGCTAATCTGATCCTTGTCCAGGTCCTCGGCGTTGCTCAGGCCGAACGGGGTCATCTTACACAGCTGGCGGCCCAGGAAGGGCACGCTGTGGGGCTTGTGGTTACACTCGCAGTGCACGGGCATTAGCATCATCCCTGCGCCGCGCTGCATATTCGGGTACAGGGCCTTAATAAAGGCCATAATCTGCTTAAAAGCTTGCTGGGCCTTGGCTCCCTCGCTAAAAAACAGGCCGCAGCTTTTCCCGCTAAACTGGTTATTCCCGCACCCGGCATCCTGCACGCAGCAGCGCGCGTCATGGCTGGTCAGTTGCACCACGCTTCTCCCCCAGCGGTTCTGGGTCACTTTGGCCTTGCTGGGTTGCTCCTTCAACGCGCGCTGACCGTTCTCGCTGGTCACATCCATCTCCACCACATGGTCCTTGTGGATCATTACCGTCCCGTGCAGACACTTGAGCTGGCCTTCCACCTCGGCACAGCCGTGGTCCCACAGGGCACTGCCGGTGCACTCCCAGTTTTTGTGCGCGATCCCGCTGTGGCTAAAGATGTAACCTTGCAACAGGCGACCCATTACGGTGCTAAAGCTCTTCTGGGTGGTAAAGGTCAGTTGCATCCCGCGGGCCTCCTCGTTTATCCAGGTCTGGCACATTTTTTGGAAGATCTCGGTCTGCTCGGGCATAAGCTTGTAAGCATCGCGCAGGCCGCTGTCAACGCGGTAGCGTTCCATCAGCACGTTCATGGTATCCATGCCCTTCTCCCAGGACGAAACCAAAGGCAGACTCAGGGGGTTGCACACGTTCAGAATACCGGGGGTCGCGGGCTGGACAATGCGTTTTCCGTCCTTGCCTTCCTTCAACAGAACCGGCGGCTGGCTGAATCCCACTCCCACGATCACGGCATCTTCCTGGGGCATCTCTTCGTCGGGGTTTACCTTGGTCACATGTTTGGTCTTTCTGGGCTGCTTCTTTTTTGGAGGGCTGTCCAGTGGAACCACGTCCTCCTCGGAAGACCCGGAGCCCACCCGCTGATACTTTCGGCGCTTGGTGGGCAGAGGAGGTAGCGGCGGCGAGGGGCTCCTTTCCTGCTCCGGCGGATAGCGCGCCGACCCGTGGCCCCGGGGCGGAGTGGCCTCTCGGTCCATAAACCGGCGCACGTCCTGACTGCCGCCGGCCATTGTTTCCTAGGGGAAGATGGAGGAGCAGCCGCGTAAGCAGGAGCAGGAGGAGGAGAACTTAACCACCCACGAGCAACCCAAAATCAAGCAGGACCTGGGCTTGGAAGAGCCGGCTCGTCTAGAACCCCCACAGGATGAACAGAAGGAAACCAACGCTGGGCTGGAGCATGGCTACCTGGGAGGACAGGAGGATGTGCTGCTAAAACACTTGCAGCGCCAGTCCTTTATCCTCCGGGACGCTCTGGCCGACCGAAGCAAAACCCCCCTCAGCGTCCAGGAGCTGTGTCGGGCCTATGAGCTCAACCTCTTCTCGCCGCGCGTGCCCCCCAAACGCCAGCCCAACGGCACCTGCGAGCCCAACCCGCGTCTTAACTTTTACCCCGTCTTTGCAGTCCCCGAGGCCCTTGCCACTTATCACATCTTTTTTAAGAACCAAAAGATCCCCGTTTCCTGCCGTGCCAACCGCACCCGCGCCGACGCGCTCCTCGCTCTGGGGCCCGGCGCGCGCATACCTGATATTGCTTCCCTGGAAGAGGTGCCCAAGATCTTCGAAGGGCTTGGTCGGGACGAGACGCGCGCGGCGAACGCTCTGAAAGAAACAGCAGAGGAACAGGGTTACACTAGCGCCCTGGTAGAGTTGGAAGGCGATAACGCCAGGCTGGCCGTGCTTAAGCGCAGCGTCGAGCTCACCCATTTTGCCTACCCCGCCGTTAACCTCCCGCCCAAGGTCATGCGTCGCATCATGGATCAGCTCATCATGCCCCACATTGAGGCCCTTGATGAAAGCCAAAAGCAGCGCCCTGAGGACGCCCAGCCCGTGGTTAGCGACGAAATGCTTGCGCGCTGGCTCGGAAACCGCGACCCCCAGTCCCTGGAGCAGCGGCGTAAGCTCATGCTGGCCGTGGTCCTGGTCACCCTCGAGTTGGAATGTATGCGCCGCTTTTTTAGCGACCCCGAGACCCTGCGCAAGGTCGAGGAGACCCTGCACTACACTTTTAGGCACGGCTTTGTCAGGCAGGCCTGCAAGATCTCCAACGTGGAGCTAACCAACCTGGTCTCCTGCCTGGGGATCCTACACGAGAACCGCCTGGGGCAGACTGTGCTTCACTCGACCCTCAAGGGCGAGGCACGGCGGGACTACATCCGCGACTGCGTCTTTCTTTTTCTCTGCCACACCTGGCAGTCGGCCATGGGCGTGTGGCAGCAGTGTCTCGAGGACGAGAACCTGAAGGAGCTGGACAAGCTTCTTGCTAAAAACCTTAAAACGTTGTGGACGGGCTTTGACGAGCGCACCGTCGCCTTCGACCTGGCCGAAATTGTTTTTCCCGAACGCCTAAGGCAGACGCTAAAAGGCGGGCTGCCCGACTTTATGAGCCAGAGCATGTTGCAAAACTACCGCACTTTCATTCTCGAGCGATCGGGGATCCTGCCCGCCACCTGCAACGCCTTCCCCTCCGACTTTGTCCCGCTGAGCTACCGCGAGTGTCCCCCGCCGCTGTGGAGCCACTGCTACCTCTTGCAGCTGGCCAACTACATTGCCTACCACTCGGACGTGATCGAGGACGTAAGCGGCGAGGGGCTGCTTGAGTGCCACTGCCGCTGCAACCTGTGCTCCCCGCACCGCTCCCTGGTCTGCAACCCCCAGCTCCTGAGCGAGACCCAGGTCATCGGTACCTTCGAGCTGCAAGGTCCGGAGAAGTCCACCGCTCCGCTGAAACTTACGCCGGGGTTGTGGACTTCCGCGTACCTGCGCAAATTTGTACCCCAGGACTACCACGCCCACGAAATAAAGTTCTTTGAGGACCAATTGCGCCCGCAGCACGCGGATCTTACGGCCTGCGTCATCACCCAGGGCACGATCCTCGCCCAATTGCACGCCATCCAAAAATCCCGCCAAGAGTTTCTTCTGAAAAAGGGTAAAGGGGTCTACCTGGACCCCCAGACGGGCGAAGTGCTTAACCCGGGTCTCCCCCAGCATGCCAAGAAAGAAGCAGGAGCCACTAGTGGAGGAGATGGAAGAAGAATGGGACAGCC

CAACAGAGCAGCCCGTCGCCGCACCATCCGCGCCGGCAGCCCCTCCGGTCAAGCCTCCTCGTAGATGGGATCGAGTAAAGGGTGACGGTAAGCACCAGCGGCAGGGCTACCGATCATGGAGGGCCCACAAAGCCGCGATTATTGCCTGCTTGCAAGACTGCGGGGGGAACATCGCTTTTGCCCGCCGCTACCTGCTTTTCCACCGCGGGGTAAACATCCCCCGCAACGTGTTGCATTACTACCGTCACCTTCACAGCTAACAAAAAGCAAGTAAAAGAAGTCGCCGGAGGAGGAGGCCTGAGGATTGCGGCGGACGAGCCCTTGACCACCAGGGAGCTAAGGAACCGAATCTTCCCCACTCTTTATGCCATTTTTCAGCAGAGTCGAGGTCAGCAGCAAGAGCTTAAAGTAAAAAATCGGTCTCTGCGCTCGCTCACCCGCAGTTGCTTGTACCACAAAAACGAAGATCAGCTGCAGCGCACTCTTGAAGACGCCGAGGCTTTGTTCCACAAGTACTGCGCGCTTACTCTTAAAGACTAAGGCGCGCCCACCCGGAAAAAAGGCGGGAATTACCTCATCGCCAGCACCATGAGCAAGGAGATTCCCACACCTTACATGTGGAGCTATCAGCCCCAGATGGGCCTAGCCGCGGGCGCCTCCCAGGACTACTCCACCCGCATGAACTGGCTTAGTGCCGGGCCCTCGATGATCTCACAAGTCAACGGGATCCGTAACCATCGAAACCAGATATTGTTGGAGCAGGCGGCGGTCACCTCCACGCCCAGGGCAAAGCTTAACCCGCGTAATTGGCCCTCCACCCTGGTGTATCAGAAAATCCCCGGGCCAACTACCGTACTACTTCCGCGTGACGCACTGGCCGAAGTCCGCATGACTAACTCAGGTGTCCAGCTGGCGGGCGGCGCTTCCCGGTGTCCGCCCAGACCCGACTTAGGTATAAAAACACTGCTGATCCGAGGCAAAGGCATACAACTTAACAACGAGTTGGTGAGCTCTTCCATTGGTCTGCGACCCGACGGAGTGTTTCAACTAGCCGGAGCCGGGAAATCGTCCTTCACTCCCAACCAGGCCTACCTGACCTTGCAGAGCAGCTCTTCGCAGCCTCGCTCCGGAGGCATTGGAACTCTCCAGTTCGTGGAGGAGTTTGTGCCCTCGGTTTACTTTAACCCCTTTTCAGGCTCCCCAGGCCTCTACCCCGACGAGTTCATACCGAACTTCGACGCAGTTAGAGAAGCGGTCGACGGCTACGACTGAATGTCCTATGGTGACTCGGCTGAGCTTGCTCGGTTAAGGCATCTGGACCACTGCCGCCGCCTGCGCTGCTTTGCCCGGGAGAGCTGCGGCCTTGTTTACTTTAAGCTGCCCGAGGAGCACCCTAACGGCCCTGCACACGGAGTGCGGATCACCGTAGAGGGCACCACCAAGTCTTACCTGGTCAGGTTTTTTACCCAGCAACCCTTCCTGGTCGAGCGGGACCGGGGCGCCACCACCTACACCGTGTACTGCATTTGTCCAACCCCGAAGTTGCATGAGAATTTTTGCTGTACTCTTTGTGGTGAGTTTAATAAAAGCTGAAATAAGACTCTACTCTGGAATCCAGTGTCGTCATAATCGCACCAAGACCATTAACTTTACCACCCAGGAACAGGTGAACTTTACCTGCAAACCCCACAAGAAGTACCTCATCTGGTTCCTCAAGAACACTACTTTTGCAGTAGTTAACACCTGTGACAACGACGGTGTTCTTCTTCCCAACAATCTTACCAGTGGACTAGCCTTCTCTGTTAAAAGGGCAAAGCTAATTCTTCATCGCCCTATTGTAGAAGGAACTTACCATTGTCAGAGCGGACCTTGTCATCACATTTTCCATTTGGTGAACGTCACCAGCAGCAGCAACAGCTCAGAAACTAACCTCTCTTCTCGTACTAACAGACCTCAATTCGGAGGTGAGCTAAGGCTTCCCCCTTGTAAGGAGGGGGTTAGCCCATACAAGGTGGTCGGGTATTTAATTTTAGGGGCGGTCCTGGGTGGGTGCATAGCGGTGCTAGCCCAACTGCCTTGCTGGGTGGAAATCAATCTTTATCTGCTGGGTTAGATATTGCGGGGAGGAACCATGAAGGGGCTTTTGCTAATTATCCTTTCCCTGGTGGGGGGTGTACTGTCATGCCACGAACAGCCACGATGTAACATTACCACAGGCAATGAGAGGAGTGTTATATGTACAGTAGTTATTAAATGCGAGCATCAATGCCCTCTCAACATTACATTTAAAAACCGTACCATGGGGAATGCATGGGTAGGCTACTGGGAACCAGGAGATGAGCAGAACTACACGGTCACTGTCCATGGTAGCGATGGAAATCACACTTTCGGTTTCAAATTTATTTTTGAAGTCATGTGTGATATCACACTGCATGTGGCTAGACTTCATGGCTTGTGGCCCCCTACCAAGGAGAACATGGTTGGGTTTTCTTTGGCTTTTGTAATTATGGCCTGCTTTATGTCAGGTCTGCTGGTAGGGGCTCTAATGTGGTTTTTAAAGTGCAAGCCCAGGTATGGAAATGAGCAGAAGGAAAAATTGCTATAAATTTTTTTTTTTTTTACAGTACCATGAATACTTTAACCAGTGTCGTGCTGCTCTCTCTTCTTGTAGCTTTTAGTGAGGCAGGAATTATTAACTTAAATGTATCATGGGGAATCAATCTAACTTTAGTGGGACCATTAGACCTGCCAGTTACATGGTATGATGGAAAGGGAATGCAGTTTTGTGATGGAAATACAATTAAAAACCCACAAATCAAGCATAGCTGTAATCAACAAAATCTAACTTTACTTAATGCTGACAAGTCTCATGAAAGGACTTACTTAGGTTACAGACATGACAGTAAGGGAAAAGTAGACTATAAGGTTACAGTCATACCACCTCCTTCAACCACTCGCAAGCCTTTGTCACAGCCTCACTATGTTACTGTGACTATGAACCATAACATGACTTTAGTGGGTCCCTTAAACCTGCCAGTTACATGGTATGATGGAGAAGGAAATAAATTGTGCAATGGAGAAAAAGTTGAGCATGCAGAATTTAATCATACATGTAACATCCAGAACCTGACACTGCTCTTTGTTAACTTAACGCATAATGGAGCATACATTGGTTATAACAAAGACGGTTCTAATAGAGAATTATATGAGGTGTCAGTCAAAACCTTGTTTCAAAACGGGGCTAAACAAAGTAAGGTTGAACAAAGTAATATTGGACAAGGTAATACTGCTCAAAGTGCAAAAAGAAAATCAACAAATAACCTTCAGCCAACACAATTGTATGTTAGACCTTTTACTAATGTTAGTTTAACTGGACCTCCAAATGGCAAGGTTATTTGGTATGATGGCGAACTTAATGATCCATGTGAACAAAAGTACAAACTTAAAACTTTTTGCAATCAGCAAAATCTAACTTTAATTAATGTAACCAGCACTTATGATGGCATCTATTATGGTACTGATGAAAAAGATAAGGCAAATCGTTACAGAATAAAAGTAAATACTACAAATCACAAAACTGTTAAAATTAAGCCACATACCAAAGAACCTCCTGCTGAACAAAAAAAACAGTTTCAATTACAAGTTGCAGAAACTGATCAAAACGAATCAAAAATTCCCTCAGCTACTGTGGCAATCGTGGTGGGAGTGATTGCGGGCTTTGTAACTCTAATCATTGTTTTTATTTGCTACATCTGCTGCCGCAAGCGTCCAAGGGCATACAATTATATGGTAGACCCACTACTCAGCTTCTCCTACTAAAACTCAGTCATTCTTATTTCAGAACCATGAAGGCTTTCACAGCTTGCGTTCTGATTAGCATAGTCACACTTAGTCTTTCTCAAATCATTAATGTAAATGTTACCAGAGGAGGTAGTATTACATTAAATGGAACTTATAAAAACACTACATGGACAAGATATCACTTAGACTCATGGAAAAATTTATGCAAGTGGAATATGACAGCTTACAAGTGTTATGATAATGGAAGCATTACTATTACTGCCACTGGTAAAATTACTTCTGGCAAATACAAGGCAGAAAGTTACAAAAATGAAATTAAGAAATCAGTATTTAAAACTAATAAAACTACATTTGAAGATTCTGGAAATTATGAACATCAAAAAATAACTTTCTATCAGCTAACAATTATTCAACTACCTACTACTAAGGTACCAACCACCACAGCCAGTACATACACTACACAGCTAAACACAACAGTGCAGAATAGTACTGTGTTGGTTAGGTACTTGCTGAGGGAGGAAAGTACTACTCAACAGACAGATGCTACCTTAAGTGCCTTTAGCAGCACTGCAAATTTAACTTCGCTTGCTTGGACTAATGAAACCGGAGTATTATTAATGCATGGCCAGCCTTACTCAGGTTTGCATATTCAAATTACTTTTCTGGTTATCTGTGGGATCTTTATTCTCGTGGTTCTTTTGTACTTTGTTTGCTGCAAAGCCAGAAAAAAATCTAGGAGACCCATCTACAGGCCAGTAATCGGGAATCCTCAGCCTTTCCAAGTGGAAGGGGGTCTAAGGAATCTTCTTTTTTCTTTTTCAGTATGGTGATCAGCCATGATTCCTAGGTTCTTCCTATTTAACATCCTCTTCTGTCTTTTTAACGTGTGCGCTGCCTTTGCGGCCGTTTCGCACACCTCACCCGACTGTCTTGGGCCATTCCCCACCTACCTTCTCTTTGCCCTGCTCACCTGCACCTGCGTCTGCAGCATTGTCTGCCTGGTCATTACCTTCCTGCAGCTTATCGACTGGTGCTGCGCGCGCTACAATTATCTTCACCACAGTCCCGAATACAGGGACAAGAACGTAGCCAAACTCTTAAGGCTTATATGACCATGCAGACTCTGCTAATACTGCTATCCCTTTTATCCCCCGCCCTTGCCACTTTTGATTACTCTAAATGCAAATTTGTTGAGCTATGGAATTTCTTAAACTGCTATAATGCTACAATGGATATGCCTTCCTATTACTTGGTAATTGTGGGAATAGTGATGGTCTGCTCCTGCACTTTCTTTGCTATTATGATCTACCCCTGTTTTGATCTCGGCTGGAACTCTGTTGGGGCATTTACATACACACTACAAAACAGTTCACCAGCCTTCACACCGCCTCCCCGCAGAAATCAGTTCCCCCTGATTCAGTACTTAGAAGAGCCCCCTCCCCGGCCCCCTTCCACTGTTAGTTACTTTCACATAACCGCCGGCGATGACTGACAACCACCTGGACCTTGAGATGGACGGCCAGGCCTCCGAGCAACGCATCCTGCAACTGCGCGTCCGTCAGCAGCAGGAGCGAACCGCCAAGGAGCTCCTTGATGCCATCAACATCCACCAGTGCAAGAAAGGCATTTTTTGCCTGGTCAAACAGGCAAAAATTACCTACGAGCTTATGTCCGGCGGCAAGCAGCATCGCCTTGCCTATGAGCTGCCCCAGCAGAAGCAAAAGTTCACCTGCATGGTGGGCGTCAACCCCATAGTCATCACCCAGCAGACGGGCGAAACCAGCGGCTGTATCCATTGCTCCTGCGAAAGCCCCGAGTGTATCTACTCCCTCCTTAAGACCCTTTGCGGACTTCGCGACCTCCTACCCATAAACTAATTGATTAAAGTTTAGAAACCAATCACACCCCATTCCCCATTTTCCCACATAAACAATCATTAGAAATAATTACTTAATAAAAATTACTTACTTGAAATCTAAAAGTATGTCTCTGGTGTAGTTGTTTAGCAATACCTCGGTCCCCTCCTCCCAGCTCTGGTACTCCAGTCCCCGGCGGGCGGCGAACTTCCTCCACACCTTGAAAGGGATGTCAAATTCCTGGTCCACAATTTTCATTGTCTTCCCTCTCAGATGTCAAAGAGGCTCCGGGTGGAAGATGACTTCAATCCCGTCTACCCCTATGGCTACGCGCGGAATCAAAATATCCCCTTCCTTACTCCCCCCTTTGTCTCCTCCAATGGATTCCAAAACTTCCCCCCTGGGGTTCTGTCACTCAAACTGGCTAACCCAATCACCATCACCAATGGAAATGTCTCACTCAAGGTTGGAGGTGGGCTAACTTTGCAAGAAGAAACTGGAAAACTAACAGTTAATACTGAACCACCTTTGCAACTTACAAATAACAAATTAGGTATTGCTTTAGACGCTCCATTTGATGTTATAGACAATAAGCTGACACTATTAGCAGGCCATGGCTTGTCTATTATAACAAAAGAAACATCAACACTGCCTGGCTTGGTTAATACTCTTGTAGTATTAACTGGAAAGGGTATTGGAACAGATTCACATAATGGTGGAAATATATGTGTTAGAGTTGCAGAAGGCGGTGGCTTATCATTTAATGATAATGGAGACTTGGTAGCATTTAATAAAAAAGAAGATAAACGCACCCTATGGACAACTCCAGACACATCTCCAAATTGCAGAATTGATCAGGATAAGGACTCTAAGCTAACTTTGGTCCTTACAAAGTGTGGAAGTCAAATATTAGCCAATGTGTCATTAATTGTCGTAGCTGGAAGGTACAAAATTATCAATAACAATACTCAACCAGCTCTCAAAGGATTTACCATTAAATTGTTGTTTGATAAAAATGGAGTCCTTATGGAATCTTCAAATCTTAGTAAATCATATTGGAACTTTCGAAATGAAAATTCAATTATGTCAACAGCTTATGAAAAAGCTATTGGTTTTATGCCTAATTTGGTAGCCTATCCAAAACCTACCACTGGCTCTAAAAAATATGCAAGAGATATAGTTTATGGAAACATCTACCTTGGCGGAAAGCCACATCAACCAGCAACCATTAAAACTACCTTTAACCAGGAAACTGGATGTGAATACTCTATTACATTTGATTTTAGTTGGGCCAAAACTTATGTAAATGTTGAATTTGAAACTACCTCTTTTACCTTTTCCTATATTGCCCAAGAATAAAAGACAAATAAACGTGTTTTTCATTTAAAAATTTCATGTATCTTTATTAATTTTTACACCAGCGCGGGTACACATTCTCCCACCACCAGCCCATTTTACAGTGTAAACAATTCTCTCAGTACGGGTGGCCTTAAATAGGGGAAAGTTCTCATTAGTGCGGGAACTGGACTTGGGGTCTATAATCCACACAGTTTCCTGGCGAGCCAAACGGGGGTCGGTGATTGAGATGAAGCCGTCCTTTGACAAATCATCCAAGCGGGCCTCACAGTCCAAGGTCACAGTCTGGTGGAATGAGAAGAACGCACAGACTCATACTCGGAAAACAGGATGGGTCTGTGCCTCTCCATCAGCGCCCTTAACAGTCTTTGCCGCCGGGGCTCGGTGCGGCTGCTACAAATGGGATCGGGATCGCAAGTCTCTTTGACTATAATCCCCACAGCCCTCAGCATCAGTCTTCTGGTGCGTCGGGCACAGCACCGCATTCTAATTTCGCTCATGTTTTCACAGTAAGTGCAGCACATAATCATTATGTTATTCAGCAGCCCATAATTTAGGGTGCTCCAGCCAAAGCTTATGTTGGGAATGATGGAACCCACGTGACCATCGTACCAAATGCGGCAGTATATCAGGTGCCTGCCCCTTATAAACACACTGCCCATATACATAATCTCTTTGGGCATATTTCTGTTCACAATCTGCCGGTACCAGGGGAAGCGCTGGTTAAACATGCACCCGTAAATGACTCTCCTAAACCACACGGCCAGCAGGGTGCCTCCCGCCCGGCACTGCAGGGAGCCCGGGGATGAACAGTGGCAATGCAGGATCCAGCGCTCGTACCCGCTTACCATCTGGGCTCTCACCAGATCCAGGGTAGCGGGACACAGGCACACTGACATACATCTTTTTAAAATTTTTACTTCCTTTGTGGTCAGGATCATATCCCAGGGGACTGGAAACTCTTGCAGCAGGGTAAAACCAGCAGCACATGGTAATCCACGGACAGAACTTACATTATGATAATCTGCATGATCACAATCGGGCAATAGAGGGTGTTGTTCAGTCAAAGAGGCCCTGGTCTCCTTATCATATCGTGATAAACGGGCCCTGCGATATGGATGATGGCGGAGCAAGGTCGATTGTTCCTCGGTGCTCATTGTAGTGCACCCTCTTGCGTACCTCGTTGTACTTCTGCCAGCAGAAAGTGGCCCTGGAACAGCAGATACCTCTCCTCCGACCGTCCTTTCGCTGTTGCCGCTCAACCATCCAACTGAAGTACATCCATTCCCAAAGGTTGTGAAGAAGTTCCTCTGTATCTGATGAAACAAAAAGCCCGTCCATGCGAATTCCCCTCAACACATCAGCCAGGACTCTGTAGGCCATCCCCATCCAGTTAATGCTGCCTTGTCTATCATTTAAAGGGGGCGGAGGCAGGATTGGAAGAACCATTTTTATTCCAAACGGTCTCGAAGGACGATAAAGTGCAAGTCACGCAGGTGACAGCGTTCACCGCCGCTGTGTTGGTGAAAACATACAGCCAGGTCAAAACCCACTCTATTTTCAAGGTGATCAACTGTGGCTTCAAGCAGTGGCTCTACGCGTACATCCAGCATAAGAATCACATTAAAGGCCGGCCCTCCATCGATTTCATCAATCATCATGTTACATTCCTGCACCATCCCCAGGTAATTCTCATTTTTCCAGCCTTGGATTATTTCTACAAATTGTTGGTGTAAGTCCACTCCGCACATGTGGAAAAGCTCCCATAGCGCCCCCTCCACTTTCATAATCAGGCAAACCTTCATAATAGAAACAGATCCTGCTGCTCCACCACCTGCAGCGTGTTTAAAACAACAAAATTTAATAAGGTTCTGCCCTCAGCCCTTAGCTGGCGTCTAAGTGTCAGCTGCAAAAAGTCACTTAAGTCCTGGGCCACTACAGCTGACAATTCAGAGCCAGGGCTAAGCGTGGGACTGGCAAGCGTGAGGGAGTACTTTAATGCTCCAAAGCTAGCACCTAAAAACTGCATGCTGGAATAAGCTCTCTTTGTGTCTCCGGTAATGCCTTCCAAAATGTAAGTGATAAAGCCAGGTAGTTTTTCTTTAATCATTTTAGTAATAGAAAAATCCTGTAAATACGTCACTAGGACCCCAGGGACCACAATGTGGTAGCTTACACCGCGTCGCTGAAGCATGGTTAGTAGAGATGAAAGTCTGAAAAACACAAAGCATGCACTAAACCAAGGTGGCTATTTTTACTGAAGGAAAAATGACTCTTTTTAGCAGCAGGGTACCCACTGGGTGGCCCTTGCGGACATACAAAAATTGGTCCGTGTGATTAAAGAGCAGCACAGTAAGCTCCTGTCTTCTTCCGGCAAAAATCACATCGGACTGGGTTAGTATGCCCGTGGCATGGTAGTCATTTAAAGCCATAAATATGCCCTGATATCCAGTAGGAACGAGTACACTGACTTTTAGGTAAACCAATACCACCCCATGCGGAAAAATGTAAAAAGATTGAGGACAAAAGAAGGTATATTTATTGCTAGCCCCTTCCTGGACGGGAGCAATCCCTCCAGGGCTATCTATAAAAGCATAAAGACATTTAGCCATAGCTTAGCCCGCTTACCAGTAGACAAAGAGCACAGCAGTACAAGCGCCAACAGCAGTAACTAACTACTTACTGACCTAGCTGAGTACTTAAAGGCAACTTACACTGACGTAATGACGGAAGGTGTAAAAACCCCGCCAAAAAAAAACACACACGCCCTGGGTGTTTTTCGCAAACACACTTCCGCGTTCTCACTTCCTCGTATCGATTTTGTGACTTAACTTCCGGATTCCCACGTTACGTCACTTTTGCCCTTACATGTGGCTGTGCCGTAGGGCGCCATTTTGTTGTGCCCAACATGGCTAAAGTCCGCTTTTTTATACGTCATCGGCCCTGCGACTTGCTTCCGGGCTAGGCCACGCCTCTGCGGCAACCGCCGGCCGTGCGTCTTGACGTCAACTGCATTACCGCTTGTTAACCAATTAACATTGGCCCTGCCCTAAAACCGTTAAAATTTAAAAGCTCATTTGCATATTAACTTTTGTTTACTTTGTGGGGTATATTATTGATGATG

>ADENOVIRUS_A_STRIPPED4

CCTATCTAATAATATACCTTATACTGGACTAGTGCCAATATTAAAATGAAGTGGGCGTAGTGTGTAATTTGATTGGGTGGAGGTGTGGCTTTGGCGTGCTTGTAAGTTTGGGCGGATGAGGAAGTGGGGCGCGGCGTGGGAGCCGGGCGCGCCGGATGTGACGTTTTAGACGCCATTTTACACGGAAATGATGTTTTTTGGGCGTTGTTTGTGCAAATTTTGTGTTTTAGGCGCGAAAACTGAAATGCGGAAGTGAAAATTGATGACGGCAATTTTATTATAGGCGCGGAATATTTACCGAGGGCAGAGTGAACTCTGAGCCTCTACGTGTGGGTTTCGATACGTGAGCGACGGGGAAACTCCACGTTGGCGCTGCCAGCTCATTTTCACGGCGCCATTATGAGAACTGAAATGACTCCCTTGGTCCTGTCGTATCAGGAAGCTGACGACATATTGGAGCATTTGGTGGACAACTTTTTTAACGAGGTACCCAGTGATGATGATCTTTATGTTCCGTCTCTTTACGAACTGTATGATCTTGATGTGGAGTCTGCCGGTGAAGATAATAATGAACAGGCGGTGAATGAGTTTTTTCCCGAATCGCTTATTTTAGCTGCCAGTGAGGGGTTGTTTTTACCGGAGCCTCCTGTACTTTCTCCTGTCTGTGAGCCTATTGGGGGCGAATGTATGCCACAACTGCACCCTGAAGATATGGATTTATTGTGCTACGAGATGGGCTTTCCCTGTAGCGATTCGGAAGACGAGCAAGACGAGAACGGAATGGCGCATGTTTCTGCATCCGCAGCTGCTGCTGCCGCTGATAGGGAACGTGAGGAGTTTCAGTTAGACCATCCAGAGTTGCCCGGACACAATTGTAAGTCCTGTGAGCACCACCGGAATAGTACTGGAAATACTGACTTAATGTGCTCTTTGTGCTATCTGCGAGCCTACAACATGTTCATTTACAGTAAGTGTGCTATGGGAGGTGGGAGGTGATTTTTTTTTCTTAAGCAGTGAAAAATAATATTTTGTTGTTTTTAGGTCCTGTTTCCGATAATGAGCCTGAACCTAATAGCACTTTGGATGGCGATGAGCGACCCTCACCCCCGAAACTAGGAAGTGCGGTTCCAGAAGGAGTAATAAAACCTGTGCCTCAGCGGGTGACTGGGAGGCGTAGATGTGCTGTGGAAAGCATTTTGGATTTGATTCAAGAGGAAGAAAGAGAACAAACAGTGCCTGTTGATCTGTCAGTGAAACGCCCTAGATGTAATTAATGGACTTTGAGCACCTGGGCAATAAAATAGGGGTAATGTGGTTTTTGTGAGTCATGTATAATAAAACTGGTTTCGGTTGAAGTGTCTTGTTAATGTTTGTTTGGGCGTGGTTAAACAGGGATATAAAGCTGGGTTGGTGTTGCTTTGAATAGTTCATCTTAGTAATGGAGTTGGAAACTGTGCTGCAAAGTTTTCAGAGCGTTCGCCAGCTCTTGCAGTATACCTCTAAAAACACTTCAGGTTTTTGGAGGTATCTGTTTGGCTCTACCTTAAGCAAGGTGGTAAATAGGGTGAAAGAAGACTATAGAGAGGAATTTGAAAACATATTGGCCGACTGTCCAGGGCTTTTGGCTTCACTAGACCTTTGTTACCACTTGGTGTTTCAGGAAAAAGTGGTCAGATCCTTAGATTTTTCATCTGTGGGACGAACGGTTGCTTCTATTGCTTTTTTGGCAACCATATTGGATAAATGGAGCGAGAAATCCCACCTGAGTTGGGATTACATGCTGGATTACATGTCAATGCAGCTGTGGAGGGCATGGCTGAAGAGGAGGGTTTGCATTTACTCGCTGGCGCGGCCTTTGACCATGCCGCCGCTGCCGACGTTGCAAGAGGAGAAGGAGGAGGAGCGGAACCCTGCGGTGGTGGAGAAGTAAACATGGAACAACAGGTGCAAGAAGGCCATGTACTTGACTCTGGCGAAGGGCCTAGTTGCGCAGATGATAGAGATAAGCAGGAAAAAAAAGAAAGTTTAAAGGAAGCTGCTGTTCTTAGTAGGCTAACTGTTAATCTGATGTCCCGCCCGCGTTTGGAAACTGTATATTGGCAGGAGTTGCAGGATGAATTTCAGCGGGGTGATATGCATTTACAGTACAAATACAGTTTTGAACAATTAAAAACCCACTGGTTAGAGCCATGGGAGGATATGGAGTGTGCTATTAAAGCTTTTGCTAAATTGGCCTTACGTCCTGATTGTAGCTACAGAATTACTAAAACAGTAACCATTACTTCATGCGCCTATATTATAGGTAACGGGGCAATAGTTGAGGTAGATACAAGCGACAGAGTTGCTTTTAGATGTCGAATGCAGGGTATGGGCCCAGGGGTGGTGGGTTTGGATGGAATTACATTTATAAATGTTAGGTTTGCTGGAGATAAGTTTAAAGGCATTATGTTCGAAGCTAATACCTGTCTTGTCTTGCATGGTGTTTACTTTCTTAACTTTAGTAACATTTGTGTAGAGTCTTGGAATAAGGTTTCTGCTAGGGGCTGTACTTTTTATGGATGTTGGAAGGGTTTGGTGGGTAGACCAAAAAGTAAACTGTCTGTAAAAAAGTGTTTGTTTGAAAAATGTGTACTTGCTTTAATTGTAGAGGGGGATGCACATATTAGGCATAATGCAGCTTCAGAAAATGCCTGTTTTGTATTATTGAAGGGAATGGCTATTTTAAAGCATAATATGGTTTGTGGGGTGTCTGATCAAACTATGCGACGTTTTGTTACCTGTGCTGATGGAAATTGTCATACCTTAAAAACTGTTCATATTGTGAGCCACAGTAGACATTGTTGGCCTGTATGTGATCATAACATGTTTATGCGCTGTACCATACATTTAGGCTTAAGGCGGGGTATGTTTAGACCTTCCCAATGTAACTTCAGCCACTCAAACATTATGCTGGAACCTGAAGTGTTTTCTAGAGTGTGTTTAAATGGGGTATTTGATTTATCTGTGGAATTATGTAAGGTTATAAGATATAATGATGATACTCGACATCGTTGCCGACAGTGTGAGTGTGGTAGCAGTCATCTAGAACTTCGTCCCATTGTGCTAAATGTAACTGAGGAGCTGAGAAGTGACCACCTTACCCTGTCTTGCCTGCGGACTGACTATGAGTCAAGTGATGAAGACGACAACTGAGGTAAGTGGGTGGAGCTAGGTGGGATTATAAAAGGCTGGAAGTCAACTAAAAATTGTTTTTGTTCTTTTAACAGCACGATGAACGGAACTACTCAGAACAACGCTGCGCTTTTTGATGGAGGGGTTTTTAGCCCTTATTTGACTTCCAGGTTACCATATTGGGCCGGAGTACGTCAGAATGTGGTAGGATCTACAGTGGACGGTCGACCTGTGGCACCTGCAAATTCATCAACATTAACCTATGCAACTATTGGACCCTCGCCTTTGGATACCGCCGCCGCCGCTGCAGCTTCCGCGGCCGCTTCTACGGCTCGCAGTATGGCAGCTGATTTCAGCTTCTACAATCACTTGGCTTCGAATGCTGTGACACGCACCGCAGTTCGAGAGGACATTCTGACTGTTATGCTTGCCAAGCTTGAAACTCTAACTGCTCAGCTGGAAGAGCTATCGCAAAAGGTTGAGGAATTAGCTGATGCTACTACCCATACCCCAGCCCAACCTGTAACCCAATAAAGAAAAAACTTAAATTGAGATGGTGTTATGAATCTTTATTGATACTTGTTTTTTCTGACATGGTAAGCTCTTGACCACCGTTCCCTATCATTAAGAACACGGTGAATGTGTTCCAGTATTTTGTAAAGATGAGCCTGTATATTAAGGTACATTGGCATTAGGCCATCTTTGGGATGAAGGTAGGACCATTGAAGGGCTTCATGTTCCGGGTTAGTGTTGTAGATAATCCAGTCATAGCAACAACGCTGGGCATGGTGATTAAATATATCTTTTAACAACAAGCTAATTGCTAATGGAAGACCTTTAGTATAGGTATTGATAAAACGGTTAAGCTGGGTGGGATGCATCCGAGGTGACATGATATGAAGTTTTGATTGTATTTTGAGATTGGCAATGTTACCTGCCAAATCTCTTCTTGGATTCATATTGTGGAGAACCACGAAAACGGTGTAGCCAGTACACTTGGGAAATTTGTCATGGAGTTTAGAAGGAAAGGCATGGAAAAACTTGGAAACGCCTTTGTGACTTCCCAAATTTTCCATACACTCATCCATTATTATGGCAATTGGACCGCGAGCAGCGGCTTGAGCAAAAATGTTTTCTGGATCAGAAACATCATAGTTGTGGTCTAGAGTTAGGTCATCGTAGGACAACTTAACAAATTTAGGACACAGCGTTCCAGATTGTGGAATAATAGTTCCCTCTGGTCCTGGGACATAATTTCCCTCACAAATTTGCATTTCCCAAGATTTAATTTCAGATGGGGGAATCATGTCCACTTGCGGAACAATAAAAAAAACAGTTTCTGGAGCAGGTGTAACCAGCTGGGCAGAAAGCAAATTACGCAACAACTGAGACTTCCCACAGCCAGTGGGTCCATAAATTACCCCAATTACAGGTTGCAAGTGATAGTTTAACGAGGTGCAGCTGCCGTCTTCGTGGAGAAGCGGAGCCACTTCATTCATCATTTGTCGGACGCGGATGTTTTGCTTGGCCAGTTCCCCTAACAGACGCTCTCCGCCTAAGGAAAGTAACTCTTGTAAAGATTTGAAATTTTTAAGTGGCTTTAGGCCATCGGCCATAGGCATGTGGTCCAGGGTTTGCTTCAGCAGTTGCAAGCGATCCCATAGCTCAGTTATATTTTCTATGCCATCTCGATCCAGCAAACTTCCTCGTTGCGGGGGTTTGGCTGGCTGTTGCTGTAAGGAACGAGGCGGTGAGCATCCAAATGGACGAGGGTTTTGTCCTTCCAGGGACGTAATGTGCGCGTCAGGGTTGTTTCGGTCACGGTGAATGGATGCGCTCCTGGTTGAGCGCTGGCCAGTGTGCGCTTTAAACTGAGGCGGCTGGTGCTGAAGCGCGTGTCTTCTCCCTGTGCTTCGGCAAGGTAGCATTTTAACATAAGATCATAAGACAAAGCCTCTGTAGCGTGGCCTTTAGCCCGTATTTTTCCTTTGGAGGTGCTCCCGCAGTGAGGACACTGAAGGCATTTAAGGGCGTACAGTTTTGGAGCCAAAAAAACAGATTCTGGAGAATAAGCATCTGCGCCACAATAACTACAAACAGTTTCACATTCAACTGACCAGGTCAGCTCAGGACATGATGGATCAAAAACAAGTTTCCCTCCGTACTTTTTGATGCGTTTCTTACCTTGCGACTCCATAAGGCGGCGTCCTTTCTCTGTGACAAAAAGACTGTCAGTGTCTCCGTATACAGATTTAAGGGGTCTATCCTTCAGTGGTATTCCGCGGTCCTCCTCGTACAGGAATTCTGACCACTCTGACACAAAAGCTCTAGTCCAAGCAAGTACAAAGGAAGCCACATGGGAAGGGTACCGATCGTTGTTAATTAAAGGGTTAGAACTTTCTAAGGTGTGTAAACACATGTCTCCTTCTTCAGCGTCCATGAATGTGATTGGTTTGTAGGTGTAAGTCACGTGTTCACAATTTTCTGGTGGTGGGCTATAAAAAGGGGCGGGTCCTTGGTCTTCATCGCTTTCTTCTGCTTCGCTGTTTACGAGCGCCAACTGGTTGGGTGAGTACACGCGCTCAAAGGCAGGCATTACCTCTGTACTCAACGTGTCAGTTTCTATAAACGATGAGGATTTGATGTTTAATCGCCCCGCTGCAATTTCTTTCATTAGGCTTTCTTCCATTTGATCAGAAAAAACTATTTTTTTGTTATCTAGTTTGGTAGCAAAAGATCCGTACAAGGCATTGGAAAGCAGCTTGGCTATAGATCTTAGGGTTTGATTTTTGTCCCTATCGGCCCGTTCTTTTGCGGCAATATTGAGTTGCACATATTCGCGTGCCAGGCATTTCCAGGTGGGGAAAATGGTGGTGCGCTCGTCAGATAGCAAGCGTAAGCGCCACCCGCGATTATGCAGTGTAACCAGATCTACGCTGGTAACTACTTCACCGCGCAAGCTTTCATTGGTCCAGGCTAAACGACCGCCTTTTCTAGAACAAAAAGGAGGAAGAACATCCAACTGATTTTCATCTGGGGGGTCGGCATCTATAGTAAAAATGCCAGGACAAAGATTTTTGTCAAAATAATCAATTTTGCAAGTGTAATTTTCCAGCGCCACCTGCCATTGCCGCACGGCCAATGCCCGCTCATAGGGGTTAAGGGGAGGACCCCAAGGCATGGGGTGTGTGAGGGCCGATGCATACATGCCGCAAATATCATATACATATATGGGCTCTTTTAGTACTCCTATGTAAGTAGGATAGCACCTGCCGCCACGAATGCTGGCGCGAACGTAGTCATATAGCTCATGTGAAGGCGCCAGGATGTTGGGCCCAAGATGTGTGCGCTGTGGTTTTTCGGCGCGGTACAAAATTTGTCTGAAAATTGCATGAGAGTTAGAGGAAATGGTAGGACGCTGAAACACATTAAAATGTGCCGCGTCAAGACCCACTGCGTCAGTAACAAACTGGGCGTATGAGCTACGCAGTTTTTCTACCAATGAGGCAGTCACAAGTACATCCAGGGCACAATAGTTTAATGTTTCCCCGATAAGATTGTAATTTTTTTCTCCTTTTTTTTTCCATAGTTCTTGATTTAGGAGGTATTCCTCCTTATCCTTCCAGTACTCCTCCAGGGGAAACCCATTTGCATCTGCACGGTAAGAACCAAGCATATAAAACTGATTTACCGCCTTGTACGGACAACATCCTTTTTCTACAGGCAGGGCATACGCTTGTGCAGCCTTTCTTAAAGATGTATGAGTAAGAGCAAAGGTATCTCTGACCATTACTTTTAAATACTGGTATTTAAAATCTTGGTCGTCACACCCTCCGTGTTCCCACAGTAGGAAGTTAGTTCGCTTTTTGTAGTGGGGATTGGGAAGGGCAAAAGTAATATCATTAAATAATATTTTGCCAGCTCTTGGAATAAAATTTCTAGAAATTTTAAAGGGTCCAGGGACGTCCAAGCGGTTATTGATTACCTGAGCGGCAAGAACAATTTCATCAAATCCATTAATATTGTGTCCTACTATATACAACTCTACAAATCTTGGCTCACCCTTAATTGCAGGGGCTCTTTTAAGATCTTCGTAGGAAAGATCTTCAAGCGCGACTAGTCCGTTTTCTTCTTGAGCCCATTGAGACAAGTGTGGATTTTTTTGTAAAAAAGTCATCCAAAGATCAGTAGCTAAGGAGGTTTGTAAGCGGTTTCTATAGGTACGAAACTGTTGACCGACCTTCATTTTTTCTGGGGTTAAGCAGTAGAAAGTAGTAGAGTCTTTTTCCCATTGGTCCCATCCAAGTTCTAATGCAAGTTGTAAGGCATGTTTGACAAGATTGTCATCCCCAGACAGTTTCATCACCAGCATAAATGGGACAAGTTGCTTTCCAAATGCCCCCATCCAGGTGTAGGTTTCTACATCATAGGTAATAAAAAGGCGCTCAGTGCGAGGATGCGAACCGATTGGGAAAAAGTGGATCTCCTGCCACCAGTTGGAAGAATGGCTGTTGATGTGATGAAAGTAGAAATCTCGTCGGCGGACAGAGCATTCATGCTGATGTTTGTAAAAGCGTGCGCAGTGTTCGCATCGTTGCACGGGCTGTATCTGTTGAATGAGGTGTACCTGGCGGCCTCGCACCAGAAAGCAGATGGGAAAATCAATACCACTTGGCAGCTGCCGTTCGTCCTCTTCCTCTTCTGCTGCATTGCCACTACCGTTTGGATCCTCGAAAGCGAGAACGGAGAGGGTGACGGTGCCCCTCGACCTGCATGTCCAGATTTCAGCACGAGAGGGGCGGAAACGGGAAATCAGGGCGTACAGCCTGGAGCTGTCCATGGTATCAGTCAGAGAGAAAAGCATGTCCGCGGGGACAGCGCGCAAGTTGACTTCGCACAGGCGGGTAAGAGCAGGCTGGAGGTGCAGGTAATACTTAATTTCTAGAGGCGTGCCGTTGGCAGAGTCTATTGCGTGAAGTATTCCATGAGCCCGGGGACTAACCACGGTTCCACGGTGCACTTTTCCAATGCGCCTGCTTAAAATCGGCGGCGCGGACGAGCTCCCGGAGGAAGCGGCGGTTCGGGTCCTGCGGGAAGCGGGGGAAGCGGTATGTCGGCCTGACGCTCTGGCAGGGGAAGGTGTTGAGCCCGAAGTTGACTGGCATGGGCGACTACCCGGCGATTGATATCTTGAATCTGTCGGCGTTGTGTAAACACTACCGGCCCTGTTGTTTTGAACCTGAAAGAAAGTTCAACAGAATCAATCTCAGTGTCATTTACTGCAGCCTGTCTTAAAATCTCCTGAACGTCGCCTGAGTTATCTTGGTAGGCAATTTCTGCCATTAATTGATCAATTTCTTCCTCCTGGAGGTCTCCATGTCCCGCACGTTCAATAGTGGCTGCAAGGTCATTAGATATCCGACTCATAAGCTGTGAAAATGCGTTTAGTCCAATTTCGTTCCAGACTCGGCTGTATACTACCCCTCCTTCGCTGTCCCGAGCGCGCATAACCACTTGCGCCAAGTTGAGTTCCACGAGCCGTGCGAACACGCCGTAGTTGCGCAAGCGCTGAAACAGGTAGTTTAAGGTGGTGGCAACGTGTTCTGAGACGAAGAAATACAGAATCCACCGACGAAGCGTCAGCTCGTTGATGTCACCTAAGGCTTCAAGACGTTCCATGGCTTCGTAAAAGTCTACTGCAAAATTGAAAAACTGGGAGTTGCGAGCTGCCACCGTCAATTCTTCTTCCAACAGACGAATAAGCTCGGCCACCGTCTCGCGCACTTCTTGCTGAAATGCGCCCGGAACTATTTCTTGTTCTTCCTCTTCTACCTCCATTATTTCTTCCTCGACCACAGGTGGTGGGGGTTGTCTTCTTCGACGCCGGCGAACGGGCAGCCTGTCTACAAATCTTTCAATCATTTCGCCGCGACGGCGGCGCATAGTTTCGGTTACTGCTCGACCGTTTTCACGTGGTCGTAACTCAAAAACTCCACCTCTAAGTTCTGTTTCATGTAAAATGGGAAATGAGGCGTTGCGAGGGGCGTTAGGTAGGGATACAGCGCTGATTATGCATTTTATTATTTGCTGCGTAGGAACTCCGCGCAAGGAGCTAAGCGTCTGCATATCCACCGGGTCGGAGAACCTTTCAAGAAAGGCATCTAGCCAGTCACAGTCACAAGGTAGGCTAAGTTTTGTTTCTTCTAAAGTACCAGGAAGCTGAGCAATGCTACTAATAATGTAATTGAAGTAAGCTGTTTTAAGCCCACGAATGGTTTTAAGAAGCACCACATCTTTGGGTCCGGCTTGTTGAATTCGCAGGCGGTCTGCCATTCCCCACACGTCACTTTGACATCGTCCAAGATCTTTGTAGTAGTCTTGCATTAACCTTTCCACCTCTACCTCGCGGTTTCCGCGATCAGCCATGTGCGTGCTTCCGTAGCCTTGCAGCGGTTGTAATAAAGCTAAATCTGCCACTACCCGTTCCGCAAGCACTGCCTGTTGAATTTGGGTAAGGGTGGTTGCAAAGTCATCCACATCTACAAAGCGGTGATAAGCTCCTGCATTAATGGTGTAGCTGCAGTTTGTCATTACTGACCAATTAACAGTTTGCGTGCCTGGCTGTACAGTTTCTGTGTATCGCAAGCGTGAGTAAGCCCGAGAGTCAAAAACATAGTCATTGCAGGTGCGCACTAGGTATTGATAGCCCACAAGGAAATGAGGAGGAGGTTCGCGATACAACGGCCAGCCAAGCGTAGCCGCAGCACCTGGAGCGAGATCTTCCAACATGAGGCGGTGGTATTCATATATGTATCTGGACATCCATGTGATGCCGGCAGCGGTAGTTGTTGCTCGCATAAATTCGCGGGCTCGGTTCCAAATATTGCGCAGGGGTAAAAAGCGTTCAATAGTTGCCACGCTTTGACCGGTCAGGCGTGCGCAGTCTTGAATGCTCTGGACATGGAAAAAATGAAAGTTGGTAAGCGACTCCCTTCCGTGGTTTGGTGGAAAAGTCACAAGGGTACCATAGCGAGGAACCCCGGTTCGAAACCGGCAGGATCCGCTATGAGCACAAGTGAGGCGCTTGCGCGTTGAACCCGGCCAAGG

AGATGCATCCTGTCCTGCGACAAATGCGACCTCAGCCCAGGGCAACCACGGCCTCAGCAGCGGTGGCGCTTTCGGGCTCTGGCGAACAGGAAGAGCCTCAATGTCCTACATTGGAGTTGGAAGAAGGAGAAGGCATAGCCCGATTGGGCGCCCACTCTCCTGAGCGTCACCCAAGGGTGCAGCTCGCCCGGGACAGTCGCGTGGCATTTGTGCCTCGTCAGAACATGTTTCGCGACAACAGCGGGGAGGAAGCTGAGGAAATGCGAGACTGCAGGTTTAGGGCCGGTCGCGAGCTGCGCCGCGGATTTAATCGCGAGCGACTGCTGCGTGAGGAGGACTTTGAGCCAGATGAACATTCGGGGATTAGTTCTGCACGGGCCCATGTATCAGCAGCCAACTTAGTAACAGCATATGAACAAACGGTTACAGAGGAACGTAACTTTCAAAAAAGCTTTAATAACCATGTGCGCACACTAATAGCGCGAGAAGAAGTAGCCATTGGTTTAATGCATCTTTGGGACTTTGTAGAAGCTTATGTACATAATCCAGCAAGTAAACCCCTAACTGCCCAGCTGTTCTTAATAGTTCAACATAGTAGAGACAATGAAACTTTTAGGGATGCAATGCTTAACATAGCTGAACCCCAGGGTCGGTGGTTACTCGATTTAATTAACATTCTGCAGAGCATTGTGGTTCAGGAACGCAGTCTTAGTTTGGCAGACAAGGTGGCCGCCATTAATTACTCCATGTTAAGTTTGGGAAAGTTTTATGCTCGTAAAATCTACAAAAGTCCGTATGTTCCCATTGACAAGGAAGTGAAGATAGACAGCTTTTATATGCGCATGGCTTTAAAGGTACTAACATTAAGCGACGATCTTGGAGTGTACCGCAATGACCGAATCCACAAAGCAGTAAGCGCCAGTCGCCGCAGAGAGCTAAGCGACAAAGAGCTTATGCATAGCTTACAAAGGGCGCTGACGGGAGCAGGAACAGAGGACGAGTCGTTCTTTGATATGGGCGCAGACCTACGGTGGCAGCCAAGCGCTCGCGCTTTGGAGGCAGCTGGAGTGGCGTCTGCTGACGTCACTGGCGATGACGATGACGAAGACCAGTACGAGGACTGATCGGCCGTACCTTTTGTTAGATGCAGCGACCGGCGATCATCGCGGAGAGGGCTCCTAACCTGGATCCCGCGGTTTTGGCGGCCATGCAAAGCCAGCCTTCTGGCGTTACAGCTTCAGATGACTGGACAGCGGCCATGGATCGTATTATGGCTTTAACGGCGCGCAGTCCTGATGCTTTTCGCCAGCAGCCCCAAGCTAACCGCTTTTCGGCCATTTTGGAAGCAGTAGTGCCGTCTCGTACTAACCCTACTCACGAGAAAGTGTTAACCATTGTAAATGCTTTGTTGGATAGCAAAGCCATCCGCAAAGATGAGGCTGGTTTAATATACAACGCTTTGCTTGAGCGCGTGGCACGCTATAACAGTACCAATGTGCAGGCTAACTTAGACCGGATGGGTACAGATGTAAAGGAGGCGCTGGCTCAACGAGAGCGCTTTCATCGCGATGGTAATCTTGGTTCGCTAGTAGCATTAAACGCTTTTTTGAGTACTCAGCCGGCTAATGTTCCGCGTGGTCAGGAAGATTATACAAACTTCATCAGCGCCTTGCGACTAATGGTTACTGAAGTGCCTCAAAGTGAAGTGTATCAGTCTGGACCCGATTACTTTTTTCAAACGTCCAGGCAGGGTTTGCAAACCGTAAACTTAACTCAGGCTTTTAAAAATTTGCAAGGTTTGTGGGGGGTTCGTGCTCCAGTAGGCGATCGTTCAACTTTGTCCAGTTTACTAACACCAAACTCGCGCCTATTACTGTTGCTAATTGCCCCCTTTACCAACACCAACAGTTTAAGTCGAGATTCATACCTGGGTCACTTAGTTACTTTGTACCGCGAAGCCATTGGTCAAGCGCAGGTAGACGAACAAACTTATCAAGAAATAACCAGTGTTAGTCGCGCACTGGGCCAGGAGGACACTGGCAGTTTAGAGGCCACACTTAACTTTTTACTAACTAACCGTCGCCAGCAAGTGCCTCCTCAGTACACTTTAAATGCGGAAGAAGAACGCATATTGCGCTATGTACAGCAATCTGTAAGTTTGTATCTTATGCGTGAGGGTGCCACCCCCAGTGCCGCCTTAGACATGACAGCGCGCAATATGGAGCCGTCCTTCTACGCTTCCAATCGAGCTTTCATTAATCGCTTGATGGATTACCTTCACCGCGCTGCGGCCATGAACGGGGAATACTTTACAAATGCAATTCTAAATCCGCATTGGTTGCCCCCTCCTGGATTTTACACTGGTGAATTTGATTTGCCGGAAGGAAATGATGGCTTTTTGTGGGATGATGTTACGGACAGTCTGTTTAGTCCTGCAGTTATTGGACACCATGGTAAAAAGGAAGCAGGTGATGAAGGTCCCTTGCTTGACTCTCGGGCGAGTTCTCCATTCCCCAGTTTAACTAGTTTACCCGCCAGTGTTAACAGCGGTCGTACCACCAGACCCCGACTAACAGGTGAAAGTGAATACTTAAATGACCCCATCTTGTTTCCAGTGCGCGACAAAAATTTTCCCAACAATGGCATAGAAAGTTTGGTAGATAAAATGTCTCGCTGGAAAACATATGCACAAGAGCGGCGAGAATGGGAGGAAAGACAGCCAAGACCAGTTCGCCCTCCTAGGCAACGTTGGCAGCGACGCAAAAAAGGGGCACATGCGGGGGATGAAGGAAGCGATGACTCAGCTGACGACAGTAGTGTATTAGATTTAGGAGGGTCAGGAAACCCATTTGCTCATTTGCGCCCACAGGGTTGCATAGGGTCATTGTATTAAATTGAATAAAAGCATACTTACCAAAGCCATGGCGACCAGTGTTCGTCTTATTTTCCTTCTTCCGTTAGCTGTGAAATGAGGCGCGCGGTGGAACTGCAGACAGTGGCTTTTCCTGAGACACCACCTCCCTCTTACGAAACCGTGATGGCAGCGGCGCCACCCTACGTGCCTCCCCGCTATTTGGGTCCTACGGAGGGAAGAAACAGTATCCGTTACTCGGAATTGTCACCGTTGTACGATACCACTCGAGTGTACTTGGTGGACAACAAGTCTTCTGACATTGCTTCATTGAATTACCAGAATGATCACAGCAACTTTTTAACCACTGTAGTGCAAAATAATGACTATTCCCCTATAGAGGCTGGCACGCAAACTATTAACTTTGATGAAAGGTCTAGATGGGGTGGAGATTTAAAAACCATCTTACATACCAACATGCCAAACGTGAACGATTTTATGTTTACCACCAAATTTAAGGCCAGGGTAATGGTGGCTAGGAAAACAAACAACGAAGGCCAAACCATTTTAGAATATGAGTGGGCAGAATTTGTGCTACCCGAGGGTAACTATTCGGAAACCATGACTATTGACTTAATGAACAATGCTATTATTGAGCATTATTTGCGAGTAGGAAGACAGCATGGAGTGCTGGAAAGTGACATTGGAGTTAAGTTTGACACCAGAAACTTTCGTCTGGGTTGGGACCCCGAAACCCAATTAGTAACTCCGGGAGTGTACACTAATGAGGCTTTTCATCCAGATATAGTACTGCTTCCAGGTTGCGGGGTTGATTTTACAGAGAGCAGATTAAGCAACATACTAGGTATAAGAAAGAGGCAGCCGTTTCAGGAAGGATTTGTGATTATGTATGAACACTTAGAGGGAGGCAATATTCCAGCTCTTTTGGATGTAAAAAAATACGAAAACAGTCTGCAGGATCAAAACACTGTAAGAGGAGACAACTTTATTGCCTTAAATAAGGCTGCTAGGATTGAACCGGTTGAAACAGACCCCAAAGGACGCAGTTACAACTTGCTTCCAGACAAAAAAAATACTAAATATCGCAGCTGGTATTTGGCATACAACTACGGAGACCCAGAAAAAGGAGTTCGGTCATGGACTCTACTAACAACTCCAGATGTAACAGGCGGCTCCGAACAGGTGTACTGGTCCCTACCCGATATGATGCAAGATCCGGTGACTTTTCGCTCCTCGCGTCAAGTTAGCAACTATCCTGTAGTTGCAGCAGAATTACTGCCAGTTCATGCTAAAAGCTTCTACAACGAGCAAGCCGTCTACTCACAGCTTATTCGCCAGTCAACCGCGCTTACGCGCGTGTTTAATCGCTTTCCCGAGAACCAGATACTGGTGCGTCCACCAGCCGCTACCATCACTACCGTCAGTGAAAACGTTCCCGCCCTTACAGATCACGGGACCCTGCCGCTGCGTAGCAGTATCAGTGGAGTTCAGCGAGTCACCATCACTGACGCCCGCCGCCGGACCTGTCCCTACGTTTACAAAGCACTGGGCATAGTTTCTCCACGAGTGCTTTCTAGTCGCACTTTTTAAAAAAGTGTGGTAACATGTCCATTTTGGTTTCGCCAAGTAACAACACGGGCTGGGGACTGGGTGCCGCCCGCATGTATGGAGGAGCTAAAACAAGGTCTAGCCAACATCCAGTGCGCGTACGCGGACATTACCGAGCTCCATGGGGCGCGCATACCCGAGGACGCACTGGTCGCACCACTGTAGACGATGTTATTGACTCGGTAGTGGCCGATGCTCGCAAGTACCGCGCGCCCGCTGAAACAGCAGGGTCTACTGTTGATGCAGTAATTGATGAGGTAGTGGCAAACGCGCGGGCTTATGCAAGGCGCCGCAGACGGCTGCGTCGCCGGCGTAGACCAACCACCGCCATGCGCGCGGCCAGAGCGTTGGTTCGACGGGCCAGGCGCATTGGGCGGCGAGCTATGATGCGGGCAGCCAGGCGGGCTGCAACGCCTGCCGGTCGAGCGCGGAGACGGGCCGCAGCTGCGGCCGCAACAGCTATTGCAAACCTAGCTGCTCCGCGACGAGGAAATGTATACTGGGTGCGCGACTCAGTGACCGGGACGCGTGTGCCAGTTCGTACGCGTCCACCTCACCCTTAGAAGACAAAGAGTGACTCAATGTCTGTTATGTATGCCCAGCATGACCAAACGCAAGTTCAAAGAAGAGCTGCTGCAGGCCTTAGCGCCTGAAATATATGGCCCATCGGATAACCTTACCAAGCGCGATATCAAGCATGTTAAAAAACGGGAAAAAAAAGAGGAAGAAGTCGCCGCGGCGTCAGCAGACGGCGTCGAGTTTGTGCGCTCATTTGCGCCCAGACGTAGGGTACAGTGGAAGGGACGGCAAGTAAAACGCATTTTGCGACCGGGCACCACAGTGGTTTTTTCTCCCGGAGAGCGAACGATTATGCGTCCCCTAAAGCGCGAGTACGACGAAGTGTACGCAGACGATGACATTTTGGAGCAAGCGGCACAACAGACTGGGGAATTTGCATATGGAAAAAAAGGGCGTTACGGAGACAAAATTGCTATTCCTTTGGACGAGGGAAATCCAACACCCAGTTTAAAGGCTGTCACTTTGCAACAAGTGTTGCCCGTCCTTGGGCCTTCGGAAGAAAAGCGTGGAATTAAAAGGGAAGCCATGGATGAATTGCAGCCTACAATGCAACTGATGGTGCCTAAGCGGCAAAAGTTAGAGGACGTACTAGAGCACATGAAGGTGGATCCTAGCGTACAGCCAGATGTAAAAGTACGTCCGATAAAAAAGGTAGCTCCAGGATTGGGAGTTCAAACAGTGGACATTCAAATTCCTGTGCAAACTGCATTGGGTGAAACTATGGAAATCCAAACTTCGCCAATAAAAACAACGGTGAACGCAAGCGTGCAAACAGACCCTTGGTACCCGCCAGTGCTTTCAACAAAAAAAAAGCGTCACTACAGACAAACAAGTTCGCTTTTGCCAGACTACGTTTTACATCCTTCCATTGTGCCCACGCCTGGGTACCGTGGGACAACTTTTCAGCGCCGAGCCACAGCCCCTAGCCGTAGACGAGGTCCATCACGCCGTAGACGTCGACGCAAAGCCACTTTAGCCCCAGCGGCAGTACGTCGCGTTGTACAAAGGGGGCGCACACTAATACTTCCATCCGTGCGTTACCACCCTAGCATTCTCTAACAAGCTGCGCTGCCGTTTTTTCAGATGGCTCTTACTTGCCGAATGCGCATACCCATTCCAGGATACAGAGGACGACCCCGCCGGAGGAAAGGGCTGACCGGGAACGGTCGATTTCGGCGGCGTAGTATGCGCAGACGCATGAAGGGTGGGGTGCTGCCCTTCCTAATTCCACTTATTGCTGCGGCCATTGGAGCCGTTCCCGGAATTGCCTCAGTAGCCTTGCAGGCTTCTCGAAAAAATTAAAATAAAATAAAACTTCCAACTTATTACTGGTACTATGACTGTTTTATGCAGACTAAATGGAAGACATCAATTTTTCGTCGCTGGCCCCGCGACACGGCACGCGGCCGTACATGGGCACCTGGAACGAGATCGGCACGAGCCAGCTGAACGGGGGCGCCTTCAATTGGAACAGTATCTGGAGCGGTCTTAAAAATTTTGGTTCCACGATTAAGACATATGGCACCAAGGCGTGGAACAGCCAAACCGGCCAGATGCTAAGGGACAAGTTAAAAGACCAAAATTTTCAACAGAAAGTTGTAGATGGTCTGGCTTCGGGAATTAATGGAGTTGTAGACATAGCCAATCAGGCTGTACAGAAAAAAATTGCCAACCGTTTAGAGCCGCGGCCCGACGAGGTAATGGTAGAGGAAAAGCTGCCACCTCTAGAAACTGTGCCCGGATCCGTTCCAACCAAAGGAGAAAAGCGGCCACGGCCGGATGCAGAGGAAACCTTAGTAACGCACACAACAGAACCGCCGTCCTATGAGGAAGCAATAAAACAAGGAGCCGCTCTGTCACCTACCACCTATCCCATGACCAAGCCTATTTTACCCATGGCTACTAGAGTGTATGGAAAAAACGAAAATGTGCCTATGACCCTTGAGCTGCCTCCTTTGCCAGAACCCACTATCGCGGATCCCGTAGGTTCCGTTCCTGTTGCATCTGTTCCAGTTGCATCGACAGTGAGCCGTCCAGCAGTGCGGCCTGTTGCCGTGGCTAGCTTGCGAAACCCACGATCCAGTAATTGGCAAAGTACCCTAAACAGTATTGTGGGACTGGGAGTAAAGTCTCTCAAACGCCGACGCTGCTACTAACATTAAAAGACGAGTGTTAATTCCCATCTGTGTATACGCCTCCTATGTTAGCGCCAGAGGACCAACGCGTGAATCGCAGTCACCACCAGCGCTTTCAAGATGGCCACTCCCTCGATGATGCCGCAGTGGTCTTACATGCACATCGCCGGTCAGGATGCCTCGGAGTACCTGAGTCCCGGTCTGGTGCAATTCGCCCGCGCCACGGACACCTACTTCACCCTGGGAAACAAGTTTAGAAACCCCACCGTGGCTCCCACCCATGATGTTACCACCGATCGCTCGCAGCGTCTGACGCTGCGTTTTGTGCCCGTGGATCGGGAAGATACTACCTACTCCTACAAGGCTCGCTTTACGCTGGCTGTGGGTGACAACCGCGTGTTAGACATGGCTAGTTCTTACTTTGACATTCGAGGGGTACTGGATCGTGGTCCCAGTTTTAAGCCCTATTCCGGAACCGCCTACAATTCTTTGGCACCAAAAGGCGCTCCTAATGCTTCACAATGGTCAGATAACGCTAAGCTTAATACCTTTGCTCAGGCGCCGTATCTTAGCGACACTATCACCGCCGCCGATGGTATTAAAGTTGGAACAGACACCGCCCAGGCAGGCGCGGCGGTGTATGCCAACAAAACTTATCAGCCAGAGCCGCAAGTAGGACCAAGTGAATGGAACACCAGCATTGAAAACGTTAAAGCTGGCGGGAGGGCATTAAAGCAAACCACTGCAATGCAGCCGTGCTATGGCTCCTACGCTCGTCCAACCAACGAACACGGAGGACAATCCAAGGATGACAACATTGAACTTAAGTTCTTTGATTCAGCTAACAATGCAGCAAACACTGCTCAAGTTGTGTTCTATACCGAAGACGTAAACCTTGAAATGCCAGACACGCATCTTGTGTTTAAGCCTACTGTTACCAATGGAACAATTGCTTCTGAGTCGCTGTTGGGACAGCAAGCAGCGCCAAATAGAGCAAACTACATTGCATTCAGAGATAATTTTATTGGCCTGATGTATTACAACAGTACAGGCAACATGGGTGTATTGGCCGGGCAAGCTTCCCAACTTAACGCAGTAGTAGACCTGCAAGACAGAAATACAGAGCTGTCATACCAGTTAATGCTGGATGCTTTGGGAGACAGAACACGGTACTTTTCCTTGTGGAATTCCGCAGTGGACAGTTACGACCCTGACGTTCGCGTTATTGAGAATCACGGGGTAGAGGATGAACTACCAAATTATTGCTTTCCTCTTAGCGCAGTAGGTGAAATAAAAAATTACAAAGGCATTAAGCCAGATAACGGAGGAGGAGGTGGCTGGACTGCCGACAACACTGTCAGTGAAGCAAACCACATAGGCATTGGGAATATAGCCGCCATGGAAATTAATTTGCAGGCTAATTTGTGGAGAAGCTTCTTGTACTCAAATGTGGGCTTATACCTACCAGACGACTTAAAATACACTCCAGGAAACATAAAACTACCTGATAACAAGAACACCTACGAGTACATGAACGGGCGTGTGACTGCCCCGGGGTTGGTGGATACCTATGTCAATATCGGCGCTCGCTGGTCCCCAGATGTGATGGATAATGTAAACCCTTTTAACCACCACCGAAACGCAGGGTTGCGCTACAGATCCATGTTGCTAGGCAATGGGAGATTTGTTCCTTTTCACATTCAGGTGCCGCAAAAATTTTTTGCCATCAGAAATTTGTTGCTGTTGCCCGGTTCCTACACTTACGAATGGAACTTTAGAAAGGATGTAAACATGATTCTTCAGAGCACACTGGGAAATGATCTTCGGGTGGACGGAGCCAGCGTTCGCTTTGACAACATTGCCCTGTATGCTAACTTTTTTCCCATGGCACATAACACAGCTTCTACTTTAGAAGCCATGTTAAGAAATGACACCAACGACCAGTCTTTTAACGATTATTTGTGTGCTGCAAACATGCTGTATCCCATCCCAGCTAACGCCACCAGCGTGCCCATTTCAATACCTTCGCGAAATTGGGCGGCATTTAGAGGCTGGAGCTTTACTCGCCTAAAAACTAAAGAAACTCCTTCCCTGGGTTCAGGGTTTGACCCCTACTTTGTATACTCTGGAACCATTCCCTATTTAGACGGCACCTTTTACCTAAACCACACTTTTAAGAAGGTGTCAATCATGTTTGACTCCTCCGTGAGTTGGCCTGGAAATGACCGTTTGCTAACCCCAAATGAATTTGAAATAAAGCGTTCTGTGGATGGGGAGGGATACAATGTGGCCCAATGCAATATGACTAAGGATTGGTTCCTAATACAAATGCTTAGTCATTACAACATTGGATACCAAGGTTTTTACATTCCAGAGAGCTACAAGGACCGCATGTATTCTTTCTTTAGAAACTTTCAGCCCATGAGTAGGCAAGTTGTGGATACCACAGAATATAAGAACTACAAAAAAGTAACCGTAGAGTTTCAACATAACAACTCAGGATTCGTGGGATACCTGGGCCCCACTATGCGGGAGGGACAAGCTTACCCCGCCAACTATCCCTACCCTCTTATAGGCCAAACAGCTGTGGAAAGCATCACACAGAAAAAGTTTCTATGCGATCGTGTTATGTGGCGCATCCCATTTTCTAGTAACTTCATGTCTATGGGGGCGCTAACGGATCTTGGGCAAAATATGCTGTACGCAAACTCAGCCCATGCTCTAGACATGACATTTGAGGTGGATCCAATGGATGAGCCTACCCTTCTTTATGTTTTATTTGAAGTTTTCGACGTGGTACGCATTCACCAGCCACACCGCGGCGTCATTGAAGCGGTCTACCTGCGCACGCCCTTCTCGGCGGGTAACGCTACCACCTAAGAAGGCACCCTCCCAGACTGCTGTAATGGGTTCAAGCGAACAGGAGCTGACGGCCATTGTTCGAGATCTAGGCTGTGGACCCTATTTTTTGGGAACCTTTGACAAACGTTTTCCGGGTTTTGTGTCTCGCGACCGCTTATCATGTGCTATTGTTAACACTGCCGGTCGCGAAACTGGGGGCGTACACTGGCTGGCTTTTGGATGGAACCCCAAATCGCACACTTGCTATTTATTCGATCCATTTGGATTTTCTGATCAACGACTAAAACAAATCTATCAGTTTGAGTACGAAAGTCTGTTGCGCCGTAGTGCGCTAGCGGCCACTAAAGACCGATGCGTTACCCTAGAAAAGTCAACCCAAACTGTACAAGGACCGTTTTCTGCAGCGTGCGGCCTGTTTTGTTGTATGTTCTTACACGCTTTTACTCACTGGCCTGACCATCCAATGGATAAAAATCCCACTATGGACCTACTTACTGGGGTGCCTAATTGTATGCTACAAAGTCCTCAGGTAGTGGGCACATTGCAACGCAATCAGAATGAATTGTATAAATTCTTAAACAATCTGTCCCCTTACTTTCGTCACAACCGCGAGCGCATAGAAAAAGCTACATCTTTTACTAAAATGCAAAATGGACTCAAATAAACGTGTACACAATGCATTAATAATAAAACCATTTTATTAGCTCATTGGAGTACAAGCTTGACTGTTTTATTAAAAATCAAATGGCTCTTCGCGACAGTCGCCGTGGTTGGTGGGCAGGGATATGTTTCTGTACTGCAAACGCTGATGCCACTTGAATTCTGGAATAACAAGCCTAGGGGGGGAGCCGTCAAAATTTTCTCCCCACAGCTGGCGCACAAGTTGCAGGGCGCCCATAACATCAGGAGCAGAAATCTTGAAGTCGCAATTAGGGCCAGCATTGCCGCGCGCATTGCGATAAACTGGATTTGCGCACTGAAAAACCAACAAACACGGATACTTAATACTGGCTAACGCTCCAGGGTCGGTTACTTCGTTGATATCAATGTTATCCACATTGCTGAGGTTAAAAGGAGTGATTTTACACAGTTGACGCCCCATCCGTGGCAGGCCATCTTGCTTGTTTAAACATTCGCAGCGCACTGGCATAAGGAGACGTTTTTGCCCATGTCGCATGTGAGGGTAGTCGGCCAGCATAAAAGCTTCAATTTGCCTAAAAGCTATTTGAGCCTTCATTCCTTCAGAATAAAACAAGCCGCAGGACTTTCCGGAGAAAGAATTATTCCCGCAGCCAACATCATGAAAACAGCAGCGGGCATCGTCGTTTTTAATTTGAACTACATTACGCCCCCAGCGGTTTTGCGCCACCTTGGCTTTCGAGGGGTTCTCTTTCAACGCTCGTTGCCCACTTTCGCTGGTTACATCCATTTCCACCAAATGCTCTTTGCGCACCATCTCCATTCCATGCAGGCATCTAAGCTCCCCTTCGCGCTCGGTACACTTATGCTCCCACACGCAGCAACCGGTGGGTTCCCAGGAATTCTGTTGGACACCGGCATAAGCTTGCATATATCCTTGCAAAAAGCGTCCCATGAGCTCCTGAAAGGTTTTTTGGGATGAAAAAGTCAGCTGCAAACCGCGCTTTTCTTCGTTGAGCCATGTTGTGCATATTTTCTTGTACACGCTGCCCTGATCCGGCAAAAAACGAAAGGTGGCGCGCTCGTCGTGATCCACATGGTACTTTTCCATTAGCATAGCCATGGCTTCCATGCCTTTTTCCCAAGCTGAAACTAGGGGCTGGCTTGCCGGATTGCGAACAACAACAACATTCTTTTCATTTTCGTCGCTGTTTTGAGCGGAAGCCTTCAAAACGTGTACCTGCCTGGTTTCCATTTTTTGAAAAGACTGAGAACCGTCTGCATGATGCATAATGCGGACGGGCGGCATGCTGAAACCCATTACTCCTAAAACTGCTCTTGGTGGTTCTGCCTCTTCTTCTTCTGCACTCTCTGGGGAAAGAGGTATCGCAGCCATAGATTTCTTGACTTTTTTCTTTGGAGGTAAAGGCACAGCTTCCAGTTCTTCTTCGCTTTCGGAATCCAGAAAGTATCTGCCCATTTTTGGCGGCGGCGGCTGAGCGCTGCGGTCTGGGGTGCGCTCCCTCTGTGAGTGCTGATTGCTGGCCATTATTTAATCCTAGGCAAAGAAACACATGATGGATCTGGAGCCACAGGAAAGCTTAACCGCCCCCACCGCTCCCGCCATTGGCGCTACGGCTGTCATGGAGAAGGACAAAAGTCTACTCATACCCCAAGACGCACCGGTTGAGCAGAACTTGGGCTACGAGACTCCCCCCGAGGAATTTGAAGGCTTTCTTCAAATCCAAAAGCAACCAAATGAGCAAAACGCTGGGCTCGAGGACCATGACTACCTAAACGAGGGAGATGTCCTGTTTAAACATCTACAGCGACAAAGCACTATCGTTCGCGACGCCATATCTGATCGCTCTTCAATACCAGTTTCAATTGCAGAACTATCTTGCATCTACGAACGCAACCTGTTCTCCCCACGTGTGCCCCCTAAACGGCAAGCCAACGGCACATGCGAGCCAAATCCTCGCCTTAACTTCTACCCAGTTTTTGCAGTGCCAGAAGCACTGGCAACATACCATATTTTCTTTAAAAATCACAAAATACCCCTATCCTGTCGAGCTAACCGCAGCCGCGCAGATGAGCTTCTTGCTTTAAGGGCTGGCGCTTCCATACCTGGGATTGTGTCCTTGGAAGAGGTGCCTAAAATTTTTGAAGGTTTAGGTCGGGATGAAAAACGAGCAGCAAATGCCCTGCAAAAAGAAAATGAACAAAATCACCATGGGAATAGTGCTCTAATAGAACTGGAAGGTGACAATGCCCGCCTGGCAGTTTTAAAGCGCAATATTGAGGTTACTCACTTTGCCTACCCGGCAGTAAATCTTCCGCCAAAGGTAATGAGCGCAGTGATGAATCAGCTACTAATTAAGCGAGCCCAACCCATTGACAAAGATGCAAACTTGCAAGACCCGGAGGCAACAGATGATGGAAAGCCGGTTGTAAGCGACGAGCAATTAACTAAGTGGTTGGGAACAGACAATTCCAACGAACTACAACAGCGGCGTAAACTCATGATGGCCGCCGTACTTGTAACTGTGGAACTCGAGTGCATGCATCGTTTTTTCTCCGACATCACCACATTGCGCAAAATTGAGGAATGTCTTCACTACACTTTCCGCCATGGCTACGTGCGCCAAGCCTGTAAAATTTCTAATGTGGAGCTGAGCAATCTAGTTTCTTACATGGGCATCTTGCATGAAAACCGATTGGGACAGAACGTGCTACACTCAACACTACGCGATGAAGCACGCAGAGATTACGTGCGAGACTGCATTTACCTTTTCCTGTTACATACCTGGCAAACTGGGATGGGTGTTTGGCAGCAATGCTTGGAAGAAAAAAACCTTCGAGAACTAAACAAACTGTTAGACAGAGCACTAAAATCCCTATGGACCGGTTTTGACGAACGGACAGTAGCTGCAGAGCTAGCTGACATAATTTTCCCAGAAAGGTTAATGATAACCTTGCAAAACGGCTTGCCTGACTTTATGAGTCAAAGTATGCTGCACAATTATCGCTCTTTTATATTAGAGCGTTCTGGGATGCTTCCTAGCATGTGTTGTGCACTTCCTTCAGATTTTGTGCCTATATATTTTAGAGAGTGCCCCCCTCCCCTGTGGAGCCACTGCTACTTACTACGACTTGCTAACTACCTAGCTTACCACTCAGACCTTATGACAGATTCAAGCGGCGAAGGCCTAATGGAGTGTCACTGCCGCTGCAATCTTTGCACCCCCCACCGTTCTTTGGTTTGCAATACTGAACTATTAAGTGAAAGTCAAGTCATTGGTACCTTCGAAATGCAGGGACCGCAGTCTGACAGCAATTTCACGACGAACCTAAGACTTACCCCTGGGCTTTGGACTTCTGCCTACCTGCGCAAATTTGAACCCCAAGATTACCACGCCCACAGTATCAATTTTTACGAAGACCAATCCAAACCCCCAAAAGCGCCACTAACGGCTTGCGTCATTACGCAGGGAAAAATTCTAGCCCAATTGCATGCTATTAAGCAAGCGCGCGAAGAGTTTTTACTTAAAAAAGGACACGGAGTGTACCTTGATCCCCAAACCGGCGAGGAACTAAACCTTCCATCACCTTTGTGTGCTACTGCGTCTCCCCATTCGCAGCATGTCCCCGAAAGCCGCAAAACAGGCTATTGCGCAGCAACGCTCAAAGAAACAGCAGCAACGGCAGGAAATCTGGGAGGAAGAATCTTGGGAGAGTCAGGCAGAGGACGAGGTCGAGGACTTGGAAGAATGGGAGGAGGAGGAGGCGGACAGCCTAGACGAGGATCCAGAGGAGGAGGAGGAAGGTTCCAAGGACGGAGCGACCGCCGCCAAACCGTCGCTTTCAACCAAGCCCTCTCCAATGAAACCCGCTGTGAGCAAATCTCAGAAAGCCAACCGTAGATGGGACACCATTGAAACCAGCGCCGCAAACTTGGGTAAGAATCGCAAGCAGGCGCGTCGGGGCTACTGCTCATGGCGGGCTCACCAAAGTAATATTGTAGCCTGCTTTCAGCACTGCGGGGGGAATATCTCATTTGCAAGGCGGTATTTGCTATACCATGATGGAGTGGCGATTCCAAGGAATGTCCTCCATTACTACCGTCATCTCTACAGCCCCTTTGAAGAGCTCGACAAGGAACCGACCTGCAACAGCCAAGCGGCCCACTAGAATCGGCAACAGCAGCAACAAGGAAAGTCCTGAGGCGCGCGAGTTAAGAAAACGCATTTTTCCCACTTTATATGCTATTTTTCAGCAGAGTCGAGGTCAAGAACACGAACTGAAAATAAAAAACCGTTCCCTGCGTTCACTTACCCGCAGCTGTCTCTACCTCAAAAGCGAAGATCAGTTGCAACGCACCTTGCAGGACGCAGAAGCTCTGTTCAATAAATACTGCTCCCTCTCGCTTAAAGAGTAAAAAAAGCCCGCGCGCGGACTTTCAACAGGCGGGAAAAGTGACGTCACAACAAGATGAGTAAAGATATTCCCACGCCTTACATGTGGAGCTTTCAACCCCAAATGGGACTGGCGGCCGGCGCGGCTCAAGACTATTCTAGCAAAATGAATTGGTTAAGCGCCGGACCCCACATGATTTCCAGGGTGAATGGGGTACGAGCCCGGCGTAACCAAATACTGCTAGAACAAGCCGCTCTCACCGCTACACCACGTAATCAACTTAACCCTCCCTCTTGGCCAGCTGCCCTGATATATCAGGAAAATCCCCCTCCTACCACTGTACTTTTGCCTCGCGACGCCCAGGCCGAAGTCCATATGACTAACGCTGGGGCACAGCTTGCGGGCGGTGCACGTCACAGTTTCAGGTATAAAGGTCGCACTGAGCCCTATCCGTCTCCAGCTATAAAAAGAGTACTCATCAGAGGGAAAGGTATTCAGCTGAACGACGAAGTCACATCGCCATTGGGAGTCAGACCCGACGGAGTGTTTCAGCTCGGAGGGTCCGGACGTTCCTCCTTTACCGCTCGTCAAGCCTACCTGACACTACAGAGCTCATCCTCAGCTCCGAGATCTGGTGGTATTGGAACTCTCCAATTTGTGGAGGAATTTACTCCATCTGTTTACTTCAATCCTTTTTCGGGCTCGCCTGGACACTATCCTGACGCCTTCATACCCAACTTTGACGCAGTGAGTGAATCTGTGGATGGCTATGATTAATGTCTAATGGAGCGGCTGACAGAGCGCGGCTGCGACATTTAGACCACTGTCGCCAACCTCACTGCTTTGCTCGAGACATCTGTGTCTTTACCTACTTTGAGCTTCCAGAGGAGCACCCCCAGGGGCCAGCTCACGGTGTCAGAATAACAGTTGAAAAAGGAATTGATACACACCTCATTAAATTTTTCACCAAACGCCCGCTATTGGTGGAAAAAGATCAAGGAAATACTATATTAACTTTATATTGCATTTGTCCTGTTCCCGGATTACATGAAGATTTCTGCTGTCATTTGTGTGCTGAATTTAATCATCTGTAGTGGCGCTGTACCGCCTGAAGAAGAACCTAACTGTCATCCGCATTTAAGCAACATTAAAATCAACCTTTCGATCCCTCATATCACTCTTCGCTGCAGTTTTTTTTCCACACATCTCACCTGGACCTTTAACGGAAAACACGTTACCAATACAGATATAAAGTTTAAACTACACAAAGAAAACATCACTCTATTTCAACCTATTAACCTGGGATACTACCGCTGCTCAGCTCCACCCTGTACGCAAGCATTTTTTGTTGCTCCAGTTATTGACAAACGCCCTGCTCCGACAACAGCTGCTGTCACTGAGCACATCACCGAGGCAGTTTCTCCTTCTAAAGGTACAGAGGAAATTGTGTACTTTTCAAACTTTACAAACCACTTAGTTTTAAATTGTTCCTGTTCTAACTCCTTAATTTCATGGTTTGCTAACAGCTCTCTGTGCAAAACTTTCTACCAAGGAAAACTTTTGTATTCTGCTAAACTCACATTGTGTAACCAGAGCACCCCTTCCCACCTTACTCTATTGCCACCTTTTGTTGCCGGTCGTTACTTTTGCATAGGAGCTGCACGTACTAGCCCCTGTCAACAGCATTGGAATTTAACTTACTGTCCCCCACCAGTGTCGCCCTTTGTGATCAATACTGAATATTTAGACTATAATCCCTTGCTTGCTTACGGCGGTCTCGCAGCTCTTATTTTATTCCTGATTTCTAACTTGTTTCTAGTGCAACATTTGTATTCATACTAACAATGCTTTCCATTTTTCTTTTATTTCTCTTTTCTTTACCTTCTGGCTTGTATGCTCAAACAGCCGAAAGACCACTAAAAGTCGTGGTGGAAGCTGGCCATAATGTAACCCTTCCCCACCTTTCTGGTTCACACCAAACTGGCCATGTTACTTGGCTAGTAGAGACATCAGATTATGGTTCAGCTTCTCCAGACAACTTCATTTTCAGTGGACAAAAACTATGCCAGTTTACTGACAGAACCATGGTGTGGCCTTATTACAATTTACATTTTAACTGTGAAAATTATGACCTTAATCTGTTTTGGCTTAAGGTGGAAAATTCGGCTATTTACAACGTTAAAAATACAGTCAATGCTTCTGAAACAAATATTTACTATGATTTAAGAGTAGTACAAATTTTTCCACCTAAATGCATCATTACTTCAAAGTACCTTACAAACGATTATTGTCACATTACAATTAACTGCACTAACTCTGATTACCCCAATAAGGTTGTGTTTAATAATGTCAGTCGATGGTACTACGGATACGGTAAGGGCAGCCCAACCCTTCCCAACTACTTTATAACTAACTTTAATGTTTCAGGTATTACTAAAAGCTTTAATCACACTTACCCTTTTAATGAGCTCTGTGATTATCCCACATCCCAATCTCAACACAGTTTAACACATACAGTAAGCACAGTAATCTTTTTAGGAATAATTGGCTTCAGCATTTTGATTATTATAGCAGCCTTTATTTATCTGTGCTGGCATAGAAAATCTTTGTGTGTTTCTAAAACAGAACCTCTTATGCCGATTCCTTACTAGTTTTCTTTTTTCTTACAGTATGGTGACGGTTCTTCTCATCTTTTTATGCCTGCCAGTCATTTTTTCTTCTTCGACTTTTGCCGCAGTCAGTGACCTTGATCCCGAGTGTTTAGCCCCCTTTGCGGTGTACCTGATTTTCACATTTGTGACTGCTACCTGCGTCTGCAGTATTATTACTCTGCTAATCACCTCGCTCCAATTTTTTGATTACTACTACGTGAGAATTGTTTACCGCAGACACCACCCCCGTTACCAAAACCCTCAAATTGCGGCTCTTTTGCAGCTCCAACCATGAAAACAGCATTAGTTCTTTTCTTTATGTTAATCCCAGTTTGGGCTAGTTCTTGTCAACTACATAAACCATGGAATTTTTTAGATTGTTATACTAAAGAAACAAACTACATAGGCTGGGTTTATGGAATTATGTCTGGCTTAGTATTTGTCTCCTCTGTAGTTTCTTTACAACTGTATGCGCGCCTTAATTTTAGTTGGAATAAGTATACTGATGATCTTCCCGAATATCCAAACCCCCAGGATGATTTACCCCTAAATATTGTATTTCCAGAGCCCCCGCGTCCTCCTTCTGTTGTTAGCTATTTTAAGTTCACCGGTGAAGATGATTGAACCTGATCTAGAAATTGATGGAAGAATCACCGAACAGAGGCTCCTCACTGATCGCGCTAGGCGACGCCAACAGGATCAAAAAAATAAAGAGTTAATTGATTTACAAACCGTGCATCAGTGTAAAAAAGGACTTTTTTGCCTGGTAAAACAAGCTACCCTTCGCTATGAATCTTTACCAGGCAAAGAACATCAACTGTGCTACACGCTGCCCACTCAGCGACAAACCTTTACTGCAATGGTGGGCTCGGTACCTATTAAAGTGTCCCAACAAGCAGGAGAACAAGAAGGCTCTATTCGGTGCCTATGTGATAACCCTGAATGTTTGTACACTTTAATAAAAACACTGTGCGGTTTAAGAAATCTTTTACCAATGAATTAAATAAATTACTTACCGGAAATCTGAAAATACATCATGGTCTCCGTGTACTCTTATAAAATTTCCCTCTTCCCAACTGTCAAACCTGACAGACTTGCAAACAGCAAACTTTCTCCAAATCTTAAATGGAAGGTCAGATTCTTCTTCCCAATCCCTACCCACCATCTTCATCTTTTCTAGATGAAGCGCAGCAGAACCCAGTATGCTGAAGAAACAGAAGAAAATGATGACTTCAACCCCGTTTACCCTTTTGACCCATTTGACACATCAGACGTACCCTTTGTTACACCCCCTTTTACTTCTTCCAATGGTCTTCAAGAAAAACCACCAGGTGTATTAGCACTTAATTACAAAGACCCCATTGTAACTGAAAATGGAACCCTTACACTCAAGCTAGGGGACGGAATAAAACTTAATGCCCAAGGTCAACTTACAGCTAGTAATAATATCAATGTTTTGGAGCCCCTTACCAACACCTCACAAGGTCTTAAACTTTCTTGGAGCGCCCCCCTAGCAGTAAAGGCTAGTGCCCTCACACTTAACACAAGAGCGCCCTTAACCACAACGGATGAAAGCTTAGCCTTAATAACCGCCCCTCCCATTACAGTAGAGTCTTCGCGTTTGGGCTTGGCCACCATAGCCCCTCTAAGCTTAGATGGAGGTGGAAACCTAGGTTTAAATCTTTCTGCTCCCCTGGACGTTAGTAACAACAATTTGCATCTCACCACTGAAACTCCCTTAGTTGTAAATTCTAGCGGTGCCCTATCTGTTGCTACTGCAGACCCCATAAGTGTTCGCAACAACGCTCTTACCCTACCTACGGCAGATCCGTTAATGGTGAGCTCCGATGGGTTGGGAATAAGTGTCACTAGTCCCATTACAGTAATAAACGGTTCCTTAGCCTTGTCTACAACTGCTCCCCTCAACAGCACAGGATCCACTTTAAGTCTGTCTGTTGCCAATCCTCTGACTATTTCACAAGACACATTGACTGTTTCCACTGGTAACGGTCTTCAAGTGTCGGGGTCTCAATTAGTAACAAGAATAGGGGATGGTTTAACATTCGATAATGGGGTCATGAAAGTAAACGTTGCCGGGGGAATGAGAACTTCTGGCGGTAGAATAATTTTAGATGTTAATTATCCCTTTGATGCGAGCAATAACCTGTCCTTAAGACGGGGATTGGGACTAATTTATAACCAATCTACAAACTGGAACTTAACAACTGATATTAGTACCGAAAAAGGTTTAATGTTTAGTGGCAATCAAATAGCTCTTAATGCAGGTCAGGGGCTTACATTTAATAATGGCCAACTTAGGGTTAAGTTGGGAGCTGGACTTATTTTTGATTCAAACAATAACATTGCCTTAGGCAGCAGCAGCAACACTCCATACGACCCTCTGACACTGTGGACAACTCCTGACCCACCACCAAACTGCAGCCTCATACAAGAGCTAGATGCAAAACTCACCCTGTGCTTAACAAAAAACGGATCTATTGTTAATGGCATTGTAAGTTTAGTGGGTGTTAAGGGTAATCTCCTAAATATCCAAAGTACTACTACCACTGTAGGAGTGCATTTAGTGTTTGATGAACAGGGAAGATTAATCACATCAACCCCTACTGCCCTGGTTCCCCAAGCTTCGTGGGGATATAGACAAGGCCAATCAGTGTCTACCAATACTGTTACCAATGGTCTAGGTTTTATGCCTAATGTGAGTGCTTACCCTAGACCAAATGCCAGTGAGGCTAAAAGCCAAATGGTAAGTCTCACGTACTTACAGGGAGATACATCTAAACCTATAACAATGAAAGTTGCATTTAATGGCATTACGTCGCTAAATGGATACTCTTTAACATTCATGTGGTCAGGTCTATCAAACTATATAAATCAGCCTTTCTCTACACCATCCTGCTCCTTTTCTTACATTACCCAAGAAT

ATACGCGTGGTTAAACTGCCTCCTTCCCATTTTACCTTGTATACCTCCCTTTCCCCCTTTGTAGCTGAAAACAACTGCACTTGAATATTTCGACTTAGGTTTTTTGGCGTTAGCGTCCACACAGTTTCTTTACGGGCAAAGCGAGGGTCGGTGATGGAAACGAATCCCTCGCCCGCACAGTCACTCAAGCGGCATTCCCCATCCAAAACCAGGTCCATGATTTTATCCTACAAAAAGTAACAACAGTCAGTGTCCATCAGCCGCCCAAGGATTCTCTCGTTGATTATAATCTCCAAATAAAATTGCTCGATGATGCATAATTAAACCCTTTAGCAGTTGCTGACGATAACGTTCATGCCGACTATGTTTTAGAGGGCGAACAGTGTTTTCAGCAATTACTTGAACAACTTTTAACATTAGCAGTCTGGTACGACGAGCGCAACAGCGCATGCGTATCTCACTTAAGTCTTTACAATAATCACAACACAGCACTAACATGTTATTTAAAATTCCATAATTAAAGGCGCTCCATCCAAAACTAACTTTTTCTAACGCTAACCAGGCATGGCCATCATACATAATTTTAAAGTAAATTAAATGGCGACCTCTAACAAAGGTGCTTCCCACATACATCACCTCTTTAGGCATTAAATGGTTAACAACCTCCCGATACCAAAAACACCTTTTGTTAATTAAGGCGCCATATACGGCCATTTTGAACCAGCGTCCCAAAAGCATCCCAGCTGACATACACTGTAGTGAACCCGGACGCTGGCAATGACAATGAATAAGCCACCGCTCATGACCATGTAATAATTGAGTAACTTCAACATTTATAGTGGCACAACACATACATACACTCATGTATTTTTTCAAAATAAACATCTCATAATCAGTTAGAATCATATCCCACGGTATTGGCCATTCCTGCAGCACTGTAAAACCTACACATGAAGGAATGCCTCTTACCTCACTTACATTATGTAAAGTCAGACTATTACACTCAGGCCATAAAGAATTTTCCGAAGTACTCAACGTAGCTTTTGACTGTTCCTCACAGGGCGGTAGTTGGTACTTGTTGTATGGTGCCAATCTGTAGCGATACCGTCTGTCGCGCTGCATCGTAAACAACAGACTTGCGAGCGTCTTCGTACTTAAAAAAACAAAACCACGTACGACCACTGGTTATCGCACCTCGTCCTTTTTGTTTGCAGCGTTGGCGTTCCGTCAAAAAAGCAAAGTACAACCACTCTCGCAGGCTTGCTAAAATGTATTCAGCTTCAGGTGTTATCTTCAAATCATGATGTTTAATAAAGCGCAGAGTATCCACACAGGATGCATGGGCTAAACCAAGCCATGCTATGCAGGCAGCCGTGTCCCGACTTACAGGAGGAGGAGGAATACAAGGTAGAGGCATAAAAACTTAATCAAGACGGTCAGCAAGGATTTGAATGCGTAAATCTCGCAGGTGGCAGCGATCGCCTCCGCTGTGCTGGTGAAAGATCACAGCCAGATCAAATTGTAAGCGATTTTCCAAATGTTCAACAACAGCTTCTAAAAGAGCCACAGCTCTGATTTCGATAAACAAAAGCAAAGCAAATGCATTATCATGAAACTCTTCTATCATCAAACTGCCTGACTGAACCATTCCCAGGTAATTTTCATTCTTCCACTGTTGTATTATTTGAACACACTGATTTTGCAGGTTTAAACCGTGAATATTAAAAAGCTCTGTAAGGGCGCCCTCCACCGCCATCCGCAGGCAGTACTTCATATTTGCTGAAAAAAGTCTGGATCTTCAAACACCTGCAGTAAATTCAGTAGATTTACATTAGGCTCCACACCTTGGTCTCGCAGCTGACATCTTAATGCCAGTTGTATAAAATCATACAAATCAGAAGCCAGCAGCAAAGAAAGTTCACCTCCAGGTACAAGTTCCGGAGTTCCCACAGAACATACAACTTGCACAAATGGACCCATATTAGTAAGCGTGGCGCCAACGTAGACATCGCGCATAGGAGGAGTTAAATAATGCATTACCAGCAGCCAAAACTCAGGTAGCACGTCTTTAAGAAACGTCACCACCTCAAAATCTAAGCCATGCAAATAGTTCCGTAAAGACTCCGGAAACAACACGGAGTAATGAACAAGCGACCTCTGAAACATGCTTTAGGTTAGCCTGAAAAATAAAAATATGTTAAATTAAAGATGCCTGGCAAACGGGTGGAAAAACAACTCTACTTAAAAGCAAGCGCGCGACTGGCTGCTTTGCGCGAACATCGCAAAACACGTCGGAATGATTAAACAACAAAACACTGAGCTCCATTCTTGAGCCTGGATAAAGCGTTTCAGCGCCAACAAAAACCCCTCTGGCGTTCATGTCGCATAATGAAAACAATGTTCCCAAATATCCAGGAGGAATATCAACTGCTATGTGCAAATATAAAAGCACAACTCCATGTGGAGGTATAACAAAATTCGCAGGAGAAAATAACACATAAGCATTAGAGTCGCCCTCTTGTTTAGGCAACATAGCCCCAGGTCCCGTAAAATACACATAAAGAGTCTCAAAAGCAGCCATAATGCCTTACCAGAAAAACAGTACAAAGCCAGGCACAGCAGACACAATCTGCCGCAAGTGCGCACCTTTAATACTGAAAAATAGTGACGTAAATGGCCAAAGTTCGCCTACACAACACAAAAAAAACCCCAAAAGCCCGCGAAAAAAATCACTTCCGCATATGACTCGGCATAATACGGTGTTCTCACGACACGTCACATCCGGCGCGCCCGGCTCCCACGCCGCGCCCCACTTCCTCATCCGCCCAAACTTACAAGCACGCCAAAGCCACACCTCCACCCAATCAAATTACACACTACGCCCACTTCATTTTAATATTGGCACTAGTCCAGTATAAGGTATATTATTAGATAGG

>ADENOVIRUS_C_STRIPPED4

CATCATCAATAATATACCTTATTTTGGATTGAAGCCAATATGATAATGAGGGGGTGGAGTTTGTGACGTGGCGCGGGGCGTGGGAACGGGGCGGGTGACGTAGTAGTGTGGCGGAAGTGTGATGTTGTAAGTGTGGCGGAACACATGTAAGCGCCGGATGTGGTAAAAGTGACGTTTTTGGTGTGCGCCGGTGTACACGGGAAGTGACAATTTTCGCGCGGTTTTAGGCGGATGTTGTAGTAAATTTGGGCGTAACCAAGTAATATTTGGCCATTTTCGCGGGAAAACTGAATAAGAGGAAGTGAAATCTGAATAATTCTGTGTTACTCATAGCGCGTAATATTTGTCTAGGGCCGCGGGGACTTTGACCGTTTACGTGGAGACTCGCCCAGGTGTTTTTCTCAGGTGTTTTCCGCGTTCCGGGTCAAAGTTGGCGTTTTATTATTATAGTCAGCTGACGCGCAGTGTATTTATACCCGGTGAGTTCCTCAAGAGGCCACTCTTGAGTGCCAGCGAGTAGAGTTTTCTCCTCCGAGCCGCTCCGACACCGGGACTGAAAATGAGACATATTATCTGCCACGGAGGTGTTATTACCGAAGAAATGGCCGCCAGTCTTTTGGACCAGCTGATCGAAGAGGTACTGGCTGATAATCTTCCACCTCCTAGCCATTTTGAACCACCTACCCTTCACGAACTGTATGATTTAGACGTGACGGCCCCCGAAGATCCCAACGAGGAGGCGGTTTCGCAGATTTTTCCCGAGTCTGTAATGTTGGCGGTGCAGGAAGGGATTGACTTATTCACTTTTCCGCCGGCGCCCGGTTCTCCGGAGCCGCCTCACCTTTCCCGGCAGCCCGAGCAGCCGGAGCAGAGAGCCTTGGGTCCGGTTTCTATGCCAAACCTTGTGCCGGAGGTGATCGATCTTACCTGCCACGAGGCTGGCTTTCCACCCAGTGACGACGAGGATGAAGAGGGTGAGGAGTTTGTGTTAGATTATGTGGAGCACCCCGGGCACGGTTGCAGGTCTTGTCATTATCACCGGAGGAATACGGGGGACCCAGATATTATGTGTTCGCTTTGCTATATGAGGACCTGTGGCATGTTTGTCTACAGTAAGTGAAAATTATGGGCAGTGGGTGATAGAGTGGTGGGTTTGGTGTGGTAATTTTTTTTTTTAATTTTTACAGTTTTGTGGTTTAAAGAATTTTGTATTGTGATTTTTTAAAAGGT

CAAGACCTACCCGGCGTCCTAAATTGGTGCCTGCTATCCTGAGACGCCCGACATCACCTGTGTCTAGAGAATGCAATAGTAGTACGGATAGCTGTGACTCCGGTCCTTCTAACACACCTCCTGAGATACACCCGGTGGTCCCGCTGTGCCCCATTAAACCAGTTGCCGTGAGAGTTGGTGGGCGTCGCCAGGCTGTGGAATGTATCGAGGACTTGCTTAACGAGTCTGGGCAACCTTTGGACTTGAGCTGTAAACGCCCCAGGCCATAAGGTGTAAACCTGTGATTGCGTGTGTGGTTAACGCCTTTGTTTGCTGAATGAGTTGATGTAAGTTTAATAAAGGGTGAGATAATGTTTAACTTGCATGGCGTGTTAAATGGGGCGGGGCTTAAAGGGTATATAATGCGCCGTGGGCTAATCTTGGTTACATCTGACCTCATGGAGGCTTGGGAGTGTTTGGAAGATTTTTCTGCTGTGCGTAACTTGCTGGAACAGAGCTCTAACAGTACCTCTTGGTTTTGGAGGTTTCTGTGGGGCTCCTCCCAGGCAAAGTTAGTCTGCAGAATTAAGGAGGATTACAAGTGGGAATTTGAAGAGCTTTTGAAATCCTGTGGTGAGCTGTTTGATTCTTTGAATCTGGGTCACCAGGCGCTTTTCCAAGAGAAGGTCATCAAGACTTTGGATTTTTCCACACCGGGGCGCGCTGCGGCTGCTGTTGCTTTTTTGAGTTTTATAAAGGATAAATGGAGCGAAGAAACCCATCTGAGCGGGGGGTACCTGCTGGATTTTCTGGCCATGCATCTGTGGAGAGCGGTGGTGAGACACAAGAATCGCCTGCTACTGTTGTCTTCCGTCCGCCCGGCAATAATACCGACGGAGGAGCAACAGCAGGAGGAAGCCAGGCGGCGGCGGCGGCAGGAGCAGAGCCCATGGAACCCGAGAGCCGGCCTGGACCCTCGGGAATGAATGTTGTACAGGTGGCTGAACTGTTTCCAGAACTGAGACGCATTTTAACCATTAACGAGGATGGGCAGGGGCTAAAGGGGGTAAAGAGGGAGCGGGGGGCTTCTGAGGCTACAGAGGAGGCTAGGAATCTAACTTTTAGCTTAATGACCAGACACCGTCCTGAGTGTGTTACTTTTCAGCAGATTAAGGATAATTGCGCTAATGAGCTTGATCTGCTGGCGCAGAAGTATTCCATAGAGCAGCTGACCACTTACTGGTTGCAGCCAGGGGATGATTTTGAGGAGGCTATTAGGGTATATGCAAAGGTGGCACTTAGGCCAGATTGCAAGTACAAGATTAGCAAACTTGTAAATATCAGGAATTGTTGCTACATTTCTGGGAACGGGGCCGAGGTGGAGATAGATACGGAGGATAGGGTGGCCTTTAGATGTAGCATGATAAATATGTGGCCGGGGGTGCTTGGCATGGACGGGGTGGTTATTATGAATGTGAGGTTTACTGGTCCCAATTTTAGCGGTACGGTTTTCCTGGCCAATACCAATCTTATCCTACACGGTGTAAGCTTCTATGGGTTTAACAATACCTGTGTGGAAGCCTGGACCGATGTAAGGGTTCGGGGCTGTGCCTTTTACTGCTGCTGGAAGGGGGTGGTGTGTCGCCCCAAAAGCAGGGCTTCAATTAAGAAATGCCTCTTTGAAAGGTGTACCTTGGGTATCCTGTCTGAGGGTAACTCCAGGGTGCGCCACAATGTGGCCTCCGACTGTGGTTGCTTCATGCTAGTGAAAAGCGTGGCTGTGATTAAGCATAACATGGTGTGTGGCAACTGCGAGGACAGGGCCTCTCAGATGCTGACCTGCTCGGACGGCAACTGTCACTTGCTGAAGACCATTCACGTAGCCAGCCACTCTCGCAAGGCCTGGCCAGTGTTTGAGCACAACATACTGACCCGCTGTTCCTTGCATTTGGGTAACAGGAGGGGGGTGTTCCTACCTTACCAATGCAATTTGAGTCACACTAAGATATTGCTTGAGCCCGAGAGCATGTCCAAGGTGAACCTGAACGGGGTGTTTGACATGACCATGAAGATCTGGAAGGTGCTGAGGTACGATGAGACCCGCACCAGGTGCAGACCCTGCGAGTGTGGCGGTAAACATATTAGGAACCAGCCTGTGATGCTGGATGTGACCGAGGAGCTGAGGCCCGATCACTTGGTGCTGGCCTGCACCCGCGCTGAGTTTGGCTCTAGCGATGAAGATACAGATTGAGGTACTGAAATGTGTGGGCGTGGCTTAAGGGTGGGAAAGAATATATAAGGTGGGGGTCTCATGTAGTTTTGTATCTGTTTTGCAGCAGCCGCCGCCATGAGCGCCAACTCGTTTGATGGAAGCATTGTGAGCTCATATTTGACAACGCGCATGCCCCCATGGGCCGGGGTGCGTCAGAATGTGATGGGCTCCAGCATTGATGGTCGCCCCGTCCTGCCCGCAAACTCTACTACCTTGACCTACGAGACCGTGTCTGGAACGCCGTTGGAGACT

CGCGGGATTGTGACTGACTTTGCTTTCCTGAGCCCGCTTGCAAGCAGTGCAGCTTCCCGTTCATCCGCCCGCGATGACAAGTTGACGGCTCTTTTGGCACAATTGGATTCTTTGACCCGGGAACTTAATGTCGTTTCTCAGCAGCTGTTGGATCTGCGCCAGCAGGTTTCTGCCCTGAAGGCTTCCTCCCCTCCCAATGCGGTTTAAAACATAAATAAAAACCAGACTCTGTTTGGATTTGGATCAAGCAAGTGTCTTGCTGTCTTTATTTAGGGGTTTTGCGCGCGCGGTAGGCCCGGGACCAGCGGTCTCGGTCGTTGAGGGTCCTGTGTATTTTTTCCAGGACGTGGTAAAGGTGACTCTGGATGTTCAGATACATGGGCATAAGCCCGTCTCTGGGGTGGAGGTAGCACCACTGCAGAGCTTCATGCTGCGGGGTGGTGTTGTAGATGATCCAGTCGTAGCAGGAGCGCTGGGCGTGGTGCCTAAAAATGTCTTTCAGTAGCAAGCTGATTGCCAGGGGCAGGCCCTTGGTGTAAGTGTTTACAAAGCGGTTAAGCTGGGATGGGTGCATACGTGGGGATATGAGATGCATCTTGGACTGTATTTTTAGGTTGGCTATGTTCCCAGCCATATCCCTCCGGGGATTCATGTTGTGCAGAACCACCAGCACAGTGTATCCGGTGCACTTGGGAAATTTGTCATGTAGCTTAGAAGGAAATGCGTGGAAGAACTTGGAGACGCCCTTGTGACCTCCAAGATTTTCCATGCATTCGTCCATAATGATGGCAATGGGCCCACGGGCGGCGGCCTGGGCGAAGATATTTCTGGGATCACTAACGTCATAGTTGTGTTCCAGGATGAGATCGTCATAGGCCATTTTTACAAAGCGCGGGCGGAGGGTGCCAGACTGCGGTATAATGGTTCCATCCGGCCCAGGGGCGTAGTTACCCTCACAGATTTGCATTTCCCACGCTTTGAGTTCAGATGGGGGGATCATGTCTACCTGCGGGGCGATGAAGAAAACCGTTTCCGGGGTAGGGGAGATCAGCTGGGAAGAAAGCAGGTTCCTGAGCAGCTGCGACTTACCGCAGCCGGTGGGCCCGTAAATCACACCTATTACCGGCTGCAACTGGTAGTTAAGAGAGCTGCAGCTGCCGTCATCCCTGAGCAGGGGGGCCACTTCGTTAAGCATGTCCCTGACTTGCATGTTTTCCCTGACCAAATCCGCCAGAAGGCGCTCGCCGCCCAGCGATAGCAGTTCTTGCAAGGAAGCAAAGTTTTTCAACGGTTTGAGGCCGTCCGCCGTAGGCATGCTTTTGAGCGTTTGACCAAGCAGTTCCAGGCGGTCCCACAGCTCGGTCACGTGCTCTACGGCATCTCGATCCAGCATATCTCCTCGTTTCGCGGGTTGGGGCGGCTTTCGCTGTACGGCAGTAGTCGGTGCTCGTCCAGACGGGCCAGGGTCATGTCTTTCCACGGGCGCAGGGTTCTCGTCAGCGTAGTCTGGGTCACGGTGAAGGGGTGCGCTCCGGGCTGCGCGCTGGCCAGGGTGCGCTTGAGGCTGGTCCTGCTGGTGCTGAAGCGCTGCCGGTCTTCGCCCTGCGCGTCGGCCAGGTAGCATTTGACCATGGTGTCATAGTCCAGCCCCTCCGCGGCGTGGCCCTTGGCGCGCAGCTTGCCCTTGGAGGAGGCGCCGCACGAGGGGCAGTGCAGACTTTTAAGGGCGTAGAGCTTGGGCGCGAGAAATACCGATTCCGGGGAGTAGGCATCCGCGCCGCAGGCCCCGCAGACGGTCTCGCATTCCACGAGCCAGGTGAGCTCTGGCCGTTCGGGGTCAAAAACCAGGTTTCCCCCATGCTTTTTGATGCGTTTCTTACCTCTGGTTTCCATGAGCCGGTGTCCACGCTCGGTGACGAAAAGGCTGTCCGTGTCCCCGTATACAGACTTGAGAGGCCTGTCCTCGAGCGGTGTTCCGCGGTCCTCCTCGTATAGAAACTCGGACCACTCTGAGACGAAGGCTCGCGTCCAGGCCAGCACGAAGGAGGCTAAGTGGGAGGGGTAGCGGTCGTTGTCCACTAGGGGGTCCACTCGCTCCAGGGTGTGAAGACACATGTCGCCCTCTTCGGCATCAAGGAAGATGATTGGTTTATAGGTGTAGGCCACGTGACCGGGTGTTCCTGAAGGGGGGCTATAAAAGGGGGTGGGGGCGCGTTCGTCCTCACTCTCTTCCGCATCGCTGTCTGCGAGGGCCAGCTGTTGGGGTGAGTACTCCCTCTCAAAAGCGGGCATGACTTCTGCGCTAAGATTGTCAGTTTCCAAAAACGAGGAGGATTTGATATTCACCTGGCCCGCGGTGATGCCTTTGAGGGTGGCCGCGTCCATCTGGTCAGAAAAGACAATCTTTTTGTTGTCAAGCTTGGTGGCAAACGACCCGTAGAGGGCGTTGGACAGCAACTTGGCGATGGAGCGCAGGGTTTGGTTTTTGTCGCGATCGGCGCGCTCCTTGGCCGCGATGTTTAGCTGCACGTATTCGCGCGCAACGCACCGCCATTCGGGAAAGACGGTGGTGCGCTCGTCGGGCACCAGGTGCACGCGCCAACCGCGGTTGTGCAGGGTGACAAGGTCAACGCTGGTGGCTACCTCTCCGCGTAGGCGCTCGTTGGTCCAGCAGAGGCGGCCGCCCTTGCGCGAGCAGAATGGCGGTAGTGGGTCTAGCTGCGTCTCGTCCGGGGGGTCTGCGTCCACGGTAAAGACCCCGGGCAGCAGGCGCGCGTCGAAGTAGTCTATCTTGCATCCTTGCAAGTCTAGCGCCTGCTGCCATGCGCGGGCGGCAAGCGCGCGCTCGTATGGGTTGAGTGGGGGACCCCATGGCATGGGGTGGGTGAGCGCGGAGGCGTACATGCCGCAAATGTCGTAAACGTAGAGGGGCTCTCTGAGTATTCCAAGATATGTAGGGTAGCATCTTCCACCGCGGATGCTGGCGCGCACGTAATCGTATAGTTCGTGCGAGGGAGCGAGGAGGTCGGGACCGAGGTTGCTACGGGCGGGCTGCTCTGCTCGGAAGACTATTTGCCTGAAGATGGCATGTGAGTTGGATGATATGGTTGGACGCTGGAAGACGTTGAAGCTGGCGTCTGTGAGACCTACCGCGTCACGCACGAAGGAGGCGTAGGAGTCGCGCAGCTTGTTGACCAGCTCGGCGGTGACCTGCACGTCTAGGGCGCGGTCTTTCCAGTACTCTTGGATCGGAAACCCGTCGGCCTCCGAACGGTAAGAGCCTAGCATGTAGAACTGGTTGACGGCCTGGTAGGCGCAGCATCCCTTTTCTACGGGTAGCGCGTATGCCTGCGCGGCCTTCCGGAGCGAGGTGTGGGTGAGCGCAAAGGTGTCCCTAACCATGACTTTGAGGTACTGGTATTTGAAGTCAGTGTCGTCGCATCCGCCCTGCTCCCAGAGCAAAAAGTCCGTGCGCTTTTTGGAACGCGGGTTTGGCAGGGCGAAGGTGACATCGTTGAAGAGTATCTTTCCCGCGCGAGGCATAAAGTTGCGTGTGATGCGGAAGGGTCCCGGCACCTCGGAACGGTTGTTAATTACCTGGGCGGCGAGCACGATCTCGTCAAAGCCGTTGATGTTGTGGCCCACAATGTAAAGTTCCAAGAAGCGCGGGATGCCCTTGATGGAAGGCAATTTTTTAAGTTCCTCGTAGGTGAGCTCTTCAGGGGAGCTGAGCCCGTGCTCTGAAAGGGCCCAGTCTGCAAGATGAGGGTTGGAAGCGACGAATGAGCTCCACAGGTCACGGGCCATTAGCATTTGCAGGTGGTCGCGAAAGGTCCTAAACTGGCGACCTATGGCCATTTTTTCTGGGGTGATGCAGTAGAAGGTAAGCGGGTCTTGTTCCCAGCGGTCCCATCCAAGGTCCGCGGCTAGGTCTCGCGCGGCGGTCACTAGAGGCTCATCTCCGCCGAACTTCATGACCAGCATGAAGGGCACGAGCTGCTTCCCAAAGGCCCCCATCCAAGTATAGGTCTCTACATCGTAGGTGACAAAGAGACGCTCGGTGCGAGGATGCGAGCCGATCGGGAAGAACTGGATCTCCCGCCACCAGTTGGAGGAGTGGCTGTTGATGTGGTGAAAGTAGAAGTCCCTGCGACGGGCCGAACACTCGTGCTGGCTTTTGTAAAAACGTGCGCAGTACTGGCAGCGGTGCACGGGCTGTACATCCTGCACGAGGTTGACCTGACGACCGCGCACAAGGAAGCAGAGTGGGAATTTGAGCCCCTCGCCTGGCGGGTTTGGCTGGTGGTCTTCTACTTCGGCTGCTTGTCCTTGACCGTCTGGCTGCTCGAGGGGAGTTACGGTGGATCGGACCACCACGCCGCGCGAGCCCAAAGTCCAGATGTCCGCGCGCGGCGGTCGGAGCTTGATGACAACATCGCGCAGATGGGAGCTGTCCATGGTCTGGAGCTCCCGCGGCGTCAGGTCAGGCGGGAGCTCCTGCAGGTTTACCTCGCATAGCCGGGTCAGGGCGCGGGCTAGGTCCAGGTGATACCTGATTTCCAGGGGCTGGTTGGTGGCGGCGTCGATGGCTTGCAAGAGGCCGCATCCCCGCGGCGCGACTACGGTACCGCGCGGCGGGCGGTGGGCCGCGGGGGTGTCCTTGGATGATGCATCTAAAAGCGGTGACGCGGGCGGGCCCCCGGAGGTAGGGGGGGCTCCGGACCCGCCGGGAGAGGGGGCAGGGGCACGTCGGCGCCGCGCGCGGGCAGGAGCTGGTGCTGCGCACGGAGGTTGCTGGCGAACGCGACGACGCGGCGGTTGATCTCCTGAATCTGGCGCCTCTGCGTGAAGACGACGGGCCCGGTGAGCTTGAACCTGAAAGAGAGTTCGACAGAATCAATTTCGGTGTCGTTGACGGCGGCCTGGCGCAAAATCTCCTGCACGTCTCCTGAGTTGTCTTGATAGGCGATCTCGGCCATGAACTGCTCGATCTCTTCCTCCTGGAGATCTCCGCGTCCGGCTCGCTCCACGGTGGCGGCGAGGTCGTTGGAGATGCGGGCCATGAGCTGCGAGAAGGCGTTGAGGCCTCCCTCGTTCCAGACGCGGCTGTAGACCACGCCCCCTTCGGCATCGCGGGCGCGCATGACCACCTGCGCGAGATTGAGCTCCACGTGCCGGGCGAAGACGGCGTAGTTTCGCAGGCGCTGAAAGAGGTAGTTGAGGGTGGTGGCGGTGTGTTCTGCCACGAAGAAGTACATAACCCAGCGTCGCAACGTGGATTCGTTGATATCCCCCAAGGCCTCAAGGCGCTCCATGGCCTCGTAGAAGTCCACGGCGAAGTTGAAAAACTGGGAGTTGCGCGCCGACACGGTTAACTCCTCCTCCAGAAGACGGATGAGCTCGGCGACAGTGTCGCGCACCTCGCGCTCAAAGGCTACAGGGGCCTCTTCTTCTTCAATCTCCTCTTCCATAAGGGCCTCCCCTTCTTCTTCTTCTGGCGGCGGTGGGGGAGGGGGGACACGGCGGCGACGACGGCGCACTGGGAGGCGGTCGACAAAGCGCTCGATCATCTCCCCGCGGCGACGGCGCATGGTCTCGGTGACGGCGCGGCCGTTCTCGCGGGGGCGCAGTTGGAAGACGCCGCCCGTCATGTCCGGGTTATGGGTTGGCGGGGGGCTGCCGTGCGGCAGGGATACGGCGCTAACGATGCATCTCAACAATTGTTGTGTAGGTACTCCGCCACCGAGGGACCTGAGCGAGTCCGCATCGACCGGATCGGAAAACCTCTCGAGAAAGGCGTCTAACCAGTCACAGTCGCAAGGTAGGCTGAGCACCGTGGCGGGCGGCAGCGGGCGGCGGTCGGGGTTGTTTCTGGCGGAGGTGCTGCTGATGATGTAATTAAAGTAGGCGGTCTTGAGACGGCGGATGGTCGACAGAAGCACCATGTCCTTGGGTCCGGCCTGCTGAATGCGCAGGCGGTCGGCCATGCCCCAGGCTTCGTTTTGACATCGGCGCAGGTCTTTGTAGTAGTCTTGCATGAGCCTTTCTACCGGCA

GCATCTATCGCTGCGGCGGCGGCGGAGTTTGGCCGTAGGTGGCGCCCTCTTCCTCCCATGCGTGTGACCCCGAAGCCCCTCATCGGCTGAAGCAGGGCCAGGTCGGCGACAACGCGCTCGGCTAATATGGCCTGCTGCACCTGCGTGAGGGTAGACTGGAAGTCATCCATGTCCACAAAGCGGTGGTATGCGCCCGTGTTGATGGTGTAAGTGCAGTTGGCCATAACGGACCAGTTAACGGTCTGGTGACCCGGCTGCGAGAGCTCGGTGTACCTGAGACGCGAGTAAGCCCTTGAGTCAAAGACGTAGTCGTTGCAAGTCCGCACCAGGTACTGGTATCCCACCAAAAAGTGCGGCGGCGGCTGGCGGTAGAGGGGCCAGCGTAGGGTGGCCGGGGCTCCGGGGGCGAGGTCTTCCAACATAAGGCGATGATATCCGTAGATGTACCTGGACATCCAGGTGATGCCGGCGGCGGTGGTTGAGGCGCGCGGAAAGTCGCGGACGCGGTTCCAAATGTTGCGCAGCGGCAAAAAGTGCTCCATGGTCGGGACGCTCTGGCCGGTTAGGCGCGCGCAGTCGTTGACGCTCTAGACCGTGCAAAAGGAGAGCCTGTAAGCGGGCACTCTTCCGTGGTCTGGTGGATAAATTCGCAAGGGTATCATGGCGGACGACCGGGGTTCGAACCCCGGATCCGGCCGTCCGCCGTGATCCATGCGGTTACCGCCCGCGTGTCGAACCCAGGTGTGCGACGTCAGACAACGGGGGAGCGCTCCTTTTGGCTTCCTTCCAGGCGCGGCGGCTGCTGCGCTAGCTTTTTTGGCCACTGGCCGCGCGCGGCGTAAGCGGTTAGGCTGGAAAGCGAAAGCATTAAGTGGCTCGCTCCCTGTAGCCGGAGGGTTATTTTCCAAGGGTTGAGTCGCGGGACCCCCGGTTCGAGTCTCGGGCCGGCCGGACTGCGGCGAACGGGGGTTTGCCTCCCCGTCATGCAAGACCCCGCTTGCAAATTCCTCCGGAAACAGGGACGAGCCCCTTTTTTGCTTTTCCCAGATGCATCCGGTGCTGCGGCAGATGCGCCCCCCTCCTCAGCAGCGGCAAGAGCAAGAGCAGCGGCAGACATGCAGGGCACCCTCCCCTTCTCCTACCGCGTCAGGAGGGGCAACATCCGCGGCTGACGCGGCGGCAGATGGTGATTACGAACCCCCGCGGCGCCGGGCCCGGCACTACCTGGACTTGGAGGAGGGCGAGGGCCTGGCGCGGCTAGGAGCGCCCTCTCCTGAGCGACACCCAAGGGTGCAGCTGAAGCGTGACACGCGCGAGGCGTACGTGCCGCGGCAGAACCTGTTTCGCGACCGCGAGGGAGAGGAGCCCGAGGAGATGCGGGATCGAAAGTTCCACGCAGGGCGCGAGTTGCGGCATGGCCTGAACCGCGAGCGGTTGCTGCGCGAGGAGGACTTTGAGCCCGACGCGCGGACCGGGATTAGTCCCGCGCGCGCACACGTGGCGGCCGCCGACCTGGTAACCGCGTACGAGCAGACGGTGAACCAGGAGATTAACTTTCAAAAAAGCTTTAACAACCACGTGCGCACGCTTGTGGCGCGCGAGGAGGTGGCTATAGGACTGATGCATCTGTGGGACTTTGTAAGCGCGCTGGAGCAAAACCCAAATAGCAAGCCGCTCATGGCGCAGTTGTTCCTTATAGTGCAGCACAGCAGGGACAACGAGGCATTCAGGGATGCGCTGCTAAACATAGTAGAGCCCGAGGGCCGCTGGCTGCTCGATTTGATAAACATTCTGCAGAGCATAGTGGTGCAGGAGCGCAGCTTGAGCCTGGCTGACAAGGTGGCCGCCATTAACTATTCCATGCTCAGTCTGGGCAAGTTTTACGCCCGCAAGATATACCATACCCCTTACGTTCCCATAGACAAGGAGGTAAAGATCGAGGGGTTCTACATGCGCATGGCACTTAAGGTGCTTACCTTGAGCGACGACCTGGGCGTTTATCGCAACGAGCGCATCCACAAGGCCGTGAGCGTGAGCCGGCGGCGCGAGCTCAGCGACCGCGAGCTGATGCACAGCCTGCAAAGGGCCCTGGCTGGCACGGGCAGCGGCGATAGAGAGGCCGAGTCCTACTTTGACGCGGGCGCTGACCTGCGCTGGGCCCCAAGCCGACGCGCCCTGGAGGCAGCTGGGGCCGGACCTGGGCTGGCGGTGGCACCCGCGCGCGCTGGCAACGTCGGCGGCGTGGAGGAATATGACGAGGACGATGAGTACGAGCCAGAGGACGGCGAGTACTAAGCGGTGATGTTTCTGATCAGATGATGCAAGACGCAACGGACCCGGCGGTGCGGGCGGCGCTGCAGAGCCAGCCGTCCGGCCTTAACTCCACGGACGACTGGCGCCAGGTCATGGACCGCATCATGTCGCTGACTGCGCGCAACCCTGACGCGTTCCGGCAGCAGCCGCAGGCCAACCGGCTCTCCGCAATTCTGGAAGCGGTGGTCCCGGCGCGCGCAAACCCCACGCACGAGAAGGTGCTGGCGATCGTAAACGCGCTGGCCGAAAACAGGGCCATCCGGCCCGATGAGGCCGGCCTGGTCTACGACGCGCTGCTTCAGCGCGTGGCTCGTTACAACAGCAGCAACGTGCAGACCAACCTGGACCGGCTGGTGGGGGATGTGCGCGAGGCCGTGGCGCAGCGTGAGCGCGCGCAGCAGCAGGGCAACCTGGGCTCCATGGTTGCACTAAACGCCTTCCTGAGTACACAGCCCGCCAACGTGCCGCGGGGACAGGAGGACTACACCAACTTTGTGAGCGCACTGCGGCTAATGGTGACTGAGACACCGCAAAGTGAGGTGTACCAGTCCGGGCCAGACTATTTTTTCCAGACCAGTAGACAAGGCCTGCAGACCGTAAACCTGAGCCAGGCTTTCAAGAACTTGCAGGGGCTGTGGGGGGTGCGGGCTCCCACAGGCGACCGCGCGACCGTGTCTAGCTTGCTGACGCCCAACTCGCGCCTGTTGCTGCTGCTAATAGCGCCCTTCACGGACAGTGGCAGCGTGTCCCGGGACACGTACCTAGGTCACTTGCTGACACTGTATCGCGAGGCCATAGGTCAGGCGCATGTGGACGAGCATACTTTCCAGGAGATTACAAGTGTCAGTCGCGCGCTGGGGCAGGAGGACACGGGCAGCCTGGAGGCAACCCTGAACTACCTGCTGACCAACCGGCGGCAGAAGATCCCCTCGTTGCACAGTTTAAACAGCGAGGAGGAGCGCATCTTGCGCTATGTGCAGCAGAGCGTGAGCCTTAACCTGATGCGCGACGGGGTAACGCCCAGCGTGGCGCTGGACATGACCGCGCGCAACATGGAACCGGGCATGTATGCCTCAAACCGGCCGTTTATCAATCGCCTAATGGACTACTTGCATCGCGCGGCCGCCGTGAACCCCGAGTATTTCACCAATGCCATCTTGAACCCGCACTGGCTACCGCCCCCTGGTTTCTACACCGGGGGATTTGAGGTGCCCGAGGGTAACGATGGATTCCTCTGGGACGACATAGACGACAGCGTGTTTTCCCCGCAACCGCAGACCCTGCTAGAGTTGCAACAGCGCGAGCAGGCAGAGGCGGCGCTGCGAAAGGAAAGCTTCCGCAGGCCAAGCAGCTTGTCCGATCTAGGCGCTGCGGCCCCGCGGTCAGATGCTAGTAGCCCATTTCCAAGCTTGATAGGGTCTTTTACCAGCACTCGCACCACCCGCCCGCGCCTGCTGGGCGAGGAGGAGTACCTAAACAACTCGCTGCTGCAGCCGCAGCGCGAAAAGAACCTGCCTCCGGCGTTTCCCAACAACGGGATAGAGAGCCTAGTGGACAAGATGAGTAGATGGAAGACGTATGCGCAGGAGCACAGGGATGTGCCCGGCCCGCGCCCGCCCACCCGTCGTCAAAGGCACGACCGTCAGCGGGGTCTGGTGTGGGAGGACGATGACTCGGCAGACGACAGCAGCGTCCTGGATTTGGGAGGGAGTGGCAACCCGTTTGCGCACCTTCGCCCCAGGCTGGGGAGAATGTTTTAAAAAAAGCATGATGCAAAATAAAAAAACTCACCAAGGCCATGGCACCGAGCGTTGGTTTTCTTGTATTCCCCTTAGTATGCGGCGCGCGGCGATGTATGAGGAAGGTCCTCCTCCCTCCTACGAGAGCGTGGTGAGCGCGGCGCCAGTGGCGGCGGCGCTGGGTTCCCCCTTCGATGCTCCCCTGGACCCGCCGTTCGTGCCTCCGCGGTACCTGCGGCCTACCGGGGGGAGAAACAGCATCCGTTACTCTGAGTTGGCACCCCTATTCGACACCACCCGTGTGTACCTTGTGGACAACAAGTCAACGGATGTGGCATCCCTGAACTACCAGAACGACCACAGCAACTTTCTAACCACGGTCATTCAAAACAATGACTACAGCCCGGGGGAGGCAAGCACACAGACCATCAATCTTGACGACCGGTCGCACTGGGGCGGCGACCTGAAAACCATCCTGCATACCAACATGCCAAATGTGAACGAGTTCATGTTTACCAATAAGTTTAAGGCGCGGGTGATGGTGTCGCGCTCGCAGACCAAAGACAATCAGGTGGAACTGAAATACGAGTGGGTGGAGTTCACGCTGCCCGAGGGAAACTACTCAGAGACCATGACCATAGACCTGATGAACAACGCGATCGTGGAGCACTACTTGAAAGTAGGCAGGCAAAACGGGGTTCTGGAAAGCGACATCGGGGTAAAGTTTGACACCCGCAACTTCCGGCTGGGGTTTGACCCCGTCACTGGTCTTGTCATGCCTGGGGTATATACAAACGAAGCCTTCCATCCAGACATCATTTTGCTGCCAGGATGCGGGGTGGACTTCACCCACAGCCGCCTGAGCAACTTGTTGGGCATCCGCAAGCGGCAACCCTTCCAAGAGGGCTTTAGGATCACCTACGATGACCTGGAGGGTGGTAACATTCCCGCACTGTTGGATGTGGACGCCTACCAGGCGAGCTTGAAAGATGACACCGAACGG

GCGGCAGCCGCGGCAATGCAGCCGGTGGAGGACATGAACGATCATGCCATTCGCGGCGACACCTTTGCCACACGGGCTGAGGAGAAGCGCGCTGAGGCCGAAGCAGCGGCCGAAGCTGCGGAGGCTGCGCAACCCGAGGTCGAGAAGCCTCAGAAGAAACCGGTGATCAAACCCCTGACAGAGGACAGCAAGAAACGCAGTTACAACCTAATAAGCAATGACAGCACCTTCACCCAGTACCGCAGCTGGTACCTTGCATACAACTACGGCGACCCTCAGACCGGGATCCGCTCATGGACCCTGCTTTGCACTCCTGACGTAACCTGCGGCTCGGAGCAGGTATACTGGTCGTTGCCCGACATGATGCAAGACCCCGTGACCTTCCGCTCCACGCGCCAGATCAGCAACTTTCCGGTGGTGGGCGCCGAGCTGTTGCCCGTGCACTCCAAGAGCTTCTACAACGACCAGGCCGTCTACTCCCAGCTCATCCGCCAGTTTACCTCTCTGACCCACGTGTTCAATCGCTTTCCCGAGAACCAGATTTTGGCGCGCCCGCCAGCCCCCACCATCACCACCGTCAGTGAAAACGTTCCTGCTCTCACAGATCACGGGACGCTACCGCTGCGCAACAGCATCGGAGGAGTCCAGCGAGTGACCATTACTGACGCCAGACGCCGCACCTGCCCCTACGTTTACAAGGCCCTGGGCATAGTCTCGCCGCGCGTCCTATCGAGCCGCACTTTTTGAGCAAGCATGTCCATCCTTATATCGCCCAGCAATAACACAGGCTGGGGCCTGCGCTTCCCAAGCAAGATGTTTGGCGGGGCCAAGAAGCGCTCCGACCAACACCCAGTGCGCGTGCGCGGGCACTACCGCGCGCCCTGGGGCGCGCACAAACGCGGCCGCACTGGGCGCACCACCGTCGATGACGCCATCGACGCGGTGGTGGAGGAGGCGCGCAACTACACGCCCACGCCGCCACCAGTGTCCACCGTGGACGCGGCCATTCAAACCGTGGTGCGCGGAGCCCGGCGTTACGCTAAAATGAAGAGACGGCGGAGGCGCGTAGCACGTCGCCACCGCCGCCGACCCGGCACTGCCGCCCAACGCGCGGCGGCGGCCCTGCTTAACCGCGCACGTCGCACCGGCCGACGGGCGGCCATGCGGGCCGCTCGAAGGCTAGCCGCGGGTATTGTTACTGTGCCCCCCAGGTCCAGGCGACGAGCGGCCGCCGCAGCAGCCGCGGCCATTAGTGCTATGACTCAGGGTCGCAGGGGCAACGTGTACTGGGTGCGCGACTCGGTTAGCGGCCTGCGCGTGCCCGTGCGCACCCGCCCCCCGCGCAACTAGATTGCAAGAAAAAACTACTTAGACTCGTACTGTTGTATGTATCCAGCGGCGGCGGCGCGCAACGAAGCTATGTCCAAGCGCAAAATCAAAGAAGAGATGCTCCAGGTCATCGCGCCGGAGATCTATGGCCCCCCGAAGAAGGAAGAGCAGGATTACAAGCCCCGAAAGCTAAAGCGGGTCAAAAAGAAAAAGAAAGATGATGATGATGAACTTGACGACGAGGTGGAACTGCTGCACGCGACCGCGCCCAGGCGGCGGGTACAGTGGAAAGGTCGACGCGTAAGACGTGTTTTGCGACCCGGCACCACCGTAGTCTTTACGCCCGGTGAGCGCTCCACCCGCACCTACAAGCGCGTGTATGATGAGGTGTACGGCGACGAGGACCTGCTTGAGCAGGCCAACGAGCGCCTCGGGGAGTTTGCCTACGGAAAGCGGCATAAGGACATGCTGGCGTTGCCGCTGGACGAGGGCAACCCAACACCTAGCCTAAAGCCCGTGACACTGCAGCAGGTGCTGCCCGCGCTTGCACCGTCCGAAGAAAAGCGCGGCCTAAAGCGCGAGTCTGGTGACTTGGCACCCACCGTGCAGCTGATGGTACCCAAGCGCCAGCGACTGGAAGATGTCTTGGAAAAAATGACTGTGGAGCCTGGGCTGGAGCCCGAGGTCCGCGTGCGGCCAATCAAGCAGGTGGCACCGGGACTGGGCGTGCAGACCGTGGACGTTCAGATACCCACCACCAGTAGCACTAGTATTGCCACTGCCACAGAGGGCATGGAGACACAAACGTCCCCGGTTGCCTCGGCGGTGGCAGATGCCGCGGTGCAGGCGGCCGCTGCGGCCGCGTCCAAAACCTCTACGGAGGTGCAAACGGACCCGTGGATGTTTCGCGTTTCAGCCCCCCGGCGCCCGCGCCGTTCTAGGAAGTACGGCACCGCCAGCGCACTACTGCCCGAATATGCCCTACATCCTTCCATCGCGCCTACCCCCGGCTATCGTGGCTACACCTACCGCCCCAGAAGACGAGCGACTACCCGACGCCGAACCACCGCTGGAACCCGCCGCCGCCGTCGCCGTCGCCAGCCCGTGCTGGCCCCGATTTCCGTGCGCAGGGTGGCTCGCGAAGGAGGCAGGACCCTGGTGCTGCCAACAGCGCGCTACCACCCCAGCATCGTTTAAAAGCCGGTCTTTGTGGTTCTTGCAGATATGGCCCTCACCTGCCGCCTCCGTTTCCCGGTGCCGGGATTCCGAGGAAGAATGCACCGTAGGAGGGGCATGGCCGGCCACGGCCTGACGGGCGGCATGCGTCGTGCGCACCACCGGCGGCGGCGCGCGTCGCACCGTCGCATGCGCGGCGGTATCCTGCCCCTCCTTATTCCACTGATCGCCGCGGCGATTGGCGCCGTGCCCGGAATTGCATCCGTGGCCTTGCAGGCGCAGAGACACTGATTAAAAACAAGTTTCATGTGGAAAAATCAAAATAAAAAGTCTGGACTCTCACGCTCGCTTGGTCCTGTAACTATTTTGTAGAATGGAAGACATCAACTTTGCGTCACTGGCCCCGCGACACGGCTCGCGCCCGTTCATGGGAAACTGGCAAGATATCGGCACCAGCAATATGAGCGGTGGCGCCTTCAGCTGGGGCTCGCTGTGGAGCGGCATTAAAAATTTCGGTTCCACCGTTAAGAACTATGGCAGCAAGGCCTGGAACAGCAGCACAGGCCAGATGCTGAGGGACAAGTTGAAAGAGCAAAATTTCCAACAAAAGGTGGTAGATGGCCTGGCCTCTGGCATTAGCGGGGTGGTGGACCTGGCCAACCAGGCAGTGCAAAATAAGATTAACAGTAAGCTTGATCCCCGCCCTCCGGTAGAGGAGCCTCCACCGGCCGTGGAGACAGTGTTGCCAGATGGGCGTGGCGAAAAGCGCCCGCGCCCCGACAGGGAAGAGACTCTGGTGACACAAATCGACGAGCCTCCCTCGTACGAGGAGGCGCTAAAGCAAGGCCTGCCCACCACCCGTCCCATCGCGCCCATGGCTACCGGAGTGTTGGGCCAGCACACACCTGTAACGCTGGACCTGCCTCCCCCCGCTGACACCCAGCAGAAACCTGTGCTGCCAGGGCCGTCCGCCGTTGTTGTAACCCGCCCTAGCCGCGCGTCCCTGCGCCGTGCCGCCAGCGGTCCGCGATCGTTGCGGCCCGTAGCCAGTGGCAACTGGCAAAGCACACTGAACAGCATCGTGGGTCTGGGGGTGCAATCCCTGAAGCGCCGACGATGCTTCTAATAGCTAACGTGTCGTATGTGTGTCATGTATGCGTCCATGTCGCCGCCAGAGGAGCTGCTGAGCCGCCGCGCGCCCGCTTTCCAAGATGGCTACCCCTTCGATGATGCCGCAGTGGTCTTACATGCACATCTCGGGCCAGGACGCCTCGGAGTACCTGAGCCCCGGGCTGGTGCAGTTTGCCCGCGCCACCGAGACGTACTTCAGCCTGAATAACAAGTTTAGAAACCCCACGGTGGCGCCTACGCACGACGTGACCACAGACCGGTCCCAGCGTTTGACGCTGCGGTTCATCCCTGTGGATCGCGAGGATACCGCGTACTCGTACAAGGCGCGGTTCACCCTGGCTGTGGGTGACAACCGCGTGCTTGATATGGCTTCCACGTACTTTGACATCCGCGGCGTGCTGGACAGGGGCCCTACTTTCAAGCCCTACTCTGGCACTGCCTACAACGCTCTAGCTCCCAAGGGCGCTCCCAACTCCTGTGAGTGGGAACAAACCGAAGATAGCGGCCGGGC

AGAGCAAAACGCTCGAGATCAGGCTACTAAGAAAACACATGTTTATGCCCAGGCTCCTTTGTCTGGAGAAACAATTACAAAAAGCGGGCTACAAATAGGATCAGACAATGCAGAAACACAAGCTAAACCTGTATATGCAGATCCTTCCTATCAACCAGAACCTCAAATTGGCGAATCTCAGTGGAACGAAGCTGATGCTAATGCAGCAGGAGGGAGAGTGCTTAAAAAAACAACTCCCATGAAACCATGCTATGGATCTTATGCCAGGCCTACAAATCCTTTTGGTGGCCAATCCGTCCTGGTTCCGGATGAAAAAGGGGTGCCTCTTCCAAAGGTTGACTTGCAATTCTTCTCAAATACTACCTCTTTAAACGACCGGCAAGGCAATGCCACTAAACCAAAAGTGGTGTTGTACAGTGAAGATGTAAATTTGGAAACCCCAGACACACATCTGTCTTACAAACCTGGAAAAGGTGATGAAAATTCTAAAGCTATGTTGGGTCAACAATCTATGCCAAACAGACCCAATTACATCGCTTTCAGGGACAATTTTATTGGCCTAATGTATTATAACAGCACTGGCAACATGGGTGTTCTTGCTGGTCAGGCATCGCAGCTAAATGCCGTGGTAGATTTGCAAGACAGAAACACAGAGCTGTCCTATCAACTTTTGCTTGATTCCATAGGTGATAGAACCAGATATTTTTCTATGTGGAATCAGGCTGTAGACAGCTATGACCCAGATGTTAGAATCATTGAAAACCATGGAACTGAGGATGAATTGCCAAATTATTGTTTCCCTCTTGGGGGTATTGGGGTAACTGACACCTATCAAGCTATTAAGGCTAATGGCAATGGCGCAGGTGATAATGGAAATACTACATGGACAAAAGATGAAACTTTTGCAACACGTAATGAAATAGGAGTGGGTAACAACTTTGCCATGGAAATTAACCTAAATGCCAACCTATGGAGAAATTTCCTTTACTCCAATATTGCACTATACCTGCCAGACAAGCTAAAATACAACCCCACCAACGTGGAAATATCTGACAACCCCAACACCTACGACTACATGAACAAGCGAGTGGTGGCTCCTGGGCTTGTAGACTGCTACATTAACCTTGGGGCGCGCTGGTCTCTGGACTACATGGACAACGTTAATCCCTTTAACCACCACCGCAATGCGGGCCTGCGTTACCGCTCCATGTTGTTGGGAAACGGCCGCTACGTGCCTTTTCACATTCAGGTGCCCCAAAAGTTTTTTGCCATTAAAAACCTCCTCCTCCTGCCAGGCTCATACACATATGAATGGAACTTCAGGAAGGATGTTAACATGGTTCTGCAGAGCTCTCTGGGAAACGACCTTAGAGTTGACGGGGCTAGCATTAAGTTTGACAGCATTTGTCTTTACGCCACCTTCTTCCCCATGGCCCACAACACGGCCTCCACGCTGGAAGCCATGCTCAGAAATGACACCAACGACCAGTCCTTTAATGACTACCTTTCCGCCGCCAACATGCTATATCCCATACCCGCCAACGCCACCAACGTGCCCATCTCCATCCCATCGCGCAACTGGGCAGCATTTCGCGGTTGGGCCTTCACACGCTTGAAGACAAAGGAAACCCCTTCCCTGGGATCAGGCTACGACCCTTACTACACCTACTCTGGCTCCATACCATACCTTGACGGAACCTTCTATCTTAATCACACCTTTAAGAAGGTGGCCATTACTTTTGACTCTTCTGTTAGCTGGCCGGGCAACGACCGCCTGCTTACTCCCAATGAGTTTGAGATTAAGCGCTCAGTTGACGGGGAGGGCTATAACGTAGCTCAGTGCAACATGACAAAGGACTGGTTCCTAGTGCAGATGTTGGCCAACTACAATATTGGCTACCAGGGCTTCTACATTCCAGAAAGCTACAAAGACCGCATGTACTCGTTCTTCAGAAACTTCCAGCCCATGAGCCGGCAAGTGGTGGACGATACTAAATACAAAGATTATCAGCAGGTTGGAATTATCCACCAGCATAACAACTCAGGCTTCGTAGGCTACCTCGCTCCCACCATGCGCGAGGGACAAGCTTACCCCGCTAATGTTCCCTACCCCCTAATAGGCAAAACCGCGGTTGATAGTATTACCCAGAAAAAGTTTCTTTGCGACCGCACCCTGTGGCGCATCCCATTCTCCAGTAACTTTATGTCCATGGGTGCGCTCACAGACCTGGGCCAAAACCTTCTCTACGCAAACTCCGCCCACGCGCTAGACATGACCTTTGAGGTGGATCCCATGGACGAGCCCACCCTTCTTTATGTTTTGTTTGAAGTCTTTGACGTGGTCCGTGTGCACCAGCCGCACCGCGGCGTCATCGAGACCGTGTACCTGCGCACGCCCTTCTCGGCCGGCAACGCCACAACATAAAGAAGCAAGCAACATCAACAACAGCTGCCGCCATGGGCTCCAGTGAGCAGGAACTCAAAGCCATTGTCAAAGATCTTGGTTGTGGGCCATATTTTTTGGGCACCTATGACAAGCGCTTTCCAGGCTTTGTTTCTCCACACAAGCTCGCCTGCGCCATAGTCAATACGGCCGGTCGCGAGACTGGGGGCGTACACTGGATGGCCTTTGCCTGGAACCCGCGCTCAAAAACATGCTACCTCTTTGAGCCCTTTGGCTTTTCTGACCAACGACTCAAGCAGGTTTACCAGTTTGAGTACGAGTCACTCCTGCGCCGTAGCGCCATTGCTTCTTCCCCCGACCGCTGTATAACGCTGGAAAAGTCCACCCAAAGCGTGCAGGGGCCCAACTCGGCCGCCTGTGGACTATTCTGCTGCATGTTTCTCCACGCCTTTGCCAACTGGCCCCAAACTCCCATGGATCACAACCCCACCATGAACCTTATTACCGGGGTACCCAACTCCATGCTTAACAGTCCCCAGGTACAGCCCACCCTGCGTCGCAACCAGGAACAGCTCTACAGCTTCCTGGAGCGCCACTCGCCCTACTTCCGCAGCCACAGTGCGCAGATTAGGAGCGCCACTTCTTTTTGTCACTTGAAAAACATGTAAAAATAATGTACTAGGAGACACTTTCAATAAAGGCAAATGTTTTTTATTTGTACACTCTCGGGTGATTATTTACCCCCACCCTTGCCGTCTGCGCCGTTTAAAAATCAAAGGGGTTCTGCCGCGCATCGCTATGCGCCACTGGCAGGGACACGTTGCGATACTGGTGTTTAGTGCTCCACTTAAACTCAGGCACAACCATCCGCGGCAGCTCGGTGAAGTTTTCACTCCACAGGCTGCGCACCATCACCAACGCGTTTAGCAGGTCGGGCGCCGATATCTTGAAGTCGCAGTTGGGGCCTCCGCCCTGCGCGCGCGAGTTGCGATACACAGGGTTGCAGCACTGGAACACTATCAGCGCCGGGTGGTGCACACTGGCCAGCACGCTCTTGTCGGAGATCAGATCCGCGTCCAGGTCCTCCGCGTTGCTCAGGGCGAACGGAGTCAACTTTGGTAACTGCCTTCCCAAAAAGGGTGCATGCCCAGGCTTTGAGTTGCACTCGCACCGTAGTGGCATCAGAAGGTGACCGTGCCCGGTCTGGGCGTTAGGATACAGCGCCTGCATGAAAGCCTTGATCTGCTTAAAAGCCACCTGAGCCTTTGCGCCTTCAGAGAAGAACATGCCGCAAGACTTGCCGGAAAACTGATTGGCCGGACAGGCCGCGTCATGCACACAGCACCTTGCGTCGGTGTTGGAGATCTGCACCACATTTCGGCCCCACCGGTTCTTCACGATCTTGGCCTTGCTAGACTGCTCCTTCAGCGCGCGCTGCCCGTTTTCGCTCGTCACATCCATTTCAATCACGTGCTCCTTATTTATCATAATGCTCCCGTGTAGACACTTAAGCTCGCCTTCGATCTCAGCGCAGCGGTGCAGCCACAACGCGCAGCCCGTGGGCTCGTGGTGCTTGTAGGTCACCTCTGCAAACGACTGCAGGTACGCTTGCAGGAATCGCCCCATCATCGTCACAAAGGTCTTGTTGCTGGTAAAGGTCAGCTGCAACCCGCGGTGCTCCTCGTTCAGCCAGGTCTTACATACGGCCGCCAGGGCTTCCACTTGGTCAGGCAGTAGCTTGAAGTTCGCCTTTAGATCGTTATCCACGTGGTACTTGTCCATCAGCGCGCGCGCAGCCTCCATGCCCTTCTCCCACGCAGACACGATCGGCAGACTCAGCGGATTCATTACCGTGCTTTCACTTTCCGCTTCGCTGGACTCTTCCTTTTCCTCTTGCGTCCGCATACCCCGCGCCACTGGGTCATCTTCATTCAGCCGCCGCACCGTGCGCTTACCTCCCTTGCCGTGCTTGATTAGCACCGGTGGGTTGCTAAAACCCACCATTTGTAGCGCCACATCTTCTTTTTCTTCCTCGCTGTCCACAATCACCTCTGGGGATGGCGGGCGCTCGGACTTGGGAGAGGGGCGCTTCTTTTTCTTTTTGGGCGCGTTGGCCAAATCCGCCGCCGAGGTTGATGGCCGCGGGCTGGGTGTGCGCGGCACCAGCGCGTCCTGTGACGAGTCTTCTTCGTCCTCGGACTCGAGACGCCGCCTCAGCCGCTTTTTTGGGGGCGCGCGGGGAGGCGGCGGCGACGGCGACGGGGACGACACGTCCTCCATGGTTGGTGGACGTCGCGCCGCACCGCGTCCGCGCTCGGGGGTGGTTTCGCGCTGCTCCTCTTCCCGACTGGCCATTTCCTTCTCCTATAGGCAGAAAAGATCATGGAGTCAGTCGAGAAGGAGGACAGCCTAACCGCCCCCTCTGAGTTCGCCACCACCGCCTCCACCGATGCCGCCAACGCGCCTACCACCTTCCCCGTCGAGGCACCCCCGCTTGAGGAGGAGGAAGTGACTATCGAGCAGGACCCAGGTTTTGTAAGCGAAGACGACGAGGACCGCTCAGTACCAACAGAGGATAAAAAGCAAGACCAGGACGACGCAGAGGCAAACAAGGAGCAAGTCGGGCGGGGGGACGAGAGGCATGGCGACTACCTAAATGTGGGAGACGACGTGCTGTTGAAGCATCTGCAGCGCCAGTGCGCTATTATCTGCGACGCGTTGCAAGAGCGCAGCGATGTGCCCCTTGCCATAGCGGATGTTAGCCTTGCCTACGAACGCCACCTGTTCTCACCGCGTGTACCCCCCAAACGACAAGAAAACGGCACATGCGAGCCCAACCCGCGCCTCAACTTCTACCCCGTATTTGCCGTGCCAGAGGTGCTTGCCACCTATCACATCTTTTTCCAAAACTGCAAGATACCCCTATCCTGCCGTGCCAACCGCAGCCGAGCGGACAAGCAGCTGGCCTTGCGGCAGGGCGCTGTCATACCTGATATCGCCTCGCTCGACGAAGTGCCGAAAATCTTTGAGGGTCTTGGACGCGACGAGAAGCGCGCGGCAAACGCTCTGCAACAAGAAAACAGCGAAAATGAAAGTCACTGTGGAGTGCTGGTGGAACTTGAGGGTGACAACGCGCGCCTAGCCGTGCTAAAACGCAGCATCGAGGTCACCCACTTTGCCTACCCGGCACTTAACCTACCCCCCAAGGTTATGAGCACAGTCATGAGCGAACTGATCGTGCGCCGTGCACGACCCCTGGAGAGGGATGCAAACTTGCAAGAACAAACCGAGGAGGGCCTACCCGCAGTTGGCGATGAGCAGCTGGCGCGCTGGCTTGAGACGCGCGAGCCTGCCGACTTGGAGGAGCGACGCAAGCTAATGATGGCCGCAGTGCTTGTTACCGTGGAGCTTGAGTGCATGCAGCGGTTCTTTGCTAACCCGGAGATGCAGCGCAAGCTAGAGGAAACGTTGCACTACACCTTTCGCCAGGGCTACGTGCGCCAGGCCTGCAAAATTTCCAACGTGGAGCTCTGCAACCTGGTCTCCTACCTTGGAATTTTGCACGAAAACCGCCTCGGGCAAAACGTGCTTCATTCCACGCTCAAGGGCGAGGCGCGCCGCGACTACGTCCGCGACTGCGTTTACTTATTTCTATGCTACACCTGGCAAACGGCCATGGGCGTGTGGCAGCAGTGCCTGGAGGAGCGCAACCTGAAGGAGCTGCAGAAGCTGCTAAAGCAAAACTTGAAGGACCTATGGACGGCCTTCAACGAGCGCTCCGTGGCCGCGCACCTGGCGGACATTATCTTCCCCGAACGCCTGCTTAAAACCCTGCAACAGGGTCTGCCAGACTTCACCAGTCAAAGCATGTTGCAAAACTTTAGGAACTTTATCCTAGAGCGTTCAGGAATTCTGCCCGCCACCTGCTGTGCGCTTCCTAGCGACTTTGTGCCCATTAAGTACCGCGAATGCCCTCCGCCGCTTTGGGGTCACTGCTACCTTCTGCAGCTAGCAAACTACCTTGCCTACCACTCCGACATCATGGAAGACGTGAGCGGTGACGGCCTACTGGAATGTCACTGTCGCTGCAACCTATGTACCCCGCACCGCTCCCTGGTCTGCAATTCGCAGCTGCTTAGCGAGAGTCAAATTATCGGTACCTTTGAGCTGCAGGGTCCCTCGCCTGACGAAAAGTCCGCGGCTCCGGGGTTGAAACTCACTCCGGGGCTGTGGACGTCGGCTTACCTTCGCAAATTTGTACCTGAGGACTACCACGCCCACGAGATTAGGTTCTACGAAGACCAATCCCGCCCGCCCAATGCGGAGCTTACCGCCTGCGTCATTACCCAGGGCCACATCCTTGGCCAATTGCAAGCCATCAACAAAGCCCGCCAAGAGTTTCTGCTACGAAAGGGACGGGGGGTTTACTTGGACCCCCAGTCCGGCGAGGAGCTCAACCCAATCCCCCCGCCGCCGCAGCCCTATCAGCAGCAGCCGCGGGCCCTTGCTTCCCAGGATGGCACCCAAAAAGAAGCTGCAGCTGCCGCCGCCGCCACCCACGGACG

TAGACGAGGAAGCTTCCGAGGCCGAAGAGGTGTCAGACGAAACACCGTCACCCTCGGTCGCATTCCCCTCGCCGGCGCCCCAGAAATTGGCAACCGTTCCCAGCATGGCTACAACCTCCGCTCCTCAGGCGCCGCCGGCACTGCCCGTTCGCCGACCCAACCGTAGATGGGACACCACTGGAACCAGGGCCGGTAAGTCTAAGCAGCCGCCGCCGTTAGCCCAAGAGCAACAACAGCGCCAAGGCTACCGCTCGTGGCGCGGGCACAAGAACGCCATAGTTGCTTGCTTGCAAGACTGTGGGGGCAACATCTCCTTCGCCCGCCGCTTTCTTCTCTACCATCACGGCGTGGCCTTCCCCCGTAACATCCTGCATTACTACCGTCATCTCTACAGCCCCTACTGCACCGGCGGCAGCGGCAGCAACAGCAGCGGCCACACAGAAGCAAAGGCGACCGGATAGCAAGACTCTGACAAAGCCCAAGAAATCCACAGCGGCGGCAGCAGCAGGAGGAGGAGCGCTGCGTCTGGCGCCCAACGAACCCGTATCGACCCGCGAGCTTAGAAACAGGATTTTTCCCACTCTGTATGCTATATTTCAACAAAGCAGGGGCCAAGAACAAGAGCTGAAAATAAAAAACAGGTCTCTGCGCTCCCTCACCCGCAGCTGCCTGTATCACAAAAGCGAAGATCAGCTTCGGCGCACGCTGGAAGACGCGGAGGCTCTCTTCAGCAAATACTGCGCGTTGACTCTTAAGGACTAGTTTCGCGCCCTTTCTCAAATTTAAGCGCGAAAACTACGTCATCTCCAGCGGCCACACCCGGCGCCAGCACCTGTCGTCAGCGCCATTATGAGCAAGGAAATTCCCACGCCCTACATGTGGAGTTACCAGCCACAAATGGGACTTGCGGCTGGAGCTGCCCAAGACTACTCAACCCGAATAAACTACATGAGCGCGGGACCCCACATGATATCCCGGGTCAACGGAATCCGCGCCCACCGAAACCGAATTCTCCTCGAACAGGCGGCTATTACCACCACACCTCGTAATAACCTTAATCCCCGTAGTTGGCCCGCTGCCCTGGTGTACCAGGAAAGTCCCGCTCCCACCACTGTGGTACTTCCCAGAGACGCCCAGGCCGAAGTTCAGATGACTAACTCAGGGGCGCAGCTTGCGGGCGGCTTTCGTCACAGGGTGCGGTCGCCCGGGCAGGGTATAACTCACCTGAAAATCAGAGGGCGAGGTATTCAGCTCAACGACGAGTCGGTGAGCTCCTCTCTTGGTCTCCGTCCGGACGGGACATTTCAGATCGGCGGCGCTGGCCGCTCTTCATTTACGCCCCGTCAGGCGATCCTAACTCTGCAGACCTCGTCCTCGGAGCCGCGCTCCGGAGGCATTGGAACTCTACAATTTATTGAGGAGTTCGTGCCTTCGGTTTACTTTAACCCCTTTTCTGGACCTCCCGGCCACTACCCGGACCAGTTTATTCCCAACTTTGACGCGGTGAAAGACTCGGCGGACGGCTACGACTGAATGACCAGTGGAGAGGCAGAGCGACTGCGCCTGACACACCTCGACCACTGCCGCCGCCACAAGTGCTTTGCCCGCGGCTCCGGTGAGTTTTGTTACTTTGAATTGCCCGAAGAGCATATCGAGGGCCCGGCGCACGGCGTCCGGCTCACCACCCAGGTAGAGCTTACACGTAGCCTAATTCGGGAGTTTACCAAGCGCCCCCTGCTAGTGGAGCGGGAGCGGGGTCCCTGTGTTCTGACCGTGGTTTGCAACTGTCCTAACCCTGGATTACATCAAGATCTTTGTTGTCATCTCTGTGCTGAGTATAATAAATACAGAAATTAGAATCTACTGGGGCTCCTGTCGCCATCCTGTGAACGCCACCGTTTTTACCCACCCAAAGCAGACCAAAGCAAACCTCACCTCCGGTTTGCACAAGCGGGCCAATAAGTACCTTACCTGGTACTTTAACGGCTCTTCATTTGTAATTTACAACAGTTTCCAGCGAGACGAAGTAAGTTTGCCACACAACCTTCTCGGCTTCAACTACACCGTCAAGAAAAACACCACCACCACCCTCCTCACCTGCCGGGAACGTACGAGTGCGTCACCGGTTGCTGCGCCCACACCTACAGCCTGAGCGTAACCAGACATTACTCCCATTTTCCCAAAACAGGAGGTGAGCTCAACTCCCGGAACTCAGGTCAAAAAAGCATTTTGCGGGGTGCTGGGATTTTTTAATTAAGTATATGAGCAATTCAAGTAACTCTACAAGCTTGTCTAATTTTTCTGGAATTGGGGTCGGGGTTATCCTTACTCTTGTAATTCTGTTTATTCTTATACTAGCACTTCTGTGCCTTAGGGTTGCCGCCTGCTGCACGCACGTTTGTACCTATTGTCAGCTTTTTAAACGCTGGGGGCGACATCCAAGATGAGGTACATGATTTTAGGCTTGCTCGCCCTTGCGGCAGTCTGCAGCGCTGCCAAAAAGGTTGAGTTTAAGGAACCAGCTTGCAATGTTACATTTAAATCAGAAGCTAATGAATGCACTACTCTTATAAAATGCACCACAGAACATGAAAAGCTTATTATTCGCCACAAAGACAAAATTGGCAAGTATGCTGTATATGCTATTTGGCAGCCAGGTGACACTAACGACTATAATGTCACAGTCTTCCAAGGTGAAAATCGTAAAACTTTTATGTATAAATTTCCATTTTATGAAATGTGCGATATTACCATGTACATGAGCAAACAGTACAAGTTGTGGCCCCCACAAAAGTGTTTAGAGAACACTGGCACCTTTTGTTCCACCGCTCTGCTTATTACAGCGCTTGCTTTGGTATGTACCTTACTTTATCTCAAATACAAAAGCAGACGCAGTTTTATTGATGAAAAGAAAATGCCTTGATTTTCCGCTTACTTGTATTCCCCTGGACAATTTACTCTATGTGGGATATGCTCCAGGAGGGCAAGATTATACCCACAACCTTCAAATCAAACTTTCCTGGACGTTAGCGCCTGACTTCTGCCAGCGCCTGCACTGCAAATTTGATCAAACCCAGCTTCAGCTTGCCTGCTCCAGAGATGACCGGCTCAACCATGGCGCCCACAACGGACTATCGCAACACCACTGCTACCGGACTAACATCTGCCCTAAATTTACCCCAAGTTCATGCCTTTGTCAATGACTGGGCGAGCTTGGACATGTGGTGGTTTTCCATAGCGCTTATGTTTGTTTGCCTTATTATTATGTGGCTTATTTGTTGCCTAAAGCGCAGACGCGCCAGACCCCCCATCTATAGGCCTATCATTGTGCTCAACCCACACAATGAAAAAATTCATAGATTGGACGGTCTGAAACCATGTTCTCTTCTTTTACAGTATGATTAAATGAGACATGATTCCTCGAGTTCTTATATTATTGACCCTTGTTGCGCTTTTCTGTGCGTGCTCTACATTGGCCGCGGTCGCTCACATCGAAGTAGATTGCATCCCACCTTTCACAGTTTACCTGCTTTACGGATTTGTCACCCTTATCCTCATCTGCAGCCTCGTCACTGTAGTCATCGCCTTCATTCAGTTCATTGACTGGGTTTGTGTGCGCATTGCGTACCTCAGGCACCATCCGCAATACAGAGACAGGACTATAGCTGATCTTCTCAGAATTCTTTAATTATGAAACGGATTGTCATTTTTGTTTTGCTGATTTTCTGCGCCCTACCTGTGCTTTGCTCCCAAACCTCAGCGCCTCCCAAAAGACATATTTCCTGCAGATTCACTCAAATATGGAACATTCCCAGCTGCTACAACAAACAGAGCGATTTGTCAGAAGCCTGGTTATACGCCATCATCTCTGTCATGGTTTTTTGCAGTACCATTTTTGCCCTAGCCATATATCCATACCTTGACATTGGCTGGAATGCCATAGATGCCATGAACCACCCTACTTTCCCAGTGCCCGCTGTCATACCACTGCAACAGGTTATTGCCCCAATCAATCAGCCTCGCCCCCCTTCTCCCACCCCCACTGAGATTAGCTACTTTAATTTGACAGGTGGAGATGACTGAATCTCTAGATCTAGAATTGGATGGAATTAACACCGAACAGCGCCTACTAGAAAGGCGCAAGGCGGCGTCCGAGCGAGAACGCCTAAAACAAGAAGTTGAAGACATGGTTAACCTACACCAGTGTAAAAGAGGTATCTTTTGTGTGGTCAAGCAGGCCAAACTTACCTACGAAAAAACCACTACCGGCAACCGCCTCAGCTACAAGCTACCCACCCAGCGCCAAAAACTGGTGCTTATGGTGGGAGAAAAACCTATTACCGTCACCCAGCACTCGGCAGAAACAGAGGGCTGCCTGCACTTCCCCTATCAGGGTCCAGAGGACCTCTGCACTCTTATTAAAACCATGTGTGGTATTAGAGATCTTATTCCATTCAACTAACATAAACACACAATAAATTACTTACTTAAAATCAGTCAGCAAATCTTTGTCCAGCTTATTCAGCATCACCTCCTTTCCTTCCTCCCAACTCTGGTATCTCAGCCGCCTTTTAGCTGCAAACTTTCTCCAAAGTTTAAATGGGATGTCAAATTCCTCATGTTCTTGTCCCTCCGCACCCACTATCTTCATATTGTTGCAGATGAAACGCGCCAGACCGTCTGAAGACACCTTCAACCCCGTGTATCCATATGACACAGAAACCGGGCCTCCAACTGTGCCCTTTCTTACCCCTCCATTTGTTTCACCCAATGGTTTCCAAGAAAGTCCCCCTGGAGTTCTCTCTCTACGCGTCTCCGAACCTTTGGACACCTCCCACGGCATGCTTGCGCTTAAAATGGGCAGCGGTCTTACCCTAGACAAGGCCGGAAACCTCACCTCCCAAAATGTAACCACTGTTACTCAGCCACTTAAAAAAACAAAGTCAAACATAAGTTTGGACACCTCCGCACCACTTACAATTACCTCAGGCGCCCTAACAGTGGCAACCACCGCTCCTCTGATAGTTACTAGCGGCGCTCTTAGCGTACAGTCACAAGCCCCACTGACCGTGCAAGACTCCAAACTAAGCATTGCTACTAAAGGGCCCATTACAGTGTCAGATGGAAAGCTAGCCCTGCAAACATCAGCCCCCCTCTCTGGCAGTGACAGCGACACCCTTACTGTAACTGCAACACCCCCGCTAACTACTGCCACGGGTAGCTTGGGCATTAACATGGAAGATCCTATTTATGTAAATAATGGAAAAATAGGAATTAAAATAAGCGGTCCTTTGCAAGTAGCACAAAACTCCGATACACTAACAGTAGTTACTGGACCAGGTGTCACCGTTGAACAAAACTCCCTTAGAACCAAAGTTGCAGGAGCTATTGGTTATGATTCATCAAACAACATGGAAATTAAAACGGGCGGTGGCATGCGTATAAATAACAACTTGCTAATTCTAGATGTGGATTACCCATTTGATGCTCAAACAAAACTACGTCTTAAACTGGGGCAGGGACCCCTGTATATTAATGCATCTCATAACTTGGACATAAACTATAACAGAGGCCTATACCTTTTTAATGCATCAAACAATACTAAAAAACTGGAAGTTAGCATAAAAAAATCCAGTGGACTAAACTTTGATAATACTGCCATAGCTATAAATGCAGGAAAGGGTCTGGAGTTTGATACAAACACATCTGAGTCTCCAGATATCAACCCAATAAAAACTAAAATTGGCTCTGGCATTGATTACAATGAAAACGGTGCCATGATTACTAAACTTGGAGCGGGTTTAAGCTTTGACAACTCAGGGGCCATTACAATAGGAAACAAAAATGATGACAAACTTACCCTGTGGACAACCCCAGACCCATCTCCTAACTGCAGAATTCATTCAGATAATGACTGCAAATTTACTTTGGTTCTTACAAAATGTGGGAGTCAAGTACTAGCTACTGTAGCTGCTTTGGCTGTATCTGGAGATCTTTCATCCATGACAGGCACCGTTGCAAGTGTTAGTATATTCCTTAGATTTGACCAAAACGGTGTTCTAATGGAGAACTCCTCACTTAAAAAACATTACTGGAACTTTAGAAATGGGAACTCAACTAATGCAAATCCATACACAAATGCAGTTGGATTTATGCCTAACCTTCTAGCCTATCCAAAAACCCAAAGTAAAACTGCTAAAAATAACATTGTCAGTCAAGTTTACTTACATGGTGATAAAACTAAACCTATGATACTTACCATTACACTTAACGGCACTAGTGAATCCACAGAAACTAGCGAGGTAAGCACTTACTCTATGTCTTTTACATGGTCCTGGGAAAGTGGAAAATACACCACTGAAACTTTTGCTACCAACTCTTACACCTTCTCCTACATTGCCCAGGAATAAAGAATCGTGAACCTGTTGCATGTTATGTTTCAACGTGTTTATTTTTCAATTGCAAAAATTTCAAGTCATTTTTCATTCAGTAGTATAGCCCCACCACCACATAGCTTATACTAATCACCGTACCTTAATCAAACTCACAGAACCCTAGTATTCAACCTGCCACCTCCCTCCCAACACACAGAGTACACAGTCCTTTCTCCCCGGCTGGCCTTAAAAAGCATCATATCATGGGTAACAGACATATTCTTAGGTGTTATATTCCACACGGTTTCCTGTCGAGCCAAACGCTCATCAGTGATATTAATAAACTCTCCGGGCAGCTCGCTTAAGTTCATGTCGCTGTCCAGCTGCTGAGCCACAGGCTGCTGTCCAACTTGCGGTTGCTCAACGGGCGGCGAAGGAGAAGTCCACGCCTACATGGGGGTAGAGTCATAATCGTGCATCAGGATAGGGCGGTGGTGCTGCAGCAGCGCGCGAATAAACTGCTGCCGCCGCCGCTCCGTCCTGCAGGAATACAACATGGCAGTGGTCTCCTCAGCGATGATTCGCACCGCCCGCAGCATAAGGCGCCTTGTCCTCCGGGCGCAGCAGCGCACTCTGATCTCACTTAAATTAGCACAGTAACTGCAGCACAGCACCACAATATTGTTCAAAATCCCACAGTGCAAGGCGCTGTATCCAAAGCTCATGGCGGGGACCACAGAACCCACGTGGCCATCATACCACAAGCGCAGGTAGATTAAGTGGCGACCCCTCATAAACACGCTGGACATAAACATTACCTCTTTTGGCATGTTGTAATTCACCACCTCCCGGTACCATATAAACCTCTGATTAAACATGGCACCATCCACCACCATCCTAAACCAGCTGGCCAAAACCTGCCCGCCGGCTATGCACTGCAGGGAACCGGGACTGGAACAATGACAGTGGAGAGCCCAGGACTCGTAACCATGGATCATCATGCTCGTCATGATATCAATGTTGGCACAACACAAGCACACGTGCATACACTTCCTCAGGATTACAAGCTCCTCCCGCGTCAGAACCATATCCCAGGGAACAACCCATTCCTGAATCAGCGTAAATCCCACACTGCAGGGAAGACCTCGCACGTAACTCACGTTGTGCATTGTCAAAGTGTTACATTCGGGCAGCAGCGGATGATCCTCCAGTATGGTAGCGCGGGTCTCTGTCTCAAAAGGAGGTAGACGATCCCTACTATACGGAGTGCGCCGAGACAACCGAGATCGTGTTGGTCGTAGTGTCATGCCAAATGGAACGCCGGACGTAGTCATATTTCCTGAAGCAAAACCAGGTGCGGGCGTGACAAACAGATCTGCGTCTCCGGTCTCGTCGCTTAGCTCGCTCTGTGTAGTAGTTGTAGTATATCCACTCTCTCAAAGCATCCAGGCGCCCCCTGGCTTCGGGTTCTATGTAAACTCCTTCATGCGCCGCTGCCCTGATAACATCCACCACCGCAGAATAAGCCACACCCAGCCAACTTATACATTCGCTCTGCGAGTCACACACGGGGGGAGCGGGAAGAGCTGGAAGAACCATGTTTTTTTTTATTCCAAAAGATTATCCAAAACCTCAAAATGCAGATCTATTAAGTGAACGCGCTCCCCTCCGGTGGCGTGGTCAAATTCTACAGCCAAAGAACAAATAATGGCATTTGTAAGATGTTGCACAATGGCTTCCAAAAGGCAAACGGCCCTCACGTCCAAGTGGACGTAAAGGCTAAACCCTTCAGGGTGAATCTCCTCTATAAACATTCCAGCACCTTCAACCATGCCCAAATAATTTTCATCTCGCCACCTTCTCAATATATCTCTAAGCAAATCCCGAATATTAAGTCCGGCCATTGTAAAAATCTGCTCCAGAGCGCCCTCCACCTTCAGCCTCAAGCAGCGAATCATGATTGCAAAAATTCAGGTTCCTCACAGACCTGTATAAGATTCAAAAGCGGAACATTAACAAAAATACCGCGATCCCGTAGGTCCCTTCGCAGGGCCAGCTGAACATAATCGTGCAGGTCTGCACGGACCAGCGCGGCCACTTCCCCGCCAGGAACCATGACAAAAGAACCCACACTGATTATGACACGCATACTTGGAGCTATGCTAACCAGCGTAGCCCCAATGTAAGCTTGTTGCATGGGCGGCGATATAAAATGCAAGGTGCTGCTCAAAAAATCAGGCAAAGCCTCGCGCAAAAAAGAAAGCACATCGTAGTCATGCTCATGCAGATAAAGGCAGGTAAGCTCCGGAACCACCACAGAAAAAGACACCATTTTTCTCTCAAACATGTTTGCGGGTTTCTGCATAAAATGACAAAAAAACATTTAAACATTAGAAGCCTGTCTTACAACAGGAAAAACAACCCTTATAAGCATAAGACGGACTACGGCCATGCCGGCGTGACCGTAAAAAAACTGGTCACCGTGATTAAAAAGCACCACCGACAGCTCCTCGGTCATGTCCGGAGTCATAATGTAAGACTCGGTAAACACATCAGGTTGGTTCACATCGGTCAGTGCTAAAAAGCGACCAAAATAGCCCGGGGGAATACATACCCGCAGGCGTAGAGACAACATTACAGCCCCCATAGGAGGTATAACAAAATTAATAGGAGAGAAAAACACATAAACACCTGAAAAACCCTCCTGCCTAGGCAAAATAGCACCCTCCCGCTCCAGAACAACATACAGCGCTTCCACAGCGGCAGCCATAACAGTCAGCCTTACCAGTAAAAAACAAAACCTATTAAAAAACACCACTCGACACGGCACCAGCTCAATCAGTCACAGTGTAAAAAAGGGCCAAGTACAGAGCGAGTATATATAGGACTAAAAAAATGACGTAACGGTTAAAGTCCACAAAAACCACCCAGAAAACCGCACGCGAACCTACGCCCAGAAACGAAAGCCAAAAAACCCACAACTTCCTCAAACCGTCACTTCCGTTTTCCCACGTTACGTCACTTCCCATTTTAAAAAAACTACAATTCCCAACACATGCAAGTTACTCCGCCCTAAAACCTACGTCACCCACCCCGTTCCCACGCCCCGCGCCACGTCACAAACTCCACCCCCTCATTATCATATTGGCTTCAATCCAAAATAAGGTATATTATTGATGATG

>COV229E_STRIPPED4

CTTTGTGTCTACTTTTCTCAACTAAACGAAATTTTTGCTATGGCCGGCATCTTTGATGCTGGAGTCGTAGTGTAATTGAAATTTCATTTGGGTTGCAACAGTTTGGAAGCAAGTGCTGTGTGTCCTAGTCTAAGGGTTTCGTGTTCCGTCACGAGATTCCATTCTACAAACGCCTTACGCGAGGTTCTGTCTCGTGTTTGTGTGGAAGCAAAGTTTTGTCTTTGTGGAAACCAGTAACTGTTCCTAATGGCCTGCAACCGTGTGACACTTGCCGTAGCAAGTGATTCTGAAATTTCTGCATATGGCTGTTCTACTATTGCGCAAGCCGTCCGCCGTTATAGCGAGGCCGCTAGCAATGGTTTTAGGGCATGCCGATTTGTTTCATTAGATTTGCAGGATTGCATCGTTGGCATTGCAGATGATACATATGTTATGGGTCTGCATGGCAATCAGACGTTGTTTTGCAACATAATGAAATTTTCTGACCGTCCTTTTATGCTTCATGGGTGGTTGGTTTTTTCCAATTCAAATTACCTTTTGGAGGAATTTGATGTTGTCTTCGGTAAGAGAGGTGGTGGTAATGTGACATACACTGACCAGTATCTCTGTGGCGCCGATGGCAAACCTGTTATAAGTGAAGATTTATGGCAGTTTGTTGACCATTTCGGTGAGAACGAAGAAATTGTCATCAATGGTCGTACTTACGTTTGTGCTTGGCTTACTAAGCGTAAGCCCTTAGATTACAAACGTCAGAACAACCTCGCCATTGAAGAGATTGAATATGTGCATGGTGATGCTTTGCACACACTACGCAATGGTTCTGTTCTTGAAATGGCTAAGGAAGTGAAGACATCTAGTAAAGTTGTGTTAAGCGATGCTCTTGACAAACTTTACAAAGTCTTTGGTTCTCCTGTTATGACAAATGGTTCCAACATCCTAGAGGCCTTTACTAAACCTGTGTTTATTAGTGCATTAGTTCAATGTACTTGTGGTACCAAGTCTTGGTCTGTGGGTGATTGGACCGGTTTTAAATCCTCTTGTTGCAACTTGATCAGTAATAAACTGTGTGTTGTTCCCGGTAATGTTAAACCTGGTGATGCTGTGATTACTACTCAGCAAGCTGGTGCTGGTGTTAAGTATTTTTGTGGCATGACTCTTAAGTTTGTTGCAAATATTGAAGGTGTCTCTGTTTGGAGAGTGATTGCTCTTCAGAGTGTTGATTGCTTTGTTGCTTCTTCCACTTTTGTAGAAGAGGAACATGTTAATAGAATGGACACATTCTGCTTCAATGTACGCAATAGTGTTACTGATGAGTGTCGTCTGGCCATGTTGGGTGCTGAAATGACTAGTAATGTCAGAAGACAAGTTGCTTCAGGTGTCATAGACATTAGTACCGGCTGGTTTGATGTTTATGATGACATCTTTGCTGAAAGCAAACCATGGTTTGTTCGCAAGGCTGAAGACATTTTTGGCCCTTGTTGGTCCGCTCTTGTTTCTGCACTTAAACAACTTAAAGTCACTACAGGTGAACTTGTGAGATTTGTTAAGTCTATTTGCAATTCAGCTGTTGCTGTCGTGGGTGGTACTATACAAATTCTCGCTAGTGTGCCTGAGAAGTTTTTGAATGCGTTTGACGTGTTTGTCACAGCTATTCAAACTGTCTTTGACTGTGCTGTTGAAACTTGTACTATTGCCGGTAAAGCATTTGACAAGGTTTTTGACTATGTCTTGCTTGATAATGCGCTTGTAAAACTTGTCACCACAAAGCTTAAGGGTGTTCGTGAAGGTGGCCTTAATAAAGTTAAGTATGCAACAGTTGTTGTTGGTTCCACTGAAGAAGTTAAATCTTCACGTGTTGAACGTAGCAATGCTGTACTTACAATCGCCAACAATTATTCCAAACTTTTTGATGAAGGGTATACTGTTGTAATTGGCGATGTGGCGTACTTTGCTAGTGACGGCTACTTCCGTCTTATGGCCAGTCCAAATAGTGTGTTGACTACTGCAGTCTATAAACCATTGGTTGATTTTAATGTGAATGTTATGGGTACTAGACCTGAAAAATTTCCAACCACTGTGACTTGTGAAAATTTAGAGTCTGCTGTTTTGTTTGTTAATGACAAAATCATTGAATTCCAATTGGATTACAGTATTGATGTCATTGATAATGAAATAATTGTCAAACCTAATATCAGCTTATGTGTTCCACTTTATGTGAGAGACTATGTTGACAAATGGGATGATTTTTGCAGACAATATAGTAACGAGTCTTGGTTTGAGGATGATTACAAGGCTTTTATCAGTGTTTTGGATATCACTGATGCTGCTGTGAAAGCTGCAGAGTCTAAAGCCTTCGTTGATACTATTGTTCCACCTTGCCCATCTATTTTGAAAGTTATAGATGGAGGCAAAATATGGAATGGTGTTATTAAAAATGTTAACTATGTTAGAGACTGGCTTAAGTCTTTGAAGTTAAATCTCACACAACAGGGTTTGCTTGGAACATGTGCAAAGCGTTTTAAACGTTGGCTTGGCATTTTGCTAGAGGCCTATAATGCGTTTTTAGACACTGTTGTTTCTACTGTTAAAATTGGTGGTTTGACCTTTAAAACATATGCTTTTGATAAACCTTACATTGTGATACGTGATATCGTGTGTAAGGTTGAAAATAAAACAGAAGCAGAATGGATTGAACTTTTTCCACATAATGACAGGATTAAGTCTTTTAGTACCTTTGAGAGTGCTTACATGCCAATTGCAGACCCTACACATTTTGACATTGAAGAAGTTGAACTTTTAGATGCAGAGTTTGTAGAACCAGGCTGTGGTGGTATTTTGTCAGTAATAGATGAGCACGTCTTTTATAAGAAGGATGGTGTTTATTATCCATCAAATGGTACTAACATTCTACCTGTTGCATTTACAAAAGCCGCTGGTGGTAAAGTTTCATTTTCTGATGACGTTGAAGTAAAAGACATTGAACCTGTTTACAGAGTCAAGCTTTGCTTTGAGTTTGAAGATGAAAAACTTGTAGATGTTTGTGAAAAGGCAATTGGCAAGAAAATTAAACATGACGGTGACTGGGATAGCTTTTGTAAGACTATTCAATCAGCACTTTCTGTTGTTTCATGCTATGTAAATCTACCTACTTATTACATTTATGATGAAGAAGGCGGTAATGACTTGAGTTTGCCCGTTATGATTTCTGAATGGCCTCTTTCTGAGTCAGATAAGGAAGAAGATGTTCAACAAGCTCAACAAGAAGCTACTCATATTGCTGAGGATGTTGTTGACCAAGTTGAAGAAGTCAATAGCATTTTTGACATTGAGACAGTGGATGTTAAACATGAGATGAGTCCTTTTGAAATGCCATTTGAAGAGTTAAATGGTTTAAAGATACTTAAACAATTGGATAACAACTGCTGGGTTAACTCAGTTATGTTACAAATACAATTAACTGGTATACTTGATGGTGATTATGCTATGCAGTTTTTTAAAATGGGCCGAGTTGCCAAGATGATTGAACGCTGCTACACTGCTGAGCAATGTATACGTGGTGCTATGGGTGATGTTGGTTTGTGTATGTATAGACTTCTTAAAGACTTACACACTGGTTTTATGGTTATGGATTATAAATGTAGTTGTACCAGTGGTAGGCTTGAAGAATCGGGAGCTGTTTTGTTTTGTACGCCCACTAAGAAGGCGTTTCCTTATGGTACTTGTCTAAATTGTAACGCACCTCGCATGTGTACAATTAGGCAGTTACAAGGTACCATAATATTTGTGCAACAAAAACCAGAACCTGTTAACCCTGTTTCTTTTGTTGTTAAACCAGTCTGCTCATCAATTTTTCGTGGTGCTGTGTCTTGTGGTCATTACCAGACTAACATCTATTCACAAAATTTGTGTGTGGATGGTTTTGGTGTTAACAAGATTCAGCCCTGGACAAATGATGCACTTAATACTATTTGTATTAAGGATGCAGATTATAATGCAAAAGATGAAATATCTGTTACACCAATTAAAAACACAGTTGATACAATACCTAAGGAAAAATTTGTTGTTAAAGAGAAGTTGAACGCCTTCCTCGTTCATGACAATGTAGCTTTCTACCAAGGTGATGTTGATACTGTTGTTAATGGTGTTGACTTTGACTTTATTGTAAATGCTGCTAATGAGAACCTTGCTCATGGTGGAGGACTTGCCAAAGCTTTAGATGTGTACACTAAAGGTAAACTTCAACGTTTATCCAAAGAACACATTGGATTAGCGGGTAAAGTAAGAGTTGGTACAGGAGTTATGGTTGAGTGTGATAGCCTTAGAATTTTTAATGTTGTTGGTCCACGCAAGGGTAAACATGAACGTGATTTACTCGTAAAAGCTTACAACACTATTAATAATGAACAAGGCACACCTTTAACACCAATTTTGAGTTGTGGTATTTTTGGTGTCAAACTCGAAACTTCATTGGAAGTTTTGCTTGATGTTTGTAATACAAAAGAATTTAAAGTTTTTGTTTATACAGACACAGAGGTTTGTAAGGTTAAGGATTTTGTGTCTGGTTTAGTGAATGTTCAAAAAGTTGCACAACCTAAAATAGAACAAAAACCAGTGTCCTTAATTAAAGTTGCACCCAAGCCTTACAGGGTAGATGGTAAATTTAGTTACTTTACAGAAGACTTGTTGTGTGTCGCTGATGACAAACCCATTGTTTTGTTTACTGACTCTATGCTTACTTTGGATGACCGTGGTTTGGCTCTAGACAATGCACTTAGTGGTGTGCTTAGTGCTGCTATTAAGGATTGTGTTGACATAAATAAAGCTATACCTTCTGGTAATCTTATTAAGTTTGATATAGATTCTGTTGTTGTTTACATGTGTGTTGTGCCATCCGAAAAGGACAAACATTTAGATAATAATGTTCAACGATGCACACGTAAGTTGAATAGACTTATGTGTGATATAGTTTGTACTATACCAGCTGACTACATCTTGCCATTGGTGTTGTCTAGTTTGACTTGTAATGTTTCTTTTGTAGGTGAACTTAAAGCTGCTGAAGCTAAAGTTATAACTATAAAGGTGACAGAGGATGGTGTTAATGTTCATGATGTGACCGTGACAACAGATAAGTCATTTGAACAACAAGTTGGTGTTATTGCTGACAAGGACAAAGATCTTTCTGGTGCAGTACCCAGTGATCTTAACACATCTGAATTGCTTACTAAAGCAATAGATGTTGATTGGGTCGAATTTTATGGCTTTAAAGATGCTGTTACTTTTGCAACAGTTGATCATAGTGCTTTTGCCTATGAAAGTGCTGTTGTTAATGGTATTAGAGTGTTAAAAACCAGTGATAATAATTGTTGGGTGAATGCTGTTTGTATTGCACTACAGTATTCGAAACCCCATTTTATTTCACAAGGTCTTGATGCTGCGTGGAATAAATTTGTTTTAGGCGATGTTGAAATTTTTGTTGCATTTGTTTACTATGTTGCAAGACTAGTGAAAGGTGACAAGGGTGATGCTGAAGACACTTTGAATAAGTTGTCTAAGTATCTTGCTAATGAAGCTCAAGTTCAATTAGAACATTATAGTTCTTGTGTTGAATGTGATGCTAAATTTAAAAACTCTGTTACATCTATCAATTCTGCTATAGTTTGTGCTAGTGTCAAACGTGATGGTGTGCAAGTTGGTTATTGTGTCCATGGTATTAAGTACTATTCACGTGTTAAAAGTGTTAGAGGTAGAGCTATTATAGTCAGTGTCGAACAGCTTGAACCGTGTGTTCAGTCTAGACTTTTGAGTGGTGTTGCTTATACTGCTTTTTCTGGACCTGTTGATAAAGGTCATTATACTGTTTATGACACTGCAAAGAAATCAATGTATGATGGTGATCGTTTTGTTAAACATGATCTTTCTCTGCTGTCTGTCACATCAGTTGTTATGGTTGGTGATTATGTTGCACCTGTTAGTACAGTGAAACCTAAACCAGTCATTTCGCAACTTGATGAAAATGCACAGAAGTTCTTTGATTTTGGTGATTTTTTGATTCATAATTTTGTTATTTTTTTCACATGGTTATTGAGTATGTTTACTTTGTGTAAAACTGCAGTAACTACATGTGATGTTAAAATAATGGCCAAAGCACCACAAAGGACGGGTGTTGTTTTAAAACGTAGTCTTAAATATAACTTAAAAGCGTCAGCAGCTGTTCTTAAATCTAAGTGGTGGCTGCTTGCTAAGTTTACGAAACTACTGTTACTTATATATACATTGTATTCAGTAGTTTTGTTTTGTGTACGTTTTGGACCGTTTAAGTTTTGTAGTGAGACTGTTAATGGTTATGCTAAGTCAAACTTTGTCAAGGATGATTACTGTGATGGTTCATTGGGCTGCAAGATGTGTCTTTTTGGTTATCAAGAGTTAAGTCAATTTAGCCATTTGGATGTTGTGTGGAAGCATATAACAGACCCTTTGTTTAGTAATATACAACCTTTCATTGTCATGGTTTTGCTGCTTATATTTGGTGACAATTATTTGAGATGCTTCTTGCTGTATTTTGTTGCTCAGATGATAAGCACAGTTGGTGTTTTTCTAGGTTATAAGGAAACAAATTGGTTCTTGCACTTTATTCCATTTGATGTTATTTGTGATGAACTGCTTGTCACTGTTATTGTTATTAAGGTTATTTCTTTTGTCAGACATGTGCTTTTTGGTTGTGAAAACCCAGATTGCATTGCGTGTTCTAAGAGTGCTAGACTTAAGAGATTCCCTGTTAACACAATTGTTAATGGTGTGCAACGTTCATTTTATGTTAATGCAAATGGTGGTAGTAAGTTTTGTAAGAAACATAGATTTTTCTGTGTTGATTGTGACTCTTATGGTTATGGCAGCACGTTTATAACACCCGAAGTTTCTAGAGAACTTGGTAACATTACTAAAACAAATGTGCAACCAACAGGTCCAGCGTATGTCATGATTGACAAAGTGGAGTTTGAAAATGGTTTTTACAGGTTGTATTCCGGTGAAACATTTTGGCGTTACAACTTTGATATAACTGAAAGCAAGTATTCTTGCAAAGAGGTTTTTAAAAATTGTAATGTTTTGGATGATTTCATCGTGTTTAACAATAATGGGACCAATGTAACGCAGGTTAAAAATGCTAGTGTTTACTTTTCACAGTTGTTGTGTAGGCCCATTAAATTAGTTGACAGTGAACTTTTGTCCACTTTGTCAGTTGATTTTAATGGTGTCTTACACAAGGCATACATCGATGTACTACGTAATAGCTTTGGTAAAGATCTTAATGCTAATATGTCTTTAGCCGATTGCAAGAGTGCTTTAGGCCTGTCTATTAGTGATCATGAATTTACTAGTGCTATTTCTAATGCACATCGTTGTGACGTGTTGTTATCTGATTTGTCATTTAACAACTTTGTCAGTTCGTATGCTAAACCTGAGGAAAAATTATCAGCTTATGACTTGGCGTGTTGTATGCGTGCAGGTGCTAAGGTTGTTAATGCCAATGTTCTGACAAAGGACCAAACTCCTATTGTTTGGCATGCAAAGGATTTTAACAGTCTTTCTGCTGAAGGTCGTAAGTATATTGTAAAAACTAGCAAAGCTAAGGGTTTGACTTTCTTGTTGACAATTAATGAAAACCAAGCTGTCACGCAAATACCTGCAACTAGCATTGTTGCTAAGCAAGGTGCTGGTGATGCTGGTCATTCATTAACATGGCTGTGGCTACTGTGTGGTCTTGTGTGTTTGATTCAATTCTACTTGTGCTTTTTCATGCCCTATTTTATGTACGATGTCGTGAGTAGTTTTGAGGGTTATGATTTTAAGTATATAGAAAATGGTCAGTTGAAGAATTTTGAAGCGCCACTTAAATGTGTCAGAAACGTTTTTGAAAACTTTGAGGACTGGCATTATGCTAAGTTTGGCTTCACACCTTTAAACAAGCAAAGCTGTCCTATTGTAGTTGGAGTTTCTGAAATTGTTAATACTGTCGCTGGCATTCCATCTAATGTGTATCTTGTTGGTAAAACTTTAATTTTTACACTACAAGCTGCTTTTGGTAATGCTGGTGTTTGTTATGACATTTTTGGAGTCACAACACCTGAAAAGTGCATTTTTACTTCTGCTTGTACTAGATTAGAAGGTTTGGGTGGTAACAATGTTTATTGTTATAACACAGAGCTTATGAAAGGTTCTTTGCCTTACAGTTCAATACAAGCTAATGCATATTATAAATATGATAATGGCAATTTTATTAAGTTGCCAGAAGTTATTGCACAAGGCTTTGGCTTTAGAACAGTACGTACTATTGCTACCAAATACTGCCGCGTAGGTGAATGTGTTGACTCCAATGCAGGTGTGTGTTTTGGCTTTGACAAGTGGTTTGTTAATGATGGACGTGTTGCCAATGGTTACGTTTGTGGTACTGGTTTGTGGAACCTTGTATTTAACATACTTTCCATGTTCTCATCTTCATTCTCTGTTGCTGCAATGTCAGGTCAAATTTTACTTAATTGTGCATTAGGTGCTTTTGCTATCTTTTGTTGTTTTCTTGTGACAAAGTTTAGACGCATGTTTGGTGACCTTTCTGTAGGTGTTTGCACTGTTGTTGTGGCTGTTTTGCTTAACAATGTCTCTTACATTGTAACTCAGAATTTAGTAACAATGATTGCTTATGCCATATTGTATTTCTTTGCTACTAGAAGCTTACGCTATGCATGGATTTGGTGTGCTGCATATTTAATTGCGTATATTTCTTTTGCTCCATGGTGGTTGTGTGCTTGGTACTTTCTTGCTATGTTGACAGGTTTGTTACCTAGTTTGCTGAAGCTTAAAGTTTCGACAAATCTTTTCGAAGGTGACAAATTTGTAGGTACATTTGAAAGTGCTGCTGCAGGAACATTTGTCATTGACATTCGTTCTTATGAGAAACTTGCTAATAGCATCTCTCCAGAAAAGTTGAAAAGTTATGCTGCTAGCTATAATAGATATAAGTACTATAGTGGTAATGCAAATGAAGCTGATTACCGTTGTGCTTGTTATGCCTATTTAGCAAAAGCAATGTTGGACTTTTCGCGTGATCATAATGACATCTTGTACACACCTCCGACTGTCAGTTATGGTTCTACATTACAGGCTGGTTTGCGCAAAATGGCACAACCATCTGGCTTTGTGGAGAAATGTGTTGTCCGTGTCTGCTATGGAAACACTGTGTTGAATGGGTTGTGGCTTGGTGATATTGTTTATTGCCCACGTCATGTTATCGCATCTAACACAACTTCTGCTATAGATTATGATCACGAATATAGTATTATGCGGTTGCATAATTTTTCTATAATATCTGGTACAGCATTTCTTGGTGTTGTAGGTGCTACTATGCATGGAGTAACTCTTAAAATTAAGGTTTCACAGACTAACATGCACACACCTAGACACTCTTTTAGAACACTAAAATCTGGTGAAGGTTTTAACATCTTAGCATGCTATGATGGTTGTGCTCAAGGTGTTTTTGGTGTGAACATGAGAACTAATTGGACTATCCGTGGTTCATTTATTAATGGTGCGTGTGGTTCTCCTGGCTACAATCTTAAAAATGGCGAGGTGGAATTTGTTTATATGCATCAAATTGAACTCGGAAGTGGTAGCCATGTAGGTTCTAGCTTTGATGGTGTTATGTATGGTGGTTTTGAAGACCAACCTAATCTTCAAGTTGAATCTGCAAACCAGATGTTAACAGTTAATGTGGTTGCATTTCTTTATGCTGCTATATTGAATGGTTGCACATGGTGGCTTAAAGGTGATAAATTGTTTGTGGAGCATTATAATGAGTGGGCACAGGCTAATGGTTTCACAGCTATGAATGGTGAAGACGCTTTTTCCATTCTTGCTGCTAAAACTGGTGTCTGTGTGGAAAGATTACTTCATGCTATTCAAGTTTTGAATAATGGATTTGGTGGTAAACAAATTTTGGGTTATTCTAGTCTCAATGATGAGTTCAGTATTAATGAAGTTGTCAAACAAATGTTTGGTGTTAACCTGCAAAGTGGTAAAACCACTAGTATGTTTAAATCCATAAGCTTGTTTGCTGGCTTCTTTGTCATGTTCTGGGCTGAATTATTTGTTTATACCACCACTATTTGGGTTAACCCTGGTTTTCTTACTCCGTTTATGATTTTGCTTGTTGCTTTGTCACTCTGTCTTACATTTGTTGTTAAACATAAGGTTTTGTTTTTGCAAGTGTTTTTGTTGCCTTCAATTATTGTGGCTGCTATTCAAAACTGTGCTTGGGACTACCATATTACAAAGGTGTTGGCAGAGAAGTTTGATTATAATGTTTCTGTTATGCAAATGGACATCCAGGGTTTTGTTAACATTTTTATTTGTCTTTTTGTTGCACTGTTGCATACTTGGCGCTTTGCTAAAGAGCGTTGTACACATTGGTGCACTTATTTGTTCTCACTCATTGCTGTTTTATACACTGCATTGTATAGTTATGACTACGTTAGTTTGCTGGTTATGCTACTTTGTGCAATTTCTAATGAATGGTATATTGGTGCTATCATTTTTAGAATTTGTCGTTTTGGTGTTGCATTTTTACCAGTGGAATACGTGTCTTACTTTGATGGTGTTAAAACTGTGCTGTTGTTTTACATGTTGTTAGGCTTTGTTAGCTGTATGTACTATGGTTTGTTGTACTGGATTAACAGGTTCTGTAAGTGCACATTAGGTGTTTATGATTTCTGTGTTAGTCCAGCCGAATTTAAGTATATGGTTGCTAATGGTTTGAATGCACCAAATGGCCCTTTTGATGCACTCTTTCTGTCTTTTAAACTAATGGGTATTGGTGGTCCTAGAACCATTAAAGTCTCTACTGTACAGTCTAAATTGACTGATCTTAAGTGCACAAACGTCGTTCTAATGGGCATTTTGTCTAACATGAACATAGCTTCTAACTCAAAGGAGTGGGCATATTGTGTTGAAATGCACAATAAAATAAATTTGTGTGACGACCCTGAAACTGCTCAAGAGTTATTGCTGGCGTTGTTGGCCTTTTTCTTGTCTAAGCATAGTGATTTTGGTCTTGGTGATCTTGTCGATTCTTATTTTGAGAACGACTCCATTTTGCAAAGTGTTGCATCTTCTTTTGTTGGTATGCCATCTTTTGTTGCATATGAAACAGCAAGACAAGAGTATGAAAATGCTGTTGCAAATGGTTCCTCACCACAAATAATCAAACAATTGAAGAAGGCTATGAATGTTGCAAAAGCTGAGTTTGACAGGGAGTCATCTGTTCAAAAGAAAATTAACAGAATGGCTGAACAAGCTGCTGCAGCTATGTACAAAGAAGCACGTGCTGTTAATAGAAAATCAAAAGTTGTTAGTGCAATGCATAGTTTACTCTTTGGCATGCTCCGACGTTTGGACATGTCTAGTGTTGACACTATCCTTAATATGGCACGTAATGGTGTTGTCCCTCTTTCCGTTATCCCTGCTACTTCTGCAGCCAGGCTCGTCGTCGTAGTACCAGATCATGATTCATTTGTGAAAATGATGGTAGATGGTTTTGTGCACTACGCTGGTGTTGTTTGGACATTACAGGAAGTTAAGGATAATGACGGTAAGAATGTGCATCTTAAAGATGTTACAAAGGAAAACCAGGAAATACTTGTTTGGCCTCTGATTTTGACTTGTGAACGTGTCGTTAAATTGCAGAACAATGAAATAATGCCAGGCAAGATGAAGGTCAAGGCCACCAAAGGTGAAGGTGATGGAGGCATTATTAGTGAAGGTAATGCTCTATACAACAATGAAGGTGGACGTGCATTTATGTATGCATATGTGACTACGAAGCCTGGCATGAAGTATGTTAAATGGGAACATGACTCTGGTGTGGTTACAGTTGAATTGGAACCACCTTGCAGATTTGTTATAGACACACCTACCGGACCCCAAATTAAGTATCTTTATTTTGTTAAGAATCTTAACAATTTAAGGAGAGGTGCTGTTTTGGGTTACATTGGTGCCACTGTGAGATTGCAAGCTGGCAAACAGACTGAGTTTGTTTCAAACTCCCATTTATTAACACATTGTTCTTTTGCTGTTGACCCAGCTGCAGCCTACCTTGATGCTGTTAAACAAGGCGCAAAACCTGTTGGCAATTGTGTAAAGATGTTGACTAATGGTTCTGGTAGTGGTCAGGCTATTACTAGTACCATTGATTCTAACACTACGCAGGACACATATGGTGGCGCGTCTGTTTGTATTTATTGCAGAGCACATGTTGCACATCCAACCATGGACGGTTTTTGTCAGTACAAAGGTAAGTGGGTACAAGTGCCCATAGGTACAAATGACCCTATAAGATTTTGTCTTGAAAATACTGTTTGTAAAGTTTGTGGTTGTTGGCTTAATCATGGCTGTACATGTGACCGGACTGCTATCCAAAGTTTTGATAGCAGTTATTTAAACGAGTCCGGGGCTCTAGTGCCGCTCGACTAGAGCCCTGTAATGGTACAGACATAGATTACTGTGTCCGTGCATTTGACGTTTACAATAAAGATGCGTCTTTTATCGGAAAAAATCTGAAGTCCAATTGTGTGCGCTTCAAGAATGTAGATAAGGATGACGCGTTCTATATTGTTAAACGTTGCATTAAGTCAGTTATGGACCACGAGCAGTCCATGTATAACTTACTTAAAGGCTGTAATGCTGTTGCTAAGCATGATTTCTTTACTTGGCATGAGGGCAGAACCATTTATGGTAATGTTAGTAGACAGGATCTTACTAAATACACCATGATGGATTTGTGCTTCGCTCTGCGTAACTTTGATGAAAAAGATTGTGGAGTTTTTAAGGAGATATTGGTTCTTACTGGTTGTTGTAATACTGATTACTTTGAAATGAAGAATTGGTTTGACCCCATAGAAAATGAGGACATACACCGTGTGTATGCTGCTTTAGGTAAGGTAGTTGCAAATGCAATGCTTAAGTGTGTTGCTTTTTGCGACGAAATGGTGCTCAAAGGAGTTGTTGGTGTTTTGACCTTAGACAACCAAGATCTTAATGGGAATTTCTATGACTTCGGTGACTTTGTATTGTGTCCTCCTGGAATGGGAATACCCTACTGCACGTCATACTATGCTTATATGATGCCTGTTATGGGTATGACTAATTGTTTAGCTAGTGAGTGCTTTATGAAAAGTGACATCTTTGGTCAAGACTTCAAAACTTTTGATTTGTTGAAATATGATTTCACAGAACATAAGGAGGTTTTGTTTAACAAGTACTTTAAGTATTGGGGACAGGATTATCATCCTGATTGTGTTGATTGCCATGACGAGATGTGTATTTTGCATTGTTCAAATTTTAACACACTCTTCGCAACCACAATTCCAAACACAGCTTTTGGACCTCTGTGCAGAAAAGTGTTTATTGATGGTGTACCCGTAGTTGCTACTGCTGGTTACCACTTTAAACAATTAGGACTTGTGTGGAACAAAGATGTTAACACCCATTCTACCAGACTTACTATTACTGAACTCTTACAGTTTGTGACAGATCCAACGCTTATAGTTGCGTCATCGCCTGCCTTGGTGGATAAACGCACTGTTTGTTTTTCTGTCGCTGCTTTGAGTACAGGATTAACATCCCAAACAGTAAAACCTGGCCATTTTAATAAGGAGTTTTATGACTTCTTACGTTCTCAAGGGTTTTTCGATGAGGGTTCAGAATTAACATTGAAGCATTTCTTTTTTACACAAAAGGGTGATGCTGCAATTAAAGATTTTGATTATTATCGTTACAACAGACCTACCATTCTGGATATTGGACAAGCTCGCGTAGCATATCAAGTGGCAGCTCGCTATTTTGACTGTTACGAGGGTGGCTGTATTACATCTAGAGAGGTTGTTGTTACAAACCTTAATAAAAGCGCTGGTTGGCCCCTTAATAAGTTTGGTAAAGCTGGTTTATATTATGAGTCTATTAGTTATGAGGAACAAGATGCTATTTTTTTATTAACAAAGCGTAACATTCTCCCTACTATGACTCAGTTAAATCTTAAATACGCCATATCTGGTAAGGAACGTGCACGTACAGTGGGTGGCGTCTCTTTATTAGCTACTATGACTACAAGACAGTTTCATCAGAAATGTCTAAAATCCATAGTAGCTACCAGAAATGCCACTGTTGTTATCGGCACTACCAAGTTTTATGGCGGTTGGGATAATATGTTAAAGAACCTGATGGCCGATGTTGACGATCCTAAATTGATGGGATGGGACTATCCTAAGTGTGATAGAGCTATGCCCTCAATGATTCGTATGTTGTCGGCTATGATCTTAGGTTCTAAGCATGTCACATGTTGTACGGCTAGTGATAAATTTTATAGACTTAGTAATGAGCTTGCTCAAGTTTTGACCGAGGTTGTTTATTCAAATGGTGGGTTTTATTTTAAACCTGGTGGTACAACTTCTGGTGATGCAACTACAGCCTACGCCAATTCTGTCTTTAATATATTTCAGGCTGTAAGTTCTAACATTAATTGCGTTTTGAGCGTTAACTCGTCAAATTGCAATAATTTTAATGTTAAGAAGTTACAGAGACAACTCTATGATAATTGCTATAGAAATAGTAATGTTGATGAATCTTTTGTGGATGACTTTTATGGTTATTTGCAAAAGCATTTTTCTATGATGATTCTTTCTGATGATGGTGTTGTGTGCTATAATAAAACTTATGCTGAACTTGGTTACATTGCTGATATTAGTGCTTTTAAAGCCACTTTGTATTATCAGAATGGTGTGTTTATGAGTACAGCTAAGTGTTGGACTGAGGAAGATCTTTCTATAGGACCTCATGAATTTTGCTCACAGCACACTATGCAGATTGTAGATGAAAATGGTAAGTATTATCTACCATATCCAGATCCTAGCCGTATTATTTCTGCTGGTGTTTTTGTGGATGACGTCACTAAGACTGATGCTGTCATTCTTTTGGAACGCTATGTTTCTCTGGCTATAGATGCATACCCATTGTCTAAGCATCCTAAACCTGAGTACAGGAAGGTGTTTTACGCATTGTTAGATTGGGTTAAACATCTCAACAAGACTCTTAACGAAGGTGTTTTGGAGTCTTTTTCTGTTACACTTTTAGATGAACAGGAGTCTAAGTTTTGGGATGAAAGCTTTTATGCTAGTATGTATGAGAAGTCTACAGTATTACAAGCTGCTGGTCTTTGTGTAGTATGTGGCTCTCAAACAGTTCTAAGATGCGGTGATTGTTTACGCAGACCGATGTTGTGCACTAAGTGCGCCTATGATCATGTGTTTGGCACTGATCATAAGTTCATTTTAGCTATTACACCATATGTTTGTAACACATCTGGCTGCAACGTAACTGACGTTACAAAACTGTATCTTGGAGGTTTGAATTATTACTGTGTAGACCACAAACCACATCTTTCATTCCCACTGTGTTCAGCTGGTAATGTCTTTGGTTTGTACAAAAGTTCTGCTTTGGGTTCCATGGACATTGACGTCTTTAACAAACTTTCTACCTCTGATTGGTCTGACATTCGCGACTACAAGCTGGCTAATGATGCAAAAGAGTCACTAAGGTTGTTTGCAGCTGAAACGGTCAAGGCTAAAGAGGAAAGTGTTAAGTCATCATACGCTTATGCTACCCTAAAGGAGATTGTAGGTCCTAAGGAACTTTTGCTCTTATGGGAAAGTGGAAAAGCCAAACCACCGTTAAACCGTAATTCTGTTTTTACATGCTTCCAAATTACAAAAGACTCCAAGTTTCAAGTTGGTGAGTTTGTGTTTGAGAAAGTAGATTATGGTTCTGATACGGTTACTTACAAATCCACTGCTACTACTAAGTTAGTACCAGGTATGTTATTTATTTTGACTTCTCATAATGTTGCTCCACTTAGAGCGCCAACAATGGCAAACCAGGAGAAATATTCTACCATTTACAAGTTGCACCCATCATTTAATGTTAGTGATGCTTATGCAAATCTTGTACCTTATTACCAACTTATTGGCAAACAGCGTATCACCACAATACAGGGTCCTCCTGGTAGTGGAAAATCGCATTGCTCTATTGGTATTGGTGTGTATTACCCTGGAGCGAGGATCGTGTTCACTGCTTGTTCTCACGCTGCTGTTGATTCGCTTTGTGCAAAAGCTGTCACAGCCTATAGTGTTGATAAGTGTACACGTATTATTCCTGCACGTGCCAGAGTTGAGTGTTATAGTGGTTTTAAACCTAACAATACTAGTGCACAATACGTGTTTAGTACTGTTAATGCGTTACCTGAAGTTAATGCAGACATTGTTGTCGTGGATGAGGTGTCTATGTGCACTAACTATGACTTGTCTGTGATTAACCAGCGTATATCATATAAACACATTGTATATGTTGGTGATCCTCAACAGCTTCCAGCTCCTAGAGTTCTTATCTCTAAAGGTGTTATGGAACCAATTGACTATAATGTTGTGACACAACGTATGTGTGCTATAGGACCCGATGTCTTTTTACACAAGTGTTACAGATGTCCTGCTGAAATAGTTAACACTGTTTCAGAGCTTGTTTATGAAAACAAGTTTGTACCTGTCAAAGAAGCTAGTAAGCAGTGCTTCAAAATCTTTGAACGCGGTAGTGTTCAGGTAGACAATGGCTCCAGTATAAATAGGCGTCAACTTGATGTTGTTAAGCGATTTATACATAAAAACTCCACATGGAGCAAGGCTGTGTTTATCTCACCTTACAATAGTCAAAATTATGTAGCTGCCAGGCTTTTAGGTTTACAAACTCAGACAGTGGATTCTGCTCAAGGTAGTGAATATGACTATGTTATATTCGCACAGACATCAGATACTGCTCATGCCTGTAATGCCAATCGTTTTAACGTTGCCATTACTAGAGCAAAGAAAGGTATTTTCTGTATTATGTCTGACAGAACTTTGTTTGATGCACTTAAGTTCTTTGAAGTTACTATGACAGATTTACAGTCTGAAAGCAGTTGTGGTTTGTTTAAGGATTGTGCACGTAACCCTATTGATTTACCACCAAGTCATGCCACTACTTATTTGTCATTGTCTGATAGATTTAAGACTAGTGGTGACTTGGCTGTTCAAATAGGTAACAACAATGTTTGTACCTATGAACATGTGATTTCATATATGGGTTTCAGGTTTGATGTTAGCATGCCTGGTAGTCATAGTTTGTTCTGTACTAGAGACTTTGCCATGCGTCATGTCAGAGGTTGGTTAGGAATGGATGTGGAAGGTGCACATGTCACAGGTGACAATGTTGGCACTAATGTACCTCTACAAGTTGGTTTTTCCAATGGTGTTGATTTTGTAGCTCAACCTGAAGGTTGTGTTTTAACAAACACTGGCAGTGTTGTAAAACCTGTTCGTGCTCGTGCACCACCTGGAGAACAATTTACTCACATTGTACCTCTGTTACGCAAGGGACAACCTTGGAGTGTGTTGAGAAAACGTATTGTTCAAATGATAGCAGATTTTCTTGCTGGCTCATCTGATGTATTGGTGTTTGTGCTTTGGGCTGGCGGTTTAGAGTTGACCACTATGCGTTATTTTGTTAAGATTGGAGCTGTTAAACATTGCCAATGTGGTACTGTTGCAACATGCTACAATTCTGTTAGTAATGACTATTGTTGCTTTAAACATGCATTGGGCTGTGACTATGTTTATAACCCATATGTCATAGATATTCAACAATGGGGTTATGTTGGTTCACTCTCCACTAATCACCATGCAATTTGTAATGTTCATAGAAATGAGCACGTTGCTTCTGGTGATGCTATTATGACTAGATGTTTGGCTGTGTATGACTGCTTTGTTAAGAACGTGGATTGGTCAATTACCTACCCTATGATAGCTAATGAAAATGCCATAAACAAGGGCGGTCGCACTGTGCAGAGTCATATTATGCGTGCTGCTATTAAATTGTACAACCCTAAAGCAATCCATGACATTGGTAATCCTAAGGGTATTCGTTGTGCTGTAACTGATGCCAAGTGGTATTGTTATGACAAGAACCCTATTAATTCTAATGTGAAAACATTGGAGTATGATTACATGACACATGGCCAAATGGAAGGCTTGTGTTTGTTTTGGAATTGTAATGTGGATATGTACCCTGAATTCTCAATTGTTTGCAGGTTTGACACACGTACACGATCTACATTGAACCTCGAAGGTGTAAATGGTGGTTCATTGTATGTCAATAACCATGCATTTCACACTCCTGCTTATGATAAACGTGCTATGGCTAAATTGAAACCAGCACCGTTTTTCTACTATGACGACGGTTCATGTGAGGTTGTTCACGATCAAGTTAACTATGTTCCTTTGAGAGCCACTAATTGCATTACCAAGTGTAATATTGGTGGTGCTGTATGTTCTAAGCACGCTAATCTCTATAGAGCGTATGTTGAGTCATATAACATTTTTACTCAAGCTGGTTTTAATATTTGGGTTCCTACCACGTTTGATTGTTATAATTTGTGGCAGACATTCACAGATGTCAATTTACAAGGTTTAGAGAACATTGCTTTTAACGTTGTTAATAAAGGTTCATTTGTTGGTGCTGATGGTGAATTACCAGTAGCCATTAGTGGTGATAAAGTGTTCGTACGTGATGGTAACACTGATAATTTAGTCTTTGTTAACAAAACATCACTGCCTACAAATATAGCATTTGAACTTTTTGCTAAGAGGAAGGTTGGTTTAACACCACCTCTCTGTATTCTCAAAAACCTTGGTGTTGTCGCCACATATAAGTTTGTCTTGTGGGATTATGAAGCTGAGCGTCCCTTGACAAGCTTTACTAAGTCTGTTTGTGGTTATACAGACTTTGCAGAGGATGTTTGTACTTGTTACGATAATAGTATACAAGGTTCATACGAACGTTTTACTCTCTCAACTAATGCTGTGTTATTCTCTGCTACTGCTGTGAAAACAGGTGGTAAGAGTTTGCCGGCTATTAAATTGAATTTTGGAATGCTTAACGGTAATGCAATTGCTACTGTCAAATCAGAAGATGGTAACATAAAAAATATTAACTGGTTTGTTTACGTACGCAAAGATGGTAAACCTGTTGATCATTATGATGGTTTTTATACCCAAGGTCGTAATTTACAAGACTTTTTGCCTCGCAGCACAATGGAAGAAGACTTTTTGAACATGGATATAGGCGTGTTTATTCAAAAGTATGGTCTAGAGGATTTCAACTTCGAGCACGTTGTGTATGGTGATGTTTCAAAAACTACTCTAGGCGGTTTACACTTGTTGATTTCACAAGTACGTCTGAGTAAAATGGGCATCTTAAAGGCAGAGGAGTTTGTGGCAGCATCTGACATAACACTCAAATGTTGTACTGTGACTTATCTTAATGATCCTAGTTCTAAGACTGTTTGTACTTACATGGATTTGTTGTTGGATGATTTTGTTTCTTTATTGAAGTCTTTGGATTTGACTGTTGTATCCAAGGTTCATGAGGTCATAATTGACAACAAACCATGGAGATGGATGCTATGGTGTAAAGATAATGCCGTTGCTACATTCTATCCTCAGTTGCAGAGTGCAGAATGGAAATGCGGGTATTCTATGCCTGGTATTTATAAGACACAACGTATGTGCTTAGAACCATGTAATTTGTATAATTATGGTGCAGGTTTGAAGTTGCCCAGTGGCATTATGTTCAATGTTGTTAAATACACTCAATTGTGTCAATATTTTAACAGTACCACGTTATGTGTTCCTCATAATATGAGAGTGTTACACTTGGGTGCTGGCTCTGATTATGGTGTTGCACCAGGAACTGCTGTTCTTAAAAGGTGGTTGCCGCACGACGCAATTGTTGTTGACAATGATGTTGTTGACTATGTGAGTGACGCTGATTTTAGTGTTACTGGTGATTGTGCAACCGTTTATTTGGAAGACAAGTTTGACTTGTTAATCTCTGATATGTACGATGGTAGGACAAAGGCAATTGATGGTGAAAATGTTTCAAAAGAAGGATTTTTCACTTACATCAATGGTTTCATTTGTGAAAAACTTGCCATCGGAGGTTCGATTGCTATTAAAGTAACAGAGTATAGCTGGAATAAGAAATTGTATGAACTTGTACAAAGATTTTCTTTTTGGACTATGTTTTGCACTTCTGTTAATACGTCATCATCAGAAGCCTTTGTTGTCGGAATTAACTATCTTGGTGATTTCGCACAAGGACCTTTTATAGATGGTAACATAATACATGCAAATTATGTATTTTGGCGTAACTCCACTGTTATGAGTTTGTCCTACAACTCTGTTTTAGACCTGAGTAAATTTAATTGCAAACACAAAGCTACTGTTGTTGTGCAATTAAAGGATAGTGATATTAATGACATGGTGCTTAGTCTTGTTAGGAGTGGTAAGTTGCTTGTAAGGGGTAATGGCAAGTGTTTGAGTTTTAGTAATCATTTAGTCTCAACTAAATAAAATGTTTGTTTTACTTGTTGCATATGCCTTGTTGCATATTGCTGGTTGTCAAACTACAAATGGGATGAACACTAGTCACTCTGTTTGCAACGGCTGTGTTGGTCATTCGGAAAATGTATTTGCTGTTGAGAGTGGTGGTTATATACCCTCCGACTTTGCATTTAATAATTGGTTCCTCTTAACTAATACCTCATCTGTTGTAGATGGTGTTGTGAGGAGTTTTCAGCCTTTGTTGCTTAATTGCTTATGGTCTGTTTCTGGCTCGCGGTTTACTACTGGTTTTGTCTATTTTAATGGTACTGGGAGAGGTGATTGTAAAGGTTTTTATTCAAATGCTTCGTCTGATGTCATACGTTACAACATCAATTTTGAAGAAAACCTTAGACGTGGAACCATTTTGTTTAAAACATCTTATGGTGCTGTTGTGTTTTATTGTACCAACAACACTTTGGTTTCAGGTGATGCTCACATACCATCTGGTACAGTTTTGGGCAATTTTTATTGCTTTGTAAATACTACTATTGGCAATGAAACTACGTCTGCTTTTGTGGGTGCACTACCTAAGACAGTTCGTGAGTTTGTTATTTCACGCACAGGACATTTCTATATTAATGGCTATCGCTATTTCTCTTTAGGTAATGTAGAAGCCGTTAATTTCAATGTCACTAATGCAGAAACCACTGTTTGTACTGTTGCGTTAGCTTCTTATGCTGACGTTTTGGTTAATGTGTCACAAACTGCTATTGCTAATATAATTTATTGCAACTCTGTTATTAACAGACTGAGATGTGACCAGTTGTCCTTTGATGTACCAGATGGTTTTTATTCCACAAGCCCTATTCAATCCGTTGAGCTACCTGTGTCTATTGTGTCGCTACCTGTTTATCACAAACACACGTTTATTGTGTTGTATGTTAACTTCAAACTTCGGAGTGGCGTTGGCAGGTGCTATAACTGTCGTCCTGCTGTTGTTAATATTACACTGGCCAATTTTAATGAAACTAAAGGGCCTTTGTGTGTTGACACATCACACTTCACTACCCAATTCGTTGGTGCCAAGTTTGATAGGTGGAGTGCTAGTATTAACACGGGAAATTGCCCTTTTTCTTTTGGCAAAGTTAATAACTTTGTTAAATTTGGCAGTGTATGTTTTTCGCTAAAGGATATACCCGGTGGTTGCGCAATGCCTATAATGGCTAATTTGGCTAATCTTAATTCTCATAATATAGGCTCATTGTATGTTTCTTGGAGTGATGGTGATGTAATTACTGGCGTCCCAAAACCTGTTGAGGGTGTTAGTTCCTTTATGAATGTTACATTGAATAAATGTACTAAATATAACATTTATGATGTATCTGGTGTGGGTGTTATTCGCATTAGCAATGACACCTTTCTTAATGGAATTACGTACACATCAACCTCAGGTAACCTTCTGGGTTTTAAAGATGTTACTAATGGCACCATCTACTCTATCACTCCTTGTAACCCACCAGATCAGCTTGTTGTTTATCAGCAAGCTGTTGTTGGTGCTATGTTGTCTGAAAACTTTACTAGTTACGGCTTTTCTAATGTTGTAGAAATGCCGAAATTTTTCTATGCGTCCAATGGCACTTATAATTGCACAGACGCTGTTTTAACTTATTCTAGTTTTGGCGTTTGTGCAGATGGTTCTATAATTGCTGTTCAACCACGTAATGTTTCATATGATAGTGTTTCAGCTATCGTCACAGCTAATTTGTCTATACCCTCCAATTGGACCACTTCGGTCCAGGTTGAGTATTTACAAATTACAAGTACACCTATCGTAGTTGATTGCTCCACTTATGTTTGCAATGGTAATGTGCGCTGTGTTGAATTGCTTAAGCAGTATACTTCTGCTTGTAAAACTATTGAAGACGCCTTAAGAAATAGCGCCATGCTGGAGTCTGCAGATGTTAGTGAGATGCTCACTTTTGACAAGAAAGCGTTTACACTTGCTAATGTTAGTAGTTTTGGTGACTACAACCTTAGCAGTGTTATACCTAGCTTGCCCAGAAGTGGTAGTAGAGTGGCTGGTCGCAGTGCCATAGAAGACATACTTTTTAGCAAACTTGTTACGTCTGGACTTGGCACTGTGGACGCAGACTACAAAAAGTGCACTAAGGGTCTTTCCATTGCTGACTTGGCTTGTGCCCAATATTATAATGGCATTATGGTTTTGCCTGGCGTCGCTGATGCTGAACGAATGGCCATGTATACAGGTTCTTTAATTGGTGGAATTGCTTTAGGAGGTCTTACATCAGCCGCTTCAATACCATTTTCATTAGCAATTCAGTCACGTTTAAATTATGTTGCATTGCAGACTGATGTTTTACAAGAAAATCAGAAAATTCTTGCTGCATCTTTTAATAAAGCAATGACCAACATAGTAGATGCCTTTACTGGTGTTAATGATGCTATTACACAAACTTCACAAGCTCTACAAACAGTTGCTACTGCACTTAATAAGATCCAGGATGTTGTTAATCAACAAGGCAACTCATTGAACCATTTAACTTCTCAGTTGAGGCAGAATTTTCAAGCCATTTCTAGCTCTATTCAGGCTATCTATGACAGACTTGATATTATTCAGGCTGATCAACAAGTAGATAGGCTGATTACTGGTAGATTGGCTGCTCTGAATGTATTCGTTTCTCATACATTGACTAAGTACACTGAAGTTCGTGCTTCCAGACAGCTTGCACAACAAAAAGTGAATGAGTGTGTCAAATCCCAGTCTAAGCGGTATGGCTTCTGTGGAAATGGCACGCACATTTTCTCACTTGTTAATGCTGCTCCTGAGGGGCTTGTTTTTCTCCATACTGTCTTGTTGCCGACACAATATAAGGATGTTGAAGCGTGGTCTGGGTTGTGCGTTGATGGTAGAAACGGTTATGTGTTGAGACAACCTAATCTTGCTCTTTACAAAGAAGGCAATTATTATAGAATTACATCTCGCATAATGTTTGAACCACGTATTCCTACCATAGCAGATTTTGTCCAAATTGAAAATTGCAATGTCACATTTGTTAACATTTCTCGCTCTGAGTTGCAAACTATTGTGCCAGAGTATATTGATGTTAATAAGACGCTGCAAGAATTAAGTTACAAATTGCCAAATTACACTGTTCCAGACCTAGTTGTCGAACAGTACAACCAGACTATTTTGAATTTGACCAGTGAAATTAGCACCCTTGAAAATAAATCTGCGGAGCTTAATTACACTGTTCAAAAACTGCAAACTTTGATCGACAACATAAATAGCACATTAGTTGACTTAAAGTGGCTCAACCGGGTTGAGACTTATATCAAGTGGCCGTGGTGGGTGTGGTTGTGCATTTCAGTCGTGCTCATCTTTGTGGTGAGTATGTTGCTATTATGTTGTTGTTCTACTGGTTGCTGTGGTTTCTTTAGTTGTTTTGCATCTTCTATTAGAGGTTGTTGTGAATCAACTAAACTTCCTTATTACGACGTTGAAAAGATCCACATACAGTAATGGCTCTAGGTTTGTTCACATTGCAATTTGTGTCTGCTGTTAATCAATCTCTTATCAAGTCGAAAGTTAGTGCTGAAGTTTCACGACAGGTTATCCAAGACGTGAAAGATGGCACTGTTACCTTCAACTTGCTAGCGTATACACTAATGAGCCTCTTTGTTGTGTATTTTGCTTTATTTAAAGCAAGATCACACCGTGGCAGATCTGCTCTTATAGTGTTTAAAATTTTAATCCTTTTCGTTTATGTGCCATTGCTGTATTGGTCTCAAGCATATATTGACGCAACTTTGATTGCTGTAATTTTGCTTGGAAGATTTTTCCATACAGCTTGGCACTGCTGGCTCTACAAGACATGGGATTTCATTGTCTTCAATGTAACCACACTTTGCTATGTGCAAGGTAAGTGTTGGTTTCTTGAAAATAAGGCTCTGAAACCATTCGTCTGTTTTTACGGAGGGGATCAATTCCTTTACATAGGCGACAGAATTGTTACTTATCTCTCAACTAACGATTTGTACGTTGCTCTTAGAGGACGTATTGATAAAGACCTCAGCCTTTCTAGAAAGGTTGAGTTATATAACGGTGAATGTGTATACTTGTTTTGTGAACACCCAGCTGTTGGAATAGTCAATACAGATTTCAAATTAGAAATCCACTAAGATGTTCCTTAAGCTAGTGGATGATCATGCTTTGATTGTTAATGTACTACTCTGGTGTGTGGTGCTTATAGTGATACTACTAGTGTGTATTACAATAATTAAACTAATTAAGCTTTGTTTCACTTGCCATATGTTTTGTAATAGAACAGTTTATGGCCCCATTAAAAATGTGTACCACATTTACCAATCATATATGCACATAGACCCTTTCCCTAAACGAGTTATTGATTTCTAAACTAAACGACAATGTCAAACGACAATTGTACGGGTGACATTGTCACCCATTTGAAGAATTGGAATTTTGGTTGGAATGTTATTCTAACCATATTCATTGTTATTCTTCAGTTTGGACACTATAAATACTCCAGATTGTTTTATGGTTTGAAGATGCTTGTACTGTGGCTTCTTTGGCCACTTGTACTTGCTTTGTCAATCTTTGACACCTGGGCTAATTGGGACTCTAATTGGGCCTTTGTTGCATTTAGCTTTCTTATGGCCGTATCAACACTCGTTATGTGGGTGATGTACTTCGCAAATAGTTTCAGACTTTTCCGACGTGCTCGAACTTTTTGGGCATGGAATCCTGAGGTTAATGCAATCACTGTCACAACCGTGTTGGGACAGACATACTATCAACCCATTCAACAAGCTCCAACAGGCATTACTGTGACCTTGTTGAGCGGCGTGCTTTACGTTGACGGACATAGATTGGCTTCAGGTGTTCAGGTTCATAACCTACCTGAATACATGACAGTTGCCGTGCCGAGCACTACTATAATTTATAGTAGAGTCGGAAGGTCCGTAAATTCTCAAAATAGCACAGGCTGGGTTTTCTACGTACGAGTAAAACACGGTGATTTTTCTGCAGTGAGCTCCCCCATGAGCAACATGACAGAAAACGAAAGATTGCTTCATTTTTTCTAAACTGAACGAAAAGATGGCTACAGTCAAATGGGCTGATGCATCTGAACCACAACGTGGTCGTCAGAGTAGAATACCTTATTCTCTTTATAGCCCTTTGCTTGTTGATAGTGAACAACCTTGGAAGGTGATACCTCGTAATTTGGTACCCATCAACAAGAAAGACAAAAATAAGCTTATAGGCTATTGGAATGTTCAAAAACGTTTCAGAACTAGAAAGGGCAAACGGGTGGATTTGTCACCCAAGTTACATTTTTATTATCTTGGCACAGGACCCCATAAAGATGCAAAATTTAGAGAGCGTGTTGAAGGTGTCGTCTGGGTTGCTGTTGATGGTGCTAAAACTGAACCTACAGGTTACGGTGTTAGGCGCAAGAATTCAGAACCAGAGATACCACACTTCAATCAAAAGCTCCCAAATGGTGTTACTGTTGTTGAAGAACCTGACTCCCGTGCTCCTTCCCGTTCTCAGTCAAGGTCGCAGAGTCGCGGTCGTGGTGAATCCAAATCTCAATCTCGGAATCCTTCAAGTGACAGAAACCATAACAGTCAGGATGACATCATGAAGGCAGTCGCTGCGGCTCTTAAATCTTTAGGTTTTGACAAGCCTCAGGAAAAAGACAAAAAGTCAGCGAAAACGGGTACTCCTAAGCCTTCTCGTAATCAGAGTCCTGCTTCTTCTCAATCTGCTGCCAAGATTCTTGCTCGTTCTCAGAGTTCTGAAACAAAAGAACAAAAGCATGAAATGCAAAAGCCACGGTGGAAAAGACAGCCTAACGATGATGTGACATCTAATGTCACACAATGTTTTGGCCCCAGAGACCTTGACCACAACTTTGGAAGTGCAGGTGTTGTGGCCAATGGTGTTAAAGCTAAAGGCTATCCACAATTTGCTGAGCTTGTGCCGTCTACAGCTGCTATGCTTTTTGATAGTCACATTGTTTCCAAAGAGTCAGGCAACACTGTGGTCTTGACTTTCACCACTAGAGTGACTGTGCCCAAAGACCATCCACACTTGGGTAAGTTTCTTGAGGAATTAAATGCATTCACTAGAGAAATGCAACAACAGCCTCTTCTTAACCCTAGTGCACTAGAATTCAACCCATCCCAAACTTCACCTGCAACTGTTGAACCAGTGCGTGATGAAGTTTCTATTGAAACTGACATAATTGATGAAGTCAACTAAACATGCCACTGTGTTGAAATTCAGGCTTTAGTTGGAATTTTGCTTTTGCTCTTGTTTTTATTATCTTTCTTTTATGAATACATTTCTTTCCTCTGATCTACGTATGATGGTACGATCAGAGCTGCTTTTAATTAACATGATCCCTTGCTTTGGCTTGACAAGGATCTAGTCTTATACACAATGGTAAGCCTGTGGTAGTAAAGGTATAAGAAATTTGCTACTATGTTACTGAACCTAGGTGAACGCTAGTATAACTCATTACAAATGTGCTGGAGTAATCAAAGATCGCATTGACGAGCCAACAATGGAAGAGCCAGTCATTTGTCTTGAGACCTATCTAGTTAGTAA

>HEPC2_STRIPPED4

ACCCGCCCCTAATAGGGGCGACACTCCGCCATGAATCACTCCCCTGTGAGGAACTACTGTCTTCACGCAGAAAGCGTCTAGCCATGGCGTTAGTATGAGTGTCGTACAGCCTCCAGGCCCCCCCCTCCCGGGAGAGCCATAGTGGTCTGCGGAACCGGTGAGTACACCGGAATTGCCGGGAAGACTGGGTCCTTTCTTGGATAAACCCACTCTATGCCCGGCCATTTGGGCGTGCCCCCGCAAGACTGCTAGCCGAGTAGCGTTGGGTTGCGAAAGGCCTTGTGGTACTGCCTGATAGGGTGCTTGCGAGTGCCCCGGGAGGTCTCGTAGACCGTGCACCATGAGCACAAATCCTAAACCTCAAAGAAAAACCAAAAGAAACACCAACCGTCGCCCACAAGACGTTAAGTTTCCGGGCGGCGGCCAGATCGTTGGCGGAGTATACTTGTTGCCGCGCAGGGGCCCCAGGTTGGGTGTGCGCGCGACAAGGAAGACTTCGGAGCGGTCCCAGCCACGTGGAAGGCGCCAGCCCATCCCTAAAGATCGGCGCTCCACTGGCAAATCCTGGGGAAAACCAGGATACCCCTGGCCCCTATACGGGAATGAGGGACTCGGCTGGGCAGGATGGCTCCTGTCCCCCCGAGGTTCCCGTCCCTCTTGGGGCCCCAATGACCCCCGGCATAGGTCGCGCAACGTGGGTAAGGTCATCGATACCCTAACGTGCGGCTTTGCCGACCTCATGGGGTACATCCCTGTCGTGGGCGCCCCGCTCGGCGGCGTCGCCAGAGCTCTCGCGCATGGCGTGAGAGTCCTGGAGGACGGGGTTAATTTTGCAACAGGGAACTTACCCGGTTGCTCCTTTTCTATCTTCTTGCTGGCCCTGCTGTCCTGCATCACCACCCCGGTCTCCGCTGCCGAAGTGAAGAACATCAGTACCGGCTACATGGTGACTAACGACTGCACCAATGACAGCATTACCTGGCAGCTCCAGGCTGCTGTCCTCCACGTCCCCGGGTGCGTCCCGTGCGAGAAAGTGGGGAATGCATCTCAGTGCTGGATACCGGTCTCACCGAATGTGGCCGTGCAGCGGCCCGGCGCCCTCACGCAGGGCTTGCGGACGCACATCGACATGGTTGTGATGTCCGCCACGCTCTGCTCTGCCCTCTACGTGGGGGACCTCTGCGGTGGGGTGATGCTCGCAGCCCAAATGTTCATTGTCTCGCCGCAGCACCACTGGTTTGTCCAAGACTGCAATTGCTCCATCTACCCTGGTACCATCACTGGACACCGCATGGCATGGGACATGATGATGAACTGGTCGCCCACGGCTACCATGATCTTGGCGTACGCGATGCGTGTCCCCGAGGTCATTATAGACATCATTAGCGGGGCTCATTGGGGCGTCATGTTCGGCTTGGCCTACTTCTCTATGCAGGGAGCGTGGGCGAAAGTCGTTGTCATCCTTCTGTTGGCCGCCGGGGTGGACGCGCGCACCCATACTGTTGGGGGTTCTGCCGCGCAGACCACCGGGCGCCTCACCAGCTTATTTGACATGGGCCCCAGGCAGAAAATCCAGCTCGTTAACACCAATGGCAGCTGGCACATCAACCGCACCGCCCTGAACTGCAATGACTCCTTGCACACCGGCTTTATCGCGTCTCTGTTCTACACCCACAGCTTCAACTCGTCAGGATGTCCCGAACGCATGTCCGCCTGCCGCAGTATCGAGGCCTTCCGGGTGGGATGGGGCGCCTTGCAATATGAGGATAATGTCACCAATCCAGAGGATATGAGACCCTATTGCTGGCACTACCCACCAAGGCAGTGTGGCGTGGTCTCCGCGAAGACTGTGTGTGGCCCAGTGTACTGTTTCACCCCCAGCCCAGTGGTAGTGGGCACGACCGACAGGCTTGGAGCGCCCACTTACACGTGGGGGGAGAATGAGACAGATGTCTTCCTATTGAACAGCACTCGACCACCGCTGGGGTCATGGTTCGGCTGCACGTGGATGAACTCTTCTGGCTACACCAAGACTTGCGGCGCACCACCCTGCCGTACTAGAGCTGACTTCAACGCCAGCACGGACCTGTTGTGCCCCACGGACTGTTTTAGGAAGCATCCTGATACCACTTACCTCAAATGCGGCTCTGGGCCCTGGCTCACGCCAAGGTGCCTGATCGACTACCCCTACAGGCTCTGGCATTACCCCTGCACAGTTAACTATACCATCTTCAAAATAAGGATGTATGTGGGAGGGGTTGAGCACAGGCTCACGGCTGCATGCAATTTCACTCGTGGGGATCGTTGCAACTTGGAGGACAGAGACAGAAGTCAACTGTCTCCTTTGTTGCACTCCACCACGGAATGGGCCATTTTACCTTGCTCTTACTCGGACCTGCCCGCCTTGTCGACTGGTCTTCTCCACCTCCACCAAAACATCGTGGACGTACAATTCATGTATGGCCTATCACCTGCCCTCACAAAATACATCGTCCGATGGGAGTGGGTAATACTCTTATTCCTGCTCTTAGCGGACGCCAGGGTTTGCGCCTGCTTATGGATGCTCATCTTGTTGGGCCAGGCCGAAGCAGCACTAGAGAAGCTGGTCATCTTGCACGCTGCGAGCGCAGCTAGCTGCAATGGCTTCCTATATTTTGTCATCTTTTTCGTGGCTGCTTGGTACATCAAGGGTCGGGTAGTCCCCTTAGCTACCTATTCCCTCACTGGCCTGTGGTCCTTTAGCCTACTGCTCCTAGCATTGCCCCAACAGGCTTATGCTTATGACGCATCTGTGCATGGCCAGATAGGAGCGGCTCTGCTGGTAATGATCACTCTCTTTACTCTCACCCCCGGGTATAAGACCCTTCTCAGCCGGTTTTTGTGGTGGTTGTGCTATCTTCTGACCCTGGGGGAAGCTATGGTCCAGGAGTGGGCACCACCTATGCAGGTGCGCGGTGGCCGTGATGGCATCATATGGGCCGTCGCCATATTCTACCCAGGTGTGGTGTTTGACATAACCAAGTGGCTCTTGGCGGTGCTTGGGCCTGCTTACCTCCTAAAAGGTGCTTTGACGCGCGTGCCGTACTTCGTCAGGGCTCACGCTCTACTGAGGATGTGCACCATGGCAAGGCATCTCGCGGGGGGCAGGTACGTCCAGATGGCGCTACTAGCCCTTGGCAGGTGGACTGGCACTTACATCTATGACCACCTCACCCCTATGTCGGATTGGGCTGCTAGTGGCCTGCGGGACCTGGCGGTCGCCGTTGAGCCTATCATCTTCAGTCCGATGGAGAAGAAAGTCATTGTCTGGGGAGCGGAGACAGCTGCTTGTGGGGACATTTTACACGGACTTCCCGTGTCCGCCCGACTTGGTCGGGAGGTCCTCCTTGGCCCAGCTGATGGCTATACCTCCAAGGGGTGGAGTCTTCTCGCCCCCATCACTGCTTACGCCCAGCAGACACGTGGCCTTTTGGGCACCATAGTGGTGAGCATGACGGGGCGCGACAAGACAGAACAGGCTGGGGAAATTCAGGTCCTGTCCACAGTCACTCAGTCCTTCCTCGGAACATCCATCTCGGGGGTTTTGTGGACTGTCTACCATGGAGCTGGCAACAAGACTCTGGCCGGCTCACGGGGTCCGGTCACGCAGATGTACTCCAGTGCTGAGGGGGACTTAGTAGGGTGGCCCAGCCCCCCTGGGACTAAATCTTTGGAGCCGTGCACGTGTGGAGCGGTCGACCTGTACCTGGTCACGCGGAACGCTGATGTCATCCCGGCTCGAAGACGCGGGGACAAACGGGGAGCGCTACTCTCCCCGAGACCTCTTTCCACCTTGAAGGGGTCCTCAGGAGGCCCGGTGCTATGCCCCAGGGGCCACGCTGTCGGAGTCTTCCGGGCAGCTGTGTGCTCTCGGGGCGTGGCTAAGTCCATAGATTTCATCCCCGTTGAGACACTCGACATCGTCACGCGGTCCCCCACCTTTAGTGACAACAGCACACCACCTGCTGTGCCCCAGACCTATCAGGTCGGGTACTTGCATGCCCCGACTGGCAGTGGAAAGAGCACCAAAGTTCCTGTCGCATATGCTGCTCAGGGGTATAAAGTGCTAGTGCTTAATCCCTCAGTGGCTGCCACCCTGGGGTTTGGGGCGTACTTGTCTAAGGCACATGGCATCAATCCCAACATTAGGACTGGAGTCAGGACTGTGACGACCGGGGCGCCCATCACGTACTCCACATATGGCAAATTCCTCGCCGATGGGGGCTGTGCGGGCGGCGCCTACGACATCATCATATGTGATGAATGCCATGCCGTGGACTCTACCACCATCCTTGGCATCGGAACAGTCCTTGATCAAGCAGAGACAGCTGGGGTCAGACTAACTGTGCTGGCTACAGCTACGCCCCCTGGGTCAGTGACAACCCCCCACCCCAACATAGAGGAGGTGGCCCTTGGGCAGGAGGGCGAGATCCCCTTCTATGGGAGGGCGATTCCCCTGTCTTACATCAAGGGAGGAAGACATCTGATCTTCTGCCATTCAAAGAAAAAGTGTGACGAGCTCGCGGCGGCCCTTCGGGGTATGGGCTTGAACTCAGTGGCATACTACAGAGGGTTGGACGTCTCCGTAATACCAACTCAGGGAGACGTAGTGGTCGTCGCCACCGACGCCCTCATGACAGGGTATACTGGGGACTTTGACTCCGTGATCGACTGCAACGTAGCGGTCACTCAAGTTGTAGACTTCAGTTTAGACCCCACATTCACCATAACCACACAGATTGTCCCTCAAGACGCTGTCTCACGTAGCCAGCGCCGGGGTCGCACGGGTAGGGGAAGACTGGGCATTTATAGGTATGTTTCCACTGGTGAGCGAGCCTCAGGAATGTTTGACAGTGTAGTGCTCTGTGAGTGCTACGACGCAGGGGCCGCATGGTATGAGCTCACACCATCGGAGACCACCGTCAGGCTCAGGGCGTATTTCAACACGCCCGGTTTGCCTGTGTGCCAAGACCATCTTGAGTTTTGGGAGGCAGTTTTCACCGGCCTCACACACATAGATGCCCACTTCCTTTCCCAAACAAAGCAATCGGGGGAAAATTTCGCATACTTAACAGCCTACCAGGCTACAGTGTGCGCTAGGGCCAAAGCCCCCCCCCCGTCCTGGGACGTCATGTGGAAGTGTTTGACTCGACTCAAGCCCACACTCGTGGGCCCCACACCTCTCCTGTACCGCTTGGGCTCTGTTACCAACGAGGTCACCCTCACACATCCCGTGACGAAATACATCGCCACCTGCATGCAAGCCGACCTTGAGGTCATGACCAGCACATGGGTCTTGGCAGGGGGAGTCTTGGCGGCCGTCGCCGCGTATTGCCTGGCGACCGGGTGTGTTTGCATCATCGGCCGCTTGCACATTAACCAGCGAGCCGTCGTTGCGCCGGACAAGGAGGTCCTCTATGAGGCTTTTGATGAGATGGAGGAATGTGCCTCTAGGGCGGCTCTCATTGAAGAGGGGCAGCGGATAGCCGAGATGCTGAAGTCCAAGATCCAAGGCTTATTGCAGCAAGCTTCCAAACAAGCTCAAGACATACAACCCACTGTGCAGGCTTCATGGCCCAAGGTAGAACAATTCTGGGCCAAACACATGTGGAACTTCATTAGCGGCATCCAATACCTCGCAGGACTATCAACACTGCCAGGGAACCCTGCAGTAGCTTCCATGATGGCGTTCAGTGCCGCCCTCACCAGTCCGCTGTCAACAAGCACCACTATCCTTCTCAACATTTTGGGGGGCTGGCTAGCATCCCAAATTGCACCACCCGCGGGGGCCACTGGCTTCGTTGTCAGTGGCCTAGTGGGAGCTGCCGTAGGCAGTATAGGCTTAGGTAAGGTGCTAGTGGACATCCTGGCAGGGTATGGTGCGGGCATTTCGGGGGCTCTCGTCGCATTCAAGATCATGTCTGGCGAGAAGCCCTCCATGGAGGATGTCGTCAACTTGCTGCCTGGAATTCTGTCTCCGGGTGCCTTGGTAGTGGGAGTCATCTGCGCGGCCATTCTGCGCCGACACGTGGGACCGGGGGAAGGCGCCGTCCAATGGATGAATAGACTCATTGCCTTTGCTTCCAGAGGAAATCACGTCGCCCCCACCCACTACGTGACGGAGTCGGATGCGTCGCAGCGTGTGACCCAACTACTTGGCTCCCTTACCATAACCAGCCTGCTCAGAAGACTCCACAACTGGATTACTGAGGACTGCCCCATCCCATGCGGCGGCTCGTGGCTCCGCGATGTGTGGGACTGGGTTTGCACCATCCTAACAGACTTTAAAAATTGGCTGACCTCCAAATTATTCCCAAAGATGCCCGGCCTCCCCTTTGTCTCCTGTCAAAAGGGGTACAAGGGCGTGTGGGCCGGCACTGGCATCATGACCACACGGTGTCCTTGCGGCGCCAATATCTCTGGCAATGTCCGCTTGGGCTCCATGAGAATCACGGGGCCTAAGACCTGCATGAATATCTGGCAGGGGACCTTTCCTATCAATTGTTACACGGAGGGCCAGTGCGTGCCGAAACCCGCGCCAAACTTTAAGGTCGCCATCTGGAGGGTGGCGGCCTCAGAGTACGCGGAGGTGACGCAGCACGGGTCATACCACTACATAACAGGACTCACCACTGATAACTTGAAAGTCCCCTGCCAACTACCCTCTCCCGAGTTCTTTTCCTGGGTGGACGGAGTGCAGATCCATAGGTTTGCCCCCACACCGAAGCCGTTTTTCCGGGATGAGGTCTCGTTCTGCGTTGGGCTTAATTCATTTGTCGTCGGGTCCCAGCTTCCTTGCGACCCTGAACCCGACACAGACGTATTGATGTCCATGCTAACAGATCCATCTCATATCACGGCGGAGACTGCAGCGCGGCGTTTAGCGCGGGGGTCACCCCCATCCGAGGCAAGCTCCTCGGCGAGCCAGCTATCGGCACCATCGCTGCGAGCCACCTGCACCACCCACGGCAAAGCCTATGATGTGGACATGGTGGATGCTAACCTGTTCATGGGGGGCGATGTGACTCGGATAGAGTCTGGGTCCAAAGTGGTCGTTCTGGACTCTCTCGACCCAATGGTCGAAGAAAGGAGCGACCTTGAGCCTTCGATACCATCAGAATACATGCTCCCCAAGAAGAGGTTCCCACCAGCTTTACCGGCCTGGGCACGGCCTGATTACAACCCACCGCTTGTGGAATCGTGGAAAAGGCCAGATTACCAACCGGCCACTGTTGCGGGCTGTGCTCTCCCTCCTCCTAGGAAAACCCCGACGCCTCCCCCAAGGAGGCGCCGGACAGTGGGCCTAAGTGAGGACTCCATAGGAGATGCCCTTCAACAGCTGGCCATTAAGTCCTTTGGCCAGCCCCCCCCAAGCGGCGATTCAGGCCTTTCCACGGGGGCGGGCGCTGCCGATTCCGGCAGTCAGACGCCTCCTGATGAGTTGGCCCTTTCGGAGACAGGTTCCATCTCTTCCATGCCCCCCCTCGAGGGGGAGCTTGGAGATCCAGACCTGGAGCCTGAGCAGGTAGAGCCCCAACCCCCCCCCCAGGGGGGGGTGGCAGCTCCCGGCTCGGACTCGGGGTCCTGGTCTACTTGCTCCGAGGAGGACGACTCCGTCGTGTGCTGCTCCATGTCATACTCCTGGACCGGGGCTCTAATAACTCCTTGTAGTCCCGAAGAGGAGAAGTTACCGATTAACCCCTTGAGCAACTCCCTGTTGCGATATCACAACAAGGTGTACTGTACCACAACAAAGAGCGCCTCACTAAGGGCTAAAAAGGTAACTTTTGATAGGATGCAAGTGCTCGACTCCTACTACGACTCAGTCTTAAAGGACATTAAGCTAGCGGCCTCCAAGGTCACCGCAAGGCTCCTCACCATGGAGGAGGCTTGCCAGTTAACCCCACCCCATTCTGCAAGATCTAAATATGGGTTTGGGGCTAAGGAGGTCCGCAGCTTGTCCGGGAGGGCCGTTAACCACATCAAGTCCGTGTGGAAGGACCTCCTGGAGGACTCAGAAACACCAATTCCCACAACCATTATGGCCAAAAATGAGGTGTTCTGCGTGGACCCCACCAAGGGGGGCAAGAAAGCAGCTCGCCTTATCGTTTACCCTGACCTCGGCGTCAGGGTCTGCGAGAAGATGGCCCTTTATGACATTACACAAAAACTTCCTCAGGCGGTGATGGGGGCTTCTTATGGATTCCAGTATTCCCCCGCTCAGCGGGTAGAGTTTCTCTTGAAAGCATGGGCGGAAAAGAAGGACCCTATGGGTTTTTCGTATGATACCCGATGCTTTGACTCAACCGTCACTGAGAGAGACATCAGGACTGAGGAGTCCATATATCGGGCCTGCTCCTTGCCCGAGGAGGCCCACACTGCCATACACTCGCTAACTGAGAGACTTTACGTGGGAGGGCCTATGTTCAACAGCAAGGGCCAAACCTGCGGGTACAGGCGTTGCCGCGCCAGCGGGGTGCTCACCACTAGCATGGGGAACACCATCACATGCTACGTGAAAGCCTTAGCGGCTTGTAAAGCTGCAGGGATAATCGCGCCCACAATGCTGGTATGCGGCGATGACTTGGTTGTCATCTCAGAAAGCCAGGGGACCGAGGAGGACGAGCGGAACCTGAGAGCCTTCACGGAGGCTATGACCAGGTATTCTGCCCCTCCTGGTGACCCCCCCAGACCGGAGTATGATCTGGAGCTGATAACATCTTGCTCCTCAAATGTGTCTGTGGCGCTGGGCCCACAAGGCCGCCGCAGATACTACCTGACCAGAGACCCTACCACTCCAATCGCCCGGGCTGCCTGGGAAACAGTTAGACACTCCCCTGTCAATTCATGGCTGGGAAACATCATCCAGTACGCCCCGACCATATGGGCTCGCATGGTCCTGATGACACACTTCTTCTCCATTCTCATGGCTCAAGACACGCTGGACCAGAACCTCAACTTTGAGATGTACGGAGCGGTGTACTCCGTGAGTCCCTTGGACCTCCCAGCTATAATTGAAAGGTTACATGGGCTTGACGCTTTTTCTCTGCACACATACACTCCCCACGAACTGACACGGGTGGCTTCAGCCCTCAGAAAACTTGGGGCGCCACCCCTCAGAGCGTGGAAGAGCCGGGCACGTGCAGTCAGGGCGTCCCTCATCTCCCGTGGGGGGAGAGCGGCCGTTTGCGGTCGATATCTCTTCAATTGGGCGGTGAAGACCAAGCTCAAACTCACTCCATTGCCGGAAGCGCGCCTCCTGGATTTATCCAGCTGGTTCACCGTCGGCGCCGGCGGGGGCGACATTTATCACAGCGTGTCGCGTGCCCGACCCCGCTTATTGCTCTTTGGCCTACTCCTACTTTTTGTAGGGGTAGGCCTTTTCCTACTCCCCGCTCGGTAGAGCGGCACACATTAGCTACACTCCATAGCTAACTGTCCC

>HEPC4_STRIPPED4

TTCACGCAGAAAGCGTCTAGCCATGGCGTTAGTATGAGTGTTGTACAGCCTCCAGGACCCCCCCTCCCGGGAGAGCCATAGTGGTCTTCGGAACCGGTGAGTACACCGGAATCGCCGGGATGACCGGGTCCTTTCTTGGATTAACCCGCTCAATGCCCGGAAATTTGGGCGTGCCCCCGCAAGACTGCTAGCCGAGTAGTGTTGGGTCGCGAAAGGCCTTGCGGTACTGCCTGATAGGGTGCTTGCGAGTGCCCCGGGAGGTCTCGTAGACCGTGCACCATGAGCACGAATCCTAAACCTCAAAGAAAAACCAAACGTAACACCAACCGCCGCCCAATGGACGTTAAGTTCCCGGGTGGTGGCCAGATCGTTGGCGGAGTTTACTTGTTGCCGCGCAGGGGCCCCAGATTGGGTGTGCGCGCGACTCGGAAGACTTCGGAGCGGTCGCAACCTCGTGGAAGACGCCAACCTATCCCCAAGGCGCGTCGACCCGAGGGAAGGTCCTGGGCACAACCAGGATATCCATGGCCTCTTTACGGTAATGAGGGTTGTGGGTGGGCAGGATGGCTCTTGTCCCCCCGTGGCTCTCGACCGTCTTGGGGCCCAAATGATCCCCGCGGGAGGTCCCGCAATTTGGGTAAGGTCATCGATACCCTAACCTGCGGCTTCGCCGACCTCATGGGATACATCCCGCTCGTAGGCGCCCCCGTGGGTAGCGTCGCCAGGGCCCTGGCACATGGTGTCAGGGCTTTGGAGGACGGGATCAATTATGCAACAGGGAATCTCCCCGGTTGCTCCTTTTCTATCTTCCTCTTGGCACTTCTTTCGTGCCTGACTGTCCCCGCTTCGGCCGTTAACTATCGCAATGTCTCAGGCATCTACCATGTCACCAATGACTGCCCGAATTCAAGCATAGTGTATGAGGCCGACCATCACATCATGCACCTTCCAGGTTGCGTGCCCTGCGTGAGAGAGGGGAATCAGTCACGCTGCTGGGTGGCCCTTACTCCTACCGTCGCAGCGCCATACATCGGCGCACCGCTTGAGTCCTTACGGAGTCATGTGGATTTGATGGTGGGGGCCGCCACTGTCTGCTCGGGTCTTTACATCGGGGACCTGTGTGGCGGCTTGTTCCTAGTTGGCCAGATGTTTTCATTCCGACCACGGCGCCACTGGACCACCCAGGATTGCAATTGTTCCATCTACACAGGGCACATTACAGGCCACAGAATGGCCTGGGACATGATGATGAACTGGAGTCCAACAACCACCTTAGTTCTCGCCCAGGTCATGAGGATCCCAACCACTCTGGTAGACTTACTCTCTGGAGGCCACTGGGGTGTCCTCGTGGGGGTGGCCTATTTCAGCATGCAGGCCAATTGGGCCAAAGTCATCTTGGTCCTATTCCTCTTTGCAGGGGTTGATGCCGAGACTCACGTGTCTGGGGCAGCAGTCGGCCGGAGTACCGCCGGCTTGGCTAACCTCTTTTCTTCTGGGTCTAAGCAGAATTTACAGCTCATCAACAGCAATGGGAGCTGGCATATAAATAGGACTGCCCTTAACTGCAATGACAGCTTAAACACTGGGTTCTTGGCTAGCTTGTTCTACACCCACAAGTTTAACAGCTCAGGGTGTTCCGAACGGCTCGCGTGCTGCAAGAGCCTTGACAGCTACGGCCAAGGCTGGGGCCCACTCGGGGTCGCTAACATCAGCGGCTCGTCTGATGACAGGCCTTATTGCTGGCACTACGCGCCTCGGCCATGCGGGATTGTGCCAGCATCCAGTGTGTGTGGCCCCGTGTATTGTTTCACTCCCAGCCCTGTCGTGGTCGGTACTACTGATCACGTCGGGGTCCCTACTTACACCTGGGGGGAGAACGAGACTGATGTCTTCCTTTTGAACTCGACCAGACCGCCGCATGGTGCGTGGTTTGGATGCGTGTGGATGAACAGTACCGGGTTCACCAAAACCTGTGGCGCCCCTCCATGCGAGGTTAACACCAATAATGGGACCTGGCACTGCCCCACCGATTGCTTCAGGAAGCATCCGGAGACTACCTACGCCAAGTGCGGATCAGGGCCTTGGATCACACCACGCTGCCTGATTGATTACCCGTACCGGCTGTGGCATTTCCCGTGCACCGCCAACTTCTCCGTCTTTAACATCAGGACATTTGTCGGCGGTATAGAGCATCGGATGCAAGCGGCATGCAACTGGACCAGGGGGGAAGTCTGTGGCTTGGAGCACAGGGATCGGGTAGAGCTATCACCCCTGCTCCTTACCACTACAGCGTGGCAGATCCTCCCCTGCTCTTTCACCACTTTACCTGCCCTCTCCACCGGCTTGATCCACCTCCACCAAAATATCGTGGACGTCCAGTACCTCTATGGTGTTGGATCTGCAGTGGTATCTTGGGCCCTTAAGTGGGAATATGTGGTGCTCGCGTTCCTGCTTCTCGCGGACGCGAGAGTCTCTGCCTACCTATGGATGATGTTTATGGTAAGTCAAGTTGAGGCGGCTCTGTCCAACCTAATTAACATCAATGCTGCTTCAGCCGCTGGTGCCCAAGGCTTCTGGTACGCCATCCTCTTCATCTGCATTGTCTGGCATGTCAAGGGCCGGTTCCCAGCTGCTGCTGCCTACGCAGCCTGCGGGCTGTGGCCCTGTTTTCTCCTGCTTCTGATGCTGCCTGAGAGGGCTTATGCATACGACCAGGAAGTGGCAGGGTCCCTTGGCGGCGCCATCGTTGTCATGCTGACCATTCTGACACTGTCTCCGCACTACAAGTTATGGCTGGCTAGGGGATTGTGGTGGATCCAATATTTTATAGCTAGGACCGAGGCTGTGCTGCATGTCTATATTCCATCCTTCAACGTGCGCGGGCCTCGCGACTCAGTGATTGTTCTTGCAGTCCTGGTCTGTCCAGACCTAGTATTTGACATCACAAAATATCTTCTGGCCATCTTAGGGCCCCTCCACATACTCCAGGCCTCGCTCCTACGCATCCCTTACTTTGTGAGGGCACAAGCGCTGGTTAAGATCTGCAGCTTGTTGCGTGGGGTAGTTTATGGCAAGTACTTCCAAATGGTCGTGCTTAAAAGCAGGGGCCTGACTGGTACTTACATCTATGACCACCTTACTCCCATGTCAGATTGGCCGCCTTACGGCCTCCGCGATTTGGCGGTGGCCCTAGAGCCAGTTGTGTTCACGCCCATGGAGAAGAAAGTCATCGTCTGGGGCGCTGACACCGCTGCGTGCGGAGACATCATAAGGGGATTACCTGTTTCGGCCAGGTTGGGCAATGAGATCTTGCTCGGACCAGCCGATACAGAAACATCAAAGGGGTGGAGACTCCTTGCCCCCATCACAGCATACGCNCAGCAGACCCGCGGCTTGTTCAGCACCATCGTAACGAGCCTCACTGGCAGGGACACCAATGAGAATTGTGGCGAAGTGCAGGTCTTATCCACCGCTACGCAGTCCTTCCTGGGTACTGCGGTTAACGGCGTGATGTGGACCGTCTACCACGGGGCGGGTGCCAAGACCATCAGCGGCCCGAAGGGACCTGTCAATCAAATGTACACTAATGTTGACCAAGACTTGGTGGGGTGGCCAGCACCCCCCGGAGTCAGATCTCTTGCTCCGTGCACCTGCGGCTCGGCAGACTTGTATCTAGTCACCAGGCACGCGGATGTAATACCCGTGCGCAGGAGAGGAGACACCAGAGGAGCTCTCTTGAGCCCTAGACCAATATCCATTCTTAAGGGATCTTCCGGAGGTCCGCTGCTGTGCCCCATGGGACACCGCGCCGGCATATTCCGTGCGGCGGTGTGTACTCGGGGGGTAGCCAAGGCGGTAGACTTCGTCCCGGTTGAATCTCTTGAGACTACCATGAGATCACCAGTGTTCACTGACAACTCAACACCCCCAGCAGTGCCCCAGACCTACCAGGTCGCGCACCTACACGCACCAACAGGAAGTGGCAAGAGCACTAAGGTCCCGGCGGCGCATGCTGCCCAAGGCTATAAAGTGCTAGTGCTCAATCCTTCGGTTGCGGCCACACTGGGTTTTGGGGTATACATGTCCAAGGCATATGGCATCGACCCGAACATCCGGTCGGGAGTCAGGACCATCACCACGGGTGCGCCAATCACGTACTCAACGTATGGTAAGTTCCTGGCTGATGGAGGTTGCAGCGGAGGGGCATACGACATAATCATCTGTGACGAGTGCTATTCCACTGACTCCACAACGATCCTTGGCATAGGCACAGTCCTGGACCAAGCGGAGACCGCTGGAGTGCGCCTCACCGTGCTCGCGACTGCTACTCCGCCAGGGTCAGTGACTACACCTCATTCCAACATAGAGGAGGTCGCCCTGCCAACAACGGGGGAAATACCCTTTTACGGCAAGGCGATCCCTCTGGAGCTGATCAAGGGGGGCAGACATCTCATCTTCTGCCATTCAAAGAAAAAGTGCGATGAACTGGCCAGACAACTGACATCTCTTGGTCTGAATGCCGTAGCCTACTACAGAGGCTTAGACGTTTCGGTGATTCCCACGTCTGGGGACGTCGTGGTATGCGCCACGGACGCCCTCATGACGGGTTTCACCGGCGACTTTGACTCAGTGATAGACTGCAATACATCTGTGATACAGACTGTTGACTTCAGCTTGGACCCCACCTTCTCCATAGAGATTACAACCGTTCCCCAGGACGCGGTATCCCGCAGCCAGCGGAGAGGCCGCACTGGTAGGGGGAGGTTGGGCACATACCGGTATGTCACCCCGGGAGAGAGACCATCAGGCATGTTTGACACTGCAGAGCTTTGCGAGTGCTACGATGCCGGGTGCGCCTGGTACGAGCTGACACCTGCTGAAACCACAACAAGGCTGAAAGCTTACTTCGACACACCAGGCCTTCCTGTGTGCCAAGACCATCTGGAATTCTGGGAGAGCGTCTTTACAGGGTTAACCCACATAGACGGTCATTTCCTATCCCAGACCAAGCAATCGGGTGAGAATTTCCCGTATCTTGTTGCTTACCAAGCGACGGTGTCGGCCAAGGTCTGGCTCGCTCCACCAAGCTGGGACACCATGTGGAAGTGCCTAATTCGCCTTAAGCCCACCCTGCACGGGCCCACGCCCCTCCTCTACAGACTGGGGTCTGTGCAGAATGAAGTGGTGCTCACCCATCCCATCACCAAATACATCATGGCTTGCATGTCAGCTGATCTCGAGGTAGTGACAAGTACGTGGGTCTTGGTGGGCGGCGTCCTGGCAGCTCTGGCTGCTTACTGTCTTTCAGTGGGCAGCGTAGTGATTGTTGGGAGAGTCGTCCTGTCGGGCCAACCTGCTGTCATTCCTGATCGCGAAGTGCTCTACCAACAGTTCGACGAAATGGAGGAGTGTTCCAAACACCTCCCACTAGTCGAGCACGGGTTACAACTGGCTGAGCAGTTCAAGCAGAAGGCCTTAGGTCTCCTAAATTTCGCTGGCAAGCAAGCCCAAGAGGCAACACCAGTGATCCAGTCTAACTTCGCTAAACTTGAGCAGTTTTGGGCGAACGACATGTGGAATTTCATCAGCGGCATTCAATATCTCGCTGGACTGTCTACTTTGCCAGGCAATCCTGCCATTGCTTCCCTCATGTCCTTTACTGCTGCCGTTACAAGCCCTCTGACCACCCAACAAACCCTCCTTTTTAACATCTTGGGGGGATGGGTGGCCTCACAGATTCGCGACTCCGATGCTTCTACCGCATTCGTCGTGAGCGGCTTGGCGGGGGCGGCAGTTGGCAGTGTGGGCCTTGGCAAAATTTTGGTGGACATTCTGCCCGGTTACGGCGCCGGCGTACGTGGCGCTGTGGTTACCTTCAAGATCATGAGCGGCGAGATGCCTTCCACAGAGGACTTGGTAAATTTGCTCCCGGCCATTCTATCGCCCGGAGCATTGGTGGTGGAGGTGGTATGCCCGGCGATTTTGCGCCGCCACGTGGGCCCGGGCGAAGGGGCCGTGCAGTGGATGAACCGTCTAATTGCGTTCGCATCGCGAGGCAATCACGTGTCTCCCACGCATTACGTCCCTGAGTCCGACGCGGCACGCCGCGTGACCACCATACTATCATCCCTCACTGTGACATCCCTTCTCAGACGCCTCCACAAGTGGATCAATGAAGATTGCTCCACCCCATGTGCCGAATCTTGGCTATGGGAGGTATGGGATTGGGTCTTGCACGTGCTGAGTGACTTCAAGACGTGCTTAAAAGCAAAGTTCGTGCCCTTAATGCCAGGCATCCCCCTCCTCTCATGGCCAAGAGGCTATAAGGGAGAGTGGCGCGGAGATGGCGTGATGCATACGACATGCCCCTGCGGAGCAGATCTGGCAGGTCACATCAAGAACGGCTCGATGAGAATCACCGGGCCGAAAACCTGCAGCAACACATGGCATGGAACCTTCCCCATCAATGCCTACACCACAGGCCCTGGTGTACCCATCCCGGCTCCGAACTACAAGTTCGCGCTTTGGAGGGTGTCCGCCGAGGACTACGTGGAGGTTCGCAGAGTGGGTGATTTCCATTATGTCACCGGGGTAACACAAGACAACATCAAGTTCCCCTGCCAAGTTCCGGCCCCAGAGCTCTTCACGGAAGTGGACGGCATCAGGATACACCGCCACGCCCCTAAGTGCAAACCCTTGCTGCGGGACGAAGTGTCGTTCTCAGTAGGACTCAATTCGTTCGTAGTGGGATCACAACTCCCATGCGAGCCAGAGCCGGACGTGGCAGTGCTAACATCCATGCTGACAGACCCATCACACATAACGGCGGAATCGGCGCGTCGGAGATTGGCTCGAGGGTCACGACCCTCGCTAGCTAGTTCCTCGGCGAGTCAGCTTTCGCCCCGTCTTCTTCAGGCCACGTGTACCGCTCCCCATGACTCCCCTGGTACTGATCTCCTCGAGGCTAACCTCTTGTGGGGGTCTACCGCTACCAGGGTTGAGACGGACGAGAAGGTAATAATACTAGACTCTTTTGAGTCATGTGTGGCTGAGCAAAATGATGACAGGGAAGTCTCGGTTGCCGCGGAAATCCTGCGTCCGACCAAGAAGTTCCCTCCAGCACTACCGATCTGGGCCCGGCCGGACTACAATCCACCTCTTACCGAGACGTGGAAGCAGCAGGACTACCAAGCTCCGACCGTCCACGGTTGCGCTCTGCCTCCGGCAAAGCAGCCCCCTGTTCCCTCTCCCAGGAGGAAACGGACGGTACAGCTCACTGAGTCCGTTGTTTCTACCGCTTTGGCAGAGCTGGCCGCAAAGACCTTTGGCCAGTCAGAGCCGAGCTCAGACCGTGATACAGACCTTACCACCCCAACTGAGACCACAGACTCGGGCCCCATCGTCGTGGATGATGCATCCGATGACGGATCTTATTCGTCAATGCCTCCACTAGAGGGGGAGCCCGGTGACCCGGACTTGACATCAGACTCTTGGTCCACTGTTAGCGGATCGGAGGACGTCGTGTGCTGTTCGATGTCATACTCGTGGACTGGGGCGCTTGTAACACCTTGCGCGGCTGAAGAATCAAAGCTGCCAATTAGCCCCCTGAGCAATTCACTTTTGCGCCATCACAATATGGTGTATGCCACGACCACCCGTTCTGCTGTGACACGGCAGAAGAAGGTGACCTTCGACCGCCTGCAGGTGGTGGACAGTACCTACAATGAAGTGCTTAAGGAGATAAAGGCACGAGCATCCAGAGTGAAGCCACGCTTGCTTACCACAGAGGAAGCTTGCGACCTGACGCCCCCCCACTCAGCCAGATCAAAGTTCGGCTACGGGAAAAAGGATGTTCGGAGCCATTCCCGCAAGGCCATTAACCACATCAGCTCCGTGTGGAAGGACTTGCTGGACGACAACAATACCCCAATTCCAACAACAATCATGGCCAAAAATGAGGTCTTCGCTGTGAACCCAGCGAAGGGAGGTCGGAAGCCTGCTCGCCTGATCGTGTATCCGGATCTCGGATCCCGGGTTTGCGAGAAGAGAGCGCTTCACGACGTCATCAAAAAAACTGCCTTAGCCGTGATGGGAGCCGCTTATGGCTTCCAATACTCCCCAGCGCAGCGGGTGGAATTTCTTCTGACTGCTTGGAAGTCGAAGAACGACCCAATGGGGTTCTCTTATGATACCCGCTGCTTTGACTCCACTGTAACCGAAAAGGACATCAGGGTCGAGGAAGAGGTCTATCAGTGTTGTGACCTGGAGCCCGAAGCCCGCAAAGTCATCACCGCCCTCACAGATAGACTCTATGTGGGCGGCCCTATGCACAACAGCAAGGGAGACCTTTGTGGGTATCGGAGATGTCGCGCAACCGGCGTCTACACCACCAGCTTCGGGAACACGCTGACGTGCTATCTCAAAGCCACGGCCGCCATCAGGGCGGCGGCGCTGAGAGACTGCACTATGTTGGTTTGCGGTGATGACTTAGTCGTCATCGCTGAGAGCGACGGCGTAGAGGAGGACAACCGAGCCCTCCGAGCCTTCACGGAGGCTATGACGAGATACTCGGCTCCCCCAGGTGACGCTCCGCAGCCAGCATATGACCTGGAACTAATAACATCATGTTCATCCAACGTCTCAGTCGCGCACGACGTGACGGGTAAAAAGGTATATTACCTAACCCGAGACCCTGAAACTCCCTTGGCGCGAGCTGTATGGGAGACAGTCCGACACACTCCAGTCAATTCCTGGTTGGGAAACATCATAGTCTACGCTCCCACAATATGGGTGCGCATGATATTGATGACCCACTTTTTCTCAATACTCCAGAGCCAGGAAGCCCTTGAGAAAGCACTCGACTTCGATATGTACGGAGTCACCTACTCTATCACTCCGCTGGATTTACCGGCAATCATTCAAAGACTCCATGGCTTAAGCGCGTTCACGCTGCACGGATACTCTCCACACGAACTCAACCGGGTGGCCGGAGCCCTCAGAAAACTTGGGGTACCCCCGCTGAGAGCGTGGAGACATCGGGCCCGAGCAGTCCGCGCTAAGCTTATCGCCCAGGGAGGTAGAGCCAAAATATGTGGCATATACCTCTTTAACTGGGCGGTAAAAACCAAACTCAAACTCACTCCATTGCCTGCCGCTGCCAAACTCGATTTATCGGGTTGGTTTACGGTAGGCGCCGGCGGGGGAGACATTTATCACAGCATGTCTCATGCCCGACCCCGCTATTTACTCCTGTGCCTACTCATACTTACAGTAGGGGTAGGCATCTTCCTGCTGCCTGCTCGGTAGGCAGCTTAACACTCCGACCTTAGGGTCCCC

>HHV1_HSV1_STRIPPED4

CACACCCCCCGGGGGTCGCGCGCGGCCCTTTAAAGCGGCGGCGGCGGGCAGCCCGGGCCCCCCGCGGCCGAGACTAGCGAGTTAGACAGGCAAGCACTACTCGCCTCTGCACGCACATGCTTGCCTGTCAAACTCTACCACCCCGGCACGCTCTCTGTCTCCATGGCCCGCCGCCGCCGCCATCGCGGCCCCCGCCGCCCCCGGCCGCCCGGGCCCACGGGCGCCGTCCCAACCGCACAGTCCCAGGTAACCTCCACGCCCAACTCGGAACCCGCGGTCAGGAGCGCGCCCGCGGCCGCCCCGCCGCCGCCCCCCGCCGGTGGGCCCCCGCCTTCTTGTTCGCTGCTGCTGCGCCAGTGGCTCCACGTTCCCGAGTCCGCGTCCGACGACGACGATGACGACGACTGGCCGGACAGCCCCCCGCCCGAGCCGGCGCCAGAGGCCCGGCCCACCGCCGCCGCCCCCCGGCCCCGGCCCCCACCGCCCGGCGTGGGCCCGGGGGGCGGGGCTGACCCCTCCCACCCCCCCTCGCGCCCCTTCCGCCTTCCGCCGCGCCTCGCCCTCCGCCTGCGCGTCACCGCGGAGCACCTGGCGCGCCTGCGCCTGCGACGCGCGGGCGGGGAGGGGGCGCCGGAGCCCCCCGCGACCCCCGCGACCCCCGCGACCCCCGCGACCCCCGCGACCCCCGCGCGGGTGCGCTTCTCGCCCCACGTCCGGGTGCGCCACCTGGTGGTCTGGGCCTCGGCCGCCCGCCTGGCGCGCCGCGGCTCGTGGGCCCGCGAGCGGGCCGACCGGGCTCGGTTCCGGCGCCGGGTGGCGGAGGCCGAGGCGGTCATCGGGCCGTGCCTGGGGCCCGAGGCCCGTGCCCGGGCCCTGGCCCGCGGAGCCGGCCCGGCGAACTCGGTCTAACGTTACACCCGAGGCGGCCTGGGTCTTCCGCGGAGCTCCCGGGAGCTCCGCACCAAGCCGCTCTCCGGAGAGACGATGGCAGGAGCCGCGCATATATACGCTTGGAGCCAGCCCGCCCTCACAGGGCGGGCCCGCCTCGGGGGCGGGACTGGCCAATCGGCGGCCGCCAGCGCGGCGGGGCCCGGCCAACCAGCGTCCGCCGAGTCTTCGGGGCCCGGCCCATTGGGCGGGAGTTACCGCCCAATGGGCCGGGCCGCCCACTTCCCGGTATGGTAATTAAAAACTTGCAAGAGGCCTTGTTCCGCTTCCCGGTATGGTAATTAGAAACTCATTAATGGGCGGCCCCGGCCGCCCTTCCCGCTTCCGGCAATTCCCGCGGCCCTTAATGGGCAACCCCGGTATTCCCCGCCTCCCGCGCCGCGCGTAACCACTCCCCTGGGGTTCCGGGTTATGCTAATTGCTTTTTTGGCGGAACACACGGCCCCTCGCGCATTGGCCCGCGGGTCGCTCAATGAACCCGCATTGGTCCCCTGGGGTTCCGGGTATGGTAATGAGTTTCTTCGGGAAGGCGGGAAGCCCCGGGGCACCGACGCAGGCCAAGCCCCTGTTGCGTCGGCGGGAGGGGCATGCTAATGGGGTTCTTTGGGGGACACCGGGTTGGGCCCCCAAATCGGGGGCCGGGCCGTGCATGCTAATGATATTCTTTGGGGGCGCCGGGTTGGTCCCCGGGGACGGGGCCGCCCCGCGGTGGGCCTGCCTCCCCTGGGACGCGCGGCCATTGGGGGAATCGTCACTGCCGCCCCTTTGGGGAGGGGAAAGGCGTGGGGTATAAGTTAGCCCTGGCCCGACAGTCTGGTCGCATTTGCACCTCGGCACTCGGAGCGAGACGCAGCAGCCAGGCAGACTCGGGCCGCCCCCTCTCCGCATCACCACAGAAGCCCCGCCTACGTTGCGACCCCCAGGGACCCTCCGTCCGCGACCCTCCAGCCGCATACGACCCCCATGGAGCCCCGCCCCGGAGCGAGTACCCGCCGGCCTGAGGGCCGCCCCCAGCGCGAGGTGAGGGGCCGGGCGCCATGTCTGGGGCGCCATATTGGGGGGCGCCATATTGGGGGGCGCCATGTTGGGGGACCCCCGACCCTTACACTGGAACCGGCCGCCATGTTGGGGGACCCCCACTCATACACGGGAGCCGGGCGCCATGTTGGGGCGCCATGTTAGGGGGCGTGGAACCCCGTGACACTATATATACAGGGACCGGGGGCGCCATGTTAGGGGGTGCGGAACCCCCTGACCCTATATATACAGGGACCGGGGTCGCCCTGTTGGGGGTCGCCATGTGACCCCCTGACTTTATATATACAGACCCCCAACACATACACATGGCCCCTTTGACTCAGACGCAGGGCCCGGGGTCGCCGTGGGACCCCCTGACTCATACACAGAGACACGCCCCCACAACAAACACACAGGGACCGGGGTCGCCGTGTTGGGGGCGTGGTCCCCACTGACTCATACGCAGGCCCCCCTTACTCACACGCATCTAGGGGGGTGGGGAGGAGCCGCCCGCCATATTTGGGGGACGCCGTGGGACCCCCGACTCCGGTGCGTCTGGAGGGCGGGAGAAGAGGGAAGAAGAGGGGTCGGGATCCAAAGGACGGACCCAGACCACCTTTGGTTGCAGACCCCTTTCTCCCCCCTCTTCCGAGGCCAGCAGGGGGGCAGGACTTTGTGAGGCGGGGGGGGGAGAGGGGGAACTCGTGGGTGCTGATTGACGCGGGAAATCCCCCCCCATTCTTACCCGCCCCCCTTTTTTCCCCTTAGCCCGCCCCGGATGTCTGGGTGTTTCCCTGCGACCGAGACCTGCCGGACAGCAGCGACTCTGAGGCGGAGACCGAAGTGGGGGGGCGGGGGGACGCCGACCACCATGACGACGACTCCGCCTCCGAGGCGGACAGCACGGACACGGAACTGTTCGAGACGGGGCTGCTGGGGCCGCAGGGCGTGGATGGGGGGGCGGTCTCGGGGGGGAGCCCCCCCCGCGAGGAAGACCCCGGCAGTTGCGGGGGCGCCCCCCCTCGAGAGGACGGGGGGAGCGACGAGGGCGACGTGTGCGCCGTGTGCACGGATGAGATCGCGCCCCACCTGCGCTGCGACACCTTCCCGTGCATGCACCGCTTCTGCATCCCGTGCATGAAAACCTGGATGCAATTGCGCAACACCTGCCCGCTGTGCAACGCCAAGCTGGTGTACCTGATAGTGGGCGTGACGCCCAGCGGGTCGTTCAGCACCATCCCGATCGTGAACGACCCCCAGACCCGCATGGAGGCCGAGGAGGCCGTCAGGGCGGGCACGGCCGTGGACTTTATCTGGACGGGCAATCAGCGGTTCGCCCCGCGGTACCTGACCCTGGGGGGGCACACGGTGAGGGCCCTGTCGCCCACCCACCCGGAGCCCACCACGGACGAGGATGACGACGACCTGGACGACGGTGAGGCGGGGGGCGGCAAGGACCCTGGGGGAGGAGGAGGAGGAGGGGGGGGGAGGGAGGAATAGGCGGGCGGGCGAGGAAAGGGCGGGCCGGGGAGGGGGCGTAACCTGATCGCGCCCCCCGTTGTCTCTTGCAGCAGACTACGTACCGCCCGCCCCCCGCCGGACGCCCCGCGCCCCCCCACGCAGAGGCGCCGCCGCGCCCCCCGTGACGGGCGGGGCGTCTCACGCAGCCCCCCAGCCGGCCGCGGCTCGGACAGCGCCCCCCTCGGCGCCCATCGGGCCACACGGCAGCAGTAACACCAACACCACCACCAACAGCAGCGGCGGCGGCGGCTCCCGCCAGTCGCGAGCCGCGGCGCCGCGGGGGGCGTCTGGCCCCTCCGGGGGGGTTGGGGTTGGGGTTGGGGTTGTTGAAGCGGAGGCGGGGCGGCCGAGGGGCCGGACGGGCCCCCTTGTCAACAGACCCGCCCCCCTTGCAAACAACAGAGACCCCATAGTGATCAGCGACTCCCCCCCGGCCTCTCCCCACAGGCCCCCCGCGGCGCCCATGCCAGGCTCCGCCCCCCGCCCCGGGCCCCCCGCGTCCGCGGCCGCGTCGGGACCCGCGCGCCCCCGCGCGGCCGTGGCCCCGTGCGTGCGAGCGCCGCCTCCGGGGCCCGGCCCCCGCGCCCCGGCCCCCGGGGCGGAGCCGGCCGCCCGCCCCGCGGACGCGCGCCGTGTGCCCCAGTCGCACTCGTCCCTGGCTCAGGCCGCGAACCAAGAACAGAGTCTGTGCCGGGCGCGTGCGACGGTGGCGCGCGGCTCGGGGGGGCCGGGCGTGGAGGGTGGGCACGGGCCCTCCCGCGGCGCCGCCCCCTCCGGCGCCGCCCCGCTCCCCTCCGCCGCCTCTGTCGAGCAGGAGGCGGCGGTGCGTCCGAGGAAGAGGCGCGGGTCGGGCCAGGAAAACCCCTCCCCCCAGTCCACGCGTCCCCCCCTCGCGCCGGCAGGGGCCAAGAGGGCGGCGACGCACCCCCCCTCCGACTCAGGGCCGGGGGGGCGCGGCCAGGGTGGGCCCGGGACCCCCCTGACGTCCTCGGCGGTCCTCCTCCGCTTCCGCCTCCTCGGGCGGGGCCGTCGGTGCCCTGGGAGGGAGACAAGAGGAAACCTCCCTCGGCCCCCGCGCTGCTTCTGGGCCGCGGGGGCCGAGGAAGTGTGCCCGGAAGACGCGCCACGCGGAGACTTCCGGGGCCGTCCCCGCGGGCGGCCTCACGCGCTACCTGCCCATCTCGGGGGTCTCTAGCGTGGTCGCCCTGTCGCCTTACGTGAACAAGACTATCACGGGGGACTGCCTGCCCATCCTGGACATGGAGACGGGGAACATCGGGGCGTACGTGGTCCTGGTGGACCAGACGGGAAACATGGCGACCCGGCTGCGGGCCGCGGTCCCCGGCTGGAGCCGCCGCACCCTGCTCCCCGAGACCGCGGGTAACCACGTGATGCCCCCCGAGTACCCGACGGCCCCCGCGTCGGAGTGGAACAGCCTCTGGATGACCCCCGTGGGGAACATGCTGTTCGACCAGGGCACCCTAGTGGGCGCCCTGGACTTCCGCAGCCTGCGGTCTCGGCACCCGTGGTCCGGGGAGCAGGGGGCGTCGACCCGGGACGAGGGAAAACAATAAGGGACGCCCCCCGTGTTTGTGGGGAGGGGGGGGTCGGGCGCTGGGTGGTCTCTGGCCGCGCCCACTACACCAGCCAATCCGTGTCGGGGAGGGGAAAAGTGAAAGACACGGGCACCACACACCAGCGGGTCTTTTGTGTTGGCCCTAATAAAAAAAAACTCAGGGGATTTTTGCTGTCTGTTGGGAAATAAAGGTTTACTTTTGTATCTTTTCCCTGTCTGTGTTGGATGTATCGCGGGGATGCGTGGGAGTGGGGGTGCGTGGGAGTGGGGGTGCGTGGGAGTGGGGGTGCGTGGGAGTGGGGGTGCGTGGGAGTGGGGGTGCGTGGGAGTGGGGGTGCGTGGGAGTGGGGGTGCGTGGGAGTGGGGGTGCGTGGGAGTGGGGGTGCCATGTTGGGCAGGCTCTGGTGTTAACCACAGAGCCGCGGCCCGGGCTGCCTGACCACCGATCCCCGAAAGCATCCTGCCACTGGCATGGAGCCAGAACCACAGTGGGTTGGGTGTGGGTGTTAAGTTTCCGCGAGCGCCTGCCCGCCCGGACTGACCTGGCCTCTGGCCGCCACAAAGGGCGGGGGGGGTTAACTACACTATAGGGCAACAAAGGATGGGAGGGGTGGCGGGGCGGGACGGGGCGCCCAAAAGGGGGTCGGCCACACCACAGACGTGGGTGTTGGGGGGTGGGGCGGAGGGGTGGGGGGGGGGGAGACAGAAACAGGAACATAGTTAGAAAACAAGAATGCGGTGCAGCCAGAGAATCACAGGAGACGAGGGGATGGGCGTGTTGGTTACCAACCCACACCCAGGCATGCTCGGTGGTATGAAGGAGGGGGGGCGGTGCTTCTTAGAGACCGCCGGGGGACGTGGGGTTGGTGTGCAAAGGCACGCGCACCCGCGCCGGCCAGGTGGGCCGGTACTCCATCCCCCCCTCCCCCGACCCTTCCCACCCCCGCGTGCCAGAGATCACCCCGGTCCCCCGGCACCCGCCACTCCTCCGTATCCTCGCTTTAGGAACAACTTTAGGGGGGGTACACACGCGCCGTGCATTTCCTTCCACACCCCCCCTCCCCCGCACTCCCCCCCCCCAGGCAGTAAGACCCAAGCATAGAGAGCCAGGCACAAAAACACAGGCGGGGTGGGACACATGCCTTCTTGGAGTACGTGGGTCATTGGCGTGGGGGGTTACAGCGACACCGGCCGACCCCCTGGCGGTCTTCCAGCCGGCCCTTAGATAAGGGGGCAGTTGGTGGTCGGACGGGTAAGTAACAGAGTCTGACTAAGGGTGGGAGGGGGGGAAAAGAACGGGCTGGTGTGCTGTAACACGAGCCCACCCGCGAGTGGCGTGGCCGACCTTAGCCTCTGGGGCGCCCCCTGTCGTTTGGGTCCCCCCCCTCTATTGGGGAGAAGCAGGTGTCTAACCTACCTGGAAACGCGGCGTCTTTGTTGAACGACACCGGGGCGCCCTCGACGAGTGGGATAACGGGGGAGGAAGGGAGGGAGGAGGGTACTGGGGGTGAAGAAGGGGGGGGGGAGAAGCGAGAACAGGAAAGGCGATGGAGCCCGGCAGAACACCGAGGAAAAAAAAACCACAGCGCATGCGCCGGGCCGTTGTGGGGCCCCGGGCCGGGGCCCCTTGGGTCCGCCGGGGCCCCGGGCCGGGCCGCCACGGGGGCCGGCCGTTGGCGGTAACCCCGAGTGTTCATCTCAGGCCCCGGGCCGGGAACCCGGAAAAGCCTCCGGGGGGCCTTTTTCGCGTCGCGTGCCGGCGAGCGGGTCCGGACGGGGCCCGGACCGCCGCGGTCGGGGGCCCCTCGTCCCGGGCCGTACGCGGCCTTCGCCCCGTGAGGGGACAGACGAACGAAACATTCCGGCGACGGAACGAAAAACACCCCAGACGGGTTAAAGAAACAGAAACCGCAACCCCCACCACCCCCGAAACGGGGAAAACGAAAAAACAGACCAGCGGCCGGCCGGCGCTTAGGGGGAGGATGTCGCCGACGCCCCTTGGCCGCCCCGGCTGCAGGGGGGCCCGGAGAGCCGCGGCACCCGGACGCGCCCGGAAAGTCTTTCGCACCACCCGCGATCGGCACGGCCGCGCCCCCGCTTTTATAAAGGCTGAGATGACGCAGCAAAAACAGGCCACAGCACCACGTGGGTAGGTGATGTAATTTTATTTTCCTCGTCTGCGGCCTAATGGATTTCCGGGCGCGGTGCCCCTGTCTGCAGAGCACTTAACGGATTGATATCTCGCGGGCACGCGCGCCCTTAATGGACCGGCGCGGGGCGGGGGGCCGGATACCCACACGGGCGGGGGGGGGGTGTCGCGGGCCGTCTGCTGGCCCGCGGCCACATAAACAATGACTCTGGGCCTTTCTGCCTCTGCCGCTTGTGAGTGCGCGCGCCGGCTCTGCGGTGTCGGCGGCGGCTGCGGCGGCTGCGGCGGCCGCCGTGTTCGGTCTCGGTAGCCGGCCGGCGGGTGGACTCGCGGGGGGCCGGAGGGTGGAAGGCAGGGGGGTGTAGGATGGGTATCAGGACTTCCACTTCCCGTCCTTCCATCCCCCGTTCCCCTCGGTTGTTCCTCGCCTCCCCCAACACCCCGCCGCTTTCCGTTGGGGTTGTTATTGTTGTCGGGATCGTGCGGGCCGGGGGTCGCCGGGGCAGGGGCGGGGGCGTGGGCGGGGGTGCTCGTCGATCGACCGGGCTCAGTGGGGGCGTGGGGTGGGTGGGAGAAGGCGAGGAGACTGGGGTGGGGGTGTCGGTGGGTGGTTGTTTTTTGTGGTTGTTTTTGTGTCTGTTCCCGTCCCCCGTCACCCCCTCCCTCCGTCCCCTCCGTCCCCCCGTCGCGGGTGTTTGTGTTTGTTTATTCCGACATTGGTTTATTTAAATAAACACAGCCGTTCTGCGTGTCTGTTCTTGCGTGTGGCTGGGGGCTTATATGTGGGGTCCCGGGGGCGGGATGGGGTTTAGCGGCGGGGGGCGGCGCGCCGGACGGGGCGCTGGAGATAACGGCCCCCGGGGAACGGGGGACCGGGGCTGGGTATCCCGAGGTGGGTGGGAGGAGGAGAAGGCGGGGGGGGGGGAGACGGGGGGAAAGCAAGGACACGGCCCGGGGGGTGGGAGCGCGGGCCGGGCCGCTCGTAAGAGCCGCGACCCGGCCGCCGGGGAGCGTTGTCGCCGTCGGTCTGCCGGCCCCCGTCCCTCCCTTTTTTGACCAACCAGCGCCCCCCCCCCCTCACCACCATTCCTACTACCACCACCACCACCACCACCGACACCTCCCGCGCACCCCCGCCCACATCCCCCCCCAACCCGCACCACCAGCACGGGTTGGGGGTAGCAGGGGATCAAAGGGGGGCAAAGCCGGCGGGGCGGTTCGGGGGGGGGGGGGGGGGGCGGGAGACCAAGTAGGCCCGCCCATCCGCGGCCCCTCCCGGCAGCCACGCCCCCAGCGTCGGGTGTCACGGGGAAAGAGCAGAGGGGAGAGGGGAGAGGGGGGGAGAGGGGAGAGGGGGGGAGAGGGGAGAGGGGGGGAGAGGGGAGAGGGGGGGAGAGGGGAGAGGGGGGGAGAGGGGAGAGGGGGGGAGAGGGGAGAGGGGGGGAGAGGGGAGAGGGGGGGAGAGGGGAGAGGGGGGGAGAGGGGAGAGGGGGGGAGAGGGGGTATATAAACCAACGAAAAGCGCGGGAACGGGGATACGGGGCTTGTGTGGCACGACGTCGTGGTTGTGTTACTGGGCAAACACTTGGGGACTGTAGGTTTCTGTGGGTGCCGACCCTAGGCGCTATGGGGATTTTGGGTTGGGTCGGGCTTATTGCCGTTGGGGTTTTGTGTGTGCGGGGGGGCTTGCCTTCAACCGAATATGTTATTCGGAGTCGGGTGGCTCGAGAGGTGGGGGATATATTAAAGGTGCCTTGTGTGCCGCTCCCGTCTGACGATCTTGATTGGCGTTACGAGACCCCCTCGGCTATAAACTATGCTTTGATAGACGGTATATTTTTGCGTTATCACTGTCCCGGATTGGACACGGTCTTGTGGGATAGGCATGCCCAGAAGGCATATTGGGTTAACCCCTTTTTATTTGTGGCGGGTTTTTTGGAGGACTTGAGTTACCCCGCGTTTCCTGCCAACACCCAGGAAACAGAAACGCGCTTGGCCCTTTATAAAGAGATACGCCAGGCGCTGGACAGTCGCAAGCAGGCCGCCAGCCACACACCTGTGAAGGCTGGGTGTGTGAACTTTGACTATTCGCGCACCCGCCGCTGTGTAGGGCGACAGGATTTGGGACCTACCAACGGAACGTCTGGACGGACCCCGGTTCTGCCGCCGGACGATGAAGCGGGCCTGCAGCCGAAGCCCCTCACCACGCCGCCGCCCATCATCGCCACGTCGGACCCCACCCCGCGACGGGACGCCGCCACAAAAAGCAGACGCCGACGACCCCACTCCCGGCGCCTCTAACGATGCCTCGACGGAAACCCGTCCGGGTTCGGGGGGCGAACCGGCCGCCTGTCGCTCGTCAGGGCCGGCGGGCGCTCCTCGCCGCCCTAGAGGCTGTCCCGCTGGTGTGACGTTTTCCTCGTCCGCGCCCCCCGACCCTCCCATGGATTTAACAAACGGGGGGGTGTCGCCTGCGGCGACCTCGGCGCCTCTGGACTGGACCACGTTTCGGCGTGTGTTTCTGATCGACGACGCGTGGCGGCCCCTGATGGAGCCTGAGCTGGCGAACCCCTTAACCGCCCACCTCCTGGCCGAATATAATCGTCGGTGCCAGACCGAAGAGGTGCTGCCGCCGCGGGAGGATGTGTTTTCGTGGACTCGTTATTGCACCCCCGACGAGGTGCGCGTGGTTATCATCGGCCAGGACCCATATCACCACCCCGGCCAGGCGCACGGACTTGCGTTTAGCGTGCGCGCGAACGTGCCGCCTCCCCCGAGTCTTCGGAATGTCTTGGCGGCCGTCAAGAACTGTTATCCCGAGGCACGGATGAGCGGCCACGGTTGCCTGGAAAAGTGGGCGCGGGACGGCGTCCTGTTACTAAACACGACCCTGACCGTCAAGCGCGGGGCGGCGGCGTCCCACTCTAGAATCGGTTGGGACCGCTTCGTGGGCGGAGTTATCCGCCGGTTGGCCGCGCGCCGCCCCGGCCTGGTGTTTATGCTCTGGGGCACACACGCCCAGAATGCCATCAGGCCGGACCCTCGGGTCCATTGCGTCCTCAAGTTTTCGCACCCGTCGCCCCTCTCCAAGGTTCCGTTCGGAACCTGCCAGCATTTCCTCGTGGCGAACCGATACCTCGAGACCCGGTCGATTTCACCCATCGACTGGTCGGTTTGAAAGGCATCGACGTCCGGGGTTTTTGTCGGTGGGGGCTTTTGGGTATTTCCGATGAATAAAGACGGTTAATGGTTAAACCTCTGGTCTCATACGGGTCGGTGATGTCGGGCGTCGGGGGAGAGGGAGTTCCCTCTGCGCTTGCGATTCTAGCCTCGTGGGGCTGGACGTTCGACACGCCAAACCACGAGTCGGGGATATCGCCAGATACGACTCCCGCAGATTCCATTCGGGGTGCCGCTGTGGCCTCACCTGACCAACCTTTACACGGGGGCCCGGAACGGGAGGCCACAGCGCCGTCTTTCTCCCCAACGCGCGCGGATGACGGCCCGCCCTGTACCGACGGGCCCTACGTGACGTTTGATACCCTGTTTATGGTGTCGTCGATCGACGAATTAGGGCGTCGCCAGCTCACGGACACCATCCGCAAGGACCTGCGGTTGTCGCTGGCCAAGTTTAGCATTGCGTGCACCAAGACCTCCTCGTTTTCGGGAAACGCCCCGCGCCACCACAGACGCGGGGCGTTCCAGCGCGGCACGCGGGCGCCGCGCAGCAACAAAAGCCTCCAGATGTTTGTGTTGTGCAAACGCGCCCACGCCGCTCGAGTGCGAGAGCAGCTTCGGGTCGTTATTCAGTCCCGCAAGCCGCGCAAGTATTACACGCGATCTTCGGACGGGCGGCTCTGCCCCGCCGTCCCCGTGTTCGTCCACGAGTTCGTCTCGTCCGAGCCAATGCGCCTCCACCGAGATAACGTCATGCTGGCCTCGGGGGCCGAGTAACCGCCCCCCCCCCATGCCACCCTCACTGCCCGTCGCGCGTGTTTGATGTTAATAAATAACACATAAATTTGGCTGGTTGTTTGTTGTCTTTAATGGACCGCCCGCAAGGGGGGGGGGGCATTTCAGTGTCGGGTGACGAGCGCGATCCGGCCGGGATCCTAGGACCCCAAAAGTTTGTCTGCGTATTCCAGGGCGGGGCTCAGTTGAATCTCCCGCAGCACCTCTACCAGCAGGTCCGCGGTGGGCTGGAGAAACTCGGCCGTCCCGGGGCAGGCGGTTGTCGGGGGTGGAGGCGCGGCGCCCACCCCGTGTGCCGCGCCTGGCGTCTCCTCTGGGGGCGACCCGTAAATGGTTGCAGTGATGTAAATGGTGTCCGCGGTCCAGACCACGGTCAAAATGCCGGCCGTGGCGCTCCGGGCGCTTTCGCCGCGCGAGGAGCTGACCCAGGAGTCGAACGGATACGCGTACATATGGGCGTCCCACCCGCGTTCGAGCTTCTGGTTGCTGTCCCGGCCTATAAAGCGGTAGGCACAAAATTCGGCGCGACAGTCGATAATCACCAACAGCCCAATGGGGGTGTGCTGGATAACAACGCCTCCGCGCGGCAGGCGGTCCTGGCGCTCCCGGCCCCGTACCATGATCGCGCGGGTGCCGTACTCAAAAACATGCACCACCTGCGCGGCGTCGGGCAGTGCGCTGGTCAGCGAGGCCCTGGCGTGGCATAGGCTATACGCGATGGTCGTCTGTGGATTGGACATCTCGCGGTGGGTAGTGAGTCCCCCGGGCCGGGTTCGGTGGAACTGTAAGGGGACGGCGGGTTAATAGACAATGACCACGTTCGGATCGCGCAGAGCCGATAGTATGTGCTCACTAATGACGTCATCGCGCTCGTGGCGCTCCCGGAGCGGATTTAAGTTCATGCGAAGGAATTCGGAGGAGGTGGTGCGGGACATGGCCACGTACGCGCTGTTGAGGCGCAGGTTGCCGGGCGTAAAGCAGATGGCGACCTTGTCCAGGCTAAGGCCCTGGGAGCGCGTGATGGTCATGGCAAGCTTGGAGCTGATGCCGTAGTCGGCGTTTATGGCCATGGCCAGCTCCGTAGAGTCAATGGACTCGACAAACTCGCTGATGTTGGTGTTGACGACGGACATGAAGCCGTGTTGGTCCCGCAAGACCACGTAAGGCAGGGGGGCCTCTTCCAGTAACTCGGCCACGTTGGCCGTCGCGTGCCGCCTCCGCAGCTCGTCCGCAAAGGCAAACACCCGTGCGTACGTGTATCCCATGAGCGTATAATTGTCCGTCTGCAGGGCGACGGACATCAGCCCCCCGCGCGGCGAGCCGGTCAGCATCTCGCAGCCCCGGAAGATAACGTTGTCCACGTACGTGCTAAAGGGGGCGACTTCAAATGCCTCCCCGAAGAGCTCTTGGAGGATTCGGAATCTCCCGAGGAAGGCCCGCTTCAGCAGCGCAAACTGGGTGTGAACGGCGGCGGTGGTCTCCGGTTCCCCGGGGGTGTAGTGGCAGTAAAACACGTCGAGCTGTTGTTCGTCCAGCCCCGCGAAAATAACGTCGAGGTCGTCGTCGGGAAAATCGTCCGGGCCCCCGTCCCGCGGCCCCAGTTGCTTAAAATCAAACGCACGCTCGCCGGGGGCGCCTGCGTCGGCCATTACCGACGCCTGCGTCGGCACCCCCGAAGATTTGGGGCGCAGAGACAGAATCTCCGCCGTTAGTTCTCCCATGCGGGCGTAGGCGAGGGTCCTCTGGGTCGCATCCAGGCCCGGGCGCTGCAGAAAGTTGTAAAAGGAGATAAGCCCGCTAAATATGAGCCGCGACAGGAACCTGTAGGCAAACTCCACCGAAGTCTCCCCCTGAGTCTTTACAAAGCTGTCGTCACGCAACACTGCCTCGAAGGCCCGGAACGTCCCACTAAACCCAAAAACCAGTTTTCGCAGGCGCGCGGTCACCGCGATCTGGCTGTTGAGGACGTAAGTGACGTCGTTGCGGGCCACGACCAGCTGCTGTTTGCTGTGCACCTCGCAGCGCATGTGCCCCGCGTCCTGGTCCTGGCTCTGCGAGTAGTTGGTGATGCGGCTGGCGTTGGCCGTGAGCCACTTTTCAATCGTCAGGCCGGGCTGGTGTGTCAGCCGTCGGTATTCGTCAAACTCCTTGACCGACACGAACGTAAGCACGGGGAGGGTGAACACGACGAACTCCCCCTCACGGGTCACCTTCAGGTAGGCGTGGAGCTTGGCCATGTACGCGCTCACCTCTTTGTGGGAGGAGAACAGCCGCGTCCAGCCGGGGAGGTTGGCGGGGTTGGTGATGTAGTTTTCCGGGACGACGAAGCGATCCACGAACTGCATGTGCTCCTCGGTGATGGGCAGGCCGTACTCCAGCACCTTCATGAGGTTACCGAACTCGTGCTCGACGCACCGTTTGTTGTTAATAAAAATGGCCCAGCTATACGAGAGGCGGGCGTACTCGCGCAGCGTGCGGTTGCAGATGAGGTACGTGAGCACGTTCTCGCTCTGGCGGACGGAACACCGCAGTTTCTGGTGCTCGAAGGTCGACTCCAGGGACGCCGTCTGCGTCGGCGAGCCCACACACACCAACACGGGCCGCAGGCGGGCCGCGTACTGGGGGGTGTGGTACAGGGCGTTAATCATCCACCAGCAATACACCACGGCCGTGAGGAGGTGACGCCCAAGGAGCCCGGCCTCGTCGATGACGATCACGTTGCTGCGGGTAAAGGCCGGCAGCGCCCCGTGGGTGGCCGGGGCCAACCGCGTCAGGGCGCCCTCGGCCAACCCCAGGGTCCGTTCCAGGGCGGCCAGGGCGCGAAACTCGTTCCGCAACTCCTCGCCCCCGGAGGCGGCCAGGGCGCGCTTCGTGAGGTCCAAAATCACCTCCCAGTAGTACGTCAGATCTCGTCGCTGCAGGTCCTCCAGCGAGGCGGGGTTGCTGGTCAGGGTGTACGGGTACTGTCCCAGTTGGGCCTGGACGTGATTCCCGCGAAACCCAAATTCATGAAAGATGGTGTTGATGGGTCGGCTGAGAAAGGCGCCCGAGAGTTTGGCGTACATGTTTTGGGCCGCAATGCGCGTGGCGCCCGTCACCACACAGTCCAAGACCTCGTTGATTGTCTGCACGCACGTGCTCTTTCCGGAGCCAGCGTTGCCGGTGATAAGATACACCGCGAACGGAAACTCCCTGAGGGGCAGGCCTGCGGGGGACTCTAAGGCCGCCACGTCCCGGAACCACTGCAGATGGGGCACTTGCGCTCCGTCGAGCTGTTGTTGCGAGAGCTCTCGGATGCGCTTAAGGATTGGCTGCACCCCGTGCATAGACGTAAAATTTAAAAAGGCCTCGGCCCTCCCTGGAACGGCTGGTCGGTCCCCGGGTTGCTGAAGGTGCGGCGGGCCGGGTTTCTGTCCGTCTAGCTGGCGCTCCCCGCCGGCCGCCGCCATGACCGCACCACGCTCGCGGGCCCCCACTACGCGTGCGCGGGGGGACACGGAAGCGCTGTGCTCCCCCGAGGACGGCTGGGTAAAGGTTCACCCCACCCCCGGTACGATGCTGTTCCGCGAGATTCTCCACGGGCAGCTGGGGTATACCGAGGGCCAGGGGGTGTACAACGTCGTCCGGTCCAGCGAGGCGACCACCCGGCAGCTGCAGGCGGCGATCTTTCACGCGCTCCTCAACGCCACCACTTACCGGGACCTCGAGGCGGACTGGCTCGGCCACGTGGCGGCCCGCGGTCTGCAGCCCCAACGGCTGGTTCGCCGGTACAGGAACGCCCGGGAGGCGGATATCGCCGGGGTGGCCGAGCGGGTGTTCGACACGTGGCGGAACACGCTTAGGACGACGCTGCTGGACTTTGCCCACGGGTTGGTCGCCTGCTTTGCGCCGGGCGGCCCGAGCGGCCCGTCAAGCTTCCCCAAATATATCGACTGGCTGACGTGCCTGGGGCTGGTCCCCATATTACGCAAGCGACAAGAAGGGGGTGTGACGCAGGGTCTGAGGGCGTTTCTCAAGCAGCACCCGCTGACCCGCCAGCTGGCCACGGTCGCGGAGGCCGCGGAGCGCGCCGGCCCCGGGTTTTTTGAGCTGGCGCTGGCCTTCGACTCCACGCGCGTGGCGGACTACGACCGCGTGTATATCTACTACAACCACCGCCGGGGCGACTGGCTCGTGCGAGACCCCATCAGCGGGCAGCGCGGAGAATGTCTGGTGCTGTGGCCCCCCTTGTGGACCGGGGACCGTCTGGTCTTCGATTCGCCCGTCCAGCGGCTGTTTCCCGAGATCGTCGCGTGTCACTCCCTCCGGGAACACGCGCACGTCTGCCGGCTGCGCAATACCGCGTCCGTCAAGGTGCTGCTGGGGCGCAAGAGCGACAGCGAGCGCGGGGTGGCCGGTGCCGCGCGGGTCGTTAACAAGGTGTTGGGGGAGGACGACGAGACCAAGGCCGGGTCGGCCGCCTCGCGCCTCGTGCGGCTTATCATCAACATGAAGGGCATGCGCCACGTAGGCGACATTAACGACACCGTGCGTGCCTACCTCGACGAGGCCGGGGGGCACCTGATAGACGCCCCGGCCGTCGACGGTACCCTCCCTGGATTCGGCAAGGGCGGAAACAGCCGCGGGTCTGCGGGCCAGGACCAGGGGGGGCGGGCGCCGCAGCTTCGCCAGGCCTTCCGCACGGCCGTGGTTAACAACATCAACGGCGTGTTGGAGGGCTATATAAATAACCTGTTTGGAACCATCGAGCGCCTGCGCGAGACCAACGCGGGCCTGGCGACCCAATTGCAGGAGCGCGACCGCGAGCTCCGGCGCGCAACAGCGGGGGCCCTGGAGCGCCAGCAGCGCGCGGCCGACCTGGCGGCCGAGTCCGTGACCGGTGGATGCGGCAGCCGCCCTGCGGGGGCGGACCTGCTCCGGGCCGACTATGACATTATCGACGTCAGCAAGTCCATGGACGACGACACGTACGTCGCCAACAGCTTTCAGCACCCGTACATCCCTTCGTACGCCCAGGACCTGGAGCGCCTGTCGCGCCTCTGGGAGCACGAGCTGGTGCGCTGTTTTAAAATTCTGTGTCACCGCAACAACCAGGGCCAAGAGACGTCGATCTCGTACTCCAGCGGGGCGATCGCCGCATTCGTCGCCCCCTACTTTGAGTCAGTGCTTCGGGCCCCCCGGGTAGGCGCGCCCATCACGGGCTCCGATGTCATCCTGGGGGAGGAGGAGTTATGGGATGCGGTGTTTAAGAAAACCCGCCTGCAAACGTACCTGACAGACATCGCGGCCCTGTTCGTCGCGGACGTCCAGCACGCAGCGCTGCCCCCGCCCCCCTCCCCGGTCGGCGCCGATTTCCGGCCCGGCGCGTCCCCGCGGGGCCGGTCCAGATCGCGGTCGCCCGGAAGAACTGCGCGAGGCGCGCCGGACCAGGGCGGGGGCATCGGGCACCGGGATGGCCGCCGCGACGGCCGACGATGAGGGGTCGGCCGCCACCATCCTCAAGCAGGCCATCGCCGGGGACCGCAGCCTGGTCGAGGCGGCCGAGGCGATTAGCCAGCAGACGCTGCTCCGCCTGGCCTGCGAGGTGCGCCAGGTCGGCGACCGCCAGCCGCGGTTTACCGCCACCAGCATCGCGCGCGTCGACGTCGCGCCTGGGTGCCGGTTGCGGTTCGTTCTGGACGGGAGTCCCGAGGACGCCTATGTGACGTCGGAGGATTACTTTAAGCGCTGCTGCGGCCAGTCCAGTTATCGCGGCTTCGCGGTGGCGGTCCTGACGGCCAACGAGGACCACGTGCACAGCCTGGCCGTGCCCCCCCTCGTTCTGCTGCACCGGTTCTCCCTGTTCAACCCCAGGGACCTCCTGGACTTTGAGCTTGCCTGTCTGCTGATGTACCTGGAGAACTGCCCCCGAAGCCACGCCACCCCGTCGACCTTTGCCAAGGTTCTGGCGTGGCTCGGGGTCGCGGGTCGCCGCACGTCCCCATTCGAACGCGTTCGCTGCCTTTTCCTCCGCAGTTGCCACTGGGTCCTAAACACACTCATGTTCATGGTGCACGTAAAACCGTTCGACGACGAGTTCGTCCTGCCCCACTGGTACATGGCCCGGTACCTGCTGGCCAACAACCCGCCCCCCGTTCTCTCGGCCCTGTTCTGTGCCACCCCGACGAGCTCCTCATTCCGGCTGCCGGGGCCGCCCCCCCGCTCCGACTGCGTGGCCTATAACCCCGCCGGGATCATGGGGAGCTGCTGGGCGTCGGAGGAGGTGCGCGCGCCTCTGGTCTATTGGTGGCTTTCGGAGACCCCAAAACGACAGACGTCGTCGCTGTTTTATCAGTTTTGTTGAATTTTAGGAAATAAACCCGGTTTTGTTTCTGTGGCCTCCCGACGGATGCGCGTGTCCTTACTCCGTCTTGGTGGGTGGGTGGCTGTGTATGGCGTCCCATCTGTGCGGGGAGGGGGGCAAGTCGGCACGTATTCGGACAGACTCAAGCACACACGGGGGAGCGCTCTTGTCTCAGGGCAATGTTTTTATTGGTCAAACTCAGGCAAACAGAAACGACATCTTGTCGTCAAAGGGATACACAAACTTCCCCCCCTCGCCCCATACTCCCGCCAGCACCCCGGTAAACACCAACTCAATCTCGCGCAGGATTTCGCGCAGGTGATGAGCGCAGTCCACGGGGGGGAGCACAAGGGGCCGCGGGTATAGATCGACGGGGACGCCGACCGACTCCCCGCCTCCGGGACAGACACGCACGACGCGCCGCCAGTAGTGCTCTGCGTCCAGCAAGGCGCCGCCGCGGAAGGCAGTGGGGGGCAAGGGGTCGCTGGCCTCAAAGGGGGACACCCGAACGCTCCAGTACTCCGCGTCCAACCGTTTATTAAACGCGTCCAAGATAAGGCGGTCGCAGGCGTCCTCCATAAGGCCCCGGGCCGTGAGTGCGTCCTCCTCCGGCACGCATGCCGTTGTCAGGCCCAGGACCCGTCGCAGCGTGTCGCGTACGACCCCTGCCGCCGTGGTGTACGCGGGCCCGCGGAGAGGAAATCCCCCAAGATGGTCAGTGTTGTCGCGGGAGTTCCAGAACCACACTCCCGCCTGGCTCCAGGCGACTGCGTGGGTGTAGACGCCCTCGAGGGCCAGGCACAGTGGGTGCCGCAGCCGGAGGCCGTTGGCCCTAAGCACGGCTCCCACGGCCGTCTCGATGGCCCGCCGGGCGTCCTCGATCACCCCGGAAGCCGCATCCGCGTCTTGGGGGTCCACGTTAAAGACACCCCAGAACGCACCCCCATCGCCCCCGCAGACCGCGAACTTCACCGAGCTGGCCGTCTCCTCGATCTGCAGGCAGACGGCGGCCATTACCCCACCCAGGAGCTGCCGCAGCGCAGGGCAGGCGTTGCACGTGTCCGGGACCAGGCGCTCCAAGACGGCCCCGGCCCAGGGCTCTGAGGGAGCGGCCACCACCAGCGCGTCCAGTCTTGCTAGGCCCGTCCGGCCGTGGGGGTCCGCCAGCCCGCTCCCCCCGAGGTCGGCCAGGGCCGCCAGGAGCTGGGCGCGAAGTCCGGGGAAGCAAAACCGCGCCGTCCAGACGGGCCCGACGGCCGCGGGCGGGTCTAACAGTTGGATGATTTTAGTGGCGGGATGCCACCGCGCCACCGCCTCCCGCACCGCGGGCAGGAGGCATCCGGCTGCCGCCGAGGCCACGCCGGGCCAGGCTCGCGGGGGGAGGACGACCCTGGCCCCCACCGCGGGCCAGGCCCCCAGGAGCGCGGCGTAAGCGGCCGCGGCCCCGCGCACCAGGTCCCGTGCCGACTCGGCCGTGGCCGGCACGGTGAACGTGGGCCAACCCGGAAACCCCAGGACGGCAAAGTACGGGACGGGTCCCCCCCGGACCTCAAACTCGGGCCCCAGAAAGGCAAAGACGGGGGCCAGGGCCCCGGGGGCGGCGTGGACCGTGGTATGCCACTGCCGGAAAAGGGCGACGAGCGCCGGCGCGGAGAACTTCTCGCCGGCGCTTACAAAGTAGTCGTAATCGCGGGGCAGCAGCACCCGTGCCGTGACTCGTTGCGGGTGCCCGCGTGGCCGCAGGCCCACCTCGCACACCTCGACCAGGTCCCCGAACGCGCCCTCCTTCTTGATCGGCGGAAACGCAAGAGTCTGGTATTCGCGCGCAAATAGCGCGGTTCCGGTGGTGATGTTAACGGTCAGCGAAGCGGCGGACGCGCACTGGGGGGTGTCGCGAATGGCCGCCAGGCGCGCCCACGCCAGCCGCGCGTCGGGATGCTCGGCAACGCGCGCCGCCAGGGCCATAGGGTCGATGTCAATGTTGGCCTCCGCGACCAGGAGAGCGGCGCGAGGGGCGGCGGGCGGGCCCCACGACGCTCTCTCAACTTTCACCACCAGTCCCGTGCGTGGGTCCGAGCCGATACGCAGCGGGGCGAACAGGGCCACCGGCCCGGTCTGGCGCTCCAGGGCCGCCAGGACGCACGCGTACAGCGCCCGCCACAGAGTCGGGTTCTCCAGGGGCTCCAGCGGGGAGGCGGCCGGCGTCGTCGCGGCGCGGGCGGCCGCCACGACGGCCTGGACGGAGACGTCCGCGGAGCCGTAGAAATCCCGCAGCTCCGTCGCGGTGACGGAGACCTCCGCAAAGCGCGCGCGACCCTCCCCTGCGGCGTTGCGACATACAAAATACACCAGGGCGTGGAAGTACTCGCGAGCGCGGGGGGGCAGCCATACCGCGTAAAGGGTAATGGCGCTGACGCTCTCCTCCACCCACACGATATCTGCGGTGTCCATCGCACGGCCCCTAAGGATCACGGGCGGTCTGTGGGTCCCATGCTGCCGTGCCTGGCCGGGCCCGGTGGGTCGCGGAAACCGGTGACGGGGGGGGGGCGGTTTTTGGGGTTGGGGTGGGGGTGGGAAACGGCCCGGGTCCGGGGGCCAACTTGGCCCCTCGGTGCGTTCCGGCAACAGCGCCGCCGGTCCGCGGACGACCACGTACCGAACGAGTGCGGTCCCGAGACTTATAGGGTGCTAAAGTTCACCGCCCCCTGCATCATGGGCCAGGCCTCGGTGGGGAGCTCCGACAGCGCCGCCTCCAGGATGATGTCAGCGTTGGGGTTGGCGCTGGATGAGTGCGTGCGCAAACAGCGCCCCCACGCGGGCACGCGTAGCTTGAAGCGCGCGCCCGCAAACTCCCGCTTGTGGGCCATAAGCAGGGCGTACAGCTGCCTGTGGGTCCGGCAGGCGCTGTGGTCGATGTGGTGGGCGTCCAACAACCCCACGATTGTCTGTTTGGTGAGGTTTTTAACGCGCCCCGCCCCGGGAAACGTCTGCGTGCTTTTGGCCATCTGCACGCCAAACAGTTCGCCCCAGATTATCTTGAACAGCGCCACCGCGTGGTCCGTCTCGCTAACGGACCCGCGCGGGGGACAGCCGCTTAGGGCGTCGGCGACGCGCTTGACGGCTTCCTCCGAGAGCAGAAGTCCGTCGGTTACGTTACAGTGGCCCAGTTCGAACACCAGCTGCATGTAGCGGTCGTAGTGGGGGGTCAGTAGGTCCAGCACGTCATCGGGGCCGAAGGTCCTCCCAGATCCCCCGGCCGCCGAGTCCCAATGCAGGCGCGCGGCCATGGTGCTGCACAGGCACAACAGCTCCCAGACGGGGGTTACGTTCAGGGTGGGGGGCAGGGCCACGAGCTCCAGCTCTCCGGTGACGTTGATCGTGGGGATGACGCCCGTGGCGTAGTGGTCATAGATCCGCCGAAATATGGCGCTGCTGCGGGTGGCCATGGGAACGCGGAGACAGGCCTCCAGCAACGCCAGGTAAATAAACCGCGTGCGTCCCATCAGGCTGTTGAGGTTGCGCATGAGCGCGACAATTTCCGCCGGCGCGACATCGGACCGGAGGTATTTTTCGACGAAAAGACCCACCTCCTCCGTCTCGGCGGCCTGGGCCGGCAGCGACGCCTCGGGATCCCGGCACCGCAGCTCCCGTAGATCGCGCTGGGCCCTGAGGGCGTCGAAATGTACGCCCCGCAAAAACAGACAGAAGTCCTTTGGGGTCAGGGTATCGTCGTGTCCCCAGAAGCGCACGCGTATGCAGTTTAGGGTCAGCAGCATGTGAAGGATGTTAAGGCTGTCCGAGAGACACGCCAGCGTGCATCTCTCAAAGTAGTGTTTGTAACGGAATTTGTTGTAGATGCGCGACCCCCGCCCCAGCGACGTGTCGCATGCCGACGCGTCACAGCGCCCCTTGAACCGGCGACACAGCAGGTTTGTGACCTGGGAGAACTGCGCGGGCCACTGGCCGCAGGAACTGACCACGTGATTAAGGAGCATGGGCGTAAAGACGGGCTCCGAGCGCGCCCCGGAGCCGTCCATGTAAATCAGTAGCTCCCCCTTGCGGAGGGTGCGCACCCGTCCCAGGGACTGGTACACGGACACCATGTCCGGTCCGTAGTTCATGGGTTTCACGTAGGCGAACATGCCATCAAAGTGCAGGGGATCGAAGCTGAGGCCCACGGTTACGACCGTCGTGTATATAACCACGCGGTATTGGCCCCACGTGGTCACGTCCCCGAGGGGGGTGAGCGAGTGAAGCAACAGCACGCGGTCCGTAAACTGACGGCAGAACCGGGCCACGATCTCCGCGAAGGAGACCGTCGACGAAAAAATGCAGATGTTATCGCCCCCGCCAAGGCGCGCTTCCAGCTCCCCAAAGAACGTGGCCCCCCGGGCGTCCGGAGAGGCGTCCGGAGACGGGCCGCTCGGCGGCCCGGGCGGGCGCAGGGCAGCCTGCAGGAGCTCGGTCCCCAGACGCGGGAGAAACAGGCACCGGCGCGCCGAAAACCCGGGCATGGCGTACTCGCCGACCACCACATGCACGTTTTTTTCGCCCCGGAGACCGCACAGGAAGTCCACCAACTGCGCGTTGGCGGTTGCGTCCATGGCGATGATCCGAGGACAGGTGCGCAGCAGGCGTAGCATTAACGCATCCACGCGGCCCAGTTGCTGCATCGTTGGCGAATAGAGCTGGCCCAGCGTCGACATAACCTCGTCCAGAACGAGGACGTCGTAGTTGTTCAGAAGGTTGGGGCCCACGCGATGAAGGCTTTCCACCTGGACGATAAGTCGGTGGAAGGGGCGGTCGTTCATAATGTAATTGGTGGATGAGAAGTAGGTGACAAAGTCGACCAGGCCTGACTCAGCGAACCGCGTCGCTAGGGTCTGGGTAAAACTCCGACGACAGGAGACGACGAGCACACTCGTGTCCGGAGAGTGGATCGCTTCCCGCAGCCAGCGGATCAGCGCGGTAGTTTTTCCCGACCCCATTGGCGCGCGGACCACAGTCACGCACCTGGCCGTCGGGGCGCTCGCGTTGGGGAAGGTGACGGGTCCGTGCTGCTGCCGCTCGATCGTTGTTTTCGGGTGAACCCGGGGCACCCATTCGGCCAAATCCCCCCCGTACAACATCCGCGCTAGCGATACGCTCGACGTGTACTGTTCGCACTCGTCGTCCCCAATGGGACGCCCGGCCCCCAGAGGATCTCCCGACTCCGCGCCCCCCACGAAAGGCATGACCGGGGCGCGGACGGCGTGGTGGGTCTGGTGTGTGCAGGTGGCGACGTTTGTGGTCTCTGCGGTCTGCGTCACGGGGCTCCTCGTCCTGGCCTCTGTGTTCCGGGCACGGTTTCCCTGCTTTTACGCCACGGCGAGCTCTTATGCCGGGGTGAACTCCACGGCCGAGGTGCGCGGGGGTGTAGCCGTGCCCCTCAGGTTGGACACGCAGAGCCTTGTGGGCACTTATGTAATCACGGCCGTGTTGTTGTTGGCCGTGGCCGTGTATGCCGTGGTCGGCGCCGTGACCTCCCGCTACGACCGCGCCCTGGACGCGGGCCGCCGTCTGGCTGCGGCCCGCATGGCCATGCCGCACGCCACGCTGATCGCCGGAAACGTCTGCTCTTGGTTGCTGCAGATCACCGTCCTGTTGCTGGCCCATCGCATCAGCCAGCTGGCCCACCTGGTTTACGTCCTGCACTTTGCGTGTCTGGTGTATTTTGCGGCCCATTTTTGCACCAGGGGGGTCCTGAGCGGGACGTATCTGCGTCAGGTGCACGGCCTGATGGAGCTGGCCCCGACCCATCATCGCGTCGTCGGCCCGGCTCGCGCCGTGCTGACAAACGCCTTGCTGTTGGGCGTCTTCCTGTGCACGGCCGACGCCGCGGTATCCCTGAATACCATCGCCGCGTTCAACTTTAATTTTTCGGCCCCGGGCATGCTCATCTGCCTGACCGTGCTGTTCGCCATTCTCGTCGTATCGCTGTTGTTGGTGGTCGAGGGGGTGTTGTGTCACTACGTGCGCGTGTTGGTGGGCCCCCACCTGGGGGCCGTGGCCGCCACGGGCATCGTCGGCCTGGCCTGCGAGCACTATTACACCAACGGCTACTACGTTGTGGAGACGCAGTGGCCGGGGGCTCAGACGGGAGTCCGCGTCGCCCTCGCCCTGGTCGCCGCCTTTGCCCTCGGCATGGCCGTGCTCCGCTGCACCCGCGCCTATCTGTATCACAGGCGGCACCACACCAAATTTTTTATGCGCATGCGCGACACGCGACACCGCGCACATTCCGCCCTCAAGCGCGTACGCAGTTCCATGCGCGGATCGCGAGACGGCCGCCACAGGCCCGCACCCGGCAGCCCGCCCGGGATTCCCGAATATGCGGAAGACCCCTACGCGATCTCATACGGCGGCCAGCTCGACCGGTACGGAGATTCCGACGGGGAGCCGATTTACGACGAGGTGGCGGACGACCAAACCGACGTATTGTACGCCAAGATACAACACCCGCGGCACCTGCCCGACGACGATCCCATCTATGACACCGTTGGGGGGTACGACCCCGAGCCCGCCGAGGACCCCGTGTACAGCACCGTCCGCCGTTGGTAGCTGTTTGGTTCCGTTTTAATAAACCGTTTGTGTTTAACCCGACCGTGGTGTATGTCTGGTGTGTGGCGTCCGATCCCGTTACTATCACCGTCCCCCCCCCCCCCTCAACCCCGGCGATTGTGGGTTTTTTAAAAACGACACGCGTGCGACCGTATACAGAACATTGTTTTGGTTTTTATTCGCTATCGGACATGGGGGGTGGAAACTGGGTGGCGGGGCAGGCGCCTCCGGGGGTCCGCCGGTGAGTGTGGCGCGAGGGGGGGTCCGATGAACGCAGGCGCTGTCTCCCCGGGGCCCGCGTAACCCCGCGCATATCCGGGGGCACGTAGAAATTACCTTCCTCTTCGGACTCGATATCCACGACGTCAAAGTCGTGGGCGGTCAGCGAGACGACCTCCCCGTCGTCGGTGATGAGGACGTTGTTTCGGCAGCAGCAGGGCCGGGCCCCGGAGAACGAGAGGCCCATAGCTCGGCGAGCGTGTCGTCGAATGCCAGGCGGCTGCTTCGCTGGATGGCCTTATAGATCTCCGGATCGATGCGGACGGGGGTAATGATCAGGGCGATCGGAACGGCCTGGTTCGGGAGAATGGACGCCTTGCTGGGTCCTGCGGCCCCGAGAGCCCCGGCGCCGTCCTCCAGGCGGAACGTTACGCCCTCCTCCGCGCTGGTGCGGTGCCTGCCGATAAACGTCACCAGATGCGGGTGGGGGGGGCAGTCGGGGAAGTGGCTGTCGAGCACGTAGCCCTGCACCAAGATCTGCTTAAAGTTCGGGTGACGGGGGTTCGCGAAGACGGGCTCGCGGCGGACCAGATCCCCGGAGCTCCAGGACACGGGGGAGATGGTGTGGCGTCCGAGGTCGGGGGCGCCAAACAGAAGCACCTCCGAGACAACGCCGCTATTTAACTCCACCAAGGCCCGATCCGCGGCGGAGCACCGCCTTTTTTCGCCCGAGGCGTGGGCCTCTGACCAGGCCTGGTCTTGCGTGACGAGAGCCTCCTCCGGGCCGGGGACGCGCCCGGGCGCGAAGTATCGCACGCTGGGCTTCGGGATCGACCGGATAAATGCCCGGAACGCCTCCGGGGACCGGTGTGCCATCAAGTCCTCGTACGCGGAGGCCGTGGGGTCGCTGGGGTCCATGGGGTCGAAAGCGTACTTGGCCCGGCATTTGACCTCGTAAAAGGCCAGGGGGGTCTTGGGGACTGGGGCCAGGTAGCCGTGAATGTCCCGAGGACAGACGAGAATATCCAGGGACGCCCCGACCATCCCCGTGTGACCGTCCATGAGGACCCCACACGTATGCACGTTCTCTTCGGCGAGGTCGCTGGGTTCGTGGAAGATAAAGCGCCGCGTGTCGGCGCCGGCCTCGCCGCCGTCGTCCGCGCGGCCCACGCAGTAGCGAAACAGCAGGCTTCGGGCCGTCGGCTCGTTCACCCGCCCGAACATCACCGCCGAAGACTGTACATCCGGCCGCAGGCTGGCGTTGTGCTTCAGCCACTGGGGCGAGAAACACGGACCCTGGGGGCCCCAGCGGAGGGTGGATGCGGTCGTGAGGCCCCGCCGGAGCAGGGCCCATAGCTGGCAGTCGGCCTGGTTTTGCGTGGCCGCCTCGTAAAACCCCATGAGGGGCCGGGGCGCCACGGCGTCCGCGGCGGCCGGGGGCCCGCGGCGCGTCAGGCGCCATAGGTGCCGACCGAGTCCGCGGTCCACCATACCCGCCTCCTCGAGGACCACGGCCAGGGAACACAGATAATCCAGGCGGGCCCAGAGGGGACCGATGGCCAGAGGGGCGCGGACGCCGCGCAGCAACCCGCGCAGGTGGCGCTCGAACGTCTCGGCTAGTATATGGGAGGGCAGCGCGTTGGGGATCACCGACGCCGACCACATAGAGTCAAGGTCCGGGGAGTCGGGATCGGCGTCCGGGTCGCGGGCGTGGGTGCCCCCAGGAGATAGCGGAATGTCTGGGGTCGGAGGCCCTGAGGCGTCAGAAAGTGCCGGCGACGCGGCCCGGGGCTTTTCGTCTGCGGTGTCGGTGGCGTGCTGATCACGTGGGGGGTTAACGGGCGAATGGGAGCTCGGGTCCACAGCTGACGTCGTCTGGGGTGGGGGGGGCAGGGGACGGAAGGTGGTTGTCAGCGGAAGACTGTTAGGGCGGGGGCGCTTGGGGGGGCTGTCGGGGCCACGAGGGGTGTCCTCGGCCAGGGCCCAGGGACGCTTAGTCACGGTGCGTCCCGGCGGACATGCTGGGCCTACCGTGGACTCCATTTCCGAGACGACGTGGGGGGAGCGGTGGTTGAGCGCGCCGCCGGGTGAACGCTGATTCTCACGACAGCGCGTGCCGCGCGCACGGGTTGGTGTGACACAGGCGGGACACCAGCACCAGGAGAGGCTTAAGCTCGGGAGGCAGCGCCACCGACGACAGTATCGCCTTGTGTGTGTGCTGGTAATTTATACACCGATCCGTAAACGCGCGCCGAATCTTGGGATTGCGGAGGTGGCGCCGGATGCCCTCTGGGACGTCATACGCCAGGCCGTGGGTGTTGGTCTCGGCCGAGTTGACAAACAGGGCTGGGTGCAGCACGCAGCGATAGGCGAGCAGGGCCAGGGCGAAGTCCGGCGACAGCTGGTTGTTAAAATACTGGTAACCGGGAAACCGGGTCACGGGTACGCCCAGGCTCGGGGCGACGTACACGCTAACCACCAACTCCAGCAGCGTCTGGCCCAGGGCGTACAGGTCAACCGCTAACCCGACGTCGTGCTTCAGGCGGTGGTTGGTAAATTCGGCCCGTTCGTTGTTAAGGTATTTCACCAACAGCTCCGGGGGCTGGTTATACCCGTGACCCACCAGGGTGTGAAAGTTGGCTGTGGTTAGGGCGGTGGGCATGCCAAACATCCGGGGGGACTTGAGGTCCGGCTCCTGGAGGCAAAACTGCCCCCGGGCGATCGTGGAGTTGGAGTTGAGGGTGACGAGGCTAAAGTCGGCGAGGACGGCCCGCCGGAGCGAGACGGCGTCCGACCGCAGCATGACGAGGATGTTGGCGCACTTGATATCCAGGTGGCTGATCCCGCAGGTGGTGTTTAAAAACACAACGGCGCGGGCCAGCTCCGTGAAGCACTGGTGGAGGGCCGTCGAGACCGAGGGGTTTGTTGTGCGCAGGGACGCCAGTTGGCCGATATACTTACCGAGGTCCATGTCGTACGCGGGGAACACTATCTGTCGTTGTTGCAGCGAGAACCCGAGGGGCGCGATGAAGCCGCGGATGTTGTGGGTGCGGCCGGCGCGTAGAACGCACTCCCCGACCAACAGGGTCGCGATGAGCTCAACGGCAAACCACTCCTTTTCCTTTATGGTCTTAACGGCAAGCTTATGTTCGCGAATCAGTTGGACGTCACCGTATCCCCCAGACCCCCCGAAGCTTCGGGCCCCGGGGATCTCGAGGGTCGTGTAGTGTAGGGCGGGGTTGATGGCGAACACGGGGCTGCATAGCTTGCGGATGCGCGTGAGGGTGAGGATGTGCGAGGGGGACGAGGGGGGTGCGGTTAACGCCGCCTGGGATCTGCGCAGGGGCGGGCGGTTCAGTTTGGCCGCCGTACCGGGCGTCTCGGGGGACGCGCGGCGATGAGACGAGCGGCTCATTCGCCATCGGGATAGTCCCGCGCGAAGCCGCTCGCGGAGGCCGGATCGGTGGCGGGACCCGTGGGAGGAGCGGGAGACGGCGGCGTCCTGGAGAGAGGGGCCGCTGGGGCGCCCGGAGGCCCCGTGGGGGTTGGAGTGTACGTAGGATGCGAGCCAATCCTTGAAGGACCGTTGGCGTGCACCTTGGGGGCTGAGGTTAGCTGCCACATGACCAGCAGGTCGCTGTCTGCGGGACTCATCCATCCTTCGGCCAGGTCGCCGTCTCCCCACAGAGAAGCGTTGGTCGCTGCTTCCTCGAGTTGCTCCTCCTGGTCCGCAAGACGATCGTCCACGGCGTCCAGGCGCTCACCAAGCGCCGGATCGAGGTACCGTCGGTGTGCGGTTAGAAAGTCACGACGCGCCGCTTGCTCCTCCACGCGAATTTTAACACAGGTCGCGCGCTGTCGCATCATCTCTAAGCGCGCGCGGGACTTTAGCCGCGCCTCCAATTCCAAGTGGGCCGCCTTTGCAGCCATAAAGGCGCCAACAAACCGAGGATCTTGGGTGCTGACGCCCTCCCGGTGCAGCTGCAGGGTCTGGTCCTTGTAAATCTCGGCTCGGAGGTGCGTCTCGGCCAGGCGTCGGCGCAGGGCCGCGTGGGCGGCATCTCGGTCCATTCCGCCACCCTGCGGGCGACCCGGGGGGTGCTCTGATAGTCTCGCGTGCCCAAGGCCCGTGATCGGGGTACTTCGCCGCCGCGACCCGCCACCCGGTGTGCGCGATGTTTGGTCAGCAGCTGGCGTCCGACGTCCAGCAGTACCTGGAGCGCCTCGAGAAACAGAGGCAACTTAAGGTGGGCGCGGACGAGGCGTCGGCGGGCCTCACCATGGGCGGCGATGCCCTACGAGTGCCCTTTTTAGATTTCGCGACCGCGACCCCCAAGCGCCACCAGACCGTGGTCCCTGGCGTCGGGACGCTCCACGACTGCTGCGAGCACTCGCCGCTCTTCTCGGCCGTGGCGCGGCGGCTGCTGTTTAATAGCCTGGTGCCGGCGCAACTAAAGGGGCGTGATTTCGGGGGCGACCACACGGCCAAGCTGGAATTCCTGGCCCCCGAGTTGGTACGGGCGGTGGCGCGACTGCGGTTTAAGGAGTGCGCGCCGGCGGACGTGGTGCCTCAGCGTAACGCCTACTATAGCGTTCTGAATACGTTTCAGGCCCTCCACCGCTCCGAAGCCTTTCGCCAGCTGGTGCACTTTGTGCGGGACTTTGCCCAGCTGCTCAAAACCTCCTTCCGGGCCTCCAGCCTCACGGAGACCACGGGCCCCCCCAAAAAACGGGCCAAGGTGGACGTGGCCACCCACGGCCGGACGTACGGCACGCTGGAGCTGTTCCAAAAAATGATCCTTATGCACGCCACCTACTTTCTGGCCGCCGTGCTCCTCGGGGACCACGCGGAGCAGGTCAACACGTTCCTGCGTCTCGTGTTTGAGATCCCCCTGTTTAGCGACGCGGCCGTGCGCCACTTCCGCCAGCGCGCCACCGTGTTTCTCGTCCCCCGGCGCCACGGCAAGACCTGGTTTCTGGTGCCCCTCATCGCGCTGTCGCTGGCCTCCTTTCGGGGGATCAAGATCGGCTACACGGCGCACATCCGCAAGGCGACCGAGCCGGTGTTTGAGGAGATCGACGCCTGCCTGCGGGGCTGGTTCGGTTCGGCCCGAGTGGACCACGTTAAAGGGGAAACCATCTCCTTCTCGTTTCCGGACGGGTCGCGCAGTACCAGGGATCGCTTGAGGAGGCCCGGGCAACGGCCCGGGCAACGGTGGGGCAACTCGTAGCAAATAGGCGACTGATGTACGAAGAGAAGACACACAGGCGCCACCCGGCGCTGGTCGGGGGGATGTTGTCCGCGCCGCACCGTCCCCCGACGACCTCTTGCAGACGGTCCGTGATGCAAGGACGGCGGGGGGCCTGCAGCAGGGTGACCGTATCCACGGGATGGCCAAAGAGAAGCGGACACAGGCTAGCATCCCCCTGGACCGCCAGGGTACACTGGGCCATCTTGGCCCACAGACACGGGGCGACGCAGGGACAGGACTCCGTTACGACGGAGGAGAGCCACAGTGCGTTGGCGGAATCGATGTGGGGCGGCGGGGCGCAGGACTCGCAGCCCCCCGGGTGGTTGGTGATCCTGGCCAGGAGCCATCCCAGATGGCGGGCCCTGCTTCCCGGTGGACAGAGCGACCCCAGGTCGCTGTCCATGGCCCAGCAGTAGATCTGGCCGCTGGGGAGGTGCCACCAGGCCCCCGGGCCCAAGGCGCAGCACGCGCCCGGCTCCGGGGGGGTCTTCGCGGGGACCAGATACGCGCCATCCAGCTCGCCGACCACTGGCTCCTCCGCGAGCTGTTCGGTGGTTGGGTCGGGGGTTTCCTCCGGGGGGGTGGCCGCCCGTATGCGTGCGAACGTGAGGGTGCACAGGAGCGGGGTCAGGGGGTGCGTCACGCTCCGGAGGTGGACGATCGCGCAGTAGCGGCGCTCGCGGTTAAAGAAAAAGAGGGCAAAGAAGGTGTTCGGGGGCAACCGCAGCGCCTTGGGGCGCGTCAGATACAGAAAAATCTCGCAGAAGAGGGCGCGCCCGGGGTCTGGGTTAGGAAGGGCCACCTGACACAGAGGCTCGGTGAGGACCGTTAGACACCGAAAGATCTTGAGCCGCTCGTCCGCCCGAACGACGCGCCACACAAAGACGGAGTTGACAATGCGCGCGATAGAGTCGACGTCCGTCCCCAGGTCGTCGACTCTATCGCGCGTGCCGCGAGCTCCGGCCCGGGAATCCGGCCGGGGCAAGGTCCCCGGGGGACCAGGCGGCGCCAGGGGCCGCCGGGGTCCCAGCTGCGCCATGCCGGGGGCGGGGGGAGGGCAAACCCCAGAGGCGGGGGCCAACGGCGCGGGGAGGAGTGGGTGGGCGAGGTGGCCGGGGGAAGGCGCCCGCTAGCGAGACCGGCCGTTCCCGGACGACACCTTGCGACAAAACCTAAGGACAGCGGCCCGCGCGACGGGGTCCGAGAGGCTAAGGTAGGCCGCGATGTTAATGGTGAACGCAAAGCCGCCGGGAAAGACAACTATGCCACAGAGGCGGCGATTAAACCCCAGGCAGAGGTAGGCGTAGCTTTCCCCGGGCAGGTATTGCTCGCAGACCCTGCGTGGGGCTGTGGAGGGGACGGCCTCCATGAAGCGACATTTACTCTGCTCGCGTTTACTGACGTCACCATCCATCGCCACGGCGATTGGACGATTGTTAAGCCGCAGCGTGTCTCCGCTTGTGCTGTAGTAGTCAAAAACGTAATGGCCGTCGGAGTCGGCAAAGCGGGCCGGGAGGTCGTCGCCGAGCGGGACGACCCGCCGCCCCCGACCGCCCCGTCCCCCCAGGTGTGCCAGGACGGCCAGGGCATACGCGGTGTGAAAAAAGGCGTCGGGGGCGGTCCCCTCGACGGCGCGCATCAGGTTCTCGAGGAGAATGGGGAAGCGCCTGGTCACCTCCCCCAACCACGCGCGTTGGTCGGGGCCAAAGTCATAGCGCAGGCGCTGTGAGATTCGCGGGCCGCCCTGAAGCGCGGCCCGGATGGCCTGGCCCAGGGCCCGGAGGCACGCCAGATGTATGCGCGCGGTAAAGGCGACCTCGGCGGCGATGTCAAAGGGCGGCAGGACGGGGCGCGGGTGGCGCAGGGGCACCTCGAGCGCGGGAAAGCGTAGCAGCAGCTCCGCCTGCCCAGCGGGAGACAGCTGGTGGGGGCGCACGACGCGTTCTGCGGCGCAGGCCTCGGTCAGGGCCGTGGCCAGCGCCGAGGACAGCAGCGGAGGGCGGGCGCGTCGCCCGCCCCACGCCACGGAGTTCTCGTAGGAGACGACGACGAAGCGCTGCTTGGTTCCGTAGTGGTGGCGCAGGACCACGGAGATAGAACGACGGCTCCACAGCCAGTCCGGCCGGTCGCCGCCGGCCAGGGCTTCCCATCCGCGATCCAACCACTCGACCAGCGACCGCGGCTTTGCGGTACCAGGGGTAAGGGTTAGAACGTCGTTCAGGATGTCCTCGCCCCCGGGCCCGTGGGGCGCTGGGGCCACAAAGCGGCCCCCGCCGGGGGGCTCCAGACCCGCCAGCACCGCATCTGCGTCAGCCGCCCCCATGGCGCCCCCGCTGACGGCCTGGTGAACCAGGGCGCCCTGGCGTAGCCCCGATGCAACGCCACAGGCCGCACGCCCGGTCCGCGCTCGGACCGGGTGGCGGCGGGTGACGTCCTGCACTGCCCGCTGAACCAACGCGAGGATCTCCTCGTTCTCCTGTGCGATGGACACGTCCTGGGCCGCGGTCGTGTCGCCGCCGGGGGCCGTCAGCTGCTCCTCCGGGGAGATGGGGGGGTCGGACGCCCCGACGATGGGCGGGTCTGCGGGCGCCCCCGCGTGGGGCCGGGCCAAGGGCTGCGGACGCGGGGACGCGCTTTCCCCCAGACCCATGGACAGGTGGGCCGCAGCCTCCTTCGCGGCCGGCGGGGCGGCGGCGCCAAGCAGAGCGACGTAGCGGCACAAATGCCGACAGACGCGCATGATGCGCGTGCTGTCGGCCGCGTAGCGCGTGTTGGGGGGGACGAGCTCGTCGTAACTAAACAGAATCACGCGGGCACAGCTCGCCCCCGAGCCCCACGCAAGGCGCAGCGCCGCCACGGCGTACGGGTCATAGACGCCCTGCGCGTCACACACCACGGGCAGGGAGACGAACAACCCCCCGGCGCTGGACGCACGCGGAAGGAGGCCAGGGTGTGCCGGCACGACGGGGGCCAGAAGCTCCCCCACCGCATCCGCGGGCACGTAGGCGGCAAACGCCGTGCACCACGGGGTACAGTCGCCGGTGGCATGAGCCCGAGTCTGGATTTCGACCTGGAAGTTTGCGGCCGTCCCGAGTCCGGGGCGGCCGCGCATCAGGGCGGCCAGAGGGATTCCCGCGGCCGCCAGGCACTCGCTGGATATGATGACGTGAACCAAAGACGAGGGCCGACCCGGGCCGTGGCCGAGATCGTACTGGACCTCGTTGGCCAAGTGCGCGTTCATGGTTCGGGGGTGGGTGTGGGTGTGTAGGCGATGCGGGTCCCCCGAGTCCGCGGGAAGGGCGTGGGTTTGGCGCGCGTATGCGTATTCGCCAACGGAGGCGTGCGTGCTTATGCGCGGCGCGTTTCTTCTGTCTCTAGGGAATCCGAGGCCAGGACTTTAACCTGCTCTTTGTCGACGAGGCCAACTTTATTCGCCCGGATGCGGTCCAGACGATTATGGGCTTTCTCAACCAGGCCAACTGCAAGATTATCTTCGTGTCGTCCACCAACACCGGGAAGGCCAGTACGAGCTTTTTGTACAACCTCCGCGGGGCCGCAGACGAGCTTCTCAACGTGGTGACCTATATATGCGATGATCACATGCCGAGGGTGGTGACGCACACAAACGCCACGGCCTGTTCTTGTTATATCCTCAACAAGCCCGTTTTCATCACGATGGACGGGGCGGTTCGCCGGACCGCCGATTTGTTTCTGGCCGATTCCTTCATGCAGGAGATCATCGGGGGCCAGGCCAGGGAGACCGGCGACGACCGGCCCGTTCTGACCAAGTCTGCGGGGGAGCGGTTTCTGTTGTACCGCCCCTCGACCACCACCAACAGCGGCCTCATGGCCCCCGATTTGTACGTGTACGTGGATCCCGCGTTCACGGCCAACACCCGAGCCTCCGGGACCGGCGTCGCTGTCGTCGGGCGGTACCGCGACGATTATATCATCTTCGCCCTGGAGCACTTTTTTCTCCGCGCGCTCACGGGCTCGGCCCCCGCCGACATCGCCCGCTGCGTCGTCCACAGTCTGACGCAGGTCCTGGCCCTGCATCCCGGGGCGTTTCGCGGCGTCCGGGTGGCGGTCGAGGGAAATAGCAGCCAGGACTCGGCCGTCGCCATCGCCACGCACGTGCACACAGAGATGCACCGCCTACTGGCCTCGGAGGGGGCCGACGCGGGCTCGGGCCCCGAGCTTCTCTTCTACCACTGCGAGCCTCCCGGGAGCGCGGTGCTGTACCCCTTTTTCCTGCTCAACAAACAGAAGACGCCCGCCTTTGAACACTTTATTAAAAAGTTTAACTCCGGGGGCGTCATGGCCTCCCAGGAGATCGTTTCCGCGACGGTGCGCCTGCAGACCGACCCGGTCGAGTATCTGCTCGAGCAGCTAAATAACCTCACCGAAACCGTCTCCCCCAACACTGACGTCCGTACGTATTCCGGAAAACGGAACGGCGCCTCGGATGACCTTATGGTCGCCGTCATTATGGCCATCTACCTCGCGGCCCAGGCCGGACCTCCGCACACATTCGCTCCTATCACACGCGTCTCGTGAGCGCCCAATAAACACACCCAGGTATGCTACGCACGACCACGGTGTCGTCTGTTAAGGGGGGGGGGGGAAGGGGGTGTTGGCGGGAAGCGTGGGAACACGGGGGATTCTCTCACGACCGGCACCAGTACCACCCCCCTGTGAACACAGAAACCCCAACCCAAATCCCATAAACATACGACACACAGGCATATTTTGGAATTTCTTAGGTTTTTATTTATTTAGGTATGCTGGGGTTTCTCCCTGGATGCCCACCCCCACCCCCCCGTGGGTCTAGCCGGGCCTTAGGGATAGCGTATAACGGGGGCCATGTCTCCGGACCGCACAACGGCCGCGCCGTCAAAGGTGCACACCCGAACCACGGGAGCCAGGGCCAAGGTGTCTCCTAGTTGGCCCGCGTGGGTCAGCCAGGCGACGAGCGCCTCGTAAAGCGGCAGCCTTCGCTCTCCATCCTGCATCAGGGCCGGGGCTTCGGGGTGAATGAGCTGGGCGGCCTCCCGCGTGACACTCTGCATCTGCAGTAGAGCGTTCACGTACCCGTCCTGGGCACTTAGCGCAAAGAGCCGGGGGATTAGCGTAAGGATGATGGTGGTTCCCTCCGTGATCGAGTAAACCATGTTAAGGACCAGCGATCGCAGCTCGGCGTTTACGGGACCGAGTTGTTGGACGTCCGCCAGCAGCGAGAGGCGACTCCCGTTGTAGTACAGCACGTTGAGGTCTGGCAGCCCTCCGGGGTTTCTGGGGCTGGGGTTCAGGTCCCGGATGCCCCTGGCCACGAGCCGCGCCACGATTTCGCGCGCCAGGGGCGATGGAAGCGGAACGGGAAACCGCAACGTGAGGTCCAGCGAATCCAGGCGCACGTCCGTCGCTTGGCCCTCGAACACGGGCGGGACGAGGCTGATGGGGTCCCCGTTACAGAGATCTACGGGGGAGGTGTTGCGAAGGTTAACGGTGCCGGCGTGGGTGAGGCCCACGTCCAGGGGGCAGGCGACGATTCGCGTGGGAAGCACCCGGGTGATGACCGCGGGGAAGCGCCTTCGGTACGCCAGCAACAACCCCAACGTGTCGGGACTGACGCCTCCGGAGACGAAGGATTCGTGCGCCACGTCGGCCAGCGTCAGTTGCCGGCGGATGGTCGGCAGGAATACCACCCGCCCTTCGCAGCGCTGCAGCGCCGCCGCATCGGGGCGCGAGATGCCCGAGGGTATCGCGATGTCAGTTTCAAAGCCGTCCGCCAGCATGGCGCCGATCCACGCGGCAGGGAGTGCAGTGGTGGTTCGGGTGGCGGGAGGAGCGCGGTGGGGGTCAGCGGCGTAGCAGAGACGGGCGACCAACCTCGCATAGGACGGGGGGTGGGTCTTAGGGGGTTGGGAGGCGACAGGGACCCCAGAGCATGCGCGGGGAGGTCTGTCGGGCCCAGACGCACCGAGAGCGAATCCGTCCGCGGAGTCCCGGCTTGGGTTTTATGGGGCCCGGCCCTCGGAATCGCGGCTTGTCGGCGGGGACAAAGGGGGCGGGGCTAGGGGCTTGCGGAAACAGAAGACGCGTGGGATAAAAGAATCGCACTACCCCAAGGAAGGGCGGGGCGGTTTATTACAGAGCCAGTCCCTTGAGCGGGGATGCGTCATAGACGAGATACTGCGCGAAGTGGGTCTCCCGCGCGTGGGCTTCCCCGTTGCGGGCACTGCGGAGGAGGGCGGGGTCGCTGGCGCAGGTGAGCGGGTAGGCCTCCTGAAACAGGCCACACGGGTCCTCCACGAGTTCGCGGCACCCCGGGGGGCGCTTAAACTGTACGTCGCTGGCGGCGGTGGCCGTGGACACCGCCGAACCCGTCTCCACGATCAGGCGCTCCAGGCAGCGATGTTTGGCGGCGATGTCGGCCGACGTAAAGAACTTAAAGCAGGGGCTGAGCACCGGCGAGGCCCCGTTGAGGTGGTAGGCCCCGTTATAGAGCAGGTCCCCGTACGAAAATCGCTGCGACGCCCACGGGTTGGCCGTGGCCGCGAAGGCCCGGGACGGGTCGCTCTGGCCGTGGTCGTACATGAGGGCGGTGACATCCCCCTCCTTGTCCCCCGCGTAAACGCCCCCGGCGGCGCGTCCCCGGGGGTTGCAGGGCCGGCGGAAGTAGTTGACGTCGGTCGACACGGGGGTGGCGATAAACTCACACACGGCGTCCTGGCCGTGGTCCATCCCTGCGCGCCGCGGCACCTGGGCGCACCCGAACACGGGGACGGGCTGGGCCGGCCCCAGGCGGTTTCCCGCCACGACCGCGTTCCGCAGGTACACGGCTGCCGCGTTGTCCAGGAGAGGGGGAGCCCCGCGGCCCAGGTAAAAGTTTTGGGGAAGGTTGCCCATGTCGGTGACGGGGTTGCGGACGGTTGCCGTGGCCACGACGGCGGTGTAGCCCACGCCCAGGTCCACGTTCCCGCGCGGCTGGGTGAGCGTGAAGTTTACCCCCCCGCCAGTTTCGTGCCGGGCCACCTGGAGCTGGCCCAGGAAGTACGCCTCCGACGCGCGCTCCGAGAACAGCACGTTCTCAGTCACAAAGCGGTCCTGTCGGACGACGGTGAACCCAAACCCGGGATGGAGGCCCGTCTTGAGCTGATGATGCAAGGCCACGGGACTGATCTTGAAGTACCCCGCCATGAGCGCGTAGGTCAGCGCGTTCTCCCCGGCCGCGCTCTCGCGGACGTGCTGCACGACGGGCTGTCGGATCGACGAAAAGTAGTTGGCCCCCAGAGCCGGGGGGACCAGGGGGACCTGCCGCGACAGGTCGCGCAGGGCCGGGGGGAAATTGGGCGCGTTCGCCACGTGGTCGGCCCCGGCGAACAGCGCGTGGACGGGGAGGGGGTAAAAATAGTCGCCATTTTGGATGGTATGGTCCAGATGCTGGGGGGCCATCAGCAGGATTCCGGCGTGCAACGCCCCGTCGAATATGCGCATGTTGGTGGTGGACGCGGTGTTGGCGCCCGCGTCGGGCGCCGCCGAGCAGAGCAGCGCCGTTGTGCGTTCGGCCATGTTGTGGGCCAGCACCTGCAGCGTGAGCATGGCGGGCCCGTCCACTACCACGCGCCCGTTGTGAAACATGGCGTTGACCGTGTTGGCCACCAGATTGGCCGGGTGCAGGGGGTGCGCGGGGTCCGTCACGGGGTCGCTGGGGCACTCCTCGCCGGGGGCGATCTCCGGGACCACCATGTTCTGCAGGGTGGCGTATACGCGGTCGAAGCGAACCCCCGCGGTGCAGCAGCGGCCCCGCGAGAAGGCGGGCACCATCACGTAGTAGTAAATCTTGTGGTGCACGGTCCAGTCCGCCCCCCGGTGCGGCCGGTCATCCGCGGCGTCCGCGGCTCGGGCCTGGGTGTTGTGCAGCAGCTGGCCGTCGTTGCGGTTGAAGTCCGCGGTCGCCACGTTACATGCCGCCGCGTACACGGGGTCGTGGCCCCCCGCGCTAACCCGGCAGTCGCGATGGCGGTCCAGGGCCGCGCGCCGCATCAGGGCGTCACAGTCCCACACGAGGGGTGGCAGCAGCGCCGGGTCTCGCATTAGGTGATTCAGCTCGGCTTGCGCCTGCCCGCCCAGCTCCGGGCCGGTCAGGGTAAAGTCATCAACCAGCTGGGCCAGGGCCTCGACGTGCGCCACCAGGTCCCGGTACACGGCCATGCACTCCTCGGGAAGGTCTCCCCCGAGGTAGGTCACGACGTACGAGACCAGCGAGTAGTCGTTCACGAACGCCGCGCACCGCGTGTTGTTCCAGTAGCTGGTGATGCACTGGACCACGAGCCGGGCCAGGGCGCAGAAGACGTGCTCGCTGCCGTGTATGGCGGCCTGCAGCAGGTAAAACACCGCCGGGTAGTTGCGGTCGTCGAACGCCCCGCGAACGGCGGCGATGGTGGCGGGGGCCATGGCGTGGCGTCCCACCCCCAGCTCCAGGCCCCGGGCGTCCCGGAACGCCGCCGGACATAGCGCCAGGGGCAAGTTGCCGTTCACCACGCGCCAGGTGGCCTGGATCTCCCCCGGGCCGGCCGGGGGAACGTCCCCCCCCGGCAGCTCCACGTCGGCCACCCCCACAAAGAAGTCGAACGCGGGGTGCAGCTCAAGAGCCAGGTTGGCGTTGTCGGGCTGCATAAACTGCTCCGGGGTCATCTGGCCTTCCGCGACCCATCGGACCCGCCCGTGGGCCAGGCGCTGCCCCCAGGCGTTCAAAAACAGCTGCTGCATGTCTGCGGCGGGGCCGGCCGGGGCCGCCACGTACGCCCCGTACGGATTGGCGGCTTCGACGGGGTCGCGGTTAAGGCCCCCGACCGCCGCGTCAACGTTCATCAGCGAAGGGTGGCACACGGTCCCGATCGCGTGTTCCAGAGACAGGCGCAGCACCTGGCGGTCCTTCCCCCAAAAAAACAGCTGGCGGGGCGGGAAGGCGCGGGGATCCGGGTGGCCGGGGGCGGGGACTAGGTCCCCGGCGTGCGCGGCAAACCGTTCCATGACCGGATTGAACAGGCCCAGGGGCAGGACGAACGTCAGGTCCATGGCGCCCACCAGGGGGTAGGGAACGTTGGTGGCGGCGTAGATGCGCTTCTCCAGGGCCTCCAGAAAGACCAGCTTCTCGCCGATGGACACCAGATCCGCGCGCACGCGCGTCGTCTGGGGGGCGCTCTCGAGCTCGTCCAGCGTCTGCCGGTTCAGGTCGAGCTGCTCCTCCTGCATCTCCAGCAGGTGGCGGCCCACGTCGTCCAGACTTCGCACGGCCTTGCCCATCACGAGCGCCGTGACCAGGTTGGCCCCGTTCAGGACCATCTCGCCGTACGTCACCGGCACGTCGGCTTCGGTGTCCTCCACTTTCAGGAAGGACTGCAGGAGGCGCTGTTTGATCGGGGCGGTGGTGACGAGCACCCCGTCGACCGGCCGCCCGCGCGTGTCGGCATGCGTCAGACGGGGCACGGCCACGGAGGGCTGCGTGGCCGTGGTGAGGTCCACGAGCCAGGCCTCGACGGCCTCCCGGCGGTGGCCCGCCTTGCCCAGGAAAAAGCTCGTCTCGCAGAAGCTTCGCTTTAGCTCGGCGACCAGGGTCGCCCGGGCCACCCTGGTGGCCAGGCGGCCGTTGTCCAGGTATCGTTGCATCGGCAACAACAAAGCCAGGGGCGGCGCCTTTTCCAGCAGCACGTGCAGCATCTGGTCGGCCGTGCCGCGCTCAAACGCCCCGAGGACGGCCTGGACGTTGCGAGCGAGCTGTTGGATGGCGCGCAACTGGCGATGCGCGCCGATACCCGTCCCGTCCAGGGCCTCCCCCGTGAGCAGGGCGATGGCCTCGGTGGCCAGGCTGAAGGCGGCGTTCAGGGCCCGGCGGTCGATAATCTTGGTCATGTAATTGTGTGTGGGTTGCTCGATGGGGTGCGGGCCGTCGCGGGCAATCAGCGGCTGGTGGACCTCGAACTGTACGCGCCCCTCGTTCATGTAGGCCAGCTCCGGAAACTTGGTACACACGCACGCCACCGACAACCCGAGCTCCAGAAAGCGCACGAGCGACAGGGTGTTGCAATACGACCCCAGCAGGGCGTCGAACTCGACGTCGTACAGGCTGTTTGCATCGGAGCGCACGCGGGAAAAAAAATCAAACAGGCGTCGATGCGACGCCACCTCGATCGTGCTAAGGAGGGACCCGGTCGGCACCATGGCCGCGGCATACCGGTATCCCGGAGGGTCGCGGTTGGGAGCGGCCATGGGGTCGCGTGGAGATCGGCTGTCTCTAGCGATATTGGCCCGGGGAGGCTAAGATCCACCCCAACGCCCGGCCACCCGTGTACGTGCCCGACGGCCCAAGGTCCACCGAAAGACACGACGGGCCCGGACCCAAAAAGGCGGGGGATGCTGTGTGAGAGGCCGGGTGCCGGTCGGGGGGGAAAGGCACCGGGAGAAGGCTGCGGCCTCGTTCCAGGAGAACCCAGTGTCCCCAACAGACCCGGGGACGTGGGATCCCAGGCCTTATATACCCCCCCCCCCGCCCCACCCCCGTTAGAACGCGACGGGTGCATTCAAGATGGCCCTGGTCCAAAAGCGTGCCAGGAAGAAATTGGCAGAGGCGGCAAAGCTGTCCGCCGCCGCCACCCACATCGAGGCCCCGGCCGCGCAGGCTATCCCCAGGGCCCGTGTGCGCAGGGGATCGGTGGGCGGCAGCATTTGGTTGGTGGCGATAAAGTGGAAAAGCCCGTCCGGACTGAAGGTCTCGTGGGCGGCGGCGAACAAGGCACACAGGGCCGTGCCTCCCAAAAACACGGACATCCCCCAAAACACGGGCGCCGACAACGGCAGACGATCCCTCTTGATGTTAACGTACAGGAGGAGCGCCCGCACCGCCCACGTAACGTAGTAGCCGACGATGGCGGCCAGGATACAGGCCGGCGCCACCACCCTTCCGGTCAGCCCGTAATACATGCCCGCTGCCACCATCTCCAACGGCTTCAGGACCAAAAACGACCAAAGGAACAGAATCACGCGCTTTGAAAAGACCGGCTGGGTATGGGGCGGAAGACGCGAGTATGCCGAACTGACAAAAAAATCAGAGGTGCCGTACGAGGACAATGAAAACTGTTCCTCCAGCGGCAGTTCTCCCTCCTCCCCCCCGAAGGCGGCCTCGTCGACCAGATCTCGATCCACCAGAGGAAGGTCATCCCGCATGGTCATGGGGTGTGCGGTGGAGGTGGGGAGACCGAAACCGCAAAGGGTCGCTTACGTCAGCAGGATCCCGAGATCAAAGACACCCGGGTTCTTGCACAAACACCACCCGGGTTGCATCCGCGGAGGCGAGTGTTTTGATAAGGCCGTTCCGCGCCTTGATATAACCTTTGATGTTGACCACAAAACCCGGAATTTACGCCTACGCCCCAATGCCCACGCAAGATGAGGTAGGTAACCCCCCCGTGGGTGTGACGTTGCGTTTAGTTCATTGGAGGCCAAGGGGAAAAATGGGGTGGGGAGGAAACGGAAAACCCAGTAGGCCGTGTCGGGAACACGCCCGGGGTTGTCCTCAAAAGGCAGGGTCCATACTACGGAAGCCGTCGTTGTATTCGAGACCTGCCTGTGCAACGCACGTCGGGGTTGCCTGTGTCCGGTTCGGCCCCCACCGCGTGCGGCACGCACGAGGACGAGTCCGCGTGCTTTATTGGCGTTCCAAGCGTTGCCCTCCAGTTTCTGTTGTCGGTGTTCCCCCATACCCACGCCCACATCCACCGTAGGGGGCCTCTGGGCCGTGTTACGTCGCCGCCCGCGATGGAGCTTAGCTACGCCACCACCATGCACTACCGGGACGTTGTGTTTTACGTCACAACGGACCGAAACCGGGCCTACTTTGTGTGCGGGGGGTGTGTTTATTCCGTGGGGCGGCCGTGTGCCTCGCAGCCCGGGGAGATTGCCAAGTTTGGTCTGGTCGTTCGAGGGACAGGCCCAGACGACCGCGTGGTCGCCAACTATGTACGAAGCGAGCTCCGACAACGCGGCCTGCAGGACGTGCGTCCCATTGGGGAGGACGAGGTGTTTCTGGACAGCGTGTGTCTTCTAAACCCGAACGTGAGCTCCGAGCTGGATGTGATTAACACGAACGACGTGGAAGTGCTGGACGAATGTCTGGCCGAGTACTGCACCTCGCTGCGAACCAGCCCGGGTGTGCTAATATCCGGGCTGCGCGTGCGGGCGCAGGACAGAATCATCGAGTTGTTTGAACACCCAACGATAGTCAACGTTTCCTCGCACTTTGTGTATACCCCGTCCCCATACGTGTTCGCCCTGGCCCAGGCGCACCTCCCCCGGCTCCCGAGCTCGCTGGAGGCCCTGGTGAGCGGCCTGTTTGACGGCATCCCCGCCCCACGCCAGCCACTTGACGCCCACAACCCGCGCACGGATGTGGTTATCACGGGCCGCCGCGCCCCACGACCCATCGCCGGGTCGGGGGCGGGGTCGGGGGGCGCGGGCGCCAAGCGGGCCACCGTCAGCGAGTTCGTGCAAGTCAAACACATTGACCGCGTGGGCCCCGCTGGCGTTTCGCCGGCGCCTCCGCCAAACAACACCGACTCGAGTTCCCTGGTGCCCGGGGCCCAGGATTCCGCCCCGCCCGGCCCCACGCTAAGGGAGCTGTGGTGGGTGTTTTATGCCGCAGACCGGGCGCTGGAGGAGCCCCGCGCCGACTCTGGCCTCACCCGCGAGGAGGTACGTGCCGTACGTGGGTTCCGGGAGCAGGCGTGGAAACTGTTTGGCTCCGCGGGGGCCCCGCGGGCGTTTATCGGGGCCGCGTTGGGCCTGAGCCCCCTCCAAAAGCTAGCCGTTTACTACTATATCATCCACCGAGAGAGGCGCCTGTCCCCCTTCCCCGCGCTAGTCCGGCTCGTAGGCCGGTACACACAGCGCCACGGCCTGTACGTCCCTCGGCCCGACGACCCAGTCTTGGCCGATGCCATCAACGGGCTGTTTCGCGACGCGCTGGCGGCCGGAACCACAGCCGAGCAGCTCCTCATGTTCGACCTTCTCCCCCCAAAGGACGTGCCGGTGGGAAGCGACGTGCAGGCCGACAGCACCGCTCTGCTGCGCTTTATAGAATCGCAACGTCTCGCCGTCCCCGGGGGGGTGATCTCCCCCGAGCACGTCGCGTACCTTGGTGCGTTCCTGAGCGTGCTGTACGCTGGCCGCGGGCGCATGTCCGCAGCCACGCACACCGCGCGGCTGACAGGGGTGACCTCCCTGGTGCTAGCGGTGGGTGACGTGGACCGTCTTTCCGCGTTTGACCGCGGAGCGGCGGGCGCGGCCAGCCGCACGCGGGCCGCCGGGTACCTGGATGTGCTTCTTACCGTTCGTCTCGCTCGCTCCCAACACGGACAGTCTGTGTAAAAGACCCCAATAAACGTATATCGCTACTACACCCTTGTGTGTCAATGGACGCCTCTCCGGGGGGGGGGGAGGGAAAGCAAAGAGGGGCTGGGGGAGCGGCACCACCGGGGCCTGAACAAACAAACCACAGACACGGTTACAGTTTATTCGGTCGGGCGGAGAAACGGCCGAAGCCACGCCCACTTTATTCGCGTCTCCAAAAAAACGGGACACTTGTCCGGAGAACCTTTAGGATGCCAGCCAGGGCGGCGGTAATCATAACCACGCCCAGCGCAGAGGCGGCCAGAAACCCGGGCGCAATTGCGGCCACGGGCTGCGTGTCAAAGGCTAGCAAATGAATGACGGTTCCGTTTGGAAATAGCAACAAGGCCGTGGACGGCACGTCGCTCGAAAACACGCTTGGGGCGCCCTCCGTCGGCCCGGCGGCGATTTGCTGCTGTGTGTTGTCCGTATCCACCAGCAACACAGACATGACCTCCCCGGCCGGGGTGTAGCGCATAAACACGGCCCCCACGAGCCCCAGGTCGCGCTGGTTTTGGGTGCGCACCAGCCGCTTGGACTCGATATCCCGGGTGGAGCCTTCGCATGTCGCGGTGAGGTAGGTTAGGAACAGTGGGCGTCGGACGTCGACGCCGGTGAGCTTGTAGCCGATCCCCCGGGGCAGAGGGGAGTGGGTGACGACGTAGCTGGCGTTGTGGGTGATGGGTACCAGGATCCGTGGCTCGACGTTGGCAGACTGCCCCCCGCACCGATGTGAGGCCTCAGGGACGAAGGCGCGGATCAGGGCGTTGTAGTGTGCCCAACGCGTCAGGGTCGAGGCGAGGCCGTGGGTCTGCTGGGCCAGGACTTCGACCGGGGTCTCGGATCGGGTGGCTTGAGCCAGCGCGTCCAGGATAAACACGCTCTCGTCTAGATCAAAGCGCAGGGAGGCCGCGCATGGCGAAAAGTGGTCCGGAAGCCAAAAGAGGGTTTTCTGGTGGTCGGCCCGGGCCAGCGCGGTCCGGAGGTCGGCGTTGGTCGCTGCGGCGACGTCGGACGTACACAGGGCCGAGGCTATCAGAAGGCTCCGGCGGGCGCGTTCCCGCTGCACCGCCGAGGGGACGCCAGCCAAGAACGGCTGCCGGAGGACAGCCGAGGCGTAAAATAGCGCCCGGTGGACGACCGGGGTGGTCAGCACGCGGCCCCCTAGAAACTCGGCATACAGGGCGTCGATGAGATGGGCTGCGCTGGGCGCCACTGCGTCGTACGCCGAGGGGCTATCCAGCACGAAGGCCAGCTGATAGCCCAGCGCGTGTAATGCCAAGCTCTGTTCGCGCTCCAGAATCTCGGCCACCAGGTGCTGGAGCCGAGCCTCTAGCTGCAGGCGGGCCGTGGGATCCAAGACTGACACATTAAAAAACACAGAATCCGCGGCACAGCCCGCGGCCCCGCGGGCGGCCAACCCGGCAAGCGCGCGCGAGTGGGCCAAAAAGCCTAGCAGGTCGGAGAGGCAGACCGCGCCGTTTGCGTGGGCGGCGTTCACGAAAGCAAAACCCGACGTCGCGAGCAGCCCCGTTAGGCGCCAGAAGAGAGGGGGGCGCGGGCCCTGCTCGGCGCCCGCGTCCCCCGAGAAAAACTCCGCGTATGCCCGCGACAGGAACTGGGCGTAGTTCGTGCCCTCCTCCGGGTAGCCGCCCACGCGGCGGAGGGCGTCCAGCGCGGAGCCGTTGTCGGCCCGCGTCAGGGACCCTAGGACAAAGACCCGATACCGGGGGCCGCCCGGGGGCCCGGGAAGAGCCCCCGGGGGGTTTTCGTCCGCGGGGTCCCCGACCCGATCTAGCGTCTGGCCCGCGGGGACCACCATCACTTCCACCGGAGGGCTGTCGTGCATGGATATCACGAGCCCCATGAATTCCCGCCCGTAGCGCGCGCGCACCAGCGCGGCATCGCACCCGAGCACCAGCTCCCCCGTCGTCCAGATGCCCACGGGCCACGTCGAGGCCGACGGGGAGAAATACACGTACCTACCTGGGGATCTCAACAGGCCCCGGGTGGCCAACCAGGTCGTGGACGCGTTGTGCAGGTGCGTGATGTCCAGCTCCGTCGTCGGGTGCCGCCGGGCCCCAACCGGCGGTCGGGGGGGCGGTGTATCACGCGGCCCGCTCGGGTGGCTCGCCGTCGCCACGTTGTCTCCCCGCGGGAACGTCAGGGCCTCGGGGTCAGGGACGGCCGAAAACGTTACCCAGGCCCGGGAACGCAGCAACACGGAGGCGGCTGGATTGTGCAAGAGACCCTTAAGGGGGGCGACCGAGGGGGGAGGCTGGGCGGTCGGCTCGACCGTGGTGGGGGCGGGCAGGCTCGCGTTCGGGGGCCGGCCGAGCAGGTAGGTCTTCGGGATGTAAAGCAGCTGGCCGGGGTCCCGCGGAAACTCGGCCGTGGTGACCAATACAAAACAAAAGCGCTCCTCGTACCAGCGAAGAAGGGGCAGAGATGCCGTAGTCAGGTTTAGTTCGTCCGGCGGCGCCAGAAATCCGCGCGGTGGTTTTTGGGGGTCGGGGGTGTTTGGCAGCCACAGACGCCCGGTGTTCGTGTCGCGCCAGTACATGCGGTCCATGCCCAGGCCATCCAAAAACCATGGGTCTGTCTGCTCAGTCCAGTCGTGGACCTGACCCCACGCAACGCCCAAAATAATAACCCCCACGAACCATAAACCATTCCCCATGGGGGACCCCGTCCCTAACCCACGGGGCCCGTGGCTATGGCAGGGCTTGCCGCCCCGACGTTGGCTGCGAGCCCTGGGCCTTCACCCGAACTTGGGGGTTGGGGTGGGGAAAAGGAAGAAACGCGGGCGTATTGGTCCCAATGGGGTCTCGGTGGGGTATCGACAGAGTGCCAGCCCTGGGACCGAACCCCGCGTTTATGAACAAACGACCCAACACCCGTGCGTTTTATTCTGTCTTTTTATTGCCGTCATAGCGCGGGTTCCTTCCGGTATTGTCTCCTTCCGTGTTTCAGTTAGCCTCCCCCATCTCCCGGGCAAACGTGCGCGCCAGGTCGCAGATCGTCGGTATGGAGCCTGGGGTGGTGACGTGGGTCTGGACCATCCCGGAGGTAAGTTGCAGCAGGGCGTCCCGGCAGCCGGCGGGCGATTGGTCGTAATCCAGGATAAAGACGTGCATGGGACGGAGGCGTTTGGCCAAGACGTCCAAAGCCCAGGCAAACACGTTATACAGGTCGCCGTTGGGGGCCAGCAACTCGGGGGCCCGAAACAGGGTAAATAACGTGTCCCCGATATGGGGTCGTGGGCCCGCGTTGCTCTGGGGCTCGGCACCCTGGGGCGGCACGGCCGCCCCCGAAAGCTGTCCCCAATCCTCCCGCCACGACCCGCCGCCCTGCAGATACCGCACCGTATTGGCAAGCAGCCCATAAACGCGGCGAATCGCGGCCAGCATAGCCAGGTCAAGCCGCTCGCCGGGGCGCTGGCGTTTGGCCAGGCGGTCGATGTGTCTGTCCTCCGGAAGGGCCCCCAACACGATGTTTGTGCCGGGCAAGGTCGGCGGGATGAGGGCCACGAACGCCAGCACGGCCTGGGGGGTCATGCTGCCCATAAGGTATCGCGCGGCCGGGTAGCACAGGAGGGCGGCGATGGGATGGCGGTCGAAGATGAGGGTGAGGGCCGGGGGCGGGGCATGTGAGCTCCCAGCCTCCCCCCCGATATGAGGAGCCAGAACGGCGTCGGTCACGGCATAAGGCATGCCCATTGTTATCTGGGCGCTTGTCATTACCACCGCCGCGTCCCCGGCCGATATCTCACCCTGGTCGAGGCGGTGTTGTGTGGTGTAGATGTTCGCGATTGTCTCGGAAGCCCCCAACACCCGCCAGTAAGTCATCGGCTCGGGTACGTAGACGATATCGTCGCGCGAACCCAGGGCCACCAGCAGTTGCGTGGTGGTGGTTTTCCCCATCCCGTGGGGACCGTCTATATAAACCCGCAGTAGCGTGGGCATTTTCTGCTCCAGGCGGACTTCCGTGGCTTTTTGCTGCCGGCGAGGGCGCAACGCCGTACGTCGGTTGTTATGGCCGCGAGAACGCGCAGCCTGGTCGAACGCAGACGCGTGTTGATGGCAGGGGTACGAAGCCATACGCGCTTCTACAAGGCGCTGGCCGAAGAGGTGCGGGAGTTTCACGCCACCAAGATCTGCGGCACGCTGTTGACGCTGTTAAGCGGGTCGCTGCAGGGTCGCTCGGTATTCGAGGCCACACGCGTCACCTTAATATGCGAAGTGGACCTGGGACCGCGCCGCCCCGACTGCATCTGCGTGTTCGAATTCGCCAATGACAAGACGCTGGGCGGGGTTTGTGTCATCATAGAACTAAAGACATGCAAATATATTTCTTCCGGGGACACCGCCAGCAAACGCGAGCAACGGGCCACGGGGATGAAGCAGCTGCGCCACTCCCTGAAGCTCCTGCAGTCCCTCGCGCCTCCGGGTGACAAGATAGTGTACCTGTGCCCCGTCCTGGTGTTTGTCGCCCAACGGACGCTCCGCGTCAGCCGCGTGACCCGGCTCGTCCCGCAGAAGGTCTCCGGTAATATCACCGCAGTCGTGCGGATGCTCCAGAGCCTGTCCACGTATACGGTCCCCATTGAGCCTAGGACCCAGCGAGCCCGTCGCCGCCGCGGCGGCGCCGCCCGGGGGTCTGCGAGCAGACCGAAAAGGTCACACTCTGGGGCGCGCGACCCGCCCGAGTCAGCGGCCCGCCAGTTACCACCCGCCGACCAAACCCCCGCCTCCACGGAGGGCGGGGGGGTGCTTAAGAGGATCGCGGCGCTCTTCTGCGTGCCCGTGGCCACCAAGACCAAACCCCGAGCCGCCTCCGAATGAGAGTGTTTCGTTCCTTCCCCCTCCCCCCGCGTCAGACAAACCCTAACCACCGCTTAAGCGGCCCCCGCGAGGTCCGAAGACTCATTTGGATCCGGCGGGAGCCACCCGACAACAGCCCCCGGGTTTTCCCACGCCAGACGCCGGTCCGCTGTGCCATCGCGCCCCCTCATCCCACCCCCCATCTTGTCCCCAAATAAAACAAGGTCTGGTAGTTAGGACAACGACCGCAGTTCTCGTGTGTTATTTTCGCTCTCCGCCTCTCGCAGATGGACCCGTACTGCCCATTTGACGCTCTGGACGTCTGGGAACACAGGCGCTTCATAGTCGCCGATTCCCGAAACTTCATCACCCCCGAGTTCCCCCGGGACTTTTGGATGTCGCCCGTCTTTAACCTCCCCCGGGAGACGGCGGCGGAGCAGGTGGTCGTCCTACAGGCCCAGCGCACAGCGGCTGCCGCTGCCCTGGAGAACGCCGCCATGCAGGCGGCCGAGCTCCCCGTCGATATCGAGCGCCGGTTACGCCCGATCGAACGGAACGTGCACGAGATCGCAGGCGCCCTGGAGGCGCTGGAGACGGCGGCGGCCGCCGCCGAAGAGGCGGATGCCGCGCGCGGGGATGAGCCGGCGGGTGGGGGCGACGGGGGGGCGCCCCCGGGTCTGGCCGTCGCGGAGATGGAGGTCCAGATCGTGCGCAACGACCCGCCGCTACGATACGACACCAACCTCCCCGTGGATCTGCTACACATGGTGTACGCGGGCCGCGGGGCGACCGGCTCGTCGGGGGTGGTGTTCGGGACCTGGTACCGCACTATCCAGGACCGCACCATCACGGACTTTCCCCTGACCACCCGCAGTGCCGACTTTCGGGACGGCCGTATGTCCAAGACCTTCATGACGGCGCTGGTACTGTCCCTGCAGGCGTGCGGCCGGCTGTATGTGGGCCAGCGCCACTATTCCGCCTTCGAGTGCGCCGTGTTGTGTCTCTACCTGCTGTACCGAAACACGCACGGGGCCGCCGACGATAGCGACCGCGCTCCGGTCACGTTCGGGGATCTGCTGGGCCGGCTGCCCCGCTACCTGGCGTGCCTGGCCGCGGTGATCGGGACCGAGGGCGGCCGGCCACAGTACCGCTACCGCGACGACAAGCTCCCCAAGACGCAGTTCGCGGCCGGCGGGGGCCGCTACGAACACGGAGCGCTGGCGTCGCACATCGTGATCGCCACGCTGATGCACCACGGGGTGCTCCCGGCGGCCCCGGGGGACGTCCCCCGGGACGCGAGTACCCACGTTAACCCCGACGGCGTGGCGCACCACGACGACATAAACCGCGCCGCCGCCGCGTTCCTCAGCCGGGGCCACAACCTATTCCTGTGGGAGGACCAGACTCTGCTGCGGGCAACCGCGAACACCATAACGGCCCTGGGCGTTATCCAGCGGCTCCTCGCGAACGGCAACGTGTACGCGGACCGCCTCAACAACCGCCTGCAGCTGGGCATGCTGATCCCCGGAGCCGTCCCTTCGGAGGCCATCGCCCGTGGGGCCTCCGGGTCCGACTCGGGGGCCATCAAGAGCGGAGACAACAATCTGGAGGCGCTATGTGCCAATTACGTGCTTCCGCTGTACCGGGCCGACCCGGCGGTCGAGCTGACCCAGCTGTTTCCCGGCCTGGCCGCCCTGTGTCTTGACGCCCAGGCGGGGCGGCCGGTCGGGTCGACGCGGCGGGTGGTGGATATGTCATCGGGGGCCCGCCAGGCGGCGCTGGTGCGCCTCACCGCCCTGGAACTCATCAACCGCACCCGCACAAACCCCACCCCTGTGGGGGAGGTTATCCACGCCCACGACGCCCTGGCGATCCAATACGAACAGGGGCTTGGCCTGCTGGCGCAGCAGGCACGCATTGGCTTGGGCTCCAACACCAAGCGTTTCTCCGCGTTCAACGTTAGCAGCGACTACGACATGTTGTACTTTTTATGTCTGGGGTTCATTCCACAGTACCTGTCGGCGGTTTAGTGGGTGGTGGGCGAGGGGGGAGGGGGCATTAGGGAGAAAGAACAAGAGCCTCCGTTGGGTTTTCTTTGTGCCTGTACTCAAAAGGTCATACCCCGTAAACGGCGGGCTCCAGTCCCGGCCCGGCGGTTGGCGTGAACGCAACGGCGGGAGCTGGGTTAGCGTTTAGTTTAGCATTCGCTCTCGCCTTTCCGCCCGCCCCCCGACCGTTGCGCCTTTTTTTTTTTCGTCCACCAAAGTCTCTGTGGGTGCGCGCATGGCAGCCGATGCCCCGGGAGACCGGATGGAGGAGCCCCTGCCCGACAGGGCCGTGCCCATTTACGTGGCTGGGTTTTTGGCCCTGTATGACAGCGGGGACTCGGGCGAGTTGGCATTGGATCCGGATACGGTGCGGGCGGCCCTGCCTCCGGATAACCCACTCCCGATTAACGTGGACCACCGCGCTGGCTGCGAGGTGGGGCGGGTGCTGGCCGTGGTCGACGACCCCCGCGGGCCGTTTTTTGTGGGGCTGATCGCCTGCGTGCAGCTGGAGCGCGTCCTCGAGACGGCCGCCAGCGCTGCGATTTTCGAGCGCCGCGGGCCGCCGCTCTCCCGGGAGGAGCGCCTGTTGTACCTGATCACCAACTACCTGCCCTCGGTCTCCCTGGCCACAAAACGCCTGGGGGGCGAGGCGCACCCCGATCGCACGCTGTTCGCGCACGTCGCGCTGTGCGCGATCGGGCGGCGCCTCGGCACTATCGTCACCTACGACACCGGTCTCGACGCCGCCATCGCGCCCTTTCGCCACCTGTCGCCGGCGTCTCGCGAGGGGGCGCGGCGACTGGCCGCCGAGGCCGAGCTCGCGCTGTCCGGGCGCACCTGGGCGCCCGGCGTGGAGGCGCTGACCCACACGCTGCTTTCCACCGCCGTTAACAACATGATGCTGCGGGACCGCTGGAGCCTGGTGGCCGAGCGGCGGCGGCAGGCCGGGATCGCCGGACACACCTACCTCCAGGCGAGCGAAAAATTCAAAATGTGGGGGGCGGAGCCTGTTTCCGCGCCGGCGCGCGGGTATAAGAACGGGGCCCCGGAGTCCACGGACATACCGCCCGGCTCGATCGCTGCCGCGCCGCAGGGTGACCGGTGCCCAATCGTCCGTCAGCGCGGGGTCGCCTTGTCCCCGGTACTGCCCCCCATGAACCCCGTTCCGACATCGGGCACCCCGGCCCCCGCGCCGCCCGGCGACGGGAGCTACCTGTGGATCCCGGCCTCCCATTACAACCAGCTCGTCGCCGGCCATGCCGCGCCCCAACCCCAGCCGCATTCCGCGTTTGGTTTCCCGGCTGCGGCGGGGTCCGTGGCCTATGGGCCTCACGGTGCGGGTCTTTCCCAGCATTACCCTCCCCACGTCGCCCATCAGTATCCCGGGGTGCTGTTCTCGGGACCCAGCCCACTCGAGGCGCAGATAGCCGCGTTGGTGGGGGCCATAGCCGCGGACCGCCAGGCGGGCGGTCAGCCGGCCGCGGGAGACCCTGGGGTCCGGGGGTCGGGAAAGCGTCGCCGGTACGAGGCGGGGCCGTCGGAGTCCTACTGCGACCAGGACGAACCGGACGCGGACTACCCGTACTACCCCGGGGAGGCTCGAGGCGGGCCGCGCGGGGTCGACTCCCGGCGCGCGGCCCGCCATTCTCCCGGGACCAACGAGACCATCACGGCGCTGATGGGGGCGGTGACGTCTCTGCAGCAGGAACTGGCGCACATGCGGGCTCGGACCAGCGCCCCCTATGGAATGTACACGCCGGTGGCGCACTATCGCCCTCAGGTGGGGGAGCCGGAACCAACAACGACCCACCCGGCCCTTTGTCCCCCGGAGGCCGTGTATCGCCCCCCACCACACAGCGCCCCCTACGGTCCTCCCCAGGGTCCGGCGTCCCATGCCCCCACTCCCCCGTATGCCCCAGCTGCCTGCCCGCCAGGCCCGCCACCGCCCCCATGTCCTTCCACCCAGACGCGCGCCCCTCTACCGACGGAGCCCGCGTTCCCCCCCGCCGCCACCGGATCCCAACCGGAGGCATCCAACGCGGAGGCCGGGGCCCTTGTCAACGCCAGCAGCGCAGCACACGTGGACGTTGACACGGCCCGCGCCGCCGATTTGTTCGTCTCTCAGATGATGGGGGCCCGCTGATTCGCCCCGGTCTTTGGTACCATGGGATGTCTTACTGTATATCTTTTTAAATAAACCAGGTAATACCAAATAAGACCCATTGGTGTATGTTCTTTTTTTATTGGGAGGCGCGGGTAGGCGGGTAGCTTTACAATGCAAAAGCCTTCGACGTGGAGGAAGGCGTGGGGGGGGGGGGAATCGGCACTGACCAAGGGGGTCCGTTTTGTCACGGGAAAGGAAAGAGGAAACAGGCCGCGGACACCCGGGGGAGTTTGTGTTCCCTTTTCTTTCTTCCCACACACACAAAAGGCGTACCAAACAAACAAACCAAAAGATGCACATGCGGTTTAACACCCGTGGTTTTTATTTACAACAAACCCCCCATCACAGGTCGTCCTCGTCGGCGTCACCGTCTTTGTTGGGAACTTGGGTGTAGTTGGTGTTGCGGCGCTTGCGCATGACCATGTCGGTGACCTTGGCGCTGAGCAGCGCGCTCGTGCCCTTCTTCTTGGCCTTGTGTTCCGTGCGCTCCATGGCAGACACCAGGGCCATGTACCGTATCATCTCCCGGGCCTCGGCTAGCTTGGCCTCGTCAAAGTCGCCGCCCTCCTCGCCCTCCCCGGACGCGTCCGGGTTGGTGGGGTTCTTGAGCTCCTTGGTGGTTAGCGGGTACAGGGCCTTCATGGGGTTGCTCTGCAGCCGCATGACGTAGCGAAAGGCGAAGAAGGCCGCCGCCAGGCCGGCCAGGACCAACAGACCCACGGCCAGCGCCCCAAAGGGGTTGGACATGAAGGAGGACACGCCCGACACGGCCGATACCACGCCGCCCACGATGCCCATCACCACCTTGCCGACCGCGCGCCCCAGGTCGCCCATCCCCTCGAAGAACGCGCCCAGGCCCGCAAACATGGCGGCGTTGGCGTCGGCGTGGATGACCGTGTCGATGTCGGCGAAGCGCAGGTCGTGCAGCTGGTTGCGGCGCTGGACCTCCGTGTAGTCCAGCAGGCCGCTGTCCTTGATCTCGTGGCGGGTGTACACCTCCAGGGGGACAAACTCGTGATCCTCCAGCATGGTGATGTTGAGGTCGATGAAGGTGCTGACGGTGGTGATGTCGGCGCGGCTCAGCTGGTGGGAGTACGCGTACTCCTCGAAGTACACGTAGCCCCCACCGAAGGTGAAGTAGCGCCGGTGTCCCACGGTGCACGGCTCGATCGCATCGCGCGTCAGCCGCAGCTCGTTGTTCTCCCCCAGCTGCCCCTCGACCAACGGGCCCTGGTCTTCGTACCGAAAGCTGACCAGGGGGCGGCTGTAGCAGGCCCCGGGCCGCGAGCTGATGCGCATCGAGTTTTGGACGATCACGTTGTCCGCGGCGACCGGCACGCACGTGGAGACGGCCATCACGTCGCCGAGCATCCGCGCGCTCACCCGCCGGCCCACGGTGGCCGAGGCGATGGCGTTGGGGTTCAGCTTGCGGGCCTCGTTCCACAGGGTCAGCTCGTGATTCTGCAGCTCGCACCACGCGATGGCAACGCGGCCCAACATATCGTTGACATGGCGCTGTATGTGGTTGTACGTAAACTGCAGCCTGGCGAACTCGATGGAGGAGGTGGTCTTGATGCGCTCCACGGACGCGTTGGCGCTGGCCCCGGGCGGCGGGGGCGTGGGGTTTGGGGGCTTGCGGCTCTGCTCGCGGAGGTGTTCCCGCACGTACAGCTCCGCGAGCGTGTTGCTGAGAAGGGGCTGGTACGCGATCAGAAAGCCCCCATTGGCCAGGTAGTACTGCGGCTGGCCCACCTTGATGTGCGTCGCGTTGTACCTGCGGGCGAAGATGCGGTCCATGGCGTCGCGGGCGTCCTTGCCGATGCAGTCCCCCAGGTCCACGCGCGAGAGCGGGTACTCGGTCAGGTTGGTGGTGAAGGTGGTGGATATGGCGTCGGAAGAGAATCGGAAGGAGCCGCCGTACTCGGAGCGCAGCATCTCGTCCACCTCCTGCCACTTGGTCATGGTGCAGACCGACGGGCGCTTTGGCACCCAGTCCCAGGCCACGGTGAACTTGGGGGTCGTGAGCAGGTTCCGGGTGGTCGGCGCCGTGGCCCGGGCCTTGGTGGTGAGGTCGCGCGCGTAGAAGCCGTCGACCTGCTTGAAGCGGTCGGCGGCGTAGCTGGTGTGTTCGGTGTGCGACCCCTCCCGGTAGCCGTAAAACGGGGACATGTACACAAAGTCGCCAGTCGCCAACACAAACTCGTCGTACGGGTACACCGAGCGCGCGTCCACCTCCTCGACGATGCAGTTTACCGTCGTCCCGTACCGGTGGAACGCCTCCACCCGCGAGGGGTTGTACTTGAGGTCGGTGGTGTGCCAGCCCCGGCTCGTGCGGGTCGCGGCGTTGGCCGGTTTCAGCTCCATGTCGGTCTCGTGGTCGTCCCGGTGAAACGCGGTGGTCTCCAGGTTGTTGCGCACGTACTTGGCCGTGGACCGACAGACCCCCTTGGCGTTGATCTTGTCGATCACCTCCTCGAAGGGGACGGGGGCGCGGTCCTCAAAGATCCCCATAAACTGGGAGTAGCGGTGGCCGAACCACACCTGCGAAACGGTGACGTCTTTGTAGTACATGGTGGCCTTGAACTTGTACGGGGCGATGTTCTCCTTGAAGACCACCGCGATGCCCTCCGTGTAGTTCTGACCCTCGGGCCGGGTCGGGCAGCGGCGCGGCTGCTCGAACTGCACCACCGTGGCGCCCGTGGGGGGTGGGCACACGTAAAAGTTTGCATCGGTGTTCTCCGCCTTGATGTCCCGCAGGTGCTCGCGCAGGGTGGCGTGGCCCGCGGCGACGGTCGCGTTGTCGCCGGCGGGGCGCGGCGGCTTTGGGGGTTTCGGTTTTTTGTTCTTCTTCGGTTTCGGGTCCCCCGTTGGGGGGGCGCCAGGGGCGGGCGGCGCCGGAGTGGCAGGGCCCCCGTTCGCCGCCTGGGTCGCGGCCGCGACCCCAGGCGTGCCGGGGGAACTCGGAGCCGCCGACGCCACCAGGACCCCCAGCGTCAACCCCAAGAGCGCCCATACGACGAACCACCGGCGCCCCCGCGCGGGGGCGCCCTGGCGCATGGCGGGACTACGGGGGCCCGTCGTGCCCCCCGTCAGGTAGCCTGGGGGCGAGGTGCTGGAGGACCGAGTAGAGGATCGAGAAAACGTCTCGGTCGTAGACCACGACCGACCGGGGGCCGATACAGCCGTCGGGGGCGCTCTCGACGATGGCCACCAGCGGACAGTCGGAGTCGTACGTGAGATATACGCCGGGCGGGTAACGGTAACGACCTTCGGAGGTCGGGCGGCTGCAGTCCGGGCGGCGCAACTCGAGCTCCCCGCACCGGTAGACCGAGGCAAAGAGTGTGGTGGCGATAATCAGCTCGCGAATATATCGCCAGGCGGCGCGCTGAGTGGGCGTTATTCCGGAAATGCCGTCAAAACAGTAAAACCTCTGAAATTCGCTGACGGCCCAATCAGCACCCGAGCCCCCCGCCCCCATGATGAACCGGGCGAGCTCCTCCTTCAGGTGCGGCAGGAGCCCCACGTTCTCGACGCTGTAATACAGCGCGGTGTTGGGGGGCTGGGCGAAGCTGTGGGTGGAGTGATCAAAGAGGGGCCCGTTGACGAGCTCGAAGAAGCGATGGGTGATGCTGGGGAGCAGGGCCGGGTCCACCTGGTGTCGCAGGAGAGACGCTCGCATGAACCGGTGCGCGTCGAACACGCCCGGCGCCGAGCGGTTGTCGATGACCGTGCCCGCGCCCGCCGTCAGGGCGCAGAAGCGCGCGCGCGCCGCAAAGCCGTTGGCGACCGCGGCGAACGTCGCGGGCAGCACCTCGCCGTGGACGCTGACCCGCAGCATCTTCTCGAGCTCCCCGCGCTGCTCGCGGACGCAGCGCCCCAGGCTGGCCAACGACCGCTTCGTCAGGCGGTCCGCGTACAGCCGCCGTCGCTCCCGCACGTCCGCGGCCGCTTGCGTGGCGATGTCCCCCCACGTCTCGGGCCCCTGCCCCCCGGGCCCGCGGCGACGGTCTTCGTCCTCGCCCCCGCCCCCGGGAGCTCCCAACCCCCGTGCCCCTTCCTCTACGGCGACACGGTCCCCGTCGTCGTCGGGGCCCGCGCCGCCCTTGGGCGCGTCCGCCGCGCCCCCCGCCCCCATGCGCGCCAGCACGCGACGCAGCGCCTCCTCGTCGCACTGTTCGGGGCTGACGAGGCGCCGCAAGAGCGGCGTCGTCAGGTGGTGGTCGTAGCACGCGCGGATGAGCGCCTCGATCTGATCGTCGGGTGACGTGGCCTGACCGCCGATTATTAGGGCGTCCACCATATCCAGCGCCGCCAGGTGGCTCCCGAACGCGCGATCGAAATGCTCCGCCCGCCGCCCGAACAGCGCCAGTTCCACGGCCACCGCGGCGGTCTCCTGCTGCAACTCGCGCCGCGCCAGCGCGGTCAGGTTGCTGGCAAACGCGTCCATGGTGGTCTGGCCGGCGCGGTCGCCGGACGCGAGCCAGAATCGCAATTCGCTGATGGCGTACAGGCCGGGCGTGGTGGCCTGAAACACGTCGTGCGCCTCCAGCAGGGCGTCGGCCTCCTTGCGGACCGAGTCGTTCTCGGGCGACGGGTGGGGCTGCCCGTCGCCCCCCGCGGTCCGGGCCAGCGCATGGTCCAACACGGAGAGCGCCCGCGCGCGGTCGGCGTCCGACAGCCCGGCGGCGTGGGGCAGGTACCGCCGCAGCTCGTTGGCGTCCAGCCGCACCTGCGCCTGCTGGGTGACGTGGTTACAGATACGGTCCGCCAGGCGGCGGGCGATCGTCGCCCCCTGGTTCGCCGTCACACACAGTTCCTCGAAACAGACCGCGCAGGGGTGGGACGGGTCGCTAAGCTCCGGGGGGACGATAAGGCCCGACCCCACCGCCCCCACCATAAACTCCCGAACGCGCTCCAGCGCGGCGGTGGCGCCGCGCGAGGGGGTGATGAGGTGGCAGTAGTTTAGCTGCTTTAGAAAGTTCTCGACGTCGTGCAGGAAACACAGCTCCATATGGACGGTCCCGCCATACGTATCCAGCCTGACCCGTTGGTGATACGGACAGGGTCGGGCCAGGCCCATGGTCTCCGTGAAAAACGCCGCGACGTCTCCCGCGGTCGCGAACGTCTCCAGGCTGCCCAGGAGCCGCTCGCCCTCGCGCCACGCGTACTCTAGCAGCAACTCCAGGGTGACCGACAGCGGGGTGAGAAAGGCCCCGGCCTGGGCCTCCAGGCCCGGCCTCAGACGACGCCGCAGCGCCCGCACCTGAAGCGCGTTCAGCTTCAGTTGGGGGAGCTTCCCCCGTCCGATGTGGGGGTCGCACCGCCGGAGCAGCTCTATCTGAAACACATAGGTCTGCACCTGCCCGAGCAGGGCTAACAACTTTTGACGGGCCACGGTGGGCTCGGACACCGGGGCGGCCATCTCGCGGCGCCGATCTGTACCGCGGCCGGAGTATGCGGTGGACCGAGGCGGTCCGTACGCTACCCGGCGTCTGGCTGAGCCCCGGGGTCCCCCTCTTCGGGGCGGCCTCCCGCGGGCCCGCCGACCGGCAAGCCGGGAGTCGGCGGCGCGTGCGTTTCTGCTCTATTCCCAGACACCGCGGAGAGGAATCACGGCCCGCCCAGAGATATAGACACGGAACACAAACAAGCACGGATGTCGTAGCAATAATTTATTTTACACACATTCCCCGCCCCGCCCTAGGTTCCCCCACCCCCCAACCCCTCACAGCATATCCAACGTCAGGTCTCCCTTTTTGTCGGGGGGCCCCTCCCCAAACGGGTCATCCCCGTGGAACGCCCGTTTGCGGCCGGCAAATGCCGGTCCCGGGGCCCCCGGGCCGCCGAACGGCGTCGCGTTGTCGTCCTCGCAGCCAAAATCCCCAAAGTTAAACACCTCCCCGGCGTTGCCGAGTTGGCTGACTAGGGCCTCGGCCTCGTGCGCCACCTCCAGGGCCGCGTCCGTCGACCACTCGCCGTTGCCGCGCTCCAGGGCACGCGCGGTCAGCTCCATCATCTCCTCGCTTAGGTACTCGTCCTCCAGGAGCGCCAGCCAGTCCTCGATCTGCAGCTGCTGGGTGCGGGGCCCCAGGCTTTTCACGGTCGCCACGAACACGCTACTGGCGACGGCCGCCCCGCCCTCGGAGATAATGCCCCGGAGCTGCTCGCACAGCGAGCTTTCGTGCGCTCCGCCGCCGAGGCTTGAGGCCGCGCACACAAACCCGGCCCGGGGACAGGCCAGGACGAACTTGCGGGTGCGGTCAAAAATAAGGAGCGGGCACGCGTTTTTGCCGCCCATCAGGCTGGCCCAGTTCCCGGCCTGAAACACACGGTCGTTGCCGGCCATGCCGTAGTACTTGCTGATGCTCAACCCCAACACGACCATGGGGCGCGCCGCCATGACGGGCCGCAGCAGGTTGCAGCTGGCGAACATGGACGTCCACGCGCCCGGATGCGCGTCCACGGCGTCCATCAGCGCGCGGGCCCCGGCCTCCAGGCCCGCCCCGCCCTGCGCGGACCACGCGGCCGCAGCCTGCACGCTGGGGGGACGGCGGGACCCCGCGATGATGGCCGTAAGGGTGTTGATGAAGTATGTCGAGTGATCGCAGTACCGCAGAATCTGGTTTGCCATGTAGTACATCGCCAGCTCGCTCACGTTGTTGGGGGCCAGGTTAATAAAGTTTATCGCGCCGTAGTCCAGGGAAAACTTTTTAATGAACGCGATGGTCTCGATGTCCTCGCGCGACAGGAGCCGGGCGGGAAGCTGGTTGCGTTGGAGGGCCGTCCAGAACCACTGCGGGTTCGGCTGGTTGGACCCCGGGGGCTTGCCGTTGGGGAAGATGGCCGCGTGGAACTGCTTCAGCAGAAAGCCCAGCGGTCCGAGGAGGATGTCCACGCGCTTGTCGGGCTTCTGGTAGGCGCTCTGGAGGCTGGCGACCCGCGCCTTGGCGGCCTCGGACGCGTTGGCGCTCGCGCCCGCGAACAACACGCGGCTCTTGACGCGCAGCTCCTTGGGAAACCCCAGGGTCACGCGGGCAACGTCGCCCTCGAAGCTGCTCTCGGCGGGGGCCGTCTGGCCGGCCGTTAGGCTGGGGGCGCAGATAGCCGCCCCCTCCGAGAGCGCGACCGTCAGCGTTTTGGCCGACAGAAACCCGTTGTTAAACATGTCCATCACGCGCCGCCGCAGCACCGGTTGGAATTGATTGCGAAAGTTGCGCCCCTCGACCGACTGCCCGGCGAACACCCCGTGGCACTGACTCAGGGCCAGGTCCTGGTACACGGCGAGGTTGGATCGCCGCCCGAGAAGCTGAAGCAGGGGGCACGGCCCGCACGCGTACGGGTCCAGCGTCAGGGACATGGCGTGGTTGGCCTCGCCCAGACCGTCGCGAAACTTGAAGTTCCTCCCCTCCACCAGGTTGCGCATCAGCTGCTCCACCTCGCGGTCCACGACCTGCCTGACGTTGTTCACCACCGTATGCAGGGCCTCGCGGTTGGTGATGATGGTCTCCAGCCGCCCCATGGCCGTGGGGACCGCCTGGTCCACGTACTGCAGGGTCTCGAGTTCGGCCATGACGCGCTCGGTCGCCGCGCGGTACGTCTCCTGCATGATGGTCCGGGCGGTCTCGGATCCGTCCGCGCGCTTCAGGGCCGAGAAGGCGGCGTAGTTGTCCCGAAGACGCCGATGGCTCCGCGGGCGGCGCTGGCGAACTTTGGATGGCGCGCCCGGAGGCGCATGAGCGTCGTGTGTACGCAGGCGTGGCGCGTGTCGAAGGTGCATAGGTTACAGGGCACGTCGGTCTGGTTGGAGTCCGCGACGTATCGAAACACGTCCATCTCCTGGCGCCCGACGATCACGCCGCCGTCGCAGCGCTCCAGGTAAAACAGCATCTTGGCCAGCAGCGCCGGGGAAAACCCACACAGCATGGCCAGGTGCTCGCCGGCAAATTCCTGGGTTCCGCCGACGAGGGGCGCGGTGGGCCGACCCTCGAACCCGGGCACCACGTGTCCCTCGCGGTCCACCTGTGGGTTGGCCGCCACGTGGGTCCCGGGCACGAGGAAGAAGCGGTAAAAGGAGGGTTTGCTGTGGTCCTTTGGGTCCGCCGGGCCGGCGTCGTCCACCTCGGTGAGATGGAGGGCCGAGTTGGTGCTAAATACCATGGCCCCCACGAGTCCCGCGGCGCGCGCCAGGTACGCCCCGACGGCGTTGGCGCGGGCCGCGGCCGTGTCCTGGCCCTCGAACAGCGGCCACGCGGAGATGTCGGTGGGCGGCTCGTCAAAGACGGCCATCGACACGATAGACTCGAGGGCCAGGGCGGCGTCTCCGGCCATGACGGAGGCCAGGCGCTGTTCGAACCCGCCCGCCGGGCCCTTGCCGCCGCCGTCGCGCCCGCCCCGCGGGGTCTTACCCTGGCTGGCTTCGAAGGCCGTGAACGTAATGTCGGCGGGGAGGGCGGCGCCCTCGTGGTTTTCGTCAAACGCCAGGTGGGCGGCCGCGCGGGCCACGGCGTCCACGTTTCGGCATCGCAGTGCCACGGCGGCGGGTCCCACGACCGCCTCGAACAGGAGGCGGTTGAGGGGGCGGTTAAAAAACGGAAGCGGGTAGGTAAAATTCTCCCCGATCGATCGGTGGTTGGCGTTGAACGGCTCTGCGATGACACGGCTAAAATCCGGCATGAACAGCTGCAACGGGTACACGGGTATGCGGTGCACCTCCGCCCCGCCTATGGTTACCTTGTCCGAGCCTCCCAGGTGCAGAAAGGTGTTGTTGATGCACACGGCCTCCTTGAAGCCCTCGGTAACGACCAGATACAGGAGGGCGCGGTCCGGGTCCAGGCCGAGGCGCTCACACAGCGCCTCCCCCGTCGTCTCGTGTTTGAGGTCGCCGGGCCGGGGGGTGTAGTCCGAAAAGCCAAAATGGCGGCGTGCCCGCTCGCAAAGTCGCGTCAGGTTCGGGGCCTGGGTGCTGGGGTCCAGGTGCCGGCCGCCGTGAAAGACGTACACGGACGAGCTGTAGTGCGAGGGCGTCAGTTTCAGGGACACCGCGGTACCCCCGAGCCCCGTCGTGCGAGAACCCACGACCACGGCCACGTTGGCCTCAAAGCCGCTCTCCACGGTCAGGCCCACGACCAGGGGCGCCACGGCGACGTCGGAATCGCCGCTGCGTGCCGACAGTAACGCCAGAAGCTCGATGCCTTCGGACGGACACGCGCGAGCGTACACGTATCCCAGGGGCCCGGGGGGGACCTTGATGGTGGTTGCCGTCTTGGGCTTTGTCTCCATGTCCTTTTGTCAATCGGTCCGCGAACGGAGGTAATCCCGGCACGACGACGGACGCCCGACAAGGTATGTCTCCCGAGCGTCAAAATCCGGGGGGGGGCGGCGACGGTCAAGGGGAGGGTTGGAGACCGGGGTTGGGGAATGAATCCCTCCCCTTCACCGACAACCCCCCGGGTAACCACGGGGTGCCGATGAACCCCGGCGGCCGGCAACGCGGGGTCCCTGCGAGAGGCACAGATGCTTACGGTCAGGTGCTCCGGGTCGGGTGCGTCTGGTATGCGGTTGGTATATGTACACTTTACCTGGGGGCGTGCCGGTCCGCCCCAGCCCCTCCCACGCCCCGCGCGTCATCAGCCGGTGGGCGTGGCCGCTATTATAAAAAAAGTGAGAACGCGAAGCGTTCGCACTTTGTCCTAATAATATATATATTATTAGGACAAAGTGCGAACGCTTCGCGTTCTCACTTTTTTTATAATAGCGGCCACGCCCACCGGCTACGTCACTCTCCTGTCGGCCGCCGGCGGTCCATAAGCCCGGCCGGCCGGGCCGACGCGAATAAACCGGGCCGCCGGCCGGGGCGCCGCGCAGCAGCTCGCCGCCCGGATCCGCCAGACAAACAAGGCCCTTGCACATGCCGGCCCGGGCGAGCCTGGGGGTCCGGTAATTTTGCCATCCCACCCAAGCGGCTTTTTGGGTTTTTCTCTTCCCCCCTCCCCACATTCCCCTCTTTAGGGGTTCGGGTGGGAACAACCGCGATGTTTTCCGGTGGCGGCGGCCCGCTGTCCCCCGGAGGAAAGTCGGCGGCCAGGGCGGCGTCCGGGTTTTTTGCGCCCGCCGGCCCTCGCGGAGCCAGCCGGGGACCCCCGCCTTGTTTGAGGCAAAACTTTTACAACCCCTACCTCGCCCCAGTCGGGACGCAACAGAAGCCGACCGGGCCAACCCAGCGCCATACGTACTATAGCGAATGCGATGAATTTCGATTCATCGCCCCGCGGGTGCTGGACGAGGATGCCCCCCCGGAGAAGCGCGCCGGGGTGCACGACGGTCACCTCAAGCGCGCCCCCAAGGTGTACTGCGGGGGGGACGAGCGCGACGTCCTCCGCGTCGGGTCGGGCGGCTTCTGGCCGCGGCGCTCGCGCCTGTGGGGCGGCGTGGACCACGCCCCGGCGGGGTTCAACCCCACCGTCACCGTCTTTCACGTGTACGACATCCTGGAGAACGTGGAGCACGCGTACGGCATGCGCGCGGCCCAGTTCCACGCGCGGTTTATGGACGCCATCACACCGACGGGGACCGTCATCACGCTCCTGGGCCTGACTCCGGAAGGCCACCGGGTGGCCGTTCACGTTTACGGCACGCGGCAGTACTTTTACATGAACAAGGAGGAGGTCGACAGGCACCTACAATGCCGCGCCCCACGAGATCTCTGCGAGCGCATGGCCGCGGCCCTGCGCGAGTCCCCGGGCGCGTCGTTCCGCGGCATCTCCGCGGACCACTTCGAGGCGGAGGTGGTGGAGCGCACCGACGTGTACTACTACGAGACGCGCCCCGCTCTGTTTTACCGCGTCTACGTCCGAAGCGGGCGCGTGCTGTCGTACCTGTGCGACAACTTCTGCCCGGCCATCAAGAAGTACGAGGGTGGGGTCGACGCCACCACCCGGTTCATCCTGGACAACCCCGGGTTCGTCACCTTCGGCTGGTACCGTCTCAAACCGGGCCGGAACAACACGCTAGCCCAGCCGCGGGCCCCGATGGCCTTCGGGACATCCAGCGACGTCGAGTTTAACTGTACGGCGGACAACCTGGCCATCGAGGGGGGCATGAGCGACCTACCGGCATACAAGCTCATGTGCTTCGATATCGAATGCAAGGCGGGGGGGGAGGACGAGCTGGCCTTTCCGGTGGCCGGGCACCCGGAGGACCTGGTCATCCAGATATCCTGTCTGCTCTACGACCTGTCCACCACCGCCCTGGAGCACGTCCTCCTGTTTTCGCTCGGTTCCTGCGACCTCCCCGAATCCCACCTGAACGAGCTGGCGGCCAGGGGCCTGCCCACGCCCGTGGTTCTGGAATTCGACAGCGAATTCGAGATGCTGTTGGCCTTCATGACCCTTGTGAAACAGTACGGCCCCGAGTTCGTGACCGGGTACAACATCATCAACTTCGACTGGCCCTTCTTGCTGGCCAAGCTGACGGACATTTACAAGGTCCCCCTGGACGGGTACGGCCGCATGAACGGCCGGGGCGTGTTTCGCGTGTGGGACATAGGCCAGAGCCACTTCCAGAAGCGCAGCAAGATAAAGGTGAACGGCATGGTGAACATCGACATGTACGGGATTATAACCGACAAGATCAAGCTCTCGAGCTACAAGCTCAACGCCGTGGCCGAAGCCGTCCTGAAGGACAAGAAGAAGGACCTGAGCTATCGCGACATCCCCGCCTACTACGCCGCCGGGCCCGCGCAACGCGGGGTGATCGGCGAGTACTGCATACAGGATTCCCTGCTGGTGGGCCAGCTGTTTTTTAAGTTTTTGCCCCATCTGGAGCTCTCGGCCGTCGCGCGCTTGGCGGGTATTAACATCACCCGCACCATCTACGACGGCCAGCAGATCCGCGTCTTTACGTGCCTGCTGCGCCTGGCCGACCAGAAGGGCTTTATTCTGCCGGACACCCAGGGGCGATTTAGGGGCGCCGGGGGGGAGGCGCCCAAGCGTCCGGCCGCAGCCCGGGAGGACGAGGAGCGGCCAGAGGAGGAGGGGGAGGACGAGGACGAACGCGAGGAGGGCGGGGGCGAGCGGGAGCCGGAGGGCGCGCGGGAGACCGCCGGCAGGCACGTGGGGTACCAGGGGGCCAGGGTCCTTGACCCCACTTCCGGGTTTCACGTGAACCCCGTGGTGGTGTTCGACTTTGCCAGCTCCCTGAGGGCCGACGCAGTGGCGCACCTGGAGGCGGGCAAGGACTACCTGGAGATCGAGGTGGGGGGGCGACGGCTGTTCTTCGTCAAGGCTCACGTGCGAGAGAGCCTCCTCAGCATCCTCCTGCGGGACTGGCTCGCCATGCGAAAGCAGATCCGCTCGCGGATTCCCCAGAGCAGCCCCGAGGAGGCCGTGCTCCTGGACAAGCAGCAGGCCGCCATCAAGGTCGTGTGTAACTCGGTGTACGGGTTCACGGGAGTGCAGCACGGACTCCTGCCGTGCCTGCACGTTGCCGCGACGGTGACGACCATCGGCCGCGAGATGCTGCTCGCGACCCGCGAGTACGTCCACGCGCGCTGGGCGGCCTTCGAACAGCTCCTGGCCGATTTCCCGGAGGCGGCCGACATGCGCGCCCCCGGGCCCTATTCCATGCGCATCATCTACGGGGACACGGACTCCATCTTTGTGCTGTGCCGCGGCCTCACGGCCGCCGGGCTGACGGCCGTGGGCGACAAGATGGCGAGCCACATCTCGCGCGCGCTGTTTCTGCCCCCCATCAAACTCGAGTGCGAAAAGACGTTCACCAAGCTGCTGCTGATCGCCAAGAAAAAGTACATCGGCGTCATCTACGGGGGTAAGATGCTCATCAAGGGCGTGGATCTGGTGCGCAAAAACAACTGCGCGTTTATCAACCGCACCTCCAGGGCCCTGGTCGACCTGCTGTTTTACGACGATACCGTCTCCGGAGCGGCCGCCGCGTTAGCCGAGCGCCCCGCGGAGGAGTGGCTGGCGCGACCCCTGCCCGAGGGACTGCAGGCGTTCGGGGCCGTCCTCGTAGACGCCCATCGGCGCATCACCGACCCGGAGAGGGACATCCAGGACTTTGTCCTCACCGCCGAACTGAGCAGACACCCGCGCGCGTACACCAACAAGCGCCTGGCCCACCTGACGGTGTATTACAAGCTCATGGCCCGCCGCGCGCAGGTCCCGTCCATCAAGGACCGGATCCCGTACGTGATCGTGGCCCAGACCCGCGAGGTAGAGGAGACGGTCGCGCGGCTGGCCGCCCTCCGCGAGCTAGACGCCGCCGCCCCAGGGGACGAGCCCGCCCCCCCCGCGGCCCTGCCCTCCCCGGCCAAGCGCCCCCGGGAGACGCCGTCGCCTGCCGACCCCCCGGGAGGCGCGTCCAAGCCCCGCAAGCTGCTGGTGTCCGAGCTGGCCGAGGATCCCGCATACGCCATTGCCCACGGCGTCGCCCTGAACACGGACTATTACTTCTCCCACCTGTTGGGGGCGGCGTGCGTGACATTCAAGGCCCTGTTTGGGAATAACGCCAAGATCACCGAGAGTCTGTTAAAAAGGTTTATTCCCGAAGTGTGGCACCCCCCGGACGACGTGGCCGCGCGGCTCCGGACCGCAGGGTTCGGGGCGGTGGGTGCCGGCGCTACGGCGGAGGAAACTCGTCGAATGTTGCATAGAGCCTTTGATACTCTAGCATGAGCCCCCCGTCGAAGCTGATGTCCCTCATTTTACAATAAATGTCTGCGGCCGACACGGTCGGAATCTCCGCGTCCGTGGGTTTCTCTGCGTTGCGCCGGACCACGAGCACAAACGTGCTCTGCCACACGTGGGCGACGAACCGGTACCCCGGGCACGCGGTGAGCATCCGGTCTATGAGCCGGTAGTGCAGGTGGGCGGACGTGCCGGGAAAGATGACGTACAGCATGTGGCCCCCGTAAGTGGGGTCCGGGTAAAACAACAGCCGCGGGTCGCACGCCCCGCCTCCGCGCAGGATCGTGTGGACGAAAAAAAGCTCGGGTTGGCCAAGAATCCCGGCCAAGAGGTCCTGGAGGGGGGCGTTGTGGCGGTCGGCCAACACGACCAAGGAGGCCAGGAAGGCGCGATGCTCGAATATCGTGTTGATCTGCTGCACGAAGGCCAGGATTAGGGCCTCGCGGCTGGTGGCGGCGAACCGCCCGTCTCCCGCGTTGCACGCGGGACAGCAACCCCCGATGCCTAGGTAGTAGCCCATCCCGGAGAGGGTCAGGCAGTTGTCGGCCACGGTCTGGTCCAGACAGAAGGGCAGCGAGACGGGAGTGGTCTTCACCAGGGGCACCGAGAGCGAGCGCACGATGGCGATCTCCTCGGAGGGCGTCTGGGCGAGGGCGGCGAAAAGGCCCCGATAGCGCTGGCGCTCGTGTAAACACAGCTCCTGTTTGCGGGCGTGAGGCGGCAGGCTCTTCCGGGAGGCCCGACGCACCACGCCCAGAGTCCCGCCGGCCGCAGAGGAGCGCGACCGCCGGCGCTCCTTGCCGTGATAGGGCCCGGGCCGGGAGCCGCGGCGATGGGGGTCGGTGTCATACATAGGTACACAGGGTGTGCTCCAGGGACAGGAGCGAGATCGAGTGGCGTCTAAGCAGCGCGCCCGCCTCACGGACAAATGTGGCGAGCGCGGTGGGCTTTGGTACAAATACCTGATACGTCTTGAAGGTGTAGATGAGGGCACGCAACGCTATGCAGACACGCCCCTCGAACTCGTTCCCGCAGGCCAGCTTGGCCTTGTGGAGCAGCAGCTCGTCGGGATGGGTGGCGGGGGGATGGCCGAACAGAACCCAGGGGTCAACCTCCATCTCCGTAATGGCGCACATGGGGTCACAGAACATGTGCTTAAAGATGGCCTCGGGCCCCGCGGCCCGAAGCAGGCTCACAAACCGGCCCCCGTCCCCGGGCTGCGTCTCGGGGTCAGCCTCGAGCTGGTCGACGACGGGTACGATACAGTCGAAGAGGCTCGTGTTGTTTTCCGAGTAGCGGACCACGGAGGCCCGGAGTCTGCGCAGGGCCAGCCAGTAAGCACGCACCAGTAACAGGTTACACAGCAGGCATTCTCCGCCGGTGCGCCCGCGCCCCCGGCCGTGTTTCAGCACGGTGGCCATCAGAGGGCCCAGGTCGAGGTCGGGCTGGGCATCGGGTTCGGTAAACTGCGCAAAGCGCGGAGCCACGTCGCGCGTGCGTGCCCCGCGATGCGCTTCCCAGGACTGGCGGACCGTGGCGCGACGGGCCTCCGCGGCAGCGCGCAGCTGGGGCCCCGACTCCCAGACGGCGGGGGTGCCGGCGAGGAGCAACAGGACCAGATCCGCGTACGCCCACGTATCCGGCGACTCCTCCGGCTCGCGGTCCCCGGCGACCGTCTCGAATTCCCCGTTGCGAGCGGCGGCGCGAGTACAGCAGCTGTCCCCGCCCCCGCGCCGACCCTCCGTGCAGTCCAGGAGACGGGCGCAATCCTTCCAGTTCATCAGCGCGGTGGTGAGCGACGGCTGCGTGCCGGATCCCGCCGCCGACCCCGCCCCCTCCTCGCCCCCGGAGGCCAAGGTTCCGATGAGGGCCCGGGTGGCAGACTGCGCCAGGAACGAGTAGTTGGAGTACTGCACCTTGGCGGCTCCCGGGGAGGGCGAGGGCTTGGGTTGCTTCTGGGCATGCCGCCCGGGCACCCCGCCGTCGGTACGGAAGCAGCAGTGGAGAAAAAAGTGCCGGTGGATGTCGTTTATGGTGAGGGCAAAGCGTGCGAAGGAGCCGACCAGGGTCGCCTTCTTGGTGCGCAGAAAGTGGCGGTCCATGACGTACACAAACTCGAACGCGGCCACGAAGATGCTAGCGGCGCAGTGGGGCGCCCCCAGGCATTTGGCACAGAGAAACGCGTAATCGGCCACCCACTGAGGCGAGAGGCGGTAGGTTTGCTTGTACAGCTCGATGGTGCGGCAGACCAGACAGGGCCGGTCCAGCGCGAAGGTGTCGATGGCCGCCGCGGAAAAGGGCCCGGTGTCCAAAAGCCCCTCCCCACAGGGATCCGGGGGCGGGTTGCGGGGTCCTCCGCGCCCGCCCGAACCCCCTCCGTCGCCCGCCCCCCCGCGGGCCCTTGAGGGGGCGGTGACCACGTCGGCGGCGACGTCCTCGTCGAGCGTACCGACGGGCGGCACACCTATCACGTGACTGGCCGTCAGGAGCTCGGCGCAGAGAGCCTCGTTAAGAGCCAGGAGGCTGGGATCGAAGGCCACATACGCGCGCTCGAACGCCCCCGCCTTCCAGCTGCTGCCGGGGGACTCTTCGCACACCGCGACGCTCGCCAGGACCCCGGGGGGCGAAGTTGCCATGGCTGGGCGGGAGGGGCGCACGCGCCAGCGAACTTTACGGGACACAATCCCCGACTGCGCGCTGCGGTCCCAGACCCTGGAGAGTCTAGACGCGCGCTACGTCTCGCGAGACGGCGCGCATGACGCGGCCGTCTGGTTCGAGGATATGACCCCCGCCGAGCTGGAGGTTGTCTTCCCGACTACGGACGCCAAGCTGAACTACCTGTCGCGGACGCAGCGGCTGGCCTCCCTCCTGACGTACGCCGGGCCTATAAAAGCGCCCGACGACGCCGCCGCCCCGCAGACCCCGGACACCGCGTGTGTGCACGGCGAGCTGCTCGCCCGCAAGCGGGAAAGATTCGCGGCGGTCATTAACCGGTTCCTGGACCTGCACCAGATTCTGCGGGGCTGACGCGCGTGCTGTTGGGCGGGACGGTTCGCGAACCCTTTGGTGGGTTTACGCGGGCACGCACGCTCCCATCGCGGGCGCCATGGCGGGACTGGGCAAGCCCTACACCGGCCACCCAGGTGACGCCTTCGAGGGTCTCGTTCAGCGAATTCGGCTTATCGTCCCATCTACGTTGCGGGGCGGGGACGGGGAGGCGGGCCCCTACTCTCCCTCCAGCCTCCCCTCCAGGTGCGCCTTTCAGTTTCATGGCCATGACGGGTCCGACGAGTCGTTTCCCATCGAGTATGTACTGCGGCTTATGAACGACTGGGCCGAGGTCCCGTGCAACCCTTACCTGCGCATACAGAACACCGGCGTGTCGGTGCTGTTTCAGGGGTTTTTTCATCGCCCACACAACGCCCCCGGGGGCGCGATTACGCCAGAGCGGACCAATGTGATCCTGGGCTCCACCGAGACGACGGGGCTGTCCCTCGGCGACCTGGACACCATCAAGGGGCGGCTCGGCCTGGATGCCCGGCCGATGATGGCCAGCATGTGGATCAGCTGCTTTGTGCGCATGCCCCGCGTGCAGCTCGCGTTTCGGTTCATGGGCCCCGAAGATGCCGGACGGACGAGACGGATCCTGTGCCGCGCCGCCGAGCAGGCTATTACCCGTCGCCGCCGAACCCGGCGGTCCCGGGAGGCGTACGGGGCCGAGGCCGGGCTGGGGGTGGCCGGAACGGGTTTCCGGGCCAGGGGGGACGGTTTTGGCCCGCTCCCCTTGTTAACCCAAGGGCCCTCCCGCCCGTGGCACCAGGCCCTGCGGGGTCTTAAGCACCTACGGATTGGCCCCCCCGCGCTCGTTTTGGCGGCGGGACTCGTCCTGGGGGCCGCTATTTGGTGGGTGGTTGGTGCTGGCGCGCGCCTATAAAAAAGGACGCACCGCCGCCCTAATCGCCAGTGCGTTCCGGACGCCTTCGCCCCACACAGCCCTCCCGACCGACACCCCCATATCGCTTCCCGACCTCCGGTCCCGATGGCCGTCCCGCAATTTCACCGCCCCAGCACCGTTACCACCGATAGCGTCCGGGCGCTTGGCATGCGCGGGCTCGTCTTGGCCACCAATAACTCTCAGTTTATCATGGATAACAACCACCCGCACCCCCAGGGCACCCAAGGGGCCGTGCGGGAGTTTCTCCGCGGTCAGGCGGCGGCGCTGACGGACCTTGGTCTGGCCCACGCAAACAACACGTTTACCCCGCAGCCTATGTTCGCGGGCGACGCCCCGGCCGCCTGGTTGCGGCCCGCGTTTGGCCTGCGGCGCACCTATTCACCGTTTGTCGTTCGAGAACCTTCGACGCCCGGGACCCCGTGAGGCCCGGGGAGTTCCTTCTGGGGTGTTTTAATCAATAAAAGACCACACCAACGCACGAGCCTTGCGTTTAATGTCGTGTTTATTCAAGGGAGTGGGATAGGGTTCGACGGTTCGAAACTTAACACACCAAATAATCGAGCGCGTCTAGCCCAGTAACATGCGCACGTGATGTAGGCTGGTCAGCACGGCGTCGCTGTGATGAAGCAGCGCCCGGCGGGTCCGCTGTAACTGCTGTTGTAGGCGGTAACAGGCGCGGATCAGTACCGCCAGGGCGCTACGACCGGTGCGTTGCACGTAGCGTCGCGACAGAACTGCGTTTGCCGATACGGGCGGGGGGCCGAATTGTAAGCGCGTCACCTCTTGGGAGTCATCGGCGGATAACGCACTGAATGGTTCGTTGGTTATGGGGGAGTGTGGTTCCCCAGGGAGTGGGTCGAGCGCCTCGGCCTCGGAATCCGAGAGGAACAACGAGGTGGCGTCGGAGTCTTCGTCGTCAGAGACATACAGGGTCTGAAGCAGCGACACGGGCGGGGGGGTAGCGTCGATGTGTAGCGCGAGGGAGGATGCCCACGAAGACACCCCAGACAAGGAGCTGCCCGTGCGTGGATTTGTGGAAGACGCGGAAGCCGGGACGGAAGGCGGCTGACGGTCAAATGCCCCCGGGGGCGCGCAGATGTGGTGGGCGTGGCCACCGGCTGCCGTGTAGTGGGGCGGCGGGAAACCGGGCCTCCGGGCGCAACACCGCCCTCCAGCGTCAAGTATGTGGGGGGCGGGCCTGACGTCGGGGGCGGGGCGACGGGTTGGACCGCGGGAGGCGGGGGAGAGGGACCTGCGGGAGAGGATGAGGTCGGCTCGGCCGGGTTGCGGCCTAAAACAGGGGCCGTGGGGTCGGCGGGGTCCCAGGGTGAAGGGAGGGATTCCCGCGATTCGGACAGCGACGCGACAGCGGGGCGCGTAAGGCGCCGCTGCGGCCCGCCTACGGGAACCCTGGGGGGGGTTGGCGCGGGACCCGAGGTTAGCGGGGGGCGGCGGTTTTCGCCCCCGGGCAAAACCGTGCCGGTTGCGACCGGGGGCGGAACGGGATCGATAGGGAGAGCGGGAGAAGCCTGGCCGGCGGCCTGGGGCCCGAGCGGGAGGGGCACACCAGACACCAAAGCGTGGGGCGCTGGCTCTGGGGGTTTGGGAGGGGCCGGGGGGCGCGCGAAATCGGTAACCGGGGCGACCGTGTCGGGGAGGGCAGGCGGCCGCCAACCCTGGGTGGTCGCGGAAGCCTGGGTGGCGCGCGCCAGGGAGCGTGCCCGGCGGTGTCGGCGCGCGCGCGACCCGGACGAAGAAGCGGCAGAAGCGCGGGAGGAGGCGGGGGGGCGGGGGGCGGTGGCATCGGGGGGCGCCGGGGAACTTTGGGGGGACGGCAAGCGCCGGAAGTCGTCGCGGGGGCCCACGGGCGCCGGCCGCGTGCTTTCGGCCGGGACGCCCGGTCGTGCTTCGCGAGCCGTGACTGCCGGCCCAGGGGGCCGCGGTGCACACTGGGACGTGGGGACGGACTGATCGGCGGTGGGCGAAAGGGGGTCCGGGGCAAGGAGGGGCGCGGGGCCGCCGGAGTCGTCAGACGCGAGCTCCTCCAGGCCGTGAATCCATGCCCACATGCGAGGGGGGACGGGCTCGCCGGGGGTGGCGTCGGTGAATAGCGTGGGGGCCAGGCTTCCGGGCCCCAACGAGCCCTCCGCCCCAACAAGGTCCACAGGGCCGGGGGTCGGGTTCGGGACCGAGGGGCTCTGGTCGTCGGGGGCGCGCTGGTACACCGGATGCCCCGGGAATAGCTCCCCCGACAGGAGGGAGGCGTCGAACGGCCGCCCGAGGATAGCTCGCGCGAGGAAGGGGTCCTCGTCGGTGGCGCTGGCGGCGAGGACGTCCTCGCCGCCCGCCACAAACGGGAGCTCCTCGGTGGCCTCGCTGCCAACAAACCGCACGTCGGGGGGGCCGGGGGGGTCCGGGTTTTCCCACAACACCGCGACCGGGGTCATGGAGATGTCCACGAGCACCAGACACGGCGGGCCCCGGGCGAGGGGCCGCTCGGCGATGAGCGCGGACAGGCGCGGGAGCTGTGCCGCCAGACACGCGTTTTCGATCGGGTTCAGGTCGGCGTGCAGGAGGCGGACGGCCCACGTCTCGATGTCGGACGACACGGCATCGCGCAAGGCGGCGTCCGGCCCGCGAGCGCGTGAGTCAAACAGCGTGAGACACAGCTCCAGCTCCGACTCGCGGGAAAAGGCCGTGGTGTTGCGGAGCGCCACGACGACGGGCGCGCCCAGGAGCACTGCCGCCAGCACCAGGTCCATGGCCGTAACGCGCGCCGCGGGGGTGCGGTGGGTGGCGGCGGCCGGCACGGCGACGTGCTGGCCCGTGGGCCGGTAGAGGGCGTTGGGGGGAGCGGGGGGTGACGCCTCGCGCCCCCCCGAGGGGCTCAGCGTCTGCCCAGATTCCAGACGCGCGGTCAGAAGGGCGTCGAAACTGTCATACTCTGTGTAGTCGTCCGGAAACATGCAGGTCCAAAGAGCGACCAGAGCGGTGCTTGGGAGACACATGCGCCCGAGGACGCTCACCGCCGCCAGCGCCTGGGCGGGACTCAGCTTTCCCAGCGCGGCGCCGCGCTCGGTTCCCAGCTCGGGGACCGAGCGCCAGGGCGCCAGGGGGTCGGTTTCGGACAACTTGCCGCGGCGCCAGTCTGCCAGCCGCGTGCCGAACATGAGGCCCCGGGTCGGAGGGCCTCCGGCCGAAAACGCTGGCAGCACGCGGATGCGGGCGTCTGGATGCGGGGTCAGGCGCTGCACGAATAGCATGGAATCTGCTGCGTTCTGAAACGCACGGGGGAGGGTGAGATGCATGTACTCGTGTTGGCGAACCAGATCCAGGCGCCAAAAGGTGTAAATGTGTTCCGGGGAGCTGGCCACCAGCGCCACCAGCACGTCGTTCTCGTTAAAGGAAACGCGGTGCCTAGTGGAGCTCTGGGGTCCGAGCGGCGGCCCCGGGGCCGCCGCGTCACCCCCCCATTCCAGCTGGGCCCAGCGACACCCAAACTCGCGCGTGAGAGTGGTCGCGACGAGGGCGACGTAGAGCTCGGCCGCCGCATCCATCGAGGCCCCCCATCTCGCCTGGCGGTGGCGCACAAAGCGTCCGAAGAGCTGAAAGTTGGCGGCCTGGGCGTCGCTGAGGGCCAGCTGAAGCCGGTTGATGACGGTGAGGACGTACATGGCCGTGACGGTCGAGGCCGACTCCAGGGTGTCCGTCGGAAGCGGGGGGCGAATGCATGCCGCCTCGGGACACATCAGCAGCGCGCCGAGCTTGTCGGTCACGGCCGGGAAGCAGAGCGCGTACTGCAGTGGCGTTCCATCCGGGACCAAAAAGCTGGGGGCGAACGGCCGATCCAGCGTACTGGTGGCCTCGCGCAGCACCAGGGGCCCCGGGCCTCCGCTCACTCGCAGGTACGCCTCGCCCCGGCGGCGCAGCATCTGCGGGTCGGCCTCTTGGCCGGGTGGGGCGGACGCCCGGGCGCGGGCGTCTAGGGCGCGAAGATCCACGAGCAGGGGCGCGGGCGCGGCCGCCGCGCCCGCGCCCGTCTGGCCTGTGGCCTTGGCGTACGCGCTATATAAGCCCATGCGGCGTTGGATGAGCTCCCGCGCGCCCCGGAACTCCTCCACCGCCCATGGGGCCAGGTCCCCGGCCACCGCGTCGAATTCCGCCAACAGGCCCCCCAGGGTGTCAAAGTTCATCTCCCAGGCCACCCTTGGCACCACCTCGTCCCGCAGCCGGGCGCTCAGGTCGGCGTGTTGGGCCACGCGCCCCCCGAGCTCCTCCACGGCCCCGGCCCGCTCGGCGCTCTTGGCGCCCAGGACGCCCTGGTACTTGGCGGGAAGGCGCTCGTAGTCCCGCTGGGCTCGCAGCCCCGACACAGTGTTGGTGGTGTCCTGCAGGGCGCGAAGCTGCTCGCATGCCGCGCGAAATCCCTCGGGCGATTTCCAGGCCCCCCCGCGAACGCGGCCGAAGCGACCCCATACCTCGTCCCACTCCGCCTCGGCCTCCTCGAGAGACCTCCGCAGGGCCTCGACGCGGCGACGGGTGTCGAAGAGCGCCTGCAGGCGCGCGCCCTGTCGCGTCAGGAGGCCCGGGCCGTCGCCGCTGGCCGCGCTTAGCGGGTGCGTCTCAAAGGTACGCTGGGCATGTTCCAACCAGGCGACCGCCTGCACGTCGAGCTCGCGCGCCTTCTCCGTCTGGTCCACCAGAATTTCGACCTGATCCGCGATCTCCTCCGCCGAGCGCGCCTGGTCCAGCGTCTTGGCCACGGTCGCCGGGACGGCGACCACCTTCAGCAGGGTCTTCAGATTGGCCAGACCCTCGGCCTCGAGCTGGGCCCGGCGCTCGCGCGCGGCCAGCACCTCCCGCAGCCCCGCCGTGACCCGCTCGGTGGCTTCGGCGCGCTGCTGTTTGGCGCGCACCACGGCGTCCTTGGTATCGGCCAGGTCCTGTCGGGTCACGAATGCGACGTAGTCGGCGTACGCCGTGTCCTTCACGGGGCTCTGGTCCACGCGCTCCAGCGCCGCCACGCACGCCACCAGCGCGTCCTCGCTCGGGCAGGGCAGGGTGACCCCTGCCCGGACAAGCTCGGCGGCCGCCGCCGGGTCGTTGCGCACCGCGGATATCTCCTCCGCGGCGGCGGCCAGGTCCAGCGCCACGCTTCCGATCGCGCGCCGCGCGTCGGCCCGGAGGGCGTCCAGGCGATCGCGGATATCCACGTACTCGGCGTAGCCCTTTTGAAAAAACGGCACGTACTGGCGCAGGGCCGGCACGCCCCCCAAGTCTTCCGACAGGTGTAGGACGGCCTCGTGGTAGTCGATAAACCCGTCGTTCGCCTGGGCCCGCTCCAGCAGCCCCCCCGCCAGCCGCAGAAGCCGCGCCAGGGGCTCGGTGTCCACCCGAAACATGTCGGCGTACGTGTCGGCCGCGGCCCCGAAGGCCGCGCTCCAGTCGATGCGGTGAATGGCTGCGAGCGGGGGGAGCATGGGGTGGCGCTGGTTCTCGGGGGTGTATGGGTTAAACGCAAGGGCCGTCTCCAGGGCAAGGGTCACCGCCTTGGCGTTGGTTCCCAGCGCCTGTTCGGCCCGCTTTCGGAAGTCCCGGGGGTTGTAGCCGTGCGTGCCCGCCAGCGCCTGCAGGCGACGGAGCTCGACCACGTCAAACTCGGCACCGCTTTCCACGCGGTCCAGCACGGCCTCCACGTCGGCGGCCCAGCGCTCGTGGCTACTGCGGGCGCGCTGGGCCGCCATCTTCTCTCTGAGGTCGGCGGTGGCGGCCTCAAGTTCGTCGGCGCGGCGTCGCGTGGCGCCGATGACCTTTCCCAGCTCCTGCAGGGCGCGCCCGCTGGGGGAGTGGTCCCCGGCCGTCCCTTCGGCGTGCAACAGGCCCCCGAACCTGCCCTCGTGGCCCGCGAGGCTTTCCCGCGCGCCGGTGGTCGCGCGCGTCGCGGCCTGGATCAGGGAGGCATGCTCTCCCTCCGGTTGGTTGGCGGCCCGGCGCACCTGGACGACAAGGTCGGCGGCAGCCGACCCTAAGGTCGTGAGCTGGGCGATGGCCACCCGCGCGTCCAGGGCCAACCGAGTCGCCTTGACGTATCCCGCGGCGCTGTCGGCCATGGCCGCTAGGAAGGCCAGGGGGGAGGCCGGGTCGCTGGCGGCCGCGCCCAGGGCCGTCACCGCGTCGACCAGGACGCGGTGCGCCCGCACGGCCGCATCCACCGTCGACGCAGGGTCTGCCGTTGCGACGGCGGCGCTGCCGGCGTTGATGGCGTTCGAGACGGCGTGGGCTATGATCGGGGCGTGATCGGCGAAGAACTGCAAGAGAAACGGAGTCTCTGGGGCGTCGGCGAACAGGTTCTTCAGCACCACCACGAAGCTGGGATGCAAGCCAGACAGAGCCGTCGCCGTGTCCGGAGTCGGGTGCTCCAGGGCATCTCGGTACTGCCCCAGCAGCCCCCACATGTCCGCCCGCAGCGCCGCCGTAACCTCAGGGGGCGCCCCCCGAACGGCCTCGGGGAGGTCCGACCAGCCCGCCGGCAGGGAGGCCCGCAGGGTCGCCAGGACGGCCGGACAGGCCTTTAGCCCCACAAAGTCAGGGAGGGGGCGCAGGACCCCCTGGAGTTTGTGCAAGAACTTCTCCCGGGCGTCGCGGGCCACCTTCGCCCGCTCCCGCGCTCCCTCGAGCATTGCCTCCAGGGAGCGCGCGCGCTCCCGCAAACGGGCACGCGCATCGGGGGCGAGCTCTGCCGTCAGCTTGGCGGCATCCATGGCCCGCGCCTGCCGCAGCGCTTCCTCGGCCATGCGCGTGGCCTCTGGCGACAGCCCGCCGTCGTCGGGGTAGGGCGACGCGCCGGGCGCAGGAACAAAGGCCGCGTCGCTGTCCAGCTGCTGGCCCAGGGCCGCATCTAGGGCGTCGAAGCGCCGCAGCTCGGCCAGACCCGAGCTGCGGCGCGCCTGTTGGTCGTTAATGTCGCGGATGCTGCGCGCCAGCTCGTCCAGTGGCTTGCGTTCTATCAGCCCTTGGTTGGCGGCGTCCGTCAGGACGGAGAGCCAGGCCGCCAGGTCCTCGGGGGCGTCCAGCGTCTGGCCCCGCTGGATCAGATCCCGCAACAGGATGGCCGTGGGGCTGGTCGCGATCGGGGGCGGGGCGGGAATGGCGGCGCGCTGCGCGATGTCCCGCGTGTGCTGGTCGAAGACAGGCAGGGACTCGAGCAGCTGGACCACGGGCACGACGGCGGCCGAAGCCACGTGAAACCGGCGGTCGTTGTTGTCGCTGGCCTGTAGAGCCTTGGCGCTGTATACGGCCCCCCGGTAAAAGTACTCCTTAACCGCGCCCTCGATCGCCCGACGGGCCTGGGTCCGCACCTCCTCCAGCCGAACCTGAACGGCCTCGGGGCCCAGGGGGGGTGGGCGCGGAGCCCCCTGCGGGGCCGCCCCGGCCGGGGCGGGCATTACGCCGAGGGGCCCGGCGTGCTGTGAGACCGCGTCGACCCCGCGAGCGAGGGCGTCGAGGGCCTCGCGCATCTGGCGATCCTCCGCCTCCACCCTAATCTCTTCGCCACGGGCAAATTTGGCCAGAGCCTGGACTCTATACAGAAGCGGTTCTGGGTGCGTCGGGGTGGCGGGGGCAAAAAGGGTGTCCGGGTGGGCCTGCGAGCGCTCCAGAAGCCACTCGCCGAGGCGTGTATACAGATTGGCCGGCGGGGCCGCGCGAAGCTGCAGCTCCAGGTCCGCGAGTTCCCCGTAAAAGGCGTCCGTCTCCCGAATGACATCCCTAGCCACAAGGATCAGCTTCGCCAGCGCCAGGCGACCGATCAGAGAGTTTTCGTCCAGCACGTGCTGGACGAGGGGCAGATGGGCGGCCACGTCGGCCAGGCTCAGGCGCGTGGAGGCCAGAAAGTCCCCCACGGCCGTTTTCCAGGGCAGCATGTTCAGGGTAAACTCCAGCAGGGCGGCGGCCGGGCCGGCCACCCCGGCCTGGGTGTGCGTCCGGGCCCCGTTCTCGATGAGAAAGGCGAGGACGCGTTCAAAGAAAAAAATAACACAGAGCTCCAGCAGCCCCGGAGAGGCCGGATACGGCGACCGTAAGGCGCTGATGGTGAGCCGCGAACACGCGGCGACCTCGCGGGCCAGGGCGGCGGAGCACGCGGTGAACTTAACCGCCGTGGCGGCCACGTTTGGGTGGGCCTCGAACAGCTGGGCGAGGTCTGCGCCCGGGGGCTCGGGCGAGCGGCGAGTCTTCAGCGCCTCGAGGGCCTGTGAGGACGCCGGAACCGTGGGCCCGTCGTCCTCGCCCGCCTCGGCGACCGGCGGCCCGGCCGGGTCGGGGGGTGCCGAGGCGAGGACAGGCTCCGGAACGGAGGCGGGGACCGCGGCCCCGACGGGGGTTTTGCCTTTGGGGGTGGATTTCTTCTTGGTTTTGGCAGGGGGGGCCGAGCGTTTCGTTTTCTCCCCCGAAGTCAGGTCTTCGACGCTGGAAGGCGGAGTCCAGGTGGGTCGGCGGCGCTTGGGAAGGCCGGCCGAGTAGCGTGCCCGGTGCCGACCAACCGGGACGACGCCCATCTCCAGGACCCGCATGTCGTCGTCATCTTCTTCGGCCGCCTCTGCGGCGGGGGTCTTGGGGGCGGAGGGAGGCGGTGGTGGGATCGCGGAGGGTGGGTCGGCGGAGGGTGGGTCGGCGGAGGGGGGATCCGTGGGTGGGGTACCCTTCAGGGCCACCGCCCATACATCGTCGGGCGCCCGATTCGGGCGCTTGGCCTCTGGTTTTGCCGACGGACCGGCCGTCCCCCGGGATGTCTCGGAGGCCCTGTCGTCGCGACGGGCCCGGGTCGGTGGCGGCGACTGGGCGGCTGTGGGCGGGTGTGGCCCCGGGCCCCCTACCCCCTCCCGGGGGCCCACGCCGACGCAGGGCTCCCCCAGGCCCGCGATCTCGCCCCGCAGGGGGTGCGTGATGGCCACGCGCCGTTCGCTGAACGCTTCGTCCTGCAGGTAAGTCTCGCTGGCCCCGTAAAGATGCAGAGCCGCGGCCGTCAAGTCCGCAGGAGCCGCGGGTTCCGGGCCCGACGGCACGAAAAACACCATGGCTCCCGCCCACCGTACGTCCGGGCGATCGCGGGTGTAATACGTCAGGTATGGATACATGTCCCCCGCCCGCACTTTGGCGATGAACGCGGGGGTGCCCTCCGGAAGGCCGTGCGGGTCAAAAAGGTATGCGGTGTCGCCGTCCCTGAACAGCCCCATCCCTAGGGGGCCAATGGTTAGGAGCGTGTACGACAGGGGGCGCAGGGCCCACGGGCCGGCGAAGAACGTGTGTGCGGGGCATTGTGTCTCCAGCAGGCCCGCCGCGGGCTCCCCGAAGAAGCCCACCTCGCCGTATACGCGCGAGAAGACACAGCGCAGTCCGCCGCGCGCCCCTGGGTACTCGAGGAAGTTGGGGAGCTCGACGATCGAACACATGCGCGGCGGCCCAGGGCCCGCGGTCGCGCGCGTCCACTCGCCCCCCTCGACCAAACATCCCTCGATGGCCTCCGCGGACAGGACGTCGCGAGGGCCCACATCAAATATGAGGCTGAGAAAGGACAGCGACGAGCGCATGCACGATACCGACCCCCCCGGCTCCAGGTCGGGCGCGAACTGGTTCCGAGCACCGGTGACCACGATGTCGCGATCCCCCCCGCGTTCCATCGTGGAGTGCGGTGGGGTGCCCGCGATCATATGTGCCCTGCTGGCCAGAGACCCGGCCTGTTTATGGACCGGACCCCCGGGGTTAGTGTTGTTTCCGCCACCCATGCCCCCGTACCATGGCCCCGGTTCCCCTGATTAGGCTACGAGTCGCGGTGATCGCTTCCCAAAAACCGAGCTGCGTTTGTCTGTCTTGGTCTTCCACCCCCCCCCCCGCCCGCCCGCACACCATAACACCGAGAACAACACACGGGGGTGGGCGTAACATAATAAAGCTTTATTGGTAACTAGTTAACGGCAAGTCCGTGGGTGGCGCGACGGTGTCCTCCGGGATCATCTCGTCGTCCTCGACGGGGGTGTTGGAATGAGGCGCCCCCTCGCGGTCCGCCTGGCGTGGGCCGTGCCCATAGGCCTCCGGCTTCTGTGCGTCCATGGGCATAGGCGCGGGGAGACTGTTTCCGGCGTCGCGGACCTCCAGGTCCCTGGGAGACTCCGGTCCGGCTAACGGACGAAACGCGGAAGCGCGAAACACGCCGTCGGTGACCCGCAGGAGCTCGTTCATCAGTAACCAATCCATACTCAGCGTAACGGCCAGCCCCTGGCGAGACAGATCCACGGAGTCCGGAACCGCGGTCGTCTGGCCCAGGGGGCCGAGGCTGTAGTCCCCCCAGGCCCCTAGGTCGCGACGGCTCGTAAGCACGACGCGGTCGGCCGCGGGGCTTTGCGGGGGGGCGTCCTCGGGCGCATGCGCCATTACCTCTCGGATGGCCGCGGCGCGCTGGTCGGCCGAGCTGACCAAGGGCGCCACGACCACGGCGCGCTCCGTCTGCAGGCCCTTCCACGTGTCGTGGAGTTCCTGGACAAACTCGGCCACGGGCTCGGGTCCCGCGGCCGCGCGCGCGGCTTGATAGCAGGCCGACAGACGCCGCCAGCGCGCTAGAAACTGACCCATGAAGCAAAACCCGGGGACCTGGTCTCCCGACAGCAGCTTCGACGCCCGGGCGTGAATGCCGGACACGACGGACAGAAACCCGTGAATTTCGCGCCGGACCACGGCCAGCACGTTGTCCTCGTGCGACACCTGGGCCGCCAGCTCGTCGCACACCCCCAGGTGCGCCGTGGTTTCGGTGATGACGGAACGCAGGCTCGCGAGGGACGCGACCAGCGCGCGCTTGGCGTCGTGATACATGCTGCAGTACTGACTCACCGCGTCCCCCATGGCCTCGGGGGGCCAGGGCCCCAGGCGGTCGGGCGTGTCCCCGACCACCGCATACAGGCGGCGCCCGTCGCTCTCGAACCGACACTCGAAAAAGGCGGAGAGCGTGCGCATGTGCAGCCGCAGCAGCACGATGGCGTCCTCCAGTTGGCGAATCAGGGGGTCTGCGCGCTCGGCGAGGTCCTGCAGCACCCCCCGGGCGGCCAGGGCGTACATGCTAATCAACAGGAGGCTGGTGCCCACCTCGGGGGGCGGGGGGGGCTGCAGCTGGACCAGGGGCCGCAGCTGCTCGACGGCACCCCTGGAGATCACGTACAGCTCCCGGAGCAGCTGCTCTATGTTGTCGGCCATCTGCATAGTGGGGCCGAGGCCGCCCCGGGCGGCCGGTTCGAGGAGGGTGATCAGCGCGCCCAGTTTGGTGCGATGGCCCTCAACCGTGGGGAGATAGCCCAGCCCAAAGTCCCGGGCCCAGGCCAACACACGCAGGGCGAACTCGACCGGGCGGGGAAGGTAGGCCGCGCTACACGTGGCCCTCAGCGCGTCCCCGACCACCAGGGCCAGAACGTAGGGGACGAAGCCCGGGTCGGCGAGGACGTTGGGGTGAATGCCCTCGAGGGCGGGGAAGCGGATCTGGGTCGCCGCGGCCAGGTGGACAGAGGGGGCGTGGCTGGGCTGCCCGACGGGGAGAAGCGCGGACAGCGGCGTGGCCGGGGTGGTGGGGGTGATGTCCCAGTGGGTCTGACCATACACGTCGATCCAGATGAGCGCCGTCTCGCGGAGAAGGCTGGGTTGACCGGAACTAAAGCGGCGCTCGGCCGTCTCAAACTCCCCCACGAGCGCCCGCCGCAGGCTCGCCAGATGTTCCGTCGGCACGGCCGGACCCATGATACGCGCCAGCGTCTGGCTTAGAACGCCCCCCGACAGGCCGACCGCCTCGCAGAGCCGCCCGTGCGTGTGCTCGCTGGCGCCCTGGACCCGCCTGAAAGTTTTTACGTAGTTGGCATAGTACCCGTATTCCCGCGCCAACCCAAACACGTTCGACCCCGCGAGGGCAATGCACCCAAAGAGCTGCTGGACTTCGCCGAGTCCGTGGCCGGTGGGCGTCCGCGCGGGGACGCCCGCCGCCAGAAACCCCTCCAGGGCCGAAAGGTAGTGCGTGCAGTGCGAGGGCGTGAACCCAGCGTCGATCAGGGTGTTGATCACCACGGAGGGCGAATTGGTATTCTGGATCAACGTCCACGTCTGCTGCAGCAGAGCCAGCAGCCGCTGCTGGGCGCCGGCGGAGGGCTGCTCCCCGAGCTGCAGCAGGCTGGAGACGGCAGGCTGGAAGACTGCCAGTGCCGACGAACTCAGGAACGGCACGTCGGGATCAAACACGGCCACGTCCGTCCGCACGCGCGCCATTAGCGTCCCCGGGGGCGCACAGGCCGAGCGCGGGCTGACGCGGCTGAGGGCCGTCGACACGCGCACCTCCTCGCGGCTGCGAACCATCTTGTTGGCCTCGAGCGGCGGAATCATTATGGCCGGGTCGATCTCCCGCACGGTGTGCTGAAACTGCGCCAACAGGGGCGGCGGGACCACAGCCCCCCGCTCGGGGGTCGTCAGGTACTCGTCCACCAGGGCCAACGTAAAGAGGGCCCGTGTGAGGGGAGTGAGGGTCGCGTCGTCTATGCGCTGGAGGTGCGCCGAGAACAGCGTCACCCGATTACTCACCAGGGCCAAGAACCGGAGGCCCTCTTGCACGAACGGGGCGGGGAAGAGCAGGCTGTACGCCGGGGTGGTAAGGTTCGCGCTGGGCTGCCCCAACGGGACCGGCGCCATCTTGAGCGACGTCTCCCCAAGGGCCTCGATGGAGGTCCGCGGGCTCATGGCCAAGCAGCTCTTGGTGACGGTTTGCCAGCGGTCTATCCACTCCACGGCGCACTGGCGGACGCGGACCGGCCCCAGGGCCGCCGCGGTGCGCAGGCCGGCGGAATCCAGCGCATGGGACGTGTCGGAGCCGGTGACCGCGAGGATGGTGTCCTTGATGACCTCCATCTCCCGGAAGGCCTGGTCGGGGGCCTCGGGGAGAGCCACCACCAAGCGGTGTACGAGCAACCCGGGGAGGTTCTCGGCCAAGAGCGCCGTCTCCGGAAGCCCGTGGGCCCGGTGGAGCGCGCACAGGTGTTCCAGCAGCGGCCGCCAGCATGCCCGCGCGTCTGCCGGGGCGATGGCCGTTCCCGACAACAGAAACGCCGCCATGGCGGCGCGCAGCTTGGCCGTGGCCAGAAACGCCGGGTCGTCCGCCCCGTTTGCCGTCTCGGCCGTGGGGGTTGGCGGTTGGCGAAGGCCGGCTAGGCTCGCCAATAGGCGCTGCATAGGTCCGTCCGAGGGCGGACCGGCGGGTGAGGTCGTGACGACGGGGGCCTCGGACGGGAGACCGCGGTCTGCCATGACGCCCGGCTCGCGTGGGTGGGGGACAGCGTAGACCAACGACGAGACCGGGCGGGAATGACTGTCGTGCGCTGTAGGGAGCGGCGAATTATCGATCCCCCGCGGCCCTCCAGGACCCCCGCAGGCGTTGCGAGTACCCCGCGTCTTCGCGGGGTGTTATACGGCCACTTAAGTCCCGGCATCCCGTTCGCGGACCCAGGCCCGGGGGATTGTCCGGATGTGCGGGCAGCCCGGACGGCGTGGGTTGCGGACTTTCTGCGGGGCGGCCCAAATGGCCCTTTAAACGTGTGTATACGGACGCGCCGGGCCAGTCGGCCAACACAACCCACCGGAGGCGGTAGCCGCGTTTGGCTGTGGGGTGGGTGGTTCCGCCTTGCGTGAGTGTCCTTTCGACCCCCCCCCCCCTCCCTCCCCCGGGTCTTGCTAGGTCGCGATCTGGGGTCGCAATGAAGACCAATCCGCTACCCGCAACCCCTTCCGTGTGGGGCGGGAGTACCGTGGAACTCCCCCCCACAACACGCGATACCGCGGGACAGGGCCTGCTTCGGCGCGTCCTGCGCCCCCCGATCTCTCGCCGCGACGGCCCAGGGCTCCCCAGGGGGTCGGGACCCCGGAGGGCGGCCAGCACGCTGTGGTTGCTTGGCCTGGACGGCACAGACGCGCCCCCTGGGGCGCTGACCCCCAACGACGATACCGAACAGGCCCTGGACAAGATCCTGCGGGGCACCATGCGCGGGGGGGCGGCCCTGATCGGCTCCCCGCGCCATCATCTAACCCGCCAAGTGATCCTGACGGATCTGTGCCAACCCAACGCGGATCGTGCTGGGACGCTGCTTCTGGCGCTGCGGCACCCCGCCGACCTGCCTCACCTGGCCCACCAGCGCGCCCCGCCAGGCCGGCAGACCGAGCGGCTGGGCGAGGCCTGGGGCCAGCTGATGGAGGCGACCGCCCTGGGGTCGGGGCGAGCCGAGAGCGGGTGCACGCGCGCGGGCCTAGTGTCGTTTAACTTCCTGGTGGCGGCGTGTGCCGCCTCGTACGACGCGCGCGACGCCGCCGATGCGGTACGGGCCCACGTCACGGCCAACTACCGCGGGACGCGGGTGGGGGCGCGCCTGGATCGTTTTTCCGAGTGTCTGCGCGCCATGGTTCACACGCACGTCTTCCCCCACGAGGTCATGCGGTTTTTCGGGGGGCTGGTGTCGTGGGTCACCCAGGACGAGCTAGCGAGCGTCACCGCCGTGTGCGCCGGGCCCCAGGAGGCGGCGCACACCGGCCACCCGGGCCGGCCCCGCTCGGCCGTGATCCTCCCGGCATGTGCGTTCGTGGACCTGGACGCCGAGCTGGGGCTGGGGGGCCCGGGCGCGGCGTTTCTGTACCTGGTATTCACTTACCGCCAGCGCCGGGACCAGGAGCTGTGTTGTGTGTACGTGATCAAGAGCCAGCTCCCCCCGCGCGGGTTGGAGCCGGCCCTGGAGCGGCTGTTTGGGCGCCTCCGGATCACCAACACGATTCACGGCACCGAGGACATGACGCCCCCGGCCCCAAACCGAAACCCCGACTTCCCCCTCGCGGGCCTGGCCGCCAATCCCCAAACCCCGCGTTGCTCGGCTGGCCAGGTCACGAACCCCCAGTTCGCCGACAGGCTGTACCGCTGGCAGCCGGACCTGCGGGGGCGCCCCACCGCACGCACCTGTACGTACGCCGCCTTTGCAGAGCTCGGCATGATGCCCGAGGATAGTCCCCGCTGCCTGCACCGCACCGAGCGCTTTGGGGCGGTCAGCGTCCCCGTTGTCATTCTGGAAGGCGTGGTGTGGCGCCCCGGCGAGTGGCGGGCATGCGCGTGAGCGTAGCAAACGCCCCGCCCACACAACGCTCCGCCCCCAACCCCTTCCCCGCTGTCACTCGTTGTTCGTTGACCCGGGCGTCCGCCAAATAAAGCCACTGAAACCCGAAACGCGAGTGTTGTAACGTCCTTTGGGCGGGAGGAAGCCACAAAATGCAAATGGGATACATGGAAGGAACACACCCCCGTGACTCAGGACATCGGTGTGTCCTTTTGGGTTTCACTGAAACTGGCCCGCGCCCCACCCCTGCGCGATGTGGATAAAAAGCCAGCGCGGGTGGTTTAGGGTACCACAGGTGGGTGCTTTGGAAACTTGCCGGTCGCCGTGCTCCTGTGAGCTTGCGTCCCTCCCCGGTTTCCTTTGCGCTCCCGCCTTCCGGACCTGCTCTCGCCTATCTTCTTTGGCTCTCGGTGCGATTCGTCAGGCAGCGGCCTTGTCGAATCTCGACCCCACCACTCGCCGGACCCGCCGACGTCCCCTCTCGAGCCCGCCGAAACCCGCCGCGTCTGTTGAAATGGCCAGCCGCCCAGCCGCATCCTCTCCCGTCGAAGCGCGGGCCCCGGTTGGGGGACAGGAGGCCGGCGGCCCCAGCGCAGCCACCCAGGGGGAGGCCGCCGGGGCCCCTCTCGCCCACGGCCACCACGTGTACTGCCAGCGAGTCAATGGCGTGATGGTGCTTTCCGACAAGACGCCCGGGTCCGCGTCCTACCGCATCAGCGATAGCAACTTTGTCCAATGTGGTTCCAACTGCACCATGATCATCGACGGAGACGTGGTGCGCGGGCGCCCCCAGGACCCGGGGGCCGCGGCATCCCCCGCTCCCTTCGTTGCGGTGACAAACATCGGAGCCGGCAGCGACGGCGGGACCGCCGTCGTGGCATTCGGGGGAACCCCACGTCGCTCGGCGGGGACGTCTACCGGTACCCAGACGGCCGACGTCCCCACCGAGGCCCTTGGGGGCCCCCCTCCTCCTCCCCGCTTCACCCTGGGTGGCGGCTGTTGTTCCTGTCGCGACACACGGCGCCGCTCTGCGGTATTCGGGGGGGAGGGGGATCCAGTCGGCCCCGCGGAGTTCGTCTCGGACGACCGGTCGTCCGATTCCGACTCGGATGACTCGGAGGACACGGACTCGGAGACGCTGTCACACGCCTCCTCGGACGTGTCCGGCGGGGCCACGTACGACGACGCCCTTGACTCCGATTCGTCATCGGATGACTCCCTGCAGATAGATGGCCCCGTGTGTCGCCCGTGGAGCAATGACACCGCGCCCCTGGATGTTTGCCCCGGGACCCCCGGCCCGGGCGCCGACGCCGGTGGTCCCTCAGCGGTAGACCCACACGCGCCGACGCCAGAGGCCGGCGCTGGTCTTGCGGCCGATCCCGCCGTGGCCCGGGACGACGCGGAGGGGCTTTCGGACCCCCGGCCACGTCTGGGAACGGGCACGGCCTACCCCGTCCCCCTGGAACTCACGCCCGAGAACGCGGAGGCCGTGGCGCGCTTTCTGGGAGATGCCGTGAACCGCGAACCCGCGCTCATGCTGGAGTACTTTTGCCGGTGCGCCCGCGAGGAAACCAAGCGTGTCCCCCCCAGGACATTCGGCAGCCCCCCTCGCCTCACGGAGGACGACTTTGGGCTTCTCAACTACGCGCTCGTGGAGATGCAGCGCCTGTGTCTGGACGTTCCTCCGGTCCCGCCGAACGCATACATGCCCTATTATCTCAGGGAGTATGTGACGCGGCTGGTCAACGGGTTCAAGCCGCTGGTGAGCCGGTCCGCTCGCCTTTACCGCATCCTGGGGGTTCTGGTGCACCTGCGGATCCGGACCCGGGAGGCCTCCTTTGAGGAGTGGCTGCGATCCAAGGAAGTGGCCCTGGATTTTGGCCTGACGGAAAGGCTTCGCGAGCACGAAGCCCAGCTGGTGATCCTGGCCCAGGCTCTGGACCATTACGACTGTCTGATCCACAGCACACCGCACACGCTGGTCGAGCGGGGGCTGCAATCGGCCCTGAAGTATGAGGAGTTTTACCTAAAGCGTTTTGGCGGGCACTACATGGAGTCCGTCTTCCAGATGTACACCCGCATCGCCGGCTTTTTGGCCTGCCGGGCCACGCGCGGCATGCGCCACATCGCCCTGGGGCGAGAGGGGTCGTGGTGGGAAATGTTCAAGTTCTTTTTCCACCGCCTCTACGACCACCAGATCGTACCGTCGACCCCCGCCATGCTGAACCTGGGGACCCGCAACTACTACACCTCCAGCTGCTACCTGGTAAACCCCCAGGCCACCACAAACAAGGCGACCCTGCGGGCCATCACCAGCAACGTCAGTGCCATCCTCGCCCGCAACGGGGGCATCGGGCTATGCGTGCAGGCGTTTAACGACTCCGGCCCCGGGACCGCCAGCGTCATGCCCGCCCTCAAGGTCCTTGACTCGCTGGTGGCGGCGCACAACAAAGAGAGCGCGCGTCCGACCGGCGCGTGCGTGTACCTGGAGCCGTGGCACACCGACGTGCGGGCCGTGCTCCGGATGAAGGGGGTCCTCGCCGGCGAAGAGGCCCAGCGCTGCGACAATATCTTCAGCGCCCTCTGGATGCCAGACCTGTTTTTCAAGCGCCTGATTCGCCACCTGGACGGCGAGAAGAACGTCACATGGACCCTGTTCGACCGGGACACCAGCATGTCGCTCGCCGACTTTCACGGGGAGGAGTTCGAGAAGCTCTACCAGCACCTCGAGGTCATGGGGTTCGGCGAGCAGATACCCATCCAGGAGCTGGCCTATGGCATTGTGCGCAGTGCGGCCACGACCGGGAGCCCCTTCGTCATGTTCAAAGACGCGGTGAACCGCCACTACATCTACGACACCCAGGGGGCGGCCATCGCCGGCTCCAACCTCTGCACCGAGATCGTCCATCCGGCCTCCAAGCGATCCAGTGGGGTCTGCAACCTGGGAAGCGTGAATCTGGCCCGATGCGTCTCCAGGCAGACGTTTGACTTTGGGCGGCTCCGCGACGCCGTGCAGGCGTGCGTGCTGATGGTGAACATCATGATCGACAGCACGCTACAACCCACGCCCCAGTGCACCCGCGGCAACGACAACCTGCGGTCCATGGGAATCGGCATGCAGGGCCTGCACACGGCCTGCCTGAAGCTGGGGCTGGATCTGGAGTCTGCCGAATTTCAGGACCTGAACAAACACATCGCCGAGGTGATGCTGCTGTCGGCGATGAAGACCAGCAACGCGCTGTGCGTTCGCGGGGCCCGTCCCTTCAACCACTTTAAGCGCAGCATGTATCGCGCCGGCCGCTTTCACTGGGAGCGCTTTCCGGACGCCCGGCCGCGGTACGAGGGCGAGTGGGAGATGCTACGCCAGAGCATGATGAAACACGGCCTGCGCAACAGCCAGTTTGTCGCGCTGATGCCCACCGCCGCCTCGGCGCAGATCTCGGACGTCAGCGAGGGCTTTGCCCCCCTGTTCACCAACCTGTTCAGCAAGGTGACCCGGGACGGCGAGACGCTGCGCCCCAACACGCTCCTGCTAAAGGAACTGGAACGCACGTTTAGCGGGAAGCGCCTCCTGGAGGTGATGGACAGTCTCGACGCCAAGCAGTGGTCCGTGGCGCAGGCGCTCCCGTGCCTGGAGCCCACCCACCCCCTCCGGCGATTCAAGACCGCGTTTGACTACGACCAGAAGTTGCTGATCGACCTGTGTGCGGACCGCGCCCCCTACGTCGACCATAGCCAATCCATGACCCTGTATGTCACGGAGAAGGCGGACGGGACCCTCCCAGCCTCCACCCTGGTCCGCCTTCTGGTCCACGCATATAAGCGCGGACTAAAAACAGGGATGTACTACTGCAAGGTTCGCAAGGCGACCAACAGCGGGGTCTTTGGCGGCGACGACAACATTGTCTGCATGAGCTGCGCGCTGTGACCGACAAACCCCCTCCGCGCCAGGCCCGCCGCCACTGTCGTCGCCGTCCCACGCTCTCCCCTGCTGCCATGGATTCCGCGGCCCCAGCCCTCTCCCCCGCTCTGACGGCCCTTACGGGCCAGAGCGCGACGGCGGACCTGGCGATCCAGATTCCAAAGTGCCCCGACCCCGAGAGGTACTTCTACACCTCCCAGTGTCCCGACATTAACCACCTGCGCTCCCTCAGCATCCTTAACCGCTGGCTGGAAACCGAGCTTGTTTTCGTGGGGGACGAGGAGGACGTCTCCAAGCTTTCCGAGGGCGAGCTCAGCTTTTACCGCTTCCTCTTCGCTTTCCTGTCGGCCGCCGACGACCTGGTTACGGAAAACCTGGGCGGCCTCTCCGGCCTGTTTGAGCAGAAGGACATTCTCCACTACTACGTGGAGCAGGAATGCATCGAAGTCGTACACTCGCGCGTGTACAACATCATCCAGCTGGTGCTTTTCCACAACAACGACCAGGCGCGCCGCGAGTACGTGGCCGGTACCATCAACCACCCGGCCATCCGCGCCAAGGTGGACTGGTTGGAAGCGCGGGTGCGGGAATGCGCCTCCGTTCCGGAAAAGTTCATTCTCATGATCCTCATCGAGGGCATCTTTTTTGCCGCCTCGTTTGCCGCCATCGCCTACCTTCGCACCAACAACCTTCTGCGGGTCACCTGCCAGTCAAACGACCTCATCAGCCGGGACGAGGCCGTGCACACGACGGCCTCGTGTTACATCTACAACAACTACCTCGGCGGGCACGCCAAGCCCCCGCCCGACCGCGTGTACGGGCTGTTCCGCCAGGCGGTCGAGATCGAGATCGGATTTATCCGATCCCAGGCGCCGACGGACAGCCATATCCTGAGCCCGGCGGCGCTGGCGGCCATCGAAAACTACGTGCGATTCAGCGCGGATCGCCTGTTGGGCCTTATCCACATGAAGCCACTGTTTTCCGCCCCACCCCCCGACGCCAGCTTTCCGCTGAGCCTCATGTCCACCGACAAACACACCAATTTTTTCGAGTGTCGCAGCACCTCCTACGCCGGGGCGGTCGTCAACGATCTGTGAGTGTCGCGGCGCGCTTCTACCCGTGTTTGCCCATAATAAACCTCTGAACCAAACTTTGGGTCTCATTGTGATTCTTGTCAGGGACGCGGGGGTGGGAGAGGATAAAAGGCGGCGCAAAAAGCAGTAACCAGGTCCGTCCAGATTCTGCGGGCATAGGATACCATAATTTTATTGGTGGGTCGTTTGTTCGGGGACAAGCGCGCTCGTCTGACGTTTGGGCTACTCGTCCCAGAATTTGGCCAGGACGTCCTTGTAGAACGCGGGTGGGGGGGCCTGGGTCCGCAACTGCTCCAGAAACCTGTCGGCGATATCAGGGGCCGTGATATGCCGGGTCACGATAGATCGCGCCAGGTTTTCGTCGCGGATGTCCTGGTAGATAGGCAGGCGTTTCAGAAGAGTCCACGGCCCCCGCTCCTTGGGGCCGATAAGCGATATGACGTACTTAATGTAGCGGTGTTCCACCAGCTCGGTGATGGTCATGGGATCGGGGAGCCAGTCCAGGGACTCTGGGGCGTCGTGGATGACGTGGCGTCGCCGGTTGGCCACATAACTGCGGTGCTCTTCCAGCAGCTGCGCGTTCGGGACCTGGACGAGCTCGGGCGGGGTGAGTATCTCCGAGGAGGACGACCTGGGGCCGGGGTGGCCCCCGGTAACGTCCCGGGGATCCAGGGGGAGGTCCTCGTCGTCTTCGTATCCGCCGGCGATCTGTTGGGTTAGAATTTCGGTCCACGAGACGCGCGTCTCGGTGCCGCCGGCGGCCGGCGGCAGAGGGGGCCTGGTTTCCGTGGAGCGCGAGCTGGTGTGTTCCCGGCGGATGGCCCGCCGGGTCTGAGAGCGACTCGGGGGGGTCCAGTGACATTCGCGCAGCACATCCTCCACGGAGGCGTAGGTGTTATTGGGATGGAGGTCGGTGTGGCAGCGGACAAAGAGGGCCAGGAACTGGGGGTAGCTCATCTTAAAGTACTTTAGTATATCGCGACAGTTGATCGTGGGAATGTAGCAGGCGCTAATATCCAACACAATATCACAGCCCATCAACAGGAGGTCAGTGTCCGTGGTGTACACGTACGCGACCGTGTTGGTGTGATAGAGGTTGGCGCAGGCATCGTCCGCCTCCAGCTGACCCGAGTTAATGTAGGCGTACCCCAGGGCCCGGAGAACGCGAATACAGAACAGATGCGCCAGACGCAGGGCCGGCTTCGAGGGCGCGGCGGACGGCAGCGCGGCTCCGGACCCGGCCGTCCCCCGGGTCCCCGAGGCCAGAGAGGTGCCGCGCCGGCGCATGTTGGAAAAGGCAGAGCTGGGTCTGGAGTCGGTGATGGGGGAAGGCGGTGGAGAGGCGTCCACGTCACTGGCCTCCTCGTCCGTCCGGCACTGGGCCGTCGTGCGGGCCAGGATGGCCTTGGCTCCAAACACAACCGGCTCCATACAATTGACCCCGCGATCGGTAACGAAGATGGGGAAAAGGGACTTTTGGGTAAACACCTTTAATAAGCGACAGAGGCAGTGTAGCGTAATGGCCTCGCGGTCGTAACTGGGGTATCGGCGCTGATATTTGACCACCAACGTGTACATGACGTTCCACAGGTCCACGGCAATGGGGGTGAAGTACCCGGCCGGGGCCCCAAGGCCCCGGCGCTTGACCAGATGGTGTGTGTGGGCAAACTTCATCATCCCGAACAAACCCATGTCAGGTCAATTGTAACTGCGGATCGGCCTAACTAAGGCGTGGTTGGTGCGACGGTCCGGGACACCCGAGCCTGTCTCTCTGTGTATGGTGACCCAGACAACAACACCGACACAAGAGGACAATAATCCGTTAGGGGACGCTCTTTATAATTTCGATGGCCCAACTCCACGCGGATTGGTGCAGCACCCTGCATGCGCCGGTGCGGGCCAACCTTCCCCCCGCTCATTGCCTCTTCCAAAAGGGTGTGGCCTAACGAGCTGGGGGCGTATTTAATCAGGCTAGCGCGGCGGGCCTGCCGTAGTTTCTGGCTCGGTGAGCGACGGTCCGGTTGCTTGGGTCCCCTGGCTGCCATCAAAACCCCACCCTCGCAGCGGCATACGCCCCCTCCGCGTCCCGCACCCGAGACCCCGGCCCGGCTGCCCTCACCACCGAAGCCCACCTCGTCACTGTGGGGTGTTCCCAGCCCGCGTTGGGATGACGGATTCCCCTGGCGGTGTGGCCCCCGCCTCCCCCGTGGAGGACGCGTCGGACGCGTCCCTCGGGCAGCCGGAGGAGGGGGCGCCCTGCCAGGTGGTCCTGCAGGGCGCCGAACTTAATGGAATCCTACAGGCGTTTGCCCCGCTGCGCACGAGCCTTCTGGACTCGCTTCTGGTTATGGGCGACCGGGGCATCCTTATCCATAACACGATCTTTGGGGAGCAGGTGTTCCTGCCCCTGGAACACTCGCAATTCAGTCGGTATCGCTGGCGCGGACCCACGGCGGCGTTCCTGTCTCTCGTGGACCAGAAGCGCTCCCTCCTGAGCGTGTTTCGCGCCAACCAGTACCCGGACCTACGTCGGGTGGAGTTGGCGATCACGGGCCAGGCCCCGTTTCGCACGCTGGTTCAGCGCATATGGACGACGACGTCCGACGGCGAGGCCGTTGAGCTAGCCAGCGAGACGCTGATGAAGCGCGAACTGACGAGCTTTGTGGTGCTGGTTCCCCAGGGAACCCCCGACGTTCAGTTGCGCCTGACGAGGCCGCAGCTCACCAAGGTCCTTAACGCGACCGGGGCCGATAGTGCCACGCCCACCACGTTCGAGCTCGGGGTTAACGGCAAATTTTCCGTGTTCACCACGAGTACCTGCGTCACCTTTGCTGCCCGCGAGGAGGGCGTGTCGTCCAGCACCAGCACCCAGGTCCAGATCCTGTCCAACGCGCTCACCAAGGCGGGCCAGGCGGCCGCCAACGCCAAGACGGTGTACGGGGAAAATACCCATCGCACCTTCTCTGTGGTCGTCGACGATTGCAGCATGCGGGCGGTGCTCCGGCGACTGCAGGTCGGCGGGGGCACCCTCAAGTTCTTCCTCACGACCCCCGTCCCCAGTCTGTGCGTCACCGCCACCGGTCCCAACGCGGTATCGGCGGTATTTCTCCTGAAACCCCAGAAGATTTGCCTGGACTGGCTGGGTCATAGCCAGGGGTCTCCTTCAGCCGGGAGCTCGGCCTCCCGGGCCTCTGGGAGCGAGCCAACAGACAGCCAGGACTCCGCGTCGGACGCGGTCAGCCACGGCGATCCGGAAGACCTCGATGGCGCTGCCCGGGCGGGAGAGGCGGGGGCCTTGCATGCCTGTCCGATGCCGTCGTCGACCACGCGGGTCACTCCCACGACCAAGCGGGGGCGCTCGGGGGGCGAGGATGCGCGCGCGGACACGGCCCTAAAGAAACCTAAGACGGGGTCGCCCACCGCACCCCCGCCCGCAGATCCAGTCCCCCTGGACACGGAGGACGACTCCGATGCGGCGGACGGGACGGCGGCCCGTCCCGCCGCTCCAGACGCCCGGAGCGGAAGCCGTTACGCGTGTTACTTTCGCGACCTCCCGACCGGAGAAGCAAGCCCCGGCGCCTTCTCCGCCTTCCGGGGGGGCCCCCAAACCCCGTATGGTTTTGGATTCCCCTGACGGGGCGGGGCCTTGGCGGCCGCCCAACTCTCGCACCATCCCGGGTTAATGTAAATAAACTTGGTATTGCCCAACACTTTCCCGCGTGTCGCGTGTGGTTCATGTGTGTGCCTGGCGCCCCCACCCTCGGGTTCGTGTATTTCCTTTCCCTGTCCTTATAAAAGCCGTATGTGGGGCGCTGACGGAACCACCCCGCGTGCCATCACGGCCAAGGCGCGGGATGCTCCGCAACGACAGCCACCGGGCCGTGTCCCCGGAGGACGGCCAGGGACGGGTCGACGACGGACGGCCACACCTCGCGTGCGTGGGGGCCCTGGCGCGGGGGTTCATGCATATCTGGCTTCAGGCCGCCACGCTGGGTTTTGCGGGATCGGTCGTTATGTCGCGCGGGCCGTACGCGAATGCCGCGTCTGGGGCGTTCGCCGTCGGGTGCGCCGTGCTGGGCTTTATGCGCGCACCCCCTCCCCTCGCGCGGCCCACCGCGCGGATATACGCCTGGCTCAAACTGGCGGCCGGTGGAGCGGCCCTTGTTCTGTGGAGTCTCGGGGAGCCCGGCACGCAGCCGGGGGCCCCGGCCCCGGGCCCGGCCACCCAGTGCCTGGCGCTGGGCGCCGCCTATGCGGCGCTCCTGGTGCTCGCCGATGACGTCTATCCGCTCTTTCTCCTCGCCCCGGGGCCCCTGTTCGTCGGCACCCTGGGGATGGTCGTCGGCGGGCTGACGATCGGAGGCAGCGCGCGCTACTGGTGGATCGGTGGGCCCGCCGCGGCCGCCCTGGCCGCGGCGGTGTTGGCGGGCCCGGGGGCGACCACCGCCAGGGACTGCTTTTCCAGGGCTTGCCCCGACCACCGCCGCGTCTGTGTCATCACCGCAGGCGAGTCTCTTTCCCGCCGCCCCCCGGAGGACCCAGAGCGACCCGGGGTTCCCGGGCCCCCGTCCCCCCCGACCCCCCAACGATCCCACGGGCCGCCGGCCGATGAGGTCGCACCGGCCAGGGTCGCGCGGCCCGAAAACGTCTGGGTGCCCGTGGTCACCTTTCTGGGGGCGGGCGCGCTTGCCGTCAAGACGGTGCGAGAACATGCCCGGGGAACGCCGGGCCCGGGCCTGCCGCTGTGGCCCCAGGTGTTTCTCGGAGGCCATGTGGCGGTGGCCCTGACGGAGCTGTGTCAGGCGCTTGCGCCCTGGGACCTTACGGACCCGCTGCTGTTTGTTCACGCCGGACTGCAGGTCATCAACCTCGGGTTGGTGTTTCGGTTTTCCGAGGTTGTCGTGTATGCGGCGCTAGGGGGTGCCGTGTGGATTTCGTTGGCGCAGGTGCTGGGGCTCCGGCGTCGCCTGCACAGGAAGGACCCCGGGGACGGGGCCCGGTTGGCGGCGACGCTTCGGGGCCTCTTCTTCTCCGTGTACGCGCTGGGGTTTGGGGTGGGGGTGCTGCTGTGCCCTCCGGGGTCAACGGGCGGGCGGTCGGGCGATTGATATATTTTTCAATAAAAGGCATTAGTCCCGAAGACCGCCGGTGTGTGATGATTTCGCCATAACACCCAAACCCCGGATGGGGCCCGGGTATAAATTCCGGAAGGGGACACGGGCTACCCTCACTATCGAGGGCGCTTGGTCGGGAGGCCGCATCGAACGCACACCCCCATCCGGTGGTCCGTGTGGAGGTCGTTTTCAGTGCCCGGTCTCGCTTTGCCGGGAACGCTAGCCGATCCCTCGCGAGGGGGAGGCGTCGGGCATGGCCCCGGGGCGGGTGGGCCTTGCCGTGGTCCTGTGGAGCCTGTTGTGGCTCGGGGCGGGGGTGTCCGGGGGCTCGGAAACTGCCTCCACCGGGCCCACGATCACCGCGGGAGCGGTGACGAACGCGAGCGAGGCCCCCACATCGGGGTCCCCCGGGTCAGCCGCCAGCCCGGAGGTCACCCCCACATCGACCCCAAACCCCAACAATGTCACACAAAACAAAACCACCCCCACCGAGCCGGCCAGCCCCCCAACAACCCCCAAGCCCACCTCCACGCCCAAAAGCCCCCCCACGTCCACCCCCGACCCCAAACCCAAGAACAACACCACCCCCGCCAAGTCGGGCCGCCCCACTAAACCCCCCGGGCCCGTGTGGTGCGACCGCCGCGACCCATTGGCCCGGTACGGCTCGCGGGTGCAGATCCGATGCCGGTTTCGGAATTCCACCCGCATGGAGTTCCGCCTCCAGATATGGCGTTACTCCATGGGTCCGTCCCCCCCAATCGCTCCGGCTCCCGACCTAGAGGAGGTCCTGACGAACATCACCGCCCCACCCGGGGGACTCCTGGTGTACGACAGCGCCCCCAACCTAACGGACCCCCACGTGCTCTGGGCGGAGGGGGCCGGCCCGGGCGCCGACCCTCCGTTGTATTCTGTCACCGGGCCGCTGCCGACCCAGCGGCTGATTATCGGCGAGGTGACGCCCGCGACCCAGGGAATGTATTACTTGGCCTGGGGCCGGATGGACAGCCCGCACGAGTACGGGACGTGGGTGCGCGTCCGCATGTTCCGCCCCCCGTCTCTGACCCTCCAGCCCCACGCGGTGATGGAGGGTCAGCCGTTCAAGGCGACGTGCACGGCCGCCGCCTACTACCCGCGTAACCCCGTGGAGTTTGTCTGGTTCGAGGACGACCACCAGGTGTTTAACCCGGGCCAGATCGACACGCAGACGCACGAGCACCCCGACGGGTTCACCACAGTCTCTACCGTGACCTCCGAGGCTGTCGGCGGCCAGGTCCCCCCGCGGACCTTCACCTGCCAGATGACGTGGCATCGCGACTCCGTGACGTTCTCGCGACGCAATGCCACCGGGCTGGCCCTGGTGCTGCCGCGGCCAACCATCACCATGGAATTTGGGGTCCGGCATGTGGTCTGCACGGCCGGCTGCGTCCCCGAGGGCGTGACGTTTGCCTGGTTCCTGGGGGACGACCCCTCACCGGCGGCTAAGTCGGCCGTTACGGCCCAGGAGTCGTGCGACCACCCCGGGCTGGCTACGGTCCGGTCCACCCTGCCCATTTCGTACGACTACAGCGAGTACATCTGTCGGTTGACCGGATATCCGGCCGGGATTCCCGTTCTAGAACACCACGGCAGTCACCAGCCCCCACCCAGGGACCCCACCGAGCGGCAGGTGATCGAGGCGATCGAGTGGGTGGGGATTGGAATCGGGGTTCTCGCGGCGGGGGTCCTGGTCGTAACGGCAATCGTGTACGTCGTCCGCACATCACAGTCGCGGCAGCGTCATCGGCGGTAACGCAAGACCCCCCCGTTACCTTTTTAATATCTATATAGTTTGGTCCCCCCTCTATCCCGCCCACCGCTGGGCGCTATAAAGCCGCCACCCTCTCTTCCCTCAGGTCATCCTTGGTCGATCCCGAACGACACACGGCGTGGAGCAAAACGCCTCCCCCTGAGCCGCTTTCCTACCAACACAACGGCATGCCTCTGCGGGCATCGGAACACGCCTACCGGCCCCTGGGCCCCGGGACACCCCCCATGCGGGCTCGGCTCCCCGCCGCGGCCTGGGTTGGCGTCGGGACCATCATCGGGGGAGTTGTGATCATTGCCGCGTTGGTCCTCGTGCCCTCGCGGGCCTCGTGGGCACTTTCCCCATGCGACAGCGGATGGCACGAGTTCAACCTCGGGTGCATATCCTGGGATCCGACCCCCATGGAGCACGAGCAGGCGGTCGGCGGCTGTAGCGCCCCGGCGACCCTGATCCCCCGCGCGGCTGCCAAACAGCTGGCCGCCGTCGCACGCGTCCAGTCGGCAAGATCCTCGGGCTACTGGTGGGTGAGCGGAGACGGCATTCGGGCCTGCCTGCGGCTCGTCGACGGCGTCGGCGGTATTGACCAGTTTTGCGAGGAGCCCGCCCTTCGCATATGCTACTATCCCCGCAGTCCCGGGGGCTTTGTTCAGTTTGTAACTTCGACCCGCAACGCGCTGGGGCTGCCGTGAGGCGCGTGTACTGCGGTCTGTCTCGTCTCCTCTTCTCCCCTTCCCTCCCCCTCCGCATCCCAGGATCACACCGGTCAACGAGGGTTGGGGGGGTCCGGCACGGACCCAAAATAATAAACACACAATCACGTGCGATAAAAAGAACACGCGGTCCCCTGTGGTGTTTTTGGTTATTTTTATTAAATCTCGTCGACAAACAGGGGGAAAGGGGCGTGGTCTAGCGACGGCAGCACGGGCGGAGGCGTTCACCGGCTCCGGCGTCCTTCGCGTTTAAGCTTGGTCAGGAGGGCGCTCAGGGCGGCGACGTTGGTCGGGCCGTCGTTGGTCAGGGCGTTGGCTCGATGGCGGGCGAGGACGGGCGAGGGGCTCAACGGCGGGGGCGGGGGCCCGGTGCGGCCCGGGGGGGAAAATAGGGCGGATCCCCCCCAGTCGTACAGGGGATTTTCCGCCTCAATGTACGGGGAGGCCGGCGCTGCATTCGCCGTGTTCACGCAGACGTTTTCGTAGACCCGCATCCATGGTATTTCCTCGTAGACACGCCCCCCGTCCTCGCTCACCGTCTCGTATATTGACTCGTCGTCCTCGTAGGGGGCGTGCCGTTCGCGGGCCGAGGCGGCGTGGGTGGCTTTGCGGCGGGCGTCGTCGTCGTCGTCGTCGGCCGTCAGATACGTGGCTTCCATCTGGTCGGGTTCTCCCTCCGGGGCGGGTCCCCACACCCGTGGCCGATCGAGGCTCCCCAGAGACGCGCGCCGGACGAGGAGGGGGCACGTCGCCGCCGGCGGTCGCCTGTCGGGTCCCGCGACGTTACGGGCCGGGAGGCGCGGGGGCACCTCCCCCATGTGCGTGTAATACGTGGCCGGCTGTGTGGCCGCAGCGGGGGGCTCGGCGACCGGGTCGTTCGCATCCGGAAGCGGGGGCCCCGCGCCGTCCGCGCGGCGCCTCCGGAACCTCCGGGTGGACGCGGGGGTCGAGTGTAGGCGAGGTCGGGGGAGGGGCGGGGGCTCGTTGTCGCGCCGCGCCCGCTGAATCTTTTCCCGACAGGTCCCACCCCCCGCGCGATGCCCCCCCGGGCCGCTGGCCATGTCGTCCGGGGGAGGCCCCGCGGACCACGTCGTCCGGCGAGACGCCACGAGCCGCAGGATGGACTCGTAGTGGAGCGACGGCGCCCCGTTGCGGAGCAGATCCGCGGCCAGGGCGGCCCCGAACCAAGCCTTGATGCTCAACTCCATCCGGGCCCAGCTGGGGGCGGTCATCGTGGGGAACAGGGGGGCGGTGGTCCGACAGAAACGCTCCTGGCTGTCCACCGCGGCCCGCAGATACTCGTTGTTCAGGCTGTCGGTGGCCCAGACGCCGTACCCGGTGAGGGTCGCGTTGATGATATACTGGGCGTGGTGATGGACGATCGACAGAACCTCCACCGTGGATACGACGGTATCCACGGTCCCGTACGTACCGCCGCTCCGCTTGCCGGTCTGCCACAGGTTGGCTAGGCGCGTCAGGTGGCCCAGGACGTCGCTGACCGCCGCCCTGAGCGCCATGCACTGCATGGAGCCGGTCGTGCCGCTGGGACCCCGGTCCAGATGGCGCGCGAACGTTTCCGCGGGCGCCTCCGGGCTGCCGCCGAGCGGGAGGAACCGGCGATTGGAGGGACTCAGCCGGTGACATACGTGCTTGTCCGTCGTCCACAGCATCCAGGACGCCCACCGGTACAGCACGGAGACGTAGGCCAGGAGCTCGTTGAGCCGCAGTGCGGTGTCGGTGCTGGGGCGGCTTGGGTCCGCCGGGCGCATAAAGAACATGTACTGCTGAATCCGATGGAGGGCGTCGCGCAGGCCGGCCACGGTGGCGGCGTACTTGGCCGCCACGGCCCCGCTCTTGAACGGGGTGCGCGCCAGCAGCTTTGGCGCCAGGGTGGGCCGCAGCAGCACGTGAAGGCTGGGGTCGCAGTCGCCCACGGGGTCCTCGGGGACGTCCAGGCCGCTGGGCACCACCGTCTGCAGGTACTTCCAGTACTGCGTGAGGATGGCGCGGCTCAACTGGCCGCCGGGCAGCTCCACCTCGCCCAGCGCCTGGGTGGCGGCCGAAGCGTAGTGCCGGATGTACTCGTAGTGCGGGTCGCTGGCGAGCCCGTCCACGATCAAACTCTCGGGAACCGTGTTGTGTTGCCGCGCGGCCAACCGGACGCTGCGATCGGTGCAGGTCAGAAACGCCGGCTGCGCGTCGTCGGAGCGCTGCCGCAAGGCGCCCACGGCCGCGCTAAGGAGCCCCTCCGGGGTGGGGAGCAGACACCCGCCGAAGATGCGCCGCTCGGGAACGCCCGCGTTGTCGCCGCGGATCAGGTTGGCAGGCGTCAGGCACCGCGCCAGCCGCAGGGAGCTCGCGCCGCGCGTCCGGCGCTGCATGGTGACGCCCGTTCGGTCGGGACCCGCCGGTCGGAGTTATGCCGCGTCCAGGGCCATCGGGGCGCTTTTTATCGGGAGGAGCTTATGGGCGTGGCGGGCCTCCCAGCCCGGTCGCGCGCCTCCCCGACACGTGCGCCCGCAGGGCGGCGGCCCCCTCGTCTCCCATCAGCAGTTTCCTAAACTGGGACATGATGTCCACCACGCGGACCCGCGGGCCCAACACGGACCCGCCGCTTACGGGGGCGGGGGGGAAGGGCTCCAGGTCCTTGAGAAGAAAGGCGGGGTCTGCCGTCCCGGACACGGGGGCCCGGGGCGCTGAGGAGGCGGGGCGCAGATCCACGTGCTCCGCGGCCGCGCGGACGTCCGCCCAGAACTTGGCGGGGGTGGTGCGCGCGTACAGGGGCTGGGTCGCTCGGAGGACGCACGCGTAGCGCAGGGGGGTGTACGTGCCCACCTCGGGGGCCGTGAATCCCCCGTCAAACGCGGCCAGTGTCACGCACGCCACCACGGTGTCGGCAAAGCCCAGCAGCCGCTGCAGGACGAGCCCGGCGGCCAGAATGGCGCGCGTGGCCGCCGCGTCGTCCCGGCGCCGGTGCGCGTCCCCGCACGCCCGGGCGTACTTTAAGGTCACGGTCGCCAGGGCCGTGTGCAGCGCGTACACCGCAGCGCCCAGCACGGCGTTGAGCCCGCTGTTGGCGAGCAGCCGGCGCGCTGCGGTGTCGCCCAGCGCCTCGTGCTCGGCCCCCACGACCGCGGGGCTTCCCAGGGGCAGGGCGCGAAACAGCTCCTCCCGCGCCACGTCCGCAAAGGCGGGGTGGTGCACGTGCGGGTGCAGGCGCGCCCCCACGACCACCGAGAGCCACTGGACCGTCTGCTCCGCCATCACCGCCAGCACATCCAGCACGCGCCCCAGGAAGGCGGCCTCCCGCGTCAAAACGCACCGGACGGCGTCGGGATTGAAGCGGGCGAGCAGGGCCCCGGTGGCCAGGTACGTCATGCGGCCGGCATAGCGGGCGGCCACGCGACAGTCGCGGTCCAGCAGCGCGCGCACCCCGGGCCAGTACAGCAGGGACCCCAGCGAGCTGCGGAACACCGCGGCGTCGGGGCCGGATTGGGGGGACACTAACCCCCCCGCGCTCAGTAACGGCACGGCCGCGGCCCCGACGGGACGCAACGCCGTGAGGCTCGCGAACTGCCGCCTCAGCTCGGCCGCCCTGTCGTCCAGGTCAGACCCGCGCGCCTCCGCGTGAAGGCGCGTCCCGCACACCCACCCGTTGATGGCCAGCCGCACGACGGCATCCGCCAAAAAGCTCATCGCCTGGGCGGGGCTGGTTTTTGTTCGACGATCCGTCAGGTCAAGAATCCCATCGCCCGTGATATACCAGGCCAACGCCTCGCCCTGCTGCAGGGTTTGGCGGAAAAACACCGCGGGGTTGTCGGGGGAGGCGAAGTGCATGACCCCCACGCGCGATAACCCGAACGCGCTATCCGGACACGGGTAAAACCCGGCCGGATGCCCCAGGGCTAGGGCGGAGCGCACGGACTCGTCCCACACGGCAACCTGAGGGGCCAGTCGATCCAACGGGAATGCCGCCCGGAGCTCCGGGCCCGGCACGCGTCCCTCCAGAACCTCCACCTTGGGCGGGGAACGGGCCCCGCCGCCGTCCTCCGGCCCGACGGCTTCCGGGTAGTCGTCCTCCTCGTACTGCAGCTCCTCTAGGAACAGCGGCGACGGCGCCACCCGCGAACCGCCGACCCGCCCCAAAATAGCCCGCGCGTCGACGGGACCCAGGTATCCCCCCTGCCGGGCCTGCGGAGGACCGCGGGGAACCTCATCATCATCGTCCAGGCGACCGCGCACCGACTGGCTACGGGCCGCATCGGGCCCGGGGCGCTGCCGGGACGCTCGGCGATGGGATGTGGGCGGGGCTTCCGACGCGCGCCGTCGTCGGGCTCGCGGGCCTTCCCGTCGACGGCGCACGGGCGGCTCGTCGCCCGCCATCTCCTCCAGAGCCTCTAGCTCGCTGTCGTCATCCCCGCGGAACACCGCACGCAGGTACCCCATGAACCCCACCCCATCGCCCGCTGGCTCGTCCGCCACGGGCGAGGCGCGGGGGCGGGTGGATGCGCGCCTCCTGCGCCCCGCGGGTTCGCGAGCCGACATGGTGGCGATAGACGCGGGTTATCGGATGTCCGCTACCCCCCAAAAAAGAAAAAGACCCCACAGCGCGGATGGAGGCCGGGGTAGGTGCCGCCGGACCCCCTCGCGATGGGAATGGACGGGAGCGACGGGGCCGGCGCAAAAAAACGCAGTATCTCCCGCGAAGGCTACCCGCCGCCCCAGCCCCCGGCCAAATGCGGAAACGGTCCCGCGCTCTCGCCTTTATACGCGGGCCGCCCTGCGACACAATCACCCGTCCGTGGTTTCGAATCTACACGACAGGCCCGCAGACGCGGCTAACACACACGCCGGCAACCCAGACCCCAGTGGGTTGGTTGCGCGGTCCCGTCTCCTGGCTAGTTCTTTCCCCCACCACCAAATAATCAGACGACAACCGCAGGTTTTGTAATGTATGTGCTCGTGTTTATTGTGGATACGAACCGGTGACGGGAGGGGAAAACCCAGACGGGGGATGCGGGTCCGGTCGCGCCCCCTACCCACCGTACTCGTCAATTCCAAGGGCATCGGTAAACATCTGCTCAAACTCGAAGTCGGCCATATCCAGAGCGCCGTAGGGGGCGGAGTCGTGGGGGGTAAATCCCGGCCCCGGGGAATCCCCGTCCCCCAACATGTCCAGATCGAAATCGTCTAGCGCGTCGGCATGCGCCATCGCCACGTCCTCGCCGTCTAAGTGGAGCTCGTCCCCCAGGCTGACATCGGTCGGGGGGGCCGTCGACAGTCTGCGCGTGTGTCCCGCGGGGAGAAAGGACAGGCGCGGAGCCGCCAGCCCCGCCTCTTCGGGGGCGTCGTCGTCCGGGAGATCGAGCAGGCCCTCGATGGTAGACCCGTAATTGTTTTTCGTACGCGCGCGGCTGTACGCGTGTTCCCGCATGACCGCCTCGGAGGGCGAGGTCGTGAAGCTGGAATACGAGTCCAACTTCGCCCGAATCAACACCATAAAGTACCCAGAGGCGCGGGCCTGGTTGCCATGCAGGGTGGGAGGGGTCGTCAACGGCGCCCCTGGCTCCTCCGTAGCCGCGCTGCGCACCAGCGGGAGGTTAAGGTGCTCGCGAATGTGGTTTAGCTCCCGCAGCCGGCGGGCCTCGATTGGCACTCCCCGGACGGTGAGCGCTCCGTTGACGAACATGAAGGGCTGGAACAGACCCGCCAACTGACGCCAGCTCTCCAGGTCGCAACAGAGGCAGTCAAACAGGTCGGGCCGCATCATCTGCTCGGCGTACGCGGCCCATAGGATCTCGCGGGTCAAAAATAGATACAAATGCAAAAACAAAACACGCGCCAGACGAGCGGTCTCTCGGTAGTACCTGTCCGCGATCGTGGCGCGCAGCATTTCTCCCAGGTCGCGATCGCGTCCGCGCATGTGCGCCTGGCGGTGCAGCTGCCGGACGCTGGCGCGCAGGTACCGGTACAGGGCCGAGCAGAAGTTGGCCAACACGGTTCGATAGCTCTCCTCCCGCGCCCGTAGCTCGGCGTGGAAGAAACGAGAGAGCGCTTCGTAGTAGAGCCCGAGGCCGTCGCGGGTGGCCGGAAGCGTAGGGAAGGCCACGTCGCCGTGGGCGCGAATGTCGATTTGGGTGCGTTCGGGGACGTACGCGTCCCCCCATTCCACCACATCGCTGGGCAGCGTTGATAGGAATTTACACTCCCGGTACAGGTCGGCGTTGGTCGGTAGCGCCGAAAACAGATCCTCGTTCCAGGTATCGAGCATGGTACATAGCGCGGGGCCCGCGCTAAAGCCCAAGTCGTCGAGGAGACGGTTAAAGAGGGCGGCGGGGGGGACGGGCATGGGTGGGGAGGGCATGAGCTGGGCCTGGCTCAGGCGCCCCGTTGCGTACAGCGGGGGGGCCGCCGGGGTGTTTTTGGGACCCCCGGCCGGGCGGGGGGGCGGTGGCGAAGCGCCGTCCGCGTTCATGTCGGCAAACAGCTCGTCGACCAAGAGGTCCATTGGGTGGGGTTGATACGGGAAAGACGATATCGGGCTTTTGATGCGATCGTCCCCGCCCGCCCAGAGAGTGTGGGACGCCCGACGGCGCGGGAAGAGAAAACCCCCAAACGCGTTAGAGGACCGGACGGACCTTATGGGGGGAAGTGGGCAGCGGGAACCCCGTCCGTTCCCGAGGAATGACAGCCCGTGGTCGCCACCACGCATTTAAGCAACCCGCACGGGCCGCCCCGTACCTCGTGACTTCCCCCCACATTGGCTCCTGTCACGTGAAGGCGAACCGAGGGCGGCTGTCCAACCCACCCCCCGCCACCCAGTCCCGGTCCCCGTCGGATTGGGAAACAAAGGCACGCAACGCCAACACCGAATGAACCCCTGTTGGTGCTTTATTGTCTGGGTACGGAAGTTTTCACTCGACGGGCCGTCTGGGGCGAGAAGCGGAGCGGGCTGGGGCTCGAGGTCGCTCGGTGGGGCGCGACGCCGCAGAACGCCCTCGAGTCGCCGTGGCCGCGTCGACGTCCTGCACCACGTCTGGATTCACCAACTCGTTGGCGCGCTGAAGCAGGTTTTTGCCCTCGCAGACCGTCACGCGGATGGTGGTGATGCCAAGGAGTTCGTTGAGGTCTTCGTCTGTGCGCGGACGCGACATGTCCCAGAGCTGGACCGCCGCCATCCGGGCATGCATGGCCGCCAGGCGCCCGACCGCGGCGCAGAAGACGCGCTTGTTAAAGCCGGCCACCCGGGGGGTCCATGGCGCGTCGGGGTTTGGGGGGGCGGTGCTAAAGTGCAGCTTTCTGGCCAGCCCCTGCGCGGGTGTCTTGGATCGGGTTGGCGCCGTCGACGCGGGGGCGTCTGGGAGTGCGGCGGATTCTGGCTGGGCCGATTTCCTGCCGCGGGTGGTCTCCGCCGCCGGGGCCGCGGGGGCCTTAGTCGCCACCCGCTGGGTTCGGGGGGCCCGGGGGGCGGTGGTGGGTGTGCGTCCGGCCCCTCCGGACCCAGCGGGTGGCGGAGGCGCCCGCGCAGGCCCCGGGCCGGACAAAACCGCCCCGGAAACGGGACGCCGCGTCCGGGGGACCTCCGGGTGTTCGTCGTCTTCGGATGACGAGCCCCCGTAGAGGGCATAATCCGACTCGTCGTACTGGACGAAACGGACCTCGCCCCTCTGGCGCGAGCGTGTCTGTAGGGCGCCACGGCGGGAGGTGTCAGGCGGACTATCGGGACTCGCCATACCTGAAGACGGGGTGTAGTACAGATCCTCGTACTCATCGCGCGGAACCTCCCGCGGACCCGACTTCACGGAGCGGCGAGAGGTCATGGTTCCACGAACACGCTAGGGTCGGATGCGCGGACAATTAGGCCTGGGTTCGGACGGCGGGGGTGGTGCAGGTGTGGAGAGGTCGAGCGATAGGGGCGGCCCGGGAGAGAAGAGAGGGTCCGCAAAACCCACTGGGGATGCGTGAGTGGCCCTCTGTGGGCGGTGGGGGAGAGTCTTATAGGAAGTGCATATAACCACAACCCATGGGTCTAACCAATCCCCAGGGGCCAAGAAACAGACACGCCCCAAACGGTCTCGGTTTCCGCGAGGAAGGGGAAGTCCTGGGACACCCTCCACCCCCACCCCTCACCCCACACAGGGCGGGTTCAGGCGTGCCCGGCAGCCAGTAGCCTCTGGCAGATCTGACAGACGTGTGCGATAATACACACGCCCATCGAGGCCATGCCTACATAAAAGGGCACCAGGGCCCCCGGGGCAGACATTTGGCCAGTGTTTTGGGTCTCGCACCGCGCGCCCCCGATCCCATCGCGCCCGCCCTCCTCGCCGGGCGGCTCCCCGCGCGGGCCCGCGTCTCCCGCCGCTAAGGCGACGAGCAAGACAAACAACAGGCCCGCCCGACAGACCCTTCTGGGGGGGCCCATCGTCCCTAACAGGAAGATGAGTCAGTGGGGATCCGGGGCGATCCTTGTCCAGCCGGACAGCTTGGGTCGGGGGTACGATGGCGACTGGCACACGGCCGTCGCTACTCGCGGGGGCGGAGTCGTGCAACTGAACCTGGTCAACAGGCGCGCGGTGGCTTTTATGCCGAAGGTTAGCGGGGACTCCGGATGGGCCGTCGGGCGCGTCTCTCTGGACCTGCGAATGGCTATGCCGGCTGACTTTTGCGCGATTATTCACGCCCCCGCGCTAGCCAGCCCCGGGCACCACGTAATACTGGGTCTTATCGACTCGGGGTACCGCGGAACCGTTATGGCCGTGGTCGTAGCGCCTAAAAGGACGCGGGAATTTGCCCCCGGGACCCTGCGGGTCGACGTGACGTTCCTGGACATCCTGGCGACCCCCCCGGCCCTCACCGAGCCGATTTCCCTGCGGCAGTTCCCGCAACTGGCGCCCCCCCCTCCAACCGGGGCCGGGATACGCGAAGATCCTTGGTTGGAGGGGGCGCTCGGGGCCCCAAGCGTGACTACGGCCCTACCGGCGCGACGCCGAGGGCGGTCCCTCGTCTATGCCGGCGAGCTGACGCCGGTTCAGACGGAACACGGGGACGGCGTACGAGAAGCCATCGCCTTCCTTCCAAAACGCGAGGAGGATGCCGGTTTCGACATTGTCGTCCGTCGCCCGGTCACCGTCCCGGCAAACGGCACCACGGTCGTGCAGCCATCCCTCCGCATGCTCCACGCGGACGCCGGGCCCGCGGCCTGCTATGTGTTGGGGCGGTCGTCGCTCAACGCCCGCGGCCTCCTGGTCGTTCCTACGCGCTGGCTCCCCGGGCACGTATGTGCGTTTGTTGTTTACAACCTTACGGGGGTTCCTGTGACCCTCGAGGCCGGCGCCAAGGTCGCCCAGCTCCTGGTTGCGGGGGCGGACGCTCTTCCTTGGATCCCCCCGGACAACTTTCACGGGACCAAAGCGCTTCGAAACTACCCCAGGGGTGTTCCGGACTCAACCGCCGAACCCAGGAACCCGCCGCTCCTGGTGTTTACGAACGAGTTTGACGCGGAGGCCCCCCCGAGCGAGCGCGGGACCGGGGGTTTTGGCTCTACCGGTATTTAGCCCACAGCTTTGGGTTCGTTCCGGGCAATAAAAAACGTTTGTATCGCATCTTTCCTGTGTGTAGTTGTTTATGTTGGATGCCTGTGGGTCTATCACACCCGCCCCTCCATCCCACAAACACAAAACACACGGGTTGGATGAAAACACGCATTTATTGACCCAAAACACACGGAGCTGCTCGAGATGGGCCAGGGCGAGGTGCGGTTGGGGAGGCTGTAGGTCTGGGAACGGACACGCGGGGACACGATTCCGGTTTGGGGTCCGGGAGGGCGTCGCCGTTTCGGGCGGCAGGCGCCAGCGTAACCTCCGGGGGCGGCGTGTGGGGGTGCCCCAAGGAGGGCGCCTCGGTCACCCCAATCCCCCCCGACCGGGTTCCCCCGGCAACCCCGAAGGCGGAGAGGCCAAGGGCCCGTTCGGCGATGGCCACATCCTCCATGACCACGTCACTCTCGGCCATGCTCCGAATAGCCTGGGAGACGAGCACATCCGCGGACTTGTCAGCCGCCCCCACGGACATGTACATCTGCAGGATGGTGGCCATACACGTGTCCGCCAGGCGCCGCATCTTGTCCTGATGGGCCGCCACGGCCCCGTCGATCGTGGGGGCCTCGAGCCCGGGGTGGTGGCGCGCCAGTCGTTCTAGGTTCACCATGCAGGCGTGGTACGTGCGGGCCAAGGCGCGGGCCTTCACGAGGCGTCGGGTGTCGTCCAGGGACCCCAGGGCGTCATCGAGCGTGATGGGGGCGGGAAGTAGCGCGTTAACGACCGCCAGGGCCTCCTGCAGCCGCGGCTCCGCCTCCGAGGGCGGAACGGCCGCGCGGATCATCTCATATTGTTCCTCGGGGCGCGCTCCCCAGCCACATATAGCCCCGAGAAGAGAAGCCATCGCGGGCGGGTACTGGCCCTTGGGCGCGCGGACGCAATGGGGCAGGAAGACGGGAACCGCGGGGAGAGGCGGGCGGCCGGGACTCCCGTGGAGGTGACCGCGCTTTATGCGACCGACGGGTGCGTTATTACCTCTTCGATCGCCCTCCTCACAAACTCTCTACTGGGGGCCGAGCCGGTTTATATATTCAGCTACGACGCATACACGCACGATGGCCGTGCCGACGGGCCCACGGAGCAAGACAGGTTCGAAGAGAGTCGGGCGCTCTACCAAGCGTCGGGCGGGCTAAATGGCGACTCCTTCCGAGTAACCTTTTGTTTATTGGGGACGGAAGTGGGTGGGACCCACCAGGCCCGCGGGCGAACCCGACCCATGTTCGTCTGTCGCTTCGAGCGAGCGGACGACGTCGCCGCGCTACAGGACGCCCTGGCGCACGGGACCCCGCTACAACCGGACCACATCGCCGCCACCCTGGACGCGGAGGCCACGTTCGCGCTGCATGCGAACATGATCCTGGCTCTCACCGTGGCCATCAACAACGCCAGCCCCCGCACCGGACGCGACGCCGCCGCGGCGCAGTATGATCAGGGCGCGTCCCTACGCTCGCTCGTGGGGCGCACGTCCCTGGGACAACGCGGCCTTACCACGCTATACGTCCACCACGAGGTGCGCGTGCTTGCCGCGTACCGCAGGGCGTATTATGGAAGCGCGCAGAGTCCCTTCTGGTTTCTTAGCAAATTCGGGCCGGACGAAAAAAGCCTGGTGCTCACCACTCGGTACTACCTGCTTCAGGCCCAGCGTCTGGGGGGCGCGGGGGCCACGTACGACCTGCAGGCCATCAAGGACATCTGCGCCACCTACGCGATTCCCCACGCCCCCCGCCCCGACACCGTCAGCGCTGCGTCCCTGACCTCGTTTGCCGCCATCACGCGGTTCTGTTGCACGAGCCAGTACGCCCGCGGGGCCGCGGCGGCCGGGTTTCCGCTTTACGTGGAGCGCCGTATTGCGGCCGACGTCCGCGAGACCAGTGCGCTGGAGAAGTTCATAACCCACGATCGCAGTTGCCTGCGCGTGTCCGACCGTGAATTCATTACGTACATCTACCTGGCCCATTTTGAGTGTTTCAGCCCCCCGCGCCTAGCCACGCATCTTCGGGCCGTGACGACCCACGACCCCAACCCCGCGGCCAGCACGGAGCAGCCCTCGCCCCTGGGCAGGGAGGCCGTGGAACAATTTTTTTGTCACGTGCGCGCCCAACTGAATATCGGGGAGTACGTCAAACACAACGTGACCCCCCGGGAGACCGTCCTGGATGGCGATACGGCCAAGGCCTACCTGCGCGCTCGCACGTACGCGCCCGGGGCCCTGACGCCCGCCCCCGCGTATTGCGGGGCCGTGGACTCCGCCACCAAAATGATGGGGCGTTTGGCGGACGCCGAAAAGCTCCTGGTCCCCCGCGGGTGGCCCGCGTTTGCGCCCGCCAGTCCCGGGGAGGACACGGCGGGCGGCACGCCGCCCCCACAGACCTGCGGAATTGTCAAGCGCCTCCTGAGACTGGCCGCCACGGAACAGCAGGGCCCCACACCCCCGGCGATCGCGGCGCTTATCCGTAATGCGGCGGTGCAGACTCCCCTGCCCGTCTACCGGATATCCATGGTCCCCACGGGACAGGCATTTGCCGCGCTGGCCTGGGACGACTGGGCCCGCATAACGCGGGACGCTCGCCTGGCCGAAGCGGTCGTGTCCGCCGAAGCGGCGGCGCACCCCGACCACGGCGCGCTGGGCAGGCGGCTCACGGATCGCATCCGCGCCCAGGGCCCCGTGATGCCCCCTGGCGGCCTGGATGCCGGGGGGCAGATGTACGTGAATCGCAACGAGATATTCAACGGCGCGCTGGCAATCACAAACATCATCCTGGATCTCGACATCGCCCTGAAGGAGCCCGTCCCCTTTCGCCGGCTCCACGAGGCCCTGGGCCACTTTAGGCGCGGGGCTCTGGCTGCGGTTCAGCTCCTGTTTCCCGCGGCCCGCGTGGACCCCGACGCATATCCCTGTTATTTTTTCAAAAGCGCATGTCGGCCCGGCCCGGCGTCCGTGGGTTCCGGCAGCGGACTCGGCAACGACGACGACGGGGACTGGTTTCCCTGCTACGACGACGCCGGTGATGAGGAGTGGGCGGAGGACCCGGGCGCCATGGACACATCCCACGATCCCCCGGACGACGAGGTTGCCTACTTTGACCTGTGCCACGAAGTCGGCCCCACGGCGGAACCTCGCGAAACGGATTCGCCCGTGTGTTCCTGCACCGACAAGATCGGACTGCGGGTGTGCATGCCCGTCCCCGCCCCGTACGTCGTCCACGGTTCTCTAACGATGCGGGGGGTGGCACGGGTCATCCAGCAGGCGGTGCTGTTGGACCGAGATTTTGTGGAGGCCATCGGGAGCTACGTAAAAAACTTCCTGTTGATCGATACGGGGGTGTACGCCCACGGCCACAGCCTGCGCTTGCCGTATTTTGCCAAAATCGCCCCCGACGGGCCTGCGTGCGGAAGGCTGCTGCCAGTGTTTGTGATCCCCCCCGCCTGCAAAGACGTTCCGGCGTTTGTCGCCGCGCACGCCGACCCGCGGCGCTTCCATTTTCACGCCCCGCCCACCTATCTCGCTTCCCCCCGGGAGATCCGTGTCCTGCACAGCCTGGGTGGGGACTATGTGAGCTTCTTTGAAAGGAAGGCGTCCCGCAACGCGCTGGAACACTTTGGGCGACGCGAGACCCTGACGGAGGTCCTGGGTCGGTACAACGTACAGCCGGATGCGGGGGGGACCGTCGAGGGGTTCGCATCGGAACTGCTGGGGCGGATAGTCGCGTGCATCGAAACCCACTTTCCCGAACACGCCGGCGAATATCAGGCCGTATCCGTCCGGCGGGCCGTCAGTAAGGACGACTGGGTCCTCCTACAGCTAGTCCCCGTTCGCGGTACCCTGCAGCAAAGCCTGTCGTGTCTGCGCTTTAAGCACGGCCGGGCGAGTCGCGCCACGGCGCGGACATTCGTCGCGCTGAGCGTCGGGGCCAACAACCGCCTGTGCGTGTCCTTGTGTCAGCAGTGCTTTGCCGCCAAATGCGACAGCAACCGCCTGCACACGCTGTTTACCATTGACGCCGGCACGCCATGCTCGCCGTCCGTTCCCTGCAGCACCTCTCAACCGTCGTCTTGATAACGGCGTACGGCCTCGTGCTCGTGTGGTACACCGTCTTCGGTGCCAGTCCGCTGCACCGATGTATTTACGCGGTACGCCCCACCGGCACCAACAACGACACCGCCCTCGTGTGGATGAAAATGAACCAGACCCTATTGTTTCTGGGGGCCCCGACGCACCCCCCCAACGGGGGCTGGCGCAACCACGCCCATATCTGCTACGCCAATCTTATCGCGGGTAGGGTCGTGCCCTTCCAGGTCCCACCTGACGCCATGAATCGTCGGATCATGAACGTCCACGAGGCAGTTAACTGTCTGGAGACCCTATGGTACACACGGGTGCGTCTGGTGGTCGTAGGGTGGTTCCTGTATCTGGCGTTCGTCGCCCTCCACCAACGCCGATGTATGTTTGGCGTCGTGAGTCCCGCCCACAAGATGGTGGCCCCGGCCACCTACCTCTTGAACTACGCAGGCCGCATCGTATCGAGCGTGTTCCTGCAGTACCCCTACACGAAAATTACCCGCCTGCTCTGCGAGCTGTCGGTCCAGCGGCAAAACCTGGTTCAGTTGTTTGAGACGGACCCGGTCACCTTCTTGTACCACCGCCCCGCCATCGGGGTCATCGTAGGCTGCGAGTTGATGCTACGCTTTGTGGCCGTGGGTCTCATCGTCGGCACCGCTTTCATATCCCGGGGGGCATGTGCGATCACATACCCCCTGTTTCTGACCATCACCACCTGGTGTTTTGTCTCCACCATCGGCCTGACAGAGCTGTATTGTATTCTGCGGCGGGGCCCGGCCCCCAAGAACGCAGACAAGGCCGCCGCCCCGGGGCGATCCAAGGGGCTGTCGGGCGTCTGCGGGCGCTGCTGTTCCATCATCCTCTCGGGCATCGCAGTGCGATTGTGTTATATCGCCGTGGTGGCCGGGGTGGTGCTCGTGGCGCTTCACTACGAGCAGGAGATCCAGAGGCGCCTGTTTGATGTATGACGTCACATCCAGGCCGGCGGAAACCGTAACGGCATATGCAAATTGGAAACTGTCCTGTCTTGGGGCCCACCCACCCGACGCGTCATATGCAAATGAAAATCGGTCCCCCGAGGCCACGTGTAGCCTGGATCCCAACGACCCCGCCCATGGGTCCCAATTGGCCGTCCCGTTACCAAGACCAACCCAGCCAGCGTATCCACCCCCGCCCGGGTCCCCGCGGAAGCGGAACGGGGTATGTGATATGCTAATTAAATACATGCCACGTACTTATGGTGTCTGATTGGTCCTTGTCTGTGCCGGAGGTGGGGCGGGGGCCCCGCCCGGGGGGCGGAACGAGGAGGGGTTTGGGAGAGCCGGCCCCGGCACCACGGGTATAAGGACATCCACCACCCGGCCGGTGGTGGTGTGCAGCCGTGTTCCAACCACGGTCACGCTTCGGTGCCTCTCCCCGATTCGGGCCCGGTCGCTCGCTACCGGTGCGCCACCACCAGAGGCCATATCCGACACCCCAGCCCCGACGGCAGCCGACAGCCCGGTCATGGCGACTGACATTGATATGCTAATTGACCTCGGCCTGGACCTCTCCGACAGCGATCTGGACGAGGACCCCCCCGAGCCGGCGGAGAGCCGCCGCGACGACCTGGAATCGGACAGCAGCGGGGAGTGTTCCTCGTCGGACGAGGACATGGAAGACCCCCACGGAGAGGACGGACCGGAGCCGATACTCGACGCCGCTCGCCCGGCGGTCCGCCCGTCTCGTCCAGAAGACCCCGGCGTACCCAGCACCCAGACGCCTCGTCCGACGGAGCGGCAGGGCCCCAACGATCCTCAACCAGCGCCCCACAGTGTGTGGTCGCGCCTCGGGGCCCGGCGACCGTCTTGCTCCCCCGAGCAGCACGGGGGCAAGGTGGCCCGCCTCCAACCCCCACCGACCAAAGCCCAGCCTGCCCGCGGCGGACGCCGTGGGCGTCGCAGGGGTCGGGGTCGCGGTGGTCCCGGGGCTGCCGATGGTTTGTCGGACCCCCGCCGGCGTGCCCCCAGAACCAATCGCAACCCTGGGGGACCCCGCCCCGGGGCGGGGTGGACGGACGGCCCCGGCGCCCCCCATGGCGAGGCGTGGCGCGGCAGTGAGCAGCCCGACCCACCCGGAGGCCAGCGGACACGGGGCGTGCGCCAAGCACCCCCCCCGCTAATGACGCTGGCGATTGCCCCCCCGCCCGCGGACCCCCGCGCCCCGGCCCCGGAGCGAAAGGCGCCCGCCGCCGACACCATCGACGCCACCACGCGGTTGGTCCTGCGCTCCATCTCCGAGCGCGCGGCGGTCGACCGCATCAGCGAGAGCTTTGGCCGCAGCGCACAGGTCATGCACGACCCCTTTGGGGGGCAGCCGTTTCCCGCCGCGAATAGCCCCTGGGCCCCGGTGCTGGCGGGCCAAGGAGGGCCCTTTGACGCCGAGACCAGACGGGTCTCCTGGGAAACCTTGGTCGCCCACGGCCCGAGCCTCTATCGCACTTTTGCCGGCAATCCTCGGGCCGCATCGACCGCCAAGGCCATGCGCGACTGCGTGCTGCGCCAAGAAAATTTCATCGAGGCGCTGGCCTCCGCCGACGAGACGCTGGCGTGGTGCAAGATGTGCATCCACCACAACCTGCCGCTGCGCCCCCAGGACCCCATTATCGGGACGGCCGCGGCTGTGCTGGATAACCTCGCCACGCGCCTGCGGCCCTTTCTCCAGTGCTACCTGAAGGCGCGAGGCCTGTGCGGCCTGGACGAACTGTGTTCGCGGCGGCGTCTGGCGGACATTAAGGACATTGCATCCTTCGTGTTTGTCATTCTGGCCAGGCTCGCCAACCGCGTCGAGCGTGGCGTCGCGGAGATCGACTACGCGACCCTTGGTGTCGGGGTCGGAGAGAAGATGCATTTCTACCTCCCCGGGGCCTGCATGGCGGGCCTGATCGAAATCCTAGACACGCACCGCCAGGAGTGTTCGAGTCGTGTCTGCGAGTTGACGGCCAGTCACATCGTCGCCCCCCCGTACGTGCACGGCAAATATTTTTATTGCAACTCCCTGTTTTAGGTACAATAAAAACAAAACATTTCAAACAAATCGCCCCTCGTGTTGTCCTTCTTTGCTCATGGCCGGCGGGGCGTGGGTCACGGCAGATGGCGGGGGTGGGCCCGGCGTACGGCCTGGGTGGGCGGAGGGAACTAACCCAACGTATAAATCCGTCCCCGTTCCAAGGCCGGTGTCATAGTGCCCTTAGGAGCTTCCCGCCCGGGCGCATCCCCCCTTTTGCACTATGACAGCGACCCCCCTCACCAACCTGTTCTTACGGGCCCCGGACATAACCCACGTGGCCCCCCCTTACTGCCTCAACGCCACCTGGCAGGCCGAAACGGCCATGCACACCAGCAAAACGGACTCCGCTTGCGTGGCCGTGCGGAGTTACCTGGTCCGCGCCTCCTGTGAGACCAGCGGCACAATCCACTGCTTTTTCTTTGCGGTATACAAGGACACCCACCACACCCCTCCGCTGATTACCGAGCTCCGCAACTTTGCGGACCTGGTTAACCACCCGCCGGTCCTACGCGAACTGGAGGATAAGCGCGGGGTGCGGCTGCGGTGTGCGCGGCCGTTTAGCGTCGGGACGATTAAGGACGTCTCTGGGTCCGGCGCGTCCTCGGCGGGAGAGTACACGATAAACGGGATCGTGTACCACTGCCACTGTCGGTATCCGTTCTCAAAAACATGCTGGATGGGGGCCTCCGCGGCCCTACAGCACCTGCGCTCCATCAGCTCCAGCGGCATGGCCGCCCGCGCGGCAGAGCATCGACGCGTCAAGATTAAAATTAAGGCGTGATCTCCAACCCCCCCATGAATGTGTGTAACCCCCCCCAAAAAAATAAAGAGCCGTAACCCAACCAAACCAGGCGTGGTGTGAGTTTGTGGACCCAAAGCCCTCAGAGACAACGCGACAGGCCAGTATGGACCGTGATACTTTTATTTATTAACTCACAGGGGCGCTTACCGCCACAGGAATACCAGAATAATGACCACCACAATCGCGACCACCCCAAATACAGCATGGCGCCACACCACGCCACAACAGCCCTGTCGCCGGTATGGGGCATGATCAGACGAGCCGCGCGCCGCGCGTTGGGCCCTGTACAGCTCGCGCGAATTGACCCTAGGAGGCCGCCACGCGCCCGAGTTTTGCGTTCGTCGCTGGTCGTCGGGCGCCAAAGCCCCGGACGGCTGTTCGGTCGAACGAACGGCCACGACAGTGGCATAGGTTGGGGGGTGGTCCGACATAGCCTCGGCGTACGTCGGGAGGCCCGACAAGAGGTCCCTTGTGATGTCGGGTGGGGCCACAAGCCTGGTTTCCGGAAGAAACAGGGGGGTTGCCAATAACCCGCCAGGGCCAAAACTCCGGCGCTGCGCACGTCGTTCGGCGCGGCGCCGGGCGCGCCGAGCGGCTCGCTGGGCGGCTTGGCGTGAGCGGCCCCGCTCCGACGCCTCGCCCTCTCCGGAGGAGGTTGGCGGAATTGGCACGGACAACAGGGGCCCAGCAGAGTACGGTGGAGGTGGGTCCGTGGGGGTGTCCAGATCAATAACGACAAACGGCCCCTCGTTCCTACCAGACAAGCTATCGTAGGGGGGCGGGGGATCAGCAAACGCGTTCCCCGCGCTCCATAAACCCGCGTCGGGTTGCGCCGCCTCCGAAGCCATGGATGCGCCCCAAAGCCACGACTCCCGCGCGCTAGGTCCTTGGGGTAATGGAAAAGGCCCTACTCCCCATCCAAGCCAGCCAAGTTAACGGGCTACGCCTTCGGGAATGGGACTGGCACCCCGGCGGATTTTGTTGGGCTGGCATGCGTCGCCCAACCGAGGGCCGCGTCCACGGGACGCGCCTGGGCCGCGGATGGGCGGGCCTACTTGGTCTCCCGCCCCCCCCCCCCCCCCCCGAACCGCCCCGCCGGCTTTGCCCCCCTTTGATCCCCTGCTACCCCCAACCCGTGCTGGTGGTGCGGGTTGGGGGGGGATGTGGGCGGGGGTGCGCGGGAGGTGTCGGTGGTGGTGGTGGTGGTGGTAGTAGGAATGGTGGTGAGGGGGGGGGGGCGCTGGTTGGTCAAAAAAGGGAGGGACGGGGGCCGGCAGACCGACGGCGACAACGCTCCCCGGCGGCCGGGTCGCGGCTCTTACGAGCGGCCCGGCCCGCGCTCCCACCCCCCGGGCCGTGTCCTTGCTTTCCCCCCGTCTCCCGTTATCTCCAGCGCCCCGTCCGGCGCGCCGCCCCCCGCCGCTAAACCCCATCCCGCCCCCGGGACCCCACATATAAGCCCCCAGCCACACGCAAGAACAGACACGCAGAACGGCTGTGTTTATTTAAATAAACCAATGTCGGAATAAACAAACACAAACACCCGCGACGGGGGGACGGAGGGGACGGAGGGAGGGGGTGACGGGGGACGGGAACAGACACAAAAACAACCACAAAAAACAACCACCCACCGACACCCCCACCCCAGTCTCCTCGCCTTCTCCCACCCACCCCACGCCCCCACTGAGCCCGGTCGATCGACGAGCACCCCCGCCCACGCCCCCGCCCCTGCCCCGGCGACCCCCGGCCCGCACGATCCCGACAACAATAACAACCCCAACGGAAAGCGGCGGGGTGTTGGGGGAGGCGAGGAACAACCGAGGGGAACGGGGGATGGAAGGACGGGAAGTGGAAGTCCTGATACCCATCCTACACCCCCCTGCCTTCCACCCTCCGGCCCCCCGCGAGTCCACCCGCCGGCCGGCTACCGAGACCGAACACGGCGGCCGCCGCAGCCGCCGCAGCCGCCGCCGACACCGCAGAGCCGGCGCGCGCACTCACAAGCGGCAGAGGCAGAAAGGCCCAGAGTCATTGTTTATGTGGCCGCGGGCCAGCAGACGGCCCGCGACACCCCCCCCCCGCCCGTGTGGGTATCCGGCCCCCCGCCCCGCGCCGGTCCATTAAGGGCGCGCGTGCCCGCGAGATATCAATCCGTTAAGTGCTCTGCAGACAGGGGCACCGCGCCCGGAAATCCATTAGGCCGCAGACGAGGAAAATAAAATTACATCACCTACCCACGTGGTGCTGTGGCCTGTTTTTGCTGCGTCATCTCAGCCTTTATAAAAGCGGGGGCGCGGCCGTGCCGATCGCGGGTGGTGCGAAAGACTTTCCGGGCGCGTCCGGGTGCCGCGGCTCTCCGGGCCCCCCTGCAGCCGGGGCGGCCAAGGGGCGTCGGCGACATCCTCCCCCTAAGCGCCGGCCGGCCGCTGGTCTGTTTTTTCGTTTTCCCCGTTTCGGGGGTGGTGGGGGTTGCGGTTTCTGTTTCTTTAACCCGTCTGGGGTGTTTTTCGTTCCGTCGCCGGAATGTTTCGTTCGTCTGTCCCCTCACGGGGCGAAGGCCGCGTACGGCCCGGGACGAGGGGCCCCCGACCGCGGCGGTCCGGGCCCCGTCCGGACCCGCTCGCCGGCACGCGACGCGAAAAAGGCCCCCCGGAGGCTTTTCCGGGTTCCCGGCCCGGGGCCTGAGATGAACACTCGGGGTTACCGCCAACGGCCGGCCCCCGTGGCGGCCCGGCCCGGGGCCCCGGCGGACCCAAGGGGCCCCGGCCCGGGGCCCCACAACGGCCCGGCGCATGCGCTGTGGTTTTTTTTTCCTCGGTGTTCTGCCGGGCTCCATCGCCTTTCCTGTTCTCGCTTCTCCCCCCCCCCTTCTTCACCCCCAGTACCCTCCTCCCTCCCTTCCTCCCCCGTTATCCCACTCGTCGAGGGCGCCCCGGTGTCGTTCAACAAAGACGCCGCGTTTCCAGGTAGGTTAGACACCTGCTTCTCCCCAATAGAGGGGGGGGACCCAAACGACAGGGGGCGCCCCAGAGGCTAAGGTCGGCCACGCCACTCGCGGGTGGGCTCGTGTTACAGCACACCAGCCCGTTCTTTTCCCCCCCTCCCACCCTTAGTCAGACTCTGTTACTTACCCGTCCGACCACCAACTGCCCCCTTATCTAAGGGCCGGCTGGAAGACCGCCAGGGGGTCGGCCGGTGTCGCTGTAACCCCCCACGCCAATGACCCACGTACTCCAAGAAGGCATGTGTCCCACCCCGCCTGTGTTTTTGTGCCTGGCTCTCTATGCTTGGGTCTTACTGCCTGGGGGGGGGGAGTGCGGGGGAGGGGGGGTGTGGAAGGAAATGCACGGCGCGTGTGTACCCCCCCTAAAGTTGTTCCTAAAGCGAGGATACGGAGGAGTGGCGGGTGCCGGGGGACCGGGGTGATCTCTGGCACGCGGGGGTGGGAAGGGTCGGGGGAGGGGGGGATGGAGTACCGGCCCACCTGGCCGGCGCGGGTGCGCGTGCCTTTGCACACCAACCCCACGTCCCCCGGCGGTCTCTAAGAAGCACCGCCCCCCCTCCTTCATACCACCGAGCATGCCTGGGTGTGGGTTGGTAACCAACACGCCCATCCCCTCGTCTCCTGTGATTCTCTGGCTGCACCGCATTCTTGTTTTCTAACTATGTTCCTGTTTCTGTCTCCCCCCCCCCCACCCCTCCGCCCCACCCCCCAACACCCACGTCTGTGGTGTGGCCGACCCCCTTTTGGGCGCCCCGTCCCGCCCCGCCACCCCTCCCATCCTTTGTTGCCCTATAGTGTAGTTAACCCCCCCCGCCCTTTGTGGCGGCCAGAGGCCAGGTCAGTCCGGGCGGGCAGGCGCTCGCGGAAACTTAACACCCACACCCAACCCACTGTGGTTCTGGCTCCATGCCAGTGGCAGGATGCTTTCGGGGATCGGTGGTCAGGCAGCCCGGGCCGCGGCTCTGTGGTTAACACCAGAGCCTGCCCAACATGGCACCCCCACTCCCACGCACCCCCACTCCCACGCACCCCCACTCCCACGCACCCCCACTCCCACGCACCCCCACTCCCACGCACCCCCACTCCCACGCACCCCCACTCCCACGCACCCCCACTCCCACGCACCCCCACTCCCACGCATCCCCGCGATACATCCAACACAGACAGGGAAAAGATACAAAAGTAAACCTTTATTTCCCAACAGACAGCAAAAATCCCCTGAGTTTTTTTTTATTAGGGCCAACACAAAAGACCCGCTGGTGTGTGGTGCCCGTGTCTTTCACTTTTCCCCTCCCCGACACGGATTGGCTGGTGTAGTGGGCGCGGCCAGAGACCACCCAGCGCCCGACCCCCCCCTCCCCACAAACACGGGGGGCGTCCCTTATTGTTTTCCCTCGTCCCGGGTCGACGCCCCCTGCTCCCCGGACCACGGGTGCCGAGACCGCAGGCTGCGGAAGTCCAGGGCGCCCACTAGGGTGCCCTGGTCGAACAGCATGTTCCCCACGGGGGTCATCCAGAGGCTGTTCCACTCCGACGCGGGGGCCGTCGGGTACTCGGGGGGCATCACGTGGTTACCCGCGGTCTCGGGGAGCAGGGTGCGGCGGCTCCAGCCGGGGACCGCGGCCCGCAGCCGGGTCGCCATGTTTCCCGTCTGGTCCACCAGGACCACGTACGCCCCGATGTTCCCCGTCTCCATGTCCAGGATGGGCAGGCAGTCCCCCGTGATAGTCTTGTTCACGTAAGGCGACAGGGCGACCACGCTAGAGACCCCCGAGATGGGCAGGTAGCGCGTGAGGCCGCCCGCGGGGACGGCCCCGGAAGTCTCCGCGTGGCGCGTCTTCCGGGCACACTTCCTCGGCCCCCGCGGCCCAGAAGCAGCGCGGGGGCCGAGGGAGGTTTCCTCTTGTCTCCCTCCCAGGGCACCGACGGCCCCGCCCGGTCGGAGGGGGGGTGCGTCGCCGCCCTCTTGGCCCCTGCCGGCGCGAGGGGGGGACGCGTGGACTGGGGGGAGGGGTTTTCCTGGCCCGACCCGCGCCTCTTCCTCGGACGCACCGCCGCCTCCTGCTCGACAGAGGCGGCGGAGGGGAGCGGGGCGGCGCCGGAGGGGGCGGCGCCGCGGGAGGGCCCGTGCCCACCCTCCACGCCCGGCCCCCCCGAGCCGCGCGCCACCGTCGCACGCGCCCGGCACAGACTCTGTTCTTGGTTCGCGGCCTGAGCCAGGGACGAGTGCGACTGGGGCACACGGCGCGCGTCCGCGGGGCGGGCGGCCGGCTCCGCCCCGGGGGCCGGGGCGCGGGGGCCGGGCCCCGGAGGCGGCGCTCGCACGCACGGGGCCACGGCCGCGCGGGGGCGCGCGGGTCCCGACGCGGCCGCGGACGCGGGGGGCCCGGGGCGGGGGGCGGAGCCTGGCATGGGCGCCGCGGGGGGCCTGTGGGGAGAGGCCGGGGGGGAGTCGCTGATCACTATGGGGTCTCTGTTGTTTGCAAGGGGGGCGGGTCTGTTGACAAGGGGGCCCGTCCGGCCCCTCGGCCGCCCCGCCTCCGCTTCAACAACCCCAACCCCAACCCCAACCCCCCCGGAGGGGCCAGACGCCCCCCGCGGCGCCGCGGCTCGCGACTGGCGGGAGCCGCCGCCGCCGCTGCTGTTGGTGGTGGTGTTGGTGTTACTGCTGCCGTGTGGCCCGATGGGCGCCGAGGGGGGCGCTGTCCGAGCCGCGGCCGGCTGGGGGGCTGCGTGAGACGCCCCGCCCGTCACGGGGGGCGCGGCGGCGCCTCTGCGTGGGGGGGCGCGGGGCGTCCGGCGGGGGGCGGGCGGTACGTAGTCTGCTGCAAGAGACAACGGGGGGCGCGATCAGGTTACGCCCCCTCCCCGGCCCGCCCTTTCCTCGCCCGCCCGCCTATTCCTCCCTCCCCCCCCCTCCTCCTCCTCCTCCCCCAGGGTCCTTGCCGCCCCCCGCCTCACCGTCGTCCAGGTCGTCGTCATCCTCGTCCGTGGTGGGCTCCGGGTGGGTGGGCGACAGGGCCCTCACCGTGTGCCCCCCCAGGGTCAGGTACCGCGGGGCGAACCGCTGATTGCCCGTCCAGATAAAGTCCACGGCCGTGCCCGCCCTGACGGCCTCCTCGGCCTCCATGCGGGTCTGGGGGTCGTTCACGATCGGGATGGTGCTGAACGACCCGCTGGGCGTCACGCCCACTATCAGGTACACCAGCTTGGCGTTGCACAGCGGGCAGGTGTTGCGCAATTGCATCCAGGTTTTCATGCACGGGATGCAGAAGCGGTGCATGCACGGGAAGGTGTCGCAGCGCAGGTGGGGCGCGATCTCATCCGTGCACACGGCGCACACGTCGCCCTCGTCGCTCCCCCCGTCCTCTCGAGGGGGGGCGCCCCCGCAACTGCCGGGGTCTTCCTCGCGGGGGGGGCTCCCCCCCGAGACCGCCCCCCCATCCACGCCCTGCGGCCCCAGCAGCCCCGTCTCGAACAGTTCCGTGTCCGTGCTGTCCGCCTCGGAGGCGGAGTCGTCGTCATGGTGGTCGGCGTCCCCCCGCCCCCCCACTTCGGTCTCCGCCTCAGAGTCGCTGCTGTCCGGCAGGTCTCGGTCGCAGGGAAACACCCAGACATCCGGGGCGGGCTAAGGGGAAAAAAGGGGGGCGGGTAAGAATGGGGGGGGATTTCCCGCGTCAATCAGCACCCACGAGTTCCCCCTCTCCCCCCCCCGCCTCACAAAGTCCTGCCCCCCTGCTGGCCTCGGAAGAGGGGGGAGAAAGGGGTCTGCAACCAAAGGTGGTCTGGGTCCGTCCTTTGGATCCCGACCCCTCTTCTTCCCTCTTCTCCCGCCCTCCAGACGCACCGGAGTCGGGGGTCCCACGGCGTCCCCCAAATATGGCGGGCGGCTCCTCCCCACCCCCCTAGATGCGTGTGAGTAAGGGGGGCCTGCGTATGAGTCAGTGGGGACCACGCCCCCAACACGGCGACCCCGGTCCCTGTGTGTTTGTTGTGGGGGCGTGTCTCTGTGTATGAGTCAGGGGGTCCCACGGCGACCCCGGGCCCTGCGTCTGAGTCAAAGGGGCCATGTGTATGTGTTGGGGGTCTGTATATATAAAGTCAGGGGGTCACATGGCGACCCCCAACAGGGCGACCCCGGTCCCTGTATATATAGGGTCAGGGGGTTCCGCACCCCCTAACATGGCGCCCCCGGTCCCTGTATATATAGTGTCACGGGGTTCCACGCCCCCTAACATGGCGCCCCAACATGGCGCCCGGCTCCCGTGTATGAGTGGGGGTCCCCCAACATGGCGGCCGGTTCCAGTGTAAGGGTCGGGGGTCCCCCAACATGGCGCCCCCCAATATGGCGCCCCCCAATATGGCGCCCCAGACATGGCGCCCGGCCCCTCACCTCGCGCTGGGGGCGGCCCTCAGGCCGGCGGGTACTCGCTCCGGGGCGGGGCTCCATGGGGGTCGTATGCGGCTGGAGGGTCGCGGACGGAGGGTCCCTGGGGGTCGCAACGTAGGCGGGGCTTCTGTGGTGATGCGGAGAGGGGGCGGCCCGAGTCTGCCTGGCTGCTGCGTCTCGCTCCGAGTGCCGAGGTGCAAATGCGACCAGACTGTCGGGCCAGGGCTAACTTATACCCCACGCCTTTCCCCTCCCCAAAGGGGCGGCAGTGACGATTCCCCCAATGGCCGCGCGTCCCAGGGGAGGCAGGCCCACCGCGGGGCGGCCCCGTCCCCGGGGACCAACCCGGCGCCCCCAAAGAATATCATTAGCATGCACGGCCCGGCCCCCGATTTGGGGGCCCAACCCGGTGTCCCCCAAAGAACCCCATTAGCATGCCCCTCCCGCCGACGCAACAGGGGCTTGGCCTGCGTCGGTGCCCCGGGGCTTCCCGCCTTCCCGAAGAAACTCATTACCATACCCGGAACCCCAGGGGACCAATGCGGGTTCATTGAGCGACCCGCGGGCCAATGCGCGAGGGGCCGTGTGTTCCGCCAAAAAAGCAATTAGCATAACCCGGAACCCCAGGGGAGTGGTTACGCGCGGCGCGGGAGGCGGGGAATACCGGGGTTGCCCATTAAGGGCCGCGGGAATTGCCGGAAGCGGGAAGGGCGGCCGGGGCCGCCCATTAATGAGTTTCTAATTACCATACCGGGAAGCGGAACAAGGCCTCTTGCAAGTTTTTAATTACCATACCGGGAAGTGGGCGGCCCGGCCCATTGGGCGGTAACTCCCGCCCAATGGGCCGGGCCCCGAAGACTCGGCGGACGCTGGTTGGCCGGGCCCCGCCGCGCTGGCGGCCGCCGATTGGCCAGTCCCGCCCCCGAGGCGGGCCCGCCCTGTGAGGGCGGGCTGGCTCCAAGCGTATATATGCGCGGCTCCTGCCATCGTCTCTCCGGAGAGCGGCTTGGTGCGGAGCTCCCGGGAGCTCCGCGGAAGACCCAGGCCGCCTCGGGTGTAACGTTAGACCGAGTTCGCCGGGCCGGCTCCGCGGGCCAGGGCCCGGGCACGGGCCTCGGGCCCCAGGCACGGCCCGATGACCGCCTCGGCCTCCGCCACCCGGCGCCGGAACCGAGCCCGGTCGGCCCGCTCGCGGGCCCACGAGCCGCGGCGCGCCAGGCGGGCGGCCGAGGCCCAGACCACCAGGTGGCGCACCCGGACGTGGGGCGAGAAGCGCACCCGCGCGGGGGTCGCGGGGGTCGCGGGGGTCGCGGGGGTCGCGGGGGTCGCGGGGGGCTCCGGCGCCCCCTCCCCGCCCGCGCGTCGCAGGCGCAGGCGCGCCAGGTGCTCCGCGGTGACGCGCAGGCGGAGGGCGAGGCGCGGCGGAAGGCGGAAGGGGCGCGAGGGGGGGTGGGAGGGGTCAGCCCCGCCCCCCGGGCCCACGCCGGGCGGTGGGGGCCGGGGCCGGGGGGCGGCGGCGGTGGGCCGGGCCTCTGGCGCCGGCTCGGGCGGGGGGCTGTCCGGCCAGTCGTCGTCATCGTCGTCGTCGGACGCGGACTCGGGAACGTGGAGCCACTGGCGCAGCAGCAGCGAACAAGAAGGCGGGGGCCCACCGGCGGGGGGCGGCGGCGGGGCGGCCGCGGGCGCGCTCCTGACCGCGGGTTCCGAGTTGGGCGTGGAGGTTACCTGGGACTGTGCGGTTGGGACGGCGCCCGTGGGCCCGGGCGGCCGGGGGCGGCGGGGGCCGCGATGGCGGCGGCGGCGGGCCATGGAGACAGAGAGCGTGCCGGGGTGGTAGAGTTTGACAGGCAAGCATGTGCGTGCAGAGGCGAGTAGTGCTTGCCTGTCTAACTCGCTAGTCTCGGCCGCGGGGGGCCCGGGCTGCCCGCCGCCGCCGCTTTAACCGCCCGGACCGCCGCCCGCCTTTTTTGCGCGCGCGCGCGCCCGCGGGGGGCCCGGGCTGCCACAGGTGAAACCAACAGAGCACGGCGCACTCCGCACGTCACACGTCACGTCATCCACCACACCTGCCCAACAACACAACTCACAGCGACAACTCACCGCGCAACAACTCCTGTTCCTCATCCACACGTCACCGCGCACCTCCCGCTCCTCCAGACGTACCCCGGCGCAACACACCGCTCCTGCTACACACCACCGCCCCTCCCCAGCCCCAGCCCTCCCCGGCCCCAGCCCTCCCCGGCCCCAGCCCTCCCCGGCCCCAGCCCTCCCCGGCCCCAGCCCTCCCCGGCCCCAGCCCTCCCCGGCCCCAGCCCTCCCCGGCCCCAGCCCTCCCCGGCCGCGTCCCGCGCTCCCTCGGGGGGGTTCGGGCATCTCTACCTCAGTGCCGCCAATCTCAGGTCAGAGATCCAAACCCTCCGGGGGCGCCCGCGCACCACCACCGCCCCTCGCCCCCTCCCGCCCCTCGCCCCCTCCCGCCCCTCGCCCCCTCCCGCCCCTCGCCCCCTCCCGCCCCTCGCCCCCTCCCGCCCCTCGCCCCCTCCCGCCCCTCGCCCCCTCCCGCCCCTCGCCCCCTCCCGCCCCTCGCCCCCTCCCGCCCCTCGCCCCCTCCCGCCCCTCGCCCCCTCCCGCCCCTCGCCCCCTCCCGCCCCTCGCCCCCTCCCGCCCCTCGCCCCCTCCCGCCCCTCGCCCCCTCCCGCCCCTCGCCCCCTCCCGCCCCTCGCCCCCTCCCGCCCCTCGCCCCCTCCCGCCCCTCGCCCCCTCCCGCCCCTCGAATAAACAACGCTACTGCAAAACTTAATCAGGTTGTTGCCGTTTATTGCGTCTTCGGGTCTCACAAGCGCCCCGCCCCGTCCCGGCCCGTTACAGCACCCCGTCCCCCTCGAACGCGCCGCCGTCGTCTTCGTCCCAGGCGCCTTCCCAGTCCACAACTTCCCGCCGCGGGGGCGTGGCCAAGCCCGCCTCCGCCCCCAGCACCTCCACGGCCCCCGCCGCCGCCAGCACGGTGCCGCTGCGGCCCGTGGCCGAGGCCCAGCGAATCCCGGGCGGCGCCGGCGGCAGGGCCCCCGGGCCGTCGTCGTCGCCGCGCAGCACCAGCGGGGGGGCGTCGTCGTCGGGCTCCAGCAGGGCGCGGGCGCAAAAGTCCCTCCGCGGCCCGCGCCACCGGGCCGGGCCGGCGCGCACCGCCTCGCGCCCCAGCGCCACGTACACGGGCCGCAGCGGCGCGCCCAGGCCCCAGCGCGCGCAGGCGCGGTGCGAGTGGGCCTCCTCCTCGCAGAAGTCCGGCGCGCCGGGCGCCATGGCGTCGGTGGTCCCCGAGGCCGCCGCCCGGCCGTCCAGCGCCGGCAGCACGGCCCGGCGGTACTCGCGCGGGGACATGGGCACCGGCGTGTCCGGGCCGAAGCGCGTGCGCACGCGGTAGCGCACGTTGCCGCCGCGGCACAGGCGCAGCGGCGGCGCGTCGGGGTACAGGCGCGCGTGCGCGGCCTCCACGCGCGCGAAGACCCCCGGGCCGAACACGCGGCCCGAGGCCAGCACCGTGCGGCGCAGGTCCCGCGCCGCCGGCCAGCGCACGGCGCACTGCACGGCGGGCAGCAGCTCGCACGCCAGGTAGGCGTGCTGCCGCGACACCGCGGGCCCGTCGGCGGGCCAGTCGCAGGCGCGCACGGTGTTGACCACGATGAGCCGCCGGTCGCCGGCGCTGGCGAGCAGCCCCAGAAACTCCACGGCCCCGGCGAAGGCCAGGTCCCGCGTGGACAGCAGCAGCACGCCCTGTGCGCCCAGCGCCGACACGTCGGGGGCGCCGGTCCAGTTGCCCGCCCAGGCGGCCGTGTCCGGCCCGCACAGCCGGTTGGCCAGGGCCGCCAGCAGGCAGGACAGCCCGCCGCGCTCGGCGGACCACTCCGGCGGCCCCCCCGAGGCCCCGCCGCCGGCCAGGTCCTCGCCCGGCAGCGGCGAGTACAGCACCACCACGCGCACGTCCTCGGGGTCGGGGATCTGGCGCATCCAGGCCGCCATGCGGCGCAGCGGGCCCGAGGCGCGCAGGGGGCCAAAGAGGCGGCCCCCGGCGGCCCCGTGGGGGTGGGGGTTATCGTCGTCGTCGCCGCCGCCGCACGCGGCCTGGGCGGCGGGGGCGGGCCCGGCGCACCGCGCGGCGATCGAGGCCAGGGCCCGCGGGTCAAACATGAGGGCCGGTCGCCAGGGGACGGGGAACAGCGGGTGGTCCGTGAGCTCGGCCACGGCGCGCGGGGAGCAGTAGGCCTCCAGGGCGGCGGCCGCGGGCGCCGCCGTGTGGCTGGGCCCCGGGGGCTGCCGCCGCCAGCCGCCCAGGGGGTCGGGGCCCTCGGCGGGCCGGCGCGACAGCGCCACGGGGCGCGGGCGGGCCTGCGCCGCGGCGGCCCGGGGCGCCGCGGGCTGGGCGGGGGCGGGCTCGGGCCCCGGGGGCGTGGAGGGGGGCGCGGGCGCGGGGAGGGGGGCGCGGGCGTCCGAGCCGGGGGCGTCCGCGCCGCTCTTCTTCGTCTTCGGGGGTCGCGGGCCGCCGCCTCCGGGCGGCCGGGCCGGGCCGGGACTCTTGCGCTTGCGCCCCTCCCGCGGCGCGGCGGAGGCGGCGGCGGCCGCCAGCGCGTCGGCGGCGTCCGGTGCGCTGGCCGCCGCCGCCAGCAGGGGGCGCAGGCTCTGGTTGTCAAACAGCAGGTCCGCGGCGGCGGCGGCCGCGGAGCTCGGCAGGCGCGGGTCCCGCGGCAGCGCGGGGCCCAGGGCCCCGGCGACCAGGCTCACGGCGCGCACGGCGGCCACGGCGGCCTCGCTGCCGCCGGCCACGCGCAGGTCCCCGCGCAGGCGCATGAGCACCAGCGCGTCGCGCACGAACCGCAGCTCGCGCAGCCACGCGCGCAGGCGGGGCGCGTCGGCGTGCGGCGGCGGCGGGGAAGCGGGGCCCGCGGGTCCCTCCGGCCGCGGGGGGCTGGCGGGCCGGGCCCCGGCCAGCCCCGGGACGGCCGCCAGGTCGCCGTCGAAGCCCTCGGCCAGCGCCTCCAGGATCCCGCGGCAGGCGGCCAGGCACTCGACGGCCACGCGGCCGGCCTGGGCGCGGCGCCCGGCGTCGTCGTCGGCGTCGGCGTGGCGGGCGGCGTCGGGGTCGTCGCCCCCCGCGGGGGAGGCGGGCGCGGCGGACAGCCGCCCCAGGGCGGCGAGGATCCCCGCGGCGCCGTACCCGGCGGGCACCGCGCGCTCGCCCGGTGCGGCGGCGGCGACGGCGGCGACCCCCTCGTCATCTGCGCCGGCGCCGGGGCTCCCCGCGGCCCCCGTCAGCGCCGCGTTCTCGCGCGCCAACAGGGGCGCGTAGGCGCGGCGCAGGCTGGTCAGCAGGAAGCCCTTCTGCGCGCGGTCGTATCGGCGGCTCATGGCCACGGCGGCCGCCGCGTGCGCCAGGCCCCAGCCGAAGCGGCCGGCCGCCATGGCGTAGCCCAGGTGGGGCACGGCCCGCGCCACGCTGCCGGTGATGAAGGAGCTGCTGTTGCGCGCGGCGCCCGAGATCCGGAAGCAGGCCTGGTCCAGCGCCACGTCCCCGGGGACCACGCGCGGGTTCTGGAGCCACCCCATGGCCTCCGCGTCCGGGGTGTACAGCAGCCGCGTGATCAGGGCGTACTGCTGCGCGGCGTCGCCCAGCTCGGGCGCCCACACGGCCGCCGGGGCGCCCGAGGCCTCGAACCGGCGTCGCGCCTCCTCCGCCTCGGGCGCCCCCCAGAGGCCCGGGCGGCTGTCGCCCAGGCCGCCGTACAGCACCCGCCCCGGGGGCGGGGGCCCGGCGCCGGGCCACGGCTCCCCGCTGACGTACCCGTCGCGATAGCGCGCGTAGAAGGCGCCGGAGGCCGCGTCGGCGTCCAGCTCGACCCGCCGGGGCTGCCCGGCCGTGAAGCGGCCCGTGGCGTCGCGGCCGGCCACCGCCGCGCGGGCCCGGCGGCGCTCGATGCGGCCCGCGGAGGCCGCGGGGGTCCTCGCCGCCGCCCGGGGCTTGGGCGCGGCCTCGGAGAGGGGGGGTGGCCCGGGCGGGGGCGGCGTCCGCCCGGGGGCTGCCGGCGCCGCGCTCGACGGACCCCGCCCGACGGCCCGCGCCTCGCGTGCGTGGTCGGCCGCGTCGTTGCCGTCGTCGTCCTCGTCCTCGTCGGACGACGAGGACGAAGAGGATGCGGACGACGAGGACGAGGACCCGGAGTCCGACGAGGTCGATGACGCCGATGGCCGCCACCGGCCGTGACGACGTCTCCGCGGCGGCTGGGCCGGCGGGCGCGGCGACAGGCGGTCCGTGGGGTCCGGATACGCGCCGCGTAGCGGGGCCTCCCGTTCGCGGCCCCGGGCCGGGGCCCGGTCGCCGGCGGCGTCGGCTGCGTCGTCGTACTCGTCCCCGTCATCGTCGTCGGCTCGAAAGGCGGGGGTCCGGGGCGGCGAGGCCGCGGGGTCGGGCGTCGGGATCGTCCGGACGGCCTCCTCTACCATGGAGGCCAGCAGAGCCAGCTGTCGCGGCGAGACGGCGTCCCCGGCGTCCTCGCCGGCGTCGGTGCCCGCCGCGGGGGCCCTCCCGTCCCGCCGGGCGTCGTCGAGGTCGTGGGGGTGGTCGGGGTCGTGGTCGGGGTCGTCCCCGCCCTCCTCCGTCTCCGCGCCCCACCCGAGGGCCCCCCGCTCGTCGCGGTCTGGGCTCGGGGTGGGCGGCGGCCCGTCGGTGGGGCCCGGGGAGCCGGGGCGCTGCTTGTTCTCCGACGCCATCGCCGATGCGGGGCGATCCTCCGGGGATACGGCTGCGACGGCGGACGTAGCACGGTAGGTCACCTACGGACTCTCGATGGGGGGAGGGGGCGAGACCCACGGACCCCGACGACCCCCGCCGTCGACGCGGAACTAGCGCGGACCGGTCGATGCTTGGGTGGGAAAAAGGACAGGGACGGCCGATCCCCCTCCCGCGCTTCGTCCGCGTATCGGCGTCCCGGCGCGGCGAGCGTCTGACGGTCTGTCTCTGGCGGTCCCGCGTCGGGTCGTGGATCCGTGTCGGCAGCCGCGCTCCGTGTGGACGATCGGGGCGTCCTCGGGCTCATATAGTCCCAGGGGCCGGCGGGAAGGAGGAGCAGCGGAGGCCGCCGGCCCCCCGCCCCCCCGGCGGGCCCACCCCGAACGGAATTCCATTATGCACGACCCCGCCCCGACGCCGGCACGCCGGGGGCCCGTGGCCGCGGCCCGTTGGTCGAACCCCCGGCCCCGCCCATCCGCGCCATCTGCCATGGGCGGGGCGCGAGGGCGGGTGGGTCCGCGCCCCGCCCCGCATGGCATCTCATTACCGCCCGATCCGGCGGTTTCCGCTTCCGTTCCGCATGCTAACGAGGAACGGGCAGGGGGCGGGGCCCGGGCCCCGACTTCCCGGTTCGGCGGTAATGAGATACGAGCCCCGCGCGCCCGTTGGCCGTCCCCGGGCCCCCCGGTCCCGCCCGCCGGACGCCGGGACCAACGGGACGGCGGGCGGCCCAAGGGCCGCCCGCCTTGCCGCCCCCCCATTGGCCGGCGGGCGGGACCGCCCCAAGGGGGCGGGGCCGCCGGGTAAAAGAAGTGAGAACGCGAAGCGTTCGCACTTCGTCCCAATATATATATATTATTAGGGCGAAGTGCGAGCACTGGCGCCGTGCCCGACTCCGCGCCGGCCCCGGGGGCGGGCCCGGGCGGCGGGGGGCGGGTCTCTCCGGCGCACATAAAGGCCCGGCGCGACCGACGCCCGCAGACGGCGCCGGCCACGAACGACGGGAGCGGCTGCGGAGCACGCGGACCGGGAGCGGGAGTCGCAGAGGGCCGTCGGAGCGGACGGCGTCGGCATCGCGACGCCCCGGCTCGGGATCGGGATCGCATCGGAAAGGGACACGCGGACGCGGGGGGGAAAGACCCGCCCACCCCACCCACGAAACACAGGGGACGCACCCCGGGGGCCTCCGACGACAGAAACCCACCGGTCCGCCTTTTTTGCACGGGTAAGCACCTTGGGTGGGCGGAGGAGGGGGGGACGCGGGGGCGGAGGAGGGGGGACGCGGGGGCGGAGGAGGGGGGACGCGGGGGCGGAGGAGGGGGGACGCGGGGGCGGAGGAGGGGGGACGCGGGGGCGGAGGAGGGGGCTCACCCGCGTTCGTGCCTTCCCGCAGGAGGAACGTCCTCGTCGAGGCGACCGGCGGCGACCGTTGCGTGGACCGCTTCCTGCTCGTCGGGCGGGGGGAAGCCACTGTGGTCCTCCGGGACGTTTTCTGGATGGCCGACATTTCCCCAGGCGCTTTTGCGCCTTGTGTAAAAGCGCGGCGTCCCGCTCTCCGATCCCCGCCCCTGGGCACGCGCAAGCGCAAGCGCCCTTCCCGCCCCCTCTCATCGGAGTCTGAGGTAGAATCCGATACAGCCTTGGAGTCTGAGGTCGAATCCGAGACAGCATCGGATTCGACCGAGTCTGGGGACCAGGATGAAGCCCCCCGCATCGGTGGCCGTAGGGCCCCCCGGAGGCTTGGGGGGCGGTTTTTTCTGGACATGTCGGCGGAATCCACCACGGGGACGGAAACGGATGCGTCGGTGTCGGACGACCCCGACGACACGTCCGACTGGTCTTATGACGACATTCCCCCACGACCCAAGCGGGCCCGGGTAAACCTGCGGCTCACGAGCTCTCCCGATCGGCGGGATGGGGTTATTTTTCCTAAGATGGGGCGGGTCCGGTCTACCCGGGAAACGCAGCCCCGGGCCCCCACCCCGTCGGCCCCAAGCCCAAATGCAATGCTACGGCGCTCGGTGCGCCAGGCCCAGAGGCGGAGCAGCGCACGATGGACCCCCGACCTGGGCTACATGCGCCAGTGTATCAATCAGCTGTTTCGGGTCCTGCGGGTCGCCCGGGACCCCCACGGCAGTGCCAACCGCCTGCGCCACCTGATACGCGACTGTTACCTGATGGGATACTGCCGAGCCCGTCTGGCCCCGCGCACGTGGTGCCGTTTGCTGCAGGTGTCCGGCGGAACCTGGGGCATGCACCTGCGCAACACCATACGGGAGGTGGAGGCTCGATTCGACGCCACCGCGGAACCCGTGTGCAAGCTTCCTTGTTTGGAGACCAGACGGTACGGCCCGGAGTGTGATCTTAGTAATCTCGAGATTCATCTCAGCGCGACAAGCGATGATGAAATCTCCGATGCCACCGATCTGGAGGCCGCCGGTTCGGACCACACGCTCGCGTCCCAGTCCGACACGGAGGATGCCCCCTCCCCCGTTACGCTGGAAACCCCAGAACCCCGCGGGTCCCTCGCTGTGCGTCTGGAGGATGAGTTTGGGGAGTTTGACTGGACCCCCCAGGAGGGCTCCCAGCCCTGGCTGTCTGCGGTCGTGGCCGATACCAGCTCCGTGGAACGCCCGGGCCCATCCGATTCTGGGGCGGGTCGCGCCGCAGAAGACCGCAAGTGTCTGGACGGCTGCCGGAAAATGCGCTTCTCCACCGCCTGCCCCTATCCGTGCAGCGACACGTTTCTCCGGCCGTGAGTCCGGTCGCCCCGACCCCCTTGTATGTCCCCAAAATAAAAGACCAAAATCAAAGCGTTTGTCCCAGCGTCTTAATGGCGGGAAGGGCGGAGAGAAACAGACCACGCGGACATGGGGGGTGTTTGGGGGTTTATTGGCACCGGGGGCTAAAGGGTGGTAACCGGATAGCAGATGTGAGGAAGTCGGGGCCGTTCGCCGCGAACGGCGATCAGAGGGTCAGTTTCTTGCGGACCACGGCCCGGCGATGTGGGTTGCTCGTCTGGGACCTCGGGCATGCCCATACACGCACAACACGGACGCCGCACCGGATGGGACGTCGTAAGGGGGCCTGGGGTAGCTGGGTGGGGTTTGTGCAGAGCAATCAGGGACCGCAGCCAGCGCATACAATCGCGCTCCCGTCCGTTTGTCCCGGGCAGTACCACGCCGTACTGGTATTCGTACCGGCTGAGCAGGGTCTCCAGGGGGTGGTTGGGGGCCGCGGGGAACGGGGTCCACGCCACGGTCCACTCGGGCAAAAACCGAGTCGGCACGGCCCACGGTTCTCCCACCCACGCGTCTGGGGTCTTGATGGCGATAAATCTTACCCCGAGCCGGATTTTTTGGGCGTATTCGAGAAACGGCACACACAGATCCGCCGCGCCTACCACCCACAAGTGGTAGAGGCGAGGGGGGCTGGGTTGGTCTCGGTGCAGCAGTCGGAAGCACGCCACGGCGTCCACGACCTCGGTGCTCTCCAAGGGGCTGTCCTCCGCAAACAGGCCCGTGGTGGTGTTTGGGGGGCAGCGACAGGACCTAGTGCGCACGATCGGGCGGGTGGGTTTGGGTAAGTCCATCAGCGGCTCGGCCAACCGTCGAAGGTTGGCCGGACGAACGACGACCGGGGTACCCAGGGGTTCTGATGCCAAAATGCGGCACTGCCTAAGCAGGAAGCTCCACAGGGCCGGGCTTGCGTCGACGGAAGTCCGGGGCAGGGCGTTGTTCTGGTCAAGGAGGGTCATTACGTTGACGACAACAACGCCCATGTTGGTATATTACAGGCCCGTGTCCGATTTGGGGCACTTGCAGATTTGTAAGGCCACGCACGGCGGGGAGACAGGCCGACGCGGGGGCTGCTCTAAAAATTTAAGGGCCCTACGGTCCACAGACCCGCCTTCCCGGGGGGGCCCTTGGAGCGACCGGCAGCGGAGGCGTCCGGGGGAGGGGAGGGTGATTTACGGGGGGGTAGGTCAGGGGGTGGGTCGTCAAACTGCCGCTCCTTAAAACCCCGGGGCCCGTCGTTCGGGGTGCTCGTTGGTTGGCACTCACGGTGCGGCGAATGGCCTGTCGTAAGTTTTGTCGCGTTTACGGGGGACAGGGCAGGAGGAAGGAGGAGGCCGTCCCGCCGGAGACAAAGCCGTCCCGGGTGTTTCCTCATGGCCCCTTTTATACCCCAGCCGAGGACGCGTGCCTGGACTCCCCGCCCCCGGAGACCCCCAAACCTTCCCACACCACACCACCCAGCGAGGCCGAGCGCCTGTGTCATCTGCAGGAGATCCTTGCCCAGATGTACGGAAACCAGGACTACCCCATAGAGGACGACCCCAGCGCGGATGCCGCGGACGATGTCGACGAGGACGCCCCGGACGACGTGGCCTATCCGGAGGAATACGCAGAGGAGCTTTTTCTGCCCGGGGACGCGACCGGTCCCCTTATCGGGGCCAACGACCACATCCCTCCCCCGTGTGGCGCATCTCCCCCCGGTATACGACGACGCAGCCGGGATGAGATTGGGGCCACGGGATTTACCGCGGAAGAGCTGGACGCCATGGACAGGGAGGCGGCTCGAGCCATCAGCCGCGGCGGCAAGCCCCCCTCGACCATGGCCAAGCTGGTGACTGGCATGGGCTTTACGATCCACGGAGCGCTCACCCCAGGATCGGAGGGGTGTGTCTTTGACAGCAGCCATCCAGATTACCCCCAACGGGTAATCGTGAAGGCGGGGTGGTACACGAGCACGAGCCACGAGGCGCGACTGCTGAGGCGACTGGACCACCCGGCGATCCTGCCCCTCCTGGACCTGCATGTCGTCTCCGGGGTCACGTGTCTGGTCCTCCCCAAGTACCAGGCCGACCTGTATACCTATCTGAGTAGGCGCCTGAACCCACTGGGACGCCCGCAGATCGCAGCGGTCTCCCGGCAGCTCCTAAGCGCCGTTGACTACATTCACCGCCAGGGCATTATCCACCGCGACATTAAGACCGAAAATATTTTTATTAACACCCCCGAGGACATTTGCCTGGGGGACTTTGGCGCCGCGTGCTTCGTGCAGGGTTCCCGATCAAGCCCCTTCCCCTACGGAATCGCCGGAACCATCGACACCAACGCCCCCGAGGTCCTGGCCGGGGATCCGTATACCACGACCGTCGACATTTGGAGCGCCGGTCTGGTGATCTTCGAGACTGCCGTCCACAACGCGTCCTTGTTCTCGGCCCCCCGCGGCCCCAAAAGGGGCCCGTGCGACAGTCAGATCACCCGCATCATCCGACAGGCCCAGGTCCACGTTGACGAGTTTTCCCCGCATCCAGAATCGCGCCTCACCTCGCGCTACCGCTCCCGCGCGGCCGGGAACAATCGCCCGCCGTACACCCGACCGGCCTGGACCCGCTACTACAAGATGGACATAGACGTCGAATATCTGGTTTGCAAAGCCCTCACCTTCGACGGCGCGCTTCGCCCCAGCGCCGCAGAGCTGCTTTGTTTGCCGCTGTTTCAACAGAAATGACCGCCCCCTGGGGGCGGTGCTGTTTGCGGGTTGGCACAAAAAGACCCCGATCCGCGTCTGTGGTGTTTTTGGCATCATGTCGCAGGGCGCCATGCGTGCCGTTGTTCCCATTATCCCATTCCTTTTGGTTCTTGTCGGTGTATCGGGGGTTCCCACCAACGTCTCCTCCACCACCCAACCCCAACTCCAGACCACCGGTCGTCCCTCGCATGAAGCCCCCAACATGACCCAGACCGGCACCACCGACTCTCCCACCGCCATCAGCCTTACCACGCCCGACCACACACCCCCCATGCCAAGTATTGGACTGGAGGAGGAGGAAGAGGAGGAGGGGGCCGGGGACGGCGAACATCTTGAGGGGGGAGATGGGACCCGTGACACCCTACCCCAGTCCCCGGGCCCAGCCTTCCCGTTGGCTGAGGACGTCGAGAAGGACAAACCCAACCGTCCCGTAGTCCCATCCCCCGATCCCAACAACTCCCCCGCGCGCCCCGAGACCAGTCGCCCGAAGACACCCCCCACCATTATCGGGCCGCTGGCAACTCGCCCCACGACCCGACTCACCTCAAAGGGACGACCCTTGGTTCCGACGCCTCAACATACCCCGCTGTTCTCGTTCCTCACTGCCTCCCCCGCCCTGGACACCCTCTTCGTCGTCAGCACCGTCATCCACACCTTATCGTTTTTGTGTATTGGTGCGATGGCGACACACCTGTGTGGCGGTTGGTCCAGACGCGGGCGACGCACACACCCTAGCGTGCGTTACGTGTGCCTGCCGTCCGAACGCGGGTAGGGTATGGGGCGGGGGATGGGGAGAGCCCACATGCGGAAAGCAAGAACAATAAAGGCGGTGGTATCTAGTTGATATGCATCTCTGGGTGTTTTTGGGGTGTGGCGGACGCGGGGCGGTCATTGGACGGGGTGCAGTTAAATACATGCCCGGGACCCATGAAGCATGCGCGACTTCCGGGCCTCAGAACCCACCCGAAACGGCCAACGGACGTCTGAGCCAGGCCTGGCTATCCGGAGAAACAGCACACGACTTGGCGTTCTGTGTGTCGCGATGTCTCTGCGCGCAGTCTGGCATCTGGGGCTTTTGGGAAGCCTCGTGGGGGCTGTTCTTGCCGCCACCCATCGGGGACCTGCGGCCAACACAACGGACCCCTTAACGCACGCCCCAGTGTCCCCTCACCCCAGCCCCCTGGGGGGCTTTGCCGTCCCCCTCGTAGTCGGTGGGCTGTGCGCCGTAGTCCTGGGGGCGGCATGTCTGCTTGAGCTCCTGCGTCGTACGTGCCGCGGGTGGGGGCGTTACCATCCCTACATGGACCCAGTTGTCGTATAATTTCCCCCCCCCCCCCCCTTCTCCGCGTGGGTGATGTCGGGTCCAAACTCCCGACACCACCAGCTGGCATGGTATAAATCACCGGTGCGCCCCCCAAACCATGTCCGGCAGGGGGATGGGGGGGCGAATGCGGAGGGCACCCAACAACACCGGGCTAACCAGGAAATCCGTGGCCCCGGCCCCCAATAAAGATCGCGGTAGCCCGGCCGTGTGACACTATCGTCCATACCGACCACACCGACGAATCCCCCAAGGGGGAGGGGCCATTTTACGAGGAGGAGGGGTATAACAAAGTCTGTCTTTAAAAAGCAGGGGTTAGGGAGTTGTTCGGTCATAAGCTTCAGCGCGAACGACCAACTACCCCGATCATCAGTTATCCTTAAGGTCTCTTTTGTGTGGTGCGTTCCGGTATGGGGGGGGCTGCCGCCAGGTTGGGGGCCGTGATTTTGTTTGTCGTCATAGTGGGCCTCCATGGGGTCCGCGGCAAATATGCCTTGGTGGATGCCTCTCTCAAGATGGCCGACCCCAATCGCTTTCGCGGCAAAGACCTTCCGGTCCTGGACCAGCTGACCGACCCTCCGGGGGTCCGGCGCGTGTACCACATCCAGGCGGGCCTACCGGACCCGTTCCAGCCCCCCAGCCTCCCGATCACGGTTTACTACGCCGTGTTGGAGCGCGCCTGCCGCAGCGTGCTCCTAAACGCACCGTCGGAGGCCCCCCAGATTGTCCGCGGGGCCTCCGAAGACGTCCGGAAACAACCCTACAACCTGACCATCGCTTGGTTTCGGATGGGAGGCAACTGTGCTATCCCCATCACGGTCATGGAGTACACCGAATGCTCCTACAACAAGTCTCTGGGGGCCTGTCCCATCCGAACGCAGCCCCGCTGGAACTACTATGACAGCTTCAGCGCCGTCAGCGAGGATAACCTGGGGTTCCTGATGCACGCCCCCGCGTTTGAGACCGCCGGCACGTACCTGCGGCTCGTGAAGATAAACGACTGGACGGAGATTACACAGTTTATCCTGGAGCACCGAGCCAAGGGCTCCTGTAAGTACGCCCTCCCGCTGCGCATCCCCCCGTCAGCCTGCCTCTCCCCCCAGGCCTACCAGCAGGGGGTGACGGTGGACAGCATCGGGATGCTGCCCCGCTTCATCCCCGAGAACCAGCGCACCGTCGCCGTATACAGCTTGAAGATCGCCGGGTGGCACGGGCCCAAGGCGCCACGCAGCCAGAACTCGCCCCGGAAGACCCCGAGGATTCGGCCCTCTTGGAGGACCCCGTGGGGACGGTGGCGCCGCAAATCCCACCAAACTGGCACATACCGTCGATCCAGGACGCCGCGACGCCTTACCATCCCCCGGCCACCCCGAACAACATGGGCCTGATCGCCGGCGCGGTGGGCGGCAGTCTCCTGGCAGCCCTGGTCATTTGCGGAATTGTGTACTGGATGCGCCGCCACACTCAAAAAGCCCCAAAGCGCATACGCCTCCCCCACATCCGGGAAGACGACCAGCCGTCCTCGCACCAGCCCTTGTTTTACTAGATACCCCCCCTTAATGGGTGCGGGGGGGTCAGGTCTGCGGGGTTGGGATGGGACCTTAACTCCATATAAAGCGAGTCTGGAAGGGGGGAAAGGTGGACAGTCGATAAGTCGGTAGCGGGGGACGCGCACCTGTTCCGCCTGTCGCACCCACAGCTTTTTTTGCGAACCGTCCCGTTCCGGGATGCCGTGCCGCCCGTTGCAGGGCCTGGTGCTCGTGGGCCTCTGGGTCTGTGCCACCAGCCTGGTTGTCCGTGGCCCCACGGTCAGTCTGGTATCAAACTCATTTGTGGACGCCGGGGCCTTGGGGCCCGACGGCGTAGTGGAGGAAGACCTGCTTATTCTCGGGGAGCTTCGCTTTGTGGGGGACCAGGTCCCCCACACCACCTACTACGATGGGGGCGTAGAGCTGTGGCACTACCCCATGGGACACAAATGCCCACGGGTCGTGCATGTCGTCACGGTGACCGCGTGCCCACGTCGCCCCGCCGTGGCATTCGCCCTGTGTCGCGCGACCGACAGCACTCACAGCCCCGCATATCCCACCCTGGAGCTCAATCTGGCCCAACAGCCGCTTTTGCGGGTCCAGAGGGCAACGCGGGACTATGCCGGGGTGTACGTGTTACGCGTATGGGTCGGTGACGCGCCAAACGCCAGCCTGTTTGTCCTGGGGATGGCCATAGCCGCCGAAGGGACTCTGGCGTACAACGGCTCGGCCTATGGCTCCTGCGACCCGAAACTGCTTCCGTCTTCGGCCCCGCGTCTGGCCCCGGCGAGCGTATACCAACCCGCCCCTAACCAGGCCTCCACCCCCTCGACCACCACCTCCACCCCCTCGACCACCATCCCCGCTCCCTCGACCACCATCCCCGCTCCCCAAGCATCGACCACGCCCTTCCCCACGGGAGATCCAAAACCACAACCTCCCGGGGTCAACCACGAACCCCCATCTAATGCCACGCGAGCGACCCGCGACTCGCGATACGCGCTAACGGTGACCCAGATAATCCAGATAGCCATCCCCGCGTCCATCATAGCCCTGGTGTTTCTGGGGAGCTGTATTTGCTTTATACACAGATGTCAACGCCGCTACCGACGCTCCCGTCGCCCGATTTACAGCCCCCAGATGCCCACGGGCATCTCATGCGCGGTGAACGAAGCGGCCATGGCCCGCCTCGGAGCCGAGCTCAAATCGCATCCGAGCACCCCCCCCAAATCCCGGCGCCGGTCGTCACGCACGCCAATGCCCTCCCTGACGGCCATCGCCGAAGAGTCGGAGCCCGCTGGGGCGGCTGGGCTTCCGACGCCCCCCGTGGACCCCACGACACCCACCCCAACGCCTCCCCTGTTGGTATAGGTCCACGGCCACTGGCCGGGAGCACCACATAACCGACCGCAGTCCCTGAGTTGGGAATAAACCGGTATTATTTACCTATATCCGTGTATGTCGATTTCTTTCCCCCCCTCCCCGGAAACCAAAGAAGGAAGCAAAGAATGGATGGGAGGAGTTCAGGAAGCCGGGGAGAGGGCCCGCGGCGCATTTAAGGCGTTGTTGTGTTGACTTTGCCTCTTCTGGCGGGTTGGTGCGGTGCTGTTTGTTGGGCTCCCATTTTACCCGAAGATCGGCTGCTATCCCCGGGACATGGATCGCGGGGCGGTGGTGGGGTTTCTTCTCGGTGTTTGTGTTGTATCGTGCTTGGCGGGAACGCCCAAAACGTCCTGGAGACGGGTGAGTGTCGGCGAGGACGTTTCGTTGCTTCCAGCTCCGGGGCCTACGGGGCGCGGCCCGACCCAGAAACTACTATGGGCCGTGGAACCCCTGGATGGGTGCGGCCCCTTACACCCGTCGTGGGTCTCGCTGATGCCCCCCAAGCAGGTGCCCGAGACGGTCGTGGATGCGGCGTGCATGCGCGCTCCGGTCCCGCTGGCGATGGCGTACGCCCCCCCGGCCCCATCTGCGACCGGGGGTCTACGAACGGACTTCGTGTGGCAGGAGCGCGCGGCCGTGGTTAACCGGAGTCTGGTTATTCACGGGGTCCGAGAGACGGACAGCGGCCTGTATACCCTGTCCGTGGGCGACATAAAGGACCCGGCTCGCCAAGTGGCCTCGGTGGTCCTGGTGGTGCAACCGGCCCCAGTTCCGACCCCACCCCCGACCCCAGCCGATTACGACGAGGATGACAATGACGAGGGCGAGGACGAAAGTCTCGCCGGCACTCCCGCCAGCGGGACCCCCCGGCTCCCGCCTCCCCCCGCCCCCCCGAGGTCTTGGCCCAGCGCCCCCGAAGTCTCACATGTGCGTGGGGTGACCGTGCGTATGGAGACTCCGGAAGCTATCCTGTTTTCCCCCGGGGAGACGTTCAGCACGAACGTCTCCATCCATGCCATCGCCCACGACGACCAGACCTACTCCATGGACGTCGTCTGGTTGAGGTTCGACGTGCCGACCTCGTGTGCCGAGATGCGAATATACGAATCGTGTCTGTATCACCCGCAGCTCCCAGAATGTCTGTCCCCGGCCGACGCGCCGTGCGCCGCGAGTACGTGGACGTCTCGCCTGGCCGTCCGCAGCTACGCGGGGTGTTCCAGAACAAACCCCCCACCGCGCTGTTCGGCCGAGGCTCACATGGAGCCCGTCCCGGGGCTGGCGTGGCAGGCGGCCTCCGTCAATCTGGAGTTCCGGGACGCGTCCCCACAACACTCCGGCCTGTATCTGTGTGTGGTGTACGTCAACGACCATATTCACGCCTGGGGCCACATTACCATCAGCACCGCGGCGCAGTACCGGAACGCGGTGGTGGAACAGCCCCTCCCACAGCGCGGCGCGGATTTGGCCGAGCCCACCCACCCGCACGTCGGGGCCCCTCCCCACGCGCCCCCAACCCACGGCGCCCTGCGGTTAGGGGCGGTGATGGGGGCCGCCCTGCTGCTGTCTGCACTGGGGTTGTCGGTGTGGGCGTGTATGACCTGTTGGCGCAGGCGTGCCTGGCGGGCGGTTAAAAGCAGGGCCTCGGGTAAGGGGCCCACGTACATTCGCGTGGCCGACAGCGAGCTGTACGCGGACTGGAGCTCGGACAGCGAGGGAGAACGCGACCAGGTCCCGTGGCTGGCCCCCCCGGAGAGACCCGACTCTCCCTCCACCAATGGATCCGGCTTTGAGATCTTATCACCAACGGCTCCGTCTGTATACCCCCGTAGCGATGGGCATCAATCTCGCCGCCAGCTCACAACCTTTGGATCCGGAAGGCCCGATCGCCGTTACTCCCAGGCCTCCGATTCGTCCGTCTTCTGGTAAGGCGCCCCATCCCGAGGCCCCACGTCGGTCGCCGAACTGGGCGACCGCCGGCGAGGTGGACGTCGGAGACGAGCTAATCGCGATTTCCGACGAACGCGGACCCCCCCGACATGACCGCCCGCCCCTCGCCACGTCGACCGCGCCCTCGCCACACCCGCGACCCCCGGGCTACACGGCCGTTGTCTCCCCGATGGCCCTCCAGGCTGTCGACGCCCCCTCCCTGTTTGTCGCCTGGCTGGCCGCTCGGTGGCTCCGGGGGGCTTCCGGCCTGGGGGCCGTCCTGTGTGGGATTGCGTGGTATGTGACGTCAATTGCCCGAGGCGCATAAAGGGCCGGTGGTCCGCCTAGCCGCAGCAAATTAAAAATCGTGAGTCACAGCGACCGCAACTTCCCACCCGGAGCTTTCTTCCGGCCTCGATGACGTCCCGGCTCTCCGATCCCAACTCCTCAGCGCGATCCGACATGTCCGTGCCGCTTTATCCCACGGCCTCGCCAGTTTCGGTCGAAGCCTACTACTCGGAAAGCGAAGACGAGGCGGCCAACGACTTCCTCGTACGCATGGGCCGCCAACAGTCGGTATTAAGGCGTCGACGCAGACGCACCCGCTGCGTCGGCATGGTGATCGCCTGTCTCCTCGTGGCCGTTCTGTCGGGCGGATTTGGGGCGCTCCTGATGTGGCTGCTCCGCTAAAAGACCGCATCGACACGCGCGTCCTTCTTGTCGTCTCTCTTCCCCCCCATCACCCCGCAATTTGCACCCAGCCTTTAACTACATTAAATTGGGTTCGATTGGCAATGTTGTCTCCCGGTTGATTTTTGGGTGGGTGGGGAGTGGGTGGGTGGGGAGTGGGTGGGTGGGGAGTGGGTGGGTGGGGAGTGGGTGGGTGGGGAGTGGGTGGGTGGGGAGTGGGTGGGTGGGGAGTGGGTGGGTGGGGAGTGGGTGGGTGGGGAGTGGGTGGGTGGGGAGTGGCAAGGAAGAAACAAGCCCGACCACCAGACAGAAAATGTAACCATACCCAAACCGACTCTGGGGGCTGTTTGTGGGGTCGGAACCATAGGATGAACAAACCACCCCGTACCACCCGCACCCAAGGGTGCGGGTGGCTCATCGGCATCTGTCCGGTATGGGTTGTTCCCCACCCACTCGCGTTCGGACGTCTTAGAATCATGGCGGTTTTCTATGCCGACATCGGTTTTCTCCCCCGCAATAAGACACGATGCGATAAAATCTGTTTGTAAAATTTATTAAGGGTACAAATTGCCCTAGCACAGGGGTGGGGTTAGGGCCGGGTCCCCACACCCAAACGCACCAAACAGATGCAGGCAGTGGGTCGAGTACAGCCCCGCGTACGAACACGTCGATGCGTGTGTCAGACAGCACCAGAAAGCACAGGCCATCAACAGGTCGTGCATGTGTCGGTGGGTTTGGACGCGGGGGGCCATGGTGGTGATAAAGTTAATGGCCGCCGTCCGCCAGGGCCACAGGGGCGACGTCTCTTGGTTGGCCCGGAGCCACTGGGTGTGGACCAGCCGCGCGTGGCGGCCCAACATGGCCCCTGTAGCCGGGGGCGGGGGATCGCGCACGTTTGCAGCGCACATGCGAGACACCTCGACCACGGTTCGAAAGAAGGCCCGGTGGTCCGCGGGCAACATCACCAGGTGCGCAAGCGCCCGGGCGTCCAGAGGGTAGAGCCCTGAGTCATCCGAGGTTGGCTCATCGCCCGGGTCTTGCCGCAAGTGCGTGTGGGTTGGGCTTCCGGTGGGCGGGACGCGAACCGCGGTGTGGATCCCGACGCGGGCCCGAGCGTATGCTCCATGTTGTGGGGAGAAGGGGTCTGGGCTCGCCAGGGGGGCATACTTGCCCGGGCTATACAGACCCGCGAGCCGTACGTGGTTCGCGGGGGGTGCGTGGGGTCCGGGGCTCCCGGGGAGACCGGGGCTCCCGGGGAGACCGGGGCTCCCTGGGAGACCGGGGTTGTCGTGGATCCCTGGGGTCACGCGGTACCCTGGGGTCTCTGGGAGCTCGCGGTACTCTGGGTTCCCTAGGTTCTCGGGGTGGTCGCGGAACCCGGGGCTCCCGGGGAACACGCGGTGTCCTGGGGATTGTTGGCGGTCGGACGGCTTCAGATGGCTTCGAGATCGTAGTGTCCGCACCGACTCGTAGTAGACCCGAATCTCCACATTGCCCCGCCGCTTGATCATTATCACCCCGTTGCGGGGGTCCGGAGATCATGCGCGGGTGTCCTCGAGGTGCGTGAACACCTCTGGGGTGCATGCCGGCGGACGGCACGCCTTTTAAGTAAACATCTGGGTCGCCCGGCCCAACTGGGGCCGGGGGTTGGGTCTGGCTCATCTCGAGAGCCACGGGGGGGAACCACCCTCCGCCCAGAGACTCGGGTGATGGTCGTACCCGGGACTCAACGGGTTACCGGATTACGGGGACTGTCGGTCACGGTCCCGCCGGTTCTTCGATGTGCCACACCCAAGGATGCGTTGGGGGCGATTTCGGGCAGCAGCCCGGGAGAGCGCAGCAGGGGACGCTCCGGGTCGTGCACGGCGGTTCTGGCCGCCTCCCGGTCCTCACGCCCCCTTTTATTGATCTCATCGCGTACGTCGGCGTACGTCCTGGGCCCAACCCGCATGGTGTCCAGGAAGGTGTCCGCCATTTCCAGGGCCCACGACATGCTCCCCCCCGACGAGCAGGAAGCGGTCCACGCAACGGTCGCCGCCGGTCGCCTCGACGAGGACGTTCCTCCTGCGGGAAGGCACGAACGCGGGTGAGCCCCCTCCTCCGCCCCCGCGTCCCCCCTCCTCCGCCCCCGCGTCCCCCCTCCTCCGCCCCCGCGTCCCCCCTCCTCCGCCCCCGCGTCCCCCCTCCTCCGCCCCCGCGTCCCCCCCTCCTCCGCCCACCCAAGGTGCTTACCCGTGCAAAAAAGGCGGACCGGTGGGTTTCTGTCGTCGGAGGCCCCCGGGGTGCGTCCCCTGTGTTTCGTGGGTGGGGTGGGCGGGTCTTTCCCCCCCGCGTCCGCGTGTCCCTTTCCGATGCGATCCCGATCCCGAGCCGGGGCGTCGCGATGCCGACGCCGTCCGCTCCGACGGCCCTCTGCGACTCCCGCTCCCGGTCCGCGTGCTCCGCAGCCGCTCCCGTCGTTCGTGGCCGGCGCCGTCTGCGGGCGTCGGTCGCGCCGGGCCTTTATGTGCGCCGGAGAGACCCGCCCCCCGCCGCCCGGGCCCGCCCCCGGGGCCGGCGCGGAGTCGGGCACGGCGCCAGTGCTCGCACTTCGCCCTAATAATATATATATATTGGGACGAAGTGCGAACGCTTCGCGTTCTCACTTCTTTTACCCGGCGGCCCCGCCCCCTTGGGGCGGTCCCGCCCGCCGGCCAATGGGGGGGCGGCAAGGCGGGCGGCCCTTGGGCCGCCCGCCGTCCCGTTGGTCCCGGCGTCCGGCGGGCGGGACCGGGGGGCCCGGGGACGGCCAACGGGCGCGCGGGGCTCGTATCTCATTACCGCCGAACCGGGAAGTCGGGGCCCGGGCCCCGCCCCCTGCCCGTTCCTCGTTAGCATGCGGAACGGAAGCGGAAACCGCCGGATCGGGCGGTAATGAGATGCCATGCGGGGCGGGGCGCGGACCCACCCGCCCTCGCGCCCCGCCCATGGCAGATGGCGCGGATGGGCGGGGCCGGGGGTTCGACCAACGGGCCGCGGCCACGGGCCCCCGGCGTGCCGGCGTCGGGGCGGGGTCGTGCATAATGGAATTCCGTTCGGGGTGGGCCCGCCGGGGGGGCGGGGGGCCGGCGGCCTCCGCTGCTCCTCCTTCCCGCCGGCCCCTGGGACTATATGAGCCCGAGGACGCCCCGATCGTCCACACGGAGCGCGGCTGCCGACACGGATCCACGACCCGACGCGGGACCGCCAGAGACAGACCGTCAGACGCTCGCCGCGCCGGGACGCCGATACGCGGACGAAGCGCGGGAGGGGGATCGGCCGTCCCTGTCCTTTTTCCCACCCAAGCATCGACCGGTCCGCGCTAGTTCCGCGTCGACGGCGGGGGTCGTCGGGGTCCGTGGGTCTCGCCCCCTCCCCCCATCGAGAGTCCGTAGGTGACCTACCGTGCTACGTCCGCCGTCGCAGCCGTATCCCCGGAGGATCGCCCCGCATCGGCGATGGCGTCGGAGAACAAGCAGCGCCCCGGCTCCCCGGGCCCCACCGACGGGCCGCCGCCCACCCCGAGCCCAGACCGCGACGAGCGGGGGGCCCTCGGGTGGGGCGCGGAGACGGAGGAGGGCGGGGACGACCCCGACCACGACCCCGACCACCCCCACGACCTCGACGACGCCCGGCGGGACGGGAGGGCCCCCGCGGCGGGCACCGACGCCGGCGAGGACGCCGGGGACGCCGTCTCGCCGCGACAGCTGGCTCTGCTGGCCTCCATGGTAGAGGAGGCCGTCCGGACGATCCCGACGCCCGACCCCGCGGCCTCGCCGCCCCGGACCCCCGCCTTTCGAGCCGACGACGATGACGGGGACGAGTACGACGACGCAGCCGACGCCGCCGGCGACCGGGCCCCGGCCCGGGGCCGCGAACGGGAGGCCCCGCTACGCGGCGCGTATCCGGACCCCACGGACCGCCTGTCGCCGCGCCCGCCGGCCCAGCCGCCGCGGAGACGTCGTCACGGCCGGTGGCGGCCATCGGCGTCATCGACCTCGTCGGACTCCGGGTCCTCGTCCTCGTCGTCCGCATCCTCTTCGTCCTCGTCGTCCGACGAGGACGAGGACGACGACGGCAACGACGCGGCCGACCACGCACGCGAGGCGCGGGCCGTCGGGCGGGGTCCGTCGAGCGCGGCGCCGGCAGCCCCCGGGCGGACGCCGCCCCCGCCCGGGCCACCCCCCCTCTCCGAGGCCGCGCCCAAGCCCCGGGCGGCGGCGAGGACCCCCGCGGCCTCCGCGGGCCGCATCGAGCGCCGCCGGGCCCGCGCGGCGGTGGCCGGCCGCGACGCCACGGGCCGCTTCACGGCCGGGCAGCCCCGGCGGGTCGAGCTGGACGCCGACGCGGCCTCCGGCGCCTTCTACGCGCGCTATCGCGACGGGTACGTCAGCGGGGAGCCGTGGCCCGGCGCCGGGCCCCCGCCCCCGGGGCGGGTGCTGTACGGCGGCCTGGGCGACAGCCGCCCGGGCCTCTGGGGGGCGCCCGAGGCGGAGGAGGCGCGACGCCGGTTCGAGGCCTCGGGCGCCCCGGCGGCCGTGTGGGCGCCCGAGCTGGGCGACGCCGCGCAGCAGTACGCCCTGATCACGCGGCTGCTGTACACCCCGGACGCGGAGGCCATGGGGTGGCTCCAGAACCCGCGCGTGGTCCCCGGGGACGTGGCGCTGGACCAGGCCTGCTTCCGGATCTCGGGCGCCGCGCGCAACAGCAGCTCCTTCATCACCGGCAGCGTGGCGCGGGCCGTGCCCCACCTGGGCTACGCCATGGCGGCCGGCCGCTTCGGCTGGGGCCTGGCGCACGCGGCGGCCGCCGTGGCCATGAGCCGCCGATACGACCGCGCGCAGAAGGGCTTCCTGCTGACCAGCCTGCGCCGCGCCTACGCGCCCCTGTTGGCGCGCGAGAACGCGGCGCTGACGGGGGCCGCGGGGAGCCCCGGCGCCGGCGCAGATGACGAGGGGGTCGCCGCCGTCGCCGCCGCCGCACCGGGCGAGCGCGCGGTGCCCGCCGGGTACGGCGCCGCGGGGATCCTCGCCGCCCTGGGGCGGCTGTCCGCCGCGCCCGCCTCCCCCGCGGGGGGCGACGACCCCGACGCCGCCCGCCACGCCGACGCCGACGACGACGCCGGGCGCCGCGCCCAGGCCGGCCGCGTGGCCGTCGAGTGCCTGGCCGCCTGCCGCGGGATCCTGGAGGCGCTGGCCGAGGGCTTCGACGGCGACCTGGCGGCCGTCCCGGGGCTGGCCGGGGCCCGGCCCGCCAGCCCCCCGCGGCCGGAGGGACCCGCGGGCCCCGCTTCCCCGCCGCCGCCGCACGCCGACGCGCCCCGCCTGCGCGCGTGGCTGCGCGAGCTGCGGTTCGTGCGCGACGCGCTGGTGCTCATGCGCCTGCGCGGGGACCTGCGCGTGGCCGGCGGCAGCGAGGCCGCCGTGGCCGCCGTGCGCGCCGTGAGCCTGGTCGCCGGGGCCCTGGGCCCCGCGCTGCCGCGGGACCCGCGCCTGCCGAGCTCCGCGGCCGCCGCCGCCGCGGACCTGCTGTTTGACAACCAGAGCCTGCGCCCCCTGCTGGCGGCGGCGGCCAGCGCACCGGACGCCGCCGACGCGCTGGCGGCCGCCGCCGCCTCCGCCGCGCCGCGGGAGGGGCGCAAGCGCAAGAGTCCCGGCCCGGCCCGGCCGCCCGGAGGCGGCGGCCCGCGACCCCCGAAGACGAAGAAGAGCGGCGCGGACGCCCCCGGCTCGGACGCCCGCGCCCCCCTCCCCGCGCCCGCGCCCCCCTCCACGCCCCCGGGGCCCGAGCCCGCCCCCGCCCAGCCCGCGGCGCCCCGGGCCGCCGCGGCGCAGGCCCGCCCGCGCCCCGTGGCGCTGTCGCGCCGGCCCGCCGAGGGCCCCGACCCCCTGGGCGGCTGGCGGCGGCAGCCCCCGGGGCCCAGCCACACGGCGGCGCCCGCGGCCGCCGCCCTGGAGGCCTACTGCTCCCCGCGCGCCGTGGCCGAGCTCACGGACCACCCGCTGTTCCCCGTCCCCTGGCGACCGGCCCTCATGTTTGACCCGCGGGCCCTGGCCTCGATCGCCGCGCGGTGCGCCGGGCCCGCCCCCGCCGCCCAGGCCGCGTGCGGCGGCGGCGACGACGACGATAACCCCCACCCCCACGGGGCCGCCGGGGGCCGCCTCTTTGGCCCCCTGCGCGCCTCGGGCCCGCTGCGCCGCATGGCGGCCTGGATGCGCCAGATCCCCGACCCCGAGGACGTGCGCGTGGTGGTGCTGTACTCGCCGCTGCCGGGCGAGGACCTGGCCGGCGGCGGGGCCTCGGGGGGGCCGCCGGAGTGGTCCGCCGAGCGCGGCGGGCTGTCCTGCCTGCTGGCGGCCCTGGCCAACCGGCTGTGCGGGCCGGACACGGCCGCCTGGGCGGGCAACTGGACCGGCGCCCCCGACGTGTCGGCGCTGGGCGCACAGGGCGTGCTGCTGCTGTCCACGCGGGACCTGGCCTTCGCCGGGGCCGTGGAGTTTCTGGGGCTGCTCGCCAGCGCCGGCGACCGGCGGCTCATCGTGGTCAACACCGTGCGCGCCTGCGACTGGCCCGCCGACGGGCCCGCGGTGTCGCGGCAGCACGCCTACCTGGCGTGCGAGCTGCTGCCCGCCGTGCAGTGCGCCGTGCGCTGGCCGGCGGCGCGGGACCTGCGCCGCACGGTGCTGGCCTCGGGCCGCGTGTTCGGCCCGGGGGTCTTCGCGCGCGTGGAGGCCGCGCACGCGCGCCTGTACCCCGACGCGCCGCCGCTGCGCCTGTGCCGCGGCGGCAACGTGCGCTACCGCGTGCGCACGCGCTTCGGCCCGGACACGCCGGTGCCCATGTCCCCGCGCGAGTACCGCCGGGCCGTGCTGCCGGCGCTGGACGGCCGGGCGGCGGCCTCGGGGACCACCGACGCCATGGCGCCCGGCGCGCCGGACTTCTGCGAGGAGGAGGCCCACTCGCACCGCGCCTGCGCGCGCTGGGGCCTGGGCGCGCCGCTGCGGCCCGTGTACGTGGCGCTGGGGCGCGAGGCGGTGCGCGCCGGCCCGGCCCGGTGGCGCGGGCCGCGGAGGGACTTTTGCGCCCGCGCCCTGCTGGAGCCCGACGACGACGCCCCCCCGCTGGTGCTGCGCGGCGACGACGACGGCCCGGGGGCCCTGCCGCCGGCGCCGCCCGGGATTCGCTGGGCCTCGGCCACGGGCCGCAGCGGCACCGTGCTGGCGGCGGCGGGGGCCGTGGAGGTGCTGGGGGCGGAGGCGGGCTTGGCCACGCCCCCGCGGCGGGAAGTTGTGGACTGGGAAGGCGCCTGGGACGAAGACGACGGCGGCGCGTTCGAGGGGGACGGGGTGCTGTAACGGGCCGGGACGGGGCGGGGCGCTTGTGAGACCCGAAGACGCAATAAACGGCAACAACCTGATTAAGTTTTGCAGTAGCGTTGTTTATTCGAGGGGCGGGAGGGGGCGAGGGGCGGGAGGGGGCGAGGGGCGGGAGGGGGCGAGGGGCGGGAGGGGGCGAGGGGCGGGAGGGGGCGAGGGGCGGGAGGGGGCGAGGGGCGGGAGGGGGCGAGGGGCGGGAGGGGGCGAGGGGCGGGAGGGGGCGAGGGGCGGGAGGGGGCGAGGGGCGGGAGGGGGCGAGGGGCGGGAGGGGGCGAGGGGCGGGAGGGGGCGAGGGGCGGGAGGGGGCGAGGGGCGGGAGGGGGCGAGGGGCGGGAGGGGGCGAGGGGCGGGAGGGGGCGAGGGGCGGGAGGGGGCGAGGGGCGGGAGGGGGCGAGGGGCGGTGGTGGTGCGCGGGCGCCCCCGGAGGGTTTGGATCTCTGACCTGAGATTGGCGGCACTGAGGTAGAGATGCCCGAACCCCCCCGAGGGAGCGCGGGACGCGGCCGGGGAGGGCTGGGGCCGGGGAGGGCTGGGGCCGGGGAGGGCTGGGGCCGGGGAGGGCTGGGGCCGGGGAGGGCTGGGGCCGGGGAGGGCTGGGGCCGGGGAGGGCTGGGGCCGGGGAGGGCTGGGGCTGGGGAGGGGCGGTGGTGTGTAGCAGGAGCGGTGTGTTGCGCCGGGGTACGTCTGGAGGAGCGGGAGGTGCGCGGTGACGTGTGGATGAGGAACAGGAGTTGTTGCGCGGTGAGTTGTCGCTGTGAGTTGTGTTGTTGGGCAGGTGTGGTGGATGACGTGACGTGTGACGTGCGGAGTGCGCCGTGCTCTGTTGGTTTCACCTGTGGCAGCCCGG

>HHV2_HSV2_STRIPPED4

AGTCCCCGTCTTGCCGCGCGGGGGCGGGCGCGGGAAAAAAGCCGCGCGGGGGCGCCCGCGGGAAGGCAGCCCCGCGGCGCGCGGGGGGAGGGGCGGCGCCCGCGGGGGAGCGGCCGGCTCCGGGGGAGGGACGGGGAAGGGGGCGCGCGGGGCTGCCCTGCCGCCCGCCCGCCGCCGCCGCCCGCCTTCGCGCCCCCCCCCAAAAAACACCCCCCCCGGGGGTTGACTCCCCGGGGGAAAAGAGGCGGGGCGGGAGTCCCCGTCCTGCCGCCGCCCCTTAAGAGGGCCCGCAACACGGCCCGGGCTGCGCACGCCAGCCGGGACGGGTGAGTTCGCTAGGCAAGCACGGACTGGCGGTTACACGTGCATGCGTGCCGAGTGAACTCTCCCGCCCCGACGCGCTCCGGCTCCGGGCCTACGCCGAGCCCAGCCGCCCGCCATGTCCCGCCGCCGGGGTCCCCGCCGCCGGGGTCCCCGGCGCCGGCCGCGCCCCGGCGCTCCAGCCGTGCCGCGCCCCGGCGCTCCAGCCGTGCCGCGCCCCGGCGCGCTCCCAACCGCAGACTCCCAAATGGTCCCTGCGTACGACTCGGGAACCGCGGTCGAGAGCGCGCCGGCCGCGTCCTCGCTCCTGCGGCGCTGGCTGCTGGTGCCCCAGGCGGACGACAGCGACGACGCGGACTACGCCGGCAACGACGACGCAGAGTGGGCGAACAGCCCCCCGAGCGAGGGCGGGGGGAAGGCGCCGGAGGCCCCGCACGCCGCGCCTGCCGCCGCCTGCCCCCCGCCGCCGCCGCGCAAGGAGCGCGGGCCGCAGCGCCCCCTTCCGCCCCACCTGGCGCTACGGCTGCGCACCACGACGGAGTACCTGGCGCGCCTGAGCCTGCGCCGGCGGCGGCCCCCCGCGTCCCCGCCCGCGGACGCGCCGCGCGGGAAGGTACGCCTCCCCTCCGACCCCCTGACGCCCCTCCGACCCCCTGACGCCCCTCCGACCCCCTGACGCCCCTCCGACCCCCTGACGCCCCTCCGACCCCCTGACGCCCCTCCGACCCCCTGACGCCCCTCCGACCCCCGTGTCTCCCCGCCCGCAGGTGTGCTTCTCGCCGCGCGTGCAGGTGCGCCATCTGGTGGCCTGGGAGACGGCCGCGCGCCTGGCCCGACGGGGGTCCTGGGCGCGCGAGCGGGCCGACCGCGACCGGTTCCGGCGCCGCGTGGCGGCGGCCGAGGCGGTCATCGGACCGTGCCTGGAGCCCGAGGCCCGAGCTCGGGCCCGAGCCCGAGCCGGGCCGTCGGGCGGTCTAGGGTTGAACCGGCGAGGGCGGCCTCGGCCGGCGGAGCCCCGGAGCTCCGAAGGTCTGCGCGAGGCCGCTCTCCGAAGAGACGATGGGAGCCCCGCGTATATATCCGCGAGGGCCCGGCGCCGCCCCGCCGCTCCGCCCGCCCCAGGGGGCGGCGCCGGCCAACCGCGCGCCGCCGCGCGGGCCCGGACTCCGCCCCGGCGACCGCCCCGCGCCGGCTTCCCGGTATGGTAATTAGAAACTTTTAATAGGCGGTCCCGGCCGCCATCCCCGCGCATGGTAATTAGCAACTTTTAATGGGCCGGCGTTCCCGCTCGCGGTAATTAGCAGCTTTTAACGGGCCGCCATTCCCGCTTATGGTAATTAAAAACGTTCGGACGGCCCCTCGCTCCCCGCGTAATTACTCCCTCGGGGTTCCGGGTTATGCTGATTACTTTCTTGGCAGAACACGCAGAGCCTCGCGCGCCGCCGGGTGGGTGGGCTGATCGGCCCCTATTGGTCCCCTGGGCTTCCTAGTATGCTAATGAATTTCTCCCCGGGGGCGGGCACCACTCAGGGCCGCGCCGGCGGGGCGCCGGGGGGACTCCCATCTGCGTCGGCGGGGGGCGGCGCATGCTAATGGGGTTCTTGGAGTACACCCGGTTGGTCCCCGGGGACGGGGCCGCCCCGAGAGGGGGGGATTCCCTCCCTCCGCCCCCGCCGGGGCGCGCGGCTATTGGGGGAATCGTAAATGCCGCCCCTTTGGGGGAGTGGATAGGCGCCGGGTATAAGGCAGCCCCGTGTGACGGTCGGGCCGCATTCGCACCCCGGCACTGCGAGCGACGGAGCGGCGGCCCGGCGGGAGGAGGAGACCCGGAGAGACAGAGACTAAAACCCGGCAAGAGAGAGACCGCGGGCCGCCGTCTCGAGTCTACCCTACCCCGGCTCATGGAACCCCGGCCCGGCACGAGCTCCCGGGCGGACCCCGGCCCCGAGCGGCCGCCGCGGCAGACCCCCGGCACGGTGAGAGGGCGACCCCCGGGTCTCAGGCCCCCCCTTTTCCCCGGACCACCCGGCTGCGGGTTGGGGGTGGTCGCGGGCGGTGGGCTCGGGGGCGGGGACGCTTGACGGGGCCGACCCCCGGCCCGCTTAAGCGGTCGGGGGACCCCCGTGGGCCGTGCGCCGCCCCCCGACCCTCTGGGGGGGCGAGGGAGGCAGGGAGGAGCCCGAGAGCGGGGGACAGGGGGGGAGACGAGGGGTCGGAATCCAAAGGACGCAGACCACCTTTGGTTACGGACCCCTTTCTCCCCCCCTTCCGAACAAAAAGCAGCGGGCGGGGGGCCGGGGTGAGGGAGGGACACGGGGGACACGGCGCGGGGGTCCCGCCTCACGCCCCGCGCCCTCTAAATCCCCCCCGTTGCTTTGTCAAGCAGCCCGCCGCCCCGCACGCCTGGGGGATGCTCAACGACATGCAGTGGCTCGCCAGCAGCGACTCGGAGGAGGAGACCGAGGTGGGAATCTCTGACGACGACCTTCACCGCGACTCCACCTCCGAGGCGGGCAGCACGGACACGGAGATGTTCGAGGCGGGCCTGATGGACGCGGCCACGCCCCCGGCCCGGCCCCCGGCCGAGCGCCAGGGCAGCCCCACGCCCGCCGACGCGCAGGGATCCTGCGGGGGTGGGCCCGTGGGTGAGGAGGAAGCGGAAGCGGGAGGGGGGGGCGACGTGTGCGCCGTGTGCACGGACGAGATCGCCCCGCCCCTGCGCTGCCAGAGTTTTCCCTGCCTGCACCCCTTCTGCATCCCGTGCATGAAGACCTGGATTCCGTTGCGCAACACGTGTCCCCTGTGCAACACCCCGGTGGCGTACCTGATAGTGGGCGTGACCGCCAGCGGGTCGTTCAGCACCATCCCGATAGTGAACGACCCCCGGACCCGCGTGGAGGCCGAGGCGGCCGTGCGGGCCGGCACGGCCGTGGACTTTATCTGGACGGGCAACCAGCGGACGGCCCCGCGCTCCCTGTCGCTGGGGGGACACACGGTCCGCGCCCTGTCGCCCACCCCCCCGTGGCCCGGCACGGACGACGAGGACGATGACCTGGCCGACGGTGAGGGCGGGCGGGGGTCGGGCGGGGGTCGGGCGGGGGTCGGGCGGGGGTCGGGCGGGGGTCGGGCGGGGGTCGGGCGGGGGTCGGGCGGGGGTCGGGCGGGGGTCGGGCGGGGGTCGGGCGGGGGTCGGGCGGGGGTCGGGCACTAACCGGGGGCTCCCGTCTCTGTCTCCCTCTGCAGTGGACTACGTCCCGCCCGCCCCCCGAAGAGCGCCCCGGCGCGGGGGCGGCGGTGCGGGGGCGACCCGCGGAACCTCCCAGCCCGCCGCGACCCGACCGGCGCCCCCTGGCGCCCCGCGGAGCAGCAGCAGCGGCGGCGCCCCGTTGCGGGCGGGGGTGGGATCTGGGTCTGGGGGCGGCCCTGCCGTCGCGGCCGTCGTGCCGAGAGTGGCCTCTCTTCCCCCTGCGGCCGGCGGGGGGCGCGCGCAGGCGCGGCGGGTGGGCGAAGACGCCGCGGCGGCGGAGGGCAGGACGCCCCCCGCGGGACAGCCCCGCGCGGCCCAGGAGCCCCCCATAGTCATCAGCGACTCTCCCCCGCCGTCTCCGCGCCGCCCCGCGGGCCCCGGGCCGCTCTCCTTTTTCTCCTCCTCCTCCGCGCAGGTGTCCTCGGGCCCCGGGGGGGGAGGTCTGCCACAGTCGTCGGGGCGCGCGGCGCGCCCCCGCGCGGCCGTCGCCCCGCGCGTCCGGAGTCCGCCCCGCGCCGCCGCCGCCCCCGTGGTGTCTGCGAGCGCGGACGCGGCCGGGCCCGCGCCGCCCGCCGTGCCGGTGGACGCGCACCGCGCGCCCCGGTCGCGCATGACCCAGGCTCAGACCGACACCCAAGCACAGAGTCTGGGCCGGGCAGGCGCGACCGACGCGCGCGGGTCGGGAGGGCCGGGCGCGGAGGGAGGACCCGGGGTCCCCCGCGGCACCAACACCCCCGGTGCCGCCCCCCACGCCGCGGAGGGGGCGGCGGCCCGCCCCCGGAAGAGGCGCGGGTCGGACTCGGGCCCTGCGGCCTCGTCCTCCGCCTCTTCCTCCGCCGCCCCGCGCTCGCCCCTCGCCCCCCAGGGGGTGGGGGCCAAGAGGGCGGCGCCGCGCCGGGCCCCGGACTCGGACTCGGGCGACCGCGGCCACGGGCCGCTCGCCCCGGCGTCCGCGGGCGCCGCGCCCCCGTCGGCGTCTCCGTCGTCCCAGGGGGCGGGGCTGGTGGGAGCGTCGCGTCCGCGTCCGGCGCTGGGGAGAGACGAGAAACCTCCCTCGGCCCCCGCGCTGCTGCGCCGCGGGGGCCGAGGAAGTGTGCCAGGAAGACGCGCCACGCGGAGGGCGGCCCCGAGCCCGGGGCCCGCGACCCGGCGCCCGGCCTCACGCGCTACCTGCCCATCGCGGGGGTCTCGAGCGTCGTGGCCCTGGCGCCTTACGTGAACAAGACGGTCACGGGGGACTGCCTGCCCGTCCTGGACATGGAGACGGGCCACATAGGGGCCTACGTGGTCCTCGTGGACCAGACGGGGAACGTGGCGGACCTGCTGCGGGCCGCGGCCCCCGCGTGGAGCCGCCGCACCCTGCTCCCCGAGCACGCGCGCAACTGCGTGAGGCCCCCCGACTACCCGACGCCCCCCGCGTCGGAGTGGAACAGCCTCTGGATGACCCCGGTGGGCAACATGCTCTTTGACCAGGGCACCCTGGTGGGCGCGCTGGACTTCCACGGCCTCCGGTCGCGCCACCCGTGGTCTCGGGAGCAGGGCGCGCCCGCGCCGGCCGGCGACGCCCCCGCGGGCCACGGGGAGTAGGGGGAGCTAACACTCGGCTTGCTGCCCAAAGGGAAGCCGCCCCCCACCGGACCACCGGCCGAGGCGCCTCGGGGGCAGGGGGAGGTGGGGGGGAAAGACGGGGAGGAGACAGGAAGTGGGGGTGGGAGTGGGGGGGGACGGACACGGCCCCGAACAGCAACACACACCAGCATTTTGTTATGGACTTTCTGGCCTTGTTGAAAACTTGAGGAAAAAAAACTTTATATTTATAAAAATTTTACAATAAAGTTTTGTGATGCTTTTGACACACTTTGTTGTTGGCCTTTGATGCAGCTCCCCCGCGCAGGGGGGCCGGGGATGGGGGGGGGGAAAGGGAGGAGGAGGAGGGGGGGCGGGCACGAGAAGCCGCCCCCACCCCCGAGGCCTGTTGGTCTTTATGATAGAACAGAGCCGGGGCCCGGCCTGCGTTCTGGCTCCCTGTCTTGGTGGGTGGGCGGGCTGGCTGGCGGGTAAAAAAAGAGTGTGTCCGTGTTGACAGGGAGGGGGGCCCGATCGTGCAGAGCACGCACGTCTGGCCGGCCAGACCCTGGGGGTGGTGGGCAGGAGTGGGAGGGCGCCTGGCTCGGGGAGGGAGGAGGGGGGGGGTCAGCCGCACCACCGGCGCGAAGCCAGGGGCCAGGGAACTTTGATAGAGAGGGGGGGAAAGTGGGGCGGGGGCGAGGGCGGTTGAATCACAACGCATGCACGCCCTCTGCCCCCGGGGACGGGTGGGAGGAAGGAGGAGGGAGAAGAGAAGACCCGAGGCATGCACCCGCACTTACGCCCGTGCCCACCCCCGCCCCGGCGCCCACCCCGCCCGCACACCTGCCCGCCACGCCCGCCCCTCCTCACCCTGGCTGGGAGAAAGGAGGAGGAGCAGGAAGAGGAGACCCGAGGCATGCAACCGCACTCACCCCACCCCGCCCGCACACCTGCCCGCCACGCCCGCCCCTCCTTACCCTGGCTGCGGGGAGACTCCCATCGGGGCGAGGGGGCTCGCGCGTTCGCAACACCACACCACACCACACGGCCCACCACAACACGGCCCACCACGACACAACACGACACGACGCGTTTTGCGGGGCATGCAAGTCGACACACCGCGCGCGTGCCTACCTTTCCCTAGCGGCCCCGGCCCCCGGCCCGTTTCCTTCCGCCACCACTACCACCACCCCCCCGCCCGCGCCCACGCGGTAGAGGAAGGGGACGGGCGCCACACCCACGGCTGTGGCTGGGCACGCGCCTTTGGGGTTGTTGGGGGGGGGTGACCGGCGCGTGGGGGCGGTGGGCGTATGGGCCCGACCCGCGCCTGCCCCCCCTGGGAACGACGCCCCCGGACGACACCACGGGGGGGGGAAACGGGGGTGGGTGGAAGGGAAGAGGAAGGAGAAAGGGGGGGTGGATCACGGGTTGGCTCGCGGGCGGTGCGGCTGACCTGCCTGCCCTTTCTGGGACCCCCGCCTCGTGTTTCTTGAAAGGGGGAGGAAGAACAGTTCTCCCCCAACCCCTGCTCTCTTCTCTCTTCCGCCCGCCCCCCCCCCCTCTCCCGCCGCCTCAGCAGAAGCTCACCTGTACGACCCTAAACCTACCTGCGAGAACGCGCGGCGTTCGAGGGGCGCGCTCTCTCACACGAGACACACGCAGGCGCCCCCCCCCCCCGGAGCCTGGGTCCCCCGGCGGACGGCTCACGCGGCGCGGCGTCTCGGTGGGACGCGGGCAGAGGGCGGCGGCGGCGGGGGGGGGGGGGAAATGTGAGGAGAGCGAGACAGAGAGAGAGAAGGAAGAGGGAAGGGGCGCGGCGGGACGGGGGAAGACGAGGAGAAGGGAAGGGGCGAGGGTCGGGCCCGGGAGCGGGGCGGCCGGGAGGGAGAAGAAACGGAACGCGGAAACGCCGCCGGCGCGGCCCGGGGCCCCGGGGCCCCCGCGCTCCGCCGGGGGCCCGGGCCGGACCGCCGGGCGGGGGACGCCTTCCGCCCGGCGCCGGGCGGCTACCCGGGACCCCCGGCCGGGAATCGAAAAAAGCCTCCGGGGGCCCCTTTCGCGCCTTTCGCGAACGCGCGGCGCCGGAGGGGGCGGCCGCCGAGGTGCGGGGGCCCCTCCGGCCGGGGCGCACCTCGGCGGCCAAGCCCCGGCCCGCCCGGGGGTCCCCGAGGCAGAGGCGGACCCTCGGAGGCGCGGAAGAAGACGGGAGGCGGGGGAAAAAAGGGGGAAGAGAGGGGGAGGTAGGGAGGGGAGAGGAGAAGGGCGCGCCGGTGCGCGGAGCAGCCTTCCTTCTCCGGAGTCCCTCTCGATCGGCGGCGGGCCCCTGCGTTCGTTGCTGCCGCGCCCCCGGTTTTATAAAGACAGGGATGACGCAGCAGAAATGCCCACAGCAACACGCGGGCGGGGCTCGGGCTCTCCGGCGGCTTAATGGATCTCCGGGCACGGCGCCCGCAACCGCAGAGCACTCAGCTGGCGCGCCCCCCCCCAACGTGGGAGTGTTTAATGGAAGGGCGTGGGGCCGGCCGCCGGATGCCCGCGGGGGCCTAATGCGGCGGGAGGCGTGGGCCGCTGGCGCCGCGGCCCGTCTGCTGGCCCGCGGCCCGTCTGCTGGCCCGCGGCCACGTAAACAATGACACAGGGGTTCTCTCCGCCGCGGCCGGCGCGGGGCGTTGCCGGCCCGGCCCGGCCCCGGAGCCCGCGGCGCTGCTCGGCTGCGGCCGCGGGCTCCGGGGGCTCCGCACTCTGCCCGGCTCGCCCCGTCCCCCCTCTTGCTGCTTTTCCGCCCGGCCGCCCACTCCCCGCTCGGCCTCTCCGGCTGCGGTGCTTGGGTCTCCTTCGTCGGGCGGCGGGGGGGGGGCGTCGGGACTCGCGGAGGGCCGGAGAATGGAAGGCGAGGGGATGCAGGAGGAGGATCGGGACTCCCCATCTTCTGCCCTTCCATCCTCCGTTTTTCCGCTTTCCACCGCCGCCGCCACCACCCCCCCTTCCTTCGCCCGCCCGCCTCGCCCCGGACCCCTCCCCCCCGTGTTCCCCCCATCGTTCACCACCACGCCCCCCACCGCGCCTTGGCTGTTTGGGGGGTGGCGGCGGTGGTCGGCGTGCTGCCGGAGGCTGCGGGCGCGGGGTAGGTGGGTGGGCGGGTGGTGGGGGGGGGCCCGGCTGCGTCTCGCCGCGATCCCGCCGGTGGGGCGCGGCGGCGGTCGGGGTGGGGGGAGAGTGTCGTGGGTGTGTTTTCGTGTCCCCCACCACCACTCCCACCCCGACCGCCGCCGCGCCCGCGTTTCTGCCGCCCGCGCGCTCCTGTGTGGACCCCGGGGTGGGCGGCGGGGGGGGGGTGCCGTGGGTGTGGCGGCGGGGCGCGGGCCGGGGCCGGGGCTCGCTGGTCCGCCGAAGTAAAGAAAAGATCGCCACCGTGTGTTCGTCTGTGTGTTCTGCGCGGCGCCGGGGCCCCCCTGCCGGGCGGGGCGGTGGGGCGGGGCCGGGGTCGCGGCGGGGAAGGAAGGAAAGACCCCGGAAGCGCCGGGAGGGGGCGCCGGCGCGACGCGGGCGGCCGGGCGGGGGCGCGCGGCGGCCGGGCGGGGGCGCGCGGCGGCCGGGCGGGGGCGCGCGGCGGCCGGGCGGGGGCGCGCGGCGGCCGGGCGGGGGCGCGCGGCGGCCGGGCGGGGGCGCGCGGCGGCCGGGCGGGGGCGCGCTTTCCCCGCGTCGCCCCTCGGGTTCCCAAGACCTATCACGTGTGCGCAGGGGAGGGGAGGACGCGGGGGAGGGGAGGACGCGGGGGAGGGGAGGACGCGGGGGATATATAAAGCGGTAGAAAGCGCGGGAATGGGCATATTGGACCCGCGTGATTCGGTTGCTCGCGGTTGTCTTGTTTGGACGTTTTTTATGCGGGAACAAGGGGGCTTACCGGTTACACTGTCCGCTCGCTATGGGGTTCGTCTGTCTGTTTGGGCTTGTCGTTATGGGAGCCTGGGGGGCGTGGGGTGGGTCACAGGCAACCGAATATGTTCTTCGTAGTGTTATTGCCAAAGAGGTGGGGGACATACTAAGAGTGCCTTGCATGCGGACCCCCGCGGACGATGTTTCTTGGCGCTACGAGGCCCCGTCCGTTATTGACTATGCCCGCATAGACGGAATATTTCTTCGCTATCACTGCCCGGGGTTGGACACGTTTTTGTGGGATAGGCACGCCCAGAGGGCGTATCTGGTTAACCCCTTTCTCTTTGCGGCGGGATTTTTGGAGGACTTGAGTCACTCTGTGTTTCCGGCCGACACCCAGGAAACAACGACGCGCCGGGCCCTTTATAAAGAGATACGCGATGCGTTGGGCAGTCGAAAACAGGCCGTCAGCCACGCACCCGTCAGGGCCGGGTGTGTAAACTTTGACTACTCACGCACTCGCCGCTGCGTCGGGCGACGCGATTTACGGCCTGCCAACACCACGTCAACGTGGGAACCGCCTGTGTCGTCGGACGATGAAGCGAGCTCGCAGTCGAAGCCCCTCGCCACCCAGCCGCCCGTCCTCGCCCTTTCGAACGCCCCCCCACGGCGGGTCTCCCCGACGCGAGGTCGGCGCCGGCATACTCGCCTCCGACGCAACTAGCCACGTCTGCATCGCAAGCCACCCTGGGTCGGGAGCAGGACAGCCGACCCGTCTAGCGGCCGGGTCGGCTGTCCAGCGTCGTCGCCCTAGAGGCTGTCCGCCGGGCGTGATGTTTTCCGCATCTACGACCCCCGAACAGCCCCTGGGGCTGTCGGGCGATGCGACGCCGCCCCTGCCGACTTCCGTGCCCCTGGACTGGGCCGCGTTTCGGCGCGCGTTTCTGATCGACGACGCCTGGCGGCCCCTGTTGGAGCCGGAGCTCGCGAACCCCCTAACCGCGCGCCTCCTCGCGGAGTATGACCGTCGGTGCCAGACCGAAGAGGTGCTGCCGCCGCGGGAGGATGTGTTCTCCTGGACGCGGTATTGTACCCCCGACGACGTGCGCGTGGTTATCATCGGGCAGGACCCGTACCACCATCCCGGCCAGGCGCACGGCCTGGCGTTTAGCGTGCGTGCGGATGTGCCGGTGCCTCCGAGTCTACGGAACGTGCTGGCGGCGGTTAAAAATTGTTACCCCGACGCGCGCATGAGCGGCCGCGGCTGCCTGGAAAAGTGGGCTCGCGACGGCGTGCTGTTGTTGAACACGACCCTGACCGTCAAGCGCGGGGCGGCGGCGTCCCACTCCAAGCTTGGATGGGACCGTTTTGTGGGCGGGGTGGTCCGACGGCTGGCCGCGCGCCGCCCGGGCCTGGTCTTTATGCTCTGGGGCGCCCATGCCCAGAACGCGATCAGGCCCGACCCTCGCCAACACTACGTCCTCAAGTTTTCTCACCCGTCGCCCCTCTCCAAGGTCCCGTTTGGGACGTGCCAGCATTTCCTCGCCGCGAATCGCTACCTCGAAACCCGGGACATTATGCCGATCGACTGGTCGGTATAAGATGCCGACATCCGGGGTCTTGATTTACGAGGGGGCAATTAATAAAGACTGTTGATGGTTAAATCTCGGGTCTCATACCGGTCCGTGATGTCGGGCGTGGGGGAAGAGAGGGTCCCCTCTGCGTTTACTATCCTTGCCTCGTGGGGCTGGACGTTTGCACCCCAGAACCATGATCCTGGCGCGTCGCCGAATACGACGCCCATAGAGTCGATTGCGGGGACCGCACCGGACGCGCACGTGGGGCCTCTCGACGGAGAGCCGGACCGGGATGCGATCTCCCCGCTTACGTCGAGCGTGGCCGGCGACCCGCCGGGGGCGGACGGCCCCTACGTCACCTTTGATACTCTGTTTATGGTATCTTCGATCGACGAACTGGGGCGCCGCCAGCTCACGGATACGATCCGTAAGGACCTGCGGCTGTCGCTGGCCAAGTTCAGCATCGCGTGTACCAAGACCTCGTCGTTTTCGGGGACGGCCGCGCGCCAGCGCAAGCGCGGAGCACCGCCGCAACGCACATGCGTACCACGCAGCAACAAGAGCCTCCAGATGTTCGTTTTGTGCAAGCGCGCCAACGCCGCGCAGGTGCGCGAGCAGCTGCGGGCGGTTATTCGGTCGCGCAAGCCGCGCAAGTATTACACGCGGTCCTCGGATGGGCGGCTCTGCCCGGCCGTCCCCGTGTTTGTACACGAGTTTGTTTCGTCCGAACCCATGCGCCTCCATCGAGATAACGTCATGCTGTCTACGGAACCAGACTAAGCACCCCCGCCGTCCCCTTTCTTTTCCCCCTACCCTTCCCCCCGTTACTGATGTGTTGTGACGTTTCAATAAATAACACGTAGCTTATTTTGTTGGATGATGGATTGATTGATTTTATTGACCGTTCGTTCGCCCGGCGGTGCCGTCGCCGCGCGCAGAGGGAATATGCAAGCGGGCGGGGTGGGGAGGAAAGAAGGTTTCAGGTTCCGGGGGTTGGGTCTGCGTCGTCCAGGGTGGGGCTGATCTGAATTTCCCGCAGAACCTCGACCAGTAGGTCTGTTGTGTTTGCTGGGAACTCGCCCGCCGTTGGGGATACGGGGGCGGGGGGTGTGGTCGGGCGGACGTCCAGGGGTGCGTTATCGCACCCCCGCGCCGCCTCGGGGGCCGTCCCGTAGATCGTTGCGGTGATGTAGATGGTGTCCGGGGTCCACACCACCGTCAGGATGCCGGCCGTCGCACTCCGGACGCTTTCGCCGTGCGATGAGCTGACCCAGGAGTCAAAGGGGTACGCGTACATATGGGCGTCCCACCAGCGCTCCAGCCTCTGGGTACTAGCGCGTCCTATAAAGCGGTATGCGCAAAATTCGGCACGACAGTCGATAATCACCAGCAGCCCGATGGGGGTGTGTTGTATCACCACGCCTCCGCGGGGCAGGCGGTCCTGGCGCGCTCGACCCCGCGTCAGAACCGCGCGCGTCCCTGACTCAAACACGTGCACCACCTGTGCCGCGTCCGGCAGCGCGCTCGTTAGCGACGCCCTGGGGTGATGTAGGCTGTACGCGATGGTCGTCTGGGGGTTCCCCATGTCTCGGGGGGGTGGGGGTGAATGTCACCCGGCCCGGGTGCGGTGGGAACGCGAGGGAATGGAGGGTTAATAGACAATGACCACATTCGGATCGCGTAGAGCAGATAGTATGTGCTCGCTAATGACGTCATCGCGTTCGTGGCGCTCCCGGAGCGGGTTTAGATTCATGTGCAGGAACTCGGATGAGGTGGTGCGGGACATGGCTACGTACGCGCTGTTTAGGCGCAGGTTTCCGGGCGTGAAGCATATGGCGACCTTGTCCAGACTGAGCCCCTGGGAGCGCGTGATGGTCATCGCGAGTTTGGAGCTGATGCCGTAGTCGGCGTTGATGGCCATGGCCAGCTCCGTGGAGTCGATCGACTCGACAAACTCACTGATGTTGGTATTGACGACAGACATGAAGCCGTGCTGGTCCCGCAGGACGATGTAGGGCAGGGGGGACTCCTCCAAGAACTCGGCCACGCCGGCCGTCGCGTGCCGCCGCCGCAGCTCCTCCGCGAACGCGAACACCCGGGTGTACGTGTACCCCATCAGCGTGTAGTTGTCCGTCTGCAGGGCCACGGACATCAGCCCCCCGCGCGGCGAGCCGGTCAGCAGCTCGCAGCCCCGGAAAATGACATTGTCCACGTAGGTGCTGAAGGGGGCGCTCTCAAACACCTCCCCGAAGAGCTCCCGTAGGATAAGGTATCGCCCCAGAAAGGCCCTCTTCAGGAGCCCAAACTGGGCGTGGACGGCCGCGGTGGTCTCAGGCTCTTCGAGGGCGTAGTGGCAGTAGAACACGTCCAGCTGCTGTTCGTCCAGCCCGGCGAAGATAACGTCAAGGTCGTCGTCGGGGAAGTCGTCCGGGCCCCCGTCCCGCGGGCCCAGGTGCTTAAAATTGAACGCACGCTCCCCCGGAGAGCGGTCGCTGGTGTCGGCGGCCCTGGTTGCCGATGCGCCGGCGGCGTCCCGGCGTAGCGACAGGAGTTCTGCCGTCAGCTCCCCTAGGCGGCCGTAGGCCAGGGTCCTCTGGGTCGCGTCCAGGCCGGGGCGCTGGAGAAAGTTGTAAAAGTGAATCAGCCCGCCGAACATGAGCCGCGACAGGAACCGGTAGGCGAACTCCACCGAGGTCTCCCCCTGGGTCTTCACGAAGCTGTCGTCGCGCAGCACAGCCTCGAAGGTCCGAAACGTCCCGTCGAACCCAAACACCATCTTTCGGAGGCGCGCGGTCACCGCGACCTGGCTGTTGAGGACGTACGTGATGTCGTTCCGGGCCACGACTAGCTGTTGCTTGCTGTGCACCTCACAGCGCACGTGCCCCGCGTCCTGGTCCTGACTCTGGGAGTAGTTGGTGATGCGACTGGCGTTGGCCGTGATCCACTTTTCCATGGTCAGCGTGGGTTGCTGCGTGAGCCGTCGATACTCGTCAAACTCTTTGACCGACACAAACGTGAGCACGGGGAGGGTAAACACAACAAACTCCCCCTCGCGAGTCACCTTTAGGTAGGCGTGGAGCTTGGCCATGTACGCGCTGACCTCCTTGTGGGACGAGAACAGCCGCGTCCACCCCGGAAGGTTGGCCGGGTTGGTGATGTAACTTTCCGGGACGACAAAGCGGTCCACAAACTGCATGTGCTCCTCGGTGATGGGAAGGCCGTACTCCAGCACCTTCATGAGGTTCCCGAACTCGTGCTCCACACATCGCTTGTTGTTAATGAAAATGGCCCAGCTGTGCGAGAGGCGCGTGTACTCGCGTAGGGTGCGGTTGCAGATGAGGTACGTGAGCACGTTTTCGCTCTGCCGGACGGAGCATCGCAGTTTTTGGTGTTCGAAGGTGGACTCCAGCGAGGCCGTCTGGGTCGGCGACCCCACGCACACCAGCACCGGCCGCAGGCGGCCCGCGTACTGGGGGGTGTGGTACAGGGCGTTAATCATCCACCAGCAATACACCACGGTCGTGAGTAGGTGCCGCCCCAGGAGCCCGGCCTCGTCGATGACGATAATGTTGCTGCGGGTGAAAGCCGGCAGCGCCCCGTGTGTGACCGAGGCCAGGCGCGTGAGGGCACCCTGGCCCAGCCCCAAAGTCTGCTCTAGGGCGGTGAGGGCGTGGAACTCGTTTCGCGCGTCTTCGCCCCCGTGCGCCGCCAGGGCCCGCTTGGTGATGTCGAGGATCACCTCCCAGTAGTACGTCAGGTCTCGCCGCTGCAGGTCTTCCAGCGAGGCGGGGCTGCTGGCCAGGGTGTACGGGTGCTGCCCCAGCTGGGCCTGGACGTGATTCCCGCGAAACCCGAACTCGTGAAAGATGGTGTTGATGGGTCGACTCAGAAACGCCCCCGAGAGCTTAACGTACATGTTCTGCGCCGCGATTCGCGTGGCGCCCGTGACCACGCAGTCCAGGACCTCGTTGAGGGTCTGCACGCACGTACTCTTTCCGGATCCGGCGTTGCCGGTGATGAGATACGCCGCGAACGGAAACTCCCGGAGCGGCAGGCCGGTCGGGACCTCCAAGGCCGCCACGTCCCGGAACCACTGCAGGCGCGGCACCTGCGTGACGTCGAGCTGCTGCTGCGAGAGCTCTCGGATGCGTGCGATGATTGGTTGGACCCCGTGCATGGACGTAAAATTTAAAAACGCCTCGTCCCTGAACCGCACGGCGGGTCTGGCCCCGGGCTGCTGTGGGGGCGGACCTGGTGCCCGGACGTCCCGCGAGCCCTCCCCGCCGGACGCCGCCATGGCCGCACAGCGCGCGCGGGCGCCGGCGATGCGGACGCGGGGCGGCGACGCGGCGCTATGCGCCCCCGAGGACGGCTGGGTGAAGGTTCACCCCACCCCCGGGACGATGTTGTTCCGCGAGATTCTCCTCGGGCAGATGGGGTACACCGAGGGTCAGGGGGTGTACAACGTCGTCCGGTCCAGCGAGGCCGCCACCCGACAGCTGCAGGCGGCGATCTTCCACGCGCTCCTCAACGCCACGACGTACCGGGACCTGGAGGAGGACTGGCGCCGCCACGTGGTGGCCCGCGGCCTCCAGCCGCAGCGGCTGGTTCGCAGGTACCGGAACGCCCGGGAGGGCGATATCGCCGGGGTGGCCGAGCGGGTGTTCGACACGTGGCGATGCACGCTCAGGACGACGCTGCTGGACTTTGCCCACGGGGTGGTAGACTGCTTTGCGCCGGGCGGCCCAAGCGGACCGACCAGCTTCCCCAAATATATCGACTGGCTGACGTGTCTGGGGCTGGTTCCCATATTGCGCAAGACGCGCGAGGGGGAGGCGACGCAGCGCCTGGGGGCGTTTCTCAGGCAGCACACGCTGCCCCGGCAGCTGGCCACGGTCGCCGGGGCCGCGGAGCGCGCCGGCCCGGGGCTTCTGGATCTGGCCGTCGCGTTCGACTCCACGCGCATGGCGGAATACGACCGCGTGCACATCTACTACAACCATCGCCGGGGGGAGTGGCTGGTGCGCGACCCGGTCAGCGGGCAGCGCGGCGAGTGCCTGGTGCTGTGCCCCCCCCTGTGGACCGGCGACCGCCTGGTCTTCGATTCGCCCGTTCAGCGGCTGTGCCCCGAGATCGTCGCGTGCCACGCCCTCCGGGAACACGCGCACATCTGCCGTCTGCGCAACACCGCGTCCGTCAAGGTGCTGTTGGGGCGCAAGAGCGACAGCGAGCGCGGGGTGGCTGGCGCCGCGCGGGTCGTCAATAAGGCGCTGGGGGAGGATGACGAGACGAAGGCCGGCTCGGCCGCCTCGCGTCTCGTGCGGCTCATCATCAACATGAAGGGCATGCGCCACGTGGGCGACATCAACGACACGGTACGCGCCTACTTGGACGAGGCGGGGGGGCACCTGATCGACACCCCCGCCGTCGACCACACCCTCCCTGGGTTCGGCAAGGGCGGCACCGGCCGCGGGTCGGCGCCCCAGGACCCGGGGGCGCGACCGCAGCAGCTTCGCCAGGCGTTTCAGACGGCCGTGGTCAACAACATCAACGGCATGCTGGAGGGCTATATCAATAATCTCTTTGGAACCATAGAACGCCTGCGAGAGACGAACGCGGGTCTGGCGACCCAGCTGCAGGCGCGCGACCGCGAGCTGCGGCGCGCCCAGGCGGGGGCGCTGGAGCGGGAGCAGCGCGCGGCGGACCGGGCGGCCGGGGGAGGCGCGGGCCGCCCGGCGGAGGCGGATCTTCTCCGGGCCGACTACGACATTATCGACGTCAGCAAGTCCATGGACGACGACACGTACGTGGCCAACAGTTTCCAGCACCAGTACATCCCCGCGTACGGCCAGGACCTCGAGCGCCTGTCGCGCCTCTGGGAGCACGAGCTGGTGCGCTGCTTCAAGATTCTGCGCCACCGCAACAACCAGGGCCAGGAAACGTCGATCTCGTACTCTAGCGGGGCGATCGCCTCCTTCGTGGCCCCGTATTTCGAGTACGTGCTTCGCGCCCCCCGAGCGGGCGCGCTCATCACCGGCTCCGATGTCATCCTAGGGGAGGAGGAGTTATGGGAGGCGGTCTTTAAGAAAACCCGCCTGCAGACGTACCTGACAGACGTCGCGGCCCTGTTCGTGGCGGACGTACAGCACGCGGCTCTGCCCCGGCCCCCCTCCCCAACCCCCGCCGATTTCCGGGCGAGCGCGTCCCCGCGGGGCGGGTCCCGGTCCCGGACCCGGACCCGATCCCGGTCGCCCGGGAGAACGCCGAGGGGTGCGCCGGACCAGGGCTGGGGCGTCGAACGCAGGGATGGCCGACCCCACGCCCGCCGATGAGGGAACGGCCGCCGCCATCCTCAAACAGGCCATCGCCGGGGACCGCAGTCTGGTCGAGGTGGCGGAGGGGATCAGCAACCAGGCGCTGCTGCGCATGGCCTGCGAGGTGCGCCAGGTCAGCGATCGCCAGCCGCGGTTTACCGCGACCAGCGTCCTGCGCGTTGACGTCACCCCCAGGGGGCGGTTGCGGTTCGTTCTGGACGGGAGTTCCGACGACGCGTACGTGGCGTCGGAGGATTACTTTAAGCGCTGCGGGGACCAGCCGACGTATCGCGGTTTTGCGGTCGTCGTCCTCACGGCCAACGAGGACCACGTGCACAGCCTGGCCGTGCCCCCCCTCGTTCTGCTGCACCGGCTCTCCTTGTTTCGCCCCACGGACCTCCGGGACTTCGAGCTCGTCTGCCTGCTGATGTACCTGGAGAACTGTCCCCGGAGCCACGCCACGCCCTCGCTGTTCGTCAAGGTGTCGGCGTGGTTGGGGGTCGTGGCCCGCCACGCGTCTCCCTTCGAGCGCGTCCGCTGCCTTCTCCTCCGCAGCTGCCACTGGATCCTGAACACGCTAATGTGCATGGCGGGCGTGAAGCCCTTCGACGACGAGCTAGTCCTGCCCCACTGGTACATGGCCCACTACCTGCTGGCCAACAATCCGCCCCCCGTCCTCTCGGCCCTGTTTTGCGCCACCCCGCAGAGCTCTGCGTTGCAGTTGCCCGGGCCCGTCCCCCGCACGGACTGTGTGGCCTATAACCCGGCCGGCGTCATGGGAAGCTGCTGGAATTCCAAGGACCTGCGTTCGGCTCTGGTGTATTGGTGGCTTTCGGGGAGCCCCAAACGACGGACCTCGTCGCTTTTCTATCGGTTTTGCTAACTCCGGAAAATAAACGTGTTTTTTATGGAACGTTCCCCACCTGTCGTGTCATCTCTCGGGGGATGGTGGTGGGCCTGTGTGTGTGTCTTGTGCACCGAAGGAGGAAAGTGGGGGGGTGGTGGTGCTGGTGGTGGAAAGACATGATAGAGGGAACAAAGAAATAGAAGAAAACCACAACCGGCGCGTGCCAGTAAATACGGACGCGCGCACACGCGGGGGGTAAGTTGGAGCACGGGGCCCCGGTTTATTGACCAAATTCAGGGAAACAGAAACCGCATCTTTTCCTCGAAAGGGTACACAAAGCTCCCGCCCTCGCCCCACACGCCTTCCAGAACCCCCGTAAACACCAGTTGAATCTCGCGCAGGATCTCGCGCAGGTGATGGGCGCAGTCCACGGGGGGGAGCACCAAGGGCCGCGGGTACAGATCCACGGGGACGCCGACCGACTCCCCGCCCCCGGGACATACGCGCACGACGCGTCTCCAGTATTGCTCCGCGTCCAGCAGGGCGCCTCCGCGGAAGGCCGTTTGGGGCAGGGGGTCGTCGGCCTCGCCTGGGGGGGTCAGAACGCTCCAGTACTCCGCGTCCAGACGCCTCCCGAAGGCATCCAGGACAAAGCGGTCACAGGCGTCCTCCATGACGCCCCGGGCCGCGCACACGGCCTCCTCCGGCGGGCCGGCGGCCGGCCGCCGGAGGATTCGTCTCAGCGCGTCGCGCATAACCTCGGCCGCCGCGGCGTACGCGGGCCCGCGGAGAGGAAATCCCTGCAGGAAGTCGGTGTCATCGCGGGAGTTCCAGAACCACGCCCCGGTCTGGCTCCAGGTGACGACGTGGGTGTAGACGCCCTCTGGCGCCAGGGAGGGGGCGAGGCGCGGGCGTATGCCGTTGGCCGAAAGTACGGCGCGCACGGACGCCTCGAGGGCCCGG

TGCCGCCGCAGACCGCAAACTTCACCGAGCTGGCCGTCTGCTCGATCTGCAGGCAGACGGCGGCCATGACCCCGCCGAGCAGCTGCCGGAGCGCGGGGCAGGCGTCGCACGCGTCCGGCACCAGGCGCTCCAGCACGGCCCGGGCCCAGGGCTCCGAGGGGGCGGCCGCCACCAGCGCGTCCAGCCTTTCCAGGCCCGCCCGCCCCCGGGCTTCCGGCAGCCCGGCCTCCCCGAGGCCCGCGAGGGCGGCCAGGAGCTGGGCCTGGAGCCCGGAGAAACAAAACCGCGCCGTCCAGACCGGCCCGACGGCCGCCGGGGGGTCGAGTAGTTGGATGGTGGTGGCCGTGGGGTGCCACCGCGCCACCGCTTCCCGAAAGGCGGGCAGGAGGCGGCCGGCCGCCTCCGAGGCCACGGCCGGCCATGCCCGCGGGGGCAGGACGACCCTGGCGCCCACCGCGGGCCAGGCCCCCAGGCACGCGGCATGGGTGGCCGCGGCGCCCCGCACCAGGTCACGCGCCGACTCGGCGGCGGCGGCGGCCGGCACGGTAAACGTGGGCCAGCCCGGAAATCCCAGCACGGCAAAGTATTGGACGGGCCCTCCCCGGACCTCAAACCCGGGCCCCAGAAAAGCGAAGACGGGGGCCAGGGCTCCGGGGGCGGCGTGGACCGTGGTATGCCACTGCCGGAAGAGGGCGACCAGCGCCGGGGCGGAGAACCCGTCGCCGGCGCTCACGAAGTAGTCGTAGCCGCGCGGCAGCAGCACCCGCGCCGTGACCCGCTGCGGGTGTCCGCGGGGCCGCAGGCCGACCTCGCACACCTCGACCAGGTCCGCGAAGGCGCCCTCCTTCCTGGTCGGCGGAAACGCCAGGGTGGTGTATTCGCGCGCAAAACGCGCGGTCCTCGTCGTGATGGTGACGGCGAGCGAGGCGGAGGACGCGCACTGGGGGCTGTCGCGAATGGCGGCCAGGCGCGCCCACGCCAACCGCGCGCCGGGGTGCTCGGCGACGCGCGCGGACAGGGCCAGCGGGTCGACGTCGACCTTGGCCTCCACGTCCAGGAGGGCGGCGCGAGGAGCGGCCGGCGGGCCCCACGACGCCCTTTCGACCCTCACGACCAGACCCGTCTGCGGGTCCCAGCCCAGGCGCAGCGGGACGAAGAGGGCCACCGGCCCCGTCTGGCGCTCCAGGGCCGCCAGAACGCACGCATACAGCGCCCGCCACAGGGTCGGGTCCCCCAGGGGCTCCAGCGGGGAGGCGGCCGGGGCCGTCGCGGCGCGGGCGGCCGCGACGGCCCCGGCGGCCGAGACGTCGGGGGAGCCGTAGAAGTCCTGCAGGTCGGACGAACCAACGGACACCTCCGCGAAGCGCGCGCGCGCCTCCCCCGCGGCGTCGCGACAGACCAGATACAGCAGGGCGTGGAGGCAGTCGCGCGTGCGCGGGGGCAGCCATACCGCGTATAGGGTAATGGCGCTGACGCTCTCCTCCACCCAAACGATGCCGGGGGCTTCCATGCCACGACGCCCGGGGGTTGCCGTGTATCGAACGAGCGCGGCCCCAGACTTATAGGGTGCTAAAGTTCACCGCCCCCTGCATCATGGGCCAGGCCTCGGTGGGAAGCTCCGACAGAGCCGCCTCGAGAATGATGTCAGTGTTGGGCTGGGCGCCGGAGGCGTGCGTGCGCAAGCAGCGCCCCCACGCGGGCGCGCGCAGCTTGAAGCGCGCGCCCGCAAACTCCCGCTTATGGGCCATCAGCAGCGCGTACAGCTGTCTGTGCGTCCGGCAGGCGCTGTGGTCGATGCGGTGGGCGTCCAGCAGCTCCACGATGGCTCGCTTGGTGAGGTTTTTAACGCGCCCCGCCCCGGGAAACGTCTGCGTGCTCTTGGCCAGCTGCACCCCGAACAGTTCGCCCCAGATGATCTTGAACAGCGACAGCGCGTGCTCCGTCTCGCTCACGGACCCGCGCGGGGGGCAGCCGCTCAGGGCGTCGGCCACGCGCTTAACCGCGTCCTCCGACAGCAAGGGGCCGTCGGTCACGTTACAGTGGCCCAGTTCGAACACCAGCTGCATGTAGCGGTCGTAGTGGGGGTTCAGCAGCTCCAGCACGTCCTCGGGGCTAAAGGTTCGCCCCGACCCCCCGGCCATCGAGTCCCACTGCAGGCACGCGGCCATGGTGCTGCACAGACGGAACAGCTCCCAGACGGGGGCGACGTTTAGGGTGGGGTGTAGGGCCACAAGCTCCAGCTCTCCGGCGGCGTTGATCGTGGGGATGACGCCCGTGGCGTAGTGGTCGTAAAGCCGCCGGAAGATGGCGCTGCTATGGGCGGCCATGGGGACGCGAAGACAGGCCTCCAGCAGCACCAGGTAGATGAACCGCGTGCGGCCGACCAGGCTGTTGAGGCCGCGCATGAGCGCGACCACCTCGGCCGGCGCGACGTCCGGCCGGAGGTACTTTTCGACGAAAAGGCCCACCTCCTCCGTCTCGGCGGCCTGGGCCGACAGGGACGTGTCGGGGTCCTGGCAGCGCAGCTCCCGCAGATCCCGCTGGGCCCTCAGGGCATCAAAATGTATCCCCCGCAAAAACAGACAAAAGTTCCTCGGGGTCAGCGCGGCGTCGTGGCCCCAGAACCGCACGTGCATGCAGTTGAGGGTCAGAAGCATGTGGAGGATGTTAAGACTGTCCGCGAGGCACGCCAGCGTGCACCTCTCGAAGTAGTGCTTGTACCGGAATTTGCTGTAGATGCGCGACCCCCGCGCCTGCGCCGCGTCGGCGTGCGACGCGTCGCAGCGCCCTTTGAACCGGCGGCACAACAGGTTCGTCACCTGGGAAAACTGTGCCGGCCACTGCCCGCTGGCGCTCACCACGTGGTTGAGCAGCATGGGCGTAAAGACGGGCTCCGAGCGCGCCCCGGACCCGTCCATGTAGATCAGCAGCTCCCCCTTGCGGAGAGTCCGTACCCGCCCCAGCGACTGGTACACGGACACCATGTCCGGCCCGTAGTTCATGGGTTTCACGTAGGCGAACATGCTGTCAAAGTGCGGCGGATCGAAGCTAAGGCCCACCGTCACGACCGTTGTGTAGATGACCACCCGGTACCGGCCCCATGTGGTCACGTCGCCGGGCGGGGTGAGCGAGTGGAGCAGCAGCACGCGGTCCGTAAACTGCCGGCAGAACCTGGCAACGACCTCCGCGAAGGAGACCGTCGACGAGAAGATGCAGACGTTATCTCCGCCGGCCAGGCGCGCCTCCAGCTCCCCGAAGAAGGTGGCGTCC

GCAGGGCCGCCTGCAGGACCTCGGGCCCCAGGCGCGGGAGAAACAGACAACGGCGCGCCGAAAATCCGGGCATGGCGTACTCCCCGATGACCACGTGAACGTTCTTTTCGCCCCGGAGGCTGCACAGAAAGTCCACCAGCTGCGCGTTGGCGGTGGCGTCCATGGCGATGATCCGCGGGCACGTGCGCAGCAGGCGCAGCATCAACGCGTCGACGCGGCCCAGCTGCTGCATCGTCGGCGAGTACAGTTGGCCCAACGTCGACATGACTTCGTCCAGGACGAGCACGTCGTAGTTGTTCAACAGGTTCGGGCCCACGCGATGAAGACTTTCCACCTGCACGATGAGACGGTGGAAGGGGCGGTCGTTCATGATGTAATTGGTGGATGAGAAGTAGGTGACGAAGTCGGGCAACCCTGACTCAGCGAACCGCGTCGCCAGGGTCTGAGTAAAACTCCGACGACAGGAGACGACCAGCACACTCGTGTCCGGAGAGTGGATCGCTTCCCCCAACCAGCGGATCAGCGCGGTAGTTTTTCCCGAGCCCATTGGCGCGCGGACCACAGTTACGCACCGGGCCGTCGGGGCGCTCGCGTCCGGGAAGGTGACGGGTCCGTGTTGCTGCCGCTCGATCGTTGTTTTCGGGTGGACCCGGGGAACCCACTCGGCCAAATCCCCCCCGTAAAGCATCCGCGCCAGCGATACACTCGACGTGTACTGCTCGCACTCGTCATCCCCGATGGGACGCCGGGCCCCCAGGGGATCCCCCGAGGCCGCGCCGGGCGCCGACGTCGCGCCCGGGGCGCGGGCGGCGTGGTGGGTCTGGTGTGTGCAGGTGGCGACGTTCATCGTCTCGGCCATCTGCGTCGTGGGGCTCCTGGTGCTGGCCTCTGTGTTCCGGGACAGGTTTCCCTGCCTTTACGCCCCCGCGACCTCTTATGCGAAGGCGAACGCCACGGTCGAGGTGCGCGGGGGTGTAGCCGTCCCCCTCCGGTTGGACACGCAGAGCCTGCTGGCCACGTACGCAATTACGTCTACGCTGTTGCTGGCGGCGGCCGTGTACGCCGCGGTGGGCGCGGTGACCTCGCGCTACGAGCGCGCGCTGGATGCGGCCCGTCGCCTGGCGGCGGCCCGTATGGCGATGCCACACGCCACGCTAATCGCCGGAAACGTCTGCGCGTGGCTGTTGCAGATCACAGTCCTGCTGCTGGCCCACCGCATCAGCCAGCTGGCCCACCTTATCTACGTCCTGCACTTTGCGTGCCTCGTGTATCTCGCGGCCCATTTTTGCACCAGGGGGGTCCTGAGCGGGACGTACCTGCGTCAGGTTCACGGCCTGATTGACCCGGCGCCGACGCACCATCGTATCGTCGGTCCGGTGCGGGCAGTAATGACAAACGCCTTATTACTGGGCACCCTCCTGTGCACGGCCGCCGCCGCGGTCTCGTTGAACACGATCGCCGCCCTGAACTTCAACTTTTCCGCCCCGAGCATGCTCATCTGCCTGACGACGCTGTTCGCCCTGCTTGTCGTGTCGCTGTTGTTGGTGGTCGAGGGGGTGCTGTGTCACTACGTGCGCGTGTTGGTGGGCCCCCACCTCGGGGCCATCGCCGCCACCGGCATCGTCGGCCTGGCCTGCGAGCACTACCACACCGGTGGCTACTACGTGGTGGAGCAGCAGTGGCCGGGGGCCCAGACGGGAGTCCGCGTCGCCCTGGCGCTCGTCGCCGCCTTTGCCCTCGCCATGGCCGTGCTTCGGTGCACGCGCGCCTACCTGTATCACCGGCGACACCACACTAAATTTTTCGTGCGCATGCGCGACACCCGGCACCGCGCCCATTCGGCGCTTCGACGCGTACGCAGCTCCATGCGCGGTTCTAGGCGTGGCGGGCCGCCCGGAGACCCGGGCTACGCGGAAACCCCCTACGCGAGCGTGTCCCACCACGCCGAGATCGACCGGTATGGGGATTCCGACGGGGACCCGATCTACGACGAAGTGGCCCCCGACCACGAGGCCGAGCTCTACGCCCGAGTGCAACGCCCCGGGCCTGTGCCCGACGCCGAGCCCATTTACGACACCGTGGAGGGGTATGCGCCAAGGTCCGCGGGGGAGCCGGTGTACAGCACCGTTCGGCGATGGTAGCCGTTTCGTTCGTTTTAATAAACCGACGTTGTGCGTTTCACCATACTTCGGCGCGCGTGTGTGTGTGTTTTTTTTTTTGTGGTGTTTATTTTCCCCCCACCCCTTCCTTTTCTTTCGGCCACCACCCCCCTCCTCCCCCGTACTATACAACAAAAAATACCACACATACGACCAAATACGGACAATCATTTCTGTCTTTATTCGCTATCAGAGAGTGGGGGCGTGAGCGTGGCAGGAGGGCGGGCCACGTCGGGGTCCCGCCGTCTGGTGTGACGCGATGGGGGGTCCGATGCGCGCCGGTACTGGGGCCCCGGCGCCCGGGTGACCACGCGCACGTCGGGGGGCACGTAGAAGTTACCCTCTTCTTCGGACTCGATGTCCACGACGTCAAATTCGTGGGCGGTCAGCGAGACGACCTCCCCGCCGTCGGTGGTGATGACGTTGTGTCGGCAGCAGCAGGGCCGCGCCCCGGAGAACGCGAGGCCCATAACTTGGCGAGCGTATCGTCGAAGGCCAGGCGGCTGTTTCGCCGGATGTCCCGGTAGATCCCCGGCTCGACGCGGACGGGGGTGATGATCAGGGCGATCGGAACGGCCTGGTCCGGGAGGATCGATGCCTTGGCGGGTCCGGGGGCCCCGCCACGCCCGGCGGGCGCTCCGCGGCCGTCCTCCAGGCGGAACGTCACGCCCTCCTCCGCGCCCGCGCGGTGCCTGCCGAGGAACGTCACCAGGTGCGGTTGCAGGGGGCAGTCGGGAAAGTGGCTGTCGAGGACGTATCCCTGCACCAAGATCTGTTTGAAGTTCGGGTGGCGGGGGTTGGCGAAGATGGGCTCGCGGCGAACCAGCTCCCCGGAGCTCCAGGCCACGGGAGAGATGGTGCGACGCTCAAGGTCGGGGACGCCAAACAGAAGCACCTCCGAGACAACGCCGCTATTTAACTCCACCAGCGCCCGATCCGGGGCGGAGCATCGCCTTTTTTCGCCGGCGGCGCGGGAATCGAGCCAGTCCCGGTCTTGGGTGACGAGCGCCTCCTCCGGGCCCGGAACGCGCCCGGGCGCGAAGTAGCGCACGCCGGGGTTGGGGATGGACCGGATGAACGCCCGGAACGCCTCCGGCGATCGCCGCGCCATCAGGTCCTCGTACGCGGAGGCCGCGGGGGCGCCGGGGTCCGCGGGGTCGAACGCGTACTTGGCTCGGCACTTAACCTCGTAGAAGGCCAGGGGGGTCTGGGGGGCGGGGGCCAGGTAGCCGTGAGGGTCCCTGGGGCACACGAGGATGTCCAGGGACGCCCCCACCATGCCCGTGTGGCCGTCCATGAGGACCCCGCACGCGTGCACGTTCTCCTCGGCGAGGTCCCCGGGTTGGTGAAAGACGAAGCGCCCGGCGTCGGCGTCGTCGTTGACGCCCGCGTCCGCGCGGCCCACGCAGTAGCGAAACAGCAGGTTTCGGGCCGTCGGCTCGTTCACCCGCCCGAACATCACCGCCGACGACTGGGCGTCCAGCCGCAGGCTGGCGTTGTGGGTGAGCCACTGGGACGAGAAGCACGGACCCTGCGCGCCCCACCGCAGCGTGGAGGCGGTCGTCAGGCCCCGCCGAAGCAGGGCCCAGAGCTGGCAGTCGGCCTGGTTTTGCGTCGCCGCCTCGTAAAATCCCATAAGCGGGCGGGGGGCGACGGCTTCGGCGGCGGACGGGGGGGCGCGGCGCGTCAGGCGCCAGAGGTGCCGGCCGAGCCCGCGGTCCACCATGCCGGCCGCCTCCAGCGACACGACGAGGGAGCACAGATAGTCCAGGCGAGCCCACAGGGGCCCGATGGCCAGAGGGGAGCGGACGCCGCGCAGCAGGCCGCGCAGGTGGCGCTCGAACGTTTCCGCCAAGATATGGGGGGGCAGTGCGTTGGGGATCGCCGACGCCGACCACATCGGGTCGGGGTCCGGGGGACCGGGGCTGCAGTCCGGGTCGATGGCGTGTGCGCCCCCCGGCGAGAGGGGAATGTCGGGGGTTGGCGGGCCGGATGAGGCCTCAGAGAGGGCCGGGGACGCGGGCCGGGCCTTTTCGCCCGGGGCCCCGCCGTCGGGTTGCCCACGTGGGGGGCTCTGGGGCCAATGGGAACCCGGGGCCCCCGGTGACGTGGGGCGGGGTGGGGCGGGGCGGGGCCCAAAGACGGTCGCCAGATCTAGGCTGTTGGGTCGGGGCCGCTTCGGGGGACTATCGGGGTCGCGGGCGGGGTCCGCGGGGCGCTTGGCGCCGGGTGTTGCGGCGGCCGCCATTTTTACGAGCAGCCGAAGAGCTCGAGGGCGGAAGGGATCCTCACGACAGAGAGTGGCGCGCGGCCGGGTTGGCGTGACAGAGGCGGGAGACCAGCACCAGCAGCGGCCTCAGCTCGGGCGGCAGCGACACCGACGACAGGACGGCCTTGTGCGTGCGCTGGTAATTTATACACTGCTCCGTGAACGCGCGCCGAATCTTGGGATTGCGAAGGTGGCGCCGGATGCCCTCCGGCACGTCATACGCCAGGCCGTGGGTGTTGGTCTCGGCCGAGTTGACAAAGAGGGCGGGGTGCAGAACGCAGCGATAGGCGAGGAGGGCCACGGCAAAGTCCGGCGAGAGCTGGTTGTTAAAGTACTGGTAGCCCGGGACGCGGGTCACGGGGACGCCCAGGCTCGGGGCCACGTACACGCTAACCAGCAGCTCCAGCAGCGTCTGCCCCAGGGCGTAGAGATCGACCGCCAGCCCGACGTCGTGCTTCAGGGGGCGGTTGTTAAACTCGGCCCGCTCGTTGTTGAGGTACTTTACCGAGAGCTCCGGTGGCTGGTTGTACCCGTGCCCCACCAGAGTGTGAAAGTTGGCCGTGGTCAGGGCGGCGGGCATCCCAAACCCCCGGGGGGACTCGAGGTCCGGCTCCTGGAGGCAAAACTGGCCCCGGGATATCGTGGAGTTGGAGTTCAGGGTCACCAGGCTAAAGTCGGCCAGGACGGCCCGCCGGAGCGACACCGCGTCCGATCGCAGCATCACGAGGACGTTGGCGCACTTGATGTCCAGGTGGCTGATCCCGCACCTGGTGTTCAGGAACACCACGGCGCGCGCCAGGTCTGTGAAGCAGTGGTGGAGGGCCGTCGCGACGGAGGGGGTGGTCGCGCGCAGGGACGCCAGCTGGCCGATGTACTTGCCGAGGTCCATGTCGTACGCGGGGAACACGATCTGGCGCTGCTGCAGCGAGAACCCGAGCGGGGTGATAAAGCCGCGGATGTCGTGGGTGCGGCCGCCGCGAAGAGCGCACTCCCCCACGAGCAGGGTCGCGACGAGCTCCACGGCAAACCACTCTTTTTCCCGGATGGTCTTCACGGCGAGCTTGTGTTCGCGAATCAACTGCACCTCGCCGTACCCCCCCGAGCCCCCGAAGCTGCGGGCCCCGGGGATCTCCAGGGTCGTGTAGCGGAGGGCGGGGTTGACGGCGAATACGGGGATGCATAGCTTGTGGATGCGCGCGAGGGACAGGATGTGCGAGGGGGGCGACGGGGGCGAGGTCATGGCCGTCTCGGACCTGCGCAGGGGCGGGCGCCTTAGCTTGGCCGCAGGGCCGGGGGCCTCGGGGGACGAGCGGCGACGAGACGAGCGGCTCACTCGCCATCGGGACAGTCCCGCGCGAAGCCGCTCCCGGAAGCTGGATCGGCGGCGGGACCCGGGGCGGGCTCCGGAGACGGCGCCGTCTCGGGGGGAGGGGCCGCTTGGGCGTCCGGACGCCCGGCGGCTGAGGGAGTGTATGTAGGACGCGAGCCAGGCCTTGAAGGAGCGTCGGTGTGCACCTTGGGGGCTGATGTCAGCTGCCACATGACTAGCAGGTCGCTGTCGCCCGGACTCATCCATCCGTCCGCCAGGTCGCCGTCCCCCCACAGAGACGCGTTCGCCGCGGCCTCTTCGAGCTGCTCCTCCTGGTCCGCAAGACGATCGTCCGCCGCGTCCAGGCGCTCGCTAAGCGCGGGATCGAGGTACCGTCGGTGTGCGGTTAGAAAATCACGTCGCGCCGCTTGCTCTTCCACGCGAATTTTAACACAGGTCGCTCGCTGTCGCATCATCTCTAAGCGCGCGCGGGACTTTAGCCGCGCCTCCAATTCCAAGTGGGCCGCCTTGGCGGCCATAAAGGCGCCAACAAACCTAGGATCTTGTGTACTCACGCCCTCCCGGTGTAGCTGCAGGGTCTGGTCCCTGTACACCTCGGCCCGGAGGTGCGTCTCGGCCAAACGTCGGCGCAGGGCCGCGTGGCTGGCGTCTCGGCTCATCTCGCCGCCCCCGCGCGCGCCCGACGTCGGACTCCTTCGCCCCGACCCCCCTGACCTCAGCCGCCCCCGCCTCGCCCGCGATGTTTGGCCAGCAGCTGGCGTCCGACGTGCAGCAGTACCTGGAGCGCCTGGAGAAACAGAGGCAACAGAAGGTGGGCGTCGACGAGGCGTCGGCGGGCCTGACGCTCGGCGGCGATGCGCTGCGCGTCCCTTTTTTGGATTTTGCCACCGCGACGCCCAAGCGCCACCAGACCGTGGTCCCGGGCGTCGGGACGCTCCACGACTGCTGCGAGCACTCGCCGCTCTTCTCGGCCGTCGCGCGGCGGTTGCTGTTTAATAGCCTGGTGCCGGCGCAACTCAGGGGGCGTGACTTTGGGGGCGACCACACGGCCAAGCTGGAGTTCCTGGCCCCCGAGCTGGTGCGGGCGGTGGCGCGCCTGCGGTTTCGGGAGTGCGCGCCGGAGGACGCCGTGCCCCAACGCAACGCCTACTACAGCGTCCTGAACACGTTTCAGGCCCTGCACCGCTCCGAAGCCTTTCGGCAGTTGGTTCACTTCGTGCGGGACTTCGCCCAGTTGTTGAAAACCTCGTTCCGGGCCTCTAGTCTCGCGGAGACTACGGGCCCCCCGAAGAAACGGGCCAAGGTGGACGTGGCCACCCACGGGCAGACGTACGGCACCTTGGAGCTCTTCCAGAAAATGATACTAATGCACGCGACCTACTTTCTGGCCGCCGTGCTGCTCGGGGACCACGCGGAGCAGGTCAACACGTTCCTGCGGCTCGTGTTCGAGATCCCCCTGTTTAGCGACACGGCCGTGCGGCACTTCCGCCAGCGCGCCACCGTGTTTCTAGTCCCCAGGCGCCACGGAAAGACCTGGTTTTTGGTGCCCCTCATCGCGCTGTCGCTCGCGTCCTTCCGGGGGATCAAGATAGGCTACACGGCCCACATCCGCAAGGCGACCGAGCCCGTGTTTGATGAGATCGACGCCTGCCTGCGGGGCTGGTTTGGCTCGTCCCGGGTGGACCACGTCAAGGGGGAAACCATCTCGTTCTCGTTCCCGGACGGCTCGCGCAGCACGATCGTGTTTGCCTCCAGCCACAACACGAACGTAAGTACGCCTTCCTCCCGCGGTGCCTGTTTCCCCGGTGCCGCCCTCCCCGAGATCGACCGACAGACAAACACAGCCAGACGCGAGTGTGGGACGACACGCCCGCAGCCCCCCCCCCGCCATGGCGGGGGGGAAGCCTTACTGTTTATTTGTAATCGGACGATGAGGCTCTGGCCACGGCCCGCGCGACCGCGGGGCAGCTCGTTGCAAACAGGCGGCTGGTATACGATGACAGAACGCAGAGGCGCCACCCGGCGCTGGTCGGGCGGATGACGCTTTCCGCGCCGTCCCGGCCCACGACGACCTCGTGCAGGTGGGCCGTGATGCGCGGGCGGCGGGTCGCCTGCCGCAGGATAACCGCGTCCACGGGGTGCCCGAAGAGGAGCTGACACAGGCTCGCGTCCCCCCGGACGGCCAGGGTGCGCTGGGCCATATTGGACCACATGCACGGGGCGACGCAGGGACAGGCCTCCGCCACGGCGGGGGCGCGCCACAGCGCGTTGGCGGAATCGATGTGGGCCGTCGGGGCGCAGGCGCCGCCTCCTCCCGGGGGGTCGGTAATCCTGGATAGCAGCCATCCTAAATGGCGGGCCCGGCTGCCCGGGGGACAGAGCGACCCCAGGTCATCATCCATGGCCCAGCAGTATATGCGGCCGCCGGGGAGGTGCCACCAGGCCCCCGGACCCAGGGCACAGCACGCCCCCGGATTCGGGGGCGGTTCCGTGGGTACCAGGTAGGCGCCGTCGAGCTCGTGGGCCACGGGCTCGTCCGCGAGCTGTTCGGCGGCGGGGTCGGGGGTTTCCTCCGGGGGGGAGGCAGCTTCCAGGTGGCCGAAGGCTAGGGTGCACAGCAGCGGGGTCCGGGGGTGCGTTACGCTGCGGAGGTGGACGGTGGCGCAGTAGCGGCGCTCGCGGTTAAAGAAGAAAATGGCAAAGAACGTGTTCGAAGGCAGGCGCAGCGCCTTGGGCCGCGTCAGGTACAGGAAGATCTCGCAGAAAAGGGCACGCTCGGGGTCGGGGTCCGGAAGGGCCACCTGGCACAGCGGCTCGGTGAGGACCGTGAGGCACCGAAAAATCTTAAGCCGCTCGTCCCCCCGAACGACGCGCCACACGAAGACAGAGTTGGCGATGCGCGCGACGAGGTCGGCTTCGGGCCCCGGGTCGGGGGCGCGCGCGTCGGGGGGGGCGCCCCGGTGACCCGGCGGGGCCGCGGCTCCCGGGGGGCCTGGCGTCGCCTGGGGACGCCAGAGTGCCCGCTGTGCCAGGTTGGTGGTGGGGAAGGGACCGGAGACGCACCAAAAGCAGAGGGGCCAGCGCGTGTATGAGTTGGGGGGGGGGTGGGTGAGCGGTGGAACAAAAGCACGCGTCAGCGGACAAGGCCGGGTCCCGTAGCCGCCCCGCGACAGAACCGGAGTCCGACGGCACGCGCGACGGGGTCTGCGAGGCTGAGGTACGCCGCGGTGTTAATGGTAAACGCAAAGCCTCCCGGAAAGACCACTAGCCCGCAGAGGCGGCGATTGAACCCAAGGCAGAGGTACGCGTAGCTCTCTCCCGGAAGGTATTGCTCGCAGACCCTGTGTGGGGCAGTGGAGGGGCTGCCCTCCATGAAGCGACATTTACTCTGCTCGCGTCCATTGACGTCACCGTCAATCACCACTGCGATTGGACGGTTGGTGAGGCGCAGCGTGTCTCCGCTGGTGCTGTAGTAGTCAAACGCGTAGTGGGCGTCGGAGTCGGCGAAGCGGGCGGGGATGTCGTCGCTGAGAGGGACGAGCCGCCGCCGCCGCCCCCGACCGCCCTGGCCGCCCAGATGCGCCAGCACGGCCAGGGCGTACGCGGTGTGAAAGAACGCGTCGGGGGCGGTCCCCTCGAGGGCGCGCATCAGGTTCTCCAGGAGCACGGGGAAGCGCCGCGTCACCTCCCCTAGCCACTCGCTCTGGTGGGGGCCAAAGTCGTAGCGCAGGCGCTGGAAGATGCGCGGGCCGCCTTGGAGCGCGGCCCGGATAGAGTGGCCCAGGGCCCGCAGACACGCGATCTGGATGCGCGCGACGAAGGCCACCTCGGCCGCGATGTCAAAGGGCTGCAGCACGGGGCGCGGGTGGCGCAGGGGTCCCTCGAGCGCGGGAAAGCGACGCAGCAGCGCCGTCTGGGCCGCGGGGGACAGCTGGTGGGGGCGCACGACGCGCTCGGCGGCACAGGCCTCCGTCAGGGCCGTGGCCAGCTCGGAGGACAGCCGCGGGGGGCGGGCGCGTCGCCCGCCCCACGCCACCGAATTCTCGTAGGAGACGACGACGAAGCGCTGCTTGGTCCCGTAGTGATGGCGCAGGACCACGGAGATGGAGCGACGGCTCCACAGCCAGTCGGGCCGGTCGCCGCCGGCCAGAGCTTCCCACCCGCGGTCCAGCCACTCGACCAGCGATCGCGGCTTGGCGGTCCCCGGCACGAGGGTGAGCACGTCGTTGAGGACGTCCTCGCCCGCGGCCCGGGGGCCCCCCGGGGTGGCAAAGCGCCCCCCGCCGGGCGGCTCCAGGCCCGCCAGCACCGCCTCCGCGTCCGACGCGCCCAGGGCTCCCCCGCTGACGGCCTGGTGGACCAGGGCGCCCTGGCGGAGCCCCGAGGCGACGCCGGAGGCCGCGTGCTTGGGGCGCGCGCGGACCGGGTGGCGGCGGGTGACGTCCTGCACGGCCCGCTGGACCAGCGCGAGGATCTCCTCGTTCTCTTGCGTGATGGACACGTCCTCCGCGGTGGCCGTGTCGCCTCCCGGGGCCGTGAGCTGCTCCTCCGGGGAGATGGGGGGGTCTGGGGTGCCGACAACGGCCGGCCCGGCCCCGCCCGAGACCGAGGACGCCTGGGGAGTGGGGGTGCCGCTTTCCCCCATCCCCAGGGACAGGTGGGCCGCCGCCTCCGTCGCGGCGGCGGGAGCCGCGGCCCCCAGCCGCGCGACGTAGCGACAAAAGTGGCGACAGAGGCGCATGAGGCGCGCGCCGTCGGCCGCGTATCGCGTGTTTGGCGGGACGAGCTCGTCGTAACTGAACAGGAGCACGCGGGCGCAGGTCGCCCACGGGCCCCACGCCAGGCGCAGCGCCGCGACCGTGTACGGGTCGTACACGCCTTGGGCGTCGCACGCGACCGGCAGGGAGACGAACAGCCCGCCCGCGCTGGGGACGCGCGGCAGGAGGTCCGGGTGCGCCGGGATGACGGGGGCTAGGATCGCCCCCACCGCATCCGCCGGCACGTAGGCGGCAAACGCCGAACGCCACGGGGTGCAGTCGCCGGTCGCGTGGGCCCGGGTCTGGGTTTCGACCCGGAAGTTCGCGGCCGCCCCACCGTCGGGGCGGCCGCGCACGAGGGCGGACAGCGGGACCCCCGCCGCCGCCAGGCACTCGCTGGAGATGATGACGTGAATCAGCGAGGCGGGGCTGCTCGGGTCCCGGGTGAGATCGTATTGGACCTCGTTGGCAAAGTGCGCGTTCATGGCCCGGCCGGCGGTGCGAGCCCTTCCCGGTGCCGGAAGGGGCGTGGGTGGGGGGTGCGTGTGCGCGTCCTCGGGGCCCGCGGGCGCACGTGCGCTTATACGCTGTGTGTTTCGTCTGTCCCCAGGGAATCCGGGGCCAGGACTTTAACCTGCTTTTCGTCGACGAGGCCAACTTTATTCGCCCGGATGCGGTCCAGACGATTATGGGCTTTCTCAATCAGGCCAACTGCAAGATCATCTTCGTCTCGTCGACCAACACCGGGAAGGCCAGCACGAGCTTTTTGTACAACCTCCGCGGGGCCGCCGACGAGCTGCTCAACGTGGTCACCTATATATGCGACGACCACATGCCGCGGGTGGTGACGCACACCAACGCCACGGCCTGTTCCTGCTATATCCTGAACAAACCCGTGTTTATCACGATGGACGGCGCCGTTCGCCGGACGGCCGATCTGTTTCTGCCCGACTCCTTCATGCAGGAGATCATCGGGGGGCAGGCCCGCGAGACCGGCGACGACCGGCCCGTCCTAACAAAGTCGGCGGGGGAGCGGTTTCTGCTGTACCGCCCCTCCACCACCACCAACAGCGGCCTGATGGCCCCCGAGCTGTACGTGTACGTGGACCCGGCGTTCACGGCCAACACGCGCGCCTCCGGCACCGGCATCGCGGTCGTCGGGAGGTACCGCGACGATTTCATTATCTTCGCCCTGGAGCACTTTTTCCTCCGCGCGCTCACGGGATCGGCCCCCGCGGACATCGCCCGCTGCGTCGTGCACAGCCTCGCCCAGGTGCTGGCGCTGCACCCCGGGGCGTTTCGCAGCGTTCGCGTGGCGGTCGAGGGCAACAGCAGCCAGGACTCGGCCGTGGCCATCGCCACACACGTGCATACCGAGATGCACCGCATCCTGGCCTCGGCGGGGGCCAACGGCCCGGGGCCCGAGCTCCTCTTCTATCACTGCGAGCCGCCCGGCGGCGCGGTATTGTACCCCTTCTTTCTGCTCAACAAACAGAAGACGCCCGCCTTCGAATACTTTATCAAAAAGTTCAACTCCGGGGGCGTCATGGCGTCCCAGGAGCTCGTCTCCGTGACGGTGCGCCTGCAGACCGACCCGGTCGAGTATCTGTCCGAGCAGCTCAACAACCTCATCGAAACCGTCTCTCCCAACACCGACGTCCGCATGTACTCCGGAAAACGCAACGGTGCCGCGGACGACCTCATGGTCGCGGTCATCATGGCCATTTACCTGGCGGCCCCGACCGGGATCCCCCCGGCCTTTTTTCCGATCACGCGCACGTCTTGAG

GCAATAAACGCCTTCCCGGAACTGTGTTTCCCCCCCTACAACAGTGTTGTCCGTTGGTTGGGTGGTTGGGGTGCGGGGGTGGGCGGGGGAAGCAAGAAAACGGTCGGCGAACACAACATCGGGAAAACGGATTCCCGCACGTGCGTCTTCCCAGATTCGACACACACACCCCCCTTCTCCTTAAATAAACACAAACCACACGCTCGTTGGTTGGTTAATGCCAGCGCTTTATTTACGTCTTGTTTTTTTTGCGTTTCCTCCGCGGGTCCCTTCCCAACACGCCTGCCCCCGCCTCAGGGGTAGCGGATAACCGGGGCCATGTCGCCGGATTGCACAACGGCGGCGCCGTCGAACGTACACACCCGAACCGCCGGGGCCAGGGCCAGGATGTCCCCGAGTTGGCCCGCGTGCGCCAGCCAGGCGACCAGCGCCTCGTAAAGCGGCAGCCTGCGTTCGCCGTCCTGCATCAGCATGGGGGCTTCGGGGTGGATGAGCTGGGCGGCTTCTCGCGTGACGCTCTGCATCTGCAGGAGCGCGTTCACGTATCCGTCCTGGGCGCTCAGCGCGAGCAGCCGGGGGATGAGCGTGAGGATGAGGGTGGTTCCTTCGGTTATGGAGTAGACCATGTTGAGGACGAGCGACCGCAGCTCGGTGTTTACGGAGGCGAGTTGCTGGACGTCGGCCACGAGCGAGAGACGGGCCCCGTTGTAATACAGCACGTTGAGGTCGGGGAGCTCCCCGGGCGTCCGGGGGTCGGGGTTGAGGTCCCGGATGCCCCGGGCGACCAGCCGCGCGACTATCTCGCGGGCCAGGGGCGTTGGGAGCGGGACCGGAAACCGCAGCGTGAGGTCCAGCGACTCCAGGCGCACGTCCGTCGCCTGGCCCTCGAAGACGGGCGGGACGAGGCTGACGGGATCCCCGTTGCAGAGGTCGACGGGGGAGGTGTTGCGGAGATTGACGGTGCCGGCGTGCGTGAGCCCCAGGTCCACGGGGCAGGCGACGATTCGCGTGGGCAGCACCCGCGTGATTACCGCGGGGAAGCGCCTGCGGTACGCCAGCAACAACCCCAACGTGTCGGGACTAACTCCTCCGGAGACGAACGATTCGTGCGCCACGTCCGCGAGCGCCAGCTGGCGGCGGATGGTCGGCAGAAAGACCACTCGACCCTCGCACCGCTGCAGCGCCGCGGCATCGGGGCGCGAGATACCCGAGGGGATCGCGATGTCTGCTTCGAAACAATCCGTGATCATGGCGCCGGGCCGCGAGACACCGGAACGCGGGGGTGCGGGAGGGCCGGAAAGCGCAACGCAACCGGGACGATGATGAAACAGAGATGGGGGGCACCGACCGTGTGGGAGAGGGGGCGGGGCAGGGCTCAGCAGCACGCACGGGGAGGTCTGTCGTGCGCAGGAGCCCCAGGTGAGAATCAGTCCCCCGGAGCTCGGGTCTGGGTTTTATTGGGACCTGCCCTCGGAATCGCGGCTCCCAGTCCAAGCCCCCCTGGGGGGGGCGGGGACAGGGGGTGTGTGTGGGTAAAAGCAACGTCGGAAAATCAAACCCAATGCCCCAAACAGGAAAAAAAAAGACGGGCGGGTGGAGGGAAAGCTGGGGAAGAAGAAGCCAATTTTACAGAGACAGGCCCTTTAGCGGGGAGGCGTCGTAGATGAGATACTGCGTAAAGTGGGTCTCTCGCGCGTGGGCCTCCCCATCGCGGGCGCTGCGTAGCAGGGCGGGGTCGCTGGCGCAGGTGATCGGGTAGGCTTCCTGAAACAGGCCGCACGGGTCTTCCACGAGCTCGCGGCACCCCGGCGGGCGCTTAAACTGCACGTCGCTGGCAGCGGTGGCCGTGGATACCGCCGATCCCGTTTCCACGATAAGACGCTCCAGGCAGCGATGTTTGGCCGTGATGTCGGCCGCGGTGAAGAACTTGAAGCAGGGGCTGAGGACGGGCGAGGCCCCGTTGAGGTGATAGGCCCCGTTGTACAGCAGGTCCCCGTACGAGAACCGCTGCGACGCCCACGGGTTGGCCGTGGCCGCGAAGGGCCGCGCCGGGTCGCTCTGGCCGTGGTCGTACATGAGGGCTATGACGTCCCCCTCCTTGTCCCCCGCGTACACGCCGCCGGCCGCGCGTCCCCGCGGGTTGCAGGGCCGGCGAAAGTAGTTGATGTCCGTGGCCACGGGGGTGGCGATGAACTCACACACGGCATCCTGCCCGTGGTCCATGCCGGCGCGCCGCGGCACCTGGGCGCAGCCAAAGACCGGGAGGGGCTGGGCCGGCCCCAGCCGGTTTCCCGCCACGACCGCGTTGCGCAGGTACACGGCGGCCGCGTTGTCTAGCAGCGGGGGGGCCCCGCGGCCGAGGTAAAAGTTTTGGGGGAGGTTGCCCATGTCCGTAACGGGGTTGCGGACGGTGGCCGTGGCCGCGACGGCGGTGTAGCCCACACCCAGGTCCACGTTTCCGCGCGGCTGGGTGAGCGTGAAGTTGACCCCCCCGCCCGTTTCGTGGCGGGCCACCTGGAGCTGGCCCAGAAAGTACGCCTCCGACGCGCGCTCGGAAAACAGCACGTTCTCGGTCACGAAGCGGTCCTGCCGCACGACGGTGAACCCGAACCCGGGGTGGAGGCCCGTCTTGAGCTGGTGATACAGGGCCACGGGGCTCATCTTGAAGTACCCCGCCATGAGCGCGTAGGTCAGCGCGTTCTCCCCCGCCGCGCTCTCGCGGGCGTGCTGCACCACGGGCTGGCGGATGGAGGAGAAGTAGTTGGCCCCCAGGGCCGGGGGGACCAGGGGGACGTGGCGCGCCAGGTCGCGCAGGGCCGGGGGGAAGTTGGGCGCGTTGGCCACGTGGTCGGCGCCCGCAAACAGCGCGTGGACGGGCAGGACGTAGAAGTATTCGCCATTTTGGATGGTGTGGTCCAGGTGCTGGGGGGCCATGAGCAGCACGCCGGCGTGCAGCGCCCCGTCGAAGATGCGCATGTTGGCCGTCGACGCGGTGTTGGCGCCCGCGTCGGGCGCCGCGGAGCACAGCAGCGCCGTCGTGCGCTCGGCCATGTTGTGCGCCAGCACCTGCAGCGTGAGCATGGCGGGCCCGTCGACGACGACGCGCCCGTTGTGGAACATGGCGTTGACCGTGTTGGCCACCAGATTGGCGGGATGCAGCGGGTGGGCGGGGTCGGTCACGGGATCGCTCGGGCACTCCTCACCGGGGGCGATCTCCGGGACCACCATGTTCTGCAGCGTGGCGTACACGCGGTCGAAGCGGACCCCCGCGGTGCAGCAGCGCCCCCGCGAGAAGGCCGGCACCAGCACGTAATAGTAGATTTTGTGGTGGACGGTCCAGTCGGCCGGCCGGTGCGGCCGGTCGTCGGCGGCGTCGGCCGCGCGGGCCTGGGTGTTGTGCAGCAGCCGGCCGTCGTTGCGGTTAAAGTCGGCCGTCGCCACGTTGCACGCCGCCGCGTAGACGGGCTCGTGCCCCCCCGCGTCAATCCGGCAGTCTCGGTGGCGGTCCAGGGCCGCGTGTCGCATAAGGCCGTCGCAGTCCCACACGAGGGGCGGCAGCAGCGCCGGGTCGCGCATCAGGTGATTCAGCTCGGCCTGAGCCTGCCCGCCCAGCTCCGGGCCCGGCAGGGTAAAGTCGTCCACCAGCTGGGCCAGGGCCTCGACGTGGGCCACCAGGTCCCGATACACGGCCATGCACTCCTCGGGGAGGTCGCCCCCGAGGTAGGTCACGATGTACGAGACCAGCGAGTAGTCGTTCACGAACGCCGCGCATCGCGTGTTGTTCCAGTAGCTGGTGATGCACTGAGTCACGAGCCGCGCCAGGGCGCAGAACACGTGCTCGCTGCCGTGAATCGCGGCTTGCAGCAGGTAAAACACCGCCGGGTAGCTGCGGTCCTCGAACGCCCCGCGGACGGCGGCTATGGTAGCCGGCGCCATGGCGTGGCGGCCAACGCCGAGCTCCAGGCCCCGGGCGTCACGAAACGCCACCGGACACAGCGCCAGGGGCAGGTTGCCGTTGACCACGCGCCAGGTGGCCTGGATCGCCCCCGGACCGGCCGGGGGGACTTCGCCGCCGGGAAGCTCGACGTCGGCCACGCCCGCGAAGAAGTCGAACGCGGGGTGCAGCTCCAGAGCCAGGTTGGCGTTGTCGGGCTGCATGAACTGCTCCGCGGTCATCTGGCACTCGGCGACCCACCGGACCCGGCCGTGGGCGAGGCGCTGCCGCCAGGCGTTCAGAAAACGCTGCTGCATGTCCGCGCCGGGGCCGGCCGGGGCCGCGACGTACGCCCCGTACGGATTCGCGGCCTCGACGGGGTCGTGGTTCACGCCCCCGACGGCCGCGTCGATGTTCATGAGCGAAGGATGACACACGGTCCCGACCGCGTTCTCCATGGACAGCCGCAGAACCTGGTGGTCCTTTCCCCAAAAAAACAGCTGCCGGGGAGGGAACGCGCGGGGCTCCGGGTGGCCGGGGGCGGGCACCAGGTCCCCGGCGTGCGCGGCGAAGCGCTCCATGGCCGGGTTGAACAGCCCCAGGGGCAGGACGAACGTCAGGTCCATGGCGCCCACCAGGGGGTAGGGCACGTTGGTGGCGGCGTAGATGCGCTTCTCCAGGGCCTCCAGGAAGACCAGCCTGTCGCCTATGGCCACCAGATCCGCGCGCACGCGCGTTGTCTGGGGGGCGCTTTCGAGTTCATCCAGCGTCTCCCGGTTCGCCTCGAGTTGCTCCTCCTGCATATCCAGCAGGTGGCGGCCCACGTCGTCCAGGCTCCGCACGGCCTTGCCCATCACCAGCGCCGTGACGAGGTTGGCCCCGTTCAAGACCATCTCGCCGTAGGTCACCGGCACGTCGGCCTCGGTGTCCTCCACCTTCAGGAAGGACTGCAGGAGGCGCTGTTTGATGGCGGCGGTGGTGACCAGCACCCCGTCGACCGGCCGCCCGCGCGTGTCGGCGTGCGTCAGGCGGGGCACGGCCACGGACGGCTGCGTCGCCGTGGTCAGGTCCACGAGCCAGGCCTCGATGGCCTCGCGGCGATGGCCCGCCTTGCCCAGGAAGAAGCTCGTGTCGCAAAAGCTCCGCTTCAGCTCGGCGACCAGGGTCGCCCGGGCAACCCTGGTCGCCAGGCGCCCGTTGTCGAGATATCGTTGCATGGGCAACAGCAGGGCCAGGGGAGGCGCCTTCTCCAACAGCACGTGCAGCATCTGGTCGGCCGTGCCGCGCTCAAACGCCCCCAGGACGGCCTGGACGTTGCGCGCGAGCTGCTGGATGGCGCGCAGCTGGCGATGCAGGCTAATGCCCGTCCCGTCCAGGGCCTCCCCCGTGAGCAGGGCAATGGCCTCGGTGGCCAGGCTGAAGGCGGCGTTCAGGGCCCGGCGGTCGATGACCTTCGTCATGTAATTATGCACGGGCTGCTCGACGGGGTGCGGGCCGTCGCGGGCGATGAGGGGCTGGTGGACCTCGAACTGCACACGCCCTTCGTTCATGTAAGCCAGCTCCGGGAACTTGGTGCACACGCACGCCACGGACAGGCCGAGCTCCAGAAAGCGCACGAGCGACAGGGTGTTGCAGTAGGACCCCAGCAGGGCGTCAAACTCTACGTCATACAGGCTGTTTTCGTCGGAGCGCACGCGGGCGAAAAAATCAAAGAGTCTGCGGTGGGACGCCACCTCGATCGTACTCAGGATGGAGCCGGTGGGCACCATGGCCGCGGCGTACCGGTAACCCGGGGGGTCGCGGGCAGGAGCGGCCATTGGGTTCCTTGGGGGATTCGCAGGCTCCATCAAGCCGAGCTCGGGAAGGCCAAGCCCCTCCCGCACAACGCCTCACCGCCGGCGGACGCGACTAACAACCCACGGGCCGCCAAAACCCCAAGGGGCAACCCGACCAACAACAGGCGAGGGGAGGAAAGGCGTAAAGGGGGCGTTGGGAGGCAAAAAGAAAGAAAACACCCAGACGTAGGCCCGAGGACCGGCCGGCGTCCTCTGTCCCCGAGCACCCACTGTGCCCAACAGGCACGGGGGCGAGCTGCCCCTGCCTTATATACCCCCCCGCCACACCCCCGTTAGAACGCGACGGGTGCCTTCAAGATGGCCCTGGTCCAAAAGCGTGCTAGAAAAAAGTTGGTAAAGGCGGCAAAGCAGTCCGCCGCCGCCACCCACATGGCGGCGCCGGCCGCGCAGGCGATTCCCAGAGAACGGGCGCGGAGGGGATCCGTGCGGGGCAGCAGCTGGCTGGCGGTGATCCAATGGAAAAGCCCGTCGGGACTGAACGTCTCATGGGCGGCCGCCACCAGGGCGCACAGGGCCGCGCCGCCCATGATCACGCACAACCCCCAAAACACGGGTGGCGACAACGGCAGGCGATCCCGTTTGATGTTCACGTACAGGAGGAGCGCCCGTGCCAGCCACGTGACATAGTAGGCGAGGACGGCGGCTATAATACATGCCGGCGCCACCGCCCGTCCGGTCCACCCGTAATACATGCCCGCGGCCACCAGCTCCAGCGGCTTGAGGACCAGGAACGACCAAGCAAACATCACCACCCGCTTGGAAAAGACCGGCTGGGTGTGGGGCGGAAGACGCGAGTAGGCCGAACTGACAAAAAAATCAGACGTGCCGTACGAGGACAGCGAAAACTGTTCATCGAGCGGCAGTTCTCCGTCCTCCCCGCCACACGCGGCCTCGTCTACCAGCTCGCGATCCAACAAAGGAACATCATCCCGCATTGTCATGGTCGGTGCGGGGAGCCGGCGAGGCAGCAAAACCGAAAGTAGTGCTGGCGGCGCGGGCCCGGGTCCGGACCCAAGCTTCAGGGATGGGGGGCGGAGGCCAAAATCAAACAAGCACCGCGCGGGTTCTACACACAACCCCCACCCGGGTAGTATCCGCGGATGCGAGTGCCTGGCGAAGTCACGTCCCAGCAGGATATAAACCTCGGCCGTTGGGCCCGGAACCCCCGAAATTCACACCCACGCCCTGACGCCCAAATCATGGGTGGATGTGGTTCGCGAGCCGCACATCCGTGCGTCCGCCCTCCCCCGCGGGCTGATGACGTGGCGGTTAGTCAGTGGGAAGGCAGGGGGAAAGATGGGTTGGGGGAGGAAACGAAGAAAACACCCAGAGGGCCACGTCGGGAATGCGCCCGGAGTTGTCCTTAAAAGGCCGGCCGTGCGTGACGGAAGCCGTCGTTTGCCCAAGCACCGACGCCGCGATCCACAGTGGGGGGAGTTCCTCCGTCCGGCCACAACCCTACGCGCGGGCGGCACGCGCGAGAGCAACCCACGGGTCCCGTTCGCGCCACCGCCAGCCCTTGCTCCCACCACCCTCCTCCCACCACCCCACTATTCCCCCCCCCAAGTCCGCCCCGTGGCTCGCCGGCCATGGAGCTCAGCTATGCCACCACCCTGCACCACCGGGACGTTGTGTTTTACGTCACGGCAGACAGAAACCGCGCCTACTTTGTGTGCGGGGGGTCCGTTTATTCCGTAGGGCGGCCTCGGGATTCTCAGCCGGGGGAAATTGCCAAGTTTGGCCTGGTGGTCCGGGGGACAGGCCCCAAAGACCGCATGGTCGCCAACTACGTACGAAGCGAGCTCCGCCAGCGCGGCCTGCGGGACGTGCGGCCCGTGGGGGAGGACGAGGTGTTCCTGGACAGCGTGTGTCTGCTAAACCCGAACGTGAGCTCCGAGCGAGACGTGATTAATACCAACGACGTTGAAGTGCTGGACGAATGCCTGGCCGAATACTGCACCTCGCTGCGAACCAGCCCGGGGGTGCTGGTGACCGGGGTGCGCGTGCGCGCGCGAGACAGGGTCATCGAGCTATTTGAGCACCCGGCGATCGTCAACATTTCCTCGCGCTTCGCGTACACCCCCTCCCCCTACGTATTCGCCCTGGCCCAGGCGCACCTCCCCCGGCTCCCGAGCTCGCTGGAGCCCCTGGTGAGCGGCCTGTTTGACGGCATTCCCGCCCCGCGCCAGCCCCTGGACGCCCGCGACCGGCGCACGGATGTCGTGATCACGGGCACCCGCGCCCCCAGACCGATGGCCGGGACCGGGGCCGGGGGCGCGGGGGCCAAGCGGGCCACCGTCAGCGAGTTCGTGCAAGTGAAGCACATCGACCGTGTTGTGTCCCCGAGCGTCTCTTCCGCCCCCCCGCCGAGCGCCCCCGACGCGAGTCTGCCGCCCCCGGGGCTCCAGGAGGCCGCCCCGCCGGGCCCCCCGCTCAGGGAGCTGTGGTGGGTGTTCTACGCCGGCGACCGGGCGCTGGAGGAGCCCCACGCCGAGTCGGGATTGACGCGCGAGGAGGTCCGCGCCGTGCATGGGTTCCGGGAGCAGGCGTGGAAGCTGTTTGGGTCGGTGGGGGCTCCGCGGGCGTTTCTCGGGGCCGCGCTGGCCCTGAGCCCGACCCAAAAGCTCGCCGTCTACTACTATCTCATCCACCGGGAGCGGCGCATGTCCCCCTTCCCCGCGCTCGTGCGGCTCGTCGGTCGGTACATCCAGCGCCACGGCCTGTACGTTCCCGCGCCCGACGAACCGACGTTGGCCGATGCCATGAACGGGCTGTTCCGCGACGCGCTGGCGGCCGGGACCGTGGCCGAGCAGCTCCTCATGTTCGACCTCCTCCCGCCCAAGGACGTGCCGGTGGGGAGCGACGCGCGGGCCGACAGCGCCGCCCTGCTGCGCTTTGTGGACTCGCAACGCCTGACCCCGGGGGGGTCCGTCTCGCCCGAGCACGTCATGTACCTCGGCGCGTTCCTGGGCGTGTTGTACGCCGGCCACGGACGCCTGGCCGCGGCCACGCATACCGCGCGCCTGACGGGCGTGACGTCCCTGGTCCTGACCGTGGGGGACGTCGACCGGATGTCCGCGTTTGACCGCGGGCCGGCGGGGGCGGCTGGCCGCACGCGAACCGCCGGGTACCTGGACGCGCTGCTTACCGTTTGCCTGGCTCGCGCCCAGCACGGCCAGTCTGTGTGAGATATCCCAATAAAGTGCAGTCGTTTTCTAACCCACGGATGCCGTTGTATGCCTATACGGGGGACTATGGGGGGGGA

GGCGGTAGCGGACGCACGGCGGACACAATAACAAACAGACCGCGGACACGGAGGGAGTCGGTTGGGTTGGGCGTGGACGCCGCTGCGTCCACACACCCGTTTATTCGCGTCTCCACAAAAATGGGACGCACGTTCGGACCACCCTAAGGATGCCCGCCAGGGCCGCGGTAATCATAACGACCCCCAGCGCGGACGCGGCCAGAAACCCGGGGGCGATGGTGGCGATGGGCAGCGTGTCAAAGGCCAGCAGATGAATCACAGTTCCGTTGGGGAACAACAACAGGGCCACGGACGGCACGTCGCTGGAAAACACGTTCGGGGTGCCCGCCACCGGCCCCTGGGCCAGCTGCTGTTGGGTGGCATCCGTGTCCACCAGCAGCACCGACATGACCTCCCCGGCCGGGGTGTAGCGCAGAAACACGGCCCCCACGAGGCCGAGGTCGCGCCGGTTTTCGGTGCGCACCAGCCGCTTCGGCTCAATCTCCCGCGCGTGCCCTTCGCAGGTGGCGGTGAGATAGGTGATAAACAGCGGGCGGCGGACGTCAACGCCCGTAAGCTTGTATCCGATCCCGCGGGGCAAGGGGGTGTGGGTGACGACGTAGCTGGCGTTGTGGGTGATGGGCACGAGGATCCGGGGCTCCGCGTTGTGCGACGGGCCGCTACACTGGTGGGTGGCCTCCGGGACGAAGGCGCGGATCAGGGCGTTGTAGTGCGCCCAGCGCGTGAGAACGGAGGCCACGCCGCGGGTCTGTTGTGCCATGACGTCCGCCGGGATGTCGGATCGGGTGGCCATGGCCAGCGCGTCCAGGATGAACCCGCCCTCGGCGAGATCGAAGCGCAGGGAAGCTGCGCATGGGGAAAAGTGGTCCGGGAGCCAGAAGAGGTTTTTCTGGTGGTCGGTCCTGGCTAGCGCGGCCCGGAGATCGGCGTGGGTCGCCGCGGCGACGTCGGACGTACACAGGGCCGTGGTTATGAGGAGGCCCCGGCGGGCGCGTTCCCGCTGCTCGGCCGAGGGCGCGCCCGCCAGGAACGGCGCCCGGAGGACGGCCGTGGCGTAAAACAGCGCTCGGCGGACCATCGGGGCGGTTAGCGCGCGGCCGCCGAGAAACTCGGCGTACAGGGCGTCGATCAGGCGGGCCGCGCTCGGGGCCACCGCGCCATAGGCCGCGGGGCTGTCCAACACGAACGCCAGCTGATAGCCCAGCGCGTGCGCCGCCAGGCTCTGCTCTCGCTCGAGGATCGCGGCCACCAGATGCCCGAGGCGCGCCTCCAGCCGCAGGCGGGCCGCCGGGTCCAACACGGACACGTTCAGGAACACCGAGTCGGCCGCGCAGCCCGCTGCTCCCCGGGCGGCCAGGCCGGCCAGCACGCGCGAGTGGGCCAAAAAGCCCAGCAGGTCGGAGAGGCGAATCGCGTCGTGGGCGTGGGCCGCGTTGACGAACGCAAACCCCGACGAGGCGAGCAGCCCCGCGAGGCGCCAGAACAGGGACGGACGCGCGTCCGTGCCGGAGCCCGGGTCCTCCCCCAAAAACTCCGCATAGGCCCGCGACATATACTGGGCGTAGTTCGTGCTCTCCTCGGGGTAGCCGGCCACCCGCCGGAGGGCGTCCAGCGCCGAGCCGTTGTCGGCGGGCGTCGGGGCCCCCAGGACAAAGACGCGATACCTGGGGCCGGCCGGAGGCCCGGGGAGCACCGCGGGGGCGTTTTCGTCGGTCGGATTTCCGACCCGAGCGAGGGTCTTGTCCGCAGGCACCACTATGATCTCGGCCGGAGGGCTGTCCCGCATCGATATCACGAGCCCCATGAAGCCCTTCCCGTATCGCGCGCGCACGAGCGCGGCGTCGCACCCGAACGCCAGCCCGCCCGTCGTCCAGACGCCCACGGGCCACGTCGAGGCCGACGGGGAGAGGTACACGTACCGACCCGGAGTCCGTAGCAGGCCCCTGGCGGCCAGCCAGGTCACGGATGCGTTGTGCAGATGCGCGATGCTCAGGTTCGTCGTCGGATGCCTCGGTGTCCCCGCGGGCGGCCCCGGGGGCGGCGCGTTGCGTCGGCCGTCCGGGTGCCTCTCGGTCGCCCCGTCGTCTCCCCGCGGGAACGTAAGCCCCTCGCGGTCCGGCGCGGCCGCGAATGTTACCCAGGCCCGGGACCGCAACAGCGCGGAGGCGCCGGGGTTGTGCGACAGTCCCTTGAGCTGGGTCACCTCGGCGGGGGGACGGGACGTGGGCCCCGCCTCGGGGAGCTCGGGCAGGCTCGCGTTCCGAGGCCGGCCGAGCAGATAGGTCTTTGGGATGTAAAGCAGCTGCCCGGGGTCCCGAGGAAACTCGGCCGTGGTGACCAACACGAAACAAAAGCGCTCGGCGTACCACCGAAGCATGGGCACGGATGCCGTAGTCAGGTTGAGTTCGCCCGGGGGCGCCAAGCGTCCGCGCTGGGGGTCGCTGGCGTCGGGGGTGTTGGGCAACCACAGACGCCCGGTGTTTGTGTCGCGCCAGTACGTGCGGGCCAACCCCAGACCGTGCAAAAACCACGGGTCGATTTGCTCCGTCCAGTACGTGTCATGGCCCCCGGCAACGCCCACCAGGACCCCCATCACCACCCACAGACCGGGGCCCATGGTCGTCCGTCCCGGCTGCCAGTCCGCAGATGGGGGGGGGTGTCCGTACCCACGGCCCAAAGAGGCTCCGCACCTCGGAGGCTATCGGAGGCCCTTTGTTGCCGTAAGCGCGGGCCAAAGGATGGGGTGGGGTGAGGGTAAAAGCACAAAGGGAGTACCAGACCGAAAACAAGGACGGATCGGCCCGCTCCGTTTTTCGGTGGGGTGCTGATACGGTGCCAGCCCTGGCCCCGAACCCCCGCGCTTATGGACACACCACACGACAACAATGCCTTTTATTCTGTTCTTTTATTGCCGTCATCGCCGGGAGGCCTTCCGTTCGGGCTTCCGTGTTTGAACTAAACTCCCCCCACCTCGCGGGCAAACGTGCGCGCCAGGTCGCGTATCTCGGCGATGGACCCGGCGGTTGTGACGCGGGTTGGGATCATCCCGGCGGTGAGGCGCAACAGGGCGTCTCGACACCCGACGGGCGACTGATCGTAATCCAGGACAAATAGATGCATCGGAAGGAGGCGGTCGGCCAAGACGTCCAAGACCCAGGCAAAAATGTGGTACAAGTCCCCGTTGGGGGCCAGCAGCTCGGGAACGCGGAACAGGGCAAACAGCGTGTCCTCGATGCGGGGCAGAGACCCCGCGCCGTCCTCGGGGTCGGGGCGCGGGGTCGCCGCGGCGACCCCCGTCAGCCGGCCCCAGTCCTCCCGCCACCTCCCGCCGCGCTGCAGGTACCGCACCGTGTTGGCGAGTAGATCGTAGACACGGCGAATGGCGGACAGCATGGCCAGGTCAAGCCGCTCGCCCGGGCGTTGGCGTCTGGCCAGGCGGTCGGCGTGTTCGGCCTCCGGAAGGACACCCAGGACCAGGTTCGTGCCGGGCGCGGTCGGGGGCATGAGGGCCACGAACGCCAACACGGCCTGGGGGGTCATGCTTCCCATGAGGTACCGCGCGGCCGGGTAGCACAGCAGGGAGGCGATAGGGTGCCGGTCGAAAACAAGGGTGAGGGCCGGGGGCGGGGCTTGCGGGCCCACAGCCTCCCCCCCGATATGAGGAGCCAAAACGGCGTCCGTCGCCGCATAAGGCGTGCTCATTGTTATCTGGGCGCTGGTCATTACCACCGCCGCCTCCCCGGCCGATATCTCGCCGCGGTCCAGACGGTGCTGCGTGTTGTAGATGTTCGTCAGGGTCTCGGAGGCCCCCAGCACCTGCCAGTAAGTCATCGGCTCGGGGACGTAGACGATATTGTCGCGCGGCCCCAGGGCCTCCATCAGCTGCGCGGAGGTGGTGGTCTTCCCCACCCCGTGGGGTCCGTCTATATAAACCCGCAGCAGCGTGGGCAGCTCCGGATCCCCGCGGGCTCCGGAGGCCCCCTGGCGATGGCTAGGACGGGACGCCGCGCGGCCGTCGGTAGGCCCGCTCGCACGAGCAGCCTGACCGAACGCAGGCGCGTGCTGTTGGCCGGCGTGAGAAGCCATACCCGCTTCTACAAGGCGTTCGCCCGAGAGGTGCGGGAGTTCAACGCCACCAGGATTTGTGGAACGCTGCTGACGCTGATGAGCGGGTCGCTGCAGGGTCGCTCGCTGTTCGAGGCCACGCGCGTCACCTTAATATGCGAAGTGGACCTCGGGCCGCGCCGCCCAGACTGCATCTGCGTGTTCGAATTCGCCAATGACAAAACGTTGGGAGGTGTGTGCGTCATCCTGGAGCTAAAGACATGCAAATCGATTTCTTCCGGGGACACGGCCAGCAAACGCGAACAGCGGACCACGGGCATGAAGCAGCTGCGCCACTCCCTGAAGCTGCTGCAGTCGCTCGCGCCTCCGGGGGACAAGGTCGTCTACCTGTGTCCTATTTTGGTGTTTGTCGCGCAGCGTACGCTGCGCGTCAGCCGCGTGACCCGGCTCGTCCCGCAAAAGATCTCCGGCAACATCACCGCGGCCGTGCGGATGCTCCAAAGCCTGTCCACGTATGCCGTGCCGCCGGAACCGCAGACCCGGCGGTCGCGGCGCCGGGTCGCCGCGACCGCCAGACCGCAAAGGCCCCCCTCCCCGACACGTGACCCGGAAGGCACGGCGGGTCATCCGGCCCCACCAGAGAGCGACCCCCCCTCCCCAGGGGTCGTAGGCGTCGCTGCGGAGGGTGGGGGTGTGCTTCAGAAAATCGCGGCGCTTTTTTGCGTGCCGGTGGCCGCCAAGAGCAGACCCCGGACCAAAACCGAGTGAGGTTCTGTGTGTTGGCTTCTGGCCAAGCATCCTCACCTGCTTAAGCGGAACCCGCGGGCGCGCGGGGACTCATTTGTCGCCGGCGACACCCACCCGACAACAGCCCCTGGGTGTCGACCGCTGTCGCCCCCGTCTGTCGCCTCTCCCTTTTTTCCCCCCCTCAAAGAACGTGGTGTTGGGCGCCGGCCAATTCTTCCCGGAGCGCCGTCGTCGCCCGCCCGCCGCCCTCGAACATGGACCCGTACTACCCTTTCGACGCGCTGGACGTTTGGGAACACAGGCGCTTCATCGTCGCCGACTCCAGGAGCTTCATCACCCCCGAGTTCCCCCGGGACTTCTGGATGTTGCCCGTGTTCAACATCCCCCGGGAGACGGCGGCGGAGCGGGCGGCAGTGCTGCAGGCCCAGCGCACCGCGGCCGCGGCGGCCCTGGAGAACGCCGCCCTCCAGGCCGCCGAGCTGCCCGTCGACATCGAGCGCCGGATACGCCCGATCGAGCAGCAGGTGCATCACATCGCCGACGCCCTGGAGGCGCTGGAGACCGCGGCGGCCGCGGCCGAAGAGGCGGATGCCGCGCGGGACGCCGAGGCGAGGGGGGAGGGCGCTGCGGACGGGGCAGCGCCGTCGCCCACCGCGGGCCCCGCCGCCGCGGAGATGGAGGTTCAGATCGTACGCAACGACCCGCCGCTACGATACGATACCAACCTCCCCGTGGATCTGCTACACATGGTGTACGCGGGCCGCGGGGCCGCGGGTTCGTCGGGAGTCGTCTTTGGTACCTGGTACCGCACGATCCAGGAACGCACCATCGCGGACTTCCCCCTGACCACCCGCAGCGCCGACTTTCGAGACGGGCGCATGTCCAAGACCTTCATGACCGCGCTGGTCCTGTCTCTGCAGTCGTGCGGCCGGCTGTACGTGGGCCAGCGCCACTATTCCGCCTTCGAGTGCGCCGTGCTGTGTCTGTATCTGCTGTACCGAACCACCCACGAGTCCTCCCCCGATCGCGATCGCGCTCCCGTTGCGTTCGGGGACCTGCTGGCCCGCCTGCCGCGCTACCTGGCGCGTCTGGCCGCGGTAATCGGCGACGAGAGCGGACGCCCGCAGTACCGCTACCGCGACGACAAGCTGCCCAAAGCGCAGTTCGCGGCGGCCGGCGGCCGCTACGAGCACGGGGCCCTGGCCACCCACGTCGTGATCGCCACGTTGGTGCGCCACGGGGTGCTACCGGCGGCCCCGGGCGACGTTCCCCGAGACACCAGCACCCGCGTGAACCCCGACGACGTGGCCCACCGCGACGACGTCAACCGCGCCGCCGCCGCGTTTTTGGCACGCGGCCACAACCTCTTCCTGTGGGAGGACCAGACGCTGCTGCGGGCGACCGCCAACACCATTACGGCCCTGGCCGTGCTTCGGCGGCTCCTCGCGAACGGCAACGTGTACGCGGACCGCCTCGACAACCGCCTGCAGCTGGGCATGCTGATCCCGGGAGCCGTCCCGGCGGAGGCCATCGCTCGGGGGGCGTCCGGATTGGACTCGGGCGCCATAAAAAGCGGCGACAACAACCTGGAGGCGCTGTGCGTTAACTATGTACTTCCGCTGTATCAGGCAGACCCCACGGTCGAGCTGACCCAGTTGTTTCCGGGGCTGGCCGCCCTGTGCCTGGACGCCCAGGCGGGGCGGCCACTGGCGTCGACGAGGCGCGTGGTGGATATGTCGTCGGGCGCCCGCCAGGCGGCGCTCGTGCGCCTCACCGCGCTGGAGCTCATCAACCGCACCCGCACAAACACCACCCCTGTGGGGGAGATTATTAACGCCCACGATGCCTTGGGGATACAATACGAACAGGGCCTGGGGCTGCTCGCCCAGCAGGCACGCATCGGCTTGGCGTCAAACACCAAGCGATTCGCCACGTTCAACGTGGGCAGCGACTACGACCTGTTGTACTTTTTGTGTCTCGGGTTCATTCCCCAGTACCTGTCCGTGGCCTAGGGAAGGGTGGGGGTGGTGGTGGTGGGGTGTTTTTCTGTTGTTGTTTCTGGTCCGCCTGGTCACAAAAGGCACGGCGCCCCGAAACGCGGGCTTTAGTCCCGGCCCGGACGTCGGCGGACACGCAACAACGGCGGGCCCCGTGGGTGGGTAAGTTGGTTCGGGGGCATCGCTGTATTCCCTTGCCCGCTTCCACCCCCCCCCCCTTCCCGTTTTGTTTGTTTGTGCGGGTGCCCATGGCGTCGGCGGAAATGCGCGAGCGGTTGGAGGCGCCTCTGCCCGACCGGGCGGTGCCCATCTACGTGGCCGGGTTTTTGGCCCTGTACGACAGCGGGGACCCGGGCGAGCTGGCCCTGGACCCAGACACGGTGCGTGCGGCCCTGCCTCCGGAGAACCCCCTGCCGATCAACGTAGACCACCGCGCTCGGTGCGAGGTGGGCCGGGTGCTCGCCGTGGTCAACGACCCTCGGGGGCCGTTTTTTGTGGGGCTGATCGCGTGCGTGCAGCTGGAGCGCGTCCTCGAGACGGCCGCCAGCGCCGCTATTTTTGAGCGCCGCGGACCCGCGCTCTCCCGGGAGGAGCGTCTGCTGTACCTGATCACCAACTACCTGCCATCGGTCTCGCTGTCCACAAAACGCCGGGGGGACGAGGTTCCGCCCGACCGCACCCTGTTTGCGCACGTGGCCCTGTGCGCCATCGGGCGGCGCCTTGGAACCATCGTCACCTACGACACCAGCCTAGACGCGGCCATCGCTCCGTTTCGCCACCTGGACCCGGCGACGCGCGAGGGGGTGCGACGCGAGGCCGCCGAGGCCGAGCTCGCGCTGGCCGGGCGCACCTGGGCCCCCGGCGTGGAGGCGCTCACACACACGCTGCTCTCCACCGCCGTCAACAACATGATGCTGCGTGACCGCTGGAGCCTTGTGGCCGAGCGGCGGCGGCAGGCCGGGATCGCCGGACACACGTACCTTCAGGCGAGCGAAAAATTTAAAATATGGGGGGCGGAGTCTGCCCCTGCGCCGGAGCGCGGGTATAAAACCGGCGCCCCGGGTGCCATGGACACATCCCCCGCCGCGAGCGTTCCCGCGCCGCAGGTCGCCGTCCGTGCGCGTCAAGTCGCGTCGTCGTCTTCTTCTTCTTCTTTTCCGGCACCGGCCGATATGAACCCCGTTTCGGCATCGGGCGCCCCGGCCCCTCCGCCGCCCGGCGACGGGAGTTATTTGTGGATCCCCGCCTCTCATTACAATCAGCTCGTCACCGGGCAATCCGCGCCCCGCCACCCGCCGCTGACCGCGTGCGGCCTGCCGGCCGCGGGGACGGTGGCCTACGGACACCCCGGCGCCGGCCCGTCCCCGCACTACCCGCCTCCTCCCGCCCACCCGTACCCGGGTATGCTGTTCGCGGGCCCCAGTCCCCTGGAGGCCCAGATCGCCGCGCTGGTGGGGGCCATCGCCGCCGACCGCCAGGCGGGTGGGCTTCCGGCGGCCGCCGGAGACCACGGGATCCGGGGGTCGGCGAAGCGCCGCCGACACGAGGTGGAGCAGCCGGAGTACGACTGCGGCCGTGACGAGCCGGACCGGGACTTCCCGTATTACCCGGGCGAGGCCCGCCCCGAGCCGCGCCCGGTCGACTCCCGGCGCGCCGCGCGCCAGGCTTCCGGGCCCCACGAAACCATCACGGCGCTGGTGGGGGCGGTGACGTCCCTGCAGCAGGAACTGGCGCACATGCGCGCGCGTACCCACGCCCCCTACGGGCCGTATCCGCCGGTGGGGCCCTACCACCACCCCCACGCAGACACGGAGACCCCCGCCCAACCACCCCGCTACCCCGCCAAGGCCGTCTATCTGCCGCCGCCGCACATCGCCCCCCCGGGGCCTCCTCTATCCGGGGCGGTCCCCCCACCCTCGTATCCCCCAGTTGCGGTTACCCCCGGTCCCGCTCCCCCGCTACATCAGCCCTCCCCCGCACACGCCCACCCCCCTCCGCCGCCGCCGGGACCCACGCCTCCCCCCGCCGCGAGCTTACCCCAACCCGAGGCGCCCGGCGCGGAGGCCGGCGCCTTAGTTAACGCCAGCAGCGCGGCCCACGTGAACGTGGACACGGCCCGGGCCGCCGATCTGTTTGTGTCACAGATGATGGGGTCCCGCTAACTCGCCTCCAGGATCCGGACTTGGGGGGGGTGTGTGTTTTCATATATTTTAAATAAACAAACAACCGGACAAAAGTATACCCACTTCGTGTGCTTGTGTTTTTGTTTGAGAGGGGGGGGTGGAGTGGGGGGGAAAGTGGGCCGAATGACACAAAAATTAGGTCGGAGGGGTGAGGGGGGGGGGCTAGGAGCCGAACCGATGGCCCCCACACGCGACGGAAGGCCCGGAAGACTACCACGGGGAGGGGGTGTGGAAAGCGACCGGTCGCAGGGAGACGGGGTTGGTTTGGGGTTGGTTTGGGGTTGGTTTTCCCGTTAGCACATGTCTGCATTTGTTTTTCTAGTCACACGCCCCCCCCCCCCAAATAAAAACCAAGGCAAAACAATACCAGAAGTCATGTGTATTTTTGAACATCGGTGTCTTTTTATTTATACACAAGCCCAGCTCCCCTCCCCTCCCTTAGAGCTCGTCTTCGTCTCCGGCCTCGTCCTCGTTGTGGAGCGGAGAGTACCTGGCTTTGTTGCGCTTGCGCAGAACCATGTTGGTGACCTTGGAGCTGAGCAGGGCGCTCGTGCCCTTCTTTCTGGCCTTGTGTTCCGTGCGCTCCATGGCCGACACCAAAGCCATATATCGGATCATTTCTCGGGCCTCGGCCAACTTGGCCTCGTCAAACCCGCCCCCCTCCGCGCCTTCCTCCCCCTCCCCGCCCACGCCCCCGGGGTCGGAAGTCTTGAGTTCCTTGGTGGTGAGCGGATACAGGGCCTTCATGGGATTGCGTTGCAGTTGCAGGACGTAGCGGAAGGCGAAGAAGGCCGCGACCAGGCCGGCCAGGACCAGCAGCCCCACGGCAAGCGCCCCGAAGGGGTTGGACATAAAGGAGGACACGCCCGAGACGGCCGACACCACGCCCCCCACTACTCCCATGACTACCTTGCCGACCGCGCGCCCCAAGTCCCCCATCCCCTCGAAGAACGCGCACAGCCCCGCGAACATGGCGGCGTTGGCGTCGGCGCGGATGACCGTGTCGATGTCGGCAAAGCGCAGGTCGTGCAGCTGGTTGCGGCGCTGGACCTCCGTGTAGTCCAGCAGGCCGCTGTCCTTGATCTCGTGGCGCGTGTAGACCTCCAGGGGCACAAACTCGTGGTCCTCCAGCATGGTGATGTTCAGGTCGATGAAGGTGCTGACGGTGGTGACGTCGGCGCGACTCAGCTGGTGAGAGTACGCGTACTCCTCGAAGTACACGTAGCCCCCGCCGAAGATGAAGTAGCGCCGGTGGCCCACGGTGCACGGCTCGAGCGCGTCGCGGGTGAGGCGCAGCTCGTTGTTCTCGCCCAGCTGCCCCTCGATCAGCGGGCCCTGGTCTTCGTACCGAAAGCTGACCAGGGGGCGGCTGTAGCACGTCCCCGGCCGCGAGCTGACGCGCATCGAGTTCTGCACGATCACGTTGTCCGGGGCGACGGGCACGCACGTGGAGACGGCCATGACGTCTCCGAGCATGCGCGCGCTCACCCGCCGGCCGACGGTGGCGGAGGCGATGGCGTTGGGGTTGAGCTTGCGGGCCTCGTTCCAGAGAGTCAGCTCGTGGTTCTGCAGCTCGCACCACGCGACGGCGATGCGCCCCAGCATGTCGTTCACGTGGCGCTGTATGTGGTTATACGTAAACTGCAGCCGGGCGAACTCGATCGAGGAGGTGGTCTTGATGCGCTCCACGGACGCGTTGGCGCTGGGCGCCTCCCGCAGTGGCGCGGGCGTGGCATTCCGGGGCTTGCGGTCCTGCTCCCGCATGTACTCCCGCACGTACAGCTCGGCGAGCGTGTTGCTGAGGAGGGGCTGGTACGCGATGAGGAAGCCCCCCGTGGCCAGGTAGTACTGCGGCTGGCCCACCTTGATGTGCGTGGCGTTGTACTTGCGCGCAAACATGCGGTCGATGGCCTCGCGGGCATCCCGGCCGATGCAGTCGCCCAGGTCGACGCGCGAGAGCGAGTACTCGGTCAGGTTGGTGGTGAAGGTGGTCGAGATGGCGTCGGAGGAGAAGCGGAAGGAGCCGCCGTACTCGGCGCGGAGCATCTCGTCCACCTCCTGCCACTTGGTCATGGTGCAGACCGCCGGTCGCTTCGGCACCCAGTCCCAGGCCACGGTAAACTTGGGGGTCGTCAGCAAGTTGCGGGTCGTCGGCGACGTGGCCCGGGCCTTCGTGGTGAGGTCGCGCGCGTAGAAGCCGTCGACCTGCTTGAAGCGGTCGGCGGCGTAGCTGGTGTGCTCGGTGTGCGACCCCTCCCGGTAGCCGTAAAACGGGGACATGTACACAAAGTCGCCCGTCGCCAGCACAAACTCATCGTACGGGTACACCGACCGCGCGTCCACCTCCTCGACGATGCAGTTGACCGTCGTGCCGTACCGATGGAACGCCTCCACCCGCGAGGGGTTGTACTTGAGGTCGGTGGTGTGCCACCCCCGGCTCGTGCGCGTGGCGACCTTCGCCGGCTTGAGCTCCATGTCGGTCTCGTGGTCGTCCCGGTGAAACGCGGTGGTCTCCATGTTGTTCCGCACGTACTTGGCCGTGGAGCGGCAGACCCCCTTGGCGTTAATCTTGTCGATCACCTCCTCGAAGGGAACGGGGGCGCGGTCCTCGAATATCCCCATAAACTGGGAGTAGCGGTGGCCGAACCACACCTGCGACACGGTCACGTCTTTGTAGTACATGGTGGCCTTGAATTTGTACGGGGCGATGTTCTCCTTGAAGACCACCGCGATGCCCTCCGTGTAGTTCTGCCCCTCCGGGCGCGTCGGGCAGCGGCGCGGCTGCTCAAACTGCACCACCGTGGCGCCCGTCGGGGGCGGGCACACGTAAAACTGGGCATCGGCGTTCTCGACCTTGATTTCCCGCAGGTGCGCGCGCAGCGTGGCGTGGCCGGCGGCGACGGTCGCGTTGGCGTCGGGGGGCGGGGTCGCCTCGGGCCGCTTGGGCGGCTTTTTGGTTTTCCGCTTCCGGGCCTTGGTGGTCGCGGGGCTCGGGACGGGGGGCGGCCGGGAGGCGGGACCCCCGTTCGCCGCGACGGTCGCGGCCACGCCGCCCGAGGCGCGGGGGGCCGCCGGGGCCGCCGGGGCCGCCGACGCCACCGCGGCCACCAGCGCCCCCACGACCAGCGCGCAAATCAAGCCCCCCCCGCGCATGGCGGGCCTACGGGGGCGCGTCGCTCCCGCCGCCCGCTAGTCTGGGGGCGAGGTGCTGCAGGACCGAGTAGAGGATGGAAAAAACGTCTCGGTCGTAAACCACGACCGAGCGGGGTCCGATGCAGCCGTCGGGGCCGCTCTCGACGATGGCCACCAGCGGACAGTCGGAGTTGTACGTGAGGTACACGCCCGGCGGGTAGCGGTACAGACCTTCGGAGGTCGGGCGGCTGCAGTCGGGGCGGCGCAACTCAAGCTCCCCGCACCGGTAGACCGACGCAAAGAGTGTGGTGGCGATAATGAGCTCGCGAATATATCGCCAGGCGGCGCGCTGGGTGGGCGTGATTCCGGAAACACCGTCAAAACAGTAGAACTTTTGAAACTCGCTGACGGCCCAATCAGCGCCCGAACCCCCCGCGCCCATGATGAAGCGGGCGAGTTCCTCCTTGAGGTGCGGCAGGAGCCCCACGTTCTCGACGCTGTAGTACAGCGCGGTGTTGGGGGGCTGGGCGAAGCTGTGGGTGGAGTGGTCGAACAGGGGCCCGTTGACGAGCTCGAAGAAGCGATGGGTGATGCTGGGGAGCAGGGCCGGGTCCACCTGGTGGCGCAGCAGCGACGCTCGCATGAACCGGTGCGCGTCAAACACGCCCGGGGCGGCGCGGTTGTCGATGACCGTGCCCGCGCCCGCCGTCAGGGCGCAGAAGCGCGCGCGCGCCGCGAAGCCGTTGGCGACCGCGGCGAAGGTCGCGGGCAGCACCTCGCCGTGGACGCTGACCCGCAGCATCTTCTCGAGCTCCCCGCGCTGCTCGCGCACGCAGCGCCCGAGGCTGGCCAGCGACCGCTTGGTCAGGCGGTCCGCGTACAGCCGCCGGCGCTCCCGCACGTCCGCGGCGGCCCGCGTCGCGATGTCGCCCCAGCTCTCCGGCCCCTGCGCCCCTGGCTCGGGGCCGCGCTCCCCGTCCTCGCTCGCGGGCGTCCCCGCGCCACGCCTCCGCCCCCCCTCCTCCGCGGCGGCCCGGGGCTCTTCCTCCTCGGCCCCCCCGGTCGCGCCGCCGGCCCCCAGCCGCGCCAGCACGCGGCGCAGCGCCTCCTCGTCGCACTGCTCGGGGCTGACGAGCCGCCGCAGCAGCGGCGTCGTCAGGTGGTGGTCGTAGCACGCGCGTATCAGCGCCTCGATCTGATCGTCGGGCGACGTCGCCTGGCCGCCGATGATCAGGGCGTCCACCATGTCCAGCGCCGCCAGGTGGCCCCCGAACGCGCGATCGAAGTGCTCCGCCCGCCGCCCGAACAGCGCCAGCTCCACGGCCACCGCGGCGGTCTCCTGCTGCAGCTCGCGCTGCGCCAGCGCGTTCAGGTTGTCGGCGAAGGCGTCCATGGTGGAGTGGCGGGCGCGATCGCCGGACGCCAGCCAGAAGCGCAGCTCGCTGATGGCGTACAGGCCGGGCGTAGTGGCCTGAAACACGTCATGCGCCTCCAGCAGGGCGTCGGCCTCCTCGCGGACAGAAGAGCTATCGGCGGGCGGCGGGCCGGCCCGGGCCCCGCCGCCCGCCGCGGTCCGCGCCAGCGCCTGGTCCAGCACACAGAGCGCTCGCGCGCGGGCGGCGTCCGACAGCCCGGCGGCGTGGGGCAGGTACCGTCGCAGCTCGTTGGCGTCCAGCCGCACCTGGGCCTGTTGGGTGACGTGGTTACAGATGCGGTCCGCCAGGCGGCGGGCGATGGTCGCCCCCTGGTTCGCGGTGACGCACAGCTCCTCGAAACAGACCGCGCACGGGTGGGACGGGTCGCTCAGCTCCGGGGGCACGATGAGGCCCGACCCCACCGCCGCCACCATAAACTCCCGGACGCGCTCCAGCGCGGCCGTGGCGCCGCTCGGGGGGGTGATGAGGTGGCAGTAGTTCAGCTGCTTGAGAAAATTCTCGACATCATGCAGGAAGCACAGCTCCATGCGGACGTCCCCGCCGTACGTCTGCAGCCGGATCTGCTGGTGGTACGGACAGGGTCGGGCCAGACCCATGGTCTCGGTGAAAAAGGCAGAGACGTCACCCGTGGTCGCGAACGTTTCCAGGTGGCCCAGGAGCCGCTCCCCCTCGCGCCACGCGTACTCCAGGAGCAACTCCAGGGTGACCGACAGCGGGGTGAGAAAGGCGGCGGCCTGAGCCTCCAGCCCCGGCCGCAGGTGCCGCCGCAGCACGCGCACCTGGAGCGCGTTGAGCTTTAGCTGGGCGAGCTTCCCCAGGCCGATCTGGGGGTCGCATCGTCGAAGCAGCTCTAGCTGAAAAACGTACGTCTGTACCTGCCCGAGCAGGGCCAACAGTTTCTGTCGGGCCGCAGTGGGCTCGGAAACCGCGGCCGGGGGCGCGGCCGCCATGGCGAGTCACCCGGCCGTGCTGTGGTTTAGTTAAGGTTTGGGGGGGGGTGGGTCAGAGGCGCGCCCCGCGCGGACTGATGCGGCGGCGGGCCCCTGACATCCCCTCTTTATGCCCGTCGCCCGCCCGCCCGCCCCGCCGGTGTGCCGTGATTCGCGGAGTCGGGGCCTTGTGTTTCTTTCTTTCCCCCCCGAATCCGTTCTTTCTTCCTCACCCCCCCCTCCCCACACACCCACCCAGGACTCGCCACCACAAGGAGGCGAGAGCCCGTCGCTAACCCAAAGACACAGTCACGAGACACGATATCGACTGTAGTTGCGATCGTTTATTTTATACACAACACCAACCTTTCCTTCGACCCCCCCCACCCCCGCCCCTAGAGCATATCCAACGTCAGGTCCTTTTTCTCCGGTGGTCCCTCCCCAAACGGATCGTCGCCGTGAAACGCCCGCTTTCGGGCGACGCCGGCCGCCCCCGCCGCCGCCGCCAAACCGCCGAACGACGCCGCGTGGTCATCCTCGTCGCCGAAATCCCCAAAGTTAAACACCTCCCCGGCGGCGCCGAGCTGGCTGACCAGGGCCTCCGCCTCGTGGGCCACCTCCAGGGCCGCGTCGGTCGACCACTCGCCGTGCCCGCGCTCCAGGGCGCGGGTGGTAAACTCCATCATTTCCTCGCTCAGGTACTCGTCCTCCAGCAGCGCCAGCCAGTCCTCGATCTGCAGCTGCTGGGTGCGGGGGCCCAGGCTCTTGACGGTCGCCACAAACACGCTGCTGGCGACCGCCGCCCCGCCCTCCGCAATGATGCCCCGGAGCTGCTCGCACAGCGAATGCTCGTGGGCCCCGCCCCCGAGACTCGACGCCGCGCACACAAACCCGGCCCTGGGGCAGGCCAGGACAAACTTGCGGGTGCGGTCAAAGATCAGCAGCGGGCACGCGTTTTTGCCGCCCAGCAGGCTGGCCCAGTTCCCGGCCTGAAACACGCGGTCGTTGCCGGCCATGCCGTAGTATTTGCTGATGCTGAGGCCCAGCACGACCATCGGGCGCGCGGCCATCACGGGCCGCAGCAGGTTGCAGCTCGCGAACATGGACGTCCAGGCGCCGGGGTGCGCGTCGAGGGAGTCCATCAGCGCGCGGGCCCCGGCCTCCAGGCCCGCGCCGCCCTGCGGGGCCCAGGCGGCCGCCGCCTGCACGCTGGGGGGACGGCGGGACCCGGCGATGACGGCCGTGAGGGTGTTTATGAAGTACGTCGAGTGGTCGCAGTACCTCAAGATCTGGTTGGCCATGTAGTACATGGCCAGTTCGCTCACGTTATTGGGGGCCAGGTTGATAAAGTTAATCGCGCCGTAGTCCAGGGAGAACCTCTTAATGAACGCGATGGTCTCTATGTCCTCGCGCGACAAGAGCCGGGCGGGGAGCTGGTTGCGCTGGAGGGCGGTCCAGAACCACTGCGGGTTCGGCTGGTTCGACCCCGGGGGCTTGCCGTTGGGAAAGATGACCGCGTGGAACTGCTTCAGCAGGAAGCCCAGCGGTCCGAGGAGGATGTCCACGCGCTTGTCGGGCTTCTGGTAGGCGCTCTGGAGGCTGGCGACCCGCGCCTTGGCGGCCTCGGACGCGTTGGCGCTCGCGCCCGCGAACAACACGCGGCTCTTGACGCGCAGTTCCTTGGGAAACCCAAGGGTCACGCGGGCAACGTCGCCCTCGAAGCTGCTCTCGGCGGGGGCCGTCTGGCCGGCCGTTAGGCTGGGGGCGCAGATAGCCGCCCCCTCCGAGAGCGCGACCGTCAGCGTCTTCGCCGACAGGAACCCGTTGTTGAACAGGTCCATGACGCGCCGCCGCAGCACCGGTTGGAATTGATTGCGAAAGTTGCGCCCCTCGACCGACTGCCCGGCGAACACCCCGTGGCACTGGCTCAGGGCCAGGTCCTGGTACACGGCGAGGTTGGACCGCCGCGCGAGGAGCTGCAGCAGGGGGCACGGCCCGCAGGTGTACGGGTCCAGCGACAGCGACATGGCGTGGTTGGCCTCGGCCAGACCGTCGCGGAACTTAAAGTTGCGCCCCTCGATCAGGTTGCGCATCAGCTGTTCCACCTCGCGATCCACCAGCTGCTTGATGTTGTTCACCACCGTGTGCAGGGCCTCGCGGTTGCCGATAATCGTCTCCAGCCTCCCCAGGGCCGTGGGCACCGCCTGGTCCACGTACTGCAGGGCCTCGAGCTCGGCCATGACGCGCTCGGTGGCCGCGCGGTACGTCTCCTGCATGATGGTCCGGGTGTTCTCGGACCCGTCCGCGCGCTTCAGGGCCGAGAAGGCGGCGTAGTTCCCCAGCACGTCGCAGTCGCTGTACGCGCTGTTCATCGTTCCGAAGACCCCAATGGCCCCCCGGGCGGCGCTCGCGAACTTGGGGTGGCGGGCCCGCAGCCGCATCAGCGTCGTGTGCGCGCAGGCGTGGCGGGTCTCGAAGGTACACAGGTTGCAGGGCACGTCGGTCTGGCCCGAGTCCGCGACGTAGCGAAACACGTCCATCTCCTGGCGCCCGACGATGACTCCGCCGTCGCAGCGCTCCAGGTAAAACAGCATCTTGGCCAGCAGGGCCGGAGAGAACCCGCACAGCATGGCCAGGTGCTCGCCGGCGAACTCCTGGGTTCCGCCGACGAGGGGCGCCGTGGGGCGCCCCTCGTACCCGGGCACCACGTGGCCCTCGCGGTCCAGCTGCGGGTTGGCCGCCACGTGCGTGCCGGGCACGAGAAAGAAGCGGTAAAAGGAGGGCTTGCTGTGGTCCTTGGGGTCCGCCGGCCCGGCGTCGTCCACCTCGGTCAGGTGGAGGGCCGAATTGGTGCTGAACACCATGGCGCCCACGAGGCCCGCGGCGCGCGCCAGGTACGCCCCGACGGCGCCGGCGCGGGCCGCGGGCGTTTCCTGGCCCTCAAGCAGGGGCCACGTGGTGATGTCGGGGGGCGGCTCGTCAAAGACCGCCATCGACACGATGGACTCCAGGGCCAGGGCGGCGTCGCCCGCCATCACCGAGGCCAGGCGCTGCTCAAACCCGCCCGCCGGGCCCTTGTTCCCGGCGTCGCGCGCGCCCCGCTGGGGCTTACCCTGGCTGGCCTCGAAGGCCGTGAACGTAATGTCGGCGGGGAGGGCCGCGCCCTCGTGGTTTTCGTCGAACGCCAGGTGGGCGGCCGCGCGGGCCACGGCGTCCACGTTCCGGGCACGCAGGGCCACGGCGGCGGGCCCGACGACCGCCTCGAACAGCAGGCGGGCGAGGGGGCGGTTGAAAAACGGAAGGGGGTAGTTGAAATTCTCCCCGATCGATCGGTGGTTGCAGTTAAACGGATCGGCGATGACCCGGCTAAAATCCGGCATAAACATCTGCAGCGGATACACGGGGATGCGGTGAACCTCCGCGTCCCCGATGGTTACCTTGTCCATCCCGCCCAGATGCAGGAAGGTGTTGCTGATGCACACGGCCTCCCGGAAGCCCTCCGTGATCACCAGATACAGCAAGGCCCGGTCCGGGTCCAGTCCGAGCCGCTCGCACAGCGCGTCCCCCGTCGTCTCGTGCTTTAGGTCGCAGGGCCGGGGCGCGTAGTCCGCGAAGCCAAAATGGCGGCGCGCCCGCTCGCAGAGCCGCGTCAGGTTGGGGGCCTGGGTGCTGGGGGCCAGGTGGCGGCCGCCGTGAAAGACGTAAACGGACGGGCTGTAGTGCGAGGGCATAAGCTTGAGGGACACCGCGGTCCCCCCAAGGCCCGTCGTGCGGGACCCGACGACCGCGGCCACGTTGGCCTCAAACCCGCTCTCCACGGTCAGGCCGACGATGAGGGGCGCGACGGCGACGTCCGCGTCGCCGCTGCGCGCCGACAGTAGCGACAGCAGCTCCAGGCCTTCGGCCGGACAGGCGCGGCCATACACGTACCCCATCGGCCCCGGAGGAACCTTGACGGTGGTCGTCGTTTTGGGCTTGGTGTCCATGGCTTTCGGGAGATCGGCGACCGGCAGGAACGGGGGCCCGGCAAGACGACCGGGGGCAGACGGGGGAGGCCGCGCGTGGTCGACGGCTGCTGCCCGCCGTCGTCTCTCCGATGGGGTCGAATGCCGGCGCTGGGGGTGGGGTCTACACCCGCCCGTTCGCCGAGCGGCCCCTGGTGGGGGTGGGATGGGTGGGATGGGGTGGGCGAGAATGGCCCGCCACCGGATCGCGCCGGACGGGGGGGCCCGGGGTTGGGCAAGGTTTGGGCGCAAGGCTCCAGCGGCGATTCGAGAGGCCTGCGGATGGCGGCCCAGAGCTGGGTATGCTCGGCCGGGGCGGCCGGTATATGTACGGCGTGCTGGGAGGGGCGGCGTCGGGCCCCGCCCACGGTCCGCCACGCCCCGCGCGTCATCGGCAGGGGGCGTGGCCGCCCTTCTAAAAAAAGTGAGAACGCGAAGCGTTCGCACTTTGTCCTAATAATATATATACTATTAGGACAAAGTGCGAACGCTTCGCGTTCTCACTTTTTTTAGAAGGGCGGCCACGCCCCCTTTGACGTCACGCTCACCCGGGCGGCCGGCCGCCCATAAGCGCGGCCTGCCGGGCCGATAAAAAGAAACCGCGGCGCCCCCGCGGACACCACACACTGGCTCTCGAACCCCGGACGCGCAGAAGGGACCCGGGCGCGGGTCCGCCGGTAAGAGCCGGGGGGAACATCGGCACCGCCATCCCACCCCGAGCTGTTGGGTGGGCGGGTGGGGGGGCTGGTGAGGCGGTGGTGGGAGGGGGCGGCGTATAGCAGGACAACGACCGGCGGCGATGTTTTGTGCCGCGGGCGGCCCGGCTTCCCCCGGGGGGAAGCCGGCGGCTCGGGCGGCGTCTGGGTTTTTTGCCCCCCACAACCCCCGGGGAGCCACCCAGACGGCACCGCCGCCTTGCCGCCGGCAGAACTTCTACAACCCCCACCTCGCTCAGACCGGAACGCAGCCAAAGGCCCTCGGGCCGGCTCAGCGCCATACGTACTACAGCGAGTGCGACGAATTTCGATTTATCGCCCCGCGTTCGCTGGACGAGGACGCCCCCGCGGAGCAGCGCACCGGGGTCCACGACGGCCGCCTCCGGCGCGCCCCTAAGGTGTACTGCGGGGGGGACGAGCGCGACGTCCTCCGCGTGGGCCCGGAGGGCTTCTGGCCGCGTCGCTTGCGCCTGTGGGGCGGTGCGGACCATGCCCCCGAGGGGTTCGACCCCACCGTCACCGTCTTCCACGTGTACGACATCCTGGAGCACGTGGAACACGCGTACAGCATGCGCGCCGCCCAGCTCCACGAGCGATTTATGGACGCCATCACGCCCGCCGGGACCGTCATCACGCTTCTGGGTCTGACCCCCGAAGGCCATCGCGTCGCCGTTCACGTCTACGGCACGCGGCAGTACTTTTACATGAACAAGGCGGAGGTGGATCGGCACCTGCAGTGCCGTGCCCCGCGCGATCTCTGCGAGCGCCTGGCGGCGGCCCTGCGCGAGTCGCCGGGGGCGTCGTTCCGCGGCATCTCCGCGGACCACTTCGAGGCGGAGGTGGTGGAGCGCGCCGACGTGTACTATTACGAAACGCGCCCGACCCTGTACTACCGCGTCTTCGTGCGAAGCGGGCGCGCGCTGGCCTACCTGTGCGACAACTTTTGCCCCGCGATCAGGAAGTACGAGGGGGGCGTCGACGCCACCACCCGGTTTATCCTGGACAACCCGGGGTTTGTCACCTTCGGCTGGTACCGCCTCAAGCCCGGCCGCGGGAACGCGCCGGCCCAACCGCGCCCCCCGACGGCGTTCGGAACCTCGAGCGACGTCGAGTTTAACTGCACGGCGGACAACCTGGCCGTCGAGGGGGCCATGTGTGACCTGCCGGCCTACAAGCTCATGTGCTTCGATATCGAATGCAAGGCCGGGGGGGAGGACGAGCTGGCCTTTCCGGTCGCGGAACGCCCGGAAGACCTCGTCATCCAGATCTCCTGTCTGCTCTACGACCTGTCCACCACCGCCCTCGAGCACATCCTCCTGTTTTCGCTCGGATCCTGCGACCTCCCCGAGTCCCACCTCAGCGATCTCGCCTCCAGGGGCCTGCCGGCCCCCGTCGTCCTGGAGTTTGACAGCGAATTCGAGATGCTGCTGGCCTTCATGACCTTCGTCAAGCAGTACGGCCCCGAGTTCGTGACCGGGTACAACATCATCAACTTCGACTGGCCCTTCGTCCTGACCAAGCTGACGGAGATCTACAAGGTCCCGCTCGACGGGTACGGGCGCATGAACGGCCGGGGTGTGTTCCGCGTGTGGGACATCGGCCAGAGCCACTTTCAGAAGCGCAGCAAGATCAAGGTGAACGGGATGGTGAACATCGACATGTACGGCATCATCACCGACAAGGTCAAACTCTCCAGCTACAAGCTGAACGCCGTCGCCGAGGCCGTCTTGAAGGACAAGAAGAAGGATCTGAGCTACCGCGACATCCCCGCCTACTACGCCTCCGGGCCCGCGCAGCGCGGGGTGATCGGCGAGTATTGTGTGCAGGACTCGCTGCTGGTCGGGCAGCTGTTCTTCAAGTTTCTGCCGCACCTGGAGCTTTCCGCCGTCGCGCGCCTGGCGGGCATCAACATCACCCGCACCATCTACGACGGCCAGCAGATCCGCGTCTTCACGTGCCTCCTGCGCCTTGCGGGCCAGAAGGGCTTCATCCTGCCGGACACCCAGGGGCGGTTTCGGGGCCTCGACAAGGAGGCGCCCAAGCGCCCGGCCGTGCCTCGGGGGGAAGGGGAGCGGCCGGGGGACGGGAACGGGGACGAGGATAAGGACGACGACGAGGACGGGGACGAGGACGGGGACGAGCGCGAGGAGGTCGCGCGCGAGACCGGGGGCCGGCACGTTGGGTACCAGGGGGCCCGGGTCCTCGACCCCACCTCCGGGTTTCACGTCGACCCCGTGGTGGTGTTTGACTTTGCCAGCCTGTACCCCAGCATCATCCAGGCCCACAACCTGTGCTTCAGTACGCTCTCCCTGCGGCCCGAGGCCGTCGCGCACCTGGAGGCGGACCGGGACTACCTGGAGATCGAGGTGGGGGGCCGACGGCTGTTCTTCGTGAAGGCCCACGTACGCGAGAGCCTGCTGAGCATCCTGCTGCGCGACTGGCTGGCCATGCGAAAGCAGATCCGCTCGCGGATCCCCCAGAGCACCCCCGAGGAGGCCGTCCTCCTCGACAAGCAACAGGCCGCCATCAAGGTGGTGTGCAACTCGGTGTACGGGTTCACCGGGGTGCAGCACGGTCTTCTGCCCTGCCTGCACGTGGCCGCCACCGTGACGACCATCGGCCGCGAGATGCTCCTCGCGACGCGCGCGTACGTGCACGCGCGCTGGGCGGAGTTCGATCAGCTGCTGGCCGACTTTCCGGAGGCGGCCGGCATGCGCGCCCCCGGTCCGTACTCCATGCGCATCATCTACGGGGACACGGACTCCATTTTCGTTTTGTGCCGCGGCCTCACGGCCGCGGGCCTGGTGGCCATGGGCGACAAGATGGCGAGCCACATCTCGCGCGCGCTGTTCCTCCCCCCGATCAAGCTCGAGTGCGAAAAAACGTTCACCAAGCTGCTGCTCATCGCCAAGAAAAAGTACATCGGCGTCATCTGCGGGGGCAAGATGCTCATCAAGGGCGTGGATCTGGTGCGCAAAAACAACTGCGCGTTTATCAACCGCACCTCCAGGGCCCTGGTCGACCTGCTGTTTTACGACGATACCGTATCCGGAGCGGCCGCCGCGTTAGCCGAGCGCCCCGCAGAGGAGTGGCTGGCGCGACCCCTGCCCGAGGGACTGCAGGCGTTCGGGGCCGTCCTCGTAGACGCCCATCGGCGCATCACCGACCCGGAGAGGGACATCCAGGACTTTGTCCTCACCGCCGAACTGAGCAGACACCCGCGCGCGTACACCAACAAGCGCCTGGCCCACCTGACGGTGTATTACAAGCTCATGGCCCGCCGCGCGCAGGTCCCGTCCATCAAGGACCGGATCCCGTACGTGATCGTGGCCCAGACCCGCGAGGTAGAGGAGACGGTCGCGCGGCTGGCCGCCCTCCGCGAGCTAGACGCCGCCGCCCCAGGGGACGAGCCCGCCCCCCCAGCGGCCCTGCCCTCCCCGGCCAAGCGCCCCCGGGAGACGCCGTCGCATGCCGACCCCCCGGGAGGCGCGTCCAAGCCCCGCAAGCTGCTGGTGTCCGAGCTGGCGGAGGATCCCGGGTACGCCATCGCCCGGGGCGTTCCGCTCAACACGGACTATTACTTCTCGCACCTGCTGGGGGCGGCCTGCGTGACGTTCAAGGCCCTGTTTGGAAATAACGCCAAGATCACCGAGAGTCTGTTAAAGAGGTTTATTCCCGAGACGTGGCACCCCCCGGACGACGTGGCCGCGCGGCTCAGGGCCGCGGGGTTCGGGCCGGCGGGGGCCGGCGCTACGGCGGAGGAAACTCGTCGAATGTTGCATAGAGCCTTTGATACTCTAGCATGAGCCCCCCGTCGAAGCTGATGTCCCGCATCTTGCAATAAATGTCTGCGGCCGACACGGTCGGAATTTCCGCGTCCGCTGGTTTCTCTGCGTTGCGTCTGACCACGAGCACAAACGTGCTCTGCCACACGTGGGCGGCGAACCGGTAGCCGGGGCACGCGGTCAGCATCCGATCGATGAGCCGGTAGTGCAGGTGGGCCGACGTGCCGGGGAAGATGACGTACAGCATGTGGCCCCCGTACGTGGGGTCCGGGTAAAAAAGAAACCGGGGGTCGCACGCCCCCCCTCCGCGCAGGATCGTGTGCACGAAAAAGAGCTCGGGCTGGCCGAGCGTATCGGCCAGGAGGTCCTGGAGGGGGGTGCTGTGGCGGTCGGCCAGCACGACCAGGGAGGCCAGAAAGGTGCGGTGCTCAAAGATCGTATTGATCTGCTGCACGAAGGCCAGGATGAGGGCCTCGCGGCTGACGGTGGCCAGCCGCCCGTCGCCCGCGCTGCACGCGGGGCAGCAGCCCCCGATCCCCAGGTAGTAGCCCATGCCCGAGAGGGTCAGGCAGTTGTCGGCCACGGTCTGGTCCAGGCTGAAGGGGAGCGACACGGGGGTCGTCTTCACCAGGGGCACGGAGAGCGAGCGCACGATGGCGATCTCCTCGGAGGGCGTCTGGGCGAGGGCGGCGAAGAAGCCGCGGTAGCGACGGCGCTCGTGCAGGCAGAGCTCCAGCCTGCGCGCGTGCGACGGCAGGCTCTTGCGGGAGGCCCGGCGCTCCACGCCGGGGTTCCCGGCGGCGGAAAAGCGCGACCGCCGCCGGGTCTTGTCGCGGCCGGGCCCGGGCCGGGAGCCGGAGCGACGGGGGGCGATGTCATACATAGGTACAGAGGGTGTGCTCCAGGGACAGGAGAGAGATCGAGTGTCGTCTGAGCAGCGCGCCGGCCTCGCGGACAAATGTGGCCAGCGCGGTGGGCTTCGGCACAAATACCTGGTACGTCTTGAAGGTGTAGATGAGGGCCCGCAGGGCTATACAGACCCGCCCCTCGAACTCGTTGCCGCAGGCCAACTTGGCCTTGTGAAGCTGCAGCTCGTCGCGATGGTCGGCGCGGGGGTGGCCAAACAGGACCCAGGGGTCGACTTCCATCTCCGTGATGGCGCACATCGGATCGCAGAACATGTGCTTGAAGATGGCCTCGGGGCCCGCGGCCCGAAGCAGGCTCACGAACCGGCCCCCGTCCCCGGGCTGCGCCTCGGGGTCCGCCTCGAGCTGGTCCACGACCGGCACTATGCAGTCGAAGAGGCTGGTGTTGTTCTCCGAGTAGCGGACGACGGACGCCCTCAGGCGTCGCATGGCCAGCCAGTAGGCCCGCACCAGCAACAGATTGCACAGCAGGCATTCCCCGCCGGTGCGCCCGCGGCCCCGGCCGTGCTTCAGCACGGTGGCCATCAGCGGGCCCAGGTCCAGGTCGGGCTGGGCCTGGGGCTCGGCGAACTGCGCAAAACGCGGGGCCGCGTCGCGCATGCGCGCCCCGCGGTGCGCTTCCCAGGACTCGCTGACCGCGGCGCGGCGGGCGTCCGCGGCGGCGCGCAGCCGGGGCCCCGACTCCCAGACGGCGGGGGTGCCGGCGAGCAGCAGCAGGATCAGGTCGGCGTACGCCCACGTCTCCGGCTCACCCCCCTGCGCCAGCGCCCCGGCGGCGGCCTCGAACTCCCCGTTGCGGGCGGCGGCGCGCGTGCAGCAGCTGTCTCCGCCCCCGCGCTTGCCCTCGGTGCAGTCGAGCAGGCGGGCGCAGTCCTTCCAGTTCATCAGGGCGGTGGTGAGGGAGGGTTGCGTTCCCGAGCCCCCGCCCGCCCCCGCCCCCGCCCCGTCATCGCCCCCGGAGGCCAGGGTCCCGATGAGGGCCCGGGTTGCGGACTGCGCGAGGAAGGAATAGTTGGAGTACTGCACCTTGGCGGCGCCCGGGGAGGGCGTCGGCCTGGGTTGCTTCTGGGCGTGGCGCCCGGGCACCCCGCCGTCGGTCCGGAAGCAGCAGTGGAGAAAGAAATGCCGGTGGATGTCGTTGATGGTCAGGGCGAAGCGCGCGAAGGAGCCGACAAGGGTCGCCTTCTTGGTGCGCAGGAAGTGGTGGTCCATGACGTAGACGAACTCGAAGGCGGCCACGAAGATGCTCGCGGCGCAGTGGGGCGCGCCCAGGCACTTGGCGCAGAGGAACGCGTAATCGGCCACCCACTGGGGCGAGAGGCGGTAGGCCTGCTTGTACAGCTCGATGGTGCGGCAGACCAGACAGGGGCGGTCCAGCGCGAAGGTGTCGACGGACGCCGCGGCGAAGGGCCCCGTGTCCAAGAGTCCCTCTGCCGTGGGGTCTGCGGGCGGGCCGCGGGCGGACCCCGGCCCCCGCCCCCCCGAAGCCTCGCGCGCGGCCCCGCGCGGCCGCGGGGGGGCGGGCGCGACGTCGCTCTCCACGTCCTCGTCGAGCGCGCTCGCGGGCGGCACGCCTACCACGTGACAGGCCGCCAGGAGCTCGGCGCACAGGGCCTCGTTAAGAGCCAGAAGGTCGGGATCGAAGGCCACATACGGACGCTCGAACGCGCCCTCCTTCCAGCTGCTGCCCGGCGACTCTTCGCGCACGGCGGCGCTCGACGGCACCCCCGGGGCGGACGTCGCCATGGCCGGTCGAGCGGGGCGCACGCGTCCGCGAACGTTACGGGACGCGATCCCCGACTGCGCGCTGCGGTCCCAGACCCTGGAAAGTCTAGACGCGCGCTACGTCTCGCGAGACGGCGCGGGGGACGCGGCCGTCTGGTTCGAGGACATGACCCCCGCCGAACTAGAGGTTATATTCCCGACCACGGACGCCAAGCTGAACTACCTCTCGCGGACGCAGCGGCTGGCCTCCCTCCTGACGTACGCCGGGCCTATAAAAGCGCCCGACGGCCCCGCCGCCCCACATACGCAGGACACCGCGTGCGTGCACGGCGAGCTGCTCGCCCGAAAGCGCGAACGGTTCGCGGCGGTCATTAACCGGTTCCTGGACCTGCACCAGATCCTGCGGGGCTGACGCGCGCTTCGGCGGGGCACCGGCACCGGGACCGACTTGTTTTACATAACAGTAGGGGGTGGGGGAACGCGCACCCTTGCCCGGTCGCGATGGCGGGGATGGGGAAGCCCTACGGCGGCCGCCCGGGGGACGCGTTCGAGGGTCTCGTTCAGCGCATCAGGCTCATTGTTCCCGCCACGCTGCGCGGCGGGGGTGGGGAGTCGGGCCCCTACTCGCCATCCAACCCGCCCTCGAGATGTGCCTTCCAGTTCCACGGCCAGGATGGGTCCGACGAGGCCTTCCCGATCGAGTACGTCCTGCGGCTCATGAACGACTGGGCCGATGTGCCCTGCAACCCCTACCTGCGCGTGCAGAACACCGGCGTTTCGGTGCTGTTTCAGGGGTTTTTTAACCGGCCCCACGGCGCCCCGGGGGGCGCGATCACGGCGGAGCAGACCAACGTGATTCTGCACTCCACCGAGACGACGGGACTGTCCCTCGGAGACCTGGACGACGTCAAGGGGCGCCTCGGCCTGGACGCCCGGCCGATGATGGCCAGCATGTGGATCAGCTGCTTTGTGCGCATGCCCCGGGTGCAGCTCGCGTTTCGGTTCATGGGCCCCGAGGACGCCGTTCGCACGCGGCGGATCCTGTGTCGCGCCGCCGAGCAGGCCCTCGCCCGTCGCCGCCGGTCCAGGCGGTCCCAGGATGACTACGGGGCGGTGGTGGTGGCGGCGGCGCACCACTCTTCCGGAGCGCCCGGGCCGGGGGTCGCCGCCTCGGGCCCGCCAGCGCCGCCCGGACGGGGACCGGCCCGTCCGTGGCATCAGGCCGTGCAGTTGTTCCGGGCCCCGCGTCCGGGCCCCCCGGCGCTTCTGTTGCTGGCGGCGGGGCTGTTTCTGGGGGCCGCTATCTGGTGGGCGGTTGGCGCGCGCCTATGAAAGGGGGCGAGCCACCGTCCCGCCCGCCAGTGCATCCCAGACGCCCGCGAGCCGCACATCCCCTCCGCTCCCGCCTCCGGCCCGATTCTTACGGCGCGACCCAAGGTCCCGATGGCCGCCCCGCAGTTTCACCGCCCCAGCACCATTACCGCCGACAACGTCCGGGCGCTCGGCATGCGCGGGCTCGTGTTGGCCACCAACAACGCTCAGTTCATCATGGATAACAGCTACCCGCATCCGCACGGAACGCAGGGTGCGGTGCGAGAGTTTCTTCGCGGGCAGGCCGCGGCGCTGACGGACCTCGGGGTGACCCACGCCAACAACACGTTCGCCCCGCAGCCTATGTTCGCGGGCGACGCCGCGGCCGAATGGCTGCGGCCCTCGTTCGGTCTTAAGCGCACGTATTCCCCCTTTGTCGTTCGCGACCCCAAGACCCCCAGCACCCCGTGAGTCCTCGGCGGGTCCCTCCGCGGCCGTCTCTCGTTGCCCCCCTTTCCCCCTTCCCGGGTGGTTCAATAAAAAACACCAACATACGATATTCGCGTTTGATACGTTTATTGGGGGGGGTGTAGGGCCCAACGATCGGCGATTAACAACACCAAACAATCGAGCGCGTCTAACCCAGTAACATGCGCACGTGATGTAGGCTGGTCAGCACGGCGTTGCTGCGCTGAAACAGCGCCCTGCGGGTCCGCTGCAGCTGTTGTTGTATGCGGCGGCATGCGCGGATCAAAACCGCCAGGGCGCTACGACCGGTGCTTCGTACGTAGCGTCGCGACAAGACGGCATTTGCCTGTACGGGCAAGGGGCCAAATTGCGAGTGTGGTGACTGGAGGTGGTCGGCGGCCAATGGGCCGGGTGGTTCGTCGGCGGGGGGCAAGTGCGGTTCCGGTGGGAGGGGGTCGAGCGCCTCGGTATCATCCGAGTCCGAGAAACGCAGGGAGTCTGCGTCGGAGTGTTCATCATCGGAGGAGATGTGCAGCGTCTGAAGCAGCGATGCGGGTGGGGGCGCGGAGTCGACGTGAAGCGCGAGAGAGGAAGCCCACGAAGTCACAGCGGACACTGGGAGGTGGGTGTTTGTATGTGTGGGAGACTCGGGCGTCGGGACCGAGTCTCGGCTCTGGGGTGTAAGCGTCCGAGTTACGGGCGGCAGGGGCGGCTGGGGCAGGGGCGGCTGGGGCAGGGGCGGCTGGGGCAGGGGCGGCTGGGGCAGGGGCGGCTGGGGCAGGGGCGGCTGGGGCAGGGGCGGCTGGGGCAGGGGCGGCTGGGGCAGGGGCGGCTGGGGCAGGGGCGGCTGGGGCAGGGGCGGCTGGGGCACCGAGCGCGCGCGGATGCGCGTCCGCGCGGCGGGTTTGGTCGCGGGTGACTGGGGTGGGGGGCGGCGGGCAACCGGGCCTCCGGGCACGACCCAACCGCACAAAGGCTCGCTCGGGGCAACCGGGCCTGGGGCCAAAGGCGGGGGGCTGGTCTGGACGGCGGAGGTCGGGGGGGCAAGGCCCGGAGAAGGCGGCACTGCCGCCGCTGCGGCGGAAACCGCGGCCGCGTGGTCGGCTGGGTCCCGGGGAGAGGGGAGGGAGTTCAACGAGGCCGAGAGCGAGGCGACCGCGGGGCGCGTGAGGCGCCGGGGTGGGCCGGCCGCGGGGCCCCGGGGGGGTGTCGGCGAGGGACCCGCTGTTGTCTGGCGGCGGCCGCGGCGGCGGTCGCCCCCGGGGACGACCGCTCCTTCGGCGGGCGGAGGCGGGATGGGCGCGAGCGTGGGGGCGGGAAAGGCCCCGCGAGCCGAGGCGGGGCCGGGCGGAAGGGGCAAAGCAGAAACCCAAGCCGGGGGCGCGGACTCCGGGGTGGGCGGCTGGTCGGGAGGACGCGCGGAAGCGGCGACCGGGGCGACCGGGGCGGGGAGTGCCGGCGGACGCCACCCCTCGGGGGGGGCGGAGGCCCGGGGCGCGCGCGATTTGGCACGCGTCCGGCGGGACCTGCGCACGCGCGGCACGGCGGCGGAGAAAGCGGCGGCAGAGCCGGAAAAGGCCGGGGGAGGAAGCGCGGCATCCGCGGGGGGACTCGGTGTGGGTGGCGAGGGCCGTGGGTCGTCGCGAGGGGCCACGGGCACGCGCCCCGTGTTTTGTTGAGGCGGGACACTCGGTCGTGTTTCGCGAGCCGTAGCTGCCGGCCCGATGGGCCGCGGTGCGTACTGGGACGTGGGGACGGACTGATCGGTGGCGGGGGGGGGAAGAAGGGCCGGGGCCGGATTGGGCGTGGGGCCGCCGGCGTCGTCGGACGCCAGCTCCTCCAGGCCGTGGATCCAGGCCCACATGCGAGGGGGGACGGGCTCGCCGGTGGTGGCGTCGGTGAGGAGAGTGGGGGCGAGGACCCCCGGGTCCGCCTGCCGTGCGGGGGGGGCAGCGGGGTCCTCGGGACCCGATCCGCCATCCCCCCCCGCAAGGTCCCGCGGGTCGCGGGCGGCGGTCGGGGCAGAGGGACCTGCCTCGTCGGCGAGGGGGCGCTGGTAAACCGGGTGTCCCGGGAACAGCTCCCCCGTCAGGAGGGAGGCGTCGAAGGGCCGCCCGAGGATGGCCCGCGCGAAGAAGGGGTCCGCGTCGGCGGCGCTCGCCGCGAGAACGTCCCCCGCGGTAGCCACAAACGGAAGCTCCTCGGTGGCCTCGCTGCCCACAAACCGCACGTCAGGGGGGCCGGGGGGCTCCGGGGCTTCCCACAAGACCGCGACCGGGGTCATGGAGATGTCCACGAGGACCAGGCACGGGGGCCCGTCGGCGAGAGGGCGCTCGGCGATGAGCGCCGACAGGCGCGGGAGCTGCGCCGCCAGACACGCGTTTTCGATCGGGTTGAGATCGGTGTGGAGGAGGCCGACGGCCCACGTCTCGATGTCGGACGACACGACGTCGCGCAGGGCGGCGTCCGGCCCGCCGGGGCGCGAGTCGAAGAGCGTCAGGCACAGTTCCAGTTCCGACTCGCGGGAGAAGGCCGTGGTGTTGCGGAGCGCCACCACGACGGGCGCGCCGAGGAGCACCGCGGCCAGAACCAGGTCCATGGCCGTAACGCGCGCGGCGGGGGTGCGGTGGGTCGCGGCGGCCAGCACGGCCACGTGCTGGCCCGTGGGTCGGTAGAGGGCGTGGGGGGCCTCGGGGAGGGACGCCTTGCGCCCCCCCGCCGGGCCGAGCGTCTGGCCAGACTCCAGGCGTGCGGCCAGGAGGGCGTCGAAGCTGTCGTACTCGGTGTAGTCGTCGGGAAACATGCAGGTCCACAGCGCGGCCAAAGCGGCGCTCGGCAGACACATGCGCCCGAGGACGCTCACCGCCGCCAGGGCCTGGGCCGGACTGAGCTTCCCGAGCGCCGGGACGTCCCGGCGCTGGGTCCCGAGCTCCAAGGCCGAGCGCCAGGGCGCCAGCGGGTCGGTTTCGGACAGCTTGCCCCGGCGCCAGTCGGCCAGCCGCGTGCCGAACAGGAGGCCCCGGGTCGGGGGGCCTCCGTCCAAAAACGTCGGCAACACGCGGATGCGGGCGTCGGGATGCGGGGTCAGGCGCTGGACGAACAGCATGGACTCCGCTGCGTCCTCGAACGCGCGTTCGAGGGTGAGGTGCATGTACTCGTGCTGGCGAACGAGGTCCAGGCGCCAGAAGTTGTAGATGTGTTCCGGAACGCCGGCCACCAGCGCGACCAGCACGTCGTTCTCGTTGAAGGCGACGCAGTGGCGCTGGGACCCCCGGGGGCCCGGCGGCGGACGCGGCGCCGCCGCTCCGGACGCCCAGCCCAGCTGGGCCCAGCGACACCCAAACTCGCGCGTGAGGGTGGTGGCGACGAGGGCGACGTACAGCTCGGCCGCCGCGTCCATCGAGGCGCCCCACGTCGCCTGGCGATGGCGCACGAAGCGACCGAACAGCTGAAAGTTGGCGGCCTGGGCGTCGCTGAGGGCCAGCTGGAGCCGGTTCACGACGGTCAGCACGTACATGGCCGTGACCGTCGGGGCCGATTCGAGGACGTCCGTCGGAAGCGGGGGCCGCACGCAGGCCGCCTCGGGACGCATCAGCAGCGCGCCGAGTTTGTCGGTGACGGCCGGGAAGCATAGCGCGTACTGCAGCGGCGTTCCGTCCGGGGCCAAAAAGCTGGTGGCGAACGGCAGATCCAGAGCGCTGACGGCCTCACGCAGCACCAGGGGCCCCGGGTCTCCGCCGGCGCGCAGATACGCCTCGCCCCGGCGGCGCAGCAGCTGCGGGTCGACCTCGTGGCCCTCGGGGGAAGAAGAGGCCCGGGCGCGGGCGTCGAGGGCGCGAAGATCAACGAGCAGGGGCGCGGGCGCGGACTCCGCGCCCGCGCCCGTCTGGCCGCCGGCCCTGGCGTACGCGCTATATAAGCCCATGCGGTATTGGATGAGTTCCCGCGCGCCCCGGAACTCCTCCACCGCCCACGGGGCCAGGTCCGCGGCCGCCGCGTCGAACTCCGCCAGCAGGCGCCCCAGGGCGTCAAAGTTCATCTCCCAGGGCACCCTGCGCACCACCTCATCCCGCAGCCGGGCGCACAGGGCGGTGTGCTTGGTGACGCGCGCGCCCAGCTCCTCCACGGCCTCCGCGCGCTCGGCGCCCTTGGCGCCCAGGACGCCCTGGTACCTGGCGGAAAGGCGCTCGTAGGCCGGCTGGGCCCGCAGCCCCGACACCGTGTTGGTGGTGTCCTGCAGGGCGCGCAGCTGCTCGTGCATGGCGCGGAACCCCTCGGGGGACTTCCAGGCGCCCCCCCGGACGCGGCCAAAGCGACCCCAGACCTCGTCCCACTCCGCCTCGGCCTCCTCCAGGGACCTCCGCAGGGCGTCGACGCGGCGCCGAGTATCAAAGAGCGCCCCCAGGCGGCCGGCGTGCCGCGCCAGGGGGCCGGGGCCGTCGCCGCGGGCGGCGCTTAGCGGGTGCGTCTCGAAGGTGCGCTGGGCGTGCTCTAGCCAGATAACCGCGGGCACGTCGAGCTCGCGCGTTTTCTCGGTCTGATCCAACAGAACCTCGACCTGGTCGGCGATCTCCGCCACCGAGCGCGCCTGGTCGAGCGTCTTGGCCACGGTCGCCGGGACGGCGACCACCTTCAGCATGGTCTTGAGGTTGGCCAGGCCCTCGGCCTCGATCTGGGCCCGGCGCTCGCGCGCGGCCAGCGCCTCCCGCAGGCCCGCCATGACCCGCTCGGTGGCCTCCGCGCGCTGCTGTTTGGCGCGCACCACTGCGTCCTTGGTCTCGGCCGTGTCCTGCCGGGTCACGAAGGCGACATACTCGGCGTACGCCGTGTTCTTCACGGGGCTCTGGTCCACGCGCTCCAACGCCGCCGCGCACGCGACCAGCGCGTCCTCGCTGGGACACGGCAGGGTGACCCCGGTCCGGACCAGCTCCGCGGTGGCCTCCGGGTCATTCCGGGCCGCGGATATCTGCTCCGCGGCGGCCGCCAGGTCCAGGGGCACGCCGCCGAGCGCCCGGTGCACGTCGGCCCGGATGGCGTCCAGGCGATCGCGGAGCTCCACGTAGTCGGCGTAGCCATGTTGGAAGAACGGCACGTACCGGCGCAGGCCGGGCACGCTCGTCATGTCGTCCGCCAGGCGCCCCACGGCCTCGTGGTAGTCGATAAACCCGTCGCCCGCCTGGGCCATTTCCAGGAGCCCCTCCGCGATGCGCAGCAGCCGCGCCAGGGGCTCGGCGTCGACCCGAAACATGTCGGCGTAGGTTTCGGCGGCGGCGTGGAACGCCGCGCTCCAGCCGAGGCGGTGGATGGCGGCGAGCGGGGGGAGCATGGGGTGGCGCTGGTTCTCGGGGGTGTAGGGGTTAAACGCGAAGGCCGTATCCAGGGCGAGGGTGACCGCCTCGGCGTTGGCCGCGAGCGCCTGCTCGGCGCGCTTGCGGAAGTCCCGGGGGTTGTAGCCGTGCGTGCCCGCCAGCGCCTGCAGGCGGCGCAGCTCGACCACGTCGAACTCGGCGCGGTTCTCGACGCGGTCCAGCGCCGCCTCGACGCCGGCGGCCCAGCGCTCGCTGCTGCCCCGGGCGCGCTGGGCCGCCATCTTCGCCGTCAGGTCGGCGACGGCGGCCTCAAGTTCGTCGGCGCGGCGTCGCGTGGCGCCGATGACCTTGCCCAGCTCCTGCAGGGCGCGCCCGCTGGGGGAATGGTCCCCGGCCGTCCCTTCGGCGTGCAGCAGGCCCCCGAACCCAGCCTCGTGCCCCGCGAGGCTTTCCCGAGCAGCGGTCGTCGCGCGGGCCGCGGCATCGATGAGGGCGGCATGGTCCCCCTCCGGCTGGGCGCAGGCCCGGCGCGCCTGGACTACCAGGTCGGCGGCCGCCGACCCCAGGGTCGTGAGCTCGTCGATGGCCCCCCGCGCCTCCAGGGCCAGCCGAGTCGCCTTTACATACCCCGCGGCGCTATCGGCCAGCACCGCGAGGAAGGACAGGGGCGAGGCCGGGTCGCGGGCGGCCGCGCCCAGGGCCGACACCGCGTCCGCCAGGGCGCCATGCGCCCGCACGGCCGCGTCCACCGTCGCCGCGGGACTTGCCGTCGCGACGGCGGCGCTCCCGGCGTTGATGGCGTTTGACACGGCTTTGGCGATTGTGGGGGCGTGATCGGAAAAGAACTGCACGAGGACCGGCGTCTCGGGGGCGTCGGCGAACAGGGTCTTCAGCACCACCACGAAGGCGGGATGCAGGCCGGCCAGAGCCGTCGCGGTATCCGGGGTCGGGTGTTCCAGGGCCTCCCGGTACTGCCCCAGCAGCCCCCACAGGTCCGCCCGCAGCGCCGCCGTGACTTCCGGGGGGGGGCCCCGGACGGCATCGGCCAGGTCGGTCCACCCCGCGGGCAGGGAGGCCCGCAGGGTCGCCAGCACGGCCGGACACGCCTTTAGCCCCACAAAGTCCGGGAGGGGCCGCAGGACCCCTTGGAGTTTGTGCAGGAACTTCTCCCGGGCGTCGTGGGCCACCTTGGCGCGCTCCCGCGCGTCGTTGAGCATCGCCTCCAGGGCGTGGGCGCGCTCCCGAAGCCGGGAGCGCGCCTCCGGAGCGAGCTCCGCCGTCATCTTGGCCGCCTCCATGGCCCTCGCCTGCCGCAGCGCGTCTTCGGCCATGCGCGTGGCCTCGGGGGACAGCCCGCCCCCGTCGACGTACGGCGCGGGGCCGGTCGCCGGGACGAAGGCCGCGTCGCTGTCCAGCTGCTGCGCGAGCGCCGCGTCGAGGGCGTCGAAGCGCTGCAGTTCGGCCAGCCCCGAGCTGCGCCGCGCCTGCTGGTCGTTGATGCCGTGGATGCTGCGCGCCAGCTCTTCCAGGGGCTTGCGTTCGATGAGCCCCTGGGTCGCGGCGTCGGTCAGGACCGAGAGCCAGGCCGCCAGGTCCTCGGGGGCATCTAGGGTCTGGCCCCGCTGGAGCAGGTCCCGCAGCAGGATGGCCTGGGGGCTGGTGGCGAGGGGGGGCGGGGGGGGGAGCGCGGCGCGCTGAGCGACGTCCCGCGTGTGTTGGTCAAAGGCCGGTAGCGATTCCAGCAACTGGACCATGGGCACGACCGCGGCCGAGGCCACGTGAAACCGACAGTCGTGGCTGTCGCTGGCCTGCAGGGCCTTCGCGCTGTATACGGCTCCCCGGTGGAAGTACTCCTTGACCGCGCTCTCGATCGCCCGGCGGGCCTGGATCCGCACGTCCTCCAGCCGCGCCTGGATGGCCTCGGGGCCCAGGGCGGGCGGGCACGGGGCCCTGCCGCCGGCGCCCGGGGCGGCGGGCACGGGCATCACGGTCAGGGGCCCGGCGCGCTGCGAGACCGAGTCGACCCCGCGGGCGAGGGCGTCTAAGGCCTCGCGCATCTCGCGGGCCTCCGCCTCGACCCGCATCTCTTCGCCCCGGGCAAACTGGGCCAGCGCCTGGATCCGATGGAGAAGCGGCTCCGGGTGCGTCGGGGTGGCGGGGGCGAACAGGGTGTTCGGGTGGGCGCGCGAGCGCTCCAGGAGCCACTCTCCGAGGCGTGCGTACAGATTGGCCGGCGGGGCGGCGCGCAGCTGCAGATCCAGGTCCGCGAGGTCCCCGTAAAAGGCGTCCGTCTCCCGAATAACGTCCCTGGCGACCAGGACCAGCTTAGCGAGGGCCAGGCGCCCGATCTGCGAATTTTCGTCCAGCACGTGCTGGATGAGGGGCCGGTGGGCGGCCACGTCCGCCAGGCTCATGCGCGTGGACGCCAGGAAGTCCCCGACGGCCGTTTTGCGGGGCAGCATGCGCAGGGTGAAGTCCAGCAGGGCCGCGGCCGGGCCGGCCACCCCGGCCTGCGTATGCGTGCGGGCCCCGTTCTCGATCAAAAAGGCGAGGACGCGCTCAAAGAAGAAGATGACGCAGAGCTCCAACAGCCCCGGGTGCGCCGGGTACGGCGACCGCAGGGCGTTGATGGTGAGCTGCGAACACGCGGCCACCTCGCGGGCCAGGGCGGCATCGCGCGCCGCGAGCCGGACCGCCGTGGCGGCCACATTGGGGTGGACCTCGAACAGCTGCGCCAGGTCGGCGCCGGGGGGCTCCGGGGGGCGGCGGGCCCCCAGCGTCTCGAGCACGGACGGCGACGACGGGCTCGCGGGCCCGTCGTCGCCGCCGCCCTGCCCGGACTGCGGGGGGGTATCCGGTGCGGGAGGGACCGTGGCGGCTATGGGCGTCGGGGAGGAGGCGGGGACCTCGGCGGCGACGGGGGCCTTCTTCTTGGGCGCGGACTTCTTCTTGGCCTTGGCGGGCGGGGCCTTGGGGGCGGGCCTCTCGCCCGAGGTCAGATCCTCCACGCTGGACGGTGGGGTCCAGGTGGGCCGGCGGCGCTTGGGCAAGCCGGTAGAATAGCGCGCCCGGTGGCGACCCACCGGCACTGCCCCCACCTCCAGGACCCGCAGGTCCTCGGCTTCTTCGGCCGCGTCCCCGGCGGGTGTCTGCGGGGGCGGGGCGGCGTGCGGTGGACCCGAGGCCGCGGCGTCCGGGGCCGAGGGCTTCGCGGGCGGGGTCCCCTCCAGGGCTGCTGCCCACACATCATCGGGGGGGCGGTTTGGGTGCCCCGCCTGCGGTGTGTCGGGTGGGCCCGAGGCCCCCCGGGGGGCCTCGGGGGGCCGGTCGGCCCCAGGGGTCTGGACGTGGGTGGGCGCGGGGAGCGCGGGGACGACCGGGCCCGAGCCTTCTCCGTCCCCCCTGGGGACCACACCGACAAAGAGCGCCCCGAGCCCCCCGATCTCGCCCCGCAGGGGGTGGGTGATGGCCACGCGCCGCTCGACGAACGGTTCGTCCTGCAGGTAAGTCTCGCTGGCCCCGTAGAGGTGCAGGGCCGCGGCGGTCAGGTCCGCCGGCGCCACGGCCCCCGGGCCGGAGGGCACAAAAAACACCATGGCGCCCGCCCACCGCACCTTGGGGCGGTCGTGGGCGTAATACGTCAGGTACGGGTACACGTCGCCCGCCCGCACCTTGGCGATAAACGCGGGCGTTCCCGCGGGCAGGCCGTGCGGGTCAAACAGATAGGCCGTGTCGCCGTCCCGGTAGAGCCCCATGCCCAGGGGGCCGATGGTCAGGAGCGTGTAGGACAGCGGCCGCATGGCCCAGGGGCCGGCGAAGAACGTGTGCGCGGGGCATTGCGTCTCCAGCAGCCCCGCCGTGGGCTCCCCGAAGAAGCCCACCTCGCCGTACACCCGCGAAAACACGCAACGCAGGCCGCCGCGCGCCGCCGGGTACTCCAGGAAGTTGGGGAGCTCGATAATGGAACACATGCGCGGCGGCCCGGAGCCCGCGGCCGCGCGCGTCCACTCGCCCCCCTCCACCAGACATCCCTCGATGGCCTCCGCGGACAGCACGTCGCGGGGCCCCACGTCGAAAAGAAGACTGAGAAACGACAGGGACGAGCGCATGCACGATACCGACCCCCCCGGCTCCAGATCGGTCGCGAACTGGTTCCGAACACCGGTGACCACGATATCGCGATCCCCCTGGCGCTTCATCGTGGGGTGAGGTAGCGCGGCCGGAATCATGTGTGCCGCGCCCGCCACGAGCGGGGCCTGTTTATGGGCCGGGCGTCCCGATGAGTACTGTTGTTTCCGCCGCCCGAACCCCCCCGCCCATCAACCGCCTGTTCGTCCCCCTAACCACACACCCGGTATCGCGTGTGTGTGGTTTCCCGGGAAGACACATCCCACCCCATGAAGTTTTGCCCTTTTTTTCCGTCCCGCACTACGCCACCTTTCCACCCCCCCCCCAAAAAAACAACAACCAACTCCCAGATGGATGGGTGCGATAATAAAGCTTTATTATTGTTTAACCAAAGGCGAGTCCTACGGGTGTACCGGTGGTGTCTCCTGCGGCGTCATCTCGTCGTCCTCCACGGGGGTGTTGGGCCAAGGGACCGTCTCGCGGCCCGCCGGGCGCGTCGACGGCGCGCGGGCCTGCGTGTCCTGTGGGCCGGGTGTCGTGGGTTCGGGGGTGCTACCGCCGGCATCTTGGGCCTCCAGGTCCCCGGGGGCCCCCGGGCCGGCGGAAGGCCGAAACGCCGAGGCGCGAAACACGCCGTCGGTGACCTGCAGGAGCTCGTTTATTAATAGCCAGTCCATGCTCAGCGTAGCGGCCAGCCCCTGGGGAGACAGGTCCACGGAGTCCGGAACCACCGTCGGCTGACCCAGGGGCCCCAGGCTGTAGTCCCCCCAGGCCCCCAGGTCATGACGGTTCGTGAGCACGACGAGGTCTGCGGCCGGGCTGGGGGGCGCGTCCTCGGTCGCGTGGGCCATCACCTCCTGAATGGCTGCGGTGCGCTGATCGGCCGAGCTGGCGAAGGGCGCCACGACCAGCGCGCGCTCCGTCTGCAGGCCCTTCCACGTGTCGTGGAGTTCCTGAACGAACTCGGCCACCCGCTCGGGGCCCGTGGCCGCGCGTGCGGCCTGATAGCCGGCCGAGAGGCGCCGCCAGCGCGCCAGGAACTGACTCATGTAACAGAACCCGGGGACCTGGTCCCCCGACATCAACTTTGACGCCCTGGCGTGGATGCCCGACACGATGGCCAGGAACCCGTGGATTTCCCGCCGCACGACGGCCAGCACGTTACCCTCGTGCGAGACCTGGGCCGCCAGCTCGTCGCATACCCCGAGGTGCGCCGTCGTCTCGGTGACGACGGACCGCAGCCCCGCGAGGGACGCGACCAGCGCGCGCTTGGCGTCGTGATACATGCCGCAGTACTGGCTCACCGCGTCGCCCATGGCCTCGGGGCGCCAGGGCCCCAGGCGCTCGTGGGCGTCTGCGACCACGGCGTACAGGCGGTGCCCGTCGCTCTCGAACCGGCACTCAAAGAAGGCGGCGAGCGTGCGCATGTGCAGCCGCAGCAGCACGATCGCGTCCTCCAGCTGGCGGACCAGGGGGTCGGCGCGCTCGGCGAGCTCCTGCAGCACCCCCCGGGCCGCCAGGGCGTACATGCTGATCAGCAGCAGGCTGCTGCCCACCTCGGGAGGCTGGGGGGGAGGCAGCTGGACCGCGGGCCGCAGCTGCTCGACGGCCCCCCTGGCGATCACGTACAGCTCGCGCAGCAGCTGCTCGATGTTGTCGGCCATCTGCATCGTGGGCCCGACGCCGGCCCGGGTGGCCGGTTCGAGGAGGGTGATCAGCGCGCCCAATTTTGTGCGGTGCCCCTCGACGGTGGGGAGATAGCCCAGGCCGAAGTCGCGCGCCCAGGCCAGCACCCGCAGGGCAAACTCGATGGGGCGGGGCAGGTAGGCAGCGTTGCACGTGGCCCTCAGCGCGTCCCCGACCACCAGGGCCAGCACGTAAGGGACGAACCCCGGGTCGGCGAGGACGTTGGGGTGGATGCCCTCCAGGGCCGGGAAGCGGATCTTGGTGGCCGCGGCCAGGTGAACCGAGGGGGCGTGGCTAGGCGGCCCGACGGGGAGCAGCGCGGACAGCGGCGTGGCCGGGGTGGTGGGGGTCAGGTCCCAGTGGGTCTGGCCGTACACGTCGAGCCAGATGAGCGCCGTCTCGCGCAGGAGGCTGGGCTGGCCGGCGCTGAAGCGGCGCTCGGCCGTCTCAAACTCCCCCACGAGCGTGCGCCGCAGGCTCGCCAGGTGTTCCGTCGGCACGGCCGGGCCCATGATGCGCGCCAGCGTCTGGCTGAGGACGCCGCCCGACAGGCCGACCGCCTCACAGAGCCGCCCGTGCGTGTGCTCGCTGGCGCCCTGGATCCGCCGGAACGTTTTCACGTAGCCGGCGTAGTGCCCGTACTCCCGCGCGAGCCCGAACACGTTCGCCCCCGCAAGGGCAATGCACCCAAAGAGCTGCTGGATCTCGCTGAGCCCGTGGCCGGGGGGCGTCCGCGCGGGCACCCCCGCCACCAAAAACCCCTCCAGGGCCGATATGTACTGGGTGCAGTGCGCGGGCGTGAACCCCGCGTCGGTAAGCGTGTTGATCACCACGGAGGGCGAGTTGCTGTTCTGGACCAAAGCCCACGTCTGCTGCAGCAGCGCGAGGAGCCGTTGCTGGGCCCCGGCGGAGGGCGGCTCCCCTAGCTGCAGCAGGCCGGTGACGGCCGGACGGAAGATGGCCAGCGCCGACGCACTCAGAAACGGCACGTCGGGGTCGAAGACGGCCGCGTCCGTCCGCACGCGCGCCATCAGCGTCCCCGGGGGCGCGCACGCCGACCGCGGGCTGACGCGGCTTAGGGCGGTCGACACGCGCACCTCCTCGCGACTGCGAACCATTTTGGTGGCCTCGAGGGGCGGGATCATGATAGCCGGGTCGATCTCCCGCACCGTGTGCTGAAACTGGGCCAGCAGCGGCGGCGGGACCACCGCGCCCCGATCGGGGGTCGTCAGGTACTCGTCCACCAGCGCCAGCGTAAACAGGGCCCGCGTGAGGGGGGTCAGGGCGGCGTCGTCGATGCGCTGTAGGTGCGCCGAGAACAGCGTCACCCAATTGCTGACCAGGGCCAAGAACCGGAGACCCTCTTGCACGATCGGGGACGGGAAGAGCAGGCTGTACGCCGGGGTGGTCAGGTTGGCGCCGGGTTGCCCCAGGGGAACCGGGGACATCTTAAGCGACATCTCCCCGAGGGCCTCCAGGGAGGTCCGCGGGTTCATGGCCAGGCAGCTCTGGGTGACGGTCCGCCAGCGGTCGATCCACTCCACGGCACACTGGCGGACGCGCACCGGCCCCAGGGCCGCCGTGGTGCGCAGCCCGGCGGCCTCCAGCGCGTGGGTCGTGTCGGAGCCGGTGATCGCCAGGACCGTGTCCTTGATGACGTCCATCTCCCGGAAGGCCGCCTCGGGGGTCTCGGGGAGCGCCACCGCCATGCGGTGCACCAGCAGCCCGGGGAGGTTCTCGGCCAAGAGCGCCGTCTCCGGAAGCCCGTGGGCCCGGTGCAAGGCGCACAGTTGCTCCAGGAGCGGGTGCCAGCACGCCCGCGCCTCCGCCGGGCCGACCGCCGCGCCCGACAACAGAAACGCCGCCGTGGCGGCGCGCAGTTTGGCCGCGGACAGAAACGCCGGCTCGTCCGCGCTGCCCGCCGGCTCGCTCGAGGGGGAGGGCGGCCGGCGGAGGTTGGTCAGGCTCCCCAACAGGACCTGCAACGGTCCGTTTGGGGGTGGAGCGGACGGGGGGGTCATGCCGGCGGGCGCCGGGACCTGGAGCGCGCTGTCCGACATGGCGACCGGCGTGCGCGCTCGGCGACGCGGCGCGGAGACCGCGGGCCCAAACGGGAATGACTGCCGCCGCCCTATACGGAGGGGCTAAGTATCGCCCGGGGACCCTTCGAAACCCCGGGCGTGTCGCAAGTACGCCGCGAAGGCGCGGCGTGTTATACGGCGCGTTATGTCCCGGCATTCCGTTCGTGGGTTCGGGCCCGGGTGCTGTCGGGTGGGAGTGTGTGTGGGGGGGGGCGGCGCGACGGCGGCCCGGACCAAGTGTATCGCGGCCGTTCCGTGGGGCGGCCCAACAGGCCCTTTAAACATTTGCGTATGCACCGGCCCAGCCAGTCGGACACCGGAACCCACCAGAGGCGGAAGCCGCCTTCGCCCGTGAGGGTGCGTGTGTTTTCTGGTGGCGTGTTTTT

CAACTCGCCCGTTGGCGATCGGCGGGAAAACCATGAAAACCAAGCCACTCCCGACAGCCCCGATGGCGTGGGCCGAGAGTGCCGTGGAAACCACCACCAGCCCGCGCGAGCTCGCGGGCCACGCCCCGCTCCGGCGCGTCCTGCGCCCGCCCATCGCTCGCCGCGACGGCCCGGTGCTTTTGGGGGACAGGGCCCCCAGGAGGACGGCCAGTACGATGTGGCTGCTGGGGATCGACCCCGCGGAGTCGTCTCCGGGAACGCGCGCTACCCGAGACGATACCGAGCAGGCCGTGGACAAGATCCTCAGGGGAGCCCGGCGCGCGGGAGGGCTGACCGTCCCCGGCGCCCCCCGCTATCACCTGACCCGCCAGGTAACCCTGACGGATCTCTGCCAACCAAACGCGGAGCGGGCCGGGGCGCTCCTTTTGGCCCTGCGGCACCCCACCGACCTCCCCCACCTGGCCCGCCATCGGGCTCCGCCCGGCCGGCAGACCGAGCGACTGGCCGAGGCCTGGGGCCAGCTCCTGGAGGCCTCCGCCCTGGGGTCCGGGCGGGCCGAGAGCGGCTGCGCGCGCGCGGGCCTTGTGTCGTTTAACTTTCTGGTGGCCGCGTGCGCCGCCGCCTACGATGCGCGCGACGCCGCCGAGGCGGTCCGGGCCCACATCACGACCAACTACGGCGGGACGCGGGCCGGGGCGCGGCTGGACCGGTTTTCCGAATGCCTGCGCGCCATGGTCCACACGCACGTGTTTCCCCACGAGGTCATGCGGTTTTTCGGGGGGCTAGTGTCGTGGGTCACACAGGACGAGCTGGCTAGCGTCACCGCCGTCTGCAGCGGACCCCAGGAGGCCACACACACCGGCCACCCGGGCAGGCCCCGTTCGGCCGTTACCATCCCGGCCTGCGCCTTCGTGGACCTGGACGCCGAGCTGTGCCTGGGGGGCCCTGGGGCGGCGTTCCTGTACTTGGTCTTCACCTACCGACAGTGCCGGGACCAAGAGCTCTGTTGCGTGTACGTGGTCAAGAGCCAGCTCCCCCCGCGCGGACTGGAGGCGGCCCTCGAGCGGCTGTTCGGGCGCCTCCGGATAACCAACACGATTCACGGGGCCGAGGACATGACGCCCCCTCCCCCGAACCGAAACGTTGACTTTCCGCTCGCCGTCCTGGCCGCGAGCTCGCAATCCCCGCGGTGCTCGGCGAGCCAAGTCACGAACCCCCAGTTTGTCGACAGGCTGTACCGCTGGCAGCCGGATCTGCGGGGGCGCCCTACCGCACGCACCTGCACATACGCCGCCTTCGCAGAGCTGGGTGTCATGCCAGACGACAGCCCCCGCTGTCTGCACCGCACCGAGCGGTTTGGGGCGGTCGGCGTTCCGGTTGTCATCCTGGAGGGCGTGGTGTGGCGCCCCGGCGGGTGGCGGGCCTGCGCGTGATCGTCTATTGACGACGGCCGCCCAACCCGAGCGACCTTCCCCTCCCACTTCCCCCCCCCTACACACCAACTCCGCCCTCGCCGTCTTGGCCGTGCGCGGCCCCGTGCGTCCGTCTCAATAAAGCCAGGTTAAATCCGTGACGTGGTGTGTTTGGCGTGTGTCTCTGAAATGGCGGAAACCGACATGCAAATGGGATTCATGGACACGTTACACCCCCCTGACTCAGGAGATAGGCATATCCTCCTTAGATTGACTCAGCACACGATCGCACCCCACCCCTGTGTGCCGGGGATAAAAGCCAACGCGGGCGGTCTGGGTTACCACAACAGGTGGGTGCTTCGGGGACTTGACGGTCGCCACTCTCCTGCGAGCCCTCACGTCTTCGCCCACCGATTCCTGTTGCGTTCCTGTCGGCCGGTGCTGTCCTGTCGACAGATTGTTGGCGACTGCCCGGGTGATTCGTCGGCCGGTGCGTCCTTTCGGTCGTACCGCCCACCCCGCCTCCCACGGGCCCGCCGCTGTTTCCGTTCATCGCGTCCGAGCCACCGTCACCTTGGTTCCAATGGCCAACCGCCCTGCCGCATCCGCCCTCGCCGGAGCGCGGTCTCCGTCCGAACGACAGGAACCCCGGGAGCCCGAGGTCGCCCCCCCTGGCGGCGACCACGTGTTTTGCAGGAAAGTCAGCGGCGTGATGGTGCTTTCCAGCGATCCCCCCGGCCCCGCGGCCTACCGCATTAGCGACAGCAGCTTTGTTCAATGCGGCTCCAACTGCAGTATGATAATCGACGGAGACGTGGCGCGCGGTCATTTGCGTGACCTCGAGGGCGCTACGTCCACCGGCGCCTTCGTCGCGATCTCAAACGTCGCAGCCGGCGGGGATGGCCGAACCGCCGTCGTGGCGCTCGGCGGAACCTCGGGCCCGTCCGCGACTACATCCGTGGGGACCCAGACGTCCGGGGAGTTCCTCCACGGGAACCCAAGGACCCCCGAACCCCAAGGACCCCAGGCTGTCCCCCCGCCCCCTCCTCCCCCCTTTCCATGGGGCCACGAGTGCTGCGCCCGTCGCGATGCCAGGGGCGGCGCCGAGAAGGACGTCGGGGCCGCGGAGTCATGGTCAGACGGCCCGTCGTCCGACTCCGAAACGGAGGACTCGGACTCCTCGGACGAGGATACGGGTTCGGAGACGCTGTCTCGATCCTCTTCGATCTGGGCCGCAGGGGCGACTGACGACGATGACAGCGACTCCGACTCGCGGTCGGACGACTCCGTGCAGCCCGACGTTGTCGTTCGTCGCAGATGGAGCGACGGCCCCGCCCCCGTGGCCTTTCCCAAGCCCCGGCGCCCCGGCGACTCCCCCGGAAACCCCGGCCTGGGCGCCGGCACCGGGCCGGGCTCCGCGACGGACCCGCGCGCGTCGGCCGACTCCGATTCCGCGGCCCACGCCGCCGCACCCCAGGCGGACGTGGCGCCGGTTCTGGACAGCCAGCCCACTGTGGGAACGGACCCCGGCTACCCAGTCCCCCTAGAACTCACGCCCGAGAACGCGGAGGCGGTGGCGCGGTTTCTGGGGGACGCCGTCGACCGCGAGCCCGCGCTCATGCTGGAGTACTTCTGTCGGTGCGCCCGCGAGGAGAGCAAGCGCGTGCCCCCACGAACCTTCGGCAGCGCCCCCCGCCTCACGGAGGACGACTTTGGGCTCCTGAACTACGCGCTCGCTGAGATGCGACGCCTGTGCCTGGACCTTCCCCCGGTCCCCCCCAACGCATACACGCCCTATCATCTGAGGGAGTATGCGACGCGGCTGGTTAACGGGTTCAAACCCCTGGTGCGGCGGTCCGCCCGCCTGTATCGCATCCTGGGGGTTCTGGTCCACCTGCGCATCCGTACCCGGGAGGCCTCCTTTGAGGAATGGATGCGCTCCAAGGAGGTGGACCTGGACTTCGGGCTGACGGAAAGGCTTCGCGAACACGAGGCCCAGCTAATGATCCTGGCCCAGGCCCTGAACCCCTACGACTGTCTGATCCACAGCACCCCGAACACGCTCGTCGAGCGGGGGCTGCAGTCGGCGCTGAAGTACGAAGAGTTTTACCTCAAGCGCTTCGGCGGGCACTACATGGAGTCCGTCTTCCAGATGTACACCCGCATCGCCGGGTTTCTGGCGTGCCGGGCGACCCGCGGCATGCGCCACATCGCCCTGGGGCGACAGGGGTCGTGGTGGGAAATGTTCAAGTTCTTTTTCCACCGCCTCTACGACCACCAGATCGTGCCGTCCACCCCCGCCATGCTGAACCTCGGAACCCGCAACTACTACACGTCCAGCTGCTACCTGGTAAACCCCCAGGCCACCACTAACCAGGCCACCCTCCGGGCCATCACCGGCAACGTGAGCGCCATCCTCGCCCGCAACGGGGGCATCGGGCTGTGCATGCAGGCGTTCAACGACGCCAGCCCCGGCACCGCCAGCATCATGCCGGCCCTGAAGGTCCTCGACTCCCTGGTGGCGGCGCACAACAAACAGAGCACGCGCCCCACCGGGGCGTGCGTGTACCTGGAACCCTGGCACAGCGACGTTCGGGCCGTGCTCAGAATGAAGGGCGTCCTCGCCGGCGAGGAGGCCCAGCGCTGCGACAACATCTTCAGCGCCCTCTGGATGCCGGACCTGTTCTTCAAGCGCCTGATCCGCCACCTCGACGGCGAGAAAAACGTCACCTGGTCCCTGTTCGACCGGGACACCAGCATGTCGCTCGCCGACTTTCACGGCGAGGAGTTCGAGAAGCTGTACGAGCACCTCGAGGCCATGGGGTTCGGCGAAACGATCCCCATCCAGGACCTGGCGTACGCCATCGTGCGCAGCGCGGCCACCACCGGAAGCCCCTTCATCATGTTTAAGGACGCGGTAAACCGCCACTACATCTACGACACGCAAGGGGCGGCCATCGCCGGCTCCAACCTCTGCACCGAGATCGTCCACCCGGCCTCCAAGCGATCCAGTGGGGTCTGCAACCTGGGAAGCGTGAATCTGGCCCGATGCGTCTCCAGGCAGACGTTTGACTTTGGGCGGCTCCGCGACGCCGTGCAGGCGTGCGTGCTGATGGTGAACATCATGATCGACAGCACGCTACAACCCACGCCCCAGTGCACCCGCGGCAACGACAACCTGCGGTCCATGGGCATTGGCATGCAGGGCCTGCACACGGCGTGCCTGAAGATGGGCCTGGATCTGGAGTCGGCCGAGTTCCGGGACCTGAACACACACATCGCCGAGGTGATGCTGCTCGCGGCCATGAAGACCAGTAACGCGCTGTGCGTTCGCGGGGCGCGTCCCTTCAGCCACTTTAAGCGCAGCATGTACCGGGCCGGCCGCTTTCACTGGGAGCGCTTTTCGAACGCCAGCCCGCGGTACGAGGGCGAGTGGGAGATGCTACGCCAGAGCATGATGAAACACGGCCTGCGCAACAGCCAGTTCATCGCGCTCATGCCCACCGCCGCCTCGGCCCAGATCTCGGACGTCAGCGAGGGCTTTGCCCCCCTGTTCACCAACCTGTTCAGCAAGGTGACCAGGGACGGCGAGACGCTGCGCCCCAACACGCTCTTGCTGAAGGAACTCGAGCGCACGTTCGGCGGGAAGCGGCTCCTGGACGCGATGGACGGGCTCGAGGCCAAGCAGTGGTCTGTGGCCCAGGCCCTGCCTTGCCTGGACCCCGCCCACCCCCTCCGGCGGTTCAAGACGGCCTTCGACTACGACCAGGAACTGCTGATCGACCTGTGTGCAGACCGCGCCCCCTATGTTGATCACAGCCAATCCATGACTCTGTATGTCACAGAGAAGGCGGACGGGACGCTCCCCGCCTCCACCCTGGTCCGCCTTCTCGTCCACGCATATAAGCGCGGCCTGAAGACGGGGATGTACTACTGCAAGGTTCGCAAGGCGACCAACAGCGGGGTGTTCGCCGGCGACGACAACATCGTCTGCACAAGCTGCGCGCTGTAAGCAACAGCGCTCCGATCGGGGTCAGGCGTCGCTCTCGGTCCCGCATATCGCCATGGATCCCGCCGTCTCCCCCGCGAGCACCGACCCCCTAGATACCCACGCGTCGGGGGCCGGGGCGGCCCCGATTCCGGTGTGCCCCACCCCCGAGCGGTACTTCTACACCTCCCAGTGCCCCGACATCAACCACCTTCGCTCCCTCAGCATCCTGAACCGCTGGCTGGAGACCGAGCTCGTGTTCGTGGGGGACGAGGAGGACGTCTCCAAGCTCTCCGAGGGCGAGCTCGGCTTCTACCGCTTTCTGTTTGCCTTCCTGTCGGCCGCGGACGACCTGGTGACGGAAAACCTGGGCGGCCTCTCCGGCCTCTTCGAACAGAAGGACATTCTTCACTACTACGTGGAGCAGGAATGCATCGAGGTCGTCCACTCGCGCGTCTACAACATCATCCAGCTGGTGCTCTTTCACAACAACGACCAGGCGCGCCGCGCCTATGTGGCCCGCACCATCAACCACCCGGCCATTCGCGTCAAGGTGGACTGGCTGGAGGCGCGGGTGCGGGAATGCGACTCGATCCCGGAGAAGTTCATCCTCATGATCCTCATCGAGGGCGTCTTTTTTGCCGCCTCGTTCGCCGCCATCGCGTACCTGCGCACCAACAACCTCCTGCGGGTCACCTGCCAGTCGAACGACCTCATCAGCCGCGACGAGGCCGTGCATACGACAGCCTCGTGCTACATCTACAACAACTACCTCGGGGGCCACGCCAAGCCCGAGGCGGCGCGCGTGTACCGGCTGTTTCGGGAGGCGGTGGATATCGAGATCGGGTTCATCCGATCCCAGGCCCCGACGGACAGCTCTATCCTGAGTCCGGGGGCCCTGGCGGCCATCGAGAACTACGTGCGATTCAGCGCGGATCGCCTGCTGGGCCTGATCCATATGCAGCCCCTGTATTCCGCCCCCGCCCCCGACGCCAGCTTTCCCCTCAGCCTCATGTCCACCGACAAACACACCAACTTCTTCGAGTGCCGCAGCACCTCGTACGCCGGGGCCGTCGTCAACGATCTGTGAGGGTCTGGGCGCCCTTGTAGCGATGTCTAACCGAAATAAAGGGGTCGAAACGGACTGTTGGGTCTCCGGTGTGATTATTACGCAGGGGAGGGGGGTGGCGGCTGGGGAAAGGGAAGGAACGCCCGAAACCAGAGAAAAGGACCAAAAGGGAAACGCGTCCAACCGATAAATCAAGCGCCGACCAGAACCCCGAGATGCATAATAACGATTTTATTACTCTTATTATTAACAGGTCGGGCATCGGGAGGGGATGGGGGCGCGCGTTTCCTCCGTTCCGGCTACTCGTCCCAGAATTTAGCCAGGACGTCCTTGTAAAACGCGGGCGGGGGCGCGTGGGCCCACAGCTGCGCCAGAAACCGGTCGGCGATGTCCGGGGCGGTGATATGCCGAGTCACGATGGAGCGCGCTAAATCTTCGTCGCGGAGGTCCTGATAGATGGGCAGTCTTTTTAGAAGAGTCCAGGGTCCCCGCTCCTTGGGGCTGATAAGCGATATGACGTACTTGACGTATCTGTGCTCCACCAGCTCGGCGATGGTCATCGGATCGGGCAGCCAGTCCAGGGCCTCCGGGGCGTCGTGGATGACGTGGCGGCGACGTCCGGCGACATAGCCGCGGTGTTCCGCGACCCGCTGCGCGTTGGGGACCTGCACGAGCTCGGGCGGGGTGAGTATCTCCGAGGAGGACGACCGGGCGCCGTCGCGCGGCCCACCGGCGACGTCCGGGGGCTGGAGGGGGGGGTCTTCTTCGTAGTCGTCCTCGCCCGCGATCTGTTGGGCCAGAATTTCGGTCCACGAGATGCGCGTCTCGAGGCCGACCGGGGCCGCGGTCAGCGTAGGCATGCTCTCCAGGGAGCGCGAGTTGGCGCGCTCCCGCCGGGCCGCCCGGCGGGCCTGGGATCGGCTCGGGGCGGTCCAGTGACACTCGCGCAGCACGTCCTCGACGGACGCGTAGGTGTTATTGGGGTGCAGGTCTGTGTGGCAGCGGACGAACAGCGCCAGGAACTGCGGGTAACTCATCTTGAAGTACTGCAGCAGGTCGCGGCAGTGAATCGTCGGAATGTAGCCGGTGCTGATGTCCAACACGATATCGCAGCCCATCAGCAGGAGATCGGTATCCGTGGTATGCACGTACGCGACCGTGTTGGTATGATAGAGGTTCGCGCAGGCGTCGTCGGCCTCCAGCTGACCCGAGTTGATGTAGGCGTACCCCAGCGCCCGCAGAACGCGGATACAGAACAGGTGAGCCAGGCGCAGGGCCGGCTTCGAGGGCGCGCCCGAGGGGGCCGCCGGGCCTGGGCCGGCGGCCCGCGTTCCCCGGTCCCCCGGGGCGAAGGCGTGCCCGCGGCGGCGCATGTTGGAAAAGGCGAAACTGGGCCTGGAGTCGGTGATGGGGGAAGGCGGCGGCGAGGCGTCTACGTCACTGGCCTCCTCGTCCGTGCGGCACTGGGCCGTCGTGCGGGCCAGGATCGCCTTGGCCCCGAACACAACCGGCTCGGTACACTCGACCCCGCGATCGGTCACGAAGATGGGGAACAGGGACTTTTGGGTAAACACCCGTAACATACTACAGAGACAGTGTAGCGTGATTGCCTCGCGGTCGTAACTTGGGTAGCGGCGCTGATATTTAACCACCAGGGTATACATGACATTCCACAGGTCCACGGCGATGGGGGTAAAGTAGCCCTCCGGGGCCCGGAGGCCCCGGCGCTTCACCAGATGGTGAGTCTGGGCAAACTTCATCATGCCAAACAGACCCATTCCGGCACGATTGTAGGTGCGGATAGGTCTCTCTACAGAGCTGTATAGGTGTGACGGTCCGGGACACCCAAGCCCGCCGCCCCTGTGTACAGTGGCTGCGGCGACGACCCCGCTCCAACAAGACGCTATCCCGGGAAAGGCACGCTCTTTATAATTCTTTTTTATTTCCCATCTACGTGCGGATTGGTGCAACCGCCGGCGCGCGCCGGTGCAGGCCGACCATCTCTCTCTTCCCCCCCTCCCCCTCCCCCGAGCCCTCAAAGAGGGTGTGGCCTAACTAGCGGAAGGCGTATTTAACCAGACTAGGGCGGCGGGTCCGCCGTAGTCCTTGGCTCGGGTAGCCACTGCTCTGTGGCTCGGGTCCCCCGGCCCCCCTAACCCCCATCCGGTCCGCGTCATCCGCCCCCTCCGCCTGCGACACAAACGGCCGCGCCTCCGGGCCCGGTGACACGACGCGCCTCGTCTCTGCGGATTGTCCCGGGAGCGTCGCGGCATGGCTCATCTTCCCGGCGGTGCGGCCGCCGCCCCCCTTTCGGAGGACGCGATCCCGTCGCCGCGCGAGCGGACGGAAGACTGGCCGCCCTGCCAGATAGTGCTGCAGGGCGCCGAGCTGAACGGGATCCTGCAGGCCTTTGCGCCGCTTCGCACGAGCCTTTTGGACTCGCTCCTGGTCGTGGGCGACCGAGGCATCCTTGTACATAACGCGATTTTCGGCGAGCAGGTGTTTCTGCCCCTCGACCATTCGCAGTTCAGTCGCTATCGATGGGGCGGACCCACCGCGGCGTTCCTGTCTCTCGTGGACCAGAAGCGATCCCTGCTGAGCGTGTTTCGCGCCAACCAGTACCCTGACCTGCGGCGGGTGGAGCTGACGGTCACGGGCCAGGCCCCGTTTCGCACGCTGGTGCAGCGCATATGGACGACCGCGTCCGACGGAGAGGCCGTGGAGCTTGCCAGCGAGACGCTCATGAAACGCGAGTTGACGAGCTTCGCGGTACTACTCCCCCAGGGCGACCCCGACGTCCAGCTGCGCCTCACGAAGCCCCAGCTCACGAAGGTGGTGAACGCCGTCGGGGACGAGACCGCCAAACCCACCACGTTCGAGCTCGGCCCCAACGGCAAGTTTTCCGTGTTTAACGCGCGCACCTGCGTCACCTTTGCCGCCCGCGAGGAGGGCGCGTCGTCCAGCACCAGCGCCCAGGTCCAGATTCTGACCAGCGCGCTGAAGAAGGCGGGCCAAGCGGCCGCCAACGCCAAGACGGTCTACGGGGAAAACACACACCGCACATTCTCGGTGGTCGTCGACGACTGCAGCATGCGGGCGGTCCTCCGGCGGCTCCAGGTCGGCGGGGGGACCCTCAAGTTCTTCCTCACGGCCGACGTCCCCAGCGTGTGTGTCACCGCCACCGGCCCCAACGCGGTGTCGGCGGTGTTTCTTTTAAAACCCCAGCGGGTCTGCCTGAACTGGCTCGGCCGGAGCCCGGGTTCCTCGACCGGGAGCTTGGCGTCCCAGGACTCTCGGGCCGGCCCGACCGACAGCCAGGACTCCTCCTCCGAGCCGGACGCGGGCGACCGCGGCGCCCCAGAAGAAGAAGGCCTCGAGGGCCAGGCCCGGGTACCGCCCGCGTTCCCGGAACCGCCGGGAACCAAGCGGAGGCACCCCGGGGCCGAAGTTGTCCCCGCGGACGACGCCACCAAGCGCCCGAAGACGGGCGTGCCCGCCGCCCCCACGCGAGCCGAGTCGCCCCCCCTCTCCGCGAGATACGGACCCGAGGCGGCGGAGGGTGGTGGGGACGGCGGCCGCTACGCGTGCTACTTTCGCGACCTCCAGACCGGCGACGCGAGCCCCAGCCCCCTCTCCGCCTTCCGGGGTCCCCAAAGACCCCCATACGGCTTTGGGTTGCCCTGACGGCAACGGGTGGTGGCCGAACGCCTCACCGCGCCCGGGCACGCGGGGTGCGTTGTGTTAAAAAAATAAATAAATGGGGTAGTGTGTCCCCCCCCCCTCCAACCAATATGGCTGTCGTGTGTGGTTCCGGGTTGCGCCTCCGTCCTTTCCACCCCCCTTCCCCCTCCTTTTTTGTTTTGCGTGCGCTTATAAGAGCGGGCCCGGGGCCCTTCGCAGCTTCACCGAGAGCGCCGTCGGGCCCCGGGTGCGGGATGTGTCGCGGGGACAGCCCCGGGGTCGCGGGCGGGAGCGGCGAACACTGCCTCGGAGGGGATGATGGGGACGACGGGCGCCCCCGCCTCGCCTGCGTGGGTGCCATCGCTCGGGGGTTCGCGCATCTCTGGCTCCAGGCCGCCACGCTGGGCTTCGTGGGGTCTGTCGTTCTGTCGCGCGGCCCGTATGCGGACGCCATGTCGGGGGCGTTCGTGATCGGGAGCACCGGCCTGGGGTTCCTCCGCGCCCCCCCCGCGTTCGCCCGGCCGCCGACGCGTGTGTGCGCGTGGCTGAGGCTGGTCGGCGGGGGAGCGGCCGTGGCCCTGTGGAGCCTCGGGGAGGCCGGCGCGCCTCCGGGGGTTCCGGGCCCGGCGACCCAGTGCCTGGCGCTCGGGGCCGCCTACGCGGCGCTGCTGGTGCTGGCCGACGACGTCCATCCCCTTTTCCTCCTCGCCCCGCGGCCCCTGTTTGTCGGCACCCTGGGGGTTGTCGTCGGCGGGCTGACGATAGGCGGCAGTGCGCGCTACTGGTGGATCGACCCCCGCGCCGCCGCGGCCCTGACGGCGGCGGTGGTGGCGGGCCTCGGGACAACCGCCGCCGGGGACAGCTTTTCCAAGGCCTGTCCCCGCCACCGCCGCTTTTGCGTCGTCTCCGCGGTCGAGTCTCCCCCGCCCCGATACGCCCCGGAGGACGCCGAGCGGCCAACAGACCACGGACCCCTGTTACCGTCGACGCACCACCAGCGATCTCCGCGGGTCTGCGGCGACGGGGCCGCACGGCCCGAAAACATCTGGGTTCCCGTGGTGACCTTTGCGGGCGCGCTCGCGCTGGCCGCCTGCGCCGCGCGAGGGTCTGACGCGGCTCCGTCAGGCCCGGTCCTGCCGCTGTGGCCCCAGGTGTTTGTCGGGGGCCACGCGGCGGCGGGCCTGACGGAGCTGTGTCAGACCCTCGCGCCCCGGGACCTCACGGACCCGCTGCTGTTTGCGTACGTCGGATTCCAGGTCGTGAACCACGGGCTGATGTTTGTGGTCCCCGACATCGCCGTATACGCGATGCTGGGGGGCGCCGTGTGGATCTCGCTGACGCAGGTGCTTGGGCTCCGGCGCCGCCTTCACAAGGACCCAGACGCCGGGCCCTGGGCGGCCGCGACCCTGCGGGGCCTCTTTTTCTCCGTCTACGCATTGGGGTTTGCGGCGGGGGTGCTGGTGCGGCCGCGGATGGCGGCGAGCCGGCGGTCGGGGTGATCGCCATTTCAAATAAAAGGCACGAGTTCCCCGAATACCACCGGCGTGTGATGATTTCGCCCTACCGCTCCGATCCCCGGGGGGAGGGGGGAAGGAAATGGGGGCGGGGGTGCCGTGGACGGGTATAAAGGCCAGGGGGGCAGGCGGGCCCATCACTGTTAGGGTGTTAGGTTGGGAGGTGGCACAAAAAGCGACACTCCCGTGTTGTAGTTGTCCGCGGGAGGCGGTGGTTTCCGGCAACCCTCCTCGCTGCGCCGGGCGCGCCCACCGGTCCTTCGCGGGGGCCGGGGCTCTTCTGGTCATGGCCCTTGGACGGGTGGGCCTAGCCGTGGGCCTGTGGGGCCTGCTGTGGGTGGGTGTGGTCGTGGTGCTGGCCAATGCCTCCCCCGGACGCACGATAACGGTGGGCCCGCGGGGGAACGCGAGCAATGCCGCCCCCTCCGCGTCCCCGCGGAACGCATCCGCCCCCCGAACCACACCCACGCCCCCCCAACCCCGCAAGGCGACGAAAAGTAAGGCCTCCACCGCCAAACCGGCCCCGCCCCCCAAGACCGGGCCCCCGAAGACATCCTCGGAGCCCGTGCGATGCAACCGCCACGACCCGCTGGCCCGGTACGGCTCGCGGGTGCAAATCCGATGCCGGTTTCCCAACTCCACCCGCACGGAGTTCCGCCTCCAGATCTGGCGTTATGCCACGGCGACGGACGCCGAGATCGGAACGGCGCCTAGCTTAGAGGAGGTGATGGTAAACGTGTCGGCCCCGCCCGGGGGCCAACTGGTGTATGACAGCGCCCCCAACCGAACGGACCCGCACGTGATCTGGGCGGAGGGCGCCGGCCCGGGCGCCAGCCCGCGGCTGTACTCGGTCGTCGGGCCGCTGGGTCGGCAGCGGCTCATCATCGAAGAGCTGACCCTGGAGACCCAGGGCATGTACTACTGGGTGTGGGGCCGGACGGACCGCCCGTCCGCGTACGGGACCTGGGTGCGCGTTCGCGTGTTCCGCCCTCCGTCGCTGACCATCCACCCCCACGCGGTGCTGGAGGGCCAGCCGTTTAAGGCGACGTGCACGGCCGCCACCTACTACCCGGGCAACCGCGCGGAGTTCGTCTGGTTCGAGGACGGTCGCCGGGTGTTCGATCCGGCCCAGATACACACGCAGACGCAGGAGAACCCCGACGGCTTTTCCACCGTCTCCACCGTGACCTCCGCGGCCGTCGGCGGCCAGGGCCCCCCGCGCACCTTCACCTGCCAGCTGACGTGGCACCGCGACTCCGTGTCGTTCTCTCGGCGCAACGCCAGCGGCACGGCATCGGTGCTGCCGCGGCCAACCATCACCATGGAGTTTACGGGCGACCATGCGGTCTGCACGGCCGGCTGTGTGCCCGAGGGGGTGACGTTTGCCTGGTTCCTGGGGGACGACTCCTCGCCGGCGGAGAAGGTGGCCGTCGCGTCCCAGACATCGTGCGGGCGCCCCGGCACCGCCACGATCCGCTCCACCCTGCCGGTCTCGTACGAGCAGACCGAGTACATCTGCCGGCTGGCGGGATACCCGGACGGAATTCCGGTCCTAGAGCACCACGGCAGCCACCAGCCCCCGCCGCGGGACCCCACCGAGCGGCAGGTGATCCGGGCGGTGGAGGGGGCGGGGATCGGAGTGGCTGTCCTTGTCGCGGTGGTTCTGGCCGGGACCGCGGTAGTGTACCTCACCCACGCCTCCTCGGTGCGCTATCGTCGGCTGCGGTAACTCCGGGGCCGGGCCCGGCCGCCGGTTGTCTTCTTTTCCACCCCTTCCGTCCCCCGTACCCACCACACCCCACCCCACCCCCCCGCCGTCCCCCGGGCGTTATAAGCCGCCGCACTCGCTTTTCCCACCGGAAAATCCTCGGCCCGATCCGAACGGCGCACGCCGCGTGGGCTCCAAACGCCTCCGGAAGAGAGCGCCCCGCCCCGATATTCAAGCCCGCGGTGGTGCTATGGCTTTCCGTGCTTCGGGACCCGCCTACCAGCCCCTCGCCCCCGCGGCCTCCCCGGCGCGGGCTCGTGTTCCGGCCGTGGCCTGGATCGGCGTCGGAGCGATCGTCGGGGCCTTTGCGCTCGTCGCCGCGTTGGTTCTCGTACCCCCTCGGTCCTCGTGGGGACTCTCGCCGTGCGACAGCGGCTGGCAGGAATTCAACGCGGGATGCGTCGCGTGGGACCCCACCCCCGTCGAGCACGAGCAGGCGGTCGGCGGCTGCAGCGCGCCGGCCACCCTTATCCCCCGTGCGGCCGCCAAGCACCTGGCCGCTCTGACACGCGTCCAGGCGGAGAGATCGTCGGGTTACTGGTGGGTGAACGGAGACGGCATCCGGACCTGTCTGAGACTCGTCGACAGCGTCAGTGGCATCGACGAGTTTTGCGAGGAGCTCGCGATCCGCATATGCTACTACCCACGAAGCCCCGGCGGGTTTGTCCGCTTCGTAACTTCGATACGTAACGCCCTGGGGTTGCCGTGAGGCGCGCGTCCGACGGTCCCGCTTCTCGCCTCTCTTCTTCCCCCACCCCACCCACCGACCAACGACGGCGTTTGGCCAATACCCTCCTTTTTTCTTTTTCTCTTCCCCCCCCCCCCAAAAAAAACAATAAACAGCTAATTGCGTACGACAAACCATGCGGAACTCGCTGTTTTTTTTCTCTGTTTGTTACTTTTTATTGAAACAGACATACGGGGAAAGGGGCCGGAAACCGAGACGGTGGGGCCGGCGGTCGCATTTTTTTAATGGCTCTGGTGTCGGCCGCGTTTGAGCTTCGTCAACAGGGCGCTGAGGGCGGCGACGTTCGTCGGGCCGTCGTTGGCCAGCGCGTTGGTCCGGGGGCGGGCGGGCATGGGCGACAGGCTTAGTCCCGGGTCCGGGGCGCGTGTGGCCCCCGGAGGGGAGAAGAGGGCAGACCCGCCCCAGTCGTACAGGGGATTTTCCGCCTCGATGTACGGGGAGTCCGGGGCGTCTCCCGGCAGGGCGGCCCCGCCGGCAAGACGCCGGCGAGGGCAGATGTTTTCGTATACCCGAACCCAGGGGATCTCCTCGTAGACGCGCCCCCCATCCTCGCCCACCGACTCGTAAATGGAATCTGCGTCCTCGGAGGGGGCGCGGGGGGCGTGGCTTTCGGCCGGCCAGGCGGCGGCGGCGGTGGTGTCGGCGGCGGGGGTGGCGCCAAGCCCGACGCCCGCGGGCATGGCGGCGTCATCGTCGGGCAGCAGATACGTGTTTTCCATCTGGTCCGGTTCGGCCTCCGCGTCCGGCCCCCAGGTCCGCACCGCGTCGTAGACCCCGGCGGCCTCGCGCTGAGCCGCGAGCGGGCGCGCCGCGGCTGCCGGCCGCTGCTCGGGGGGCGCGGGGTTGCGGGGCGGGAGGCGCGGGGGCGCCCCGGCCATATGCGTGTAATACGTGGCCGGCCGGCCGGCGCAGGGCTCGGGACCCCGGTCGGCCGCGTCGACGTGCGGGGGCTCGGGGAGGTCCTCGCGGTGGCGCCTGAACCTCCGAGGGGCCGCGGGGGTCGAGTGGGGGCGAGCCCGGGGGAGCGGCGGGGGTGCGTTGTCGCGCCGGGTCCGTTGTATCTTGTCCCGGCAGCTCCCGCCGACCGCGCCGCGGCCCCCCGGTGGGCCGGACGCCGCGAGGCGCAGGATGGACTCGTAGTGGGGCGACGGGGTTCCGCTCCGAAGCAGGTCCGGGGCCAGGGCGGCCCCGAACCAGGACTTGATGCTGAGTTCCATCCGGGCCCAGCTCGGGGCGGTCATCGTGGGGAACAGGGGGGCGGCGGTCCTGCAGAAGCGCTCCTGGCTGTCCACCGCCGCCCGTAGGTACTCGTTGTTCAGGCTGTCGGAGGCCCAGACGACATACCCGGTAAGCGTCGCGTTAATTATATACTGGGCGTGGTGGTGGACTATGGATAGAACCTCGACGGTCGAGACGATGGCGTCCACGATCCCGTACGTGCCGCCGCTGCGCTTGCCGGTCTCCCACAGGTGGGCCAGGCGCGTCAGGTGGCCCAGGACGTCGCTGACCGCCGCCCGCAGGGCCATGCACTGCATCGAGCCCGTGGTGCCGCTGGGCCCGCGGTCCAGGTGGCGCGCAAACGTCTCCGCGGGCGCCTCCAGACTCCCGCTGAGCGCCACGAACCGGCGATCGGCGGGGCCCAGGCGGCGACACACGTACTTGTCCGCCGTCCACAGCATCCACGAGGCCCAATGGTACAACACGGAGACGTAGGCCAGGAGCTCGCTCAGCCGCAGTGCGGTGTCCGTGCTCGGCCGGCTCGGGTCTGCGGGGCGCATAAAGAACATGTACTGCTGGAGCCTGTGGGCCGCGTCGCGCAACCCCGCCACCGCGGCGGCGTACTTGGCCGCGGCGGCCCCGCTCTTGAACGGGGCGCGCACCAGCAGCTTCGGGAGCAGGGTGGGCCGCAGCAGCACGTGCAGGCTGGGGTCGCAGTCGCCCGCCGGGTCGTCGGGGATGTCCAGGCCGCTGGGCACGACCGTCTGGAGGTACTTCCAGTACTGCGCTAGGATGGCGCGGCTCAGCTGGCCGCCCGACAGCTCCACCTCGCCGAGCGCCTGCTTGGCGGCCGACGCGTAGTGCCGGATGTAGTCGTAGTGCGGGTCGCTGGCGAGCCCGTCTACGATCAGGCTCTCGGGGACGGTGTTATGGTGCCGCGCCGCCAGCCGGACGCTGCGATCGGCGCCGGTCAGAAACGCCGGCTGCAGGTCGTCGGCGCGCTGCCGCAGGACGCCCACGGCCGCGCTGAGGAGCCCCTCCGGGGTGGGGAGCAGACACCCGGCGAAGATGCGCCGCTCGGGGACGCCCGCGTTGGCGCCGCGGATGAGGTTGGCCGGCGTCAGGCACCGCGCCAGCCGCAGGGAGCTCGCGCCGCGCGCCCGGCGTTGCATGGCGGAGACCGTTCGGTCGGGGGCCCCGCCGGTCGGAGGTATGCCGCGTCCCGGGATATAGGGTTGCTTTTTATGGGGAGGCGCCTATGGGCGTGGCGGGCCGCCCAGCCCGGTCGCGCGCCTCCCGGACACGTGCGCCCGGAGGGCGGCGGTCTCCTCGTCGCCCATGAGCAGTTTCCGAAACTGCGCCATGATGTCCACGACGCGGACCCGCGGCCCCAGCACGGACTCGCTATTCAGGGGGGCGGGGGGGAAGGCCGCCAGGTCTTCGAGCAGGAAGGCGGGGTCTGCCGTCCCGCTCACGGGCGCCCGGGGCGCCGAGGACGCGGGGCGAAGGTCCACGTGTTCCGCGGCGGCGCGCACGTCCGCCCAAAATTTGGCGGGGGTGGTCCGCGCGTACAGGGGCTGGGTCGCGCGGAGGACGCACGCGTAGCGCAGGGGGGTGTACGTGCCCACCTCGGGGGCCGTCGACCCGCCGTCAAACGCGGCCAGGGCCACGCACGCGACCACCGTGTCGGCCAGGCCCAGCAGCCGCTGCAGGATGAGCCCCGTCGCCAGCACGGCGCGCGCGGCCGCCGCGTCGTCCCTGCGCCGGCGCGCGTCCCCGCAGGCCAGGGCGTATTTCAGGGTAACGGTCGCCAGGGCCGTGTGCAGCGCGTACACGGCCGCGCCCAGCACGGCGTTCAGCCCGCTGGTGGCGAGCAGGCGGCGCGCCGCGGTGTCGCCCAGCGCCTCGTGCTCGGCCGCCACGACCCCGGGGCTGCCCAGGGGCAGGGCGCGAAACAGCGCCTCCTGCTCCACGTCCGCAAACGCGGGGTGGGCGGAGTGCGGGTGCAGGCGCGCCCCCACGACCACCGAGAGCCACTGGACCGTCTGCTCCGCCAGGACCGCCAGCACGTCCAGGACGCGCCCCGCAAACGCGGCCTCCCGCGGGAGCACGCATTTGACGGCGCCGGGGTTGAAGCGGGCGAGCAGAGCCCCGGTGGCGATGTACGTCATGCGCCCCGCGTAGCGGGCGGCCACGCGACAGTCGCGCCCCAGGAGCGCGCGCACCCCGGGCCAGTACAGCAGGGACCCCAGCGAACTGCGAAAGACCGCGGCGTCGGGGCCGGGGTGGGGGGGCGCGGCCCCTCCCGCGCTGAGCAGCGGCACGGCGGCGGCCCCCACGGGCCGCAACGCCGTGAGGCTCGCGAACTGCCGTCGGAGCTCGGCCGCCCTGTCGTCGAGCTCCGAGCCGCGCCCCTCCGTGTGCAGGCGCGTCCCGCAGACCCACCCGTTGATCGCCACCCGCACGATGGCGTCCACCAGAAAGCCCATCGCGCGGGAGGGGCTGGTTTTTGCCCGCCGATCCGTCAGGTCGAGGATCGCGTCGCCCGTGACGTACCAGGCCAGCGCCTCGCCCTGCTGCAGCGTCTGGCGGAAAAACACCTTTGGGTCGGCCGGGGAGGCAAAGTGCATGACCCCCACGCGCGACAGCCCGAACGCGCTATCCGGACACGGGTAGAACCCGGCCGGATGTCCCAGGGCCAGGGCCGAGCGCACGGACTCGTCCCACGCGGCGACTCGGGGGGTCAGGCGGTCCAGGGGGAATGCCGCCTGCAGCTCCGGGCCCGACACGCGGCCCGCGAGAATCTCGACCGTCGCGGAAGGCCGCGCCCCGGCGCCGTCATCGTGCGCGACGGCGGCGGGGTAGTCGTCCTCCTCGTAGTTGAGCTCGTCCAGGAACAGCGGCGAGGGCACCACCCGCGAACCGCCCACCCGCCCCAAAACGTCGCGTGGGTCCATCGGGCCCAGGTAGCCTCCCCGCGGGGCCCGCGTGATGGCGCTGTCCCGGCGTCCGCGAACGGACTGGCTCCTGGCCGTAACGGACCTGGGGCGCGGAAAGGACGCCCGGCGGGGGGGCGCCGCCGCCCGGGCCTCGGACGCGCGTCGGGACCCGGGGTGACCGCGGGCCTCCCGGCGACGGCGCGGGGGCGGCTCTTCGCTCGCCATCTCCCCCGCGGCCTCGACCTCGCTGTCGTCGTCCACGTTAAACACCGCCCGCAGGTACCCCATTAACCCGACTCCACCGCCCTCGGGCTCGTCCTCCACGGGCGAGTCGGCGCGATGCGCGGACGGGGCATGGGACCGGGTGGAGGCGCGCCTCCGGCGTACGGCATGCCCGCGCACGGACATGGTGGCCGGAGGCCCGATTTTTTACACACGCCCTCCCCGCAGACGGACGAGGAAAGGGGTGGTGCGAGGGGGGAGGCCCAAACGGGGAGGTGGGGGGTAGGGGGCGGTCCCAGGGAGCGGGGGGTAGGAACCGGCACGACGGGAACAGAGAAAACGCGACCGCTCCAACAAGGGTGGGGGGGTGGGCCTCGTCCCCACGCAGACCCGCGGGCAAATGCGAGAACGGGACCCGCGCGCCTGCCTTTATACGCGGACCCCAGCACCACGAGCCGTTCTGTGACGCGAATCTACACGACCGCGGGCTCGTAGGCGCGACTAACGCCCAACCCAACGGCACACACCCCCCACCCCGCGCGTAACCCCATTTCTTTCATGGTCCCGTAATAAACAGCCAACGCACGCCGCGTATGATGAGTTGCTTGCCAATGTTTATTGCTGTGGTTGCGAACCCTCTATCGCGATACAGACGGAGGTGAGGCGGGGCGGTGGTGGGGGGGGGCGCGCCGCCCGGTCGCACATCCTACCCCCCAAAGTCGTCAATGCCCATGGCATCGGTAAACATCTGTTCAAACTCAAAATCGTCCACGTCCAAAGCCCCATACGAGACGGGGTCGTGGGTCATTCCCGGGGAGGGGGACTCCACGTCCCCCAGCATCTCCAAGTCGAAGTCGTCCAGGGCGTCGGCGGGCGTCATATCCACCTCCTCGCCGTCCAGGCGGAGTTCGTCTCCCAGGCTGACGTCGGTAATGGGGGCGGTGGTGGACAGTCTGCGGGGGCGTTGTCCCGCGGAGAGAAACGACATGCGCGGCGCCACCAGCCCGGCCTCCGCGGGAGCGTCATCGTCGTCCGGGAGGTCGAGCAGGCCCTCGATTGTCGATCCGTAATTGTTTCTGGTCCGCCCGCGGCTATACGCGTGCTCCCGCATGACGGACTCGCCCTCCGAGGTCGCGACGCTGGAGTACGAGTCCAACTTGGCCCGGATCAGCAGCATAAAGTACCCAGAGGAGCGGGCCTGGTTGCCCTGCAGGACGGGCGGGGTCGTGAGGGGCGCCCCGGGTTCCTCCGCCGCCGCACTTCGCACCAGCGGGAGGTTCAGGTGCTCGCGAATGTGGTTTAGCTCCCGCAGTCGCCGGGCCTCCACGGGAACTCCCCGCACGGTGAGCGATCCGTTGATAAACATCAGGGGCTGAAACAGACACGCCAACTGGCGCCAGCTCTCCAGGTCGCAGCAGAGGCCGTCGAACAGATCGGGCCGCATCATCTGCTCGGCGTACGCGGCCCATAGGATCTCGCGGCTCAGAAAGAGGTATAGATGCAGAAACAGGACGCGCGCCAGGCGCGCGGTCTCGCGGTAGTACCTGTCCGCGATCGTGGTGCGCAGCATCTCCCGCAGGTCGCGGTTGCGGCCCCGCATGTGTGCCTGGCGGTGTAGCTGCCGAACGCTGGCGCGCAGGTACCGGTACAGGGCCGAGCAAAAATTTGCCAACACGGTCCGGTAGCTCTCCTCCCGCGCCCGCAGCTCACCGCGGAAAAACTGCGCCATGGCCTCGTAGTACGAAGGCAGCTCGTCGCGGGTGGCGGGCAGGGTGGGGAACGCCACGTCGCCGTGGGCGCGAATGTCGATCGGGGAGCGCTCGGGGACGTGCGCATCCCCCCAGTCGATCACGTCGCTGGGCAGCGTCGACAGAAACTTGCACTCCCGGTACATGTCGGCGTTGGTCGGGAACCCAGAGAACAGGTCCTCGTTCCAGGTATCTAGCATGGTACACAGCGCGGGACCCGCGCTGAAGCCCAGATCGTCGAGGAGACGGTTAAACAGGGCCGCGGGGGGGACGGGCATGGGCGGCGAGGGCATCAGCTGGGCCTGACTCAGCCGACCGGTGGCGTACAGCGGAGGGGCGGCTGGGGTGTTCTTGGGACCCCCGGCTGGCCTGGGGGGCGGTGGCGAAACCCCGTCCGCGTCCGCAAACAGATCGTCGACCAACAGGTCCATGGGGGCGGTTGGGTCCGGGAATAACGATCTCGAGAGGCGAATGAGACGTGCCCGAGCGCCCGGCGGCGGAGAGGGGGGGAGGGATCCGGGACCCGCGACAGAAAAAGGCCGGGGCCCTCGCGAAGGGAATCGCCGGGGGTGCCGTGCGTCCCCGAGGACTGACATCTCGCGTCCACCACCCCGCATTTAAGTATCACCCCAGTGCCGCCCCAAACCTCGTGACTTCCCCACCGCTCCGGGCGGCCCGTCCCCCGCGCTCGGAAGGGAGGCGTGTCCTTCGTGATGCTTTATTGTCTGTCGTCTGAACGCGGTCGGGGTCGCTACTCGAGGGGGCGGCGGGGACGGGAAGCCGAGCGGGCGGGGGCCCGTGCGGTCGCGGCGGCACGCCCCGCGGGGCGGCCCCGGGCGGCCGCGGTCGCGTCGACGTCCTGCGCCGCGTCGGGATTCACCAACTCGTTCGCGCGCTGCAGGAGGTTCTTGCCCTCGCAGACCGTCACGCGAATGGTGGTGAGGTCGAGGAGCTCGTTGAGGTCTTCGTCGGTGTGCGGCCGCGACATGTCCCACAGCTGTACCGCCGCCAGCCGGGCGTGCGTGGCCGCCAGGCGCCCGACCGCGGCGCAGAAGACGCGCTTGTTGAACCCGGCCACCCGGGGGGTCCACGGCGCCGTGGGGCTCGGTGGGGCGGTGCTGAAGTGCAGCTTCTTGGCCAGTCCCTGGGCGGGTGTCTTGGTTCTTCCCGAGGCCGTGGGAGCGGGGGCGTCTAGGAGCACGGCGGAGTCGGCCTGGGCGGGTCGCCTGCCGCGGGCGGGGTCGGTCGCCGGGGTCGCGGAGGCCTTAGGCGCCCCGCGCGTCATTTTGGGGGTCCGCGCGGGAGGGGCGTGCGAGCGCCCGCCGGCGCCCACGGGGCCCCCGGGGGGTGGAGGAGCGCGCGCGGGGCCGGGGCCGTGAGAGCCCGCGACGGACGCCGAACGACGCGGTCGCGCGGTATCCCGGGACTCGTCGTCGTCCGAAGACGAGTCCCGGTAGAGGGCATACCCAGCCTCGTCATAATGGAGAAAGCGAACCTCGCCCCTCGGGCGCGCGCGCATCGGGCCAGCGCCGCGGCGGAAGTCGTCGCGCGGACTCTCTGGGTCCGCCGGGGAGACCGGGCCATAGTACAGCTCCTCGTGGGTCCCGCGCGGCGCTTCCCGCGGACACGACTTGACGGAGCGGCGAGAGGTCATGGTCTATCGGAGACACCGGGGACGCCCGTGCGGATCACAGGGAAGGCGTCGGCGAAGGAGGCAGAGAGCGTCGGAAGGCGGCGAGGGAGGGAAAGAGGGAGACCGGCGGGGTACGGGAGAGCAGCGAGGGCCTGCGTAACCCACGGGGGCCGCGGGAGTGGCTCCCTGCGGGTTGCGGGGGAGAGTTTATAGGAAGTGGATATAACCGCAGGCGACGGGACTAACCAATCCCCGGGGGGGCAACGGACAGACACGCCCCGAACAGGCCCGACTTCCGCGAGGAAGCAAAGGCCGGGGGCCGCCCAACGACACGCCCACCCCTTCCCAACAGGGCGGGCTCAGGCTGACCCGGCGGCCAGTGCCCGCTGACATATCTGATACACGTGCGCGATCATACATACGCCCATCGAGGTCATGCCTAGATAAAAGGGCACCAGGACCCCCGGGACGGACACCACACCGGCGCTGTCGCCCCGGCATTGCGCGTCCCCGATAACGCCGCGTGCGCCTGCCGCGTTCGGCGGCTCCCCGGGCACGCCCGCGACGAGCGCGACGAACAACAGCACCACCCAGCGGCCCAGTCTTGCGGGTTTCCCCGTCATCGCGGCGATGAGTCAGTGGGGGCCCAGGGCGATCCTTGTCCAGACGGACAGCACCAACCGGAATGCCGATGGGGACTGGCAAGCGGCCGTAGCTATTCGCGGGGGCGGAGTCGTTCAACTGAACATGGTCAACAAACGCGCCGTGGATTTTACCCCGGCAGAATGCGGGGACTCCGAATGGGCCGTGGGCCGCGTCTCTCTGGGCCTGCGAATGGCAATGCCGCGGGACTTCTGCGCGATTATTCACGCCCCCGCGGTATCCGGCCCCGGGCCCCACGTGATGCTCGGTCTCGTCGACTCGGGCTACCGCGGAACCGTCCTGGCCGTGGTCGTAGCCCCGAACGGGACGCGCGGGTTTGCCCCCGGGGCCCTCCGGGTCGACGTGACGTTTCTGGACATCCGGGCCACCCCCCCGACCCTCACCGAGCCGAGCTCCCTGCACCGGTTTCCGCAGTTGGCGCCGTCCCCGCTGGCAGGGTTACGAGAAGATCCTTGGTTGGACGGGGCGCTCGCGACCGCCGGGGGGGCGGTGGCCCTGCCGGCCAGACGGCGCGGGGGATCGCTGGTCTACGCGGGCGAGCTAACGCAGGTGACCACCGAGCACGGCGACTGCGTGCACGAGGCGCCCGCCTTTCTGCCAAAGCGCGAGGAGGACGCAGGCTTTGACATTCTCATCCACCGAGCCGTGACCGTCCCGGCCAACGGCGCCACGGTCATACAGCCGTCCCTCCGCGTATTGCGCGCGGCCGACGGACCAGAGGCCTGCTATGTGCTGGGGCGGTCGTCGCTCAATGCCAGGGGCCTCCTGGTCATGCCTACGCGCTGGCCCTCCGGGCACGCCTGTGCGTTTGTTGTATGTAACCTGACCGGAGTCCCGGTGACCCTACAAGCCGGGTCCAAGGTCGCCCAGCTGCTCGTCGCGGGGACCCACGCCCTCCCCTGGATCCCCCCCGACAACATCCACGAGGACGGCGCATTCCGGGCCTACCCCAGAGGGGTTCCGGACGCGACCGCCACCCCCCGAGACCCGCCGATTTTGGTGTTTACGAACGAGTTTGACGCGGACGCCCCCCCAAGCAAGCGGGGGGCCGGGGGGTTTGGCTCCACTGGCATCTAGACCGCGCCTCGCGTCGGGCCAGATGGGGCCCCGGTCAATAAAGAGCTCTGTTTCGCATATGCCCTGGTGTTGGCGGTTTTTTTTGTTGTCTGTCTGCCCGGCGCTCGGTTGTCCGTTCTGTCGTCGCTATCACATACGCACAAACACACGGGTAGAGTGGAACCGAAACCGGTCGACGTTTATTCACCACACAGAAACACAAGCTAAGCGAGAAGGAGGGGGGCCTCGGTCGACGAGGCCTGGCGTTTGGGGGCGGACGTGCGATGACGTGGGTCCGGTGTAGGGTCCGCGGGGGGCACGGGCCCGGGGCGAACGGGGGATCTGTCGCCGGCGTGGGTGACTGGGACCGACGCAACCTCCGGGGCTTGTGCCCTCGTAGGCCCGGGGGGGGCCTCGGTCGCTCCAAGCCCCGCGGTGCGGGTCCCTCCGGCCAGAGCCGAGGTGGAGAGACCAAGGGCCCGCTCCGCGATCGCCACGTCCTCCATGACCACGTCGCTCTCGGCCATGCTCCGAATGGCCTGGGAGACGAGCACGTCCGCCGACTTGTCCGCGGCCCCCACCGACATGTACATCTGCAGGATGGTGGCCATGCACGTGTCCGCCAGGCGGCGCATCTTGTCCCGATGCGCCGCAACGGCCCCGTCGATGGTGGAGCCCTCGAGTCCCGGGTGGTGGCGCGCCAGCCTCTCGAGGTTGACCATGCAGGCGTGGTATGTGCGGGCCAGGGCGCGCGCCTTCACGAGGCGCCGGGTGTCGTCCAGCGACTCTAGGGCGTCGTCGAGCGTGATGGGGGCGGGCAAAAGCGCATTGACCACCGCCAGGGCCTCCTGCAGCCGCGGCTCCGCCTCCGAGGGCGGAGCCGCGGCCCGAATCATCTCATATTGTTGTTCCTCGGGGCGCGTTCCCCAACCGCACAGCACCCCGAGCAGGGACGCCATCCCGGAACACGCGCGCGGCTCTGCGCCGGCTTTCCCCCACCCCACCCCCTCCGGGTTCGCAGGGGCGATGGGGACGGAAGACTGCGATCACGAAGGGCGGTCGGTTGCGGCTCCCGTGGAGGTTACGGCGCTGTATGCGACCGACGGGTGCGTTATCACCTCCTCGCTCGCCCTCCTCACAAACTGCCTGCTGGGGGCCGAGCCGTTGTATATATTCAGCTACGACGCGTACCGGTCCGATGCGCCCAATGGCCCCACGGGCGCGCCCACCGAACAGGAGAGGTTCGAGGGGAGCCGGGCGCTCTACCGGGATGCGGGGGGGCTAAATGGCGATTCATTTCGGGTGACCTTTTGTTTATTGGGGACGGAAGTGGGCGTGACCCACCACCCGAAAGGGCGCACCCGGCCCATGTTTGTGTGCCGCTTCGAGCGAGCGGACGACGTCGCCGTGCTCCAAGACGCCCTGGGCCGCGGGACCCCATTGCTCCCGGCCCACGTCACAGCAACTCTGGACTTGGAGGCGACGTTTGCGCTCCACGCTAACATCATCATGGCTCTCACCGTGGCCATCGTCCACAACGCCCCCGCCCGCATCGGCAGCGGCAGCACCGCCCCCCTGTATGAGCCCGGCGAATCGATGCGCTCGGTCGTCGGGCGCATGTCCCTGGGGCAGCGCGGCCTCACCACGCTGTTCGTGCACCACGAGGCGCGCGTGCTGGCGGCGTACCGCCGGGCGTATTATGGGAGCGCCCAAAGCCCCTTTTGGTTTCTGAGCAAATTCGGCCCGGACGAAAAGAGCCTGGTGCTGGCCGCTAGGTACTACCTACTCCAGGCTCCGCGCTTGGGGGGCGCCGGAGCCACGTACGATCTGCAGGCCGTGAAAGACATCTGCGCGACCTACGCAATCCCCCACGACCCACGCCCCGACACCCTCAGTGCCGCGTCCTTGACCTCGTTCGCCGCCATCACTCGGTTCTGTTGCACGAGCCAGTACTCCCGCGGGGCCGCGGCCGCTGGGTTTCCGCTGTATGTGGAGCGCCGCATCGCCGCCGACGTACGCGAGACCGGCGCGCTGGAGAAGTTCATCGCCCACGATCGCAGCTGCCTGCGCGTGTCCGACCGGGAATTCATTACGTACATCTACCTGGCCCACTTTGAGTGCTTCAGCCCCCCGCGCCTGGCCACGCATCTCCGGGCCGTGACCACCCACGACCCCAGCCCCGCGGCCAGCACGGAGCAGCCCTCGCCCCTGGGTCGGGAGGCGGTGGAACAGTTCTTCCGGCACGTGCGCGCCCAGCTGAACATCCGCGAGTACGTAAAGCAAAACGTCACCCCCAGGGAAACCGCCCTGGCGGGAGACGCGGCCGCCGCCTACCTGCGCGCGCGCACGTATGCCCCGGCGGCCCTCACGCCCGCCCCCGCGTACTGCGGGGTCGCAGACTCGTCCACCAAAATGATGGGACGTCTGGCGGAAGCAGAAAGGCTCCTAGTCCCCCACGGCTGGCCCGCGTTCGCACCAACAACCCCCGGGGACGACGCGGGGGGCGGCACTGCCGCCCCCCAGACCTGCGGAATCGTCAAGCGCCTCCTCAAGCTGGCCGCCACGGAGCAGCAGGGCACGACGCCCCCGGCGATCGCGGCTCTCATGCAGGACGCGTCGGTCCAAACCCCCCTGCCCGTGTACAGGATTACCATGTCCCCGACCGGCCAGGCGTTTGCCGCGGCGGCGCGGGACGACTGGGCCCGCGTGACGCGGGACGCGCGCCCGCCGGAAGCGACCGTGGTCGCGGACGCGGCGGCGGCGCCCGAGCCCGGCGCGCTCGGCCGGCGGCTCACGCGCCGCATTTGCGCCCGGGGCCCCGCGCTCCCCCCGGGCGGCCTGGCCGTCGGGGGCCAGATGTACGTGAACCGCAACGAGATCTTCAACGCCGCGCTGGCCGTTACGAACATCATCCTGGATCTGGACATCGCCCTGAAGGAGCCCGTCCCCTTTCCCCGGCTCCACGAGGCCCTGGGTCACTTTAGGCGCGGGGCGCTGGCGGCGGTTCAGCTGTTGTTTCCCGCGGCCCGCGTAGACCCCGACGCCTATCCCTGTTATTTTTTCAAAAGCGCCTGTCGGCCCCGCGCGCCGCCCGTCTGTGCGGGCGACGGGCCCTCGGCCGGTGGCGACGACGGCGACGGGGACTGGTTCCCCGACGCCGGTGGTCCCGGCGACGAGGAGTGGGAGGAGGACACGGACCCCATGGACACGACCCACGGCCCCCTCCCGGACGACGAGGCCGCGTACCTCGACCTGCTACACGAACAGATACCAGCGGCGACGCCCAGCGAACCGGACTCCGTCGTGTGTTCCTGCGCCGACAAGATCGGGCTGCGCGTGTGCCTACCGGTCCCCGCCCCGTACGTTGTGCACGGCTCCCTGACGATGCGTGGGGTGGCGAGGGTGATCCAGCAGGCGGTGCTGTTGGACCGCGACTTCGTGGAGGCCGTAGGGAGCCACGTAAAGAACTTTTTGCTGATCGATACGGGCGTGTACGCCCACGGCCACAGCCTGCGCTTGCCGTATTTCGCCAAGATCGGCCCCGACGGCTCCGCGTGCGGCCGGTTATTGCCCGTCTTCGTGATCCCCCCCGCGTGCGAGGACGTTCCGGCGTTCGTCGCCGCGCACGCCGACCCGCGGCGCTTCCACTTTCACGCCCCGCCCATGTTTTCCGCGGCCCCGCGGGAGATCCGCGTCCTCCACAGCCTGGGCGGGGACTATGTCAGCTTTTTCGAGAAGAAGGCGTCGCGCAACGCCCTGGAGCACTTTGGGCGACGCGAGACCCTGACGGAGGTTCTGGGCCGCTACGATGTGCGGCCCGACGCCGGGGAGACCGTGGAGGGGTTCGCGTCAGAACTGCTGGGGCGAATAGTCGCGTGCATCGAGGCTCACTTTCCCGAGCACGCGCGGGAATATCAGGCCGTGTCCGTTCGCCGGGCCGTCATTAAGGACGACTGGGTCCTGCTGCAGCTGATCCCCGGCCGCGGCGCCCTGAACCAAAGCCTCTCGTGTCTGCGCTTCAAGCACGGCAGGGCAAGTCGCGCGACGGCCCGGACCTTTCTCGCGCTGAGCGTCGGGACCAACAACCGCCTATGCGCGTCCCTGTGTCAGCAGTGCTTTGCCACTAAATGCGATAACAACCGCCTGCACACGCTGTTTACCGTCGATGCGGGCACGCCATGCTCGCGGTCCGCTCCCTCCAGCACCTCACGACCGTCATCTTCATAACGGCCTACGGCCTCGTGCTCGCGTGGTACATCGTCTTTGGTGCCAGTCCGCTCCACCGATGTATTTACGCGGTGCGCCCCGCCGGGGCGCACAACGATACCGCCCTCGTGTGGATGAAGATAAACCAGACGCTGTTGTTTCTGGGCCCGCCGACCGCCCCCCCCGGCGGGGCATGGACCCCCCACGCCCGCGTCTGCTACGCCAATATCATCGAAGGTCGGGCCGTGTCCCTCCCGGCCATCCCCGGCGCCATGAGCCGCCGGGTCATGAACGTGCACGAGGCCGTAAACTGCTTGGAGGCCCTCTGGGACACCCAGATGCGCCTGGTGGTCGTCGGTTGGTTTCTGTATCTAGCGTTCGTCGCCCTTCACCAACGACGATGCATGTTCGGCGTCGTGAGTCCCGCGCACAGCATGGTGGCCCCGGCGACCTATCTTTTGAACTACGCCGGCCGCATAGTGTCGAGCGTGTTCTTGCAATACCCCTACACGAAAATCACCCGCCTCCTCTGCGAGCTATCCGTTCAACGCCAGACCCTGGTGCAGCTGTTCGAGGCGGATCCGGTCACCTTCTTGTACCACCGCCCGGCCGTTGGCGTCATCGTGGGCTGCGAGCTGCTGCTCCGCTTCGTGGCCCTCGGTCTCATCGTCGGCACCGCTCTCATCTCCCGGGGCGCCTGCGCGATCACACACCCCCTGTTTCTAACAATCACCACCTGGTGTTTCGTGTCCATCATCGCCCTGACGGAGCTGTATTTCATCCTGCGGCGGGGCTCGGCCCCCAAAAACGCGGAACCAGCGGCCCCCAGGGGGCGCTCCAAAGGGTGGTCGGGCGTCTGCGGGCGCTGCTGTTCCATCATCCTCTCCGGTATCGCCGTGCGCCTGTGCTATATCGCCGTCGTGGCCGGGGTGGTGCTCGTGGCGCTTCGCTACGAACAGGAGATTCAGCGGCGCCTGTTTGATCTGTGACGTAACGCCTCTTCCGTTGGAAGAGGCGGACCCAGTCGCCCATGCAAATTAAATACACGACCCGCCTCGGGCCTACGCACCCTCGCACGTCGCATGCAAATTAAAATCGTGCACAGAGCCGATCCGGCCTCGGGTCTGCTTGCCCCTCCCCCGGCCCAGCACAGGCAGGCTCGTCCGACTTCCGCATACACCCCACCCTACCGCGTGCTTCCGCACCCCCGCCTACGCGTGTACGCGAAGGCGGACCCAGACCTGCCGTATGCTAATTAAATACATAAAACCCACCCTCGGTGTCCGATTGGTTTCTGGGGACGGCGGGGGCGGGGGCGGTGACGCCCGACGGGGAGGGACAAGGAGGAGTTTCGGAAAGCCGGCCCCGGTCGTGCGGGTATAAGGGCAGCCACCGGCCCACTGGGCGCTGTGTGCTGCCGTGTGCCGACCCCGGTTGCGCGTCGGTGCCGCTCCTCGATTCGGACCCGGCCACTCTCTTCCGACACGCGCCCCCTCGGAGGACACCCGCCATCCCAGCCCCGGCGACCTACAACATGGCTACCGACATTGATATGCTAATCGACCTAGGATTGGACCTGTCCGACAGCGAGCTCGAGGAGGACGCTCTGGAGCGGGACGAGGAGGGCCGCCGCGACGACCCCGAGTCCGACAGCAGCGGGGAGTGTTCCTCGTCGGACGAGGACATGGAAGACCCCTGCGGAGACGGAGGGGCGGAGGCCATCGACGCGGCGATTCCCAAAGGTCCCCCGGCCCGCCCCGAGGACGCCGGCACCCCCGAAGCCTCGACGCCTCGCCCGGCAGCGCGGCGGGGAGCCGACGATCCGCCACCCGCGACCACCGGCGTGTGGTCGCGCCTCGGGACCAGGCGGTCGGCTTCCCCCCGGGAACCGCACGGGGGGAAGGTGGCCCGCATCCAACCCCCGTCGACCAAGGCACCGCATCCCCGAGGCGGGCGGCGAGGTCGCCGCCGGGGCCGGGGTCGATACGGCCCCGGCGGCGCCGACTCCACACCAAACCCCCGCCGGCGCGTCTCCAGAAACGCCCACAACCAAGGGGGTCGCCACCCCGCGTCGGCGCGGACGGACGGCCCCGGCGCCACCCACGGCGAGGCGCGGCGCGGAGGGGAGCAGCTCGACGTCTCCGGGGGCCCGCGGCCACGAGGCACGCGCCAGGCCCCCCCTCCGCTGATGGCGCTGTCCCTGACCCCCCCGCACGCGGACGGCCGCGCCCCGGTCCCGGAGCGAAAGGCGCCCTCTGCCGACACCATCGACCCCGCCGTTCGGGCGGTTCTGCGATCCATATCCGAGCGCGCGGCGGTCGAGCGCATCAGCGAAAGCTTTGGACGCAGTGCCCTGGTCATGCAAGACCCCTTTGGCGGGATGCCGTTTCCCGCCGCGAACAGCCCCTGGGCTCCCGTGCTGGCCACCCAAGCGGGGGGGTTTGACGCCGAGACCCGTCGGGTTTCCTGGGAAACCCTGGTCGCTCACGGCCCGAGCCTCTACCGCACATTCGCAGCCAACCCGCGGGCCGCGTCGACAGCCAAGGCCATGCGCGACTGCGTGCTGCGCCAGGAAAATCTCATCGAGGCCCTGGCGTCCGCGGATGAGACGCTGGCGTGGTGCAAGATGTGCATTCACCACAATCTGCCGCTCCGCCCCCAGGACCCTATCATCGGAACGGCGGCCGCCGTGCTGGAAAACCTCGCCACGCGCCTGCGCCCCTTTCTGCAGTGCTACCTGAAGGCCCGAGGCCTGTGCGGGCTGGACGACCTGTGCTCGCGGCGACGCCTGTCGGACATTAAGGATATTGCCTCCTTTGTGTTGGTCATCCTGGCCCGCCTCGCCAACCGCGTCGAGCGCGGCGTGTCGGAGATCGACTACACGACCGTGGGGGTTGGGGCCGGCGAGACGATGCACTTTTACATCCCGGGGGCCTGCATGGCGGGTCTCATTGAAATACTGGACACGCACCGCCAGGAGTGTTCCAGTCGCGTGTGCGAGCTGACGGCCAGTCACACTATCGCCCCCTTATATGTGCACGGCAAATACTTCTACTGCAACTCCCTATTTTAGGCAAGAATAAACATATTGACGTCAACCCAAGTGGTTCCGTGTGATGTTCTTGGCGCGCGCGGCGGGTGGGGCGGAGACTCCGGGGCGATGCCGGCGTGCGCGTGGGAGGAGGGCGATGACCCACCGGATAAATGTGGGGCCCCGGCCCGGCCCGCTTCATAGCGCGTCCAGGAACTCACGGCAGACGCGTATTCACCGACCCCCCCCTCGCAACATGACAACGACGCCCCTCTCGAACCTGTTTTTACGGGCCCCGGACATCACCCACGTCGCCCCCCCGTACTGTCTGAATGCCACGTGGCAGGCCGAAAACGCCCTGCACACGACCAAAACGGACCCCGCGTGCCTGGCCGCGCGGAGTTATTTAGTCCGCGCCTCCTGCTCGACCAGCGGCCCCATCCACTGTTTTTTCTTTGCGGTGTACAAGGACTCGCAGCACTCCCTTCCGCTGGTTACCGAGCTCCGCAACTTCGCGGACCTGGTCAACCACCCGCCCGTCTTGCGCGAACTAGAGGATAAGCGTGGGGGGCGGCTGCGGTGCACGGGCCCATTCAGCTGCGGAACCATCAAGGACGTCTCCGGTGCATCCCCCGCGGGGGAATACACGATAAACGGTATCGTGTACCACTGTCACTGTCGGTATCCGTTCTCCAAAACCTGCTGGCTCGGGGCATCCGCGGCCCTACAACACCTTCGCTCTATAAGCTCAAGCGGCACGGCCGCTCGCGCGGCAGAACAGCGACGCCACAAAATCAAAATCAAAATCAAGGTATAACCCACCCCCTTCCCTCCGAGTCCGTATGCAACCTCATTAATAAAGAGTGAGAACCAACCAAAACAGACGCGGTGTGAGTTTGTGGGTTATAGGAACCCGGTAAATACCACGCGACGAACCAGCATGTGTGTTAACGCAACTTTTATTCGTTGTATCGCGGGAGGGGGGAAGCTTACCGCCAAAGGAAGGCCAAGATGATAACGACGACCACCGCGACCACCCCAAAAACCGCATGACGACACGTCCCGCCACACCACCCTGGGGCTTGGGGCGTGTCGGAGCTCGACGCACAGCGGGCCGCGCGTTGGGCCCGGTACAGCTCTCGCGAATTGACAAGCGGGGGTCGCCACGTGCGCGAGCTTTGCACGCGGGGTTGGTCGGCCGGCCCCACGGACCCGCCCGGTGGCTCGGTCGGACATGCGGCCATGACCATGGCGTAGGTGGGGGGGCGATCCGAGGTCGCCTCTGCGTAAGTAGGGAGGCCCGACGGGAGGTCGCCTCCCACGCCAGGGTGGGCCCCAATCATAGTTTCCGGTAGAAACAGGGGGGTCTCCACAAACAACCCCCCTGGGCCAAAGCTCCGGCGCCGCGCCCGTCGTTCGGCGCGGCGCCTGGCGCGCCGAGCGGCCCGCCAGGCGGCGCGGCGCGAGCGGCCACGCTCACACACCTCGCCGTCACCGGAAGAAGCCGGTGAAACAAGCCCAACCGGCGACGTCCCTGCAGAGTACGGTGGAGGCGAGTCCGTGGGGGTGTCGATATCAATAACGACAAACTGGCCCGCGCTCGCGCCGGCCACACTCTCGTATGGGGGCGGGGCGTCAATCACGCTATCATCTCCGTCATCCCTGCATGCGTGGGCATGCCCAGCCCCCAACGCCATGGTGGGGATTCGCGGCTCAGAAGCCTGCATGTCGTGTGGTCGGTCGTAGTCCAACGTGCCTCCCCCACCCACCACACAGCCGGTCCCCACGCCGACCACTAGACCGCAGACGTCGCCCAACCGAGGTCCCCGTGCACAGACCGCGCCTTTTATAGCCCCAGGGGTTGCTAATTAACGCACGCATGCAGACGCAATTTATTTTGCTCCCCCGCGTCCTCCCCTCCCCTGCGCACACGTGATAGGTCTTGGGAACCCGAGGGGCGACGCGGGGAAAGCGCGCCCCCGCCCGGCCGCCGCGCGCCCCCGCCCGGCCGCCGCGCGCCCCCGCCCGGCCGCCGCGCGCCCCCGCCCGGCCGCCGCGCGCCCCCGCCCGGCCGCCGCGCGCCCCCGCCCGGCCGCCGCGCGCCCCCGCCCGGCCGCCCGCGTCGCGCCGGCGCCCCCTCCCGGCGCTTCCGGGGTCTTTCCTTCCTTCCCCGCCGCGACCCCGGCCCCGCCCCACCGCCCCGCCCGGCAGGGGGGCCCCGGCGCCGCGCAGAACACACAGACGAACACACGGTGGCGATCTTTTCTTTACTTCGGCGGACCAGCGAGCCCCGGCCCCGGCCCGCGCCCCGCCGCCACACCCACGGCACCCCCCCCCCGCCGCCCACCCCGGGGTCCACACAGGAGCGCGCGGGCGGCAGAAACGCGGGCGCGGCGGCGGTCGGGGTGGGAGTGGTGGTGGGGGACACGAAAACACACCCACGACACTCTCCCCCCACCCCGACCGCCGCCGCGCCCCACCGGCGGGATCGCGGCGAGACGCAGCCGGGCCCCCCCCCACCACCCGCCCACCCACCTACCCCGCGCCCGCAGCCTCCGGCAGCACGCCGACCACCGCCGCCACCCCCCAAACAGCCAAGGCGCGGTGGGGGGCGTGGTGGTGAACGATGGGGGGAACACGGGGGGGAGGGGTCCGGGGCGAGGCGGGCGGGCGAAGGAAGGGGGGGTGGTGGCGGCGGCGGTGGAAAGCGGAAAAACGGAGGATGGAAGGGCAGAAGATGGGGAGTCCCGATCCTCCTCCTGCATCCCCTCGCCTTCCATTCTCCGGCCCTCCGCGAGTCCCGACGCCCCCCCCCCGCCGCCCGACGAAGGAGACCCAAGGAGTGCGGAGCCCCCGGAGCCCGCGGCCGCAGCCGAGCAGCGCCGCGGGCTCCGGGGCCGGGCCGGGCCGGCAACGCCCCGCGCCGGCCGCGGCGGAGAGAACCCCTGTGTCATTGTTTACGTGGCCGCGGGCCAGCAGACGGGCCGCGGGCCAGCAGACGGGCCGCGGCGCCAGCGGCCCACGCCTCCCGCCGCATTAGGCCCCCGCGGGCATCCGGCGGCCGGCCCCACGCCCTTCCATTAAACACTCCCACGTTGGGGGGGGGCGCGCCAGCTGAGTGCTCTGCGGTTGCGGGCGCCGTGCCCGGAGATCCATTAAGCCGCCGGAGAGCCCGAGCCCCGCCCGCGTGTTGCTGTGGGCATTTCTGCTGCGTCATCCCTGTCTTTATAAAACCGGGGGCGCGGCAGCAACGAACGCAGGGGCCCGCCGCCGATCGAGAGGGACTCCGGAGAAGGAAGGCTGCTCCGCGCACCGGCGCGCCCTTCTCCTCTCCCCTCCCTACCTCCCCCTCTCTTCCCCCTTTTTTCCCCCGCCTCCCGTCTTCTTCCGCGCCTCCGAGGGTCCGCCTCTGCCTCGGGGACCCCCGGGCGGGCCGGGGCTTGGCCGCCGAGGTGCGCCCCGGCCGGAGGGGCCCCCGCACCTCGGCGGCCGCCCCCTCCGGCGCCGCGCGTTCGCGAAAGGCGCGAAAGGGGCCCCCGGAGGCTTTTTTCGATTCCCGGCCGGGGGTCCCGGGTAGCCGCCCGGCGCCGGGCGGAAGGCGTCCCCCGCCCGGCGGTCCGGCCCGGGCCCCCGGCGGAGCGCGGGGGCCCCGGGGCCCCGGGCCGCGCCGGCGGCGTTTCCGCGTTCCGTTTCTTCTCCCTCCCGGCCGCCCCGCTCCCGGGCCCGACCCTCGCCCCTTCCCTTCTCCTCGTCTTCCCCCGTCCCGCCGCGCCCCTTCCCTCTTCCTTCTCTCTCTCTGTCTCGCTCTCCTCACATTTCCCCCCCCCCCCCGCCGCCGCCGCCCTCTGCCCGCGTCCCACCGAGACGCCGCGCCGCGTGAGCCGTCCGCCGGGGGACCCAGGCTCCGGGGGGGGGGGGCGCCTGCGTGTGTCTCGTGTGAGAGAGCGCGCCCCTCGAACGCCGCGCGTTCTCGCAGGTAGGTTTAGGGTCGTACAGGTGAGCTTCTGCTGAGGCGGCGGGAGAGGGGGGGGGGGCGGGCGGAAGAGAGAAGAGAGCAGGGGTTGGGGGAGAACTGTTCTTCCTCCCCCTTTCAAGAAACACGAGGCGGGGGTCCCAGAAACCCCCGTTTCCCCCCCCCGTGGTGTCGTCCGGGGGCGTCGTTCCCAGGGGGGGCAGGCGCGGGTCGGGCCCATACGCCCACCGCCCCCACGCGCCGGTCACCCCCCCCCAACAACCCCAAAGGCGCGTGCCCAGCCACAGCCGTGGGTGTGGCGCCCGTCCCCTTCCTCTACCGCGTGGGCGCGGGCGGGGGGGTGGTGGTAGTGGTGGCGGAAGGAAACGGGCCGGGGGCCGGGGCCGCTAGGGAAAGGTAGGCACGCGCGCGGTGTGTCGACTTGCATGCCCCGCAAAACGCGTCGTGTCGTGTTGTGTCGTGGTGGGCCGTGTTGTGGTGGGCCGTGTGGTGTGGTGTGGTGTTGCGAACGCGCGAGCCCCCTCGCCCCGATGGGAGTCTCCCCGCAGCCAGGGTAAGGAGGGGCGGGCGTGGCGGGCAGGTGTGCGGGCGGGGTGGGGTGAGTGCGGTTGCATGCCTCGGGTCTCCTCTTCCTGCTCCTCCTCCTTTCTCCCAGCCAGGGTGAGGAGGGGCGGGCGTGGCGGGCAGGTGTGCGGGCGGGGTGGGCGCCGGGGCGGGGGTGGGCACGGGCGTAAGTGCGGGTGCATGCCTCGGGTCTTCTCTTCTCCCTCCTCCTTCCTCCCACCCGTCCCCGGGGGCAGAGGGCGTGCATGCGTTGTGATTCAACCGCCCTCGCCCCCGCCCCACTTTCCCCCCCTCTCTATCAAAGTTCCCTGGCCCCTGGCTTCGCGCCGGTGGTGCGGCTGACCCCCCCCCTCCTCCCTCCCCGAGCCAGGCGCCCTCCCACTCCTGCCCACCACCCCCAGGGTCTGGCCGGCCAGACGTGCGTGCTCTGCACGATCGGGCCCCCCTCCCTGTCAACACGGACACACTCTTTTTTTACCCGCCAGCCAGCCCGCCCACCCACCAAGACAGGGAGCCAGAACGCAGGCCGGGCCCCGGCTCTGTTCTATCATAAAGACCAACAGGCCTCGGGGGTGGGGGCGGCTTCTCGTGCCCGCCCCCCCTCCTCCTCCTCCCTTTCCCCCCCCCCATCCCCGGCCCCCCTGCGCGGGGGAGCTGCATCAAAGGCCAACAACAAAGTGTGTCAAAAGCATCACAAAACTTTATTGTAAAATTTTTATAAATATAAAGTTTTTTTTCCTCAAGTTTTCAACAAGGCCAGAAAGTCCATAACAAAATGCTGGTGTGTGTTGCTGTTCGGGGCCGTGTCCGTCCCCCCCCACTCCCACCCCCACTTCCTGTCTCCTCCCCGTCTTTCCCCCCCACCTCCCCCTGCCCCCGAGGCGCCTCGGCCGGTGGTCCGGTGGGGGGCGGCTTCCCTTTGGGCAGCAAGCCGAGTGTTAGCTCCCCCTACTCCCCGTGGCCCGCGGGGGCGTCGCCGGCCGGCGCGGGCGCGCCCTGCTCCCGAGACCACGGGTGGCGCGACCGGAGGCCGTGGAAGTCCAGCGCGCCCACCAGGGTGCCCTGGTCAAAGAGCATGTTGCCCACCGGGGTCATCCAGAGGCTGTTCCACTCCGACGCGGGGGGCGTCGGGTAGTCGGGGGGCCTCACGCAGTTGCGCGCGTGCTCGGGGAGCAGGGTGCGGCGGCTCCACGCGGGGGCCGCGGCCCGCAGCAGGTCCGCCACGTTCCCCGTCTGGTCCACGAGGACCACGTAGGCCCCTATGTGGCCCGTCTCCATGTCCAGGACGGGCAGGCAGTCCCCCGTGACCGTCTTGTTCACGTAAGGCGCCAGGGCCACGACGCTCGAGACCCCCGCGATGGGCAGGTAGCGCGTGAGGCCGGGCGCCGGGTCGCGGGCCCCGGGCTCGGGGCCGCCCTCCGCGTGGCGCGTCTTCCTGGCACACTTCCTCGGCCCCCGCGGCGCAGCAGCGCGGGGGCCGAGGGAGGTTTCTCGTCTCTCCC

ACCGCGGCCTGGGACGACGGAGACGCCGACGGGGGCGCGGCGCCCGCGGACGCCGGGGCGAGCGGCCCGTGGCCGCGGTCGCCCGAGTCCGAGTCCGGGGCCCGGCGCGGCGCCGCCCTCTTGGCCCCCACCCCCTGGGGGGCGAGGGGCGAGCGCGGGGCGGCGGAGGAAGAGGCGGAGGACGAGGCCGCAGGGCCCGAGTCCGACCCGCGCCTCTTCCGGGGGCGGGCCGCCGCCCCCTCCGCGGCGTGGGGGGCGGCACCGGGGGTGTTGGTGCCGCGGGGGACCCCGGGTCCTCCCTCCGCGCCCGGCCCTCCCGACCCGCGCGCGTCGGTCGCGCCTGCCCGGCCCAGACTCTGTGCTTGGGTGTCGGTCTGAGCCTGGGTCATGCGCGACCGGGGCGCGCGGTGCGCGTCCACCGGCACGGCGGGCGGCGCGGGCCCGGCCGCGTCCGCGCTCGCAGACACCACGGGGGCGGCGGCGGCGCGGGGCGGACTCCGGACGCGCGGGGCGACGGCCGCGCGGGGGCGCGCCGCGCGCCCCGACGACTGTGGCAGACCTCCCCCCCCGGGGCCCGAGGACACCTGCGCGGAGGAGGAGGAGAAAAAGGAGAGCGGCCCGGGGCCCGCGGGGCGGCGCGGAGACGGCGGGGGAGAGTCGCTGATGACTATGGGGGGCTCCTGGGCCGCGCGGGGCTGTCCCGCGGGGGGCGTCCTGCCCTCCGCCGCCGCGGCGTCTTCGCCCACCCGCCGCGCCTGCGCGCGCCCCCCGCCGGCCGCAGGGGGAAGAGAGGCCACTCTCGGCACGACGGCCGCGACGGCAGGGCCGCCCCCAGACCCAGATCCCACCCCCGCCCGCAACGGGGCGCCGCCGCTGCTGCTGCTCCGCGGGGCGCCAGGGGGCGCCGGTCGGGTCGCGGCGGGCTGGGAGGTTCCGCGGGTCGCCCCCGCACCGCCGCCCCCGCGCCGGGGCGCTCTTCGGGGGGCGGGCGGGACGTAGTCCACTGCAGAGGGAGACAGAGACGGGAGCCCCCGGTTAGTGCCCGACCCCCGCCCGACCCCCGCCCGACCCCCGCCCGACCCCCGCCCGACCCCCGCCCGACCCCCGCCCGACCCCCGCCCGACCCCCGCCCGACCCCCGCCCGACCCCCGCCCGACCCCCGCCCGACCCCCGCCCGCCCTCACCGTCGGCCAGGTCATCGTCCTCGTCGTCCGTGCCGGGCCACGGGGGGGTGGGCGACAGGGCGCGGACCGTGTGTCCCCCCAGCGACAGGGAGCGCGGGGCCGTCCGCTGGTTGCCCGTCCAGATAAAGTCCACGGCCGTGCCGGCCCGCACGGCCGCCTCGGCCTCCACGCGGGTCCGGGGGTCGTTCACTATCGGGATGGTGCTGAACGACCCGCTGGCGGTCACGCCCACTATCAGGTACGCCACCGGGGTGTTGCACAGGGGACACGTGTTGCGCAACGGAATCCAGGTCTTCATGCACGGGATGCAGAAGGGGTGCAGGCAGGGAAAACTCTGGCAGCGCAGGGGCGGGGCGATCTCGTCCGTGCACACGGCGCACACGTCGCCCCCCCCTCCCGCTTCCGCTTCCTCCTCACCCACGGGCCCACCCCCGCAGGATCCCTGCGCGTCGGCGGGCGTGGGGCTGCCCTGGCGCTCGGCCGGGGGCCGGGCCGGGGGCGTGGCCGCGTCCATCAGGCCCGCCTCGAACATCTCCGTGTCCGTGCTGCCCGCCTCGGAGGTGGAGTCGCGGTGAAGGTCGTCGTCAGAGATTCCCACCTCGGTCTCCTCCTCCGAGTCGCTGCTGGCGAGCCACTGCATGTCGTTGAGCATCCCCCAGGCGTGCGGGGCGGCGGGCTGCTTGACAAAGCAACGGGGGGGATTTAGAGGGCGCGGGGCGTGAGGCGGGACCCCCGCGCCGTGTCCCCCGTGTCCCTCCCTCACCCCGGCCCCCCGCCCGCTGCTTTTTGTTCGGAAGGGGGGGAGAAAGGGGTCCGTAACCAAAGGTGGTCTGCGTCCTTTGGATTCCGACCCCTCGTCTCCCCCCCTGTCCCCCGCTCTCGGGCTCCTCCCTGCCTCCCTCGCCCCCCCAGAGGGTCGGGGGGCGGCGCACGGCCCACGGGGGTCCCCCGACCGCTTAAGCGGGCCGGGGGTCGGCCCCGTCAAGCGTCCCCGCCCCCGAGCCCACCGCCCGCGACCACCCCCAACCCGCAGCCGGGTGGTCCGGGGAAAAGGGGGGGCCTGAGACCCGGGGGTCGCCCTCTCACCGTGCCGGGGGTCTGCCGCGGCGGCCGCTCGGGGCCGGGGTCCGCCCGGGAGCTCGTGCCGGGCCGGGGTTCCATGAGCCGGGGTAGGGTAGACTCGAGACGGCGGCCCGCGGTCTCTCTCTTGCCGGGTTTTAGTCTCTGTCTCTCCGGGTCTCCTCCTCCCGCCGGGCCGCCGCTCCGTCGCTCGCAGTGCCGGGGTGCGAATGCGGCCCGACCGTCACACGGGGCTGCCTTATACCCGGCGCCTATCCACTCCCCCAAAGGGGCGGCATTTACGATTCCCCCAATAGCCGCGCGCCCCGGCGGGGGCGGAGGGAGGGAATCCCCCCCTCTCGGGGCGGCCCCGTCCCCGGGGACCAACCGGGTGTACTCCAAGAACCCCATTAGCATGCGCCGCCCCCCGCCGACGCAGATGGGAGTCCCCCCGGCGCCCCGCCGGCGCGGCCCTGAGTGGTGCCCGCCCCCGGGGAGAAATTCATTAGCATACTAGGAAGCCCAGGGGACCAATAGGGGCCGATCAGCCCACCCACCCGGCGGCGCGCGAGGCTCTGCGTGTTCTGCCAAGAAAGTAATCAGCATAACCCGGAACCCCGAGGGAGTAATTACGCGGGGAGCGAGGGGCCGTCCGAACGTTTTTAATTACCATAAGCGGGAATGGCGGCCCGTTAAAAGCTGCTAATTACCGCGAGCGGGAACGCCGGCCCATTAAAAGTTGCTAATTACCATGCGCGGGGATGGCGGCCGGGACCGCCTATTAAAAGTTTCTAATTACCATACCGGGAAGCCGGCGCGGGGCGGTCGCCGGGGCGGAGTCCGGGCCCGCGCGGCGGCGCGCGGTTGGCCGGCGCCGCCCCCTGGGGCGGGCGGAGCGGCGGGGCGGCGCCGGGCCCTCGCGGATATATACGCGGGGCTCCCATCGTCTCTTCGGAGAGCGGCCTCGCGCAGACCTTCGGAGCTCCGGGGCTCCGCCGGCCGAGCACGGTCCGATGACCGCCTCGGCCGCCGCCACGCGGCGCCGGAACCGGTCGCGGTCGGCCCGCTCGCGCGCCCAGGACCCCCGTCGGGCCAGGCGCGCGGCCGTCTCCCAGGCCACCAGATGGCGCACCTGCACGCGCGGCGAGAAGCACACCTGCGGGCGGGGAGACACGGGGGTCGGAGGGGCGTCAGGGGGTCGGAGGGGCGTCAGGGGGTCGGAGGGGCGTCAGGGGGTCGGAGGGGCGTCAGGGGGTCGGAGGGGCGTCAGGGGGTCGGAGGGGCGTCAGGGGGTCGGAGGGGAGGCGTACCTTCCCGCGCGGCGCGTCCGCGGGCGGGGACGCGGGGGGCCGCCGCCGGCGCAGGCTCAGGCGCGCCAGGTACTCCGTCGTGGTGCGCAGCCGTAGCGCCAGGTGGGGCGGAAGGGGGCGCTGCGGCCCGCGCTCCTTGCGCGGCGGCGGCGGGGGGCAGGCGGCGGCAGGCGCGGCGTGCGGGGCCTCCGGCGCCTTCCCCCCGCCCTCGCTCGGGGGGCTGTTCGCCCACTCTGCGTCGTCGTTGCCGGCGTAGTCCGCGTCGTCGCTGTCGTCCGCCTGGGGCACCAGCAGCCAGCGCCGCAGGAGCGAGGACGCGGCCGGCGCGCTCTCGACCGCGGTTCCCGAGTCGTACGCAGGGACCATTTGGGAGTCTGCGGTTGGGAGCGCGCCGGGGCGCGGCACGGCTGGAGCGCCGGGGCGCGGCACGGCTGGAGCGCCGGGGCGCGGCCGGCGCCGGGGACCCCGGCGGCGGGGACCCCGGCGGCGGGACATGGCGGGCGGCTGGGCTCGGCGTAGGCCCGGAGCCGGAGCGCGTCGGGGCGGGAGAGTTCACTCGGCACGCATGCACGTGTAACCGCCAGTCCGTGCTTGCCTAGCGAACTCACCCGTCCCGGCTGGCGTGCGCAGCCCGGGCCGTGTTGCGGGCCCTCTTAAGGGGCGGCGGCAGGACGGGGACTCCCGCCCCGCCTCTTTTCCCCCGGGGAGTCAACCCCCGGGGGGGGTGTTTTTTGGGGGGGGGCGCGAAGGCGGGCGGCGGCGGCGGGCGGGCGGCAGGGCAGCCCCGCGCGCCCCCTTCCCCGTCCCTCCCCCGGAGCCGGCCGCTCCCCCGCGGGCGCCGCCCCTCCCCCCGCGCGCCGCGGGGCTGCCTTCCCGCGGGCGCCCCCGCGCGGCTTTTTTCCCGCGCCCGCCCCCGCGCGGCAAGACGGGGACTAGCAGGCTGTGCCGCAGACCACCACACACTCCCAAGCTCCCCGCCCCCCCGAAGACGCCAGTCGCACCACCGCTCGCCCTCGCAGACCAGACAGTTGCACCAAGCACCCGCCCGCCCGCACACGGTTCCCCGCCACCCCCTCCCTCCCCTCCATCCCGCCGAGCTCGCGGCAGCCCCTCCCCCCCGCGCGCCACGGGGCTGCGGTCCCGCGGCCGCCTCCCCCGCGGCCGCCTCCCCCGCGCCCCGCCCCGGGGGCTTCCCCCGCCCCTCCCCCCGCGCCCGCGGCCCCGAGCTCGCAGCAGCCCCTCCCTCCCGCGCCCCGTGCCTTCCCTCCCGCTCCTGCGGGGGGGCTCGGGCCACCTGACCTTCGTAACCTGCACTCAGGTCAGAGCCCCAGACCCCCCGCGGGCGCGGGAGACGTGCCGCCCACCCGACCCCCGCCCGCCCGACCCCCGCCCGCCCGACCCCCGCCCGCCCGACCCCCGCCCGCCCGACCCCCGCCCGCCCGACCCCCGCCCGCCCGACCCCCGCCCGCCCGACCCCCGCCCGCCCGACCCCCGCCCGCCCGACCCCCGCCCGCCCGACCCCCGCCCGCCCGACCCCCGCCCGCCCGACCCCCGCCCGCCCGACCCCCGCCCGCCCGACCCCCGCCCGCCCGACCCCCGAATAAACCACACAAGGCGGTACGTTTTCGTCTGTCTCGTTCTTTATTTCTCACACACGCGCGCGGCCATCGCCGCGTCTGTCTTAAAGGCGCACAGACGCCCGATTCCTTCCCCCTCTCCCCATCTCCCCCCTCCCCCGCTCCCGGAAGTTTCCCCCCCGTCACTCCCCAAACAGTCCGTCGTCGTCGTCCTCCAGCTCCGCGTCCATGTCCACGGGCTCGCGCCTCGGCGGCGTGGCCAGCCCCGCGGCGGTCCCCACCACCTCCACGCCGCCGCCCGCCGCGGCCAGCACCGTCCCCGCGCGGCCCGCGGCCGACGCCCAGCGTATCTGCGGGGGCGGGCCCGCGTCCGCGTCGTCGCGCAGCACCAGCGGGGGCGCGTCGCCGTCGGGCTCGAGCAGCGCCCGCGCGCAGAACTCCCGCCGCGGCCCGCGCAGCTCCGCCGGGCCGCCGCGCACGGCGTCGCGCCCCAGCGCCACGTAGACGGGCCGCAGCGGCGCGCCCAGGCCCCAGCGCGCGCAGGCGCGGTGCGAGTGCGCCTCGTCCTCGCAGAAGTCCGGCGCGCCGGGCGCCATGGCGTCGCCCGCGCCCGAGGCGGCGGCCCGGCCGTCCAGCGCCGGGAGCACGGCGCGGCGGTACTCGCGCGGGGACATGGGCACCAGCGTGTCGGGGCCGAAGCGCGTGCGCACGCGGTACCGCACGTTGGCCCCGCGGCAGAGGCGCAGCGGCGGCGCGTCGGGGTACAGGCGCGCGTGCGCGGCCTCCACGCGCGCGAAGACCCCCGGCCCGAACACGCGGCCGGAGGCCAGCACGGTGCGGCGCAGGTCCCGCGCCGCCGGCCAGCGCACGGCGCACTGCACGGCGGGCAGCACCTCGCAGGCCAGGTAGGCGTGCTGCCGCGAGACCACGGGCCCGTCGGCGGGCCAGTCCGCGGCGCGCACGGCGTTGACGACGATGAGGCGGCGGTCGCAGGCGCCGGCCAGCAGCCCCAGGAACTCCACGGCGCCGGCGAAGGCCAGGTCCCGCGTGGACAGCAGCAGCACGCCCTGCGCGCCCAGCGCCGAGACGTCGGGGGCGCCGGTCCAGTTGCCCGCCCAGGCGGCCGTGGCGGGCCCGCAGAGCCGGTTGCCCAGGGCCGCCAGCAGGCAGGACAGCCCGCCGCGCTCGGCGGACCACTCCGGGGGGGGCCCGCCCCCGGCGCGGCCCGCGGCCAGGTCCTCGCCCGGCAGCGGCGAGTAGAGGATCACCACGCGCACGTCCTCCGGGTCGGGCACCTGGCGCATCCAGGCCGCCGCGCGGCGCAGCGGGCCCGAGGCGCGCAGCGGGCCGAAGGCGGCGGGCGCGCCGCCGGGGGGCGGGGCGGCGCAGCGCGCGGCCAGCGAGGCCAGCGCGCGCGGGTCGAACATGAGGGCCGGGCGCCACGGCGCGGGGAAGAGCGGGTGGTCCGTGAGCTCGGCCACGGCCCGCGGGGCGCAGTAGGCCTCCAGGGCGGCGGCCGAGGGCGCCGGCGTGTGGCTGGGCCCCGGCGGCTGGCGGCGCCAGCCGCCCTGCGGGTCGGGGCCCTCGGCGGGCCGGCGGGTCAGCGCCGCGGGGCGCGGCGGCCGCGGCGGCGGCGTCGGCGGGGCGGGGGGCGCGGCCCCCGCGGGAGGGGCGGCCGCGGGGCGGGGGGCGTCCGCGCGGCTCTTCTTCGGGGGGCGCGGGGCGCCGCCCGGCGGCGCCCTGGCCGGGGCGGGGCTCTTGCGCTTGCGCGCCTCCCGCGGCGCGGAGGCGGGCGCGGCGAGCGAGTCGGCCGCGGCGACGGTGTCGGCCAGCAGGGGGCGCAGGCTCTGGTTCTGGAAGAGCAGGTCCGCGGCGGCGGCGGCGGCGGAGCTCAGCAGGCGCGGGCTCCGCGGCAGCGCCGGGCCCAGGGCCCCGGCGACCAGGCTCACGGCGCGCACGGCGGCCACGGCGGCCTCGCTGCCGCCGGCCACGCGCAGGTCCCCGCGCAGGCGCATCAGCACCAGCGCGTCGCGCACGAACCGCAGCTCGCGCAGCCAGGCGCGCAGGCGGGGCGCGTCGGCGTGCGGCGGGGCGGCCGCGCCCGCGGGCCCCGGGCGCGGGGGCGCGGCGGGCCGGGCTCCGGCCAGCCCCGGCACGGCCGCCAGGTCGCCGTCGAAGCCCTCCGCCAGCGCCTCCAGGATCCCGCGGCAGGCGGCCAGGCACTCCACGGCCACGCGGCCCGCCTCCGCGCGCCGGCCGCCGCCACCACCGCCGGCGCCGTCGTCGTCGTCGTCGTCGTCGGCCCCGGCCGGCGCGGAGGCGGGCGCGGCGCTCAGGCGCCCCAGGGCGGCGAGCACCCCCGCGGCGCCGTAGCCGGCGGGCACCGCGCGCTCGTCGGCCGGCGACGCCGCCGCCGACGGCAACGGGGCGGCGGCGGCGGCGGGCTTCCCGCGGGCGTCGTCGCCGTCGTGGCGGTTGGCGTCGCCGCCGTCGTCGGGGGTTCGCGCCCCGGTCAGCGCCGCGTTCTCGCGCGCCAGCAGGGGCGCGTAGGCGCGGCGCAGGCTGGTCAGCAGGAAGCCCTTCTGCGCGCGGTCGTAGCGGCGGCTCATGGCCACGGCGGCCGCCACGTGCGCCAGGCCCCAGCCGAAGCGGCCCGCCGCCATGGCGTACCCCAGGTGGGGCACGGCCCGCGCCACGCTGCCGGAGATGAAGGAGCTGCTGTTGCGCGCCGCGCCCGAGATCCGGAAGCAGGCCTGGTCCAGCGCCACGTCCCCGGGCGCCACGCGCGGGTTCTGGAGCCACCCCATCGCCTCCGCGTCCGGCGTGTACAGCAGCCGCGTGATCAGGGCGTACTGCTGCGCCGCGTCGCCCAGCTCGGGCGCCCACACGGGCGCCGGGGCGCCCGAGGCCTCGAACCGGGCCCGCGCCTCCTCCGCCTCGGGCGCCCCCCAGAGGCCGGGGCGGCTGTCGCCCAGCCCGCCGTACAGCACGCGCCCCGGGGGCGGGGGGCCGGCCCCGGGCCACGGCTCCCCGCTGACGTACCCGTCGCGGTAGCGCGCGTAGAAGGCGCCGGAGGCCGCGTCGGCGTCCAGCTCGACCCGCCGGGGCCGCCCGGCCGTGAAGCGGCCCGTGGCGTCGCGGCCGGCCACCGCCGCGCGGGCCCGGCGGCGCTCCAGGCGGCCCGCGGTCGCCGCGGGGGTCCGGGCCGGGGCGGGCTCGGCCCTGGGCGGGCTCGGCCGGGGCGCCGCCCCCGGGGCCCTCGCGGGCACCCCCGCCTCCTCGTCGTCCGCGCCGAGGGTCCCGCCCGCGGCGTGGTCTCTTCTTCCGCGCCGTCGCGCTCCGGGGGGGGCGACGGGATCGTGCGAACGGCCTCGTCCACCATCGAGGCCAGCAGGGCCAGCTGCCGCGGCGAGACGACGCCGTCCGCGGCAGGCTCGTCGACGGCCTCCCCGGACGCCGGGGCCGCCTCGTCGGCATCGGCATCGGCGGCGGCGTCGTCGGCCTCGTCTTCGTTCTCCTCCGGCCCACCGTGCCACCCGAACCCGGGCCGCGCGGCGGGGCGACGGTCCGGGTTCGGGGTGGGCGGCGGTCCGTCGGCTGGATCCGGAGATCCGGGGCCGCCGGTCGTCTCCGCCGCGGGGGGGGGTGGTTGGCAGTCTCTCTCCCCCCCGTGCGGTGCGTGCGTGTGCCTGTGTCTTTTCGCCTCTCCGCGCCGATCGGGTAGATCCTGGCGGCCGCGTCGGTAGCCGCGCTCCGTGTGGACGATCGCCCCGTCGCCTGGCTGATATAGTCCTCGGGGCGCGCGGGGCGGGGGGAAAGGAGGAGGACGCGGAGGAGGAGCGATCGACGCCGCCGCGCCCCGGCTCGCCGGGGTTCCGCCCCCAGGTGGAACCGCATTATGCGCGGCCCCGCCCCGACGCCCGCGCGTCCGCGTCCGTGGCGGCGGCCCGTTGGTCGCGCCGCCGCCGGCTCCGCCCGCGCGGCATCTCATTAGCGCCCGGCGCGGGCGGCTTCCGCTTCCGCCCGCGATGCTAATGAGACCCTCGTCGCGGGCGGGCTCGCTCCCCTGCCCTTCCGGGTTCGTGGTAATGAGATGCCGGCCCCGCGCTCCCGTTGGCCCCCGCCGGCCCCAAAGGGGCCGGCGAGGTCGCCCCGTTGGTCCGCGGGCGGCTCCGCCCCAAAGGGGGCGGGGCCGCAGGGTAAAAGAAGTGAGAACGCGAAGCGTTCGCACTTCGTCCTAATAGTATATATATTATTAGGGCAAAGTGCGAGCGCTGGCGCCCTGCCCGGGGCCCGCGTCATCCCGCGGGCTCCGCCCCAAAGGGGGCGGGGCCGCAGGGTAAAAGAAGTGAGAACGCGAAGCGTTCGCACTTCGTCCTAATAGTATATATATTATTAGGGCAAAGTGCGAGCACTGGCGCCCTGCCCGGGGCCCGCGTCATCCCGCGGGCTCCGCCCCGAGGCGGGCCCGGACGGGGGGCGGGCCGTTCCTCGCGCACATAAAGGGCCGGCGTCCCGGTCGCCGCCGCACCAGGGGCACACCGGCTGCGCGGCGGAGACCGGGACGGCAGCGGCGGCATCGCGAAGGGGGCCACAGCGAGACAGAGACGCCGGCGGCGAGCGGGGCACCGACGCACCCGGATCGGATCGGATACAGAGACGCGGGCGCATCGGTTCCTTTTCGTTCTGCCTTTCCCTCCCCCCCCCCCCCCCACCCTGTACGTACCGCGAGGACCCATCCACCCACTGCAGCCTTATCGCAGGTACGGTGACCCGGGGGGCCGGGCCGGGGGGACGGGCCGGGGGGACGGGCCGGGGGGACGGGCCGGGGGGACGGGCCGGGGGGACGGGCCGGGGGGACGGGCCGGGGGGACGGGCCGGGGGGACGGGCCGGGGGGCCGGGGGGCCGGGGGGCCGGGGGGACGGGGGGACGGGCCGGGGGGACGGGCCGGGGGGCCGGGGGGACGGGGGGACGGGGGGACGGGGGGACGGGGGGACGGGGGGACGGGGGGACGGGGGGACGGGGGGACGGGGGGACGGGGGGACGGGGGGACGGGGGGACGGGGGGACGGGGGGACGGGCCGGGGGGACGGGGGGACGGGCCGGGGGGACGGGGGGACGGGGCCCCGATCCCAACATCCGCGCTTTCTCGCAGGCCGGGCGCCGCCTTCGTGGACGGGACACCGGTGTGGTAACTGGCGACAAGGCGTTGCCACTATGGCAGACATCCCCCCGGACCCGCCCGCGCTCAACACGACGCCTGCGAATCATGCTCCCCCATCCCCACCCCCGGGTTCACGGAAGCGCAGACGCCCCGTCCTCCCCAGCTCGTCGGAATCTGAGGGTAAGCCCGACACAGAATCGGAATCCTCCTCGACCGAGTCGTCCGAGGATGAGGCGGGAGACCTACGCGGCGGGCGCCGTCGCTCCCCGCGGGAGCTCGGGGGGAGGTATTTTTTGGATCTGTCGGCAGAATCGACCACGGGGACGGAATCGGAGGGAACGGGGCCGTCGGACGACGATGATGATGATGCGTCAGACGGCTGGTTGGTTGACACACCCCCCCGCAAATCCAAGCGACCCCGAATCAACCTGCGATTAACGAGCTCCCCCGACCGGCGTGCGGGTGTGGTTTTCCCCGAGGTGTGGAGAAGCGACAGACCTATCCGCGCGGCGCAACCCCAGGCCCCGGCCCAGTCTTCCGGGGATCGCGCAGCCGCACCGCGGCGCTCTGCTCGCCAGGCCCAGATGCGGAGCGGAGCCGCCTGGACGCTTGATCTGCATTACATACGCCAGTGCGTCAACCAGCTCTTTCGGATCCTGCGTGCCGCCCCGAACCCGCCCGGCAGCGCCAACCGCCTGCGCCACCTGGTGCGAGACTGCTACCTCATGGGCTACTGCCGGACCCGCCTGGGGCCGCGCACGTGGGGCCGCCTGCTGCAGATCTCGGGCGGAACCTGGGACGTGCGCCTGCGAAACGCAATCCGGGAGGTCGAGGCGCATTTTGAACCCGCCGCCGAGCCCGTGTGCGAGCTGCCCTGTCTGAACGCCAGGCGTTACGGCCCCGAGTGTGATGTTGGCAATCTCGAGACCAACGGCGGCTCGACGAGCGATGATGAGATATCGGATGCGACGGACTCGGACGATACCCTCGCGTCCCATTCCGACACGGAGGGGGGGCCCTCCCCGGCCGGCCGGGAGAACCCGGAATCCGCGTCCGGCGGGGCTATCGCGGCTCGGCTGGAGTGTGAGTTTGGGACGTTTGACTGGACGTCCGAGGAGGGCTCCCAGCCCTGGCTGTCCGCGGTGGTCGCCGATACCAGCTCCGCCGAACGCTCTGGCCTACCCGCCCCGGGCGCGTGTCGCGCAACGGAAGCCCCAGAACGCGAGGACGGGTGCCGAAAAATGCGCTTCCCCGCCGCCTGCCCCTATCCCTGCGGCCACACATTTCTCCGGCCATGAGCGCGGGACCCCCAGCCCGGTGTGTTTGCCAAACGAAAAATAAACGCCCTACAAGAAAGCTTTTGTGTCTGAGTGTCTGGTTTTTCTGGGGGTGGAGGAAGGAACGACAAAAAAAAGAAACAAACGCGACACCGCTCGTACGTGTAATGGGGCGCAGTGTTTTTTATTAGCATCGGGGGGGGTTAGAGGTTGGTGATTGGATAGCAAACGTGGGATGACGGAGGCCACTCGTCGCCAACGGCCAGCGGGGGCCCGGGGTTCTGGGGGTCATCGTCCCCCGTCTGCCAGGAGGGCTCATCGGGAATCTCGGGTCGCCCCATGCACGTAAAACACGGGCGCTGCGTGGGGTGGGTCGCCGGATGCGGGCGGGATGATGCGGGGCGGGGTTTGTTGTGAGGAGCCACGAGGGACCGTAGCCAGCGAAGACAGCTGCGTTCCCGGTCGCCGGGCACCACCACGCCGTATTGGTATTCGTATCGGCTAAGGAGATTTTCCAGGGGGTGATTAGGCGCTGCGGGGAACGGGGTCCACGACACGGTCCGCTCGGGCAAAAACCGATCGGGCAGGGGCCACGGTTCCCCCACCCACGCGTCGTTGGTCTTCGTGGCGATGAAGCGAAACCCCAGCCGGGTTTTTTGTGCGTACTCGAAAAACGGCACACACAGGTCCGCCGCCCCGACCACCCACAGGTGGTATAGCCGGTGGGGGCCGGGGCGCTCTTGATGCAGGAGCCGAAAACACGCAGGGGCATCCAGAATCTCGATGCTTTCCAGGGGGTCGTCCTCCGCAAACAGGCCCGTCGTGGTGTTTGGGGGACAGCGACAGGAGCGGGTTCGCACGATCGGTCGGGTGAATTTGGGCAAGTCCATCAGAGGCTCGGCCAGCCTGCGAAGGTTCGCCGGGCGAACCACCACCGGGGTTCCCAGAGGCTCGGAGGCCAGGATCCGGCATTGCCGAAGCAGAAAACTCCACAGAGCCGGGCTTGCGTCAGCGGAAGTCCGCGGCAGGGCGTTTCGTTGGTCTAGGAGGGTAACCACACTTACAACAACAACGCCCATGTCGGTATATTAGGCCCGTGGTCCGATCTTCACTCACTCGCCTGTCTGCGGACCTATGCACGGCGGGACGGCGCGCGGACCCGGGGGGGCTGCTTGCTATCACACGGCCCGTTCGCACGTTCGATTTTTTCAGCCTTGTTTGGTTGGCTAGGTATCCCGGATAATCTGACGTTCCGGATATAGGGGGCGGGGGGTAGTGGGGGGTGTGTCGACAAACTGCCGCTTCTTAAAACACCGGGGCCCGTCGCTCGGGGTGCTCGTTGGTTGGCACGCGCGACGCGGCAAATGGCCTGTCGTAAGTTCTGTGGGGTCTACCGTAGACCCGACAAGAGACAGGAGGCGTCCGTCCCGCCGGAGACAAACACGGCCCCGGCCTTCCCGGCGAGCACCTTTTATACCCCCGCGGAGGATGCGTACCTGGCCCCCGGGCCCCCGGAAACCATCCACCCTTCCCGCCCACCGTCCCCCGGCGAGGCTGCGCGCCTGTGTCAGCTGCAGGAGATCTTGGCCCAGATGCACAGCGACGAGGACTACCCCATCGTGGACGCCGCGGGTGCGGAGGAGGAAGACGAGGCCGACGATGACGCCCCGGATGACGTGGCCTACCCGGAGGACTACGCGGAGGGGCGTTTTCTGTCCATGGTTTCGGCCGCCCCCCTGCCCGGAGCCAGCGGCCATCCTCCTGTTCCGGGCCGCGCAGCCCCCCCCGACGTCCGGACCTGCGACAGCGGTAAGGTGGGGGCCACGGGGTTCACCCCGGAAGAGCTCGACACCATGGACCGGGAGGCACTTCGGGCCATCAGCCGCGGGTGCAAGCCCCCTTCGACCCTGGCAAAACTGGTGACCGGGCTGGGATTCGCGATCCACGGAGCGCTCATCCCGGGGTCGGAGGGGTGTGTCTTTGATAGCAGCCACCCGAACTACCCTCATCGGGTAATCGTCAAGGCGGGGTGGTACGCCAGCACGAGCCACGAGGCGCGGCTGCTGAGACGCCTGAACCACCCCGCGATCCTACCCCTCCTGGACCTGCACGTCGTTTCTGGGGTCACGTGTCTGGTCCTCCCCAAGTATCACTGCGACCTGTATACCTATCTGAGCAAGCGCCCGTCTCCGTTGGGCCACCTACAGATAACCGCGGTCTCCCGGCAGCTCTTGAGCGCCATCGACTACGTCCACTGCGAAGGCATCATCCACCGCGATATTAAGACCGAGAACATCTTCATCAACACCCCCGAGAACATCTGTCTGGGGGACTTTGGGGCGGCGTGCTTTGTGCGCGGGTGTCGATCGAGCCCCTTCCATTACGGGATCGCAGGCACCATCGATACAAACGCCCCCGAGGTCCTGGCCGGGGATCCGTACACCCAGGTAATCGACATCTGGAGCGCCGGCCTGGTGATCTTTGAGACCGCCGTCCACACCGCGTCCTTGTTCTCGGCCCCGCGCGACCCCGAAAGGCGGCCGTGCGACAACCAGATCGCGCGCATCATCCGACAGGCCCAGGTACACGTCGACGAGTTTCCGACGCACGCGGAATCGCGCCTCACCGCGCACTACCGCTCGCGGGCGGCCGGGAACAATCGTCCGGCGTGGACCCGACCGGCGTGGACCCGCTACTACAAGATCCACACAGACGTCGAATATCTCATATGCAAAGCCCTTACCTTTGACGCGGCGCTCCGCCCAAGCGCCGCGGAGTTGCTGCGCCTGCCGCTATTTCACCCTAAGTGACCCCGCTCCCCCCGGGGGGCGTGGAGGGGGGGGCTGGTTGGATGTTTTTGCACAAAAAGACGCGGCCCTCGGGCTTTGGTGTTTTTGGCACCTTGCCGCCCGGCGTCATGCACGCCATCGCTCCCAGGTTGCTTCTTCTTTTTGTTCTTTCTGGTCTTCCGGGGACACGCGGCGGGTCGGGTGTCCCCGGACCAATTAATCCCCCCAACAGCGATGTTGTTTTCCCGGGAGGTTCCCCCGTGGCTCAATATTGTTATGCCTATCCCCGGTTGGACGATCCCGGGCCCTTGGGTTCCGCGGACGCCGGGCGGCAAGACCTGCCCCGGCGCGTCGTCCGTCACGAGCCCCTGGGCCGCTCGTTCCTCACGGGGGGGCTGGTTTTGCTGGCGCCGCCGGTACGCGGATTTGGCGCACCCAACGCAACGTATGCGGCCCGTGTGACGTACTACCGGCTCACCCGCGCCTGCCGTCAGCCCATCCTCCTTCGGCAGTATGGAGGGTGTCGCGGCGGCGAGCCGCCGTCCCCAAAGACGTGCGGGTCGTACACGTACACGTACCAGGGCGGCGGGCCTCCGACCCGGTACGCTCTCGTAAATGCTTCCCTGCTGGTGCCGATCTGGGACCGCGCCGCGGAGACATTCGAGTACCAGATCGAACTCGGCGGCGAGCTGCACGTGGGTCTGTTGTGGGTAGAGGTGGGCGGGGAGGGCCCCGGCCCCACCGCCCCCCCACAGGCGGCGCGTGCGGAGGGCGGCCCGTGCGTCCCCCCGGTCCCCGCGGGCCGCCCGTGGCGCTCGGTGCCCCCGGTATGGTATTCCGCCCCCAACCCCGGGTTTCGTGGCCTGCGTTTCCGGGAGCGCTGTCTGCCCCCACAGACGCCCGCCGCCCCCAGCGACCTACCACGCGTCGCTTTTGCTCCCCAGAGCCTGCTGGTGGGGATTACGGGCCGCACGTTTATTCGGATGGCACGACCCACGGAAGACGTCGGGGTCCTGCCGCCCCATTGGGCCCCCGGGGCCCTAGATGACGGTCCGTACGCCCCCTTCCCACCCCGCCCGCGGTTTCGACGCGCCCTGCGGACAGACCCCGAGGGGGTCGACCCCGACGTTCGGGCCCCCCGAACCGGGCGGCGCCTCATGGCCTTGACCGAGGACACGTCCTCCGATTCGCCTACGTCCGCTCCGGAGAAGACGCCCCTCCCTGTGTCGGCCACCGCCATGGCACCCTCAGTCGACCCAAGCGCGGAACCGACCGCCCCCGCAACCACTACTCCCCCCGACGAGATGGCCACACAAGCCGCAACGGTCGCCGTTACGCCGGAGGAAACGGCAGTCGCCTCCCCGCCCGCGACTGCATCCGTGGAGTCGTCGCCACTCCCCGCCGCGGCGGCGGCAACGCCCGGGGCCGGGCACACGAACACCAGCAGCGCCTCCGCAGCGAAAACGCCCCCCACCACACCAGCCCCCACGACCCCCCCGCCCACGTCTACCCACGCGACCCCCCGCCCCACGACTCCGGGGCCCCAAACAACCCCTCCCGGACCCGCAACCCCGGGTCCGGTGGGCGCCTCCGCCGCGCCCACGGCCGATTCCCCCCTCACCGCCTCGCCCCCCGCTACCGCGCCGGGGCCCTCGGCCGCCAACGTTTCGGTCGCCGCGACCACCGCCACGCCCGGAACCCGGGGCACCGCCCGTACCCCCCCAACGGACCCAAAGACGCACCCACACGGACCCGCGGACGCTCCCCCCGGCTCGCCAGCCCCCCCACCCCCCGAACATCGCGGCGGACCCGAGGAGTTTGAGGGCGCCGGGGACGGCGAACCCCCCGAGGACGACGACAGCGCCACCGGCCTCGCCTTCCGAACTCCGAACCCCAACAAACCACCCCCCGCGCGCCCCGGGCCCATCCGCCCCACGCTCCCGCCAGGAATTCTTGGGCCGCTCGCCCCCAACACGCCTCGCCCCCCCGCCCAAGCTCCCGCTAAGGACATGCCCTCGGGCCCCACACCCCAACACATCCCCCTGTTCTGGTTCCTAACGGCCTCCCCTGCTCTAGATATCCTCTTTATCATCAGCACCACCATCCACACGGCGGCGTTCGTTTGTCTGGTCGCCTTGGCAGCACAACTTTGGCGCGGCCGGGCGGGGCGCAGGCGATACGCGCACCCGAGCGTGCGTTACGTATGTCTGCCACCCGAGCGGGATTAGGGGGTGGGGGTGGGGGGCGAGAAACGATGAAGGACGGGAAAGGGAACAGCGACCAAATGTCACGATAAGAACAATAAACCTGTGACGTCAATCAGATATGTGAGTTTGGTTGTGTTTTGTGGGACTGGGGGCGGGGGGTGGGAGGTATCAGTGGGTGACAGAGTCTTTTAAAAGACGTGTCCCGGGGCCCTCGAGACGCGCAACTTTTGGCCACACAGAGAAAGGCCCCCAGACGAAGTCACCCGGGTCCCCGAACAAAAACAAAAACCTTGACCGCCGCCGGGGGGCGTGCCTGTTGTTTTGGTCTCAATGGATCGGTATGCCGTTCGGACCTGGGGGATTGTGGGAATCCTCGGGTGTGCTGCTGTTGGGGCCGCACCCACCGGCCCCGCGTCCGATACAACAAACGCGACCGCACGCCTCCCCACGCACCCCCCACTCATCCGTTCCGGGGGCTTTGCCGTCCCCCTCATCGTGGGGGGGCTGTGTCTCATGATTCTGGGGATGGCGTGTCTACTCGAGGTCCTGCGTCGCCTGGGTCGCGAGTTGGCGAGGTGCTGCCCCCACGCGGGCCAATTTGCCCCATGATTTTTCGCCTTTCTGGCCTTGCCCCCACCCCATCGCCCCGATTGTGTGTCGGGTGCCCGGGGTACAGCAGCTATGGAGCGGTCGGTAATATAACTTTGGTTGTCGCCACACGCCCCGTGCCGGGCATGGGTTGTGCGGGAAGGACGAAATAATCCGGCGATCCCCAAGCGTACCAACTGGGGGGGGGGGGGGGAAAAGAAACTAAAAACACATCAAGCCCACAACCCATCCCACAATGGGGGTTATGGCGGACCCACCGCACCACCATACTCCGATTCGACCACATATGCAACCAAATCACCCCCAGAGGGGAGGTTCCATTTTTACGAGGAGGAGGAGTATAATAGAGTCTTTGTGTTTAAAACCCGGGGTCGGTGTGGTGTTCGGTCATAAGCTGCATTGCGAACGACTAGTCGCCGTTTTTCGTGTGCATCGCGTATCACGGCATGGGGCGTTTGACCTCCGGCGTCGGGACGGCGGCCCTGCTAGTTGTCGCGGTGGGACTCCGCGTCGTCTGCGCCAAATACGCCTTAGCAGACCCCTCGCTTAAGATGGCCGATCCCAATCGATTTCGCGGGAAGAACCTTCCGGTTTTGGACCAGCTGACCGACCCCCCCGGGGTGAAGCGTGTTTACCACATTCAGCCGAGCCTGGAGGACCCGTTCCAGCCCCCCAGCATCCCGATCACTGTGTACTACGCAGTGCTGGAACGTGCCTGCCGCAGCGTGCTCCTACATGCCCCATCGGAGGCCCCCCAGATCGTGCGCGGGGCTTCGGACGAGGCCCGAAAGCACACGTACAACCTGACCATCGCCTGGTATCGCATGGGAGACAATTGCGCTATCCCCATCACGGTTATGGAATACACCGAGTGCCCCTACAACAAGTCGTTGGGGGTCTGCCCCATCCGAACGCAGCCCCGCTGGAGCTACTATGACAGCTTTAGCGCCGTCAGCGAGGATAACCTGGGATTCCTGATGCACGCCCCCGCCTTCGAGACCGCGGGTACGTACCTGCGGCTAGTGAAGATAAACGACTGGACGGAGATCACACAATTTATCCTGGAGCACCGGGCCCGCGCCTCCTGCAAGTACGCTCTCCCCCTGCGCATCCCCCCGGCAGCGTGCCTCACCTCGAAGGCCTACCAACAGGGCGTGACGGTCGACAGCATCGGGATGCTACCCCGCTTTATCCCCGAAAACCAGCGCACCGTCGCCCTATACAGCTTAAAAATCGCCGGGTGGCACGGCCCCAAGCCCCCGTACACCAGCACCCTGCTGCCGCCGGAGCTGTCCGACACCACCAACGCCACGCAACCCGAACTCGTTCCGGAAGACCCCGAGGACTCGGCCCTCTTAGAGGATCCCGCCGGGACGGTGTCTTCGCAGATCCCCCCAAACTGGCACATCCCGTCGATCCAGGACGTCGCGCCGCACCACGCCCCCGCCGCCCCCAGCAACCCGGGCCTGATCATCGGCGCGCTGGCCGGCAGTACCCTGGCGGTGCTGGTCATCGGCGGTATTGCGTTTTGGGTACGCCGCCGCGCTCAGATGGCCCCCAAGCGCCTACGTCTCCCCCACATCCGGGATGACGACGCGCCCCCCTCGCACCAGCCATTGTTTTACTAGAGGAGTTTCCCCGCTCCCGTGTACCTCTGGGCCCGTGTGGGAGGGTGGCTGGGGTATTTGGGTGGGACTTGGACTCCGCATAAAGGGAGTCTCGAAGGAGGGAAACTAGGACAGTTCATAGGCCGGGAGCGTGGGGCGCGCACCGCTGTCCCGACGATTAGCCACCGCGCCCACAGCCACCTCGACCCGTCCGATCCCGGTATGCCCGGCCGCTCGCTGCAGGGCCTGGCGATCCTGGGCCTGTGGGTCTGCGCCACCGGCCTGGTCGTCCGCGGCCCCACGGTCAGTCTGGTCTCAGACTCACTCGTGGATGCCGGGGCCGTGGGGCCCCAGGGCTTCGTGGAAGAGGACCTGCGTGTTTTCGGGGAGCTTCATTTTGTGGGGGCCCAGGTCCCCCACACAAACTACTACGACGGCATCATCGAGCTGTTTCACTACCCCCTGGGGAACCACTGCCCCCGCGTTGTACACGTGGTCACACTGACCGCATGCCCCCGCCGCCCCGCCGTGGCGTTCACCTTGTGTCGCTCGACGCACCACGCCCACAGCCCCGCCTATCCGACCCTGGAGCTGGGTCTGGCGCGGCAGCCGCTTCTGCGGGTTCGAACGGCAACGCGCGACTATGCCGGTCTGTATGTCCTGCGCGTATGGGTCGGCAGCGCGACGAACGCCAGCCGGTTTGTTTTGGGGGTGGCGCTCTCTGCCAACGGGACGTTTGTGTATAACGGCTCGGACTACGGCTCCTGCGATCCGGCGCAGCTTCCCTTTTCGGCCCCGCGCCTGGGACCCTCGAGCGTATACACCCCCGGAGCCTCCCGACCCACCCCTCCACGGACAACGACACCCCCGTCCTCCCCCCGAGACCCGACCCCCGCCCCCGGGGACACAGGGACGCCCGCGCCCGCGAGCGGCGAGATAGCCCCGCCCAATTCCACGCGATCGGCCAGCGAATCGAGACACAGGCTAACCGTAGCCCAGGTAATCCAGATCGCCATACCGGCGTCCATCATCGCCTTTGTGTTTCTGGGCAGCTGTATCTGCTTCATCCATAGATGCCAGCGCCGATACAGGCGCCCCCGCGGCCAGATTTACAACCCCGGGGGCGTTTCCTGCGCGGTCAACGAGGCGGCCATGGCCCGCCTCGGAGCCGAGCTGCGATCCCACCCAAACACCCCCCCCAAACCCCGACGCCGTTCGTCGTCGTCCACGACCATGCCTTCCCTAACGTCGATAGCTGAGGAATCGGAGCCAGGTCCAGTCGTGCTGCTGTCCGTCAGTCCTCGGCCCCGCAGTGGCCCGACGGCCCCCCAAGAGGTCTAGGTCCAAGCGGGCCGTTCGGCAGGCCCGCCCCACCGCCCCCATCGTGGTTATTTCCCCCCCCCCCCAATAAACCGATGTTATTTGCCTATATGCGTGTGTTGGATCCCTTTGTGATCGTTCGTCATTCCCCGGATGGCATGGGAGGCGGGTAATGGATGGGCGGGGCCCGGGGGGGAGGAAAAAGAATAAAGGGGGTAGTGTCGGAGAGGCCCGCCGCGCATTTAAGGAGTCGCCGCCCCGACTCTGTGTCTTCGGGTGACTTGGTGCGCCGCCGTCAGCTAGTCTCCGATCTGCCCCGACCGACGGCTCCTGCCACCCGAACATGGCTCGCGGGGCCGGGTTGGTGTTTTTTGTTGGAGTTTGGGTCGTATCGTGCCTGGCGGCAGCACCCAGAACGTCCTGGAAACGGGTAACCTCGGGCGAGGACGTGGTGTTGCTTCCGGCGCCCGCGGAACGCACCCGGGCCCACAAACTACTGTGGGCCGCGGAACCCCTGGATGCCTGCGGTCCCCTGCGCCCGTCGTGGGTGGCGCTGTGGCCCCCCCGACGGGTGCTCGAGACGGTCGTGGATGCGGCGTGCATGCGCGCCCCGGAACCGCTCGCCATAGCATACAGTCCCCCGTTCCCCGCGGGCGACGAGGGACTGTATTCGGAGTTGGCGTGGCGCGATCGCGTAGCCGTGGTCAACGAGAGTCTGGTCATCTACGGGGCCCTGGAGACGGACAGCGGTCTGTACACCCTGTCCGTGGTCGGCCTAAGCGACGAGGCGCGCCAAGTGGCGTCGGTGGTTCTGGTCGTGGAGCCCGCCCCTGTGCCGACCCCGACCCCCGACGACTACGACGAAGAAGACGACGCGGGCGTGAGCGAACGCACGCCGGTCAGCGTTCCCCCCCCAACCCCCCCCCGTCGTCCCCCCGTCGCCCCCCCGACGCACCCTCGTGTTATCCCCGAGGTGTCCCACGTGCGCGGGGTAACGGTCCATATGGAGACCCCGGAGGCCATTCTGTTTGCCCCCGGGGAGACGTTTGGGACGAACGTCTCCATCCACGCCATTGCCCACGACGACGGTCCGTACGCCATGGACGTCGTCTGGATGCGGTTTGACGTGCCGTCCTCGTGCGCCGAGATGCGGATCTACGAAGCTTGTCTGTATCACCCGCAGCTTCCAGAGTGTCTATCTCCGGCCGACGCGCCGTGCGCCGTAAGTTCCTGGGCGTACCGCCTGGCGGTCCGCAGCTACGCCGGCTGTTCCAGGACTACGCCCCCGCCGCGATGTTTTGCCGAGGCTCGCATGGAACCGGTCCCGGGGTTGGCGTGGCTGGCCTCCACCGTCAATCTGGAATTCCAGCACGCCTCCCCCCAGCACGCCGGCCTCTACCTGTGCGTGGTGTACGTGGACGATCATATCCACGCCTGGGGCCACATGACCATCAGCACCGCGGCGCAGTACCGGAACGCGGTGGTGGAACAGCACCTCCCCCAGCGCCAGCCCGAGCCCGTCGAGCCCACCCGCCCGCACGTGAGAGCCCCCCATCCCGCGCCCTCCGCGCGCGGCCCGCTGCGCCTCGGGGCGGTGCTGGGGGCGGCCCTGTTGCTGGCCGCCCTCGGGCTGTCCGCGTGGGCGTGCATGACCTGCTGGCGCAGGCGCTCCTGGCGGGCGGTTAAAAGCCGGGCCTCGGCGACGGGCCCCACTTACATTCGCGTGGCGGACAGCGAGCTGTACGCGGACTGGAGTTCGGACAGCGAGGGGGAGCGCGACGGGTCCCTGTGGCAGGACCCTCCGGAGAGACCCGACTCTCCCTCCACAAATGGATCCGGCTTTGAGATCTTATCACCAACGGCTCCGTCTGTATACCCCCATAGCGAGGGGCGTAAATCTCGCCGCCCGCTCACCACCTTTGGTTCGGGAAGCCCGGGCCGTCGTCACTCCCAGGCCTCCTATCCGTCCGTCCTCTGGTAAGGCGTCTTCCGACGACGCGGACGTCGGCGATGAACTGATTGCCATCGCGGACGCACGCGGGGACCCGCCAGAGACCCTGCCCCCCGGCGCGGGCGGCGCCGCGCCCGCGTGCCGCAGACCACCTCGCGGCGGCTCCCCCGCGGCCTTTCCCGTGGCCCTCCACGCCGTGGACGCCCCCTCCCAATTCGTCACCTGGCTCGCCGTGCGCTGGCTGCGGGGGGCGGTGGGTCTCGGGGCCGTCCTGTGCGGGATTGCGTTTTACGTGACGTCAATCGCCCGAGGCGCATAAAGGTCCGGCGGCCAGCCCCGCCGCAGCTCATAAAAATCGTGAGTCACGGCAACCGCACCTTCGCCTCCGGCCCTCCGCCAGCGCCCTTCCGCGTCCGCGATGACCTCCCGGCCCGCCGACCAAGACTCGGTGCGTTCCAGCGCGTCGGTGCCGCTTTACCCCGCGGCCTCGCCCGTCCCGGCAGAAGCCTACTACTCGGAAAGCGAAGACGAGGCCGCCAACGACTTCCTCGTGCGCATGGGCCGCCAGCAGTCGGTCCTAAGGCGCCGACGGCGGCGCACGCGGTGCGTCGGGCTGGTTATCGCCTGTCTCGTCGTGGCCCTCCTATCTGGAGGGTTCGGGGCACTTTTGGTGTGGCTGCTCCGCTAAATGACGCCTCGATGTATGGCGCCTTCTTCGCCCCCACCCCTCGCCGCGACCCACGTCCGTATGTTAATTGCAATAAAGTGGTTGATTGTCATTACGGTCTACTAGGTTGTCTTTTTTTTTTGGGGGGGGGGGAGGAAATGCAGAAAAGGGTAAGAAATTCTCGGAATTTCACCCCCGGGGGGGGGCAAGTGCAGTAACCCAGTTCCTCAGTGTTTGGGAAATCTATTGAACTCTCCCGGCTCCTCCGTGTTAGGGAAGTCTCTTGGGGAAATCTATTGACCTCTCGCCCCCCCCCCCCAGGAGGGGGGCAGTGCAGTACCCCAGTTCCTCCGTGCTGGGGAAATCTCTCTGCCGGGTACGGGCTCCAGACGAAGGACCCATACATTTCCCCATCCGCACCCCACATCTGGCGTTCTAGAGTCACGACGCATTTGCCCCCGTCCCCGCAGCAACACACAAAGCGATTTCAATTTTCACGATTTTATTATTAATTACACCAACCACCCTGTCCCCGGGACGTGGTCAGGACCGGGGGTCCGCACCCAAACGCACGAAACAAATGCTGGCAGTGTGCCGAATATAACCCCGCGTAGGAACACGTCGACGCGTGCGCCAAACAGCACCAGAAGGCGCATGCCATCAGCAGGTCGTGCATATGGCGATGTGTTTGGACGCAGGGCGCAGCCGCGGCGATAAAATTCATGGCGGCCGTCCGCCAGGGCCACAGCGGCGAGGACTCCCTGTTGGCCCGAAGCCATTGGGTATGAACCAGCTGCGCCTCCTGTCCGACCCTGGCTCCCGCCAGCGGGGGCGGTGGGTCGTGGGTGTTGAGAGCACACAGGCGGGACACCTCGATCACCGTCCGAAAAAAGGCCCGGTGGTCCGCGGGCAGCATCTGCAGGTGCGCCAGGGCCTGGGCGTTGAGAGGGTACAACTCGGAGCCGGGGGACTCCGGGGGCCGGTCCGCGCGGTGCCGCGAGTTGGCACGCTTTGGGGCCCGGGTGTCGGACGCGGGCGCGTTATGGATCCCGACGCGGGGCAGAACGTACGTGCGTTGGCGCGGCGATGAGGGGTCCGGGCTGCCGAGGGGGGCGTAGGGGACCGGGCTAGGCAAGCCCGCGGGTTGCGCGGGGTTCCCGTGGGGGTCTAGGCTCCCTGGGCACCCGTGGGGGTCGTGGGGGTCGCGGGTCCCTGGGTATGCGCGGGACCCTGGGTTCTCTGGGAGATCGTGGAACTCGCGGTTCCCTGGGCTCTCGGGGAACCCGGGGCTCCCTGGGGACACGTGGTGCCCTGGGAATTCTTGATGGTCGGACGGCTTCAGATGGCTTCGGGATCGAGAGGGCCGCACAGACTCGTAGTAGACCCGAATCTCCACGTTTCCCCGCCGCCGGATCATGGTCGCCGCCCCGGTGCGGGGGCCCGTCGGTCGGAAGCGAGTGCCCTTCAAGCGTGTCCGCTCCTCTGGGCTGCATGCCGTCGGATGGGGTGCCTTTTAAGGAAAGGTCTCGGCTGCCCGCCCCAACCGGGGTTTGGGGGTGGGCCGGGGAAACCCCGGATGCCATGGGGGGGTCACACCCTAAGCGCCGGCGCGCTGGTTGGGTGGGGGTAGAGGGGAGTCCCCGGTCGACGAGATCGTATCAAGGGGCCAGCACGCGATCCTGCCGCTCGTTCGATCTAGCACACCCACGGGTCTGCTGTGTGGGATTTCGACTCGCGGGATCCGATCGCACGTCCGGAGGACACAGCAGCGGGAGCTCCGGGTCGGTCACCGCAGTTCTGGCCGCCTCTCGGTCCTCCCGTTCCCTTTTATGGATCTCCGCGCAGACATCGCCATACGTCCGGTGTGTGCACCGCGAAGAATCCAGAAACATGTCCGTCGTTTTCAGGGCCCAAGACATGGTGTCCCGTCCACGAAGGCGGCGCCCGGCCTGCGAGAAAGCGCGGATGTTGGGATCGGGGCCCCGTCCCCCCGTCCCCCCGGCCCGTCCCCCCGTCCCCCCGGCCCGTCCCCCCGTCCCCCCGTCCCCCCGTCCCCCCGTCCCCCCGTCCCCCCGTCCCCCCGTCCCCCCGTCCCCCCGTCCCCCCGTCCCCCCGTCCCCCCGTCCCCCCGTCCCCCCGTCCCCCCGGCCCCCCGGCCCGTCCCCCCGGCCCGTCCCCCCGTCCCCCCGGCCCCCCGGCCCCCCGGCCCCCCGGCCCGTCCCCCCGGCCCGTCCCCCCGGCCCGTCCCCCCGGCCCGTCCCCCCGGCCCGTCCCCCCGGCCCGTCCCCCCGGCCCGTCCCCCCGGCCCGTCCCCCCGGCCCGGCCCCCCGGGTCACCGTACCTGCGATAAGGCTGCAGTGGGTGGATGGGTCCTCGCGGTACGTACAGGGTGGGGGGGGGGGGGGGAGGGAAAGGCAGAACGAAAAGGAACCGATGCGCCCGCGTCTCTGTATCCGATCCGATCCGGGTGCGTCGGTGCCCCGCTCGCCGCCGGCGTCTCTGTCTCGCTGTGGCCCCCTTCGCGATGCCGCCGCTGCCGTCCCGGTCTCCGCCGCGCAGCCGGTGTGCCCCTGGTGCGGCGGCGACCGGGACGCCGGCCCTTTATGTGCGCGAGGAACGGCCCGCCCCCCGTCCGGGCCCGCCTCGGGGCGGAGCCCGCGGGATGACGCGGGCCCCGGGCAGGGCGCCAGTGCTCGCACTTTGCCCTAATAATATATATACTATTAGGACGAAGTGCGAACGCTTCGCGTTCTCACTTCTTTTACCCTGCGGCCCCGCCCCCTTTGGGGCGGAGCCCGCGGGATGACGCGGGCCCCGGGCAGGGCGCCAGCGCTCGCACTTTGCCCTAATAATATATATACTATTAGGACGAAGTGCGAACGCTTCGCGTTCTCACTTCTTTTACCCTGCGGCCCCGCCCCCTTTGGGGCGGAGCCGCCCGCGGACCAACGGGGCGACCTCGCCGGCCCCTTTGGGGCCGGCGGGGGCCAACGGGAGCGCGGGGCCGGCATCTCATTACCACGAACCCGGAAGGGCAGGGGAGCGAGCCCGCCCGCGACGAGGGTCTCATTAGCATCGCGGGCGGAAGCGGAAGCCGCCCGCGCCGGGCGCTAATGAGATGCCGCGCGGGCGGAGCCGGCGGCGGCGCGACCAACGGGCCGCCGCCACGGACGCGGACGCGCGGGCGTCGGGGCGGGGCCGCGCATAATGCGGTTCCACCTGGGGGCGGAACCCCGGCGAGCCGGGGCGCGGCGGCGTCGATCGCTCCTCCTCCGCGTCCTCCTCCTTTCCCCCCGCCCCGCGCGCCCCGAGGACTATATCAGCCAGGCGACGGGGCGATCGTCCACACGGAGCGCGGCTACCGACGCGGCCGCCAGGATCTACCCGATCGGCGCGGAGAGGCGAAAAGACACAGGCACACGCACGCACCGCACGGGGGGGAGAGAGACTGCCAACCACCCCCCCCCACTGCCGCCCCTGAAGAAGGCCCCGGATCTCCGGATCCAGCCGACGGACCGCCGCCCACCCCGAACCCGGACCGTCGCCCCGCCGCGCGGCCCGGGTTCGGGTGGCACGGTGGGCCGGAGGAGAACGAAGACGAGGCCGACGACGCCGCCGCCGATGCCGATGCCGACGAGGCGGCCCCGGCGTCCGGGGAGGCCGTCGACGAGCCTGCCGCGGACGGCGTCGTCTCGCCGCGGCAGCTGGCCCTGCTGGCCTCGATGGTGGACGAGGCCGTTCGCACGATCCCGTCGCCCCCCCCGGAGCGCGACGGCGCGGAAGAAGAAGCGGCCCGCTCGCCTTCTCGACGCGGGCCGCTGGGTCCGCGGACCGGAGACGACGTCCGCGGTCCGCGGGGCGTACCCGGACCCCATGGCCAGCCTGTCCCTCGGCGCGGACGACGAGGAGGCGGGGGTGCCCGCGAGGGCCCCGGGGGCGGCGCCCCGGCCGAGCCCGCCCAGGGCCGAGCCCGCCCCGGCCCGGACCCCCGCGGCGACCGCGGGCCGCCTGGAGCGCCGCCGGGCCCGCGCGGCGGTGGCCGGCCGCGACGCCACGGGCCGCTTCACGGCCGGGCGGCCCCGGCGGGTCGAGCTGGACGCCGACGCGGCCTCCGGCGCCTTCTACGCGCGCTACCGCGACGGGTACGTCAGCGGGGAGCCGTGGCCCGGGGCCGGCCCCCCGCCCCCGGGGCGCGTGCTGTACGGCGGGCTGGGCGACAGCCGCCCCGGCCTCTGGGGGGCGCCCGAGGCGGAGGAGGCGCGGGCCCGGTTCGAGGCCTCGGGCGCCCCGGCGCCCGTGTGGGCGCCCGAGCTGGGCGACGCGGCGCAGCAGTACGCCCTGATCACGCGGCTGCTGTACACGCCGGACGCGGAGGCGATGGGGTGGCTCCAGAACCCGCGCGTGGCGCCCGGGGACGTGGCGCTGGACCAGGCCTGCTTCCGGATCTCGGGCGCGGCGCGCAACAGCAGCTCCTTCATCTCCGGCAGCGTGGCGCGGGCCGTGCCCCACCTGGGGTACGCCATGGCGGCGGGCCGCTTCGGCTGGGGCCTGGCGCACGTGGCGGCCGCCGTGGCCATGAGCCGCCGCTACGACCGCGCGCAGAAGGGCTTCCTGCTGACCAGCCTGCGCCGCGCCTACGCGCCCCTGCTGGCGCGCGAGAACGCGGCGCTGACCGGGGCGCGAACCCCCGACGACGGCGGCGACGCCAACCGCCACGACGGCGACGACGCCCGCGGGAAGCCCGCCGCCGCCGCCGCCCCGTTGCCGTCGGCGGCGGCGTCGCCGGCCGACGAGCGCGCGGTGCCCGCCGGCTACGGCGCCGCGGGGGTGCTCGCCGCCCTGGGGCGCCTGAGCGCCGCGCCCGCCTCCGCGCCGGCCGGGGCCGACGACGACGACGACGACGACGGCGCCGGCGGTGGTGGCGGCGGCCGGCGCGCGGAGGCGGGCCGCGTGGCCGTGGAGTGCCTGGCCGCCTGCCGCGGGATCCTGGAGGCGCTGGCGGAGGGCTTCGACGGCGACCTGGCGGCCGTGCCGGGGCTGGCCGGAGCCCGGCCCGCCGCGCCCCCGCGCCCGGGGCCCGCGGGCGCGGCCGCCCCGCCGCACGCCGACGCGCCCCGCCTGCGCGCCTGGCTGCGCGAGCTGCGGTTCGTGCGCGACGCGCTGGTGCTGATGCGCCTGCGCGGGGACCTGCGCGTGGCCGGCGGCAGCGAGGCCGCCGTGGCCGCCGTGCGCGCCGTGAGCCTGGTCGCCGGGGCCCTGGGCCCGGCGCTGCCGCGGAGCCCGCGCCTGCTGAGCTCCGCCGCCGCCGCCGCCGCGGACCTGCTCTTCCAGAACCAGAGCCTGCGCCCCCTGCTGGCCGACACCGTCGCCGCGGCCGACTCGCTCGCCGCGCCCGCCTCCGCGCCGCGGGAGGCGCGCAAGCGCAAGAGCCCCGCCCCGGCCAGGGCGCCGCCGGGCGGCGCCCCGCGCCCCCCGAAGAAGAGCCGCGCGGACGCCCCCCGCCCCGCGGCCGCCCCTCCCGCGGGGGCCGCGCCCCCCGCCCCGCCGACGCCGCCGCCGCGGCCGCCGCGCCCCGCGGCGCTGACCCGCCGGCCCGCCGAGGGCCCCGACCCGCAGGGCGGCTGGCGCCGCCAGCCGCCGGGGCCCAGCCACACGCCGGCGCCCTCGGCCGCCGCCCTGGAGGCCTACTGCGCCCCGCGGGCCGTGGCCGAGCTCACGGACCACCCGCTCTTCCCCGCGCCGTGGCGCCCGGCCCTCATGTTCGACCCGCGCGCGCTGGCCTCGCTGGCCGCGCGCTGCGCCGCCCCGCCCCCCGGCGGCGCGCCCGCCGCCTTCGGCCCGCTGCGCGCCTCGGGCCCGCTGCGCCGCGCGGCGGCCTGGATGCGCCAGGTGCCCGACCCGGAGGACGTGCGCGTGGTGATCCTCTACTCGCCGCTGCCGGGCGAGGACCTGGCCGCGGGCCGCGCCGGGGGCGGGCCCCCCCCGGAGTGGTCCGCCGAGCGCGGCGGGCTGTCCTGCCTGCTGGCGGCCCTGGGCAACCGGCTCTGCGGGCCCGCCACGGCCGCCTGGGCGGGCAACTGGACCGGCGCCCCCGACGTCTCGGCGCTGGGCGCGCAGGGCGTGCTGCTGCTGTCCACGCGGGACCTGGCCTTCGCCGGCGCCGTGGAGTTCCTGGGGCTGCTGGCCGGCGCCTGCGACCGCCGCCTCATCGTCGTCAACGCCGTGCGCGCCGCGGACTGGCCCGCCGACGGGCCCGTGGTCTCGCGGCAGCACGCCTACCTGGCCTGCGAGGTGCTGCCCGCCGTGCAGTGCGCCGTGCGCTGGCCGGCGGCGCGGGACCTGCGCCGCACCGTGCTGGCCTCCGGCCGCGTGTTCGGGCCGGGGGTCTTCGCGCGCGTGGAGGCCGCGCACGCGCGCCTGTACCCCGACGCGCCGCCGCTGCGCCTCTGCCGCGGGGCCAACGTGCGGTACCGCGTGCGCACGCGCTTCGGCCCCGACACGCTGGTGCCCATGTCCCCGCGCGAGTACCGCCGCGCCGTGCTCCCGGCGCTGGACGGCCGGGCCGCCGCCTCGGGCGCGGGCGACGCCATGGCGCCCGGCGCGCCGGACTTCTGCGAGGACGAGGCGCACTCGCACCGCGCCTGCGCGCGCTGGGGCCTGGGCGCGCCGCTGCGGCCCGTCTACGTGGCGCTGGGGCGCGACGCCGTGCGCGGCGGCCCGGCGGAGCTGCGCGGGCCGCGGCGGGAGTTCTGCGCGCGGGCGCTGCTCGAGCCCGACGGCGACGCGCCCCCGCTGGTGCTGCGCGACGACGCGGACGCGGGCCCGCCCCCGCAGATACGCTGGGCGTCGGCCGCGGGCCGCGCGGGGACGGTGCTGGCCGCGGCGGGCGGCGGCGTGGAGGTGGTGGGGACCGCCGCGGGGCTGGCCACGCCGCCGAGGCGCGAGCCCGTGGACATGGACGCGGAGCTGGAGGACGACGACGACGGACTGTTTGGGGAGTGACGGGGGGGAAACTTCCGGGAGCGGGGGAGGGGGGAGATGGGGAGAGGGGGAAGGAATCGGGCGTCTGTGCGCCTTTAAGACAGACGCGGCGATGGCCGCGCGCGTGTGTGAGAAATAAAGAACGAGACAGACGAAAACGTACCGCCTTGTGTGGTTTATTCGGGGGTCGGGCGGGCGGGGGTCGGGCGGGCGGGGGTCGGGCGGGCGGGGGTCGGGCGGGCGGGGGTCGGGCGGGCGGGGGTCGGGCGGGCGGGGGTCGGGCGGGCGGGGGTCGGGCGGGCGGGGGTCGGGCGGGCGGGGGTCGGGCGGGCGGGGGTCGGGCGGGCGGGGGTCGGGCGGGCGGGGGTCGGGCGGGCGGGGGTCGGGCGGGCGGGGGTCGGGCGGGCGGGGGTCGGGTGGGCGGCACGTCTCCCGCGCCCGCGGGGGGTCTGGGGCTCTGACCTGAGTGCAGGTTACGAAGGTCAGGTGGCCCGAGCCCCCCCGCAGGAGCGGGAGGGAAGGCACGGGGCGCGGGAGGGAGGGGCTGCTGCGAGCTCGGGGCCGCGGGCGCGGGGGGAGGGGCGGGGGAAGCCCCCGGGGCGGGGCGCGGGGGAGGCGGCCGCGGGGGAGGCGGCCGCGGGACCGCAGCCCCGTGGCGCGCGGGGGGGAGGGGCTGCCGCGAGCTCGGCGGGATGGAGGGGAGGGAGGGGGTGGCGGGGAACCGTGTGCGGGCGGGCGGGTGCTTGGTGCAACTGTCTGGTCTGCGAGGGCGAGCGGTGGTGCGACTGGCGTCTTCGGGGGGGCGGGGAGCTTGGGAGTGTGTGGTGGTCTGCGGCACAGCCTGCTAGTCCCCGTCTTGCCGCGCGGGGGCGGGCGCGGGAAAAAAGCCGCGCGGGGGCGCCCGCGGGAAGGCAGCCCCGCGGCGCGCGGGGGGAGGGGCGGCGCCCGCGGGGGAGCGGCCGGCTCCGGGGGAGGGACGGGGAAGGGGGCGCGCGGGGCTGCCCTGCCGCCCGCCCGCCGCCGCCGCCCGCCTTCGCGCCCCCCCCCAAAAAACACCCCCCCCGGGGGTTGACTCCCCGGGGGAAAAGAGGCGGGGCGGG

>HHV3_VZV_STRIPPED4

GCCCAGCCCTCTCGCGGCCCCCTCGAGAGAGAAAAAAAAAAAGCGACCCCACCTCCCCGCGCGTTTGCGGGGCGACCATCGGGGGGGATGGGATTTTTTGCCGGGAAACCCCCCCCGCCAGCCTTTAACAAAACCCGCGCCTTTTGCGTCCACCCCTCGTTTACTGCTCGGATGGCCACCGTGCACTACTCCCGCCGACCTGGGACCCCGCCGGTCACCCTCACGTCGTCCCCCGGCATGGATGACGTTGCGACCCCCATTCCCTACCTACCCACATACGCCGAGGCCGTGGCAGACGCGCCCCCCCCTTACAGAAGCCGCGAGAGTCTGGTGTTCTCCCCGCCTCTTTTTCCTCACGTGGAGAATGGCACCACCCAACAGTCTTACGATTGCCTAGACTGCGCTTATGATGGAATCCACAGACTTCAGCTGGCTTTTCTAAGAATTCGCAAATGCTGTGTACCGGCTTTTTTAATTCTTTTTGGTATTCTCACCCTTACTGCTGTCGTGGTCGCCATTGTTGCCGTTTTTCCCGAGGAACCTCCCAACTCAACTACACGAAACTACTGTCCGGAAGGGGAAGGTATTTATTCTCGCTTGCAGCTTGTCGCGCGTGTATGCACAACAAAAGCTATATATGTCACCAAAGCCAACGTCGCCATCTGGAGTACTACACCCAGTACATTGCATAACCTGTCCATTTGCATTTTCAGTTGCGCGGACGCCTTTCTCCGGGATCGTGGCCTTGGGACATCAACCAGCGGAATAAGAACCGCCGGTGGTCTTGCCCGAACGACGAGTGGCGACGCGTTGTTCTGCATAAGCTCTGTATGCTGATACATAAACACAGAGTCTGTATCGCTATCAGATTCCCGAACACCTTCCGGTACCCCATACTCCGATACCCTGGACATTGCGGATCCCAAAAATATAATATTAACAGGATTTGCTTATACTTTGCTACAGCTTATATAAATTTATGTGCGATACATCTTAAGTGCATCCGTACGTTATTTATACATTGCCTGTCACGTGAAAAGACTGTGTTACCCAATAAAGGTTCTACAAAAAATGCTTTATTGGGTGTTTGTTTAATAGCTATTATCGTAACCCACCCCCGTAAAATCATAAAATGCATGTAATTTCTGAGACACTTGCATATGGGCATGTTCCCGCATTTATTATGGGCTCCACTCTGGTGCGTCCCAGTTTAAACGCCACCGCCGAGGAAAATCCCGCGTCAGAAACGCGATGTTTATTACGAGTGCTTGCGGGGAGAACTGTAGACCTGCCAGGCGGAGGAACGTTACACATTACCTGTACCAAAACCTATGTAATTATTGGCAAATATAGCAAACCCGGCGAACGTCTTAGCCTTGCCCGTCTAATAGGGCGTGCAATGACGCCTGGAGGTGCAAGGACATTTATTATTTTGGCGATGAAGGAAAAGCGATCCACAACGCTTGGGTATGAATGTGGTACGGGCTTGCATTTACTGGCTCCATCTATGGGTACATTTCTCCGCACACACGGTTTAAGTAACAGAGATCTCTGTTTATGGCGGGGTAATATTTATGATATGCATATGCAACGTCTTATGTTTTGGGAGAATATCGCGCAAAATACCACTGAAACACCTTGTATAACGTCGACGTTAACATGCAACTTGACAGAAGACTCTGGTGAAGCCGCACTTACCACGTCAGACCGACCCACTCTCCCAACCCTAACAGCCCAAGGAAGACCAACAGTTTCCAACATTCGTGGAATATTGAAAGGATCCCCCCGTCAACAGCCGGTCTGTCACCGGGTTAGATTTGCCGAACCTACGGAGGGCGTATTGATGTAATCACTAAATAAAATACACCTTTTTTCGATTGTACGTATTTTTATTTAAATGTGTAGTTCATAGTCCGCCGACAGCCGCTCGGGCTTTTCCCCCACATACAACATGATCGTATGCCTCGGATGCACCGGTCCAACACTCCGCCGAGAAGGGGGATTTACAATGACAGTGATACCCAATAGCCGCCAGATGTACACCCAGCTGTCCGGACTCCAGCATCATCTGCTGAGTTGCGGCGCTGAAGGGTGCATCGCATAGGGTGTTATAATTAGCCATTTCCGGTAACAGTCGTTGGGAATTTAGGAGGCTGCAAAACGGCTGTAGGTCAACATACATTGGGGATTCAGATGGTTTATCTCGACGTCCAAGTCCAATCAAAAAAGCGTGTAAATCATCAGCCCGGCCGCATGTTGCTCGAAGAGCACATAACCTCTTAACACCGTACAGAGGGGATGGCGTCGGTGCATGTGAGTTGGCAGGGCATGTCCACGTTGTTTCCAACGCCAGTGGCGGTATAACTTGTGTAAACGACGCCAACGGGTCAGGTTTAAGATTCACTCGGATGGGTTGACTGCTTTCGGAAGCTCCCGTTGTATCCATTAATTAAACGTTCGGTACACGTCTGGTGTGTGTTTTACCCGAATCAGAGACGGAATTGCAAAGATATTGGTTTGAAAGCAATGTAATCCCGCCCATATATCCCCAACGTCGCCTTAAAAACTCCCACAATATTACATTTTTATTAGTCTTTTATTAATATAGAATCACATAAACAATTGATAAAATCAAGGGGTGGTGTATAATGATTAAAAATATAAATTGATATGTTTTACAAGCATGAAATAGGTATTTACTATTCTAACAGGTAAATATGCTTAATGATTAAAAATACAAATTAGTATGTTTTGACAAGCATGAAAAAGGTATTTTTTATTTTAGCAGTTAAAGGTACTACACTTAAAATATTTACCGTATGGACGGGCGTCAGAAAGATGCCCGGCCCAAGTTGAGAGGGTACATTCAACACGACCACACTCGCGTTGGTGGGTGATTAGGGCCTCTAAAACACCGGCCAGACATGACCCGGGTGTATATTCTTGTAACACTTGAACGTTACAACTGATATCATCATATTCCACAAATTTAGAGCCACGGACAACTATATTAGCAATGCGGGCAATCATAACAAACATATAAGTAGTAATACACGTGATATCACTAAAACGTTGCTGGCGCAACAGTTCGGGGAGAGTACGAGACCCCAAATCGTTGTCCCTGTTTAGAAGAAGACATCTTACAAAAGGCCCCAGCTTTAACTTTAAATTCTCCAAAAGTGACTTCGAGGTTGCAACAATGGGATTATTTGTGTAGATGGGCAAGTTTTTTGCCGCTAACATTTTAATCCACGTTAACAGTTCATCCGCAGACTCCAACGCTTCAATCAAAGATTCTCCACGTATGACTCTCTCACGCAACGCGCGGGCAATACGTGAGTCCATTTTATATGACTCAAAGGTACGATAAAGTTCATGTCCGTACAACATCAACTCCGGCCAAGATGTGTTTTGTTTTATCCCCGGAAAACATCCACCGGAAGCCCATGAATCACCCTCTTGTATTGTGGCATATCGGACTACCAGTTTTTCAATTGTTTCATCTAAATGGCGTACCGAGTCAATGGTCACGCTGGCTCCCGCGGTGGAGACGACTTCAATAGCACGGCCCGTAATTCGATCGACCGGGATATCATACTCTTTTCGAATACGCTCTCGGCGGGCGTCTCTCTTGGAAAATCGCAACCTGTACGATTCGTCATGTGTCTGATCATTTCTTTCTCCCGTGGTCATTGCAGGAGGCGTTGTAGGACGCCGTCTTCGATTTGACAGGGATCGATCACGGTGTTTTCTTGAACTTTGGGTGTTATAAGATCTGGATGATCGTCGATGTCCCCGTTCGATGCGTGCATATCCAGTCTCCACGTCTCTTCCTCCATGATGGTTTGAATCGGGTAATACAACAACCAAAGTTTTCGGGCGATTGTGGTGGTAGCTTTCACGCCTTCCGTGCCTTCGTTTGGAATACCGTGGATTATATGCTGTATCTGCAGTACGCTCCACATACACAGTTCTAGACGTTGTGGAGTCCTCGCCTGGAGTGGAGCCAATAGCTTCATCATTTGCCCAATCGGTGACTTCCAATGCAAAGTCATCCGAAGGTTCGTCTGGTAGCAAATTCATAAAGTCTTCACAAATAGTAGACACGTCTGGGTCGGTTGGAATTGAAGCAGAGGCCATGGCTGCAAAATATCTGACAATTGCGTGTTTGCAGTTGCCTGTATCTTCCGCCAATGTTGTAGAATTTATAGGCTCACCCAACCCCGCAATGGGCGTGTTTAGTCACATGATTAATGTTTCTGGGAGTTTTCACTTTCCCCAAACAAGCTTACCTGCACCCTTTGTTCGTAATGCATAAAAATAACCACTGCTATAGCAAATATGACGATATAAAAACATTTTATAGCAAGGCCGGACATTACTGTAGCGCAACATGTTGTGCATATACCACGTATTCCCCCCGTATTGATATGATTTAAATGATTATCCTTGGTTGGTTTTGGTCTAACATAAGATATAAGCTCTACTATAGCGAGCGTGCATACAACAACCCAGGCCAGAATCCGAATGTATGTGGGGTATAATAACGCGCATGGTGTATATGCAACGCCAAGCGTTAAAAGCACAATACATCCAGATGATATATGAGCGATAACCTCCAAAAGCATCAATAACGTAACACCTTTATGCATATATAAAAAACTTATAGGGTCAGCATTAAATACTTTACTCATACCATCCCGTCGCATGGAAACATCACATAACAACCTTGCCAACTTTGTATATGGGTAACCAAGAAGAATGTTCGAAATAACCCGTGTTACGTAATTCAGTGAATATGATGTGGGGGATATTAACTCACAGGATGATCGGAATGGCCCAAACATACGACGTATTCGTCGAAATTGTAAATACATACCATATACAAACCATGCAAAAAAAATCATTTTTAGCTGCACGCACCAAAAATAAGCGTGACAATTACGTGTTCCCAGAACAATTCGAATTTTGTCATGCAAAGGTGTAGAAATAGCGGTTTTTACCATAGTATCTCCTGATAATAGATTTTCCCGGCAGCTGTAATCGTATCCAGATAGGCCATCCAAAAACGTTGAGTGGTTTACAAACGTTACATATATAAGAGAGTTGTTATAAGACCCCCATACAACCGGTCCACCATTAATCACCGTGGTTGCATACACACACTCATGTTCAAACTTTACACGAGCGGTATACCATAGGGTAAAAACAGCATGTCCGCTAAGTAGACACATAATTATAAAATGTTCTGTCTTGATTCCTAAAGCCTGCATGACCCGTGGAAGATGGCAATTCAAGCACGATGTAGTATCACACGGTTGGTGTTAACTCGAAGTTAAATTTGGATAATTAGGTACTTCTAGAGTAAAGATTGTATGCATGCGATTGCTATCGCACTTTGTAGCAAAACATTGTTGTGCAAGCGAAATACACAAACGGTTGTGATGATCCACTCGCAGAGACACAAATGTCCGGGGAGCCGTTCTTCCTCCGCGATGGGGATATCGAAGACAAGTGAACCCTTTTGTTCCGCATATGAGCTGAAATAACACCCAGTCCCTTTTGATGGCGATACACTTTGATGATGTTAAGGTATATTCGCGATCACGCCCGGGGAAATGAACAGCAATATGCTCCACAATAGATTCTAATATTGTGCTGTCGACAAAGGCCTCCAGTGTAAATGCGTCCAGACAAGTTACCCCGCGCTCTTTTAGAGCCTTTGTTAAAGATATTTGCGGGGAGCTAAATATTTGTTTATTACGCGCAACCTTACGTTCAAAAAACTCTGCGTATTCCCCCCCAAGGTTATGTAAAATAAATTGCACTGGAACATTCGACTGCGGTCTTGAATGAAAATGAAAGTTTGCCGGGTTTCTATGTGATGTCACAAACGCTAATATATCAATACACTGCTCAGGTACAACATAAAATGGGAGTAGTTGTCCAACCGCCGTCCCTGTGGTTGTTACTTTGGAGAAAAAAGGCAGTCTTAAACTATGTCCGTGGCTATAAACACCAGTATCTATAAACGAAAAGTCCCGTAAATACGGACCAATATATTCAACAAATTCCCGTTCCAGCAACACCGCTTGCTGTAATATTTGTGCAAACCCCTTTAAAGTGGAAGACCCCACTAACGCATAGGGATTTGGGATTGGTACGCATACCCTGAAACCTATTTTCTCTTTACAGTTACAGGGTAGAGTTTCATGCAAGTTTTCATTGTTTGATACATCGGCGTGTGTATGGACTTCAGACGTTGTCTGTGTATCAAAAAACCATACATCCTCTGTATAATTCTCTTCTACACACGTGTATAATTCGCCATTTTCTATGTAAAAATCGATGTCAGAATGGCTGGTTATATCCAATAAATTATCATCATCCAACACCTCAACGGTAGGTTCAGGACATGCAGTTTTATAAAAATAACATGGGTCTTTGTTAGGGTTTACCACGGCCTTTGGAAAAAGTAATTGCATGGCCGTTAAAATACCATGACGAAATGCTCGCATGCCGGCATGTAAAATACCCAATGGGATGGGTTTTCTTATATGAAAGTCTACATCAAGTATGAGGTTTGTGATTATAAGATTTGTATTAAATAGCTCATTCCTGTTTATATAAAGCTGATCTTTGGGTATGTTTGATGAAATTTTAGAAACGTTTTTAACAGACGTAGATAATAGTAAAGTCAACTGCATATCTCGTAGTGAAGCGGCAACAAAATTACATGGATTAATTTGTTTAAGGTCCTCCGCAATTAATCGAGCCTCGTGCGGTAAAGTGTAACGGTTTGTTATTGATGACCACGTATCATTAGCAATAACAGCAAATGCTTGGGCGCCTTGAGGCAAGGCTACCCGATATACAGGCATTGGTCCAGTTACCTCAGAATGGCCGATGAGGGCTTCTAATGGAGTTTTATAACTCAGGATGGATACATCATGTGTGGCTATCCCAGTGGCAGCAGAGAAAAACAGTAATAGTTTTGTAATCCCCGGGCTCGTATCAAAACCAGTACGACCACTTTGGTTAGGTGTATCGTTTGCAAAGTTGGCTGCTCGTAACGCCTCCGCGGAAACAACCGAATCCTCAAAATTAGACAATTCGTCAAAACCGGGTGGATTTGAGGGAATAGTGGAGGACCATCCATATGGACTAAATTGTTTTTCAATGTTTTCCACACGACGAGTTAGCGTTGTAGCTAGGTCACATACGCCTATAAACTTGCTAGGTTTTGCGGCATACGTAAGACTTAAAGTATATGTTTTAGTAATTGTATATTTATGTCCAATCTCAGGTCCAAGTTCAGTGACATCACAAATTACGTTCTTTTTTATATAGTCACGCATGTTGAGACGAGAACGTACATGATTAAAAAAATTAGCAGTAGCTCTTTTTCCCAGGTTGGATGATTTTAAGAGGACCGGTTTATTCACAAAATCTGAGTATGTAACCGCTTGTAGGTGGTCTGCGATCTGTTTCCGATTGAAACATTCAAAATGTGCCAGATAAATATAATCAACAAATTCACGGTCTGGAACTTTAAGGCCTTTTCTATCGTTGGTAATATACTCCGATACTGCGTGTATTTCCGTTGTGTCTGTATGTATTCGCTGTAAAATGTACGATAGAGCATTTTTGGCTGTCAAACCTCGTGTATATGTTGAGGAACAACAAAACATGGAAAGTTTATCAAAAGACAACAAGTCCGAAATATTGTACCCACTACAATTAGGTAATGCTGGGACTTGGTAAGTTAAAAACAAATCTTTAATTGCCTGTAAGTCATATAAGGGGGTTTCCAACGTATTGTAACTTGTGTCCGTTTGTAACAAGTAATAGCGTGTAGCCAACACTAGCGTTTTTTCAGAGGGTCCAAATCGAACAATATACCAAAACGGCGAGCATCCATACCCCCAGTAGAGTCGTCGATATGCAGCCAATACTTGACGTTCGTAATGGGCATATAATGATGTTAGCTCCTGACGACCAACGGATTTTTTAACTAACTTGCAGAGTGTTGCCTCTGTGATGCATAGGCCGTTGTCCGATAATCCCTTTCGGTTTAAATGGTGTGTTGTTACCATCAGAGTTTGTATAACTTCCGAGTGAATGTCAAACGTCTCCGATATACATAGGGTATCAGATATTATATGCGGATTTAGGGGTGCTCCATACCATAACGCCTTATATAAAGCTTTAAAATCAGTTTGGGTTTTAAAACAACAAAAAAATATAGGCCAGACCCGGGATCGTACATCTCCAGTTGAAAATCCACCAATTAAATAAAAAATAACGTTGACGTCCCTACTACAAAATAAATGCATTATTTGGTTTTCTTCATCGTTTTCAGTTACTTCACGTGGGCGTTTAGTTGGGATTACTTGCGTGATCTCTTCCCTCCCATTTTTGACAAAGACGTCATCTAAGTCGGGAGTCCAAGTATAACTCACCACATACAGAGGTTCTGTGCTTATCTGCCCGGTAAGCAACAACAGCGAGTGGGAGATTGCACATCCCTTTGTGGCAAATAATAACCGAATCGTCGGTTTGGAGGATTTATCCATAGTTCAATACGTTGGAAAGCCAGTCAATCATGCAGACGGTGTGTGCCAGCTTATGTGGATATGCTCGAATACCAACTGAAGAGCCATCTTATGAAGAGGTGCGTGTAAACACGCACCCCCAAGGAGCCGCCCTGCTCCGCCTCCAAGAGGCTTTAACCGCTGTGAATGGATTATTGCCTGCACCTCTAACGTTAGAAGACGTAGTCGCTTCTGCAGATAATACCCGTCGTTTGGTCCGCGCCCAGGCTTTGGCGCGAACTTACGCTGCATGTTCTCGTAACATTGAATGTTTAAAACAGCACCATTTTACTGAAGATAACCCCGGTCTTAACGCCGTGGTCCGTTCACACATGGAAAACTCAAAACGGCTTGCTGATATGTGTTTAGCTGCAATTACCCATTTGTATTTATCGGTTGGCGCGGTGGATGTTACTACGGATGATATTGTCGATCAAACCCTGAGAATGACCGCTGAAAGTGAAGTGGTCATGTCTGATGTTGTTCTTTTGGAGAAAACTCTTGGGGTCGTTGCTAAACCTCAGGCATCGTTTGATGTTTCCCACAACCATGAATTATCTATAGCTAAAGGGGAAAATGTGGGTTTAAAAACATCACCTATTAAATCGGAGGCGACACAATTATCTGAAATTAAACCCCCACTTATAGAAGTATCGGATAATAACACATCTAACCTAACAAAAAAAACGTATCCGACAGAAACTCTTCAGCCCGTGTTGACCCCAAAACAGACGCAAGATGTACAACGCACAACCCCCGCGATCAAGAAATCCCATGTTATGCTTGTATAAATATTGAAATAAAAACTAAAAACGTTTCTGGTGTATGTTTTTATTTTGTATATAAAATTAAAACATTGCTGGCCGGCGTGGTTATTACATTTAATGTTTTAGTAGAAAATCGACATCGTTTGTTTCTTTATCAGTTGAACCAAATCCACGCGTTCCCCGTTCGCTGGGTGTGGCTATTAGATCTAACGTTTTAGTAAAATACCATTGTACACCCGGTATGCCACATTTACCGCGGATAGCATAAGGAAATGCAATATTACTTAAAACGTTGTGTTTTAAGTGTATTTGGGTGTTGTGATCTATTAACAGGACCTGTGCAAGACGATCTCCCGTTTTTATACGTATGTCATCACCCGTGAGATTATATACGTAGAATTTACAGTGTTCTCCTGCAGGCCATGCCGTTGGACACACGATAATGCCTGATCGGCTTTTCGATGATCTTCCAAAAATATAAGCGTTTATACTCGGATGTTGTAAGTCCCAGTCTCTTATAATCGGTAAGACAATTTTTATAAATTCATTCCTTTTTAAATATAGGTTATATGGTACACAAATATCATATCCCGCGTCTTCTTGGCGTTTTGGATTGATGATATGTTTGTAGGTTAAGGGAACATCGATATGGTATTCTGCAGAATCCCTATGTAAAGGTTGCCCCTGCTGTACCGTGGAAATATCAGCAAATTCAGATATAACGGGTTTTTCATAATTTGACGGCGAGTTTGATAAGGGTTGAACTTGTATCGATTTAAAAATTGGATCCAGATGTTTAAGAACGTTTTTTGGGAGAAGGCGACTTTGTCTTAATTTTACCGGGAACAAGTAGATTGTTAAATGTCCGGGTAAAATAACGGTTACTCCTGGCCGGTAATACAAAAGGGCTGAAATTACTCCTCTGTAACCCGCATCAATAACTCCGTTGGCGACAAAAAAATTGTCTTCATCAGCAAGGGCAGTATCTTTGCATTGAATTAACAACAGTGCGTATTCATTGGGAGGCGCCGACTTAACCAACAGCTCCAACTGCTGCATATAAAAACCGCCCCGTGTTACAGATTTTTCAGATGGCAGTTCGAGTTTCTTGTGGTTCCGGAGTAACAACGGTTGATGTCGACTTACTTTATCGTCTAACACGCATTGCAGCGTATCTGCACATTCAGGTTGAACTTCTATTAAAATTGTATCTTTTAAACACCGATTCGGAATAGTTTGGCTACAAAACATATCACCTGTATTTACTGCCGTTTCCAAGATGGGATCAATTACCGCTTCGTTCATATTAATAACGATGCAAATTTTATTTTTTTGTGAAGACAGCAGTGGGGAGCCAAACTTTGCAGAACGGAATTTTTGGCATGCCAGCTGTTCGGCTCGTGGAGTTTATATCGACGGATCAATGATCACCACCCTTTTCTTCTACGCATCCCTTTTGGGGGTGTGTGTAGCCCTTATTTCGTTAGCTTATCATGCGTGTTTCCGGTTATTTACTCGTTCTGTATTACGCAGCACGTGGTAAACCCGTTTGCCTATAAAAGGGGCAGGCGTGTATAAGAGGGCCCCTGTTTAATACGCGGTCTGCCGTGTTTGGATATTTCACGACCCTATCGTTTATTTACGTAATGGCATCTTCCGACGGTGACAGACTTTGTCGCTCTAATGCAGTGCGTCGTAAAACAACGCCTAGTTATTCCGGACAATATCGAACCGCGCGGCGAAGTGTGGTCGTAGGACCCCCCGATGATTCAGACGACTCGTTGGGTTACATTACCACAGTTGGGGCCGATTCTCCTTCTCCAGTGTACGCGGATCTTTATTTTGAACATAAAAATACGACCCCTCGCGTACATCAACCAAACGACTCCAGCGGATCGGAAGATGACTTTGAAGACATCGATGAAGTAGTGGCCGCCTTTCGGGAGGCCCGTTTGAGACATGAACTGGTTGAAGATGCTGTATATGAAAACCCGCTAAGTGTAGAAAAACCATCTAGATCTTTTACTAAAAATGCGGCGGTTAAACCTAAATTAGAGGATTCACCGAAGCGAGCTCCCCCGGGAGCAGGCGCAATTGCCAGCGGGAGACCAATTTCCTTCAGCACTGCACCAAAAACCGCAACAAGCTCGTGGTGCGGTCCTACGCCATCATATAACAAACGCGTCTTTTGTGAAGCGGTCCGGCGCGTAGCCGCCATGCAGGCACAAAAGGCTGCCGAAGCGGCTTGGAATAGTAATCCCCCAAGGAATAACGCCGAATTAGACCGTTTGTTAACCGGAGCCGTTATTCGTATTACGGTGCATGAGGGTTTAAATTTAATACAAGCCGCTAATGAAGCAGACCTAGGTGAAGGAGCATCGGTATCCAAACGTGGACATAATCGAAAAACTGGAGATTTACAGGGGGGCATGGGTAATGAACCTATGTACGCACAAGTTCGTAAGCCAAAAAGTCGAACGGATACACAAACGACTGGGCGTATAACTAATCGAAGTAGGGCCCGTTCGGCATCAAGAACTGATGCGCGAAAATAGGGATATAATTACGCAGTAACGGTTTACCCGGTATTATGTATAATAAATAAACGTATAAAAGACAGTCGTGGTTTGTGTTTATTATAAATGTGTATTATATGTCACATATTATAAACTGTTTAAATAGTACCACGTGGTATTATGAACAGTTTATAATCAGTTGCTACCAAACAAACCCCATTAGACGGCGGGTTTTGATAAAGGGAATCGCTTATTTAAACTAAAGATTTTACTCTATAAGTATGGAGTGTAATTTAGGAACCGAACATCATAGTACAGATACGTGGAATCGTAGTAAAACGGAACAAGCGGTTGTGGACGCATTTGATGAATCGTTGTTTGGTGATGTAGCATCGGATATTGGATCCGAAACGTCGTTATATTCACATGCAGTTAAAACTGCTCCGTCTCCGCCTTGGGTAGCTAGCCCTAAAATTTTATATCAACAGTTAATACGGGATCTTGATTTTTCAGAAGGGCCGCGTTTACTATCATGTCTTGAAACCTGGAACGAGGATTTATTCTCATGTTTTCCTATTAATGAGGACCTATATTCCGATATGATGGTTTTATCCCCGGATCCAGATGACGTTATCTCAACCGTTTCAACCAAAGACCATGTTGAAATGTTTAATTTAACAACCCGGGGTTCCGTTCGATTGCCTAGTCCACCAAAGCAACCGACGGGGCTTCCAGCTTACGTTCAGGAGGTCCAGGATTCGTTTACCGTAGAACTACGCGCCCGGGAAGAAGCATACACAAAACTACTAGTTACTTATTGTAAATCGATTATACGTTATCTCCAAGGAACGGCGAAAAGGACGACAATAGGTCTTAATATACAAAACCCTGACCAGAAAGCTTACACGCAACTCAGGCAAAGTATTCTACTTAGATATTATCGTGAGGTGGCAAGTTTGGCGCGTCTTCTGTACCTACATTTATATTTAACCGTAACGCGTGAATTTTCCTGGCGTTTGTACGCCAGTCAATCTGCACACCCGGACGTGTTTGCGGCTTTAAAATTCACCTGGACCGAACGTCGACAGTTCACGTGTGCGTTTCATCCTGTATTATGCAACCACGGCATTGTGTTATTAGAAGGGAAACCACTAACAGCGTCTGCCTTGAGGGAAATAAATTACCGCCGCCGAGAACTGGGACTGCCTCTAGTTAGATGTGGTCTTGTTGAAGAAAACAAATCTCCGTTGGTTCAACAACCCTCATTTTCGGTTCATTTACCACGGTCGGTGGGGTTTCTTACCCACCACATTAAGCGTAAGTTAGACGCATATGCGGTCAAACATCCTCAAGAACCGAGACATGTACGAGCGGATCATCCTTACGCAAAAGTTGTTGAAAATAGAAACTACGGTAGTAGCATCGAAGCTATGATTTTAGCACCTCCGTCCCCATCCGAGATCCTGCCGGGGGACCCACCACGCCCACCCACGTGTGGGTTTTTAACGCGTTAAACGTCATTGGGGTAAAGGGTGTAAATAAATTACGAAAACGTGCATGCGTTTTTTATTTTTACAATGCGCCGTATATGGTATGTCTGTCATGTGCTCTAAAGTCCCATATATAAAAGAAGCCCCAACGAGTGTATGCGTATTGCGTACCGCGACCCTGGGATGTTTTACAGGCGCGTTTGTTTGTCTCGGTTATAAGTATGCAGTCGGGTCATTATAACCGGAGGCAATCCCGCCGACAGCGGATATCGTCTAATACCACAGACTCCCCCCGTCACACACACGGAACACGTTATCGGTCAACCAATTGGTATACACACCCACCCCAGATATTGTCCAATTCAGAAACATTAGTTGCGGTTCAAGAACTACTGAACTCCGAGATGGATCAGGACAGCAGTTCTGACGCATCGGATGATTTTCCGGGATACGCCTTACATCATTCTACATATAATGGATCCGAACAAAATACATCAACTTCCAGACATGAAAATCGCATATTTAAATTAACGGAGAGGGAAGCTAATGAGGAAATCAACATCAATACGGACGCGATCGACGACGAGGGAGAGGCGGAGGAGGGAGAGGCGGAGGAGGACGCGATCGACGACGAGGGAGAGGCGGAGGAGGGAGAGGCGGAGGAGGACGCGATTGACGACGAGGGAGAGGCGGAGGAGGGAGAGGCGGAGGAGGACGCGGCGGAGGAGGACGCGATCGACGACGAGGGAGAGGCGGAGGAGGACGCGATCGACGACGAGGGAGAGGCGGAGGAGGACGCGATCGACGACGAGGGAGAGGCGGAGGAGGATTATTTTTCTGTAAGTCAAGTTTGCAGTCGAGACGCGGATGAGGTTTATTTTACGTTAGACCCGGAAATAAGTTACAGTACCGATCTTCGCATTGCAAAGGTTATGGAGCCTGCGGTATCAAAGGAACTTAATGTATCAAAACGTTGCGTTGAACCTGTTACCCTAACAGGCTCTATGTTAGCGCATAATGGGTTTGATGAGTCCTGGTTTGCTATGCGCGAATGTACCCGTCGCGAATATATTACGGTCCAAGGATTATACGACCCAATTCATTTACGGTATCAGTTTGATACTTCCCGGATGACACCCCCACAGATTTTGAGAACTATACCAGCCCTTCCTAACATGACACTTGGTGAACTTTTATTGATTTTTCCTATTGAATTTATGGCCCAGCCAATTTCTATAGAACGTATTTTAGTTGAAGATGTATTTTTAGATAGGCGGGCTTCCAGTAAAACACATAAATACGGCCCGCGTTGGAATTCCGTCTACGCACTTCCATATAATGCGGGTAAAATGTATGTACAACACATTCCTGGGTTTTATGACGTGTCCTTACGTGCTGTGGGCCAAGGAACGGCCATTTGGCATCACATGATATTATCCACAGCAGCATGCGCTATTTCTAATCGCATTTCACATGGAGATGGATTAGGATTTTTGTTAGACGCGGCAATTCGTATTAGCGCAAACTGTATTTTTTTGGGACGTAACGATAATTTTGGCGTGGGGGATCCATGTTGGTTAGAAGACCATCTTGCCGGATTACCACGAGAAGCCGTACCCGACGTACTCCAAGTGACACAGTTGGTTTTGCCAAATCGGGGTCCAACGGTTGCCATTATGCGTGGTTTTTTTGGGGCGTTGGCATATTGGCCCGAACTAAGAATTGCTATAAGTGAACCATCTACATCTTTGGTGCGATATGCTACCGGTCACATGGAACTTGCCGAATGGTTTTTATTTTCACGTACACATAGTTTAAAGCCACAATTTACCCCAACGGAACGGGAAATGTTAGCGTCATTTTTTACGTTGTATGTTACTCTTGGTGGAGGAATGTTGAACTGGATCTGTAGAGCAACTGCAATGTATTTAGCTGCTCCTTACCATTCCCGTTCGGCTTACATCGCGGTCTGTGAATCTCTGCCCTATTACTATATCCCGGTTAATAGTGACCTGTTATGTGATTTAGAGGTATTACTGTTAGGCGAGGTCGACCTCCCAACTGTTTGTGAATCCTACGCAACTATTGCACACGAATTAACCGGATATGAGGCTGTTCGCACAGCAGCCACAAATTTTATGATAGAGTTTGCCGATTGTTATAAGGAAAGTGAGACCGATTTAATGGTAAGCGCGTACCTGGGGGCCGTTTTATTGTTACAACGGGTGTTGGGTCATGCAAATCTTCTTTTGTTGCTTCTCTCCGGTGCTGCGTTGTACGGAGGATGTTCAATTTACATCCCCCGAGGTATTTTAGATGCATATAATACTTTAATGTTGGCAGCAAGTCCTCTTTACGCTCACCAAACTTTAACATCCTTTTGGAAAGACCGCGATGATGCAATGCAAACTTTGGGGATTCGACCGACAACGGACGTTTTACCCAAAGAGCGAGACAGGATAGTTCAGGCATCACCTATAGAGATGAACTTCCGTTTTGTGGGATTGGAGACCATCTATCCCCGAGAACAGCCCATTCCCTCCGTGGACCTAGCCGAAAATCTTATGCAATACAGGAATGAAATTCTGGGTTTGGATTGGAAAAGCGTAGCCATGCATTTACTACGAAAATATTAAGGGTTGTGATTTTTTTCATTAGGATGAAAAGAACGTTTCCTAGCCACACCCACAAAGGAGTTTGTAAAATAAAATCTCTGTTTAGACCTTAAAATTTGTTGTGTGTGTTGTGTGGGGGGTCCGTGAGGATCGACCTTTACAAGATATAATTTGTCCATATCGCAATGTTTTCTCGGTTTGCGCGTTCCTTTTCCAGCGATGATAGAACGCGTAAATCTTATGATGGTAGTTACCAAAGTTTTAATGCCGGCGAACGTGATTTGCCCACACCTACCCGGGACTGGTGTTCTATTTCCCAACGCATAACCAGCGAGCGCGTGAGGGATGGATGTCTTATTCCAACGCCCGGCGAGGCTTTGGAGACGGCGGTAAAGGCTTTATCTGAAAAGACCGACAGCCTAACATCGCCGGTTTTACAAAGTACCGAAAGACACAGTGTTCTGCTTGGATTACACCATAATAATGTTCCTGAATCGTTGGTGGTCTCGTGTATGTCTAACGATGTTCATGACGGGTTTATGCAGCGTTATATGGAAACAATTCAAAGATGTTTGGATGACCTGAAACTTTCTGGGGATGGACTTTGGTGGGTTTATGAAAATACATATTGGCAGTATCTCAAATACACCACAGGAGCCGAGGTACCGGTGACTTCAGAGAAGGTAAATAAAAAGTCTAAATCCACGGTTTTGTTGTTTTCATCCGTAGTTGCCAATAAACCAATATCCAGACATCCTTTTAAATCTAAAGTTATAAATTCGGATTACCGGGGAATATGTCAGGAGCTACGTGAGGCGTTAGGAGCTGTGCAAAAGTATATGTATTTTATGCGTCCAGATGATCCTACAAACCCCAGCCCGGATACAAGAATACGTGTACAAGAAATTGCGGCTTACACGGCTACTGGCTACGGGTGGATGTTATGGTTCTTGGACGTTGTGGACGCCAGGGTATGTCGCCATCTCAAACTTCAATTTCGACGGATTCGAGGGCCGCGCGCGTCTGTTATTCCAGATGATTTGCTTAGACGACATTTAAAAACGGGTCCTGCGGTCTCAGCGGGCACAGGAGTTGCGTTTATTTTAGCAGCAACAACTGCCAGCGCTCTTACTGCGCTTTTGCGTATTAGTGTATTATGGCGAAAGGAAGAGTGGCGGGATGGTTTAAATGGAACCGCAGCTGCAATTGTTGCGGCGGTTGAACTTATTACGCTTTTGCACCACCATTTTCAATACTTAATTAATATGATGCTTATTGGATATGCATGTTGGGGGGATGGGGGATTAAACGATCCTTATATATTAAAGGCGCTACGTGCCCAGGGACGGTTTTTATATTTTGCGGGTCAGTTGGTTAGAACAATGTCAACACACAGTTGGGTTGTGTTAGAGACCAGCACCCATATGTGGTTTTCCCGGGCCGTGGCGCAGAGTATTTTAGCACATGGGGGTAAACCCACAAAGTATTATGCTCAGGTTCTTGCCGCCAGTAAACGGTATACTCCGTTACATTTAAGACGTATATCCGAACCATCGAGTGTGTCTGATCAGCCGTATATTCGTTTTAATCGACTGGGATCTCCAATAGGGACAGGTATAGGGAATTTGGAATGTGTCTGTTTAACGGGAAATTATTTATCTGACGACGTAAATGCAAGTTCGCATGTAATTAATACAGAAGCACCGTTAAACAGTATAGCACCCGATACAAATAGACAGCGGACTTCTCGCGTTTTAGTTCGTCCAGACACGGGTTTGGATGTAACTGTCCGAAAAAACCACTGTTTGGACATAGGCCATACGGACGGTAGTCCAGTTGACCCAACGTATCCTGATCATTACACCCGGATAAAGGCGGAATATGAAGGTCCGGTTCGGGATGAATCAAACACAATGTTTGACCAAAGATCGGATTTACGTCACATAGAAACCCAAGCATCTTTAAATGATCACGTATATGAAAATATACCACCCAAGGAAGTGGGTTTTAACTCATCTTCAGACCTGGATGTGGATAGCCTTAACGGGTACACTTCCGGAGACATGCATACAGACGATGACTTATCACCAGATTTTATACCCAACGACGTTCCCGTTAGATGTAAAACCACGGTTACGTTTAGGAAAAATACGCCTAAGAGTCATCATTAAGTACAGCGGTTAATAGATAGTTATGGACTAGGCACTTTGGCGGTCATTTCCACAACCAGGTTAAAATTGGGGGATTTGGGAGAAAATAGTCTATTGCGTATTTTCTGTTCAATAATTGGACTGCGTTATTTAAAGGTCTGATTGGTTGATTGGGTTATAAAAGGAATTACTCCTTTAAATTTTACTTAATGTACCCACAATATCAAGTGGTCGTTTGTATTTAACGATTATTACCGGTACCATGGGAGACTTGTCATGTTGGACAAAAGTGCCGGGTTTTACGTTAACCGGCGAACTTCAGTACTTAAAACAAGTGGATGATATTTTAAGGTATGGAGTTCGGAAACGCGATCGAACAGGAATCGGAACGTTATCTTTATTTGGAATGCAAGCTCGATACAATTTGCGAAATGAATTTCCTCTTTTAACTACAAAGCGTGTTTTTTGGAGGGCCGTCGTGGAAGAGTTGTTATGGTTTATCCGCGGGTCAACCGATTCCAAAGAACTCGCCGCTAAAGATATACACATATGGGATATATACGGATCGAGCAAATTTCTAAATAGGAATGGCTTCCATAAAAGACACACG

CTGTCAATCAAACTATTTACAGCAAGGAATCGATCAGCTGCAAACTGTTATAGATACAATTAAAACAAACCCAGAAAGCCGACGAATGATTATATCGTCTTGGAATCCAAAGGATATCCCCTTA

AATGGATTTTTAAATGTTGTCAAGACAGTAGATGTGTTGCGAATGTAATAAAATGATATACACAGACGCGTTTGGTTGGTTTCTGTTTATGAACAGCAACGGATGCATAGGGTTGCGATAACTGCGATAAGACCCAATGTCCCAAGGATAGATATCACACCAATTATAACTGCTACAACGGAAAATGTAGTGGCGTAGGTAGATGCATCGTAGGTATAAACGGCCGAAAACGGAGGGAATTTTTTAGGGTAACCATCTAGATGACACGAATAGGTGATAGGTCCGTCGAGTTCCGATGTTGGACAAGAACTTTGCATGTTTACAAACCGTTTGTTTTGATCACACACCCCAGTAATCTCACTGTTTTCGTGGTTGATGGGAGAATCGTTAACCCACCATACGAAATGTACAACGCCACGTGGCACACATTTTGCCGTACATACTATGTGTCCATCAATAATACCTATAGACACGTTGGGAAATGGATAGACGTCAGGGGTAACGACAGCAGAATATTTCATATTAGAGACGCCATCCCGAATCCATAAAACATTACATTGGATGGCTGGGGGTGGGTAATCCATTTGTTTTTGCTGTGGAATTCGTACCGCCGAAACATAACTAAATAATCCATTGGCATATTCTTGTATTGCATCGGTTATAAAATTTTTTCCGATGTTACCAAACCTTGAAGTCCACCGAACACGTACCGAGTGCGGTGGATAATACTTTGATACGTTACAGTAGGCTGCGTATGTCTGTCCGGTTAAGACTGGATCGCCGACAACGGTAATATTTGGACGATAATACGTTGTAACTGTAATACTGTGTTCCGATATGACGTTCTTAGTTTTTGTATTAACGACTCGCCAAATATACGTTCCCTCCGTGGTAGCATCCATAGATAAAATTGTTACAGAAAAATCAGACGTTGTTTTAACATCTGGTATTACATAATTTTCCTTAGCGTGTGTAAATATCTCAGGGTTGTTTATTAAGTTTAAATCGGCACTGTTGCTATATAACATAACCGGTAAATCTGGCATGCGTATTAACGCATTGCCCAGTTGACGGTGCGGATCTATAAGGTGACGCGTAAACCAAACTTCAATATGAAGATCGGGGCGTATAAGCGACTTCCACCTTGTTATATTTGAACCTTCCGGATCTAAAGAATATTGTTCATATGTTTTTTGTTGCTGCTTAAAGGCCGCCTGTTGTCCGGTCGTTAGACGCATGTAACAAGGCATGATAAATGTGTGAAAATAGGGTATGGATTGTATTCCGCCGTGAACGCATTGTATATTTTCAAATAGAAAAGGTGGTTGTGAATGTTGGGCGTTGGCTGCGGGATCGGGCTTTCGGGTAGCGGCCGAGGTGGGCGCGACGGCGGGATCGGGATTTCGGGAAGCGGCCGAGGTGGGCGCGACGGCGGGATCGGGATTTCGGGAAGCGGCCGAGGTGGGCGCGACGGCGGGATCGGGATTTCGGGTAGCGGCCGAGGTGGGCGCGACGGCGGGATCGGGCTTTCGGGAAGCGGCCGAGGTGGGCGCGACGGCGGGATCGGGCTTTCGGGAAGCGGCCGAGGTGGGCGCGACGGCGGGATCGGGCTTTCGGGAAGCGGCCGAGGTGGGCGCGACGGCGGGATCGGGCTTTCGGGAAGCGGCCGAGGTATATAATTCAGTTATACTTACGGGTGTGGGTTGAGATTCAGTCGATAATTGTATACACGCGATCGTTAAAATTAAATTTATTTGTATCCGCTTCATCCTGGTTTTTATTGACACATCCACGCTCCCCTTAAATAAAAGATTAAAACACCCACCGCGGAATTTAAATGATGGAAACGTTTTTTTCGACATTGGGAATAATAAAAACGGCTTTTGCAACTTTAAAAACTTTATTTATCTCGATTACGATACATATGTACCACATAGATAGCATAGATTTATTATAATATAAACACACACGTGATATACTTTAGTGATATGAGATGCCATAAAACAGTCAATAGGTTTAACGCTTAATCTCATCATCTGAATACACGTCAAACCCGCCGCAACTGTTGATGTTAGAATTATAATAGCTCCCCATGAAATGCCGGCAAATGTTACAGCTATACCCGTCACCGAGGTCGTTGTATATAATACAATTACCCATAGGTTTTTTTTTTCTTGATATAAAACGGCAAAACCCTGTAACCCAAATGCTATAATATGACCTCCTATTGAAACTGCTAACGTTACTTGTGTAAGTTTGATAAAATGATTTAATTTAATTATATGTGAGATTGCCCACATTAATGGGGTAACTATATATAACACCGGGGGTATAACAGACATTATACGAATTCCTTTAAACACGCGTTTAAGGGTCCGGGAACTTTCTCTATGGTCACATACTCTCCCGCGGTCATTTTGTGTATATACAACGGCAAAACCTAAATCTGTATAAGTGTTTAATTGCTTATGGCGATTTTTACGATATATACACGTATCTTGCAAATCGGTGGCGGCATCGACAATTGAAACTAGTGTGACAATAGATATACACAATCCAATAAGAACCTCATATTTACTGACATACATATATAAAATAACGGTTAGTAAACCTCCCAACCCAGTTCCCAACATCATAACATAAAAATAAATATGCGGTCCATTGAATGTCGTAACAAAGTTGTAGTAATGGATATGCACAGCAGCCACTGTTCCGGTAATCGCGGATATGGAAATTCCCAGTAATTCTACAAATGGAAGATCCCGGGATATTGGGCAACCAACCGCCCATAACACAGCAAAACCCAACACGACCACCGTCTGCAAACATCGTCCCAATTTTGCTAATGTGCGTAGAAATTTCACGGATGTTGGCCATAACCCCGAAACGACGATCAACCCCATAATAGTTGCATTGACGGCAGCTTCGCAGACGTGATATTGTAAAATTAACCCGGACGTGATAACACTTGCTTGTAGTCCCACGAGAAACAACCGCGATGCTGAGGTTATTGCACACGAATTACATTCTTGAGGGTTTCCGACACATCCTTGGATTGATTGAGCGCGGATTAATTCTCTGTCTAACACACCCAGGTTTTCATCATGGACAGCTCTTTCACCATTCACGGCCATGTCTTAAGTTTAATAATTCAAAACAGATAAAAATGTGTTCATCTATGGTACACACAAGTTTGTATGTAAAATATAAGCAAAAGTTGCACTTATTTAACTGTACATATTACGTCAGATTCACGTGATAATTCAGAATAATCCAGGGTTCCTGCAGGGTCCACTGGAGGAGCCACACAATATTCGCGAATTCCGATTCCCTCCTGCCATGTGGTTTCGGGGAGTTTCCCCCCCATTTTATTTCCGGTATTTTTTTCGTTTCTTTTTGTTAATAAATTGCGTCTTTTTTTTAATGGTGGTTCATCCTTCACAGATTCCGTGTTCGCAAATAATTGCATCGAGGTTAATTTTTCTTTAAGGTCTTTGGGACTTAAGAACGTTGCATAAAAAAAAGAATGCACGGGTGCGGAACGTTGGATATACAATCCAACCATGGGGGAGTTAGTTAAGGCGAGATAAAAATTAATATAACACGTCTCATCCCGTGTTAACTTAAGATTTTGTACGGCAGAACGGAATCCACTGTGTGTTTCCAATAATACTCCAAATTCACGCATACTCCCGCTGCCATAAACAACATTATTAAGGATCCTTTTTGAATTTGTGATTGAGCGTATTAAATTATATGGTGTAGGCTTGCTTCCGTTTATATCCAAGGAAACATTAAATGAGATAAAACCACCCCCGGCGGTCTGGATGTACATATCCGTGGCTGTTAGAATGAAGCATGTTGTAAACCCAAAAGTTTTAAGTAGTCGCTGTAAACGGGTGAATTGATCGCGTTTTAAGCAAATGCTTATATCTGGAGTTAGATTTGGGAACATCATTGTATAACAAGCGAGTTCACGTTTTACAACTTGTTTGTAACATTGTACTTGATCATCTGGACCACAATCACCCGGGCGTTGCCATACCATCGTTTGGATAATACTCCGCTCGGGGGGTTGTCCGGTAAATTTAAAATATAACCGTGTTGGGGTCGACGGATCTTTTGTATGGCGAAACGCGTCAATAAGCGAGGACCGTCCCTCCGTTGCCGCGAGTACAACCATTCTCGGCCCAGTCCAATTATACTGGTCAAACATATTTGCCGGTATAGGAATATACAGTTGTTCTGTTTCCAAACTACAGTGAATAATTAATCCTTCGTCGCTGAATATTAAAATAGAATCCCTTAGTCTATTAACCAGAGGTGATATAGACGAAATTAAACCAGTAAGCGTTTTTTCCGTTAAAACAGCTCTGGCGATTTCTGGGGCGTCAAAACCCGCATGCAATTCCATGTCCAAAGCATCGTCTGTACGCGACCTCAAATCCATAATTTACTACTTAAAATGTTTACTATAGAAAAAGTAATCATATGTAAACACACGAGTTTCGTTAATATGTTTGTTTAACCCGATCCGGTGACTTAAGTACATAAACAGGCATGATATTTGAATAGTACGGCCCATGGGAGGGAACATTTCCACGTGTTCCAATACAGGGGGTGTTCCTTAATAGGGACTGTGCAATAAAATACGTAAGAAGTTACCAGATTTGATGTAATGTTTGTCATAAAAAATATGTACATCATTATATACGTCTGTAATTAACACAAGATCACATCGAAGAATTACTGAAGCCGCTGTGAAACCTTTCACAAGACGATATAAACTTGGTTAAGTGTATTGATGGGGCTCTTTGGACTGACACGCTTTATCCATGAACATAAACTGGTTAAACCCAGCATCATTTCAACGCCACCCGGAGTTTTAACCCCCGTGGCGGTAGACGTATGGAACGTCATGTACACATTGTTGGAACGTTTATACCCTGTGGGTAAACGCGAGAATTTACACGGACCATCTGTAACGATACATTGTCTTGGAGTCTTATTGCGGCTATTAACACAACGGTCATACTATCCGATATTTGTATTGGAACGTTGTACAGACGGCCCATTATCACGTGGAGCCAAGGCAATTATGTCACGGGCCATGAACCACGATGAAAGGGGAACCTCGGACTTAACCCGTGTTCTACTATCATCCAACACATGTTCTATCAAGTATAACAAAACATCGGAAACATATGACAGTGTGTTTCGAAACTCTTCCGCGAGTTGTATTCCTAGCGAAGAAAACAAATCCCAGGATATGTTTTTGGACGGTTGTCCACGACAAACTGACAGGATGATCTGCCTGCGCGACCAAAACGTATGCAGTCTTACCTCTACAATGCCATCCCGAGGACATCCTAACCATCGATTATATCACAAATTGTGTGCAAGTCTTATTAGATGGATGGGGTATGCATACGTCGAGGCGGTTGACATTGAGGCGGACGAGGCATGTGCAAACTTATTTCATACGCGTACAGTGGCTTTGGTTTATACGACAGATACTGATTTACTCTTCATGGGCTGTGATATTTTGTTAGATGCAATTCCTATGTTTGCTCCAGTAGTACGATGTCGCGATTTGCTTCAATATTTAGGAATTACATACCCTGAATTTTTGGTTGCCTTTGTTCGCTGTCAGACCGATTTGCATACAAGTGACAACCTAAAATCTGTTCAGCAAGTTATTCAGGATACCGGCCTGAAAATTCCACATCAAATGGACACTTCAACGCGCTCCCCCACTTACGACTCGTGGAGACATGGCGAGGTTTTCAAAAGTCTTACCGTAGCCACGTCGGGTAAAACAGAAAACGGAGTGTCCGTTTCCAAATATGCATCTAACCGATCGGAGGTGACAGTAGACGCCAGTTGGGCTTTAAACCTTCTGCCACCCTCATCCTCCCCATTGGATAATTTGGAACGCGCATTTGTTGAACATATAATCGCCGTGGTAACTCCATTGACCCGCGGTCGCCTAAAGTTAATGAAACGTGTAAATATTATGCAAAATACGGCAGACCCATATATGGTTATTAACACCTTATATCATAACTTAAAGGGGGAAAAAATGGCTCGCCAATACGCACGTATTTTTAAACAGTTTATTCCTACTCCACTCCCACTAAACACTGTATTAACAAAATATTGGAATTAAAACACACATAAGAGCGACTTAATGGTTCATTGTTTTATTTTGCTCGTATATACATGTTATAAATCGTTTATCACTGTGCCCGCATAAGATGTACTGTGTCTCTCAAAAAAATTTGTGTTTTTATCTGCAATCATAAATGCAAGTGGAAAGTCCGAATCGGGAGGTGGGGTGTTAAATAGTTTTGGTACATTAATCGCTGATAAAAGCCTGTCCGCGCTGAATTTCACGTATTGTGTAATTGCATCGACGTTCACCAAACGGGTTTTGGGTGCATGGGATTTTAAAAACGCACACTCGATTTCAACGGCTTCCGAAAACAGTTGATGTATTCTGGTGATAGCGGGTTTTTCGGGTACATAGTTATTGTATATACAACACGATGCGCTGGTATGTATGGCTTCATCTCGGCTTATAAGGTCGTTAAATTGACAAGTTACAACAAATAGTCCGTTATTGCGTAAATATGCAATAGCCGCGAACGATGATACAAAAAAAATGCCCTCTATAAGAATCATTAGTATATATTTTTCTGCAACGGATGGGTTGTCCCGTACCTTTTCTTCCAACCATTGTACTTTTTGTTGGATCGACGGATTATTAATAGTGACATTTACGTATTGTACCCGCAACGATTCATCCCCTCTGAACAACATTAGTTGAATTTGACTATAGACACGCGCGTGGACAACCTCGATGCACTCTTGTTCAATGTAGTAATGGTGAATATCCTTTTGGGAAAAGAGTTGGGTTAGAGAGCCCAAATTAACATTTACCAGATCATCTGCCGCCGATAAAAATGTAAAAATAAATCTGTAGAATATTAGTTCATCTTCCGTTAAACAGTCCAAGTATTGATAATCATCTTCAATGATAAAATCGCTTTCTAACCAACGATTCGAAATGCTCAGGGCACGTAAATTGTTTATATCTGGACACTCCGGCCTGTAAAAAAAATGACTGCAATCTTTCTGATCCATTTTGGAATAGTTTCCCGTGTAAATTTATAAAGCACAACTGGTACAGGTTAATTCGCCTCCCGCAAACAGTCCGCTGTTCGTAGCTTTACGAATTTTACAGTAGTACATACCCGTTTTAAGGCCGGCTTTATAGGCACGTATAAGCAAATTCATTATTTTGGAGGCGGGAATTGTCCCGTCTGGGCGTTCCTCAATAAATAAAGTCATTGATTGACTTTGGTCAATAAATGGCGCCCTTTCTGCACACATATCAACGAGATCCTCTTGCTCATATTCAAACGCTGTTTTATATTTTAAGAGTGGGTGACTATTAGATAAACAGCCAAACGAACGTATTACTGACCATTGGTTTTTCTCAAGTATGTTTATAACTTCCAGTCGTTTTTCTTCACATGAATACATATCTCTTAGTTCGTCCATAAGGTCTAAGTTGGGTCTAAGTAACTCACCCGAGGTGGTGACCTTACTAAACATATTATTATAAATTGGAGAGAAACCCTCACTGCACTCCGTTACCTGTGCAGATGAAACTGTGGGCATTAACGCTAAGAACTGCGAGTTGTATAACCCATAAGCGCAAATATCATCTCGCAGGGTACACCATGGTAAATCTAAATAACTTATCGTAGAAAACCCATCTTGGTGTAACCATCCCTTAGCATATTTACTTTCGGTAAAACCCTTAAACGGGGCTAAGCCGCCAATCTTACACATTTCCATGCTTGTTTTCATTGTCTCATACAACATTAACTCCGCTATTTGTACATTTAACCGTCTAGCTGGTTGGGAAGTTAAATCAAATCCTAAGCGGAGACAAGTTGTATGTAACCCTTGTATGCCAATGCCAAGTGATCGGTTGTTTTTTACACCTTTACATGATTTTTTACATGGAAAGTTCCCAGCCGCCAGGACCCCGTTTAAAAAAATAACAGTCGTTCTTGCTGTCAATTGAAGGTCGTTTAAATTAAATGACACTGGGCCTTTGGATAAGCACGTTGTAAGATTTATGCTGGCAAGATTACATACGCCGTGTTGATGAGCGTCTGCCTTTTGAACAATTTCCGTACACAAATTTGACCCCGTGATAGCATTTCCTTGGGTATTCATATGATAATTACGATTACAGGCATCTTTGAACATTAAAAAGGGGCTTCCTGTTACAGCAGCACTGCGTATGATTGTGAATGCGATATCTTGAATGGGAACAGAAGAAACGCCTAATCCTTCTCTCTCTAAACGTAAATAGGTTGAAGTGAATGCCTCCCCGTGTAATGTTCGAAGGATATCGGCTCTGTTATCAAAAAGAGTCCACTGAACATTACTAGCCCCTTTTAGATAGCTTAGGTATCTTTCAAAAAATAAATCTGGGGTCCATAAACAACAAAATATGTTATCACATCGAAATATTTCATCACGAACCAACATTCCACGTGTGGCCAAAACAGTTTGTAGATCGACGTGCCATGGTTCTATGTAAACACAAACTCCAGTTGGTCGTTCACAATCACTGTTAATTGCCATAACCATGCAATCTAAAAGTTTTAAAACTGCAAGAAGACCTTTCGTTTGATTTTCCGTAGGTATTAAATTCAGACTCTGTAGAGAAATTCCCACTCCACCTCGACTTTGTAATACCGTTCCCACATCGCCTGTGATAGCTCGAACAGCTCTCCCAACAGTGATGGATTCCGGGTCCATTAAATAACAACTGGCCGTTGCCCCGGTCTCTCGACCTAAAAACATCATAACCGGTGTAGCCGGGACAATTTTCTGACATGCCAACGCTGTGAAAAATACCCGACAGACATCAGTCCATGTATAACCATCATTTATTCCGGGAATAAGAGTTGCGATTTTAGGCAGGTTTACGATTTCTGTTGTCACGGTGGCCGCCAGTCTTAAAAAGAATTGGCAAAGCGACTCTAATTTACCTTCCTCTAACTTAGTTAAATAAAAGTCTTCGTACTTTAAAGCAGACTGTAGTCCAAGGGTAGCTAAAGCGGGGTATTGATCTTTCAAAAACGGTTCTAATATAGCCCGACGAATTTCGTCCCTCCGCCCTTCAATTGCTTGGCGGACTCGGGGAGTTAAACAGAGAATTGGGGAAGTCAACCACGTTTCCATGGAAACGGATCGTAGGTTAATACGGCAATGGATAAGTTCTCCACAACATCGGTACACTCGCTCATCTTGTCGCGTCACCGCCTTAAGTTTTGAGACGATAGTGCTAATATACTCCATTAATTCCACCGGTGTGGTTGATTCGGGCGGAATGATGTATTCCTTGTAGCCATGTTGACATAATCGGTTTATAATGTCATGAACCGTATTAAAAATTCTTTTGAACTCCATAACGGATAACGTATTTAGGCTCCGGAATAAACCTTTAAACCCTAAACTCACAGCTGAGTTAGTTCTACAATATTGTAGACTCCCTTATATATGGTTACGTACAGCCTGCCCCTCCCCAGTATATAATATCACGCAAAACCCACGCTATGTTAAATTCAGTTTATTTTACATACATGCTTTAATAATAACATTCGTTCCATGTATTTGTACCCCCCCACACAACCCCCTCTAACCAAATAGTTGGCACGTTATAACCTCCGAACCGTTCCATGCGTCTTGTATAACGCACAGACTCTGATGGAATTGTTCCAATTAACGTATATGCCGCATACATGCAGGATAATTGTGTGGGAAGGCCCCGAAAATCGCCGGTCCATTGATACAATCGCTGTCTAGCCAAGTTCCAATTTACTCCTGTAATTTCGCCAATACTACATCGAGGGCTTGTCGGGTCATTGGATAACTGCACAAGCGGCAACGCCCTTGTGTTATATGGCTGGTGGGTATTTGCAACCCCTTCAGTCCCCCAGGCGGCATTTTCAGCTCGTATGCGTCCTAACAGGAAGCCAATACCACGACCAAAACATTGTTCGTTTAGTTGGCTTAATGCAAGATGCAGTCTTACACCTTCTCGTTGGCGTCGCTGTGTATATACAAAAACCAAGAACACATGCTTCAGTCCGTCCGCGGAAAGATGTAAATCTTTGTCAACGTCCCAAAATACGCAGGCCGGGATGTTGGCTGTGACCCTGCGAGTTGAAGTTTTGTCTGTACGTGCAGCTTCTTGGGGACCTTTGGCCACGGCGGTTATATTGCATAAATTATCCTGAATGGTATATTCCAGCAGGGACCCAAAAAAACTTATAAATCGATGTGGAAATACATGACATTGTACCATCGCACGTAAACACTCCGAAAACCTTATGAGCCGCGTTTCCATACGACTGCATCCATAGGCAGAAACAATTGCTGTTCTGTTGGCATCCGCTGCCTGTTTATCCGTATATTCTTCTGCCCGGCATGCGGCGATGAAACTTAATGACGTTACATATGCTCTAAGCCCCCCACCTTCTCCAACGGTCCAAGGAGCCGTGCAGGCATTGAATAGGTTTCGTAAACCCTCTAGTAGTACATCGGGGTCACGTCCAGCCTGTGTAAGTGTATTAGCTTCTCCAATCATGTCAGATGGATGACGAAGGATTAAGACGATTGACCCAGCATGCTCAATGTCCGGACGAAAAAAATCGGTTAATGACACTTGTTGGATTAGCTGTGTCGTTGATTTAAAATTATTTAACGGGAGTCTAATGGTAACTTGCGGGTTACCAATTGAAGTTGGATTTATTTGAATGTTGTTCATACGATTAATAACAATTGAACGGGGGGTTACTTGAATAGACGCGGTTTCTGTACGTTTTGGTGGTACATGTATCGGTTGTTTGTTCAGACCTCCAAAGCGAGGGCCAATTGTTAAATCGCGACTCCAATTTCCGAAGAAGCCCGGAGCATAAGTCATATGAAGCCCGTTCCCTATTTGAATAAAACGGTTATTTCCTAAAAGACTGATATTAGTTCCACATAGCGTTTGTTCGTTTAAAGTAAAATGCGAGTTGGTTGGTTGACTCCCCATAGCTGAGGGGTTAAATTCACACAATGCAATCGTGACGTGGTACTATCTGAAATGTTGCCTGGGGTATGTGTACACATTATACAGTCGTAGTACCGTTTATATAATGTTAGGTAGGAGGAGCCTATAAAAATATTTTGATTGGCGTTAAAAGGTTCTTCAACTTACCGTGACGTCCTTTTTATTAACATGCGTTTTTATTGATGTTACATTTATGTCTTTTCATTCCGGACGGATGTAGCTTTTTCATATCACGTTATAAAGTTAAGTCAGCGTAGAATATACCATGGAAGAACCAATTTGTTATGATACACAAAAACTTTTGGATGATTTAAGTAACTTGAAAGTACAAGAAGCGGACAACGAAAGACCATGGTCACCAGAGAAAACAGAAATCGCCAGAGTTAAGGTAGTTAAGTTTTTACGATCTACCCAGAAAATTCCAGCTAAACATTTTATTCAGATATGGGAACCCCTGCATTCTAATATCTGTTTTGTATATTCCAATACATTTTTGGCGGAGGCTGCTTTCACGGCCGAAAATTTACCCGGACTGTTGTTTTGGAGACTAGATCTAGACTGGACGATAGAGGAGCCAGGTAATAGCTTAAAAATTTTAACCCAGCTATCAAGTGTAGTACAAGATTCCGAGACGTTACATCGTTTATCGGCCAATAAATTACGAACCTCGTCTAAATTTGGACCCGTTTCGATACACTTCATTATAACGGACTGGATAAATATGTACGAGGTCGCCTTAAAGGATGCAACAACAGCCATTGAATCACCATTCACTCACGCTCGTATTGGAATGTTGGAAAGCGCCATTGCAGCTTTAACACAACATAAATTTGCGATCATTTACGATATGCCATTTGTTCAAGAGGGGATTCGTGTTTTAACACAATATGCAGGATGGCTTCTTCCGTTTAATGTTATGTGGAATCAGATTCAAAATAGCTCACTCACTCCTCTAACACGAGCCCTTTTTATAATCTGTATGATTGATGAATATCTCACGGAAACGCCAGTACATAGCATATCAGAATTATTTGCAGATACTGTAAATTTAATTAAAGATGAGGCGTTCGTATCCATCGAAGAAGCGGTAACGAATCCACGAACGGTGCACGAGTCACGAATTTCCTCAGCTCTGGCTTATCGAGACCCTTATGTTTTTGAGACATCCCCGGGAATGCTTGCTAGGAGACTTAGATTAGACAATGGTATATGGGAAAGCAACCTCTTATCGTTGTCCACCCCCGGAATTCATATTGAGGCGCTGTTACATTTACTAAACTCCGACCCGGAAGCGGAAACCACATCTGGAAGTAATGTAGCAGAACACACCCGTGGCATTTGGGAAAAGGTTCAGGCTAGTACATCGCCTAGTATGTTAATAAGCACCCTTGCCGAATCCGGGTTTACAAGATTTTCATGCAAATTGCTACGTCGGTTTATTGCTCACCACACACTCGCCGGTTTTATTCACGGAAGCGTTGTAGCAGACGAGCATATTACAGATTTCCAACAAACACTAGGATGTCTCGCTTTAGTGGGTGGACTGGCATACCAATTAGTGGAAACGTACGCTCCTACTACCGAGTATGTGTTAACATATACACGGACAGTAAACGAGACCGAAAAACGGTATGAAACGCTATTACCCGCCTTAGGATTACCACCGGGAGGCCTGGGACAAATTATGCGGCGCTGTTTTGCTCCACGACCCCTTATTGAAAGTATACAAGCGACACGCGTAATACTACTTAATGAAATTTCACATGCAGAAGCTAGAGAGGCAACATATTTTAAGCAAACACATAATCAATCCTCAGGTGCGTTATTACCACAAGCAGGACAAAGTGCCGTACGCGAAGCCGTACTAACCTGGTTTGACCTACGTATGGATTCAAGATGGGGTATTACTCCCCCGGTGGATGTGGGTATGACACCTCCTATTTGTGTTGATCCACCGGCTACAGGGTTGGAAGCTGTCATGATAACAGAAGCACTAAAGATTGCATATCCTACCGAATATAATCGCTCTAGCGTGTTTGTGGAACCGTCGTTTGTGCCTTATATTATTGCAACAAGCACGCTTGATGCCCTTTCGGCAACAATAGCTTTGTCTTTTGATACACGGGGAATACAGCAAGCCTTGTCTATTCTTCAGTGGGCTCGCGATTATGGATCCGGAACCGTGCCCAATGCAGATGGATATCGCACAAAACTATCTGCTCTTATAACAATATTAGAACCTTTTACCCGTACACACCCCCCAGTACTTTTACCATCTCACGTTTCTACTATAGATTCCCTTATATGCGAACTTCATCGGACTGTTGGCATTGCCGTTGACCTGCTTCCCCAGCACGTCCGTCCTTTGGTTCCTGACCGTCCTTCTATTACAAATAGCGTTTTTTTAGCAACTCTCTATTATGATGAACTTTACGGTCGTTGGACCCGACTGGATAAAACATCGCAGGCGTTGGTTGAAAATTTTACATCCAACGCGTTAGTGGTTTCTCGGTACATGTTAATGTTACAAAAATTTTTTGCGTGTCGTTTTTATCCAACGCCAGATCTTCAGGCTGTTGGTATCTGTAACCCAAAGGTTGAACGCGATGAACAATTTGGGGTATGGCGTTTAAACGATCTTGCTGATGCGGTTGGTCATATTGTTGGGACAATACAAGGAATCCGAACGCAAATGAGAGTGGGAATATCCAGCCTGCGCACAATTATGGCCGATGCTTCCTCAGCCCTTAGGGAATGTGAAAATTTAATGACTAAAACCTCCACTTCTGCTATTGGGCCTCTTTTTTCAACGATGGCTTCCCGGTATGCACGGTTTACACAGGATCAAATGGACATTTTAATGCGTGTTGACAAACTAACAACAGGAGAAAATATACCCGGTCTTGCAAATGTAGAGATTTTTTTAAATAGGTGGGAACGAATAGCAACAGCTTGTAGGCATGCCACGGCAGTCCCGTCGGCCGAATCTATTGCAACCGTGTGTAATGAATTGAGGCGCGGTTTAAAAAATATACAAGAGGATCGTGTAAATGCCCCAACCTCATATATGAGTCACGCCCGAAATCTGGAAGATCACAAGGCAGCAGTTTCATTCGTTATGGACTCCAGGCAACAGTTTATTGTGGATTCTGGACCTCAGATGGGCGCGGTTTTAACTTCACAATGTAATATAGGAACATGGGAGAATGTAAATGCAACGTTTTTACACGACAACGTTAAAATAACGACAACGGTCAGAGACGTAATTTCAGAGGCTCCGACGCTGATAATAGGACAAAGATGGCTTCGTCCAGATGAGATTTTATCTAATGTAGATTTGCGTCTTGGCGTACCCGGGAATACAAGTGGGAGTGACCCTTAATATAAAACAGGCGTGTTTATGTACATTAAAGTATTTGTGGTTTTTATTGACTGGGCGTTTCGTTTGTATAACGCTGTTGTTGCTAGTATTTTCATAACCTCCTAGGTTTTTGGAGCTACACGTGCTTATTCAACGCTCTTTGGGATTTGAATCATCGTAAACGTAGCGTCCCTACCAGTTGAGCGCGTAATTTTCGTAAGCAATAAAATGGATATAATTCCGCCTATAGCTGTCACTGTTGCGGGAGTGGGAAGCCGTAATCAATTTGACGGTGCCCTGGGACCGGCGTCAGGTCTGTCATGTTTAAGAACATCTTTATCGTTTTTGCATATGACATATGCGCATGGAATTAATGCAACCCTGTCATCAGACATGATTGATGGATGTTTACAAGAGGGTGCAGCATGGACTACGGATCTGTCTAATATGGGGAGGGGTGTCCCAGATATGTGTGCTCTTGTTGATCTCCCCAATCGAATTTCATATATTAAACTGGGGGACACTACCAGTACGTGCTGCGTTTTGTCTAGAATATACGGCGATAGCCATTTTTTTACCGTTCCAGACGAGGGTTTTATGTGCACACAAATTCCCGCTAGAGCGTTTTTCGATGATGTGTGGATGGGACGTGAAGAGTCGTATACAATTATAACTGTAGACTCAACGGGAATGGCCATCTATCGTCAGGGAAACATATCTTTTATTTTTGATCCACATGGCCATGGGACTATAGGACAGGCTGTAGTTGTTCGGGTGAATACCACGGATGTGTACTCTTATATCGCATCGGAGTATACCCACCGCCCCGATAACGTAGAATCCCAATGGGCCGCTGCATTAGTTTTTTTTGTCACCGCAAACGACGGTCCCGTAAGCGAAGAAGCGCTATCTTCGGCAGTAACGCTTATATACGGAAGCTGTGATACATATTTTACAGATGAACAATATTGCGAAAAACTGGTTACAGCTCAACATCCGTTGCTTCTTTCACCTCCTAATTCCACGACAATTGTGCTTAATAAATCGTCTATAGTACCTCTTCACCAAAACGTTGGTGAAAGTGTATCCTTGGAAGCAACCCTACATTCAACGTTAACCAACACGGTTGCACTGGACCCTAGATGTAGTTACAGCGAGGTTGATCCTTGGCATGCGGTTCTAGAAACAACCTCGACTGGGTCTGGCGTTTTGGATTGTCGTCGTAGACGCCGTCCTTCATGGACTCCTCCTTCAAGCGAGGAAAATTTAGCTTGTATCGACGATGGCTTGGTAAATAATACACATTCCACGGATAATTTACATAAACCCGCTAAAAAGGTTCTCAAATTTAAACCAACTGTAGACGTGCCGGATAAAACACAAGTGGCACATGTATTACCCCGCCTACGAGAAGTTGCTAACACCCCAGACGTTGTGTTAAATGTATCCAATGTAGATACGCCTGAATCCAGTCCCACTTTTTCACGGAACATGAATGTAGGAAGCAGTTTGAAAGATCGGAAGCCATTTCTATTTGAACAGAGTGGTGATGTCAACATGGTTGTCGAAAAACTACTACAACATGGGCATGAAATTAGCAATGGATACGTACAAAATGCGGTGGGTACGTTGGATACTGTTATTACCGGTCATACAAATGTTCCCATTTGGGTAACAAGGCCCTTGGTCATGCCAGACGAAAAGGATCCATTGGAGCTTTTTATTAACCTCACCATTTTGCGTTTAACGGGATTTGTGGTGGAAAATGGAACACGTACACATCATGGTGCTACAAGCGTTGTATCAGACTTTATAGGTCCCCTTGGGGAAATTTTAACAGGATTTCCCTCCGCCGCGGAACTTATACGCGTTACAAGTTTGATATTAACAAACATGCCGGGGGCGGAATATGCTATTAAAACTGTTCTCCGGAAAAAATGTACAATTGGCATGCTCATTATCGCTAAGTTTGGTCTAGTTGCCATGCGGGTTCAGGATACAACCGGCGCTTTACATGCCGAACTAGATGTGTTAGAAGCGGATCTAGGAGGTTCGTCGCCCATAGACCTCTATTCTAGACTGTCGACAGGTCTTATAAGTATACTAAATTCGCCTATTATTTCTCATCCCGGACTTTTTGCCGAGCTTATTCCAACCCGTACAGGGTCCCTGTCTGAACGAATACGTCTTCTTTGTGAATTAGTCTCGGCCCGGGAGACACGCTATATGCGTGAACACACCGCGCTTGTTTCTAGTGTAAAGGCTTTAGAGAATGCATTACGGTCTACCCGCAATAAAATTGATGCCATTCAAATACCAGAAGTTCCCCAGGAACCCCCGGAAGAAACCGACATTCCACCCGAAGAGTTAATTCGGCGTGTATATGAGATACGATCCGAAGTTACAATGCTATTGACCTCGGCTGTTACAGAATACTTCACCCGCGGAGTGTTATATAGCACACGGGCCTTGATCGCTGAACAATCCCCTAGGCGTTTTCGGGTCGCGACCGCAAGTACGGCACCCATTCAACGGCTTTTAGATTCTCTTCCGGAATTCGACGCTAAATTAACGGCAATCATATCGTCCCTGTCTATACACCCTCCTCCTGAGACTATACAAAATCTCCCCGTCGTATCTCTGTTAAAAGAGCTTATTAAAGAAGGGGAAGATTTAAACACAGACACGGCTCTCGTATCGTGGTTATCTGTAGTCGGGGAAGCTCAAACCGCAGGTTACTTATCCAGACGAGAGTTCGATGAATTATCACGTACAATTAAAACCATTAATACACGCGCAACGCAACGGGCTTCCGCGGAAGCAGAGTTGTCTTGCTTTAATACGCTAAGCGCGGCCGTAGACCAAGCCGTAAAGGACTATGAAACATATAACAATGGTGAGGTCAAGTATCCTGAAATAACACGGGATGATTTATTAGCAACAATTGTACGTGCTACAGACGATTTGGTGCGACAGATAAAAATTTTAAGTGATCCAATGATCCAATCCGGTTTACAACCTTCGATTAAAAGACGATTGGAAACAAGGCTTAAAGAGGTTCAGACGTATGCAAACGAGGCCCGAACCACACAGGACACAATAAAGAGTCGAAAACAGGCGGCATATAATAAACTCGGGGGGTTACTTCGCCCGGTAACCGGTTTTGTGGGACTTAGGGCTGCAGTAGATTTATTACCGGAACTTGCTTCTGAGTTAGATGTCCAAGGAGCCCTGGTAAATCTCAGGACCAAAGTCTTAGAGGCGCCGGTAGAGATCCGTTCTCAACTTACGGGTGATTTCTGGGCGTTATTTAACCAATATCGAGACATTTTAGAACATCCCGGAAACGCACGCACATCTGTCTTAGGAGGACTGGGAGCTTGTTTTACAGCTATTATCGAAATTGTGCCGATACCTACGGAGTATAGACCATCATTGCTTGCGTTTTTTGGTGACGTGGCAGATGTGCTTGCATCCGACATCGCGACCGTATCTACTAACCCGGAAAGTGAGTCCGCCATAAACGCTGTTGTTGCAACTCTTAGTAAAGCGACGTTAGTTTCATCTACAGTGCCAGCCTTATCCTTTGTGTTGTCGTTATATAAAAAATATCAGGCTTTACAACAAGAAATTACGAATACCCATAAGTTGACTGAATTACAAAAACAACTTGGAGATGACTTCTCCACCCTAGCTGTCTCATCTGGACACTTGAAGTTTATATCATCTTCAAATGTAGATGATTATGAAATAAACGATGCGATATTATCAATACAAACAAATGTGCACGCCCTGATGGATACGGTTAAACTTGTTGAAGTTGAACTGCAAAAGCTACCCCCCCATTGTATTGCTGGGACATCTACCTTATCTCGAGTAGTAAAGGATCTTCATAAACTCGTCACAATGGCACATGAGAAGAAGGAACAGGCAAAAGTGTTAATTACCGATTGTGAACGTGCACATAAACAACAAACGACTCGGGTTTTGTATGAGCGTTGGACACGTGATATTATAGCATGTCTGGAGGCAATGGAAACGCGCCATGTATTTAACGGGACAGAACTGGCACGGTTGCGAGATATGGCCGCTGCGGGAGGGTTTGATATACACGCAGTTTACCCACAAGCACGTCAGGTTGTAGCGGCATGTGAAACTACAGCCGTTACGGCATTAGATACCGTGTTTCGCCACAATCCACATACCCCCGAAAATACAAATATTCCCCCACCTTTGGCTTTGTTAAGAGGGTTAACATGGTTTGATGATTTTTCGATTACGGCTCCCGTATTCACCGTTATGTTTCCAGGTGTTAGTATTGAAGGACTCCTTCTGCTTATGCGTATTCGCGCGGTTGTGTTATTATCCGCCGATACGTCTATTAATGGAATACCTAACTACCGAGATATGATATTACGAACCTCGGGGGATCTATTACAAATACCCGCATTGGCTGGGTATGTTGATTTTTACACACGGTCTTATGATCAGTTTATAACCGAAAGTGTAACGTTAAGTGAACTTAGAGCAGACATCAGACAGGCTGCCGGGGCTAAACTTACAGAAGCAAATAAGGCTTTGGAGGAAGTAACTCATGTTCGGGCACACGAAACGGCTAAACTTGCACTTAAAGAAGGTGTCTTCATTACATTACCAAGCGAAGGTTTATTGATTCGGGCTATAGAGTATTTTACAACTTTCGATCATAAACGATTTATAGGAACGGCATATGAAAGAGTTTTACAAACAATGGTAGACCGCGATCTAAAGGAGGCCAACGCAGAGCTTGCACAGTTTCGTATGGTGTGTCAGGCAACAAAGAACCGTGCAATACAAATTTTACAAAACATTGTTGATACTGCTAATGCCACTGAGCAACAAGAAGACGTGGATTTCACTAACCTGAAGACGTTATTAAAACTAACCCCCCCTCCCAAAACAATTGCATTGGCCATTGATAGATCTACTTCCGTTCAGGACATTGTCACGCAGTTTGCATTGCTGTTAGGGCGTCTGGAAGAAGAAACTGGTACGTTGGACATTCAGGCGGTTGACTGGATGTACCAAGCTCGCAATATTATTGACTCCCATCCACTAAGTGTGCGTATAGACGGTACCGGCCCCCTGCATACTTATAAAGATAGGGTGGATAAACTTTATGCGTTACGGACTAAATTAGATCTCCTACGACGACGAATAGAAACCGGTGAGGTTACGTGGGACGATGCATGGACAACATTTAAAAGAGAAACGGGGGATATGTTGGCATCGGGGGACACGTACGCTACTTCCGTAGATAGTATAAAGGCACTCCAGGCATCGGCGTCTGTGGTTGACATGCTTTGTTCCGAACCCGAATTTTTTTTATTGCCTGTGGAAACGAAAAACCGTCTCCAAAAAAAGCAACAAGAACGTAAAACGGCGTTGGATGTTGTGTTGCAAAAACAAAGACAGTTTGAAGAGACCGCGTCTCGCTTACGAGCTTTAATTGAACGTATTCCAACGGAGAGTGACCATGACGTTCTTCGTATGTTATTACATGATTTCGATCAATTTACACATTTGCCTATATGGATAAAAACACAGTATATGACATTTCGAAATTTACTCATGGTACGGTTAGGCTTGTATGCAAGTTATGCTGAGATTTTTCCACCCGCGTCTCCAAACGGAGTGTTTGCTCCTATTCCCGCCATGTCGGGTGTATGTCTAGAAGACCAATCCCGATGCATTCGCGCGCGGGTGGCCGCGTTTATGGGGGAGGCGTCTGTGGTGCAAACGTTTAGGGAAGCCAGATCTTCTATAGACGCTTTGTTTGGAAAAAATTTAACCTTTTACTTGGATACTGATGGGGTTCCACTTCGATATAGAGTGTGTTATAAATCAGTTGGGGTTAAACTTGGAACCATGCTATGCAGTCAGGGTGGATTATCTTTACGACCGGCACTTCCCGATGAAGGTATTGTGGAAGAAACTACACTATCGGCATTACGCGTGGCCAATGAGGTCAATGAGCTACGCATTGAATACGAATCCGCTATAAAATCCGGGTTTTCTGCCTTTTCCACCTTTGTTAGGCATCGCCACGCCGAATGGGGTAAAACCAACGCACGCAGAGCCATTGCAGAGATATACGCCGGCCTTATAACAACAACATTGACACGACAATACGGGGTTCATTGGGACAAGCTTATTTATTCTTTTGAAAAACACCACCTAACTTCTGTAATGGGCAATGGACTAACTAAACCAATCCAGAGAAGGGGTGATGTACGCGTATTAGAGTTAACCCTATCTGATATTGTAACTATTTTGGTTGCCACAACCCCGGTACATCTTCTCAATTTTGCTAGATTGGATTTAATTAAACAGCATGAGTATATGGCCCGTACCCTCAGACCCGTAATCGAGGCCGCATTTAGAGGTCGTTTACTCGTTCGCTCATTGGATGGAGACCCGAAAGGCAATGCCCGGGCCTTTTTTAATGCCGCCCCATCCAAACATAAACTCCCGTTAGCTCTTGGATCAAACCAGGATCCTACCGGCGGGAGAATATTTGCATTTCGGATGGCAGATTGGAAACTTGTTAAAATGCCACAGAAAATAACGGATCCTTTTGCGCCATGGCAACTTTCCCCCCCCCCCGGGGTAAAGGCCAATGTCGATGCAGTTACCCGTATAATGGCAACAGATCGTCTTGCGACCATTACTGTACTTGGGCGCATGTGTCTCCCGCCAATTTCCTTAGTGTCAATGTGGAATACGCTGCAACCGGAGGAATTCGCATACAGAACACAAGATGATGTGGACATTATAGTTGATGCGAGACTGGATTTGTCATCCACGCTTAATGCAAGATTTGATACCGCTCCCAGCAATACCACGTTAGAGTGGAATACAGACCGTAAAGTAATTACAGATGCTTATATTCAAACCGGGGCAACGACAGTTTTTACAGTAACGGGGGCGGCACCAACTCACGTTTCTAATGTAACAGCGTTTGACATAGCAACTACGGCTATTTTATTTGGGGCTCCTTTGGTTATTGCCATGGAACTTACATCCGTTTTTTCACAAAATTCCGGACTTACTTTGGGGTTAAAATTATTCGATTCCCGGCATATGGCTACAGATTCGGGTATATCCTCAGCCGTATCTCCCGATATTGTTTCTTGGGGGTTACGTTTACTGCATATGGATCCCCACCCAATTGAAAATGCATGTTTAATTGTCCAACTAGAAAAACTGTCCGCGCTCATTGCAAACAAACCTCTTACAAACAATCCCCCGTGTTTACTGCTATTGGACGAACATATGAATCCCTCTTATGTTTTATGGGAACGAAAAGACTCGATTCCAGCTCCGGATTATGTGGTCTTTTGGGGGCCAGAATCTCTTATTGATTTGCCGTACATCGACTCCGATGAGGACTCTTTCCCCTCGTGTCCCGATGATCCATTTTACTCGCAAATTATTGCCGGTTATGCGCCCCAAGGCCCCCCAAACCTCGACACAACTGATTTTTACCCAACGGAGCCACTATTTAAGTCTCCCGTTCAAGTTGTTAGAAGTTCCAAATGTAAAAAAATGCCCGTCCAGCCCGTCCAGCCCGCGCAGCCCGTCCAGCCCGCGCAGCCCGCGCAGCCCGTCCAGCCCGCGCAGCCCGTCCAGCCCGCGCAGCCCGCGCAGCCCGCGCAGCCCGCGCAGCCCGCGCAGCCCGTCCAGCCCGTCCAGCCCGCGCAGCCCGTCCAGCCCGTCCAGCCCGCGCAGCCCGCGCAGCCCGTCCAGCCCGTCCAGCCCGCGCAGCCCGTCCAGCCCGTCCAGCCCGTCCAGCCCGCGCAGCCCGTCCAGCCCGTCCAGCCCGCGCAGCCCGTCCAGCCCGTCCAGCCCGCGCAGCCCGTCCAGCCCGCGCAGCCCGTCCAGCCCGCGCAGCCCGTCCAGCCCGCGCAGCCCGTCCAGCCCGCGCAGCCCGTCCAGCCCGCGCAGCCCGCGCAGCCCGCGCAGCCCGCGCAGCCCGTCCAGCCCGCGCAGCCCGTCCAGCCCGCGCAGCCCGTCCAGCCCGCGCAGCCCATAGAACCGGGCACACAAATAGTGGTACAAAATTTTAAGAAACCCCAAAGCGTAAAAACAACCCTTAGCCAAAAAGATATTCCCTTGTATGTGGAAACTGAATCAGAAACGGCTGTGCTTATACCTAAGCAATTAACCACCTCCATTAAAACAACCGTTTGTAAAAGTATTACCCCACCAAATAACCAATTGTCGGATTGGAAAAATAATCCACAGCAAAACCAAACGTTAAACCAAGCGTTCAATAAACCAATACTTGAGATTACCTCCATTCCGACAGATGACTCGATATCTTACCGGACTTGGATTGAAAAATCAAATCAAACACAAAAACGGCATCAAAATGACCCTCGAATGTATAACTCCAAAACAGTATTCCACCCTGTAAATAACCAATTACCTTCTTGGGTTGACACGGCAGCCGATGCCCCCCAAACGGACCTATTGACAAACTATAAAACAAGACAGCCGTCGCCAAACTTTCCGCGGGACGTACACACATGGGGCGTATCTTCTAACCCGTTTAACTCACCGAACAGAGACCTATATGAAAGTGATTTTAGTGAACCTTCTGACGGCTATAGCAGTGAGAGTGAAAATTCTATCGTACTAAGTCTCGACGAACATCGGTCATGTCGCGTTCCTAGGCACGTACGTGTTGTTAATGCCGATGTAGTCACCGGTCGACGTTATGTCCGAGGGACCGCCTTGGGAGCACTGGCCCTGTTAAGCCAGGCATGTCGGCGTATGATCGACAACGTTAGATATACACGTAAACTTTTAATGGACCACACGGAAGATATATTTCAAGGCCTGGGGTATGTTAAATTGTTATTAGATGGAACATATATATAAAGTAGCGCCTATTAAAGAAAAAAAAAAAACAACGATTATTTTCTGTGTATTTTTATTTACACCCTACGACTTCTTGAAGCGTTTCCAGATTGTCCCGTGTGGGACAAGGTCTGTCCCTTACCCCCCTGGGGGGTATTTTGGGTTGGGGGCGGGGTAGACTGTGGCACGCCTTGGGCCGCGGGCGGTGATCCGGTTGTTGGCTGGACAGTGCTTGACTGTGCTCCCTGTTGCGGTTGTTGTCCAGAAGACCCCGACACCACGTGTTGCTGTTGTCCAACGGATGCCGACGTCGTTTGAGGTGGGGGGTGTTGCGGGGATGATCCCGAAAACGCCAACGCGGCGGGCTGTTGTAAAGCAGACTGATCGGCGCTCTGTGTTTTTTGCGGCAATATAGTAGGCCCCGAGATTCCCAAACTCATGGATGGATTTGGGGGTTGTGGTCGTATAATACGCGGGTTAAACGTACGTTTTAAGCCAACCGTTGGTCTTAACCATGTCATAGGGTCAGTCTCGGCAAACATGGCCGTTCGGCGTATCGTATTTGCATTATGGTTAGCGCGTGCACGCGCGGCACTGGCCGCGGCTCCCACGGTGTAAATGCTTCTGGCATCAGCGATGTCCACACGGTGACCAGGTTGCAAAGGTCCACTGGCGTTTAAAAGTCGTATTAAAGCAACGGGGGTGTAAGCCGCAATTGCTTCCACCGAAAATGTGGTGGGGTTGCTGGGATCAAAGACTACACGAGACGATGCGGGTTGTGTCATCGTTTATTAGTTTACGGGACAATCGATAACAGCATACACGTACATCTGCGCAGGATATGTACGGAAAGGCAATTTATTTCCAGAAAAGCACCGCCCCTAATACAACTACCAGTACAATTACAACGAACAGGGCATATGTCACGTTAGCTACGGGTAGAGCAAGTTTCCAGACACGCGTAGTTTGGGTATCGGGTAACGCAGGTTTACTGTCACTTTGCATTTGAACAGACGTGTTTGGACTTCCGTTCTCGGGTGGGGATCTGAATGAAGGCCGCCAGCGTATATATTCATCCAAATTATTGCCAGTTTCCTTATACATGTATGCATCCGTGGCGCGGGCCATAAGTTTAATGGTGCGAGATGGATCTTCCGGTCCCATAAAACGAAAGGATAACTGAACATATGGCATTCGCACAAAGCAGTTCACCCACATTAAAGCCTGGAGAGGTCGGCGGTCAATACCCCCACCTCGTTTAATTGATTCCAAAGCAGATAGGTTGATACCGGTACTTAACGTTGAACTAAGAATCACGTTATTACTGTCAATGGACACTTCAGCCACTGGTGCGTTAGTCGGACGAAAAAAAAAACCTTGAAATAGCACAGACACCCCCGTATTTTGAATTTTTATGTAAGGGTCACAATCTACTTGCGCCCAATTCGCCATTAAACGCATAATATACTCTACCGGAAAGGCTTCGGATACGTTGTCTTCGCCGTTAAACTGAAAAACACAACGGGCGGGGGGGCGTTGTGGATCAAATATTGGAAGATCCCCATCGCAACATTGAAGAGCGCTTGGTACCACCAACCGAATACGTTGTAAAAGATTATCTCCGCAACCCCTCCTGCGTTCACTCCGTACATACGTTCTCCGTGACATATTGATCTAAGGTTGCAAACCAAGGCACACGCGTGAAGTATTTAGACCATTTATCGTGGGATATAGGAGGAGTTTGGAGTGATCCACCCCCTGACGACTTATTAATGCGTTTATTTTCCCCATGTATTAAGCATCCTTCAATATTTCATGCAAATCTAGAAATTTGGCCATGACTCCCGCAAAGCGTTCACGGCGACGGGTCACGCTGGCACTATGTTCACATGGAACAACATAAGCAGATTTTTCTGAATCGTTACTTTCTTTATGTTTTAAAACGGACGCCAGGCGACTGGTAAATGATATATAATTTAATTGAGCGTCAGTTGTAGGTAGAATTGCTTCTATTTCCGGGGGAATTAAATTTTCAAACCAAACGGAAAGAGTAAAGGTGCTATCAGCAGGAAAATACTTTGACTCCAGTGCATCGATATTTAATAGATTAACATCGGTGTCTGTAATTAAATCGCGGGCCCTCATCCCAGAGATGGATCGGGTAGAATCAGAAGAACCCATGGATGGATTCGAATCGCCCGTATTCTCCGAAAATACATCTTCTAATTCCGGATGGTGTTCCGACGCATTTTCCGATTCGTACATCGCTTATAATCCAGCCCTTCTGCTAAAAAACGATTTGTTATTTTCAGAATTGTTATTTGCCTCCCACTTAATAAATGTTCCCCGTGCAATAGAAAACAACGTCACTTATGAGGCCTCTTCGGCGGTAGGTGTGGATAATGAAATGACCTCAAGTACCACTGAATTTATAGAAGAAATTGGAGACGTTTTGGCGTTAGACAGAGCCTGTTTGGTCTGTAGAACGCTTGATTTGTATAAACGTAAATTTGGACTGACACCGGAATGGGTTGCGGACTACGCCATGTTATGTATGAAAAGTCTGGCATCCCCGCCCTGTGCAGTTGTCACTTTTAGCGCTGCCTTTGAATTTGTGTATCTTATGGATCGTTACTACCTGTGCCGTTATAACGTTACTTTGGTTGGGTCCTTTGCCAGGCGCACGCTTTCCCTGTTAGATATACAAAGACATTTTTTTTTGCATGTATGTTTTCGTACCGATGGAGGGTTACCAGGTATACGACCGCCCCCCGGTAAGGAAATGGCCAACAAAGTAAGATATTCCAATTACTCCTTTTTTGTACAGGCGGTAGTTAGGGCTGCATTACTATCGATCAGCACGTCTCGTTTAGACGAAACCGAAACGCGTAAGTCATTTTACTTTAATCAGGACGGACTGACTGGAGGCCCTCAACCTTTAGCGGCCGCCTTGGCTAATTGGAAAGATTGCGCGCGGATGGTTGACTGTTCATCATCGGAACATCGCACAAGTGGGATGATTACCTGCGCGGAACGTGCATTAAAAGAGGATATAGAGTTTGAAGATATATTAATAGACAAACTTAAAAAATCGTCTTACGTAGAAGCAGCTTGGGGTTACGCAGACTTGGCTTTATTATTACTGAGTGGGGTTGCTACTTGGAATGTAGACGAGCGTACAAATTGTGCTATAGAAACTCGCGTTGGATGTGTTAAATCATACTGGCAGGCGAACCGGATTGAAAACTCCAGGGACGTTCCAAAACAATTTTCCAAATTTACGAGCGAGGATGCCTGTCCCGAAGTAGCATTTGGGCCTATTTTGTTAACTACCTTAAAAAACGCAAAGTGCCGTGGTCGCACGAATACCGAATGCATGTTATGTTGTTTATTAACCATAGGGCACTATTGGATCGCTTTGCGGCAGTTTAAAAGGGATATATTAGCATACTCAGCAAATAACACAAGTTTATTTGACTGTATCGAACCTGTAATCAATGCATGGAGCCTAGATAACCCCATTAAACTTAAATTTCCATTTAATGATGAGGGTCGATTCATAACCATTGTAAAAGCAGCAGGTTCCGAGGCCGTATATAAACATTTATTTTGCGATCTCCTATGCGCTCTCTCGGAATTACAGACAAACCCTAAAATTTTATTTGCCCATCCTACAACCGCGGATAAGGAAGTGTTGGAGTTATATAAAGCCCAACTGGCTGCACAAAACAGATTTGAAGGTCGTGTATGTGCTGGCCTGTGGACATTGGCGTATGCATTTAAAGCCTACCAGATTTTTCCACGCAAACCAACCGCCAATGCCGCATTCATACGAGATGGAGGACTTATGCTTCGACGACATGCAATATCGCTGGTCTCCCTCGAACACACCCTATCGAAGTATGTCTAGGCGATATAAATCCGTATCTCGGAGCGGGCCTTCGATGCGTGTACGCTCCAGAACGCCATGCCGCCGTCAAACCATTCGAGGAAAACTTATGTCAAAGGAGCGGTCTGTGTACCGCCATTATTTTAATTACATCGCAAGGTCCCCCCCAGAAGAACTAGCTACCGTTAGAGGCTTAATCGTGCCAATTATTAAGACGACCCCTGTCACCCTTCCGTTTAACTTGGGTCAGACAGTGGCGGATAACTGCCTGTCGTTATCCGGAATGGGTTATCATTTAGGTCTCGGAGGTTATTGTCCGACATGCACTGCATCTGGAGAACCGCGTCTATGTCGAACCGATCGGGCGGCTCTGATACTAGCATATGTTCAGCAGCTTAACAACATATACGAATATCGTGTGTTTCTTGCATCCATTTTGGCGCTATCAGACCGAGCCAACATGCAAGCAGCGTCCGCTGAACCCCTATTGTCGAGCGTATTGGCACAACCGGAATTATTTTTTATGTATCATATTATGAGGGAGGGGGGCATGCGAGATATACGCGTACTTTTTTATCGTGATGGAGATGCCGGAGGGTTTATGATGTATGTTATATTTCCGGGGAAATCTGTTCACCTCCATTACAGACTAATCGATCATATACAGGCCGCGTGTCGGGGGTATAAAATAGTCGCACACGTTTGGCAGACAACATTTTTACTGTCGGTATGTCGCAACCCAGAACAACAAACAGAGACTGTGGTGCCATCCATTGGAACATCGGACGTTTACTGTAAAATGTGTGACCTTAACTTTGATGGAGAATTGCTTTTGGAATACAAAAGACTCTACGCATTATTTGATGACTTTGTTCCTCCTCGGTGATTTCAGCTTCAGTGTTCATTTTATTATCCCAGCGCGGGGCGTGTATACAAACAAAGCCTGCCGCCTGCAAGCGGTTTAGCATTTTAACGTTAACAACTCGTGTCTCTGGAATAAAACGTTTTAAAAGCCGTTCTGTGAGTTTAGTGTCGTTTCCAAATAACGCCTTAAAAGTTACACTCGCCGTCCCAATGAGATGAGAAAAATAATAGTCAATGTTTAAAGACAGCCCGTGTGATGTTACGTGAATGGGATCTTCCGCTAAGTCAGATATTATTAACTTACGCTTTGCTTCCCCACACCGTTTACCTGCGGTATTCTGTAAAGGATCTCCACGTAGCAAAGCTACACTTTTTGCATCAGCCTCCACTTCGTCTGTGGGGGCCACAATAACATAAGGGATGCGTTCTCGAACGTTTGGGATTTGACCCTGTCTCATTACTAATTTATAATATACTGTTAAGTGAGCCAAGCGACGGTTTATGTAGGCGGATGGTGGACGACTAAGCTCGGCCGTCATAACAAACTTATTAATATCCAATTTGGGTGATGTAATCTGGCGATGTGCATCTGCAATTATGCGTCCAAACCCGGCCATCCCAGACGGCATGGCCCGTCTATTCCATTCAGCAATGGAAACACACGACGCCTCCGCCGCAGCACGCGAGACGGTGTCGTCATATAACAACAGTTCTACAAGTTTGCGGGCATAATCGTTAATAAATTGACAGTTGTTTTTTCTAACCAAGTCGACTCCCTTCATTAAAACCTTTCCGCCGTAAATTACCCCAATGTACTTTTTCTTTGTTATAAGTAAAAGTTTTATAAAAGTTTTTTCACACTCCAACTTTATAGGAGGACAAAACAGAGCCGTTGAAATTATATGTGCCATTTTCTCGCCGATTTTAGCTATCCCCTCAACACCAACACCCTTGAATCGGATAAACACAGAATCCGTATCTCCATATATAACCTTTACCTCGTACGCTTTTTGGGAGAGAACGCTACTTTCAATGTCTGGAAACGCTGTAATAAAACGTTCAAATGCGGCCCAGTTATTATGAATATAATCTCTGGTACTTAATAACATTTGACGGCCAATTGTAGTGACAGTGGCCGCTACGTATAAACATGGCAGAAATCCCTGCGCAACTCCAGTAAAACCGTACACGGAATTACAAACTACTTTTATCGCGGCTTGTTGTTTGTCTAATAACACTGCTTCATCTGAAGAACTTCCGGGTATGCGCGCTCTAATAGCCTTGCGCATAGCCAACCAGTCTTTTAAAAGAACACCCAGCAGACTTTCTCGAACGTTAGAGCGCACAAAAAAAAGACGTTTTCCTCCAACTGTAAAGGTGGCATAATCGGATGGATTCAAACGTTTAACCGTCTCAAAATTTAACGTTAGCGTGGTAAAACATAAGTTATGGGCCTGAATTATACTTGGATATAAACTTGCAAAATCCAATACGACCACCGGATCGATATAAAATCCCGTATCAGGGTCAAAAACCCTGGCTCCTTTATATCCTACATTTCGCCCACTTGACGTACCAGTGGGAGAAACGCTCTCGTCTTCATCCATCTCTTCCTCAACATCCCCGACATCGGGAATAACATCCTTATATTCAAAAGTAGCTGGGTATCCCCCATCGGGTAAAATAAATCCTCGAGACGAAGCCAGTCCTAATAAACAAGTGTAAATCCTAACCTGCTGTCCGTCGTAAATAGCCTTGGTTAAAGTAATTCTAGCTAGCCTTGCAACCGCGGATAACTCAAGGTGTGGTAAATATTTAAAAAACAGTTTCCCCACAAGAGCCGAGTCTTGTATACAATATTCACCAATAATTCCTCGTGTATTCGGTCCACTAGCGTAATATCCCGGAATGTCTTTGTAGGGCAAATCTCTCTTGGACTCATTTAGAGCTTCACGTGCAACCGAATCTAATTTATAACTCGAGAGTTTTAATTTTTCAGTTGCAATTGCATACATATCCAGAGATATGAGACCGTTGATCTTTACCTTGCTTCGTCGCTGAAATCCGGATTTGCCAACATCCCATATCTTAAACAGACCCCCACGGTTTATACTGCCATAACCATCAAGCTTGAGACTGTATATAGAATTAAGTTTCTCCATAATAAACGCCCAATCAAAATTAACAATGTTATAACCTGTGGCAAACTCGGGAGCGTACTGTTTTACGAGGGTCATAAATGCAATTAATAGCTCGAATTCACTATCAAACTCCAGCACAGTCGGCTCCGGTAACCCCGCGTCCTTCATTTCTTGTACATACCTTTGTGGTAAGTCACAAGAGCCAAGGGAAAACAGTAAAATGTGTTCTAAAGACTGTCGAGGGATTGAATATAATAGACAAGAAATTTGGATTACAAGATCCTCCAAATGTGTTGCATCGGGAAACGCCAGCTCATTAGATCCTCCTGATTTACATTCAATATCGAAACATAACAACTTGTAGTCAGGCCATGAGTCATCGTTTGGTATAGCCTGCAGATTATCCGACATGCAGTCAATTTCAACGTCGCTTAACGTTAATTGGCGACTTGCCGGTCGAACTCGAACACGTTCCCCATCAACTCCAGGTTTTAGTTGATACCAACCAAAACTAACAAAGCCGGGATTATCCATTAGAAAACGAGTGGTAGCGTCTACCCGACCTTCATACTTTTTCAACTCCGGGTGAAAGTTATCACAAAGATAATTTGTAAATTTAGATGAGGGAGAATACACCCTGTAAAACGCACATGGCTGTGTATCGTAGTAATAAACATCTGTGCGCTCAATAACCTCAACGCGAAAGCTTTCTGGAGATGCGCTTTTAAACGAGGTACCATGAAAAGCGTTCTTGTCTCCATTTAACGTTGCATCATTTTGTGTTATCATAGAACTGCGTAAACACTCGGCAAGTAATACAGATAACTCGCTACAGGAACGTATGCCACAAGCGGTATCCACCTCGGCTTTGTTTATATAAAAATATTGACAGATGCCGTATACATGAACTGCCACCCTTTTTCCACATCGGGACATGCCAAGTAAAGTAATAACGGTACCAAGCGGTCGTGTTGCAGTTGCAAACCGGGATACATCTCCATTAGACGCGGCTTCTGTTGTTTCGACAATATCATATACATGGAATGTGTTAAAGCGGGGGTCAAACTTATCCCCACGAAAGTCGATTTCCCCCCAAATATTCACGCGTCTAGGCCAGGGGCTGGAACAACGAAAGTCCAGAATCGGAACTTCTTTTCCATTACAGTAAACTTTAGGCGGTCGACTAAGTGTACCGACGTGAACCCCCTTTCGTTCTTCCATGGGCACATCTTCATCTAAACATTTAGGGGCCAAAAATTGAAACGATGACATGGTAGTTTTGTAACTATGAAGAAATTCTCTGTTACTACCGCGCCCGGTTCTTGGGTTATATTTAATCCCTGATGCTTGGGTTAAAAAGGGATTACAAAACCCCGTTCTGATCGCCATTTTATGTTAACGATTGATAATCTTGTAAAAAGCCAGTGTTACTGAGTAACACAACCCCACGCCCTTCTAATACATAAAGTGTAATCACGTGATTTGTTGTGGTTTCCGCATATGTAATACCCGTTTAAAAGCCTCTCTTCTTAATGTATCGACAGACTGGGTTTTGGGTGGTCATTTGACCCTGCCAACAACCCCCCATTATTACGAGTACTTCACCAAAATGGAAAATACTCAGAAGACTGTGACAGTGCCCACGGGGCCCCTGGGTTACGTTTATGCGTGCCGGGTTGAAGATTTGGATCTGGAGGAAATTTCATTTTTGGCCGCTCGTAGCACGGACTCTGATTTGGCTTTATTACCTTTGATGCGTAATTTGACCGTGGAAAAAACTTTTACATCCAGCCTGGCGGTGGTTTCTGGAGCACGCACTACGGGTCTTGCCGGAGCTGGTATTACCTTAAAACTCACTACCAGTCATTTCTATCCATCTGTCTTTGTCTTTCACGGAGGCAAACACGTTTTACCCAGCTCTGCGGCCCCAAATCTCACACGCGCGTGTAACGCGGCTCGAGAACGGTTTGGGTTTTCACGCTGCCAAGGGCCTCCTGTTGACGGTGCTGTTGAGACGACCGGCGCTGAGATATGCACCCGCCTTGGATTAGAGCCAGAAAATACAATATTATACTTGGTGGTCACGGCATTGTTTAAGGAAGCCGTATTTATGTGCAACGTGTTTCTGCATTATGGAGGACTCGATATTGTTCATATTAACCATGGGGATGTTATACGTATACCGTTATTTCCGGTACAACTTTTCATGCCCGATGTTAACCGTCTGGTACCCGACCCATTCAACACTCATCACAGGTCTATCGGAGAGGGTTTTGTATACCCAACACCCTTTTATAACACCGGGTTGTGCCATTTAATACATGACTGTGTTATTGCTCCCATGGCCGTTGCCTTGCGCGTCAGAAATGTAACTGCCGTCGCCCGAGGAGCGGCCCACCTTGCTTTTGATGAAAATCACGAGGGGGCAGTACTCCCCCCTGACATTACGTACACGTATTTTCAGTCCTCTTCAAGTGGAACCACTACCGCCCGTGGAGCGCGTCGAAACGATGTCAACTCCACGTCTAAGCCTAGCCCATCGGGGGGGTTTGAAAGACGGTTGGCGTCTATTATGGCCGCTGACACAGCCTTGCACGCAGAAGTTATATTCAACACTGGAATTTACGAAGAAACTCCAACAGATATCAAAGAATGGCCAATGTTTATAGGCATGGAGGGCACTTTGCCAAGGCTAAACGCTCTGGGGTCATATACCGCTCGTGTGGCCGGGGTCATTGGTGCGATGGTTTTCAGCCCAAATTCTGCGTTGTATCTAACTGAGGTGGAGGATAGCGGGATGACCGAAGCCAAGGATGGGGGACCGGGTCCATCATTTAATCGATTTTACCAGTTTGCCGGACCTCATTTAGCTGCGAATCCCCAAACAGATCGAGATGGCCACGTTCTATCCAGTCAGTCTACGGGTTCATCAAACACAGAGTTTAGCGTGGATTATTTGGCACTCATTTGTGGATTTGGAGCACCCCTGTTGGCGCGACTGCTTTTTTATCTAGAACGCTGTGACGCTGGTGCGTTTACAGGGGGTCACGGGGATGCGTTAAAATATGTTACGGGGACCTTTGACTCTGAAATTCCATGTAGTTTATGTGAAAAACACACGCGGCCGGTATGCGCTCACACAACAGTACACCGACTTAGACAACGCATGCCGCGATTTGGACAAGCCACCCGTCAACCTATTGGGGTGTTTGGAACAATGAACAGCCAATATAGCGACTGCGATCCTCTAGGAAACTATGCTCCATATTTAATCCTTCGAAAACCCGGGGATCAAACGGAAGCAGCAAAGGCAACCATGCAGGACACTTATAGGGCTACACTAGAACGCTTGTTTATCGATCTAGAACAAGAGCGACTACTGGATCGCGGTGCCCCATGTTCTTCCGAGGGACTATCGTCTGTCATTGTGGATCATCCAACGTTTCGTCGCATATTAGACACACTGCGTGCGCGTATAGAACAGACAACAACACAATTTATGAAAGTGTTGGTTGAGACCCGCGATTATAAGATCCGTGAAGGATTATCCGAAGCCACCCATTCAATGGCGTTAACGTTTGATCCATACTCAGGAGCATTTTGTCCCATTACCAATTTTTTAGTTAAACGAACACACCTAGCCGTGGTACAAGACTTAGCATTAAGCCAGTGTCATTGTGTATTTTACGGACAGCAAGTTGAGGGGCGGAACTTTCGTAACCAATTCCAACCTGTTTTGCGGCGGCGTTTTGTTGACCTGTTTAATGGGGGGTTTATATCAACACGCTCTATAACCGTAACATTATCTGAAGGTCCTGTATCCGCCCCAAATCCGACATTGGGACAAGACGCGCCCGCGGGGCGTACCTTTGATGGGGATTTAGCGCGCGTAAGCGTGGAAGTTATTCGGGATATACGAGTTAAAAATAGGGTCGTTTTTTCAGGTAACTGTACAAATCTCTCTGAGGCAGCCCGGGCAAGGCTTGTAGGCCTTGCAAGTGCGTACCAACGCCAAGAAAAAAGAGTGGATATGTTACACGGGGCCCTAGGGTTTTTGCTTAAACAGTTTCACGGCCTGTTATTTCCTCGGGGTATGCCACCAAACAGTAAATCCCCCAACCCGCAGTGGTTTTGGACCCTGTTACAACGCAACCAGATGCCGGCAGATAAACTTACACACGAAGAGATTACCACTATTGCAGCTGTTAAACGGTTTACCGAGGAATATGCAGCACTAAACTTTATTAATCTACCCCCAACCTGCATAGGAGAATTAGCCCAGTTTTATATGGCAAATCTTATTCTTAAATACTGCGATCATTCACAGTACCTTATAAATACCTTAACTTCTATAATTACGGGTGCCAGGCGCCCGCGTGACCCATCATCCGTTTTGCATTGGATTCGTAAAGATGTCACGTCCGCCGCGGACATAGAAACCCAAGCAAAGGCGCTTCTTGAAAAAACGGAAAACTTACCGGAATTATGGACTACGGCTTTTACTTCAACTCATTTAGTCCGCGCGGCCATGAATCAACGTCCCATGGTCGTTTTAGGAATAAGCATTAGTAAATATCACGGAGCGGCAGGAAACAACCGCGTCTTTCAGGCAGGGAATTGGAGCGGTTTAAACGGGGGTAAAAATGTATGCCCGCTATTTACATTTGATCGCACTCGCCGTTTTATAATAACATGTCCTAGAGGAGGTTTTATCTGCCCCGTAACAGGTCCCTCGTCGGGAAATCGAGAAACCACCCTATCCGACCAAGTTCGCGGTATAATTGTCAGTGGCGGGGCCATGGTTCAATTAGCCATATACGCCACGGTTGTGCGTGCAGTGGGCGCTCGAGCACAACATATGGCATTTGACGACTGGTTAAGTCTTACAGACGATGAGTTTTTAGCCAGAGACTTGGAGGAGTTACACGACCAGATTATCCAAACCCTGGAAACGCCCTGGACCGTAGAAGGCGCTCTAGAAGCAGTAAAGATTCTAGATGAAAAAACGACAGCGGGAGATGGGGAAACCCCCACAAACCTAGCATTTAATTTTGATTCTTGTGAACCAAGCCATGACACCACATCTAACGTATTAAACATTTCAGGGTCAACTGTCCCTGGTCTTAAACGACCCCCCGAAGATGACGAACTCTTTGATCTTAGTGGTATTCCCATAAAACATGGGAACATTACAATGGAAATGATTTAACCTCCCTCTTTATCCAATTAAAGCCCACACGCGGGTGAGTGTACGTAATAAACAAGTCAATATTACATATTCTGTTGTGTTTTCTTTTTTGCGTGTAGTCCTTACCCATATGACCTGTAATATAGTGTGTCTCCAACCATTCAGCTTACAGTCCAGTGGACAGTAACAGCCCGATAACATGGAATTGGATATTAATCGAACATTGTTGGTTCTACTGGGTCAAGTTTATACGTACATCTTTCAGGTTGAACTGCTACGTCGATGTGATCCAAGGGTGGCGTGTCGCTTTTTATATCGGTTAGCGGCTAACTGTTTGACAGTTCGTTATTTATTAAAGCTGTTTCTCCGGGGATTTAATACCCAGCTAAAATTTGGAAACACTCCCACGGTTTGTGCACTGCATTGGGCATTATGTTATGTAAAGGGAGAAGGTGAGCGTTTGTTTGAGTTGCTACAACATTTTAAAACGCGTTTTGTTTATGGTGAGACTAAAGACTCAAACTGTATCAAAGATTACTTTGTCTCAGCGTTTAACTTAAAAACCTGCCAATATCACCATGAGCTGTCGTTAACAACATACGGAGGTTACGTATCGAGTGAAATTCAGTTTTTACACGACATTGAGAATTTTTTAAAACAGCTTAATTACTGCTATATTATCACGTCTTCTCGTGAGGCGCTAAACACATTGGAAACCGTGACGCGGTTTATGACAGATACTATAGGAAGCGGTCTAATACCACCCGTGGAGTTGTTTGATCCGGCGCATCCATGTGCTATATGTTTTGAAGAATTATGTATAACAGCTAACCAAGGTGAGACCTTACATCGTAGATTATTAGGATGTATCTGCGATCACGTTACTAAGCAAGTTCGGGTTAACGTGGATGTTGACGATATTATTCGGTGTTTACCATATATCCCTGATGTACCGGATATCAAACGTCAATCCGCCGTTGAAGCGTTACGAACACTTCAAACCAAGACGGTAGTCAATCCCATGGGAGCAAAGAACGATACGTTTGACCAAACATACGAAATTGCGAGCACCATGCTTGATTCTTATAATGTTTTTAAACCTGCCCCTCGGTGTATGTACGCCATCAGCGAGCTTAAATTCTGGTTAACGTCTAATTCCACTGAAGGACCCCAACGTACTTTAGACGTGTTTGTTGATAATTTGGATGTATTAAACGAACATGAAAAACACGCAGAACTTACAGCCGTAACGGTTGAGTTGGCGTTATTTGGAAAAACTCCCATACACTTTGATAGGGCGTTTTCTGAAGAACTCGGATCTCTGGATGCAATTGATAGTATTTTGGTTGGCAATCGCTCATCCTCACCGGACAGTCAGATAGAAGCATTAATTAAAGCCTGTTATGCCCATCATCTATCGTCGCCTCTCATGCGTCACATTTCTAACCCGAGTCATGATAACGAAGCCGCCTTACGCCAACTTTTAGAAAGAGTTGGGTGTGAGGATGATTTAACCAAAGAGGCGAGTGACAGCGCTACAGCATCCGAATGTGATCTGAACGATGATAGTAGCATAACTTTTGCTGTTCATGGATGGGAAAACCTGTTATCCAAAGCAAAAATTGACGCTGCGGAAAGAAAACGAGTATATCTTGAACATCTGTCTAAGCGCTCTCTAACCAGCCTCGGTAGATGTATCCGCGAACAGCGCCAAGAGCTAGAAAAAACACTCAGGGTAAACGTTTATGGAGAGGCCTTATTGCAGACATTTGTTTCGATGCAAAATGGGTTTGGGGCACGAAACGTGTTTTTAGCTAAGGTTTCCCAGGCAGGGTGTATTATCGACAATCGCATTCAGGAAGCGGCCTTTGATGCACATAGATTTATAAGGAATACCTTAGTTCGACATACAGTAGATGCGGCTATGTTACCTGCACTTACACATAAATTTTTTGAGTTGGTCAACGGCCCATTGTTTAATCACGATGAACACCGTTTTGCACAACCCCCTAACACCGCCTTATTTTTTACCGTGGAAAACGTTGGCCTATTTCCGCACTTAAAAGAGGAATTGGCAAAGTTTATGGGCGGTGTCGTTGGTTCCAACTGGCTTCTCAGTCCATTTAGGGGCTTTTATTGCTTTTCTGGGGTAGAAGGCGTTACTTTTGCACAGAGACTTGCCTGGAAATATATTAGGGAGCTTGTGTTTGCAACCACACTATTCACCTCTGTTTTCCATTGTGGGGAGGTGCGGTTATGTCGCGTTGACCGTCTAGGTAAGGATCCACGCGGGTGCACGTCTCAACCTAAAGGTATAGGCAGTTCCCACGGACCCTTAGACGGCATTTATTTAACGTACGAAGAAACATGTCCCCTTGTGGCTATTATTCAAAGTGGAGAAACAGGGATCGACCAGAATACCGTCGTAATCTACGATTCAGACGTTTTTTCTCTTCTATACACCCTAATGCAGCGGCTGGCTCCGGATTCAACGGACCCGGCGTTTTCATAACCTCCGTTACGGGGGTGTGGTTATGCTTTTTATGCATATTTTCTATGTTTGTTACGGCGGTTGTGTCGGTCTCTCCAAGCTCGTTTTATGAGAGTTTACAAGTAGAGCCCACACAATCAGAAGATATAACCCGGTCTGCTCATCTGGGCGATGGTGATGAAATCAGAGAAGCTATACACAAGTCCCAGGACGCCGAAACAAAACCCACGTTTTACGTCTGCCCACCGCCAACAGGCTCCACAATCGTACGATTAGAACCACCTCGGACATGTCCGGATTATCACCTTGGTAAAAACTTTACAGAGGGTATTGCTGTTGTTTATAAAGAAAACATTGCCGCGTACAAGTTTAAGGCGACGGTATATTACAAAGATGTTATCGTTAGCACGGCGTGGGCCGGAAGTTCTTATACGCAAATTACTAATAGATATGCTGATAGGGTACCAATTCCCGTTTCAGAGATCACGGACACCATTGATAAGTTTGGCAAGTGTTCTTCTAAAGCAACGTACGTACGAAATAACCACAAAGTTGAAGCCTTTAATGAGGATAAAAATCCACAGGATATGCCTCTAATCGCATCAAAATATAATTCTGTGGGATCCAAAGCATGGCATACTACCAATGACACGTACATGGTTGCCGGAACCCCCGGAACATATAGGACGGGCACGTCGGTGAATTGCATCATTGAGGAAGTTGAAGCCAGATCAATATTCCCTTATGATAGTTTTGGACTTTCCACGGGAGATATAATATACATGTCCCCGTTTTTTGGCCTACGGGATGGTGCATACAGAGAACATTCCAATTATGCAATGGATCGTTTTCACCAGTTTGAGGGTTATAGACAAAGGGATCTTGACACTAGAGCATTACTGGAACCTGCAGCGCGGAACTTTTTAGTCACGCCTCATTTAACGGTTGGTTGGAACTGGAAGCCAAAACGAACGGAAGTTTGTTCGCTTGTCAAGTGGCGTGAGGTTGAAGACGTAGTTCGCGATGAGTATGCACACAATTTTCGCTTTACAATGAAAACACTTTCTACCACGTTTATAAGTGAAACAAACGAGTTTAATCTTAACCAAATCCATCTCAGTCAATGTGTAAAGGAGGAAGCCCGGGCTATTATTAACCGGATCTATACAACCAGATACAACTCATCTCATGTTAGAACCGGGGATATCCAGACCTACCTTGCCAGAGGGGGGTTTGTTGTGGTGTTTCAACCCCTGCTGAGCAATTCCCTCGCCCGTCTCTATCTCCAAGAATTGGTCCGTGAAAACACTAATCATTCACCACAAAAACACCCGACTCGAAATACCAGATCCCGACGAAGCGTGCCAGTTGAGTTGCGTGCCAATAGAACAATAACAACCACCTCATCGGTGGAATTTGCTATGCTCCAGTTTACATATGACCACATTCAAGAGCATGTTAATGAAATGTTGGCACGTATCTCCTCGTCGTGGTGCCAGCTACAAAATCGCGAACGCGCCCTTTGGAGCGGACTATTTCCAATTAACCCAAGTGCTTTAGCGAGCACCATTTTGGATCAACGTGTTAAAGCTCGTATTCTCGGCGACGTTATCTCCGTTTCTAATTGTCCAGAACTGGGATCAGATACACGCGTTATACTTCAAAACTCTATGAGGGTATCTGGTAGTACTACGCGTTGTTATAGCCGTCCTTTAATTTCAATAGTTAGTTTAAATGGGTCCGGGACGGTGGAGGGCCAGCTTGGAACAGATAACGAGTTAATTATGTCCAGAGATCTGTTAGAACCATGCGTGGCTAATCACAAGCGATATTTTCTATTTGGGCATCACTACGTATATTATGAGGATTATCGTTACGTCCGTGAAATCGCAGTCCATGATGTGGGAATGATTAGCACTTACGTAGATTTAAACTTAACACTTCTTAAAGATAGAGAGTTTATGCCGCTGCAAGTATATACAAGAGACGAGCTGCGGGATACAGGATTACTAGACTACAGTGAAATTCAACGCCGAAATCAAATGCATTCGCTGCGTTTTTATGACATAGACAAGGTTGTGCAATATGATAGCGGAACGGCCATTATGCAGGGCATGGCTCAGTTTTTCCAGGGACTTGGGACCGCGGGCCAGGCCGTTGGACATGTGGTTCTTGGGGCCACGGGAGCGCTGCTTTCCACCGTACACGGATTTACCACGTTTTTATCTAACCCATTTGGGGCATTGGCCGTGGGATTATTGGTTTTGGCGGGACTGGTAGCGGCCTTTTTTGCGTACCGGTACGTGCTTAAACTTAAAACAAGCCCGATGAAGGCATTATATCCACTCACAACCAAGGGGTTAAAACAGTTACCGGAAGGAATGGATCCCTTTGCCGAGAAACCCAACGCTACTGATACCCCAATAGAAGAAATTGGCGACTCACAAAACACTGAACCGTCGGTAAATAGCGGGTTTGATCCCGATAAATTTCGAGAAGCCCAGGAAATGATTAAATATATGACGTTAGTATCTGCGGCTGAGCGCCAAGAATCTAAAGCCCGCAAAAAAAATAAGACTAGCGCCCTTTTAACTTCACGTCTTACCGGCCTTGCTTTACGAAATCGCCGAGGATACTCCCGTGTTCGCACCGAGAATGTAACGGGGGTGTAAATAGCCAGGGGGTTTGTTTTAATTTATTAATAAAAATGTGTATTACGTTACTCATGTGTCTCCATTACGCATCACAGGGGGTATTTATACCCGATAATATACAAAACGCGTTTTGTACCTCTACCGCACCCGATATCTTAACGGGGGTTATTATGGAATCGTCTAACATTAACGCGCTACAACAACCGTCGTCTATCGCACATCATCCGTCCAAACAGTGCGCTTCAAGTCTCAATGAAACAGTAAAAGATTCTCCCCCCGCGATTTATGAAGATAGGTTAGAACACACGCCGGTACAATTACCCCGCGACGGTACACCCCGAGACGTATGTTCTGTGGGACAGCTAACCTGTCGAGCATGTGCAACGAAACCTTTTCGCCTTAACCGCGACAGCCAATACGACTACTTAAACACATGTCCAGGGGGCCGTCATATTTCACTGGCACTGGAGATTATAACGGGTCGATGGGTTTGCATCCCGCGTGTGTTTCCGGATACCCCAGAGGAAAAATGGATGGCGCCATATATTATTCCAGACCGAGAACAACCATCATCAGGGGATGAAGATTCTGACACCGATTAAATTTAACTTAAATAAAACCTTACCACCCATAAAAACGCCTTCTGTTTGTTTAACACGACACCGCTTAACAAAAAAAAAAACCCAAACACGCCTTTTATGAATGTAATACTTTTATTTGTTGGTTAACACCGCCCCACCATCATCTGATTTGCAAACATATCGGCGTCGTCTGCCGTGGACCCCTGTATTAAAGGGGCCTTGGAACTAGCCTCCACTGCATTTACATCTTGTCCAACTGTATCTGTATGTGGGGTGCTTGTTGTATTTTGGGATGAGCATAGACCCGAAACGCTTTGAAGCTGTTTTAATAAAATCGATATTCGAGGATCCCGTGTCCCCTCTGGTATATTTGTATGGTGCGACAAAGGCATTTGTGTCCCATTTTGTGATTTTAGCTCTGTAACCTCCTGTTGCAGTTTTGCCACAACCCCAGCAAGCTCTTCGTGCTGACCATTAGAAACTCTGTGTCTCCTCTGCCAATATGATGGAGAAACTCGACGTCTCCGATGCGTTATATACGTTGGTTCACCGGGAAAATATATATTTGAGGGAAACTCTCCGTCCATTTGAGACTCCCCACTAGAAAAAGAATCCAATTCCCTTTGATCCATGCTCTTGAAATCCCGTTTTCCTGGACGACGGACATCGGTTTTGTCTGGAAAATTTACACACGGGGTCTGCAAGTCAATACCCCGTTCGGCGGCCAATGCGTTCATAAATGCGGACATTTGCATTTCCAAACGATTGGGTGGTGGATATCCCGGAAACCCGTACGGTCCCCCGAAGTGTCCCGGAGGGCAACCATAACCCCCTGTATTAAATGGGAAGGCGGGCGGGTGTGGAGATCCATATGGCCCGACGATATACTGTCCGTTATTTGGAGCTCCAATTGATACCTGCGGATTTTTAGTCTGCCCGGTTAACAGCTGTGAATAATACGCGGTAGGTATCAGTACAAATTCCCCTCCGGTTGGAACGCCCGACGGGGGCTGTGGTGAGATACCACTAGCGTTACCTGCTACAGAAGCCATATCGCTGTCGTTCCTACACAACTGCGTAACCTTTAAATGCGGAACAGTCTTTTCACAATCTTCATTTGATTCCCCAACACCCAACGCGAGATCGTATATGGGCCCGCCGGGGTGGAATGTGGCGTTTATAACACCCGCGTTGGGTAATTTAGACTCCACCCCATTAACGTTGGTTATCCGAGCAAGTCCATATCCGGTGCTAGCCTGAAGATAAACGTGACCCATAATTCCGGCTTCGCGTCTACGTTTTGCAACCACGTCCCATCTATCTCTTAAAAGCATATTGTTCACGGCTGTGGATAATAACACCTTGGCGAGTTTATCTTCGCTAACCTTCCATACTTTATTTAAACCCGCGTAGTCTTTAACCAGCGACAATAACCGCGCTTTACTTTCCATCGATAAAACCCGGAATGGTTCAATTGAAGATTCCGGGGTACAGTCATAATTGACCACTGTTCCAACGCGTCTTCCAACAACACATAACGCAACATGGGTAAAAAAATTACCGTCTGGTATCTCATTCGGGGACAATCGTTTTGAAGACAGGGATACGGAGGGTAAGTAATTTGTGACCAAGTATAACGCACGTTCTAGCGGAGATAATACAGAATCTCTATTTCCAAAAAAATTCGAATGGGCCGCTTCAAACAGCACCGCATGTAGTTGAGGGCATCTAACGATACCCAAAAAAAAAGGTCCGCGTATGTCCTCAATGATTGCGATTACTTCACCCACGACACAGTCTTTTCGATGATCGATGTTTATTGGTATTTTACTAGTAGGCGGCAAAGCGGACCGCACAATCTCTGGGGTAATATTTAATTCCCCTTCGTCCTTTGAATATAAGGCTAAATACCCAGCCACGTATAACGCTTCACAGTTCTCTTCGTCAGCTTCAGCAGCCATTATAAACACCCCACGGACCGGATAGTGAATACTCACGGTGTGGAGGCAAACTGAGGAATGACACCCAAACAGACAAAATATAGAAGATCATAGTCACTGTTAACGTTGAACTGCGCAAGGCGGCGACTTTCTTCCAATGCCGCCCTTACACGCGGTTGGTGCATTAACATTCCAAGTCCCCGTTCATATTGCAACATAACACTGTCATGTATTGATACCACGGCGGCTATGGGTAGGGATGTAACATTTTGTCGGCGGTGTTCTAATTCCAATGCAATTAAGCTTATGAGCCGATCTTGGTACTGTCCAGAAGAAATATCTATTACGGTTCTTCCTAAACTTCCACGACTAAGCTGGGTATGCGCGTCTAAACAAAGAGCAACTAATCCAGGAAACATTTCAGTCAGCTCTGTGGTCCGATTTAACGTATACAGTGGTGCTATATATCGTTCACATAAAAATTGAAAGTTATTATTACCGCTTTTAAACTTCCCATCAAACCCCGTCGCTCCGCGCAAGATTACATTGTTGGTAGGGGTTCCTGTTGCTTCTGACACAATCAAACCCAGTTGAAAATTATTTTTTAGTTTATCTCCGTATACGTTCCCGTTCCATAATAAGCGCCTTAATAATAATAACGCCGTAATCGTGTCAATTGTTAACCTTAATAGAGTTTGGTCTTCCATAAGAAACACGTTTTGGGCCCGTTCTAAATACGCCGCGGCCGCCTGTTGAATCTTGTCCACATATGCGGTATGATTGCGATCAATAATGTCATTAACCCCAGGATTAAACTGTCCAGGTGCAGGCGGTAGGACCTGCAACCGTATAAGCGCATCCATAACAGAATGTGACGTTAAGGCGCCTTGATCATACCGCCCCCCACGAGCATGAAACTGGTCGCGTGGTAGACGATCATAGCAAAATTGATAACTGTTTTTATTTTCGTGTGTTGTCATATAATTCACAAATGTCTCAGTATATTCCGGTAGGTGCTCTATAAGGTTCCCGAAGGACGAAACTTGAGGTTCGTGGACACTATTAGATGTCCTATACATTAAATATAAACATAATACCGCACACTCGAACGCGGAGTACGCTCTATCTCCAACATACATTCTCCCGGCGGACTGTAGACATGTTACCGTTGTGTTCATAAACGTACGGGAAATGCGCCCGTCTTTACAATCAACTCCGCGTGCAGCTACGGGCCTATCTAACACAAGCCGTTCCTGCAGAGTACGATACCATGGCCCGAAAACAATCCCTGGAGAGTTATTGCCCCTTGCCCTTCCCAAGTACACCAGGGTGATAAAATCCACTTGAAAGTTTGTATCGTACTGCAACGGTGCATCATTTTTGGCAATCTGTACCTCGGGGTGTATAGACTCATTGCGTATTATTTCTGTACGTGTACATTCCTCAGATTGTGCATCTGCTTCTTCCGCCTCGGCAGCAGCCGTCTCCAGGGAATCCAAAACCTTGGCCATGCGCGTTAGTTGTTCTTCGAGGGGCTTTAAACGACGATCTATTTCCGTTGGTAACGTAATCGTTTCCCCGCGAAGGTTGTCTAATGCGGCAACGACCGCCGCATTTTTTAACGTTAACGTATTTTTTTCCAAATCGGGATTCATACGCCCTCTTAACTCAAACGCGGGAGCCGTCCAGTAGTGTATGGGGAAGTTGGGGGCTATAAAGTTCTTAGTGGTAGACAAAAATATCCCACATTTATTCGGAAACGAGATAGATCCGAACCCATATCTCGCCGTCATGGTGTCTGCAGCAAACAAAGTCAACTGGCGTGAATATAAACCGGTACTGCTTTAAAAGCTGTTTTCTTACCCATGGGAAAACATCCCGGTTATACTTTGTAAAATTCCACCACAAGCACCTAAAGAAGGCCTTCTAAGGGGTAAATCCACCCCACAGGCTGCATTTTCTTCAAACTTTGTTAAAGCGGAACGATGGCATGATTTCGCACGCTTTTTCGCAAGAGAGCATACGTGAATTTTCTTTTTGCATAGACGTCTTCGCTCTCTAACGGACCTTATCGGGGGGGTATATTCCGCTACATTCTCCAAATGCGACGCTAGCATAACAAGGTTTCCATGAATCACCTTCGGGGGTAACCGAGTTACCTGTAACAGGTTCAGACCCCGTTGAGATACAAACACAAGGAGGGGGGTCACCATTATTTCATCAGATCCCGTGGGTGTGGTTTCCTTTATTAAAGCCACGGTATCCCTCAGCTGGCGCATACCCTCGCAAAACTGGTGATACTTAGTAGGGGTATGTATATTAGCGCTAAAACGGCAAGATTTTAATTCCACTATAAAACAAACGGTCTTTCCGGCACCACTGGATTCCGTTTGTATAATACAAACACAATCGGGGCGTCGGCGTCCCAAATTTACTTCAAACGACATTGATATGCGTACAGCCCTTTGAACATCCACGTGGGATAACGGCGACAGGAGTTTTGCCAGCCTCGGGTTGAACGCGTCCGCGAAACCTCGACGTACGTTATCAATATCCTTTTTGAGTACATCGTAAAAACGAGTGTGGCAACGTTGTCCCAAACGAAAACACTTGGCCCGAATTCGACTAGCGGACATATTTGAAGTTCCGTCCCAGAAGATAACCTAAGACGCGTTTGTCTACAATAAACATGTCAACGGATAAAACCGATGTAAAAATGGGCGTTTTGCGTATTTATTTGGACGGGGCGTATGGAATTGGAAAAACAACCGCCGCCGAAGAATTTTTACACCACTTTGCAATAACACCAAACCGGATCTTACTCATTGGGGAGCCCCTGTCGTATTGGCGTAACCTTGCAGGGGAGGACGCTATTTGCGGAATTTACGGAACACAAACTCGCCGTCTTAATGGAGACGTTTCGCCTGAAGACGCACAACGCCTCACGGCTCATTTTCAGAGCCTGTTCTGTTCTCCGCATGCAATTATGCATGCGAAAATCTCGGCATTGATGGACACAAGTACATCGGATCTCGTACAAGTAAATAAGGAGCCGTATAAAATTATGTTATCCGACCGACACCCAATCGCCTCAACTATATGTTTTCCCTTGTCCAGATACTTAGTGGGAGATATGTCCCCAGCGGCGCTTCCTGGGTTATTGTTTACGCTTCCCGCTGAACCCCCCGGGACCAACTTGGTAGTTTGTACCGTTTCACTCCCCAGTCATTTATCCAGAGTAAGCAAACGGGCCAGACCGGGAGAAACGGTTAATCTGCCGTTTGTTATGGTTCTGAGAAATGTATATATAATGCTTATTAATACAATTATATTTCTTAAAACTAACAACTGGCACGCGGGCTGGAACACACTGTCATTTTGTAATGATGTATTTAAACAGAAATTACAAAAATCCGAGTGTATAAAACTACGCGAAGTACCTGGGATTGAAGACACGTTATTCGCCGTGCTTAAACTTCCGGAGCTTTGCGGAGAGTTTGGAAATATTCTGCCGTTATGGGCATGGGGAATGGAGACCCTTTCAAACTGCTTACGAAGCATGTCTCCGTTCGTATTATCGTTAGAACAGACACCCCAGCATGCGGCACAAGAACTAAAAACTCTGCTACCCCAGATGACCCCGGCAAACATGTCCTCCGGTGCATGGAATATATTGAAAGAGCTTGTTAATGCCGTTCAGGACAACACTTCCTAAATATACCTAGTATTTACGTATGTACCAGTAAAAAGATGATACACATTGTCATACTCGCGTGTACGTGTTTTTCTTTTTTATATATGCGTCATTTATTACCACATCCTTTAATCCCGCCTTTATCTCCCTAAAACGGAGTGGTAATATTAAAAGCCGCCAAGCCTGTTGGTGGGTGAGGAGGGGTAAAGGCACGCTGTGTGCATAACGTTGCGGTGATATTGTAGCGCAAGTAACAGCGACTATGTTTGCGCTAGTTTTAGCGGTGGTAATTCTTCCTCTTTGGACCACGGCTAATAAATCTTACGTAACACCAACCCCTGCGACTCGCTCTATCGGACATATGTCTGCTCTTCTACGAGAATATTCCGACCGTAATATGTCTCTGAAATTAGAAGCCTTTTATCCTACTGGTTTCGATGAAGAACTCATTAAATCACTTCACTGGGGAAATGATAGAAAACACGTTTTCTTGGTTATTGTTAAGGTTAACCCTACAACACACGAAGGAGACGTCGGGCTGGTTATATTTCCAAAATACTTGTTATCGCCATACCATTTCAAAGCAGAACATCGAGCACCGTTTCCTGCTGGACGTTTTGGATTTCTTAGTCACCCTGTGACACCCGACGTGAGCTTCTTTGACAGTTCGTTTGCGCCGTATTTAACTACGCAACATCTTGTTGCGTTTACTACGTTCCCACCAAACCCCCTTGTATGGCATTTGGAAAGAGCTGAGACCGCAGCAACTGCAGAAAGGCCGTTTGGGGTAAGTCTTTTACCCGCTCGCCCAACAGTCCCCAAGAATACTATTCTTGAACATAAAGCGCATTTTGCTACATGGGATGCCCTTGCCCGACATACTTTTTTTTCTGCCGAAGCAATTATCACCAACTCAACGTTGAGAATACACGTTCCCCTTTTTGGGTCGGTATGGCCAATTCGATACTGGGCCACCGGTTCGGTGCTTCTCACAAGCGACTCGGGTCGTGTGGAAGTAAATATTGGTGTAGGATTTATGAGCTCGCTCATTTCTTTATCCTCTGGACTACCGATAGAATTAATTGTTGTACCACATACAGTAAAACTGAACGCGGTTACAAGCGACACCACATGGTTCCAGCTAAATCCACCGGGTCCGGATCCGGGGCCATCTTATCGAGTTTATTTACTTGGACGTGGGTTGGATATGAATTTTTCAAAGCATGCTACGGTCGATATATGCGCATATCCCGAAGAGAGTTTGGATTACCGCTATCATTTATCCATGGCCCACACGGAGGCTCTGCGGATGACAACGAAGGCGGATCAACATGACATAAACGAGGAAAGCTATTACCATATCGCCGCAAGAATAGCCACATCAATTTTTGCGTTGTCGGAAATGGGCCGTACCACAGAATATTTTCTGTTAGATGAGATCGTAGATGTTCAGTATCAATTAAAATTCCTTAATTACATTTTAATGCGGATAGGAGCAGGAGCTCATCCCAACACTATATCCGGAACCTCGGATCTGATCTTTGCCGATCCATCGCAGCTTCATGACGAACTTTCACTTCTTTTTGGTCAGGTAAAACCCGCAAATGTCGATTATTTTATTTCATATGATGAAGCCCGTGATCAACTAAAGACCGCATACGCGCTTTCCCGTGGTCAAGACCATGTGAATGCACTTTCTCTCGCCAGGCGTGTTATAATGAGCATATACAAGGGGCTGCTTGTGAAGCAAAATTTAAATGCTACAGAGAGGCAGGCTTTATTTTTTGCCTCAATGATTTTATTAAATTTCCGCGAAGGACTAGAAAATTCATCTCGGGTATTAGACGGTCGCACAACTTTGCTTTTAATGACATCCATGTGTACGGCAGCTCACGCCACGCAAGCAGCACTTAACATACAAGAAGGCCTGGCATACTTAAATCCTTCAAAACACATGTTTACAATACCAAACGTATACAGTCCTTGTATGGGTTCCCTTCGTACAGACCTCACGGAAGAGATTCATGTTATGAATCTCCTGTCGGCAATACCAACACGCCCAGGACTTAACGAGGTATTGCATACCCAACTAGACGAATCTGAAATATTCGACGCGGCATTTAAAACCATGATGATTTTTACCACATGGACTGCCAAAGATTTGCATATACTCCACACCCATGTACCAGAAGTATTTACGTGTCAAGATGCAGCCGCGCGTAACGGAGAATATGTGCTCATTCTTCCAGCTGTCCAGGGACACAGTTATGTGATTACACGAAACAAACCTCAAAGGGGTTTGGTATATTCCCTGGCAGATGTGGATGTATATAACCCCATATCCGTTGTTTATTTAAGCAAGGATACTTGCGTGTCTGAACATGGTGTCATAGAGACGGTCGCACTGCCCCATCCGGACAATTTAAAAGAATGTTTGTATTGCGGAAGTGTTTTTCTTAGGTATCTAACCACGGGGGCGATTATGGATATAATTATTATTGACAGCAAAGATACAGAACGACAACTAGCCGCTATGGGAAACTCCACAATTCCACCCTTCAATCCAGACATGCACGGGGATGACTCTAAGGCTGTGTTGTTGTTTCCAAACGGAACTGTGGTAACGCTTCTAGGATTCGAACGACGACAAGCCATACGAATGTCGGGACAATACCTTGGGGCCTCTTTAGGAGGGGCGTTTCTGGCGGTAGTGGGGTTTGGTATTATCGGATGGATGTTATGTGGAAATTCCCGCCTTCGAGAATATAATAAAATACCTCTGACATAAAAAACATGTATAATAAAAAGTCACTATAAACGTATTCTCTACAATACTTTATTCGCGAATAATACACACTACCTTTGGGTTTTTTTCCCGTCCCCAAATGGTGTTTGGTGCACTCTACCAAAAAATAGAGCGCCTAAATATGCTATATAACGCCTCCCAGCAAAATACGGTTCAAAGGCATTACCCGATATTGTATTGTAGTACAGGGCAATGGGAATTGATGATCCCAATAAACGGCATAGACGCACAGCGCCGTTATAGCAGGGGTCTCCAGAGTACAGGGTATCTAAGTACCGGGATATCTCATACTCATGCCTTTCCGTGACAGAAACATCAACCGGAACAGTATCCGATAAACCAACTCCTGTTTTTGCAAGGCGTAAAATTCGCACACCTTCCTTTTTTGCAAGATGTGACGTTTCCTTGTAACAGGGAAGCTGGGGGAGTGGTAAGAACAACAAAGTTTCAGCCAACGTGCCAATAAAGCCCACTTCCCTCAAGAGGCTGTTTGCTGTATCCACAATGGTCCGTATTAAATCTTGAGCAACTTGATCCGTGTCATCATCACTGGGTAACGCGTTAACATAACTACGCGTTAAATCTTCAATAACGGCATAACAATTAAACGCTTCCCACCGAGACAGTATATATTGAACAATCACGAACCGTTGACAGGACGTCAGATCACGTCCGTAAGCATGCCCGAAAAATGGAAGTTCCCCCCGTTCGCCATATACCGCAACAACTGCGGTATATATCGTCTCACGGGCTTCATTAAGTTCATCTTCAAGTCCAGGCCATTTTCTGGCTTTAAATATAACCTCGTCCGCAAAAAAAACCGCACATGATAACGCGCGGATACAATGAGTAGTGGCTTTATGGCGAGGATCCCAAATGTCCATTACCCGGGGGATGGTCCTAATCTGTACAAAGTTACTTAGTGTAATATGATCGGACTTCTTACGCCGTCTAGGCTGTTTCTCAGAATACGGTTCACCCGAAATCGGCACATCATCTGCTTTTACGTCTTCCGTAACCACATCAGCAGCGCGCCGACTAACAATTATACTTGTTTTTTCATCGTCGTTACTTCCGTTAAGCGCGTCTCGTATCTCGGGCGTCCCGTCGAATAATCCACTCACTAGCTCCTGCAAACCTTCTGGTAACTCCAACATACGCATATACACCAATGAAAAACTGGCTTCGTTTGGTACGTACATAAAGCCATTTGTGGTATTAATGGCGGTGGGTGTTGGAAACAATTTTAGCTTATTCTCGCGCGTAACATCTACCCCCGCCACCAATGTTAAATGCGTCACGGGGAGGGACACGAGATAATCTGCGAGCGTAGGGTCCTCCACTTCAACATCAAATGTTCCGCAAAGGTCGCGATCCACCGCCCCCGATCCCGCTGCAAGTAAGGCCACTCGATCCAAAAACACGCAGTTATTATTGGATGATACCGCCCATGTCTTCCCGGTGCGATTGAGCTCACTTCGAACGTAACTGGCAACAGATCTGTCACCGGGTCCGACCCCGCGAACAACATGTCCAAATTTTGCGATCTCGCCTCCATGTTTGCGGGGTATGGAAATTAAGCATCCCCCGCATATAAAATACGCCCTGGTAGCACGCTCGTTAAAATAAAACGTTACGCCGTTATAAGATACGGTTGAATGATATGGAAATTCCATATTAAAGCGTTTATCGGAACATTAACCTCGAACTTGCCGTCCCGTGATCGTGTGATCGCCAACCTTAGGTCCACACCGAATATGAGAAATATATAACTACACGCAAACATTCAAAACACCGTGGTATCATTAACGTCATATGAAAAGATCCAATCAATCCAATCAACCACACCTCCTACCGTTTAGCACGTCAGCTATGTGACATGCTCCAAACATACGTAAACATTTAGAGAGGGTGTTATAACAGTCTGTCAGGCGGGGTATATTCTACATAATACAAGGATCGGCTTTAACTTTGTCAACATTTTTACTTTGGACTATAAACTGCGACTGAACGTTATGAACCCACCCCAAGCCCGCGTCTCGGAACAGACAAAGGACTTGCTTAGCGTTATGGTTAACCAGCACCCCGAAGAGGACGCAAAAGTGTGTAAATCCAGTGATAATTCACCGCTTTATAACACCATGGTTATGTTATCGTATGGGGGTGATACGGACTTACTATTAAGCTCTGCATGTACCCGCACATCTACCGTAAACAGGTCGGCGTTTACGCAACACTCCGTGTTTTATATTATATCCACGGTGTTGATTCAACCAATATGTTGTATCTTCTTTTTTTTTTACTATAAAGCGACACGCTGTATGCTCTTATTCACAGCCGGGTTACTTCTGACGATTCTACATCACTTTCGACTTATTATTATGTTATTGTGTGTCTACAGAAATATACGATCAGACCTGCTACCCTTATCTACATCCCAGCAACTGCTGCTTGGAATTATTGTTGTGACTCGAACAATGCTATTTTGTATTACGGCGTATTATACTCTTTTTATAGACACCCGGGTGTTCTTTTTGATTACCGGACACTTGCAAAGTGAGGTTATTTTTCCAGATAGCGTTTCAAAAATACTTCCTGTGTCGTGGGGTCCAAGTCCAGCCGTGTTACTGGTAACGGCGGCAGTTATTTACGCTATGGACTGTTTGGTGGACACGGTATCCTTTATTGGGCCAAGGGTGTGGGTCCGTGTTATGTTAAAAACATCTATTTCGTTTTAGTCCATTTCAATAAATGTACTATAATTGTTCAGTCTAAAAATAATGTTGGGTATTTATAATTACCGCCCCCGTGTTACTTGGAAACACCCATACATATGTTCCACTCTACATCAAACTTCTCGCAGTTTTCTTGTTCCCGCACACGTTTACACGTCCGGATTCAAGTCGCAACGCTGCTGACAAAATGACAACGGTTTCATGTCCCGCTAACGTGATTACTACAACGGAATCTGATCGTATTGCTGGGTTATTTAACATCCCAGCGGGGATCATTCCAACTGGAAATGTGCTGTCAACCATAGAGGTGTGTGCACACCGTTGCATTTTTGATTTTTTTAAACAAATACGATCAGATGATAACAGCCTTTACTCGGCTCAATTCGATATTCTTTTGGGGACATACTGCAATACATTAAACTTTGTGCGTTTTCTAGAACTTGGACTGTCTGTCGCTTGCATCTGTACTAAATTTCCGGAGCTGGCTTACGTGCGAGATGGCGTTATTCAATTTGAGGTACAACAACCCATGATAGCACGTGATGGCCCACATCCCGTCGATCAGCCTGTTCATAATTATATGGTTAAGCGGATACACAAGCGTTCGTTAAGCGCTGCGTTTGCAATTGCATCGGAAGCGTTGAGTTTGTTAAGTAACACATATGTCGATGGGACAGAGATTGACTCATCGTTACGTATAAGAGCTATCCAACAGATGGCTCGTAATTTACGCACCGTTTTGGACTCATTTGAACGAGGCACTGCCGATCAACTTCTTGGTGTTCTATTGGAGAAAGCCCCACCGCTATCGCTGCTTTCACCAATTAATAAATTCCAACCCGAGGGACATCTAAATCGTGTTGCACGCGCGGCCCTACTTTCGGACCTCAAACGTAGAGTCTGTGCGGATATGTTTTTTATGACCCGACACGCCAGGGAACCTAGGCTGATCTCTGCGTATCTGTCGGATATGGTTTCGTGCACCCAACCATCGGTGATGGTATCACGAATAACTCATACAAACACTCGCGGACGGCAGGTTGACGGTGTGTTGGTAACAACAGCAACCTTAAAACGGCAACTATTACAGGGAATTTTACAAATTGACGACACCGCCGCTGACGTACCAGTAACATATGGCGAAATGGTTCTACAGGGGACAAACTTGGTAACCGCCCTTGTGATGGGAAAGGCCGTCCGCGGAATGGATGATGTAGCCCGCCATCTCCTTGATATAACCGACCCTAACACGTTAAACATACCGTCTATACCCCCACAATCCAACTCCGATTCAACGACAGCTGGGCTTCCGGTTAACGCCCGTGTTCCTGCGGATTTAGTGATTGTTGGGGATAAACTTGTATTCTTAGAAGCATTAGAACGGCGGGTCTACCAAGCTACGCGCGTTGCCTACCCTCTTATTGGAAATATAGATATTACGTTTATCATGCCAATGGGAGTGTTTCAGGCAAACTCCATGGACAGATATACACGACACGCCGGCGATTTTTCAACTGTATCCGAACAGGATCCACGTCAATTTCCACCCCAAGGGATTTTTTTTTATAATAAAGATGGGATATTAACACAGTTGACTCTTCGTGATGCAATGGGTACCATCTGCCACAGTTCATTGCTTGATGTTGAGGCCACACTTGTTGCCCTCCGCCAACAACATTTAGATCGTCAGTGTTATTTTGGTGTATACGTGGCCGAGGGTACAGAGGACACATTGGATGTTCAAATGGGGAGGTTTATGGAAACGTGGGCAGATATGATGCCTCATCACCCTCATTGGGTAAACGAACATTTAACAATTCTACAGTTTATAGCTCCGAGCAACCCGCGTCTAAGGTTTGAATTAAACCCCGCCTTTGATTTTTTTGTTGCACCGGGGGACGTAGACCTTCCCGGACCGCAGCGTCCCCCGGAAGCCATGCCAACCGTTAACGCAACATTACGGATTATCAACGGAAACATTCCCGTGCCTCTATGTCCCATTTCATTTCGAGACTGTCGCGGAACCCAACTCGGTTTGGGAAGACATACAATGACCCCGGCAACCATTAAAGCCGTAAAGGATACATTTGAAGACCGCGCATACCCAACTATTTTCTACATGCTAGAGGCTGTTATTCATGGAAACGAAAGAAACTTCTGTGCGTTACTGCGACTGTTAACACAGTGTATTCGCGGGTATTGGGAGCAATCCCACAGGGTGGCATTTGTAAATAACTTTCACATGTTAATGTACATAACTACATATCTCGGAAACGGTGAGCTTCCCGAAGTCTGTATTAATATATATCGGGATTTACTGCAGCATGTAAGAGCATTACGCCAAACTATAACCGATTTTACAATACAAGGAGAGGGCCATAACGGCGAGACCTCGGAAGCGCTAAATAACATCCTTACGGATGACACGTTTATTGCACCTATTCTATGGGATTGTGATGCGTTAATATACCGTGATGAAGCCGCCCGAGACCGACTCCCCGCAATTCGTGTAAGCGGGCGAAACGGATACCAAGCCCTTCACTTTGTGGATATGGCCGGGCATAACTTCCAACGACGCGATAATGTGTTAATCCACGGGAGACCCGTTCGGGGAGACACGGGTCAGGGTATTCCCATTACCCCACACCATGACCGTGAATGGGGTATTCTCTCCAAGATTTACTACTATATTGTCATTCCTGCATTTTCCCGCGGTTCCTGTTGTACAATGGGCGTGCGTTATGATCGCCTATACCCTGCGTTACAGGCAGTTATCGTTCCGGAAATTCCCGCTGATGAAGAAGCCCCAACTACCCCAGAAGATCCAAGACACCCTCTTCACGCACACCAACTCGTTCCGAACTCTCTTAACGTTTACTTCCATAATGCACACCTAACCGTTGATGGTGATGCATTGCTCACACTACAAGAGTTAATGGGAGATATGGCTGAACGAACGACGGCCATTTTAGTATCAAGCGCCCCCGATGCGGGAGCCGCCACGGCAACAACCAGAAATATGAGAATATATGACGGAGCGCTTTACCATGGCCTTATTATGATGGCATATCAGGCGTACGATGAAACCATTGCAACGGGTACTTTTTTTTATCCCGTTCCGGTCAACCCTCTGTTTGCATGTCCGGAACATTTGGCATCATTGCGTGGAATGACAAATGCTAGGCGGGTTTTGGCAAAAATGGTACCACCAATCCCTCCTTTTCTGGGAGCCAACCACCACGCAACTATACGCCAACCCGTTGCCTACCATGTAACGCATAGTAAGTCGGATTTTAATACTCTTACATATTCTCTTCTTGGAGGGTATTTTAAGTTTACACCAATATCTCTTACACATCAACTACGAACGGGATTTCACCCCGGGATTGCCTTTACCGTAGTGCGCCAGGATCGCTTTGCCACAGAGCAACTTTTATATGCCGAGCGTGCTTCTGAATCGTACTTTGTCGGACAAATCCAAGTACACCATCATGATGCTATTGGGGGGGTAAACTTTACCCTAACCCAACCCAGAGCTCACGTGGACCTGGGAGTCGGGTATACAGCTGTATGTGCCACAGCAGCCCTGCGATGCCCTCTCACGGATATGGGCAATACTGCCCAAAATCTTTTTTTTTCACGAGGAGGAGTGCCAATGTTACATGATAACGTTACCGAATCGTTGCGTCGTATAACAGCATCGGGGGGTCGCTTAAATCCCACCGAACCCCTACCCATCTTCGGCGGACTACGTCCTGCTACATCGGCAGGAATTGCACGAGGGCAAGCCTCTGTGTGTGAGTTTGTGGCCATGCCGGTGTCCACTGACCTACAATATTTTAGAACTGCATGCAATCCTAGAGGTCGAGCATCTGGAATGTTATATATGGGTGACCGTGACGCCGACATAGAGGCTATAATGTTTGATCACACACAATCGGATGTTGCTTATACAGATCGAGCAACTCTTAACCCATGGGCATCACAAAAACATTCATACGGTGACAGGCTATACAACGGAACATACAACCTTACAGGCGCTTCTCCTATCTACAGCCCATGCTTTAAGTTTTTTACACCAGCGGAGGTTAACACTAATTGTAATACACTGGATCGGCTTCTAATGGAGGCAAAGGCTGTGGCGTCGCAAAGCTCCACCGACACTGAATATCAATTTAAACGCCCTCCCGGTTCTACCGAAATGACACAGGATCCGTGTGGCCTTTTTCAAGAAGCATATCCACCACTATGCTCAAGCGATGCGGCCATGTTACGAACGGCTCACGCGGGAGAAACCGGGGCAGATGAAGTTCACTTAGCCCAATATCTGATTCGAGACGCGTCGCCCCTTAGGGGATGTCTTCCTCTTCCGCGATAATTTCACCACGCCCACATACCCACTCCCAATAAAAGCCCTGTAGAGCGCATTGGCATCTTACTTGAGATTTGGATACGCTCGGCCGACTTGGTCTGTTTCACGCTTCCTTAAACAACATGGCTATGCCATTTGAGATAGAGGTATTGTTACCAGGAGAACTATCCCCGGCGGAAACATCTGCATTACAGAAATGTGAGGGAAAAATTATTACCTTCTCAACCCTGCGTCATCGAGCTTCACTGGTGGATATAGCGCTGTCGTCATATTACATTAACGGTGCTCCACCAGACACGCTCTCGCTGTTAGAGGCATACCGAATGCGATTCGCGGCAGTTATAACACGGGTCATCCCGGGAAAGTTGTTGGCGCATGCCATTGGCGTGGGTACTCCTACACCCGGGTTGTTTATTCAAAATACATCCCCCGTTGATCTTTGTAATGGCGATTACATCTGCTTACTTCCTCCGGTTTTCGGGTCCGCAGACTCAATTCGCTTGGACTCTGTAGGACTGGAAATTGTTTTCCCTTTAACCATCCCCCAGACCTTAATGCGAGAAATCATCGCCAAAGTGGTTGCACGGGCCGTTGAGCGCACGGCCGCGGGTGCTCAAATTTTACCCCACGAAGTTCTACGAGGCGCGGATGTCATTTGTTACAATGGAAGGCGTTATGAACTCGAAACAAATTTACAACATCGGGACGGATCGGATGCGGCTATTCGCACATTGGTTTTAAATCTAATGTTTTCCATAAACGAGGGATGTCTGCTTTTATTGGCGCTGATTCCAACTTTGTTAGTTCAAGGAGCACACGACGGTTATGTAAATTTATTGATACAAACGGCCAATTGCGTTAGAGAAACCGGCCAGTTAATTAATATACCGCCAATGCCGCGGATTCAAGACGGCCATCGCCGATTTCCCATATATGAAACTATTTCATCTTGGATATCAACATCATCTAGACTGGGGGATACCTTGGGAACTCGCGCAATTTTACGCGTCTGTGTGTTTGATGGACCCTCTACTGTTCATCCGGGAGACCGCACGGCCGTGATTCAAGTGTAAACAGGTGTTAATAAAAACACAACCAGTCTAGTTACATTTCACGCGTCTTGTTTTTATTTAATAGGCATAAACACGGAATCCGGTATACATGAACTGCCAATATACACGGACATAATTAATGCAACCATCAGATCATCTGACATTGTTCCCGTGGTACCTTTACCCGTGTAAGTTTTTGTGTCTAGATTACCCATACCGCCTTTAATTACCTCTGTCAGGTTATCCAACTGTTTACATAGATACTCCACGGGGTCTACACCTAACTTTACTGTTAGGGATACAAGCTCCTGTGAGGCTATTATATTTCCGGAGTTAAATCGTTTAACAAAATAGTCTACGGCCGGCGTTTTTTGTTTTTGTAATAAAAAAAAAGGGTACGCCACGCTACATCCGGGAGGTATGGAATGATAAAACAGTAACACTGGAGCGGAAGATAGCACGTTTCCCTTTTCGAGGACAGCAAACTGTTGTGCTATAGCCAACGATATGGCAACTGCAGAATCCTGGCTGCTGTTTCCCTCTATAGAAACGTGTACGTTTGTAAATGTATTGGGGTGTAAAGCGAGTATGTGGCCTAAGCATTGAGTAACGCAACGCCCTATCTCACTGGAAGACGTGCCAGTTAAAGCTCTAAGAAAAAAGTGCTCCAATCCAAATATAATCCAATCCGACTTATAACGACCAACAATCGCTACACCAGTACCAGACGCTCGTGTATTTGAGGTAAATGCAGGGTCTACGTAAACGTACAACACTGACGATAATATAGCACAATTCGCAACGGTTGACGGCCGATATAAAATAAACCTCTCACGGGCAGTTTTTGTAAATAATGGCCGGTCAAACCCCACACCCCCAGAATTCTGTTTACGCCCACCTACAATTTCCTGCACGAAGGAGTCGGCCATAAATAAATCTGCAGTGCGCCGCATGGCTCCATCCATTGTGATGAAAACCGGCTTATTTAATACATAACACGAACAAGCTGTGACATCGCTATGTGCTAAAACACGCGGCATGTGATCGTCGCATACATATGTAACAACGTTTAACAACTGATCCGACGATCCACGTAAGTTATACAAAAAACTTGTACTTGCTTTTCCGGTATTTGTTGATGAAACAAAAATAATTTTACAATTGGTTTGATTTAAAAATCCGACTATAGTTTGTACAGCATCAGGTCGAATAAAATTAGCTTCATCCACAAACAGAAGATTAAAATCTTGACCTCGGATACCCTGGAACGATAGAAAGATATATAGTTACCCCACCAAAGTTTAAATGTATCCTTAAATACCACGTACGTAAAAAATGTTTGAATACGTACATATTTCTTTTTTTTTCCAGTACAACCATATCCGGTGTATAATGGAAGCCCATTTGGCAAATGAAACCAAACATGCACTTTGGCATAATGATCACACAAAAGGATTACTACACGTTGTGATACCTAACGCGGGGCTTATTGCGGCCGGAATAGATCCCGCATTACTGATTTTAAAGAAACCCGGACAACGCTTCAAGGTTGAAGTACAAACAAGATATCATGCTACAGGTCAATGCGAACCGTGGTGTCAAGTTTTCGCCGCGTACATTCCCGATAACGCCTTAACAAATCTCTTAATACCAAAAACGGAACCATTTGTTTCACACGTTTTTTCGGCCACGCATAATTCAGGGGGATTGATTTTATCATTGCCTGTTTATCTTAGCCCCGGTTTATTCTTTGATGCATTTAACGTTGTAGCGATACGAATAAATACTGGAAACCGCAAGCACCGTGATATTTGTATTATGTATGCAGAACTAATCCCAAACGGAACGCGTTATTTTGCTGATGGACAACGGGTACTTTTATTATGCAAACAGCTGATTGCGTATATCCGATGCACCCCTCGTCTTGCATCGTCTATAAAAATATACGCAGAGCATATGGTGGCAGCCATGGGTGAATCACACACGTCAAATGGGGACAATATTGGACCCGTTTCATCCATAATCGATCTTGATCGACAGTTAACTTCTGGAGGTATTGATGACTCCCCTGCTGAAACACGCATACAGGAAAATAATCGGGACGTCCTTGAGCTAATAAAACGGGCCGTAAACATTGTTAACTCCAGGCACCCCGTCCGACCTTCTAGTTCCCGCGTTGCATCTGGGTTGCTTCAAAGTGCAAAGGGCCACGGAGCGCAAACTTCCAACACAGATCCGATCAATAACGGTTCCTTTGATGGCGTCCTTGAGCCGCCTGGACAAGGGCGATTTACGGGAAAGAAAAACAATTCGTCCGCCAGCATCCCACCTTTACAAGACGTTCTATTGTTTACCCCAGCTTCGACAGAACCCCAAAGTCTTATGGAATGGTTCGACATCTGTTATGCCCAATTAGTTAGCGGGGACACTCCAGCAGATTTCTGGAAACGGCGTCCCCTATCAATTGTACCGCGACATTACGCAGAATCCCCCAGTCCGTTGATTGTAGTATCTTACAACGGATCCTCTGCCTGGGGAGGACGTATTACCGGAAGTCCAATTTTATATCACTCTGCACAGGCTATTATTGATGCTGCGTGTATAAATGCCCGGGTTGACAATCCCCAAAGCCTACATGTGACAGCTCGCCAAGAGCTAGTCGCGCGTTTACCGTTTTTGGCTAACGTCCTAAATAATCAAACCCCCTTACCCGCCTTTAAACCAGGCGCCGAAATGTTTTTAAACCAGGTATTTAAACAAGCGTGTGTGACATCGCTAACCCAAGGTCTTATAACGGAGTTACAAACGAACCCGACTCTACAACAACTCATGGAATATGATATTGCAGATTCTTCCCAAACGGTTATTGATGAAATTGTAGCCCGCACACCAGACCTGATTCAGACTATAGTTTCGGTGTTAACGGAAATGTCAATGGATGCGTTTTATAACAGCTCCTTGATGTATGCGGTTTTGGCGTATCTGTCATCTGTATATACACGACCACAAGGTGGGGGGTATATACCCTACCTTCACGCTTCCTTCCCATGCTGGTTAGGTAATCGTTCTATATATTTATTTGACTATTATAATTCAGGAGGGGAAATACTTAAGCTTTCCAAGGTCCCCGTTCCCGTAGCCTTAGAAAAGGTTGGTATTGGTAATTCCACACAACTGAGGGGTAAATTTATACGCAGCGCGGATATTGTTGATATTGGAATTTGTTCTAAGTATTTACCCGGTCAATGTTACGCGTACATTTGTCTAGGATTTAACCAGCAATTACAATCCATTTTAGTTTTACCGGGGGGATTTGCGGCATGTTTTTGTATTACCGATACCCTACAGGCAGCACTACCTGCATCGTTAATCGGACCTATTCTAGACAGATTCTGCTTCTCTATTCCCAACCCCCATAAATAAATTAGTGTCACTATAAAAACATAACACCAGAATCTCTTCATATTTAATTTTACGTCATTTCTCCCGTTTCCACCCCCTCTTAAAATATAAAATAACCGGGTGGGTGGCATTAAACCCACAAGTACCCGGGCGGCAATCCGCTAGACTGTTTTTCTGCTCATGGAATTACAACGCATATTTCCGCTGTACACCGCTACGGGTGCAGCGCGCAAATTAACCCCCGAGGCAGTTCAGAGACTCTGCGATGCATTAACGCTGGATATGGGATTATGGAAGTCCATCCTGACCGATCCCCGGGTGAAAATAATGCGATCAACTGCTTTTATAACTTTAAGGATCGCTCCGTTTATCCCCCTTCAAACGGATACTACTAATATTGCCGTTGTTGTAGCCACAATTTACATCACGCGCCCACGTCAGATGAACTTACCTCCGAAGACTTTTCATGTAATTGTAAATTTTAATTACGAGGTCTCGTACGCAATGACGGCGACTTTAAGAATTTATCCGGTTGAAAACATAGACCATGTTTTTGGAGCAACGTTTAAGAACCCGATCGCGTACCCCCTTCCAACATCTATTCCGGATCCTCGAGCAGATCCCACCCCCGCAGATCTTACACCAACGCCAAACTTAAGCGACTACTTACAACCCCCGCGGCTTCCGAAAAATCCATACGCATGTAAAGTTATTTCTCCGGGAGTGTGGTGGTCAGACGAACGAAGGCGTTTATATGTACTGGCTATGGAACCTAATTTAATAGGGTTATGTCCCGCCGGATGGCATGCTCGGATACTTGGCTCTGTATTAAATCGACTCCTCAGCCATGCGGACGGATGTGATGAATGTAATCATAGAGTTCACGTGGGGGCACTGTATGCGTTACCCCATGTCACAAATCATGCGGAAGGTTGTGTGTGTTGGGCTCCGTGTATGTGGAGAAAGGCCGGTCAGCGGGAATTAAAAGTGGAGGTAGACATTGGCGCTACGCAGGTTCTTTTTGTAGATGTCACCACCTGCATTCGAATTACGAGTACTAAAAATCCTCGCATTACCGCAAATCTTGGCGACGTTATAGCGGGAACCAACGCCAGTGGTCTCTCTGTACCAGTAAATTCATCTGGGTGGCAGCTTTATATGTTTGGAGAAACATTAAGCCGGGCTATTATTAACGGCTGTGGTCTGCTTCAGCGAATTTGCTTCCCCGAGACACAAAGATTATCGGGTGAACCGGAACCTACAACCACCTAGTATACCTTAACTCAACCGCCGTTGTGGAAAGGTATATGTCAACATTTACAGTAATATATTAAAGGTTAAATTTATAAAACACTCACGTTTGTGTTGTGACTTGACGCGAACACCGCTGTGCTGTAAGACCCGTCGGTAAATGAAAACGTAATAGATTCGCCTTTTACATGATCCACGTAATTTGCCCCAAACCACTGTTCCAGGCGAGACTTGATACCCTCAAACACGGGTTCCGTTGCTTTGCGTATATGAGCCGTATAACCCACTTTAATTCCTCTAAACGTGGCCATTACTAAAGCTATTAATGGTACAAGAAACCATGTTTTCCCATGTCTACGTGGTACCAAAAACACAGTTGATTTTTGTTTGAAGTGTTCTAAAACACTGTCAGAAACACTTGGCGTGTTAAACACTGTACGCAGAAAGCAGTCAACTCTGTCGGCATGATCGCCCAATAGCACCGATGAAATAAAATGCGTGGTGTGCATGAGGATCATTTTTTGAAACAGTTCCAACGTCCCCTTATATCTGCCATAGATTGGAACGTCAACCTTTGCGCGTTTGCCATGACTTCCACACTCTTCAATACTCTCAAAAGATGTTTCCACAAGGTACGAAAACCGTTGTGTAAAGGTAGACAACTGACAGAAACTATCCGACAGAGAAAACGCGCGAAATGTGTTCATAACACCGCTATACGCATTTCGATGAGGTGCTGCTTCTTCCGGTGAATATTCATAAAACTGTACACTACTGACAGCCTTTTTTAATTCAGGGCTTACGTTTGCATTTACCGAATATCGCCATGGTTTCAAAACTACATTGGGGGTACAGTTGTACCCTGTTGACGATAGAAACGCGCCAAACATTGCCCGTCGAGCAGTAGCCGAGAACAGTGGAATATATTCACAACAGTTGTGAAGCGTTCCAATTCCGGGAATAACGGCCTGATGACGTCGGGTTACATCTATAGCAAAATTCAGAAACGGGATTTGGGTTGCGTTTCCCAGAGACCCTTGCCGCGTGGAACACGGGGTAGGGGACTCCAACGTCCCAAAGCGTTCATCCCTACGACGCTTTAGACGTTCAAAATATCTTACAGATTCTTCACCAAGCGTACGACCAAACATTATCAATGACATTTAACATCAATTCACGGAATCCGCCTCATCTCTTGTAAGCAGTAAAACAGGAAGCCGCGTCATCTTACGTACTCGTTACGTATATATCATAAACATTTTCAGGGCCGCATTCATTCACTTTGGTCATGTCAGGCCACACTCCAACCTACGCTTCTCATAGGCGTAACCGTGTCAAACTAGTTGAGGCGCATAACCGCGCGGGGTTATTTAAAGAACGGACCCTCGATCTAATCCGTGGGGGTGCGAGTGTACAAGATCCAGCATTTGTGTATGCCTTTACTGCTGCAAAAGAGGCCTGCGCCGATTTAAATAACCAGCTCCGCTCTGCAGCTCGCATAGCTTCAGTTGAACAGAAGATTCGTGATATACAATCCAAGGTTGAGGAACAAACAAGTATTCAACAGATTTTAAATACAAACAGACGCTATATAGCACCCGATTTTATTCGCGGTTTGGATAAAACAGAAGACGATAATACCGATAATATAGACAGACTGGAAGACGCGGTAGGACCGAACATCGAACACGAAAATCATACTTGGTTTGGAGAAGACGACGAAGCGTTACTTACACAATGGATGCTGACGACACACCCCCCAACCTCCAAATATCTCCAACTGCAGGACCTTTGCGTTCCCACCACAATACCGACGGACATGAACCAAATGCAACCGCAGCCGATCAGCAAGAACGAGAATCCACCAACCCCACACACGGATGTGTAAATCATCCATGGGCCAATCCGTCAACTGCAACATGCATGGAATCACCAGAACGATCACAACAGACAAGCTTATTTTTATTAAAGCACGGCTTAACGAGAGATCCAATACATCAACGCGAAAGGGTGGACGTTTTTCCACAATTTAACAAACCCCCATGGGTTTTTAGAATTTCCAAATTATCCCGTTTAATTGTACCCATCTTCACGCTCAATGAACAGTTATGTTTTTCTAAATTACAGATTCGAGATAGACCCAGGTTTGCGGGACGGGGAACGTATGGGCGTGTTCATATATACCCATCGTCAAAAATAGCTGTAAAAACCATGGACAGTCGTGTTTTTAATAGAGAGTTAATTAACGCGATTTTAGCGAGTGAGGGTTCTATACGAGCAGGGGAAAGGCTAGGTATTTCTAGCATAGTTTGCCTTTTAGGTTTTTCGTTACAAACCAAACAGCTACTGTTTCCGGCATACGACATGGATATGGATGAATACATTGTTCGCCTGTCCAGACGGTTGACAATACCTGATCACATAGACAGAAAAATTGCCCATGTATTTTTAGATTTGGCTCAAGCGTTGACGTTTTTAAATCGAACGTGCGGCCTGACCCACCTAGATGTGAAATGTGGCAATATTTTTCTTAACGTCGACAACTTTGCCTCGTTGGAAATAACCACAGCAGTAATCGGAGACTATAGCCTAGTAACATTAAATACGTATTCCCTTTGTACTCGAGCGATATTTGAAGTTGGAAATCCATCCCACCCGGAGCACGTACTACGCGTACCCCGGGATGCATCGCAGATGTCATTTCGTTTGGTGTTGAGTCATGGAACAAACCAACCCCCTGAAATCTTGCTTGATTATATTAATGGAACGGGCCTTACTAAATATACTGGAACCTTGCCCCAAAGAGTTGGACTTGCGATTGATCTTTATGCATTGGGCCAAGCACTCTTAGAAGTTATCCTGCTAGGACGTCTTCCCGGACAACTGCCCATTTCAGTACATCGGACCCCGCATTATCACTACTACGGTCATAAGTTATCACCAGATTTGGCGCTTGATACGCTGGCATATCGATGTGTCCTGGCGCCATATATACTCCCATCTGACATCCCCGGGGACTTAAATTATAATCCCTTTATACACGCCGGAGAGCTGAACACCCGTATTTCCCGGAATTCTTTACGCCGGATATTCCAGTGTCACGCAGTGCGTTACGGCGTAACGCACTCAAAGCTTTTCGAAGGCATACGCATTCCGGCCTCATTATACCCAGCCACTGTTGTTACGTCGTTGTTGTGTCACGATAATTCAGAAATACGCTCGGATCACCCTTTATTATGGCACGATCGGGATTGGATAGGATCGACATAAGCCCCCAGCCAGCCAAAAAAATTGCCCGTGTGGGAGGTCTACAGCACCCTTTTGTAAAAACGGATATTAACACGATTAACGTTGAACACCATTTTATAGACACGCTACAGAAGACATCACCGAACATGGACTGTCGCGGGATGACAGCGGGTATTTTTATTCGTTTATCCCACATGTATAAAATTCTAACAACTCTGGAGTCTCCAAATGATGTAACCTACACAACACCCGGTTCTACCAACGCACTGTTCTTTAAGACGTCCACACAGCCTCAGGAGCCGCATCCGGAAGAGTTAGCATCCAAATTAACCCAAGACGACATTAAACGTATTCTATTAACAATAGAATCGGAGACTCGTGGTCAGGGCGACAATGCCATTTGGACACTACTCAGACGAAATTTAATCACCGCATCAACTCTTAAATGGAGTGTATCTGGACCCGTCATTCCACCTCAGTGGTTTTACCACCATAACACTACAGACACATACGGTGATGCGGCGGCAATGGCGTTTGGAAAAACCAACGAACCGGCGGCACGAGCGATAGTTGAAGCATTGTTTATAGATCCGGCTGATATCCGTACTCCTGATCATTTAACGCCAGAAGCTACAACTAAGTTTTTTAATTTTGACATGCTCAATACCAAATCTCCAAGTCTCCTTGTGGGTACACCAAGAATCGGAACGTATGAATGTGGACTTTTAATCGACGTTCGAACGGGACTTATAGGCGCGTCGTTGGACGTTCTTGTATGTGACAGGGACCCTTTAACTGGCACCCTAAATCCCCACCCTGCAGAAACCGACATTTCATTTTTTGAAATTAAATGTCGTGCTAAATACCTCTTTGATCCAGATGATAAAAATAACCCGCTCGGTCGGACGTACACCACGTTAATAAATAGACCTACAATGGCAAATCTACGGGACTTTTTATATACTATAAAAAACCCATGTGTAAGCTTCTTTGGACCCTCAGCAAACCCAAGTACACGCGAGGCCTTAATAACGGATCACGTTGAATGGAAACGTTTAGGATTTAAAGGTGGGAGGGCCCTTACAGAACTCGACGCCCATCATTTGGGCCTCAATCGGACAATCTCATCCCGAGTGTGGGTATTTAATGATCCGGACATACAAAAGGGGACAATTACAACCATTGCATGGGCCACTGGAGATACGGCTCTTCAAATTCCTGTATTTGCCAATCCGCGGCACGCTAACTTTAAACAAATTGCCGTACAAACCTATGTATTATCCGGTTACTTTCCAGCGCTAAAACTACGGCCCTTCCTTGTCACCTTTATAGGACGTGTGCGCCGACCACACGAGGTGGGAGTCCCATTGCGCGTCGATACACAAGCGGCTGCCATTTACGAATATAACTGGCCGACTATCCCACCCCACTGTGCGGTTCCGGTTATAGCCGTTCTAACGCCTATCGAAGTTGATGTGCCTAGAGTGACAAAAATACTTAAAGACACAGGAAACAACGCGATTACATCAGCATTGCGGTCATTGCGATGGGACAATCTTCATCCAGCGGTCGAGGAGGAATCTGTGGATTGTGCAAACGGTACAACGAGCTTGTTACGTGCAACGGAGAAACCGTTGCTTTGAACTCAGAGTTCTTTGAAGACTTTGACTTTGATGAGAATGTAACAGAGGACGCCGATAAATCCACACAACGCCGCCCACGAGTGATCGATGTAACACCAAAACGAAAACCTTCGGGAAAGAGCTCCCATTCCAAATGCGCAAAATGTTAAACCCTGATAAACGTTCTAATAAAAACATCAAATCATGGTTGGTTACTGTGAATGTTTGTTTTATTGCTTGGGGGGTTACAAGTACAACCCACGCTACTCCCACCCACTGTTTGATCGCTCGTATAACAGCTCATCCTCGCGGTCCGTTTCATATGTTGAGTCATTTTCATAGACGTAGCCGTAGCCTTGTGATGGGTAATTTGTGCGGCGAGAATTTCTATGTGCAGGTTTTACTTTTCGTATGTATCCCCGTACCCGCTCGGGTACTCTTCTTACGGCACCGTAGAACCGACTGCGTTTCTGTCGATGATACACATATGCACGCATCAATCTGAGAAGCAACATGACAACGGAAAACACGGCCAGGCAAGCCAAGGTTCCCCGAGTTGTGGGAATTAACCGTGGAGATTGAACCGATATAGGGTCATATAATCGGTCCATATACGAGTGCGCGGCGGTTCCCAACGTAGCACAGGCCACGAGCGTTCCCAGGGACGGTCCTATTAACACGTGTATATAATGCGCCAAAATTAATTCTGATACTATAAGATATACAACTGACAATGTACTAAATGTAGACATGGCCACGGACACCGATGACCACAGTCCCGTATGTAGATGATTCGCCACCACAAGTTCCAGCATTAATGATACAAATAGGATACATATCGCCATCAACGCAGCCATCAAATTCACGAACACTGCGCGCGTAGGCCCCGCAAGGCGATATAAAAAGACGCTCTGCTGTCGTAAATTTGCGACCGCTTTTATGTTCGTTTCGTCCAATTTTCCGCGTCCACAAAAATACGTTGTAAATATTACACTTGTCGCAAAATGTCCAAGATATAATGTAGCAGCCACGCCGATTTGCTTGTAAGCTAATAATAACACAACGGCGTTTAATAACCACAATGACAAAAGACCCCAAAAAAGTGTTGTGGGATCTACAACTAACCATGCAACACCGGAGCTTTGCCGGACACGTTGATTTTTCGTTTCTCGGTGTATAATCGCGGCCGTGATCAGTGTATATACCGCCATGGCCATTGCCGTTAAAGCCGTGTAGTAAGTAAATGCCACAACGCTATGTGGTTCCAAAAACAAAACCGGGGCGCTGTATCCACCTCTATTTCCGGACCATACCCCCCCATCTAGGGTGGCGTTAAATAACTCATAATCAACTACGGCAGCATAAAAACAAGGGATCCCGGTATATTCAGAAGAGGCGGCAATTAACGTAGCCAGGAGCATTACCGCACCCAAAGTGAACATCATCACCTGAATTATCCAAATTCGCCAATTAAGCGTATCCATTTGATGATCTAACGCTTCCACCTCGGGTGTCGTGGTGTCGTACGGTGAGACTTTTTCAGAACGCGGCCCCTTCTTTTGAGTTCCCATGTCTCCCAACACCGGGGAGAGCAACGCCGCCGTCTATGCGTCCAGTACACAGCTCGCGCGGGCGTTATATGGAGGGGATCTGGTTTCGTGGATTAAACACACCCACCCGGGAATTAGCCTGGAACTGCAATTGGATGTTCCAGTAAAACTAATAAAACCTGGTATGTCACAAACTCGCCCGGTAACCGTCGTACGTGCCCCTATGGGCTCTGGTAAAACAACAGCCTTGCTTGAGTGGCTTCAACACGCGTTAAAGGCAGATATTAGCGTACTGGTTGTCTCATGTCGCCGTAGCTTTACCCAGACGTTGATTCAACGGTTTAACGATGCAGGCCTCTCCGGATTCGTAACATATTTGACATCCGAGACATATATTATGGGTTTTAAACGTTTGATTGTGCAACTTGAAAGCCTACACCGCGTATCCAGCGAAGCTATCGACAGCTACGACGTATTAATACTGGATGAGGTAATGTCAGTGATTGGACAATTATACTCCCCCACAATGAGACGTCTTTCCGCGGTTGATAGCCTATTATATCGTCTTTTAAATCGCTGTTCTCAAATTATCGCGATGGATGCTACAGTAAACTCTCAGTTTATTGATTTAATCTCCGGATTGCGTGGAGATGAAAACATACACACAATTGTGTGTACATACGCGGGAGTTGGGTTCTCCGGAAGAACTTGCACGATCCTGCGTGATATGGGCATCGACACGCTTGTGCGAGTCATTAAACGATCTCCTGAACACGAGGATGTACGTACCATACACCAACTACGTGGAACATTTTTTGACGAACTAGCACTACGATTACAATGTGGGCATAACATCTGTATATTTTCATCAACTTTATCGTTTTCGGAGCTAGTTGCTCAGTTTTGTGCAATATTTACAGACTCTATTCTTATTTTAAACTCAACTCGGCCCCTATGTAATGTAAACGAATGGAAACATTTTCGCGTGTTGGTGTACACTACCGTCGTGACCGTTGGATTGAGTTTTGACATGGCTCATTTTCATAGCATGTTTGCTTACATAAAGCCAATGTCATATGGGCCGGATATGGTATCGGTCTACCAGTCATTAGGGCGTGTACGTTTATTGCTACTTAATGAAGTTTTGATGTACGTCGATGGCTCAAGGACCAGATGCGGACCCCTGTTCTCGCCAATGTTACTAAACTTTACCATCGCAAATAAATTTCAATGGTTTCCTACACACACCCAAATAACTAACAAACTGTGCTGTGCATTTAGGCAACGATGTGCAAATGCATTTACACGCTCGAACACCCATCTCTTCTCAAGATTTAAATACAAACACCTTTTCGAGAGATGCTCTCTTTGGAGTTTAGCCGATAGCATTAATATCTTACAAACTCTTTTGGCCTCTAACCAAATTTTGGTTGTATTGGATGGCATGGGTCCAATAACGGACGTTTCCCCAGTTCAATTTTGTGCATTTATACACGATCTCAGACATAGCGCTAACGCCGTAGCTTCCTGTATGCGTTCTCTTAGACAGGACAATGACAGCTGCTTGACCGATTTTGGCCCTTCCGGATTTATGGCCGATAACATTACCGCGTTTATGGAAAAGTATCTTATGGAGTCAATTAATACCGAAGAACAAATTAAAGTATTTAAAGCCCTTGCATGTCCAATAGAACAGCCTAGACTAGTCAATACGGCAATATTGGGGGCGTGTATACGAATACCTGAAGCGTTGGAAGCATTTGACGTATTTCAAAAAATATACACGCACTACGCTTCCGGTTGGTTTCCCGTCCTGGACAAAACCGGGGAATTTAGCATCGCGACTATAACTACCGCCCCAAATTTAACCACACATTGGGAGCTGTTTCGCCGTTGTGCCTATATTGCAAAAACACTCAAGTGGAATCCGTCCACCGAAGGCTGTGTAACACAAGTTTTGGATACGGACATTAATACACTTTTCAATCAACACGGGGATTCGCTGGCTCAACTAATATTTGAGGTTATGCGCTGTAACGTTACCGACGCTAAGATTATATTAAACCGCCCGGTTTGGCGAACAACCGGATTCTTAGATGGATGCCATAATCAATGCTTCCGTCCAATCCCTACAAAACACGAATATAACATTGCTCTATTTCGTTTAATTTGGGAACAATTATTTGGCGCCCGCGTAACTAAAAGTACCCAGACCTTTCCGGGAAGTACTCGTGTGAAAAACCTAAAAAAAAAAGATCTAGAAACTTTACTTGATTCAATTAACGTGGATCGTTCTGCATGTCGTACCTACCGCCAGTTGTATAACCTGCTTATGAGCCATCGCCATTCGTTCTCCCAACAGCGTTACAAAATTACTGCCCCCGCTTGGGCACGCCACGTGTATTTTCAAGCACATCAAATGCACTTGGCCCCGCATGCCGAAGCCATGCTACAATTAGCGCTATCGGAACTGTCCCCGGGATCGTGGCCGCGGATAAACGGGGCGGTAAATTTTGAAAGTTTATAACCCATTAATACCATATATGGACATCCATAGGGGGGGTTACATAAATACTAAGCCTCTGTACAACACAAAGGGCCTCTAACAATGCACTGAACCACAACCAAGCTATGGACGCAACGCAGATTACCTTGGTTAGAGAAAGCGGACACATTTGTGCCGCAAGCATATACACATCCTGGACACAGTCCGGACAATTAACACAGAACGGTCTTTCCGTGTTATACTACTTATTATGCAAAAACTCATGTGGGAAATACGTCCCTAAGTTTGCCGAAATTACCGTACAACAAGAGGATTTATGTCGCTACTCCAGGCATGGGGGGAGTGTTTCTGCGGCAACGTTTGCGTCTATCTGCAGGGCGGCGTCCTCGGCTGCGTTAGACGCCTGGCCCCTTGAACCACTGGGTAACGCAGACACCTGGCGTTGTCTCCATGGCACTGCCCTGGCCACTTTACGGCGCGTATTAGGGTTTAAATCGTTTTATTCGCCAGTAACATTCGAGACTGATACGAATACAGGTCTTCTGTTAAAAACAATCCCCGATGAACACGCGTTGAATAATGACAACACGCCATCTACCGGAGTATTGAGGGCTAATTTTCCCGTGGCCATTGATGTTTCAGCAGTCAGCGCATGTAACGCCCACACGCAAGGTACGTCGCTAGCCTACGCCCGCCTGACCGCACTTAAATCTAACGGTGACACCCAGCAACAAACACCTTTAGACGTGGAGGTAATTACACCAAAGGCCTACATACGTCGGAAATATAAGTCTACGTTTTCCCCCCCTATAGAGCGGGAAGGTCAAACCTCCGATTTGTTTAACCTTGAAGAACGCCGCTTGGTTCTTAGTGGCAATCGCGCAATTGTGGTAAGGGTACTCTTACCGTGTTATTTTGACTGTTTAACAACGGATTCCACCGTTACATCTTCCCTTTCAATATTAGCAACATATAGACTGTGGTACGCGGCGGCGTTTGGAAAACCCGGGGTTGTCCGTCCAATCTTTGCGTATTTAGGCCCGGAACTCAATCCGAAGGGTGAAGACAGAGACTACTTTTGTACTGTCGGATTTCCCGGATGGACCACTCTTCGGACACAAACTCCAGCCGTCGAATCTATTCGCACGGCTACGGAGATGTACATGGAAACGGATGGGTTGTGGCCAGTAACCGGTATTCAGGCCTTTCATTATCTAGCCCCCTGGGGACAGCATCCCCCCTTACCTCCGCGGGTGCAGGATCTTATTGGGCAAATCCCTCAAGATACTGGACATGCAGATGCAACTGTCAATTGGGACGCGGGCCGGATATCTACCGTCTTCAAACAGCCTGTACAACTACAAGATCGTTGGATGGCAAAGTTTGATTTCAGCGCCTTTTTTCCCACGATATACTGCGCTATGTTCCCCATGCATTTTAGATTAGGCAAAATCGTCCTGGCTAGAATGCGTCGAGGAATGGGGTGCCTAAAACCCGCGTTGGTGTCTTTTTTTGGGGGGTTACGGCACATACTCCCGAGTATATACAAAGCTATTATTTTTATAGCCAATGAAATTAGCCTTTGCGTCGAACAAACGGCCTTGGAACAGGGCTTTGCTATATGTACTTATATAAAAGATGGATTTTGGGGAATCTTCGCCGATTTACATACGCGCAATGTATGTTCAGATCAGGCACGTTGTTCGGCCTTAAATTTAGCGGCCGCCTGCGAAAGAGCAGTCACGGGCTTATTACGAATTCAACTAGGTCTTAACTTTACACCCGCCATGGAACCGGTACTCCGGGTCGAGGGTGTGTACACTCACGCATTTACCTGGTGTACCACGGGAAGCTGGCTGTGGAATTTACAAACAAACACGCCTCCGGATTTAGTTGGCGTGCCATGGCGAAGTCAGGCGGCGCGAGATTTAAAGGAGCGTCTTTCAGGACTCCTATGTACCGCAACAAAAATTCGAGAACGGATACAGGAAAATTGCATATGGGACCGTGTCCTATACGACATATGGGCCGGACAAGTTGTGGAGGCTGCCAGAAAAACATACGTCGATTTTTTTGAACATGTTTTTGATCGCCGTTATACTCCGGTATACTGGAGTCTTCAGGAGCAAAATTCGGAAACAAAAGCAATACCGGCATCTTATCTGACATACGGACACATGCAAGATAAGGATTATAAACCAAGACAGATAATTATGGTTCGTAATCCCAACCCACATGGACCTCCTACTGTTGTTTACTGGGAATTGCTACCATCGTGTGCCTGTATTCCCCCCATAGACTGCGCTGCTCATCTCAAGCCCCTTATACACACGTTTGTCACTATTATTAACCATCTTCTAGATGCTCATAATGATTTTTCAAGTCCATCATTGAAATTTACTGACGATCCCCTTGCTTCATATAACTTCTTGTTTTTATGACAAAAAAACACGCCGCAACAACCCATCCTTAAAATAAAAGGTTTATTTACTTTACAACCCGTGGTGAATTTTTATACGTTTCAAATAACTGAACATTTTTCGGTGTTACCATGGTGCGATTTAACCACCAAAAATATACGCTCTTCTGATATTCCGAATCTCGTAAAGGTCCATTTAACAATCCCGGGGGCACTTGCACCACACCATCTGGACAGGGGGGGGTTCCGTGGGGCAGGTCAAAACGCTGACCCACCCCACATGAATATATAGCCTTTATAATATTGGGGGCCGTTCCAGGCTGAGGGTTCAGTAACTTAACAAACATATAATGCGGCAATACGCGGGTTTTTGTAAAGGGGTTGTTATCAACGACATACATTAGAGTGTTTAACAACCATAAAACTCCCTCATATAAAAACCGACGCATTTTTTCCAAAGGTCCTATTTGACACTCAACGCGTCTAAGATATACAGACAATTGTACAAACAGCGATGGAGATGCCCCGGAGGGCCCAATGCCTTCCAGATACATTAAAATAACACATAAGGTAAAATCTAGGACATTATCCGGGCGGAATAGAGTCATCCGATAGATTAACAGGCGCGGAGGCACCCCCACCGTATACACCCTATCTTCAACCGCAGTTAATACGGAAAAAATAAATCCGCGGAACGCTGGTTGAGTAACACACTCCATGTAGTAACGATCACAGGACACCTCACTTGAATCACCATTCAACACTACTAAAACGGTCTCTTGGTGTTCCGGTTTTACGCGCAGTGATACAACAGAGTTTGCCAAAAAGCGTGGCTTCAAACCGGTTACCTCCCGCGCCTCGCATACGAATCTTGGTATTGCTTGTATTCTAAGATCTTCGATCACGTCGCTCACATCCAACCCCTCTTCGGCTCGTGTTAGTAAGTTGTCGATCGTTACGCTGCAACCTAAAATGCTGGGTATATTTATTCCGGACATCCCATCGGCCATCCCCGCGCCTCCGGTTTGCTCGAATTTTATCCAGTAAGGTCGAATCCGCTGCATTTACCTTGTGTACCCGTAACCTCTCAGGGGGGTGTCCTTTCATAAAATGGGATAGGTTTTTATATCCAACATGCATGTATTGGTTATTTATTTTATTGGGTTCCGGGATTCTTTCGTCATCTTCTGTAGGGTCAGGCAAACCCCAGGAAGGACTTGGTGTTCTCCGTGGGCCCCGTTTTATTACCTCTGCGCGAACCTGCATTTCATATAATATTCGGATTTGGGATAAATAGGACTCTGTTCTCGCCTTTTTAAAAATAGCCTGGCATAACTCTTCCTCTGACCTATGTACCTCGCTTTGAGTTACCAAGAATCCCAATCGGGTGGCCCGTAATATGAATGAAAAATACGGCGCAACTAGTAATGAGATTGACGCATTTGAATATGATACAGAAATTTCCTGGCCTTGATTATTGTTTACCCGGTGAAGCTTAAAACAGCGAACAAGTTCCTGTTTCCATAGCTCAGACAAACGTTTTATATCATCTCCATAAGGGGGGATATAACGAGATTGAAAACTATTGGCAATATATGCATCATCCCCTATTATGCCGGTAAGATCTATAACCTCGTGATTTAAATCGGCAATACGTGTTTCTTCTGCCATTGTAATATGTGACCCTTTAGATGGCTTTATTTTTACCCTCTCTTCCCGTAACCGTTTCAGCTCTCCTTCTTTGAACTGGAGCCTTTCGGTCAGATCGCTGTTCACATCCTTGAGACCCTCAATGGTTTTGAATAAATTATTCACATAACCCTCGAGCATGCCGTTGATACTGTTGACCACCGATGTTTTAAACGCACTTTGAACGTTTGTTGTTCCGGACATTGCCCCCCCGTTAAAGGATTGGTTGGCCTTGCCAAACCCCGGTTGTGATGTGTCCACCGATCCACTTCCTTCCAGAATGTGATTGCCCGTTTCTTCTAGATAGGAACGTACGGTTTCGGTAATATCTCCAACATGTCTCATGTTTTTTAAGTTAACTATTAGCTTTACAAGTCTAGACGCGGCCGATCCAGCCCGTGTTGTATCGTTCTCGCCCATTATACGATCAACCGCACGTGTGCTGTGAGATCTATCATCTTCATTCCGGCGACCTATTAACACGCGCAAAGGGGCTGTATTTAAAACTTGGCAGACGCGAGCATGTTCACGTAATGCATAACAGGCCAACACCTCCCCAGAAAGCCGCTGTAAGGGTGAGTCAAATACTACACCCTCCCCACATACAACGGGCGGCCACACGACCAAACACTCTCCCTTCATGCCCGTTACATCATCCTTTGCCATAATTAATCTTCGGTTATAATTATAATAAAGACGCGTCCTATCATAATCCATAATAGCAACATTTTGCATACACTCAACTAGGCTTGTGACAACCGCCGCCCCTCTGGCCAACGTTGCATCGGCAACTTTTAACATCTGGGACAGTTCTGCCGCTTGACCCATATACGTATTTAATGGTGCAGGGGTTCCATTCTGTTCTGATCGTACCTTTCTTACAACGGGCACAATACCTACACAGGCTATCCAGTCCACGTATTTGGCAAAACCGACCCTTCCATTTAAACCACTGGTATAGAGACAACCGGTTATTCCACGCAGAAACTCAAGTAACGATGACTGTAATGTTTGACGCCAGGTTTCAAAAACCTGATGTGCAAGCCGTACGGCTTCTGATTCTCCACATAACCCATAACGTTCCGCTAGAGCCCCGGCATGCAGGTTACATTGTTGGATGTGGTGGTCCCAATCTGCTGCTAGGTCCTCATACCGAGTTGCATCCAACGCGTTCATCAAAACGGTTGCCTGAACTTGGCGAATTACAGTTTCCGTAGACCGTACAGCGCTATATATGCCTTGTCCATCGGTATATCCAAAGTCACCGGCTAGGATTTTTCGAAACAACATACTTTGCGTGGTTGGGTGTATTAACATCCAGCCATCTTCCTCCGGAAATGTACAAAACCCTATATCCGGGGCGTACTCATTCCAGTATATATCGAACATGTTCTTGTATTGGTCATTTGGGTTACTTCCATTCAAGCCCTGGTCAATAGAAACAGAACTTGCTATCCTTTTTTCTTCACTACCGGAACTGTTATTAAAAAGAGACGTTATTTCGGCCATTGAAAACCACGATGAAAAGATCAATTTCTGTAGACAGTTCTTCACCCAAAAACGTTTTTAATCCAGAGACGCCCAATGGATTTGATGACAGTGTATATTTAAACTTCACCTCTATGCATAGCATTCAACCTATCCTCTCACGGATTCGAGAACTTGCCGCAATTACGATTCCAAAAGAACGTGTTCCGCGGTTGTGTTGGTTTAAACAGTTACTCGAACTGCAAGCGCCTCCTGAAATGCAGAGGAATGAGCTCCCCTTCTCCGTTTATTTAATTAGCGGAAATGCCGGCTCCGGAAAAAGCACGTGTATCCAAACGCTTAACGAAGCTATCGATTGCATTATTACCGGATCCACCAGGGTTGCTGCCCAAAATGTTCATGCTAAGTTATCAACGGCTTATGCGAGTCGTCCGATAAACACAATCTTTCATGAATTTGGTTTTCGCGGAAATCACATTCAGGCTCAGCTGGGCCGTTACGCATATAACTGGACTACGACCCCCCCTTCTATTGAGGACCTGCAAAAAAGAGATATTGTATACTACTGGGAAGTTTTAATTGATATAACAAAACGAGTGTTTCAAATGGGGGACGACGGTCGCGGAGGAACATCGACATTTAAAACCCTGTGGGCAATTGAACGTTTGCTTAATAAACCTACAGGCTCAATGTCCGGAACCGCGTTTATCGCATGCGGTTCCCTTCCGGCTTTTACCCGGAGCAACGTTATTGTTATTGATGAAGCAGGATTGCTAGGGCGTCATATTCTCACGGCCGTTGTTTACTGTTGGTGGCTTTTGAATGCTATATATCAAAGCCCTCAGTACATAAACGGTCGAAAACCGGTCATAGTATGCGTCGGTTCGCCCACCCAAACTGACTCGTTAGAATCTCATTTTCAACATGACATGCAGCGTTCACACGTAACTCCTAGTGAAAATATACTCACGTATATAATCTGCAATCAAACTCTGCGTCAATATACTAACATCTCACATAACTGGGCAATCTTTATTAATAACAAACGATGTCAAGAGGACGATTTTGGAAATCTTTTAAAAACGCTTGAGTACGGGCTACCTATTACCGAAGCACATGCGCGTCTGGTCGATACATTTGTTGTACCTGCATCCTATATTAACAATCCTGCTAATCTCCCCGGATGGACGCGTCTGTATTCGTCGCATAAGGAGGTGAGCGCGTATATGAGTAAGTTACACGCGCATTTAAAACTATCGAAAAATGACCATTTTTCTGTGTTTGCCTTACCGACTTATACATTCATCCGGCTAACGGCATTTGATGAATACCGCAAATTAACGGGACAACCCGGACTTTCTGTTGAACATTGGATACGGGCAAACTCCGGTCGTTTGCACAATTATTCCCAAAGCCGAGATCATGACATGGGAACAGTTAAATACGAAACACATTCAAATCGCGACTTAATTGTAGCCCGTACAGACATCACTTACGTGCTAAATAGTCTCGTAGTTGTAACCACAAGACTACGTAAGTTAGTTATTGGATTCAGTGGTACATTTCAATCGTTTGCAAAGGTTTTACGTGACGACTCCTTTGTGAAGGCTCGAGGAGAGACATCCATTGAATATGCTTACCGGTTTCTGTCAAACCTAATCTTTGGAGGCTTGATTAACTTTTACAATTTTTTGTTAAATAAAAACCTACATCCCGATAAGGTATCGTTAGCATACAAACGGTTAGCTGCCTTAACCCTGGAGTTATTGTCTGGAACAAACAAAGCCCCCTTACACGAAGCAGCGGTTAATGGGGCGGGTGCCGGGATTGACTGTGATGGTGCAGCTACTTCTGCCGATAAAGCCTTCTGCTTTACCAAAGCCCCCGAGTCCAAAGTAACGGCCTCCATACCCGAAGACCCGGATGATGTAATTTTTACGGCACTTAACGACGAGGTTATTGACTTGGTATACTGCCAGTACGAATTTTCCTATCCCAAATCATCCAATGAGGTCCATGCTCAGTTTCTGTTAATGAAAGCTATTTACGATGGTCGATATGCCATATTAGCAGAGCTTTTCGAAAGCAGCTTTACAACCGCCCCCTTTAGCGCGTATGTCGATAATGTTAATTTCAACGGAAGCGAGCTTTTGATCGGCAATGTGCGGGGGGGGCTGTTATCTTTGGCATTACAAACAGATACGTATACCCTTTTGGGGTATACTTTTGCACCCGTGCCAGTCTTTGTAGAGGAACTGACCCGAAAAAAGCTGTACCGCGAAACTACCGAAATGTTATATGCTCTACACGTACCTCTTATGGTCTTACAGGATCAACATGGGTTTGTGTCCATCGTAAACGCTAACGTATGTGAATTTACCGAGTCTATAGAGGATGCAGAATTGGCAATGGCCACCACGGTGGACTATGGCCTTAGTTCTAAACTAGCCATGACAATTGCACGCTCACAGGGCCTGAGTTTAGAGAAGGTAGCTATCTGTTTTACGGCGGATAAACTGCGCCTAAATAGTGTGTATGTTGCCATGTCGCGTACGGTCTCCTCTAGGTTCTTAAAAATGAATCTAAACCCTCTACGGGAACGATATGAAAAATCCGCAGAAATTAGCGATCACATTCTTGCCGCTCTACGTGATCCCAACGTACACGTTGTGTATTAAAGCATTGTATAAAAACACGCATGCGGGCTTGCTGTTCTCATTTCTAGGTTTTGTCTTAAATACACCCGCCATGAGCATCTCTGGACCCCCAACGACGTTTATTTTATATAGGTTACATGGGGTCAGGCGGGTTCTTCACTGGACTTTACCGGATCATGAACAAACCCTCTACGCATTTACGGGTGGGTCAAGATCAATGGCGGTGAAGACGGACGCTCGATGTGATACAATGAGCGGTGGTATGATCGTCCTTCAACACACCCATACAGTGACCCTGCTAACCATAGACTGTTCTACTGACTTTTCATCATACGCATTTACGCACCGGGATTTCCACTTACAGGACAAACCCCACGCAACATTTGCGATGCCGTTTATGTCCTGGGTCGGTTCTGACCCAACATCTCAGCTGTACAGTAATGTGGGGGGGGTACTATCCGTAATAACGGAAGATGACCTATCCATGTGTATCTCAATTGTTATATACGGTTTACGGGTAAACAGACCTGACGATCAGACCACACCAACACCAACCCCGCACCAGTATACATCGCAAAGGCGGCAGCCTGAAACCAACTGTCCTTCACCACAACCGGCCTTTTTCACATCAGACGACGACGTTCTTTCGTTAATATTACGGGACGCCGCAAACGCGTAAAGACAGATTCAAGACTAACATTTATCCCAACTGATTACATTTCATACGCGAATAAACGACACAAAAAATTTATATTTAACGGCTTTTAATTTGAAGACACCTATCCTCTTAACGTTGAGGAGCCTTGCAGGTTGGGTGCCGCGCTTCACCGGTATTATACATAACCGATTTACCGTGTTTACGGCAGTCTGACCATTTACCAGTGTATGTCTGTAATACGACGTTGTTGTGTCCCGACAAAATTAACTCGCGTACAAATTTCTGATGTTCCCCCGGCGTGGCAACGCTGGCATTTCCAAACACATTACGTTCTCGTACGTCCATGACCGCTATTTTCAGTATTAATTGGTTGGTCGGTCAAAGTATTTTCCTTATGTAAAAGGACACGATCTAAAGCCGTAAACTCGTACACAAACACTGGTACCAACGGACGCGATTTTCCGTCCGTTGAGCGGGTGTAATATCGGCGAGGTCTTCTTGCACGAATACTCTCGTACAGTAGGTTTCTGACACGGGGTGCATGGGTTTTTTGACACAACACAAACATTTGCAGGCTCTTATGACTGGATGGATTGAATTTATTTTTAGATAGGGTCACGTGTTTTTGTCGTGACACGCCTCGACCAGAAAAGGCTGCGGTTTTCGTACACGCGACCGTTATTTCACAGGCGTTCATAATCAAGCTGCGGCGGATGGTGTCGGTTAATTGTCTCCGCCCAAGTTCGTCAATAGATGATACCATGAACAACGTATCAAATGGTACATAGTCGTCTTTGGTTTTCTCAATACAGCCCGCGTGCCCAATCGGAAAATTTTCATTTGCATCAACGCTATTTTCTGTAAAATCGGTCTGAACACTGTGTTGGCTGGCTACCTGTTTAAAATTTGGGATCGAACACGGTCCACGATGCAATCCCCAACCCCATTGAAGCAATGCCGTCGGTACGGAAGGAGGCAACTCCGAAAACATTATGGTACGCGAGAGGGTCGATTGGAGTGTTATATAACACTCCAATCGATCTCGGGTTCGCCTTTACGCGTAAAATACTCATTGGCTTGAACGAAATGTCGACAATTCCGAAATGGAACACGGGACAATGGCGACGGATGCGCGTGTGTTAGCACCAGATGACATCTTGAATTCGGTTGGGTTGTCTTCTGTGCATGCGCACCCCACAGCATAAAAACTAACCCTGTACGGTTCTCGCATAACCTCTGTAGCACGCGTTGCACCAGCCGCCCCCAGCCTAAGTATACATGCGACCCCGGAGTCCCGCGACGAACCGTAAGCGTGGTATTCAGCAATAACACCCCCTGCCTTGCCCAACTCTCCAGGCATCCGTGAGTGGGCGGAGTCATATTTGGGTATGATTCCATGAGGGCCGCAAAAATATTTTTAAGACTAGACGGTGGTGTTATGCCACGTTTTACACTAAACGCTAGCCCATGTGCATGTCCCGCGGTAGGGTATGGATCTTGACCAATAATTACAACGCGAATGCTCTGGGGTCCGCAAAATCGCGTCCATGCAAAAATATCGCCTGTAGATGGAAGTATTTCTTCCCCTGAATTTAAAAGACGATTGTATTCTAAAAAAATACCTTTCGCGTACGGCTCTTTAAGTTCGTCCGACAACAGGTCATACCACTCAGGGGAAATGTTAAACTTGCTGAAAACTTCAACCGAATCCAGTTGCGAAGAGACGGGGGTGAACGTTTCCGTGTCGTAATGATGTGACATGTTATTTAACTTGAAGGTTGGGGGGTCTAGCTTAACCCCCAAAGGCAGCCCGCGGGGTCGCTTGCGGGTTTTTTTGGTAACCGGATGGGCCAAAACATAAATGTCCTTTGAATCCGATAGTTTCATTTCATTGGCATACGCGTTGGAACAAACGGTCGGCTCCCCAGACACATCCATTTTCCGGGATATTTGTGGAAGATGGAGTAGAGTCTACCCATACACCGGAAAGGGCATCCAACAAAGCATCGCGTATGTCCCCGCTTTTATGTTCTTCACCAACAGATTGTGTCAGCCCCTTTAAGGTGACGTATGGATTTGTCCAGTACGCCATTTGTTTGTCTTTAAACCAAAGTATAACTTCCGGTACTGGACATTTTGTCTTAACCACGATTCCCGATAGCGCCTCGCTGAGGTTTGATACCGGGGGTGCCGCATAGTCCCACGCCTCATATACCGATGACACGCACGGTTCCGTTATAATCAAACTCACATCCGATAGCGGTTTGGCTCCAAAAAACAACGGAGTGTCGTCTTGGAGATGAAGACAATACGCGATTGTGATAGTTTTTAAAAAAACTATCATCTGCAGTAACCATTTATGTGATGCCATGACGCTTGTGTTTTCCCTTCACTACGACGTTGTCGTATCCTTTGAAAAACTTGACCACTCTAATGGAAGCATGGACAAGTATGAGTTTTATATATACAGTTGGCCTTTAGTTAAACTCTTGGTGTCATATCTCATTTTCCTAAAAAGGGCGATCTTAATATGTCAAACGTCACGGCGTGCCGACAAAGCGAATTTCCATGCAAGATTTGGATGTAGTATTTATACACCCAATCACACGTCACGTATTAAGCTTTACAGTCCCCCGTTATCTGATATAATCACTTTTCTTAACACGTCATCGGGAAAACAGATGTTTATATTATACCTCTCGCGGTCATTTATGGCAAATACTTAGACCGTTTTCAAGCGGACTGAAAACGCTCAAATTGCCTTTTGGAGGCCTGCCCAACGGCCATTATCCCTTGGATCTAAGATTGATTTGCGGTAACGTTTGCCAATCAAGCTTTAAAAACGTACCCCAAACTTAAAACGCTCAAATTGCCTTTTGGAGGCCTGCCCAACGGCCATTATCCCTTGGATCTAAGATTGATTTGCGGTAACGTTTGCCAAACCCACGCATTTCAGTTTAAATATTTCTAAGCATTCTTAGTGCGTACTTGGCAGCGTGCTTAAAATATCAACCAATATCCATTATGCTAAACGTTTCCTTCTATCCGTTTCAATCCATTAAAAGTCCATTACCAAAAATGATGCATCATACCTAATTCACCTAAAAACCTGACTCATTGCAGCAGCGTTTCCTCCTTGCAGACTATCCAGTTGGCATTTTAAACGGGTCCGGCTGCCTGAACCGAAAACACCGTTGCCTTTACTGTAAGTACAAAACTAAAATTTATATTTGCGTGCGTATTTTGTAACATATATGCCTTTTATCCCCCCGCAAGTTTGCTTTACCCTCGCCTTCACCACCCCCGCCACCTTCCGGCCATTGTAATAACTTTAATTGCTATAAGACATACCCAAACCGGATGATTTTTGCCGCTGGAAAAACAGCTTCTAATTTTCCCGTCTCAACTCGGCCTTGGTTGCATCTCCAAGTATACCTTTAGTTTGCTCCCGTAGAGGTGTATAAATACAAACGGTGACAAGTATTGAGCGTAATCTCAAATTTTTGTAATTTAGGGCGGAGCGCTTACGACAGCACATGCGTACTGTTAGACTGTTATGTTTATTGTATTTGCAGAGCAGGATGCCCCGGTTACTCCGAGACCGGATTGCGGGCATTCCGAATCGTGTACGGACTTACCAGGGGGCAGTATTTACACCTTGGGTTCCAGATATACCAACCCTTACGACCAATAGCAACACTCAGGTATTTTTAAAATGCACGTTTAATGATCATAATTTACATACAGTTGGTAATAAAGCAGACCGTGGATGTTTAAGGCATTTCCTTCCCCCTCCCAACAAACTAGGACTTCTTCATCTTGTTTGGAATACCTTTACCCGCTTTACCGGCAGAGCTTTTTTTGGTAAGGTGTTTCAGTGAACCTGATGTTGATCCGGAGGTGGAGGGGGTATTGGACTCCCCCTGTGGAGAGGCAACTTTGCGGGTTTTACTTCCCTTACATGCCGAATCAGACTCAGATGTCAGGTCTATTGTTAAGCATCGTTTAACGTCTCTGCCGGTATGAAATAAACGGCGCTTAGCACCCCTTGCGCTTCCCGGTTTAATCCCCGGTAACACAGAAAAAAGCCTGACTTTTTGGGGTGTATTTACCAATCGGGTATCCCTTTCATCGCCACGAGAGGTCTCCCCGGTTGAGGTGGTTTCTGGTCTTACAATTGGACCTGTAATTAGTTGGATGGCTGTATCTTTCCAGGTCCAGGTTTGCATGGTTAGGCGGGTTGGATCGGTACATCGATCCAACAAGAATAACATGTTTGTTACAAACGGTCCTGTTGAATCATGCAAAAGACAACGCAGGGATGTTTTTAATCCCGCCTCATCACGCCCGTAAATACCTATATAGTTTAATATCAACATTTTTGTAGGCTCTACAATTTCGGGTTGATACAGTTCCGCAAGTTGATCATCAAGCCATCCGAGTAAAGGTTGCATGTAACACGGGAATCTCGCGTTTCCCTCTGTTCCTCTATCCGTGGCTCGAAAAGGCAGTCTGTCCATGGTTCGTGGGTCTTGATTAATTCCCACAGATACTGGACGATCACGGTAGTCCTGCCCCCCGGTCCGGGGTTGCTGTGCAGATTCAATCGAGCCATACACCACCGGGGTCGCCGATCGAACAGCAGGTTGGTCTTTAAAAAATACCTTCCGTAAAAATGATGCGGTAGAGCATGTTTTGGTTACACCAGGGCTCGAGTCTCGGGTCGGTGGTTGTATAGAATCCTGTTGAGAGTCACTTGGTGACTCTGCTGTGGGCTCTCTAGCCGACGATTGAAGGGGCCCAGGGTTTGGTGATTGAATGGGCTCCCGACTCGATCTTGATGTTGGCTGTTGGATGGACTCCCGACTCGGTCCTGGGCTTGGTGGCAGAAGATCTATGACATCTCCCGGTAGGATGTCGATGGAATCTTCAAATGACGGCTCAGAAAAACCATCGTCGTCGGATGGGTGCACTTCATATTCCTTGTAACTTGTATCACTTACGATCTTATGCAGGATGGATTGCACTGGACACCGGCAGAGAGGACACTGGACGCTGGTGGAGGTCCATGCCCGAATACAAACAAAGCAGAAGTCGTGCAAACACGGCATGGTTTTTCCGAGATCGGAAACGGTGCTCATGCATATGGTGCAGGTATTATCCGAAGCGTCGGAGGTGCCGCTACCGCCCGCTAATATGGTATCCATGGTAACAACTGGCTGTATTCTAATGTCCGGGCATCCAAACACGTAGCAGAACTGCCATGCGTTCTAAATTGTGAGTTGTGGCGAGTACATTTTTATAATTGGTACCAACGAAGACACACCCCTATATCCCTCCACCCATTTCTTTTAAGTCCCACCCACTAAAACGTGGGTATAAAATGTGTATTGGGGTAGGCGGACAGTCCCAACAAACAGGGAAGTTGATTGGTATAACCTTGGGCCGGGTATACAGCTAAGTGACATTTTAGATTCTGTCTTTATTTAGATAAAGAGCGATACGAAGACATTTCTCCACCCCCCTGTAATACCCGTAAATAAAGGTAAGTCCACAAACAAAAGCACTGTATATAGGAAGTCGGGTGTATTGGGACAGTTACTCCATTAGAGGCGTACAGACAATACTGGGATAGGGTAATGCAAGTCCCCCCCGATGGTCGCCCCGCAAACGCGCGGGGAGGTGGGGTCGCTTTTTTTTTTTCTCTCTCGAGGGGGCCGCGAGAGGGCTGGGCCCCCCCCCGGGGTCCGCCGGGCGCCCAGAAACCGGGGGGGGTTATTTTCGGGGGGGGTCCGACCAGCCCGCCCGTCGCCCGCCCGCACAGACAGACAGACACTTTTTTCATAAAAACCGTTCCGCTTTTATTAACAAACAACAAACAGTCCGCGCGCCGGTGGCGCTCACGAGAAAAGGAGGGGACTCCGTCACCCCCGACTCTGCGGGGGGCTCCTCCCCCCGCGCCCTCCCCACACATCGTCCTCGTCCTCGGAGGACGAGGACGAGGACAACAGCTCCACCTTGACCGCCGGGCGCAAGCCCACCCGGCGGTCTCGCAGCACACCCGGGGCCACCGACACGATGCTCACCCCAAAGGATGACCCCGGTGCGTCCCCGTCGTCCCCGCCCCCCTCCTCGCTGTCCCACGCGTCTTCACACCCCACCTCCCAATCGTCCGGCTCCAAAGCGTGTTCTCTGTCGTCTGCGGTGCGCCGCTGTCGCCCCGCCTGGGTTTCTGCCGGCCGTTCCGAGCCCCCGTGGTGTCCGAACGCGAACCGTGTTCCGTCGCTCCCCTCCAACACCGTCTCCGCGGCCCCAAAACCGGGCGGCCACATTACTCTGGGAATCGGGGGGAGGGCATTCCGAGCCTCGTCCGCCGACGCATACAGCGCCACCGACCGACCGGCCACGGGTGGAAGCACGAGTGGTTCCGCGGCAGGGTCGGGTTCCAGCAGGGCGTGGCGGCAAAACACCCTCGCCCAGGTGGGTACGTCGCCGGCCTCCGGCCCGGCGGCCCCCGGTCTCCGTCCCTCGGGAAGGAAGACGGGTCGAAGCGCGGCACCCAGGCCCCATCGGTTTGCTGCGCGGTGGCTATGTGCCGCCTCGTCCACAAAGTCGGCTGCCCCGAGCCCCAGACCCCGAGACTGTCGCGCGAGGTCCTTGCAACCGTCAAAACCCGGCAGCACGTACTGCCGGTATTCACGGGGCGACAGGGGGACGCGGGTCTTGGGGCCCGCGCGGGTACACACGGTGTATGCGACGTTCCCACCGCGGCACAAACACAGGGGTTGTTCGCCCGGGTACAGGTTGGCAAACGCAGTCTCGATACGAGCAAAACTCGCTGGCCCAAAGGTGCGCGACGATGCAAACACGGCCCGGGCGAGTCCTTCTGTGACCGCCGAGTCTGGCCATCGGACGACGGCCTGGGCGTCCGGTCGCGCCGGGGCCCGGACGTACACGTGATACTGAGACAAAGCGGGTCCATCCCCGGGCCACCTCTCGAGGGCCACCGCGTCCAACACCAGCAACCGGCGCCGGGCAGAGGCCAACCGCGAGCCTAGATACTCGACGGCCCCGGCAAAGGCCAGGTCTCGGGTCGACAGTAATAAAACGCCCCGGGCGTTCAAAGCGGACACGTCCGGCGGGCCGGTCCAGTTCCCGGCCCAGGCATGAGTGCTCGGCAGGCACAACCGGTTACTCAGGGCTGCCAGGACCACAGACAGTCCCCCTCGGGATGGACTCCATGACGGTCCCGGATCTGTCGCGAGGGTGCTCTCGAGGGGGCCGTTGATGTCCTCTCCGGGCAACGGATCGTAGATGATCAGAAGCCTCACATCCTCCGGGTCTGGGATCTGCCGCATCCAGGCGCACCTCCGCCGCAGCGCCTCCACTCCGCTGGGTGGACCAAACCGTCGGTCTCCTCCGCCCGGACGCCGAGCGGCGATTTCCGCCAAGGCGCCGGGATCAAAGCTTAGCGCAGGGCGCCAGGCCGTGGGAAACAATGGGTCGTCGACCAGACGGGCGATGGTTTCGGGGGTACAGTACGCCTTGCGAGCCTGGTCCGACGGGACCGGGGTATGCAGGGCCCCCCGGGGAATACGCCGAAATCCCCCGTTTGGGGCCGGTCCGTCAAGTGGCATCGTTATTACGGCGGGGGGATCCACCACAGGGCCCGAGGTGATGGTCACGGGCTCGGATACCCGCCTCTTGGCCTTGGAAACCACATGATCGTCTGCAACCCGGGCGTCCGCGACGGGTGTCTCCCTAATCTTGTCGAGGAGGCTTCTGCTCTCGACTGGCTGGGACTTGCGCTTGCGCGGAGTTCGTAAACGATCATCCGGTGGACACACAGAAAGAGAGCGTGCGGCGGCCGACGGCTGAGGGTCGGGAGTCTGTGTGGCCGGGGTTGTTGGAGAAGGGTGACCGCGGGAGATCCGCGCCGCCGGACTGGAGCCCGTTGCCTCGGGGTATGCCATGCCGGCAAAGGCTCTGCGGAGACTCTGTAGGATAAAGTGTTTTTGGGCCCGGTCGTACCGACGGCTCATAGCCACGGCCGCGGCCGCGTGGGGGAGAGCCCAGAGGGCCTCCCCCGTGGCCATGGCTTCGCCTACATGCGGAACGGGAGACGCTACGCTCCCCGTAACGGCGGTACCCGCCCGTCCCGGTGGCAACAGCTTTTGGTAGAACTGGTTCAGGGCCGAGTTGACACCGGTCAGCTTGGGGTTCTGGAGCCATGCTATAGGGTCTCTGTCTGGACAGTAGATCAGGTTAATCAGCGCGCGGTACTGTCTAGCCGGATCTCCCAACTCCGGCACGTAAAGCGGCGCGGGTTCAGTTGAGGCCTCGTAACGAGCCCGCGCCGCTCTCACAGCCTCATCCTCCCAGTGACCCTCTCTGGTCTCCCCGGACGGTCCAAACCGCACCCTGTTGGATGGGAGGGGCGCCGATCCGGGCCAAGGGCTTCCGTCGGGCATCATGAGCGGCCCCGACACCGGGGGAATTATCGGGGTTCTGGATCGCGGCAGGGAAAATGATTTCTGTCTCTGGCGCCCCGGTTCCCCCGCAAGACGTTTGGTCTTACGAATCCTCGGATCGGGACCGCTGATGGATCGATATCCCGGTTGGATATTTTGTTTCGTCGACCCACCATCATTTGAGTCCGAATCATCCGAATTTGACGGGGAAGGGGCGTGTTCGCGTCCGGACCTGCTGCCTGTAGTTTCACTTCCCACCGAAACGCGCCGGGGTTCATCGTCTTCATCCTCCGATGACGATCCCCACGACGAGGAAGAGGATGAAGACGAAACAAACTCACGACTCTTTGGCTTTTTCTCCACCGGGCTGTCATCCTCAATCGGGTCTGGTGCGTGGGATCTTCCCGGCAGGGCCAAAAACGCTCTAGGTTTGCCCCCCGACGAACGTCCAGGGACGCGAGGTGTTATACCCCGGGCATCATGTTTCCTTGGGCGGGTATCATCGGTCTCAAACGGCAGGTCCGCCTTTGCCCCCTTAGCGGGAACGCTGTCCGAAAGGACGTGGTACAATTGCTCAACCGGGCCGGGTACAGGTCCACCGGGTTTCCGCGCCGGGAGTGGGACCTTAACCTTCAAAGTCTTTTTCTTCGGGCTCTTTCCCTGAGCGGGCCGTTGAGTTTTCTGGAGAACTACTCCGTCCCCCGATGCATGCGCATGACCCGCTTGCTCATCGCCCGGCTTTTTACCCGAGATGGACCGAGTTTGTCTGTCTCGATGGACCACCGACGGCAAACCTGGTGAATTTCCTCTCGTCGTTTGTCGGGGTATAGACCGCTGGTCTTCCCGTTGATCGTTCCCGGCGGCGTCTCCAACAGGAGACGCGGGGGATACAGGGGAGAAGGCCTGCGGGAACGGAGGGGTCGTACCTCTGCCCGTTTCCCCATCGTTCATCGGTGGTTTTGGAGACCTAGCAAGCTTCGTTCCGAGAGAGACTGTCTCGAGGGAGCGATCGGCTCCTGTTGGTTCTCGCGCGCCGGCCTCCGAGAATCGGGTGTGGAAGACCTCGGCCAGCGGGATTACAGGCGAGCCCGTTAGATCCTGACCGTCCTCGCATACGTAGTCGTCTTGTGTTAGCTCTTCGCCAACATCTTCCGTTCTGGGTTCTGGTTGAAGTCCCGATACGGAGGGAATTGAAACGATCTCATGTTCCCGTCCCACCATGACCCCGTTCTCTCCAAATAGTAGATCGTCAGGCTGACTCGAGGTGACCACCCGGGCCCTGTGTTCGGCGGCCGCGGCGGCCGCGTCCAACAGGTCCATTAACTCCAAAGTATCAGGCGACCCCGCGCGTTGGGGTGTAGAGCGCTGCATCGGCGGCGTATCCATCGCACTGGGGTGAATTTAGACGTACCCGAGTTTTCCAAACGCTCTCGCAGCCTTCAAAGGATTGCGATTGCGGTTGGTGAGGGAGTTCCAACAGTACTTAAAACGTGTTGTGCCCCCCCCTCGACCGCATATTTCCTCCCCGTGTCGTCACCGTGTAAATATTCTTAATGATAAGACGATGTAGTGATTGGACGAGACTCGAGGCGGGAAGTTCATGGACCATAGTATGCGTTTAAGGAGAGACCGCTGGTTGGCGATGTACGCCCGGTGTCTATTTCCGCATACCTTACAACATCATAACAAGGGATACCAGACATGTGAATTTCATTTACATATGTTTAAATAACAACCAATCATCGTGTGTCTACAGACGATATATAATATACATAAACACAATTGGGGTTGTCTCACATGCAAAACATCTTATATAACACGGGTTGTTTCCACCCATCCGGCATCTAGTTAATCAAATGCACGTCGACGGTGTGTTTGGGTCCCTCTCCGTCGTCATTACGTTCGCTCAATCAACAAGCGTATACACCACCACCCCTCCCAACGATTATCATGTCAGGCGGCACGAAGCCCGCGATAACCCATAAAATACACACGGGGTTGTGGTGTTCACGTAACCCCCCGCCGATGGGGAGGGGGCGCGGTACCCCGCCGATGGGGAGGGGGCGCGGTACCCCGCCGATGGGGAGGGGGCGCGGTACCCCGCCGATGGGGAGGGGGCGCGGTACCCCGCCGATGGGGAGGGGGCGCGGTACCCCGCCGATGGGGAGGGGGCGCGGTACCCCGCCGATGGGGAGGGGGCGCGGTACCCCGCCGATGGGGAGGGGGCGCGGTACCCCGCCGATGGGGAGGGGGCGCGGTACCCCGCCGATGGGGAGGGGGCGCGGTACCCCGCCGATGGGGAGGGGGCGCGGTACCCCGCCGATGTTTATAACCATAATTCTCTAAACCGTTGTAGAAAATCACAAAAAAATTTATTCAAAAACAAGTCGAAGAACTTCATATCTGAGGCATGTAAACCCATTCGCACTTCCTGGGGTGGAATGGGGTGGGGTGGGGGGGTGAAAAAGGGGGGGGTTAAATTGGGCGTCCGCATGTCTGTGGTGTACGCCAATCGGATACACTCTTTTAATCTGCATTCGCACTTCCCGTTTTTTCACTGTATGGGTTTTCATGTTTTGGCATGTGTCCAACCACCGTTCGCATTCTTGTTCGCGCGTGTTCCCGCGATGTCGCGGTTTTATGGGGTGTGGGCGGGCTTTTCACAGAATATATATATTCCAAATGGAGCGGCAGGCTTTTTAAAATCGATTTGACGTGATAAAAAAAAAACACACGGGCCCCCCCTTTTTTTTGGTGTTATAAAGGCAACCCAATCGAAGGTCTCCCGCCCCGGAATCCCCCATTGCCATTTTACCCAAGTAGCCTTATTCATAGATGTAAACGTTTGGGTGTGTGTTTTGTTGTGCAGGGTTCGTCCGATTCATAACGCGACAGCGTCGAGTCGGTTTTAAGGGAAAAGGTTACTACGGCCCCAAGGACATGTTTTGCACCTCACCGGCTACGCGGGGCGACTCGTCCGAGTCAAAACCCGGGGCATCGGTTGATGTTAACGGAAAGATGGAATATGGATCTGCACCAGGACCCCTGAACGGCCGGGATACGTCGCGGGGCCCCGGCGCGTTTTGTACTCCGGGTTGGGAGATCCACCCGGCCAGGCTCGTTGAGGACATCAACCGTGTTTTTTTATGTATTGCACAGTCGTCGGGACGCGTCACGCGAGATTCACGAAGATTGCGGCGCATATGCCTCGACTTTTATCTAATGGGTCGCACCAGACAGCGTCCCACGTTAGCGTGCTGGGAGGAATTGTTACAGCTTCAACCCACCCAGACGCAGTGCTTACGCGCTACTTTAATGGAAGTGTCCCATCGACCCCCTCGGGGGGAAGACGGGTTCATTGAGGCGCCGAATGTTCCTTTGCATAGGAGCGCACTGGAATGTGACGTATCTGATGATGGTGGTGAAGACGATAGCGACGATGATGGGTCTACGCCATCGGATGTAATTGAATTTCGGGATTCCGACGCGGAATCATCGGACGGGGAAGACTTTATAGTGGAAGAAGAATCAGAGGAGAGCACCGATTCTTGTGAACCAGACGGGGTACCCGGCGATTGTTATCGAGACGGGGATGGGTGCAACACCCCGTCCCCAAAGAGACCCCAGCGTGCCATCGAGCGATACGCGGGTGCAGAAACCGCGGAATATACGGCCGCGAAAGCGCTCACCGCGTTGGGCGAGGGGGGTGTAGATTGGAAGCGACGTCGACACGAAGCCCCGCGCCGGCATGATATACCGCCCCCCCATGGCGTGTAGTCTTTATAAATAAATACAATGGTTTGGCTCGTGTCTTTTTTTGATGTCTGTCTGTGGGGGAGTGGGGTGTTGTGGATATTAGAGGGTAGAGGGTGCTGGTTTGAACGTCTCCATTAACCCACGGGGTCCCCACACGGGCCGTGTGGTATGAATCTCTGCGGATCCCGCGGTGAGCACCCGGGCGGTGAATATGCCGGACTTTACTGCACACGACACGATACCCCCGCGCACCAGGCTCTCATGAACGACGCCGAACGGTACTTCGCCGCCGCGCTATGCGCCATATCTACCGAGGCCTACGAGGCTTTTATACACAGCCCCTCCGAGAGACCGTGCGCGAGTTTGTGGGGGAGGGCAAAGGACGCCTTCGGACGGATGTGCGGGGAGCTCGCAGCGGATAGACAACGTCCACCCTCGGTTCCGCCGATCCGCAGAGCGGTGTTATCGTTATTACGCGAGCAATGCATGCCGGATCCACAATCGCATCTGGAGCTCAGCGAGCGGCTGATATTGATGGCATATTGGTGCTGTTTGGGACACGCCGGACTTCCGACTATTGGATTGTCGCCCGATAATAAATGCATCCGCGCCGAATTATATGACCGCCCCGGGGGAATTTGTCACAGGCTTTTTGACGCGTACCTGGGCTGCGGGTCCCTTGGAGTCCCAAGAACCCACGAGAGATCCTGACACCCCATCCCTTTATATAGAAAAAAAAAAAATAAATTTAAAACATACACCGGATAAAAGCGTACTGTTTTTTATTTAAATTTACACGCTCGACGTTGCCCCGGTTCGGTGATCACCGGGTCTTATCTATATACACCGTGTAACTCGAACCCCCGTGACTCCCTCCAATCGCGTTACCAAACTCTTCTTCCGTATCCGTAGATTCCGAGTCCTCGAAATCGTCCACTTATCCAACAAATTGTGACGTTATATATCCCAAGGCAAAGGCCGCTCCCGTCATAGCAAATACAAAGACAATTATTAGCGTAATATAACAGAATTTTTTACGATGATATATTTTATGTTGATATTTTCCAATTCGACGCAAAAATTCATCTGCCGTTTCATTTTCGCTATCACTATAATAACACTTTTCAGCCGAACGGCTCGGTTGTATGGCTGTTATCGTTGTATTATTTGGTTGCGCTCGCGGGGTTACCACCGCTTCCATCAGTAAGGCCACGGCCTCACCCTCCATGGTGTTTTGTCCGGCCATAGAAATCCAGATTGTAAGGCCAGCAGGCTAGTTTAAAAGTGTTTAATACCACACCTTTTGATATTTATATACATGCAAGATTCTAGATTATTCATCAATAGGTCGTTTAAAGCGCGTTTTCATAAACGTTGTCAGCTATACCGACATTCTCACAAAGAGGTAAAGTTACCTTACGTTATTATTAAATAAAACATGTAGACATTATTAATAATCCTAGGAACAATCAAATCCATATTTGTAAGTTATGTTTAACCCCTCCCCTTTTTGTCATTATCTCCGCCCTCTTATAATCGGATCACTTTATAAGTGTGTCGGTGAGTATATTTTGTACAGTTGTTGGACAACAGGTTTTTGGTTCATTAACACTATCAACATAAGTCGGGGTATACAAGTATAATGAACGACGTTGATGCAACAGACACCTTTGTTGGACAAGGAAAGTTCCGTGGCGCCATCTCAACATCACCGTCACATATTATGCAAACATGTGGGTTTATACAACAGATGTTTCCAGTTGAAATGTCGCCCGGCATAGAATCTGAGGATGATCCCAATTATGACGTTAACATGGATATACAGTCTTTTAATATATTTGATGGTGTACACGAAACTGAAGCCGAAGCCTCTGTGGCATTGTGCGCAGAAGCACGCGTTGGAATTAATAAAGCGGGATTTGTAATATTAAAAACGTTTACACCAGGGGCGGAAGGTTTTGCGTTTGCGTGTATGGACAGTAAAACATGTGAACATGTGGTCATTAAAGCGGGTCAACGTCAAGGAACGGCCACCGAGGCAACCGTGTTAAGAGCGTTAACCCACCCATCCGTTGTACAGCTTAAAGGAACGTTTACGTATAACAAAATGACATGTCTTATATTACCACGTTACCGAACAGATTTATACTGCTATCTAGCTGCAAAGCGCAACCTCCCCATATGTGACATTTTAGCAATTCAGCGATCTGTATTACGCGCGTTACAGTATCTTCATAATAACAGTATTATTCACCGTGATATAAAATCTGAAAATATATTTATTAACCACCCAGGTGATGTTTGTGTGGGAGACTTTGGAGCAGCGTGTTTCCCCGTGGATATTAATGCCAACAGGTATTATGGCTGGGCTGGAACAATCGCCACAAACTCTCCTGAGTTATTGGCTAGAGATCCATATGGACCTGCCGTGGACATATGGAGTGCCGGGATTGTATTATTTGAAATGGCTACAGGACAGAACTCGTTATTTGAACGAGACGGTTTAGATGGCAATTGTGACAGTGAGCGTCAAATTAAACTTATTATACGACGATCTGGAACTCATCCCAATGAATTTCCCATTAACCCTACATCAAATCTTCGTCGACAATACATTGGTTTGGCAAAACGGTCTTCTCGAAAACCCGGATCCAGGCCATTGTGGACAAATCTATATGAGTTGCCAATTGATTTGGAGTATTTGATATGTAAGATGTTATCGTTTGACGCACGTCATCGACCATCGGCAGAGGTGTTGCTTAACCACTCTGTTTTCCAAACTCTTCCCGATCCATATCCAAATCCAATGGAAGTTGGAGATTAAAATTCATTAAGCCTGTTAATAAAATATTGTATAAATTGTGTTTATAACGTATAACCCGTTAAGGCAAATAGGGTACAAACGCGCAATGTTTTGAAATACTAATATAAATAACATAACCAATAGAAACTTAATACAGAGTCACGCCCCATTACAACAAGGATAAAACACGGGATCATTTTCTTAACATTGTAGTAGCGCTGAAAAGCGTCCCCTCCCCCGGCTCACAGAGCTGCTCTTCGGTGTAGTTGGGTATACTGGTGCGCCTCATTTAATCGCGATGTTTTTAATCCAATGTTTGATATCGGCCGTTATATTTTACATACAAGTGACCAACGCTTTGATCTTCAAGGGCGACCACGTGAGCTTGCAAGTTAACAGCAGTCTCACGTCTATCCTTATTCCCATGCAAAATGATAATTATACAGAGATAAAAGGACAGCTTGTCTTTATTGGAGAGCAACTACCTACCGGGACAAACTATAGCGGAACACTGGAACTGTTATACGCGGATACGGTGGCGTTTTGTTTCCGGTCAGTACAAGTAATAAGATACGACGGATGTCCCCGGATTAGAACGAGCGCTTTTATTTCGTGTAGGTACAAACATTCGTGGCATTATGGTAACTCAACGGATCGGATATCAACAGAGCCGGATGCTGGTGTAATGTTGAAAATTACCAAACCGGGAATAAATGATGCTGGTGTGTATGTACTTCTTGTTCGGTTAGACCATAGCAGATCCACCGATGGTTTCATTCTTGGTGTAAATGTATATACAGCGGGCTCGCATCACAACATTCACGGGGTTATCTACACTTCTCCATCTCTACAGAATGGATATTCTACAAGAGCCCTTTTTCAACAAGCTCGTTTGTGTGATTTACCCGCGACACCCAAAGGGTCCGGTACCTCCCTGTTTCAACATATGCTTGATCTTCGTGCCGGTAAATCGTTAGAGGATAACCCTTGGTTACATGAGGACGTTGTTACGACAGAAACTAAGTCCGTTGTTAAGGAGGGGATAGAAAATCACGTATATCCAACGGATATGTCCACGTTACCCGAAAAGTCCCTTAATGATCCTCCAGAAAATCTACTTATAATTATTCCTATAGTAGCGTCTGTCATGATCCTCACCGCCATGGTTATTGTTATTGTAATAAGCGTTAAGCGACGTAGAATTAAAAAACATCCAATTTATCGCCCAAATACAAAAACAAGAAGGGGCATACAAAATGCGACACCAGAATCCGATGTGATGTTGGAGGCCGCCATTGCACAACTAGCAACGATTCGCGAAGAATCCCCCCCACATTCCGTTGTAAACCCGTTTGTTAAATAGAACTAATTATCCCGGATTTTATATTAAATAAACTATATGCGTTTTATTTAGCGTTTTGATTACGCGTTGTGATATGAGGGGAAGGATTAAGAATCTCCTAACTATAAGTTAACACGCCCACATTTGGGCGGGGATGTTTTATGAAGCCTTAAAGGCCGAGCTGGTATACACGAGAGCAGTCCATGGTTTTAGACCTCGGGCGAATTGCGTGGTTTTAAGTGACTATATTCCGAGGGTCGCCTGTAATATGGGGACAGTTAATAAACCTGTGGTGGGGGTATTGATGGGGTTCGGAATTATCACGGGAACGTTGCGTATAACGAATCCGGTCAGAGCATCCGTCTTGCGATACGATGATTTTCACATCGATGAAGACAAACTGGATACAAACTCCGTATATGAGCCTTACTACCATTCAGATCATGCGGAGTCTTCATGGGTAAATCGGGGAGAGTCTTCGCGAAAAGCGTACGATCATAACTCACCTTATATATGGCCACGTAATGATTATGATGGATTTTTAGAGAACGCACACGAACACCATGGGGTGTATAATCAGGGCCGTGGTATCGATAGCGGGGAACGGTTAATGCAACCCACACAAATGTCTGCACAGGAGGATCTTGGGGACGATACGGGCATCCACGTTATCCCTACGTTAAACGGCGATGACAGACATAAAATTGTAAATGTGGACCAACGTCAATACGGTGACGTGTTTAAAGGAGATCTTAATCCAAAACCCCAAGGCCAAAGACTCATTGAGGTGTCAGTGGAAGAAAATCACCCGTTTACTTTACGCGCACCGATTCAGCGGATTTATGGAGTCCGGTACACCGAGACTTGGAGCTTTTTGCCGTCATTAACCTGTACGGGAGACGCAGCGCCCGCCATCCAGCATATATGTTTAAAACATACAACATGCTTTCAAGACGTGGTGGTGGATGTGGATTGCGCGGAAAATACTAAAGAGGATCAGTTGGCCGAAATCAGTTACCGTTTTCAAGGTAAGAAGGAAGCGGACCAACCGTGGATTGTTGTAAACACGAGCACACTGTTTGATGAACTCGAATTAGACCCCCCCGAGATTGAACCGGGTGTCTTGAAAGTACTTCGGACAGAAAAACAATACTTGGGTGTGTACATTTGGAACATGCGCGGCTCCGATGGTACGTCTACCTACGCCACGTTTTTGGTCACCTGGAAAGGGGATGAAAAAACAAGAAACCCTACGCCCGCAGTAACTCCTCAACCAAGAGGGGCTGAGTTTCATATGTGGAATTACCACTCGCATGTATTTTCAGTTGGTGATACGTTTAGCTTGGCAATGCATCTTCAGTATAAGATACATGAAGCGCCATTTGATTTGCTGTTAGAGTGGTTGTATGTCCCCATCGATCCTACATGTCAACCAATGCGGTTATATTCTACGTGTTTGTATCATCCCAACGCACCCCAATGCCTCTCTCATATGAATTCCGGTTGTACATTTACCTCGCCACATTTAGCCCAGCGTGTTGCAAGCACAGTGTATCAAAATTGTGAACATGCAGATAACTACACCGCATATTGTCTGGGAATATCTCATATGGAGCCTAGCTTTGGTCTAATCTTACACGACGGGGGCACCACGTTAAAGTTTGTAGATACACCCGAGAGTTTGTCGGGATTATACGTTTTTGTGGTGTATTTTAACGGGCATGTTGAAGCCGTAGCATACACTGTTGTATCCACAGTAGATCATTTTGTAAACGCAATTGAAGAGCGTGGATTTCCGCCAACGGCCGGTCAGCCACCGGCGACTACTAAACCCAAGGAAATTACCCCCGTAAACCCCGGAACGTCACCACTTCTACGATATGCCGCATGGACCGGAGGGCTTGCAGCAGTAGTACTTTTATGTCTCGTAATATTTTTAATCTGTACGGCTAAACGAATGAGGGTTAAAGCCTATAGGGTAGACAAGTCCCCGTATAACCAAAGCATGTATTACGCTGGCCTTCCAGTGGACGATTTCGAGGACTCGGAATCTACGGATACGGAAGAAGAGTTTGGTAACGCGATTGGAGGGAGTCACGGGGGTTCGAGTTACACGGTGTATATAGATAAGACCCGGTGATCACCGAACCGGGGCAACGTCGAGCGTGTAAATTTAAATAAAAAACAGTACGCTTTTATCCGGTGTATGTTTTAAATTTATTTTTTTTTTTTCTATATAAAGGGATGGGGTGTCAGGATCTCTCGTGGGTTCTTGGGACTCCAAGGGACCCGCAGCCCAGGTACGCGTCAAAAAGCCTGTGACAAATTCCCCCGGGGCGGTCATATAATTCGGCGCGGATGCATTTATTATCGGGCGACAATCCAATAGTCGGAAGTCCGGCGTGTCCCAAACAGCACCAATATGCCATCAATATCAGCCGCTCGCTGAGCTCCAGATGCGATTGTGGATCCGGCATGCATTGCTCGCGTAATAACGATAACACCGCTCTGCGGATCGGCGGAACCGAGGGTGGACGTTGTCTATCCGCTGCGAGCTCCCCGCACATCCGTCCGAAGGCGTCCTTTGCCCTCCCCCACAAACTCGCGCACGGTCTCTCGGAGGGGCTGTGTATAAAAGCCTCGTAGGCCTCGGTAGATATGGCGCATAGCGCGGCGGCGAAGTACCGTTCGGCGTCGTTCATGAGAGCCTGGTGCGCGGGGGTATCGTGTCGTGTGCAGTAAAGTCCGGCATATTCACCGCCCGGGTGCTCACCGCGGGATCCGCAGAGATTCATACCACACGGCCCGTGTGGGGACCCCGTGGGTTAATGGAGACGTTCAAACCAGCACCCTCTACCCTCTAATATCCACAACACCCCACTCCCCCACAGACAGACATCAAAAAAAGACACGAGCCAAACCATTGTATTTATTTATAAAGACTACACGCCATGGGGGGGCGGTATATCATGCCGGCGCGGGGCTTCGTGTCGACGTCGCTTCCAATCTACACCCCCCTCGCCCAACGCGGTGAGCGCTTTCGCGGCCGTATATTCCGCGGTTTCTGCACCCGCGTATCGCTCGATGGCACGCTGGGGTCTCTTTGGGGACGGGGTGTTGCACCCATCCCCGTCTCGATAACAATCGCCGGGTACCCCGTCTGGTTCACAAGAATCGGTGCTCTCCTCTGATTCTTCTTCCACTATAAAGTCTTCCCCGTCCGATGATTCCGCGTCGGAATCCCGAAATTCAATTACATCCGATGGCGTAGACCCATCATCGTCGCTATCGTCTTCACCACCATCATCAGATACGTCACATTCCAGTGCGCTCCTATGCAAAGGAACATTCGGCGCCTCAATGAACCCGTCTTCCCCCCGAGGGGGTCGATGGGACACTTCCATTAAAGTAGCGCGTAAGCACTGCGTCTGGGTGGGTTGAAGCTGTAACAATTCCTCCCAGCACGCTAACGTGGGACGCTGTCTGGTGCGACCCATTAGATAAAAGTCGAGGCATATGCGCCGCAATCTTCGTGAATCTCGCGTGACGCGTCCCGACGACTGTGCAATACATAAAAAAACACGGTTGATGTCCTCAACGAGCCTGGCCGGGTGGATCTCCCAACCCGGAGTACAAAACGCGCCGGGGCCCCGCGACGTATCCCGGCCGTTCAGGGGTCCTGGTGCAGATCCATATTCCATCTTTCCGTTAACATCAACCGATGCCCCGGGTTTTGACTCGGACGAGTCGCCCCGCGTAGCCGGTGAGGTGCAAAACATGTCCTTGGGGCCGTAGTAACCTTTTCCCTTAAAACCGACTCGACGCTGTCGCGTTATGAATCGGACGAACCCTGCACAACAAAACACACACCCAAACGTTTACATCTATGAATAAGGCTACTTGGGTAAAATGGCAATGGGGGATTCCGGGGCGGGAGACCTTCGATTGGGTTGCCTTTATAACACCAAAAAAAAGGGGGGGCCCGTGTGTTTTTTTTTTATCACGTCAAATCGATTTTAAAAAGCCTGCCGCTCCATTTGGAATATATATATTCTGTGAAAAGCCCGCCCACACCCCATAAAACCGCGACATCGCGGGAACACGCGCGRAAAAAAAGTGCGAACGGTGGTTGGACACATGCCAAAACATGAAAACCCATACAGTGAAAAAACGGGAAGTGCGAATGCAAATTAAAAGAGTGTATCCGATTGGGGTACACCACAAACATGCGGACGCCCAATTTAACCCCCCCTTTTTTCACCCCCCCACCCCACCCCATTCCACCCCAGGAAGTGCGAATGGGTTTACATGCCTCAGATATGAAGTTCTTCGACTTGTTTTTGAATAAATTTTTTTGTGATTTTCTACAACGGTTTAGAGAATTATGGTTATAAACATCGGCGGGGTACCGCGCCCCCTCCCCATCGGCGGGGTACCGCGCCCCCTCCCCATCGGCGGGGTACCGCGCCCCCTCCCCATCGGCGGGGTACCGCGCCCCCTCCCCATCGGCGGGGTACCGCGCCCCCTCCCCATCGGCGGGGTACCGCGCCCCCTCCCCATCGGCGGGGGGTTACGTGAACACCACAACCCCGTGTGTATTTTATGGGTTATCGCGGGCTTCGTGCCGCCTGACATGATAATCGTTGGGAGGGGTGGTGGTGTATACGCTTGTTGATTGAGCGAACGTAATGACGACGGAGAGGGACCCAAACACACCGTCGACGTGCATTTGATTAACTAGATGCCGGATGGGTGGAAACAACCCGTGTTATATAAGATGTTTTGCATGTGAGACAACCCCAATTGTGTTTATGTATATTATATATCGTCTGTAGACACACGATGATTGGTTGTTATTTAAACATATGTAAATGAAATTCACATGTCTGGTATCCCTTGTTATGATGTTGTAAGGTATGCGGAAATAGACACCGGGCGTACATCGCCAACCAGCGGTCTCTCCTTAAACGCATACTATGGTCCATGAACTTCCCGCCTCGAGTCTCGTCCAATCACTACATCGTCTTATCATTAAGAATATTTACACGGTGACGACACGGGGAGGAAATATGCGGTCGAGGGGGGGGCACAACACGTTTTAAGTACTGTTGGAACTCCCTCACCAACCGCAATCGCAATCCTTTGAAGGCTGCGAGAGCGTTTGGAAAACTCGGGTACGTCTAAATTCACCCCAGTGCGATGGATACGCCGCCGATGCAGCGCTCTACACCCCAACGCGCGGGGTCGCCTGATACTTTGGAGTTAATGGACCTGTTGGACGCGGCCGCCGCGGCCGCCGAACACAGGGCCCGGGTGGTCACCTCGAGTCAGCCTGACGATCTACTATTTGGAGAGAACGGGGTCATGGTGGGACGGGAACATGAGATCGTTTCAATTCCCTCCGTATCGGGACTTCAACCAGAACCCAGAACGGAAGATGTTGGCGAAGAGCTAACACAAGACGACTACGTATGCGAGGACGGTCAGGATCTAACGGGCTCGCCTGTAATCCCGCTGGCCGAGGTCTTCCACACCCGATTCTCGGAGGCCGGCGCGCGAGAACCAACAGGAGCCGATCGCTCCCTCGAGACAGTCTCTCTCGGAACGAAGCTTGCTAGGTCTCCAAAACCACCGATGAACGATGGGGAAACGGGCAGAGGTACGACCCCTCCGTTCCCGCAGGCCTTCTCCCCTGTATCCCCCGCGTCTCCTGTTGGAGACGCCGCCGGGAACGATCAACGGGAAGACCAGCGGTCTATACCCCGACAAACGACGAGAGGAAATTCACCAGGTTTGCCGTCGGTGGTCCATCGAGACAGACAAACTCGGTCCATCTCGGGTAAAAAGCCGGGCGATGAGCAAGCGGGTCATGCGCATGCATCGGGGGACGGAGTAGTTCTCCAGAAAACTCAACGGCCCGCTCAGGGAAAGAGCCCGAAGAAAAAGACTTTGAAGGTTAAGGTCCCACTCCCGGCGCGGAAACCCGGTGGACCTGTACCCGGCCCGGTTGAGCAATTGTACCACGTCCTTTCGGACAGCGTTCCCGCTAAGGGGGCAAAGGCGGACCTGCCGTTTGAGACCGATGATACCCGCCCAAGGAAACATGATGCCCGGGGTATAACACCTCGCGTCCCTGGACGTTCGTCGGGGGGCAAACCTAGAGCGTTTTTGGCCCTGCCGGGAAGATCCCACGCACCAGACCCGATTGAGGATGACAGCCCGGTGGAGAAAAAGCCAAAGAGTCGTGAGTTTGTTTCGTCTTCATCCTCTTCCTCGTCGTGGGGATCGTCATCGGAGGATGAAGACGATGAACCCCGGCGCGTTTCGGTGGGAAGTGAAACTACAGGCAGCAGGTCCGGACGCGAACACGCCCCTTCCCCGTCAAATTCGGATGATTCGGACTCAAATGATGGTGGGTCGACGAAACAAAATATCCAACCGGGATATCGATCCATCAGCGGTCCCGATCCGAGGATTCGTAAGACCAAACGTCTTGCGGGGGAACCGGGGCGCCAGAGACAGAAATCATTTTCCCTGCCGCGATCCAGAACCCCGATAATTCCCCCGGTGTCGGGGCCGCTCATGATGCCCGACGGAAGCCCTTGGCCCGGATCGGCGCCCCTCCCATCCAACAGGGTGCGGTTTGGACCGTCCGGGGAGACCAGAGAGGGTCACTGGGAGGATGAGGCTGTGAGAGCGGCGCGGGCTCGTTACGAGGCCTCAACTGAACCCGCGCCGCTTTACGTGCCGGAGTTGGGAGATCCGGCTAGACAGTACCGCGCGCTGATTAACCTGATCTACTGTCCAGACAGAGACCCTATAGCATGGCTCCAGAACCCCAAGCTGACCGGTGTCAACTCGGCCCTGAACCAGTTCTACCAAAAGCTGTTGCCACCGGGACGGGCGGGTACCGCCGTTACGGGGAGCGTAGCGTCTCCCGTTCCGCATGTAGGCGAAGCCATGGCCACGGGGGAGGCCCTCTGGGCTCTCCCCCACGCGGCCGCGGCCGTGGCTATGAGCCGTCGGTACGACCGGGCCCAAAAACACTTTATCCTACAGAGTCTCCGCAGAGCCTTTGCCGGCATGGCATACCCCGAGGCAACGGGCTCCAGTCCGGCGGCGCGGATCTCCCGCGGTCACCCTTCTCCAACAACCCCGGCCACACAGACTCCCGACCCTCAGCCGTCGGCCGCCGCACGCTCTCTTTCTGTGTGTCCACCGGATGATCGTTTACGAACTCCGCGCAAGCGCAAGTCCCAGCCAGTCGAGAGCAGAAGCCTCCTCGACAAGATTAGGGAGACACCCGTCGCGGACGCCCGGGTTGCAGACGATCATGTGGTTTCCAAGGCCAAGAGGCGGGTATCCGAGCCCGTGACCATCACCTCGGGCCCTGTGGTGGATCCCCCCGCCGTAATAACGATGCCACTTGACGGACCGGCCCCAAACGGGGGATTTCGGCGTATTCCCCGGGGGGCCCTGCATACCCCGGTCCCGTCGGACCAGGCTCGCAAGGCGTACTGTACCCCCGAAACCATCGCCCGTCTGGTCGACGACCCATTGTTTCCCACGGCCTGGCGCCCTGCGCTAAGCTTTGATCCCGGCGCCTTGGCGGAAATCGCCGCTCGGCGTCCGGGCGGAGGAGACCGACGGTTTGGTCCACCCAGCGGAGTGGAGGCGCTGCGGCGGAGGTGCGCCTGGATGCGGCAGATCCCAGACCCGGAGGATGTGAGGCTTCTGATCATCTACGATCCGTTGCCCGGAGAGGACATCAACGGCCCCCTCGAGAGCACCCTCGCGACAGATCCGGGACCGTCATGGAGTCCATCCCGAGGGGGACTGTCTGTGGTCCTGGCAGCCCTGAGTAACCGGTTGTGCCTGCCGAGCACTCATGCCTGGGCCGGGAACTGGACCGGCCCGCCGGACGTGTCCGCTTTGAACGCCCGGGGCGTTTTATTACTGTCGACCCGAGACCTGGCCTTTGCCGGGGCCGTCGAGTATCTAGGCTCGCGGTTGGCCTCTGCCCGGCGCCGGTTGCTGGTGTTGGACGCGGTGGCCCTCGAGAGGTGGCCCGGGGATGGACCCGCTTTGTCTCAGTATCACGTGTACGTCCGGGCCCCGGCGCGACCGGACGCCCAGGCCGTCGTCCGATGGCCAGACTCGGCGGTCACAGAAGGACTCGCCCGGGCCGTGTTTGCATCGTCGCGCACCTTTGGGCCAGCGAGTTTTGCTCGTATCGAGACTGCGTTTGCCAACCTGTACCCGGGCGAACAACCCCTGTGTTTGTGCCGCGGTGGGAACGTCGCATACACCGTGTGTACCCGCGCGGGCCCCAAGACCCGCGTCCCCCTGTCGCCCCGTGAATACCGGCAGTACGTGCTGCCGGGTTTTGACGGTTGCAAGGACCTCGCGCGACAGTCTCGGGGTCTGGGGCTCGGGGCAGCCGACTTTGTGGACGAGGCGGCACATAGCCACCGCGCAGCAAACCGATGGGGCCTGGGTGCCGCGCTTCGACCCGTCTTCCTTCCCGAGGGACGGAGACCGGGGGCCGCCGGGCCGGAGGCCGGCGACGTACCCACCTGGGCGAGGGTGTTTTGCCGCCACGCCCTGCTGGAACCCGACCCTGCCGCGGAACCACTCGTGCTTCCACCCGTGGCCGGTCGGTCGGTGGCGCTGTATGCGTCGGCGGACGAGGCTCGGAATGCCCTCCCCCCGATTCCCAGAGTAATGTGGCCGCCCGGTTTTGGGGCCGCGGAGACGGTGTTGGAGGGGAGCGACGGAACACGGTTCGCGTTCGGACACCACGGGGGCTCGGAACGGCCGGCAGAAACCCAGGCGGGGCGACAGCGGCGCACCGCAGACGACAGAGAACACGCTTTGGAGCCGGACGATTGGGAGGTGGGGTGTGAAGACGCGTGGGACAGCGAGGAGGGGGGCGGGGACGACGGGGACGCACCGGGGTCATCCTTTGGGGTGAGCATCGTGTCGGTGGCCCCGGGTGTGCTGCGAGACCGCCGGGTGGGCTTGCGCCCGGCGGTCAAGGTGGAGCTGTTGTCCTCGTCCTCGTCCTCCGAGGACGAGGACGATGTGTGGGGAGGGCGCGGGGGGAGGAGCCCCCCGCAGAGTCGGGGGTGACGGAGTCCCCTCCTTTTCTCGTGAGCGCCACCGGCGCGCGGACTGTTTGTTGTTTGTTAATAAAAGCGGAACGGTTTTTATGAAAAAAGTGTCTGTCTGTCTGTGCGGGCGGGCGACGGGCGGGCTGGTCGGACCCCCCCCGAAAATAACCCCCCCCGGTTTTTGGGCGCCCGGGGGACCCCGGGGGGGG

>HHV4_EBV_STRIPPED4

AGAATTCGTCTTGCTCTATTCACCCTTACTTTTCTTCTTGCCCGTTTTCTTTCTTAGTATGAATCCAGTATGCCTGCCTGTAATTGTTGCGCCCTACCTGTTTTGGCTGGCGGCTATTGCCGCCTCGTGTTTCACGGCCTCAGTTAGTACCGTTGTGACCGCCACCGGCTTGGCCCTCTCACTTCTACTCTTGGCAGCAGTGGCCAGCTCATATGCCGCTGCACAAAGGAAACTGCTGACACCGGTGACAGTTCTTACTGCGGTTGTCACTTGTGAGTACACACGCACCCTTTACAATGCATGATGTTCGTGAGATTGATCTGTCTCTAACAGTTCACTTCCTCTGCTTTTCTCCTCAGTCTTTGCAATTTGCCTAACATGGAGGATTGAGGACCCACCTTTTAATTCTCTTCTGTTTGCATTGCTGGCCGCAGCTGGCGGACTACAAGGCATTTACGGTTAGTGTGCCTCTGTTATGGAATGCAGGTTTGACTTCATATGTATGCCTTGGCATGACGTCAACTTTACTTTTATTTTAGTTCTGGTGATGCTTGTGCTCCTGATACTAGCGTACAGAAGGAGATGGCGCCGTTTGACTGTTTGTGGCGGCATCATGTTTTTGGCATGTGTACTTGTCCTCATCGTCGACGCTGTTTTGCAGCTGAGTCCCCTCCTTGGAGCTGTAACTGTGGTTTCCATGACGCTGCTGCTACTGGCTTTCGTCCTCTGGCTCTCTTCGCCAGGGGGCCTAGGTACTCTTGGTGCAGCCCTTTTAACATTGGCAGCAGGTAAGCCACACGTGTGACATTGCTTGCCTTTTTGCCACATGTTTTCTGGACACAGGACTAACCATGCCATCTCTGATTATAGCTCTGGCACTGCTAGCGTCACTGATTTTGGGCACACTTAACTTGACTACAATGTTCCTTCTCATGCTCCTATGGACACTTGGTAAGTTTTCCCTTCCTTTAACTCATTACTTGTTCTTTTGTAATCGCAGCTCTAACTTGGCATCTCTTTTACAGTGGTTCTCCTGATTTGCTCTTCGTGCTCTTCATGTCCACTGACCAAGATCCTTCTGGCACGACTGTTCCTATATGCTCTCGCACTCTTGTTGCTAGCCTCCGCGCTAATCGCTGGTGGCAGTATTTTGCAAACAAACTTCAAGAGTTTAAGCAGCACTGAATTTATACCCAGTGAGTATCTATTTGTTACTCCTGTTTAGTTGAAGAAAACAAGCTATTGGATTGTAACACACATTTTACGCTTTGTTCCTTAGATTTGTTCTGCATGTTATTACTGATTGTCGCTGGCATACTCTTCATTCTTGCTATCCTGACCGAATGGGGCAGTGGAAATAGAACATACGGTCCAGTTTTTATGTGCCTCGGTGGCCTGCTCACCATGGTAGCCGGCGCTGTGTGGCTGACGGTGATGACTAACACGCTTTTGTCTGCCTGGATTCTTACAGCAGGATTCCTGATTTTCCTCATTGGTAAGTGTGACACCAACAGGTGTTGCCTTGTTATGTCACCGTTCTGACACATGACTTACATGGGTTTGGCTTTTGTAGGCTTTGCCCTCTTTGGGGTCATTAGATGCTGCCGCTACTGCTGCTACTACTGCCTTACACTGGAAAGTGAGGAGCGCCCACCGACCCCATATCGCAACACTGTATAAAGGTAAGTATTATTAAATTTTAGAGACACTATCACGTGTAACTTGACGTGCAAGGATGGAGGAGAGGGGCAGGGAAACGCAAATGCCGGTTGCCCGGTATGGGGGCCCATTTATTATGGTAAGGCTCTTCGGGCAAGATGGAGAGGCAAACATACAGGAGCAAAGGCTATATGAGCTACTCTCTGACCCACGCTCCGCGCTCGGCCTAGACCCGGGGCCCCTGATTGCTGAGAACCTGCTGCTAGTGGCGCTGCGTGGCACCAACAACGATCCCAGGCCTCAGCGTCAGGAGAGGGCCAGAGAACTGGCCCTTGTTGGCATTCTACTAGGAAACGGCGAGCAGGGTGAACACTTGGGCACGGAGAGTGCCCTGGAGGCCTCAGGCAACAACTATGTGTATGCCTACGGACCAGACTGGATGGCAAGGCCTTCCACATGGTCCGCGGAAATCCAGCAATTCCTGCGACTCCTGGGCGCCACGTACGTGCTTCGCGTGGAGATGGGCAGGCAGTTTGGCTTCGAGGTGCATAGAAGCCGGCCATCCTTCCGTCAGTTCCAGGCCATCAATCACCTTGTCCTGTTTGACAACGCCCTTCGCAAGTACGATTCCGGCCAGGTGGCGGCGGGCTTCCAGAGGGCCCTTCTGGTGGCCGGGCCAGAGACCGCTGACACGAGGCCGGACCTCCGCAAGCTGAATGAGTGGGTGTTTGGTGGCAGGGCTGCTGGTGGCAGACAGCTGGCCGACGAGCTAAAGATCGTGTCCGCGCTGCGAGACACTTACTCGGGCCACTTGGTCCTTCAGCCCACGGAGACCCTTGACACATGGAAGGTGTTGAGCAGGGACACACGAACCGCTCATAGTTTGGAGCACGGATTCATTCATGCCGCGGGGACCATCCAGGCCAACTGCCCACAGCTGTTTATGAGACGCCAGCACCCCGGCCTCTTTCCCTTCGTTAATGCAATAGCATCATCGCTGGGCTGGTACTACCAGACCGCCACCGGCCCCGGAGCAGATGCCAGGGCGGCGGCCCGGCGCCAACAGGCCTTTCAGACCAGGGCGGCGGCTGAATGCCATGCCAAAAGCGGGGTTCCGGTCGTGGCCGGCTTCTACAGGACCATCAACGCCACGCTCAAGGGAGGAGAGGGCCTACAGCCCACTATGTTTAACGGGGAGCTGGGGGCCATCAAGCACCAGGCACTTGACACTGTGAGGTATGACTACGGCCACTATCTCATAATGTTGGGGCCATTCCAGCCATGGAGCGGACTGACGGCCCCTCCGTGCCCCTACGCCGAAAGTTCATGGGCACAGGCGGCCGTGCAGACGGCCCTCGAGCTGTTCTCGGCCCTGTACCCGGCCCCGTGCATCTCGGGCTACGCGCGCCCCCCGGGCCCCAGTGCTGTGATCGAGCATCTGGGGTCCCTAGTCCCAAAGGGGGGTCTGCTGTTGTTTCTGTCTCACCTACCGGATGATGTTAAGGACGGGCTCGGAGAAATGGGGCCGGCCAGGGCCACGGGACCTGGAATGCAGCAGTTTGTCAGCAGCTACTTCCTCAACCCCGCCTGTTCCAACGTCTTCATTACAGTGAGGCAGCGAGGGGAGAAGATCAACGGCCGTACCGTCCTCCAAGCGCTCGGACGCGCATGCGATATGGCAGGCTGCCAGCACTATGTGCTGGGCTCCACGGTTCCCCTCAGTGGACTCAACTTTGTCAACGACCTGGCGTCCCCGGTTTCCACCGCCGAGATGATGGATGATTTCTCTCCCTTCTTCACCGTGGAGTTTCCCCCGATTCAAGAGGAGGGCGCAAGTTCTCCGGTACCCTTAGATGTGGACGAGAGCATGGACATCTCTCCGTCTTACGAGTTGCCCTGGCTCTCGCTGGAGTCATGCCTCACAAGCATCCTGTCACACCCCACCGTGGGAAGCAAGGAGCACTTGGTCAGGCACACGGACAGGGTCAGCGGAGGACGCGTGGCACAGCAGCCCGGGGTAGGTCCCCTGGACCTGCCGCTGGCGGACTACGCCTTCGTTGCCCACAGTCAGGTCTGGACCAGGCCCGGTGGGGCTCCTCCCTTGCCCTATCGTACCTGGGATCGAATGACAGAGAAGCTGCTTGTCTCCGCAAAACCCGGCGGAGAGAACGTTAAGGTTTCAGGTACCGTGATTACATTGGGAGAACAGGGGTACAAAGTGTCGTTGGATCTGAGGGAGGGAACCAGGCTGGCAATGGCTGAGGCGCTGCTGAACGCAGCATGTGCCCCAATCTTGGATCCGGAAGACGTCTTGCTCACCCTGCATCTACACCTGGATCCGCGCCGGGCAGACAACTCGGCCGTGATGGAGGCTATGACGGCGGCGAGTGACTACGCGCGTGGCCTGGGCGTGAAGCTGACCTTTGGCTCGGCCTCCTGCCCCGAGACCGGCTCGTCCGCCTCCAGCTTCATGACCGTGGTGGCCTCTGTCTCCGCCCCAGGGGAATTCTCGGGTCCTCTGATCACGCCAGTGCTTCAGAAGACGGGCAGTCTCCTGATTGCGGTGCGTTGCGGGGATGGCAAGATCCAGGGAGGGTCGCTGTTTGAGCAGCTCTTTAGCGACGTGGCCACGACCCCACGGGCGCCCGAGGCGTTGTCTCTGAAGAATCTCTTCCGGGCAGTCCAGCAGCTGGTCAAGAGCGGCATCGTGCTG

GCAGCGGGGAGTGACCATCACTATGCCGGTGGCCTCCGACTACCTCCCGGAGATGTTTGCAGAGCACCCCGGCCTGGTGTTTGAGGTGGAGGAGCGCAGCGTGGGTGAGGTGCTGCAGACCCTGCGCTCCATGAACATGTACCCGGCAGTCCTCGGTCGAGTGGGCGAGCAAGGTCCAGATCAAATGTTTGAGGTGCAGCACGGCCCAGAGACGGTGTTGCGCCAGTCGCTGCGCCTGCTACTGGGAACCTGGTCCTCCTTTGCCAGCGAGCAGTACGAGTGCCTGCGACCAGATCGGATTAACCGGTCCATGCACGTGTCCGACTACGGCTATAACGAAGCACTGGCAGTCTCCCCGTTGACAGGAAAGAATCTCAGCCCACGCCGGCTGGTGACAGAGCCTGACCCACGATGTCAGGTGGCCGTGCTATGCGCCCCGGGCACCAGGGGCCATGAAAGCCTCCTGGCGGCCTTCACGAATGCCGGATGCCTGTGCCGACGGGTGTTCTTTCGCGAGGTTAGGGACAACACGTTCCTCGACAAGTACGTGGGTCTGGCCATCGGAGGAGTTCATGGGGCCAGGGACTCTGCCCTGGCAGGCCGTGCCACCGTGGCGCTGATTAATCGTTCCCCCGCCCTGCGTGACGCTATTCTAAAGTTCCTCAACAGGCCAGATACGTTCTCGGTGGCCTTGGGGGAGCTGGGGGTGCAAGTTTTGGCTGGCCTGGGGGCCGTGGGGTCAACAGATAATCCACCCGCCCCTGGCGTGGAAGTTAATGTCCAGAGATCACCTCTGATTCTGGCCCCCAACGCCTCTGGCATGTTTGAGTCCCGCTGGCTGAACATTAGCATCCCGGCGACCACCAGCTCTGTCATGCTGCGTGGCCTCCGGGGCTGCGTCCTGCCTTGTTGGGTGCAAGGCTCGTGCCTGGGCCTGCAATTTACTAACCTCGGGATGCCATATGTTTTGCAGAATGCCCACCAGATCGCCTGCCACTTCCACAGCAATGGCACGGACGCCTGGCGCTTTGCTATGAATTATCCAAGAAACCCCACGGAGCAGGGCAACATTGCAGGGCTCTGTTCACGCGATGGTCGTCATCTGGCTCTCCTGTGTGACCCCTCACTTTGTACAGACTTTTGGCAATGGGAGCACATTCCCCCCGCCTTTGGGCACCCCACGGGGTGCTCCCCCTGGACACTTATGTTTCAAGCAGCTCACCTATGGTCACTCAGGCACGGTCGCCCCTCCGAGTGACCAGTCACCTTCCAGACTATGCATACACTGAATTTAGCCTGATATTGTCCCCCGAGCCCCGGGCCCAGCCCTCCTCAGAAAACTCTGCATGGAGAAGCTGGACGTGAACCTCCCCCCCCCGACCTGTGTGCTGTATTTACAAACACTACAATAAACCCAATGTGCAAATGTGGTTTGTATGGCTACTTTGTGTTCCTAAAAAATGCAACAATAGAAGTGGAAACCCTCAGTCACGGGACATTAACCTCAACCACAAAATGGGGGTTGGAGAAAGTAACCACATATACTGGAGATGATTCATGGGCTGGGGGTTCCCGGACAATACACCCATCTGGAGTTCAACCTAATTACATGGTAGATAAATTAAGAGTCCCTTCTCACCACTCGAAACTATGGCAGACATTCTATAAGATAACGCGGAGAGATGAGGTGAGGGCAGAGGACATTGGGCAGGTGTGGGCCACGGGGCAGCTGGCCATATCCCCCGCACCACAGAAGTGTAAGCAAAGTGAAGGGCTAGGAAGGCAGGCGGGGCCTAGCAATGTCACAGCTAAATGCCCACCGGGGCACACACTCAAGCGGGGTCTCGGAGCTCCCAGGTCAGACCACGAAAGGTCAGCCTGCAAGGTGGATGGCGTGTTTTCTGAGGTTATCCCCGCTACGTGCAGTGCTGGGTGAGAGAGACCCTGGAATGTGTGTCGAAATGACCAAGCGTCCCCGCAGCGGGGCTCCCAACACGGGTTCCCAGAGAGGGTAAAAGAGGGGGCCATAAAGCCCAGGGTGTAAAACACCGACCGCGCCACCAGATGGCACACGTGGGGAAATGAGGGTTAGCATAGGCAACCCCCGCCTACACACCAACTATCGCAAACCCCGCCCCGTCACGGTGACGTAGTCTGTCTTGAGGAGATGTAGACTTGTAGACACTGCAAAACCTCAGGACCTACGCTGCCCTAGAGGTTTTGCTAGGGAGGAGACGTGTGTGGCTGTAGCCACCCGTCCCGGGTACAAGTCCCGGGTGGTGAGGACGGTGTCTGTGGTTGTCTTCCCAGACTCTGCTTTCTGCCGTCTTCGGTCAAGTACCAGCTGGTGGTCCGCATGTTTTGATCCAAACTTTAGTTTTAGGATTTATGCATCCATTATCCCGCAGTTCCACCTAAACGGGGCTTAACGTTGCATCCCAGAAGATGCACACGTAACCCCGCCTACAACCGTGACGTGGCTGTTTACCAGCATGTATAGAGTTACGGTTCACTACATCAAACAGGACAGCCGTTGCCCTAGTGGTTTAGGACACACCGCCAACGCTCAGTGCGGTGCTACCGACCCGAGGTCAAGTCCCGGGGGAGGAGAAGAGAGGCTTCCCGCCTAGAGCATTTGCAAGTCAGGATTCTCTAATCCCTCTGGGAGAAGGGTATTCGGCTTGTCCGCTGTTTTTTTTGTGGCTAGTTTTGCACCCACAACATGTAAGGGCCCGCTACCCCTACAACACAAAACAAACTATCTCCCCTAACCATCCCTTTGCCAATCAATTCTGTGACAGGGTTTCCTGGACACACAGTCTTAGTTCAGGTAGACACCCAGTTATGCAGTGCCACCAATTCCAACCATTTTTAAACCTCCTGGAATTCTATCATTAAACGGCATGCAGGAAAAGGACAAGCAGCAAAAATTCACGCCCCCTTGGGAGGTGGCGTCATATGCAAAGGATAGCACTCCCACTCTACTACTGGGTATCATATGCTGACTGTATATGCATGAGGATAGCATATACTACCCGGATATAGAATAGGATAGCATATACTACCCGGATATAGATTAGGATAGCATATGCTACCCGGATATAGATTAGGGTAGCATATGCTATCCTAATCTATATCTGGGTAGCATATACTACCCAAATAGAGATTAGGATAGCATATGCTACCCGGATATAGATTAGGGTAGCATATGCTATCCTAATCTATATCTGGGTAGCATATACTACCCAAATAGAGATTAGGATAGCATATGCTACCCGGATATAGATTAGGATAGCATATGCTACCCGGATATAGATTAGGATAGCATATGCTATCCATACATAGATTAGGATAGCATATACTACCCTAATCTCTATTGGGTAGTATATGCTACCCAGATGTAGATTAGGATAGCATATACTACCCGGATATAGATTTGGGTAGCATATGCTATCCTAATCTATATCCGGGTAGCATATACTACCCAAAGAGAGATTAGGGTAGTATATGCTATCCGAATATCTATCTGGATAGCATATGCTATCCAATCTATATGCGGGTAGTACATGCTATCCTAAATCTATATTCGGGTAGTATATGCTATCCTAATCTCTATGTGGGTAGCATATGCTATCCTAATCTCTGTCTGGGTAGTATATGCTATCCTAATCTGTATGCGGGTAGTACATGCTATCCTAATCTCTATGTGGGTAGCATATGCTATCCTAATCTCTATCTGGATAGCATATGCTATCCAATCTATATGCGGGTAGTACATGCTATCCTAATCTATATCCGGGTAGTACATGCTATCCTAATCTCTATGTGGGTAGCATATGCTATCCTAATCTATATCTGGGTAGCATATGCTACATATGCTATCCAACGCAACATTATTCCACCGTGCTATCAGCGACCTCGTGAATATGAGGACCAACAACCCTGTGCTTGGCGCTCAGGCGCAAGTGTGTGTAACTTGTCCTCCAGATCGCAGCAATTGCGCCCCTATCTTGGCCCGCCCACCTACTTATGCAGGTATTCCCCGGGGTGCCATTAGTGGTTTTGTGGGCAAGTGGTTTGACCGCAGGGGTTAGCGAGGTTACAATCAGCCAAGTTATTACACCCTTAGTTTACAGTCCAAAACCGCAGGGCGGCGTGTGGGGGCTGACGCGTGCCCCCACTCCACAATTTCAAAAAAAAGAGTGGCCACTTGTGTTTGTTTATGGGCCCCATTGGCGGGGAGCCCCGTTTAATTTTCGGGGGTGTTAGAGACAACCAGTGGAGTCAGCTGCTGTCGGCGTCCACTCTCCTTCCCCTTGTTACAAATAGAGTGTAACAACATGGTTCACCTGTCTTGGTCCCTGCCTGGGACACATCTTAATAACCCCAGTATCATATTGCACTAGGATTAAATTCGTGTGAGATGGACATCCAGTCTTTACGGCTTGTCCCCACCCCATGGATTTCTATTGTTAAAGATATTCAGAATGTTTCATTCCTACACTAGTATTTATTGCCCAAGGGGTTTGTGAGGGTTATATTGGTGTCATAGCACAATGCCACCACTGAACCCCCCGTTCAAATTTTATTCTGGGGGCGTCACCTGAAACCTTGTTTTCGAGCACCTCACATACACCTTACTGTTCACAACTCAGCAGTTATTCTATTAGCTAAACGAAGGAGAATGAAGAAGCAGGCGAAGATTAAGGAGAGTTCACTGCCCGCTCCTTGATTTTCAGCCACTGCCCTTGTGACTAAAATGGTTCACTACCCTCGTGGAATCCTGACCCCATGTAAATAAAACCGTGACAGCTCATGGGGTGGGAGATATCGCTGTTCCTTAGGACCATTTTACTAACCCTAATTCGATAGCATATGCTTCCCGTTGGGTAACATGTGCTATTGAATTAGGGTTAGTCTGGATAGTATATGCTACTACCCGGGAAGCATATGCTACCCGTTTAGGGTTAAAAAGGGGGCCTTATAAACACTCTTGCTAACGCCCTCTTGAGGGTCCGCTTATCGGTAGCTACACAGGCCCCTCTGATTGACGTTGGTGTAGCCTCCCATAGTCTTCCTGGGCCCCTGGGAGGTACATGTCCCCCAGCATTGGTGTAAGAGCTTCAGCCAAGAGTTACACATAAAGGCAATGTTGTGTTGCAGTCCACAGACTGCAAAGTCTGCTCCAGGATGAAAGCCACTCAGTGTTGGCAAATGTGCACATCCATTTATAAGGATGTCAACTACAGTCAGAGAACCCCTTTGTGTTTGGTCCCCCCCCCCGTGTCACATGTGGAACAGGGCCCAGTTGGCAAGTTGTACCAACCAACTGAAGGGATTACATGCACTGCCCCGCGGGAAATACGTCCTACCCAGGAACCCGAAACAGTGTTTCCCAGAAGCTGTAAAAATAGAACGCCCTGGAACTGCCCCACTGTGCAATGCAGCTTTTAGCCATGCCATGCTCTATAAATCACTCCCCTATCTCAGGTAGGCCTGCACACCTTAGGTATGGAGCGAAGGTTAGTGGTCACTCTGCAGTGCCTGGTGCTGCTTTACCTGGCACCTGAGTGTGGAGGTACAGACCAATGTGACAATTTTCCCCAAATGTTGAGGGACCTAAGAGATGCCTTCAGTCGTGTTAAAACCTTTTTCCAGACAAAGGACGAGGTAGATAACCTTTTGCTCAAGGAGTCTCTGCTAGAGGA

CCGCCTGCGCAGGTGCCACAGGTTCCTGCCGTGTGAGAACAAGAGTAAAGCTGTGGAACAGATAAAA

AGCCAGGTGATAATTCCATACCCTGGAAGCAGGAGATGGGTGCATTTCACCCCAACCCCCCCTTTCGACTGTCATTTACAATAAAATGAAACCTTTTATTCTTGATTGCTTCTTGTGTTCTTGCCGCCCAGGTACCTTCCTGTGTTCTCCCCACGGGAAAAAGAATAGCTTCTGCAGAAGGCCATTGACGCAAGTTTTGCCCGTGGGGATTACCCGACCCAGCCACTTACAGCACATTTTGTTCTAGGTCCATCTTAGGAGCCCGGGCCAGCATTCTATCAGCTTAACGGGAAGAGAAGTGGGGAGGGCACTCGCCCACTAACCTTAACACCTGCAGCCTACAAAAGTACACTAGCTGTTTGCTCTATTCGCCACTAGAGACCGCCAAAATGCGAAACTACAGGCCCGGGCCCAGGCCTTGCAGGGCAGACGGTTAGGCTGACAAGTGGACAAGTGTGGCAGGTGGGCGGGAAGGGGCACAAGAATGCCGGCGAAACTGGACCACGGTCCACCCCGCCCTCAAGCGTCCGGGAGCCGGGCGGCTCGGCCAAGGAGGGCGGCCTTGCGAACAATCATTAGTAGCTACCAACAAGGGCCCCCAGATGCCCCCACCAGTCACCCCGTCCGTGTCCACTCACATATTCCACTCTTATTTTTAAATTCATGTGTCCCAATTAGAAACCCAAGCGCAGAAATTAGTTGAGAGGCTAGTGTTTTAAACATGCACTCTAGGCCAGCCAGAGATAATTTCACAAGACTATCAAGTTGGTGTAAACACGCCGTGGGAAAAAATTTATGGTTCAGTGCGTCGAGTGCTATCTTTGGAACAGTAGAAAATTGAACCTTGTTGGCGGGAGAAGGCATAACGCCTTATCTGGGAGGAGCGACGGATTATAGCCAATAAGAGAGCTCAAGACGCAGGGCTCGCAAAGTATAGTGGCCCCGTGGGACCTTAGAGGTGGAGCAACGTCTAAAGTGGTAATAACACCAGGCGGGGCTGGGCAAAGGGGTCCTACGGGCGGGATTAATTACGCCTTGCTTACGCAAGCTCAGTTAATTCGCCCACGACTTGAAAAATGTAGCCCTTAACCAATTGGCGGCCCCTAAGGGGGGGACTAAGGTCCCACTACAAAAACTCTGTGTTCTTCTGCAAATTTTAGATCAGATGGCATAGAGACAAGGACACCGAAGACCCCCAGAGCCCTCATCGCAGGGTTCTTACCATGTGGCCATGTAGGCCCACTTAACACTACAAGACCTACGCCTCCCCATTCATCATGTAACCCGCAAATCATCTAAACCGTAAGTCTAAGGGCCTCCTGAGGTTTTCTCAGGAGGCCCTAATGTATAATTAATCATGCATTTGATTTAAAAAAAGTAGGTTATACTCATTTTAGGCCAGTCTTTATTTGCAGATTAATAATTTATGTGATTCTCCTTCCCTCTAGGACTGAAGAAACAGCCTCCTGCACGTGAGCATGTATCTGAAATAATTATTATGTCATAAGTGTAATGATTAGAAAGTCATAAACCCACTTCCCTTTACATGAATCTGGGCACTGAATTTTGGGGTACCTCTAAAGACTAACGTGTTCGATTTCGGGGTCACTTCCCCTTTTATAAGTGTGTGAACAGTGATTTCAGTAAAACCTAAGAGATATTTGGTGTCACTTCCGCATTTTAAGTTTCAGAAAATTTTAAAATTAAAATTGAAATTTCTCTCAAAATAATTCCAATGAAAACTTCAAAGAATCTTATGTATATAATTCTTTTGCCCCAAACTGGGCTTCAGATGCCTTCTATTGCACTCTCACAAAAACATTCTGGACACATGTGCCAGACGCCTGGGCCTCTAAGGCCCTCGGGTCCCCCTGGACCCCGGCCTCAGCAACCCTGCTGCTCCCCTCCTGCCACCCCAGCCTCCCCCCCCCCCGTCCCCCTTCGCTCCTGATCCTCCCCCGGTCCCCAGTAGGGCCGCCTGCCCCCCTGCACCCAGTACCTGCCCCTCTTGGCCACGCACCCCGGGCCAGGCCACCTTAGACCCGGCCAAGCCCCATCCCTGAAGACCCAGCGGCCATTCTCTCTGGTAACGAGCAGAGAAGAAGTAGAGGCCCGCGGCCATTGGGCCCAGATTGAGAGACCAGTCCAGGGGCCCGAGGTTGGAGCCAGCGGGCACCCGAGGTCCCAGCACCCGGTCCCTCCGGGGGGCAGAGACAGGCAGGGCCCCCCGGCAGCTGGCCCCGAGGAGGCGCCCGGAGTGGGGCCGGTCGGCTGGGCTGGCCGAGCCCGGGTCTGGGAGGTCTGGGGTGGCGAGCCTGCTGTCTCAGGAGGGGCCTGGCTCCGCCGGGTGGCCCTGGGGTACGTCTGGGAGGCAGAGGGTCGGCCTAGGCCCGGGGAAGTGGAGGGGGATCGCCCGGGTCTCTGTTGGCAGAGTCCGGGCGATCCTCTGAGACCCTCCGGGCCCGGACGGCCGCCCTCAGCCCCCCAGACAGACCCCAGGGTCTCCAGGCAGGGTCCGGCATCTCCAGGGGCAGCAGGCTCACCACCACAGGCCCCCCAGACCCGGGTCTCGGCCAGCCGAGCCGACCGGCCCCGCGCCGGGCGCCTCCTCGGGGCCAGCCGCCGGGGTTGGTTCTGCCCCTCTCTCTGTCCTTCAGAGGAACAAGGGACCTCGGGCACCCCAGAGCCCCTCGGGCCCGCCTCCAGGCGCCCTCCTGGTCTCCGCTCCCCTCTTAGCCCCGTTAAACCCAAAGAATGTCTGAGGGGAGCCACCCTCGGGGCCCAGGCCCCAGAGTCCAGAGGTCAGGGGCACCTCAGGGTGCCCCAGAGGCCGGTTCCTCGCCCCTTCCCCGGGCTTCAGAGCCCAGGATGTCCCCCAGAAGGGACCCTAGGCGTCCCCTCTCCTCCCCTCCAGGCTCGAGCCTCTCCCTCGCGGAGAGGGGCCTCTTTGGGCCCTCACGTCCAGCCCCACCGAGACCCGAGTGGCCCGGATCCCCCCACCGGCCCTTCTCTCTGTCCCCCTGCTCCTCTCCAACCTTCGCTCCACCCTAGACCCCAGCTTCTTGCCTCCCCACCGGGTCCATCAGGCCGGCCGGAGGGACCCCGGCGGCCCGGTGTCAGTCCCCCCTGCAGCCGCCCAGTCTCTGCCTCCAGGCAAGGGCGCCAGCTTTTCTCCCCCCAGCCTGAGGCCCAGGCTCCTGTGCACTGTCTGTAAAGTCCAGCCTCCCACGCCCGTCCACGGCTCCCGGGCCCAGCCCCGTCCACCCCTCCCCACGGTGGACAGGCCCTCTGTCCACCCGGGCCATCCCCGCCCCCCTGTGTCCACCCCAGTCCCGTCCAGGGGGGACTTTATGTGACCCTTGGGCCTGGCTCCCCATAGACTCCCATGTAAGCCTGCCTCGAGTAGGTGCCTCCAGAGCCCCTTTTGCCCCCCTGGCGGCCCAGCCCGACCCCCGGGCGCCCCCAAACTTTGTCCAGATGTCCAGGGGTCCCCGAGGGCGAGGCCCAGCCCCCTCCCACCCCTGTCCACTGCCCCGGTCCCCCAGAAGCCCCCAAAAGTAGAGGCTCAGGCCATGCGCGCCCTGTCACCAGGCCTGCCAAAGAGCCAGATCTAAGGCCAGGAGAGGCAGCCCCAAAGCGGGTGCAGTAACAGGTAATCTCTGGTAGTGATTTGGACCCAAAATCTGACACTTTAGAGCTCTGGAGGACTTTAAAACTCTAAAAATCAAAACTTTAGAGGCGAATGGGCGCCATTTTGTCCCCACGCGCGCATAATGGCGGACCTAGGCCTAAAACCCCCAGGAAGCGGGTCTATGGTTGGCTGCGCTGCTGCTATCTTTAGAGGGGAAAAGAGGAATAAGCCCCCAGACAGGGGAGTGGGCTTGTTTGTGACTTCACCAAAGGTCAGGGCCCAAGGGGGTTCGCGTTGCTAGGCCACCTTCTCAGTCCAGCGCGTTTACGTAAGCCAGACAGCAGCCAATTGTCAGTTCTAGGGAGGGGGACCACTGCCCCTGGTATAAAGTGGTCCTGCAGCTATTTCTGGTCGCATCAGAGCGCCAGGAGTCCACACAAATGTAAGAGGGGGTCTTCTACCTCTCCCTAGCCCTCCGCCCCCTCCAAGGACTCGGGCCCAGTTTCTAACTTTTTCCCCTTCCCTCCCTCGTCTTGCCCTGCGCCCGGGGCCACCTTCATCACCGTCGCTGACTCCGCCATCCAAGCCTAGGGGAGACCGAAGTGAAGTCCCTGGACCAGCCCGGCCCGGGCCCCCCGGTATCGGGCCAGAGGTAAGTGGACTTTAATTTTTTCTGCTAAGCCCAACACTCCACCACACCCAGGCACACACTACACACACCCACCCGTCTCAGGGTCCCCTCGGACAGCTCCTAAGAAGGCACCGGTCGCCCAGTCCTACCAGAGGGGGCCAAGAACCCAGACGAGTCCGTAGAAGGGTCCTCGTCCAGCAAGAAGAGGAGGTGGTAAGCGGTTCACCTTCAGGGGTAAGTAACCTGACCTCTCCAGGGCTCACATAAAGGGAGGCTTAGTATACATGCTTCTTGCTTTTCACAGGAACCTGGGGGCTAGTCTGGGTGGGATTAGGCTGCCTCAAGTTGCATCAGCCAGGGCTTCATGCCCTCCTCAGTTCCCTAGTCCCCGGGCTTCAGGCCCCCTCCGTCCCCGTCCTCCAGAGACCCGGGCTTCAGGCCCTGCCTCTCCTGTTACCCTTTTAGAACCACAGCCTGGACACATGTGCCAGACGCCTGGGCCTCTAAGGCCCTCGGGTCCCCCTGGACCCCGGCCTCAGCAACCCTGCTGCTCCCCTCCTGCCACCCCAGCCTCCCCCCCTCCCCGTCCCCCTTCGCTCCTGATCCTCCCCCGGTCCCCAGTAGGGCCGCCTGCCCCCCTGCACCCAGTACCTGCCCCTCTTGGCCACGCACCCCGGGCCAGGCCACCTTAGACCCGGCCAAGCCCCATCCCTGAAGACCCAGCGGCCATTCTCTCTGGTAACGAGCAGAGAAGAAGTAGAGGCCCGCGGCCATTGGGCCCAGATTGAGAGACCAGTCCAGGGGCCCGAGGTTGGAGCCAGCGGGCACCCGAGGTCCCAGCACCCGGTCCCTCCGGGGGGCAGAGACAGGCAGGGCCCCCCGGCAGCTGGCCCCGAGGAGGCGCCCGGAGTGGGGCCGGTCGGCTGGGCTGGCCGAGCCCGGGTCTGGGAGGTCTGGGGTGGCGAGCCTGCTGTCTCAGGAGGGGCCTGGCTCCGCCGGGTGGCCCTGGGGTACGTCTGGGAGGCAGAGGGTCGGCCTAGGCCCGGGGAAGTGGAGGGGGATCGCCCGGGTCTCTGTTGGCAGAGTCCGGGCGATCCTCTGAGACCCTCCGGGCCCGGACGGCCGCCCTCAGCCCCCCAGACAGACCCCAGGGTCTCCAGGCAGGGTCCGGCATCTCCAGGGGCAGCAGGCTCACCACCACAGGCCCCCCAGACCCGGGTCTCGGCCAGCCGAGCCGACCGGCCCCGCGCCGGGCGCCTCCTCGGGGCCAGCCGCCGGGGTTGGTTCTGCCCCTCTCTCTGTCCTTCAGAGGAACAAGGGACCTCGGGCACCCCAGAGCCCCTCGGGCCCGCCTCCAGGCGCCCTCCTGGTCTCCGCTCCCCTCTTAGCCCCGTTAAACCCAAAGAATGTCTGAGGGGAGCCACCCTCGGGGCCCAGGCCCCAGAGTCCAGAGGTCAGGGGCACCTCAGGGTGCCCCAGAGGCCGGTTCCTCGCCCCTTCCCCGGGCTTCAGAGCCCAGGATGTCCCCCAGAAGGGACCCTAGGCGTCCCCTCTCCTCCCCTCCAGGCTCGAGCCTCTCCCTCGCGGAGAGGGGCCTCTTTGGGCCCTCACGTCCAGCCCCACCGAGACCCGAGTGGCCCGGATCCCCCCACCGGCCCTTCTCTCTGTCCCCCTGCTCCTCTCCAACCTTCGCTCCACCCTAGACCCCAGCTTCTTGCCTCCCCACCGGGTCCATCAGGCCGGCCGGAGGGACCCCGGCGGCCCGGTGTCAGTCCCCCCTGCAGCCGCCCAGTCTCTGCCTCCAGGCAAGGGCGCCAGCTTTTCTCCCCCCAGCCTGAGGCCCAGGCTCCTGTGCACTGTCTGTAAAGTCCAGCCTCCCACGCCCGTCCACGGCTCCCGGGCCCAGCCCCGTCCACCCCTCCCCACGGTGGACAGGCCCTCTGTCCACCCGGGCCATCCCCGCCCCCCTGTGTCCACCCCAGTCCCGTCCAGGGGGGACTTTATGTGACCCTTGGGCCTGGCTCCCCATAGACTCCCATGTAAGCCTGCCTCGAGTAGGTGCCTCCAGAGCCCCTTTTGCCCCCCTGGCGGCCCAGCCCGACCCCCGGGCGCCCCCAAACTTTGTCCAGATGTCCAGGGGTCCCCGAGGGCGAGGCCCAGCCCCCTCCCACCCCTGTCCACTGCCCCGGTCCCCCCAGAAGCCCCCAAAAGTAGAGGCTCAGGCCATGCGCGCCCTGTCACCAGGCCTGCCAAAGAGCCAGATCTAAGGCCAGGAGAGGCAGCCCCAAAGCGGGTGCAGTAACAGGTAATCTCTGGTAGTGATTTGGACCCAAAATCTGACACTTTAGAGCTCTGGAGGACTTTAAAACTCTAAAAATCAAAACTTTAGAGGCGAATGGGCGCCATTTTGTCCCCACGCGCGCATAATGGCGGACCTAGGCCTAAAACCCCCAGGAAGCGGGTCTATGGTTGGCTGCGCTGCTGCTATCTTTAGAGGGGAAAAGAGGAATAAGCCCCCAGACAGGGGAGTGGGCTTGTTTGTGACTTCACCAAAGGTCAGGGCCCAAGGGGGTTCGCGTTGCTAGGCCACCTTCTCAGTCCAGCGCGTTTACGTAAGCCAGACAGCAGCCAATTGTCAGTTCTAGGGAGGGGGACCACTGCCCCTGGTATAAAGTGGTCCTGCAGCTATTTCTGGTCGCATCAGAGCGCCAGGAGTCCACACAAATGTAAGAGGGGGTCTTCTACCTCTCCCTAGCCCTCCGCCCCCTCCAAGGACTCGGGCCCAGTTTCTAACTTTTTCCCCTTCCCTCCCTCGTCTTGCCCTGCGCCCGGGGCCACCTTCATCACCGTCGCTGACTCCGCCATCCAAGCCTAGGGGAGACCGAAGTGAAGTCCCTGGACCAGCCCGGCCCGGGCCCCCCGGTATCGGGCCAGAGGTAAGTGGACTTTAATTTTTTCTGCTAAGCCCAACACTCCACCACACCCAGGCACACACTACACACACCCACCCGTCTCAGGGTCCCCTCGGACAGCTCCTAAGAAGGCACCGGTCGCCCAGTCCTACCAGAGGGGGCCAAGAACCCAGACGAGTCCGTAGAAGGGTCCTCGTCCAGCAAGAAGAGGAGGTGGTAAGCGGTTCACCTTCAGGGGTAAGTAACCTGACCTCTCCAGGGCTCACATAAAGGGAGGCTTAGTATACATGCTTCTTGCTTTTCACAGGAACCTGGGGGCTAGTCTGGGTGGGATTAGGCTGCCTCAAGTTGCATCAGCCAGGGCTTCATGCCCTCCTCAGTTCCCTAGTCCCCGGGCTTCAGGCCCCCTCCGTCCCCGTCCTCCAGAGACCCGGGCTTCAGGCCCTGCCTCTCCTGTTACCCTTTTAGAACCACAGCCTGGACACATGTGCCAGACGCCTGGGCCTCTAAGGCCCTCGGGTCCCCCTGGACCCCGGCCTCAGCAACCCTGCTGCTCCCCTCCTGCCACCCCAGCCTCCCCCCCTCCCCGTCCCCCTTCGCTCCTGATCCTCCCCCGGTCCCCAGTAGGGCCGCCTGCCCCCCTGCACCCAGTACCTGCCCCTCTTGGCCACGCACCCCGGGCCAGGCCACCTTAGACCCGGCCAAGCCCCATCCCTGAAGACCCAGCGGCCATTCTCTCTGGTAACGAGCAGAGAAGAAGTAGAGGCCCGCGGCCATTGGGCCCAGATTGAGAGACCAGTCCAGGGGCCCGAGGTTGGAGCCAGCGGGCACCCGAGGTCCCAGCACCCGGTCCCTCCGGGGGGCAGAGACAGGCAGGGCCCCCCGGCAGCTGGCCCCGAGGAGGCGCCCGGAGTGGGGCCGGTCGGCTGGGCTGGCCGAGCCCGGGTCTGGGAGGTCTGGGGTGGCGAGCCTGCTGTCTCAGGAGGGGCCTGGCTCCGCCGGGTGGCCCTGGGGTACGTCTGGGAGGCAGAGGGTCGGCCTAGGCCCGGGGAAGTGGAGGGGGATCGCCCGGGTCTCTGTTGGCAGAGTCCGGGCGATCCTCTGAGACCCTCCGGGCCCGGACGGCCGCCCTCAGCCCCCCAGACAGACCCCAGGGTCTCCAGGCAGGGTCCGGCATCTCCAGGGGCAGCAGGCTCACCACCACAGGCCCCCCAGACCCGGGTCTCGGCCAGCCGAGCCGACCGGCCCCGCGCCGGGCGCCTCCTCGGGGCCAGCCGCCGGGGTTGGTTCTGCCCCTCTCTCTGTCCTTCAGAGGAACAAGGGACCTCGGGCACCCCAGAGCCCCTCGGGCCCGCCTCCAGGCGCCCTCCTGGTCTCCGCTCCCCTCTTAGCCCCGTTAAACCCAAAGAATGTCTGAGGGGAGCCACCCTCGGGGCCCAGGCCCCAGAGTCCAGAGGTCAGGGGCACCTCAGGGTGCCCCAGAGGCCGGTTCCTCGCCCCTTCCCCGGGCTTCAGAGCCCAGGATGTCCCCCAGAAGGGACCCTAGGCGTCCCCTCTCCTCCCCTCCAGGCTCGAGCCTCTCCCTCGCGGAGAGGGGCCTCTTTGGGCCCTCACGTCCAGCCCCACCGAGACCCGAGTGGCCCGGATCCCCCCACCGGCCCTTCTCTCTGTCCCCCTGCTCCTCTCCAACCTTCGCTCCACCCTAGACCCCAGCTTCTTGCCTCCCCACCGGGTCCATCAGGCCGGCCGGAGGGACCCCGGCGGCCCGGTGTCAGTCCCCCCTGCAGCCGCCCAGTCTCTGCCTCCAGGCAAGGGCGCCAGCTTTTCTCCCCCCAGCCTGAGGCCCAGGCTCCTGTGCACTGTCTGTAAAGTCCAGCCTCCCACGCCCGTCCACGGCTCCCGGGCCCAGCCCCGTCCACCCCTCCCCACGGTGGACAGGCCCTCTGTCCACCCGGGCCATCCCCGCCCCCCTGTGTCCACCCCAGTCCCGTCCAGGGGGGACTTTATGTGACCCTTGGGCCTGGCTCCCCATAGACTCCCATGTAAGCCTGCCTCGAGTAGGTGCCTCCAGAGCCCCTTTTGCCCCCCTGGCGGCCCAGCCCGACCCCCGGGCGCCCCCAAACTTTGTCCAGATGTCCAGGGGTCCCCGAGGGCGAGGCCCAGCCCCCTCCCACCCCTGTCCACTGCCCCGGTCCCCCCAGAAGCCCCCAAAAGTAGAGGCTCAGGCCATGCGCGCCCTGTCACCAGGCCTGCCAAAGAGCCAGATCTAAGGCCAGGAGAGGCAGCCCCAAAGCGGGTGCAGTAACAGGTAATCTCTGGTAGTGATTTGGACCCAAAATCTGACACTTTAGAGCTCTGGAGGACTTTAAAACTCTAAAAATCAAAACTTTAGAGGCGAATGGGCGCCATTTTGTCCCCACGCGCGCATAATGGCGGACCTAGGCCTAAAACCCCCAGGAAGCGGGTCTATGGTTGGCTGCGCTGCTGCTATCTTTAGAGGGGAAAAGAGGAATAAGCCCCCAGACAGGGGAGTGGGCTTGTTTGTGACTTCACCAAAGGTCAGGGCCCAAGGGGGTTCGCGTTGCTAGGCCACCTTCTCAGTCCAGCGCGTTTACGTAAGCCAGACAGCAGCCAATTGTCAGTTCTAGGGAGGGGGACCACTGCCCCTGGTATAAAGTGGTCCTGCAGCTATTTCTGGTCGCATCAGAGCGCCAGGAGTCCACACAAATGTAAGAGGGGGTCTTCTACCTCTCCCTAGCCCTCCGCCCCCTCCAAGGACTCGGGCCCAGTTTCTAACTTTTTCCCCTTCCCTCCCTCGTCTTGCCCTGCGCCCGGGGCCACCTTCATCACCGTCGCTGACTCCGCCATCCAAGCCTAGGGGAGACCGAAGTGAAGTCCCTGGACCAGCCCGGCCCGGGCCCCCCGGTATCGGGCCAGAGGTAAGTGGACTTTAATTTTTTCTGCTAAGCCCAACACTCCACCACACCCAGGCACACACTACACACACCCACCCGTCTCAGGGTCCCCTCGGACAGCTCCTAAGAAGGCACCGGTCGCCCAGTCCTACCAGAGGGGGCCAAGAACCCAGACGAGTCCGTAGAAGGGTCCTCGTCCAGCAAGAAGAGGAGGTGGTAAGCGGTTCACCTTCAGGGGTAAGTAACCTGACCTCTCCAGGGCTCACATAAAGGGAGGCTTAGTATACATGCTTCTTGCTTTTCACAGGAACCTGGGGGCTAGTCTGGGTGGGATTAGGCTGCCTCAAGTTGCATCAGCCAGGGCTTCATGCCCTCCTCAGTTCCCTAGTCCCCGGGCTTCAGGCCCCCTCCGTCCCCGTCCTCCAGAGACCCGGGCTTCAGGCCCTGCCTCTCCTGTTACCCTTTTAGAACCACAGCCTGGACACATGTGCCAGACGCCTGGGCCTCTAAGGCCCTCGGGTCCCCCTGGACCCCGGCCTCAGCAACCCTGCTGCTCCCCTCCTGCCACCCCAGCCTCCCCCCCTCCCCGTCCCCCTTCGCTCCTGATCCTCCCCCGGTCCCCAGTAGGGCCGCCTGCCCCCCTGCACCCAGTACCTGCCCCTCTTGGCCACGCACCCCGGGCCAGGCCACCTTAGACCCGGCCAAGCCCCATCCCTGAAGACCCAGCGGCCATTCTCTCTGGTAACGAGCAGAGAAGAAGTAGAGGCCCGCGGCCATTGGGCCCAGATTGAGAGACCAGTCCAGGGGCCCGAGGTTGGAGCCAGCGGGCACCCGAGGTCCCAGCACCCGGTCCCTCCGGGGGGCAGAGACAGGCAGGGCCCCCCGGCAGCTGGCCCCGAGGAGGCGCCCGGAGTGGGGCCGGTCGGCTGGGCTGGCCGAGCCCGGGTCTGGGAGGTCTGGGGTGGCGAGCCTGCTGTCTCAGGAGGGGCCTGGCTCCGCCGGGTGGCCCTGGGGTACGTCTGGGAGGCAGAGGGTCGGCCTAGGCCCGGGGAAGTGGAGGGGGATCGCCCGGGTCTCTGTTGGCAGAGTCCGGGCGATCCTCTGAGACCCTCCGGGCCCGGACGGCCGCCCTCAGCCCCCCAGACAGACCCCAGGGTCTCCAGGCAGGGTCCGGCATCTCCAGGGGCAGCAGGCTCACCACCACAGGCCCCCCAGACCCGGGTCTCGGCCAGCCGAGCCGACCGGCCCCGCGCCGGGCGCCTCCTCGGGGCCAGCCGCCGGGGTTGGTTCTGCCCCTCTCTCTGTCCTTCAGAGGAACAAGGGACCTCGGGCACCCCAGAGCCCCTCGGGCCCGCCTCCAGGCGCCCTCCTGGTCTCCGCTCCCCTCTTAGCCCCGTTAAACCCAAAGAATGTCTGAGGGGAGCCACCCTCGGGGCCCAGGCCCCAGAGTCCAGAGGTCAGGGGCACCTCAGGGTGCCCCAGAGGCCGGTTCCTCGCCCCTTCCCCGGGCTTCAGAGCCCAGGATGTCCCCCAGAAGGGACCCTAGGCGTCCCCTCTCCTCCCCTCCAGGCTCGAGCCTCTCCCTCGCGGAGAGGGGCCTCTTTGGGCCCTCACGTCCAGCCCCACCGAGACCCGAGTGGCCCGGATCCCCCCACCGGCCCTTCTCTCTGTCCCCCTGCTCCTCTCCAACCTTCGCTCCACCCTAGACCCCAGCTTCTTGCCTCCCCACCGGGTCCATCAGGCCGGCCGGAGGGACCCCGGCGGCCCGGTGTCAGTCCCCCCTGCAGCCGCCCAGTCTCTGCCTCCAGGCAAGGGCGCCAGCTTTTCTCCCCCCAGCCTGAGGCCCAGGCTCCTGTGCACTGTCTGTAAAGTCCAGCCTCCCACGCCCGTCCACGGCTCCCGGGCCCAGCCCCGTCCACCCCTCCCCACGGTGGACAGGCCCTCTGTCCACCCGGGCCATCCCCGCCCCCCTGTGTCCACCCCAGTCCCGTCCAGGGGGGACTTTATGTGACCCTTGGGCCTGGCTCCCCATAGACTCCCATGTAAGCCTGCCTCGAGTAGGTGCCTCCAGAGCCCCTTTTGCCCCCCTGGCGGCCCAGCCCGACCCCCGGGCGCCCCCAAACTTTGTCCAGATGTCCAGGGGTCCCCGAGGGCGAGGCCCAGCCCCCTCCCACCCCTGTCCACTGCCCCGGTCCCCCCAGAAGCCCCCAAAAGTAGAGGCTCAGGCCATGCGCGCCCTGTCACCAGGCCTGCCAAAGAGCCAGATCTAAGGCCAGGAGAGGCAGCCCCAAAGCGGGTGCAGTAACAGGTAATCTCTGGTAGTGATTTGGACCCAAAATCTGACACTTTAGAGCTCTGGAGGACTTTAAAACTCTAAAAATCAAAACTTTAGAGGCGAATGGGCGCCATTTTGTCCCCACGCGCGCATAATGGCGGACCTAGGCCTAAAACCCCCAGGAAGCGGGTCTATGGTTGGCTGCGCTGCTGCTATCTTTAGAGGGGAAAAGAGGAATAAGCCCCCAGACAGGGGAGTGGGCTTGTTTGTGACTTCACCAAAGGTCAGGGCCCAAGGGGGTTCGCGTTGCTAGGCCACCTTCTCAGTCCAGCGCGTTTACGTAAGCCAGACAGCAGCCAATTGTCAGTTCTAGGGAGGGGGACCACTGCCCCTGGTATAAAGTGGTCCTGCAGCTATTTCTGGTCGCATCAGAGCGCCAGGAGTCCACACAAATGTAAGAGGGGGTCTTCTACCTCTCCCTAGCCCTCCGCCCCCTCCAAGGACTCGGGCCCAGTTTCTAACTTTTTCCCCTTCCCTCCCTCGTCTTGCCCTGCGCCCGGGGCCACCTTCATCACCGTCGCTGACTCCGCCATCCAAGCCTAGGGGAGACCGAAGTGAAGTCCCTGGACCAGCCCGGCCCGGGCCCCCCGGTATCGGGCCAGAGGTAAGTGGACTTTAATTTTTTCTGCTAAGCCCAACACTCCACCACACCCAGGCACACACTACACACACCCACCCGTCTCAGGGTCCCCTCGGACAGCTCCTAAGAAGGCACCGGTCGCCCAGTCCTACCAGAGGGGGCCAAGAACCCAGACGAGTCCGTAGAAGGGTCCTCGTCCAGCAAGAAGAGGAGGTGGTAAGCGGTTCACCTTCAGGGGTAAGTAACCTGACCTCTCCAGGGCTCACATAAAGGGAGGCTTAGTATACATGCTTCTTGCTTTTCACAGGAACCTGGGGGCTAGTCTGGGTGGGATTAGGCTGCCTCAAGTTGCATCAGCCAGGGCTTCATGCCCTCCTCAGTTCCCTAGTCCCCGGGCTTCAGGCCCCCTCCGTCCCCGTCCTCCAGAGACCCGGGCTTCAGGCCCTGCCTCTCCTGTTACCCTTTTAGAACCACAGCCTGGACACATGTGCCAGACGCCTGGGCCTCTAAGGCCCTCGGGTCCCCCTGGACCCCGGCCTCAGCAACCCTGCTGCTCCCCTCCTGCCACCCCAGCCTCCCCCCCTCCCCGTCCCCCTTCGCTCCTGATCCTCCCCCGGTCCCCAGTAGGGCCGCCTGCCCCCCTGCACCCAGTACCTGCCCCTCTTGGCCACGCACCCCGGGCCAGGCCACCTTAGACCCGGCCAAGCCCCATCCCTGAAGACCCAGCGGCCATTCTCTCTGGTAACGAGCAGAGAAGAAGTAGAGGCCCGCGGCCATTGGGCCCAGATTGAGAGACCAGTCCAGGGGCCCGAGGTTGGAGCCAGCGGGCACCCGAGGTCCCAGCACCCGGTCCCTCCGGGGGGCAGAGACAGGCAGGGCCCCCCGGCAGCTGGCCCCGAGGAGGCGCCCGGAGTGGGGCCGGTCGGCTGGGCTGGCCGAGCCCGGGTCTGGGAGGTCTGGGGTGGCGAGCCTGCTGTCTCAGGAGGGGCCTGGCTCCGCCGGGTGGCCCTGGGGTACGTCTGGGAGGCAGAGGGTCGGCCTAGGCCCGGGGAAGTGGAGGGGGATCGCCCGGGTCTCTGTTGGCAGAGTCCGGGCGATCCTCTGAGACCCTCCGGGCCCGGACGGCCGCCCTCAGCCCCCCAGACAGACCCCAGGGTCTCCAGGCAGGGTCCGGCATCTCCAGGGGCAGCAGGCTCACCACCACAGGCCCCCCAGACCCGGGTCTCGGCCAGCCGAGCCGACCGGCCCCGCGCCGGGCGCCTCCTCGGGGCCAGCCGCCGGGGTTGGTTCTGCCCCTCTCTCTGTCCTTCAGAGGAACAAGGGACCTCGGGCACCCCAGAGCCCCTCGGGCCCGCCTCCAGGCGCCCTCCTGGTCTCCGCTCCCCTCTTAGCCCCGTTAAACCCAAAGAATGTCTGAGGGGAGCCACCCTCGGGGCCCAGGCCCCAGAGTCCAGAGGTCAGGGGCACCTCAGGGTGCCCCAGAGGCCGGTTCCTCGCCCCTTCCCCGGGCTTCAGAGCCCAGGATGTCCCCCAGAAGGGACCCTAGGCGTCCCCTCTCCTCCCCTCCAGGCTCGAGCCTCTCCCTCGCGGAGAGGGGCCTCTTTGGGCCCTCACGTCCAGCCCCACCGAGACCCGAGTGGCCCGGATCCCCCCACCGGCCCTTCTCTCTGTCCCCCTGCTCCTCTCCAACCTTCGCTCCACCCTAGACCCCAGCTTCTTGCCTCCCCACCGGGTCCATCAGGCCGGCCGGAGGGACCCCGGCGGCCCGGTGTCAGTCCCCCCTGCAGCCGCCCAGTCTCTGCCTCCAGGCAAGGGCGCCAGCTTTTCTCCCCCCAGCCTGAGGCCCAGGCTCCTGTGCACTGTCTGTAAAGTCCAGCCTCCCACGCCCGTCCACGGCTCCCGGGCCCAGCCCCGTCCACCCCTCCCCACGGTGGACAGGCCCTCTGTCCACCCGGGCCATCCCCGCCCCCCTGTGTCCACCCCAGTCCCGTCCAGGGGGGACTTTATGTGACCCTTGGGCCTGGCTCCCCATAGACTCCCATGTAAGCCTGCCTCGAGTAGGTGCCTCCAGAGCCCCTTTTGCCCCCCTGGCGGCCCAGCCCGACCCCCGGGCGCCCCCAAACTTTGTCCAGATGTCCAGGGGTCCCCGAGGGCGAGGCCCAGCCCCCTCCCACCCCTGTCCACTGCCCCGGTCCCCCCAGAAGCCCCCAAAAGTAGAGGCTCAGGCCATGCGCGCCCTGTCACCAGGCCTGCCAAAGAGCCAGATCTAAGGCCAGGAGAGGCAGCCCCAAAGCGGGTGCAGTAACAGGTAATCTCTGGTAGTGATTTGGACCCAAAATCTGACACTTTAGAGCTCTGGAGGACTTTAAAACTCTAAAAATCAAAACTTTAGAGGCGAATGGGCGCCATTTTGTCCCCACGCGCGCATAATGGCGGACCTAGGCCTAAAACCCCCAGGAAGCGGGTCTATGGTTGGCTGCGCTGCTGCTATCTTTAGAGGGGAAAAGAGGAATAAGCCCCCAGACAGGGGAGTGGGCTTGTTTGTGACTTCACCAAAGGTCAGGGCCCAAGGGGGTTCGCGTTGCTAGGCCACCTTCTCAGTCCAGCGCGTTTACGTAAGCCAGACAGCAGCCAATTGTCAGTTCTAGGGAGGGGGACCACTGCCCCTGGTATAAAGTGGTCCTGCAGCTATTTCTGGTCGCATCAGAGCGCCAGGAGTCCACACAAATGTAAGAGGGGGTCTTCTACCTCTCCCTAGCCCTCCGCCCCCTCCAAGGACTCGGGCCCAGTTTCTAACTTTTTCCCCTTCCCTCCCTCGTCTTGCCCTGCGCCCGGGGCCACCTTCATCACCGTCGCTGACTCCGCCATCCAAGCCTAGGGGAGACCGAAGTGAAGTCCCTGGACCAGCCCGGCCCGGGCCCCCCGGTATCGGGCCAGAGGTAAGTGGACTTTAATTTTTTCTGCTAAGCCCAACACTCCACCACACCCAGGCACACACTACACACACCCACCCGTCTCAGGGTCCCCTCGGACAGCTCCTAAGAAGGCACCGGTCGCCCAGTCCTACCAGAGGGGGCCAAGAACCCAGACGAGTCCGTAGAAGGGTCCTCGTCCAGCAAGAAGAGGAGGTGGTAAGCGGTTCACCTTCAGGGGTAAGTAACCTGACCTCTCCAGGGCTCACATAAAGGGAGGCTTAGTATACATGCTTCTTGCTTTTCACAGGAACCTGGGGGCTAGTCTGGGTGGGATTAGGCTGCCTCAAGTTGCATCAGCCAGGGCTTCATGCCCTCCTCAGTTCCCTAGTCCCCGGGCTTCAGGCCCCCTCCGTCCCCGTCCTCCAGAGACCCGGGCTTCAGGCCCTGCCTCTCCTGTTACCCTTTTAGAACCACAGCCTGGACACATGTGCCAGACGCCTGGGCCTCTAAGGCCCTCGGGTCCCCCTGGACCCCGGCCTCAGCAACCCTGCTGCTCCCCTCCTGCCACCCCAGCCTCCCCCCCTCCCCGTCCCCCTTCGCTCCTGATCCTCCCCCGGTCCCCAGTAGGGCCGCCTGCCCCCCTGCACCCAGTACCTGCCCCTCTTGGCCACGCACCCCGGGCCAGGCCACCTTAGACCCGGCCAAGCCCCATCCCTGAAGACCCAGCGGCCATTCTCTCTGGTAACGAGCAGAGAAGAAGTAGAGGCCCGCGGCCATTGGGCCCAGATTGAGAGACCAGTCCAGGGGCCCGAGGTTGGAGCCAGCGGGCACCCGAGGTCCCAGCACCCGGTCCCTCCGGGGGGCAGAGACAGGCAGGGCCCCCCGGCAGCTGGCCCCGAGGAGGCGCCCGGAGTGGGGCCGGTCGGCTGGGCTGGCCGAGCCCGGGTCTGGGAGGTCTGGGGTGGCGAGCCTGCTGTCTCAGGAGGGGCCTGGCTCCGCCGGGTGGCCCTGGGGTACGTCTGGGAGGCAGAGGGTCGGCCTAGGCCCGGGGAAGTGGAGGGGGATCGCCCGGGTCTCTGTTGGCAGAGTCCGGGCGATCCTCTGAGACCCTCCGGGCCCGGACGGCCGCCCTCAGCCCCCCAGACAGACCCCAGGGTCTCCAGGCAGGGTCCGGCATCTCCAGGGGCAGCAGGCTCACCACCACAGGCCCCCCAGACCCGGGTCTCGGCCAGCCGAGCCGACCGGCCCCGCGCCGGGCGCCTCCTCGGGGCCAGCCGCCGGGGTTGGTTCTGCCCCTCTCTCTGTCCTTCAGAGGAACAAGGGACCTCGGGCACCCCAGAGCCCCTCGGGCCCGCCTCCAGGCGCCCTCCTGGTCTCCGCTCCCCTCTTAGCCCCGTTAAACCCAAAGAATGTCTGAGGGGAGCCACCCTCGGGGCCCAGGCCCCAGAGTCCAGAGGTCAGGGGCACCTCAGGGTGCCCCAGAGGCCGGTTCCTCGCCCCTTCCCCGGGCTTCAGAGCCCAGGATGTCCCCCAGAAGGGACCCTAGGCGTCCCCTCTCCTCCCCTCCAGGCTCGAGCCTCTCCCTCGCGGAGAGGGGCCTCTTTGGGCCCTCACGTCCAGCCCCACCGAGACCCGAGTGGCCCGGATCCCCCCACCGGCCCTTCTCTCTGTCCCCCTGCTCCTCTCCAACCTTCGCTCCACCCTAGACCCCAGCTTCTTGCCTCCCCACCGGGTCCATCAGGCCGGCCGGAGGGACCCCGGCGGCCCGGTGTCAGTCCCCCCTGCAGCCGCCCAGTCTCTGCCTCCAGGCAAGGGCGCCAGCTTTTCTCCCCCCAGCCTGAGGCCCAGGCTCCTGTGCACTGTCTGTAAAGTCCAGCCTCCCACGCCCGTCCACGGCTCCCGGGCCCAGCCCCGTCCACCCCTCCCCACGGTGGACAGGCCCTCTGTCCACCCGGGCCATCCCCGCCCCCCTGTGTCCACCCCAGTCCCGTCCAGGGGGGACTTTATGTGACCCTTGGGCCTGGCTCCCCATAGACTCCCATGTAAGCCTGCCTCGAGTAGGTGCCTCCAGAGCCCCTTTTGCCCCCCTGGCGGCCCAGCCCGACCCCCGGGCGCCCCCAAACTTTGTCCAGATGTCCAGGGGTCCCCGAGGGCGAGGCCCAGCCCCCTCCCACCCCTGTCCACTGCCCCGGTCCCCCCAGAAGCCCCCAAAAGTAGAGGCTCAGGCCATGCGCGCCCTGTCACCAGGCCTGCCAAAGAGCCAGATCTAAGGCCAGGAGAGGCAGCCCCAAAGCGGGTGCAGTAACAGGTAATCTCTGGTAGTGATTTGGACCCAAAATCTGACACTTTAGAGCTCTGGAGGACTTTAAAACTCTAAAAATCAAAACTTTAGAGGCGAATGGGCGCCATTTTGTCCCCACGCGCGCATAATGGCGGACCTAGGCCTAAAACCCCCAGGAAGCGGGTCTATGGTTGGCTGCGCTGCTGCTATCTTTAGAGGGGAAAAGAGGAATAAGCCCCCAGACAGGGGAGTGGGCTTGTTTGTGACTTCACCAAAGGTCAGGGCCCAAGGGGGTTCGCGTTGCTAGGCCACCTTCTCAGTCCAGCGCGTTTACGTAAGCCAGACAGCAGCCAATTGTCAGTTCTAGGGAGGGGGACCACTGCCCCTGGTATAAAGTGGTCCTGCAGCTATTTCTGGTCGCATCAGAGCGCCAGGAGTCCACACAAATGTAAGAGGGGGTCTTCTACCTCTCCCTAGCCCTCCGCCCCCTCCAAGGACTCGGGCCCAGTTTCTAACTTTTTCCCCTTCCCTCCCTCGTCTTGCCCTGCGCCCGGGGCCACCTTCATCACCGTCGCTGACTCCGCCATCCAAGCCTAGGGGAGACCGAAGTGAAGTCCCTGGACCAGCCCGGCCCGGGCCCCCCGGTATCGGGCCAGAGGTAAGTGGACTTTAATTTTTTCTGCTAAGCCCAACACTCCACCACACCCAGGCACACACTACACACACCCACCCGTCTCAGGGTCCCCTCGGACAGCTCCTAAGAAGGCACCGGTCGCCCAGTCCTACCAGAGGGGGCCAAGAACCCAGACGAGTCCGTAGAAGGGTCCTCGTCCAGCAAGAAGAGGAGGTGGTAAGCGGTTCACCTTCAGGGGTAAGTAACCTGACCTCTCCAGGGCTCACATAAAGGGAGGCTTAGTATACATGCTTCTTGCTTTTCACAGGAACCTGGGGGCTAGTCTGGGTGGGATTAGGCTGCCTCAAGTTGCATCAGCCAGGGCTTCATGCCCTCCTCAGTTCCCTAGTCCCCGGGCTTCAGGCCCCCTCCGTCCCCGTCCTCCAGAGACCCGGGCTTCAGGCCCTGCCTCTCCTGTTACCCTTTTAGAACCACAGCCTGGACACATGTGCCAGACGCCTGGGCCTCTAAGGCCCTCGGGTCCCCCTGGACCCCGGCCTCAGCAACCCTGCTGCTCCCCTCCTGCCACCCCAGCCTCCCCCCCTCCCCGTCCCCCTTCGCTCCTGATCCTCCCCCGGTCCCCAGTAGGGCCGCCTGCCCCCCTGCACCCAGTACCTGCCCCTCTTGGCCACGCACCCCGGGCCAGGCCACCTTAGACCCGGCCAAGCCCCATCCCTGAAGACCCAGCGGCCATTCTCTCTGGTAACGAGCAGAGAAGAAGTAGAGGCCCGCGGCCATTGGGCCCAGATTGAGAGACCAGTCCAGGGGCCCGAGGTTGGAGCCAGCGGGCACCCGAGGTCCCAGCACCCGGTCCCTCCGGGGGGCAGAGACAGGCAGGGCCCCCCGGCAGCTGGCCCCGAGGAGGCGCCCGGAGTGGGGCCGGTCGGCTGGGCTGGCCGAGCCCGGGTCTGGGAGGTCTGGGGTGGCGAGCCTGCTGTCTCAGGAGGGGCCTGGCTCCGCCGGGTGGCCCTGGGGTACGTCTGGGAGGCAGAGGGTCGGCCTAGGCCCGGGGAAGTGGAGGGGGATCGCCCGGGTCTCTGTTGGCAGAGTCCGGGCGATCCTCTGAGACCCTCCGGGCCCGGACGGCCGCCCTCAGCCCCCCAGACAGACCCCAGGGTCTCCAGGCAGGGTCCGGCATCTCCAGGGGCAGCAGGCTCACCACCACAGGCCCCCCAGACCCGGGTCTCGGCCAGCCGAGCCGACCGGCCCCGCGCCGGGCGCCTCCTCGGGGCCAGCCGCCGGGGTTGGTTCTGCCCCTCTCTCTGTCCTTCAGAGGAACAAGGGACCTCGGGCACCCCAGAGCCCCTCGGGCCCGCCTCCAGGCGCCCTCCTGGTCTCCGCTCCCCTCTTAGCCCCGTTAAACCCAAAGAATGTCTGAGGGGAGCCACCCTCGGGGCCCAGGCCCCAGAGTCCAGAGGTCAGGGGCACCTCAGGGTGCCCCAGAGGCCGGTTCCTCGCCCCTTCCCCGGGCTTCAGAGCCCAGGATGTCCCCCAGAAGGGACCCTAGGCGTCCCCTCTCCTCCCCTCCAGGCTCGAGCCTCTCCCTCGCGGAGAGGGGCCTCTTTGGGCCCTCACGTCCAGCCCCACCGAGACCCGAGTGGCCCGGATCCCCCCACCGGCCCTTCTCTCTGTCCCCCTGCTCCTCTCCAACCTTCGCTCCACCCTAGACCCCAGCTTCTTGCCTCCCCACCGGGTCCATCAGGCCGGCCGGAGGGACCCCGGCGGCCCGGTGTCAGTCCCCCCTGCAGCCGCCCAGTCTCTGCCTCCAGGCAAGGGCGCCAGCTTTTCTCCCCCCAGCCTGAGGCCCAGGCTCCTGTGCACTGTCTGTAAAGTCCAGCCTCCCACGCCCGTCCACGGCTCCCGGGCCCAGCCCCGTCCACCCCTCCCCACGGTGGACAGGCCCTCTGTCCACCCGGGCCATCCCCGCCCCCCTGTGTCCACCCCAGTCCCGTCCAGGGGGGACTTTATGTGACCCTTGGGCCTGGCTCCCCATAGACTCCCATGTAAGCCTGCCTCGAGTAGGTGCCTCCAGAGCCCCTTTTGCCCCCCTGGCGGCCCAGCCCGACCCCCGGGCGCCCCCAAACTTTGTCCAGATGTCCAGGGGTCCCCGAGGGCGAGGCCCAGCCCCCTCCCACCCCTGTCCACTGCCCCGGTCCCCCCAGAAGCCCCCAAAAGTAGAGGCTCAGGCCATGCGCGCCCTGTCACCAGGCCTGCCAAAGAGCCAGATCTAAGGCCAGGAGAGGCAGCCCCAAAGCGGGTGCAGTAACAGGTAATCTCTGGTAGTGATTTGGACCCAAAATCTGACACTTTAGAGCTCTGGAGGACTTTAAAACTCTAAAAATCAAAACTTTAGAGGCGAATGGGCGCCATTTTGTCCCCACGCGCGCATAATGGCGGACCTAGGCCTAAAACCCCCAGGAAGCGGGTCTATGGTTGGCTGCGCTGCTGCTATCTTTAGAGGGGAAAAGAGGAATAAGCCCCCAGACAGGGGAGTGGGCTTGTTTGTGACTTCACCAAAGGTCAGGGCCCAAGGGGGTTCGCGTTGCTAGGCCACCTTCTCAGTCCAGCGCGTTTACGTAAGCCAGACAGCAGCCAATTGTCAGTTCTAGGGAGGGGGACCACTGCCCCTGGTATAAAGTGGTCCTGCAGCTATTTCTGGTCGCATCAGAGCGCCAGGAGTCCACACAAATGTAAGAGGGGGTCTTCTACCTCTCCCTAGCCCTCCGCCCCCTCCAAGGACTCGGGCCCAGTTTCTAACTTTTTCCCCTTCCCTCCCTCGTCTTGCCCTGCGCCCGGGGCCACCTTCATCACCGTCGCTGACTCCGCCATCCAAGCCTAGGGGAGACCGAAGTGAAGTCCCTGGACCAGCCCGGCCCGGGCCCCCCGGTATCGGGCCAGAGGTAAGTGGACTTTAATTTTTTCTGCTAAGCCCAACACTCCACCACACCCAGGCACACACTACACACACCCACCCGTCTCAGGGTCCCCTCGGACAGCTCCTAAGAAGGCACCGGTCGCCCAGTCCTACCAGAGGGGGCCAAGAACCCAGACGAGTCCGTAGAAGGGTCCTCGTCCAGCAAGAAGAGGAGGTGGTAAGCGGTTCACCTTCAGGGGTAAGTAACCTGACCTCTCCAGGGCTCACATAAAGGGAGGCTTAGTATACATGCTTCTTGCTTTTCACAGGAACCTGGGGGCTAGTCTGGGTGGGATTAGGCTGCCTCAAGTTGCATCAGCCAGGGCTTCATGCCCTCCTCAGTTCCCTAGTCCCCGGGCTTCAGGCCCCCTCCGTCCCCGTCCTCCAGAGACCCGGGCTTCAGGCCCTGCCTCTCCTGTTACCCTTTTAGAACCACAGCCTGGACACATGTGCCAGACGCCTGGGCCTCTAAGGCCCTCGGGTCCCCCTGGACCCCGGCCTCAGCAACCCTGCTGCTCCCCTCCTGCCACCCCAGCCTCCCCCCTCCCCGTCCCCCTTCGCTCCTGATCCTCCCCCGGTCCCCAGTAGGGCCGCCTGCCCCCCTGCACCCAGTACCTGCCCCTCTTGGCCACGCACCCCGGGCCAGGCCACCTTAGACCCGGCCAAGCCCCATCCCTGAAGACCCAGCGGCCATTCTCTCTGGTAACGAGCAGAGAAGAAGTAGAGGCCCGCGGCCATTGGGCCCAGATTGAGAGACCAGTCCAGGGGCCCGAGGTTGGAGCCAGCGGGCACCCGAGGTCCCAGCACCCGGTCCCTCCGGGGGGCAGAGACAGGCAGGGCCCCCCGGCAGCTGGCCCCGAGGAGGCGCCCGGAGTGGGGCCGGTCGGCTGGGCTGGCCGAGCCCGGGTCTGGGAGGTCTGGGGTGGCGAGCCTGCTGTCTCAGGAGGGGCCTGGCTCCGCCGGGTGGCCCTGGGGTACGTCTGGGAGGCAGAGGGTCGGCCTAGGCCCGGGGAAGTGGAGGGGGATCGCCCGGGTCTCTGTTGGCAGAGTCCGGGCGATCCTCTGAGACCCTCCGGGCCCGGACGGCCGCCCTCAGCCCCCCAGACAGACCCCAGGGTCTCCAGGCAGGGTCCGGCATCTCCAGGGGCAGCAGGCTCACCACCACAGGCCCCCCAGACCCGGGTCTCGGCCAGCCGAGCCGACCGGCCCCGCGCCGGGCGCCTCCTCGGGGCCAGCCGCCGGGGTTGGTTCTGCCCCTCTCTCTGTCCTTCAGAGGAACAAGGGACCTCGGGCACCCCAGAGCCCCTCGGGCCCGCCTCCAGGCGCCCTCCTGGTCTCCGCTCCCCTCTTAGCCCCGTTAAACCCAAAGAATGTCTGAGGGGAGCCACCCTCGGGGCCCAGGCCCCAGAGTCCAGAGGTCAGGGGCACCTCAGGGTGCCCCAGAGGCCGGTTCCTCGCCCCTTCCCCGGGCTTCAGAGCCCAGGATGTCCCCCAGAAGGGACCCTAGGCGTCCCCTCTCCTCCCCTCCAGGCTCGAGCCTCTCCCTCGCGGAGAGGGGCCCTTTGGGCCCTCACGTCCAGCCCCACCGAGACCCGAGTGGCCCGGATCCCCCACCGGCCCTTCTCTCTGTCCCCCTGCTCCTCTCCAACCTTCGCTCCACCCTAGACCCCAGCTTCTCCGTCCCCGTCCTCCAGAGACCCGGGCTTCAGGCCCTGCCTCTCCTGTTACCCTTTTAGAACCACAGCCTGGACACATGTGCCAGACGCCTGGGCCTCTAAGGCCCTCGGGTCCCCCTGGACCCCGGCCTCAGCAACCCTGCTGCTCCCCTCCTGCCACCCCAGCCTCCCCCCCTCCCCGTCCCCCTTCGCTCCTGATCCTCCCCCGGTCCCCAGTAGGGCCGCCTGCCCCCCTGCACCCAGTACCTGCCCCTCTTGGCCACGCACCCCGGGCCAGGCCACCTTAGACCCGGCCAAGCCCCATCCCTGAAGACCCAGCGGCCATTCTCTCTGGTAACGAGCAGAGAAGAAGTAGAGGCCCGCGGCCATTGGGCCCAGATTGAGAGACCAGTCCAGGGGCCCGAGGTTGGAGCCAGCGGGCACCCGAGGTCCCAGCACCCGGTCCCTCCGGGGGGCAGAGACAGGCAGGGCCCCCCGGCAGCTGGCCCCGAGGAGGCGCCCGGAGTGGGGCCGGTCGGCTGGGCTGGCCGAGCCCGGGTCTGGGAGGTCTGGGGTGGCGAGCCTGCTGTCTCAGGAGGGGCCTGGCTCCGCCGGGTGGCCCTGGGGTACGTCTGGGAGGCAGAGGGTCGGCCTAGGCCCGGGGAAGTGGAGGGGGATCGCCCGGGTCTCTGTTGGCAGAGTCCGGGCGATCCTCTGAGACCCTCCGGGCCCGGACGGCCGCCCTCAGCCCCCCAGACAGACCCCAGGGTCTCCAGGCAGGGTCCGGCATCTCCAGGGGCAGCAGGCTCACCACCACAGGCCCCCCAGACCCGGGTCTCGGCCAGCCGAGCCGACCGGCCCCGCGCCGGGCGCCTCCTCGGGGCCAGCCGCCGGGGTTGGTTCTGCCCCTCTCTCTGTCCTTCAGAGGAACAAGGGACCTCGGGCACCCCAGAGCCCCTCGGGCCCGCCTCCAGGCGCCCTCCTGGTCTCCGCTCCCCTCTTAGCCCCGTTAAACCCAAAGAATGTCTGAGGGGAGCCACCCTCGGGGCCCAGGCCCCAGAGTCCAGAGGTCAGGGGCACCTCAGGGTGCCCCAGAGGCCGGTTCCTCGCCCCTTCCCCGGGCTTCAGAGCCCAGGATGTCCCCCAGAAGGGACCCTAGGCGTCCCCTCTCCTCCCCTCCAGGCTCGAGCCTCTCCCTCGCGGAGAGGGGCCTCTTTGGGCCCTCACGTCCAGCCCCACCGAGACCCGAGTGGCCCGGATCCCCCCACCGGCCCTTCTCTCTGTCCCCCTGCTCCTCTCCAACCTTCGCTCCACCCTAGACCCCAGCTTCTTGCCTCCCCACCGGGTCCATCAGGCCGGCCGGAGGGACCCCGGCGGCCCGGTGTCAGTCCCCCCTGCAGCCGCCCAGTCTCTGCCTCCAGGCAAGGGCGCCAGCTTTTCTCCCCCCAGCCTGAGGCCCAGGCTCCTGTGCACTGTCTGTAAAGTCCAGCCTCCCACGCCCGTCCACGGCTCCCGGGCCCAGCCCCGTCCACCCCTCCCCACGGTGGACAGGCCCTCTGTCCACCCGGGCCATCCCCGCCCCCCTGTGTCCACCCCAGTCCCGTCCAGGGGGGACTTTATGTGACCCTTGGGCCTGGCTCCCCATAGACTCCCATGTAAGCCTGCCTCGAGTAGGTGCCTCCAGAGCCCCTTTTGCCCCCCTGGCGGCCCAGCCCGACCCCCGGGCGCCCCCAAACTTTGTCCAGATGTCCAGGGGTCCCCGAGGGCGAGGCCCAGCCCCCTCCCTCCCAAGCTGCTTTGATTCCTGGGATATTTTTGGGAATGGTGTTAACTTTCTCCCCTTGTATTTGCTATTCAATCAACCTGATTCCCCCTGCTCATACCTCCACTTACAACCAAGCCACTACGGCCACGTCCCCGACCTCCCGCTCGGGTAAGTGCTTTTTCATTTTTAGCCCCAGCCCCTCCTCTATAAGTTATAGGCAAACCTCCAATCACCAGCCACCTTCCACTGTAGTCTCTTAGAGAGTGGCTGCTACGCATTAGAGACAGATTTGAGCCACCCCCAGTAACCACCCAGCGCCAATCTGTCTACATAGAAGAAGAAGAGGATGAAGACTAAGTCACAGGCTTAGCCAGGTGATTTGTGAATTTCAGTTTATTTACTTTCTTCCAATCAAGCTTTCCCAGCCTCCGCTTGTTAGGTCCTAGTTATGGGTTTTCCATGGGGGACTTAGTATCCGTTCTATTAGATTAACGTGCAAGACGCTAAACTTAACCAAGGTCAGCCAAGGGACGCGTGTTATCCCAGGCTGCCCACCCTGAGGATTTCCCCCCAAAATCCTCCTACCCTCTCTTTATGCCATGTGTGTTGTTGGCTTGTGTTAGTGCTATGTAATGCGTTGCCGCCAGGTGGCAGCCTGTTTATAGATGTGCAGTACCCCTTAATGTTAGGTCTGCTTTAGGGCTGCCAGGTGGCGCAATCTAGGATTAATTCACCTGTATCCCTTTCCCTCCACCCGCAGTAACCCAGCACTGGCGTGTGACGTGGTGTAAAGTTTTGCCTGAACCTGTGGTTGGGCAGGTACATGCCAACAACCTTCTAAGCACCCGCGCTTGTGTTTTGCTTTATCTGCCGCCATCATGCCTACATTCTATCTTGCGTTACATGGGGGACAAACATATCATCTAATTGTTGACACGGATAGTCTTGGAAACCCGTCACTCTCAGTAATTCCCTCGAATCCCTACCAGGAACAACTGTCAGACACTCCATTAATTCCACTAACAATCTTTGTTGGGGAAAACACGGGGGTGCCCCCACCACCTTCACCACCTTCACCACCACCCCCGCCCCCACCACCCCCACCACCTCAGCGCAGGGATGCCTGGACACAAGAGCCATCACCTCTTGATAGGGATCCGCTAGGATATGACGTCGGGCATGGACCTCTAGCATCTGCTATGCGAATGCTTTGGATGGCTAATTATATTGTAAGACAATCACGGGGTGACCGGGGCCTTATTTTGCCACAAGGCCCACAAACAGCCCCTCAGGCCATGTTGGTACAGCCACATGTCCCCCCTCTACGCCCGACAGCACCCACCATTTTGTCACCTCTGTCACAACCGAGGCTTACCCCTCCACAACCACTCATGATGCCACCAAGGCCTACCCCTCCTACCCCTCTGCCACCTGCAACACTAACGGTGCCACCAAGGCCTACCCGTCCTACCACTCTGCCACCCACACCACTACTCACGGTACTACAAAGGCCTACCGAACTTCAACCCACACCATCACCACCACGCATGCATCTCCCTGTCTTGCATGTGCCAGACCAATCAATGCACCCTCTTACTCATCAAAGCACCCCAAATGATCCAGATAGTCCAGAACCACGGTCCCCGACTGTATTTTATAACATTCCACCTATGCCATTACCCCCCTCACAATTGCCACCACCAGCAGCACCAGCACAGCCACCTCCAGGGGTCATCAACGACCAACAATTACATCATCTACCCTCGGGGCCACCATGGTGGCCACCCATCTGCGACCCCCCGCAACCCTCTAAGACTCAAGGCCAGAGCCGGGGACAGAACAAGCAACGCAAGCCCGGTGGACCATGGAGACCAGAGCCAAACACCTCCAGTCCTAGCATGCCTGAACTAAGTCCAGTCCTCGGTCTTCATCAGGGACAAGGGGCTGGGGACTCACCAACTCCTGGCCCATCCAATGCCGCCCCCGTTTGTGGAAATTCACACACGGCAACCCCTAACGTTTCACCAATACATGAACCGGAGTCCCATAATAGCCCAGAGGCTCCCATTCTCTTCCCCGATGATTGGTATCCTCCATCTATAGACCCCGCAGACTTAGACGAAAGTTGGGATTACATTTTTGAGACAACAGAATCTCCTAGCTCAGATGAAGATTATGTGGAGGGACCCAGTAAAAGACCTCGCCCCTCCATCCAGTAAAAACCCTTGCCCTCTCCAGCAAACAATGTATCCCAAATAAATGTTACTTCTTTTGCTCTTAACCATTGACACGCCTGTCATTCTATCAATTAAACAAGGGAAAAGGTTTAGCTATTCCACCAACACGACCCCAAGGAAGGCTTGCCAAAATTGGTGCCTTGCTCTCAGCACTTTGCCAGCAACTTATAGCATGGTAGGCAGCTCAACTCGGCCCGTCTTACTGCCCAGCCTACTCTCCACTCCCAGTCCATGTTCGCACTCCTATGCATTTCCTGCCCTCCCACTTTTACCCCAGTCCCAACCCAAAACCACACACAACACATAGAATTGTTAGTTTAAACAGTTTATTGATACATGGCTGCTTTTAGCCTAATTGTGTATTGCTCTCGTTGCCAAAACCTGGTGTAAGGGCCGGCACCCGCAACATGGGGAAAACATAACCGCCGCCATCCCATGGGGAGGGTAGAGGCGGTTGACATGTAGGTGAGTAGTGTAAGAAGCATGGCGAAGTAGACAGGTTACTTTTAGAGTGTAGTGTACAGGGCCGGGCGCAACAGTGCCACCAACCCGGGGTCTGAGCATTCCATGGGCAGCAGGGACACTGCACTACCGCCAGGTCCAGGGGCAGCCGGGGTTCCTGGCGCTCCGGGGGCAGCAGGGCGGCCGCCGGTGGGTCCGCTGGGCCGCTGCCCCGCTCCGGGTGGGGGGTGGCCCCGCTGGGCACCGCTGCGCCGCCGCCAGGTCCCGGGGCAGCCGGGGTTCCTGGCGCTCCGGGGGCAGCCGGGCGGCCGCCGGTGGGTCCGCTGGGCCGCTGCCCCGCTCCGGGTGGGGGGTGGCCCCGCTGGGCACCGCTGCGCCGCCGCCAGGTCCCGGGGCAGCCGGGGTTCCTGGCGCTCCGGGGGCAGCCGGGCGGCCGCCGGTGGGTCCGCTGGGCCGCTGCCCCGCTCCGGGTGGGGGGTGGCCCCGCTGGGCACCGCTGCGCCGCCGCCAGGTCCCGGGGCAGCCGGGGTTCCTGGCGCTCCGGGGGCAGCCGGGCGGCCGCCGGTGGGTCCGCTGGGCCGCTGCCCCGCTCCGGGTGGGGGGTGGCCCCGCTGGGCACCGCTGCGCCGCCGCCAGGTCCCGGGGCAGCCGGGGTTCCTGGCGCTCCGGGGGCAGCCGGGCGGCCGCCGGTGGGTCCGCTGGGCCGCTGCCCCGCTCCGGGTGGGGGGTGGCCCCGCTGGGCACCGCTGCGCCGCCGCCAGGTCCTGGGGCAGCCGGGGTTCCTGGCGCTCCGGGGGCAGCCGGGCGGCCGCCGGTGGGTCCGCTGGGCCGCTGCCCCGCTCCGGGTGGGGGGTGGCCCCGCTGGGCACCGCTGCGCCGCCGCCAGGTCCCGGGGCAGCCGGGGTTCCTGGCGCTCCGGGGGCAGCCGGGCGGCCGCCGGTGGGTCCGCTGGGCCGCTGCCCCGCTCCGGGTGGGGGGTGGCCCCGCTGGGCACCGCTGCGCCGCCGCCAGGTCCTGGGGCAGCCGGGGTTCCTGGCGCTCCGGGGGCAGCCGGGCGGCCGCCGGTGGGTCCGCTGGGCCGCTGCCCCGCTCCGGGTGGGGGGTGGCCCCGCTGGGCACCGCTGCGCCGCCGCCAGGTCCCGGGGCAGCCGGGGTTCCTGGCGCTCCGGGGGCAGCCGGGCGGCCGCCGGTGGGTCCGCTGGGCCGCTGCCCCGCTCCGGGTGGGGGGTGGCCCCGCTGGGCACCGCTGCGCCGCCGCCAGGTCCTGGGGCAGCCGGGGTTCCTGGCGCTCCGGGGGCAGCCGGGCGGCCGCCGGTGGGTCCGCTGGGCCGCTGCCCCGCTCCGGGTGGGGGGTGGCCCCGCTGGGCACCGCTGCGCCGCCGCCAGGTCCTGGGGCAGCCGGGGTTCCTGGCGCTCCGGGGGCAGCCGGGCGGCCGCCGGTGGGTCCGCTGGGCCGCTGCCCCGCTCCGGGTGGGGGGTGGCCCCGCTGGGCACCGCTGCGCCGCCGCCAGGTCCTGGGGCAGCCGGGGTTCCTGGCGCTCCGGGGGCAGCCGGGCGGCCGCCGGTGGGTCCGCTGGGCCGCTGCCCCGCTCCGGGTGGGGGGTGGCCCCGCTGGGCACCGCTGCGCCGCCGCCAGGTCCCGGGGCAGCCGGGGTTCCTGGCGCTCCGGGGGCAGCCGGGCGGCCGCCGGTGGGTCCGCTGGGCCGCTGCCCCGCTCCGGGTGGGGGGTGGCCCCGCTGGGCACCGCTGCGCCGCCGCCAGGTCCTGGGGCAGCCGGGGTTCCTGGCGCTCCGGGGGCAGCCGGGCGGCCGCCGGTGGGTCCGCTGGGCCGCTGCCCCGCTCCGGGTGGGGGGTGGCCCCGCTGGGCACCGCTGCGCCGCCGCCAGGTCCTGGGGCAGCCGGGGTTCCTGGCGCTCCACTGCACCTGGAATGCAGGGTGGGGGCGTGGTCCCCTGGACCCCAGCCCCGCCGATCCCTCCCCCAGGGCGTACCCGGCTTGCCTGGTTCTGGGGCTCCTCTGGGGGTCGCTGCATCCGCCGGTAGGGTTCGAATGGGCGTGGTCCGCTTGCTCTGCTGGCCCGGTACGCCTGGATTGCCGGCTGGGGGCTGGGGTCCCGGGACGCCCCCTCCCTGCTCCCACCCGGTTCCCTCCCCCAGGGCGTGCCCCGCTTGCCTGGTCCTGGAGCTCATCCGGGGATGCTGCATCCGCTAGTCCGACCTGGGTGGGTGCGGTCCGCTGGCCCCACCCTGGGGGTCGCCGCCGGGTCTGCTGGTCCGGTGCACCTGGAAGGCAGGGGGGGGGGGCAGTAAGGGAGGGGCGTGGTCCTGGGACCCCGCGCCGACTGGCAGGGGGTCCCCATGGCACAGGGCTAGGGGTCCAGGGGGCAGCCGCGGCCCAGCGCGCCCCGTTCACGGGGGAGGACCGCGGCCGAGCCGCCAGGGGCCCGGCGGGGGTGGGGGGTGCGCTCCCAGGCCGGACCCTGGTGCCAGGCAGGGACCCCGCGCCACCCGCTTCATGGGGGGGGAGGCCGCCGCAAGGACGCCGGGCCGGCTGGGAGGTGTGCACCCCCCGAGCGTCTGGACGACGCTGGCGAGCCGGGCCAGCTCGCCTTCTTTTATCCTCTTTTTGGGGTCTCTGTGCAATACCTTAAGGTTTGCTCAGGAGTGGGGGCTTCTTATTGGTTAATTCAGGTGTGTCATTTTAGCCCGTTGGGTTTCATTAAGGTGTGTCACCAGGTGGGTGGTACCTGGAGGTCATTCTATTGGGATAACGAGAGGAGGAGGGGCTAGAGGTCCGCGAGATTTGGGGTAGGCGGAGCCTCAGGAGGGTCCCCTCCATAGGGTTGAACCAGGAGGGGGAGGATTGGGCTCCGCCCCGATATACCTAGTGGGTGGAGCCTAGAGGTAGGTATCCATAGGGTTCCATTATCCTGGAGGTATCCTAAGCTCCGCCCCTATATACCAGGTGGGTGGAGCTAGGTAGGATTCAGCTAGGTTCCTACTGGGGTACCCCCCTACCCTACCTTAAGGTGCGCCACCCTTCCTCCTTCCGTTTTAATGGTAGAATAACCTATAGGTTATTAACCTAGTGGTGGAATGGGGTATTGCAGCTGGGTATATACCTATAGGTATATAGAACCTAGAGGAAGGGAACCCTATAGTGTAATCCCTCCCCCCCCTACCCCCCCCTCCCTTACGGTTGCCTGAGCCCATCCCCCACCCCAGCACCCCGGGGTGACGTGGCCCCCCGCGTGCCTTACTGACTTGTCACCTTTGCACATTTGGTCAGCTGACCGATGCTCGCCACTTCCTGGGTCATGACCTGGCCTGTGCCTTGTCCCGTGGACAATGTCCCTCCAGCGTGGTGGCTGCCTTTGGGATGCATCACTTTGAGCCACTAAGCCCCCGTTGCTCGCCTTGCCTGCCTCACCATGACACACTAAGCCCCTGCTAATCCATGAGCCCCGCCTTTAGGAAGCACCACGTCCCGGGGACGGAAGGGGACTTGGGGTGATTTTCTATGTGGGGGTGGAAATATGAGCAAGAATAAGGACGGCTCCTTATTAACCTGATCAGCCCCGGAGTTGCCTGTTTCATCACTAACCCCGGGCCTGAAGAGGTTGACAAGAAGGGTCAAGGTTTCGTCTGTGTGTTGAAGGGCAGGGGCTGTTGGGTGCATCTGGAACGGCTTACCTCGGGTAACTGTTTGCCATTAAAAGGTTGGGGATTAGGTTTAGCCCCTTTAGCTGCCATTTCGAACCGGGGTGTGCAGATGCAGGTCTCCGGGTGGGCAGGCAGTACGAGATGTCACGTTGTGTTGTCTTTCCTCCCACCCCTGTCCTGGCTGTGGCAAATGCGACCCTCATAGAGTTGTGTTTCAGGTCTGTGTCCTGTTTTGCGGTGGGTTATTTCCCCCCTCAGTGTTCGCCAGCTTATTTCCCCAGTCCCCACGTACTGGGGCCTGTGGACACCTGAGGGTGCGGTCGTTGGTGGGCATGTGTTGGAATTGCTCCCACCCTCAATTTTCGCTTGCCTTCTTCCCTTGTTAACCTGATAGCATAGCCTCTAGGTTTCCTTGTAGGTCTGTTTGGGTTTGGTGGTTCACGTGGTGCTAACTTGAATTTTTTGGTTTTCTAGTTCCCTCTTAATTACATTTGTGCCAGATCTTGTAGAGCAAGATGGCCTATTCAACAAGGGAGATACTGTTAGCCCTGTGTATACGGGACAGTCGTGTGCATGGAAATGGTACCCTGCATCCTGTGTTGGAGCTAGCAGCAAGAGAAACACCTCTCCGCCTTTCGCCAGAGGACACTGTAGTTCTGCGTTATCATGTGTTGCTTGAGGAGATAATTGAACGAAATTCAGAGACATTTACAGAAACTTGGAACAGATTTATAACACACACCGAACATGTGGACCTGGATTTTAACTCAGTATTTGTAGAGATATTTCACCGTGGAGACCCAAGCCTTGGGCGCGCGTTGGCCTGGATGGCCTGGTGCATGCATGCCTGCAGGACATTGTGTTGTAACCAGTCTACTCCTTACTATGTTGTGGACCTGTCAGTTCGTGGGATGTTAGAAGCCAGCGAAGGCCTGGATGGTTGGATTCATCAACAGGGCGGCTGGTCTACATTAATTGAAGACAACATTCCTGGATCCAGAAGGTTTAGCTGGACTTTGTTTCTTGCTGGACTGACTTTGAGTCTGTTAGTTATATGTAGTTATTTATTTATCTCCAGAGGAAGACACTAATCTATACATTTTCTCAGCACTTTATATGAATCAGGGTCATTGGGCCTGCGGGGAACTGAGCCAGTAGGATATTAGGCAAGGGTGACACAGTGCCCATGCATTATAATTTAACCAAACAGTGGTCGTGAGTTTTAGGCCGGCCATGGGGGCTTACAAGAATAACATGCCAATGACCCGGCCCCCACTTTTAAATTCTGTTGCAGCAGATAGCTGATACCCAATGTTATCTTTTGCGGCAGAAATTGAAAGTGCTGGCCATATCTACAATTGGGTGTCCTAGGTGGGATATACGCCTGTGGTGTTCTAACGGGAAGTGTGTAAGCACACACGTAATTTGCAAGCGGTGCTTCACGCTCTTCGTTAAAATAACACAAGGACAAGATACTAAAGAAATAACTGAGGTGAGTGTGGGAAGATGGGACTACTATGTGTTATGTTAACGGGTGAGAGCCTATACTGCAGCCCAGACTCGGGGGGAGGAGGAAATGGTAAGAGTTATACTCTACTTATCTTTTTTGACACTACAGTTAACTGTTATGTAACAATGTTTGCTTATTTTCATGTTCAATAAATGCTATGTTAATGATGAAGAACCTGTGTTGTTCTTTGGAAGTGGGCCCGATGGGGTAGTAGGTTTTGGGAGGGTGCCGTGTTAGATATTTCAACTGCCACAGACCCCATTTTGTCCCACCTGTTACCACATTCTAGGTCCTGCATCCAGTGGGCCAGGTGTCTCACCATGGCTCTTTCTAGGTGGATACCACAGTCCAGGCCCCCAAGGCTACCGTGCTAATTACCTCCTCATGTCCACCCCCACCCTGTGTTACTGTCGCCTGATTATCCTGGCTTAGCAGCCTCCAAGTTTTACAAGACGTCCCATTGCCCTGCCCTTGGTCCAAGTCTCGCCGGTTTTCAGCAGCCTGTTGTAGCCTGCCCCCAAGTTTCGCAGGTCTCCCCCATGCTTCCACCCGTTAACCCAATAGCATGACAGCCAATCCAACACGAGGCAAGTTTCAAGAGTTAAAAGCAACTACTGTTTATTTTCCAAAATGAGCTGGGTATAGTTGATGATCTGTAGGCGCAGCTCATCCCCACATTCCAGGTCCTTGATGGCCTCGTAGATGGCATCTTCGTCGACATTGACAGCCTTCTCATATACTGTGTCTCTGGGGCTGACCTTTATGCAGAAGGCGTCCCCTACTAGGTCCACGGCCAGCTCGTAGGTGGGGCCTATGTTTTCACATAACAGTTTCAAGCAGGTCTCTGGGATGTGGAGGGAGGTGCCCTGGAGCAGGAGATGCATGATTAGGCGCCCTTTTCCATTTGTGCTGAAGATGGGGCAGATGGTGCCACAAAAGTGTCCGGTGACCAGGTAAGCGTAGAGAAGGCTGGGTTGGGAAAGTCCAGCCTTTACTGCACTGGGAGAGCTGCTGAGCAGAGACACATAGAAGGTCTTGTTGGGGATTATCTTGTGGACATTGTTGAAGAAGGAGAGCTGGGTGGAGCTAAACTCCTGAGGCACATGAACCTGGGACCTATTGATGCAGATCTCGCAGTGAGACCCCAGAGTCAGGCTGTGGCCAAAGGGAGACAGGCGAAGGCAGCGCCCGGGGGAGAGAGTGCACAGTGACAGTGGGAGAAACACGGCCTCTGAGACATGTATGGGGGTGTTCATCTCACGCAGAAAATCTTTGCCCAGCTCAAAGTTGGCAGAGATTCCCCTGAAGAAGTCCCGTAGTGAAAAATGGGATCTGTCTACACCATGTCTGGTGTGCCGGGAACATATTGAGCGGGCCACACTGCCAACCCTTTCCATTCTTCCCAGCTCTGAGCGAGATTTTCCACACCTGGACACCGACTTCACGCTATGCGCCGAGGCCTTTGAGGCCGTGTAGTTTCTGTGGTGCGGATGCATTAGGCGGCGCAATGCGGGATCTGCCGGTCGCTGTTGGCGTGCATTCACGGCCTCTGGGGTGACCGGGGCCATCGGGTTTACTTTTCACACGTAGACCTGGGAAGTTTGATAGGACTGTACCAGGTCAAGGCCGTGGATGCGCAGGACCACGTCCAGTTCCTTAGTGACATCCACGAGGATTGTTTTGCCCACTCTGGCCACTTGTGTGGATTTAAATATGTACACAAGCGTAATTAACGAGTCACAGACCCCCTGTTCCAGATTCTGACCGGCTGCAAGCGCTGCCTTAAAGGCCTGGAAGCTGGGTGGGTAAATCTGACCAAACAGCACGCTCGGATTCGTGATGCTGTGGTTGATGGCACACAGGGGGTCGCAGAACAGGTGCTTGTGGAAGTCTTGCGGGGTGCACATCTGCAGCCAGGCCCTTAGCCTGGGGCATGGCACATCCAGCAGCGTGTTTTGGGTCTTGATGAGGAACACGATCCTGTCTAGGATTTTGATGTTGTTGCCGAATGAGTCCAGAATCAGGCTCTTGAAGCGGTCAAGGGTGTCCTTGGCGTCCGGGTGGGCCCCGAGGCTCTCGCAGAGTGGGCAAATGGTCCGTGAGGCATTCTTGTGCCTTAGTCCAAACATGGGGGCCAGGAGGCAAGGGCCCTGCGAATGGTCGCCAGCCTCCGGTCTGGTGATGGCCAGGGCCAACTCCGCCAGCTCATCGCCACTGTATTCCGCGTTTAAACCGATAGCATGGTGGCCTGGCCCCCCGAGCAGGTCCGTCCCCTGCCACGTACCTAATAGTAGTCCACAGTAGTCGGCCTTGGTTGTAATTTCAGGAGAGAGTCCTCCCTTTTCGGCCCTGAGAAATGGATGCTGAACTCGGTTTCTGGTAGGCAGGTGGCAGCACAGGGCGGTGTACTGGCCCCTGCCGATGTCCCCTGGGACATCCTGGGAATCTTTGCAGGTTCTGGGTCCAGGGAGGGTAAGAAAAGTGGGGGTGGTTCTGGGCCACATGGACTTGAAGCAGAAGTTGGCCGGGGACTGGCCAGTGAGGATGGATTTCAGAAACTCCAATTTGTAGTAGCCGAGGTTGGCATTTCTAATCATGTCAGAAGAGGACACGGGGAGGAAGCACCGGCAAATGTAAAAGTGAAGCTGGATGTCAATGGCCAGAATCCTGGAGGGCATGAAGAGGGAATCCAACCCCCCGGCCATGGGGAAGTATTTTATCAGGATGTGTAAAAAGTCCATGCCCGTGATGAGGCTAGAGATCCAGGCTCGTGGGGCATTTAGACAGTAGTAGCAGAGCAGGGCATAGTCCTCAAAGAAGGCCACGGGGGCATCTGAGTGATTGACCAGGGTGTCGAGCAGATCACAAACTCGGCAGGTGCTGGCTGGAGAGAGGGACTCGTAGGTGTGGACGAGTGGTGGGTAGGCTATGCCTTCTTCCGCGTTGGCTGGAAGATAGGAGTGGGCCATCAAAAGGCCGACTGCCTCGAACTGGCTTTTCAGATTGTCCACGGTCCAGGGCACAAACTCCTCCATCTTTGGAGTTCTGCCCGCGGTCTGTGCCACCTCTGTTACGCCACTCCTCGTGAGGGGGCAGCTGGACAGTCTTTTTCCGGTCAGGGGGTTTGGCTCGTTTGCGCTCGTGACTTTGTGAGCCATGACACATCTGGGTGGCAAAGTGAGGTCTTCTGGGTTTTTAATACCGGGGTCGGCACCAGTTTCTGGGACACCGCCACAAGGACAAGGTGGGCTAGCAAGTTCTCGAGTCTATGAAGACTCCGGGGGCAGTTTTTTGAGTTTCTCGCCTATGATCCACCCCAATCTCGCCCCCATAATTGCGCCATCTGCCTACGCGAGGCTGAACCTCCTGAATCACTGCATCTTTCTCGAGGCGTTTAAAGAAGAGAATAGTGGCCAGGGCCTCGGTGGGGTCCAGCGTGAGGTCTTATTTTTGAAAAGGGATATTATAAAACAAGTCATTGCTCGGATTGTGGCAGCCGATAGCACCCTAGATCTAGTGAATCATGGCGAGTCCGGAAGAGAGGCTCCTAGACGAGCTCAATAACGTAATTGTGTCATTTCTGTGTGACTCTGGGTCTCTGGAAGTGGAGAGATGCTCCGGGGCGCATGTGTTCTCCAGGGGCAGCTCCCAACCCCTCTGCACCGTGAAGCTGCGCCACGGACAGATTTACCACCTGGAGTTTGTCTACAAGTTCCTGGCCTTTAAGCTGAAGAACTGCAACTACCCCTCCTCGCCTGTGTTTGTGATATCCAACAACGGCCTGGCCACCACCCTGAGGTGCTTTTTGCACGAGCCCTCGGGTCTCAGATCGGGCCAGAGCGGCCCTTGCCTGGGTCTCTCAACGGATGTTGACCTACCAAAGAACTCCATCATTATGCTGGGCCAGGATGACTTCATTAAGTTCAAAAGCCCCCTGGTCTTCCCTGCTGAGCTTGATCTCCTGAAATCTATGGTGGTCTGCCGGGCCTACATCACGGAACACCGGACGACGATGCAGTTTCTGGTGTTTCAGGCCGCCAACGCCCAGAAGGCCTCGCGGGTCATGGATATGATTAGTGATATGTCTCAGCAACTGTCTCGGTCTGGTCAAGTCGAGGATACGGGCGCCAGAGTCACAGGTGGAGGAGGTCCCAGGCCTAGCGTCACGCACTCGGGGTGTCTTGGGGACTCACACGTTAGGGGGCGCGGTGGTTGGGACTTGGATAACTTTTCAGAAGCTGAGACCGAAGACGAGGCGAGTTACGCTCCTTGGAGGGACAAAGACTCGTGGTCGGAATCCGAGGCGGCGCCGTGGAAGAAGGAACTCGTGAGGCACCCCATCCGCAGGCACCGGACACGCGAGACTCGCCGTATGCGCGGGAGCCATTCACGGGTGGAACACGTGCCCCCTGAGACCCGGGAGACGGTGGTGGGGGGAGCATGGCGTTATTCTTGGCGCGCCACACCTTATCTGGCACGGGTGCTGGCGGTCACGGCCGTGGCCCTGCTCCTGATGGTTCTGAGGTGGACCTGACGTTGCCGGCCCTTGGGGAGCGGGAGTTCTCCAGGCTCCTGGATCTGGGGCTGGCCTGCCTGGATCTGAGCTATGTGGAAATGAGGGAATTTGTGGTTTGGGGCAGGCCCCCAGCTTCTGAGGCGGCTGTGGCCTCTACGCCAGGCTCGCTTTTCCGAAGCCACTCGTCCGCCTACTGGTTGTCGGAGGTGGAGAGGCCCGGGGGCCTTGTCCGCTGGGCCAGGTCACAGACCAGCCCCTCATCCCTGACCCTCGCGCCCCATCTTGGCCCGTCCCTCTTGTCCCTTTCAGCGTTCACCGGTGGTGGGTGTGGAGCCGTGGCCTTTTGCAACGCCTTTTTCCTAGCTTATTTTTTGGTTGTGCGGTCTGTTTTCCCCGCGTTTTCTGATAGAATAGCTGCCTGGATCTGCGCCCGGTCCCCTTTCTGCGAAAACACCCGGGCCGTGGCCAGGGGTTACCGAGGCCTCGTGAAGAGGTTCTTGGCATTCGTGTTTGAGCGTAGTAGCTATGACCCCCCCTTGTTGAGGCAAAACTCTAGGCCTGTGGAGCGCTGCTTTGCCATCAAGAATTATGTCCCGGGCCTGGACTCACAAAGCTGTGTGACGGTCCCGAGCTTCTCCCGCTGGGCCCAGTCTCACGCCAGCGAGCTCGATCCCCGGGAGATTCGCGACAGAGTTACACCAGCGACTGCACCTTCGTTCGTGGCTGATCATGCCTCGGCTCTATTGGCCTCCCTCCAGAAGAAGGCCTCCGACACCCCCTGTGGGAATCCCATTCAGTGGATGTGGTACCGCCTGTTGGTAAACTCGTGCCTGAGGAGCGCCCACTGTCTTTTGCCTATACCTGCCGTCTCTGAGGGGGGGAGAAAGACGGGCGGGGGCGTAGGGGAGGAGCTCGTGGGGGCCGGGGGGCCCTGCCTGAGCCGGGATGTTTTCGTGGCGATCGTAAGCCGCAATGTTCTCTCGTGTCTGCTGAACGTGCCTGCCGCGGGTCCCTGGGCCTACAAGTGTTTCAGATCCCACGCCTCCAGACCGGTGTCTGGCCCGGATTACCCTCCCTTGGCCGTGTTTTGCATGGACTGCGGTTACTGCTTGAACTTTGGAAAGCAGACAGGTGTGGGAGGCAGGCTCAATTCCTTTAGACCCACTCTCCAGTTTTATCCCCGTGACCAGAAGGAGAAGCATGTGCTGACCTGCCATGCCAGTGGCCGTGTGTACTGCTCCAACTGCGGCTCTGCGGCGGTGGGCTTCCAGAGGCTGGCTGAGCCACCGAGCGCCCGCTCGGGCTGGCAGCCCCGAATCCGGGCGGTGCTGCCGCACAACGCGGCCTATGAGCTCGACCGTGGCTCCCGCCTCTTGGATGCCATCATCCCCTGCTTGGGACCCGACCGCACATGCATGCGGCCGGTGGTCCTGCGGGGGGTGACGGTCAGGCAGCTCCTGTATTTAACTTTGCGGACAGAGGCCAGAGCCGTTTGCTCCATCTGTCAGCAACGCCAAGCTCCAGAGGACGCCCGCGACGAGCCTCACCTGTTCTCCTCCTGTTTAGAGGTAGAATTGCCACCTGGTGAGCGGTGTGCGGGCTGCCGTCTCTATCAGACGCGTTATGGCACGCCGGCTGCCCAAGCCCACCCTCCAGGGGAGGCTGGAGGCGGATTTTCCAGACAGTCCCCTGCTTCCTAAATTTCAAGAGCTGAACCAGAATAATCTCCCCAATGATGTTTTTCGGGAGGCTCAAAGGAGTTACCTGGTATTTCTGACGTCCCAGTTCTGCTACGAAGAGTACGTGCAGAGGACTTTTGGGGTGCCTCGGCGCCAACGCGCCATAGACAAGAGGCAGAGAGCCAGTGTGGCTGGGGCTGGTGCTCATGCACACCTTGGCGGGTCATCCGCCACCCCCGTCCAGCAGGCTCAGGCCGCCGCATCCGCTGGGACTGGGGCCTTGGCATCATCAGCGCCGTCCACGGCCGTAGCCCAGTCCGCGACCCCCTCTGTTTCTTCATCTATTAGCAGCCTCCGGGCCGCGACTTCGGGGGCGACTGCCGCCGCCTCTGCCGCCGCAGCCGTCGATACCGGGTCAGGTGGCGGGGGACAACCCCAAGACACCGCCCCGCGCGGGGCACGTAAGAAACAGTAGAGGGCACGAAACATGGTGTATGCACTTTATTAATAAACAATTACAGATACAAAAACTTGAGTCTCTCGAGGTCTGCGATGAGGCGGTGGGTGGAACGCTCCAGCTTGCGGCGAAGCTGGCTCACGAAGCGAGACAGGACTCGGCTAGCCTGACTAAGGGTGAGGCTATAACGCAGGTCCTGTTCCGGAGCGGCGGTGGATAGAGAGGAGGGGGATCCGGAGGGGACCACTAGGTCGCCGGAGGTCGACCCTCCTGTCACCACCTCCCTGATAATGTCTTCAATAGACAGAAATTGGGTGACCACTGAGGGAGTGTTCCACAGTAATGTTGTCTGGTCGCTAGATGGCGCGGGTGAGGCCACGCTTTGCGAAAACGAAAGTGCTTGAAAAGGCGCGGGATAGCGTGCGCTACCGGATGGCGGGTAATACATGCTATCCTTACATTTTGGCATTTTGGGCAGCTGGGAGGCGGCGGATGGGGGTGCTTCTTTTTGCACGGTGTATGTTTGGGGACCCGCATGCCGGTACTGGGATAGGCGCACCTCGGGCCGCGCGCCAGGCTCCGAGCCGGAATGCATTGGGGGCAATGGGATTGCGGGGGATTGTTGCTGTCTGCTCCTGACAGGGAGAGACACGCGCGGTGGAGATGCAGCCGACGGCGGGGCCGCGGTGGGCTGCCCCCGAGGACGGGCGCCGGCCGCCAGCGCCCCCGTGGCCTTTGGCACGGGCCTGGCCCCCACCGCTTTAATTGTGGGGGTGGGCAGGGCAGCTGCATCTTGGGGCCTCTGTGCTTGCGTTTTTTGGGGGCTTGGTGCCAATGCACCAACTGGGGTGTGCGCGGGGGCGGCCAAGCCGGACCCCAGGGCGGGTGCCTGGGGGATGGGAAGGCCGGACGGCGCTTCTCCCGGGTCGAACGCTGGAGTAGCGGAGGCTGCTGCGCCGGCGGCCACCACGGGCGCACGGGGTCGCAGCCCGACGGCCGTGGGGAGGCGGGTGGCGGAGGGCCGAATCTCCGCGGCTTCTTCCCGGCCCCCCTGCTGTTTCTTCTCCCGTTGCATGATAGAATGGCCATAGGGTGGGTCCTGAGAGGAGGCTTGTGTGTCCTGGGGCTGGAGCCCAAAAGTCGTTAAAGACGCTGCTGATGGTGTGGGAGCTATGCCTCCCGTCGACTGGCCGGGCTTGTAGGGGGCTGAGGGTGGATAACTGGGCTTCTGTGAAGGCACCAACCCTGGAATCTGGATGGTATGTTTCTTCTGTGACCCCGAGGCAGTCGATGGTGTAGAGTGTGGAGACAATGTGTAGACGATGGGCCCTTGTTCAGAAGCCCAGGGACTTGAGGGGGGCTGTTGTGGTGCTGGTTGGGGAAGGAGCTCCAGGGAATCTTTGGGCCATGGCCTTGGGGAGGTTCCCGGAGACCGGTCTGGGCTCTCGGAAGCCCTCGTTTCGGCCCCGAAATAGGGCCTTGCCATCAATCGGGGGCCTGGGAGAGTGATGGGGGCGGCCAATCCCGGGGTAACTGTCACGTCCCGGGGGGAGGAGGTAGGAGACAGCCAGTCCCTGGGCCTGCCAGGGGCCACCTTCTCTAAGAGGGGGCTCTGTGGGCTGGGAGGGCCAGAGGCCTCAGATTCAGCCGTAGTGCTCCCCTCTTCCCCCTGGTCCGTCTCCCCTCCTCCCAACTGCTGGAGCCGGTCGGAGGAGGCCGGGGTGTTATCTGCTGACTGAAACCCGTCCCCGCTGACCAGTCCGTGCCCCACCCTTGGGGGGAAACCGGAGAACAGCTCCTGGACGTTGCGTGGATTCGGGGGAAGCTGGTATCCAACCGGCAGTGGAGGATCTTCGTGCTCGTAGAAGGAGGGGTTGAGTACATCGGTCGGCCATCGTGAGGCCCCGGCCGCGTTAAAGTAGAACTGCACGTCCGGCAGATTGTGCCGATAGGTGAAACACTTCCAGATGATGTTTTTTCTGTTGGCCAGGATGGCCACGGTGGGGGGCCTGGCCTCCTTAGGTTTGGCGGCCCTGGCCTCGGTGAGAAGCTCGCGTAGCCACACGGCCTGGCGTGCAAAGATGGACATCTCTGGCTCGAAAGACTCGGAGTAGCCGTCCAGGTCCTGCAGAAAATTCAGCGAGATGGTCTCCACCAGGGACCGGAAGGGCTCGGAGTGCCCGTCGCAGTAGAGGAGGGGAGCAACGACCCTGACCTGTCCCAGGGTCTTCAGGTTAAACAGATATTGAGAGGAGACAAAGAGAGTTAGGGGCCGACCGAGGAAGGCCGCCGCCACGGCCGCCTCAAAAACGGAGACGGGGATGGTGTCACCGGAGCCCCTCTTAGGACCGGTAATGGGAGTGCCATAAGGCATAAGATTTCTCAGGGCCCGGCCGGTAACGGTGCCGTAGGAAGACGGGGTTTCGCGGGGGACCTCGAGGCCCTCCGCCCTGGGAAGCTCTTCTCCGCGTGTATAGGCCTGCTTCACAAAGTCGCGCAGGTAGTCCTGAAACGCGACCGGGCCCTCCAGCGGGCGCAATGAGTGCCAGAGCTGCTGAAGGGCCTCGGGGGCGAAGCACCGGCGTGCGAGGAGCAGCATGCAGGCTCGGGCGCGGGCCGTACTTTGGTTGTGGACCAGGCCCAAGAACTCGGGGTGCGGCCAGAGGGCGGCCCGGGTATCCATCTCCTCCCAGGCGTCCTGGAAGAAGATGAAGCCGGTGGGTGGACCGGCGATGCGGTGGCGGGTGAGGCGGCGCGCGTCTTCCCCGTCGTCGCTGCCGCGGGTGGTTGAGGGCATGCCCCCCCTCCCGGAGGCTGGACTCCTGACCAGCCTGTAGGTGAGGACCGAGTCCGACAGGAGGTCTCCCAAACCCCCATCTCTCGCTAGAGCCGAGACCAGGCCGAGTCCTGCGTAGAACGATGGTGCGCCCAGGAAGGCGGCAGCGTAGGCCGGATGTGTGCCGACCAGCAGCGCCATTATCTCCCGTTGTTCCAATAGAATAACTTCCCGGTCTGTGGCCGGGGCTGGATAAGGGGGGTGATTCCTAGAGGCGATGAGACTGGCGTGCGCTAGAAGCGTCATGGCCACAATGGGGTTGTCTGCCAGGTCTTCCATCAGGGCTTTGGGCGCAGAGACGTATTCCCGAAGCAGCTCCCCGGCGTTGGACTCCACGTCGGGCCAGGTGTCCCAGTAGGAGTCGGCGGCGGCGGCGCTGAGGCGGGCGGAAGCTACACTGGCCAGGGTTCTTCTCCTTCTCTCTTGGTCATCCTGCGGGGGACCAATAGCTTGGGGGCGTCCGGCTGGGGTCAGGGAAAAGGCCTCTGGGTTCTCCAGCACGGTGGGCATGACATATTCCAGAAAGTTGTGGTAGACGGGGATGTAGTTGAGCGGCTCCTGGGTGTCTGCGGAGACGTAGGCCGGGTTAAGGGGGTCGCAGGGAGACTCTGTTTCCAGCCAGAGGGTGCCGGCGTATTTCGCCGGCCCTGCCGCCGCCAGAAATTGTGCCCGCCGGGTCGGGGCTCCATTGCCCCATCCAGTTGGTGGTGCCGAAATCGTGATGAGGAGGGGCAGGTTGTTGGTCAAGGGATGCTTAACGAAAACGGTAGGCTGGGCGGTCTCGTAAAAAGCCAGGAAACTCTGCTTGGCCGAGGCATAGCGCAGCAGCTTGTCCTTGAGGAGGGCATACTGGGAGCCAGCCGAGGCCCCAAGCGCCAGGCCCCTGGCAGCCTCCACCACGATCTTGAGCTGGCGCGGGTCGGTGTGGCCCCTGGCCTGGGTGACCAGGTCCTGCAGCGTTCCCTGCAGCTGGGACTCTTCCTGGGCCTCCTGGATGATGGCCTCCAGTCGGGAGAGGCGCCTTTTCCAGTCTGCGACGGTCTCCTTGCCCCCCGCGACCCGCTTGGGGTCCAACGTGGCCAGAGCCAATCTCAGCTCCTCCATGCCATCCATGGAGTTCTGGGCCATGCCCTCGACTTCCAGGAGCCGTGTTAGCTCATGAATTTCACCGTCAGCCGCAGCGGCTAGGTTCAGCCAGGCACCGGCGCCCCCAGCTAAGGCCAGGGCTCCTTCGGAAAGACCCCGCACGGCCTCGCAGATGCCCCGGATCCACTTGGCGGCTGCCAGGGATTTCCGGTAGGGCCACGAGCCGTTCCCGGCCGCTGCCCGGGCCAGGGCGGCCTCGAGGGGAGCCTGGACAGGGGCTTTGGGCGGGGAGGGGAGCAGGCTCCGGAGTTCATCGTCGGGGGCTTCGTCGCGTGACCTGGAGAGGACGGCCTCCAGAGCCGTGTGAAAGCCCCGCCGAGTGCTTGCCGCCATCTCGTGGGCCTTCGCCATCAGGGTCTGGCTCTCCCGGACCTGCTCTTCCAGCGCCCGGACCTCGGCCGCCTCGGCCTCGGTCAGCAGCTCTGAGAAGAAGTCCCCCGTGGCCTGGAGGAGATCGTCCCGCTCTCGCCTTGTCAGCAGCTGGGGCTTCTTAGGCCAGAGCGCCAAGTCCGAGGCCAGCCTGGGCGGGGCGGTTGCCTGGGGGATAGTTGGAGGAGGAGGCAGGTTAGCCTGGCCTGGGTCATTAGTGGCTTCGGGTAGCGTCCGATCCACGTACTCGCTCACGATGGCCGTCAGGGCAGCCTCGGCTGCTCGTCTTTTTTCCAGAAGCCCGGCCAGCCCCCGCTCGTACTCCGCGTAGGGGGCCTCCAGATCCGTGTTGACCACCGCTGATTTCATGTCCGGGGACTGCAGGGCCTGGCGCGTCTGCGCGAGGGCCGAACGGATGGCATCGGCCGCCGTCCTGGCGCGAAAGAGGGCCCCGGCCGCTTCCTCCGCTCCTCGCCCTCCTCCTCCTTCTTTGGCGGTAGCCGCGGGGGTGGCGGGCCAAGCGTCCAGTCTGGCCAGAGGGCCGGTCTCGATATCCGTGAACCAGCCGGGTTCCGCGGCCTCCATTCTCTCCGCCGCACCACCATCGTCCACGAGCAGGGATCGCAGTCTCTCCCTCCTCACCCTCGTTATTCCCAATAGCATAGCGGCAAGGATCTGTGTGAGGGAGTCCAAGATGTCCGTGTTTCTGGCTACTGCCGCCGCTGCTGCCGCGGCTGAGTCCGTATTGTCTGGCAGCAGGGAGGCCAGCAGGGTGTTCCAGTCATCGGGTGAAGTGGGAGCGGGCTCTGGGCGTGCCCCCAGCGCCTTCCTAATTCTGGCCCAGGCCTCATTCGCCTCTCGCGCTCGCCGCTCCCGCCTCTCCTTGTCTTCCTGTTCTCGGAGCTTCTCCTTTTCCTTGCGCCCGGTCTCCATAAGCTGCCGCAGCTTCTTCTCATACTGTCGCTTGAGCTCTTTGTTGGGGGCAGTGTCCAGAAAGGCCTCGAGCTGTTCCTCGGTGGCGGGCTTAAAGCCTTCGGCCTCCAGGCGCCAGGCCTGCACCTCCTTCTGTCTGAGCTGATCGTTGTTGTTATTCTTCAATTTCTGCAGGTAACTTAGGAAGCGTTTCTTGAGCTTCCCTGGGATGAGCGTTTGGGAGAGCTGATTCTGCAGCCCAGAGAGTAGTCTCAGGGCATCCTCTGGAGCCTGACCTGTGACCGTCGCATCATAGACCGCCAGTAGACCTGGGAGCAGATTCACCGCCGCGGCCGTCTCCTTTAAGGTGCTGTGAGTAGCAAAATTCTGCAAGGCCACTAGGCGCGCTGGCTCCAGCGTCAGCCGGTTGCCCATCTCGAATGTGTGCAGGGCCTCTGAGACCATGGGGTCCAGGATGCGGTCAATGCCATCCTGCACCTCAGGGTCAAGGACCGGCAAGTCACGATAGAGGTGGTCTATGCTCTCCTCGAAGGAGGCAATGTAGTTATCGATGGTGTAGAAGGTGATGGATTTCAGGATGTTCATCAGGTACTTTTTGGAGCGAACAATCTGCTGTATAGTGTCACGTAGGCGGATGTACGTGGGGTTCTTTGCGGCCCCGACTATCGACCCTGCATTTGCGATGTACTTTTCTATGACGGGGATGGTGAGGGCCGCGGTGTCGGCCAGCGGTGGCGTGGCTTCGGGGTTGTCGTGGTTGGCGGGTGTCGCAGAGGGAGAGGCGGGAGAGATGGGGGCGCCTGGGGCCGAGGCCACACCGGCCAGGCCCAACATTGCCTCGATGTCGTCCAGGATGGTGCGGAGGCGCTTTTCGTTTTCTCTGGTGGTCTCGAGCTCCTTCTGTTTTTTCGCGACTGTCTCAAACTCTGGAAGGGGGGCAATGCTGGGGTCGTCCTCCTCAACTCGCTCCAGGGGCCAGGGGATACCGCTCATATCACTAAGGGCGGTGCCCAGGTAGAGGAGCTCACGATAGTCCCATTCAATGGACGTGTACCGGATGTTTAGGAGAGGCAGGGAGGCGATGATCTGGCATGTGTGCCGCAGGTGTGTCAGGAGGTCGTCAAAATCCATCACTGTTGGGAGGCTTGGGTCCTCAAGGTAGGAGAGATAATCGGAGGCCGCCGAGGCCACCTTGTCCCTGATGTCCGCCGTACACCTGCGCACGTGCAGGGCCGCATTCTTGGACCGGACGGCCACGTTGTGGACAAAGGGGGGCACTGAGGCGGCGGGAGGGGCCCCATACTCTATCGCTGTCAACAGCGCCAAAAAGCGGACGTCCTCCTCATCTACCCCAGCCTGTTGTCTGGCCACGGCCGTTCGGGCGGCCTCCGCCAGGGATAGGAGGCGCTTCCAGCTCTCGTCGTCCAGGACCAAGGGGACGTCCACGTGCGGGCCCCTGTAGATGGAATGATCCTCGGGTTCTCCTCCTCCTTCCTCCCCCTCCTGATCTCCGCCCGAGAGCAGGTCGGTCAGGCGTCTGCGGGCCGCCTCCAGGTCAAATTTTCCGTCGTCGCTCTCGGCCAGCTGGGGAATTTCAGCCAGCATCTTAGCACCGGCATCTACACGGACCGCGTCCTTCGTGGCCAGGGACGGCAGGCAGGCCTCCAGCTTTGCGGCCAGGTGCTCATGGAACTCTCCCGCTCTTCCCTTGTTTTCTGATAGCATGTTTGCGAGGTTTTGGATGTTAAGTTCGGAAGTGAGCAGTTGCTCCAGGTCCAGCGTGGGGACCTGCAGATGTCCCGACCAGTCCTTTAAGAATTCCAGCAGATTTAGCACAGCCGATCGGTCCCTACTCCTTATTAGCCCCTGCTCGAGGACCACTGTCACAAGAAGATAGTCTATCATGCTCAAGGCATCTGCCTCTGGCACTTCCCGGTTAGAGGCCGGGTCGTAGACGATGGCCTGTTCCTGGTAGGTATGTCCGGCTATTCTCGCAATGTTGCTCTCGAGGGGCACAAAGTCCATCTCAGGAGTCTCTATGTCAAAGGTGGTCTGATAGTATTGGCTCCTGGCGGTGTCCAGTGTGATGGGGGACGTGGGGGCACTGGATCCCGATTCCAGGCTGTTGGAGAACACTTCATCTTCGAACATGTCTTCATCCTCTGTGGTGGGGATATCGGAGGCTAAGTCGCTCTCCGCTTCTTCAGAGTCGGACATGGATAGGAAAGGCTCCTCTAGGTCAGACAGGTAGCGGACGAGGCCAGAACCCCCAGATGCATCATCCCCAAAGGAGGGCTGCTGCCCGAAGGGAGGTGATGGGGATATCTCCGTTCCAGCCCTGTCAGCGGCCGAGGGATTGTTTTTTTCTGGTTCTAGTGTCGTGGCTGATGGTGGGAGCTGCTGAGCAGGAGGAGGAGCCGGGGTAGCTGATGGCAGGGGCTGCTGCTGAGGAGGAAGAGGAGAAGGAGCCCGGGCGGCTGACGGCGGGGGCTGCTGCTGAGGAAGAAGTGGAGAGGGAGCCGGGGCGGCTGATTGCGGGGGCTGCTGCTGAGGAAGAAGTGGAGAAGGAGCCGGGGCGGCTGATTGCGGGGGCTGCTGCTGAGTTGGAGGAGGAGAACGAGCCGGGGCGGCTGATTGCAGGGGCTGCTGCTGAGTTGGAGGAGGAGAAAGAGTCGTGGCGGTGGGGGCTGCTGCTGCAGTCGGGGAAGGGGATGGGGTGGTCAGAGGGATTTCTGGGTTCGAGGGAGCTGCCTGTGGCAGAGGGATGGGTATTTGCAAAGGGAGGCGAGGAGATGGAGTCACTGAAGGAGCGATAGTTGAGACTGGCGCGCGGCGGGGTGTCGGGGAGGCGGGTGGTGATTGGTGAGGGATGGGGATTACTGGAGGGGGAAGGCGAGCTGCTGAAGGGGGGCGATGGGGCGGAACGGGGGGGCGTGGCAGCTGATCATCCTCTGTGTCAGTGGTGGAGGACAGAGGGAGGCGGCGGCCGGAGGTGGGCTTCTTGTGGGGGCTATCTTTGCCCAATCCCTTTTTCCTCTTGGGAGTCTGAGGCGCTGCGCTGCCCGACGCCCTTGGTGGCGTGGAGGGAGCGGGGACCCCGGGGGTGTGACCTAGGCCGGGGATGGGGATGAAGAGGGGAGGGCTGGAGGCCGGGGCCGCGGAGTCCGGAGCCGCAGAGGCCGGAGCCGCAGAGGCCGGAGCCACAGAGGCCGGAGCCACAGAGGCCGGAGCCGCAGAGGCCGGAGCCGCAGAGGCCGGAGCCGCAGAGGCCGGAGCCGCAGAGGCCGGAGACGGCGGCGGGGAGTTGGTCTTTGCAGGACTATACCTGGCGGCAGGGAATGAGTCGGATGTGAAAGATCGAGAGGGCAGTGGCCTGAGGTTATACGGTATTATTCGCCGTTCAAACGGTAGCATGACGGGAGGGCTGCTATCAGCATCGGGCGTCCCCGCCGCCTCCCCATCACTGGACACAAGCTCGGGCCCCACCAGGTCAAAACCGCTGCCGTTGGCCTCATAAAAGTCATACACGCCATAGTGTTCCAGCATAAAGATGCGGGGGTCCTCTGTCTCAAAGGCCTCGGGTAGAAAATAGAGATGCACGCAAGTGTACTGGGCCCCTGGTGCCCCCACGTACTGCAGGATGTCGTGCGCATAGGTGCTGACTCTGACATGGGCGGGGGTGCCCGGGGCCGCATCCTTCTGGCAGTGGGGGTCAAACAAGTAGAAGGAGCCATCTGTCTCGATGATGATGGCCCCCGCGTAGATGTCGCAGATGTAGAGGATGAACTGGGCCACCCCGTTGTAACTGCCGTGCAGGACCTCGGCCAGGGACTGAACAACTGCCGAGTTTGCGATCTGGGCAGGGAATAGGACGAGGCCAAAGATCTCCGCCGAGCGGTATATGTGCACGCGCCCACCGCCCCTCAGGACCACGGAGCTGGGCACGTCCGTCAACTGGGCCATCTCGTGCCCCTTGAGGATGCCGCTCTGGCGCATGAGGGCATCCAGCCGCGCCCCCTCGTCCAGGACCTCGTCCAGCTCAGGGCGGGAGGTCAGGGGGCGGCCGGCCAGGAAGCTCTTGACCAGGTAGAGGACGCAGTTGCTGACACACTGGATGCCGGCAAAGCGGCCAAACTTGCAGTGGGCCTGGTTGCACGAGGCCGTGCCTAGGATGCGGAGGGCCGAGCCTCCACTCCCGCCCCCGGGGGCATTCACATCCATGGTCCTGATTCCGCGCATGGGGCCGGTTCCCCGGGGGCGCTGGCTTTGCCCCCAGTCGCCGTTACTCATCTTCGGCGGTGGGACGGGGAGGACGCCTTGTCGCCCCCCTTCTGGTCCGGGGTCTTACGCGGCTGGCGGCGGCAGCCGCCGAGAGATAAGGGGGGCACGTGTGTGCCTCCGCCTCTCCTCTGTCTGGGCCGCCGCCGCCGCCGCTTGCCCGCCTTGAAGGAGAGGGGGTAGTCCGCGGACTGCGTCTGCGGGGGCAGGAGGTCTCAACCTTCCGGGCTCGGGCCGCGGTGTCGATATCCGATGGCCTTTCTCTCTCTTCCTCGTATGCTCCTTCTCCTCCTCCTCCTCCTCCCGGCACGCCCCTGAGATCTGCCTTCCCTCCCTCTCCCTCGTCCTGGTCGGAAAAGTCTGAGGAGGAGAAGGAGAATGGGGAGGAGTCCAAAACGGCACGCCACCTGCCGTGGGGCGGTGGTGACAGGTCCCGGCTGGCCCGGCGCTTGCTCGCGTTCCTGCCGTTACCCAGGAGAATGGCCGCGAGTTTTTTGGCGGGGAGGATGCGGAATGGCGGGGGCGTTTGTCCCACGGGTGAGGGGGAATCGTCGGTTAGGGCCGGCACGAGGTGGTGGGTCTGGACCCGGGCCGTGCGAGCAAAGGCGGCGAGAACCGAGGGGCTTCTGGGGGTGACTGTGATCTGTTCCGGATTTAGGTCCATGGCGGGTGTGTATGTTTTAATAGGGGTGGTCTCTGGCGCGGCAGGATGATGGTCGAGGACGTCCACCAGGGCCTTGCAGATGCTCTTGCCTAGATACATGATGTCGTCCATGCTGAGGGGAGGTGGGGTGTCTGCTCCCCCCTGCGGAAGCCGCCTGGGTGCGGGGGTGAAGACAGGTGGTGGGCGGGCGTCTCGCCGGACTATGGCCTCGGCACGCTCGGCGTCGATGGCGGGCGGCTGGAACAGACGGGCGAATGTGTAATCCCGGAACCGGTAGGCGACGCTGCGCCTGAGGGCGCCCGTCAGGCTGTATCCCAGCTCCAGGGCGTGCTCCACCCGCTCGTTGAGCTCCTCGAGGTCCGGACGCAGGGGCTCGCTGGTGTGGGCCCAGAGGGGGTGATCCGCGATGCCCCGGCTCTCCCTGAGGGCCGGCACCAGGAGGCGCCTTCTGAGGGTGGCCGTGTCGGCCGTGGCCAGGGCCCACCTGGCGGCGGCGTCCCGGCACACATCCTGGATGCCCTCCACGACGCTCTTTAGCGTCTGGAGGTCCGTGGAGTAGTGGCGGGGGGAGGATGAAACGCTCTTTTCCTTCACCGCTACCACCGCCTCCTCCTCCTCTTCCGTCGCCAGAGGGATCTGCACCCTCCCGGTCTCTGCGTCGTACAGGAGCGGGCGGGAGCACAGCCTCCAAGCTGCCCCCGTCAAGCGCGAGATGTCCTCCGAGAGGGTCTCACCCGAGACCAGAAAGCGGCGGGTGGCCAGGCCCAACGACTCCGCCGTCGTGCTGTATCTCAGGGTGAAGAGGAGTGAAAAGAGGGAGGTGGGCCAGGCAAGCGGTGGTGCTTCCGCCGCCCGCTCTGAAGCTGAGATAGTCTCGGAGATGATGCCTGAGACCTCTCGGACGGCGTCCATGATCCTAAGGACTGCGTCGTGGGACGACAGCCCCCAGGGGCCCCCGCCCTCTTCGTCTTCTGCACCATCGGCTCCTGCATCCCCGGCCTTGCCTTCCCCCTCTAAGTTGAGGGGGCGCAGTCCGACCGCCTGGGGGGACTCCCCAGGCATCGGAGGGGCCCCGTCATAGATCTCCCAGACGGTGGCGTATATGAGCTCGAGAGGACGGCGGGCCCGGGTCAGCTCGGGGGAAGGGAGGGCCAGGTCGCTGCCGAAGGAGACCAGCCAGCGCAGGGCGCCCAGAGAGCGGGTTTTGGGCAGCTCGTTGGAGAGGACCCGGCGAAGGGCGGGCCAGATTTGGAACTCGATAAAGGCGGCCGGGAAGAATGGGCTGCGGACGTAGGCCGGATCCGCGCGCGCCGTTTGGCCGGCCCTCAGGGACCGGCAGTATGCCTCGACGTCTGTCCGCGGGACCGCCGCCACCGCCGCCGTCCACTGCCTTCTCCCCTGCTCGCCGGGGAGGAGGGGGGGCTTACAGGGGAGGGCCGGAGCCGGGGCCGGGGCCTGCCACAGGCGGCTGTAGCGGACCCATAGCAGAGACCTGAGGAGTTCGGATGAAAGGTCCCCCGCCACCTGCTCATACTCGGCCGCGGGAGGGGGGACGATGAAGATGCGCAGAGGGGTTACGGCGTCCCAAGGGTCCGCCACCGCCCCCACACCCACAGCCGTCGCGGCGGGCGTAGAGGGGCCGCTGGTGCGCCGGGCTCGTCTGTCCACGGCCTCGGCCTCCGCCCGCAGGTAGGCCGCCCGGGCCACACGGGCGAAGCGGCTCGTGGGGCTCGCGGTGGGCAGCAGTCGGAAAAAGTGCAGGGCAAAGCCCGATAGACTCTCTAGGAGGGCGGCGGTGGCCTCGAGCCATCTCCACCGCGAGCGGGACACCCGGGGCACAGAGGCCAGCATCATGGCGTAGTCCCCCGCCACGGTCTCGTTGAGCCCGGCCGAGAGCAGAACCGTGGCCACCTGCTCGATGGCGGCTGGAGAGAAGGATGCCCGGCTCCCCGCCGCCTCCTGCACACGAGCGGCCAGGGCCTCCATCTCTGCCGCCATCCCGGCCAGGAAGGCCTCGATGACCGAGTCTGGGACGCCGTAGGTCTGGTCCCAGAGCAGGGCCTCGTACACATAGTCGTAAAAGAGGGCCCCTGAGGGCTCCAGAAGCCGGAGCCGGGCGGCGTCAAAGGCCAGGACGGGCACGGCCGCAACGGGGGGCGTTTGTCCCCCGCTGGCCTCCGCGTACACGCCCAGGATCTCTACCGCCCGCCGCCGGGCCAGGGGCAGCGAGGCCACCACGCTGGAAAGCGACTCGGGGCGGTGAAAGAGACCGCCACCGCCGCTTTCTTCACCCTCTCCCCCGCCGGCCCTGCCCCCACTGTGCTCCACCAGCTCCACGGCCATGGCCTTGATGTCCGCGGCCGTGGGCTGACCCTGCCCTGCAGCCGCCCAGGGGTAGCGGTTGGTCTCCGCGTATACGGTGACCAGCCATCTCCCCAGCGTCGTTTTCGCCGCGTTAAAAGCGTAGAATGACAGCCCCTCCCGCGGGAAGGCGTCCCACCGGGCCAGATAAGTGTCGGCCACCAGCTCTTCCACGAAGGCAAAGGTGGCCGTTGGGCCAGAGACTGCGAGCACCTCCCCGCTGCCCTCTTCGATGATGCGCCGGTACTCGGCCGCCAAGGCCCGGGTATCTGCGATGAGCCGAGAGCCGTCCAGCGGATCGTCGGTGGCCGGAGAGGCTGTCGTGGGGGGCAGTGAGGAGGCCAGAACGTCCAGGGCCGCCTCCAGATGGCCGAGGCCGAAGCTGCGCCTGGAAAAGGAGGCCGCCCGGAGTAGGTAGTAGGCGTGGTGGCGGAGGACCGCCGCCGGGTAAGCGTGGCCGCTCATGAGGGTGAGAGTATTTAAAAAATCACGCACCAGCACCGGCTGGGCCAAATCTCCCAGTCCAAAGATTCCCAGCTCCAGAGGCATCAGCGCGCGCAGGCGGGCGGCGGGGTCGTCCCCAGACAGCAGCAACTGACGCGTCACGCGGGCGAGCCCCCCGTCCACCTCTGCCAGGGGCGGCTGGGCGTCTGCCCCTCCGCTACCGCCGCCGCTGTCACTCTCCATAGCGGACGCCATGAAGGTCCAGGGGTCCGTCGATCGCCGCCGTCTGCAACGCCGAATCGCGGGGCTGCTCCCCCCTCCGGCCCGGCGTCTAAATATTTCCCGGGGGTCCGAATTCGCGCGGGACGTTCGTGGGCTGGTTGAGGAACACGCGCAGGCCTCCTCGCTGAGTGCGGCGGCCGTCTGGCGCGCAGGGCTGCTGGCCCCGGGGGAGGTGGCGGTCGCCGGGGGTGGCAGTGGAGGGGGGAGCTTCAGCTGGTCTGGGTGGCGGCCGCCAGTCTTTGGGGACTTTCTGATACACGCCAGCTCCTTCAACAACGCCGAGGCCACTGGAACGCCCCTTTTCCAATTCAAGCAGAGTGACCCTTTCTCGGGCGTCGACGCGGTATTCACTCCTCTCTCCCTGTTTATCCTAATGAATCACGGCCGGGGTGTAGCCGCCCGGGTCGAGGCGGGTGGGGGCCTGACGCGGATGGCCAACCTGCTGTACGACAGCCCCGCAACCCTGGCTGACCTGGTCCCGGACTTTGGGCGGCTGGTGGCCGACCGCCGCTTCCACAACTTCATCACCCCCGTGGGCCCCCTGGTGGAGAATATAAAGAGCACCTATCTGAATAAAATCACCACGGTGGTCCACGGGCCTGTGGTCAGCAAGGCCATCCCTCGCAGCACCGTCAAGGTGACGGTGCCCCAGGAGGCCTTTGTGGATCTGGACGCGTGGCTCTCCGGCGGCGCCGGGGGTGGCGGTGGAGGATGCTTCGTCGGGGGGCTGGGCCTGCAGCCGTGCCCCGCCGATGCGCGCCTCTATGTCGCTCTGACCTATGAGGAAGCCGGGCCGCGGTTTACGTTTTTCCAGTCGTCCCGCGGCCACTGTCAGATCATGAATATCTTAAGAATTTATTACTCACCATCCATCATGCACCGCTACGCTGTGGTCCAGCCCCTACATATAGAGGAGCTGACCTTCGGGGCGGTCGCCTGTCTGGGGACATTTAGTGCTACCGACGGTTGGAGGAGGTCTGCCTTCAATTACCGTGGCTCTAGCCTCCCCGTGGTGGAGATTGACAGCTTTTATTCCAACGTCTCTGACTGGGAGGTGATTCTCTAGACTTAACGGGAGGAAACAGGAGGAGGAGGGGGGACAAGAGCACAAAAGTGGTCCAGTGGACACCCACCACACAGCATGGCAACGACCAATCATGTCGAGCATGAGCTCCTCTCCAAATTGATTGATGAATTAAAGGTCAAGGCCAACACAGACCCCGAGGCTGATGTCCTGGCAGGGCGCCTGCTCCACCGACTTAAGGCCGAGTCAGTTACACACACAGTAGCCGAATATCTGGAGGTCTTCTCTGACAAATTCTACGATGAGGAATTCTTCCAGATGCACCGGGATGAGCTGGAGACCCGAGTCTCTGCTTTCGCGCAGAGCCCGGCCTACGAGCGCATCGTCTCCAGCGGCTACCTGTCGGCCCTGCGCTACTATGACACCTATCTGTATGTGGGGCGCAGCGGGAAGCAGGAGAGTGTGCAGCACTTTTACATGCGGTTAGCCGGCTTCTGTGCCTCAACCACCTGCCTCTACGCGGGTCTCAGGGCAGCCCTGCAGCGGGCCAGGCCGGAGATTGAGAGTGACATGGAGGTGTTTGATTACTACTTTGAGCACCTAACCTCCCAGACGGTGTGCTGCTCCACGCCCTTTATGCGCTTTGCCGGGGTGGAAAACTCCACTCTGGCCAGCTGCATCCTCACCACCCCCGACCTCAGCTCCGAGTGGGACGTGACCCAGGCCCTCTATAGGCACCTGGGGCGCTACCTCTTTCAGCGAGCCGGGGTGGGTGTAGGGGTGACGGGGGCTGGCCAGGATGGGAAACACATCAGCCTCCTGATGAGGATGATCAACAGCCACGTGGAGTACCACAACTACGGCTGCAAGCGGCCGGTCAGCGTGGCGGCCTACATGGAGCCCTGGCACAGCCAGATTTTTAAGTTTTTGGAAACGAAGCTGCCGGAGAACCACGAGAGGTGCCCGGGCATCTTTACGGGGCTCTTTGTCCCCGAGCTCTTCTTCAAGCTTTTTAGGGACACGCCCTGGTCGGACTGGTACCTGTTTGACCCCAAGGACGCCGGGGACCTGGAGAGGCTCTACGGGGAGGAGTTTGAGCGCGAGTACTATCGGCTGGTGACAGCGGGCAAGTTTTGTGGGCGGGTCTCCATCAAGTCCCTGATGTTCTCTATCGTCAACTGCGCCGTCAAGGCCGGCAGCCCCTTCATCCTTCTGAAGGAGGCCTGCAACGCCCACTTTTGGCGCGACCTGCAGGGCGAGGCCATGAACGCCGCCAACCTGTGCGCCGAGGTGCTGCAGCCCTCGAGGAAGTCTGTGGCCACCTGCAATCTGGCCAACATCTGCCTCCCGCGCTGCCTGGTGAATGCGCCTCTGGCTGTGCGGGCACAGCGGGCTGACACGCAGGGGGATGAACTCCTGCTGGCCCTCCCTCGTCTCTCAGTCACCCTACCTGGAGAGGGGGCAATCGGTGATGGATTCTCGCTAGCCCGCCTCAGAGATGCCACCCAGTGTGCCACCTTTGTGGTGGCCTGCTCCATTCTTCAGGGATCCCCCACTTATGATTCCAGGGACATGGCCTCCATGGGCCTCGGGGTGCAGGGCCTGGCCGATGTCTTTGCGGACCTGGGCTGGCAGTACACTGACCCTCCCTCTCGCTCGTTAAACAAGGAAATATTCGAACATATGTACTTTACGGCCCTCTGCACCAGTAGTCTGATTGGACTTCACACCAGGAAGATTTTTCCGGGTTTCAAACAGAGCAAGTATGCCGGGGGGTGGTTTCACTGGCACGATTGGGCAGGAACAGACCTTTCTATTCCCAGGGAAATTTGGTCTCGCCTCTCTGAACGCATTGTGAGGGATGGGCTTTTCAATTCACAGTTTATCGCCCTGATGCCCACCTCAGGCTGTGCCCAGGTGACGGGCTGTTCGGACGCCTTCTACCCCTTCTATGCCAATGCGTCCACCAAGGTCACCAACAAGGAGGAGGCCCTTAGGCCAAACCGGTCTTTTTGGCGTCATGTGCGTCTGGATGACAGGGAAGCTTTGAATCTTGTCGGGGGCCGCGTCTCCTGCCTCCCGGAGGCTCTGCGGCAGCGCTACCTGCGTTTCCAAACGGCCTTTGATTACAACCAGGAGGACCTGATTCAGATGTCCCGGGACAGGGCCCCCTTTGTGGACCAGAGCCAATCTCACAGCCTGTTTTTGCGTGAGGAAGACGCCGCGCGGGCCAGCACGCTAGCTAACCTACTGGTGCGCAGCTACGAGCTGGGCCTGAAGACTATCATGTACTATTGTCGCATTGAGAAGGCCGCCGACCTGGGGGTGATGGAGTGTAAGGCCAGCGCGGCTCTGTCGGTGCCGCGGGAGGAACAGAATGAGCGGAGTCCCGCTGAGCAGATGCTGCCTCGTCCCATGGAACCGGCGCAGGTTACGGGGCCGGTTGACATCATGAGCAAGGGCCCAGGGGAGGGACCAGGTGGGTGGTGTGTGCCCGGGGGATTGGAAGTGTGCTATAAGTACCGTCAGCTCTTCTCAGAGGATGATCTGTTGGAGACTGACGGTTTTACTGAACGAGCCTGTGAATCTTGCCAATAAACGTTTATTGCCATGTCCAAGTTGTTGTACGTGCGTGATCATGAGGGCTTTGCCTGCCTAACGGTCGAAACCCACCGCAACCGCTGGTTCGCGGCTCACATTGTCCTCACCAAGGACTGTGGGTGTCTCAAGCTACTCAATGAGAGGGACCTGGAGTTTTACAAGTTCCTCTTTACGTTCCTGGCCATGGCCGAGAAGCTTGTGAACTTTAACATTGATGAACTGGTCACCAGCTTCGAGAGCCACGACATTGATCACTACTACACCGAGCAGAAGGCCATGGAGAACGTCCACGGGGAGACTTATGCTAACATTTTAAACATGCTCTTTGATGGGGACAGGGCGGCGATGAACGCCTACGCCGAGGCCATCATGGCCGACGAGGCCCTGCAAGCCAAGATTTCCTGGCTCCGTGACAAGGTGGCGGCCGCCGTCACCCTGCCGGAGAAGATTCTCGTGTTCCTGCTGATTGAAGGCATCTTCTTCATTAGCTCCTTCTACAGCATAGCCCTGCTGCGGGTCCGGGGCCTAATGCCTGGCATCTGCCTGGCCAATAACTACATAAGTAGGGATGAGCTGCTCCACACCCGCGCTGCCTCCCTGTTATACAATAGCATGACAGCCAAGGCTGACCGACCAAGGGCCACCTGGATCCAGGAGCTGTTTCGCACTGCGGTGGAGGTAGAGACTGCCTTCATCGAGGCTCGTGGAGAGGGGGTTACCTTGGTGGATGTGCGAGCCATAAAGCAGTTTCTGGAGGCCACGGCCGATCGCATCCTGGGTGACATTGGTCAGGCTCCCTTGTATGGCACACCACCCCCCAAGGACTGCCCGCTCACCTACATGACTAGCATCAAGCAAACTAATTTCTTTGAGCAAGAGAGTTCCGATTACACCATGCTGGTGGTAGATGACCTTTGAGTCAGGGTGGCTACTTGCTCAGGTTTCTGGGCATAAATTCTCCTGCCTGCCTCTGCTCTGGTACGTTGGCTTCTGCTGCTGCTTGTGATCATGGAAACCACACAGACTCTCCGCTTTAAGACCAAGGCCCTAGCCGTCCTGTCCAAGTGCTATGACCATGCCCAGACTCATCTCAAGGGAGGAGTGCTGCAGGTAAACCTTTTGTCTGTAAACTATGGAGGCCCCAGGCTGGCCGCCGTGGCCAACGCAGGCACGGCCGGGCTAATCAGCTTCGAGGTCTCCCCTGACGCTGTGGCCGAGTGGCAGAATCACCAGAGCCCAGAGGAGGCCCCGGCCGCCGTGTCATTCAGAAACCTTGCCTACGGGCGCACCTGTGTCCTGGGCAAGGAGCTGTTTGGCTCGGCTGTGGAGCAGGCTTCCCTGCAATTTTACAAGCGGCCACAAGGGGGTTCCCGGCCTGAATTTGTTAAGCTCACTATGGAATATGATGATAAGGTGTCCAAGAGCCACCACACCTGCGCCCTGATGCCCTATATGCCCCCGGCCAGCGACAGGCTGAGGAACGAGCAGATGATTGGGCAGGTGCTGTTGATGCCCAAGACGGCTTCCTCGTTGCAGAAGTGGGCACGCCAGCAAGGCTCAGGCGGCGTTAAGGTGACACTCAATCCGGATCTCTACGTCACCACGTATACTTCTGGGGAGGCCTGCCTCACCCTAGACTACAAGCCTCTGAGTGTGGGTCCATACGAGGCCTTCACTGGCCCTGTGGCCAAGGCTCAGGACGCGGGGGCCGTTGAGGCCCACGTTGTCTGCTCGGTAGCAGCGGACTCGCTGGCGGCGGCGCTTAGCCTCTGCCGCATTCCGGCCGTTAGCGTGCCAATCTTGAGGTTTTACAGGTCTGGCATCATAGCTGTGGTGGCCGGCCTGCTGACGTCAGCGGGGGACCTGCCGTTGGATCTTAGTGTTATTTTATTTAACCACGCCTCCGAGGAGGCGGCCGCCAGTACGGCCTCTGAGCCAGAAGATAAAAGTCCCCGGGTGCAACCACTGGGCACAGGACTCCAACAACGCCCCAGACATACGGTCAGTCCATCTCCTTCACCTCCGCCACCTCCTAGGACCCCTACTTGGGAGAGTCCGGCAAGGCCAGAGACACCCTCGCCTGCCATTCCCAGCCACTCCAGCAACACCGCACTGGAGAGGCCTCTGGCTGTTCAGCTCGCGAGGAAAAGGACATCGTCGGAGGCCAGGCAGAAGCAGAAGCACCCCAAGAAAGTGAAGCAGGCCTTTAACCCCCTCATTTAACACCATGTTCTCGTGCAAGCAGCACCTGTCCCTGGGGGCCTGTGTCTTCTGTCTCGGCCTACTGGCCAGCACCCCCTTCATTTGGTGCTTTGTCTTTGCCAACCTGCTCTCTCTGGAGATCTTCTCACCGTGGCAGACACACGTGTACAGGCTTGGATTCCCGACGGCATGCCTAATGGCCGTCCTCTGGACGCTGGTACCCGCCAAGCACGCGGTGAGGGCCGTCACTCCAGCCATCATGCTGAATATTGCCAGCGCCTTGATCTTCTTCTCCCTCAGAGTCTACTCGACCAGCACGTGGGTTTCTGCCCCCTGTCTCTTTCTGGCCAACCTGCCTCTCTTATGCCTGTGGCCCCGGCTGGCCATCGAGATTGTTTACATCTGCCCGGCTATACACCAAAGGTTCTTTGAACTTGGGTTGCTCTTGGCCTGCACCATCTTTGCCCTGTCCGTGGTCTCCAGGGCCCTGGAGGTGTCGGCTGTCTTCATGTCTCCATTTTTCATCTTTCTGGCTTTGGGCTCTGGAAGCCTGGCCGGTGCTCGGCGTAACCAGATTTACACCTCGGGTCTCGAGCGGAGACGCAGCATTTTCTGCGCCCGGGGGGATCATTCGGTGGCATCCCTGAAGGAGACCCTCCATAAATGCCCGTGGGATCTGATGGCCATATCTGCCTTGACCGTTCTTGTCGTCTGTGTGATGATTGTGTTGCATGTGCACGCAGAGGTGTTCTTTGGACTCTCTAGATACCTGCCCCTCTTTCTCTGTGGGGCGATGGCCTCCGGGGGGCTGTACCTGGGCCATTCCAGCATCATTGCATGTGTCATGGCCACCCTCTGCACCCTGACATCTGTTGTGGTATATTTCCTCCATGAAACCCTTGGACCCCTGGGCAAGACCGTGCTGTTTATCTCAATCTTTGTCTATTACTTTAGCGGGGTAGCGGCCCTGAGCTCAGCTATGCGCTACAAGCTTAAGAAGTTTGTGAACGGACCCCTGGTCCATCTCCGTGTGGTATACATGTGCTGTTTTGTCTTTACTTTTTGTGAATATCTGTTGGTGACATTCATTAAATCCTAACGACCGGAGTCCTGTCTCTTTGTGTTCTTGGGGGACTTGAGTTAGCTGTCTTTCCTCTTATTACATTGGGCTAACGGGAGGAAATGAACACAGTGGTGGCAGTGGATGGGGTCATTTATGGGCAAAACTCACAGGACGTGTTTGGGGGAGTTAGCATTGGCGTCGGGAAACACGGCTCTGGCAGTTATAACCGCACCAGCTAACAGGACATGTTTGGGGGAGTTGGCATTGGCGTCAGGAGACACGGCTCTGTCAGTTATCACCGTACCATGAGTGCCATGTGTGTCCAGTGCCTAATCACCGTTCCTCATTTTGTGTGCCTCCTCAAATGTTCCAGAAGTCGGCCACGGGGGAGGTGGCTGAATTAGGGCCTTTTCCCTCATTCCCCCATGAGACCCACGTGGCAGGCCTAGGGGCTACATTCGCCTCCCACGTTTCCCTTCGCGTGAGGCGTCCGATATGACTGAATTTTCGCAGTCTCTTTTCCCTCTTCCCTTGTTATTCCCATAGAATTACAGTGAGGTTACACAGGTGGAGATTCAGTTTAACCATTTATTGATTTAATCCAGGAACAAAAAACAGTCCTAGTGATCCAGTGGCCGGAGAGAGAATGGCCCTGACAAGTCGGCTGCATGATGCACTTCGGCAGTCACGTGTGTGAGTCTCCACGGCCTCTGTCAAAAGGGAGCTTAGCGTGCCAGGGTTGTAATTCTTGATGTAGTGGCCCAGGAATTCAACTTCATCGTGTCTCCGTCTGCAGTTGGCGTTAATGTAGGCTGGGGCTACTGCCGCATATGCTGCCAAGAGACAGAGGGGCTGCTTCACATATGAGCTGCTCAGGGTCTCCACCACCTTGTTTTGACGGGCCGTGGCACAGGTGATGTAGAAGAGTTGCTTCACAAAGTTGTAGTCTCGCGTGTTAGGAAGGAAGCAGGGTGCCAGCTCTTTGAGCTTGGTCAGGATCACCTTGCTAAGACTCATGGCGCAGGCCAGGAGGATGTCTTCCGCGGGAGCTAGGGGCAGGTCGCCGTGGTAGGTGATCTCCTGGAGCCAAAAGATGGTCTCTTCTAGCATGGCCACCAGGGTGCAGAGCCCCGCGTTCTGGATCGCCTGCATGCGTGCATCCAGCCATGTGTCCTTGTTGGTTGACTTGGTGAAAAACTCACGTAGTGTCTTGTAGCTCCTGCGCAGCTCGTGTCTGGGTTGCACTTTCTGCCAGGCTCCAATCTCTGGATGGGCGGCCACCGCCAGCATCGACTGTAGGAACGGGTCTTGGATGGGCTCTAGGGTCAGAGAGGCCAGGGGGCTGGGCAAGGTGACAAATGTAATCTTGGAGACAGGCTTAACCAGACTCATGTCAAACCACGGTTTGTTGGGCAGGGGCCTCTGGCTGCGTTCTTGCCTCGCCTGCTTCCTTGTGCTCCTGCCGGCCCCTCGAGATTCTGACCGGGGACCTCTGGTTGCTCTGTTGCTTCGGGGAGCTCTTGGAGACCTCGGTGCTCTAGGCACCCTGGGGGCCCTTGGGGCTCTGGGCGCTCTTGCTCCCGGGGGCAGGTGTCGGCGCTTGCCATAACTTTCATCGGTGCAGCCATGGACCTCTCCGCGTCGCCTTTTGTGGCCTCTGGTGTAAGAGGAGTTGCCAGTCTCCTCCTTCTCGTCCTCGTCCCTGCACAGGGGTGAGCGATGCAGCGTGACCGTCTTGTCCTGTAGGTCCCACTTCTTTCTGGGAATCACAAACGATGCCGAGGTAGGGGTTATGACCACGCTGGAGGGCTGTGCAGGTACGGCGTGGGCCGGAGTTGGATCTTCATCCTCCTCCTCTGAGGATGAAATCTCTCCATCTGTGGAGTGTTCTTCGCTGCCCTCCATAGGGTCCATATCGCAGTCTGTGTTGGTGTCTGAGACCGCTTCGAGTTCCAGAATGTGGCTCTCTGCAGAGGGGAGACAAAAGGTGGAGACTGCCTTGAGCACCTCTATCTCAGGCACCGGATGCCCCCGGCTCCACGGCCCCGGCCCCTGGCCGGTGTAGCTTCTTACCTGCGGGATCCTCGTTGGAGGAAATGCTGCTAGTTCGGGAGAGTCTCTGAGAAGGAACCATCTTGTCTGTCTCTACGACGGGCTAGCTGGGATGTAGTGCTGTCTTGACTGGCCTCAGCCCTATTTATGATTCTGGAGGCGGGCACGCTGATGGAGAAATGGGCGGTCGGTTGATTGGCCCCACAGCGACCGGCGAAGCACTGACTCATGAAGGTGACCGTGATGGCCTGTGATGTGTAGTAGAGTACCAGAAACACCCTCACATTCTTGGAGCTGGCCCTGTGGGTATGCCTCAGGCACGCAAAGTTCCTGCCCCGGGCATGGCACACCTGAACTAAGTTTGGCCCGGTTTGCTCAAATGTGACATGGAGAAACTGGGGGAATTTGTCTTCTGGCACAGCTGTTGCCAGGGTGCTCATGAGCGAGGGCCAGATGCAGGAGCTGACCCAGGCGACGAGATCCAGGCCCAGATGTCCCTCTATCATGGCGCAGACATTCTCCACGGTGGGGGGCAGGGTCTCGCGGGTCCTCTGGATTAGATAGTCACGCCCATCATCCGCGATGTGGTAGCAGAAGGTTTTGGGGGCCGGCCAGCCCACGTGCAGTGAGTGATGTAAGAGGTTTTGAATGTTGAGGGCATTCTTAACATAGCTGTGCTTGTCTTCCTCTTCCGGATGACAGACAAAGAGGCGCAGCTGCCGGCTAAGACCACCGCCCCTGTCCACCTTGTAGGTATGCGGCAGCCGGATGCACCGCCCGGCGTGATACACGCCGCTGTCAAAAAGCGGGGCCCCAATCTCTTTGATCTTGTGACGCATGCGGCGCAGGCAGGCCGTTAGGCCCATGAGCTTCTGCAGCACAGACACAAACCCTTGTACTGCGCTTGTTCCCACAATAGCATGGCCTCTAGGTAGGGGTGTGATGACGCGAAAGCCCAGTTTTCCCGTGCATATGCAAAAGGGGAGCACATCTTCCATATTATCCGGGTCGGCGGGTGGACAAGCTGATTTGAAAAAATAGACTGGGTGGGCCCTGGACACTGGACCCAGGCGGCGCATGAGGCGCAGTACCTCACGCCGCACGGTCCGGCACAGGTCATAGATTTCCTCCAGCGACCAGGGGGGGCCCTTGATCTTTAGATCCAGGTCCAGGACCAGGTTGCAGACCGGAAGCCGGGGATTAAAGTATTCATGCCGGGAGACAAAGAGCTGCTCGCTCAGGCTGTTCTGTGAATAGTACACTGGGGTGTAGGAGAGGGCCCTGGTGAGACACGTGTCTGGGAGGCGGCAGTTGGTCGGGGTGGAGACGACCTCCGCCAGGTGGGACGAGAAGGGGTCAGCGGCTGTCATTACAAAGTAGTGCCTGTCTGCAAAATGGCAGAGGAAGACCGGTAGCCGCTGCACCCTTCGAAGGACGGTGGGTGGGAGGAATTGTTCCTTGGGATTCCACTGGCCCCGGCAGGTGGCCTGGCCGGCCAAGCATAGAAACCCTTGAAGCGTGGGGGGGTATGTGGGACCCTCATCCGCGTGCCAGCGCGCGAGCTCCACCAGCTCCCGGGCCACATCCACACTGAGCCCGGCCCAGGCCCGCATGAGTCCGTCATCGGGGTCGGGGTCCCACGTGTATGGGGCCGGGGGCTCCATGCGGATTTTCAGCTGCTGGACACGCACATGCTCAGCCAGGTAAGTCTCCCGGGTGAAGTAGGTGCGCATGTGCTCCGCAAAGCCCCTGTCCAGGAGCGAGGGGAGCACGACGCCCCCCGAAGGCAGACACCCAATTTCTCCCACGCTCGTTAACTGAGAGTATCGCTTAAAGGTTCCCTCGTTGAAGCACTGTGCGTGGGCCAAATAGACGTAGCGCACGAGATCGGCCGAGGCCAGGGGAAGGCGCCCCCTGTAGGCGTCTATCGTCCTTGCCACAGTGCGGATCTCTCGCGAGTCCCGCCGCAGCTTCTCGTGTGCAAAGTGGGCAAAAGCCTCTGTCTGCTCCGCCCATGCCGAGGAGCCAAAGACCTCCCCCAGCTCGGCCAGGGACGTGACGGCGGCCAGGCTCTGACCAGACTCGGAAGTAAATAGCTCCGTGAGGTGCGCCAGGGTCTCAATCGTACAAGGAATGCCCCAAAAATAGTAAGCAGCCGTGACTAGCACGAACTGGGCCTCGTGGGAGCCAAAGGTGCTAATGAACCACCTGGCCGAGATGTTAACGCGGTAGATGCGGCGCAGACAGCCCACGATCTTGGGACGCAGCCACGCCACGCGGCCTCTGGCATCCCCCTGTGGCTGCTTCTTGGCGCTCAGTGTGAGCAGTTCCACGAGGGGCGTGAGCGAGCGCAGGGCCCCCGCGCGATCTAGGTAGGTGGATAGACGGTCCGCGGTGAGCGGCGTGAGGCCGCGCAGGAAGGGGAAGGCCTCCTCCGCCGGCAGGCGCAGCGTCAGAACCAGGCCGCAGCGGCTCTGTGAGGTCAGCCGCTTCTTGGGCAGGTGAAGCTGCAGTTCCACGAGAGAACCCGCCACGTGGTGGAGGGGCGAGGCGTTGTGGCACAAACAAAACAGGCGGAAGCCCTCGTCAGGCCGCGAGAGGATGGCATCCAGGATGGCCTCCGCAATGTCAGTGTTTGAGGCCACAAGGGCCTTGATGACGACGGGGGTGGACATTATTTAAGACCGGGAGGCCCCAACGGCGGGCTAAACAGAACGATGGCCTTCTATCTCCCAGACTGGTCGTGCTGCGGGCTCTGGCTCTTTGGCCGGCCCAGGAATAGATACAGCCAGCTCCCTGAGGAGCCGGAGACCTTTGAGTGCCCGGACCGCTGGCGAGCCGAGATAGATCTGGGCCTGCCCCCTGGTGTGCAGGTGGGAGATTTGCTAAGAAATGAGCAGACGATGGGCTCACTGAGACAGGTTTATTTGCTCGCCGTTCAAGCCAATAGCATCACGGATCACCTGAAGCGCTTTGACGCCGTCCGCGTCCCTGAGAGCTGTCGTGGGGTGGTGGAGGCCCAGGTGGCCAAGCTTGAGGCCGTGCGCTCAGTCATCTGGAATACCATGATCTCTCTGGCCGTAAGCGGCATCGAGATGGACGAGAATGGGCTTAAGGCCCTGCTGGACAAACAGGCTGGCGACAGCCTGGCCCTGATGGAGATGGAGAAGGTGGCCACGGCGCTCAAGATGGACGAGACCGGTGCCTGGGCGCAAGAGATCTCGGCCGTTGTCTCATCGGTGACCGCCCCCTCAGCCTCGGCCCCTTTCATCAACTCCGCCTTTGAGCCCGAGGTGCCCACCCCCGTCCTTGCACCGCCTCCCGTGGTGCGGCAGCCGGAGCACTCTGGGCCCACGGAGCTCGCGTTAACGTAGCAACCAGACTCCACACCAAATAAACATTTTATTGGTAAAACAAGGGATATGAAGGTGTCATTGACCCGAGGATCCAAACCCCCTCCCCTGTCTCCCCTCGAGCGCCTCGCTCAGCCCACTATCACCCATGGCCAGGCCCGGCACCTCCTCGAAGGCGCAGCTGGCCCACCTAAAGAGAGATCTGGGGCCAATGACCCCCGCGTCACTGTGGGGGCTGTAGAAGGAGGTGAGGTGGTGCTTGTGAAGGTAAACAAGCTGACAGAAGCGCCGGTACTTGTTAAGGAACACGGTCTGGTCACTAAAGTTGGTCAGGCTGACGTCCACCCCACCCCGGCGCCACCTGCAGGGCTTCACTAGAATACCCTGCATGGCCAGGCCCGACCTGCCAAAGATTGTCGGCCTGTGGTGAGGGATAGAAGGGGGGGGCACGGTGAGTGTCACTGAGACGGTCTGATGGGGGAAGAGGGCCAGGTCCTTTGGCAAAGAGACGTCCAGGCCCACGTCCCCGGGGTACTGGGGGTGGTTGATGGGACCCTTGTCCTCCTCCATCTGGGGGGTGGCATATCTGAAGGCAGCCAGGTGGATTTTGAGCTCCGATGGACGCAGCGTGGAGTTGTAGCGCCGCTGATTCTGGAGGATTAGCCGGAGTTCCCCCGTGTAGCCGGGATCGATGATGCCAACATGAGACGTGACCGGACGGGAGGTGCTGCCCCACAGCATGAGCCCGTGACCCTCGGGTGGGCGGGCATAGAGGCCTAGGTCCACAGTTGTGGTCTTCATCGGGCGCAGCAGGATGGTGGTCTTGTTGACCAAGGTGAGCCGCCCTACACTAGCCTGCTGGAGCAACAGCTTGTCATTCTGGAAGGCGTAGCGTATGTGTGGACAGGCCTCCATGGTGATGATCTAACAGACAGGGACGGCGGCGCTATATATAAGAGCCCAAGACCCGGCTCTCTTTACTGCGAAATGGGGAAGGTCCTAAGAAAGCCGTTTGCAAAGGCTGTGCCACTGCTCTTCCTCGCCGCCACCTGGCTTCTGACCGGGGTGCTGCCGGCCGGCGCTTCCAGTCCCACAAACGCGGCGGCGGCTTCCCTGACTGAAGCCCAGGACCAGTTCTACTCCTACACATGTAATGCGGACACATTCTCGCCTTCTTTGACCAGCTTTGCCTCCATCTGGGCACTTCTGACGCTTGTCTTAGTCATTATAGCCTCAGCCATCTACCTGATGTACGTCTGCTTTAACAAGTTTGTGAACACGCTGCTGACGGATTAGATGGGGATATTTAAAAGGGGCAGCAATCTCGGCTGTCTGTACTTCTTCTCTGCTCGTTAAACCAATAGCATGTCAGCTCCACGCAAAGTCAGATTGCCTTCTGTTAAGGCTGTTGACATGAGCATGGAAGATATGGCCGCCCGCCTGGCTCGCCTGGAGTCTGAGAATAAGGCTCTGAAGCAACAGGTCCTCAGAGGGGGTGCCTGTGCCTCGTCTACCTCTGTTCCTTCCGCTCCAGTGCCTCCGCCTGAGCCGCTTACAGCTCGACAGCGAGAGGTAATGATTACGCAGGCCACGGGCCGTTTGGCGTCTCAGGCTATGAAGAAGATTGAAGACAAGGTGCGGAAATCAGTTGACGGTGTAACTACCCGCAATGAAATGGAAAATATATTGCAAAATCTGACCCTCCGCATTCAAGTATCTATGTTGGGTGCAAAAGGCCAACCCAGCCCTGGTGAGGGAACACGACTACGAGAATCAAACGACCCCAACGCCACCCGACGTGCCCGCTCCCGCTCCCGGGGACGTGAAGCAAAGAAAGTGCAAATTTCTGATTAATAAATCTTTATTGACTTTATACATAGGTCTCGGCGTCATCATATGGTGGGGTGGTGTAGGTATGGGATGTCGACAAGTTACGCCTGAAGGCGCAGTCCGCCATGACCAGCAGCAGCAGAAGGGTCAGCACCGCCAGAGAGGCCCACTGCAGTACTAGCATGGAGAGGTTTGAGAATCTGGGCTGGGACGTTGGCGGGACTGGCACGGTGGCTTGGGCTGTGGTAACCGGTGGGCTCGTAAAAGTCCAGCGGGGCCGCAGTTCGCTAGAAGTGCTGGGAGGTAGATAGGTGGTCGCATTGTATCTCGTTCTTGGCGTAGTTGAATCACCGCCGTAATCTGTGGTGGGCTCTGTACTTGTCCGGGCTCCATGTCCTGTGGTGTGCTTTCCACCGGTGGTAGAATTGGCCTTTCCACCTGTTGAGGTGACCGTGGGAACCGCCGTCTTTTGGCCACTGGGGGCCTGGGGCGACGTTGCATTTTTGGGGGGCGTGCCTTTGGTGACATTAACCTCCCCCGGTTTTGTGGATGTGGAACTGTTTCCAGGGCCTGACGCTTGGCTGGTGGTGCCTGGGCGGGGCGCTGGCGAACTGGTGGACACATGATGTGTGCTGGTAGAGGCTGGTGTCACCTGTGTTATATTTTCACCACCTGTTGGGTGAGCGGAGGTTAGTAAAGGCATATGTGACGTTGAATTGTCACTGGTGGAGGGGCTGAGTGTCTCTGAGATTGAACTGGGTCTCAGTGACATGGAAGAGGTTGAACTTGAAGTTATGTTATGTTGGCCTGTGGTAACAGCACTGGTTGCATTTTTTGGTGGGCTGGTAACTACTGGGGTGGAACTTGTTCCTCCTAATGTGTGGTTGGTGGTATTTGCCTGTGGACTTGTTTCTCCCACAGTAGGGCTGGTGGCATTTGGGGTTGGGGTAGTCACTGCTGAGGTGGGACTTGTTTTTCCCAAGGTGGGGCTGGTGGCATTTGGGGTTGGGGTAGTCACTGCTGGGGTGGGGCTGGTGGCATTTGGGGTTGGGGTAGTCACTGCTGAGGTGGGACTTGTTTTTCCCAAGGTGGGGCTGGTGGCATTTGGGGTTGGGGTAGTCACTGCTGGGGTGGGGCTGGTGGCATTTGGGGTTGGGGTAGTCACTGCTGAGGTGGGGCTGGTCATGTCGGGGGCCTTACTTTCTGTGCCGTTGTCCCGTGGAGATGGACTTGGTGTCACCGGTGATGCGCCTGACGTTGTGCCGGCTGGTGTTGGGCTGGTGACATCCGCGGTGGATACAGTGGGGCCTGTGCTTGCAGGTGCGGTGAGGTTGGTAGGCACGTGAGTAGAGCTGGGTAGACCTGTCGTTGTATTGGGAGCAGCAAATCCAGTTGTATTCGAGGTAGGGGAGGTGGTGGTGCTCTCGGGTGCCTTGGAGAATATAACCTTGTGGGTTGTTGTGGTGGCATTGGTAGCCGTTCGTGTGATAATGAGTGTCTTGGGGGCCGTGCCAAGACCCGAGACAATAATGTCAAATGTCCGATTGCTCGCAAATGCACCAGAAATATTTTCACAACCCGAAGGTGTCCCCGAGGTGAGAGTCCATTTGCACTTAAAGTCAGTTTCAGTGTTGTTTGGCCAGGCCCAAAAGGCAGTCACTGTAACATTTGGCGAGTTTGCGTCCTCAGAAGTGACCATTGGCACTGAATAGGTAGCATTGTCACCCACATATGTGATGTCTGTGGTGTTTGTCGGCATGTCCTGTGAAGCTGGAATCTCATCAGAGAACACAATGTTGGACTGAATGCAGTAATCTCCCCCGCTCGCCTTTGGTCCATTCCCAGAGTAAAACACGTACAGGATACTGTTATTGCCAAGAAATCGTGACACTGGACGTGGTGTCAGACGCAGGCTGTATGCATACCCTGTACCAGGTATTGGGGTGACCACGGGACTCGTTGATGTGAGAATTCCGCCGCTGGGAACATGGCTCTCGTATCCACTGCAGGTGATGTTAAATTTGTTGTCTCCGGGCAGAACTTGTGAAATTTCGCCATCCTCCATAATACACTCAATATCTATCTCATTACCGAGCATTTGTGTTTTTACGCTGAAATTCGAGTCTTGAGCTGACGTTGGCAAACTTAAGGGTAGCGTGACATCCAGCCCCTGTGCCCTCACTACTGCCGTTATATTGGTAGAATTACAGTTATCCCACTTTATGTATGGCACTGTTTCTGGTATCAGGTACACGGGGTTTTGCATTTCTGCATGGTGGCACCACATGGTTCCAAACACATCTTGAAAGTAGACATCTACAGATTCCAGGCTTACTTGTTGCTCCTCTCCAGTGGTGACGTTAATTGGAAGCTTCTTAGACCGCATAGTTAGAGCCAATTCTCCTGCACCAAGGAGCTCCAGTAGAAAGAGATTGGTGGCATTTTCTGAGCCACCAAATGCACCTCGAGGTTGGTAGACAGCCTTTGTATGGGGTGTCAGCTGGCCAAAGTCAAGATCAAGTTGATGCTTTTTGCCCCCGACATCGAAATTGATAGTTACATTGACATCTGCCGTGCAAACATTGCATGTGGGGTAAAATGGGAATTCCGGAATCTCAACATTGAAAAAACCAGGATCTTCACCCGTGAGATGGATCAGGCTCTGGATGGTGTACTGACACACAAGCAAGGCTGCCTCCATTGTCTCGGCACCGATTTCTAGGCAGCATCCTCTTTAATAGGTACAAGGGGGGTGCGGTGTTGGTGAGTCACACTTTTGTTGCAGACAAAATGGACAAGGACAGGCCGGGGGACCCGGCCCTGGATGACAACATGGAAGAAGAAGTCCCATCTACCTCGGTTGTGCAGGAACAGGTATCGGCGGGAGATTGGGAAAACGTCCTCATAGAGTTATCAGATAGCAGCTCAGAAAAGGAAGCAGAAGATGCCCAGCTGGAGCCGGCCCAGAAGGGTACGAAGAGAAAGCGGGTCGATCATGATGCCGGTGGGTCAGCTCCAGCACGACCCATGCTCCCACCCCAGCCGGATCTCCCTGGGCGAGAAGCCATTCTCCGCAGGTTTCCACTAGATCTAAGAACACTTCTTCAAGCGATTGGAGCCGCGGCTACGGTGAGCATCCCTATGGCCTAAGTGTGTGATGTGTGTTTTTACCCATCACACAACAACAAGGTAAGTAATTTGTTGCCGTTGGTTTCAGCGCATCGACACACGAGCCATAGACCAGTTTTTCGGATCCCAGATTTCAAATACCGAGATGTACATAATGTATGCCATGGCCATTCGACAGGCCATTAGAGATCGTCGGAGAAATCCAGCTTCTCGTAGAGATCAGGCCAAATGGAGACTGCAAACCCTGGCCGCCGGATGGCCTATGGGTTACCAGGCATACAGCAGCTGGATGTACAGCTACACAGATCACCAGACGGCTCCCACATTCGTACAACTCCAGGCGACACTTGGGTGCACAGGTAGCCGTAGGTGTCACGTGACCTTTTCCGCCGGCACCTTTAAGCCGCCGCGATGTACGCCCGGGGATCGCCAGTGGTTGTATGTTCAGAGCTCCGTGGGTAACATTGTACAGAGCTGTAATCCCCGCTACAGTATTTTCTTTGACTATATGGCTATACACAGGAGCCTCACGAAAATCTGGGAGGAAGTTTTAACACCTGACCAGCGTGTTTCATTTATGGAATTCCTAGGATTTTTGCAGAGAACGGATTTGTCCTATATCAAGAGCTTTGTCAGCGTTGCCCTGGGCACCACTAGTATCCAAACACCGTGGATCGATGACAATTCTAGCACGGAGACGGCACAGGCTTGGAATGCCGGCTTTCTCCGGGGTCGTGCGTATGGGCTAGACTTGCTTAGAACTGAAGGGGAACATGTTGAAGGTGCTACCGGTGAAACGCGGGAAGAAAGTGAGGACACGGAGAGCGATGGAGATGATGAAGATCTTCCTTGTATAGTGTCCAGAGGTGGACCTAAGGTCAAACGACCCCCTATATTTATAAGACGTCTGCACAGGTTGCTGCTGATGAGAGCGGGCAAACGAACGGAACAGGGCAAGGAGGTACTGGAAAAGGCCCGTGGGAGCACTTATGGCACACCTAGGCCGCCTGTTCCGAAACCAAGACCAGAGGTCCCACAAAGCGACGAGACAGCTACCAGTCACGGGTCGGCGCAAGTCCCAGAACCCCCAACCATCCACCTAGCAGCTCAGGGAATGGCATACCCATTACATGAACAACGCGGCATGGCCCCGTGTCCGGTAGCACAGGCCCCACCTACGCCCTTGCCCCCTGTATCTCCAGGGGATCAACTCCCAGGTGTTTCTAGCGACGGGCGAGTGGCGTGTGCACCAGTACCCGCCCCGGCTGGGCCTATTGTCCGGCCCTGGGAGCCATCCCTGACACAGGCTGCGGGGCAGGCCTTTGCACCCGTTAGACCACAACACATGCCAGTAGAACCCGTCCCTGTCCCGACAGTGGCACTTGAGCGGCCAGTTTACCCCAAGCCAGTTCGTCCGGCACCTCCTAAGATTGCTATGCAGGGCCCCGGGGAAACTTCTGGCATTAGACGCGCGCGGGAGCGTTGGAGGCCCGCACCTTGGACGCCAAATCCACCCCGTTCTCCCAGTCAGATGTCCGTGCGTGACCGTCTGGCTCGTTTGCGTGCTGAGGCACAGGTCAAACAGGCTAGTGTTGAGGTGCAGCCACCCCAGTTGACCCAAGTATCCCCTCAGCAACCAATGGAGGGGCCGTTGGTACCAGAGCAGCAGGTTGCTGATGTGGTCCACACACCTGGGGTACCGGCGATGCAGCCACAGTACTTTGACCTCCCCTTAATTCAACCCATTAGCCAGGGGGCACCCGTGGCCCCGTTGAGGGCTAGTATGGGCCCGGTACCTCCGGTACCGGCAACACAGCCACAGTATTTTGACATCCCCTTAACTGAACCCATTAACCAGGGGGCATCCGCGGCCCATTTTCTACCTCAGCAACCGATGGAGGGGCCGTTGGTACCCGAGCAGTGGATGTTCCCAGGTGCCGCCCTGAGCCAGAGTGTTAGGCCAGGGGTAGCGCAGTCACAATATTTTGACCTCCCCTTAACTCAACCCATTACCCATGGGGCACCCGCAGCCCATTTCCTACATCAGCCACCAATGGAGGGGCCGTGGGTACCCGAGCAGTGGATGTTCCAAGGTGCCCCCCCTAGCCAAGGCACTGACGTGGTCCAACATCAGCTGGATGCTTTGGGGTATCCACTCCATGCTCTCAACCATCCCGGGGTTCCCGTGTCTCCTGCCGTTAACCAATATCATCTCAGCCAGGCTGCCTTTGGGTTACCTATTGATGAGGATGAGAGTGGCGAGGGGTCCGATACCTCCGAGCCGTGTGAAGCTCTTGATTTGTCAATCCATGGCAGGCCCTGCCCTCAGGCCCCCGAGTGGCCTGTTCAAGGGGAGGGTGGCCAGGATGCCACCGAGGTTCTTGATTTGTCAATCCATGGCAGGCCCCGCCCTCGGACCCCCGAGTGGCCTGTTCAAGGGGAGAGTGTCCAGAATGTCACAGACCATGAACCTAGAAGGGTGGTGGTGTCAGCTATTGTTCACATGTGTCAGGATGACGAGTTTCCGGATCTACAAGATCCTCCAGATGAGGCCTAAGCAAAGGTGTAGAAGTGTGTCCCCCTCCATTCCACCCACTGATAATACGCCCGACAATAAAGTTGATGATATTGAATTCCACACCTGCTTGTGTTTGTGATTTTATTTCATATTCCATGAGAGAGACCTCGCATATTTGCAGAAGGGTCACTGAAACATCTTATCTTAAAACAGTTACACCTGAAAAATGAAGAAAGCGTGGCTCAGCAGAGCACGGCAAGCCGATGCCGGGGGGGCATCTGGCTCCGAGGACCCACCAGATTATGGAGATCAAGGTAATGTGCAACAGGTGGGATCTGGTCCTATTTCACCTGAGATTGGCCCCTTTGAACTCTCTGCGGCCAGTGAGGATGATCCTCAGTCTGGGCCAGTGGAAGAGAATTTAGATGCCGCTGCAAGAGAGGAACAGGAACCTCATGAGCAGGAGCACAATGGTGGTGACGATCCCTTGGATGTCCATACTCGCCAGCCTAGATTTGTGGATGTGAACCCAACGCAGGCTCCAGTTATCCAACTAGTCCACGCTGTCTATGATTCCATGTTGGTAAGAGGCACCTAGAACATTTCCAGATGTTTCACTTGGATTTTTTGGCCAGTCTTAATTGATTGTCATTGGTTTCAGCAATCGGACCTCCGGCCCCTAGGCAGTTTATTCCTTGAGCAAAACCTGAACATCGAAGAATTTATATGGATGTGCATGACCGTGCGTCACAGATGTCAGGCCATCAGAAAAAAACCATTACCGATTGTTAAGCAGAGGCGGTGGAAGCTCCTGTCATCTTGCAGATCCTGGCGTATGGGTTACCGCACGCATAACCTCAAAGTAAACAGTTTTGAGTCGGGGGGGGACAATGTCCACCCGGTCCTTGTGACTGCTACGCTAGGATGTGATGAGGGCACGCGGCATGCAACAACGTACAGTGCTGGCATTGTACAGATACCACGAATATCAGACCAAAACCAAAAGATCGAAACAGCCTTCCTGATGGCACGTCGTGCTAGGTCACTTTCGGCAGAAAGATATACTTTGTTCTTTGATTTAGTATCCTCCGGAAACACCCTGTATGCTATATGGATTGGGCTGGGCACGAAAAACCGAGTTTCATTTATTGAGTTTGTAGGATGGTTATGCAAGAAGGACCACACTCATATACGCGAATGGTTCCGCCAGTGCACCGGGAGACCCAAAGCAGCCAAGCCATGGTTAAGAGCGCATCCTGTCGCCATTCCTTATGATGATCCGTTAACAAACGAGGAGATTGATCTGGCCTATGCCCGTGGGCAGGCCATGAATATTGAGGCTCCTAGACTGCCAGATGATCCTATAATTGTTGAGGATGACGACGAAAGTGAGGAAATTGAAGCTGAAAGCGACGAGGAGGAAGACAAGAGTGGAATGGAATCTCTTAAAAATATACCGCAAACACTGCCGTACAATCCAACAGTATACGGCAGGCCCGCGGTGTTTGACCGAAAGTCAGATGCAAAATCAACCAAAAAATGCAGGGCCATAGTAACTGACTTTAGTGTAATCAAGGCCATTGAAGAGGAACACAGAAAGAAGAAGGCAGCCATAACAGAGCAGCCAAGAGCCACGCCTGAATCCCAGGCCCCCACAGTGGTCCTACAGCGACCACCCACGCAGCAAGAGCCTGGCCCCGCCGGCCCACTGAGTGTCCAGGCTCGGCTGGAGCCATGGCAACCTTTGCCTGGGCCCCAAGTGACAGCAGTTCTACTTCACGAAGAATCCATGCAGGGTGTCCAAGTACATGGTTCGATGCTAGACCTTCTTGAAAAAGACGATGAAGTCATGGAGCAGAGGGTTATGGCAACCCTACTGCCACCAGTACCACAACAGCCCCGGGCTGGCAGAAGAGGCCCTTGTGTCTACACCGGTGTCCTAGACATAGAGAGTGATGAGCCCGCTTTGACAGAGCCGGTTCATGATCAGCTACTGCCTGCCCCAGGACCTGACCCTCTTGAAATCCAACCACTAACGTCCCCCACCACGTCTCAACTTAGCAGTTCGGCACCAAGCTGCGCACAAACTCCATGGCCGGTGGTTCAGCCAAGTCAGACTCCAGATGACCCAACGAAACAGTCCCAGCCACCGGAAACAGCTGCCCCACGCCAGTGGCCAATGCCACTGCGACCTATCCCTATGCGCCCCTTGCGGATGCAGACAATCCCATTTAATCATCCAGTGGGACCCACTCCCCATCAGACACCTCAAGTGGAACTAACACCATATAAGCCCACTTGGGCTCAGATGGGGCACATTCCATATCAGCCCACACCAACGGGTCCTGCTACCATGCTCTTGCGCCAATGGGCACCCGCCACCATGCAGACACCACCGAGAGCGCCCACTCCCATGTCACCACCTGAGGTGCCGCCCGTTCCCCGGCAGAGGTCTCGGGGGGCGCCTACTCCCACGCCACCTCCTCAGGTGCCGCCCGTTCCCCGGCAGAGGCCTCGGGGGGCGCCCACTCCCACGCCACCTCCTCAGGTGCTACCCACTCCCATGCAGCTGGCACCAAGGGCTCCTGCTGGTCAGCAGGGGCCGACAAAGCAAATTTTGCGCCAATTGTTAACGGGGGGCGTTAAGAAAGGGAGACCATCACTTAAGTTACAGGCCGCCCTTGAGCGTCAAGCCGCTGCGGGCTGGCAGCCTTCAACAGGGTCCGGCACCAGTGCCAAGATTGTGCGGGCACCTGTTTTCTACCCACCCGTTTTGCAGCCCATACAGGTTATGGGGCAAGGGGGTTCCCCAACGGCCATGGCCGCCTCAACGGTGACACAGGAACCCACGGAATATACCAGGGAAAGGAGGGGAGTGGGGCCTATGCCTCCCACCGATATTCCGCCGTCTAAACGAGCGAAGATCGAGGCCTATACAGAGCCCGAGATGCCGCACGGGGGGGCCTCGCACTCTCCCGTCGTTATCTTGGAGAATGTCGGCCAGGGGCAACAGCAGACTCTGGAGTGCGGAGGAACTGCTAAACAGGAAAGGGACGTGTTGGGGCTGGGGGACATTGCAGTTTCTTCCCCTTCCTCTTCTGAAACATCGAACGATGAGTGATTTTGCCCATGTAACAAGAACTGGGATGAACCCTGGGGCAACAGACTGCGGGGAGGAGGGGGGCAGTGATAAGTCATGACAATTTTAGATGAGGTAGAAATTTTGCATATTTTCAGACCCACCATGGAATCATTTGAAGGACAGGGGGACTCTAGACAGTCACCCAACAATGAGCGGGGAGATAATGTACAGACTACCGGCGAGCATGATCAGGACCCTGGGCCGGGGCCTCCATCCAGTGGGGCTTCTGAGAGATTGGTACCAGAAGAGTCATACTCAAGAGATCAGCAACCTTGGGGGCAAAGCAGGGGTGATGAAAACAGAGGCTGGATGCAGCGCATCAGGCGAAGGCGGAGAAGACGGGCTGCCTTGTCCGGCCATCTGTTAGACACGGAAGACAATGTGCCGCCATGGTTGCCTCCACACGACATCGCACCATATGTCGCAAGGAATATCAGGGATGCCGCCTGCCGGGCTGTCAAGGTGAGTATGCCTCTAACTGGGTTCATGGGGGCCATCTAAGGCCCACGTGTGACCCATGTTTCCATTAATTTTAGCAATCGCACCTGCAAGCGCTATCAAACCTGATACTCGATAGTGGGTTAGACACACAACACATCTTGTGCTTCGTGATGGCAGCCAGGCAGCGTCTTCAGGACATTCGACGTGGACCCTTGGTTGTGGAGGGCGGTGTGGGTTGGCGACATTGGCTTCTAACATCTCCCAGCCAATCCTGGCCCATGGGATATCGTACAGCAACACTACGCACATTAACTCCCGTGCCTAACAGGGTTGGGGCTGACAGCATCATGTTAACTGCCACATTTGGATGCCAAAATGCGGCACGAACTCTAAACACCTTCTCTGCCACCGTGTGGACACCACCCCATGCTGGACCAAGAGAGCAAGAAAGATACGCTCGGGAAGCCGAGGTGCGCTTCCTTCGTGGTAAATGGCAGAGGCGGTACCGAAGAATCTATGATTTGATAGAACTGTGTGGCTCTCTGCACCACATCTGGCAAAACTTGCTCCAGACCGAGGAGAACCTTTTAGATTTCGTGCGTTTCATGGGTGTCATGTCCAGCTGCAATAATCCAGCTGTGAATTACTGGTTTCACAAGACAATCGGAAACTTTAAGCCATATTACCCGTGGAATGCACCACCTAATGAAAATCCATATCACGCGCGGAGAGGCATAAAAGAACACGTAATCCAGAACGCATTTCGAAAGGCACAAATACAGGGTTTATCAATGTTAGCAACGGGAGGTGAACCCAGAGGTGATGCTACTAGTGAAACGAGCAGTGATGAGGACACCGGTAGACAGGGTTCGGACGTGGAGCTAGAGTCCTCGGACGATGAGCTGCCATATATCGATCCCAATATGGAGCCGGTTCAGCAGAGGCCCGTCATGTTTGTGAGCCGTGTGCCTGCAAAGAAACCGAGGAAACTGCCTTGGCCCACGCCCAAGACGCACCCAGTGAAGCGCACAAATGTTAAGACCTCTGATAGATCTGATAAGGCAGAAGCACAAAGCACCCCTGAAAGGCCGGGCCCTTCCGAACAATCATCAGTGACCGTGGATCCCGCCCACCCGACCCCGGTGGAGATGCCAATGGTGATTCTCCATCAACCACCTCCAGTGCCCAAACCGGTTCCAGTCAAGCCTACGCCACCGCCTTCCCGTAGGAGAAGGGGAGCGTGTGTTGTGTACGACGATGATGTCATAGAGGTGATTGATGTTGAAACCACCGAAGATTCATCGTCAGTGTCACAGCCAAATAAGCCACATCGGAAACATCAAGACGGCTTTCAACGTTCAGGCCGACGTCAAAAACGAGCCGCGCCTCCCACCGTGAGTCCTTCGGATACTGGGCCTCCTGCCGCGGGGCCTCCTGCCGCGGGGCCTCCTGCCGCGGGGCCTCCTGCCGCGGGGCCTCACATACTGACGCCTCCTTCCGCCCGGCCTCGTATAATGGCGCCTCCCGTCGTACGTATGTTTATGAGGGAGCGACAGCTCCCCCAGACCACCGGCCGTAAACCTCAGTGCTTCTGGGAAATGCGGGCTAGTCGTGAAATTACACAAATGCAACAAGAACCAAGTTCACACCTGCAGTCCGCCACTCAGCCTACAATGCCTCGCCCATCATGGGTCCCATCAGTCTGTGCCCTCTCGGTGATGGATGCTGGTAAGGCCCAGCCCATACAAAGTTCACACTTGAGTTCCATGTCGCCCACACAGCCGATATCGCACGAAGAACAACCCCGGTATGAGGATCCTGACGCTCCTCTGGATTTAAGTTTACATCCAGACGTTGCTGCTCCACCAGCTCCCCGGGCTCCATACCAGGGATACCAGGAGCAGCCGGCCCCCCAGGCTCCATACCAGGGATACCAGGAGCAGCCGGCCCCCCAGGCTCCATACCAGGGATACCAGGAGCAGCCGGCCCCCCAGGCTCCATACCAGGGATACCAGGAGCAGCCGGCCCCCCAGGCTCCATACCAGGGATACCAGGAGCCGCCGGCCCACGGGCTCCAATCATCTTCATATCCAGGATATGCGGGTCCCTGGACCCCAAGGTCTCAACATCCATGTTATAGGCACCCCTGGGCACCATGGTCTCAAGATCCTGTGCATGGGCACACCCAGGGTCCATGGGATCCCAGGGCACCACATCTCCCACCTCAGTGGGATGGATCTGCAGGACATGGCCAGGATCAGGTCTCCCAGTTCCCACATCTGCAATCGGAGACAGGCCCACCACGTCTTCAACTTTCATCGGTGCCACTGGTCTCATCCTCTGCACCATCATGGTCATCTCCCCAGCCCCGAGCCCCCATACGCCCCATTCCAACAAGATTCCCCCCTCCCCCTATGCCGTTACAAGATAGCATGGCCGTGGGGTGTGACTCATCAGGTACAGCATGCCCAAGCATGCCCTTTGCCAGTGATTACAGTCAAGGTGCATTTACCCCACTGGACATTAATGCCACCACGCCAAAAAGGCCTCGAGTAGAAGAAAGTTCTCACGGACCCGCCCGGTGTTCCCAAGCTACTGCTGAAGCACAGGAGATTCTCAGTGACAATTCTGAGATCTCCGTGTTCCCAGAGGACGCGAAGCAGACTGACTACGATGCATCCACTGAAAGTGAGCTAGATTAAGGGGATCCAAGGTGACCCCTGTTAGCTATTTGATCTTTGACTGACACATAAACATGGTTTAAGGAATGAACACTCATGGTGTGAGACTGGAACTGTACTAAATTTGCTGACATATGTACAATGAGAGCCAAAAATTTGATAAACCTTAAAAGTCCCCCCATCTAATGATGTCCAGTTCCCTTCTCCCACCCTGTACACCCCGACCCAAAGGGACTCAATGGCATTCAGATTTCTAGTTACCACAGGTAGAATATCGGGCGTTGGCCCATAAAAATAAGTGCATGGATATAGCTCTGCACAGGCTTGGAAACACCCATTCCAGGTGTGCTTCTTTTTGGTGAAATAAAAACAGCATCCTTTATATGAAAATGTGTATTCTCTGGTGTTGCAGTATGTACAGTTAGCTTTGGTATAGTTTTGGGGTACCTGAAATGTGTGCAGGGTGGGTGTCCAATGTGGCAGTTTTACCTCTTTGTCCCCATACTCCTGCTCGGCCGTCTTGTTAAAGTTAACCGGCGGTGGAGGATCCACCGGCCAGACCTCTACATTTGGTTTGGGTACCCAGGTGATGGCCGCGGCTGCCACCCGCCCTCCTCCTCTTACCCTGGGTGGCAAAAAGTATGCCAGGAGTAGAACAATAACAAGTGCGATGGCGGTAAACAATGGCACCCTCACCTGCTTAAATGAAACCATGGCAACCACTTCAAAGAGAGCCGACAGGAAGATATTTATTAATATTCCATTAGTAAACGAGGCGTGAAGCAGGCGTGGTTTCAATAACGGGAATTAGAAATTTAAGAGATCCTCGTGTAAAACATCTGGTGTCCGGGGGATAATGGAGTCAACATCCAGGCTTGGGCACATCTGCTTCAACAGGAGGCGCAGCCTGTCATTTTCAGATGATTTGGCAGCAGCCACCTGCGGACAAAAATCAGGCGTTTAGATGGGGCATTTATGTTTGGGACGCTAGCCGCCTGGGCATTCGTGTTAGTATATACTGACCTCACGGTAGTGCTGCAGCAGTTGCTTAAACTTGGCCCGGCATTTTCTGGAAGCCACCCGATTCTTGTATCGCTTTATTTCTAGTTCAGAATCGCATTCCTCCAGCTGCGAGCAAGGGAATGCGTTACTACAAGTGGTGCCTAGTCAGTTGAAACAAGCCCCACCGTCCGCTGCCGCCCCTCCTTGAGCCCCACCGTCCGCTGCCGCCCCTCCTTGAGCCCCACCGTCCGCTGCCGCCCCTCCTTGAGCCCCACCGTCCGCTGCCGCCCCTCCTTGAGCCCCACCGTCCGCTGCCGCCCCTCCTTGAGCCCCTCGTTACCGATTCTGGCTGTTGTGGTTTCCGTGTGCGTCGTGCCGGGGCAGCCACTGGTGCAGGCTGTGGAACACCAATGTCTGCTAGCTGTTGTCCTTGGTTAGCCCCGGGGCAAGCAAACACCACTGCTGCTGTTTGAACAGTAGAATTGTCTCCAGGTTGAGGTGCTTCTCCCCCGGCTTGGTTAGTCTGTTGATTCTGGGTTATGTCGGAGACTGGGAACAGCTGAGGTGCTGCATAAGCTTGATAAGCATTCTCAGGAGCAGGCTGAGGGGCAGAAAACCACGACCCAGTTGGAGCGGTTGAAACATGATAGGCAGTTAGCTGGCCTTGTGGCAGAGGCTCTGGCAGCACCGGCCACAGCACACAAGGCAAAGGAGCTTGCGATGGCCCTCCCAGGTCCTGATAGACTCTGGTAGCTTGGTCAAAAGCTTGTACAAAAGGCACCTGGTATGGGTCAGGTGTAAATTTTACATCTTCAGAAGTCGAGTTTGGGTCCATCATCTTCAGCAAAGATAGCAAAGGTGGCCGGCAAGGTGCAATGTTTAGTGAGTTACCTGTCTAACATCTCCCCTTTAAAGCCAAGGCACCAGCCTCCTCTGTGATGTCATGGTTTGGGACGTGCTAAATTTAGGTGTGTCTATGAGGTACATTAGCAATGCCTGTGGCTCATGCATAGTTTCTAAAAGAGGAGGAGGCAGTTTTCAGAAGTGTCTAAAATAAGCTGGTGTCAAAAATAGACAGCCCAGTTGAAATATGCATGGCATGCAGCAGACATTCATCATTTAGAAATGTATCCAAGATTTCATTAAGTTCGGGGGTCAGGGGGGAGTCCAGATTCAAATCCTCTGTCATGGACTCTAGTGTTGTGGTCAGTTCGTCCAAATGGCCACGAGGGGGCGGGTGGTTCAGGTCCATCTGTCCACATATGGCTGCTTCCTCCTTCTGGGGAATAACAGTGTCAGCCATCTCCCTTAGGGCCTTCACGGCCTGACTGGTTTCCTCATCAGGGTCCTCCAACAGATGACTTGCCTCGGGGGTTACTGCGGGGGCCGGGTCAAGTGGCCGGGGCACTGGGGCTGGCGTTAGGGATCCGATCGGTTCATGGACAGGTCCTGTGGGGGTGGGAGCCAAAGAGGCAGGCAGGGGCCGGTTGGCCCACGGGGATCCGGGTGGATGGAAGGGCCTGATCCTCTTTGGCTGACACACCTCTCGCCCCTCGAACACGTCAGATATGGCACTGCCCGCTTCCGGCTTTGGCAGGAACATACCTTCCCGGCTATCCCTGAGGCCCTTCTTCCTTTTAACGGGAGGAAGAAAGGTGGGCTTTGAGGGGTGGGGGAATATGGGTCTCTCATCGCTCTCTTGGTGGACCGCTGCTATCCAAGGCTGTTCAGGTTCCGCCGCGTTGGAAGGACATGGAGTTTGACCACGGTTGGGCCTGGATGTACGGCGCGACTTTGGGGCCCGCAGGCGCGGGGCCTCGGCCCTGGCCTCTTCCCGCTCGCTCTGCTCGGTGTCACTGTTGCCCGAGTCACTGCTGCTGGAACTGCTGTCACCGCAGTCGGCGCTTTGGGCACCGGGCTCCAGGGGCATGGTCGGGCTAGGGAGGCTTTCGAGTTCATCTGTAAAAGCATGAAACTGTCCGGACTCCGAGTGGCGGGCCTCGGTGTGAGAGGCACCCCCATCATTCCCCATGAGCTCCTCATCCATCATGTCAGCTCCGGATACGAGGATAGGAGTTTCCACTGCCTTGGACTTGGTTGACAGCAGGCAAGCGGGAAGCACGCCGCTCACGTAGCTCCTCTGTCCGGCGTGGCTGGAGTAGGAGGCCCGGGGCAGTGTCTTAATCAGAGCCCTGACATCCTTGACATCGTCCGTCAGATGGCCTGTCTTGGACGAGACCATAGTCTGGAACATCTCCTCGAGGACGGGATAGGTGAACACCCACTTGCAAAAGGCCTTGAACTTGGAGCTTAGGAGGCCTTCCTTCTCCATCCTGTTCAGGTGTTCCACTACCTGCTTGCCGGAGGCCATGATGGCCGCGCGGTCCACGCCCAGCACCTTGCTGTAGGTGTAGGCCCGCACCCGACTGTGTTTTAGGAGCTTGTACATAGCGGTGCCTATGGTGGCAGGAATCATCACCCGGTTGCTGGGGGCCTGGATGAAGAATCTGTCAGTGACCACTATCAGGTGGTCTAACACGTAGCGCATCACTATAGGGCACGCGATGGAACATGCGTCGTTGCCGGCATTCTCGGCCCGTCTTCTTACCCTGTTGTTTCGGAGAATGGCCCAAAAATTGCAGATGTTGAGCGTGGCCATTAGCCCGCCCCATTCTCGCCCGTGGGCCTTGGCCTCATTTATAAATGCCTTGCATATTTTGTAGGATCTCAGAGTAATCTCCACACTCCCGGCTGTAAATTCCTTGTTGAGGACGTTGCAGTAGTCAGAGACCAGAGAGCCCAGCTGCTTTTTGATTTCAGGAGTTAGCCTCAGAAAGTCTTCCAAGCCATCCTTTTTAGGCCTCATGGCTAGTAGTAACAGAGGAAATGCCCGACCATTAAAATCTTTCCTCCATGAGCTTTACCTGAAACACTATCCCGAAGTGGGGGATGTGGTGCATCTACTGAACACCATCGGGGTCGACTGCGACCTCCCACCTAGCCACCCACTCCTGACAGCCCAGAGGGGGCTGTTCCTGGCAAGAGTCTTGCAGGCTGTGCAGCAGCACAAGCTGCTGGAAGACACCATCGTCCCCAAGATCTTAAAGAAGCTGGCTTATTTCTTAGAGCTGCTAAGCTACTACTCCCCCAAGGATGAACAGCGTGCCATCGCCGAGGTTCTTGACCACCTCAAGACGAATCGGGACCTGGGGCTGGACGACAGACTCTGGGCCCTGATTAGGAAACTGCGCCAAGACAGACACCACGCCTCTGTAAATGTCCTCATGCCAGGAAGCGACTACACAGCCGTGTCGCTGCAGTACTACGACGGCATCTCCCTAGGTATGAGGAAGGTAATCGCGGATGTCTGCCGCAGCGGCTATGCCTCCATGCCCTCCATGACGGCCACGCACAACCTCTCCCACCAGCTCTTGATGGCATCCGGGCCCAGTGAGGAACCGTGCGCCTGGCGCGGGTTCTTTAACCAGGTCCTGCTCTGGACTGTGGCCCTCTGCAAGTTTCGCAGATGCATTTACTATAACTACATTCAGGGATCTATAGCCACCATCGCCCAGCTTCTGCACCTCGAGATCAAGGCCCTCTGCAGCTGGATAATATCCCAGGATGGCATGCGCCTCTTTCAATACAGCAGGCCTCTCCTCACCCTCTGGGAGAGCGTGGCCGCAAATCAGGAGGTCACAGATGCCATTACCCTGCCTGACTGCGCTGAATACATAGACCTACTGAAGCACACAAAACATGTCTTAGAAAACTGTTCTGCCATGCAATATAAATAAATTTCTCTTACCTGCGTCTGTTTGTGTAGTGAGGTGTTGTGTCCTGTATGGTATTCTACTTTAAAAATGCTGGCTGACATGGATTACTGGTCTTTTATGAGCCATTGGCATGGGCGGGACAATCGCAATATAAAACCCTGACCATCACATGGGGCATTAGGCGACTCTGCATCAGCATCGCTTAAGTATGAGTGGGCAGCAGAGAGGCTCGGTTATTTTGGTTCCTGAACATCTGGCTGGGGCATTAACTAAGCTTATGAGCGATTTTATCACAGGACAAGATGACACTCTTTCTGGAGGAAATATTGCAGTCAAAATTCGCGATGCTATAAACCAGACCCCCGGGGGTGGTGATGTAGCTATACTTTCTTCCCTGTTTGCTTTATGGAATGCCCTCCCAACATCTGGTAGACAATCCTCCAGGGACAATTTAATCCCAGCCGCCGTGCAGGCCTTAACCACGGCCCACAACTTATGTCTGGGTGTTATTCCAGGTGAGACCTCACACAAGGACACACCCGAGTCATTGCTCCGGGCTATCGTGACGGGTCTCCAAAAATTGTGGGTGGATTCGTGCGGATGTCCAGAGTGCCTACAATGTCTTAAGGGATTGAAGGCAATTAAGCCCGGCCTTTATGAAATCCCTAGGATAATACCACACACTAAGCAGTGTAGTCCTGTCAATCTCCTGAACATGTTGGTCCACAAGCTTGTGGCTTTACGTGGTCATGTGCAGCCTGCATACGACGCCCGTGTCCTGACGCCTGACTTTCATGAAATCCCTGACCTCAATGACTCTGATGCTGTTTTCGCACGCACCTTATTGGCAGCCTTATTTCACCTCAATATGTTCTTTATTCTCAAAGATTACATAACACAAGACTCCATGAGCTTGAAGCAAGCCCTTAGTGGTCATTGGATGTCTGCCACGGGCAACCCCCTGCCTGTAGCACCGGAAACCCTGCGAGACTACTTGGAAGCTTTCCGAAATTCGGATAATCACTTTTATCTCCCGACGACAGGGCCTTTAAACACCTTCCAATTTCCCGAAGAGCTTCTGGGGCGCGTTGTTGTTATTGATTCCTCTTTGTGTGCCGCCAGTCACGTTCAGGACGTTATCACCCATGGTGTTGGGGCGGGTGTTCCTCGTCCTCGGTTTTCGGCCCTGCCTCCGGCCCCATCCCGTGAGCCCCAGCAGACATGCTCTCAGTTAACGAGCAGAGGGAATGAAAGCTCACGGCGAAACTTGGGCCAGCCCGGGGGGACCTCCCCCGCTGTTCCCCCAGTTTGCCCCATCGTTTCCCTGGCAGCCTCCGGGGCCAAGCAAAACCGCGGGAGCACGGGATCCTTGCACTTAGCCCAGCCTGAGGGGACCTCCCCCGCCGTCTCCCCAATATGCCCCATCGCTTCCCCAGCGGCCTCCAGGTCCAAGCAGCACTGCGGGGTCACTGGATCCTCACAGGCCGCACCCAGCTCTTCTTCCGTTGCCCCAGTAGCATCTCTGTCTGGTGACCTTGAAGAGGAAGAGGAGGGGTCCCGAGAATCCCCATCCCTACCGTCCAGCAAAAAGGGGGCCGAGGAATTTGAGGCCTGGCTTGAGGCTCAGGATGCAAATCTTGAGGATGTTCAGCGGGAGTTTTCCGGGCTGCGAGTAATTGGTGATGAGGACGAGGATGGTTCGGAGGATGGGGAATTTTCAGACCTGGATCTGTCTGACAGCGACCATGAAGGGGATGAGGGTGGGGGGGCTGTTGGAGGGGGCAGGAGTCTGCACTCCCTGTATTCACTGAGCGTCGTCTAATAAAGATGTCTATTGATCTCTTTTAGTGTGAATCATGTCTGACGAGGGGCCAGGTACAGGACCTGGAAATGGCCTAGGAGAGAAGGGAGACACATCTGGACCAGAAGGCTCCGGCGGCAGTGGACCTCAAAGAAGAGGGGGTGATAACCAT

CCCCGGGCGGCTCAGGATCAGGGCCAAGACATAGAGATGGTGTCCGGAGACCCCAAAAACGTCCAAGTTGCATTGGCTGCAAAGGGACCCACGGTGG

GGCCGGGGTCGAGGAGGTAGTGGAGGCCGGGGTCGAGGAGGTAGTGGAGGCCGCCGGGGTAGAGGACGTGAAAGAGCCAGGGGGGGAAGTCGTGAAAGAGCCAGGGGGAGAGGTCGTGGACGTGGTGAAAAGAGGCCCAGGAGTCCCAGTAGTCAGTCATCATCATCCGGGTCTCCACCGCGCAGGCCCCCTCCAGGTAGAAGGCCATTTTTCCACCCTGTAGGGGATGCCGATTATTTTGAATACCTCCAAGAAGGTGGCCCAGATGGTGAGCCTGACGTGCCCCCGGGAGCGATAGAGCAGGGCCCCACAGATGACCCAGGAGAAGGCCCAAGCACTGGACCCCGGGGTCAGGGTGATGGAGGCAGGCGCAAAAAAGGAGGGTGGTTTGGAAAGCATCGTGGTCAAGGAGGTTCCAACCCGAAATTTGAGAACATTGCAGAAGGTTTAAGAGTTCTCCTGGCTAGGAGTCACGTAGAAAGGACTACCGAGGAAAGAAATTGGGTCGCCGGTGTGTTCGTATATGGAGGTAGTAAGACCTCCCTTTACAACCTCAGGCGAGGAATTGCCCTTGCTGTTCCACAATGTCGTATTACACCATTGAGTCGTCTCCCCTTTGGAATGGCCCCTGGACCCGGCCCACAACCTGGCCCACTAAGGGAGTCCATTGTCTGTTATTTCATGGTCTTTTTACAAACTCATATATTTGCTGAGGTTTTGAAGGATGCGATTAAGGACCTTGTTATGACAAAGCCCGCTCCTACCTGCAATATCAAGGTGACTGTGTGCAGCTTTGACGATGGAGTAGATTTGCCTCCCTGGTTTCCACCTATGGTGGAAGGGGCTGCCGCGGAGGGTGAT

AGGGTGAGGAAGGGCAGGAGTGATGTAACTTGTTAGGAGACGCCCTCAATCGTATTAAAAGCCGTGTATTCCCCCGCACTAAAGAATAAATCCCCAGTAGACATCATGCGTGCTGTTGGTGTATTTCTGGCCACCTGTCTTGTCACCATTTTCGTCCTCCCAACATGGGGCAATTGGGCATACCCATGTTGTCACGTCACTCAGCTCCGCGCTCAACACCTTCTCGCGTTGGAAAACATTAGCGACATTTACCTGGTGAGCAATCAGACATGCGACGGCTTTAGTCTGGCCTCCTTAAATTCACCTAAGAATGGGAGCAACCAGCTGGTCATCAGCCGCTGCGCAAACGGACTCAACGTGGTCTCCTTCTTTATCTCCATCCTGAAGCGAAGCAGCTCCGCCCTCACGAGCCATCTCCGTGAGTTGTTAACCACCCTGGAGTCTCTTTACGGTTCATTCTCAGTGGAAGACCTGTTTGGTGCCAACTTAAACAGATACGCATGGCATCGCGGGGGCTAGACCTCTGGCTGGATGAGCACGTGTGGAAGAGGAAACAGGAGATTGGTGTGAAAGGAGAAAATCTGCTTCTCCCCGACTTATGGCTAGATTTCCTACAACTCAGCCCCATCTTCCAGCGCAAGCTTGCTGCCGTTATTGCCTGTGTCCGACGCCTGCGGACTCAGGCCACCGTCTACCCGGAGGAGGACATGTGCATGGCCTGGGCCCGCTTTTGCGACCCCTCTGATATTAAGGTGGTTATTTTGGGCCAGGACCCCTATCACGGGGGTCAAGCAAACGGCCTGGCATTCAGCGTCGCATACGGCTTTCCAGTCCCCCCCAGCCTGAGGAACATCTACGCGGAGCTGCACCGGAGTCTGCCGGAGTTTTCTCCCCCAGATCACGGCTGTCTAGACGCGTGGGCCTCCCAGGGGGTGTTGCTACTCAACACCATCCTGACCGTGCAAAAGGGCAAGCCCGGCTCGCACGCAGACATTGGCTGGGCGTGGTTTACTGACCACGTAATTTCATTGCTCTCTGAGCGGTTAAAAGCGTGCGTGTTTATGCTGTGGGGTGCGAAGGCGGGAGACAAAGCTTCACTAATCAACTCCAAGAAGCATCTGGTTCTGACCTCTCAGCATCCCTCTCCCCTGGCCCAGAACAGCACCCGAAAGAGTGCCCAGCAGAAGTTCCTGGGCAACAACCACTTTGTCCTCGCTAACAACTTTTTGCGTGAGAAGGGGCTCGGTGAGATAGATTGGAGGCTGTAGAGGGGTCATAACTATGGCCATGTTTCTGAAGTCGCGTGGGGTCCGGTCTTGCAGGGACCGGCGCCTCTTGTCGGACGAGGAGGAAGAGACTTCACAGAGCAGCAGCTACACTCTGGGGTCTCAGGCCTCCCAGTCTATCCAGGAGGAGGACGTGAGTGACACTGATGAGTCTGACTACTCAGATGAAGACGAGGAGATTGATTTGGAGGAAGAGTACCCCAGTGATGAAGACCCATCCGAGGGCAGTGATAGCGACCCCTCGTGGCATCCTTCAGATTCAGACGAGTCTGACTACAGCGAGAGCGACGAGGATGAAGCAACCCCCGGCTCTCAGGCCTCACGATCTTCAAGAGTCTCGCCATCTACCCAACAGTCTTCAGGTCTGACACCCACGCCTTCGTTCTCCCGACCACGCACCCGGGCACCTCCGAGGCCGCCGGCTCCCGCGCCGGTCAGGGGACGGGCCTCAGCACCTCCCAGGCCACCAGCCCCAGTTCCGCAATCCACCAAAGACAAGGTTCCCAATAGACCTACGCGACCTGTACTTAGAGGCCCAGCTCCACGCCGCCCCCCTCCACCTTCAAGTCCCAATACATACAATAAACACATGATGGAAACCACCCCCCCCATTAAGGGCAATAACAACTACAATTGGCCATGGCTGTAAATAAAATGTCATAACCTGGAGTCTGCATGTCTGTTGTTTTATTCAGTAAACCAGTAGTGCGCGTGAGTTCTTTAGGGCATCCACGATGTAGCCGCTCGCGGGGTTCCCCTCCCCAGTGATCATCTCGGATAGGGGATTCCTGTCCATGACCACGCAATTAGAGTGCCGGGCCCGGGACAGCGCCACATACACATGGCCGGGTTTGATGTTTCTGTGGCTGCCGAAGCAGATGGCGACTTTGTTTAGGGACAGACCCTGGGCTTTGGCTATGGTCATGGCCAGCTTTGAGCTAATGCCATAGTCACGGATGCTGCAGAGGTTCAGGGACTTGTCCTCTATAGTCTCATACAGCTTGTTAGTATTGTGTTCCAGGCAGCACACGAAGCCTGCCTCATCCTTGACCATTAGCCTGGGCATGCGTGAACTGCCAGCGTCCTGAGGCTGCTGCTTTCCTCGGATGCCAAAGAAGACGTTGAGATGCGTGTAGCCCAGAAGCGTGTAGTTCTCGGTGGTGGAGGCGTAGTCCAGGAGGCCGTGAAGGAGAGGCTCGTCTGAGGTGAACTCTATGTTGTCGCGAATCAGCATGTTGTTGGTAAATGTGCAGAAGGGGAGGTCCCTGAACTCCCTTCCGCCATAGCGGACGGCCACATCCAGGCATTGCCTGAAATAGGCCCTGAGGTCATTATATATGTTTAACAGGGAGCAGAGGGGGGCAGAATTTGCGGCCGGGGGAGCCAGTACTCGGGCATAGAAGACAGCGGCGGGGCTCCGCTCCCCATCCCAGGCAACCTCCAGCGGCAGTTCGCCCAGCTCCATCCCAGCAGTCACCTCCGGATCCCACGTACGCCCGGGCAGGGGCACAGCACCAAGCTCCGCCACGTATTCCCCGTTTTCACAGAGAGAATGTCCTCCGTGGCTAAAAGCGTAGATGCCGCCGTAGATGAGTCGGGCCAGGAAGCTGTAGACATACTCGGGCTGCTCATGCCCGTGGGCCTCCACGAAGCTGTCCGCCTCGAGCGTGTCCATAAAGTCCCCGAAGGTGCCGGTATAGCCACAGATGGACTTTTTGGTCTTGCAGTTGACCGACACTGAGCTGTGCTTCACGTAGGTGACATTGTAGGTGACCTTGACCCGTTCTTCATCCTGCTCGGTTCCCACCGGGACCATGTCTTGGTCGGCGAACTGCGAGTAGTTACCGAGGCGTGCATAATTCTTTTGGAGCCAGGTGTGGGCCGTGAGGCCCGGAAGACCGACCAGGGTCTTGTACTGGGCCAGGGGATCGAGGAAGACCTCGCACTCCACCGGGCAGGTAAACATGGTCACCCCGCCCCCATCTCCCCCAGTTCCCCGCGCGGCGCGCCCCTGCCCGGCAGTCTTGAGCGTGGCGTGGAGGGTGGTGAGGAAGGTCTTGACCTCGGCGTGGGAGAGGAAGAGCCGGGTCCAGCCCACGTACTGCGTGGGGTCCATTATGGCCGCCCTGGGGACGACGAAGCGGTCGACGTAGGCCAGGATGTCCGGCGAGAGCTCGAGGCCGTACTCGAGGGTCTTCATGAGGTGTCCAAACTGGACGTCGGTGCAGCGCTTGTTGTTGATGAAGAGGGCCCAGTTGCGGGCCACGTCCACGTAGGTCGCGGCCCTGGGGTTGCCCACCAGGAAGGTGAGGATGTTGTCGCACTCGCGAATCTTGTTTACCTGGGTCTCGTGGCTAAAGGAGGACTGAAAGGCGTCTGTCTGGGTGGGAGAGCCCACGCAGACGATGCAGGGAATGCGGCCCCGGCGGTAGAGTGGGGTGCGCAGCCAGGCGTTGAAGAACCAGTAGCAAAAGACCACGGCTGTTAGAATGTGCACGGAAAGCGTTCCAGCTTCGTCCACCACGATCACATTGGTGGTCCATAGCTGCCCCTGGTGCATGTCTCTCAGGACCTCAAAGGCGGGGCCAGAGACTCCCGAGTAGAGCCCCCTGGGCTTGGTTCGCCTGAACTCGGCGGCAATGTCGGAGAGTACCGGCCAGTATTTGGCCAGGTCCCGCCGCTGGAGTTCCTCTAGGGCGGCGTCCGTAGAGCGACCATGACTGCTGACCCGCTGCGTCATATTTATGTGGCGGCTCTTGAACCCAAAGGCGCTATAGACGGTTGGGCAGTAGGCTCGGAGTGTCTGGGAGAGGTTCTGTGCGGCCACGGTTGTGGCTCCCGTGACCAGGCAGTCCATCGTGTGGTGGAGGCAGCTAACGCTGGTGCTCTTGCCAGCCCCCGCCGTTCCCGTAATTACATAGGCTGAAAAGGGCAGGAAGGGGGGCTCCGAGAGCTCCGGGTCAAACTCGGGGGAGAACGTCTCCATATCCGGGAGTTGTTGGACGCGGCGCTTAGCCAGGGTCCCTATCCTCCTGACTATACGCCTCACGGAGGCGTCTGAGGTCATGTTCAACATGAACGTGGACGAGAGCGCCTCTGGCGCCCTCGGCTCCTCGGCCATTCCTGTTCACCCCACGCCGGCCTCGGTCCGACTTTTTGAGATCCTGCAGGGAAAGTACGCCTACGTCCAGGGACAGACCATCTACGCCAACCTCCGCAACCCCGGAGTCTTCTCGAGGCAGGTGTTTACCCATTTGTTTAAACGAGCCATCTCTCATTGCACGTACGATGACGTGCTACATGACTGGAACAAGTTCGAGGCCTGCATCCAGAAGCGATGGCCGAGCGATGACTCGTGTGCGAGCCGGTTTCGTGAGTCCACCTTCGAGTCGTGGTCCACGACCATGAAGCTGACCGTGCGTGACCTGCTGACCACCAACATCTACCGAGTGCTACACAGCCGCTCCGTGCTCTCCTATGAGCGTTATGTGGACTGGATCTGCGCCACCGGCATGGTGCCCGCCGTTAAGAAGCCCATAACCCAAGAGCTCCACTCCAAGATAAAGAGCCTGAGGGACAGGTGCGTCTGTCGGGAATTGGGGCACGAGAGGACCATCAGGAGTATCGGGACGGAATTATATGAGGCAACGAGGGAAATAATGGAGTCGCTCAACTCCACGTTCATCCCCCAGTTTACGGAGGTGACCATCGAGTACCTTCCGAGGAGCGACGAGTACGTGGCCTACTACTGTGGCCGCCGCATCAGGCTGCATGTGCTCTTCCCCCCGGCCATCTTTGCCGGAACGGTGACCTTCGACAGCCCGGTGCAGCGCCTCTACCAGAACATTTTCATGTGCTACCGCACGCTGGAGCATGCCAAGATCTGCCAGCTCCTGAACACAGCCCCTCTCAAGGCCATCGTGGGCCACGGGGGGCGAGACATGTACAAGGACATCCTGGCCCATCTGGAGCAGAACTCACAGCGCAAGGACCCCAAGAAGGAGCTGCTGAACCTGCTGGTCAAGCTCTCGGAGAACAAGACCATCAGCGGGGTCACGGACGTGGTGGAGGAGTTCATAACGGATGCCTCCAACAACCTGGTGGACCGCAACCGTCTATTTGGCCAGCCCGGGGAGACAGCTGCGCAGGGCCTAAAGAAAAAGGTCTCCAACACGGTGGTCAAGTGTCTGACTGATCAGATAAACGAGCAATTTGACCAGATTAATGGCCTAGAGAAGGAGAGGGAGCTCTATCTAAAGAAGATCCGCTCCATGGAGTCTCAGCTGCAGGCCTCCCTGGGTCCCGGCGGCAACAACCCAGCGGCGTCAGCCCCCGCCGCAGTTGCGGCAGAAGCCGCGTCTGTAGATATACTGACGGGCAGCACCGCCTCCGCAATCGAAAAGCTGTTCAACTCCCCGTCCGCCAGCCTGGGTGCCAGGGTGTCTGGTCACAATGAAAGCATCCTAAACAGTTTCGTTTCTCAATACATCCCCCCTTCGCGGGAAATGACTAAGGATCTGACTGAACTTTGGGAAAGCGAGCTGTTTAACACCTTCAAGTTAACACCCGTGGTTGATAATCAGGGGCAGCGTCTCTACGTCAGATACTCGTCAGACACGATCTCTATATTATTGGGCCCCTTCACCTATCTGGTGGCAGAGCTTTCACCGGTGGAACTCGTGACAGATGTCTACGCCACCCTAGGCATCGTGGAGATCATCGACGAGCTCTACCGGAGCAGTCGCCTGGCCATCTACATCGAGGACCTCGGTCGAAAATACTGCCCCGCAAGCGCGACCGGGGGAGATCATGGCATCCGGCAAGCACCATCAGCCCGGGGGGACGCGGAGCCTGACCATGCAAAAAGTAAGCCTGCGCGTGACCCCCCGCCTGGTGCTGGAAGTTAACCGCCATAACGCCATCTGCGTGGCCACCAACGTCCCTGAGTTCTACAATGCCAGGGGGGACCTTAACATCCGAGACCTCCGGGCCCACGTCAAGGCCCGGATGATCTCGTCCCAGTTTTGCGGCTACGTCCTCGTGAGTCTGCTGGACTCCGAGGACCAGGTCGACCACCTCAACATATTCCCCCACGTGTTCTCCGAGAGGATGATCCTGTACAAACCCAACAATGTGAACCTTATGGAGATGTGCGCCCTGCTCTCGATGATTGAGAATGCCAAGAGCCCCTCCATAGGCCTCTGCCGGGAGGTGCTGGGTCGCCTGACCCTCTTGCACTCCAAGTGCAACAATCTGGACTCTCTGTTTCTGTACAATGGGGCCAGGACGCTGCTGTCCACCCTGGTCAAGTACCACGACCTGGAGGAGGGGGCTGCCACCCCCGGGCCGTGGAATGAGGGCCTGAGTCTCTTTAAGCTGCACAAGGAGCTGAAGCGCGCCCCATCCGAAGCCCGGGACCTCATGCAGAGCCTCTTTCTGACCTCGGGGAAGATGGGGTGCCTGGCCAGGTCACCCAAGGATTACTGCGCGGATCTAAACAAGGAGGAAGATGCCAACTCGGGCTTCACATTTAACCTGTTTTATCAAGATTCTTTATTGACCAAGCATTTCCAGTGCCAGACCGTCCTCCAGACCTTGAGACGCAAGTGCCTCGGGAGTGACACGGTCTCAAAAATAATTCCCTAGAATAAACTGAGAACAGTCATCAGTAAATCTGTCTCTCGCGTGATTTCCATAGGAATGGTGTAGCCGGGGTGGAGGGCCGATATCACATCAAGCAGAAAGGCCATAATCTCTCGAAAGTAGGCGGTGGGGCTGAGACCATGCTCAGTGGCCGTCTGGCAGGGGGCCGGGCGCGCTCCGTCCTTGTCCAGGAGACACACGTGGCTTCCAGAGAGGCGCAGCCCAGCCCTCCGCAGCCGCTGAAGCCAGGCTCGCGGAAGAGCCCAAAACCTGTTTCGGCGCCGCCCGGGGGCCAGTCTCCGGGTCAGGTCGCGGACCAGGGTCAACAGGTGGTCGTGGGATGGCGGGGCCTTGTCTGCCTCGGGTCTCGCCGCTAGTTGGTCCAGGGTCCAGGAGAAGGCTTCGTGCCAAACCAAAAAGGGCCCCGAGTGCTCCCTACATCCACCCACGTAAAGATCCCCCTGAAAGATGGCCATCAGTAGGCACCCGGGCCCGCGTCGAGCCTTCACCCGAATGTGTCTGCGGGCCACGGTGGCCTCTCCACCCATCACATCCCGGTCGAGCCGGCTGGCATCCTCCGAGTCTTTCACGCCTTGCAGGAAAGCCTAGGAGATACAGCAACAGAAAGCTATTAGCCGGTGGTTCCCCCACCATCATTCTTCCTGTTAACGGGAAGAATAAGGAGTTGGGCAAACCCCGGGGGCCGCGCTCTCCCACCCAGCCCCGCTTCTCACCTGTGCTAGTGGCTCCTCTGAAGGATGGGCGGAGGTTGGTGCCACAAAGCCCAGGATGAACTCGTCTGCATAAGCCCAGGTCAGTCCTAGGTCAGCGGCCGCGTGCAGGAGAACCCGGGTGACGGCGGTGTAGAGGCCCCCGAGTGCCCGTCGCGTGTCTGAGGTGCCATAGCGGTGAAGGGCCCGCAGCCAGGTTTGCGCGTCCCGCGCCTGCCCTCCGCCATCAGGCGTTCCCACGGGGGCGCCCCTGGCAGAGAGGTGGCAGCGGGCCAATTCGTAGAGCCACCAAGTGGCATCAGCCTCAAGGATGGCTGTGGCCTCCGCGCGCCCGACCACCGTCGTCTCGTCCTCCCCCCCTCCCTCGCCGCCCTCCCGCGTGCAAACGTGGCGAGGGTTAATCTCCTTTCGGGTCGGGGGCCAGATTTGTTGTAGGAGCAGCGAGCCGCGTCGTTGCCCTGACCGCGCGTCGAGGCCCAGGAGGGCGTCTGCCAGGGGCGTCCCAGAGACTCCCAGGTTCAGGTCCAGTAGCAGGAGACCCTCGCTGTGTGGCGCCCGGTGCCAGAAGGCCGGCCTCGCCCGTCCCACATAATGGATGGGCAGGAAGGGAAAGCCCGGGACATAGGGCTGGAAATCTGAGCCCCCTGGGCAGAGTTCGGGGTCCAGGAGGTAGAAGATGGGCTTGGTGCCTCTGTGGTTGGCGTAGCAGGAGGCATAGATACTGCGGAGGAAGGCGTAGAGCCCGCCCCCGGCCATACTCCAAGAGTTGACAAGCCAGGACTCGAATCCCCCAGCCGGCTCGAGAATTTTCAGGCTGACGCGGTGCCGTCGGGCGTCCCCACCACGGCCGGTGGCCCCGTCGGACGACACCAGATCTACTTCATAAGTGACCGGTCGCAGGATGTCCCTAAAGGGGACGGGAGAGGGGTCGTCGGGAGTCTCGGTGGAATAGGTGAAAACATCCCCACGCGGTGTCCTGATGTATACGTCCAACTGTCCGGGAGACTCAGAGTGCCTCTGAGCATGGGGGCATGTCTGTTCCCCCTCCACCTCGGACCCGAAGCCATCAACAGGTGGGGGTTGTTGGTCCCGCCCATCATCCCCCGAGCAGCTTTGGCAGACCACCTGTGCTGGAAAGAGAGGCTGGAAGAGGAGGCCCTGCTCATCCTCCACCCGGGCGGCGGACAAGAGTCTGCGGTCTCGGGTTCTAAATGAAAGGTCAAATAGGTCCTTCTCGGCGGCATCAGCGAGCATAGCAATGAGCCCCCCGCTGCGCCTGAGCTCCCGCTCCCATCGCAAAAAGTTGAGTTCGGTAGTCGAGGGCGCGTTGACCACGGGGGGCTCCAGGGAGCCTCCAAGCGGCGGCTGGCAGGCCTGCACCACGATCAGAGTCTCAACGTCCTCCCTTTTGATGGGCACGATGCCCACGACCCAAATCGCCCACCACCGCCCTGCGGTCTGGGTGACATTATAAAAGGTAACCGAGCTGACCCGGGCCCTGACGCTCTCCGCGGGTGTTTCCATCATTGTTTGAGATCTGAGGAGGACTGGACCCTTTAAAACATCCGGTCACGCCCTTTGCAAATTATTTAAAAGGTGAATGCTCAACTGAGACCATCGCAATCATGAAGTCCTCCAAGAATGACACGTTCGTCTATAGAACGTGGGTCAAAACGCTTGTTGTGTACTTTGTGATGTTTGTCATGTCGGCGGTGGTCCCCATCACCGCCATGTTCCCCAACCTGGGGTACCCCTGCTACTTTAACGCACTGGTTGATTACGGGGCACTTAACCTGACCAATTACAACCTGGCCCACCACCTGACCCCCACGCTCTATCTGGAGCCGCCGGAGATGTTTGTCTACATCACACTGGTCTTTATCGCGGACTGCGTGGCTTTCATCTACTACGCCTGCGGCGAGGTGGCGCTAATCAAGGCCCGAAAAAAGGTCTCGGGTCTTACAGACCTCTCGGCCTGGGTCTCGGCAGTGGGCTCCCCAACCGTGCTGTTTTTGGCCATCCTCAAGCTCTGGTCCATACAGGTCTTCATCCAGGTCCTTTCCTACAAGCACGTCTTTCTCTCGGCCTTTGTGTACTTTTTGCACTTTCTGGCCTCAGTTCTACATGCCTGCGCATGTGTGACCCGCTTCTCCCCGGTCTGGGTGGTCAAGGCCCAGGACAACTCTATTCCCCAGGACACCTTCTTGTGGTGGGTGGTCTTCTACCTGAAGCCCGTAGTTACAAACCTGTACCTGGGGTGCCTTGCCCTGGAGACGCTGGTCTTCTCGCTCAGCGTGTTCCTGGCCCTGGGCAACAGCTTTTACTTTATGGTGGGGGACATGGTGCTGGGAGCCGTGAACCTCTTCCTCATCCTGCCCATCTTCTGGTACATTCTGACGGAGGTGTGGCTGGCCTCCTTCATGCGGCACAACTTTGGCTTCTACTGCGGCATGTTCATCGCCTCCATCATCCTGATCCTGCCCTTGGTCAGGTACGAGGCCGTCTTTGTCTCAGCCAAGCTGCACACCACTGTGGCCATCAATGTGGCCATCATACCTATCCTGTGCTCGGTGGCCATGCTCATCAGGATATGCCGGATTTTCAAAAGCATGCGCCAGGGCACTGACTATGTCCCTGTCTCGGAGACGGTGGAACTGGAGCTAGAGTCAGAGCCGAGGCCTAGGCCCTCGCGCACGCCATCACCCGGGCGCAACCGCCGCCGCTCTTCTACGTCCTCATCTTCCTCCAGGTCAACCAGGAGACAGAGGCCCGTCTCTACCCAAGCCCTCGTCTCCTCCGTTTTACCGATGACGACGGACAGCGAGGAGGAGATCTTCCCCTAATGCAATAAAAACTTAAAACACTGAGGTTACTTTCCCGTCATTCTTTCGGGGGAACGAGGGGAGGCGGGAATTGGGTTAAGATAGGGGCGAAGGGTGGGGGTGGTTGCAAGAATTGGGGCTGGGAATGGAGAGGGGAGTGGGCTAGGTGCCGACGCCGGGGTGCCAAGATAATGGATTGAGTAAGCATGGTGCTCTGATCGGGTCCGCCGGGTTCTCGGGGGTGTAGTGGGTGGGCATTGCATATTTTTGCCGCGGTGCTGTTGGGCATTGGACTCGGGGTGATCATCCGTACCATCACCCGCACCCGCACCCCAGTCCACAGCCACCGGCCAAGGTCCTGGGCCTCCCACCACCGTTATGCCTCCCCCTTTACCCATTAATTACAAGAGATGTTAGTTTGGTTTTTTATTTGGCAAAAACAGCAATTCATCATTTTCAGAGTCCTCATCATATTCGAGCCCCTCGTTGGTTTCCCCGCAGGCCCTCCCTTCTTCGACCGCTATTAGCTTAGTAGTCTCCAGGTTAAACTCCTCATAGTCATTATACAGGTTGATTATTCCCCCGTCCACGTCGCCTATGGAGTTGACTCGTCGTCGGCAAAGAGACCAGAGGGCACCCATGGCGCGGTGTCAAAAGTATTGTCTGCGTACGCTTTCCAGGAGCCAGCCGCGGTGCTCAAGGTCTTACGGATGACAGAGTCCGGCAGGACCACGGGTGTCACCAGCACCGCCACGGGAATCTCCACCGAGGCGTCCAGAAGCAGGTCTGAGCCGAGCGTGCAGGTCGCCGGGTCTAGAGGCGACCGTTTTCGAAAGAAGGCCGTCACAATGTTCACCCGGGGTGAGCAGTCTCTCCCGGGCTTGCCACCCCCACTGTGGCGGACGTAGTCTCCAACAATTTTGTATTGGAGGAGCACCTGGTAGAAGTAGTTGTGCCGTGGATTGATGAAGATGTTGACTGGGACCCGGTCTTTAATACCAATGCGCCCCGCATTTTCGCTTGGGTCCGTCATTACGTAGAGCATAGACTCCACCCCCCTGTTGGCAGCTAGGCTGTCTGCCACCAGGTCATGACCGGGGCCCAGTTTGCGCTTACGGACATCTTTAAGATTCCAGGCCTCATCCTGCGTCAACAGATAGTCACCCTCCGAGGGCAACCGCCCATCCGGGACGTACTCCACGGTAGGACGAGCTATAGAATTGATAAATCTGATAAATGACCTCTTGCATGGCCTCTTGTAAAGCGCAGTGTAGGATGGGTAGATGGGGTCAAACTCTGACTTGGAAAAGAGGTACTTGAAGCGGCACTTAATCTCATAAATGCAGCTCCGGTCGGTGAACAGTATAAAGTCTCCCTGTGACTCCACATTGACGCAAAGATCCAGAGACACCCCAAAAATGCCATCCGTGGGACTAATCATAAAGCCAAATTGACGGTTGGCGGATGCGTCCCCGCAGATGAGCTTACAGACAATGTCCTTGACCGTGTCCTCACACCGCAGGCCAAAGGCAACAGGTCCCCCAAAGTAGTGATTTGTGGAGATGGGAGCTGGCTCAAACACCTTGGTGGGTCCATTCTTAATGGTGGAGAGCAGCTTGGAAGAGGAAATTATGCCATTTCGCAATATGTCCCACATCAGGTTCTCAGACTGCCCCCTGGTCATGGACTCCACGTACGAGCAGAGAACAGTCCTCTGCTCGTCGGTGGCCTCCTGTAGCCCCCAGTAAATGGATTTCAGGGAGGGACCGTCCTGGCTGTCATTCTCTTGGACTAACGAGGAGACAAAGTCACAGAAGCCAGTTTCACCAGAGAACTCTTGTATTTGTTTACAGAGGCAATAGAGATAGACATAGCGCATGGCCGGCATCTGAGGTGGACGGTCAAGGTTACGGACAAAGGCCTCAGTCTCCGGACTGCGGAGGAAGCGGGCAAACGTGTAGGAGGTCATCTCCTCCATGGGATCCTCGAGCTCATCCACGTCGGCCATCTGGACCAAAGAAGTCGTCTGCCAAGAGTTCAGCTACCAGACCTGGAAGATGAGGGTGCTCAAACCGTGGGCGACAGTTGAAGAAGTAGCTCTCCTTGAACCTCTTTTTAAGGCTCCGGCACCACTGCAAGAATTGACTCATATGCTCCGCCGTGACATCCACGCACGGACTCTCGCCACACGAGGTCAGGCCCATGTCTAAGTTCAGGTTCCACATCTGCGACAGCACCTCCAACAGCACCACCTTTGGGGCTGCAAATTGCAAAAAGTAGAGCGGGTCGGATCGGTCAAATCCCATGTCAGGGTTGGGGTAGGGGATTTTGTGGGTGGAGTCAGCGAGGTGCATGATACCATAGAGCAGCGAGTAGCCGAGCGACTGCAGATCCAGGCGAAGGGCCGTCTGCGCCCCCACGGGGCCACACGCCGAGGGGTCAGGGATGTGCCCAGCCCCCCTCAAGATGTAGCACTTGCTCAAAAGGCAGAGGGGCTTATAGGTGTCCTTGGCTATAGAAAATGGTTCCCTCTGGCAATAGAGGCGATAGAGCTGCCGGCCCTTAGAAGACTTTAGCCGCACATCCAGCATCTTGTTGCGGTCGTGGAGGGAAGCAGTCCCATAATCAGTCAGGACCAGCCTCCCCATGCCCCACATGGTGTCTGTGAAATCCACCAGGATGTTGCTGGGGCTAATGTCCGAATGGAAGAGGCCGCAGTGCCGATTCAAAAAGTAAACGGCATCTTTGAGGCCCTGAAAGCCCCGCACCAGGGGCTCAATACTACCATCATGCCAGTGGCCATAATCCTGGAGACTGCATCTGAACTGAGGCATAAACAGGGCGTGGCAGGACGTGCAGGCCGACAGGTAGTCCACCAGGGCCTTGTCCTGCCCGTCCTCGGCCGTGGCCTTCCCAATCTGAATCATGTCACACACCATGAGCTCGTGATACAGCTCCGTCACAGAGTCATAGAGTTTGACTGTGGCATTATCTGCATGTGCATACACGGCCCCGTAGCTCCCCCGCCCCAGCAGATACTCGCAGGTAATGGGGAGGTGATCACAGCGCGTCATGTTCTCCGGCAGCTTTACATAGAGGGTTTCCGTCATGTCATCAATGTTGGTCACCTTCAGGTGTTTGTGCTGAAAGGTGAAGTAATCAATGACAGTCACCTTCCCCAAAAAGGCCTGGGTCTCTCGAGGGGGTTCTGGGGAGACACTCAACTCGCCACTGCTGGAGGAGTTCGTCGGGCTCAACTCCGCAGCCATATTCACATCCATGTTCCTCAAATGGCTCGAGGGCCTGTCGCAGCTCGTCTCTGGCCTCAAGCTCCTGCTCACGGAGCTCCTCCACCCGCTCTAGCTGCTTGTAGTTGATTTTTGGAAATTGAGTCTTGGTCGCGGTGACCACCCTCTGATAGGTAGAAATTAGCTGTTTGGACTCAAACGTCTCCCTTGCGCGGCGCAGGGACTCTAAGGCACCCCGAGCAGATGTAAACTGTGTTTCAAACAGAGCGTGGTCCCTCCCAAATCTGTCACGTGCGCTCACAGCCGCTCTCTTTTCTACCGAGGCTCTTAGTTGCTGGGCCACCAGATCTCGCTTAGAACTACTCATCTTCATAAGTCACCATGTCCGCAACTATGGAGCCGAGATCATACGTGGGGTAGAGTACGGTAGTTCCAGTGGAGGCTTCCCGGTAATTTCCCACAGCGTCCACCATATATCTTTCTGCCTCTCCCGTTAGAATTAGGCAAGGATCATACGTGTCCACCGGCCTTTTATACTGAGCGTTTAGGTTTTGTTTATGTAGCAAGCACAAAAGGCACACACGAGTGATGCAAAAGGGTTCCTGAGGCAGCAGGCAGAGCTGTTTTGCCATTTTATTCAGGCGGCTAACGTCAAAGGGAGGAGCTATATCCTCACCCTTCCAGTCACGCACGTCCAAGTACAGGGCATACACACACCTGGTGAGGTGTGCCAGGAATGCCTCTATGTTGGCACATGGTGTATAAACCGCAGTGGGTAGCAGAATAGGGCCCCTCTTGCCCCGTGCTGCAGCGTAAACACAGTGACGCTCTTCGCAGTGGGACCTGGGGCCGTAGAAGAGGGCCCACATCCAAGGGAGTGGGTCTTCAGGCACCAGGGAGGTCCAGGTATGGGAGTGGGCCAATATTTGCAAGGCCTGACCTATAACCTCATCTTTGTTCCAGGCCAGCGCAATTCGCATAAGGTCCCCATCAAACACCTCAAAACACAGACCCATGCCCATTTCAGGCTGAGAGGGCTCCATCCGGCTCGACCACCCTTGTCCACCAAACTGCCATTCTTCTGGTAAACGGGGGTTGAGGGGCAAGAGCTCCAAAGCCAGGCTCGAGAAGTCATAGTCATCCTCGGCCACACGGCCGGAGCTCCGGGCCTCGTGCCAGGGCCTGTTGTCCTGAGGGAGGATATTGGACACGAGCAGGAAGCTCTTGAGTGGCGTCTCCACCAGCTTAAATTGCTCGGGCGTGTCCTGGCAGGCCTCCAGTGCCAGTTCCAGACACTGCCCATACCTGCGGGCGAGCATCGGGTCATCGGGCATATCGGCCTTGACCGCGTTGAACATGCTGTATGCCTCGCAGCGCGGCCGTCTGACCGAGAACCTAAGAAACGCCCTTCAGCAGGACAGCACCACGCAAGGCTGCCTGGGTGCCGAGACCCCGAGTATTATGTACACGGGGGCCAAGTCAGACAGGTGGGCTCACCCTCTGGTGGGCACAATTCACGCCAGTAATTTATATTGCCCAATGCTTCGAGCATACTGCCGCCACTATGGCCCCAGGCCCGTGTTTGTAGCTTCTGATGAATCATTACCCATGTTCGGTGCGAGCCCCGCCCTTCACACCCCAGTCCAGGTCCAGATGTGCCTACTACCAGAGCTACGCGACACGTTACAGCGCCTGCTGCCCCCACCCAATCTTGAAGACTCCGAGGCCTTGACGGAATTCAAGACCAGCGTGTCCTCTGCCCGTGCCATCCTTGAGGACCCCAACTTTTTGGAGATGAGAGAGTTTGTCACCAGCCTGGCCAGCTTCCTGAGTGGTCAGTACAAGCACAAGCCCGCCCGCCTAGAAGCATTCCAGAAACAAGTAGTGTTACATTCTTTTTATTTTCTGATCTCAATCAAATCTTTAGAGATTACAGACACCATGTTTGACATCTTTCAAAGTGCTTTCGGGTTGGAAGAAATGACGCTGGAGAAGCTGCACATTTTTAAGCAAAAAGCCAGCGTGTTTCTTATCCCCAGGCGCCACGGCAAGACCTGGATAGTCGTGGCCATCATCAGCCTCATCCTCTCGAATCTCTCCAACGTGCAAATAGGCTACGTGGCTCACCAGAAACATGTCGCGTCCGCCGTTTTCACTGAAATTATTGACACCTTGACCAAGAGCTTCGACTCCAAGCGTGTAGAGGTCAACAAGGAGACCAGCACCATCACGTTTAGGCACAGTGGGAAAATCTCCAGCACCGTAATGTGTGCCACCTGCTTCAATAAGAATGTAAGACCTGACGTTTCAGTACTTGGCAATTGTAGAGCATAGCCCGGCTGTAAAGGTCAGAAAATCGCAGCAGGGTCCAAGGTTGTGCTGTACATGGGACCTCTTTCCCATTAGCAAGAACCCCCTGCAGGACACGTGACATGTCCGGGTGCATTTTGGGTGGGTTAAATCTCAGTCCCACCACAAAGGGGGCATCCTCCGGTTTGAACATCAGACCCAACAAAGCCCGATGCCCAGTTATGGGTACGTAGTCGTTGTTCAGGGCCGTGCATGGCAGCAGACAAGGACAGGTGCCAGATGTGCCTGGGCTATCGTCCTCCGTCCAGCCACGCAGGATGTTCACGTGGGCCCCGGCACCATAGCATGTCACACATTCCCCGTTATCACATCTGGTTAGCAGGTTGATAAAATGGGTCAGTGATGGAAAGGTTGGCATATTGGGGCAGCACATCAGCATGTCCATGTTAACGAAAAACATGTACAGGGCCCCTTCTGCATACCAGGCACCACCCCGTCCCAGTGGGATGATCTCCGAGGGTGTGATATCTTGCAGTTCTTCTACTGTTTTAACGGCGGTTGAGGTGGTAAAGACGTGGGCCGTGGTCAGATCTGTGCAGGTGACTACAGGGTTACCCCTAATCTCCACAGGCACCGCCTCACCCACTGCATCTGAGAATACCCCAAAGTACATGAGAGTCAGGCTGTGTGGCCCCTGGACTGCCTTAGTGAAGAGAACCTCGGGCCTGGCCACGGTGGCTAGGGTTCCATTGATGTAGACGGTCACATAGGTGGGCTTCTTCTTGGGCTTCAGCACAATGAGGGTAACATTCATGTAGGTTTTAGGAGGTCCGGCTATCTGAGGCACGTACACAGCTGACACGGCAGTTGTGGCCGTATAGACTTTCATCTGGGGCGTAGAGGCATCGCTCAGCACCCAGAGGCACTCCTTGTTGAGGAACTTGCGAAGCTGTTCCCGGCTACTGTTCGCGGCGGATGCCATGACGTGCCAGAATATATCCCCTCTCCTCGGGGGTGAGTGCCAATTGGCCTTTAATAACAAAGCCCCCAGGCAGCACCAAAAATGCCTGCCCGTCCGATGTGGTGGCCAGGTGGACGCAGTGCCCGTCAGTTCCAAGGGCTACTAGCTGGGAAGCAGCCCCAACCAGCCCACCGGGGGGCCTGGAGTCGATCACCTTACCCCAGGCCGAGGCCCCTTCCTCATACAGTGGGTGGCTATCTATCCATAGGCAGGCATCCGGCGTCTTTGGTGCATTGGAGATAGCTTTCACCCAACAACTTTCCCAACTAACCCGTGTCTGGACAGTGAAGAACGCTTCCCTGATCAGGTCTGAATTTTTATAGATACGGGAGTAGGAGGTGGGAATAACAACTGGGATTTCTTGTTGTGCTGTCCAGGCCTGCATGGCCAGTTTTTCCCTGAAGCTAGCAGAAATTCTGAGGGCCACTGAAATGAGGAAGCGAAACTCCCTCTCTGGAGCTCCCAAAATTGAAACCTCAGCAAGATCTGTTGCTGGGGAGGCATGGGTGACAGCTGTCATCCTGTGCAGTCTGCCCTGGGCACTCAGCTCTGGATATGTGACAACATAGAGAGCGTGGGGGCTAAAAATATGAGCAATTCCCCTGACCAGGGCCCTGGACTCACGAATGGCCCGACGGGTCTTAGAGAAAGAAACAGGCACCCTCGAGGGTGCCCCCGACCCGACACCCACAGTGCCGCCAGTCCCTGCTCGGCCTCCGCCGCCTTCCCCACCGGCGCTGCCCCTGATGTTGCTGGGGTTCTCGAGGGCTGGGTGGTGCTTGGGCACAGAGGTCTCAGCAGCCGCCTTGGTCTCGGCCCCGGCCCTAAGTCTGAGCCCCAGGCAAAGGGCCGGACTCCCAGCGTGGCCCAACCTCTGCTCCCCTCTATTCTCCTCTTGCGTTATCTCCAATAGAATTTGCTTGAGGTCATACGTTTTAGGGTGCTCGGCCTGGGCCGCGGCCACCGGCATATGCTCTATACCCGCCCCTCCGGGGGGCCCAGGATCTATAGGCATGGGCTGCATAGCCGCAGCAGACTCCTGGACCCCAGAGGCCTCTCTGATCAGATGCCCGTCTGTCAGAGCCCTTTTGGCCCCCTCGAAGAGAGACAGGTAATAAATCTGTAGCTCCCCAACCAGCCCTCCTTCATCGTAAAATCGAAGGGCGGCCACGTGGAAGGGGTTGTAGAGCTCTGGAAGGCCCTCCTCGCAGTACACTGGCACACTGGTAAACGTGCCCCGATGGCTAGGCCGTCCGGGCAGCATGCCCCGAGCAGCAAACACGCGGCAGACCCTCGTGAGACCCGTCCGGTCACTGAAGAGAGTCTGGCACCAGGCCCCCTCGCAGTTTGGCACGCGATTGGGGCAAAGCTCTGCCATAACCGTGTCGGGAACAAATAGGTGCACGAGGAGGGGGGTCCCGAGGCCACTCAGCACTTGGTTGTCAATGTGGACATCCATAGCTCTCTCATGCGTTTGGCTACAGCATCATAGCGCTTGTTTCTGGTGGATTTAAATAACAGGGCCCCGTAGACAGTCTTTTGTGAGTAAATAGAGATGATGACATGGATGTAGAGACTGAGGACCACATCCACCACCTTCTCGGAGGAGGCCCCCCTAAACAGCATCAGGCAGCAAGGGAACACAAAGGAAACCAGGGCCGGGATGTGAGGCCTCAGGGCCCCATCCTGATCAAAGAGGGCCTCGCTGACCCCGGAGATGACATTCTCATTCAGAAAGTAGTGATAGAGGTGATTGACCACAGTCTTAACCAGGCCCTGGACTTGTTCAGGCTCCCACTTGTCCCGCTGGTCCTGTGTGTCTTGTCGGATCTCGGTCCAGGGCCTCAGCGCCGGCTGGAAATGCGGCCCCATGTAGTTGCCTGTAAGGGCGCACACCACTCCCTCATGGGTCTCAATCAGGGTGCACTCGCTGGATCCATCACATACGTGGTACTCGCCACAGCCCCAGCAGGCAAACACGGAGGCCATGCTCTCAGGTAACGGGAGATGGAACTCCAGCTTACTATACGAGCACAGATGGCGAGGATTGGGCTCATCCGTGCCCCCCTCCCCTCGCGGGAGGCTCAATCGGCCTTGGTCTGACATTCCACCCCGGCCAGGTCCAGGAGGGTGCAAATATTCTCCAGGCGCTGCACCTCAGAGACCTCCTGCTCAAAGAGGCCTCCCACCGCCACGTAGACGCGGGCCACCGTCCGGGGAAGGTCAGTGGGGTCCCAGCTCAGCAATTCTCCAAATTCTCTCTCCCCAATAGTGACTCGCTTCTTATCCTGTCTTTCAGAGCATCCGAGGGCAGACATTTCACCTCTTGTTTGTGGACGAGGCTAACTTTATCAAGAAGGAGGCCCTGCCGGCGATCCTGGGCTTTATGCTTCAGAAGGATGCTAAGATTATCTTCATCTCGTCTGTGAACTCGGCTGACCAGGCCACCAGCTTTCTTTATAAGCTGAAGGATGCTCAGGAGCGGCTGCTGAACGTGGTAAGTTATGTGTGTCAGGAGCATCGGCAAGATTTTGACATGCAGGACAGCATGGTCTCATGCCCCTGCTTTCGCCTGCACATCCCGTCCTACATCACCATGGACAGTAACATCCGAGCAACCACCAACCTCTTTCTGGACGGGGCCTTTAGCACCGAGCTGATGGGTGACACCTCCTCGCTGAGCCAGGGTAGCCTGAGCCGCACTGTGCGTGACGATGCCATCAACCAGCTGGAACTCTGCCGGGTTGACACCCTCAACCCCCGAGTAGCCGGACGCCTAGCCTCCTCCCTCTACGTGTACGTTGATCCGGCCTATACCAACAACACATCCGCATCAGGCACCGGAATCGCCGCCGTGACTCACGACAGGGCGGACCCTAACAGGGTCATCGTCCTGGGCCTGGAACACTTCTTCCTCAAGGACCTAACAGGGGACGCTGCCCTCCAGATCGCCACCTGCGTCGTGGCCCTCGTCTCCTCGATCGTCACCCTGCACCCCCACTTGGAAGAGGTGAAGGTAGCCGTGGAGGGCAACAGCAGTCAGGACTCTGCGGTGGCCATTGCCTCAATCATTGGGGAATCCTGCCCCCTCCCCTGCGCCTTCGTGCACACCAAGGACAAGACGTCCAGCCTGCAGTGGCCCATGTACCTCCTGACTAATGAGAAGTCCAAGGCCTTTGAGAGGCTCATCTACGCAGTGAACACGGCCAGCCTTTCTGCCAGTCAGGTCACCGTCTCCAACACCATCCAGCTCTCCTTCGATCCGGTCCTCTATCTCATCTCCCAGATCAGGGCCATCAAGCCCATCCCTCTCCGCGACGGGACCTACACCTACACCGGCAAGCAGCGCAACCTCTCTGACGACGTGCTGGTTGCGCTAGTCATGGCTCATTTTCTCGCAACAACACAGAAGCACACGTTCAAGAAAGTTCATTAAACTTTATTGACTACACCAGTCCCTTGTAAAGCGACGGGTCTCGCGTGACGGCATTCGTGAGCAGGGCTTCGTCCAGGGGCTTGTTCTTGGTGGACATCATTAGCCCAGCTGCAAATATCAGAATTAGCATTAGAAAAGTTAGTCCCACAAACACCAGTGTCCAGAGAGGAAGACCGTAAGATAAAGATGGCTGCCTCTCATCTGGAACGGTGGGAAGCTCAGCAGTTGTTTTTGTGGCATTGGACGTCCCTTTGGAGGACAGCGTGGGGGCCAAGGTGGTAGCGTTGGTAATCCGGGTAGTAGCACTGGCGGTGGTGGAGGACCTGGTGGTGACATTGCTAGTCACACCCGTGGAGGTTCCTGTTCCGGCCTCGGTGGCAGTGATGTTCTGTGCAGTAACCTTAGTGGTGACATTGATGGTGGATGCGTTGGAAGTTGTTGGAACTGGTGTGACAGTTGTCCCAGTGGATGTCACCGTGGTTGTGTTGGTGCTCAGGAGAGCAGTCGTGGTTATAGGGGCGCTGGTCGTGGTCAAGGTCGTAGACTGGTTTGTGCTAGGACCCGATGCCGATGGTGATGGTGTAGTCACAGCCGTTGTGCCTGTCACGTTCCCCGCCGAGGCCGTCGAACTGCCACTAGATGTCCAGATAAGGCTTGTCTCACAGATGAGTATCATGGCCAGGACAGCGCCTGCCTTGTCTCTGGCGTGTGCCATCGCGTCTGGACGCAGAAGGCCTCCCGGCCTCTTTTATAGCTAGTCTCCACACCCAATACTCTACTGAACCATCACATACATGACCTCCTCGAGGTATGCAGGGAATGAGCGGTCCGTGAGCCGGTCAACACGACATTGCTTCCGTTTCATGCCTCCAGCTGCCCCTGACCAGTTAGGACCCTTGACGGATGTCTTTAACGGCGCGGTGCAGTTGGTCACCAATGACGGCCTAAAGGCCAACACATCCTTGAAGGAGGGCGTAGGAATGGTACCAAACTCGGGGCCCACCCCATCAAAGACATAATATGTCTCATAGTGGCAGTGATGATGCATCACCACCACAGCACTCGCCAGGACCCTCTGCATATCTTGTACAAGGCGCCTTTCAACTCGGCCACTGGCTCTGGTGACGTTAAATGTCCTGTTCCTATTAGTCACAGCCTGTAGATTTGGGCACCCAGACTCAAAAAGTGCAGCTACATGAAGGGCAGCCGCCTCAAATCCACCATGACCCCCAAGGCTGTCCGTGTTGTTGGGGTAATAAGTCACATTGTTAATGACCACGGCCGGGATAAGGGTGTAAACCTTGCAGAATGGATTGGTAGGACACCCATAAGACAGGGGCACCCCAAAATCGCGCCCCTTACCCCGAAGCACCTTGGCCCCCACCGGCATAAAGCTGGGCAAAAAGAGTGGGTTAAAACCAAAGGCGAGTAGGGCCAGGAACGCCAAATAGCAGCAGTAATAGATGAAAACAAAGCTCAGCATGAAACAGCGTGGAGGCTCAGCTAGGGTCTCTGCCTCTCCATCATAGACATCTTCCTTGAATCTCATTCTCTCACCGCATACCTCGCTCTTCATCCAGGAGGGGGCCATGGCTGCCATTCTACCAGTTAACGAGGAGAGAGAGAGTAGGTCCGCGGAAATTGGTGCCCCTCTCTGCCCTCCTGACGAGGCCATGGTGTCATCCATCTCCGCAGTCCGTTCTTCAGCTTTGGCATTGGTCCGGGCCCGGGTGGTCTGATTCTGATTCTGATCCTGGGTATTGGTCTTGGTCTCTCCTCCCCCATTGGCATGGATTGGCATAGCTGGGTGTGGCTCAGGCTCAGGTTCCGGCCCTGGGACGGCAGCAGCCGCCGGGACGGTGAAGTCGTGGAAGGTAGAGGCCCGTCCCTCCCGAGGTCGTGGGGCCGGAGCCTTATAAAAGACTTCCACCCTCTCCCCGCTGGCCAAGACACGCCGCTCGTGGACCACGCCATCTTCCTCCCGGCTGATTGTGTGGCTGACGGTGCCGTGTTCCACCGCCACTTGTTCATCGACCATGGTACCCCCTTTATCTTAACCAGCAAGTGGCCGTCAGGGTCTCTTGAGAGTATGCCGCTGTGGCCAAGCGAGGCCCCAAATTAAATAGTGATGCCAAAGACTGTAGGTAGGTCATCATCACACGCATGCGTGATAAATCATCCGCCACTGACAGGTCATCCAGGTCTATCCGGGCTATCTCATCCGGCACCATCTCCTGGAAGAGATTCAAGAGGTCATGATGCTCATGCCGGATAAGGCCTCGGACCAGGCGCATACTGGCCCTGGGCAGCAGGGTCACCATGATGCAAAAGTAGAGACTCAGATTGTCCAGCAGGGCCAAGCCAAGGGGCCCCGGAACCTCTGGGAGGGCCAACTCGTAGTGGTGCCCCAGGTATGAAACAGAGCCAAGATGCATGTGTACATCGAGCATGTCTGCGTTCCCGGGAGCCTGCATGACAACCCGGGAGTACACGTTAAACAGGAGAATCTTCTGCAGCACCTCCTCTGCTATGGGCGTAGGCAGCACCATGGGGAAAACAATGTCCACATCATTGGACTCTAACTTCACGGTGGCATGCTCTCGTCCAAACACCGGGGGCATAACACTGAGGCTCCCGGTCCCATGCCACTGGAAAAAGGGCTGGTACTTGTTCTTAATGGCGTAGGTCTGACCTGGAACAATCTTGGTGAGTATCAAACTGTCCACGCTAACCTCATCCAGCACGGCCAGGGTGCAATCAGACAGGTAGTTGTACATGGACACGTAGTCCGGGACCGTCTCTATAGAGTACACCTGACCCAAGCCCAATCCCTGCACATTCTGCGTCCCGTGAGTGGAAGCCAGGGGTAAGATGCAGCCAATCCTCTGTTGCATCTTGGCAATCTCATCGGTATACAGACGAGAGGAGAGAGACACTACCACTTTCAAATCCATCTTTATTGACAATTATCAAAAAACCACCTTATTTCCAAACTTTAATATTCTTCGTACCGGCGCCACCTCTTCAATTATATAGTGTCCGTAATGGATGGGGGCGTGGGTCTGTTTGACAGACATAAACTCATCGATGAGTGCCCGGGAGGAGGCTGAGAGTGCGGGGAATGCCTCCTGCAGAAAGCTGCAGGGCTGCTCCAGAAACACGTCAGTTCCAGCAATCACTACAAACTGCACCTCTGTGTTGCTGGTGGCTGGGTGCCCTCCAAGTCGCTGGCTGTACTCGTTGACCATGTTGTAGAGCCCCCTGTTGTTGCGCAGAAGCTCCTCCTTGTTGAAAAATGCCCGGCAGGGGCTGTAGAGGCCCGGGACGGCCGTCTGGCGATAGGAGGAGTTGTACATGATGTCGCCCAGAGAACCCAGCTGAGATGCCCAGGGATTCACAGTGCTCCGGTACTCATAGGCGGCATCCGGGCGAGAATGGTCATAGATGAGCCCCTCGGCAACCTCCTGATTGTAGTTTTCACAGGAGACCACACAGGCGGCCCGTCCCCTTGGAGAGTTGGACTTTTGAAAATAAGCCACGTCTGCCGTGACCGGTGTTACTATAATCTCACAGGTGGCCTGCTGGCCGTGGCAGAGTCCTGGAGCTCCATTAACATTAGTCATACCCGCCAGGTATGTCCTGGGGTCCCGAAGCAGCGTCCCATTGCGCTGAGCACCCACCTTGGCCTTGATGTAGTCGTTGACTTGCTGGTTTCCAAAGGCCTCGGCCGGAAAGACGCTAAAGAAGTCTTGGGTGTGGATACCCATGTCAGTAGTGATGGCCGCCACCCTGGCCGGAGTCATGGTCGAGCTATAACTAAGCCCGGTGTCGATGGAGGCCATCTCGTGATGCACCTCAAAGGTTACCGCGTCCACCCTGGCCTCCCGGCGGCTGACATTTGGGGTCCCAATGAACATGGATGTTGAGGCCCTGGAGCTAAACAATATGTTTTCAGAGAGGATCTCATCGGTCCTGACCACGGTCATGGCCACCCCTGGGTGGATCTTGAGCTTGGCCTGGGCAATATAGGCCATGGGGGACATCTTGATGTGCATGGCGGTCATTCCACTGATTGAAACGAGGGAAGGAAGACATTCGGCCGCGTATTTGCCCATGGGCGAGCGGTGCCACTCCCGGTACTCTGCGAAGAGCTGCTCTGGCCGGTTGAAGGCTTCCACGGCCCGCTGCTGAGGATTGCGCATAACAAAGGTGGCAACATCCTGGTGCATGGTGGCAGCCACTCGCGGGTCCCCGTAAAACATATGGAAAGGAATGGCGTGAAAGAGACACTGGGTGACGGCCCGGGTCCTCTCGGAGAAGGCAAAGGCCACCAGCCCGTTCACCAAAACAGTCTGCTCTGTCCGCTTGTCGGCGGGATTCGGGGCCAGCTGCTGCGTAACGTCATTGTCCACCGACACGCGCACGGCGCGGGTGAAAGTGGGGCAGGTCATGAATGAGGCGCTGAGGTCCCTGATCATGCCCACGGTGGGGCGGAGGTCGGAGATCTCCAGCAGATCCCTGAGCGTCCCATTCTCCAAATTGTCGAGTATGTCCTCGTCCCTGGTAAAATGGTGGCTGAAGGCTGGCCCGTTGTAGGCCAGGGTCTGGGCCACGTGCTGAAAGTCCACCCCGAGGCCGCACATGTGGGCATTGGTGCAGGTCGGGAGGAAAACGTAGTAAAAGATCTTTTCCAGCACATCCGCATGCCCCTCATCTACATAAGGGCCTAGGTGCAGACGGAAATCGTGGTCGTGGTCTCCGTTAACCCGGTAGCCGTACAAGGCCACAAATTGGGCAGCCATCTCATCCATGTTTCCAACCCTCTCAATAAACTGGGGCGCGGCCAGGGTGTCAGCGTAAACCTCATTTCCGATAATAATCTGGGGGGCCCGGTCACTAACGGTGAGAAGATGGGTGAAAATGTCTGTGTAGGCCACCGGGGGGAGCAGGTTAGGGTCCAGGAGTGCACAGACATACTGACCCACGCTCTCATCCCCCACAACATCTGACCCGGCCAGGCGCATCAGGGCCTGCTCTAGGGCTATAAGTTCCCCATAGATTTTTCTATACATGGAATAGGCCTCCTTGGAGATGGCGTTATTTCCCAGGTGGCGGCAGATGAACTTGATCATGGAAAAGCTGTTCACAAAGGCAAGCCTCCCTGCCCGTTCCCAGTAGGTGTTGATGCACAGGGACACCAAAGGCACGTTCATGACAAACTTTTCCTCAAACCCGTGGATCATAGCCTCAACTACGTAGAAGAAGGCTGGATAGGCAGTGTCATAGGCAGTATCCTGCACAGTCTCAATAACGGCCTGATCCACCACGTGGGCCAGAGATGTGGCGGTCTCAAACTGCTGCCCCCGGGCCTCTTGGAATGCAGCTGGGGCCAGGGGAGTCGGCAGGTTACCCACCATTAGCCGGTGCACGGCCCTGTGCCTGGCCCTCTCCCCGGCATCCCTGCCAATGTAAATATCATAAAGGGGGTGCAGCTCCAGCCGCAGCAGGTCATAATTGGACGGGTGGAGGAAGTCTTCGGTGGGCAGCCCGCACTTGAGAGCTATATCTGTCACGGGGGCTGCATACTTGTTATCATAGAACTCGTCCACAATAACAAGCACATTCATGTGATTGGGCCTCCTGTGTTGCAGGGAGTAGGTCTCGCGCCTGTCTCGCGGGGCCGGGGCCGCGTTGAGGCTGTTTAGGGTATGGGCGGGTGTGTGGAGTCGGGGGTGACAGAGAACCTTGAGAGCATTCTGTAGGTTAAACGCGAGGAGAAGGTTATTCTTGTTTACGATCCATGCCTCCACCGGTAGCTGCTGTGTGGGGTTGTCCAGCATTTTGATGGCGGCGGAGGTCGTATACTTGGGATTGGGCATAAACAGGCCCACTGGGAAATAGTAGCTGTACTGCATTCTTCTGTTGAGGGGGTATGGGGACTGAGTGTCATTGTACATCTTTTGCAGGCTTTCCACGGCCACCGCGTGGTTGCCCAGCTTGATGACGGCGGCTGAGATCGGCACCCGGGGCTGATCCTCGACCCCTGCGGCCACAGCCGGCAGGTCAGACTTGGTGCTTCCGGCTTTTTCCGGTGAGTCCACGATCCTAGCCATGAAATGCTCAAACGTACGCATCACGCGCCCGTAGCTCACGGCAGTGACCAGGTTCTCCCCCCGTACCACAAAAGAAGCATAGCTCGAGGGCCCCATGATCTGGTTGTCGGCCTCCTCACCCAGGAAGGTCAAGAGCTGGCGCAGAACGTTGTCGGTGACAATAAACACCCCCCCCACTGGCTCTCCCCCCTTGGCGGTCGTGTAGGTACTGACCCCCTTGAGCACGCTCTCCCCGGACACGGCCGCTACCATCTCAGAGAGACGGCTTCGCACGTACTGAGAAAACCCGGAGCCCATGTTCTCGGCCCGGTCCAGGAAGAAGGAGTGCTCCAGCAGATGCCTCTTGAACATGGCAATGAGGTCAGACTTGACAGTCTTGGAGAACCCCCTCTCAGTGAAGGTGGGATCCGCCAGGGTCTGCAGGATAAACATGGGAGGGGCGTGGCGAAGCTTCACACTCAGGACGGTGTTAATGAGGCCCCTCTCCAGGGCATCGACCCCAAACTGTAGGGCCGAGGCCACGGTCTTGACAGCCCCCACGTACTCTGCGTACTCGACCGGGGTCTCGGGGATACTATGCAGGATCTCCAGATCCAGCATGGACAGTTCCATTTCCGTACTAATGTGGTGTTTGTGGCAATTTTTGACCACAATGAATGTCCGCTGCTTGCTGGGTCTCCTTCCGTCCCCGTGAGCAATGGTGGGGACGGAGATTCGAAATTGAATCTTGCCATCCGTCATACGACTCAGGTCTTTGAATTCCGTGTTCACACAGGACACGGCCAGTGCCGTCTCCAGGAAGCGAACATATTGGATGGCGTTCGTGTAGACCCCGAGTAGCACCTCAAACTTGATGCCCGCCTCTCTGGCATCCTTGCCCACCAGCAGGTCAAAGCTATGAAACAGCCCCTCGGCCGCTGACTGCCGCAGGTTCGAGAGCAGGTCGGCATCCACCGTCAGATAGGGGAAGGGTCTGTTTTCCACACCCTCATTTGAGGCCATGACACAAGGTAAGAGGGAGATGGGGGGAGGTCTCGAGGGCTTCTCTTCACAGCTGGGTCTCTTTTACGCCCTGGCCTGCAACCGCAGCCCACCCGCACTTCCCGAGGATGCTACCCTTCTAATCAAATGGTTGGACACGGCCCTGGGCAGGGAGGCCACCTTTTACGCGTGTCGGGCTATGCGTCGTCTTCTACTCGGCGTTATCCGAATAAATGACTGCCAGGAGCTGCCACCCGGTTTAATAATTCTGAGTCCGGGCACCGTCCCTGGCCCCCTTGGAGTCCAGAGTCTGGAGCATACAGACTGCGAAATATGGTCCTCTGCCCACCCTGACCACGCTGCCCACCTCCCGGTGCCCAGGGTCATCACATACACCGACTGCCCGGGTTCCATAAGCACGAGCTCAATGTTTCGCCTTATCATCCGCTACTTGTCTCATCACCAATTTGAGCGCTGCTTCGAGCAGTTCTGCCGCGTGGTCCCGCGTCGCTTCCTAGGGACCTGTAAGCAAAACTCTGCAAAGATGCTGGCTCATCTGAAGCAGGTTACCAGGATCCCCCCCTGTCCGCCCTTCAGCGGGCGGGAGGCCAGACTCAAGTTCCACTTCTTCTCCTGGAGCACATTCATGCTGTCATGGCCAAACAATGCCACACTCCGCGAGATCAGGACGAGGGCCGCCACCAACCTCACCCACCACCCACATCTAGTGGACACTCTGTACCACGCCTCTCCGCAGACCCCATTTCTGACACGCAGCGGTGCTCTATACCGCTTCGTCACCTGTTGCAACTGCACCCTGCCCAATATCTCCATCCAGCAGTGCAAGGCCGGGGACAGACCGGGGGACCTGGAGATCATTCTACAGAGTAACGGCGGAGGGAGGCCTGCGAGCTTCCAGTTCCCCTCCTCCCCAACCGGCGCCCTATTGCGATGCATAGTTGCTGCGGCCCTGCTGCCAGAGGTGTCCGTGGGGCACCAGGAGCTGTCTCCGCTCATGTCCAGAAGCCATGGAGGGCAGACGGATGTCAGGTCGGGCCCGGACCCGGCCCGGAGGCTGGTGGCCCTCCTGCGAAGGGAAGATGGGGCACCTAAGGACCCCCCTCTGGGACCGTTTGGACACCCCCGGGGGCCCGGCCCGGCCAAGAGCGAAGACGAGGAGTCTGAGCGTCGAGACGCCCCTCCACCCCCGCTCGATTTCAGCTTCCAAGCTTCCCGGTTGGTGCCCGTGGGGCCTGGGTTTCGCCTGCTCGTGTTCAACACCAATCGGGTGATCAACACTAAATTGGTGTGCTCAGAGCCCCTGGTGAAGATGCGAGTTTGCAATGTCCCCCGCCTCATCAACAACTTTGTAGCCCGCAAGTACGTGGTGAAAGAGACGGCGTTCACCGTCAGTCTATTCTTTACGGACGGGGTGGGGGCCAACTTAGCCATCAATGTCAATATCAGTGGCACCTATCTGAGCTTCCTATTGGCCATGACGTCACTGCGGTGCTTCCTGCCTGTAGAGGCTATTTATCCCGCGGCCGTGTCAAACTGGAACTCGACTCTAGATCTCCATGGGCTGGAAAATCAGAGCCTAGTCAGAGAGAACCGAAGCGGGGTCTTTTGGACTACCAACTTTCCCTCGGTGGTGTCCTGCCAGGACGGTCTCAACGTGTCCTGGTTTAAGGCCGCAACTGCCACCATATCTCGAGTGCACGGGCGGACATTGGAGCAGCACCTGATCCGTGAAATCACCCCCATCGTGACGCATCGAGAGGCAAAAATCTCCCGGATTAAAAACCGGCTCTTTACCCTGCTAGAGCTACGCAATCGGAGTCAGATTCAAGTGCTGCACAAGCGTTTCCTGGAAGGCCTGCTAGACTGCGCCTCCCTCCTGCGCCTGGATCCCAGCTGTATCAACCGAATCGCCTCCGAGGGCCTGTTTGATTTCTCCAAGAGAAGCATCGCCCACTCCAAAAACCGACACGAGTGCGCGCTTCTGGGTCACAGACATTCGGCGAACGTGACAAAGCTTGTGGTAAACGAGCGCAAGACCCGCCTGGACATACTGGGCCGTAACGCTAACTTTTTAACGAGGTGTAAGCATCAGGTTAATCTAAGACAGTCACCTATTTTCCTGACCCTCCTGAGGCACATCCGCCGACGTCTGGGCCTGGGCCGTGCTTCCGTAAAACGAGAGATTACCCTTCTCCTGGCCCACCTGCGCAAAAAGACAGCCCCCATCCACTGCCGTGATGCTCAAGTGTAAGCAGCCCGGGGCCCGCTTCATTCACGGGGCCGTGCATCTGCCATCGGGACAGATTGTCTTCCACACCATCCACAGCCCCACTCTTGCCTCGGCGCTGGGACTGCCTGGGGAAAATGTACCCATCCCGGCCCTCTTCCGTGCCTCGGGCCTCAACGTCCGTGAGAGCCTGCCCATGACCAACATGAGAGCACCGATCATCTCGCTGGCTCGCCTCATCCTGGCCCCCAACCCCTATATCCTAGAGGGACAGCTGACGGTGGGCATGACACAGGACAACGGCATTCCCGTGCTTTTTGCCAGGCCTGTCATTGAGGTAAAAAGCGGGCCTGAGTCCAACATTAAAGCCTCCTCGCAACTTATGATAGCAGAAGACTCCTGCCTGAATCAGATCGCCCCCTTTTCCGCATCAGAGCACCCCGCCTTCTCCATGGTTGAGTCCGTAAAACGAGTCCGGGTCGATGAGGGAGCAAACACCCGGCGCACCATCCGGGATATTCTGGAGATCCCCGTGACTGTGCTCTCATCCCTGCAACTGTCTCCCACCAAGTCCATCCTGAAAAAGGCACCGGAGCCCCCACCTCCGGAGCCCCAAGCCACCTTCGATGCCGCCCCCTATGCCCGCATCTTTTACGACATCGGGCGACAGGTGCCCAAGCTGGGCAATGCCCCCGCCGCGCAGGTCAGCAACGTGCTCATCGCCAACCGCTCCCACAACTCTCTAAGGCTGGTGCCCAATCCGGACTTGCTGCCTCTCCAGCATTTGTACCTCAAGCACGTAGTGCTAAAGAGTCTGAATCTGGAGAATATAGTGCAGGACTTTGAGGCCATCTTCACCTCCCCGTCTGATACCATCAGTGAGGCTGAAACCAAGGCCTTTGAGAAGCTGGTGGAGCAAGCCAAAAACACCGTAGAGAACATAGTCTTTTGCCTCAACAGCATCTGTTCCACCTCTACACTCCCAGATGTCGTCCCCGATGTCAATAACCCAAACATTAGCCTGGCTCTAGAGAAGTATTTTCTCATGTTCCCTCCCTCAGGCACCATTATGAGAAATGTCAGATTCGCCACCCCCATCGTCCGGCTCTTGTGCCAAGGGGCTGAGCTTGGCACCATGGCACAGTTTCTAGGAAAGTACATCAAGGTCAAGAAGGAAACCGGAATGTACACACTGGTCAAGCTTTATTACCTGCTGCGCATCTAAAGGAAAAACATAACAATCTTGTGGACCAGAAAGATACCCAGAGCAAAAGCAATAAAGTACAGGATTATTGCCAAAACAACGTGTGCTCTTTCTTCATACAGGCCCGCAATTTCCATGACAGTCCCGTTGGTGGTCAGCAGCAGATAGTGAACGTGGAGGTTGTCAAAATCAAAGTAGTTGGAGCTCAAGATGGAGTTTTGGACTTCCTGGGAGGTGATGTAGGTTGTAGTTTCCAGGCCTTCCTTTTCATCATAACTGAGCAGGGCAAAGCCACAAAAAATGCAGGATTTCTGCGTCCTGGTAAAATTCTGGATCTTTGGAATCTGGCGGGGCTCCCCAGCCACAGCACCCTGCGAACATTTATTCATTATAACGGGGGAGAGAAAGAGAGAGCTGCTGAGATAGGTGGTGCTGGCCTCGTATAGCGCCGAGCCTCGGACCTCACGGTCACTAGAGATTATGAATGTCACGTTGATGAGCGGGATAATCATCAGAACTTTGTCGAGCCTGTCCACGCATTTGTAGGCGGGGAGATGCCACGCATCCCTGTCTTCTCGCTCCAAAGAGAGACGCCCAAGAAACCCATCCACAGCATTTGAAACGGCCGCCTGGTCCAGCATTGCCTCCTGGGGGGCCATGCTCAGCAGCTTGTCTCGTGTGAGGTCAAATCGTAGGCTGAGGTAGCACGGTGAGAAGAGTCCGCTCTCCGTCCCCAGGGCTAGCCCCCGCAAAACCTCCCCAATCTCTAGGGCCGAGCACAGGGCGGTGGACAGCAGTTGGTATAGGGCAAGGTTGGGCCCCTGGGTAGTCACGTTCAGCCGCAACTCGCGTAGCACCACGTGGCTGCCGATAAACAGGGTCTCTCTCATCACGGTATGCAGGGGCTGGAAAAGGGGGTGGCGGTTGTAGGCCGAGAGAAGCACAGATGTGGCGCCTCCAATGAGGCCACTGTAAACCCCGGCCTTGGGGTAGCCGACGGTGGCTAACCTCAGCGCGTACTCCTGTTTCTCAGTCGTCAGGTGACCAAGCTCCTCCATCTTGACCGTGGCCATCAGCATGGCGGCCAAGCGCTCCAGCCCGTAGGATTGCATGCCCTTGACAGTGGCCCCATAACATATGCCGATGATGTCTTTCAGGACAGTCAGCTCAAAGAAGCTCTTGGCCAACCAGCGGAGGTCCACGCAGCCATTGCCAGTCTCACCCACGGCATGACCCACCTTAAAGAAGGCCACAGAAACCTCAAACATGGTAGTCAGCGTTTCCGTGTCCAGTTCTGGCTCCCGGCAGCCTCCCTTCATCTCCAGCAGGACCAGTTTCTGGAGAACGTAGCGAGCGTAGCTGGCGGCTGTCATGGTGACGGCTCGGGAAAACATATCCTTCAGGTTGGGTACAAAGTAGTTGTGAAAGTTGGCATAATGCACAAAGGTTGTAACAATCACCAGGGAATAGTCCCCGCTTTGGGCACTGGTTAAGGATGGGTAACTAAAAGGCCCCCTCAGATCCGGCAGGTCCTTCGTCTTGCCAAAGATCAGGCTCAACACATGCTCATCTCCCTTCTCGGTCACTCGCTTGTAGGTGCCCATCAGAAATTTAGAAGTCATGGCCCCCGTGTACTGAAACTTGTCCCCGTTGATGGACAGGGCCACATAAGACAAGTGACAGCGCAGCTGATAAAAGACATAGCTGTGTGGCCGCGTGTTGGGCAGCATGGTGCCAATATAGTAGAAGAGCTGCTTCTCAAGGGGGGCACTAAGCATGCAGGCAGGGGAATTCAGGCCGCTAATGACTCCGGGATGGACCTTAGATGCATCCACTTGCATGGATCCTTCAGAGACAGCAGGGATATCGACAGGCTCGGCCAGCGCAATACCAAGGGTACCAGACGTCTTGTAAATTAACTTGTAGCGGTTAAGCATAGACGCCAAATCTTCGGTGACATTTGCCTCTCTCCACAGCGCCTCTGGGCTAAGGCCCGGGACCTTTGCCATCAGTTCGGTCCATGGGATGGTGTAATGCGAAGCATGCCCCTCTATGTCCAGGTGCAGCTTAACCTCGCTGAGGCTGGCAGCCCCCACCTCCCATAGCAACACCAGGCAAAAAACACAGAGCAACTGCATCCTAGTCCCGATTTCCCCTCTCAAAATCAGAGATCACCTTGCTCAGACCAGCCCAATCGAAAAACTGAGATCGTATTGCCGGATTCTTCAATGCCTGCATGTAAATCTCCGTCCAGCATCCAGGTAAATCGTCCTGAAACTCTGAGAGGTCCACAAGCACAAACTGAAGGTAGGCTAGCGTTCGGGTGAACGCAAGACAAACTTCCAACAACACCGCGTCGGCTCGGAAAGGCTGTATGACTTCCTTAAGTACACTAAAGATGCTGTTCTTATACAGCTTCTCGGCCACACCACTTCGAATTATGGGGGTGTGGCTTTGATGACATACTGTCGTGATTGTTGTTAGACCGGCACATACCTTCACAATGTCCTCGGGGGCAAAATACTGTGTTAGGAGCCAGGCACAGTAAACGGCGTGATATGCATCGTTGACACTCTTCAGGTAGCCAGCATCCAGTCCTGACTCATGTTTCCTCCCTCGCTTCTTCAGGCGGCGCATGTTCTCCTCCACGTTTAACTTCATCCAGACTATGGTGTCCCCCGGGTCTGCGGTAAACGTGGCCAAAACTTGAATAAAGTCACTATAGGAGAGAAGCTGGCTCCGGAGCAGCATTAGAGGGAAAACCACGGAGGCCGACAGCAAATGGCGATCATGCAAAATCCAACAATCCAGGGGCGCGACTGACCTGGCACCAGACTCGGTAACCAGCAAGCTCCGCTTCCTAGAGGCCAAGACTCTGAAAGGGGTGGTAAATTTCATCTGGCATGCTAAAACCTCAGCCGACGTGTCTTCCCTTCCATGCCTCGCCCGAGTCACATTCTTGTGCATGGCCTTAATGGCATTTTCATACACATGAGTCCAGTACCGCATCGGTTCAGGGACTACAATGGTCAGGTCCCCAAAGACAGCCTTCAAATGATTCAGCATAGTAGTCTTTCCCACACCAGGGGCACCTTCCAAAAATAGGGAACAGGCAGGTTTGATTACTGGTACATGATTTGTTAGGTGGGTCACAATTGGAACCCGCATGCTCTCCTTCCTCTGAGCCTTGGCCTGGCGGGTGTCTTGGGCATCATCCAGATTCAGAACATTCATCACACTCCCACTTAGCCGCTTCAGCTGGGCAGCATGCTTGGATAACTTACTAAACTCGCGCCCATGGGCGGCCAGGTGTTCGAAGAGACCAGAAGGCTTACCCTTGCCACCATTCTTTTGTTTTAACGCGGAATGAGAAGAGGGCCTGCGGAAATTAGACTCATCCTCAGACTCACAGTCAGATTTGTCATCGAGCCCAAGGCCGGCCAGGCCCTCCTCAAAGCCTTTCTGGTACATGAAGCTCCGGCTCGTGGAGTCCGCACCTCCTTCTGTGCACGAAGTTTTGCGGAACCAGGAGAAGGGGTCTGGCGTCTTGCTGGGGCCACACTCCCGGCTACGTGGCTTCGGGGTAGGGGCAGTAGGCTTTTGGTGTGCGGGTGCTGGTGGCTGGGCTCCCCTGGGTAGGGTAAAGGGGCACGATGTGTGCCGGCTACCCGGAGAGTTTCCAGTATTAGATGTCACGGCAGCCTGGGTCCGGCACGGCACCCTCTCCCCAGACAGTCCGGTCGGAGCCATCAAGGGGGGCCAGTGGGTGGGCACCTGGTAGAGTCCGTCATCATCTTCCTCACCTGCCCCTGAGTCACTGCCGGTTGGGGTAAGAACTGAGGGGGCAAAGTCATCAATCTCAGCGTAAAAGTTTTCGTGTCTTTCGTTTTCAGGGGACTCATCCTCCTGACATTTTCGCCAGCCGCCGGGCGGGCCGGCCTCCTTTCCTGGAAATCCAGCCATGGATCCCACCCGAGGTCTGTGTGCCCTCTCCACGCACGACCTGGCAAAATTTCACAGTCTCCCCCCGGCTAGAAAGGCGGCAGGTAAGCGAGCGCACCTTAGGTGTTACTCCAAGCTGCTCTCTCTTAAGAGCTGGGAGCAACTAGCCTCTTTTTTGTCTCTGCCCCCGGGACCCACGTTTACGGACTTTAGACTATTTTTCGAAGTCACCCTGGGTCGGAGAATCGCAGATTGCGTTGTGGTAGCTCTGCAGCCTTCCCCCCGGTGTTATATTGTAGAATTTAAGACGGCCATGAGCAACACGGCCAACCCGCAAAGCGTTACTCGCAAGGCACAGAGGCTAGAGGGCACCGCCCAGTTGTGTGACTGTGCCAATTTTCTTCGCACGTCCTGCCCCCCCGTGCTGGGCAGTCAGGGCCTGGAAGTCTTGGCGGCGTTGGTATTTAAAAACCAGCGATCCCTGAGAACGCTCCAGGTAGAGTTTCCAGCCCTGGGCCAAAAGACCCTCCCCACCTCCACCACCGGCCTGCTAAACCTCCTCTCCCGCTGGCAGGATGGCGCTCTCCGGGCACGTCTTGATAGACCCCGCCCGACTGCCCAGGGACACAGGCCCCGAACTCATGTGGGCCCCAAGCCTTCGCAACTCACTGCGCGTGTCCCCCGAAGCGCTCGAGCTGGCAGAGCGGGAGGCCGAAAGGGCCAGGTCGGAGCGGTGGGACAGGTGTGCCCAGGTGCTCAAAAATAGGCTGCTCCGCGTGGAGCTGGACGGCATCATGCGTGACCACCTGGCCAGGGCGGAGGAGATCCGCCAGGACCTGGATGCTGTAGTGGCCTTCTCTGATGGCCTGGAGAGCATGCAGGTCAGGTCCCCCTCCACGGGAGGGCGCTCTGCGCCAGCCCCGCCCTCCCCATCCCCAGCCCAGCCGTTCACTCGGCTCACCGGGAACGCCCAGTATGCAGTCTCAATCTCTCCCACGGACCCCCCTCTGATGGTGGCCGGCAGCCTGGCTCAAACGCTGCTTGGTAATCTGTACGGGAACATCAACCAGTGGGTACCGTCCTTCGGACCCTGGTACAGGACCATGTCGGCTAATGCCATGCAGCGGCGCGTGTTCCCTAAGCAGCTGAGGGGCAACCTGAACTTTACCAACTCCGTCTCCCTAAAGCTGATGACAGAAGTGGTGGCGGTGCTTGAGGGCACCACCCAAGACTTTTTCTCAGACGTCAGGCACCTGCCAGACCTCCAGGCTGCCCTGATCCTCTCGGTGGCCTACCTGCTACTCCAGGGGGGCTCCTCACACCAGCAGCGCCCCCTCCCTGCCTCACGGGAAGAGCTGCTGGAGCTGGGCCCGGAGAGCCTAGAGAAAATCATCGCCGACCTCAAGGCCAAGTCACCCGGCGGAAATTTTATGATTTTAACAAGCGGAAACAAGGAAGCGCGCCAGTCAATAGCCCCTCTCAACCGACAGGCGGCATATCCACCCGGCACATTCGCGGACAATAAGATTTACAACCTGTTTGTGGGAGCGGGACTACTGCCCACGACGGCCGCGCTGAACGTGCCCGGGGCGGCGGGTCGGGACCGAGACCTGGTGTACCGGATCGCCAACCAGATCTTTGGGGAGGACGTGCCCCCCTTCTCATCTCACCAGTGGAACCTGCGCGTAGGTTTAGCCGCACTCGAGGCCCTGATGCTCGTCTACACGCTCTGCGAGACCGCCAACCTGGCCGAGGCGGCCACCCGGCGTCTACACCTATCGTCCCTGCTCCCCCAGGCAATGCAGCGGCGCAAGCCTGCCATGGCGTCAGCTGGTATGCCGGGCGCCTATCCAGTCCAGACGCTTTTCCGCCATGGGGAGCTCTTCCGCTTCATCTGGGCCCACTACGTGAGGCCCACGGTGGCGGCAGACCCCCAGGCCTCCATCAGCTCTCTTTTCCCCGGGCTGGTTTTGCTGGCCCTGGAGCTGAAGTTGATGGATGGGCAGGCTCCCTCCCATTATGCCATAAACCTGACCGGACAAAAGTTTGACACCCTCTTTGAGATTATCAACCAGAAGCTTTTATTTCACGACCCGGCTGCCATGCTGGCGGCGCGCACACAGCTGCGTCTAGCCTTCGAGGACGGCGTCGGTGTTGCCCTGGGGCGGCCCTCGCCCACGCTTGCGGCGCGGGAGATCCTGGAGCGTCAATTCTCCGCCTCGGATGACTACGACCGGTTGTACTTCCTGACGCTGGGCTACCTGGCCTCCCCTGTGGCCCCAAGCTGAGCCAGTTCCTCGCACTGGAGTGGGTCATTGGCAAAAAGGTAAATAAAGTCATCGCACGGGGGTTTTGCCTCCTTCTCGTCTCTTGTTTCGGGTAGGGGAGTAAGGCCGTGCCAGGCCGCCATGCTCAGGGCCACGGCGTGCCAGAGGCCCTCGTAGTCGTGCGCATCCGAGAGGATGGCACGGTCCAGAAGCAGATAGCCGGCCAGGCAGAGGAAGGCCACAAAGAGGGGGCGAAGGCGTGCCCGAACCCGGGTTTCATGCTCATCTGCCCCCCAGTGAACAAGGCAGTAGAGGACACCCACCACCAGGCGGTTAGGGAGGACACTGCCAAGGTTGAAGAGCAGATTTCCGTCAGCCAGGGTGACCTGGCTCAGGTCCGGCGCCCTGCGCAGTCCAAGCTGCGCCCACACACATGCACAGACGGCCCCTGTGACATCAGGCCGGTCATGCAAAAACAGACAAAGAGACCGTGAGCGGTTACCGGGGCGCAGGGCCTCTGCCGGGAAGCCCACCCGGGCCAGGGCCCGGTAAAGCAGGTACCAGTATTCATCCGGCACCTTGCGTGCCAACACACGATTCGTGCGGTTTCCAGTATTTATCACGGCTTCCCGCCACAGGTAAAAGTTAACACTTAGGGTCAGCAGCTTGGTCAGGGATAGGTGCAAAAACCTGAGCTCGTCCTCGCGCAGAGCGCAAAGCGGCCAGTTCTTTAGCATCTTCAGGAGGAGCCCGTGAATCCCAGGTGTCATTCGCGCGTCATCCCCGCGAACCCCCAGTCCCATTAACATAGCGGGCACAATGGTGCAGGCACCGTCTGTATACGTCTGCGGCTTCGTGGAGCGCCCGGACGCCCCACCCAAGGACGCCTGCCTTCACCTGGATCCCCTCACCGTCAAGAGCCAGCTCCCTCTGAAGAAGCCCTTGCCACTCACGGTGGAACACCTGCCGGATGCTCCGGTCGGCTCAGTCTTTGGCCTTTACCAGAGCCGAGCGGGTCTCTTTAGCGCAGCCTCGATTACCTCTGGGGACTTCCTGTCCCTGCTGGACTCAATTTACCACGATTGCGATATTGCACAGAGTCAGCGCCTGCCCCTCCCTCGAGAACCCAAGGTGGAGGCTCTGCACGCCTGGCTCCCCTCACTGTCACTGGCCTCCCTCCACCCAGACATACCCCAAACCACCGCAGATGGAGGCAAGCTGTCCTTCTTTGACCATGTGTCTATCTGTGCCCTGGGTCGCCGGCGCGGCACCACGGCAGTCTACGGTACAGACCTTGCGTGGGTCCTGAAGCACTTTAGTGACCTGGAACCGTCTATCGCCGCCCAGATTGAGAATGACGCCAATGCCGCAAAGCGTGAATCCGGATGCCCGGAAGACCACCCTCTGCCCCTCACGAAGCTCATAGCTAAGGCAATCGATGCTGGATTTCTGAGAAACCGCGTGGAGACTCTGAGGCAGGACAGGGGTGTGGCCAATATCCCAGCCGAGTCGTATTTAAAGGCCAGCGACGCCCCGGACCTACAAAAGCCGGACAAGGCACTTCAGAGCCCACCACCGGCCTCCACAGACCCAGCCACCATGCTATCAGGTAACGCAGGAGAAGGAGCAACAGCCTGCGGAGGTTCGGCCGCCGCGGGCCAGGACCTCATCAGCGTCCCCCGCAACACCTTTATGACACTGCTTCAGACCAACCTGGACAACAAACCGCCGAGGCAGACCCCGCTACCCTACGCGGCCCCGCTGCCCCCCTTTTCCCACCAGGCAATAGCCACCGCGCCTTCCTACGGTCCTGGGGCCGGAGCGGTCTCCCCGGCCGGCGGCTACTTTACCTCCCCAGGAGGTTACTACGCCGGGCCCGCGGGCGGGGACCCGGGTGCCTTCTTGGCGATGGACGCTCACACCTACCACCCCCACCCACACCCCCCTCCGGCCTACTTTGGCTTGCCGGGCCTCTTTGGCCCCCCTCCACCCGTGCCTCCTTACTACGGATCCCACTTGCGGGCAGACTACGTCCCCGCTCCCTCGCGATCCAACAAGCGGAAAAGAGACCCCGAGGAGGATGAAGAAGGCGGGGGGCTATTCCCGGGGGAGGATGCCACCCTCTACCGCAAGGACATAGCGGGCCTCTCCAAGAGTGTGAATGAGTTACAGCACACGCTACAGGCCCTGCGCCGGGAGACGCTGTCCTACGGCCACACCGGAGTCGGATACTGCCCCCAGCAGGGCCCCTGCTACACCCACCCGGGGCCTTACGGATTTCAGCCTCATCAAAGCTACGAAGTGCCCAGATACGTCCCTCATCCACCCCCACCACCAACTTCTCACCAGGCAGCTCAGGCGCAGCCTCCACCCCCGGGCACACAGGCCCCCGAAGCCCACTGTGTGGCCGAGTCCACGATCCCTGAGGCGGGAGCAGCCGGGAACTCTGGACCCCGGGAGGACACCAACCCTCAGCAGCCCACCACCGAGGGCCACCACCGCGGAAAGAAACTGGTGCAGGCCTCTGCATCCGGAGTGGCTCAGTCTAAGGAGCCCACCACCCCCAAGGCCAAGTCTGTGTCAGCCCACCTCAAGTCCATCTTTTGCGAGGAATTGCTGAATAAACGCGTGGCTTGAAAGTAAACTTTATTGCGTGTTAGTACCTGTCCATTCACAGGGGTATCCAGCCCTTGCGCCGCCTCCCCCAGCCCGCCAGCCACCCCAGACAGGAGATGATAATGATGAGGAGCACCGGAGCCACCACAGCACAAGTGATTAGGAGCAGGGCCCAGTGCACCCAGGTGGTCTTAGGGCGCCAGGGATCGATTGGAAAAGGGCCCAGGGTCACTGGCTTATGCGTGGGACGTTTAGAAACAGGCCGCCTATGGGGCCTGTGACTGGTGCTTGTGGTGTGGGAGACTAATGTGGTGGGGGCTATGGTAGTGGCTGGGATAACAGTAAGATGCATACGCTGAGTGAGCGTCCGGTTGGCATGGTATTGGTCGTCTTCTTCCCCTGCAGAGTAATTGCAGTGGACCCCGGAGGCCACACTGCAATTTCTCAGTGTCACATTGCACGTGTAGTAACCTGCATGCGCAAGGGTCACATTGGGGATTATCAGAGAGACGGAGGTGTTGGAGTCATTTACCCATTCTAGGGTAAGGCTATAATTGTAACCCCCGTTAGTTATATGAGTTCCGTTGTTGGAAGTAGCTACGGCCAAGGGCAGTTGTCCATCCCCGGGAGTGTATCCCCGGCCCAACTCGATCCGAGAGACCGACTCATTGCTAGGAACGCTGCAGGTGAGATTCACTCTAGCACCTGCATGGGCGGTGACATTTTCAAATTTAACCAGATCTGAGAAAAATGCACAAACAGACCCCACACAGCAGCACAATAGAAGCACTAAATGAGTCATTCCTAAACTGTCAGTTTTAAAACTCCCTGCTTCTCAGGCCTAAATACGTGGTGGGGTGTGCTTAGGATCACTTTCATATTCTGCAACAACAGCCATACCCGGAAGAGGAGCTGCCGGTTGCCATTTTTCAAGCTGCTAAACCACGAGTGGCAGCAGGCCTAAGAAGCTCCTCAGCAACATGGAGACCTCGAAGGGAAACTGGCAGGAGCAGGGAGTCACGTAGGCACTAGCCTCTTCATGTGAGGTAAGAGATCGCTAAAAATGGGATCAGGGTATGTAAACCGAGTTTTGCGGGGGATGGTGAGCCAGACACGGCGGGTGGGGGGAAGGAGCTGACACGAGTGCGTAGAAAGGGCCAAAAATACACCAGCTATAAGGAATTGCTCAGGCCAAAGTTGTTCCTCAGGTGGCTTTAGGCCTAATGTAGGCAATTGCGTGCCTAGAACATTGCTAATGTGCCCTGTGTTTCCTGCCTTCATGCAAATATCCTACCTCCCCCGGCCTGGTGCAAAATGTCTGCCTCAGAATACTAACAGCTAATCCAAGCTAACATTCTATCAGTAAACGGGCAGAAAACGGATAAGGACCGCGGAGTTTGGCCCTCCGCGGTGTCCGGTGGTCCTCACACGTGCCCTCCCCCGGGCCGATGGCTGAGGCCCGGAATATGCAAGTGCATCTTTCTAACCAGTAGGGGCCTCCACCTAGGTGCTTTGTTAATCTTTAGTGGGAACTAGTGGGAGTGCTGTGCCTAGGGTACCCCTATCCTAGAGGTCCTACCGGAGCTCCTTGTCTTGATAATCCCTGTAAACACACACCACCTAAGAACAAGGCATCGTTAACCTTTGGTGGAACCTAGTGTTAGTGTTGTGCTGTAAATAAGTGTCCAGCGCACCACTAGTCACCAGGTGTCACCGGAGGCTACTTGCCTCAGTGCCACTTTTACCTTCTCAAATCTATACGGGGGGGGGGGATCTGTAACATTTGGTGGGACCTGATGCTGCTGGTGTGCTGTAAATAAGTGCCTAGCACATCACGTAGGCACCAGGTGTCACCAGGGCTACTTGCCTCGGCATCTCCTCACCGGAGAAGGGGTTAACAAACCCGTGGGGGGTCTTAGTGGAAGTGACGTGCTGTGAATACAGGTCCATAGCACCGCTATCCACTATGTCTCGCCCGGGCTATATGTCGCCTTACCTCCCCTATATAGTCACGACCCCACCGAACCAGGCATGATGTAGAATAAAATTTTATGCATCATCTTCTAATCTGTGCCGCTTGGAGGGAAACATGACCACCTGAAGTCTGTTAACCAGGTCAGTGGTTTTGTTTCCTTGATAGAGACACAAGGACTGCCAGCCCCGTTGGGGAGGGGGTGGTGGGTACGGGAGAATTTGGGCTCGTTTAAACAAAGCCTCCTCGGATGCTCTGTGGCACCTCAAGGTGAATATAGCTGCCCATCGACGTATCGCTGGAAACCGGTGGGCCGCTGTTCACCTAAAGTGACGCAAGGTCTGTCAGCCGCCAGGGTCCGTTTACCAGGCTTTCAGGTGTGGAATTTAGATAGAGTGGGTGTGTGCTCTTGTTTAATTACACCAAGATCACCACCCTCTATCCATATCCCACAATTGATAAACCTCCGCATGTCCAACCACCACGTTGAACAGGATGTGGCACCCTAAGAGGACGCAGGCATACAAGGTTATTACCCAGTCCTTGTATGCCTGGTGTCCCCTTAGTGGGACGCAGGCCTAGGTAGCATCATTGACACTAAAAGCAGTGACCTTGTTGGTACTTTAAGGTTGGTCCAATCCATAGGCTTTTTTTGTGAAAACCCGGGGATCGGACTAGCCTTAGAGTAACTCAAGGCCAAGCATTTCACACCTGCAAATGCACCATGTAACCACAGATCTAAACTGAAAGTTGCAGCTTTAGATGGCAAGGAAACTTGGGTTTCAGGCATAGAAAGCCTGGCTCACTATAGCAGCCCATGTTTGTTCCAGGGTGGGGGAAAGGCACGTGCCCTTAGAAAACTTAGCTGCAAAAATTCTATTGTGTTGGGAGAGCCTCTATATCTAAAGGCCTTTCCTCACAATACAAATGTTACTAACGTCTGCCCTCTGGAGACCTGCTATGTGGCTAGACGTATGGCCTACCCAAGACGTTGGGGGTCTCGGGTAGGCCATGATTCTTCCAGGCATAGGTTACAACCAGTCACTGCTATCAAGCCTACTCAGTTCCCAACGCAGCACATACCCCCCGCCTCTCCTGCCATGAGGACTTATGGCAGTGTTTACTGTTCTGCTTTTACTCTTGGACCAGGCTGTCATTCTATCAGAATAACAGGGGAAGCAAGGCCCCCTGCGTCAGCGGGACACGTGTTTCTAGAATCTCGGAGCCAATAACTACCTGCCCCTCTAATCTGTATGCTGCATGAAAAACCACATACACGTAATGTAAGTTTAGCCAGTTTATTGTTACACCAATGCCCCGAAAGTCCCCCCTGTCCCTTTGGGTCTCAGGACCCAGCCCTGGAGCTCGGGGGGCGGCCGGGTGGCCCACCGGGTCCGCTGGGTCCGCTGCCCCCGCTCCGGCCGGGCGCCGGGGTGGCCGGCTGCAGCCGGGTCCGGGGTTCCGGCCCTGGAGCTCGGGGGGCGGCCGGGTGGCCCACCGGGTCCGCTGGGTCCGCTGCCCCGCTCCGGCGGGGGTGGCCGGCTGCAGCCGGGTCCGGGGTTCCGGCCCTGGAGCTCGGGGGGCGGCCGGGTGGCCCACCGGGTCCGCTGGGTCCGCTGCCCCGCTCCGGCGGGGGGTGGCCGGCTGCAGCCGGGTCCGGGGTTCCGGCCCTGGAGCTCGGGGGGCGGCCGGGTGGCCCACCGGGTCCGCTGGGTCCGCTGCCCCGCTCCGGCGGGGGTGGCCGGCTGCAGCCGGGTCCGGGGTTCCGGCCCTGGAGCTCGGGGGCGGCCGGGTGGCCCACCGGGTCCGCTGGGTCCGCTGCCCCGCTCCGGCGGGGGTGGCCGGCTGCAGCCGGGTCCGGGGTTCCGGCCCTGGAGCTCGGGGGGCGGCCGGGTGGCCCACCGGGTCCGCTGGGTCCGCTGCCCCGCTCCGGCGGGGGGTGGCCGGCTGCAGCCGGGTCCGGGGTTCCGGCCCTGGAGCTCGGGGGGCGGCCGGGTGGCCCACCGGGTCCGCTGGGTCCGCTGCCCCGCTCCGGCGGGGGGTGGCCGGCTGCAGCCGGGTCCGGGGTTCCGGCCCTGGAGCTCGGGGGGCGGCCGGGTGGCCCACCGGGTCCGCTGGGTCCGCTGCCCCGCTCCGGCGGGGGTGGCCGGCTGCAGCCGGGTCCGGGGTTCCGGCCCTGGAGCTCGGGGGGCGGCCGGGTGGCCCACCGGGTCCGCTGGGTCCGCTGCCCCGCTCCGGCGGGGGTGGCCGGCTGCAGCCGGGTCCGGGGTTCCGGCCCTGGAGCTCGGGGGGCGGCCGGGTGGCCCACCGGGTCCGCTGGGTCCGCTGCCCCGCTCCGGCGGGGGTGGCCGGCTGCAGCCGGGTCCGGGGTTCCGGCCCTGGAGCTCGGGGGGCGGCCGGGTGGCCCACCGGGTCCGCTGGGTCCGCTGCCCCGCTCCGGCGGGGGGTGGCCGGCTGCAGCCGGGTCCGGGGTTCCGGCCCTGGAGCTCGGGGGGCGGCCGGGTGGCCCACCGGGTCCGCTGGGTCCGCTGCCCCGCTCCGGCGGGGGGTGGCCGGCTGCAGCCGGGTCCGGGGTTCCGGCCCTGGAGCTCGGGGGGCGGCCGGGTGGCCCACCGGGTCCGCTGGGTCCGCTGCCCCGCTCCGGCGGGGGTGGCCGGCTGCAGCCGGGTCCGGGGTTCCGGCCCTGGAGCTCGGGGGGCGGCCGGGTGGCCCACCGGGTCCGCTGGGTCCGCTGCCCCGCTCCGGCGGGGGGTGGCCGGCTGCAGCCGGGTCCGGGGTTCCGGCCCTGGAGCTCGGGGGGCGGCCGGGTGGCCCACCGGGTCCGCTGGGTCCGCTGCCCCGCTCCGGCGGGGATGGGGGTGCGCTCCCAGGCCGGACCCTGGTGCCAGGCAGGGACCCCGCGCCACCCGCTTCATGGGGGGGGAGGCCGCCGCAAGGACGCCGGGCCGGCTGGGAGGTGTGTACCCCCCGAGCGTCTGGACGACGCTGGCGAGCCGGGCCAGCTCGCCTTCTTTTATTCTCTTTTTGGGGTCTCTGTGCAATACCTTAAGGTTTGCTCAGGAGTGGGGGCTTCTTATTGGTTAATTCAGGTGTGTGATTTTAGCCCGTTGGGTTACATTAAGGTGTGTAACCAGGTGGGTGGTACCTGGAGGTCATTCTATTGGGATAACGAGAGGAGGAGGGGCTAGAGGCCCGCGAGATTTGGGGTAGGCGGAGCCTCAGGAGGGTCCCCTCCATAGGGTTGAACCAGGAGGGGGAGGATTGGGCTCCGCCCCGATATACCTAGTGGGTGGAGCCTAGAGGTAGGTATCCATAGGGTTCCATTATCCTGGAGGTATCCTAAGCTCCGCCCCTATATACCAGGTGGGTGGAGCTAGGTAGGATTCAGCTAGGTTCCTACTGGGGTACCCCCCTACCCTACCTTAAGGTGCGCCACCCTTCCTCCTTCCGTTTTAATGGTAGAATAACCTATAGGTTATTCACCTAGTGGTGGAATGGGGTATTGCAGCTGGGTATATACCTATAGGTATATAGAACCTAGAGGAAGGGAACCCTATAGTGTAATCCCTCCCCCCCCTACCCCCCCCTCCCTTACGGTTGCCTGAGCCCATCCCCCACCCCAGCACCCCGGGGTGACGTGGCCCCCCGCGTGCCTTACTGACTTGTCACCTTTGCACATTTGGTCAGCTGACCGATGCTCGCCACTTCCTGGGTCATGACCTGGCCTGTGCCTTGTCCCGTGGACAATGTCCCTCCAGCGTGGTGGCTGCCTTTGGGATGCATCACTTTGAGCCACTAAGCCCCCGTTGCTCGCCTTGCCTGCCTCACCATGACACACTAAGCCCCTGCTAATCCATGAGCCCCGCCTTTAGGAAGCACCACGTCCCGGGGACGGAAGCTGGATTTTGGCCAGTCTTCAATTTTGGGGAGTGGTTTTGTGTGAGCCGGAAGTTGGCAATGGGGTGAGGGCGGCGCTGGTTAAGCTGACGACCTCCCAAGGTCTCTCACCCTGGGTACACAGGTGGGGCGGCAGCCTCTAACTTTGGCTGTGGCCTCTATTTCCTCCCTTTCCTAGCCAGGGCCATGTGTTCCTGCATGTCTACTTGCCTCCTGTGGTGGCAGAGCTTGGCCCTGGGCCCAACCCCCGCCTTGGGAGCCTGTAGGGGCCAACACCCTTGGTTTGTTTGTGTTCCTGTTTGCTGGCAACTTACTGGCAGCTGAGCAAATTCTAATGGGCGCCCGCCTTCTTTCTCTCTTGTTTTATTAATAGAATCTCAGCCAGGACCTATACCTGAGACTTCAAAGTCTGGTCCTGGGTTCTGAGACCCCCAAGATTTGTCATGCACACCTGCACACCTGTTGGTATTGGGTTTCTATTCTTGAGTGTGAAAGTTTGTAAAAAAATTCATAAAATGTCACTAATTCCTCTTACCTGTTTAGGGTATTGTGCAATTCTTCAGCCTGCCTATTTTCAATTTGCCTAAGGTGGCAATTTAAGATGTGGTTAATTAACCATTTTCCTGTCTGACACCACTGCATGGGCAACCGGGTTCCATGGCACATTTAGAGATAAACATAGATGTCTTGTCTTGCTCATGTGCAGAGGAGGGGGTGTTGGTGTGCAATATAGTTTCTGGATTCCAAATTGAGTTGGGGGTGCTATTTTCACTATGGAATTAAATTACTGACATTAGACAGTGGACACCGGGCTATATGTGGGGATGTCTGTGGCTTGTCATTTCCTCTTAGAAGGTAATCCCCCATCTTAACTTCCCTTTAAATTGTGATGCAAGCCCTGGGTTATTTATAGAATGATTATCTAGGTTTGATAGTCTGAAGGCTGGGCAGAGAATGTTTGTAATTTTTATTCACCTTCTTTACCCCCCACGAGTATCCAGTTCTAGAAGATCTCCTGATATCCCGGGCTGCCATTATTCCCTTGAGTGTTATAGCTTCCTCTTAACTTAAGCAAGAGCTCCAGGATGTTAGCTTTTTTGGTGGGGCTGGTTGTCAGGAAGAGGTTCCAGTGTTGTCCTTTATTTTTAGATGTTAGCTTTGTGTTAGGTTAGTATGGGCTGGGTATTCACTAGTGAAGGCAACTAACACAGTTAGACGTGCTAGTTGTGCCCACTGGTGTTTATCCGGTCCCAAATGTCACCACAGAACACAGGGGGCTGGATTTGGCAGCAGCACTTGTGCTTTTGTTGATTTTTACCCGTGTATCAGAGTGGGGGATGCTAGCCAATTTAGCTTCCCCTCCCCTTAACAGGGGGTCTCGCGGGGTGCCAATTGTCGCCTGCCTTCCCCCGCTTCCCCTTGTTAACTTATAGCATGATAGGTAGGTCACCTAACGTGGAAGCCTGGTGGGTGATCCTTCCTCGGTAGGGAGCGCTTAGGGCTGTTGAGCTCAACAGCCCCACCTGGGTAAAATGTATGTTCTAAAGAGTTACCCAATTATAACAAAACTGTTGTAGGGTAACGAAGACCTGATGGAAGTGGTATTGTTGCCGTTGAAAGACGGGTGTCCTGGCTCAAGTTCGCACTTCCTATACAGTGTTAAAGCCTTGTATCGGAAGTTTGGGCTTCGTCCCAGTGTACTCGATAATGTCGACTGCTGCGAAGGTTTGGACCGTCTTCCAGTAGGTGTTGGGGGTCCCAAATCACGAGGTTAGGCAGGTGCACTTGGCTCTTTAGGAGGGACCCTTAAGCCAGACAATGTAGTGCCCCTTTTTTTTGCAAATTGGCCTTATTATTAATTTCTTGTTAACACTAATTCTGTTCTATGACCCTGGGTTTTTCAGATGCCGTTGAACGTGTCACTGAGCTGAATTTGGACGCAGCTACTTGACCTTTGCCCCCGTGCCTCCAGCGCTGATAAGTGCTGCGTCCACTTTGTGTTACAGGTGGGCCAAACCTCCAGAATATCAATTGGTGGGGCCTTGGTGGGCTGCATAAGGCAGTAGGTTTGAGGTGACCTACTTGGACCATGTGGATCCAGTGTCCTGATCCTGGACCTTGACTATGAAACAATTCTAAAAAAAATGCATCATAGTCCAGTGTCCAGGGACAGTGCACTCGGAAGTCTCATCATCTCCGTTTGTGTGTTTAGTGTGGCCAGTACGGCCACCCCTGTGCCACGCCCTGGCATGCTGCTGACATCTGGCCGCCAATTTCAGCGGGCCCTTTTCCCCCTTGTTCACCCCATAGCAAGAAGGGTAGGTTACATGGGTATTTTCCCATCAGCACCTGACTGGCCGGTGCAATTAGAGGAGAGGGCAACAACGCAAGGCTGTTGTCTTATTTGGGTTACAAGAGCTGCGGCGGTCGATGGGTTCACTGATTACGGTTTCCTAGATTGTACAGATGAACTAGAACTGTCACAATCTATGGGGTCGTAGACAGTGTGCTTACCAGACTTCCATGGAAGATGTGAATTTGCTGCTAGCTATATGGGTGGTGCTATGGGCTCCCTAGGGACTCATGTAGTGGGGCTTTGTGATAGCTAATGAATGTGGCAGCTGTTGTTTGTACTGGACCCTGAATTGGAAACAGTAACTTGGATTCTGTAACACTTCATGGGTCCCGTAGTGACAACTATGCTGAATATCTTGAATATGGGAGGAGGGGGGCTTTGGGTTCCATTGTGTGCCCTTTCCTGGCCAACGTGAGGGTCCTAGTGTTATAGGGCGTGGCAGTTTTCTTGAGGGCTAATAACCCGGGTGAGGCGGTTGTCACAGGTGCTAGACCCTGGAGTTGAACCAGTACCACTCGGTTACAAAGTCATGGTCTAGTAGTTGTGACCCTGCAAAGCTACGTGGGGATGAGCAGCCAGGGACTTTGGTTGGCAAGCAGACAGGCGGCGCATTGGAACCCCAGAGGAGTGTCCCGGGGCCACCTCTTTGGTTCTGTACATATTTTGTTATTGTACATAACCATGGAGTTGGCTGTGGTGCACTCCATCTGGTAAGGGGGCTGGTGCGGACGCCTGTGTTTAGTCTATGCCAATGTTTACCTGCCTTGGGTTACTATTCCAAACGACCACACCTTTGAGGACACCTGGAGCCCTGATCATTCTCGGCTTTTACTGCCACCTGGCTTCTGTTGGGTCAGACAGTTTGGTGCGCTAGTTGTGTGCTTAGCAGCAACGCACACCAGGCTGACTGCCTTAGCAGTGTGGCCCTTTATTGTGGCATCCTAAGGAGGGATTCTGGAGTGCCTTTCGCGTGAAGCATGCCCTGAGACGTACTCGAGTTAGGACTTAATCGCTCCTGTGCCGCTGGATGAGGGAGCGCCAATTTGTACATCCTAGCTCTGGCCATAGAGTTAGCCCACCCTTGTGTCTCCCTTTGGCCTTTGCGGTGCCAATTTCCGGTGGTTTCCCTTTTCCGCCCGTTTATCCAATAGCATGTAAGAGAGGTTGCCTAGATTTGGCAACTTTGAGGGAACGTTCCGTGTAGCTGGTGACCTAACACCCGCCCATCACCACCGGACAGATTCTGAACTTGTCCTGTGGTGTTTGGTGTGGTTTTGGGGTACGCAGGAGTACGTTGGAATGCTTTGGAGCCGAGAGGGATGGGCCCGCTTGTGCGCTTATGTGTTACACGGTGCCAATAACCGGCCCGGTGCGGCTGCCCCGTGACCCGTGGGCCTTACCTTCCTGGCCATCGGGGGACCCTGGTGCTAGGGTCCCTTGTGTTGCTTTCTGCCATAGGGGGGAAAGCATCGCCTTCAGAATTGGCTGCTCCGTTGGAACATTTGAGGCCTACTGTATCCGTGTCCTGACAACATTCCCCGCAAACATGACATGAGTTAATTTAAACATGTTTTGTTTGCTTGGGAATGCTCTTAGGGCCTGGAAGCTTGTCATTGGATTCATCGTTTCCTGAACTACAGGCGTAGGGCCTATTGTAGCAGGCATGTCTTCATTCCTGCGTACCGAATGGCATGAAGGCACAGCCTGTTACCATTGGCACCTTTTTTCCATGTAAACCTCCGTGATCCTGGGTCCTTTGGAGACTCAGGTGTGAATTTGTTTTGGTGTTCGGCGCCAGGGCATCTCGACGTTGGAATGTCAACTCAACTTGGGCACCTCGATAACCGGCTCGTGGCTCGTACAGACGATTGTTTGGCTCTGTAACTTGCCAGGGACGGCTGACGATGTGTTTAGTCTGCCACTTGCATCCGGCGCTTTGGTTACTCGGGAGACTAATGGGGGGTGTGGTATGGCACAGGCTGGGGGTGAGTCTGGGGATGTCCCTGGGCGTTGCTGCAGCCCATTCGCCCTCTGGGGATGAGATGTTCAGGGGTGGCCGGTACCCTACGCTGCCGATTTACATAATATAAATTGTAAATGCTGCAGTAGTAGGGATCTGGACGCGCGACCTGCTACTCTTCGGAAACGCCAACCCAGGAGCGTCGCCTCTGGCCCCATACTCCCGCCATGCGACTGCTCGCCCCCTCCCAGGCCTCCCTGGTGAGCCCTTGCCGCTCCCCGCATTCCTGCTTTCGGCGCCCCTGCGGATCCCGATGACAGCAGGCCTTTCCTTCCCCCGTTAATGAAAAGAATGACAGTGAGGTTGTGACAGAAGGACAGCTTTATTCAGTTTACAGAGTGCCCTCGGAGGCTACGATATTCCCGTTAAATGTCTTGTTGATTCTCTCAAAGGTGGGGAGGGAGGAGCTCTCCACAACAATGTTCCCTGGCAGCGTGAGCGCGCAGCCCTGCCGTTGGATGTATCTTCTCATGATGGTGCTGATAGAGGGGTCTCCGGCGTAGATGAAAAAGGCCTGGGCCATGCTCTGGCCGGTCACGATCGTTATGGGGTTGTTGGAAATGTTCCGGACCGTCAGCTTGAGGGTCTGGCCCGGCTTCCACTCCTGTGGGTAGACGTAGAAGACCGGGTTGGAGGAGTGGGACACGACAACGGCCGTAATCTTGGAGCTCAGGGGGGCCTCGTAGGTGTTGTTGTATTCCAGCTCCGTGATGAAATTAGGAGGAATAATCACAGGGGAGCCAAAGTAGCGGATGTCTGTGGATTCCCCGTCCCAGCGCCAGTGGCTCTTAGGGTAGGGGTTGTAACGGAAGGCAATAATCACATCATCCAATAGGGTCATGCCCACCTTGACGTTCAGCGGGCCCTCTCGTTTCAGGTCCGGCGTGTCCACGGAGACTCGGACGTAGCCCTTACCGCGGCGTATGGCGTTGACCGGACACACCTTCCCCGGGAATGTGTGAATACGGGCGTATGACTTTAGAAATGGGGGCGTGTGCTGCGCCAGCAGGTAAGGCAGGCACTCGTCCTGGCTGGTGACGGGAGAGCCACTGAGGAAGATCTGGGGCTCGCTGGTGTTTAGCTTGTCCCCACTCTGGGTGCAGGAGCGTGTCAGCTGAATGTCGCTCTGCCCGGGCAGAATCTGCAGGTAGAGGTAGGGGTTCTTGACCAATCTGATGGGCACAATGTACCAGGTAAACTTCCCTTTCTCTATGAACAGGCTGCGCGGATTCAGGACGCTTAGCACGATGTCCTGGTCAGAGTGCATAACGAAGAAGGGCTTGAGGAATACCTCGTTGTCTTCCGCTCCAAAGAACAAGAACGCAACCGTAAAGTAGCGGCTGCCGTAGGTGGTCGTGTTGAAGGAGAAAGAAGGTAACTTGAAGCTGAGTATCTGGCCCACCGAGGGGCAGGGAGGCAGCTCTTGGCACTGCGCGTCCAGCTGCAATACCTGCTTGTTGGTGACGCGAACGTATGAGGGGAAGATCTCGTACTTCCACACGCCTCTCATGAACGACGTGTCTGGTTTTTCAGTGGGCCGCAGGCGGCGGAGGCTGTTCCTGAACGACGAGCGCCGGGACGCTAGTGCTGCATGGGCTCCTCCGGGGTAAGCTTCGGCCATGGCCGGAGCTCGTCGACGGGCAAGGTGAGAGTCGGGGGGCGGGCGACGGTGCGGCCCCAATACAACTCTCCGCTCGTTAGCTGGTAGAATATCCGCCCGGCGTCTAGGTTGTCACTTCGCTCGGCCGGCCAGAAGAGCGCAAGTCCAAGTCTGGTGCTGGGGCCGATGTGCAGCGGTTTGTGCCCGCAGTTGTAGACTGTCATTTTTATGGGCGAGTGGGCGGTCCACACGCGCGGGCGCAGCACCCATTGGTCGCACGCCGCCTCCTGGAATGTAAACCCCCAGAGAGAGGGCGTGCCGCCCTGGAGATGGCCCTGTGCCATCACATGTATTTCCTCCTTGGGTGGAACGACGGCGTCGTGCTCCGGGTGGAGGGGGAATAGCGTCCAGGCATCTTTCAGGGTCACGAGACCGGGGTCCATGCTTAGAAAACAGCCCTCCCGGGCGGTGGGCGGCCCGGGCTCCAGCAGAACGTCGCAGACCCAGCCCTCCTCGGCCCTGTCCACCTGTATGTCCAGGTGCACGGACCCGGAGGCTGCGTCTCGTGACATGGCCAGGCCTGGTGCCAGCCGACCACGTCCCGTGTCCCAGCCGAGGCCGCGCCAGAGCAGAGCCCGGGACTGACTCAGGGCCACATCCCCTCGGCCCGCGGACGCCGCCTCGCCAGCCCCCGGGCCTTCATGGGCCCGCTTTCTACCTCTCTCCGGCACCCCAGCCTGGTCAGCCGCAGAGGAAGCATGACCTTGGGGTGGGACGGGGCAGGCGTGATCCTGGGCGCAATCTTTGCCGATCCCCACACCTTCACTCCTTGTTAGGTTGATAGAATGTCGGTACCACGCCACGGGGGGCGGGCCCGCATAGGGAAAAGCCAGGGAGAGCGATGTGGGCGAGGATGGGCTCAGGCGGCCCCAGACACGCAATTTGCCCCCCTGGGCGGCCGCAGCCTGCCCCTCGGCGGCCCGTGCCCCAGCTCCGTCACGGGGGGCGCATAGGAGGGGTATATCTAGGATAGCCGCACCTACACAAATGAGACACAGACACAGGTCATGAGGATTTAGGCAACGCAGGCTTGTCTTTATAGTTACAAACATGGGAGCGTGCACCTGGAAGATGCAGCTGGGGTAGATCTTTACATCTTTACAGGGCGCAGCAGCCGCCAGACACTGAAGGGCAGAGTTCACGGCGGGCACCTCCCAGAGGGAGCCCACCAGCCCGTACCTGGCCACGGCCAGGGCCTCGTAGGCCGAGACGGGCAGCCGGAGCTTGTGGTACTGTCCCTCCGGCAGGTGGAGTGGGACACAGTTAGAGAACATTAGTCCTCTGGTCCCTATCTCCACCCGCCAGGCCTGTGTGTCAGTTTGCAGGGCCATCCTCGCGCTCAGGTGGACTGGCTAGGCACCCTTCTGAAGTATCTGGCGGTGACTGTCACCTGGTTCTTGAGAGAGTCCATAAAATGGCTGAAGCTCCAGGCGTATAGTATAATGAGCAACAGGGCCAAACAGGCGGCGGGGCCTGGGTAGTAGCGGGCAACGAGAGACTCTGTGCAATCAAACCCCAGGCTCCCGGCCTCACCCAGGAAGAGCAGCGGCAGGGACAGCATAAACCAGGAGAAGGCGCAAATGAGTCCGGTGAAGGTGACGTTGCATATCAGGCGCGGCTTCCTTCCGAATTTTGTGCGCAAAAGTTTCCAGATGATGATAACTGTGAGGAGGACGATCAGGACTGCCGCCAGTAGGTAGCAGCCGGCTTTCAGTCCTTGGACGGCCGTGTGCATGCCTTTGGTGGGGCCTTCCCTGCACATGTTGGGGCCTCTGTTGAGATTGGCGTCGGGGCCCATGGTAATGAGGAGGATGATAATCAGCAGGAGTACCAGACAAAACACGCCCATCAGGTACAGGCACACATTTCTGTGGGAGGTTCTCTTGGGCGTTCGGCTGAACAATGCTAGGGTCTTCTCCAACGCCATACCCAAGTGAGTCCATACGGAGCACATCAGGCCCAAGAACATCATGTTCTGGGTCAAAAGGCAGAGACCGGTAGACGAGAACTCCTGAATCATTTTTCCCAGCACCCAGAGCAGCAGTTCTATGAGAAGAGCTATCAGCCAGACATCCATTCGGTGAACCAATTTTCTTACAAAGATGATAAACAAGATGCCAGCCAGTGTTAGCAGAATCAGCAGGACGAGCAGCAGGCTTGTCATGCCGCTGAGGAAGGCGCTGTAGGATTTAGTGCACGCATCTTCCGTTGCATTGACGGAAGTCATGTTGGCCACCAGGGTCCCCACGGTGGACCCGGGGGCCATGGTGGAGAGCATCTTGCTGGTCAGAGCCAGACTGGGTGGTGTCTGCAGCAAAAGAGGAACTTGCCCAGGCAGTCAGTTATTTTGCATGCCACCTCCCTGCCTGGTGGACTTCCAGACTATTTTCTGCATTCGCCCTTGCGTGTCCATTGTTGCAAGGAGCGATTTGGAGAAAATAAACTGTGAGTTTCACAGATCCACGGGCCACGCTCCCCTGGGGGCTTCATGATCCCACCGCCTTTCCCGATGATGATGACAACCGCGGCTGTCTGAAGCGGCTGACGAAATCGGTTGAGATTCTGATGAGAGGCTTGGGGGGGTCTTTGCCCTCAAGGCGAGGCTCCTTCTCCTAGGAATGCCGAGCCCCCTGCACTAGCTTCGCTCCACTGGGGATCTTTGCCAGCCTTCATACTAGATTCAGCGATCCCCCGGTTGGGAATCTTCGCCAGCCCCCCGTCCTGCTATCCCGCTCGTCGCCGCGCCTCCCATGCTAAGGGCCCCCTTCCTTTCCCTTGACTTTGGGGATATTCGGAGTCTGCTCTCGCCGCTCTCTTCTCTCGTTTAAACGAGAGAATAGTAGTAGGGTCCAGTCTCAGGCCCCCTCACTTTGGGTCTTAGAATGGTGGCCGGGCTGTAAAATTCTGGAGGACGGAGAGGGCGGCCCCGGAGTTGTTATCAAAGAGGCACTGGAGGATGTTGGCCGCTCCTTGGAGCAGCTTGTCGAAATAATGATCCACGGCCACGGGAACGCCGTGCCGCTCGGCGTAGGCCGGGTCCTCGGCCATCTCCGTCTTTCTCGCCCCCTTCACTCCCCCCTTGGGCTCCACAAAGACGTACTGGATGCGGTCGTGGATCTGGGGCAGTTCCTCGTTGCGCTCGACGAACTTCTGGTAGACGGCCAGGTGAGGCATCTGGGTGCTCTTGTAGGCTGAGAGCTTGCGGCTGAGCTCCGTTGAAAAGCAGAGCTCCCCCATGGGGACCCTGCCTTCACGGAGGTCTGTGTAGGCCTGGTTTAGGATGTCAATGACGGGCAAAAAGCCCACAGGTAGCCCTTGTGTAAATGACTCTTGGAAGGGCCGGTGGGAGAGGAGGCTGGCCGCCTCCTTTACCCGGGCATCCGCCAGCACCAGGTCGAGCACGCGCCGGCAGCGTGTCTGCACAAACTTGCAGGCCGTCTTCCGGACGAGCTCCACCCCCTTCATCAGGGTCTTGCCGTCCGTCAGCACCCCCACATATCTCTTCTTTGTAATCAGCATCAGGCAGGAGAAGGTCTTCTCGGCCTCCAGGGAGATGGGGGCCACAAACAGGCTCCGGGTGGTGTGGGCGGCCAGGGCATCGGCAAAGCGCAGGGTCTCGCTCTCTGAAAACCCCCGGCACTCGATAAACAGCGAGTCCGTGTCCCCGTAGATGACTCGAAGCTGGCCCTCGGGGTTGAGGGGCGCCCAGGCGTCCGGGGAGGGGGCCAGGGCCTGCAGGTTGGCGGGGCTCAGGGCCTCCACGAAGGCCTTGGCCCGCTCCAACATCGTGCGGCCCTGCAGCGTCACCGTCTCGGCGATGGAGAGGCAGGGAAAGAGGCCGTTGGCCACCCCGGTGAAGCCGTATACGGCGTTGCACGTGCACTTGATGGCCAGCTGCTGCTTGTCGAGGATGGTCCTTTGGCGCGGATCCTCGCAGGCCGCCAGCAGCTTCTTGATGGCCTTGCGCTTGGCCAGCCAGGAGGTCAACAGACTAGCCAAGAAGGACTCGTGCACGTGCTTCTTTACAAAGTGGTAGACGCCCCCCGTGAGCCTGAAGGACTCATAGTCTTCTCCCGGGCGCAGGCCGGCTAGCCTGTGCTCTTCTCCCGGCGTTATCATGGTAGAATAACAGAGATTATGAGCCTGAATGATGCTCGGGTAGAGGCTGGCAAAGTCCACCACCAGAACCGGGGAGTTGTAGAATCCGGACAGGGGCTGGATGACGGTGGCCCCCTGGTAGCCGTCCCGGTCAGAGGCCGAGGGCATGGGCAGGATAAAGTTTTCCTTTTGGGCGGCCGCCAGGAGGCAGGAGAACACGCGGATCTGCTGCCCATCGTCCAGCACCCGCCTGCAGGGGATGTGAGCGATCTTGGCAATCTCTGCCACCTCCACGTGGATCACGAAATGGTTTAGCAGATCCATGACCAGGGCCGAGTCCTGCACGCAGTACATGCCGAGCCGCCTGCGCCCCTCGGGGCCCGCTGCAAAGAGGCGAGGAATCTCCTTGTAATGCACATCCTCCTTCTTGGCCCCCAGTAGGTGCCTGGCTACTGTGTCCAGCTTGTAGTCTGAGAGGCTGAGCTTGTCCCGGCACACGGCGTACATGTCGATGGGGATGAGGCCGGTGATGCGGACCTTGGTGTTGGCCCGCAAGAAGCCCTTGCCCGCATCATGGGGTCGCCTGACCTCGCAGACGCCCCCAGCCCTAATTTTGCCCAGAGAGGCTGGGTTGATGCTGTAGATGTGCCTGGCTCTGTCCAGAATGTAGGGCCAGTCAAAGTTGGCCACGTTGTAGCCGGTCACAATCTCCACGCTGAGGTCTCTGATGAGCTGGAAGAAGGCGTAGAGCATGTCCAGCTCCGATGGGAACTCGTAGACCTCAACCCCCTCTATGTCTTCGCAGGTGCCCAGCGTCAGCAGGATGCGCCTATAGCGCCCGGCCTCCTCCCCTGTCGACCAGAGGACGCAGGATATCTGCAGGATCAGGTCAGCCTCGTTGGTGGCCGTGGGGAAGCCCTCCTCCCCCAGACACTCGATATCGAAGGCCAGGGCCTGGTAGGAGGGCCAGGAGCTGTCTTCACGCAGGACCGAGAGGTCGCCCACCTCACAGTCGTACTCGAGCTCGGCGTACGAGTCCCGGTGCTGGAGGCGGGGGATGGCGCGGCGGCAGCTGTACCAGCCAAAGGTGACAAAGTCATTGTCCAGGACAAAGCGGCGCGTGGCATCCACGTTGGCCTCAAAGATCCGACACCCGTGCTTGTCTTGCAGCCACGTGGCCACGTGACACACACTGTTGGGATGGGAGAGGGTGATCTTGTGGTAGTCGCCGGCATGGTTGCCGTAGCCCATAATGGAACGGCGCGTGACCTTCTCCACCGAGACCCGGCAGGGGGTCCTGCGGTCGAAGGTGCTGGCCTTGAGGGCGCTGAGGACTGCAAACTCCACGTCCAGACCCTGAGGCGCGCTGGCGTAGAAGTAGGCCTGCTGCCCAAACACGTTCACACACACGCTGGCCCCATCGGCCTTGCGCCGGCCCAGTAGCTTGATGACGATGCCACATGGCACCACATACCCCTGTTTATCCGATGGAATGACGGCGCATTTCTCGTGCGTGTACACCGTCTCGAGTATGTCGTAGACATGGAAGTCCAGAGGGCTTCCGTGGGTGTCTGCCTCCGGCCTTGCCGTGCCCTCTTGGGCACGCTGGCGCCACCACATGCCCTTTCCATCCTCGTCACCCCCCACCACCGTCAGGGAGTCTTGGTAGAAGCACAGGGGGGGCTGAGGCCCCCGCACATCCACCACCCCTGCGGCGCCTGGTGTCTGGAAACACTTGGGAATGAGACGCAGGTACTCCTTGTCAGGCTTTTTCAGAAGGCCTTTATTAGGTCTTAGGAAAGGGTTATAGAAGAGTCCCCCGGACATGGTTAAAACTCAGTCTCTGCCTCCCCAAGCAGTGCGGCGGCGGTCTCTGGATCGTGATAGCGTCTTCTGCGTAGGCCTGGAAAACGGTCCCTGGCTGCCTGCAATGCTCTGCTGGCCACTGAGGGTCCGGCCGCCCTCTGAGCTGCTCTCTTTTGCTCCTGGTTTTGCTCATGCAGCGCTAACATGATGGCTTGTAATTCTGTCTTACTAATGGGATTAATGCCTGGACCCTCACCAGAGGCATGTTGCTGAGCGAGCTCGTCGATCCCGGGGTAGAGCATCTGCACCGGCTGCTGCGACATCTGGCGCGTGCGCCTCGTGAGGGAAATAACCAGGATCACCACCCCCGCCACCAGGACCAGAATGAGCATGCCGCCGAAGGGGTTTTTGAAGAAGGAGATGAAACCCGAGACCAGGCTGCTAAACAAACCCCCCACCGTGCTGACTAGGTTGGTGATGGACTGACCCACGCTACCCAGACTGTCCATAAGTTCCCCCAGGCCGTCCACAAATTGATTTCTGCCGTTTGACACTGCATTGTCCAAATCCTTCCGCAGGCCGGCGATGTTTTGCGCCTGGAAGTTGTACTCCCGGAAGATGCCCTCCAGGTCAAAGACGTTGGAGGCACGCTGTTCGTCCCGTGAGTACAGCTCCAGGGAGGCAAAGTCAATGTTCTCGATGAGGGAGGTGTTTAGTGAGATGAAGGTCTGCAGGGTGGCAATGCCGTCCAGCTCGATGGTTTTAAAGTGGTGGTAGTCGTTGTAGACGTGGATCTCGTTGCCGGACTGGAAGTAGTACTGGCTGGTCGCCTGGCACACCTCCGTCATCTTTTTTGTGAGGAAGATCTCGTTGTCGGTGCCCAGCTGTCCCTCGTAGGTCTTGGTGTCGTTGATAAAGCTGAAGGACACCAGGGGGCGCGAGTAGCACATGGTCTCGGAGCCAGGGACCCTCATGCTCTTGCGCAGGGTGACGGTGGCCTGGTTAACGGGCACGCACTGGGAGACTGAGATGACATCCCCCAGGCGCTTGGCCGCCACCGCCTTACCGTAGATGCTGGACATGACGGTGGTGGGATTAATCTTGGTTAGTTCTCTCAGCACCATGTTCTGCCTCTTCTGCTCCAGGCACCAGGCCCGCGCAAGGTCTCCCAGCATGCGGTTGATCTGGCGGCGCAGGGAGTCGTAGGCAAATTGGATCTGGACGGTGGCGGGATTGTTGAGGGTGCCCAGGGACTTCCCGGGGGCCGCGGGGGGCACCGGTGTGGTGGCATTCCCCGCATCCCGCCTCCGGCGCCTCAGAACGGCGGCGGAGGTGCTCCCGCGGGCCGCGGGTGGGGCGGGGGGCGATGGACTGCTGGGGGGTGAGGAAGTCGGAGTGGTAAGCTCCGTCAGGTTCTTGACGGTGGCCAACGAGCGCGGGGTCAGAGGTAGCCAAGCTAATAACAATCCTCCGCTCGTTATAAAATATGTAATGGCTTCCTGGCCCTTCGTGTAACGATCCTGGACGGCCTCGTACTTCTCATGCATGGTCTTGTTCACCTGCTCTTCGATGCACTTGAAGGCGTCCGGGAGCTCTATGCCCACGGTTGTGTTGGTCACGAAGCTAGAGGTGCCCTCGTCAGTCACAAAATGTATTGACTTCCCTGTTTCTGTGGCGATGGTCGAGTCAAAGGTTTGCCAGTGTTGAAGCGGGCAGTAGGCTGTCCTGTTCTCGAGCTTCCAAGATAGGGTGTAAGTGCCCTTGTCCAGGAAGGCTCGGCGTTCGCCTTGCGGGTTCGTCCCTCGGTTGTCGTAGTCCACTATCTTGTAGTTAGTTCTCACGTGGAAGGAGTCTGCCCGCTCATGGAAGGTTTCCTCATTTTTCCCGTCATAGAAAGGGGACATTTCCACAGTCTGCCCGGTGGTGGTCACAAAGAAGTCGAAGGGGCTGTTGGACTTGGCCATCATGTCAGTTATCAGGCAGTTGACGGTAGTTCTTGTTCTGTAAGTCCATATCAACCACCCGGGGGCGTCATAGAGCTCCGTCTGGCTGGCGTAGCGGCGCACCCCGTTGGCCAGGCCCCCGGTGGGCTTTAGGTTGACGGTGATGTTAACTCCGTCGCGGTCTACATACACGCGCGTCAGCCCATCTTTTGTCATCTTGACCGCGTTGTAGCACTGGTAGATGGTATCCATCTGGTCAGTTTCGTAGCTGTCAACGGAGAACTTCTCCTCGTGCCGGTTGGTCACGGAGTCCGCGTACCAGCCATTGTAGATGAGAATGTTGGTCACTATCTTGGTGTAGGAGCGGACCTTAAACGAGTAGGGAATAATGTTGTCTTTAAACACCATCAACAGGCCCTCCGTGTGATTCTCCCGCGTGCCAAACGAGGGACACTGGATGTCCGAGGAGAAGCGGAACAGGTCGCCGTGGCTGGAGAGCTCGCAGACTCGGAAAGGAAAGCTGGTTTGCTGACGCGTGGCGGTAGGCTGCACCGTGGTGGCGGGGGGTGCGGGCTGCTCTGGGGTCTGCGCACCGAGGCGGCACGCCAGGGCGGCTAGCAGCACGACCACGCTTAGCACCCTACGCCGAGTCATCTCTCATTTGGAGGTGCAGGTAGAGAAGGGCATATAGATCCTTAAATACCCACCCCCTGCCCTTATACAGAAGAATTAGGGGGCGGTCAGAGTCGTACGTGAGGTAAAGCCCATCCGGGGGCAGGGCCTGGCCGGGGCTGACCGCGTCCGCCCGGCGCAGGATCAAGGACCGCCCCCAGGTCTTGTTGTAGAGGGACACGGTTAGGACGGCCTCGCGCAGCGCCCGGCACAGAATTTGCTGGCTAGATGCCAGTGAGCCCCCGGGTACGCTGTAGAAGCTGTTGAAGGAGGTCTCTATCCAGTCGCTCGGCTCGATGCCTGGCCATATCAGGGAAGTCAGGAACGCCTTCTGGTGGGGCAGCGTACCTGCGGCGTCACAGCAGCGAGCCAGGGCCACGTTGCTGGGTGGGGGAAAGAGCCCGCTCTCCTCCGCCAGGGGCCCCGTGATGAAGGTGTACAGGCTGTGCGTCAGCGCGTGCAGGTGCTCCGAGCTCAGGGTCTGGGTAAACAGGTGTGTTTTGATGTACTTGGAATTCTCAAAGGCGGCACCCTCGCCGGCGCGCCTGTCCTCCCAGGGACCCGAGACGAAGGCCCGTCTGTAGAGGAAGTGGTTGCGCATGCGGGCCAGCTCCCAGTAGACCACGTCCCCCCAGACGCGCAGGCACAGGGTCTC

CTTGGCCAGACCCTCGGTGGCCACCTGGCGCAGGTACTGCTCCTTGCGCTTGAGCGCGTCCGAGAGGGCGCCGGACGGGCCGGGCTCTCGTGCCCCAGCCGGCCGGGGCACCTCCGGGCTCTCCCGGGACGCCTCCTCCTCGCCTCGGCCCAACCGCTGCATGGCTCGGTTGAGCCGCGTGTAGAGCTCGTTCCTCTTTTGCAGGATGGCCCGGTACTGGGGGTGCGCCGTGAAGGCGGCGGCGCAGTCCGCCTTCAGCGCCTCCACCGCGTCGCCCGAGGAGCTGTAGACCCCGCCGCAGAAGAGCCGCTCTGTGGCCCCGGGAGCCACGGCATCAAACAGGTGAGTCAGCCTTGCCCCCGCCAGCGCCTCCTCGCAGGCCCGCCGCACCAGGGCCAGGCGACGCTCCCGGGCAAACAGGGCAGAGAGGCGGGAATGGCCGCCACCCTCCCCCTGCCCCGTTGCACCGATAGCATGGCCGCCAGAGTTCCAATAGAGGAGCTCCGAGAGTTCCGCCACCTCCGGGGGCACTGTCGAGAAGACGTTGTAGGTGTCCAGCGCTCTGGTCGCCCCCTCTGCCTCCGGCCGCCCCGGGCCCGGGACCGCGCCCTCCTCTGGGCCGCCCGGCCTCGCCTTCTCCTCAGCCTCCAACAGGTGCCCGAGCCCAGCCTGCCGGACTTCATTCTCAAACAGTCCCGAGACCGGCTCCGGATTCACCGGCACCGCCAGGTGGTTACAGGAGACGTGGGTCCCCTCTGCCGTGGAAGGGTTGCCGTGGTTGGGCAGAACCATCAGCTCGCCCACACAGCGCCAGCAGGGCACAGAGGTGATGTAGAGGCGCGGGTCTGGGATGGGACTTACGCCCCGAAAGCGGCCCAGCAGATCCAGGGCCCGTTCCAGGCTCTCCAGCCCCATGGTGTGAGACATGCAATAAAACACGCTATTGATTCTCTTCATTAAAATCTCTATGTCATTTATTAGGCACAAACTTACATCGACTTTATGCCCCCCGTAAAACTCCACAGAGTACGCGACTGAGGGGGTGCGGAGAGGCGGGACCCGGGTACCCTTTCTACCAGGGGCGAGCAGCGCGGCAGAGGCCTCTCTCGAGTTCTCTAGCAGGTGCACCAGCTCCAGGGACAGGGCGCTGCATGCACGGTCATTCTGCCGTCTCAAACGGGGAAGGAGGATGGCCTCCAGCTCGGCCAGCAGGCCGGCGTTGCGCACTACCGCAGCCACGTCCAGACTCCGGGGGTCCAGCCGGGCGCACACGCTCAGCTCAACCGCCAGGGCGTACACCTGGCTGTACGCCGCCGCCAGCAGCCCCGACATCGCCGCCCCAGGGGTCTCTAGACCTCGAGTCCGGGGAGAACGGTGGCCAGACGGCGCTTGCGTCTGCCCCCGGAGCCCTGCCCTCCTCCACCCAGCAGCAGCCCGGCCGAGGCCTGCGACGCGGTGCTGACCGGCTCGGCCACGCTGATAAAGTTGTCCTGGGCTGCCCCGGGCCCACCCCACACTCCCTCCAGAAAGTCCCGAGCGGCCCCCGCCGTCCACTCTATCCCGCTGGAGGCAATGGTCGCCAGGGTTTCTAGGACGCTGTCCGCCAGGACGGAGAAGCGGCCCAATAAGTACTCCGCGTCGTCCCTAGTCAGCGAGGCGCATGCCTCGCCCATGGCATCCACAAGGTTGCACACCACATCAAACACACAGTCTTCCTCCTGTTTTTGTGATATAATGGCCTCCAGGCCAGCCCTGATGTTCTCAATCTCATATGTGGTCGCGGCTTGGGTCCGGCGCTTCACGGTCAACCCTAGGGTGGGGGTGGCAAAGACAAACTTCTTCCGCATGGAAGAGCCCCCGGCCTGCTTGCGCAGCCCAGCCCCGGGGGCCTGCAGCAGGTTCCTGTCCACGCCCCGGCCCATAAAGTATCCCAGGTTCCCGGCCTGGAATATCTGGTTGTTGCCGTTGACCCCCGTGTACTTGTTGATGGTCACTGGCAGCGTGACAACCGGACGGGCCTTGCAGACCTGGCTAAGACAGTCTGTGGCCGCGCAGACCACCGTGGTCGCAGTAAGGGAGGAGGTGGCCTCCGCGTAGGCCGCTGCCGACTCCACCGCCCGCGTGCCCAGTACGTGGGGGTAGTCACGGGCGGGCACCGACTGCGTCCTCGGCACCAGTCCCTGAATCAGGCTGATGTAGAACTGGGTCTGGCCGCACGCCTTCAGGATGGCGTTGTTGAGCCTCTGCTTGGCGTAAGTGACCAGGTTGCCAGGCACCACATCTATGACGTTGCTCTCTTCGTGGGCCCGGGAGCCCCCGTCCACAAAGAGGGCCAGGTCAGAGTACTCCTCCGCGCTGGCCCCGCTGGGGACAGGGACCGAGCGCCGCCTGGAAAAGTTGTGCCACAGGTACAGGCTTGAGAGCTTAGTGTCCGGGAATAGGGTCTTGTGGTAGGTGTTGAGGAATTTCATGTAGGGCCCGTTGATGATGTAGTTCTCCCTCCTGGTAGTGGACTTGATGAAGCTGTTCTGGAGGGCGGCATTCTCCCCCGTGAAGACCACCCTGTTCTTGATCTTGATGTTCCTGGGGCACAGCATCAGCACCTTGGACATGCGCACAGGCAGCCGCCGGCCGTACACCCGGCCCTGCAGGGCCGCGTCCAGGTCTGGCAGGTCGCAGGTGGGCTCCCCATGCACTACCTTGGCCTCCTTGGCCGTGAGGACCCCCTTGTCGATGGCCAGGCTCCTAAAGTTGGTGCACAGCGTCTGGTAGTGACCCTTTAGCCACTCTGGGGGGCTCTGGCCAAGCCCGGGGTTGTCATTCTCATAGCACATACAGATGGGCAGGGAGATGTCCTGCAGGATGGTCAGCAGTGAGCGGTAAAACAGCTGGGTGAAGATGGGGCAGGCGGGCTGCGCAAAGGGGTTGCACGAGTACTGCATCACGTGGTAGCAGCTCTTGACCAGGTCCTTGTAGGTGATGTTGTTCTTGGCCATGCTGTTCATAAACTGGACCACTTCGGCGTCCACCGCCGCATCCACGTCCTTGAACATCTTGACAAAGTCACGCGGGCCATGGGGCTCCTTCTCTAGCTTTCCCTCAGCGTCTATGCCCAGCCGAGACAGCCGCTCCAGCAGGTTCTGGTTCAGCTGCCAGTAGGTGTAGCGGGGCTCGTCGTCCGGCCGCTGCCCGTCGTCCTCCTTATCGATGAAGTTGAGAAAGTTGCCCAAAAAGTCCGTCTCGTTGTAGGAGCCCGAGGCCCCCGAGATCACATAGGGGTCCCTCCGCTGCGTGGACATGACGGGGGGAAAGCGGTCCCTCAGCCTAAAGAAGAGCGTGTTCAGGCACACGGCCGGGGCCCGGCCCTCGCAGAGCGAGCACATGGGGCTGGCGGCCGCCCCCGCCACGTAGCTGCCCGTCTCCGGCACCGGGGTCAGAGAGCTCTTCTGTCCCTGGCAAAACTGCAGGTAGTAGGCATAGCGGGCAAGAAGGTTGGGCGAGAAGGAGGCCGCATAGACCAGGTGCTCCACAGCGTAGTTTCCCGGACCGTTGGTTCCGGTCACGTCTGGCCCACCCCAGCCCGAGAAGCAGGGTCGGCGGCAGGGGTCCCAGGTCCCCTCCTGCAGGGTCCCCAGGCCGTGGGTCATGTAGAAACTGTTAAAGAGACTCTCCTTGCCCTGACCGGTTGACTTCGAGACCCCCGAGACGTAGAGGACGGAATTGGTGGCAAAGATCTGCGTGGACACGTGGGGGGCCAGGCTGGCATTATATCGGTGTAACGCAGCCACACGGGCCTCTGGACCCTCACAGTCGGCAAACAGGGGCCACGAGTCGTAGTTGAGGCTGGCCGGGGTCTCGTGCGAGGCCTCCAGCATGGCGGGTGCGTAGCTCACCGCCAGCTCGCATGCCGCGCTGTCCACAATCATTAAGGCTCCCGAGTCCGGGTGACTGATGGTTGAGGCTGGGAACTCCTTGAGGGGGGCCACCTTGGCCACCTTGGCCTGGTCCTGCAGGCTCTGCTTCTCCAGCAGCTCCACCAGCTTGCCCACCCGTCGGACGCGCAGCGCCTGTGCCAGCCCGGTGTACAGCGCCTCGTGCATGCAGCGGCTGAGGTCCGAGTTGTAAAACTGGCGGAGCTGGGGCACGCCCTCTGGGAACACCTCCTTGTCGTAGAGCGGGACCCTAACGCTCGCAGACTGCCCCACCGCTACCTCCTGTTTTAACGATGGAATGGCCACCAGGTTTCCGCTGTAGAGTCGCTCCTTGAAGGCCTCGGTTATTGCCACCGCCCCCAGGTAGGCAGAGGGATCTAGCCCTTCGGGGAAGAAGTCCCCCGGCTTGGAGCTTTCCCTCGGTAGGGCGCTGTAGGCGTCGTACCCAAACACCTCCCTGGTCTCGCCACAGAGGGCCTCGAGACCCGGCCCCTCAAAGATGGGGGGAACCATATGGGCATTGTGGAACACGTAGATGTCCCTGTGATAGGAGGTAGCGCGTAGGAGCCCGCAGTTGGGGTCGGGCCTCCTGTGCAGAGCCTTGACATTGATGCTGAAGCCCGGCTCCACGGTGATGCCGCAAAGGAGCGGCACCGTCAGGCACCTGTGGCCCGCGTAGCCGGTCCCCAGTGTGGCCACCTCCCTAAGAGGGTAGGTGGCCAGGGGGTAAAAGTAGATGTAGCCGCACGGACCCGGCTGGCTCTGGCTGCCCAGATTATCCTCGCTAGTCTGTGCACCCTGCATGATGCCCAAGGTATCGCCCCGGCCTCCCAGTCCCACATTAAATGTTACACTTTACTCATCACGCAACACCCACTGTTTATTCATTTACAAAGATTTCAGGAAGTCAGTCAGGCTGGCCAGGGCCCACGTCACGGGGAACTGACGTCTCAGCGATCTTGGCATGCCGCCCAGCCTCGCAAACCAGAGTCTGCGATAGAGGGCCAGGTAGTGGGCGATTGCCCCCAGCACGAAGGCGGCGCTCTTGTGGTCATCCAGGTAGTTTCGCACCGCAAACACCACTGTGTAGCACAGCACCACCCTGAGCCGCGACCAGTAGTCGTAGTGGTCGTTGTACACTGCGCGCAGGACGCTGATGATGAGCCGTACGTGCGTGTCTTTGCCCCCGATGTCGGCTGTCCTGCAGGCCAGCTCCGCGTACAGCTTCCTATCCTTCCTCAGGGAGGCCTTGATGAGCCGGCAGAGGACCAGGGCTGGCAAAGGCAGGTCTTTCTCATCCCGGGTGAACACCGCGTACATGGCCCTGAACATGAGGTAGCTGGACTCAGCCACCTTGTCGTCCGGCGGCGAGGGCGCGACCCACGCCTCGACCGGGGTCCTCACAAACACAGAATCTGTAGACTTGGCTGGCCTCATGGTCTCGTCAGGCCAGCTCACGGGCTTCAGGCTTATATGATAAAATGGGCGTGGCAGAATAGTATAAGACGCGAGGCCTGGGTGAGGAGAGTCCAGAGCAATGGCCAGGTTCATCGCTCAGCTCCTCCTGTTGGCCTCCTGTGTGGCCGCCGGCCAGGCTGTCACCGCTTTCTTGGGTGAGCGAGTCACCCTGACCTCCTACTGGAGGAGGGTGAGCCTCGGTCCAGAGATTGAGGTCAGCTGGTTTAAACTGGGCCCAGGAGAGGAGCAGGTGCTTATTGGGCGCATGCACCACGATGTCATCTTTATAGAGTGGCCTTTCAGGGGCTTCTTTGATATCCACAGAAGTGCCAACACCTTCTTTTTAGTAGTCACCGCTGCCAACATCTCCCATGACGGCAACTACCTGTGCCGCATGAAACTGGGCGAGACCGAGGTCACCAAGCAGGAACACCTGAGCGTGGTGAAGCCTCTAACGCTGTCTGTCCACTCCGAAAGGTCTCAGTTCCCAGACTTCTCTGTCCTTACTGTGACATGCACCGTGAATGCATTTCCCCATCCCCACGTCCAGTGGCTCATGCCCGAGGGCGTGGAGCCCGCACCAACTGCGGCAAATGGCGGTGTTATGAAGGAAAAGGATGGGAGCCTCTCTGTTGCTGTTGACCTGTCACTTCCCAAGCCCTGGCACCTGCCAGTGACCTGCGTTGGGAAAAATGACAAGGAGGAAGCCCACGGGGTTTATGTTTCTGGATACTTGTCGCAATAAACGCACTTGCCTATTTCACCTTGTTTTAGTGTGGCATTGGGGGGGTGGCATTGCGGGTGGATAGCCTCGCGACTCGTGGGAAAATGGGCGGAAGGGCACCGTGGGAAAATAGTTCCAGGTGACAGCAGCAGTGTGTGAAGATTGTCACAGCTGCTGGTTTGGAGAAAACGGGGGTGGGCGGTGATCAGGGAGAACAATTCCCCGGGGACACCTGCACGAGACCCCTGGGCTCTCAGGAACTCCGCCCAGGTCTCGCCAATTGGGGTGATCCTGTAGCGCCGCGGTTTCAGCATCACAGGTTATTTTGCCTGAAGCTTGCTGGGGCGTAAATCCCTCTCGCCTTGTTTCTCAGATAGCATTTCAGGCCGGTTTTGCAGTCGCTGCTGCAGCTATGGGGTCCCTAGAAATGGTGCCAATGGGCGCGGGTCCCCCTAGCCCCGGCGGGGATCCGGATGGGGACGATGGCGGAAACAACTCCCAATATCCATCTGCTTCTGGCTCTGATGGGAACACCCCCACCCCACCGAACGATGAGGAACGTGAATCTAATGAAGAGCCCCCACCGCCTTATGAGGACCTAGATTGGGGCAATGGCGACCGTCACTCGGACTATCAACCACTAGGAAACCAAGATCCAAGTTTGTACTTGGGATTGCAACACGACGGGAATGACGGGCTCCCTCCCCCTCCCTACTCTCCACGGGATGACTCATCTCAACACATATATGAAGAAGCGGGCAGAGGAAGGTAAGAGTGCCATCTATCTGTACTTTTATTTATTGCATCACAAGTCACATCAATAATAAGGGCGCCATCTAGCGGGAGATGTTATCCACACCATCCCAATTCACATCTCAGGGACAACAGGTCAAAGTTCTTTGTTGACACCCCCAGCGCTGGCTCCAGGGGGTGGCAGCGTTGGATGCAGTCCTCCGCATCGGGGCGGACGCCTCCTCCCAACGCGTTTCTGCGGATCAGTCGCTGGCTGGTGGGCATCGGAGTCGGTGGGCGGTCCTCCACGGGGACACGCTCCTTCTTGGCCTTGTTCTTTGACCTTTTGGACATTCTTCTGAAGGAACGGCGGAGAGTAGCGTAGAATCCAGCCAGTGGTCTACCCGATCGCATGGTGGCTTCTTAGATGAGGAGCAGGCATAAAAGTCCAAACAGGACACAGAGTACCACCAGGAGTAGTCTTAGTCTGCTGACGTCTGGGTCCTCGGGGCAGGGGTGGCTAGGCCTGGTCTCCGTAGAAGAGCCGGGCAGGCCGCAGGCAGAGGACTGCTGCTCTAGCAAAGCACGCTCCAGGACGTGTACCATCTCGAGAGTGAGGCACAGCTGTTTTCGTGGACTTTTATACAGTAAGGACAAGGAAAGAAGGCCAGAGGAATGTGGAAAGATGAGCGAGGACAGGTGTGGAGGTTTTGGGCTAGCTCTTAGTTTCTGGGTGTGAGAGAGGGATTAAAGTGCTTATGCGCAAAGAATGTGTCAACAACAGGTGTTCCTGCCTCTGCTGGCATGAGTTAGGTGTGGCTTGGGCTGAATCCAAATGTGTATTGGCACAAGATGGAAAGCAAAGTTGCTGGAGTTACTGGGTGGGAGACAGGGATGTATGTGGTCCCCCGCTGGTATGCCAGTACCCTGTGGAAGTAAGGGGCCTCATCTGCCCGGTAGTTGTGTTGTGCAGAGGTCTGATGTGTGTAGGAGGGGTGGGTTCAACGCAGGGGCGTTGGTGGCGGAGTCTGGCAACGCCCGGGTCCTTGCTACCTGTGTGGTGTGTGAAGGGCTGGGTAAAGGTGTCTGCCAATTCTCGCATGTCCTCCTTTCCCCTTGTTTTGAAATAGAATATGAATGTGGCTTTTCAGCCTAGACAGACAGTGTGGCTAAGGGAGTGTGTGCCAGTTAAGGTGATTAGCTAAGGCATTCCCAGTAAATGGAGGGAGAGTCAGTCAGGCAAGCCTATGACATGGTAATGCCTAGAAGTAAAGAAAGGTTAGTCATAGTAGCTTAGCTGAACTGGGCCGTGGGGGTCGTCATCATCTCCACCGGAACCAGAAGTACCCAAAAGCAGCGTAGGAAGGTGTGGATCACCGCCGCCACCGTCTGTCATCGAAGGCGGGCCCCGGTCACCTCCTTTGTTTTCAACCTGTTCCGTCAAATTTGGAGGGCCTCCATCATTTCCAGCAGAGTCGCTAGGGTTATGAGGCAGCGGGTCATGTGGGCCATTGTCATCAGTGTTGTCAGGGTCCTGTGGGCCATTGTCATCAGTGTTGTCAGGGTCCTGTGGGCCATTGTCATCAGTGTTGTCAGGGTCCTGTGGGCCATTGTCATCAGTGTTGTCAGGGTCCTGTGGGCCATTGTCATCAGTGTTGTCAGGGTCCTGTGGGCCATTGTCATCAGTGTTGTCAGGGTCCTGTGGGCCATTGTCAGGACCACCTCCAGGTGCGCCTAGGTTTTGAGAGCAGAGTGGGGGTCCGTCGCCGGCCCCACTCACGAGCAGGTGGTGTCTGCCCTCGTTGGAGTTAGAGTCAGATTCATGGCTAGAATCGTCGGTAGCTTGTTGAGGGTGCGGGAGGGAGTCATCGTGGTGGTGTTCATCAGTGTGTCGTGGTCCATGATAATACATCCAGATTAAAATGGCCAGAAACAGGAGGAGCCAAAGGAGATCAACCAATAGAGTCCACCAGTTTTGTTGTAGATAGAGAGCAATAATAAGCAGGATGATGGCTAGGAAGAAGGCTAGGATGAAGGCCAAAAGCTGCCAGATGGTGGCACCAAGCCGCCAGAGAATCTCCAAGAAGTAGATCCAGAGACCTAAGACTGCGTTGAAAAAAAGAGTGTTAGGGCTGGAAAGGTGAGGGTGTGGTAAATAAGTCCTAGGGAATGTTAGATCTTACCAAGTAAGCAGCCAAAGATGAACAGCACAATTCCAAGGTACAATGCCTGTCCGTGCAAATTCCAGAGAGCGATGAGTAGGAGGGTGACTGGGGAAAGAGGAGAAAGTGCGTTAGAGAAGGAAGCGTAAGGGAAAGGGGGTGGGGGGCAAAGGGTGTAATACTTACTCATCAGTAGGAGTAGACCAAGGCCTCCAAGTGGACAGAGAAGGTCTCTTCTGAAGATAAAGATGATGAGAATGATAATAATAAGCATGAGAGCAAAGGAATAGAGGACAAGGAGCGCTCCTCCAGTCCAGTTACTCATAACGATATACAGCCAGAACAGTAGCGCCAAGAGCAGGAGAAGGAGAGCAAGGCCTATGGAAGAGGAGAGGGGGGGTCCTAGAGGGGGCCGTGGCGGGCCCGGTGGGCCCCTCTCAAGGTCGCGTTCCATGCTCAGGGTAGTGTGGCAGGAGCAAGGCAACTGAGGAAAGAAGCTGACAGAGCAGTGAAAGAGGCTTATGTAGGCCGCCTACGAGAGAGTAACGCGTGTTTCTGGGGATGGAGGCCCGGGGGGATTTGCGGGGTCTGCCGGAGGCAGTACGGGCGCCGCTTTCCCGAAAGCGGCGTAGTGTGTGTGCATGTAAGCGTTAAAAGAGGAAGTAGAAAGCGTGTGTTTGTGTTAGAAAAGCGAGTCCCCGAGGGGCAAGCTGTGGGAATGCGGTGGTAAAGCGTAGCAGGAAATGGAAAGGCAGTGCGGCAATCACAAGGGGGAGTGAGCAGTGTTGTGGGAAGCGGCAGTGTAATCCACAGAAAGAGGCGTAGGGCGCGCAACGTTGGGAGGTCGTTGTCGGCAGGCGGGAGGCTGTGCTTTAGGGGGGTTCAGGTGAGGCAAGGCTGTGGGGTAACCGTAGGGGAGGCGGGTGAGGCGGATAAGAGGGCTAAGGGTCGGCGGGTGACGAAGCAGCAGACGGGGGATATGGGAATTTCAGAATGAGGTGGCGGATTCCGGCGAAAAGGGTGTGGGCTGTGCAAGTGTCATGAGGCAGGCGCGGAAAGTCGCTGCGGCTTGCTGGCGTGAGGCATGGGGGGCGCATTCCTGGAAAAAGTGGAGGGGGCGTGGCCTGCCCCCCGCGGCCCCCCTAGCCCCCCCGCACAGCGCGGCGCAACGGCCGGCGGGCGGGGGGGGTCGGGGTCCGCGGGCTCCGGGGGCTGCGGGCGGTGGATGGCGGCTGGCGTTCAGGGGGTGGGGGGGTCGGGGGGCGCTGCGCCCGCCCAGCCATGCGTGACCGCGAGGAGGGGACAGGGGCGCAGGGGGCGTGTCTGGTGGGGGCGGGAGCGGGGGGCGGCGCGGGAGCCTGCACGCCGTTGGAGGGGAGAATGACAGGGGGGCGGGGACAGAGAGGCGGTCGCGCCCCCGGCCGCGGCAGGCAAGCCCCCGGGGGGCGGGGCGCGGGCAGCGGAGCGTGACGAAGGGCCCCAGGGCTGACCCCGGCAAACGTGACCCGGGGCTCCGGGGTGACCCAGCCAAGCGTGACCAAGGGGCCGCTGGGTGACACAGGCAACCCTGACAAAGGCCCCCCAGGAAAGACCCCCGGGGGGTATCGGGGGGGTGTTGATGGAGGCATGGGGGGAGGCATGGGGGGCGCATTCCTGGAAAAAGTGGAGGGGGCGTGGCCTGCCCCCCGCGGCCCCCCTAGCCCCCCCGCACAGCGCGGCGCAACGGCCGGCGGGCGGGGGGGGGTCGGGGTCCGCGGGCTCCGGGGGCTGCGGGCGGTGGATGGCGGCTGGCGTTCAGGGGGTGGGGGGGTCGGGGGGCGCTGCGCCCGCCCAGCCATGCGTGACCGCGAGGAGGGGACAGGGGCGCAGGGGGCGTGTCTGGTGGGGGCGGGAGCGGGGGGCGGCGCGGGAGCCTGCACGCCGTTGGAGGGGAGAATGACAGGGGGGCGGGGACAGAGAGGCGGTCGCGCCCCCGGCCGCGGCAGGCAAGCCCCCGGGGGGCGGGGCGCGGGCAGCGGAGCGTGACGAAGGGCCCCAGGGCTGACCCCGGCAAACGTGACCCGGGGCTCCGGGGTGACCCAGCCAAGCGTGACCAAGGGGCCGCTGGGTGACACAGGCAACCCTGACAAAGGCCCCCCAGGAAAGACCCCCGGGGGGTATCGGGGGGGTGTTGATGGAGGCATGGGGGGGAGGCATGGGGGGCGCATTCCTGGAAAAAGTGGAGGGGGCGTGGCCTGCCCCCCGCGGCCCCCCTAGCCCCCCCGCACAGCGCGGCGCAACGGCCGGCGGGCGGGGGGGGGTCGGGGTCCGCGGGCTCCGGGGGCTGCGGGCGGTGGATGGCGGCTGGCGTTCAGGGGGTGGGGGGGTCGGGGGGCGCTGCGCCCGCCCAGCCATGCGTGACCGCGAGGAGGGGACAGGGGCGCAGGGGGCGTGTCTGGTGGGGGCGGGAGCGGGGGGCGGCGCGGGAGCCTGCACGCCGTTGGAGGGGAGAATGACAGGGGGGCGGGGACAGAGAGGCGGTCGCGCCCCCGGCCGCGGCAGGCAAGCCCCCGGGGGGCGGGGCGCGGGCAGCGGAGCGTGACGAAGGGCCCCAGGGCTGACCCCGGCAAACGTGACCCGGGGCTCCGGGGTGACCCAGCCAAGCGTGACCAAGGGGCCGCTGGGTGACACAGGCAACCCTGACAAAGGCCCCCCAGGAAAGACCCCCGGGGGGTATCGGGGGGGTGTTGATGGAGGCATGGGGCGTGAGGCATGGGGGGCGCATTCCTGGAAAAAGTGGAGGGGGCGTGGCCTGCCCCCCGCGGCCCCCCTAGCCCCCCCGCACAGCGCGGCGCAACGGCCGGCGGGCGGGGGGGGGTCGGGGTCCGCGGGCTCCGGGGGCTGCGGGCGGTGGATGGCGGCTGGCGTTCAGGGGGTGGGGGGGTCGGGGGGCGCTGCGCCCGCCCAGCCATGCGTGACCGCGAGGAGGGGACAGGGGCGCAGGGGGCGTGTCTGGTGGGGGCGGGAGCGGGGGGCGGCGCGGGAGCCTGCACGCCGTTGGAGGGGAGAATGACAGGGGGGCGGGGACAGAGAGGCGGTCGCGCCCCCGGCCGCGGCAGGCAAGCCCCCGGGGGGCGGGGCGCGGGCAGCGGAGCGTGACGAAGGGCCCCAGGGCTGACCCCGGCAAACGTGACCCGGGGCTCCGGGGTGACCCAGCCAAGCGTGACCAAGGGGCCGCTGGGTGACACAGGCAACCCTGACAAAGGCCCCCCAGGAAAGACCCCCGGGGGGTATCGGGGGGGTGTTGATGGAGGCATGGGGCGTGAGGCATGGGGGGCGCATTCCTGGAAAAAGTGGAGGGGGCGTGGCCTGCCCCCCGCGGCCCCCCTAGCCCCCCCGCACAGCGCGGCGCAACGGCCGGCGGGCGGGGGGGGGTCGGGGTCCGCGGGCTCCGGGGGCTGCGGGCGGTGGATGGCGGCTGGCGTTCAGGGGGTGGGGGGGTCGGGGGGCGCTGCGCCCGCCCAGCCATGCGTGACCGCGAGGAGGGGACAGGGGCGCAGGGGGCGTGTCTGGTGGGGGCGGGAGCGGGGGGCGGCGCGGGAGCCTGCACGCCGTTGGAGGGGAGAATGACAGGGGGGCGGGGACAGAGAGGCGGTCGCGCCCCCGGCCGCGGCAGGCAAGCCCCCGGGGGGCGGGGCGCGGGCAGCGGAGCGTGACGAAGGGCCCCAGGGCTGACCCCGGCAAACGTGACCCGGGGCTCCGGGGTGACCCAGCCAAGCGTGACCAAGGGGCCGCTGGGTGACACAGGCAACCCTGACAAAGGCCCCCCAGGAAAGACCCCCGGGGGGTATCGGGGGGGTGTTGATGGAGGCATGGGGCGTGAGGCATGGGGGGCGCATTCCTGGAAAAAGTGGAGGGGGCGTGGCCTGCCCCCCGCGGCCCCCCTAGCCCCCCCGCACAGCGCGGCGCAACGGCCGGCGGGCGGGGGGGGGTCGGGGTCCGCGGGCTCCGGGGGCTGCGGGCGGTGGATGGCGGCTGGCGTTCAGGGGGTGGGGGGGTCGGGGGGCGCTGCGCCCGCCCAGCCATGCGTGACCGCGAGGAGGGGACAGGGGCGCAGGGGGCGTGTCTGGTGGGGGCGGGAGCGGGGGGCGGCGCGGGAGCCTGCACGCCGTTGGAGGGGAGAATGACAGGGGGGCGGGGACAGAGAGGCGGTCGCGCCCCCGGCCGCGGCAGGCAAGCCCCCGGGGGGCGGGGCGCGGGCAGCGGAGCGTGACGAAGGGCCCCAGGGCTGACCCCGGCAAACGTGACCCGGGGCTCCGGGGTGACCCAGCCAAGCGTGACCAAGGGGCCGCTGGGTGACACAGGCAACCCTGACAAAGGCCCCCCAGGAAAGACCCCCGGGGGGTATCGGGGGGGTGTTGATGGAGGCATGGCGTGAGGCATGGGGGGCGCATTCCTGGAAAAAGTGGAGGGGGCGTGGCCTGCCCCCCGCGGCCCCCCTAGCCCCCCCGCACAGCGCGGCGCAACGGCCGGCGGGCGGGGGGGGTCGGGGTCCGCGGGCTCCGGGGGCTGCGGGCGGTGGATGGCGGCTGGCGTTCAGGGGGTGGGGGGGTCGGGGGGCGCTGCGCCCGCCCAGCCATGCGTGACCGCGAGGAGGGGACAGGGGCGCAGGGGGCGTGTCTGGTGGGGGCGGGAGCGGGGGGCGGCGCGGGAGCCTGCACGCCGTTGGAGGGGAGAATGACAGGGGGGCGGGGACAGAGAGGCGGTCGCGCCCCCGGCCGCGGCAGGCAAGCCCCCGGGGGGCGGGGCGCGGGCAGCGGAGCGTGACGAAGGGCCCCAGGGCTGACCCCGGCAAACGTGACCCGGGGCTCCGGGGTGACCCAGCCAAGCGTGACCAAGGGGCCGCTGGGTGACACAGGCAACCCTGACAAAGGCCCCCCAGGAAAGACCCCCGGGGGGTATCGGGGGGGTGTTGATGGAGGCATGGGGGGAGGCATGGGGGGCGCATTCCTGGAAAAAGTGGAGGGGGCGTGGCCTGCCCCCCGCGGCCCCCCTAGCCCCCCCGCACAGCGCGGCGCAACGGCCGGCGGGCGGGGGGGGGTCGGGGTCCGCGGGCTCCGGGGGCTGCGGGCGGTGGATGGCGGCTGGCGTTCAGGGGGTGGGGGGGTCGGGGGGCGCTGCGCCCGCCCAGCCATGCGTGACCGCGAGGAGGGGACAGGGGCGCAGGGGGCGTGTCTGGTGGGGGCGGGAGCGGGGGGCGGCGCGGGAGCCTGCACGCCGTTGGAGGGGAGAATGACAGGGGGGCGGGGACAGAGAGGCGGTCGCGCCCCCGGCCGCGGCAGGCAAGCCCCCGGGGGGCGGGGCGCGGGCAGCGGAGCGTGACGAAGGGCCCCAGGGCTGACCCCGGCAAACGTGACCCGGGGCTCCGGGGTGACCCAGCCAAGCGTGACCAAGGGGCCGCTGGGTGACACAGGCAACCCTGACAAAGGCCCCCCAGGAAAGACCCCCGGGGGGTATCGGGGGGGTGTTGATGGAGGCATGGGGGGGGAGGCATGGGGGGCGCATTCCTGGAAAAAGTGGAGGGGGCGTGGCCTGCCCCCCGCGGCCCCCCTAGCCCCCCCGCACAGCGCGGCGCAACGGCCGGCGGGCGGGGGGGGTCGGGGTCCGCGGGCTCCGGGGGCTGCGGGCGGTGGATGGCGGCTGGCGTTCAGGGGGTGGGGGGGTCGGGGGGCGCTGCGCCCGCCCAGCCATGCGTGACCGCGAGGAGGGGACAGGGGCGCAGGGGGCGTGTCTGGTGGGGGCGGGAGCGGGGGGCGGCGCGGGAGCCTGCACGCCGTTGGAGGGGAGAATGACAGGGGGGCGGGGACAGAGAGGCGGTCGCGCCCCCGGCCGCGGCAGGCAAGCCCCCGGGGGGCGGGGCGCGGGCAGCGGAGCGTGACGAAGGGCCCCAGGGCTGACCCCGGCAAACGTGACCCGGGGCTCCGGGGTGACCCAGCCAAGCGTGACCAAGGGGCCGCTGGGTGACACAGGCAACCCTGACAAAGGCCCCCCAGGAAAGACCCCCGGGGGGTATCGGGGGGGTGTTGATGGAGGCATGGGGCGTGAGGCATGGGGGGCGCATTCCTGGAAAAAGTGGAGGGGGCGTGGCCTGCCCCCCGCGGCCCCCCTAGCCCCCCCGCACAGCGCGGCGCAACGGCCGGCGGGCGGGGGGGGTCGGGGTCCGCGGGCTCCGGGGGCTGCGGGCGGTGGATGGCGGCTGGCGTTCAGGGGGTGGGGGGGTCGGGGGGCGCTGCGCCCGCCCAGCCATGCGTGACCGCGAGGAGGGGACAGGGGCGCAGGGGGCGTGTCTGGTGGGGGCGGGAGCGGGGGGCGGCGCGGGAGCCTGCACGCCGTTGGAGGGGAGAATGACAGGGGGGCGGGGACAGAGAGGCGGTCGCGCCCCCGGCCGCGGCAGGCAAGCCCCCGGGGGGCGGGGCGCGGGCAGCGGAGCGTGACGAAGGGCCCCAGGGCTGACCCCGGCAAACGTGACCCGGGGCTCCGGGGTGACCCAGCCAAGCGTGACCAAGGGGCCGCTGGGTGACACAGGCAACCCTGACAAAGGCCCCCCAGGAAAGACCCCCGGGGGGTATCGGGGGGGTGTTGATGGAGGCATGGGGCGTGAGGCATGGGGGGCGCATTCCTGGAAAAAGTGGAGGGGGCGTGGCCTGCCCCCCGCGGCCCCCCTAGCCCCCCCGCACAGCGCGGCGCAACGGCCGGCGGGCGGGGGGGGTCGGGGTCCGCGGGCTCCGGGGGCTGCGGGCGGTGGATGGCGGCTGGCGTTCAGGGGGTGGGGGGGTCGGGGGGCGCTGCGCCCGCCCAGCCATGCGTGACCGCGAGGAGGGGACAGGGGCGCAGGGGGCGTGTCTGGTGGGGGCGGGAGCGGGGGGCGGCGCGGGAGCCTGCACGCCGTTGGAGGGGAGAATGACAGGGGGGCGGGGACAGAGAGGCGGTCGCGCCCCCGGCCGCGGCAGGCAAGCCCCCGGGGGGCGGGGCGCGGGCAGCGGAGCGTGACGAAGGGCCCCAGGGCTGACCCCGGCAAACGTGACCCGGGGCTCCGGGGTGACCCAGCCAAGCGTGACCAAGGGGCCGCTGGGTGACACAGGCAACCCTGACAAAGGCCCCCCAGGAAAGACCCCCGGGGGGTATCGGGGGGGTGTTGATGGAGGCATGGGGCGTGAGGCATGGGGGGCGCATTCCTGGAAAAAGTGGAGGGGGCGTGGCCTGCCCCCCGCGGCCCCCCTAGCCCCCCCGCACAGCGCGGCGCAACGGCCGGCGGGCGGGGGGGGTCGGGGTCCGCGGGCTCCGGGGGCTGCGGGCGGTGGATGGCGGCTGGCGTTCAGGGGGTGGGGGGGTCGGGGGGCGCTGCGCCCGCCCAGCCATGCGTGACCGCGAGGAGGGGACAGGGGCGCAGGGGGCGTGTCTGGTGGGGGCGGGAGCGGGGGGCGGCGCGGGAGCCTGCACGCCGTTGGAGGGGAGAATGACAGGGGGGCGGGGACAGAGAGGCGGTCGCGCCCCCGGCCGCGGCAGGCAAGCCCCCGGGGGGCGGGGCGCGGGCAGCGGAGCGTGACGAAGGGCCCCAGGGCTGACCCCGGCAAACGTGACCCGGGGCTCCGGGGTGACCCAGCCAAGCGTGACCAAGGGGCCGCTGGGTGACACAGGCAACCCTGACAAAGGCCCCCCAGGAAAGACCCCCGGGGGGTATCGGGGGGGTGTTGATGGAGGCATGGGGGGTCGGATTTCGCCCTTATTGCCCTGTTT

>HHV5_CMV_STRIPPED4

TTGCGTGTGTGGACGGCGACGGCGACTAGTTGCGTGTGCTGCGGTGGGTACGGCGACGGCGAATAAAAGCGACGTGCGGCGCGCACGGCGAAAAGCAGACGCGCGTCTGTGTCTGTTTGAGTCCCCAGGGGACGGCAGCGCGGGTCCTTGGGGACACACGCAAAACAACGGCCAGACAAGACGCGGGCGCAAGGGAGGAGTCGCGGGCCCCGGGGCACACTGCACAACCCGCGTCGAGGACACACGCAGACACGGCCCGCCAACACACCCCGACACACCCCTGACACACCCCGCCGACACACCCGGCACACGCCCGCGACACACCCGGCCAACACACCCCGACACACCCGGCACACGCCCGCGACACACCCGGCCAACACACCCCGACACACCCGGCACACGCCCGCGACACACCCGCGGCACACCCTGACACACCCGCCACACCCGGCACACACCCACCCCGCCGCGCCCCCGACACACCCCGACCGCCGCCGGTGCGGGACAGGGCTAAGCGCCTTTATGGCGCCGCAAGCGCTCCGCCGCTTCTGCGGCTTGCTGTCCACGGCGCTTTCTGCGCGCTGCCGGTGCGTGTGGCTCCACAGCACTTGTCGTTCCGCCAGTCCGACACTGGACAGGGCTGAGGGAGTGTTGCGTGACATAAGTAAGCGCGTCGGCGTCCTCAGCGCGTTTGCGGCGTGCTGTGTCCGGCGCTTTGTGCGCACTGCCGTTGCGTGGCGTCCCGCGGTGTTTTTTTTCGCGGACCAGCGCCGGGGACGGGGTGTTGCGGGCGCTCGGGGGTTGGCGGCGGGTGTTTCTACGGTGTTTGTGCGGCGTTTCTACGCAGCTTTTGCGCAGCGCTTCGCTTTTCGGGGCGGCGACGGCGTTGTTTTCGGGCGTGCTGGGCGCCGGCGCCGGTAACGGGAGTTACGCTGGGGACAGGGACGGGGGTTGCGCCGGGGACGGGGGGTGTGCGGGGACGGGGGGTGTGCGGGGACGGGGGGTGTGCGGGGACGGGGGGTGTGCGGGGACGGGGCGTCGCGGGATGGCGGGCTGTTGCGTGCCGGGGACGGGGGACTCTTGCGGCGGGGACGGTGGTGAGGACGGGGACAGGGGCATTTCGCGGCGGGGACGGGGAGTTGCGGGATGGCGAGCTGTTGCGTGGCGGGGACGGGGGACTCTTGCGGCGGGGACGGGGACGGGGGCCTTTCGCGGCGGACACGGGGAACACAGACGGCACGCACACGCAGCTCGCCTATTTAACCTCCACCCACTACAACACACACATGCCGCACAATCATGCCAGCCACAGACACAAACAGCACCCACACCACGCCGCTTCACCCAGAGGACCAACACACGTTACCCTTACACCACAGCACCACACAACCTCATGTCCAAACTTCGGACAAACACGCCGACAAACAACACCGCACGCAGATGGAGCTCGACGCCGCGGACTACGCTGCTTGCGCACAGGCCCGCCAACACCTCTACGGTCAAACACAACCCCAACTACACGCATACCCCAACGCCAACCCACAGGAAAGCGCTCATTTTCGCACAGAGAATCAACATCAACTCACAAATCTACTTCACAACATAGGCGAGGGCGCAGCGCTCGGCTACCCCGTCCCCCGCGCGGAAATCCGCCGCGGCGGTGGCGACTGGGCCGACAGCGCAAGCGACTTCGACGCCGACTGCTGGTGCATGTGGGGACGCTTCGGAACCATGGGCCGCCAACCTGTCGTCACCTTACTGTTGGCGCGCCAACGCGACGGCCTCGCTGACTGGAACGTCGTACGCTGCCGCGGAACAGGCTTTCGCGCACACGATTCCGAGGACGGCGTCTCTGTCTGGCGTCAGCACCTGGTTTTTTTACTCGGAGGCCACGGCCGCCGTGTACAGTTAGAACGACCATCCGCGGGAGAAGCCCAAGCTCGAGGCCTATTGCCACGCATCCGGATCACCCCCATCTCCACATCTCCACGCCCAAAACCACCCCAGCCCACCACATCCACCGCATCGCACCCACATGCTACGGCTCGGCCAGATCACACGCTCTTTCCTGTCCCTTCTACACCCTCAGCCACGGTTCACAATCCCCGAAACTACGCCGTCCAACTTCACGCCGAAACGACCCGCACATGGCGCTGGGCACGACGCGGTGAACGTGGCGCGTGGATGCCGGCCGAGACATTTACGTGTCCCAAGGATAAACGTCCCTGGTAGACGGGGTAGGGAGATCTACCAGCCCAGGGATCGCGTCTTTCGCCGCCACGCTGCTTCACCGATATCCAATAAACCCATCCCCTCGCCACGACGTCTCCGCGTATCTTTGTAGCCTCAGGAATCCGTCCCCACGTCCACCCATCCCGAGCACTCCACACGCTATAACAGACCACGAACACGGCAAAAAATGCATGCAAACTTCTCATTTATTGTGTCTACTACTCTGTGTTGCTACAGGGAGTGAAGGGGGTGAAGGCAAAGAAAAAAAAAAGGAACAAAATAATAGATTAGCAGAAGGAATAATCCGTGCGACCGAGCTTGTGCTTCTTTTCTTATAAGGAGGCAAATATACTAGGGAAAACATAAGAATAGGAAGAAACCGAGGTTTGGGAGAAAAGCTGAGATAAAATAGCGCATTTTCCATACAGAGGTTGTTGTTTTTGTGGATCCTAAGAGGTTTCAAGTGCGAATCTCAAAGTTCTCACGAGAATATTGTCTTCAAGAATCGACAACTGTGGTCCAAGATTTTTTTTTGGTCTTTTTAGGTTCTGCGAGGGACATCACGATGGATCGTTGCGATGAAGTCACGCGTACGCCTCTGGTGTGGCGCGGTGTCGTGACAGGAGAGTGTGTTTTCAGTGCAGAGCTGTCTTGATTCCTATATCCGAGTATCTGTTTTCTCGTAAGGACGGTAATCTTCTTTGGTGTAAGTACATCTAAAAGCTGCAAACTATATTTTAAGGGCTGTCTCTAGGTGTACTTTGATGCTGGAGTTTTTCGCTGTGTTGATGTGAATAAATCTACTACTACTATTATATGCAGAAAGAGTGATTATGCCGAGACAAGATTGCATTGGCTGAACTGTTTCAAAAACGCCTACACTCTACTTATCCGTAAACCTAAGGTAATACTATGTGTAAGTTGTTTTTTTTTCTTTTTGTAGTAAAATGGTGATACGTGCAATTAAAACTGTATTCCATGTTTCCATCCTTTCATTTCAACTTTAAAGGCGGCTTTGAGAGCGAAGAAGTGCGAGGATAAAAATGGATGACTCCTTCGTGTCCAGGGAGTCGACTACTGCAACGCTGATTGATTAAAAGATGGTCTCCGATGATGATGTTGTTATTGATCGAATCATGGTGCAGAACGGCGACGGAGAGGAGCGTGTCCGCCGCCGGGAAGGTGGTCTCTTTCTCTTTTCTTTTTTCAAGAAATCTTCCATGTGTTTATCGTAGTGATCGAAATCGACTGATCTCGGGTTCTTTTTGTTGGTTTCTTTTCGGTTAATCATGTATTGTTTTCTTTTTTTACAGAAAGATACTTTTTTCATGAGCAATTCCTCGCCCGGCGCCGGCATGCCGAGGTGGGGCCACTGCGATCAGCGGCATGCCGACGCCGACCCGGGGATCTTGGATTCACCGTTTTCTCTCTTCTCTCTCTACATACAGACCGGGTGGCAGGAGCGGTAAGGAATCATCGTCGTCTTTCATTCTTCGATGATTATGGTAATACTAAATCTTATCTAGGAGCATATACATCTAAGATTGGAGTACTAGTAGTCGTTTGTGGTTTCTATTTTTTTTTTATATTTATCTATGACAGTTTTTCTGTTTTTCGTTTTGATAATAATATAATAAAAACTCATGGACGTGAAATCTGGCTTGGTTGTGGTGATTTCATTCTCATTATTGTTGTTTTCTTTCCGTCTTGCGGATGAAGATGTTGCGATGCGGTTGTTGTTGGTGTTGCTATACACCGAGAGAGATGATCTTTTTGTTCTTCTGGTTCATTTCCTATGATTGTTTGGCTGCTGACCGACGCGTCAGGATGTGCAGGGCATGCGGGGAATCAGGACCGGACACGGGATAATTTCATCTACCTATACGGAGATCGCGGTCCTCGCCATGAGGATCGCGACAGGCGCGTCGAGGGGGCAGGAACACCCTTGCGGATTGACATTCTTGGTGGTATTTCGTTGTTGTCGGTAGTTGTTGTTGACGATGAGGATAAATAAAAATGACCTTGTTTTTGTTCTGTTTTCTCTTGTTGGGAATCGTCGACTTTGAATTCTTCGAGTTATCGGAAAGCTGAGGTACCCAAATGTCTGTAGCTTTTTTCTTTTTACCCTCTTGTTTATCATCTGCGATTCGTGGTAGGTAGGAGAGGGAAATGATAATCCGAGATT

CAGAAGCCGACCGGCCGCCGACCCGTTCCCCAGGACCAGCCTACGAGGAACGGATAACGCGGTGGCGACGGCAGCGGTGGTGGCGCTGGGGGTGGCGGTAGTGGTACTGCTGATGGTAGTCGGGACGGAGGAGAGACGATGCATACATACACGCGTGCATGCTGCATGGGTGGATGGTCCGACCGGGAGACGCGGAAGAGAAACTCACATAAAAAGGTGACAAAAAGAGCGGTTGAAAAAAGAAAACGAGATTCGACCAGACAGAAGAGAAGGACCGGGGCTTGGCGACCCTTCCACGACTGCTGTTGTCATCTCGGCTCCTCCGTCTTCTCCCGGCCACGGGCGGCTAAGTCACCGCCGTTCTCCCCATCCGTCCGAGCGCCGACCGACCAGCCGGCCGATTCGCCCGCCGGGGCTTCTGGAGAACGCCGGAGCAGCAGCGATCTGGGGAAGCCGCTAAACCCCTGCGTTTTTATATGGTAGCTCTGCCGAGCGCGGGCTGACGCGTTGAGTAAGCGGAAAGACGTGTGTGACGAAAAGGGGTCCCATGGTATTTCACGTGACGATGAGGAGATGCGGTTTGGAGCACATACGGTTTAAAAAAAGGGAGTTGTCGTGACAAGGGCTGAGGGACCTCTGTCTCCATGTGTGTATAAAAAGCAAGGCACGTTCATAATGTAAAAAAGAACACGTTGTAAACAAGCTATTGCTGTATCATTCGGCTGACTATGCTTCATTCGGACTGATTTTCTTTTCCTAACGGCGTAACTTAAAGTGATTAACGTATGATATTTGTTCCCCAGAGTTATACTATAGTCATCATCCTAAAATTCAGATATAAATGAACACACGTCGTATGGGATTATTAAGAAACCGAAACTCTCCACAGTTCACCATCTTCTTCGTCATTCAACCGATGACCCACTCCGTACAACGAATCAGTCTGCTGCGTCATATTGCAAAGCACAAGCGACGTATGCGAACAACTTGAAACACAGGCTGTTGTATTGATGACCGTTGTACCATTATTAGTCACATCGTATAGAGACTCTCCACCGTCATCCCATGTTTCCCACCCGATGGAAAACCGTCTTCTATCATCAACTGTGGTAAGATTTCGACCCTGCGAGGTATTCAGTTTCCTCATATCCATAACCTGGATTTTATCATTAAACCCCAATATTAAATACTTTTTTAGTACCCCCCACCCACCAAAAAATGTGACTGGACCGGTTCCTAGCAGCTCTGGGAGCCATGTTCAGGTTGAACCACAGCTACAGCGAAACCGAGTCCAGTGACCGGTAACCACGTCCAGCCCCTGCGTATGTACCAGTCCAAGCACGTCCGGTCATTGTTCTACACAGGAAATCTAACTAGGTCAACGCAATTTTATTCCACCGTTACGCAGAATACTAACAAAAAAACACACAAATTTAACGAATTACACGTAGTTTATTACATGAAAACTGTAAGAACACCAATTCACTAAGCGATACAACATTTAGCTGACTTCCAAGTGCCACACATCACCACTGTATTCATCCATGTTTTCACCGAACCAACGAGACAGATCGAAGAAGCCAGAATCTCCCGACTTTAAATTACATAAATCCAACGTATTATGACCACAGCTCGACACACAAATAGTTGCGTTACCATTCACAGTAGCATTACCTATACCCGTAACGTTGCACAACCACTGATCACCATTGTTACCAAAAACGGTTTTCCACTTAGTTGTCAACGGATCTTTCCTATGCGTAATGGTAAAATTACTCCCAGTCGTCGCTTTTAGCTCATTACGAGTATTATCCGCATCCACATATATCAACGTCATAGCTAGGCACGCTATAAGTACCCCCCCCCCACAATGGAATGTTGCCAAACCGGTTCTTTCCCGTTATAGCCATAGCGTTCCCAGGCAAAAGCAAACGCCAAACCTAATGCAGTGAAAAGCGCTTGCAGCCAGAACCAGCTTATGTACCAGCCACAATCACATCCGGTTATTGTTTCCACAGGAAATCCTACCAGGCAAAGCCCCGCTTGTTGTGTTCCTGACCATCTTGTTTAGCAATTCGTAAACTGTCAGCCTAGCGACGTCCGTTTAGATCAAAAGTCACGTATATAGCGACGCTGTTTCCACCCGTTTCCCCGTCCCGCCGTTTCCGAACAACCCACCCGGGTTCAGACAACCGACCACCAACAGAAATATACACACAGACCACCGGGAGTTCAGTTAAAGATTTCATCAGGTTTATTTTGGCTGCTGCTAGTCTTTTGCTTCTTAGAAAAAAAATACCCATATAGAGAAATAATGATAGTTTGACAACACATATGGCAGGGATTTCTTCTTCATCAATAAGATATGCAATTCCCCCAGGGAGAGACTTTCAACAATTGAATTTACAAAAACAAAATTACATCAGGAGAAAGAGAGGATACATTAATAAATATATTATATCTGGTGTATATACTGAATGCTGCTGGTTCATAAGGTAACGATGCTACTTTTTTTAATTCCAAGATGGTTTTTCTTTGTTAGTCTTTTGTTGACTTGCTGGTTCCTAAAAGTTCGCAAAAACGATTGTGTGAAGATTTTATGACGTTGGTTGACTAGTTCATGAGATTCTGCTGTACGTGTGATGGTTATTCGCTGGTTCGTTCTAAGATGAGTATCGTACTGTGTCTGCGATGGTCGTCTCTTACTGGCATTCTCTCGGCTGCCTCTTGCTTTCATGATTGAAAAGGAAAAAAGGACTCCGAGGGCGCGGTCATCTTTTACTTTTCGGTTTTCTCGTTGGCGGGTCAGAGGTAGTCAGATCATGAGACTGTCGTGGTCGATGAAACTGTGTCTGCTCAAGTGACGTCCATTTCTTGTACGGAGAAAAAAGTCATCGGGATAAATAAGGCTATACAAGGCGTTGTCAAGCGTGCGGCTCTAAACAAATTAAGCGATACAAAATTACAGTAATACGAATAATAAATTACCCCCCTCCCCCTGTGGTCCCCCGAGACGAGAGCCACCCATCGTGTACTCTCGCACCACCCACGACCACAGAGGGAGACGGGACGAAGAGACGACGCACAGCGCCATCTCCTCCTGGAGGCCGGCGACGTTAACTGCTACAGCTGCGGCGGCGAAGACAGCTGCGATTTGTCGGCCGACATGCCGATGGTAT

AGAGAAGAGGAGCGGGGCGTCCGAAGGCGAGGATGGCATGGTCTCGCCGGAGCGCCCGGCTTTTATGGAACACTCGCGTCCGGTTGGGTATCACCCACAGGAAGATGAGTCACAACTTCCAAACCATCTTGAGACCCGAGTAACGGTTTACAGGTCGCACGCCAGTCTCAGCTAAAAACAGCGGACAGTCCCACGCTGTTTCTGTTGTGGCTCTCTCCAGTTTCCTCATCACCGTCCCGGTCTCCGTCGTCATCGGAAGAATACCACCCGCTCTCATGCGGCAGTCGATCGGCCTCGACGAACGAGACGCGGCGACGCCTCTCCACGGCCGACTGGTTGTGGTGGTGAAAGAAGAGCACCAGCAATCCCAGGAGGAGCAACAAGCCCTCACATGTCCAGGAGGTCGGGGAGAGGGCCTGTCGGAGATGGCCGTGAGGCATCACGTACGGCAGCTGAGGAGAAACGGAGAAGAAAGGAAAATTACCGTCAGGGGCCGGGGTTCTTATTAGAGAAACAGCACGTAGGTCAGGATCCAGATGCTAATGGCGATCATGATGACGATGATCATGCAGGCCAAGACGCGGCGCACCAATGCCGAATCCAAGAGCCGCCGTGCCGCCGGTTGGTGGCTGGCGGCATCTAGAGACATGGTTTGGGGGGGACCGGCGGCGCGAAAAGACAGGGAGATGGACAGTGACACGGTGTTTTGTTATGATTAGGACATGGGAACCGGAAGCCGAGACAGAGTACTACAGAGTGTTGAAGGGTAACGTGAGGGAGATCATGTCATGGGCGGGCTGAAGACCGTGCGGGAAGGATTGACGTGTGCGGTGCTTGTGGAACACGGTGTTTTAATATGTATCCGCGTGTAATGCACGCGGTGTGCTTTTTAGCACTCGGTTTGATAAGCTACGTGGCCGTTTGCGCCGAAAACACGGTTACCACCAACTGTCTCGTGAAAGCAGAAAATACCCACCTGACATGTAAGTGCAATCCGAATACCACATCTAATACCAACAATGGCAGCAAGTGCCACGCGATGTGCAAATGCAGGGTCACAGAACCCATTACCATGCTAGGCGCATACTCGGCCTGGGGCGCGGGCTCGTTCGTGGCCACGCTGATAGTCCTGCTGGTGGTCTTCTTCGTAATTTACGCGCGCGAGGAGGAGAAAAACAACACGGGCACCGAGGTAGATCAATGTCTGGCCTATCGGAGCCTGACACGCAAAAAGCTGGAACAACACGCGGCTAAAAAGCAGAACATCTACGAACGGATTCCATACCGGCCCTCCAGACAGAACGACAACTCCCCGTTGATCGAACCGACGGGCACAGACGACGAAGAGGACGAGGACGACGACGTCTGATAAGGAAGGCGAGAACGTCTTTTGCACCATGCAGACCTACAGCACCCCCCTCACGCTTATCATAGTCACGTCGCTGTTTTTGTTCACAACTCAGGGAAGTTCATCGAACGCCGTCGAACCAACCAAAAAACCCCTAAAGCTCGCCAACTACCGCGCCACCTGCGAGGACCGTACACGTACTCTGGTTACCAGGCTTAACACTAGCCATCACAGCGTAGTCTGGCAACGTTATGATATCTACAGCAGATACATGCGTCGTATGCCGCCACTTTGCATCATTACAGACGCCTATAAAGAAACCACGCATCAGGGTGGCGCAACTTTCACGTGCACGCGCCAAAATCTCACGCTGTACAATCTTACGGTTAAAGATACGGGAGTCTACCTCCTGCAGGATCAGTATACCGGCGATGTCGAGGCTTTCTACCTCATCATCCACCCACGCAGCTTCTGCCGAGCTTTGGAAACGCGTCGATGCTTTTATCCGGGACCAGGGAGAGTTGTGGTTACGGATTCCCAAGAGGCAGACCGAGCAATTATCTCGGATTTAAAACGCCAGTGGTCCGGCCTCTCACTCCATTGCGCCTGGGTTTCGGGACTGATGATCTTTGTTGGTGCACTGGTCATCTGCTTTCTGCGGTCGCAACGAATCGGGGAACAGGACGCTGAACAGCTGCGGACGGACCTGGATACGGAACCTCTGTTGTTGACGGTGGACGGAGATTTGGAATAAAAGATGCGTGTTGACCGTCAAAAACGCAACAACTTCACGTATCGACAAACGGTATATATAATCCTGACCTTCTACATTGTATATAGGGGCAAATGTAACAGCACTGATACCAATAATTCTACGTCTACTTCAGATAGCACCGTCTCTGATACTAGTGTGTACTCTACTCCAAATCTTCCCAGTACATTTTTTACAACTCTTGATACATCTACTGGCTCACAGATATCAACCACCTCAAACACCATATCCAGCACTACAAATACATTGGCCGCATCTTCTATAACTACGTTAAACACCTCGACTAACACTACGTCTTATACAGCTTCAACTCTTACTGCTCTCTCCAGTACCCACACAAATTCCCCAATACTCTCCAACAACGCAACATTATACACCACATCGTTGGATAATACGACTACAGACATAACGTCCAGCGAAAGTTCGATTAACGCGTCAACCATTCATAACACTACGTACAGTCCTGTAACATCGATTGCTGTTAATTGTACTGTCGCAGCTAATGAAACAAACAATTCAAGTTCAAAAAATTGCCAACAAGATATTGGAACAATACGTGTTAAATCAACTACATTAACGGCAGAAGAAGGAAAAAACATTACGATACAGGGCAATTCAACATGGAACTGCCCCAATGTAGTTTGGTACCGACATTACAATTGGTCTACACATGGACACCACATTTATCCCAATAGACGTTACCAAACGCCAACGTATCAACACAAGATCTTAACATCACATCCCATATGTCATCCTGACGTCTCATCACCTGCGGCGTACCACGATCTATGCCGTTCGTGTAATAAGACAGAACTACGTATATACGATTTAAACACTACAAATTCAGGCAGATATAGTCGACGGTGTTACAAAGAGTACAACCACGATGGACCACACGAGGATGAAAATTTCGGACTAACTGTAAATCCCAGAAACGACACTGACAATCATACCACCCCACTATGTCCCAGATACGTAGGAACACAATCAGAAGAAGACGAAGACGACGATTATACACTAAGCACTATCACAAATAATAACATGCGCAAAACAAGTCACCGTGACATCTCACATGGCACGCGCACTACATGGGCTCTTACACTAATCTGCATAGCCTGCATACTTCTGTTTTTTGTTCGACGAGCCCTCAATAAAAGATACCGTCCATTACGGGACGATATTAGTGAATCTAGCTTTGTTGTACGATATCATCCTGAACATGAAGACTGACGTTTCCGGACACGCAACACATGAAATTAAGTAACATATCTACCATGAAATACAGCAAAGATATACTAATGTCTATCCATCCAATAGCGGTACCATGCACTGGCATCTTGCGATTACATGGACGGTAATCATATTAACGTTTTCGGAATGTTACAACCAAACTTGTCCGTGCCCCTGCATTTGTGTCAATTCTACAACAGTTAGCATATCCACCAGTGAAACAACGTCTAAAAACATCACTCCAACTACCACGACAAATTCCAAAAAAACAACGTCAAGTATTGCTACCACTACACCCTCGTTAGTAACGACTGGCAAAGTCGTTTCTACTGCAGCGTCGTCAACCATAATATCTCAAACCAACCGCAGTCATACGAGTAATGCTATCACAACCCCGAAAACGCGATTTGAATATAATATCACGGGATATGTTGGCCAAGAAGTGACTCTAAACTTCACTGGATCATGGAATTACATTCAATGGTTCCGGTACGGTTCTCCAGGCTGGCTTTATTCCTCGGAACCAATATGCACCGTTACCAGCAATTATCATCATACTTTCCCTCGTGGTGCTCTTTGCTTTGATTGTGACATGACAAAACTTCTAATTTACGATCTGACGTTAAACGATTCTGGAAAGTACGTTGTCAAGAGAACGCGCCATGACAATCAATACGAAGAAGCATGCTACAGCCTCACGGTGATTTTTGCCAACACGACATCCATAGTCACCAACAGAACGTGTGATAGAAAACGAACAGAAAATACAGACACTACTAACCATGAAATCGGGAAACATATTATTGAAACTATTAAGAAAGCCAACATTCCTCTGGGAATTCACGCTGTATGGGCGGGTATAGTAGTATCGGTGGCACTCATAGCGCTATATATGGGCAACCGTCGCAGACCCAGGAAACCGCGTTATACCAGACTTCCTAAATACGATCCAGATGAGTCTTGGACTAAAACCTAATATGCACATCAATAAAATTTTTTATCTTTAGTCATTAATGTTTGGGTGTGTTTATTCTGAGTTAATCACTTATAAGTCGGGATATCCATAGACATTTAAAAACATGGGTGTACAATGTAACAGTAAACTGCTGCTACTAGCCGTACTAATAACAATTATCCTATCTAGCATTCTGGTACAGGCAATTCCACATAAACAAAAAACATCCTACCAGCAGCTCTTGCTGCAAAGTGAACATGTACAAATACCCATCACAGTAGCCGAAGGAGATACAATTTGCTTTAACGTTAGTGATAACCCCTGCAACTTTTCTAGTTACTGGAATCACAATAACTGCGAACTTTGCGGTTGGACACCATTTTACTCGGAATATGCTGGATATTCCGAAAACAAGTCGTGTCACCCACGATTTACCTGTCTTCACGATACTAAAGGTCTGAAACTACACAACGTAACTACAAATGATTCAGGAATTTATACCCGAAACGTTTATTACTGTGACATTCCATGCAACATCAGCGATGATCATAAACATAACGTAGAGGACTTTGACAACTGTAACACCACTATAAATAGAACTCACTATATTATTACTGTCTCGTCTTCACGTTATTCTAAACGCACCAATTCCCACGTAGCCACTCACGTTGGTTGGACAGCCACCGTGGTGATAATTATCTGCGTTTTAACTTACGTTAACGTTACAACAACCCTGAAGCACAGACTACGAACTAGAAACAACGTCAACCACACAATGTGATCACAAAGTACCAACGCTAATTTATTTAGGGTACATTTGTACTACTTTGTGTAGTTCTCAAAAACTGTAAGGTCCCGCTTTTCCACTCCGTCATGAAGGATCGCAATAAAATACTTCTATGTATCATCTTTATTTGCATCATGTGCCTCATTTGTATTTACTTTAAACGTCATTGTATTCCTACTCCGTCTCCGGACAAGGCGGATTTCCGAGTAGAATTTCCTTCGTTACTCCCGTGTATCGGCGTACAGTGCGCTGCATAGAAAGACGCGTGACACATACCGTACCCCTGGACGGTACAGTCTATGATAACGTAATTCAAATAAAGTACAGGTTCCTAGTGACATGTTATCATAAAACATAGATTTTTCTACGTGTTTTTACAAAAGAGCGTCTCGAAGCAGCTTGAGCCACACTGCGGTCCAAATGACAAGCATGATCAAAAATATGCTGCGTAGCAGTCGAAAGCCGTACTGAGCGTGCGAGGCGGGTAGGCTGCCGAACGACGGATATGCGTCGTCATCTTCGACTATAAGGATCGTGACCGAGTCTTCGGCCATGCTAAACGCTACCCTGTGTGGCTGGTATGTAGTGTATCCGGTTTGGAATTGTTCTGTTCCGGCTCGGGGGATAGTGAGGAATTCTCAAGGGACAATACGGGACCCAATGACTGGATATGAGAAGGGTTTTTCCCCGTAAGATGATCCTGGTATCACATGAGGTCTGGATATGTATAAATGAGGAGTGAAATAGGTAAAGGGAATCAGATGCCGCCTTCGTGATGCAGCCGCTGGTTCTCTCGGCGAAGAAACTGTCGTCTCTACTGATTTGCAAATACATCCCGCCTTAAGCGATAAGTCCATAAAACGCCGTTGTCCGGATACGGTGAAGGTGACCCGGATTATAGCACGTTCCTTTTTTTTTGTTTTTGCATCACTTATCGCCACTATCAGTGCAATATTTTGATTGTGAGACTGAAAGAGTATCGTTATGATGCTTAGAACGTGGATATCATTACCGATGGTACTGCTTGACGCGTACTGTTTTTGTATTTTTGTGAGTTGTTCAATCAGCACGACGACTGCTCCCGTGGAATGGAAGTCTCCCGACCGTCAGATTCCTATGAATATTACTTGCGCTAATTACTCAGGGACCGTCAACGGCAACGTTACATTTCGAGGTCTTCAGAACAAAACGGAAGATTTTTTGCACTGGCTGTTAGGGTGGGGTCATAAGTCCATTTGTTCATTTTTTCCGAAACTCCAAGGCAATAAAGAACAACATTATATGTATGGAATAACGAATCTCACGTATAACTGTACCTATGACCGCCTGACGTTGCTGAATTTGACGACGGAAAACAGCGGAAAGTATTATTTCAAACGAGAAGATGTGAATTCCACCTTTTATTACTCTTGTTACAACCTGACCGTGACCTAAACGCATGTAAAGTTCCACAGAGCCGCGTGGCTGTAGCTATTGTGTTTACGTTGCTTTTGAAATGTTAAGCGTCCCTACGGCGCTAACATGTTTCTAGGCTACTCTGACTGTGTAGATCCCGGCCTTGCTGTGTATCGTGTATCTAGATCACGTTTAAAGCTCATGTTGTCTTTTGTGTGGTTGGTCGGTTTGCGTTTCCATGATTGTGCCGCGTTCGAGTCCTGCTGTTACGACATCACCGAGGCGGAGAGTAACAAGGCTATATCAAGGGACGAAGCAGCATTCACCTCCAGCGTGAGCACCCGTACACCGTCCCTGGCGATCGCGCCTCCTCCTGACCGATCGATGCTGTTGTCGCGAGAGGAAGAACTCGTTCCGTGGAGTCGTCTCATCATCACTAAGCAGTTCTACGGAGGCCTGATTTTCCACACCACCTGGGTCACCGGCTTCGTCCTGTTAGGACTCTTGACGCTTTTCGCCAGCCTGTTTCGCGTACCGCAATCCATCTGTCGTTTCTGCATAGACCGTCTCCGGGACATCGCCCGTCCTCTGAAATACCGCTATCAACGTCTTGTCGCTACCGTGTAGCTAGTTAGCCAGCTGTGTGTAGTTGTATTTTGCTTTTGCATATTTGTTTTCAGTCAGAGAGTCTGAAACGGGGTGGGAGGGACTTTTGCGGGTAGTGCATGCTAAGATGAACGGATGGGCTGGGGTGTGCTTGATAACTCACTGTTTGAATACGCGCTCACGCACATATGTAGCACTCAACATGTTAGCTTTTGCCCGCACGCCCCGGGGCGTGCCGAGCTGCCTTTTTAATAAAGTCTGGGTTTCCAGATACGCGCTGGTTCTGATTTTGATGGTTTGTGCCTCTGAAAGCTCTACGAGCTGGGCCGTGACATCCAATGGACTGCCTAACTGTAGCACGGTAACTAGAACAGCGGGTCAAGACGCTGAATTGCACGGTCCGGCACCGTTAAGCTGTAATGTGACCCAGTGGGGACGTTACGAGAATGGAAGCACACCCGTATTATGGTGCACTTTACGGGGATCACGCATGCGAGTCTCATTAGGACACCGTGTAGCGTTTGGCTGTTCTTGGAAAACATTTTTTATTTATAACGTTTCTGAAAGTAGCGGTGGCACTTACTATCAAAAAGGTTACAACTGCACCGACAAACATATAACACTATCTTGTTTCAACCTAACGGTGGTTCCTCGAGCGGTTCAAAGCACAACCACCGTAATGACACCCACGCTGGTTACAAACTCCACATTCAGTGTGTCACTTGTTGCGTTGAGACTGACGACAAATTCCAGCGCGTTTGGACACGCTATTTATCAACGACAACAGCGTGTTGAAAACGGGACGTTATCCAAGAACATAACTAACTTGGCATTCACCTATGGCAGCTGGGGCGTTGCGATGCTGCTGTTTGCCGCCGTGATGGTGCTCGTTGATTTGGGTTTGCCTCAATCGGCTTGGCGACGCTGGCGAAGCCACGTGGACGATGAAGAACGTGGTTTGTTAATGTAGGAAATAAAAGGCAGTTTGAGCATGACTGTTTCCAAACCGTAACGTGGTAAATAAATCATGGCTTCTGACGTGGGTTCTCATCCTCTGACGGTTACACGATTCCGCTGCAGAGTGCATTATGTGTACAATAAACTGTTGATTTTAACTTTGTTTGCCCCCGTGATTCTGGAATCCGTCATCTACGTGTCCGGGCCACAGGGAGGGAACGTTACCCTGGTATCCAACTTCACTTCAAACATCAGCGCACGGTGGTTTCGCTGGGACGGCAACGATAGCCATCTCATTTGCTTTTACAAACGTGGAGAGGGTCTTTCTACGCCCTATGTGGGTTTAAGCCTAAGTTGTGCGGCTAACCAAATCACCATCTTCAACCTCACGTTGAACGACTCCGGTCGTTACGGAGCAGAAGGTTTTACGAGAAGCGGCGAAAATGAAACGTTCCTGTGGTATAATTTGACCGTGAAACCTAAACCTTTGGAAACTACTCCAGCTAGTAACGTAACAACCATCGTCACGACGACATCGACGGTGACCGATGCGAAAAGTAACGTTACAGGGAACGTCAGTTTAGCACCACAACTACGTGCCGTCGCTGGATTCTCCCATCAGACGCCTTTGGAAAACAACACGCACCTGGCCTTGGTAGGTGTTGTTGTGTTTTTAGTTCTGATAGTTGTTTGTATTATGGGGTGGTGGAAGTTGTTGTGTAGTAAATCAGAGTTATAGTAATGTCCTTTTTATCAGGGAGAAGGTTTTGTTCCCACAATGACTAGCTCGAGACTATCTGCGTCGGAAAATTATGACGGAAATTATGAATTCACGGAAACTGCCAATACAACGCGTACAAATACCAGTGACTGGATAACGTTAGGAAGCAGTGCATCGCTGTTGAAAAGCACGGAGACTGCAGTCAACCTCAGCAACGCGACTACGGTCATCCCACAACCTGTAGAATACCCGGCTGGGGGAGTACAATATCAAAGAGCGGCAACGCATTATTCTTGGATGCTAATCATTGTCATCATTCTCATCATTTTTATTATCATCTGTCTACGAGCACCTCGAAAAATCTACCATCACTGGAAAGACAGTAAACAGTACGGACAAGTGTTTATGACAGACACGGAACTGTGACAGTGATGTCTAAGCGTTTGCAGGTATTTCCATGGATAACAATTTTATTTTACACATCAAAATCCCAGTATTGGAACTATATGACAATACCATGTACCCCTACAGTTGGATACGGCAGTCATAATATTAGCTTGCATCCGCTTAATAACTCATTATTTCAAGACGATGTTTTTGAATGGTACATAGACAAACCAATGGTTACTAACAAGTTATGTCTTTATCAAAGTAATGAACGCATAAAATCCAATCTAGACTCTCCAAATATTATGTGGCAGTGCACAGATAATCGTACACTAATTCTCATGAACTTAACCACAACATACAGTAGAAACTATTATTTTCAATCTTTTAAATATCTCGGACAAGGAGTACCAAAACCGAATAACTTGTGTTATAACGTTAGTGTACACTTTACCCACCAAACACATTGCCATACAACTACATCATCCCTGTATCCACCTACATCTGTACACGATTCATTAGAAATATCACAGTCATTCACCTCAACCAACTTCACACATACCGCGGTCCACTACGCCGCCGGTAACGTTGAAGCACAACACGACACTGCCACTTCACATACAATGTGGATCATACCCCTAGTTATCGTTATAACAATCATCGTTTTAATTTGTTTCAAGTTCCCCCAAAAAGCTTGGAATAAATTCACACAATACCGATACAGCGGTATGCTCGCCGCCGCTTAAAGAATCAACGCCAAGGAAATCAAAACGTAAAAAGAATAGATATGTACGTTTATTTTTCAGCTCACTGTTTGAATACCGTAAACATAATGACGTACATATACGTGGTTATACAACAGGTGTTTGTGTTATGCGGCAACTGATTAACCATATCGTGAACCATGATCTTTTCCGATGGTCCGTCGTGACCGCAATGATATTTTACAGATATTCCGAAACCTGTATGGAGGTCACTGTCAGAGTAGGTGATCCAGTTACCCTCGGTAGTGGACATGGTTATCATCCAGGACAAAAAGTACACTGGTATAACCAGTCATGCGTCGGCATCGGCAACGGCGAAAATACGCATCCTATCTGCACCTACGACCCTCCTAAACCTGGTAGACAAAAGACAATGAAAACTACTCCGTTGCCATCACCATTGTTGTACGAGTGTCACAATTCCACATTAAGCATTCTTCATGTAAACGTCTCAGATCCCAGAAACTATTGCAGGCGAAAATGTCCACCAAAGGGTAACTGTGAGTTTCCCACATGTTTTACATTATCGCTGATTTCTAGAACTACGACCAGAAAACCCGAACAAAAAACTACGTTGTTGCGATTAAAAACCACGCCAAATAAACATACGCAGCACAAAAGATCCACGCGGAGAACGTCACCTAAAGATTACAATGTAACGGGTCTTCCAAAAGGCTTTGCGGACTCGTTTACCGGTAACGTAGAGGCACATCGAACCAAAGATGCCGCACACAGCGCATGGATTCTCATTATCATCATCATTATCATAGTCGTCATTCTATTCTTCTTCAAGATTCCTCAAAGACTCCGAGAGAAATGGGACACCAAGGGATACCTTTACAAAGGAACCGATGGCCTGCCCACTACGGACTAATTATCGTGAGCGGACGGATATGTTCGGTTTCAAACTCACTGTTTGAATATAGGGACAGTCCCTACGGAACCTGAGAACATGTGGAAATCACCTGTGGTAGAATGCTGTTCAGGTACATTACCTTTCATCGCGAAAAGGTACTTTACCTAACGGCTGCATGCATCTTTGGTGTCTACATCAGCCTCCACGATGCCTGCATACCGGTGGTCGGCAAAATAGGTACCAACGTTACGTTAAACGCGGTAGATGTTCTTCCCCCTCGCGATCAAGTTCGTTGGTCATACGGTCCAGGTGGGCAAGGCTACATGTTATGCATTTTCACTGGCACATCAACAACAACATTTAACAATACGCGCTTTAATTTTTCATGTCTAAGCAATTACAGCCTTCTCCTCATTAACGTTACCACGCAGTATAGCACCACCTATCGTACTATGACATCACTAGACCATTGGCTTCACCAACGACATAACCATGGTTCTCGATGGACTTTAGACACATGTTACAATCTGACAGTGAACGAAAACGGTACATTCC

GAAGACGACGATAAGCACTACCCATCATAAACACCCCAGTCCCAAAAAATCCACCACCCCTAACAGTCACGTAGAACATCACGTTGGTTTTGAAGCCACAGCAGCGGAAACACCGTTACAACCAAGCCCACAGCACCAACACTTGGCTACACACGCCCTCTGGGTTTTAGCGGTCGTAATCGTTATTATCATCATTATCATTTTCTACTTTCGAATACCGCAAAAGCTGTGGCTGCTCTGGCAGCATGACAAGCACGGCATCGTGCTCATCCCCCAAACCGATCTGTAAGCAAGTCGCGTAGGAAATGATTGCATGAAATCACTGTGAAACGCCAACTCCGTGCCAGCTGGCGCGGCGGACAGGCCTTTGACGTATTTGAAGCCAGGCGCGCTCTCGATACCGAAAGGATCCAAGGGGGCTTTCCAAAGCCGACGTCCCTGATTCCCTTCATAAAGCTGTTGACCGGCCCTAGAAAGACCAAGAGCATGCTGTGGGCCCACTGCGGTCGCTTCTTGCGTTATCATCTGCTCCCGCTGCTACTGTGTAGACTGCCATTCTTACTCTTTTTTCAGCGGCCGCAGTGGGCCCACGGCTTGGACATTGTCGAGGAGGACGAGTGGCTACGGGAGATACAAGGAGCGACGTACCAGCTGTCCATAGTACGCCAAGCTATGCAGCACGCCGGATTCCAAGTCAGAGCGGCGTCGGTTATGACACGGCGAAACGCCGTTGACCTGGACCGACCGCCGCTTTGGTCTGGATCGCTCCCGCATTTGCCCGTCTACGATGTGCGTTCCCCGCGGCCGTTGAGACCGCCGTCATCACAGCATCACGCCGTATCACCCGAACTGCCGTCGCGAAACGGGATACGTTGGCAGTACCAAGAGCTGCAGTATCTGGTGGAAGAACAACGGCGGCGAAATCAGTCGCGTAATGCGATTCCGAGACCCTCGTTCCCCCCTCCGGATCCACCATCGCAGCCGGCAGAGGATGCACGAGACGCGGACGCAGAACGTGCCGAATCACCACACACTGCAGAAAGCACCGTCAGCCACGACGCGAGTGACAACGCAGTGCGGCGACGGCACGAAAGACGGCGCTATAACGCTCTGACGGTCCGCAGCAGGGACTCGCTGCTCCTAACGCGAATACGCTTCTCCAACCAACGGTGTTTCGGACGCGGGCGTCTGAGACATCCCGCGGGAAGCGGTCCCAACACCGGCGGACCGCGACCCGGCGGTGCGGGACTCCGTCAACTACGCCAACAACTGACGGTCCGCTGGCAGCTGTTCCGCCTACGGTGCCACGGTTGGACACAGCAAGTCTCTAGCCAGATCAGAACCCGCTGGGAGGAAAGCAACGTCGTGAGCCAGACGGCCACGCGAGTACGTACGTGGTTTGTGCAAAGAACCACGTTGTGGCGTCGCACGTGGGTTCCGGGACAGAACCCGGCGGCCGAAGCGCAAGAACTGGCGATCATACCGCCGGCACCCACGGTGCTCCGGCAGAACGAGGAACCACGTCAACAGCTTACGGGAGAGGAGACAAGAAATTCAACGCACACTCAACGTGAAGAAGTGGAGGACGTTTCGAGAGAGGGCGCGAGAGAAGGGAATGATGGGAGCCGAGCAAGTGGAAACGACGAGAGAAGGAATAATGCGGGAAGATATGATGATGATCATGAGGTTCAAGAGCCGCAGGTCACTTATCCAGCGGGACAAGGAGAACTGAATAGGAGGTCACAGGAGGAGAACGAGGAAGGTGGACCGTGTGAATCGCCGCCAATGACGACAAATACGCTGACCGTGGCCTGTCCGCCCCGCGAACCCCCGCATCGTGCCCTGTTTCGTCTATGCTTAGGACTGTGGGTCTCGAGCTACCTGGTTCGACGGCCCATGACGATTTAGAATACACCGAGCCATTCCTTTATTTCCCCCCATCCCCGGTCGCTTATGCGTGTCAAACACTACCAATAAAGATAATCTGCCAATCGCACCTTATATATAATATGTGGTCGCGTGTGGTCTTTTTAAGGAGCTCTGAAACACAGACAGGTATGGGGGGTGGCCGGCTGCCGCCGCTGTGGCTGCCGCTGCTGATCGCCTGGAGCGAGTGGGGCAACTGCTGTCTCGATGCGCCTCCGGTGGTGCGTTCGCCCTGTCTGCAGCCGGTGCGCGACCGCAACCGCGAGCGGAACCCGGGCTCACCGCAGTTGCTGCCTTACGGCGACCGTCTGGAGGTGGCCTGCATCTTCCCCGCGCACGACTGGCCAGAGGTCTCTATCCGAGTCCACCTCTGCTACTGGCCCGAGATCGTGCGTTCGCTGGTGGTGGACGCACGCAGCGGTCAGGTGTTACACAACGACGCCAGCTGTTACATCGCCGGCGGGCGCTGGCGTTTCGAGGACGGCGGCGCGGCGCAGCGGCTGAGCCTCTCGTTTCGTCTCATCACCGAGACCGCGGGCACCTACACCTGCGTGCTGGGCAACGAGACCCACAGCCTGGCGACCGAGACCACGGCGCTGGTGGCCGACGTGCACGACCTGCGCCACTCGGACCGCTCCTGCGACCTGGCTTTCGGATCGCGCTCACAGACGCGGTACCTGTGGACGCCCGATCCCTCCAGGTTGCGCAGTATAAACTGCGGTTGGGAGGGTGAACGGCACCGCGTAGTCCACTACATCCCCGGCACCTCGGGTCTGCTGCCCTCGTGCGAGGAGGACGAGCGCGAACTGTGCGTGCCCTTCATCAGCCAGAGCATCGCGGACAACAACTGCAGCCGCCGGCATCGGGTAGACGGCGCTAGGCGGCGCTATCATCTGCGGAGGGATTACTGGCTGACGGATCCGAAGATCGGGCTGCTGGCCGCGGGATCGGTGGCCCTGACCTCCCTCTGCCACCTGCTGTGCTACTGGTGTTCCGAATCGTACCGGCGTCTGAACACCGAAGAGGAAAACGAGGCGGCGGAGGAAACTGCCGCGGGAGAAGCCTCTGCGGTAGCGGCGGCGGCCGTCTCTGAGGAAGAGCAGCAGCGGGAGTAAACGAGGAGAGCCATGAAGCGGATGATTCGCAGTCACGGCAGGAAAACGGAGTGTCAGATGACGGGCGCCGGCGAGCGACGCGGCTCCGCCGTCGGTGCGCTCATCTGCGGCAGCGGTACCCGACGCGGCAGCGGCGCCAACGAACGCCGCGACTCCGACGTCGGTCCCATCGCCCACAGTAGCGGTACCAGACGCGGTTCGGCGAATGAAACGTCCGCCTGTACGCGGACCGATCACCAGAAGGCGGACATTGGGCTGTGGTTCATGTTTCTGGTTTTTGGACTGTGTTCGTGGTTGGCGATGCGGTATCGCGCACAATAAATTTTGAATCGATGTCAAGGAACGCGTGTTTTGTATTTTATTGGGAATATTGGCGGGGACAAACCGGTTTCGGATGTTTACCCTTAATCTTACCGGGGACCTCGTTGTCCTCTCCTCCTTCTTCCTCGGACACGGGGCTTCATGCTGACGTAGGTACCGACTGGGGTCAAAAGCCTGGGTACTTATGGGGAGCGCGCACAAAGGACCGTCAGGCGCCGGCATGGAGCGTCGCCGAGGTACGGTACCGCTGGGATGGGTGTTTTTTGTTCTTTGCTTATCTGCCTCTTCCCCGTGTGCTGTTGATCTGGGTAGCAAGTCCTCCAACTCGACCTGCCGCTTGAATGTGACTGAGTTGGCCTCGATCCATCCTGGGGAAACGTGGACGTTACACGGGATGTGTATTTCTATCTGCTACTACGAGAATGTGACCGAGGACGAGATAATCGGCGTGGCTTTTACTTGGCAGCATAACGAGTCTGTGGTTGACCTGTGGTTGTACCAGAACGACACGGTGATCCGCAATTTCAGCGACATCACCACTAACATCTTGCAAGACGGACTGAAAATGCGAACCGTCCCTGTGACTAAACTGTACACCAGCCGCATGGTCACTAATCTTACCGTGGGCCGCTATGACTGTTTACGCTGCGAGAACGGTACGATGAAAATAATCGAGCGCCTCTACGTCCGATTGGGCTCGCTATATCCGAGACCGCCCGGATCCGGGCTCGCCAAACACCCCTCCGTAAGCGCCGACGAGGAACTGTCCGCGACCTTGGCGAGAGACATCGTGTTGGTCTCAGCCATCACTCTGTTCTTCTTCTTGTTGGCCCTACGGATCCCCCAGCGACTGTGTCAGCGGCTGCGCATTCGCCTGCCGCATCGATACCAGCGGTTACGCACCGAGGACTGAACGGATAACCGCAAAGGCCACGTGCAACGTTCACGCTGCTATAAGAAGGCCATGTCCCCCGTGGACGGGTCTCTTTGACACGAGCGCGGCACGCCGTTGCCACGAGCATGGATCACGCGCTCTTCACACACTTCGTCGGCCGACCCCGTCACTGTCGGTTGGAAATGTTGATTCTGGACGAACAGGTGTCTAAGAGATCCTGGGACACCACGGTTTACCACAGGCGCCGCAAACATCTACCTCGACGTCGCGCTCCGTGCGGCCCCCAGAGGCCCGCCGAGATTCCCAAAAGAAGAAAAAAGGCGGCCGTCCTTCTATTTTGGCACGATTTGTGCTGGCTGTTTCGACGACTTTTCTTTCCTCGGGAGGACTCAGAGCCACTGATGTCGGATCCGGCACGGTCTCCCGAAGAGGAGGAGTAAACAACACACGGCTAAGAGGATACATCATCAAAGAAGATAGGAGGGGTCAAAACGCGGACTGAAAGTATATAACGCCGATCATGTTCGAGGAACTGTTAATAAAACGCCATGATGACAATGTGGTGTCTGACGTTGTTTGTGCTGTGGATGTTGAGAGTGGTGGGAATGCACGTGTTGCGTTACGGGTACACGGGGATTTTCGATGATACATCGCATATGACGTTGACCGTTGTGGGGATTTTTGACGGGCAACACTTTTTTACCTATCACGTTAATTCCAGCGATAAAGCGTCAAGTCGGGCCAACGGTACCATTTCTTGGATGGCTAACGTCTCGGCGGCCTACCCCACCTACCTGGACGGGGAAAGAGCCAAAGGTGACCTTATTTTTAACCAAACCGAGCAAAACCTGTTAGAGCTGGAAATTGCGTTGGGTTACCGGTCACAGAGCGTGCTGACGTGGACGCACGAGTGTAATACCACGGAAAACGGTAGTTTTGTAGCCGGTTACGAGGGATTTGGGTGGGACGGGGAAACTTTAATGGAGCTCAAGGATAACCTGACACTATGGACGGGCCCCAATTACGAAATTAGTTGGTTGAAGCAAAACAAAACGTACATCGACGGTAAAATTAAAAACATCAGCGAGGGGGATACTACAATACAAAGGAACTATCTCAAGGGTAATTGCACTCAATGGTCCGTCATTTATAGCGGGTTTCAAACCCCCGTCACCCACCCAGTGGTAAAGGGCGGTGTCCGAAACCAGAATGACAACAGAGCTGAAGCATTCTGTACATCTTACGGGTTCTTTCCAGGGGAAATTAATATTACTTTTATCCATTACGGTAATAAGGCGCCCGATGATAGCGAGCCTCAATGCAATCCGCTACTTCCCACCTTCGATGGGACTTTCCATCAGGGATGTTACGTAGCCATCTTTTGCAATCAAAACTACACCTGCCGCGTTACACACGGTAATTGGACGGTGGAAATCCCCATCAGCGTTACCTCACCTGACGACAGTTCCTCGGGGGAGGTCCCTGATCACCCGACAGCTAACAAACGCTATAACACCATGACCATCAGCAGTGTCCTCCTAGCCCTGCTTTTATGCGCTTTGCTATTCGCGTTCCTGCACTACTTTACCACCTTGAAACAATACCTACGTAACCTGGCCTTTGCGTGGCGCTATCGCAAGGTCCGGTCGTCATGACCAGCAACGCCCTGTATGAGCTGTTTCGACGTCGGTTACCGCGTGCCCCCGTCAACACGGTCATGTTTCTCACGCGACGCACTCGTGATGGGTTCTGCGGTCGGTTGACGTCCATCGCCACGAATTCCCACTACACTATGTTCGTGTTAGATCACGGATCCGTGCGCATCGAGCGACCGAGTCAGTCAGAAGTGGATTGCGCCAGTTTAATGGAAACGCTGAAGCGGATTCGGTTACGAAATTCGTGGGTAGCGTCAGAAGACGAGCTAGATGTGAGTCGCAGGGACGCGTGACACGAAACGCGTTCAGGATTAACGTAGGTTTTCGAAATAACCTACGTCCGTGAGTGACGCGGTTTCGTGTTGAAACCCGCGCCCGCTTCTCACGGCGGTTTATGATGAAACCGGCGTTGCGGATCCACGCGGGTTCCTCATTCAACCTGCGAAAAGAGGAAGTTGCGGTAAAACCACGTCAATAAAGACGTCAATGACACCTCAATGTTGCGTTGGAACGGTCTTTATATATACAAACGCCGTTATGATCAGTGTCCGGCAAGATGCTCGGGATACGGGCTATGCTGGTGATGCTGGATTACTACTGGATACAGTTGATAACGAACAATGGCACTCGAAGCAACAATACCGATACCATCTTTGTATCTCTCCTTACCGGGCCCAACGGAGTTACTCGCACAGCCATCGGAGGTCTGTATTCAAACTACACCAACTTAACTGGAGCATTTGGCTTCACTTCAACAAATATGTCAGCAACCAACTCTTCCGCTGAGGATAATTGGAGCGTAACCAACCTGACGGAGAGTTGCATCAACCGCGGTGAGTCCTATGTGACTACCATCTGGCTTCTGGACTGCACTAAAAACGATACTTATTGGTACTATGGAAATGCCTACAATCATACATGTGAAGGTACAATTTCGGGATATCTCCTGGGCATGTGCAAGCTATGGAAAAGTTGGGTCAATAATATTACTTCTTATAACACTGTCAGAGTCGAATCGCTGGGAAATGAAACACGGTGCATGCTGCTCCCTAGACAGTATACTCTCAACGCCACGGTGGAATGGTACAACAAATCTGAAGGTGACGTACCAGAAGAATTCATGGACTATGTTATCCTGACCCCCTTGGCTGTGCTTACATGCGGACTGCAGGAAGCTTATATACTCGACAAAGGTCGTAGATACATGTATTTGTTTTCCGTGTCCTGCGTGGGAATCACAGGTACCGTATCTATTATACTCGTCTCCCTATCGCTGCTCATCCTCATCTGTTACTATCGCTGTGGCCGGCTTCTGATATGCCCACGCGGCTTTGAACTCTTGCCAGAATTCACTGAGGAAGAGGAGGAAAAAGAAAAATTGTTAACGCATAATGACATTGAAGTCCAAGTGCCTATTCGCACGCGGCGACTACTCGTCCCTTGGATCCGGGAGAGCAAAATGTGGGTACTACCACCCCCGTTGCCTCCACGACCTCCCCACTTAATAGAATTCCCGCCGTCTCCTCCGCCATCGCCTGGGCCCATGCACATGGTGGTCTGCATGCCAGCATGACGAACTTTGGACTCTGAGCCCCAAGCGGTACGAACTACATATTTTCCATAAATCTACACTGAACTTGAGCACAAAGATACTGACAATAGACTGGATATACAGACTTTTATATGATCCCTGTACAGATGTAAATAAAATGCTTTTATTTAAAACTGGTCCCAATGTTCTTCGGGAATCATGGGGTGGGGACGGGGGACGCGGTAGGGAGCAAAACCGGGTACATGGGGGGGAACATCGTCCAGCAGTAGCACCAGCGGATTGGGTAGGGGTTGCTGCGGAGGTCGGTCGATGACGATGTCGATCTCCATCGGCAGATCCGGCAACATCTCTTCATCTCCCTCACCGACCAGCACTCGGCGCTGTTCTGGATGTATATGATTCTGGAAAAGCCTCCGACGAGCTCGCGGCGCGTAGAAAGCCAAGCGGCGCAAGGGCCGGCGAGCCCGAAAGTCCATGCGCACAGATGGCATGAGTCCTTGAGTGACGGTGGTGAGCTGGGGAACAGGGCTACCTCCCATCGCGACGGTGACAGTGGATCCATGAGAGAGGCGCCGCACGCTGCATGGCTAAATACCGTGAATCCCCTGACGTCGTCTTTCGTCCCGAACGCGTCATGTTGGGGGCGAGGCGTAAACCGTCGAGGTTGAAAAACCGCGTATCTGCGACCCGTCCGGACTACGTTGTTTTTTAGAAGCGGCCACATGACCTCGAGATGTCGTCACCCAAGGTATTTAACGGCACACAGCCAGACGCGTTCGTCAGCAGCGACGCCGACAAGACCTCAGCATGGCTCGGAGGCTATGGATCTTGAGCTTACTAGCCGTGACCTTGACGGTGGCTTTGGCGGCACCTTCTCAGAAATCGAAGCGCAGGTAAACGGAATCTGGGGAATTCAACACAGGTAAGAAATACAAAAAAATAACGTGATTGTGAACGCGGTTATCGTGTTTTGCAGCGTGACGGTGGAACAACCCAGTACCAGCACTAACTCCGATGGTAATACCACCCCCAGCAAGAACGTAACTCTCAGTCAGGGGGGGTCCACCACCGACGGAGATGAAGATTACTCCGGGGGAGACTATGACGTTTTGATTACGGATACAGATGGAGGTAACCATCAGCAACCACAAGAGAAGACCGACGAACACAAGGGAGAACACACCAAAGAAAATGAAAAGACCCAGTAGCAGCAGCAGATCCCAAGGGTTAAAGACCATGTTGACTATTTTGTTTTTTATTAAAAAGCTGTAAGGTTTTGCTCTAAAAACACCCCGCCTCCGGTCTTTTTTCTTTTGTATTCGGCACGCGAAACACGGTTTCTTCCCATAGCCTGTCTAACTAGCCTTCCCGTGAGAGTTTATGAACATGTATCTCACCAGAATGCTAGTTTGTAGAGGCTATGCGGGATGCTGCGGCGGCGCGACCTTCCCTCTCCACCCAGCCCCGTCAAAACACACGCGACTCGAGCGGTTCGTATGAAAAATAAAAAACAGCTTTTTATTTACAGGAACGGGGAAAAAAAAAGGCACACGGTCCGTGGGAGACGCGGGTTCACGCGTCGTCAAAAAGTTGGTGGTCCACTCCGTAAGGACAGGTAGGCTTATTTAGCTTCCGCATGCTCCTGGTTCCGTAATAAATGCCGTTTTCGTGGCAGCGTGTCATGCCGCGAGTCACAAACTCCATCAAACTGTCGGCCACGATGCAAACGTGCTGATTGTTGGCAGCAAAGACGCGCATACAGTCGTCCACGAAGAGGTTGATCACGTCGTAGGGGCTCACCAACCAGCCTAAAGGTTCCACGTGGTTACTGCCGACCATGACCCTCCAGTCGTTAATCTCGCTCCAGTCGTACAGCCGAATCGTGGAGACGCGAATGACGCTGTAATCACCCATGACCATGAGTCGGCCGCGATACGTAGCACGCCACTGCGCGAACGCGTGGATGTGCATGCAGCCGGCCAGCGCTCTAAGCGAGGCGGTGTGCGGCAGCTCCTCTGGGACGGTGATGAAGTTGCAGCGTCGCAAACCGATGTTGAGAAATTCAGTGATGCTCTCGGCCACAAAGGTCAACGAGTCAGAGTAGATGTGGTCGGTCCATAGGTACATGGCGCCCGAGGCGCCCAGGTACAGTTCAGACGGCACGTTGTGATCGCCCTTGTGTTTAAGAAAGTTGTAGGTGCAGATGCTGCCGACGAAACGCAGCGGCTCGGGGCAGCAGAGGTAGCTGGCCAGACGCTGTGCATCCCGTCCTTCGTCGCGCACCAAGCGCCAGCGACGCCGGATAACGAGGCAGCGGTCTTTGGGCCAGACCAGGGCCACGCGTTGCCCGGGTTTCCACGGTCGCGACGTCTTAGGAGGCCTCCAGCGGTCGAGCAGATTGAGAAAACAGTCCTTGATTACCGACATCGCGGTCGCGCGTCGGTGGACAAAAAGAAATCGGGCCGATCCGGAAAAAAAAAACGACGGCAAAACACCGCCGTGCTCGAGCGAAGGGTGGCGGAGGGCCAGAAGAGGCGGCCTTGACGGCGTTGGCAGCGAAAAAATTGGCACGCGAGTCAAACGGGAAGTAGCGTCGGTGTTTTATGCCCCAAGCAGCGTCGTCGTCACTCGTGGCGTCACAGTCAACGGTGCTGACGTCCTTTGGGGCAGTCGGGCACGCGATCGTAGATGCCGTTGTGGCCGCTGAAACGTCGGTTTTCAAACAGCAGGTTAAGTCCCAGACACATGAACGTGTTGAGATTATCTCCCACCCGGATGTAGCGGTCGTCGCGCACGTCGCAGGCGTAGACGGCCCCGGTATAGGCGACGACGATGGGGATAAGGTCGACGGGCCAGCGCAAGTGAGGAAAGGGCGCGTTCTCGCCCTTGAGGCTGACGGTTCCCAGGCCGAGAACGCGCATTCCGAAAGCGGTTTTGATGTTGCGCAGCAAGTGACCGCCTTCCACGCTGTTTTCGAAACACCTGAGGTTGCATAGACGCAGTTCCGTTCCCGGCGGGTACGTCAACGGCATGAACTGCCCGTGGTGGCGGATGATGAATCGCGCCATGGTATCCAAACCGAGGCTCCAGGCGCGCAACAGCGGGCGAAAGTAGCGCTTAACCAACGACGAGGTCAGGTAGCGCATGCAGTGCAGGGTCTCGACGGCGCGCAGCCCGACGCGCGCAAACTCCATGAGGTTGCGGGCCAGGTAGTAGACGGCGGTGTCCTCGCGTACATAGCAAAAAACATAGCCCTCGTCCGAGATGAGGCACACAGCGGTCTTCTTCTGCTGATCCGGCGACAACACGCCCTCGTTCACGAAGCGACCCACGAAGGCCAGGCGCGTCTGGCAACACAGGTAGTGACTCCAAGCCTTCACGTCCTCCGGTTTGAAGTCCTCGTCCGTCTCGATCTCCTGCAGCACTAGGTTCCAGCCCGGCGGCCAGACCACGGGCAACACCTGGCCTGCGTTGATGCGCACGTAAGCTTCCAGACAGCCCAGGCCGAACTCGGCCGTGAGCGCCAGGCTAGCCAGATCGCTCATGTGACGCGCCGAGTCAGTGGGCGAGCCCGGGGGCCCGTCGCACACCACGCTCCGTCTTCTTGTCCTCACCGCGGCCAGCGTGGCGAGGACACTTTCCGCGCCCGAGGCTGTATCTTCGGTTTGCCCGCCGGAGCCGGCCCTCACTATATAACGTCCCGCCCGGGTCTCCTCCATGTATGCAGGTAAGCAACTGAGCCGAACGCACCTCAGCAGACGAGAGGATGTCGTCGCGGCGTCGCAGCTCGTCACGTCGCTCTGGCGAACCCTCGACGGTGATTTATATCCCCTCGAGCAACGAGGACACGCCGGCGGATGAGGAGGCGGAGGACAGCGTTTTCACGAGCACGCGGGCGCGCAGCGCCACGGAAGATCTGGATCGCATGGAGGCCGGTTTGTCGCCCTACAGCGTCTCCTCGGACGCTCCGTCGTCCTTCGAGCTCGTGCGCGAGACCGGCGGCACCGGCGCCGCCAAGAAACCGAGCGAAAAGAAACGATCGTCGTCGCGTCGGCAACCGCAGATCGCAGCGGGCGCGCCTCGGGGCTCGCCGGCGACACCCAAGGCCGGCAAGTCGCCTAAAGTCTCGCGACCGCCTAGTGTGCCCTCGCTGCCCGAGAACGGCGCCGGCGGCGGTGGCGACGAT

TCCCGTGGCGCCAGGTGAGCCGTCCGCTGCCGAGGGCGATGAGTTTTCCTTCTGCGACAGCGACATCGAAGACTTTGAGCGCGAATGTTACCGGGTCAGCGTGGCCGACAATCTGGGCTTCGAGCCCAGCGTGGTCGCGCCGCAGCACGTCGAGTATCTCAAATTCGTGCTGCAAGACTTTGACGTGCAGCACCTCCGCCGCCTCAACGAATGCATACCCATGCCGGCCTTCGCGCTCACCAGCCTCGTCGACCCCGTCTTAAACAACGTAGCGCCTGGCGAGCGCGATCTCACGCGTCGGATAATCACGCACGCGGTGATCATCAACTATTACTACGTGGCGCAAAAGAAAGCGCGCCACATGGTGGAGGCCATACGGACCACCGTGCGGGGCGACACGGTACGCCGGGTAGCCGCGCAGGTCAACAACCAGAGCCGTTCGGGGCGTGCGGCCGCGCTAGCGCTTCACTTTCTCACGTCACGAAAAGGAGTGACGGACGGCCAGTACGCCACGTCTCTGCGGCGGCTGGACGAAGAGCTGCGGCATCGCGGCACGCCCGAATCGCCGCGGCTCACCGAGGTCTACCAGACGCTACGCGATTACAACGTGCTCTTCTATACCGCCCACTACACCTCGCGCGGCGCGCTCTATCTCTATCGGCAAAACCTGCAGCGGCTCAACGAGAACCACCGGGGCATGCTCCGGCTGCTTTCGGTCGAAGAGATATGTGAAGAGCACACGCTCAACGATCTGGCGTTCCTAGTAGGCGTCGAGCTTATGATCACGCACTTTCAACGCACCATTCGCGTGCTGCGCTGCTATCTCCAGCACCAGCTGCAGAGCATCTCGGAGCTGTGTTACCTCATCTATGTACAACTGCCGTCGTTGCGCGAAGACTACGCGCAGCTTAGTGACGTGATCTACTGGGCCGTCAGTCAAAACTACGACTACGCGCTCTACGCGAGCACGCCGGCGTTGTTTGACTTTTTACGCGTCGTGCGTCAGCAGGACGCCTTCATTTGCACCGACTACGTGTACTGCGCCCTGCGTCTGCTGGCCTGTCCCGACAGACCTATTATCGGTGACACCGGCGGCAGCAGTAGCTCCCAACGCCTCGTAGGCGAGTTTATGGTGCGCGATCCGCTGTTGCGCGACCCGCGCGCCACCCACCTGCGCCAGAAACTCATCACCCGCGACATATGCGTGGCGCGGTTGCAAGCGCAGCCCTCGAGTCGACACATTCCGGTCGAACACACGGGTGTCTCCTCCGTCACCCTGCTCAAAATCTTTAGCCAGGTCCCCCCCGACGAACGCGAAGAAGACACGTTACGCGAGATGGCTCTTAAAGCGTTTATGGAAGCGAACGGTAATCACCCCGAACAAATCTGCCGATCCCCACCACCCCCGCTGCCACCGCGCGACTATCCTCAACGCGACGAGCGGGACCGTCACCGTCGCGACCGCCGCGACAGCGGGGAATACTGTTGCTGATGGTGGGACGAAACAGCAGGGCGGAACAGTTTATGATAGAAAGTCACAGGAAAGTATGTGTTGTTTTTTTTTTTAATGTACCAAGAATAAAAAGTGCGTCTACGACCAAAGCGGTGTGTGGACGCTCGTCCTCTGTCTTCTCCGGTTTTTTTTTTATGTGTGTGTTTTTCTTTTCCTTCCTATTTTGTTACGGCAACAGCGCTGATGGCACGTTGCCGGCTTCGAACATCGCGTCGGTGATTTCTTGCTTGCCCGGCGTCACACGGTGACGCAGCAGCGCGCGGCTCACGTAGCAGGCCGACTCACGGATGACCTGGCCGTCGGCGTCGCGTCGCAGGCCCGAGCGGTTGCCGTGACGCAGTCTGCCCTGCGCAGCGCGCTCCACGTCTTCAAAGTAGCTGTGTAGCAGGCCGCGCTCCAGCAACTGCGGCAGCGAGTCGGCGGCGCGCACCACAAAGTTCTCACGGCTGATCTCGTAGCACAGCACGCTGCCGTCGGCCGCCACGCCGGCCACGCTGCGGTCCCAACTGAAAAGGTTGGCGAGTCCGATGGTGCCGATGACGCGCAACTGACCCTGGGTCACCACCAGCAGCTTCCAGTATTCTACGTCGCGCGGGGTGAGGATGGTCTCCTCCACGTCGCAGACAAACAACGTGTAGCCGCGCGGATAGGGCAGATCCAGGTGGCGACCGCGCTGGCGGCGCATAAAATCGTCTAAATTCAAACCGCCGTCGGGTGCGCGCCTGCTCGTCATCGCCGCGCCTCGTCGGTCGATGACCCCACGGTGCTTATAACGCGCCGCCGCGGCTTCATGTGGCGTGACCTCCGACCTCGTGAGGCCGAAAACGGCGTACATGAAGACGCTCAAACTTTTGAATGTGGGCCCGGTAGCGCACCGAGGGCCCCGGGGCGGCGACGACGGCGGGTCCGAGTTCCAGCGGGGCCTTGCGGCGGCAGCGGTTGGCGTGGTTGCTCAGCTCGGCGTCCGAGAGCGCCGAGCTGAACTGCGGCAGCCGCGTGCGATCCTGCGGCGCGTCCCCGTGTCGCAGCGAGTGCCAGAGTAGGCGCTGGACGCGCACCGCCTCGGGCGTCGGCGGCGCGCGACAGCCCCGGCGCAGCTTGAAAACGTGCAGGCACAGCAGCTCGCGCTTGATGCGCAGCGACACGCTGCGGTAGTCGGGAATCCGCTGCACCAGCTCGAGAAAGTCGCAGAAGGTCTCCACGAACGTGTCCTCGGTGAAGCGAATGCGCTTCAGATCGTGGACGTGTTTGCGAAACCGCGACAGTTCTCGACGTTGCACGGGGTTCTGAGCGAGTCCCTTGCGCAGCAGCGCAGCCTCGCCTTTAAACAGCCTGATGAGCCGCTGCACGTCCCCGCTCAACATACGTATACACGCCGTGTACTCGTGACGTATACTGGCGCGCAGCAGCCGAATGATACGCAGGGCCAGCACGGCGTTGGAGGCCAGGTACATGGCGTAGCCGCGACGCGGGTTGGCACAGGCCCAGCCCGCGGGGAGCAGAAAGTAGTCGTCGACCAGCGTCTGCGACCAGTCGGCGAAGCCCAGGTCACGTGATACGCTGTCCTGGACGCGGGCCACGTCGCCGGCTGTGAGGTGGCGGATCGCCGGCAGGTGAAACGCGCCCAGGTGTCGATTGCGCTCCAGCCTCAGCTCGGCGTGCTCCAAACGGGAATGGTGGGACGCCACCGCGGAGGGCGACAAAGAGGAGTGGTCGCCGCCGCCGTAGTTACCGTTGTGATTACCGCCGTCGTCGCGCCCGTCGCCGCACTCGCAAAAGGCCGCGTAGAGGTCCTTCAACGCCGCTTCGGCTCGCGCCATAAACGTGGCGTGGAAAAAAACGGCGGCGCGGTGCGTCCGGTACTTGACGGGCAACCCGCGGCACAGGGCCGCCGGCAGGCAGCGGCCGATGAGTTCGCGCTCCTCGGGCTCCAGAAACAGGCACAGGGTGCCGTCCAGGCGCAGGTACAGCTCCTCGGTCATCGAGCATAGCTGCCGCAAGTAATGGGTGCGCGTCCCAAAGGTCTTGTAATCGAGCAACGTGCACACCACGTATTGCCCCGTGGCCACGGCCAGAGCGATGCGTTTGGCGGCGCGACTGATCTCTGGCAAGTACTGCGCCTCGTGCACCAGACGGCGGAAAGCGCCGGCGTTGAGCCAGCGAAAATGCTGCGGATCGGGCGGCAAGGGCACGCCTCGAAGCGCGGCCCAGACAGCGAGGTCCGACTCGAGCGTCAGACCGCGGATGTCGTACTTGCCGTGCGCCGTAGCGCAGGCTGAATGGACCAGACAGCTGCGGCGAATGTACACCATGGCGTGCTTGGGATGTTTGGGCGCCGGCGTTTTCCTTTTCTGACCGCCGGCGGCCGCCAGATCCTCGGGCGTGCGACACAACAGGCCGGCGCGCACAGCCTCCTGTCGATTACGAATCGGCGTCAGGTAGGCGCGCAGGAACTGGTGACAAAACTCCTCATCATCACGACAGTCGTCGAGATACTCGTACGTGGTGAGCGGATCGCGAAATAGGCGCTCGTCACCGTCGTCATGGTCTTCTTTAGCCTGCTCCTCCGGCTGCTGGGTTAGCGGTGGAGGTGGCGGCTGATCCACGGGGTTCATGACTGAGAGGAAGAAGAAGGTGGCGGCGAAGCGACGCGGAGCGACGGCGGTAAAGCCAGACAGCGGCTATATAGCTAGTCATCACAGTCTCCTCCTTCACGACGCCCCCGTGCCGCTCACGCTATCCAGCACGCTACGACCCGAAAACACGTACTCGCTGACGTCGTACGCGGGCGATGTATGGCTGCTCACCGGTTTCGCGGCAACGGTTGCGCTCGAGTCCAACGGCGAGAAGCAAAAACGCCGTGGGCAACGAAACCAGAAGGAGCCCTGACGGATAAAACCGCGCAGCGTCTCGGCCAACTTAACCAGCATCGTACCGTACAGCAGTACGTGAATGCCGCCATGCGCGTCCATAAATACGGCTTTGTTCACGGGTTCCATCCATCCGATGACTACAAAATGGGCCTGTTCTAGCACGCCGATCACGAAATTGTTGGCCTCGTCGGCCTCGGCCACGTTCCACGAGCCGAAAGTGAAAGTACAAGCGGGCGAGCCGCCCAGGCGGATCTTGCTACCGGCGTGGAGCTGACATACGCGCAGCAGATTGGCGCGGTCGTGCAGTATCTGGGAGAGTTCGTACATGCCCGCAAAGGTGTGCTTAAACCACGCGCCCTCTACGATCTCATCCACGTAGTCGCGCTCAAAGAAGCTGTACACGGCAAAGAGGCCGTTCTCAAAAAACTCGCCGAACGAGAGCCCCAGCACGTACACCTTGTCCTCGCCGGGCAGGTACGCAAAGGCGTGCCCGTGCCCGGAGACCCAGATCTCGGGCGCCGTGTTTGCGTCCGGCACGCATTCGTACACACTGACGAGGCCGATAAAGTACAAGCGGCCAGCCTGGCGCAGGCACGAGAAGCGCCGGTAGGTCTTGTGATCGCGCACCACCCCAAAGTACTGAGTGTCGCCCAGCATGATGCCGTGCAGCGGCGGCCAGCACAGCGGGAGCCAACGACCCGCCGTGGCGCGCACGTAGCGCTGCAGGTGAACCCCGCTCGCACGCTCGCGCGGCTTCGGGCGCTTGTGGGTCCAGGCATCACGCAGACCGCGCCAGATGCTGCTGAACTTGGGCTGCCCGCGCAGATAGAGCGACGAGAGCGAGTCAAAGTAGCCCACGACGAGCCTGTCGGGAGACACAAGAGCGCGAAAATCAAACCTAGAGCGACGACGGTGAAAAAACCGACCAGAAGCGCGTGTCTCAAACACGCTACTTTCGGTTATAAAAACACCGTCGCCCTATTTCTGGGCGCGTGTACACTGATGACTCACCTACGCTTTTTGAACGGCAGTCTCAGCTCGGGATTGGCCTCGTACAGCGAGCTGCGGTCCACGGGGCCGATGCTCTCGTAGCGAAAGTCGTCGATGAGCAGCGCCAGCCCCACGCGCACGAAGCCCCTGAGGTCGCGCGACAGCCGCACCAACTTATCCTGCCCCACCAGCGCCGCGTACACGGTGCCCGTGTCGCCGCAGAGAATCCGCACGCGGTGAAAGAAGGTCTTGTCCTCGGCGCCCTCAATTTCGCCCAGCGGCATGACGGGCTCGCGCGTGTACAACGAACGTTGAAAGCGGCGCAGCATCGAGGCCGAGAGCCCCAGATCGCGCGCCGTGCGCAGCACTAGGGAATGCTTCTCGGGCCAGATGAGGGTCAGTTGCGCCTCGCGGTGCGCCTCTACGTAGGCGCAACGAGCGGCGGTGTCCTCGCAGGCCAGCAACTCGCGGAAAGCCAGCAGCGAACGTAGGTAGCGGCCGCGAGCGGAGGCGCGCGAGCGGCGGCACAGCTCGGCCCGATGGTCGGGATGCACCAAGGGCACGTTAGGTTGCAGACGCGCGCAGATGGATTCGTGCACCGGGTCGCAGCGGATCATGCCCTTGGCAAAAAATCCGGCCAGATCCGAGGCCAACTCGTACAGGCAGTCCTCTTGCGCGTCGTAGGCGAACACGGCGCCGTACGCGTCCACGAACACCTGGTACCGGCAGGTGGCGTGCGAGACCGTGCCAATGAGATGCAGAGCTCGGAATTCGCCGAAAAAGTCGTTCTGGCAGTGCTCCAGATCGATCTCAGTCAGCGAGTGCGGCGAATGCTCGCCCCCGACCACGTAGATGCACTGCGAGGGCCAGCCCAGCGACACGCACGAGCCCTCGAAGCGCCGCAAGTAACGCCGCAGGCCCTCATAGTCGCGTCGCACGCACAGGTCGGCCAAGTCGCGCGTGCAAAAGACCTCGGGTACCAAGCAGCGTTTGCGACGCGGCCGACGCGCGTGCCCAGGCA

GCCGAGCAGCCCTTGCGACGGCCGGACATGCCGGCAGTCCGCGACGATCCACAGGAGACAAAAAAGCAGAAGCAGTAGTAGTCTCGGCGACCCGCTCCACCCCGTCCTCCACACGCTCAGCCGCGACTGAGCGCCGGGGCGCGCCGCTACTTGGGTTTTTATAGCCATCTGCCCCCCGTCTCGGGCACCCGGGAGCGATCTACGGAGACCTGACAGCAGTTGGGCAACACAAGACAGGGAAATACAAAGACACTTTTAATAAAAAACGAGACTACTTTGTGTGTGTGCTCCGTAAACTGTTTATTCTCCCCCTCCGTCTCGCTCTGGATGGGCTCCGGGTCCGTCAACACGCGACCCGCGCGGCAAAAGGCACGCTGTTGACGGCGCGAGAGCCCGTCGTGATAGTCCATCATGCCCCGGAGATCGTGCACAAAGCAGCTGTCGCCGCGTAGAAACCGACGCAGCGTCTCCACGTGCTGCAGCTGTCGGCGCGTATCAGGAGCCGTCATCGCCGATGTCGTCATCGCCCTGACAGGCGCGTAGATGGCTCCGCGAGATCATGCGCGTTTTCAACCGCCGTGACACATCAGGTCCATCTTGAGCTGGCGCCGGGCCTCGCGCAGGTCTCGCACGCGTTGTGAGCGGGAGGCGAGTTCGGCTTCTTGCTCGAACTCCTGCTGCTCACTGTCCGAGAGGGTGCGATAAAAGGCGGCAAAGTCCTCCAAGTCGGCTACATGCGCCCTGGGTCTGACGCTCCAAAGCGTACGCAGTCTGATGAAGCGGACCCATCGAGCGTCACGGCACGCCGTCTTGAACGCAGGGCCCGGGAAGAGATTCTTCTCCCCGGCGCGCTCGGGCCGGCGAGGCCGACGCGGTTTATATACACCGTCTCGGACGGCGGGACGCCGAGCCCGCGCCGCGGCCGCTCATCCGGAGACGGCGGAAACCGCGGCGCCGGAGGAAACGGGGACCGGCAACGACGGCGGTGGCGGCGACCAGATTATGGGGGACAAGCCCACGCTTGTGACCCTGTTGACCGTCGCCGTGTCGTCGCCGCCACCGTCGTCGCCGCTGCCGCTCGTCAGCTTCACGGAGCTGCTGTTACCGCCGCCGTCCGTCGCCGCCGCTGCGGTGGCGGCAACAGCGACGAGCGAGGTGGGCGAGAAAACCGCGGAGCAAGAGGTAGCGGCTGCGGGTCCGGAGACCAGGAATGAGAGAAGAGAAAACAGGGAGGACGAAGGAGGGGAGACGAGGACGACGGGCACCACCGCGGTCAAAAGGTCGCACGACGGTATCCCTCGCCAACTGGCAGAGCGCCTGCGGCTGTGCCGCCACATGGACCCCGAGCAGGACTATCGTCTGCCGGCGCAGGACGTGGTGACCTCGTGGATCGAAGCGCTACGCGACGCGGACCGCGACAACTACGGCCGCTGCGTGCGCCACGCCAAGATTCACCGTTCGGCCTCGCACCTGACGGCCTACGAGTCGTACTTGGTGTCCATCACCGAGCAGTACAACACGGCCTCGAACGTGACGGAGAAAGCTTCGTACGTGCAGGGCTGCATCTTTCTCTCGTTTCCCGTCATTTACAACAACACGCAGGGCTGCGGCTACAAGTACGACTGGTCCAACGTGGTGACGCCCAAGGCGGCGTACGCCGAGCTCTTCTTTCTGCTCTGCTCCACCAGCGAGAGCTCCGTGGTGCTGCAACCGCTCATCACCAAGGGCGGGCTCTGCTCGTCCATGGCGGTTTACGACGAGGAAACCATGCGGCAGTCGCAGGCGGTGCAGATCGGTTTTCTGCACACACAACTGGTCATGGTGCCCTTCGTGCCGCACGCCTGCCCGCATTACGCCGTGCCTTTCACGACGCCGGGAAAGCCGGGCTGCGGCGGTGCTCCGAGCGGCGTTGCGGGGTTGGAGGAGACGGCGCCCTTTGGACGGGTCAGCGTCACGCGGCATGGCGCGACGCTGCTGTGTCGCGTGGACCATCTGACCTGGATCAGTAAACGCGTAACCACGTACGGACACAAAAAAATTACGCGCTACCTCGCGCAGTTCCGCGGCACGATGGACGACGACGAGGCGGCGCTACCCGGCGAGGACGAGGCGTGGATCGCGTCCAAAAACGTACAGTACGAATTCATGGGTCTCATTTTCACCGTCAACGTGGATTCACTATGCGTGGACGCGGAACAGCGCCAACTGCTGGGCACCGTGGCCACCTCCTTCTGTCACCGCGTCTCGGACAAGATCACGGCGCGCAACATGCCGCGCGCCTTTTCCTTCTACCTGCTGACGAGCGCGCAGCGCGGGTACGACCTGCGATTTAGCCGCAACCCGTCACTCTTTTTTAGCGGCGACGCGCTCAACTGTCCGCTTCTCAACGAGCCCAACGTGTTTTCGCTCACGGTGCACGCGCCTTACGATATCCACTTCGGAGTGCAACCGCGGCAGACGGTGGAGTTGGACTTGCGCTACGTGCAGATCACAGACCGGTGTTTCTTGGTGGCCAACTTGCCACACGAGGACGCCTTTTACACGGGGCTCAGCGTGTGGCGCGGCGGCGAGCCGCTCAAAGTCACGCTGTGGACGCGCACGCGTTCCATCGTGATCCCGCAGGGCACCCCCATCGCCACGTTGTATCAAATCACCGAGGGCGACGGTAACGTGTACTCGTACAATCACCACACGGTGTTTCGGCAGATGCACGCCGCCGGAGCAACCACGTTCTTTCTGGGCGACATGCAATTGCCCGCGGACAACTTTCTCACGTCTCCCCATCCCTGACCCTCCGTCCGTCCTCCTTTCCCGACACGTCACTATCCGATGATTTCATTAAAAAGTACGTCTGCGTGTGTGTTTCTTAACTATTCCTCCGTGTTCTTAATCTTCTCGATCTTTTGGAGGATGTTCTGCACGGCGTCCGACGGCGTTTTGGCGCCCCCCATGCCGGCAGAACCCGGTTGCGGCCCCGTACCGCTCTTCTGGGGCGACGATAAGTCGAAAGCCACCGTTTTCATGCCCGTCGTGCTCTTGACGGGGGAACCTACGGCGGCGATCCCCGTCGAGCGGCGTGATTGCAAAGCCGCGCTCGCCCCCGGTTTCAGGATGGAGGGGGAGGCCACAGGCGGCGCATTCGATACGCTGCTTTTGGCCGTAGACGACGGTGGGTAAACGGTGGTTACCGCGGGATACGTCGGCGTGGTCGAGGCGGCCCGGCTGCTGCCGGACAGGCGACCCGGCGCGCTACCGCTCACGGGGACCGAGGGCGGTCGACCTACCACCGCCTTGCCGCCCAAAGTAGGTTTCAAGGAAGGAACACCGACGCGGCTGCCCCGGCCTTTCACCGGAGACGGGGGGGCACTCTTGGCCGGGGACGGAGAGGCTGACGAAAGCATGGACAGCGGCGATGTGGCAGGGGACACGACATCATCCTCCGTGGGCGACAAAACGGACGCCGAGGCTGACGGCTGTCGAGCCGAAGAAGCGGAAGAGGTTCCCGCGCCAGAAGTCACGTTCCTTGATGACGTCGTTTTAGACGAAGCCGGTTGAGGTTGCAACAGCGTGGCGGGTACCGTCGACGGCGTGCCCGACACCTGTTTCTCTAGCCTTCCCTGAACCGGCGTCGACGTCACCGTCTGCGCTCGGGCGGACGCGTGCGGCGTCGCGACTCGCTTGCCCAGCACCGGTTTCTGGCTCGTGGATGTCGTCGTCATTGGAGACGATAACTTAGCTTTACGTATTCTGGACGGCGTCGACTGCTCGGGCGTCTGACTGGGAGGCGAAATGACGTCGTTGTTGTAATCGGACGACGGTGTTGTGTGTCCCAGGCTGACGACGGAGCCGGTGTCCGAGGAGTCGTCGTCTTCCTCCTCGCTGTCTTCGACCGGTGACTCTGCAGTTTGGTCCCTTAAAGCCCAAACCTCATCAGCGGCGTCCCGAGACGCTGTTTGTGTCACCGCGGCGCGTGGAGTCGACGGCCTCCGAGGGGTGGTGGACACGGTGTTTTGAGAAGCCGTGGAAGTCGTAGGCATCCTGAAGGGATTGTAAGCCAGGTGAGGATTCTTGAGGGCCCACGCGCGTTCGCGCGGCCAGTTGGCGGGGTTCATATCCCCGGGCAACGGCGCCGTCGGAGCCCAGGGCGAGTTACCGTTGACCGGGGTTTGGGTACCCGCGAAGGTAGGTGTCGGGGCCGGAGCGGGGGCCGTGGAAGGATTGACAGGCGTCGGCGTGAGGATGGCAGCGCCGGCGCCAGCAGGAACGTTAACTCCGGCGCCGAACGTCAACGTCGGTTGCTCGAACTTGTACGCGGTGGTGACGGGCGGTTTGGCGCTCGTCTCGGTATCCGTGATGTCCACCAGCGTGTCGGTGAAACGCGGATCTTGACGGTTGGGGGGATAGCCATCCGAGCTGTCGGAATCCTCGTCGCCCGAGAAAAGATCCCCTCTGGTCTCCGTGAGCGGCCTCACGTCCCACGCGCTGTCCCGACGGACCCTTCCCGGGCTGGCCTTGGTCACCTGCGGGGAAACGAGACTGAAAGCCGCGTGACGCTGTTGTTGCTGCGGGATGTTCAAGGGACCGCTGGTCGGTTTCTGACTGCCCGAGGATAACAGGCCGCTGAAAATGCTGGAAACACCGCCACCACTAGCGGCGCCCTTGCCGCTAGTTCCCGGTTTCTTGATGGGCGTAAAGATGTTTTTCTCGTCATCATCATCGTCGTCGTCGTCCTCATCGGCACTGGAGCCAAAGAGCCTCCGGGAGGCGCCCGGTTTACGTGTCGGGGGCGGCGGTTGCTGCTGACGTTGCTGCAGGTTCTGCTGCCTCTCCTCCCAAGCCTTCAGCTGCTGTTTCTCACGCTGCACCACCTCGTCGTCCACCCGTTTCTGCCGCTCGCGACGCTTTTCCTCTTCGTCGTAATAGCCGACGGCCGCCGAACGGGCAGCGTGGGCTTCGGCGGCCGGTGCCAGAGAACCATGGGCCTCGAAGCGGAACGGTTTGTGTCCCTTCCAGGGACTGGCGATCCAGCTCCAGCCGTCCAGCGGCTGCGTGGGGACATGTTTCTTGGGTACCGACGAGAAGGCTGAACCGCCGCCGAGCGAGAGGAGATTGGCGTCATCATCAAACTCCAACGACGGCGAGCGCGCGCCCAAAAAGGTGTGCGCCGACTGCGGGAAGCTGTCCACGTAGATGTCAAAGTCCTCGATGAGCAGCTCCAGCAGCGTGTCGGCCGAGTCGCCGTTTTCCACGGCGTGCTTGAGGATATTGCGACAGTAGTTGGAATCAAAGGAAAGGCACATGCGCAGCTCCTTGACCAGCAGCTTGCAACGCTCCTGAATGCGCGCCAGACATTTGCGCTCCAGCTCCTCCCAAGACCTGCGCACGTTCATGATGAGACGGCCCGTGTACACGAGCTTGTTGACGGCGTTGACCAGCGCCGTGTTGGCGTGCCGGTCCAGGTTAAGGTCGAGCGGTTTCGCACAGAACATGTTACGGCGCACACCCTCCAGGTTTTCTTCAATGCGCTGCACCTCCGTATCCTTGAGGTGCACAAAGGCGATGGGTTCCGTCTGGCCGATGGCTGTGACCAGCGTCTCGCGCACCGACATCTTGGCCAGAATGACCGCGCTTACGAGCGCGCGCTCCACGATCTCGGCATCGTGGCGCACGTCCGTATCGAATTCGGTACGGTCTAGCACAGCCAGGTGGTCACGCGCCTTACCACGATCACCGAACGGGTAAGTGTAGCCGCGACGCGCCACGGCCGCGCAACGCACCTCGAACTCCTCGAGCACCGAGGAAAGGTCGGGGTTGTGGAAACGCAGCTCGCGGTAGTATCCCAACCAAAGCATGAGCTCGTTGAACAGCACCGTACGCCGGTGCAGGCGTTTTTCGCCGCATTTTTTCAGGATCTTGGGGTGTGCCTCGAGATCCACGTCGGGCTTTTGCGTGAGATGGCGCAGAAAGTTGACCAGGGCTACCACATCGCGCCGCTGTAGACCGATAAACTGCAAACTCATGCTGGCTTTTCTCCAGAACCCGGAAGCGTCGTCGCCCCGGACTGCGCCCGCGGTCTGCTATTCGTCCACGATGGACACCATCATCCACAACACCACGGTGAGCGCCCCACCTAGAGGGAGGGGGGGTAGTTTAATAGCGGAGGCGGATACGCGGTTTTCACCCGGGCGCCGCTGACTTGTTTCTTCTGTTTTTTACTCCGTATGCTGTTCCGCCCAGAACCGCAGTACCGATACTCCGCATGTCAACATCACCTGTAACATAACGGAGCCGCTATCCGCCATCCGTACCACAGAAGCCGTGATCAACACGTTCATCATTTTCGTAGGCGGCCCGCTCAATGCCATAGTGTTGATCACGCAGCTGCTCACGAATCGCGTATTGGGCTATTCAACCCCCACCATCTACATGACCAACCTCTATTCCACCAACTTCCTCACGCTCACCGTACTGCCATTTATCGTGCTGAGCAACCAGTGGCTACTACCCGCTAGCGTGGCTTCGTGCAAATTCTTGTCGGTGATTTACTACTCGAGCTGCACAGTAGGCTTTGCCACCGTAGCCCTGATCGCCGCCGACCGATACCGCGTTCTTCATAAGCGTACCTACGCGCGGCAGTCGTACCGCTCCACCTATATAATTTTGCTATTGACCTGGTTTGCCGGGCTGATCTTTTCCATGCCCGCGGCTGTCTACACTACAGTAGTGATACATAATGGTACAAATGGACAAAGTAGCAATGGACACGCTACCTGCGTATTGTACTTCATAGCCGACGAGGTGTACACGGTACTACTCTCGTGGAAAGTGCTGCTGACGCTAGTGTGGGGCGCCGCGCCCGTTATCATGATGACGTGGTTTTACGCCTTCTTCTACTCAACCGTACAGCGTGCGTCTCAAAAACAAAGGAGTCGCACTTTAACCTTCGTCAGCGTGTTACTCATCTCCTTCGTGGCGCTACAGACGCCTTACGTGTCCATCATGATTTTCAACAGTTACGCCACGGCCGCATGGCCCATGGACTGCGAACACCTGACACTGCGACGCACCATTGGCACGCTGTCACGCCTGGTACCCCACCTACACTGCCTCATCAATCCCATTCTGTACGCGCTGCTGGGTCATGACTTTCTGCAGCGCATGCGGCAGTGTTTCCGCGGCCAGTTGCTGGACCGCCGCGCTTTCCTGAGATCGCAGCAGAATCAGCGAGCTACAGCGGAGACAAATCTAGCGGCTGGCAACAATTCACAATCAGTTGCTACGTCATTAGACACCAGTAGCAAAAACTGCAATCAGCACGCCAAACGCAGCGTGTCTTTCAATTTTCCCAGCGGTACGTGGAAAGGCGGCCAGAAAACCGCGTCCAACGACACATCCACAAAAATCCCCCATCGACTCTCACAATCGCATCATAACCTCAGCGGGGTATGAGCTTTCCTGTTACTTTATTCAGAAAGCACCAGAACCCGTCGCCATTTCCCCTCATATACGGTACACGTCCCCCTGATCTGTCATCACGGTACACAGATTTCGCCCGACTGCGGACGCCGACGGCCAATCGCGTGGCGTAGGAGTGGCGCCCCGGCTTCATTATAACGCCACGTCGGAGCCCCTGCGCGCCACAACGCCGTCCGGCGCAACTTCTGTCTCGGCACGGTACGATAAAAACGACGTCCCCCGTCGACGTTGTTTTCTCCGAGCGGTGATCGTTCCCGTCCCTCTCCTCCCTCCGCGGCCCCCACGGCGGCGGCCTGCTCGCACGGACCTATACTATTACCGCCCCACCGCCGTCGTCGTCATGAACTTCATCATCACCACCCGAGACTTCTCCAACGACGATTCAGTCCTGCGAGCCGCCGAGATGCGTGACAACGTGGCAGGCTCGATTTCCAAAGCGTACAAGGGCACGGTACGCGCCGAAGGCAAGAAGAAGCTGCTGCTGAAGCACTTGCCCGTGCCGCCCGGCGGCTGCTCGCGCCGCAACAGCAACCTCTTCGTTTTCTGCACCGAGCGCGACTACCGCAAGTTCCACCAGGGCATCGCACAGCTCAAGCGCGCGCCGGCCGAACTGGACCCCCACGAGATCCAGCAAGTCACGGCCAGTATCCGCTGCCGCCTGCAGCCCAGTCTCCGCGAGCCGCCCACGCCGGCCGACGAGCTGCAGACGGCTGTGTCGCGCGTGTGCGCGCTCTTCAACCAGCTGGTTTTCACGGCCCAGCTGCGCCACTACTGCGAGCACCAGGACAAGGTGGTGAGCTACGCGCGCGACGAGTTGACCAAACGCTGCGGCGAAAAATCGGCGCTGGGCGTAGAGGTGCATCAACTGGTAGCCTTGCTGCCACACGAGCGCCACCGCGAACTGTGCCACGTCCTCATCGGCTTGTTGCACCAGACGCCGCACATGTGGGCGCGCTCCATCCGTCTCATCGGACACCTGCGCCACTACCTGCAGAACAGCTTCCTACACCTGTTGATGAACTCAGGTTTGGATATCGCACAAGTCTTCGACGGCTGTTACCACAGCGAGGCCTACCGCATGCTCTTCCAGATCGGTCATACGGACTCGGTGTCGGCGGCCCTGGAACTCTCACACAGCGCGGCGGCCGGGCCGCCCGAGGCCGATGAGAACAACGACGAGGGAGAGGAGGACGACGACGAGCTCCGTCACAGCGACCCGGCGCCGCTTCACGATTCCAAGAAGCCCCGCAACGCCCGTCGTCCCCGCACACGCGTGCCGCCTCACGAGCAAAAGCCCGAAGAAAACGAGGAGGAAGAAGAGGAGCTGTTTCCCTCCTGCAAGGCAACCGCAGCATTCCTGCGGGCAGAACCCTCCGTCTCCAACGACGACGGCAACGGTGGCGAACGCTGCGACACGCTAGCGACCGCCCTGCGGCATCGCGCCGACGAAGAAGACGGACCTCTAGCCAGCCAGACCTCTGTGCGAGTCGCCGCGACCCCCTCACCTTCAGTCACCTCAGCCCTTACCCCCGTCACGTCCCCCATAACCCCGTTGTGTATTTAACGTCACTGGAGAACAATAAAGCGTTGATTTCTCAAGTTCCGCTCTGGTTTTGGTTTCGTTTTCAAAGGGGGCCCCATCATGGCCCAGGGATCGCGAGCCCCATCGGGCCCGCCACTGCCCGTTCTCCCCGTGGACGACTGGCTCAACTTTCGGGTTGACCTGTTTGGAGACGAGCACCGGCGCCTGCTGCTCGAGATGTTGACCCAGGGCTGCTCCAACTTTGTGGGGCTGCTCAACTTTGGCGTGCCCAGCCCCGTATACGCACTCGAGGCCTTGGTGGACTTCCAGGTGCGCAACGCTTTTATGAAGGTGAAGCCCGTGGCCCAGGAGATTATCCGTATCTGCATTCTCGCTAACCACTACCGCAACAGCCGCGACGTATTGCGGGACCTGCGCACGCAGCTCGACGTGCTGTACTCGGAGCCGCTTAAGACGCGGCTGCTTAGAGGGCTCATCCGACTCTGCCGCGCTGCGCAAACCGGCGTTAAGCCCGAGGACATCAGCGTGCACCTGGGCGCCGACGATGTAACATTCGGCGTGCTAAAACGAGCGCTGGTCCGGCTGCACCGGGTACGCGACGCGCTGGGGCTGCGCGCGTCTCCCGAGGCCGAGGCGCGCTATCCGCGCCTCACCACCTACAACCTGCTGTTCCACCCACCGCCCTTCACCACGGTCGAGGCGGTGGATCTGTGCGCCGAGAACCTGTCCGACGTAACACAACGTCGCAACCGACCGTTGCGCTGCCTCACCTCCATCAAACGCCCGGGCTCACGCACCCTGGAGGACGCGCTAAACGACATGTATCTGTTGTTGACACTGCGACACTTGCAGCTGCGACACGCGCTGGAGCTACAAATGATGCAGGACTGGGTGGTGGAACGCTGCAACCGTCTTTGCGACGCGCTTTACTTTTGTTACACGCAAGCCCCCGAAACGCGGCAGACTTTCGTCACGCTGGTGCGTGGGCTGGAACTTGCGCGGCAACACAGCAGTCCGGCCTTCCAGCCGATGCTGTACAATCTGTTGCAACTACTGACGCAGCTGCACGAGGCCAACGTGTATCTCTGCCCGGGATATTTACATTTCAGCGCGTACAAGCTGCTGAAAAAGATCCAATCGGTCTCGGACGCCCGCGAGCGCGGCGAGTTCGGGGACGAGGACGAAGAGCAGGAGAACGACGGCGAGCCGCGCGAGGCCCAGCTCGATCTCGAAGCCGATCCCACGGCGCGCGAAGGCGAGCTCTTTTTCTTCTCCAAGAACCTGTACGGCAACGGCGAGGTTTTCCGCGTGCCCGAGCAACCCAGCCGCTACCTGCGTCGACGTATGTTCGTGGAACGGCCCGAAACCCTGCAGATCTTCTATAACTTCCACGAAGGCAAGATCACCACCGAGACGTATCACCTCCAGCGCATCTATAGCATGATGATCGAGGGCGCCTCTCGGCAGACGGGCCTGACACCCAAGCGCTTCATGGAACTCCTCGACAGAGCGCCTCTGGGCCAGGAGTCGGAACCCGAGATCACAGAACATCGCGATTTATTTGCCGATGTTTTTCGCCGTCCTGTGACCGACGCGGCTTCTTCGTCGTCCGCGTCTTCGTCGTCGTCCTCAGCATCTCCGAATTCTGTTTCGCTGCCGTCTGCCAGGTCGTCATCCACACGAACCACCACGCCCGCGTCCACGTACACCTCGGCCGGGACTTCTTCTACCACGGGTCTCTTACTCTCCTCTTCTTCTTTGTCGGGGTCGCACGGCATTAGCTCCGCGGACCTGGAGCAGCCGCCCCGGCAACGACGCCGCATGGTCAGCGTGACCCTCTTTTCGCCCTACTCGGTAGCCTACAGCCACCACCGACGTCACCGAAGGCGACGCAGCCCGCCACCCGCACCCCGAGGGCCGGCCCACACACGCTTCCAGGGACCCGACAGCATGCCGAGCACTAGCTACGGCAGCGACGTCGAAGACCCGCGGGACGATCTGGCCGAAAACCTACGGCATCTCTGAACGCGGTTTTTCCTCTTTTTCTACGTGTCTGTCTCAGGACGAGACGTCGATATCAATAAAAATACCGTCGACGTGGTTTTCTAACAGCGTGGTTTTCTTTATTGACCAGCGGAGTACACAGTTTACGAGTAAAAAAGACAGGGAAAGGTTATATAAAATGCTGTGTTATATACAAAAACATGCACATAGACAGACAGAACCACCGTGCTCGTCGTCCCCTCCTTAATCAGTTGTTCATGTAGGCGTGTGGCGGGGTGAGGGGCGGCATGCCGTTGGCGGCGCCGGGAATAATGTGCCGTCGACCGACGTCGCACACCTTGAAACGCCGTCGGCGCACGCAGCGGTCGCAGGACGGGATATCCCAGAGGAAGCCCATGTAGGTCTCGGGGTCCTCGTCGTGAAAGCGGTAAGAGAGTTCAAAGTGGTGCAACGAGCCCGTCCGAGCTCGCAGCTTCTGGCGAACACCCTCCACGTCATCGGTGCACAGCGACAGTGCTGGGCTGTCACACAGGGCCTGAAGCTCCTGCGGCCACAGGTGCGTAGCCAGGGGCGAGTCCGTCGTCACCAGTTTGACGCAGTGCATCAGGTTCTCGGTGATGGCGTCGTACAGGCGACTCTCGGCCTCCTCGTGCGTCATCACGTTTCGAGGCAGCGACAGCTCGTCGTCGTCATCCTCGTCAAACATGATCATGGGGTCAGGGGTTTTTTTGGGATGTTGACAGGTGGGTGTCTTTTCCAGACGCACGATGGCCTCACGCCGGCCGCTGAAACGGTGGTTTCGGTGTCCCTTCTTTCCCATGACGCAGGTGAACATAACCACGTCCTCGGCCAGACGGTAGACGGCGTCCATGGCGGGGTCGTAGCCGTAGACGACGCCGAAAGTGTCCACCAAGACGTACTGGCGTACGAGGAACTCTTTGCGTTCTGGCACCTCGTGGCCCAGCGCGCCCAACAACTGGTGGTAACAGGTGATGCGCGGCACGGTACGGATCATGAGCTCCATGGTCTGGATGCTGCCGCCCGCGCGGACGACGCTGAAAGATGTTTCCTTGAACTTCATAACCTCTGTGTTGTGGGTCCAGAAGGCGAAATGGGTGTCGGGACACTCATCGAAAGGGTCGTCGATGCTGTAGGAAGCGTAGCCCCGCTTGGTCACCTCGGCCGACAGGCTCTCCACGTCGCCGCGGTAGAGCATAACGGCGTTCCAGTAGTCGTCATACTGCACCATGGGCCGCTGGTAGTCGCGCATAGTGTGGAAGTGGTCGCAGTGACGAAAGCCATGCCGCAGAAAATCCTTCATGGTGGGCGCCAGCTCGTAGATGCAGTCGCGCAGGTCATCGTAGCAGTAGATGCCGCCGCGCTGCCCGATGAGCACGATGAGTTGGTAGCGCATAAAGCCCGGACCCTCGACGAAGCCAAAGGGGTGCAGGTACTCCTGACAGCAGACGTAAGCACCTGGTAGAGAATAGAAAAAATCCACGCACGTTGAAAACACCTGGAAAGAACGTGCCCGAGCGAACGTCCTCTTTCCAGGTGTCTTCAACGACGTGGGGCTTACCTTGCGAACAGACGGTGCCCATCTTGCCCACGAAGGGCCCCAGGGCGCTGCGCGAACGGAGCTGGATGAAGCAGCGTTCGGGCCAGGCCACGTGCAGCCGGGTGCCGCATTCCTGCTCCAGAAAGTCGTTGAGACCGTTGAAGTCCCCGGCTCGGATGGCGATGCAGCCGTAGGCCATCAGCGTGTCCCGTAGGTCGTCCATGACGGACTCCTCTACCTTCGCTCGCCGACGCTGCGCTTCTCCAGCCACCGCTGCGGTCGACAGACTCCTCCGTCCGCCTTCGGAGAACTACGGCGCGGCGGCACGGCCTTTATAGACACTATCAGCGTTGACGTCAGACGATCCAATGAACGTCGTTTTTTGTGCTGGAACTTCCCTCGTCCCGACAAATGTAGCGGAAATCTTCAAGCAAATCGCGACGAAGTCCGATGAGGAGGATGCAGAAGAGGCTGAGCAACGCGATGCTGCCCGCCGCCACAGTACATATGCTCAACAACGCCCAGTGTCCCAACGCGCGACTTTTGGCTCGGAGCAGAGCCGAACGGCGGTTTTTCCACATGATGGATAACGTAGTCCAATATTTCCACCCCTGGCATTGTTGTAGTGTCCACTCGGGAAGCGACGTCACGTTAGTTCCCGTAAAAGTTGTGTTTCTTCTGTTGTTTTTCGCAAGCTTAACGCTCCTCTTAAGAAACCGCGGACACATGTCTTGTAGAAAAATGTAATCACTTTCCGCATATACCGTTAAGACTGACATCACGGTGACGGTGTTGTTTTCCGAAGACGTCGCGTTGTCGGTGACGTTGGTTTCCTCCCAATTTACGGATGACTCAAACGGACTCGTGCGCGCCAGTGCCCGTAATACGTAACTGCGGCCGGTAAGGTTAAGCGTCAGCTGTCCCATGGTAATGTTAGTGTCATTTGTAAAACACGCAACCTCCCCGTAGACCTCGGTGACGTTGATATCGCGGCTCTCGTTCAGGATGCGCAGGGGAAACCAGCCTTCCAGGTGGTACTGAAAACCAAACGTGAGCATGACGCTGTGCCACTGCCGACGTGATTGCCGAAACGTTACGTTCAAGGGCAGTTTGGATTCGGCTCCGGCGCAGGGGCCGTTGTAGATTTGCGTATGATTGCACGTGCAGTTTAACCGGCAGTTCATACTCGTGGCGTTGGAAGTGACGTTAATGTCCGTACCGTGGTACGTACATCGAACAGAAACACCGTATCCCGTGCTCCAAAACAGCGTCAACAACAGCCACACAGACACCTACGTGGGGACGACACGGGACTTTTTATTGACGGAGACTCACGTTTCTGCCCTCCCCTTTCCCGTAGGTAAAAACCCACGTTTATCACACACGTTGTTTTTACCTGAAACCCGCGCAGCCCGTGGACGCGACAAAAAACCGCGGCACTAGAAAGAAAATGAAACAAGTATGTTTATTAAGCAGCATGTGGGGCTAATAGGGGGGATAACTGAGGTATAGCAACTATGAAAAAATACTACAAAAAAAAAGCTGAACATGGTCATCTAGCAGCAAAGTTCTCCTTCTAGACCACGACCACCATCTGTACCACGTCGCCCTCCCCGGCCGTGTACATCACATCCTTCACCACGACCGGCGGCAACGGCGGCGACGAGGACAACTCGCTCTCGACGGAGGCCGGGACGACAGA

GGTCTTCTTCCGACACGGGCGACGGCAGGCTCGGCGGCGCGGACAGCACCCGTTGCGCCGGAGCGTGAGAAGGCTGAGCCCCGGTGGCCTGGATGTGGGCCAACGAATTGGCTCGCAGCGAGTCGCGATCCACGAAGGTCATAGGAATTTTCCCTTCGCGGATTCGCCGCTCAGATTCCAGGATGGCGCGCACGTAGCTGTTCACCGACTTGGCAAAAGTGCGCGGCCCCTCCGTATTCTTGTCGCGACGCGCTTCCAGCACCTGCTTTTCGTAGTCCAGCTGGTGGAAGACCATCACCAGGTCGTCCATAGTGTGCGCGTGCTGACGGACGTGGGAGCGCACCTCCACCGGGAACAAAGCGTTCCAATACTCCAGCACTATGGCACCGTGCCAGAACTGCGCCATGCTGGGCGCCAGGAAAAACAGGATACCGGAGTCGTAGGCGAACACGTCCCACTTGGGCGTCATGAACAACACCAGCTGACGCGTGGGCCGCACCGAAGCTTCCTCCCAGGCCTCGATGACCCCGAACATGATGAGCTCCTGGTCCAACGGGGGGCAGTGTCGCTCCAGCCAACTGATCTTGCTCAGGTTCATCTGCAGAAACTCGTAAGAGGGGTCGCAGATGCACACGTAGAGACCCGAGTCGTGCCGCAGCCTGGCTCCGCGCTTCATCAGTTTCCTCACCGCGTAGCGAAGCGCCACCTTGCCCAACGCCGACGCCTGGATCAGTCCCCCCACGTCCATCTGCGTCTGTCGCCACTCGGCCTCGTCCAGCAGGCTCATGATAGCGGCGGTGCTATGCGTGGTCGTAGTCATCCTTTCTATCCTTCTCTATGAATAGCAGCAATAGCGGTAAAGTCCCTTCTTATACTATCCCGGAGTCTGTGGTTTTTTTGTTTACCCCTGCTTACTGGTGAGACTGCTGGGGGCCGTTGTGCTGCAGCAGCCGAGCTCGTCGCCGCCGTTGCCACAGGAACCGGTGCCTCCGCAGGGCCTTTTTGAGAGCCTCGCAGGCTTCTCGCGCAAGTCCTGAGAGGCCCTCGGCGTCCATGGGGTTCACCTCGGGCGTCCGAGCCTCGTTTTCTTCTTCTTCATCCTCCCTTTCCTCCTCCGTGTCCTCCCGCTCTGTGTCCTCCGTTACGCTCTCCTCCCCGGCCTCGGCCAAGAGCGCGGCCACCAAGTCCACGGACCGCTCGGTCTCCGAGTTCTCACCGTCAATTACGCCATGTTGGCGGCGTAACCGGTGCCGAGAACGCCGGGTGAGCGCACATGCTTTTTTCTTTCTTAACCAAGGCGGGAGAGGATCTTCAAGGCGTTTTCGCTGGATCCAGCGGTAGCTAAAGTACCAAAAGGCCAGCAGGCCCACGCTACCTAACAGATTCACGTAGACTGGAGACATAATTAAAGAAAGAAGTGAAACCCGCGTGTGGGTCTCACGTCGTCTTGAAACACCGTCTTATATACATGAAGATGCCGGACATGACGCGCCCAAGACACGTGGGGTTTTCCCCTTAGGCGACCCGGTTTCTTAAGATGTTTTTCATCTTCGCACGCGATGTACTACATCAAAGGGTCGGCTGACCGACCGCATTGACGCACAGTTTCCGAGTACGCGCGTCTCGGAGCACCTGACGGTGAACCACCCAACTCACGCGGATAGGGGACAACACTGACGTGAGGGGCGATTCACGTCACTGACGGCTGACGGGAATAAGACGGGTGAGGGATTTCCACATTTTTCTTAAGTGTGACTCTCCTTACGGTAAATCGCACCTGTGACTTCTTAACCCCTCCTCCCTGGTACCCGATAACAGTGAAAAACACACACCACACGTCACGACACCGATCGATTTTCTTTATTCTTAGTGTGATGATAGGTAAGGGCACTCGTGAGGATGTGCAGTTATCATTATCAAGCCTTCTTCAAGGCGTAGTGATGATCGTTGGGCAGAACCCCCAGGCTCCTAGCGATCTGGGAATAGAAGGAGGAGAACGACCCCAAGGCCAGAATGCCCACAGTGTACATGGCCCAGGTCTCCAGACCGAACGTGGCGGGTCGCAGCTTCAGATGGTAGGCCACCCGCTCCGAGAGTTGTGAATGCTCGTTCAGGCAACAGGACTGCAGGTGGGTGAGCCCAAAAGCGCTTTCGTTTACGCCGCGCACGTGCACCGTCTGGGCCGGGCAATCCTGGTGTTGCGCGCGAAAATGGTCCTGACAGGAGATTCCGTCTACGTGGCGACGCGTGTTGTTACCCACTTCGATCAGCAACGTGTTATCGGCAGGATGATGCGAGAACGCGACGACGGTGTTGCTGGAGGTCTGGCGGCAGCAGTACACGTCGAGCGTCATGAGGACCATGTCGCCTTGGTGGTACACGGCGTACGCCCAACCCTGGAACACGAGCGGACATAGCGGACCGTGAGCGGACGTCACGCCGGCGGTTGTTACCGTCGTCTCGACAGGAGAAGACAATAAACTCCTGATCCTCATACACAGGAGTCCAACCGTCAGAATTAAAGTCCGCGGAGCCATAACCGCGCAAGTGAAGCCGATACGAGTGTTGCTGAATTTGTTCATTCTGCCGACTGTTGCTCACGAGCGTTCGGAGGCGGTGCCACAGGCTGTTGGCCATTAAAAAGTCCTGGCCCGAATGACGACGAGACAGAGCCCGAGGCGAAGAAAAAGGCGCCCGTCATGAAGACGTAGGCAGGGGAATTCCCATATTTTTATGGCTTCTTTTAAAAGTCTGTATCCGACTCCATCCGGCGCTTTTCCCAAACCGTGGTCTCCTCGTCGTCCGACTCGGTACCCAGGAGGTGGTAAGTCTTTTGCCGCACGTAGAAAGCTTTCAACGTGGAGCAAAAGATGAGAATAAAGACCCCGAAAACGAAACAAACCACGCCGATCATGCCGATGCAGACGTTCATGTCGACGTAGCCGGCGGTGCTGTTGGCGGTGCGGCAAAAGAGTGTCATGTCGTGCGTGCACAAAAAACAACACACACCACAGGCCAGGTCGTAGCGTAGTTATTATTCCGTAGCAGCAATGATGGTACAGTCAAGCACATGCTCTATCCCCGTTACCCCGATGATGCTTGCGTCCCCGTTGTTATATTGGCACTGTCCCGGTTAATCACCACGGTGAACACCACGGCCAAGAAAATGATCCCTAATATAGCGACCACTAAGAGAGCAAAAGTCCATTTCCAGCCGTTGTCAAAGTACGCCCCCGTGGTGGGATGCATGGTGGCGGGCATTTCCATCATATCCATGTCGAACGTGTGTCGCGGCGACGGCGAACTAACCAGGCAGTACGGGGGTCGATAGGGCGGTGGGCTGCAGTCGGGTGGTGGCGGCGGTGGCGTGGAAACCGTCGTCGGGCACAGACCCATGGCCTGCTCGTAGGTGGGGGGCGCGTCGTCGTGATCCCGGTCGCGGAGCATCGGCGTGGGCTCCATGTCGGTGGCAGTGACGGCGACGGTGGTAACTGTGGTGGAGACGGTACCGACGGCGTCCGCGGCTCACCTTCGAGCAAAGAGCCCCTTCTTTTTGCGCAAACGACGACAAAACAGTTCTCTGGGACAGCCGGTGGCGCGGTAAGCGGGTGCCACGCTTTCAGGGTGGGTAAAACAGTCGCGGGCGAAGCAGTAGTTGTTGCAGAACCGCAAGAACCCGACGCGAAAGAAGCCCAGGAGTCCGCGCGCCAGAAAGTGCGCCTGCCGCGTATCGGGATGCACGCCGAAGACGGCGCCGCTCTCGTTCACCAGTATGGAGATGTCCAGGCGCTGCTGCGACTCCACCGGCACGGCCCGCACCACAAATACCTGCAGCACGTTCAGCGAGCACGTCTCTTTTAACCAGTTGCCGTGGGCCGGATCCTCGTAAGTCTGGCTCCCGTTCAAGACGACCGTCGTCAGCGCCTCGTTACCGTCTCGCCAGCTGAAGATAGAACCCTCGCGCTTCATGCACAGGCGCCACAGGGCCAGCAGGTCGCGCGCCAACATGAACTCGCGACCCACGTCGCCGCCGGTCTCGAAGCGGACATAGCCCAGTTCTTCGCGCAGCGGCGCGTAGTTGCGCAGGCCCTCCTGCACGAAGCCGCGGAAACCGGACCGCGACACCAGGTACAGCGATTCCACCACAGGCGAGTAGACGTAGACGCGACCGCCCTCGCCGATGAGTACGGGTAGCGGTGGGCGGCCGATGGCTTCGCAACGACTCACAGTGCCCACCGGCAGCAAGAACTTGTCGCAGCACAGGAAGGTCTTCTCCAAACCTTTAATATTGAGATGTCCAAAGTAGCCGACGCGTAACAGGTCGCAGTAGGTGAAGAACCAACCGTTTGGCCAGCTGAGACGCAGCACCGTGCCGCTGACGCGACGAACCAGCTTCTGCAAGTCCTTGCGGGCGTCGGAGGTGACAGAGCAGCGGAAGGTCTCGTTGACCAGCTCGACAGCCAGCGCGTCCTCCAGCGTGCGTTCCTTCATCTCGTCGTTGATGCTCTGGCGGCGCCGCCGGATTTCGTCGAAACGGGCCGCGGAGGCGGCGACCGACGCGGAGGTCGTCCGAACGCCCTCTGTGACGCTGTCGTCCGGCCAGTCAAGAAAGCTAAGGCTGGCGCTGCGCCGCCTAAAGTGTCCGATCCGCGCGGGACGTCGCTGAGGGACGGTGGCTGGTCTGCTGGGGCGGGTACGGCCGCGGGTGTCCGCGGACACGTTAGTTATACACGGAATTGAGTCACGTGGCACGTTGCCAGCTGAAACCGCCGTCGTCTCCGCCGGCGTTTTCTCCATCGCGGGACCGCGCCGTGCGCACGTTCCCAGGCACGCGGCCCACGCTCTAGCCGCACTTTTGCTTCTTGGTGTTAGGGACGAACTCGAACGTTACAGAATCCTCGCTGTCGCTCTCCTCTTTCGCGTCGTTGAAGTAATTGCCGGAGTTGCGATCCAAACCGCCGCCTCCTCCTCCGCCGCCGCCGCCCGATCCACCTTTGGACGTCAGGTAGCTGGTGATCTTGTGCTGCTCGTATTTTTCCTTGGAGGAAAGACCGTGGTCGTGATCACCGCCGCCGCCACCGCTGCTCATTTTCCGCGTACCGGAACCACCGCCGCCACCGCGGTCGTGCTTCTTGCCGCCACCGCCGCCACCTCCTCCCAGACCGCCGAGACCCATGGGCTCGTTCATAATATCGTTATCCAGACCCGGGCCGTCGTCGTGCAGACCGCCGGCATTAGCCAGCGAAGAGAGGCTGCCGCTACCACCGCCGCCGCCACGCGATTTGCCGCTGTTCCCGACGTAATTTTTGTCGAAGGGATCGCCACGCTGGAAAGGTTCCTCGGTGAGAAAATTCTCCACGGCGAACAGACCGTTGCGGCTGGCCACGTACAACAGCGTGTCGTGCTCCGTAACTATACGCAACGTGCACGGCAGTTTGGTGACGGCGCAATTGAGCAGCGTCTGGTAGAAGTTCTTCAGCTGCACGTTGATACGCATGTTTTTCACGCCGTGGAAACTGACGCGGTTATTGGCTGTGAATTCCAGCTCGCTGCCGTTGGTCAGGATAAACTTGATGGCCGGTGGACCGGCGTGCACCAGAATCTGCACGGTGCCCGTAGGGCAGGGCGCTTTTTTAACGTTACGCTTGACGCGGGTATGCGGCCCGATCCACTTAAGCAGGTCGGCCACCACGCCGAAATCTAGATCCACGTGCACGGCCGAATTCTCGCTTTCGCGCACAATGTCTTGGCCGTGCACGCAGGCCGAGCTGAACTCCATATTGAAATCGGGCGCGCACATGGAGATCTTGGCCGACAGGTCCGAGATGTCCTGCACGTAGAACTTGGTCAGGTCCTTGCTGGAAGTCAGGTACATGAAATTACCCAGCAGCGGCGTGGAATTGTTAATGGTCTTGGGCTGAAACGACTTGTCAGTGATGTAGAGGCATGAGCTGTTAAAAGTGATTTTTGACACGCAGTGACTGCGTACCGTTTGCAAGATAAGCGACGGCGTGGGCAAGAAGGTAACCGTGGTGTTCTCCTTGAGCGCACGGATCACAGATCGCAGCTGCTGGATAGCCGTCTTGTACGGCTTCAGCCGCAGCGCCAGCGTCGGCGGCTCCGAGAGGCGCGTCTTGCGATCCATCCCGGACAGCGTGCAAGTCTCGACTAAGGAGCGGGCGCGAGCGAGCGAAAGTTTTATAGAGAGCACACACGACGACCGGGAACGCTGCGAAGACGCCCGGCGTCTAATAATACAGCCGCGCCGAGCCAGCGGGCCCCCGACTAAGAGGCACAGTACTTATATACTCCGACCTTAAAGCGCCAGTGGTACCACTTGAGCATCCTGGCCAGAAGCACGTCGGGCGTCATCCCCGAGTCATAGTAGAAAACCAGGGCCACGCACTGGTCCACAAACACGCTCAGGTTCACGGCCGCCATTTCCACGTCGTTTTGGATCGCCGGTGCCGCCTGGAACAGACACTGCGTCGCCTTGCCCTCCTCCTGGTGCTGCTCCAACCACGCGTAATTCACCACGGGCACGCGCAGCGGCCTCCGCACCACGGTGGGGAAGTAACACTCACGGTTGGGCGGGCACAATGACCACACCGTCTCCTCCTCGAACACGGTGCCGCGCGAAGCCCACACTGACGGCGTCACGCCCCACAGATGCGCCACCTCGTCGTCGGGACCCACCGCCAGAAACTGACAGTTGCGCAATCCGAACTCGAGCATGTCGGCGCGCAGCGCTTCCCAGCGCGCGCTGGCGATGGAGAGCCGCGGCAACCGATACAATTCGAAAATGAATTTGCCCTCTTGATAGATGGTGCGTTCGAACCACTCGCAGCGCGGCAAACCCGACTTGCACAAATCGACGCTAGCGCGCACCGCGGCAAAGTACATGTGCTCAAAGATGCGCTCGATCAAGTCCCAAGAGGCAAAGTACGTGAACCCTAACCGCATGAGCGCCGTGTGCAAGCCAGCCACGCCGATGTGCAGCGGACGCAGTTTTTCCAGCGCGCTCTCTACCCACCATTCGGACGCCGACATTAGCGCGTCCAAGCGCGCGTTGCCCCAAACCACCGCCTCGGTCACCAACTCGCGCAGCACGCTCAAATCAAAGTAACGTCGCGTGTTCCCCAAAACCACGTCGGGTAGATGCAGCTTCTGCTCGTCGCTACGCGCAAACACGCAGCGAGCCACGTTCACCGTCAGCCGCTGCACCGGCATGTCACACTCGCCAAAGTGGCACGACGCCATATCGGGACTCAAGCACGGCGGCAGGCACACGCTGTCGGCCATAATCGAGTACTTGACTACGTGATGGACAAAGACCACCGAGGCACGGCCCTTGAGCGCGCACAGCAACATCTTTTTCAGAAAATCGTCCGTGTTCACGACCACCTTGGGGCACGATTGCTCGCAGCGCGAATACTCTTTCTCGAAAGCCGACTCCTGACCCAGGTCCGAGAGCCGCCGGGAGACAGGCCGCCCAAACAGCGAGTAGCGCTGCTCACGCGCACGGTAGCGCTTCATTAACACGCTAGGCACGTTGAAAGCGTAGCAAACCCCCGTCAACTCCGACGTGCTTTCTTTGAGAATAAAGTTAATCACGCGGATAGCGGCCACGTCCCACATGTCCACAAACACACGTACCACGGGTCGATGCACCTCCTTCTCGCGTATCAAATCGCAGTATCCCCCCAGGCAACGAATCACGCTGTTCACATCGGCGTTAAGTCGCGTTACGTTCACCGACACAGAAACGCCGCAACTCAAGGTACTCATCCACTTGCACATGGCCGCCCAACTGGCGTCACGCGAGAAAGGGTCGGCCGAGATCAGAAAGTCGTACTGCGGCACGCGATCGAAACCCACGGTAGACATGGTGAAGGTGGACAGCGACAGCTGCCCATCGCGACAGCGCTTCAACACCGATTCCAACACCTCGCCCTCGAAACGCGCATCCAGATGGAAACGATAGATGCGCGAGTGCCTACTGTTCTCGATAGCGGCCGTCAACGCCACGGCGATGCGCAAAAACACGCCGCCCGGGCTCTCGTCCTGTCCGTGCAGTTGGCGACACACCTTATCCAAACACAAAATGGCCGCGTACAAGCCCCAGCAACCGGCCAATTCCACAAAACGCGCCGTCTCCTCGGCCAGCTTGGGTAGATCCTCCATGTGACGCAGCACAAAACGGCGCACCGACTCATCGCACAGCTCCGAAGCGTAACACAGTGGCGTGCGGCTTTCACGCGCCCAGTTGGCTTTGAAATAAAAGCGACCCAACAGCAGGTCGCAACGCGGCGAGTGACGAATCAGACAGGGACCGTGGCGCATAATGAGCTGAAATAGCCTGAAACTGCCCAAACCGGCACTGTGCCGCGACACGGTGTCCATCTCGCGCCACAGCGCGTTCCTGTCGGACGGCAGCTCCCGCGCCGGCTCCTGTACGCCGCAAAAGCGAAACTTGCCCCAGTAGCCGTGACAATGACACTTTTTGCCCATCAACATGCGCATAGCTTGTATCGGCGGCGATACTTTGCAGAGCGAAGCCCCGAAATCGTCCTCCTCCTCGACACTGTCCAGCTCCATCCTGGTCGCGCCGGCCGGATTAAAGGTGCTCAGACCGCTACTCACGCGTCCACCGCGACTGGGCACGGCGGGACCGCTGTCACGCGTCAACGACAGCACAGACGGCGTGCCGTCGGGAGACGGCGACTCGGGACGCCAACTGACGACGCCGCCACCACTCGTAAAACCCGCTACACATGCTACACCGCTCGATACGTTGGTATTTCCAGCGGACGCTTCCTTGTCACCCCCGGGCAGCGGCCCCTCCTCGAGCTCGCTGTCATCTCCCCCGATAGTATCAGCGGCGACCTCTGCCGACGATTCCTCCGTCTCGGTTTCCGCGCTGCGGCTTGGAATCCTACCTGGCCGGCACCGATGTGCGGGCACCGAGGACACCCGCTGTTCCTCGTCCGCGTCAGCCGGATTCATAAGTTTACGAGGAAAATAACAAAGAAATCAGGTAGATTTCAATAAAGTGAGTCTAGATGGCGCCGACAACTACGGTTTATAAAGTCTGTGTGCGATGTGTTTTTTTCTTCTGTGTCTCCTCCCCGTATGCTGTCAGCGCCGCTCAGACGAATTCTCGAAAGTCTCCCAATTCGACGCTAAAGTTGTCCAAACGGACGACGGACAGTTTGAGTTCTTTGTGTACCAGGAACGAGGTGTGAATGTCGTCAGCCAGGCACCAGCCCAGCTTTTGTATGACCCCGGTACACAGAGGGATCTGGCGCGGGCGCGTGATGCGACGGTTGACAAAGCTACAGCGCTCGCGGGCGAACTTTCCGCGTGCAACGTCGACCAAGGTCTGCCAGTGTGCGATGCTGGAGGTGAGCACGTAGATGCCGGGACGTGTTTCGGGCCCGTCATAGTCATAGACGATGATTAAATACACGTATTGCAGCCGTCCCCGGGTCTCTTCCCACGTCAGGTACATGTCTTTCGGTATCATCAACGCGAACACCTCCGTTTTGAGCGTGTTGTAAAGGTAGCCGCGCATGACGCAGGTGAGCAACGAGGTGATGCCCAGCGAGACGGTCTTGACGCAGCCCAGCGTCTCGAGGCGGCGGTGCAGCAGATGCGGGCCCAGGTCCAGCCACTGCAGCGCGGCGCGCGCGGCCGAGGCCGTGTACACGCTTTCGAGCAGGCAGCGCGTGCTGGCCGAGACGTTGGAGGCGCGAATGCCTAACAGGTAAAGGCTAATGTAGAGGTGTCGCGGCGAGTCGCAACCCGTCTCCATGCGGATGAGCAGCGCGCCCGGCTGCGCCTCGAACTCTACCAGGCCCTCGGGCACGAAGAAACGCGCCGTGAGCGCCTGGTGATCGGCGTGGTAGAGATAGCGAACCGATATAGTATTTACCTCGCGTTTGGCTTTGAGCGCCGTCACTAGTTCATTGTCCTCGTCGGCCGGGTCGCGCGGCCGTTTGGCCACCGCGCGCGCGTCCATGATGGCGAGGCGCACGGTAGATTTCAAAAAGTTGATAGAGCAGCTGCGGGCACGGGCCACGGACAAAGCGGAGGCGTTAAATACCGTGAGCCAATTGGAGATCGGCGCGGTGGATGCCCAGGACGTGACCGCGAGCGCCGTGCGCGCCTTCGTGGGTGCGTTGCCGAGCTCGGGCTACCACTTTGGCTTCGTGCGTCAGAACGTGGTCTTTTACCTCCTAAGCCACGCCACGGTACAGACGGCGCGCGACCCGCTGTACGCCGCCGAGCAGTTGCACGAACAGCTGGACCGCTTCCTGCGACACCAGCACGACGGCGGCGGAGACGAGGACCGGTTGCCGTTCTACCACAACGGGGCCACGCTGACGGCTTTCCAGAAGCTGTTGCAGACCCTGCGCGAGATCCAGACCGTAATAGCCGAACAGAGCGGCGGCACCGCGGCGGCGGCGGACTTGATCGCCAGTAACAACGCGTCGACCGAGCGCCGCGGCAAGAAGGGCGGTTCGAGTTCCGGGGGCCAGCAGCCGCTGGTCCGCCGGGTGATCACGCAGCTGGAAACGGCTGCCACGGAGGCGCGGCCCTACGTCAATTGTCGCGCCGTGGCCGAACTCCTGGACCTGACCTACCAGCGGCTCATCTACTGGGCCTGCACGCTCATGCCCTACGTGTTGTTTCGGCGCGACACCGACACCGAACTGGACACGGTGCTTCTGATGCATTTTTTTTACACACACTACCGTTCGGTTAACGGCGATTTGGCCGTGGAGTTTCAAAACTACGTCAAGAACAGCGTGCGGCACATGAGCTCTTTCGTCAGTTCCGATATCGACGGCGACCAGAAGCCCGGTGCCGAACACATGCGTGACGTCAGCTACAAGCTGTTCGTGGGTAATCTGCAAGCGCGTGACGCCAGCGGCCTCATGTTTCCCATCATTAGCACGCGCATCTCCACCGTGAACCTTTACCTGTCGCCCGAACGTATGTTTTTCCACCCGGGTCTGATCTCGCGTCTGTTGAGTGAGGAAGTTTCGCCGCGCGCCAACCTAGACGCTTACGCGCGCGTGTGCGATCGCGTGCTGGAAGACCACTTGCATACGCCGCGACGCGTGCAACGGCTACTAGATCTGACGCAGATGGTAATGCGACTGGTGGAACTGGGTTTCAATCACGATACCTGCGCGGCCTACGCACAAATGGCGCTGATCCAGCCGGCCAGTCAGAAGAGCTCGCTCTTTGTCAGCGAGATTCGCGAGAAACTCATACAGATCATCTACAATTTTTACACGTTTTTCATGTGCCTCTATGTGTACAGCCCCACGTTCCTGTTCGACCACCGGCGGCGGTTGATTTTGGAGCAGCATCGATCCACGTTGATCGGCTCCAAGGAGGAACTACAGCACGTCTGGAGCAACGTGACACTGAACGTCAATACGCACTTTGCGGTTCAGTACACGGAAGAAGACTTTGAGGCACATACGAAGGGTGCCACGGAGGCGGAGCGCGAGTACCTGTATCGGGACCTGCACAGCAAGTGGGGCGTGCACCTGTTTACCTTGCGTCCGTCTCGCGGCGCGGCCGGCGCGGCCTCGCCTTTGCCTCCGCTTGACGGCGTCACACGCTCCGACATCTTACGCGAATGCGCGCTCGTTAATCTGAACGAAGGCCGCGTCAACTACGCCTCCCTGCTAGCCTTCAGCCATCATCCCGAGTTCCCCAGCATCTTCGCGCAGTTGGTGGTGGTAACTGAGTTCTCGGAGATCTTTGGTATCCCGCAGGGCCTGTTTCAAGCCGTGGGTTCGCCGCGTCTTTTCGCACTCATTCAGCTGTGCCGTGTATTGTTGCCCGAGCAGGTGACGCTGTACCAGAACCTGGTCTCCATCTACAACCTGACCACCTTCGTCAAGCACATCGACGCCGCGGTTTTTAAGACGGTACGCGATTGCGTCTTCGACATCGCCACGACTCTCGAGCACCTCAGCGGTGTACCCGTCACGCCCAATGTGGACCTGCTGGCCGAGCTCATGGCGCGCTCCGTAGCGCATAACCTGTACACCACCGTCAACCCGCTGATCGAGGACGTGATGCGCAGCAGCGCCGGCAGTCTGAGAAACTATCTGCGACATACGCGACTCTGTTTCGGTCTGGCGCGTGGCCGGGCGCGCCTCTCGGAGGACGGCGTGACGGTGTACGTGGAGGTACAAGGTCAATACGGACTACGCGTACCCACCACGCGTTTCGTAGAACAGTTGCGCGAGCTGGTTCGCCGCGATCGGCTGTTGGCCGAGAATCTGCGCGGCTTGAACGAGCGCCTGCTGAGTGTTCGCGTGCGCGTACGTCAGATCAGCAGCGACACAGAGGAAGTAAGCCGACACGCCAAGGGTCACCGCACGGTGGCCCAGATGAGCAAGGCGCTCAAAAAGACGGCCTCCAAAATCAAAATGTTGGAAACACGCGTGACATTGGCGCTCGAGCAGGCGCAACGTTCCAATGGCGCCGTCGTTACCGCGGTGCAACGCGCGCTAGCCGTCTTTGACGTACTAAGTCGCGAGAACTTGGAACGCCGCGGCGCACAGCTCTGTCTGACGGAAGCGACGAGCCTACTGCACCGACATCGCGCGCTAGCGCCGATGACCTGGCCCGCGGGCACGGGCGTTGCGGCGGCGGCCGAAGCGGATGGCGCCTTACGCGAGTTCTTGGAGGCGCCCTGGGAATCGGCGCCCCAACCGCCGCGACTCCGCATGACGCCCGACACCGATCACGAAGAATCAACGGCAGGCGCGACGTCCGTACCGGAGGTCCTGGGTGCGCGCTACGAACCCGCACACCTGGCCGCGAGCGACCTATTAAACTGGTACATCGTCCCCGTAAGCCAGGCGCAGCAGGACATCTTGTCTTCGATCGACCCGCCCGCCGGCTCGACATCGGTGTCCCTGCCGCCGGCCTCGCCATGAAAGTCACACAGGCCAGCTGCCACCAGGGCGACATCGCTCGCTTTGGAGCGCGAGCGGGCAATCAATGCGTCTGCAACGGCATCATGTTCCTACACGCCTTGCACCTGGGTGGAACGAGCGCCGTCCTGCAGACCGAGGCGCTGGACGCCATCATGGAAGAGGGCGCGCGTCTGGACGCGCGGCTAGAGCGCGAGTTGCAAAAGAAGCTGCCCGCCGGCGGGCGGCTGCCGGTCTACAGACTGGGCGACGAAGTGCCGCGCCGCCTGGAGTCGCGGTTCGGCCGGACCGTGCACGCGCTCTCGCGGCCCTTCAACGGCACCACCGAGACGTGCGACCTGGACGGCTACATGTGTCCGGGCATCTTCGACTTTCTGCGGTACGCGCACGCCAAACCGCGTCCCACCTACGTACTCGTCACCGTCAACTCGTTGGCGCGCGCCGTGGTCTTCACCGAGGACCACATGTTGGTCTTTGATCCGCACAGCTCCGCGGAATGTCACAACGCCGCCGTGTATCACTGCGAGGGTCTCCATCAGGTGCTGATGGTGCTCACGGGCTTCGGCGTGCAGCTGTCGCCCGCTTTCTACTATGAGGCCCTTTTTCTCTACATGCTGGATGTGGCGACCGTACCAGAGGCTGAGATCGCCGCGCGTTTGGTCTCCACCTATCGCGACCGCGATATCGACCTCACCGGCGTCGTCCGAGAAAGCGCGGACACGGCAGCGACAACGACCACCGCCGCACCTTCCTTACCTCCGCTGCCCGACCCCATCGTCGACCCGGGTTGCCCTCCTGGCGTGGCGCCCAGCATTCCCGTCTACGATCCCTCGTCCTCACCCAAAAAAACACCCGAGAAACGCCGCAAGGACCTCAGCGGTAGCAAACACGGAGGCAAAAAGAAACCCCCGTCCACGACGTCCAAAACACTGGCCACCGCCTCCTCCTCCCCCTCAGCGATAGCGGCGGCCTCTTCTTCGTCCGCGGTACCACCGTCCTACAGCTGCGGCGAAGGGGCCCTGCCGGCCCTGGGCCGCTACCAACAGCTGGTCGACGAGGTAGAGCAGGAGTTGAAGGCTCTGACGCTGCCGCCGTTGCCTGCCAACACCAGCGCCTGGACGTTGCACGCGGCGGGTACCGAAAGCGGCGCTAACGCGGCAACGGCCACGGCGCCGTCCTTCGACGAAGCTTTCCTCACCGATCGTCTCCAGCAGCTCATCATCCATGCCGTCAATCAGCGCTCGTGTCTGCGTCGCCCCTGCGGTCCGCAATCGGCGGCGCAGCAGGCGGTACGCGCCTATCTGGGCCTATCCAAGAAACTGGATGCCTTTCTGCTCAACTGGCTGCACCACGGCCTGGATCTGCAGCGCATGCACGACTACCTGAGCCACAAGACCACCAAAGGCACGTACTCGACGCTGGATCGCGCACTGCTGGAGAAAATGCAAGTCGTCTTCGATCCCTACGGACGTCAGCACGGCCCGGCGCTCATCGCCTGGGTGGAGGAGATGCTGCGCTACGTGGAAAGCAAGCCCACTAACGAACTGTCTCAACGACTGCAACGTTTCGTAACCAAGCGACCGATGCCCGTTAGCGACAGCTTCGTCTGCCTGCGACCCGTAGACTTTCAGCGTCTGACGCAGGTCATCGAACAGCGACGTCGGGTGTTGCAACGTCAACGCGAGGAATACCACGGCGTTTACGAGCACTTGGCCGGCCTCATCACCAGCATCGACATTCACGACCTAGACGCCAGCGATCTGAACCGACGCGAAATTCTGAAAGCGCTGCAGCCGTTGGACGACAACGCCAAGCAGGAACTCTTTCGCCTGGGCAACGCCAAAATGCTAGAGTTGCAGATGGACCTGGACCGTCTGAGCACGCAGCTGCTGACGCGCGTGCACAATCACATCCTTAACGGCTTTTTGCCGGTAGAGGACCTGAAGCAGATGGAACGCGTCGTCGAGCAGGTACTGAGACTCTTTTACGACCTGCGCGACCTGAAACTGTGTGACGGCAGCTACGAAGAGGGATTCGTCGTCATACGCGAACAACTGAGCTACCTCATGACGGGCACTGTGCGCGACAACGTACCGCTACTGCAAGAGATCCTGCAGCTGCGACACGCGTACCAGCAAGCCACGCAGCAAAACGAGGGTCGCCTCACGCAGATCCACGACCTGCTTCATGTCATCGAGACGCTGGTGCGCGACCCGGGCAGCCGCGGCTCGGCGCTGACACTGGCCTTGGTACAGGAGCAGCTAGCTCAGCTGGAAGCGCTAGGCGGCCTGCAGCTACCCGAAGTGCAGCAGCGCCTACAGAACGCGCAACTCGCGCTAAGCCGCCTCTACGAAGAGGAAGAGGAAACGCAGCGTTTCCTCGACGGACTCTCGTACGACGATCCGCCCACCGAACAGACCATCAAGCGACACCCACAATTACGCGAGATGTTACGTCGCGACGAACAGACGCGTCTGCGACTCATCAACGCCGTACTGAGCATGTTCCACACATTAGTGATGCGACTGGCGCGCGACGAGTCGCCGCGACCGACGTTTTTTGACGCCGTCAGTCTGTTATTGCAGCAACTGCCACCCGACTCGCATGAACGTGAGGATCTGCGTGCCGCCAACGCCACGTACGCGCAGATGGTCAAGAAACTGGAGCAGATCGAGAAAGCCGGTACCGGCGCATCCGAAAAACGCTTCCAAGCGTTACGGGAGTTGGTTTACTTTTTCCGTAATCATGAATATTTCTTTCAACATATGGTTGGACGACTGGGCGTCGGACCTCAGGTAACGGAACTCTACGAGCGATATCAACACGAGATGGAAGAACAGCACCTGGAACGGCTGGAACGTGAATGGCAAGAAGAGGCCGGCAAGCTCACGGTAACTTCTGTGGAGGACGTGCAGCGTGTCTTGGCCCGGGCACCGAGCCATCGTGTCATGCATCAAATGCAACAAACGTTAACCACCAAGATGCAAGACTTTTTAGACAAGGAGAAACGTAAACAGGAAGAACAGCAACGGCAGCTACTGGACGGCTACCAAAAAAAGGTACAGCAGGATTTGCAACGCGTGGTGGACGCCGTTAAGGGCGAGATGCTCTCCACCATCCCGCACCAACCACTGGAGGCCACACTCGAGCTGCTCTTGGGCCTAGATCAACGCGCCCAACCGCTACTGGACAAGTTCAACCAGGACTTGCTGTCGGCGCTGCAGCAGCTGAGCAAAAAACTAGACGGGCGGATCAACGAGTGTCTGCACGGCGTGCTAACGGGTGATGTAGAGCGGCGCTGTCACCCGCACCGAGAAGCGGCTATGCAAACCCAAGCCTCGCTAAACCACTTGGACCAAATTTTGGGTCCACAACTCCTGATCCATGAGACGCAGCAGGCCCTGCAACACGCCGTCCATCAAGCGCAGTTCATCGAGAAGTGTCAACAGGGCGATCCAACTACAGCCATCACGGGTAGCGAATTCGAGAGCGACTTTGCACGCTACCGCAGCAGTCAACAGAAGATGGAGGGACAATTACAAGAGACTAGACAACAGATGACCGAGACTAGCGAGCGGCTAGATCGCTCGCTGCGCCAGGATCCCGGGAGCAGCTCCGTCACGCGTGTACCCGAGAAACCTTTCAAGGGTCAGGAGCTGGCGGGTCGGATCACGCCCCCGCCCGCCGACTTCCAGCGGCCCGTCTTCAAAACGCTGCTAGATCAGCAGGCCGACGCGGCCCGGAAAGCGCTCAGCGACGAGGCCGATCTGCTGAATCAGAAAGTACAGACGCAGTTGCGACAACGCGACGAGCAGCTGAGCACGGCGCAGAACCTGTGGACTGATCTGGTCACGCGCCACAAAATGAGCGGCGGACTGGACGTGACCACCCCCGACGCCAAGGCGCTGATGGAAAAGCCGCTGGAGACACTTCGCGAGCTGTTGGGCAAAGCCACGCAACAACTGCCGTACCTGTCGGCGGAGCGCACGGTGCGCTGGATGCTGGCTTTTCTGGAGGAAGCCCTTGCGCAAATCACCGCGGACCCTACGCACCCGCATCACGGAAGCAGGACCCACTACCGGAACCTGCAACAGCAAGCTGTCGAGAGCGCCGTGACGCTAGCGCATCAAATCGAACAAAACGCAGCCTGTGAAAATTTTATTGCACAGCATCAAGAGGCGACTGCCAACGGCGCGTCCACGCCGCGGGTCGACATGGTCCAGGCGGTGGAAGCGGTCTGGCAGCGACTGGAACCCGGACGCGTAGCCGGCGGCGCCGCGCGTCATCAAAAAGTGCAGGAACTGTTGCAGCGCTTGGGTCAGACGCTAGGCGACCTAGAACTGCAGGAAACGTTGGCGACGGAATACTTTGCGCTGTTACACGGCATCCAGACCTTCAGCTACGGGCTGGACTTTCGGTCGCAGTTGGAAAAGATCCGCGATCTGCGGACTCGTTTTGCGGAACTGGCCAAGCGATGCGGTACACGTCTCTCCAACGAGGGAGCCCTGCCCAACCCACGGAAACCGCAGGCGACGACTTCGCTGGGCGCCTTTACACGCGGGTTGAACGCACTGGAACGACACGTCCAGCTGGGTCACCAGTATCTGCTCAACAAGCTCAACGGCTCATCGCTAGTCTATAGGCTGGAAGACATTCCTAGCGTGCTTCCGCCAACACACGAGACCGACCCCGCGCTGATCATGCGCGACCGCCTGCGTCGTCTATGCTTCGCGCGTCACCACGACACCTTTCTTGAAGTGGTAGACGTCTTCGGCATGCGGCAAATCGTCACGCAGGCCGGCGAGCCCATTTACCTGGTCACCGATTACGGCAACGTAGCCTTTAAGTACTTGGCGCTGCGAGACGATGGCCGACCCCTGGCATGGCGGCGCCGCTGTAGCGGCGGAGGACTCAAGAACGTCGTCACCACACGTTATAAAGCCATCACGGTAGCCGTGGCCGTCTGTCAGACATTGCGCACTTTCTGGCCGCAGATCTCGCAGTACGACCTACGGCCCTACCTCACGCAGCATCAGAGCCACACGCACCCCGCGGAGACTCACACGTTACATAACCTTAAGCTCTTTTGTTATCTGGTGAGCACCGCCTGGCACCAGCGCATCGACACGCAGCAGGAGCTGACGGCCGCCGATCGCGTAGGCAGCGGCGAAGGTGGTGACGTAGGGGAACAGAGACCGGGCCGCGGCACCGTGCTGCGTCTGAGTCTCCAAGAGTTTTGTGTACTCATAGCAGCTCTGTACCCCGAGTACATCTACACCGTCCTCAAGTACCCGGTGCAGATGTCGCTACCCTCCCTCACAGCTCACCTACATCAGGATGTAATACACGCGGTAGTCAATAACACACACAAAATGCCCCCCGACCACCTCCCCGAACAGGTCAAGGCTTTCTGTATCACCCCCACCCAATGGCCCGCCATGCAGCTCAATAAACTGTTTTGGGAAAATAAACTGGTGCAGCAACTGTGCCAGGTAGGCCCGCAAAAAAGCACACCATCCCTAGGCAAGCTATGGCTCTACGCCATGGCCACGCTGGTCTTTCCACAAGACATGCTGCAGTGTCTGTGGCTAGAACTGAAACCCCAGTACGCCGAGACCTACGCCTCGGTGTCCGAATTGGTACAGACGTTGTTTCAGATTTTCACGCAACAATGCGAGATGGTGACCGAGGGGTACACGCAACCGCAGCTCCCCACCGGAGAGCCGGTGCTTCAGATGATCCGCGTGCGACGCCAAGACACAACCACCACAGACACAAACACGACCACAGAGCCAGGACTTTTAGATGTTTTTATTCAAACAGAAACCGCCCTAGACTACGCGCTGGGCTCCTGGCTTTTCGGCATACCCGTGTGTCTCGGCGTGCACGTAGCCGACCTGCTGAAAGGCCAACGTGTATTAGTAGCGCGCCACCTCGAATACACGTCGCGAGACCGCGACTTCCTCCGCATCCAACGCTCCCGGGACCTCAATCTCAGTCAACTGCTCCAGGACACGTGGACCGAAACGCCGCTGGAGCACTGCTGGCTACAAGCCCAAATCAGACGGCTACGCGATTACCTGCGTTTCCCCACCCGCTTAGAGTTTATTCCCCTAGTCATTTACAACGCACAGGACCACACCGTTGTACGCGTGCTGCGACCGCCCTCCACGTTCGAACAGGACCACAGTCGGCTGGTGTTGGACGAGGCCTTCCCCACCTTCCCGCTGTATGACCAAGATGATAACACATCCGCGGACAACGTCGCTGCGTCTGGCGCCGCTCCAACACCGCCGGTACCTTTCAACCGCGTACCAGTCAATATTCAGTTTCTGCGTGAAAACCCGCCACCCATCGCACGAGTTCAGCAGCCGCCGCGCCGACATCGTCATCGAGCGGCCGCGGCCGCAGACGACGACGGACAGATAGATCACGCACAAGACGATACATCAAGGACAGCCGACTCTGCATTAGTCTCCACCGCCTTTGGCGGGTCCGTCTTTCAAGAAAACCGGCTGGGAGAAACACCACTATGTCGAGATGAACTTGTGGCCGTGGCACCCGGCGCCGCCAGCACCAGTTTCGCCTCGCCGCCTATCACGGTGCTCACGCAGAACGTCCTCAGTGCTCTAGAAATATTGCGACTAGTGCGATTGGACCTGCGACAACTGGCGCAATCCGTACAGGACACTATTCAACACATGCGGTTTCTCTATCTTTTGTAACCGACACTGACAGTAGCGGGCAATAAAAACAAGAGGATTGTTATCGTTTTTTATGATACAAAACAACGTGTCACTTTCACGGTGATTTATTCTTGCTATTACTTTTCCCCATGGGCTGTCAGCGTCGGGTGCGCGACACGGCTATCATGCGCAACAGGTCCAGCTTAAAGGCGCACTTGTCGTTAAACAGACTGGACATGCGCGTATACTTGCTCAGCATGGTGGCCAGCACCGGGTGGGTGGCCTCTGAGATCTCGGTCGGCAACTCCAAAACGACGTTGACGACGTGACGGTGTTTTTCGTCCCGCTTGTTGGCCACCGTGGGTCCCGGCGCGGTGTTAGACATGGGGCAGGCCGTGGGGGGAGGACGAGGAGGACGTCGCTGCTAAACCGCCGCGCGCCTGCTGCACAATGTGGCCGCCGACGTGGCAGGCGGTCTGTTTAACCAGCGCGCAGCCTCGACACAGCGGGGCGCCGTCTTCGCTTTCCAAACAGCTGTCGCGGTACTCGCCCGTCTGACAGCGCGCGCACAGCAGGCCGTGCCCGTGCGAAGTGAGACGCAGGAGACGCGGGACCGTCACGCCGCGTACCACCACAGTGGAGTCGCAGGTGCGTGCCGCGCAGGGCAGAATGACGTCGAAAGCCAGCCGGTGATCGTACACGGCACAAGCCGCGTTGAGGCCCAGCACGGCTTTCCAGCCCACGCGTACGCAGCGCTGTCCAAAGAGCGTCTCGGAGACGAGCTCGTAGACGCGCTGCCGCACTACCCGCTGACTGCCGCAGAGCGAGCAGTGTACGAGCTCGGCGTGCGTGTTGAAGATGACGCTCTTTTCTTGACGGTCCCGATAATAGAACATCGAGTTGAGCGGAAAATTTTGCTGGCAGTGTAGCTTTTCCTTACCCAGGTTGAGGCAGTGTCCGCACTGCCGACAGACCACGGCCACCAGCGAGCGCGCATCCAGATGGCGCTCGCACTTGAGTCGACACAGACACCAGAGCGGCAGGTCGATGACGCTGCCGATGAGGCCGCCGCGCAGCGCGGCGCTGAGTGCAAAGAGGACGATCTTGGTGGGCTCTACGTGATGCGCCTGCTGTCCGGCGCCCGCGTGTCCTACCGCCGCAGCTGCCGCCGTCGAGCCTCCTCCGCGCGTCTCGTCGTGCAGACCCAGTGCCCGCAACGGCACCAGGTATCGCGGACACGTGTCGCAAAACGTCTGCACCGCTTGTCGGGCCAGTACGTAGAGCGGGTTTCCGCAGGGTACCTTCCCGGCGTGCCGACGCAAGGCTGCGATGAGGCCCCGCAGCTGCGGCGACCGCGGCTGCCGTTGGTGACACCACTGGTTACGGTGGTATACGGCCAAATCAGCGCGGGCGTCGAAGCGCTTGGCGCGTAGTAGTGCTAGGCACGGCGAGCTGGTGGGGTGAAGCACGGGCAGCCGAAGGTCCACCCCGAAAAGGAAACGGTGAAGGTCACCTAGCAGCGAGACGGTGACACCGTCCAACAACGCGTGCAGCCGCTCGGGCGGGTAGAGCCGCAGACGGCGCAGCAGGTAGTCGGTGTCGTAGCGTTCGAAACGCAGAAAGGCCATCGTGCGGACGGCCACGGTGTGCAGACAGTCCATGCTGTAGACGTAAGCGAGAAACACAAAGTAGGGCTTGGTCATAACCATACGCTGAAAGAGCGCCGTCACCGCCTCCCGCTCGGCCTGCCGACACACCAGCCATTCGCGCAGGAAGCGTTGGTAGAGACGGTCGCCCAGCTCGCGATTCAGAAAGCGCTTATCCGTCACGAAGAGATGAAGGACGCAAGAACGTGGCACGTGATGCACCAGCTGCTGCTGGAGGACCGCCGACGTCTGCGCCGCAAACTGCGCCGGTGGCTGCGACGTTTCTACCGCCGCTTCCTCCGGCTGCAGCGCACCGCGGCCGATCACCAGCTGCACATGGAAATGGTCCTCGTGAACGCAGAGGGGCGCGAAGAGACGGCGCAGAGCCTGGTGGAACTCATCAGTCGCGGTGTGCGGAGCGTGTCGGAGACGACGATTGGCCATGACCGCGCCA

AGAGTCGCAAAGCGCGCGGGCAGCCACGGCCCAGACTGCGGTCGCGATGGCCCGGAGCGCGCTCGCCACCACGATGACGGTGCCCAACGATAACCAGTCCGCTCCAAGGACGGCGTGCACGGCGGAGACGGCGGATGACGGTGATGGGTCGACACCCCTCGCCGACGACTCACGTGCTCCTCCAGAGGCCGACGCGCGGACCCTCCGATGTCCTGGCCCGCCGCTGCCGCTGCCGCCTTCCCTTCTCCCGCCAGAGCCAGCAACTCCTCCTCCTCTTCATCAGCGTCTCCCTCGCTTGCGCATCCGCATCGTCCCATACAGGCCTCACAACGACACAGCCGCCACGACCCCGCCGCCATGGGTGGCGGCGGCGGCCGAGGCCCGGCAGCGGCGCCGCCAGCGGCGACCATGGTGGGAGAGCAACTCGGATGACGAGGAGGAGGAGGAGGGGGAGATGCGGTCCGAGGGGACCGCTTTCCCGCCGTTCGCGTGAGCGCGGCCGACATGCGGGCGCGCCACAGAGATGGACCACTGCCGCTGTGACTGCTTACGGTGACGTGGTTCCGGACCGCCAACGACGTCGACGCGGCTTTCTTGGCGTACAGCTCGCGCAGCAGATTCTCGTACTCGCCCTCGTTTTCGGGTCCGAAGGCGATGAGCTCGATGTTGAACACCGACGCCGAATTGGATTTGCGCACCACGCACTTCGTCAGCACTCCGTAGGCCGAGGGCTTGATCTCCTCGATGTCCTTGAGCGTGACGATGAGCGACTCGTTCACCTTAAGCACATTGAACTCACCTACGTGGCGCGCCGGCGAGACGAGCTTGACGGGCGCTCGCACAAAACAGCAGAGGGAGACGGCGCAGCCAGTGTTTTTAAAGATAAAACAAGGCACGTGGTCTGTGCGGCTCTCCCAGTAGCTGAGCAGATATTCGACACAATAGACCGTGTCTGTCTTGAGCATGGCGTCGCACACCGAGTAATTGGGATTTTTACAGATAAGGCCGGCGTCGGTGACGCGCAGCTCGCTAGGACCCAACTTGAGGATACGCCGCGTGGCCTGCACCAGATCCTGATGGAGAACCTTGTTCATCTCCATCGCACCGACGCCACCGCCGATTTATTTACCCGGCGCCGGCTCGTCTTTTCCCTCCAGGATTCCGTTAATGTCCATGAGCTTGCTGACGATCGCCGTTAATAGTTGCGTCTTCTCACGGAGGATCTCTCCGTGACTGCAGGTCGCGCAGTCGCCGTGCACGTACTTGAGGAAGGCGGCGTACTTCTGACCCGCGTTCACGAAATTTAAGCGCGCGTCCAGAGAGGGCAGCAACAGATCGTAGACGCGCGGCAGCATCGGCTCGAACTGTAATAGCAGATCGTCGTCAAGATCGGGTAGCGCGTGTCCGTCTTCACCGTCCTCGTCGTCACCACCTCCCCCCTCGAGCCCACCGCTCGTACCAGCCGCGGGCTCCGCGTCCTCGTCGATCACCAGCGGTCGCGTCGGCACCGGAGAATCCACGTCATCCTGCACGTCGTTTTCCTCCTCTCCGTCGTCATCGTCCAGAAACGGCACCCGCTGCTTAGCCCAGGACATTCTTTTTCCGCGTCCTCAATCAGCGGCGCCGATCGCCATGAATCCGAGTACCCACGTGAGCAGTAACGGCCCAACGACTCCCCCTCACGGGCCCCACACCACGTTTCTTCCCCCGACCAGCCCGGCCCCGTCCACCAGCTCCGTCGCCGCCGCTACCTTGTGCAGTCCGCAACGACAGGCCGTTTCGCGTTACAGCGGCTGGAGCACCGAGTACACCCAGTGGCACTCGGACTTGACAACTGAGCTGCTATGGCACGCGCACCCGCGTCAAGTACCTATGGACGAAGCGCTGGCCGCCGCGGCGGCCGCCTCATACCAGGTGAATCCTCAACACCCCGCCAACCGTTACCGTCACTACGAATTCCAGACGCTCAGCCTCGGCACCTCGGAGGTAGACGAACTGCTCAACTGCTGTGCGGAAGAAACCACGTGCGGCGGCACGCAATCCACCGTACTCACCAATGCGACCAACACCACTAGCTGCGGCGGAGCCGTCGCCGGCAGTAGCAACGCAGGACCCGCCGGCGCTTCGGCCGCCTGCGACCTAGATGCAGAACTGGCCGGCCTCGAAACCTCGGCGGCCGACTTTGAACAACTGCGGCGACTGTGCGCGCCGCTGGCCATCGACACGCGCTGTAACCTATGCGCCATCATCAGCATCTGCCTCAAACAGGACTGCGACCAGAGCTGGCTCCTCGAGTACAGCTTGCTGTGCTTCAAATGCAGTTACGCGCCCCGTGCGGCGCTCAGCACGCTCATCATCATGTCCGAGTTTACGCATCTGCTGCAGCAGCACTTTTCCGATCTGCGCATCGACGACCTGTTCCGACACCACGTTCTCACGGTCTTCGATTTCCACCTGCACTTTTTCATAAATCGTTGCTTTGAAAAACAAGTGGGCGACGCGGTTGATAACGAGAATGTCACCCTGAACCATCTAGCCGTGGTGCGGGCCATGGTCATGGGCGAAGACACGGTGCCTTACAACAAGCCTCGGCGCCACCCGCAACAGAAGCAAAAAAACAACCCTTATCACGTCGAAGTGCCGCAAGAACTGATCGACAACTTTCTAGAACACAGCTCACCTAGCCGCGACCGCTTCGTGCAGCTGCTTTTCTATATGTGGGCCGGCACCGGCGTCATGAGCACCACGCCACTCACGGAACTTACGCACACTAAGTTCGCGCGACTAGACGCGTTATCCACGGCCTCGGAAAGAGAAGACGCAAGGATGATGATGGAAGAAGAGGAGGATGAAGAAGGAGGAGAAAAAGGAGGAGACGATCCGGGCCGTCACAACGGCGGTGGCACCAGCGGGGGGTTCAGCGAGAGCACGCTAAAAAAGAACGTGGGTCCCATTTACCTATGTCCCGTACCCGCCTTTTTTACCAAGAACCAAACTAGTACCGTGTGTCTGCTGTGCGAACTCATGGCCTGCTCCTATTACGATAACGTCGTCCTGCGCGAGCTGTACCGCCGCGTCGTCTCGTACTGTCAGAACAATGTGAAGATGGTGGACCGCATTCAGCTGGTATTGGCCGACCTGTTGCGCGAATGCACGTCGCCGCTCGGCGCGGCGCACGAGGACGTGGCGCGCTGTGGACTCGAAGCGCCCACCTCGCCCGGAGGCGACTCGGACTATCACGGCCTGAGCGGCGTCGACGGCGCACTGGCGCGACCCGACCCGGTATTTTGCCACGTCCTGCGTCAGGCGGGCGTCACGGGCATCTACAAGCACTTTTTCTGCGACCCGCAGTGCGCCGGCAACATCCGCGTCACCAACGAGGCCGTGCTCTTCGGACACCTGCACCCCCACCACGTCCAGGAGGTGAAACTGGCCATCTGTCACGACAATTACTATATAAGTCGACTTCCGCGACGTGTGTGGCTCTGCATCACACTCTTCAAGGCCTTTCAGATTACAAAACGCACCTACAAAGGCAAAGTGCACCTGGCGGACTTTATGCGCGATTTCACGCAGCTGTTGGAGAATTGCGACATCAAGCTGGTGGACCCCACGTACGTGATAGACAAGTATGTCTAGCGTGAGCGGCGTGCGCACGCCGCGCGAACGACGCTCGGCCTTGCGCTCCCTGCTCCGCAAGCGCCGCCAACGCGAGCTGGCCAGCAAAGTGGCGTCAACGGTGAACGGCGCTACGTCGGCCAACAACCACGGCGAACCGCCGTCGCCGGCCGACGCGCGCCCGCGCCTCACGCTGCACGACCTGCACGACATCTTCCGCGAGCACCCCGAACTAGAGCTCAAGTACCTCAACATGATGAAGATGGCCATCACGGGCAAAGAGTCCATCTGCTTACCCTTCAATTTCCACTCGCACCGGCAGCACACCTGCCTCGACATCTCGCCGTACGGCAACGAGCAGGTCTCGCGCATCGCCTGCACTTCGTGCGAGGACAACCGCATCCTGCCCACCGCCTCCGACGCCATGGTGGCCTTCATCAATCAGACGTCCAACATCATGAAAAATAGAAACTTTTATTACGGGTTCTGTAAGAGCAGCGAGCTACTCAAGCTCTCCACCAACCAGCCGCCCATCTTCCAAATTTATTACCTGCTGCACGCCGCTAACCACGACATCGTGCCCTTTATGCACGCCGAGAACGGCCGGTTGCACATGCACGTCATCTTCGAAAACTCCGACGTGCACATCCCCTGCGACTGCATCACGCAGATGCTCACGGCGGCGCGCGAAGACTACAGCGTCACGCTCAACATCGTGCGCGACCACGTCGTTATCAGCGTGCTGTGTCACGCCGTCTCGGCCAGCAGCGTCAAGATCGACGTGACTATTTTGCAACGCAAGATTGACGAGATGGACATTCCCAACGACGTGAGCGAGTCCTTTGAGCGCTACAAAGAGCTCATTCAGGAGCTGTGTCAGTCCAGCGGCAACAACCTATACGAGGAGGCCACGTCGTCCTACGCAATACGGTCTCCCTTAACCGCGTCGCCGTTGCACGTAGTTTCCACCAACGGCTGCGGCCCCTCCTCCTCGTCTCAGTCCACGCCGCCTCATCTCCACCCGCCGTCGCAGGCGACGCAGCCCCACCACTACTCTCACCACCAGTCTCAGTCTCAGCAGCATCATCACCGTCCCCAGTCACCACCGCCGCCGCTGTTTCTCAACAGCATTCGTGCGCCTTGACACTGTACGGCAGAAAAGCCGGCTCCAAGTGCAAGCGCCGCGGCAGCACCATGTGCAAAAACTTGTCCTTGCGCGCGGTTTCGCCGCCGGGAAAGACGGGCGACAGCACGTTGGTTACAGCCTTGAGAACCTGCTCAAAGTACTTGTCGGCGTGAATGGGCACGCCGTGCTCGCGCACGTAGCTCGGATCTTCGGCTACCTCGTAGTTGCACACGGCCGACGGTGGTTTCCGCGCCCTCTTCTTTGCCGGCTCTCCTCCTCTCCTGTTGCTCTCCTCTACCCCGCCGCCGTCAGCGTCGTCGTCCGTGCCATCAATCGCGTCCGACCGGGAAACCACGCCGGTGGTTACAGAATCACCGTTGTCGGAGGAACCCTGCGGCGCCGCCCGGACACCGGGCGCCGTCAGGACGTAAAAGACCCGATCCCCGACCGAGGGTAGCTCCTCAGAACGGGCCGCCAATCGCTTAATGACGGCAATGTGCGGCAGGTTAGATTGACGGTACAACGAGATGTCCTTAGAAAGCACCGACGAAAGCACCAGGTCCTCAACACGCACACGGTGCAGGTACAGATCGTCGCGGGCCTGCACCAGGCGGCGCAAGATACGCCAGAAACCGCGTGGCACACCGTATTTCTTAACTTCATCGAGTGAGAGGCGCGACAGGCGCACGGCTGCTTCCGAGACCTCGCGATCCTCAAAGAGCAGCGAGAGGACGTCACGCGTGACGCCCTTGACGAACTCGCAGGCCGTCTTGCGCACCAGATCCACGCCCTTCATGCTCAGACCCGAGGCGCCCTCCACTTTGCCGATGTAACGTTTCTTGCAGATCATCATAAGAGAGACGAAGACCTTTTCAAACTCCAGCTTGACGGGCTCCACAAAAAGACAAGCCGTCACGTAGTGCGCCAGGCTGGGCCCACGCGCCACCAGAGCCTGCGGCGTCAGGCCACGAAAGCGGACAAACACGCTGTCCGTGTCCCCGTAGATGACCCGCGCCTCCACCCGCCGTTCGTTTAAGCCCCCTGATGATGTTTCGAGCCCCTCCGGTAACGTGCTGCTCTCCTCCGAATCCCCCTCCCGCGTTCCTACTACATAGTCTTCCTGATTAAAAAAATTGTGCAAAAAACACGGCTCTGAAAAGTTGTCTTTGATGAACCGCGCCGTGCGCTCTAGCATGTCGCGACCGATGCGCGTAATGCTGGCGGCGATGGGCAGACACGGCATCATGCCGTTGACCACGCCGGTAAAACCGTAGAAAGCGTTGCACGTTACTTTGAGCGCCATCTGTTCCTTGTCGAGCAGCATACGGCGCACGGGGTCTTGACACTCGCGCATGCATTCGCGCACGGCACGCCGCTGCGAAACCCACTTGTTGAGCAGTTCCGAAAGCACCGAGACGCGCACCGAAGCACGCACAAAGCGGTGTGTCACGCCGTTCTCTAGCGTGACGCTGTATACGTCGGCAGGGTCCACGGGGTACTCGCCACCCGGCACCAGCAGGGTGGAGTAGCAGAGGTTGTGAGCCATGATGATGGAAGGGTAGAGGCTGGCAAAGTCGAACACGGCCACGGGGTCGTTGTAGTAACCCACCTCGGGCTCAAACACCGTGGCGCCCTGGTACGAAACCGCCGCAGTACCACCGGCGCCGTGATTGTCGTTGGAAACGCCGACGCCGCCACTACTGCCGGAGCCGACGCTGAAAACGCCGACGCTGCTACTACTGTTACTGCCGGAGCCGGGTGAAACGCCGTCCTGACTGGACGGCGCAGATTGCAAGGGCGGCGACATCTGAAACATAGCCGCCACAGAACCCGCGTCGCCGGGCACGGCGGCGGTAGAGATGATAGCGGCGTTAGGTGACACGGCAACACTATTCGTTTCGGGCACCGTCGTACCTTTGCTGTAGTGGTTGGGCAGGATAAAATCGCGGCAGGCGCACTCGTCCAGCAGCGAGGTGTAGATACGGATCTGCTGTCCGTCAAAGATGACACGCCGCAACGGAATTTTAGCCAGCCGCGCGATGGCCCCGGCCTCGTAGTGAAAATTAATGGTGTTGAACAGATCGCGCACCAATACGGCGTCCTGCAGACAGTAACGGCCTACCTGGGCGCGGCCCTCGGCATTAGCCACGAAACAACGCGGGATGTCCTTGTAGGACAGGTCATCCTTGCGTTGCCGCAGGTAAAGCTCGGCCATAGTGTTGAGCTTATAGTTGGGCGAGTTAGTCTTGGCCATGCATACGGGGTACATGTCGATAACCACCGAACCCGCAATATACACCTTGGTGGCGGCCGTGCTGGCCGGATTGTTGTGAGAAGCCGAGGGAAAGGCGGCGGCGTACTGCCGCTTAAAACCCACGGCGGGGCTGTGTAAAAAGAAACGGCCGCCCTGCGCCGTAGGCAACTTGCAGAAGCGCTGCGAGTCCACCTTATACAGGTACTCGAGGCGCGTGAGGATGTACTTCAAGTCAAAAGAGTTGATGTTGTAACCGGTCACAAAGGCCGGCGCGTACCGTTGAAAGAAAAGCATAAAGCCCAGCAGCAGCTCGTATTCGGAAGGGAACTCGTAGACGTCCACGTCTGGGCCCACCTGCCCGCAGGTGCCGATCGTGAAGAGATGAAGACCCGAGTGCCCAAAGATCACACCCTCCGAAGTGCAGCCCCGGCCATCGTTCCCGTTTGGGATCCCCTGATCCACGGCGGTGTTTCCCCCCGTCTCGTAGCACACGCACGAGATCTGAATGACAATGTCATCGGACTTCTCGGCGCAGGGAAAACCACCCTCGCCGCTCATGCACTCGATATCGAAGGACAGGCATCGATAGCGCGGCCACGAGCTGTCGTCGGGCACGGCCACCAGGTCAGAGACATCGCAGTCGACCTCGATATCACAAGTCGACGCGCGACCCTGCTGCCGCCAGTCGTAACGATTCACGGAGCACCAGCCGAACGTGGTGATCCGCCGATCGATGACCAAACGCGTCAGCGGATCCACACGGACCTCGTACACGGGAAAACCCTGCTCCAGCAGATACTCGCCGATTTTTCTGGCCATGGTCCAGTTGCTGATAGACACACACTGCAAATCGGGCACGGGTCGCGTCCCGTACCCGTAGATCGAGGTTTTGGTGGCCGGCGTGACAGACACGGCGTATGGCGTCCGCGGTTCGGGCACTAGTTCGCCCACGCTGGCAATGACCTCACGCAGCCTATCGGTGTCGCTGTACTCACAGTAAAAGTAGCTGCGCTGCCCGAAAACGTTGACGCAGATACTGTAGCCGTGTTCTGTGGCCCCGAAGAAACGCAACACGTTCCCCGAAGGCACCAGATGCTGACGATAGCGCGGCGACACGTTTTCGGGCGAGTCGAAGAAGAGCACGGCGTCCGTCTGATCGTAGGTGTGAAAACGAATAGGTCCCACCACGCGACCCACCAGGGTCTCGCGCCAAGGACACGGCCAAACCATGTCATGACTCAACAAATGTTTAATCTCTCGATAGAACATGAGAGGCAGCCGTCCCGTTTTATGCTTGATCAACCCCGTCTGACCGTCGAACATGACGCCTCGCGGCACGATCTGCAAAAACTGTTTCTGTGGCGGCCGCTTGCCCGAGCCCTGCGCGGAGCCGGGCTGCGAACGCTGACGCCGGCCACCCGCGACCGCACCGCCGGTCACGCCGCCGCTCAGATACGGGTTGAAAAACATAGCGGACCGTGAGAGGCTGACAGCTTACGAAGCAAAATCACAAAGAAAATACACATGCAGCACCTAGATATCCAGTTTAACCCCGTATATCACAAG

TCGTCAGAGTCTTTCAAGTGTCGGTAGCCGTTTTTGCGATGTCGCAGTCGGTCTAGTAGGTTGGGCTTCTGTCCCTTGTCCTGCGTGCCAGTCCGTCCGTCCAAAGAATCTGTACCGTTCTGCTGCGCTCGCTGCTCTGCGTCCAGACGGGCCAGGGCCAGAAGCATCTGGTAAGCCTGCTCGTTGGTGTAAGGCGGAGCCGCCGTGGATGCATCAGACGACGGTGGTCCCGGTCCTTTGCGACCAGAATTATAAACACTTTCCTCGTAGGAAGGCGGAGCCTGTAACGACGTGTCTTTGGTGCTGCCCGACGTCACGGTGGTCCCGTCGGCGGACACCAGATAGGGAAAGAGGTTCTGCAGCGGCTGCGTGCACAAACGCCGCTGTCGAGTATAGATCAAATAAGTGATAATGACTACAGCTATGGCCACGAGGATGATGGTGAACGCTCCGAAGGGGTTTTTGAGGAAGGTGGCAACGCCTTCGACCACGGAGGCCACCGCGCCACCCACGGCCCCAATGGCTACGCCAACGGCCTTTCCCGCGGCGCCCAGGCCGCTCATGAGGTCGTCCAGACCCTTGAGGTAGGGCGGTAGCGGGTCGACTACCTTGTCCTCCACGTACTTTACCCGCTGCTTGTACGAGTTGAATTCGCGCATGATCTCTTCGAGGTCAAAAACGTTGCTGGAACGCAGCTCTTTCTGCGAGTAAAGTTCCAGTACCCTGAAGTCGGTATTTTCCAGCGGGTCGATATCCAGGGCGATCATGCTGTCGACGGTGGAGATACTGCTGAGGTCAATCATGCGTTTGAAGAGGTAGTCCACGTACTCGTAGGCCGAGTTCCCGGCGATGAAGATCTTGAGGCTGGGAAGCTGACATTCCTCAGTGCGGTGGTTGCCCAACAGGATTTCGTTGTCCTCGCCCAGTTGACCGTACTGCACGTACGAGCTGTTGGCGAAATTAAAGATGACCACGGGTCGTGAGTAGCAGCGTCCTGGCGACTCCTTCACGTTCATATCACGCAGCACCTTGACGCTGGTTTGGTTGATGGTCACGCAGCTGGCCAGGCCCAAGACATCACCCATGAAACGCGCGGCAATCGGTTTGTTGTAAATGGCCGAGAGAATGGCTGACGGGTTGATCTTGCTGAGTTCCTTGAAGACCTCTAGGGTGCGCCGTTGATCCACACACCAGGCTTCTGCGATTTGCGCCAGCGCCCGGTTGATGTAACCGCGCAACGTGTCATAGGTGAACTGCAGCTGGGCGTAGACCAGATTGTGCACCGATTCCATGTTGGATAAATGAGTTGCATTGTTGCCATCTGTACTTCTTTTGGTTCTATTATGAGTAAGATTCAGACTGGAGCGGTTGGCCAAACGTTCGAGTTCCACCAGAGATTTTTGCTTGATACCTTGCCAGAACACTACCAAACCACCAGTGGTTTCAAAGACGGACACGTTTCCATATTTTTCATATGTTTGATTGTATGAAGTATTGAAAATCTGCTGTAACTTATTTATAGCCTCATCACGTACGCAGTCCAGCGCAGAGTCGGACATGTTCACCTCTTGCTTCTTAGATAAGAAAGTGGCGGTCATTTTGGCAGAAGAAAAGTGATACGAGTCCTCGGCTTCGGAACGAATGGTGCGTTCCGAGGCTTCCCAGAAAGTGAGTTGACAAGTGACATTCTTTTCGTCCTGTATATCCCAGGAGATCACCGAGTCCGCACGTTCAAGAAAAGCCACCAACCTGTGGGTCTCTAACGCAGAATTCGGTCTTCCAAAGTCGGAGACGATAGTGTAGTTCGGAAAAATGAAAAACTTGTCGGCGTTTTCTCCAAAGTAGCTGGCATTGCGATTGGTTCCGTTGTAGAAAGGAGAAATGTCAACCACGTCACCCGTGGAAGTGGCGAAAAAATGATAAGGATATTTGGAGCGCGCAGTAGTGATGGTCACCATACAATTCAGATTACAGGTCTCACGATAGAGCCAGGTGCTGCCGCGGCTGTGCCATTGATCCTTGACCGTCACGTAACGGGTACTGTGGGTGTTGGAATAATCGTCGGGCATTAATTGCATGGTTTTGTTTTCATAGCTGTCCCTATGATAAGCCACGAAAACCGTGCCTGCTATAACGCGGCTGTAGGAACTGTAGCACTGACTGTGGCTGTTGATATGATGAATCTCCCACATAGGAGGCGCCACGTATTCCGTGTTGCTGCCCAGCAGATAAGTGGTGTGGATGTAAGCGTAGCTACGACGAAACGTCAAAACCTTCTGGTAGACTCGTACCTTAAAGGTGTGCGCGACGATGTTGCGTTTGTAGACCACCATGATGCCCTCGTCCAGGTCTTCATTGATGGGCTTCATCGAGGTGCAGACGATATTACGTTCAAAGCGAATAAGATCCGTACCCTGGGCCATAGAACACACGCGATAGGGGTACTTGGTGGTATTGACCCCCACCACATCTCCGTACTTGAGGGTAGTGTTGTAGATGGTCTCGTTAACACCATGGCTGACCGTTTGGGAAGAAGTTACGCGTTGAGAGACTGAACCGGATCGAGAGTGAGCAGCAGACGTCGTATGAGAGGAATGGTGACTGTGAGTAGCAGAAGTTCCACGAGTAGAAGATGAGGAAACCGCAGCACCCAGACAGACGATACACAAGTTAACGCAGACTACCAGGCACCAGATCCTGGATTCCATGTTCGTCGCGGGCCAAATCCAGCAGCGATGAGGCGCGTCGTGGTCTCTTGCGTGTTGCGCGGACCCTCCGGGAAACGCCCGCGGTCGAGGAGGAGGGGTACGGACTTGGCAGCCAAGGTCGGTCCGGCTCCCTGAAGGCACCCGAGACGGCCGCGGCGGCCGTCAGGGTGGAGGGCTTGGCCACGGGAGCTGTTGGCACGTCGCCACTCTCATCCGGTCTGGACAGATGCCTGTAGAGGAGGAGATATAGATCTTTGGACTTATAAAGACTTCCTTCGTGACGAAGCAGCAGCGGCCACTCTTTGTTATACGTGAGAATCACATCTCTGTCCGGGTGCAGTTCGTCGCGCAGGCACGCGATCGAGAGTTGTTTCCCGAAAGTTTCATTATATAGTGCGACGGAGAGCACGAGCTCCCGCACGTGCATCCACATCTCCTTCTGCAGCACGTTTAGGTCCTGACAGTCCGAAAAATTGAAAAAACCCATGTACTTCACCACCATCCACTCACTGGGATACACGGTACCTTCCGCGCATTTGACCAAATCGTCCTTGACGTGGGGTAGTACGCCCGCGTTGTCGCAGGCATAGGCCATGTCCACATTGTGAGAGAGGGGATAGCGATCGGTACAGTGTGTGAAGAGGGGCCCGTTACACAACTCGTAGATCTGCTGACCCAGTAGCGGGAGGGATTCCACAGGCAGACTCTTGTGGATCAGGTTATTGACCACATACAGGTGCTCATCGTACGTGAACTGATTCCCCACGTCCACCACGTCTTGGTCCTGGTGGTATTGGCTGCGGTATAGAAACCCATTCATGAGCTTAGAGATAAAGTCCAGACACAAGGGCCCCACTAGGTTGACATCGATGAGTTTGCTAGTCAGACGCTCCTGCGTTTTGATGCAACGGATCACCTTGCCATAGCCCACCTCCGAGACCTTCTGCAGGTAGGCGCGTTTGCGCACGTTCACCTCGCGGGTGACGTTGTGGATGCGGGAACGCGCGTCCACCAAGTCGAGAGCTTCGTGTTCGTCGCAGTTGCGCACCCGTAAGCCGTTTTCGCTGCCGTCGCCGTCCTGCCCATTCGCCCCTCCCCCTACAGCTTTCTTGCCTCCTCCACGGGCCCGGCCGCCGCCACCGTTATTCCTCTGACTGTGAGTACTGCTGTTGCTGCTGTTGCTGGCCGTCATCAAAGTCGTACCCGTCCCCGACATCGCCTCCCGTCCACGCAGGTGAATAACCTCGCCCTCGGGGCCGTCGCCCCCCGTGCCATCGGGCAGCGGACGTCGAATCTCCTCGAGAATATGCTTGATTTTGGTGTACATCTCGTTGCTTTCGTGGAGCTTGTTGAACACCGGGTTGTCCTCGAAAGCTTGAATGCTGAGGGATGTGATGAGGTCGATGATCCTGTTGGGGGCGGCAAAGACCGACCCCACGAACATGCGCTCCTCCCCGTCCAACGCCTTTTCCCCGAGCACGAAGATGTCCTCCACGTCCTCCCCGTACAGATGGCGACTGATGCCGTTCATGAGCGCCCGGCACAGCTGGTGATACACATTTAGCTGCTGGATGGTGATGCCCACCCGCTTGACGATAACCTCCGAGGTACGGGACCAGTAGGTAAAATCCGACAAGGAATATATTCGTTCCGGTATATCCGTAAACAGGTTGTACTCCCTCAGCGCCTCCTCCGCCTCCTGGATGTAGCTGTGGTAGGCCGATGAAGAAGAGAATAGGCTTTTGAGGGCCGAAAGGACTCCAGCCAAGTGGGGGATGCGCGTTGTCAGGTCCAGCAGGTCCTGCTCCACCGTCTGGATATTCACATCGGACTGGCTTGACGGACGGTGGACCGCTATATGGTTGCACAGCAAGCCCTGCAGCCGCTTGTTCAGCGAGCGGCCCTGATTCGGGATGATGGTCAGCTCCTCGTAGCATTGGGCGCATGTCGTCCCTTCGACGTACACTTCCTGACGCGCCACCGGCGAGATGCCGCATAGGCGACGGAGGAGCTCCAGCAGCTGCGCGCAGACCTCCAGGCCGGCCTCCGGCGCCAGGATCCCGTACACGTAGTTCATTTTGCACAGGAAGCGCTCGATGTCGTTGAGTGTGGCCAGACTGACGCTGAAACGGACGTTGTCCGTAAACTGGAGCTCCACGGTGTGATGGCGATCGCAGCGATCCAAACGGAGGACGGTACGGTAGAAGGCCGCCCGGTCCGGCTGGCGCGAGTAGGCCATCAGCGCCCGATCCAGCAAAGCCGTATCCTCGTGCAGCGCCTTCAGCAGCATCTCCAGATAGAGAGTCAGCAGCGAACTCTGCGTACGATTCTGCGCCACCACCTCCGGGTAGATCTTCCGGTACAGATACACTATAGCCGCCGCGTTTCTCTTGAACGGCGTGGACTCCGCCAGTAACACGTTCGGATCGCAGTACTTTAGACACTCCAGCTCCATGGCGTATTCGTTGCATTTCGAACACACTACGCATAGTTTCTGTAACAAATTCATCTCCATGACTCGACTCGCTCACGTACGAGACGCTGTCGTCCGGTCTGGCGCCGGCCAGAGACATGGAGTCGGTGCACAAATAACTCGCGGGCCGCTCGCTATGCCGACTGACGTTGACGTTAATATATAACGACGTCGTCGACGACGCGGGTTCTGCTCCCGAAGCTGTTGCCGCCGCTTGCGGCGCAA

AGATGACCGGCTGTGTCTGACACTCCTCCCCTTCCTCAGGCGGCCCGGGCGCCGACGCGAATGTCGGAGTTTGCCAGCGCGGCGGCGGTCTCTGTCTCTGGTGCCGCGGCGCTAACCTTCGGGGCTGTTGCTGCTGTTGATGATGCGACGCCGTCCGTCGCCGCTGT

CGATGCTCGTGGCTGCGGCGGCCGTTGTCGCGGCGTTTGCTGGCGGTTACAACCGGCTGCGTTTGGCCGGCAATAACCCGCTGCCCCCGCCGCCCCCGCTGCTCCCGCCGACGCCGCCAGCCTCGTCTTCGCCGGCGTTCACGAGAAAGCAGCCACCTCCCGTCTCGCCGGGCACGCCGAAGCAAATGGAGTTGCCCGCGACGGACTCGCCGAGAAGAAGACCGCCACCCCCGACGCCGGACGCCGCGCCGACGCCACTGGGCGCGAAGAGCGCCGACAGGTCGTGCACCTCCCCCCCGGCGGCGTCCGTTAATCGCTGGGCGTCGGCGTCCAGCACGCGTCGCAAGTTCTCCAGCGAAAAGTCCTCCACGCCCTGCTCCTGCAACGCGGCAAACTTGTCCATCAGCGACGCGGCCAGCGCCTCGCAGCCATCCACGAAGAAGAGCACATCGTCGGACGCGGGGATCTCCTCACGCACGCTCAAAATCTCGTACACGGCCATCACTTCGGGGTCGCAATCCAAGTTCTCGGCGTCCAGCGCCAGCATGACGCGGTTTTTTATAAGATCCGCGTCAAAAAGCACGTTCTCGCGGCGCGAGCGTTTGATGAGCACGTCGGCCAGACGCGTAGCCAAGAGATAGCGCTGGCGCATGAAACGATAATCTTGACCGCTCATAGAGCTCACGTTAAGGCTGCGTTCCACACCGTTGCCCGAAAAATAGCCGATCTGCCCAAACTGATAGATCTCCTTGCTGTTGTTGATACCAGCATATTTTTCCACGCTCACGGGCACGGTCACCAAGGAACGATGCTCAAAAACGCTCCGTACCAACGATTCACGCGCCACAGTGGCGGCCATGGGCGCCGGCACGCCTGCGGTCTTCAAGCCCTTGACATGCAACGCAAATTCGGCGGGCGACGAGAAACGCGGACTAGCACCTAACACGTGAGGAAACTGCGCGTGGTTCTGCGTCGTTAAGCGCGTCGTTAACCCGTGCAGCGAGCCGATGTAGTCCTTGAAGCCGTAGTAGCAGAGGAATTTGTTATGGAAACGGCTTTCCACGTAACTCAGCACACAGTCTGGCGCCACATCCAGCAGATCGTGCTCCTGATAGTCAGCCGTCACAGCCACCAGAAATTTGACGAAAGCATTGAACTCGCCCATGTCACCTATGGGCACATTCTTGGGCAACGCGTTGGAACAAACCTTCTGCCAAAACTGTAAGCAGGGGAGACCACATTCAGGAAAGAGTCGCTCGTGATGTCGATACAGCAGAAATCCCAAGCAGCCCTTAGCCGGATTACGACGCGGAACGTGATCGCGGCGAAAAAACACGCTACCCGCGTTGCCCTTGCCCGCGCGGTAGATGGGTCGGTTTTTCACCCGCACCATGATCAACGTGGGTACCGATAGCCGCGAGAGCTTGATCTCCATGGGCACCACGGCGTACGTGCCCTGCGCGTACAGCCTAAAGTCCAGCAGGCGGTCGTGATCCGAATTCTTGGACGACTTGATCTGCTTGGTGAAGAGAAAGCCCTTGCGCGACGACGTGGTGGAGAACGCGCCGTGAATGGATTGAAAATGCTGCGTCATCCATTTGGATACCAAGTTGGTGGTCAACGGATTGTCCACAATGTATGAGGTAGCGGTAATAAGCGCCACGTTCTGGATCACGTAAAAGACGGATCTGAAATAGGCGTAGGCCAGCAGCGGCTGGAAGGCCACGGCGTAGGGATTCAGATCCAGGTTGAAGGCCTGCGTGGCGCCCGCCACCTCGTCGCGGCTGCTCTTGAGGCGCACCTCCGAAACGAAACCCAGGGCCTCGTCGTCCACAAACTTGTTGAGCGCCGAAAAGACGGCCACAAAGTCGCTTTTGCCGTGCGCGCTAAAGGTATCCTCGCCCGTCACGGGGTCGATGAGCCGCATCTTGCGGCAGTAATCCAAGATGCGATTGAGCCGATAGGTACGGTCCACGCTAGCGCCCAGCATGCGACCGCCGCGCCCCATCATTCCCCCGGAATCCCCGCCACCCCCACCACCACGACCGCCGCCCAGACCGTCGCTCGGGCCCCCGCTCACGTCCCGTCCACCACCCCCGCCAGCACCGCCGCCCGGAACCCCGTCGTCACCTTTGCCGTCCAAACCCCCGTCCTTGGCGTCGACGTTGTAACGCCGACCGAAGCTGCCCAAAATATCCACGTCGTTGAGAAAACGCGACTGCACGGTGATCACGCAGGGCTCCTTCTTGGGCTGCTTGGGCACCACGGGCAAGCGGGTGCGCACCCGCACGAAGGCCGTCTGATAACACGTGTGGCAACAAGTACCCCCACAGGCCTCGCACAGCCCCGCGGCGCAGCCCACCAGGTGATTCGTGAGCGTCGACGAACCCGACAAGCCCGTGTTATACACCGAGACACGATTTAGATACCAGACGAAGCCCGAAACTAGCTGCGGACACGTGCCACACACCAACGCCAAATGCTGCGGCCCATAGCGTTCGTCCTTGAGCGGCGCGCCTTGAAACTTGAGCACCTTGCGCGCGTCGTTGTAGACGTCTTCGCAGGCCGCCGACAACCCGTTGGTGAACTGAATAGCCTTGAGCAACGTCTCCTGACTGGCCGTACCGCCGGCGCTGGGATGCCGCGCCGACGACTGGAGATACACCAGCCTGTGCTGGTAGAGCACCGAATTAGCGCTGAAGACCAAGGCGGCCACGTGCGTCGAGAGATGCAACTTGAGCTCGGTCAGCGCGCGGATCAGATCGCGGTGATCGGTTGCGTTGGTCACTAAAGGCCACTCGGAAAAGAGCATAGACTCGGCAGGTTGGTAGGCCGAATCGAAAAATACCGAGGCAAAACTGAAGGCCAACTCGCAAACCACCGCGTCACTCAGCATCAGATGATCCTTTTCCAGACTGCTGAGTCGCTGGCTCATGTACCCCAAGTAGCGCTTATGTGGCGCCAGCTTCACCGACTGCTGACTGTCGTGCACAAACTGCCGCAACGCCGCCTCGATCAGCACACGCGGCTCCGAGAAGCGCAGCGATTGACACCATGACGTGTACACGTAGTAGAAAAGCGTCTCGCTTACGGCCGGCACGTAGAGCCCTCGCGCCTCCACAAAAGCGCTGCGCGCATCCAGCGAGACCTCGTCGGCTTCGGCGTCAAGCTGCAGCGAATTAAAGAGCGTAGGCGGGTACAACGGCACGCGCACCGCCTCGCCGCCGTGCAGTCGCACCGTGGTCGCCTCCTCCACGCATGGAATCAGCTGACCGGCAAAGAGAAACTCCTTCAAGCCGTTGCCCACCACCACGTGCACAGTCGTCTCGGACGCCTGACAGCCCACCGCCGCGCACAACGCCGCCAGATCGGTAGGCACGCGATCCGCCTCGGGCGTGTAGGCCTCCAACGCGTACTTCTGGCGGGCGTCCTCGCACAGCCGATGCACGTCTCCGTGATCCTCGGTAAAAGCCACGATGCCTTGCGTATGATGAAAGTAGAGCGCAAAAGGACAGAAGGACGTGACTTTCGTGAGCACCCCGCCGTCGTAACAAAGCACAGGCGTACGCACAGAGACGCCGAAATCCGCCTCCACCGTGAGCCCCGCCAACAGAGGAGCGATCACCACGCTCGAGGAACGGTCGCATAGCGAGAGAGTGGCCAGAATCTCCTGCGTTTCTGCGTTCAACCTGCTGAAGTAGAGAAAAGCCGCGGGCCCCACCGGCGCTAGCGCGGTTAGTTCCTCGTGGCTCATGGTGGATGAACGGAAGACAATGGCTACGCCGCCACTGAGTGAATTTTATACCAAGGAAAAGTTCAGCACGTCATGTTTGACGCACGACGTCTGAGACACCACCGTGGCCACCACTGCGGTCTGGCTGCGGTTGCGGACCACCAAAGGCGACAACCGCAACGATCCCAGCAATTCGTAAGAAAAGCTAACCGCTACGGTCGGGTAGCCTCTCGCAGCCAGACCGCTAGCCGACGCACCCGCCCGCGAAAATAGCGTGATGTTCGGGACGGCTTTGCGTCACCGCCAACTAACGTCGGTAGTCGAGCACGTCGTTTATCCTCAGCACACCGTCCGATCACAATCCGTTTTCCCACTCAGTCGCACAAGCAGCACATAAAAACCCCACACAGGGCACGTGAAAACACCGTCCCTAGAAAACGGTGTTTTCTGTCCTACCGTCACCGGTATACACAGGCAAATCCCAAGCCCGATCCCCGAAAACACCGTACGGTGTTTGTGACCTCCAAAATCACATCAGCTAACAAACCGTGAAAAGTCACGTTTCACGAACACGGTGTTTTTAAATCACAAAGAACCGCCTAACGGTTTACAAGCAGAAACACCGCACCACGGTGGTACAAGCGCGATGAATCTGGTCTCGCAACCTCAATCGCCGCTATCACCACCGATTTTCGCTGCGCTCCGCCGACAAAACGTCGTACAAGCTACACACCCCAAAAACCCGCGCGCCTACGGGCGCCAAACCTGTGTATTATCTCAACGTCACAACACGACACAAACCGCGTAACGTGGTTTCCCGAACACGTACGCGGCACAGACCCCCGACACGTACTCGAAGACCTTACAGTTTACGAGTCAATAAAACAGGAAAAGATCCGAACTTTAAAATTGTGTATTTTTATTTTCCCATCCCCCTCTTTTTACCAAAAAACACATTTTTCGTCTTGTAAAAAGTAACTTTCGCCCATTGCCATGAAACACCGTGATGGGGAACGGTGTTGTGTGTCGACTGACGTCACTACGGCGATCAGTATCGACGTCGTGTATACATAACGGTGCCCGGTGTTTTTATTCGGGGCGTTGTCGCGTCTTGATGTAGTGTAACCTGAAACCGCCGTGCCCAAGAATGCGGAAGCCAGCGTGTAATCATAACGGGGTTTTGGGTACAATCTGACGACATCTGGCGGCGAGCGTACACCATCGAATGTGGCGATCGCCGGCTCTACGTCACAATGACGCAAAAACACACTGTAAAACCCGCGTAGACAGCTTTCCTGGTCAACGAGCGCCATCTGGTGTCGGCATAAGAACAGGCATCAACCCCGTGGCCGGCGAGGCGGTGAGCACTTTTCCTGGTCACGTGACCATCAGTGCAGGAAGCGAGGCCCGTAGAACCGCCCAAGAGGCGGTGCCAGATGCCAACGTCATAATCACAAGGTGATTTGTTACGTCACGCGTGTGCGCACACGCGCACGCGCGCACACGCGCGCGGTAGAATACAGCGATCCCTAGTGAAGCCACACCCATTACGTGTAGCCATATCCGCTTACGTATACAACCACACCCCTAGGTACGCCACCTTATCTACCAATCACAGAAACGGATATACAATGAGCCCTCCCTAGACTCCACCCCTTGTACGGAAATTTCAGATAGGTGGAACCCGTTAGGGTTCCACCGTCCTCGGTGTACGTACAGGCTTCTCCGTCTACCGGAAATATACACCTGCTGACGTAGACGCTACTCCCGGATACGCGTCATAAGCTACTGGACCCTAGGGGGGAGTGTCTACAGGGCTACGTGCACGTCCCCTTACCTAGGGTATCCGCCCCCTTCCGCTGTTTTGGCCTAGTAAACTTAACGCCGCCGCTTCTCACGTGACCCCTGACAAGCCCACGTCACACTCGCGTAGCCACACCCACTCCGGATATACGTCATCCTGTGGACTTCCGGACATACGGTGACGTAGCGAGCGTAGCGAGCTACGTCACGTATGCGTACGTCATCTCCGGCGGAAATCATCTCTGATGACGTAGCGAGCGAAGCGAGCTACGTCATCAGTCCGTTTTACGTATACCGGATGCTAGGCGACGCCCCGTAGGGGCGGAGCCTAGCTTCCACCCCTAGGATGCATACCCTATATAGCATAATTCTTCTAACGAAACGTTCTACGAAAACGGACTGGCGGAACGGGAACCACCGTAACCCCCCCCCCTCACCCCCCCCCTTCTCCTCCGGAACCGGGGGGGGCAAATTTTTACCAAATTTGGGCAACCATGATTTCCAATGGGACGGCGTTTCCGTGCGCATGCGCAGTCGGGGCGAATTTTCGGTTGTTAGGGCGTTGCCACGCGGATTATGGGATGTGGCCTCGAGTGCGCATGCGCCGGGGATGTCGTATAAAAAGCCTATATATAAAGAGGGGTGAACCAGGGGACCCGGTGCGCATGCGCGGGCCAGGACCCGCGGGAGGCGCGCCCTGCGCATGCGCCGGTAAAATTCCACTGGGCATGCATCGTGCGCATGCGCCGGTATTTTTCCACTAGAGGCGGTCAGTGCGCATGCGTCGGTAAATTTCCACTGGATGCGCGTCGTGCGCATGCGCCGGTATTTTTCCACTGGGCGGCCGCACCTAGGGAGCGCGAGCCCCGTGCCGGGCATGGGTCGCGGCGGTGGAAAATTACCGCTCCGCCCACCTAGGCGGGGCCTCTGAAAACCTATAAAACCCGGCGTGCCCGCCGCCCCCCGGCGCAGTCCGCGGCAGGGTTCCGGCCGTGCTGCGGTCCGCACGCTGCGCCCGCTCCCGCCTGCCTCCCGCCCTACCCCCCACCCTCCCCGGCCGAGGCCCGGCGCCGGTCCGTCCGCGGGCCCGTCCCACCGCCCTGGAGCACCATCCGGGGCCGTGGGCCGGGCACCGGGCGCGGCCCGCTCCGGACCTCGGCCGGGGGTCCCTGCCCTCCCCCCGCTCGACCCCCCATCCGACGGCCCGGCCGGGCTGGGACCCCCGCACCGGGGTCCCGGTTCCCGTCCGCGGCCCGGGGGGACCCGAGCGGGGGCTTCCCACCCCCACCCCGCTCCTCCCCGGGCTCCGGCCCGGGATCCCTCGCTGCTCCCGGCGACCTCCGCCGGCTTCCCGGTCCACCCGCCGCGGAACGGACGGGACCCGGGGTCCGCGCCCTTCCCCTCCCCCCACGGGGGGCTGGGTCGCGGACCCCGGTTCCTAGGCTCGTTCCGCGGTGGGCGACCGGGGATCCCCCACCCAGCTCCCCTTCCCGGCCCGCCCCGCTGGCTTTTGGGCCCCTCCGGGCTTTTTTTTTCCGGCTGGGGGTCGCGGCGGTCGGCCGACGACGACGGTAGGTGGGCCGGGTGGACGGTGGTGGGGACGGGCGACGCCCCGGCTCGACGGCAGTCGGTCCCGGAAGGTTGGGGGCTGGGGGCCCGGTCAGGAGCTTCGGGAGCGGGGTCGACCGCGACGGCTTCCGGGTCTCGCGGCGGCTCCCTCTCGGCGGCTCCGGTTGGGCTCCCCTCCCCCCTCTCGAGGGTCCGGCCGCCAGTCGTGACCGGGGGTCCCTCGGCCTAGCCGCCGGCTCTCGGTCCGCCTTATCCTGGGCGTTGGCCGGTCCCGTGACGCTCCCCTCCCCCACTGCTCCCCAAAAAAACTCCGCCCGAACCGTCGCGGCTTGCTGGCCCTGGGCGTGGTCCCCCACTCCCCTCCCCCCATCGGCCGCCCAGCCGGGGTCGGCGCCTCGGACCCCACCAGGCTGTGGCGTGTGTGCTGGCCGATGCGGCGGCGAGGTTGGGTGTGGCCGGAAGCGCTCGGGGTCGACGGTGGGCCGCCATGACACCTCAATTGCCGTCAGTACGCCCCTCCACAATCACCGTCCCCACACGATGGGCCCGGCAGGTCACCCAACGTTGGTTCAGGCCCAGTCGAGTTTTTCCCCGGCACGAACGCACGTCCCCGTGGGCTCCACGCGTTTTCCACCCTTTCCTGGAGGGGTCCGGAACACCGTGAATCCACGGGGAGGGTCCCGGCACGGGCCGAGGAGACCACGACCGTCCCACCCGGCGTGTCGACTCGTCCGAGACCCGGGAAGGGAACAGGCCCCACCTTTTTTTCCCTTCTCCGATTTTGCCGTGGAAAACCCGTGAACCGATACGGGTGCAGACGGCCGAAAAAAATCGAGACGACAATATGACGGCAGGGCGCGATCTTCTCCCCCATCCGACAAAACCGTGTCCCTTAAAATTCCCCACCTTTCTCTGTTCAAATGGGCCCGAAACTGTAAAACACCGTTTGACCGCACCCCAACCGGCGCCATCTTGGTGACTTTCTCGACGGTTCTCTCGCTCGTCATGCCGTTCTGAGCTCCGACATGGCGGACGAGAGAAAATGGCGTCGAGAGCCTAGGAGCGTTTTCGCTCCAGGCGGGTAAAAAAATAGCACGATAACTTTTCTGTGCTTTTTTTTGAGACGTTTTAGAAGAGCTTTTTTCTGCTCAGAGCGAAAAAATGATAGCCCTGAAAATCTCGACGAGTCTGGCCGAGCGGCGCCATCTTGGAGGAGGGGCGAGTCGCGGGCACCGCCTCGGTACCCCCTGGCCGAGGCGAGTCCGCGGTCGCCGCCTGTTCCGTGATGCTACCTAGAGGGCGCTGTCGAGGCGACTCTTCCTGTTTTCGCCCTGAGGGCTAACGGTCGCTGACGTCAAACCATCTCGTGCTCGCTGAGTCACATCCGGTTGTTGACAAGCGATGGAGGACCGCACCCAAAGTGCGCCCTCTAGTCATCGCGCCTGACCCCTTTTATAAACTGCTCGAAGAAAAGAACACCTTATGTGAAAAAATACAGAATGATGACAAGTTCATCCAACACAACCGCTCAACAACGCCATATCTATCAGTGTCCAAAAACTATCTTCTATCCTTTGAAACTATAAATGCTGCCTATATACATATTTAGTATCCAAGACTCTTACCACGTAGACGAAAAGAAGTGATACAATGATCTTGACGTGTATCGTCTATATCGTGCTAGATATATTCAGATAAGACGCGCAAACCATAGATTTCTCATCAGTATCATGAAAGACCTATAGCTCTATATACGAACCTAGTCATTTTAGGACAGCCGCCGGAGAAGCCGACGAGGGATCGGGCGGGTGCAGCCAGAACCTCACGCCCGATCCCGCCTCCGGTAGGCGATTTGCATCTGTTTGGTAAAAAGCTCATAAGTCTGTATGTGACCTATATATATATTATACGCTATGTACACCGAACTGTCGCTGTTGTATAAGAAGAAAAAACTCTCCATATTTATATCGTCTGAATTTTTGCTTGATAGACACGTGTTTGGAACTCTGTCCCCCCACGTTTTCACTGTGTATAACAAAAATATGTGTTTCTCAAAAGATCTTGAGGTGTTTGAAAACGGGGGAAACCTGCGTTTGGGTGCGCTAAGCCCCGGACTGGGACGTAGCCGGCGTCCGGCACCTATATTTTTCTATTTTTTTACAAAATATATGATGAACCAAGAATAAAACTCTAGCTCTCGTCTATTTTTAATATGCTCTACTTAGAACCTTTTTAATGACAGAATGAACTCCATGTTATACGCTCTTTATATAGTTTCTCTGCACTAACCTTTAAAACCGTATCCTTCCCTGTTGTACAAATCATCTTTTGATACACAATGATGACCTGATATCCCTCCATATATATGATCGGATATTATTCCGTTAGACTTGTCCTCCTTTTTTTTCCTCATCTCCTATATCTGGAGATATATGTTGACCACCACCGCCATGACCACCAAAAAGCTAGCCGTCACGACTAGAAATGTGTAGGATTCGGACTTTCCGTTCGAGAAGAAAGAGACCGCGTCTCTGGACGCTCTTTTTGTCGGTCTGAATCGACCCGGGATACGTAAGAGAGCGGCCCTACATCGGGGGGCGCTCGAGACCGACGACGTTCCATCTGACCAGAAAAAAAGGCACCCCTCGGTAGCGACCTCTCACCATCGTTTGCCCGTCCGCCCGTCCTTCGTAGCCATCATCATCATCTCAGGCTCTATCGGTACCATCGTTGTCATCTGAAAAAAAAAACTGCCTCACCCACCTGCGTAAAAACACCATCTTTCCGGAGGTGCGGTAAGACGGGCAAATACGGTCGTGCCGAGGCAAAAAAAAACGCACCATCGACACCACACCCTCATGAGCACCACCTGTCGGTGTTGGTCGTCCTCCATCGTTCTCTACGAACATCTCGACGCCCGGGTGACGGACGACGGCAAGACGTCCCGGAGAAGACGGTGTTCTCTCGGGCGGTACGCTCTCTGGATCTATAATATCTATAGTAGCTAAACGAGACTGTGAGTACGACGAACCACATCATCTTTTTTTTATGTTGCTTCTTTAGAAAATGACTTATGTCGACGACACTCGGCATCAGCCATCTCGTGAAACACGCTCGCTTTTCGTCTCTCCAAGGAACACTGGGTCCGCTGAAAGGGACCGTGTACCGACCAAAGCAAAAAACACACACGTAGTAACATGATCAACCACGTCTGAATGACACGAAAACACAATCGTATAACGCTCTATTCATGGAACGAACTTGGAATAAAAAAAACCATCGCAGGCCAGAGGCTAAGCCGAAACCGTCCGGGGAAGCGGGCGCGAGTTTTCCGACTTAGCCTTTGGTGCTCGTTGAGCCTCTTTTTTTTTTTCTGATTCTCTGAAGAATCACCGTCACAGCCCTATGACGCGAAATCAATTGCTAGAACATAAACGTTCTCAACAGGTATGAAATGAACAAACTAGATGATGCTATAACCTTATATTGTGTGTATATAGATAGGTGTGAAATTTGTAGGATAAAAAGTGTCGTTGTATGATGCACAACGATCGTGAAACTGGAGACTGTAGCTCTCTACCGAATGCAAATACACAAATGACATCGATTCCCGTCCCCACATAAAGAAATGTGCTTTACTGTGAAAGAATGAAGAAGATTCTTGTTCCTCGTACGACGGGGCCCTCGCTCGTCGTGCCTCTTCCCCCCTCCGGGAGAGGGGACGTCGGGGCCCTCCGTCGCACCGGGCCGAAGCCAGTGAAATGTTTACTACACTGTCATCAGAATATATGATGTATATTATTTCCTCCAAACTCCTCACCATAGCCACCAATTCGCATCACTTAAGAAAGTAGTAGCAACCGCGGCGGCGGCGACCGGCCGGTCGTCGTCTCCTCGTCCTCAAATGTTGTACATGTGCAGAAAAATGTGTAAATACGTGTTATTTATCCCATGCGTCTTGTACATAGATATATGTTTTTATATACGCTATTTATACTTTATATATCCTTTTGCATAACCATAGACAGTCAAGGATTTTAATGATTTGCTCATCCGCCTTTGAGCCATCGCTTAGGAGTTAGTTCCTCTATGTTCTCGGCCCACCTTTTCGACTACAGTAGCAAACCCTTGTACTACCACCCCGATAAAAACCACATCATCATCGTCACCACGACCTGGAAACGACACACGTTCCCCCCCAATCTTGGGCATGTGTATATATATATAAAGAATGGGAGGGAGAGGACGTGGGGCTCGAGAAGAAATAAACGCCAAGCTCGATTCGAACCAAAAAACCACATGTGTATTGTGCTTTGTTTTTTTTTTTTACGGTGGGGAAAAGGAGGGGGCCGTCATTAACGGAAACCGTGTATGGGGTCCGGACACGAACAGTACACAGCTTATGGGGAAAAAAGCTCACAGAGAGAAAAAAAAACACCAAGCTCAGGCACGCGTACATCATTATCATCATCGGATATCTCACCACGAGTCATAGTAGTACCAAGGAGTGTGTAACACCATTTTTTCTTTTTTCTTTGTAACGGGATAAGGGACAGCAATCATCACGCACAACACCCTTCACTCTCTTTTTAGTCATCCATATCATCGCTGTAACACAGCATGTCCTCGTAATCGGGCGTCTGGCAGCGCATTACCACCGAGTCGTCTTCTTGCGGTACCGGTGGTGGCGGCGGCGGCTGCTGCTGTTGGGTTGCCGTCGTACTGTGATTACCGTTGGCGGACTGCACCGGGATGATGGGCTGCTTGTGGGGAACCTGGGGTGGACTGCCGCCGTGAGAAGGCGACGGCGTCATCAAGTTAAGCTCACCACGGTGACTCCGGACACCGGCGAGGGGCGCCGGGGGACTGGGAGGGACCGCGGTCGTCTTGTAGACGACGGTGTCCCCGTGTCGATCCGTGGCTCGTACCAGATCTTGACTGCTAGCGTCGTCACTGTCTTCGTCCTCTTCCAGCTCGCCCTCAGAGTAGTGCTGCTGTGGTTGCGACGGTGGCTGGGCGGGAGGAGCGGCGGCGGCGATCATTGGAGAGGGATGTCGATGACTCCCTTCTCTGTCCTTTTTATCGTAGGCTGTCAGCGTTGCTGGGTCCGTCCTGCTTTCCATACTTGCGCATTGCTCATCGGTGGGATGAATTTGGTCTCCTCCCCGCTGTTGTCCGCCGGCAGTGGCGTGGTTGCTGGCGGTTGTCGTTGTCGTACCGGCAAAGACGGTGAGATCCAATAGCGACTGCTCGTCGAAGGGACAGTACGCTATCATGAAACGATAGGGTGCCAACGCGCGTTGGATGCGCAGTTCGCACATCTCGTTCTGACACTCGTGGCACTGCAGGGCGCCTAGGATCAGGTCCGAGACAGCGCCGCAGCGGTAGGTACCCATGGCGTTGTTAGTATCGAACTGGTCAAAAAATTGGGGCGTACCGGTGACTTGCAACGCGCGACGGCGTAGCGAGACGGCCACGCGCGAGAAGGAGCACACGTAGGCCATGGCGCGGTGCATGGGTTGCGAGAAGGTCTCGGGCGGACGCTTCTGCAGATCGCAGACGTCGTCGCGTAGCCAGGCGCTCATTTGACCGGGCTTCTTGACTAGCCGTTTGAGCGTGCTGCAATGGTCGCCCCAGCCGTCCTGGTGGTCCAGGATGCAGCCCAGGTCCAGGTTGTTGAGTTTGTTGAAAAGCAGCTGACGCATGCCGCCCACCGTCTCCAGATAGGGATCGTGCGGGTTGACGGGTAGCCCGTGCAGGTGGTGGTACTTCATGTAGCTGAGCGTTTCGTCGATGATGGCCAGCAACGTGTGCAAGTTGGGAGCGTTGTATACGGCGAAGATCTTTTCCACCACCAGCTTGCGCAGCAACGGTTCCTCCAGCCAATCGAACTGTTGACGGATGTGCAACAGGTAGTCGGTGTGCATGAGCTCGTCGTGTGACAGCAGGATGCGACCGCGCGGCTGATGATCTTGCGGGAAGGCGGTGGGGACCTTGAGGTCGGCGGGGTAGGGTGCCAGACGTAGACTCTCGGCCGTGTAGCGCTGAAGGTCGTAGACGGGCGAGGTAGAACTCGGTGAGGTACCCGACGAGGCGGCGCCGCGCTGCAGACGCGCTCTTTTTTTCTTTTCGATCAAACGGCTGAGTTGCTGTAGTTCGTCCTCGTCCATGGCGTCCAGTTCGTCGTCAATAAGCGCCAGCATCTGTTGTTGTTGCGGTCCGGCGGACGATCCGTGATGATTATTGGCTGAGGAGGGGTGAGAAGAACCGAAAGTCGTAGGACAACTGGGAACTCGGCGACGAAGATGCGTCGAATCGCCGCCGTGATGGTGCGGTTCGCCGTCATCGTTGTCGTAAGACTTACCGTAGTGGGGGTTAAGGGGCACCGAGGCGGACGCGGCCACGCGTCGCTTGAAAGAGGAGGACGCCCTATGTCCGCCACGGAAGCCCGCAGTGCCCATGATGATGTGTCCGCCGGTGCCCCCGAGTGCGTGGCGGGAGGAGGGTGGAAGGGGAGGAGGATAGTGGTCCGGATCGCCTTCGGTATCATCGTCTTTGCTGTAGCGGGGTCGTCGTGCGGGGACGCAGGGTCGGTGATGATGCGAGGCGGCGCCGACGGTATCTTCCGCGAGATGGTATTCGCTGGCGGCTGCTCCGTTCCGTGTCGACGGCGAGGTTGGACTTCGCTCGCGTCGGAACTTCCGTGGCACGGGTTCGTAATCCAGACAGAAGCGCCGTGCGCGACGGGCGCGGCGTTCGCGCTCGCTCAGGGAAGATAACGACGGAGCGTCGTGACGGCCGCGTGAGTGCAGCTCCATGGCCGCCGTCGCTAGGAAGGTCACGTTCTGGCACGCTGATGTATATATAGATGAGACCGCTGCCGGGGGGCGGGTCACCGGCGCCGTGGAAAGTGAGGCTCAGACGGCGGTCGCCGGCGGCATGGGCGCGTCGGGCGGTCTGATTTTGATGGAAATGTGGACGTTTTTGGCGTTGGAGTGACACTTTTTGGTGAAACAGCGGCTCCAGAGGCTGGCCCAGAGCGCGTAGCTGTGCTCGGTGCGCAGGTCGATGAACACCTGCACGGTCTCTTGCGGGTTGCGGTGCGTGTAGTTGAGACAGCGAAAATCCCGCGTGCGCGCGCCGTCGCGTCGCTTGACGGCCACGCAGCAGGCGCCGTGGGGCTGAAAGAGGAGGACGTGGGGCGCGGTAAACTGCTCGCTGACGTGCGGTTCGTAGTGTTGCGTGAGGTGCTCGAGCAGCGGCGGCCACACGCGGGTGACGACGAGCCGCTGCAAGTCCGTGTCGGAAATCGCAGCGGCAGTGGCGCCGTCGCCACCGTACAGGTGATAGGCGAGCACCTCGGTGAGACCGCGGCGTCGATAACGCGTCACGTTAAGCGAGCGCGTCTCGATAAAGTTGGCTTCGGTCGAGGGGCAGATTTTGTCGCGTACGCTGAGAATGACGCGTGGCGGCGGTGACAGGGGCAACGCGGGCAGGTCGTGCGGCGGGTGGTGGTGAAGCAGGTTACGCAGATCCAGTTGGGCGCGCACAAAGCCTAGCGGGTGTTCGCGGTAGGCGTCGGGCACGATGAACAGCGGCAACAGACGGCGATGCATGAAATAGCCGTCGTCTTGGTCCATTTTATACATGTAGGGCAGACGTACAGAGCGTCCATGGTGGTAGATGCCTGTGTCTAGGCTGCTCTCGGGATGCGAGATGGGGTCCAGCAGCGTGTGCAGTTCGGCGTCGAGACAGACGGCGTGATTGAGCACCTGCGCCACGGCGCGTAAAACGCTGGGGTGTACGGCTATGGTACAGGCGGGGAACGGCGTGATGATGCGCAGCCCCAGTTTGCCCTTGCAGCGGCAGTAAGGGGGTGACGTGTCAACAGAGGACGTTGTTTTTTGGAAAACGCCGTTACCCGGGACGTTATTTTTATCCTCTTTCCCGTCTTCGTCTTCCTCTGTGTCGCGCTCGTCCCGGTAATCGAGATAGTCGTCGTCATCGAAAGGCGCGCCGGCCGCGTCCACGGGCACGCTGTTGGGTGGGCACGCGCTTTTGAAGAAATAGACCGGGTGCCGGTCGGGGTGCGTGTAGCCAAAGAGGCTCGCCCATACGGTCATCCAGACGCGTCGTAGTCCGCGACATAACTCAAAGACGGTGTGTCGCGCCAGACCGGAGACGCCGTCGCGCAGCCGTAAATCAAAGTCGGCCACAAAATTGAAGACGGGCAGACGTTCGTTGAAGACTTCGTGTCGCGTGTAGTAGAACTGTGTCTCGGGGCTGGTGCTGGCCACGTCGTCGTCGTGTAGCCACACGGTCTCGGTCAGGGCCTCGTCCGAGAAACGGCTGTCGGGTACGTGACGGAGCAGGTCGCGCGGAAAGAGGCTGCGATGCCAGGTTTCGGAGGCCACGGCGCAGAAGACGTGCTGGTCATTGGGCAGGTGTACGCGGTAGACGGGCAGCGGTCGCTCCAGCAGCGGTGCCAGCGCGGGCTCGGGTAGCAGGTAGCGACGTTGCGAGTAACGCGTTAGCGTGCCGGTGGTGTAGGTCTGAGCTGTGCGCAGCGAGGCGCATAGACGTAACAAGCCGGACAGGGAGCGTTCCAGCGGCGAGAAGACAGACTCGGAAAGCGTGTTGATGCGTTCGAGCTGGCGCGCCAGCTGCGTGGAGGTGCCGAAGAAGCCCGCCAGGTGCGTGCCGTCGATGCGGCCGCCGTAGCCGGCCAGCCCCAGGCCGTGCGGGCTGGTCGCCGAGTGGGGGGATTCGTCGAGACGTAGTAGATGCGTCTCCACGTAATCGTGTAGAAAGTTGTCGAGCGAGAAGTATTTTTGCATGACGTCCAGCAGCTCGGTGGAAAGCCGGCGGCCCAGAAAACCCGGTTCGCGCGTACACTGCGCTTCGGGCGCCGCGTCAGCGTCGTAAGCCACCACGCGCCGGTACTCGAGCAACCGCGCGCGTGCCAGCGCCGTGCGGTAGGCCAGGTAGACGTAGTGCACGCAGACCGTGTCGGGCAGACGCGCACGTTCGCGGAACGCGTTGATCTGCGTGTCCACCTGCTCTAGCTCGGTGTAGTCGCGGCGGTTGCGCGCCACGGCGTACGCCACGAAAGCGGACACGCGCTGACGGAAGGGCGAGCCCAGTAGCAGACGCGCGAACTCGCCCATGGAGGCGTGCGTGGGGATGATGGTGCCCAGGTCGCGCGTGCAGAAGCTGCGCACGTACTCCTCCACGGTGGAGATGGTGCTGTACTGGCCCTCGAATAGGTAGTAGGCCATGGTCAGTAGCACCTGGCCCTCGGTGTGCCCGAAGACGCTGATGAACCACGAGGGCGAGGTGGGGCAGAGGAAGACCTGGTTGAGATGACGTAGCACGGCCGCGTGGTGAAAGTACACCAGGTGCTTGAATTCGCGCACCTCGCCGCCGTGTTCGGGCGAGAGCACGGGCGTGCGGAAGAGATGCCGGTAGAGCGGCCGCGTCTCGGCCTCGTCCAGACTGGCGATGAGCGCCGAGAGGGGGATGGGCTGGCGCGCGGCCAGGTAGCGCGAGAGTTGCAGCGTTTCGTTGTTCACGGCGAAGACGGGCGCCACCCGCCGCGAGTCCGAGCACTTTTGCGTCTGTAGGCAGAAATAAACACGTCGCGAGACCTGGTGTTTGACCAGCAGGGGGAAGACGCAGTGGTCCGTCGGTGTCTGCGAGAGTACGTTGGCGACTATATGAGCAGAATCATACTCTGTTGCGAACAGAACGAGCGTCATCGTCGCGCCGGCACGATGCAGCTGGCCCAGCGCCTGTGCGAGCTGCTGATGTGCCGTCGCAAAGCCGCGCCTGTGGCCGATTACGTGCTGCTGCAGCCTAGCGAGGACGTGGAGCTGCGCGAGCTGCAGGCGTTTCTGGACGAGAACTTTAAGCAGCTGGAGATCACCCCGGCCGACCTGCGAACCTTTTCTCGCGACACGGACGTGGTGAACCACCTGCTGAAGCTGCTGCCGCTCTATAGGCAATGCCAGAGCAAGTGCGCGTTCCTCAAGGGCTATCTCTCGGAGGGCTGTTTGCCTCACACGCGGCCGGCGGCCGAGGTGGAGTGCAAGAAATCGCAGCGTATCCTAGAGGCCCTGGACATTCTCATCCTCAAACTGGTGGTGGGCGAGTTTGCCATGTCCGAGGCCGACAGTCTGGAGATGTTGCTGGACAAGTTCTCCACGGATCAGGCCTCGCTGGTGGAGGTGCAGCGCGTTATGGGCCTGGTGGACATGGACTGTGAGAAAAGCGCGTACATGCTCGAGGCCGGCGCGGCTGCGACGGTTGCACCACCGACGCCACCGGCGGTCGTTCAGGGGGAAAGCGGCGTCCGCGAGGACGGGGAAACGGTCGCCGCCGTGTCGGCCTTTGCCTGTTCCTCGGTTTCGGACTCGCTGATCCCCGAGGAAACGGGGGTCACGCGTCCTATGATGAGTTTGGCTCACATTAACACCGTCTCCTGTCCCACCGTTATGAGGTTCGACCAGCGGCTGCTGGAAGAGGGCGACGAGGAGGATGAAGTGACCGTGATGTCGCCGTCACCCGAGCCCGTGCAACAGCAGCCGCCGGTCGAGCCCGTGCAGCAGCAGCCCCAGGGACGCGGGTCTCACCGTCGGCGCTACAAGGAGTCGGCGCCGCAGGAGACGCTGCCTACGAATCACGAACGCGAGATTTTGGATCTCATGCGACACAGCCCCGACGTGCCTCGGGAGGCGGTGATGTCACCGACCATGGTCACCATACCTCCTCCCCAGATACCCTTTGTGGGTTCCGCGCGTGAACTCAGGGGCGTGAAGAAAAAGAAACCCACGGCGGCGGCCTTGCTGTCCTCCGCGTGAACAGCCTGGCACGTTTTGGAAAACGTACGTGATCACGAACACGACGAGTACGAGGTTTCTCATAGACGTACTTTATTAGGTCAGGGATGACGGGGAGGTTTCGGACCGACGTCAAAAATAACGTCACTCGTGTTGACAGGGCTTTCTGCGTCGGAGCTCTTTTCATCTTCTTCTGTCTCGTCGACGTCATCGTCTACCGGCGAGGGTGTCCGTTGCAGCAACGCGTGCTCGGGTGTGTGGGTGAAACCGATGTCGGGGGTGGGCGGCACGATCATCTGTCCTAGGGGGTGACTGCCCACCGGCAGATAGGTAAAGCGGTGGGTGGTAAAAACCGCTTTGGCTACGGTGGTGTGTGGGGAGATGCAGACGGTGGTGTGCGAAGTGTTGACCACCGTCACGCCGGCCGCGGTACCCGGGAGCCAGATGGTGGGTCGGATGATGAGATCCGATTGACTAAACTGGCGCACGCCCACTATGAGGGCGCAGATACCGGGCGCGTGCACGTAGGCCGCGTCAAAATAGACGGTTTGCGTGTGACCCGGACCGATCACCAGCGTCTGACGGGTACGTAATGAAAAGAAACGGTGTTCGTTGGGCGGCGGCAAGTTCATGAGCTGCCAGGGTTCTGGCACAAAACAGGGGAAAACGCCGATATCGCCTTCGATGGTGCCCGGAAAGATGGACTGAAAAGTGTCGTTGAGGTTGACGACATCCAACTGCGGGACTTGCAGCCCGGATTCCAGCAGCTCGGGCATGCAAACGAATTGCGCGTCCAGGCATTTGTAAAAGGTAATGCCGAAAAAACCTTCGGGGATATAGAGGCTGACGCCCAGCGAGGTGGGCACTTTGCGCTCGCGTGATAGCCAAATGATGTGTTTATTGTAAAAGGCCAGCTGCGTGTGGCATTGTTTGACGATGAAACTGGAAGGCATCCACTTGTAGGGAACTTTGAGCGGCGACGGTAATGGCGACGACGCTTCATCCTCTCCCGGATGCTGCTCTTTGTCGTATTTCTCCTCGGTCGATTGGGGCAGCGTAAATGTGGTTTGAAAATCGCTATCGCTAGCGAAACGCACGCAGTAACGCATATTGACGGATTTCTCGGCTAGGATGATGGAGCCTGATGACGGTGCGGACTCTTCCTTCATTATTAACGTAGGGGTCTCCCAGAATCGCTGAAAACGGGAGCGCGGCAGCCGCGACAGTACCAGTTGAGAGTCGATTCGGTCGGTTAACATCGTAAGCATCGTGGCGGTGGTGTGATGGAGTGGAACACACTAGTATTAGGTCTTTTGGTTTTATCGGTAGTGGCAAGTTCCAACAATACGTCGACTGCTAGCACACCGCGTCCCTCTAGTTCTACTCACGCCTCAACAACCGTGAAGGCAACGACTGTTGCGACAACTAGTACAACTACGGCGACAAGTACTTCATCGACGACTAGTGCCAAACCTGGTTCCACTACTCACGACCCCAACGTGATGAGACCACATGCTCACAATGATTTTTACAATGCGCATTGTACATCGCATATGTATGAGCTTTCACTGTCCAGCTTTGCAGCCTGGTGGACTATGCTTAACGCTCTCATTCTGATGGGAGCTTTTTGTATCGTATTACGACATTGCTGCTTCCAGAACTTTACTGCGACCACCACCAAAGGCTATTGAGGGTGGATAGATTTGCAGCCCGGCGGTGTTCCGGCGGGGTAAGATTTCCATACGCGGGTAACTGGAGGCTAAAGTTACGGATTTTATCTAGAAACAGCAGCGAGTCTAGATAGTCCCATAGGGGATCTATGAACGTTCTCTGAAACCTCGTCGATGGTGACGTAGGTGTAGTTTCGTTATTATCGGAAGCCGTTTCGTTTTCCACGGACATGGTTTCGTTGTAATATAAGGAGCTCATGTCAAGAGTGCCATAAATAGTGTACGGCGTTTCGTTACGAATCAGTACGTGCGTGTTTTTCATAAATTCTGACACGGCGGTACGGTTACGGTCTGGTTTACAAAAGGGTTCATTCCGATACCGCAGAGTAGTATATACCCATGTTGCGAGATCTCTTAACTGCGTGGCCATAATGGATTTCATAAAGTTACTATCAGGACGATAACCTGTCGTAGATGTGGCAACCCGCGTTACAGCAGCGGTAGCACTATAAGTCACGTTAGTGGTGACGTTGAAAGCGGTAGACGTTGTATAGGAAAGATATGGCGTAGTAGTACTTTGAGATTTCTTATTCTTTTTTTGTGGTTGTTCTTTGACAAGCGCTTGTTTACGTTTGAGTTTTCGCATAGTGTTTTTCAACTTGGTGCCGTTAATATACTTGGGAACGCGGAATAAATTTCGGCTCATGGCGTTAACCAGGTAGAAACTGCGAGTGCAGTTACGTTGCGCGTATCGTAAAAGTAGGGCGGTTAGGCCCAAAAAGTAGATTGTCTGACTATCCACGTTGACTTTGTTGGAACCTATGTACAGTTTTGTGTTCCAACGTGGTACGTTGAAAAACATAGGATTAAACGTGGTGAAATTACCGCAACCTTTTTCGCCGGTATCATTGCGTTTGGAAACATTTAACATTTCGGATAGGCAGTTCATAGAAGGCACTGTGCCGCAAGGTGGGGGTCGTAACGTTATTTTGTGGGCCGTGTGATTATATTCGGAATATACGTACTTGGCTGGTTTTCGGAGTTGAGTACTGTAAAAGTCAAACCACATATGGGTAATGCTATCATTTCTAATGGGACCCGCTAAAATGTAGTCTTGAGGAAATTTATCCATGCTAATAACGATGGCATGAAATTTTTGCTGACTGGGGTACATTAAAAACCGGTATCCGTCAGATGATGTAGACATCAATCGTTTTAAGATATCTTCCTTCTGTTTCTTCAGAATCTCTTTACCTCGATAAGATAGCACGGTATACGGCCAGGAACGTCTTGTTCCTCTGGAGTTTACCAATACATTACAATTTATGAGGGAAAGGAGCAAGAACGTTATAGAGATCAGGAGCATAATTTTAGGAATGCCTTTCACCATTATCATCTCTTTTTTCCCCATTACAGAGGAGGAGACCCCGCACCGTCCGTCTGCCTTGTGGTTTGGTTTGCCTGCGTGTACTCACTGCTGATTCTGGTCGTTTTGCTGCTCATCTACCGTTGTTGCATCGGCTTCCAAGACGACCTAGTCTCCCGCACCTTGGCTGTGTACCGAGCTTGTATACAGGGCCCGATATGTAACCAAACCCACAACAGTACCTCGTAAATAAAGACGCACAAACCTCACGCATATAGTATCATCACACCGTGTGGCGTGTACTTTATTACAATGAGCAAGAGTGCCCCTAAGTATTGGGGCCCGTACCGTTTTAGAAGGTTTTGTGCGAATGTCTTTAACTTCTCTGTCCCTTTTTTCATAAACTGTCAGGTCCTACAATCAGCATGTCTTGAGCATGCGGTAGAGCAGATAGATGCCGATGATGGCCGATAGCGCGTAGACGGACATCATGAGGAGACGACTGTCGGTGGCGTCCACGACGACGTCAGTTACTTCTAGTACCGTACCGTTTTTCAAAAGCATGAGGTAGTGAGTTCGCGGAGATGAGACCACCACTTCGTTGTAGGGATCCAGGGCGAAAAGGACGTCGTCCGAGTCGTGCATGTACATGATGTTGATGACGCCTTGCGTGTCGTCGTATTCTAGCAGGGCGCTTTGGCAAAAGGCGCAGTTTTCTAGCGAAATGTTGAGCGCCACTGTGATGCTGTGTGTGGTATGCATGTTGCGCGTCAGTTCGCATTTAGTTTGACTGTCCGTCTGGGTGATGATGAGGCTCTGGCCTACGACGGTGGTGGAGACAGGGTAGGAGATACCTTTGATCAGGTACTGGTTTGTTACGATATAACTGACGTGTTCGGAGACGGTCAGCGCGGAGAAGGATTCGCCGAGCGGCAAGCAAAACAGGTCGGGGAAGGTTTCCAGCGTGCTTGGTTGCATGGTAGATAGGATGGAGAGGGCGGCGGGAACGGTAGCGGGGACGGTGGCATCGGGGAAGAGACGCGTGAGGCGTTCGAGCGAGTGATCGCGTCGCCCGCTACTGGAACAGGGTGTGTACAGGTCGCTGAGGTATTCGTGGTGTGGATGAGCTAACAACTGCGTAAAGTGTGATAGCTCGGCCAATGAACAGAGGCCCGTTTCTACGATGAAGATTTCGCGTCTCTCCGTCGTATGTACCAGCATGGAGTGGACGAGGCTGCCCATGAGGTAGAGTTCTTGGCGTGCGAAGGCTGAAAGAAAAGAGGCCAGGTGCGTTTTGTGTAGTTTTAGGGCAAAGTCGGCGATCTGTCGTAGTGCCCATTGGGGGATGAGATGTTGCTGATTCTGTTTAGAGAGTATGTAGACCAGGCGTACGAGGCTGGTGATGTCGGTGATCTGATTCGGTGTCCAAAGGGCTCGTTTGGCCAGGTCCACGGCCGTGGGATACAGCAGCAACGTGGTGCGTGGTGGTGTTTGTGAGAGGCAGGTGATCATAAATTCTTGTATTTGTAAGAGTGCGGCCTGGCGGTCTAGGGCCCGTGGGACGGAGACTTGGGCGCCGGCCTCTTCTTGTCGGGCTGCTGCGAACAGTGCTAATGCGTAGGCGAAGGCCATTTCTACCGTGCGGCGGTCCAGCATCTGACATCGACCGCTCTTGAGTACATCCACGGCGTAACGGTGAAAGCTGTTACGTAGTAGTGCGCTGAGGTCTAGGTAGTTGAAGTCAAGTGCGGCGTCAAGAAAGTCCGGGTCTTTGAGATAAGAGTGACGGTTCAGTTGATCTTTCTTAACTAGCACCAGGAGCTCGTGTTTTTCAGTTTGTCGTAGTATAAAGTTGTCGCGTTGATAGGGCGCTTTGAAAAGTACGCGTGGAAGATGGCCGAAGATAAGCAGCATGGGTGTGTCGTCGTCTATGGACACCGTAACTACGAAGAAGTCCTCGGTCAGTGTTATTTTAACGTAACGTAGTTCGTCGATGAGGTAAAAGCCTTGGTGCAAACAAGGTGTGACGGTGCTGAATAGTAGATCGTGTCCATCAAAGAGGATACAGGTCTGGTTAAAGTGTGGTCGGTGTAGTCCTGAGGTGGTATGTGATTCTGTCCAGCCGTGTGGAGTGGTTTGCGGTGGCATCCAAACGTGAGGTATTGACAGGTCAATGGGCGGTGGCACAGTGGTGGGCTGTTCACCTAGGCTGTCTTGTGCCTTTAGCTGCTGCGAAAAAGATCGGTAGCTGGCCAGGTCTTTGGATACCAGCGCGTAAGTGTTAAGTCTCTGTTGGTATCTTTCCAGGGTTTCGGTCAGATCTACCTGGTTCAGAAACTGCTCCGCCAGAGGACCCGCAAAAAGACATCGAGGCATATGGAATACATAGTATTGATTATAGCTTTGGAAAAAGTTGAAACTGATGGCGTTTTCCCTGACGACCGTGCTGTTACGGAGGCTGCTGTTGTAGGTACACTGGGTGGTATTTTCACGCAGGAAGCGGATGGGTCTCCCGTAGGTGTTGAGCAGTAGGTGAAACGCTTTGTCCAGCGGTTCGGATACGGCTTCTGCGCCATATCGTGACGAAAGTAGGTGGCTGAAGAGACAGACGGCGAGGATGATGAGGTAGGAGGGGAGGCCTGGCCGCATAGCGCGGCCGCGCCGCTGGGTTCAGCGGCGTGATCCAGGTGGTGGTTGGCGTTACACCCGAGAGAAGGAGAGAAAGGATCCCAGGAAGGAGCACCCGGGTGTGGCGCTACGGGTTACAAAAGTCGCGTCTCCGTCTATTTAATACGATGTCATTGGCCGCTGCGAAGGGAGAAGAGGGGACACGCGAGTAAGCCATGCCGTCCGGGTGTGGGGACGACGCTGATTCGACGGGGAACGCTCTGCGGAGATTGCCTCACGTGCGTAAGCGGATCGGTAAGCGCAAGCACCTGGACATCTACCGTCGCCTGCTGCGGGTCTTTCCCTCGTTTGTGGCGCTCAACCGCCTGTTGGGAGGCCTTTTCCCACCCGAGTTGCAAAAGTACCGTCGCCGTCTTTTCATCGAAGTACGATTAAGTCGGCGGATTCCCGACTGCGTGTTGGTGTTTTTACCGCCGGACTCTGGGTCACGCGGCATCGTGTATTGCTACGTGATTGAGTTCAAAACCACGTACTCAGACGCCGACGATCAGTCCGTGCGGTGGCACGCCACCCACAGCCTGCAGTACGCCGAGGGCCTGCGCCAGCTCAAGGGCGCCCTGGTGGACTTTGATTTTCTGCGTCTGCCGCGCGGTGGCGGTCAAGTCTGGAGCGTAGTGCCTAGTTTGGTTTTTTTTCAGCAAAAGGCCGATCGCCCATCTTTTTACCGGGCTTTTCGCTCGGGCCGTTTCGACTTGTGTACCGATTCTGTTCTGGACTATCTGGGACGGCGTCAGGATGAGTCTGTTGCACACCTTTTGGCGGCTACCCGTCGCCGTCTTCTTCGAGCCGCACGAGGAAAACGTGCTGCGCTGCCCCGAGCGCGTGCTTCGGCGGTTGCTGGAGGACGCGGCGGTGGCAATGCGCGGCGGGGGCTGGCGCGAGGACGTGCTCATGGACCGGGTGCGCAAACGGTATCTGCGTCAGGAGCTGAGGGATCTGGGTCACAGGGTACAGACTTACTGCGAGGATCTCGAAGGGCGCGTGTCCGAGGCGGAGGCGCTGTTGAACCAGCAGTGCGAGCTCGACGAAGGACCGTCGCCGCGGACGCTGCTACAACCACCGTGTCGTCCGCGTTCGTCGTCCCCAGGGACCGGCGTGGCAGGAGCTTCCGCCGTCCCACACGGTCTTTATAGTCGGCACGATGCCATCACGGGACCCGTCGCCGCCCCGTCTGACGCGGTCGCCGCGTCAGCGGCCGCCGGTGCTTCTTCTACCTGGCTGGCGCAGTGCGCCGAGCAGCCGTTGCCCGGGAACGTACCTAACTACTTTGGAATCACGCAGAACGATCCCTTTATCCGCTTTCACACCGATTTTCGCGGCGAGGTGGTCAACACCATGTTCGAGAACGCCTCCACTTGGACTTTCTCCTTTGGTATCTGGTACTATCGGCTCAAGCGGGGGTTGTACACGCAACCACGGTGGAAACGAGTGTACCATCTGGCGCAGATGGACAACTTTTCCATTTCGCAGGAGCTGCTGCTCGGCGTGGTCAACGCTTTGGAAAACGTGACGGTGTATCCGACGTACGACTGTGTACTCTCCGATTTGGAAGCCGCCGCCTGTCTGCTAGTCGCCTACGGACACGCGCTTTGGGAGGGCCGCGATCCGCCGGACTCCGTGACGGCGGTGTTGAGTGAGCTGCCTCAGCTGTTACCGCGTCTGGCCGACGACGTGAGTCGTGAGATTGCCGCTTGGGAAGGCCCCGTCGCCGCGGGTAACAACTATTACGCGTATCGCGACTCGCCCGATCTACGCTACTACATGCCCCTAAGCGGTGGTCGCCACTATCACCCGGGCACTTTTGATCGTCACGTGCTGGTGCGGCTTTTCCACAAACGCGGCGTTCTTCAGCATTTGCCGGGCTACGGGACGATAACGGAGGAGCTGGTGCAAGAGCGTCTGTCGGGCCAGGTGCGCGACGACGTGCTTTCTCTCTGGAGTCGACGTCTGCTGGTCGGCAAGCTGGGTCGCGACGTGCCCGTCTTTGTGCACGAACAGCAATATCTGCGTTCGGGCCTGACCTGCCTGGCTGGCCTGCTGTTGTTGTGGAAGGTGACCAACGCGGATAGCGTCTTCGCTCCGCGCACGGGCAAATTTACGTTGGCCGACCTGCTGGGTTCGGATGCCGTAGCCGGCGGCGGGTTGCCCGGGGGGCGCGCGGGCGGCGAAGAGAAGGGCTACGGGGGACGGCACGGGCGGGTACGTAACTTTGAGTTTCTGGTACAGTACTACATCGGGCCGTGGTACGCGCGCGACCCCGCGGTCACGCTGTCGCAGCTCTTTCCCGGCCTGGCTCTGTTGGCCGTGACCGAGAGCGTGCGCAGCGGCTGGGATCCCTCACGTCGCGAGGACAGCGCCGGAGGTGGCGACGGCGGCGGCGCCGTGCTCATGCAGCTCAGCAAGAGCAACCCCGTGGCCGACTACATGTTCGCGCAGAGCTCCAAACAGTACGGCGATTTACGTCGCTTAGAGGTACACGATGCCCTGCTCTTTCACTACGAACACGGGCTAGGGCGGCTGTTGTCAGTGACCCTGCCGCGTCACCGTGTGTCCACTCTGGGCTCGTCCCTCTTTAACGTCAACGATATTTACGAACTGTTGTACTTTTTAGTGTTGGGGTTTCTTCCGAGCGTGGCGGTGTTGTAATTTCCACCACGTGTCGCTCGCTGCATAAAGGGCGAACGTCCTCGGAGAGGGTATATTCGTTCGGCGAGAGCGGGCGGCGGTGGTGGGTATGTCCCCTTCTGTGGAGGAGACTACCTCAGTCACCGAGTCCATCATGTTCGCTATCGTGAGTTTCAAACACATGGGCCCGTTCGAAGGCTACTCTATGTCGGCCGATCGCGCCGCCTCGGATCTACTCATCGGCATGTTCGGCTCCGTTAGCCTGGTCAACCTGCTGACTATCATCGGTTGCCTCTGGGTGTTGCGTGTTACGCGGCCGCCCGTGTCCGTGATGATTTTTACTTGGAATCTGGTACTTAGTCAGTTTTTTTCCATCCTGGCCACCATGTTGTCCAAGGGTATCATGCTGCGTGGCGCTCTAAATCTCAGCCTCTGTCGCTTAGTGCTCTTTGTCGACGACGTGGGCCTATATTCGACGGCGTTGTTTTTCCTCTTTCTGATACTGGATCGTCTGTCGGCCATATCTTACGGCCGTGATCTCTGGCATCATGAGACGCGCGAAAACGCCGGCGTGGCGCTCTACGCGGTCGCCTTTGCCTGGGTTCTTTCCATCGTAGCCGCTGTGCCCACCGCCGCTACGGGTTCACTGGACTACCGTTGGCTAGGCTGTCAGATCCCTATACAGTATGCCGCGGTGGACCTCACCATCAAGATGTGGTTTTTGCTGGGGGCGCCCATGATCGCCGTACTGGCTAACGTGGTAGAGTTGGCCTACAGCGATCGGCGCGACCACGTCTGGTCCTACGTGGGTCGTGTCTGCACCTTCTACGTGACGTGTCTCATGCTGTTTGTGCCCTACTACTGCTTCAGAGTCCTACGCGGTGTACTGCAGCCCGCTAGCGCGGCCGGCACCGGTTTCGGCATTATGGATTACGTGGAATTGGCTACGCGTACCCTTCTCACCATGCGTCTTGGCATTCTGCCGCTCTTTATCATTGCGTTCTTCTCCCGCGAGCCCACCAAGGATCTGGATGACTCCTTTGATTATCTGGTCGAGAGATGTCAGCAAAGCTGCCACGGTCATTTCGTACGTCGGTTGGTGCAGGCGTTGAAGCGGGCTATGTATAGCGTGGAGCTGGCCGTGTGTTACTTTTCTACGTCCGTCCGAGACGTCGCCGAGGCGGTGAAAAAGTCCTCCAGCCGTTGTTACGCCGACGCGACGTCGGCGGCCGTTGTGGTAACGACAACCACGTCGGAGAAAGCCACGTTGGTGGAGCACGCGGAAGGCATGGCTTCCGAAATGTGTCCTGGGACTACGATCGATGTTTCGGCCGAAAGTTCCTCCGTCCTCTGCACCGACGGCGAAAACACCGTCGCGTCGGACGCGACGGTGACGGCATTATGAGCGGCGGCGCTGTACGGCAGCGGGGAGAAAAGTGGCAGATAAATCACGTCAGGTTCACACGTCGTTAGCCAGCGTCGGCATATGAAGGGCGCGGGCGGCCAGTACGGCCTCTGGGCTGAGACAGGACGAGGCAGGGTGAGAAAGAGGAGGATGGGGGGGACCGGGGTGGTGGTGCTGCTGCTGTTGTGGGTGCGGACGGTGCGGGTGCCGGGACAGCGTGCCGGCGAACGTTCTGTAATCTTCCATAATAAAAGTAAAAATGCCCGTCTCGTGTCGACTCCGCTGGATGTCGAAGGCGTCGGGGGTAATGCGCATCTTGCCGGTGCCGATGAGATAAAAGTACCACATTTTTTGACAGATGATGCGAATCAAGGGTTCGTACGCTTCGGCACCCCAGTGGCGCGTGAAGAAGGCCGCCAGACGAAACAAGCGGTGTCCGTAGAGCGTGCCTAGGGAGAAGAGGATGTTGCCGTTGCGCGCCAGGTCTTCGGGGAAAACGACCGGCAGGCCGGTGTGGCGCTGCACAAAGCGCGTCAGCAGTCCGCCGCTCAAGCGCGGGTGACATAGGCGCTGGCTGAGACGGGCGGCGCGCGTTTCATCGAACACGGCCGCCTCAAAGTCCAGCCCCGGGAAGGCCTGGCGCAGTTCGCGGTACAGATGAGGCCAGTAGGGTTGCGGCGTCTTGCGACTAAGCACGGCGTGGTCCGAGACACCCAGGTTGTTCATGGTTTCGCGCAGTAGCAGCGTTTCGAGACCGCGGTGAAAGAGGAGGACGCAGATGAGGCGTACGATCTTGAGTTCTTCCAAACGCAGCGAGCTCAGCGGCTGTCCGCGCGACATCTTCTCGCTAATCTGTAATATTAGATGATTGGCGCAAGTAAAGGAGAATTTGCCCGTGCGGACCCGCGGGACGGCGGGGTTCTCTTCGTCGCGGGCCATCATCGTTCGCTCGGTGAGCGGGTAGCGACGGTGACGACAATGACGATGGACGAGCAGCAGTCGCAGGCTGTGGCGCCGGTCTACGTGGGCGGCTTTCTCGCCCGCTACGACCAGTCTCCGGACGAGGCCGAATTGCTGTTGCCGCGGGACGTAGTGGAGCACTGGTTGCACGCGCAGGGCCAGGGACAGCCTTCGTTGTCGGTCGCGCTCCCGCTCAACATCAACCACGACGACACGGCCGTTGTAGGACACGTTGCGGCGATGCAGAGCGTCCGCGACGGTCTTTTTTGCCTGGGCTGCGTCACTTCGCCCAGGTTTCTGGAGATTGTACGCCGCGCTTCGGAAAAGTCCGAGCTGGTTTCGCGCGGGCCCGTCAGTCCGCTGCAGCCAGACAAGGTGGTGGAGTTTCTCAGCGGCAGCTACGCCGGCCTCTCGCTCTCCAGCCGGCGCTGCGACGACGTGGAGGCCGCGACGTCGCTTTCGGGCTCGGAAACCACGCCGTTCAAACACGTGGCTTTGTGCAGCGTGGGTCGGCGTCGCGGTACGTTGGCCGTGTACGGGCGCGATCCCGAGTGGGTCACACAGCGGTTTCCAGACCTCACGGCGGCCGACCGTGACGGGCTACGTGCACAGTGGCAGCGCTGCGGCAGCACTGCTGTCGACGCGTCGGGCGATCCCTTTCGCTCAGACAGCTACGGCCTGTTGGGCAACAGCGTGGACGCGCTCTACATCCGTGAGCGACTGCCCAAGCTGCGCTACGACAAGCAACTAGTCGGCGTGACGGAGCGCGAGTCGTACGTCAAGGCGAGCGTTTCGCCTGAGGCGGCGTGCGTTATTAAAGCGGCGTCCGCCGAGCGTTCGGGCGACAGCCGCAGTCAGGCCGCCACGCCGGCGGCTGGGGCGCGCGTTCCCTCTTCGTCCCCGTCGCCTCCAGTCGAACCGCCATCTCCTGTACAGCCGCCTGCGCTTCCAGCGTCGCCGTCCGTTCTTCCCGCGGAATCACCGCCGTCGCTTTCTCCCTCGGAGCCGGCAGAGGCGGCGTCCATGTCGCACCCTCTGAGTGCTGCGGTTCCCGCCGCTACGGCTCCTCCAGGTGCTACCGTGGCAGGTGCGTCGCCGGCTGTGTCGTCTCTAGCGTGGCCTCACGACGGAGTTTATTTACCCAAAGACGCTTTTTTCTCGCTACTTGGGGCCAGTCGCTCGGCAGCGCCCGTCATGTATCCCGGCGCCGTAGCGGCCCCTCCTTCTGCTTCGCCAGCACCGCTGCCTTTGCCGTCTTATCCCGCGTCCTACGGCGCCCCCGTCGTGGGTTACGACCAGTTGGCGGCACGTCACTTTGCGGACTACGTGGATCCCCATTATCCCGGGTGGGGTCGGCGTTACGAGCCCGCGCCGTCTTTGCATCCGTCTTATCCCGTGCCGCCGCCACCATCACCGGCCTATTACCGTCGGCGCGACTCTCCGGGCGGTATGGATGAACCACCGTCCGGATGGGAGCGTTACGACGGTGGTCACCGTGGTCAGTCGCAGAAGCAGCACCGTCACGGGGGCAGCGGCGGACACAACAAACGCCGTAAGGAAACCGCGGCGGCGTCGTCGTCGTCCTCGGACGAAGACTTGAGTTTCCCAGGCGAGGCCGAGCACGGCCGGGCACGAAAGCGTCTAAAAAGTCACGTCAATAGCGACGGTGGAAGTGGCGGGCACGCGGGTTCCAATCAGCAGCAGCAACAACGTTACGATGAACTGCGGGATGCCATTCACGAGCTGAAACGCGATCTGTTTGCCGCGCGGCAGAGTTCTACGTTACTTTCGGCGGCTCTCCCCTCTGCGGCCTCTTCCTCCCCAACTACTACTACCGTGTGTACTCCCACCAGCGAGCTGACGAGTGGCGGAGGAGAAACACCCACGGCACTTCTATCCGGAGGTGCCAAGGTAGCTGAGCGCGCTCAGGCCGGCGTGGTGAACGCCAGTTGCCGCCTCGCTACCGCGTCGGGTTCTGAGGCGGCAACGGCCGGGCCCTCGACGGCAGGTTCTTCTTCCTGCCCGGCTAGTGTCGTGTTAGCCGCCGCTGCTGCCCAAGCCGCCGCAGCTTCCCAGAGCCCGCCCAAAGACATGGTAGATCTGAATCGGCGGATTTTTGTGGCTGCGCTCAATAAGCTCGAGTAAGAGAGACGCTATATTTAGGGCTTCCCTCTCTTTTTTTTCTACACCGTGATACCCTAATAAAGCACACCGCGGTTATTATCAACGTCTCTGTGTTTTTATTATTTAGAAATAAATACAGGGAATGGGAAAAACACGCGGGGGAAAAACAAAGAAGTCTCTCTCTAGATGCGGGGTCGACTGCGTGGGGTGCTGGAAGTGGAAGCGGTGCTGATGGGTGAGGGTCGTGGCGCGGGCACGGACCGCAACGTGCTGCTGATGTCTGCCGCGGTACGCACGTCGCCGTCCATGTCGCTGCGCAGATAAGAGGTAGGTCGTAGTGCGGCGTGCTGCACGCTCACCGTTAATGGTACCAAGTCGTCAAGGCTCGCAAAGACGTGCCACGAGGGGATGACGAGCGTGAGAGCCCCGTTGTTACCGCTTCGACGTCTTTGTCCGGTCAGGATCAGTGCCCGGGACAGTCCGGCTTGGGTGTCCGAGTCCTCGTCGCCGCTGGCTTCCTCGAAGCCGGCAAACATGGCTTCGGACAGGGGGGTCGGCGTCGGTGTGGAGGAGAGGGGTGGGTGGTAATCCGGGGGACTGCGGGAGAAACTCGGAGACGGCGCCGCGCATGACGTTGCTCCGTGGAAAGAGACCGGCGCGCAGCTGCACCTGGGGACGCTTGATTTTGTCCGGTTTACCGGGTGTGAGAGTCCAAAACCCACGGCGGAAAAAGTGGATGCGGCCTAGCGGCTGTCGGTGTTCCAAATGAACGGCCTGATCGCCGGTCAGCGTGACGCGGAGGGTGATTCGCACACGATCGGGTAGCGGGCCGGCTTCTATGGAGACGCCCGGGATGTTTTCCGGGAAAAAGATGGTGTCGTGAGTCTGATTGGTCTCGAAAGCATTCTGGATCTGCACGATGTACTCGGGATGTATGCGCGTCAGCGTAAAACTTTTGGGAATCAACAGCTGGAAGCCGTTGTCCGGCAAGCGTCGTAGGTGCGGGTACGGATTGTGTCGCGCCACCACCTCGGCGCGATGCGTGTAAACCGAAAAGTGCAGAAACACGCTGGTCGGCGGGTGCGGTGAGTCGTGATGCAGAAACAGCATGATCCATTGGCCTCGTTCGTCCGTCTCCGTTTTGTGGATGTACGTGTTAGGGTCCGAACAGGCCAGCTGCTCCAGGGCGTCTACCAGCGTCAGCGGGATGGCGCCGGCGCGAAAGGCGAACTGGCTGACAAAGATCTGCCCTGCCTCCAAACTGCTGTCGGTTCTGCGGCGCCAGTTCGGCGTTACGGTCAGTCGCACGGCCCAGTGGTGAGCCGTGCGGCGGATGATGGCGCGCGCTTCCATTCGCGGCCGATTTTCTTCGCCGCCGCGCCGCTGGCTCTGAAAGAGGTGCAGTCCGCTAACGGGCACGCGGTCCAGCGGCAGCGCAAAGGCCAGTACCGAGACCGTGTTGTTTTCTGAGCCTGGCGTCAGGCGTCGTGGGCCAAAGTTGTTGAGGTCCACCAGCAGTCGGTCCTGTTCGCCCACCACGCAGCGGCCCTTGATGTTTAAGTCGGTCAGGTCTACGGTGTCGTGCGGAGATTTGTTCTCCTGAAAACAGCAGAGAACCGAGGGCCGGCTCACCTCTATGTTGGTACGCAGGTCCAGGAGTCGCAGACGACCGGCTTCCAGCGAGCCGCCTTCCACGTTGGTGATGAGCCGAAGCACCTGGCAGTGCAGGCGACCAAAGCTTCCGCTGGCGGCTTCGGCCTCGCTGATCGCGGCCGCTTCCGACGAGGGTCCCTCACCGGGCGAGGACGATGCCTGAGACATTGCGAAGGCGGGATGGGGGGAGGGTCAGGGGATGCGCAAAGGTGAACGGGTCTTCGTGGGAGGTCGGGAAGGGTTCCGGCAACTGTCGCAAATATAGCAGCGGTGACAGGTGTGGCGGCCAAAAGTTGCGTGTCTGAGTGGACGTGGGTTTTTATAGAGTCGTCCTAAGCGCGTGCGCGGCGGGTGGCTCAACCTCGGTGCTTTTTGGGCGTCGAGGCGATGCATGGCCCGGGCAAGGCGTCTTGCCGGTGGCGGCGACGTTTGGGTTGCGCAGCGGGCTGCCATACGCCTTCCAATTCGGCGAAGATGCGGTAGATGTCGTTGGCGTCCCAGAAGAACTCCTGGTACTTCAGATTCTGACCCTGAACCGTAGCCACCATGGGCACCAGGTTGCGGGCCAGGATGCCGGCCTGCCAGGGCGGCCAGGTGAACACGGCCGGATTGTGGATTTCGTTGTCGGAATCCTCGTCGGTGTCCTCTTCGGGCGCGACGGTGGACTCGGCCTTAAGGCGGCCGCGTGTCATAACGCCCGCCGTGCACGCCGTCGCCGAGGATGCTGATTTGCGTTTGCGGCCCGCGGAAGTGGAGGCGCTCGCCATGGCGCCGCCGCCGGTGACGCGGGGCGTCTTACGCTCGGTGGTTACGAGTTCTTCGTCGGAGTCCGATCCGCTGGTCCAGACGTCGTCGTCGCCCTGGGCGGCACCCTCGTCGTGCCGGTCCCAGGTGTGTCGGTACTCAAGCTTGCCCTGGATGCGATACTGGCTGGTGAAGGTGGGGTGCTCGCTGTACTGAGGCCCGCGCTGCAGCAACAAGTCGATATCGAAAAAGAAGAGCGCAGCCACGGGATCGTACTGACGCAGTTCCACGGTCTCGCGTATCGCTTGTACCTCCAGGAAGATTTGCTGCCCGTTCATCAACAGGTTACCTGAGATGCTCAGGCCCGGGATGCTCTTGGGACACAGCAGCCCAAAATGCTCGTGTGAGGTAAAAGCCACATCCAGCATGATGTGCGAGATCTTGCCCGGTTTGATTATCATATTTTTGGGACACAACACCGTAAAGCCGTTGCGCTCGTGGGGGCGCATGAAGGGTTGCGGGTTGCGGGTCATCGTTAGGTCCTCTTCCACGTCAGAGCCCAGCGTGACGTGCATAAAGAGCTTGCCGGAGGGCACGTCCTCGCAGAAGGACTCCAGGTACACCTTGACGTACTGGTCACCTATCACCTGCATCTTGGTTGCGCGCGTGTTCTCCATGGAGCAAACCAGCTCGTGCGCGCACACCACGTGCCGCAGTGCCACGTCCTTGGTGGGAAACACGAACGCTGACGTGTAGTAGACGTCGGGCTCTTTCCACTGGTTCTGCTGACGCGTCCAGGCCAGTCCCGAGACCGTGAGACGCGCCTGCCACATCTGCTTGCCCGACGCGTGAATAACAGCGTCGGCTACGGGCAGGTGTCGGTGTTTGCGCTCGGCCGCCGACGGGTAGTGGTGCACGTTGATGCTGGGGATGTTCAGCATCTTGAGCGGCAGCGCGTACACATAGATCGACATGGGCTCTTGGCTGGGGCAGATGCTTCGGCCCGTGGGGTTGTGCACGTTGACCGACACGTTCTCCACCTCGCTGCCCGTAAAGTACGTGTGCTGCACCTGCAGCTGATTGTCGCCGCGGTGGCATGGCGTCGAGTCGGGCGTGTACTGCGACACCAGGATCAGCGAGGGCTGGCTCACGCGTACGTGGATACCCGTCTGCAGGAGTCGCGTCTCGTGCGGCAGCACCGGCGTATCGCCGCGACTAAACACGGCTTTCAGCACGTGCCCCGAAATGGGACCCAGTACGGATATCATTTCGGGACAACGGCGACCGCGCGACTCCATGCTGCCTGCGCGTACGGGTGTAGGCGACTGAGCGGCGCGCCCTCTGCGGCCGCCGCCTTACATAGGCAGGCGACCAAACGCGGAACCCGAAATAAAAACGTTATACACAGAGACAACCGCGGATTATTGAGTGTCTTTTTTTATTACAAAAAAAAAGAGGCAAAGCCCCACCGTCACCACACCCCATCACACACCACCACCGATTTTTTTTGTTTTAATCCCGTATTGCGCGGACGCCTAGTGTCCGTTTTCCATCACCAGGGTCCTCTGTTTAGAGATCGCCGCAGACCATGGCTAGAGTGACAGGACTCGTTTTCTCTGTCGTATTTTCCGTAAGCTTACAGTCTTGCGGTTCCGTCTCCGGGGACGCCAGTCGCATGGGCAGCAGGTCCTCCAGCGCGATGGAAGCGCCCAGCACCGAGAGCTGCTGTTGCGACGGCGAATGGGACGTGGACCGCGAGTGTAGCGTGGATTTGACTTGGTGCGTCATTGCTGACAGGCAACCGCGATTCAGCGTATGCTTTGACGAGATAAAATAGAGGCGTCCCAGGAGCGCGTCCCGTGGGAACGTGGCGCCGTTCTCGTCGCTTACCAGTACGGTTAATTCCAACCAGGAGCGCGGTAGCCAGACCGTAACGGGCATTTTGAGTCCCTGACGGTTGTGTGGTACAAAAACACCCAGATAAGGCCCGTAAAAGCGGCGGTAGATACGTAACGTGTGCGAGTTTTTCAGCGTCAATTCGTAAGGGACGCGCACCTCCAGTCCCTCGTCCGCCGCGCCGGAGCGTGGCGGTACAAAGTAAGGCAGTGGCGCGTCCGAAAAGAAGGGTCGTCGCACCGTTTCGCGTCGCAGCCGCAGGCGAAACGCCACTGGGTCGGCTGGCGCCTCGGTGCGGTCGCAGGTCACGTTGAAACGTAATATGCCGTCTTGGTATAGCGTGAGTGACGACAGCGTCAGGTCCGGCGGTGATTCGTTCGGGTCTAGCTCCAATCGTCCAAAGACGGAGGGTCCCAATGTCTTGGCAGTGGTTTCCGAGAGGCGCGCCGAAATACGGCTGGTGAGTCCACGCGGCCCCGAGATGCCGCCTTCCACTCGATGCCAGCACAGCGCGTGTCGTACGCGCACCGTCAGTGTGGGCGTCAGATCCGCGTCCGTTGATTCCGCGGTATCAGCGACGGAAGCCGCGTTCTCCGTTACGTTGTTTATATCCAGCGTCGGCTCGAACGTGAGTTCTGGCAGATGCAGCGCCAGACAGTCGTGTAACGCCGTGTGATGCGCGGCTTTACGTCGTAGCGGTAGCCGTTTCAGCAGCGGCGTGATGATACGGAGCGCGAAGAGATTGAGTGATAAGCGCACGATGGCCATGCGCGTCAGTTGTTGGTCAATTACCGAGCGCAGGATATGGCAGCCTGGGCGTGCGGGAAAGAGAGAGAAGGCCGGGCGCACGTCAGAATCCTCGTTAGAGACCACGCATAGAATGCCGCGTTCACGATCGTCGTTGCGGTCATCCTCGTCCTCTTCTTCTTTCTTCTCTTCTTTTTCCTTTTTTTTCTCGGGCTCGTGGGAAGCCGCCGTTTCTTCTTCTTGCGACGTCGCGGGGGCGGTTTGAGACTCGCCGTTCGCTTCCCCCAATTGCAGCGGCGTAGAGAGCAAAATCTGGAAGGGATCCCGCAATTCTTCGGGTCGGAGGTCGAGGTGCAACTGGATCAGATGGTAGGTGCCGCGGTGCACCCGAGGCTGACGGATGTCGTGTTTATCCGTCAGTGTGAGGATGGTCTGCGGTGAGCCGCTGTGCTTGTCCAGCTCGTCCGGCGTTTTCAGGAGGAGACTGTCGTCGTCGGTACTGGCGACACCCATCATGGTCGTGGTGGTAGTGGTGGCGAGGAAAGTGAGCGGCGGCGTCGACAGAGCTCGGCGTTGGCGGCGGCATTTGCCGCTGTGTCGGCTGCTATTGCTGCCAACGCCACCGCCGCCGCCTCGTCTGGCTCGTGGCCGACGGGCCCGATTCCGAAGGTTGGGGTCGACGCGTGGCATGCTTGGTGTCTGCGGGCGCGAGAGGGCCGGCTCAGCCTTTAAATATGCAGGTCGCGGATTTGTTATCGGGTGAAACGTCACACACCGTGAAGACGACCTGTTCGCGGATGAGGTCATCCAGCTGTCGCAGCATGACGAAAAGCGCCGACAGCCGCGCGATCTCGTCGTCGGGCGACACGTGCTGCGGCCGCGCGGGCGTGCGCGGCTCGCCGACGCTGCGCTCGCGGTCCAGCCGCATCAGCAGCTCCTGGCACTTGACGAGCAGCATGGAGCTGTCCTCTAGCGCTAACTTGCGCACGTAGGTCATGGTCAGCTCCGAGGCTAGGTTAGCCACCATGGACATGGAGAGGCAGGCGGTCTTCATGTCGATCAGCAGGTGCTGGTCGATGACCGGATCGGGGATGGTGAAGGTGGCGTCGCGAAAAGTAATGGTCTGCAGCTGCTGCACGGCAGCCTTTACCTCCTCGTACGAACGGTCGAGCGAGAAGAGGCCCATGATGAGTAGTCGCTGGTTGATTTCCAGCGCCAGTGGCATGGGTACGATCCAGGGCAGCACCAGCTCCCACTGGCCCAGCGTCAGCAGGTTCTCGCGCGCCAGCGGTCCGTGGAAGAGCGGCGGCAGCACGCATAGCGCGTCGCCCTTCTCCCAAGTCACGGGTCCCGTGTTGAGGACGGTGTAGAGCAGTCCGTGCGTCGGTACGTGTAGGAGGATCTGGTTGCCTTCTACGCGCCGCATCAACGTCAGCGTCATATTGCGCAGCAGGCCGCGCAGTCGTACGTAGCCGCGGGTGTGATCTACGAACTGGTGTAGGCCCAGCTGGTAGTGCTTGATGAGATGTAGACGCTGCGGAATGGGCACAACGGCCGCTACTAGTTTGGTCAGTTTGCCTACGTCGGCGATGCTGAGCTTGTGGTCGAAAGTGCAGAAGATGTTGGCCTCCATGGCCGCCATAGCGGCGGTGAAATCCTGGCCGCGACGGAGGAGAAGCGGAGACGAACAACGTCTGCACCGGGCGCGGCGTCAGAGCGAGCGTGGCGCGTCCGGGCCCGCGTTTGCGTCTAGGTGACTCGCCGCTAACCTGCGGTCGTCGCCGTCCTCCTCACCGGACGGCCTCACGAGTTAAATAACATGGATTGCTGCAGCGGGATGATTTCGCCTACGACGTAGTTACCAAAGTGCGTTTCGGACGTAGCAAAAGCCCCGGCGCCACCCTTGAGTTTGGTCTCCATCAGCGCCAGCGTGGTGGTGCTGAGGATCGGTAGCGCTTCCTGCGTCAGACGGCACGGGTTTTCGATGAGTTGTTCCGTGCCTTCGACGCAGACGTACTGCGTGTCCGTGTCGCCGCGGATGCAGTCCTTGGCGCGTAGCAGGTACTCGTCGATGGTTTTGAAGAGCGTTTTGTTGGCCGCGATAATCTCTTCTGTGTTAAAGTACTGCGCGCAAGGGCTGTAGAATTTGGAGTTGTAGCCTAGACGTTCGCGATGTCGGGTGTTGTAGAGTACGTCGCTCAGACAGCCGGCTTGCGAGGCCCAGGGGTTGTGTGTGGCCGCGAAAGTCTGTGCGTCCGCTTCGCGATGGTCGTAGATGGCCTTGGTGGCGGCCTCCGTGTCGTACGGATCGACGGCCAGCATGCAGGAGGCACGCCCGCGCGGGTTGTTGGGGATCTTAAAGTAATTAACGTCCATCGTCACCGGCGTAAGGATTAGTTCGCACGCGGCCTTTTGTCCGTGCACCGTGGCGGCGGCATTGCGCTCGGACATGCTGCCGAACGTCAGCATGGAGATGGTCTCCGTGTCTAACAGTTGCGGCCGTTCTACGCCGGCCGCGTGCCGGATCCAGCGGTCCACCTCGTCGTGCCGGTACACGTTCATAGGGAAGACGCGAAAGAGGTCCTGCACGCGGACGCCCATGTCGGTTCGCACGCGGTTTACGTAGGCTACGCAGGTATTTGACGTGTAACCCAGACCCATGTCTACGGTGTTAATGTTCTGCGTGACGTGGTACGTGGTGCTGATGTCGCGTTCCTCCTTGGTCACGATAGGGTTGTTGATGATAACTGACGTGCATGATTTGCCGCTGTAGAGCAGCATGTCCACCTCGAAGGTGTCGGTGCGTACGGCCGTGAGTGCGAATCCCGGGTGGATGTGCGCCTTGGTCTGCAGCACCAGTGAAACTGGTGAGATTTTGTATAACATGGCGGCCAGCGTCATGACTGAGTGCAACACGTTGGGACAGGTGGCCGAGTAACGCGAAAAGGGCGAGCGCAGCCAGTTGTGGTACTCGTGCGCGAAGGCTGTGGGTAGCGGGAAACCACCGTCGTGACGGTGATAGTGCGGGAACTCGGTCACGTAGCGTTTAATGTCGTCGCTCAACGCCGCGCAGATGGTGGGGTTTGAGTAGAAACGGTGGAAAGGTACGGGTAGGCTGTACTCGATCAACGTCTTAGGCGCCGTCACGACGCAGCAGCCGTTGTAAAGCACGTGCTGACGTGAGATAAAGTCCGGCAGGCCCTGACGCTGCGCGTGGTCCAGAGGCGCGCGCACTTCGAGCACCTTGACGTGCTCGCCCACGAATTGCACGGCCAAAAACAGTTCACGACAGGCCTGCAGCAGCGGCGTATGTGCGTCGGTGGCGACGTCCTCCACCAGCTCGGTCAGCATCTCGCCTACGGCTTGACGTTGCGCCGCTATCGAGTCTTCGGGGGTGACGCCGCTTGTGCTCTCTTTCGACGTCGTACCTGACGTGGAGACCGCGGTGGCGGCCGGCATCAGGAGAAACGCCGGTCGGTAAAAGAGGTCTACTAGCAGCGTCTTGAGGTTGAGTCCCAGGCCGCAGGCCCGGTTGTTGGTCATGGCGGGCATGAGGCAGAGATAAAAGACCTTTTGTAACGTCCATTCGTCGTCGGTGGCACGGTAATCGTCCACAAACAGCGGCTCGTCGGCATCCATGGCGCCCAAACGCGGTACGTCCGAAACGCCGTGGTGTCGCGCCTCGATGTTGGCCGGGTTCAACGGTTGCCGGTCGGCCACTACCTGTACGCCTTCCATGTTACGCGGCAGGTGCGTAACGAAGGGGGGCCACAGCCGGTGGTCGTGCAGCGCGTTCACGTAAGCCGATAGCGGTTCCTCAGCCAGTTGACCGTTGTTAAGTCCCGGCAGCGCTGAGATGCGCGTTACCAGACGCAGCACGGCGACCAGATTGCGGTAGTGAAAGAGCAACTGCGGTGGTAGGGCGCCATCAGCCAGGTGTTCGGCGATCAACGTCACCAGCGCGTAGCTGTGCGCAAAAACCAGCAGCTGACGTGTGTGAAACATGTTGACGATACAACGTGCTACGAAAGTGCGGATTAGCAAAAAAGCGTCGACGTTGCCGTGTACCAGCACGTCGACCAGGTAGCAAAGCTCGGGGTAATTGGGGCTTGTAACGGTGGTTTTGAAAAGTCGCAACGTCTCTTCGTAGTCGGGTGGTGGCCGCAGTCGCATGTGTTCCATGATCTCCCAGGTGCGCAGTTCGTGGAAGGGGCCCGGTGCCAGTCCATCTGGCAAATTACCGATGACGATACGCGGTGTACACAGCGCCACCGTTTCGCTGTTTTCCTGGCAGTGCGTAAAGTCGAAGAAGGGGTGCAGCTCGGTGTAGAGCGTGATGTTGCCCACCTTGTAGAAGTCGGTGACCACAAAGTCCTGCTTCATTTCGTTCACCGTGCGCGGGACCTCGCGTCGTACGCGGTAAAAATGCGGTATGCGGCGCGCCGCACCGCCCATGGGTTCCTGCTGAAAACGACACTCGAGCAGTCGTTGCATGGCGGGTTCCGAGGGTGGTCCGCGTTCCGTGAAGGTCTGTAGACAGGGCGCGGGCTCGTGCAGCACCGGGTGGCACAGCGTCTTGAGCGCGTCCACAAAGTCTATTTTTTGTACGGCACGGTCCCGGTTTAGCAGGTAGGCCGTGGTGGGCAACGCGTTGCGAACGGTGTCGTTAAGCTTAACTTTGCTTTCCACCGTGGTGTAACCGCGATCCTCGGGCAGATACAGCCCTACGGGGAAGAAAAACGTCAGGTCCACGTTACGTTCTAGCGGATCTTTGGTATCGGTGTTTTTGTAGACGCGCCGCAAGTTTTCCATAATCACCGTTTTTTCGCCCAGTCGGATCACGTCCATGCTCAGCGGCGTTAAGCTGTGCGCCCCGGCCTGCGAAAGCGAGTCGTTGGGCAAATGCGGTTGGCCCGAAGTCAGATGAGCCTTGTACGAGTTGAAATCGGCCAGGATCGAGTGATAGGATATGGCAGTGACGGCATTTTCGGGACTGAGTACAAAATTGCCGTAGGTGGCCGGCGCCGAGACCGTTTCTTTGGTGATGTGGCTTGAGAGCAGCGACATGATGATCTGCATAACGTTGGCCGTGCTTACCATCACGCCACTGATCTTGGCCCCCGAGCTCGTGGTGTACGTGGTGGGGTTGTCTAGGATGCTATCGGTGGCCGCTTCGGCCAGACGCGTGAGGAACTTGAGCACATAGTCGCGATCGCGCGTGCGATTCAGCAAAAAGAGCGTGGCCAGCATTTTGGCCTTGAAGCTCTGCAAGATGTTGCTTCGCTGGATGCGGTTCAGCGCCTGTCGCGCCAGCGTGGCGTTCTCTACCAGCGTCTGCACCACAAAGTACGGCGGCGCCTTGCGTAGCAGTGTCTGTAAAAAGCTGTGAATCAAGCCGCGTTCCATGGCGTCGGCCGTGTTTTTGAGCGCGCGCAGCACCGTGTGCATAGCTTCCACGTTGAGGATCTTGTCCAGGATGGTGCCTTCGAACGTCTCGCGCAGATACGTGAGGCAGGCTGCGCTGAGCTCAAAGGGGATGGTGATGGGGGATTTTTCACTGTATTTGGTGACCATAATGGTGGTCTGACGACTGGTAGGCAAACCGGCGCCGCTGGCCACCCGCGGCACCTGTACGTGGAACAGCATTTTTCCCGTAGTCAGTTTATTGAGGTCGTGGAACTTGATGGCGTGCGCCGCCGCGGCCAAGCCGCTGGTCAAAAAATAAACCCATTCCAGGCGATTGCAGAAGGTGCCGAAGATGGCTTCGAAGTGAATATTGTAACGCTCGGGGTCATCGCCGTAGTAGATGCGTAAGGCCTCGAACATCTCCTCGCCGGCGCTGGTCTTGACGTGCGTCAGAAAGTCAGTGGGAATGCCTACTTTAGGCAGGAGCTCGAGCGCCGACCAGTTCTCCATCGCGGCGGCGGCGTGAGCGCGAGGCGTCGGAGCTCGGGGAAAGCAGCGCGACCCGGAGAATGGCCGGCGCTGCGCCGCGCCGCCTCGGCTGTGACGCTCTAATAGTCGTCGGCGGCTCCGCTATGCCGCGCCGGGTTTTACACGTCCCCGTGCACGTTCGCGCCTGCAACCTCACCCAAGAGCTATCGACGGGCGAGGACGCCCGCTTCTGTCGTCCGCGACCCGTTAACGTCGAACGGGTGCGCGCTGTTTTTGCGGCTCTCTACCGTGCCTGTCCGATACACGTGAGGACCGAACCCGAGCGTGTCAAGCTGGTACTGGGTCGTCTGTTACTGGGACCCGTGGCCGTACCCTGTTTTTGCGACGGTGAAGTGGAGGGCCACGGTGAACATCTGGTACCTACGACGCAGTTTTGTCGCGGGCCGCTGCTCTACGTGCACCGACGTTGTTGTTGCGGATCCGTGACCGCCGGGCGCGCGCTGTCCTACCACGTTCTCGAAAACCACGTGGCCACGCATGTGCTACGCGGATTGCTCTCGCTGACGGAATGGAATCGAGAATTGCCGAGCCTCTTTTGCGACTGTCCTGGCGGCGGTGGCGCCTCGGGAACCGAGGAACGCTACGCTATGGCCTGCCTGCCGCGCGACCTCAGCCTGCACCTGGACGACTATCCTTACCTGATGGTGGAAATCGGACGCGTACTCAGTGTCAGCGAGGTAGACGACTACGTAACCGCCGTCTCCGGCTACCTGGGCGAGGCCGCGGCGCCGCGCATCCAGGTTCACTACAAGCTGCTCTTTGGACTCAACGTGCGTCCGCAAGCGCCGTGCGCGTTGGACGCTACACGCGACTTTTTTCTGCTGGAGCTGCAAAAGCTTTGGCTGGGCGTTGAATATCACCACGAAGTGACGTCGGAGTTTTTCGGTCGCGTGCTGGCTCAGCTGCATCGCGACCGCGCCCGCGTCATGATGGCGCTTCGCTTGCCCGAGCAGACGGTGTGCCACCTGAGCACCTTCGTTCTCAGTCGCTTCAAGCGACAGGTACTGTACTTCAAGTTACAGGTGAGCTACGGCAAGTGCCGGACTGGCCACGCTGACAGAAGTGGGGGAGGGGGAAACGGTGGAAATCAGGGACACCACAACCTGCTGTGTTATCGACGCCTTAGCGTCACGTTTGCCGACACGGACACGGTGTGGAGAAACCTTTTCTACGTTTATTATGAACTAGCTCGGGATCTGGGGTCCCATGGGACAGAGAACCGACCCGTAAACCGCGGTTACGGTGTTTCTTGCGCTCCGAGGACGTCGCGGCTATCACCGTCAGAATCGACGGTGGTTTCGGCGAACGGACACGCGCTGTCTTCCACCGCGCTCCCGACGACGAGCGCGGGTCACAAGCTGTCACTGCCGCGCGACCCGGCCGCCGATCGCGTTCGACGTTACGTGTGCATCATCTCGCGTCTCATGTACGCTCGGTACGGGGAGAGATGGCGTAAACACCGTCAACGGCGGTCGGAGACGGGAGAAGAGGAGGAGGAAGAGACGCTGGAATCGGGGGAGACTGACGCCACGCCGCCATTTGACTTTACGGGGCAGCAGCTGCGCCGGGCCTATCAGGAACACCGACGTCGTAAACATCTAGCCGTGCAGCGTTACGCGCCGTGTCGTCGTAAGCTCATCGGCGGGATGGAGTTTGCCGAGGTGACGGGCGTGAGTCTGGACCGCATCGCCGTCAACGCTTTCAACACCAACCGCGTTATCAATATGAAGGCCGCGCTCTCGTCCATCGCCGCGTCGGGTCTCGGCGTGCGCGCGCCGCGGCTTCCCAAGAACATGACCCACAGTTTTGTGATGTACAAGCACACCTTTAAGGAGCCCGCTTGCACCGTCAGCACCTTTGTTTCCAACGACGCCGTCTACATCAACTCGCTCAACGTCAATATTCGCGGTTCCTACCCCGAGTTTCTGTACTCGCTGGGCGTGTACCGGCTGCACGTTAATATCGATCACTTTTTTCTGCCGGCCGTGGTGTGCAACAGCAACTCCTCGCTGGACGTGCATGGGCTGGAGGACCAGGCGGTGATCCGCTCGGAGCGCAGCAAGGTGTACTGGACCACCAACTTTCCGTGCATGATCTCGCATACTAACAACGTCAACGTGGGCTGGTTCAAAGCGGCTACGGCCATTGTGCCGCGCGTCTCGGGCGCTGACCTGGAAGCCATTCTGCTCAAAGAACTCTCGTGCATCAAGAACATGCGCGACGTGTGCATCGATTACGGTCTGCACCGCGTTTTCACGCAACTAGAGCTGCGCAATTCGTACCAGATCCCCTTCCTGGCCAAGCAGTTAGTGCTGTTTCTGCGTGCTTGCCTGCTCAAGCTGCACGGTCGAGAGAAGCGGCTGCAGTTGGACCGCCTAGTATTTGAGGCGGCACAGCGGGGTCTCTTTGACTACAGCAAGAACCTCACGGCGCACACCAAGATCAAGCACACTTGTGCGCTCATCGGCAGTCGTCTAGCCAACAACGTGCCCAAGATCCTGGCCCGGAACAAAAAAGTCAAATTGGATCACCTGGGCCGGAACGCCAACGTGCTGACGGTGTGTCGGCACGTGGAAGCCCACAAGATCCCTCGCACGCGCCTCAAAGTGTTAGTCGAGGTGCTGGGCGTGTTGCAGAGTATCAGCGGTACGCCGCACACGCGCGAAGTGATCCACCAGACGTTGTTTCGATTGTGCTCGGCGGCCGCAGCCACATCGGGCCTGTGTTCATCCCCTCCCCCATTGTGTGTGTCCTCATCTTCCTCCGTCCCTTCTGTCCCAACCTCCGTCAGCGTTGACGGCAGTTCTGAACCCACGTCGCCGCGAGTGCGGTTTGCATCACGATGATGGAAGCCGCGGCCGCTGCCGCCGCGGCGTTTCGTCCGGAGGAGCGTCCGACGCCGGGTTGGCACGACGCGGCGTTGTTAATGGACGACGGTACGGTGCGCGAGCACGCGTTTCGCAACGGACCGTTGTCGCAACTGATTCGCCGTGTGTTACCGCCGCCGCCCGACGCCGAAGACGACGTGGTTTTTGCTTCCGAGCTGTGTTTTTATTGCAGCGGTCGTTTTAACCGCAGGTCGTCCGTCTTCTCCATCTATTGGCAGAAGCATAGCGATCTGGTGTACGCGCTTACGGGCATTACCCATTGCGCCAAGTTGGTGGTGGAATGCGGTCAGTTGGGGAGTAGTAGGCTACGGTGGCGCGACGGTGATGCGAGTGGTGAGGAGCGCCGGGGAGACGACGACAGCAGGGACGAGCTGTACGACGTGCCGGGCATTTATATGATTCGCGTCAACGACGGCGGCAGCACCGGCCCCAGACACGTTATTTGGCCGGGTACCAGCGTGCTGTGGGCGCCGGACGTTGTGATCACTACGGTGCAGCGACGAATCTCGGCGGCGCGCGCCCTGGTGAACACGTTCCGCCAATATTTTTTTTTGCTGGAACGGCGCTCGCACGAGGAGCTGGTTCTTTGTCCGCCCGAGATGGAGGAGCGTCTAGCGCCGTTGTTGCAGAGTGCCACGCGAGGTGATTCGGACATGTTTGACGGTGTGGTGGCCAGCGCTTATCACCGTTTGCGAATGAGTAATATTCCGCGTTCATCCGCCCGTCTGCTGGAGCACTGCGTGGGGCTGGCGGGTGCTAAGAAGCTGCTCTTGCTCGACGTGCCGCGTCTGGAGAACTATTTTCTTTGTCAAGTCTGTCTTTACGAGTTGGACGAGGACGAGATGGGCGAGGAGATGCTGGGCATGTTGGCCGGAAAGCCCGAGGATGCCGCCGTCTCGGGCGCAAGCGGCGGTTTTCTGCTACATCGCAAGACGATGAAGCTGGCCGCCTGTCTGTGTTTGTTGCTCAATTCGCTGCATTTGCACCAGGAGGCGCTGGAGGCCTTGGATCCTCCGCCGCCGCGCGTCGAGGAGAACGACCTTGTCAACGTGGTGCTGCGCCGTTATTATCGCAGTCACGGCGGCGTGCAGGCGCGGACGCTGGCGGCGGCCCGGGCTTTGTTAGCCGACTACGCTGAAACGTTTTCGCCTTTGGGGAGTTTTACGCGCCTGGGTTACGATCGTCTCGTTTCTGCCGATGCCGGCGTCAGTCGCCGGCACCTGGTGGCTCTGCTGCGTGCCTAGCTGACCCTGAAACGGATGGCGTGTATATCGTCACACAGGTAGGTGGCCATGATGACGGCGATGATAAGATCGTCCGAGATACGATTCTGGCGCTTGGCCGAGTAGCGTGCCGTCGTGCCTTCGGCCAGCGTGACGCGGTGCAGGTTCTGAATCTGCTCCAGAAGATACTCGATGGGGTCGTGGCTCAGCTTGATGGTGTAGGAGACGAGCTCTTGCGAGGCTTTGATGTAGCCCGAGTTGAAACGCGAGATGAACTGTTCCACGGCCAGCGCCTTGTCGCGGCCCATGAGGTAGAAGGGCTGTTCGATGTGGTTCTGGTCGGGCGTGTGGTAGAAGAGCACGCGGATGAGCGTGCTGCTCTGCACGCTCTGTCGGATGAGGCAGGCGATGCGCACGGCCGCCGCCTGGTTGGTGTTGCCCTCCACGGCGATACGCAGTTCGTCCAGGTAAGGGTGCAGGCTCAGCACCGAGATGATCATGTGCGCCGCGCACTCGGCGATGGCTACCTCAGAACTCTCGGAGAGGTCGCGCAAAAAGAAATGCTCTAGGCCGTAAATGAGAAACTGGTGTCGGTAGGCGCCTACGGCCGCCACGCCCGTGCCCGAGGCCTTGCGGTTGGTGGTGAAGGCCGGGTCCAGATACACGTAAAGCGTCTTGCCGAAATAATCGTAGGCGTTGGTGTTGAGCGTGCTGTAACGCAAAATATCGAACTCTTCGCGGCTCTGGTCCGTGATGAGCACGGTGTTCTGCGAGATTTTATTGGTACCGCCGATGATCTCGTCCATGAAAGCGCCCGGCATAAACATGTTGGCCGTCTTGCGCACCTGCGAGTTGAGGCTGATGAAGGTGGGCTTGTGCAGTCGGTAGCAAGGACACGCCGTGGCGTCGCCCTTCTCCGTGAAGCTGTGCAGGTGCTCTTCGCACACGTAAGAGACCACGTTGAGCATGTCAAAGGGCGCATTGTTGAGGCGCGTCAAGAAACACGTGGCGTCACTGGTAGTGTTGGTGGACGATATGAAGATGATCTTGGTGGTATTCTGGGCCAGGAACCCCAGAATGGTGTTGAAGGCCTCTTTCTTGATGAAGTGCGCCTCGTCCACCAGCAGCAAATGGAAGTTTTGTCCTCGGATGCTCTGTGTAGAGAGGAGACAGAAAAGGGACTCTTATGATTACGCACGCTCGGCTGGAAGCCTACAGAGTCGGGGTGGGGCCGGACAGGTGAGCCAGGTGAGCCGCCAGGTGAGGCGGGATCGCCGTGTGCCAACCGGGCTGCGACCTGAAAACCGGAACCAATCCGCCGACACCGGCGCCGCGTGACGCGCGCCCATAAAAACGAAAGTGTCGTCGTCGCGACCCGCCACAGCCGCCATGAACTCGTTGCTGGCGGAACTCAACCGACTGGGGGTCGCGCACGCCACTACGGAGGATGTTTTTATCTTTGTCGACCGCCTCTTTCAACACTTTTCCTTCCTTTTCCAGGCCGAGGAGTCAGGCCCGCGCCGCTTGGAACTGGTCGCGTCCGTGTTCGAGCACCTGACGGTGGAGTGCGTCAACGACATCCTGGACGCCTGCAGCCACCCGGACGTGAACGTCGTGGAGACAAGCAACACCTGTCGTCCCTGCCCTTCTCCTGTTCCCTCCGCCCCCAAAACTGTCAGCGACGCTCAGACGTCATGTGCGACGCCTCGGGCGCCTGTGACATGAGGCACGTCCAGAACGCGTTTACCGAGGAGATCCAGTTACACTCGCTCTACGCGTGCACGCGCTGCTTTCGCACGCACCTGTGTGATCTGGGCAGCGGCTGCGCGCTCGTCTCCACGCTCGAGGGCTCCGTCTGCGTCAAGACGGGCCTGGTATACGAAGCTCTCTATCCGGTGGCGCGTAGCCACCTGTTGGAACCCATCGAGGAGGCCGCACTGGACGACGTCAACATCATCAGCGCCGTGCTCAGCGGCGTGTACAGCTACCTCATGACGCACGCCGGCCGTTACGCCGACGTGATCCAAGAGGTGGTCGAGCGCGACCGCCTCAAAAAGCAGGTGGAGGACAGTATTTACTTCACCTTTAATAAGGTTTTCCGTTCTATGCATAACGTCAATCGTATTTCGGTGCCCGTCATCAGCCAACTTTTTATTCAGCTTATCATCGGTATCTACTCAAAGCAGACCAAGTACGACGCGTGTGTCATCAAGGTTAGTCGTAAGAAGCGTGAGGACGCGCTTCTGAAACAGATGCGTTCCGAATATGGAAACGCACCTGTATTCGGATCTGGCGTTTGAGGCGCGGTTCGCTGACGATGAGCAATTGCCTCTACACCTGGTGCTTGACCAGGAGGTGCTGAGTAACGAGGAGGCCGAGACGCTGCGCTACGTCTACTATCGTAATGTAGACAGCGCTGGCCGATCCGCGGGCCGCGTTCCGAGCGGAGATGAGGACGACGCACCGGCCTCCGACGACGCCGAGGACGCCGTGGGCGGCGATCGCGCTTTTGACCGCGAGCGGCGGACTTGGCAGCGGGCCTGTTTTCGTGTACTACCGCGCCCACTGGAGTTGCTCGATTACCTACGTCAAAGCGGTCTCACTGTGACGTTAGAGAAAGAGCAGCGCGTGCGCATGTTCTATGCCGTCTTCACTACGTTGGGTCTGCGCTGCCCCGATAATCGGCTCTCAGGCGCGCAGACGCTACACCTGAGACTGGTCTGGCCCGACGGCAGCTATCGTGACTGGGAGTTTTTAGCGCGTGACCTGTTACGAGAAGAGATGGAAGCGAATAAGCGCGACCGGCAGCACCAGTTGGCTACGACCACGAATCACCGTCGGCGGGGCGGACTGCGTAATAACTTAGACAATGGGTCGGATCGCCGTTTGCCCGAAGCGGCTGTGGCTTCTCTGGAGACGGCCGTCAGTACTCCATTTTTTGAAATTCCGAACGGAGCAGGAACCTCCTCCGCGAACGGCGGCGGCAGATTCAGTAACCTGGAGCAGCGGGTAGCGCGTTTGTTGCGCGGCGACGAGGAATTCATCTATCACGCGGGTCCATTGGAGCCGCCTTCCAAGATACGCGGTCATGAGTTGGTGCAGCTGCGCCTGGACGTAAATCCAGACCTCATGTACGCCACCGATCCGCACGACCGCGACGAGGTCGCGCGTACGGACGAGTGGAAGGGTGCCGGTGTCTCGCGTCTCCGCGAGGTCTGGGATGTGCAGCATCGCGTGCGCCTCCGTGTGCTGTGGTACGTCAATTCCTTTTGGCGCAGTCGCGAGCTGAGCTACGATGACCACGAAGTCGAACTATACCGGGCGTTGGACGCTTATCGGGCGCGCATCGCCGTCGAGTACGTGCTGATTCGCGCCGTGCGCGACGAGATCTACGCTGTACTACGACGGGACAGCGGCGCGTTGCCACAGCGTTTCGCCTGCCACGTGCCACGGAACATGTCCTGGCGCGTTGTTTGGGAACTTTGCCGTCATGCCTTGGCGCTCTGGATGGATCGGGCGGACGTGCGTAGCTGTATTATTAAGGCGCTAACGCCTCGTCTGAGCCGGGGTGCCGCCGCTGCCGCTCAGCGAGCTCGTCGCCAGCGCGAGCGCTCGGCGCCCAAACCGCAGGAGCTGCTTTTCGGACCGCGGAACGAGAGCGGTCCGCCCGCCGAACGGACTTGGTACGCTGACGTGGTGCGCTGCGTTCGCACGCAAGTGGATTTGGGCGTGGAAGTGCGCGCGGCGCGTTGTCCTCGCACCGGGCTTTGGATCGTCCGTGATCGCCGCGGACGCCTGCGACGTTGGCTCTCGCAGCCCGAGGTGTGCGTGCTCTACGTCACGCCAGACTTGGACTTTTACTGGGTGCTGCCGGGCGGCTTTGCCGTCTCTTCGCGCGTCACTCTTCATGGCTTGGCGCAGCGGGCTTTGCGAGACCGATTCCAGAACTTTGAAGCAGTTCTTGCAAGAGGAATGCATGTGGAAGCTGGTCGGCAAGAGCCGGAAACACCGCGAGTATCGGGCCGTCGCTTGCCGTTCGACGATCTTTAGTCCGGAGGACGACGGCTCGTGTATCTTGTGCCAATTGCTGTTGTTCTACCGCGACGGCGAATGGATCCTCTGTCTTTGCTGCAACGGCCGTTATCAAGGCCACTATGGCGTGGGCCACGTACATCGGCGTCGTCGACGCATCTGTCATTTACCTACCTTGTACCAACTGAGCTTCGGAGGTCCTTTGGGTCCAGCCAGCATTGATTTCTTGCCAAGCTTTAGCCAGGTGACCAGCAGTATGACGTGCGATGGTATTACGCCCGACGTGATTTACGAGGTCTGCATGTTGGTGCCCCAGGATGAAGCCAAGCGCATCCTGGTCAAGGGTCACGGTGCCATGGACCTGACCTGTCAGAAGGCAGTGACGCTAGGCGGCGCCGGCGCCTGGTTGCTGCCGCGTCCCGAAGGCTACACGCTTTTCTTTTACATTCTGTGCTACGACCTGTTTACCTCATGCGGCAATCGGTGCGATATCCCTTCCATGACGCGGCTCATGGCGGCGGCCACGGCCTGCGGGCAGGCGGGTTGCAGCTTTTGCACGGATCACGAGGGACATGTAGATCCCACTGGCAATTACGTGGGTTGCACCCCCGATATGGGCCGCTGTCTTTGTTACGTGCCCTGTGGGCCCATGACGCAGTCGCTCATCCACAACGAGGAACCCGCGACTTTTTTCTGTGAGAGCGATGACGCCAAGTACCTATGCGCCGTAGGTTCTAAGACCGCGGCGCAGGTCACACTGGGAGACGGCCTGGATTATCACATCGGTGTCAAGGATTCTGAGGGCCGATGGTTGCCCGTCAAGACCGATGTGTGGGACCTGGTCAAGGTAGAGGAACCTGTGTCACGTATGATAGTGTGTTCCTGTCCGGTGCTTAAGAACCTAGTGCACTAACGGGGTCTGACAGTTCACGGGGAGAAGAAACAAGAAACAACAAAAAAAAAAGAGGACATGGACTCGCCACGGTTTGTGGCAAGGCGTATATTATCATCATGGAGCTACTCACGTTGGTGTTGTAGCAACTGGCAAAAAGCGCCGTGCTCTTGGCGCCGCGGTGGTCGATGCTGATCACGTTGTCCTTGTTCTCGACCACGTAGTCGCGCGCGAAGGTGTGGCGGCAGCGGAACTCGACCTCTTTGAGCACAAACTGCGACACGTGCTTTTGGTGCGCCACGTAGCCGATGCTGATGCCGATCATGTGCTTAAGCAGAAACGAGATAATGGGGATGATGAACCAAGTCTTGCCGTGACGTCGCGGCACCAGGAACACGGTGGCTTTCTGCTTAAAGATGTCGATGGAGGTCTGCGAGAGGAAGTCGATCTGGAAGGCGTGGATGAGGTACTGCAGCACGCGATTGGCCAGCACGGGGATCTTGGTCACGGCTATAAAAAAGATGACGTGTATCAATAAATTCTTTTGAAACGGTTCGAGTCGGATGGCTTTTGCGTCGCCCTCGACGGCGGTACTGAAGCCGCCGTCGAGCCACTTTTTAAAGTCGGTCATGAAGTTGTTGATCTGCTGAAACTGCGGATCGCGGTAGAGCTCGGTCAACGCGTCCAGCTTCTGGTAGGAGGCGCGCTGCTCCTCGGAGCACGGGCGAAACGTCAGTTCATCGAGCGCACTCTTGAGGCGCTCGTGAAACAGCAGCTCGCGCTGGCTTTCCTCGGGCGAGTTGTAGTCGCGGTGGCGGCCGCAGAAGGCCATGAGCGGCAGGAAGGCCTCGTTGCACGAGTGGGCCAGCCCGAGTTCGGGGTGCATCATCTGGTAGCGCTTGCGGCACAGCGCCGCCACATTGGTGAAGGCCGTGGAGATGCAGGAGGTGGGGTGGCTCTTGCGCTTCTGCAGCTCCGCGTAGCGCTCCTGGATCTTGGCGGCCGAATCTCCGCGCAACATGATGGCGGCGGCGGTGGTGCGAGCGGAGGTTAGGCGGCAGCGGCGAGAGGAGAGGAAAAAGATGGCGTCCGCGAGGACGACGGAGGATCCACCTGAAAACCACGTTGTCGCGGACGTGGCTTGTGGGACGGGCGCCGTCACTCGTTCGTCTTCGTCGTCCCTAGTGGTGTCGTCTTCCTCGGCGTCAGGCTCAGACGAATCTTCCTCCGCCTCTCCTCTCAGTTTCCCCGTCTCCTCTCCCTCAACTGCCGTCAGGTCTCCGGGGTCCGCCGGGGTTTCGACGTCCCTGTGCTCGGTGGAACGGATGGTCGAGCTGTCGGCGCAGTCTCCGGCCGCCGATTTCTCGGTCTCCGAGGCTTGGCGCTTCGAGGAGGCCGTAAATATGGCGCTGGTGGCCTGCGAGGCCGTGTCACCTTACGATCGCTTTCGTCTAATTGAAACGCCCGACGAGAATTTCTTGTTGGTCACCAACGTAATTCCGCGCGAGTCGGCCGAGGTGCCGGTGTTGGATAGCAGTAGCAGTGGTGGCGATAGCGGGCCGGAGGACAAAAAGAAAAACGTCGGGAATAAAACCGCGGGGGAAAAGAACGGCGGTGGGTCTCGGGCCAAACGCCGTCGTAGACGACGCGCTCCGAAAAACGACGCCGCCACGCCGTCTTTTCTACGTCGACACGACGTGCTGGAGCGTTTCGCGGCCGCGGCTGAGCCTTTGCCGTCGCTTTGTGTGCATGATTATGCGTTACGCAATGCTGACCGTGTTACCTACGACGGCGAATTAATCTACGGCAGTTACCTGTTGTATCGCAAGGCTCACGTGGAGCTGTCACTCTCCAGCAACAAGGTGCAACACGTGGAAGCCGTGCTGCGACAGGTGTACACGCCGGGCTTGTTAGATCATCACAACGTGTGCGACGTGGAGGCCCTGTTGTGGCTGCTGTACTGTGGACCGCGCAGCTTTTGCGCGCGTGACACCTGTTTCGGTCGCGAAAAGAACGGTTGTCCTTTCCCCGCGTTGTTGCCCAAACTCTTTTACGAACCCGTGCGGGACTATATGACCTACATGAATCTGGCTGAGCTGTACGTCTTTGTTTGGTATCGCGGCTACGAATTCCCTGCGCCAACGCCGCAGGCGACGACGGCGGGCGGGGCCGGCGCTTGTGCGGTCGAGACGAGCGCGTCAGCAGGCCGGGTCGATGACGCCGGCGACGAGGTGCATTTGCCTTTAAAGCCCGTCTCGCTGGACCGTCTCAGAGAGGTGTTACAGGCGGTGCGCGGCCGCTTCTCGGGGCGCGAGGTGCCCGCCTGGCCGGCCTCGTCGCGCACCTGTTTGTTGTGCGCGCTCTACAGTCAGAACCGTCTCTGTTTAGATCTCGCGCGTGACGAGGCGCGGACCGTGAGTTATAGCCCCATCGTCATCCAAGACTGCGCCGCGGCTGTTACCGACGTCACTTTGAGCCACATCTTGCCCGGCCAGAGCACCGTCTCGCTTTTCCCCGTCTACCACGTCGGCAAGTTGCTGGACGCTCTCTCGCTGAACGACGCGGGTCTCATCACGTTGAATCTATGACGTCGGTCAACAAACAGCTCTTAAAGGACGTGATGCGCGTCGACCTTGAGCGACAGCAGCATCAGTTTCTGCGGCGTACCTACGGACCGCAGCACCGGCTCACCACGCAGCAGGCTTTGACGGTGATGCGTGTGGCCGCTCGGGAACAGACCCGATACAGTCAGCGAACGACGCAGTGCGTGGCCGCACACCTGTTGGAGCAACGGGCGGCCGTGCAGCAAGAGTTGCAACGCGCCCGACAGCTGCAATCCGGTAACGTGGACGACGCGCTGGACTCTTTAACCGAGCTGAAGGACACGGTAGACGACGTGAGAGCCACCTTGGTGGACTCGGTTTCGGCGACGTGCGATTTGGACCTGGAGGTTGACGACGCCGTCTAACAGGTATAGCAATCCCCGTCACGCCTCTGTTCAGATTTTATTAAAAAAAAAACACACCATAACGACAGTGTCGGTGTGGTAGCTAGTGCAGCCCTAGGAACAGGGAAGACTGTCGCCACTATGTCCTCCGCACTTCGGTCTCGGGCTCGCTCGGCCTCGCTCGGAACGACGACTCAGGGCTGGGATCCGCCGCCATTGCGTCGTCCCAGCAGGGCGCGCCGGCGCCAGTGGATGCGCGAAGCTGCGCAGGCCGCCGCTCAAGCCGCGGTACAGGCCGCGCAGGCCGCCGCCGCTCAAGTTGCCCAGGCTCACGTCGATGAAGACGAGGTCGTGGATCTGATGACCGACGAGGCCGGCGGCGGCGTCACCACTTTGACCACCCTGAGTTCCGTCAGCACAACCACCGTGCTTGGACACGCGACTTTTTCCGCATGCGTTCGAAGTGACGTGATGCGTGACGGAGAAAAAGAGGACGCGGCTTCGGACAAGGAGAACCAGCGTCGGCCCGTGGTGCCGTCCACGTCGTCTCGCGGCAGCGCCGCCAGCGGCGACGGTTACCACGGCTTGCGCTGCCGCGAAACCTCGGCCATGTGGTCGTTCGAGTACGATCGCGACGGCGACGTGACCAGCGTACGCCGCGCTCTCTTCACCGGCGGCAGCGACCCCTCGGACAGCGTGAGCGGCGTCCGCGGTGGACGCAAACGCCCGTTGCGTCCGCCGTTGGTGTCGCTGGCCCGCACCCCGCTGTGCCGACGTCGTGTGGGCGGCGTGGACGCGGTGCTCGAAGAAAACGACGTGGAGCTGCGCGCGGAAAGTCAGGACAGCGCCGTGGCATCGGGCCCGGGCCGCGTTCCGCAGCCGCTCAGCGGTAGTTCCGGGGAGGAATCCGCCACGGCGGTGGAGGCCGACTCCACGTCACACGACGACGTGCATTGCACCTGTTCCAACGACCAGATCATCACCACGTCCATCCGCGGCCTTACGTGCGACCCGCGTATGTTCTTGCGCCTTACGCATCCCGAGCTCTGCGAGCTCTCTATCTCCTACCTGCTGGTCTACGTGCCCAAAGAGGACGATTTTTGCCACAAGATCTGTTATGCCGTGGACATGAGCGACGAGAGCTACCGCCTGGGCCAGGGCTCCTTCGGCGAGGTCTGGCCGCTCGATCGCTATCGCGTGGTCAAGGTGGCGCGTAAGCACAGCGAGACGGTGCTCACGGTCTGGATGTCGGGCCTGATCCGCACGCGCGCCGCTGGCGAGCAACAGCAGCCGCCGTCGCTGGTGGGCACGGGCGTGCACCGCGGTCTGCTCACGGCCACGGGCTGCTGTCTGCTGCACAACGTCACGGTACATCGACGTTTCCACACAGACATGTTTCATCACGACCAGTGGAAGCTGGCGTGCATCGACAGCTACCGACGTGCCTTTTGCACGTTGGCCGACGCTATCAAATTTCTCAATCACCAGTGTCGTGTATGCCACTTTGATATTACACCCATGAACGTGCTCATCGACGTGAACCCGCACAACCCCAGCGAGATCGTGCGCGCCGCGCTGTGCGATTACAGCCTCAGCGAGCCCTATCCGGATTACAACGAGCGCTGTGTGGCCGTCTTTCAGGAGACGGGCACGGCGCGCCGCATCCCCAACTGCTCGCACCGTCTGCGCGAATGTTACCACCCTGCTTTCCGACCCATGCCGCTGCAGAAGCTGCTCATCTGCGACCCGCACGCGCGTTTCCCCGTAGCCGGCCTACGGCGTTATTGCATGTCGGAGTTGTCGGCGCTGGGTAACGTGCTGGGCTTTTGCCTCATGCGGCTGTTGGACCGGCGCGGTCTGGACGAGGTGCGCATGGGTACGGAGGCGTTGCTCTTTAAGCACGCCGGCGCGGCCTGCCGCGCGTTGGAGAACGGCAAGCTCACGCACTGCTCCGACGCCTGTCTGCTCATTCTGGCGGCGCAAATGAGCTACGGCGCCTGTCTCCTGGGCGAGCATGGCGCCGCGCTGGTGTCGCACACGCTACGCTTTGTGGAGGCCAAGATGTCCTCGTGTCGCGTACGCGCCTTTCGCCGCTTCTACCACGAATGCTCGCAGACCATGCTGCACGAATACGTCAGAAAGAACGTGGAGCGTCTGTTGGCCACGAGCGACGGGCTGTATTTATATAACGCCTTTCGGCGCACCACCAGCATAATCTGCGAGGAGGACCTTGACGGTGACTGCCGTCAACTGTTCCCCGAGTAACCTGGACGCGGAACGTGACGGTTGCCGAGGGGAAAGGCGACAGAGAAGGTACAAACCCACCGGCGGGGAAAATACTGAGGCGCCGCCATCATCATGTGGGGCGTCTCGAGTTTGGACTACGACGACGATGAGGAGCTCACCCGGCTGCTGGCGGTTTGGGACGATGAGCCCCTCAGTCTCTTTCTCATGAACACCTTTTTGCTGCACCAGGAGGGCTTCCGTAATCTGCCCTTTACGGTGCTGCGTCTGTCTTACGCCTACCGCATCTTCGCCAAGATGCTGCGGGCCCACGGTACGCCAGTAGCCGAGGACTTTATGACGCGCGTGGCCGCGCTGGCTCGCGACGAGGGTCTGCGCGACATTTTGGGTCAGCGGCACGCCGCCGAAGCCTCGCGCGCCGAGATCGCCGAGGCCCTGGAGCGCGTGGCCGAGCGGTGCGACGACCGGCACGGCGGCTCGGACGACTACGTGTGGCTCAGCCGGTTGCTGGATTTGGCGCCCAACTATCGGCAGGTCGAGCTCTTCCAGTTGCTGGAAAAGGAATCGCGCGGACAGTCGCGCAACTCGGTGTGGCATCTGTTGCGTATGGACACGGTCTCGGCCACCAAGTTCTACGAGGCCTTCGTCAGCGGCTGTCTGCCCGGCGCCGCGGCGGCGGACGGTTCGGGTGGCGGCGGCTCGCACTACACGGGCTCGCGCGCCGGCGTCTCGCCGGGCATCCAGTTCGGTATCAAACACGAGGGCTTAGTCAAAACGCTGGTGGAATGTTACGTGATGCACGGACGCGAGCCGGTGCGCGACGGCCTCGGTCTGCTCATCGACCCCACGTCGGGGCTGCTGGGCGCTTCCATGGACCTGTGCTTCGGCGTGCTCAAGCAGGGCAGCGGTCGCACCTTGCTGGTGGAACCGTGCGCGCGCGTCTACGAGATCAAGTGCCGCTACAAATATTTGCGCAAAAAGGAGGACCCCTTTGTGCAGAACGTGCTGCGGAGGCACGACGCGGCGGCCGTGGCCTCGCTGTTGCAGTCACACCCGGTGCCGGGCGTGGAGTTTCGCGGTGAACGCGAAACCCCGTCGGCACGCGAGTTTCTGCTTTCGCACGACGCGGCGCTCTTCAGGGCCACGCTCAAGCGCGCGCGCCCGCTCAAGCCGCCCGAACCGCTGCGCGAGTACCTGGCCGATCTGCTGTATCTCAATAAGGCCGAGTGTTCGGAAGTGATCGTGTTTGACGCCAAGCACCTGAATGACGACAACAGCGACGGGGACGCCACGATCACTATTAACGCGAGTCTCGGCCTAGCCGCGGGCGACGCC

GATTCGCCGCCGCCGATACCTTTCGAGGACGAAAACACGCCCGAGCTGCTGGGCCGGCTCAACGTGTACGAGGTAGCGCGCTTTTCACTGCCGGCTTTTGTCAATCCGCGTCACCAGTATTACTTTCAGATGCTCATTCAGCAGTACGTGCTCAGCCAATACTATATAAAGAAGCATCCGGACCCGGAGCGGATCGATTTCCGCGACCTGCCTACCGTCTACCTGGTCTCGGCCATCTTCCGCGAGCGCGAGGAAAGCGAACTGGGCTGCGAGTTGCTGGCCGGCGGTCGCGTTTTCCACTGCGACCACATCCCGCTCCTGCTCATCGTCACGCCCGTGGTCTTTGACCCTCAGTTTACGCGCCATGCCGTCTCTACCGTGTTAGACCGTTGGAGTCGCGACCTGTCCCGCAAGACGAACCTACCGATATGGGTGCCGAACTCTGCAAACGAATATGTTGTGAGTTCGGTACCACGTCCGGTGAGCCCCTGAAAGATGCTCTGGGTCGCCAGGTGTCTCTACGCTCCTACGACAACATCCCTCCGACTTCCTCCTCGGACGAAGGGGAGGACGATGACGACGGGGAGGATGACGATAACGAGGAGCGGCAACAGAAGCTGCGGCTCTGCGGTAGTGGCTGCGGGGGAAACGACAGTAGTAGCGGCAGCCACCGCGAGGCCACCCACGACGGCCCCAAGAAAAACGCGGTGCGCTCGACGTTTCGCGAGGACAAGGCTCCGAAACCGAGCAAGCAGTCAAAAAAGAAAAAGAAACCCTCAAAACATCACCACCATCAGCAAAGCTCCATTATGCAGGAGACGGACGACTTAGACGAAGAGGACACCTCAATTTACCTGTCCCCGCCCCCGGTCCCCCCCGTCCAGGTGGTGGCTAAGCGACTGCCGCGGCCCGACACACCCAGGACTCCGCGCCAAAAGAAGATTTCACAACGTCCACCCACCCCCGGGACAAAAAAGCCCGCCGCCTCCTTGCCCTTTTAACTCATAAACTTTCAGGTCTCGCGTACGATTCGCGAGTCGGGAATGGGACACCCGTGGGTGTTTCTCCGTGTGTATATTATTTTTTTTTTTTGTGTGTTTGCGCCCCCGTGTGTCTAATGTGCTGTTTGAAACACGTAAAGTAGCTGGTGGAAGAACAGATAAACCTTTAATAAAAAAAAAAGTATGTGCTCCCGACCCACGGTCTGCGTGTCTCTTTTTTATGTCCATGTCTCCAAGTCTGGTGCGGGTGGCGGCGGGGTTAAGCGTCCTCGAAGTCTTCATCATCGTCGTCCTCTTCTTCGCGGAGGCGACGGCTTTCCAAGCTGTCGTGGTGACTGAGCGCAGCGACTTCTTCGCCGGAGGCTGTGGCCAGCGCCTGGTACTTGACACTGCCGCTACCGCGTCCGCGAAAGTAGCGGACGGCGCGACACGTCGTAAACATGGCCCATATGAAAAAGAGCATGCCGAACGACCAGCTGATGCCGGTGCGGTATTCGTTGCTGAGGAAGGTATCGTACTGCACGATGGGGTAGATGAGGCCGCAGAGTCCAAAGAAGGCGCCCAGGTGGTAGCCGAATTGCACCTTGACGTATTGAAAAAAGACGGCCTCGATCAGTAAAAAGTAGATGATGGAGATGATAGCGTAGACCACGAAGACGGCTAACACCATGTGGCCTGTACGCACGAAAAAGTTGTTTCCGAAGCCGTAGCACAGGGCCATGGCTACCACGGTGGTGTTGAAACCAAGCGCTACCTCTACCAGGTTGACGATGAGCGTGCGGAACTGCACCGTACCTTTGAGCTTGGGGTGCAGACGCGAGAAGAAAAAGAGTGAGCGTTTGTAGCTGCGGTACTGCGTGACCATGCTCACGTTGAAAATGGTCAGGCAGAAAAAGTGTACGGCGGCCATGAAGGCGATCATGCTGGGCAGCCGAAATGACATGGTCAGTGTGAATAGTTGGAACGTGTCCATGCTGAGAATGAAGAGGAAGGCTGTGAGGCTGTCGCCCATGTACGAAATGTCGCGTGTCGACTGGTTTAGGCTCATGCCTTTGTCCTTGCGCATGCTGATCTTGATCCAGCATACCAGGTAGTAGATGGTCACGGCTAAAAAGACGAGCTGCATGAACACGGCGTAGCACACCAACTGCACCGAGTCTAAGAAAAGCATAGGCGTGTGCAGGTGCATTACGTTGTAGGCCGACATGTTGAGCCTTTCAAAGTCCACGACGTGATAGTAGACGCAGGGGTAGCCCAGGTGCGGAAAATTGCTCAGCACTAGATGCACGCTGACGTTGACAAAAGTCAGCACCATGAAAACGATAGAAGCGCTCCATGTCCGTGTATTCACCTTATCCACGTGCGAGGGGGCCATGGCGATAGCGGCGGCCCGCTCGCTCGGGAGGCGATGGGGGCGCGCCGATGACGACAGGCTCGCGGGTCGTTAAATACTACGATGGGAGCCGCCGCGGCTCACGACGCGGTTTGAGCACGTCCGGGCGGTCGGTGAAAAAAGACCCCGCGGGCCTTCGCGACTCTCTTCTGTCCGAGGATGACCGCTCAGCCGCCGCTGCACCACCGCCACCACCCGTACACCCTGTTCGGGACCAGCTGTCATCTCAGCTGGTACGGCCTTCTAGAGGCCTCGGTGCCTATCGTACAATGTCTGTTTTTGGATCTGGGTGGCGGCCGTGCCGAGCCGCGGCTTCACACGTTCGTGGTGCGCGGTGACCGTCTGCCGCCGGCTGAGGTGCGTGCTGTGCATCGCGCCAGCTACGCTGCGCTGGCCTCGGCCGTGACTACGGACGCCGATGAGCGTCGGCGCGGCCTAGAGCAGCGTAGCGCCGTGTTGGCGCGCGTGTTGCTAGAAGGCAGCGCGTTAATCCGCGTGTTGGCGCGCACCTTCACGCCGGTGCAGATTCAGACGGACGCTAGTGGCGTGGAGATTTTGGAGGCCGCACCGGCACTGGGCGTGGAAACCACAGCGCTGTCGAACGCGCTTAGTCTTTTCCACGTAGCCAAGCTAGTGGTCATCGGCTCGTATCCCGAAGTGCACGAGCCGCGTGTGGTCACGCATGCCGCGGAACGCGTCTCTGAAGAGTATGGCACCCACGCGCATAAAAAATTGCGTCGCGGTTACTACGCCTACGATTTGGCCATGTCGTTTCGCGTCGGCACTCACAAGTATGTGCTGGAGCGCGACGACGAGGCCGTCCTGGCACGCCTCTTTGAGGTGCGCGAGGTGTGTTTTTTGCGCACCTGTCTGCGTCTGGTCACGCCTGTCGGTTTCGTGGCCGTGGCAGTGACCGACGAGCAGTGTTGTTTATTGCTGCAGTCGGCCTGGACTCACCTTTACGACGTGCTTTTCCGTGGTTTCGCTGGGCAGCCGCCGCTACGCGACTACCTGGGGCCGGACCTCTTTGAGACGGGCGCCGCCCGTTCTTTCTTTTTTCCCGGTTTCCCGCCCGTGCCCGTCTACGCGGTCCACGGTCTGCACACGTTAATGCGCGAGACGGCGTTGGACGCGGCGGCTGAGGTGCTCTCGTGGTGCGGCCTGCCCGACATCGTGGGCTCGGCCGGCAAGCTGGAGGTGGAACCCTGCGCGCTCTCGCTCGGCGTGCCCGAGGATGAGTGGCAGGTCTTCGGTACCGAGGCCGGCGGCGGCGCCGTGCGTCTCAATGCCACGGCTTTTCGCGAGCGACCGGCCGGCGGCGATCGTCGCTGGCTGTTGCCGCCGCTGCCGCGTGACGACGGCGACGGTGAAAACAACGTCGTGGAAGTCAGCAGCAGCACCGGCGGTGCGCACCCGCCGAGCGACGACGCTACTTTCACCGTGCACGTTCGCGACGCCACGCTACATCGAGTGCTCATCGTGGATTTGGTCGAGCGCGTGCTGGCCAAGTGTGTACGCGCGCGCGACTTCAATCCCTACGTGCGTTATAGTCATCGACTCCACACTTATGCGGTTTGTGAAAAGTTTATTGAAAATCTGCGTTTTCGCTCGCGACGCGCCTTCTGGCAGATCCAGAGTCTGCTGGGTTACATCTCCGAGCACGTTACGTCAGCCTGCGCTTCGGCCGGCCTTTTGTGGGTTCTGTCGCGTGGCCACCGCGAGTTTTATGTCTACGACGGCTATTCGGGTCACGGACCCGTCTCGGCCGAAGTGTGCGTGCGGACTGTGGTCGACTGTTATTGGCGCAAACTTTTTGGCGGCGACGATCCGGGTCCCACCTGTCGTGTTCAAGAGAGCGCGCCCGGCGTGCTGTTGGTCTGGGGCGACGAGCGGTTGGTGGGTCCCTTCAACTTCTTCTACGGCAACGGCGGCGCCGGTGGTAGTCCGCTCCACGGGGTGGTGGGTGGTTTCGCGGCGGGACATTGCGGTGGCGCTTGTTGCGCGGGCTGCGTCGTCACTCACCGCCATTCTAGCGGCGGCGGTGGTAGTGGCGTGGGCGACGCGGACCACGCGAGTGGCGGCGGTCTAGATGCCGCTGCCGGGAGTGGTCATAACGGCGGTAGTGATCGGGTTTCTCCCTCCACGCCGCCCGCGGCGTTAGGTGGCTGTTGCTGCGCGGCCGGTGGCGACTGGCTCTCGGCCGTGGGTCATGTCCTGGGCCGGCTGCCGGCGCTGTTACGGGAGCGCGTGAGCGTGTCCGAGCTGGAAGCCGTGTATCGCGAGATCCTCTTTCGCTTCGTGGCTCGCCGCAACGACGTGGACTTTTGGTTACTGCGCTTCCAGCCCGGTGAAAACGAAGTAAGGCCGCACGCTGGGGTGATTGATTGCGCGCCCTTCCACGGCGTGTGGGCCGAGCAGGGCCAGATCATCGTACAGTCACGCGATACGGCGTTGGCGGCCGATATCGGCTACGGCGTCTATGTGGACAAGGCCTTTGCCATGCTCACGGCTTGCGTGGAGGTCTGGGCGCGAGAGTTATTGTCGTCCTCCACCGCTTCCACCACCACTTGTTCTTCTTCTTCCGTTCTCTCCTCCGCCTTGCCGTCCGTCACTTCGTCCTCTTCGGGCACGGCGACGGTGTCTCCTCCGTCTTGTTCTTCTTCGTCGGCAACTTGGCTCGAGGAGCGCGACGAGTGGGTGCGCTCGCTGGCGGTTGACGCGCAACACGCTGCTAAGCGGGTGGCTTCCGAGGGCCTGCGGTTTTTCCGGCTCAACGCTTAACGAGTCACGTAGGGGAACTACGTGGGTAAGTGACGTGGATACTAGTAAAAAAAAGTGCGTCAAAGTTCTCAGCGTGTGACGTGGATACTAGTAAAAGGGACGTCAAAGCTCACTACGTGTTGCGTGTTTTTTTTTCTATGATATGCGTGTCTAGTTCGCTTCTCACTCTTCCTCTCCCCGTTCCCAGCGCGGTGGCAGCTTGGGGGGTGAGGGCAAATTGGGGTAGTTGGCGTTGAGCACGTCTAGCAGGCCCAGGCCCACGGGCCAACCGTCCACGGTCTTACGCTCGGTCAGCTTGAGGCTAAACGAGTGTGCCTCGTCTTGACCGGTAAGGCGGAAAAAGAAGCGTGCTACCAGCTGCAGGCAGGTATGCCGCGTCTGCTGGAAAAGCACGAAGGTAGCGGGCACGTACTGCACAATGTGCGGTTCTTTTTCCTCAAAGAGTAGGTAGAGCGCGCTGCAGATCAGCCGCCGGGCGCTGTGGTGCAGCAGCCGGCCGAAGCTTTCGCGCACGTTCACCGCGTCTAGGTACTGGAGCAGGTCGTGCAGGCACTTGCGCGTTAAGTTGCAATTTTCCACGCATGAAATAACGGTACAGAGCGCGAAGTGCAGCAGGTTGTCGGCCTTGACGATGCCGCAGCGGTGTTTGAGCCGCAGATCCGAGAGCCTCACCTGCGTGACGGCGTCTTCGGTCTCGAGCAAAAACACGGCGGAGTAGCCCAGAAAGGCCGAGGTGCACAGCAACTCGCTGCGGTACTCGGCCATGGAAACCAGCAGCCCGTGCTCCGTGTGCAGCCACAGCTTGTCGCCGCGCACCGTAAAGTCGAGCACTTGCGGCTCCATGATCATCACATTCTGTCTAGTGAAATCCGTATGGACCTCCAGCACGCCGCGGATCATCAGGGCCTCCATTTCGAAATCGGCCGACACGCTCTGGGCCGCGCCGCTCCTCGTCTGCCGTGATCAAGCGGCGCGGCGCGGACCTTTCAAGCGTTCCTGGGCCGCCGCTCGAGGCAGTTCCCCTTTCTGGCACTCCGCCCGCCGCTTCGCGGCTCATTTGGCGCCGGCGCGCCTTCTCGCGGCTGCAAATCAACTCCACGTATCGGCAAAACTTGCTGTCGTCGTAGGCGGCGGCTACGATCTCGCCGAAGGAGAGCTGCAGGTAGGCCTCGGGTACGGGGTCCAGCGTGCCCAGCGCCAGGATGTGACACAGATAGGGCAGGGTCACGCGCTCTACCGTGTAATTGGAGTATACGATGGCCTCTTCGGCCCCCTGATGCGTGACCAGACGCCGCAGGCGAAAGGTGCGGAAATACTCGTTTTCCCACAGCTGCGTGAGGAAGCGTTCCAGCGACTCGGTGCCGGGCACGAACTGCGAGAAGAAGCTGTTGGCCACCAGGCGGTTGTCCTCCACCGCCAGCGGACGGAAGGGCGCCGCGTCGCGCGCCTTGCGCACGGCCTCCAACACGGGCAGGTGGTACAGTTCGGCGTCGCGCGCGCCCAGGCTCATGGAGTCCTCGCGCCGCGAGGCGTAGCGCGTGAGCAGGTCGCGCAGCTCGCGCACGCGATTCTCCCAGGTCTGGTTGAGCGTGCGCAGGTCCTGGATCTCGTCCACCTGCGACTGGATCTGCTCCTCCAGGCACTTGATGACCTGCTTCTTAAACAGGTCGCGGATGTCCCGCTCGGGCGCCGCCGGGCCGGGTGGCGGCGGCAGCAGCCCGACGTGGCCCGCGGGTCCTCCCACCACGGCGCCGCCGGGTCCCACCACGCCGGGTCCACCCGGACCACGCGCGGGTAGTAGACGGTTTTGGTCCACCAGCGAGGGGGTCAGGTCCTGCAGAAAGGACTCGACGCTGTCCTCGATGCCGATGCGCGATTTGCTGTCCGAGACGTTAAGCAAAAACTTCATAATGGACTTTTTGGCGTCGCTGCCCCGGTCGTGCTGCTCCATCATCTCCACCAGCTTCTTGCAGTTGAGCTCGTGGCGGCTGGCGGTCACCACTTTCACAGGAAAGGTATTGAGCAACTGGCAGATCTTTTGGTGGCGGCAGAGCCCGTCGTAGCGCAGAATCTCCTCGTGCAGGTGTGCCACCGGCGTGGTGAACAGCAGCTTGTCGCGCTCATAAGCCAGCGGTTCGGCCGCCACGTACAAGCGGATGTGCTTGCCGCGCAGCTGCGCCTCCAGCCGCTCCGAGCGCACCTTCTTGAAGACGCGTACCTCGGGCGCGTTGGCTATGCGCACGGCGCCCAGGCGCTCGGCCACCTGCAGCAGTAGCGCCAGGTTAGCCTGCAGCAGGTCCTGCGCCAGCGGGTGTGTCTCGGTGGCCCGCTGCACGGCCGCGCGTACAAATTGCGCCCGCTCGGCCGCCTCGCTCGGCTTGGTCTTCACGTCCAGCAGCGGTACCAGTCCCACCGTTACGCACCAATCCACGTAGAGACCATAGTCGTCGTTATCGGCGTACTGATATAAAATGTCGCGGAGCGCGCCCAGCACGCCCGTTTGCACGCTCTGGCGCAACGAGGCGCTCCACACCAACAGATACTGCTCCAGGTCCTCTTCGTCCAGCGCGCGGTAGGGAAACAACGCCGCGTGTAACTTCCACTCCTCGGCCACGCGCCGCACCGTGATGGTGTCAAAGAGCGTCTTGCACACTCCGTAGAGCAGCTGCTTGCGCAGCACGCACGGGTCGCGCAGCACCTGGTGCATGCTCTGGCCGCGACACGTCCCCAGAAAGCCGTGCAGCAACCGCAGGAAGCTCATCGTCTGGCCCGTGGGGAAAATGTCGATGACGGCCTCGTCATCCACGCCGCGGCCCACGCCCAAGTACGACGACGCCTTGATCCTCAACCTCTCGTCGGCCGCCAAGATCGAACGGATCGTCGACAAGGTCAAGTCCCTCTCGCGCGAGCGCTTTGCGCCCGAGGATTTTTCGTTCCAGTGGTTTCGCTCCATCAGTCGCGTTGAACGAACGACAGATAACAACCCCTCTGCCGCAACTACCGCCGCGGCAACGGCGACCGTTCACTCCTCCGTCTCCTCTTCTGCCGCCGCTGCCGCTTCGTCCGAGGCCGGCGGCACGCGCGTGCCCTGCGTCGACCGTTGGCCCTTCTTTCCCTTCCGCGCGCTGCTCGTCACCGGCACGGCGGGCGCCGGCAAGACTTCCAGCATCCAGGTGCTGGCGGCCAATCTAGATTGCGTGATCACCGGTACCACGGTGATCGCCGCGCAGAACCTCAGCGCGATCCTCAACCGCACTCGCTCGGCGCAGGTCAAGACCATCTACCGCGTCTTCGGCTTCGTCAGCAAGCACGTGCCGCTGGCTGACAGCGCCGTTAGCCACGAGACGCTGGAACGCTACCGCGTGTGCGAGCCGCACGAGGAGACCACCATCCAGCGCCTGCAGATCAACGATCTGCTCGCCTACTGGCCGGTCATCGCCGACATCGTGGACAAATGCTTAAATATGTGGGAGCGCAAGGCCGCTTCGGCCTCCGCCGCGGCCGCGGCCGCCGCCTGCGAGGACCTCTCGGAGCTGTGCGAGAGCAATATCATCGTCATCGACGAGTGCGGCCTTATGCTGCGCTACATGCTGCAGGTGGTGGTGTTTTTTTACTACTTTTACAACGCCCTGGGGGACACGCGACTTTACCGCGAACGCCGCGTGCCCTGCATCATCTGCGTCGGTTCGCCCACGCAGACCGAGGCGCTGGAGAGCCGCTACGACCACTACACGCAAAACAAGAGCGTGCGCAAGGGCGTTGACGTGCTCTCGGCGCTGATTCAGAACGAAGTGCTCATCAACTACTGCGACATCGCCGACAACTGGGTCATGTTTATTCACAACAAGCGTTGCACCGACCTGGACTTTGGCGACCTGCTCAAGTACATGGAGTTCGGTATCCCGCTCAAGGAGGAGCACGTGGCCTACGTGGACCGCTTCGTGCGGCCGCCCAGCTCCATCCGCAACCCCTCGTACGCCGCCGAGATGACGCGGCTTTTTCTCTCGCACGTCGAGGTGCAGGCTTACTTCAAGCGGCTGCACGAGCAGATCCGCCTGAGCGAGCGCCACCGTCTCTTTGATCTGCCCGTCTACTGCGTGGTCAACAACCGCGCGTACCAGGAGCTCTGCGAGCTGGCCGACCCGCTGGGCGACTCGCCGCAGCCCGTCGAGCTCTGGTTCCGCCAGAACTTGGCGCGCATCATTAACTACTCGCAGTTTGTCGACCACAACCTCTCCAGCGAGATCACCAAGGAGGCGCTGCGCCCCGCGGCCGACGTCGTTGCCACCAACAACCCCTCCGTCCAGGCTCACGGAGGGGGAGGATCTGTAATCGGGAGCACCGGCGGCAACGACGAGACGGCGTTTTTCCAGGACGATGATACCACCACCGCGCCCGATAGCCGTGAGACGCTGCTCACCTTGCGCATTACCTACATCAAGGGCAGTTCGGTGGGAGTCAACTCTAAGGTGCGGGCCTGTGTTATCGGATACCAGGGCACGGTCGAACGTTTCGTGGACATCTTGCAAAAGGACACGTTTATCGAACGCACGCCCTGCGAGCAGGCGGCCTACGCCTACTCGTTAGTTTCGGGCCTGCTCTTCTCGGCCATGTACTACTTTTACGTGTCGCCCTACACGACCGAGGAGATGTTGCGTGAGCTGGCGCGCGTTGAGCTGCCCGACGTGAGTTCGCTCTGCGCCGCTGCCGCCGCCACGGCCGCCGCTCCCGCTTGGAGCGGGGGAGAGAATCCGATAAATAATCACGTCGACGCGGATTCTTCTCAGGGCGGCCAGAGCGTGCCGGTATCTCAACGGATGGAACATGGCCAAGAGGAGACCCACGACATCCCCTGCCTGTCCAACCACCATGACGACTCGGACGCCATCACGGACGCCGAACTCATGGATCACACCAGTCTGTACGCGGATCCCTTTTTTCTCAAATACGTCAAGCCACCTAGCCTGGCGCTGCTTTCTTTCGAGGAGACGGTGCACATGTACACTACCTTCCGCGACATTTTTCTCAAGCGCTACCAGCTCATGCAGCGTCTCACGGGCGGTCGCTTCGCCACGTTGCCGCTCGTTACCTACAATCGCCGTAACGTGGTGTTCAAGGCCAACTGTCAGATCAGCTCGCAGACCGGCTCCTTCGTGGGCATGCTTTCGCATGTGTCGCCGGCGCAGACGTACACGCTCGAGGGCTACACCAGCGACAACGTGCTCAGTCTGCCCAGTGACCGCCACCGCATCCACCCCGAGGTGGTGCAGCGCGGCCTTTCGCGGCTGGTGCTACGCGATGCGCTCGGGTTCCTCTTTGTGCTCGACGTTAACGTCTCGCGCTTCGTCGAGTCGGCGCAGGGCAAGAGTCTGCACGTGTGCACCACCGTGGACTACGGCCTCACTTCGCGCACGGCCATGACCATCGCCAAGAGTCAGGGCCTGTCGCTCGAGAAGGTGGCCGTGGACTTTGGGGACCATCCCAAGAACCTCAAGATGAGCCACATCTACGTGGCCATGTCGCGAGTCACGGACCCCGAGCACCTCATGATGAACGTCAACCCGTTGCGACTTCCCTATGAGAAGAACACCGCTATCACCCCCTATATCTGTCGCGCGCTCAAAGACAAACGCACCACGCTTATTTTTTGACACAACACCGTGTAAGGAAAACGTGACTTTATTGAGCAGGGTAAAAACCACGTACAAGAACCACGTTGTCTATCCCCAAAAAACACACACCGTCAGGGAACACATCGCCTATAGATAGCGGCACTTCACATAAAACCACCGTACCTGCATCACGGTGGCTCGATACACTGGAAATTCAATAAAAACCACCGTGTCTCCGTGACGGTACTTATCGGGTCAGCATCTTTCTCTTGAGATTTTTGTTCGTAAACTTATCCGTTTCCCCGGTCCGCGGTGTCTCCTCGTAAGGCTGACAGTCTACGAGTGGTACCTGCAAGAGAAGAAACCCGGGTGGGAGCGACGCCGTCGCTGGGTATCAACCCCGCGGCTGACCGTCGTCCGGTAAAGGAACAACCCGTCGTCGCAAGCCGGGTTCGACCAAGAGAAAAACCCGGGTGCGGGGGGGAGACGGGTCGTCCTTTGGTTGTTCGCGGACGGCGTACATCCCGCGTGGGTCAGTCGACGGCGTCGCTCCGTGCGGTCGGTCATCATTCTGCTTCACATATATGGGTTGTTTGTGTTTTTTTTTTTATAATGAATACGCACTGATCCTATCCGTGACTGCGCGTGTGGCAGAGAGGATGCCTTATAACATGTATTTTGAAAAATTGCCAACAGCTATAATTTCTCTCATGTAGCAGAATAGAGACCTTTTGTCGTCTTTTTGTTTGTCATTACTTGTTTTCCAGGGAATTAGAGAGAGGGAACCGCGCCTCCGGCGGCGGTGCCCGCGGACCCCGGCCCCTTCTCGCGTGCGCGGTGTGACTGGTTGAGCGAATGAGCAGCTAGGCTTGGTGGTGCTCCGCGTGCGGGGGAGAAGACGATTAACAACAAAAAATAAGTGGAAGTGGCCGGTGGGTCTTTGTCCGCGTGCGCGCCCATCCGTCGCCGGGACCGAGCAGAAAGTGATGTGGTGGTACATTGATTTTTTCCTTGACAGGAAAGAAAAAAAAAGAGTTTTGTTTTCCTATGTGAGAGGAGAAAGGTATGTGAGGAGATGTTCGATGATCGTATGTTACAGTTATGCTGTAAGGAAGCTTTTATCGTGCGTCCTGTTTTTCATTTGATGTATATGACACAATTGAAACCTATCGATAGGCGTATATCGAGGATTCATCAATTCTTAGAATCGTCGTCTTTTTGGCTAATTGGACTTTGCCCATGTTGGTTGTCATTCGTGGCCTGAGGTCATCGTCGTCCATGACGACGTGTCTATAGCGTGCGGTGTGATCATTGTGTCGAGCCAGAGAAAGCGCGCCTCGCACGACGTTTGCGGATCGGCTCGCGGGTGTGTGGAATTCCTAAGAACATAATCAGCTGGTCGTCTTTCTTTGATGTGTTGTTGTCGTCGAGGTCTTGCTTCGTTTTCTTTTTTCTTTTTAGTCGATGGAACTTTTCTTCGGTACGGGTTCTTGTTATGGAAGCTTGTGTTTTCGAACATGAATTCGAAAAAATAAAAAGGCCTATCTTCGTTTCAAAAAAAGGACAGATATCAATCTTCTTAACTTATATCATGGTAAATTCAGAATCCTATGGTGTCTTATTATCTCTAAAGTAGTCAACATTATGGTCTAATTTGTATTTCCCTGACGAGATATATATGATCCTTATAACCTGGCTACTATCATGAACAACAATATCCTTACTTACAGTCATCTTCGTGAGTTAATGAAGTATAATATCGGTCATCTATCAACTTATCTGCTATGTAACGTACCCTTTTAGGTATTTTGCGTTTCTTAACGAGTGTACCCGCCTGTGTGAGGCGAAACTCTGAGAAGTCTACCGAGTCGAGTTACAAGTCACTAAAACACTTACACGAGTTATCTATACTAAAATCACTATCTATGTTGTTTGCTTACCTAATTATTATCCTACATGACGAAGCTACCTCCCAACGTAAGGTAGGGGGAGAGGAGACAGAACAATAAAAAGTAACTAATGTTTCTTAGAACTTACCCGCTAAGGACTTACCAAACTATATTCACCAAAAAACAACAGCTACGTGTTTCATTTGTTTTAATCTACCGAAGTAAAAAAAAAAAGATGATTAGCTATCCAGAACCTACTTACTTCTTAATGTTTTAACTAAGGATGCCTATGGGATTGGAAAAAAAATCACAGCAACTTGCTACTAATCAGTTGACAGCGAAGAGACTCATAACAAAGATTTCTGGGTAATACGGTTATAATAATGCTTATGGACTAAAGGATACTTGGAAAAAAAGAATGGGCTATGACTATAGAGATTCGTTGAGATATCAAACTTCAAATAGGCGGCTATCATTCATGGTTGTGGTGACTATATCGTGGAGAAAAAATGTGATCGTTAGTTAGCTAGGTGAGACTTACAGCTATCCATCCGTCTAGTTTTTCGTTGTAATGATGATAGTACGTCTATGGTGGTGATCGATTTTGGTTAGCAATTTGTTCGTTTAAAGGCTTAATGTACTTATGCTACATGATGTATTATTCTTTGATTCATCGTTCCTCCTAAGGGGGTGTATGTATGTATGTACTAGTCGTATAGTGTTCCTAACATCATGACTATTCAGACTATGGCTTCATCTATCGTGTCTAAAGTTCACCTATTCTACTATTACTATATATATGCACTACTATGTAACTAGGATATGGTCCTATAAGGTGTCTTCTATCACGGTGGCTTGTTTATCGCTTGGCGGTTACGAGCAAGAGTTCATCACGGACCAGCCGTGAGGCAGGGCACACGCGGGTCGGCGGCGATGATGTTCCCCGCGAAGGGGACAACAAAAACAAGACAAGAGGCCGCCGGCCGCGGCCACGGACGCGTAGCGGTTACACAATGTTTGGTTGAGCGTTTTGTTTCATCGTCGTGGTGGTGGTTTTGTTGTTCTCTGTATATATCGTGTGGTGGCTTTATCGTCATCATTATTATCATCATTCTTGTTTCCATCATCACGATGAGTTTTCTCCGTTTTCCTCTCCTCCAGTGGTAGTCGTGTATCATCATCAATCATCGTAGTGACGTCGTTGCTGCTGCTGCTCTTGCCTTCATGGCGGTATTTCTCTTCCTCCCCCCTAACCCCATATTAACTCGTGAGTGTGATGGTTAGAGTGGCTGCTTGTTTTTTTTTTCTTTTCTCTTTGGAACAACAAAAGAGGATAAAGATGGTCGGTGAATGTATTATTATTATCATCATCATTATGATACGGTCGCGGTCTTCTTCTCCGATGACGAAACCTGCGCACATCGAAGAAAAGACGAGCGCGCGAACCGATAGCCGTCCGTCTGGGACGAAGGAGAAGATGATGGGGAGAGGAGGAGAGCCCCAGAAGCCAGAGCGAGAAGGGAGACGACAGACATACGTCGTCACCGTCCTCTGGAGGAGGCACGGCGGCGCTGTTTGTTGTTTGGATGCTTGATTATATCCTGTTCTATGGGGTAGATTATTATCAATAGGCTTGGTTTTCAAAGGTCAGCCTGTGTATTGTCGTGTCTTTTTTTTCGTTCTCATGATCGCGGAGACCACACAGACGTGCGCGTCTCCCAATGGCTAGGCGTTCTTTTTAGGTAGTAATTTTTTGATCTTTTTTTTTTCTTAACAAGTCTGGCTTGATTTCTTTTATCTATGATCGATTCTTCTTTTTCTCGGGGGTTGCATCTTCCGTGAAAGTAAAGTGACACTACTCTAAATGGTAACCATATTATCTGTTGATTAGGAGAAAAAATAATTTTTTCGCACGAAATCGATCCTAAGTGAGGTGATTTACTTGCTATCACACGAAATGATTATCTTTTGCTGCTAACGTACTGAATTTTTTAACAGAATTGCTTCTCCGTAACTATTTCCGCAGATTCAGACAGATTGTCAAAAAAAAAATACGGCACAGAAATAGTGGGTCTGTGGCTTTTGGTTCGTGTACATTCGCGTTTGCGTGTCGAGATTTCTACGGTATGTTTATTCTTCCTGCGATGATGTAGGGTCCTTGGTGTAAGTAGGATTTCGAGTATCTCTCTTAGAGCGAACAAAATAATCAAAAAACAACAGCTAGGAAATCGAGGGTTACTCTACGATAAAGTGTCTCTACAAAGTGAAGAATGTTACGTTGTGGTGGAATAATAAGACTCGCGTGATCGATGAGTGATCGAGAGCGGCTCGAACCTTCTTTAAGAGCTTTGTTTAGTGCAACTTTAAATTACAAGGAGTAGAAAGCTGAAATGAATCTATGAAGGTGCTATTCTTTGAATATCTTACTTTGTACGCTTCACATTCGTTATTTGGATAGAGAGTTGTCTAGAGAAAATCTGTGATTCTCTATGAGTGTTATTTTTATTATCCTTTTGGGGACTACGATTTTTCTTCTTGTTCTACATACCACTACTACTCGTAATCACATACATGGACGAAAAAAAAATTCGTCAGGCAGTAGATACCAGATTCTCCGACGTTACGGCGTCTTTTTTTCTTTTGAGAGAGTATCTGCTGAGATTGTCCGTGGTGTATCTAGTCGCTATTTTTGTTGTTACTAGTAGTTTTGCACACAGTTTATTCAGTATAGTTTTTCTTCTTGCCATGATCAATTGAGCCCACCACCTTTTTTTTTAGAGAGGAGGAATTTCGTCTTGATCTCCAGCCGGAGACAACGGCGGCGGTGGTGGTGGTGGCGGGAGAGACTTCAAGGCAATGAAAAAAAAATTTCGTTTTGCCATCAAGTGGTGACGATAACCCGTCAGATTGATAATTGGTTCCTACAGAAACTATTCTAACCGCGGAAGAAAGAAATTGAAAAAAAAAATTGACAAAAACATCATAACATAAAGGACCACCTACCTGGGACGCGCAGTTGGGCGGCGGATTGGGGCGGCATGCTGCGGCGATGCTGTCGGTGATGGTCTCTTCCTCTCTGGTCCTGATCGTCTTTTTTCTAGGCGCTTCCGAGGAGGCGAAGCCGGCAACGACGACGACGATAAAGAATACAAAGCCGCAGTGTCGTCCAGAGGATTACGCGACCAGATTGCAAGATCTCCGCGTCACCTTTCATCGAGTAAAACCTACGTTGGTAGGTCACGTAGGTACGGTTTATTGCGACGGTCTTTCTTTTCCGCGTGTCGGGTGACGTAGTTTTCCTCTTGTAGCAACGTGAGGACGACTACTCCGTGTGGCTCGACGGTACGGTGGTCAAAGGCTGTTGGGGATGCAGCGTCATGGACTGGTTGTTGAGGCGGTATCTGGAGATCGTGTTCCCCGCAGGCGACCACGTCTATCCTGGACTTAAGACGGAATTGCATAGTATGCGCTCGACGCTAGAATCCATCTACAAAGACATGCGGCAATGCGTAAGTGTCTCTGTGGCGGCGCTGTCCGCGCAGAGGTAACAACGTGTTCATAGCACGCTGTTTTACTTTTGTCGGGCTCCCAGCCTCTGTTAGGTTGCGGAGATAAGTCCGTGATTAGTCGGCTGTCTCAGGAGGCGGAAAGGAAATCGGATAACGGCACGCGGAAAGGTCTCAGCGAGTTGGACACGTTGTTTAGCCGTCTCGAAGAGTATCTGCACTCGAGAAAGTAGCGTTGCGATTTGCAGTCCGCTCCGATGTCGTTCACCCAGTTACTTTAATAAACGTACTGTTTAACCACGTTGCGTCGTGACGTTGTTTGTGGGTGTTGCTAGGCGGGCTGGAAAGATGATGTATAAATAGAGTCTGCGACGGGGTTCGGCGCTCTGCCGGCTGCGGCGGCACTCGCTCCACGGCCTCCGACGAGCGTTGCGCTCGCGCTTTGCGCCGCCGCGTCATGGATCTGCCTACTACCGTCGTGCGAAAATACTGGACTTTTACGAATCCTAACCGCATCCTGCATCAGAGCGTTAATCAGACTTTCGACGTGCGCCAGTTCGTCTTTGACAACGCCCGTCTGGTCAACTGCGTGGACGGCGATGGCAAGGTGCTGCACCTCAACAAGGGCTGGCTCTGCGCTACCATTATGCAGCACGGCGAGGCTTCGGCTGGCGCCAAGACGCAGCAGGGCTTCATGTCCATTGACATTACGGGCGACGGGGAACTTCAGGAGCACCTCTTTGTACGCGGCGGTATCGTCTTTAACAAATCCGTCTCCTCGGTGGTGGGCTCCAGCGGACCCAATGAGAGCGCGCTGCTCACTATGATTTCCGAGAACGGTAATTTGCAAGTGACTTACGTGCGGCATTACCTGAAAAACCATGGCGAATCCTCCGGCGGAGGCGGTGGTTGCGGCGCCGCGTCTACCGCCTCCGCCGTCTGCGTGTCCTCGTTGGGTGGCAGCGGCGGGACTCGCGACGGCCCTTCTGCGGAGGAACAGCAACGGCGAAGGCAGGAACAGCGTCACGAAGAACGGCGCAAAAAATCGTCCTCGTCTGCCGGTGGTGGTGGAGGCGGCGGCGCTAGTGGTGGCGGTGGCGGCGGCGGGAGCGGCGGTCAGCACTCCTCGGACTCCGCCAACGGACTGCTGCGGGATCCCCGGTTGATGAACCGGCAGAAGGAGCGGCGGCCGCCTCCCTCCTCCGAGAACGACGGTGAGTCCCGGCCCTCCTCGCGTCACGGTGCTTTCCGAGTGGACTCGTGAGCCCCCCGTAGCGCACGAGCGAGCAGGCGAGCGGTGTTGGTGCGCTGGTGGTTGTGTGGATGATAACCATGTGCTTTTTCGTGCGCTATGTGTCGTCCCGTCTGTAGGCTCTCCTCCCCTCCGGGAGGCGAAGAGACAAAAGACCACCGCACAGCACGAAGGCCATGGCGGCGGCGGCAAGAACGAGACGGAGCAGCAGTCCGGTGGTGCTGGCGGTGGTGGTGGCGGCGGCAGCGGCCGCATGTCGCTGCCGCTGGACACGTCTGAAGCGGTGGCCTTTCTCAATTACTCGTCCTCATCCTCCGCGGTCT

CGCCGTGACGGACGTGGCCGCCGGCACCGACGGTGCGTTACTTCTACCCATTGAGCGCGGAGCGGTGGTTTCGTCGCCGTCGTCGACGTCGCCGTCGTCACTTCTTTCGCTCCCTCGACCCAGCAGCGCCCACAGCGCGGGCGAGACGGTGCAGGAGTC

ATGAGGAGGATGAGGAGGGCTCCGGCTGAGGCGGCGGAGGCACCACCGCAGTCGGAGGAGGAGAATGATTCCACCACTCCAGTCTCTAACTGCCGTGTTCCTCCGAATTCGCAGGAATCCGCGGCGCCTCAGCCTCCTCGCAGTCCGCGTTTTGATGACATTATACAGTCATTGACCAAAATGCTCAATGATTGTAAGGAGAAAAGATTGTGCGATCTCCCCCTGGTTTCCAGCAGACTCTTGCCAGAGACGTCGGGCGGGACTGTCGTCGTCAACCACAGCAGCGTCGCGAGGACCGCCGCAGCTGTCTCCACAGCCGGCGTTGGCCCCCCAGCAGTCGCATGTCCGCCACTCGTCACCACCGGTGTTGTACCCTCAGGTTCCGTCGCCGGTGTCGCGCCCGTTGCCGCCGCAGTCGAAACACCAGCTGCTCCTCCCCGGCCCGTGTGTGAAATCAAGCCCTACGTGGTAAACCCCGTTGTCGCCACCGCCGCGGCTGCCAGTAACTCTTCCTCGTCTTCTTCGGCTCCACTGCCGCCGCCACCACCACCGCCGGGCGGACGTCGGGGTCGGGCCCGGAACAATACCCGAGGAGGCGGCGGCGGTGGTGGCGGTGGTAGAAACAGCCGGCGGCAGGCCGCATCGTCGTCGTCTTCCTCCTCTCGGAGATCGCGACGGAGAAACAACCGCCATGAGGACGAGGACAACGATCCTCTGCTCCGGTTGTCGCAAGTCGCCGGCAGCGGCCGCCGGCGAGGGCCCTCGTTCCTCGAGGACGGACTCGAAATTATCGATCCCAGCGAGGAGGCTGCGATCGCCGCCGCCTCGATCGCGGCGTTTTTCGACGATTAAAAAACAGAGCCGAGACCGGAAAAATTATGAAACAGGACGCGCTTGGACATTTGGGTTTCCACCCCTTTCGGTGTGTGTCTATATATATTGTGGTCACTGATTTTTTTTTACAATAAAGTAGACATCACAGTTCACCACCTTGTCTCCCCGGTGTATCTATTATCATCAATCACCCACAGAGTCGCCAGTCCATGGTCTCTCGGTAATGCGTGTCCAGATACGCGTTGGCCAGTATAAAGTGGTCGTTGCCCACGAAGGCGCGGGTGGTGTTGCGCGGCGACGGGTGGCAGGACTTGAGTACCAAGTGCCGCCGTCGGTCGATCAGGTACTCGCAGGTGTGCGCGTCGGCGCCCCACAGCATGAACACCAGATGCTCCCGGCGCTCTGACAGCCTCCGGATCACATGGTTACTCAGCGTCTGCCAGCCTAAGTGACGGTGAGATCCAGGCTGTCCGTGCACCACGGTGAACACGGTATTGAGCAGCAGCACGCCGCGTCGCGCCCAGGCGTCCAGGCAACCCGAGGCCGGACGCTGAAACCCGTCCACCGTACGCGCCAGTTCGCGAAACACGTTGTTGAGGGAGGGCGGCGGCGGTCGGCCCGCCAGCGTGCCGAAGGCCAGGCCGCTGGCGCTGCCGTCGCAGTACGGGTCCTGGCCCACGATCACCACGCGCACCTGCTCGGGCGGACACAGATAGCTCCAGCGGTGTACGTGCTCGGGTGCCGGGTACACCATCTCGAGTTGCCGCGCGCCCTCCACCGCCGCCACCGTGTCGCGCAGCAGCACCGTGTCGTGGTCGGGCAAGCTGAGGAAGCGGATCCAGTCGGCGCTCAGACAAAACACGCGAGCCTGCTCGTCGGGGGTTAACAGAGAGCCTTTATTATCAGCAATGTTAGCGAGCATCCACTGCTTGAGGGCCATAGCGCGAGTGAGCCGGCAGGTTGACGCGCGTCTGCTTCAGCTCGGGCGGCAGTCCGGCGTAGTATTTATCTAGGTGGCGTAGCAGCGGCGGGTCCAGCTGGTGACGCAGGCAGAATTCCTTCACTGCGTTGTACAGGCCGTAAAAGAGCGTGATGCCCTCGGGCGCGGCAGCGGTGCTCACGGGCAGACGCACGGCGCGGTTGGTACGCGTGGCTTCGTTGCGTATGGCCACCACCACGTTAAAGAGAGACGGTGGCACCAGCTCGAAGCCTAACACGTGTTCCGTGAAGATGCTGCGCCCGTATGACAGTCGCGTGAGGTCGTAGCCGCGGCACAGGTCGTCCACGCACGTGTACACGGCCGGCGAGCCATCGCCGCACTCGCTGTAGCCGCGCATCACCGTCATCCAGCGCGGCGCTGTGTCCGAGCTCAACAGCGTCAGCAGGGCCCGCAATTGATCCGGATTGTTGTACAGCAGGGCCAGAGTGTCCAGGAAAGCCTCGTCCAACAGCACGGAGTTGGCGGCCTCCGGCGTAACGGGACGGTAACGGATAAGTTGCGATAGCGGGCCATCGCGCCCGGTAACATTCACCAACGGGCGCAGCCAACTTTCATACTTGTCACCCTCAAACACCTCACCCAACAAGCATCGGCGCGTTAGTTCGGGGCACTCCGCGGGGACTTTCTCGGCGGCGGTAGGAGCGACGCTGACGGCGGCTGAGGAAACAATGGGCAGCAGAAGGCAACACCACAGCAGTATCACCGGTCCAGGTGAGAAAGAGAAGCCGCAATCCGGGCGGCGGCACATCAAGTCTGCGGCACGATGAGAGTGTGACGGTAAGGAGCCAGTTGGCGCCAAAAGTTGGCACTCAGGTCTTCGATCCCTAAAACGTTATATATTGCATCCAGCAGGTGAGCCAGGCTAAACGGATTCACGTACCAGGTTTGGTTACCCGCGACGATGACGGCCAGACCGTGGGCGCTACAGTTGGAGAGGTTCCTGGGTACGAAGGTAACTGAGTCGATGTCGCGCCACGGGGGGAATGAGACAGACGACTGGCGCACGCTGTAATCACAACTGTGATTGACGTATTGTAGCGTGTAATTTAGGTTGCACTCAGCCTCGAAGTAGAGGGGGAACCACAGTTCGTCGTACTCGTCGTCGTCCTCCAGTTCTGGCTCTTCTTCATCCACCGCAATGTCTACGCTGCTCTGAGATTCCTCTTCGTACAGGATGATTGACAGGTTATGGCTACAAAGGTCCTGGGCGGGAGGACGCGTGGGAGCGCGGGTGGTGGTAATGTTTTCCAGGTCAAAAGTTGGAGTGTAGTCGGATGTTACATCCCCGTTGTTGGAGGTGGTAGAAGTCGCGGCCGGTGTCACGGTGGTAAGTATGGATACAGAAGAGGAGGGGGAAGTAGCGTTCGTACCGATGGTTGTGGTATTATTATTCCTTGTATTTCTTGTTCCAGAAACCGTTGACGTTGAGATGAGAATCGACGTGGCGCTGGACGTCAGATTGCTGACCGAGGAAACCGTGGTGGGAGTGGTGACGGTGTTACTCGTGGTTGAAGTGACGTTAGGGGAGGTAGTAGTGGTACCGGTGGTGGCGACGGTAGTGTTTGTCGTGGCGGCGGCAGCGGTGGTACTGGTAACGGTGGTCGCGTTGGTTTCCACCGCTTCACACAGTAAGCAAAAGCACAGGGCCAGGAAAAGCAACCAGCCCCGCCATCGCCGCCGCCGCTTCATGAGGTGGGCAGGCGAAAGCTGGTGAATTCGTTGTACAGCGGCAAGTGGGGCGCCGCGATCGAAGGGTACGTCAACAAGCTGACGTTGATATTAAATACGTCTGGCTGCTTTTCTACGATGGAAGCGCACAGGGTTACGGCGTCGAACAGGTCTTTCTTGGTGGCGCCCGAGACCCACATCTGGTATACACCCGTCTCGTGGTACGAAGTAGAGCGCGGCACCACCGGACGGATGCAGTCCAGAACGCGGTTGGGATCCTGGTGAAAGAATTTGAACGTGGCTACGGCCTGTGGCGTGTGCGGCATCGTCTGCGTGATGAGCTGCTGGCCCGCTAACACGGTGACGTTGTGCAACTTGAGCAGGGCACTCTTGAGGGCCTGGAAAGCGTTGCCGCACGAGGCGCTGATTTGCAGCTGCACGGCCGTGGAGTCGTGCAGCCGCATGAGACGTGACACCTCTTCGAAGACGTACTTATACTTACTGGCAAAGAGTGGCGCGTACCGACAGTCGGCCGGCAAAATGTAGGTGGCGTTGCCGCCGTTGGTGGCCACGGCGGGCGCAGCGGCCGCGGAGGCCGGCGTAAACAGCGTCAGCGGCCGGTGGTGGCTGGTAAGGTCGATCATGGGCGGCGTGGTGACCGTGGCGGTGGCGGGCATGACGGGGTTTGCGGCGACGGGCACTCCGGCCACAGCGGCGGCAGCGGCGGCCACGGCGGCGCTGGCCGAGCCCACACCTGCCGGCAGTCCTCCGCCACCCATGACGCCGCCGGGCAGAGCGTCGCCCAGACAGACTTCCACAGTGGCGGGCGCGCTCTCGGCGGTCAGTACGGTTTGCCGATCGACCTCGCGACGAAAGCTGGTGAGGAACTCACTATGATCCATGGCCGCAGGGCCCGAGATCCCGGGATTCTGCGGGTGCTGACCGAGTGCGGGCCGAGTTATATGGAAGACGATTAGCTTGGAGCGGAGTTTTGCGTCCCTAGCTGACCTGCGGATCAGCGACGTGCCATAGGGATAGACTGTGAGCGGCGGCCGCAACGGCGGGGTCGGCCGCCGTTCGTCGTCACGGGGCGGCGCGAGGGAGGAGGAGGTGGTGGTGGGTACGATCTTGACGTGGTTAACGTCCTGCCCGTCCGGGGGAATACGCAAAAAACCCCGCCGCGGCGCTACCACGATGGTGCGATGGGTCTTTCTCTTGTTGGCCGGGGCCAGGGACTTGCAGATGCGTGTGGAGCCGTAGACGATCTGGACGTGGTCCTGGGAGAACATGACCATCGCCGCCAACGCTCAGCGGGGGGACGGGTTGGGAACACAGAGGCTGAGGGGAAACCCCGTAGAAGTCAGCGAAATAAAAACAACACAGCAGCCGCTCCTCTCGTTTCGGGCCCTACCACTGCTTGAAGTAGGGCACCGGGTGTTTCTTTTCTTCAACGGGCTCCTCCAGTCTCTTATAGGACCAGTCCCGCCGGCGCGCCAGCATGTAGGTCACGTACAAAAGAATAATTACCATGAACACCAGGAAAGCCAGCACGCCGTAGGCCAGCAGCCGGTCCTCGAACAGCGGGTCGCTCTTGATAAACACGTAGGTGGTGGTAAAACTTCGGCCCGCGATCTGGACGTGGAGACGCACGACAGTATACGTGCCGTTGAGGTAGAAGACAAACTCGCGTAACCGTTGTCCGTTATACGTCACGTTACTAATATTCCACGGCGGAATGAGCTGGTCGCCCTGATGCAGATGCACGGTGCTGTTGGGGTGATAGAGGCTGCTACCGTTGAGCAAGCAGTGTTCGTGTTCCTGAAGCAGCACGCGGACCCGCATAGTGGTAGCGTTCAAGCGAGTCCCGTACACGGCGTAAATGGGATAGGTGAAAAGGTCCCAAGTGGCGTTGTGATGGCGGCCCCAGCTGAAGAAAGAGCACGTGTACTCAGTGGTCTCCTGCGGCCTGAGTCCCGAGATAAGCAGCTCTTGAGCAGTAGCGTTGTAGGAGAGATGTAGTTTTCCTGTGGAAAAAATTAATGAGTTGTTTATTTTGTTAGCAGGTTGGCGAGGGAGGAAGGGGAACAAAACAGAAAGGTACGTGTTACTTACCTTTATCGTTGGAGGGAAAAGCGCTAAGATATCCCACCTGAGTGAAGGGACCCTTGCAGTCTGTCCGTGCATAACAAGTAACTGATAAAATGTCTGGATTTTTGGTATTATTCAACAGGATAACTTTGCAGGTGGCGTTTAGAGACACTTGGTCGTAGCTGTAGCTGGCTTCGCAATTCACAGTATACAGGTGCCCCTCTTTCTGCGTCGTGGCTATCACGGAGGTGGAGGCGGACGAGGTAGAGGTTTGTACCGTGGTGGTGACAGCAGAACTGACGTTGTTAGAGGTACTTATTGACGTAGTAGACGTGACGGTGGTATTACTAGGGGAAGTGACGGCGCTTGTGGTGCTACTTTTCACTCCCGGGTGCATGTCGCCCAAGAGCGCAACTACGAGCGCGATCGCCAGCACGGAACACATGTTGCCGTGTGACGAGACGGCGTGTGGACGAGCTATATGTGGCAGGAGGTCGCGTCACCTCTTGTGACGCCTAAACGTCCAGCTCCAGATAAAAGAGGCGTTAATAATGAACACTACAAAAACCACTTGCGTCAATATGACGATCATAAAGGCTCGGTGATCGCTGCGCCTAAAGTATGCGGGATTCTCCACCAGCTCACTATCTTCGACAAAGTGGATGAATGTGTTAGTGTTACCGGCCGTTTTGTTGACCATGGATCGTACTATGAAAGTCCCGGCGCCAAAAGTTCCATTAGAGCCCCAGCAAGTAACGCTGCCGTTCACGTAGGTTCCCGGCTCTCCTGTCAGCATGTGTGTCAGTTGGTGGGTATAATTCTGTTTAATGTTTTCCATGTCCTCGCTGTAATTAACTTTTCTGGTGAGAAACTGTGTACGGTGCGGGAGAACGATCATCATCCCTGAGGCCAAAAAGGGCGAATCATAAGCTGTTGTGTTACAAAAAATAGTCAGGTTAGTATCATTGTGCTCATAGATATAAGCCATTTTTACTTGAGGTTCATACCACCACCCTACCCTAATTGTAGTTGCCACCGTCACCGAGTCCCATCTCCCGAAACTTACCACCGCCACCACTAATAGCGTCACCCCCGCACGGTACATAGTTACCCTCTCGACGTCGCCGGCTGTCAATGACGTGCCTGCGTCAGTGGCTATGATTTATAGCTTTTGGACACAACCGCAACGGATCTGTCGTAATCTACCTTCCACAGGGCCGCCGTGACGATGCTGAACGACAGGATCAGACAGACGGCGTACAGGAGTCCTAGGTCGGCGTCGACACGGCAGGTGCGGATGTCTCGCAGGGTGGGTAGATGGGCGATGCACAACTCTTTCTCCCCCCGCCCGTACATCCCATCTCGTATCAGCAGCCGTAGCGTGGCATTGATGGTCAGCGGGGTAACCAAAGAAATCACATAGGGATGTGTACAGGAAGTACAGTGATGGGTATCCGTGAGATGTACGTCATCACCCTCCTCACCCTCATCATGAAAGACCAGGACTCGGGTGAGACGACCCGATGAATACTGGATCTCCCACCACAGTCTTTGGTCCAACACCGAGAGGGCGCAAGAGATTCTAAGTCTCCCTGGGTTGGGGGAGCAGATGTAAGCCCCGTATGTGCCTCTCGCCATCAGGGCCATACACATGAGGAGGAGAAGGACAAGTATCCGGGACCACCCGCACCCCCACATCAGGAGACCAGAGACGGAGATGTATAAAAAAGCTACTTTTATTAAACAGCATTCTCACCACACGTTAATACTGTCACGGGAAATCACTATGTACAAGAGTCCATGTCTCTTTCCAGTTTCTCACTTACTGAGATTTGTTCCTCAGGTCCTGGATGGCTGCCTCGATGGCCAGGCTCAGGGTGTCCAGGTCTTCGGGAGGGGTCTCGGTGGGCTTCTCAAACTGCCCCACGGCGTAGGCCTTCGCGGCCGTCTCGTAGATAGGCAGCATGAACCCACCCTGGTTGGTGGAGAAGATGCGCACCATGACCTGTTTGGGAAACTTTTGCATCAGGGGCAGGCACAGGTTGAGAGCGCCCAACAGGTCCACGGGGGTGGCAGCGTGGATAATCATGTTGCGGTAATCAGAAGAGCGGGGGCACAATTGGTGGGTGTGCAGTTCTTTGAGGTTCCACACGGCCTTGACACCGTCGTTACAAGCATCGGCCGTGCGCTGCGCCACATCAGGAGGGTGTGTCACAGGCATAGTGTGCTCCATGAGGAAGGGAGTGGAGAGGGCCAGGTTGCACATGCTACCCAGGCGACACCGTACCTGATCCACCTCATTCTTCACTTCATGATTGCGGGTGTAGATGATCTGAATGCCCTTGTTGTTCACCTGCATGGTTTTGCAAACTTTGATGGCCTCTTCTAACACTTGGTGCATACTAGGGATCATGAATGGCAGATTCTTGTACTCAAGAGAACGATTGGTGTGACGGAACATGCGGCTCACCTCGTCAATCTTGACGCGACCCCGCCGAGTCTGCACGTTGGGTGTGCAGAAGGGGGTGTTCTTATCTTTCATGATATTGCGCACCTTCTCGTTGTCCAACTCGGAGATGCGTTTGCTCTTCTTCTTGCGAGGTCCGGTGCTCGCCCCGCCGCTGCTCTGATGGCCGCAGCTCAGCAGAGAGGAGGAGGCCGCGCCACCAAAACCGCCGCGCCCATGGTGGCTCGAGGTCACGGATGCTCCTCCGCCACTGCTGCATTTCATCTCCTCGGACTCACTCTCCGAGTCCGAAGCCGAACTGCAGGAGGAGGAAGACGAAGAGGAACTATCTTCATCGGGCCGGCCCAAAGGATCGGGAAGAGGAGGGTGGTTCATCTGGGAGAGCGGGTGCGTGGGAGAGGTCACTCGCGGCGTGCCGCTGCCGGTGGAAGGGGAAGACGCGGTAGCACCGCGGGTCTCGACTTCTTCACCCTGTTCTTCCTCGCTATCAGAGATCACGATACAGCCGGCGGTATCGATAATCTTGTTGCGGTACTGGATGGTAAAGTCGGGCTCGGGCTTGATGTCTTCCTGTTTGATGAGGGGCAGCATGATAGGCGCGGGAGGCACGGGCGGTTTAATAATCACCTTGAAAGGACGCGTGGTTTTGCGCGGTTTCTTACGCGGGCTGAGCTCGGGAGTAGCGGATGCCCCGGGGAGAGGAGTGTTAGTAACCGCGACGCTGGTGGGGGTCGGCTTGTTAAGAGGGGCGCTGCTAACGCTGCAAGAGTGGGTTGTCAGCGTGGGGCCGGTGCTACTGGAATCGATACCGGCATGATTGACAGCCTGGGCGAGGATGTCACCTGATGGTGATAAGAAGACACGGGAGACTTAGTACGGTTTCACAGGCGTGACACGTTTATTGAGTAGGATTACAGAGTATAACATAGAGTATAATATAGAGTATACAATAGTGACGTGGGATCCATAACAGTAACTGATATATATACAATAGTTTACTGGTCAGCCTTGCTTCTAGTCACCATAGGGTGGGTGCTCTTGCCTCCAGAGGCGGTGGGTTCCTCAGCACCATCCTCCTCTTCCTCTGAGGCAACTTCCTCTATCTCAGACACTGGCTCAGACTTGACAGACACAGTGTCCTCCCGCTCCTCCTGAGCACCCTCCTCCTGTTCCTCATCACTCTGTTCACTTTCTTCCTGATCACTGTTCTCAGCCACAATTACTGAGGACAGAGGGATAGTCGCGGGTACAGGGGACTCTGGAGGTGACACCAGAGAATCAGAGGAGCTGGCACCAGCGGTGGCCAAAGTGTAGGCTACAATAGCCTCTTCCTCATCTGACTCCTCGGCGATGGCCCGTAGGTCATCCACACTAGGAGAGCAGACTCTCAAAGGATCGGCCCCCAGAATGTACTGGGCAAAGACCTTCATGCAGATCTCCTCAATGCGGCGCTTCATTACACTGATAACCTCAGGCTTGGTTATCAGAGGCCGCTTGGCCAGCATCACACTAGTCTCCTCTAAGACATAGCAGCACAGCACCCGACAGAACTCACTTAAGAGAGAGATGCCCCCGTACATGGTCATCATACAAGCGTCACTAGTGACCTTGTACTCATTACACATTGTTTCCACACATGTAGTGAGGATATCCATAAATATGTGATCAATGTGCGTGAGCACCTTGTCTCTCTCCTCATCCAAAATCTTAAAGATTTTCTGGGCATAAGACATAATCTCATCAGGAGAGCACTGAGGCAAGTTCTGCAATGCCGCCATGGCCTGACTGCAGCCATTGGTGGTCTTAGGGAAGGCTGAGTTCTTGGTAAAGAACTCTATATTCCTGTAGCACATATACATCATCTTTCTCCTAAGTTCATCCTTTTTAGCACGGGCCTTAGCCTGCAGTGCACCCCCCAACTTGTTAGCGGCGCCCTTGCTCACATCATGCAGCTCCTTAATACAAGCCATCCACATCTCCCGCTTATCCTCAGGTACAATGTAGTTCTCATACATGCTCTGCATAGTTAGCCCAATACACTTCATGTCCTCGAAAGGCTCATGAACCTTATCTAAGATATCTAAGGCATTCTGCAAACATCCTCCCATCATATTAAAGGCGCCAGTGAATTTTTCTTCCGTCTGGGTATATTTTTTCAGCATGTGCTCCTTGATTCTATGCCGCACCATGTCCACTCGAACCTTAATCTGTTTGACTGTAGAGGAGGATAACAACACATATAAGTATCCGTCCTCCTGACTCATTTATCGCTATCTCGATGCCCCGCTCACATGCAAGAGTTAATCTTCACTCTATCTGACATACACAAGTAAATCCACGTCCCATGCAGGTTAGTATATATCACATACATGTCAACAGACTTACCGAGTTCTGTCAGGACATCTTTTTCGGGGTTCTCGTTGCAATCCTCGGTCACTTGTTCAAAGGTTTTGAGGGATTCTTCGGCCAATTCTGGGAACAGCGGGTCTCCCAGGCTCAGCTGACTGTTAACCTCCTTCCTTAACATAGTCTGCAGGAACGTCGTGGCCTTGGTCACGGGTGTCTCGGGCCTAAACACATGATAAACAAAGTCATAAGCACATGGATCACATACAGGAAATATGTATATAACATTAAAGATATAACTTTTTATTAAAAAAAGGGGAACACAAGTCCCGACACGTACCGTGGCACCTTGGAGGAAGGGCCCTCGTCAGGATTATCAGGGTCCATCTTTCTCTTGGCAGAGGACTCCATCGTGTCAAGGACGGTGACTGCAGAAAGGATCCATGGAAAGGAACAGTCTGTTAGTCTGTCAGCTATTATGTCTGGTGGCGCGCGCGGCAGCAACGAGTACTGCTCAGACTACACTGCCCTCCACCGTTAACAGCACCGCAACAGGAGTTACCTCTGACTCTCAACAGAACACAACTCAGCTGCCTGCTTCTTCTGCTGCTGCCTTAAGTCTTCCATCTGCGTCAGCGGTGCAAGCCCATTCCCCGAGCTCATTTTCAGACACATACCCTACCGCCACGGCCTTGTGCGGCACACTGGTGGTGGTGGGCATCGTGCTGTGCCTAAGTCTGGCCTCCACTGTTAGGAGCAAGGAGCTGCCGAGCGACCATGAGCCGCTGGAGGCATGGGAGCAGGGCTCGGATGTGGAAGCTCCGCCGCTACCGGAGAAGAGCCCATGTCCGGAACACGTACCCGAGATTCGCGTGGAGATCCCACGCTATGTTTAATAAAAACTGCGGGTACTGGGGACGGTGTTGTTGTATATGTGAATTTGTAAATAATAAATGGGACCCCATCCTGTAAAAATACAGAGTCCGTGTCAGTCTCTGAAGGACAGAGTATTGGCATATAGCCAATAAAGAGAGTTGTGGCAAAGAGCCATGTTATGGATTAGTAATGGAAAGTATCGCCACCAATAGAGGAGTGGTCAATAATGGTCAATAACCCACACCTATAGGCTAAGCTATACCATCACCTATAGCATAAGGAAGCGGGGGTGTATAGACCCCAAGCCAAAAACAGTATAGCATGCATAAGAAGCCAAGGGGGTGGGCCTATAGAGTCTATAGGCGGTACTTACGTCACTCTTGGCACGGGGAATCCGCGTTCCAATGCACCGTTCCCGGCCGCGGAGGCTGGATCGGTCCCGGTGTCTTCTATGGAGGTCAAAACAGCGTGGATGGCGTCTCCAGGCCATATTGGACATGAGCCAATATAAATGTACATATTATGATATAGATACAACGTATGCAATGGCCATTAGCCAATATTGATTTATGCTATATAACCAATGACTAATATGGCTAATGGCCAATATTGATTCAATGTATATATATCGATATGCATTGGCCATGTGCCAACTTGGTATCGCCTCTATCGGCGATATGGCCTCATATCGTCTGTCACCTATATCGAAACTGCGATATTTGCGACACACAGAATCGCCCAAGTCGCCAAAGTCGTCTATCGCCATCCCCCGTAAACGATATAAGCGTTATCGCCAGATATCGCATATGCCCAAAAATCACTTTTTAAAAAATGGCGATATCAGTTACACAGAAACTCACATCGGCGACATTTTCAATATGCCATATTTTCAAATATCGATTCTTCCAATATCGCCATCTCTATCGGCGATAAACACCACTATCGCGCGACATGAATTTAGTCGGCGACAGAAATCTCGAAACGCGTATTTCGGACAAACACACATTTTATTATTCACTGCAGCATATAGCCCATTTTAGCGCGGCACACATCCAGCCGTTTGTGTTTCTTAACGCTCTCCAGGTACTGATCCAGGCCCACGATCCGGGTTATCTTGTCGTATTCCAGATTGATCCATCAATAGGGAACGCTGCCAGCGGCGCCCAGCAGGTACTGCGCCTTGTCGTTTACTTTGCCGCAGCGTATTCGCCCGTCAGCTTCGAGGTATAACCTACAACACGGAGGGGAAGGGGGGTACAAAACGTGAAATTAGACTTTTTTTTTTAATGATGTTTTGTCCCTCTCTGTCTTACTCTCCCATAGGCTGTAAGGCCCTCAGGAAGAGACTTACGGATTGTAGTTGCAGCTCGTCAGTTTGTTGTGTACGACCTGGCGTGTCAATGAATGGGTCATGGTGGTGACGATCCCGCGAATCTCAGCCGTTTTCTCGGGACTGTAGCAGACTTCGCCGTCCGGACACCGCAGCCTGTGGATTCATGAAAATCTACTCTGGCATTCCCGAGGATCGTCGATGGAACATGGCTATCAGAAACGTCGAGAGACAGATCCAGACGCACCACAGAACGCAGACAATCATGAAAATACGTACGCGACGGTGAAGCGATTGCACATTTTGAAATCGTAACAGCGTTCCGGCGGGTGGTTGACGTTTATGAATTCGCAACATTCTTCTGCGCGCACCCGCGGCACGCGGCTGTGACCCAATAGCAGCCACAACGCCGTCAAGAACGGCGTCAGATCTTTGGGACTCATGACGCGCGGTTTTCAAAATTCCCTGCGCGCGCGACGGGCTCAAACGATGAGATTGGGATGGGTGCAGAAGGTGTAAGTCTGGTTATTGGCCTCGGTGAACGTCAATCGCACCTGAAAAGACACGCTGTAGTCCCGGAAGACGTGAGCCCAGCTCTCCAGCTTCATCACACACATCTGATAACGTGTGCCATCGTTGACGACGAAGCGTAGCAGCTTGGTCTGCTTGGGCACCATGTGCGCTCCAAAAATCTTGGCGTCTTCCACGCTGATCTGCACGTTTCCGTCGCTCGGTTTCGAAGCCGTTCGGGGCATCCGTTGGAGGATGGTTTGGTTCCGACCGCTCAGGTACCAGATCACCTTTTTCACCCAGGTGGAGCTTCTCTCCACCAAGGTCTGGCCTTCCCGGTTGTACAGCAGATACAGGGTCTCGTTGCGACACTCGGGACCCGTTGATACCCGCTGGAACCCCGAGAATTGCAAGGGGGATCGTGGGGGCGAGGGATAGAGAAAAGGACAGTAAAACGTCGCCGCGTCATGCGGTTTGGAATACGTCAGTTTAGACCATGGCGGGGACGGATTCTGGTTTGCTGTTAGCGTCGACCACGGAGACGCCAGACAGGGCGTTGCCCAAACCGCGCACAGAAGCAGGCAGTGAAAGTGGTGACGAAGCAGAAGCCGCAGCATATTATTTCCCGTGACGCAGGCTAGTTGGCAAAGAGCCGCACGCTGAACTCGAGGCTCCGGGCGTGTGGCGCCAGCGAACCGGCGGCGTTGAACGTGGTCCTTTTGTTGGTGCCGCCGCGACGGTTCTGACGTCTAAAGTCGCTGATGAGCAACGACACCTCGGTCACGTTGATTCTGCAAGCACAGGTTCCGAACGTCATTTCATACCCCATGCGGTTACTTACCCGTTACCCGTTCGCCCTTACCTTCCCGTTGTCATGCATCTTTAGCGCGTACCCTCACCTCTTGAGCACGTCAAAGTTGTCCAAGCCGTGGCTCGCATCGTAGTGGTAGTTCAACGTGAGGTCCACGAGCTGTTCCACATACTTGTAACGGGTTTGGTCGGGCAGCGCGCGAGAGCACGCGTCCCAGTAATGCGGTACTCGGTAATAATCGTTTTTTTCCGCGGTTTCCCGCTGGCACTGACCCAGCACCACGGCGCACAGACAAACAGACAGCCACACCCGACACAGCCGCATGTTGCAGACTGAGAAAGAAAGCTTTATTATGAGACATCATACACATAGTATAGGCGAGGTGATGGGGCGGGGAAAGAGTTGGAACCGAAAGACAAAAAAAAAAGCCTAGTCGTACTCGGGATCTCTGAGCGAGACGGGTTGCATGGCAACTTTCATTAGTTTGGGAATCTGCCAGCTGGTGCTGTTCGAAGGTTCTTCCATTTCCGAGGCGGTCAGTTCATCGTACACCGAAACGTAGTACCTGATGGGGTCCTCCTCATTGTCCGAGAGGTGAGATTCGATGGTCAAAGGCGAGCCTCTCCCATAATTGGGATTCACGAACGACGTGTCCAAGTTGCCATCCTTTCTGAAATAGATGACGTTCTCAGGATCATGTTTCATGCGCTCGCGGGCCGCGGACGCCTCCTCCTCCTCGTCCCAGTCCCGAGTTTCCAACCGCTGATAAGGGCTCGAGGAACAAAATCCGGCGGGGATCTGAGAACCTCGTCGGGAACCGCTGCCAAACGGGCTGCTGCCGCCACTGTCGTCCGTGTCGTCCAACAGGTTGACGGCTTCTTCGTCGGCGAAACGAAAGCGACCCGGGTGCTTGCAACACGAGGAGTAAACTACCGCGACCAGTACCGCTATGAAGCTGAAAATGGAGGTGCCTGTCACGATGTAGAAGAGGATAGCCAGCACTTTCATGATCTCGTTATTGCGCACGTTGTGAACGGAAGACTCGTGGGTGGTGGTCATGTTGATCCCGGTCGTGGGTCCGCTACTCGTGGCGTTGTCGACGCTATTTCTGTTGCTGGTGCTAGTAGGGACTTTTGTGCTGCTGGTCACATTCGTAACGTCGCTGAGGTCTATCTGAAGCAGCAACCCGAACGCGACCAGGGCCAGGAATGTTGCGCGAAGGGGACCCCGCGGGGCCGGCATTCTCGAGACGTGGCGACGTGGATTTCTTGCTATGTCCGCGAACGACGTGTGACGAGGACGTGGTTTCCGCAAGCCTCTACCGACGCCGCGACACCAGGTAGGTTATCAAAACGCGAGCCCATATCGCCGCCATCATTGTAATCAGCAATGTGTTGAGGTACTGCACGATGAATCTGTCTAGTGACACCAGCCAACCCTCTGCTTTTGCGGGCAAGCGCGCTTTCGGTGACAGGGTGTATCGTACGTAGCCACGGGTCAGGCGCGCGTTGTAGCGGTACACGCAGAAATCTATCCACAAGCCAACGCCCGGTTGTAGCTTCGGATGGTGGATAATAGCGCGGTGACGTACGCCGCGGGGCTTTAGAATCTCCACCTGTAAGGCCATCTCCTCCAGGTAGTGGGTCTGACTGCGACGCAGCGTCCAGTTCATGTAAAAGTCGGTCTCGCCGTGTCCGGCCACGAAGAGGCTGCTTACTAAATCGGGCGCCAGAGCTAGGTCAGGCGTATCAAATTCCACTGTCAGGCGACCTGATTCTAACGGTTCCACGATCCGGGAGAGCGTTTCTAGATATAGAGCAAAGCGTACCACGTCTACCTGCGGTGTAAAAAACTGTTGTGGGCGTTCACCGTCGTTGACCACGTAGGCCACGTAGAGGCCAACATTTTCCACCACGGGTTCTAGCTGCAGGCGGCACGTAAAGCTTAGAAACGACGGCTGTACGGTTTGGTTCCCGTGAAGCTGAAGCGTCACTTCCTTGCCGGGGCTCACCGTGCTGTAACGCCGCACCGAGTCGGTCATCTGCTCCAGATCGGTAGACCAGAAGGGCGTGCAATGCATACTGTCCCAGTCGCGACACGCAGCCCAGCCTAGCTCGGTGAAGGGTCGACGCACACCCGAGAAAGTGTGCTTGAAGACCAGGGGGTCGCCTCGGTAGCTCAGTAGTCGAACATGCACATAGTCGCGGCTAGCGTCGACAGACGGTCCGTAGAGGGCCAGCAGTACGAGCGTGAACAGCAAGCGCAACATGCTGCGCGGGTTAGGAAATGCGGCGTGCCGGCCACCGCCCGACTCATAAACGCTACCAGCATGACGTCTCAGATCACACAAGTGACGAGGAGCGTACCGCAAATCACTAGGGAAAAGGCCAGCAGAGCCCGATAGTCTTGCTCTTCGCGAACGATCTCGTCCGGTTCCTCGCAATCTTCGTGGTCCACAGAAGATGAGGAGCAGGAATTTTCGTTAATCTCTGCGAGGATACTAGTGCTGTACCACACCAGAGCGCTTAGTGTGCCCAGAGCTACCGCACGGTAAAATAGGGACATGATCACCAGCGCAGTCTAAAGTAGTGGTAATTCAGTTTCTTGGCGTATTTCCAGAGAAAGGCTTTGTAGGCCGTAGGGACTGGCCAGGCACCGAACTCAATATTTGTAGACACTACGTCGTAAATGCGTTGTTCCTCGTCTAAGACTAACCGAAAAAATAGCCGGTTGATGTGACGACGCACGGCTTGCGCGTTAGGATTGAGACACTTGGTGCCCTTGTCCTTTAAAATAGCCAGCACTTCCTGACGATTGCAGCTTTCGCTCGCTGCGATTGGCTTAAGCAATTCGGTTCCGACCGGCAGGGTATTCAACAGAATTTGGTTATTACAGCGACACCGCTTGTCGTAATCTTCTAGTTCTAAAAGACGGACGGCTATGGGACATATGGCAAGTAACATATATGCGGTCAATGACAGGTATCGTACCGATAAAAGTTTAATGCGCGAATTCAGAATCGGATGATGTAACCATGTTAGCAACATATTGAAAACATGATATTTTCTGCTCTAACAAACTTGTCTGTTTAAAATATATACTAAATATTATTCCTTCAGACCTACTAGGGTTACAGTTATTTTTCCCGGCCCTCGTTCCGGTTGTCTTGTTACATTAACATTATACCACCTGTTATCGTTGCGTTTGTCTAGCCATTTTGAAAAAGGGTGATGGGGCGATAAACATACTCCAGGTCCATGTTTTTTTCCTCCGCCTAGATACAAGAAATATTCATTGTTATTGCATCTGGGACCACCAGATTCGCGATGAAACCAAAAATTACCCAACATAATTGTTTTGGGCGGCAGTTTTTTACCATCAAGACATTTGCAGCGTAATTCCGTTCCATGCACTTCATAATAATACATATACGTTAAAGAAATAATCAACGCACCAAAAATTAATCGCATTATAATTTTATTATTTACGTCACTACCAATAATTCGTAATATCCGGTATTCCCGGAAAATCACTCAAAACTGCGTCCATGACACATCAATTCCCGATAAGTACCCCCCTTTGAAATCGGATCCCCCCACATACCAATCAATCATACAACACACAGGTTTAAAAATCGATCACACGTCAATTAGGTTTCAAAATCGATACCATTTATTATCAGGAATCTAGACTAATTCTACAATGACAGCTCTGAATTTCTCTCTCGTCTTTCTTGTCAGGTTCTCATCATCAATCTTCACTTCCACCCATCGGGGAGTCATCGTCGCTCCAAAACCCTTTGGGGTCACTGGTTGGAAAAGTCTCTGACACGATCCAGGCACCCCGTACCCAGTCCGACTGATCTAGCCTACGGAGCATCTCAACAGGCATGAGCTGCAGGGCCACGGCTGTCACGGCACTGTATCGATGTAACACTAGGGACTTTCTTTGCGATGTAGCCATCAACACGGCGTATGCCCCATAGTTCGCGTGATACGACGCATGATGGGTTAAACGTTCCCATCCGGCAGTGCCGTCTCGGGTCCGTGCACACAACAGCTGCACAGCGTTATGATGCTTAAAATTAACCATAACGCTGGGACTACTGATGAAGGAGTAGTAATGAGCCAGGACGCCGTACATCGAAGGCAACAAGAAAGAGTGACAGCACGATAGCACCGGGCTCTTATGTAGGCGACAGCTTATTTTTCCTGACGTCGGCAAAAAGTACCTAAATTCCCCACAGATATTCAGACACGGTTCCGTAAAGTGCTTCTTTTTTTAGTGCAGGAATTGGAAAAAATAATAAAAAATATGAACAGCTCATCTGTAATTATCTGTGTGACTTCATCGTACCGTGATGTAAAAACAACAACAGGAAGCTTACAGGGTGCGGTAGAAAATTTTGCCGATTGAGCAACACTGTTGGCATCTCTCACTCCGATAGGCGGCTATAAGATAGAGAATTAAAAGTATGATACCCACAAGAAAGATAAAGAGAGACAACCAGGCTAGAGTATGACGACCGCTTTTTCCTTGTTTGACGGTTATATGTGCGGTATGATTTTGCTGTCGTTGCTTGTGATGTTGGACGCCTGGAGTGGAAAAGTACGTATGGTTCTTAGGCGCGCATACGGTATTATTGGTGGAAGTGCAGTTACGAATCATGACTTGAGTGACGTTACATTGAGTGCAATCGGTACAGTTGTAAAGTCCCGATACATACGTGCCGTTGGGGCAAGGGGTACACGTTACGCTGGTACGCTCCGTGCATACTTTAGTAACTCTTTGTCCCAAACCACACGGAGGACAGCATTCGTTGCCCAGTTGCACTTCATTATGCTGACACACTTTAGTCACCCCAAGCTGCAATAATATCACACCGAAGCAGATGAGCATCACCAGAGGCTTCATGCCTCCTACCGGAAGAATAAAAATAACTCATGGGGCCGAACGGTGTCATCCTCTCCGCGGTTTGTAATACGAGATCGTAAACGTAAATAAATGACATAACTTCACTAACCCGCATACTACAAAGTCCACCTACGACGCTGAAAGTTTTTCCAGGACAGAACAGGATAGTCAGCCATCTTCACAGTCTACCTCTTAGGCCGTATCTAGGAGCATAGGTAATCAGTTTGCAGCCACAGTACAGCGAGCCCAAGAGACCGCACACGGTCCCTGCTGGGAACACGTACCACCACATCGATTCGTCGTGCCGTATAATCGTAGAGTTTTCCGAACTTTTATACACGCCGGTGGCGTTAGGGCCGTGTGTGCTGCTGTGATTGGACGTTTTGTGAGCTAGGTAACAGCTGTGATTTCACCTGTCGCCAAGACTGACAGCGATTACCCAGGTGGAGCACAATCACATAGCTGATAGACGTTAATTGATCCGTTGATTCCCATGGACATTTTAACGGTAACAATACAGCTCCCGTTAAACATTAGATTAATAGACGTTAGTGGATAACAGCATGTTATTCGCCCGTGATCGTGGTTATGCACTTTCTTGTTTTTTGCTCATATGCTGTAAGGTGTTCGAGAATCGTGGGGAGTATATGTGTTGAATCAGAGTCATCTTTACTGACCGCGCCATACTTCGTATACGAACTTAACCGGCGTAAAGTGTTTCCCGATATATAAACCGGCGCCTATTGTAGCTGTAGCGCCCATAGGTATGGCATATACCCACGGTGATGTTGTGTTATTCGTTTTTTGTGATAAAACGTAGCTTATGTTTAGTGTGTGTTCCGTCACGTTATGTGTGTCGTTAAAAGACGGCGTCTGTACAGTATGGCTTTGAGTTGTATCTTTAATTGTTATTGCATTTGGAGGTGTTGTGTACAGAGTTGTTATTGCGTGTTCAGGTGTTACGTTTTGAGGTACAGCTGTGGTGTACACGGGCTCCAAGGTGTAGTTACGGAGTCTTTCTATGCAGGTAGTGTTGAGATATTTGTGAATGCTGGTTATGTTCGATTCTGTGAGGTTAAAGTGTGTACTATTTATGGCGGTGTAATTTAGACGGTCTTGCCATCCTGAGGATGTTAGTGTTAGGTAATTCGTGTTGTTCACGTTTGCTTGATATGTATAAGTAGGTGTACTGTTTGTGAGGTCGCAAGTGTGATTCTCTTGCAGAGATTTTATCCATCTTGTGTGAAAATATTGAGATACGCGATGAATGTTTTCGCTATCTATATTGTAAAGCGTTTCGGTGGTACTTAGGGGTTGTTTGCTGTAACTCTTATTTTGGACCCAGGGTGTGAACCATGACTCCAATGTTTGTATAGTAAGGTGTCCTATTAATAAAGACGAACTGATTCCTACCGTAATGTTATATCGCACACCTAGGGTGCCGTTTACAAACACGGAAATGTTTCCGTTACAAACCACGTTGGCAGATGAATTAGATTCCAGGTGGTAACGATAGGATAATGACCGTTCGCTCCCAACGGATGACACAAAGTATCCGAATAACCAACACGCCCATTCAATCCGCATATTTTAATCACACTATTCACATTTCACACACTGCATTTTTTAACATCTTATTTTTTTATTTTATGCGTGTTCTCACCTCTTCATCTTTTTAACACCGGGGTAACTATCGTAAGTCGGTAGGCGTCGATAGCCCTCACCGCCTCGTCGTCCCCTTCCCGGCGTGGGGCACCAGCGTCCACAGCACTGCAGGTAACACAGGTAGCATAGGAAACATACGGTGAAAATACTCCAAAATCCCAAAAATGCCGCGATTCCCCGAGTGGCCCAGGGAGACATCCCGGTGTCTATGTCGGCCGGCGGTGCTGGCGTCACCGGTAAAAATTTCGGCGGGTGTGGCTGCGAACGGTAGCAGTCGCCGGGGAGCCGGTAACGCTGTATCACTGTCCAACAGCGGTCGGGTTCCTCGTCCGGACATGCGGGTTTCCAGCAATCCTCGGCGTCGGCGCGGCCGATATAGAAGTAGTTGCGTTGAAAACCGCGGTACATCCCGCAGTCGTGATTCCGTAGACGCCAGGGCGTCGGCGACCAGATCTGGTCTCCCAGCGAGTAGCGACCTAACGCCGGCGTGCAGCAAGGTTCGTCGGGCCGGCTGAGCGTCTCCAGTTGCGTCAGAATTACGAAGCGTTGCATGATGAGGCCGTGGCTATAGTTGCGCAGCACGCATTCGTACATGCCGGCCGTGTCCGTCGATACGTTGAAAGTCAGCGAGAATATTTGGCCGAGATGCAACTGCGAGAAATTCCAAGTGGCGTACGGCAGGCGGTATTGAAGTCCATTCATCAGCCTATGGCCTTTGACGGCGTCCAGGATGAGCTCGTCGCTGCCGTCGTGGGAACGACAGAAACGTGCGCGAATGGAAACCATGGGCCAGGAGTGTGTCATGACCGTGCAGGGGATGGTAACTTGCTCTCCCTCGGCGACCAACACCGGCGCCGGCGACGTGGTCTCATAGTTCTCGGCCCACATCTTTTCGGCGATGTCAGCGGTGGCGAAGGGGAACGAAGAGGAAGAATATTCGAGGAGTCGCGGGCAGCTCAACAGCACCCAGAACAGCCACGGCAGAGTTCGGAGCGACTCCCGGCGGCACATGATGATTCTTTTCTTCCCTTTTTCGCAGAGACGCTGCCCGCCTGTTCCTGCTCCGTGTGTCGGCCGCTCAAACGTCGGGCCGGCGTGGTGGTGACCACCGTGCGACGCAGCTTCTCGCCCGGGATGCCCGCAACTGAGCGTCCGGTTTTTTTACAGGTCTTTTTTGCTGCCTCTCCCTCGCCGTCGCGGCCGACGTGGTGGACCAGCACCGCGCAGGAACTCTCGCGTCGCCGGCGGTACGCGACCTGTCTCATTGCTACCTCGGATGTTTAAGAAGGAACGTTTATCTGCGTCACAGGGTCTGATGAAGCTGCCAAGAGTCGTGGCTGTGGCGTAGCGCGTTTTGTACAGCGCGTTTCACCGCTTTCTGCATGGCCGCTACCACATCGGGTGGGAGCGGCTCCGGCGGAAGCTCGATGAGCAGTTGCTGCGAATCTCGGCGTTCGGTGTCCGCCGTTTCGTCGGACGTGGCGTAAAAAACCGAGGTGGTCGCCCAGTCGTCCACGCTGTCGACGGCTTCTGTCAGTGCCGGGTTGTCAAAACCGCCATCGGACGCGGGTGATAAAAGAACGTACGATGACACGCTGTTAGTACGACTCTCGTCGTCGCTTTGGGAACGACGTGATGGACGACGGTAGATGACCTCGTCTTGCCACGCGTCGAAGCGGTCGCAGCAGCGCTGGATCCAAGCGCAGCGAAGCAGCTTACGGAACACGTCGTTGTTCCAAAAGTAGAGCATAAAGAGAAAGAAAAGTAGCGTAACGATGAAGCCGAAAACGACGAGGGTCGGCAGGGCACTACCGCCGCTGCCGTTTTTTGCGCCGTGCGGGTGCACGGTGGTAGTGCCGTTAGTCTGAGCGGGGGTCATGACAAGTCTGAAGAGATGAGAGCGCGGGTGCTCATCAGGAACAGTTGAGGTGTCTCCCTACCGAAGCCTTAGCCTCTACGGTGTTTTATGATCAACGTGTCTACGAACGTCATTGTGAAAGTGACGTCTCAGGCTTTCCGAAACCGCGTCAAGTTCAACGTTGGTTTCGGTTTAGCCTGCGTCACCGAGGCGGAGGTGGAAATGAGCCGTCCTGTGGGGGAGTGTACGACCCTGTAGTGCCCATGGGTAACGTTGCATCGGAAGAAGTGAATGCGGCATTGGTGTACGCGTGAGTGGCTTTGCTCTCTGACTCGGAGGAGTTGCCGCAGCAGCTGCAGACTTTACGTACTAGCCAAAAACAGCAAAGGCAGCAAGTAAATATGAGAAGGAGTCCAGATAATGTCCAGCCGCTAGCGGCAAGTAGCGCGAGCTGTGGCGCCCCCAAATCACTGCCGTTAGAAGCATTAATACATGCTGTCGATGGTGTAGTCGTGTTAGTGGTACCAGCAGAAGTAGATTGACTGGAATTAGAGCTGGTACCTGTAGTGGTTTCACTCGCCGATGCGGCAAGTGCAAATAAAACTAATATCCACAGCATGTTCGTTACTATATAATTGATATACGAACCCCTTTGTCGTGACAATCAGCGTTATATACGCTGTATCGGCATCGTTTTACCGGAAAGTTTATCGTAATGTAACCCGCGTTGTGTACATTCGTACTGAAAGGGAACCCCCGGTGATGTGCACATTATACTCTTTCATTCTGGGGTTTCCCAATGACGTAAAAATTTCCACTACACAATAAAATTACTGACTCATGTGAAAAGTGTGCTTTTTATTAACAGAGCAGAGGGTTTACAGTAGATATATGTTTGCCAGGGCCACCGTTTTCTAACACCGATCACCGCCACCATTACCACCCGTTGAACTCCACACCCGGGAGCCGCCTGATCGCCAGGGACTCCTCACCGTCCATCGTCCGAACAAGCTCCCGTCACCGATGCTGCCACCATCACCGAGAGAAAGAACCGCTTGCTGCAGATACGCTTGGGCTCGCCTCCGTGCGGACGCCGTTTCGTGCAGACGCTGAGTAGATCGAGCAGAGAATGTCAAAACGACATTACCGCGATCCGCTCCCCTCTTTTTTCTTTTTCTCATTCACGTGTATTCTTGATGATAATGTACCATGGCTACGGTGGTGAACTGCGTCGCGGATCCCGTCACGGGTTTCAACAGATCGACGTCGGTCAGCGGCGCCGTCACCGCCATGTCCGGCGGAGGCACGCTGTTTCTCTGGTTAGCGACGTGGACCGACGACGAAGACGATGAACCCGCGCGGCGGTCTGTTATCCGCGACGACGCGTAGCTGCACTGGGAAGACACTTCCTCCCAACGGACCAAGATCTCATCGGGTCGTTCGGAGAAACGGTATCGTCTGTCCGACTCCCGCCGTACGGCGCCGAGGCCCAGCGACGACAGGTCCGCGAACCGGCGCTCGTATTCCCCGTACAGCTCGCAACAGCGGATCAGCCAGCGGTAGCTCAAAAACATGCGCACCAGTTTGAAGGTGTCGTGCCAATGGTAAGCTAGATAGCAGAGAATGGCCACGATCAGCACGAGCATCACGCCGATGATGGGTAACCCGACGTTCAGCGGCAGATCGTCCATGGTGACCGTCCTCTGTCCGGATCTACGTCCCAGTCTCTCTCTTTTGTACAGCACTCGCGCGGGAACGGCCCCCTCAACCCTCTTACGTAGCGGGAGATACGGCGTTCTCCCGCGGGCCACTTACTTGCACGGTCGCTTGAACGGCGGCTTGGACCGCCACATGTACCGCATCCATCCATTCTGGCAGCAGCGCGTTCGACGACGTCGTACGAGTCGCGGATGATGTTACCCCGCCAGCACCTCCGCCGGCAACCGCGTCGTCGTTGCTATCGTCGCCGGTTTCGGGCGATGACAGCGCCGGCGGCGCGGGTCTCGTCTCGTCCACCATTTCCACCGTGTCGAAGCGACAGCCGCTGCCGTAGTACATGGCCCCGTTCAACGGCCGGCGGGCCGGGTCGCCGAGTTCCGGGTCGGGCACATCCATGGCTCGCCGTCTGCTTCTCTGCCGCTCGTGGTGCCGACGGCACTTCTCAGGATAATGACAGCCGCAAAATAGATCGTGGAGCATGTCTCGCCAACTGTCCTGGTGGTAATATCTTAAGTACGCGATGAGCGCGCCGATGGCCATAATCATAAGCGTAAGCAAAACGGCACAGATAACGTGAAACACCGCGGTCATCCAAGTCGGGCGGCGTCGGGGACGCGGTGGGTCGGTTTCTCTTACGCCGGCGTCACTCAGCCACCACACCCGTAGTCGACATTCCCAGAACCGGTGAATGCGACTCAGGACCTTTCGACGCCGCCATTTATTTCCAACGTCCAAGTCCCACGTCATTTCTGGCATCTCCACGCCCTTGACTGACATACTCTCTTTCTCTCTCTTAGCTGCGGTGAAAAAGAGGGAAGGCGTGTGCTGCTATACAACTGTACAACGGACGCGCTCGCTGTTTCGGTCTCAGGTCATCTGCATTGACTCGGCGTCCTTCATGACGCTCTGCACCGCCTTTTCCAAGAGTTCCTCGATGTCCGACCATCGAGGAGGCGGGGCTAACTCGGAAACCGACACGATAGGCAGCGTGGTCGGCTCCGTCGGCGTGCGGGGTCGGGGACAGGGACACGAGAGTCCCACCTTCGAGAGATTCTCCAGCCCGACGGTGCGCGGCAGTCTCGGATTCCGCGGTGGCTTTTGTGGCGTCGGCGTTTTCGGGAAGGGCCTGGGCGTCACCGGCGGTGTCCAGCCGACCGGCTTGGGTTTCGTGGGCGGCGGTGTTTTCTTGGTGGGCGGCGTGCTCAGGTTCTTACGCGGCGCGGGTATCGGCGTCGGGGGCCTGTGCGACGACAGCCGCGTGGTGGGGGCCCGGACCGGCGGCGTAGGCGGCCGCTTCTTGCGCCCGGGCGGCGGAGGTGGCTTCCAGGATGGCGGCGGCTGATGCAGTACCGTGTCGACGCTGGCCGAGGACGACAAAGAGCTCGACGAGGAGCAATGCGACGGAGATCGGCCGATGCTGGTCGGCGTTCCCGGCGTGGATACGTCGGGGATCTCGAATCGCGCCGGAGGAAACTCGGGTTTATCTATCGGCAGACCATCCTCTCCTATGTAGAGCGACGTACACCGCGGCACCTGCGGCGTCGGCGGGTGGGTGGCCACCCGCATGAGCCCCAGTTCCAGATCCAGCGGCTCAACGACGTCTTCTTTCGGAATTCGATAGCAGCACGCGCAGGCACCACGCTTATCAGAAGCAGCACCCGGGAGCCGGCCTCGCGACGAAGTCTCGTCGGATCGCTTGCGGCCTCGGCGCTGGGTAAATAAGGAAATGGCCAGGACCAGGGAAGCCAGTCCGGTACCGCCGAGGAGCCCGACGCCGAGCCACAGCCACACCATGATCTTCTCTCCTGCTTGGAATCTCAAACTCCGTGTCGGGAAGGGCCGGTGTACGGACATTTATGCCTTGGATTTCTGGAAACGTCATTTTTTGGCAAGGAATGTGTTTATTGTCCAAACACTGAGGAAGGAGATGTGAGCCAAGTCGGAAAATTCCTTATCACACCGGGGGCGGGTTACGTTCCGGTCTGATGCTGCTGCTGTTGTTGTAGAGCCGCGGCCATGGCCGCCTGCACGGCAGCTTGTACCGCCTCGGCCACGCCGGGTGGCATCTGCGGCATGGCGGGGGGAGACGCGTCGGGCGGACCGCCGGGCATCGCCGTCGGCTGCGACGGTGGTTGTGAACTCACCGTCGGCTCGCACGGAGGTTTGTTCTTCGGTCTACCCTTCGGTTTGTCTTTCGCCCTACCTTTCTTCGGTTTGGGTTCCGATGTCGGTGTTGGCGGTTGCGGTGGGATGACGGGCTGGTGGGACTCCTCCGACGGCGGGGGGACGAACACCGTCGGCGCCGAAACCGGGGGACTCTCGACTATCTCGCAGATCACCCTGTCAGGATCGTCGCCGTGTCCGGGACGCCGTCGATGACCGTATTGGACCATGTCGTAAATCATCGTCTCCTTGTAACACGCTGAACAGCAGCGGCTACAGGGGCCCGAGATGCATTTGCAGCTGCACTTACAGCTGCAGCTGCAGTAGCGAACCCATCGGCAGGTAAGGAGGTCGATTACGGAGTCTTTGAAGAATTCCCGGTAACGGATGAGATACGCGCAGAGGAAAATCATGAAAACAGAACAGCCGACTACGGCTGCGATGCCGGGTCCCGAAGAGACATCCGCCGACGATCCCGCCAAACACCAAATTCCTAAAGCCGCGCATGTTATCCAGGCCACAATAATCGTGGGAACGCCCCATTGGCATTGCCACGAAGGATCGTGCACGTCGCAACCCATTGCTACTGCGTTCTCCCACAAACGCCATCGCACTATTTATCTCTACAGCGGCTGCCGAGTCACGTCCGCCGGCGCCCATCGGCCGCGGCGATCTCCTAGTAACACTCGTCCGACACTTCCACCATCTCCAGCTCGGCCGGCGGTTCGGCGTCCTCCACCAGCGGCGTCGTCTCATCTTTGCCACAGCAGCGGACGCACACCTTCTCCAGGCAGAACGCCACCAGCTGCCGCCGAACGTACCACAGGTACACGTGCAGACCTGCGAACAGGACTACGGAGGTCATGACCACCACGACACACACGGGAATCCAGGGATCAAAATTGCTATCGGAACTCATGGCTATCGCCGCCGACGTGCGCGCGTGTGTCTCACCGCCGCTCGCCCGTTGTCGCGCGGCTTGTTATACGCTAGCCCGTCGCCGCCTCGGGGCACGGTGCCCTCCTACCCACGTAACTTCCTCCGTGACTTAAAGTCGCGTGTGGTAGATCTCCTGCTCCGTGGACGAACCGTCCGGCAGGATAGCGGTTAAGGATTCGGTGCTAAGGCCGTGTCGCCAACGTCGAATGCTACGTTGCAACAGCTTCGACGGACGGCCATCCTCCCTCTCATCGCAATAATAAAACACCAGCAGCGCGCACGACGCGATCACGGTAACCCCCATGACTAGACCCACGCAGATAGCCAGCCCCGCTAGCGTATCCAGCGCCATCCCGTTCGCTCCCGTCGTCGTCTCCTGAACAAAGCAACAACTCCGCAGTCCCCGTTTTCAACCGTTTTTGTTTCCTTCTCCGCGAGCAAATGTTAACTCCCGCGGTCTTTCCGGCCGTGCTCTACCTCCTGGCGCTTGTCGTCTGGGTTGAGATGTTCTGCCTCGTCGCCGTAGCCGTCGTCGAGCGCGAGATCGCCTGGGCGCTGCTGTTGCGGATGCTGGTCGTTGGCCTGATGGTGGAAGTCGGCGCCGCCGCCGCTTGGACCTTCGTGCGTTGTCTCGCCTATCAGCGTTCCTTCCCCGTGCTTACAGCCTTCCCCTGAAACCCACGTTAACCGACCGTCCCGAAAACGCCGGTGTTAACACAGGAAAAAAAGAAACCACGCAGGAACCGCGCAGGAACCACGCGGAACATGGGACACTATCTGGAAATCCTGTTCAACGTCATCGTCTTCACTCTGCTGCTCGGCGTCATGGTCAGTATCGTCGCTTGGTACTTCACGTGAACCACCGTCGTCCCGGTTTAAAAACCATCATCGACGGCCGTTATAAAGCCACCCGGACACGCGCCGCGGCACTTGCCTACGGCGCTGCTCCAGGGAAACTCCTCTTCCTTCTGCTCTTCCTCCTTCACCGCAGGGATCGTTTCCCTCGGCCAGGGACATACCGAAGCAACTACCGGAACAACCTGGAGGAGTCGCGGCATGACGGCGCCCAAGTGTGTCACCACCACGACCTATCTGGTCAAGACCAAGGAGCAGCCCTGGTGGCCCGACAACGCCATCAGGAGATGGTGGATCAGCGTTGCCATCGTCATCTTCATCGGAGTCTGTCTGGTGGCCCTGATGTACTTTACGCAGCAGCAGGCACGCAACGGGAGCGGCAGCGGCTAGACAAGTCCCAGGCGGCTACAGCTCCAAGCGCCGTAGCCGGCCCGCCTGCCGATCGCGACGTCGTGGACCATCGAACAGAGAGTCACGCGTACGAGACCCCGAGGTACGCCACGCGGTGCCTAACGCGGTATACCACACCCGTACGGTCTGCAGTGCGGCGTACAACGTGTGGAAAACGCGTTGCGTCGCAGAGTCCGCCACGTCCCTGTCTTGTCGCTCCCCAATCGTCTCCCGCACACCCCCCGCGGCACCCAGAGGGCGGGTGAGGCAAGTATTCTTAAGGCCGTTCTCCGTTCCATAGCCCATAAATTGTTGATTCCGGAGCTCGTTGGCGCGGAAATAGCCGGATAAGGGGAGCAACAACCGTCGGCGAAAGCCGTCCCGGTCATTCAGTCCGGGTTTCGCGTCCAGTCGGACGTGTGACCGTTGGGCAACGGAACGGCGTTTCACTGCCAAAATTGTATCGGGTAGTGTACGAGACGTCGGCGGTGCAGAATGCGACTCGCGGCGTAGCTCGCCGTCGCTATGCGGCTCGTCGCCGTGTGGCGCGGCCTGGCCGGCTGTCTGCGCCCAGATCTGTTGGCCTTTTGGTTCCTCTGGCTGCTGCTGCGTGTGTGCTTTGGCAGACGCGGTGGCAGTTTGCGGTCTGCGGTAAGTGAGGATGTTGCCGAGCAAGCGCACTTGCGGCGCGTGATCGGCACGCGTGTTATTGTAGGTTCGTTGCCAGATGGCAAGTGCTGTCAACAGCAGACGTTGTGGGCGGTCGGTGTATTTTTGTGGGTTGCGGTGAGAGTCGGCACTCGGTGTTTTGTGAGTGATCTCAACAGTTTGTGTTGCTTTTAGCAGCGTCCAAAACAGCGACGCGACTTTGGGGATGGCCTCGTGCTCACCGCCGCGGAGAGTGTCGCCGGACCTGCTCGTCAGCAGCGAGCTACGCAGACGGAATATCTGGAGGAGAGTTACGTGTGTCACAGGAGAGCGCGGGTCTCCGGCGGTAACGACGGCGGTGTCGTCGACACGTGTGCGGCCTGCTGTGCTCTGCGGAAAAGCGCCGGTCTCGGAGACCGTGGACGAAAAAGAGAACGGAGCAGCTACCGCTGGCGGCGGCGGCGTTAATGCTGCCGTTGATGTTAGACGTTGTGAGTACTCGGAAACAGCGGTGAGGCAGAAGCTCGATCCTTCAGGGAACGACAGTCGATGTGTGGTAGCCGCAGCAGGTGAGGTTGGGGCGGATAACGTGTTGCGGATCGTGGCGAGAACGTCGTCCTCCCCTTCTTCACCGCCCCACCCACCCTCGGTTGGTGTTTCTTTTTTCTTGTGTCCTGCAGATAGTTCCACGGACAGCGACGGCAAGTCCATAATCGCCGGTGTGCAAGTGGTGGACCACGACGAAGATATCATCGCGCCGCAGAGTTTGTGGTGCACGGCGTTCAAGGAAGCCCTCTGGGATGTGGCTCTGTTGGAAGTGCCGCGTTGGGCGTGGCAGGGCTGGAAGAGGTGGCGCAACAGCGAGTCTGGGCGTCGATGGAGTGCTGGGTCTGCGTCGGCTTCCAGCTTGTCTGACTTGGCGGGCGAGGCCGTTGGAGAATTGGTGGGATCGGTCGTCGCGTACGTGATCCTTGAACGTCTGTGGTTGGCAGCCAGGGGCTGGGTGTGCGAAACCGGTGTGGAAGCCGAGGAGGCTATGGCGCGGCGGCGACAGCGCATGCTGTGGCGTATGTTCTCTCGTGGAGGCGACGGCGAATGCAGCAGACGGTGTTCGATGGAGATGGCGTGCGAGGAAGAAAGCGCCGTGTTGTGAGCAGACGACGTTGGATGCGGGACGTCGGAGCACATGGGCCATGTGTGGTGGCAGATGGCGGTGTCCACTTGTGCTTGTCGCGGCAGTGCACAGACGAAGCAACATGTCGCTGTGAAGAGATAGAGTGTGAGCATAGCTGTATGCAGCGTTGCGTGTAGAAGCGGGGGGATGAAGACGTTAATAAAGAGTAGCGGCGGTTGTGATAGGGCGACCGCTGAGGCGAGCTGCGTGTGCGTGCCGTCTGTGTTCCCCGTGTCCGCCGCGAAAGGCCCCCGTCCCCGTCCCCGCCGCAAGAGTCCCCCGTCCCCGCCACGCAACAGCTCGCCATCCCGCAACTCCCCGTCCCCGCCGCGAAATGCCCCTGTCCCCGTCCTCACCACCGTCCCCGCCGCAAGAGTCCCCCGTCCCCGGCACGCAACAGCCCGCCATCCCGCGACGCCCCGTCCCCGCACACCCCCCGTCCCCGCACACCCCCCGTCCCCGCACACCCCCCGTCCCCGCACACCCCCCGTCCCCGGCGCAACCCCCGTCCCTGTCCCCAGCGTAACTCCCGTTACCGGCGCCGGCGCCCAGCACGCCCGAAAACAACGCCGTCGCCGCCCCGAAAAGCGAAGCGCTGCGCAAAAGCTGCGTAGAAACGCCGCACAAACACCGTAGAAACACCCGCCGCCAACCCCCGAGCGCCCGCAACACCCCGTCCCCGGCGCTGGTCCGCGAAAAAAAACACCGCGGGACGCCACGCAACGGCAGTGCGCACAAAGCGCCGGACACAGCACGCCGCAAACGCGCTGAGGACGCCGACGCGCTTACTTATGTCACGCAACACTCCCTCAGCCCTGTCCAGTGTCGGACTGGCGGAACGACAAGTGCTGTGGAGCCACACGCACCGGCAGCGCGCAGAAAGCGCCGTGGACAGCAAGCCGCAGAAGCGGCGGAGCGCTTGCGGCGCCATAAAGGCGCTTAGCCCTGTCCCGCACCGGCGGCGGTCGGGGTGTGTCGGGGGCGCGGCGGGGTGGGTGTGTGCCGGGTGTGGCGGGTGTGTCAGGGTGTGCCGCGGGTGTGTCGCGGGCGTGTGCCGGGTGTGTCGGGGTGTGTTGGCCGGGTGTGTCGCGGGCGTGTGCCGGGTGTGTCGGGGTGTGTTGGCCGGGTGTGTCGCGGGCGTGTGCCGGGTGTGTCGGCGGGGTGTGTCAGGGGTGTGTCGGGGTGTGTTGGCGGGCCGTGTCTGCGTGTGTCCTCGACGCGGGTTGTGCAGTGTGCCCCGGGGCCCGCGACTCCTCCCTTGCGCCCGCGTCTTGTCTGGCCGTTGTTTTGCGTGTGTCCCCAAGGACCCGCGCTGCCGTCCCCTGGGGACTCAAACAGACACAGACGCGCGTCTGCTTTTCGCCGTGCGCGCCGCACGTCGCTTTTATTCGCCGTCGCCGTACCCACCGCAGCACACGCAACTAGTCGCCGTCGCCGTCCACACACGCAACTCCAAATTTCACCCCCCCGCAGAAAACACCCCCCCGCCCCTCGGGGACCCAGCACACGGCCCGGAATGGATGTCGGGCGTCCACCTAGGAGGGTGCGCGCTCGGGGACCCAGTCCGCGGCCCTGTTGCGCGTGTTCCTCCGGGTTCCTGCCTTTTCCAGTCGAGTGCGTCGTCGGCTGCCGGGTGGTTTTCCACGGCCTTCCAGACTGCACGGCCCCAAGGCGGCGCCAGCAAGCGCCATACACGTCGCTGCCTATAAAAGCCAGGTGCGTGTCTGCCGTGGCACACGGACGACGGAGCCGTCCGCGTGTGTAAACGGCGTGGTCGCTGACGCGGGTTTGCTTCCTATATAGTGGACGTCGGAGGTGTCCGGCGGCCATGGCCCAGCGCAACGGCATGTCGCCGCGCCCCCCGCCCCTCGGTCGCGGCCGCGGGGCCGGAGGGCCTTCGGGGGTTGGTTCCTCTCCTCCTTCTTCTTGTGTGCCGATGGGAGCGACGTCAACAGCGGGCACTGGTGCGAGTGCTGCGCCTACGGCGACGCCGGGCCACGGCGTCCACCGGGTAGAACCCCGCGGGCCGCCGGGCGCCCCTCCGAGTAGCGGCAACAACAGCAACTTTTGGCACGGCCCGGAGCGCCTGTTGCTGTCTCAGATTCCGGTGGAGCGCCAGGCGCTGACGGAGCTGGAATACCAGGCCATGGGCGCCGTGTGGCGCGCGGCGTTTCTGGCCAACAGCACGGGCCGCGCCATGCGCAAGTGGTCGCAGCGCGACGCGGGCACGCTGCTGCCGCTCGGACGGCCGTACGGATTCTACGCGCGGGTGACGCCGCGCAGCCAGATGAACGGCGTGGGCGCGACGGACCTGCGTCAGCTGTCGCCGCGGGACGCGTGGATCGTGCTGGTGGCGACCGTGGTGCACGAGGTGGACCCCGCGGCCGACCCGACGGTGGGCGACAAGGCCGGCCATCCCGAGGGTCTGTGCGCGCAGGACGGACTGTACCTGGCGCTGGGCGCCGGGTTCCGCGTGTTCGTCTACGACCTGGCGAACAACACGCTGATCCTAGCGGCGCGCGACGCGGACGAGTGGTTTCGGCACGGCGCGGGCGAGGTGGTGCGGCTGTACCGCTGCAACCGGCTGGGCGTGGGCACCCCGCGCGCGACGCTGCTGCCTCAGCCGGCGCTCCGACAGACGTTGCTGCGCGCCGAGGAGGCGACGGCGCTCGGACGGGAGCTGCGCCGGCGGTGGGCCGGCACGACGGTGGCACTACAGACGCCGGGAAGGCGACTGCAGCCGATGGTGCTGCTGGGCGCGTGGCAGGAGCTGGCGCAGTACGAGCCGTTCGCGTCGGCGCCGCACCCCGCGTCGCTGCTGACGGCCGTGCGTCGGCACCTGAACCAGCGTCTGTGCTGCGGCTGGCTGGCGCTGGGCGCGGTGCTGCCCGCGCGGTGGCTGGGTTGCGCGGCGGGGCCGGCGACGGGGACGACGTCGCCGCCAGCGGCGAGCGGCACGGAGACGGAGGCCGCCGGCGGGGACGCGCCGTGCGCGATGGCAGGAGCCGTGGGGTCTGCTGTGACTATACCTCCGCAGCCGTACGGAGGCGCCGGCGGGAGCGCGATTTGCGTGCCAAACGCGGACGCGCACGCGGTGGTCGGGGCGGACGCGACGGCGGCGGCAGCGGCAGCAGCGGCGGCGCCGACGGTGATGGTGGGTCCGACGGCGATGGCGGGTCCGGCGGCGTCGGGGACCGTGCCGCGCGCCATGCTGGTGGTGGTGCTGGACGAGCTGGGCGCCGTGTTCGGGTACTGCCCGCTGGACGGGCACGTGTACCCGCTGGCGGCGGAGCTGTCGCACTTTCTGCGCGCGGGCGTGCTGGGCGCGCTGGCGCTGGGGCGCGAGTCGGCGCCCGCCGCCGAGGCCGCGCGGCGGCTGCTGCCCGAGCTGGACCGCGAGCAGTGGGAGCGGCCGCGCTGGGACGCGCTGCACCTGCACCCGCGCGCCGCGCTGTGGGCGCGCGAGCCGCACGGGCAGTGGGAGTTCATGTTTCGCGAACAACGCGGTGACCCCATACATGATCCCGTCGCATTTCGTCTTTCGGACGCTCGAACTCTCGGTCTCGACCTCACCACCGTCATGACAGAGCGTCAAAGTCAATTGCCCGAAAAGTATATCGGTTTCTATCAGATTAGGAAACCTCCTTGGCTCATGGAACAACCTCCACCCCCATCTCGCCAAACCAAACCGGACGCTGCAACGATGCCCCCACCGCTCAGTGCTCAGGCAAGCGTCAGCTATGCGCTCCGATACGATGATGAGTCTTGGCGCCCGCTCAGCACAGTTGACGACCACAAAGCCTGGTTGGATCTCGACGAATCACATTGGGTCCTCGGGGACAGCCGACCCGACGATATAAAACAACGCAGACTGCTGAAGGCCACTCAACGACGAGGCGCCGAAATCGACAGACCCATGCCTGTCGTGCCTGAAGAATGTTACGACCAACGCTTCACTACCGAAGGCCACCAGGTCATCCCGTTGTGCGCGTCCGAACCCGAGGATGACGACGAAGATCCTACCTACGACGAATTGCCGTCGCGCCCACCCCAGAAACATAAGCCGCCAGACAAACCTCCGCGCTTATGCAAAACGGGCCCCGGCCCACCTCCGCTGCCGCCAAAGCAACGGCACGGTTCCACCGACGGAAAAGTTTCTGCGCCCCGACAGTCGGAGCATCATAAAAGACAGACCCGACCGCCAAGACCGCCACCGCCCAAATTCGGGGATAGAACCGCGGCCCATCTCTCGCAAAATATGCGAGACATGTACCTCGATATGTGTACATCTTCGGGCCACAGGCCACGGCCGCCAGCACCTCCGCGGCCGAAAAAATGTCAAACACACGCCCCTCACCACGTTCATCATTGAAAGTCTCTCCAGTCCCTATGTTGTCAGGACGTGCTGTCGTTCTTCGCTTGCTGCGAAGCCCGTTCTTCCGAGTCGTGTCGCTGCGTCCAGCGTCGCGCCCAAGATGGGAATTTGGGTCTTTTCACGCGTAGCCTCCTCCACCACGGCTGCTGATCGCCGTCACTAAGGACCGACACGGAGGATGACGAGGAGCTTCTCCCCGACTCCGCGGTCCGCGACCGGCTACGTAGCGCGTGTCCCTGCCAGTCTCCGCAGTTACACCACACGTCGTGAGCAGCGTGCACCTGCTGCCGCCACTGGGCCTCGGCGTGCTCAGGCCACCCGCCGGAGCCCGGTCTGAGCTCCGACGCAGGATGCGCGTACTCAACGTGCGCCTTCCAGTCCATACAGCAACACCATAGGTCGTGTGAGTCGTCGGCTACCCGCCGCCAGGCCAGTTCCCGCATGGGAAGGCTGGACACGCCGACCGAGAGGTCACCGAGCCCGGACGCCATCTCTTCTTCCTCTCCGTCGCTGTCATTAAGCAGCCAGGTCACCTCCTCCGCTCCGCGGTCCGCCGGTCTCGACGGACCGCGCCGCCGTCGGCAACACGGAAAACAGCACGCCAGCCCGAGCCGCTAAGGCCGCATGCCCCTGCCGCCCAACTGAACACGCATACCCCGCTCAACTGCGTTTTGCCACCCCTGCCAGTGCTCTCGCTCGAGCACCACCCCGTATCTCCCAACCTTTTTCCAATAAACGAAACCGACATGACATACGTAATGGGTGCTCGTGGCTACATTTATTGAAACAAACCGCGATCCCGGGCGTCTTAGCACACGAAAAACCGCATCCACATCATAGACAAGTTACAGTCCACAGTCACATACACGATAAACAATACCAACAGGGTAATGTTTATGGAGTAAAACACTATTGTCCAGGCCACATGCGTGTATGACTTCCGCACCATCCCGTACTGCATGTTCCACATGTACGCGCTAGACGTGTAATCCACTCGCAGTTCGGGGACGCAACGCAGCCAGATCACATCCCCTTGCAGTACCAGACGCAGGGCTAGCGTCTCGAAGATCGGCATCACATCTAAGTTCCGCACGTTCCACTTTAACGACTCCCCGGGAACGAACTCCACGTCGTCGGCGTGTACGTACAGGTTCTCTCCCACGCCGCCATAATCGGCCTTCGGATCGAAGACGAACCGACTCATGTTGCCCACGATGCTCCCCCGAGCAAACAACTTGCCGTTGTCAATGTAGCACCGGTTGTCCTCGATCTCAAACCAGGGGTGCTTGGCCGTGGACTTCCAGGGCCGGAGCGCGTCTTCCCCGGCTTTAGTGATGCCATCGGGCAGGCGGATCAAGGGACCCATGGAGGTCCAAAGACCCACCCAGGCTTTCCAGAGATTGTTCATGGTGAAACAGCGTGTGGACTGTACGCTCTTTCCCAAGTTATATCCCAGAGTAGTGACGTGAGCCCAGCCACCTCCCAGATTCCTGACGTTTTGGTTAGCTTTCCTGCCAATTCCTCCCGTAGACTTATGATTCTCCTAGCCCATTCCCGATAAAAATACACGGAGACAGTAGATAGAGTTACGAATAAACCGGTTTATTTATTCAAGTGTCTCAGGAGATTATTGAACGAGCGTGGATACCACGCCATCGTCAGTTCATGGTGGCATTGAGCAGCCATAGCACCAGAGTCCCGGCGCCCGGTATCAGACATGCTGACCTACCGGGCGCCTCCGAGTCCATATCCCGCGGCCTGGGTGTTAGAGTCCGTACCTGGCAGCCCAGGTAGGTTTCAGGTACCAGCTGGTTCGTACCTGTTAAATAAATCGCAGACGGGCGCTCACCCCTACGGTCAGGAGCACAAGAACAACCAGAGAGAACAGATATACGAGCAGGGTTCTGAACAGCAGACCCCAATTGTCGTCTCTCATGCTTCGCTGAAGGTACCAGTTGATGGTCTGAGAGCTATAGTCCATCCTCACCTGGGGAACACACGCGGCATATTTCTTGGGGTCTCCCCACCTCGTAGACAACGTGATGTCCACCATATCCACGGTGTGCGACACCGGGTACCCACCGATGTTCCACTCGAAATAGGCTCCGCGCTCATCATGGTGGTACTGCTCACCGGACACTTGCAATCTGTCCATGTAAGATTGAGAGACGATACCCACGTTCACAAAGTGTTTCTTGGTGAAGTTGCCCGACATCCTCCCCTTGAAGTACAGCATGCCCATATGGAACCAGCATTGGTTCTCCTCCACCCGAAAGTGGGCCGATTTGATCTCCGATACCACCACATTCAGGGGCCGGGGCACCGAGTCCGCGAGTCTCAGGAACAGGACGGCCAGGATCGCGAGCACCAACACCGGCTTCATGGCTCCGAAGGTCCGCTGCTCGGCTCCGCTCACCGCTCCGGTCTGGCTGCAGCAGTGCTTCGCTGAGAAGTAGCGTGTGGACTGCACGGTGTTTTTGAATATATAGCGTTTCTTGGTGACGTTGTTTCCCCTACGTAGTAGGCAACTACGTGCCAAAAGAGGCGTTACGGTACTTTCCGTACCGGGATTTCCAAACCGCGACTTTCCACACGGCGGTTTCAACACCGGGACTTTTCACACGGTGATTTCGGCACCGGGACTTTCCACACGGCGGTTTCGCCACCGCTGACGTTCTCATCGCCGCCCACGTCAACGGTGGCGACACCGTACTTTCCCATGCGGTTTATAAACGTCAAGAGTCACGTCAGTCGCCCACCCCCATTACACGGCGATATCCCGATAGGGCATGAGGGGACCCGGGTGTCGCGACATGTCGACGACAGGTGCGGATTAGTGGTCGTGTCGCGACATGGACGTGCAGGGGGATGTCTGTCGCGATAGAGTTGATGTGACAGCCCGCTACACCTCTCTGTCGCGACATGCATACACAACGGGCCGGCTTGTCGGCGATTGTCGCGACATATCGTTATCAGTTAGCGACCGGAGTTGTCTATCGCGACATATCGTCGACTATCGCGACAGAAAAAATACCGTTCGTAGAGAATGCCGTGTTGAAGGAACGCGCTTTTATTGAGACGATAAAACAGCATCAGGAGCCACAACGTCGAATCCCACGTCCAGTCGATTCGTATGTTATGCTGCACAGCAATGCTAGAATAACAACCAGCAGGGTAATCCCGCAACATAAATACAAAGTCACAGCGAAGAATCCGTGTCGTTCTATCAAGCGAAACGCGTTCCAAACGGCCCCGTCACAGACGCAGTTATTCATAAGCGTTAACAACCGGTGGCTAGGATGAATATCCAAATCACAGGGCAGTAGCCGACGGACTCGTTGACAGGTCAGCCTACCCTCAAGGTTCCTATCGTTCGGACGGGATTTGTGCGTTTTAGGCCTCTTTTTCGCCGCCTGCAAGCATTGGTGCGCAAAGTCCTCACCCAGCTGTTTCCAGCTATCATCTGCATCTGTGCAGTCCCCTGTATCGTTGTAACAAACGGGTCTGTGCGACTTTGTTCTCGGAACACAAGCTTGTCGCGGAGACAGAGAGAGAAGGGTTTTCGGGTCACGCGAAGACCGCTCACCGGGGGTCGGCAACGCACACATCAACAGAAAACCGAGACGGATCAAGAGGTCCATAGTGAAGAAGGAGCGATATCGACGTACTTACGAAACGGCGATTATATATGTTCTCAACAATACCGCCCTACGTTGTATGATGTAACGTGTGACGTGAGTCTGATCCAACACTGAACGCTTTCGTCGTGTTTTTCATGCAGCTTTTACGGACCATGACAAGCCTGACGAGAGCGTTCATCGGGGCATGAAGTACGCATTACACAAACTCCATATATTTGTTACGATAGAATACGGAACGGAGGAGGCTTTCGCCACACCTATCCTGAAAGCGTTGCATTCTTTATGATAGGTGTGACGATGTCTTTACCATTCCCACGGCTGCTTTGCGTGATGATGACATTCATCATGTATTTCCATTCACACATACCTTTTGTGCATACGGTTTATATATGACCATCCACGCTTATAACGAACCTAACAGTTTATTAGCCCTTGACAGGATAGGTCAAAAGATTATATGTAGGTTTTCCGGTAAACCGAATTGTGATATTTCTCTGCAGGAAATAGAACAGCCTGGTACCTATAAAACGGACAATGCAGTACTGTAGCAGCGTAACCAAGTAGGTCCACATGAACACGTACAAAATTATGGTAAGCCATCGTTTTTCATACCACAGCCTGTAGCTGTCGTACATGAATGAGGACGGTCGAGGAACCCAGGGTAGTTGTAATTGGGGGCGACATTCGTACTGTCCAGAAGACAATTGCACGGGTTTCAGTGAGATGAGTACTTTAGCGATGTCGGCGGGGGCGCTACGTTTCACCGTGACGGTGAGAACTTGACCGTCGTTTTGTACTTCATGAGGCACGTTATACAAGCCACTGGTATCATGAAGGATGACCTCTGATGCGATGTGAGGATTAAATTGTCCCTCAAACCGCCAAACGCTGGTCATGTTTCCACCGTCAATTACGCAGCTGACGGTGTGAGATACCACGATGTTGGACTTAGGTTTGGGGGCTAATTGCCTTTTTACAAATTCCCTTCTGTATTGCAGGTCCTGCTGCCACTGCTTTTCCGTGCGGAAAGTCGCCATGTCTTCCACACGTGTGGCGACGATAGACGCCACCAAGGTAGATACCAGAAGCAGCTGGATCCGCATGGTATTACCGTATGTCAATTAGAAAGTTGAGCGGACACGGTTATCGTTCCTGGCGGATATAAGTATATAAACGCGAGTTAGCCTTTCCCGTCCGTTTTGTACACCCGTTCCCCACACAAATGACGAATACGACCTTTTTTTTTTATAAAAATAAACCACGTGTATTATAGAAAAACATTTACATAGAAAAGAGACACACGGATCAACATAAGGACTTTTCACACTTTTGGGGTACACAGGCGTGCCACCGCAGATAGTAAGCGCTGGATACACGGTATACAGTCCTGGCCAGCACGTATCCCAACAGCAGCACCATCGCCATCTGTATGGCGATCACGACCCCGAGCTCTAAGTGTCTGTATTCATAGTGTAGTCGTCGCAGGTTATCCACTGAATTCCCGTAACTGAAATAACGTATATGGTACCGAGGCTGGCACCACATGGGTTTGCATTTGGTGCACGGCACCAAATGCAGAGTGAGATGGTCCAAGTCCGTGGGCACCCACTGGCGCAAACGGAATACGGCTTCGGTGGTCTCCACGAGGCACTCCGGGGCGTGCAGACGGCCCCACTTTCGTCCGCGACGGCCCGACCAGCCGACCCGAGCCACTATCCCTTTCTCGGGATAGAACGTACCCTGTACACGCCACACAGCGTCCAACACGCCGTCCTTGACGACGCAGCTGGCCTGATAGCTGGACACGTTGTTAAGCGGCGGAAAGCGAAACTGACGTGCCGGCGGAGCCACATAGTTCGGTTCACCGTGTTGTCGCGGTTCGTCCTCCCTATAGTAATAGTAGTCGTCGTCCTCATAGGGGTTGCCGGCGTGAGCCAGCGTTACCCAACAGCAGCCCAGGCCGACGAGGAGGCGCAGCCACCGCCTCATGGCGGCTTCGCCAGTCAATCGTCTTTAGCCTCTTCTTCCCGTGAAGTCCTTCCGGTGGCGCGGTGCCGACCTCGGACCCAGGGACGTATCCACCTCAGGTACACACAGCAGGCTACCTGGACACCGAAGCTGAACAAGGCTACGTGTTTCACAAACTGCACCAGTACCACATAGAGGAATGTCAGGTAGCGTCTCTCCGCAAACAGCCGTTCCAAGTCTGAGGGCGTTACCCGCAGCGGCAACCAGGGCAGCCTGGACGCCGGCCGGCAATGGAGCACGCTCCGGTTACAGGCACTGCAGGGGTAAACGGTTAACATCACGTAAGAGAGTCGTGCGTCCACCTGTGGGAGCTCAGTTTCGTAACGTAGAGCCCCGTCATTTTCCAGCTGGGGTGCGCCGACCTTGAAATGGGTCGCGCTCCGCTCGTTACCCCAGGTGCCGTAGGCTCTCGGGGCCGTATCGGAGAAGTTGCCACGCACAAGCCAGGCGGCCACGAGTACCCCGTGCTGGACGTAACATTCGGACACGGAACTGGAGACACGGTAGCCGGACACGTCCCCAAACCCGCGAGGGTACTGGGGCAGACGAACGGACTTGCTATTTGACAACGGACAGATACGAGACGACGAGGACGCAGACGACTCGTCGCTGGACCACGACAACCGGAGCGACTCCTTGGAGCGGCTCGAGAGTACACTTACTGCGATCAGACACCAGTGCCAGAAGAAGGAACAGGTGGACGGGGACCACAGGATCATAGCCGCCGGCACCGCGGCCGGCCGCAGGAAGCCGCCCGGCGCGTCGTCTGTGTGCGGGAGCCGAAACACCGTGCCTCTTTATATCGTCCCGACGTGACGCGAGTATTACGTGTCAGGGGAAACCCCCGTCACGACGAACGTGATTTGTAAGTGACGCGGGGTGCTGACGGGGTTCGGCCCGAGAGGTGACGGAGCGCCTCACGTCAGTATGATGTCCGATCCGCGTCAGCCCCGACGTGGTTGTGGTCACCGAAACCCACGTTTATATGGACGTTGAGAGCAGCGCCTGACCACATGATTCATCATACCATTTCTCGGAATCGGGCCCATGCCGGGAAAGCACATTCCTTTTCAGTAAACAACAATGACATCATAACAAATCATTTTATTCGCGAGGTGGATAATAACCGCATATCAGGAGGAGGGATCGGGTGATGACGCAGGCCCCGCAGAACAGTCCGAAATAAATTTTTAGTATTGCCCCATAGTCGCCTAGATACCAGAGGTACGTCAAGTTCATCAAAACGCCCATCGGCGTCCCGGAATCGTATACCGGGCACACGAAGCGTTCATAACAATCCCGGGAGGCGAGTGTTAGGGTAGCAGAGTAGTTTCGGGGTCGGTTTCCTTCCGGCGACGACAGTTCCGTGGGCAGCAGAATGTACAGCGCCTCGGTAGCTGTCGCGGTGCCTTCCACGAGGATGGGCTGCCGGTGCCTTTCGTGATTTTCCCCGTCGTGTAGCCAAGCCGAGGCCCGCAAAGTCTTAGGCGAGGGGAATTGTCCATAGAGTTTCACCGCACCCTTCAGTACATGGTTCTGAATAACACAGCCGCACGTGAAGTAGGTAGGTTCTCTCGTCTCCTCCGTGGCTGCCGCCACCACTCCCAGCCACCACAACAGGCAGATCGCCAGAGGGTTCCGGAGGCTTCCCCGGCGTAGCATGGTTTTGGGTTAAAGCAAAAAGTCTGGTGAGTCGTTTCCGAGCGACTCGAGATGCACTCCGCTTCAGTCTATATATCACCACTGGTCCGAAAACATCCAGGGAAAATGTCGGTGCAGCCAACCTTTCACATACAGCCCCCAAAACACTTGAATCACTGCCACCATCATCAGCGTATACTGCGCCGACTTAATCGTGAGCGCGTAGTACGCCATTAGACGGCGATCTTCGAACAATAGTCGTTCGATGTCCTCTAACGAGCTCCACAGGGGAACCCAAGGCACGAGGCACCGGGGTTCGCACTCTACATAATAAGTTTGGCATTGATGACAGGGGGAAAAGTAGAACAACACGAGTTTTGTGCGTTGGGGAACACGATAGTCCCGGAGCCAGTAGCGTTTTGCGACGAGGCTTTCGGAGATGTCCTCCACCGGCGTCGGCACTCGATCCGCGTAGCCCTCCAGCGTCTGGTAGTACACCCGGGGTGTCGGCGTGGGCACGGACAGGTTCCCGCGCAGGGTCCACAGAGCCTCCAGTCGACCGCCCGATCGGAGCACGCAGCGCGCCTCGGAATACTCTACTCGGTACTCCGAAACATCGGGCAGAGGCGGTAACGGCTCCGTCTCCACCAAGGGCGGAGGTTCATCGAAAAGAGTCAAGGATAATTCAGGCATACTACCCGCGACCGGGGCCCAGAGGGCTAGAATAAGCATTACAAGATTCATTCTGTCTTACAAGGGAAGGCTGTTCCCCTGTCTAGACTCAAAAGCTGTAAGGCTATCTTATAGCATGTAGTCTTGCACGTCACGGGGAACAGGGTGGTGATCTAGTGACGTCGGGAGAACACGGTGTTTTAGGGTGCGGGGGACAAAGGACAGTACGACAGATTAGGTGATAGAAACGTTTTTTTTTATTTATGAAAAAGCCAGTGTGCCGTGCGGCCTAGGGCCCCGGCGTAGTTTGGATACCAGATGGGGGCCGTCAGGGGTACTACCACGAGCAGAAACATAATGACTTGGTCCATATATAGCAGCATAGCGGTGCGCAGCAGGTCGCCGTCCGTGTAGCAATTTGACGGTGAGCGATAAAGCACCGTTAATGTGTCGCGGATAAGCACGATCTTGAGGCCGTAGATGAAGCTCACAGTCAGTGCTAAAATGATGCGTTGGTATGGTTCCCAGGACTGCACGGCGATGAAGAGCCAGAGTATGGGAAGCATGAAACTTAGCAAACAGAGGATGGCTAACCGTCGTTGCATGTTCCAGGCCATGAGCCAGGCTAGGCCCGTACACCAGACGCAGAGCATGGATGACAGGACATAGGCCTGGATTACCACGGTGCGATCGAAACACAGCCCGATGGTGGACACGGATATCGTAGTGAGGGTGGTATATACCATGACCAGCATCAGGGTCCCGGGTCGGCGCTGACGTTCCAGCCAGTACGCGTGGCAACGCAGAGCACAGGGTAGCAGTGTGCTCCAGAAGGGCAGTGTATCGCGCAGGTAGGGGGTTGTCACGCGCCACGGTATGAGCATGAAAAGGATGGTAGTGGCTATGGTGGCGCTGGTCTGGAACACGACGGTGCCGTAGAGACGTACCATCCAGAGAAAGTGTTGAACGCTCCGCAGGGTGTCTTCATCTTTGGTGATTACGGTGACTCGACGGATCGGCGGTGGTGACGGCGGCGACACGGGTGGGGGTTTCTCTTTCTTATGGCCGAGTGGCTCGCCTTGGTGAAACTGGATCTGTACCATGACGGGTGCTCGACGAACAGTCGTCGGGGCTTCAGGTACCCGGCAAGTTTTATAGAGAAAGGGGGACGATGGGTGGTGGCTACGAGCCACCGCCACCTTCGCAATACGAGGATCTGAAGGCGGCAAAGACGGTCGTCCAGGGCAGGCGCCAGAGGTTGGGACTGAGCACGATCAGCGTGATTTTAAACATGGTCACCAGTCCTACGTAGATCAGCAGCGAGCCGCGTAACGTCTGAGCAGCCGGCAGTTCGTCGCGGATGTAACGCGTGCCGTAGAAAGTCACGGTCATCATAAGGAAGACGATGGCGCCGTAGCCGTAGAGTAGAATACGCTGATGATGGAACACGGTCTGGTCGCCGATAACCCAGAGCGTGATGAAAAAAACGCTGGTGAGCACCCGTGAGCATATGAGCTCCCAACGCTTAGCGCGAAAACTGTCCCCAACCATGACAGCGCCGGTGCAAGCTATCCACAGCGTGAGGACCAGTGTGTAGTCGATGAGGATGGCGGGCAGGTCGGAGCACCAGGTGTAGAAAACCGTGGTAACGGAGAGGAGGCCTACGTAGCCCATGGTCAATACCACGTCGTCGGGGTGCCTTTCGCCCTGTATCAAGACCAAACACCAGAGAAGGGAGGGGGCAAAAACCAGCAGCAGAGGGGAAGATTCATGTTGACATATGTTGTGGGAATCGGGGATACCCAGCCAAATCATTCCGCAGAAAGCCGTACTGATGGCGATGTGAAAGACCACTAGGGCGTAGACCCGGACGAGGACAGCAAAACGGCGCAGCCACATAAGGCCGTGGTGCAGCTGCAGGAGGGAAGCCCATTGCGGCGAATGTAGCGACGGCAGCGGCGGGTCCATGAGGCGGGTGATGCGCCCGAGTGAACGGGTGAGCGTCTCGGTGGAGTCTTCTTATAAACCAGCGGGTCTCAAGCAACCCTGCTCTGGAACGTCGCGGTGGTGCTGTTGAGGATGACGCTGAGCGTGCCGTTGTCAACCCGGTAATGATGATAGGTGCCCAGCTTGGCCAGGTAGCTGAACATTTGGTCCCAGCGTGCCGACCACACCACGGGCGTGAGCATCAGGAGTGTGGTGTGATAGATGAGTGTCTCGGTGGCGTAAAGTACCAGCGAGCTGCGGATGATGTGGCTCACGGGCATTTTGGTGGCGATGTAGCGCACGTCTTGGAAAAGGACGGCCAGGATGCAGCCCACGAACACGGTGTAGAGACACAGCAGAGTCTTATGCAACCAGGTGTAAGTAGAAGCCAGGACGCTGACCATCACCGTCAAAAGTGTGGAGGTGAAAAGCGCGTCACGCCACACAGAGCTGAGGCGGTGTTCCCAAGCCACGCCGTTGCAGGCCACGAACAACGTCCACGTTAGGATGAGGCTGGAAACGCCGATGGGCGCTGTGGCGCACAGGTTGAGCCCGGCGGTGGTGAACGAGAGAAGCGCCACATACAGTGCAAACACCAGGCCGTTGCTGGGGTGTCTGTGGTCGGTGAGCTCCAGCGCGCCCAGAACCAACACCGGTGTGCAGCTAAGCAATAGCGGCGAGGGATCGTCGCGGCACTCGTAGCCCAGTGAGGGGTAACCCAGCCAAACCAGCGCGCTAATGAGTACGCTGAAAGCGGTTTCCAGCGTCAGCAATCCGTAGACACGCATGACGATCGCGGTCCGACGTAGCCAACACACCGCATCTTCGGAAGCTGTGGACGCTGTTTCCGAATACCGGGAGGAGATCGTGCTTCCCTCTTCCAAGGATCGGAAAGTAGCGTCCGTCGTTTCCGCGGACGCGGCTTCCCTGGTACGCTCCGTTTCCGACGACGCGGTTTCCCGCTGCGTGGAAACTGTCTCCATGTCGGGACCGCAGCGCCCGGCGGCGTATCCGCAAGGTCTCGAAGCTACAGCTTGTCAGAGGAAAAGTAGGTTTGCAAAAAGGTGCGCAGGGTCATGATTCTCAGCACCATCAGCAGAGTGAAAACCAGGCTGAGAAACACCTTGACGGCCGCCAAAAGCGCGCGTTCCAGCGGCGTCTCGTAGCGTACAGCCAGGGCCGCTTCGTGGAAATGCGAGACGGCTAGACAGGTAATGAGCACGCTGAAGGACAAGACGATCTTAAAGCACCAGGACCAACCACGCCTCAAGATGACCACCACGATTGCCGTGAAGGTCAACGTGATCAAAGCATGGATGACCACGATCTGACGGCGGACGGTACGTTCGGGAGCCAACAACGCTACGCCGGTGCAGCTGAGAAAGGCCAGTAAGGTGAACAACGCGGCCGAGATGACCAACGTACCGTCCAGGCAGAGACATATTACGATCAACGGCGGCACGTGAAGCAGCGTGTAAAAGAGCAGAACGCCGATATTGCTGGGATGCGATGTTTCGTAACAGTGAATGAAGATCACCGACGTGACGGGTATGACAAAGACGAGGCTGGGCGAGGACTCCGTGAGACACAGACGGGAATGGTGAAACCACGTCGCGGGCGCCGCGTAGCAGAAGGCGCTCAACAACGCGGTCAAGCCGGCCAGCTGCCAACCCACGGCGCCATAGGTGTGCAGCGCCACGCGGCAACAGTCGACCCAAGCCAGACTGCGGGTCGCCAGCCGGGTCTCTTGGATCCCGGGGGGCACGTAGATGACCGTGCCATCGGTGGGTACTTGAAACCCTTTTTCTCTTCTCATGGTGCGCTGCGTTCTCTGGAAACGGCTGCTCTGTCCGAAAACCAGTTCCGAACGAAAATCTAGGGCGAGAGGGTGGACAACGGCGTTGACGACGAAGCATGGGACAGGTCGTTCGGCGTTAACGTCATCGCGTCGGACGACGGTAGTTCTAAGAGACGTAGATCGCTCAGCAGGTCCTGACAGTTGCGGATTCGCAAGATCAGAAAAAAAAGGGAAATGAACGTAATAAAGAGCTGTAGCGACGTATGCGCCACATCGCGTGGCATAAGAACGTGACGGACGAAAAGGACCTGCTGCGAAAAGTGGCCGGCGAAGATAAGGCCCACCGTGCTGTAGAAGCCCAAAAGCAGCCGCAGGGGCCAAGTCCAGGGCCGCGTGAAGACGATGAGAACGTTAGCCAGAAAGACCACGACCCAGACGCCGTTGATGAGGGCAAATTGATCGGACAGGGTGCAGTTGTCGCGACAGATGAAGACTACTTCCGCGCAGAGCAAGGTGATGACCAACGTGAGCACAAACGACGTCAACACCTCGCGGGGCTCCTGGCAGGCACACGTGACACCTAGCGCCGGGATGTGCGCCAGGAGGCCGGCGAGTAATAGCACCAGCTGTCGGAACGGACGACGGCAGCGCGGGTGCCGGTTTCGCTGAGCGAGAACCGGTCGCTCATAGCGGAAATACACGAAGAGCGCGGAGGCCACAGGCACCAGGAGGAGCACCTCGGGCGCCCAGACAACGTGACAAGGAAAGCCCGGACGCGACTTGAGAGTCGCTGTAGGGAAGACCAGAGAGAAGCTACCCAAGACGGCCACCGCCGTGGAGATTTGGAAGAGGAGCAAGCCGGCGATTCGGACGACAACCTCGAAGCGATGCACCCAGCCCAGCACGGCCACCACGGCCGCTTCATCATAGTCGTCGTTGTTGCCGCTGTCGAACAGCCGCCGAAACACGATCTGTCGCTGGGTCGCGGTGGGAAAGCGCAGACCCATGACAGCCGGAGGCTATATGACCGCGCGTCTAAGACGCGAGATCCGTGGGGGGACTTTTAGATGTTTGGGCGGCCCGCGGTTCTAACAGGCTTGATTGGTGGAGACGGCCGGCGCGGCGGGTGGGGGAAACGACGAGTTTTTCCGTTACGCCATGGTTCGCGTGAGGTTTCTCTGTACCTCCCGCAAAAGGTCACAGCCCGAAATGGAGGCCGCGTTGGTGGCCCCGGTGGCGCGTGACGATAACCAGGTCATCCAAGCGATGAGTTTGTCTAATGAGTCCTCGGTGGTGAAAAGGATGAGAATGAGCAGGTACAGGTACACCAGGTTCTCATAGAGACACAAGGTGAGCAGGTCGGCCTCGGACCACGCGATCTCAAACAGGCGCGTGGTGTCAAAGACCGTGACGACCAGCATGAAGCTGAGCGCCATGGCGTAATAGCCCAAAAAAAGTTTGTGCCCCAACGGTACGGGCTGCAGGTAAAGTGCGATCAAGAACGCGATAACGCCGATCACAAACAGCGTGACGATGACCTGCCATCGACGGTGATTATGGCCGGCTAGACCCGTGACGCAGCTGCAGAGGCTAAAAAGCACGCAAGCCAAGAGGCCCGAGAAGGTCACCAGCGTAGAGGAGGAGCAGGCGCTGGCCACGATCACCGAAAGCGTCGTGAGCACGCTATAAATGGTGAGCAGGCCCGGGCTCGGCGGCGACGTGAACGATCCTTCATCGCGTTTGCCGTGCAGCAGGGCCAAACAGATGGTGGGCACCATCAAACTCAAGGGCGGCATAAAGCCGGTGCAACAGAGAAAGACGGTGCCTTTAAGATGCGGAAAAGCCAGCACCAGGCCCAGACAGAGCAAGAAGGTGCAGGTGCCCTGCACGGCCACGGTGCTGTAGACCCGCATACAAAGTAAAAAGCGACGTACGTCGTTCGTCGAGACGGAGGAAATCATAATGACTCCGCGCGAGGGTCGCGGGGGTGGGGGCGCCCAGGCCGTCCCGGTGGCCTCTGAGTTCGGAGACATGACGGCGGTGGCTATCAAAAGGCGCGTATGAGAAACCGTTTATAGAGTGTAATATAATCACCGTCATTCCCACACGGCGTTCCCCCATAAAGTCACGTCACACTCGAGTAAGCGTGAAAAAGCTTTATTGTTGAATAAAAAACACGAGTACAACACCGAGTTGCGGTGTTCTGTCTGTCTACTGGGTGGGGGAGGTTCATCGTCTGTCTCTAGAGGGAAGGTGGGGAACGTCTAAGCGAGCGGGAGCGTGTCATCTCCCCCATCTTTTTACAACAAGCTGAGGAGACTCACGCCGTCGATGCGTCCGCCGTGTTTCTCGGCGTACTGCTGCACCCAGACGTGGCCGCTAAAGATGGCGACGCTCATGTTTAGGAGACTCATGACGATGGTGTACAACACGACGCTGACACAGACGCTGTTTTTAGACAACGTTCCACGCTGGTAGATGAGATCCAGGGTCTCGTAAATAAGCACGGCCGAAGCGGCGGTCACCACCAGGACGTAGAGTCCGCTGTAGATCTTGCTGACCCACAGCACGGGCGAAAAGTAAAGCAATAGGTAAAAGACGATGACGGACCAGCCGTAGCCAATCCCGATGACTTTCCAGCGCGTGGGATTGTTGCCGGCCAGGTAGGTGAGACCGCTGCAGAGAACGAAAAAGACCATCACCAGGGCAAACGACAGACCGATGACGCGCCTTTCTCCGCAAAAGCCCGTGCACACGGTGATGCCGGTGTTGATCAGCAAGCACGCCACTGTGAGATGAGCAAAATTGGTGGTGTGTGGGCGAAACTCGGCGAAACCGCGTAGCATAGCCAGCGTGGACACGGGTACGATGGAGGATAGGGCTGGCACTATGCCGTTGGCGCACTGTCCCTGCACATCGGGGAAGGCGAGCCAAGCCAGCAAGCAGACCGTGAGGGTACAAGCCAGCTGCCACACGAGCCCGTGATAGACCTCCATGAGCAGCTTAAAGCGTTTCAACCATTGGAAGAGCTGCTGTTCGGCCACCAGCGCGTGGCTGCGATGGAGCGGCACGATGGTGACCGTCGGCGACTCATGGTGTTCGGAAACCGAGGCGGTGTCGCCCATGCTGCCGCTTACGACCGCTGTCGGTCTAAGGTAGGCGTCGATGAAACAGTCCGTCTTATCAGCACCCGGTTACCGCGGATTTGATTGACGTCACGAGTGTGGTCAAACCGTGGCGGCACCCTGTATCCGACCCGTCGTCATGGGCTCCACAACCAGAGCCTCAGAAGATGGTACATGCCGATGAATAAAGCCACATTTTCGACATAGAGGCGTAGCGAGGGCTGAAAACTCTCCGGGAAAGAACTCTGACAGGTGATCAGGGACAGATCGTGAATTAGCATCAGCGTCACCGTCAACAGCGTCGTCGCGTGTAAACCGAGAAAGAACGGGGCCGCGGCCCGCAGCAGCCAAAGTCCCAGCGCCGTAGCGCAGAGCAGAGACAGGACCGACGGTAGCCACAGCCGCCGGAGAGACGCGCCAGGATCGCAACCCAAAAGCGAGGCCCCCAGGCAGCTGAGATCTACCGCCAGGGCGAGAAGAGCCGCGCCGACAAAGGCCTGCGGCGACGGCTGGCACATCAGCAAGGTCAGAAAGGCTAGCGCGTGCGGCAGGCAGTAAGCCAACAGGAGTGGGAGTTTGCGGGGACAACGGTCGATCGACGGACCGCGTAGCAGCAGGAACAGGCAGCCGACGGGCACGACGAGGCTGAGATGAGAAAGCGACGGTGGGTCGTCGTCCCGTCCCCGCTCGCATAGCTCGGCCACCGGTGGCGGCATGAGCCACCAGCTGAGCACGCTGAGGGCGACGGTGGCGGTAAGCTGGAAGGCGACGAGGACGGAGGCGCGCAGCCATACCGCCAGCCTCTCTAGGTAGGGGACTACCTCCTCGACGGTCCATTCTAGCGGGACGACATGAAGCATGGCGACAAGCGCGGCTGCTGTGAAAACGGGCGCGGTTTTATAGGCATTAGGACTTCCCCGTCGTACTGGCGGCTGTCAAAGTCCCGTTGTCCAAAGGCGCGCCGTCCGAAAGACTAATCCAACGGGGACCCGAGAGCATGAGCAACAACGTGAGAAAGATGGCCATGCTGTCCAGGTAGAGACAGACGGCGTGACGGATGCATTGGTTAGGTGGGCAGAAAAAGATGACCATGAGACTGTCGTAGGCCAGAATACCCAAAAAGAAGCTGATAGAGAAGGCGCACAACGTCACCACTATCTTCTGCAGCCAATCGGCGTCGCTTAGCAGAGCGAGCGTGAGGAACGAAAGCAGCATCACCACGTAGACGCAGCTGATGCATTTCCAGCGACGTCGGTCACGGCCACCTAGAAACGCCAGCCCCGTAAAGGAGATAAACAACGCCAGGGTCATCACGTAGGAACCTACTAGTACGCGGCTTTCAGAGCACATTTGGAAGATGGCCGCCGTCAGGCTGTTGGCCAACAGATAGATGAAAAGCACCGTGGCGTTACTAGGGTGTTCGTTGCCCAACGTGTACGTGATGAACATGCAGACGATGGGCACGAGCACGGTGAGAAAGAAGCTGTAGTTCTCGACGCAAAAGTTGCGGTTTTGTGGGAACCCCAACCAAAAAACGCTTCCCAAGCCGAAGCTGAAAGCCAGCTGAAAGATGAAGATGGCGTACACGCGCAGCCATACGGTGAACTTTTTGAACCACTCGAGAGCCTCCATGCGGGAGAGCAGCAGCGCGTTAGCCTCCTGCGCCTGCATGGTGGCGACGGTCTCGGCACAAAGCCGCTGCGGCGCACCTACCCTTCTCTTATACACAAGCGAGCGAGTGGGGCACGGTGACGTGGTCACGCCGCGGACACGTCGATTAGGAGACGAACTGGGGCGACGCCGCTGCTGTGGCAGCGACCGTCGCAGCGACCGTCGTCTGAGCAGTGTGGGCGCTGCCGGGCTCGGAAGGCATGAAGTAGAGCACGGAGACAAAGAGGTACATGAGGTCCATGTACAAGCAGAGCGCGCCCGGGATATAACTCTCATACTCGATGTCGTGCAGGATGTCCTGCGTATCGCACACCACCGAGGTCACGATGACGGCCAAACCGGCTATCATCACCAGGATCTCACTTACCGCCTCGGGAAAAAGAGAAAATACGGCGAATAGTAAGAGAATCAGCGTGGATGCGCCCGTCAATAGGGAACGCTGTAATTCCACGTCGCGGGCAAACAGATACGTAGCGAGCGTGAGGAAACAAAATAGCGTCACTGTGGCCACCATGGCATAAATGACTGAACGATGACTAAAGTGGAAGCCTGACGCCGTGACAGCCACGCTGGTAAGCAACGTGTACGTCAGTAAGATCCATACGTTTTTGGGAAAGTTGGGCTCGGCCCAACGCAACAGACCTAGGCACACGATGGAGATCATTAAGCAAGACAGCGTCAGACGCACGCTGGAAAAGAGCTGCTCCAGCCGGTGCGGCAACACCAGCCAGCAAAAGGCGCAGACGCTCATAAGGATGAGGCATTGCACCCAGATAAGGATGTAGATGCGCAACAGGAAGACCGACCGGGCTATCTGGACCTGACCGCGGAGCGACATGGCGGCAACGCCGGCGGTTATCGCCGAGATTCGTCTAAATACACGAAGCGAACTAGAAAACGCACACACGTGATTTGCAAAAAGAAAGCAGCTGCCGGCTTATTATTTTATTAAAAATTTATCTGTGCAGAATCATAAGTTTATGATGAATAAAAACGGGGAAAGGGAATCTGCTTTTAGGGACCCGGGTCTGGTCCGTCGTCTCCCATCTGGTCGGGTTCGGGGATGGGGACCTGTTTCAGCGTGTGTCCGCGGGCGTGCATGGCTTTTGCTCGCCGGCCGCGCTGTAACCAGGCCTCTTTCTCTGTGGTCGGCGAGTCTTCCGACGGGTAGGGAGCCTGGGAGTCCATCGCTTCAGGCCCACCGCTCGTTCCCTCGACCGTCGTGTCGTCCTCGTTTTCGCTATTACACGGGGTTTCTGGAGTATCGCCTATACGGTTGGCAATTCTCCGGGGGCGGCCGCTTTCGTCCTCGTCGCTGCTATCGCCGCCCGGTAATTCGACGCCGCATTCGTTGTACGGAGCGCGGCACATGGGCGGCGGAAAGAACTTGGGCATGCGAAAGCAGCGTTGTCCATCCACGGTCTGCGTGGTTTCATCGTTATCCTCCCATAATCCCCCCTGTAGCGCCGGCAGCGTTTCGACGCTGTGAGAGGGGAAGGCCCAGTTCTGGTTGTCTTGCAGCGCGCCCGTGGGCAGTAGGTCCGTGCGGCCCCAGGCGCTGCTGTTGTTGGGTACCTTGTCAGTGCCGCGAGTAGGTCGCAGAAACCAGTCCAGAGCGCTCTCTAGCTGCGAGCGTGTGATGGTGCCCAGTGCGCCGTGCCAGCGCAGCACGTCTCTTTTCAGCGTGTGGTGACAGACGGGCAGCTCCTCCAACCGACACTCGCCGCGCAATCCGCGGTCGAAGCGGCAGAGACCACGCAGTTTAAGCAGACCGCACTTGAGAAACATGTGAAAATTATCGGCAATGCGATACAGGTCTGAGTCCTCGATCTTGTGTAGGTAGACCACGCCAAACTTGTCGAGCAGCACCATGCCGCTGGGCACAAAAGGCCCGTATGCCAGGTAATAGCCCACGAGGCCGACGACGTACCACTCGCAGCACAAGCGTTGACGAATAAAGTTCAAAAGATCGCGAAAGTCCGCGGCCGGCATGTGGTCAAAAGGCCGGCAGGCGCGCAGGCCCTCGATGGAGCCCAGCATGAGCAACGGCTCCACCTCGGTGCGACCCGGCGTGCGGATGACCAGGTTGAGACCGTTCATTTCGCGGGCCGTCTTGGCCACGGCCGCAGCGTCAGTGGGGTCGGTGCAGAGGAATTTTTGCACATGATAGCGCGGTTCGGTGGTGGCGAACGGCGTTTGTGGGTGCCGATACACATATTCGCACCAGAGTAGGCCGTTCTTGGAAAAGGCTTTGATATCACTGGCCACCTCGTAGAGCCCGTCGGTCTCCCAGTCGTAGACGTAGACGGTGCCGTAATGACTTAGCATGAGCACGCAGGGCAGTTCCTGCGCCTGCTTGGTGTTTCGTGTTAGATCGCTGTCGGGTGGACGTACGGCTAGTACACCGACGGCTTCCAGGGTGTCATCGCAGCAGAGATAGTCGGCGGCCAGAGAACGTGCGTAAATCTGCGGGATAGCGGCCTGTTCGCGCATCACTAGGAACCAGTTGGCGGGGTTGCGCAGTGCTACGGTGGTTCCCTGGTGGCGCTGCACGTAGGTTCTCAGCGCCGGAGGATCGTACTGGCGCAGATAGAGGCCTTGCAGCATCGATAACGTCTTTTGAAAGACGGTGTTTCTAAATTGGAAAACGCCGTAGTCGCAGCGGATAGCATCTTCGCAGCGCTCGTCGCGCTGTCGGAGATAGGTGCCCCAGGCTTCGGCGGCGGCTTTGGTGAGTAGGGACATGCCGGCAAAGCCGTCTCGACAGCGAGTCGGATAAAGCGCGCTGCGCGAAAGCTTAATATAGGAGCAGCGTCAGACGAATCGCGGCTGGTGGCCCAGGGGGTGGGACGCGCCGCCTACACAAAGTGCTCCCGAAAATCGAAACTCTTGACCCACTCCGGAGACAAATCCGTATTCAGATTGATGCGTCGCGCTTCCACTTCGGCTTCCGAAACCTCGGCCTCCGTCCGGTAGGCGTTAACGATACGCTGACCCAGGTGCCAACGTTCTTTCTCTGCCAAACGCCGTTGCTCAAACCATTCGTCTACGTCCTTGAGGTCAAAGACAGTGTCCTCCTCAAGGTCAAAGCCTAGGTCTTCCCACTCGTCGTCATCGCTCTCGTGGCCGGCGGCCATACGCGCGGCAACCGCGTCTTCCCCTCCTCTTCTATCAACGTTGGGTACCACGTTGTTTTCTTCGGGTTCCATAGGTTCTGCGCCACTGTCGTCATCATCCTCTCCCTGCTCCTCATCGTCCGCCAAGGCGTCGTGGATCACCTCCAGGTTCTGATTGTCGGGTACGACGTGGTTATCTTCGTCGTCGTCGCGTGGCATGGGCGGCGGCCGACGGCGGACGACCGGCATGGCGCGGCCGTCGTTTCCTTCGTCTTCCTCTTCTCCGTCTCCCAGGGAACGCGGTCGACGGCGTTCCGCGAAGTCGCCGCGGACCACGCGCGCCTGCCAGATGGTAAACGCGTCCCAACCATCCCAGTTATTTAGCATTTCGGCGCGGAAACGGTCGCCTCGACAGAGCCAGCGAAACTGCCGCGCGTAGTCGCGGTCTACGCCGCTGTCGAACATGGTAAAGTGAAGACGCGCCGCCTCGCCCATGTGTACGCAGCCTCCGTTGCGTTCCAGCCTGGCCGCGCGCCGCAGACCGTGTTCGTAGCGGCGACGCACGTACACCTTCATGAGGCCGGCGCGAAAGAGTTCCTCTAGGCTGTCGGCCAGACGGTAGATTTCACCGGCTAGACGCTGCAGGGGCGGCGAGCGGTCCAGGTGCGACTTGACGATTACCACGTAAAAACGACAGAAACGGTCGAAGATGATGAGGAATGACGTGTCAAAAAAACCACCGGCGCGGTAGGAGCCCACGGCGCCCAGCAGGTACCAGCGGCAACGCAGTTGCAGCGTGACGTACATTTCGCACTCGGCCAAGCGGGCGGCTGGCGCTACCTCGAAGGGCCAGCAGTCCGTCAAGCAGCCGAAACTGGTCAGGAGTTTCAACGTTTTGGCATGGCGTCCAGGTGTATGAAAGTTCACGTCGCGTCCGTGGTGTTCGCCAACGCAGGCGGCCAACGCGTCGGTGTCATGACCGTGACGCAGCAGCATCGCTACCACGTCGTGCGGTACCCGCGTAGCAAACGGCGTCTGTGGCTGACGGTATACGGCTTCGGTGTACATCATACCGTAACGCGCCAGTTCGTCCAGATGACGCGCGCACAGCAGCAGAATCTCTTGCGAGGGTTCGTAGATGTAGAGGCGCGTACCGCCACCCATGCAGAGCACCAGCTCCGTCTCTTCATAGTGATCGTCCACCATGATCACGCATTTGCCTAGCACGATAAGGCGTTCGGGGCAACAAATTACGTCGTCCAGCAGTTGGTCGCGCAGCTCCGGCATGGTGCTGCCGGGCCGCACCTGCAGGAACCAGTTGTGCGGAATGCCGAGCGACAACACCTGGTCGACGTGGTTACGGACCCAGTCGCGAAGCACGTCGGCGCTGTACTGGCACTCAAAGATGCCCTGAAAGTCGCCCATGACCCGCAGAAAAGTTTCGTAGCGCGTGTGGCAATAGAGGAATTCATCGTTTCGCGTAAACGTGGGAGCTCCGTCTTCCCAGCGTGTACGCCACATGTCAAAAGAGGCCGCCAGCTAGACACCCCAGAAAAGAAGCAGAGAAAGAGAGTTCTTTGTGCGACACGTTTTATTCCGCGTCCTCCGCTCGACGCTCAAATCTGGATGTACTCGCGCACACCCGTCAGGCTCTTTAAGGGAAAAGGGTCCGAGTACGTCACTAACCGCGACTGATGCACCAGGGCGGTAATCACCCGCTCTGCGCCCTCGCGCGTCGACGAACGCGTCGTCACCAGGCAGTGCAGCCGCGGGCCCGTATCGTCCTGATGACCAGCGGCCTCGCGCTCGGCTGCTTCCACACCGACAATGTCGGGATCCAACACGTAGCTCTGCGAGTTGGTGTCGTAGCGGTGTAGCACCAACGTGTTGGGGTCCAGACGCTCCCACGCGCCCTCGTGCGGGTCAAAACGCTCCGTTAAACAGAGCCAGTCATACTGCTGCTGCAGAATACGCCGCTCGCGCTCGCGTCGCTCATCGGGCAACGCGGCGTCTTCGTTGAAGAGAATGTCCCGCTTGTGGTCTACGGCACGCTCGTGGTGGTGCGGGCACAGATGACGGTGTTCCATACGCGTCTGACGTTGACGCTCGCGCTCAAAACGCCGGTGTCGAAAGACCATTTTCAGCAACCCCATGCGGAAAAACTCCGTGATGGTGTTGGCAACGCGCCGCACGTAGTGGTTGGGGTCGTCCATCTGGATGGCGTACACGGCACCGAACCAGTCCAACAGTACCAGCACTTCGGCCACAAAACTGCGTCCCGGCCGCGGACGTCCCGTCACGCCTAGCACATACCACGGCGTGGCCAGATTAGCACGGACAGCCCACCACCAACGACGGCTCTCCACCTCGGTGAGCGCACAAAAGGGCCAAATGCGGTGTAACTGCTGCACCGTTTTCATCAGCCGCATAATCACCGTGCCGTAACCCGGTGTATGCAACTTCACGTCGCAACCCAGGATTCGTTCGGCCGTGGCGTACGAGCCCTCAGGCGTTGTGTCATTGAGAAACAAAACATGCATGGTACGCGCGCCCTTAGGGTATCGTCGCGGAACAGGTACCGTCATTCTCCGCAGAGTGGTGTGAATCACGTCGCGATACGCAATCTCCGAACGTGACACACCGTAACGTGCCAGTTCGTCCAGGTTGTGCGATACCAACACCATGTACTTTTCACGAGTGTCGTAGGCGTAGACGCGAGAAAAGCGACCCATAAAAACCACGTACGGGGTAGCCACCATGCCATCATGGTGATCGCGACGTGGCTCGGGCAACAAAATAACAGCGTATCCCAACGGCGTCAGCGGCTCGCGGCAACAGATGAGCTTTGACGCCGCCTGTTTGGCGGCGGTAATGATTCCGTCCTCCGTACGTAACATCACATGCCAGCCCTTGGGGGGACCCAAGGACAGACAGCGTCCCTCGTTACGATGAACGTAACGCGTGATTTCCATTGGCTCCAGGCAAAAGAACAGTTCCTTAAAATCCCGCAACACTTGTCGGTATAACGCCATGGGATCCTCAGCCGCCACAGGCAGCGCGGGGAGCTCCGGCGGCACAACTGCAGCGCCGTCAGGGCCAGAACCCGCAGCCGGATCCATCATTGCGCGACACTCTCAGCCGGACAACCGGCGTCACTGACAGAAGCCGAGCCAAATACAGAGAAAGCAACGCTACACCGTCACCCCGCTCCCAAGCGCCGCGGAAAGTGCTCCGATTTTTCACCGTCGTTCGCGACGTTGATTTGCCTCGGTCTGAGAACCGACCTAGCGTTCGGACCGGTGCGCAGAAACAGCCGGCGGTCCGAGCCACTGAGCGGTTCACAGCCCCGGCCGCCGATAGTTACCGGAGAGACGTTCGAGCTGCAGGTACATCGGCGCTCCCCGCTCCGCCACCCCGCGCCCGCCCCAGTTTATACTCTCCGACGCCCCGTCCAACGCGCCTGTGGAGGGCCAATCGGACCGCGGGAGCTCTCCAAGTGGATGACAGGCACAGCCGGGTGCCCGACCGTGAAGAGCCCTCATCCACCTGAACAGACCGCTAACCGAAGGACCCCGAATCGCGTCCGTCGGTCCCGACGTCCGTCGCCATCTGGTTCCCTGCTGTTGGCTACCTCTCGGATTTCAAAAAAGAGCACGTGCCGATGACGGTGCACAGGAAAGAGCCAAAGTGTCACGGCGTCTTTTTTTATTTGTGTTCCTTTCCTGTTTTGTACTCGTAAACTGTTGACGTTGTTTTTACATCCAAAAGGGCAAGTAAGAAACAGGATGAGGCATGGTAGGTTTGGGCGTGGGGCGGCCCTCCAGCACGGCGGCCCGGGCCGCCCGGCGGGTGAGCACCCGGCGTTGCGCCGTATCTATCTTGTGTTTCTTCTGTGTCTTTTTCCTATCTTGTTCCGCGACGGCCTCTTTCATCACGTTCAGCATGCGTTCCTCGACGCCCTCCAGGGATCCTGGGGAGGAGGGAGTCCTAGTGAGGCTTCCAATGTTGTTTTGTGGATTTTCGGTTTCCTTTTCTTGGTCGTCATCGTCGGACGTGTCGTCTTCCTCTTGATCCTCTTCTTCGTCCGAGTAGTAGACGCATAGTCCCTGGTTCATCAGGCTGGGATTCATCAGGTTCTGACGGGGAATCCGCTGTTGTAGACGTTTAACCGCCCGTTCCAGGCGAGAGCTCATGCCGCACCAGACGCTGTAACGCCGCACGGGCCCGTAGCGGGCTGTTTGTTCGCGTACATGATCGTTGAGCTCTTGCCAATATTGTTTGGCACACTCCAGATCGGAGGTTTGTGGATAGTCGGGTCGGATCCGCGGATCCCAACTGACATCGGCGGTGCCAGAGACTTCGTCCAGACTGTTACGCATAGAGCACCAGTCGGGTCGGACGATAAACCTGTCCTTGCGGATTAACCATTTATAACGTAGTTCGTGATGGCGTGTAGAGGCCCGTACACGCTCCACGGTCCCAAAGCGGTCCCAGAAGGGAAAGTTTTCGTGAGGGCAGCGACCCGGCACTTCCAGACGTTCGGCGTCGTCCACGGCGTAGTGAAAACGCCGGCCGGCCTGGTAAATTTTGAGCAGACCCACGGTTAACAACATATCCACGCTGTCAGCCAACCGCCAGATCTCGCGTCGAGACACGTCAAAATAGAAAAATTCACAGGCTCGGTCGACCAGGATCACGAAATCGGCGTGAAAGACGCCGGAGGGTAGCGACTCGCCCACCACACCCATTATCATGGTTTCGCAGCATAAGCGGTCCACAAAGAACTTCAACAGGTCGTTGAATTGCTCCGTCTCCATACAGATGAAGGGCCAGACGCCTTTGAGGTTCTCGGCCTGGCCGCAGAGCAGCAACGGACGCGTCATCTCGCCTGGAGTGCGCAGAGGCACGCATTCGCCGCGATAACGACAGGTCACACGCTGCAGTTCGCTGATGCTGTTGTCGTGCAGGCGAAGGTCGCAGATAATATGATCCGGTTGCGTGGTTAGCAGCGGCGTGCGCATCTGCTCGCCGTAGATGGCCTCGCAGTGCAACAGTCCGTGTCGCGCAAAATCATCCAGACTGTGCGCCAGGTAGTAAAGCACCCCGCAATCGCGGTCTAAACACCACACGGTTTCGTAACGTCCTAGCAGGAGCACCAGACGGGCCTGGCTAGGTGGCTCAATTTCCTCTACATATACGAAAAAGTCGTCATCGTCCGAGTCCTCGTCCTCAGAAGAGGACCGCGGCCCGTGTACTCTGGGCAACACGGTGGTAGAGAACTGCAGGACGCCCAGAGACTCGAGCGACTCTTCGCAGCAGATGAGCTGACCCCAGGGCGTTTCGGGCCCGTCGGTGACAGCCGCGCTGCCAAAGATGTCCTCAAACTCTACAAAATCTAGACGCCATCCGGGTGGCGCTGAAACGGGAAGGCTAATGTTCATATCAGCATAGCTACGAACTAAGTGGCGGATGTCCTGCCGCAAGTCTTGGCAGAGAATGAGCTTTCGTAAACCCTTGAGGGTCCTCCGAACAACGGCCCCAGACGCGTAGCGATAGGACTGGCGCATGGTGCCGCGGCGTGGAGCGGCACTTGGCAGCCTATTTTATGGAGTTTCTTCAGTGACGTGGCTTGTTCACGTCGTTCGTGGGCTGCGGTTGGCAGCTCCGGTCTGTAAACCACCCGAAAAGACTGACATCGACGTCAAAGACTCACGTAATTTGGAACATGTGCGACCGCAAAGTGCGTCAGAATAGCACGTGGCTTTAGGACATAAAAAGTACCGTGAGGTCTAGACGTGGTTTTTGTGATTGACACTTACACCAGGTAAGCCAAGGGACGGTGAAACTGTATGTGAGGAACCTGGGTGCTTAGACAACTAACGTGTAATGCTTTTTACAGGACCGTTCAACAGGTGATACTACCTGCAAGGTAATGACTACATCTACTACAACTACCACTAATATCATGCTACAGGTGAGCAACGTAACGAATCACACCTTGAATAGCACCGAAATTTATCAGTTGTTCGAGTACACTCGGTTCGGGGTATGGTTGATGTGCATCGTGGGCACGTTTCTGAACATGCTGGTGATTACCACCATCCTGTACTACCGTCGTAAGAAAAAATCTCCGAGCGATACCTACATCTGCAACCTGGCTGTAGCCGATCTGTTGATTGTCGTCGGCCTGCCGTTTTTTCTAGAATATGCCAAGCATCACCCCAAACTCAGCCGAGAGGTGGTTTGTTCGGGACTCAACGCTTGTTTCTACATCTGTCTTTTTGCCGGCGTTTGTTTTCTCATCAACCTGTCGATGGATCGCTATTGCGTCATTGTCTGGGGTGTAGAATTGAACCGCGTTCGAAATAACAAGCGGGCTACCTGTTGGGTGGTGATTTTTTGGATACTGGCCGCGCTCATGGGGATGCCACACTACCTGATGTACAGTCATACCAATAACGAGTGTGTTGGTGAATTTGCTAACGAGACTTCAGGTTGGTTCCCCGTCTTTTTGAACACCAAAGTCAACATTTGCGGCTACCTGGCGCCCATCGTGCTGATGGCGTACACGTACAACCGTATGGTGCGGTTTATCATTAACTACGTGGGTAAATGGCACATGCAGACGCTCCACGTTCTTTTAGTTGTGGTTGTATCTTTTGCCAGCTTTTGGTTCCCCTTCAATCTGGCACTATTTTTAGAATCCATCCGTCTTTTATCGGGAACGCAAAACGAGACTCTCCAAACCGTTATTACTTTCTGTCTATACGTCGGTCAGTTTTTGGCCTACGTTCGCGCTTGTCTGAATCCTGGGATCTACATCCTAGTAGGCACTCAAATGAGGAAGGACATGTGGACAACCCTAAGGGTATTCGCCTGTTGCTGCGTGAAGCAGGAGATACCTTACCAGGACATTGATATTGAGCTACAAAAGGACATACAAAGAAGGGCCAAACACACCAAACGTACCCATTATGACAGAAAACATGCACCTATGGAGTCCGGGGAGGAGGAATTTCTGTTGTAATTCGATCCTCTCTCACGCGTCCGCCGCACATCTATTTTTGCTAATTGCACGTTTCTTCGTGGTCACGTCGGCTCGAAGAGGTTGGTGTGAAAACGTCATCTCGCCGACGTGGTGAACCGCTCATATAGACCAAACCGGACGCTGCCTCAGTCTCTCGGTGCGTGGACCAGACGGCGTCCATGCACCGAGGGCAGAACTGGTGCTATCATGACACCGACGACGACGACCGCGGAACTCACGACGGAGTTTGACTACGATGAAGACGCGACTCCTTGTGTTTTCACCGACGTGCTTAATCAGTCAAAGCCAGTTACGTTGTTTCTGTACGGCGTTGTCTTTCTCTTCGGTTCCATCGGCAACTTCTTGGTGATCTTCACCATCACCTGGCGACGTCGGATTCAATGCTCCGGCGATGTTTACTTTATCAACCTCGCGGCCGCCGATTTGCTTTTCGTTTGTACACTACCTCTGTGGATGCAATACCTCCTAGATCACAACTCCCTAGCCAGCGTGCCGTGTACGTTACTCACTGCCTGTTTCTACGTGGCTATGTTTGCCAGTTTGTGTTTTATCACGGAGATTGCACTCGATCGCTACTACGCTATTGTTTACATGAGATATCGGCCTGTAAAACAGGCCTGCCTTTTCAGTATTTTTTGGTGGATCTTTGCCGTGATCATCGCCATTCCACACTTTATGGTGGTGACCAAAAAAGACAATCAATGTATGACCGACTACGACTACTTAGAGGTCAGTTACCCGATCATCCTCAACGTAGAACTCATGCTCGGTGCTTTCGTGATCCCGCTCAGCGTCATCAGCTATTGCTACTACCGCATTTCCAGAATCGTTGCGGTGTCTCAATCGCGCCACAAAGGTCGCATTGTACGGGTACTTATAGCGGTCGTGCTTGTCTTTATCATCTTTTGGCTGCCGTACCACCTGACGCTGTTTGTGGACACGTTAAAACTCCTCAAATGGATCTCCAGCAGCTGCGAGTTCGAAAGATCGCTCAAACGTGCGCTTATCTTGACCGAGTCGCTCGCCTTTTGTCACTGTTGTCTCAATCCGCTGCTGTACGTCTTCGTGGGCACCAAGTTTCGGCAAGAACTGCACTGTCTGCTTGCCGAGTTTCGCCAGCGACTCTTTTCCCGCGATGTATCCTGGTACCACAGCATGAGCTTTTCGCGTCGGGGCTCGCCGAGTCGAAGAGAGACATCTTCCGACACGCTGTCCGACGAGGTGTGTCGCGTCTCACAAATTATACCGTAATAAAAAAGCGCTACCTCGGCCTTTTCATACAAACCCCGTGTCCGCCCCTCTTTTCCCCGTGCCCGATATACACGATATTAAACCCACGACCATTTCCGTGCGATTAGCGAACCGGAAAAGTTTATGGGGAAAAAGACGTAGGAAAGGATCATGTAGAAAAACATGCGGTGTTTCCGATGGTGGCTCTACAGTGGGTGGTGGTGGCTCACGTTTGGATGTGCTCGGACCGTGACGGTGGGTTTCGTCGCGCCCACGGTCCGGGCACAATCAACCGTGGTCCGCTCTGAGCCGGCTCCGCCGTCGGAAACCCGACGAGACAACAATGACACGTCTTACTTCAGCAGCACCTCTTTCCATTCTTCCGTGTCCCCTGCCACCTCAGTGGACCGTCAATTTCGACGGACCACGTACGACCGTTGGGACGGTCGACGTTGGCTGCGTACCCGCTACGGGAACGCCAGCGCCTGCGTGACGGGCACCCAATGGAGCACCAACTTTTTTTTCTCTCAGTGTGAGCACTATCCTAGTTTCGTGAAACTCAACGGGGTGCAGCGCTGGACACCTGTTCGGAGACCTATGGGCGAGGTTGCCTACTACGGGGGTTGTTGTATGGTGGGCGGGGGTAATCGTGCGTACGTGATACTCGTGAGCGGTTACGGGACCGCCAGCTACGGCAACGCTTTACGCGTGGATTTTGGGCGCGGCAACTGCACGGCGCCAAAACGCACCTACCCTCGGCGCTTAGAACTGCACGATGGCCGCACAGACCCTAGCCGTTGCGATCCCTACCAAGTGTATTTCTACGGTCTACAGTGTCCTGAGCAACTGGTTATCACCGCCCACGGCGGCGTGGGTATGCGCCGCTGCCCTACCGGCTCTCGTCCCACCCCGTCCCGGCCCCACCGGCATGACTTGGAGAACGAGCTACATGGTCTGTGTGTGGATCTTCTGGTGTGCGTCCTTTTATTAGCTCTGCTGCTGTTGGAGCTCGTTCCCATGGAAGCCGTGCGTCACCCGCTGCTTTTCTGGCGACGCGTGGCGTTATCGCCGTCCACTTCCAAGGTGGATCGCGCCGTCAAGCTGTGTCTTCGGCGCATGTTGGGTCTGCCGCCGCCACCGTCAGTCGCACCACCTGGGGAAAAGAAGGAGCTACCGGCTCAGGCGGCCTTGTCGCCGCCACTGACCACCTGGTCACTACCGCCGTTTCCGTCCACGCGGATACCTGACAGTCCGCCGCCACCGTACCAGCTTCGTCACGCCACGTCACTAGTGACGGTACCCACGCTGCTGTTATATACGTCATCCGACATCGGTGACACAGCTTCAGAAACAACGTGTGTGGCGCACGCTACTTATGGGGAACCCCCGGAGCCCGCTCGATCGACGGCTACGGTTCAGGAATGTACCGTTCTTACCGCCCCGAATTGCGGCATCGTCAACAACGACGGCGCGGTCTCTGAAGGCCAAGACCATGGAGATGCGGTTCACCATAGCCTGGATGTGGTTTCCCAGTGTGCTGCTGATACTGGGGTTGTTGACACCTCCGAGTAACGGGTGCACCGTCGATGTTGGACGAAACGTATCCATTCGAGAACAGTGCCGCCTTCGAAACGGTGCGACGTTCTCCAAGGGAGACATCGAAGGTAACTTCAGTGGGCCCGTCGTCGTGGAGTTGGACTACGAAGACATCGATATTACTGGCGAACGGCAGCGACTTCGGTTCCATCTCAGCGGACTCGGGTGTCCTACAAAGGAAAATATAAGAAAAGACAATGAAAGCGACGTCAACGGTGGAATTCGCTGGGCTCTATATATACAAACCGGCGACGCCAAGTACGGTATTCGTAACCAGCATTTGAGTATACGGTTAATGTATCCTGGGGAAAAAAATACACAACAGCTGTTGGGTTCTGATTTCAGTTGCGAACGTCACCGGAGACCGTCCACGCCGTTGGGAAAGAACGCCGAAGTGCCTCCCGCGACCCGCACGTCTTCTACATACGGCGTCCTCAGCGCTTTTGTAGTGTGGATCGGATCCGGCCTCAATATCATCTGGTGGACCGGCATCGTGCTTCTGGCGGCGGACGCTCTCGGACTTGGCGAGCGTTGGCTGAGGTTGGCACTGTCCCACCGGGATAAACATCACGCATCGAGAACCGCGGCGCTCCAGTGTCAACGCGACATGTTACTTCGGCAACGTCGACGGGCTCGGCGGCTGCACGCCGTTTCTGAAGGCAAACTGCAGGAAGAGAAGAAACGACAGTCTGCTCTGGTCTGGAACGTTGAGGCGCGACCCTTTCCGTCCACACATCAGCTGATTGTGCTGCCCCCTCCTGTAGCGTCAGCTCCTCCTGCGGTTCCCTCGCAGCCCCCCGAGTATTCGTCTGTGTTTCCGCCTGTATAAAAATAAAGAGACGGGAGGCTGATCGCGGCCTTCAGCGTCTCATTTGTCTTTACTCTCGAGTGCGGTCGGTGTCTCATCGGTGAGACGAGGCCGCCGCCCGACAAGTTCGATCTCATGTCGCTCTTGGAGCGCGAAGAGAGTTGGCGTCGCGTAGTCGACTACTCGCACAACCTGTGGTGTACGTGCGGTAACTGGCAAAGCCACGTTGAGATTCAGGACGAGGAGCCCAACTGCGAGCAGCCGGAGCCCGCACACTGGCTAGAATACGTGGCGGTCCAGTGGCAGGCCCGGGTTCGCGATTCTCACGATCGCTGGTGTCTCTGCAACGCCTGGCGTGATCACGCCTTGCGCGGCCGTTGGGGTACGGCGTATTCCTCGGGTTCCTCGGCCTCTTCCTCCGGTTTCGTCGCGGAGAGCAAGTTCACCTGGTGGAAACGACTGCGCCACAGTACCCGGCGCTGGTTGTTTCGCCGCCGGCGAGCTCGATACACTCCGTCTAACTGTGGGGAAAGTAGCACTAGCAGCGGCCAGAGTAGCGGTGACGAGAGTAACTGCAGTCTACGCACCCACGGCGTGTACACACGGGGTGAACAACACTAATCGATAAGTCGCGTGTAGGCGACTGGCTACATCAACCGGATATCTGCGGGGATTTAAAAAGACGACCCGTTGTCATCCGGCTTAGAGCAAACCGTCCTTTTATCATCTTCCGTCGCCATGGCTATGTACACATCCGAATCCGAACGCGACTGGCGTCGTGTAATCCACGACTCGCACGGCCTGTGGTGCGATTGCGGCGACTGGCGAGAGCACCTCTATTGTGTGTACGACAGCCATTTTCAGCGACGACCCACGACCCGAGCCGAACGGAGGGCCGCCAATTGGCGGCGACAGATGCGGCGGTTACACCGTCTGTGGTGTTTTTGTCAGGACTGGAAGTGTCACGCGTTATACGCCGAGTGGGACGGCAAAGAATCCGACGACGAGTCGTCGGCGTCTTCCTCGGGCGAAGCGCCAGAGCAACAGGTCCCCGCTTGGAAGACCGTGCGAGCCTTCTCGCGGGCCTACCACCACCGCATTAACCGGGGTCTGCGGGGCACGCCCCCACCGCGCAACTTGCCGGGATACGAGCACGCTTCCGAGGGCTGGCGGTTTTGCAATCGACGGGAACGGCGAGAGGACGATCTTCGCACGCGGGCTGAGCCGGACCGCGTGGTGTTCCAGTTAGGGGGAGTACCTCCTCGCCGTCACCGGGAAACTTACGTGTAAGAACACGGCGTGACAATAAACAACATAGCGTAAATCCCCGTGTGATGTGTGTGATTGACGTTCGGGAAACATGTCCCCATCATCAGCGTCACAACTGACGTGGGTTGGTCACTGACGTGCAGGATGTTACGCGAGTCAGAGAATCGCATAAGAACGGGGTGGTGAGCGGGTTCCCACAGGAGTCTCTGGCGCAAAAGCACCATGAGCCTCAGGTTCCCCGAGAGGGCGGGTTACGAGAAACTGGGATACCGCCCGCATGCCAAACGCGTGTGGGTGCATGACCCGTTGGGATTGACGCGGTTTATCATGAGGCAACTCATGATGTACCCGCTGGTGTTGCCGTTCACTTTTCCGTTTTACGTGCCGCGGTCCTAGCACGTCAGTGGTGACGCTGATAATTGCAACATGGCCCATGACGAACCCGCTTGGGACGAACGTCAATACCACGTCAAACCGCCGTGACTTGGCTGAACGTTGAAACATAAAGCCAAAGCGCCGTCGGCACTTGGCTTCAGAGCAGCGCCTCGGGGCGATGCGACGGCGATGAACTTAGAGCAACTCATCAACGTCCTTGGTCTGCTCGTCTGGATTGCCGCTCGTGCTGTCAGCCGCGTTGGTCCGCATGGCTCCGGACTCGTTTATCGTGAGCTTCATGATTTCTACGGGTATCTGCAGCTGGACCTTCTGGGACCAGTGGTGGCGGGGAATCGCTCAGTCCGGACCTGGAGAGAGCAGGCGGACCGAGCCAGAGGGACCTTCGTTCGGCGTTCAGGCCTTAATACTAGCCACATCTTACCTGTCGGCGGCCTGTCTGGGGGCTCCGGTACCTTACCCGCCGGCCTGTATCGTCCCGAAGAAGAGGTGTTCCTCCTCTTGAACCGCTGCCATGGGCCACTGTCAACGCCGAAAAGCGCTTGTCTGGCTGAGGTTGGTGTCGCTAATGCCAGTTTTTTATCTCGCTTCAATGTCGGTGATTTTCACGGAGCGTCATGGGAAAACGGTACCGCTCCCGATGGAGAGCCCGGGGTATGCTGAAATTCTTCTTAAAATTACGTAAACGACGTCGTCCAGTCGTTGTGCCGCGATTCGTACGGTTCATCGTCTACGTCGTTTTGTTCACCGTCGCTGTGCAACGCGTGAAACAAGAGCGTGATGCGCACCTTCGGCGGTATGAAGAACGATTGCAGAAAAACCGTGCACGGCGTCGGCAGAGTTTTCCGTGACTTGGGGCGGTGGGTCCGAGCTGCGGTATGGGTCACGGCGGCGTGCGTCTTATTGACAAAGATGCCGATGTGTGACTAAAAAACGTCCCAGCCCCAGAGCGATGTGTTTCAATAAAAATTATGTAGTATCATATTATGCGTGTCCTGGTTTTTCATTTTTTGGATGTATTTGTCGCATAAAAGGCGGTGGGATGTGGGGATGAAATATATCCAGATACGCAGTTTTGTTATCCTAACAAAACCCGTGTCATGCTAAAAACGGTAATGCAGGATGAAAGTCCCGTGTGGGGGGGGGGGGCATATAGTAGTCGCTTTTACCGCTGGGCATACGCTATGCTTGTATTTGTGACTATACTATGTGCAGTCGTGTGTCGATGTTCCTATTGGGAAGGGTGTGAATGTAGGAGGTATAAAGAATGGTGGGACGCAGGAAGACATCGCTAGACACAGCTGTATTGTGCTAGCCCCGCTTAGCGTCATGGGTAAAACGGTGATAAAGCGTCAAAACACCATGGGGGGGGGGGGCAGGAAGCTTGGTGGCGGTGTTCATTAAATGCATTCTGTGTATTTATTGGTACATTTGCAAATCGCCGAGTATACCGGTATAATGTGGCAATAATCGTATTGTGTGTACTATGCAGCGAAGAGGACAATATCAGTGTAACGTGAAGCAAGTGTGGGAACAATGCCAGTGAAGAATATATAAAAGCCAGAAACATACATAAATGAATTGCTAGATACAGGGTTCTTTGTGTACTAGCCCAATCATGTCAATACAATGTGTACAGAAAAAAGACGCAACAACGACGGGGGGGGGGGGGGCGTACGATTAATTCTACATATTACAGTACAGGCTACCGCGTGGTGTTGTGTATGTTACGATGCGGTGTGCCTGTCGGTGTAATTGGATGTATGTTACTTATCCGTGGCGTTGTGATAGTGTTGTGAAAAATAATTGTCGTGAGCATAATGACAGCTGCAATATAAAATCACTTTATTAAGCATTGTAATGATAATGCGTGGCTACATTAGAAAACGTGACGCGTCGCATGTCGCGGCACAATCTGGCAGCGGGGTCGGGGTAGGGTACGGTGGGAGGCATGTACACAGATGGAACAAAAGCAGAAGTAACGTGAGACGGAGCATATAGTCCAGTATCCAGCGGTTCCTGAGTAGCACCACCCATCAACTGAATGCCCTCATGAGTAAAAGTCTGCGGGCGGCAGCCCTTGGGGACCGTTGGCATGGGACGATCAATCTCCAAACCACAGCGTAACACCGTTTTCTTCCAACGTCGTTGATACACGTCGTTTTTACGGTTACTCCCCAGAACCCAGAAAGTCTCGTCCAAGTCGTACCAGGAATCTTCTCCAGGGACACGCGACGGTTTCCAATCCTCGTCGTCTCGTCTCAAAGCACGTCCCAAACTGGCTTGAGGAGTCAACGGTGGTTCTGTGGGTCGGGTGTAGCGCGAGTGCTTTCCCTTCATGACCGATTCGTCTTCCTTGCCTTTAGGCTTTTTGGTCTTTTTGTGTATCATCTGGCCACCGGCTTCCATAACCACCGTGGCCAAGTCCAGTCCCAGAGCTTGAGCGTCGGCGCGGCGTCTGGCGTCTTGCAGATAGTCTTCCACATTTGCACAGATGACCGGATGTTTGGTGGCTAGGGTGAGCACCTCAGCCTCGCCGCGGCCCGGACGTAGCAAAAAAGCTAACTGCCCGTGCGGCTCGCGCGCCCACAGCGCGGCGCGCGGGTGCAGGTGCAGCGCGTCCCAGCGCGGCCGCTCCCACTGCTCGCGGTCCAGCTCGGGCAGCAGCCGCCGCGCGGCCTCGGCGGCGGGCGCCGACTCGCGCCCCAGCGCCAGCGCGCCCAGCACGCCCGCGCGCAGAAAGTGCGACAGCTCCGCCGCCAGCGGGTACACGTGCCCGTCCAGCGGGCAGTACCCGAACACGGCGCCCAGCTCGTCCAGCACCACCACCAGCATGGCGCGCGGCACGGTCCCCGACGCCGCCGGACCCGCCATCGCCGTCGGACCCACCATCACCGTCGGCGCCGCCGCTGCTGCCGCTGCCGCCGCCGTCGCGTCCGCCCCGACCACCGCGTGCGCGTCCGCGTTTGGCACGCAAATCGCGCTCCCGCCGGCGCCTCCGTACGGCTGCGGAGGTATAGTCACAGCAGACCCCACGGCTCCTGCCATCGCGCACGGCGCGTCCCCGCCGGCGGCCTCCGTCTCCGTGCCGCTCGCCGCTGGCGGCGACGTCGTCCCCGTCGCCGGCCCCGCCGCGCAACCCAGCCACCGCGCGGGCAGCACCGCGCCCAGCGCCAGCCAGCCGCAGCACAGACGCTGGTTCAGGTGCCGACGCACGGCCGTCAGCAGCGACGCGGGGTGCGGCGCCGACGCGAACGGCTCGTACTGCGCCAGCTCCTGCCACGCGCCCAGCAGCACCATCGGCTGCAGTCGCCTTCCCGGCGTCTGTAGTGCCACCGTCGTGCCGGCCCACCGCCGGCGCAGCTCCCGTCCGAGCGCCGTCGCCTCCTCGGCGCGCAGCAACGTCTGTCGGAGCGCCGGCTGAGGCAGCAGCGTCGCGCGCGGGGTGCCCACGCCCAGCCGGTTGCAGCGGTACAGCCGCACCACCTCGCCCGCGCCGTGCCGAAACCACTCGTCCGCGTCGCGCGCCGCTAGGATCAGCGTGTTGTTCGCCAGGTCGTAGACGAACACGCGGAACCCGGCGCCCAGCGCCAGGTACAGTCCGTCCTGCGCGCACAGACCCTCGGGATGGCCGGCCTTGTCGCCCACCGTCGGGTCGGCCGCGGGGTCCACCTCGTGCACCACGGTCGCCACCAGCACGATCCACGCGTCCCGCGGCGACAGCTGACGCAGGTCCGTCGCGCCCACGCCGTTCATCTGGCTGCGCGGCGTCACCCGCGCGTAGAATCCGTACGGCCGTCCGAGCGGCAGCAGCGTGCCCGCGTCGCGCTGCGACCACTTGCGCATGGCGCGGCCCGTGCTGTTGGCCAGAAACGCCGCGCGCCACACGGCGCCCATGGCCTGGTATTCCAGCTCCGTCAGCGCCTGGCGCTCCACCGGAATCTGAGACAGCAACAGGCGCTCCGGGCCGTGCCAAAAGTTGCTGTTGTTGCCGCTACTCGGAGGGGCGCCCGGCGGCCCGCGGGGTTCTACCCGGTGGACGCCGTGGCCCGGCGTCGCCGTAGGCGCAGCACTCGCACCAGTGCCCGCTGTTGACGTCGCTCCCATCGGCACACAAGAAGAAGGAGGAGAGGAACCAACCCCCGAAGGCCCTCCGGCCCCGCGGCCGCGACCGAGGGGCGGGGGGCGCGGCGACATGCCGTTGCGCTGGGCCATGGCCGCCGGACACCTCCGACGTCCACTATATAGGAAGCAAACCCGCGTCAGCGACCACGCCGTTTACACACGCGGACGGCTCCGTCGTCCGTGTGCCACGGCAGACACGCACCTGGCTTTTATAGGCAGCGACGTGTATGGCGCTTGCTGGCGCCGCCTTGGGGCCGTGCAGTCTGGAAGGCCGTGGAAAACCACCCGGCAGCCGACGACGCACTCGACTGGAAAAGGCAGGAACCCGGAGGAACACGCGCAACAGGGCCGCGGACTGGGTCCCCGAGCGCGCACCCTCCTAGGTGGACGCCCGACATCCATTCCGGGCCGTGTGCTGGGTCCCCGAGGGGCGGGGGGGTGTTTTCTGCGGGGGGGTGAAATTTGGAGTTGCGTGTGTGGACGGCGACGGCGACTAGTTGCGTGTGCTGCGGTGGGTACGGCGACGGCGAA

>HHV6A_STRIPPED4

CGCATAACTCTGTTCACAACCCTAACACTAATCCTCGCATCCGCACCTAACGCTGCCCCTCTTTCACACCATCACCCCCTGCCGCTTCAACTTCACCTTCTTCCTCCATCTCGCCCCGCTTGTTTCTACACTTGTCCACCCCTCTATTCTTACTCTAGGATGCCGCTAACGGTGCCATATCGTCTTCCGCTCTCCAACTATTGGTGGCTACTCTTGGGTCGACATTCCCTTCGTCATGTTCATTCCTACGTGCGTCTGCACAAGGGTCTACGCATTCCTTTACCTTGGCCCGAGCAGGAATGCCTACATTTACATCCTAAGCCTTACAAGTGTCTCCTGCGTTACCCCTGTATAACAACACAACCGCATCTTCTTCAGGGCTGGCCTACGAAGTCTTCTCTATGTGAGTGTCATATACATTTCCACTTTTTCACATGATTTATTGCGTTTTGTCTACTACACCGGGTACATTTTGGTTTCCCTCAGGGTTCGACCCTAAACCCTACCATCCTTCGGCCGACAGCAAGTTGCTACCGCTGGGCCTGATCACGCTGTCCGCCTGTTCCACGCGCGTGTCGCAGCCGACACACCGGAGCGGCTTCGACGTGACAGATCTGTCTCTCAGCTGGCTGACGGGATCGTCCCCTTGGCTCGTAATCCTACAAGGGCAGGGGGGGTCTCTGTTCTGCCACGACGTGCTGCAAGGCCGACTCTATGTCCTGTCGCACTCCGTGTCGCTCTTTCTAAAGACGGGCCTCCGCCACTGCGAGGCCATCTATCGCGCACCGCTGTGGCGCGACCGACCCCTGCCGAGCCTGTGGACGTGCCGGGACCCCGACAAGGCCTTCTTACCGACATTACTGGCCAGAAGCGCCCGACGCGGCCTGGCCGCGTTCTACGCCCTGTGGAGACTGCATCTGGGATCGCGCTCGGAGCTCTCCCACCCCGTGTTGGAGTGGGAGAGAACAGAGCTAGTCCTGACGGACTGGAGACGCGGGCGGCCGTGTACGCACCTCCCGTCCGACTCGCGGTCCCAGCGCGCTACGTCGAGTGGCGCCACGAACACGCGGGACGCGGCGACAGAGGAGGCGGCGGGGGGGAAAAAGGAGGCAGAGGGAGGCGGGCCCAGCGTCGCGGAACACTTCGCGAGGTGCAGACCGCTGCTGGACGAACTGTGCGGAGAGGGCGGCTGGCTTCCCTTCGCGTTTCTCTCGACATCTGCGCACGTCTGGCTGATCCTAACGGAAGGAGGCCCCGTCCTGGCGGTCGACCTGAACGACACCTCCGTGTGGCGCATCGCGGACGACCTGGAGCTGCTGCGTCGCCTGGGGAACCTGCTCCTCGTCTCAGGCCTCCGGCTTCCTCTCCATCTCCCGAGCGGGAGCGGCGAGGCGGCCGGAGAGCCGGGGTACGACCAAGAAGAGAGAAGAGGGAGAGCGTCGACGGCGAGCGCGACGGCCGCGACGTCGACGCGCGGACCGACCCGTCCGACACGGGTGACGCGTAAGGGGCGTGTGGCAACCGGAGGCGTCCATCTCCCCGCACGCCCCGAATCTGAGGAACAGACAGACGGCCACCACGGGCGCCAGGAGAGCAGCCACGACGACCAGCGCGGCGGGAGCGAACGAGGACACCGCGATGACGGCGCGCACCGCCACGCGAATGAGAAAACAGAGCCCCAGCAGCGCGGAGAGCACGAGGAAGGGAAACAGACCAACTCCGGGCGCCACGAGCACGGACAGGAGAGCCACGTCGCAAGAAGAGACGAGGAGGGAACGGAGCAGGGCGGTAGCGGAAGGAGCTGCGGGGGAGCGACGCAGACGTACGGCGGCAGAGGCCGACATGATTCCTGCCCGTCGATCCCCCTGTCCGTCCCCGGGCCCGATCCCCGCCTGTGGGTCCCGCCCGCTCATCTGTTATTCCCTTCCCCTCTGCCACCGATGACGCCCGTCGACGACGAGCCGTCCGTCCGCCCTCGGCGCCCGCCGAGTCCCGCGGAGGAACCCCCCACGTGTCGTCCCCGTCCGCCCCGCCCGTCGTCCGACACCCCGCTGTCCGCTGTCTCGCGCCCCTCCGCTCCGCCCGTCCCCCCGCCGTCCACTGCGCGTGTCCGTTTCTTCCTCTCTTCCTCCTCCCCCTCCCCGTCCTACTCTCCCTCCCCGTTGTCTCCGCCCTCTCCCGTCTCTCCCTCGTCTCCCAGATCTCCGTTCATCCCCCCCATCAGATCTCCGGGACTCCGAGCGAAACCGCGAGTGTCCTCCGGGCATCCCGCGACGTTCCCGCCGGCGCCCTCGTCCGCGCCGGCCCGTTTCGAAACAGTCCCGTCCGTTCCCTCATCGGAATCTCCGTCCGCGCCGTGCGTCGGAAAGTCGCAGCCGCCCGCCGCACACACGGCGTGAGACGCCGCGCGAGGCGACGGAGGCGACGAAAGAGGACGGGAAGAACAGGCGGAAGAACACGAAGACGAAGAGGAAGAAGACACGAGAACGAAATGAGGGGAACACGCCGGGGACCGAGCGGCGGATGGAGCCCGCTCGGTCTCGCACTTCCGCGATACCCAGCGGGCTCCGTCGCCGCTTCGGACCCGAGGTCCGACACGCCCCCACCTCGCGCACCGCCTCCGCCACCACCGCTGACAACCTCCGCATATCGCCCCCATACACACCCAGCAGCAGAGTCCGGCGCACGCACACGCGCGGAGCACGCACGAGAACACGCCAGACATCGACCGCCGGAGGTAACGGAGCCCGTGCGCCGTCCCGGTTTCAGCGGGAGTGTGTGCGGGCGTACCGTCACACGGAAAAGCCAGCGGGACGAAACGATCGACCCACTCCCGCTATTCGCCCGATCCGCCCCGATAAAAACAAGTCATCTCACTCCTGCCAGTTCACACGGACGCGACCGATGACAATAAAAAAAAAACACACCCCCCACCAGCGAACGCCCGCCGGCCCATCCACCTCCCCTCCCACCCCCGCTCGAGTCGCGCAGGCGCGTCGACGGGCAGTCCCTCGGACGCCTCCACGGCAACGCTCCTCCCCTGTCCCCTCGGTCCAACCCCTCCCATCCCCCTCCCCGGAGCACACACGCCGACTCCGTCCGCAGAGGCGGCGACCGCAGCGGCAGCGGAAACTGTAACGGCCACGTTCTCCGAACAGCGACAGTAACGGCGGCGGCGGCGGTTGTCGCCGTCACGGTAGTCGTGGCGGAGCACCCGGGGAGGCGACGACGGGCACTACCGGCGCCGAGATCATCGCGCTACGAAAAGAGTAGCCAATGTAAAAAGTTAAAGATTCTTTGTATATCAAAAAAAAATACTACGTGGAAATAAAAAATAAAAAAAAATATACAATATAAAAGTGTATTATAAAATAAAAACAAAATAAAGACTAGACACCGACTAAACGCTCATCCTTCTCCGTACGTCCCCTTTCCGTAAGTGCCAAATGCCGCATATCCATGAACGGACACACGCCGGTAGAATCCGACCTTCCTCACAACAGGTAGCCCGCGACCTAAGCACAGGACGGTACCTGAAACCGAAAGTATAAAGGGCGGCCTCCCATGTTCCGCCCATGAGGCGGGACTTCGAGACGAGCCACCCTTCGTATACGTCCGGCCCCTCCCACGCGCGCCTATAGGACGCACCCCCTCGTTCCGAAACCGCTAAAGGGTACAGTCGCCGTCACACCCCCTGTCCCTAGCTCGCGACGGGCCGGGGCAAACCCTGCCGCGGCGGCAAAGCCGAAACGAGACCGCGACGTACGCGGGCATAAAGAACGAGAAGTCAACGCGCAATCCCAAATTATCGCGAGACTGTTTGCTCGGGCCCGCCCTCGTCCTCATACCCTCTCCCCGCCCTCATGCCCTTCCACACCCGGTAGACAAAAAAAAAACACGAAACATACATTAGGTAAAGGTAATTTTCGCGGTTAAAAATGTTTCCACGTGACGTCCCCGGCGATTCCCGGAGATGCGACACGCGACGCATTGGCCCGTTGACATCGGGGTCCGCATACGCGGTTCCCCCGGGGCGTCGCGACGTCGGTGCCGCAGAAAACACCGCGGTCCGCCGTCACCCTTCAGTCACGCGCTATTTCCGCGATCGTATAATTTATCTCCACCGACACGGGTTCTGTCTGTCACTTCTCACCGAGCCGCTCCACCACCAACAGTGTCACTGACTACATCGCATACGTGGTGTTTTGCCTGTTTTTTGTTTATCTCTCCACCCCTCCCCGTCGCAGACCAGCGAACACCTTGCCACATCGACACCGACTACACCCGACCTCGCATGACAACGGTCGCCGGTCGCAACGCAGCTGCAGCGGGCGGCGAATGAAAAAAAAATAACATAAATATACTAAAAGAGAGGCCCCCGTCCCACATTAGGACATAAGAGCAGGCAAACAAAAAACCCGTCCGTTATTCACGCAGCATCCGTAAAGCCATGTCGACGCGACACACAGAGAGGACGGGCGGACGCATCCCGGTCCGACGCGACAGGGCGCGACTGGCCCAGGCGCGAGCCCGTGCTCGCTTCGAGTGGCTTCTCCTGGCCCGCGGCAGGCCGTCCAAACTCTACGGCTATGCGAGTCGGCATCGCGGAGAACTGATCCACCTACCGTGGCCGCCGTACTGGTGTCTAGAACTCCACCACGATCCGTACAGAGACGCCCGAAGTACCACCGTGTGGGGCCACCGCTGGGGTTGGCCGCCGACGCACGTGAGACCCAGATGCGTTCAAGACTGCGGTGAGTGGACGTACGGGCGAGCCGCGGGGAGGGATGTCGGTCAGAGAGTGAGTGGGTACAAGGTTCGTCGACAACCACCAGAGACACTGGCCGGTGGACAGTGGGGACAAAAAAAAAGTCACGTCAGGGGCGGAGAAATATAGGGTACGCAGACCGTCAAACGGCGAGTAACGGAAAACGAGTATAAAAACAAGGAGGGGGGAAGAAACATAGTCAAGTCTGTGGCGCGCGAGATCGTCAGCCGGCAAAGTCGGTGACGGCCTCCGGCCCCAGAGACGGCTCGCGGAGTGGAGTCCAGTTATTGTTATGTTTTTGTAATTTTTTTTTATCCACAATCCGGTATCCGTGAATCCCCGCGGAGGTACTTTCCATTAACGATGGTACAGGCGGTATGACTTCCGGAACACGGTGATTACGGATATCCTGTCTCGTTTACGCGAGACCTCTTTGTTTTAATCTCCTATAACGGTAAAAAAAACACAAAAGAAAGATTAGAGCTCCTCCTTTTTTATGCGTTATGGCATCCTCGCATCAGTCACCATTCCTGTGGCGTGTGCTTTTCGCTTTGAATTGTACGCGACAGGATCCGAATCCGTTATACTCCGCAAGGGCATAGCGACCACGGCTGTATGCGGCATCTACCGTTGCACGGGATGCCTTTGCGTGTGCAGATGTGTTGTGCTTTTTTTATACGGTCAGAGACCACAGACAAAAATAAGGCAACGCCCGCCATAACGTTTATGGTTTCTTGTTGTTTTGTTTGGGTTATGTTCTACGCCTCTTCTATCGTGTCGGCCGGATCCATCATTTTCAATCGTTGACGTACGCGCGTCCCATCACAGCTCTAGACTCGTGTCTGTACGTATGTTGCGGATACGGAGAGAAACTTCAACCCGTGGGTTTCGTAAAGTCGTATGTAACCAACTCCCAGCTCGACACGCTCCGCGTGCTCCTGGTGGGCAAAGACGGAGCCGTGTACGTCCACCACATGAGGGCGGCAAGACTCTGCCGACTGGCGTCGAGCACAACGGAGTTTACAAGACGAGGGCTGCAACGCGACGCCGTGACGTATGAAGAAGACCTAGAGCTGCCGGACCAGCGTATGTGCGGAACGAACGCCCGACATCTGTTCGACGTGATCGCCGCGGCCGCCGACGAACACAACCTGCTGACCGTCGGCGGCCTGTGTCAGACGCACGCCGGAGTGTGCTGCAACTTACTAGAGACCGTGGGAGATCCGTGGACGGCGGTTCCGGGCGCGCGCATGACTCTGACCGTGCCGCAGGTCCAGTACCGATTATGGCCCGAAGCCCGGAGAGACCTCCGCCGGCACCTGTACGCGGGACATCCCCTGGGACCGTGGCTCGTGTGCGGCGTTCTCTCGAGAGAGAGAGAGACGCAGAGGCCGTCGCCTCCCATATGCAGCGGCGGCGCGACTGTAGGAAACGTGGCCACGCCGGGGCCACGCGAGGTGGAGATCGTTTGGGTGGTCTTGACTTTGGCGGGACCGCTGTTAGCGTTCTGGCCCGATACCGGCAAGATCTGTCGTCTGGCCAACTCGTTCTCCACTCTGTGGAGGATGGGACCGCGGGCCATGAGAGGACACTGGACGTATTCGGCTCCGGGTAGACATCTCCCCGGGGATGCGTGGCCACTGTGCGAACACGTGAGACCGCAGGTGGGAAAGCTTCCCAGGAAGAGAGCGTACCTGGATTAGACGTCACAGAGGTGACGTAAGCATGTCACCTACGGCCAGAGACGTGACCGGAGGAAAATCA

ACTCGTTTTCTTCCCCCCTTCCCTTTAACCCCACGAAAAATAAAATCCACGAACGTAGACAGTCACACACAGGGTGCTTACGCTCCGTGTTACTGATGGCAGGTAACCAAAAAAAGTATGTGTCGGGCGTGATGCACCGCCGCTCTATAGCATAGTCGCTATATCAGCGCCCGATGCCAACGCGACAGGGTGAGTCACATAGATCGGGACTGCTTCAAAGCGCGTTGCGTTCGCCTCTTATATAGGCACCCCGGGGGGTGGGAGGAGTGAACATCCAAAGGAGGTGCGTCCGGGATTGGATGCGAACGTACAAAACCAACATCGTCAGAAAAAAATAAGAAAAGGCGGAGACACATAGCCTTGGCGGGAAGACGACAACAGGTTTAAAAATAGACAGTGAAAAAAATAGCAGACTGTGACGTCCAATACACATGTGGCGATCATCCAATCAACGGGGTGTATCGAGGCGCAGAAACAAAAGTATGCGGAAGTACACACGACACGGTAACGCGGACCGAAGACAGAGGGCGATCGCTAGCATGGCAAGTATAAGAAAAAAAAAAACCACGTTCCAAAAACACCTATACGGGAAACATCTCTTCACTCCCTCCCCGTCTCAAGCACCGCAACATTCACCTCCGTAATCCGTTGTCTCCGCGTTTCTACCACAGACGCGTACACACGCAGACACACAGACACGACAACCCACCCATGTGGTAGTCGCGGGTGGGTACGTAGATGGGGCATACGGGGGTCAGACTTGGAGACAATTCAGAACGACATCACGCGCGAGGTGCCACCGTGTTGGACCCACGATCGCCTGGCACGGTGCCAAAGGAAACCGCCGGCTGACCCATCCCCCAACGCGCGCGCGCACGCCGCTATGGGAGGCGCCGTGTTTTTCACCAACACGCGCGCCGCTGCGAGAGACGCGTGGAAAAAACTCCCCTCTCGGTGCGGCCCGAGTCGCCCGCGCGCGGAAGACACGCGTGTCGTGGATGCGCGAGCTCCCCCCCCCCGCACCCCCGGAAGAGCGGACGACGGTACAGGGGCGGACGGCGCGTAGTTTAAAGGCGAGGATGAGCGCGAAGAACCGATGGCGCCGGCGAGAGAAAGAGAGAAAGAGAGAGACGGGAAGCAGAGCCGCAGAGGGCAGACGAGGAGACGCCGGAGGAGAGAGAACGGCCGCGGTCGTTCCCGAGGAGGGCCCGCGCGCGGCGCAAGAGAGGGTGGAGAGCGGACGCGCGTAGAGACGCCCCGCCGGGCGGTTGGTGACCTTTCCGCAGACATTTTTGCAGACCCCCGACCCGTGTCACTGATGCAAACACGCGCGCGAACGTACACAGACACGCCGACGCGCCCCCACCGAGCAGACCGCCACGTGAGCGAAAACACACACGCGCGCGAAAAAAAAACACACAAAAAAATACACACACGCAAAAAAATACACACACGCAAAAAAAAATTATGTATGCATTGTTATCACGATGT

ACGAGGACGGAATCACCAGATGTCGACTCCTCCGTCTCCGTCTCCTCGTAATCCCCACGAACTACGGTAGAAGCTCCGCAAGACGCCACGGGCATTCCTCGTTCTACCATGTGCGGAGAAAGTTGATAGACGGCGGGCAGATCTATCACAGGCGCGTGAACGCAAGTCACGGGCAGCTGCATGGTCCACAGATGTCCCGGAGGGAATCGATGGTTCTTGAAGAGTTTCCTCACACCCATGGCGAGAAACGCCCTGAAACTATCGGAGAGACGCACCACGGCTCCCCCGTCGAGACTGGCCGCGTACACCTTGCCGTCCTGATCCATGAGCACCCGCTGTCGTCTAAGCAGAGGTGAAGTGACGCCCCGTCCGAAGAATCTACCGATCACCACCGCGACGCCGCCGCCGGGTATGAGCCGCCACCTCTTCTCCGGCCTCCCGGGTACGGCGAACTCAAAGAAAGCCCCGCGCGGCCAAGGCAACGGCACGAGAGCCTTATCGAAAGACACGGCGAAAGCCAGGGCGTCGTCAACTCCACTCATGCCGCCGAAGAAAGAGGCGCCGGTTTCGGTGAGACACTTGGAACCGTAGACGGGCTCGAAGCGACGCAGGCCGTGTTTCCAGAGACCGAACGCGTCCTCGGCGAGACGATACAGCGCGTCGTCGAACACACCGTTAAACGCGTAGATCTCCCCGCACTCTCCGACCAGCACCAGCGCGGAAACTTCTGCGAAGATAGTGACCCCGATGACCGTGAGATCCATACCGGGACAGCACCCGTATCGGCGAGACAGCTTGGCCACCTCGGCCTGCGTGATCCCGAGCATGTCGGAGGTGGCGAACCTCAGTTCGGCCCCCACGGGCCACGGGAGCGCCAACCTGCAATGTGCAAAAGTAGAGACGAAGTCCCACACGTCGTCCGCCGACAGCGGCCGGGTGACGAGAGTGCGAACGAACGCCGTCCATTTAACCTCCGGGACCTCCGCATCGCACCGCAAGTCACGTCCCGCAGAGAAAGAGCACGAAGGAGGACACAGAGCCGCAGACGCCAGACTCCAAGAAGATATGCCGAGAAACGGAGAGCCACAAAACATTTTATTTTCTGTGCGGAATCCGAGATAAAATTAAAAATGGAAAACAAAAATAAAAAATACAACGATAACGACAGCGTTACCGGAAGTCTCGCATAAACGTCTACGATCGCGTTAAGGATACTACGACTGTTAGGGAAAAAAGACAGCGTTGAACCGACACATTCCCGACGTTATCCACGAATATAAAAATGAACCGATCGCCCGCAAAAGTGTGAACACATCCCCGGGTAAAATATACAACGATCCCAGACGTTAGAATCGCCCGTAAAAACTCGATCTATCGCGTTTCCATTCTCTTTATTTTTTCTTTTACTCAACCACCCACATTCGGGCGAATCATCGAACCACGTCGTGTACGGCACTCCTAGTACCAGCGTCCGGAAATTTCTTGCGCTCGTCGTTCCTAGCTATTAAAAACAGCACATCAGCGTCGGCCTGCCTCCAAGTTTTCTGATAAAACATTCGCGATAGATGACAGGTAGGAAGAAGCGCCCATTCGGGCTGCTCCCTACGGAAAACGTAGCTCTTATAATACTGAGTGACTCCTATCCGTCTAAACTGCTGAAAATCTTCCGCTACGAAGCGCGGCCGTCCGTCTTCGTCGTTGTCGTTGAATCCGAAAATCTGGCCGTCGTCGGTCACGTAGAGAGAAATCCGACCTTCGTCCCCAAAGTACTTGTATCCGAAGCTGCCGAAGAAACACATCTCCGCGAGCCTGCGCCGCGCCTCGTCGCACATGACGAAGGCGAAAGAATCCCGCCGCCGATGAAAGAGCAGAGTCTCGTTCTTGGGCCACCTCAGGGCGAAAGAATGACCTATGTGCCGGTCTCGAAAACACAGCACGTCCCGACAACTGCGGACGCCGTCCACCTCGGGGTCCTCCACCACAAACTGCAGCGATGACACCAGCTGATGTGCGTAATAGAACCTCTTCAGACCTTTACGTAGAAAATCGTCAATGGTGTCACCGACGCGGCACAGTTGCTGCGGCAATAACCACGTGTGCGCATAAACACGACCGGCGCCGCCGAGCAGAACGACTATGTCACCTTTCGAAAACCAGTAACACGGCTCCGATCCGATCCCCCCCCTTTTCTTCACGTATCCCAGTACGACGAGGCGTTCCGGGCAACACACCAGACCGGCCAGTTCGTCCAACTTTTCATCGGCATATTCATTCTCGTCTCTACAGTTATTGATGACCAGCTGGTATCCTTCAGGCCAAGCCAAAGGAATTCGCCTGCCGAAATTTACGAACGTAAACATTCGAACGCTATTCAGGCACGTATAGACGCGCAAGAGATCTACGAGATCTCTCTTCGACTCGTTATAGACACGTTGACGTGTCTTGGTCCTCTCCCGAGACTGCAGCTCAGCATTCCCACACATCGTAGAATCTTGCGCTGGCTCATGAAGGCCTCCAGTAAATCGCACACGCCGGTTCTTTGTCGTTTCCATTACGGGCTCGCAGTAACAGAGAAATAGCACCCAAAACTTACAGACTAAGCAAAAGTTGCAACTCACAAAAAAAATGAAAGATAATATAGTCACAAAAGAATTCTGTCACAATGCACACCCAAGGACAACGATATGGATGATCACGACAACAATAATATCGTTTTTTCTTTGTGAAAACTAATCAGTAAAAGCGTACCGGTTTTCCGGATTCGTCCGTATCAGATAAAGGTCACACAAGCCGGCTTAGGAACGCGGCGCGCTTCACGAATGTCTAACCCTCGCTGCTGCACGCGTCTTATAAGCCTACGTCTGACGACTATTCGCTTCTGACGTCTCCGGTGCTCCTGCAGCCTGATGTCAAACGAGCCGCCGCCGCGCACAGCGTAAGGAACGACGTTCGGATACACCCCACTCCACATATGGGTCCCGTAAAGGTTGCAATCGTGAAAACGAAAAAACCTAACCGGATTCGACATGTAAGATAACTCCAGAGTATTGAGGAAGCCCTCCAGGACGTCGTGCCTTATCTCGCGAGCGAGTCGCATGGATGCACACAAGTCGCCGAGACAGATCTTTTTAAGGCACAGATGCTCTAAGAGAGCACGAACGCGATGCCGTTCCTTGTCATAACCCGATACGAGAATCGCCACGATCTCAAGAAAGAAAAAACACAACCTAGGGACGCGCATGGGATCTTTAATCTCGAACTGAGAACTGACAACTAACAGTTTGATCCTGACACCGCGGAGGCCAGAAACGGTATCGATGCAACGCTCCATTTCGGAGATCGCTTTTACGAAGAAACGAGCAATGGCAGTGAGATCACTCACGATGCGCTGTTTGACGCGTCTCAGTAGAAACAGACACAGAACGATGTTGCAGTACACCTTCAAGATGTTACGTTCAAAATGCCCTCTCCCCAACTGCTTATCATGAAGAATGGGCAAATGCCGCCTTTCCATAGAGTTAAGGATGAGAGAAGAGCCGAGATCTCGGTCGATCTCCGCCCGGAGAGCGACCGCCTCACGCGCGTCGAGGTGCAGCTGGCCTAAAATGAGATCAGCCGGATCGAATTCTGAAATATCGGATATGTGCACGGCCCGGGAATCTTTCTTACCCAGATAGATCCGAAAGAGAGACGCCCGGCACTGATCGCAACTACAGAGCACAGAAGACACAAAGCGATAAGAAGAAGCGTACGCGACAAAGATGTGATCGGACAAAAAGTGTTTCAAGAGCACACTCGTCGGAGACGCCGCGTCGCCATAGATCTCCTTGGGATTGGGCAAAATATCGAACAGGGTCAACAGAGTATTGGACTCGCTCCTGTTCCTCAACATGAGGAACTGCAGGAAGAGGGAGTCCAACAACTCCAAAAGACGGTTAAAATGCGCCGATATAGTATCCCTCTTCTCCCCATCGACGATGTACTCCAGAATCGAGAAGTGCGCCAGCGCGGTGCACTTGGTCAGGAGTCGATAAAAAACTGACGTTTCGTCCTCAAAAGTTAACCGTTCTAAGAGAGACATAAAAGCCGAGAGATCCATGGTCGTCTTTCCACGCCTCTGGAATTCAACAGAAACTTTCTCCGCCAGAGCCGCCCAGAGAACACTATCGTCACAAAATCGTAAGCCGCGGGTCATAAACAGATCCTCTGCGGTCAACGTGCCGCTATCTATAATCATCTCCTCCGTTATGTCTTTCAGATCAACATCAAGTTCCCTGGAATGATGAACGGTAAGCGAGAGATAGGGATGGTTGGGAATGGTATACGTGACCCGTTCTCGGACGGGTCCCTTATAAATGTCGTGGTCCAATAACTCCATCACTCAAGTAAGTATATATCCGAGACAGCGTTTATTTTCCGTCGCGGAAAAATAAAGATAAACGAGCAGAATTCAAATTACAAGAGACGGCCACGTACATCACAATCGAGAAAGCACACAATCACTCATCCCCACCATATCCATTCAGTTCGCCGGGACAATCCGGTGGTGTGTATTCATAATACTTGTAATCATCCCTGAGGACGTCCAACGACCCCAATTTATATGCGATAGCTCTGACGTCTTTCTCGGGACTTATTTGAAAACCGCGATGATTGCCACTCCCAAAATATTTCAAGCCACTGAAAAAAAAAGCCATAGTCCCGTTTATGCACCTGGTGTTAGTTAAATAATCTTCGGCGACGGGCAACACTACCCGTTGTAGCCGCTGTAAGAAATTCTCTCCATCGGTATTAACGATAAAAAACCCCGGAATGAGATTATCACAAGACACCAGGTCACGATCATTGTCCTCGTGAAAGCGTTTTACATGAAGATGATGCATAAAAAACATTTCCATGAACGGAGTCTCGTCGTAATCTTTGTAGGCAGGAACGGAAAAACCGATTCTCAGAGCTTCCCAACACTTTAAGATAAACTGACTCTTTTCCCCGGGACACCTAACAATTTCTTTCATCTGTTTCATCGGAGAAAAGTAACACAGGTCTCCCAGACACCCATTCCGCCCCAGCTTCATCTCTTCCTCCATGGCCACACACAATTTCCCCACTTCCTCCGTGAAGAGGTCGTAATATATGTCCAACTCAGCATTTATGACACGGTAGAGGGTGTTCACAACGAAGACAATGAAGACAATATTAGTTAAGAAGGTTAGCAATGCCTTCTTATCCGGAGTGACAGAGATCTGGAGCGAAATAGGCAGCCTACTGAGCTCTATCTGACCTTCGATCATGCTCAACCCTAAATCCTTCGCTATGAGACTCGACAGACTCACATCGTAGGGTTCACCAAGATGCAGAAAAGCCGGAAGGCGAATTGAACCTATCATAGGAAAACACTGGATTCGGACACTGTGGCCAAAATCAAACTTCCTACCTCTCTTGTTATACATGATAAACATGCGCCTACACTCGACACAACCACAGAGGAGCATAAAGTGTTCGATAAGACAGGATTCGATACGCTCGAAGAAATCGTACCTGAAGAAAACATCGGATTTGTTTTCCAAGCAGTCCTTCAATTCTTGACCCCAATGACAAAGATTATAACCTCCCAGTTGCCGAGTAGATTCCTTGGTCAACTCTTTTAACTTGCTTTCAAACTGAAATAATAACATGGAGGCATACTCACTTAGATAACGACACCAGTAGCCCAGCGTTGGTTTGGACGTGAAGTTAAACAGAGACGCTCCGGCATAGAGACCGAGAAAAGCCAGTTTAGTGATGCACTGCCACTTCTCATTAAAATTGTCCATAAATTTTAAGCCTTTCACCATGTCCGCAATCTGATCGGCTCTGATGTAGGTGGAAGGATACTTGTAAGTTTCATACTCATAATCCAGCAGTATGTTCCACACCAAGCAGTCCGAAGAGGGATGTAGTCCTTTAATGCCAAATCTAGCTGCTGCGGTCCCCTGCAGACCGTTCGTCAACCTGGCGCGGGAGACGGGAACAACAAACCTGTTGGTAGCGGGCAGTCGACCGACGAATACCGGCGTGAGAAATTTTGTGATAAATTTAACCGTCGGTGAATCCTCTTCATCCAGACGCACCGCAAACAGAATTTTGCTACGGTCTTTCACAAGCCTGTTGTAAGAAAAAAAAAATTAGCGGGGAGTCTCCATCCTCGAATAATTATCCTCTCTGAAAAACGACACAGAACACATCCACCCACCTGTCCTTTCGCACGCACATATGCCGAGGCTTACTACGGAGAACGGGCAACTCCCCATTCAACATCCTATTGAGATTCTCCGTCTCAGTGTCACTTATATAAAATAAGGTGTCGTCACTCCAATCCAACGAATACTTCTCTTTATAGAGCAACTCATCAAATCCTCTACGTATGAAAGTCCTCAAACTTTTAGAGACTTTGCTGATTCTGTTTTCTACGTAGATATAAATGTGACAGTTCGAGCCCATAACGATAGCAGCTCTGCAATTCGGTTCAGCCTGAGAACGGGCACACGTCCCTATGATAGCATAATCCTTTTCTGTGATTTTCTCTAGAATGCCAGGTCCAGTCAGATAATAAATATCCGACAAGGAACAAAAGGAAAAGTCAAAGTCCTGGAAAGCATCCACCCTGATTCTCTCGCCGGAATGTTTTGCCACGTAATCCTTCATAGCAGGGAGATTCCTCTGTAAAAGGATAAATTCCTCCCGGCCGCATTTAGTCTCTCCGAACCACATCTTTTCCTGCTCTGGATCTAACTCCGCTTCGGCAAAAGGCGGAAATTGTCTAAGTCCAATATTGAAGAACTGAACCAATGATTCTGCTATAATGTACACTTTCTCCGTCACCGTGTCCACGGCAATGGTCTTTCCGGTTGAAGTATGCATCACGTATGGATCGTAGTCAGGATTTGGATCACGATATATCGGGTGAGCATACCCAACAGCGGACATAAATTCATCAGCATCCAGCAGACTGCTCCAGTCATAAGCACTGACCCACCTATGCGTGTGAAAGTGATAAAACTCACCCAGGGTTAATGCATACATGGGAGGCCAGGCGATACAACACCGCAAGCCCGGAGAACTGATTTCCAACCCGTCTCCGTAAGCTGCAGACAGAACGTCCACATCAAAATCCAAAGTAGGCACACATGATGACAAGGCGAACTCGTCGTTGCAGACCGGTACGATCTCTGTTTGAAACATAAACACAGAATAAAGTAAAATACTTTCACACAAGACTCGCTCTCCGCCCTCCGTTTCAAGTATCAGTCAAAAATTTCACACCAACTACTTACTGAGACTCATGCACGATTGATAAGTTTCCGACATGCCCAACGTCGACATCCGTCCGAAGCGCATGGAGATTTTTCCAATCTTCTCATAACCGCAGTCCGCAAAAAGTCTGGCAAACCCCACGCGCAAAAAAGTCAGTAGACAATCGCCCAGCTTAACCAGCACGTTGTCTATCCAATCGTAATAATATATTTTTCCAGATTTCCCAATACAAAGAATAGGTCTTGTACATTTAGACTCGTAATCATGGTCATTCAGGTAACCTAACACTACTACTTTTTCTACTAAGTCTTCCTGTTCTTTTAGATTTGCCAAGCGACATGTCGCTGTCCAGACACTGTCACGAGTCCCTAGAGTCAAACTCTTAAAAACCGGCATATCCAATCTAAGATCTCGGCCGATGTTGAGAGACACGAAACGTTCCACATCGTCCAAACTGGCACAACGGGCCAGCTCATCAAACTCCCGCAACATGGCAATGTAATGTTTCCTCATAGCCTTGATGCCCAAAACGCCTTTCCTATTCAACTTCACATGGACATTTAGAGTATAGGGACTCCTTAACACATCATTATTCATTAAGTTTTCAAGCGATTCGAATCTGGGGCCTTCATACAAGCCATGACGGTACACAGGCTCCGCGCATGTGATGATATTAGTACCCAGGACGCCGTATCTCACAAATTGCTCAAAATTATCACACACAAAGTGGAGAGTATCCGTCTCTAAATCGTGCAAGAAAATTCTTTTAGATCGACCAATCAGAATTGGAACATCTCTGTAAGGAATCTGAATAGTCCCCAGTAGACACAGTTTTTCCACAAAACCCTGATAGTGATCATCCATATCTTCAAACATCTTTTCAGTATATCCAGCTACATCTTTACGCGGAGTGAATCTCAGCCAGCAGTTCCGAGGCCAGATAATAGACAGACACCAGTTCTTATACCTGCCGACAAAATTCTCGATCTGAGAGAGATCGGCAGATTCGCATAAACGGCACAATTCGGCAAGAGCGTTATCGTTCGGAATATCACCTGAAATCTCCTTCGCCTCGAGGTCCATCTTGAAAACTCCGATTAACACTAGAAGTGAAACAATGAGGTATCTGAAATATAAGACCTGGATACGAGACGCCGTCACATGACAGGAAATTGCAAAAAAACAGAGTGGTGTTACAAACTTTATTAAATTTCAGCCTCCGTTTTGACTCACACGCTTTCCTCCGCATGTACAAATAAATCCGTTCCATCGACCATTTCTTGCGAATGTTGAACAACAAACACCTTCATTTCCTCGGAATTATGAAAATCTTCCTTACATACATCAGAAGCGAAAGTATATAAACACTCGCATTCCGAAAGTTTTATACATTGCGATTTGACGTAGCTGCAATCCGCATTCAAACCTCGCACTCCGTACTTTAAGAAGGAAACCTCCATCGTTCCGGCTTCTGTATTGCACTCCTCGGATTTCGATTTAGAAAACAGCTGTACGACTGTCTTCCATAACTTTCTAACAGCCATATCGGCAGATCCACTTCAGAGAAAACGAACTCGAAACACCACTAAAAACAGATGGAAATAGTCACATACAAGACGGTTTCAGCGAGATCTCCGACCGTCACCTGGTCATCGGGATTCGGACGAGCTATAGCCTCGATTCAAAAACGGAATCAAGATAATATCAGGAAACCCCTACGCTTCTACAGCGGTCTCCTACACTGCCTTATAAAACAATATGAGCATTGCCTCGTCCCGCCAAATAAATCAATACGTTTCGACAAAGGGAAGATTGAAGTCGCTGCCCTAATTCTAGATCTCGGACACCAGGTACTTGGTCGTCAGATACACGTACGACAACGTATCTACAGCTGGACTAGCATCACACTCCCTAAACTCTTTACCCCCAAAGAACTTTACTTTTTAGTCGCCTCTCCAGGAGAAGAAAACATCGCATTTAATCCAACTATAACAAAGGGAGGTTGGATCTCGGGGAGTTTTTCGTATCCCGTTGATTACAGATCAAACTTTTCGCTAACCGGAATGTCTGCGAACATATTAATGGTGCCGTTCGTGCCGTACAAATACCCACTAAACTATGCTCGCTTCATATCGTCAATCGACCTGATGATACTAAACGAACAATTTCCGGAACACGAATGCGGAGATATACAGATTTTAAAACAGCGAAACTACCTCTACCTGGGAGTCATCAAAAATCTTACGTGGAAAAAAAGCGTCACCGGCACAGGTCAGACGGCTCCACATAGAATCCTAAAAGCTTCGTTCATAGGAAGTTGGCCGGACACGTCCCTGCCTGACAGAGTGGCACTGCGTTTCTTTAATAACACTAGGTTTACAATACACTGCCACGAATTCTCGATTAACATTGAAAATCTCGGTCTCGTCAAAAACAAAGAAAAAGTTTTTGGGACACTAGCCACCGTATGCTGCGAGCAAATCCCTTCTTTACTAACAACGGAAAATCTACCGAGATATTTAATAGTTCAGTTTGAAGTGGTAACGCAAATCGAAGAGCCCGAACCGCTGTTATTTTCCAGTAACCCGAAATTATATTTTACAGGAGACGTACTAAACGCAACGATACAACTGCAACACAACCCAAGTTATTACGAGCTACTTGTACACGCACCATATGACATCCATTTCTATCCAAGCCGATGCCACATAGTAATTCTACCTATCCGATATTTCACAAAGCCCGATAAGCAAATCCTCATATCCGGCTACCAGAACGAAGGATTCTTCGAAACCCAGGTGATGCTGTGGGCCCCGGGAACACCTTTGCACATTACGCTGCGTTCATTTTCTCCAAATCTGATCCTGCCTCAAAGCACAAAAAGACGTAATCGCAAAGCTGTCTGAGAATGGGCATTTTATTGGCAACCTGAAACTCCCCAGAGAAAATTTTTTACATCACGACGCGATCGCTGACTCGCCTTTGGCTTCTATATCCAAAGACTCAGCGACTCCCGGCCCAGAGACGGTGTTTTCATCAGTTTCCCCGTCTTGACGTATCCTCAGAACCATCTGTTCAATTATATCATCTTTTTCTTCAGGTGTCTCAACCTTCATTAATTGCGCAGGAATAAAACTTCCATCCTGACCGACTTTGAAATCTGAGGTTTCCGTCTCGCCTGCCTTCACTTTTTCAAGAGCGAGATCTATTTCTTGTTCTCCGTTACCCGGAAGCTTCAAGTCTAACGCGTCCTTAAATGACGCGGAATCTAACTCGCGAGACAAATCAGCATCCGGTAGCCTAAACGGGGACTGAACGGAATCGAGATCGTTTTGCAACCTAAAATCTTTAAACATATCTCGAGGATTACTAGCTTCAAGAATTTTTTCTACGTTAGCTTCCTCCGATTCGCGTCCCGACTTGTTTTCTGTAGCCAACTTTAAATCGGTCCCGCGCTGATTCTGCAAAATGTCTTCGATGCCCTTCTCGCCTAAACTAATCAGATTTGTAAATTGACTTAGAAGATTGTTAACTCGCTGCCCGAGACCCGCTTGAATATATTGTTCATCTCCGGCATTTCTAGCATCTCCCATTAAACTACGTGATGGCTCTTGCCCGTTTCGCGTCATTTCTTTATCGACATTAAACTTTCCCGACATTTCTTTTTTTTTTGCATTGCCGCCAAAATGCAAGAAGTCCTTGCTATTTCCTTTCCCCCCTCTTAATACGTCACGATCATTTTTCTTATTTAACCCGGGAAAAAAACCATTCGCTTGCTTAATTTTTCTCAACGCCGGGGAATTCCGAGCAGCTACAGTTTCAGTCGCACCGCCAGAATCTCCGGTCACCTCGCGTGAGTGCATTATAGCTTTATCATTTCTTTCCTTAGTTAAGCTTTTCGGAATATCTGACTGCTTCTGTTTCCTAAAACCGTCTTTGCCTCTCAAATCTTTAATGAGAAAAACATTACGGGGTGACACGCCGGTCTCCGTGCTGTAAGTCGTATGCTTCGCTTCTGATCCCGCCACGTCGGAATTATTCAACACGAAGGGTGTCCCTGTCTGCTCAGGAACAAAGCGATGCACCGGATCTAAAAAATCCGCATTCGCCGCTGTGTTTCTGCTCCACGGTGGCAAAACTTCCGATTTATTCTGATGCAGATTGAGATTAGCTAAACCATTTACCACATCGGTAATACCGCTACTGGGACTCCCGATCGAAGTTTTATCCTGTAGTAAATCCACGCCGTTCGTATTCAATTGCGCATTAGGTGGAAATTTAACATTAAAATCTAAAATCCCCGAAGGGCCAATTTTATAAATACCGTGCGACTTATCTCGCAGATCCAGATTAGTAGAAGTGAAAGACGACAACGGCAGATCCAGGTTGGTAGAAGTGAAAGACGACGGCGACTCAACTCCGGACATTAAAGAGACAGACGAAGCAGGCGTTGTTGCTTTAACTTCTTCACCCCCGAGTGTTGCTTCCTTTATTAAAATTTTTCCCCCGGCATTATCAACGCTATCCAACTCTAGAAATTTCGGTTGTCTCTGCATTCTTAAGAAAGTATCCAAACTTCTGGATTTACTTTTTTTCAGACGAGCCGGATCTACAGATGATTTCTTTGTATCTATAGACATACTAGGTAAAGTCAAATTTGAAGGTATATCGCCGTGTAAAGGGACGAAATCCATTAAAGAATAAGGCCCAATCTCAGACATCGAAAATGCTTTTGATGCGCTGTGTGATTCCAGACTACTTTTGATAATATCATAATCACTGGTGATTATTTTCAGAAAACTCTCTACTCTTTGACCAGTCAGAGGAGTCTGCAAAACCCTATGAGAATATACAATATTAAAATTGTCGTGCCTCCTCAAATGACCCGAGAGATGATTTTGCGTGATTACAATTTGACTCATAGCAACCTTTTCTAGCTTTGACCAGGACTGAGTTAACGCTAAAAATAACTTACCGAGATAAATTAATTTATTAATACTCGTTATTAAGTAACCGTTTTCGTCCCAGGGGTCATTTATGTTATAGACGGTATACCACAGCATGTTGTTTTCAGCATCTTGCAAATTATTTTCTATCTCGCTGCTGTCTTCTGCAGTAAGATTTACATAAGGAGTATCGATCGCAACATTGCTACCCCACTTAATAGAGTTTCCTAAACACAGTAAAGCGCGATTAAAATCACTGAATTGCGATTCAAACTTTTCTTTGTTCGTGAACATATTTTCAAAAATTCCTTTATTCAGACCTTTGGATTTTAAGTACTCGTTTACGACACTTTGGAGTTTCGTGATGTCTTGCCATATAGCATCATAATCCGGCTTCCTTTTAGATAAGACGTAGTGATACATAAGCCATAACACTAGTAGGTTATACAATGTTTTTAAATCGTCTACATCTTCTCCCTCTCTCACGACACAAGAATTAGTCACATGGGGATGCTCTCGCAAATCTACATTATCCAGTCTTTCCAAATTGCTGAGAAAATTTGTAAGATACTCAACTTTGTCTCGATCTAGCCACGCAAACGGAATCGGATGTCTTTGCAGATCCATATTTTAGAAAACAAACTCAAAAACAGAACTGCTCATTTCCGCCAACATTCAATGTTTAATAGTCTTTTTTTTACCCACACAGAAAACTGACAAGCGACGAGATGGACACTGTCATTGAGCTGTCCAAACTTCTGGTAAGTCTCCAGACATCGTTGTTACCGAATATTTTAATGTAGCGTAACATAAAAATAACATTCAATATCTTTAATAGCGCAATGAAGAGTTCAAAGGCAATGCTTCCTGTACCTCGACACCCACGCTAAAGACAGCACGAATCATGGAGAGCGCAGTTACCGGAATCACGCTCACCACATCTATCCCCATGATCATTATAGTGGTTACCACCATGATTCTCTACCACAGAGTCGCAAAACATAACGCTACTTCATTTTACGTCATCACACTCTTTGCTAGCGATTTTGTGCTCATGTGGTGTGTCTTTTTTATGACAGTGAACAGAAAGCAACTGTTCTCATTCAACCGTTTTTTCTGTCAGTTAGTTTACTTCATCTATCATGCAGTCTGTTCATATAGCATAAGCATGCTAGCTATAATCGCAACTATCAGGTACAAAACCTTACACCGGCGTAAAAAGACCGAAAGTAAAACATCTAGCACGGGGCGAAACATAGGAATTCTCCTACTAGCTTCCTCAATGTGCGCGATTCCCACCGCTCTCTTTGTGAAGACTAACGGAATGAAAAAAACGGGAAAATGCGTCGTTTATATATCCTCAAAGAAAGCTTACGAACTGTTTCTAGCGGTGAAAATTGTCTTCAGCTTCATTTGGGGAGTCCTTCCAACCATGGTGTTCAGCTTCTTCTACGTTATATTTTGCAAAGCTTTGCACGACGTCACCGAGAAGAAATACAAAAAGACTCTATTTTTTATTAGAATCCTTCTCCTGTCATTTTTACTCATACAGATCCCCTATATAGCTATCCTTATATGTGAACTCGCATTTCTCTACATACCGCAGAACACCTGCTTTTGGTTAGCCCGTGTAGAGATACTGCAGTTAATCATTCGATTAATGCCACAGGTACATTGTTTCTCTAACCCGTTAGTCTACGCATTCACTGGAGGCGAACTCAGAAACCGATTTACCGCCTGTTTTCAATCATTTTTTCCCAAAACATTATGTAGCACACAGAAACGCAAGGAGTCAGACGTATCCGAACATGATCAGAACTCAAAATCGAAAGCGTCAGTGGAAAAAAACCAACCCCTTTAAACACGTTTTACAAATTTTTAACTAATAAATGCTACCCACATGAAATCACGTACTATCTTTTTCTGTGTTTTATCTTCCATAGAGCATGGCGCACGCTAAAAAACGGGTACGACGAAAACTTTTAACTTCGACAGACGACCCCATATTGTCCAACACTTTTACCATGCGCCCGACAAGTAAGATTGCAGACGCTGAAATCATTTCAAGAGAACATGATTATATCGCAAGCAAAACACAGACGGATTCAAAAAAAATATCGCCCCTGTCTGTGATTTTAGACAAAACTGCCCTCTTTGAATTTTACGGTATAGGAGACAACAACGAAAAAGCTATCGTCTACCCTATCGATCCAGATTTCTTATTATGTGACTCGGAACATAATTGCACCCTATCACCTTTCTTATAAAAATTTTTTTCATGATCTCACGTCACAGTTTTTTTTCATATGTCTCGCGTTGATACATCCCGTATGAAATATTTAAGGTGGTTGAAATCACACCAATGGTCACACACGCGATGGAAGAACCAAAGACATTCAACATTCCAACTTTCGTCTTAGACGAAAATTGTAACTTCATACCCGAAGTTCTCTCACGCGCCAACGCTAAATATATCAAAGATGTACTCATCCAAGACTCCTACAATACGGTTTGTCTCGCAAACAGCTTTATTCCCATGACGATTCAAACCACAGAGCAAATTATGGTTATCGTCACAAAGTTTAAGTTTTCACGTGCACGAAATCTGTTCGAAAAAGTACTCCGCCTCGGCGTACATATAAACAGATTTTATGCCGGAAAAAAACAAGTCAAACATATGATTACAATGATGAAAAGTCTTTTCGACACCGAAGAAGCCATGCGACGTCTCGACAGAGCTCTTATGGGGCTGTTTGTCGATGCTCGTGGTAGTTCATATATGCCTCTCATAGCCTTGTCCTTGCACGAAAACGGCCTTCCAGATTCCAAATTCGCAAAAGCCGTCAAATTAATAAACACAACCTTAAACTCCTTTCATAATCGACCGGACGCAGATATCGAACAGTATGCACAAAAATTACGAGCATACAATTACCTCTATAAAATACCCCATTATACTTTGAAAGAAGCTGTTGACGTTTATTCCGAAAATCTTAAAGATCTCTCTATTGGAGTTAATAAAAAGCCCACATTACTGTTTACTTCTTCAGACGACGGGTATTTATCTCATATTTTTAACGACATGCTATTCTTAACTTCTACTTGGAATATGATTTATAACTCCAAAGTACAAATCAGGCGTTTGACCACTTGGATCAAATACGAAATCAACTCCATATTAGAAGATTGCGTATTGGTGGGATTCCAACTTCCAGATCTAAAGGACACAATACTAGATCTAGCGGCACTCACATCAAATATGAATCTCGTCGACCCAGATAAAGACCTTTTTTCTCACTACAAGCTCATACTGGAAAAACTTTTTGAAATCTCTATTTTCGCGACTAAGGCAAACATTTGCATTTTACCAACCTTCATAAAGAGTCACCTAATAGAGTTCGAGGATGTCTTAAAAAGAAGCAATGATGATGAAGACCTCAATTATCTGCTTCTGAAATCTAACGATTCTGATGATGAATACGACGAAGACAAACTACCAATACAAGTCGATCCAGGCAGAGTAGACAACGTGCTTACAGATTCTAACTTTTTTAACGTAACCGCAGATAATGCTTTCTCGTCTATAGCGATCATGCCAATTTCTTATGATAAGATCATAGATGTGGAAGAAAATGTAATTCAAGTCCTAGAAGTTGAGATGCAGAGCCTCTCAGCAGTTGTCTACGGAGCAGTTGCAAACAAATACGGCTTAAGCCTACCACAAGTCATCAAAAGGTTAAATCAAAATGAAGGACGGGCCTCTTCTCGTGCATCCTCCAGCCACAGTACTTCTACCATCCCATACTCCCCCCCGCAAAGCGGTCGCTCCACTCCAACTTCTATCCTAAGGCAAAGAGCACCAATACGTTCCAACAGCAGATCATCATCGGTCTCTTTCTCACAAGACGACGATAATCGTAGTCATTACTCTGACGAAACAAATATCAGTGATTATTCATATCCCATGGCAGATCTAGATTTAGAAGACGAAGAACCCATGGAGGACCATCCACACTCCCCTCAATCAGCATCATCTAACAACTCCATGTCTCGTCGAAGCCGAGCTCTACAAAACGAACAAAGAAGACGTACGCCCACAATGGCCCCACCATCTCCGGCAAGAGGACAAAATAATGCCCGCCTCAGACGCAGAACCAGACGCTCAACGGAAACGAACGACGAAAGGTTATAATCACAAATAATTTTATTAACTGTAAAAAGTGCTTTATCTAGCCAATTCATTGTACGATAATAAAAGTTCCATTGTTTGATTGATTTCCGTATTCTTGAGCTTGTAGATCATATCCAATAAAATATCCCGCGTTAGGTGTAAATCATCAACGTAAGAAAAAAAGCGAATAGCATTCTTTTCTTCATCATCACCAAGTCTAAGGTGACTCTTCTCTAAAAACTAAAAAAAAAAACACAAACCATCAATTTTATCCATCCAATCCAAGATAACTTTACATTAAACACTAAATTACCACCATAAAAGTATAGCCTAGCAACCTAATGGACCAAGCACCTAATTACAATTTATCTTGAAATCTACCGTACAAAATCAAACTCACCGCATATATCATATTGAGGAAAAGCTGTTTTTCTTGTCTACTGGTTAACATATACAACAACACAATTAACTTCCTGTAAAAGAAAAACACATTAATCGGCATACATTAGCCCGCTCGCTTCTACGTATACTTAAATCAGTACCCACCTTTTTATAGGAATATGGGTGAACTGAGCTACGACCATACAGTCGTTGACTTTCCGGATGTTCAATCTAAAAAATTGATCAATATCTTCCCATCTTTTTAAAATAAATAACATTTCAATAACTACGGATTTTGTAACCGTCATACTCAACGTAAGAGCCGGTAAAAGAAATCTATACATAGTATAAAAGTCCATTTTAAATAGATATTTAACGTATACGATTTCGATTTTTGACAACATATTCGACAGTGACATTAATACAGGCAAAGGACACAATTCACGGCATTCTTGAAGCCGTTGACGCTTCCACACATCCATCTTCGTCACAGCCAAAGCTCTGTTCCGCTCAGCACGGCAGCTCTCCGTATTATAAAAACAGATACTGAATCACACGCTTTAAGAATTAAGAATCATTGGCTACTTGCCCATCCCTTAGAACATCTCATAACACATACAATGGTACAAAACAGGATTTAAAAACTACAACGACAAATATTATCATCGGCTAAAATTTTTATTCTGTATACTAACATCTCCATACATCAAGTTAAATACAACTCAAAGTATGACGTATCTATTTACTCGTCATCCTCAGATGATAAATCATAATTGTAATTATTCGAGAATGTGGGCCCTAGAGAAGTATGGGGACACAACGGCAAAATTTCTTGGACGGTGCTAATGTTCCTATGGAAAAACTTATAGCGACGATATCCCCTAACAATCCCTTGTCTTAAAAAAGATTCAAAATCGTCTGCTATTTTAACACACGATCCAATTCCCGTTCCGATCGTATCAATACCATAAATTGCCCCCGATAAATCCATCATCACAAACATCGTTTTAGGATACAGTAATGTTTTTCCAATACTTCCGATAACCTGCCCGTGATACGGCATACTCTCGTAGACATCCGAAAAGAAATTCCATTCCATCCTACTAGGAAAATCTTCTTTCCTAGTAAGAAATAAAACAAATACTCCATCGTTATTTTCCAAGCAAGACTTCATAATGTAATGCCTCCCACTATTTTTATAAACGATAGTAGCGAATTTCCATAAATTATCCTGCGCATCAATCAACGATTGAAGAACATCATCAATTTTCGTTAAAAAGCGAATGTCCTTTTCTCGATATACCGGATCATAGCGACGCAATCCGAGAACTGCAAATCGTTCCGTCGTTTCCGCCAACAGATACAGTGTCTCATTCACAAAATCGAGACCGTAAAATTTTTCGTCTTCTCCCGCAAACACCAACGTTTTAGTTAGGCACACATCACGAGTCTCCTCTCCCCAGACGGAAACATATCCAAGTAGTTGTAATCTTTCCGGGCAGCAAAGTAATAACGAACCTTATTTAAAAAGAAAAATAACTTTATTTTCACCACAAAAACGAGAATGACACATCACAGAACTGACACGAAACACTGATACTTACACATTTCTTGCAACTCTGCGGCAGACTTTGTGGAAAATGGAATTTTACACAGTGTCGAAATCACCAAGCATCGACTTTTCGGCCAATTAAACTGTATAGTTAAGCCGGTCTTCTGTTCTACGTATGTCGATAGAGAATCACGAAAAACTGCAATTTTTATATCGTTTAAATTCATCAGTGCAATATCATAACGAGTTTTAACAGAATCGCCGGTTCGTTCGTCTGCCATATCTACGTTATGGAACCGTCAACTGCCCCTTTGAACCCGAAACTACAAATAAACCAGCTCGCATTTCTTATCCATCTTTTATATCACACAGAAATAAAAAACTCAACTTAATCAACTACCATCCAAAAACAAGAAATGTCATAGTGTTGAACTGGAACTACCATCTCGTCCACCCATGGAAAAAACAAAGACAACCGAACAATTCCCTGCGTTAATAATATCCATTCTTATGTATGTAAAATAATACAATGTAAACAATAATGATTCAACAATATCCTTAGTTTATTCTCATAGAACTGATTTCCCGTAAAGGAAACGAATGATAAAAACCACCGTTGCGGAAAATTCAAATACTCGTCATATGCAAAAATTCCCTAAAATCTTATATATAAGAAAGCCACAAACCCTCAAAACCTCATACATACCAAACTTTCCGAAGATCAAAAAGAGCAGCAAATCGCTGCCACCATTACAACCATGATCGCCTTAAACATCAACTTTTTCTTCATCTATTCCAACGGAAGGAACAATCCAGAGATTACCTCTCAAGATAGACAGATCCATAAGCGGCAGCCATCGAATGATACCGACATTGAAATGGTAAGAAATATTGAAAACGTATATACAAATTTACCGATAGAAACGCGATATTTATTTAAGCACTGTTAAATCATTTCCATAGGCAGACTTCTTACCTGAAAACTACAGTAGTCGGATCCGTAATTCCCATCGACAAAACAAAATAGTAAGTACTTTTATCGTTTCGTTATACACTAATGCCCAGAAGAGCTCAGATTTTGACCGATCTGCACATTACGAACACCCCAGGGTATTTACACCCGTTTGAAATTTTTTATAGGTCCCACTAATTCATATTCTAACGACACTGGGAATGAACGCTATCCGCGCTTCTCTATCAAATACGCATTGACGCAGGTGAGTAAGTCTTTTCACTAATCCATTTTAAATCCGACAAAAAAAACAACAACATTGACTATATGTAATGAATATTTTTTATTTCCGCAGATATGATTTAATCCAACGACCACTACACGTTTTACTGTCCACGACCACCTTTTCCATTGTTTTAATGTAATTATTTTAATAAACTTTAATATCCTAAATCTTTTACAATATGTTTCCGCGTAATCATTGACAAACAGCATAGCAATAACAGCATCGTAAATGCGCCCCCGGCCAAAAAGAACAACCCAGCAGCCGACATTGAATACGTAATTTCAGATAAATTCTCGTAATCGCTTGAAAAATCCAAAAACGCTTCGATCTCCTTATATTTATACCAATTACTCCATGCAGACCTCCATGTATTTCTTAAGAATTTTTTCGGCTTCGTATCATTTTTGATTAATGAAAAATTTTTCGGATGCGCAACCTTTTTCTTGTAAATTTCAGACGACAACTGAGGACAGATCCTGCTCAGAAAATAGCTATCCAAAGACACTCTCTCATTTAAAATGTTCAAAGTGCCATTGGCATCCTTCCCGTCACTTATCCATCGACTCGCATTGATTTCAGTGCTGGAATTGAGAACAATCCGTTCCGTTCCATAAAATAGAGTCATCACACCTCCTGCGCTATGGTCATCTAGCCAGCAATTTACAGTACTCGATAAATGAGCGGCATTTGGAAGCCTTTTAGCGATCAAAGCTCTAAGCATCACTCCTCTAAAATAATAATTAATAATCACACGCAGAAAAGCCGTTTTTTCAGAGATAGATGTAATATTTCCAACTCTCATGTCTTTAAAGCCATTTGTTAACATCGCAAGGTTGATCAGATATGAAGTTTTGACAGAAACACAGCTAGAATTCCCAGCTAACACTCCATTCACATGACAAGTAGAGATGCATATAGATCTAAGTTTATGTTCCTGAATTAAGACTATTTTTTGCTCGCATGTGAAATTCGATGTAAAGCAGACTCCCATTACGAAGATATAAACTACCAAGGACTCCATAGTTAACTTCTTCATGACATTTAGTATTTACAAATCGTTTGCAATTTCTTCCAAAAAATCTGCCGCGTCTCTGTGGAATACAACAAAACAAACAAATTTTAAAATGTAGAAAACAACAAACCCATTTAAGACACGGTAACGCAATTCAGTCATCTATAACCCACTTTCGTGCACTCTTGTACCAGCAATTGCCACATGCATCAAGCGAAAACATCCACCATCCCTAAAATTACCGTTTGCGCGCACGCTCTCTCCATACGTGAAACAATTAAAAGACCTACCTCAGCACAGCTGCCGATTCCTGAACATCATTTTCTGGGCTCTTAAGCCAATGAATGTTCGGTTTGCATTCTGATGGAGTAGCAATGATACAATAACCTTTTAAAGGTTTTTCTTGCGCCAAAGACGCACCGCCCTGAACATTTTCACTACCTACATCATCGGTTGCCATAGCACCGAACACTGTCGTATCGTACACCGAGTCGATTCGAGGATCAGAGACATCAAAAGTATTTTTTGCGGTCGTTTTGACTTCCGTTTCCAAAATTGACTGATGGACCAAATTTTCACCGAGTAACAGAGTTTCTGAAGAATTATCCACCACGTCTCCGGTTTGGGTGGCAGCGTTACACATCACCTCAGACCGATCTGTCTGTGTATGCCTCGAGAACATAATTTTTATACCACGAGTTTTGGTCAGTCCATAGATTGTGCAATGTCGAGCGGAGTTGTCTTCTTGACACGATAAAGCCATACACACCTTGCCCCCAACATCTTTATTCGCAGATGGCTGCAGCAATTCTTCAGTCGGCCGACAAAATGTATCCTTAACACCAAAAGAGTCACACTGAATTGGTCTCATGGAAGCACGAGCATCTTCTTCCACACAATCACCGCTCTCTACCTTCTGAAACCGTCTAGGCAATACACCTGTCAAAAAAATTGGGGTATGTCTCATTCTCGTAAAATATTTTTGAAATTGATCACTGTATTCATCGCGGGACTTCTGTGCGATAAAATTACTCAAGATCGCGTTCTGACACGAGAACTCCATGGCAGTTGTCCAAAAATCTGAAAACGTAGGTGACAAATAAAATACCACACCATCTTCATATCCAAGCACTTGACCAGTTGACGATAACAAAAAAACAACCTGTCTCTCTGGAGCCGCAGACGCACCTTCCCATTCTTCCGTTACACCAAACATACTCATCCTACCCTCAAAAGGATACAACCCGTTTATTAATAAACATCTCAACTGACGGTCAGACAAGATATCGGAATTGTCACAAATCCGAACTTTAATACAAGTGATATGTTGAAGAACTATTCCGGAAGCCATAGCCTCCTGTACAACTTTCTCAAATTTCAGAGCGAACCCCGCCGAAGAAATCATCTTATCCTTAAACTGCAACATGCGCGCCAGTTCATCACCGGTGAATGCCATTACGATGTTTTTCTCGTTAATGATTATTGTAACCGGCCATCCAAAAATGTAATAACTGCACAATCTTAGTTAAATTTATACGAAAAAAACACACTGCAACTCCGGAAATGGGTGTGGCATAATTTTAAAAGCACCTTACGAAAAAAAAAACAGACACTGTTAACAAAAAACCAATTTATTAAAAAAATCTCTATTCAAATAACCTCACAGTCATGGAAATATTTTCCAAAGGTAAAATTTCTAACTTCACATTGCTAAAAATTTCGTGAGATTTTAGACTGTCGTTCTATATCAAACGAATTATTTCCGCAGACAACCGCTATCGAATTGTCACGGACCTTTTGAACGCTATGGAGTTCCATGTAATCATCAGCTTCGTAATTGCAGTATTCCACTTCCATAATCACCATAGGCACTTCATCATTTCTCCTACGCCTTTTTAGCACTCTATTTGGATTCAATGAACATCCCCCAGAAACAAAAAACAGAATATACCGCGTTAACATATATACAAACATACACCCAGCAGCAATTGGTATCACAGCAAAAATCCACAGCAGCTGCAATTTATTAAATTGTTTTCTGTGCGGTGCTGGAAAATTATGACGTGGAAACATGTCTTCGGATTGCTTCACGGGATTCACAAAACCTTTAAAACTATAAGCATCTTCTCTTGTTAAGAGAGCTGGGGTAACTAATAATTTATGCGTAATTTCACATGAAAAATTACGTTGTGTCATGCTCAAAGCTCTACTCAATGCTCTTTCATAAGAGTATAGAAGTTGACCGGCAATTTCGGTTCTAATGAAATCGTCAGTCATATGATCATCTGAGACTGTCTGATTTTGAAGACTCCATTGAATATTCGTTCCAATGGGCCGATATCGCCGAGCTGTACAAACGAGCAAACCTGTATCTCTCTTGCTGAAAACTTGCGGAGGACGATGTCTAGAGTCTCCGAAAACAACCTTACCATAGAAATTTAAGATGTCTTCTACACAAAAACGTCTTGCATAAAACGACAATACGTGCAAAGACCGATTTTGTAAAAAACTGCTCAAATTTATAAGATCGTGAACAAAACCACGATTTTCATTAAACAGATCTATCTGTACATAGGTTGTATTACCCGCTAAAAAAACGTAGCTGCCAGACAACTGCGTATTATTGTTAACGAAGCAAACCAATTCGCTTACAGAAAAATTTTGAAGAGAGACATATTGAAGCCCAGCGTGCAGTCTATGAAATTCACGTATCACCACATGTCCTAAGAAACGGTCTACGGTAGAATTTTTTCCTTCTTCCAAGTTCCCCGTAAGGTCAAACAACGTCTCTGCAACAACCGTATGGTTCAGCAATGTCAAATCTTCATAGAGCCTCTCGTAAGCAAAAACTATATCATTATCAATGCCAATAACGGTCTGAAGTTTACCGATTCCTTCAGCCAGAGTAGTCTTGATGTAAAGTTTAGCCGGCAACGAAGAAACGCATTGAAATAAGCATGCGACAAAGACAGTTATCATTTTTAAATTTTTAATTCACGTCGAAGAAAGGTACGGCCCTTGAAGAAACAATTAACCGCCGTAGTCAATAACGTATCACAAAAAGGCCACATGTAGTCAACACTAACAAAGATAAAGTATATTATTTCCTTCAATCCTACAAAACAGGAAATACCCAGGATACCCACAGAAACCGCCACCGCACCCATGAACATAAGGTTTAATATGATAGATGTCATCTCTGCAAATGATAACATAGCCGAACCTTCGTCATAAATCTGAATAGACTTTTCAACATTCTTCTCCGGAAGCGTAACGATAACATTATCGCCACTCTCCTTGTTCGTGCACATGCAGAAATATTTATCCCCTACCCATTTATCCAATGGAATAAGTCCCCACGTCACATCGTCGCGAAATAAAGACGTATATGCACCGGACGATCTCATCTTTACCGTTTTCCATGCCATATCAAACGTCAGTGGCTTCTCCGCAAAGATTTCGATCTTTCCCAG

CGCCCAGCCACCTAGAGAACGAACATTTCAAATCATCAGCTCTGAACAATCTTTCTGAAGAAGATCTGATAACCGGCATAAAATTTAGGTTCAACTTCTCGTCGTAAGGCAGAGCTATTTGTCTAAATTCACCGTACAACTCAGATAGGATTTTAACGTCGTTGTTGCACGAAGATATCAAGTACTGTTGATATGTCGAGAGTTCAGTCCAAAGACGTAACATCGTTCTGTCTTCACCCTGAAAATTCTTATCTGCAATCCAAGAGTTATTGTTCCGATCAAGAAAATAAGCAACACGTAGTACTTGTTTCTTGAGACTTGCTTCACTCATTCTACTCAGTCGTATCGATTTTTTATTAACAGAGCATTGGACTTGAATGCTAATAAATGTCATTTTTCCATGACCACATTTCTGAACACGCTTTCTTGCAAGATGCATAGTTGCACTACTCACGGTATTAAACAATAGTTTATGTACGTTATTAAAAACCTGAAAACATCGAAAAACCAAAAACTTCCCGAAATCATAATCAATTTGACTGTTTTCCATTCCTCCATAAAACACTTGGTTGTTGACCGCCGCACTGCCCCACAACTCCACTTTACCGCTTCTTTGATTTCTGTATGCAGATATCTCTAACAGCAACTCCCCAGCTCTAACCGCTTCATCGTTCCCTACGCCAGGGGCCGGACAAATGGGAGGATAGATTTCACCCCGGGCTAACGTCAAGACGCATAAGAAAACAAAGCAGATCATCATGCTGCGTTTTCCTCCTCTTTTGCGATCACTAGCGTCCACTAACTCCAGCGAGGCAATCTTTAATATAAGATCGCCGTCATACCACAACGTCGAAGGGTTACGCCCACATATTAAACAACGGATCCAAAGCAAACCAGCAAGAACAAAGAACCACAACAGCAAAAAGGTAAACCGAGATACAGTAATAAACAAAGCGCTCACAGCGGGATAAAAAGCGTCGTCTCTCTTTTCGGCAGAATGCTACAGAAATGAAGCAATCACCCCTCTCTGTAGTTTTTCTAGCGTAATCCACCACATACGTTTTAGTTCTAATTGACTCCATCACCGATTGATTTAGTAATTGTAACTGCACACCGCTCGGAAAGGAAACATTACACTTAGTATCATTGTCCACGAACACAACACCAATATTTTTATTGTATCGACATAATATCTTAGACCCCATGGCGGTCTCGATAACGCATCGAGGGTTAAACAAAAAGGCTATATCTCTTTCGAAATAAGCATTAATAGGCACACTAGCCATCCGCCACCAAGCAGTTCCGTTTCTCTGAGTCATTTTTACAAAAATCAACCAATGCCTTAACTCCGTTTCACTGTGTGTTGCGATAAAAACTGAATTTTCATTGTTAATAATATGCAAAGACGCTATCACCGACACGTATAATGCACTCACCCATACTAAACTACAACCCTGAGGCACCATAATCACTAATAACAATCTATGAGAACCTCATCTGCAAGTCTTTGCATCCCTCCTCATTTGTACATCCGGTTTTTTCTCTTCATCCCAAAACAAAGACCTCTGCTTGTCTTTCTTATACCAATTACACAAAATGACTATCAGAATTACGAACGCCCCGATAAAACTAATCCCGCCGATCCAAATTACTAATGAACTTAGAAAATCTTTAAAATACTCCTCATCCCCCTCATGTGTATCTGTAAACTCAAGGACTTGAACAGTATCAGTTTCCCAAAGAGCCTGAACCGTCTTTTTCGGTGTGGATTTCGTCGGCAAACCCTGAGACGTCTTTTCAGACACATCGGAAAATGTCGACGCCGATATTACTGTGAAAGACGTCTCAATGTTCTGCGTCTCACCACCCTTGACCGTCGGAAACGAACTTGTAACATTTACATTATTGCCAATGGATCGATCTGTGGTTTCCACGGCTAACGTAGAGGTCACCATAAAAGACTGATTTAAATATGTCTCAGAGTCTATAGATATTTCGGTAACATTTTTACCCCACGTTTCAGAATTCGCCGAAAACGTGGTTTCGGATTTCCCAGTTAAATAGTCGCTGGATGTCGATACATCCGTTTCCATGTGTTTAGACGCAATTGATGCAAATTCAGTTGCCGAAACTTCTGTAGTCATTAGGTTGAGCATTCGCACCTCTTCACAAAGACACACGAGTAAAAAAGACAAGAACAACATGTTTTTGCGATTGCCCAAGAGCAGCGGTTTTTTAATCACACCCACACCAAGATAGCAGAATAAAAATCTTTAGAACAATGTTCCGAATTAGTTTTTATAATACAACTCGACGAATTCAAATTAAAAACACAAATGTCGATCATAAAAAACACGATTTTGCATTAAATTCAGATTTCCTCATACGTTCAAATTCGCTATGTTAGTTTAATAGTCAATGCCTGCTATTCCTTTTAACCAATAGAGATTTTTCAATGTTCATGTTTCCGCTCCACATCGCATCAGCAAATGTTTCTAAAACAGGTAATTCATCGCCTTTGACGATTCACATGGAAAACGTAAAGAACAAAGACAATAAACAAAATCATTGCGATAGCCAAGCCCGTCAAAACTAAAAAAGAAAACGTCTTTCGTCTATTGTTCCACAAATTCCACGCCGGCCGAGACTGCGGAGGCGGTATGCATTCAACAAACGATGTATCATTGCTAACGTGCGGAGAAACCTGTCCACACATGACGTCCATCATCAGCACCTCTGAATAGGACGGCGGCGGCGTCCGAGGGGGATCCATCCTCTTCACGACGAAGATTCACGTTGAAATACAAGGCGAAGATAAAATTTCTCCAAACGGACTTAAATTACTAAGAAAATCGGAGTAAACGAGAAATGCGAGCACGAATGCAACTACGAGAATGTATGTTCCTTCCCAGAATTCATCGGCACCTCTAGGAATGGGGAATAGAGTTTTGCTTATAAGCAACATGGTAGAAGAATACAACCTACCCTCTTTCAATACAATTAACCCCCCTGACTAGAGACGTACATATCTTTAATAATCCTCGCTCTCGACGCGACCTCTGACAGTGCTCACGGAGAGGACAAAGGACGTCTCTAGAAAACATCTCATCCACCTGCTTAATATAGTCCGTATCTTGCGTAAACCATTGCGTATGCAACCGACGATTCTCATTATAACGCAGACATCCAATTTTTAAAAAATCGGACATATTGCGAGCTATGAAGAGAAGGCTCTCCTCACGGTCGACGCAAAAAAGCATACCGTGGCCGTCAAACACTACTACGAGCTCCCTCCAGTCACCGAAAAAATTATGCTGTACAATAAACACTACATCTAGTAGACTCGTCTGCAGTTTCCTTCGCCACTGAATCAAATGTCTACTAGAAAATTCAGTCATGCTCAGATTGCAAAAGCGAACAAAGGTCTTGTACGGATATGTTTTCAAGGTAAAAAAAGCATTCTTTCTTTCATTTCGGTAATGTAAGAGCTCAGAAAACGATTTACTGTGAGCAATCATATCCAGCGCCACAGTACTGTCCAGTGCATAATCACACGTCTCATAAATCGGATAAAAAAACCGCAAACCATTAGAAAACAAATCGTGAAAAGAAGAAGCCACGTGATAAAGACTTCTGTCAAAAAAACCATTATACACATAGATGTGCCCCCGCGCCCCCTGAAGGACAATCGGTCCCCTGCAGGTTCTCGTTTTTGCCTTATGTATAACCCCAACCACAACCAATTCGCCTCCGCAACACATATAGGTCTTCGCCCAATTTTGAACATCTTCACGTGAAATTTCCGGGATGTTTTCTACACTATGCAGAGTCAATCGATAATCCTCGGGCCAGGGTAAAGCTAACGTGGCGCCCTGGCGATTTTCGATAAAATTTACCAGCAAATCGATGTCTTTGCTTGAAGATAAACCCATAACGATGAACTGCCCGGCTCAGGGCAAATAAACACAAATTGGGAAAACATTAATACTGCAAAAAATTTTATTGGTGTTCACCAAAACAGGCACGATAAATTGTCTAATCACACACCAAAATACATGCAACACACGACTAAACAAAAAAAACCGTTCAATAAATCACCCGCATAGAAAGATCTGTCTTTCAGATCGCTGCTCAAAGTCAACATAATCGAACTAAACCCGAGACCTACAAAGGCTTCAAAGATAATTCGATATAGGCAGACGTTCATCATAAGTCCCGATAAGATCGCCACCACCGCGCACGCAGCGGCTTTGAATACGTTTAGTTTAAAAAACACGTTGTTGACCACAGTCAGGAAAAAAAGATGGTTAGATTTTGGCATACTCAAGAACATTTTTCCGATCCAGAGTATCGTAAATAATAACAAAAATTCGACCGTAAACTTTACAAGGCATTTCAAATCAACTTCACGAAACGACGTGGACTTCAAAATCGGACGATATTTTTGCAAACCAAGAAGGAATCCAGACGATCGTATCATCCATCCATATCTCACAGTAATCGGCAGGAGAGACGCAGCCGTTATCAAGACAGAATAATCATCGAACATTTGACTCTTAAAAAACAAAGACACCATGATTCCCGTAAACACAGCTGGGGAATAAGACGGCATAATCATGACACAACGACTTAAAATCATTCTCAACCTCATCTCGGAAAATATCAAGCAGATCATGACCACTGCAGACAATAATGATGGCAGTTTCGGCGCCATTAAAGACAGAAACACAATCTTGAAATCACCTATGGGTTTATAAAAGCGTGGATCAAATCTTTTAAAAGTCAACAAAAAGGAAATCGCAACGGTTATCAAAACGCCGATCTGTCCGAGGGGGGACCTCCCCTCTGTCATTCTAAACGTATACAACCCCGGAATAACGGCACCCTTAGCAAGACCGAGAATAAAACTATCTGTCAGACGACGCATCTCTGCCTCTTGGGATTGGGATTAGAGCTGTAGTCAAAATGCATGGAATCGTCACTTTCGCTATCTTCCTTCTCGTTAAATAAGCTAGAGCCCGCAGTTCCGTTTTTAGCGGGGACCATGTAGTTGGTTATTTTATATTGCATTTCCTGTTTTGATGCCAACGCACTTCCGTCTTCGTTCTTATGACTCCTATCCCCTTTTCCGTCATCTTGTTTACCCATTCGTTCAAAGCGAGATTCCTCTTTGAGCTGTTCCTGAGTTAAAAAGGCTTCCACCGTCAACAACGTGCTTTTGGAAGTTAATATCAATTTACAGTCCTTAGCGGCTACCGTACGTAAACTACACGACGTCTTGATTACAGCACACATACTTAAAGCCTGCTGTAAATTTTTGGCAGATAGATTAAATCGCATGCTCTTAACATCGTAAAATATGACCTTATTGCTGTTCGAAAACTCCAGTTCATTCATCTCTGTCACGAATTTTATAGTCGGAGGATTAACCTGTACTATAATGTGCGCCATTAAAGCGTCAGATTTACCAGAGCGTTTAGTCTTAGTTACGGGCGAAAGCCATTTCAACACATCGGAAACAGTCGAATGATCAAGGTCAATCCTCAACGGCGATCTCCCGCTGTCTCGCACCACTTCTTGTCCGTGCACACAGGGAACCGACGCCTGTGTACATGTATCGGAAGCCGTTACCAAAACCCTCGTATACAGATCACTATCATGCTGAATGTACATCTTCGTAACCTCAGGATTCGAGATGATGTTCATAAAACTTTCAAACAGTGGAATATGATTATTAATGGTTTTCGTTAAAAAACGATCCGTATCTGACAAAAACAAACATTCCGCCTGAATAGTCAGCTTTTGCACGAGATGATTTTTAGCAGACTGAATTATTATCGACGGCTGCGGTGTGAAAGTCACTGTAGTATTCTCTTTTAACAGTTTTGCAAACGCTTTAAGCGATTTATTAATAGTTTTCCAGCTTTTCATATGAAAAGCTAGGGTCGGCGGTTCTCTCGTTTCTCTATGTTCCCGATGATCACGGTGATCACGATGATGATCTCGACTACCGCGCTCCATCTCTGTCAAGAACGAAGTCCTAGCACCAACTCGCGCCTTATGTGCTTTAAAAAACAAATGAAATGACCAACACATTATATACTGTATTTTTTTGGCGCCAACATTTTATTACATCACTACAAAATGTTCTTTACGTGCATAACCACACCATCCGAACATTTCCCGATCAACGGTAACGGATTTGATAAATCAAGGTGTTTATACATCTCAGAATTACACAAAACCTTATACATAGCGACTTTAAATCCGGCTTTATACAGCAACGGTAGCAACAGTCTCAAACTCACAGTATCGTTGAAATAAAACACATTTGCCTGCCCCTGATCTAAAAAAGGCAATCCACTCTTATACATCTCAATCAAGTCATTCAAATGATAACTAAAACCATCTTTGAAAACGGCTAAATCATCCAACTCCTTGTCCGTAAATAGGTTCGTGTCCACGTTTAATACCCTATATCCCACAGTCTTCAAATATTGCTGATGCTTGTTAAATCTGTTGAGCAACAGATTGTTATACACGGGGATATATAACGATTTTAATTCCGACGAATAAACTGAAGTGACATCATTTAAACCGTCTTTTCCAATTACGACTTTGATATTTGACCGCCTTAAGATTTTATTGTCTTCCATCGGCCAGAACGATTCAGAAGAGTTGATGATATCGAATTCTTTCTGCATGGCAGTACCGGCGGTGAAATGAATATTGCGAACTCCGTACATCAGCACGTCTTTCCTCAGCGTTGTCCACAATTCCTCAGGGAGCGTGCATTCGACATTATCAAAATGATCAAAGTATAACATCCCCAGTGCGTATTTTGACCTGTCAAATAAATTACATGGCTCTGCTCCTTTCATGCAACAATCCACACTCGTTCGAACACAAGTATAATAAATGTGTTCACATATCATCCGATATAATCGACGAGCATCTGGATGTGCATAGGATAACCCGACAGTCATGAAAACAGAATGAAGACCCGTGACACAGATACCTAGACTTCGTCCATCTTCGATTCCCTCACACAGAAAATCTTTATTTTCTACAGCATAATCAATCACAGCATTAGCGACCAACACCATTTTTCGAACCACCCGCCTTAATTCATTAAACATAAAAAATTTACACTGCAAACGCACGAAATCAATCTCCTCAGTTAGCTCATCGCTTACTTCGTCACCCAAGAAATGTACCACATTAAGCAACATTCTCAAACAAGTGTTAATACCGTCTCTGACGGGTAAAACATCCAAAAAATCACCCCCGCAATACAAAGGCACATGAGGAATCATAGAATATTGATATACGTTTTTTCGGAAAATAAGTCCCATCCTGCCCCCTTTTAAACAAAGGCAAATTTTTTCTATAAAACTAGACATAGTAATTTTCGCATGATCTGTTGTCCGCTCAAACTCCAAATATTTTGTCGAGAAAGTTATCTCGTCACACTTTCCCAATTCAAAAGCTATATTTTTCTGAAAAAGAGACCACTGCGCCCTTTTTAGCTCGTACCTGTCCATGAAAATCCCAGGAATGATCAAGCAAAATGTGACCCGAGAAGTCTGAGGCAAAGCTTCATACAAGTCCAGCCACATTAGTGCAGACACATGCCAGACTTCCACATAGACTGTCAACTGAACCGTCTTATCGGTATCACGTTCCACCACCCGAATTTGAGACAGCAATATCTCAAAAATATTCTTGGCTTCCTGTTGATAACGAGTCAAATTCATGCTTATATTAGAACCGTTTAACAAAGCCGGAAAAAGATGTTCGCCGTTTACTGTTGTAAAATCCACAAGCGAATATAAACATGTATTGTAAATCGCCACGTCAAAAACCGTGCTATTAGCCTTTTCCACGAGGCCCAAATTTGTCATACAGGAAAACGGTAAAACCCATTTTTGAGTAAAAAGGGAAAACGCCAGTTCTTCGAACATCACGTAAGGATTCTCCTGATATATCTCCCGCAGATAACCAATTCTTAACACGATCTCTGTCAAAGTAGCACACATACGAAGATAGATGCATGGAATACTCTCTTCGACACCGTTTAAAGAATTGGAAATTTCTTCTAAAAAGCGAGATGCGGCCAGAACTCCGGGCATATACTGGTTTTTCCAACTCACGCTTACTTTATAGAGTACGCTGCGAATAAAATCTCGATACTTCTTTACAAAGTTCAATACATTTGGCATAATCACATTCGGGCATGATAAAATTAAATCGTCCGGACAAAAATCGTAAAAAACACTCGTCTTCAGATACCATCTTCCTAGCATTCGATCCACTCGTGGAACGCAATGTGTAGCTCTCAAAACAGAAATTATGGCATCCCGCAAACACCAAGCATCTAAACCTGAGTCGAGACGATCGATTATCTTAGCGATGTCACGAACCCGTTCATACAAAACAGAAGCGCATGAATATTGAGAATAACAAAAACGTTTTTGCGAAGCTTCGTAATGACAAACGCATTTTCTGTTGTAGCCATAATCTTTATTAATACGACGCTCTTTTCTCTTCATTTTCAAGCAGAAGGCTCAGAAGCTATACAAACACTTTTTTCTCAGAAAGGTCAACGAATATGTTCTCTAGCTTTATCACTGACAGGGTAATTCCACGATACTGCAGAAAACCCGATTTAATGTCCTCCGTCGCGCAACGACCAATACGCTGAACGCTTCCAAAGTAACTCAGCGTGGTATTGCTATTAATAAGATAGTTCAAGAACAGAAACCGCTCCTTTTCGAAATACTGTCGCAAGATGTCAACTATCAGATCTTCGTTCTGAAAACTAGTCAGAACTACGTAGATCCCATACTGGAGCTTTGCTAAAACCTCCTTGTAAACATGAACCACGTAAACATACTGGGTGGAATGCTGCCGCGGTTCCCAGTCTGGTTCTAGTATAATCTCTTTTAATACCGTGAGCCCGTAAATGTTTTCTTTGTTAACGTCATAGACATTTCCTTGCATGACGCATGACAACAGACAACTGCTGCCGCAAGAAACTAAGCTCGTCACACCAAACAAAGACACTTTGTCTTCCAGGTACCTACACGTATTGATCATCGATAACACGTTATCAACCATTGAGTTTGTATACATGGCAGTTAAAGCGGTTCTCACACAAGTAGACACATTGAATGCGTTAGAACAAAGGAAAAACAGACCCACAAACAGAATTTTAGGGTTGTCCACATCAGTCTCAAAACGATACATCAACATTCCCAAATTTTCCAGCTCCGGTGCCAGTAATTCACAAGGAGTGAAAACCCTTGCCGCTATACGTAAATTCTGCCTGCCGGACAAAAACTGTTTCAGAAATTTCCTTTCTTTTTCTACAACATTATTTAAGGTTCCCTCATCGATAGATATATATTTTCTCTTCTTCACAGCTTCCACAGTATCCACAATGGCAGCTCGTGCACTCGATTTAGAGTTCATCCTTAGTTCACTCAAAAAAAGCACGTCTCCAGAATCATTACTAGCCGCTACGGCAAAAATCGAACTTTTATCCCTGGCAGCCGATAGCGTAACACACAACAGGGTAATAGCTTTTATTAATCGACTAACCCCGAAAAAATATCACTTTGACCTCATCAGACAGTATGCGGTATTCTACTTTTTAAACTCTACGACCTTAACATCAGAAAATAAATTGCTCTCCGCAGAACTTTTATACGAAGAACTCAGCCAAATTCCCTCTTCGAGTTCGTCGGGCACAGAACTTGAAAACTTAAATAATGCAGAGGTCCTTTACGTGTTCGATCGAATACTCAATTCCATTAAAATGCTAAAAAATGAGTTATCGTCCCCAATCGGCAAGCTTCGAGCACAACCCGCCGCCAATGACCCAGGTACAGATAAAACGATAAAATACTCCAAGATTCAGTCGATCATACAAAAGATAAACAGCTTTACACACGAAAATTCACTTCTTGCCAACTGCCGCGCCGTCGTAGAGCTGATAGACGATCTATATCGGAAACTTTACTCATGGTTTCTGCACGTTCTCACTTTCGAAGACATTCAGTTTCCAGGCGACACATTTCTAGACAGGCTGTTGAAAATGGACTACTGTTTTACATATTACCCTTCTTCAAATCGCCATCTAATAGATCTGTTCGAAAAGGCGTTGGACAATCAAACGTTCACGAACCTCGATAAATTTTTTGACACCTCAGGAAACAGCCCAGAACTACTTTATCAAAAAACGTTTAGCTTGAAAATATTTTCAAAAAATCTCACAGCCCAGGACAATGGATTATATATCTATCCACTACTTAAAACCGACCTGTCCATCCTCGACTTCCTCGGAACCGAAAATATTCTCTTCCATCGAGGCCTCATCTATCACATACTTCATCAAAAAACAATCCCTCAAGAACGGGAGAATGATCTCAATAAAATCAATCAATTTTTCGCTACTGTCATCCAGCAGGTCATCGAAACGAAAAGCTCATGCTTACCCGCATCGTTATCCCAACTGCTAGATACAATTTTTCACTTCAACAGAATCGGTTTAAACATGGAAACGTGCAGAACCTACATCGAGATCCTCTCAAACCACATGGCAACGCCAGACACCCAACCCATAATCAATACATTCACCATTAACTTAACCCATATTGTTTTCACCGCGCACGTGTTTTTTATCTGTATGGAAAATTTTAGCCCAACATTCATATTCTATAATAGGAAAAAACTCATACTAGAACAACAGAGAGCCATCTTGATCATCGAACGAAATGATTATTCCACACTCTGGAAGCAAATATCTGATCACATCGACTGTCTCTTCAACGTCTCTCTCTCCGAATCCTTTTTTAAAGAATATACCAAAGGAGGAAACGAAGATCAAAAACAGTTTCTATACAAAAACCTATTCGAAAAATGGGGTAACGTATTTTTCCCTTTCACGTACTCGGTATCCACCTCAAACAACTCTACGGCATACCATATTACAACCCTTGAGCTAAGGGATATCTGCAAAGAGGTCTATCAGAGCGATTCCCCGGACGCATATGAATCTTTACTTCCCTACAGCACTCATCCATCTTTCAAAACATTATTCGTTAAAATCTACGTTATACCTATGGTTTCACACATTACCAATCTTACCTTTGATAAATTACAATCTGATTGCAGACTCTTAACTCTAATCCACGCCTGCAAACTCTTACTCCCCTCGCAACATCTACTACTACACTACATGGCGTGGCTATATGCCTTCTCCATAAACGTGGACCATATAGATCTAGGCACTTTCACCGTAATAAAGTCCGTAATCTTTAAAATCGCTGACCACATAAACGTAATGACGCATACGATCTACTCCCCGGAGACAAACCTTCTCGTAAGCATTCTACTAAACGCGTATACCAACTACCTCCAAAAATACGTAAACCCTTGGATCAAACAAACCATAACGGCAAACTTCTCCCTGATACAGACGTACATAACTTTCACGAAACAATGCGCCAGCATCTTGGCGACCAAATGCAACATAAACTTAGACAATCTATTTATCTACATAACTATCGGAACAGACAAAATTGTAACCACTTCGTTTTGCTCCTTCATCGCTACATGCAGAAATTTAGTACGTCAACACGAAGAATTCGAAAAATCACTACAAACGATCCAAATTTCCGAAACCACACTCACGGGGATGCTACGAAACATAATCACGAGCGTCTCCTCCTCCAAAGAGCTGCTAACCAACGAAGCACTACAAAAATTCATCGACACTGTCCAACGCATCTCCCAACACGTAAACGAAACATACCAGTCTATTTCCATGAACCTCGAAAAATGTAAAACCTCAAACGACATCCTAATCGAATCCCTGAAGAAGATCATCTACATAGTTGATGTACTCAGCTCCAATGCAATCCTAAACACGTCGTTAGCTTCCAGATGTCTGGAAGCCGCCAACCTCGCGGTTTCGAACAATTCTTTCACAATACTCGAAATTAAAAAGGACGCAGTTGCCGTTTTCAAGCCTTTTATAACACAACTATTTGAAAGCATGAAACCCACCACGAGTTTACATAAGAAATTGATGTCTACCCAAAAACTAACCACCGACCACATTCCATTTCTCGATACATTTGACGATAGGTACAACCTGGTCAGACACGTCGAACGACAATTAAACTGGTACGCTGCACATGCTGAGGCAGCGCAGCAAGATCTTATTACTCCTCTTAAATTTTAACGGTCGCGTTCTAACCCATGAAAATCATTACCAGTAGCACAAACCAAAATGACTCTAAATACGGACCGAGAGCCGGAAAACAATGCATGTCGAACAGCTTTTCATTTCTCCATACCGTTTACTTAAACGGAATTAACAATTCTCTCAATGCGGGTACTATAGACGCCATAATGGAAGAAGGCTATCACTTAGACACGGCCGGCACCTTAGCCCTAATGTTAAATAACTCAGATTCGCAAGACTACAGACTCCCCACCGAGATACCCAAGAGGATCCATTCTAGATACGGTGTCACACAGCATGAACTCTCACGGCCATTTAACGGAACCCTAGACACACAAAAAATTGACAATGAGGTATATCTCGGACTCATAGATTTCATACTGTATGGCAAAAGCAAGAATGGTCCTACTTTTGCAGTCATCACGATCGGCGTGCTATCGCGAGCACTTTTTTTTCTTAACAATACTCTCTACTTATTCGACTCTCACCCTACTGAACGAGAAGCCACGGCAGCCATCTACATCTGTCAAAACATTGAAGAAGTTTATGAACTGCTCACCACCCACGGCACCGAAGGCTTCTACTACGACGCCAGCCTCATTTTTTTCATAGAAACCTCCAATTTGTCGCTCTCTAGTCACGACGCCGAGCTTCTCATCCTAAAAACATATAAAGACCCCGACATAGCGATTGCGCTAGATAAATTTTCTTCCACGGAAATCCATGAGATAAAAAAAACAGATGATATCGGATCGCAACAAGACCTCGTTGCGGATAAAACGACAGATCTAGAACACGCGCCTCATAAAAGAAAGAAAAACTCTCATAGTCTAGAACTAGAACTAAATGACAAAAAGAAAAAAGATACCGCATCCTTGACATATTACGCAACAGAAGTTGACCTCATTCCGAGTTTTTATGAACTACGATCACAATTTCAATCTTTATTTCACGATCTCAAATCTTTTCCTATAATGAAATCTAATTTCAACTGGACCATATACTTACAAGATTCTCCTATTAATCCTAATCAGCCCTTCGCAACACCCTTTCTCTGGAACAGAGTTTTCCACTTATTGTGTCAAATTATCGACGTGTTTGTAGGCGTCGGATCCACAAATGACGATTCTAGCAAACAACAACAACAAACTATTTTCATAAATTATTTATTGCCTTTCAAGGACTTTTCCGAAGTATTCAACGAGGCGTTGGCAGCTTGTCAAGAAAACAATCTGGACATCATCTTCATTTACAATAATTACCTGTGCAAAACCACGACCTTTCGAACACTCGAGAGAATCTTACTAAGTAAATTTCTGGCAATAGTTGATAATGATCACAAAAAACATTATGAATGGGTCAAATCGTGGACCACGCAAATGTTCCAAGGAATGCCAAAAAAACTAGATGACATAGAAAATTATCTAAAAGCCTACGTGGACCATAACCCTGTGAAACACTTTCATGAATTCATTTGTCTGAATAAGGCAGAAAAATACAAAGTAGCTGTTCTGCTAAACGAAAAACGAAAAGAGATTCAAGAGGCTATTGAACGAGAAAAGAACAGTTTCGCACAACTCTCTAATTTTATTGACAAACTTGGCGAAACTCCCGCACTTCCTATAGAATCAGAAAACGTGCACAAAGTTCACACGAGCGACATCACTGAAGGCATCGTTCCGCGTTTCATAACCGAATCGATTGAACTACCAAACATCTCCACGCTAAACAACACCCAACAAATATCCCTAGATAAACAACTCAATGAAAAACTCACCAACACCATTCACACGCTCACAAACAAATTCACCAAAATCGTACAAGATAACTATAACAATATCGCTGCCGGTTTCATGCCCGTTACCGAACTGAATTGCCTATTTGCCTACCTAGTAAACCTTTATTTTAACATTGAAGTTTTAAAACACAGTGGCCTAAACATTAACACTGTGTTGCTTCAAGAGGTGGAAAAACTGTATGACAACACGCAATTTCTACGCTTCGGAACTTCTCACTTCAATATAAACAACCTTTCAAACTTTACCCTATCTATCAGAAAAATGTTCGTAGATTTCTATAACAGTCAGAAACCCTCGGACAGAGCTTCCGAAATCCTAGCCGCAATCGAATCCATCCTAGCGGATCCCAGCAAAAACAAGACAGTCGTAAATATAGAAATGATCAAATCGCAACTTGAAGAACTGGGGAAAATGGAAATCTCCACCACCGAAAACAAACAGACAGCTGCAATCACAAAACAAATCCTCGGGGACCAAGAGTTAACCCCCATTTACGATTTCCTGCACCACCTCTCTGCTTATAACCTACCTAATACTACAACCGTAAAAAATTTACACCTCCATTTCATCTTAGAACAACGACCAGATATCGCTATGACTCTACACGATAAAATACAATCCATACTTGACATATATGTAGACGACATGCTCAATGACATAACGGTCCCTGAACAGACGTTTTCAACCGTTCTATTTCTTGTAGATCTCTTCCCAAACAGCACCGAAAAAACAGCACTGTTCGAATCCGTTCTCACGCTACGGCAATTAGCAAAGAAATGCGCTAACTTAAAAACTCTAGATGAATTCGACGATCTAGCCCAATTCATCACGACGAATAGCGAACAACTTCAAAACATGATGAGACAGCATTTTGGAAAAAAAATACCAACGCTGATGGGACATATCAAATTTCTCTACTCTCAAAAAATAATCACCACTGAAGAGAAAAACTGGATACAGAGAGCAAAAACGGTCGTCATCACATCTCCCGAAGAACTGACAGCCTTCTTAGCCACTGCACCCACTAAACACGCCTTACAAACTTGCAAACCTGACCTCGATAAAGCACTACAACGACATATGGAAGAGCAAATGAAACAAACTGCCGAGAACGACAAAAAACACATCCTCACAATCAGAAGCACCCTCGAAAAAAGACTGAACGACATCTTACTTATTCTAAAGGATGGTCAATTTTCGTCTCTGGAAACAATGCATTTGAATCTTTTAGAAACCTTTCTGAAACAACTGCAAGATAACAATGTCATCATTCACTTTACCCATGCACTACTGCCAGTGTTAAAAGACATAGAAACGACCATCAGCAAGATCATCTCCGACGTTATTGAAAAGATATTAATCAAAACTCCTCTAAACCCCGAACAGATGTCCAAGGAAGAACAAAAATATACCCCTTTACTCAGTTTCTTATCCAAGTTCAAAAAAACAACATTCTGCACAGAAGATATAAAAACAGAAATCGACCAAATGCAAAAGAGTATCACGTTCCTCAAGAAAATTGCTACCTCCGCCAATAAACATACTAGACTCAGCCACTCCATCTACGGACAAGAATTAAACCTCTACGAAGAACGCATTACAGAGCTGAGAAAAGAGACGAACAAAATGAAAGAACAGCTCTCCAAAGAATACGCATTAGCTGAAAAAAAAATTCTTCTCTCGTCACAAGATGCAAAAACTGACAAAATTTATCTAGTTCTAAATACGCACACGCTGAAAGAAATAAAAAACACTCAATTCAGAGAAACAGCATTCGCTAAAGCTCTCACGGTGGAAGTCAACAATAAAGAAAATCAACTCCAGGAATTGTTAAACCACTTTAACGCACACCTAAAGGCGAAAATGGATCAGAACCATATAACAAAACTTTCATTCGATACGAAATGGACAGCCTTCGTTTCGGATTCCAGGCTCTACATCCCAGACTTCATTAACATCAAACTGCAAGACTTTATTTCTGATCCCTTTAAAGTTATAAGTCAGCTAATGAACAAAGCAACCAACGAGATGCCTTACATTCAAGCAGAGATAACTCTCAAATGGTTAACAAAATTAATACACGACATCAACAAATTCTGCCTGTCAGCTATAAGCGAATTCGGTAAAGAAGCAATCCCATTCAATTACGCAGCCCTACGAGATTTGGAGTACCAGATCAACACCAAATACGTAGAAATTGAAAACAAAGTCATTTGCAACGAAACGGTCGAAAACACGAAAAACATTCCAAAACTGACTAAGCTCTTGAAGCAGTTAGATCCTAAACGCGTTGCCGGTGGACAGGAACAGTATCAAACTCTCATGAACAAGATTCTCAGTTCCGAAACTTCTATGCAACAGACCTACGAAAAAGAACAACTCAAAAAAGAATACTTTGAGATCGTCAATAATGTCGCCAGCTTCAAGTTAGCATTCAACTTTCCTCAGCAACTACAAAATGTGGAACGGCTAATAGAGAAGTTCAAATCGCTGCCCAAAAGCCAGCCATTTGAAAAATTTCCACAGGAAAATGATCTACTTTCTGACTCACTCAACACAGAAAATTATATTAACGGTCTGCGCGCACTCCTCAACTTCATAACGGCAGCGCAAAACTACATCCAGAACACACTTCTGAAACAGTGGGCGGTCTTTCAGCAACAAAATTTCATCCCGATTGATTACTCAGTAGCGAATGTCAAACCTATTTCGGATTTATATGCCCGCCTCAGGATTGAACGGGAACGTCAAGTGTTCTACCAAGTAAACTCCGTCTTTGGAACTCACCTAATCGTTGACGATACAGGAGTTCCGCTGCAATTCCATAACATCTTTAACAACGCCATAGTAAAGTTTTTCTCCCTGAACTACAAGCAGATTCATGTACCAGAAGACACCCCCCGCTTGGTATCTAGCCAATATAAACTCCTGTCCGTATGTAAATCTTTCATAATGATACTACAGCAGTTCTGGGAGAATATAATCACCCTGGACCTAGGCCCTTACCTCAGAGACGGTACACAAAATTTTAAAAGAGAACTCATACCGATCGTGAACTTGAAATTATTTATATACTGCATAACACAAGCCTGGACAGCCTCTGAAGACAGTACTGTTTCCACAGCGTTCGAATTACCCATCAAGCAATTCACACTTTTGATACTATGCTCACATCCTGAATACCTCTACGGCTGCCTCAGTCATTCCACAGATCTGGTTATAAATTCATTAGCCAAAAGCATAGACAAAAACAGTCTCTATAACACATTCGTCGTTAGCCACAACCCTCCCGAGAAGCCCATGCATTTAATGAGAAATATATGTATCGACACCCAGCTATGGCAACCCGCCAAACTTATGAAAGATACCTTCCAACAAACATTTTTTACACAACTGTGCCCCAAAAACGAAAAGTTTTTTATCTATCTCACTGCCTTTTTAATTCTACCCTACAAATTTATGAACTATATCTGGATTCAATACAAACCGGCCATTTTTACCCAAAGATCTTATCAAAACCTAATTAAGGACTTATGCTCCGAATACGTACACCAAAATAAGATCACTACGTCCTCGGTAACTCCCCACGAACCAGACACAATAAAATCCGGAGAAAGGATCACCTCAAAAATCACCGTACACAAAGCACAAAACACACCGACACTCACTCGCCTGCAGGCTCAGGAATATGTCTTCGACTACATTCTTTACTCTTTCTTGACAGGGTATGAAATGACATTCGCTATGTACATCGATACCATCGAAAAAACATACCTACTTTGCATGAGACACCTGGAAAACGTACTCCACGATAAAGACTTCCAATCTGTCCTCAGAGCACGCACATTCGACATCGACTACATCCTGAAACAGTCATGGACTAAAAACATAGTCGAACATTCACTCTTTTCAGTACAGCTCGATAAAATCGTCTCCTACCTCAACCACACAAACAGAGCGACACCCAATATTCCTTTGATATTATTTAATTATGACAACGAAGTTGTCAACGTTTATCTACCGCCGATGTCCAACAATCCAAAAAAAGTCGCCTTTTACATCAAAAATCCTTTCCACTTTCCCGTGCAAGAATACGAAGCGACAAATTTAATTTCCTTCCATCTCTACCCAAAAACAACAGATATTTTAAACCAACTTCCGCCCAACAATACTGAGTCTACTCGCCCGGGTAAACAAACCTCTGAAACTTTAACCACCAAAAACCTCTCCGAACCAAAGTTTAAAAAGCCTGCCGTTACTGGACTTGTGCCCAAGAGCCAATCGATCATCCTATCCACTGATACCAACGTACCGGAAACCAGTCCAGACGTAAAAGCTAACACGGCCAGCGCAGCAATAAAAGATGTCACTCTAGCTCGAGAAAAAATCAACGAGTTCTCCGAATCTATAAATACAACTATTTCAAAATTAAAATCTATGTATCTGTAACATTCAACACACAGTCATACTCCTTTTATAAATGTTTATTATCCAAACATACGAAATAAATGAAGCTTTCATCAATCACTCCCTGTTTTTGTGTTCAGTCTATGCAGCGCCAGCATTCTTATCAAATCGAGTCGGAACGCCGTTTTATCCACGTTACCGCCGTTCTGCTTAGCATATTTTGGCAAAAAAACCCCAAGAACGGGATGACTGGCCATATTAGGCTGCAGCCCCAAAACACCGGTAAGCATCTGCTGCTTTTTCTTTTCTTCTTCTTTTTTTGAAGAACCTGAAATCTGAGTGATCTGATTCGACAAATCATCACTACGTATGGTCGTCATTTCAAATACACCTCACGTTTTGGGAAATCTGGCAACCTTGACAGATATTAACACAGTCTTCCAGATCTAAGCAAGTCTCTCTAAACGAATGCTGACAGCGCTGACACTGAAAATCAGAACCGTGTGAAACAAGCCGTAACAACTTCTTGACGGTCACGTTGTAGAGATGCACAGTAGAATAACAGGATCTACACGAACAAGGAACAATAACATCGAACTTCACATTGTCATTTAAAATAGTGCAAGCAGAATTTGTTCCGATCACCGCCTTCCATGAAACGGTTTTCACCTTCATCCCCAATACACAATGCTCAGACATCTCATATATCCGCTCGAACACCAACTGCTGACTCCCACACAGCGAACAATACATTACATCCGTGTGCATGCTGTATATCAAATTTTTCTCCTGCTTGTCTCTAAAATAGAACATGGAGCTCAGCGAAAAGCCCTGCGGCGAACACAGCTTTTCTTTTCCGGAGTTTAAGCAGTGACCACATTTTGCGCAAATAACGCAAATGTAACTCTCTTCGAGTATAAAGCGAGAACATTTAGTCTTACAATAGCACATAACGGGTAACGAAATAACACTGGTAGCTAAGCCATCCTTTAAGGACGTCGCCAACGCAAAGGTTAGAATCTTTGGAAAATCACCCCGTTCAGGTTTGCTCGGCACCATAGGTACTAGATTATTATGCTCCACTGGAATAAGATATCTGTCTGCACTAAAACAGAAGTTTTCCACCAAAGCCTTTGCCATAGTAAACATGAAATTCCCCGCCGGAGATTTCAAAGCATACTTTCGCAACATAGAGGGAAATGACAAAACGTGCTCTCTCTGCCTATATCGCAACATCACCGTCTCGGTCTGCTCATAGTCACGTAAACAGAATAGCCTCATACACGGTAAGTTCACGTGCGTCGGAATCGGAAGCAACAACTGTTGCTCGAACAGGAACATGTGCAACTCATTGATTTCGGAAAACGAGGGCATCTCCAACAACTTATTAAGCTTGTACGGGGGATAAACCCTGAATCTAGTCTCGACGTCCACCCATTTGATTGTTTTAAGTTTATAAAGAGACAACAATTCGAGAACTTTACACTGCCGATACACGAAATACAGATAGGCGAGCAAAACAAAAAGACCCTTTGTCATAACCATTTTGTTAAAAAGTCGCTTAGTGATTAATCGCGTCTCCGGCCTGCAACGCAACCAAATTGCGAAAAACTCATTATAAAATTTTTCCGTTACTTCCTTATTCAAGAATTTTTTATTTGTCAAAAACATTGTAAGAACACACTTTCCTGGCACACAGGCCTCACCCATCACAAACAATTGCAAATGCGTGTACGGCCCATGTTTACACAACGGAGCCAGAAGCTGGCGTAGGAGTTTTTTTAAGTAGAACATAACAAAAATACAAGGCTAAAAGAAAACAGATGCCGACAAGCGCAAACCGACAATGTCTTTCACTTAGTCTCCAAAACTTCACACCGGACAGTAATGACTTCTCTTTTTCATAGAACTGCTTTATACTCTCCTGTGATGTCACAACCGCAGAAGCTTTATTTACAACTTTCCCGCTACTTTCGAATCGCCCCTTTTCCAAAACATGTGAAGACCTTCTTCTCAAACCTTGACAAAACCTATTTGATTCGAGATGCTGTTTCCCCATAGAACACTTCTTTAAGAAAATTGCCTTCAAAAGCGAAATGTATTCAGATTCGTTTTCAGGTCCGAACGCCACAAACTCAACAACGTAAGTGTCGTCTCGATTACGCCTGATTATACATTTCGTGAACACTGCAAAAGTTGAAGGCTTCATAGCCACTATGTCGCTCATGCGGACAGTCAAACATTCATTAACACGCAGGATGTTGCATTCTCTCACAGCATGATGACCTTCTACTTTAACCGGGGCATGCAAATAGCAAGACATCGACACAGCTCCGCCTGTGTTCTTGAACACAAATCTGGGTTCCTGCTTAGTTACATGTTCCCAAAGACTTATCAGATATTCTAGATTATAAGCGTAGTCTAACTTGAGAATGATATCACACAAAGGAAACTTCGGGTTCTTCGACAATAGATTCCGCTCAGTTATCCTATAGTCATGAGACCCCAACTTTAAGATACGACAGGTCGCACTCAGTAGTTCATCGTACATCTTTTCTTTGAGTACGTTAGCCATTTCACCTATTTAAAAGCTATTCCGTATTTAAATATTCTTGATGTTCTAATATTCCATTTACATTTAACAGCTTTGACAATATCGAAGTAAGAAGTACCATTTTTTGAGCCATGACCTCTCTATGCACGCAATCGACATCCCCGTGAACGTATCTAGCAAAAGCAACGTGTCTATATCCAACACTAAGAAAATTCAGTCGCGCCTCCAAAGAAGGAAGCATCATTTCATATAATCGTGGCAACATTGGATAAAACACGACCTCAAACTCCCTTCCCTGCTCACAGACTCGCTCATACAAAAGTTCAAGATTCGTCTTTTCAGGTGAGGAATCCATCATGTACAAAGGATGGGACTCAGAAGCGTTGCCTATACAATCTGAGATACTAAACGAGATTCTGTTATATTGCTACCTCACTCCTCAACCACCGATCCCTTCCGAGACGACAACAGCCACCAGCCCCACAAACATAGAAAACGAAATATCCAATTTAGACAACTCTGAAAACCTAGAGGAATTAAAGCAACTATCCGTCGCATTAAATATAGACAGACGATGCAACATCTGCTCCATCGTAAATCTGTGCCTCAAACAAAACAAATCATGGATCTACGACTACAGTCTACTGTGCTACAAATGCAACTACGCGCCAAAAACTCCACTGTCTCTTCTGATCGTTTCCACAGAATTTTTAATGTTAATACGGGAACGATTTCCAAACATCAATTTCGACGGTCTTTTTCTAAACAACATTGTCTCCATCTTTGACTTTCACGTTCATTTTTTTATTCACAGATGCTTCGCAAATACAGTCAACGATCACATCCAAAGCGAAAACATAACCCTGAATCACATGGCAATAATCCGATCGACCCTGCTAAAAGAAGACACTATACCGCACATCAAAATAAAAAAATTTCTAACCAAAAAAATGAACCCGAAAAAAACACAAAGCTCCGAACTGAATAAAAAACTCACAGTGCCCATGAAGACTCGCTTCACGACACTCTTATTCTATATGTGGTCTGGCACGAACGTCTTCGACCGAGTTCCCTTCACGGACCTAACTATCCGCAAACATCGCTTCATCAAAAACCTGTATTCCAATAAAACCGACATCGAACTCACAGCTGGTCCCATTCTTTTGGCACAGATTCCATTTTCTATCACCAAAAACAAAACAACCAGCGTCTGTCTCCTATGTGAACTGATGACAGCTTCCAAACAAGATTACATTTTTTTAAAATATCTACATCAGAGCATCATGGACTACTGTCAAAACAACCTAAAGATGATAGATAGAGTTCAGTTCGTCATCGCCGATATCTTCGAGAAGACAAAAATACACACGCACGTCAAAAATCTTTCCGACTACAGCAAGGCGATATTCGATAACGAATTCTCATTCTCAGATGATAACTTCACTCTCGATACCCACGTTTACCTGATACTCCGCCAGACTGGAACAGTGGGAGTCTATAAGCACTTCTTTTGTGATCCCCTGTGTCTAGCAAACTGTAAAACCATCAACCCGGAGGTTCTCTTCAACACTACAGACGCCGGAGAAATCCAAGACTTGAAAGTCACCATATGCTACCGTAACGAATATTTAAGCATCGTCGAAAAACATGTTTGGCTAGCGATACATCTTTTCAAAGCTTTTCAGATTATTAAACCAAACCATAAAAATAAAACTCAAATTACAGAGTTTCTCAAAGACTTCACTAACCTATTAGCACTTCACCACTTCGATATCATCGATCCCATTTTCACCGTCAACTACTACGTTTGAAGAATGACTGTACACAAGAGCCGTGTTCGACGATCGCGAAGCCTGTCCGTGACACATAGAATACAAAAACGACCTGATCACAGAGAAAAAACAAAGCTTTACTTACAACTGAAACTTCACGATCTACACACCGTCTTCAACATGTTTCCAGAATACGAACAAAAATTTCTAGCGATCATAAAACTGCCAATTACCGGTAAAGAACCCATCGACGTCCCATTCAGTCTCAGCAATCACCACCAACACACCTGCCTAGAATTCTCCCCCTACGCTAACGAACAAATCTCCAAAAGCGCCTGCCTACACTGCGAATCCGTCTCCGTTCCAACCTCCTCAGATGCAATGGTCGCCCACTTGAACCAAGTCAACAACGTCATGCAAAACAGACTTTACTTCTACGGTTTTCGAAAGGACATGGAGCTCATACGAATGTCCGCCAAACAACCCACTATCTTTCAAATCTTTTATATTGTCCACAACACGATCAACAACATATTCCCTATCATGTTCGAAAGAAAACAAAAGCTCGGAATGCACATAGTTTTCCAGTCTCGCACTCTGCACATTCCTTGTGAATGCATTAAACAAATAGTAGCGGTATCCTCGGGCTACAACGTTTATCTAGATATACTACAAGAAAGTGTAATCCTAACTGTCCTCTGCGAAACACTAGATACCAATACTAATATCCACATCGACATAGGGATGCTTCAAAAAAAACTCGAAGAAATGGACATCCCAAACGAAATAAGCGACAGACTCGAAAAATACAAAGGACACCTAATAGGTTTCCATTAAACCACTTTATTATTAACATGTACAAACGACAAAACCTAAATACATTCACATTACCTCTGCAATAGCAGAAAATGTTTCATCCAAATACACTTTCATAGGAAGTAAATACAACAGTAACTTCTCTTTTTTTATATCGGTTTTCGGAAAAATGGGTGAGATCGCATTAGTCACAGCCTTGATAATCTGATCGAAATACTTCTCCGCATGTATAGGAATCTTGTGTTCTATCACATAGTTGGGATCTTCTGCTAATTCATAGTTATGCGTCTGTTTATTGCCAATAGATGGTGCTATTAACACGTACATAATTCGGTCACCTACATTCGGAATTTCTTCCTTTCTCTGCGCCAACCTTCTAATGACGCTAAGGTGAGCCAAATTCGGTTGCTTATATGCAGCCATTTCTTTGGAAAGCACAGAGGACAACATTAAATGTCTCACGTCTGCCCGATTCTGAAAAAGCTCCTCCCGCGCTTCGCACAGACGACGCAAAATTTTATGAACACCCACAGGCACTCCTTGCTCACGCAACTGTGTTTGTGTCATGTGAGAAAACTCCACAGCAGCAGTCTGAACCTCTTCATCAAAAAACAACAAGTCCACGATATCTTTCACCACACCCTTCACAAAATCGCAAGAAGTCTTTCTCACCAGATCTACCCCCTTAAAAATTAAAAGCGAATCATCCTGTCTACCAATGTATCTTTTTTTACAAATCAAAATTAACGGACATAAAATCTTTTCAAACTCGAGCTTGATAGGCGACTTGAACAGACGATCTGTGATATGTTTGGCGATCATCGGCGCAATCCTTCGCAGAGACTGATTAACCATATTTCTGACAGACATAAAGATGCTATCCGTATCACCATAAATTACCTCCACTTCCAAATCACCAGTAAAATCTGATGACGTTAAACCGAATTCCTCGCAAAAAAATTGCTCGGACTGCATCTTGGAATTAACATAATCCACCGTGGAACAAAGCATCTCTCTTCCAAGACACGTTACAGAAGCAGCAATCGCAACACACGGCAATAACCCGTGCGCCGCTCCCGTAACACCGTACACCGAGTTACATGTTGTTTTCAGAGCGAGCTGCTTTTTATCCAGAAGAAGTTTCATCATCGGATCCGAACAGTTCTGCATCTCCGCCTTCACTTCTCGTCTCTTGGCCAGCCAGTCCTTTAGTAGACTTCCAAGCACAGACTCACGGATACAAGGCTTCACAAACCGATGAGTCTCATTCCCCAACTTCACGGTTAAGATGTCACTCTCTGACAATCCAGCTATCTGTCGTTCATCCAAAACTAAAGTACTATAACACAGATTATGCGCCATCATAATGCTCGGATACAAACTTTGAAAATCAAACACCACGGTAGGCACAGCATAATAACCCGTCTTAGGCTCCAAAACGGTAGCCCCTTTGTAACCTATCCCTTGTCTGTTGTGCGAAGACACCATACTCGGAAGAATCATATTACGGCGTTTTGCTTCCGTAAGAATGCAGGGAAATATCTTCTTCTGCTGACCCTCGAACACCACACATCTAGCCGTGACGTGTGCCAATCTGGCGACCTCGGCAACCTCAAAATGATAATTAATCTGTTTAAAGAGACGCACAACTAAGACAGAGTCCTGCAGACAATACTTACCGACAACAGCCCTGCCACTGGGTCCACTAATAAATTTCTTTGGTATTTCCTTGTACGATAACTGCTCCTTTTCTTGCTGGAGACAGATCTTAGCAATAGTATCCAGTTTGTAATTCTGCGCCGTTATCTTACTAGAATAGACGGGATACATATCCAGATACAACACTCCGGAAGTAAACACCTTGGTTTGCGCCTGAAGGAACCCCTTCTTGTACTGTTCGTGAGGGACAGAGATTCCAATCTTTCCATTCTTCAGTTTAGAAAAACAACCAATATCATAATGGTAAATCTTATCCATCCTTATACACAAATATTTTAAATCAAAATTATTAATATTATAACCGGTAATAAACTCCGGAGATTCAATCCTTAAAAATATGAAAAAACCCAAAATTAATTCAAACTCTGACGCAAATTCATATATATGCACGCCGTCAATTTTTTCACATGTTCCCAGAGTAAACAGATGTCGCTCATCACGGTCGCCTTCCGTATCGAAACTAATTACAGAAATCTGAATAACTATATCGCCTAAATTTTCGGCATCCGGGAAATTCCCATTTTGGCCCAAACACTCTATGTCGAAGGACCAGCATCCATATAAGGGCCAATTAACGTCTTCCAAAGAAACTAAATCAGAGACATGACAATTAATTTCCACCTCAAGATTACTCCCTTTTCCCATATCTTGAGGAATATATTTTTTTACATTATACCATCCGAAACTCAAAAAACCATTATCGACGAAGAAACGAGTCAAAACATCTACGTCGATTTCATAAACTACGAATCCCTCATTCTGCAGAATCTTTCCAATACGTTGAGATACATAAAAATTTCCAAAACTCACTTTAAACAGATTAACGACAGTGTTGGCATTGTACCCATACAACGACAACTTATCAGCCGGCTCAATTACAAAAGAACACGACATTTTAACCTCGCCGGTTAACATCAAATTGTTTATAGTAGCCTTCAGGCTTTTTCCGTCGACGCATTCGCAGTAGAAATATTGCTCCTGCCCAAACACGTTCACGCAGATCTTCTCACCGTCCTCAGTTCTCCCAAACATTCTAATCACTGTCCCCGAAGGAATCACAAAATGTCTATACTGAAAAGGAAGATTCTCGATCGAATCCGTAAACATTAACGTTTCTACAGCATCATATATGTGAAACTTCATCCTCATATGATCATAATCCTTGGACCGAGCTATGTCCAAACTCGGCCAGGTCATTTCTTTGCTCAGTAAATACTGTCGATCTCGATAAAACATACGCGGTTCAGAGTCACAAACATCCTTTATTAAGCCCGCCGCACCATCATGCATTATACCGCGAGGAAGTATACGAATGTAACTCGATCTGCTTTTTTTCTTTAAGCGATTCGCTTCCAAATATGGATTAAAAAATGACACCGAATCCATCACGCTTCTTCTACATTTACACTCTTATAACCTCTATACCTGATCCTGTCTATCAAACTAGGTTTTGAGGCATGAGACTCGGAAGACGAAGGTTTTCTTCTGTAGGACTCGTCTAAGGACTTTATGGCCTTGAGCATTTTTAAAGCATCTTCTTGTGAATATTCATCGTCACTTACTTGCCCACTGACGTCAGCCATACCCTCCTCCTTTTCCGAAACCGCAACAGATGTGCCCCCGTCAACATCTTTAACACTAGGTGTCTTGACGACAGTGGTCCCCGTAACACTGGACACAGTAGTCACCGGATTGGTGGCATAAGGAAACATCATGTCAATAGGCTTACTGAGCACATGTCTTTGTCTAACGAAAACCACAATTATTATCACAACAACTACTATCGCTAAAATCAACATGAGACCACCCCCGAATGGATTTTTTAGAAAAGACACCACTCCACCCACAATATCCCCAAGCGCTCCTGCCGTAACACTTATAACCGAGCCCAATCCAGTGCCTATAGCCCCAAGCCCTTGTAAAAAAGAATTAATCCCATTAACATACGAAGGTGTATTCGTAGCAATTTTAGCTTCTATAGTATATAGTGCGCTCTTATATGAGTTATATTCACGAAGAATATTCTCTAGATCGAAAACGTTTGCTCTACTCAATTCGTCCGGCGAATATAAATCGAGTACTTTAAAATCGGCATTTTCAAGAGGATCGATTTTTAGTCTAATAAAAGCATCCAATACCTCAATGTCTTCTATGGGCGTCGAATTCGTATGCGTATAATCGGTATACACGTGCGCATGATTTCCAGATAAAAAGATCTTCGTACTAGGTATTTCACATTCCTCTGTCCTATGATCACCCAACAGAATTTCATTATCTAACCCTAGCTGCCCAGGGACAACCTCAGGCGTCGAGTTCACAAAGCTAAACGTCACCAAAGGCCGATTATAACACATCGTTTCACTCCTTACTCCCTTTGCATCAACAACCCGCATACTCTTATGAAGCTGAACGGATGACTGATTAACCTCTATGCATTTCGAGATAGCTAACACGTCACCATGCAACTGTGCAGATATCGGACGACCGTAAACCTCAGACACGATACTCGACGGACTAATCTTACTAAGTTCGTGCAACATCGTTATCGTTCGCTTTTGATCGAGACACCAAGATTCTGCCAAATTCCCCAATGCATCATTGATATAATCTTTCAAAGTATCATAAAGATACTGTAATTGCACATAGAGAATGTCATGTCTAGACTTAACATCCACCACATCTCGCCTCCTACGTATTTTTTCCGAACCCTGCTCAAGAAACATAAGAGATTTTTGCACAAGAGGCTGCCAAATCAAAATCAAATCTCCCGTAGTCTTAAAAATTTGATAACTACCATTCATGCTATATGTATCATTATAATCTGACATATAGACTTCATTAATTATTTTTTCAAATTCATCCTTAATACACGTTTGTTTCGGATCGGTAAGATTTAAGGACTCCTTGGGGGCGACGAAAGCGGCTGTCAACTCCTTAGAGATAAAGTGATAAGTCTCATTCGTCTCCGCTCGAAGCCCGTGAGTCACCGTGGTCCAGTGCTTTAGCATACACACCGATTCATTCTCTTCCTTGATTTCCCAAGAAAACAAAGTATCCGCTTTCTCGAGAAAAGCGATTTTCCTCACTAACGTAGTGGCCCCATTCATACCATTCATTAGATCTTCTATCATAGTATAGTTTTCCAAGATCGTCAATTTTTCTAACGGCTCTGCAAAATGTTTACCGTTTGAACCGTTGAAGAACGGAGACCCTTCCACGATTTCGCCAGTCGTCAAAGCAAAGTAACTAAACGGGTATTTCGCCTTAGCCGTAGCCTCCGTCACAATACAATTGAGAGACGTTGATGTAGAATATAGCCACAAAGGACCCCTTGCAAAGTAGGGTTCTTTCGTAGTGATAAATCTTTTATTAGTTATAGATTTGAAATTCAGAGGAAATAAATTTAGAGTATTGTTTTTATTATTATCCTCATGAAAGGCACTAAACACCGTACCATCGGGTCGTTTCATCGCTACGGCGGAATAACACTGCGCATGAGAATTAACTAAATTTGCTTCGTACACCGGCATGGCCAAACCCATCACCGTCCGATCCAGAAAATAAACCACACCCACATCACGGTAACTACTTTGGAACGTCAACTCTTTTTTATACGTTCTCACTGGAAAAGTGTAGGTCTCGATATTTGTTTTGTAAATGATGAAAAAACCCTCCGACATCTTTGCATTAGACTTATACGGCGAGCACGAAATATCTCTGTCGAACCGCATTAAATCCGTGCCTTTGGCAATCGAACAAATCCGAAAAGGATATTTGTGATTATAGCCCGCTCTGATATAATGATCCGGATCACAATATATCATTAAAACACTATTCATCAAAAAGACAGCCAGGAATAATACTGCCATCTTGCTCATTTTCCCTAACGAGATCTAGTAGATTAGGCGTTCGAACAGAGTAACTTACTCCTTCCGGCACGTCTACAGTCTCTCTCGACAACTGAGCTTGCAAATCACAATATAACAGCGAATACACGTCTTTAAACTTTGTCATGCCACCACCAGTGTGAAATAATAAAGGAGACTCGTGATTAAATGTCAAAACGACATTTCCACTCAAATCCCCCTCCTCATCTATACGCACAATTTTTAATCGCTTTCCAAAAACATCATTATATAGTGCAACAGAAAGTGTTAACTCTCTGATAAAATTCCACATTTTTTTCTGCATGTCATTTACATCAGTTACACCGGAAAAGTTAAAAAATTCATTGTATTCGCACACCATCCACACACTAGGATGAATAGTGCCCTCTATGCATCTAGCTAAATCTTCCTTCATATGCGGTAACATCTCCGCGTGGTCGCAACCATACGCCATTGAGATATTCGCCGGAAGTGGATAGGCAGATTTATGAAAATCCGATAACGGACCCGTTAACAATGTGTACATAATCTCACTTAAATCCGGCAAGAGCTCCAAAGGGAGCTTCTTCGGCATCAGATTGTTTTTAATATAAAGATGTTCATCGTATGTACAGCTATCTACACGGAGAGAGCCATCCAAGTACATTTTTCTGAGAACGAAACCGTTCATAATCTTCGATAAGGCCTCAAAAATAAAATTCCCATACACATTCACACTCACTACCTTATTGATGAGCGATTCCTGCTCTCGAATACATGCCATCACCCTATTATAACCAGATTCAGAAACCTTTTGAAAATACGCCTTTTTCCGGGCCAAGACTTCCTGCTGAAGATTATGAGTTTGAAGCATGCAATTGATCGTGTTCAGCCTTCCCGGCGTTTCTGTCGTCTGTGGATCCCGAATGTCATGCAAGAGACTCTTAATTTTATGTTGAACCTCATCTTCCTCAGCCAATCTTTTAAATATTGGACTTTCCTCGAGATTTTTAATGCTCAGGCCAGTGACAAGATCTATCAGCCTCGAAGGAGACACATAAATGGAGCCAACGTACAAGCGCTCGTCATGCGTTAACAAATTTTCACTCAAAGCCAAGAAGTCTTCAGACACTTCACCGTAAAGATAAAGACTAACATCATTCTGTAGTTGCTTATACAACGTATGACAACTATTTAACTGCTGAAGCGTAATAGCGACATTTCTCACTATCGTTTCCGATATTTTAGACCAATACGTAAACTCACTTAAAGAATATATCACGTCAGGTACCTTGCTAAAAATATTATACTTTTTCAGAGTTTTCTCAGCATCACTAACATGCAACGACTCGGCTGAACGCTGCTGCTTAAAAATCTTTTTGATTTCAGTCGCAACAGACTGAACAGTACCATAGTTCAAAATAGCCTCCCCTAAGTCTTTCTCTATCGTCTCAATGTTTTTTTCAATATTAAAAAAAGCTTTTTGTTCGGTCAAATGATTACAAAACTTCCCATGTAATCTTCTTCGAAGTGATTTTCCTTGATTTGGAGTCAAAGTCAATTCTTCGTAACACTTCATGCAAGTCTTGGTTTCGAGATAACTCTCCGGTTTCGCAACCATACATGCACCACACATGATAGTTAACAAGTCTATAATTTTTCCGCAAATCTGCAACCCTTCTTGGGGCAAAAGTAAAGCATATACGGAGTTGATCTTACTGATTAATTGTTCTATATCATTTAGCGTAGATAATTGCACGATAAACTCAACACCGTTATTCAATGAAATGTTTATTGACAAGTGCTCAGCACAACGTCTCAAACACAACACCTTTTCAAAATATTTTTTTCTATCCTGGTCTACCATAAAGCTTTCCAACGCTGCATCCAAAAGAAGCACCTGTTCATACATAGCTTTTAACAGTACCTGCATATAAACCGTCAAAGCTGACGCACAAAGAAGATTTTGTTCTTTGATACCTTCAAAAAAAGTTTGATATAAATGCAGAATCACAATCCCATTCTTTTTGAAATTTGCCATATCAGACAATACTATTACAGGATCACAAAACTTTAAACATTCAAGGTCCAAAGCACATTCATTAAGCCTAGCGCACAACACACACAGAGATTGTAACGAATTCATACTCCGATTATAATCGGTTTTTCTTAGTCATCGGCTCATCTGTTTCGGTTTCCGTTTCATCACATTTTAATACACCGTTTCCTTGATCATCCTCCTCCGCTAGAATTTCAGAAAAGTCATAGACCGGAGCATCGTTTGAAGACACCGTCTCGAATACCGACTGCAGATTACTTATTGAAAAGTCTTTCACATTCATATCAACTAAGTGATTCAATTTAAGCATTATGGATGCCGCTAAGGCTTCCCTACCATCCACAAAAAAAAGCATATCATCCATAGTCGGCTTGTCTTCCCTATCCCTCATCACCTCAGCTATTATTAATAATTCTGGATCAATATCGTTAGTTAAATTCTCAACAATGCTGAGAACCTTGCCCTTAACTACTTCCGTATCAAACATAATTGCCTCACGCCGAACCTTTTTCACTATAACTTCTGAAAACTTTGTAGCAATAATCGTTTTCTGTCTCATAAATCTAAAATCCTGCAATGCAGAAGAGGTAGGGTTTAAGTTCCTATCCACGCCACTTCCGCCTATCCAACCTAACTGGCCAAACTGAAAATATTCCCTATTAGCCAAACTCACAAATTTCTCTAATGTCAAACCAAATGTAACCAAAGATCGACTTGTGAAAATTGTACCAAAATTGGGTTCTTTGGACAGTGACGCAATCACAGGATTCGGAACGCCATCCAAAACTAACTTTTTAACATAAACTAAATATTCCTGAATAGAAGAAAATTTAGGCTGCTTGTCTAACACCACGGGAAACAACACAGGATTCTGCGTAACCAGCTTAGATGTAAGACCGTGTAAGCTAGTCAAGTAGTCATGAAAACCTAAAGCCGACAAAAATTTGTTATGAAAATAACAATCTATAAAAGTTGAGAGACACTCTGGCTGTATGTCGATAATATCTATCTCATCATAGGTATTCGTAATACTGAATGCAAATTTAATAAAAGTTTTAACTTCTTCGGGGTTACCAATATCTAAAGTTTTCGGAACATTGTTGAGCAGAAAACGTTGCCATAGCTCTAAGCACGAAATCTTCACATTCGGAAACAATTTATCATGATATTTAAATAAAAGAAAGGAGATACAGCCAGACAAAGGATTCTTCTTATGTTTCACGTTTTTTTTAAAATAAGGATTGTTGTGTGCTGTCTTACTAGACTTGTTGAACGGTCTATTTTTTATTCTAAAATCTCGAAGACATTGCATAGACAAACGGCAAAGTTTCGCCTGAATCGTAGTTTTCACATATTTACCCGATTTATATAGATCGCAATCTAAGATTTGCTCCATATCAACATTTGAAGCGACTTTAACTGTTCTCGTATTCAGAAAACCCTTTTTGAGATAGCTGCTGTGAAACGTTCCGTATAGTGACTGGTACTGCTGAACGAGCCATTTAGAAATTGAATTACCGGTACAAGGTCTATCGACAACATGACCTGAAGCCACTATCAATGCGAGGTTCTGCAAAACCGTCAAAATGACCTTATAATATGAAAACGTCAAAAAAGGAGAAAAGGCAGTTGCATAAGGGGTCGTATCTACATTAAATGACTGTAGACAATTTTCTATCTGTTCTCTAGGCGTTTGTGTCCTTCTCATTTCAACAATGCATCTAGAAACACATTCCTCGATACACTGTGTTAATCCGTGAATTATAGAAACAAAATCTTTTTTACTTCGAACATTGAAAGTATCTTCGCCTGTTACAGGATCAATCAGAGAATTTTTTTTACAGTAATCGAAGATCTGAGATACGAATTTACCCCTGTCCAGTGAGAGCGTGTTTTGTTGATCTTTTCCAATCTCTTTGAGCTCGCTGACAGGCTTCCTGCCAAAAGAGCCTAATATATCAACCTCGGCATAATACCGAGAAAACATGCTCATAACAAGTGGTTCCTTTTTAACATTCTTTGGTATTGCCGGCAAACGACTACCGACCCGCACCATGGCAGTTCCAATACACGATTGACAACATTTCCCATCACAGAATTCACATAAATTCGCCGAACAATTTACCAAATGATTGTAAATCTCAGTATGCGCATTACCCGCGTTATAAACGCTCATACGATTTAAATTCCACACTACATAAGAAAGCAGCTGTGGAGAAGTCGCACAGGCATAAGCCAAGTGATTAGCTGAAAATTTGTCATCTTTGCCTGATGTCGAATCACATTTGATAATTTTTCTATTATCGTTATACACATCCTCAGTCAAAGACGACAACGCATTACAAAAAGACAAAGATCTCAGCAGTAACTCCTGAGTCATCACCGAATTAAACGCTTGTTTCACGTTTGGTATAAATGCCAATTTTGAGCAATATAAGATAGAATTTGGGGCAAAGATCAAAACAGCAAGATGACTAGACAGATGTAACTTTAAATTCGTGAGCTGCGCCATGCGTTCAGCGTGTGTCTCTGAGTTTCTTATGATTGGCCAGTCATTAAAATTCATTATCTGACTAGGGTCATACACCGAATCCAGATATTCGGCTACATGACTAAACGATAATTCTGTCACAACGGAATCAACCAACATCAAAAAATCTTTCTCGACCGCTGAAATTTTCTGTCCGGAGATACCCAGATAAGTTTTATGGGGTGCTAATTTGACAGTTTGCTCCCCATCCTGTGTAAATTGTTTCAGGCCAGCATCGATTAATTCCTTAGTATTAGAAAATCGTAATGTTGTACCCCACGATGTAAAAACATAATAAAAAAGCGTCTCACTTAACGCAGGCAGAAAAAATGCTCGTTCCTGAATAAATTCTGTACAGGAAGATAAAGAAATCGTTTCTTCAGTTTCAAATAAAGTTGCGGAATATAGAGGAATTTTCACACACGAATACTCCCCAACTTGTACTTGAATTTGTTCTTCAACACAAGGAATTAACAGACCAGCGAACAACAACTCTTTGAAACCGTTTCCACACGCAACATGACAAATAACAGAATCTGCATCTTTGCCGACAGCAGAACAAAGAGCTTTGATATCGATTACTTTGCGTGCTGTAGGCACCTCAAACGACTGCAAGTGAAATTTCTGTCTGGTTTGCTCACATAATCGAATAAGATCACCATGATCTTCGGCCATACCAACTAACTGCTCAGTTCCATGAAAAAAAAAACACACGGGCATAAAAGACGTAATTTTAGTTAGAATTGTGCCTCCAAAATTAGTGATAGGTGTTTTAACCGTGGTCGAGAAATCGTTTTCTACAGTCAAATTCATTAACAACGGCGAAATAACTACAGGAGAATTTCTCTCCATTAACGATAAAACAGATAAAACATCCAAAAGTTCTTTATCTTTTGGGAAAACATAGATCCAAGCAGCAGTAGAAACGGGAGCAGAAACTACCGTTTCGTTTTCATCAGCCATCTTTGTGGATTTGATCACAAAAACAAAGATAATGGGGTTTTGTGGTGAAATCCTTATATATTATGTTTGACGTAACATAACACGCGTCATCAAACATAAGAGTAAACCACAAGTTGGATTATACCGTTTTCTATATGAGGTTTACGGACAAAAGAAAAACGATTTTTTTATGCAAATATTTTCCACGCAGATGATATGACACGCCCCTTAATTTAAATTTATGCAAATCGTCGTCCACCTCAGGTACAATAGTATATTATATATATATTATTTTAATAAACTTATTGAGGACGGGAGAACGAGGGCGTGGCGTTTACGTCATAGTCTAATTATGCATTCTCAGAACAGGATTTAAAAAGGCTGCGAGCGGCTGACTGTTCAGAGGGACGCTGGGGTACGGCTTGAGACGTTTGATTGAAAATGATTCTTCGTGTGCTATTTTCTGCAAAAAAATTAATTCGCCGGCGACAGTAAACTTTTCAGCGGAATTTCAAAAATTAATTCCACATGTAATTTAAGCATTTTAAAACGTATAACTCACAACGTGAAAACATATTTACGAATACAGTAGTTTCCGTGATATTTTTTCGAAATTAAAAAATTTTAAATCGGGTAAATGATAAAGCATACTAAGCTAAGTTTTTTGAGGTTTTCGGTATAATGTTAACATTTAGTTCCACATGTAATTTAAGCGTTTTAAAACGTATAGGTCACAACGTGAAAACATTTACGAATACAGTAGTTTTCGTGATATTTTTTCGAAATTCAAAAATTTTAAATCGGGTAAATGATAAAGCATATTATTAGATTCCACATGTAATTTAAGCATTTTAAAACGTAGAATTCACAAAGTGACAAAACATTCACAAATACAGTAGTTTTCACGGTATTTTTCGGGATTAAATTTTAAATCGGGTAAATGACAAAGCATGCTGTTAAATTCACGTTTTTAGGGCAAGTTCTTCGAGGTTTGCTCTGTGATTATCAGTGAATATTCAGCGTTTCAATTTTAGATTATGGTTCGGTAAAAATATAGGTCTTTGAGGTGTTTGAAGTCGAAGTCGTTAATATCGTATTCAGAATTAAGGAATTGGTATTTAGGATAGTTTTTCAGTATTTTTAGTCGCGACGAGGTTATGACGATGTTTTCGTTTGCATAGTTTAAACGGTAAATTCAGAATCCGAAAAAAGTTCAAAGAGTTTCATGGATAGACTCTAAAACAGGCTTTCAAATTTTAGCTAGTATTTATTTTGAATTTCACTTAGGGTACATAAAGTTTTGGCATCAAAATTTTTTTGATTTTAGAAAGAAGTAGTTCAGAGTGTGACAGCAAAAAGTTTGTACAAAATGTAAGAATTTGTTTACGGTAAGATCGTTATTCTGAGTCAGAAGAACATGCGTTTAAGTTAGTTTCACAGGTGTCAGCTTCATAGTCTAAATCTAAGTCCATTTCAACACTCGATTCACTTTCATGTTTAGAGTTGACGTTTTCATTAATGTTAATAATCACAATTTCTTCTTGTTTTGCTTGATTGAAGTTTAGTTCTCTAGGATTTGGTATTTTGTTGTCGGTTTCCATTGATTCTTCTATGAGAATTTCGGTAGTTGTTTTTGTCGTTGTTAGGATTGAGGTGTCTGGTTTTTCGACACAAACTTTGGACTTGAGTTTAGGCGTATCATGTTTTTTATGTGAACGATGAGAGGTTTGAGGTAGGTCGTGATTTGTTGAATAGCTGCATGAAGGTTCCGTGTCGACCTTTGGACAAGTAAAAGAAAGATTTTGGTATTCTTCAGGAAAATTTGGAAATAGCTTAAACTTTGGTGCGTCTTTGGCGAAATAAGTGCAAAAAAATAGACTCGGTTTCCAAGGAGCCATGATTCGTTCGATTCTATTTTGACAGCTGACATCATCGCATTTATGGTTACTGAATTGTTTTCCGATGAGTCTGCTCATCAGACCGCATTCGTATCGCTCGGCTACTCGTCCCGTGTCGAAATCGCGGATAAATTGCGGTTTCTTTGTTTGTATGATTTGCTCTTTAATTTGTCGTAGTATTCGAGCAAACGTAACAACGATGGACATGGCTTGTTGAATTGGGTGTCTAAACGAGTCTGGAGTTTGTTGTTGTACTTCAAGCTCGGAGTTTGGCATCTCTCGATCTAGTACAGGTTTACCGCAGAGTTGCCTGAGTTTTCGGTATTCGATGTCACCGTGTCCGTTGTCGTTGAAAACACAGGTTAACTTTGCCATGTTGAGTTTGTTGAAAAATTCGAATTTCATGACGGTGATGGTATACATGAATAAGTCTTGTGGGTTGACCGGCAGGTTGTGAAGATCGTGAAATTTAAAATAACTGCAGGCTTCTTCTACTGTAGCAACTGTAAAAGCAAGACAAGGTGAGTTGGTCAGGGAGAATATCCGGTCGCCCATCAGTTGCTGATTTTCTTCTCGTGTGAGCGAGTCTAGACATTGTCGGTAGCGAAGCAGCAGGTCGGTTTCCAGTATATCATTAGTCAGGAGGCAAATTCGTTCGTGAGTCTGTGGGAATCTGTCTCGGATGCGTTGTTTTGGATCACGGGCGTCTCCGTTGTAAGGTCTCAGGCCGAGAGATTTTAGCGAAAAGTTTGTGTCGGCAAAGGGGACTGTTGGAAGATTAGGCAGAAAGGTGATCGCATTGTTTTGAGCGCGTTTTTCTTGTTGTTTCTCTTCGATGATGGTCTTCAGCTGTTCTAACTCTGTCAGACTTAGTGCGTCTAGTCTGGATTCATCCAGTTGTTTCAGAGGTGCGAGTTTTCCTCGAAAATTTTTGGCGAAATGGCTGATGAACTTGCTGGTTTGTCTATGCGTGGAACGTTTTATCGTTCTAAAGGCGGTTTGTCTATGATAATCATGATGTGAGCGTTTTACTCCACGAGGATACATGTTTGCTTATAATAGATGAAATGACATAAATTGGTCTCTTTATATGTTTTTTGAGATGCTTTTCGTTGGCGCTTTTTTGCGGTTAAGTTCTGCGAAAAAAGGAAGGACGGTGTTACTGATGCTTTAATCTCCGGAAATACTAGGATATGGCGCGATTTAGTTAGCGTGTGCGTTGGAGTCAAATGGTAAAGACTAAAAGAAGGTCTGGGTTTAGAGGTAGTAAGTTAGTTTTTTAATTGCTCCAAGGCGATGGAACAATGTACTTGTTTGCTGTTGGACTGGCATTTAGTAGCAAAGCACGTGCTCCACAAGATGAGAATTAACCGATTTCCGTCGGCTTTGAGTTGTATGTAAACTAGGACGTTATCTCGATTTCCTCTATGTTGTCTGTTCAGGCAGCTAAAATCCGAAACTCTTCCAAATTGTGTTTTTTTGATCTGGAGGTTCTTTGAATCTATTTTAATAAAAGTTACTTGTGAAAACTGTTGCGCTATCCTAGTCTCGTAATGAGTAATTAGTTGCGTTTTAATGACTGGCCATCCTATTAATCTGATAAACGATAAAAGGGAGTCATCCGCTGATGTTGCATTGTTGTTAAAACCATGCGTGTTTAGGTACTTGGTGATTAGAGTCTCTGTAGCTATGTTAACTTTGTTCCACAGTTGTCTGGATTTTAAATCCATGAAACTTAGGTTTTCTTTAGGGCAGCCTTTATCCGTTATGTTATAGATTAATTCTTCGATGGGAGCCTTCTTAGTACCGTGATGTAGAAGGTTGTTTAAATCCATTTGCATCTGAATAAACGTCTTGCAAGAATTTCGGTAACCTGCTGGGACGATAAATATGGGAGTTAGACGTCCATGTAGCATTTTCCCTTTGTCCATGTCTGTCTTGTACATATAACCTATTCTGATGCAGTGACCTGTGTGGTAAATGCCGATGTCGAAACATTCCCCGGGAAATGTTATGGAATTGAGGATCTGACTAAGCTCTTGATCTAAACACATAACGTGATTTAAAATCTTGGATAATTGTTTTAATGGTTCTCCACCGGCGATCGCTGTGCCATTAGGTAATGGGATTGATATTCTAAGACCGATCTTTTTCCGACAAACACAGAATTGTTTGATTTCGGTTGGATCTTCAGTATAGTCTAGTGTGTCGTTAGTTGTGTCGCACTGGGTTTTGAAGAAGAAGATGGGATGTGTGTCTGTGTCGATGCTTGGAAAGATTTTTTTCCAGGCGTTGATGAGGGTAAGTCTTATAAGGCGACACATCGAGAAAAATGTTTCCTCCGTAATAGTTGCTGTATCTTGCAGCGGAAGATCCAAGTCTCCTATGTAATTTGTGACTGGTATGCGTTCGTTGAATATTTCGTGTCGTGTGGTATAGAATTGTTTTTCGACGTCGTCGCAGAGAAAATGTGTGTCTTGCAGCCAGATGGCGCGAGTTAGAGCTTCATCAGAGATATATTCGTCGGGGAGATACTGTAAGATGCGGTTAAATCCCATGTTTTCGTACCAATCCTCAGAGTTTCCGAAGCAAAAAATATTTTTATGTAAAAGCTGCACCCTGAATACAGGAAAGGGACGGTGATAATCTTTGTGTAGGTGTTGTCTTCTGTAGATGCGCTTATCTTGTATCTGAACGTATCTATCGGAAGCGGAGATTTCTAATAATCCTTGTAATCCTGGTTTTACTGGAGAAAAAAGACCTTCTCCGTTTTTATTACATTTCGCTATGGCACGCGTTATTTGTTCGGAAGTTAATGCTTGGATGGTCTTCTTTTTAGAGACCAGAGCATACTCGTATTCTGTAACGTTTAGATTTGGATTTTTTAATCTTTTTATGTCTATGTATGTTTTAAAGTAACAATCCGCATTAAAGTATTGCTTCATGATACACAGTAAATTTTCGTGCAGGGAATTTCCAAGAATGTGGAGATTTCTCTGACATTGTTGCCCTATTGGTAAATTAGAGTCGTAAGAAGTGAGATTGCAGTAATTGACGAATTTTGTTGTCTCTAGGGCCGTGTTGTAAGCGATATATATGTAATGGACACGGAATGTGTTGGTTAGATGTGAACTCTTTCTAAATAGATTGATTTGAGTGTCTAGATGTTTAAATTCTTCCTTATCGCGAAGATTTTTTTGTCTGGCGTACGTTGAGAAGTCTGCAAATAGTTTTCTAAATTTTGATTTTAAAGTGATGTTTATAAATTCGGACATGGAAGAATACGTGAACACCATGTTTCCGGTGTTTTCCGTGAAGCATTGAACATAATTTTTGGTAGTCGTGATGGTACTCTTTTGTTCCTCGAACAAATAGTAAAACATTGTTAATAAAAGCATACCTTCTGTTTGCCCGTAGGTCGATAAAAACCAAAAGGGGGAAGTGGGCAATAGGAATTTACCGGTGAGATATTTGATAATGAGCGATTTGTGGAAATAGGCCATGTATTTAAATTCGACGTCCTCTCTTTCTTTTCCAGTTTGAGATAATATCATGTCGACATATAACGATTCGGTTTCTTTGTCGTTGAGGCTGTTGGCGACATCTTCAGCCTTTATCGGACTTGCAGTTTGTATGTAGTGGCTGAGATTTAATTTTTCGGCATGGCAGACAAATACCGTGTCGATTCGCTTTGAATTTTTGCATTTTTGAGTTTGAAGGCAGAAACTAACGTTATTTGATGGTTTATATTTAACAATAATCGGAAAGATTAAATGTTCACTGGGACTTCTACATAGGATACTAAAAATTACGTTGGCGGCATCGTATTCAGTCGCAAACACTACGATGGTCATTGTGTAGAGCTCTGTTGATTACAGATGAATTTAGCTTTAAATGCCCTTAGGGTATTGGGGTGTTATGGGAAATTGTTTTCGACGATTATCTTTGTTTGGACAACCGACTCAGGCAAAGTATGAAATGTTGTGCTCTTCCGACGATTTAGAGACCGAAGAGCTGACGTATTTTCTGGATATGACGTATAAAGATTTTGGAGTTTATCAGAATGATATTATAAGTCATCAAAAAGACACGGAAACTATGAAAACTCTGTTAGGCTTATTACCAATGTACAAAAAAACTAAGCTCAGGCATACGGTTATGGAGCGTTGTTTATCTAATTGTCCTAACCATGTTAAAGATGCGCTCTGTGTAGAGCTTATGAAAGCTGAAAAAATATTACAAACGATGGATGTCGTTTTTATGAAAACTTTAATTGGCGAATTTTCCATGTGCACCGAAAATCTTAATCAGTTGCTTAACAAATTCGCTACAGATCAGTCAACACTTAGCGATGTCGAGAAAATAAACAGTCTCATAGAGATTGATGGCGAAAACAGCAAACGTCTTTTAGTCGAGTTGGATCCGATTCTCCATGAGGAGACAGGATTATATCAAGCGCTGCCAAATGTTGTGACGGAAGCTCCAAGTGAAAAGGTTAAATCGATACGCGTTGAGTCTGAAGGTGAGAGTGTATAGTTAAGCGTTATTGGAGGCGGCATAATGAAACAAGAAAGAGGAATTGGCGTCTAGTACCTGTAGCCGGTTTATTGCACGTTCAGGCATTAAACTGAGGCAAAAATGTCCGCAAGTAAAAATTATCCTGGCGATGCAGATGCCTGTAGGGATGTAGATAGTTTTGCGTGTGGAGTTGAAGATCTTTATGGAGGGAATTGTGTCTTTGTTCCAGATGTAGGTTTCGATCAATAAGCCGAGTTTGGCAATTTCTTTTGAGGGAATCATGAGAGCCTTCAGTTCATCGGCGCAAACGTACATGGCGTCGAAGTATTGTATCGTATAGCTGTTCGGTTTAGCTATAAATTCTCTGGATGCTTTTAGATGGAAAATTGCAAATTCGGGGGGAGACAGATTGATCATCTCCCATGGTTCCGGGATGTAGCAAGGGAGAAAGTTGATTGAGAATTCCACATCTCCTGGGAGAAGCATTAGAGAGTGCTCGGATAAGTTCATTAGAAAAACTGAAATGTCGGTTTCGTCGGTTATTATTTCAGTGACGCATTCGCAGAATACGTCGTTGCTGTGGCCAGTAATAACTCCGAAGAAACCATCTGGGATGTACAGGGAAATATCTAGTTTTACGATTCGAACTTGATCGTGCGGCACCCATATGAGTTCTCTATTTACGAGTGTGATTTTGTTGGTGTCCATAAAAACGTCCATTTTACTATTCCTGTGATGGCTTACTAAAATAGATGGTTCTTCCGAAATACAAGTGTCCGTATTTGCATTTCTTTTTGCGCGGTGCCTTGGGTAGAAAGTTACTGGGTTGTCGTGTTTCCTTTCATAGTTAACACAATCGGTTTCTCTTTGCTTCTCTTTTTTGCAGGTCTTCGACACTGTTCCATTTTGCGGAATACCTAGAAGCGGGATTGGGCGTTGATAAGAGGTTAGTTCGTTTTCTAGAAATTTTTTAAATGATTTCTTATTTACAGCCGGCCACACATCGTGCGTTTTGTCTTCAGATATGTCTGTAGTCCAAAGTTCATTGATGTCCACATTTCGAAAGACGAAAAATTCAATGTATCTCGAGCTCGTTTGTCTCTGTAGGGTAATCGTTTCGGAGATCTTTTCTGAAATTGCGCTGTACATTTTTCCAGGTGACAGAGAGCTGTCTGCTGTTAATCACGTCGACGCGAAGACTAGTTGAAAATAACGCTTAAGATGAGTTGTAAGAAAAGCGAAAGGCAACGCTTATATGTCAGTCTCTGGCTCTTTTCCATTCTTGTTTTTGCGGCCGCTACGGAAGTGGACTTTTATTCTCCAGAGTGCCATTCGCATACCTATGAGATCGTCTTGAATTCGTTTTCTTCGATCTGGCTTTTGATAAATCTTTTTTTATTGTTATGTTCTTTCGCGATTTTCTTGAAATACTGGTGTTATAAGACTTTTGCTTCAGAGACGGTGAAGGGTTATTAGATCGCGTGAGACTTCTATTTTAAGTATAAATGTAGCATACGACAAATAAAAGCATTTTTGTTGTGATTGGATCTTGTTTTATTATCTTCAATAGATTTTATAACGACAAAAGACAAAACTCATTTCAAAATTGAGTTTTTATTGAAGACTGAGGATGAGCAGCTTTGGCAAACGTATGCAGTTATGTGCTGTGTTGTTTAAATCGCGTCGTTCGTTATATGTGTTATGCGTTGCATTTTGGGTTGAGTATGCTTTGGTTTCAGTAGTGAGGTTATCTGGATGGTTGTGTGAGTTTTTATCGGTGATGCGGTAATCGTTGTTTCCAGGTAGAGTTGCGTTAGCCTCCCGACTTTTTGTGGAACTTTTTTTTTTAAACCTCTGTAGATCGTCCCGTGATTGTTTGGGGGTAGATGTTAGTCGTTTGTGACGAGATGTTAGCTTTTGTTAGTTTGGCTGATTGAGTTCTATTGCTAGTCGTTAGCTGTGTAGTGATATGATCTGTCCACCTTAGACTTGTATTCGTTAATGGTTCCTTAGGCGTTGTTGATAATAAATTTGACCAGGTTAGGATGGCGGGCTTTGAAGATGTCGTTAAGAACGTTTCAGCGGTGAAGGACCGTTGAGGTGTAGTTGATTTTTCCGATGTGTGAGCGTTAAGAATTGGTGTCATTTGTGCTATTTCTGTGTGGTTTATCTCTTCGTGAGTGGACGCTGACATTCTAGTATGCTTTACCTCTTTAGTGACTTTAGTGATTGAGTATGATGAATTACTAGATGGTAAAACGTTGTTAGTTATGTGTACTTCAGTTTTGGCAGTTACATTCGACGGTAGTTTTTGTTTTGGAGTGACAGACAACGTCTGAATGGTAGGTAGCTCGTTTGAGGATTTCAAGGTTGTCCTCTGGAATTTGCCGGTCATGTTTTGTACTTCTGTGTTGTGACTCATGTATGTGCGTGCCGTGTTTTGCCAAGTGGTAAACTTTAAAATCGGAGTGGTGTGTTCGGCGTCGGTGAATTTTGTTGTACTCTGCTGTGATTTGCTCTTAAGATAAATATAGTTTTTTGGTGAAGAATAGGTATTGCCGTTAATTTCCTTGATTCCTATTGTTGTGAAGGTGGTGCTTTCGATCTTTTGGGTTGCGTTTGTTGTTACTGTAGCGCTCTCAAAGGTTTTGGGAATTCTGATTGTGGTATTTTCAAAATTTGTTGGTTTTGGTGGACTTTTTGGGTTTTCGGTGTTTTGTTCGGTTTTAATAGTGGCGTTAAGACGGATTGTAAGAGTTGGAATGTCGGTTGGGGTGTACATGGGTCGAGGAAAATCTGAACGTTTAACTATTTTTTTGATGCTTTCCGTTGTTTGCGTTGTTATGGTGGGTGTGTTCGGTGTAACTTTTATGGTGTCTGTATGCAGTTTTGTAGAGTTTTTTGTTACGGGGGGAGGTTCTGGGGTTGGTATGTTGGTACTAGGGATATGTGGTGATGTGAGAGACGCTGACGTCGATGATGGTGTCGTAGTTAGTATCGAATCAAAGTTTACTATGGTTCGTCCAAAGGAAGTAGTAACATTTATAGGTTTTGGCGACTGTTTAAAATATTGATCATTTGAAGGGACGCATGATGTTGTGTTATAGAGCAAGAAGTAAACAAAGCTGGTGTAATTAAAGAAGATTTCTGACGTGAACGAAGTCAATGACCGTGTCAGATTCGAGTTGGAAAATTTGCTCATTGCCATCCGCAAGGATTCTTCGGTCAGTTTTATGTCAAATAACTGTGCTCCCAGAATTTTAAATGGTAGTAACTCTACGCATGGCGGATCTGCGGTATATGTAGATAGTGTGGCAAACAGAAGATAAAATAGAGAAAAACTGCTAGCGATGATAGATTTGTTTGCAGCCGTCGACGATAGCACTATTTTGGTTTCTGTCCGCGGTAAATTCTGTAATGGAGACAAGTTAACCGCAGACTTGCATTGTGAATCTCGAATGTCCCAAGAGAAAACGTTAGTTAAAATTTGAGTGAAGCAGTCGAGGGATAGATTTGTCTGACAAAAACTTGTATACAAGTTTCGGAGGTCGTAAAAATGTTCTCTTTGTTCGTCGCTCCAATTGTGGCGGTTGACGGTTTTGAAAGCTTCTAAGGGATCAGACGTGTATTTTATTAGATTAGCGGAGTGTATGACTATCGCGAAAAGGCCCAGAAAAAGGCCGAGTTGCGAGATGTGAAGCATGTCGGGTGAGTTGACCACAATGAATTTGTACAAGTAGAATGTGTGACTAAAATAGTATTTGCTTTTTTTATAGGATTGGACGATGACCTCCAAATGCATGCTGTGTTGGACCGCGATTACGAATGCCCTGGCTATTTTAATGTTATTACTAGTAATTTACCGGGCGATAATCGGATTTAATGGCGATATTTATACGAGCACGTTGTCGGCGTTTAGCTGTATACGGACAAATTCATCTAATACCTAAGATAAATAAAACGTTTATTCAACACAATCTGATAAGTCTATATAATCCGAATAAAGCAATTATTGCGATCAGAATATAAATGATAACCAATATGATAGACACTTGGTCTATATCGATTCCGACTTCAGACATCTCAAAAACAGAGCCGTTTTTGGCTAACAAAAGATAGTGCGTCCTGGTGTTGGGAACAAGTAAATTGTTGTTGGGATCTGTCAATGTTTTTAGTTCGTCTATGTTTTTTATGTAAATGTATTGTAAGGGACCGTCGATATCATCATATTCCATGACTACGCTCTGACAGAACTCGCATGTTTGAGATGAGATGTTTCGTAGCACAAGCAGATCTTGTCCTGCATATTTATAGTTGGTTGAAACGTAGGTGGAATTGAGAGGTATGGCTGTGATTATTATACTCGTAGCCACAATTGTCGTTGTAATTGTTAGGCTCACTCCTTTAATCACGTAAGAGGGAGAAATTACAAATGTCACATTCGGCAGAGTCAGAGAAGCTGCTGGTTCACTTGGGGAGAGACACTGAATAGCCGCTACTCTCTCCATATTTGGCGGTCTGTATACGGATAGCATCTCCATCATGTCTGATCGATAACGGTCAAGGGAAGCGTACGTGAGAACTGTTTTTAGAAAAGAATTTGTTACATCTCTGCGTACGGGGGAGGCACAAGGCGAATAAATATTTTCTAGATTCGGTGCCCTGTCAGCTGTAAGCATGCGGGCCCATGAAACTATTTCTACGGGGTTACACAGGTTTCCAATTAGTAGTTGCATGTGCCTCTGCATGCGTTCATCGGTAGGGATGATATTCGCTATTTCGGCTAGCATGAATAGAGTTTCCCTATCGGAAAATGTCAGATTAGTAAAAAACATGTGTTTTTCATAGATGTTTTGTAGAGTGTTGGCAATGTCAGTCCACTTGTACTTGGAAATCGTTACATTGGATTCGTACGCTGTCGCTAAGTACGTGAGCAGCGTAGATACGGAAATGGATTTGTTTTTGGTTATGTAGCTATAGTCCGAGGGCTCGTATTTTAAAAATTCCTTCATTTTTGGGTATTGTAACGTACCTTCCGATACGTTCACACATCGGTTGACAAATGAGTCGATGGACAGGAGATTTTCGTGTGTCTTTAACACTCTTAACACGGGCATTGGTCCTGTGTTTTTTGTGCTATATGAGAAATGTACGATACCGTAAAGAAACATCATCTGCGGTATGCCGGCATAGTTGGGTTTTTTACAGTCTCCTCTTAATAGGGTTTGGATTCCTAGGTTATTAAATTTGAGTAGTAGAGCTTCTACTTCATCTACGTCATTCAACGTTTCGTCTACGAATGTCGCATCTGATATGAAACGGTATTGTACATCTATTACGTCTTTTTCGACTATCAATAGTAAATCGTCTGTCAATGTCTGTTTGAATATGAATGAACTTTTCTTATAAGGGGCTTTAAAGTTTATGCTGGTGGTGTTTCCGTACATCATAGTCAGATCTCCCGCGGGATTCTGGATGTACAGTATCATAAAGTCCGGAGTAAAGAAATGGGTATAGATGTCTTCGCCCACATAGCGCGTAATTTTAGCGCATCTGACTTGGACTGGAAATATTAGTTCCTTGAGAGAATTAGGCAGCTTACAGGAAACGTTGTCGTATTGCATCTGTCGAACTTCTACGACCCTGACGTAATAAATTCCCTGTGAAGGGTACATGTTTAAGGAATACACAACCTGCGGTTGGATAGAGCGATCTAAGTTAAATTGTTTGACCGAAGGTACAGGAGAAAGTCTTAAAATATCGTTCATTGGCGGCTCATAAAAACGTGTAACGCGTTTTTCATAATTTGTTAACGATTCTGTCGTGTTGATGGCATCAAACAGGTGGTATGTGATATCGGAGCCGAGTAAGCATTTAGGGACTTGATATATCCGAGTGTCGTTTTTTGTATAAAAGTTAAATGTTATAAAGCCGGGGCGAACGATTGGATTTTCAAAGTTTCCATTTCTACAATGGCTCGAGTTCGATATGTTCAACGGTCTCCAACCGTAACAGGGAGTCAACAGGACAAAGACCCAGAGTCGGAGGAGCATAGTTTTTCTTCATCGATGAAGTGTGAAACGGTTCTGCTATTCTTTGAAAGTTGAACACTTTTTGATCTTTCCATATTAAATAGATTACAAAAATGATGTGTAATGGAACTCGGAATTGTTTCTAAATTCTCCGTGTCTTTTTTTCCCCGTGAAATAACATGTCTCTGACAGGTTTGCCTGATATCCGGAAAAAAATTGGACAGTTTCACCACTTGAGGATATATAAACAGATCTTATCTTTTCAAGGAAATTTTGCGCGACTAAATTATTTTTTAGGAGACGTGTTTCCGGCTAATTTGCGATCGGCATCTGTCTCGGTGTTTTTCGAGGTGCGTCTAGGGCCGCGCATTCCGGATTGTATCGTCTTGCTAAAGAGTGTAGATGTCAAAGATGAGTTTGCTTTTCATTGTTATTTTTTCGAGTTTAAGACGACTTTGGGGAAGTCGACGATGCAGTCTGTGCATCACAACTGCATTCACCAGGCGCAATATTTGCAAGGTTTGAGACAGTTGCAACAGTCTATTTCGTTCTTGGATCAATATTTAATCGCAGACGAGGTTTCGTGGAACGTTGTTCCCGTAATCTGTTTTTTCAGGCAGTGGGGACTTAAGTTAGATTTTTTTAAAAAGTTTTCGGGGAAGACCAAGCGGTTGTCATTTTCGTTTATACGTGATTTGTTCGCCAGGTCTCAGGATGGCGCAGTGCAATCTCTTTTATCAATACCCAATTACACCAATTTTAGAAGGGCATGTCAGAAACATACTGATTTGTACAGAAAAAGATGTCGAAAAGCTCCAAAGTCAGTCCTCACTAAGACTTCGGGAGAAAATAGATCAAGGGCATCGCGACAAGTTGCTAAGAATGCGCCTAAAAACCGAATTAGACGCACTGCAAAAAAAGATGCAAAAAGACAGTGACGTTTTGAATTCTCATTTGAAAGCGATAGAGGATGCTTTGCTGTTTACGAATGATGGGGAGGTAAACGTCGAAACGAAAGCTGATGCGCAACTGATACCGAAATCTCCTGAGAAGCTGGAAAAATTCAATCAGGTCGCTATAACCCCACTCGACCCCTTTATAAGATTTACAGACGATTTTAGAGGAGAAATGATCAATACATTTTTCAATAATGCCCAGATGTGGAATTTTACGTTCGGGTCTTGGTTCTATAAGCTCAAGAGGGTTTTTTACAATGAGCCCGGTTTAAGAAGGGCTTTAAAACTTACTAATGTGGATTCTCTGACTATTTCAAAAGAGTTACTAGCAGTTACCGTCAATGCGTTGGAGCAAGCGACGGTGTATCCTATATTCGGAAGTGAAATGTCAGACCTGGAAGCAGCCTTGTGTATCCTGGCAGCTTTTTATTCCACTTATGAGAATTCACAGATCGACGAACGTACAACCCTAGAAGATGTTATAACCCTCTTGCCTGTGATATTTAGATTACTCGGCAGTGAAATTACAGCATTGAAAAATGTTTCTCCTTCTGGCACCTATTTTGGATTTAATGATCCTTCTTGTATGAAATTTTTTGTTCCCATGAGAAAAGGCAAGCATTATGCAGAAAACACATTCGGGAATCATGTGCTTATAAAGATGTTGTTAGGTAGAGGGGTAATGCAAAAAATTCCCGGTGAGAAAATTAGTCAAAATTTTGATGTTGAAGCTCGTTTACACGGGGCCATTAAGAATGATGTTTTGGTGTATTGGACATATCAGCTAATGAGGCCAAAATTGGGAAATAATGTTCCAATCTTCATACACGATCAACATTATTTACGGTCTGGCTTGGTTGCAATTGAAAGTCTTTTTTTATTGTGGCGGATCCTGAACTCAGAAAGTTTGTTTAACAAGAGAGTTGGGAAGTTTCTCTTAACTTCGGTTTTTCCACAGTTGGAGAATGTCGATTTCGCCGAAAATAACTTTGAAGCTGGAAATATTCAAAATTTTGAATACTTAATGCACCATTACGTTGTGCCTATGTACAACCTGCAGAATGACATATCAATTTCGACTCTTTTTCCTGGTCTCGTGGCCGTGTGTGTAAATGAGAGTGTGAGATTGGGATGGGAACATAAGTGTGCGGGGGCTCCGTCCGATGCCGTTCAGGTTCAATCTAAGGAAAATCCGTTCGTGGAGTATATCCGCGCTCAAATGGAACAACAGGCGGATGTGGCGATACTTGAAAAGCATGACTGCATTCTCTTTCATCTTGAAAACGGTTTAAACATCACTCTATCTTTTACATTACCGAGACAGAGATTGTTTGCAATGGCTTCTTCATTATTTAATGTAAATGATACTTATGATTTCATCTATTTTCTAGTTTTGGGTTTTCTGCCAATACCGGCCGTTATATAAGGCGGGATGTTGTGTTGATTACGTCAATACGGATGGGGTTTTGGATATAAAGACTTACAGAGCTTTTAAAGAATCATTACTTCGTTTATTCAGGATGGGGAAAGAAACGAAGTCTTTGGCTTGGCCAGCCACTGCGGAGTTTTATGGTTGGGTATTTATTTTTTCGTCCATACAGTTGTGTACAATGGTTTTGCTGACGGTTAGGTTTAATAGTTTTAAAGTCGGTCGAGAATACGCTGTGTTTACATTTGCCGGGATGAGCTTTAATTGTTTTTTACTTCCGATAAAGATGGGATTGTTGAGTGGACATTGGTCTTTGCCGCGAGATTTTTGCGCAATTCTTCTCTACATCGATGATTTTTCTATCTATTTCTCCTCGTGGTCATTGGTGTTTATGGCGATCGAGCGCATCAACCACTTCTGTTACAGCACACCGTTGTTGAACGAGAATTCCAAGGCTCTGGCAAAGGTGTGTTTCCCGATAGTTTGGATCATATCGGGAGTGCAAGCTCTTCAGATGCTGAATAATTACAAAGCAACAGCTTTGCAGAACGAGACCTCACAGTGTTTTCTCGCGTTCTTAAGGAGCGGACACGACATGTGGTTAATGTTGGTTTATTCGGTCATGATTCCAGTTATGCTGGTGTTTATTTACCTATACAGCAAAAATTTCATGCTTTTGAAAGATGAACTTTCGACCGTGACTACGTATCTCTGTATTTATTTGCTGCTAGGTACAATAGCGCATCTGCCGAAAGCTGGCTTAAGTGAAATCGAGAGTGACAAGATTTTTTATGGTTTGCGCGATATTTTTATGGCGCTTCCTGTTTTAAAAGTTTATTATATACCCGTTATGGCCTATTGCATGGCATGTGATGATCACACTGTGCCCGTTCGTTTGTGTAGTATCTGGCTGGTCAATTTGTGTAAGAAATGTTTTTCGTGTACGCGGCGCGAAAAGGAGTCGGATTTAGAGGTTGGAATAAAAATGTTAAAATGAGTAAGGAGTTTATCGTTTTTATTCATAAGAGAATAGATCTTCGGCGCTGATTCCTGTAAGGTCAAATATGGCAGAGCTGTCGAGTAATTGTGGATTTTCAGACATACCGCAGAAGACTTTAAAGTCGTTTTGGAGAAATGAAAACAGTCCGGCTTTGTGCTCGGCTCTTTGCTGATTGTACGCCAGTGTGGATATCTCAAATTTTCCAAACATTATAAGATAGGCAAACCATAGGTGTTTGCAGATGAGCCGCACTGTTGGTTCTCGTTCTTGATCCCCCCAAAATCGGTTAAAGAATCTGCAGATTTTTAAAAGTCGGTGATTATAGATAGCTCCAACGGAGAAAACTATATTGCCTTCACAGATTAATTCTTCCGGAATTTTGAGATTTACACCAAACTCGTCCATGAAAAGAAAATTAATCATGTGACTAACATTTCCGCTGTAGTGCAGCTTTTTTGAGATATATGCTGAGTGACTTTCGTTAAACATATGTCGGAGTAGTTCCGCGTCAGAATGCTGTTTCAGAATAGAAAATATTCGAAACCAGTAGTTGTTGCTGATTTTACGATTCAAAATCACTATGTCTCCGATTCCAGAATTGTACAAACTTTCGCGTAGTAGTAAGATCTTGATTCCGTGTGACAGCATTGATGTACATACGTGTTGAATTAAAAGTAATTCCTCTTGTTTAAAGCTAGTCAGATTCTCGTTTCTAAGCAGTTTTGTGGTTATGTGTAATATTAAATTAGGGACGGCGTCGTTCAATTTTATGTACTGTCCTATCTGGGTCATCTCAAAATGTCTAAAGTTTGGGTAGGTGGATTCCTTTGTGTTTATGGTGAAGAACCGTCGGAAGAATGTTTAGCTCTGCCCAGGGACACGGTTCAAAAAGAACTGGGGTCCGGAAATATTCCTTTACCGTTGAATATTAATCACAACGAAAAGGCCACTATTGGAATGGTACGCGGTCTTTTCGATTTAGAGCATGGGCTTTTCTGCGTTGCGCAGATACAGTCTCAAACGTTCATGGACATCATAAGAAATATTGCTGGCAAGTCAAAGTTAATCACAGCAGGCTCGGTAATTGAACCTTTACCACCGGACCCGGAGATTGAGTGTTTAAGTTCAAGTTTTCCTGGTCTATCGCTGTCTAGCAAAGTTTTGCAGGATGAAAATTTAGACGGTAAACCGTTTTTTCATCATGTGTCTGTATGTGGAGTCGGTCGGAGACCGGGAACTATAGCGATTTTCGGGCGAGAAATAAGCTGGATTCTAGATAGGTTTTCATGCATTAGTGAGAGTGAGAAGAGACAGGTTTTGGATGGGGTCAATGTTTATTCTCAGGGTTTCGATGAAAATTTATTTTCGGCGGATTTATATGACTTGCTCGCGGATAGTTTGGATACATCTTATATTAGAAAACGGTTTCCAAAGTTGCAGTTGGATAAACAGCTATGTGGTTTGTCTAAATGTACGTATATTAAAGCTAGTGAACCACCCGTGGAGATTATTGTCGCAGCGACCAAAGTTGCCGGTGATCAAGTTCAGTTAACTACGGAACCTGGGTCTGAATTAGCGGTTGAAACGTGTGACGTGCCTGTTGTGCACGGGAATTACGACGCCGTTGAGTCTGCGACAGCTACAACGGCTATGAGTAATCAAAATTTGCCAAATACTACTCCTTTGCTGTCGAGTCCACCGTTTTCGGATTGTGTTTTTTTACCGAAAGACGCTTTTTTTTCTCTTTTAAATGTTACAACCGGACAGCAACCGAAAATAGTTCCGCCTGTTTCTGTTCATCCGCCCGTAACTGAACAGTATCAAATGCTACCGTATTCTGAGTCGGCTGCTAAGATTGCAGAACATGAGTCGAATCGATATCACAGTCCTTGTCAGGCAATGTATCCCTATTGGCAATATTCCCCCGTTCCCCAGTATCCGGCCGCGTTACACGGTTATCGTCAATCGAAAACGCTTAAAAAGCGGCATTTCCAGAGTGATTCGGAAGATGAATTAAGTTTCCCGGGAGATCCGGAATACACAAAAAAAAGGAGGCGCCATAGAGTTGACAACGACGACGATAAGGAGATGGCTCGAGAAAAGAACGATTTAAGAGAATTGGTGGATATGATAGGAATGTTAAGACAAGAGATTAGTGCGTTGAAGCACGTTCGCGCTCAATCGCCGCAGAGACATATCGTTCCGATGGAGACTCTGCCTACGATCGAGGAGAAAGGCGCCGCGTCCCCAAAGCCATCTATTTTAAACGCTTCTTTGGCGCCTGAAACCGTAAATAGGAGCCTTGCTGGTCAGAACGAATCCACGGATCTGCTGAAACTCAACAAGAAATTGTTTGTTGACGCGTTAAATAAAATGGATAGTTAAAATGTGTTTTTATGTTGTGATCAAGTGGTGTTTAGCTGTGTGTTACAAGGAAGATTCAGAGTGAATTCCTGGACATGGTGAAACTTGACACATAATTGTTTACCGACGCGTTCAATAAAACGAATTGTTAAAAGTTGTTTTTTGTTTTTTTTATTTTCGTATAAAATGGTATTTAGGCGCTAGTTACGGGGAAGATCTATGGGAGTGATTGTGACAAGGAATGAAATGAATGCCGACGTCTCCTTCTAATATCGTTAGGTTTTGTGGGGTTCCAGGCGGCATGAATTCCATGCGAGTAGATTCTCCGGGTTTTAGAGAAAAGTGCAGGGGTACCATATGTGCGGTGTCGAACGCAACGCGGAGTGAACTCAAAAAAATTCCCATGTTGATGTCTGGCGTTAAGTTTGCAACCGTGCTTAGACTTGGTGTTCGCAATACTGCTTGGTTTGTGAAAGTTACGATGTTTCTTACGGGACCATTTGGAGATTCTGTGTTTACATAAAGTTTGCTTGTTTCTGTCCAGTTGAATTCGACATTATTCCCCGGTGGTAAAATTTGTTTGGGGAAGAAGTATACTACGCCTATAGGTTGCGAGTAATTGATGCGACGGTTCTTCTTAAATGCTTTCATGGTGATGTAGAGAGGCCGTGTCTCATCCCAAATGCCGGTGGTTGTAGAGATTCCGAGCAAGAGTTTGGGTATAAATAAACCCGTAAAGTTTGAATTTGAATTAAAGATAGTGTTAAAAGAACAATGGACAGGAGTAACCGTGTTAGGGATGATATTTTTAGTTGCGCAGACAGTTATTGAATGGGTGTTGCATTTTAGAAGTGTTTCTTGATTCTGCGACAATGTAATGTCTGCGGTTTGGGGGGTCAGTGAAATTTGTATGGCAAGTTGTTCAGGATAAAAATTATCGTCAAGGAGATAGTTGTTTTCGTATTTTGCCTTCAAAATTAATAGATTTCCGCAGAGTAAGATTCTAGAAATATAGACGTTTTTGTGTGATATAAATTCGATGTTAGCAGTTTTAAAAATTTGGTCGAGCGGAGTGTTTTGGAGATTAACCCATATTTCTGCCTTAAATGAATATCGTGTTGGCTCTTCTGGTGTTTGTTCTATCCATCGTATATCAGCCACTTTAATTTTAATACGCGTGAGAGGGCCATATATGACAAACTTCGCGTCGCACTGTCCGTGGGGTATAATGTGGCGATCGTGGGCTTCGATGCGTTGCACCGGTAAATTTGATATATTCACGTTTGCGTGAGGAAGTGCAAATATATTAAGGACGATAGGTAATCTACTGAGATCCAGATCTAGATCTGAGATGTTCTGCACCGCGAAGGGTATGTTGCCGTAATCTTTTGGATCGATGTAAGTAAAAGGCGATGATGTAAAACTGCTTTCGTCTCTACAAATGCACACTACGGCGGATGAAGGGGGGGTGATACCTAATCCGCATTTAAACAATCTCAGTTCGCCGGGTTTTAATATGGCGGTAGTTGTTACTCTTAGCTGTAGTACGTACGAAGACCATTGTAGAGTGGCGGGTTGCATGTTTGCTTTTCTGGATGTCTGAAGGGAATCCTGTTAGTGTATTTTGGATGTTAAATAGTTGATGTAAACGTAGAGAGTCATATGCGTATTTTTATAATAGAGTGTTTTTCCACGGACGGGAGAGTCTGCGGGTTTCCACGCGCAGGCTTGTCTTCGTATTCTAAGGGATGTAATGTGAGTTCCGGTAGCAAGTATGCAGGAAAACAATTGCCCATGAAGTGTACGTCGAATGGTTTATCGGTATTCTCCGTGTGTGTTAAAAGAGATTTCATGTCGCTGTTTGAGAGGGAGCGAAACGGCTTTCTAAACAACTTGTTCGTCACAAAAAAGACACGACCTAACATGTCGTGTTCGGTTACGGAAACTGTTCTTTCGCACGAAATCCGCAATTTTAACGGTTCTTGGGCAAACCATAAAAATGGGTACAGCTTGAAGACATGCGTCTGGACAGGGATGAAGATGCCTACGATTTTTTTGTTAGTGAACTCGCCACGTAGCGTGATCTCTTTGTTTGTATTACTTACTAAGGACATATCGCTGGGAAAGAAAACCTCTAGATTCTTCCCGTCTGCGCAGATTTTAAAATACGGCATATTCATATTTAGCGTGACGGTGCTCCGATCAGGGGGGGAAAAGGTCAAGCGAAGATAACATTCTTTCGTGATTGGAAACCGTCGCTTGTTTTCCGGCAACACTACGTTACGCAAAAAGATTGTGAGAAAAGGATCGTTAAATTCTATTTTGTAGATAGAGATGCCGGGTTCGGAAATCATGTTGACTCTGCAGAGTGGGATGATACTTTTGAATAGGTTTTCCCAATAGGGACATTCTCTGGTATCGATGCTGAACACGCTAAAAACTATTAGCAGCTTTCTGCCATAGCGGTCTAATTTTTGAGACTGATAAGGACTTTCTGTCCAGTTTAGGTTAAATAAGATCAAAGACATTTCAACGCTATCATTCTGAACTACTTTAAATTGATTTATCACGTTGGTTGTCGAGATAGGCATATTGGTTAGGCCATATTGTTCGTATACGGTCGGATCGATGTTTTCTTTTTGACATAGCGCATTGATGTCGTCAATCTCCAGTGCATGTGACGGCAGATAAATGGGTATGGAAAATAAATGAATCCGTACGGGAACCGTCCTAGAAATAGGTAAAATTTTTATTTTGATAGTCGTTGTTTTTTCGAGAAACTGTACATCGAACAGAACGTCCTCAGTTTCCGATTCAGTGCCATCTGTTATGAATAGGATTCCTGGTTTTGTGCTTGAGATTCGACATTTCAAAATGATTTCTTGGTAAGAATTCGAAACTATGTGATTGCAATCAAAAACGAGTTGCAAGACATGGTTTAGTTGAGGAGAAAACATGCTGGTACAAAAAACATTTTATGTTTCATCTCCCTTTTTAAATACTGTGTTTTTTTAAGGGATGAGGTCATTTTGTATGACGGATGGAGGACGCGGGTGTCCCGGCGGGGTGTGTTTTTATAATCCTGCGTATGAGTGATAGAAAAGTCATAGTAGGCTAGTGTTTTTTTAAAAAGCATTTAATTTTTATAAATACATGTAGCCATTCTGTTATCTGCGGAAACGTCACAGACAACAAAATACGTTTTCTCGTTGGTCAAAGAATTAATTTGGTCGATCATAGTTAAGACTGATTTGACTTTTTTTAATTCATCTTTAATTTCCGTAGCGTGAAGTTGTCCGCAGCGCTGTGTGTTTAACGTTGTTCTTTTAGATAATAGTTCTTGGCATTTGATCAAAAGCATTGAATGGTCTTCCAGCGTTAGCCGATCTATGTAAGTTCTCACGATGTCTGGGGCCATGGCCGTGATCATGGACAGCGAGATGCAAGCTGTTTTCATGGAGTACATTGATTGTTTGTCGTTGACTATGTCTGGTAATTTAATGAGTACGTCTCTGTATTGGATAGAGCATAACTCATCGATGATTGTTTGCATCTCATTGTATTCTCTGTGAACTGCTATAAGACCAATGCATAGCAATCTGATGTTGATCTCGGCCGCAATGGCTGTCGGAACGACTAAAGGTACAGTGAGCTCCCAATCACCTAGTTTCAATAGCTTTTCTTCCGAGTGCGTCGATAAAGGTGGAATCAATGTCAAGGTGTCACCCTTTTCCCAGGGGAAGGGTCCTGTGTTCTTTATGGCATACTGATGGCCGGTCACCGGCTTTCTCAAAACCAGTTGATTGCCTTCCACCTTTTGCAGAATGGTTACGACCATGGTCCGTAATACGTTTCGGACGTGGACGTAATCTTTGTTGGAGGAGACGATGGGATAAAGACCTAAATTGCCGCTACCTATTAGATGGTGGTGAGCTGGGATCGGTATGACGATGTTCATGAGCTTGCATAGGGTGCTGATATCGGAAAGTGACAGTTTGTGATCGAAAGTGCAGTAGACGGTTTCCATTTTATATAGATGATTCGATAATGAGTTGGAAAGGTATGGTTTCTCCTATGGCGTAATTACAATAATGGGTTTCGCTGATCTGCGTATTGCCGGTTTTAGACCGACTCTCTAATAGAGCTTGGTTGGATGAACAGTGAAGAGGCAGAGCGTTTTGTAAGAACTGGCATGGTCTGCTTACGAAGCTATCCGTGCCCTCAGAGCACGAGTATTGTATTTCGCTATCGCTGTTTAAACACGAGTTCGATTTCATGCAGTATTCGCTTATCACTTTGTACATCATCTTGTTTGTTTTTAAGATGTCAGATTCGGTAAAAAATTGAGCGTTGGGACTGTATGTCTTGGGGTTGTAACATAGTTGTTCTCTGTGTGCCGTGTTGTATAGAATATCGCCTAAGGAGCCTGGTAGAGATGCCCAAGGATTTGTGGTTGCGGCGAATGTATCGCTGTCTGTTTGGGTGTGGTCGTACAATGCTTTTCTAGCCGCCTCTTCGTTGTGCGGGTCCGTTCCCATCATACACGATTCCCTGCCTCTTGGGTTGTGGGGCGATTTGAAAAAGTTAATGTTTGTCGTGACCGGGGTCAGTATAACTTCGCAGATAGCTTGTTGACCATGCAGTAGTATGGAGGGTGGGTTTTTGTTAATTCCGCCGAATGTTATGATGTTTAGTGCTTCGTTCTCGGAGGGATTAGGTTTTTCTATCCCGACGTGATGTCGAATCCACGTATTTATGTCCGCGTTGGTAAACGCGTGTATCGGGAAGGCGGAGAAAAGGTTTTGTATCTTGCTCCCCATATCTGATTTAATTCGCTTAAGATTGGCAGTGGCGGTGGTAGAGCTAAAACCTAAGCCCATATCTACAAAACTTAGGTGCTGGGTGAAGTTGTAAGTGGTTGCGATATCTTTAGCTTCCGCGGTGACCGTGGGATCGTCTAAAATTATAGATGTGGCTGCTCTGGAACTGTATAATAGGCACTCGACGTCAAAATTATCCGTCCGGACTAGTGTGGCCGCAAAGCCCGGATGGATTTTGTTTTTGCTTTGGATCGCTATGGCAACGGGAGACAATTTTGAGTGCATGGCGGCAAGCGTCATAATGCTCAACAGGGAATTGGGGCAGAAGGCTGAGTACACGGAAAACGGTGTTCGTTGCCAATTATAAAATTCTAATGCTAATGGGGGCGGGAGTGGAAAACCGCCGTCGTTTCTTTGATAGTGAGGAAATGTATTTAGGAAAACTTGGATATCAGCATTCATGGTGCCACAGATTCCTGGGTCGGAGAAGAATCGATGAAATGGGATTGGTTGATAGTACCGTCTTAGGGTGGTGATTGGTGAGATCAGGCAAAGCCCGTTGTATAGAATGTGTTGTAGTGATTGAAAATGAACGGAGTTGCCGTGTCGCTGCGCAGGATCTAAGGAGACCTCCACTTCCAGTATTTTCGTATTTTCATTAATGTAGATTAGCGATTTAAACAGCTGTTTTGCGATACAGGATGTGTTGGCTACGGTGTAGCCAGGGCCCATACCTTCTCGGATCAGAGACATTAGAACGTCGCTGGTAATCGGATTCTCCTGGAAATAATCATCTGGACTGATGAACGGTTCCGTGTAGAATAAGTCTAGAACTAGTTCCTTTACGTTGACGCCAGCGCCACAGGCCTTGTTATTTGATAGTGCCGGGAGTACGCAGAAGTAAAATATCTTGCTCAGGATGGTGGTTTCGTTCGATGGTCTGTCATTGTCGGTGAAGACGACGCTTGAATCTATTAGATTCATTCTTTGCACATCGGATATTTCGTAATTTCTAACTCTTACGGTGTTCTGTGTCAGTGGTGTATCATCCGCTGTTATTTTTGCATTCGTGTCGTTTCTGGGCATGGTATGGACGAACGGGCAGAACAGACGTCCGTCGAACAACGCGTTGGCGAAATTCACCAGAGGTTCGCCGCAAAGTTGCTCGTTGAGGTTGGAGATAGAGATTGTTCTCTTCACTAGGCGAATTAGCGACACAAGATTTCTGTAGTGAGTGAAAGCTGCTCCCGGGATCAGTTCGTCGCCCATGTGGTTAGAGATTAGCATGATCATCTCGAAGCTGTTGCAAAAAAGAAGTATATGTTTCATGTTAAACCAATAAGAAATACATTGGCTAATTACTTGTTTTAGGATCATAAAAGCATGCTTGTTTCCATGCACTAAGGTCTCGATAACGTAAGCCAATTCTGGGTACGAGCCACTCGTCAAGCTTTCGGTGACAATTTTGAGGGTGAAGTCGTAGTTTTGGCAGTTCGTCTTTGCATGTTCCAGTATTTGATTTGTACGGGCTTCGTGGAAAGAGACTGGAGCTAACGGTAAAGGTATGTTGCCTATCATTATTCTTGGTGTGCAGAGTACTTCTGTGGCTCGGTTTTTCTGGATATACGTGAGATCGAAGAAGGGGTGTCTTTCGGTCGGCAGTGTTGGATTGTCCGGTTTGTAAAAATCTTCAATGTTTAATTCGTTTTTTAAGACGTTGGTGTTTTTGGGTACTTCTTGTCGACTTTCGTATAAGTTGTATAAGGTCTGTAAGATCCTGGCTGGGTTTTCTCGGGTAATTTTTAGTTGACATAGTTGGGAAAAGCGGTGGCCGGTAGGCAATGGTTCGCTTTTCATGAGTCGTTCGACGATGGTCGAGTCGTGGACAATGGGATGGCAAACCGACGGCAATATGTCGGCAAAATCAATTCGCTGCACGATTTGGTCTTTATTGTGGAAAAAAACGCTGGTGGGTAGGTTGTTTTCCATGGTGTCGTTTAATTTCACACGTGTTTCCATTGTACTGAAACCGGTTTCGCTCGGTATGTAGATACCCAAAGGGAAAAAGAATGTCAGTTGTAAGCTCTGTTCTAGCGGATCGTTGACGTCTGTGTTTTTGTAAACTTTGTGTAGGTGGTCGAATGCGACTAATTTATCTCCCAATTTCAACGTGTTCAGCTTTAGGTCGGCGTACGCGCGGTTTTTGTCGAATATTTCTGATTTGTTGTTAGTGTCTTGTTGTTCTGTCCCGGCGTTCACGGTGAATTGAGTAAAATCGGCCATGATTGCACGGTATGCAATCGCAGTCACTGCGTTTTCTTTGCCCATAATAAAGTGACCATATGAGATTGGGGCGGAGACGACTTGTGTGGAGATGTACTGGGAGAGTATGCTGGTGAGTTTTTGGATAGAGCCGATCGGTCCTAATAAAACGCCATCTAGGGGGACGTTTTCTTTGGAAGTGTAGTTGTTCGTATCATTTAGAATGCTTTCCGTAACCGATTCCATCATGTCGTTTAGAATTCTATAGATATATGAAGTATTATTATTTCTGTTCAAAAAAAATAATGACGTTAACAATCTGTTCTTTAGGCTTTGAAACATGTTGCTGCGCTGTACTCGGCTCAGAGCTTGTTTATTATGTACTTTGTTCTCATTCATGGTCAGGACGATAAATTGCGGGGGGGATTTCCTCAATAAGGTTTGCATGAATGCGTGAATCAGACCTCGCTCGAGCGAATCGGCTGAATTCTTTAAAGATCTTAAGACTGTATGCAACGCATTGATGTTTAAAATCTGATCGATGACTGTGTTTTTAAATGTGTTTTCCAGATGTTCCAGACAGGCAGCGCTCAAATCGAACGATATGTTTATGGGGTGTTTTTCTGAGTGTTTGGCTACCATGATGGTGGTTTCTTTTGAAGCGGTTACGTCGTTTCCTGTGGCGACTCTGGGTAATTGAATAAAGAAGAGAACCTTCCCGAGGGTCATTCGACTCACATCATTGAAGCGGATTACGTTCGCTGCAACGGCGATCGGGGTGGTGAAAAAGTTAATCCATTCTATTTTGTTGCAGTAGATTCCGAGTAAGGCTTCAAAACTGATGTTGTAACGGCTCGGATCGTCACCGAAATAAATTCGCAAATTGTCAAAAAGTTGTTCGGCTGTGTATGTTTTAATGTCATTGAAAATATTTAGAGGTGCTTCGATCTTAGGTAAAATTTCGGTCGCCTGCCAATTTTCCATGTTTCGGCGTTATGCAGCACACCGGTAATTGTGAAACTTTAATAGTTAATTCATGTTTTGGTTCCACTTGTGCACGGTCGATTCCAGTTTTTATAGATTCGTGTGATCTGACCGCTGAAGTGTCTCGCGATGAGGAAACTCGTTTAGCGCGATCCGTGCCTGTAGTTCTGGAAAAGATCGAGTCAATCATAGAGAAAATTTTTCAAACGTCCGGGCCAAATATCGTTCATGATAAAGACAGGGCTAAAATTGCCTTGTGTCGTCTGTTGTTAGGACCAGTTGCGGTGCCATGCTTCTGTGAAGAGTGGGACACCAACGACTATTTGTCGAAATCTGGCTGTAAATGCATAGGTCCAATCTTATATATTCATACCAGTCGATGTCGTTGTAGCGACATTCCGATTTTTAAGTTTTCTATTATGAAAGATTACTATGCTTCACACGTGTTCAGAGGTTTATTATCTCTGAAAGAGTGGAATACACATCTACCGAATGTATTGTGTACGTGTGAGTTGTCGATGAGCGATAGATATGTGGCGACAGTGTATCCTAAGCAAAATTCTATTTATCTAGAATACTATCCGTATTTTTTGTGCTACCTGTGTCGCCATCTTACTGTCATTGAGATTGAGCAGTGTACAAATGACTTAATTTCGCTTCTTGGCCCTAAAGTAGCTCAGCGAGTCATAATTCATTTTAAATTGCTTTTTGGTTTTCGTCACAAGCCGCATATTGGTACTGTTGATTCGTGGTTCTGGGAAAATTTTTTTATGTTAGAATTGCATAAGCTTTGGTTAACCGTAGTCAAACATAATCGGGTGACGACAGATTTTTTTAATGTAGTCTACGAGAAAATTCAAAACTATAAACAATACGCAATCAAGACCCTGAGGATGTCGTCTAAGGCGGTTCCTGCAATACAGAGGTTGTGTTTGGCAAAATTTAAGCAGCAACTGTTGTATCTAAATATTAAGGTTACGGTAAAAAAAAATAAGCGAGAAATGTGTTTGAATGGCTTTGTTTACGGTAAAACATTGTATGTCGTTGAATCTTCTCAGTTAATCTTCCGGAATTTGCTTCTGTTGTATTACGATTACAGTTTGCCGGACGAATGCAAGACAAACGAAGAAAACGTTTTGACGGCTCATTACATACGAGTAATTTCGAGATTGTCGTTTAAGCGGTCTCGGAGTGCGCTCCCGCCAGGCGTGAGATCAGATTTTATCTTTGTGGCACAACAACCTAAACGTAAAGAGTTACCTAATGTTCCCGGTGGTATCGATTTTGCTGAAATTACCTCAGTGAGGCATGGCGCGGTAACTCTTAACGCGTTTAATACGAACAAAGTCATGAATTTAAAAGCAACCATTTCAAAAAGGGCTAACTTTGTATATCATCGCATTCCTAAGACGATGACCCACAGTTTTGTCATGTACAAGCATACGTTTAAAGAACCTGCGTTTACCGTAAGCACGTTTGTTTCAAACGATGATTTAGATATGAGTTCGTTGAATATCAACATACGTGGACCGTACTGCGACTTTTTATATGCTTTAGGCGTTTATAAGATGCATGTTTCTATCCGAGATCTATTTTTACCGGCGTTCGTTTGCAATAGCAATAATTCAGTGGATTTACAGGGACTGGAAAATCAGGATGTTGTGAGAAATAGAAAGAAAAAGGTGTATTGGATCACTAACTTTCCGTGCATGATTTCTAATGCTAACAAAGTGAATGTGGGATGGTTTAAAGCAGGAACGGGTATTATTCCTCGGGTGTCTGGGGAGGACCTTCAAAATGTTTTGCTTCAGGAATTAAATAACGTTCGAGAGATTCCCGGGTTAGTCTTTGATATGGATTTGCATCAACTGCTTGTTTTACTGGAACAACGAAATCTACATCAGATTCCGTTTCTCGTTAAACAGTTTCTTATTTTTTTACGTCTCGGTCTGTTAATGGGTTACGGGCACTCTCGGCGCAACAAGGTGCATGATATCATGTTACATTTAATTTCGAATGGTCTGTTTGATTTTAATAAGAACTCCGTAGCAAATACAAAAATCAAACACGGGTGTGCGTTGGTTGGGACGCGGCTCGCCAACAATGTTCCGAAAATCATTGCTAGGCAGAAGAAAATGAAGCTAGATCACATGGGACGAAATGCTAATTCGCTCGCCGTGTTGCGTTTTATCGTTAAAAGTGGGGAACAGAAAAATAAAACTGTTTTCATTAAATTGTTGGAATATTTAGCGGAAACCTCAACTGCCATAAATACGCGGAATGAAGTCGCCAGATTACTTCAGACTCTGACGGCTAAGGTGAAAACATGAATGTACTCGTGGCCGACGAATGGTTCGATTGCGCGATTAGGTTAGATTCGGAAACCATAGCTGTCCATGAGATTTTCAATCCGGAGTTAAGTAAACTGCTTAACTTGCACTCGAAAACAGTCTACATGTCCGACCTATGCGCTTTTATTTCTGGTTGTGTTAATCGGAATGTCGGTAAACTTACCATATATTGGCATGTGAACGGGGACATAATCTACGCATTGACTGGTATTTTACATTGTGTAAAAATAAAGATAGAGTGCGGGGAGAGAATTGCCGATGGTCGATATAGATTATACGAAATTCCTAAATTATTTTTAATGAGAGGACAGTCAACACCCATGGAATTGAAGTGGAAGCACGCCGTGGGTATCGCGACGACGAATAAGCCTTTGCTGACGCACGTTTTAACAGATGTGTTGGAAACGTCTCCTTTTACCTTGCCAGATACGCTTCTGTCGGTGCAGGAGTTGTCTATTTTCAGAGAGAGATTGTCGTACATTTACTATGTGCTGGGATCAGATGTTGATATCGTAGCGCGGACAGAGAGAGAGATTTTTCAAAAATGTGCAGAACTAGCTCGCCTACAGCAAGTGTTCCTTATTCAAGGAAATATTATGGAAAACTTTGTCCTCGTCCAGGCTTGTCTATTTCAGCTGGGGGCTGATGGTTTGTGGGAAGAGATATCTGGTTCTGTACGTCCTAGGCCGGAGTTGATGTCCAGTGCGTTCATTCAACACAGAGTAATGTTGAATAATTGTTATTGTATCGCTGTCATCTTCAATGCCATTTACAAACACAAACTTTCCCTGCCTACCGTAGAAAGAAGCCACGAAACCGTTAATCGTGTAGTTCAGGAATATTATAAGTCTTATGTGAATGCTCCTCTCTCTGTTCTTGTGTGTGCGACTAAGGTGCTTACTTTGTTTACAGAAGAATATAACTTTAAGTCAGCTCTCGTATTTGTCAGTCAGTTTTTCCAGGTGGACGTCGAGGCTTCGAGAGCGGATGTGATTCGTCTGTTTTTAGCGTGTCTAAAGGGTGATTAAATCTCTCGGAAAGAGGCTGAACTGTTTCCAGAGCATACATAAATCGCCATAATTATAGCGATAATTAAGTCGTCGGAACAGGCTTGTTTTTTAGCGCTGTATGTTATGTAATTGTTAACGGAGATCTGGTGAATGTTTCTGATTTGTTCTAGTGCGTATTCCACGGGATCGTAGGTAATTTTTATTGTAAACGATATCAATTCCTGTGACGCCTTGATGTTTCCTGAATTAAAATTCGAGATAAAAAACTCAACTGCTAGTTTTTTTTCTTTACCGAGTAGGTAAAATGGCTGTGCTATCTGATTTTGGTCTGGGGTGTGGAAAAAAGTCACCTGTATAGATTTATTAGCCGTGATGTTTTCCTTTATGATACATGCAATTTTTACGGCAGAAGCTTGATTGGAATTCCCTTCTATGATGATTTTTACTTCCGTGAAGAAAGGATGTAGATCTAGGATTGACAATATCATATGCGCGGCACATTCAGCTATTGCCGTGTCGGAACTTGTCATTAAACTTTCTAGAAAGTAGTGCTCCATGCCGTAGACGATATATTGATCTAGATAGGTGCCTATTGCTGCTATGCCTGTGCCAGAAGCACGGCGGTTGCCTGTATAGGCAGGATCTAGGTATACGTACAGATCTTTACCTAAAAAGGGAATTAGATTTTTATTGATGGTGCTGTATCGGAAAAACTCGAATTCGGTTTGGCCTTGTTCCGTAATTAAAACGTCGTTGATCACATTGCAAGTGGCTCCGCCCATGATTTCATGGATGAAAGCTCCTTCTAGGAAGAGGTTGGCCGTTTTTTTAACTTCGGCGTTAATGCTGATGAACTTGGGTTTGTGGAGTCGGTAGCAAGAACAGGCTGTGGCGTTACCGCGCTCGTTTAACATATGGGCGTGATCTTCGCATACGTAAGAAACTACGGAGAGCATTTCAAACGGAGAGTTGTTCAGCTTCATTAAAAAAGATGTTGAATGGTTTCCGGAATTGGTCGAAGATATAAATAGGATCTTGGTAGATGCTTGGGGCAGGAAACCTAAAATCGTGCTGAATGCGTCTTTCTTTATAAAGTGGCTCTCGTCGACAATAAGCAGATTGAAGCTCTGTCCGCGTATACTCTGAGAGGGGAATGAATTCGGCGTTAAACGATATAAAAGACGATTTTGAGAACTGTGAAACGAAAGACGACCTTTTTAAAATAATTGATAAAATAATCAAAAATTGCGATTTTATAGTGGAACAGGTCGAGTCTTTGCCTCGGAGGGTGGATTCAGCGGCCATCCTATTTGATAATCTCGCGGTGGAGATATTTAACGATGTAATATATCGACAAAATGGAGTTGCCGCGAAAATACGACAGGGTAACGGGCAGGATATTGACACATAAGAATAACCAGATGTGTACAACCGAATGTTCTCAGATGTATAATTTACACAATCCTATCACGTTTGAGTTGGGACTTGGAAACGTGTTTGTCTGTATGCGGTGTTTGACGGTTCACCACTGTGATATGCAAACTGACTGTACCATTGTCAACACGCATGAGGGGTATGTTTGTGCAAAAACGGGTTTATTTTATAGCGGTTGGATGCCTGCGTATGCCGACTGCTTCTTAGAACCAATCTGTGAGCCGAATATTGAAACTGTTAATGTCGTGGTGGTCTTGTTATCTTATGTATATAGTTTTTTGATGGAAAACAAGGAACGTTATGCTGCCATTATTGATAGCATTATTAAAGATGGGAAATTTATAAAAAACGTGGAAGACGCTGTGTTTTATACTTTTAACGCGGTTTTTACTAACTCAACTTTCAATAAGATTCCTCTGACGACGATAAGTCGGCTTTTTGTTCAGTTGATTATAGGAGGACACGCTAAAGGAACGATTTATGACAGTAATGTAATTCGTGTCAGTCGTCGGAAACGAGAAGACAGTTTACTAAAAAAGATGAGATTGGAGTATGGAAACGCACTTATACTATGACATCCTGTATCAATATCAAGGCGGGGTGTATCCGGCTCATATTTGCCTGCCGACAGATGTGTGTCTTCCGATGAGAGTGGATTGTATCGAGTCTTTATATTTTCGGTGTGTATTTTTTAAGAATGGGATGCATTATACTGAATGGAGTAAATTAAAGTTTACTGTGATTTCACGTGAAATAAAGTTTAAAGATGTGTTAAAGGATGCAGACTCTGACGAAGTTTTTACCGGTTTGGTGGTAATGACTATCCCAATTCCGATAGTAGATTTTCATTTTGATATCGATTCTGTAATTTTGAAATTGGTTTATCCGCAGTTAGTGCACCGGGAAATAGTGCTGAGACTCTACGATATTATATGCGTCAGACCTCCGTCAGACCGGCCGTCAGAAGCATCAGCTAAAAATATTGCTAATGATTTCTATCAACTAACCTCACAGGGAAATAAACAGACACCCGATGAGGAAAAACGTTGTCTATTTTTTCAGCAGGGACCTTTAGAGCCACCCTCTACCGTCAGAGGCTTAAAGGCGCCCGGTAATGAAAAGCCAATACAATTTCCCGCCCATGCTAACGAAAAAATGACCGAATCTTTTTTAAGCGATAGTTGGTTCGGACAAAAAGTCAGATGCAAAAAAATTTTGGATTTTACGCAAACGTATCAAGTCGTGGTATGTTGGTACGAGCTTTCGTTTTCCCGCGAGATGCAGATCGAGAATAATTTACTGTCCGCTTCCCAGCTAAAGCGGGTTAACGCTGCGGATTTTTGGGATAGAACTAATCGGTATTTGCGAGATATTGGAAGCAGGGTATTGACACACATCGTGAAAACGCTTCAGATTCATAATAGGCAATTTAAACAGAAATTTAATTGCAATTTTCCAGATAATTTCAGCTTTGATCGTCTATTATCATTTATGCAGCTCGGGAAAGATTTTTGGATTTTAAACTTAACTTTAGACAGCTGCATTATTAAGGCAATTATCTGTTTCCTAGGTTTTCAAAACGGGGGAAAATCTTTTTTAGCCCAAGATGAAGTTTGGGGGGATTTAATAGACTGTTCTAAAGGATCGGTGATCTACGGGGAAAAGATCCAATGGATTTTGGACTCGACTAACAATTTATATTCGACGTGTCGGGAAAAACAGAATAAGTCGTGGGAATTATATGTTGATTGCTGTGCTTTGTATGTATCTGAAAAGTTAGAGTTGGATTTTGTGCTACCCGGCGGATTTGCAATCACCGGTAAATTCGCGCTTACTGATGGCGATATCGACTTTTTCAATTGGCGATTTGGGTTATCTTAGAAATTTTCTGCAGAATGAATGTAACTGGTTCAGAATTTGTAAAAAAACATTCTATCGCGAATATCGTAGCGTTGCGACATCGTCTCCTACATTCTCGTTGAATAATAAGCCTAAGAAATTTTGCATGCATTGCGAGATTGTAATACTCAAGCGAAGTGAAGAATTTATGTTCAGCCTTGCGGTAAATGGCATACATTTTGGGCAGTTTTTAACCGGAAAAATGAAATTTAATAAGAAAGCAGTTCCGGAAGGGCTCTATTACTATATATTGGAATTGGGAAGCATAACCCCTATCGATTTGGGCTTTATTCCGAGATATAATTCCGACTGTGTTACAAACATGCGTTGTGTTACACCGGAGGTTATTTATGAAAATTGCTCTATTGTGTGTCCCGAAGAGGCAAATCGCCTCACGGTAAAAGGGTCCGGGGACAATAAATTGACTCCCTTAGGTGGGTGTGGAGCATGGTGTCTGAAAAATGGTGGCGATCTGTATATCTATACTTTTGCACTCGCTTACGATCTTTTCCTAACTTGTTATGACAAATCCACCTTTCCATCTCTGGCGAAAATTATTTTTGATATGATAGCTTGCGAATCCGAAGATTGTGTCTTTTGTAAAGATCACAACAAACATGTATCGCAAGCTGGACAGATTGTAGGGTGCGTCTCTAATCAAGAAACCTGTTTTTGCTACACACCGTGTAAGAAAAAAATGGCTAATATTAACAATCCGGAGTTAATCTCTCTGCTCTGTGATCAGGAAATTAATAAGATAGATATTATGTATCCCCAAATAAAAGCATCGTTATCACTGGACATTAATTCTTACGCTCATGGGTACTTCGGTGACGACCCTTATGCGTTAAAATGTGTTAATTGGATACCAGTCAGGATCAGCGCAGCTCTGAGCAGGCTGATTGTTTTGTCGTGTCCTGTGTGTAAGCGTGTGGTAATGGACTAATTGTGTGTTGTTTTATGTATTAATTTTTTATTTCTGAAAATAAAGTTAAATTAATAGTACTTACGTGTGTATTGTAGCAGCTGGCGAAAAGTGCTGTGCTCTTTATATTTTGATGGTCGATTGTAATTACATTATCCAGGCATGTGATTGTCTTTTCTGGAAACATTCGGCGGCATTTAAACTCGACTTCTTTCATCACAAAGTGAGATACGTGTTTTTGATGTGCCACGTAACCGATGCTGATTCCTTCAATGTTCTTCAATAAAAAACTTATAATAGGAACTATGAACCACGTTTTTCCATGTCTTCTTGGGACTAGGAATACGTTAGTTTTTTGTTTCAGCGTATTCAGAGTACTTTCGTTTACGAATTCAATGTCGAACACGTGGGTCAGATAGTTGATAACACGATTGGCTAAAGCTGGAAGTTTGGTAACGGCGATGAAGAAGATTACATGTATGAGAATGTTCTTCTGAAATGGTTCCAGTTGTATTTTGCGCGTGTCTCCAAAGTCGTTGAATTCTCCTTTGATCCATTTTCTGAAATCTTTGATAAAACCGTCTATTTGCAAGAACAGCGGTTCACGATATAGAATCTTAAAGGATTCAAGAAATTCCGTGTATTGTGATTCAAAGGATTTGTCGGAAGCGGGTAAAAACTTCATTTCTTGTAGCTTGTGCGAAAGGCTCGGTAGTATAGTAAGAGGCTTACGGTTTTTAAGATGTCGCTGTTTTTCACAAAATGTATATAGAGGTTTTACCTGTTGGTTATATGAATGGGCAAAGCCGAGTTCTGGCGTGAGCATTATAAAACGCTTCTTATAGAAGATGGCACTGTTGGGGTACTTGGTAGATATTGTCGAGCAGTCTCTTTCGCCTTTCCATATGATCGCTTCATAGTTATTTTTTATGTGGGTTATGTCGCAGGTACGTAGCATGGAGCCCGACCTTACGTTGGCGGCGGTCTATCAGGCGGCGGCGAACCTCACAGAGCAAGATAAGGAGATTTTTGCCGAAGCGGTAAAAACTGCGTTTTCAGTGTGTAGTTCGGCAGCCCCGAGCGCTAGGTTGAGGATGATTGAAACGCCTACACAGAATTTTATGTTTGTGACGAGCGTTATTCCTTCGGGTGTGACGTCTGGTGAAAAAAAAACAAAGTTAAATATCGATGCCGCTCTGGATAATTTGGCTTTGTCGTTTGCGAACAAAAAATCAAAGAAGATGGCTAGAACGTATTTGCTGCAGAACGTTTTGCGGACTCAAGATCAACAAGTTGCCATTTCGGGGAAGTACATTTTGTATACAAAAAAACACATTGAAACGTCTTTGATGATCGATAAGACGAAGTTAGTTAAAAAAATTCTCGAGTATGCCGAGACCCCTAATCTGTTAGGATATACCGATGTGCGTGATCTTGAATGTTTACTTTGGTTAGTGTTTTGTGGTCCTAAAAGTTTTTGCCAGTCAGACAGTTGTTTCGGATACAGTAAGACGGGATATAATGCCGCGTTTCCAAATCTATTGCCTCCGTATCTGTACGAATGCGGCCAAAATAATGGACTGTTTTTTGGCATTGTGCAAGCTTACGTGTTTTCTTGGTACTCAGATTTTGATTTTTCGGCGCTTGAGATTTCAGAACGCGCTCGTCGTCGAATCAGGTCACTCCTGTACGACTTAAAACAGAAGTTTGCGGAGCAAGAAGTTTCTGTTTTACCGGTAGCGTCACAGATGTGTATCTTTTGTGCATTATATAAACAAAACAAACTTAGTCTAGAATACGTTTCTGGTGACTTAAAGACTTCTGTTTTTAGTCCAATTATAATAAAGGATTGTTTATGCGCGCAGACGACAATTTCTACAACTCAGATGCTGCCAGGTACAAAGAGCTCAGCCATATTTCCGGTATATGACCTTCGTAAGCTGCTCGGTGCGCTTGTCATTTCGGAAGGTAGTGTCAAGTTCGACATATAATGTCTTTGAAGGACTATCTGAGACAGTCCATTTCTAAAGATTTGGAGGTGAGACATCGAGATTCTTTAAAGATTAGATTAGGGGAGAGACATCCATTGAGTGTGCATCAGCATATGATCGCCGCTAGGCAGATCATCAAATCGGACAATGCAGAACAACAGCATGTAATATCTTCTTTGAGTGGTTTTTTGGATAAGCAGAAGAGTTTTTTAAGAGTGCAACAAAAAGCTTTAAAGCAGCTAGAGAAATTGGACGTCGACGAAATAATTGATACGGCAGCGGAAGTGAAAGCAGTCAGTAATGATATAAAAGAAACTCTTATAGCAAGCACTGAATTAGAATAATTATGGACAACGGTGTGGAGACACCTCAGGGTCAAAAAACTCAGCCGATAAATTTGCCACCTGTCAGGAAAAAGTTAAGAAAACATGAGGGACTCGGAAAAGGTGTTAAACGAAAACTTTTTGCCGAAGATAGCCCTCCCTTAAAGAAACAGATTTCCGCCTGTAGTGATATGGAAACACTTTCTTCGCCTGTAAAGTCTGGATGCGAGTCGCGAAGTGCTTCTCTCGATGAAAGTTTCGGAAAATGTAAACACGAAACTGCTTGCGATTGTTCTGCGATAGAGGAATTGCTTTGTCACGAGTCGCTTCTAGACTCGCCGATAAAACTGTCGAATGCCCACACCATCTTCAGCTCAGACAAACGGAAACTGGAGCTAGAGAAAATTATAGCTTCAAAGCAGATCTTTCTAGACATGAGTGAGAATGCTGAACTTGCGGCCTACGGCGAAACTTTGTGTAACCTGAGAATTTTCGAAAAGATCAGCTCGCCGTTTTTGTTTGATGTGCAAAGCGAAGAGCGTTCGTATTCAGTGGTCTACGTCCCTCACAACAAAGAACTCTGTGGACAGTTTTGTCAACCTGAGAAAACTATGGCTCGAGTTCTCGGAGTGGGTGCTTACGGGAAGGTGTTTGATCTAGATAAAGTGGCCATAAAGACGGCCAATGAAGATGAGAGTGTCATTTCGGCTTTCATAGCTGGTGTCATCCGTGCAAAATCAGGAGCCGACTTATTATCTCACGAGTGTGTTATTAATAACCTATTGATTTCAAATTCCGTTTGTATGAGTCATAAAGTGTCTTTGTCACGTACTTATGATATTGATCTCCATAAGTTCGAAGATTGGGATGTCAGGAATGTAATGAATTACTACAGTGTGTTTTGTAAGTTAGCTGATGCTGTAAGGTTTCTAAATCTGAAATGTAGAATTAATCATTTCGATATCTCACCTATGAATATATTTTTAAATCATAAAAAAGAGATCATCTTTGATGCCGTGTTGGCGGATTACAGCTTGTCCGAGATGCATCCCAATTATAACGGCACGTGTGCTATTGCTAAAGAGTATGACAAAAATCTTCAACTTGTGCCAATTAGTCGTAACAAATTCTGTGACATGTTTAATCCTGGATTTCGACCACTTGTCGCCAATGCAATGATATTGGTCAATGTGTGCGGGGCTTTTGATGGTGAAAATAATCCTCTTAGACATTGTAACTTGGATCTGTGTGCTTTTGCCCAGGTTGTATTATCGTGTGTGCTAAGAATGACAGATAAACGTGGATGCCGCGAAGCTCAGTTATACTACGAGAAAAGGTTGTTTGCCTTGGCTAACGAGGCCTGTCGATTGAATCCTCTCAAATATCCATTTGCTTACAGGGATGCTTGCTGTAAAGTATTGGCTGAGCATGTAGTATTACTAGGGTTATTGTTTTACCGAGACGTAGTTGAGATATATGAAAAACTATACGATTTTCTAGATGAGAGAGGGGAATTTGGGTCACGAGACCTTTTTGAGGCAACTTTTTTAAATAATAGTAAACTTACCAGACGTCAGCCAATCAGAGAGGGTCTCGCGTCTCTACAGTCGTCCGAGTATGGAGAAAAACTTTTACATGACCTTAGAGAGTTGTTCTTGATCAATTCTACTGCGGATCTGGATAAAGATACATCATCTCTCTTTCATATGTGATATAGTAATGGATCTTGATCAAATATCTGAAACACTGAGTTCTGTGGCCGAAGAAGAGCCTTTAACCATGTTTTTACTTGATAAACTGTATGCAATACGGGAAAAGATCAAGCAAGTTCCATTTTCGATTGTTCGCTTGTGTCATGTTTACTGCATGCTAATAAAATATAACGCTTCTAACAATAATTGCATTCTGGGCCGTAAACTTATTGAGGAAATGCAGCAGTTTTTGTGCGGCACAAGAGTGGATGGATCGGAAGACATTTCTATGGATCTGAGTGAATTGTGCAAGCTCTATGATTACTGTCCATTATTGTGTTCTGCTCTGTGTCGTGCGCCTTGTGTATCTGTGAACAAGTTATTTAAAATCGTAGAGCGTGAAACTCGGGGGCAGTCGGAAAATCCCCTCTGGCACGCGTTACGGAAATATACCGTGACGGCGACTAAGTTGTATGACATCTATACGACAAGAAGTTTTTTAGAGTATAAAGGACAGCAGTTTTTTGGGGAAGCGGTGATTTACGGCGCGAAACATGAGCGTGTTATTAGACACCTCGTAGCGACCTTTTACGTTAAAAGGGAAGTTAAGGAAACGTTGGGATTGCTGCTTGATCCTTCTTCTGGAGTTTTTGGTGCGTCTCTAGACGCGTGTTTTGGAATTTCATTCAATGAGGATGGATTCCTGATGGTTAAGGAAAAAGCGCTGATTTTTGAGATTAAATTCAAATATAAATATTTACGGGATAAAGAAGATCACTTTGTTTCTGAACTATTAAAAAACCCCACGGAGAAATCCTTTTCGGATTTTATTTTATCTCATCCGGTGCCTGTCATAGAGTTTCGAGAAAGAGGGAAGATTCCGTCATCTAGAGAATATTTAATGACGTATGATTTTCAATATCGTCCTCAGAGAAAGTTGCGTACTTGCCCCACTCCAGCTATTTTGGCACCTCATATCAAACAACTGCTGTGTTTGAACGAAACACAGAAGTCTACGGTAATCGTTTTTGATTGTAAGAGCGACTTGTGTGAGCAGAAGCTGTCTGTGTTTCAGAAGGCTGTGTTTACTGTGAATGTATTCGTAAATCCGAAACACAGGTATTTTTTTCAGAGTCTGTTACAACAATATGTAATGACTCAGTTTTATATTAATGATCATAATAATCCAGAATATATCGAAAGTACGGAAGTGCCTTCTGTTCACATTGTGACGGCTTTCTTCAGGAGAAGAACAGAGGAAGAAAGGAGTTTGCATTTGGTGATTGATGAGACGGAATATATAGAAGAAGAAATACCTTTGGCCTTGATTGTGACTCCGGTAGCACCGAATCCGGAATTTACTTGTTGTGTTATAACAGACATATGCAATCTGTGGGAAAATAATATTTGCAAGCAGACCAGTTTACAGGTATGGGCGCAAAGTGCTGTAAACCAGTATCTTGCGGCATGTGTAAGAAAACCGAAAACACCTTGATTGATTACAAGGGAAATCCTATATCACTAGCGGATGAATTTACTGTTTTAACCGATACCGAATCGGAAGAGGAAGGGATGGCAGATCTTGAAAAGCCCTTACTTGAAAAAGTACAGACCACATTAGTTGCTAAGTGTGATACGGAAGCTGAAAAAAAATTGCCTTGTAAATCAAAAAAATGATAGACGACGCAGATTTACGGCACGCTCGTTTTTTTTTTATTTTTTTGGCTATTTGACAATAAACATGATTGTAATAAAGTGTCACTCGTTTTCATCCATTAGGCTCGAGCTTTCAGTATTAAGTAGTTGCTTGTACCGACGCTTCTTTTTAACTAGGTAGCGTACAAGTCGACAAATTATAAAACCGATCGAAATTATGGCGATGACCGCTAAATTGATAATAAGTCCTGTGCTCCACTCGGATTCAAAGAAAGTTTCGTACTGGAGAATAGGAAAAGATAGGCCTACGGCGCCACAAAACATACCGACGTAATAGCCGAACAAGGGTTTAACGTAGCGAACCAGGACGGCTTCTATTACTATACTATATATGCAAGCCATGACAAGAAAGGTATTGATGGAAGAGTAAGCAACGGTTATGGAACGTATGTAGATACTATTTCCGACGCCCAGGCAAGTAGTAATGCCGAGAAACATTGTAGAGTAACCTAGCATGAGTTCGGTTAGATTGATAACAACGGTCTTGAATTGTACGGTCCCCTTTAGGTTAGGATGAATTTTCTGAAAGGCGAAAAATGACTTATCCGCTGATTGAAATTGCGTTATCAGCGTAACGTTAAAGGTGGTGAAACACACGAAGAAGATGCAATAGGCAAATGCGCCTAGGGCTACCAGTCTGAAAGACAAGGCGGTGACGAATAATTGAAATGTATCCATGATTAAGATGAATTGAAAGCAGGATGACGTGTCCCCCATCCAGGTAATATCCCTCGTTGTTTGGTTAACGTTAGATGTTTTGTTAGAGGAGAACACTTTTGCACAGCAGACAATGTAATAAATAATAATCAGGAGGAATAACATAGCCATTATCAGTACGTAGCATATAATCTGCACGGGGTTTATGTAAAGTTGATGCGTCATCTGATATATTTGATTGTTAGCGGAGAGATTAACCGCTTTGAGGTCATTAATTTCAAAGTAGGCGCACGGAAATCCTAAATTTGGAAAGTTTATGGCTATCAGATGGACCGTGACGTTTATGAATGACAAGGCTGCACAGATTATAGAGACCAGCCAGATCCGTAAATTAATGGTGTCTACACGACTGGAGGCCATACCAGCTCTGTGATGGAGAACAGGTTTCAGAACGATACTCTGTTCTCAGAGTGGTTCGGTCAGAGTTTGTCCGATGTAAGATTTCCGGACAATGTCACCGTTTATTCTCAAGCCGACTCGGCGGTGAGTTTTGAAAACGTCAGACAGCCGATAAAACTGGTTCGCGCAGCGATGGGTTCAGGAAAAACAACAGCCTTGATTCATTTTTTGAAACAGGTACCCAGGGAACTATCTGTCCTTTTGATTTCTTGCCGTAAAACGTTCGCTGCTGAAATTTTGCACCGTTTTACTCTCAATGGGTTGAAGGATTTTGAATTATATTGCGATATTACGGAGCGGCAGATAAACAACAGAAAAGTCATTGTCCAGATAGAAAGCTTGCATCGTCTTACAGAAAATTACGATGTTTTAATATTAGATGAAATTATGTCGATTATAAAGCAGTTTTATTCCAAAACGATGACAAAAACCAAAGAAGTTGATTGTAAGTTTTTATCGCTGATCAAAAATTCCAGTCATGTAATAGCCATGGATGCGACGCTGACTCGGCATGTGGTGGAGTTTTTTGCCGCGTTCAAGCCAGATACTCAAATCGCCCTCATCAGAAATACATTTGTGTCCGCGATGTTTTCCAATAGGATCGCTTACTTTTGTGACACGTTTTTTGGTAAAGAGTTTTCTTTTTTTGCACGCTTGGAAGACAAGCTACGATGGGATAAGAAATTGTGTTTGTTTTGCAGCACAGTGTTGGCAGCGGAATACATGCATGATTTGATACGGTCTCGGTTTCCACTCAAAAAAGTTCTCTTGTTGACGTCTAAACAAGGGAAATGCTCTTCCATAGAATCGTGGATACGGTATGACGTAGTGATTTATACGTCCGTCGTGACTGTCGGTCTCAGTTTCGAACCGGTTTATTTCAGCTCTTTGTTTGTTTACATTCAGTTGGCCAAAGGAGGGCCGGACATGGTTTCAATATTTCAATCCATTGGTCGTGTGAGGCGAGTTATCGATGAAGATATATACATTTACATGAATCCCGTATTAATTAGAAGCTATGATCCTTTGGCACCTATTGCCATACCTCCGTGCAGTGATTGGAGTGTCGCTGAGCAGTCTATCATCTCAGAGAGTTGCATCGACTTCCGCGGAAAGTGCAGCGGAGCGCATAAATATAATTTTTGTTCCGTACTGAAGTCTCTTTTTCGGTACAGGCATTATATCGAAAAGACTACTATTACAAGTTTATCTGATAGTTTATTTCTGCTATGCAGTCTGTTATGTGAGAATTCTATTAAGGTGGACATCGTTGGAAATGGATTCCCGATGCGCAAAGAGGTTTTTCTGAGTTTCTTGCAGATTCTAGTTGAGGAGTGTCATTTTATCGAAAAGAAGATTACTTTGCCGGGTGACGACATGACGTTCCAAGAGATTATAAGTAGCAGAGAAACTATCATGAACGGGGATTTTTATGAAAACGGTGATCAACTATTACATAAGGACTATATTACCGACATGGGAAAGTTCAGAGCGACTTTCTTATCTCCGGGCGTTGACATTTTCATCGCATCGGACATTGTATATGATCTAAAAAATGAAAGCAAACGCTATGTTTTTGTGAACGTCTGGTTGCAAAAATGTGTATCTGCCGGTGTGGAAAGCACGGAAATCGAAAGAGTTTTTTGTGAGCGGATTAAGTGCTATATGCTGCCGAAGAGTTTTCTGTGCGACGAGTACTTTGTTCTAGGTGACATTTCTGGGGTTTACGAGTGGGGAATGTTAATAGATCTGGCCTTCCTGGCGGAAATGATCCGAAAAGATTTGAAACTGAAATCGTGCACGGATACTACTACTGACATTTCTGAAGATGATCTCCTTCTGTGTGCGGCCAGGAGGTCAAGTGACATCTTACAAATAATGCAATTAGTGTTTACGGTGCATGTGCAATTTTTTCAAAAATACAGTCTGCAGACTCTGCAGCTTTTTAACAAGTTGAGAGGCATGCGGATTGTGACGGGTGTGTTCTCTATAGAAAAATTCAGCATTTCCATCCTCAGGCTTTTCTTTAAATGTGCATTTAATATGACTCTGTCCGCCAGCAGACCTCGGTACATTCCCGGGAAGGCGTATCGCAATCTAACAAAAAATGATATGGAGAACATGCTAGACAATTGGGAGATTTCGCGCACCAATCTTAAGACTTGCAAAGAACTACGTAAAGCCCTCACCGAGGCCTCGAGAGCAAGGCGGAAACAAACAATTTATAAACTGCAAGGCTCGGATATAAGCCTCTCCGTTAGTGAAGTTGGAGTTTTCGGGCAACATGCATCTCCGGGGGTGTGCGTGTCATCTTAGCCTTTATTGTGTGTACAATGACTGGGAGAATAAGATCTACAGAGTACCGATTTTTCAGTGTCTGTTTTTAGAAGCGGAGACTCGTTCGTTAAAAACGTTTTTGATTAGGGGACAGAGCCTAGACCAAGAATCGTTAAATGAGATAGAAGTTACAAGAAAAGAAACAATGCTTTGGGACCTTCAGGAACAAAGTAATATGATGGACAAAAAAATAGCAGCAATCAGCAACCTCATTATGAATAACGGAGAGCTTGTTAGGAAACTTTCAAAATTTTTCGTGCCTCTAACCGTTGTTCTTGGAGATGACGGGTTAGAGATTTTGGAAGCATACGTCTGTGGCGAGGAACTCATGTTGCCTCTGGACACGGTACCTGTTATCTTACGGTGTATCGGAGACTACGCGGCCTTGGACACGAAGCATCTTTTGAGTAACGAATGTACACAGGCTTCCAAAAAAATTCGCTTTGGATACAGTGTGATGGATTTTCACTTTTCGTTGACTGTGTCTGACGTGAAAATTTGTTTTTCACACACGGACACCGGTGAGGCCGTATGTGAAAAAATGAAACAAATTTTTTCTTTTTCTGTGTGTGCTTTCGGCGGTGAGCAAGTTCTCTTAGTGACGCCAAAAAATGCTTATGCCTTATTGTTCGACGATGATTTGTGTTTGCTTTTACTGCAGAGCGTGTTTGCTTTCCTGCACGAGAAGATATTCGGAGTTTATAAACAGGTGTTAGTTCAGCTTTGTGAATACATCGGTCCCGACTTATGGCCCTTTGGGAATGAAAGATCAGTTTCTTTTATAGGATATCCAAATCTGTGGCTTTTGTCTGTATCGGACTTAGAAAGGCGGGTTCCGGATACAACTTATATCTGTCGCGAAATTCTCTCATTTTGCGGTTTGGCGCCTATCCTGGGACCTCGTGGTAGACACGTGGTCCCCGTAGTCAGGGAGCTAAATATTGAAATGCCTGGAAGTGAAACTTCTTTGCAGCGTTTCAGGTTCAATAGTCAGTATGTTTCAAGCGAGTCCCTGTGTTTTCAGACAGGTCCCGAGGACACACATCTATTCTTTAGTGACAGTGATATGTATGTGGTGACTTTGCCAGATTGTCTGAGGCTATTATTGAAGTCCACGGTTCCTAAGGCCTTTTTACCATGTTTTGATGAAAACGCCACGGAAATCGATCTGCTTTTAAAATTTATGTCAAGGCTGCAACACAGATCGTACGCTTTATTCGATGCGGTGATCTTTATGTTAGATGCTTTTGTTTCCGCTTTTCAGCGAGCATGTACCCTAATGGGAATGCGATGGCTGTTAGTGCGGGACTTACACATGTTTTATTTAACCTGTGATGGTAAAGATACACATGTTGTCATGCCTTTACTGCAAACGGCCGTAGAAAACTGTTGGGAAAAAACTACGGAAATCAAACAAAGACCTACATTTCAGTGTGCGGAAATTTCGCGCTGTGGATTTATCGTCTACGCCAGATTTTTTTTGAGCAGTGGGTTGTCACAATCTAAAGAAGCGCACTGGACAGTAACGGCGAGTAAATATTTATCTGCATGTATTCGGACCAATAAGACAGGTCTTTGCTTTGCCAGTATAACTGTTTATTTTCAGGATATGATGTGTGTTTTTATAGCTAATAGGTATAATGTTTCTTATTGGATCGAAGAGTTCGATCCTAATGATTATTGTCTGGAATATCATGAAGGACTTCTGGACTGCAGTAGATATACGGCCGTGATGTCTGAAGATGGACAGCTTGTGAGACAAGCACGTGGAATTGCGTTGACTGACAAAATAAACTTTTCTTATTACATTCTCGTTACACTGAGAGTGTTGAGGAGATGGGTGGAGAGTAAATTTGAAGACGTTGAACAGACACAGTTTATTAGGTGGGAAAACAGGATGCTCTGTGAACACATTCATTTACTGCATTTGAATTAACTTTATTTCTTTACATAACATAGAAGAATGAGACTTAGCTAGATGTGTCTCCATTCTTCGAATAAAAAGTTTGTGCGAATTGTGTTGCTAGTTGTATGACTTGGAGAGAACTGTCTTGCAGCTGTCTAGATTCTAATGATAAATTGATGAAATTGTGACTACGTTTTGTGTTAAAATAAAGTTCCAAAACTTCTATATGCGTGTTTTTCATTTCATTGTATAGTCTAATCATCAGAGGCACCTTGTGGAGGATTTCTGAGGCGTGTTCGTTAAAAAAAATGTGTCTTAGTGTACAAACCAACGTGTCACACGTGTTAAATAATATTTTCGTTATCGGAGATAGGGCTGGAAAGAGAACGAGCTTAGCGATTAGTTTGTGTAGGGTGCTTTGCTTCAGTGGAAGATTTTCTAATGCGAACACTAGTCCGCAAAGCATAAATTCTTCTGGCGTATCAACTTTCATATGGATTAGTCTGCGTTTGAACTGAAAGGGATTGAGTCGCAGTTCTTGCAGTGTATCTTCCGAGTCTAGTATTATGATGCCAATGAAGCCTTGAAATCCCAATTCTTTTTCGCAGATGGTCTTGTACTCCAGGCCGTTGATCATGGTTCCGTCTTCCAAGTGGAACCATATTCCGTCATCTCCTACTGTTATATTTACTAACGGATGTCGTGCGAGTGATATACACTTATCAATCTTGATCACCTTTAGGATAGTTCGGACGTGTAAGATGTTGAATACGTCTGAGGTTTCTAATTCTTTTAATTTTTTCAAGGATTTCATTCTCCGGGTTTGGGGAGAAAATGTTCGAATCGAAAATCGGTTCGCTGTTTTCGGGGTGCGTTTGGGTTAAATTAAACACTTCTGATATCTGATTTAGCTCTCGGACGTATACGAGTCTTAAATAATGCTTGATTTTGCTGCTTTCGTAGATGGCTAATAAGATTTCACCGAGGGATAGAAAGAGAAATTCTTCGGGAATCGATTCGAGGTTGTAAATGTTTAAGAATCGCCTTAGGAAAGGCACGATGAGCAGCTCGATGCTATAGTTAGAGTATGTGATACTTTCTTCCTGTCCTTGATTATTAACGTTTTTTCTTAACCGGAAGGTTCGCATGTATTCCATTTCCCATAGCTGGTCTATTCGTTTGTCGGCATATTGCGTGTCTGGGATATACTGTGAAAAAAAACTGTTCGCGACTGATCGGGTGTCATCTATGGAAACGCTAGTGAATGGGAGCCTTTGAACTATGTTTAGCGCTCGAGATAAAGAGAGATTCTCGAGGTCATGGTCCAGATTCGGGTTATAGCGATCACAATCGTTATTCGTCTCGAGCAAGGAGTGTAGATCCTTGATTTTCCTTTCGAACATTTGGTTTGTTGTTTTGAGTGTTTCGATTTCATCCATCTGTGTTTGTATCTGCTCCTCCATGCACTTGATGATTTGTTTTTTGAACAGATCTCGAACGTCTCTCTCTCCTGTGTCGATCGCAGAGCGTTTTCGGAACTGTCCTCGGTTCAACATGAGTTTATTTTGGTCTATTATAGAGGGAGTGATTTCTTGAATAAATCCTTCAACGCTGTCCCTAATGCCTATTTTAGATTTGCTGTCCGATAAGTTGAGTAAAAATTTGATTAAACTTTTTTTCGCGTCGCTGCTTTTCTCTTCTTTTTCTATCATGTCCATTATCTTTTTGTTGGTTACATCTGTCTTTGCTGTGGTTAGCACCTTTATGGGAAATGTATTCAATAATTGGCACATTTTTTGATGACGCCGCAGACTATTGTGTTTTTTTAGTTCTGGAGAAAGGTGTGCGAGGGGCGTAGTCAGCATTAAGCGATTTCTAATAACATATGGGGTGCTGTACGTGAAAAACGCTATAGCCTTCTTGTTTTTAAAACAAGAAATCTGCTCCGAATTTCGGTCTCGGAAAATCTTAATATTGTGAAAATCCGGAATGATCACGGAAGATAGACCCAGTAAAACGGGAGTTACAATGTTTTGTTTATGCCGCACTATGTCGTGGATTAGTTTAGATGACACTTGTTGTAGGAGATTGTATAAGTTCGGGGCCGTAGGGGTGGTTCTCATGAACGGAACGATTCCTACAGTACAGATCCAGTCTACATATCTGTTATAGTGACTGTTGTCTATGCTCTGAAGAATAAAATTAATTATATTAGATATATCACCCAAGATCACCTGTTTAATCGCGTGGGCCCATACGCTAAAATAATCTTGAAATGACAATTTTTTGTTGTGGAACGGAAATGACCTCACATGACGTTTCCATTCATCGTGGATTTGTTGAATGGAAACGCTGTTTAAAAGGGCTTGCGCTACTCCATAGGCTATTTGTTTTTTCAGTACGGCGGAATCTCGGAGAGCGTTGTGGATTGTCTGACCTTTGAAAAAATTTTTGTGTCCGTGTAGCATTTCAATAAAAGTCATTATCTGCTCCGAGGGATAAATCATGTCTATCTCTTCGTTGTTCGGAGGTAGATACGATAATAAGTTTTTGCTTAACATGTCTTCGGCTCCCAAAATAGAGTTGATTGTAGATAAGGTGGCTTCGTTATCAGAAGGACGCTTGGAGGGGCGCCTGCCGGAAGATTGGTTTCGCCACATCATGGATCCCGAAACCGAGTTCAACAGCGAATTTGCTGATGCTCTGTGCATAGGTATTGATGAATTCGCTCAACCTCTGCCTTTTTTGCCGTTTAAGGCCTTGTTAGTAACCGGTACGGCCGGGGCGGGGAAAACGAACAGCATTCAGACCTTAGCGGCCAACTTAGATTGCATCGTTACTGCAACGACATCTATCGCCGCTCAGAATTTGAGCGTTGTTTTAAATAGAAGTAAATCCGCGCAAGTTAAAACAATTTTTAAGACTTTTGGGTTTAATAGTTCACATGTATCTATGAGCGAACGTCAAAGTTATATTGCAAATGACGAGAGGTCGATTCAAATTCAACAGAAGCAAGACCTGTCCATCTATTGGAATGTTATCTCCGACATAGCGGATAGGGCGTTGGGTGCTGTCGCATGCAAAACTAAAGAGTTACCTGATTTGTGTGAGAGTAGTGTTATAGTTATCGACGAGGCGGGTGTAATATTACGACATATTCTGCACACTGTTGTTTTTTTTTATTGGTTTTATAACGCGTTGTATAAAACCCCTTTGTATGAGGACGGAATTGTTCCGTGTATCGTGTGTGTGGGTTCGCCCACGCAGAGTAATGCTTTGGTGACTTCATTTAATCCGCTGACTCAAAATAAGGACGTGAAGAGAGGAATAGACGTACTTTCGGCCTTGATTTGCGACGACGTGCTGTCTAAATATTGCGAAGTCGATAACAACTGGATAATCTTTGTAAACAACAAAAGGTGTGCGGATCATGCGTTCGGAGATTTTTTAAAGCACATAGAATTTGGTTTGCCGTTGAAGCCGGAGCTTATAGAGTATGTCGATCAGTTTGTGAAGCCCGCTTCGTACATCAGAAATCCCATGAACGAAATAGAAACGACCCGCTTGTTTTTATCGCATAACGAGGTAAAGAACTATTTCCGTTCACTGCATGAGCAGGTTGAAGTTACCAACAGAAATAACTTGTTTGTGTTTCCGGTTTATTTTCTGATAAAAAACAAGACTTTCGAGGACTACAAAAGTGAAATTGGCAATTTCTCCTTAGAAATTGAACCGTGGTTTAAATCTAATATTCATCGGCTGAATACTTATTCTCAGTTCGCGGATCAGGATTTATCGAAAACGGTTCAATTGGAGGAGATTGTTTTGGAAGATGGGTCGGTGGAAGAGACTTTGATAACCTGTCATCTGAAACATATCAGAAACAGTTCAATAGGTGTAACGTCCAAGATCAAGGCTTCGACCGTGGGTTTTTCCGGAACTTATGAAAAATTTGTAGAACTGTTACAGAGCGATTTATTTATCGAAAAAACGTCATGTGATCAAACTATCCACGCATATTCTTTTTTATCCGGTCTTATGTTCGGAGGGATGTATTCTTTTTGCTGTTCGAAATTCACCACCCCTGAAGTGTTAATGGAAATCAAAAACATAAAGATGCCCAGTATTGAATTCTTAGAAAGTGAAATGTCGAGGATGTCTCCGGACGTACAAACCGTAGAGACAGATGAAAGGTATGATTTCGGATTAGTCGATGATGGGTTATCGGATGTGGATCTATTAGAGATCGATCCTTGCGGAGATCCATTTTTTACTAGGTATAGTAAGTTGCCTTTAACTAATTCTCTGTCGTTCGAGGAAATCAGTTTGCTTTATACGACGTTTAAGGATATCTTTATATCTAGGTTTGCTATTCTGCAGAAGCATACAAAAGGGAAGTTCGGAAAGACTCTCCTAGTTACTTATAACAGAAACAACGTTTCTAGAAAACAGTGCGGTGAGATCTATTCCCATTTAAAGAGTTTTTATGGTATGCTCACTTACGCTATCCCTGCCAATAATTATACTCTGGAGGGCTATACAAATGATAACGTAGTTCATTTAGGAACGGATAAACAATTACCGCAGATATTATATAAAAAAGGATTACCTAGATTAGTTATAAAAGATGAAATGGGGTTCATTTCTGTTTTAGATAATAACGTGTCGAAATTTATCGACGTCGTAAATGGTCAGAGTTTTCATCTATGTACAACGGTGGATTACGCCACTGTTTCTAAAGTTTCTATGACCATCACGAAAAGTCAAGGGCTTTCCATACAAAAGGTCGCAATAGATTTCGGGAGTGATCCGAAAAATTTAAAATTGAGTTCTATTTATGTTGGGATGTCACGAGTAACCGACCCGAATAATTTAATAATGAATGTTAATCCTTTGAGACTGAATTATGAGAATGATAACTTTATTGCTCCGCATATTGTAAAAGCGTTAAAGAACGAAAATACCATGTTAATATTTTAGGGAGGTTTTCTATGAGTATATAAAAAACTAAAACATCAGATGAATTGTGCGTGTAATTGTTAAAATCCCATACGGTCGAATGAGCTCATTCCCGTCGGTCCGTTTTTCTCTGCAAAACAAAGGTACCACGGGGATTTAGTACAGTATGTCTCCAAACAGAGAAGGGTATGAAACTTTATGAGGAACTGGTGAGAAATAACTTACGGTTATGATCTCGGTTTCGTCGCAAAATTGAATACAGCTACAGGGGTGTGGTCTGATATGACATCTGCAAAAAAACAAATGAAAGTCTCCCCCGGAGTACACGTATGTACTCCGGGGGGAAACCTCAAAAATGCACAATTTAGCCTATTAAGCAGCCGCGAACTCGTTAATAGTTACTTTGATGTTAACTCAGGTGGTGGCTTGGATGGTGTCCTAAGTATCAATCGAAGCTCCTTTATTTTTTGCACTACTGAAGTTATTTTAGGTATCGGGGTAAGAATAAGATGACGAAAAAAATATGCTAGATCCGTCCATTAAATCCTAGAGTTGTTTTTAGTAATAGGTTATTTACAAAAGCCTGCATATGATCCAATGTTTCTGTATGAATCCAGATAAACAAAAATCCAGGAGTAATGATTGGAATCACAAAATGATTAGATGCTTAACTTACTTAATAACTATACTTAACTATACTTATAGACTAAACTTAACTTAACTATACTTAATTTTTAGGTCCTTATAATGTGAACTAAATAATCAACTGTTTTGTGCCCAACCCCCCACTACTAAAGCAATCGTCTTTGATTTATGAAACAACGAGGCCCACTTCACGTTATTACATAGATCAAGGTCTATCTCTTGGCGGTTTTTGAATTCTTTCGAGAGCGTAACGGTTTCTAGGTTTCAGACACGTTTATCCATTCGCCGGGTATCGATCGGATCATATCATTAGCCGTGAATAATCTCTGACTTTAGATATAACGTGTGAAAACTAGTAAAAAAGGACAAGAGCAGCCGCCTTTGCCGTCTTCATGAGCGTTAAACACTCATGAGGTTCGTATCTTACAACACAATATGTGAAATATTGTGAGCGAGGTTACCGGTGTCCAAGTCGTAATCCGAAAATCATATATTGAAACTTGTAACAATGTTATGCAGTTGTACTCAAAGTATATGGTGATGAATTGAACATGTTTTTGATGTTTTCGATGGTATTCGAAAATAATTTTAGATATCCTGTAGTTTTTTTCTGATGACTTTTCTGATGACTTTTCTGATGACAATATAGAAGATGGCAAAACAACAAGTTACTGGTAGTTCTTGATGTTAAATAAAATGAATCGGAGATATGACAAGTAGAGAGAGGTAGGTGATACGAGTAAAATCGAAGGGTGTTTCCACACAGACGGGGGGGAAATTCTATGAGAACCACAGCCAGAAGCAATTGACGACAATAGAGAAAATAAGCAAGCATATTCATCTGTTATAGAAAGAACCAGTGACCTACGACTTTCCATTCAGGCTAGCATGGTGTGAACGAGGGTTTTTTCCGAAAAAACTAGATGACTATTTCTTTAAGCAAGTGTAATTTTTAGCAGTCCAAGTTTTTATGCAACGCAGATTTTAATGATGTGTCCCTCGATAACTATACGCTGGAAACTAACTTACATCTTAGTTGATAAACGATTTTCTATTAAAAAGACTAAAGGGATTTTTTGATATATACAAAATTGTATTGAGCTCTTGGAAGTGCGTAGATGCAAAATAGGTTAAATCTTACCTTGAAGTTACAACAGGTCAACGTTAAAGTAGACGTAAATTCCATGTGTCGAATGCGCTGTAAACTTTTTCACGGAAAATTAAAAACACCGAATTTGTTCACGATTATATAAGCATGAATTTGCAATTTTAATAGTCCTATAAAGTTTTCATTTCGATCTAACTACATTTTAGTATTTATGTTCCATAATGTATCAAAGTGTATACGTTCAATCTGTATAAGAGTCTGCATCAAGCTTCACGTGATTTGTAGTTCGCGGTTTAGTATTCGCTGTTTTGCCGTGTATGAGACTTACATAGTTTGATCCCTAATACTTCTGACAGGCGACGAGCCTTTTTATATGCATTTTACGACAAAGTCCACGCCCTTGTCGCTGACGCAATACGTAAAGGTTTTCGGTTTGGATGAGAATGTGGTTAACCGTTAATAAAAAAGTCGATACGGAAAAAAAGTCGAACGGTATTTTTAGATATCGGGTCACATATCGATTTAATAGATTTTTATATCGTGTTTGTTGTCCGACTTAGATATGTCGTTTCATGGGAAAAATAAATCGTTTTAGTTGCGTGCTGTGGTTTTTGGATCGCAAACGCTTGAGCCCGGAAGTGTGATTCGTGCGCATAAAAGGACCTTGGTAACTGTGTTCGCTAGCTCATTTGTGTTAGATGAGCGGCAAATGACTCATCTTTCTGGGATTTATATGGGAATATTCTGGTAAAAGTGCATGTATCAATTTTAGTCGTTTTTGGATTTCTGTAATGTTACGATTTGCTATGTGTATTTTCATGATGTCGAGCATGTTTATTTTATTTTTTACACAGATAAGATGTATGCTGAAGAACGTGCATATGGATCATTTGACGATGTTATGGAAGTTTATCAACAAATTGTTACCGAGTCTATTCAGCTGAAAAGGCTTCATTTCGGCAGTGGCTGTTTAATTGAGTTCTTAGGTGACTCTGGAACGTGTGAAACATTTTGTAGTGGATGGATATCAATGATTTGTTGGACATCCGATACAAATTCAATGGGTTCTTTAACGGTGGATATTGGGATAGATGATGGAAAATATAAAACGTACAGTAGTCGGGGTTTTTTACTGTGTTCGAAATCGATCACGTCGATTTCTCAGAATACTGAAGGTAGAGATAGGATTTTGACTGTTTCCCAAGAAGGCAACAAACTTCAAATAACGTTTGTTACTCTCACTAAAGTTTTCAAAGAGCAGGATATACGAAATCTGGGCGATGTGAAATGTATGGAAAAGTTTGAAAAAGAATGTCGAGCTTTAGACAGAAAAAAACACGACGATGAACACAGAAAACGCTCGGGGA

GCAACCGGACAAAAAGGAGGAATCGGATGGTAAATATGAATGTATAGTTGTTGTAAATTTTTATTTTTCGTGTGTCACGATCGTGGATTTTTTTTTAAATAATATCTGTTTTGTTGCAATTTCAGAACTTCCTAAGGAGAAGAGACCGAAGTACCATGACATGAAACGTAATTTGGAAGAACAAAGTCACGAGGATGGCATAACACCAACTTCTACGACACTCGTAAATGGAGCGGTTGAGGGTGCGTTACCGCCCTGTATTTCTATTGATAATCACGAAGATCAACAACATGATGAATTAGACAAGCGCGCTTATGCGCAGGTGGGTGGAGTTTCGGGTTCGCCAAAACCTAGGTCTTCGGGGTCTTTGTTGTGCGTATCTAAAGCTGATCTTTTTATTTTAGGGGACGAACCGAGAAGGTCTATCCAATGAAGATAATTATGGGAATTTTCGGCTTAATAAGTCTTTAGAACAGCTTAGGGCCAAACTTGTTGCAAGCAGCGGCGACATTGTAGAAAGATCGCTTTTGAAATTGAAAGAGTGTTTGGATAATGTAAAGGATAATTTAATAAAAAATGAATGTGCTGATGTTACTGGTCCAAGTAAATGTTTAAGTAAAACAAAACATATCGAGCCAAAGAAACAGATAGTGTTTTCTGATTGTGTCAGGCCGGTACCCGTGTGTGAGATTAAGCCGTTTATCGATGTGCGACTATTTGAAACGGCGCAGAACCCAAGAAGACTTCGACAACGGACCAGGACAATCGTCGGATCAACAGATGGTGCGATCGAGCAACAGCGTGTTATTTCGGGACAAAACCGAGGCAGAGCACGAGGACGTGGCCGAGGGAGAGCTCCCAGGAGACGAAATTCCAATATAAATAATTCAAGGACACAAACTACAATTGTTATAGACGATAGCAGCGAAGCCGAAAACTTTGAAAATGAGGGGAGTTTTAATGAAGATTTACTGGCAACCACAATATTGGAAACACTGTAAATTTTATTGTTGTATTATAATTATTAGGTAAACTGAATAGGTTTTATTCGTTCAGAATGTTCCAATTGATTGGGTCTTTCCCGTGCTCCGTAAGAAATAAATTTGCTCGCACGAAATGATTGCAACCAATAAATGGCATCCTTGCAGATTTCACTTTGGGCGATGGATGTGCACTTTCTAAGACGAGATGTTTCCGTGCGTCTATTAGATAAGAGAGTTTCCGGGCATGGTTTCCCCACAATAAGAAAACGAGAGAGTCCATTTCTATCGATAGTTTGCTGATAATCTTATAGCTCAGTGTTTGCCAGCCAAATGCCTCGTGAGACATTGGTAATCCATGAACTACAGTGAATATCGAGTTTAGCAATAGGACTCCTTCACTACACCAGGAGTTTAAACAGCCGTTGTCAGGGGGGGAAAAATTCGGCACGCTTCTTTCCAGTTCTTTAAATATATTCTTTAGAGATTCTGGAATCGAGCACTCTTTCACTGTACCGAAGGCCAAGCCGCAACCTCTGCCGTCGGGATACGGATCTTGTCCCAAAATCACAACTTTTACATCGTCGGGTTTGCATAAAAAACTCCAAGCGTGGACGTTACCCGGAGAGGGGTGGACCGTTAGATGAGCCCTATCGTTGTCCACCAATTTATATACATGTTTAAGGTGTGTAATTTCTAACTTGGAGAGTTGCAGGAAACTCAACCAGTCTCTGTTTATGCCGAAGAGTGAATGCTGATCATCTATTGAAAGGTTTTCACAGTTTTTTTCTTCATCCTGAACGTGATCTAACATCCACTGTAGTAGGGCCATGGTCGGGGAATGAAATGCAGTGGATTAAATTCCCTAGGAGGATTTTATGTGAATGGGACGTGATGTCATAGCCAATTATGTGTTTCTAATCAGAATTGTAGGGCGGGAACTTCGGAAAGGTAGTTTAGAATTGTAGGTTTCCATATGTTTTAGCAGGTCGAGCTCTACTCCGGTTTTCAAGTGGAAATACTTTAAACAATTCAAAATCGTATCAAAAAGAGTCAAAGAGTTAAAAGGGAACCTAATAATCTTTGTGACGCCTCTAGCAGGATTCTCCAAGTATAATAAAAAACCAATGTTGCTGTAAAAACTGTCTGTGTTTTCTGAAATGTTAAACTCGAGACCCGCAATGTTATCAACAAAAAAGTTATTGACGTACGTAATTTTTTCGGGCGAATATTTTCTACAGTTATCTCTGATGCAGGTTGTTATTCCGACGGAAGGTGAACATGTCTGGAAGCCATTGAAAAAGTTTAGCCAGTTGTCCCGAGATTCTGGGTCCCTTATTAGCGCAAACAATGTGCGTAGTTGTTCCGGGTTATCCTGTAGGAGTAAGAAGTCGCTGAGAAATTCATCGGTTACCATTATACGGCTAAAAGGCTTTTTTTTTGTCGGGGGTTGTAGCAGTATTGACATAACGTCTTCATTATATACCGGAGGCTGGTAGATTGGTTCTAGTAATAGAGGTACTTCGGATTTCGTGCAGTTTATTACAGCTGCGATACAGTCGTCAAGTTTTTCAAAATAGAAAGAATTATGGTTGAAAAGAGGTATTGCTTTTGAGAAGGTTAGTAATATGAGTGACATTACAAATAGTAAAAGTTCCATGGTTATTTTTTATTTGGAAAAGTTAACACGACGGGAACAACATCTTATATATAGTAGGGAAAAAGTCTTGTCGAAATATCCATTCGGCTTTTTATTGGTTTTTTTTATACGGCATATATTGGTATGGCTATCGGATTTATATGTAGTTCCCCCGATGCGGAGCTGTTTTCCGAAAAATCACGTATTTCGTCTTCTGTCTTGTTAGGATGTTTGTTGTGTTGCATGGATTGGTCCGCTGCCGTACCTGGGAAAACAGAGTCTTTTAGAAAACTTTTTGATGCAGTCATGATTAAAAAGCTAAAAAGTTGTTCTGCTGCTTACCCGTCTGATTTGGAGCAGGGCTCGATGTGTGATATGGCAGATGCATCGCCGACAAGTCTTGAATTAGGATTGTCGAAATTAGACAAAGAATCATGAAAGTATTCGAAGACATTAATACTGGTTTTCTTTTTCGTTACGACGAACAATATAGAAGACGCAAAGAGAGACCAATTTAGATTCGAGCCAAAAAAGTTTATTTAGTCAGTTTAATAGTTTGCCATAATCATCCCAATAAATATATTTGTTGAAAGAGTTGTATATGGTGATGTGTTCTTGAAGGAGCATCGGTTGTGTTAGCTTTGCGATATTCAAAGTAATCTCGTTGGAATAGCAGATCGCAAAATTGATACAAACGTGAGCGGCGCATAATAAATCCTTTTCCGTTGGCGCGTTTATCATGAGAGAGCAAATGCCGACAAGCTTTTTTTTTGAGGCGTTTACCGGTATAGGAGTTACAGCGTTTTTTCGGAGTGCGTCCGTTTGTGGGACGGTCCCTCTGAAAAACTGATTGATTGCAATAGCGACTACCGGTTCATGGTGAACCGATTCAGAAATTATCTGCTCGCCCACGTTAATAACCATGTTGCCGATGGACATCAATTTTTCTTGCATGGCGTTTATTAAGATCTTATTTCGGGAATAAACCTGTATTTGCACAATTGAAGCGTTGTAGCAGCGGAATAATCTGTATATTTCTGACATAAAATTTTTAGGTGAGCACGGGGAGTAAGGTGGAGATGTTTCGTAAGATTGAAAAATTTTGAAAGCGTTGACTGTATTTCGATCTTGCCTTTGGCATTCCGTAGACCTGACAATTTCATTTTTTTCCGTTGTCGATGTATTTACAGGCATGTTGAATGACAGGTCGGCGAGAGGTTTGTCCGTAAATACAGTAACACGATTAATTTCTTTTTCTTGACGAAACACGGCGTTTGAGATTTTCCGTAGTTTGGAATGGGCGTAGATCGCGTTATTTCGATATTTTTCCTTATTAAATTTTATATCGGATGGGGTGTGCTGGGGAAAGACTCTTGCAGTTGCATAATCGACGGGTTTTTCGGTTGGGGCGGTTTGCGGAGATAGATTGTTTTGTATTCGATTATTTCGTATAGTCGTTTTAGTGGGTAGATTTTTTTTCCGTTCTACGAACAGTTTCGTTGTTTTTCTAGTTCGGCGAGATTTGACGGTATGTCTTGTTTTAGAGGTGTTTTTATTTCCTCTGTCTTTCATCTCTTCTGAACTTAAGCTATATAAATTTTAACTTTTAAATGCAACGATACACACCGAAATCAGTAGCACTATAACAGATATCAATATCAGAATTAATGGTACGATCCAGGTAAATACGGTTTCGTCAGAATTTGAGCCTCTTACCGCTTCTTTACTGTGTTCGTCGTGATTCTGCACAGTGTGTTCGCCGGAAGTTACAAGAGCGCTAGCCTTTTGGTTGGTAAACCAACTCGACGCTAGACATGAGACGGTTTTTCCGACAAATTCCAGCTTCGTTCGTCTTTTGAAAGTAAAGTTCACACTAACGGTTGACGATCCGTTCTCATTCTGTCTAACTATTGGTATGTCTCTCTTATATACTTCTCCAAGAAATTTAATAACCACATTTGGTTTTGGATAAGACGTCACGGTACACGTAACGTCTAAAAAATTGTCTAAGTAACGAAAGTACAAAACGACGATGGGTTTCATAATGAGTTTTAAGCAACTAGTCGCAGACGTTTTCTCGTTATCTACTGTGAATGTACAGGTTAAACATGTCGCGTCATATGCTGATTTGGCTTTAATTTTCAACAGAGATGTAGTTCTATTGTGTTTTATGTGGAAATATAATTTACTGTCCACGGCGAGATTGTTGATCTTATTTAGTTCGTGTATAAGGACGTGGCCATCGTCAGTGAACGATAAGTTGTCCATGTTTTGTACCTCATGATTCGATATAAGTCGGCTTTTATTGTACCACGAGACGCTTCGGATGTTTGTGGTCGTGTTGAGGCGACAATGGAAAGTGACAATTGACCCTATTTCGTATATTAAAGAAGGACTCGTCTCCGGGTGGAGTACAAAATGTGCACTTATGATATATAGAAGCGGCAATAACCGAAGCATCAGAGAACTCATAGTGTACACCTGTGTCAAATAGCAGTTTCTGTGCGAGATAGCGGTTTCTGATATATATAAGTCACGCTTAAAATAAAAAAAAACTTAAGGATTTCCGTTATACTGTTTTATTTTTTTTTAAAAAAAAAGTTGGGAGTTAACATTTTGAAAGTGTACTAAATTTCGATTTAAAATCTTTGCTGATAGGATTATAGATGGAGATAGGATTGTCACCGTCGATGATTGAACACACCCGAAGGACAATTTCTTTAGGGTTATCTTTGGCATACTTATTGCATGTTTTAACAGCTCCTACAAAGTCGATGGGCGTAGCGGCGTAAATTATCATAGTCTTTACGTCCGAATGTCTCGTAAATAGATCATGTGTTGTGTTGTTTTCAAATTTATCCCATAATTCTTGCGGTCCTTGTTTTTGTAGTTGGATAAATCGTTTAATTTCATCTTTGTCATAATACTTAGGCGGTGCATGATTCATTTTAAATGCCTCTGAAACAGTGATGTTGGGTCGAATCATTAATTCTTTTTGCATTATCTTTATGATTTGAGCTACTTTTTTTGATCTTGTAAAGTACATAATGATGGCTGCGTTGTTAGAGAGCCCATTACAGCAGATGCTGTATACAGCTTCTTTTAGATTTTTCTCACTGCAAGGTTTTTTGGGGATCTGTTTGTATTCAAATGCTCTCATAGTATTTCTAAATAGTTTTTGAAAATCTTTTGTAGCCACCCTCCCGCGGTGAATTTTAAATTTTTCTGATACTTTTCTTTCTTTTAGTCTAATATAATCTCTATAACACAAATCGTCAGTTTCACTTACTTCAGACTCTGTTTTCTGCGCTCCGGATATTCTTTTCTTGCAGAAATTTTTTATGCTGGAATCTGAATCATCATCGCGGGGGCGTTTTTTTTTGCATTTTTTTGTGTTGTGTAATTTGACATTTTGTTTTTGATGTAGGTGAGAAGTCGTGATATTAGGTGCCTTTTTTTGTTCTTCATCAATGGCGTTTTCGGAATGATTGGATTGTTCATGTGTGTTAAATGGACTAGAATTGTGTAGGGGAAATTTTTCACCTGCTTCCGGGGAGTTTCTAAACACATCTGCATGTCTATTCGATTCGTAATCTGAAGACATGTTTGTAGTTTTGTGTTTTGAATCTGATTTAGATTTTATTTGTTTATTGTCTTTATATTGAGTTTTGCGGCATGTTTTTAATGTGGATTTGGAACATGATAAAGAAGCCTAGTTCTGGAAGACTGATAGTAAAATGGTGTAAACTTCTCTTTTCCGGTTACTATATGTGAATTAGATCTGCAATTTTCTTCAATACTGGAAGAGATGCTTGATGATCCAGATTTGTGTTTTTTTCTTCTTACAATGGTCAGTGAATCTGGTGAATTTCGACAGTGAGATTTGCATCTAGACATAGAACTTTCTGAATCGGTGTCATTGGTGGATTCTGATTTTTTTCTGTTGGTTCTTATATGAGGTCTGAATCGGGGGTTGTAAAATTGATCATTGTGATTAAAGCACTGGAGTCGAGCTGCAGTATTTTTGTAGAAATTTAGATCTTCTCCACATCGAAATTTTATTTCTGATATTTCAAATTTTGTATTAATTGAGGTGTCATGTGGAGGCGTTTGTCTTTTATTAGAATCGCAATAAATCTGCCTCAGCTCCCTGCTGGAAGCC

AGGCTCTGCTAGAATCCCTGCTGGAGGCTCTGCTTGAGGCTCTGCTTGAGGCCCTGCTAGAATTCCTACTGGAGGCTCTGCTGGAGGCTTTGCTTGAGGCTTTACCGGAGGCTTTGCTTGAGGCCCTGCTGGAAGCCCTGCTGGAGGCTCTGCTGGAGGCTTTTCTTGAGGCTTTACTTGAGGCTCTGCTAGAATCCCTGCTGGAGGCTCTGCTGGAGGCTTTGCTTGAGGCCCTGCTAGAATCCCTGCTGGAGGCTCTGCTAGAATCCCTGCTGGAGGCTCTGCTGGAGGCTTTGCTTGAGGCCCTGCTAGAATCCCTGCTGGAGGCTCTGCTGGAGGCTCTGCTGGAGGCTCTGCTGGAGGCTTTGCTTGAGGCCCTGCTGGAGGCTCTACTGGAGGCTCTGCTAGAATCCCTGCTGGAGGCTCTGCTGGAGGCTCTGCTAGAATCC

GAGCCCTTTTAGAAGCCATGGTGCAACTTTTACTCAGATGTTTATCAGAGTTTTTGCCACAAGTTCGGTTAGGATCTTTAGATTCATTTTTGGTGGAATTGCAGTTAGGTTTCTTGTCAGTACACTCATCACTGTTAGAAGTGCTATTCGTGATGCTTTTGTTATTGTTGGATTTGTTAAAAGCAGTAGGATTTAAATTACTCCGAATAGGTGAATTGTCTGTTAAATTTTTGTCGGCGCTTGCAGTGTGGTTTTTAGCACTGTTTTTGTCCATAATTGAAGGATTTTTTAACTTGCAATATGGAGAAGGTGATGGGATAATAGTTTTTACCGGAGATTTGTATTGACCTTCTTCATCAGAGCTACTGGAATAATGGTGTGTTTTTGATTTTGGTGATGTAGATGTGGCTTTCCCTGAAACATTCTTAACGGATACTTCCTTAAAGTTTTGGTGTCCGTTGGAGTTTGTAGTATTTTTTTTAGATGTTGTACGTCCAGGAGAATGTTTCATTTTAGGATAATTGTCTTTGGAGTCATGATGTTTTTTGTAAGAATTATCTTTGGTCGGGGCTCTTGGAGTATGTTTTGATATGGGAACTGTGTCTTGTAGAGAAGTTTTTGATCGGTGTAGATTTTGTTTTATTTGGTTGAATTCTGGTGTCGAATAGAATTTATCTGTATGGTCCGCAAAGTCTATGGCGGGTTCAGGTGTCAACGGAAGAGGAGATAATAGGGGATCTAATTTTGAAAAATCTCTAGCTTTAGTAACGGCTTTATCTTCATGCTCATTAGATTTTCTCTCTTTAAGATCAGAACGTGATGAGGCATGGTTTTCATCATGTTTGTTTGTTTGAGAACTGCGTTCTTTTTTCTTCTGGACTGATCTACAACGGATATGTGCTTTTTTGTACTTCTGAATATCCACGTCACGATTTGAGCCATTGCCTGTTAACGCTTCCTTCGGTAACGGAATAACGTCAGTGTCATATATTTTTTTAATAATATTCAGTTTTGTGGGACTAGGTCTGTATGATAAACTGATCGCGTCATTAGATGTTAAAACTTGATTTTCTCTGGATGTTTCGTTATCCTTAGTAGCCTCATGTTTTTTAGTTTTTGTAGTTTCTGGTATTGTCTGTGCTTTTGTTGGATGATTGTAATTGTCAGATTGTACGTGTGTGGAGTCTGTTGAAAATGCCATGCTATAGTCATTGGCTCCAAAATTGAACTTGTTTGTTAGTTGTGTAAAATCTTTATAGTTATTCATACGGGGGTCTGGATTCTGTTGTTCGTAGTTTGGATACGTGTAATGTTCATATGGTAAATCCGCGGATCTGTCTGAGCAATCGTATGATCTGTGGCCTCTTCCGCGTCTGTTATAAGTTCTGGTAAAACATCTTTTGTATGGCTGTTGATACTTTGAGTTTGGATTGTAGTAATTATATTTTCCATTTCCTCCTCTGGGCCTATACGAATGATGTCTAAAGTTATAGTCATATCCTCTATTGGTATTTCTAGATTCTCTGTAGGAACTCTCTATATCTCTGTATGGATCATGATAAGGTTGATACGGTAACGAGGTGAAATTGTTCTGTAGATTTGCATTTTGGTTATGTGACGTGTACATGGGCTGTAGGAGTTGATTCGGTAGTTCCGAAGCAAAGCGCACCTGGGTCGAACCGCTTTCGTTTGATGGTAGATTGGAAGCAGAGGATGTGCATTGTCTGTGGGTCTGAGCTACTATCCGTGGATCCTGGATAGTTTTTGAAAATATTCTGGATTCTTGAGAATATATAGGATCTTGATGCGTATTATCACTCAACGGTTGTGTGTGTGTGATGTCCGCATCACAGTATAGTCCGGATAATTTCATGGCGCTTTGAATGACGTCGTTATCATTTCCTTGTTTGCATTTGTGTTCTGGAAAACATGTTTATAAAATTGAAGCTTAATAGACAGTATTTGCTTAATGTATTTAATAAATTACGGATAATCATTTTCATTATGATACAGCCCGTGCAGACGATGATGTTCATACCGATTGTGCGTGGTGTGCAAATGAAGACATTATAGTCTGTAGTAAAAATTCACTAAATCGAATTACTAACAATGGGGGGGATTATTTATATAACAATAGTATATTAGGTGTCATATATTAATTTCATTTAAAACTAGGATGGACAATGACATAAGTTATATATGAAACCCGGTGAAGTTAATGACAATTGAATTGGATGGTCTATTAATTTATTTTTAAATATTTGCATTAGATTATATTATAATAAAATTGTTTTAATGTTAAAAATTTAAATGATTGATGTATTCATTATTGACATGCAGTAAATCCAGTATATTGGATAAAAGTGGGATTTATTAGTCGGGGGGGCGTATGTATAATATTCATGTTTTTAAGATATGGTATTTATCAATAATCTACTTGTCTAATTGTGTTGGTTGTGTGCAAGAAACCTGTGGATATATTAAGGAATAATTGAAATTGAATTGGGTGAAGTTTGTGACTATCAAAGGGTTAATATGCATAATCAAATAAATACTGATGCTAACATGGGGATGGATATAATTTAATAATTAGAATATAATTATTAAATTATGTATTATGAATATAACATAATTTAAAGTTTATAGTAGACTAGTTTTATTATGTTTCTAATGTGATAGCGCCCTCTGCTGTTGAGGTAGATTATATGATATTGAGCATTTTAAGTATAGTAGTTTGAATATGTTTCTGATGTGATAGCACCCTCCGTTGTTCAGGTGAAAAATTAATAGTGGTAAATAGACATTTTTAAAGTACTTTAAACCTAGTAATTTCATCTATTGTGCTTTTTAAACAAATAGATAGAGGATAGAGAGATAGATATGGGGGGGGATATAAATATGGTAATTTTCAATCTGTGTTTCCAAAATTGGGAATCATGTGTAGCATCTTACCTTCAAAGTGATTACAGTTAATAAGATGTTGGAATAAAAAATGGATTTGGTATTTTAGATATATACTTTCAAGTGTTTGGTGTGACATTCAAAGTCTAATGGTTGAAAATGTTTTTCATAAATGGTAGCTATTATGTGAATGTTAAGCAAAAACAGAATATACCTGAAAGAATAATATTTTGTAGAGTGTTATGATCTAGTTATAGCTAATGTAATATATTTTTTAACAGTACTATGATTTATAGAATTAAGATGTCTCGGGGGCATTAGTCTTAATCAACTGGTTTAGTTTGGGAAAGTGAAAGTAAAAATCTGAATACTTGACATCTGTATAAGGTTTTTCAAGAATACAATTGTTTATAGTATCACGTCTCTAAGAAAGCTGGAATTAACTTATGGAAATATTATATGATGACTTATAAGTCAGTTGATGTACATGTCTTGGAATTAAAAAGAGAAAGCAATGTCTATATTACTTTTTATTATTTGTAAGGTGATATTGAAAATAAATTCATTTGAGGAATCACGTGTTTGTTGATGTATTACATAATACAAAAAGTTACATTTGCTTATGTAATTTCTAATCTATAGCAACAAAAAGAGTTACACAATATGGGGAAAGTCTATATTTTACAACAGTATAAGCATATTTACATAATACACATCTGTATGCTAATGATTGCTAATCAATTTACATCTCGGTTTTATCTAGCTAATAGATTTGAGCATTTTCTATGAACGGATCATAATCAGAAGGGTAGCCATCTAATTTAAAGGCCTCTGTACCATCACTGTTGCAATCACTTATATTGGTACATTTTTCATTAGAATTACTTGTACCTTCAGTGCATCTGTTCTCATCATTTTGACTTGTTGTGTCATTGATAATATTTAGTATGGAGGAATTATTTCCTGTTACCGGTGGGTCAAACACTTGAGTTGCGGTTGTTGCAAAAGTTTGGGAATTTGCCACATTTAAATCTTTCTCACATGTAGTAGGGGTGACAACATTATATTTATTGCAAAGATCCTGGCAATGTTTGATTATGCCCTGTTTAATGATATCATTTAGATCCATAAAATTATCCATAACCATATCTATGATAAGTCTCTTAGTTGGTGTTATGACTCCCGAGTCTATTTTGCTATTCTGTATTTTATTATAAATATATGAGACATTGTCCTTATCATAGTCTTCATCATCCGTTTTGATGGGGACAAAGTGATTCATAAAGCAGTGTTTGGCTTCTGTCCCATCATTGTCATCTTTTGCATCCACATTTTCAGAACTGTGAATAGGACAATTGTCTACTTGGTGAGTTTTTTTCATATACCAAATCCAATTTTTTCTGGGAATAAATTTGGATTTGATACCACTGATACCCTGCATTGACCTTATTGGGATGGTAAACACTGGAGTTGGAAAACCTTTACTTGGACAATCACCATTATTGGTAACTTTATCATAATCACTTAAATGTAACTGTTCATGTTCAGATGTACAGTCATCTGACTCGCTGCTCGATTCAGAATCAGATTCTGATTTAATAGCTTTTATTAAGATTGAGTTACCAATAATATTGTCACCATCCTCTTCATCTTCACTCTCTGAACTTATTACAATTACGTCTGCAGCCTTAGGTAACTTATCTGTCTTAATCTTTTTATGTTTGGGTGATTTACTTGAAACATGTTTCCTTTTAGCTACCCTCTTTTTAGGTTTGCTTCCCGGGGTCATAGACTTAGGTTTGACATTTTTCTTAGAAGGGGTGACCTCTGGTGGTGAACATATAGAATTGCTAAATGGTGTATTCTCATCAGTGCTATTCATATGAATGTTATTAGTCCTCGCAGCTGAATCAGAGTTTGCAATGTAATCAATTTCATCATTGTTATCGCTTTCACTCTCATAGGGTGCCGAGTGATCAGTTTCATAACCAAACATCAACATTTCTGATATTAAAGGGTTGCCATTGGAACCATCTTGTTCTGTCCCTTCAACTACTGAATCACATTGAGTGTCAAGTAGTCTTTCTAGCAACATGTTTTTACAACTCTGGAGGCCGCTGATGGAATTCAAGTTATCAGGGCCGCCCAGATCTGTCACTGAGGCTGTACCTACACAGTTAGGGTTTGTTTGTTCGTCCCCTGACTGTCTGCTGTTTTCAGTGAATGTTCCTACATATTTGGATTTCCCTGGCACTTCTGGAGAAGGAGTGCATGGATACATTTTATTGTCATCATCTTCATGGATAGATTCGATAGAAGATTCCTCTGAATATCTTCCATGGTCATCAAAATCAGCTAAGTCTAATATCATTGAGTCTGATGGGTCTGTGTTTCCATCACAAATGGAAGACTGTCTATTTTTATCCATATTAATGTAGCCTTCCATTATTTGAAGGGGAGCTAAAGGTTTAATTAAACCAGTATCATCATAACTTGATTCTAAGCGTTCACATGCTTGGGTAATCATAGTCAGTATGGTAGCTTTAAATCCCTGTCTCAGAAGTTTGAGGTGTTGCCATGAAAATTCATTGCTGCATATAATAATACTCTGGAGAATAGCATTGGCAAGATTTTTGGCGGCAGTAATTAAATTTTTACAATTCACATTAAGTTTAAGGAATGCTTTATCACAACCATCCATGAAGATTTGATTCATCTGCTTTATAGCAAATGTCTGTAAAATTTGCATATTTTTAGCATTTGTTTCTACTATCTCATTTTTACCTTCCAGTAATGTCTGTTTAAGTACATTTATTTTTTCACAAAAGTCCTTACTGGAGATGTCAGGATTGGACATCTCTTTGTTGTGTGCCATCATCATCAACATGGTGGCGCCCTGGTTAAGTTTCTGACAATCTAATGTCACAGTAGCTTTAGAAAAAATGTCTTTGACAGCCTTAATAATGTCATTGTTCATATTAGCTCTCAAATCCAGGAGCAAGTTTCTGGATTCTGATGCCACCTTTTTCACTAACCCATCGAATTTATCACATGCAAAAACAATGAATCTAGGTTCGCACCAATTAAAAAAATCCAACTCTTTTCCATGCAACATTTTTGAATAAGATTCAACATATTTAGTTGAAGCACTACTTAATAAATTAATGTTTATCAGCTGTGATAATTGTTCTAATCCTAGTCCTATGAACATGGATTGTTGCTGCGTTAATAAGCCAGCGCACTTAATTAATTTGTCTTCCATGTCAGACTGTTCCTGTTTCATGGCAGCCTTCACTGATGCACCAATACTTTGGATGCAATTCCCAGCGGACTGAGCTAGGATGTAAAAGAAGATATTCTGAGTATGTTATATGTCACATGCTTAAAACTATGTTTTTTCTTGTTTTCTATCTTGGGACATATAATATAATTCATGTACTTACCAGCTAAAGTTTTCAAGTCTTCATTTGATCCTTCTTCTACATTATTGGACTCTATGTTGTTGGAAGACTCTGGTATAATAAGACCAAAGCTTTCAAGGCCTGGAAGAATCAGGTCATCGAAGGATGTATCACACATGAGACTCTCTTCCAATATCTGTTGGATAGATTCTTCCATCATTGGATCGGGACGGTAGCTGTAAGTATAAATATTTTTTAAGTACTTAGAAAAATTCCCAAATAGTCTTAAATAAAATAGATCTAATAAGGACTAATCATACTTACTCTTGCATACGTTCATCATTGGATCCCATGTTGTTTCCAGAGGGTTTTGCTGGCTCCATTGTATATTAAGATGCAAATTGAGACACCGCCTACTGTGCTGATAGCCAATGAAAAAATATCTCCCGTCAGCACATATAATACTATATAAGGACATTACCACCACCTCTTTCTTTGCAGAGATTCTCTGCTTGCACATCTGTAACACTGACCATAATGGGGAAAATGTCATCTACTGGAAATGGAAAAACTAATCTAAAGATACTGGCATGTATGCTATTGATATTTCTAATAGCAACAATATCTTTGTTAATACTGGAAATTATATCGGGCCAAAGATATTCCAATGATGATTCCGAAGGTGTGACTGCGGCGCTGAAGCATGTAAGCACACCTACTACCAACTGCACTGAAACCACTACTCCTGGTTAGTGAAGGATCTGTCTTTTTTATTTTAATGTATTGATTATATATTTGCATACATTTCTTAAGAATGAAAAAAAAAATATCACGGATGGTTTGTTTTTAGATTTCGTAACGAGCCAAGCCACGGGAAACAAAGAATCAATGAAAAAAAATGAAGGGGAACCTCCAGTTTGGATTCAGGCTTTAACTACAACATTATCAATTATCCTATTGGTTTGCATAATAATGGCATGCATTATATGTTCCCGGACCACGGAAGAAGAGAAGAGCGGAATGCAATCTTCTGCTTCTTCAGTAGAGACTTTACAGTCTTTAAATGAGGCTATTTTACCGAAAGGTGAAATGAATGTGTAATGTCTGTATTTTTCTTTGCAGAGATGTATGGAGAGCTTATATTGGGGGAAAATACCTGACTGTTCTGCCTATATTCGAATGTTGAAGTATGTACAATATAATTTCTTACCTTTTAAGAGTAATTCAAGGTGGAGGTTTCTTTGGAGATGATTCCAGGTGGTGGTTTTGGGCGCAATCAATCTTCTGGGTGGGAAGAAAATCCAGCAATGTAATAATTGATGGGATGCAATCGATGTGACAAATCTGTAAGATTAAATGTGAACATTATAAATTATTTTGTGATTAACAAATTACAATCATGTGCATGTGAAAATATCATTAAATTGTTTTACAATTTTTAAATGCAATTTAAAAGAATTTTTGTAGCAATCTTAAATATTAATCGTTACAAAATTGACTTACCAGACTGCAACGATATAAGAAAGAATGTCTGTGATGAGAATGTTCTCCGGAGCCACTTCTTGAGAATAGTGGCACTGAACTGGCTGTAACTTCTGCTTATATACAATTCTGTAGCGGGTGTGATGTACAGGAAAATGACGAAGATGAAGGAACCAATCGCTGTATAACGTTAAACCGCAAGTGCAGAAATACACCCCTTTTAGACAAATATGGAATTGAAATTAGTTAATGCTTGCAAAAATTTAGGGTTTTGTTATATTTTACAAAAACAGGAATTTGTGGTCTGGATTTTTTGAGGTTTTCTATAATAAACAACATTTTACTGATAAATTGTCATGTTTTTTCTAAGAACATCTGTTACACATCATAAGACATGTGGTTTTGGTGTTAGCGTTTCTCAATAAATAAATGACTCAGTCAGGGGAAAATGTTGCAATCGCGGTCCCCTCTAACCTTTAATTTTATGAGAAAAACAGGAACTGCGGTTGCCCCGTTAGCAAATTTTAGGGTTCTTGTTAGCTTGGGCAGGAAAGACCCCAACCGCGATACCCACTGACCTTTAATTTTAAGAGAAAAACAGGAACTGCGGTTGCCGCGTTAGCAGATTTTAGAGTTCTCGTTTGCCTGGGCAGGAAATATTTTCCGAGAAAAACAGGAACTGCGGTTGCCGCGTTAGCAGATGTTAGAGTTCTCGTTCGCCTGGGCAGGAAACACCTCAACCGCGGTACCCACTGACCTTTAATTTTCCGAGAAAAACAGGAACTGCGGTTGCCGCGTTAGCAGCTTTTAGAGTTCTCGTTCGCCTGGGCAGGAAACACCTCAACCGCGGTACCCACTGACCTTTAATTTTCCGAGAAAAACAGAAACTGCGGTTGCCCCGTTAGCAGATTTTAGAGTTCTCGTTCGCCTGTGCAGGAAAGACCCCAAACCGCGGTACCCACTGACTTTTAATTTTCCGAGAAAAACAGAAACTGCGGTTGCCCCGTTAGCAGATTTTAGAGTTCTCGTTCGCCTGGGCAGGAAAGACCCCAACCGCGGTACCCACTGATCTTTATCTTTATGAAGAAAAACAGGAACTGCGGTTGCCCCGTTAGCAGATTTTAGAGTTCTCGTTCGCCTGTGCAGGAAAGACCCCAACCGCGGTACCCACTGATCTTTATCTTTATGAAGAAAAACAGGAACTGCGGTTGCCCCGTTAGCAGATTTTAGAGTTCTCATTCGCCTGGGCAGGAAAGACCCCAACCGCGGTACCCACTGACCTTTAATTTTCCGAGAAAAACAGAAATTGCGGTTGCCGCGTTAGCAGCTTTTAGAGTTCTCATTCGCCTGTGCAGGAAAGACCCCAACCGCGGTACCCACTGACCTTTAATTTTCAGAGAAAAACAGAAATTGCGGTTGCCGCGTTAGCAGCTTTTAGAGTTCTCATTCGCCTGTGCAGGAAAGACCCCAACCGCGGTACCCACTGATCTTTATCTTTATGAAGAAAAACAGGAACTGCGGTTGCCCCGTTAGCAATTTTTAAGGTTCTAACCCGCTTGGGCAGGAAAGACCCTAACCACAACACCCACTGACCTTTAACTTTATGGAAAAACAAATTATTTTGCTCACCATGCAGTTTTTTATATATCGTTACATCTCTACCCAATCAGCACCCTTGATGATGCATACATTAAGGGAATGTTGATTTTTTTTTCATTGTACCCACTTACGAATAACGAATCAGAAGCCAGAAAGTAGAATATTTTAATGATGTATTAATCATCATTTCCTACCACACCTATTAACTTCAGTATTTATAGGAGAGGCATTTTTCCGTTATGCGCCATTAGCTGTTCTTCTGCTAACTCGGACGCAGCGGTACAGGGTGCGTTATCAGTGTCTTATAATGTGATGTCTTTTTTTTATTTTCTTGTCGGTATTTTAAAAGTAGTCTTTTAAGATGTTGTTAAATATATAGTTATAAAATTTTCGGAACCGTGTAGTCAATAAAACAATTTATAGAACGGTTCCTGGCCCAAAACAGAAATTGATTTATGTCATCTTTCTGAATTACAGGAAAATTGCGAGGGATGACTTTATCAAATGTAAAATTATACACCCTCTGTGATAGATTTATTTTGTGGATATTGGAATGTACCATCGAGCGTATATCAACGCAAGATGTGGCTATGATGGGACACGGTCCTATCTGTACATGGTCGTTTTCATTAATTGGGAATATTATTTTTTGACCTCCAAGGAGGGATTTTATAATATTAAAATTTTCAAAGTTGATATTTATGTCTTTCCACCACAAGACTAAAACCTGTCCGTAAGCTCTTAAATTTTTTGTATTCAAAGATCCATGTAATATTCCATGTAACGGTATATTTTCAAGAATGGCTCCCGTTAACATCGATTTTCCACATCCGGGTGGGCCGATAAAACTAACGGTGTTTTTTTTTCCTCCTCTGTGGCTTAGCCACTGATATAATATGGTTCCTGCTAATATAGGGTCAAAAGACTGCAGACAGAACAGCTGGTAAACCGGATTTTCATAAATCTGAAAGTCAGTGTCTATGTGCGCTATAAAATCGATAGCTTTCCTAGTTGCTCTTATTCTTTTTTTAGCTATAGATAGAGCCCTATGGATTAGCACGTCCCCGTGTGTTAGCGTCAACAATTGCATGTATGATCTAATGTCTCTTCGTATCCACGCGTCTTCCGTGACTATTCCAAGATCAGACAGCCATTCGATGGTTCCCAGAAATTTAGATTTTAAAGATATCCCGTTAATCCCATCCTTTGGAAACAGCATAAGTTTGACATTAGGTTTTTTATTAAAATGGGATATGATGTGTTTTCGATGTATTTCATCGCATACGTCTCCCAGTTCGTTAATATTTGTCCACGCCCATTGGAACTGTGGTCTAATAATTTGAAGAAGGGCAAAGGGGATGTAACTTAGACTGAAGACCTGGGTTCCGCCACCATTCTTCTTTGTTTTGGCATACGTGCACCAATCATCTATTTTAGGGGCGTTCCCGTCGAAGAAATCTTTAATTAATACGCCCCTGATTTCCGTTGTGTGTTTTCCTATCGTTGCGCTGGGAATTCCATCTAAAGCTATCAATAATATTAGGGAGGGTCTGCCAGCTTTATTCTCCGCTTGAGCGTACCACTTTGCAGGGGTGGGGACGGCAGTTTTATTATTCCAGTAATTGAGTATGACATCGATCATTTTGGTACATGCAGTTATCCATTTAATATCGGGCGCTCCCGGTGAGTGCATATCCCTGAAATGTGGCACGGACACGTTAGAGAATTTACAGAAAAAATCCGTAGATATTCCAGGGATTTCTGCCTCCCACTCCATGGGGCCTTTGATAGTCAACATGATATATTTGTCCTTAGTCCAGAAATCATCACTCGGATTTATTATGGAAAACATACCGTTCGCGCCTCCCAAGTTTTTTATAGTTGTATACAGAAAATAAGGTAATTCCCCTGGCTTGTCACCTCTAGATGTCTGCAAGTCCGGACGATTTCTTTTGGCAACAAATTCTCAATATGGATCCGACGGAACTTTTGTCGGACGATGCAATCTCGTCCACGTGTGACGAAAACACTGCAGCTGGAACTGATATTATGCAGCCTTTGTGCGTTGATATGTCTGTCCAACCGACGACATCGGGGTGTTATACCGGAACGATGATGTCAGACTCGTATTCCTCCAATGACACACCGGATCAAAACTGCCAATCACATCCGACTTTGACGGACCTTTCGCCGGAAGACATGAACAATCAAATCAATGTTCAACCCAATCAGATGACCTTTCAACCCATTTCCCCACCGATACAGGATCGAAATTATGGCTATAGCAATAATATGATTAATCCCATCAAACCTGCATCAATTATCAAATTACATGGTCATTCGATAGGAGAAATGACATCTTCAGACCCTTCCTCTCATGTTAATGCTCAACACTTTGTCCAACAGTCTAATCCTAAATTCTTTCTAAATATGCATCCGTGCATGACCGCTAACTCACAAGGACCCGGGGAATGTCAGTCTGTCCATAATCAATCTTCTGGAAGCGGGTCTAATTCTTATGACATTACTACGGCACCCCGCGATGAATGGATATTGACTACATCCGGCGGAGAATCCTGGATTCTGAAACGGAACACCCCTAATCCTCCAAATAGCAGAACTAATTTAATTTTCAACAACGCACAACCGATTTTCCATATCCAACCTCAAGTATCTGGGTTTAGCGACTGTTCCTATCAGAGGGGTGGTCTCAATTCACGTGAGTATGTAAATTTGGCAGGTTACACTCCCGTGAGCAACATGCAGAATATGAAAAACTCATGGGCGACACAACTAGGAGCATCCATTGACTGCATGACTGCGTCACCAACTGGTATAGATGCGAATGTCAACTCTGCTTTCCAGCCGGTGGGGGTGGTATCAAATAATCGGGGTGCTAATGTTCCAATGGGAGGCATGGGTAATTATATAGAGAATAACAGTCCATGGAACCAATACTATAAATCGGAAATGGCTAATAATTCCATAAATGTCAACGAGTCGAATGGTTATGGCCATGTGGGGTGTGTTAATGACGCCATTTCCGATAAACAAGGTGGCACCGCTAACGTCCCATCTTCTCTATTGAATCCGGATCACCAAGATTGGATGAGGATGACTGGTACGAGCACACATATGTTAAACAATATAAATGCTATAACAGAAATGAAAAACTATGATTTCCCGGAAAATGATGGCAATGTCCACGGAGCCACCGGTGTATATTTATCATCAACCCTGTCCAATGGTCAGCAGCATAGCGCATTTGAAGGAAATGGAACATTTCCCGTGGTAAATACCGGCAATCCAAGAACGACGTTGAACTGTGCTCCTCAGTTGTTGTGGCTTGGAGGAAACCCGACAATTGCACATCCAATGAATGGGAATAATGAAACCCCAGATAAAAATGTATGCAAGCCTACCCCCAGCACAGGTGTAATAAAAAAAATAAACTTTGACTGTGTTGACCGAGGGGAAGGAATAGGAAATTTTCCCAAACTGGCGTCTTTTGTAGCTATAAGAGAAAAAATGTCAAACACGGTCAATCCTTCTTGTGGAACTTTTCTAGCACAGTTGGAGGGATTACGCCAGCAGAATTCATTTGAAAAAAATATATCCCTTACTGATGTACAGTTTTCTGAAGAAGATGATGTGCTTTCCAGTGCATCCTCGGTCAGCTGTAATAGCAACTGCGACATGAAAATCGGAGTTTCGCAGCAAGGTACCACTGTGTCTAATTTACAACAAGGGTTTAGGCAACAAATCAACATGAACTCATCCGTGGTTAAAATGGACGGGAGCTATAAAACACAAGAGATGTCAAATGACTGCGCGACAGACATCACGGATAATGCAGGCATCATTCAAGAAAATAAACGTATACACTCTCAAATTGAGAATCCAGAGGATGGCCGGGTACGGGAGGGGGGGTGTTCAGACGACAAAAATCAACCTCCGCGGAAAAGTGCGCGTATTCATAACATGAAGTCCGAAGGCGTGACTTGCGGTATGTGCATGACGGCTGCGGATTCCACACGGCAGGATGCGTCTGGAGGTTCAAGCAGCGGAACCAAGAATGAATATTACGATGATGAATCGGAATTAACCGGACTGTCGGATACAGACAGCGACAACGAAGTCCAATGCTGTCAAAAAGTAACAAAGGTCGGTCCCAAGACGTACTCCTCAGAAAATTTTAATCCTGATTATCGCCAAGCCAAGAGATTGCTTGCCGACATTCCATACAGAGGATGGATTCCAGACCCAGTCAATATGGAAGAAAACGACGGTCCGTTCATTCCAATAGTGACTAGACCACCCACTGTGTGTATGAACGGTAGACGCCGGAGGACTTTCTTCCGGCAATGTGTTACCGCTTTCGGTCCTCTGAGTAAGTTAATTTATTTCAAAGAATTACTGCAGAGCTACGTGTCCAAAAATAACAATAGTTATTTAAGTATCAGTTGGCCTCCCAAACACGGGGTGTATGTCATGTCAGAGAAGAAATTAGGGTATGAACATATACCTACCTTGAAGGACAAGTTTCCACTGCCATGTGGGTGGATGATGGTCCTTGGAGTTGTCGGTGCAGAACCGCCCGCGGCTCTGAATAAAAACATGGTGATTCTCCTGTGTGAAAATAGATGGGTGCTTCTGCATAATTACTCAAACTCTACACACGAGCTGTTTTTAGCCGCCTCGGATTTGAAACAGTTTATGGAGGAGGGTCTGTCTAGATGTGATCCTATATATGAAGAACCAACAGTGCCGTACGGTGTGGCCATGGAAAACTCTCTTCGAGATTTCTTGAGAAACAGTAGAACGTTTCAGGATCTTATGGATCAAAGGGACAACATGCACGGATGTAACTGGACCTTTAACGGAATGCCTGGTAGACTAGGTGACAGAGTCATACATCTCTGCAATCCTGAATCGGTTGATTCCATACCAGGAGACGAGGCCGTCGTATGTGAAGGAAGACCCTTGTATTTCTTTGCATATGTTACCACTTTCAAATCAAACCCCGCTACTAAGGCTACCGTGTTAATAGCCGCGGATAAAGATTTAAAAATATATGGCTATCATAAAGGTCGCCCCAGGATACGGTACTTGTGTAAGAACGTAAAAACTTTCTTTAAAGCCGGGGCTAGAAAGTTCTATTTAGATTTTCAGATTACACCTAAAAGCTTGCTTGCCGTCAACGAAGAGGAGTATCTGAATACTCTACAAAACGCTCCTTGTCTGCTCTTAAAACCATCTGTGTTTAGAAAGACTTACTCTCAGGAAGGTAAATAAGAATTCCTATGTCAATAGTTTTACTTCACAGTCCCCACCCTCTTTTTTTAATTAAAACGGAAAGGCCGTTGGATCCAATCTGCCGAGAGATTTACTGTTTCTTTGACATTTATCAGGGATACGTATATTGTGTACCTTGTAATTTCTATGTAAACTTCTGCTATTTTTTTTAATGTTGAGTAATTGAACATTGTAAATAGGTAAACTGTTTTTTCAAAATGGCTAGAAATTCTATGTACTTATCTTTTTTTCTGTGTATTGTAAAAGGGAAGATTTGTAAGGACAAAGTTCAGTAACATTTAATAAAGAACATGTGACACGATTTCTGACTGTCTCATTAATTTTTGTATTTTAATAAGAGCCAATACCAAGGTGAGGGAAACGTAGGTTCCGGCCAAGGTGAGGAAAACGGAGGTTCCTGCCAAGGTGAGGGCAACGTAGCTTCCGAGGGATAAAAAAGTAATTCGTTACTATGAGTAGTCGGATAAAACATTTGTAGTTTTTTGTCTAACACATAATAATTATCTTGATAACATTCATGTCGGAATAAATCCGATATAAACGTTTCCATCTGTAGATATCTTGGCCCGTTTTCCATCTGTAGATATCTTGGCCCGTTTTTGCGTGCCAACATAGCACTTTTGGTTAAATTGTTTATCACATCTCTTCCGTAGTCACTTAAAAAAAAACAAAAAAGACATGAAATGTTATAGTCGGCCTACCTGCAATTTATATAAGTACAAATAAACACAGTAAAAATAAAATGATTCACATATGGATTAATACCTAATGACTAGATTGAAATCCATCCACGTTACGGACAAGGGTTTTTGAACTTGGTCTTGCATTGTATATACAATTGTTCTATTGTCCGTGAGAGGTCGACAAAACTTCTGGCCTTCATTTTGAAACAGACTGAACAAATATCAAGTTATAAATTAACCGGCGATGTCCTGTGTTATTACGCAGACTATACACGTATACATCCACATGCACACACGATAAAATGGTATAGTAATCATTCAGGTTTACCCGAGGTGACGTTTTCCCCGAGTAAACCGAACGATACTTACATTTCACCGTCTAGGCAGGTCGAAATCAAGGAAGAGACAGAAGTCGATGAGTTAAGAACGACATAGCTATCACAGCACGAAGTAATCTTCGGTAACGTGCTAAAAAGCGGTAGCGCAGCCATTCCACGATATGCATGGAAATGTATTAAAATATACACAACCAAGAAATGCATAATTATCTAGCAGCAATATAGTTTGTTATTCAAACAATGATTCTTTTATCTTCATTTAAATATAATATGAATATTTACAGCTATATATACAATTGAATATACACATGGTCGATAACGCACTATTATATATATTCCAAAAAACAACAAAAATTTATCGGGGGTTATATTTATCTACATCGATAAAGTGTACAAATGTATATGTAAACACAACAAGAAAATATACATGTCCCAAGGCGTGTACATTCTAACACGCGCACATAACGGTCAGGTCCTCACGACAGTAGGTTATATAGTTCTCATTTTGATGTTTTCAGGTTTTACGTCTTTCGGATTGTAAAATACGTGCCCACCCTGACATAAAGCTTGTCCGAGGTGTTTATATACATATTCTTTTTTTTCGAACATATTTTGCGTAGTATATCTCCCTTGGACTGTGAAGATGTTGTTAACCATCAGTAGTCCCGCATTTCTAAAACGTTGTTTTGAAGTTCCGGTCTGTACAAATATGGTGCTGCAAATTAACAAAAACTGCATAAATATAAAAAAAATTCCGGAAAAGTCATTTATTTAATTACCATCTTTTATAAACAGCACTCACCATAATGCGGGAGCCATGTGTGTCGGACTGGCTATATCGTATTTTATATTAGTCGTTGTCAGGTTTTCTGGCAAAGAGTGCCACAATGCGCTTACGATGGCAGGATCTATGTCGCTGTGCATAACCGGAAAATCGGAAAAAAAATTAACATAAAAGATTACTCCAAAAAATTTGTTCCTACCAATCCCCTGTTATTTAAACTCACCGTATAGTTGTTCCTTGCTGTCCGACCATTGACAATATTTGTGAGGCAAGTCGCATTGCGATGGTTTCCATCAGTTCCTCAGTTTCAAATAGTGCATTTCTAAACCCTCTGTTAGAAACATAAGTTAAGGAATCTCAAACGATACATCGCACACAATATAAATTCAAACTTGCGTTAAAAAAAAAGAAAATAAGCTTACTTACAGTACGGAAGATAAATAATTCTTCGTCTCGTTTTTATTTAAACGTGAACACCATTCGAAACCTTGCAGTGTTTCAAACCTGACAAAAAACGAAATTCACAATTACAATTTTAGAATTGTGTTCCACCTCCATGTGGCAAAGCTAATAAAAAAACATAAATGTAGTTTTTTAGTAAAAATCACTAAAATTACTTACACGACGTAGCTATCGTTTGGACAATGAGTATCGTTTATAGGCACGTATACGTCAACGATATCTTGCCACGGTTCGAAATATTGAGTCTGAGTCCACGAGTTAGAAGATTCTAACGTACGACGACCATGCTGCACGTCTCGGTGACGACAACACGTGTCATTAGCGACACGTTTATACCTCAATATGTCCTCTATATTAACGGGCGCAAAGCGCGTGCAGGTTTCCCAATCCTGCCCGTCGTTGTCTTTTCGAACGATCGCAAACGAGGTTAACGTCTTCATAGTCTGTCCGCCATTGTTTCGTATTTTCAAAATCTCGATACTGACGATCAGTTCACTTGAGTGAATAAATACATGTGTGCCCCACGGAGAGGCAAGACACGGGAAACGAGAAAAAATTTTATGGTTACAAAGGCTTCCAGCCCCATAGTTTCTACTCTGAAAGGATGTAGGTATGCGTGCGGGGTTGATACGTATGCGCGTACAGGGGTGTGGTCTGTCTTCTAAGGGGGGGGGTGGAGTGGAGAGTGTAAGACAAGTACTGCAACGTTTCTAGAGTAACAGGTGCATCCGAAGACGCGGCAGGGTGTATAGATGGGACTCTTTTTAGTAAACTCGGGGTGTTTATGTAACTGGGATCTTACACTTTTTTAAGTCCATAAATACAAAATGCTTGTTTTTCCTTGTCAATAAAGGAGACGTGATAGAAAGGCACACAAACTTGTACAGGTACTCACCATAGAGTTTCTTTCACTTTGTAGTTTTGGCGTAGCTCTAATTTAGTAGTCATTGGACAGAAAGCTATCGCCGTCATCGCGGTCAGTGGTTTCAGGCGCCTCATATAGGCCAAACTGTTATAGTTGCTCCCAAAAGCGCCATAAAGCAGAGACAGGTGAAGGCCGAGGCTGCTAGAGAGAATTTGCGATAGACCCTTAGGCATTATTCTATCTTTAAAATTTCTCACTCCTTTGTTGTCGGCCGAGAGAGGAACGCAGATTATGATATCTCCGAACATGATGCTGCGATAAATTAGATATGATTATTATCTATTCATTTATTTATGATCTCAGTAGATTCCGACATCGGATAAACAAGATGAAATGAAACGCTGCATCCTTACCGTGCTTGTGTCTCTATGGTACAACCGCAGCTATTGTCATCGCAAACGGCGTAGTTCAGGATGTCTGCTATATCTTCTAAGGTAGTAGTCGGGTTTTCATCTACGGGGACGAAAGCTTTAACTCTTTTTAAACTTAACATTTCGGAGGTAGAAAATTTAGAGATGATTTCCGATAGCGTCCGTCTCCTCGGCGTAGAATTTCTACCTTCGTAAATGTTAAAATAAATGTCTTTGTCATAGTATTTTGCGGTATAGTTATGTTTGTCCTCTGGCGGCGGTGTGGATTTCACGGTTCCCGCACGTCGGTGGGCCGTAGCGTTCGTCGGAGCCGTCGCCATCGAAAACGCACACAAAAATATCAGCGCACAAGATCTCGGTGGTTTCATAACACCCAACCTTGCGGTTGCCATTCTCCGTCTCAGTCCTGTGAAAATTTTGTATGCGTGGGAGAAACAGGACATGGAGTAAAGGAAGATGATGTTAAATAGACTATACACGACAACAAACGCGAAACACATCCTATGACGACGGGAGACAATAATAAAACGGTTCGCGTCGGGAAACATTCGCGTGAATCTAAAACCATGAGAAATTAATGCAACTAGAGAGTTCACAAAAATCATATATGGCTGCCGACAACGTCGGGCGCGTTCATAGTCGTTTACCGTGTGCGACTGACACACGTAGTCTTCAAACTCTGCCTTCTAAAGTCACACCTCGATGGAAAGATGCTGCGGCGTACCGTTGTCAAACAGCGAACTCAAAGATGAGTCGGGGCCGATCATGTTCATCTGCGGCAATATAAAGCGGGGAGAGGAGCGGTATAAATCCATAGCGAGGAAAAATACATCCAATCGCCATTTGGCGATCAGTACTCTGTCGCTCATTTTTTTCCGTAACCGGCGTGTGTGCAGACTGTCGTTTTGGTGGTCACATATTAAGTCTGCGTCATTTTTTCCTCTCTTTCTATCTCTTCCTCTCTATTCTTTTATCTGTCTCTGTCTGTTTATGACCGCGCCCGTTGCACCACTTATCACACAACGCGCGCCGGTGCGCGCTTTTACCCGTTTCTCCGGCCTCTTTTACTCACCTCTGAGGCACTCAACGGCACTCCGGCCGGGTCACTTTTTCTCATAAAAGCGGTCTACCTCCACTCCGCTAAGCCGGACCGTACCCGCACCTGAACAGTTATTCCCATATAGCCGCCGTTTTTCTGCAGCGGCGAGTCGGAGTGCGCTCGGGACGGGATCCTTTTTTCGTTTCGTTTTCCTAAACCACCGTCGCCCCGCGGCGCGAAGTCAGACCGCGCCAGGGCCGGCACACTTTTTTCTCCAACACCGTTTCTCCGGGAGACAGAATACCTCGCGTTTTAAAAATTACGTTAAATCCCCCGGGGGGGCTAAAAAAAGGGGGGGTAATAACCCTAACCCTAACCCTAGGTCTAGCCCTAACCCTAACCCTAACCCTAGGTCTAGCCCTAACCCTAACCCTAACCCTAGGTCTAGCCCTAACCCTAACCCTAGGTCTAGCCCTAACTAAAACATTAACCCTAA

CGCATAACTCTGTTCACAACCCTAACACTAATCCTCGCATCCGCACCTAACGCTGCCCCTCTTTCACACCATCACCCCCTGCCGCTTCAACTTCACCTTCTTCCTCCATCTCGCCCCGCTTGTTTCTACACTTGTCCACCCCTCTATTCTTACTCTAGGATGCCGCTAACGGTGCCATATCGTCTTCCGCTCTCCAACTATTGGTGGCTACTCTTGGGTCGACATTCCCTTCGTCATGTTCATTCCTACGTGCGTCTGCACAAGGGTCTACGCATTCCTTTACCTTGGCCCGAGCAGGAATGCCTACATTTACATCCTAAGCCTTACAAGTGTCTCCTGCGTTACCCCTGTATAACAACACAACCGCATCTTCTTCAGGGCTGGCCTACGAAGTCTTCTCTATGTGAGTGTCATATACATTTCCACTTTTTCACATGATTTATTGCGTTTTGTCTACTACACCGGGTACATTTTGGTTTCCCTCAGGGTTCGACCCTAAACCCTACCATCCTTCGGCCGACAGCAAGTTGCTACCGCTGGGCCTGATCACGCTGTCCGCCTGTTCCACGCGCGTGTCGCAGCCGACACACCGGAGCGGCTTCGACGTGACAGATCTGTCTCTCAGCTGGCTGACGGGATCGTCCCCTTGGCTCGTAATCCTACAAGGGCAGGGGGGGTCTCTGTTCTGCCACGACGTGCTGCAAGGCCGACTCTATGTCCTGTCGCACTCCGTGTCGCTCTTTCTAAAGACGGGCCTCCGCCACTGCGAGGCCATCTATCGCGCACCGCTGTGGCGCGACCGACCCCTGCCGAGCCTGTGGACGTGCCGGGACCCCGACAAGGCCTTCTTACCGACATTACTGGCCAGAAGCGCCCGACGCGGCCTGGCCGCGTTCTACGCCCTGTGGAGACTGCATCTGGGATCGCGCTCGGAGCTCTCCCACCCCGTGTTGGAGTGGGAGAGAACAGAGCTAGTCCTGACGGACTGGAGACGCGGGCGGCCGTGTACGCACCTCCCGTCCGACTCGCGGTCCCAGCGCGCTACGTCGAGTGGCGCCACGAACACGCGGGACGCGGCGACAGAGGAGGCGGCGGGGGGGAAAAAGGAGGCAGAGGGAGGCGGGCCCAGCGTCGCGGAACACTTCGCGAGGTGCAGACCGCTGCTGGACGAACTGTGCGGAGAGGGCGGCTGGCTTCCCTTCGCGTTTCTCTCGACATCTGCGCACGTCTGGCTGATCCTAACGGAAGGAGGCCCCGTCCTGGCGGTCGACCTGAACGACACCTCCGTGTGGCGCATCGCGGACGACCTGGAGCTGCTGCGTCGCCTGGGGAACCTGCTCCTCGTCTCAGGCCTCCGGCTTCCTCTCCATCTCCCGAGCGGGAGCGGCGAGGCGGCCGGAGAGCCGGGGTACGACCAAGAAGAGAGAAGAGGGAGAGCGTCGACGGCGAGCGCGACGGCCGCGACGTCGACGCGCGGACCGACCCGTCCGACACGGGTGACGCGTAAGGGGCGTGTGGCAACCGGAGGCGTCCATCTCCCCGCACGCCCCGAATCTGAGGAACAGACAGACGGCCACCACGGGCGCCAGGAGAGCAGCCACGACGACCAGCGCGGCGGGAGCGAACGAGGACACCGCGATGACGGCGCGCACCGCCACGCGAATGAGAAAACAGAGCCCCAGCAGCGCGGAGAGCACGAGGAAGGGAAACAGACCAACTCCGGGCGCCACGAGCACGGACAGGAGAGCCACGTCGCAAGAAGAGACGAGGAGGGAACGGAGCAGGGCGGTAGCGGAAGGAGCTGCGGGGGAGCGACGCAGACGTACGGCGGCAGAGGCCGACATGATTCCTGCCCGTCGATCCCCCTGTCCGTCCCCGGGCCCGATCCCCGCCTGTGGGTCCCGCCCGCTCATCTGTTATTCCCTTCCCCTCTGCCACCGATGACGCCCGTCGACGACGAGCCGTCCGTCCGCCCTCGGCGCCCGCCGAGTCCCGCGGAGGAACCCCCCACGTGTCGTCCCCGTCCGCCCCGCCCGTCGTCCGACACCCCGCTGTCCGCTGTCTCGCGCCCCTCCGCTCCGCCCGTCCCCCCGCCGTCCACTGCGCGTGTCCGTTTCTTCCTCTCTTCCTCCTCCCCCTCCCCGTCCTACTCTCCCTCCCCGTTGTCTCCGCCCTCTCCCGTCTCTCCCTCGTCTCCCAGATCTCCGTTCATCCCCCCCATCAGATCTCCGGGACTCCGAGCGAAACCGCGAGTGTCCTCCGGGCATCCCGCGACGTTCCCGCCGGCGCCCTCGTCCGCGCCGGCCCGTTTCGAAACAGTCCCGTCCGTTCCCTCATCGGAATCTCCGTCCGCGCCGTGCGTCGGAAAGTCGCAGCCGCCCGCCGCACACACGGCGTGAGACGCCGCGCGAGGCGACGGAGGCGACGAAAGAGGACGGGAAGAACAGGCGGAAGAACACGAAGACGAAGAGGAAGAAGACACGAGAACGAAATGAGGGGAACACGCCGGGGACCGAGCGGCGGATGGAGCCCGCTCGGTCTCGCACTTCCGCGATACCCAGCGGGCTCCGTCGCCGCTTCGGACCCGAGGTCCGACACGCCCCCACCTCGCGCACCGCCTCCGCCACCACCGCTGACAACCTCCGCATATCGCCCCCATACACACCCAGCAGCAGAGTCCGGCGCACGCACACGCGCGGAGCACGCACGAGAACACGCCAGACATCGACCGCCGGAGGTAACGGAGCCCGTGCGCCGTCCCGGTTTCAGCGGGAGTGTGTGCGGGCGTACCGTCACACGGAAAAGCCAGCGGGACGAAACGATCGACCCACTCCCGCTATTCGCCCGATCCGCCCCGATAAAAACAAGTCATCTCACTCCTGCCAGTTCACACGGACGCGACCGATGACAATAAAAAAAAAACACACCCCCCACCAGCGAACGCCCGCCGGCCCATCCACCTCCCCTCCCACCCCCGCTCGAGTCGCGCAGGCGCGTCGACGGGCAGTCCCTCGGACGCCTCCACGGCAACGCTCCTCCCCTGTCCCCTCGGTCCAACCCCTCCCATCCCCCTCCCCGGAGCACACACGCCGACTCCGTCCGCAGAGGCGGCGACCGCAGCGGCAGCGGAAACTGTAACGGCCACGTTCTCCGAACAGCGACAGTAACGGCGGCGGCGGCGGTTGTCGCCGTCACGGTAGTCGTGGCGGAGCACCCGGGGAGGCGACGACGGGCACTACCGGCGCCGAGATCATCGCGCTACGAAAAGAGTAGCCAATGTAAAAAGTTAAAGATTCTTTGTATATCAAAAAAAAATACTACGTGGAAATAAAAAATAAAAAAAAATATACAATATAAAAGTGTATTATAAAATAAAAACAAAATAAAGACTAGACACCGACTAAACGCTCATCCTTCTCCGTACGTCCCCTTTCCGTAAGTGCCAAATGCCGCATATCCATGAACGGACACACGCCGGTAGAATCCGACCTTCCTCACAACAGGTAGCCCGCGACCTAAGCACAGGACGGTACCTGAAACCGAAAGTATAAAGGGCGGCCTCCCATGTTCCGCCCATGAGGCGGGACTTCGAGACGAGCCACCCTTCGTATACGTCCGGCCCCTCCCACGCGCGCCTATAGGACGCACCCCCTCGTTCCGAAACCGCTAAAGGGTACAGTCGCCGTCACACCCCCTGTCCCTAGCTCGCGACGGGCCGGGGCAAACCCTGCCGCGGCGGCAAAGCCGAAACGAGACCGCGACGTACGCGGGCATAAAGAACGAGAAGTCAACGCGCAATCCCAAATTATCGCGAGACTGTTTGCTCGGGCCCGCCCTCGTCCTCATACCCTCTCCCCGCCCTCATGCCCTTCCACACCCGGTAGACAAAAAAAAAACACGAAACATACATTAGGTAAAGGTAATTTTCGCGGTTAAAAATGTTTCCACGTGACGTCCCCGGCGATTCCCGGAGATGCGACACGCGACGCATTGGCCCGTTGACATCGGGGTCCGCATACGCGGTTCCCCCGGGGCGTCGCGACGTCGGTGCCGCAGAAAACACCGCGGTCCGCCGTCACCCTTCAGTCACGCGCTATTTCCGCGATCGTATAATTTATCTCCACCGACACGGGTTCTGTCTGTCACTTCTCACCGAGCCGCTCCACCACCAACAGTGTCACTGACTACATCGCATACGTGGTGTTTTGCCTGTTTTTTGTTTATCTCTCCACCCCTCCCCGTCGCAGACCAGCGAACACCTTGCCACATCGACACCGACTACACCCGACCTCGCATGACAACGGTCGCCGGTCGCAACGCAGCTGCAGCGGGCGGCGAATGAAAAAAAAATAACATAAATATACTAAAAGAGAGGCCCCCGTCCCACATTAGGACATAAGAGCAGGCAAACAAAAAACCCGTCCGTTATTCACGCAGCATCCGTAAAGCCATGTCGACGCGACACACAGAGAGGACGGGCGGACGCATCCCGGTCCGACGCGACAGGGCGCGACTGGCCCAGGCGCGAGCCCGTGCTCGCTTCGAGTGGCTTCTCCTGGCCCGCGGCAGGCCGTCCAAACTCTACGGCTATGCGAGTCGGCATCGCGGAGAACTGATCCACCTACCGTGGCCGCCGTACTGGTGTCTAGAACTCCACCACGATCCGTACAGAGACGCCCGAAGTACCACCGTGTGGGGCCACCGCTGGGGTTGGCCGCCGACGCACGTGAGACCCAGATGCGTTCAAGACTGCGGTGAGTGGACGTACGGGCGAGCCGCGGGGAGGGATGTCGGTCAGAGAGTGAGTGGGTACAAGGTTCGTCGACAACCACCAGAGACACTGGCCGGTGGACAGTGGGGACAAAAAAAAAGTCACGTCAGGGGCGGAGAAATATAGGGTACGCAGACCGTCAAACGGCGAGTAACGGAAAACGAGTATAAAAACAAGGAGGGGGGAAGAAACATAGTCAAGTCTGTGGCGCGCGAGATCGTCAGCCGGCAAAGTCGGTGACGGCCTCCGGCCCCAGAGACGGCTCGCGGAGTGGAGTCCAGTTATTGTTATGTTTTTGTAATTTTTTTTTATCCACAATCCGGTATCCGTGAATCCCCGCGGAGGTACTTTCCATTAACGATGGTACAGGCGGTATGACTTCCGGAACACGGTGATTACGGATATCCTGTCTCGTTTACGCGAGACCTCTTTGTTTTAATCTCCTATAACGGTAAAAAAAACACAAAAGAAAGATTAGAGCTCCTCCTTTTTTATGCGTTATGGCATCCTCGCATCAGTCACCATTCCTGTGGCGTGTGCTTTTCGCTTTGAATTGTACGCGACAGGATCCGAATCCGTTATACTCCGCAAGGGCATAGCGACCACGGCTGTATGCGGCATCTACCGTTGCACGGGATGCCTTTGCGTGTGCAGATGTGTTGTGCTTTTTTTATACGGTCAGAGACCACAGACAAAAATAAGGCAACGCCCGCCATAACGTTTATGGTTTCTTGTTGTTTTGTTTGGGTTATGTTCTACGCCTCTTCTATCGTGTCGGCCGGATCCATCATTTTCAATCGTTGACGTACGCGCGTCCCATCACAGCTCTAGACTCGTGTCTGTACGTATGTTGCGGATACGGAGAGAAACTTCAACCCGTGGGTTTCGTAAAGTCGTATGTAACCAACTCCCAGCTCGACACGCTCCGCGTGCTCCTGGTGGGCAAAGACGGAGCCGTGTACGTCCACCACATGAGGGCGGCAAGACTCTGCCGACTGGCGTCGAGCACAACGGAGTTTACAAGACGAGGGCTGCAACGCGACGCCGTGACGTATGAAGAAGACCTAGAGCTGCCGGACCAGCGTATGTGCGGAACGAACGCCCGACATCTGTTCGACGTGATCGCCGCGGCCGCCGACGAACACAACCTGCTGACCGTCGGCGGCCTGTGTCAGACGCACGCCGGAGTGTGCTGCAACTTACTAGAGACCGTGGGAGATCCGTGGACGGCGGTTCCGGGCGCGCGCATGACTCTGACCGTGCCGCAGGTCCAGTACCGATTATGGCCCGAAGCCCGGAGAGACCTCCGCCGGCACCTGTACGCGGGACATCCCCTGGGACCGTGGCTCGTGTGCGGCGTTCTCTCGAGAGAGAGAGAGACGCAGAGGCCGTCGCCTCCCATATGCAGCGGCGGCGCGACTGTAGGAAACGTGGCCACGCCGGGGCCACGCGAGGTGGAGATCGTTTGGGTGGTCTTGACTTTGGCGGGACCGCTGTTAGCGTTCTGGCCCGATACCGGCAAGATCTGTCGTCTGGCCAACTCGTTCTCCACTCTGTGGAGGATGGGACCGCGGGCCATGAGAGGACACTGGACGTATTCGGCTCCGGGTAGACATCTCCCCGGGGATGCGTGGCCACTGTGCGAACACGTGAGACCGCAGGTGGGAAAGCTTCCCAGGAAGAGAGCGTACCTGGATTAGACGTCACAGAGGTGACGTAAGCATGTCACCTACGGCCAGAGACGTGACCGGAGGAAAATCA

ACTCGTTTTCTTCCCCCCTTCCCTTTAACCCCACGAAAAATAAAATCCACGAACGTAGACAGTCACACACAGGGTGCTTACGCTCCGTGTTACTGATGGCAGGTAACCAAAAAAAGTATGTGTCGGGCGTGATGCACCGCCGCTCTATAGCATAGTCGCTATATCAGCGCCCGATGCCAACGCGACAGGGTGAGTCACATAGATCGGGACTGCTTCAAAGCGCGTTGCGTTCGCCTCTTATATAGGCACCCCGGGGGGTGGGAGGAGTGAACATCCAAAGGAGGTGCGTCCGGGATTGGATGCGAACGTACAAAACCAACATCGTCAGAAAAAAATAAGAAAAGGCGGAGACACATAGCCTTGGCGGGAAGACGACAACAGGTTTAAAAATAGACAGTGAAAAAAATAGCAGACTGTGACGTCCAATACACATGTGGCGATCATCCAATCAACGGGGTGTATCGAGGCGCAGAAACAAAAGTATGCGGAAGTACACACGACACGGTAACGCGGACCGAAGACAGAGGGCGATCGCTAGCATGGCAAGTATAAGAAAAAAAAAAACCACGTTCCAAAAACACCTATACGGGAAACATCTCTTCACTCCCTCCCCGTCTCAAGCACCGCAACATTCACCTCCGTAATCCGTTGTCTCCGCGTTTCTACCACAGACGCGTACACACGCAGACACACAGACACGACAACCCACCCATGTGGTAGTCGCGGGTGGGTACGTAGATGGGGCATACGGGGGTCAGACTTGGAGACAATTCAGAACGACATCACGCGCGAGGTGCCACCGTGTTGGACCCACGATCGCCTGGCACGGTGCCAAAGGAAACCGCCGGCTGACCCATCCCCCAACGCGCGCGCGCACGCCGCTATGGGAGGCGCCGTGTTTTTCACCAACACGCGCGCCGCTGCGAGAGACGCGTG

>HHV6B_STRIPPED4

CGCATCTGGCCCTACCGCTACCCCTCTTTCAACCCCTCACCATCACCCAACCCGCTGCCCCACCCACATCCCCCCCACCGCCACCGCTACCGTCACCTCTGAACTTCACCTTTTTCCTCCATCTCGCCCCACTTCTCTCTACATTTCTCCGCCCCTCTATTCTTACTCCTGTTTTCTAGGATGCCGCTGACGGCGCGTGCCGGCCACACCCTGCATCGTCTTCCGCTCTCCCACTATTGGTGGCTTCTGTTGGGTCGACACTCCCTTCGTCATGTCCATTCCTACCTTCGCCTGCGCAAAGGTCTACGCCTTCCTTTACCTTGGCCCGAACAAGAATGTCTGCATTTACATCCTAAGCCTTACAAGTTTCTCCTGCGTTACCCTTGTCTAACAAGACAACCGCATCTTCTTCAGGGCTGGCCCGCGGATTCTTCTCTATGTGAGTGACATTTACACTTCCACTTGTTCACATGATTTATTGTGTTTTGTCTGGTACACCAAGCACATTTCGGTTTCTCTTTTATACATTTATCTCTATTCTCTCGGTCTCAGGGTTCGACCCTAAACCCTACCATCCTTCGGCCGACAGCAAGTTGCTACCTCTGGGCCTCATCACGCTGTCCGCCTTTTCCATGCGCGTTTCGGAGCCGACACACTGCAGCGGCTTCCACGCGGCAGATCCGTCTCTCAGTTGGTTAACGGGATCGTCCCCTTGGCTCGTGCTCCTACAAGCGCCAGGAGGGTCTCTGTTCTGCCACGACGTGTTCCAAGGCCGGCTCTATCTCCTGTCGCACTCCGTGTCGCTCTTTCTAAAGACGGGCCTTCGCCAGTGTGAGGCCATCTATCGCGCACCGCTGTGGCGCGTACGGCCCCTGCCGAGCCTATGGACGTGTCGAGATCCCGACACGGCTTTCTTGCCGAAATTGCTGGCGAGAACCGCCCGACGCGGCCTGGCCGCTTTCTATGCCCTGTGGAGACTGCATCTGGGATCCCGCTCGGAGCTCTCTCACCCCGTGTTGGAGTGGGAGAGAACAGAGCTGGTCCTGACGGATCGGAGACGCGGGTGGCCGTGTACGCACCTCCTGTCCGGCTCGGAGTCCCAGCGCGTTTCCTCGAGTGACGCCGGAGACACATGGAACGCAGCGACAGAGAAGGCGGCGGGGGGAAAAGAGGAGGCGGAGAGAGGCGGGCGACAGCAAGCCACTGACAGACTCGCAAGTCCGCACCTGACGCGGGGCCGCCGCGACTGCGGTCGGTCCCTTCAGGGTGAGGAGCCCAGCGCCGCGGAAGACTTTGCGAGGTGCAGACCGCTGCTGGACGAACTGTGCGGGGAGGGCGGCTGGCTTCCCTTTGCGTTTCTCACGGCATCTCCGCACGTCTGTCTGATCCTAACGGAGGGAGGCCCCGTCCTGGCGCTTGACCTGAACGACACCTCCCTGTGGCGCATCGCGGACGACTTGGAGCTGCTGCTGCGCCTGGGGAGCCTGCTCCTGCTCTCAGGGCTCCGGCTTCCTCTCCGTCCCCCGAGCGGGAGCGGCGAGGCGGCGAGAAAGCCGGGGTACGAGAAGGAAGAGGGAAGAGGGAGAGCGACGACGGCGAGCGCGACGGCCGCGACGTCGCCGCGCAGACCGACCCGTCCGAGGGGGGTGACGGAGAAGGGACGTGTGACAACCGGGGACGTCCCTTTCTCCGCACATCCCGAATCTGAGGAACAGACAGACGGCCACCACGGGCGCCAAGAAAGCGGCCACGGCGACCAGCGCGGCGGGGACGGACGAGGACACCGCGATGACGGCGCGCGCCGCCACGCGAATGACGAAACAGAGCCCCAGCAGCGCGGAGAGCACGAGGACGGGGAACAGACCGACTCCGGGCGCGAGGAGGACGCACAGGAGAGCGAGGTCGCAAGAAGAGACGAGAAGGGAACGGAGCAGGGCGGTAGCGGAAGGAGCTGCGGGAGGGCAACGCAGACGTACGGCGGGAGAGGCGAACATGGTGCCTGGTCGTCGATCCCCCTGTCTGTCCCCAGGCCCGATCCCCGCGTGTGGGTCCCTCCTCCTCATCTGTTATTCCCTTCCCCGCTGCCGTCGATAACGCCCGTCGAAGACGAGCCGTCCGCCCGCCCTCGGTGCCCGCCAGGTCCCGCGGAGGAACCCTCTAAGTGTTCTCCGTGCCCGCCCTGCCCGTCGCCCGACGCCCCGCAGTCCGCTGTCCCGCGCCTCTCCGCTCTGTCCGTCCCCTCGCCGTCCACCGCGCGTGTCCGTTTCTCCCTCTCCTCCCTACTCTCCCTCCCCGTTGTCTCCGCCCTCTCCCGTCTCTCCCTCGTCTCCCAGATCTCCGTTCATCTCCCCCATTAGATCTCCGGGACTCCGAGCAAAGCCGTGGGTGTCCTCCGGGCATCCCGTGGCGTTCCCGCCGGCGCCCTCGTCCGCGCCGCCCTTTTCCAAAAGAGTCCCGTCCGTTCCCTCATCGGCATCTCCGTCCGCGCCGTGCATCGGCAGGTCGCGGCCGCCCTCCGCACAGACGGCGTGAGACGCCGCGTGAGGTGACGGAGGCGGCGAAGGGACACGAGAAGAACATGCAGAAGAACATGAAGACGAAGAAGACGAAGAAACGAGGACGAAAGGAGGGGAATACACCGGAGACCGAGCGGCGGATGGAGCCCGCTCGGTCTCGCACGTCCGCGATACCGAGCGGGCTCCGTCGCCGCTCCGGACCCTCCACGCCCCTCCGTCCAGGACCCGAGGTCCGACACGCCCCCACCTGGCGCACCGCCTCCGCCACCACCGCTGACAGTCACCGCATATCACCCCCATACACACCCAGCAGCAGAGGCCGGCACATACACACGCGCGGAGCACGCACGAGAACACGCGAGACATCGGCCGCCGAGATTAACGGAGTCTATGCGCGCGCCGTGACACGGAAAACCAAGCGGAGCGAGACGATCGACCGACTCCTGCTATCCGTCCTCCCTGGGCACGGTCCACACGCCAGTCTGCGGAGTCACCTGAGGGCCAGATCCGCTCTGCGCCCCCCGCCCGATCCGCCCCGATAAAAAAAAATCATCTGACTCGTGCCAGTTCACACAGATGCAACCGATGACAAAAAAAAAACACACCACCGCAAGCAAACGCTCGCCGGCCCGTCCACCGTCCCTCTCACCCCCACTCGAATCGCGCAGGCGCGTCGGCGGGAAGTCCCCCGAACGCCTCCACGGCAACGCTCCTCCCACGCCCCCTCGGTCCCCCCTCCCTCCATCCCCCTCCCCGGAGCATACGCGCCGACTCTCTCCGCAGAGGCGGCGACCACAGCGGCAGCGGAAACTGTAACGGCCACGTTCTTTGAACAGTGACAGTAACGGCGGCGGCGGCTGTCGCCGTCACGGTAGTCGTGGCGGAGCACCCGGGGAGGCGACGACGGGGACTACCGATGCTCTCAGATCATCGCCATAGTGAAAGAGTACCGAATAAAACAAGTTAAATTTCGTTGTAAATAAAAAAAAATACTATGTGGAAATTAAAAATAAAAAAAATGAAAATAAAGGCTAAACACTGACTAAACGTACATCCTTCTCCGTACGTCCCCTTTCAATAAATGGCGGACCCGACCTCTCCATAAACGGAGACACGCCGGGAGACTCCGGCCCTCCCGTTCCAGAGGTAGCCCGAGACCTAATCACAAAACTGTACCTGAAACCGAAAGTATAGAGGGCGGCCTCGAATGCCCCACCTATGAGGCGGGACTTCGCGACGCGCCACCCTTCCTATACGCCCGGCCTTCCGCGTGCGCGCCTATAGGACGCATCCCCTCGCCCCGAAACCGCAACGCGGCAGAGTCGGCGCCACACCCCCGTCCCCCGAGTTCGCGATGGGCTGGCGCCGACCCTGCCGCGGCAACGAAGGCGAAACGAGACCCCGACGCATGCGGGCATAAAGAGCGAGAAGTAAACGCGGGGTCCCGAATTATCGCGAGACTATTTGCCCGGG

TGACACACCCGGTGGAAAAAACGAAAGACACATAAATAAAACGAATTTTGTTGTTAAAAATGTTTCCACGTGACGTCCCCGGTGATTCCCGGAGATGCGGCACGCGGCGGATTCGCCCGTTTACATCGGGGTCCGCAGACGCGGTTCTGCCAGGGCGTTGCGCTGTCGGTTCCGCACAAAACACCGCGGTCCGTCGTCACCCTTCAGCCACGCGGTAGTTCCGCGATTCTATGGTTCATCTCCGCTCACATCGTTCTTTTTTTTTTATTCTTTCACTGAGCCGCTCCACCACCAAGAGTGTTACTATCTACACCGTATACATGTTTTTTTTTTATCTCTCCGCCCCTCCCCATCACAGACCATCAAACACCTTTTGCCGTATCGACACTGACTATACTCGACCGCGGATGACAACGGTCGTTGGTCACAACAGAGCTACAGCGGACAGCAAAAATAACATAAATACAGTAAGAGAGAGGCCCCGGTCCACATAGGAAGCCAAGGGCGGACGAAAATAAAAACCCGTCCGTAATTCACACACGAATCGGGGGTTATGACAACGCGACACACGCAGACGAGGGACGGACGCATCGCGATCCGGCGCGACGGGGCGCGATTGGCCCACGCGCGAGCCCGTGCCCGCTTCGAGTGGCTGCTCCTGGCTCGCGGCAGGCCGTCCAAACTGTACGGCTATACGAGTCGGCACCGGGGAGAACGGATCCACCTACCGTGGCCGCGGTACTGGTGCCTAGAACTCCATCCGGATCCGTACAGGGACGCCAGGAGCGCCACCGTGTGGGGTCACCGCTGGGGTTGGCCGCCAACGCACGTGAGACCCAGATCCGTTCAAGACTGCGGTGAGTGAGCGCCGCCGCGACCGGCGACGCTATGCGCACACGGGCGAGCGGCGGGGAGGGACGTCGGCCGCAGGGCGAGCCGGTGGAGGGCGAGCCGGTGGAGGGCGAGCGGGTGGAGGGTGAGCGGGTGGAGGGTGAGCGGGTGGAGGGTTCGTCGAAAACCACCGAAGACATTGGCTGGCGGAGAGTGGGGACAAAACAACGTCACGTCAGGGGCGGAGAAATATAGGGTGCGCAGACCGTCAAAGCGCGAGTAACGGAAAACGAGTGCGGATACGGGGGGGGGGGGAAGGAAACATGGTGAAGTCTGTGACACGCGTGACCGTCAGCCGGAAACGCCTGCCACGGCCTCTGGCCTCACAGACGACTCACGGAGTTTCCTTCAGACATTGTTTTTTTTTTTGTATTCATCGGTAATGCGGTATACGTAAATTCCCCGCAAAGGTACTTTCCGTTAAGGACGGTACAGGCGGTATGACTTCCGGAACACGATGATTACGGATATCCTGCATAGTGTACAGGAGACCGCTTTTTTTTTAGGTTCCGTATAAGACGTACGTCGGCGACGTACACACAACAGTTAAAAAAATAAAAAAAAGAGGAAACAGTAAAGCTCCTCCTTTCCTCTATGTTATCGCGTTCAGTTAGTAGGCGCCATTGCTGTTGCGTATGCTTTTCGCGTTAACGGTACGCGACAGGATACGAATGCGTTACACGCCGCAAAGGCTCCCTACAATGACTCTATGCGGCATCTCCCGTTCGCGCAGAAACGCGAGTTCCCGGGACGCCAGTCTTGACGTCCGGCCGATGGGATGCCTTTGCGTGTGCGGATGTTTTCTGCTGTTTTATAGGAACAGAGACAAGAGACGAAAATAGCGGAACGCCCACTATAACGTGCATTTTTCTCTGTGTTTTGTGTTTTCATGTTTTATGATTCTTCTACGGTATCGGCCGGAACGGTCATCCTCGATCCTTCTCGTACGCGTGTCCCGCCCCAGCCCTGGACTCGAGTCTGTACGTATGTTGCGGATACGGAGAAAAACTTCAACCCGTGGGCTTCGTAAGCTCGTATCTGACCCACTCCCCGCTCGACACGCTTCGCGTGCTCCTGGTCGGTAGAGACGGAGCCGTGTACGTCCACCACATGAGGGCGGCCAGACTCTGCCGTCTGGCGTCGAACGTAACGGAGTTTGCAAGGCGAGGGCTGCAGCGGGACCCCGTGGCGTATGAGGAAGACCTAGAGCTGCCGGACCGGCGTATGTGCGGAACGAACGTCAGACATCTGTTCGACGTGATCGCCGCGGCCGCCGACGAACACGACCTGCTGACCGTCGGCGGCCTGTGTCAGACGCACGCCGGAGTGAGCTGTGAATTACTAGAGACCGTGCGAGATCCGTGGACGGCGGTTCCGGGCGTACGCATGACTCTGACCGTGGCGCGGGCTCAGTATCGCTTGTGGCCCGATGCCCGGAGACAGCTCCGCCTGCACCTGTACGCGGGACACCCCCTGGGACCGTGGATAGTGTGCGCCGTTCTGTCTCGAGAGAGGGAGACGCAGACGCCGTCGCCTCCGATAGGCAGCGGAGGCGTGATTCTGGGAAACGTGCCCACGCCGGGGCCACGCGAGGTGGAGACGGCTTGGGTGATCGTCACCGTGGCGGGACCGTTGTTATCGTTCTGGCCCGATAACGGCAAGATCTGCCGTCTGGCGAACTCGTTCGCCGCCCTGTGGAGGATGGGGCCGCGGGCCATGAGAGGACATTGGACGTATTCGGCCCCGGGTAGACATCTCCCCGGGGACGCTTGGCCACTCTGTGAACACGTGAGACCGCCAGTCGGAAAACTTCCCCGGCAAAGAGCGTACCTGGATTAGACGCCACGGCGGCGACGTAAGGATGCGACCTACGGCCAGAGACATGACCGTAGGAAGATCATGACAAGGATCTCGAAATATAAAACCAAAAAAAAAGTGAGGAGTGCGTAATCGCAAGAAAAGAAAAAACAGAGCCGCGGTGGAAATGTCTATCCGCCTGCGTGTATGTGTGGGGTGTGTATATGTGCGCGTGACCGTATCCCTCCCTGCCTTTAACCCGATGAGAAATAAAATCGGCGAATGTACACAGTCACACACAATGTGCTTACGTGCCGTGTTACTGACACCACATAACAAAAAAAAAAAACATACTTCCTGTCCGTGAGACACCGCCGCCCTCTAGCGGGGTTGCTATAGCAGCGCCCGATGCCAACGCGACACGGTGAGTCGCATAGATCGGGACTGCTTGAAAGCGCGCCACATTCGTCTTTTATATAGACACCCCGGGGAGTGGGAGGAGCCAACATACACACGAGGTCGGTCTGAGGTTGGAGACGAACGCGGAAAATCAACGGCGTAAAAAAAATAAGAAAGGCGGAGACACATAGCCTTGGCGGGAAGACGATAACAGGTTCAAAGATAGACAGTGAAAAAAAAAAGAGGAGACCATGCCATTGAATACACGTGTGGCAGCAATCCAATCAACGTGACGTGCCGGCGCGCAAAAAAAAAAGTATACGGAAGTGCATGCGACAGACAGTCACGCGGACCGACGACAAAGGCCGACCCCTAGCATGGCAGCTAGGAAAAAAAATAAACAACGCTGAAATATACTAACAAAGGAAACATCCCTTCATCCCTCCCTACCCCATCTACCACAACACCCCCCTCCGTAATCCCTTTTCTCCGCGTTTCTCCCACAGACGCGTGCACACGCAGACACGCAGACACGCACACACCACCTCTATGGCAGTCGCGGGTGGGCAGGCGGGGAGCATACGGGGGGCAGATGTGAAGACAATGAGGAACGGCATCGGGCGCGACGTGCCGCCGTCTCGGACCCATGCTATCCTGGCACGACGCCAAGGGAAACCGCTGGCGCAATTAACCTAACCCTAACCCATCCCCCAACGCGCGCGCGCGCGCCTCTATGGGAGGCGCCGTGTTTTTCACCAAAAACGCGCGCCACTGCAAGAGGGGGGTGAAAAAACCTCCCCTCCCCGACCGGCCCGAGTGGTCCGCGCGTGTCGCCGGTGCGCCCCGCGCCCGGGCCCCCCCCCCCCGGGAACGGGCGAGGGTATATGGGTGGATGGCGTGTAGTTTAAAGGCGAAGGTGCAGCCGGGCGCCGGAGCCCGGTGAGAGCGAAAAAACGATGGCGCGCGCGAGAGAGAAAGAGAGGCAGAAGGCAGAGCCGCAGAGGGACAGACGAGGAGACGCCGGAGGAAAGAGAGAACGGCCGCGAGGGCCATGAGCTCCACCTAGCGTCAGACTACGCCACCGCGGCCGCGGTGCGCTCGTTCCCGAGGAGGGCCCGCATGTGGCGCGAGAGAGGGAAAAGAGCGGGCGCGTGTAGAAACGGCCAACGGGCGGATGCGCAGAGCGCCCCGCCGGGCGAATCACCTTTTTATAGACCCCCGACCCATTTCGGTGATGCAGATACGCACGCGAACACGCACAGACATGCCTACGCGGCCTCACCGAGCAGACTGCAACGTGAGCGAAAGCATACACACACACGCCATTTTATTTATGCGCTGTTCTCACAATGTTAACATAGGACAAAGACAACCACG

GGCGGGATCACGAAGTGTCAACTCCCGCATCTCCGTCGCGCAACGTGTCCTCGCAGTCCCTACGGACTACGGTAGAAGCCCCGCAAGAGACTGCGGACATTTCCCGCTCTACCATGTGCGGAGAAAGTTGATAGACGGCAGGTAGATTTATCACAGGCGCGTGCACGCACGTCACGGGCAGCTGCATGGTCCACAGATGTCCCGGAGGGAATCGATGGTTCTTGAACAGTTTTCTGACACCCATGGCGAGAAACGCCCTGAAGCTATCGGACAGGCGCACCACGGCTCCCCCGTCGAGACTGGCCGCATACACCCTGCCGACCTGATCCATGAGCACCCGCTGTCGTCTGAGCAGAGGTAAAGTAACGCCGCGTCCGAAGAATCTGCCGATCACCACCGCGACGCCTCCGCCGGGTATGAGCCGCCACCTCTTCTCCGCCCTCCTGGGTACGGCAAACTCAAAGAAAGCTCCGCGCGGCCAAGGCAACGGCACGAGAGCCTTATCGAAAGACACGGCGAAAGCCAGGGCGTCGTCAACTCCACTCATGCCGCCGAAGAAAGAGGCGCCGGTTTCCATGAGACACTTGGAACCGTAGACGGGCTCGAAGCGACGCAAGCCGTGCTTCCAGAGACCGAACGCGTCCTCCGCGAGACGGTACAGCGCGTCGTCGAACACGCCGTTAAACGCGTATATCTCCCCGCACTCTCCGACCAGCACCAGCGCGGAAACTTCTGCGAAGATAGTGACCCCGATGACCGTGAGATCCATACCGGGACAGCACCCGTATCCGCGAGACAGCTTGGCCAACTCGGCCTGCGTGATCCCGAGCATGTCGGAGGTGGCGAATCTCAGTTCTGCCCCCACGGGCCACGAGAGCGCCAACCTGCAATGGGCAAAAGTAGAGACGAAGTCCCTCACGTCGTCCGCCGATAGCGGCCGGGCGACGAGAGTCCGAACGAACGCCGTCCATTTAACCTCCGGCACTGCCGCATCGCACCGCAGATCACGTCCCGCAGGGAAAGAGCACGAAGAAGGGCAGAGAGCCGCAGAGGCCAGACTCCAAGAAGATATACCGAGAAACGGAGAGCGACAAAACATTTTTTTTTCTGTGCGGAATCCGAGATAAAATTAAAAGGAAAAAAAAAATAAAAAAAACGATAACGGCAGCGTTACCGGCAGTCTCACATAAACGTCTACGATCGCGCTAAGGATGCTACAACTGTTAGAAACAAACAACGTTGAATCGACATATTCCCGACGTCTTCCACGAAAATGGAAGTGAACCTACCGCCCACAAAACTGTGAACACATCCCATCCCCGGGTAAAATATACAATGATCCCAGACGTCAAAAGCGCCCGTAAGAACTCGATCTACCACGCTTCCATTCTTTTTTTTTTTATACCTAACCTCCCACGTTAGGGCGAACTATCGAACTACGTCGTGCACGGCACTCCTAGTGCCGGCGTCCGGAAATTCCTGACGCTCATCGTTTCTAGCCTTTACAAACAGCAGATCTTCGTCGGCCTGCCTCCAAGTTTTCTGATAAAACATTCGCGAGAGATGACAAGTCGGAAGAAGCGCCCATTCGGGCTGCTCCCTACGGAAAACGTAGCTCTTATAATACTGAATGACTCCTATCCGTCTAAACTGCTGAAAATTTTCCGCCACAAAGCGCGGCCGTCCGTCTTCGTCGTTGTCGTTGAATCCGAAAATCCGTCCGTCGTCGGCCACGTAGAGAGAAATCCGACCGGCGTCCGCAAAGTACTTGTAGCCGAAGCTGCCGAAGAAACACATTTCCGCGAGCCTGCGCCGTGCCTCGTCGCACCTGACGAATACAAAAGAATCCCGCCGCCGATGAAAGAGCAGAGTCTCGTTCTTGGGCCACCTCAGGGCGAAAGAACGACCTATGTGCCGGTCTCGAAAGCACAGCACGTCCCGACAACTGCAGACGCCGTCCACCTCGGTGTCCTCCACCACAAACTGCAGCGATGATACCAGCTGATGTGCGTAATAGAACCTCTTCAGACCTTTACGAAGAAAATCGTCAATGGTGTCACCGACGCGGCACAGTTGCTGCGGCAATAACCAAGTATGCGCATAAACGCGACCGGCACCGCCGAGCAGAACGACTATGTCACCTTTCAGAAACCAGTAAGCCGGCTCCGATCCGATCTCCCCCCTTTTCTTCACGTACCCCAGTACGACGAGGCGCTCCCGGCAACACACTAGATCGGCCAGTTCGCCCAACTTTTCATCGGCATATTCATTCTCGTCTCTACAGTTGTTAATAACCAGCTCGTATCCTTCAGGCCAAGCCAAGGGAATTCGCCTGCCGAAATTGACGAACGTAAACATTCGGACGCTATTCAGGCACGTATAGACGCGCAAGAGATCGACGAGATCTCTCTTGGACTTGTTATAGAGACGTTGACGTTTCTTCATCCTCTCCTGAGACTGCAGCTCGTCATCCCCACACATCATAGAATCTTGAGCCGGCTCATGAAGGCCTTCGATAAATCGCACCGTAGCCGTAGCCAAAATCTGCCGTTGCGCCGAAGAAACACGCCGGCTGTTTGTCGTTTCCATTACGGGCTCACAGCGACAGAGAAATAGCACTCAAAACTTAGTAAGCAAAAGTTGCAACTCGCAAAAAAAAAATGAAAGATAATATAGTCACAAAAGAAACTTGTCACAATGTACACCCAAAAAGAACGATATGGATGATCACACGACAACGGCAACAATAATATCGTTTTTTTTTTTAAAGCTAAACAAAGTGAAAGAAGCGTACCGGTTTTCCGGATTCGTCTGTATCAGATAAAGGTCACACAAGCCAGCTTAGGAACGCGGCGCGCTTCGCGAATGTCTAACCCTCGCTGTTGCACGCGTCTTATAAGCCTACGTCTGAGGACTATTCGCTTTTGACGTCTCCGGTGCTCCTGCAACCTGATGTCAAACGAGCCGCCGCCGCGCACAGCGTAAGGAACGACGTTCGGATACACCCCACTCCACATATGGGTCCCGTAAAGATTGCAGTCGTGAAAACGAAAAAACCTCACCGGATTCGACATGTAAGATAACTCCAGAGTATTGAGGAAGCCCTCCAGAACATCGTGCCTTATCTCGCGAGCGAGTCGCATGGATGCACACAGGTCGCCTAGACAGATCTTTTTTAGGCACAGATGCTCCAAGAGCGCACGAACGCGATGCCGTTCCTTGTCATAACCCGATACGAGAATCGTCACGATCTCAAGAAAGAAAAAACACAACCGAGGGACGCGCATGGGATCTTTAATCTCGAACTGAGAACTGACAACTAACAGTTTGATCCTGACACCGTGGAGGCCAGAAACGGTATCGATGCAACGCTCCATTTCTGAGATCGCTTTTACGAAGAAACGAGCAATGGCCGTGAGATCACTTACGATGCGCTGTTTGACGCGTCTCAGCAGAAACAGACACAGAACAATGTTGCAGTACACCTTCAAGATGTTACGTTCGAAATGCCCTCTCCCCAACTGCTTATCGTGAAGAATGGGCAAATGCCGCCTTTCCATAGAGTTAAGAATAAGAGAAGAACCAAGATCTCGGTCAATCTCCGCCCGGAGAGCGACCGCTTCACGCGCGTCGAGGTGCAGCTGGCCTAAAATGAGATCAGCTGGATCGAATTCGGAAATATCAGATATGTTCACGGCCCGGGAATCTTTCTTACCCAGATAGATCCGAAAGAGAGACGCCCGGCACTGATCGCAACTACAGAGCACAGAAGACACAAAGCGATAAGAAGAAGCGTACGCGACAAAGATGTAATCGGACAAAAAGTGTTTCAAGAGCACACTCGTCGGAGACGCCGCGTCGCCATAGATCTCCTTGGGATTGGGCAAAATATCGAACAAGGTCAACAGAGTATTGGACTCACTCCTGTTCCTCAGCATAAGGAACTGCAGGAAGAGGGAGTCCAACAACTCCAAAAGACGGTTGAAATGCGCCGAGATAGTATCCCTCTTCTCCCCATCGACGATGTACTCCAGAATCGAGAAGTGCGCCAGAGCGGTGCACTTGGTCAGGAGTCGATAAAAAGCTGACGTTTCGTCCTCAAAAGTTAACCGTTCTAAGAGAGACATGAAAGCCGAGAGATCCATGGTCGTCTTTCCACGCCTTTGGAATTCCACAGAAACTTTCTCCGCCAGAGCCGCCCAGAGAACACTATCGTCACAAAATCGTAAGCCGCGGGTCATGAACAGATCCTCTGCGGTCAACGTGCCGCTATCTATAATCATCTCCTCCGTTATGTCTTTCAGATCAACATCAAGTTCCCTGGAATGGTGAACGGTCAGCGAGAGATAGGGATGGTTGGGAATGGTATACGTGACCCGTTCTCGAACGGGTCCCTTATAAATGTCGTGGTCCAATAACTCCATCACTCAAGTAAGTATATATCTGAGACAGCGTTTATTTTTTTGTCGCAGAAAAAAACAAACGAGCAGAATTCAAATTGCAGGAGACGGCCACGTACATCACAATCGAGAAAACACACAATCACTCATCCCCATCATGTCCATTCAGTTCGCCGGCACAGTCCGGTGCATATTCATAATACTTGTAATCATCCCTGAGGACGTCCAACGACCCCAATTTATACCCGATAGCTCTGACGTCTTTCTCGGGACTTATTTGAACACCCCGATGATTACCACTCCCAAAATATTTCAAGCCACTGAAAAAAAAAGCCATAGTCCCGTTTATGCACCTGGTGTTAGTTAAATAATCTTCGGCGACGGGCAACACTACCCGTTGTAGCCGCTGTAAGAAATTCTCTTCATGGGTATTAACGATAAAAAAACCCGGAATGAGATTATCAGAAGACACCAGGTCACGATCATTGTGCTCGTGGAAGCGTTTTATATGAAGATGATGCAGAAAAAACATTTCCATGAACCGAGTCTCGTCGTAGTCTTTGTAAGCAGGAACGGAAAAACCGATTCTCAGAGCTTCCCAACACTTTAAGATAAACTGACTCTTTTCCCCGGGACACCTAACAATTTCTTTCATTTGTTTCATCGAAGAAAAGTAGCACAGGTCTCCGAGGCACCCGTTCCGCCCCAGCTTCATTTCTTCCTCCATGGTCACACACAATTTTCCCACTTCTTCCGTGAAGAGGTCGTAATATATGTCCAACTCAGCATTTATGACACGGTAGAGGGTGTTCACGACGAACACAATGAAAACAATGTTAGTTAAGAAGGTTAGCAATGCCTTCTTATCCGGGGTGACAGAGATCTGTAGCGAAATAGGCAGCCTACTGTGCTCTATCTGACCTTCAATCATGCTCAACCCTAAATCCTTCGCTATGAGACTCGACAGACTCACACTGTAGGGTTCACCCAAATGCAGAAAAGCCGGAAGACGAATTGAACCTATCATAGGAAAACACTGAATTCGGACACAGTGGCCAAAATCACACTTCCTGCCTCTCTTGTTATACATGATAAACATGCGCCTACACTCGACACAACCACACAGGAGCATAAAGTGCTCGATGAGACAGGATTCGATACGCTCAAAGAAATCGTATCTGAAGAAGACATCGGATTTGTTCTCCAAGCAGTCCTTCAATTCTTGACCCCAGTGACAAAGATTATAACCTCCCAGCTGCCGAGTAGATTCCTTTGTCAACTCTTTTAACTTGCTTTCAAACTGAAATAATAACATGGAGGCGTACTCACATAGATAGCGACACCAGTAGCCCAGCGTAGGTTTGGACGCGAAGTTAAACAGAGACGCTCCAGCATAGAGACCGATAAAAGCCAGTTTAGTGATGCACTGCCACTTCTCATCAAAATTGTCCATAAATTTCAAGTTTTTCACCATGTCAGCAATCTGATCGGCTCTGACGTAGGTGGAAGGATACTTGTAAGTTTCATACTCATAATCTATCAGTCTGTTCCACACTACGCAGTCCGAAGAGGGATGTAGTCCTTTAATGCCAAATCTAGCTGCTGCGGTTCCCTGCAGACCGTTCGTCAACCTAGCGCAGGAGACGGGAACGACAAACCTGCTGGTAGCGGGCAGTCGACCGACGAATACCGGCGTGAGAAATTTTGTGATAAATTTAACCGTCAGCGAATCCTCTTCGTCCAGACGCACCGCAAACAGAATTTTGCTGCGGTCTTTCACGAGCCTGTTGTAAGAAAAAACAAGTTAACGGAGAGTCTTCATCCTCGAAGAATTGTCTTTTCGGGAAAAACGACACACAACACATCTACCCACCTGTCCTTTCGCACGCACATATGCCGAGGCTTACTGCGGAGAACGGGCAACTCCCCATTCAACATCCTATTAAGATTCTCCGTCTCAGTGTCACTTATATAAAATAAGGTATCGTCATTCCAGTCCAATGAATACTTCTCTTTATAGAGCAATTCATCAAATCCTCTACGTATAAAAGTCCTCAAACTTTTAGAGACTTTGCTGATTCTGTTTTCTACGTAGATATAGATGTGACAGTTCGAGCCCATAACGATAGCAGCTCTGCAATTTGGTTCAGCTTGAGAGCGGGCACACGTCCCTATGATAGCATAATCCTTTTCTGTGATTTTCTCTAGAATGCCAGGTCCAGTCAGATAATAAATATCCGACAAGGAACAAAAGGAAAAGTCAAAGTCCTGGAAAGCATCCACCCTGATTCTCTTGCCACAATGTTTTGCTACGTAATCCTTCATAGCCGGGAGATTCCTCTGTAAAAGGATAAATTCCTCTCGGCCGCATTTAGTCTCTCCGAACCACATCTTTTCCTGCTCTGGATCTAACTCCGCTTCGGCAAAGGGTGGAAATTGTCTAAGTCCGATATTGAAGAACTGGACCAGTGATTCTGCTATGATGTACACTTTCTCCGTCACCGTGTCCACGGCAATGGTCTTTCCAGTTGAACTATGCATCACGTATGGATCGTAGTCAGGATTCGGATCACGATATATCGGGTGAGCGTACCCAATGGCGGACAAAAATTCATCAGTATCCAGAAGACTGCTCCAGTCATAGGCACTTACCCATCTATGCGTGTGAAAGTGATAAAACTCACCCAGAGTTAATGCATACATGGGAGGCCAGGCGATACAACATCGCAAGCCCGGAGAACTGATCTCCAACCCGTCTCCGTAAGCTGCAGACAGCACGTCTACATCAAAATCCAAAGTAGGCACACATGATGACAAGGTGAACTCATCGTTGCAGACCGGTACGATTTCTGTTTGAAACATAAACGCAAAATGAAGTACAATACTTTCACACAAGACTCGCTTTCCGCCCTCCGTTTCAAGTATCAGACGAAAATTCGACATGAACTACTTACTGAGGCTCATGCACGATTGATAAGTCTCCGACATGCCCAACGTCGACATCCGTCCGAAGCGCATGGATATTTTTCCAATCTTCTCATAACCGCAGTCCGCAAAAAGTCTGGCAAATCCCACACGCAAAAAAGTCAGTAGACAATCGCCCAGCTTCACCAGCACGTTGTCTATCCAATCGTAATAATATATTTTTCCAGATTTCCCTATACATAGAATAGGTCTTGTACATTTAGACTCATAATCATGGTCATTCAGGTAACCTAACACTACTACTTTTTCTACTAAGTCTTCCTGTTCTTCTAGATTTGCCAAGCGACATGTCCCTATCCAGACACTGTCACGAGTCCCTAGAGTCAAACTCTTAAAAACCGGCATATCCAATCTCAGATCCCGGCCGACGTTGAGAGACACGAAACGTTCCACATCGTCCAAACTGGCACAACGGGCCAGCTCATCAAACTCTCGCAACATGGCAATGTAATGTTTCCTCATAGCCTTGATGCCCAAAACGCCTTTCCTATTCAACTTCAGATGGATATTCAAAGTACAGGGACTCCTTAACACATCATTATTCCTTAAATTTTCCAGCGATTCGAATCTGGGGCCGTCATACAAGCCATGACGGTACACAGGCTCCGCGCATGTGATGATGTTAGTACCCAGGACGCCGTATCTTACAAATTGCTCAAAATTATCACACACGAAGTGGAGAGTATCCGTCTCTAGATCGTGCAAGAAAATTCTTTTAGATCGGCCGATCAAAATTGGAACATCTCTGTAAGGAATCTGAATAGTCCCCAGTAGACACAGTTTTTCCACAAAACCCTGATAGTGATCATTCATATCTTCAAACATCTTTTCTGTATATCCAGCTACATCTTTACGTAGAGTGAATCTCAGCCAGCAGTTCCGAGGCCAGATAATAGACAGACACCAGTTCTTATACGTGCCGACAAAATTCTCGATTCGAGAAAGATCAGCAGATTCACAGAAACGACACAATTCGGCAAGAGCGTTCTCGTTCGGAATCTCACCTGAGACCTCCTTCGCCTCGAGGTCCATCTTGAAAACTCCGAATAACACTAGAAGTGAAACAATGAAATATCTGAAATATAAGACCTCGATGCGAGACGCCGTCACATGACAGGAAATTGGAAAAAAGAGAGTGGTGTGACAAACTTTATTAAATTTCAACCCCCGTTTTGACTCACACGCTTTCCTCCGCATGTACAGAAAAATCCGTTCCACCAACGATTTCCTGCGAATGTTGGACAACAAACACCTTCATTTCCTCGGAATTATGAAAATCTTCCTTACATACATCAGAAGCAAAAGTATATAAACACTCGCATTCCGAAAGTTTTATGCATTGCGATTTGACATAGCTGCAATCCGCATTCAAACCTTGCACCCCGTACTTTAAGCAGGAGACCTCCATCGTTCCGGCTTCTGTATTGCACTCCTCGGATTTCGATTTAGAAAACAGCTGTACGACTGTCTTCCATAACTTTCTAACAGCCATCGGCAGATCCACTTCAGAAAAAACGAACTCGAAGCGCCACTAAAAACAGATGGAAATAGTCACATACAAGACGGCTTCAGCGAGATCTCCGACCGTCACCTGGTCATCGGGATTCGGACGAGCTATAGCCTCAATTCAAAAAAGGCATCAAGAAAATATCAGGAAACCCCTACGCTTCTACAGCGGTCTCCTACACTGCCTTATAAAACAGTATGAGCATTGCCTCGTCCCGCCAAATAAATCAATACGTTTCGACAAAGGGAAGATTGAAGTCGCGGCCCTAATTCTAGATCTCGGACACCAGGTACTTGGTCGTCAGATACACGTCCGACAACGTATCTACAGCTGGACTAGCATCACACTCCCTAAACTCTTTACTCCCAGAGAACTTTACTTTTTAGTTGCCTCTCCAGAAGACGAAGACATCGTATTTAATCCAACTATAACAAAGGGAGGGTGGATCTCGGGAAGTTTTTCGTATCCCGTTGAATACAGATCAAACTTTTCGCTAACCGGAATGTCGGCTAACGTATTAATGGTGCCGTTCGTTCCGTATAGATACCCACTAAACTATGCTCGCTTCATATCGTCAATAGATCTGATGATACTAAACGAACAATTTCCGGAACACGAATGCGGAGACATACAGATTTTAAAGCAGCGAAACTACCTCTACCTGGGAGTCATCAAAAATCTTACATGGAAAAAAAGCGTCACCGGCACAGGTCAGACAGCTCCACATAGAATCCTAAAAGCTTCGTTCATAGGAAGTTGGCCGGACACCTCCCTGCCTGACAGAGTAGCACTGCGTTTCTTTAATAACACTAGGTTTACGATACACTGCCACGAGTTCGCAATTAATATTGAAAATCTCGGTCTCGTCAAAAATAAAGAGAAAGTCTTTGGGACACTAGCCACCGTATGCTGCGAGCAAATCCCTTCTCTACTAACGACTGAAAATCTACCGAGATATTTAATAGTTCAGTTTGAAGTAGTAACGCAAATCGAAGATCCCGAACCCCTGTTATTTTCCAGTAACCCGAAACTATATTTTACAGGAGACGTACTAAACGCAACGATGCAACTACAGCACAACCCAAATTATTACGATCTACTCGTACACGCGCCATACGACATCCATTTCTATCCAAGCCGATGTCACATAGTAATTCTACCTATCCGATATTTCACGAGGGGTGACAAGCAAATCCTCATATCCGGCTATCAGAACGAAGGTTTCTTCGAAACCCAGGTGATGCTGTGGGCTCCTGGAACACCGTTGCATATTACGCTGCGTTCATTCTCTCCAAATCTGATCCTGCCCCAAAGCACACCTATCGCAACCCTGTTTTATGTAGAAAGAATGACAAGCCAGAACACTGAACAAAAAGACGTAATCGCAAAGCTGTCTGAGAATGGACATTTTATTGGCAACCTGAAGCTCCCCAGAGAAAATTTTTTACATCACGACGCGATCACTGACTTGTCTTTGGCAGCTATACCCAAGGACTCAGCGACTCCCGGCCCAGGGACGGTTTCTTCATCAGTTTCCCCGTCTTGACGTATCCTCAGAACCATCTTTTCAATTACATCGTCTTTTTCTTCAGGTGTCTCAACCTTCATTAATTGCGTGGGAATAAGAGTTTCATCCTGACCGACTTTGAAATCTGAGGTTTCTCTCTCACCTGCCTTCACTTTTTGAAGAGCGAGATCTATTTCCCGTTCTCCGTTACCCGGGAGCTTCACGTCCAGCGCATCCTTAAATGACACAGAATCTAAATCGCGAGACAGATCAGCATTTGGCAGCCTAAACGGAGACTGCACAGAATCGAGATCGTTTTGCAACTTAAAATTTTTAAACAGATTTTGAGGATCACTAACTTCAAGAATTCTTTCTACGTTAGCTTCCTCCGATTCGCGTCCCAACTTGTTTTCTGTAGGCAACTTAAGCTCGGTCCCAGACTGATTGTGCAAAATGTCTTGAATGCCCTTCTCGCCTAAACTAATCAGATTTGTAAATTCGGCTAGAATCTTGTTAACTCGCTGCCCAAGACCCGCTTGAACATACTGTTCCTCTCCGGCATTTCTAGCAGCTTCCATTAAATTAGGTAATGGCTCTTGCTCGTTTTGCGTCATTTCTTTATCGACATTAAACTTTCCTGAGGTTTCCTTTTTTTCTGCATTGCCGCCAGAATACAAGTCCCTGCTATTTCCTTTCCCCCCTCTTGATACGTCACGAGCATATTTCTTATTTAACCCGGCAAAAATATCATTCGCGTGCTTAGTTTTTCTCAACACTGGGGAATCCCGAGCAGTCACAGTTTCAGTCGCATCGCCAGAGTCTCCGGTCACTTCGCGTGAGTGTACTATAGCCTTATCATTTTTTTCCTTAGTCCAGCTTCCAAGCAGATCTAACTTCTTCTGTTTCCTAAAACCGTCTTTGTCCCTCAAATCTTTAATGAGAGAAACGTTATGGGGCGACACTCCGGTCTCAGTACTGTGCGTCGTAAGTTTCGCCTCTGATCCAGCCACATCGGAATTATTCAACACGAAGGGTGTCCCTGTCTGCTCAGAAACCAAACGATGCACCGGATCGAAAACATCTGCATTCGCCGCTGTGTTTTTACTCCACGGCCGTGAAACATCCGATTTATTCTGACGCAGATTGAGATTAGCGAGACCATTTACCACATCAGTAATACCGCTACTGGGACTTCTGATCGAAGTTTTATCCTGTAGTAAATCCACGTCTCCCGTATTAGATTGCGCCTCAGCTGGAAGTTTAACACCAAAATCTAAAATCCCCGAAGGGCCAATTCGATAATTACCGTGCGACTTATCTCGCAGATCCAGATTGGTAGAAGTCAAAGACGACAACGGCTCAACTCCGGACATTAAAGAGACAGACGAAACAGGCGTTGTCGCTCTAACGTCTCTACCCCCGAGTGTTTCTTTCTTTATCAAAATTTTTCCCCCGGCATTATCGACGCTATCCGACTCTGGAAATTTATGTCGCCTCTGCGTTTTTAAGGGCGCGTCCAAACTTTTGGGTTGACTTATTTTCAGACGAGCCAAATCCGCAGATGGTCTCTTTGTATCTATAGATATACTAGGTAAACTCAAATCTGAAGGCATGCGGCCGTCTAAAGAGGCGAAATCCATTAAAGAGTGAGGCCCATTCTCAGGCACCGAAAATGCTTTCGACGCGCTGTATGATTCCAGACTACTTTTGATAATCTCATAATCACTGGTGATTATTTTCAAAAAACTCTCTACTCTCTGACCAGTTAGAGGGGTCTGCAAAACCCTCTGAGAATACACAGCATTAAAATTCTCGTTCTTCCTCAAATGACCCGAGAGATGATTTTGCGTGGTTACAATTTGAGACATAGCAACCTTTTCTAGCTTTGACCAGGACTGGTTTAACGTTACAAATAACTTACCAAGATAAACTAATTTATTAATACTCGTCACTAAGTAACCGTTTTCGTCCCAGGGGTCATTTATGTTATAGACGGTGTACCAGAGCATGTTTTTTTCAGCGTCTTGCAAATTATTTTCTATCTCGCTGCTGTCTTCTGCAGTAAGATTTACATAAGGAGTATCGATCGGAACATTGCTACCCCACCTAATAGAATTTCCTAAACGCAGTAAAGCGCGATGAATATCACTGAATTGCGATTCAAACTTTTCTTTGTTCGTGAACATATTTTCAAAATTTCCTTTATTCAGACCTTTGGATTTTAAGTACTCGTTTACGACATTCTGGAGTTTCGTGATGTCTTGCCATATAGCATTATAATCCGGCTTCCGTTTAGATAACACGTAGTGATACATAAGCCATAAAATAAATGTGTTATACAGTGTTTTTAACTCGTCTACATCTTCTCCCTCTCTCACGACACAAGAATTAGTCACATAGGGATGTTCTCGTAAATCTACATTCTCCAGATTTTCCAAATTGCTAAGAAAATTTGTAAGACGCTGAACTTTGTCTCGATCTAGCCACGCAAACGGGATCGACTGCGCTTTCAAATCCATCTTTTAGAAAACAAACTCAAAAACAAAACTGCTCGTCTTCGCCAAGACTCAATGTTTAATAGTCCTTTTTTTACCCACACAGAAAGCTGACAAGCGACGAGATGGACACTGTCATTGAGCTGTCCAAACTCCTGGTAAGTCTCCAGAGATCATTGTTACCGAATATTTTATTGTAGCGTACCATGACAATAACATTCAATATCTTTAATAGCACGATGAAGAGTTCAAAGACAACGCTTCCTGTACCTCGACCCCCACGCTAAAGACAGCACGAATCATTGAGAGCGCAGTTACCGGAATCACGCTCACTGCATCCGTACCCATGATCATTATAGTGATTACCACCATGATTCTCTATCACAGAGTCGCAAAACATAACGCTACTTCATTTTACGTCATCACACTCTTTGCTAGCGATTTTGTGCTCATGTGGTGTGTCTTTTTTATGACAGTGAACAGAGAGCAGCTGTTCTCATTTAACCGTTTCTTCTGCCAGTTAGTTTACTTCATCTATCATGCAGTCTGTTCATATAGCATAAGCATGCTAGCTATAATCGCAACAATTAGGTACAAAACTTTGCACCGGCGTAAGCAGACCGAAAGTAAAACATATAGCACGGGGCGAAACATAGGAATTCTCCTACTAGCTTCCTCAATGTGCGCGATTCCCACCGCTCTCTTTGTGCAGATTAACGGAGCAAAAAAAACGACGGGAAAATGCGTCGTTTATCTATCCTCTCCGAAAGCTTACGAACTGTTTCTAGCGGTGAAAATTGTTTTCAGCTTCATTTGGTGAGTCCTCCCAACCATGGTGTTCAGCTTCTTCTACTTTATCTTTTGCAAAGCTTTGCACGGCGTCACCAAGAAAAAACACAAAAAGACTCTATTTTTTATTAGTATCCTTCTCCTGTCATTTTTACTCATACAGATCCCCTATATAGCTATCCTTATATCTGAAATCGCATTTCTCTACATGCCACAGAACACCTGCTTTTGGTTAGCCCGTGCAGAGATATTGCAGTTAATCATTCGATTAATGCCACAGGTACATTGTTTCTCTAATCCGTTAGTCTACGCATTCACTGGAGGCGAACTAAGAAACCGATTTACCACCTGTTTCCAATGTCCATTTTTTCCCAAAAGACTATGTAGTACACAGAATCGCAAACAGTCAGACGTCTCCGAACATGATCAGAACTCGCCATCAGAATCGTCAGTGGATGAAAACGAACCCCCTTAAACACGTTTTCCAACTTAATAAATGCTACCCACATAAAATCACGTGCTAGCTCTGTTTTCTTTGTTTTGTCTTCCATAGAGCATGGCGCACGCTAAAAAGCAGGCACGACGAAAACTTTTAACTTCGACGGACGATCCCATATTGTCCAGCACTTTTACCATGCGCCCGACAAGTAAGATTGCAGACGCTGAAATCATTTCAAGAGAACATGATTATATCGCAAGCAAAACACAGGCGGATTCAAAAAAAAAATTATCGTCCCTGTCTGTGATTTTTGACAAAACTGTCCTGTTTGAATTTTACGGTATAGGAGACAACAACGAAAAAGCTATCGTCTACCCTATCGATCCAGATTTCTTATTATGTGATTCGGAAAATAATTGCACCCTATCACCTTTCTTATAAAATTTTTTTCTCCTGATCTCACGTCACAGTTTTTTTTTCATATGTCTCGCGTTGATACATCCCGTATGAAATATTTAAGGTGGTTGAAATCACACCAATGATCACACACGCGATGGAAGGGTCGAAGACATTCAACATTCCCACTTTCGTCTTAGACGAAAATTGTAACTTCATACCCGATGTTCTCTCACGCGCCAACGCTAAATTTATCAAAGAGGTACTCATCCGAGACTCCTACAATGCGGTTTGTCTTGCAAACAGCTTTATTCCCATGGCGACTCAAACCGTAGAGCAAATTCTGATTATTATCACAAAGTTTAAGTTCTCACGTTCACGAGATCTGCTCATGTCAGTATTTCGCCTCGGCGTACATATAAACAGATTTTATGCCGGAAAAAATCAAGTCAAACATATGATCACAATGATGAAAAGTCTTTTCGATACCGAAGAAGCCATGCGACAACTCGACAGAGCTCTTATGGGGCTGTTTGTCGATGCTCGTGATAATTCATATATGCCTCTCATAGCATTGTCCCTGCACGAAAACGGCTTACCGGATTCTAAATTCATAAAAGCCGTCAGATTAATACAAACAACCGTAAACTCCTTTCATAATCGACCGGACGCAGATATCGAACAGTATGCAGAAAAATTACGAGCATACAATTACCTCTATAAAATACCCAAGTATTCTCTGAAAGAAGCTGTCGACATTTATTCCGATAATCTTAAAGATCTCACTATTGGAGTCAATAAAAAGCCCACCTTACTGTTTACGTCTTCAGATGACGCATATTTATCTCACATTTACAACGACTTACTATTTTTAACTTCTACTTGGAATATGATTTATAATTGCAAAAAAGAGATAAGGCGTTTGAATACTTGGATCAAATACGAAATTAACTCCATAATGGAAACTGCTGTATTGGTAGGATTCCAACTTCCAGATCTGAAGGAAACAATTCTAGACCTAGCGGCACTCATATCAAATATGAATCTCGTCAGCCCCGATAAAGAACTTTTCCCTCACTACAAACTCATATTAGCAAAATTGTTTGAAATCTGTATTTTTGCAACTAAGGCAAACATTTGCATTTTACCATCCTTCATAAAGGGTCACCTAATAGAGTTCGAGGATGTCTTAAAAAGAAGCAATGATGACGAAGACCTCAACTATCTGCTTCTGAAATCTCGAGATTCTGATGACGAATACGATGAAGACAAACCCCCAATACAAGTCGATCCAGGCAGAGTAGACAACGTGCTTACAGATTCTGACTTTTTTAACGTAACCCCGGAAAATGCTTTCTCTTCTATAGCGATCATGCCAATTTCTTATGATAAGACGATAGATGTGGAAGACAATGAAATTCAAGTCCTAGAGGTTGAGATGCAGAGCCTCTCAGCAGTTGTCTACGGAGCCGTTGCAAGCAAATACGGCTTAAGCCTGGAGCAAGTTATCAGAAAGTTAAATCGAAATGAAGGACGGACCTCTTCTCGTGCATCCCCCAGCCACAGTACTTCTACCGTCCCATACTCCCCCCCGCAAAGCGATCGCTCCACTCCAACTTCTATTCTAAGACAAAGAGTACCAATGCGTTCCAACAGCAGATCATCATCGGTTTCTTTCTCACAGGAAGACAGCAATCGTAGTCATTACTCTGACGAAACAAATATTAGTGATTATTCATATCCCATGGCAGATCTAGAATTAGAAGACGAAGAACCCATGGAGGACCATCCGCACTCCCCTCAATCAACATCATCTAACAACTCCATGTCTCGTCAAAGCCGAGCTCTACAAAACGGACAAAGAAGACGTGCTCCCACAATGGTCCCATCATCTCAGACACGAAGACAAAATAATGCCCGCCCCAGACGCGTAGCCAGGCGCCTAACGGAAATGATGAACGACGCAAGGTTATAATCACAAATAATTTTATTGACTGTGAAAAGTGCTTTATCCGGCCAATTCATTGTACGATAATAAAAGTTCCATTGTTTGATTGATTTCCGTATTCTTGAGCTTGTAGATAATTTCCAATAGAACATCCCGCGTTAGATGTAACTCATCAACGTAAGAAAAAAAACGAATAGCATTCTGTTCTTCATCATCACCAAGTCTAAGGTGACTCTTCTCTAAAAACTAAAAAAAAAAACACACCATCAATTTTATTCAACCAATCTAAGATAGTTTTGCATTAAACAATAAATTACTGCCAGAAAAACTATAGCCTAGCAAGCTAGTGAATCAAATACCTAATTCCAATTTAATATATCTTGAAATCTACCATAACGCGTACAAAATCAAACTCACCGCATATATCATATTAAGGAAAAGCTGTTTCTCTTGTCTACTGGTTAACATGTACAACAACACAATTATCTTCCTGTAAAAGAAAAACACATCAATCAGCATACATTAGCTTGTCATTCTCTCTTCCTGTAACAAAATAAACAATTTATGCAATCCAATTAAAGTCCCTTGTCTTTGTATCGAAAGAAAACTATTAAAGCCCGCTTCCACGTATACTTAAATCAATACCCACCTTTTTACAGGAATATTGGTGAACTGAGCTACGACGAAACAGTCGTTTACTTTCCGGATGTTCAATCTAAAAAATTGATTAATCTCTTCCCATCTTTTTAAAATAAATAACATTTCAATAACTACGGATTTAGTAACCGTCATACTCAACGTCAGAGCCGGCAAAATAAATCTATACATAGTAGAAAAGTCCATTTTAAATAGATATTTAACGTATATAATTTCGGTATTTGACAAGATATTCGACAGTGACATTAATGCAGGCAAAGGACACAGTTCACGGCATTCTTGAAGCCGTTGACGCTTCCACACATCCATCCTCGTCACAGCCAAAGCTCTGTTCCGCTCAGCTCGGCAGCTCTCCGTATTATAAAAACAGATACTGAATCACACGCTTTAAGAATTAAGAATCATTGGCTGCTTGCCCATCCGTTAGGACATCTCATTCTTAACACATATCCAATACAATGGTAAAAAAACAGGATTAAAAAAACAAACAACGACAAATCTTATCATCGGCTGAAATTTTTATTCTGTATACTAACATCCAACATCTCCATACATCAAGTTAAATACAACTCAAAGTATGACGTATCTATCTACTCTTCATCCTCAGATGATAAATCATAAATGTAATCATTCGAGAATGTGGGCCCTAGAAAAGTATGAGGACACAACGGCAGAATTTCTTGGACGGTGCTAATGTTCTGATGGAAAAACTTATAGCGACGATATCCCCTAACAATCCCCTGTCTTAAAAAAGATTCAAATTCGTCTGCTATTTTAACACATGATCCAATTCCTGTTCCGATCGTATCAATACCATAAATTGCCCCCGATAAATCCATCATCACAAACATCGTCTTCGGATACAGTAATGTTTTTCCAATGCTCCCAATAACATGCCCGTGATACGGCATGCTCTCGTAGACATCCGAAAAGAAATTCCATTCCATCCTACTAGGAAAATCTTCTTTCCTAGTAAGAAATAAAACAAAAACTCCGTCGTTATTTTCCAAACAAGACTTCATAATGTAATGCCTCCCACTATTTTTATAAACGATCGTAGCAAATTTCCATAAATTATCCTGCACATCAATCAACGATTGAAGAACATCATCAATTTTTGTTAAAAAGCGAATGTCCTTTTCTCGATATACCGGATCATAACGACGCAATCCGAGAACTGCAAATCGTTCCGTCGTTTCCGCCAACAAATACAGTGTCTCATTCACGAAGTCGAGACCATAGAATCGTTCGTCTTCTCCCACAAACACCAACGTTTTAGTTAGGCACACATCACGAGTCTCCTTTCCCCAGGCAGAAACATATCCAAGTAGTTGCAATCTCTCTGGGCAGCAAAGCAATAACGAACCTTGTTTAAAAGGAAAAATAACTTTATTTTCACAGCAAAAAACGAGAAAGACACATTACAAAACTGACACGAAACACTGATACTTACACATTTCTTGCAACTCTGCGGCAGACTTTGTGGGAAATGGAATTTTACACAGTGTCGAAATCACCAAGCACCGACTTTTCGGCCAATTAAACTGTATAGTTAAGCCGGTCTTCTGTTCAACGTATGTCGATAAAGAATCACGAAAAACTGCAATTTTTATATCGTTTAAATTCATTAGTGCAATGTCATAACGAGTTTTAACATAGTCGCCGATTCGTTCGTCTGCCATCTCTACGTTATGAAACCGTCAACTACCCCTTTGGACCCAAAACTACAAATAAACCAGCTCGCATTTCTTATCCATCTTTTATAGCACACAGAGATAAAAAACTCAACTTAATCAACTACCATCCAAAAACAAGGAACGTCATAGTGTTGGACTAAAATTACCATTTCGTTCGCCCATAGAAAATCAACAGAGACTACCGGACAATTCCCTACGTTAATAATATCCATCCTTGTGTATGTAAAATAATACAATGTAAACAATAATGATTCAACAATGACCTTGGTTTCTTCTCATACAACTGATTTCCCGTAAAGGAAACGAATGATAAAAAATCACAGTCGCGGAAAATTCAAATACTCGTCATATGAGAAATTCCCTAAAATCTTATATATAAGAAAGCCACAAACCCTCACAACCTCATAGACACCAAACTTTCTGAAGAGCAAAAAGAGTAGCAAATCACGGCCACCATTACAACTATGTTCGCCTTCAACATCAACATTTTCTTCATCTATTCTACCGGAAGAAACAATCCAGAGATTATCTCTCAAGATAAACAGATCCATAAGCGGCAGCCATCGAATGATACTGACATTGAAGTGGTAAGAAATATCAAAAAACGCGGATACAAATTTTTACCGATAGAAACGCGATATTTATTTAAGCACTGTTCAATCCTTTCCACAGGCAGACTTCTTACCCGAAAACTACAGTAGTCGGATCCGTAATTCCCACCGACAAAACAAAACGGTAAACACTTTTATCGTTTTGTTATACACTAATACCCAGGAGAGCTCAGATTTTGACTGATCTGCACATTACGAATACCCCAGAATATTTACACCTGTTTGCGATTTTTTTATAGGTCTCACTGCTCCACATTCTAACGACACTGGGAATGAACGTTATCCGTGCTTCTCTATCAAATACGCATTGACGCAGATGAGTAAGTTTTTTCACTAATCCATTTTAAATCCGACAGAAAAACACACCATTGACTATATGTAATTAATATTTTTTTATTTCCGCAGATATGATTTAATCCGACGACCACTACATGTTTTACTGTCCACGATCACCTTTTCCTTAATGCAAATAGCACTCACGGCACTTTATAGAAAATGTCTCACGATAATGCACACCAATTTTCTGAATGTTTTCATGTAATTTAATAAACATTTATTTGTATCCTAAATCTTTCACAACATGTTTCCGCGTAATCATTGATAAACAGCATAGCAATAACAGCATCGTAAATGCACCCCCAGCCAAAAAGAACAACCCAGCAGCCGACATTGAATGCGTAATTTCAGACACATTCTCGTAATCCCTTGAAAAATCCAACAACGCTTCAATCTCCTTGTATTTATACCAATTAGTCCATGCAGACTTCCATGTATTTCTTAAGAATTTTTTCGGCATCGTATCATTTTTAATTAATGAAAAATATTTCGAATGCACAACCTTTTTCTTGTAAATTTCATCCGACAATTGAGGACAGATCATGCTCAGGAAATAGCTATCCAGAGACACTCTCTCATTTAAAATGTTCAAAGTGCCATTGGCATCTGGCCCGTCACTTGTCCATTGACTCGCATTCATTTCAGTGCTGGACTTGAGAACAATCCGTTCCGTTCCATAAAATAGAGTCATCACACCTCCTGCGCTATGACCTTCTAGCCAGCAATTTACAGTACTCGATAGCTGAGCGGCATTTGGAAGCCTTTTAGCGATCAAAGCTCTGAGCATCACTCCTCTAAAATAATAATTAATAATCACACGTAGAAAAGCCGTTTTTTCAGAGATAGATGTGATATTTCCAACTTTCATGGCTTTAAAGCCATCTGTTAGCATCGCAAGGTTGATCAGATATGAAGTTCTGACAGAAACACAGCTGGAATTCCCAGCTAACACTCCATTCACATAACAAGTAGAGATGCATATAGCTCCAAGTTTCTGTTCCTGAATTAAAACTATTTTTTGCTCGCATGTGAGATTCGATGTAAAGCAGACTCCCATTACAAAGGTATAAACTAACAAGGACTCCATAGTTAACTTCTTCATGATATTTAGAATTCACAGATCGTTTGCAATTTCTTCCAAAAAATCTGCCGCATCTCTGTGGAATACAATAGAAACAAATTTGAAACTTTATAAAAGAACAAACCCATTTAAGACGCGGCAACACAATTCAGTCATCTATAATCCACATCCACTTTCGTGCACTCTTGTACCAGCACTTGCCACATGCATCAAGTAAAAACATCCACCATCCCTAAAACTACCCTTTGCGCGCACGCTCTCCCCATCCGTGAAACAATTAAAAAACCTACCTCAGCACAGCTGCCGATTCATGAACAGCATTTTCTGGGCTCTTAAGCCAATGAATGTTCGGTTTGCATTCCGATGGAGTAGCAATGATACAATAACCTTTTAAAGGTTTTTCTTGCGCCAAAGACGCACCACCCTGAACATGTTTACAACCTACATCATCGGTTGCCATAGCACCGACCACTGTTGTATCGTACACCGAGTCGATTCGAGGATCAGAGACATCAAAGGTATTTTTTGCGGTCGCTTGGACTTCCGTTTCCAAAATTGACTGATGAACCAAATTTCCACCGAGAAACAGAGTTTCTGAAGAATTATCCACCACGTCTTCAGTTTGAGTGGCAGCGTCACACATCGCCTCAGACCGATCTGTCTGTGTATGCCTCGATAACATAATTTTTATACCAGGAGTTTTGGTAAGTCCATAGATTGTGCAATGGCGAGCGGAGTTGTCTTCTTGACACGATAAAGCCATACACACCGTCCCCCCAACATCTTTTTTCGCAGATGGCTGCAACAATTCTTCCGTCGGCCAACAAAATGGACCCTTAGCACCGAAAGAGTCAGACTGAATTGGTCTCATGGAAGCACGAGCATCTTCTTCCACGCAAGCACCACTCTCTACCTTCTGAAACCGTCTAGGTAATACACCTGTCAAAGAAATTGGGGTATGTCTCATTCTCGTAAAATATTTTTGAAATTGATCACTGTATTCATCGCGGGACTTCTGTGCAATAAAATTACTCAAGATCGCGTTCTGACACGAGAACTCCATGGCAGTTGTCCAGAAATCTGAAAACGTAGGTGACAAATAAAATATCACACCATCTTCATATCCAAGCACTTGACCAGTTGACGATAACAAAAAAACAACTTGTCTCTCCGGAGCCGCAGATGCACCTTCCCATTCCTCCGTTACACCAAACATACTCATCCTACCCTCAAAAGGATACAACCCGTTTATTAATAAACATCTCAACTGACGGTCAGATAGAATATCGGAATTGTCACAGATCCGAACTTTGATACAAGTGATATGTTGAAGAACAATTCCGGAAGCCATAGCTTCCTGTACAACTTTCTCAAATCTCAGAGCGGACCCCGCCGAAGAAATCATCTTATCCTTAAACTGCAACATGCGCGCCAGTTCATCACCGGTGAATGCCATTACGATGTTTTTTCTCGTTAATGATTAAGATAATCGGCCATCCAAATATGTAAGAACTGCACAATCTTAGTTAAATTTATACGAAAAAATACTGCAACTCCGGAAATGGGTGTGGCATAATCTCTAAAAGCACCTT

CTCTGTTCAAATCACCTCACAGTCATGGAAACATTTTACAAAGGCAACATTTCTAACTTGACATTGCTAAAACTTTCGTGAGATTTTATTGACTGTCGTTCTATATCAAACGAATTATTTCCGCAGACAACTGCTATTGAATCATCACGGACGTTTTGAACGCTATGAAGTTCCATGTCGTGATTTTCAGCTTCGTAGTTGCAGTATTCTACTTCCATAATCACCATAGGCACTTCATCATTCCCTCTACGCCTTTTTAGCACCCTATTTGGATTCGATGAAGATCCCCCAGAAACAAAAAACTGAATATACCGCGTTAAAATATATAGAAACATACACCCAGCAGCAATTGGAATTACAATGAAAATCCACAACAGCTGCAATTTATTAAATTTTTTTCTGTGCGGCGCTGGAAAATTATGACGAGGAAACGTGTCCTCGGATTCCTTCACGGGATTCACAAAACCTTTGAAACTAAAAGCATCCTCTCTTGTTAAGAGAGCTGGGGTAACTAATAATTTATGCGTAATTTCACATGAAAACTCACGATGTGTCATGCTCAAAGCTCTACTCAATGCTCTTTCATAAGAGTATAGAAGTTGACCGGAAATTTCGGTTCTAATGAAATCGTCAGTCGTATGATCATCTGAAACTGTTTGGTTGTGAAGACTCCATTGAATGTTCGTTCCAATGGGCCGATATCGTCGAGCTGTACAGACGAGCAAACCTGTATCTCTCTTGCTGAAAACTTGCGGAGGACGATATTTAGAGTCTCCGAAAACAACCTTACCATAGAAATTTAAGATGTCTTCTACACAAAAACGTCTTGCATAAAACGACAATACGTGCAAAGAACGATTTTGTAAAAAACTGCTCACATTTATAAGATCGTGAACAAAACTACGATTCTCATTAAACAGATCTATCTGTACATAGGTTGTATTTCTCGCTAAAAAAACGTAGCTGCCAGACAACTGCGTATTATTGTTGACGAAGCAAACCAATTCGCTTACAGAAAAATTTTGAACTGAGACATACTGAAGCCCAGCGTGCAATCTATGAAATTCACGTATTACCACATGTCCTAAAAAACGGTCTACGGTAGAATTTTTTCCTTCTTCCAGGCTTCCCGCGAGGTCAAACAACGTCTCTCCAACAGCCGTATGGTTCCGCAAAGTCAAATCTCCATAAAGCCTCTCGTAAGCAAAAACGATATCGTTATCAATGCCGATAACGGTCTGAAGTTTACCGATTCCTTCAGCCAGAGTAGTCTTGATGTAAAGTTTAGCCGGCAACGAAGAAACGCATTGAAATAAACATGCGACAAAAACAGTTATCATTTTTAAATCTTTAATTCACGTCGAAGAAAGGTACGGCCCTTGAAGAAACAGTTAACCGCCGTAGTCAATAACTTATTACAAAAAGGCCACATGGAATTAACACTAACAAATATAAAGTATATTATCTCCTTCAGTCCTACAAAACAGGAAATACCCAGAATACCCACACAAACCGCCACCGCACCCATGAACATAAGGTTCACCATGATAGATGTTATCTCTGCGAATGCTAACATAGTCGAACCTTCGTTATAAATCTGAATAGACTTTTCGACATTCTTCTCCGGAAGCGTAACGATAACATTATCGCCACTCTCCTTATTCGTGCACATGCAGAAATATTTATCCCCTACCCACTTATCCAATGAAATAAGTCCCCACGTCACATCATCGCGAAACAAAGACGTATATGCACCGGACGATCTCATCTTTACCGTTTTCCATACCATATCAAACGTCAGCGGCTCCTCCGCAAAGATCTCGATCTTTCCCAGCTTGCTAACACCCCAACCTGAATATTCACAAACTGCAAATTCTCCTTCTGCGCCCAGCCACCTAGAGAACGAACATTTCAAATCATCAGCTCTGAACAATCTTTCTGAAGAAGATCTAATAACCGGCATAAAATTTAGTTTTAACTCCTCGTCGTAAGACAGAGCCATTCGTCTAAATTCGCCGTACAACTCAGATAGGACTTTAACGTCGTTATTGCACGAAGATATCAAGTACTGTCGATATGTCGAGAGTTCAGTCCAAAGACGTAACATCGTTCTGTCTTCACCCTGGAAATTCTTATCTGCAATCCAAGAGTTATTGCTACCATCGAGGAAAAAAGCAACACGTAGTACTTGTTTCTTGAGATTTGTTTCATTCATTCTACTGAGTCGTATCGATTTTTTATTAACAGAGCATTGGACTTGAATGCTAATAAATGTCATTTTTCCATGACCACATTTTTGAACACGCTTTCTTGCAAGATGCATGGTTGCACTACTCACGGTATTAAACAATAGTTTATGTACGTTATGAAAAACCTGGAAACATCGAAAAACCAAAAACTTTCCAAAGTCGTATTCAATTTGACTGTCTTCCATTCCTCCGTAAAACACTTGGTTGTTTACCGCCGCACTACCCCATAACTCCATTTTACCGCTTCTCCAATTTCTGTATGCAGATATCTCTAAGAGCATCTCCCCAGATCTAACCGCTTCACCGTTCCCTGCGCCAAGGGCCGGACAAGTGGAAGGATAGATTTCACCCCGGACGAACGTTAAGACGCATAAGAAAACAAAGCAGATCATCATGCTGCGTTTTCCTTCTCTTTTGCGATCACTAGCGCCCACTAACTCCAGCGATGCAATCTTTAATATAAGATCGCCGTCATACCACAGCGTCGAGGGGTTACGCCCACATATTAAACAAAGGATCCAAAGCAAACCAGCAAGAACAAAAAACGGCAACAGCAAAAAGGTAAACCGAGATGCAGTAGTAAACGAAGCGCTCATAGCGGGGTAAAAAGCGTCTTCTTTCTTTTCGGCAGAATGCTACAGAAATGAAGCAATCACCTCTCTCTGTAGTTTTCCTAGCGTAATCCACTACATACGTTTTAGTTCTAATTGACTCCATCACCGATTGATTTAGTAATTGTAACTGCACACCGCTCGGAAAGGAAACATTACACGTAGTATCATTGTCCACAAACACAACACCAATATTTTTATTGTATCGACATAATATCTTAGACCCCAGGGCAGTCTCGATAACGCATCGAGGGTTAAATAAAAAGGCTATATCTCTTTCGAAATAAGCATTAATAGGCACACTAGCCATCCGCCACCAAGCAGTTCCACTTCTCTGAGCCATTTTTACAAAAATCAACCAATGTCTTAACTCCGTTTCACTATGTGTTCCGATAAAAACTGAATTTTCATTGTTAATAATATGCAAAGACGGTATCACCGACACGTATAATACACTCACCCATGCTAAACTCCAACCCTGAGGCACCATAATCACTAGTAACAATCTATGGGAGCCTCATCTGCAAGTCTTTGCATCCCTCCTGATTTGCACATCCGGTTTATTCTCTTCATCCCAAAGCAAAGACCTCTGCTTGTCTTTCTTATACCAATTATACAAAATAACTATCACAATTATGAATGCCCCGACAAAACTAATCCCGCAAATCCAAATTACTAACGAGCTTAGAAAATCTTTAAAATACTCTTCATCTCCCTCATTTGTATCTGTTAACTCAGGGACTTGAACAGTATCAGTTTCCCAAAGAGCCTGAACCGTCTTTTTTGGTGTGGATTTCGTCGGCAAACCCTGAGGCGTCTTTTCAGACACATCGGAAAGAGTCGACGCTAAGATTACTGTGAAAAACGTCTCAATATCCTGCGCCTCATCACCCTTGACCGTTGGAAACGAACTTGTTGTAACATTTACATTATTGCCACTGGATCGATTTGTGGTTCCCACGGCTAGCGTAGAGGTCACCATAGAAGACTGGTTTAAATATGCCACAGAAGCTATTGATATTTCGGTAACATTTTTACCCCACGTTTCGGGATTCGCCGAAAACGTAGTTTCAGATTTCCCAGTTAAATAGTCGCTGGATGTCGATACATTCGTTTCCATATTTTTAGACGCAATTGAGGCAAATTCAGTTGCCGAAACTTCTGTAGTCGTTAGGTTGAGCATTTGCACCTCTTCACAGAGACACACGAGTAAAAAAGACAAGAACAACATGTTTTTGCGATTACCCAAGTGCAACGGTTTTTTAATCACACCCACACCAAGATAGCAGAATGAAAACAATCTTTTAGAACAATGTTCCGCATTAGTTTTTATAATACAACTCGACGAATTCAAATTAAAAACACAAATGCCGATCATAAGAAAACACGATTTTGCATTAAATTCAGACTTCCTCATACATTCAGATTTGTCATGTTAGTTTAATAGTCGATGCCTGCTATTCCTTTTAAACAATAGAGATTTTTCAACGTTCATGTTTCCGTTCCACATCGGGTCAGCAAATGCTTCTAAGACAGGTAATTCATTTTTTTCGACGATTCACATTGAAAACGTAAATAACAAAAGCAATAAACAAAATCATTGCGATAGCCAAGCCCGTCAAAACTAAAAAAGCAAACGTCTTTCGTCTCTTGTTCCACAAATTCCACGCTGAGCGAGATGACTGCGGAGGCGGTAAGCATTCAACAAACGATGTATCATTGCTAGCGTGCGGAGAAACCTGTCCGTACATGACGTCCATCATCAGCACCTCTGAATAGGACGGCGGCGGCGTCCGAGGGCGATCCATCCTCTTCCCGACGAAGATTCACGTTGAAATACACGGCGAGGACAAAATTTCTCCAAACGGACTTAAATTACTAAGATAATCGGAGTAGACGAGAAATGCGAGCACAAATGCGACTACGAGAGTGTATGTTCCTTCCCAGAGTTCCTCGGCACTTCTAGGAATGGGGAATAGAGTTTTGCTTATAAGCAACATGGTAAAACAATACAACCTACCCTCTTTCAATACAATTAATTCCCCTGACTAGAGACGTACATATCTTTAATAATCCTCGATCCCGGCGCGACCTCTGACAGTGCTCACGGAGAGGACAGAGGACGTCTCTAGAAAACATCTCATCCACCTGCTTAATATAGTCCGTATCTTGCGTAAACCATTGCGTATGCAACCGACGATTCTCATTATAACGCAGACATCCAATTTTTAAAAAATCTGACATATTGCGAGCTATAAAGAGAAGGCTCTCCTCGCGGTCGACGCAAAAAAGCATACCGTGGCCGTCAAACACTACTACGAGCTCCCTCCAGTCACCGAAAAAATTATGCTGTACAATAAACACTACATCTAGTAGACTCGTCTGCAGTTTCCTCCGCCACTGAATCAAATGTTTACTAGAAAACCCAGTCATGCTCAGATTGCAAAAGCGAACAAAGGTCTTGTACGGATACGTTTTCAAGGTAAAAAAAGCATTCTTTCTTTCATTTCGGTAATGTAAGAGCTCAGAAAACGATTTACTGTGAGCAATCATATCCAGTGCCACAGTACTGTCCAGTGCATAATCACACGTCTCATAAATCGGATAAAAAAACCGCAAACCATTAGAAAACAAATCGTGAAAAGAAGAAGCCACGTGATAAAGACTTCTGTCAAAAAAACCATTATACACATAGATGTGTCCCCGCGCCCCCTGAAGGACAATCGGTCCCCTGCAGGTTCTGGTTTTTGCCTTATGTATAACCCCGACCACAACCAATTCGCCTTCGCAACACATATAGGTCTTCGCCCAGTTTTGAACATCTTCACGTGAAATATCCGGAATGTTTTCTACACTATGTAGAGTCAACCGATAATCCTCGGGCCAGGGCAAAGCTAAAGTGGCGCCCTGGCGATTTTCGATAAAATTTACCAACAAATCGATGTCTTTGCTTGAAGACAAACCCATAACGATGAACTGCCCGGCTCAGAGCAAATAAACACAAATTTGAAAAACATTAATACTGCAAAAAATTTTATTGGTGTCCACCAAAACAAGCACGATAAATTGTCTAATCACACACCAAAATACATGCAACACACGACTAAACAAAAAAAGCCGTTCAATAAATCACCCGCATAAAAAGATCTGTCTTTCAGATCGCTGCTCAAAGTCAACATAATCGAACTAAACCCGAGACCTAAAAAGGCTTCAAAGATAATTCGATATAGGCAGACGTTCATCATCAGTCCCGACAAGATCGCCACCATTGCGCACGCAGCAGCTTTGAAAACATTTAGTTTAAAAAACACGTTGTTGACCACCGTCAGAAAAAAAAGATGATTAGATTTTGGCATACTCAAGAAGATTTTTCCGATCCAGAGTATCGTAAATAATAACAAAAATTCGACCGTAAACTTTACAAGGTATTTCAAATCAACTTCACGAAACGACGTTGACTTCAAAATCGGACGATATTTTTGCAAACTAATAAGAAATCCAGATGATCGTATCATCCATCCATATCTCAGAGTAAACGGCAGGAGAAACGCAGTCGTCGTCAAGACAGAATAATCATCGAACATTTGACTCTTAAAAAATAAAGACACCATCATCCCCGTAAACACAGCGGGGGAATAAGACGGCATAATCAGGACACAACGACTTAAAATCATTCTCAACCTCATCTCGGAAAAAATCAAGCAGATCATGACCACTGCAGACAAGAATGATGGCAGTTTCGCCGCCATTAAAGACAGAAACACAATCTTGAAATCACCTATGGGTTTATAAAAGCGTGGATCAAATTTTTTAAAAGTCAACAAAAAGGAAATCGCAACGGTTATCACAACGCCTATCTGTTCGAGGGAGGACCTCCCCTCTGTCATTCTAAACGGATACAGCCCCGGAATAACGGCACCCTTAGCAAGACCGAGAATAAAACTATCTGTCAGACGACGCATCTCTGTCTCTTAGGATTGGAGCTGTATTCAAAATGCATGGAATCGTCGCTCTCGCTATCTTCTTTCTCATTAAATAAGCTAGAGCCCGCAGTCCCGTTTTTAGTGGGAACCATGTAGTTGGTTATTTTATATTGCGTTTCCTGTTTTGATGCCAACGCACTTCCGTCTTCGTTCTTATGATTCCTATCCCCTTTTCCGTCATCTTGTTTACCCATTCGTTCAAAGCGAGATTCCTCTTTAAGCTGCTCCTGAGTTAAAAATGCTTCCACCGTCAACAACGTGCTTTTGGAAGTTAAGATCAATTTACAGTCTTTAGCGGCTACCGTACGTAAACTACACGACGTCTTGATTACAGCACACATACTTAAAGCCTGCTGTAAATTTTTGGCAGATAAATTAAATCGCATGTTATTAACATCGTAAAATATAACCTTATTGCTGTTTGAAAACTCCAGTTCATTCATCTCTGTCACGAATTTTATAGTCGGGGGGTTAACCTGTACTATAATGTGCGCCATGAAAGCGTCAGATTTACCAGAGCGTTTAGTTTTAGTTACGGGCGAAAGCCATTTTAACACCTCGGAAACGGTCGAATGATCAAGGTCAATCCTCAACGGCGATTTCCCGCTGTCTCGCACCACTTCTTGTCCATGCACACAGGGAACCGACGCCTGTGTACATGTATCGGAAGCCGTCACCAAAACCCTCGTATACAAATCACTATCATGCTGAATGTACAGCTTCGTAACCTCAGGATTCGAGATGATGTTCATAAAACTTTCAAAGAGTGGAATATGATTATTAATGGTTTTCGTTAAAAAGTGCTCCGTATCTGATAAAAACAAACATTCCGCCTGAATGGTCAGCTTTTGCACGAGATGATTTTTAGCAGACTGAATTATTATCGAAGGCTGCGGAGTGAAAGTCACTGTAGTATTCTCTTTTAACAGTTTTGTAAACGCTTTAAGCGGTTTATTAATAGTTTTCCAGCTTTTCATATGAAAAGCTAGGGTCGGCGGTTCTCTCAGTTCTCTATGTTCCCGATGATCACGGTGATCACGATGATGATCTCGACTACCGCGCTCCATCTCTGTCAAGAACGAAGTCCTAGCACCAACTCGCGCCTTCTGTGCTTTAAAAACAAATGAAATGACCAACACATTATATACTGTATTTTTTTGGCGCCAACATTTTATTACATCATTATAAAATGTTCTTTACGTGCATAACCACACCATCCGAGCATTTCCCGATCAACGGTAACGGATTTGATAAATCAAGGTGTTTATACATCTCAGAACTACACAAAACCTTATACATAGCGACTTTGAATCCAGTTTTATACAGCAACGGTAGCAAGTGTTTCAAACTCACAGTATCGTTGAAATAAAATACATTTGCCTGCCCCTGATCCAAAAAAGGCAACCCACTCTTATACATCTCAATCAAATCATTCAAAGGATAACTAAAACCATCTTTGAAAACAGCTAAATCATCCAACTCCTTGTCCGTAAATAGATTCGTGTCCACGTTTAAAACCCTATATCCCACAGTCTTCAAATATTGCTGATGCTTATTAAATCTGTTGAGCAACAGATTGTTATACACGGGAATATATAACGATTTTAATTCTGACGAATAAACCGAAGTGACATCATTTAAACCGTCTTTTCCAATTACGACTTTGATGTTTGACCGCCTGAGAATTTTATTGTCTTCCATCGGCCAGAACGATTCAGAAGAGTTGATGATATCGAATTCTTTCTGCATGGCAGTACCGGCAGTGAAATGAATATTGCGAACTCCGTGCATCAGCACATCTTTCCTTAGCGTTGTCCACAATTCCTCCGGAAGCGTGCATTCGACATTATCGAATTGATCAAAGTATAACATCCCCAGTGCGTATTTTGACCTGTCAAATAAATTGCATGGCTCTGCTCCTTTCATGCAACAATCCACACTCGTTCGAACACAAGTATAATAAATGTGTTCACACATCATTCGATATAATCGACAAGCATCTGGATGTGCATAGGATAACCCGACGGTCATGAAAACGGAATGAAGACCCGTAATACAGATACCTAGACTTCGTCCATCTACAATTCCCTCACGCAGAAAATCTTTATTATCTACAGCATAATCAATTACAGCATTAGCGACCAACACCATTTTTCGAACCACCCGCCTTAATTCATTAAACATAAAAAATTTACACTGCAAACGCACAAAATCAATCTCCTCGGTTAGCTCATCGCTTACTTCGTCACCCAAGAAATGCACCACATTAAGCAACATTCTCAAACAAGTGTTAATACCGTCTCTGACAGGTAAAACATCCAAAAAATCACCCCCGCAATACAAAGGCACATGAGGAATCATAGAATACTGATATACGTTTTTTCGGAAAATAAGTCCCATTCTGCCCCTTTTTAAACAACGGCAAATTTTTTCCACAAAACTAGCCATAGTAATTTTTGCATGATCTGTTGTCCGCTCAAACTCCAAATATTTTGTCGAGAAAGTAACTTCGTCACACTTTCCCAGTTCAAAAGCTATATTTTTATGAAAAAGAGACCACTGCGCTCTTTTTAGCTCGTACCTATCCATGAAAATCCCAGGAATGATCAAGCAAAATGTGACCCGAGAAGTCTCAGGTAGAACTTGATACAAGTCCAGCCACGTTAGTGCGGACACATGCCAGACTTCCACATAGACTGTCAACTGAACCGTCTTATCGGTATCACGTTCCACCACCTGAATTTGAGACAGTAATATCTCAAAAATATTCTTGGCTTCCTGTTGATAACGAGTCACATTCATGCTTATATTTGAACCGTTTAACAAAGCCGGAAAAAGATGCTCGCCGTTTACTGTTATAAAATCCGCAAGCGAATATAAACATGTATTGTAAATCGCCACGTCGAAAACCGTACTATTAGCCTTTTCCACGAGGCCCAAATTTGTCATACAAGAAAACGGTAAAACCCATTTTTGAGTAAAAAGGGAAAACGCCAATTCTTCGAATATCACGTAAGGATTCTCCTGATATATCTCCCGCAGATAACCATTTCTTAACACGATCTCTGTCAAAGTAGCACACATACGAAGATAGATGCATGGAATACTCTCTTCGACACCGTTTAAAGAATTGGAAATCTCCTCTAAAAAGCGAGATGCACCCAAAACTCCCGGCATATATTGGTTTTTCCAACTCACACTTACTTTATAGAGGACGCTACGAATAAAATCTCGATACTTCTTTACAAAGTTCAATACATTTGGCATAATCACATTAGGGCATGATAAAATTAAATCGTCCGGACAAAAGTCGTAAAAAATACTCGTCTTTAGATACCATCTTCCTAGCATTCGATCCACTCGTGGAACGCAATGTGTAGCTCTCAAAACAGAGATTATGGCATCCCGCAAACACCAAGCATCTAAACCTGAGTCGAGCCGATCCATTATCTTAGCGATGTCACGAACCCGTTCATACAAAACAGAAGCGCATGAATATTGAGAATAACAAAAACGTTTTTGCGACGCTTCGTAATGACAAACGCATTTTCTGTTGTAGCCAAAATCTTTATTAATACGACGCTCCTTTCTCTTCATCTTCAGACAGAAGGCTCAGAAGCTATACGAACACTTTTTTCTCAGAAAGGTCAACGAATATGTTCTCTAGCTTTATCACCGGCAGGGTAATTCCACGATACTGCAGAAAACCCGATTTAATGTCCTCCGTTGCGCAACGACCAATGCGCTGAACGCTTCCAAAGTAACTCAGCGTGGTATTGCTATTAATGAGATAGTTCAAAAACAGAAACCGCTCCTTTTCGAAATACTGTCGCAAGATATCAACTACCAGGTCCTCGTTCTGAAAACTGGTCAGAACTACGTAGATCCCATACTGGAGCTTTGATAAAACCTCTTTGTAAACATGAACCACGTAAACGTACTGGGTGGAATGCTGCCGCGGTTCCCAGTCTGGCTCTAGGAAAATCTCTTTTAATACCGTAAGCCCGTGAATGTTTTCTTTGTTAGCGTCATAGACATTTCCTTGCATGACGCATGACAACAGACAACTGCTGCCGCAAGAAACTAAGCTCGTCACACCAAACAAAGACACTTTGTCTTCCAGGTACTTACACGTGTTGATCATCGATAACACGTTATCAACCATTGAGTTTGTATACATGGTAGTTAAAGCGGTTCTCACGCAAGCAGACACATTGAAAGCGTTCGAACAAAGGAAAAACAGACCCACAAACAGAATTTTAGGGTTGTCCACATCAGTCTCAAAACGATACATCAACATTCCCAAATTTTCGAGCTCCGGAGCCAATAATTCGCAAGGCGTGAAAACCCTTGCCGCTATACGTAAATTCTCCCTGCCGGACAAAAACTGTTTCAGAAATTTCCTTTCTTTTTCTACAACATTATTTAAGGTTCCCGCCTCGATAGATATATATTTTCTCTTCTTCACAGCTTCCACAGTATCCACAATGGCAGCTCGTGCACTCGATTTAGAGTTCATTCTTAGTTCACTCAAAAACAGCACGTCCCCAGAATCATTACTAGCCGCTACGGCAAAAATCGAGATTTTATCCCTGGCAGCCGATAGCGTAACACACAACAGAGTAATAGCTTTTATTAATCGACTGCCCCCGAAAAAATATCACTTTGACCTTATCAGGGAATATACAGTTTTCTACTTTTTAAACTCTACGACCTTAACATCAGAAAATAAATTGCTCTCCGCAGAACTTTTACACGAAGAACTCAGTCAAATTCGCTCTTCGAGTTCGCCGGGCACAGAACTTGAAAACTTAAATAATGCAGAGGTCCTTTACGTGTTCGATCGAATACTCAATTCCATTAAAATGCTAAAAAATGAGTTATCGTCTCCAATCGGCAAACTTCGAGTACAACCCGCCGCCAACGACCCAGGTACCGATAAAACGATAAAATACTCCAAGATTCAATCCATCATACAAAAGGTACACAGCTTTACACGAGAAAATTCACTTCTTGCCAACTGCCGCGCCGTCGTAGAACTGATAGACGATCTATATCGGAAACTTTACTCATGGTTTCTACATATTCTCACTTTCGAGGACATTCAGTTTCCAGGCGACACATTTCTAGACAGGCTGTTGAAAATGGACTACTGTTTTACATATTACCCTTCTTCAAATCGCCATCTAATAGATCTTTTCGAAAAGACGTTGGACAATCAAACATCCACAGACATCGATAAATTTTTTGACACTTCAGGAAACAGTCCAGAACTACTTTATCAAAAAACGTTTAGCTTGAAAATATTTTCAAAAAATCTCACAGCCCAGGACAATGGATTATATATCTATCCACTACTTAAAACCGACCTGTCCATCCTCGACTTCCTCGGAACCGAAAATATTCTCTTCCATCGAGGTCTCATCTATCACATACTTCATCAAAAAACAATCCCTCAAGAACGGGAGAATGATCTCAATAAAATCAATCAATTTTTCGCTACTGTCATCCAGCAGGTTATCGAAACGAGAAGCTCATGCTTACCCGCGTCGTTATCCCGACTCTTAGATACGATTTTTCACTTCAACAGAATCGGTTTAAACATAGAAACGTGCAGAATCTACGTCGAGATCCTCTCAAACCACATGGCGACACCAGACACCCAACCCATAATCAATACATTCACCATCAACTTAATCCATATTGTTTTCACCGCGCACGTGTTTTTCATCTGCATGGAAAATTTCAGCCCGACATTCTTATTCTATAATAGGAAAAAACTCATATTAGAACAACAGAGAGCCATTTTGATCATCGAACGAAATGAATATTCCACACTCTGGAAACAAATATCTGATCACATCGACTGTCTCTTCAACATCTCTCTCTCCGAATCCTTTTTTAAAGAATATACCAAAGGAGGAAACGAAGAACATAAACAGTTTCTATACAAAAACCTATTCGAAAAATGGGGTGACGTCTTTTTCCCTTTCACGTACTCGGTAACCACCTCAAAAAACTCCACGGCACACCATATTACAACCCTTGAGCTAAGGGCCATCTGCAAAGAGGTCTATCAGAGCGATTCCCCGGAGGCATACGAATCGTTACTTCCCTACAGCACTCATCCAGCTTTTAAAACATTATTCATTAAAATCTACGTTATACCTATGGTCACATACATTACCAATCTTACCTTTGATAAATTACAATCTGATTACAGACTCATAACTCTGATCCACGCCTGTAAACTCCTACTCCCCTCGCAACATCTACTACTACACTACATGGTGTGGCTATACGCTTTTTCCATAAACGTAGACCACATAGATCTAGGCACTTTCACCGTAATAAAGTCCGTAATCTTTAAAATCGCTGACCACATAAACGTGATGACGCATACGATCTACTCCCCTGAGACAAACCTTCTCGTAAGCATTCTACTAAACGCGTATACCGACTACCTTCAAAAATACGTAAACCCCTGGATCAAACAGACCATAACGGCAAACTTCTCCCTGTTACAGACGTACATAACCTTCACGAAACAATGCGCCAGCATCTTAGCGACCAAATGCAACATAAACTTAGACAATCTATTTATCTCCATGACTATCGGAACAGACAAAATTGTAACCACTTCGTTTTGCTCCTTCATCGCTACATGCAGAAATTTAGTACGTCAACACGAAGAATTCAAAAAATCACTAAAAACGATCGAAACTTCCAAAACCACACTCACGAATATGCTACTAAACATAATCACAAGCGTCTCCTCCTCCAAGGAGCTGCTGACCAACGAAGCACTACAAAAATTCATCGACACTGTCCAACGCATCTCCCAACACGTAAACGAAACATACCAATTAATTTCCGTGAACCTCGAAAAATGTAAAATCTCAAACGACATCCTAATCGAATCCCTAAAGAAGACCATCTCCATAGTTGATGTACTCAGCTCCGATGCAATCCTAAACACGTCGTTAACTTCTAGATGTCTGGAGGCCGCCACGCTCGCGGTTTCAAACAATTCTTTCACAATACTCGAAATTAAAAAGGACGCAGTTGCCGTTTTCAAGCCTTTTATAACACAACTATTTGAAAGCATGAAACCCACCACGAGTCTATATAAGAAATTGATGGCTACCCAAAAACTGACCACCGACCGCATTCCATTTCTCGATATCTTCGACGATAGGTACAACCTAGTCAGACACGTCGAACGACAATTAAACTGGTACGCCGCATATGCCGAAGCAGCGCAGCAAGATCTTATTGCTCCTCTTACGTTTTAACGGTCGCGTTCTAACCCATGAAAATCATTACCAGTAGCACGAACCAAAATGACTCTAAATACGGACCGAGAGCCGGAAAACAATGCATGTCGAACAGCTTTTCATTTCTCCACACTGTTTACTTAAACGGAATTAACAATTCTCTCAATGCGGGTACTATAGACGCCATAATGGAAGAAGGCTATCACTTAGACACGGCCAGCACCTTAGCTCTAATGCTAGATAACTCAGATTCGCAAGACTACAGACTCCTCACCGAAATCCCCAGAAGGATCCATTCTAGGTACGGCGTCACACAGCATGAACTCTCACGGCCATTTAACGGAACCCTAGACACACAGAAAATTGACAATGAGGTCTATTTTGGCCTCATAGATTTCATACTGTATGGCAAAACCAAGAATTGTCCAGCTTTTGCAGTCATCACGATCGGCGTGCTATCGCGAGCAATTTTTTTTCTTAACAATACTCTCTACTTATTCGACTCTCACCCGACTGAACGAGAAGCCACAGCAGCCATCTACATCTGTCAAGACATTGAAGAAGCTTATGAACTGCTCACCGCCCACGGCACTGAAGGCTTCTACTACGACGCCAGCTTCATTTTTTTCATAGAAACCTCCAATTTGTCGCTCTCTAGTCACGACGCCGAGCTTCTTATCCTAAAAACTTATAAAGACCCCGACATAGCGATTACGCTAGATAAATTTTCTTCCACGGAAATCCATGATATAAAAAAAACAGATGATATCGGATCGCAACAAGACCTCGTTGCGGCTAAAACGACATATCTAGAACGCGCGCCCCAGAAAAGAAAGAAAAACTCTCATAGTCTAGAACTAGAACTAAATGACAAGAAGAAAAAAGATACCGCATCCTTGACATATTACGCAACGGAAGTCGACCTGATTCCGAGCTTTTATGAACTACGATCACAATTTCAATCTTTATTTCACGATCTCAAATCTTTTCCTATAATGAAATCTCAATTCAACTGGACCATATACTTACAAGATTCTCCCATGAATCCTAATCAGCCCTTCGCAACACCCTTTCTCTGGAACAGAGTTTTTCACTTATTGTGTCAAATCGTCGACGTGTTTGTAGGCGTCGGATCCACAAATGACGATTCTAGCAAACAAAAGCAGCAAACGATTTTCATAAATTATTTATTGCCTTTCAAGGACTTTTCCGAAGTATTCAACGAGGCGTTGACAGCTTGTCAAGAAAACAATCTGGACATCCTCTTGATTTACAATAATTACCTGTGCAAAACCACGACCTTTCGAACACTCGAGAGAATCTTACTAAGCAAATTTCTGGCAATAGCTGATAATGAGCACGAAAAACATTATGAATGGGTCAAATCGTGGACCACACAAATGCTCCAAGAGATGCCAAAGAAACTAGATGACATAGAGAATTATCTAAAAGCCTATGTGAGCCAGAACCCTGTGAAACACTTCCATGAATTCGTTTGTCTGAATAAGGCAGAAAAACACAACATAGCTGTTCTGCTCAACGAAAAACGAAAAGAGATTCAAGAAGATATTGAACGAGACAAGAATATTTTCGCACAACTCTCTAATTTTATTGACAAACTCGGCGAAACTCCCGCACTTCCTATCGAATCAGAAAACGTGCACAAAGTTCACACGAGCGACATCACTGAAGCCATCGTTCCGCGTTTCATGACTGAATCGATTGAATTACCAAACATCTCCACACTGAACAACACCCAACAATTATCCCTAGAAAAACAAATCAGTGAAAAACTCACCAACACCATCCACACACTCAGAAACAAATTCACAAAAATCGTACAAGATAACTATAACAATCTCGCTGCCGGTTTCATGCCAGTTACCGAACTGAATTGCCTATTTGCCTACCTGGTAAACCTTTATTTTAACATTGAAGTTTTAAAACACAGCGGCCTAAACATTAACACTGAGTTGCTTCAAGAAGTGGAAAAACTGTATGACAACACGCAATTTCTACGCTTCGGAACTTCTCACTTCAATATAAACAACCTTTCGAACTTTACCCTATCTATCAGAAAAATGTTCGTCGATTTCTATAACAGTCAAAAACCCTCGGACAGAGCTTCCGAAATCCTAGCCGCAATCGAATCCATCTTAGCGGACCCCAGCAAAAACAAGACAATCGTAAATATAGAAATGATCAAATCACAACTGGAAGAACTGGGGAAAATGGAAATCTCCACCACCGAAAACAAGCAAACAGCTGAAATCACAAAACAAATCCTTGGGGACCAAGAGTTAACCCCTATTTACGATTTCCTGCACCATCTCTCTGCTTATAACCTACCTAATACTACGACCGTAAAAAATTTACACCTCCATTTCATCTTAGAAAAACGACCAGATATCGCTGCGATTCTCCACGATAAAATACAATCCATACTTGACATATGTATAGACGACATGCTCAATGACATAACAGTCCCTGAGCAGACGTTTTCAACCGTTCTATTTCTTGTAGATCTCTTCCCAAACAGCACCGAAAAAACAGCACTGTTCGAATCCGTTCTCACACTACGGCAATTAGCAAAGAAATGCGCTAACTTAAAAACTCTAGAAGAATTCGACGATCTGGCCCAGTTCATCACGACGAACAGCGAACAACTCCAAAACATGATGAAACAGCATTTTGGAAAAAAAATACCAACGCTGATGGATCATATCAAATTTCTCTACTCTCAAAAAATAATCACCGCTGAAGAGAAAAATTGGATACAGAGAGCAAAAACAGCCGTCATTACATCTCCCGAAGAACTGACAGCCTTCTTAGCCACTGCACCCACTAAACACGCCTTACAAACTTGCAAGCCTGAGCTCGATAAAGCACTACAACGACATATGGAAGAGCAAATGAAACAAACTGCTGAAAACGACAAAAAACACATCCTCACAATCAGAAACACCCTCGAAAAAAGACTGAACGACATCTTACTCATTCTAAAAGACGGTCAATTCTCGTCTTTGGAAACAGTGCATTTGAATCTTTTAGAAACCTTTCTAAAACAATTGCAGGATAACGATCTCATCATTCACTTCACTCATGCACTACTGCCAGTGTTAAAGGACATAGAAACGACCATCAGCAAGACCATCTCCGACATTCTCGAAAAGATATTAATCAAAACTCCTCTAAACCCCGAACAGATGTCCAAGGAAGAACAAAAATATACCCCTTTACTCAGTTTCTTATCCAAGTTCAAAAAAACAACATTCTGCACAGAAGATGTAAAAACAGAGATCGACCAAATGCAAAAAAGTATCACGTTCCTCACGAAAATTGCTACCTCCACTAATAAATATACTAGAATCAGCCACTCCGTCTACGGACAAGAATTAAACCTCTACGAAGAACGCATCACAGAGCTGAAAAAAGAGACGAACAAAATAAAAGAACAGCTCTCCAAAGAATACGCAGTAGCTGAGAAAAAAATTCTTCTCTCGTCACAAGATGCAAAAACCAATAAAATTTATCTAGTCCTAAATACGCACACGCTGAAAGAAATAAAAAACACCCAATTCAGAGAAACGGCATTCGCTAAAGCTCTCACGGTCGAAGTCAACAACAAAGAAAATCAACTCCAGGAGCTGTTAAACCACTTTAACGCACACCTAAAGGCGAAAATGGATCAGAACCATATAACAAAACTTTCATTCGATACAAAATGGACAGCCTTTGTTTCGGATTCCAGGCTCTACTTCCCAGACTTCGTTGACATCAAACTGCAGGACTTTATTTCTGATCCCTTTAAAGTTATAAGTCAGCTGATGAACAAAGCAGCCAACGAGATGCCTTACATTCAAGCAGAGATAACTCTCAAATGGTTAACACAATTAGTACACGACATCAACAAATTCTGCCTGTCAGCTATAAGCGAATTCGGGAAAGAAGCAATCCCGTTCAATTACGCGGCCCTACGAGATTTGGAATACCAGATTAACACCAAGTACGTAGAAATTGAAAACAAAGTCATCTGCAACGAAACCGTCGAGAACACGAAAAACATTCCGAAACTGACTAAGCTCTTGAAGGAGTTAGATCCTAAACGCGTTGCCGGTGGCCAGAAACAGTATCAAACTCTCATGAACAAGATTCTCACTTCCGAAACTTCTATGCAACAGACCTACGAAAAAGAACAACTCAAAAAAGAATACTTCGAGACCGTCAATAATGTCGCCAGCTTCAAGTTAGCATTCAACTTTCCTAAGCAACGACAAAATGTGGAACGACTAATGGAGAAGTTCAAGTCGCTGCCCAAAGGCCAGCCATTTGAAAAATTTCCAGAGGAAAATGACCTATTTTCTGACTCACTCATCACAGAAAATTATATTAACGGTCTGCGCGCACTCCTCAACTTCATAACGGCTGCGCAAAACTACATCCAGAACACACTTCTGAAACAATGGGCGGTATTTCAGCAACAAAATTTCATCCCGATCGATTACTCAGTAGCGAATGTCAAGCCTATTTCGGATTTATATGCCCGCCTCAGGATTGAACGGGACCGTCAAGTGTTCTACCAAGTAAACTCTGTCTTTGGAACTCAACTAATTGTTGACGAAACAGGAGTTCCGCTGCAATTCCACAACATCTTTTACAACGCCGTAGTGAAGTTTTTCTCCCTGAACTACAAGCAGATTCATGTACCAGAAGACACCCCCCGCCTGGTATCTAGCCAATATAAACTCCTGTCCGTGTGTAAATCTTTCATAATTATACTACAGCAGTTCTGGGAGAATATAATCACTCTGGACCTAGGCCCGTACCTCAGAGACGGTACACAAAATTTTAAAAGAGAACTTATACCGATCGTGAACTTGAAATTATTTATATACATCATAACACAAGCCTGGACAGCATCTGAAGACAGTACTGTTTCCACAGCGTTCGAATTGCCCATCAAGCAATTCACACTTTTGATACTATGCTCACATCCTGAATATCTCTACGGCTGTCTCAGTCATCCAACAGATCTGGTTATAAATTCATTAGCCAAAAGCATAGACAAAGACAGTCTCTATGACACATTCGTCGTTAGCCACAACCCTCCCGAGAAGCCCATGCATTTGATGAGAAGTATATGTATCGACACCCAGCTATGGCAATCCGCCAAACTTATGAAAGATACCTTCCAACAAACATTTTTTACACAACTGTGCCCCCAAAACGAAAAGTTTTTTATCTATCTCACTGCCTTCTTAATTCTCCCCTACAAATTTCTGAACTATATTTGGATTCAATACAAACCGATCACTTTTACCCAAAGATCTTATCAAAACTTAATTAAGGACCTATGCTCCGAATACGTACACCAAAATAAGATCACCATGTCCTCGGTAACTCCCCACGAACCGGACACAATAAAATCCGGAGAAAAGATTACCTCAAAAATCACCGTACACAAAGCACAAAACACACCGACACTCACTCGCCTGCAGGCTCAGGAATATGTCTTCGACTACATTCTTTACTCTTTCTTGACGGGGTACGAAATGACATTCGCTATGTACATCGACACCATCGAAAAAACATACCTGCTTTGCATGAGACACCTGGAAAACGTACTCCACGATAAAGACTTCCAATCCGTCCTCAGAGCACGCACATTCGACATCAACTACATCCTGAAACAGTCATGGACTAAGAACATAGTCGAACATTCAATCTTTTCAGTACAGCTCAATAAAATCGTCTCCTACCTCAACCACACAAACAGAGCGACACCCAATATTCCCCTGATACTATTTAATTATGACAACGAAGTTGTCAACGTTTATCTACCGCCGATGTCCACTGATCCAAAAAAAGTCGCTTTCTACATAAAAAATCCTTTCCACTTTCCCGTGCAAGAATACGAAGCGACAGATTTAATTTCCTTTCATCTCTACCCAAAAACAACAGATATTTTAAACCAACTTCCGCCCAATAAAACTGTGTCTACTCGCCCATACAACCTTAGCTCTGAAACTTTAACCACCAAAAACCTCTCCGAACCAAAGTTTAAACAGCCTACCGTTACTGGACTTATGCCCAAGAGCCAATCAATCATCCTATCCACTGATACCAACGTACTGGAAACCAGTCCAGACATAAAAGCTAACACGGCCAGCGCAGCGATAAAAGATGTCACTCTAGCTCGAGAACAAATCAGCGAGTTCTCCGAATCGATAAATACAACTCTTTCAAAATTAAAATCTTTGTATCTGTAACATTCGACACACAAAGTCATGCTTCTTTTATAAATTTTTATTAGTTAAACATACGAAATAAATGAAGCTTTCATCAATCACTCCCTGTTTTTGTGTTCAGTCTATGCAGCGCCAGCATTCTTATCAAATCGAGTCGGAACGCCGTTTTATCCACGTTACCGCCGTTCTGCTTAGCGTATTTTGGCAAAAAGACCCCAAGGACGGGATGATTGGCCATGGTAGGCTGCAGCCCTAAAACACCGGTAAGCATCTGCTGCTTCTTCTTTTCCTCCTCTTTTTTTGAAGAACTTCCTGAAATCTGAGTGATCTGATTCGACAAATCATCACCACGTATGGTCGTCATTTCAAATACACCTCACGTTTTGGGAAATCTGACAACCTTGACAGATATTAACACAGTCTTCCAGATCTAAGCAAGTCTCTCTAAACGAATGCTGACAATGCTGACACTGAAAATCAGAACCGTGTGAAACAAGCCGTAACAACTTCTTGACGGTCACGTTGTAGAGATGCACAGTAGAATAACAGGATCTACACGAACAAGGAACAATAACATCGAACTTCACATTGTCATTTAAAATAGTGCAAGCAGAATTTGTTCCGATCACCGCCTTCCATGAAACAGTTTCCACCTTCATCCCCAATACACAATGCTCAGACATTTCATATATCCGCTCGAACACCAACTGCTGACTCCCACACAGCGAACAATACATTACATCCGTATGCATGCTGTATATCAAATTTTTCTCCTGCTTGTCTCTAAAATAGAACATGGAGCTCAACGAAAAGCCCTGCGGCGAACACAACTTTTCTTTTCCGGAGTTTAAACAGTGACCACATTTTGCACAAATAACGCAAATGTAACTCTCTTCAAGTATAAACCGAGAACATTTAGTCTTACAATAGCACATGACGGGTAACGAAATAACACTGGTAGCTAAGCCATCCTTTAAGGACGTTGCCAGGGCAAAGGTTAGAATCTTTGGAAAATCACCCCGTTCAGGTTTGCTCGGCACCATAGGTACTAGATTATTATGCTCCACTGGAATAAGATATCTGTCTGCGCTAAAACAGAAGTTTTCCACCAAAGCCTTCGCCATAGTAAACATGAAATTCCCCGCCGGAGATTTCAAAGCATACTTTTGCAACATGGAGGGAAATGACAAAACGTGCTCTCTCTGCCTATATCGCAACATCACCGTCTCGGTCTGCTCATAGTCACGTAAACAGAATAGCCTCATACACGGTAAGTTGACGTGCGTCGGAATCGGAAGCAACAACTGTTGCTCGAACAGAAACATGTGCAACTCATTGATTTCGGAAAACGAGGGCATCTCCAGCAACTTATTAAGCTTGTACGAGGGATAAACCCTAAATCTAGTCTCGACGTCCATCCATTTGATTCTTTTAAGTTTATAAAGAGACAACAATTCGAGAACTTTGCACTGCCGATACACGAAATACAGATAGGCGAGCAAAACAAAAAGACCCTTTGTCATAACCATTTTGTTAAAAAGTCGCTTAGTGATAAATCGTGTCTCCGGCCTGCAACGCAGCCAAATTGCAAAAAACTCATTGTAAAATTTTTCCGTTACTTCCTTATTCAGGAATTTTTTATTTGTCAAGAACATTGTAAGAACACATTTTCCTGGCACACAGGCGTCACCCATCACAAACAATTGCAAATGAGTGTACGGCCCATGTTTACACAACGGGGCCAGAAGCTGGCGTAGGAGTTTTTTTAAGTAGAACATAACAAAAATACAAGGCTAAAAGAAAGCAGATGCTGACAATCGCAACCCGACAATGCCTTTCACTTAGTCTCGAAAACTTCACACCGGACAGGAATGACTTTTCTTTTTCATAGAACTGGTTTATACTCTCCTGTGATGTCACAACCGCAGAAGCTTTATTTGCAATTTCTCCGCTACTTCCGAGTTGCCCCTTTTCCAAAACATGTGAAGACCTTCTTCGCAAACCTTGACAAAACCTATTCGATTCGAGATGCTGTTTCCCCATAGAACACTTCTTTAAGAAAATTGCCTTCAAAAGCGAAATGTATTCAGATTCGTTTTCAGGCCCAAACGCCACAAACTCCACAACGTAAGTTTCGTCTCGATTACGCCTAATTATACATTTCGTGAACACTGCAAAAGTTGAAGGCTTCATAGCCACTATGTCGCTCATGCGAACAGTCAAACATTCATTAACACGCAGAATGTTGCATTCTCTCACAGCATGATGGCCTTCTGCTTTAACCGGGGCATGCAAATAGCAAGACATCGACACAGCTCCGCCTGTGTTTTTGAACACAAATCTCGGTTCCTGCTTAGTTACATGTTCCCAGAGACTCAACAGATATTCTAGATTATAAGCGTAGTCTAACTTGAGAATAATATCACACAAAGGAAATTTCGGATTTTTCGACAATAAATTCCGCTCAGTCATCCTATAGTCATGAGACCCCAACTTTAAGATACGACAGGTCGCACTCAGTAGTTCATCGTACATCTTTTCTTTGAGTACGTTAGCCATTTCACCTATTTAAAAGCTATTCCGTATTTAAATATTCTTGATGTTCTAATATTCCATTTACATTTAACAGCTTTGACAATATCGAAGTGAGAAGTACCATTTTCTGAGCCATGACCTCTCGATGCACGCAATCGACATCCCCGTGAACGTATCTAGAAAAAGCAACGTGTCTATATCCAACACTAAGAAAATTCAGTCGCGCCTCCAAAGAAGGAAGCATCATTTCATATAATCGTGGCAGCATTGGATAAAACACGACCTCAAACTCCCTTCCCTGCTCACAGACTTGCTCATACAAAAGTTCAAGATTCGTCTTTTCAGGTGAGGAGTCCATCATGTACAAAGGATGGGACTCAGAAGCGTTGTCTATACAATCTGAGATAGTAAACGAGATTCTGTTATATTGCTACCTCACTCCCCAACCACCAATTCCTTCCGAGACGACAACAGCCACCAGCCCCACGAACATAGAAAACGAAATCTCCAATTTAGAAAGCTCTGAAAACCTAGAGGAATTAAAGCGATTATCCGTCGCATTAAATATAGACAGACGATGCAATATCTGCTCCATCGTAAATCTGTGCCTCAAACAAAACAAATCATGGATCTACGACTACAGTCTACTGTGCTACAAATGCAACTACGCGCCAAAGACTCCATTGTCTCTTCTGATTGTCTCCGCGGAATTTATAATGTTAATACGAGAACGATTTCCAAACATCAATTTTGACGGCCTTTTTCAGAACAACATTGTCTCCATCTTTGACTTCCACGTTCATTTTTTTATTCACAGATGCTTCGCAAATACGGTCAACGATCACATCCAAAGCGAAAACATAACCCTGAATCACATGGCCATAATCCGATCGACCCTACTAAAAGAAGACAGTATACCGCACATCAAAATAAAAAAATTTCTAACCAAAAAAATGAACCCGAAAAAAACACAAAGCCCCGAACTGAATAAAAAACTCACAGTGCCCATGAAGACTCGCTTCACGACACTCTTATTCTATATGTGGTCTGGCACAAACGTCTTCGACCGAGTTCCCTTCACGGACCTAACTATCCGCAAACATCGCTTCATCAAAAACCTGTATTCCAACAAAACCGACATCGAACTCACAGCTGGTCCCATTCTCTTGGCACAGATTCCATTTTCCATCACCAAAAACAAAACAACCAGCGTCTGTCTGCTATGTGAACTGATGGCAGCTTCCAAACAAGATTACCTATTTTTAAAATATCTACATCAAAGCATCATGGACTACTGTCAAAACAACCTAAAGATGATAGATAGAGTTCAGTTCGTCATTGCCGATATCTTCGAGAAGACAAAAATACACATGCACGTCAAGAATCTTTCCGACTACAGCAAGGCGATATTCGATAACGAATTCTCATTCTCCGATGATAACTTCACTCTCGATACCCACGTTTACCTGATACTCCGCCAGACTGGAACAGTGGGAGTCTATAAGCACTTCTTCTGTGATCCTCTGTGTCTGGCAAACTGTAAAACCATTAACCCGGAGGTTCTCTTCAACACTACAGACGCCGGAGAAATCCAAGACTTGAAAGTCACCATATGCTACCGTAACGAATATTTAAGCATCGTCGAAAAACATGTTTGGCTAGCGATACATCTTTTCAAAGCTTTTCAGATTATTAAACCAAACCATAAAAATAAAACTCAAATTGCAGAGTTTCTCAAAGACTTCACTAACTTATTAGCGCTTCACCACTTCGATATCGTCGATCCCATTTTCACCGTCAACTACTACGTTTAAAGAATGACTGTACACAAAAACCGTTTTCGACGATCGCGAAGCCTGTCCGTGACACACAGAATACAAAAACGACCTGATCACAGAGAAAAAACAAAGCTTTACTTACAACTGAAACTCCACGATCTACACGCCGTCTTCAACCTGTTTCCAGAATACGAACAGAAATTTCTAGCGATCATAAAACTGCCAATTACCGGTAAAGAACCCATCGACGTCCCATTCAGTCTCAGCAATCACCATCAACACACCTGCCTAGAATTCTCTCCCTACGCTAACGAACAAATCTCCAAAAGCGCCTGCCTACACTGCGAATCCGTCTCCGTTCCAACCTCCTCAGATGCAATGGTCGCCCACTTGAACCAAGTCACCAACGTCATGCAAAACAGATTTTACTTCTACGGCTTTCGAAAGGACATGGAGCTGATACGCATGTCCGCCAAACAACCCACTATCTTTCAAATCTTTTATATTGTCCACAACACGATCAACAACATATTCCCTATCATGTTCGAAAAAAAACAAAAGCTCGGAATGCACATAGTTTTCCAGTCTCGCACTCTGCACATTCCTTGTGAATGCATTAAACAAATAATAGCGGTATCTTCGGGCTACAACGTTTATCTAGATATACTACAAGACAGTGTAATCCTAACTGTCCTCTGCGAAACACTAGATACTAATACTAATATTCACATTGACATAGGGATGCTTCAAAAAAAGCTCGAAGAAATGGACATCCCGAACGAAATCAGCGATAGACTCGAAAAATACAAAGGACATCTAATAGGTTTCCATTAAACCACTTTATTGTTAACATGTACAAACGACAAAACCGAAATACGTTCACATTACCTCTGCAATAGCAGAAAACGTTTCATCCAAATACACTTTCATAGGAAGTAAATACAATAGTAACTTCTCTTTTTTTATATCGGTCTTCGGAAAAATGGGTGAAATCGCATTAGTCACAGCCTTGATAATCTGATCGAAATACTTCTCCGCATGTATAGGAATCTTGTGTTCTAACACATAGTTTGGATCTTCTGCTAATTCATAGTTATGCGTCTGTTTATTACCAGTAGATGGTGCTATTAACACATACATAATTCGGTCACCAACATTCGGAATTTCTTCCTTTCTCTGCGCCAACCTTCTAATGACGCTAAGGTGAGCCAGATTCGGCTGCTTATATGCAGCCACCTCCTTGGAAAGCACAGAAGACAACATCAAATGTCTCACGTCTGCCCGATTTTGAAAAAGCTCCTCCCGCGCTTTGCACAGACGACGCAAAATTTTATGAATACCCACAGGCACTCCTTGCTCACGCAACTGTGTTTGCGTCATGTGAGAAAACTCCACAGCAGCAGTCTGAACCTCTTCATCAAAGAACAACAAGTCCACGATGTCTTTCACCACACCTTTCACAAAATCGCAAGAAGTCTTTCTCACCAGATCTACCCCCTTAAAAATTAAAAGCGAGTCATCCTGTCTACCAATATATCTTTTTTTACAAATCAAAATGAGCGGACATAAAATCTTCTCGAACTCGAGCTTGATAGGCGATTTGAACAAGCGATCTGTGATATGTTTGGCGATCATCGGCGCAATCCTCCGCAGAGACTCATTAGCCATATTTCTGACAGACATAAAGATGCTATCCGTATCACCATAGATTACCTCCACTTTTAAATCACCAGTAAAATCTGATGCCGTTAGACCCAATTCTTCGCAAAAGAATTGCTCGGACTGCATCTTGGAATTAACATAATCCACCGTGGAACAAAGCATCTCTCTTCCAAGACAGGTTACAGAAGCAGCAATCGCAACACACGGCAATAACCCGTGCGCCGCTCCCGTGACACCGTACACCGAGTTACATGTTGTTTTGAGAGCGAGCTGCTTTTTATCCAGAAGGAGTTTCATCATTGGATCCGAACAGTTTTGCATCTCCGCCTTCACTTCTCGTCTCTTGGCCAGCCAGTCCTTTAGTAGACTTCCAAGCACAGACTCGCGAACACAAGGCTTCACAAACCGATGGGTCTCATCCCCCAACTTCACGGTTAAGATGTCACTCTCTGATAATCCAGCTATTTGCCGTTCATCCAAAACTAAGGTACTATAACACAGATTATGCGCCATCATAATGCTCGGATACAAACTCTGAAAATCAAACACCACAGTGGGCACAGCATAATAACCCGTCTTAGGCTCCAAGACGGTAGCCCCTTTGTAACCTATCCCTTGTCTATTATGCGAAGACACCATACTCGGAAGAATCATGTTACGACGTTTTGCTTCCGTAAGAATGCAGGGAAATATCTTCTTCTGCTGACCCTCGAACACCACACATCTAGCCGTGACGTGTGCCAATCTGGCGACCTCGGCAACCTCATAATGATAATTAATCTGTTTAAAGAGACGCACAACTAAGACAGAGTCCTGCAGACAATATTTACCGACAACAGCCCTGCCACTGGGTCCGCTAATAAATTTCTTTGGTATTTCCTTGTACGATAACTGCTCTTTTTCTTGCTGGAGACAGATCTTAGCAATAGTATCCAGTTTGTAATTCTGCGCCGTTATCTTACTAGAATAGACTGGATACATATCCAGATATAACACTCCGGAAGTAAACACCTTGGTTTGCGCCTGAAGGAACCCCTTCTTGTACTGTTCATGAGGAACAGAGATTCCAATCTTTCCATTCTTCAGTTTAGAAAAACAACCAATCTCATAGTGGTAAATCTTATCCATCCTTATACACAAATATTTTAAATCAAAATTGTTAATATTATAACCGGTAATAAACTCCGGAGACTCAATCCTTAAAAATATGAAAAATCCCAAAAGTAATTCAAACTCTGACGCAAATTCATATATATGCACGCCGTCAATTTGTTCACAGGTTCCCAGAGTAAACAGATGTCTCTCATCACGGTCGCCCTCAGTATCAAAACTAACTACAGAAATCTGAATAACTATATCACCCAAATTTTCGGCATCCGGGAAATTCCCATTTTGGCCCAAACACTCTATGTCGAAGGACCAGCATCCATATAAGGGCCAATTGACGTTTTCCAAAGAAACTAAATCCGAGACATGACAGTTAATTTCCACCTCAAGATTACTCCCTTTTCCCATATCTTGAGGAATATATTTTTTTACATTATACCATCCGAAACTCAAAAAACCATTATCGACGAAGAAACGAGTCAAAACATCTACATCGATTTCATAAACTACGAATCCCTCATTCTGCAGAATCTTCCCAATACGTTGAGATACATAAAAATTTCCAAAACTCACTTTAAACAGATTAACAACAGTGTTGGCATTGTACCCATACAATGACAACTTATCAGCCGGCTCAATGACAAAAGAACACGACATCTTAACCTCGCCGGTTAACATCAAATTGTTTATAGTAGCCTTCAGGCTTTTTCCGTCGACACATTCGCAGTAAAAATATTGCTCCTGTCCAAACACGTTCACACAAATCTTCTCACCGTCCTCAGATCTCCCAAACATCCTAATCACTGTCCCCGAAGGAATCACAAAATGTCTATACTGAAAAGGAAGATTCTCGATCGAATCCGTAAACATTAACGTTTCTACAGCATCATAGATGTGGAACTTCATCCTCGTATGATCATAATCCTTGGACCGAACTCTGTCTAAACTCGGCCAAGTCATTTCTTTGCTCAGTAAATACTGTCGGTCTCGATAAAACATACGCGGTTCAGAGTCACAAACATCCTTTATTAAGCCCGCAGCACCATCATGCATTATACCGCGAGGAAGTATACGAATGTAACTCGATCTGCTTTTTTTCTTTAAGCGATTCGCTTCCAAATATGGATTAAAAAATGACACCGAATCCATCACGCTTCTTCTACATTTACACTCTTATAACCTCTATACCTGATCCTGTCTATCAAACTAGGTTTTGAGGCATGAGACTCAGAAGACGAAGGTTTTCTTCTGTAGGACTCGTCTAAAGACTTTATGGCCTTGAGCATTTTTAAAGCATCTTCTTGTGAATATTCATCACCACTTATTTGTCCACTGACGTCAGCCATACCCTCCTCTTTTTCCGAAACCGCAACAGATGTGCCCCCGTCAGCATCTTTAACACTAGGCGTCTTGACGACAGTGGTCCCCGTAACACTGGACACAGTAGTCACCGGATTGGTGGCATAAGGAAACATCATGTCAATAGGCTTACTAAGCACATGTTTTTGTCTAACGAAAACCACAATTATTATAACGACAACTACTATCGCTAAAATCAACATGAGACCACCCCCGAATGGATTTTTTAAAAAAGACACCACTCCACCCACAATATCCCCAAGTGCTCCTGCCGTAACACTTATAACCGAGCCCAATCCAGTGCCTATAGCCCCAAGCCCTTGTAAAAAAGAATTAATCCCATTGACATACGACGGCGTATTAGTAGCAATTTTAGCTTCTATAGTATATAGTGCGCTCTTATATGAGTTATATTCACGAAGAATATTCTCTAAATCGAAAACGTTTGCTCTACTCAATTCGTCCGGCGAATATAAATCAAGTAGTTTAAAATCAGCATTTTCGAGAGGGTCGATCTTTAGTCTAATAAAAGCATCCAATACCTCAATGTCTTCTATGGGCGTCGAATTCGTATGCGTATAATCGGTATACACGTGTGCATGATTTCCAGATAGAAATATCTTTGTACTAGGTATCTCACATTCCTCTGTCCTATGATCACCCAACAGAATCTCATTATCTAACCCTAGCTGACCAAGGACAACCTCAGGCGTGGAGTTCACAAAGCTAAACGTCACCAAGGGCCGATTATAACACATCGTTTCACTCCTTACTCCCTTCGCATCGACGACCCGCATACTCTTATAAAGCTGAACGGATGATTGATTAACTTCTATGCATTTCGAGATAGCTAACACATCACCATGCAACTGTGCAGATATCGGACGACCGTAAACCTCAGACACGATACTCGATGGACTGATCTTACTAAGTTCGTGCAACATCGTTATCGTTCGTTTTTGATCGAGGCACCAAGATTCTGCCAAATTCCCCAAGGCATCGTTGATATAATCTTTCAAAGTATCATAGAGGTACTGTAATTGCACATAAAGAATATCATGTCTAGACTTGACATCCACCAAATCTCGCCTCCTACGTAAGTTTACTGAACCCTGCTCAAGAACCATAAGAGATTTTTGCACAAGAGGCTGCCAAATCAAAATCAGATCTCCCGTAGTCTTAAAAATTTGATAACTACCGTTCATGCTGTATGCATCATTATAATCTGACATATAGACATCTGTAATTATCTTCTCAAATTCATTCTTAATACACGTTTGTTTGGGATCGGTAAGATTTAAAGACTCCTTGGAGGCGACGAAAGCGGCTGTCAACTCCTTAGAAATAAAGTGATAAGTCTCATCCGTCTCCGCTCGAAGCCCGTGAGTCACCGTAGTCCAGTGCTTTAGCATACACACCGATTCATTCTCTTCCTTGATTTCCCAAGAAAACAAAGTATCCCCTTTCTCCAGAAAAGCGATCTTCCTTACTAACGTAGTAGCCCCATTCATACCATTCATTAGATCTTCTATCATAGTATAGTTTTCCAAGATTGTCAATTTTTCTAACGGCTCTGCAAAATGTTTACCGTTTGAACCGTCGAAGAACGGAGACCCTTCCACGATTTCACCAGTCGTCAAAGCAAAGTAACTAAACGGATATTTCGCCTTAGCCGTAGCCTCCGTCACAATACAATTGAGAGACGTCGATGTAGAATAGAGCCACAAAGGACCCCTTGCAAAGTAGGGTTCTTTCGTAGTGATAAATCTTTTATTAGTAACAGACTTGAAATTCAGAGGAAATAATTCTAGAGTTTCGTTTTTATTATTATCCTCATGATAGGCACTAAACACCGTACCATCGGGTCGTTTTATCGCTACGGCTGAATAACACTGCGCACGAGAATTAACTAAATTTGCTTCGTACACCGGCATGGCCAAACCCATCACCGTCCGATCCAGAAAATAAACCACACCCACATCACGGTAACTGGTTGGGAACGTCAGCTCGTTTTTATATGTTCTCACTGGAAAAGTGTAGGTCTCGATATTTGTTTTGTAAATGATGAAAAAACCCTCCGACATCTTTGCATTAGACTTATATGGCGAACACGAAATATCTCTGTCGAACCGCATCAAATCAGTGCCTTTGGCAATCGAACAAATCCGAAAAGGATATTTGTGATTATAGCCCGCTCTGATATAATCATCCGAATCGCAATATATCATTAAAACACTATTCATCAAAAAGACAGCCAGGAATAATACTCTCATCTTGCTCATTCTCCCTAACGAGATCTAGTAGATTAGGCGTTCGAACAGAGTAACTTACTCCTTCCGGCACGTCTACAGTCTCCCTCGACAACTGAGCTTGCAAATCACAATATAACAGCGAATACACGTCTTTAAACTTTGTCATGCCACCACCGGTGTGAAATAATAGAGGAGACTCGTGATTAAATGTCAAAACGACATTTCCATTCAAATCCTCCTCCTCATCTATACGTACAATTTTTAATCGCTTTCCGAAAACATCATTATATAGTGCAACAGAAAGTGTTAACTCTCTGATGAAATTCCACATTTTTTTCTGCATGTCATTTACATCAGTTACACCGGAAAAGTTAAAAAACTCATTATATTCGCACACCATCCACACACTGGGATGAATAGTGCCCTCTATGCATCTCGCTAAATCTTCCTTCATATGCGGTAACATTTCTGCGTGGTCGCAACCATACGCCATTGAGATATTCGCCGGAAGTGGATAGGCAGATTTATGAAAATCCGATAACGGACCCGTTAACAATGTGTACATAATCTCACTTAAATCCGGCAAAAGCTCCAAAGGGAGCTTCTTCGGCATCAGATTATTTTTAATATAAAGATGTTCATCGTATGTACAGCTATCTACACGAATAGAGCCATCCAAGTACATTTTTCTGAGAACGAAACCGTTCATAATCTTCGATAAAGCCTCAAAAATAAAATTCCCATACACGTTCACACTCACTACCTTATTGATGAGTGATTCCTGCTCTCGAATACATGCCATCACCCTATTATAACCAGATTCAGAAACCTTTTGAAAATACGCCTTTTTCCGAGCCAAGACTTCCTGCTGAAGATTATGAGTTTGAAGCATGCAATTGATCGTGTTCAGCCTTCCCGGTGTTTCTGTCGTCTGTGGATCCCGAATGTCATGCAACAGACTCTTAATTTTATGTTGAACCTCATCTTCCTCAGCCAATCTTTTAAATATTGGACTTTCCTCGAGATTTTTAATGCTCAGACCAGTGACAAGATCTATCAACCTCGAAGGAGACACATAAATAGAGCCAACGTACAAGCGCTCGTCATGCGTGAGCAAATTTTCACTCAAAGCCAAGAAGTCTTCAGAGACTTCACCGTAAAGATAAAGACTAACATCATTCTGTAATTGCTTATACAACGTGTGACAACTATTTAACTGCTGAAGCGTAATAGCAACATTTCTTACTATCGTTTCTGATATTTTAGACCAATACGTAAACTCACTTAAAGAATATATCACGTCAGGTACCTTGCTAAAAATATTATACTTTTTCAGAGTTTTCTCAGCATCACTAACATGCAACGATTCGGCTGAACGCTGCTGCTTAAAAATCTTCTTGATTTCAGTCACAACAGACTGAATAGTCCCATAGTTCAAAATGGCCTCTCCTAAGTCTTTTTCTATCGTCTCAATATTTTTTTCAATATTAAAAAAAGCTTTTTGCTCGGTCAAATGATTACAGAACTTTCCATGTAATCTTTTCCGAAGTGATTTTCCTTGGTTTGGAGTCAAAGTCAATTCTTCATAACACTTCATGCAAGTCTTGGTTTCGAGATAACTCTCCGGTTTCGCAACCATACATGCGCCACACATGATAGTTAGCAAGTCTATAATTTTTCCGCAAATCTGCAACCCTTCTTGGGGCAGAAGTAAAGCATATACGGAGTTGATCTTACTGATTAATTGTTCTATATCATTTAGCGTAGATAATTGCACGATAAACTCAACACCGTTATTTAATGAAATGTTTATTGACAAGTGCTCAGCACAACGTTTCAAACACAACACCTTTTCAAAATATTTTTTTCTATCCTGGTCTACCATAAAGCTTTCCAACGCTGCATCCAAAAGAAGCACCTGTTCATACATAGCTTTTAGCAGCACCTGCATATAAACCGTCAAAGCTGACGCACAAAGAAGATTCTGTTCTTTGATACCTTCAAAAAAAGTTTGATATAAATGCAGAATGACAATCCCATTCTTTTTGAAATTTGCCATATCAGACAATACTATTACAGGATCACAAAACTTTAAACATTCAAGGTCCAAAGCACATTCATTAAGCCTAGCGCACAACACACACAGAGATTGTAACGAATTCATACTCTGATTATAATCGGTTTTTCTTAGTCATCGGCTCATCCGTTTCGGTTTCCGTTTCATCACATTTTAATACACCGCTTGCTTGATCGTCCTCCTCCGCTAGAATTTCAGAAAAGTCATAGACCGGAGCATCGTTTGAAGACACCGCCTCAAATACCGACTGCAAATTAGCTATTGAAAAATCTCTCACATTCATATCAACTAAGTGATTCAGTTTAAGCATTATGGATGCCGCTAAAGCTTCCCTACCATCCACAAAAAAAAGCATATCATCCATAGTCGGCTTGTCCTCCCTATCCCTCATCACCTCAGCTATTATTAATAATTCTGGATCAATATCGTTAGTTAAATTCTCAACAATGCTGAGAACCTTGCCCTTGACCACTTCCGTATCAAACATAATTGTCTCACGCCGAACCTTTTTCACTATAACTTCTGAGAACTTTGTAGCAATAATCGTTTTCTGCCTCATAAATCTAAAATCTTGCAATGCAGAAGAGGTAGGGTTTAAGTTCCTATCCACGCCACTTCCACCTATCCAACCTAACTGGCCAAACTGAAAATATTCCCTATTAGCCAAGCTCACAAATTTCTCTAATGTCAAACCAAATGTAACCAAAGATCGACTTGTAAAAATTGTACCAAAATTGGGTTCTTTGGACAGTGACGCAATCACAGGATTCGGAACGCCATCCAAAACTAACTTTTTAACATAAACTAAATATTCCTGAATAGAAGAAAATTTAGGCTGCTTGTCTAACACCACAGGAAACAACACAGGATTCTGCGTAACCAGCTTAGATGTAAGACCATGTAAGCTAGTCAAGTAGTCATGAAAACCTAAAGCCGACAAAAACTTGTTATGAAAGTAACAATCTATAAAAGTTGAGAGACACTCTGGCTGTATGTCGATAATGTCTATCTCATCATAGGTATTCGTAATACTGAATGCAAATTTGATAAAAGTCTTAACTTCTTCGGGGTTACCAATATCTAAAGTTTTCGGAACATTGTTGAGCAGAAAACGTTGCCATAACTCTAAGCACGAAATTTTGACATTCGGAAACAATTTATCATGATATTTAAATAAAAGAAAGGAGATACAGCCAGACAAGGGATTCTTCTTGTGTTTCACGTTCTTTTTAAAATAAGGGTTGTTGTGTGCTGTCTTACTGGACTTGTTGAACGGTCTATTTTTTATTCTAAAATCTCGAAGACATTGCATAGACAAACGGCAAAGTTTCGCCTGAATCGTAGTTTTCACATATTTACCCGATTTATATAGATCGCAATCTAAGATCTGCTCCATATCAACATTCGAAGCGACTTTAACCGTTCTCGTATTCAGAAAACCCTTTTTGAGATAACTGCTGTGAAACGTTCCGTACAGTGATTGATACTGCTGAACGAGCCATTTAGAAATTGAATTACCAGTACAAGGTCTGTCGACAACATGACCTGAAGCCACTATCAATGCGAGGTTCTGCAAAACCGTTAAAATGACCTTATAATATGCAAACGTCAAAAAAGGAGAAAATGCAGTTGCATAAGGGGTCGTATCTACATTAAATGATTGTAGACAATTTTCTATCTGTTCTCTAGGCGTTTGTGTCCTTCTCATTTCAACAATGCATCTAGAAACACATTCCTCGATACATTGTGTCAATCCGTGGATTATAGAAACAAAATCTTTTTTACTTCGGACATTGAAAGTATCTTCGCCTGTTACAGGATCAATCAGAGAATTTTTTTTACAGTAATCGAAGATCTGAGATACAAATTTACCCCTGTCCAGTGAGAGCGTGTTTTGTTGATCTTTTCCAATCTCTTTGAGCTCGCTGACAGGCTTCCTGCCAAAAGAACCTAATATATCAACCTCGGCATAATACCGAGAAAACATGCTCATAACAAGTGGTTCCTTTTTAACATTTTTTGGTATTGCCGGCAAACGAGTACCAACCCGCACCATGGCAGTTCCAATACACGATTGACAACATTTCCCATCACAGAATTCACATAAATTCGCCGAACAATTTACCAGATGATTGTAAATTTCAGTGTACGCATTACCCGCGTTATAAACGCTCATACGATTTAAATTCCACACTACATAAGAAAGCAGCTGTGGGCAAGTCGCACAGGCATAAGCCAAGTGATTAGCTGAAAATTTGTCATCTTTGCCTGATGTCGAATCACATTTGATGATTTTTCTATTATCGTTATACACATCCTCAGTCAAAGACGACAACGCATTACAAAAAGACAAAGATCGCAGCAGTAATTCTTGAGTCATCACCGAATTAAACGCTTGTTTCACGTTTGGTATAAATGCCAATTTTGAGCAATATAAGATAGAATTTGGCGCAAAGATCAAAACGGCAAGATGACTAGACAGATGTAACTTTAAATTCGTGAGCTGCGCCATGCGTTCAGCGTGTGTCTCTGAGTTTCTTATGATTGGCCAGTCATTGAAATTCATTATCTGACTGGGGTCATACACCGAATCCAGGTATTCGGCTACATGACTAAACGATAATTCTGTCACAACGGAATCAACCAACATCAAAAAATCTTTCTCGACCGCTGAAATTTTCTGTCCGGAGATACCCAGATAAGTTTTGTGGGGTGCTAATTTGACAGTTTGCTCCCCATCCTGTGTAAATTGTTTCAGGCCAGCATCGATTAATTCCTTAGTATTAGAAAATCGTAACGTTGTACCCCATGATGTAAAAACATAATAAAAAAGCGTTTCACTTAACGCAGGCAGAAAAAATCCTCGTTCCTGAATAAATTCTGTACAGGAAGATAAAGAAATCGTTTCTTCAGTTTCAAATAAAGTTGCAGAGTATAGAGGAATTTTCACACACGAATACTCCCCAACTTGTACTTGAATTTGTTCTTCAACACAAGGAATTAACAGACCAGCGAACAACAACTCTTTGAAACCGTTTCCACACGCAACATGGCAAATAACAGAATCTGCATCTTTGCCGACAGCAGAACAAAGAGCTTTGATATCGATTACTTTGCGTGCTGTAGGCACCTCAAACGACTGCAAGTGAAATTTCTGTCTGGTTTGCTCACATAATCGAATAAGATCACCATGATCTTCGGCCATACCAACTAACTGCTCAGTTCCATGGAAAAAAAAACACACGGGCATAAAAGACGTAATTTTAGTTAGAATTGTGCCTCCAAAGTTAGTGATAGGTGTTTTAACCGTGGTCGAGAAATCGTTTTCTACAGTCAAATTCATTAACAACGGCGAAATAACTACAGGAGAATTTCTCTCCATTAACGATAAAACAGATAAAACATCCAAAAGTTCTTTCTCTTTTGGAAAAACATAGATCCAAGCAGCAGTAGAAACAGGAGCAGAAACCACCGTTTCGTTTTCATCAGCCATCTTTGTGGATTCGATCACAAAAACAAAGATAATGGGTTTTTGTGGTGAAATCCTTATATATTATGTTTGACGTAACATAACACGCGTCATCAAACATAAAAGTAAACCACAAGTTGAAATATACCGTTTTCTATATGAGTTTTACGGACAAAAGAAAAACGATTTTCTTATGCAAATATTTTCCACGCAGATGACATGACACGCCCCTTAATTTAAATTTATGTAAATCGTCGTCCACCTCAGGTACAATAGTATATTATATATATAGTTTTTTAATAAACTTATTGAGGACGGGAGAACGAAGGCGTGGCGTTTACGTCATAGCCTAATTATACATTCTCAGAACAGGATTTAAAAAGGCTGCGAGCGGCCGGCTGTTCAGAGGGACGCTGGGGTACGACTTGAGACGTTTGACTGAAAATGATCCTTCGTGTACTATTTTCTACAAAAAAATTAATTTGCCGGCGACAGTAAACTTTTCAGCGGAATTTCAAAAATTAATTCCACATGTAATTTAAGCATTTTAAAACGTATAACTCACAAGGTGAAAACATATTTACGAATACAGTAGTTTTCGTGATATTTTTTAAAAATTAATAAATTTTAAATCGTGTAAGTAATAAAGCATACTATTAGATTCTTCACGTTAACAGAGCAAGCTCTTCGAGGTTTTCAGTAGAATTTCAATATTTAATTCCACATGTAATTTAAGCATTTTAAAATGTAGAATTCACAAAGTGACAAAACATTCACAAATACAGTAGTTTTCACGGTATTTTTGAAATTAATAAATTTTAAATCGGGTAAATGGTAAAGCATGCTGTTAAATTCACGTTAACAGAGCAAGTTCTTCGAGGTTTGCTATGTGTTTATCAGTGAATGTATTTAGTATTTCAATTTTAGATCATGGTTCGGTAAAGATATAAGTCCGTGTAAAATTTTTTGGTTTAGTTTTCATATCTACTAGACTGTGAGGTGTTTGAAGTCGATGGTTTAGCTCATGTCAAAACGCAATGTCAAATTGTAATAAGTCGTTCATTTCGTGTTTAGAAATAAGGAATTGGTATTTAGAATAGCTTTGTCGCATTTTTAGCCGCGACGAGAAGGTTATGACGACGTTTTCGATTGCATTGTTTGAACCTTTTTCGGAGTTTGAATTTACCGTTTAAAGAAAAACAGTTTCACGAATAGAATCTAAAACAGGCTTCCAAATTTTAGTTAATATTTATTTCGAATTTCACTTGGGGTACAGAAAGCTTTGGCATCGAGATTTTTTTGATTTTAAAAAGAAATAGTTCAGAGTGTGACAGCAAAAACCTTTGTACAAAACATACTTGTAAGAATTTGTTTACAGTAAGATAGTTATTCTGAGTCAGAAGAATATGTGTTTAAGTTAGTTTCACAGGTGTCAGCTTCATAGTCTAAATCTAAGTCCATTTCAACACTTGATTCACTTTCATGTTTAGAGTTGACGTTTTCATTAATGTTAATAATCACAATTTCGTCTTGTTTTGCTTGATTGAAGTTTAGTTCTCTAGGATCTGGTATTTTGTTGTCGGTTTCCATTGATTCTTCTATGAGAATTTCGGTAGTTGTTTTTGTCGTTGTTAGGTTTGAGGTGTCTGGTTTTTCGACGCAAACTTTGGATTTAACCTTAGGCGTACCTTGGTTTTTATGTGAACGATGAGAGGTCTGAGGTAGGTCGCGACTTGTTGAATAGCTGCATGAAGGTTCCATGTCGACCTTTGGACAAGTAAAAGAAAGATTTCGGTATTCGTGAGGAAGATTTGGAAACAGCTTAAACTTTGGTGCGTCTTTGGCGAAATAAGTGCAGAAAAATAGACTTGGTTTCCAAGGAGCCATGATTCGTTCGATTCTATTTTGACAGCTAACATCATCGCATTTATGGTTGCTGAATTGCTTTCCGATGAGCCTGCTTATCAGACCGCATTCGTATCGCTCGGCTACACGTTCTGTGTCGAAATCGCGGATGAATTGCGGTTTCTTTGTTCGTATGATATGTTCTTTAATTTGTCGTAGTATTCGAGCGAACGTCACAACGATGGACATGGCTTGTTGAATTGGGTGTCTAAATGAGTCTGGAGTTTGTTGTTGTACTTCAAATTCGGAGTTTGGCATCTCTCGATCTAGTACAGGTTTACCGCAGAGTTGCCTGAGTTTTCGGTATTCGATGTCGCCGTGTCCGTTGTCGTTGAAAACACAGGTTAATTTCGCCATGTTGAGTTTGTTGAAAAATTCGAATTTCATGACGGTGATGGTATACATGAATAAGTCTTGTGGGTTGACCGGTAGGTTGTGCAGATCGTGAAATTTAAAATAACTGCAGGCTTCTTCTACTGTAGCAACTGTAAAAGCAAGACAAGGTGAGTTGGTCAGGGAGAATATCCGGTCGCCCATTAGTTGCTGATTTTCTTCTCGCGTGAGCGAGTCGAGACATTGTCGATAGCGAAGCAGCAGGTCGGTTTCCAGTATGTCATTAGTTAGGAGACAAATTCGTTCATGCGTCTGTGGAAATCTGTCTCGGATGCGTTGTTTTGGATCACGGGCGTCTCCGTTGTAAGGTCTCAGGCCAAGAGATTTTATCGAAAAGTTTGTGTCGGCAAAGGGGACTGTTGGAAGGTTAGGCAGAAAGGTGATTGCATTGTTTTGAGCGCGTTTTTCTTGTTGTTTCTCTTCGATGATGGTCTTCAGCTGTTCTAACTCTGTCAGACTTAGTGCGTCTAGTCTAGATTCATCCAGTTGTTTCAGAGGTGCGAGTTTTCCTCGAAAATTTTTGGCGAAATGGCTGATGAACTTGCTGGTTGGTCTATGCGTGATGGAACGTTTTATCGTTCTGAAGGCGGTTTGTTTATGACGATCATGTTGTGAGCGTTTTACTCCACGAGGATACATGTTTGCTTATAATAAATGAAATGACATAAATTGGCCTCTTTATATGTTTTTTGAGATGCTTTCCGTTGGCGCTTTTTTGCGGTTAAGTTGTGTGAAAGAACGAAGGACGGTGTTGCTAATGCTTTAATCTCCGGAAATGCTGTGATATGGCGCGATTTAGTTAGCATGTGCGTTGGAGTCAAATGGTAAATACTAAAAGAAGGTCTGGGTTTATAGGTAGTAAGTTAGTTTTTTAATTGCTCTAAGGCGATGGAACAATGTACTTGTTTGCTGTTGGACTGGCATTTAGTAGCAAAGCATGTGCTCCACAAGATGAGAATTAACCGATTTCCGTCGGCTTTGAGTTGTATGTAAACTAGGACGTTATCTCGATTTCCTCTATGTTGTCTGTTCAGGCAGCTAAAATCCGAAACTCTTCCAAATTGTGTTTTTTTGATCTGAAGGTTCTTTGAATCTATTTTGAGAAAAGTTACTTGTGAAAACTGTTGCGCTATCCTAGTCTCGTAATGAGTAATTAATTGCGTTTTAATGATTGGCCATCCGATTAATCTAATAAACGATAAAAGGGAGTCATCCGCTGATGTTGCATTGTTGTTAAAACCATGCGTGTTCAGGTACTTGGTGATTAGAGTGTCTGTAGCTATGTTAACTTTGTTCCACAGTTGTCTGGATTTTAAATCCATGAAACTTAGGTTTTCTTTAGGGCAGCCTTTATCCGTTATGCTATAGATTAATTCTTCGATGGGAGCCTTCTTAGTACCGTGATGTAGAAGGTTGTTTAAATCCATTTGCATCTGAATAAACGTCTTGCAAGAATTTCGGTAACCTTCTGGGACGATAAATATGGGAGTTAGCCGTCCATGTAGCATTTTCCCTTTGTCCATGTCTGTCTTGTACATATAGCCTATTCTGATGCAGTGACCTGTGTGGTAAATGCCGATGTCGAAACATTCTCCGGGAAATGTTAGGGAATTGAGGATCTGACTAAGCTCTTGATCTAAACACATAACGTGATTTAAAATCTTGGATAATTGTTTTAATGGTTCTCCCCCGGCGATCGCTGTGCCATTAGGTAATGGGATTGATATTCTAAGACCGATCTTTTTCCGACAAATACAGAATTGTTTGATTTCGGTTGGATCTTCAGTATAGTCTAGTGCGTCGTTAGTCGTGTCGCACTGGGTTTTGAAGAAGAAGATGGGATGTGTGTGTGTGTCGATGCTTGGAAAAATTTTTTTCCAGGCGTTGATGAGGGTAAGTCTTATGAGGCGACACATCGAGAAAAATGTTTCCTCCGTAATGGTTGCTGTATCTTGCAGCGGAAGATCCAAGTCTCCTATGTAATTTGTGACTGGTATGCGTTCGTTGAATATTTCGTGTCGTGTAGTATAGAATTGTTTTTCGAAGTCGTCGCAGAGAAAATGTGTGTCTTGTAGCCAGATGGCGCGAGTTAGAGCTTCATCAGAGATATATTCGTCGGGGAGATACTGTAAGATGCGGTTAAATCCCATGTTTTCGTACCAATCTTCAGAGTTCCCGAAGCAAAAAATATTTTTATGTAAAAGCTGCACTCTGAATACAGGAAAGGGACGGTGATAATCTTTGTGTAGGTGTTGTCTTCTGTAGATGCGCTTATCTTGTATCTGAACGTATTTATCGGAAGCGGAGATTTCTAATAATCCTTGTAATCCTGGTTTTACTGGAGAAAAAAGACCTTCTCCGTTTTTATTACATTTCGCTATGGCACGCGTTATTTGTTCGGAAGTTAATGCTTGGATGGTCTTCTTTTTAGAGGCCAGAGCATACTCGTATTCTGTAACGTTTAGATCTGGATTTTTCAGTCTTTTTATATCGATGTATGTTTTAAAGTAACAATCCGCATTGAAGTATTGCTTCATGATACACAGTAAATTTTCGTGCAGGGAATTTCCAAGAATGTGGACATTTCTCTGGCATTGTTGTCCTATTGGTAAATTAGAGTCGTAAGAAGTGATATTGCAGTAATTGACGAATTTTGTCGTCTCTAGGGCCGTGTTGTAAGCGATATATATGTAATGGACACGGAATGTGTTGGTTAGATGTGAACTCTTTCTAAATAGATTGATTTGAGTGTCTAGATATTTAAATTCTTCCTTATCGCGGAGATTTTTTTGTCTGGCGTACGTTGCGAAGTCTGCAAATAGTTTTCTAAATTTTGATTTCAAAGTAATGTTTATAAATTCGGACATGGAAGAATACGTGAACACCATGCTTCCTGTGTTTTCCGTAAAGCATTGAACATAATTTTTGGTAGTTGTGATGGTGCTCTTTTGTTCCTCGAACAAATAGTAATACATCGTTAATAAAAGCAGACCTTCTGTTTGCCCGTAGGTCGATAGAAACCAAAAGGGGGAAGTGGGCAATAGGAATTTACCGGTGAGATATTTGATAATGAGCGATTTGTGGAAATAGGCCATGTATTTAAATTCGACGTCCTCTTTTTCTTTTCCAGTTTGAGATAATATCATGTCGACATATAACAATTCGGTTTCTTTGTCGTTGAGGCTGTTGGCAACATCTTCAGCCTTTATCGGACTTGCAGTTTGTATGTAGTGGCTGAGATTTAATTTCTCGGCGTGGCAGACGAATACCGTGTCGATTCGCTTTGAATTTTTGCATTTTTGAGTTTGAAGGCAGAAGCTGACGTTATTTGATGGTTTATATTTAACAATAATCGGAAAGATTAAATGTTCACTGGGACTTCTACATAGGATACTAAAAATGACGTTTGCGGCATCGTATTCAGTCGCAAACACTACGATGGTCATTGTGTAGAGCTCTGTTGATTACAGATGAATTTGGCTTTAAATGCCCTTAGGGTATTGGGGTGTTATGGGAAATTGTTTTCGACGATTATCTCTGTTTGGACAGCCGACTCAGGCAAATTATGAGATGTTGTGCTCTTCCGACGATTTAGAAAGTGAAGAGCTGACGTATTTTCTGGATATGACGTATAAAGATTTTGGAGTTTATCAGAATGATATTATAAGTCATCAAAAAGACACGGAAACTATGAAAACTCTGTTAGGTTTATTACCAATGTACAAAAAAACTAAGCTCAGGCATACCATTATGGAGCGATGTTTATCCAATTGTCCTAACCACGTTAAAGATGCACTCTGTGTAGAGCTTATGAAAGCTGAAAAAATATTACAAACGATGGATGTCGTTTTTATGAAAACTTTAATTGGCGAGTTTTCTATGTGCACCGATAATCTTAATCAGTTGCTTAACAAATTCGCTACAGATCAGTCAACACTTAGCGATGTCGAGAAAATAAACAGTCTCATAGAGATTGATGGCGAAAACAGCAAGCGTCTTTTAGTAGAGTTGGATCCGATTCTCCATGAGGAGACAGGATTATATCAAGCGCTGCCAAATGTTGTGACGGAAGCTCCGAGTGAAAAGGTTAAATCGATACACGTCGAGTCTGAAGGTGAGAGTGTATGGTCAAGCGTTACTGAAGGCGGCATAATGAAACAAGAAAAAGGAACTGGCGTCTAGTACCTGTAGCCGGTTTATTGCGCGTTCAGGCATTAAACTGAGGCAGAAATGTCCGCAAGTAAAAATTATTCTGGCGATGCAGATGCCTGTAGGGATGTAGATAGTTTTGCGTGTAGAGTTGAAGATCTTTATGGAGGGAATTGTGTCTTTGTTCCAGATGTAGGTTTCAATCAATAAGCCGAGTTTGATAATTTCTTTGGAGGGAATCATGAGAGCCTTTAGTTCATCGGCGCAAACGTACATGGCGTCGAAGCATTGTATCGTATAGCTGTTCGGTTTAATTATAAATTCTCTGCATGTCTTTAGATGGAAAACTGCAGATTCGGGGGGAGACAGATTGATCATCTCCCATGGTTCCGGGATGTAGCAAGGGAGAAAGTTGATTGAGAATTCGACATCTCCTGGGAGAAGCATTAGGGAGTGCTCGGATAAGTTCATTAGAAAAACTGAAATGTCGGTTTCGTCGGTTATAATTTCAGTGATGCATTCGCAGAATACGTCGTTGCTATGGCCAATAATGACTCCGAAGAAACCATCCGGGATGCACAGGGAAATATCTAGTTTTACGATTCGAACTTGATCGTGCGGCACCCATATGAGTTCTCTATTTACGAGTGTGATTTTGTTGGCGTCCATAAAAACGTCCATTTTACTATTCCTGTGATGGCTTACTAAGATAGATGGTTCTTCCGAAATACAAGTGTCCGTATTTGCATTTCTTTTTGCGCGGTGTCTTGGGTAGAAAGTTACTGGGTTGCCATGTTTCCTTACATAGTTAACACAATCGGTTTCTCTTTGCTTTTCTCTTTTGCAGGTCTTCGACACTGTTCCATTTTGCGGAATACCTAGAAGCGAGATTGGGCGTTGATAAGAGGTTAGTTCGTTTTCTAGAAATTTTTTAAATGATTTCTCATTTATAGCTGGCCACACATCGTGCGTTTTGTCTTCAGATATGTCTGTAGTCCAAAGTTCATTGATGTCCACGTTTCGAAAGACGAAAAATTCAATGTATCTTGAGCTCGTTTGTCTCTGTAGGGTAATCGTTTCAGAGATCTTTTCTGAAATTACGCTGTACATTTTTCCAGGTGACAGAGGGCTGTCTGCTGTTAATCACGTCGACGCGAAGACTAGTTCAAAATAACACTTAAGATGAGTTGTAAGAAAGGCGCAAGGCAACGCTTATATGTCAGTCTCTGGCTCTTTTACATTCTTGTTTTTGCGGCCGCTACGGAAATGGACTTTTATTCTTCAGAGTGCCATTCGCACACCTATGAGATCGTCTTGAATTCATTTTCTTCGATCTGGCTTTTGATAAATCTTTTTTTACTCTTGTGTTCTTTTGCGATTTTCTTGAAGTACTGGTGTTATAAGACTTTTGCTTCAGAGACGGTGAAGGGCTATTAGATCGCATGAGACGCCTATTCTAATTATAAATGTAGCACACGACAAATAAAAGCATTTTTTGATGTAATTGGATCGTGTTTTATTATCTCCAATAGATTTCATAACGACAAAAGACAAACCTGATTTCAGAATTGAGTTTTTATTGAAGACTGAGGATGAGTAGCTTTGACAAACGTATGCGGTTATGTGCTATCTGTGTTGTTTAAATCGTGTCGTTCGTTGTATGTGTAATGCGTTGCATTTTGGGTTGAGTATGCTTTGGTAGTGAGGTTATCTGGATGGTTGTGTGAGTTTTTATCGGTGACGCGGTAGTCGTTTCCAGGCAGAGTTTTGTTAGCTTCCCGACTTTCTGTGGAACTTTTTTTTTTAACGTCCGTAGATCGTTCCGTGATTGTTTGGGGGTAGATGTTAGTCGTTTGTGACGAGACGTGAGCTTTTGTTAGTTTGGCTGATTGAGTTCTGTTGCTAGTCGTTAGCTGTGTTGTGATATGATTTGTCGACCTTAGACTTGTATTCGTTAATGGTTCCTTTGGCGTTGTTAGTAAGTTTGACCAGGTTAGAATGGCAGACTTTGAAGATGTCGTTAAGGACGTTTCAGCGGTGAAGGGCCATTGAGGTGTAGTCGATTTTTCCCATGTGTGAGCGTTAAGAATTGGTGTCATTCGTGGTATTTCTGTGTGGTTGATCTCTCCGTGAGTGGACGCCGACATTCTATTTTGCTTTGGTTCTTCGGTGACTTTAGTGATTGAGTGTGATGAATTACTAGATGGTAAAGCATTGTTAGTTACCTGTACTTCAGTTTTGGCAGTTACATTTGACGGTAGTTTTTTTTTTGGAGTGACAGACAACGTCTGAACGGTAGGTGGCTCGCTTGAGGATTTCAAAGTTGTCCTCTGGAATCTGTCCTCTTTGAAGGTTTTGACGGTCTGCACTTCTGTGCTATGGGTTTCGTTTGCGTATTCTGTTTGGTTTTTTACGTTGAGAAGTTGGGTAGGATTTGTGGGTTCTTTAGGCGTGATTCCCATCGTTTCTCCAAGAGAAATTTTAATAAAATTTTCGGTCATGTTTTGTACTTCCGTGTTGTGGCTCATGTATGTGCGCGCCGCGTCTTGCCAAGTGGTAAACTTTAAAATCGGAGTGGTGTGTTCGGTGTCGGTGAATTTTGTCGTGCTCTGCTGTGATTTGCTCTTAAGATAAATAGAGTTTTTTGGTGAAGAATAGATATTGTCGCTAATTTCCTCGATTCCTATTGTTGTTGCGAAGGTGGTGCTTTCGAGCTTTTGAGTCGTGTTTGTTGCCACTGTAGTGCTCTCAAACGTTTCGGGAATTCTGATTGTGGTATTTTCAAAATTTGTTGGTTTTGGTGGACTTTTTGGGTTTTCGGTAGGGTTTTCGGTGTTTTGTTCGGTTTTAATAGTGGCGTTACGACGGATTGTAAGAGTTGGAATGTCAGTCGGGGTGTACATCGGTCGAGGAAAATCTGAACGTTTAACTACTTTTTTGATGCTTTCCGTTGTTTGCGCTGTTATGGTGGGTGTGTTCGGTGTAACTTTTATGGTGTCTGTCTGCAATTCTGTAAAGTTTTTTGTTACGGGGGAAGGTTCTGGGGTTGGTGTGTTGGTACTAGGGATATGTGGTGATGTGATAGACGCTGACGTCGATGATGGCGTCGATGATGGCGTCGTAGTCAGTATCGAATGAAAGTTTACGATGGCTCGTCCAAAGGAAGTGGTAACATTTATAGGTTTTGGCGAATGTTCGAAATATTGATCATTTGAACGGATGCATGATGTTGTGTTATAGAGCAAGAAGTAAACAAAGCTGGTGTAATTAAAGAAGATTTCAGGAGTGAACGGAGTCAATGACCGTGTCAGATTCGAGTTGGAAAATTTACTGATTGCCATCTGCAAGGATTCGTCGGTCAGTTTTATGTCAAATAACTGTGTTCCCAGAATTTTAAACGGTAGTAGCTCTACGCATGGTGGATCTGCGGTATATGTAGATAGTGTGGCAAACAGAAGATAAAATAGAGAAAAACTGCTAGCGACGATAGATTTGTTTGCAGCTGTTGAGGATAGCACTATTTTGGTTTCTGCCCGCGGTAAATTCTGTAATGGAGACAAGTTAACCGCAGACTTGCATTGTAAATCTCGAATGTTCCAAGAGAAAACGTTAGTTAAAATTTGAGTGAAGCAGTCGAGGGATAGATTTCTCTGACAAAAAGTTGTATACAAGTTTCGGAGGTCGTAAAAATGTTCTCTTTGTTCGTCGCTCCAATTGTGGCGGTTGACGGTTTTGAAAGCTTCTAAGGGATCAGATGTGTATTTTATTAGATTAACGGAGTGCATGACTATCGCGAAAAGGGCCAGAAAAAGGCCGAGTCGCGAGATGTGAAGCATGTCGGGTGAGTTGACCACAGTGAATTTTTACAAGGAGAATGTGTAACTAAAATAGTATTTGCTTTTTTTATAGGGTTGGACGATGACCTCCAAATGCATGCTGTGCTGGACTGCGATTGTGAATGCCCTGGCTATCTTAATGTTCTTACTAGTAATTTATCGGGCGCTAATCGGATTTAATGACGATATTTTCACGAGCACGTTGTCGGCGTTTAGCTGTATACGGACAAATCTATCTAATGCCAAATATAAATAAAACGTTTATTCAACACAATCTGATGAGTCTATATAAGCCGAATAAAGCAATTATTGCGATCAGAACATAAATGATAACCAATATGATAGATACTTGGTCTATATCGATTCCGACTTCAGACATCTCAAAAACAGAGCCGTTTTTGGCTAACAAAAGGTAGTGCGTCCTGGTGTTGGGAACAAGTAAATTGTTGTTGGGATCGGTCAGTGTTTTTAGTTCGTCTATGTTTTTTATGTAGATGTATTGTAAGGGACCGTCGATATCATCATATTCCATGACTACGCTCTGACAAAACTCGCATGTTTGAGATGATATGTTTCGTAGCACAAGCAGATCTTGTCCTGCATATTTATAATTTGTTGAAACGCAGGTAGAATTGAGAGGTATGGCTGTGATTATTATACTCGTAGCCACGATTGTCGTTGTAATTGTTAAACTCACTCCTTTAATCACATAAGAGGGAGAAATTACAAATGTCACATTCGGCAGAGGCAGAGAAGCTGCTGGCTCACTTGGAGAGAGACACTGAATGGCCGCTACTCTCGCCATATCTGGCGGTCTGTATACGGATAGCATCTCCATCATGTCTGATCGATAACGGTCGAGGGAAGCGTACGTGAGAACTGTTTTTACAAAAGAATTTGTTACATCTCTGCGTACGGGAGAGGCACAAGGCGAATAAATATTTTCTAGATTCGGTGCCTTGTCAGCTGTAAGCATGTGGGCCCATGAAACTATTTCTACGGGGTTACACAGGTTTCCAATTAGTAGTTGCATGTGCCTCTGCATGCGTTCATCGGCAGGGATGAAATTCGCTATTTCGGCTAGCATGAATAGAGTTTCCCTATCGGAAAATGTCAGATTCGTAAAAAACATGTGTTTTTCATAGATCTTTTGTAGAGTGTTGGCAATGTCAGACCACTTGTACCTGGAAATCGTTACATTGGTTTCGTACGCTGTCGCTAAGTACGTGAGCAGCGTAGATACGGGAATGGATTTGTTTTTGGTGATGTAGCTATAGTCCGAGGGCTCGTATTTTAAAAATTCCTTCATTTTTGGGTATTGTATCGTACCTTCCGAGACGTTCACACATCGGTTGACAAATGAGTCGATGGACAAGAGATTTTCGTGTGTCTTTAACACTCTTAACACGGGCATCGGTCCTGTGTTTTTTGTGCTATATGAGAAATGTACGATACCGTAAAGAAACATCATCTGCGGTATGCCGGCATAGTCGGGTTTTTTACAGTCTCCCCTTAATAGGGTTTGGATTCCTAGGTTATTAAATTTGAGTAGTAGAGCTTCTACTTCATCTACGTCATCCAACGTTTCGTCTACGAATGTCGCATCTGATATGAAACGGTATTCTTCATCGACTACGTCTTTTTCGACTATCAATAGTAGATCGTCTGTCAATGTCTGTTTGAATATGAATGAACTTTTCCTATAAGGGGCTTTAAAGTTTATGTCGGTGGTATTTCCATACATCATAGTCAGATCTCCTGCGGGATTTTGGATGTACAGTATCATAAAGTCCGGAGTAAAGAAATGGGTATAGATGTTTTCGCCCGCATAGCGTGTAATTTTAGCGCATCTAACTTGGACTGGAAAGATCAGTTCGTTGAGAGAATTAGGCAGCTTACAGGAAACGTTGTCGTATTGCATCTGTCGGACTTCCACAACCCTGATGTAATAAATTCCGTGTGAAGGGTACAGGTTTAAGGAATAGACAATCTGCGGTTGGATAGAGTGATCCAGGTTAAATTGCTTGACCGCAGGTACGGTAGAAAGTCTTAAAATATCGTTCATTGGCGGCTCATAGAAACGTGTGACGCGTTTTTCATAATTGGTTAACGATTCTGTCGTGTTGATGGCATCAAACAGATGGTACGTGATATCGGATCCGAGTAAGCATTTAGGGACTTGATATATCCGAGTGTCGTTTTTTGTATAAAAGTTAAAAGTTATAAAGCCCGGGCGAACAATTGGATTTTCAGAGTTTCCATTTTTACAATGGCTCTCGTCCGATATGGTCCACGGTCTCCAACTGTAACAGGGAGTCAACAGAACAAAGACCCAGAGTCGGAAGAGCATAGTTGTTCTTCATCGATGAAATGTGAAAGGGTTCTGCTATTCTTTGAAAGTTGAACACTTTTTGATCTTTCCATATTAAATAGATTACAAAAATGATGTGTTATGGAACTCGGAATTGTTTCTAAATTCTCCGCGTCTTTTTTTCCCCGTCAAATAACATGTCTTTGACAGGTTTGCCTGATATTCGGAAAAAAATTGGACAGTTTCACCATTTGAGGATCTATAAGCAGATCTTATCTTTGCAAGGAAATTTTGCGCGACTAAATTATTTTTTAGGAGATGTGTTTCCGGCTAATTTGCGATCGGCATCTGTCTCGGTGTTTTTCGAGGTGCGTCTAGGGCCACGCATTCCGGATTGTATCGTCTTGCTAAAGAGTGTAGATGCCAAAGATGAGTTTGCTTTTCATTGTTATTTTTTTGAGTTTAAGACGACTTTGGGGAAGTCGACGATGCAGTCTGTGCATCACAACTGCATTCATCAGGCGCAATATTTGCAAGGTTTGAGACAGTTGCATCAGTCTATTTCGTTCTTGGATCAATATTTAATTGCAGACGAGGTTTTGTGGAACGTTGTTCCCGTAATTTGTTTTTTCAGGCAGTGGGGACTCAAGTTAGATTTTTTTAAAAAGTTTTCGGGGAAGACCAAGCGGTTGTCATTTTCGTTTATATGTGATTTGTTCGCTAGGTCTCAGGATGGCGCAGTGCAATCTCTTTTATCAATACCCAATTACACCAATTTTAGAAGGGCATGTCAGAAACATACTGATTTGTACAGAAGAAGATATCAGAAGGCTTCAAAGTCAGTCCTCACTAAGACTTCGGGAGAAAATAGATCAAGGGCATCGCGACAAGTTGCTAAGAATGCGCCTAAAAACCGAATTAGACGCACTGCAAAGAAAGATGCAAAAAGACAGTGACGTTTTGAATTCTCATTTAAAAGCGATAGAGGATGCTTTGCTGTTTACGAATGATGGGGAGGTAAACGTCGAAACGAAAGCTGATACACAACTGTTACCGAAATCTCCTGAGAGGCTGGAAAAATTTAATCAAGTTGCCATAACCCCACTCGACCCCTTTATAAGATTTACAGACGATTTTAGAGGAGAAATGATCAATACTTTTTTCAATAATGCTCAGATGTGGAATTTTACGTTCGGGTCTTGGTTTTATAAGCTCAAGAGGGTTTTTTACAATGAGCCTGGCTTAAGAAGGGCTTTAAAAATTACTAATGTGGATTCTTTGACTATTTCGAAAGAGTTATTAACAGTTACCGTCAATGCGTTGGAGCAAGCGACGGTGTATCCTATATTCGGAAGTGAGATGTCAGACCTGGAAGCAGCCTTGTGTATCCTGGCAGCTTTTTATTCCACTTATGAGAATTCACAGATCGACGAACGTACAACCCTAGTAGATATTATAACCCTTCTGCCCGTGATATTTAGATTACTCGGTAGTGAAATTACAGCATTGAAAAATGTCTCTCCTTCTGGCACCTATTTTGGATTTAATGATCCTTCTTGTATGAAATTTTTTGTTCCCATGAGAAAAGGCAAGCATTATGCAGAAAACACATTTGGGAATCATGTGCTTGTAAAGATGTTGCTAGGTAGAGGAGTGATGCAAAAAATTCCCGGTGAGAAAGACAGCCAAAATTTTGATGTTGAAGCTCGCTTACACGGAGCCATTAAGAATGATGTTCTGGTGTATTGGACGTATCAGCTAATGAGACCAAAATTGGGAAATAATGTTCCAATCTTTATACACGATCAACATTATTTACGGTCTGGCTTGGTTGCAATTGAAAGTCTCTTTTTATTGTGGCGGATCTTGAACTCAGAAAGTTTGTTTAACAAGAGAGTTGGGAAGTTTCTCCTGACTTCGATTTTTCCACAGCTGGAGAATGTTGATTTCGCCGAAAATAACTTTGAAGCTGGAAATATTCAAAATTTTGAATACTTAATGCACCATTACGTTGTGCCTATGTACAACCTGCAGAATGACATATCAATTTCGACTCTTTTTCCCGGTCTCGTGGCCGTGTGTGTAAATGAGAGTGTGAGATTGGGATGGGAACATAAGTGTGCAGGGGCTCCGTCCGATGCCGTTCAGGTTCAATCTAAAGAAAATCCGTTCGTGGAGTATATCCGCGCACAAATGGAACAACAGGCGGATGTGGCTATACTTGAAAAGCATGACTGCATTCTCTTTCATTTTGAAAACGGTTTAAACATTACTCTATCTTTTACATTACCGAGACAGAGATTGTTTGCAATGGCCTCTTCATTATTTAATGTAAATGATACTTATGATTTCATCTATTTTCTAGTTTTAGGTTTTCTGCCAATACCGGCCATTATATAAGGCGGGATGTTGTTGTGTTGATTACGTCAATACGGATGGGGTTTTGAATATAAAGACTTACAGAGCTTTTAATGAATCATTGCTTCGTTTCTTCGGGATGGAGAAAGAAACAAAGTCTTTGGCTTGGCCAGCCACTGCGGAGTTTTATGGCTGGGTATTCATTTTTTCGTCCATCCAGTTGTGTACGGTGGTTTTCCTGACGGTTAGATTTAATGGTTTCAAAGTCGGTCGAGAATACGCTGTGTTTACATTTGCCGGGATGAGCTTCAATTGTTTTTTACTTCCGATAAAGATGGGATTGCTGAGTGGACATTGGACTTTGCCGCGAGATTTTTGCGCAATTCTTCTCTACATCGATGATTTTTCTGCCTATTTCTCTTCGTGGTCGTTGGTGTTTATGGCGATTGAGCGCATCAACTACTTCTGTTACAGCACACCGTTGTTGAACGAGAATTCCAAGGCTCTGGCAAAGGTGTGTTTCCCGATAGTTTGGGTCGTATCGGGAGTGCAAGCTCTTCAGATGCTGAATAATTACAAAGCCACAGCTTTGCAGAACGAGACTGGACAGTGTTTTCTCGCGTTTTTAAGGAGCGGACACGACATGTGGTTAATGTTGGTTTATTCGGTTGTGATTCCAGTCATGTTGGTGTTTTTTTACCTATACAGCAAAAATTTCATGCTTTTGAAAGATGAACTTTCGTCCGTGACTACGTATCTCTGTATTTATTTGCTGCTGGGTACAATAGCGCATCTGCCGAAAGCTGCCTTAAGTGAAATTGAGAGTGACAAAATTTTTTATGGTCTGCGCGATATTTTTATGGCGCTTCCTGTTCTAAAAGTTTATTATATATCCGCTATGGCCTATTGCATGGCATGTGATGATCACACCGTGCCCGTTCGTTTGTGCAGTATCTGGCTGGTCAATTTATGTAAGAAATGTTTTTCGTGTACGCGGCGCGAAAAGGGGTCGGATTTAGAGGTTGGAATAAAAATGTTAAAATGAGTAAGGAGTTTATCGTTTTTATTCATAAGAGAATAGATCTTCGGCGCTGATTCCTGTAAGGTCAAATATGGCAGAGCTGTCGAGTAACTGTGGGTTTTCAGACATGCCGCAGAAGACTTTAAAGTCGTTTTGGAGAAATGAAAACAGTCCGGCTTTGTGCTCGGCTCTTTGCTGATTGTACGCCAGCGTGGATATCTCAAATTTTCCAAACATTATAAGGTAGGCGAACCATAGGTGTTTGCAGATAAGCCGTACTGCTGGTTCTCGTTCTTGATCCCCCCAAAATCGGTTAAAGAATCTGCAGATTTTTAAAAGTCGGTGATTATAGATAGCTCCAACGGAGAAAACTATATTGCCTTCGCAGATTATTTCTTCCGGAATTTTGAGACTTAGACCAAACTCGTCCATGAAGAGAAAATTAATTATGTGACTGACATTTCCGCTGTAGTGCAGCTTTTTTGAAATATATGCTGAGTGACTTTCGTTAAACATATGTCGGAGTAGTTCCGCGTCAGAATGCTGTTTCAGAATAGAAAATAGTCGAAACCAGTAGTTGTTGCTGATTTTGCGATTCAAAATCACTATGTCTCCGATTCCAGAATTGTATAAACTCTCGCGTAGTAGTAAGATCTTGATTCCGTGTGACAGCATTGATGTACAGACATGTTGAATTAAAAGTAGTTCTTCTTGTTTAAAGCTAGTCAGATTCTCGTTTCTAAGCAGTTTTGTGGTTATGTGTAATATTAAATTAGGGACGGCGTCGTTCAGTTTTATGTACTGTCCTATCTGGGTCATCTCAAGATGTCTAAAGTTTGGGTAGGTGGATTCCTCTGTGTCTATGGTGAAGAACCGTCGGAAGAATGTTTAGCTCTGCCCAGAGACACGGTTCAAAAAGAACTGCGGTCCGGAAATATTCCTTTGCCGTTGAATATTAATCACAACGAAAAGGCCACTATTGGAATGGTACGTGGTCTTTTCGATTTAGAGCATGGGCTTTTCTGCGTTGCGCAGATACAGTCTCAAACGTTCATGGACATCATAAGAAATATTGCTAGTAAGTCAAAGTTGATCGCAGCAGGTTCGGTAATTGAACCTTTACCACCGGACCCGGAAATTGAGTGTTTAAGTTCAAGTTTTCCTGGTCTATCGCTTTCTAGCAAAGTTTTACAGGATGAAAATTTAGACGGTAAACCGTTTTTTCATCATGTGTCTGTATGTGGAGTCGGTCGGAGACCGGGAACTATAGCGATTTTCGGACGAGAAATAAGCTGGATTCTAGATAGGTTTTCATGCATCAGCGAGAGTGAAAAGAGACAAGTTTTGGAGGGGGTCAATGTTTATTCTCAGGGTTTCGATGAAAATTTATTTTCGGCGGATTTATATGACTTGCTCGCGGATAGTTTGGATACATCCTATATTAGAAAACGGTTTCCAAAGTTGCAGTTGGATAAACAGCTATGTGGTTTGTCTAAATGTACGTATATTAAAGCTAGTGAACCACCGGTGGAGATTATTGTAGCAACGGGCAAAGTTGCCGGTGATCAAGTTCAGTTAACTACGGAACCTGGGTCTGAATTAGCGGTTGAAACGTGTGACGTGTCGGTTGTGCACGGGAATTACGACGCCGTTGAATCTGCGACAGCTACAACGGCTATGAGTAATCAAAATCTGCCAAATACTACCCCTTTGCTGTCAAGTCCACCGTTTTCGGATTGTGTTTTTTTACCGAAAGATGCTTTTTTTTCTCTTTTAAATGTTACAACTGGACAGCAGCCGAAAGTAGTTCCACCTGTTTCTGTTCATCCGCCCGTGACTGAACAGTATCAAATGCTACCGTATTCGGAGTCGGCTGCTAAGATTGCAGAACAGGAGTCGAATCGATATCACAGTCCTTGTCAGACAATGTATCCCTATTGGCAATATTCCCCCGTTCCCCAGTATCCGGCCGTGTTACACGGTTATCGTCAACCGAAAACGTTTAAAAAGCGGCATTTCCAGAGTGATTCGGAAGATGAATTAAGTTTCCCGGGAGATCCGGAATACACAAAAAAAAGGAGGCGCCATAAAGTTGACAACGACGACGATAAGGAGATGGCTCGAGAAAAGAACGATTTAAGAGAATTGGTGGATATGATAGGAATGTTAAGACAAGAGATTAATGCTTTGAAGCACGTTCGCGCTCAATCGCCGCAGAGACATGTCGTTCCGATGGAGACTCTACCTACGATCGAGGAGAAAGGCGCCGCGTCCCCAAAGCCGTCTATTTTAAACGCTTCTTTGACGCCTGAAACGGTAAACAGGAGCCTTGCTGGTCAGAACGAATCCATGGATCTGCTAAAACTCAACAAGAAATTGTTTGTTGACGCGTTAAATAAAATGGATAGCTAAAATGTGTTTTTTTATGTTGTGATCAAGTGGTGTTTAGGTGCGTGTTATGGGGAAGATCTATGAGAATTATGACTGACAAGGTATATGGCGAATGCCCACATTTCCTTCAAAGTGAATTCCTGGACATGGTGAAACTTAACACATAATTGTTTACCGACGCGTTAAATAAAACGGATAGTTAAAAGTTGTTGTTTTGGTTTTTTATTTTCGTATAAAATGGTATTTAGGAGCTAGTTACGGGGAAGATCTATGGGAGTAATTATTGTGACAAGGTATGAAACGAATGCCGACGTCTCCTTCTAATATCGTTAGGCTTTGTGGGGTTTCAGGCGGCATGAATTCCATGCGAGTAGATTCTCCGGGTTTTAGAAAAAAGTGCAAGGGTACCAAATGTTCTTTGTCGAACGCGATGCGGAGTGATTTCAAAAAAATGCCCATGCTGATCTCTGGTGTTACGTGCGCGACCGTGGTTAGACTTGGTGCTCGCGATACTGGCTGGCTTGCGAGAGCTACAATGTCTTTTACGGGACCATTTGGAGATTCTATGTTTACATAAATGTTGCTCATTTCTGTCCAGCTGAATTCGATATTATTCCCCGGTGGTAAAATTTGCTTGGGGAAGAAGAATACTTTGCCTAGTTCTTGCGAGTAATTGATGCGACAGTTCTTTTTCTGTGCTTTCATGGTGATGTAGAGAGGCCGCGTCTCGTCCCAAATGCAGGTGGTTATAGAGATTCCGTGCAAGAGTTTGGGGATAAAGAAACCCGTAAAGTTTGAATTTGATTCGAAGATAGTGTGGAAAGAGCAATGGAAAGGCGTAATCAGGTTAGAGACGATATTTTTAGTTGCGCCGACAGTTATTGAACCGACGTTGCATTTTAGAAGTGTTTCTTGATTCTGCGACATTGTGATGTCTGCGGTGTGGGGGGTCAGTGAAAGTTGTATGTTAAGTTTTTCGGGAAAAAAAGTATCATCAAGGAGATAGTCGTTTTCGTACACTGTCTTCAAAATTAATAGATTTCCACAAAGTAAGATTCTAGAGATGGAGACGTGTCGGTGTGATATAAATTCAATTTTAGCAGAGTTAAAAATTTGGTCGAGCGGAGTGTTTTGGAGATTGATCCATATATCTGCGTTAAATAAATAACGTGTTGGCTCTTCTGGTGTTTTTTCTGTCCATCGTATATCAGCCGCGTAAATTTTAATGCGCGTGAGAGGGCCATATAGAATAAACTGCGCCTCGCACTGTCCGTGGGGTATGGCGTGGCGATCGTAGGCTTCTATGCGTTGCACCGGTAAATTTGATACAGTCACGTTCGCGTAAGGGAGTGCGAATATGTTAAGAACGATGGGTAATCTGCCGAGATCCAGATCTAGATCTGAGATGTTATGCACCGCAAAGGGTATGTTGCCATAATCTTTTGGATCGATGTAAGTAAAAGGCGATGCTGCAAAACTGCTTTCGTCTCTACAAGTGCACACCACGCTGGATGAAGGGGGGCAGATACCTAATCCGCATTTAAAATATCTCAGTTCGCCGGGTTTTAATATGGCGGCAGTTGTTAATCTTAGCTGTAGTACGTACGAAGACCATTGTAGAGTGGCGGGTTGCATGTTTGCTTTTCTGGATGTATGAGGGGAATCCTGTTAGTGTATTTTGGATGTTAAATAGTTGATGTAAACGTAGAGAGTCATATGCGCATTTTTATAATAGAGTGTTTTTCCACGGACGGGAGGATCTGCGGGTTTCCACGCGCAGGCTTGTCTTCGTATTCTAAAGGATGTAGTGTGAGTTCCGGTAGCAAGTATGCTGGAAAACAATTGCCCATGAAGTGTACTTCGAATGGCTTGTCGGTATTCTCCGTGTGTGTTAAAAGAGATTTCATGTCGCTGTTTGAGAGGGAACGAAACGGCTTTCTAAACAACTTCTTCGTCACAAAAAAGATACGACCTAACATGTCGTGTTCGGTTACGGAAACTGTTTTTTCGCACGAAATCCGCAATTTTAACGGTTCTTGGGCAAACCATAAAAATGGGTACAGCTTGAAGACATGTGTCTGAACAGGGATGAAGATGCCTACGATTTTTTTGTTAGTGAACTTGCCGCGTAGCGTGATCTCTTTGTTTGTATTAATTACTAAGGACATATCGCTGGGAAAGAAAACCTCTAGACTCTTTCCGTCTGCGCAGATTTTGAAATACGGCATATTCATATTCAGCGTGACGGTACTCCGATTCGGAGGGGAAAAGGTCAAGCGAAGATAACATTCTTTCGTGATTGGAAACCGTCGCTCGTTTTCCGGCAACACTACGTTACGTAAAAAGATTGTGAGAAAAGGATCGTTAAATTCTATTTTGTAGATAGAAATGCCGGGTTCGGAAATCATGTTGACTCTGCAGAGTGGAATCATACTTTTGAATAGGTTTTTCCAATAGGAACATTTTCTGGTATCGATGCTGAACACGCTAAAAACTATTAGCAGCTTTCTGCCATAGCGGTCTAATTTTTGAGACTGATAAGGACTTTCTGTCCAGCTTAGGTTAAGTAAGATCAAAGACGTTTCAACGCTATCATTCGAAACGACTTTAAATTGATTTATCACGTTGGTTGTCGAGATCGGCATGTTGGTCAGGCCATATTGTTCGTATACGATTGGATCGATGTTTTCTTTTTGGCATAGCGCATTGATGTCGTCAATCTCCAGTGCAGGTGACGGCAGATAAATGGGTATGGAGAATAAATGAATCCGTACGGGAACTGTCCTAGAAATAGGTAAAATTTTTATTTTGATAGTCATTGTATTTTCGAGAAACTGTACATCGAACAGGACGTCCTCAGTTTCTGATTCAGTGCCATCTGTTATGAATAGGATTCCTGGTTTTGTGCTTGAGATTCGACATTTCAACGTGATTTCTTGGTAAGAATTCGGAACAATGTGATTGCAATCAAAAACAAGCTGTAAGACATGATTTAGTTGAGGAGAATACATGTTGGTACAAAAAAACATTTTATGTTTCACCTTGCTTTTTAAATACTGTGTTTTTTTAAGGGATGAGGTAATTTTACATGACGGATGGAGGACGCGGGTGTTCAGGCGGGGTGTGTTTTTATAATCCTGCGTATAAGTGATAGAAAAGTTATAGTAGGCCAGTGTGTTTTTAAAAAGCATTTAATTTTTATAAATACATGTAGCCATTCTGTTATCTGCGGAAACGTCACAGACAACAAAATACGTTTTCTCGTTGGTCAAAGAATTAATTTGGTCGATCATAGTTAAGACTGATTTGATTTTTTTTAATTCGTCTTTGATGTCCGTAGCGTGAAGTTGTCCGCAGCGCTGTGTGCTTAACGTTGTTCTTTTAGATAATAGTTCTTGGCATTTGATCAAAAGCATTGAATGGTCTTCCAGCGTTAGCCGATCTATGTAAGTTCTCACGATGTCTGGGGCCATGGCCGTGATCATGGACAGCGAGATGCAGGCTGTTTTCATGGAGTACATTGATTGTTTGTCGTTGACTATGTCTGGTAATTTAATGAGTACGTCTCTGTATTGGATAGAGCATAACTCATCGATGATTGTTTGCATCTCATTGTATTCTCTGTGAACTGCTATAAGACCAATGCATAGCAATCTGATGTTGATCTCGGCCGCAATTGCGGTCGGAACGACTAAAGGTACAGTGAGCTCCCAATCACCCAGTTTCAATAGCTTTTCTTCCGAGTGCGTCGATAAAGGTGGAATCAATGTCAAGGTGTCACCCTTTTCCCAGGGGAAGGGTCCTGTGTTCTTTATGGCATACTGCTGGCCGGTCATCGGCTTTCTCAAAACTAGTTGATTACCTTCCACCTTCTGCAGAATGGTTACGACCATGGTCCGTAATACGTTCCGGATGTGGACGTAATCTTTGTTGGAGGAGACGATGGGATAAAGACCTAAATTGCCGCTACCTATTAGATGGTGGTGAGCTGGGATCGGTATGACGATGTTCATGAGCTTGCAGAGGGTGCTGATATCGGAAAGTGACAGTTTGTGATCGAAAGTGCAGTAGACGGTTTCCATTTTATATGGAAGATTCGATAATAAGTTGGAGAGGTATGGTTTCTCCTATGGCGTAGTTACAATAATGGGTTTCGCTGATCTGCGTATTGCCGGTTTTAGACCGACTCTCTAATAGAGCTTGGTTGGATGAACAGTGAAGGGGCAGAGCGTTTTGTAAGAACTGGCATGGTCTGCTTACGAAGCTATCCGTGCCCTCAGAGCACGAGTATTGTATTTCGCTATCGCTGTTTAAACATGAATTTGATTTCATGCAGTATTCGTTTATCACTTTGTACATCATCTTGTTCGTTTTTAAGATGTCAGATTCGGTAAAAAATTGAGCGTTAGGACTGTATGTCTTGGGATTGTAACATAGTTGTTCTCTGTGTGCCGTGTTGTACAGGATATCGCCTAAGGAGCCTGGTAGAGATGCCCAAGGATTTGTGGTTGCGGCAAATGTATCGCTGTCTGTTTGGGTGTGATCGTACAATGCTTTTCTAGCCGCCTCTTCGTTGTGTGGGTCCGTTCCCATCATACACGATTCCCTGCCTCTGGGGTTATGGGGCAATTTGAAAAAGTTAATATTTGTCGTGACCGGAGTCAGTATGACTTCGCAGATAGCTTGTTGACCATGCAGCAGTATGGAGGGTGGGTTTTTGTTAATTCCGCCGAATGTTATGATGTTTAGTGCCTCGCCCTCGGAGGGATTAGGTTTTTCTATCCCGACGTGATGCCGAATCCACGTATTTATGTCCGTGTTGGTAAACGCGTGTATCGGGAAGGCGGAGAAAAGGTTTTGTATCTTGCTTCCCATGTCTGATTTAATTCGCTTAAGATTGGCAGTGGCGGTGGTAGAACTAAAACCTAAGCCCATATCTACAAAACTTAGATGCTGGGTGAAGTTGTAAGTGGTTACGATATCTTTAGCTTCCGCGGTGACCGTGGGATCGTCTAAAATTATAGATGTGGCTGCCCTGGAGCTGTACAACAGGCACTCGACGTCGAAATTATCCGTCCGGACTAGTGTGGCCGCAAAGCCCGGATGGATTTTGCTTTTGCTTTGGATCGCTATGGCAACGGGAGACAGTTTTGAGTGCATGGCGGCAAGCGTCATAATGCTCAACAGGGAATTGGGGCAGAAGGCTGAGTACACGGAAAACGGTGTTCGTTGCCAATTATAAAATTCTAATGCTAATGGGGGCGGGAGAGGAAAACCGCCGTCGTTTCTTTGATAGTGAGGAAATGTATTTAGGAATACTTGGATATCAGCATTCATGGTGCCGCAGATTCCCGGGTCAGAGAAGAATCGATGAAATGGGATTGGCTGATAGTACCGTCTTAGGGTGGTGATCGGTGAGATCAGGCAAAGCCCATTGTATAGAATGTGTTGTAGTGATTGAAAATGAACGGAATTGCCGTGTCGCTGCGCAGGGTCTAAGGAGACCTCCACTTCCAGTATTTTCGTATTTTCATTAATGTAGATTAGCGATTTAAACAGCTGTTTTGCGATAGATGATGTATTGGCTACGGTGTAGCCAGGACCCATAGCTTCTCGTATCAGAGACATTAGGACGTCGCTGCTGATCGGATTCTCCTGGAAACAATCATCTGGACAGATAAACGGTTCCGTGTAGAATAAGTCTAGAACTAGTTCCTTTACGTTGACGCCAGCGCCACAGGCCTTGTTATTTGATAGTGCCGGGAGTACGCAGAAGTAAAAGATCTTGCTCAGGATGGTGTTTTCGTTCGATGGTCTGTCATTGTCGGTGAAGACGACGCTTGAATCTATTAGATTCATTCTTTGCACATCGGAGATTTCGTAATTTCTAACTCTTACCGTGTTCTGTGTCAGTGGTGTATCATCCGCTGTTATTTTTGCATTCGTGTCGTTTCTGGGCATGGTATGAACGAACGGGCAGAACAGACGTCCGTCGAACAACGCGTTGGCGAAATTCACCAGAGGTTCGCCGCAAAGTTGCTCGTTGATGTTGGAGATAGAGATTGTCCTCTTCACTAGGCGAATTAGCGACACAAGATTTCTGTAGTGAGCGAAAGCTGCTCCTGGGATCAGTTCGTCACCCATGTGGTTAGAGATTAGCATGATCATCTCGAAGCTGTTGCAAAAAAGAAGTATATGTTTCATGTTAAACCAATAAGAAATACACTGGCTAATTACTTGTTTTAAGATCATGAAAGCATGCTTGTTTCCATGCACTAAGATCTCGATAACGTAAGCCAATTCTGGGTATGAGCCACTCGTCAAGCTTTCGGTGACAATTTTGAGGGTGAAGTCGTAGTTGTGGGAGTTCGTCTTTGCATGTTCCAGCATTTGATTTGTACGGGCTTCGTGGAAAGAGATTGGAGCTAGTGGTAAAGGCATGTTGCCTATCATTATTCTTGGTGTGCAGAGTACTTCTGTGGCTCGGTTTTTCTGGATATACGTGAGATCAAAGAAGGGGTGTCTTTCGGTCGGCAGCGTTGGATTGTCCGGTTTGTAAAAGTCTTCAACGTTTAGTTCGTTTTTTAAGACGTTGGTGTTCTTGGGTACTTCTTGTCGACTTTCGTATAAGTTGTATAAGGTCTGTAAGATCCTGGTTGGGTTTTCTCGGGTAATTTTCAGTTGACATAGTTGAGAAAAGCGGTGGCCGGTAGGCAATGGTTCGTTTTTCATGAGTCGTTCGACGATGGTCGAGTCGTGGACAATGGGATGGCAAACCGACGGCAGTATGTCGGCAAAATCAATTCGCTGCACGACTTGGTCTTTATTGTGGAAAAAAACGCTGGTGGGTAGGTTGTTTTCCATGGTGTCGTTTAATTTCACACGTGTTTCCATTGTACTGAAACCGGTTTCTGTCGGTATGTAGATACCCAAAGGGAAAAAGAATGTCAGTTGTAAGCTTTGTTCTAGCGGATCGTTGACGTCTGTGTTTTTGTAAACTTTGTGTAGGTGGTCGAATGCGACTAATTTATCTCCCAGTTTTAACGTGTTCAGTTTTAGGTCGGCGTACGCGCGGCTTTTGTCAAAGATTTCTGATTTGTTGTTAGTGTCTTGTTGTTCTGTCCCGGCGTTCACGGTGAATTGAGTAAAATCGGCCATGATTGCACGGTATGCAATCGCAGTCACTGCGTTTTCTTTGCCCATAATAAAGTGACCATATGAGATGGGGGCGGAGACGACCTGTGTGGAGATGTACTGGGAGAGTATGTTGGTGAGTTTTTGGATAGAGCCGATCGGTCCTAATAAAACACCGTCTAGGGGGATGTTTTCTTTGGAAGTGTAGTTGTTCGTATCATTTAGAATGCTTTCCGTGACCGATTCCATCATGTCGTTCAGAATTCTATAGATATACGAACTATTATTATTCCTGTTCAAGAAAAATAATGACGTTAACAATCTGTTTTTTAGGCTCTGAAACATGTTGCTGCGCTGTACTCGGCTCAGAGCTTGTTTATTATGTACTTTGTTCTCATTCATGGTCAGGACGATAAATTGCGGGGGAGATTTTCTCAATAAGGTTTGCATGAATGCGTGAATCAGACCTCGTTCGAGCGAATCGGCTGAATTCTTTAAAGATCTTAAAACTGTATGCAACGCATTGATGTTTAAAATCTGATCGATGACTGTGTTTTTAAATGTGTTTTCCAGATGCTCCAGACAGGCAGCACTCAAATCGAACGATATGTTTATGGGGTGTTTTTCTGAGTGTTTGGCTACCATGATGGTGGTTTCTTTTGGAGCAGTTACGTCGTTTCCTGTGGCGACTCTGGGTAATTGGATAAAGAAGAGAACCTTCCCGAGGGTCATTCGACTCACATCGTTGAAGCGAATTACGTTCGCCGCAACGGCGATCGGCGTGGTGAAAAAGTTGATCCATTCTATTTTGTTGCAGTAGATTCCGAGCAAGGCTTCAAAACTGATGTTGTAACGGCTCGGATCGTCACCGAAATAAATTCGCAAATTGTCAAAAAGTTGTTCGGCCGTGTATGTTTTAATGTCATTGAAAATATTTAGAGGTGCTTCGATTTTAGGTAAAATTTCGGTCGCCTGCCAATTTTCCATGTTTCGGCGTTATGCAGCACACCGGTAATTGTGAAACTTTAATAGTTAATTCATGTTTTGGTTCCACTTGTGCACGGTCGATTCCAGTTTTTATAGATTCCTGTGATCTGACCGCTGAAGTGTCTCGCGACGAGGAAACTCGTTTAGCGCGATCTGTGCCTGTAGTTCTGGAAAAGATCGAGTCAATCATAGAGAAAATTTTTCAAACGTCTGGGCCAAATATCGTTCACGATAAAGACAGGGCTAAAATTGCCTTGTGTCGTCTGTTGTTAGGACCGGTTGCGGTGCCATGTTTCTGTGAAGAATGGGACACCAACGACTATTTATCGAAATCTGGATGTAAATGCTTAGGTCCGATCTTATATATTCATACCAGTCGATGTCGCTGTAGCGATATTCCGGTTTTTAAGTTTTCCATTATGAAAGATTACTACGCTTCACACGTGTTCAGAGGTTTATTATCTCTGAAAGAGTGGAATACACATCTACCGAATGTATTGTGTACGTGCGAGTTGTCGATGAGTGATAGATATGTGGCGACCGTGTTTCCTAAGCAGAATTCTATATATCTAGAATACTATCCGTATTTTTTGTGTTATCTGTGTCGCTATCTTACTGTCATTGAGATTGAGCAGTGTACAAATGATTTAATTTCGCTTCTTGGTCCTAAAGTAGCTCAGCGAGTCATAATTCATTTTAAACTGCTTTTCGGTTTTCGTCACAAGCCGCATATGGGCACTGTTGATTCGTGGTTCTGGGAAAATTTTTTTATGCTAGAATTGCATAAGCTTTGGTTAACCGTAGTCAAACATAACCGGGTGACAACAGATTTCTTTAATGTAGTCTATGAGAAAATTCAAAACTATAAACAATACGCCATCAAGACTTTGAGGATGTCGTCTAAGGCGGTTCCTGCAATACAGAGGTTTTGTTTGGCAAAATTTAAGCAGCAACTCTTGTATCTAAATATTAAGGTTACGGTAAAAAAAAATAAGCGAGAACTGTGTTTAAATGGTTTTGTTTACGGTAAAACATTGTATGTCGTTGAATCTTCTCAGTTAATCTTTCGAAATTTGCTTCTGTTGTATTACGATTACAGTTTGCCGGACGAATGCAAAACAAAAGAAGAAAACGTTTTGACGACTCATTACATACGGGTAATTTCGAGATTGTCGTTTAAGCAGTCTCGAAGTGCGGTCCCGCCAGGCGTGAAACCAGATTTTACCTTCGTGGCACAACTGCCTAAACGTAAAGAGTTGCCTAATGTTCCTGGTGGTATTGATTTTGCTGAAATTACCTCAGTGAGGCATGGCGCGGTAATTCTTAACGCGTTTAATACGAACAAAGTCATGAATTTAAAAGCAACCATTTCAAAAAGGGCTAATTTTGTGTATCATCGCATTCCTAAGACAATGACTCACAGTTTTGTCATGTACAAGCATACATTTAAAGAACCTGCGTTTACCGTAAGCACGTTTGTTTCAAACGATGATTTAGATATGAGTTCATTGAATATCAATATACGTGGACCTTACTGCGACTTTTTATATGCCTTAGGCGTTTATAAGATGCATGTTTCTATCCAAGATCTGTTTTTGCCGGCGTTCGTTTGCAATAGCAATAATTCAGTGGATTTACAGGGACTGGAAGATCAGGATGTTGTGAGAAATAGAAAGAAAAAGGTGTATTGGATTACTAACTTTCCGTGCATGATTTCTAATGCCAACAAAGTGAATGTGGGATGGTTTAAAGCAGGAACAGGTATTATTCCTCGGGTGTCTGGAAAAGACCTTCAAAATGTTTTGCTTCAGGAATTAAATAACGTTCGAGAGATTCCAGGGTTAGTCTTTGATATGGATTTGCATCAATTGCTTGTTTTGCTGGAACAGCGGAATCTACATCAGATTCCGTTTCTCGTTAAACAGTTTCTTATTTTTTTACGTCTTGGCCTGTTAATGGGTTACGGAAACTCTCGGCGCAACAAGGTGCATGATATCATGTTACACTTAATTTCGAATGGCCTGTTTGATTTTAATAAGAACTCCGTAGCAAATACAAAAATCAAACACGGGTGTGCTTTGGTTGGGACGCGGCTCGCCAACAATGTTCCGAAAATCATTGCTAGGCAGAAGAAAATGAAGCTAGATCACATGGGACGAAATGCTAATTCGCTTGCCGTGTTGCGTTTTATCGTTAAAAGTGGGGAACATAAAAATAAAACTGTTTTCATTAAACTGTTGGAATATTTAGCGGAAACCTCAACTGCCATAAATACGCGGAATGAAGTCGCCAGATTACTTCTGACTCTGACGACTAATATGAAAACATGAATGTACCCATGGCCGACGAATGGTTCGATTGCGCGATTAGGTTAGATTCGGAAACCATAGCTGTCCATGAAATTTTCAATTCGGATTTAAGTAAACTGCTTAACTTGCACTCGAAAACAGTCTATATGTCCGATCTGTGCGCCTTTATTTCTGGTTGTGTTAATCGGAATGTTGGTAAACTTACCATATATTGGCACGTGTACGGAGACATAATCTACGCATTGACGGGTATTTTACATTGTGTAAAAATAACGATAGAGTGCGGGGAGAGAATTGCCGATGGTCGATATAGATTATACGAAATTCCGAAATTATTTTTAATGAGAGGACAGTCAACACCCCTGGAACTGAAGTGGAAGCACGCCGTGGGTATCGCGACGACGAATAAGCCTTTGCTGACGCACGTTTTAACAGATGTGTTGGAAACCTCTCCTTTTACCTTGCCAGATACGCTTCTGTCGGTGCAGGAGTTGTCTATTTTCAGAGAGAGATTGTCGTATATTTACTATGTGCTCGGGTTAGATGTTGATATCGTAGCGAGAACAGAGAGGGAGATTTTTCAAAAATGTGCAGAACTAGCTCGCCTACAACAAGTCTTTCTTATTCAAGGAAATATTATGGAAAACTTTGTCCTCGTTCAGGCGTGTCTATTTCAGCTGGGGGCTGATGGTTTGTGGGAAGAGATGTCTGGCTCTGTACGTCCTAGGCCGGAGTTGATGTCTAGTGCGTTCATTCAACACAGAGTAATGGTGAATAATTGTTATTGTCTCGCTGTCATCTTCAATGCCATTTATAAACACAAAGTTTCCCTGCCTACCGTAGAAAGAAGCCACGAAATCGTTCATCGTGTAGTTCAGGAATATTATAAGTCTTATGTGAATGCTCCTCTCTCTGTTCTTGTGTGTGCGACTAAGGTGCTTACCTTGTTTACAGAAGAATATAACTTTCAGTCAGCTCTCGTATTTGTCAGTCAGTTTTTCCAGGTGGACGTCGAGGCTTCGAGAGCGGATGTGATTCGTCTGTTTTTAGCGTGTCTGAAGGGTGATTAGATCTCTCGGAAAGAGGCTGAACTGTTTCCAGAGCACACATAAATCGCCATAATTATGGCGATAATTAAGTCGTCAGAACAGGCTTGTTTTTTAGCGCTGTATGTTATGTAATTGTTAACGGAGATCTGGTGAATGTTTCTGATTTGTTCTAGTGCGTATTCTACGGGATCGTAGGTGATTTTTATTGTAAACGATATCAATTCCTGTGATGCCTTGATGTTTCCTGAATTAAAATTCGAGATGAAAAACTCAACTGCTAGTTTTTTTTCTTTACCGAGTAGGTAAAATGGCTGTGCTATCTGATTCTGGTCTGGGGTGTGGAAAAAAGTCACCTGTATAGATTTATTAGCCGTGATGTTTTCCTTTATGATACATGCGATTTTTACGGCAGAAGCTTGATTGGAATTCCCTTCTATGATGATTTTTACTTCCGTGAAGAAAGGATGTAGGTCTAGGATTGACAATATCATATGCGCGGCACATTCAGCTATTGCTGTGTCGGAACTCGTCATTAAACTTTCTAGAAAGTAGTGCTCCATGCCGTAGACGATATATTGATCTAGATAGGTGCCTATCGCTGCTATGCCAGTGCCAGAAGCACGGCGGTTGCCTGTATAGGCAGGATCTAGGTATACGTACAGATCTTTACCTAAAAAAGGAATTAGATTTTTATTGATGGTGCTGTATCGGAAAAACTCGAATTCGGTTTGTCCTTGTTCCGTAATTAAAACGTCGTTGATCACATTGCAAGTGGCTCCGCCCATGATTTCATGGATGAAAGCTCCTTCTAGGAAGAGGTTGGCCGTTTTTTTAACTTCGGCGTTAATGCTGATGAACTTGGGTTTGTGGAGTCGGTAGCAAGAACAGGCTGTGGCGTTACCGCGTTCGTTTAGCATATGGGCGTGATCTTCGCATACGTAAGAAACTACGGAGAGCATTTCAAACGGAGAGTTGTTCAGCTTCATTAAAAAAGAAGTTGAATGGTTTCCGGAATTGGTCGAAGATATAAATAGGATCTTGGTAGATGCTTGGGGCAGGAAACCTAAAATCGTGCTGAACGCGTCTTTCTTTATAAAGTGGCTCTCGTCGACAATAAGTAGATTGAAGCTTTGTCCGCGTATACTCTGAGAGGGGAATGAATTCGGCGTTAAACGAGATAAAAGACGATTTTGAGAACTGTGAAACGAAAAACGACCTTTTTAAAATAATTGATAAAATAAGCAAAAATTGCAATTTTATAGTGGAACAGGTCGAGTCCTTGCCTCGGAGGGTGGATTCAGCGGCTATCCTATTTGATAATCTCGCGGTGGAGATATTTAACGATGTAATATATCGACAAAATGGAGATGGCGTTGCCGCGAAAATACGACAGGGTAACGGGCAGAATATTGACACATAAGAATAACCAGATGTGCACAACCGAATGTTCTCAGATGTATAATTTACACAATCCTATCACGTTTGAGTTGGGACTTGGAAACGTGTTTGTCTGTATGCGGTGTTTGACGGTTCACCACTGTGATATGCAAACTGACTGTACCATTGTCAACACGCATGAGGGGTATGTCTGTGCGAAAACGGGTTTATTTTATAGCGGTTGGATGCCTACATATGCCGACTGTTTCTTAGAACCAATCTGTGAGCCGAATATCGAAACGGTTAATGTCGTGGTGGTCTTGTTATCTTATGTATATAGTTTTCTGATGGAAAACAAGGAACGATATGCTGCCATTATTGATAGCATTATTAAAGATGGAAAATTTATAAAAAACGTGGAAGACGCTGTGTTTTATACTTTTAATGCGGTTTTTACGAACTCAACTTTCAATAAGATTCCTTTGACGACGATAAGTCGGCTTTTTGTTCAGTTGATTATAGGAGGACACGCTAAAGGAACGATTTATGACAGCAATGTAATTCGCGTCAGTCGTCGGAAACGAGAAGACAGTTTACTAAAAAAGATGAGATTGGAGTATGGAAACGCACTTATACTATGACACCCTGTACCAATATCAAGGCGGAGTGTATCCGGCCCATATTTGCCTGCCGACAGATGCGTATCTTCCGATGAGAGTGGATTGTATCGAGTCTTTATATTTTCGGTGTGTTTTTTTCAAGAATGGGATGCATTATACTGAATGGAGTAAATTAAAGTTTACTGTGATTTCACGTGAAATAAAGTTTAAAGATGTGTTAAAGAATGCTGACTTTGACGAACTTTTTACCGGTTTGGTGGTAATGACTATTCCAATTCCAATAGTAGATTTTCATTTTGATATCGATTCTGTAATTTTGAAATTGGTTTATCCGCAGTTAGTGCACCGAGAAATAGTGCTGAGGCTCTACGATCTCATATGCATCAGACCTTCGTCAGACCGGCCGTCGGAAGTATCAGCTAAAAATATTGGTATTGATTTCTATCAACTAACTTCACAGGGAAATAAACAAACACCCGATGAGGAAAAACGTTGTCTATTTTTTCAGCAGGGACCTTTAGAGCCACCCTCTACCGTCAGAGGCTTAAAGGCAGCTGGCAATGCAAAGCCGATGCAGTTTCCCGCCCATGTCAACGAAAAAATGACCGAATCTTTTTTAAGCGATAGTTGGTTCGAACAAAAGGTCAGATGCAAAAAAATATTGGATTTTACGCAAACGTATCGAGTCGTGGTATGTTGGTACGAGCTTTCGTTTTCCCGCGAGATGCAGATCGAGAATAATTTACTGTCCGCTTCCCAGCTAAAGCGGGTTAACGCTGCGGATTTTTGGGATAGAACTGATCGGTATTTGCGAGATATTGGGAGCAGGGTATTGACACACATCGTGAAAACGCTTCAGATTCATAATAGGCAATTTAAACAGAAATTTAATTGCAATTTCCCAGTTAATTTCAGCTTTGAACATCTATTATCATTTATGCAGCTCGGGAAAGATTTTTGGATTTTAAACTTAACTTTAGACAGCTGCATTATTAAGGCAATTATCTGTTTCCTAGGTTTTCGAAACGGGAGAAAATCTTTTTTAGCTCAAGATGAAGTTTGGGGAGATTTAATAGACTGTTCTAAAGGATCGGTGATCTACGGGGAAAAGATCCAATGGATTTTAGATTCGACTAACAATTTATATTCGACGCGTCGTGAAAAACAGAATAAGTCGTGGGAATTATATGTTGATTGCTGTGCTTTGTATGTATCTGAAAAGTTAGAGTTGGATTTTGTGCTACCCGGCGGTTTTGCGATCACCGGTAAATTCGCTCTTACTGATGGCGATATCGACTTTTTCAATTGGCGATTTGGGTTATCTTAGAAATTTTTTGCAGAATGAATGTAACTGGTTCAGGATTTGTAAAAAAACATTCTATCGCGAATATCGCAGCGTTGCGACATCGTCTCCTATATTTTCGTTGAAGAATAAGCCTAAGAAATATTGCATGCATTGCGAGATGGTAGTGCTCAAGCGAAGTCATGAATTTATGTTCAGCCTTGCGGTAAACGGCATACATTTTGGACAGTTTTTGACCGGAATAATGAAATTTAAGAAGAAACAAGTTGCGGAAGGGCTCTGTTACTATGTATTGGAATTGGGAAGCATAAGTCCTGTCGATTTGAGCTTTATCCCAAAATATAATTCTGACTGCGTTACAAGCATGCATTGTGTTACACCGGAGCTTATTTATGAAAATTGCTCTATTGTGTGTCCCGAAGAGGCAAGTCGCCTCACTGTAAAAGGGCTCGGGGACAATAAATTGATTCCCTTAGGTGGGTGTGGAGTATGGTGTCTGAAAAATGGTGGCGATCTGTATATCTATGCTTTTGTACTCGCTTACGATCTTTACGTAGCTTGTTATGACAAAACCATCTTTCCATCTCTGGCGAAAATTGTTTTTGATATGATAGCTTGCGATTCCGAAGATTGCGTCTTTTGTAAAGATCACAACAAACATGTATCGCAAGCCGGACATATTGTAGGGTGCGTCTCTAATCAAGAAACCTGTTTTTGCTACACACCGTGTCAAAAAAAAATGACTGATATTAACAATCCGGAGTTAATCTCTTTGCTCTGTGATCAGGAAATTAATAAGATAGATATTATGTATCCCGAGAAAAAAGCATCGTTATCACTTGACATTAATTCTTACGTTCATGGGTACCTCGGCGACGAGCCTTGTGCGTTAAAATGTGTTAATTGGATGCCAATCAGGATCAGCTCAGCTCTGAGCAGGCTGATTATTTTGTCGTGTCCTGTGTGTAAGCGTGTGGTAATGGACTAAGTGTGCGTTATTTTCTGTATTAATTTTTTGTTTCTGAAAATAAAGTTGAATTGATAGTACTTACGTGTGTATTGTAGCAGCTGGCGAAAAGTGCTGTGCTCTTTATATTTTGATGGTCGATTGTAATTACATTATCCAGGCATGTGATTGTCTTTTCTGGAAACATTCGGCGGCATTTAAACTCGACTTCTTTCATCACAAAGTGAGATACATGTTTTTGATGTGCTACGTAACCGATGCTGATTCCTTCAATGTTTTTCAATAAAAAACTTATAATTGGAACTATGAACCACGTTTTTCCATGTCTTCTCGGGACTAGGAATACGTTAGTTTTTTGTTTCAGCGTATTCAGAGTACTTTCGTTTACGAATTCAATGTCGAACACGTGGGTCAGATAGTTGATAACACGATTGGCTAAAGCCGGAAGTTTGGTGACGGCGATGAAAAAGATTACATGTATGAGAATGTTCTTCTGAAATGGTTCCAGTTGTATTTTGCGCGTGTCTCCAAAGTCGTTGAATTCTCCTTTGATCCATTTTCTGAAATCTTTGATAAAACCATCTATTTGCAAGAATAGCGGTTCACGATATAGAATCTTAAAGGATTCGAGAAACTCTGTGTATTGTGATTCAAAGGATTTGTCGGAAGCGGGTAAGAACTTCATTTCTTGTAGCTTGCGCGTAAGGCTCGGTAGTATAGTAAGAGGCTTACGGTTTTTAAGATGTCGCTGTTTTTCACAGAATGTATATAGAGGTTTTACCTGTTGGTTATATGAATGGGCAAACCCGAGTTCTGGCGTGAGCATTATAAAACGCTTCTTATAGAAGATGGCACTGTTGGGGTATTTGGTAGATATCGTAGAGCAGTTTCTTTCGCCTTTCCATATGATCGCTTCATAGTTGTTTTTTATGTGGGTTATGTCGCAGGTACGTAGCATGGAGCCCGACCTTACGTTGGCGGCGGTCTATCAGGCGGCGGCGAATCTCACAGAGCAGGATAAGGAGATTTTTTCCGAAGCGGTAAAAACTGCGTTTTCAGTGTGTAGTTCGGCAGCCCCGAGCGCTAGGTTGAGAATGATCGAAACGCCTACACAGAATTTTATGTTTGTGACGAGCGTTATTCCTTCGGGTGTGCCGTCTGGTGAAAAAAAAACAAAGTTAAATATCGATGCCGCTCTGGATAATTTGGCTTTGTCGTTTGCGAACAAAAAATCAAAGAAGATGGCTAGAACGTATTTGCTACAGAACGTTTCGCGGACTCAAGATCAACAAGTTGCCATTTCGGGGACGTACATTTTATATACAAAAAAACACATTGAAACGTCTTTGATGCTCGATAAGACGAAGTTAGTTAAACAAATTCTTGAGTATGCTGAGACCCCTAATTTGTTAGGGTATACCGATGTGCGTGATCTTGAATGTTTACTTTGGTTGGTGTTCTGTGGCCCTAAAAGTTTTTGCCAGTCAGACAGTTGTTTCGGATACAGTAAGACGGGATATAATGCCGCATTTCCGAATCTATTGCCTCCGTATCTGTACGAATGCGGCCAGAATAATGGACTGTTTTTTGGCATTGTGCAAGCTTACGTGTTTTCTTGGTACTCAGATTTTGATTTTTCAGCGCTAGAGATTTCAGAACGCGCTCGTCGTCGAATCAGGTCACTCCTGTACGACTTAAAACAGAAGTTTTCGGAGCAAGAAATTTCTGTTTTACCGGTAGCGTCACAGATGTGTATCTTCTGTGCATTATATAAACAAAACAAACTTAGTCTAGAATACGTTTCTGGTGACTTAAAGACTTCTGTTTTTAGTCCAATTATAATAAAGGATTGTTTATGCGTGCAGACGACAATTTCTACAACTCAGATGCTGCCAGGTACAAAGAGCTCAGCGATATTTCCGGTATATGACCTTCGTAAGCTGCTCAGTGCACTTGTCATTTCGGAAGGTAGCGTCAGATTCGATATATAATGTCTTTGAAGGACTATCTGAGACAGTCAATTTCTAAAGATTTGGAGGTGAGACATCGAGATTCTTTAAAGATTAGATTAGGGGAGAGACATCCGTTGAGTGTGCATCAGCATATGATCGCCGCTAGGCAGATCATTAAATCGGACAATGCGGAACACCAGCATGTCATATCTTCTTTGAGTGGTTTTTTGGATAAGCAGAAGAGTTTTTTAAAAGTGCAACAAAGAGCTTTAAAGCAGCTAGAGAAATTGGACGTTGATGAAATAATTGATACGGCGGCGGAAGTGAAAGCAGTCAGTAATAATATAAAAGAAACTCTTATGGCAAGCACTGAATTAGAATAATTATGGACAACGGTGTGGAGACACCTCAAGGTCAAAAAACTCAGCCGATAAATTTGCCACCAGATAGGAAAAGGTTGAGAAAACATGACGGACTTGGAAAAGGTGTTAAACGAAAACTTTTTGCCGAAGATAGCTCTCCGTTAAAGAAACAGATCCCCGCCTGCAGCGATATGGAAACACTTTCTTCGCCTGTAAAGTTTGGATGCAAGTCGCGAAGTGCTTCTGCTCTCGATGAAAGTTTTGGAAAATGTAAACACGAAACTGCTTGCGATTGTTCTGCGATAGAGGAATTGCTTTGTCACGAGTCGCTTTTAGACTCGCCGATGAAACTGTCGAATGCCCACACCATCTTCAGCTCAGACAAATGGAAACTGGAGCTAGAGAAAATTATAGCTTCAAAGCAGATCTTTCTAGACATGAGCGAGAATGTTGAACTTGTAGCCTACGGCGAGACTTTGTGTAACCTGAGAATTTTCGAAAAGATCAGCTCGCCGTTTTTGTTTGACGTGCAAAGCGAGGAGCGTTCGTATTCAGTGGTTTACGTCCCTCACAACAAAGAACTTTGTGGACAGTTTTGTCAACCTGAAAAGACTATGGCTCGAGTTCTCGGAGTGGGTGCCTACGGGAAGGTGTTTGATCTAGATAAAGTGGCCATAAAGACGGCCAACGAAGACGAGAGTGTCATTTCGGCTTTTATAGCCGGTGTCATCCGTGCAAAATCGGGAGCCGACTTATTATCTCACGACTGTGTTATTAATAACCTTCTGATTTCAAATTCCGTTTGTATGGATCATAAAGTGTCTTTGTCACGTACTTATGATGTTGATCTCTATAAGTTCGAAGATTGGGATGTCAGGAATGTAATGAATTATTACAGTGTGTTTTGTAAGTTAGCTGATGCTGTAAGGTTTCTAAATCTGAAATGTAGAATTAATCATTTCGATATCTCACCTATGAATATCTTTATAAATCATAAAAAAGAGATAATCTTTGATGCCGTGTTGGCGGATTACAGCTTGTCCGAAATACATCCCGAGTATAACGGCACGTGTGCTATTGCTAAAGAGTATGACAGAAATCTTCAACTTGTGCCAATCAGTCGTAACAAATTCTGTGACATGTTTAATCCTGGATTTCGACCACTTGTCGCCAATGCAATGATATTGGTCAATGTATGCGAGGCTTTTGATGGTGAAAATAATCCTCTTAGACACTGTAATTTGGATCTGTGCGCCTTTGCTCAGGTCGTATTATTGTGTGTCCTGAGAATGACAGATAAACGCGGATGTCGCGAAGCTCAGCTATACTACGAGAAAAGGTTGTTTGCGTTGGCTAACGAGGCCTGTCGATTGAATCCTCTTAGATATCCATTTGCTTACAGGGACGCTTGCTGTAAAGTATTGGCTGAGCATGTAGTGTTGCTAGGGTTATTGTTTTACCGAGACGTGGTTGATATATATGAAAAAATATACGATTTTCTAGATGAAAGAGGGGAATTTGGGTTACGAGACCTGTTTGAGGCAACTTTTTTAAATAATAGTAAACTTACCAGACGTCAGCCAATCAGAGGAGGTCTTGCGTCTCTACAGTCGTCCGAGTATGGAGAAAAACTTTTACATGACCTTAGAGCGTTGTTCTTGATCACTTCTTCTGCAGATCTGGATAAAGATACATCATCTCTCTTTCAGATGTGATATAGTAATGGATCTTAATCAGATATCTGAAACACTAAGTGCCGTGGCAGAAGAAGAGCCTTTAACCATGTTTTTACTTGATAAACTGTACGCCATACGGGAAAAGATCAAGCAAGTTCCATTTTCGATTGTTCGCTTGTGTCATGTTTACTGCATGCTAATAAAATATAACGCTTCTAACAATAATTGCATTTTGGGCCGTAAACTTATTGAGGAAATGCAGCAGTTTTTGTGCGGCGCGAGAGTAGATGGATCGGAAGACGTTTCTATGGATATGAGTGAATTGTGCAAGCTGTACGATTACTGTCCGTTATTGTGTTCTGCTCTGTGTCGTGCGCCTTGTGTATTTGTGAACAAGTTATTTAAAATCGTAGAGCGTGAAACTCGGGGGCAGTCGGAAAATCCGCTCTGGCATGCGTTACGGAGATATACCGTGACAGCAACTAAGCTGTATGACATCTACACGACAAGAAATTTTTTAGAACATAAGGGACAGCAGTTTTTTGGGGAAGCGGTGATTTACGGCGCGAAACATGAGCGTGTTATTAGACACCTCGTAGCGATCTTTTACGTTAAAAGGGAAGTCAAGGAAACGTTGGGATTGCTGCTTGATCCTTCATCTGGAGTTTTCGGTGCGTCTCTAGACGCGTGTTTTGGAATTTCATTCAATGAGGATGGATTCCTGATGGTTAAGGAAAAAGCCTTGATTTTTGAGATTAAATTCAGATATAAATATTTACGGGATAAAGAAGATCACTTTGTTTCTGAACTATTAAAAAACCCCACGGAGAAATCTTTTTCGGATTTTATTTTATCTCATCCGGTGCCTGCCATAGAGTTTCGAGAAAGAGGGAAGATTCCGTCATCTAGAGAATATTTAATGACGTATGATTTTCAATATCGTCCTCAGAGAAAGTTGCGCACTTGCCCCACTCCAGCTATTTTGACACCTCATATCAAACAACTGTTGTGTTTGAACGAGACACAGACGTCTACGGTAATCGTTTTTGATTGTAAGAGCCACTTGAGTGAGCAGAAGCTGTCTGTGTTTCAGAAGGCTGTGTTTACCGTGAACGTATTCGTAAATCCGAAACACAGGTATTTTTTTCAGAGTCTGTTACAACAATATGTAATGACTCAGTTTTATATTAATGATCATAGTAATCCAGAATATATCGAGAGTACGGAAGTGCCTTCTGTTCACATTGTGACGGCTCTCTTCAGGAGAAGAACAGAGGAAGAAAGGAGTTTGCATTTGGTGATTGATGAGACGGAATATATAGAAGAAGAAATACCTTTGGCCTTGATTGTGACTCCGGTGGCACCGAATCCGGAATTTACTTGTCGTGTTATAACAGACATATGCAATCTGTGGGAAAATAATATTTGCAAGCAGACCAGTTTACAAGTATGGGCACAAAGTGCTGTAAACCAGTATCTTGCGGCATGTGTAAGAAAACCGAAAACACCCTGATGGATTACAAGGGAAATCCTATATTACTAACGACGGAATTTACTGTTTTGACCGATACCGAATCGGAAGAGGAAGGGATGGCAGATCTTGAAAAGCCCCTGCTTGAAAGAGTACAGGCCACATTAGCTGCTAAATGTGATACGGAAGTTGAAAAAAAACTGCCGCTTAAATCAAAAAAATGATAGACGACGCAGATTTACGACACGCTCGTATTTTATTTTGTGGCTATTTGACAATAAACATGATTGTAATAAAAAAAAGTGTCATTCGTTTTCATCCATTAGGCTCGAGCTTTCAGCGTTAAGTAGTTGCTTGTACCGACGCTTCTTTTTAACTAGGTAGCGTACGAGTCGACAAATTATGAAACCGATCGAAATTATGGCGACGACCGATAAATTGATAATAAGTCCTGTGCTCCACTCGGATTCAAAGAAAGTTTCGTACTGGAGAATAGGGAAAGATAGGCCTACGGCGCCACAAAACATACCGACGTAATAGCCGAACAAGGGTTTAACGTAGCGAACCAGGACGGCTTCTATTACTATACTATATATACAAGCCATGACAAGAAAGGTATTGATGGAAGAGAAAGCGACGGTGATGGAACGTATGTAGATACTATTTCCGACGCCCAGGCAAGTAGTAATGCCGAGAAACATCGTAGAGTAACCTAGCATGAGTTCGCTTAGATTGATGACAACGGTCTTGAATTGTACGGTCCCCTTTAGGTTAGGATGAATTTTCTGAAAGGCGAAAAACGACTTGTCCGCTGACTGAAATTGCGTTATCAGCGTAACGTTAAAGGTGGTGAAACACACGAAGAAGATGGAATAGGCAAATGCACCTAGGGCGACCAGTCTGAAAGACAGGGCGGTGACGAATAACTGAAATGTATCCATGATTAAGATGAATTGAAAGCAGGATGAGGTGTCCCCCATCCAGGTAATATCCCTCGTTGTTTGGTTAACGTTAGATGTTTTGTTAGAGGAGAACACTTTTGCACAGCAGACAATGTAATAAATAATAATCAGGAGGAATAAGATAGCCATTATCAGTACGTAGCATATAATCTGCACAGGGTTTATGTAAAGTTGATGCGTCATCTGATATATTTCATTATTAGCGGAGAGATTGACCGCTTTGAGGTCATTGATTTCAAAGTAGGCGCACGGAAATCCTAAATTTGGAAAGTTTATGGCTATCAGATGGACCGTGACGTTTATAAATGACAAGGCTGCACAGATTATAGAGACCAGCCAGATCCGTAAATTAATGGTGTCTACACGACTGGAGGCCATACCAGCTCTGTGATGGAGAACAGGTTTCAGAACGATACTCTGTTCTCAGAGTGGTTCGGCCAGAGTTTGTCCGATGTAAGATTTCCGGACAATGTCACCGTTTATTCTCAAGCCGACTCGGCGGTGAGTTTTGAAAACGTCAGGCAGCCGATAAAACTGGTTCGCGCAGCGATGGGTTCAGGGAAAACAACAGCTTTGATTCATTTTTTGAAACAGGTACCCAAGGAACTATCTGTCCTTTTGATTTCTTGCCGTAAAACGTTCGCTGCGGAAATTTTGCACCGTTTTACTCTCAATGGGTTGGAGGATTTTGAATTATATTGCGATATTACGGAGCGGCAGATAAACAACAGAAAAGTCATTGTCCAGATAGAAAGCTTGCATCGTCTTACAGAAAATTACGATGTTTTAATATTAGATGAAATTATGTCGATTATAAAGCAGTTTTATTCCAAAACGATGACAAAAACCAAAGAAGTTGATTGCAAGTTTTTATCGCTGATCAAAAATTCCAGTCATGTAATAGCTATGGATGCGACGCTGACTCGGCATGTGGTGGAGTTTTTTGCCGCGTTCAAGCCAGATACTCAAATCGCCCTCATCAGAAATACATTTGTGTCCGCGATGTTTTCCAATAGGGTCGCTTACTTTTGTGACACGTTTTTTGGTAAAGAGTTTTCTTTTTTTGCGCGCTTGGAAGACAAGCTACGATGGGATAAGAAATTGTGTTTGTTTTGCAGCACCGTGTTGGCAGCGGAATACATGCATGATTTGATACGTTCTCGGTTTTCACTCAAAAAAGTTCTCTTGTTGACGTCTAAACAAGGGAAATGCTCTTCCATAGAATCGTGGATACGGTATGACGTAGTGATTTATACGTCCGTCGTGACTGTCGGTCTGAGTTTCGAACCGGTTTATTTCAGCTCTTTGTTTGTTTACATTCAGTTGGCCAAAGGAGGGCCGGACATGGTTTCAATATTTCAATCCATTGGTCGTGTGAGGCGAGTTATCGATGAAGATATATACATTTACATGAATCCCGTACTAATTAAAAGCTATGATCCTTTGGCGCCTATTGCCATGCCTCCTTGCAGTGATTGGAGTGTCGCTGAGCAGTCTATCATCTCAGAGAGTTGCATTGACTTCCGCGGAAAGTGCAGCGGAGCGCATAAATATAATTTCTGTTCCGTACTGAAGTGTCTTTTTCGATACAGGCATTATATCGAAAAGACTACTATTACAAGTTTATCTGATAGTCTCTTTCTGCTCTGCAGTCTGTTATGTGAGAATTCTATTAAGGTGGACATTGTTGGAAATGGATTCCCGATGCGCAAAGAGGTTTTTTTGAGTTTCTTGCAAATTCTAGTCGAGGAGTGTCATTTTATCGAAAAGAAGATTACTTTGCCGGGTGACAACATGACGTTCCAAGAGATCATAAGTAGCAGAGAAACTATCATGAACGGAGATTTTTATGAAAACGGTAATCAGCTATTACATAAGGACTATATTACCGACATGGGAAAGTTCAGAGCGACTTTTTTATCTCCGGGCGTTGACATTTTCATTGCATCGGACATTGTATCTGATCTGAAAAATGAAAGCAAACGCTATGTTTTTGTGAACGTCTGGTTACAAAAATGTGTGTCTGCTGGTGTAGAAAGCACGCGAATCGAAAGAGTTTTTAATGAGCGGATTAAGTCTTATGTGCTGCCGAAGAGTTTTTTGTGCGATGAGTACTTTGTTCTAGGTGACATCTCTGGGGTTTACGAGTGGGGAATGTTAATAGATCTGGCCTTCCTGGCGGAAATGATCCGAAAAGATTTGAAACTGAAATCGTGCACAGATACTACTACTGACATTTCTGAAGATGATCTCCTTTTGTGTGCGGCCAGGAGGTCGAGTGACATCTTACAAATAATGCAATTAGTGTTTACGGTGCATGTGCAATTTTTTCAAAGATACAGTCTGCAGACTCTGCAGCTTTTTAACAAGTTGAGAGGCATGCGGATTGTGACGGGTGTGTTTTCTATAGAAAAATTCAGCATTTCCATCCTCAGGCTTTTCTTTAAATGTGCATTTAATATGACTTTGTCCGCCAGCAAACCTCGGTACATCCCGGGAAAGGCGTATCGCAATCTAACAAAAAATGATCTGGAGAACATGCTAGACAATTGGGAGATTTCGCGCACCAATCTTAAGACTTGTAAAGAACTACGTAAAGCCCTCACCGAGGCCTCGAGAGCAAGGCGGAAACAAACAATTTATAAACTGCAAGGCTCGGATATAAGCCTTTCCGTTAGTGAAGTTGGAGTTTTTGGGCAACATGCATCTCCGGGGGTGTGCGTGTCATCTTAGCCTTTATTGTGTGTACAACGACTGGGAGAATAAGATCTACAGAGTACCGATTTTTCAGTGTTTGTTTTTAGAAGCGGAGACTCGTTCGTTAAAAACGTTTTTGATTAGGGGACAGAGCCTAGACCAAGAATCGTTAAATGAGATAGAAGTTACAAGAAAAGAAACAATGCTTTGGGACCTTCAGGAACAAAGTAATATGATGGACAAAAAAATAGCAGCAATCAGCAGCCTCATTATGAATAACGGAGAGCTTCTTAGGAAACTTTCAAAATTTTTCGTGCCTCTAACTGTTGTTCTTGGAGACGACGGGTTAGAGATTTTGGAAGCATACGTCTGTGGCGAGGAACCCATGCTGCCTTTGGACACGGTACCTGTTATATTACGATGTGTCGGAGACTACGCAGCCTTGGACACCAAGCATCTCTTGAGTAACGAATGTACACAGGCGTCTAAAAAACTTCGCTTTGGATACAGTGTAATGGACTTTCACTTTTCGCTAACTGTGTCTGATGTAAAAATTTGCTTTTCACACACGGACACCGGTGAGGCTGTATGTGAAAAAATGAAACAAATTTTTTATTTTTCTGTGTGTGCTTTTGGTGGTGAGCAAGTTCTCTTAGTGACGCCAAAAAATGCTTATGCCTTATTGTTCGACGATGATTTGTGTTTGCTTTTACTGCAGAGCGTGTTTGCTTTCCTGCACGAGAAGATATTCGCAGTTTATAAACAGGTGTTAGTTCAGCTTTGCGAATACATCGGTCCCGACTTATGGCCCTTTGGGAATGAAAGATCGGTTTCTTTTATAGGATATCCGAATCTGTGGCTTTTGTCTGTATCGGACTTAGAAAGGCGGGTTCCGGATACAACTTATATCTGCCGTGAAATTCTCTCATTTTGCGGTTTGGCGCCTATCCTGGGACCTCGTGGTAGACACGCGATCCCCGTAATTAGGGAGCTAAGTGTTGAAATGCCTGGAAGTGAAACCTCTTTGCAACGTTTCAGGTTCAATAGTCAGTATGTTTCAAGTGAGTCTCTGTGTTTTCAGACAGGTCCCGAGGACACACATCTATTCTTTAGTGACAGTGATATGTATGTAGTGACTTTGCCAGATTGTCTGAGGCTATTATTGAAGTCCACGGTTCCTAGGGCCTTTCTGCCATGTTTTGATGAAAACGCCACGGAAATCGAACTGCTTTTAAAATTTATGTCGAGGCTGCAACACAGATCGTACGCTTTATTCGATGCGGTGATCTTTATGTTAGATGCTTTTGTTTCCGCTTTTCAGCGAGCATGTACTCTAATGGAAATGCGATGGCTGTTAGTGAGGGACTTACACGTGTTTTATTTAACCTGTGACGGTAAAGATTCACATGTTGTCATGCCTTTACTGCAAACGGCCGTTGAAAACTGTTGGGAAAAAATTACGGAAATCAAGCAAAGACCTGCATTTCAGTGTATGGAAATTTCGCGCTGTGGATTTGTCTTCTACGCTAGATTTTTTTTAAGCAGTGGATTGTCACAATCTAAAGAAGCGCACTGGACAGTAACGGCAAGTAAATATTTATCTGCATGTATTCGGGCCAATAAGACAGGTCTTTGCTTTGCCAGTATAACTGTTTATTTTCAGGATATGATGTGTGTTTTTATAGCTAATAGGTATAATGTTTCTTATTGGATCGAAGAGTTCGATCCTAATGATTATTGCCTGGAATATCATGAAGGACTTCTGGACTGCAGTAGATATACGGCCGTGATGTCTGAAGATGGACAGCTTGTCAGACAAGCACGTGGAATTGCATTGACTGACAAAATAAACTTTTCTTATTACATTCTCGTTACATTGAGAGTGTTGAGGAGATGGGTGGAGAGTAAATTTGAAGACGTTGAACAGGCAGAGTTTATTAGGTGGGAAAACAGGATGCTCTATGAACACATTCATTTGCTACATTTGAATTAATTTTGTTTCTTTACATAACATAGAAAAATAAGACTTAGCTAGACGTGTCTTCATTCTTCGAATAAAAAATTTGTGCGAATTGTGTTGCTAGTTGTATGACTTGGAGAGAACTGTCTTGCAGCTGTCTAGATTCTAAGGATAAATTGATGAAATTGTGACTACGTTTTGTGTTAAAATAAAGTTCCAAAACTTCTATATGCGTGTTTTTCATTTCATTGTATAGTCTAATCATCGGAGGCACCTTGTGGAGGATTTCTGAGGCGTGTTCGTTAAAAAAAATGTGTCTTAGTGTACAAACCAATTTGTCGCACGTGTTGAATAATATTTTCGTTACCGGAGATAGGACGGGGAAAAGAACGAGCCTAGCGATTAGTTTGTGCAGGGTGCTTTGCTTCAGTGGAAGATTCTCTAATGCGAACACTAGTCCGCAAAGCATAAATTCTTCTGGCGTATCCACTTTCATATGGATTAGTCTGCGTTTGAACTGAAAAGGATTGAGTCGCAGTTCTTGCAGTGTATCTTCCGAGTCTAGTATTATGATGCCAATGAAGCCTTGGAATCCCAACTCTTTTTCGCAGATGGTCTTGTATTCCAGGCCGTTGATCATGGTTCCGTCTTCCAAGTGGAACCATATTCCGTCGTCTCCTACTGTTATATTTACTAACGAATGTCGTGCGAGGGATACACACTTATCAATCTTGATCACCTTTAGGATAGTTCGGACGTGTAAGGTGTTGAATACGTCTGAGGTTTCTAATTCTTTTAATTTTTTCAAGGATTTCATTCTCCGGGTTTGGGGAGAAAATGTTCGAATCGAAAATCGGCTCGTTGTTTTCGGGGTGCGTTTGGGTTAAATTATACACTTCTGAGATCTGATTTAGCTCTCGGACGTATACGAGTCTTAAATAATGCTTGATTTTGCTGCTTTCGTAGATGGCTAATAAGATTTCACCGAGGGATAGAAAGAGAAATTCCTCGGGAATTGATTCGAGGTTGTAAATATTTAAGAGTCGCCTTAGGAAAGGCACGATGAGCAGCTCGATGCTATAGTTAGAGTATGTGATACTTTCTTCCTGTCCTTGATTATTAACGTTTTTTCTTAACCGAAAGGTTCGCATGTATTCCATTTCCCATAGTTGGTCTATTCGTTTGTCGGCATATTGTGTGTCCGGGATATACTGTGAAAAAAAACTGTTTGCGACTGATCTGGTGTCATCTATGGAGACGCTAGTGAATGGGAGCCTTTGAACTATGTTCAGCGCTCGCGATAAAGAGAGATTCTCGAGGTCATGGTCCAGATCCGGGTTATAGCGATCACAATCGTTATTCGTCTCGAGCAAAGAGTGTAGATCTTTGATTTTCCTTTCGAACATTTGGTTTGTTGTTTTCAGTGTTTCGATTTCATCCATCTGTGTTTGTATCTGCTCCTCCATGCACTTGATAATTTGTTTTTTGAACAGGTCTCGAACGTCTCTCTCTCCTGTGTCGATCGCAGAGCGTTTTCGGAACTGTCCTCGGTTCAGCATAAGTTTATTTTGGTCTATTATAGAGGGGGTGATTTCTTGAATAAATCCTTCAACGCTGTCCCTAATGCCTATTTTAGATTTGCTGTCCGACAAGTTCAGTAAAAATTTGATTAAACTTTTTTTCGCGTCGCTGTTTTTTTCTTCTTTTTCTATCATGTCCATTATCTTTTTGTTGGTTACATCTGTCTTTGCTGCGGTTAGCACCTTTATGGGAAATGTATTCAATAATTGGCACATTTTTTGATGACGCCGCAGACTATTGTGTTTTTTTAGTTCTGGAGAAAGGTGGGCGAGGGGTGTAGTCAGCATTAGGCGATTTCTAATAACATATGGGGTGCTGTACGTGAAAAACGCTATAGCCTTCTTGTTTTTAAAGCAAGAAATTTGCTCCGAATTCCGATCTCGGAAAATCTTAATATTATGAAAATCCGGAATGATCACGGAAGATAGACCCAGTAAAATGGGAGTGACAATGTTTTGTTTATGCCGCACTATGTCGTGGATTAGTTTAGATGACACTTGTTGTAGAAGATTGTATAAGTTCGGGGCCGTAGGGGTGGTTCTCATGAACGGAACGATTCCTACGGTACAGATCCAGTCTACATATCTGTTATAGTGACTGTTGTCTATGCTCTGAAGGATAAAATTAATTATATTAGATATATCTCCCAAGATCACCTGTTTAATCGCGTGAGCCCAGACACTAAAATAATCTTGAAATGACAATTTTTTGTTGTGGAACGGAAATGACCTCACATGACGTTTCCATTCATCGTGGATTTGTTGAATAGAAACGCTGTTTAAAAGGGCTTGAGCTACTCCATAGGCTATTTGTTTTTTCAGTACGGCAGAATCTCGGAGAGCGTTGTGGATTGTTTGACCTTTGAAAAAATTTTTGTGTCCGTGTAGCATTTCAATAAAAGTCATTATCTGCTCAGAGGGATAAATCATGTCTATCTCTTCGTTGTTCGGAGGTAGATACGATAATAAGTTTTTGCTTAACATGTCTTCGGCTCCCAAAATAGAGTTGATTGTAGATAAGGTGGCTTCGTTATCAGAAAGACGCTTGGAGGGGCGTCTGCCAGAAGATTGGTTTCGCCACATCATGGATCCTGAAACCGAGTTCAACGGCGAATTTGCTGATGCTCTGTGCATCGGTATTGATGAATTCGCTCAACCTCTGCCTTTTTTGCCGTTTAAGGCCTTGTTAGTAACCGGTACGGCCGGGGCAGGGAAAACGAACAGCATTCAGACCTTAGCGGCCAACTTAGATTGTATCGTTACTGCAACGACATCTATCGCCGCTCAGAATTTGAGCGTTGTTTTAAACAGAAGTAAATCCGCGCAAGTTAAAACAATTTTTAAGACTTTTGGGTTTAATAGTTCACATGTATCTATGAGCGAACGTCAAAGTTATATTGCAAATGACGAGAGGTCGATTCAAATTCAACAAAAGCAAGACCTGTCTATCTATTGGAATGTTATCTCCGACATAGCGGAGAGGGCGTTGGGTGCTGTCGCGTGCAAAACTAAAGAGTTACCTGATTTGTGTGAGAGTAGTGTTATAGTTATCGACGAGGCAGGCGTAATATTACGACATATTCTGCACACTGTTGTTTTTTTCTATTGGTTTTATAACGCGTTGTATAAAACCCCTTTGTATGAGAACGGAATTGTTCCGTGCATCGTGTGTGTGGGGTCGCCCACGCAGAGTAACGCTTTGGTGACTTCATTTAATCCGCTGACTCAAAACAAGGACGTGAAGAGAGGAATAGATGTACTTTCAGCCTTGATCTGCGACGACGTGCTCTCTAAATATTGCGAAGTCGATAATAACTGGATAATCTTTGTAAACAACAAAAGATGTGCGGATCATGCGTTCGGAGACTTTTTAAAGCACATAGAATTTGGCTTGCCGTTAAAGCCGGAGCTTATAGAGTATGTCGATCAGTTTGTGAAGCCCGCTTCGTACATAAGAAATCCCATGAACGAAATAGAAACGACCCGCTTGTTTTTATCGCATAACGAGGTAAAGAACTATTTCCGCTCATTGCATGAGCAGGTTGAAGTTACCAACAGAAATAACTTGTTTGTGTTTCCGGTTTATTTTCTAATAAAAAACAAGACTTTCGAAGACTACAAAAGTGAAATTGGTAATTTCTCCTTAGAAATTGAACCGTGGTTTAAATCTAATATTCATCGGCTGAATACTTATTCTCAGTTCGCGGATCAGGATTTATCGAAAACTGTTCAATTGGAGGAGATTGTTTTGGAAGATGGGTCGGTGGAAGAGACTTTGATAACCTGTCATCTGAAACATATCAGAAACAGTTCAATAGGTGTGACGTCCAAGATCAAGGCTTCGACCGTGGGGTTTTCCGGAACTTATGAAAAATTTGTAGAACTGTTACAGAGCGATTTGTTTATCGAAAAAACGTCATGTGAGCAGACTATTCACGCATATTCTTTTTTATCCGGTCTTATGTTCGGAGGGATGTATTCTTTCTGCTGTTCGGAATTCACCACCCCTGAAGTGTTAATGGAGATCAAAAACATAAAGATGCCCAGTATTGAATTCTTAGAAAGTGAAATGTCGAGGATGTCTCGAGACGTGCAAACCGTAGAGACAGATGAAAGGTATGATTTCGGATTAGTCGATGATGGGTTATCAGATATGGACCTATTAGAAATTGATCCTTGCGGAGATCCATTTTTTACTAGGTATAGTAAGTTGCCTTTAACTAATTCTCTGTCGTTCGAGGAAATTAGTTTGCTTTATACGACGTTTAAGGATATCTTTATATCTAGGTTTGCCATTCTGCAGAAGCATACAAAAGGGAAGTTCGGAAAGACTCTCCTAGTGACTTATAACAGAAACAACGTTTCTAGAAAACAGTGCGGTGAGATATATTCCCATTTAAAGAGTTTTTATGGTATGCTCACCTACGCTATCCCTGCCAATAATTATACTCTGGAGGGTTATACAAATGATAATGTAGTTCATTTAGGAACGGATAAACAATTACCGCAGATCTTATATAAAAAAGGATTACCTAGATTAGTTATAAAAGATGAAATGGGGTTCATTTCTGTTTTAGATAATAACGTGTCGAAATTTGTCGATGTCGTAAATGGTCAGAGTTTTCATCTATGTACAACGGTCGATTACGCCACTGTTTCTAAAGTTTCTATGACCATCACGAAAAGTCAAGGGCTTTCCATACAAAAGGTCGCAATAGATTTCGGGAGTGATCCAAAAAATTTAAAATTGAGTTCTATTTATGTTGGGATGTCACGAGTAACCGACCCGAATAATTTAATAATGAATGTTAATCCCTTGAGACTGAATTATGAGAATGATAATTTTATTGCTCCGCATATTGTAAAAGCGTTAAAGAACGAAAATACCATGTTAATATTTTAGGGAGGTTTTTTATGAGAATATATAAAAAAAACTAAAACATCAGATGAATTGTGCGTGTAATTGTTGAAATTCCATACGGTCGAATGAGCTTATTCCCGTCGGTCCGTTTTTTCTGCAAAACAAAAGTAGCACGGGGATTTAGTGCAGTATGTCTCGAAAAAGACAAGGGTGTGAAACTATGAGGAACTGATGAGAAATGACTTACGGCCATGATTTTGTTTCGTCGCTAAATTGAATACAGCTGCAGGGATGTGGTATGAGCTGACATCTGCAAAAACAAATGAAAGTCTCCCCCGGAGTACACGTATGTACTCCGGGGGAAACCTCAAAAATGCACAATTTAGCCTATTAAGCAGCCGCGAACTCGTTAATAGTTACTTTGATGTTAACTCAGGTGGTGGCTTGGATGGTGTCCTAAGTATCAATCGAAGCTCCTTTATTTTTTGCACTACTGAAGTTATTCTAGGTATCGGGGTAAGAATAAGATGACGAAAAAAATATGCTAGATCCGTCCATTAAATCCTAGAGTTGTTTTTAGTAATAATAGGTTATTTACAAAAGCCTGCATATGATCCAATGTTTCTGTATGAATCCAGATAAACAAAAATCCAGGAGTAATGATTGGAATCACAAAATGATTAGTTAGATGCTTAACTTACTTAATAACTACACTTATAGACTAAACTTAACTTATCAACTATACTTAACTTTTAGGTCCTTATAATGTGAACTAAATAATCAACTGTTTTGTGCCCAACCCCCCACTACTAAAGCAATCGTCTTTGATTTATGAAACAACGAGGCCCACTTCACGTTATTACATAGATCAAGGTCTATCTCTTGGCGGTTTTTTAATTCTTCCGAGAGCGTAGCGGTTTCTAGGTTTCAGACACGTTTATCCATTCGCCGGGTATTGATCGGGTCATATCATTAGCCGTGAATAATCTCTGGCTTCATTTCGATACAACGTGTGAAAACTAGTAAAAAGGACAAAAGCAGCCGCCTTTGCCGTCTTCATGAGCGTCAAACACTCATGAGGTCCGTATCTTACAACACAATATATGAAATACTGTGAGCGAGGTTTACCGGTGTCCGAGTCGCAATCCGAAAATCATATATTGAAATTTGTAACAATGTTATGCAGTTGTACTCAAAGTATATGGTGATGAATTGAACATGTTTTTGATGTTTTCAATGGTATTCGAAAAATATTTTAGATATCCTGTAGTTTTTTTCTGATGACTTTTCTGATGACTTTTCTGATGACAATATAGAAGATGGCAAAACAACAAGTTACTGGTAGTTCTTGATGTTAGATAAAATGAATCGGAGATATGACAAGTAGAGAGAGGTAGGTGATACGAGTAAAATTGAAGGGTGTTTCCACACAGACGGGGGAAATTCTATGAGAACCACAGCCAGAAGCAATTGACGACAATAGAGAAAAATAAGCAAGCATACTCATCTCGTATAGAAAGAACCAGTGACCTACGATTTTCCATTCAGGCTAGCATGGTGTGAACGAGGGTTTTTTCCGAAAACTAGATGACTATTTCTTTAAGCAAGTGTAATTTTTAGCAGTCCAAGTTTTTATGCAACGCAGATTTTAATGATGTGTCCCTCGATAACTATACGCTGGAAACTAAACTAACTTACATCTTGGTTGATAAACGATTTTCTATTAAAAAGACTAAAGGGGTTTTTTGGCATATACAAAATTGTATTGAGCTCTTGGAAGTGCGTAGATGCAAAATAGGTTAAATCTTACCTTGAAGCTACAACAGGTCAACGTTCGAGTAGACGTAAATTCCATATGTCGAATGCGCTGTAAACTTTTTCACGGAACATCAAAAACACCGAATTTATTCACGTTTACATAAGCATGAATCTGCAATTTTAATAGTCCCATAAAGCTTTCATTTCGATCTAACTACATTTTAGTATTTATGCTTCATAATGTATCAAAGTGTATACATTCAATCTGTATAAGAGTCTGCATCAAGCTTCACGTGATTTGTAGTTCGCGGTTTAGCATTCGCTGTTTTGCCGTGTATGAGACTTACAGTTTGATCCCTAATACTTCTGACGGGCGACAAGCCTTTTTATATGCATTTTACGGCAAAGTCCACGCCCTTGTCGCTGACGCAATACGTAAAGGTTTTCGGTTTGGATGAGGATGTGGTTAACCGTTAGTAAAAAGTCGTTACAGTAAAAAAGCCGAACGGTATTTTTAGATGTCGGGCCACATTTCGGTTTAATAGATTTTTATATCGTGTTCGTTGTCTGACTTAGATATGTCGTTTCATGGGAGAAATAAATCGTTTTAGTTGTATGCAGTAGTTTTTGGATCGCAAACGCTCGAGCCCGGAAGTGTGATTCGTGCGCATAAAAGGGCGCTGGTGACTGTGTTCGCTAGCTCATTTGTGCTAGATGAGCGGCAAAGAACTCATCTTTCTGAGATTTTTCTGGGATTATTCTGGTAAAAATGTATGTCTCGTTTGTAGTCGTTTTTGGATTTCTGTAATGTTACGATTTGCTATGTGTATTTTGATGGTGTCGAGCATGTTTATTTTGTTCTTATTTTCACACAGATAAGATGTATGCTGAAGAACGTGGATATGGGTCATTTGACAATGTTATACAGGCTTATGAACAAATTATTAGTCAGTCTCTTCATTTGAAAAGGTTTGAATTCGACAATGGCTGTTTCATTGAGTTCTTAGCTGACTCTGGAACGTGTGAAACGTTTTCTAAAGGATGGATATCAATGATTTATTGGACATCGGAGACAGATTCAATGGGTTCTCTAACGGTGGATATTGGGATGGATGAAGGAAAATGCAGAACGTACAGAGCTCGGGGCCTTTTACTGTGTTCAAAATCAATCACGTCGATTTCTCAGAATACTGAGGGCAGAGAGAGGATTTTGACTGTTTCCCATGAAAACGGAAAACTTCAAATAACGTTTGTTACTATTGCTAAAGTTTCCTCGGAGCCCGAGCTACGAAATCTGGGCGATTTGAAATTTATGGAAAAGTTTGAAAAAGAATGTCGAGCTCTAGACAGGAAAAAACACGACGATGAACACAGAAAACGCTCGGGGAAGCAGAAAGAAAAAAGAAAAGTGGAGGACATTGACAAAAAAAAAGAAGATGAGAAACGAAAACAAGAAGAAAAAAAACGAAACGACGAAGACAAGCGGCCGGACAAAAAGGATGAATTTGATGGTAAATATGAATGTGTAGCTGTTGTAAATTTTTTTATTATTGGTGTGTCACGGTCGTGGACTTTTTTAAATAGTATCTGTTTTGTTGTGATTTCAGAACCCCCTAAGGAGAAGAGACAGAAATCTCACCACGAAACGAAACGTAATTTGGAAGAACAAAGTCACGAAGATGGCATAGCACCGACTTCTACGACATTCGTGAATGGAGCGGTTGAGGGTGCGTTATCGCCCTGTGTTTCTATTGATAATCACGAAGATCAACAACATGATGAATTAGACAAGCGCGTTTATGCGCAGGTGGGTGGAGTTTTGGGTTCACCAAAACCTAGGTCTTTGGAGTCTTTGTTGTGCGTATCTAAAGCTGATCTTTTTCTTTTAGGGGACGAACCGAGAAGGTCTATCCAATGAAGATAATTATGGAAATTTTCAGCTTAATAAGTCTTTAGAACAGCTTAGGGCCAGACTTGTTGCAAGCAGCGGCGAGGTTGTAGAAAGATCGCTTTCGAAATTGAAAGAGCGTTTGGATTATGTAAAGGATAATTTAATAAAAAATGTACTTGAATGTGCTGATGTTACTGTTCCAAGTAAATGTTTAAGTAAAACAAAACATATCGAGCAAAAGAAACAGATAGTGTTTTCTGATTGTGTCAGGTCGGTACCTGTGTGTGAGATTAAACCGTTTATCGACATGCGAGTATTTGAAACTGAAACAACGCAGAACGCAAGAAGAGTTCGACAACGGACCAGAACAACCGTCGGATCAACAGATGGTGCAATCGGGCAGCAGCGTGTTATTTCAGGACAAAACCGGGGCAGAGCACGAGGACGTGGCCGAGGGAGAGTTCCCAGGAGACGGAATTCCAATCTAAATAATTTAAGGACACAAAATTCTGCAATTGTCATAGACGATAGCAGCGAAACCGAAAACTTTGAAAATGCTGGGAGTTTTAATGAAGACTTACTGGCAACCACAATATTGGAAACACTGTGAATTTTATTGCTGTATTGTAATTCTTATGTAAACTAAATAGGTTCTATTCGTTCAGAATGTTCCAATTGATTGGGTCTTTCCCGTGCTCAGTAAGAAATAAATTTGTTCGCACAAAATGATTGCAACCAATAAATGGCATTCTTGCAGATTTCACTTTGGGTGATGGATGTGCACTTTCTAAGACGAGATGTTTCTGTGCGTCTATTAGATAAGAGAGTTTCCGGGCATGTTTTCCCCACAACAAGAAAACGAGAGAGTTCATTTGTTCCGATAGTCTGCTGATAATCTTGTAGCTCAGTGTTTGCCAACCAAATGCCTCGTGGGACATTGGTAATCCATGAACTACAGTGAATATCGAGTTTAGCAGTAGGACTCCTTCTCTACACCAGGAGTTTAAACAGCCGTTGTCAGGGGGGGAAAAATTCGGGATGCTTCTTTCCAGTTCTTTAAATATATTCTTTAGAGATTCTGGAATCGAGCACTCTTTCACTGTACCGAAGGCCAAACCGCAACCTCTGCCGTCGGGATACGGATCCTGTCCCAGAATCACAACTTTAACATCGGTGGGTTTGCACAAAAAACTCCAGGCGTGGACGTTATCCGAAGAGGGGTGGACGGTTAGATGAACTCTATCGTTGTCCACCAATTTATAAACATGCTTAAGATGTGTAATTTCTAACTTGGAGAGTTGCAGGAAACTCAACCAGTCTCTGTTTATGCCGAAGAGTGAATGCTGATCATCTATTGAAAGGTTTTCATAGTTTTTTTCTTCATCCTGAACGTGATCTAACATCCACTGTAGTAGGGCCATGGTTGGGAAATGAAATGCAGTGGATTAAATTCCCTAGGAGCATTTTATGTGAATGGGACGTGATGTCATAGCCAATTATGTGTTTCTAATCAGAATCGTAGGGCGGGAACTTCGGAAAGGTAGTTTAGAATTGTAGGCTTCCATCTGTTTTAGCAGGTCGAATTCTACTCCGGTTTTCAAGTGGAAATACTTTAAACAATTCAAAATCGTATCAAAGAGAGTCAAAGAGTTAAAAGGGAACCTGATAATTTTTGTGATGCCTGTAGCAGGATTCTCCAAGTATAATAAAAAACCAATGTTGCTGTAAAAACTGTCTGTGTTTTCTGAAATGTTAAACTCGAGACCTGCAATGTTATCAACAAAAAAGTTATTGACGTACGTAATTCTTTCAGGCAAATATTTTCTACAGTTATCGCTGATGCAGGTTGTTATTCCGACGGAAGGCGAACATGTCTGGAAGCCATTGAAAAAGTTTAGCCAATTGTCCCGAGATTCTGGGTCCCCTATCAGCGCAAACAATGTGCGTAGTTGCTCTGGGTTATCCTGCAGAAGTAAGAAGTCGCTGAGGAATTCATTGGTTACCATTATACGGCTAAAAGGCTTTTTTTTTGTTGGGGGTTTTAGCAGTATTGACATAACGTCTTCATTATATACCGGAGGCTGGTAGATTGGTTCTAGTAATAGAGGTACTTCGGATCTCGTGCAATTTATTACAGCTGCGATACAGTCGTCAAGTTTTTCAAAATAGAAAGAATTGTGGTCGAAAAGAGGCATCGCTTTCGAGAAGGTTAGTAATATGAGTGACATTACAAATAGTAAAAGTTCCATGGTTATTTTTTATTTGGAGAAGTTAACACGACGGGAACAACATCTTATATACAGTAGGGAAAAAGACTTGTCGAAATGTTCATTTGGCTTTTTATTGTTTTTTTTTATGCGGCATATATTGGTATGGCTATCGGATTTATCGGTAGTTCCCCCGATGCGGAGCTGTCTTCAGAAAATTCACGTATTTCGTCTTCTGTCTTATTAGGATGTTTGTTGTGTTGCACAGATTGGTCCGCTGTCGTACCTGGGAAGACAGAGACTTTCAGAAAACCTTTTGTTGCAATCATGATTAAAAAGCTAAAAAGTTGTTTTGCTGCTTACCTGTCTGATTTAGAGCAGGGCTCGATGTGTGATATGGCAAACGCATCGCCGACAAGTCTTGAATTAGGATTGTCGAAATTAGACAAAGAATCATGAAACTATTCGAAGACATTAATACTGGTTTTCTTTCTCGTTATGACGATCAATATAGAGGACGCAAAGAGAGACCAATTTAGATTCGAGCCAAAAAAGTTTATTTTGTCAGTTTAATAGTTTGCCATAATCATCCCAATAAATATATTTGTTGAAAGAGTTGTATATGGTGATGTGTTCTCGAAGGAGCATCGGTTGTGTTAGCTTCGCGATATTCAGAGTAATCTCGTTGGGATGGCAGATTGCAAAATTGATACAAACGTGAGCGGCGCATAATAAATCTTTTTCCGTCGGCGCGTTTATCATGAGAGAGCAAATGCCGACAAGCTTTTTTTTTGAGGCGTTTACCGGTATAGGAGCTACAGCGTTTTTTCGAAGTGCGTCCGTATGTGGGACGGTCCCTCTAAAAAACTGATTGATTGCAATAGCGACTACCGGTTCATGGTGAACCGATTCAGAAATTATTTGCTCGCCCACGTTAATAACCATGTTGCCAATGGACATCAATTTTTCTTGCATGGCGTTTATTAAGATTTTATTTCGGGAATAAACCTGTATTTGCACAATTGAAGCGTTGTAACAGCGGAATAATCTGTAAATTTCTGACATAAAATTTTTAGGTGAGCACGGGGAGTAGGGTGGAGATGTTTCGTAAGATTGAAAAATTTTGAAAGCGTTAACTGCATTCGGATCTTGCCTTTGGCATTCCGTAGACTTGACAATTTCCTTTTTCTGCGTCGTTGATGTCTTTACAGGCATGTTGAATGATAGGTCGGCGAGAGGTTTGTCCGTAAATGCGGAAAAACGATTAATTTCTTTATCTTGGTGAAACACGGCGTTTGAGATTTTCCGTAGTTTGGAATGGGCGTAGATTGCGTTATTTCGATATTTTTCCTTATTAAATTTTATGTCGGGCGAGGTGTGCTGGGGAAAGGCTCTTGCAGTGGCAGAATCGACGGGTTTATCGGTTTGGACGGTTTGCGAAGATAGATTGTTTTGTAATCGATTATTTCGTATAGTCGTTTTGGTGGTTACATTTTTTTTCCGTTCTGTGGACGGTTTCGTTGTTTTTCTAGTTCGGCGAGATTTGACGGTCGGTCTTGTTTTAGAGGTGTTTTTATTACCTCTGTCTTTCATCTCTTCTGAACTTAAGCCATATAAATTTTAACTTTTAAATGCAACGATGCACATCGAAATCAGTAGCACCATAACTGATATTATCAATATCAGAATTAATGGTACGATCCAGGTAAATACGGTTTCGTCAGAATTTGAGCCTTTTACCGCTTCTTTACTGTATTCGTCGTGATTTTGCACAGTGTGTTCGCCGGAAGTTACGAGAGCGCTAGCCTTTTGGTTGGTAAACCAGCTCGACGCTAGACATGAGATGGTTTTTCCGACAAATTCCAGCTTGGTTCGTCTTTTGAAAGTAAAGCTCACACTAACGGTTGACGATCCGTTTTCATTCTGTCTAACCATTGGTATGTCTCTCTTATATACTTCTCCAAGAAATTTAATAACCACATTTGGTTTTGGATAAGACGTCACGGTACACGTAACGTCTAAAAAATTGTTTAGGTAACGAAAATACAAAACGACGATGGGTTTCATAATGAGTTTTAAGCAACTAGTCGCAGATGTTTTCTCGTTATCCACTGTGAATGTACAGGTTAAACATGTCGCGTCATATGCTGATCTGGCTTTAATTTTCAACAGAGATGTAGTTCTATCGTGTTTTATGTGGAAATACAATTTACTGTCCACGTCAAGATTGTTGATCTTATTTAGTTCGTGTATAAAAACGTAGCCATCGTCAGTGAACGATAAGTTGTCCATGTTTTGTATCTCATGATTCGATATAAGTCGGCTTTTATTGTACCACGAGACGCTTTGGATGTTTGTCGTTGTGTTGAGGCGACAATGGAAAGTAACAGTTGACCCTATTTCGTATACTAAAGAAGGACTCGTCTCCGGGCGGGGTACAAAATGTGCGCTTATGATAATACATAGAAGCGGCAATAATCGAAGCATCAAGGGACTCATAGTGTACACCTGTGCCAAATAGCAGTTTCTGCGCCAAATAGCGGTTTCGGATATATATAAGTCACACCCAAAATAAAAAAAGTCAAGGATTTCCGTTATACTGTTTTATTTTTTTATTTAAAAAAAAAGTTAGGAGTTAACATTTTGAAAGTGTAAGATATTTCGATTTAAAATCTCTGCTGACAGAATTATAGATAGAGATAGAATTGTCACCATCAATGATTGAGCAGACCCGAAGGACAATTTCTTTAGGTTTATCTTTGGCATACTTATTGCATATTTTAGAAGCCATTACAAAGTCGATGGGCGTAGCGGCGTAAACCATAAGAGTTTTTACGTCCGAATGTCTCGTAAATAGATCATGTGTGTTGTTTTCAAGTTTATCCCACAATTCTTGAGGTCCTTGTTTTTGTAGTTCTATAAATTGATTAATTGCATCTTTATCATAATAGTTAGGCGGTGCATGATTCATCTTAAATGGCTCTGAAACAGTGATGTTGGGTCGAATCATTAATTCTCTTTGCATCGTCTTAATGATTTCTGCTACTTGTTTTGATCTTGTAAAGTACATAATGATGGCTCCGGTGTTAGAGCATCCATTAGAACAGATGTTGTATACAGCTTCTTTTAGCTTGTTTTCAGGGAAAGGTTTTTTGGGAATCTGTTTGTATTCAAATGCTCGTATGGTATTTCTAAAGAGTTTTCTAAAATCTTTTGTAGACACCCTGCCGCGGTGAATTTTATATTTTGCCGATTCTTTTTTTTCTTTTCGTCTAACATATTCTCTATAAGACAACTCTTCTTCAATTTCACTTATTTCAGAATCTGTTTTCTGCGTTCCAGATATTCTTTTCTTGAAGAAATTTTGCATACTGGAATCTGAATCATCATCCCGGGTGCGCTTTTTTTTGCATTTTGTTGTGTTGCGTGATATGTTACTTTGGTTATCCCGTGGGCGATAAATTGTGTTAGTGGTGGTGTTCGTGGTCTTAGGTGTCTTTTTTTGTTCTTGATCAATGGCGTTTTCTAAATGATTGGATTGTTCATGTGTGTTAGATGGACTAGAATTGTCTAGGGGAAATTTTTCACCTGTTTTCTGGGAGTTTCTAAACATATCTGCATGTCTATGTTTTGATTCGTGATTTGGAGATTTGATTTTGGTTTTGTCTCTTGAAGCTGATTTAGGTCTTGTTTGTTTTTCTAGTTGTTGTAGTCTAGATCTAGGTGAATATGTGGCAGGATCTTCACTGTTTTTATATCGACTTTTGCGCCAGGATTTTGATCTGGAACTGGAACATGATGAAGAGACTGATGATGATGATGATGATCTAGTTCTGGAGTGCTGATAGTTAAATGTTATAAGCTTGTCTGTTCCGGTTTCTGTACGTGAATTAGATCTGGAATTTTCTTCAATACTGGAAGAGATGCTTGATGATCCAGATTTGTGTTTTTTTCTTTTTGGAGTATTGAGTGAATCTGGTGAATAACGAGAGTGAGATCTGGATCTAGATGTAGAACTTTCTGAATCGGTGTAATTGGTGGATTCTGATTTTTTTCTGTTGGTTCTTATATGAGGTCTGAATCGGGGGTTGTAAAATTGATCATTGTGGTTAAAGGACTGGAGTCGAGCTGCAGCATTTTTGTAGAAATTGAGTTCAGGCCCGCACCGAAATTTGATTTCTGATACTTCAGCTTCTGTACTAATTGAGGTGTCATGTGGATATATTTGTCTTTTATTAGAATCGCAATAAATCTGCCTCGGTTTACGTTTGTTTGAAGTTTTTTCATAAGTTTGAGGGGAAGGTATGTTGGACATTAATTTATTGTTAGGGGTTTTACCGGAGGCTCTGCTGGAGGCCCTGCTGGAAGCTTTGCTGGAAGCCCTGCCGGAGGCCCTGCTGGAGGCCCTGCTGGAGGCCCTGCTGGAGGCCCTGCTGGAGGCCCTGCTGGAGGCCCTGCTGGAGGCCCTGCTGGAGGCTCTGCTGGAAGCTCTGCTGGAGGCTCTGCTGGAGGCTCTGCTGGAGGCTCTGCTGGAGGCCCTGCTGGAGGCCCTGCTGGAGGCCCTGCTGGAGGCCCTGCTGGAGGCTCTGCTGGAGGCTCTGCTGGAAGCTTTGCTAGAGACTCTGCTGGAAGCTCTGCTGGAGGCCCTGCTGGAGGCCCTGCTGGAGGCCCTGCTGGAGGCCCTGCTGGAAGCTCTGCTAGAGACTCTGCTGGAAGCTCTGCTGGAGGCCCTGCCGGAGGCCCTGCCGGAGGCCCTGCTGGAGGCCCTGCTGGAGGCCCTGCTGGAGGCCCTGCTGGAGGCTCTGCTGGAGGCTCTGCTGGAGACTCTGCTGGAGGCCCTGCTGAAGGCTCTGCTGGAGGCCCTGCTGGAGGTCCTGCTGGAGGCTCTGCTGGAGGCTCTGCTGGAGGCTCTGCTGGAGGCTCTGCTGGAGGCTCTGCTGGAGGCTCTGCTGGAGGCTCTGCTGGAGGCTCTGTCAGAGACCTCGGTGAAAGTTTTACTCAGAGGTTTATCAGAGTTTTCGCCATTAGTTTGGTTAGAAGTTTCAGATTTATTTTCGGTGGAACTGCAGTTAGGTTTCTTGTCAGTACATTCATCACCGTTAGAAGTGCTATTCATGGTGCTGTTGCCACTGTTGGATTTGTTAAAAGCAGTAAATGAGCTAGGATTGGAATGACTCCGAATAGGTGAATTGTCTGTTAAATTTTTGTTGGCGCCTGCAGAGTGTTTTTTAGACGCACAGTTTCTGTTCATAGTTGAAGGAGTTTCTTGCGTGGGGCATGCAGAAGTTGATGGAATAATAGTTTCATTACAAAATAGTTGTGAAAATGGCTGAATAATATTAGAGTCCTCTATTTCTGTTTTATTTGTATTTTCATTGGAAAAACTGTTTAAGTCCGTATTTTCGGTACTTTCTTCTGTTTTATGGTTTGTTTTGCTGTTGGGGGTTGTTTTAGTGAAATTATCATAGAATTTATCTGATAAACAATTTCCATAATTGCTTTCACTGTTGTTGGGGGATTTGTATTGGCCTTCTTCAGAGCTACTGGAATCATGTATACAATGGTGCGTTTTTGATTTTGGTGGTGTAGATATCTTTTCATTGTCGGGTGAGGTTTTATATTGTTCATCAGATGTTTTGCCGTGTAGGTTATGTGAGGGATCAGACTTTTTTGATGTATTTTTGGCGGCTTTCCCTGAAACATTTTTAACGGCTACTTCCCTAACGTCTTGGTGTCCGTTGGAGTTTGCAGTATTTTTTTTAGATGTCGTACGTCCCCGAGAATGTTTCATTTTAGGATCATTAGTGTCTTTTGGGTCATGATGTTTTTTGTAAGAGTTATCTTTGGTCGGGGCTCTTGGAGCATGTTTTGATATGAGAACTCTGTCTTGTAGAGAAGTTTTTGATCGGTGTATATTTTTTTTTGTGTGGTGGAGTTCCGAGTCAGAATGAATTTTAGTTTTATGAACCGCAAAGTTTAAGGTGGGCTCAGGTGTCATCGGAAGAGGAGATAGCAGGGGATCTAATTTTGAAAAATCTCTAGCTTCAGTAACGGCTTCATCTTCATGCTGATTAGATTTTCTCTGTTTAAGATCAGAACGCGATGGAACATGGTTTTCATTATGTTTTTGTACTTTATGTTTGTTTATTGTTTGAGAACTGTGTCCTTTTGTCTTTTGGATTGATCTACAACGGATATGTGTTTTTTTGTACTTCTGAGTATCCACGCCACAATATGAGCCATTGTCTATTAGTGCTTCCTTCGGTAACGGGATAACTTCAGTGTCATATATTTTTTTAATGAGATCCATTTTTTGGGAACTAGGTCTGTATGATAAACTGATTGCGTCAGGAGACGTTGAAACTTGCTGTTTTTTGTGTTCTTTGTTATCCTTAGCATTCTCATGTTCTTTAGTTTTCGTAGTTTCTGGTGTTGTCTGTGTTTTTGTTGGATAATTGTGATTGTCAGATTGTACGTATGTGGGGTCTGTTGTTATTGAAGTCTGTGAAAATGCCATGCTATAGTCTTGGGCTTGAAAATTGAAGTTGGTTGTTAGTTGTGTAACATCTTTATAGTTATTCATACGGAGATCTGGATTATATTGTTCATAGTTTGGATGCATGTATTGTTGATATGATAAATCTGCGGGTCTGTTTGAGAAATCGTGTAGTCTGTGGCCTCTTCCGCGTCTGTTATAACTTCTGGGAAAAAATCTTCCATATGGCTGTTGATACTTTGAGTTTGGATTGAAGTAATTACGACTATCGTATCTTCCCTTTCCTCCTCTGGGCCTATGGGAATGATGTCTAAAGTTAGAGTCATATCCTCTATCGGTATTTCTAGATTCTCTGTAGGAACTCTCTATACTCCTGTATGCATCATAATTAGGTTGATACGGCAACGAGGTGAAATTGCACTGTGAATTTGTAGTTTGGTTCTGTGATGCGTACATGGGCTGTAGGACTTGATCTGGCAGTTCGGAAGCAAAGCGCATATGGATCGAATTGTTTCCGTTTGATGGTAGATTGGAAGCAGAGGATGTGCATTGTCTGTGGATCTGAGCTGCTCTCCGTGGATCCTGGGGAGTTCTTGAATTTTTTCTGGAATCTTGAGAAGATATGGGATCTTGATGCGTATTCTCACTCAATGGTTGTCTATGTGTGACGTCCGCATCATGGTATAGTCCGGATAATTTCATGGCTTGGTGGACAACGTCGTTATGAGCATTTGCTTGTTTGGATTTTTGTTCTGGAAAACATGTTTATAAAATTCAAGCTTAATAGATAGTATTTTTCCGTCGATTAGTGATACGCTTAATGTATTTAATAAATTATGGATAATCATTTTGATTATGATACATCCCGTGCAGAGAATGATGTTTATACAGATTGTGCGTAGTATGCAAATGAAAGTTATTCCATCGTTTTGTAATATGAAAAGTTTCAAATATAGTAAAGAAAAGTACATATACGTTTTCAAGGTGTGTTCTAATAAAATGATGAATAAAATAGTAGTTTTCTATAGAACGTATTATAGACTTTTATTGCATAATAATCATTGAGTAAATACGTGGAGACATTAAATTCGTATTTTGTTTTTTAGATTGTTGAGCTATATAGATAAATTTGGATGATAAAGACGTTATAATTTGTAGTGAAATTCACTAAATCGTTTTAAGCTTTTATATTTATCAATTAAGTAGTAACTTTCCGTGGAAATGTCGCAGAAGCGGAATAATGCATTTACTAATTGGAAAAGATGTAGGAGAGTGGTAAAAGCCTATTGGTTTCTTAAGTATGTATATCGTGCGATGTGTTATTAACTAGTTAGATGTGGATTACTAATAATGGGGGGGGGGGAAATTATTCGCATAACATGCAATTAAAATTCAATTCAAAATTTATGAAATTGACATTATTTGGAGAAATTTTATGGGACGCATGATTTATATAATTGATCTATTAACAGGCCAAGCGACGGGATTTAGTGAGCATGATATTAAATCTTTTCATTCAGTAGTTGAAGTTATTTTCGTGGTTGATTACATTTTTGAATAATTAATGGTATATAATTAATAATAAGTAGGGCTAATAGTACAATTTATTATCACTATTCTTATGCGATGAGTTCAAGTTCGGGCGCTGAAAAAATACGTGTAATGAACACTTTTTTTAGAGAACTTATGTTATTAGAGAATTTATGTTAATTAGTAGTTCAATAATAGAATTTATGATTTATCAATCACGTGATATGCCATATATTTGGAAATCGCGTGTAATAGTATATTAGGTGTCATATATTAATTTCATTTAAAACTAGGATGAACATTAACATAAATTATATATAATACCTGATGAAGTTAATGGCAATTGAATTGGATGGTATATTAATTTATATTTACTTATTTGCATTAGATTATATTATAATAAAATTGTTTTAATGTTAAAAATTTAAATTATTGATGCATTCATTATTGGCGTGCAGTAAATCCAGTATAATGGGTAAAAGTGAGATTTATTGGAGGGGGGGGGGATGTATAATATCCATGTTTTTAAGATATGGTAATATTTATCAATTATCTAATTGTCTAATTCTGTTGGTTGTCTACAGGGAACCTATAGATATATTCAGGAATAATTGGAGTTGAAATTGCTGAAGTTTGTGACTACCAAAGGGTTAATATGCATAATCAAATAGATACTGATGCTACCGTGGGGATGGATATGATTTGATAATTAGGATACAATTATATATTATTTATTATGTATTATGGAGATGACATAATTTAATATAATACATTATATGATAATTTAAAGTTTATAGTAGACTAGTTTTATTATGTTTCTAATGTGATAGCGCCCTCCGCTGTTCAGGTAAATTATATGATATTGAATGTTTTGAGTAGACTAGTTTGATTATGTTTATGATGTGATAGCGCCCTCCGCTGTTCAGGTAAATTATATGATAGTGAAAGTTTTGAGTAGACTAGTTCGATTATGTTTCTGTTGTGATAGCGCCCTTCGCTGTTCAAGTAAATTATATGATATTGAAAGTTTTGAGTTGACTAGTTTGATTATGTTTCTGATGTGATAGCGCCCTCCGCTGTTGAGGTAAATTATATGATATTGAAAGTTTTGAGTAGACTAGTTTGATTATGTTTCTGATGTGATAGCGCCCTCCGCTGTTCAGGTCAAAATTTAAGGGTTGTAAATAGACATTTTTAAAATATTTTAAACCTAAATAATTTCATTTATTGTGCTTTTTGTTAAGATGCTGAGTTGTTGTGTTTGTGTGTTGTGTTTGTGTATGTGAGAGAGAAAGAGATAGAGAGAGATAGATATATGGGGGGGGGGGAATATATAAATATAGTAATTTTGAATCTGTGTTTCCAAAATTGGGAATTATGCGTAGCATCCCACTTTCAAAGTGATTTCAGTTAATAAGATGTTGGATTAAAGATGGATTTGGTATTTTAGATTTATACTTTCAAGTGTTTGGTGGAAAACAGATAGAACAATGGAACGTTGTCTCTGAAAAAAAAAAACTAGTAGTGTGACATTTAGGAAATGTGGTTTGGATACTTTCATCGTGTAAAAAGTCAAAAGGTTGAAAATGTTTTTTTTTTCATGAATAGGAGCTATTATGTGAATGTTGAACAAAAACAGATTAAATAGACCTGAAAGGATAATATTGTGTAGAGTGCTACGGTCTAGCTACAACTAATGTAATACATTTTTTAGCAGTACTATGATTTATAAAATTAAGATGTCCCCGGGGCATTAGTCTTAATAAACTGGTTTAGTTTGGAAAAGAGAAAGTGAAAATCTGAATACTTGGCATCTGTATAAGGTTTTCCAAGAATACAATTGTTTATAGTATCACATCTTCAAGAAAGCTGGAATTAACTTATGGAAATATTGTATGATGACTTATAAGTCAGTTTATGTACGTGACTTGGAATTAAAAAGAGAAAGCAATGTCTGTATGACTTTTTATTATTTGTAAGGTGATATTGAAAATAAATTCCTTTGAGAAATCACATGTTTGCTGAGGTTTTACATAATAGAAAGAGTTACATTTGCTTATGTAATTTCTAATCTATAGCAATATAAAGAGTTACACAGTATGGGGAAAGTATGTATTTTACAACAATATAAGCATATTTACATAATACACATCTGTATGTTAATGATTGCTAATCAATTTACATCTCAGTTTCATCTAGCTAATAAATTTGAGCATTTTCTTCGAATGGATCATAATCAGAGGGGTAGCCATCTAATTTAAAGACCTCCATTTTATCACTGTTGCAATCACTTCTAATGGTACATTTTTCATTATCATTACTTGTACCTTCCGTACATCTGTTCTCATCATTTTGACATGTTGTGTCATTGATAATATTTAGTATGGGGGAGTTATTTCCTGTTCCCTGTGGGTCAAATATTTGAGTTTCGGCTGTTACAAGATTTTGGGAATTTGCCACATTTAAATTTTTCTCACATGCGGTATGGGTGATAACAGTATATTTACTGGAAAGATCTTGGCAATGTTTGGCTATGCCGTGTTTAATGATATCAGTTAGATCCATAAAATTTTCCATGACCATTTCTGTGATAAGTTTCTTGGTTGGTGTTATGTCTTCCAAGTCTGTTTTGCTATCCTGTATTTTACTATAAGTATATGAGACATTTTCCTTCTCATATTCTTCATCATCCGTTTTAATGGGAACAAAGTGATTGATGAAGCAGTGATTAGCTTCTGTCACATCACTGTCATTTTTTACATTCATTTTTGCAGAACTGTGTATAACACAATTGTCTACTTTGTGAGTTTTCCTCATAAACCACAACCAATGTTTTTTGGGAACAAATTTGTTTCTGATATCATGGGTACCCGGCATTGACCTTATTGGGATGGTAAACACTGGGGATGGAAAACCTTTACTTTGACAATGACCATTATTGATCACTTTATCATAATCACTCAAATGTAAGTAATTATCTTCAGATGTACAGTCATCTGACTCGCTACTCGATTCAGAATTAGGTTCTGATTTAATAGTTTTTTCTAACATTGACTTATCAATAATATTGTTACCATCCTCTTCATCTTCACTCTCTGAACTTATAACAATTACGTTTGTGGTTTCAGGTAACCTATCTAGCTTAATTTTTTTAGATTTGGATGGTTTAATTGGAACATTTTTCCTTTTAGCTGTTCTCTTTTTAGATTTGCATTTTTGAGACACAGCCACAATTTTCTCAGTTTTGTGATCTTCCTTAGAAGGAGTGACCTCTGGTGGTGAATATAGAGACTTGCTAAATGGTGTATTCTCATTAGTGTTATTCATATGAATGTTATTAGTTCTGACAGTTGAATCACAGTCTGCAATGTAATCAGTTTCATCATTGTTATCGCTTTCACTCTCATAAGGTGCTGAGTGATCAGTTTCATAACCAAATGTTATCATTTCTGATATTAAAGGGTTGTCATGGAAACCATCACATAGATTCACTAGACTCTCTACAGCTTCCATTTCTTTGTCTAATTTGCTTTCTGGATTTAGAGTATCCTGAATTGCACCATGACTTTTGACAGGAGTTGCTGAGTTGGTAAAGGTCATACCAGGAAGCGTTTCGGTACACTTGGAGTTAGAACCTTTTTCCTGAATCCATTCTTTTGTCTGTGACCTTTTATCGGGGCTGTAAGCCAAAATGTCTTGTTGTCCAGACCCGGTCTCTACAACTACTGAGTCGGGTTGAGTTTCAAGTAGTTTTTTTATCAAGAAGTGTTTACCACTTTCAAGGCCAGTAATGGATTTAAAGTTATCAGGGCCGCCCAGATCTGTCACTGAGGCTGTACATACACAGTTAGGGCTTGTTTGTTCTTTCCCTATCTGTCTGCTATATTCAATGAATGTTTTTGCCTCTTTGGATTTCCCTGGCACATCTGGAGAAGGAGTGTCTGGATACATTTTTCCTACATCTTTTTCATGTATGTTTGTAAGTTTTTGTCTTGTTGGGGATTCATTGTTCTTTGACGGAGTGGGAATATTATTATCAGTAGGAGATTGTACTTCCATGTATTTTAAATAGTCTACATTGTCATCATCTTCATGGATAGATTCGATAGAAGATTCCTCTGAATATTTTCCATGTTCATCAAAATCAGCTAACTCTAATAATATTGATTCTGACGGGTCTATGTTTAAATCACAGATGGAAGACTCTCTATTTTTATTTATAGCAATGTAGCCTTCCATTATATCAGAGGAAGTTAAAGGTTTAATCAAACCAGTATCATCATAAATTGTTTCTATGCATTCACATGCGTGGGTGATCATATTCATCATGGTAATTTTAAACTGGTGTCTGCGAGGTTTGAGGTATTGCCATGAAAATTCATTGCTGCATAGAACAATACTTTGGAGAACAGCAATGCCAATATTTTTGGCGGCAGTAATTAAATTTTTACAATTTATATGAATTTTGAGGAATGTTTTATCACAGTTATCCATAAAGATTTGATTCATCTGCTTATTAGCAAATTCCTGAAACATTTTCATATTTTTAACATGGTTTTCTATTATCTCATTTCTACTTTCCAGTAGTTCCTGTTTAAGTAAATTTACTTGCTTACAAAATTCCTTATTGGAGATTTCAGGATTGGACATCTCTTTGTTGTGTGCCATCAACAACAATGAGGTGGCGACCATGTTCAATTTTCCACTATCTAATGCCACATAAGCTTTGGAAAAAATGTTTTTGAGGGCTGTAATAAATTGATTGTTCATATTAGCTTTCAAATCCATGAGCAAATCTCTGGATTCTGATGCTATCTTTTTCACTAACCCATCAAATTTATCACATGCAAAAACAATGAATCGCGGTTCGCACCAATTAAAAAAATCCAAGTTTTTTCCAGACAACATTTTTGCATAACATGAAACAAATTGAGATGAAGCACTACTTAGTAAATTAATATTTATCAACTGTCCTAATTGTTCTAATACCAGCCTCTCCAACATGGATCTGTGCAGCGTTAATAAGCCAGCGGAGTTAATTAAATTGTCTTCCATGTTAGACTGTTCCTGTTTCATGGCAGCCTTCACTGATGCACCAATACTTTGGATGCAATTGCCAACGGACTGAGCTAGGATGTAAAAGAAGATATTCTAAGTATGCTATATGTTACATGCTTAAAACTATGTTTTTTTCTTGTTTTCTATCTTGGGACATATAATATATAATTCATGTACTCACCAACCAAAGTTTTGAATTCGTCAGATGCTCCTTCTTCCACATTACTGGAATAGGACACATTCTTGGAAGCGATGTCGTTGGAAGACTCTGGGATGAAAAGATCAAAGGCTTCCAGTTCTGGAAAAAGCAGGCTTTCAAAGGACACATCACACTTGAGACTCTCTTCCAATATTTCTTTGATGGATTCTTCCACCACTGGATCGGGATGGTAGCTGTAAGTATGAATATTTCTTAGTACTTAGAAAAACTCCCATATAGTTTTAAATAAGATAGATCTAATAAGGACTAATCATACTTACTTTTGCATACGTTCCTCATTGGATTCCATGTTGTTTCCAGAGGGTTTTCCGAGAATGAACATAGAAGTAGAGGTTGTATCTTTTGCTGACTCCATTGTATATTAAGATGCAAATTCAGACACCGCCTACTGTTCTGTTAGCCAATGAAAAAATATCTTCTGTCAGCAGATATAATACTATATAAGGAGATTACCACCACCTCTTTCTTTGCAGAGATTATTCTCTGCTTGAAAATCTGTAACACTGATCATGATGGGATATGAGGAAAAAGTGTCAGCTACTGGAAAGACTCGTTTAAAGATACTGGCATGTCTGATCGTTTTAATACTAGCTGCGGCAATAACTATGTTAACGCTGGAAATTATATCGAACCAAAAACGTACCACTACTGATCTCGAAGCTGTGACTGTGGCGCTGAAGCATGTAAGCACATCTCTTGCCAACTGCACTGAATCCACTACTTCTGTACATACCGGTTAGTGAAAGATATGTCTTTTTTATGTTAATGTATTGATTATATATTTGCATACATTCCTTAAGAATGGAAAAATCACGGATGGTTTGTTTTTAGATTCTGTGACGAGCCAACCCACGAAAAAACAAAGAATCGAGGAAAAAAATTGAAGGGAAATCTCCAAGTTGGGTTCAGGCTTTAACTACAGCATCTGGAATTATCCTACTGTTTTGTATAATGATGATATTCATTACATGTTCCTGGACCACAGAAAAAGATACAGAGAAGAGTGAAGTGCAATCTTATGCTTCTTCAGTAGAGACTTTAGACTCTTTAAATGAGGCTATTATACCGAAAACTGAAATGAATGTGTAATGTCTGTATTTTTCTTTACAGAGATGTACGGAGAGTTTATATTTGGGGAAAATACCTGACTGTTCTGCCTATATGCGAATGTTAAAGTATGTATAATATAAATTCTTACCTTTTAAGAGTGATTCAAGGTGGAGGTTTCTTTGGAGATTGATTCCAGGTGGTGGTTTCGGGTGCAATCAATCTTTCTTCTGGGCGGGAAGAAAATCCAGCAATCCAATAATTGATGGGATGTAATCAATGTCACAAATCTGTAAGATTAAATGTGAACAGTATAAATTCTTTTGTGCTTATCAAATTACAATTATGCGCATGAAAATATCATTAAATTGTTTTAAACATTCTTAAATTCAATTTAAAATAATTTTTGTAGCAATTTTAAATATTAATAGTTAAAAAATTGACTTACCAGACTGCAGCGATATAAGAAAGAATCTCTGTGATGAGAATGTTCTCCGGAGCCACTTCTTGAGAAAAGTGGCACTGAACTGGCTGTAACTTCTGCTTATATACAATTCTATAGCGGGTGTGATGTACAGGAAAATGACGAGGATGAAGGAGCCAATCGCTGTATAATGTTAAACCGCAAGTGTAGAAATACACCCCTTTTAGACAAATATGGAATGGAAATTAGCTGATGCTTACAAAAATTTAGGGTTTTGTCATATCTTACTCAAAAACAGGAATTTGTGGTCTGGATTTTTTGAGGTTTTCTTCTTATGGCTGACTTGTATTTTTTTATGAGAAAGACGGAGTTTGCGGTTAAAGGCGGGATAATAAAGAACACTTTACTGATAAATTGTCATGTTTTTTTTTAAAAAAAAACATCTGCTACACATCATGAAACAGGAATATGGTTTTGGTGTTAGCGCTTCTCACGAAATAAATGACTCAGTCAGGGGAAAATGTTGTAACCGCGGTACACACTGACCTTTAACTTTATGAGAAAAACAGGAACTGCGGTTGCTCTGTTAGCAGATTTTAGGGTTCTCGTTAGCCTGGGTAGGAAAGACCCCAACCACAGTACCCACTAACCTTTAACTTTATGAGAAAAACAGGAACTGCGGTTGCTTCGTTAGCAGTTTTTAGAGTTTTAATTCGCATGAACAGGAAAGACTCCAACCGCAATATCTGCTAATTTTTAACTTTATAAGAAAAACAGGAACTGCGGTTGCCGCGTTAGCAGCTTTTAGAGTTCTCATTCGCCTGGGCAGGAAATACTTCAACCGCGGTACCCGCTGATCTTTAAATTTATGAGAAAAACAGGAACTGCGGTTGCCGCGTTAGCAGCTTTTAGAGTTCTCATTCGCCTGGGCAGGAAATACTTCAACCGCGGTACCCGCTGATCTTTAACTTTATGAGAAAAACAGGAACTGCGGTTGCCGCGTTAGCAGCTTTTAGAGTTCTCGTTCGCCTGGGCAGGAAATACTTCAACCGCGGTACCCGCTGATCTTTAACTTTATGAGAAAAACAGGAACTGCGGTTGCCGCGTTAGCAGCTTTTAGAGTTCTCGTTCGCCTGGGCAGGAAATACTTCAATCGCGGTACCCGCTGATCTTTAACTTTATGAGAAAAACAGGAACTGCGGTTGCCGCGTTAGCAGATTTTGAAGTTCTCATCCTTATTAGCAGGAAAGATTTCAACCGCGGTACCCACTAACCTTTAATTTTATGAGAAAAACTGGAACTGCGGTTGCTCCGTTAGCAGATTTTAGGGTTCTCGTTAGCCTAGGTAGGAAAGACCCCAACCGCGGTACCCACTGATCTTTAACTTTATGAGAAAAACAGGAATTGCGGTTTCCCCCATTAGCAGATTTTGAAGTTCTCATCCTTATTAGCAGGAAAGATTTCAACCGCGGTACCCACTAACCTTTAATTTTATGAGAAAAACTGGAACTGCGGTTGCTCCGTTAGCAGATTTTAGGGTTCTCGTTAGCCTAGGTAGGAAAGACCCCAACCGCGGTACCCACTGATCTTTAACTTTATGAGAAAAACAGGAATTGCGGTTTCCCCCATTAGCAGATTTTGAAGTTCTCATCCTTATTAGCAGGAAAGATTTCAACCGCGGTACCCACTAACCTTTAACTTTATGAGAAAAACAGGAACTGCGGTTGCTCCGTTAGCAGATTTTAGGGTTCTCGTTAGCCTGGGTAGGAAAGACCCAACCGCGATACCCACTGATCTTTAACTTTATGAGAAAAACAGGAATTGCGGTTTCCCCCATTAGCAGATTTTGAAGTTCTCATCCTTATTAGCAGGAAAGATTTCAACCGCGGTACCCACTAACCTTTAACTTTATGAGAAAAACAGGAACTGCGGTTGCTCCGTTAGCAGATTTTAGGGTTCTCGTTAGCCTGGGTAGGAAAGACCCCAACCGCAGTACCCATTAACCCTTTAACTTTATGAGAAAAACAGGAACTGCGGTTGCTCCGTTAGCAGGTTTTAGAGTTTTAATTCGCATAATCAGGAAAGACTTCAACCGCGGTACCCACTAACCTTTAACTTTATGAGAAAAACAGGAACTGCGGTTGCTCCGTTAGCAGATTTTAGGGTTCTCGTTAGCCTGGGTAGGAAAGACCCCAACCGCAGTACCCACTAACCTTTAACTTTATGAGAAAAACAGAAACTGCGGTTGCTCCGTTAGCAGCTTTTAGAGTTTTAATTCGCATGAACAGGAAAGACTTCAACCGCGGTACCCGCTGATCTTTAACTTTATGAGAAAAACAGGAACTGCGGTTGCCGCGTTAGCAGATTTTGAAGTTCTCATCCTTATTAGCAGGAAAGATTTCAACCGCAGTACCCACTAACCTTTAAGTTTATGGGGAAAAACAGGAACTGCGGTTGCTTTGTTAGCATCTTTTAGAGTTCTCATTCGTATTGGCAGGAAATACTTCAACCGCGGTACCCACTGATCTTTAACTTTATGAAGAAAAACAGGAACTGTTGTTGCTTTGTTAGCAGATTTTAAAGTTCTCATCCTCATTATCAGGAAAGACTTCAACCGCAGTTCCCACTGACCTTTAAGTTTATGAGGAAAAACAGGAAATGCGGTTGCTTTGTTAACAGCTTTTAGAGTTCTCATCCGTATGGGCAAAAAAAATTTCAACCGCGGTACCCACTGATCTTTAGCTTTATCAGAAAAAACAGAAACTGCGGTTGCCCCGTTAGCGAATTTTAGGGTCCTCACCCACTTGAGTAGGAAAGACCCTAACCGCGGCACCCACTGACCTTTAACTGTATGAAGAGAAACAGAAACTGCGGTTGCCCAGTTAGCAATTTTTAAGGTTCTCACCCGCTTGGGTAGGAAAGACCCTAACCGCGGCACCCACTGACCTTTAACTTTATGAGGAAAAACAGAAACTGCGGTTGCCCCGTTAGCAATTTTTAAGGTTCTCACCCGCTTGGGTAGGAAAGACCCTAACCGCGGCACCCACTGACCTTTAACTTTTTGATGAAAAACAGAAGCTGCGGTTGCCCCGTTAGCAATTTTTAAGGTTCTCACCCGTTTGGGTAGGAAAGACCCTAACCGCGGCACCCACTGACATTTAAATTTATGGAAAAACAAACTTTTTTGTTCATCATGCACTTTTTTATATATCATTATATCTCTATCCAATCAGCACTCTTGAGGGTGCATACATTAAGGCAGTGTTGATTTTTTTTCATTGTACCCACTTACGAATAACGAATCAAAAGCCATGAAGTAGAATATTTTAATGATGTATTAATCATCATTTCCTACCACGCCTATTAACTTCAGTATTTATAGGATAGGCAATTTGCGCTATACGCCATTAGCTGTTCTTCTGCTAGCTTGGACACAGCGGTACAGGGTGAGTTATCAGCGTCTTATAATGTGATGTCTTTTTTTTTATTTTCTTGTCGGTATTTTAAAAGTAGTCTGTTAAGATGTTTTTAAATAGATAGTTATAAAATTTTTGGAACCGTGTAGTCAATAAAACAATTTATAGAACGGTTTCTGGCCCAGAACAGAAATTGATTTATGTCGTCTTTCTGAATTACAGGAAAATTGCGAGGGATAACTTTATCAAATGTAAAATTATATACCCTCTGTGATAGATTTATTTTGTGGATATTTGAATGTACCATCGAGCGTATATCAACGCAAGATGTGGCTATGATGGGACACGGTCCTATCTGTACGTGGTCGTTTTCATTAATTGGGAATATTATTTTTTGACCCCCAAGGAGGGATTTTATAATATTAAAATTTTCAAAGTTGATACTTATGTCTTTCCACCACAAGACTAAAACCTGTCCGTAAGCTCTTAAATTTTTAGTATTCAAAGATCCGTGTAATATTCCATGTAACGGGATATTTTCAAGAATGGCTCCCGTTAACATCGATTTTCCACATCCGGGTGGACCAATAAAACTAACGGTGTTTTTTTTCCCTCTTCTGTGGCTTAGCCACTGATATAATATGGTTCCTGCTAATATAGGGTCAAAAGACTGCAGACAGAACAACTGGTAAACCGGGTTTTCATAGATTTCAAAGTCAGTGTCTATGTGCGCTATAAAATCGATAGCTTTTCTAGTTGCTCTTATTCTTTTTTTAGATATAGATAGAGCCCTATGAATTAGCACGTCCCCGTGTGTTAGTGTCAATAATTGCATGTATGATCTAACGTCTCTTCGTATCCACGCGTCTTCCGTGACTATTCCAAGATCAGACAGCCATTCGATGGTTCCCAGAAATTTAGATTTTAAAGATATTCTGTTGGTCCCATCCTTTGGAAACAGCATAAGTTTAACATTAGGTTTTTTATTGAAATGGGATATGATGTGTTTTCGATGTATTTCATCGCATACGTCTCCCAGTTCGTTAATATTTGTCCATGCCCATTGGAACTGTGGTCTAATAATTTGAAGAAGGGCAAAGGGGATATAACTTAGACTGAAGACCTGGGTTCCGCCACCATTTTTCTTTGTTTTGGCATACGTGCACCAATCATCTATTTTAGGGGCGTTCCCGTCGAAGAAATCTTTAATTAATACACCCCTGATTTCCGTTGTGTGTTTTCCTATCGTTGCGGTGGGAATTCCATCTAAAGCTATCAATAATGTTAAGGAGGGTCTGCCAGCTTTATTCTCCGCTTGAGCGTACCACTTTGCAGGGGTGGGGACGGCAGTTTTATTATTCCAGTAATTGAGTATGACATCGATCATTTTGGTACATGCAGTTATCCATTTAATATCGGGCGCTCCCGGTGAGTGCATATCTCTAAAATGTGGCACGGGCACGTTAGAGAATTTGCAAAAAAAATCCGTAGATATTCCAGGGATTTCTGCCTCCCACTCCACGGGGCCTTTGATAGTCAACATGATATATTTGTCCTTAGTCCAAAAATCATCACTTGGATTTATTATGGAAAACATACCGTTTGTGCCTTCCAAGATTTTTATAGTTGTATACGGAAAATAAGGTAATTCCCCTGGCTTGTCACCTCTAGATGTCTTCAAATCTGGAAGATTTGTTATGGCAACAAATTCTCAGTATGGATCCGGCGGAACTTCTGTCGGACAATGCAATCTCGTCCACGTGTGACGAAAACATCGCAGCTGGACATCATTTTACGCAATCTCCGCACGTTGAAATGTCAGTCCAATCAACGACATCGGCGGGTCATACTGGAGTGATGACGACACAGTCGCAGTTTTCCAATGGCGTACGGGATCAAAACCGCGAATCACTGTCGACTCTGACGGGCCTCTCGCTGGAAAGCATCAACAATCAAATCAATGTTCAACCCACTCAGATGACCTTTCAACCCATTTCCCCGCCGATGCAGGGTCAGAATTATGTCTATAGCAATAATATGATCAATCCCATCAAACCTAGATCAATTATCAAATCACATGGTCATTCGATGGGGGAGATGTCATTTGCAGACCATTCCTTGTATGTTAATGCTCAACCGCCTGTCCAACAGCCTCAACTTAAATCCCTTGTAGGTATGCATCCATGCATGACCGCTACCTCACAGGGTAAATATCAGACAAATAAAACTGTGGGGCCACCCTCTATTTCAGCTTCCCAAATTACTACGGGAAATGCCGGTATAAGACCCGGGGAATATCAGTCTGTCCATAATCAATCTTCTGTAAACGGGTCTAAGTCTTATGAGATCACTACGGCATCTGGCGATGAATGGATACTGACTACACCCGGCGGGCAATCCTGGACTCTGAAACGGAACCCCCCTAATCCTCCGAATAACAGAACTAATTCAGTTGTCAACAAGGCACAACAGGTCTCGCATGCCCAACCTTATGTCTCTGGGTCTAGCGACGGTTTCTATCAGGGGGCTGCTCTCCAATCATGTGCGTATGTAAATACGCCAGGTTTCACTCCTGTGTGCGAAACACAGAATATGAACAACTCACAGGCGACACAACTAAGCGCATCCATGAACTGCATAAATGCGTTACAAACTACTATGGATGCGATTGTCACTTCTACTTCCAAGCCGGTGGGGGCGGTATCAAATAATCGGGGTGCTAATTTTGGAATGGGAGGCATGGAGAATTATATGGATAATAACAGTCCATGGAACCAATACTGTAAAGTTCAAGACATTGTATCTCAAAACTGCTCCCAGGGAAAAGTTGTTTCTTCGACACCGGGAATCGCTCCTAATCTGATGAAGGGCAACGGGTTGAATGTTTATGGCCATGTGGGGTGTGTAGATGCGGCCATTTCCGATAAACAAGGTGGCACCGCTAACGTCGCATCTTCTCTATTGAATCCGGAACATCAAGATTGGATGCGGGTGACCGGGACGAGCACAAATCTGTTAAACAATATAAATGCTGAAACAAAAATGGAGAACTATGGTTTTCCGGAAAATGGGAATGTCCACGGAGCCGTCAATACAGCTTTACCACTAACTCTATCCAGTGGTCAGCCCTACACATCTGTGCCACAACATGGCGCATGTGAAGGAAATGGAACAATTCCCGTGGTACAGATCTGCAGTCCCAATACAGCGTTCAAAGCGCACTACAGTTTGCTCGGTACAGTGGATGAAAACAACCCCTTGTCCGTCAGAGAGAGCATACAGGACACAAGTTTTAGTAATGGATGTGCTCCTCAGTTGTCCTCGCCTGGAGGAAACCCGACAATAATTGCACATTCAATGATTGGAAATAACGGAACCCCGAATAAAGATGTATGCAAGCCTACCCCCAGCCTACGTGCAATAAAAAAATTAAACTTTGACTATGATGACCGAGGGGAAAATATAGGATTTCCTTCCAAATTGGCAGCCCTTCTGTCTATGGGAGAAAACATGTCAAAGATGGACAATCCTTGTTATGGAACTTCTCTAGCGCAGTTCGAGGAATCACATCAGCAGAATGCATCGGAGGGTAAAATATCCATTGCGGATTTAGAGTTTTCTGAAGAAGATGATGTGCTTTCCAGTGCAGCCTCTGTCAGCTGTAATGACAACTGCGTCATGAAGATCGGAGCTTCGCAGCAAGGTACCACTGTGGCTGATTTACAACAAGGGTTTAAGCAACAAATGAACGGAGAATTCTCCATGTTTGCAGTGGATGGGAACATTAAAACACAGGAGATGTCGAATGACTGCGCGTCAAACGTCACGGATAATGCATGCGCCATTCGACAAAATAAACGTATGCACTGTGAAATTGGGATTTCAGAGGATGGCCGGGTGCGGGAAGAGGAGAAATGCTCAGACGTCGCAATTCACGTTCCGCGGAAAAGTGCGCGTATTCATAACATGAAGTCCGAAGGCGTGACTTGCGGTATGTGTGTGACGGCTGCGGATTCCACACGGCAGGATGCGTCTGGAGGTTCGAGCAGCGGAACCAAGAAAGGCGAAAAGTTGCAAGGACTTTGGAAGGGATACCAGGATGACGACGATTCGGAATTAACCGAACTGTCGGATACAGACAGCGACAACGATGTCCAAAACTGTCACGGAGTAAGAAAGACCGGTTCCAAGACGTACTCCTCAGTATTTTTTAATCCTGACTATCGCCAAGCCAAGAGATTGCTTGCCGACATTCCATACAGAAGATGGATTCCGGACACATTCAACATGGAAGAACACGAGGGTCCATTCCTTCCAATAGTGACTAGACCACCCACTGTGTTTATGGGCGGTAGACGCCGGAGGACTTACCTCCGGCGAAGTGTCACCTCTATCGGTCCTCTGAGTAAATTAACTTACTTCAAGGAATTACTGCAGAGCTACGTGTTGAGAAATAGCAACTGTTATTTAAGTATCGGTTGGCCTGCCAAACACAGGGTGTATATCATGTCAGAAGAGAAATTGGGGTATAATCATATACCTACCTTGAGGGAGATGTTTCCACTGCCACCCGGATGGATGATTGTCCTTGGAATTGTCGGTTCAGAGACGCCCGCGGCTCTGTATAAACACATGGTGGTTCTCCTGTGTGAAAATAAATGGGTGCTTCTGCATAATTACCGGGACTCTAAACACGAGCTGTATTTCGCCGCCTCGGATTTGAAACAGTTTATGGAGGAGGGTCTGTCTAGATGTGACTGCATATATTATGAAAAGTCAGTGCCGTACGGCGTGGCCATGGAAGACTCTGTTCGAGAGTTCTTGAGAAACAGTAAAACGTTTCAGAGTCTTATGGAGTATAGGAAGAATATGCACGGATCTACCTGGACCTTTAACGGAATGCCTGGTAGACTGGGCGACAGAGTTATACATATCTGCAATCCTGAATTGGTTAACTCAATCCCGGCAGACGAGGCCATCAGATATGAAGGAAAACCTTTGTATTTCTTTGCATTTGTCACCACTTTCAAATCGCACCCCGGTAGCAAGGCCAACGTATTGATCGCTGCGGATAAGAACTTAGGGATATACGGCTATCATAAAGGTCGCCCCAGGATACGGTACTTGTGCAAGAACGTACAGGCTTTTTTTAGAGCCGGAGTTAGAAAGATGTATTTGGATTATGAGATTCCATCGAAAACGTTGCTTGCCGTCAGCGAAGATGACTATCTGTGTATTCTACAAAAAGCTCCTTGTCTGCTCTTAAAACCAGCTGTGTTTAGAAAGACTTTCTCTCAGGAAGGTAAATAAGAATTGCTATGTCAAGAGTTTTCCTTGACAGTTCCCACCCTCTTTTTTAATTAAAACAGAAAGGCCATTGGATTCCATCCGCTAAGAGATTTACTGTTTCTTTGACATTTACCAGAGATACGTGATACTCGGTCTACTGTGTATTGTGTACCTTGTAATCTCTAGGTAAACTTCTGCTATTTTTTTAATGTTGAGTAATTGGACATTGTTGATAGGTAAACTGTTCTTTCAAAATGACCAGAGATTCTATGTACTGATCTCTTTGTTTTTTTTTTTCTGTGTATTGTGATAGGGAAGGTTTGTAAGGACAAAGGACAAAGTTCAGTAAGATTTAATAAAATAACATGTGACACAATTTCTGACTCTCTTTCAGTAATTTTTTTATTTTAATAATGTCCGTTGCCATAAGGTGAGGGCAACGTAGGGTCTGAGGGATAAAAAAGTAATTCGTTCATATGAGTAGTCGGATAAAACATTTGTAGTTTTTTCTCTAAAACATAACGATTATCTCGATGACATTCATACCGGAATAAATCCGATATAAACGTTTCCATCTGTAGATATCTTGGCCCGTTTTTGTGTGCTGACATAGCACTTTCGGTTAAATTTTCTATCACAGCTCTTCCGTAGTCACTTAAAAAAAAGCAAAAAAAAAAGACATATGAAATTTTATAGTAGGCCTACCTGCAATTTATATAAGTACGGATGAAAACAAAGAAACAAACATAGTGGAAATAAAATGATTCGCATATGAATTAATACCTAATGACTAGATTGAAATCCATCCATGTTACGGACCAGGGTCTTTGAACTTGGTCTTGCATAGTATATACAATTGTTCTATTGTCCGTGAAAGGTCGACAAAATTTCTGGCCTGCATTTTGAAACAGACTGAACAAATATCAAGTTATAGATTAACCGGCGATGTCCTGTGTTATTACGCATACTATACGCGTATACATCCACATACACGCACACACGAGAAAATGGTGTATTAATCATTCAAGTTTACCCGTGGTGACATTTTCCCCGAGTAGATAGAACGACACTTACATTTCACCGTCTAGGCAGGTCGGAGTCGAGGAAGATACAGAACTCAGTGAATTAAGAACGACATAGTGATCACAACACGAAGTAATCTTCGGTAACGTGCTAAAAAACGGTAGCGCAGCCACTCCAGGATATGCATGGAAATGTGTTAAAATATACACAGCCACGAAATGCATAATTATCTAGCAGCAATAGTTTGTTATTTAAACAATGATTTTTTTATGTTCATTTAAATATAGTATGAATATTTACAACTAAATATACAATCGACTATACACATGGTCGATAACGCACTATTACATGCGTTCCAAAAAACAGGTTTATCGGGGGTTATCGTTATCTACATCCATAAAGTGTACAAGTCCACATGTAAACATCACAGGAAAATATAGAAGTCCGAGGCCGTGTACATCCAAACGCGCGCAAATAACGGTCAGGTTCTCACGACAGTAGGTTCTGTAGCTGCCATTTTGATGTTCTGAAAGTATACATCTCTCGGGTTGTAAAATACGTGCCCACCCTCGCATAAAGCTTGTCCGAGGTGTTCATATCCATATATTTTTTTTTCGAACATATTTTGTTTACTATATCTCGCTTGCACAGTGAAGATGTTGTTAACCATCAGTAGTCCCGCATTTCTGAAACGTTGTTTTGAAGTTCCGGTCTGTATAAAGATGGTGCTGCGGATTAAGAAAAACTGCATAAATATAAAAAAAAACTCTGGAAAAGTCATTTGTTTAATTATGGCCTTTCGTCTTCTAATCCAAAGAAAGTGAAATTAAACGTAAAAAAAAACTCTACATTTAGATTCATAGTTTAACAGCGCTCACCATAATGCGGGACTCATATGTGTCGGACTGGCTATATCGTATTTTATATTAGTCGTTGTCAGTTTTTCTGGCAAAGAGTGCCACAATGCGCTTACGATGGCCGGATCTATGTTGCTGTGCATAACCGAAAAAAGCGAAAAAAATAAATTAATATAACTGGTTACTAAAAAAAAAATTCGCTCCTACCAATCCCCTATTATTTAAACTCACCGTATAGATGTTCCTCGCTGTCCGACCAGTGACAATATTTGTGAGGCAAGTCGCATTCCGATGGTTTCCATCAGTTCCTCAGTTTCGTACAGTGCATTTTTAAACGCTCTGTGAGAAACACAAGTTAAGAAATCTCAAATATAAACTCTTTCAAACGATACATCGCAGATAGATAAACTCCAACTTACGTTCAAAGAAGAGAAAATAAGTTTACTTACAGTACAGAAGATAAATAATTCTTCGTGTCGTTCTTATTCAAACGTGAACACCATTCGTGACCTTGCAGGGTTTGAAATCTGACGAAAAAAAAAGCGAATTTCGAAATTAGAATTTCAGAATTGTGTTCCACTGCTATGTAGCAAAACCAATAAAAAACATAGATGGAGTTTTTTATTAAAATTGGCATGAATCACTCACACGACGTAGCTATCGTTTGGACAATGATTATCGTTTATAGGGACGTATGCGTCAACGACGTCCTGCCACGGTTCGAAATACTCAGTCCGAGTCCATGAGTTAGAAGATTGTAACGTAGGACGATCATGGTGCACGTCTCGGGGAGGACAACATGTGCCATTGGCGGCAGGTTTAGACCTCAATATGTCCTCTATGCTAACGGACGCGAAGCGCGTGCAGGTTTCCCAATCTTGCCCGTCGCTGTCTTTTCGAACGATCGCAAACGAGGTTAACGTTTTGATAGTCTGTCCGCCATGGTTTTGTATCTTCAAGAGTTCGATATTGACGATCAGTTCGCTGGATTTTGAGTGGATGAATACATGTGTGCCGCACAAAGAGGCAAGATACAAGGAACGAGAAAAAATTTTCTTGTTACGAAGGCTTCCAGCCCCATAGTTTCTGCTCTGAAAGGATGTAGGTATGCGTGTGGGGTTGACACGTATGCGCGTACATGGGTGTGGTCTGTCTTCTAAGAGGGGGTGGAGTGGAGGGTGGAGGAGAAGTGCTGCGACATTTTTAGAGTGACAGGTGCATCCGAGGGCGGGGCGGGGTGTATAAATGGGACTCTTTTTAGCGAATTCGGGATATTTATACGGCGGGGATCTTACGCTTTTCTATGTGAATAAATACACAATGCATGCCTTTCCTTGTCGATAAAGGAGACGTGGCGGGAAGGCACAAAAACTTGTACAAGTACTCACCATCGAGCCTTTTCCAGTCTGTAGTTCTGGCGTAGCTCCAATTTACTAGTCATTGGACAAAAAGCTATCGCCGTCATCGCGGTTAGTGGTTTCAGGCGTTCCATATAGGCCAAACTATTATAGTTGCTCCCGAAAGCGCCATATAGCAGAGAGAAGTGAAGGCCGAGGCCGCTAGAGAGAATCTGTGATAGACCCATAGGCATTATTCTGCTTTTTAAATTTCTCACTCCTCTGCTTTCGGCCGAGAGGGGAACGCAGATTATGATATCTCCGAACATGATGCTGCGGGGAATTAGATATGATTATTATATATTCATTTATTTGTTATCTCGGCGGATTCCGACATCGGTGTCCGGGAGAATGAGCGCGTGGTAAAGAAACAAGATGAAATAAAATAAAACGCTAGATCCTTACCGTGCCTCTGTCTCTATGAGACAACCGCAGCTGTTATCATCGCAAATGGCGTAGTTCAGGAGGTCTTCTATATCTTCTAAGGTAGTAGTTGGGGTATAATCTACGGGGACAAAAGCTTTAAGGCCTCCTAGACGTGACATTTCGGAGGTAGAAAATTTAGAGATGATTTCGGGTAGCGTTCGTCTTCTGGGCGTAGGATTTGTACCGTCGTAAATGTTAAAATAAATGCTGTCGTCATAGTATTTTGCGGTATAGTTGTCTTCGTCGTCCGGCGGCGGTGTGGATTCCACGGTTCCCGCATCTCGGTGGACCGTAGCATTCGACAGGGCTGTCGCCATGGAAATCGCGCACACGAGTATCGGCGCAGAACGTCTCGGCGGTCTCATAGCGCTTAGCGTTGCGTTCGCCGTTCTCCGTCTTAGTCTTGCGAAAATGCGTATGCGCGGGAGGAAGAGGACAGAAAGGAAGATGGTGTTAGATAAGATCTACAGGAGGAGAAACGGGAGACAGATCCTACGAGGACGGGAGAGCATAATAGAGCGGTTTGCATCCGGAAACATTCGCGTGAATGTAAAGCCACAAGAAATTAATGCAACTAGAGACTTCCCAAAGATGATATATGGCTACGAGCAACGTCTGGCGCGTTCACACTCGTTTACCGTGTGTGACTGAGACACGTAGTATTGAATCTCCGCCTTGTAAAATCACACGTGGGTGGAAAGATCGTGCGGCGTATCGTCGTCGGGGAGCGTACTCGGAGATAAGTCTGGGGCGATCATTTTTATCTGCGACGATATAAAACGGGGGAGGGGGGTGGTATAAATCGATAACGAGGAAGAAATACATCTGATCGTCATTTGGCGATCAGTAGTTTGTCGGTGATTTCTTCCGTAACCGGCGTGTGTGTAGACTCTCGTTTTGGTGGTCAGATATTAAGTCTTGATCACGCGCGTACCCGCGCGTTGTAGCGGCTTCTCGGGTGTCTTTTATTTTCACCCCGCAGGCTGAACCGCACTCCGGCCGGGCCACTTCTTTTCATACAGGCGTTGTCTGTCTGCTCCGGGGAGCTGGGCTGCACTCGGGCCGGGACATTTATTCCCATACAGGCGCTGCTTTCTCTGTTTTGGGAGGTCGGACTGCGCTCGTGCCGGGACACTTATTCCCATACAGGCGCTGCTCTTCTGCTTTGGGAGGTCGGACTGCGCTCGGGCCGGAACACTTATTCCCATACAGGCGCTGCTTCTCTGCTTTGGGAGGTCGGTCTGCGCTCGGGTCGGGACACTTATTCCCATACAGGCGCTGCTTCTCTGCATCGGGAAGTCGGACTGCGCTCGGGCCGGGACACTTTTTTGTGCGATACTGTTGCTCTGGAAGACAGATATTCCTCGCGTTTCAAAAATTACTTTAAACTCCCCGGGGGGGTTAAAAAAAGCCCTAACACTCATCCTCGCATCTGGCCCTACCGCTACCCCTCTTTCAACCCCTCACCATCACCCAACCCGCTGCCCCACCCACATCCCCCCCACCGCCACCGCTACCGTCACCTCTGAACTTCACCTTTTTCCTCCATCTCGCCCCACTTCTCTCTACATTTCTCCGCCCCTCTATTCTTACTCCTGTTTTCTAGGATGCCGCTGACGGCGCGTGCCGGCCACACCCTGCATCGTCTTCCGCTCTCCCACTATTGGTGGCTTCTGTTGGGTCGACACTCCCTTCGTCATGTCCATTCCTACCTTCGCCTGCGCAAAGGTCTACGCCTTCCTTTACCTTGGCCCGAACAAGAATGTCTGCATTTACATCCTAAGCCTTACAAGTTTCTCCTGCGTTACCCTTGTCTAACAAGACAACCGCATCTTCTTCAGGGCTGGCCCGCGGATTCTTCTCTATGTGAGTGACATTTACACTTCCACTTGTTCACATGATTTATTGTGTTTTGTCTGGTACACCAAGCACATTTCGGTTTCTCTTTTATACATTTATCTCTATTCTCTCGGTCTCAGGGTTCGACCCTAAACCCTACCATCCTTCGGCCGACAGCAAGTTGCTACCTCTGGGCCTCATCACGCTGTCCGCCTTTTCCATGCGCGTTTCGGAGCCGACACACTGCAGCGGCTTCCACGCGGCAGATCCGTCTCTCAGTTGGTTAACGGGATCGTCCCCTTGGCTCGTGCTCCTACAAGCGCCAGGAGGGTCTCTGTTCTGCCACGACGTGTTCCAAGGCCGGCTCTATCTCCTGTCGCACTCCGTGTCGCTCTTTCTAAAGACGGGCCTTCGCCAGTGTGAGGCCATCTATCGCGCACCGCTGTGGCGCGTACGGCCCCTGCCGAGCCTATGGACGTGTCGAGATCCCGACACGGCTTTCTTGCCGAAATTGCTGGCGAGAACCGCCCGACGCGGCCTGGCCGCTTTCTATGCCCTGTGGAGACTGCATCTGGGATCCCGCTCGGAGCTCTCTCACCCCGTGTTGGAGTGGGAGAGAACAGAGCTGGTCCTGACGGATCGGAGACGCGGGTGGCCGTGTACGCACCTCCTGTCCGGCTCGGAGTCCCAGCGCGTTTCCTCGAGTGACGCCGGAGACACATGGAACGCAGCGACAGAGAAGGCGGCGGGGGGAAAAGAGGAGGCGGAGAGAGGCGGGCGACAGCAAGCCACTGACAGACTCGCAAGTCCGCACCTGACGCGGGGCCGCCGCGACTGCGGTCGGTCCCTTCAGGGTGAGGAGCCCAGCGCCGCGGAAGACTTTGCGAGGTGCAGACCGCTGCTGGACGAACTGTGCGGGGAGGGCGGCTGGCTTCCCTTTGCGTTTCTCACGGCATCTCCGCACGTCTGTCTGATCCTAACGGAGGGAGGCCCCGTCCTGGCGCTTGACCTGAACGACACCTCCCTGTGGCGCATCGCGGACGACTTGGAGCTGCTGCTGCGCCTGGGGAGCCTGCTCCTGCTCTCAGGGCTCCGGCTTCCTCTCCGTCCCCCGAGCGGGAGCGGCGAGGCGGCGAGAAAGCCGGGGTACGAGAAGGAAGAGGGAAGAGGGAGAGCGACGACGGCGAGCGCGACGGCCGCGACGTCGCCGCGCAGACCGACCCGTCCGAGGGGGGTGACGGAGAAGGGACGTGTGACAACCGGGGACGTCCCTTTCTCCGCACATCCCGAATCTGAGGAACAGACAGACGGCCACCACGGGCGCCAAGAAAGCGGCCACGGCGACCAGCGCGGCGGGGACGGACGAGGACACCGCGATGACGGCGCGCGCCGCCACGCGAATGACGAAACAGAGCCCCAGCAGCGCGGAGAGCACGAGGACGGGGAACAGACCGACTCCGGGCGCGAGGAGGACGCACAGGAGAGCGAGGTCGCAAGAAGAGACGAGAAGGGAACGGAGCAGGGCGGTAGCGGAAGGAGCTGCGGGAGGGCAACGCAGACGTACGGCGGGAGAGGCGAACATGGTGCCTGGTCGTCGATCCCCCTGTCTGTCCCCAGGCCCGATCCCCGCGTGTGGGTCCCTCCTCCTCATCTGTTATTCCCTTCCCCGCTGCCGTCGATAACGCCCGTCGAAGACGAGCCGTCCGCCCGCCCTCGGTGCCCGCCAGGTCCCGCGGAGGAACCCTCTAAGTGTTCTCCGTGCCCGCCCTGCCCGTCGCCCGACGCCCCGCAGTCCGCTGTCCCGCGCCTCTCCGCTCTGTCCGTCCCCTCGCCGTCCACCGCGCGTGTCCGTTTCTCCCTCTCCTCCCTACTCTCCCTCCCCGTTGTCTCCGCCCTCTCCCGTCTCTCCCTCGTCTCCCAGATCTCCGTTCATCTCCCCCATTAGATCTCCGGGACTCCGAGCAAAGCCGTGGGTGTCCTCCGGGCATCCCGTGGCGTTCCCGCCGGCGCCCTCGTCCGCGCCGCCCTTTTCCAAAAGAGTCCCGTCCGTTCCCTCATCGGCATCTCCGTCCGCGCCGTGCATCGGCAGGTCGCGGCCGCCCTCCGCACAGACGGCGTGAGACGCCGCGTGAGGTGACGGAGGCGGCGAAGGGACACGAGAAGAACATGCAGAAGAACATGAAGACGAAGAAGACGAAGAAACGAGGACGAAAGGAGGGGAATACACCGGAGACCGAGCGGCGGATGGAGCCCGCTCGGTCTCGCACGTCCGCGATACCGAGCGGGCTCCGTCGCCGCTCCGGACCCTCCACGCCCCTCCGTCCAGGACCCGAGGTCCGACACGCCCCCACCTGGCGCACCGCCTCCGCCACCACCGCTGACAGTCACCGCATATCACCCCCATACACACCCAGCAGCAGAGGCCGGCACATACACACGCGCGGAGCACGCACGAGAACACGCGAGACATCGGCCGCCGAGATTAACGGAGTCTATGCGCGCGCCGTGACACGGAAAACCAAGCGGAGCGAGACGATCGACCGACTCCTGCTATCCGTCCTCCCTGGGCACGGTCCACACGCCAGTCTGCGGAGTCACCTGAGGGCCAGATCCGCTCTGCGCCCCCCGCCCGATCCGCCCCGATAAAAAAAAATCATCTGACTCGTGCCAGTTCACACAGATGCAACCGATGACAAAAAAAAAACACACCACCGCAAGCAAACGCTCGCCGGCCCGTCCACCGTCCCTCTCACCCCCACTCGAATCGCGCAGGCGCGTCGGCGGGAAGTCCCCCGAACGCCTCCACGGCAACGCTCCTCCCACGCCCCCTCGGTCCCCCCTCCCTCCATCCCCCTCCCCGGAGCATACGCGCCGACTCTCTCCGCAGAGGCGGCGACCACAGCGGCAGCGGAAACTGTAACGGCCACGTTCTTTGAACAGTGACAGTAACGGCGGCGGCGGCTGTCGCCGTCACGGTAGTCGTGGCGGAGCACCCGGGGAGGCGACGACGGGGACTACCGATGCTCTCAGATCATCGCCATAGTGAAAGAGTACCGAATAAAACAAGTTAAATTTCGTTGTAAATAAAAAAAAATACTATGTGGAAATTAAAAATAAAAAAAATGAAAATAAAGGCTAAACACTGACTAAACGTACATCCTTCTCCGTACGTCCCCTTTCAATAAATGGCGGACCCGACCTCTCCATAAACGGAGACACGCCGGGAGACTCCGGCCCTCCCGTTCCAGAGGTAGCCCGAGACCTAATCACAAAACTGTACCTGAAACCGAAAGTATAGAGGGCGGCCTCGAATGCCCCACCTATGAGGCGGGACTTCGCGACGCGCCACCCTTCCTATACGCCCGGCCTTCCGCGTGCGCGCCTATAGGACGCATCCCCTCGCCCCGAAACCGCAACGCGGCAGAGTCGGCGCCACACCCCCGTCCCCCGAGTTCGCGATGGGCTGGCGCCGACCCTGCCGCGGCAACGAAGGCGAAACGAGACCCCGACGCATGCGGGCATAAAGAGCGAGAAGTAAACGCGGGGTCCCGAATTATCGCGAGACTATTTGCCCGGG

TGACACACCCGGTGGAAAAAACGAAAGACACATAAATAAAACGAATTTTGTTGTTAAAAATGTTTCCACGTGACGTCCCCGGTGATTCCCGGAGATGCGGCACGCGGCGGATTCGCCCGTTTACATCGGGGTCCGCAGACGCGGTTCTGCCAGGGCGTTGCGCTGTCGGTTCCGCACAAAACACCGCGGTCCGTCGTCACCCTTCAGCCACGCGGTAGTTCCGCGATTCTATGGTTCATCTCCGCTCACATCGTTCTTTTTTTTTTATTCTTTCACTGAGCCGCTCCACCACCAAGAGTGTTACTATCTACACCGTATACATGTTTTTTTTTTATCTCTCCGCCCCTCCCCATCACAGACCATCAAACACCTTTTGCCGTATCGACACTGACTATACTCGACCGCGGATGACAACGGTCGTTGGTCACAACAGAGCTACAGCGGACAGCAAAAATAACATAAATACAGTAAGAGAGAGGCCCCGGTCCACATAGGAAGCCAAGGGCGGACGAAAATAAAAACCCGTCCGTAATTCACACACGAATCGGGGGTTATGACAACGCGACACACGCAGACGAGGGACGGACGCATCGCGATCCGGCGCGACGGGGCGCGATTGGCCCACGCGCGAGCCCGTGCCCGCTTCGAGTGGCTGCTCCTGGCTCGCGGCAGGCCGTCCAAACTGTACGGCTATACGAGTCGGCACCGGGGAGAACGGATCCACCTACCGTGGCCGCGGTACTGGTGCCTAGAACTCCATCCGGATCCGTACAGGGACGCCAGGAGCGCCACCGTGTGGGGTCACCGCTGGGGTTGGCCGCCAACGCACGTGAGACCCAGATCCGTTCAAGACTGCGGTGAGTGAGCGCCGCCGCGACCGGCGACGCTATGCGCACACGGGCGAGCGGCGGGGAGGGACGTCGGCCGCAGGGCGAGCCGGTGGAGGGCGAGCCGGTGGAGGGCGAGCGGGTGGAGGGTGAGCGGGTGGAGGGTGAGCGGGTGGAGGGTTCGTCGAAAACCACCGAAGACATTGGCTGGCGGAGAGTGGGGACAAAACAACGTCACGTCAGGGGCGGAGAAATATAGGGTGCGCAGACCGTCAAAGCGCGAGTAACGGAAAACGAGTGCGGATACGGGGGGGGGGGGAAGGAAACATGGTGAAGTCTGTGACACGCGTGACCGTCAGCCGGAAACGCCTGCCACGGCCTCTGGCCTCACAGACGACTCACGGAGTTTCCTTCAGACATTGTTTTTTTTTTTGTATTCATCGGTAATGCGGTATACGTAAATTCCCCGCAAAGGTACTTTCCGTTAAGGACGGTACAGGCGGTATGACTTCCGGAACACGATGATTACGGATATCCTGCATAGTGTACAGGAGACCGCTTTTTTTTTAGGTTCCGTATAAGACGTACGTCGGCGACGTACACACAACAGTTAAAAAAATAAAAAAAAGAGGAAACAGTAAAGCTCCTCCTTTCCTCTATGTTATCGCGTTCAGTTAGTAGGCGCCATTGCTGTTGCGTATGCTTTTCGCGTTAACGGTACGCGACAGGATACGAATGCGTTACACGCCGCAAAGGCTCCCTACAATGACTCTATGCGGCATCTCCCGTTCGCGCAGAAACGCGAGTTCCCGGGACGCCAGTCTTGACGTCCGGCCGATGGGATGCCTTTGCGTGTGCGGATGTTTTCTGCTGTTTTATAGGAACAGAGACAAGAGACGAAAATAGCGGAACGCCCACTATAACGTGCATTTTTCTCTGTGTTTTGTGTTTTCATGTTTTATGATTCTTCTACGGTATCGGCCGGAACGGTCATCCTCGATCCTTCTCGTACGCGTGTCCCGCCCCAGCCCTGGACTCGAGTCTGTACGTATGTTGCGGATACGGAGAAAAACTTCAACCCGTGGGCTTCGTAAGCTCGTATCTGACCCACTCCCCGCTCGACACGCTTCGCGTGCTCCTGGTCGGTAGAGACGGAGCCGTGTACGTCCACCACATGAGGGCGGCCAGACTCTGCCGTCTGGCGTCGAACGTAACGGAGTTTGCAAGGCGAGGGCTGCAGCGGGACCCCGTGGCGTATGAGGAAGACCTAGAGCTGCCGGACCGGCGTATGTGCGGAACGAACGTCAGACATCTGTTCGACGTGATCGCCGCGGCCGCCGACGAACACGACCTGCTGACCGTCGGCGGCCTGTGTCAGACGCACGCCGGAGTGAGCTGTGAATTACTAGAGACCGTGCGAGATCCGTGGACGGCGGTTCCGGGCGTACGCATGACTCTGACCGTGGCGCGGGCTCAGTATCGCTTGTGGCCCGATGCCCGGAGACAGCTCCGCCTGCACCTGTACGCGGGACACCCCCTGGGACCGTGGATAGTGTGCGCCGTTCTGTCTCGAGAGAGGGAGACGCAGACGCCGTCGCCTCCGATAGGCAGCGGAGGCGTGATTCTGGGAAACGTGCCCACGCCGGGGCCACGCGAGGTGGAGACGGCTTGGGTGATCGTCACCGTGGCGGGACCGTTGTTATCGTTCTGGCCCGATAACGGCAAGATCTGCCGTCTGGCGAACTCGTTCGCCGCCCTGTGGAGGATGGGGCCGCGGGCCATGAGAGGACATTGGACGTATTCGGCCCCGGGTAGACATCTCCCCGGGGACGCTTGGCCACTCTGTGAACACGTGAGACCGCCAGTCGGAAAACTTCCCCGGCAAAGAGCGTACCTGGATTAGACGCCACGGCGGCGACGTAAGGATGCGACCTACGGCCAGAGACATGACCGTAGGAAGATCATGACAAGGATCTCGAAATATAAAACCAAAAAAAAAGTGAGGAGTGCGTAATCGCAAGAAAAGAAAAAACAGAGCCGCGGTGGAAATGTCTATCCGCCTGCGTGTATGTGTGGGGTGTGTATATGTGCGCGTGACCGTATCCCTCCCTGCCTTTAACCCGATGAGAAATAAAATCGGCGAATGTACACAGTCACACACAATGTGCTTACGTGCCGTGTTACTGACACCACATAACAAAAAAAAAAAACATACTTCCTGTCCGTGAGACACCGCCGCCCTCTAGCGGGGTTGCTATAGCAGCGCCCGATGCCAACGCGACACGGTGAGTCGCATAGATCGGGACTGCTTGAAAGCGCGCCACATTCGTCTTTTATATAGACACCCCGGGGAGTGGGAGGAGCCAACATACACACGAGGTCGGTCTGAGGTTGGAGACGAACGCGGAAAATCAACGGCGTAAAAAAAATAAGAAAGGCGGAGACACATAGCCTTGGCGGGAAGACGATAACAGGTTCAAAGATAGACAGTGAAAAAAAAAAGAGGAGACCATGCCATTGAATACACGTGTGGCAGCAATCCAATCAACGTGACGTGCCGGCGCGCAAAAAAAAAAGTATACGGAAGTGCATGCGACAGACAGTCACGCGGACCGACGACAAAGGCCGACCCCTAGCATGGCAGCTAGGAAAAAAAATAAACAACGCTGAAATATACTAACAAAGGAAACATCCCTTCATCCCTCCCTACCCCATCTACCACAACACCCCCCTCCGTAATCCCTTTTCTCCGCGTTTCTCC

TCTATGGCAGTCGCGGGTGGGCAGGCGGGGAGCATACGGGGGGCAGATGTGAAGACAATGAGGAACGGCATCGGGCGCGACGTGCCGCCGTCTCGGACCCATGCTATCCTGGCACGACGCCAAGGGAAACCGC

>HHV7_STRIPPED4

CCCCCCCGTTTCGTATTTCAAATCCTAAATAACCCCCGGGGGGTAAAAAAAGGGGGGGAGCTCTCACTGTCACCCTAACACTAGCTCCAAGTCATCTGTTCTAGATCCTATCCATATCTGCCCTGACTCCTGGTTCCTTACCGCTCCGAGCCCCACCCTCCGTCCCGCCCTCCTCCTGTTCTCCATGCCCTGCCTTCTCAACCCTTCCTCTTCCACGCCCACATTGCCTCTGCACTCCGCGCTCTCTTGGCTGTGCGCCCTGCCTTTCCGTGACCTACTGGGAGCGCCGCCAAATCTGTTTTGCCCCGCCCCTGCGCGCGCGGGAACTGTCGGCGCCGCGCTGCTGCTAGCCCGCCTTCCAGAGCTCCCTCCCTCCGTCTGCCTCCTCACCCTTGCCACTCACCCTTCCATCTCTTCTATCACAGACTCTGTGTTACACCACCTATGACTGCTGCAACCACAGAACATTTTGCTCTCCGCGCGGCACTCAATCGTTACTGGTGGCTGCTTCTGGGACGACACAAGCTCAGTTTGGTATGCAACTACGTCACAGCTCATCGCCAACAGTTACTGCCGCTGCCGTGGCCCGAACAGGAATTTCTCCAACTTGACCCGGCCCCCTACTCCAATCTCCGCAACCGTGTCGCTCACCATCTCCATCGCGGCTGGCCAGCGGCACACAACACATGTAAGCTACCGTACATCTCTTTCACAAACCCAGGGCTCACATAGAGACAAGCACAAGCTCGCGCAATGACATTAAAACCTCCCATCATTGTCCTTTCCTGTCGCTTTGCCGATAACGTCTTTGCTCTATCGCAGGTTTCGACCCCCGTCCTTACTTCCCCAATGCTAAAGTCAAGCTGCTTCCGCTCGGCTCCATCACCCTTACCAGATCATTCTCCAGTGACGAGCCTCATCCTATTGGTGATGATGTGCATCACAGTCATGACCGGGGTGACTACCATACTGTTATCTGCAGCTGGCTCACAGGAACCTCCCCGATCCTAGTGCTGCTTCAAGGACCGGACGGCAGCATCTATTGCCACGACGTGTACCGCGGCCGATTGTATCTCGTGGCCCACTCTGTATCGTTGTTCGCCAGGCTAGGCCTTCGCCACTGCGAACCTTTATATGCGGCACCCAGATGGAAGCACGTTCCTCTGCCCAACATGTGGGTGGCGAGCCCGCCAGCGTCCGCCACCCTCACGCAAACACTCGCCGTGAGTGCCACGCACGGTCTGGACGCGTTATACTCGCTGCTAAAAATCCACAGAGGAACTCCGTGTTCGCTAATCCACCCCGTGAACGGCTACGTCCTGGACATGATACTGACGGGCCGCTCATTCCAAGAAGCACCCTGCCAAAACACTCGCACGTCCGTTAAAACAACGCCACATGTAATGGACGCAGTCTGCGGTGGCCGCGGGTCATGGCTGTCCATCGGCTACCTAGTAAAGATGCCGCACATTCACCTGGCGGTGACCCGAACATGTCTGGTCACCGCCATAGATGTCCGACAAAACTTTCTGTGGCGCGTGGCGGACGACGCGCTGCTATTCCTGGTCACCGGTAGTCTTTTACTACTGTCGCGGCCGACCGCAGACTTGACGTCTTGGTCATGTTTACAGCAAGAACCTGTGTGGAGGAACTGTCTAGATACGCGCGGAGAACAGGATGAGACAGAAGACCAAGAGATGAAACAAAGCACAAGCAAAAAGCAAAATGAGAATAAAAAACTCAACACCTCAAAAAAACACACCCGCGTATCGTCGGCAATTCCGACCTTTCCCCTCAGTCTCCGAGAAACGCCGCCAGAAGCCAGGAGCCCAGCCGTCCTCGCCGCCGCCACCCAGTCTCACAAAACTCGAGCGATCTCGACGCATAATGCCACGACAACAATAAGAATACCGCGCCTTCCCAGTTACCTGCTGGAAGCGCGTCTCTTGTCCGTGACAGCTATCCTGAAAGACACAAAGAAAAAAAAAACCCAGCCTCAGGCGTAGCAGCTGCGACGTCAGCGCGGTGTCTGAAAGCTCGCCAAGGTCTCGCGTAAAAGAACAGATGTGAACTTCAGATGTACCAACCAAATAATACGGGTTCCGCTATAAAAAGTGCACCTCTATTCCCGTTCTTATCCCCGTTCTAACTCTTCCTTGTATCATACCTTGCATGTTAACCGGATCCCGTGGATCTTACACACATACACACACACACAAACTTGGTGAGGTAAACACAGAGATCTCACTAACTCATAATCCCCTACACGCTTACCACCACCTAAAAATGGTTACAACCAAAACTGGCAATAGTCTATCTTCTTTTTCTTTCCATTCACAGCCAATGTGCAGTACTCGTGGGTCCACAACAACGAAAGAGACTGTAGAGACAGTTCCTTTAAGTAGACCTTAGAGACACACCAAATACAACCACAACCAAAAAAAAAACAGAAAACAACACAAAGCCAATGAGTGCAGAAATGCTCCGCGCTGTTCAGCTCCAGCCAAGACGCCGGGGACATTCCTCATCTCCCACTTCCCCTCCACTCGAAGGAGAGCCCAGTCCCAAGAGACTCCAATCGAGCGACAGTCACCAAGGGCGTAGAGGCAGACCTAAACCCAGAGCTAAAACATGGAGCGAAGCTTTATCCCACCGGTCCTTCCTCAACATTTACGCGTGGCTGTCTTTGAGTCGAGGGTCTCCGCGAAAAGTGTACGGATATGCCTTCAGGCACAGAGGAGAACTCGTAGCATTGCCATGGCCGCCTAACTGGAGCCTGGAACTTCACCACGATCCCTATCGAGACGCCAGAGCACAAACCGTTTGGAGTCACCGCTGGGGATGGCCTGCAACACACGTGACAGCTCGCACGGTGCGGGACTGCGGTGAGTGTAAGCAGTGTGACACATTGTTATCGCAATTGTCTTACCCGATTAACTTTTTATTAATGTATTAAGCACTCTTTCTTCACGTGTGACTGTTGTGTTTTTTGTTGTTATCTACATCCCGGCAGCCCTCGACACGCATATGTACGTGTGCTGCGGACGCGGAGAAAAGTTGCAGCCCGTCGGATACGTACGCAACAGAGCCGCGCCTTCAGACCTGAACTCGTTACGCGTCCTCCTCATAGCCAGGGACGGAGCAATGTATGTGCATCACATGAGAACGGCGCGACTGTGCCGCCTGGCCAGCAGTGTGACCGAATTCGCGCGACGAGGGCTGCAGCGAGAATCCGAGGTTTATGAAGATGATGTTTCCTTGCCAGACCGTCGAGTAGGTTCGGCAACGGCCATTCACCTGTTTGACGTAATTACCCAGGCAGCCGATGTCCACGACCTACTCACCGTGGCCGGACTGTGTCAGACTCACACCGGCGTCAGCTGCCAACTGTGGTATACAGACCACGATCCCCACACCGTCGCTGGGGCGGCACGCTTCACACTGACGGTCGCACGGCAGCAGTATCGATTGTGGCCAAACGCACGACGCAAACTGCTGCAGCACCTACATCCGGACCACCCACTTGGGCTGTGGCTGTTGTGTGCCGTGCTCACGTACGATGCAAAAGAGACGAATCGCGCAGTGCCACCCGTAACGCCAGGGGCCGAAACCGTGTGGGTGATAGTTACTGGCAGGGGTGCCATTCTAGGATTCTGGCCAGAGAGCGCCAAAATGTGCAGATTGGCCTCGTCTATGAAAGGACTCTGGAAAAACGGAGCGCGGGCGCTAAAAGGTCACTGGACATACGCAGCACCCGGCCGGCATAGAGCGGGAGAGGCCTGGCCTTTGTGTGCACACTACCAATCTCCTAGATAGAACAAAATTAAAAAGATTAAAAAAAAAAGAAAAAAAGTACAAGAGTGTTATCGCGAAACAGCGTGTCAAAAAAAAAACAATCCACATACTCTAGAACAAACTGTACCCAAAAATAAGTCCGTGTGCAAAACTGGGAAAAAAAAATCACCTTCCTCGTTGCCACTAGAGGGAGTACCGAAAGTGTAGGCAAGAAGGCCACGCTGTAAATGACTGTCAGCGTTTGGCGCTGAAAACATTGCTGTTCTTGCTGGCTCAAGCACAATCACGTGATTAAGATTCCTTTCGTTTTCAAAGTGTGCCCGGGAGGCAGACATGCCCTTTCTCGTGAGACATTATGAGATTTGCCTGCCAGAGAACCACGTGACTTGGACTTACTTTCGTTTTCTAAACGTGCCCTCTAGGCATGAATGCTCTTTAGCGTTAGCCATGAGGCTAGCGTGATCCTGTATAGTACATAAGTTTCTAAGAATATGTTTTTAACAATAATCATGTCCCAAAAAGTCGCGAGTGACTAAAATTCTCTGTAAATGAAGGCAAATTAAACAGGATACAGACAGTTGTGGCAGTGGTCCGTTTCGTCTTTCTGTGTTTTCCTTACGCGGCTGACGAGGTAAAGTGTCTCAGTCCATATTGTTGTCTGTGCCACCGTAGTTAGCGGTGGCATACTAAAAACTCCGATAGATGCAGAACAATAACACCGAAAACCACGCTGTGGAACCAGACCACACTTTATAAACAAAACGGCCTTATCACCTGGAAAAAAAACTAAAAATAAGGCAATGATACACCTGACTTTCCATTGGAAACCTGCCGTAACCCTGACCACAAATCCCATGCTAAATCCCCTGAAACACTGCCAAACGTCGCTACAAGGTTTTTCCGGGATCGAGCCGCAGCAAGCTTAAACTGAGGTCACACACGACTTTAATTACGGCAACGCACAGCTGTAAGCTGCAGGAAAGATACGATCGTAAGCAAATGTAGTCCTACAATCAAGCGAGGTTGTAGACGTTACCTACAATGAACTACACCTCTAAGCATAACCTGTCGGGCACAGTGAGACACGCAGCCGTAAATTCAAAACTCAACCCAAACCGAAGT

CCTAACCATGACCCTGACCCTAACCCTAGGCTGCGGCTAGGGCTGCGGGGGCTGCGGCCCTAACCCTAACCCTAGGGCTGCGGTAGGGCTGCGGGGGCTGCGGCCCGAACCCTAGCCCTAACTAACCCCGCCCCCACTGGCAGCCAATGTCTTGTAATGCCTTCAAGGCACTTTTTCTGCGAGCCGCGCGCAGCACTCAGTGAAAAACAAGTTTGTGCACGAGAAAGACGCTGCCAAACCGCAGCTGCAGCATGAAGGCTGAGTGCACAATTTTGGCTTTAGTCCCATAAAGGCGCGGCTTCCCGTAGAGTAGAAAACCGCAGCGCGGCGCACAGAGCGAAGGCAGCGGCTTTCAGACTGTTTGCCAAGCGCAGTCTGCATCTTACCAATGATGATCGCAAGCAAGAAAAATGTTCTTTCTTAGCATATGCGTGGTTAATCCTGTTGTGGTCATCACTAAGTTTTCAAGCTTTTGGCAAGGCATGAAAAATAACATTACTATTGGACTGTTTATACTTATCTTCAAATGTTCACTCACAGCAGCGAAGGGGACACTAGAAAACACTCCCAAGTAGAACTACGAGGCGGAACAGCAATGGAAACTCAGACGTGTTTTTACTTTTATTTTCTGAAATCATTTAATAGCATACAAGTACGGCCTCTCCGAATCGAAGCTAATCTGAGAGCAAAGACAAAGAGCACTGTAAGACTGAAGGCAAAAAAACCCCCCGCTTAAAAAAGAATTTCATAATTCCGCAGCTCTTCCGAAGCCCCCCGAGAACAACACAGATCGCTAAGGGTGTCTGGACTGAGCTCAGTATCCCGGTAAGCCTCCAGAGCAGATGGACATAAACTATATTCTCGTGGTAATTGAACTACCGGAACGTGGATGCAAGTGACGGGTAACTGCATGGCCCAAGCGTTTCTATCAGGAAAGCGATAGTTTTTAAAGAGTCTCCTGGCGCCCATGGCCAAAAAAACCCTAAAACTACAAGCCAAGCTAACGACGGCTCCTCCGCGAACATCCAGAGCGTAGACAGCACCCAAGCGATCCATAAGAACCCGTTGCTTTCGAAGAAGACCACGACCGAGGCATCTTCCGACAAACTTTCCGATAACCACGGCAACGCCACGCCTAGGAATGAACCTCCACCGCTCTCCATGGTCCTCCGGGACGGAAAATTCGAACAGCACGCCTTTAGGCCACGGAAGAGGCACAAGAGCCTTGTCGAAAGCAACAGCGAAATTCAAAACATCCTCCACCCCGCACATAGGGCCGAAGAAAAAACCCCCCGAAACGATGGACTCGAAAAAAGCGTACACAGGGTCAAATCTGCGCAGACCACGCTTCCAGAGACCATAAGGGGTGTCCGCAACTCTGTAAATGGCGTTGTCCACGGAACGGTTAAATGCATAAAATTCACCCCGATCTCCAATAAGCATCAAAGCAGATAAATCAGAATAGAGCACCAGACCGATGACCCAAACATCCACAAAAGGACAACAGACAAAATTTCTGGTTAACTTGCAGACTTCGTCTTCCGAAATGCCAAGCATCTCCGAAGAGGCAAACCTCAACTCAGCGCCGGAAGGCCACACGAGAGGAAGTCGATGGTGAGCATATTGCAAAACAAAATGAGCAACGTCTCCGAAAGATACGGTTTCCGCAGCGAAGCTCTGCAAAAACGTGGTCCAGTAGACCTCCGGAACAGGTGATTCAACTGTATTCGATTCCAGATTAAACAAACCGTTCGATGCATTCGACTCAACAGCCGTTTCCACACCGGAATCCAAAAGCGGAGAATCTGACATACTAGCAACATGCAAATGATCAGAGAAAAAAAACAAGTGAAATGCACAGAGACAAATACAATCAAACTCATACTCTAAAGCACGAACGCTGTTTATTAATTACCATGTCAATTTCAAAACCCACCAGCAAACAAGGCAATAAAAAAAAAGAGTCACAAACTTAGCAACGAAAAATCTGGGTTCCAACATCAGGAAACGCTGCCCGCTCATCTGCTCTTGCAGCCAAGAAGAGAAGGTCACAGTCGCCCTGTCTGGCAGTTTTCAGATAAAAGCGCGACGAAAAATGACACAATGGTTTCTGGCACCATTTGGGCTGAGCCGCGTAAAAAGTGTGATGTTTATAGAAGTTACGCACACCGACAAACCTAAAAACCTGAAAACTCTCTGCGATGAAAGTCAAGTTTCCGTCGGGATCGTTATCATTAAACCCATAAATTTTTCCCGAGCAGTCAGCGTAAAGGGAAATTCTAGCTCCGTCTGAAAAACATTTCAGTCCGAAGCTCGCAAAGAAACACATATCAGCTAAAACACTTTTTATCTCCGGGTCAGAAACAAAATGAAAACTGTCTTTCCTGTGATGAAACAGGATATTCTCTCTCGCCGGCCATCGCAACTCAAATTTTTCACCAAGATGCCTGTCCCGAAATGCCAAAATTTCAGCGCATGTTGTGATAGCTTCAATCTCTGGATCTGATACCTCAAATTGCAGACACTTACACAAAACGTATGCGTACAGACATCTTTTGAGACCTTTCCTCAAAAAACTGTCAATAGTGTCACCCACGCGACATAGTTGATCAGGAAATAACGTTGTATGTGCATAAACGCGGCCAACTTTCCCAAGTAAGATAACCACATCATCAGTCTCATACTCAAGTTCGTAAACAGTCGTCACAGTATGTTTTATGTAACCAAGAACAGTAAGTTCTTCAGGACAACACACCAACGCGGCGTACTTTTTGAGCTTATCTTCCTCATATCCCTCCACACCATAAGTCAAAACAAGTTCAAACCCCTCTGGAAAAACCAAAGGCACCCTCAAATGCGGATTCAGAAACAAAAACATTTTTAGACTATTAAAATTTGTATAAACACGTAGAAAATCCACGAGTTTAGCCTTAGCAGATTCAAAGATTTTCTCTTTTCTTTTTTTATCAGATAAAAACGTTTTCTCAACGTTGGCAACCTTCAAATCCTTTTTCCCGTCAACATGAGCAACATATTTTCGTATAAACGAATTTTCCTTTTTTAAATCTTTTGCCACAACTCTCAAAACACGCTCTTCAAAATTGAAGTCAGACCCCAAATCTGCCATACTTAAATCGTACCTAGCGTATTAATAATCTTTGTGCACCGGTTTCGCAAAAGGGCAGAAAGCCAACCGCGGAATGCGACGCGACTGGCGAATGGTAAATCCGCGGTGCTGAGACTCTTTAAAAAGCCTTTTCCTCACCGAAACTCTCCTTTGTCGCATCCTGTGTTCAAAGATCCTAACCTCAAAAGAACCTCCACCCCTAACCGCATGCGGCACTACGTTTGGAAACAAACCACTCCAAAGATGCGTCCCATATAAATTGCAATCTTGACACCGAAAAAAACGCACTGGATTGTCCATGTAGTGCAGCTTTAAAGGATTCGTAAATCCTTCCAAAATATCGTGCCTTAACTCCCGAGCTAAGTGTAAAAGAAAGCACGAACAAGTTAAAGCTACCTTCTTTTGACACAAGTGCTCTAGGAGAGCTCTCACTCTATGATTCTGTTTACTGTAGGAAGCTACTAGAATAGAAGCCACTTCAAGAAAAGCGGAACAGTGCCGAGGAATGGACATCACATTGTGACTGCTAAAGTGACTACCTAAAACTGCTAACTTCCTACAAACGTTTTCAACTCCTTCAAAAGGTAGTATCCTTATTTCTAAAAAGACTAAAGACTGCGCGAAAAAACGCGCTATTTTGTCCAAATCACGAGAGATGCGTTCTCTTACTCTGCGCGCCAGGAAAAGACATAAAATGATATTACAATAAATCTTTAAAATGTTTCTTTCTAGATGACTATTCCCTAAAATTTCATCATGTAGAATTGGCAATGTTTTCGATTCCATTGCTTTCAGTATTAACTCCGCGCCGAGATCTTTGTCAATACTCTCTCTAACGCGTAAAGCTTCATCATTAGTTAAATGTAATTGCTGGAGAATCAAGTCCTGTGGACTAAAGTCCTGTATATCGGACAAATGTAGGACGCCCCTTTCTCTCCGCTTCAAAAAACGAGAAAATAGCGCCTTGCGACATTCCCCGCAACTACAAAGATTAGATACAACAAAGTGATAATTCATCACGAATGAAAAAAAGATTTTGTCGTCTAAGAAATTTTTTAATAGGAGATTGGAAGGCGCTACGACTTCACCAGCTATTTCCTTCGGATTTGGAAGTAACTCAAACAAACTCAACAATATATTTGAATCACAGCGATTTCTAAGCATAAAAAATTGTAAAAACAATGAATCTAAGAGCTCCATTAAACGATTAAAGTGAGACGAAAGAGTGTCGCGCTTTTCTCCAGCTACAAAATATTCCGCTATTGTAAAATGAGCCAAAGCTGTAGCTTTAGTCAGAACTCGATAAAACGGAGAAGTGCACTCTTCAAACCTTAGACTTTGAAGAAACGCGAGAAAACCGTCTAAAGTCATTGTTGCCTTGTCCCTCCTTTTAAATGAAACGGAGTTTTTCTCCACTAAAACAGCCCATAGTAGACTGTCATCGTAAAATTTTAAGCCTCGAGTTTGGAACAAATCTTCTGAAGTGAGATGACCGCTATCCGCAATCATCTCCTCTGTCAGCTCATTCAAGTTGACGTCTAACTCGCATGAGTTAAATGCCGTCCATGACAGATAAGGGTGATTCGGTATAACATACGTAATGCGATTCCTTAGCGGCCCTTTACAAACATCATAATCGATTAAATCCATTACGCAAATCCTTGTCAAAAATGTTTATTCATTTCCATCACTAGCATCGACTCCAACACAGGCTAACGTTTCTACATTGGCAAAGTAACACAAGTCATTCTGAATCTTGTCAAGACCCCCCAGCTTATACCCTACAGCTCTTACATCTTTTTCTGGACTGGTCTGGAAATTTCCATTCCCAAAATTTCCAAAATATTTGAGACCACTGAAAAAAAGTGCCATTGCTCCATTAATGTTTTTTGTGTTACTTAAATATTCAGCTTCGATGTTCACGATCGATCTCTCCAGCACATCTATGAAGCTCTTTTCCTTAGCATTCACTATGAAGAAACCTGGTGCTAGATTATTTGCAGCAACCAAGTCCGTGTGCTTCGCAGGCTGCCTTTGGATGTAAAGATGATGCAAATAAAAGCTTTCCATGAAGTTTGTTTCATCATAGTTTTTGTAATACGGAATTTCAAAGCTCATCTTTATCACTTCTAAGCAGTTGAAAATAAAGTTTGAGGATGTGCCAGGATTTCTGACAATATCTTTAACCTGCTTCAACATATTGAAATAAACGATATTGTTCATACAACCCTTACTGCCCAGTTTCATTTCACATTCCATGCTAGACTCCAAGTTCTTTAATTCATCTAAGTACACATCATAATACATTTTCAGTTCAGTAAACAAAACACTGTTAAGAGTTTGAATTATGAAAAGCAAAAACACAATGTTACTAAGAATATTCAACAGATTTCTTTTGTCCTGACCCATATCCACAGTAACCGAGATAGGAAACCTGGAATGTTCAATCTGCCCCTCAATAAAACTTAAACACAAATCTTTGGCTACAAACATAGAAAGATTAGAATATTTCTCAGATAAATGTGGAAAAATAGGTAAATCGATCTTACCCAAAGCTGGAAAGAAATGTAATTTGACAGAGCCAGGATTCTTTTTCATTGTGCCTACATTTTTAAACCTGAAAAAATTGTATCGACATTCTTCGCAACCACAAAATAGTAAAAAGTGCTCAAGTAAGTACAACTGAAAACGATCAAAAAAATCGCGTCTGAAGAATGATTCTGTTTTGTTTTGCATTTCTGTCTTAAAAGATTCGCTCCAAAATGCACAGCTAAAGCCCCCTAACTTCTGCTTAGATTCTTTACTCAATTCCTTCAATTTTCCATCTAGTTTAAAAAATAACATAGAAAGGTACTCACATAGGTAGCGACACCAGTATCCAATTCCAGGCTTAGCACCCAAATTAAACAATGATGCCCCAGAATAGAACCCTACAGCTGCAAGTTTAGTAATGCACTGCCATTTGGGGTCAAAATCTTCCATAAATTTTAAATCGCGCAACTGCAAAAGAAATTTATCAGCCCTTGTGAAAGTTGATGGATACTTGAACATTTCGTACTCATAGTCTATCAACCTATTCCAAATTACACCATCTGAACATAATCTTAGCCCTTTTATTCCAAAGCGTCCAGCAGCTGATGCTTGAAGGCCGTTGATTAGTCTGGATTCTGTCACCGGAACAATGTACTTCAGTTCCGTGTCTCCGTTTGGAATGATGATTGGATGTAGAAACTTTGTAACACTTTGAACAAGAGGATTACTGATTTCAGATGAGTGCACTGCAAACAGAATACTTGGCATGTTCTTCAATAATCTGAAAAAGAACATCCATGGAACCATCAGTAAGAGTATTGTGTAGAGAATACCGAAAAGAAGAACTTTGATCTTTACATACCTATCACACCTTGGATACATATGCCGTGGTTTCTTCCGAAGAATCGGAGACTCTCCATTTAACATTCTGTTCAAATTTTCCGCCTCACTTTCACTCACATAAATCAAACTGTCATCATGCCAGTTCAGGGCATATCTTTCTTTGTATAAGAGTTCTTCAAACCCTCGCCTGATAAATTCTCTAATAGACCTTGCAACTTTGTTGATCTTGTTATCACAATACACATAAATGTGAAAATTGGGACCTAGTAGGATGACAGCTCTACAATTTGGCTCTACCTGACATCTCGCGCAAGTTCCTATGACAACATACTTTCTTCTTAGTATTTTTTCGAGAATACCAGAACCTGTGATGTAATGGATGTCATTCGAAGAACAGAAAGACAAATCAAAATTTTGAAAAGCATCGATACGAATTTTTTGACCACATTGGCGATTGACAAAATTCTTTAAGGCTGGAATATTTTTTTGTAAGAGTACGAACTCCTCACCGCTGCATTTGGTTTCTCCGTACCACAGTCTGTCTAGCTCAATATCTAATTCAATCCTTGCAAAAGGTGGGAAATTTCTCAGCCCGATACTGCAAAAATGTGCTGGAGATTCCGCTATGATGTACAATTCATCTGTGACCGTATCTAATGCAAGCATCTTATTTGATGAGCAGTACATCACAAATGGATCAAAGTCCGGGTCCGGTTCTCGGTAATTTGGATGTGCATAACCAACAGCGCAAAGAAATTCGTCTGCTCCTACCAAATTACTCCAATCATAAGTGATGACACTACGTCTACATTTGAAATGATAAAATTCGCCAAAAATAAGCACATAGTTTGGCGGCCAAGCAAGACAGCACTTTATACCATTGCGAACTATCTCCATGCCTTCTCCATATGAAGACAGCAGCAACTCAACGGCAAAATCAAAAGACGGTAAATCCGGAGTCGGTTCAATGCAAACATCATTGCACACAGGAACAATTTCTGCAAAATTAACTTGTGATTAGTACTTCCGTCACTTTCAGAAAAATTAAAAACCATAAAAAGTACTGTTACTTACTTAGATACCAGGAGTACTGTTGAACCGGTGCACTTCCAAGAGTTGATAATCTTCCAAATCTGGCAGTGACTTTTCCAATTTTATCATAACCAAAATCTCCACAATATCTCGCGAAGCCGATCCTTGCAAAAGTCAGTAAACAATCAGCAATCCGCGTCAACACTTTATCAATCCAATCATAATAATAAATTGCTCCTGTTTCTCCAATGCATAGAATTGGTCTCAAACCATAGTCTTCGGCTGAAATATTTAAAAAACCTAAAACAACTACTTTTTCAAACAGTGCCTCTTGTTCTTCAAATCTTTTTTGGTCTGAGGTGCCACAGTGTATGTTTTTCCTGTCTTGCAGGATTAACGCTGTAAAGATTGGAGTTTCCAAACGAAGTTTTAAACCAGTGTTTATAGAAACGAAGTGTTCAATCTCTGCTAACGTTTTACACCTTGCCAATTCATCTAATTCGGAGAGCATTGAAATATAATTTCTCCTCAAAGCTTTTAGTGCCAAAGCTGTTTTCCTGTTAAATCTTAGACTAGAATTCAAATTAAGTGGTTCCTGTAATAATCCTAAGTCTTTAAGAGTCTCCAATTTTTCAAACTTTGGACCATCATAATAAAAGCGAGTATAAACAGGTTCTGAGCAAGTGATTACATTTGTACCTAGAATACCAAATCTAACAAATTTGTCAAAGTCTTCAGCTATGTAATACAAAACATCATCCTCCAAATCGTGACAAAAAATTCGACTCGATTTTCCCACAAAAATTGGCACGTCTTTCCTTCCAATTTGAATCGCTCCAATCAAGCACAAATTTTCCTTAAATCCTTGGTAATGGTCATTCAGTTCTGCCAATTGTTGTTCTGAGTAACCAGCAACGTCAACTGGCTGTGCAAGCCTTAACCACAAATTCTTTGGCCAAATCAAAGACAAGCATTCATTTCGATGTCGTTCAGTAAGAAGGCGAATTCCAGGGAAATCAGTAAAACGACATAAATTCTTCAATTCCCTTAACAATCCATCATTTAGTTGTCCAACACAAGGCAAAGTTTTATATTGTTCTGCCATCGTCTCAAAATTTTTCATTGAAGTGAATGTGGTAAGAAGTTTGTATTCTCATCCATTTCTCAAAAACTCAACCAGATAAATATAAGAGAAAAAAATCACATGACAGGAAATAAATTTTAAAGCTTTGCAGATTTTTTATTTCCCATTACAGGAAACATTAGAGTGATTAAAACCATAAGCCTGGTGTTATCGGATTAAAAACCTCTTGTCGAAAGTAGGAATCGGATTTTTACCTTTTTTAAAGATCGATTTTTAGAACATTCACGGTAACAATACAAAACAAACAGTGTCATTAATATTACAAACTCAGAATCTGCCACTGAAAGGAAAATCCCTTTTCGCCCTAAGTAACTACACTTAAAAATGGCTATAGCAGAAAGCGGAGCGTTCGAAATAAATGTCAATCTTGGAAAATCCATCGCTTCCATACAAAAAAATCCTATAATCTACATGCGTAGACATCTCTCTTTTTATGTGGAGTTATTAAAATTTATCATTCATCAATATGAGCAATGTTTTTTACCACCGAAAGGAACGATACTTTACCACAATGGCTTAATCGAACTAAATACTTTAATTATCGATCTGAATCAACAAATTACATCGAAACAGCAGATCTATAGTTGGACGAGCATTACTTTGCCAAAAATATTTTCGACTAAAGAATTATATTTTATTGTCGCGTCACCAGAGTCGGAAAACATAACTCTCAATCCAGCTGTTACTAAAGGAGGATGGTTATCTGGGAGTTTTAGCTTTCCATTGAGTTTAAGTTGTGCATACGCCCTCACTGGCGTATCTTCAACAATCTATATGTTACCATTTATTCCATATAAATTCCCAATGACTTACGTAGACTTTTCGACACTTCGAACATACGAGGTAACAAGTGAATATGGATCAATCCAAATTATAAAACAGCGGAATTTTTTATTTTTGGGAATTATACGGGATCTGTCATGGAAAAGTCAGAGGGATAACAAGAATTTTATCCTGAAAGCAATGTTCGTTGGAAACTGGTTAGGGATACAAATTCCAGAAGCTTTTGCATTGAGACTTTTTAATAACACACGGTTTTCTATTCAGGATTTTGAATTCTCCATTAACATACAAAATATAAACCTTACTAGGGACAATAAAATTTTGGGCTCTCTTTCCACGGTTTCTTGTGATCAGATGCCACCAAACTTGTCTCCAGAGAATCTCCCAAACTATCTAGTTATTCAGTTCGAATTGGTTTCAACTCTCGCAAATCCTGATCATCTACTGTTTTCTTGCAATCCCAAATTGTTTTTCACAGGAGATATTCTGAACAGCGCTATAAATTTACAACATAGTCCTAATCATTATGAGCTTACAGTGTACGCACCACATAATTTACATTTCTATCCCAGCTGTTTTCATATAGTAACATTACCAATTCAGTTTTCATCCAGAAATGATAGACAGATGTTGGTGTCAAGCTATCCTAATGAAGGCTACTTTGAAGTACAAATGTGCCCATGGGTACAGAATTCTCCTCTTCAAATTGTTATTAAATCTTTTTCAAAAAATCTAGTGCTACCGCAAGGCACCCCTATTGCAATTCTCTTATATATGGAAAAAATGACAACAGGAAAAAATTCTTTACGGGATCAAGAATTAAAAATTAATAAAGACATTACCCGAATAGGAAACGTAAACCTTCCAAAAGAAAATTTCTTACATTATAATAGCTAATCTTTATTCAACTGTGTTTTAGACGACGTCTTCTTCTGAGTGTGTTAAATGCGATACTAGTTTATTAACTATATCATCATCTTTTTGAACAGAATTTTCAAGATCGATTAAATCTTTATCGCCATTTTCTTGAGCCTTGCTAGCACCGAAAATGGTATTTTGCGTCTCTTTGCCTGTTAAAAGTGAACCTGAATCAAAATCCTGGATTTCAGCATTTGGTAAAAAAAATGGAGACCGCACAGGCTGCATCTTTTCAGTGAAGCTAAAATTTTGAAATAAAGGATCTCTCTTTCTGGAGTTCAGTAAATTCTTCAGGTTGTTGTCATTGAAAGTCGCAATGTTAGAAACATCTGTTTTTTTCGGGGGTTCGAGTACGTCTTTTAAGCTTCGCTCTTCTAAACTAATTGTATCCGTAAAATCCGTTAATAATTTCTTAACTTTTTCACCTAGTCCTGATTCAAAAAGTTCTTTTTGAAGAAGATCGTTCCTTCTACTAAGATCCAGTAATGAAGAACTCGGAGTCCACTTCACATCTTTGACTAATTGATTTTTTTTAGCTGAAGAAGCACTCTCTTTTTCCTTCATTGAAGCGTTTACGTCATTAGTACCTATCATATTTAAAGTCTTGGTGGAAGAATTTGCTTTATTTCCATTTTTTTGCTTAATGCTTTTTTGATCTTGTGGAGGCAATGACATTTCATTATGCTCATCGTCAAAACTTTGTAAGTTCTCGTGGACAATTTCTTTTCTGCGTTTTTCTGAAGGAAGTTTGAAAATGTCTTCATTATTTTGTTTAATTGTTTTGATTCGTTCTCGTAGCTCCAAAATTTTCTGCAAGTCATTGGTCGAGTTTTTAGACAGTTGATCGCGTGATTGCACTCCGTTTTGTCTGTTTACTTCGTTTTCTGGCAGAAAAAAGGTAGAGTGATAATCAATTTTATTTTTGGGTAGCTGAACTACGTTAACTTTTGGTGAGCTAACAATGTCTATTACTTTGTTTGAGTTGTGTAGATCTAGTTTGCTCAAGGTATCTATAACTTGGTTAATACCGTCTGGTGCCACAGCAATTTCATTGAATTGGTTTACTTTGTCCTCTGGTCTGTGCATAGTTTGATCAGTTAAATCAAGTATTTCAGTCTGTAGTATTCTGTGCATTTTTTTAGAATTGTTTATAGACGTGGAATTTCTTGGCTCGTCCAATATCATGGAATCCTTTTTTACCGAGGATAACAACCCATCTTCAGAGCCTACAAATAAATCTTTGGATAGCGTGTTGTTACTCCGTTGTTCAGTTTTTATAAATGGTTCTGGATTAAATGATGAACCATGCGAAGTTTTTTTAGAACTACTCTCAAATTGATTGCTTAAAGACGATTTCGACAAATCTGTTAAAACTTTGAATGTCTGAATCGGTTCAGGAGTAAATTTTACACGATCATCCCATGTACTCTTTACGGGAAGAGCATGTAACTCAAGACTTTTTGTTATAACATCAAAGTCTTCGGTTAAAATTTTAAAAAAAGAAGTTACTCTATGACTAGTTAATGGCAAACTTAAAATTTGATAAGAATATATCGAAACAAAATTTTTGTTGTTTTCAATCGCCTTTAAAATTGCATTTTTCTTTTCATTAATGGTATTTAATGCGAGTTTCTCCATGTTCATCCAGGATCCATGTAATGTAATTAATAAATTCCCAAGATAAAGCAATCGATTGATGTTAGTAACATAGTAGCCATTTTCATTCCACGGATCAACTATTTGGTAAATTGTGAAAGAAAGCATATTGTTTTTTGCCTTTTGTAAATTCTCGCCAATTTCCGCTCTTTCTTCTGTGGTAAGATTGACGTAATCGGCTGCGTGTGTCACAGTGCCCCAACGAAGAAAACTGCCCAGTTTAAGCAAATCTTTTGCTACGTTTTTAAATTCTGTTTCAAAGCCAACTTTGTTAAAAGATGTTAAACTAGAATAATCAGTAATCATCTGTCTTTGCTCTAAGTAATCTTTCAGAATTTTTTGCACCTTTAGTATTTCTTGCCAAACTTCCTCATAGTCTGGTTTCTTTTTACACAAGGTTTGATGAAAATAAATCCAAAGAATCAAATTGTTGTAAAGTGTTTTTACATTGTTAATGGAATTTCCAGTTTTAACGATACATTGACTCAAAATCCATGGATTTTCGCGAATATCAACAACTGGTAAAGACGATATGTTTTCAAAAAATCGAGATAAAAAACACTTTGCTTCATCCGAAATCCAAGCAAAGGGTAAATGTGACATTTTCATGACTCGTTTTTTCTGTTGAGCATTATTGAACACTTCTTAAAAAATTAGTTTAATAGTCTCTATTTTCTCTTTGATTAGATACATGCGCTGCCGACATGGACACTCTAATTGATTTCCAAAAAATCCTGGTATGATAATCTAATGTTATGATTTGCTACAGCTTTGCTAAAAATGTTACCTTTGCATTTTTAATCATCTTGCAAAACTTTTTTTCACAGCATGATGAAGAGTACAAGTACAATTATACGTGTATTACGCCAACAGTACGGAAAGCCCAAAGACTTGAAAGCGTAATTAACGGAATTATGCTAACGCTGATACTTCCTGTTAGTACTGTTGTCATATGCACTCTGCTAATCTACTACAAATGGACAAAACAGACAATTACTTCTCCATATCTTATCACACTCTTTATTAGTGATTCTTTACATTCATTGACTGTGTTACTTCTCACATTGAACCGAGAAGCTCTCACAAACCTTAATCAGGCTTTGTGTCAATGTGTGCTTTTTGTATACAGTGCGTCCTGCACATACAGTCTGTGCATGCTAGCAGTAATATCCACAATACGCTATCGAACCCTGCAAAGAAGGACATTAAACGACAAAAACAATAATCATATTAAAAGGAACGTTGGAATTTTATTTCTGTCTTCTGCCATGTGTGCCATTCCAGCAGTATTATATGTTCAAGTGGAAAAGAAAAAAGGCAATTATGGAAAATGTAATATACACATCTCAACGCAAAAAGCATATGACTTGTTTATAGGAATTAAAATTGTCTATTGTTTTCTCTGGGGAATTTTTCCAACTGTCATTTTCAGCTATTTTTATGTGATTTTTGGTAAGACCTTGCGTGCCTTGACCCAAAGTAAACATAACAAAACTCTGTCATTCATTAGCTTACTGATACTATCCTTTTTATGTATTCAAATACCAAATCTCCTAGTAATGTCTGTGGAAATTTTTTTTTTGTATATAGCAAATACTTCCTGCTTAGGCACCATACAAAGAGAAATTGTGCAAATAATATCTAGATTAATGCCAGAAATACACTGCTTGTCTAATCCGCTAGTATATGCATTCACTAGAACAGATTTCCGATTACGATTTTACGATTTCATTAAATGTAATTTGTGTAATTCATCTTTAAAGAGAAAGAGAAATCCTCTGACAATAAAAAATTGAACAGTGAAGCTTGCTTAAAACTTTGAAAATTTTTTGTGATTTTAAATAAAATTTAAAATCAATTTATCCTAAGCTTTTCAGGCTATGACTTTGAATCAACAGCCATCTAATTGCAGACTAATTACAGCTAACGATCCAGTACTTGCATCGAATTTTACAATGCAGCCTACTTTTAAAATAGCCGATAAGAAAGTTGTACTCAGAGATCATAATTACATTGCAGTCAAAGATTTTGTTCTATCAAGGTCTTTCATGCATTGCATTCGATGTCAGGAAAAAATTGAAAAGAAGACATCCGATAGCATTAGAGCATATTCCATTAAGCCAGATTTCATGTTTTATGACTCAGACGACGAAGTTCGTTCGTCACCATTTTTATAACAATATTTAAAGTAGCTGAATTGTACGTTCTGAAACAGTCTGAACGGATCCGCTGAACGCAGACACAATGGAACAGCAAAAAGGATTTTCGATTCCATTTTTTGTCACTGACGAAAACTGCAACTTTGTGCCTGAAATATTACCTCGTATACATACTAAATTTCTTAAAGATGTCTTAATTGCCGATTCCTACAACTCTGTTAGTTGGGCAAACAGTTTTATTCCCATGCCTATTCAAACGCTTGAACAAATTATGGTTCTTATAACAAAGTTCAAGTTTTCTCGCTCGCGTGATTTTTTATTCCCAGTAATTCGATTAGCTGTTCATATCAATAGGTTCCACACAGGGAAGAAACAGCTGAAAACCATGATAGAAATTATGAAAAGCTTGTTTAACACCGAAGAGGCTATGCGACGATTCGATGAAGCATTGATGATTCTATTTTCTAATGAGCAAACCAATACTTACATGACAAACATAGCTTTATCGATGCATGAGAATGGTCTTCCAGATTCAAAATTTATGAATGCTCTAAAAATGATTTACAGAGCTGGAAATTCTTTTGATAATCAACCAGACAATGATATAGAAAGCTACAACGAGAAATTAAAAATCTACAACTACCTAATTAAAATACCTAAGTACACACTAAAAGCTGGAGTTGATTTATATAATGAAAATATAAAAGATCTTTCGATTGGAATCCAAAGACAACCTACTTTATTATTCACATCTCGTAATGATTTTTCATTGAAAGCTATTTATAATGACGTGTTATTTCTAGTTTCTGCATGGAATATGATCATCAACTATAAAAAAGAACAAAGAAGACTCTTTAGTTGGATAACATTTGAAATAAACTCTTTAATGGAGAATGTTGTGCTTGCAGCCTTTCAGTTACCAGATTTAAAAGAAATGACACTTGATTTAAGCGCTTTGATTGCCAACATGAATCTTTTAAAACCAAATGATGATTACAGCCCCCATTTTAAACTAATTATCAACAAATTTTTTGAAATCGGAATTTTTGTCACGAAATCATATATTTGTATTTTGCCTTCTTTTGTCAAAAGCCAACTTATTTCGTTTGAAAACGTTTTAAGTTCAAACAGACATGCGGAAGATGTGACTTTCATACTAACATCTTCCAAGGAATCTGATGATGAATATGATGAAGATAAACCTCCACGACAAGTAGATCCAGACAGAGTGGACAACATTTTAATGGAATCAGATTTTTTTAATGTGAAACCGGAAAACGCCTTTTCAGAAATCGCATTAATGCCAATTTCACATGACAAAATTATAGATGTCAACAACTCAAACATACAAGTTCTTGAAACTGAATTGGCACATACAAATTTATTTGTGTATAGCGCAATAGCTCAAAAATATGATTTGCCTTTAAAAGAATACGTAGAGCGCTTAAACGTCTACAATCCTGATCTATCATCTGGAAACTCTACACCCGCGCGGAATTCTAATTCTATTCATACAACTCCAGTTCTAAATATATCCAGACCAGGAAGCACTACACCCTCTGGGAACTCTGCAAGATATGGGAATAACACACCTAGAAGTATAACTCCGGTTTTAGAGATTTCAAGATCTAGAAGTGCTACCCCTTCTGGAAATTCTGAAATCTATGAGAATAGAACATCCCCAACGTTCCGTGTTTCTAGGAGTGCAACTCCAATAGAAAGAAGTTCGAGATCTGCTAGTATAATTTCTGGAGAATCTGTTCCTGGTTTTTTTAATGACCAAGAACGTTTGAGCACCAATTCGCCTATTTCCATAAATGGTAATACTCCAAGACAGCAAAGCCATGGGGACAATGAAATACAAACTATAGATTCCACGGATGAAGACTCAATGAATGCCCCACAATCACCCCAATCCATCTATTCAATTTCTAGCTATGTTTCAACAGACGATCAACTTCTACATTCACCGACAAATAGCCCATTTAATCTTTTTGACTCTGTCTCGGAAATGCAAGAAGACACCGAATAATTAATTGTTTTTTATTTAGCTAATTCATTGTATGACAACACAAGGGCAATTGTTTGATTAATTTCCACGTTTTTTAGCTTTTGAATAATCTCAATTAAAACATCTCTGGTCAGTTGGAGTCGTTCTATGTATGAAAAAAACATTATCGCGTTTTCATGCTCATCGTCTCCAAGCCTCAGATGACTTTCTTCTAGAAACTAAAGAAAAAAAAAGTTAAAAGATCTTTTACAGAAGAACATTTATTATTTTTAAAAATGATTTTTCTTACCGCATAAATCATGTTTAAGAAAAGTTGCTTTTCATGCTTTGTTGTCATCATATACAATAATGCTGTGATTTTCCTGAAAATAAAAGAGCGGGGTTAGACAATAAAATTTTGAAACTCGTTTGTGTAGAAATAATAGAGGTGGACTCACCGTTTTACAGGTATGTGTGTGAATTGAGCGACGATATAACAATCCTCAGTTTTGTGAACATCTAGATTGAAAATCTGCTTGATTTCTTGCCATCTCTTAATAATAAACAAAGCTTCAATCACTACAGACTTTGTCACAGTTGTGGTTAATGTTAATCCAGAAAAAATAAATCTATATGTTGTATTAAAATCCATTTGAAAAAGATATTTTATATAGATTGTCTCCACTTTAGATATAATATTAGATAACGTCATCAATATCTGTAGTGGACACAGCTCGCGAAATTCCTGTAGTCGTTGTCTTCTCCAGGTTTCCATTTCTCACAAAGAAGAGTCTACCCTCGTAGAAGAACACTAACTAAATGAATTTTTTTAGGCGAACGCTTAAAAAACTCTTTTACTGGCACACCGCCTATAAAAACCCCCAAAGGAAATTTTTATGTAACCACACAGAACCAAAAAAAGATTAACCGTTATTAGAAAGTGTTCGTTTTTAACGATTTTTATTGGCACATTTTGACCGGGTTAAATTTCGTCTTCATCAGATGTATCCACTTCTCTGAAATCCTCACTTAAATATATAGCTAATCCGGCAGCGTGTGGGCAGGTCGGATAAATTTCATGAGTAATTGTTAAATTCCTTGGAAAAAATTTGTATGTGCGATATGTTCTAACTATCCCCTGTCTTAATAAACATTCAAAATTTTCTGCAATTTTGACACAGGGACCGATCCCGGTTCCAATTGCGTCAATACCATAAATAAAACCTTTTAAATCCATCATTAAAAATAAACTTTTAGGAAACATTAAGTTTTTACCGACGCTGCCAATGATATGTCCATAGTATGGCATATTGTCATAAACGTCAGCTAGAAAATTCCATTCTGTGCGATTAGGGAATTCTTCTTTTCTGGTTAGAAACAAAACTAGTTTTTCATCCATCTTTTCCCTCTTCAAACGAAGAGTGTACACTTTCCCACTGTTAGCCGCTATTATTTCAGCAAACTTCACAAGATTATACTGTGCATGAATGAGACTTTGCAAAACGTGATCGATAGACGTTAGGAAGGGAATGTCACTATCTTGATAAATCGGATCAAAACGGTGCAACCCGAAATAAGCAAAGCGCTCTGTTGATTCAGCAAGCAAATATAAATTTTCTTTTTCGAAGTCCAAAGCATAGAAACGCTCTTCTTCTCCGAGAAGAATGATGGTTTTAGCAGCTGCTGTTGTTACGTCGTTTTTAGCACTCAGAAAACCGATGACTTGTAACCTTTCATTGCAACATAGGAGATAGCAACCTATGAAAACAACAAAAACTTATTAGAACATCATGACACATTGGAAATGTTTTGACTGCTTTTGCAACTTACATAGGTCTTGCAGCTCGGTTTCACTAGTAGTTCTGAAAGGCATATCCCGCAAAGGCGAAATGATTATTTTCCTACTCTGAGGCCATCTTATGAAGATTTCACACCCACTATTTTGAGTAACGAAGTCACTAAGAGAATCGCGATAAAAGGCTTGTTTAACAAGGCTTAAATTTGCGAGTGCATGCCTAAATCGAACTTTTTGTTCACTTTCGGCCATGTTGAAGCACACTCTAAGCTTATGAAAACAGCAGTTGTGACTAGGTTTTTATATATTACTGAAGCATGACGTCGCTTTATGCTTAAGCTGGAAAGTTCTGAAAATATACAGCTATCTTAAACAAGTCTGTTTACAATAGGGATTTCCTTTTCCTGTAAATGCTGCCTGATACTAGAAATCATGTCCGTGAATTCCAGTATGGAAATCTGGGAAATTCCAAAATATATGATATATAAATTCCTACTTTTCCATGCAGCATCATTAACGCCGAATCGAATATACACATTTTCAGAGCTGCAGTTGACATACTAAAATATGGCTATTAAGATCACCGTATGTCTTCGATACTTTAATCGAGATCGTAACGAACTTGGAAACATTACTTTTAATATGGAATACAAAGACAGGAGAGTCGCTGTGACATCGAGTTATATTCGGGTACATTTTGTTTTTAATCAGCGTTAACTGATCAAACTTAGAAATGTTTCCTTTAATACAAAATGCGGAAGAAAAAGCTTACACTTACCTCTTCTTTATATTGAGTAAAAATGGACCGCGGGGGGAACAGATTTTAACAGTTCTAGATTACACTCGGTTTAAATTTTTTGATGGCTCAGTGACCATTGCACACTCAAAAAAAAATTATTTTCTTTCAGGTTCTCTCAGATATAAGAAAGATGATTATGACGTTATTCAAAAACAAAAAGGTAAGCGAATTACATGTAAACATTCAAAATTTATTGTTTGTTAAACATGTAGAAACTCACAATGTTTTCTCTATGATCCAGCAAAGACTTGCAAAGACAGAAATTGAAATGAGCCTGACAGTCACGTAAAAGTGTTCAAAAAAGATTCCTGATGATAATCGAGAATAAATCAACCAACATTCTTGTCAACTTTGCGCCGATTTACTATCGACAAACAGAAAAACAAAAAAAGTACCATTGCTGATCCGCCGACAAGAAACACTACACCAATAGTTGTCGCTTGGGTTTGATCGAGGAAGCTTTCGTAATCATTCAAGAAGATGACATTTTTACCTCCATATGAAGCGCTGAGATCTTCCAATTCTTTGTACTTGTACCAATCAAGCCACGATGACTTCCAAGTTTTGCGCAAAAATCTTTTATAAATAGTTTCATTGCGTACTATTGTAAAGTTTGGATTTAAGACTTTCCTTTGATATATTTCATCAGATAAATGAGGACATATCATTGCTACGAAATAACAATCATAATTAAATTTTTGATTTAGAATTTCAAGTGTTTTGTTTTTATCTTTTCCGACGCTTCTCCACTTAACTGCAACTATGCCAGATCTTAGCTGCAAAAGAAACTTTTGACCCCCTGTCATAATAGTGATGGAACCACCCGAATTATGACCTTCTAACCAACATTCAAGTGTACTGTTAGCCCGAGAAGCAAAGTGCCATTTTTTGGCAATTAGTGCTCTTAACATAACTCCTCTAAAGTAATAATTGATCAGCACTCTCAAATAGGTTAATTTATGAGATGTTGTAGTAAGATTTACTAGACTCATTTCGCGAAATCCTTCAGTTTTTAAAGACAAAGAAATCAGAAAAGCTGTTCTTACAGATAAACAACTAGAATTCCCTATTAGCATTTCATTAACATAACACGTGGTGACACAAACAGCTTCTAATTTATTATCTAGAAATATTATATTCTGGTGACATTTCAAATTTGACATGAGTGTGAGAGATAAGCAAATCAAAAAGACAAAAAACATCATCTCTTTTCCCATGGTCCTTTATAAAGACTTCACAATTTCTTCAAGAAAGTCCACGGTACTTCTATAAAACAAAAAAAAACACAATTAACTATTGATGAACATTGCAGCATTTTTCCAATGCATTGCCATACATACCCCGAAGAAATTTTTCCTTTGAAAAGATACTCTAGATGTGAAAATTGTGTTGAAGAAAGAAGTTCCGTCTTAGTGGACATACATTCAAACGCAGGCTCAGGGAGTTTTTGAATGGTTGATATTCTGCTATCTTGTTCTTTCCCCCTTTAAATGGCCATAAAAAAGAAATTATCAGTTAAAGACAGCGAAAAATTTAAAATAAGTAAAAAACAATAAGGCACCTACTCGGTAAATTGTTCGAAATTCACCGGGAAACCCGAAGTTGAAGTGTGTTCCAAGTTTTTCAGTGGCGAGTTTGCTATTTCTTCATAGGTTTCCAATGAGAAGTTTGAACTCTCTTCTAGCATATCTAAAGGTGCTGTCTCTGTCTCGTTAGCTGTGTTTACCTCTTTATAGGGATACATTTCCGCTGTGGTTTGCGGTTTGTAACACCGTTCTTTAAAAAAAATGTCATTTTGTGATGTTTGAGTACTGCGAGTAAAATGTCCGAGTCCCGTTTGCGTAGATCTGGACACTAAAATTGTCACACCAGGTGTATGACACATTTTGTAAATCTTGCAATTATTTGGAAGATTGTCATTTTCTGAGTATCCTATGGACATATACATTTTTTCTGCCTGTGTCTCTCTATCATTCGAAAGCTTGTGACCAGGAAGAACTCTTTGAAATCGCGCAGGTAGTTTTCCACTAATATTGATTGTTTTTTGTCGTATTCGATGATAGTAATTGATAAATCTTTCATGGCATTCATCTCTCGACATCTTCTTTGACATGCCATGTGTAATTGCATTTTGACAGCTAAATTCCAATTTCGTGGTCCAGAAATCTTCAAATGTGGGCGATAAATAGAACATCACACCATCTTCATAAGCAAACACATGCTGAGACAAGGACAGTAAAAAAACTATTTGTCTTCGCGGCGTCCCAGAAGCTGACACCCATTCTTCGGCAACCCCAAACATCACCATCTTTCCCTCAAATGGATAAAATCCGATGTTAACTAAACGTTTAAGTCTCCTTTCTGGAAGAATGTCAGTCATATCACAAATTCGAATATTGATACACATTATGTGTTGAATGGACACGCCATCCTTCATTATTTCTTGAACTGTTTTTTCAAAAAACTTTAAATCTTTCGAAGCTGCAATTAATTTTTCTTTGCAGCGCAGCATCGTCGTTAAGCCACATTCCTTAGACATAATCGTCTCTTCATAAAAAATACTGAATTTTAAGCGTATATATTCAACCCCCCGGATATGGGCGTGGAAATTAATAAAAACACTTCAAAAAGCAAACTTTTTATTGAAACTCATTAATGACACATGAAATCTAAGGTCCCGTGAATTTGAAGATCTGGAGAATCCTTAGACAGGCAGTCTGAATCTGAAATTTCCATTTTTTCAGTTGCACACAAATTTTCCACAGATATATAATCACAGGGATCAATTTCCATGATTACTTTAACACTATCAGATCTATCAAGGTCCTTACGCTTATTAAGAACAGCCATAACATGCAGCCAAATTTCAAAAAACATTTTTTTAACAGTGATTATAGAAAAAATAAAGGCAAGTATAGGAATGATTAAAAGTATCCAAAAAACTTCAAAGTAATTCCCGTGTTTGTAAGTTTTGTGAAACAAATTTTGAAAATATTCATCTGAATTATTAAATTCAAGGACGTTAAAAGAGTAATTACATTTTTGATGAATTACCACACATTGATAATTAGCGGACTCCGTGTTAATAGATTTAAAAAAAAAGTAGAATTTTTTCCCTTTTAAGATGTCCGATGCCAAATATTCTTCTGTCAAATCATCAGGAACCAGTTTATTTTCTAGCTTCCATTGAATACTCAATCCCAGTGGACTATAAAGCTGCGCCGAACACGTTATGTTAGATCCACGTTTTGTGATGGACACCACAGGTGGCCGATATCCATCTTGACCGAAGGTCACATGTGCAGAAAAATCTGCGAGTTGTTTAACACAAAACTGCTGCACATAAAATTTGAGAATAAGTAATAATTCAACATTAGCATACATGTTTTCGTCAAAACCACTGCTAGAAAAATCCAACAGGTTGACATTCAGTGTCGTGTCATTATGAACTTTTAACAAATAATGACTTTTCAAAACGGTGTCAATTTCACAAATGAATTCAGCGTTAAAATTGTGGTGATAATGAACACTGCGATTCCTATGTGACCAGACAAAATAAGAGAAAATGCTGTTTAATGTAGATTCGTCCTTGTTGGTAGAAAAGACGTAAGACAAAGCATTAAATTTCGTTGGAAAATCTGTGAGGAAAGACGAAATACTAGTATTGCCGACAGACAGATTCTTATAATGTGCCAGTTCATAGTTATCCATCATCAATTCATATTTCTCAAGATGTTCTTTATTGATTATTGACATTGTCAACTTATGTGCCACATATTTGGTAAAATGTACAATGAAAACTAAAAGACATGTTTTTTTCACAAACATTGCTGTTTGAGAGATTCTTTTTTAGGTAACGGTTCTTGTCCAAACATCAAGTTTGTTAGATTGATTATTTTCTCTGATACTGGTTCTTTTTGTATGTGCTGTAAAGGCCACGTCAGAACTTTCTGAACACCGTCTAAACAGGAAACTCCTAATAAAACTACCGCTGTAACAAATGCAAGGAATAATATAGCAATCAATGTGTAGCAAAGCATGTCTACGAAAACATTACTAGACGTTTTTTCACTATCTAATAGATCCAAATCCACATCAGGTAATGTTACCTGTATTTGTGTTCCTGTGTTCTTATTCGTACAGGTACAAACATATGTGGTCATTTCTACTCTTAGAAAAATTAGACCCCAAAGCACATCACTACCATACAAAGGAGTAAAAGCACCCGTAGAGTTGAAACCAACTCTTTTCCACTCTTCTCCAAACGGACTTTTTATCTTTTGCAGAACTTCTAAGGAGCCGAAACCGCTAACACCCCATCCTGTATATTTACAAACTGCTACCTTTACACCATTCTGTCTAAATTTCGAAAATGAACAATCTATGTTATTAGTTCTATATCGTTGTTCATTTCTTTGTTCAATCATAGGATTAAAGCCTTGCTTAAATGTAGCTTCGAATGTTTTCGCGAGTGATAGGTACTTTCTTTGCCAAAAAAAAAGTTGATTGAAGTCATTTTCACAAGAATTGATCAGAAAGTTTTTAGCGTGTGTCAAAGTGTGCCAAATTCGATAAGTGAAGGGATCATCTTCACCTAAATAGTTTTTAATCGCTACCCACGTGCTGTTTCTTTTATTTTGCAAGTAGAATGTGGTTTCAAGAATTCTAGGAGAAATTCCAGTTTCATTCATACGTCTCAGACGGATAAAACTTTTAAAGGGTGAACAGATAATTTGAATATGAATGTAAGACTCGACATCAAGACAGAGTTTATCATATCGCTTCATCAGTCCTTTTGTTTTTTTAAAGTTCTTAAACAAAAAATGATTTAGTGTAGAAAATACTTGATAACAGTTGAAAGCCAAATATTTGCCAAAATCGGTTTCAATTTGCGTTTCGCTGTTGCCAAAAAAGAAAGCATTGTCTGTCATTGCCGCGACTGCAGAGTGTCTGATTTTATCGTCTGAACATTGTTTGCTCAAAGATATGTCGAACAATAGCAAATCATCCACTGTAGCATTCACATCAAAATCCACAACTGCTAAGGGACAGAAATCCGGATAGAGTTCTCCATAAATCACAGGAACGCAAAACAGCAAGATAGTCCACATGTTGAATTAGAAAAGAGTGATGAGAAAGAAAGCTTTTATTTCTTAATATATATTTGACGACTGAACGTCACAGTTACGCCCATTTTACGGTGATAAACGTTAGGGGTTCCGCAAAAATGTGTGCATTGATCTTTCTGTGATGACACAGTGGTTAGCAAAGCACATCTAACATAATAAGTCTGTTTTTCTCAGTCATAGGATTCCACAACAGGATAAGACATTTGTGCTGGTCTGATTCCTAATTACATGAGCTGTATTTACGCCAGAATTTTGTTTATTTCCTTTAATTACCCTCAAAAAATGAAAACATTGATGAAACATACTTCCCAGTGAAACGCTTTCAACGGAGATCATGCGTTGATGGTAGATAAAACACAACAAGGAGAACTTTGCCCAAAAAAACATTTATTAATCCCAGACACCAATAAAGGCTTGGTTATTGTATGCATTTTTCGGTTTCTTAGAATGGTTGCGTTTGGGGTTTTTAATTAGTAAAACTAACAACGCTAACGCTACAATACCAATAAAACTAACTGCTGCAATTAAAATCACAAGGAACAAAACGAACTTTTTTAAAGGGCTATTCAAAAGTTGAGGTTTTGTCAGTTCTGGAGTTGAAATTGTCAGTGTGAAAAATTCAGATGCATTCCCACTGTTTTCATGATTAGTAGTACTGCCACTGGAGAATGTCGAGGTCTTTGTAAAATTTAAGGATGTAGATTCGACATTCTCTTTAGTTGTTGCGATAGTCGTAGGTATACTCATAGACACAGACATTAAATCGCTCGTACTCTCTGTATCTGATGTGATTGACGTGGAACTAAAATCTGAAGTTCTTGACATTTCCAGAGTGGGCGTACTAGAAACAGTACTCATGTTAACAAAGGTAACCATTTCTAAAAAAAATAAAATTCCAAATAACATTTTAAACACTGCGCAATTAGAAGAAGCCTAGTTTAATATTGTACGTGTGCCGACTAAATTACTGTTTTTAAGCAACGCAAAAAAGTGCTGGAATCGGTGTAAAATTCAGGGCTTCGTACCCCCATATCGAACATTGAAGATGTGTAGAATAAGAATCAGACATAAGATAACCGATATTATGAGACATGCCAGTATTATAAAAGTCCCTCTTCGGCGCACATTTTTGATTTCAGAACTAGAAGTTGAATTTATTGTTCTTGGTGATAGATTTTCCTGATGCAAAAAAACGGAATGATCGTGGAACATTTGCAACATTACATCATTATAAGAAGGTGGCGGTGTCTCATGAGTCATCTTCTAGGGATATTTAGAGCACCTGATAAATGATAACATATTCTCTGCTATTGAAGGACTTATATAATCATGATATGCAATGCAAGCCAAAATAAACAGAACAAACAAATAATAAATCCCTTTCCAACAATCTTCATAGGTCTCTGGGAAAAAATGGTTGTACTTATAAAACATTATGTGCAGAATTATTTTTTCTACCAACACTTTTCCATAGTAATGGTTGTTTAAATATGTTCCTTTTTTTCCTTTTACAAGTATTTCCCCATGGACAGTTGATCTGCCGTGAAAGCTCTTCTTCCACATTTCTAACATCGTTTGTTCTTTCAAACCAATACCCATGTAGTCGACGATTTTCATTAAATCGTAAACAACCGATTTTCAAAAATTCAGTTATGTTATGTGCGAGAAAAATGATTTGTTCATCATCTACACCAAAAATTTTCCCATTCACATCGAATATTATCACAAGTTCAAACCACTTCCCAAAAACGTTGTGTTTTACACAAAAAAGAACATCTACAATGTTGCAACGAATGACCCTTTTCCAACGGTGAAGGTCTTGATCAGTATAAGGTGTCATGGGAAGTCTGCAAAATCGTGTGTATGTTTTATAAGGACTACTTTTCAATATAAAACAAACGTTTATATTTTCATCCCTATAAATGATCATATCAGAGAAAGATTTGCTATGCTCCACGATTTTGCTACCAGTTTCAAAGTCCACGATAAAATCACACAATTCGTAGATGGGGTAGAAAAACTTCAATCCTACTAAAAAGAAATCTTGTAAGTTTGATGAAACATAGTAAAGACTTTTGTCAAAAAAACCATTGTAGACATACAAATGCCCTCTGTCACCTTGTAAAAGAATTGGTCCTCTGCAAGTTGATTTTTTAACATGATGTAACACACCAACAACGATCAACGTTTTTTCACAACACAAGTAAGTGTTTCGAATTTCTTCGATGTCTTCCTTTGAAATTTCTGGGATTGTTTCTAATGAGTGAAATGTAACTCGCCATTCTTCTGGGAAAGGCAATACGAAAGATGTACCTTCCTTACTTAAGATGAATTCTTCGAGAAGAGTAAGTTTATTGACACAGCACAAATTCGCTAAGAAATCCATGCTTCAGTTTTAAAATTACCCAAGAAAAAACAAAGGCATAAATATTTCAATTTTTTTTATTTAGCACATTTAGAAGCTGCAAATTTTCTTTAGAAAAAAATTACAAAGCAAGCTGAACAATAAAAAAGGTTCAACAAATCGCCTGTAAAAATATCTTTTCTACCCACTGTTTTTGAAATTTGAGCAAATAGAGCGCTATAACCTAAGCCAAGAAAGAACTCAAACAACAATGAAAAGAAACAATGTTCTGTTATTACCCCTGCTAACAAACACAGAATACTACACAGAACAAGTTGATATAGTTTAGTGATAAACAAATTACTATGTAAGACTGCCAAGAAAAATAAATGCTGGCAACTTTTTCTTGGTAAATACAATTTTCCAAACCAAACCAGCAGCGTTAAGGTTAAAAATTGCAGCAAATAAACAATAACATCTTTTATAGCCATTTGGTACGGGCCATTCGGTTTATAGATCGGTTTGTATCGCTGAAGACACATGGCAAACTGATGAGAGTTAACCAGAAACTTATAACTGGTTACATATGGCGTTATCAGAAACACTGTAACAAGCAATCTGTAATGAACCAAATCTGATATTTTTAACACAATGGCAATTAGGGTGCCGAAATAAGATGCCATTGAATGCGTTGGAAATATTAGCATGCATCTACAGACTATGGCTCTCAAGCGGATTTCAGAAAAAAATAAAAACGAGCAGATTACCAAAAAAAATTCATCGGTGATTTGCAACACGAGTAATGATAAAGTTATAATTTTGAGATCGTTGAAAGGACAATAGTTAAACAAATTCACCCGACGGTAAGTCAATAGCAAACCTCCGCTAAAAAGGAATATTAGCACTACTTGTTCAAACAGCGTTTTCTTTATCATAAGATGGAAACAGCTTTCTCCCGGAATTACATTCCCTAAAACAAGGCCCATAGAAAAACTTATGATTACATTCCACATCTTTGTCTTTTCGAATTTGGGGTATAGTCAAAATGTGCAGAATCGTCACTTTCGCTGTCCTCTTTCTCATTAAAAAAATTTGTTCCTGTGCCACCATTTTTAGTGGGCACCATGTAACTTGTAATTTTGTGTTGCGTTTCCTGTTTACAAAAAAAATTATTTCCTTCCTCCACCTTGTGATTTTTCTCTCCTTTTTCCTCTGTTTTTGCTTGACGTTCAAATTTTGGGTCGTCTTTAACCTGCTCTTGACTTAAATAAGCTTCTACGGTCAGAAAAACATTTTTAGCGATTAAAAATAATTTGTATTCTTTGCCAGCTATTGCTCTAAGACTACACGAAGACTTTAAAACTGCACAAGTACTAAATGCTTGCTGTAGATTTTTGGAAGATAGTACAAGTCTCATGCATTTTGCGTCGTGAAACACAACTTTCCCGCTATGAGCAAATTCTATTTCGTTTAAGTCGGTTAGAAATTTTATACTTGGCGGGTTAACTTGCACAACTATTTGTGCCAACGTCATTTCATTTTTATTACTGCGTTTGTTCTTTGTGACCGGAGCAAGCCATTTTAAAATCTCCGTTACAGTTGAATGGTCCAAGTCTATTCGAAGTGCAGATTTTCCGCTTTCTCTTACAATTTCTTGACCATTGACACAAGGAATAGATGCTTGTGCACAAATATCTGATGCCGTGACTAAAAACCGCGTATAGAGATCGCTATCATGTTGAATATACAGTTTAGTGACATCAGGATTTGATATAATGCCCATAAAACTATCAAATAATGGAACAAAGTTATTTATCGTTTTAGTCGAAAAATGATCTGTATCGGTTATATATAAACATTCTGCATGAATAATCAGCTTTAAAACCAAATGGTTTTTTGCAGATTGAATTATGATTGCCGGTTGAGGAGTAAAAGTAACTGTAGTATTTTCTCGCAAAAGCTTTGCAAAAGCTCTTAGAGGCTTATTGATTGTTTTCCAAGTTTTCATGTGAAAAGCCATGGTGGGAGGCTCTTTGGAATCTCGATGTTCGCGATGATCTCGGTGACTATGGTGTTCTCTGTTGCTACGATCCATGCTGCACCAAAAATAGATATTAACAGGAAGAGTTGTATGAGAATACTAAAAAATCACAGAGATATTCAAACCATTATATACTGTGTTTTTTTGGCGCCAAATTTTTATTACATCACTGAAATACATGCATAGTACTCATCACCACACCATCAGAAAAACCACTCAGCAAATCAAATTTTTTTGATGGATTGACACCGTCACACTTTTGATTTTTATACACAGCTTGATAAATTCCTGTTTTAAACCCATTTTGATACAAACATACTAAAATGTCCCTCATATTTTCTGTCTTATTTATGAAAAATACATTTGCTTGTCCATGATCTGTAAACGGCAACGCACCGACGTACATATTTAGCATTTCATTCACAGAATAGTCAAAAGCATTGAGAAAAATTGTCATTTCCTGCATTTCTTCTTCGGAAAATAAATTTTTGTTGACTTTGGACACATCATAATTTACAGAGCTTAAGTACTCTAAATGGTTTTTAAATCTGTTTAAGAACAGGCCATTGTACACTGGTATGTATAATGTCTGTAATTCAGTAGAATAGACAGACAGATCACTATGGAAAACAGGATTAGGATGCAAAACTTTTATACTAGATCGCTTTAAAATCTTATTTCCCTCTATGGGACAAAAAGATTGTGAGCAATTTAACAAATCGAACTCTTTTGACATGGCCGAACCAGACACAAAGGTTAGATTTCTAAGCCCATATTTTTTTATGTCGCTTCTTAAATTCGTCCATAAAACATTTGGTAATGTATATTCAACATTATCATACAAATCGCAGTGCAAAATCCCTTGTGCATATTTGGATCTTTGAAAAAAACTGCAAGGTTCCGCTCCTTTTTTGCAACAATTCATACTTGTTCTAACACTAGAATAGTAAATGTTCTCGCATACTATTCGATATAATTGGCATCCAAGTTGAGAATTAAATGGCAGATTCATTGACATTAATGCTGAATGTAATCCAGTAACACAGATAGCCAAGCTTCTTGCATTGACTATTCCATCCATTAAAAAATCCCTATTTTCCAAAGCATAATCTATAACAGCATTCCCAATCAAAACTAACTCAGTCACTGTTTTTCTCATTTTCTTTAAATTGAAAATTTTATCTCTATAATGAAACATATCTATGTTAGTATGGGACTTTTGTTCTTCTGAAACGTTTTCGACAAAATAAGAAAGATTTAAGGCAATTCTGAAAGCTGTATTCAGGCCATATTCAAAAGGAATTACATCCATAGAATTTCCAAGACAGTAGGAAGCTATTTGAGGTAACATGGAGTACTTATGTATATTTTTCCTAAAGATGAATCCCATCTGTCCTCTGCGTAGGCAGCGACAAATATTATCCAGCAACGTTTCAGTTTTGATTCTGGCATGTTCAGCGGTGCGTTCACATTCAAGATATTTACTTGTAAAATCAGATTCATCATATAACCCTAAATTAATTGCCAGAGCTTTGTGGAAAACTGACCAATAAGAATTTTCAGTTTTCAGCCGTTCCATAAAAATTTCTGGCAGTATTAGACAAAACTTGACTTGAACAGTTGTTGGCAAAATTTCACAGAAGTCCAGCCACATTAATAATGACAGGTGCCAAATTTCAAGATAAATTGTTAGACCAGTTGTTTTATTTGTGTCCTTCTCTACAATTTGTAACTGAGACAATAGCAATTCCATAAGAGACTCTGCCTCGTAGTGATAACGAGTCAGATTTACACTAACATATGATCCATTTAACATCGCAGGCAAGAAAAAACCACTGTTAATGTCCAAAAAATCTTCCAGAGAAAAGTTACTCACATTATGAAAGACAGTGTCAAAAACACTCTTTTTTTCAGACACTAAACCCAAATTACTCATACAAGGAAAAGGTAACATACAAACTCTCTTATACAATAAAATCAAAATTTCTTCCACTAGAGGGCATTTGTGACTAACAAAACTTTTTTGTAAATATTCGATTTTTTTTATTTGACTGGCCAATGTCACACTGATTCTTAGAAAAATCGTTGGGATGTTTTCTTCGATTCCGTTAAATGAATTTGAAATCTCATCGAGAAATCTCGATGCAGCTAATACTCCTGGTGAAAACTTCGCTCTCCAGACATGGATAGCTGCTTGCAAGATTTTTTCAAGTTCTACTTTGGAGTCACGTACAAATTCAATCAATTCTTTTTGTAAAATATTTTCACTTTCTAAAATAAGTGAGATGGAATCAAATTCCGAAGCAGAGTTTGTTTTGAGATAGAACTGGCCTAACAAACGATCAACGTGCGGTGCATCATGCATTGCTCCCAGTGCTGAAATTATGGCATCACGTAAGCACCACATATCTAAGCCCGAATTTAAGCGCCGGAGAATTTTTGAAATTTCATATAACTGCTTATAAAGAAGAGTTTTGTGATGGAAGCGTGAATAGCAAAACCGTTTTTTGTTATATTCATCATGACAAAGACATTTCCTCAAGAGACCCATGTTTTTATTTATGGAAACTTTATTGTTAGGATCCATAATCACAAGAAAATTTTTCTTTCACACAATTCGACAAAGACGTCTTTGAGTCTTATAATGGTTAATGAGATACCCTGTAAGTCTAAAATTCCTGATTTTATGTTTTGGGTTTTACAATGACCAATTCGTTGAAGGCTTCCAAAATAGTTTAGATTTCCATCACCGTTGATCAAATAGTTCATAAACATAAATCGTTCTTTGGAAAACTTGTTTCTTAATAAATCTATCAATACTTCTTCATGTGTCAGCTCAGATAAAACAATGTAGATCCCGTATTCTATTTTATTAAATGCTTGCCTATAGATATAACAGAGGTAAATGTACTGAATTGCATTTTGTCTTGGTTCCCAGTCAGGTTCCAATAATAAATCTTTCAATATGACCAATCCATATATATTTTCCTTTCTTACGTCATATACATGACCTTGCATTACACAGGATATTAGACAATTAGTCCCTTGAGAGATATAATTAGTTACTCCAAACAAAGACACCTTTTCTTCTAAGTAAGAACAACTATCTAACATTGTGTTGATACTGTCAACTAATGAAGTTTTATACAGATTAGCTAAAGAAGTCCTCGTGCAGAAAGACACATTGAAGGCATTCATGGCTAAAAACAATACACTAATCAACAAAAATTTTGGACTCTCCGCGTCTGTTTCAAGTCTGAACATAAGCATTCCTAAATGGTCTCGTTCAGGTCCTAAAAGTTCGCAAGGAGTAAAACTTCGCGAAAGCACACACTCTTTGGGAACACCAAAAGCTAGCAAACTTTTGATCTTTTTTTTCTCATTTTCAATGAGATTATTTAAGGAAGTTTCATCAATTGTCTGATGTTTCCTTTTTTGAAGCATTTCCATTGTTCGCAAGCGAAATGGATTGTACAAATTTACAATTTGAGTCAATATTGAATTTACTTAAAACAGATGTGTCCGATAATACATTCTTAACTATAACAGCGAAAATTGAAATTTTTTCTCTTGCGGCTGAAAGTATCACAGCAGATAAGATATTGTCATTTGCGAAATTATTACCAATACACAATTATCATTTCAACTTTATTAAAAATCATATGGTGTTTTATATATTAAATTATAGTACTCTAGGATTTGCCAAAAGATTTTCAATAGCTGAAACACTATGTAGAGAATTAAAAATTTTCCACAGAACATTCCAATCAACGTCTAAATTACAAAACTTAAATAATGCAGAAGTCCTTTATCAATTTGAAAAACTTATTGAATCCATTCAAGTATTCAAGGCGGAATTGGCATCGCCTATTGGTAAACTTCTACGTCAAGAAACAATGATCTATGACAAACCCACAGAAAAATCAGTTAAATATGTCATTATTCAGCAAAATTTGATAAAAATTAATAACTTAATTCAAGACTGTGAATCGATAAAAAACTGTAGACTCATAGCGGAGCTGATAGATGAATTATATCAAAAAGTTTATAGGTGGTTTATGCAAATTTTCACATACGACGATATTATTTTCCCCAATGACAATTTCTTAGATAGACTTTTAAAAATGGATTTCTGCTATACATATTACACGGCATCTAATCAGCATTTATTATCACTTTTTGAACAGACAATTGACAATCAAATTTTTATCGACCCTTCTCCATACTTCGAAATAAATCCAGTATATTCTCCCGAATTACAATTTATGAGCACATTTAGCTTAAAGATATTTTCAAAAAATATTTCAGCGAAAAACGAAGATCTATATATTTATCCACTTTTAAAAACAAACTTTTCCATACTTACCTTTCTGAGTTTAGAAAATATTTTTTTCCATCATGGATTTATATACCACATCTTGCATAGAACAAATATCACACCAACTGAAGAAAAAAAATTAGGAAAAATAAATGGATTCTTAAACACGGTTATTCAGCAAGTCCTAATTAAAAAGAATAACTTACAAATCACTTTCCCGGAGTTGCTTGATAAAATCTATCATTTGCATAGAATAGGCTTGAATATCGAAACGGCACAAATTTTTTTACAAATGTTAACAACATACAAACCAGCGACGACAAATAATAAGTTGCAGACATTTTTCACGAATTTTTCAATAATAATTTTTTCGGCATATATTTTTTTTCTATGTATTGAGTTATTCAGCCCAACTTTTATTTTCCATAATAAAAAAAAACTTATCCTCGAGAAACAAAAATCCATCATACTAATTCTCGGTGAACAGTTCTCTTTCATTTGGAAAGAGGTAAATGAAATTGTAGATTTGTTATTTAGTAGCACAGTTACAGAAACCTATTTCAAATTTTATTCTAAAGGTGCCGATGACTACGAAAAAGATTTTCTTTATAAAGACTTAATGGAAAAATGGGGAGAATTGTTTTTCCCATTAACTTATTCAATGACAACGCCACAGAAATATACCGACAAGCATGTATCAAGTACAGTTTTGAAAAATTTATGTGATACAGCATATCAAAGTAAGATGGAAACAGCGTATGAAAGTCTATTACCATATATTACTCACCCAGAGTTCAAATTCATTTTTATTACACATTACGTCAGACCTTCTTTGTCATTAATCACAAACTTAACCTTTGAAGAAATAAAAGATAATCGAAGATTACTAATACTAATATTCGCGTGTAAACTGCTAATGCCATCAAATTATCTTTTATCACATTACTTATTATTGTTACACGCTTTCACACTACAAATTTTCAAAGTAGATCTTGGCCATTTTTCAATTATACATGCAATTACTCAAAAAATTTTTGACAATATTAATTCTCTGACCCAAACTATTTTTATTCCAAAAACAAATTTTTTAGTCAGCCTTTTATTAACAGCATACACTGTGCATATGCAAACTTATGTGAATCCTTGGATACAAAAAACAATCAGTGAAAATATAGCCCTTCTTAAAGAATACATCGATTTCACAAAGAAATGTTCAAGTACTTTGGCAACTACGTGCTATTTGAACTTAGAAAATTTTGCAGTAAATATGTATTTTGGGAAAAACAAGGTTGGAAGTACTTCACTATCAGCTTTTTATAGAACTTGCTCTAAGCTAATTGAGGAATCTAAACTGTTTAAAGATAGGCTTCAAGAAATCAAAGTTTCAAAAACATTGTTTATAGAGATGCTACAAAATGTTGTGAAAAACATCACAAAATTTAAGGATTTAGTCTCAAATCAAACTTTGCAAAATTTTATAATTATTGTTGAAAGAATCTCCTCTCATGCAAATACAACATATCAAGATGTTCTCAATAGCATAGATGAATGCCATTTTTCAAACATGCAACTTATCCAATCTTTTAAAAACATCGTGTATGTCATTGATGTTCTGAATACTAAAAACATCTTTAACTTTTCTCTCGCATCACAATTAATTGAAGCGAAAAAACTTGTAAAAAAACAGGACACCTATAATCAATTAAATGTGCAAGATGATTTTGTCACTGTATTAAAGTCACATCTAAATAATCTGTTTGAAAAGCAGAAGCCTACAATTAATATTGAAAGAAGATTTATGTTAGAAGGAATACCCGACATAAAACAGATTCCATTCTTGGATGTTTTTGATGAAAGATATAGACTAATACCACAAATTGAAAAGTATCTGCATTGGTACATTGCATATAGTGAAGCTGCGCAGGCTGATTTGGTCGAGCCTTTACTCTTAAAACTTGGTTAGATGAGAATCATAGCAGGAAGCACAAATCAAAACGATCCTAAATACGGACCAAGAGCCGGAAAGCAATGTATGTCAAATTGTTTTTCTTTCTTGCATACAGTTTATTTGAACGGAATAAACAATGTGTTAAATAAAGAGTCTATTGACATAATCATGGAAAATGGAGCATTATTGGATAATATCAGTACAACGACATTGAAACTCGAAACTGGCAATATCCCAGAATATCGATTTTTCACAGAAATCCCAAAAAAAATTAGTTCTAATTTTGGCGAAACAATACATGAATTATCTAGACCCTTTAATGGTACCTTAGAATCACAACATATAGATAATGAAGTTTATCTTGGACTGTTAGACTTTCTATTGTATGGGAAAAATAAGAAACCAGCTTTTATTGTCATCACTATAGGGGTAATGGCACGAGCTATATTTATAGTTGATGAATTGTTTTACCTTTTTGATTCACATGCATCAGACACAGAAAACTCTGCAGCCATCTATATCTGTGAGGATATTGACGAATTATATGCTCTATTGGCCATAGAGAATGTTGCGGAATTTTACTATGATGCAGTTTTTTCATATTTCATTGAAACGACTGATTTATCTCTTGAAGACGGAGATGCAACAATTTTAATTTTAAAGACTTACAAAGATCCAGATATAGCTCTTAGTTTGAATGATTTTTTAACAATGTATTCATCTACATCCTCAACAAAGACAGCGGAAACAAACACTTTAATTTCAAAACAATCACCAAGCAAACGCAAACAAGAAAAAACCAGTCTAAATTCAAATTCTCTAGAAAAAAAAAGGAAGCAGGGCTCATCACTCAAATACTATAACAATGAAGTAGATTTAGTACCAAGTTTTTATGAGCTTAGACCTCAATTTAACAATATTTTATTTGAGCTTTCTAATTTCCCAATTGTAAAGGAAAATGTAAATTGGACCCTTTACATACAGAAATTTGCAACAAAGTCTACACAGCCATTTACAAAACCTTTTATATGGAATAGGGTATTCCATCTATTTTCTCAAGTGGTTGACGCCTTAATTATGATTAAAAACGATCATTGGGATGAGACACAACAGCAAAAACAATTCTTCACACATTTCTTGCCGTTCAAAGAATTTTCTGAGGAATTTGAAAACGCTATAGAGGCTTGCCGAGAAAATAATCTTGATCTAATATTGCTTTATAAAAACTATCTTTCGAAAACTACTGCATTCAAAAACCTTGAAAGAATTTTATTAACGAAGTTTAGCGCCATTGTCAGTCCGGTGCATGAAAAACACTACACGCTTGTAAACACATGGCTAACTAACTTAATACAAAAACTAGTCAAACATCCCGAGGATACCAATGCTTTCATCAATGACTACGTGTTAAAAAACCCCTTAAATCATTTCATTTGTTTGAATAAGAAGGAAAAGCAGAGCATCGCTCTACTACTGAATAAAAAAAGAATGAGTATGCTAAAAGATGTGGAAATCGAAAAGAATGGTTTTGTTCAACTCCAAGCATTCATCGAGAACATAGGAGAAGCTCCAGCAAATTATTTAGATCCGGAAAATGCACGCAAAGTGAATGTTGAAGAAGTCTCAGAAAAAGACATCCCAACATTATCCACAGATAAAGTTTCCATACCCAATGAAAGTATGTTCACATCAAATAAAAAACACAGCATAGAAAAATTAATACATGCTAAGCTGAAAGCCATTTTGAGTACAATGGGGCAAAGATTAACTAGAATTATTCAAGAAAATTATAATAACATCGCTGCAGGTTTTCTGCCGGTGAACGATCTTAATAATCTGTTTGCCTATTTGGTCAAACTCTATTTTGATGTCTATAGCATCACCATTAACGGATTTGTGGTGGAAAACGAATTGATAAAAAATATTGAACAAATTTACGACAATACGCAATATCTGAGATTTGGATTGACACGCTTCAATATGCAAAATTTGACACCGTTTACTATATCTGTCCGCAAAATGTTCCTGGATTTTTTTCTATCACAAAAAACTCTGATAGATAGAGCTGAAGAAATTATAGAGAACCTTGAGTTTAAATCAGTTACACCAGAAGGAAAACAAAAACTTGCCACAAAGAATATGCTCAGAGAACAATTAGAACAGTTGAATGCTATGGATGTGGATGATACAATAAATCTGAAAACAGACACATTAACACATCAAGTATTATTTTCAGACCAAGAATTACGCATGATACAAGACTTCATTTTACAACTCTCCATTCACAATATTCCAAGCATTAACTTTGTGAAATCTTTGAAATTACATATTATTTTAGAAAAAAGACCTGATATACTATTAGCTCTACAAGAAAAAGTCCAGAATATTCTATATTTTTATTTTCAAGATCTAGTTAACGAGATACCTGCTCAAGAAAATGTTTTGTCAACAATGTTATTTATAATAGAGCTTTTTCCAGCCGACAGTAGAATACATCTACTAGAAACCGGATATATTTCCAGACATATTGTAAAAAAATGGCTAAACATGAAATCATTGCAAGATGCTGAGGATTTAATTCGATTTATAAATATTAATAAAGAACAACTAGGAAAATTTGAACATCAGCCATTTGGAAAAGAAATTCAAAAACTAATTGAAAAAATACACTTGTTCTATAAGCAAAAAGTAATCGAGTATCAGGAAGATGTCTGGAGTGAAATGGCTAAAAATATAATTTTAACTTCACCCTCTGAATTATCTCAATTTTTAGCTTCAGCTCCCACTCAACGTATCATACAAAAACACAAAAATAATTTAGATCAGAAACTTTTAATACATATGGAAAACCAAGCCAAGCAAGCAATGGAAGATGACAAAAAAAGAGTTGCCTGTTCTAAAATTAATCTGGAACGACACCTGAATGATTTACTGCTCTTATTGAAAGACAGACAATTTGCTTCCATACAGGCTTCTGTTTTGATTGTGTGCGAAAATATATTTAAAACGATACCAGATGATAACCTAATTATTCAATTTTCACATGCTCTGCTTTCAGTTTTACTTGACATTGAAAAGGATTTAAAAAGCTATTCATCAGAAATATTAGAGAAAATACTAATAAATAGGCCCCTCGAAACCAGTAGATTATTAGTGTTTAAAGACGCTTATGGTAATCTGAAAGAGTTTTTAAACGCCTTAAAACAATCACTTTTTGCCACAGCGGATGTTCAAAACAAGGCTGATTTTCTTATCCAAATTTTAGATTTTACCTATAAATTTAGACATAAGACAAATAAAGGTAAACTTCTACATTCCATTTATAATGAGGATTTCAAACTATACGAAGAAACATTAACAGAGTTAAGAAAAAAAGCAACAGATGCAAAAGAGTCGTTAACTAAACTTTTTAAAGCATCCGAACAAAAGATCGAGCTGTCGCGTACGATTCCGTTAAAGGAAATCTACCTGAACATAGAAACTGTTAATTTCCAAGGTTATGGCAACGTAGTTTTTCGAGAAAGTGCTTTTAAACGCGCAATAGAGGTAGAAATAAAAAATTACGAAATGAAATTAAACGATCTAATAAAACACTTTAATTCACACTTAAAAACAAAAATTGACCACATACAGATTCTTAATCTATCTTTTGATAACAAATGGAAAGATTTTGTCTCCAAGTCAAAAATATCTTTCCCACCAGAACTGACAATAAGTTCACAAGAGTTGATCAAGGATCCCATTAAAGTTATAACTGAAACTCTAAACAAAGCCTCAAACGATTTAGCGTATGTGATTAGTGAGAAAATATTGAAGTGGTTAATAGTTTTTGTCAAAGAACTGAATACCTTTTTTGTAGCTACAATGTCAGAATTTGGAGAAGTTATCCCCTTTGACTATAAACATTTCAGAGCTTTGGAATACGAAATTAATTCTAAGTACATAGAGATTGAAAATAAAATAATCTGCAACGAAATTATCGAAAATACTGACAATATAGAAAAACTCTCAACCTTGATAAAACAAATAGATCCAAATCGTATTGCTGGTGGTAAACAGAAATTTCAGGATTATCTGAGCAAAATTCTAACAGCTGAAACGAACCAGCAACAAACACGCTATAAAGAACAGTTAAAAAAACAGTACTTTGACCTTTTAGATAACATCGCCCATTTCCGGTTCGCATTCGATTTTAACCATCAACAAAATTTAATTTTAAAACTGAAAGACAAATTCAAAACTCTTAGAACAGACACTGTATTTGAAAGATTTCCAAATTTAGATGATACTTTTGTCAGTTCAATGAATGTCGAGAACTTTTTGCAGGCACTTGAGGCTTTAAGCCATTTCGTGCAGGCAGCACAAAATTTTCTACAAAACGTTTTAACAGAGCAAGCGGATTTATTTCCACAGACGAATTTCATTCCTGTCGAACTTTCCACCGTCAAAACAATTCCAAAATCAGATATAAATTTACGTATGAAAATACACACACCCCAAACTTTTTTTCAGGTTGATTCAGTTTTTAATACACAGTTGATAGTTGATGAGAAAGGAATTCCAGTCCAATTTTACAATGTTTTCCACAATATTGTTTTCAAGTTTTTTGCTCTAAATTATAAGAAAATTATCGTACCTGATAAAGTGCTGAACTTAGTATCAACCAAGTATAAGATCTTAACCACATTAAAAAGCATTCTGAGTGTTGTAAAAAGCTTTTGGAAAGAGATTATAAATTTCGATTTAACTTCTTATTTCCAAGGGAAAGCAGAATTTACTTTTCAAAATGTTTTCCCAATAATTAATCTTAAAATATTTATTTACATTATTACTCAGGCCTGGTCAGTCACATCTGATGAAACACAGCATTCGTTTGAACTGCCACTAGAAAAATTTTCTCTTTTAATTATAGCAAATAATCCAGAGTTTCTTTTTGGTTCTCTGCAGTGTCCAGTGGACCTAGCTATTAATTCTCTAATACCCTTATTGGAAAAGAAAAAATATTTCACTGCATTTACCATCTCTGACAATCCACCCAAGCTATCTATGGATGAATTAAAAATTGTGTGTTTGGATTTGAACACCTGGAGTGAAATAACATTAGAAAAATACACTTTTAAAAAGAACAGTTTGATGCAGTTATGTATGGGCAAAGAGAAATTTTTTATTTACCTTTTATCAGCGTTGGTTCTTCCTCAAAATTTTTTGAATTACATTTGGATTCAATACAAACCTTCGTGCTGTGCTCAGGATTCATTCCAACAACTCATTCAAGATTTATGTTTCGAGTATACACACCAGAATCACATAAAGCCCATTTCATTAAATCTCCAAGAACCAAATGCATTAAAACATGGTGAAAGAATCCTTTCGAAATTCGTGCTGGAAAAAAATGCAAACACTTCTCTTTTTAGTATCTTTTTAGGCAAGCAGTTTTTATTAGATTATCTATTATTTTCATACTTAACCGCAACTGAAATGACTTTTTCATATTATGTGGATTCCATCAAAAATTTTCTTCTTACGATCCGACATTTGGAAAATGTCCAGCAAAACGTAGACTTTAGAACGATATTGCAATCTCGAAATTTCGATCTAAAATATCTTTTAACACAATCATGGACACAAAATGTTTTAGAACAGTCCATCTTTCACGTGCAACTTGATAAAATCATTGCTGACATCAAACAACCACAACTAAGTCTGAAGAAAATTCCACTGGTTTTGTTTAACGGTGACAACGAAGTCGTGTCAACTTATGTGCCCCCTGAACAAGCAAGCCAGACAGAGCAGAGCTTTCGCATTAAGAATATTTTCCCAAATCCTGTGCAAGAGTATAGTAGCAAAAATGTGATTCTTTTTACGAACTATCCAAAAAACACCAAATTTTTATTTAACTCTCCTCCACCTAAAACAGCGGCAAAAAGTTACAAACTACCAGACACTACCGATGACATAAACACGGAAACATTATCAAGTCCCACGATTCAAAGAATCCCTATCAAAGGACTTGTACCCAAAGAAAACGAAATTGTCTTTTTACCAGAAAAAAACACTGCACACACAGACTCTAAAGAGACAAAAACGCATTTAATAGACACCTTCAATATATTGTCTCAAACAAAGGGTGAAATCAAAACATTTTCTACAGATTTTGATCAAACAATTTCCAAATTAAAACATTTGTACTTTTAAGATGATTCGCTAACTTTGCTATTTAATCTATGCAAAGCAAGCATGCGTAAAAAATCAAGTCTGAAGGCTGCTTTTTCAATGTTTCCGGTATTTTGTTTTGCATATTTTGCTAGAAACGTCCCTGTGACAGGGTGTGAACCCATAGAAGAATGCAAGCCTAGAATATTCGTAAACATTTGTTGCTTTTTCTTTTCTTCTTCTTTTTTTGAACTGTTGCCAGAAATCTGGTTGATTTGAGCAGTGATTTCATCACCACGTGTCGCAGCCATGTTGCGTGCACTTAGCTAATTGAGAAATTTTACAGCCTGTACAAATTTCTCCACAGTCCTCTAGATCAAGACACGTTTCTCTGTAGATATTTTGACAATCTTGGCATTGAAATTCTGTGGAGTGAGAAATTAGTTTCAACAGTTTATTTATCGTTAGATTCTGCAGATGGATCATAGAGTAACAAGTTCTGCAAGAACATGCAACAATGGCATCAAATTTAACACTATCGTTTAAAATAGTGCAAGCGGAATTAGTTCCAATTATAGCTTTCCATGATACACTTTTTACTTGAATGCCTGAAACACTATATTCATACAATTCATATACTTTTTCAAGTGTCAAACGTTGCCCGCCACATAATGAACAATAAAGCAAATCTGTGTGCATACTGTAAATAATATTTTTTTCCTGTCTATCTCGATAATAAAACATCGAGCTTAAAGAAAAAGTTTGTTTCCCTTTAAGCCTCTCTTTGCCGGAATTTAAACAATGTCCACATTTAGAACATATCACAGTGAGATAACTATCAATTTTAGCATATCTGAGACACTTTGTTTTGCAAAAACATAATACCGGTAAAGAGATGATACTTGAAGTTAAGCTTCTTTTCAAGACTGTCGTCAAAGCAAAAGTTAGAATTTTTGGAAGTTGATGTTTTTCTTGTTTGTTCAAAACAATCGGCACCAAGTTATTATTTTCTAGCGGGATTAAAAAACGATCGAAACTGTAACAATAATTTTCTATCATGGCCATGGCCATAGCAAAGATAAAATTTCCACATGGAACTGTTAAAGAACAACCATGAAATATATCTTGATATGTCAGGTTGCTTGCTCTATGAGCGTATCGCAGTGTGATGTCATTACATTGTTCAAAGTTACGTAAACAAAAGAGTTTCATACAAGGAATATTACAATGAGTTGAAATTGGCAACATTAACTGTTGCTGAAATAAATAATAATAGAGATCATTAAAAGACACAAACGACGGTATTTCCAACAACTGATTTAACTTATGTATTGGGTATTGTTTGAATCTCTCCACAATTTGTTTCCAAGTAAGACGTTGAATTTTGTAAAGAGCCAGAATTTTTAGAACCTTGCATTGCCTGTACAAAAAATACAAATATGCCAAAAGCAAAAAGAAATTTTTTGTCATCATCATTTTATTAAAAATAGCTTTAATCTGATTTCTTGAATCCAGATCACACTTTAACCAAACTTTTAAAAACTTCCGGTAGAAATTATCAGTTAAATCTTTACTCAAAAACTTTTTATTCGTTACGAACATCGATAATACGCAATTTTCAGATTTGCTGACATCTCCAATAAGAAATAACTGCAAATGACTGTACGAACCATGTTTACACAGCGGAGAAATCAGCCGACAAAGCAAATTCCTTAAAGATGACATAGCATACAATTAGTAAGGCGGCAACCAAAAAACCACAAAGAATATTATTTCTCCGTGCAAAAATAGGAACAGTAACTGGGAATACTGCTGCGTCTTTTGTGTTTCCCATGTAATTTATACTCCTGAATGATGTCATCTCTCCTACTGACTTCAACTGTAACCTCAATGGTTTTTTTATGTGCCTTGCAGCTCTATGTTTCTCTAAATCTTGGCGCTCTAAAAATTTTTTCTTTAAGATTATCGCTTTAAGAAGAGCGATATACTCAGATTCACTCTCGGGACCAAAAGCCACAAATTCAACTATAAAAGCCGCATCTCGGTTTCGTCTAATAATGCACTTTGTCAGTACACCACTTGAAGAAGGTTTAATAGCTTCAATGTCATTCAAACACACAGATAAACACTCATTGACATTTAAGATGTTGCATTCTTTGACAATGTTCTGACTTTCTTGTTTTATGGGAGCGTGGAGATAACAAGACATGGAAACAGCACCTCCCGTATTTTTAAAAATAAATCTCGCTTCTTGTTTTGTCAAATCTTCCCAGAGACTCAGCAAATATTCCAGGCTATATGCAAAATCCAATTTTAAAAGTATATCACATAAAGGAAATTTAGGATTTTTAGAAAATAAATTTTTATCTGTGACACGAAAGTCTGCGGGACCCAATTTTAAAACGCGGCACGTGGAAAGAATTAATTCATCGTACATCTTTTCTTTCAACATGTTTATTTTCTCTTTAAAAATTTCTGCAGTTGTATATTATGTATTTAAATATTCCTGATGTTCTAAAATTCCATTCACATTTAACAGTTTCGATAAAATTGAAGTGAGTAGTTTGATTTTTTTTTTGATAATCTCCGAGTGACTGCATTGATTTGCTCTGTGATGTAAATATTTTAAATAGGTTAAGTGTCTGTATCCAACATTAATAAAATGTAGTCGGGCATCTAATGAAGGCAGCATTATATCGTACAGTTTTGGTAACATAGGGAGAAAAGCTATTTCAATTTCAACTCGGGAAGATAATTGTTCATAAAGAATTTCTATATCTGTTTTGGCATTGTCGTTAGACATGGCTTATAAGGGATGGAACTCCGATTCCTTTTCTATGAACTCAGAACTTTTTAATGAAATTCTTTTATATGCACACTTAGATTCAAGTGGAATAGATTCTGATGATCTTAACACAAATCCCAATACACTTGAAAATGAAATTAACTCCGTTGAAAAAACTTTAAATATTGAAGAACTGAAAAAAATTACAACTGCTTTAAATATTGACAACCGATGCAATATATGTTCAATTATAAACATCTGTTTACGACATGAAACCGATAAAATGTGGATCTATGACTATGCTCTTTTGTGTTACAAATGTAATGCTGCACCTAGAACTCCCTTGGCTGTCGTAATAATCGCCACCGAATTTATGCAGTTGATTCAAAAGCATTTCTTAAACATAAATTTCGATGGGTTATTTTTAAACAACATTTTGTCAATACTCGATTTTCATGTACACTTTTTCATAAACAGGTGTTTCTCAAACACCAACGATGATCTATTACATAATGAAAACATAACCTTATATCACATGGCCATATTAAAATCACTTCTATTGGAAGACGAATCTATACCAAATATAAGAATAAAGAAATTTAAATTAAAAGGAAAACCAACGAAAAAACAGCATGGAAATGCTATCCTTGAAAAACAAACTCTTCCACTTAACACGCATTTTACACATTTAATTTTTTATATGTGGGCTGGGACAAACATATTCGATCGCATTTCACTAACTGATCTAGCCATCAAGAAACGCCAAATTTTAAAAGCCATTTACTCTACTAAAAATGAGCTCAATTGTTCTGCGGGACCAATCCTACTATCTCAAATACCGATCTCCATCACTAAGAACGCCACCAGTAGCGTATGCTTATTATGTGAACTAATGACATCTTCTCAGAAAAATTTCGACTTGCTACAGTTCATCTACACCAGTGTCATTAATTACTGTCAAAATAATTTGAAGATGATTGACAGAATTCAATTCGTACTCGCAAATCTTTTAGATTTAGCTAGAATATATACTAACGTTAAAACCACATCAGATTGCTCAAAAATTGTATTAGCCAATGAACAAGAATTTTCAAACTCTGATTTTGTAATTGATTGTCATAGTTTTTTAATTCTAAAGCAGGTTGGACCTGTGGGATTATACAAACATTTCTTTTGTGATCCACTGTGCATAGCAAACATTAAGACAATAAAACCCCATATTTTATTCTATACAACAGAAAGCTGTATACTACAAGACTTCAAAGTTGCAATTTGTTATCAGAATGAGTATTTAAACAGTGTTGAAAAACATGTTTGGTTAGCCATTCATTTTTTTAAAGCATTTCAGGTTTCAAAATTAAACCACAAAAATAAAACGCTTATATCTGATTTCTTAAAGGATTTCACACAGCTCTTAGCAGATCAAAACTTTGAAATTGTCGATCCTACATTTACCATTCATTATTACGTTTAGCATGGCTATTCAAAGTACACGTAGACTGAGACGAGCATCTAGCTTGTTAAAGAAAAGCAAACCTTACAACAAAGAAAAAACTAACTTATCTTTATCTTTGTCACTTAAAGAACTCCATTCGGTTTTCAAATTATTTCCAGAGTATGAATTGAAATTTCTAAATATGATGAAACTTCCAATAACCGGTAAAGAACCTATCAAAATTCCATTCGATCTAAGTCTACATCATCAACATACGTGCTTAGACTTATCACCATATGCCAATGAACAAGTTTCAAAAAGTGCATGTGTTAATTGTGGTACAACAAACATTCCAACAGCTTCAGATGCTATGGTGGCATATATGAATCAAATTTCAAACGTAATGCAAAATAGATTATATTACTACGGTTTTCAGAAAAAGGTTGAACTGATTCGTATGTCAGCCAAGCAACCAACGCTTTTTCAAATTTTTTATATTCTTTCAAGCATAGCAAGTAATTTTTTACCAATCATGTTTGAAAATAACGAGAAATTAAATATGTATGTTGTTTTCCAAACAAGAACTCTTCATATTCCTTGCGAATGTATCAATCAGATTATGACAGTCTCTTCCGGATACACTGTACTTTTAGACATCTTACACGACAGCATTGTACTACATGTTCTTTGTAAAACTATTGAGACTAGCAATATTCAAATTGATATTAATGTTCTTCAGCGAAAAATTGAGGAAATGGACGTACCCGATGAAATAGGTGACAAGTTCGAAAAGCTGAAACACATTCTACCGTTTATTTAAAACAGATCACAGAACATCTGTCAGGTCACAAAAGCTTTGATCTACATATACTTTTAAAGGTAGGATTGAAAGTAAAAACTTTTCTTTCTTAATGCCCGTTTTCGGAAATATTGGAGAGATAGCATTTGTTACAGCTTTCATTATTTGTTCAAAATATTTATCTGCATTGATTTTTAAGTTGTTCTCAATAGCATATGCTGGATCTTCTGCTAACTCATAATTATGAAATGTTTTTTTTACGGTTTCTGTGGGTGCAATTAAAATGTACATAACTCTATCTCCTATGTTTGGCAACTCTTCCTTTCTTTGAGCTAACCTTTTAACAACAGCCAAATGTGGTAAATTTGCTTGCTTGTAGGCAGAAATGTCTTTTGATAAGACTGAAGACAAAACCAAACTTTTAATGTTAACCCTGTTTAAGAAAAGTGCGTCTCGTGCGTCACACAACTTTTTAATTATTTTTTGGATACCATCGGGAACTCCATTTTCATAGATTTCTTGTATTGTCATATTAGAGAGCTTTTCAGCAGACTTTTGTACATCGACATCCCAAAAGAGAAGATCGATGATGTCTTTAACTACCACTTTAACAAAATCGCAAGAAGTCTTGCGAACTAACTCCACACCTTTAAAAACAAGCGTTGCATCATCTAACTTTCCAATGTAACGTTTCTTGCATATTAAAATTAATGGAAATAGGATCTTTTCAAATTCTAATTTGATAGGATGTTTAAATAACGTGTTTGTTATGTGGCTTGCAATAGAAGGAGCTATTCTTTTCAAAGCTTTGGGACAAACATTTTTGAAAGTAACAAAAAGACTGTCAGTATCACCATAAATCACCTCTATTCCAAATGTCCCTGAAAAATCACCGCGTGTCAATCCAAATTTCTCAATGAAAAAAGTGTCCGAATACATGGCGCTGTCAACGTAATCAACAGTTTTACAAAGCATTTCACGCCCAAGACATGTGACAGAAGCTGCTATTGCCACACATGGAAGTAAGCTATGTGTTGCCCCGGTGACTCCGTAGACTGAATTACAAGTAGTTTTTAATGCAAGCTGCTTCTTATCTAAAAGCATTTCCATCACGGGGTTGTTACACATTTTCATCTGCATTTTTACTTCTTTTCTTTTATCCAGCCATTTTTTCAATAAACTCGCTAAAATTGATTCCCTGACTGTCTTTTTAACAAACCTGTGCGTTACAGGTCCAACATGCACAGTGAGAATGTCATCTGCATGTAAACCAATTACTGCATTTTCATCAACAACCAGGGTACTATAACACAGATTATGAGCCATCATAATACTTGGATACAAACTTTGGAAATCAAAAACTACTGTTGGAGTAGCATAATAACCTATTTTCGGTTCTAAAACTGTAGCACCTTTATAGCCCACATTTTCTTTGCCTTTATTAAAGTTAGTGTTCATACTTGGTAAAATCATATTTAAATTTTTAGCTTCGTGTAAAATGCATGGGAAAATTTTTTTTTGTTGTCCCTCAAATACGGCGCAACGAATAGTAATATAAGCGAGACTTGCAACCTCAGCCATTTCATAGTGATAATTAATCTTTGTAAAAAGCTGAACAACCAATAGAGAATCTTGTATACAATATCTGCCAACCACAGCTCTACCTTTGGAACCATCAATAAATTTTTTAGGAATTTCCTTGTAAGAGAGATTTTCTTTTTCCTGATTTAAACAAAGTTTGGCTATAGTGTCTAATTTATAATTCTGAGCTGAGATTTTAGATGAATACACATTATACATGTCGAAGCATAAAATCCCAGACATGTTAACTTTAGTTAAAGAATTCAAAAACTTCTTATGCTGTTCGTAGGGAACGATGACAGAAAACTTTCCACGTTTTAGTTTCGAAAAACCCCCTATCTCGAAATTATATATTTTTTCCATTCTCGTACATAAATACTTTAAATCGAAATTGATGATGTTGTATCCAGTAAGGATTTCTGGTGATTTACATTTTAAAAAAAGGAAAAAAGCATATAGAAGTTCAAATTCTGATGCAAACTCATAGATGAATACCCCTTCGATTTGCTCGCAAGTGCCCAAAGTAAACAAATGTTTACTTTGGTAATTTCCTTCAGAATCAAAATCGATGACCGAAATTAATGATGATGTCACCATTTGCTCAGCATCTGGAAAGTTTCCATTTTGGCTTAGACATTCAATATCAAAAGAACAACAATCATATAATGGCCAAGAATCTTCCTTTAACAAAAATAAATCTGACACATGACAATTTAGTTCAATTTCAACATTACTGCTTTTTGCAAATTCTTGAATAGATAGATAATTAATTTGGTACCAACCAAAGCTTTTTAAGTTGTTATCAATGAAGAAACGATTGAGAATCTCAACTTCTGCTTCATAAACAGAAACGCCCTCATTTAGTAAAATTTTTCCAATTCGATTACTGATATAAAAGTTTGAAAATGATAATTTAAATAAATTTTTGATAGGTTCCGTGTTATAACCGTAAAAATTGTATTTAGTTACAGATTCAATAGAAAATGAGCATGACATTTTTATTTCAGAACTATTCAGAAGTGAACAGATTCTAGAATTCAGCTCTTTTTTACATTGATATTCACAGTAGAAATAACTATTTTGTCCAAATACATTGATACAAACTTTTTTACCACATTCTGTTTTTCCAAAAAGTTTTATGACATTACCAGATGGAATAACGAAATGTCTATATTGGAAAGGGATGTTTTCTATAGAATCAGTAAATAATAAAGATTCGGATGCATCATAGATATGAAATTTTAAAGGCGCACGCAATTCTTTTTCGGCTACTTGAGAAAGCGATGGCCATGTCATATCATTTTTGAGAATGTATTGCTTATCTTGATAGAACATTCTCGGTTCCGAGTCACAAAGAGTCTTCATTAAGCCGGGTGCTCCATCGTGCATTATACCGCGCGGAAAAATCCGGAGAAAAGTAGATTTTGTTTTTTTCTTAGTTCGTACATTCTCTAGATACGGATTAAAGAAGGACACCAGATCCATCACAGTTCTTCTGTTGAAAGTTTCTGATAGCCCCTGTATTGAATCCTTTCAAGTAGGCTTGGTCTCTGTGATTTTTTTGCCTCAGCGATTTCCGCTTTTTTATATGACTCATCTAATAATTTAATAGCTTTCAACATTTTTAAAGCTTCCACTTGTGAATATTCATTAGTGTGTGTTATGTCATCACCTTGCGGTTCTGTGTCTTTGTTAGTTTTTAAATAAGGCGGAGATGAATCTAAAGGTTCTTTCACTTGCGCTTGAACCGATTGCGTTGCTTGAAGTACCGAATTTGTTGATTTTGATGTATAAGGAAATAAAATATCAATCGGTGTTTGTGCTAATCGTTTATGTCTTAAAAATAAGAAAATAACTAAGCCAATCACTCCCAAAGTTAGTAAGATAGTTAGTGCTCCACCAAATGGATTTTTAAGAAAAGAGAAAACTCCATTCACCACATCTCCCAAAGCACCAGCCGTTACACCCAGCACAGCCCCCAAACCAAGACCAAGAGCGCCAAGACCTTTGAAAAATGTGTCAATCCCTCCTATATATGTTGGAGTAACAGTTGCAATTTTTGTTTCTATATGGTGTAGCGCACTTTTGTAAGAATTGTATTCTCTAAGAATGGTTTCCAAATCAAAAACGTTCGCTTTGCTCAGTTCATCTTTTGTATACAATTCCAATAATGTAAAATCTGCATTTTCTAAAGGATCTATGTTTAAGTCAACAAACGCATCTAAAGCTTCTATTTCTGTTATCAAACTAGAATTAACGTACGTATAGTCTTTAAAAATATGAGCGAATTTACCAGACAAAAAAATTTTCGTGTTAGATTGTTCACATTCTTCTGTTCTGTGATTTCCCAGTAATATCTCATTGTCTAAACCAAGTTGTCCTAAATAAGTTTCCTTCGAAGAATTTGCAAATGAATATGTTAACAATGGCCGAGAATAACATCTTAGAGCATCGTAACTTGAATCTTTTGTCAATCTCATACTTTTATGTAGCTGAACAGAAGTCTGGTTGACTTCAATGCACTTTGAGACTGCTAATACATCACCAATTAATTTAGCGGACATGGGTTTACCGTACACTGCTGAAATGATTCCAGAAGGACTTATTTTACTAAGCTCGTGCAACACTGTGATAGTGCGTTTTTGATTTAAACACCAGGCTTCAGCAAGCTTACCTAGTGCTGTGTTAATGTAATCTTTCAGAGTGTCGTATAGGTATTGTAGTTGAACATATACAATGTCTTTGTTAGTCTCTAATTCCCTCTTCCTCCTTTTTCTAGAAGCATTAGAAAAATTTTCCAAAACCGTTAATTTCCGTTGTATTAACGGTTGCCAAATCAGAATTAAATCTCCCTCAGTTTTAAAAATTTCATAACTACCATTTTTGTCATGCGAATTGTTGTATTGCGTTTGGTAAATCTCATCCAAAATTGAAGTATAATTGCTGTTAATACAATTATATTTTGAATCAGAAAGCGTATAATTACTTTTTCCTGTGACAAAAGAAGCAGTTAGTTCCTGCGCAATAAAGTGAAAACTGTTAGCATTTTCTGCCCGTAAAGCGTGTGGGATTGTCATCCAATGCTTTAACAAACAAATGGAAGACTCTTCATTCTGCACTTCCCATGAAAAAATAGTATTTCCCTTTTCCAGAAAAGCCATTTTAGTTAACGTCACAAAATTTCCTTTTGATCCATCATCAAACGTTTTCAGCATAGTATAATTATTTCTAAACAAAATTTTTTCCGTTGGTTCATTAAATGTTTTAGAATTAATACCATTATAAAACGGTGACCCTTCCACGGTTTCCCCTGTTGATAAAGCAAAAAAATCAAAAGGATATTTAGTCTTTGCAATGCAGTCTGTAACTATGCAATTTATCGATGTAGATGTTGAATAAAACCAGAGGGGACCATTTCTTAAAAATGGTTCTTTAGTAGTTATATATCTTCTGACAGTATCAGATTTAAAATTTAACGGAATCAAATCCAACGTTTTATTCACATATTCATCTTTATGATATGCAACATACTCCTCTTCCTCTGAACGTTTTACAGAGATAGAGGACAAACAACGCGCCTCGGTGTTTACCATGTGCACTTCTTCAATTGGCATTGGCAAAGTTGTAACAGTTCGATCTAAGAAATACACAGTGCCAACATCGCGATATGTTGTTTGAAATGTAAGTTCTTTTTTAAAAGTTCTGACAGAAAACGTGTGTGCTTCAATCTTTGTTTTGTAAATTATCAAAATACCTTCGGTAGTTTTAATGTTAGAGCCGTAAGACGCGCAAGAAACCTCCCTGTCAAAACGCACTAAATCTGTCCCGGTGGCAATTGAACAAATTCGAAATGGTAAATGCTGATTGTGTCCAGTCATGACAAAGTCAGCTTCTGTTTGTAGAGATAGCTGTAAGCTAAAAGTTATAAAAACACTCAGGAATAGAATTTTCATTTTGTCTACATAGATCTAACAGATTTGGAGCCGGTATATGGGAAACTTGCTTAGTTTGTGAAATTTGAACAGGATCGCATTGAGTCTTTAAATCGAAATACAATAAAGAGTACAAATCATTAAATTTTGTCGTGGTCGTTCCACGCTTGAATAGTAAAGGAGAACCACTATCAAAAGTTAAAAGTATTTTTTCGACAGTTTCTGTCTCTTCGCCAAACTTGACGATTGATAATTGTTTTCCAAATACATCATTATATAGTGCAACAGACAAAACTAATTCCCTGATAAAATTCCACATTTGTCTTTGCATATCGTTGATATTATGCAAACCATCAAAATTGAAAAATTTATTATATGTACACAGCATCCAATTTTCCGGGTAGATCGTGCCTTCAACACATTTAGCCAGATCTTCTTTCATATGTGGTAAAAAATCTGCAACATCGCAAGCATACGCCATGGATATGTTGCTGGACAATGGAAAAGAAGCGGTGTGATAATTCGACAATGGACCAGTCAACAATCGATACATTTCTTGACTTAAATTAGGTAAAAGTTCCTGAGGCAATTTTTTTGAAAGTAGATTGTTTTTAATGTATAAGTGATCATCATAAGTAAATGGATCAGCAGAGAAATTTTCTAGTGACTGCAGTTGTCTATTTGCAAATGCGTTCATCATTTTCGAAAGACTTTCGAAAATGTGATTTCCTAGGATATTAATATTCATTAACTTATTTATTAATGCTTCTTGCTCTTTGATACACAGCATTACTTTACTATAACCCGATTCGGAAATTTTTTGGTAATAAGCTTTTTTTCTAATGTGAATCTCTTGTTGTAAATTGTGATCTTGTGTCATTGAATGCGTTACGTATTTTTTTGGGACAGTTTCATGAGCTGAATTTTCAAGTTCATTTAATAAAGTTTTTATTTTAGTTTGTATTTCATCTTCCTCTGCCAGCTTTGTAAAAACCGGATCTTCTTCTAAGTTTTTTATACTAACATTGGTAATAATATCAACGAGTCTGCAAGGGGAAATAAATCTCGATCCGATGTATAATTTGTCATCATTTGTAACATTTTCTTCGTTAAAATTGAAAACATCTTGAATAGTTTCACCATATAAAAACTTACTAATTTCATTTTGGAGATCAGCATAAAGTGAATGACAAAGATTTAGTTGATTTAGAGTTATTGAAGCTTTTTGAATAACAGATTCTGAAATTTTAGACCAATAGGTGAATTCACTCAACGAATAAATTGTATCTGGAATTTTGCTAAAAAGGTCAAAGTTGATTAAAGTGTTTTCAGCTTCTTGTAAATGTGGAGGTTTTGAATATAGTTGCTGTTGAAAAATATTTGTGATTTTCGCCATATACTCTTTTACAAGACCATATTGTTTTGTGGAAAAATCCAAATCTTTCTCAATGATGTCAACGTTTTTTTCTAAATTGGATACCATATGTGTTTCAGTTAGATGATTACAAAATTTGCCAGCTAGTCTTTTACGAATAGACTTTCCTTGATTGGGCACTAAAGATAATTCTTCATAACATTTTAAACATGTAGTAGTTTCAGAAAAAACTTCTGGCTTCATCACTACGCAAACGCCACACAAAATTGTTAGAAATTCAATAATTTGACTGCAAGCATTAATGCCATCCAGTGGTGAAATTAAGCCAAAAATACAATTCATCTTACATAAAAGTTTTTCAATATCATTGATCGTAGAAATGTCTACAGAAAAAGTCAAATAATTATTAAGAGCCAAGTCCAATTTTAAGTGTTGCTTGCATTCATTGAGTTGGAATATATTTTCAAAATATTTTTGTTTGTCATTATCTAATAGAAATTGGTGAATTGAAGCATCGAGTAATAAGACTTGATTATACATAGCTTTTAATAAAATCTGTAAATAAATGGTTACAGGAGATGCACAATTAAAATTTTGATGACAGAGTTCAGCAAATAATGTCTTAAACAAATGAATTATTACAACACCGTTTTTCCTGAAATTTGTCATATCTGGAATAAGTGTGACAGGGTCACAAAATCGTAAGCACTCTAATTCTAAAGCACATTCACTTAACCTTGAACACAAGACACACAATGATTGCAAAGAATTCATTTTTACAATCTAGCTTTTTTTAAGGAGGGTTCCTCATTTTCATGCTCAGTCTCTTCACATTTTAATATACCAAAACCTTGCTCATTACCTTCTACCAAAAGTTCTGAAAAATCATAGATGGCAGCACTATCTTCTACTTGTTCTTCAAAAACTGACAATAAAGTTGAAAGAGAAAAATCATGTACATTTAAATCCGTTAAATGCTGAATCTTGTTCATTACGGATTTTGCTAGAGGCTCTCTACCATCCACGTAGAATAACATATCATCCATAGTAGGCTTACTATCTCTATCTTTCATCACTTCAGCTAATATGAGTAGTTCTGGATTTACAGAATTGGATAAACTTTCAATAATGTTGAGCACCTTTCCTCGAATTACTTCCACATCATAAACAATGGCTTCTCTCTTAACTTTCTTCACAATAATGTCACACAGTTTTGTAGCAATCACAAATTTCTGACGCATAAAACGGAAATCTTGCAAGCCGGAGGAAGGAGGGTTTAAATTGCGATCTACACCACTTCCAGCTATCCAACCTAACTGACCAAAGTGGAAATAATCTCTACTAGCAACACTCACAAATTTTTCGATCATTAAACCAAAGGTAATAATAGATCTTCCTGTGAAAATGCTCCCAAAATTTGGTTCTTTTGTTAATGATGCAGTCATAGGTTCTTTAAGTCCATTCCCTACCAACTTTTTTACGTGCATGACGTATTCCTGCACAGAAGAAAATGTTGGATGTTCTTTAAGAAAAACTGGGAATAACATAGGGTTTTGAGGAACTAGTTTAGAAGTTAATGCATGCAAACTTGTTAGATAATCTCTATAACCAAGAACTGATAATAATTTATTATGAAAGTAATACTCGATAAAGGATAGTAAGCATTCTGGTTGAATATCTAACAAATCTATTTCATCATATGAATTGGTGACACGGAAAGTAAACTTGATGAAAGAACGCATGTCTTCAACATTACCTATATCTATGGTTTTTGGCATGTTATTAAGCAAAATACGCTGCCAAAATTCTAGGCAGGATATTTTTAAATTTGGAAAGAGCCGTTCATGATATTTAAACAGCAAAAATGATAAGCAACCAGAAAGTGGGTTCTTTTTCTGTTTGACCTTTTTGAAAAAAGGATTATTTTGTGTGTTTTTGTTTGGCTTATTGAATGGTCTATTTTTTACTCGAAAATCTTTCAAACATTGCATGGAAAGGCGACAGAGTTTAGCTTGGATAGAGGTTTTAGCATATTTACCCGATTTAAACAAATTGAAATCAATGTATTGTTCCATGTCTACATTTGAGGCTATTTTTACAGTTTTCATATTCAAAAAACCTTTTTTAAAATGACTGTTGTAAAATGCTCCGTAGAGAGATTGGTATTGTTGCATTAACCATTTAGAAATTAAGTTTCCAGTACAAGGCCTATCTACAACATAGCCCGTACCGATTATTAAAGCCAAGTTCTGTAAAACAATTAGTATAACTTTGTAATACGTGAAAACAAAGAATGGAGAAAAAGCCAAAGACAGAGGTGTCGTATCAATGTTGAAAGATTGTAGACAGTTTTCGATTTGTTCTCTGGATGTCTGCGTTTTTCTCATTTCTGAAATACATTTTGAAACTGCTTCCTCAATGCATTGAATTAAATCATTAATCATATTCACAAAATCATTTTGACTTTTAAAATTTAAATTGTCTTCCCCAGTTATTGCATCAATCAAATTATTTTTCTTACAATAATCGTGAATCATTCCTAAGAATTTGAATCTATCTAAGGAAGGTGTGGTTTGAGCTTCTTTCATTGGATCTTTGCTTTCATTAACTCCCTTTTTCCCAAAAGAACCTAGAACATCTACATCTGCATAAAAACGAGAAAACATTGTCATAACAATAGGTTCTTTTTTAGTCGTTTTAGAAATCTGAGGTAATCTGCTGTTGATTCTGATCAATGCTGTTCCGATACACGAATGGCAACACTTACCTTCACAAAACTCACAGAGATTAGAGGAACAATTTACGATGTGGTTATATATTTCAGAATTTCCACAGTTCGTGTTATAAATAGACATTCTGTTCAAATTCCATATTATATGCGATAAGATTTGTGGACATGTCGCACATGCGTAAGCTAGATGAAAAGCGGAAAATTTATCTTCTTTACAAGGTGAAGAATTACACTTTATCAATTTTCTGGCATCGTTATAAAAATCTTCGTTAAGAGATGACAAGGAATTACAAAACTGGATTGAACGCAGTAATGTTTCTTGAGTGATCGCAGAGTTAAAAGCTTGTTTTGTGTTAGATATATAGGCCAATTTGTTACTGTAAAGTATCGAATTCGCAGCAAAGACTAAAGCAGCTACATGGCTAGATAGATGGAGCCTCAATTTTTTCAGTTCCACGATTTTCTCTTCGTGGGTTTCAGCCGATTTGATGATGGGCCACTCTGAAAAATTCATGATTTGATTATTTTCATAAATAGAATCAAGATACTCTGCTGTGTAACTGAAGGTTAGTTCCGTGATTACAGCGTCCACTAACATTAAATGATCTTTTTCAATCGGTGATAGTTTTTGACCAGGAATACCGTGATATGTTTTATTCGGTGCAAGCTTGACAGTTTGTTCAGTATCTTGGATAAATTGTTTCAGACCGGCTTTAATCAATTCCAGGGTATTATTAAATCGCAAAGTCATTCCCCATGATGTAAAGATTAAGTAGAACAATACTTCGCTGATCTGAGGCGCATAAAAGCCGTTTTCTTGAATGAATTCAGTGCAAGTATCGATGCACAAAGTTTCTTCCGATTCAAACAAGGTAGCCGAATATAACGGAATTTTTATTGCCAAGCATTCACCAACCTGGACTTGAATCTGCTCTTCTACACATGGGATTAATAATCCAGCAAATAAAAGTTCTTTGAAACCATTCCCAACAACTATATGACATAGAACGTCATCTGCATTTCTTCCAACACTTTCACAGATCTTTCCTATATCCACAGACTTTCGATTTCCGTTAACGACAAATTCTTGTAGATTGAATTTTTCTCTTGTTTGTTTGCACAATTTATCAAGATTTCCATGGTCTTCTGCTTCTTTTAAAAAAACATCTGTACCGTGGAAAAAGAAGCATACAGGCATAAACGATGTGATTTTGGTAATGACTGTTCCGCCGTAATTAATAATGGGTGTTTTAACGGTTGGGAAGAAGTCATTTTCAACCGTTAGATTAAGTAACAAGGGTGAGATCACTACTGATTTTCTTTTTTCCATTAACGATAATGTAGTCAGAATTTCTATTAATTTTTGTTCTTTCGGCAGAATGTACAGCCATGCTGCGGTACATATTGGGGCCGAAACAACAGTTTCATTATCATCAGCCATTCTTGAAACATGAAGACCCCAGTTTCTGCAAGGGGTGTTTCTGCTAAACCTTATATATTTTAGTTGACGTAACAAAACTTAGCGTCATCATACAACGGAAATTAAGTTTTCTCGTTCTACTGTTTTAGTGCGGAGAACTAAAAACGATATACTGACTCGGATTTGATAGTTTATGCAAATTAGCGTCCACCTCACTCGTAATAGTATTTATTTTTCATGGTTTTTAAAATAGAATTGCAGGCAAGGAGGATCAATGAACCCTCCCACTTCCGCAAAGTAAGCAACGACCAATCAGAAGTTTGCATTGCAAGTATTTAATTTGTTGCAGCAAAGCATTAGCTAGAGGACAGCCACAGAGATGCTACATATTGGGAAATTAATCCGAATCATCAGAATCAGTGTCATTAATGTCGGTCTCACAAGTTTCTACATCTTCATAATCTAAATCCAGTTCCATTTCATTATCAGAGTTTTCCTCTCCATTGTTTTCTTTAATTTTCGCTGATTTCATAGAAGTATCATTTTCAATAAATACAAAGCGAGTTTCATCTTCCTGTAGAGTCGTGTTTGTTTCAAAATCTATAGAATCAACTTCTTTACTCCAGTCTTTTTTCAGTCTGTTTTCGCGTAGCTCATTTGTTTTCTGTCCTTTATTCGTTTTATCCGCAGTAAGAAGCTTTTTACATTTGGCTTGCTTTTTGGTTTGTAAATTCAATTGGACGGTTTGCTTAGATTGCTGTGAAGCAAAGCTGCAAGACGGAGTTGTAGAACATGCAACATTAAAGCTGTTGTACTCTTCAGGCATGTTAGGATGCAGACCAAACTCCACAAATTCTTTGGGAAGATAGGTACAGAAATATAAGCTTGGTTTCCAAGGTGACATCATGCGTTGGATTCTAGTTTGGCAACCGATGTCATCACATTGATGATTGTTAAATTGGTCTCCGACTAATCTGGAAATCATGCCACATTGATATTGTTCACTTACACGATCTTGGTCAAAATCGCGTATGAACTGAGGTTGTTTAGTTTGAAGAATACGTTCCTTTATTTGACGTAAAATCCTTGCAAAAGTTACTATAAATGATAAAGCTTGCTGTGCTGGATATTGAAAAGATCCAGGAGTCTGTTGCTGTACTTCATATTCCGTGTTAGGCATATCACGGTCATATACCGGTTTTCCACAAAGTTGTCTGAAAATACGGTATTCTATGTCACCATGTCCATTGTCATTAAAAACACAACAAAGTTTTGCCATATTCAACTTGTTGAAGTATTCAAACTTCATTATGGTAATAGTATACATAAACAGATCTTGAGGGTCAATAGGTAAGTTGTGCACAAAATGATATTTATAGTAAATGCAAGCTTCCTCAATAATCGCAAGAGAAAATGCTAACGATGGAGAATTCGTCAATGAAAAAACTCGGTCTCCGAGTATCTGTTGATGTTGTTCTCTATTGAGGGAGTTTAAGCAGTTCTTATATCGTAGTAACAAGTCTGTTGTCATTAGTTCTTCTTTTTCCAAATAGATTTTTTCATGATTCCGGGGAAATCGATCTCGAATCCTATGAGCTGGATCTCGTGCTTCACCGGAATACTTGCGTAAACCAAGGGCTTCCGCGGTGTAGCTAGAGCCAAAAGGTGCTGTTGGTAAATTGGCGAAAAAAGTCAATGCATGCGTCTGTGCACGCTTTTCTTCCTGCTTTTCTTCAATGATGAGTTTCAGCTGTTCTAGTTCTCTGAGACTCAAAGCGTCTAATTTTGATTCATCAAGCTGCTTAATAGGAATGATGCGTCTGTGAAATTGTTTCGTAAAGCGGCTGACATACTTGTTAGCAGGTTTATGAACAAGAGTTCGTTTAATGGTTTTCAAACCATATCGTTGCCGCCCTAATACATTCTTTTTAACCCCGCGTGGATACATGTTTCTGTCGAAAATGAGAACCTTGTTCATAAACGGCCTTTTATATTATTTTTGTCTCGTGTAGCGGTGACGCCTCTGTGGTTTTTAATATAAGGAACATTTGACATATAAAGCTAGACACGAATTAAGGTGCGTCTAAAATTTTATTTACAAAACACAAATAAATAACATAAAAATGTCACATATTCAAAGAATCTAAAACGACAGATGAATGCACTTGCTTACTATTAGATTTACATTTCGTAGTAAAACATTTGCTCCATAATATTATCAAAATGCGATTGTTATCTACCTTAAATTCTAAGTACACTAAGACATTTTCACGGTTTCCCTTATGATTCCTTGTTAAACAACTGAAATCACTTAGTTTTCCGTTTTGCAATTTTTTTAGTTGTATATTTTTGTGATCAATTTTTAGAAAAGTTACATGTTGAAATTGTGTAGCTGTTCGTGCTTCACAGTGTTGGATGTTCTTTTTTAACATTTGTGGCCAGGCAATTGAACGAGTAAAAGTCATTAACAAATCATCACGGGAAAGACAACTTGTTTCCGAAAAACCATTGACATGTAAATATTTCAAACATAAAGTCTCCAGTGAACTTTGTTTTCTGTGATACAGTTGCTTAGTCCTAGAATCTATAAAAGAAAACTCTGTTGGACAGGCCTTATCAAAAATGCTGTAGATTATCTCTGTAATGGTTTGGTCTTTAGTTCCATGATGTAAAATATTATTGAGATCTAGTTGCATGCTAACGAAATCTTGAAAATTCTTTTTCATTTTTTCCGGGACAATAAAAATAGGCTTCAGACGACCATACAAAAATCTGTTATTCTCTTCGTCAACTTTATACATGAAACCTAACCGTAAACAACGTCCCGTATTATAGATTCCTGTATCAAAACATTCACCAGGAAAAATAACCGAGTTAAGAATCTGCATTAAATCGTGGTTTAGACACATTAAATGATTGAATATTTTAGAAAGTTGTTTTAATGGCTCACTTCCAATTATTGCAGTACCTTGAGGGATTGGAATAGCAATTCGAAGACCAATTTTTTTTTTGCAAACACAAAAAGCTGTTTCAACTTCATTGTAACATACATTGTGGTTCAGGGGATTCTCCGGATTACTACAGGTAGTTTTGAAAAAAAAGATGGGGTAGGCGTCTTTGTCAATAAACGGAAATATTTTTTGCCATGCATTGATCAGCACATTCCGCATCAATTTACACATAGAGAAAAATAACGATTCCGTTATTATAGCTGAATCCTGTAATGGCAGATCTAAATCACCAATATAATTTGTAACCGGAAGGTATTCATTAAAGATTTCATGCCTGGTTACGTAAAACTGTTTTTCTATTTCGTCGCTTAAAAAGGTCGTGTCCTGCAACCAAAATGTACTTGTTAAAGATTCATCCGAAATGTACTCATTGGGTAAAAATTGCATAACCTGGTCGAAACCCATATTTTTGTACCAACTTTCTGCACTTCCAAAACAATAAAGATTTTTGTAAGACATTTGAACTCTAAACATAGGAATGGGGTTGCTGAATTGGTCATGTAGATATTGTCTCCTTGTAACAGGTTTACCATCTATCAAAATAGATTTATTAGATGCCAAAATTTTTAAAAATCCTTGTAAGCCTAATTTAATTGGTGTAAAATACCCTTCACCGTGTTTATTGCATTTGTTAACCATCTTTGAAATTTGGTCTGAATTTATTACAAGCATGATATTTCTATTACTGTTTATGTCATAAACGTACTGTTGCATTTGAAATTTGTTGTTTAACATCCTTTTTATTTCTACATAATTTTGAAAATAACTGTCTTCGTTAAAGTACTGTTTCATGATTGAAATGAGATCCGAGTGTAAAGAATTTCCTAGGATGTACATATTTTCCTGACATTGAGAAGTATCAGATCTGGTAGGATCATAAGATGTCAGTTGACTATACTTGATGAATTTATTTTTTTCTAATGCTGTACTGTAAGCAATGTAGATATAGTGGACTCGAAAACTGTTTGTGAGATGCACGCTGTTTCTAAAAATATCTATTTGCTTATCAAGATAAAGAAATTCTTTTCGATCTCTTATGTTTTTTTGTTTTGCATATTCAGAAAAACTAACAAATTTTTTCCGATAGTTAGATTTCAAAGTAATATTAATGAATTCAGACATGGAGGAATAAGTAAATACCATATCTCCTAATTTATCAGTGAAACACTGAACATAATTCTTAGTTGTTGCTATGGTGCTTTTCTGCTCTTCAAATAAATAATAATACATCGTTAATAGTAGCATTCCTTCTGTTTGACCGTATGTTGATAAAAACCAAAACGGTGACGTGGGTAAGAGATACTTATTTGTTAGATGTTTAATGATTATAGTCTTGCAAAAAAAGACTAAGCTTTTAAATTCTATATTTTCACTTCCTTCTGGAACATGAGAAAGTAAAAAATCTTTATACAGATTTTCTGTTTCTTCCGTATTTAAACAGTGAATAATATCCTCAGACTTAATGGGTAATGCATTTTTTACATAATAGCTGAAATTCAAAGATTCTGCGTCGCAAACAAATACCGTCTCAATTCTTTTGGAAAACTTGCATTTCTGTGTCTGCATACAAAAATATACATTTTTTGATGGTTTATATTTAACGATTATTGGAAAAATTAAATGTTCACTAGGGTTCTTACATAAGATATTTACTATCACGTGCGCGGGGTCATATTCCGTTGCAAACACAGTAATAGTCATTGTGAATGTCTTTCAGTTTATCTTCCAACAGCTTTTAATATAAGACAATAGGAATCATGGGAAATTGCTTTGTAAAAAAAATCTCTTCTGACATCTTCAACTCTAACTACCAGATGCTCTATTCTGAGCTCTCAGAGCAAGAGGATTTACTGGATTTTTTAGAGACAAAATATACAGATTTTGGAATTTTAAAAACCGATATTCTCAACTATGAAAGAGACTCCGAAACATTCAAAACTTTGTTGCAAGTGTTACCTATCTATAAAAAAACAAAGCTGAGGTATAATTTGATTGAACGCTGTTTAAATAATTGTCCCCCTCACGTTAAAGATGCATTGATTATTGAAATCATGAAAGCTAAGAAAATTTTAGAGACTCTGGATGTTGTGTTCATGAAAATTATGATTGGAGAATTTACAATTTGCAGTGACAATGTGAATCAATTGCTCAATAAATTTTCAATAGACCAAACAACATTATGTGACATGGAAAAAATAAATACTTTAATTGACTTAGACGAAGAAAATAGCAAGCGTCTTTTGACAGAGATCGATCCTTTGTTACATCAAGAAACAGGCCTGTATCAAGCGCTGCCTAATGCAGTTACGGATCCACCAAGCGAACAGAGAGCCGCAACTAAAAAATGTTACGAAGGATTTACCAAATAAAATTTTATTCCGTGCTGTACTGAAACCAAAGCATCGATGTTTTATCTAACACTGCAAGCTGATTCAACACTCGATTAGGTAATAGCTTTAAAATAAAATGACCGCATGTGAAAATGATGGTTGCAATACAAATTCCTGCTTGCACAAAGATCCTCTGAGAAGTGGGATTAAAAATCTTTACACTTGGAGTTGTGTTCTTTAACCAGATATTAGTTTCAACCACTAATCCATGACAGAGAATTTCTTTGTTTGCTATTATTAAAGCTTTCAATTCATCAGGACAATCAAACATGTAATCAAAATTTTGGATTGTGTAATTATTGGATTCTACATAGAAATCTTTATTGGTAATTAGTGAAAACTTGATAAAATTTGGAGAAGGAAAATTATAAGTTTCCCAGGGTTCCGGTATATAGCATGGAAGGATGTTAATCACTAATTCTATATCACCAGGTAACAACATTACTGATTCACTTGACAGGTTTATTAAAGAAATGGAGATAACGTTTTCTGTTGTGATGAGTTCTGTTACACATTCGCAGAGAGTGTCGTTAACTGTTCCAGTCAAAATCCCAAAAAGTCCGTCGGGAATGTTTAGTGACACATTTAAATTAAAAATGCGTACTTGTTCACAAGGCACCCATATAAGTTCTCTATTTACGAGTACAATTTTATTTGTATTTAGAATTACATCCATCCGACTTTTCTCAAAATGATTTATTAAAATTGGAAGTTCGTTTACTCGAGGAACATTTTCAAAACCAAATCTGCTAGATCTGTTTCTTGGAAAGGTGAGATATTTCTTTTCATTGTCCATATGTTCTGCAAAGAACTCATCGTAAATTTTTTCACAGGATTTGAATAAACCGTTTTGAGGAATTCCAAGTAAAGAAAATGGTCGTTGATAAACCGTCAATTCAGTGTTCACATATTCATTTAATTGCTTTGCATCGATTTTAGGCCATGTAAGAGAGCTCTCTTTTGTTTCGCTAAAGTTCCAGATATCATCCACATCCGTTACAGTAAACAGGAAAAAGTGCAAGTATCTGGACATAGGCAATTTGTTAAGTGTAATTGTTTCAGAGATTTTTTCAGAAATTATTTTCTTGTTTGTTTCAGATCTTCTGGAGGGTAGACTTTGCATTTTCGTTGAAGCCTTCGGATGATTAGGAAAAATGACTCTATACAAGATTGTTTCCAAGCCAATTATATTGCTGGCATTTTTTTTTACCCGAGTTGTTTTCACAAATGAAGTCGACGGGGAGGAGCTGTTTTATAAGCCTACTTGTCATTCAGATACATATGAAATTATTTTAAAAAAATTTTCTTCTATTTGGATATTGGTCAACACATTTATTTTATTATGTTCATTTTCATTATTTTTAAAGTATTGGTGTTTTAAGACTCTCGCCAAAGAAACTGTAAAAGGGTATTAAATCTATATGAAGAATGTATGAAAATGCCCTCAGAACTGTTTTAATGTATTTTTCTCCATGCATATTTGAACATAAATATTCTAAGCCAAATATTTTTCTCGTGATTAGTCTGCGTTACTTCTGTCACCACACTCATATTTTTCATGGTATTTGTTAGAATATCTGTGGCTTTTGCTTTCCATCATCACGTCATGCTGTATGTTGAAGTTGAACAATGGTGTGTCTGTGGTTGAATTTGTTACGAACGCTTAATAGAAATTATTGTACATTTTTCTATTAGTATGAGTGCTATGAAAATTTCCTACAAAAGTTCTGTTTTGAGTGTTAATTAAAGTTGCATTGAAAAGAATGGCGGTTGTACTGTAATTTCTCAAATCAAAGTTTTAAAGTATGCTTTGTCACGGAGATACTCTACACTACGAAGTTATAAGTCCGTGTTTTGCTGATTTATGTGCTTGTGTTACTATGGTTTGTCGTGAAAAAGGTCGTAGGAAATGATGAAGTCTGTTGTGAAGCTTGCATGCTTGAGGTGCCTCGAGTAGATGGAAAAAATTTTGTTGGTTCGGAAGTAACAGCGTTCGGAATTGCTGTAGGCTTAGTAACTTTGGTCTTATTTTCTAAGAACATCAAGTTCCCCTTAAAACTTTGTATTGGTGACATGATTCCCCTTACCAAGGAGAAGTTGGTAACACCAAATGGTGTTGACACATTCCGCGTGTTCACAGAATTATAGAAAAATTTTGAAATAGAATTCTTGCAATCTGTTCGATTGTAAACAAAGTAGTAAATAAAATTCGTAAAAGAAAAAAGTTGCTCAGAATTTTCGCGTGAAAATAATTGAGCTTCAGTATGGTTTTGGAGATCTTGGAAAATGGTTTCAATCTTGTTTTGGCTAAAGTTAATTTTAAACAAATGTGAAGCAAGTACTTCAAACGGTATTTTATTGACACAAGAGTCATCCATCGTTTTGAATGCTAGTGTAGCAAAAACTAAATACAAAAAGGAAATATTATCTAAGTAAACGTGACGTTTTCTGTTTGTTTTAGTGGGAAATTCAAAATGTAATGTCCATCGCGGAAAATTATCTAGATAGAGCATATTTATTTTAGAAAGGCAAATATTCTTTTTCTGGGATAGACGGAATGACCAAAATAAAATCTGTTTAAAACAGTTCTTAGAGATGGGTATGTCGCAACTCTGAATATATGTTTTTTCAAAAGACTCATAATGAAATTTTGCGAATTTCGGCCACATGGAAAAATTCGTTAAATTTACAAACGAGATTTTTTCAGAGCAAACACTTAATACAGGAGTCCATATGATCAGATAACTATAAAAAAGAAAAGAAATCAACGTGAACACTAGCATTGAATACATTTTGTTTTTCATAAATGTGAATTATTTAGGAACTGTCAATATCCTAAAATAGTTATTCATTTTTTTAACCAGGATCATGATTGCAAAATGTACCTTGTGTTGGACAGTATTTGTTCACGCGCTAATCATTTTAATGATACTTTTATTAATTCTTCGTTCTCTAATTGGATTTAACAACGATCTTTGGTCGCAATCACAACTAGCTTTTACTTGTCTGAATAAAAGTTTATCTCATGTTCCAGCATAATATCAAAACAATCTAAATACATGATAAATACCAAACAAAACTATAATTATGATAATTAAATACAGAAGAACTAACATGATTGAAACTTGACTTGATTTTAGATCTAATGCGGTAATTTCGAAAACAGAGCCATTTCTTAAAAACAATAAATAATGTGTTCGTGGATTCACATCGATAAAATTTGTGTCCGGATCTATTAATTTTAATAGTTGCTTGTCATCCATTATGTATACAAATTGGATAATTCCGTCTATGTCGTCATATTCCACAACCAAGCTTTCACAAAACACACAATCGTGGGATGAAATATTATAGATTGGTTTTATATTTTTCACCGAATATTTATAATTAGTAGGTGTGCAGGTAGAGTTTAAGGGAACAACAGTGATAATAATTGTAGTGCTTATGACAGTACTCGTGACACTGAAACTAGTACCGGCTAAAATATATTTTGAAGAGATGGTGTAAGTAATATTTGGTAATGTGAGGAAGGCAGCTTGTTCATTTGAAACACAGGAAATGCTTTTAAACATATCTAATCTCTTTTTTCTATATACAGAAAGTGTGTTCATCACCTTGTTTGTGTATGCGTCTAGGTTTTTATAGGATAAAATGTTGTTGACAACTCTTTGAGTTAAATCTTTGCGCGTGGCTGTTGCACAAGGAGAAAACATCGTTTCAATGTCCATGACATTATCAAGCATATGTGTCCAAAAATACATTTCCAAAGGATTGCACAGACTTGCGAGCAAAACATGAATCTTTCGCTTGATCTTTTCTTGGAAGACTAGACTGTTTCCAATTTCTGAGAGTAAAAACAAATTTGCACGTTCCGGTTGGGTTAAATTTGTAAACAATTTATGTTTTTCATATAATGTTTCAATATTGTTTTCTATTTCTTTCCATTTGTTTTGTGGAACTGTAACGATTCTTCCATCTGCTAAAGATAAAAATGCTAGCTTAGCAGAGATAGGAATTGTCAGATCAGATGTAATATAAGAATAATCTACTAACTGGAAATTCTGAAGTTTTTCCATCTCAGGATATAATAAATGACTCTTAGTCATTTTGAAGCACTTTTCTAAAAACCTACCCATCGTAATGATGCTTTGGTGAGTATTAAGGATTTCCAACAGAGGGAGGAGTCCATCTCCTCGAGTACTATATAGAAAATGAGTTAAGCCGAATAAAAACATTAAATGGAATGTGTCTTTGTTAACTGGTTTTCCGCAAAATCCCTCCATGATATAAACAGTTGCTAACTTATTGAATTTGGCTATTAAAAACGAGATGTCATAATTTTGTTTCCAAATATCATCCAGAAAGTCTATTTTGAGAAACGGATAAAATCTTTCAATTGTTGTTTTTTTTGTAATTAACAGGAGATCGTCTGAGACAGTCTGTCTATATATGAATGAACTTTTTCGAAAAGGTGCTTTAAAAAAAATACGACTAACATTTCCAAAGATAAAAACGACATCATCCATTCCTGTTTCCACTGAAATTATAAAAAACGATGGTGTCAAAGCTAGCATGAACACAGTATTTAAGTAATTATAATGTCTCATAACACATCTTTCTGTTATTGGGAATAGATAATCAGTCAAGCCATTTGAAAGTGTGCAAATGTATTGGTTGTAATAGAGTTTGCGAATTTCCGAAACATTTGCAAAGTAGACATCCTTTTCATAAAATAAAGGTAAGAAAGACACTGTAATCGTTTTTGTATGCGAAACAGGTTTGATATTTGTCTGAAATTTTTGTGCATAGATTTTCACAGCTTTCTCTGTTGATGGGTTAAAAAAACGTGTAATCCGATGTTTATATTGATCAAATGATTCATCGAAATTCACAGAATCAAATAAATTCGAGACAAAAGTATATCCGAATAAGCATTTGGGAATCTGATAAACCCGAGTTTCGTTATAATCATGGAAATTAAACGTTATTAAACCAGGTTGAATAATAGTTTGATTTGTTTTTTCATTTACGCAAATTGAAGAATTGAGTATATTCCAGTGTTTCCATCCATTTATTGACACGATGAGAAGTAGACTATTTATGTAAAAATACATTTTCTCCAATTTAGAGCCTGGAAGATAAACGGGAAGTTTGTTGTAGGCTACGCCTTAAACTTTACATATTAAATACATAACGGGATAGGAATCATAGAGATATTTTGGGTAATATGTCTTTAGAATACTTGCCCCCTGTTAGAAGACGTATTGGTCAATATAATCATTTGAGAATCTACAAAAAAATTCTGTTGTTAAAATCCAATTTTGAAAAATTAAATTTTTTTTTGGGCAATCTTTTTCCAGAAGAACTGCATGATTCAAAAATACATGTATATTTTGAAGTTAGATTAGGGTGCCGTATTCCGGATTGCATTATTGTTTTTAGGCATTTTGGTGAGAAATTATTAAAAACTTTCCATTGTTATTTTTTTGAATTCAAAACTACTTTTGCCAAATCAAATCTGTTTTCAATTCAAAAAAATAGGACACAAAAAATTCAATATTTACAGGGACTAAGGCAACTACGACAAGCAACAGATTATCTACAGCAATTTGTCATCAAAAATGAAAGCTTATGTAAGGTAAACCCTGTAATCTGCTTTTTTAGACAGCATGGTTTAAAATTGGACTTTGTGAAAACTTTCATAGCTAAAGAATTGCAACTGTCTTCCACGTTTCTTTGCAACCTTTTTACGAAATATCAAAATGACACAGTTAAGTCTATTTTATCAATTTCCAATCCAACCAATTTTCGAAGGGCATGTCAGAAATACTCTAATTTGTACCGAGGAAGATATGCAACAACTCCAAAACTTGGGAATTCGAAAACTTCGAAAAGAAAAAGAAGAAATTCAAAAAAACAAGATTTTAAAAAACTTGTTAAAAACTGAACTTGATGTTTTGCAAGCACATGTACAGACAGAATGCCAGAAATTAAATACAAATTTAAGAGACATTGAAAATGCATTGCTTTTAGAAAATCAAAAAATAATTCCTTCTAGTGAAACACATTCTGTCTTGGAAGAGTCTCTCCAGGCTAAAACTGTCACGCAAGTCACAATTACACAAATTGATCCTGCTATTCATTTCACGGAAAATTTCCGACCTGAGATGATAAAAACTTTTTATAATAATACTCAGATGTGGAGCTATACGTTTGGAGCTTGGTTTTACAAATTAAAGCGAGCTTTCTTTACCGATTCTAAATTAAAAAGAATGCTAAAGTTAACATATGTTGATTCTCTCTCTATTACACAGGAATTGCTGTCTATTTCAATTAATGCATTGGAACAAATTACTATTTATCCTATGCATGATAATTTAGTGTCCGATTTAGAAGCTGGTTTGTGTCTACTGACTGCTTTTTTTGCATCGTATCCTGGAACTTTCTTAACCGAAAATATTAAATTCGTGGATGTCATTCAAAATTTGTCTCAAATTTTTAGATATCTAAACACCGAAATTTTAGCTACGAAAAACGCGTCACCACAGGATTTTTATTTTGGATTCAATGATCCAGACAAAATGAAATATTTTATTCCGTTGTGTAAAGGGAGGCATTATGCAATAAATACATTCAGTAATCATATCTTAATTAAAATTTTTATAAAAAAAGGTGTGATAAAGCAAGTTCCCGGAGATCAAATGTCGAAAGGACATGTTGTAATAGAATCGAAGTTGACAGGCACGTTAACAGATGATAAATTATTATATTGGACTCAAATTTTATTGCAACCGAAATTGGGAAAAGAGGTGCCGATATTTGTCCATCAGCAGCAGTATTTGCGTTCTGGAATTGTTGCCATAGAATCCTTATATTTACTTTGGCAAATTCTAAACTCAGAAAGCATTTTTGGAAAGAGAACTGGGAAATTTTATCTAACAACAATTTTCCCCCATGTAAATGCAGAAGATGTTACAGAAACAGAATTTTCTTCTGTCAATATCCAGAATTTTGAGTTTCTCATGAAAAACTATGTCGTCCCCACATATTTAGCCAATAATGAAAGTACTATTTCCACTTTATTTCCGGGGTTAATCAGCATCGTAGTTAACGAAAGTGTTCGTTTAGGCTGGGATCATAATCAAAATACTTTAACACAAACTAATGCTTTGCATAGCCAAACAAAAGACAATCCGTTTGTTGAATATATCCGTTCACAATTAGAAGAAACGGCAGAACTTGCGGTACTCGAGAAACATGATAAAATTCTTTTTCATTTTGAAAATGGCTTGAATGTGACACTCTCATTAGCACTTCCACGACATAGGTTGTTCGCTATGGCATCCTCATTATTTAATGTGGCTGATTTGTATGATTTCTTGTATTTTTTGGTATTAGGTTTTATTCCAGTAGCAACTGTTATTTGAAGACCAGGTGACGTCTAGACATTTGTTTTGGATATAAATACGCGTGGAAGTGTTAAGGAGCTTCATTTTAAAAAAGACACCTTTCTTAAGCTTTTGTAAACATGAAAAATATCGATTTAACAAATTGGAAATTACTGGCAGAAATATATGAATATCTGTTTTTTTTCTCGTTTTTCTTTCTTTGTCTGCTGGTAATAATAGTGGTGAAATTTAATAACAGCACCGTTGGTCGAGAATACACATTTAGTACTTTTTCTGGAATGTTGGTTTATATTTTATTGTTACCGGTTAAAATGGGAATGTTAACCAAAATGTGGGATGTATCCACCGATTACTGTATAATCTTAATGTTTCTGAGTGATTTTTCATTTATCTTTTCGTCCTGGGCGTTAACACTGTTGGCACTGGAACGTATCAACAATTTTTCTTTTTCTGAGATTAAGGTAAACGAAACTAAAATTCTAAAACAAATGTCTTTCCCAATTATCTGGGTTACTTCCATCTTTCAAGCTGTACAAATTTCAATGAAATATAAGAAATCGCAAATGAATTTAGAAGATGATTACTGTTTATTGGCTATTGAACGCTCTGCAGAAGAAGCCTGGATCTTATTAATGTATACCGTTGTCATCCCAACTTTCATTGTTTTTTTCTACGTGTTAAATAAGCGATTTCTCTTTTTAGAAAGAGACCTGAACTCAATTGTTACACATTTAAGCTTGTTTTTATTTTTTGGTGCTTTATGCTTTTTCCCTGCTTCGGTGCTTAACGAATTCAACTGCAATCGATTGTTTTACGGTCTGCATGAATTGCTCATTGTTTGTTTAGAGCTAAAAATTTTTTATGTTCCCACTATGACATATATAATAAGCTGTGAGAATTATAGGCTAGCTGCCAAAGCTTTTTTTTGTAAATGTTTTAAACCTTGTTTCTTGATGCCTTCGTTAAGAAAATTGCAACAACCTACAAAATCTACACAGTTTTAAAATTGTAGAGGTCCAATAGAAAACAAATCTGATAAATTACACTCTGTTACTTTTGCTTCTTTCTCCACTATGCCACAAAAAACTTTGAAATCATTCTGAATAAAAGTAAATAATCCATTCCTGTGTTCTGGTTTCTGTTGACTAAAAGCACCGTGAGAAATTTTGATCTTGTCAAAAAAAATTAAATAACCAAACCACAGATGCTTACAAATTTTTCGGATAAAAGGTTCATATACTTCTTGCCCCCAGAACTTGTTGAAATAGCGACATAATTTTAATAAACGGTGATTGTAGATGGCACCAATAGAAAACATTATATTCCCATCGTGAATAATTTCAGCTGGAATTGTAATTGAAAGACCAAATTCATCCAAAAAGAAACTTTCTATGATTTGTTTAACAATTCCAGTATGATGTAATTTTTTCGAGAGCTGAGCTGCATTTTCTTCACTGAAGATATGTTTAAGGATTTCTGATCCAGATCTTTGTTTAATCATGCAAAAGATCTTAAACCAAAATTCATCAGGTACCTTTCGATTCAGAACCACTAAATCACTCACTCCGATATTGTGCAAAGTCTCTCTCAACAGTAAGATTTGGATGCCGTGAGAAAACATTAGGGTACACACATTCTGGATGATCAAAATCTCTTCTTGTTTTAGATTAAATAAAGATTCACCGCTTACCAATTTCTTAGTGATATGTAATATTAAATTGGAGGTTGAGTTGTTCGAGATTATGTATTGACCTACTTGTGTCATCGTAATAATGGAAACTGTGCTAGTGGCAGGCTTCCTATGTGTGTATGATGATAATGATATAAATGATAACTTTTATCTGCCAAGGAGGACGATACAAGAAGAAATCAATTCCGGAAATGGTTTGAATATTCCATTGAATATAAACCACAATGAAAATGCCGTTATAGGCACAGTCTCTTCTTTAAGTGTTTACAGCACGGTTTGTTTCGTTGCCCGTGTTCAATCAAAGGAATTCCTTACAATAATTAAGAAAATAGCTGCAAAATCTAAGCTGATAACCAACACGGAAGAAAAAACTCTGCCACCAGATCCCGAAATAGAGTGTTTAAATTCAATTTTCCCAGGTTTGTCATTATCGAACAGGGTTGGTGGCAATGAACGTGATCCGTTTTTTAAACATGTCTCTATCTGTGGCGTTGGTCGTCGACCTGGAACAATAGCTATTTTTGGACGAAATTTAAATTGGATTCTGGATAGATTTTCTTCTATTACAGAAGCGGAAAAAGAAAAGATTCTGAGCACAGATCAAAGCTGTGTTCAGTTTTTTGCCGAGGAACAATTTAAAGTGGATTTATATGATCTGCTAGCGGATAGTTTAGACACTTCGTACATAAAAGTACGTTTTCCAAAATTACAGTCTGATAAGCAACTTAGTGGAATTTCAAAATCTACTTATATTAAAGCGAGTGAAAATTTAACGGCTAACAACCATACTATTAACGTGAATTCAAAAGTCACAAAGGAGACAGAAGCAACAGACAGCGTTTCACAAGATGATTGCGCAGTCCATGCACCAGATTTGATAAGTACGATTTGCTCGACAACGCACACAACGCATCACGACCTAGTCAGAATGAATGGCTCAGCTACTGGCAACTCAGCTAGTCTTCCCGCTCCTCAGTTTTCCGAATGTGTTTTTTTACCGAAAGATACATTTTGCTCCTTACTGAATGCTACAGCTGGGGCCCAAAATAAAAACGTAACGCCAGCTGCTCCGATATTTAAAACTGATGAATATATAACTCCGTACCCAGAATCTCTGAGCAGAGTGGATTATGGAAATAGAATGAATTATCACATACCACCTCCATATTGGTATCCCTCTATGCCTGGATTTAATTATAAATCTTATCGTGGTTCCCAAAAACGGTGCGCTCCAACAGATTCGGATGATGAAATGAGCTTTCCAGGAGATCCTGACTATACGACAAAGAAAAAGAAGCGGTATAGAGAAGATGACGATCGTGAACTCACTAAAGACAAAAATGATATTAAAGAACTAGTTGATGCAATAGGGATGCTGCGTCATGAAATTTCAGCGCTAAAGTATATTCGGTCTCAGTCTCCTCAGAGACAGCATTGTACAGCGGTAGATACAATGCCAACGATTGAAGAAAAAAACGTGGCATCACCTAAACCATCGGTTGTAAATGCCTCGCTAACTCCAGGTCAAGACAGGAATCAAAATTTGATGCAAAGTGATCAATCTTTATTGAGTTTGAACAAAAAATTGTTTGTAGAAGCTTTGAATAAAATGGACAATTGAATACAAATTATGCGTTAATGTCTCTGTTCCCACTGGGACAAGGAACCGTTTGCAGCCCAATGTTGCCTTCTAAAACAGTTAATGTGTGTGGTAAGGCAGTGATTGGTAGAAATGTGCGAAGGTTGTCTGTTCTCATGCGATATCTGACTGGTACAAGGTGTTCCTTAGCTATGTTTAAATGAAGAGAATTTAGATTAATAGATACGTTAGTTGCAGCTGTGATATTTGGTACTGTAGATAGTACAGGACTATCAGCGACAGGTGTTGTTTCCGATCTAAAGACACTTATTTGATTATTTTGATTGGAAATCAAAATTCTGCTTGTCTCTGTCCAGCCAAAATCTATATTTGATTGATCGTGTGTTACTATTTGACTGGGTAGGAAAATAAGTTTTCCCAGTTCCTGAGAATAATCAATACGGCAGTTTTTTTTGTAGGCTCTCATGGTTACTTGAAGATGAGTTCTCTCTTTCCAAGTGAAAGTGTTTAATGAAATCCCGTTTATTAATCTCGGAATAAAAAGTCCTGCAAATTTCTGTTTAGAATCAAAAAATGTTGGAAATGTACATGAGAAGGGGTCAGTGGTATTCGAATTAATGTTTTTTGTAGAACCTACGGTTATGCATGTGCCATCGTACGTCATTACAGGTTCTTGATTTCTTTCCATCACAATGTGAGGAATGGTTTGGATAAAATTTACTTGGAAAAAGATGTCGCTAGGAAAATTATCAGGATTTAAAAGATAATTGTTTTCATAAGATGCTCTTATAACAAGGATTTTTTCATGCAGCATTATTTGCTTAAAATAAACATTTCTGCTGGATATAAATTCCACTTTTGCACTGTTAAAAATTTGATCTGGTGGAGTTGTTTGTAAATCTATCCAAAATTCACACGCGTACATGTAATGCGTTGGTTCTTCGTGTTGTAATTCTGTCCAGCGTATTCTCTTAATATGGGCTCTTAATTTAGTTTGAGATCCATAAAGAATAAATTGAGCACGGCACTCTCCATGTGGAATGTAATTGTGCTCCATGCGCTCAACTATTTGGACAGGAAGTGATACCAAATTAATGCTAGGAAGAAAAAAGGCAAAAAGATTAATAATTAAAGGAAATTGTTTTAAATCTAAATCTACATCAGATATATTTTGAACAGCCAAGGTTACATTGCCACAATCTCTTAGATCGATATACGTAAAGTAGGCTGCTGTAAAGCTTCCTTCATCTCTGCACATACACACAAATGTTGATTGTTGCGAAAGTATGCTTAGCCCTGTCCTTAAGCATCTTATTTCACGAGGTTTTAAAAGGATTGGTGCTGTCAATTTTAGTTGTAGAAAATATGAAGACCAGACCACATAACAGGATTCCATTCTTACTTTAAATCTTTTATTGTTGTAAAATACTTAAATAGTTGATATTACAATTGCATACGTTTGAAGCGGGGTGTTATGTTCATAGAGTTATGTCTAATGTTTTCTGTCTCTTGTATTTGTTCGTAGGGAAAGTCTGTCATGGGATGAACTGTCAAAATTGGGAGAGAGTCTGAAAAAAAATCGTTCCCTAAAAAGTTAATTCGAAATGAATTGAAAGCGGCTTCAACTTTTGATTTGCAATCTGCGTGTGCTGTAGGATGGAAAGAATGTGGAAATTGTTCTCTACTGATAAAGTAGACATGACCAATTATATCATGCTCCGTTACAAATGTCTCATTTCGGCAGTACACGTAAATTCTAAAAGTCTCACGCGGTTGCCAAATGAAAGGAAATGAGAGTACATTAGATTTCTTTGGAATGAATAGCCCTACAGCAGCAGGATTCTCAAAGGTTCCTCGTAAATTGATTTGTGTGATGTGATTAGGTGTCAGTGTCATTCTTTCAGGGAAATAAATTTCCATTGTTTTGAATTCTTCAGAAATATTGAAATACGGCATGCAAATGTTAAGTGCAACTGATTGCATATTTGGTTTAGAAAGTCTTAAGCGTAAAGAACACTCATCCACACAGAAACGTGAATTCATTACTTTAAGATACCGTAGAAAGACAAATAACACCGGTGATCGATATTCAATCTTGTACACCGAAAGATTGTGTTCTGATATCATTTCCACGTTGCAGATTGGCGTTGCGCTCGCGAAAAAAACATTCCAATATTTGCAGTATGTTGTATTGAAGGTGAAGATGCAGTACATGGTTTCCAGTTTTTGATCATTGGAGTTTTTCAGTAAAGGTTGAAATTCATTCTTTTCCCAAGTAATTTTAGTTACTGTGGCATTTACGATCAGATCACCATTTTTCACAGAATATAAAATTATAGGAGTTGTTCCTAAACCCTTTTCAAGATAGGCAAATTGATGAAATCTAAGAGGATCCATATTTTCTTTAAAGTAACAATTATCTTGTATCAGCAGGGATGACGTAATGTTAAAAATTGGCAGGGCAAAAATATTGACAAAGTTTATCGATTGTATCATAGGAAAAACATTAATTTTGATCACTTTTTTGTTTTTTAGAACGTGACCCACCAAAGTTACGCATTCTGCATTGTTTTCAGTTCCATCGGTAATTAGCAACAGTACAGGTCTTTCGTGCGAAAGTATGCAGGTAATCTCTGCGGGAGAAAAATTGAGTTCTGGCTGCAAATCATTAAAATCCACAGTAAACTGAATTAATTGCGATAATTCAGAGACTGGTGTTGAATTCATTTTTACAAAGAAAACTAGAATATACAAATGATTCTTAATTGTTTTCACACGTTTTATAGAATCTAGCAAGCTTCTAAGGCTTTTTAAAACCGCAATCTACTTTTTGAAGGGGCGTGGTTATTTATATTTGATCCAGATTGCAATTTTTTTTTACGAGCGCTCCAACCTTTTCTTAAGATTAAATCTGGCAAATCAGAGATAAAAATTTTGTCTGCTAAAAAATACAGTGCGTCAGATTCTGGATCTTCTGAGTTAAGCTCAATTTCAGCAGCAAAACTATCCGTATAGTTGGGATCAAATTTAGTTCGTAAAATATGCGTTGGGATAAAATAAATGCTGCCTATTATGTCATTTGTAGTCACTCTCTGCGATCTATCACTAGTCACTTTTGTGGTAAAACTTGTAGTGGGAGACCAGAAAGAAGCAATAGACTGCACTTTGGAATTTGGGTCTGGAACGTAGAGAGCATTAGCATTAAAATTATAAAAAGTGCCTTTAAAATGAAGTTTTTTAGTTTCGCCCTCATCGAAAAGCATCGTGTCTTGGAAAAAAACGTCAATTTTTCTTTTATCGCTACAGATTTTAAAGTAATGCATATGCACAGTTAGAGAAATTTCGTTTGGGATTATTTCGTTGACAAAACCAAGTTTAAGCATGCAATCGTTTGGAACCATATACGTGTTATTTTTAGTTACCTCTGTATAACATCGAAGATAGATTTCGAGTAGAGGAGGAGAAAAGATCATTTTAAGTACGGAAACTGTTGGGTCTGAAATAGAAAACACTTTGCACGTTGGAAAATCTGAACCACTCCGAACATCGTGAAACCAAAAAGGTAAATTGTTTATGTTAACAGAGCAGACTGTACATAGAGTTGTTAATGGATGGCCAGGTGTTCTTTCAATCGAACGTTGATAAGTACTTAGTTTCCATTCAATGTTAAACATCGTGACGGTTATTTCGTTTACTTTATGTGGGGTTTGCGTCACTATGACCTTTTCGTTTTTGAATCTTATCAGACTAGGAAAAACTCCCATCTGCTCTTGTTCTTTAACATCATGGTTTGGGATGATGACGATAGAGCTGTTAATTTTTAACAACGGAAATCCAAAAAACAAGACTTTTGATGGATAAATGTGTCTATTCAGATTTTTTAAATGTAGTTTTAGTTGTTGTTCTTCCATTAAAGGACGAGCAGAAAAAAAGGAGTCAGAATTCCTTGAATTTATTTCAGCGCTCAAAAGTAAAACTGGTTCAGTTTTTGATTGGACATACTCGTCAAATAATAATGTAACATTATCTTTACCCTGGAATTCTTCTTTGTATGGAATTTCAATCTGAACATATTTTAAGATATCAGAAGTTGCGAGAGACATGGAGCTGAAATAAAATCTGTTACAACAGACACTATTTCAAAGAACTTTTTATTCACTCTTTAAATATACAAGTTGCCAGCCTATTGTCAGCAGAAACATCAGCCACAATGAAAAACGTTTTTTCTTGTGTAAGGGAATTGATTTGGTTAATCATGGTGAAAACCGTTTTAATTTTCTGTAATTCTGTTTTAATTTCAGTGGCCTTGAAAGAACTAGGTTCTTGAGAAAAATTTTTTTTTGCTAGGAGTTCTTGACATTTAATCAATAACATAGACTGATCTTCTAAAGATAATCTTTCTATATATGTTTGAACTATATCAGGAGCCATGGTGGCAATCATAGAAAAAGAAATGCATGCTGTTTTCATGGAAAACGTGGACTTACAATCATTGATAACATCTGGTAACTTAATTGTTACATCTCTGTACTGCAAAGTGCGCAGCTCATCTATAATTATTTGAACTTCTTCATATTTTCTATGTATGGAAAGGAGGCTGATGCAAAGCATTCGCACGTTTATTTCCGTTGCGACGTCTGTAGGGATCAGTAATGGCAGAATCAGCTCCCAGTTAGGTAAGACAAGTAATTGTTGCGAAGGTTTCAGAAATGGTGGCAGTAAACTCAATTGATCTCCCTTTTCCAGAGGAAATGGTCCGGTATTTTTGATTTCATACTGGTGTCCAATTTTTGGACGTATTAATATTAATTGGTTTCCTTCTACTTTTTGTAAGATTGTTAACACCATTGTTTTTAAGATATCCCTTATTTGAATATAATCGGTGGTTGTAGATAAGACCGTGTAAAGTCCTAAATTTGTGTTGCCAATTAAATGACGGTGCGACGGTATTGGGATGACGGCATTAGTGAGTTTGCATAAAGTTCCAATGTCAGAAAGAGTAAGCTTTTGATCAAAAGTACAGTAGATTGAATCCATTTCTATAGCAGGGACTCAATAATATTTTGTAACGGAATACACTCCCCTATAGCGAAATTTGCAAAATGCGTTTCACTTAACTGTGTCATTCCGTTCTTAGATCGACTCTCTAATAGAGCTTGGTTAGAGGAGCAATGGATTGGAAACGCAATTTGTAAAAACTGACAAGGACGATGAGTGACATCGTCTGTTCCTTCGGAACAAGAATACTGAATATCAGTGTCGCTGTCCAGGCATGTTTTAGTTTTTATGGAATAATCACTGATAAGTTTAAACATAAACTTATTTGTCTTAAATATTTCAGTGTCTGTGAAAAAGACTGCGTTAGGACTGTAACTTTTTGGATTATATCCTAATTGATCTCTGTGATTTATATTATATAAGACATCAGATAAAGATCCTTCTTGAGATGCCCAAGGATTTGTTGTTGCCACAAAAGCATCGCTATCAACTCGAGAATGATCATACAAAGATTTTCTAGCTTCTGACTCATTATGGGGATCGACACCCATCATACACGAAGCCCTTCCTCTAGGATTTTTAGGTGTTTTATAGAAATTAATATCGGAAGTCACTGGAGTAATGACAACCTCGCAGATTGCTTGTTGGCCATGTAAAAGTATTGATTGTGATTGCTTATTTATCTTACCGAAAGAAAGGATGTTTAAAGCATCGGTTTCGGAAGGATTGGGTTTTTCAATGCCAACGTGATGCCTAACCCAACTATTTACAGTAGGGTTGGTGTATGCGTGCATTGGAAAGACCGAAAATAGATCTTGCACTTTACTTCCCATATCAGTTTTTACCCTTTTCAAATTTGCGATTGCAGTGCTGGAACTAAATCCTAATCCCATATCAAGAAAACTTATATGCTGCGTAAGGTTATATGTGGTTGAGATGTCTTTTACTTCTGTAGATACCGTTGGGTCATCAATCATTATGGATGTTGCAGATTTGGAGCTATACAGTAAGCAATTAATGTCGAAACAATCTGTTCTTACTAAAGTCGCAGCGAAACCAGGATGAATTTTTTGTCTGCTTTGAAGAATGATAGCTATTGGAGACAATTTGCAATGCATAGTAGCTAAAGTCATGATACTTAGCAATGAATTTGAACAACAAGAAGCATAGACAAAGAACGGTGTTCTTTGCCAATTATGGAATTCATGAGATAATGCTTGTGGTAGAGGAAAACCACCGTCATTTCTTTGATAGTGGGGAAATATGTTTAGATAGGTTTGTACTTCAATATTCATTAAGCCACAAATAATAGGGTCTGAGAAAAAACGATTAAAAGGAATTGGCTTGAAATACCGTTTCAACACATTAATTGGTGATACTAAACACAGTCCATTATATAAAACATGTTGTAGAGATTTGAAGTTTGAAGAACTACCATGTCTTTGAGAAGGATCTAAAGAAGATTCAATTTCCAAAATTTTTGTATTCTCCGTTAGGTATAATATAGATTTAAAGAGTTGTTTACCAATGCAAGATAAATCTGTGAGTGCATGTGAGGGACCAACAGCTTCAGTTATCAATTCAATCAATACATTGTTCGTAATCGGTAATTCGCAAAAGTTGTCATCAGGTAATATAAAGGGTTCTGTGTAGAAGAAATCTAGAATGAAAGATTTCACATCAAAACCTGCACCACACATCTTGTTATTTGTCAGTGCTGGTAGAAAGCAGAAATAAAAAATTTTGCTTAAAACAATTGTTTCAAAAGATGGTCTATCTACGTCTGTGAAGATTTCTGTTGAATCCAATAAATTCATTCTATTCAGGTCAGATACTTCATAGTTTCTTAATTTCACTGTATTTTGGGTAAGGGGAGTGTTACCAGCTGTGATTATTGCATTTGCTTCATTTTTTGGAAGACTGTTTAGAAATGGAGGGAATAGACGATTATCAAACAAAGCGTTCACAAATCCAACCAATGGTTCCCCACACAGTTGCTCATTAAAGTTTGAAATAGAAATTGTCCTTTTCACTAATTTTAAGATTGAAATTATATTTTTGTAATGTGTATAAGCAATTCCTGGAATTGACTCATCTCCAAGATAGGTTGTAATTAACCAAATCATTTCAAAATTATTGCAAAACAATAATATGTGCTTTATATTATACCAATACGTAATACATTGGCTGACAACGTCTTTAAGAATCTGAAACGCTGTCTTATTACCATGGATGAGAAGTTCTATGATATATGCTAGTTCGGGATAAGCTGTGTTAGTTAAACTTTCAACGACGAGTTTCAGGGTGTAGTCATAATTTAAATTATTTGTCTTGGCTTGTTCGATCATTTGATTGGTCCTAGCTTCATGAAAAGATGAAGGTGCTAAAGGCAGAGGTATATTGCCTAACAAAATTCTTGGTGAACACAAAACATCAGTTGATCTGTTTTTTTGAATATAGGTGAAATCAAAAAATGGATGTAGCTCTGTCTTTAAAGTAAAATTTTCAGATTTATAGAAATCTTCTGTGGTAAGTTCATTTTTAAGCATATTCGTCGTCTTAGGTATTTCTTTTTTCATTTCATATAAGTTGTATAAATTTGATAAAAAGGTGCTTGGAGGTTCTCTAACAAATTCAACCTGACACAGTTGGTTAAAAGATTCCCCCGTAGGTTTTGCAGCATTTTTAAAAATTCGCTCTGAGACACTACAATCAAAAATAATTGGATGGCATAATGATGGCAATAGATCACTATAGTCTATTCTCTGCAAAAGCTTATCCTTATTATAGAAATAGATAGATGTGGGTAGATTGTTTTCCATTGTATCATTTAGTTTAAGCCTGCTATCCATAGTGCTAAAACCACTATCCTTCGAAATGTATAAGCCCATTGGGAAAAAAAACGTCAATTCCAATTTTTGTTCCAAAGGATCCTCAATGTTAGTGTTTTTATACACTTTCTTTAAATGATCTAAGACAACTGTTTTATCACTCAGTTGAATAATGTTTGTCTTTAGATCAGCATGTTGAGTTTGGTTTTCAAAGATTTCAGATTTTTTGTTTTGGTCTTGTGTTTCAGTCGCAACGTTTTTGGTATAATTTGAAAAATCCGCCATAATAGCCTGGTATGCTATTGCAGTAACAGCATTTTCTTTCCCCATAACAAATGTGCCATAAGAAACAGGTACAGACATTGTCATTTGTGAAATGTGTTGCGATAGGGCGTTGGACAAAATCTGAATCGTATTAGGAGTTCCGAGTAAAACGCCGTTAATAGGTGTTCCATCTTTTAAGACATAGCTATTGGTGTTATTTAAAATGCTTTCAGTGGTTGAATCAACCATTTCACACAGATATCTGTATATAAAGCTTACATTAGAAGTTCTGTTCAAAAAAAATAAGGAATTGATGAGTTTGTTTTTAAAGTTTTGAAACATATTGCTTCGCTGTACTTTAGAAAGAATCTGTTTACTGTTGACTTTGTTTTCTAACATGGTTTTCAGGATAAACTGTGGCGGTGCCTTCTTAAGAATAGTTTTAATGAAAGCATATATTAACCCTCGTTGTAACGAATCAGCAGAATTCTTCAGCGATCGTAACACCGTATGAATCGCATTGATGTTGAGCATTTGATCTAAAATTGTGTTTTTAAAGGTGTTTTCTAAATGCGCTAAACAAGCAGCACTTAATTCAAACGATATATTGATAGGATGCTTTTCTGAATACTTGGTAACCAGTACGGTTGTTTCTTTAGGCGCAGTTACGTCGTTTCCTGTAGCGACTCTAGGTAATTGTATATAAAACAGAATTTTTCCCAGTGACATTTTGTCTAGGTCGTTGAAACGGATAACATTTGCAGCAACCGCTATGGACGTATGAAAAAAATCTATCCATTCAGTCCGATTACAGTAAATCCCAAGTAATGCTTCGAAACTTATGTTATAACGATCAGAGTCGTCACCGTAATACAATCTTAAGTTTTCAAAAAGTTGTTCAGCAGTTTGTGTTCTGATATCATCAAACACGTTTGGAGAAACATCTAGTTTTGGGAAAATTTCAGCTGTGCGCCAATTTTCCATGGTTAGTAATAATGATAGACTCAATATCTGAAGAAACTTTAATAGTAAAGAGCTACACCGTTAATCACTGTGCTAAAAATGTTCCAGTGTTTATTAACTCCTATGATTTAACCGCAGAAGTGGCCAAAAATGAAGACGTGCGATTAGCGCGACAAGTTCAAATTTCATTAGAGAAAATAGATGAAGTTATAGAATCAATTTTTTCTGCGTCTGGTCCTAGCGTTGAAAATGTAAAAGATCAGGCAAAGTTTGCTTTGTGTCGTTTACTGCTTGGTCCTGTGAGTATTCCGTGCTACTGCGAAGAATGGGATGTCAATTTTTATCTGACAAAATGTAGTTATAATTGCGAAGGCCCGGTTCTATATATCTATAAAAATGCTTCTCAATGCTGTGAAAGCACATATCGTTTTTCTATCATGACTAATTATCATTCCACTCACATCTTTAGAGGATTATTATCATTACAAGAATGGAATAGTCATCTATCAAATATCTTATGTACTTGTTCGAACGTAACAGGTGATAAATATACTGCAACAATCTTTCCAAACAATGCTTCAATTTACTTGGAATATTATCCGTATTTTCTATGCTATCTATGCAAGCATCTGTCTATCATTGATATTGAGCAATGTACTAATGAATTGATAGCTTTTCTTGGTCCAAAGACTTCTCAAAGGATTATAATTCACTATAAACTGTTATTCGGGTTTCGATCTAAACCAATGAATTTCACTGTTTCTTTGTTAGAACAGGTTTTTACCCTTGAAATCCAAAAACTCTACTATTCCGTTAGTAAGCACAACAGTACAACAGCAGATTTTTTCAATGTCATTACCGCTAAATTTGCAGAGGACAAATATTTTGTTCTACGAACATTTAAATTGTCTGCGCAAATCACTCCTGGTATTCAAAGTTTTTGTTCATTGAAATTCAAACTCCAGACCTTATATCTAAATTTGAAAATTATGAAAAACACAAAATTATCCATTTCTAATAGTTTTTATCATGGTAAAACTTTATATACACTGGATGAAAAGCAACTTGTTTGGAGAAATTTATTGTTAATTTACTATGGTTACAATTTAAAAGACAATGTAAAACAAACACAAGAAGAGAGTTTGTTGTCGATGCATTACATACGAATATTGGAAAGATTGTCTCTAAAAAGTTTTCGCGAAATTAATCAACAATTTAGCTTTGAAATTCCGAGTTACCAAGAGAAAACTTTGCAGTTTATTCCAGGTGGAAATGATTTCGCAGAAATCACATCGGTCACGCATGGAGAAACAACTGTGAATGCATTTAATACAAATAGGGTCATGAATGTGAAAGCTGCTCTTTCAGGAGAAATACACTGTGTTTTACATCGTATTCCTAAAAGCATGACACATAGTTTTGTGATGTATAAGCGCACTTTTAAAGAACCCTCTTTAACAGTGAGTACCTTCATTTCAAATGATGATTTCACCACAAGTTCATTGAACATTAACATTCGAGGTCCCTACTGTGATTTTTTATATGCATTGGGTGTGTATAGATTACATGTTAACATACAAGATTTTTTTTTACCTGCTTTTGTTTGTAACAGTAATAATTCGATGGATTTGCATGGGCTAGAAAATCAAGGAATTGTGCGAAAGCGTAAAAAGAAGGTTTACTGGATAACTAATTTTCCTTGTATGATCTCCAATTCTGAAAAAGTTAATGTTGGTTGGTTTAAAGCGGGAACAGGCATAATACCCAAGGTTTCTGGAACAGACTTAAAAAATGTTCTTTTGAAAGAACTCATAAGCATCGGAGAAATTCCCAATATTACTTTTGATATGGATTTACATGCTTTGTTAACTCTTTTAGAGAAACGAAATATGCATCAGGTTCCATTTCTTATTAAACAATTTTTTATGTTTCTTCGTTTAGGTCTTTTAGTGGGATATGGACGTAAACAGGAAAGAAAGGTCCATCACATTATGCTATTCTTAATACAAAAGGGTTTTTTCGATTTTTCGAAGAATTCAGTTGCCAACAGTAAAATTAAACATGCATGCGCTCTAGTTGGAAGTCGACTTGCCAACAATGTGCCAAAAATTCTGTCTAAGCAGAAAAAAATGAAACTGGATCATTTGGGTCGAAACGCAAACGCTTTAACCGTTTTACGTTTTATTGTAGAAAATGGTTATTATAAAAGGAAGACAATTTTTCGCAAACTTTTGAAGTATTTAGCTACTACATCTTTTAATGCTCATGTTCAAACTGAAAGTAATCGTTTACTCAACTTGATGCACAATGACAGCAAAACAAATTTTTCCAGTTTGGAAAGACTGTACACTTTACGTTAACAATGAGACAGCAACTGTGCATGAAATCTTGAATTCCGATTTAAGTGAACTGTTACAGTTAAAGACGGAATTTGTATCTATGACAGACCTATGTGTTTACATTACTGGATGTATAAATCAGAATATTTCCAGCATCACGATATATTGGCATGCTTACAGTGAAGTAATTTATGCTTTAACTGGAATTATACACTGTGAAAAGATTTCTATTGAATGTGGAATTAAATCCACGGACAATAACATTTTGTATGAAAAGCCCAAACTGTTTTTACTTCGAGAAAATTTAGCACCAACTGAATTAAGGTGGAAATCTTTAATAAAAACAAAGACTATAAAAAGTGCTCTGTCTCCAAATCAAAATGAGATTTTTCCCAAAATAGCACACAAGCCGTCAATCCTTTTAGAGATTGAAGAAGCACCCCGATTTAAGGAAATGGTGTCATGTATTTGGAAGTTAGTTGCCGAAGAGGCGACCATAACCTCAAAAAGCGAGAATGATATTGTCAAAACATGCAAAAAGCTTGCTGAATCACAAAGATATACTTTGACAAATGGTACTGTGTTGCAAAATTTTATATTAGTCCATGCCTGCTTATTTAAGCTTGGAGCTGTTAATTTTTGGGAGGAAATGAATGGAAAATTACGTCAACGGCCAGAACTAATGTCAAAATCATTCACTGGCCATGAGGAATGTTTCTATAATTGTTATTATTTATGCACTTTGTTAAATTCCATTTATAGTTACAAAACTTTATTGCCAGAAATTGTAGACAATACCAGATCCATTCATGTAGTAGTGAAAGCATATTATTCAGAGCACATAGATGTTTCTTACAAAATTCTTTCGTACTCAACAAACATGATGAACTTATTCTCTCAGTATTTAAATTTTACAGATTTATTGCCATATATAAATAAGCACATTAAAATTGATGTTTCAGCATCTAAGCAAGATATGATTAAATTCTTAAATGCCTGTTTGGGACTTTAGATTTCTTTAAATGAAGTTTGTTTACCTTCATGGCACATATAAATAGCCATTATAATAGAAATTAAAAGGTCATCTGAACAGGTTTGTTTTTTGGCATTATAGGTTACGTGTTCATTTATATTAATTTGGTGAAGATTCTTAATTTGTTCAATCACATACTCTATTGGGTCATACGTTATTTTTATTGTAAAGGAAATTAGTTCTTGTGACGCTTTAATGTAACCTGAATTAAAATTCGAAATGAAATATTCTACGGCTAATCTCTTTTCTCTCCCCAGTAAATAAAATGGTTGTGCAATTTGGCTCTGATCTAAAGTATGGAAAAATGTTATGTGCTTATATCTTATAACCGATATAGTTTGTTTCAATATGCATGCAATTTTCACCGCCGATGATTGATTGGAATTACCCTCGATAATAATTTTGAGCTCAGTAAAAAAAGGGTGTAATTCTAAAACAGCTAGTATCATATGTGAGGCACATTCAGCAATTGAAGCATCGGAGTTTGATAGTAAACTTTCTAGAAAATAGTGCTCCATTCCATATATTATATATTGATCACCATATGTTCCGATCGCTGCTACCCCTGTTCCAGAAGCACGTCGATTGATTGTGTATGCTGGATCAATATATATGTAGAGCTCTTTGCCTAAGAATGGAATTATCTGTTTGCTAATTGTGCTATATCGGAAAAGGTCGAATTCAATGAGTCCTTGCTCTGTTATCAAGGTATCATTTACCACATTGCAGAGAGAACCACCCATTATTTCGTGTTTGAAAGCACCTTCTAAAAATAGGTCGGCGGTTTTTTTGACGTCAGCGTTGATGCTGATAAATTTCGGTTTATGTAGTCGGTAACATGCACAGGTTGTTGCATTTCCTCTGTCATTTAGTATGTGCACATGATCTTCACAAACATAAGATACTACAGTTAACATTTCAAAGGGGGAATTGCTTAGTTTTGTCAAGAAGGAAGTAGAGTGATTTCCGGAATTTGTGGATGATATAAAAATAATTTTAGTCGAAGATTGTGGCAGAAATCCAAGAATTGTGCTAAACGCGTCTTTTTTGATAAAATGGCTTTCATCAACGATGAGAAGATTAAAGCTCTGACCACGTATACTCTGAGAAGAAAATGAACGGAGTTTTAAACGATATAAAGACTGAGTTTTTATGTAATACTAAAACAGATCTTTTAACGTTGATACAAAAAATTTGTCTGAACTGTGATTTCATTCTTGAACCGGTAGAATCTTTTCCTAAAAAAACCGAGTTGGTTGCGGTGATGTATGATACGCTCGCGGTGGAAATTTTTAACGATCTTTTAAAATATAATGAACAAAAAAAAGATGGACTTGCCTAAATGTCAGTCAATCACAGTTGCTTGTGAAGGAGAATGTTCGCAAATGTACAATTTGCATAATCCTCTGACATTTGAAATGGGTTTGGGAAACATCTTTATATGTGTTCGGTGTTTTAAGATACATTTTTGCAATATGCTAGAAGACTGCAACCTGATAAATACTCATGAAGGATGCGTGTGTTCAAAAACCGGGCTTTTTTATAACGGATGGATGCCAGCCTATTCACATACCTGTATGGAACCTACTGAAGAGCCAAATATGGAGACCGTTAATGTAGTTGTAGTGCTGTTATCATACGTTTACAGTTTTTTAATACAAAATAAAGCCAGGTATTCAAACATTATTCGCGACATTATAAAAGATGGAAAGTTTATAGAACAAGTAGAAAATGCTGTTTTTTGTACATTTAATAAGGTGTTTAAGAACTCCACCTTGAATAAGTTACCTCTTACTACTGTCAGTCAACTTTTTGTTCAGTTAATAATTGGAGGCCACGCGGAAGGCACTATTTACGATAATAATGTCATTCGTGTTAGTAGAAGAAAGAGGGAGGATAACATACTTAAAAAAATGAGGATTGAATATGGAAATGCACTTGCTCTGTGAAACAATGTTTACTTGCAGAAAAAATAATATTTTACCGGTACATTTATGTATTTTACTGGATGATGTTATACATAAAGAGAAAGTAAAAGCTATAGAAGGGATCTTTTTTCAGTGTGTATTTTTTAAAGAAAAGCTTGTATATACGGAATGGACAAAAATAAAGTTTACTTATGTGTTACATGATCTTGTAATTTCTCAAATCTTTAAGAATGCCTGTATTAAAGAAGTAATACATGGGGCATTAATTCTTTCAGTTCCCATAAATATTGATAACCTACATTTTGATACAGATATTTTAATTCTAAAAATTATTTACCCACATTTTTTGCACGATGATATTGTCATAAAATTATCGGAAATTTTGTCTGGAGCACCTCGCATGCAAAAAACAGTGGAAAAAAAACAAGAGGTGGAAAAACCTTTTTTCCATATTCCTGCAAAACTTGGAGATCTCACAAAGGAAGACCCTATTTCGTTCAACCATCATGGTCCGTTAGAACCTCCATCAACTGTTAGAGGATTAAAACAATCTGCGAACGTTAGGCATAGTCATCCAATATCAAGGCCTGAAAAAGCGAACGTAACCTTTCTAAGTGATTCGTGGTACAGCCAAAATCTAAAGTGTGACTTCATATCTGACATTCAACAAAGACATGTGCTTGTCATTTTTTGGTATGAGTTATCGAAAGGGATACAAATGCAAATTAAAAATATTCAAATTCCTCCTGAAAATTTGTTTTCATCAATAACGAATTATTTAGATAGAGTCAACACATATCTAGACGAGATTGCTGAAAGAACTTTTCGATGTATTACTACTAACATGGAAATTCAGAATAGACATCTTCCACAAAAATTTAATAGTCATTTTCAAATAGAGTTTAATTGTACTCACTTAATTTCTGGTATGGAATTGGCGAGGGATTTTTGGATTTTGTCTTTAGATAGAAATAGTTGTGTTTTAAAAGCTATGGCCAGTCATTTTCTTCATAAAAAAAAAGGGAGAAGCTCACTTAGTTCGAATGAATTTTGGGCTGACTTAATTGATTGCACTACGGGAAAAACCTTATATGGAGAGAAAGTACGGTGGCAATTAAATTCTGAAACGAGCTTATACTCCACATTCAGAAAAAATCAAAACATTTCATGGGAATTACAGCCTAATTGTTATGCACTATATATGTCTGAGAATTTAAAGCTGTATTGGGTATTACCCGGGGGGTTCTGCGTGTCTGGAACTTTTAAATTAAAAGAGAACGATGAATTTTTCTTCGATTGGCAATTTGGGATGTCTTAGGAGTTTTTTACAAAATGAGTGTAATTGGTTTTCGGTGTGTAAAAAAAAGTTGTACCACGAATATCGTTGTGTGGCAACATCTTCTCCTGTTTTCGCTGTTGATAAATTTAAAGATTGCTTGCACTGTAATATAATTATTTTAAAAAAGAATTTGGATTTTGTTTTTAGCTTGGCCATAAACGGAATACATGCCGGACAGTTTGCAACAAATTCCATTAAACTAAAAAAAATTATAATAACAAACGATTTGGTGTACTACATATTAGAATTGGGATCTTTAACGGTAACGGATTTACATTTCATTCCAAAATACAATAGTGAACATGTGCTGAATGTGCGACCTATTACGCCAAATCTAATTTATGATACTTGTTCAATTGTTAGTTATGACGAAGCTAAACTTTTAACTGTTAAAGGACCTGGAGAAAATAAATTAATTCCTTTGGGTTGTGGTTCTTGGTGTCTCAACAACATGGGACGTTATTATGTGTACACTTTTGTCCTGGTGTACGATTTATATCTGGCTTGCTTTGAAAAAAACACTTTGCCATCATTATCTAAAGTAGTTTTTGATATGATTTCCTGCAATAATAAACATTGTGTTTTTTGTAAGGATCATAGTAAACACGTAGAACAAACTGGCAAAACCGTTGGATGCACTGATAATCAAGAAACATGTTTTTGTTACACCCCATGCAAGAAAAAAATGGCTAAGATTTCCAACCAAGATCTGTCTTCATTATTATGTGATCAAGAGCTCGATTTACTTGATCTAATTTACCCTGAAAAGCCCACTAGTCTTTCAACTGATATAAATGCTTACGTACATGGACATAAGAATCAAGAACCAGTCGTTTTAAGGAATACAAACTGGATATTAATTCGTCTTGACCCAGCAATTAGCAGATTAATACTCCTTTCCTGTCCAGTCTGTAAACGCATAGTAAGTAGGTAAGAAAAATTTGTTACTTACGTGAGTGTTGTAACAACTAGCAAACAGTGCTGTACTTTTTATTGTTTCGTGTTCGATAGTAATCACATTATCTTGACAGGTGATATTCTTTTGCGGGAAAAAACGTCTACATTTAAATTCAACATCTTTCATAACAAAGTGCGAAACGTGTTTTTGATGTGCAACATAACCAATGCTGATTCCTTCTAAATTCTTTAATAAAAAACAAATAACTGGTATCATGAACCATGTTTTACCGTGTCTTCTGGGAACTAGAAAAACACTAGCTTTCTGTTTAAGTATGTTTACGCTGCTTTCGTTTATAAACTCTATGTCAAATTTATATTTTAAGTAATCAAGGACATGATTGGCCAGTGTGGGTAATTTCGTAACAGAGATGAAAAAAATTATGTGTATAAGAATGCTCTTTTGAAATGGCTCTAGTTTAATTCTTTCCTTTTTATTGGTGTTCTCAAAATCACCGCAGATCCATCTTTGAAAATCTTGAATAAATTTTTCGATCTGTAAAAACATGGGATTTCTGTAAAAACTTGTTTGTAGCTCTAGATGTTTTTGATATTGTACTTCTGTGTTTTTCTCTATGATTGCAGTAAATTTGAGCTTTGACAAAGCGCAGTTCAAGGGGTCGCATATTGCAATTTTAGATTTTACGTGCCGTTGGCGATCACAAAAGAGATATAAAGGTTTAACATGCCTGCAATATGCATGTGTAAAGCCAAGTTCTGGAGTTAATATAATAAATCGTTTTTGGCAAAATATCGCGCTGTTTGGAAAAGTTGAAGAAATCTTTACGTCCTGTTCATGTTTCCAAATTATAGACTGATACGCTTTCTGAATTGCATCTATATCGCATGATCGCAACATGGACACAGATATTGCTCTAGCTGCAATTTATAAAGAAACGACTAAATTAAATGAAAAGGATGCTAAAATTTTCTCGGAGGCAGTGCAGACCGCACTAACTGTGTGTAAAGCAACCGCTCCTAATACACGTCTAAAACTCGTTGAAACACCAACTAATAACTTCTTACTAGTAACAAATGTTGTTCCATCAGAAACTTCGAAAGCAACGACTGAAGCAAATCTTAATATTGATGCAGCGTTGGAAAAACTGGCGTCTTCCTTTAATACAGCGGTACCTGTAAAATCATCCAAAAAGTATTTGTTGCAAAATGTGAGAAAAATGACCAGTGAGAACATCGCTCTAACTGGATCATATATCATCTATACGAAAAAACACATCGAGGTGGCGTTTCTGTTAGATAAGTCTGATTTTGTTCAGGATATTTTACGTTATGCTGAAACACCCAGTCTTCTAGGACATACCGATGTACGTGATTTAGAATGTTTGTTATGGTTAGCTTTTTGTGGTCCTATGAGTTATTGTCAGGCTGATAATTGTTTTGGACTAAATAAGGCGGGGTATAACGCCCCTTTCCCAATATTGTTTCCACCATGCATGTATGAAAGAAATATGAACCTTAGTGTATTTTTTGGGTTATTGCAAATTTATGTGTTCTCTTTGTATAGAGATTTTAGTGTCGAAAATTCAAATTTACAGCAAGGTATTAAAAAGCGTATTAAGTTGGTTCTGTCAGATTTACGGGCCAAAGAAAGAATTTGTGAGGAAGAGATAGGAAATTTTCCATTGGCGGCTCAAATATGCCTGTTTTGTGCATTATATAGACAAAATAGGCTCTGTATGGAATATGCCGCAAACAATCTAAGTATGAGTGTGTTCAGTCCAATAATATTAAAGGACTGTACATTTATGCAAACAACAGTTACCATAACTCAAATCTTGCCAGGTTCTAAGGAAGCAATAATTTTTCCAGTTTACGATATAGGCAAATTACTATCAGCTCTTGTTTTTTCAGAGAACGGTGTACTTTTGAAACTATAATGTCACTGCATGAATTAATAAAACAAACTATGTCCAAAAATTTAGAAAAAAAACATTATGAGTTGTTAAAATTAAAACTTGGTGAAGATCATCCTCTTAGCGTTCGACAGCAAATTCACGCTCTCAATCAAAATCTTGTATCAGAAAATCTCGAACAGTCCCAGATAATTACTTCTTTGACAAAAATGTTAAAGGATCAAAAGCTGCAGCTGAAAGCGCAAAGGAAAAATGCTGCTCAGCTAGAATGTGTAGATTTGGATGACATTTTGGATACGGCAGCGGAAGTGAAATCCGTCACCGACAATATAAAAGAAACTTTACTGGCCGGATTAGAATCAGACTAAATATGGAGCAGCTTAAGACACCCCAAAATCAAAAAACACGTCCAAGAAATATGCTTCCTAAAAAAAAAGGAAAAGAACTTAAAAAAAGGCCTTGTAAAGTAAAACGTAAATTATTTGGTTCCGAAAACATCAGACCTAACAAAAAAATACCTCTGGCTTCAGACGTGGATAACGAATTGGAAAAAAAACGGGGCTCGATGATACGAAAACGGTCTGAGACGGACTTATGTCCAGATCCATCTGTAACAGACCTCCTATGTCATGAATCTTTGACTGTATCTCCAAAGTTTGAACGAGATGGATTGAGTGCATGCACGGAATTTGAGAATTTTATGGATACAAGGAAAATCGTGTTAAGTCGAAATGAAAAGTCTGTGACAGATTTAAGTGCACATTACCCCGTTTTATGTAATCTTGGAATTTTTGAGCGTATTCATTCACCCTTTTTGTTTTCAATACACATTGATACTCAGTCATTTTCAGTTGTCTATGTTCCACATAAGGAAAGTTCCTGTTCTCAGTTTTGCGAGCCAGAAAAAAACATGGCACGGATTTTAGGAAGCGGATCATATGGAATGGTATATGATTTGAACAATGTTGCAATTAAAGCTTCTGATGACTTAGAGAGCTGCATTTCTTCTTATGTGTCTGGAGTAGTTCGTGCAAAAGCCGGAGCTCAATTAACCTCACGCGAATGCGTGTTTAAAAGTCTTTTGATATGTAATTCTGTCTGCCTGAACCATAAAATCTCCCTTTCCAAAACTTATGATACAGATTTATATAAATTTACAGACTGGAAATTGGAAAACGTTGAAAATTATTACTCTATTTTTTGCAACCTTGCAGAAGCTGTTCGTTTTTTAAACATGGTGTGTAAAATCAACCATTGTGATATTTCACTAGCAAATATTTTGATACACCACAAGGAAGGTATTATTTTGGAGGCTGTGTTAGCTGATTACAGTTTAGCTGAAGTACACCCACAGTATAATGGAAAATGTGGAATACTAAGACAATTTGATCATAGGATCCAGATTGTGCCTAAAAGTTATAATAAATTGTGTGACATGTTTAATCCAGGTTTCAGACCCATGATAGCTCACAAAATAATTTTGGTCGAAGTTTATGCAGAATTTGATGGTAAGGGCAATCCAGTGAGACATTGTAATCTAGATCTTTGTGCACTAGCGCAAGTATTTTTATTATGTGTCATCAGAATGTTGGATGAACGCGGATGCCGTGAGGCGCAAAAATATTATGAAAATCGATTGTTCACGTACTCAAATGAAGCTTGTACTTTGAATCCAATCAAATACCCTTTAGAATATAAAGATGCTTGTTGCAAAGTTTTAGCTGAGCACTTAGTTTTATTTGGCATTCTTTTTTATCGTGAGGTGGTGGATATGTTTGAAAACTTGTATGACTTTCTGCACGCAAGTGGTGATTTAAGCGTAAGAGATTTACTCGAGGAAACATATGTAAATGACAGTAGAGATGTTAGAAGACAACCAATCAGGTATAGGCACGCCCAATTACAAAGACACGAAATTGGTCAAATACTTTTAAATGATCTGCAACAATTGCTTTCCATTATAACTATTTCAGATTTAGAGAAGGATCCATATTCTGTATTTCGGGTGTAACATGGCAATAGATTACGCACAAATTTCTTGTAATTTGGCTTCTATTATAGAAGAGGACTCGGTCTTTTTATTTCTAATAGACAAATTAAACAATCTGGACATTTCAAGAAGGAAAATTTCATTTAATTTTATCCGTCTGTGTTATACTTATTATATCTTAATAAAGTTTAATTCTCGCTTCAAAGATACCTTCTTAGCCAGATCGTTCATTGATTATATGCATCAAAACATATCAGATTTTATCGATGAGAATGTTGAGCTATCTGATTTATATAGCAATATTTATGTCCGCTTACAAGATGCGAGTCCAAAAGTTGTTAAGAATCTATTTAAAATATTAGAACGAGAGACAAGAGGACAGTCAACAAATCCACTCTGGCACGCTATGCGAAAAAATTGCATCACGGCAACTAAAATTTATGACATCTATATCTCTAAATCTTTTTCGGGTATACAGGAGCATTCTTATTTAGGAGATGCGGTTTTATATGGAATTAAACATGAACGCATCATAGAACACCTGTTAAAGACATTCTTTGTGAAAAAGCCCTGGATATCTAAAACACTTGGTTTATTATTAGATCCTTCATCTGGAGTGTTTGGTGCATCCATAGATTCTTATTATGGAATCTCTTTTAATGACAACAACCTGATAGAAGTAGGGGATAAAGTTGTTATATTTGAATTGAAATTTAGATATAAATATCTGAGAGAAAAAAACGATCTATTTGTTTCCGAGCTGTTACAAAATCCGTCAGAAATTGCTTTAGCTAAATTCATCTTATCACATCCAATACCAGCTATAGAGTATAGAGAAAATGGAAAGATGCCCTCGGCAAGAGAATATTTAATCACTAACAATCCTCTATACGATTCTGGTAAAAAACGTCGTGCTTGCTTGACTCCCAAAAATTTGACGTTCGACATTACACGACTAATTCCCATGAACGAAAAAAATGTGTCAACGGCAATTATTTTTGATGTCGTTAAAGACTGTATATTAAACACATTGGTTGCATACCAAAAAGCTATTTTTACTATTGATGCTTTTATAAATCCTAGGCATAGATATTATTTTCAGAGTATTTTACAGCAATATGTAATGACTCAATTTTATATACAGGATCACGATAATCCAGAAAATATTGAAAAAGAGAATTTACCCTCGGTTTATATTGTATCTGCCATATTTCGAAAAAGAGAAGACGACGAAAAAAACTGTCGCTTGCTAATTGAGGACACAGAATATTTAGAAGAAGAAATCCCTTTAATTTTATTGATTACTCCAATTACTATTGACGCTGAATTTACTTCACGAGTAATCAAAGATATATGCTGTATCTGGGAAAATAAAATTGCACAACAGACAAATTTAAAAATATGGGCTCAAAGTGCTGTAAGACAATACATGGCGGCATCTTCAGCAAGGCCGAAGACACCTTAGTAGACTATAAAGGAAAATATATTAATCTTGAAAAAGAATTTTCTGCTTTAAGTGATACTGAATCTGAAGAAGAGTTGCAACTAGAGAAGCCACTTCTAAATAAACAAGATTCTAGCGTTTCGTTAACCCAGAAGAAACTTGAAAATCAATCCAAATAAACGTCCATTGATTAAAAACCCTTTATTCATTTGCTTCTTCTAATACATCTAGGTCTTCCACTGTTGTAGGTAATTTCTTATAATTATGATGTTTCCGCATAAAAAATCTAATTAATCTGCATATAATAAAGGAAAGACAAACTATGGTGATCACAGCTAGATTGACTATGAATTTGACAGTCCATTCCGTTTTAAATAGTGAATCATATTTTAGTATTGGGTAGGTTAAGCCTAGGATACCAAACAAAATTCCAAAATGTAAACCAAATTGAACCTTAACATACTGATGTAAAATTAATTCAATCACCAAAGAGTATACAAAAGACATAACAAAAAAGGTGTTGATAGATGCGAAAGCCACTGTTGATGATTTAATATAGAAATTGTTACCTAAACCTAGACAAATCGTAATTGCAAACACCATACTGGAAAAACCCAACATTAGTTCTATCATATTGATTATGATAGTTTTATATTTTATTGTGCCTTTGAGTTTTGGGTGGATCCGTTTCAATACGAAGAAAGATCTTTCAGAGGATTGATACTGTGTTATCATAGTCAACGTGAAAGCTGTGAAGCAAATGAAAAACAGGCAATACGTAAAAGCTGCGAGGGTGACTAGTCGAAAAGAAAGACAGGTGACAAAGAACTGATATGTATCCATAGTTAAAACAAATTGGAAACACGATAAACTATCCCCCATCCAAGTTATATCCCTTGTAGATTGATTGATGTTGGGAGTTTTTTCGGAAGAGAATACTTTTACGCAACAAACTATATAGTATACTAAGATGAAGAAAAAGCAGATATCCATGAAAACAACATAGCAGATAAGTTGGATAGGATTTAGGTAGAGTTGTGGCGTTAGGCTGCGGATGTCATTCCGTATAGAGAGGTTGATGGCTTTCATATCATTGATTTCATAATATGCGCATGGGAATCCCAAATTGGGAAAGTGAACAGCCACTAAATGCACTGTGACGTTGACATACGTTAAACAAGCACAGATTATACTTAAGACCCAAATTCTCATATTAATCACATCTACTCTGCTCAATGCCATTGTGGGGATCTGTCAAATGGAAACTCAGCTTCAAAATGATCAATTATTTTTGGAATGGTTTGGACAAAATTTGTTGGATTGCCACTTTGCACAAAACGTTTCAGTGTATTTGCAAGATGCTTCAATGGTTCATTTTAAAACCTTTTCTGAACAAATAAAAATTATAAGAGCTCCAATGGGTTCTGGCAAAACCTCTGCATTGATAGAATTCTTGAAAACTGTTTCATATATTGATTCTGTCCTTGTTATTTCCTGTCGTAAAACTTTTGCTGCAGAACTTTTAAATAGATTCAAGAAGAATGATTTGAACGATTTCTATCTGTACAGCGAAATTAAAGAGCGTCAAATCAACAAGAACAAACTGATAATTCAAGTAGAAAGTTTACATCGTGTTACCAGGAATTATCATGTTTTAATACTAGATGAAATAATGTCTATTATAAAACAGTTCTATTCAAAAACCATGACTAAAGTCAAGGAAGTAGATGCTAAGCTTTTAACGCTTATTAGAAATTCAACACAGATCGTAGCTATGGATGCAACAGTCAATCGTTATGTGGTGGATTTTTTCTCTCTCTGTATGCCGCATTTTAAGTCTGCATTGATCATAAATACGTTTGTAAGCGCGAATTTTTCTAATAGATCTGCTTATTTTTGTCCAACTTTTATAGATGGGAATCTTGCATTTTATGGAATTCTAAAACAAAAATTAGGCTTAGGAAAAAACATTTGCTTATTTTGTAGCACAGTTACATCTGCCGATTTCATGTCAGAATTGTTAAAAACTGATTTCCCGGATAAAAAAATTTTGCTTTTAACTTCTAAGCAGGGCAAATGTCATTCTGTCGAGAGTTGGATAAACTATAATATTGTTATTTATACATCTATTGTAACGGTCGGGCTCAGTTTTGACTTTCTACATTTTTCAGCTATGTTTGTTTACATTCATTTGGTAAAAGGTGGCCCTGATATGGTTTCGGTTTTTCAATCAATGGGTAGAGTTAGAAAGGTCACAGACAACGAAATTTTTATTTATTTGAATCCTGCATTAATTCAGGTGCCTCTTTCTGTATCCCCTATTTCGATTCCACAATGCTATGATTGGACACTTTTTGAAAAGTCAATCCTACAATGCAGCTGTATGGATTTTAATAAAAAATGTCTCAGTGCACAAAACTACTTGTCTAATTCCATGATAAAACAATTTTTTAGAATTAGGCATTATATTGAAAAAACTACGCTATTAAATCTTCCTGATAGTCTCTATCTTTTATGTCTTTTATTGGATAGTAATTCAATTAAAGTTCACATCGATGGAGACGTCTTTCCTATAGCTAAAGAAAAATTCTACGCATTTACAAAAATGTTGGTTCAAGGTTGCCATTTTTTTGAAAAGAAGAAAACAGACTTTGTAGAAAATACTATGACACTGAAAGAATTGTTTTCTAATACAAATATTACTGTTAACGGAGAATTCTATGAACTTGGAAATTTCCAAGTTCATAAAGATTACATTGTTAATTTAAATAATTTCCAGAATTTATTTTTGAAAAATGATGTTGACATTTTTGTAATTGAAGAGATTATGCTTACTTTAAAATCTGAAATTAGGAGGTTTGTTTTTATAAATGCTTTGCTACAAAAATATGTTGCCACCGGTATCGATGTGGAAAAGATTAAAGCGTTTTTTAAATCCCGAATCAAAACATTTACTCTACCTGAAAACTATATATGTAGCAAGTTTTATTTATTAAGTGATATTTCAGGCGTACATGAATGTGGTATGTTGATGGATGTAGCTTTTTTAGCAGAATCTATTAGAGCGGATTTAAATTTACAGTCCTGTACAGATACGCAAACTGACATCTCAGAAGATGCTATCTTACTTTGCGCTGCAAGAAGATCATCTGAAATCCTTAGGATTTTACAAATTGTTTTTACAACGCACGTGCAATTATTTGAAAAATACAACAGTTATACGTTATATTTGTTCAATAGGTTAAAGGGAATGCAATTAAATACCTGGTCACTTTCGATTGCAAAATTCAGTGTTTCAATAATAAGGATGTTTTTCAAATGTGCCTTTAATATGAATCTGGTTAAGAGTAAACCCAGATATATCGTTGGGAAGCCATTTAGAAGTCTAACGAAAAGGGAAATAGAAACTTTACTTGACATGTGGCACGTATCAAGAACCAATCTGAAAACCTACAAAGAGTTACGTAAGGCTCTGACAGAAGCTTCAAAAAAAAGACAAAGAAAAAAAATTTATAAATTACTAGGTCACAATATAAGTTCTTATATCAGTGAAACTGGTTGTCTCTTCCAGCATGCAGATGCGGGGATGTGTTTGTCATCTGGGTGTCTATTGCGTTCATAACGATTGGAAAAATAAACAATATAGAGTACCAATTTATCAATGCCTGTTTTTTAATGCTGAGACTCACTCCTTGCATACTTTTTTGGTTATTGGAAATGAGATTTCTGAAAATCTCCTTGATAAGATTTCCATTTCAAAAGAAAAAATCTTTATGTGGGATTTGAATGAACAAATAATGAGTATAACTCAAAAGACAGAGTCAATCTGTGAACTGATGTTTGGCAACAAACAAATAATTCAAAAACTATCCAGAACATTTATTTTCTTAACAATTATTTTAAATTCTAATATGTATGAAATAATAGATGTCTGCATAGACAGTAATGCGGTTTTGTACATGCCAGACTTAAGACCAATGATAATAAGATGTGTTGGAAACTATTCGAAAATATCTTCTGAACTATTGTCAGATTCAGACTGCACACAGGCGACAAAGAAAATGCGATTTGATTATCACGTCACAAACTTTTGTTTCTCATTAAATGTCAATGGTAAAAAAGTACTGTTTTCTAGTACAGAGTCTTCAGAAAGTATTTTAGAAAAATTGCTTGAGTTTTTCACAATACAAACATATAAATTGGCAGAAGAACAATTCTTAATGGTGACACCTAAAAACTTTTTTACCGTCTTATTTGATGAGGACATGTGTCTACTTTTGCTGCAAACAGTTGTTAGTTTTTTGTATGACAATCTATTTCGAAATAAGTTAGTTGTGAAACAAGTTCATGATTACATTGGTCCAGATTTGTGGCCGCAAGGACATGAGAGGGCAGTTTATTTTGTAGGGTTTCCTAATATGTGGTTTTTATCGATTTACGATTTGGACAACAAGATACCTTGTATTAAAAATATTTGCAATAGAATTTTACTTTACTGTGGCCTGCCAGATAGTCTAGGACCAGATGGTTGCCAGAGTGTTCCAAGTACGCAGTGTGTGGATGAATTCGAAGACCTTCCCAATTTGGGAGCATTACAATATTTGAAATATAATAGTTTGGTTGTTACAATGGAAACAGTAGAAAATTCTGAGAATGTGTATTATTTTTTTGGAGAGCAGGATCTGTTTATTGTCAAATTGGTAGACATTATTCAATCTTTGTTGGAACTATATGTTCCTAAAAGTTTTCTCCCAGATTTTAGCGATGCTTATATAAAAAGTGAAATCTTATTGAAATTTATATCTCGACTTCACAAAAAATCTAACAACATTTTTTGTAAGATCGTAAAAAAAATCACAGAGTTTTTGAGATGTTTTTTAAACGCGTGTCGATTAATGGATCTTAATTGGATGTTTATTAGAAACATGCATATTGTGTATTTTATAGGTCCAAAAAAGGATCCTTCTGTAGTACTTCCTCTGTTAAAAACGTCTGTGGAAAGTTGTTGGAAAAAAATTCTCAATTCTTCAAGAACTCCAGTAGTGAACATAAATTACATCCCTGGTTACAATTTCCTACACAGCTCATGTAGCTTTTTGACAAGCGGAGACATCAATAGTGAAGATTCTCATTGGACTATCACAGCTAGCAAGTGTTTATATAATTGTTTAGGGGCAAGTCAGATTACAGTAGATTTTAAAAATATTTACACAAATTTCCAGCAGATGGTTAGCGTTTTTCTAAGTCACAGATACGAAAATAAATATTGGATCGAATATTTTGAGCCCAACAACTATTTTCTAGAGACACACGAAGGTTTACTAGACTGTAATAGATATACAGCTGTCTGGACAACAGAAAACAAACTAATAAGACAATCTGTTGGTTATCCATTAACGGATAAAATAGATTTTATTCATTACATCCAGGTTGTGATAGAAATTTTCAAAAAGTGGCTCTTAACAAAATATAGTCAACAAGAGTATGCAGAAACTGTTAGATTAGGAAGTAAGATCATCTCTGATCATTTACATTTGTTTAATGTAAACTAGTTTTTTCTATTTCTGTCATCTTATATTCCTTTGAAAAGGCATCGCATATAGCGCTTTTTAAAAAATCTATTAAATATTTAGAGTCGTCTTGCAAACGTCTCGTGTTTAGTGATAGAGTACATGTGTCGTCATTGTCGTGAGATTTGAAAAAAAGATTTAACATGTGTATATTTGCTGTTTTTGACTCATAATAGAGCTGAATAATGGGTGGAACTTTATCCAGTACGTCTTGCTGTTTGTCATCGTAAAAAATGTATCTTAAAATACTAATCAATTTTTGACAGGTGCAGGCTAGAAGTTTAGAGACTGGCAGAGTTACACTGATTTTTTTAATAATATCTCGTAATTTAAGTAGTGTCTGCCCTTTTATTGGTAGATTTTCTAGAGCATATACAATACCACAGAGTAAAAACTCCGTGTCTTCCTTTAAAATCAAATAAATGATTCTTCGTTTACATTGAGTTTTACTCAGCCGAATCTCTTCTAGCGTATCTTCGGAATCAAGAAGAATAATGCCAATGAAGCCTAGAAACCCTAATGTTTTCTCACATATGCTTTTATAGGATTTGCCATTTACAATTGTGCCATCTTCCAAATGAAACCATACATCATCATTTCCAACCGAAATATCAATTAAAGGTAAATTTTTAATGGAAACGTGTTCGGCGTGTTTGTTTAGTTCACAGATATTCCGGACGCTTGATATATTGAATATGTCTAGAGTTTCGAATCCTTTTAAGCTTGCTGGCAACGTCATCTTGCGGATATTGAAAATCTTCTGGGTTTGTGGATCCTAAATCAGGTGTCAAAGTCAAATCACTTTGTTTCTGAGTATGTATTAAATGCGAAAGCTCGTTAATCTGCCGTATATAGATTAATCTTAAATAATGCTTAATTTTACAGTTTGAGTACACAGCTAACATAATCTCTTTTAGAGAAAGGAACAGAAACTCTTCTGGGAGCAGTTCAAAATGTCTTAATTTTAGAACATGTTTCATGAAGGGTATAAGAAGCAATTCTAGCGTGTAATTTGAATATGAGATGCTGTCTTCCTGTCCTTGATTGTTGACATTTTTTCGTAGACGGAATGTCCTTGTGTATTCAGTTTCCCATAATTGATCAATTTTTTTGTCTGCGTATTCAATATCTGGAATATATTGTGAAAAAAAACTGTTTGCCACAGCTCTGGTGTCGTCTATTGATACACTAGTGAATGGGAGACTTTGAACTTTGTTCAGAGCTTTAGCGAGAGACAAGCTTTCAATATCTGAATCTAAAGTAAAGTCATGTTTAGAAGTTTCGGATAAATTTATTAATGAATGCATGTCTTTAATCTTATTTTCAAATAGCCGATTTGCAGTTTTCAGTGTTTCGATTTCGTCCAGTTGTGATTGAATCTGTTCCTCCATGCATTTTATGATTTGTTTTTTGAAGATATCTCTAATATCGCGATCATTATTGTCCTGCCCACGACGTTGCAAATTTCCTTTGCTGATTAATAATTTATTCTGGTCAATTATAGACGGCGTGATGTCTTGTAAAAAACCCTCAATACTATCTGTTATTCCAATTTTAGATTTACTATCGGACAAGTTTAATAGAAACTTTATAAGACTTTTTTTCGCATCGCTGGTTTTTTCCTCTCTTTCTATAAGTTCTAGAATTTTTTTGTTATCAACACTGTTCTTTGATGTAGTTAAAACTTTAATTGGAAAAGTATTCAGCAATTGACACATTTTACGGTGCCTGTGTAGGTTTTCGTGTTTATTCAATTCTGAGTATAAGTGAGCTAGGGGTGTTGTAAACAGCAATTGATTATTTATGAAATATGGTGCACTGTAAACAAAGTACTCTAGTTCCTTGTTATTGGAGAGACAGGTTATATTGCCGTCTGACTTGTTTCGATAAATGTTTATGTTGGTGAAATCGGCAATAGATGTTGAGGAGATCTGTGTTAAAATGTGCAGTAGAAAGTTCTGTTTGTTTCGTATGATTTCTTGAATCAATCTAGAGGAAATACTTTTTGCTAAGGGTGTCAGCGTAACCTTGTTTTTTGGTAATGTGACAATTGGTGTTATTCCAATCGTACAAATCCAATCAATGTATTTATTATAAATGTCGTTATCGAGATTTTGAAGGATGTAGTTTATCGTTTGCAAAATGCCTTCCAGGATTACCTGTTTGATTGCATAATACCAGATATTAAAATATTCTTGAAATGACATGCCTTTGCTGCGGAACGGAAATGCTTTCAGATGACGTTGCCATTCAGCATTGATCTGCTGGATGGATATACTATTCAAGAGAGTGTGTGACAGTGTGTAAAACATTTGCTTCTTAAAGACAGCCGAGTCTCTAAGAACACTGTGTATAGATTGTCCCTTAAAGAATTCTTTTTTACCGTGTAGCATTTCTATAAATTTTATAGTCTTAATAGAAGGAAATAAAATGTCATTGTCTTCATTATTCAATGGTAAATATGACACAAAATTCCTTTTAAACATGTCATCTGCTGCTAAGGTGGAGTTGATTGTTGAGAAAGTTGCAGCTTTAGCTGATGCCTGTTTAGAGACGCCGTTACCAACTGACTGGTTTCGTAACATTCTTGATCCTGAATTAGAATTTAATAGCAATTTTGAAGAAATTCATTCTATAGGTGATGAAGAATTTGCTCAACCGTTGCCATTTTTACCTTTTAGAGTATTATTAATAACCGGTACTGCGGGTGCGGGCAAAACAAGCAGCATTCAAACCTTAGCAGCTAATAGTGATTGTCTTATAACTGCTACCACTTCCATTGCTGCTCAAAATCTTAGTGGTTTATTGAACAGAACCAAATCTGCGCAAGTGAAAACAATTTTTAAAACATTTGGTTTTAATAGTTCACATGTATCTATGAATGAAAGAATTAGTTGTTCAGTAACAACTTTAGATTCGATTGCGGATCAGCAAAAACATGATTTATCTACATATTGGAACGTCATCGCAGATATAGCGGAAAGAGCTTTGAATGCGGCAAATGGGAAAACCAAAGTAATACCTGATCTATGTGAAAGCAGTGTGATTGTGATTGATGAAGCAGGAGTGATCTTAAGGCACATTTTGCATACAGTTGTCTTTTTTTATTGGTTTTATAACGGTCTTCATAAAACACAGCTCTATAAAAATAGAGTTATTCCTTGTATTGTATGTGTTGGATCTCCTACACAAAGTGGGGCTTTAATTTCATCGTTTAATCCGTTAACACAAAATAAAGACGTAAAGAAAGGATTTGATATCTTATCCGCTTTAATATGTGACGACATTCTATCAAATTACTGTAAAATATCAGAAAATTGGGTGATTTTTGTTAATAATAAACGATGCACTGATGTGGAATTCGGCGAATTTTTAAAACATATAGAATTTGGTTTGCCATTGAAACCTGAATTGATTGAGTATGTTGATAGGTTTGTTAGACCGGCAACTTATATTAGAAATCCTACAAACGAAATTGGAATGACGCGTTTATTTTTATCACATTACGAAGTTAAGTCATATTTTAAAGTTTTACATGAGCAGGTCGAACTGACAAATAAAGATAATCTTTTTACTTTTCCTGTTTATTTCATAATTCAGAATAAAGCATTTGAAGATTACAAAAATGAAATTTCAAATTTTACTTTAGAGATTGAACCATGGTTTAAAACTAATTTACACAGATTAAATACTTATTCCCAATTCGCTGATCAAGATTTGTCTAAGACCATACAAATCGAAGAAATTGTATTAGATGATGGTTCGGTAGAGGAAACTTTGATAACGTGTCATTTGAAACATATAAAACATAGTTCTATTGGCGTTACTTCCAGAACAAAATCTTCAACTGTTGGGTTTTCAGGGACATATGAAAAATTTGTGGAGCTTCTGCAAAGTGATTTATTTATTGAAAAAACAGCATGCGAATATAGCGTGCACGCCTATTCCTTCTTGACAGGTTTAATGTATGGGGGTATGTATTCTTTCTGTTTATCCGAATTCACGACTTCTGAAGTAATGACAGAAATAAGAAAGATCAAATTGCCCAATATTGATTTTCTACAAACAATGACAGCTGAAGTTTCTTTGCAAACCTTCGATGAATCAGACGAATACTATGATCTACACATTGCACCTACAGATGAAGAAATGTTAGCTTCGGATCCGTGCCCAGATCCTTTTTTTTTAAAGTACAAGCAACTTCCATTAACGAATGTTCTAACATTTGAAGAAATCAGTTACCTTTATACAGTATTTAAAGAGATTTTCATTTCTAGATTTGCAATTCTACAAAGGCACAGTAAAGAAATGTTCGGCAAGAGTAATTTAATCACATATAATAGGAATAATGTTTCGAGCAAAAGATGTGGGGAGATATGTTCACATGTTAAAAGCTTCTACGGCATGTTAACATATGCTGTGCCTGCTAATAATTATACTTTGGAAGGATATACTTATGATAATGTGATTTTCTTAGGGACGGATAAAATGCTTCCTCCGATAATTTACAAAAGAGGTTTACCCAAAATTGTTATCAAGGATGAAATGGGCTTTATTTCGATCTTAGACAACAATGTATCAAAACTTACTGACACTGTCAATGGTAACAGTTTTCATATCTGTACAACCATAGATTACGCCATAGTTTCTAAAGTTGCAATGACTGTGACAAAAAGTCAAGGCTTATCTATACAAAGGGTTGCCTTAGATTTTGGAAATGATCCTAAAAATTTAAAGTTGAGTTCTATATATGTTGGTATGTCAAGAGTTGTTGATCCAAATAATTTAATTATGAATCTGAACCCTTTACGGTTGAACTATGAAAATGATAATATTATTGCTTCACATATTGTAAAGGCCTTAAAAAATAAAGATACTATGCTTATTTTTTAAATGCCAGAGTTTGTGTTGATTGACGTATTTTTTTAAACTAAGTTGAAATTAAGTTTTTCATGTATCGATGTGCTTTTTTCTGGTATGTTTTTAATGGTTGGCAATTGTAAAAATAAAGACATGAATCAATGATTTGGTATACACTATTTTATTGCGAAAATGATAAATACTCTGGGTCCGGAAGTTGAGGCTTTTAGAAATCTTTCATCTTCTGAATCTGCAAAGAAAAAAATGCTTAGAATCTTTTGCACATCTAGATAAAGTAGGTAAAAAATAAATATAAACAAAATTTACCGTCTGCGTAGTTATTTGTTCCAGTTGAATGAGGACTGCAGTATTCTTATGCCAGGTTTTTCAAAGACGCAGTCTAAAAGTAAAGGGAAAAACTTTTCCGGAGTATACAAATGTACTCCGGATTGCGCAGGCGTGTTTATTCTACATTTAAAATTGATGGCAGTTAGCGGTGTCAGGTGGTATGTATGGTGTCTATTTTAAGTATGTCAGAAATCAACTAAAGTTCCTTTTCCACACACTACTGAAGGTTGTATGAATATCACAATTCACAATTTTATAACAATCTAGAGTTGCTTTTTAAGGTATGGAAAAAGCCTATGAAATCATGCAAAATGCTGATAATTGTACATTCAGAAAGCTAAAACTACTTATTCTTAATATCTATGACTTAACCTTAAAAATCTATGACAAAACCTATTGATTATTCTTAACAGCTATTATTAAAATTAAAAGTGATTAACTATTCTTAAAAACTATGATTTTACCCCATTCCCCACCCCCACCCCCACCCTTCTTGATTATATCCCGGACCCCACTAAATGGATTGTCTTTGTATTATGTTGGTGTGTTGCCCACTACACACTGAAACATAGTACAAGGGCTAACTCTTAATGGTTACAAGTATGCAGCTCAAAATCGTAATGGTTCTGATCTTTCTTGTACATGTTCAAGTTTTCGGCGGTTAAGATGCTTTAGAGATAGTTCAAGATTTTAGATTCAAAACATCACTTATCTCCACAGATCATTTCCAAGCAATTGTACAGAAAGATAGAATGTCAATGAGAACAGCCGTTCTCATTGTCTTCACAAAGTGAGGTTCAGATCATGACATAGTAGCCGTAAATGGCTACTGTGGTTTACATTATAAATGAGAACAGCCGTTCCCATAGTTTTAAAATGAGGTTTAAAACATACAGTAGCCATTACATGGTTACTGTGGTCCAAAACGCGGTATCCGAATCGCAATCCACAATACACAGTCCCGTTGTTCAAAAAGTTATGCAGTTGGCGGTCTTAATCAGGTATGAGATGGTATGGCAATACACGTTGTAAAAATTCTGAGATTTGAAACAATGATGTCACTGTTGTAGGATATCCTGTAGTTTTTGATGGTTTTTTGGTTGATGACTTACACAAGATCAAAAGTTACTGGTAATCCTTAGTGTTAGATCTTACATGATATGACAAGAGAAAAAAAGCTATATATGGGAAGGGATAACCCATATCAAACAAGAAAAGTTAAAGAGAAGAAACTATGAGATGGGAGAACCTATAACAAATACACTTTTAAGATTGGGGTGGGTGAACAGGGCATAACTGTAGCAATAAAACCAGTGACCGACAGTTACATTCCTTCATAAAAACTAACGTGATAGCAAAAAAAATATACATAAATAAAGAAGATGGCTTTTCAAGTGATAATGGCAGTCTAGATACACTTATATTCGTGAGAGGTGTGTCAGTCCATATACAACTTTTCCTATTACCTCGTCTAAAAAAGGCAACCTAAAACTGGCTGTTTTACCTGGGATGATTTAAATATAGAATAAACATTCACCTACCTTGTTTTCTTCTCTGAAGAGTAGTAGTGTTGACTGCATTGTCTCCGTGTATCTTGATCCAATAGAGCTTTATCTAATAAATACAAAAAAGGAAAAATCCGTATATTCCATAACTTGTTAAGTACAATTTCCATAAAAATTGAAAAAATATATCGTTTAGATGTTATACAAAAGTTTATCGTTTTAACTAATCATTGTCTATTTTCAGACATCGATACAGAGTTTGATATTTACGAGTTTTTCGAAAGATTAAATTTCAATCTCACCTCTTTCGCTGTCTTAACAGTTCATGTAGATCGCGGGCTTTATAAACAAATGCTTTCCAGTTTTCCACGCCCTGGGGGATATGACGCAATTTCCTTCATCCGTGAACTTGTCGTGTTTGCGGTTAAACTCGCGGAAGTAAACCGTTATTTTTTCTCGAACGGTAAAATACAAAAACGGTTTATGCGAAACAATTTCAGGCAAATATCATTGCGACTTTTTAAAAACGATTTAAGTTATGAGGGTTTTTGTTGGCTACATTCCGGAAATCAATTTTCATGTCAGAAAGCATAAAAGGGAGATGCTTTTGTGTCAGGAAGCAACTTTATCAGTCAAGAACTGTGAAGTACATTCCTTGAGAAATCACTTAGACTCGTAGTAAGAAGTGAGTTTTTCAGTGAGTGGAAGCAGGTAAGTATAAAATTTCCCTATTCTATTTCAACCTGTACTCGACTCTGAATACGTTATTTCTTTTATTGACAGAATGATTGCGGAGGATAGAGAATATGGAACGTTTGAATCTGTAACCCAGGCTTATCAGCAGATCATTAGTCATACTTTACAGCTGAGACGATATGAATTTGAAACTGGGTGCATGATTATGTTTTCTGCTAATTCTGGAAAATGTGAGATGCTCTCTAATGGTTGGATTTCAATGATTTCATGGACTTCAGAAACCGATACGGCCGGCTCATTGACATTGGATATTTGTACTGAGGGAGGGCAGTGCAAAACTTACAGTGCCAGAGGTCATATATTATGTTCTAAAAACATCACTTCAATTTCTCAGAAAAACGAGGGAAAGGAAAAAGTTTTGACGATTTGTCATGACAATGGAAAATTGCATTTAACTTATATCACAGTTCTAAAAAGCGGCCTTGATTGTGATATCAAAGACCAAAAACTGGAGAAACTGTTTGAAAAAGAACATGCAGAACGAAAAAAGCAAGATGATGATTATAAAAAAAAAGCTCTTAAACAAAAAGATAAACGCCGTTCTGAGCAAAAAATTTTGGAAGACTGTGATAAAAAAGATGAAAAAAAAAGAATGGATGACACCGAAAAAAGAAAACTACAAGAGGACCGTCGAAACGAAAAACAAGACCTAAAAAAAAGAGTAGATGACACCGAAAAAAGAAAATTAGAAGATGACCGTCGAAACGAAAAACAAGACCTCGAAGGTTAGTTGCCTTTTTTCTAAATCACTAGTCCTAAATATGACTTGTTGAATTTTTGTAGTGTCTAAGGTTTTTACATAAACATGTTTTCTTAGATGCATCCAAAGAAAAAAGGATGAAAGTGCATCACGAAAAACGTCATGCGGAAGAACAAGCAAACGAAGAGGTTGCTTCTTCGAGTCAGTTATCAAGTAGAATACCAGAGGGTGCGTTATCGCCCACTATTTCTATTGATCTTCAGGAATATCAAGAATTTGAGGATTTTGACAAGCGCATTTGTGGGCAGGTGGGTGGAGTTTTGGGTTTATGATGCATTTTTATTGCAGTTTTTTATGTGGTTAAAGATGTTTCTTTCTTTTAGGGGAAGAATCAAGACGCTGTATGCAAGAAAGTCCAAAGTGATGAAAGTTTTTGTATAAATAAACCGTTAGAGCAGTTTAGAGAGAAACTAATAAAAATTACTCATGAAGCTGTACAACAGTCACTGTTACAATCGCGGGGAAAAAATGAGGATAATAAAAAAGATGTTACCCAAAATGTAAAATTCGCTGATGAAAATATGAATTTCGCAGGGGGTTCTAAATGTACTTCTAAAACTAAGCATATTGAAGATCAACAAATACAGTTTGGGGCACAAAATAGATTTGTTCCTATATGTGAAATAAAACCATTTATTGATGTCAACCTTTTAAAATCAAATGTAAGAGGTAGAAGGAGTACTCGTGGAAGAAGAAACTCAAACTCCGCTAGATTTTCGGCCTCTACTTTTGAACAACCAATTGAAACTTCTGAGATGCTGACGGTTTCGACTCGATCCAGAGGACGTTCCAGGGGAAGGCCAAGAGGCAGAGGCAGATCCAGAAACATGTCAATGAGACAAACTCCAAGAGAAGTTGAAGACATGTTACCGATTGTTTTGGACAGTGACAGTGACACGGAGACTTTAAGGCGTAATGAAGATTTATTGGCGTCTTCCATATTACAGACTTTATAAATCGTTTGTTATGTTCCAATCAATTGGACATTTGTTGTGGATTTGTAAATATTTATTTGCTTTTAAAAAATGACCACAACCGATAAAAGGTGTTTTTGATCCTTTTGTTCTCGGAGATGGATGTGCACATTCCAAAATCAAGTGTTTCTTTGAATCTATTAAAGACACGAGTTTGCGAGCCTGTGATCCCCACAACATAAAAACTAAGTTTTGCATTTTTTCTGATAGTTGGTTAATAATTCGGTTGCTCAAAACTTGCCATCCAATCGCTTCATGGGACATTGGTACTCCCCTTATAACTGTGAAAACAGTGTTGAGTAACAAAACGCCTTGGGCACACCAAGATTTTAAAGATCCGTGGGCTGGAGCTTTAAAATTCTCTATTGTTCTTTCAAGTTCTGCAAAAATGGTTTTTAAAGAATTCGGAGGGGAACATCCTCTCACAGTGCTGAAAGCTAATCCGTGACCTCTACCATCGGGATAAGGATCTTGGCCCACTATTATCACCTTAATCTCTTCAGGCGAACATAAATAACTCCAGCTGTGTACATTCTGAGGATCGGGGTAGATAATTAATCTTTCTCTGTCACGCTGAACCAATTTGTATACATTTTGTAATTGTACAATGTCAGAATCTGATAAGTTTAAAAACTTAAGCCACTTCACATTGATTTGAAACGTTTCATGTTGTTCTTCAAGAGACATTTTTATTGAGTTGTCATCTGAAATGTTTTCCAGCATCCACTGTAGTAATGCCATTATTGAATGGGAGAAGCAGTGTTCATGGGCTTTTTATTAATGTTGCCTGAACACGTGACACATAAGTTGAGCGATCTATAAAGCATGCGTCAGTAAGTTCTAATGAGCAAATTCGAAAACTCGCTTCGGAATGGAAGCACTTCATTATATTTTTCCATTTCTTGAATTAACGGGAAACTAAAGTTGTAATACCTAGAAAAATATCTCAAACAATTTAACAAAGCATCAAAAACATTAATTCCATGGTGATCAATTCTCACAATTCTCTGTGTGCGAGTCTCTTCATTTTCAAGGTAAATCAAAATTGACATGTTAAAATTTATCATATTTGATGGTATATTGAATTCAAATCCGATTATATTTTCTACAAAGATGTTGCCCGTGAATTTGAGTTTTTCATAAGAGTATGATTTACACACATTATCTCTACAGGTTAAAAGTGTGTTATCTGAAAGACATCTTTCAAAGTTATTTAAAAAGCCAAGCCAGTTGTGCCCTGATTCTGAATCTTTAATTAAGGAAAGTAAGGTGTGTAGTTGATTAGGATTATTATACAGTAGGTAAAATGTTTCTAAAAAATCTTCATCAATTAAAACTTGATCTCTCCTTGAGATATAGGGAGTATGATATAATAAGCGAGAAATTATATCCGATTTTGGTATAGGTGCCTGATCAATAGGTTCCAGAAGCGACAGAGTTTTTGATTGGGTACAATTTAGAATGTTTTTGATACATTCTTGTTCTAAATTAGAATAATAAGAGTTATTAAATAAAGCATATATTTGGTTTAGAATGGAGATAAGAAATATAAAAAAGATGTTAGTTTTCATGTTCCATAAAGTTGTATACAAAACACCAGGAACTTAAATATGGAAGACACATTTAAATGGAATCTAATAATTTACATACCGTTTTTGAAGTGAGCTTTCTAATTATTTCGTGATTATAGAAATTATCATTCTGTTCTTAAATACTCTGAAACAGAAAATCTGGAATTCTTAACAGTAGGTTTTCCTTACCTGTATTAGTAGAAGTTGTATTTTTAGAAGTCGAATCTGAGTCAAGATCTATAGAAAATATAAACTTTATATCATTCCAAAAGTTAAGCAATAAATATATAACGTAATTCGAGTCAATCAGAGTACATAAGTTTTAAAATATACTATGTCTTTAATAGTTTACCATAATCGTCCCAGTAGATGTATCTATTGAAAGTATTATAGATGATTAAATGCTCTTTAAGTAATAAAGGTTGTGTTAACTTTGAGACATTTAGTAAAATGTCAGCAGGGTAACGAATTGAAAATTGGACACAGAGATGAGCAGCACAAAATAGATCTTTTTCAGTTGGGGCATTTATGTACAGAGAGTAGACACCACTATTTTTCCTGTGTTTGCTGTTGACATTCACAGGCATCATAGCTTCTTTTCTAGTCAGTTCAGTATGTGGTACTGTTCCTCTGAGAAATCTGTTAATTGCTATGGCAGTTTTCTGTTCATGGAAAAAGGTCTCAGAAACGGTTTGTTCGCCTTTGTTAACAATCAGATTAGCTATTTCCATCAATTTTTCGTGAAGTGAGTTTACAAGTAATCCGTTACGTGAATAAACTTGAATTTGAACTACAGTCGAATTATAAAATCGAAGTAATTTATAAACTTCCGATAAGAAATTTTTCGGCGAACATAAACCGCATGGATGCATAAAATCACAATTAAAATCTGTATAATTTTTTATTTCAGGAGAGTGGATAGGTTTGGAATCGTTAAAATTGCTGTGAACAATACCCTTAGATTTTGTTGCAAAGTTTGTCGTTGAGAGATTTTCTTTTACAGCCTTAAATATTTTTCTCAATTTAGAATGTTTATAGATGGCATTCGTATATTTGTCCGCCTTAAGTAACATTTCTGATTTTGAAATGTTTATTTCAGAATTTATTTTTTCAGGAATTGTACATTTTATGGCTTTTTCATTAAACGGTAACGTTTTTGAAAAACAGTTACATGTATATCGAGCATTGGCTTTAGTACACATGCAGCTTTTAGAAGTGAGTTTACCTTTGGCCTTACAAACAGTTTTACGTTTTTTGCCCCTCATATTCAAGTACTGAAGCTTTCAAAATTTTCAATGTTGATGAGTTCCTAGTTTACACAGAAGGGTTGAAGCTATTATAACGAAAAGCAGGACACTGGAGATTATTAAAATTATCCACGCCAAATCGTTGTTAATTAACTCTGACTGTTTAAGAGAATCATCACTTAATATTGTATCATTATAGGGGTTTTCAAATGGTTGAATAATCGAGGTAACATTCTCGTCTATCTCTCCGCCGTAAGACAGACAAGTGATCGGTTTGCCAAAGAACGTATTGCTGCCTTGTCTTTTAAAAAGAAAGCTGAGTGTCGTTGTCTGCGAGCCGTTGTAATTTTCACGCACTGTTGGACTGTCCTGTCTATATATCTTATCTAAAAATAAAATGGCTACTAATGGCTTTGGATACGACGTCACAGAACATGTTACATCCAAAAAATTTTGCAGATGGCGATAGTACAAAGCAATTATTGGCTTCATTGAAAGATGCACACAACTCATAGTAGACAATCGAGTCTTGGCGAAGAATGCACATGTTAAGCAACCAGCATCGTTAATTGTTCGAGATTTAAATTTCAGCAATGCTGTGATATTGTTAGCATTGAAGTAAATAATGCTTTCTTTGTCACGATGTTTAAATTCATGAACTATTTTTTCACTCATGTTTTTCAGGTTTTTGGTATTTATGGTATGATTTAATATCAGTTCGTAATGTTTAAACCATATTACAGAGCTGAGATTTTTAGTTGTATCAACAGTACACTGAAATACAATTGTGTTTCCTAGTTCTACTCTATGATGCATTATTGGATATAATGCATATATGATTACAGAAAATCCAATCATCTTTATGAAAATTGCACCTAAGAAAGGCATTGTGTGAAATTGTCTGATTTTGGATTTTTCTGCATCTATATTATCTGGACACACCCTAAAAAACCACTATTTTTTCTCTTTTACTTTGATTTTTGTGGTTTTTTTTTTAAATGTGGACCCACCCATTCAAAAATAAGGCGTTTGGG

TTTACAAAGTGCTGTGTATTTTGAAAGAAAATCTCTACTTACAGGATTATAGATGGAAATCGGATTATCCCCATCGGTGATAGAGCTTAATCGCAGAACAATTTCTTTTGGTCTCTTTTTTGCATAATTGTTACAGATTTTAGCTGCCATGAAAAAATCTATTGGTGTTGCAGCGTATATTATAATTGTTTTGAGATCTGAATGTCTTGTATACAGAGTATGTGCACTATTACTAATGTTCTCCCACAATTCATGCGGTCCTTTTTTTTGTAATTCGATGAAATTCTTTATGGAATCTTTGTCATAATATCGCGGGGGGGCGTGGTTCATTTTAAAGGGTTCAGACATGGTGATATTAGGTCTTACCATTAGCTCTTTTTGCATGGCTTTTATGTCTTCGGCAACAGTTTTTGACCTGGTAAAATATATTATAAGCGCACCGGATTTAGAGCTTCCATTGCAGCACAAAGCATAAACCGATTCTTTCAATTTGGCTTCCGGAAACGGTTTTTTTGGAATTTGTTTATATTCAAAAGCGCGGATTGTTTTTCGAAACAGTTTTTCAAAATCTTTAGTGGGAACACGACCACGTTGTACTTTAAATTTAGCTGATTGCTTTTGCTCTTGTAATTTTACATACTCCCTGTAGGATAAATCATTATCAGATAGTTCTGAGTCAGATTTTTGGGTACCAGATATCCGCTTTTTGTAGAAAAGCTGCATGCTTGAGTCCGAATCATCAGAAAAACGTTTCCTCGCATTTCTGCGTTTATTTTTAAAAAATTTATTAGTACTTTTCTTTTGTTCATACTCATTCGGAATACTTTTGGCCCCATGAGTAAAATCTATATCTACACGCTTGCTTTTTTGTGTAGATCCAGAAGACCACGAATGATTAGAACAGCTTCTTTCCGATCTTTTTTTGCAATCTGATTCAGAAGACCTGCTTCTACTTCTTAGAGTTTCTGAATTCTTATTTTTACAGGATTGATAAGTGCTTTCAGAAATACTGTGTTCTGAATTTGATCTAGATATAGATATCAAATGTTTGCATTGTGTTTCCGACTGAGAATCTTGTTTATTGTTAGAACACACTTTACATGTTCTGGATTTAGATGCAGATCTAGATCTCGATGTATCAGAGCTGCTTCTAGATCTGTGTTTTTGGCGATGTCTTGTTTTAGTACTTTCTGAACATGATTCTGACTCAGTGTTTCTGTGTTCGTGCCGTCTCTTTTTAGAAGAGGAAAAGGATTGGGATTGTAAATCTGAATTGGTATCCGATTCTGACTCTGTGCTCGGTGGTCTCTTTTTCCTTATGTGAGGGCGAAATCTTGGATTATACAAATGTTCATTGTTTTGATCTAATTGAAGTCTAGATTCAACATGTTTATACAAATTTAGCTCTTTTCCACACCTGAATTCAAATTCTGACATATCAACTTTGCTAACAAAATCACACTGTCTATTGCCATTAGAATTAGGAGATTCTGTATATGAAGGTTTTAACTCTGTAGAGGAACAACTATTTCTACTTAATACAGAATTTTTAGAAGATGCTGAGCTAGATGACTTACAACTGCATTTACTGCTTGAACTGCTGCTTGAACTCGATGTATCCGTATCAGAGTCACTGGATGAACTTCGTGTAGATTTTGGACTCATAGATCTATCACTTATAGGAAACTTTTTTGTTTCAGAGGAGTTCTGTGTGCATTGTAATTCATTATCAAAGAATTCAAATAGCCCATTCTCTGTGTCTGATTTAGCTAAAAATTCTTGTTCAGGACTTACAAAATTTTGTGCTATTTCATCTGTGGTTTGAGAAATGATCTGTTCCATGTGTTGATTTTCTATGCTGTGTACATGTTCAACTTCCGACTGTGAATAATCATTTACTGGTGATTTATATTGTCCTTCCTCAGTACTACTTGAATCAGATCGTGTCCTTTGTGTAAGATTGTCGTTACACATGGAATCTGTTGTATTTGATTTTTCAGATTCTACAGGGGGATTATCGATAGAAGGTATATTACATGGTGCTGTGACTTTCCCCTCAAAATCGTTTTCCTTAGCATCTATTTTGGAATTAACAGTAGTTTTTGCTTGTCTAGGTCTACCTACACGATATGAATTTTTTCCTGTACATTTTGTATCAGAGAAAACAGTACTAGAATTACAATTTAAGGCAGTACTTACTGTACAGCGTGAAGATTGCTTAATGAATTCTATATCAGAAGCAGAAATGTCTTTGTCATTTTTTTCTAGGAGTCTTTTAAAATTAATCAACTTAGTTGGAGCTCGTTGATTTGATTCCTGTACGTCTGGTTTAGGAGTTAATGGCAGTGGAGAAAGCATTGGTTCTAGTTTTGATAATTTTCTGAGATTTTCTATAGCTTCATCTGGTGATTTGTCCTTTTTCTTTAAGTTTGAAGAACCTTTGTCCAAGTTGGGACAATGCTTAGAATCTGTAGTCGATGTGATTTGTTTTTCTTTTGATGTACTAGGTCTTGTTCTAGCTTTCATAAAAGTCTTTTCCTGTGAAGACTTGTTATATTTACATTTGTCTACATCTGAAGTCGAAGATAGACTGTCTTTAGCCAGGGGAACTATATCAGCATTATAAATTCTTTTAATTAGATCCATCTTATGAGAACTTGGAATATATGACAAGCTTACAACATCCTCATTTTGTGGTGTTTTGTCTTTGTCTGTCTTTTTCCTGGCAGGTTTTTCCAAATTGTTCACGTTTGAGATTAATTTGGTTTCCTTGTGATTTGTCTGAATTGGTGTATTTTGACTGTATTCACTTTTCTTTGATAATGTAAGACCTGATGAAATACATTCTTTTTGAAGGTTTTGACTATCTGTGATCATAGAGCTGCTTGGAATTTGTGAAAAAGCCATGCTGTAATCTCTGCTTTGAAAGCCATAGTTAGGACAAAGTTGTTGATAATTGTCTCTGTAAGGATTATGTCTAAAATCTGAATTGTATGAGTCATATGACTGATCTCGTTGCATATATTCCGGAAAGGTAAATTCATTTCTGTTGGAAAAGTCATGAAATTTAGCATAGCCACGTCCACGTCTGTTGTAAGGTCTGTGAAAATGTTTACCATAATGATTTTGGTATTTTGAGTTAGGGTTACAATAATTTCTACTGTCATATTTTCCTCTGTTGTTGCGACCCCTGTAATGATGTTGTCGATAGGTTGTATCGAACGAATTGTGACAAAGTTCTCTATTTTTATAGTCCAAATTGTTAAAAATATCAACTGAAGATTGGGGTTGATATGGTAAAGAGATAAAATTTTCTCCACTTGCTTGAGGATAATTATAATACAATGATTGCAGTCCTTGATGTGGCAATTCTGATGCAAATCGAAATTGTCCAGTTTCAGTTGTATTTGGAGAAGATTCAGAAGTAAAAGTACTTCTTGATTTAAGAGGAATAGCACGTCGCGGATCTTGAAGAGTTTTTGATGTGACTATTTCTTGGTTGTTAAAATTTGAATAAGTTATTTCATTTTCTGGTTTTGTATGATCGTGAATTTCATTATATAAACCCGAAAGAGTCATGGCTTCACGAACCACGTCAGCTGTAGTGCTCATGGGTGCATGCAATGGTCTTTTTTCTGAAAATGAAAAATAAAAATTGTTTATTTTAAAATGTTATTAAATTAGCGATAATACTAAGATGATATATACCTTTAAATAGAGTGGAAATGTACTTTTAATTGTCTAGAAAATATAACGCAATATGTTAGTTAAAAATGTAACTTGTTAGATTGATATTTCATGGGCCCTGAAACCTGCATATGAGCTAATGTGTGTACAAATTTGTGACAATAAATATTAAAGTGGAAACATTCCAAATGTAGCAATTTACATGTAAACGGATATGTAAATGTAATAAAGATGTGTATGGTTATGTCAAAAGAGCTAATTTGCAAACATGCAGTTATTAATTGTAATGGATGTACAAATGTTGAT

AATATTATATGTTGGTACATATCAATGTATTTATGTTGATAAGCAGATGTTAGTATGTATTAGTATCAATGGATTGTAGTATGCTACTGCATGCTATTAAACAAGTAGAGTGTTTACTTGTATTAGATTATATTTACATGACATTGTCATGTAAATATATAGTTGCATACATTTTTCATGTAACAACACATATGTGTTCATGTACATATTACATAATATATGCTAGTAAATGATTACATGCACTAGTAAATATTTGCACATGCTAATGTGTTCATGTGGGTATATGTACATATTACATAATATATGCTAGTAAATGATTACATGCACTAGCATATATTTGCACATACTAATGTGTTCATGTGGGT

GCTAGTAAATGATTACATGCACTAGTAAATATTTGCACATGCTAATACATAACAGTACATGTTAATAACTTTTGATCTATGCCGCTACATGTTAGTACATGCTATACTGATAATGCGTGGAAGTTTATTATTTGGATTTCTGTAAGGTCATGTGTATTCTATGAATGAGTATATAAATGGGTATGTTAATATATGTTAGTATGGTTGCAAGTAAAGATATGTTAATGTGTATTAGTAAGTACGTAATGTATGTGAATGTATTCAATTTTCAAAGAAATGATATAGAAATTTCTAAACATGTTATTGTGGAAAATGTAGATCTTGTGATCTATTATAATAATATAAACATAAATTAAATTTTTAAGTTATAGCTACCAATCTTTGCCTATTTTTCTTGGGGGGCATACTTTTTATATGATTTTATATGTACGGAAATATATGTACGTAGAATAAAGAATAAAATACTTGAAATCAAACCCTGTTATAAGTAAATTTACTTAGTATCATATATGTATAAATTTTGATAATTTATGAAGATGGCATAAATGTTGAAAATATTCATGTGTTTGTTATAAGAATTTCAATGTTACTAAAATAAAACCAATCACTAATTATCTTTTATATGCAAAAATGTGTCTTTGTATATATTCAAATTAAAATTTAAAACTTATGTTTTAATCATGTGAAATACAGATGTATGAATATGTAGCATGGAAACTTCAAAAATATAAATCATATAGACCTATTGCTTTAACTATATTTAAGATTTACATATTTAAATATTACATAATAAAAATTTTAATATTTAAATAAAAATATGTATTATAAATATGTTATTTACTAGTACAGCTTTATAAATTAAAGAACATATGTATTTAAAGTTTAAAAACTAATGAAATGTGCTTCTATGAGTTAATTGTAAATGTCAATTTTGTGATTTTAATATTTTTCTAAATAATGAGATTCAATGTTGTTAATTAGATGTGTGCAGTTTGAGAACATGTTTATATTTACAAAAATTTGTATATATTTAAAAATATGTTAATCAGCATAGCCTTATTTATAATGATTAATTTTAAATTCTATGTAATTAAATCTTATGGACAAAATAAATTTCAAACCATTTATTTTGAAAAACATAAATGCAGTTTAGAACTGATACAATTTTTTTTTTCAAAAGGGGTACATACTTAAAAGATTTTTTACAGTGGTTATAAAAGTATTAGAAAAGGAATTAAGATATATATGAAAAAATTTCCATCCTGCTTATGGAACTATAATATATCAAAGCAATTTATTTATGAAAATTCCAAGTCTATGAAAGTAAAGATACCCATTCAAGTGAAAGGCTATTATAGTTAGGAGGGCTAGCAATATAAGAAAACTTATATTCAATGTATTTTTTTTTAAGCTATGAAAGTAAAGATACCTATTCAAGTGAAAGGCTATTATAGTTAGGAGGGCTAGCAATATAAGAAAACTTATATTCAATGTATTTTTTAAGCTATGAGATAAATTTGATAGGTCTAAAACATTTTATTTATAAGAAAAAAATGAAAAATTTTATTTCATAGATACAATAACACAGATGAAAGTTACATTTATAATGTATCTATCCAAATTCTTATAATGTGTAAACAGTAAGTTAAACTAATATGTAAAATATTTAAGTAAAAAAAGTATAAAAAACTTAAGGTTTATTTAAAAGTCCATATTTTATAAAAGTTAATGAAAAAGAAACTGAATAGAAATAGATTGATAAGTCCATATGCAAAAACTACAATTTCAACAAAAGGAAAAGTGATGTTTAATAGATTTGAGCATTTTCCAAAAATGGATCATATCCCTGTGGATAACCTTCAAGAAAGTCAACATAAGTTTGATCAGAATCTGTGTTTTCTTTCCCATAATTTTGAGTGTCAACACTCTGTTCTAAAATAAATGAGTTCATCTCACTTACATTTTTATTTGCATATCTCTTTTTTAATTCTTCATAATGTCTATTAATACCATTTTTAATCATTTCTGTGATTTCCATAAAATTATCCATGATAATATTTGTTACCATGGTTTTAGTCTCTGAAATGCTGTCAACCTCAATAAAATTTTCTTGGAGTTTTGGAAATATATATGAGACATTTTCCCGCCTATATTCTTCATCATTACTTCTTATTGGAATAAAATGATTATTAAAACAATAACTTGCTTGTTTGGTCAGTTCTTTATTTTCATCTTTTTTCATGCCTGTGTCTGGAATGTTAACTTTATGAGTCTTACGTAAAAACCACAGGCAGTTTTTTTTAGGAACAATTTTAACTCTGACACCTCTTGTCACCCCCCGTATGGATCTGATAGGAATTGTGAAAACAACACTAGGGAATCCATTGTTTTCACAGTTATCAGAATTGTTAATTAGTTTTGATCTGTAAGGAATAGAATCTGTAGACATCTCTGTATTAAAATTACTCATGTTTTCATTGTCTGTATCAGTGCAAGAATTTAACTCCATATTATTGCAGCCCTCTTTAACAATTAGTTTTTTTTTTTTTGCTTTGCTAGTTTTCAACTTGCTTTCACTTTTTCTCTTTAATTGTTTTTTTTTAACAGCACTGAAATCATGTGCTGTAGTCTGTGAGTCACTGAAGGATGTTAGACTGACACTACTGAATGTAGAATTTGTATCAGTGCTGGAGCTCTCACTTTCATATATGGTTGATTTATCTGTATCATAACCATATGTTTCCATCTCAGAAATCACCAGATTTGTATTATCTGTATCTCTACTGTTATTATTGGAATTCTGCTCCAATAAAATCAATCCTTCAGCGGCCACTTTTTCCACATCACTTTCTGTGTCAACATCAGCTGTTGATCTTTCTACAGCTCTATTAGTTGAAATATCCTTTTTCTTATCTCTGATGTAAAAGGTTTCATTAGGAAATTTATCTGGAATAATTAAATCTTTTCCATCTTTATATTTGTTTTCATTTCTGTTAAGGTTATGAAAATTTACATTGGAGGTTTTATTGCCCTGACTAACACTCTGTTTATTTTTAATCATTTCTTCTTGCCCTGATAAAGACTTATCTGTTTTAATTTTAACATCTGTTTTGTCTTCAGACAGATTGTGACCTTTGTTCTGATTATCTAATGCATGATTTGTCATGGTAAAAAGTTCAAAGTACTGTTCTTTATCTTGGGTGTCCTTATTAAATATTGACCTTTCCATGAGTATTCCAGCTGGGCTATTTGTTTTTTTATCTGTTCTGGAAAAGGTAGAAGGAAATTCATTTGAAAATGTCAAAGAGTTATTGACGCAAATTTCCTCAGTTACTGACCTTTCCATGAGTATTCCAGCTTGGTTATCTGTTTTTTTATCTCTCCTGGAAAAGGTTGTATGAGGAAATTCATTTGAAAAGTTCAAAGAGTTCTTATCTTGATAAGTTTCCTCATTTCTTTTAAGGTTATACAAACTCATGTTAAAGGTTTCATTGTTCTTACTGACTTTCTGTTTGTTTTTAATAGTCTTTCTCTGTCCTGTTAGAATTTGATCTGCTTTAGTATCCCTGCAGCTTTTCATAGTAGCCTCTTTGAAAAAATCCTGTGTGGCTGCTGCTTCGGAAAGCCCCGTCTCATTTTTTATAGCATTCACTTCAGAATTTTTCTCAACTGAGATTATAGGATTATCAGATAAGAATCTGCAAATAGTATTCCTGGAAAAATCCTGTGTGTCTGTTCCTTCAGAAACATTTACCTCACTTTCTTCAGTACTCATTCCAGGATTTGTTTCAGCTGGATACATGAGACAGTCAGGTGAGTCTTTTGAAACAACCTTTTTGGCAAGATTCTGTGTATCTATCTCTTCAAAGAAATCTAATTTATATTCTCTGGTACAAACTTCAGGATTTTTTCCAGTTGTGTTTTTAGAGTGTGATTCATCTGATGCTTGTGAAACAGCCAGAGTTGTTTTAATGGTTTGGCATATGTCTGAAAAATTTGATTGAACATTGTACTGTTCAGACTTTGAAACACACTGTGTAGGACTGACATTCTGATGTACACTTTGGTAGGATTGTTCAGATGTTTCAGAACATTCCTCAGAATACTTTCCATAATCATTAAAATCTTCAATTTCTGGTGGAGTCGAGTCAGAATTATATAAGTGTGACTCAAGCAAAGTGGAAGAGTGATGTCCATTTCCTGTGACATATCCATTTCTAATGGATATTGAATTTAACACTGTAACTTCTCCTGTGACATCATATAGCTTTTCTATTTGATCACAAGCATCATTAATCATATGCATAAGTGTGATCTTAAATTCTTTTCTATTTGCCCTGAATTTTTTGCATGAAAATTCTTTAGAGCATATTACAATACACTGCAGTACAGCAGCTCCTAGGTTTTTTGCTGCCTCTATTAAATTTATACAATTGAAATGTATTTTAAGGAATGTTTCGTCACACATTGACATGAACATCTGATTCATCTGTTTGATTGCAAAGTCTTTCATAGTTTGAGCTATTCTGCTGTTTTTTTCAATTATTTCAAATTTACATTCCAACAAATCTTTTTTAATCTTATTTGTTTTTATGTAGAATGCCTGATTGGAAATGTTTATATCTGTTATGTCTCTGTTTTTTCCCATCATTAATAACATTGTTGCACATCTGTTAAGTTTCTCATTACTGCTTATAAAGTTGCCCATACTTATGATCTTTTTTATACATTTGATAAATTCTCTATCCATATTATTTCTCAAATCTGATAAATATTCTCTACAATCTGCAATCACTTTTTTAACAATTTCATCAAATTTATCACAGGCATATACAGTGTATCTTGATTCACACCAAAGAAAAAATTCTAATTGTTCTCCAGATAAGATATCTTCATAAAAAGATATAAATCCAGAGGAAGCACCACTAATTAAATTAACATTTAGCATCTGAGATAATTGTTCTAAAGCAAGATCTGTAAGCATGTGTCTGTCATTCGTTAAAATTCCAGCGTGGTTAATTAAACAATCTTCAATGTCTGCTTTTTCCTGATCCATGGCAGCATTCACGGATTTAAGAATACCTTGGATGCAGGCACTAATGGACTGAGCTAGAATTTAAAGAAAAAAGTCTAAATATGAAATCTGTACACATATATCATATTTTATATGTAAGAAAATGAAAAAAAAATACCTGCTGCCAGTTTAATATCCGAGGAAGCTCCTTCTTCAACATTTGTGGAATAGGAAGCATTTGCCGTTGCAATATCATTAGATGATATTGAGTCAAGGAAATCAAAAGAATCCAATTCTTGGAAGAGAGTATTATCAAAGGAAGGGTTGCATTTTAAACTGTCTTGCAATATGTCTCTGATGGTCTCTTCCACGGTGGTATCTGGGTGATATCTGTAATAAAATTTATAAATTTTATTACAGAGAAATTAAATCTGTCTTTTTTGTATTAAAATATTTAGGTTTACCTACTCAATATGTCTTTCGTCATTCGATGCCATGGTTTCAGATGGTTGTCCAACAATCAACATAGGTGAAGTAGCTCCACTTCTTTCCATAGTTTTATCTGTATGTTTAGCCACACCTACAATAACAAACCAATCAGAAATATTAACCCCATTCCAAACTCAATATAAGGCCAGCCTAAAAATACTTTCAAGCAGAGTTTTAAAACTCTGCTTGAAAAAAATGTATACTCTGGAATATGAAAAACGTGTATCAAGGCCAAAACTTACTTATTGGATCATTTTGGCAATATTATTTGTTTTTTTAATAATAACTGGATCTGTATTAATTGTAATTGAAACTCTGTCAATTCAAAGAACGACTTTGAATGCTCAAAATGATAAAACAAGTACTGTTGTTCCAGAATTAACATCTAATTCTCCAGGTTTGTTCAGCTATAAAATGTCATTGTTTAAAAAAAAAGTTTGTAACTCTGTTAATATGAATATTTTTTCTTGTAGATCAAACAACTGTGACAAATTTTTCTGCAAGTTCTAAACCAACTCTTAGCAGTAAACAACCCGGATGGATACAGGCACTAACTACAGCTTTTGGAATTTTAACACTGTTTTCAGTTATGATGATTATTATAACTTGTAATTTTTGGCTAACTGAGAAAAATGACAAAACTGCAAATCCAACAGAATACTACTCAGAAGACATATTAGATTATACAAATCCAAGTTTTACAGAAATTGATGAAGACAGCTCAAAAGTATAACATTGCTTCAGAAATAACAAAATTGTAAGTAAATTTACTTGATTTTCTTACACTATGAGATATTTAATGGTTTTAAAATAGAAAATGTCAGAGTTTAAAGTAAAATTAATGTAATTTATGATTTTTTAAATGTGTCTTACAACTAGAAAATAACTTTAAATTATTATTAGTATTGTAATAATTTAAAATTATTAATATTAGTATTGTAATAATTTAAAATTAATAATATTAGTATTATAAACAGTTTTTAAAATATAAAAAAAATAAATTTAAAATTTATATTAAATGATTTTAAATTTACTTACCAGATGGCAGCGATATATTGGAAATTCTGTGTAGCAGAACTGTATCTTCAAGCTCTTGAAGAAAAAGTGGCAAAATAGTTGATCTGTCTTCTTATTATATACATTTTGAAGCGGATGTGATGCACAGGAAAATGACGTTAATATATCAACCAATTAAAAAAAGCCAAAAAATTATAATACTAAAAAAATTAGGGTTTAACCTTATTTGTACATGTATTTTTTTAAAATATTATTTTTCCAATAAAAACCACAATGGATTTATATTTTACGTAAACATGATTTTTGCGGTTATTTTATTTCGAATTGGTATTTAAATCTTTGAAACAACAGGAATTGCGGTTGTCTTCTCAGCAAGTTCTACACATTCCCACTCGCTTTAGGCAGGAAAGACCCCAACCACATAACCCACTGACATTTAAACCCGTGAAAACAACAGGAACTGCGGTTGCCTTCTCAGCAGGTTTTACGGGTTCTCACCCGTTTAGGCAGGAAAGACCCCAACCACATAACCCACTAACATTTTAATATGTGAAAACAACAGGAACTGCGGTTGCCTTCTCAGCAGGTTTTACGGGTTCTCACCCGTTTAGGCAGGAAAGACCCCAACCACATAACCCACTGACATTTAAACCCGTGAAAACAACAGGAACTGCGGTTGCCTTCTCAGCAGGTTTTATGAGTTCTCACCCGTTTAGGCAGGAAAGACCCCAACCACATAACCCACTGACATTTAAACCCGTGAAAACAACAGGAACTGCGGTTGCCTTCTCAGCAGGTTTTATGGGTTCTCACCCGTTTAGGCAGGAAAGACCCCAACCACATAACCCACTGACATTTAAACCCGTGAAAACAACAGGAACTGCGGTTGTCTTCTCAGCAGGTTTTATGAGTTCTCACCCGTTTAGGCAGGAAAGACCCCAACCACATAACCCACTGACATTTAAACCCGTGAAAACAACAGGAACTGCGGTTGCCTTCTCAGCAGGTTTTATGGGTTCTCACCCGTTTAGGCAGGAAAGACCCCAACCACATAACCCACTGACATTTAAACCCGTGAAAACAACAGGAACTGCGGTTGTCTTCTCAGCAGGTTTTATGAGTTCTCACCCGTTTAGGCAGGAAAGACCCCAACCACATAACCCACTGACATTTAAACCCGTGAAAACAACAGGAACTGCGGTTGCCTTCTCAGCAGGTTTTATGAGTTCTCACCCGTTTAGGCAGGAAAGACCCCAACCACATAACCCACTGACATTTAAACCCGTGAAAACAACAGGAACTGCGGTTGCCTTCTCAGCAGGTTTTATGGGTTCTCACCCGTTTAGGCAGGAAAGACCCCAACCACATAACCCACTGACATTTAAACCCGTGAAAACAACAGGAACTGCGGTTGTCTTCTCAGCAGGTTTTATGAGTTCTCACCCGTTTAGGCAGGAAAGACCCCAACCACATAACCCACTGACATTTAAACCCGTGAAAACAACAGGAACTGCGGTTGCCTTCTCAGCAGGTTTTATGGGTTCTCACCCGTTTAGGCAGGAAAGACCCCAACCACATAATCCACTGACATTTAAACCCGTGAAAACAACAGGAACTGCGGTTGCCTTCTCAGCAGGTTTTATGGGTTCTCACCCGTTTAGGCAGGAAAGACCCGAACCACATAACCCACTGACATTTAAACCCGTGAAAACAACAGGAACTGCGGTTGCCTTCTCAGCAGGTTTTATGGGTTCTCATCCGTTTAGGCAGGAAAGACCCCGACCACATAACCCACTGACATTTAAACCCGTGAAAACAACAGGAACTGCGGTTGCCTTCTCAGCAGGTTTTATGGGTTCTCACCCGTTTAGGCAGGAAACACCCAGACCGCATAACCCACTGACATTTAAACCCGTGAAAACAACAGGAACTGCGGTTGACTTCTCAGCAGGTTTTATGGGTTCTCACCCGTTTAGGTAGGAAAGACCCAGACCGCATAACCCACTAACATTTTAATATGTGAAAATAAACAGGAACTGCGGTTATAGTTTCAGTACATTCTAGAGATTACATCCTGCTAAGGCGAAAAACACTTTAACCGCAAAAGCCACTGATTTTTAAACTTGTGAAAATAACAGGAAACCACAGAAACGTCAGGAAAAAAACGTTGTTTTATAATTATGTCCATAAAACATGCAACATAATCCAAACCGGCCAATGATTTTTCTGCTTAAATGTAATTTAAATAATTTATTCATACGTAAGTTGTTATAGCCACGCCTACGCAGCAATGTATATAAGCAGACACCGTCATTTCGCAGTTAGTCTGCTGGAAAGCTTGTGAAGACTACATAAAGGTAAAAATTTGACATTTGCAAATGTATTTTATAATTACATTATTTTAGTTTAAAAAATCCAAAATTCTTAATAGAAATTTATTGTGAAATTTAAAAGATTTTGTAAATATGCATTAATATCTATTAAATAATTGTTATTTGACAGATTTACTAAATGTAGTGTAAAATATCAAAAAAATTTAAAATTAATTTATTCTGAAGTTTAACATTTTTTAAATATGTATTTAATTATTACTGTTTCATCTATTTTTAGTTTCATTTGATACATTGCGTAGGTTTGGAAATGTGTTTCGAAAATTGCTGTAAAAATTATTGACTCTTTTTTAAATTTATAGAACATTGGATGTAATTGCACATTCCTGGAAAATTCAAAAAAAGAAAATACAGTAAGTCAATTTATATGTATTGATTTTAAATGGCCTCCAATAAATATTTTTAGCCAGTTTAATGTAAAACAAAGCATAAAGTTTATGTAGTTTTAAAATGATGCCATATTTTAACGAAATTCATTTAAAGACAGTATCTATTAAGAATTTAAATGCAGTTCTATGGCTTTAAAAATTTGTACGTTTTATTTGAAAAGTTTGCTCTATTTGAAATCAAATGTTTTAGATATTTCTTTTCATAAATTCAGTTGAAATATGTTTCATTTATAATAGGTATTTTTCTGTTTCTAAAAATAGATGATAAAAGTCACTAGATAGAAAATATCTTATAAATTGTTATTTAGTAGAATAAACATCTTTTTATATAACAAAAACACAAAGTACATGACTTTAGAAAGCCAACTAAATGCATTATTATTTAAAAATTACAGAATCAACGAGTAATGTCACTATAGTTTTTTAAAGTAACTTTTAATTGTTCACTTCTAGTTGTAATCAAAAGAAGGATGGAGAGTGGGGATAGTTTCGGAAATAATCACCAAGTCTCATCGAATTCAGATGCTTTTCAATTCCGGCAATATTCAACAACTGTAGATACTTTTGCATATTCATCAATGGATCCTTCAAATTGCATGCTTAATGAACAAACTCATTTAGAAACGGTACTAGTTTATCCTGTTTACAACATGTCTTCCCAGCAAACAGTGGAGAGAAATCTCTATGCCAATAATATAGGAAATGGAAATGTGCTGAGTTATTGTAGACAAGATAGTTTGCAAGGAAACCAAGGAACATATTTTCCAACATATCAGGGATTTCACTCAAACACTCCTCATAACTTTTCTATAGAGAATTTTAATTTTGGCACCAATGTCATCAGGCCTATACCATTTAGAAATGAGGGAGTAGACGCATCAACATCCAAAGATTATGGCTTTAAACCTTGCATTACAACGATGCAAATACAGTTTAGTCAGCCATCTGCTTTTCAAACTTATTCTTTAATGAATGGAAATTTTAGTGAAAATGGTTATGGATACAATACTAAGATTGGTGAAAATTGTGCTATGTCTTATGTTGAGCAAGAGTCTGTAGACTCAAAATATCCAAACAAAGAAGTTAGTATCTCTTATCAAAGAAATGAACATTATGATGAGAATCTCCCGACGAGAGAGCTTAATACAGATTTTCCGCAATATAATCAAAGTTCTGTGTTACAGCACACCTTTGCGAATGTCCAAAGCATTATTTCACAGCAAAGTCCATATCAGTTGATTGGAAAAGAAAACAGCTTTAACAACTATCTGGAAACAGCTAACGTTGATAGTGATTGTCAATCTAACGGGGGGCCTAAAATTGGCCTAGACACAGATCAATCTGTTTTTTCAGATGAAGTGACTGGGGCGTGTGTAGAAAATGTTCATTTTCCAATGAATCCTAAAAATGAGTATGTAAATAACATATCACTTGATGTACAAACTGAATTTCCTGTGACTGAGAATCCTCTTGTAGGAGAAAGTCAGGCTACAAAGAACAAGGATGTGGACAGTAACGCAGAGGTTAATAATCATTCTAAATATCGTCTATTGAAACGCAATATTACACCGACCATGGGTAATATTAAACATTTAAATTTTAGTTGCAAGATATCTACCGAAGAAGAAAGGAAAACATTTTTTAACAGGTTGAGTGAGCTATTGAAAATAAGGGATGATATAAAAAACACTAAAATTTCTTCAAAAGTGGATTTTATTCAATCTGAAGCTTCTACATCATTGGACATTTGTAAAAATACATTCAATAACAATTCGGATTCATCGGATGTGGACACTGATATCCTTGCAGACAACCCATTAGTGATATGTGAAAATGAACTGATTTGTGATAACAATGAGGAAAACATCAAGTTTCCGCCTAACGTTGAAAAAGAGGCTGTCCCCATGCAGACGGTTAAACGCAGCTTTCCAGAAATCTGTCCAGAGCATTTTAAAAAAAGAAGATTTATAAATGGTGATGTGATTTATGAAGATTTAAATTCTGTTTATAAAGTGATGCCAGCAAGTGCGACATATGACGATGTGCGTTTCGGAGAAGTGGATTATCAAACCTCTTCGGCTCAGACAAAAATTTCCAACCACCAGTTAGCGTTGTTGCCAACAAATTATCAGCACATGATAGGGCAGGAAACAGACATTTCATCGCGGGATTATCACAATGACAGTGCTCAAATTATTTACAATTGCTCATTTCAACGACAGGGAAAAAGGCTTCTTGCTGATATTCCATATAGACCTTGGTTAAAAGAAAATGTTCCCACAGGGGAATATGGAAAAAGATTTGTCAAAACAGTCCCTAGACCAGCCAGTGTTTTTATTTTTGGAAGATGTCGAAAAAAAATGCTGAGGGAAAGCGTTAAATCTTTTATGCATTTAAGCAAGTTACAGTATACTAAAGAAAGTCTAGAAGCATATGTACTTAGAAATTGTAACAAGTTTTTAGATTTGAGTTGGCCGATACGACACAAGATCTATATTATGCCTGATGTGAGTAGAAATTATAACATTGAAGAGGTTAAGAATTTATTTCCCGTACCGGAAGGCTGGATGGTAACTCTAGGTTTTGTAGGAACAGAAGAACCGGTGACCAAAATTTATAACATTGCTGTATTGTTATGTGAAAATGGGTGGGTCATGATTCACAAGAATGATAAAGTGGAACCTGAACTTTATTTAGCTGCTTCGAATCTCAATGAACTTATAGAGGATGGACTGGCACGCTGTGATTGTATTTACGAGAAAAGGTCTGTACCATATGGAATTGTCATGGAAGGAAAATTAAGACAGTTTATGGACAATTTTGGCTCACTGCAAAGTGTGCTAGCCTACAGAAAGTATTTACATGGTTTTCTGTGGGCTTTTAATGGCACACCTGGAAGATTGGCAGACAGAGTGTTCCATACTTGTGTACCTGGGGTTCATAATGCCCTTCCCTTAGATGCTGTGATTAAACATGAAAATAATCCCTTGTATTTCATTGGATATGTAACCACCTTTAAACAGCAAAATGATTTCAATGCTAATGTTTTCATCGCGGTAGATGGAAATTTAAGCATTTATGGCTATCATCTTATATCTCAAAAAACTTGGTTTCTAGCAAAAACATTTTCAACTTTTCTGAAAATGGGAACTAGAAAGATGTACTATGATTATGAGATTCCACTTAAAATTCATTTAGGAGATAGTGCAGATTCATTTCTGTCTTGTTTTAAAAATGCCCCTTGTCTGCTGCTTAAGCCAGAAGTACTTAGAAAGCAATTTCCTAAACCTTAGATTGTTAAAACTTGGATAACCAAACTATATTGAATTATGTATATCTTTTTATTGAATGGAACATGTTTGTAAACATTTTTGAAGCTTAAATAAAGAGTGTTTTCAAATATCTCTGTTTTGTCTCTTTACAGAGGACTTGAAAAATTTTTTACTGTCTTTAGAACTTGCGAATACTTGTTGAGCTTTTCTTTCCGGGTAACGCATCTCCACAGTGGTGCAGGTCGCTTCGTAAATATAATCAAAAAATTCTTCTACTTCTAGTAGTTTTGGTGTCGGATCTTCATTGGCGCCATAATTTGAATTAATTCTAGCAGCTTTTATTCCTTCCCAACTAAAAGAGAAAATGTTTAAATTACAGTAAATTGCAACTGGCATTCCTTGGAAAAATGATGCTCACCTGAAGGCTTTGTTTAAATCAATCCACGGTGGCATTGGTTGAGTCTTTGTAGGTTTGTTGTTAGGCGTAGGTTTAATTTCATGTATGATTGTTGTACTCGTATATATAGGAGTGCAGTATGTAATACCTCCGGCCTGATATATGCTGCGAACGAGAAATTAAGAAATGAGTTTGTTTCTAAGACGAAAAGTATTGTCCCATACAGTAAAAACTCACATGTTTCCGTCGTCACATGTCAGTTCTATCACATCACCGTCTGTAAATCGCAAGTATTCATCACAGCAGGATTCTAGTTTAGGTAATTGAAGTTCGCTAGCTAGAATTAAAAAAATTCTATACAATATACAGATTAATCTATATATGCACATCATGGACGCATTTTATGGCTTCGAGCTATACACTCTTTTGTTTTATTCATTTCTTTAATACTTTGTATTTTATAGAAACTAAATAAGAAGTTTTAATGAAATGCATTTATTAACCACCCATAACATTTTGTAACTTTTCGGATACAGTATTGTTAAAGGCTTGAGTTATATTTCCACTGTTACACAGTTGAGCATTTAAAGATTTGTTCGAGATGAGTTTTTTTGTTGTCCGGTCATCTTTTTTTAAGAAAGTCAATAACAGCGTATCAATAGTGAGTAGTCTTCCGTTGTCAAACTCTGTTTTTGCACTGTCGGTTTTAATTTTAATTGAGCTGGTATGAAAAAAAAACACATTAAGTTTTCTTGTTAGATTCTTATCCCCCCCCCCCCCGACAGGAGAGTTTTCAAATTACCAAGGTCCGAAATCTTTAGATGGTGTTAGTAGTACATCGTATTGGAGATCCGTTGTAGATATCTTAGATGGAAAGTCTTGCCACATAGCACTTACGATGGCTGGATCGAATTCTCTAGGATGAAGAAGCGAAAATTAGATTATATCTAGTTTTGCTTTCTAAGTTAATAATATTTACAATTTACCTGTTACTAAACGACTGTCGGTGCGGATGTGGAAACATGTCTTGTACAGTCTTTTGTCCAATAGTGTCGAATAAGCGTTCTATTTCTAGTAGTGCTATTGGAAAAAGACTGCGAAACACAATTTATTTGCTTATTCTCTCCAGTTTTTAAATTTCGAGTAATAAAAGACACTTGCTTTTCTTACATTACGGTGGTGACGTAGTTTCGTGCAGTTGTATCGTTTTTAGGCGAGCACCAAATTTGTCCATATGTAGACTGGAATCTAAAAAAAAAAATATTGTCAACTCAACCTTTTTGTCATTTTGTTGATTGCATGTATGTGCGGTTTTCCTCTTTTTTAGTGTGCTTGTTAATATGAGCAGTTTTGTGCGACAGTACTTACACTGTAGTGTTTGTTAAGCACCTTTCGTCCGTGGGTATCTGTCTAATAATGTCTATGTCTCTCCAGGGTTCGATGGTTGTCTCTGTCCAGGGATGTTCTGACCATCGCCACGCGTATGTCGTGTTTTCTCGACTTTTGTTCTTATCCGGGCAACAAGCGTCATACAATGGTTTTTCTGAAGTCATGTTCACTTTAGGTATGTTAAAATCTTCTTCTTTCCATTGTGAAAATTGCATGCAAGTTTCCCATTCGTAGCCCTTGTTGTCTTTTCGGACGATTGCCAGTGATATTTGTGTCCGAAAGGCTATTCCGCTGTAGCTACGCATCTTTTTAAAATCTGCGCGTATCTGTAATTCACTAATAAGCGGGTACTTTACATTCAAATTCTTGGAGAAAAAAATAACTATAGATAGTACCTTTTTTTGCTTACCATTGAGCAGACGACTGGGTGTATTGCTCACGTCTTTCCCAACGGTGGCGAGCAGGGAACAAAACCAGGCTCGTCAAAATGTTTGCGGTGTTCATTCTTTTCACATACAACAAACTGTTTTGTTTACTTCCGAATCCTCCATTAAGTAGAGAATAGTGTAAGCCTGTAGTTACTGACAAGACTTCAGACAGCCCTTTACTCATAATTTTTTCTCTTAATCCGCGCACTCCCAATGAGTTCTCTTTTAGTGGAATGCAGAGTGTTGTTTCTAATAATATAACCCTTAGAAGAATTAAAAGTAAGGTGTGAGAAATTTACTTAAAAAGCTCTTCAAAGTCGTCATATTGTAGAAATCAGTGTAACCATTTTTACTTACTGTGCTTCGTCTGGCCATTCACATTCGCAGGAAAACGATGTACATCTTGTGTATTCTAACAAGCTTTGTCTTTCCTCGGAACTTAGACCAATATATCTAGTAGAGCTTGCGTTGTGCATGTTTTCATTTATTAAGTTTTCGATTATATCTTCTAGAGTTATTGCCGTGCGTGCTTTTTCAAGTGGGACCGTATTGGTACATATCTTTATGAGTGATGCGAAAATTATATAATGCAGTTGCACCATTATCTGCGAAGAGATTTTTTTTGCAGCGTCTTTATGTGCAGGCTTATTCCCCCCCGTTTCGTATTTCAAATCCTAAATAACCCCCGGGGGGTAAAAAAAGGGGGGGAGCTCTCACTGTCACCCTAACACTAGCTCCAAGTCATCTGTTCTAGATCCTATCCATATCTGCCCTGACTCCTGGTTCCTTACCGCTCCGAGCCCCACCCTCCGTCCCGCCCTCCTCCTGTTCTCCATGCCCTGCCTTCTCAACCCTTCCTCTTCCACGCCCACATTGCCTCTGCACTCCGCGCTCTCTTGGCTGTGCGCCCTGCCTTTCCGTGACCTACTGGGAGCGCCGCCAAATCTGTTTTGCCCCGCCCCTGCGCGCGCGGGAACTGTCGGCGCCGCGCTGCTGCTAGCCCGCCTTCCAGAGCTCCCTCCCTCCGTCTGCCTCCTCACCCTTGCCACTCACCCTTCCATCTCTTCTATCACAGACTCTGTGTTACACCACCTATGACTGCTGCAACCACAGAACATTTTGCTCTCCGCGCGGCACTCAATCGTTACTGGTGGCTGCTTCTGGGACGACACAAGCTCAGTTTGGTATGCAACTACGTCACAGCTCATCGCCAACAGTTACTGCCGCTGCCGTGGCCCGAACAGGAATTTCTCCAACTTGACCCGGCCCCCTACTCCAATCTCCGCAACCGTGTCGCTCACCATCTCCATCGCGGCTGGCCAGCGGCACACAACACATGTAAGCTACCGTACATCTCTTTCACAAACCCAGGGCTCACATAGAGACAAGCACAAGCTCGCGCAATGACATTAAAACCTCCCATCATTGTCCTTTCCTGTCGCTTTGCCGATAACGTCTTTGCTCTATCGCAGGTTTCGACCCCCGTCCTTACTTCCCCAATGCTAAAGTCAAGCTGCTTCCGCTCGGCTCCATCACCCTTACCAGATCATTCTCCAGTGACGAGCCTCATCCTATTGGTGATGATGTGCATCACAGTCATGACCGGGGTGACTACCATACTGTTATCTGCAGCTGGCTCACAGGAACCTCCCCGATCCTAGTGCTGCTTCAAGGACCGGACGGCAGCATCTATTGCCACGACGTGTACCGCGGCCGATTGTATCTCGTGGCCCACTCTGTATCGTTGTTCGCCAGGCTAGGCCTTCGCCACTGCGAACCTTTATATGCGGCACCCAGATGGAAGCACGTTCCTCTGCCCAACATGTGGGTGGCGAGCCCGCCAGCGTCCGCCACCCTCACGCAAACACTCGCCGTGAGTGCCACGCACGGTCTGGACGCGTTATACTCGCTGCTAAAAATCCACAGAGGAACTCCGTGTTCGCTAATCCACCCCGTGAACGGCTACGTCCTGGACATGATACTGACGGGCCGCTCATTCCAAGAAGCACCCTGCCAAAACACTCGCACGTCCGTTAAAACAACGCCACATGTAATGGACGCAGTCTGCGGTGGCCGCGGGTCATGGCTGTCCATCGGCTACCTAGTAAAGATGCCGCACATTCACCTGGCGGTGACCCGAACATGTCTGGTCACCGCCATAGATGTCCGACAAAACTTTCTGTGGCGCGTGGCGGACGACGCGCTGCTATTCCTGGTCACCGGTAGTCTTTTACTACTGTCGCGGCCGACCGCAGACTTGACGTCTTGGTCATGTTTACAGCAAGAACCTGTGTGGAGGAACTGTCTAGATACGCGCGGAGAACAGGATGAGACAGAAGACCAAGAGATGAAACAAAGCACAAGCAAAAAGCAAAATGAGAATAAAAAACTCAACACCTCAAAAAAACACACCCGCGTATCGTCGGCAATTCCGACCTTTCCCCTCAGTCTCCGAGAAACGCCGCCAGAAGCCAGGAGCCCAGCCGTCCTCGCCGCCGCCACCCAGTCTCACAAAACTCGAGCGATCTCGACGCATAATGCCACGACAACAATAAGAATACCGCGCCTTCCCAGTTACCTGCTGGAAGCGCGTCTCTTGTCCGTGACAGCTATCCTGAAAGACACAAAGAAAAAAAAAACCCAGCCTCAGGCGTAGCAGCTGCGACGTCAGCGCGGTGTCTGAAAGCTCGCCAAGGTCTCGCGTAAAAGAACAGATGTGAACTTCAGATGTACCAACCAAATAATACGGGTTCCGCTATAAAAAGTGCACCTCTATTCCCGTTCTTATCCCCGTTCTAACTCTTCCTTGTATCATACCTTGCATGTTAACCGGATCCCGTGGATCTTACACACATACACACACACACAAACTTGGTGAGGTAAACACAGAGATCTCACTAACTCATAATCCCCTACACGCTTACCACCACCTAAAAATGGTTACAACCAAAACTGGCAATAGTCTATCTTCTTTTTCTTTCCATTCACAGCCAATGTGCAGTACTCGTGGGTCCACAACAACGAAAGAGACTGTAGAGACAGTTCCTTTAAGTAGACCTTAGAGACACACCAAATACAACCACAACCAAAAAAAAAACAGAAAACAACACAAAGCCAATGAGTGCAGAAATGCTCCGCGCTGTTCAGCTCCAGCCAAGACGCCGGGGACATTCCTCATCTCCCACTTCCCCTCCACTCGAAGGAGAGCCCAGTCCCAAGAGACTCCAATCGAGCGACAGTCACCAAGGGCGTAGAGGCAGACCTAAACCCAGAGCTAAAACATGGAGCGAAGCTTTATCCCACCGGTCCTTCCTCAACATTTACGCGTGGCTGTCTTTGAGTCGAGGGTCTCCGCGAAAAGTGTACGGATATGCCTTCAGGCACAGAGGAGAACTCGTAGCATTGCCATGGCCGCCTAACTGGAGCCTGGAACTTCACCACGATCCCTATCGAGACGCCAGAGCACAAACCGTTTGGAGTCACCGCTGGGGATGGCCTGCAACACACGTGACAGCTCGCACGGTGCGGGACTGCGGTGAGTGTAAGCAGTGTGACACATTGTTATCGCAATTGTCTTACCCGATTAACTTTTTATTAATGTATTAAGCACTCTTTCTTCACGTGTGACTGTTGTGTTTTTTGTTGTTATCTACATCCCGGCAGCCCTCGACACGCATATGTACGTGTGCTGCGGACGCGGAGAAAAGTTGCAGCCCGTCGGATACGTACGCAACAGAGCCGCGCCTTCAGACCTGAACTCGTTACGCGTCCTCCTCATAGCCAGGGACGGAGCAATGTATGTGCATCACATGAGAACGGCGCGACTGTGCCGCCTGGCCAGCAGTGTGACCGAATTCGCGCGACGAGGGCTGCAGCGAGAATCCGAGGTTTATGAAGATGATGTTTCCTTGCCAGACCGTCGAGTAGGTTCGGCAACGGCCATTCACCTGTTTGACGTAATTACCCAGGCAGCCGATGTCCACGACCTACTCACCGTGGCCGGACTGTGTCAGACTCACACCGGCGTCAGCTGCCAACTGTGGTATACAGACCACGATCCCCACACCGTCGCTGGGGCGGCACGCTTCACACTGACGGTCGCACGGCAGCAGTATCGATTGTGGCCAAACGCACGACGCAAACTGCTGCAGCACCTACATCCGGACCACCCACTTGGGCTGTGGCTGTTGTGTGCCGTGCTCACGTACGATGCAAAAGAGACGAATCGCGCAGTGCCACCCGTAACGCCAGGGGCCGAAACCGTGTGGGTGATAGTTACTGGCAGGGGTGCCATTCTAGGATTCTGGCCAGAGAGCGCCAAAATGTGCAGATTGGCCTCGTCTATGAAAGGACTCTGGAAAAACGGAGCGCGGGCGCTAAAAGGTCACTGGACATACGCAGCACCCGGCCGGCATAGAGCGGGAGAGGCCTGGCCTTTGTGTGCACACTACCAATCTCCTAGATAGAACAAAATTAAAAAGATTAAAAAAAAAAGAAAAAAAGTACAAGAGTGTTATCGCGAAACAGCGTGTCAAAAAAAAAACAATCCACATACTCTAGAACAAACTGTACCCAAAAATAAGTCCGTGTGCAAAACTGGGAAAAAAAAATCACCTTCCTCGTTGCCACTAGAGGGAGTACCGAAAGTGTAGGCAAGAAGGCCACGCTGTAAATGACTGTCAGCGTTTGGCGCTGAAAACATTGCTGTTCTTGCTGGCTCAAGCACAATCACGTGATTAAGATTCCTTTCGTTTTCAAAGTGTGCCCGGGAGGCAGACATGCCCTTTCTCGTGAGACATTATGAGATTTGCCTGCCAGAGAACCACGTGACTTGGACTTACTTTCGTTTTCTAAACGTGCCCTCTAGGCATGAATGCTCTTTAGCGTTAGCCATGAGGCTAGCGTGATCCTGTATAGTACATAAGTTTCTAAGAATATGTTTTTAACAATAATCATGTCCCAAAAAGTCGCGAGTGACTAAAATTCTCTGTAAATGAAGGCAAATTAAACAGGATACAGACAGTTGTGGCAGTGGTCCGTTTCGTCTTTCTGTGTTTTCCTTACGCGGCTGACGAGGTAAAGTGTCTCAGTCCATATTGTTGTCTGTGCCACCGTAGTTAGCGGTGGCATACTAAAAACTCCGATAGATGCAGAACAATAACACCGAAAACCACGCTGTGGAACCAGACCACACTTTATAAACAAAACGGCCTTATCACCTGGAAAAAAAACTAAAAATAAGGCAATGATACACCTGACTTTCCATTGGAAACCTGCCGTAACCCTGACCACAAATCCCATGCTAAATCCCCTGAAACACTGCCAAACGTCGCTACAAGGTTTTTCCGGGATCGAGCCGCAGCAAGCTTAAACTGAGGTCACACACGACTTTAATTACGGCAACGCACAGCTGTAAGCTGCAGGAAAGATACGATCGTAAGCAAATGTAGTCCTACAATCAAGCGAGGTTGTAGACGTTACCTACAATGAACTACACCTCTAAGCATAACCTGTCGGGCACAGTGAGACACGCAGCCGTAAATTCAAAACTCAACCCAAACCGAAGTCTAAGTCTCACCCTAATCGTAA

>HHV8_STRIPPED4

TACTAATTTTGAAAGGCGGGGTTCTGCCAGGCATAGTCTTTTTTTGTGGCGGCCCTTGTGTAAACCTGTCTTTCAGACCTTGTTGGACATCCCGTACAATCAAGATGTTCCTGTATGTTGTTTGCAGTCTGGCGGTTTGCTTTCGAGGACTATTAAGCCTTTCTCTGCAATCGTCTCCAAATCTCTGCCCTGGAGTGATTTCAACGCCTTACACGTTGACCTGTCCGTCTAATACATCCTTGCCAACATCCTGGTATTGCAACGATACTCGGCTTTTACGAGTGACGCAGGGAACATTGACTGTTGACACCCTTATCTGCAATTTTAGTTGTGTGGGACAATCTGGGCATCGATACAGCCTTTGGATTACATGGTATGCACAACCTGTCTTACAAACCTTCTGTGGACAACCATCAAACACAGTCACTTGTGGTCAGCATGTTACTTTGTATTGTTCTACCTCTGGAAATAATGTTACCGTTTGGCATCTACCAAACGGACAAAATGAAACCGTGTCACAAACTAAATACTATAATTTTACGCTCATGAACCAAACTGAGGGGTGTTATGCTTGTTCTAACGGGCTGTCGTCTCGCCTGTCAAATCGTCTATGTTTTTCGGCGCGTTGTGCCAATATAACTCCAGAAACTCATACTGTATCTGTCAGCAGTACTACAGGCTTTAGAACATTTGCAACCGCACCTACTCTATTTGTAATGAAAGAAGTAAAATCTACATATCTATATATTCAAGAGCATTTGCTTGTATTTATGACACTCGTAGCTCTGATAGGAACCATGTGTGGTATCTTAGGAACTATTATCTTTGCCCATTGTCAAAAACAACGTGACTCAAACAAAACAGTGCCACAACAATTGCAGGATTATTATTCCCTACACGATTTGTGCACGGAAGACTATACGCAACCAGTGGATTGGTACTGACATTCAGGTAAGATAATCTAAATATTCTCTATAACATAATTGTAATGTGTTTTATGTTTATAGCTACAAATGTTTTATGCAAAATACATTTTATGAGGTCGGATACTTATTAAAAGCATTGTCTTAAGTACATTAAAAGGACATTGTATAACCGTGCTACTTACAGCATGGCCTTTTTAAGACAAACACTGTGGATTTTATGGACATTTACCATGGTCATTGGCCAGGACAATGAAAAGTGTTCCCAAAAAACCTTAATTGGATATAGACTTAAAATGTCTCGTGACGGTGACATTGCAGTTGGAGAAACAGTGGAATTACGTTGTAGATCTGGATACACTACTTATGCCCGCAATATAACAGCAACATGTTTACAAGGTGGGACGTGGTCTGAACCAACGGCAACATGTAACAAAAAGTCCTGTCCAAACCCAGGTGAAATACAAAATGGAAAGGTTATATTTCATGGTGGACAAGATGCCTTAAAATATGGGGCAAACATTTCATATGTTTGTAATGAAGGATATTTTTTGGTTGGTCGAGAATACGTGCGATATTGTATGATTGGAGCATCTGGCCAAATGGCGTGGTCATCTTCTCCTCCTTTTTGTGAAAAAGAAAAGTGTCACAGACCGAAAATCGAAAATGGAGATTTTAAGCCTGATAAAGATTATTATGAGTATAATGATGCAGTTCATTTTGAATGTAATGAAGGATATACTCTAGTTGGACCACATTCCATTGCATGTGCAGTTAATAACACGTGGACATCTAACATGCCAACCTGTGAACTCGCAGGCTGTAAATTTCCATCGGTGACTCATGGTTATCCAATCCAAGGTTTTTCTCTTACTTATAAACATAAGCAAAGTGTTACTTTTGCATGCAATGATGGATTTGTTCTCAGAGGATCCCCCACAATTACGTGTAACGTTACTGAATGGGACCCACCACTTCCTAAGTGTGTTTTGGAAGATATAGATGATCCAAACAATTCAAATCCTGGACGTTTGCATCCAACACCCAATGAAAAACCAAATGGTAATGTCTTTCAACGCTCAAACTATACAGAACCTCCAACAAAGCCTGAAGACACCCATACAGCAGCTACTTGTGATACCAACTGTGAACAGCCACCTAAAATCCTGCCAACATCCGAAGGTTTTAATGAGACTACCACATCTAATACAATTACAAAACAATTAGAGGATGAGAAAACTACATCCCAGCCAAATACACATATTACATCTGCCTTAACATCCATGAAAGCGAAAGGTAACTTTACCAACAAGACCAATAACTCTACTGATCTACATATAGCGTCTACACCCACTTCCCAAGATGATGCTACGCCTTCAATACCTAGTGTACAGACACCCAATTATAATACTAACGCACCGACACGTACACTAACGTCTCTCCATATTGAAGAAGGCCCATCCAATTCTACTACTTCAGAAAAGGCCACTGCCTCTACTCTCTCACACAACTCACACAAAAATGACACCGGAGGCATATACACAACATTAAACAAAACAACACAGTTGCCATCCACTAATAAACCTACAAACAGTCAAGCCAAGAGTTCCACTAAGCCACGCGTTGAGACACACAATAAAACAACCAGTAATCCTGCCATTTCTTTAACAGATTCTGCAGATGTGCCTCAGAGACCGCGAGAACCAACACTCCCTCCCATTTTCAGGCCACCGGCGTCTAAAAATCGCTATCTGGAAAAGCAACTAGTTATTGGACTACTAACCGCTGTCGCCCTAACGTGTGGACTGATTACCTTATTTCACTATCTGTTCTTTCGTTAGCCTAGACTTGCTCCAGTGTTAGACAGGGCTATGATTGCTTCTCCACGCTGTCCACCTTAACACTTCCCAATAACAAATCCGGTATGCAGCAGCGTGACACTACTAATGTAACCTAAAAAATGTGCATGTGGTATGTATTGTACTAAAGATACCGACCAATACAAGACAACTAATATTAACCATAGTGTGCGTTTCTTTGTATAAAATACGCGTGTGGGAAAGCGACAGAAGGGGGCGGCGTTTCCATATGAGGCCAAGTGCATTGGCTATTTTAGGGGCGGTGACCACGCACTATAGTGCGCGGTGTGGCAGAAAATTCACACCGTATATAAACAAGGAAAGGGGACTCTGCGCGCTTAAGCGCCAAGCCATTATACACACGGGTTTTTTGTTGTCTTGGCCAATCGTGTCTCCATGGCGCTAAAGGGACCACAAACCCTCGAGGAAAATATTGGGTCTGCGGCCCCCACTGGTCCCTGCGGGTACCTCTATGCCTATGTGACACACAACTTCCCCATAGGGGAAGCCTCCCTGCTGGGCAATGGCTACCCGGAGGCAAAAGTATTTTCACTACCTCTTTTGCACGGGCTCACAGTGGAATCCGATTTCCCCTTAAATGTAAAGGCGGTGCACAAGAAAATCGATGCAACCACAGCTTCTGTGAAATTAACTTCATACCACAGGGAGGCCATCGTCTTTCATAATACTCACTTATTTCAGCCAATCTTTCAAGGAAAGGGACTGGAAAAGTTATGTCGAGAGAGCCGAGAGCTGTTTGGATTTTCAACGTTTGTTGAGCAACAACACAAAGGGACGCTCTGGAGCCCAGAGGCATGCCCTCAGCTACCCTGCGCGAATGAGATTTTTATGGCGGTCATAGTTACAGAGGGATTCAAGGAGAGACTGTACGGCGGCAAACTGGTGCCCGTGCCCTCTCAGACAACGCCCGTACACATTGGGGAACACCAGGCGTTCAAGATACCCTTGTATGACGAGGATCTGTTTGGTCCAAGTCGCGCCCAAGAACTATGTAGGTTTTACAACCCCGATATCAGTAGATACCTACATGACTCCATATTCACTGGAATAGCACAGGCTCTAAGGGTAAAGGACGTTAGCACGGTCATCCAAGCCTCAGAAAGGCAATTTGTGCACGACCAATACAAGATACCAAAGCTGGTCCAAGCCAAGGACTTCCCCCAGTGTGCTTCCAGGGGAACCGACGGGTCTACCCTAATGGTGATAGACAGTCTGGTGGCTGAACTTGGTATGAGTTATGGTCTGTCCTTTATTGAGGGACCCCAGGATAGCTGCGAGGTTCTAAATTATGACACGTGGCCCATCTTTGAAAACTGCGAGACGCCAGATGCCCGCCTTCGTGCACTAGAAGTTTGGCACGCAGAGCAGGCCTTGCATATTGGCGCCCAGCTGTTTGCGGCCAACTCTGTGCTCTACCTGACCAGAGTGGCAAAGCTGCCTCAGAAGAATCAGAGAGGAGACGCCAACATGTACAACTCATTCTACCTACAGCATGGCCTGGGATACCTCTCAGAGGCAACTGTAAAGGAAAATGGAGCCTCTGCCTTCAAGGGCGTGCCAGTGTCTGCACTGGATGGGTCATCTTACACCCTCCAGCACCTGGCCTACGCGTCCTCTTTCTCCCCACATCTCCTGGCAAGGATGTGTTACTATCTGCAGTTCTTGCCCCACCATAAAAACACCAACAGTCAGTCATACAATGTGGTGGACTACGTGGGCACCGCGGCACCTAGTCAAATGTGTGACCTGTGTCAGGGGCAATGTCCAGCTGTATGCATCAACACGCTGTTTTACAGGATGAAGGACAGGTTCCCACCTGTTCTGTCAAACGTTAAGAGAGACCCATATGTGATCACGGGCACAGCGGGAACGTACAATGACCTAGAGATTCTCGGAAACTTTGCCACCTTCAGGGAGAGAGAGGAGGAGGGGAATCCTGTGGAAGATGCTCCAAAGTATACATATTGGCAACTATGCCAGAATATAACCGAGAAGCTAGCGTCCATGGGCATCTCGGAGGGCGGCGATGCCCTAAGAACCCTCATTGTGGACATCCCCAGCTTCGTCAAAGTGTTCAAGGGGATAGACAGCACGGTAGAGGCAGAGCTCCTAAAGTTTATTAACTGCATGATCAAAAACAATTACAACTTCAGAGAGAACATCAAATCCGTCCATCACATCCTTCAGTTTGCATGCAACGTATACTGGCAGGCGCCGTGCCCGGTTTTTCTGACCCTTTACTACAAGTCACTGCTGACGGTCATACAGGACATATGTCTGACGTCATGTATGATGTACGAGCAGGACAACCCGGCCGTGGGAATTGTACCATCCGAGTGGCTTAAAATGCACTTTCAGACAATGTGGACCAACTTCAAGGGTGCCTGCTTCGACAAAGGAGCAATCACGGGCGGGGAACTAAAAATAGTCCACCAGTCCATGTTCTGTGACCTCTTTGACACCGACGCTGCCATAGGAGGGATGTTTGCACCCGCTCGGATGCAGGTCAGGATAGCCAGAGCAATGCTCATGGTTCCAAAAACCATAAAAATAAAAAACAGGATCATCTTTTCCAACTCCACCGGAGCAGAGTCGATCCAGGCAGGTTTTATGAAGCCGGCCAGCCAAAGGGATTCATACATCGTCGGAGGACCCTACATGAAATTCCTAAACGCCCTGCACAAAACACTTTTTCCTTCCACAAAAACTTCTGCCCTGTACTTGTGGCATAAGATTGGCCAGACCACAAAAAATCCCATACTACCAGGTGTCTCGGGGGAACACCTAACGGAGTTATGTAATTATGTAAAGGCAAGTAGCCAGGCTTTCGAAGAGATAAATGTTTTGGACCTTGTGCCAGACACCCTGACCTCATATGCGAAAATAAAACTAAACAGTTCCATTCTCCGGGCTTGCGGACAGACACAGTTTTATGCAACTACTCTCTCTTGCCTTTCGCCAGTGACTCAGCTGGTTCCGGCCGAGGAGTACCCCCACGTACTGGGGCCAGTGGGGTTGTCATCTCCAGATGAATACAGGGTAAAAGTCGCCGGCAGGTCTGTAACCATTGTACAGTCAACACTGAAGCAAGCTGTTTCCACCAACGGACGACTCCGGCCTATCATTACCGTGCCACTGGTGGTCAACAAATATACAGGGAGCAACGGGAACACAAACGTCTTTCACTGTGCAAACCTGGGATACTTCTCGGGGAGAGGGGTGGACAGAAATCTCAGGCCAGAAAGCGTCCCCTTTAAAAAGAATAATGTCAGCTCTATGCTAAGAAAACGCCACGTGATTATGACCCCCCTGGTAGACAGGCTGGTAAAGAGAATAGTTGGCATCAACTCTGGGGAATTCGAGGCAGAAGCGGTTAAGAGAAGTGTGCAGAATGTCCTGGAAGACAGAGATAACCCAAACCTGCCGAAGACAGTTGTATTAGAGTTGGTTAAGCACCTAGGGAGCTCCTGTGCAAGTCTCACAGAGGAGGACGTGATTTACTACCTGGGCCCTTATGCCGTACTTGGGGACGAGGTCCTGTCATTACTGAGCACAGTGGGCCAGGCGGGGGTGCCATGGACGGCCGAGGGTGTGGCCTCGGTCATCCAGGACATAATAGATGATTGCGAGTTACAGTTTGTGGGCCCAGAAGAGCCTTGCCTTATCCAAGGACAGTCGGTAGTGGAGGAGCTTTTTCCGTCCCCGGGCGTCCCAAGCCTGACAGTGGGTAAAAAACGAAAAATCGCATCCCTGCTCTCTGACCTGGATTTGTAGTTGTGTACCCGTAACGATGGCAAAGGAACTGGCGGCGGTCTATGCCGATGTGTCAGCCCTAGCCATGGACCTCTGTCTTCTTAGTTACGCAGACCCGGCAACACTGGACACTAAAAGTCTGGCCCTCACTACAGGGAAGTTTCAGAGCCTTCACGGCACACTACTCCCCCTCCTCAGACGACAAAACGCACACGAATGCTCAGGTCTGTCACTAGAATTGGAGCACCTTTTGGAAAACGTGGCTGATGCTCTGACCACGTTGGGAGTGTGCACTAGCAGAAAACTGTCTCCAGAAGAGCATTTTTCCCTCCTGCATTTGGACATAACATGCAACAAGCACCGGAGCGTTAGGTTTAATTTTTACGGAAATTGGGCCTTGGAGTTAAAGCTGTCACTAATAAACGACGTTGAAATTTTCTTTAAACGTCTTAGTAGCGTTTTTTATTGTATAGGATCGGGCAGTGCTCTGGAGGGTTTGGGGGAGGTATTGCGTTTCGTTGGGAAGCTGAGGGGTATCTCACCCGTACCTGGGCCGGACCTATATGTCTCAAATCTGCCCTGCCTAGAATGCCTTCAGGAAGTGTGTCTGACTCCCAACCAGGGCACCAGTCTGCAGGCCATGCTCCCAGACACGGCCTGCAGTCACATATGTACCCCCGCATGCGGTGAGCCTGTCCGGGGCCTCTTTGAGAACGAGCTAAAACAGCTCGGGCTTCAAACCCCTGAGTCCATACCTACTACCCCCTGTCAGTCCCGGGTAAGGCAAGATGATGAAATCAGACAGAGCTCTCTAATGGCGGTAGGAGATCACCACATTTTCGGAGAGGTGACCAGATCTGTCCTGGAAATCTCAAACCTGATCTATTGGAGCTCTGGCCACTCGGATGCCACCTGCGACGGAGACAGAGACTGCTCTCACCTGGCCTCGCTGTTTACTCACGAGGCTGACATGCATAAAAGGCGCGTCGACCTGGCCGGATGCTTGGGCGAACGCGGCACGCCCAAACACTTTTTTGACTGCTTTCGCCCAGACTCCCTAGAAACCCTTTTCTGTGGTGGTCTTTTTAGCTCCGTGGAGGACACCATAGAAAGTCTCCAAAAGGACTGCTCTTCTGCCTTCTACCAACAGGTAAACTACACTACTGCACTGCAAAAACAGAACGAGTTTTACGTCCGACTCAGCAAACTGCTGGCAGCTGGTCAGCTAAATTTGGGCAAATGTTCCACTGAAAGTTGCCCATCCGAGGCCCGTAGGCAGCTGGTAGGTGGGGACAAACCAGAGGAAGTGCTGAGGGATGCAAAACACCGGCAAGAACTATACCTTCAGAAAGTGGCACGCGACGGTTTTAAAAAACTCTCTGATTGTATAAGACACCAGGGCCACATCCTGTCTCAGACCCTGGGTCTAAGACTGTGGGGGTCTGTCATCTACAACGAGGCATCTGCCCTACAAAACCACTTTTTACACAGAGCACAGTTCATATCCCTCCCCTGGCAGGACCTGACGGTCGACTGTCCAACGCGGTTTGAAAATTCTAAATATATCAAAAATTCTCTGTACTGCCAGCGTCTGGGGCGGGAACACGTAGAGATCCTGACACTGGAGTTCTACAAACTTATCACGGGCCCGCTGTCAAAGCGACATACTTTATTTCCCAGTCCTCCAAATGTGACGCTGGCTCAGTGCTTCGAGGCTGCGGGCATGCTTCCCCATCAAAAGATGATGGTATCAGAGATGATCTGGCCCAGCATAGAGCCGAAGGACTGGATAGAGCCCAACTTCAACCAGTTCTATAGCTTTGAGAATCAAGACATAAACCATCTGCAAAAGAGAGCTTGGGAATATATCAGAGAGCTGGTATTATCGGTTTCTCTGTACAACAGAACTTGGGAGAGGGAGCTAAAAATACTTCTCACGCCTCAGGGCTCACCGGGGTTTAAGGAACCGAAACCCGCAGGACTCACAACGGGGCTGTACCTAACATTTGAGACATCTGCGCCCTTGGTGTTGGTGGATAAAAAATATGGCTGGATATTTAAAGACCTGTACGCCCTTCTGTACCACCACCTGCAATTGAGCAACCACAATGACTCCCAGGTCTAGATTGGCCACCCTGGGGACTGTCATCCTGTTGGTCTGCTTTTGCGCAGGCGCGGCGCACTCGAGGGGTGACACCTTTCAGACGTCCAGTTCCCCCACACCCCCAGGATCTTCCTCTAAGGCCCCCACCAAACCTGGTGAGGAAGCATCTGGTCCTAAGAGTGTGGACTTTTACCAGTTCAGAGTGTGTAGTGCATCGATCACCGGGGAGCTTTTTCGGTTCAACCTGGAGCAGACGTGCCCAGACACCAAAGACAAGTACCACCAAGAAGGAATTTTACTGGTGTACAAAAAAAACATAGTGCCTCATATCTTTAAGGTGCGGCGCTATAGGAAAATTGCCACCTCTGTCACGGTCTACAGGGGCTTGACAGAGTCCGCCATCACCAACAAGTATGAACTCCCGAGACCCGTGCCACTCTATGAGATAAGCCACATGGACAGCACCTATCAGTGCTTTAGTTCCATGAAGGTAAATGTCAACGGGGTAGAAAACACATTTACTGACAGAGACGATGTTAACACCACAGTATTCCTCCAACCAGTAGAGGGGCTTACGGATAACATTCAAAGGTACTTTAGCCAGCCGGTCATCTACGCGGAACCCGGCTGGTTTCCCGGCATATACAGAGTTAGGACCACCGTCAATTGCGAGATAGTGGACATGATAGCCAGGTCTGCTGAACCATACAATTACTTTGTCACGTCACTGGGTGACACGGTGGAAGTCTCCCCTTTTTGCTATAACGAATCCTCATGCAGCACAACCCCCAGCAACAAAAATGGCCTTAGCGTCCAAGTAGTTCTCAACCACACTGTGGTCACGTACTCTGACAGAGGAACCAGTCCCACTCCCCAAAACAGGATCTTTGTGGAAACGGGAGCGTACACGCTTTCGTGGGCCTCCGAGAGCAAGACCACGGCCGTGTGTCCGCTGGCACTGTGGAAAACCTTCCCGCGCTCCATCCAGACTACCCACGAGGACAGCTTCCACTTTGTGGCCAACGAGATCACGGCCACCTTCACGGCTCCTCTAACGCCAGTGGCCAACTTTACCGACACGTACTCTTGTCTGACCTCGGATATCAACACCACGCTAAACGCCAGCAAGGCCAAACTGGCGAGCACTCACGTCCCTAACGGGACGGTCCAGTACTTCCACACAACAGGCGGACTCTATTTGGTCTGGCAGCCCATGTCCGCGATTAACCTGACTCACGCTCAGGGCGACAGCGGGAACCCCACGTCATCGCCGCCCCCCTCCGCATCCCCCATGACCACCTCTGCCAGCCGCAGAAAGAGACGGTCAGCCAGTACCGCTGCTGCCGGCGGCGGGGGGTCCACGGACAACCTGTCTTACACGCAGCTGCAGTTTGCCTACGACAAACTGCGGGATGGCATTAATCAGGTGTTAGAAGAACTCTCCAGGGCATGGTGTCGCGAGCAGGTCAGGGACAACCTAATGTGGTACGAGCTCAGTAAAATCAACCCCACCAGCGTTATGACAGCCATCTACGGTCGACCTGTATCCGCCAAGTTCGTAGGAGACGCCATTTCCGTGACCGAGTGCATTAACGTGGACCAGAGCTCCGTAAACATCCACAAGAGCCTCAGAACCAATAGTAAGGACGTGTGTTACGCGCGCCCCCTGGTGACGTTTAAGTTTTTGAACAGTTCCAACCTATTCACCGGCCAGCTGGGCGCGCGCAATGAGATAATACTGACCAACAACCAGGTGGAAACCTGCAAAGACACCTGCGAACACTACTTCATCACCCGCAACGAGACTCTGGTGTATAAGGACTACGCGTACCTGCGTACTATAAACACCACTGACATATCCACCCTGAACACTTTTATCGCCCTGAATCTATCCTTTATTCAAAACATAGACTTCAAGGCCATCGAGCTGTACAGCAGTGCAGAGAAACGACTCGCGAGTAGCGTGTTTGACCTGGAGACGATGTTCAGGGAGTACAACTACTACACACATCGTCTCGCGGGTTTGCGCGAGGATCTGGACAACACCATAGATATGAACAAGGAGCGCTTCGTAAGGGACTTGTCGGAGATAGTGGCGGACCTGGGTGGCATCGGAAAAACGGTGGTGAACGTGGCCAGCAGCGTGGTCACTCTATGTGGCTCATTGGTTACCGGATTCATAAATTTTATTAAACACCCCCTAGGTGGCATGCTGATGATCATTATCGTTATAGCAATCATCCTGATCATTTTTATGCTCAGTCGCCGCACCAATACCATAGCCCAGGCGCCGGTGAAGATGATCTACCCCGACGTAGATCGCAGGGCACCTCCTAGCGGCGGAGCCCCAACACGGGAGGAAATCAAAAACATCCTGCTGGGAATGCACCAGCTACAACAAGAGGAGAGGCAGAAGGCGGATGATCTGAAAAAAAGTACACCCTCGGTGTTTCAGCGTACCGCAAACGGCCTTCGTCAGCGTCTGAGAGGATATAAACCTCTGACTCAATCGCTAGACATCAGTCCGGAAACGGGGGAGTGACAGTGGATTCGAGGTTATTGTTTGATGTAAATTTAGGAAACACGGCCCGCCTCTGAAGCACCACATACAGACTGCAGTTATCAACCCTACTCGTTGCACACAGACACAAATTACCGTCCGCAGATCATGGATTTTTTCAATCCATTTATCGACCCAACTCGCGGAGGCCCGAGAAACACTGTGAGGCAACCCACGCCGTCACAGTCGCCAACTGTCCCCTCGGAGACAAGAGTATGCAGGCTTATACCGGCCTGTTTCCAAACCCCGGGGCGACCCGGCGTGGTTGCCGTGGACACCACATTTCCACCCACCTACTTCCAGGGCCCCAAGCGGGGAGAAGTATTCGCGGGAGAGACTGGGTCTATCTGGAAAACAAGGCGCGGACAGGCACGCAATGCTCCTATGTCGCACCTCATATTCCACGTATACGACATCGTGGAGACCACCTACACGGCCGACCGCTGCGAGGACGTGCCATTTAGCTTCCAGACTGATATCATTCCCAGCGGCACCGTCCTCAAGCTGCTCGGCAGAACACTAGATGGCGCCAGTGTCTGCGTGAACGTTTTCAGGCAGCGCTGCTACTTCTACACACTAGCACCCCAGGGGGTAAACCTGACCCACGTCCTCCAGCAGGCCCTCCAGGCTGGCTTCGGTCGCGCATCCTGCGGCTTCTCCACCGAGCCGGTCAGAAAAAAAATCTTGCGCGCGTACGACACACAACAATATGCTGTGCAAAAAATAACCCTGTCATCCAGTCCGATGATGCGAACGCTTAGCGACCGCCTAACAACCTGTGGGTGCGAGGTGTTTGAGTCCAATGTGGACGCCATTAGGCGCTTCGTGCTGGACCACGGGTTCTCGACATTCGGGTGGTACGAGTGCAGCAACCCGGCCCCCCGCACCCAGGCCAGAGACTCTTGGACGGAACTGGAGTTTGACTGCAGCTGGGAGGACCTAAAGTTTATCCCGGAGAGGACGGAGTGGCCCCCATACACAATCCTATCCTTTGATATAGAATGTATGGGCGAGAAGGGTTTTCCCAACGCGACTCAAGACGAGGACATGATTATACAAATCTCGTGTGTTTTACACACAGTCGGCAACGATAAACCGTACACCCGCATGCTACTGGGCCTAGGGACATGCGACCCCCTTCCTGGGGTGGAGGTCTTTGAGTTTCCTTCGGAGTACGACATGCTGGCCGCCTTCCTCAGCATGCTCCGCGATTACAATGTGGAGTTTATAACGGGGTACAACATAGCAAACTTTGACCTTCCATACATCATAGCCCGGGCAACTCAGGTGTACGACTTCAAGCTGCAGGACTTCACCAAAATAAAAACTGGGTCCGTGTTTGAGGTCCACCAACCCAGAGGCGGTTCCGATGGGGGCAACTTCATGAGGTCCCAGTCAAAGGTCAAAATATCGGGGATCGTCCCCATAGACATGTACCAGGTTTGCAGGGAAAAGCTGAGTCTGTCAGACTACAAGCTGGACACAGTGGCTAAGCAATGCCTCGGTCGACAAAAAGATGACATCTCATACAAGGACATACCCCCGCTTTTTAAATCTGGACCTGATGGTCGCGCAAAGGTGGGAAACTACTGTGTTATTGACTCGGTCCTGGTTATGGATCTTCTGCTACGGTTTCAGACCCACGTTGAGATCTCGGAAATAGCCAAGCTGGCCAAGATCCCCACCCGTAGGGTACTGACGGACGGCCAACAGATCAGGGTATTTTCCTGCCTCTTGGAGGCTGCTGCCACGGAAGGTTACATTCTCCCCGTCCCAAAAGGAGACGCGGTTAGCGGGTATCAGGGGGCCACTGTAATAAGCCCCTCTCCGGGATTCTATGACGACCCCGTACTCGTGGTGGATTTTGCCAGCTTGTACCCCAGTATCATCCAAGCGCACAACTTGTGCTACTCCACACTGATACCCGGCGATTCGCTCCACCTGCACCCACACCTCTCCCCGGACGACTACGAAACCTTTGTCCTCAGCGGAGGTCCGGTCCACTTTGTAAAAAAACACAAAAGGGAGTCCCTTCTTGCCAAGCTTCTGACGGTATGGCTCGCGAAGAGAAAAGAAATAAGAAAGACCCTGGCATCATGCACGGACCCCGCACTGAAAACTATTCTAGACAAACAACAACTGGCCATCAAGGTTACCTGCAACGCCGTTTACGGCTTCACGGGCGTTGCCTCTGGCATACTGCCTTGCCTAAACATAGCGGAGACCGTGACACTACAAGGGCGAAAGATGCTGGAGAGATCTCAGGCCTTTGTAGAGGCCATCTCGCCGGAACGCCTAGCGGGTCTCCTGCGGAGGCCAGTAGACGTCTCACCCGACGCCCGATTCAAGGTCATATACGGCGACACTGACTCTCTTTTCATATGCTGCATGGGTTTCAACATGGACAGCGTGTCAGACTTCGCGGAGGAGCTAGCGTCAATCACCACCAACACGCTGTTTCGTAGCCCCATCAAGCTGGAGGCTGAAAAGATCTTCAAGTGCCTTCTGCTCCTGACTAAAAAGAGATACGTGGGGGTACTCAGTGACGACAAGGTTCTGATGAAGGGCGTAGACCTCATTAGGAAAACAGCCTGTCGTTTTGTCCAGGAAAAGAGCAGTCAGGTCCTGGACCTCATACTGCGGGAGCCGAGCGTCAAGGCCGCGGCCAAGCTTATTTCGGGGCAGGCGACAGACTGGGTGTACAGGGAAGGGCTCCCAGAGGGGTTCGTCAAGATAATTCAAGTGCTCAACGCGAGCCACCGGGAACTGTGCGAACGCAGCGTACCAGTAGACAAACTGACGTTTACCACCGAGCTAAGCCGCCCGCTGGCGGACTACAAGACGCAAAACCTCCCGCACCTGACCGTGTACCAAAAGCTACAAGCTAGACAGGAGGAGCTTCCCCAGATACACGACAGAATCCCCTACGTGTTCGTCGACGCCCCAGGTAGCCTGCGCTCCGAGCTGGCAGAGCACCCCGAGTACGTCAAGCAGCACGGACTGCGCGTGGCGGTGGACCTGTACTTCGACAAGCTGGTACACGCGGTAGCCAACATCATCCAATGCCTCTTCCAGAACAACACGTCGGCAACCGTAGCTATCTTGTATAACTTTTTAGACATTCCCGTGACTTTTCCCACGCCCTAGTGACTCAGACGCGGAAACAGCGCCTAGAAAGTTTCCTCTTGCGCTATGTGGGACAACTAGAGTCCAACCTGGCAAGCAGTGGAGCAAGACGCCAGACAGCCGATCTCGAAAAAAATAATGCAGACAGAGGCAACGTTCATCCTAGGTGACTGGGAGATAACGGTGTCTAACTGCCGGTTTACTTGCAGCAGCCTAACATGTGGCCCCCTTTACAGATCTAGCGGCGACTACACGCGGCTAAGAATCCCCTTCTCTCTGGATCGACTAATACGTGACCATGCCATCTTTGGGCTAGTGCCAAATATTGAGGATCTGTTAACCCATGGGTCATGCGTCGCCGTAGTGGCCGACGCAAACGCCACAGGCGGCAACGCGCGACGCATCGTCGCGCCTGGCGTGATAAACAATTTTTCAGAACCCATCGGCATTTGGGTACGCGGCCCTCCGCCGCAAACGCGCAAGGAAGCTATTAAGTTCTGCATATTTTTTGTCAGTCCCCTGCCCCCGCGGGAGATGACCACATATGTGTTCAAGGGCGGCGATTTGCCCCCCGGAGCAGAGGAACCCGAAACACTACACTCCGCCGAGGCACCCCTACCGTCGCGCGAGACGCTGGTAACTGGACAGCTGCGATCCACCTCGCCACGAACGTATACGGGATACTTTCACAGTCCTGTCCCGCTCTCTTTTTTGGACCTCCTGACATTCGAGTCCATGGGGTGTGAAAACGTGGAAGGTGACCCCGAGCCATTGACACCCAAGTACTTGACGTTCACGCAGACGGGAGAACGACTTTACAAAGTAACCGTTCACAACACCCATTCGACAGCATGCAAGAAGGCCCGTATTCGTTTCGTCTACAGACCGACGCCCTCCGCCCGTCAGCTTGTCATGGGTCAGGCTTCACCCCTCATAACAACCCCTCTGGGAGCCAGGGTATTCGCAGTCTATCCAGACTGTGAGAAAACTATCCCACCTCAGGAAACCACCACCCTGAGGATTCAATTGCTGTTCGAGCAGCATGGTGCCAACGCCGGAGAATGCGCCTTTGTCATCATGGGGCTCGCCCGTGAAACAAAGTTTGTCTCATTTCCCGCAGTACTCCTTCCGGGCAAGCACGAACACCTTACTGTATTCAACCCACAGACACATCCTCTGACCATTCAACGGGACACAATAGTGGGCGTGGCAATGGCTTGCTATATCCACCCCGGTAAGGCAGCCAGCCAGGCACCATACAGCTTCTACGACTGCAAAGAAGAGAGCTGGCACGTGGGGCTCTTCCAGATCAAACACGGACCTGGAGGGGTCTGTACACCACCTTGCCACGTAGCGATTAGGGCCGACCGCCACGAGGAACCCATGCAATCGTGACTGTCCGAGCACATATGGCGCAGGAGTCAGAGCAGTGCTCCCGTGCGTTTGCAGTATGCAGTAGTAAACGACAGCTCGGGCGCGGCGAGCCCGTATGGGATTCCGTCATTCACCCTAGCCACATCGTCATCTCTAATCGAGTACCCCTCTTACTAAGAGAACAGAACATATGTCTCCCTTCGTGCCCCAGCGTCGGCCAGATCCTCCACAGAGCCTACCCCAACTTTACATTCGACAACACGCACCGCAAGCAGCAAACGGAGACCTACACTGCATTCTACGCTTTTGGGGACCAAAATAACAAGGTTAGGATCTTGCCCACTGTTGTGGAAAGCTCCTCGAGCGTGCTGATTTTTAGACTACGCGCGTCGGTCTCTGCGAACATCGCCGTGGGAGGGCTCAAAATAATAATACTTGCTCTCACCCTGGTGCATGCCCAAGGAGTGTACCTGCGTTGCGGTAAGGACCTTTCTACACCACACTGCGCACCGGCTATTGTTCAGCGTGAGGTGCTGAGCAGCGGGTTTGAGCCGCAGTTTACCGTAACTGGCATTCCAGTGACATCCTCGAACTTAAACCAATGCTACTTTCTGGTAAGAAAGCCAAAAAGCCGGCTGGCAAAGCCGTTTGCACGCCTGTCCGCGGAGACGACTGAGGAGTGTCGCGTCAGGTCTATCCGCCTCGGGAAGACACACCTGCGGATATCGGTGACTGCGCCTGCGCAGGAAACGCCCGTCTGGGGGCTCGTGACCACGAGCTTCAGCCTTACCCCCACCGCACCGCTGGCCTTTGATCGTAACCCGTACAATCACGAGACATTTGCCTGTAACGCCAGGCACTACATCCCAGTAATCTACAGCGGACCAAAAATTACGCTGGCCCCGCGCGGCCGCCAGGTAGTCTGGCACAACAACAGCTACACGTCCTCCCTGCCATGCAAAGTCACAGCCATCGTGTCAAACCACTGCTGTAACTGTGACATATTTTTAGAGGACTCGGAATGGCGCCCAAACAAGCCAGCACCCCTGAAACTGGTGAACACGAGTGATCATCCCGTCATATTGGAGCCGGACACACACATTGGAAACGCCCTCTTCATCATCGCACCCAAGGCCCGAGGTTTACGCAGACTGACTCGCTTAACCACAAAAACCATTGAACTTCCTGGCGGGGTAAAGATAGACAGCAGGAAATTACAAACATTCAGAAAAATGTATGTTGCCACCGGACGCAGTTAGGTGTCCGGTTCCCACCCACACATTTGTCTTTATTGCTTTCAAATAAAACGGTGTTCTCTCAACCTCCTACGGGCTCACTAGTATTGTGTTCCCATACGCGCCTGTCGCCCCAGGATCAACACTTCGTCCCCTATCCACCCGAATACATAACACACACAAAGACATAGTGACTGTAGACAGTTAATCTTTATTGTCTAGACACGCAAAGTATATTAGTGTTATAAGAAATTTTATGTCACGTCGCTCTTTACTTATCGTGGACGTCAGGAGTCACGTCTGGGATAGAGTTCAAAACACGCACCGCTTGACCTGCAAACTTTTCCATTGCACTCAGAACATAAAACGAAGCAAAGTGTCTCACCCAATACTTAAGTCCCTGAAGCCTCCCTAATAGACCGCGGTCAAATTTGGGTGGACTGTAGTGCGTCTTAGTCAGCTTATTGAGCTCTTCCTGTATGTCCCATCCTAAGGTCTTCGTCAGAAGCTCCATGACGTCCACGTTTATCACTGATTTTCCAAACTCCGTCGTTAAAAACTTAAACAACACCTCGAATTCGAAAAAGCCATCGGCGAGCTTTTTAAGGCAGCTAGTCTCATTAAATCCTATTAACCCGCAGTGATCAGTATCGTTGATGGCTGGTAGTTTCAGATGAAAAATAGCAGCGGGCTCTAGAATACCCTTGCAGATGCCGGTACGGTAACAGAGGTCGCGGAAGCATTCATCGATCACCCATAGCATCCAATTGAGTCTCTGAATGAGAAGATCCTTTTCAAACTCGGGGGCGTCCGGCAACTTGCCCCGCGTTCCAGATACCAGCAGTGAACCGACCAGCAAGATAGACCACAACTTGAACCAGCGCATGGCTGCTAACGCGGCATACACTAGCCGGTGGTGCCCGAGCGGGAGTTACGAAGTCTCACTGAAGGGCGGGGTCGCGGGTCGGGGTCGCTCCAAATCAGGCAACGCCGTATCCGAACTCTGAGTCACTTTTATGTAGGTCTCAAACATGTAAAAGATACCACGTTCTTGAAAAACCCTCTCTTGCTCGCCAGGCTTGGGGTTCACGCGGGCATACGCAGCCAAGCTATCATGCGAGAGAAACACGTCACAGGCAAAGTCATGTAAAACCCGGGTTAAAAATAGCCTAACTGGCCAGGGGCCAGTGAGCGCCTCCCGGTACAAGTTCCCACCCCCGATGACCCAAACCTTGTCAATTTGCTGTGCTAGCTCTGGGCTTCTCGCCAACCCAAGCGCGGCATCGAGCGAACTCGCCAAAAAGTGAGCACCAGGGGGCGGGGTTTCTAACGTGCGACTTAGAACCACATTGATTCTACCCGCCAATGGTCGACAGCCCGCGGGAATCGAAAGCCATGTGCGCCGCCCCATAACAACCATGTTTTGTTTTCCAGGGGCACAGTCGGTAGTCAGCTGTCGAAAACGCCTCATGTCTCCCCGCAATGCAGGCCACGGGAGACATCTGTTTTTTCCGATCCCGAGTTTGGTATCAACCGCAACTACACAGTAAAGTGTAGGATCCATGCAGCGAGGGTATAGGTAAACACCACCAACCACACAGTGTGCTCTTATATACTTTTAATGAAACATAAGGGCAGACGAAACAGCCGAACGTTTCCTAATCACGCCCATGGAACCATAGCCACCCCCAAGCAAACCCTGTGGAAGGATATCAACTAGAGAGGAGGGTCCAGCCTTATTATGGCAGGAGACACTATAAGCCCCATCGCCCGACTGGGCACCAACATAACCGCCACAGTAAGTGGCCCTATACCGCTCAGCGCCCAAGTTGTTACAGTCACACCCAACCGCGGTTGGCTCTACATTGTCATCACGTCCATCATTATGTGTTGGTTCTCCCGCTTCCTTGTACCCTGCAGCTTCATCCACGGATTCTTCTGAGTCGCGATGCACAGGAGCGCCATCCGCGGGGCCATCTTGGTCGCCTGGAGCTGCCCCCGCGGGCCCCTCCTCGTCCTGGTTATCCCCACGGGGAAGAATTTCCTGAAGCTCGATCTCCTCTACCGCACACTCTGGTGATGTCGGCCGAGGTCTATATGGAAACACTTCAACCCGGGTGTTTACAGCAGCGTATGCCCGCCCCACGTGGCGCATCATGTGGAAAAACGCACCCAACCCAAAAACGACAAACAATTGGTAAAACACGAAAAAAACGTAGTACGCGGCTGCAGCGACGTGATCTATCTCTGGGTCATGACCGCCCACTATATATAGCCAAACCCACGTCGCAGCGGCAAGGCCAGCGGCCCCCAATGTCATGATGAAAATAAAAACAATCAGTTCCAGACCCTCCTGGTAAGTCAGCCGAGGCAATAGCGTCATTTCGCGCAAGGGTCGCCAGACCACGCGCGTGTTGTATACGACGCCACATATCTGACAGGCCGTGTTTCTAGAGATAGTGAGCCAGGTGCTTAAACAACTTCTATGGACGTTCTCGAGCTCTCCTGTGCATCCACAGGCTCTAAATCTCTCATTTCCGAGCTCCTCGTTGCAAATCCAGCAGACAGGAACATCCTCATCTTCCATATCCTGAGAGAGAACCCACAATAAAACATGGCATTAACCCCTGCAACAAGTGACCGTACCAGGGCACGCGTCCAGGCAACCGGGGTCCCCCTCGTTGGTCTATACAATTCCATGACTACCTACTGGTAATGCTACAGCCACTCACTGTACAAGCCGGTTAACTGGGAGGCGACGCTGGCGTGGTATCGGCAACTGAAACACACCACTCCACTCCAAACACTTATGTACTTTGTGGCTCGGCTTTATTGTAACAGCCAAGAGGGGCGTTTGTGGCTCAGCTTTATTGTAACAGCCAAGAGGGACGTATGTGGCTATCTCACAAAAAGTCACCGATTCATGTAGACAACCCGCTCCCACGAATTCGGTTTTTAAAAAGCCCTCACGTATACAGACGGGCCACTAAACACGCACATGAGCGGGCATCCTGTTTCCGCCTTGACGCCCACCACTCTGACCGCACGCTAAACATCGCCCTACCTGCTATACTGCCATTTCCATACGAATGGTAGGATGCGGGCAGTAGTCCACCAGTCTAAAATCATCAGGTGTAAACTCTTCCATGGAAGAAACAGACCGGAGTATCTCCAGGCGCGGAAAGGGACGTGGAGTGCGCGTCAGCTGCAGCCGTAGTGGCTCTATATGCGTTTT

CCTTCTGTGCGCCAGGCCGCGCCCGGCCAGGAACTCCCTGGAGCCATTTTTGTCCCATATCTTGACTCCTGTTCTTGAAAGCTCCCTGGAGTCAGTACTCCCCTTCAGAAACCAAAGCAGCTCTTGCACTACGCCTCGCCAAAACACCCGCTTTGTGGTTAGTAAGGGAAAGTGGTCCCGCAGACTATACCTGGCCTGCATGCCAAATAGAGAGAGGGTGCCTATGCCGGTGCGGTCGAGTCGATCGCTGCCACGGCACAAAATTTCCCTCAACTGCCTGAGATACTGAAGTTCCTCGTGGGGCGTCTCAGCCCCAGTTACCTCATGCTGAATCGAACAAGGGTCAACCTCGGGGGCCAAAGCCAAGACGCCAGGCTTTTGACAGAAGCGAAACCCCCTGGCACGGAATAACTTTTTGGCGACATACAAGCTTAAAGGTACAAACGGAAACATGATAGATCCTGGAAGTTTGTGAAGCCCTGTGCCCGGAGAGACACCCCTCAACTCGCAGTGCTCGGAGACCTACATGTATACTCAGGCTCTTCTATAAACCCTCCCCAAAAGTTTATAAAACACCGTACGTAATACACATTACTCACAGTTCCCACGGTGACGCCCAAACCCATGCACACGGGCGTGATCGATACCAGAAAACATCACAAGAACAAAAAGTGTGTGTCTGACATTCACATTTATTTTTACAAGACAATTTTGTGCAGTAGAGTTGTGCCTTCCGACACCCCGCGCCGTTCGCTGTTCTCCTGTAATTGGGAGATCCCACTCCTTGGCAGGCACGTTTCACGAAACGCTCTTGTCTCGCTGGCCTTAGACTTGTGGACCCAACATGGGTATCGTTAGAGATCCGTCGCGTAAATGCGCAGCTGGCAAAGCATTCTTCAGCGAGCAGTGACTGGTAATTGCTGCATCAGCTTCTTCACCCAGTCTTTCGATTTGTCGGCACACACCTGGCGACCACGCTTTGTCAAAAATATCACACCCGGCTTGCTGCACAGTTGGGAGGTGGGGTACCAGCTGGACAGAAGCACCTGTGGTAATGGTCTTTTCTGGTAACCGAGACAGCACTTGTCCGGTCTATGCCAGGACGCTCCCAGCGTGTCCCCAGATTGCAAACAAAGCAAGGCAGTCAGCACAGCGACGAGCAGGATGCCCTTGGTGTCCATAACTCCCCTCGTGTGTCCTCGTGTAAATGCGAAACGGCGATGTTAGGTCAGGCGCGGTAAACAGCTCAACTCGGTTCAAAACACGTACGTGATGTAGTGCTGGTTCTACGACGCCTACCTGTAAACTCCAGGATCCTGGGCTTTTATTACGAAGGCCAACACCCCAAAAAATCCACGCCCCCGTGACCGCAGGGGCGGTTACTAACGACGGTTACAGGTCCCTCCCGAGCCACGCACCTGCCATGTAACCTGCAAGGTAACCAGACAAACATCTAGGAAGCGTAAATATCCCCAGGTAGGAGAAGTATTGCATATGTCACAGACTCAACACACACGGGCCGTTACGCAACGGCTAGGGGCATAACCCTTTACCGGCGCGAAGCGCTACGCGCTTCGCGAGAGGTATCTCCGTGTGCTTCTCCATCAGAAGACGCGTGCGCCGCTTCGCAGGCGACCCGCATACTTTCCGCCCCGAGTGCGTTACAAAAATGACTGCCTTCTGGCGACAATACACGGTGGACGTCCAGTACCACCCGCATATCAGCTTATCCGGTGGCAATCTGGCACTGGACAGGGAATTCTCGCAACAATCCGAGGCCATGATGGTGGCAGGACCGCTGGCCGCACATAGCTCAATCACGGCCACCCAGAAGAGCAGCCCCAAATGTGCGCGCAACACCCAGCACATGCTCCACATACAGTTCTGGCGCCACAACGATGATGCGCAAAGGGGTGCATTACCCTAAATCCCAGCCTAGTTATAAATTATTGAAGCCCAGGCGACCAGGGGTCGCCGCGCTTTTCCTCCCCAAACGCGACGATAAAGACCAGCGTTGCCAAATGTAACTTATGTATAACCCAAAATATTGCGCATCGATAAGGTTTGCCAAAACACCCGAAAGTACACACACAAAAAAACAGCAACAAGACGCTCACTAGACATTCACCCCTTCCCCCACCCCCGAAAACAAAACAACTTGACACAGGGGAAACACCAGGGGCGGCGGAGGTTGTCAATAGTGTCCAGTATTTCGTTAGACGCGGGTTCTTGGACCCGATGTCCCAGGTCATTAAAGTCTCAAATGGGATTAAAGGATCATAGTTCCCAGGTTTAATACTCCAAGCTATCCCAGAACAGGACCCCGGCAGAACCCCGCTAACAGCACCAAATCCACTTGCGGTCCCAGAAAAGGTCGCCGAGGTGGCAAGGTGACTGAAAAGGTCATAGAGAGGACACCGGTCCCATTTCCCACGGTCCAAAAATCCAGCGCGCCCCACCGGCTTTCCGAGAACTTCGGCAAAGCTAATTTGCATGCGCTAATCCTTTTATGTGCATAAATTATGTAGATGAGGAGTCGCGCATGCGCAGAAAAATTCAGAGCGCCGGGTGCACGGGGTCACCTCCAGGTCACGCCGCTAGGTGGGACCGTGAGCGACTCGAAAAATTATAATTTTTGGCCATTTTATGGGCGCCGCCATCTTGAATTTGCTAATCCCCCGTAATCCTCTGCCCCTCTCCCATTGGTCCGCCGGCCCGTCAATCAAAGTTTTCCGAGCCGCCATTGGCCCATTCGGCCGACCAATCCCGTTCGAGCTAGGCGACCGCGCCATTCCATTGGACGCCCCAGCCGTCAATCAAATTCGGAGGCCTCCCATTGGCCCCTATCCCTAGAACTCCCAAGCTGATTGGCCCACAGCGGGAACCAATCAGCGATTAGAGTTTTGTTTTGATTTTTCCTATATATATATATATAATCCTTTAATCCTAGCGCAGCTGAGTCATCGCAGCCCCTATTCCAGTAGGTATACCCAGCTGGGTAATCCAGTAGGTATACCCAGGTGGGTGAACCCAGCTGGGTATACCCAGCTGCAATTCTATAATTAAACAAGGTAGAAACCAACGGGGTCCTCAGGTGGTATTTCCGGAAGCATTACCAAATAAGGCAACCTCAGCTGGGAATACCAGCGGACTACCCCCAACTGTATTCAACCCTCCTTTGTTTTCCGGAAGTATATCCATTTATGGAAATCAGCTGGGTCACTCTACTGGGTTATTCTTTATAATAGGGCCCGATGAGTCATGGGGTTGGGATTTTTCTACTAGGTCGTTTCGGTGGATGGGTGCCAGGATTATAGGGGCCCTGTCCACGGGGTTGTTCGGTGGCGGGGGGGGGGGGGGGGGCTAGTGAGTCACGGGCCTGGAATCTCGCCTCTGGGTGGTTTCGGTAGATGGGGGCCGGGAGGATGGGGCCCCGCCCACCGCTGGCGCGCCCCAGAACATGGGTGGCTAACGCCTACATGGGCAGCTTGTCCTACGGTTACGCCCATTTGAGACGGGTTAACCAACTGTTACACCCCTTCGCCGGGAACGCTATAAAAACGAGGGACAGCAGCCCCCCTCGCGCACTGCGCGCGCGGCGGCACGTGGGACGGATCTCTTGGATTTACCCGTAACGAGGAGCGGCGCACCCCAGGAGCCCCGGCAGCACCCCAGGAGCCCCGGCAGCACCCCAGGAGCCCCGGCAGCACCCCAGGAGCCCCGGCAGCACCCCAGGAGCCCCGGCAGCACCCCAGGAGCCCCGGCAGCACCCCAGGAGCCCCGGCAGCACCCCAGGAGCCCCGGCAGCACCCCAGGAGCCCCGGCAGCACCCCAGGAGCCCCGGCAGCACCCCAGGAGCCCCGGCAGCACCCCAGGAGCCCCGGCAGCACCCCAGGAGCCCCGGCAGCACCCCAGGAGCCCCGGCAGCACCCCAGGAGCCCCGGCAGCACCCCAGGAGCCCCGGCAGCACCCCAGGAGCCCCGGCAGCACCCCAGGAGCCCCGGCAGCACCCCAGGAGCCCCGGCAGCACCCCAGGAGCCCCGGCAGCACCCCAGGAGCCCCGGCAGCACCCCAGGAGCCCCGGCAGCACCCCAGGAGCCCCGGCAGCACCCCAGGAGCCCCGGCAGCACCCCAGGAGCCCCGGCAGCACCCCAGGAGCCCCGGCAGCACCCCAGGAGCCCCGGCAGCACCCCAGGAGCCCCGGCAGCACCCCAGGAGCCCCGGCGCGCCCCCCCCCCCCCCCCGGAGGGGGATCCCGGCGCGCCACCCTCCCCCGGAGGGGGATCCCGGCGCGCCACCCTCCCCCGGAGGGGGATCCCGGCGCGCCACCCTCCCCCGGAGGGGGATCCCGGCGCGCCACCCTCCCCCGGAGGGGGATCCCGGCGCGCCACCCTCCCCCGGAGGGGGATCCCGGCGCGCCACCCTCCCCCGGAGGGGGATCCCGGCGCGCCACCCTCCCCGGCAACAACCTGTTGCCATGTATGGCGATTTGTATCAGTCACAAGCACACAACCCCTGCTAGTATTAATGGTGTTTAAAACGTTCTACACGTACGGCGGACCGCATCCGTCGCAAGCACGCGCATATAACCCCCAAATGCACCATGATGAGAAGCACAGCCACGCGTCAAAAAACTTTAAAAACATCGTTATCCAATATCATTAAAAACCACACCGAAATTTACACAGGTAGCACGTCACCGTGTTAGTGTCACCCACTGTACACAAGGCGTGTCGTATATGTAGTATAGGTATTTGATGAGGCGGAAGCATATCCCGCTTCCAGCGAACGGAAATAAGAATCATCCATTCCAGCATTTATTCAAAGGGGGCACAGAGGATTCACATTGTTTAGAGAGAGTTTTTCTTAGTCACCATTCCATACTTGGGCAGTATTGGCCTACGATTTGGGCGACGTTTCAGGCTGGTCTATTCTCCGTCCACTTTTCCCCGGCTATTCTGTCCCAGCATAGGCTCTTGAAATAAACAATGTTTACCGAGTAAAAGGTTCCACTCACCCTCATTTGTCGTTGCACCCATCCCCCCTTTGCTTAATCACCCGAAAACTAGAGGACACGGATGGAAAACATATCGCACGCGGGTTGTTTGAAAGTCAACAGCTACTTGTTTTTAATGAGGACAGATTTGGGCACAGGCCAGAGGGTAAAGCCCTACGTGTGCGCGGGGGGGGGGGGGGGGGGGGTGTATACGCTGCGAAAACCTGCACGGTGCATAACACCCAGGGCGTCACGTCACATATCTCTGTGCACCCAAGTGGTTGTTCAACCGTTGTTTTTTGGATGATTTTTCCGCACCGGCTTTTTTGTGGGCGCGCATAGGTCGGTACGCGCTGTCCCCCTAAGTCCCGCACGGTCGTTCGGGCCCCCGTCCGGCTCGTCTCCGGATGAACCGTCACGTTCTTTGTCTCCAGAGGCGACGTCTCCTTCAGATGACTCGTCCGTGGGCTCCTCGTCCGTCCCGCCCGCGGGTCCGACAAGGACCGTCAATTCGATGTTATCTTCGTTCGCGGTTGGCCGGCGCGGCCGTCGGTATGGCAGTACGGTCACCCGGGTGTTATTTGCCGCGTATAATGCCCTCACAGTGCCACTTACGCGGCATATGCCGCCAAATGCAAACACAATAAATATTTGGTAAAACCCAAAGAAGCAGAGAAAACCGAGCACGGCCCCGGGGGAGAATGTTCCCGCAGGAGCAGTTAGGATGACCAGGAGCGTCCAGGTGCACAACGCCACGCCGACAAGCCCAGCCACCACCACAGACATCAGCAAAAACAGTTCAAAAATTTCTTGGCGCTCCATCTCCGGCCACAGGTTAAGGCGACTACGCCACTGCGTGCGCGTGCGGTATATAACGCGACACATTTGACAGGCCGTGTTTCGAGACACTGTTAGCCAAGTGCTTAAACACTGCGGGTGGACGACATCCAGCTCTCCGGTACAGGCGCAGGGGTGTATGCCCTCGTTCCCCACCTCTTCCCTACATATCCAGCAGATGGGTCCCTCTACACCCTCTTCTACGTCCTTGGACGCCATCTCTGCAGCTGGGGTGGAAGTCTGAAAAAGGGAAAGGGGAGGTGAGCAGAGTGCCCAGTTAGTCTCCGACCCGCCGTCCGCCCTACTGTCGCTATCCCGCCTTGACAGATGTCTAACGTATTCACGGACGCCACATGTGTGTCTATTTTCCTACATCCAGGCTTTCCCTGGAAAACTGTCACAACCCACCCTGCTTTAGCTCTACATCTGTATTTTTGTTTACGCACAGGATCAACGCTTCGTGCCCGTCCACCCCCGCGCTCTCCGCCTGTGTTTGGAGGTTTTATGAGTGGTTAGTTCTAGGCAGCTCCGGACAAGTTGTCCAAAACACGGCGCGCCCCGCCCTTCCTTCCCTCCGGATCCGCCCACACCGGACCTATGAAATAAGGGACACGCGTCATCACTAGTTATGAGAGAAAAACCACAACAGCTTTATTGGAAAACACCTGAGTGGATCCCCCACCCCCCGCGTACGACAGGCGTTTCTGTGGTGCGCTTCTGGGAAAAACGTTTTTCCCCCATTTCTTCCTCGACAGGTCTTCTAAGGTAGATAAATCCCCCCCCTTTGCGCGTCTCCTAGAATGGCCTAGGCGCACGATGGCGTTGTCGCCTCGAGCAGTTGGGCCGCAGTGATATCTTCAACTTTCGACCGTCTAAGCTATGGCAGGCAGCCGCTGCATCAGCTGCCTAACCCAGTTTTTGGAAGGGTCTGCGCAGATCTGACGCCCTCGCTTGGTCAGCAAAATAACTCCGGGTTTTGGGCACGCTGGGGACGTGGGATACCACTCTTTTAGAATTTGGACGGGCGGTGGGTGCTGCTGGAACCCGTAGCAGCAGCTATTAGGCGTGTACGACACGAGTGACCCCGCGCTTTCTGTGGGCGTCAGGTAAAACGTGGCAAGCAGTACGCTAACGCAGCATAAAACGTGGACGGGGGCCATCTGGAGGTGCCAAGTTCGCAACAGTCTAAAGAAAACCGTAAAGGCTATTTGGGGTTTCTGTTCTGTCAGATGTAACGCCGAGTTCCTTATATGCTTACCTGATTCTGGTCTCACCTGTTTATTTATAGTGGCGTATGCTAACCGCCAGCTTACATGCGGGATAAGTTGGCCTAACTCACCAAAAACGGGTTGCAGACAAAAGTGATTGTTGGGGCGCTTACTTAGAAGGTGTGAGGGTTTCTAAGAAACCCCGCCAACGCCCGGAAACCGCATGCGTTCCAGTCGGTGCGGCCTGCGCCGGCGTCGCTGTGGCGCCTTTGTGGGCTTTGAGTTCTGTCATTAAGCCAGGTTTCCATTGCCACCCGGGCGAAAACAAGCCGGGTAGTTTCAGGGGTCATCTGGCGATCAGTGTACCATATTCCCACGACCCATCAACACCGCTGCTTGAGGCGTGTCTCTGTATGTGTCACCGGAGACTGCATGTATCGTGCATATCTGTATTGTGCGCTTGCGCGGAGACAACATACCGACGACCAAGTCAGGGGTCACCTCCAGTGCACGCCGCTAGGTGGGACCGTGGGCGAGCCGAAATAATTATATATTTTTTTGGCACGGTTGTGAGCAACGCCATCGTGAGTTGGTTAATACCCTCTAAACGCATAGTCTTTTTTTATTTGTCAACCAACCAGTCAATCACCTGTCATCGCCGCTCAGAAGCACACGTCTTCGGCCAATGCCGTGTTGGCGGGTTTGACCACGGTTACTGATAGGTAGACGAGTCCGACAATCACACACGTCCGCCAGCGATTTGCAGCGCAGCTAAAATCGCGTGGCCGGGTTGGTAGAAGCAAATTATCCAATGGTCGTGTTTGGGTTTGTTTTGGGGTTATCTACATATTATATTCCTTATCCCGACTGGTTGCGGAAGTATTCGCAGCTTGGCTACTCTGCTCGATTACCCCGTGAATAACTGGGCGGGGGGTGACCCAACATAGTGATTCGGTAGATTTGGGGGACTGGATGAACATTAATGAAAGTTTATTAATGTTCATCCGTATTGTGTATATGTAATTTGGTTTCCATATTTGGTAGGAGTATGGAGTTTTCTTATGGATTATTAAGGGTCAGCTTGAAGGATGATGTTAATGACATAAAGGGGCGTGGCTTCCAAAAATGGGTGGCTAACCTGTCCAAAATATGGGAACACTGGAGATAAAAGGGGCCAGCTTGAGTCAGTTTAGCACTGGGACTGCCCAGTCACCTTGGCTGCCGCTTCACCTATGGATTTTGTGCTCGCTGCTTGCCTTCTTGCCGCTTCTGGTTTTCATTGGTGCCGCCGATTGTGGGTTGATTGCGTCGCTTTTGGCAATATACCCATCCTGGCTTTCGGCTAGGTTTTCCGTCCTACTTTTCCCACATTGGCCTGAGAGCTGTAGTACAAAAAACACCGCGCGGTCTGGAGCTCTCCATAAGCCCGCAGAACAAAAGCTGCGATTTGCCCAAAAACCTTGCCATGGCAACTATACAGTCACCCCTTGCGGGTTATTGCATTGGATTCAATCTCCAGGCCAGTTGTAGCCCCCTTTTATGATATGCGAGGATACTTAACGTGTCTGAATGTGGAATATAATGTGAAAGGAAAGCAGCGCCCACTGGTGTATCAGAACAGTGGTGCACTACCTATCTGCTCATTCGTTGTTTCGGTTCTGTGTTTGTCTGATTCTTAGATAGTGTTGAGGTAATTCTAGAAAGCGGATTGAGTGTAAATCGGGCCACTTTGCCCTAAATGTGACAATCTGGATGTGTATCTTATTGGTGCGTTGTGAAGCATTTTAAAATGCGTTTTAGATTGTATCAGGCTAGTGCTGTAATGGTGTGTTTATTTTTCCAGTGTAAGCAAGTCGATTTGAATGACATAGGCGACAAAGTGAGGTGGCATTTGTCAGAAGTTTCAAAGTCGTGTAAGAACATTGGACTAAAGTGGTGTGCGGCAGCTGGGAGCGCTCTTTCAATGTTAATGTTTTAATGTGTATGTTGTGTTGGAAGTTCCAGGCTAATATTTGATGTTTTGCTAGGTTGACTAACGATGTTTTCTTGTAGGTGAAAGCGTTGTGTAACAATGATAACGGTGTTTTGGCTGGGTTTTTCCTTGTTCGCACCGGACACCTCCAGTGACCAGACGGCAAGGTTTTTATCCCAGTGTATATTGGAAAAACATGTTATACTTTTGACAATTTAACGTGCCTAGAGCTCAAATTAAACTAATACCATAACGTAATGCAACTTACAACATAAATAAAGGTCAATGTTTAATCCATATTTCCTGACTTGTGTCTTGACTTGCGTCGATTGGGATGGGGGTGTGGGATGGGGGTGTGGGATGGGGGTGTGGGATGGGGGTGTGGGATGGGGGTGTGGGATGGGGGTGTGGGATGGGGGTGTGGGATGGGGGTGTGGGATGGGGGTGTGGGATGGGGGTAAATGACAATGGGGCGCTTGGTGACACATTTGCCCCACCGTCGCCTGCCCGGAACCAGCTTGGTGATGTGCTGTCTGGCTCTCAGGTGCACTTTATGCAAAGCAGTTGAGGCGCATTAGATATATAAAACTTGGGTACACACCCTTGGTGCTGTGCGCGTGCTATGTGCCCTGGTGACCGTCCACAATGGACGAGGACGTTTTGCCTGGAGAGGTGTTGGCCATTGAAGGGATATTCATGGCCTGTGGATTAAACGAACCTGAGTACCTGTACCATCCTTTGCTCAGCCCTATTAAGCTATACATCACAGGCTTAATGCGAGACAAGGAGTCTTTATTCGAGGCCATGTTGGCTAATGTGAGATTTCACAGCACCACCGGTATAAACCAGCTTGGGTTGAGCATGCTGCAGGTTAGCGGCGATGGAAACATGAACTGGGGGCGAGCCCTGGCTATACTGACCTTTGGCAGTTTTGTGGCCCAGAAGTTATCCAACGAACCTCACCTGCGAGACTTTGCTTTGGCCGTTTTACCTGTATATGCGTATGAAGCAATCGGACCCCAGTGGTTTCGCGCTCGCGGAGGCTGGCGAGGCCTGAAGGCGTATTGTACACAGGTGCTTACCAGAAGAAGGGGACGGAGAATGACAGCGCTATTGGGAAGCATTGCATTATTGGCCACTATATTGGCAGCGGTCGCGATGAGCAGGAGATAACGCGTAATTCGAGGTCCCCGGAAGAGTAGAGGGTTGCATGTTATACAAACAACATAAACATTAAATGAACATTGTTCAAAACGTATGTTTATTTTTTTTTTCAAACAGGGGAGTAGGGTAGGAAGGGTACGTCTAATACGTAACTGTTCGCTACTGCTTGTTCAGGAGCTCCTCGCAGAACATCTTGCGAATTTTAGATTTTGGACTAGAGCGACTGCTGGCTTCAACGCGGTTCGATGTAGGGTTCGGCGTAGGAGCGTCTTTCTCCACCGCCGCGCATGGTGTATGCGTGGTCTCCGGTGCCTGTTGTTGGATGCTCTGCGTGCTGGAGGCGGGGGTGGGTTCAGCGGGTGGTGCGCCAACTACCGCGAGTCCTGTAGAGACTGGCGGGTGGCTCACATGTGGCTGAGCAAAAAGGATGGGCGCCGCTTGCTGGAACTGACCGTGTGGCGCCTGCACGTAAATGGGTGGGTGTACGTAGGTTCCTCCGTGCTCCTTCATTGTCGGGAATTGACACGGGGCCGCTGAATTGGCGTGGGGCCTGTAGTGTGGATCTACTGCGGCTGCTGCTGCAGAGGAGGACGGCGGTGGCCCTGCGTGCCAACCGTTCAGTTTCATCTCTTTGAGTTCAGACTGTATTTCCGCTATGTTCTTTGACATGGACAAGATATCCTTGTGATACGCCGGCTCCTCTCCTGGAAAGAGGTGTCCTTCGTCGTCCTCTGCGCCGCGCTTGCGCTTCCCCGTCCTATATCCAGGCAGCTGTGGCGAGTAATACCATGGATCGTATGGGTTCTTGTAAGCGTAGCCGTATGGTGGCGCTGGGTTTGAAACATACGAAGGTAGGTGATGGTCGGTGGGGAACATCTGGCCCCCACACCCCATTAGGCCTGGCCCTGAAAGTGTATGTGACATTTTTGCCGCTGTGGTCTTCATTCCATCGATGCTGCTTTGTAGCATGCTCAGGAAGGCGGATTTGGGGATGGATATGATATCCTCTTGACCAGAGCTGTTCATGGCTGGTCTGGGTGGTGTGACGGCTTGGATGCCGACCGGGAATTGGCTGGCCTTTAAATACGCCGGGCTCAATATGCTGGCCACACCTCTGTCAGTTTTCAATAGGTCGAGGCGGTCCCGTATGAAGCTGGCATCTATAGCTTTTGCCATTAAGGTCTCCAGGGGACTGACGAAATTTGGTGTGGAAAGGTCCTCCAGCCTGCAGCTACTTACGTGCTGGAGGATGTGGGCGCGCTCCGACTTAGATACTGATGAGAATCTGGAAACCACCCACTCGGCGTCGTGTCCGTACACGGCCACTGTGCCGCGTCGGCGCCCCAGGGCGCATAGTGATACGTGTTGAAACACGGGACCGCTGGGAGTCTGGGATAACTCGCGGGGATGTATAGACGATAAAGACAGCCCCGGGAGCCACGTGTGGAGTATCTCCAACAGTGGTTCCTTAGGGAGATTTTTCACGGGGGCTCTGGCCACGTGGGAGGTGTCCGCCAGCCTGGATGCCAGCTCTAGGAAGGCTGGCGACGTGATGGCTCCGGTGCAGAAAATACCGTGGGACACTTGAAATAGACCCAGTGTCCAGCCCACTTCTGTCTCTGGTAGGTGTTCGATTGTTATTGGAAGGGGTTCTGTGACTGGGAGATAATCCGTCACCTGATCCGGATCGAGATAGAGCTCTTGCTCCAGCTTGGGGCAGGACACAACATCTACAAACCCTCCGACGTACAGGCCCTGTGCCATGCTCGGAAAATACGTGTGTGAGACCGAGCCGCTGAGCCCGGGGCTTAGGAGGCTCATGTGGCGCTTTTTGCAAAATAAGAATTTAAATACATTCCACGCCCAAGAGCTGCGTTTTATCCACTTGGTTCTCTGCAAGATGTACAATTTCGGTCTAAATGTGTACCTGTTAAGGGAGGCTACTGCCAATGCCGGGACCTACGACGAGGTGGTCCTGGGACGCAAGGTTCCTGCGGAGGTGTGGAAGCTCGTGTACGATGGGCTCGAGGAGATGGGCGTGTCCAGCGAGATGCTGCTGTGTGAGGCATACCGGGACAGCCTCTGGATGCACTTGAACGATAAGGTGGGGCTCTTGAGGGGCCTGGCGAATTATCTGTTTCACCGGCTAGGGGTCACCCACGACGTTCGCATCGCCCCGGAAAACCTGGTGGACGGAAACTTTTTGTTTAATCTGGGAAGTGTGCTCCCCTGCAGGCTGCTCCTTGCGGCGGGCTACTGTCTCGCCTTTTGGGGCAGCGATGAACACGAACGCTGGGTGCGCTTCTTCGCCCAGAAGCTTTTCATTTGCTACCTGATAGTCTCCGGGCGTCTTATGCCACAGAGGTCTCTGCTAGTTTGGGCCAGCGAAACGGGCTATCCCGGTCCGGTGGAGGCAGTCTGTCGCGACATCCGCTCCATGTACGGCATACGAACGTATGCGGTCTCGGGTTATCTTCCGGCTCCGTCTGAAGCGCAGCTGGCCTACCTTGGTGCGTTTAACAACAACGCGGTTTAAACGACCGCGAGGACCACCGGCAGGCAGCCAAGAACCATAAAGTACGCTCTATCGTAGTCATCGCCGCCGCCAAACTGGGACTTGATAATCTCCTGGAGGAGGGTGGGTGGGGATGGGTGTGAAAGCAGGACGTCCAGGCCCTCTTCTGTTGCCAGGCGGAGGGCTGTTCTCGCCTGGAGCAGCGCCAGTGGATCTCGGAATGTAAGCTGCTGGTTCAGGATTTCGAATATCTCATTAAACCTACTGCCTGTCAGATTTACAAATGGTCCGGGTTGTTTGTGGGACACGGTCGATCGCGCCTCGAGGGCGGCCAGTATTATGCCAGGGAAGATGAAGGACACGGGGGCGTTTGGATTAGCCTGCAGTGTGGGGATTATGTAGTGCTCCGATATGAACGAAAATAGTTGGCCCCTTTTCAGCATGGGGGCGTTTGGATCCGGTAGGGCACCGGGCTGAAATTTGGGTCCCAGCAGGGATACCAGGTTCAAGCGGCGGTTCGGGTGCCCTCGCGCGACTTGCCCAAACTCCAGCAATCCATACGCGAGGATAAACACCTCCAGCGCAACAATCCCCGCTCGCAGGTTCCACTGGTATGCGGAAAATGGTGGTATATCGGACCCAAACATGGCGCTCGTAATGGCGAATACCAAGTCCATGGCGGGCGCTGTCCCTGGCGCGCCCGTACCCTTGTTGTGGGGAAATAATCCAGCCTTAGCCATCATTGCGTGAAGCTTGTGGCGCTGGAAGAAGGCTGTCGGATAGCGGCTCTCCTTATTGAGAGGCGCCAGCGAGGCGCGCTCCTGGGGGTTTGAGTATGTGAAGCTGAAGTCCCCAGGACCGCTTTCCTGTTTTAGCTGAGTGATTAGCAGGTCTAGCTTTTGAGGCAGGTCTGCTAACAGGTCATCGGGAGTAGCGGGCAGTTGCCTGGATGTCTTTTGACAAAAGTACGCGTTGACGAGGCAAAGCGCGGCCTGGGTGTCCGTGAGATGCCTGGCGTCGGCGAAAAAGTCAGCGGTGGTCGAGGCGACCGTCGTCAGGGTGTGAGAGATGAGTTTGAGCGATGTGGAATTCTGAAAGTTAACAGTCCCCTTTAGTTCTTTAGGGAAGACGCGCCGCTGCATGGCGTTGTCCGTGAGGCTGATGAACCACGGCCCAAAGGATGGCAACCACTGATTCTGGTTCATGTACAGGGTGGGCATGAGCTCGCCGCGCAGGTCCCTGTCAACGGAGAAGTGAGGGTCCCCGGGGACGATCGCCACGGTGAAGTTACGGTGGCTGGCCTGCGGGGGGGATGTCACTAAGGGAGGCTCATGGGAACGGCTTTGGGGCATGTCTATGTTGTCAGACCATGTCATGTTGCCTATCATCTGTTTCACCGCGTCGATATCTGCGTTAATGACGCGGACGCGTGAGTCATGGACCTGAACAAGCCGGTCCAGCTCTAGGGAAAGCAGGTGTGCCTTTGTCTTTCGTTCTCGATTTCGCACGAGTTGGCTGCGCAGTCCAAGGGCGACCCTTCTTGTTTCTTCCATGGTGGGCTTGTGAATAAACAGCACGTTTTCCGGGTGTGGGGCCCAGAATCTTCCCGCCTCTGTCCATCTTCGGTTTTTTGGGTACCTTAGATAGGACCTTTCTGATGTCAGCATTTTCTCTAGCAGTGAGAAAGGCGCACAATTTTCCTTCGGTGGTGTGCACCGGCGTGGGAAACGCCCCGGGTGATTCAGAGTATACTGTCTTTAGTGTTTTCTGATTCTTAAATATCAGCAGGGGCGTGATAGTCCACGCCTCGGTACCCGGAGGGGCCGAGTGAGCGATGTAATGGATCGAGTCGGAGAGTTGGCACAGGCCTTGAGCTCGCTGTGACGTTCTCACGGTGTTGGTTGGGATCAGCTGGTGACTCAGACAAGTCTTGAGCTCTACAACGTAACATACGGGCTGATGCCCACCCGATACCAGAATTACGCAGTCGGCAATTCTGTGCCCTAGAGTCACCTCAAAGAATAATCTGTGGTGTCCAAGGGGAGGGTTCTGGGGCCGGCTACTTAGAAACCGCCATAGATCGGGCAGGGTGGAGTACTTGAGGAGCCGGCGGTAGGTGGCCAGGTGGGCCCGGTTACCTGCTCTTTTGCGTGCTGCTGGAAGCCTGCTCAGGGATTTCTTAACCTCGGCCTCGGTTGGACGTACCATGGCAGAAGGCGGTTTTGGAGCGGACTCGGTGGGGCGCGGCGGAGAAAAGGCCTCTGTGACTAGGGGAGGCAGGTGGGACTTGGGGAGCTCGGACGACGAATCAAGCACCTCCACAACCAGCACGGATATGGACGACCTCCCTGAGGAGAGGAAACCACTAACGGGAAAGTCTGTAAAAACCTCGTACATATACGACGTGCCCACCGTCCCGACCAGCAAGCCGTGGCATTTAATGCACGACAACTCCCTCTACGCAACGCCTAGGTTTCCGCCCAGACCTCTCATACGGCACCCTTCCGAAAAAGGCAGCATTTTTGCCAGTCGGTTGTCAGCGACTGACGACGACTCGGGAGACTACGCGCCAATGGATCGCTTCGCCTTCCAGAGCCCCAGGGTGTGTGGTCGCCCTCCCCTTCCGCCTCCAAATCACCCACCTCCGGCAACTAGGCCGGCAGACGCGTCAATGGGGGACGTGGGCTGGGCGGATCTGCAGGGACTCAAGAGGACCCCAAAGGGATTTTTAAAAACATCTACCAAGGGGGGCAGTCTCAAAGCCCGTGGACGCGATGTAGGTGACCGTCTCAGGGACGGCGGCTTTGCCTTTAGTCCTAGGGGCGTGAAATCTGCCATAGGGCAAAACATTAAATCATGGTTGGGGATCGGAGAATCATCGGCGACTGCTGTTCCCGTCACCACGCAGCTTATGGTACCGGTGCACCTCATTAGAACGCCTGTGACCGTGGACTACAGGAATGTTTATTTGCTTTACTTAGAGGGGGTAATGGGTGTGGGCAAATCAACGCTGGTCAACGCCGTGTGCGGGATCTTGCCCCAGGAGAGAGTGACAAGTTTTCCCGAGCCCATGGTGTACTGGACGAGGGCATTTACAGATTGTTACAAGGAAATTTCCCACCTGATGAAGTCTGGTAAGGCGGGAGACCCGCTGACGTCTGCCAAAATATACTCATGCCAAAACAAGTTTTCGCTCCCCTTCCGGACGAACGCCACCGCTATCCTGCGAATGATGCAGCCCTGGAACGTTGGGGGTGGGTCTGGGAGGGGCACTCACTGGTGCGTCTTTGATAGGCATCTCCTCTCCCCAGCAGTGGTGTTCCCTCTCATGCACCTGAAGCACGGCCGCCTATCTTTTGATCACTTCTTTCAATTACTTTCCATCTTTAGAGCCACAGAAGGCGACGTGGTCGCCATTCTCACCCTCTCCAGCGCCGAGTCGTTGCGGCGGGTCAGGGCGAGGGGAAGAAAGAACGACGGGACGGTGGAGCAAAACTACATCAGAGAATTGGCGTGGGCTTATCACGCCGTGTACTGTTCATGGATCATGTTGCAGTACATCACTGTGGAGCAGATGGTACAACTATGCGTACAAACCACAAATATTCCGGAAATCTGCTTCCGCAGCGTGCGCCTGGCACACAAGGAGGAAACTTTGAAAAACCTTCACGAGCAGAGCATGCTACCTATGATCACCGGTGTACTGGATCCCGTGAGACATCATCCCGTCGTGATCGAGCTTTGCTTTTGTTTCTTCACAGAGCTGAGAAAATTACAATTTATCGTAGCCGACGCGGATAAGTTCCACGACGACGTATGCGGCCTGTGGACCGAAATCTACAGGCAGATCCTGTCCAATCCGGCTATTAAACCCAGGGCCATCAACTGGCCAGCATTAGAGAGCCAGTCTAAAGCAGTTAATCACCTAGAGGAGACATGCAGGGTCTAGCCTTCTTGGCGGCCCTTGCATGCTGGCGATGCATATCGTTGACATGTGGAGCCACTGGCGCGTTGCCGACAACGGCGACGACAATAACCCGCTCCGCCACGCAGCTCATCAATGGGAGAACCAACCTCTCCATAGAACTGGAATTCAACGGCACTAGTTTTTTTCTAAATTGGCAAAATCTGTTGAATGTGATCACGGAGCCGGCCCTGACAGAGTTGTGGACCTCCGCCGAAGTCGCCGAGGACCTCAGGGTAACTCTGAAAAAGAGGCAAAGTCTTTTTTTCCCCAACAAGACAGTTGTGATCTCTGGAGACGGCCATCGCTATACGTGCGAGGTGCCGACGTCGTCGCAAACTTATAACATCACCAAGGGCTTTAACTATAGCGCTCTGCCCGGGCACCTTGGCGGATTTGGGATCAACGCGCGTCTGGTACTGGGTGATATCTTCGCATCAAAATGGTCGCTATTCGCGAGGGACACCCCAGAGTATCGGGTGTTTTACCCAATGAATGTCATGGCCGTCAAGTTTTCCATATCCATTGGCAACAACGAGTCCGGCGTAGCGCTCTATGGAGTGGTGTCGGAAGATTTCGTGGTCGTCACGCTCCACAACAGGTCCAAAGAGGCTAACGAGACGGCGTCCCATCTTCTGTTCGGTCTCCCGGATTCACTGCCATCTCTGAAGGGCCATGCCACCTATGATGAACTCACGTTCGCCCGAAACGCAAAATATGCGCTAGTGGCGATCCTGCCTAAAGATTCTTACCAGACACTCCTTACAGAGAATTACACTCGCATATTTCTGAACATGACGGAGTCGACGCCCCTCGAGTTCACGCGGACGATCCAGACCAGGATCGTATCAATCGAGGCCAGGCGCGCCTGCGCAGCTCAAGAGGCGGCGCCGGACATATTCTTGGTGTTGTTTCAGATGTTGGTGGCACACTTTCTTGTTGCGCGGGGCATTGCCGAGCACCGATTTGTGGAGGTGGACTGCGTGTGTCGGCAGTATGCGGAACTGTATTTTCTCCGCCGCATCTCGCGTCTGTGCATGCCCACGTTCACCACTGTCGGGTATAACCACACCACCCTTGGCGCTGTGGCCGCCACACAAATAGCTCGCGTGTCCGCCACGAAGTTGGCCAGTTTGCCCCGCTCTTCCCAGGAAACAGTGCTGGCCATGGTCCAGCTTGGCGCCCGTGATGGCGCCGTCCCTTCCTCCATTCTGGAGGGCATTGCTATGGTCGTCGAACATATGTATACCGCCTACACTTATGTGTACACACTCGGCGATACTGAAAGAAAATTAATGTTGGACATACACACGGTCCTCACCGACAGCTGCCCGCCCAAAGACTCCGGAGTATCAGAAAAGCTACTGAGAACATATTTGATGTTCACATCAATGTGTACCAACATAGAGCTGGGCGAAATGATCGCCCGCTTTTCCAAACCGGACAGCCTTAACATCTATAGGGCATTCTCCCCCTGCTTTCTAGGACTAAGGTACGATTTGCATCCAGCCAAGTTGCGCGCCGAGGCGCCGCAGTCGTCCGCTCTGACGCGGACTGCCGTTGCCAGAGGAACATCGGGATTCGCAGAATTGCTCCACGCGCTGCACCTCGATAGCTTAAATTTAATTCCGGCGATTAACTGTTCAAAGATTACAGCCGACAAGATAATAGCTACGGTACCCTTGCCTCACGTCACGTATATCATCAGTTCCGAAGCACTCTCGAACGCTGTTGTCTACGAGGTGTCGGAGATCTTCCTCAAGAGTGCCATGTTTATATCTGCTATCAAACCCGATTGCTCCGGCTTTAACTTTTCTCAGATTGATAGGCACATTCCCATAGTCTACAACATCAGCACACCAAGAAGAGGTTGCCCCCTTTGTGACTCTGTAATCATGAGCTACGATGAGAGCGATGGCCTGCAGTCTCTCATGTATGTCACTAATGAAAGGGTGCAGACCAACCTCTTTTTAGATAAGTCACCTTTCTTTGATAATAACAACCTACACATTCATTATTTGTGGCTGAGGGACAACGGGACCGTAGTGGAGATAAGGGGCATGTATAGAAGACGCGCAGCCAGTGCTTTGTTTCTAATTCTCTCTTTTATTGGGTTCTCGGGGGTTATCTACTTTCTTTACAGACTGTTTTCCATCCTTTATTAGACGGTCAATAAAGCGTAGATTTTTAAAAGGTTTCCTGTGCATTCTTTTTGTATGGGCATATACTTGGCAAGAAATCCGAGCACCTCAGAAAGTGGATTGCCGTCACATATCAGTTCGACCACCCCTGCACCTAGCCATGCGGCGCTTTGACGGTCTTTGGGGCTACACATCATAAAGTACTTTTCCATGGCTTCTATAAGCACCTTGGAACAATCTGGGGGTTGGCGAATGGGTTCCCTAAACGGGAAATCCTCTATGGTATTCAGGCAGAAGACCGCGTCCTCCACCCGACGTTTGAGTCTTTCTAGCAGAGCGCCGAAGAACTCCCGCTCGTGTGTTTTCGCAGGGGCAAGTTCTGCGCCGTACAGCGATGAGAAACACGACACGATGTTTTCCAGCCCCATGCTGCGCAGCAACACGTGCTTCAGGAACAGGTGTTGTAGCCGGTTCAGTTTTAGCTTGGGTAGAAAAGTTATCGAGTTGTTAGCACGCTCCATGATGGTAACGGTGTTGAAGTCACAGACCGGGCTTTCTCCGAGTCTCGGCCGCCTGAGTCCAATCATGTAGAACATAGACGCGGCCTCGTTGTCTGTGTTAAGTGACACGATATCCCGTTCGCAAACCTGTGCGATGTTGTGTTTCAGTATAGATCTGGTCTGACCGGCACGGGGTGTTATGGGGTGACGCGGTAAAGGCGACTCTGGGTCAAACACCTTTATGCGGTTGGCGGCCTCGTCGATGACGACACGCTTGTTCGCGGCGTGTATGGGGACGCGACGGCATCCCGCTGGCAGATCTATAATCTTAAAGTTGGTATAAGACTGGTCGCTCGTTATGGCCAGCCGGCACTCCGGTAGTATCTGCGTGTCCTCGAATTCGTGGCCGCGTACGACTGGCTTGGAGTGCAGGTAAACGCCAAGAGATGCGGTCTCTTCGCCTACGCACAAGTGGCTTCTTAACGCGTAGGGGTGCGGTGAGAGCATGATCCGTAGCAACGATAGTTCCGGGTGCCTAGCCGCGTAGAGTGGCAGGGTAGACGAGTCCGGAGTCCCAAACTTTTCGAACAACAGTGGCATCGGGACTTCAGGATTAGAGACTCCCACCATGGCCGCCACCGCCGGAGAGGTCAAGACGTGAAACACGCGCTCGCCTGTCGACAGGCGCGCCGCGCCCTCTACTAGACTAGCCTTCACGTCCGGAACTCGTAACATAGCTTAGACCAGCGGACGGACGCAACGTACGCGGGGATCGGCTGGCGGTGTCTGCTCGTTGGACGCGGCCGTTCGGTGGCGCCAGTGCAGGCCTAGTTTGCGAATGGCGTGACGGACAATTTGTGGCTTTAGAGCGGCGAACCGATGACCCGTGGTGGCGACGAACGAAATGAAGTTTGCATTGCGGCCCAACTCGTCTAGCCTGGTCTTCTTGTTTCGGGCATAGATTTTCGGGATTAGGTTACACTTTTTATATCCCAGTACTGCGCACTCGTGTTTGCTTTTAGTGTGACTGATTATCTTCTTTGAGAAGTCAAACAGGCCCCGGGCGGCGGCTCGCCTAATGCAAGCCACGTCAAGCCTGAGAAACGAACAGCATTCCACCAGACACTCCAGGAACCTTTTGTGTAGCGTCTGTATTTGGGAACGGTTTCTGTGCTCAAGTAGGGAGAATATTCTATTTTTGTTTCCGTCGATGCGCGCGTGCTGGTCCGTGAGAATGGGCGCCAGCTCGTGGCGAATCTGTTCCACAAGAGGCTGCCCGTACACTTTAGAAATCGTGGCTGTCGCGGCCTTAAACCAGGACACGTTTAGCCCATCCTTGCTGGAGACCACAGATGGAAAGTTTGTGGTCCAAAATACGTTTTTTCGCCCCATTCTCACCATGTACTGGTTTTCCAGTCCGTGCAGGTCCAACGTGGAGTTCCAATTTGCTATCGATACAGGAAATATGTGCCTGATTGGCAGAAAGCATTTCAGCGTACCCATTGCGAAGAGAAAGTGCAGCATGTCCCCACTGATGTTGATGTTTATTGCGGTGCCTTGACACATGTTGTCGGAAAAAAACACGCTTATGGTAAAAGAAGGTTCCTTTACGGAGTACTTTCGTATAACAAAATTGTTGGTCAATCTGGGGATGTTTAAAATAGTCTTTTGCAGGGTGTTAGGAACGTGGCAGCTTATCTTAGTGTTAATCACCATGTTGGTGTTGAATATGGTGATCTTGAAGTTTTCCAAACTGACGTGTTTTGTGGGTTCCAGCATGTCTGACACTGTGGAGCTGCCCAGAGTCCGCGCGTCCGTGGCCGCGTATCGTTGGAAGCACGCCTGCAAATTTCCTTTCATGGCTGCTCGCCGGTCTTTCGGCGCGTACCGGATTCTTGAAAGCGTCGCCGCCAGGAGACGCGGTGTCTCGTGGGTGCCTAAAAAGTTTGCGCAGGGGTGCAGTCCGCTGCACGAGTGGCCGATGCAGTCTGCCACTGCCATACACATGACGAGTCTGTAGATGGCCGGTGTGCCCGGATACACTAGATAGTAGGTACAATCTGGGGTACTGACGACCACCCTGTATGGCTTTGGTCCGGGGTCCTTGCGTTGGATTTTTACGTGCAGACGGGACACGAGCTGGTTTAGAGCCAGCTGAAAGCCCACCAGATCCCGTCCGTTAACCTTGACGTCCTGGTGCTTACTCTGTTTCGACAGGTTCTTCAGCACGGTGGGCAGTCGCTCTACGTTGTGAGCGATGGCACGGCGCAGCGAGACCAGCTCTCCGTGCCACCCCCACGTGGCCATGAAGCTGCTGATGTTAAACTTTAAAAAATGTAGCTGTGCATCTGGGGATGCGGGTGGCATTATTGAAAACGAGAGATGCTTCAGGCTCTCCAGGAGTGCAAAATAATTTTGATAGATTGTGGGTTGTAGACTATGGGGCAACACCGCCAGAAACGCATGAAAACACTGTTCGAACTCCCAGAACTCCAGGTACCTGCACACTATCCTGAACATGGCTTTGTAACATATGGTGCACGTTAGTAGCGCGGGAAGATACAGCGAGCGTAGCTCCCTGAATTCGCAGGGTTTATCACAATCATCGGTAAGTTCCCATGATCCCACCGCAGGTAGGTAGTTGTCGGTGTCTATCTGTCCGCGCGTAAACACTCCACCACCGTCAATTATTAAACCTTCGCCGCTGTACCGTCGACCCACTTTTCCCAAAAGAGTCCCTTCTTGATGTATAAAAGGGTGGAGGCGTTCCCCCAGGAGTAGTCTGCGTATCGCTCTGCAGGCGAAAAAGGTGGGCTCGGGCTGCATCATCTTATCAAGACCTTCTAAGGTCAGCTCTGCCTGCAGGTGCGAGTTGGTGGCCAGACAGCAGAATATTTCCAGCTGTGATTCCCAAGTCGCTTGATAACACGTGGTCTGCGGACTCGTCGTCAGGGAGGCGCTCGGTGGCAGTAGTAGGGGGCCCTCGAGCGCTGCCATGGAGGCGACCTTGGAGCAACGACCTTTCCCGTACCTCGCCACGGAGGCCAACCTCCTAACGCAGATTAAGGAGTCGGCTGCCGACGGACTCTTCAAGAGCTTTCAGCTATTGCTCGGCAAGGACGCCAGAGAAGGCAGTGTCCGTTTCGAAGCGCTACTGGGCGTATATACCAATGTGGTGGAGTTTGTTAAGTTTCTGGAGACCGCCCTCGCCGCCGCTTGCGTCAATACCGAGTTCAAGGACCTGCGGAGAATGATAGATGGAAAAATACAGTTTAAAATTTCAATGCCCACTATTGCCCACGGAGACGGGAGGAGGCCCAACAAGCAGAGACAGTATATCGTCATGAAGGCTTGCAATAAGCACCACATCGGTGCGGAGATTGAGCTTGCGGCCGCAGACATCGAGCTTCTCTTCGCCGAGAAAGAGACGCCCTTGGACTTCACAGAGTACGCGGGTGCCATCAAGACGATTACGTCGGCTTTGCAGTTTGGTATGGACGCCCTAGAACGGGGGCTAGTGGACACGGTTCTCGCAGTTAAACTTCGGCACGCTCCACCCGTCTTTATTTTAAAGACGCTGGGCGATCCCGTCTACTCTGAGAGGGGCCTCAAAAAGGCCGTCAAGTCTGACATGGTATCCATGTTCAAGGCACACCTCATAGAACATTCATTTTTTCTAGATAAGGCCGAGCTCATGACAAGGGGGAAGCAGTATGTCCTAACCATGCTCTCCGACATGCTGGCCGCGGTGTGCGAGGATACCGTCTTTAAGGGTGTCAGCACGTACACCACGGCCTCTGGGCAGCAGGTGGCCGGCGTCCTGGAGACGACGGACAGCGTCATGAGACGGCTGATGAACCTGCTGGGGCAAGTGGAAAGTGCCATGTCCGGGCCCGCGGCCTACGCCAGCTACGTTGTCAGGGGTGCCAACCTCGTCACCGCCGTTAGCTACGGAAGGGCGATGAGAAACTTTGAACAGTTTATGGCACGCATAGTGGACCATCCCAACGCTCTGCCGTCTGTGGAAGGTGACAAGGCCGCTCTGGCGGACGGACACGACGAGATTCAGAGAACCCGCATCGCCGCCTCTCTCGTCAAGATAGGGGATAAGTTTGTGGCCATTGAAAGTTTGCAGCGCATGTACAACGAGACTCAGTTTCCCTGCCCACTGAACCGGCGCATCCAGTACACCTATTTCTTCCCTGTTGGCCTTCACCTTCCCGTGCCCCGCTACTCGACATCCGTCTCAGTCAGGGGCGTAGAATCCCCGGCCATCCAGTCGACCGAGACGTGGGTGGTTAATAAAAACAACGTGCCTCTTTGCTTCGGTTACCAAAACGCCCTCAAAAGCATATGCCACCCTCGAATGCACAACCCCACCCAGTCAGCCCAGGCACTAAACCAAGCTTTTCCCGATCCCGACGGGGGACATGGGTACGGTCTCAGGTATGAGCAGACGCCAAACATGAACCTATTCAGAACGTTCCACCAGTATTACATGGGGAAAAACGTGGCATTTGTTCCCGATGTGGCCCAAAAAGCGCTCGTAACCACGGAGGATCTACTGCACCCAACCTCTCACCGTCTCCTCAGATTGGAGGTCCACCCCTTCTTTGATTTTTTTGTGCACCCCTGTCCTGGAGCGAGAGGATCGTACCGCGCCACCCACAGAACAATGGTTGGAAATATACCACAACCGCTCGCTCCAAGGGAGTTTCAGGAAAGTAGAGGGGCGCAGTTCGACGCTGTGACGAATATGACACACGTCATAGACCAGCTAACTATTGACGTCATACAGGAGACGGCATTTGACCCCGCGTATCCCCTGTTCTGCTATGTAATCGAAGCAATGATTCACGGACAGGAAGAAAAATTCGTGATGAACATGCCCCTCATTGCCCTGGTCATTCAAACCTACTGGGTCAACTCGGGAAAACTGGCGTTTGTGAACAGTTATCACATGGTTAGATTCATCTGTACGCATATGGGGAATGGAAGCATCCCTAAGGAGGCGCACGGCCACTACCGGAAAATCTTAGGCGAGCTCATCGCCCTTGAGCAGGCGCTTCTCAAGCTCGCGGGACACGAGACGGTGGGTCGGACGCCGATCACACATCTGGTTTCGGCTCTCCTCGACCCGCATCTGCTGCCTCCCTTTGCCTACCACGATGTCTTTACGGATCTTATGCAGAAGTCATCCAGACAACCCATAATCAAGATCGGGGATCAAAACTACGACAACCCTCAAAATAGGGCGACATTCATCAACCTCAGGGGTCGCATGGAGGACCTAGTCAATAACCTTGTTAACATTTACCAGACAAGGGTCAATGAGGACCATGACGAGAGACACGTCCTGGACGTGGCGCCCCTGGACGAGAATGACTACAACCCGGTCCTCGAGAAGCTATTCTACTATGTTTTAATGCCGGTGTGCAGTAACGGCCACATGTGCGGTATGGGGGTCGACTATCAAAACGTGGCCCTGACGCTGACTTACAACGGCCCCGTCTTTGCGGACGTCGTGAACGCACAGGATGATATTCTACTGCACCTGGAGAACGGAACCTTGAAGGACATTCTGCAGGCAGGCGACATACGCCCGACGGTGGACATGATCAGGGTGCTGTGCACCTCGTTTCTGACGTGCCCTTTCGTCACCCAGGCCGCTCGCGTGATCACAAAGCGGGACCCGGCCCAGAGTTTTGCCACGCACGAATACGGGAAGGATGTGGCGCAGACCGTGCTTGTTAATGGCTTTGGTGCGTTCGCGGTGGCGGACCGCTCTCGCGAGGCGGCGGAGACTATGTTTTATCCGGTACCCTTTAACAAGCTCTACGCTGACCCGTTGGTGGCTGCCACACTGCATCCGCTCCTGGCAAACTATGTCACCAGGCTCCCCAACCAGAGAAACGCGGTGGTCTTTAACGTGCCATCCAATCTCATGGCAGAATATGAGGAATGGCACAAGTCGCCCGTCGCGGCGTATGCCGCGTCTTGTCAGGCCACCCCGGGCGCCATTAGCGCCATGGTGAGCATGCACCAAAAACTATCTGCCCCCAGTTTCATTTGCCAGGCAAAACACCGCATGCACCCTGGTTTTGCCATGACAGTCGTCAGGACGGACGAGGTTCTAGCAGAGCACATCCTATACTGCTCCAGGGCGTCGACATCCATGTTTGTGGGCTTGCCTTCGGTGGTACGGCGCGAGGTACGTTCGGACGCGGTGACTTTTGAAATTACCCACGAGATCGCTTCCCTGCACACCGCACTTGGCTACTCATCAGTCATCGCCCCGGCCCACGTGGCCGCCATAACTACAGACATGGGAGTACATTGTCAGGACCTCTTTATGATTTTCCCAGGGGACGCGTATCAGGACCGCCAGCTGCATGACTATATCAAAATGAAAGCGGGCGTGCAAACCGGCTCACCGGGAAACAGAATGGATCACGTGGGATACACTGCTGGGGTTCCTCGCTGCGAGAACCTGCCCGGTTTGAGTCATGGTCAGCTGGCAACCTGCGAGATAATTCCCACGCCGGTCACATCTGACGTTGCCTATTTCCAGACCCCCAGCAACCCCCGGGGGCGTGCGGCGTGCGTGGTGTCGTGTGATGCTTACAGTAACGAAAGCGCAGAGCGTTTGCTCTACGACCATTCAATACCAGACCCCGCGTACGAATGCCGGTCCACCAACAACCCGTGGGCTTCGCAGCGTGGCTCCCTCGGCGACGTGCTATACAATATCACCTTTCGCCAGACTGCGCTGCCGGGCATGTACAGTCCTTGTCGGCAGTTCTTCCACAAGGAAGACATTATGCGGTACAATAGGGGGTTGTACACTTTGGTTAATGAGTATTCTGCCAGGCTTGCTGGGGCCCCCGCCACCAGCACTACAGACCTCCAGTACGTCGTGGTCAACGGTACAGACGTGTTTTTGGACCAGCCTTGCCATATGCTGCAGGAGGCCTATCCCACGCTCGCCGCCAGCCACAGAGTTATGCTTGACGAGTACATGTCAAACAAGCAGACACACGCCCCAGTACACATGGGCCAGTATCTCATTGAAGAGGTGGCGCCGATGAAGAGACTATTAAAGCTCGGAAACAAGGTGGTGTATTAGCTAACCCTTCTAGCGTTGGCTAGTCATGGCACTCGACAAGAGTATAGTGGTTAACTTCACCTCCAGACTCTTCGCTGATGAACTGGCCGCCCTTCAGTCAAAAATAGGGAGCGTACTGCCGCTCGGAGATTGCCACCGTTTACAAAATATACAGGCATTGGGCCTGGGGTGCGTATGCTCACGTGAGACATCTCCGGACTACATCCAAATTATGCAGTATCTATCCAAGTGCACACTCGCTGTCCTGGAGGAGGTTCGCCCGGACAGCCTGCGCCTAACGCGGATGGATCCCTCTGACAACCTTCAGATAAAAAACGTATATGCCCCCTTTTTTCAGTGGGACAGCAACACCCAGCTAGCAGTGCTACCCCCATTTTTTAGCCGAAAGGATTCCACCATTGTGCTCGAATCCAACGGATTTGACCTCGTGTTCCCCATGGTCGTGCCGCAGCAACTGGGGCACGCTATTCTGCAGCAGCTGTTGGTGTACCACATCTACTCCAAAATATCGGCCGGGGCCCCGGATGATGTAAATATGGCGGAACTTGATCTATATACCACCAATGTGTCATTTATGGGGCGCACATATCGTCTGGACGTAGACAACACGGATCCACGTACTGCCCTGCGAGTGCTTGACGATCTGTCCATGTACCTTTGTATCCTATCAGCCTTGGTTCCCAGGGGGTGTCTCCGTCTGCTCACGGCGCTCGTGCGGCACGACAGGCATCCTCTGACAGAGGTGTTTGAGGGGGTGGTGCCAGATGAGGTGACCAGGATAGATCTCGACCAGTTGAGCGTCCCAGATGACATCACCAGGATGCGCGTCATGTTCTCCTATCTTCAGAGTCTCAGTTCTATATTTAATCTTGGCCCCAGACTGCACGTGTATGCCTACTCGGCAGAGACTTTGGCGGCCTCCTGTTGGTATTCCCCACGCTAACGATTTGAAGCGGGGGGGGGGGTATGGCGTCATCTGATATTCTGTCGGTTGCAAGGACGGATGACGGCTCCGTCTGTGAAGTCTCCCTGCGTGGAGGTAGGAAAAAAACTACCGTCTACCTGCCGGACACTGAACCCTGGGTGGTAGAGACCGACGCCATCAAAGACGCCTTCCTCAGCGACGGGATCGTGGATATGGCTCGAAAGCTTCATCGTGGTGCCCTGCCCTCAAATTCTCACAACGGCTTGAGGATGGTGCTTTTTTGTTATTGTTACTTGCAAAATTGTGTGTACCTAGCCCTGTTTCTGTGCCCCCTTAATCCTTACTTGGTAACTCCCTCAAGCATTGAGTTTGCCGAGCCCGTTGTGGCACCTGAGGTGCTCTTCCCACACCCGGCTGAGATGTCTCGCGGTTGCGATGACGCGATTTTCTGTAAACTGCCCTATACCGTGCCTATAATCAACACCACGTTTGGACGCATTTACCCGAACTCTACACGCGAGCCGGACGGCAGGCCTACGGATTACTCCATGGCCCTTAGAAGGGCTTTTGCAGTTATGGTTAACACGTCATGTGCAGGAGTGACATTGTGCCGCGGAGAAACTCAGACCGCATCCCGTAACCACACTGAGTGGGAAAATCTGCTGGCTATGTTTTCTGTGATTATCTATGCCTTAGATCACAACTGTCACCCGGAAGCACTGTCTATCGCGAGCGGCATCTTTGACGAGCGTGACTATGGATTATTCATCTCTCAGCCCCGGAGCGTGCCCTCGCCTACCCCTTGCGACGTGTCGTGGGAAGATATCTACAACGGGACTTACCTAGCTCGGCCTGGAAACTGTGACCCCTGGCCCAATCTATCCACCCCTCCCTTGATTCTAAATTTTAAATAAAGGTGTGTCACTGGTTACACCACGATTAAAAACCACTCACTGAGATGTCTTTTTAACCGCTAAGGGATTATACCGGGATTTAAAACCGCCCACTGATTTTTTTACGCTAAGAGTTGGGTGCTTGGGGGGTTTTGCATTGCTCTGTTGTAAACTATATATAAGTTAAACCAAAATTCGCAGGGAGACAAGGTGACGGTGGTGAGAACTCAGTTGAGAGTCAGAGAATACAGTGCTAATCAGGGTAGATGAGCATGACTTCCCCGTCTCCAGTCACCGGAGGAATGGTGGACGGCTCCGTCCTGGTGCGAATGGCCACCAAGCCTCCCGTGATTGGTCTTATAACAGTGCTCTTCCTCCTAGTCATAGGCGCCTGCGTCTACTGCTGCATTCGCGTGTTCCTGGCGGCTCGACTGTGGCGCGCCACCCCACTAGGCAGGGCCACCGTGGCGTATCAGGTCCTTCGCACCCTGGGACCGCAGGCCGGGTCACATGCACCGCCGACGGTGGGCATAGCTACCCAGGAGCCCTACCGTACAATATACATGCCAGATTAGAACGGGGTGTGTGCTATAATGGATGGCTATGGGGGGGCTGTAGATAATTGAGCGCTGTGCTTTTATTGTGGGGATATGGGCTTGTACATGTGTCTATCATCGGTAGCCATAAAATGGGCCATGACAACTGCCACAAGTAAGTCGTCCGACATGTGCTTTTGCTTGGCGCTGTATGACTGCCCTCCATCCCTAAGCGGGACGCACTTGATCGCGCGGACCTGTTCTACCAGGTAGGTCACCGGGTCAAATGATATTTTGATGGTGTTGGACACCACCGTCTGGCTGGCGCTCAGGGTGCCGGAGTTCAGAGCGTAGATGAATGTCTCAAACGCGGAGGATTTCTCGCCTCCCAACATGTAAATTGGCCACTGCAGGGCGCTGCTCTTGTCAGTATAGTGTAGAAAATGTATGGGGAGCGGGCATATTTCGTTAAGGACGGTTGCAATGGCCACCCCAGAATCTTGGCTGCTGTTGCCTTCGACCGCCGCGTTCACGCGCTCAATTGTGGGGTGGAGCACAGCGATCGCCTTAATCATCGTGCATGCGCAGGACGCTATCTCGTAAGCAGCTGCGCCAGTGAGGTCGCGCAGGAAGAAATGCTCCATGCCCAATATGAGGCTTCTGGTGGGAGTCTGAGTACTCGTGACAACGGCGCCCACGCCAGTACCGGACGCCTCCGTGTTGTTCGTATACGCGGGGTCGATGTAAACAAACAGCTGTTTTCCAAGGCACTTCTGAACCTGCTGGGCGGTGGTGTCTACCCGACACATGTCAAACTGTGTCAGCGCTGCGTCACCCACCACGCGGTAAAGCGTAGCATTTGACGACGCTGCTCCCTCGCCCATTAGTTCGGTGTCGAATGCCCCCTCCATAAAGAGGTTGGTGGTGGTTTTGATGGATTCGTCGATGGTGATGTACGTCGGAATGTGCAGTCTGTAACAAGGACAGGACACTAGTGCGTCTTGCAGGTGGAAATCTTCGCGGTGGTCCGCACACACGTAACTGACCACATTCAGCATCTTTTCCTGGGCGTTCCTGAGGTTAAGCAGGAAACTCGTGGAGCGGTCTGACGAGTTCACGGATGATATAAATATCAGCTTGGCGTCTTTCTGAAGCATGAAACCCAGAATAGCCGGCAGTGCATCCTTTTTAATAAAATTCGCCTCGTCTACGTAGAGCAGGTTAAAGGTCTGTCCCCGAATGCTCTGCAGACACGGAAAGACACAAAAGAGGGGCTCATAAGCGGCTAACAGTAAAGGAGAGGAGGCGAACAGTGCGTGGCTCTTGTTCTTGGGAATAAAAGGGGGCGTGTGTGCCGATCGTATGGGTGAGCCAGTGGATCCTGGACATGTGGTGAATGAGAAAGATTTTGAGGAGTGTGAACAATTTTTCAGTCAACCCCTTAGGGAGCAAGTGGTCGCGGGGGTCAGGGCACTCGACGGCCTCGGTCTCGCTGACTCTCTATGTCACAAAACAGAAAGACTCTGCCTGCTGATGGACCTGGTGGGCACGGAGTGCTTTGCGAGGGTGTGCCGCCTAGACACCGGTGCGAAATGAAGAGTGTGGCGAGTCCCTTATGTCAGTTCCACGGCGTGTTTTGCCTGTACCAGTGTCGCCAGTGCCTGGCATACCACGTGTGTGATGGGGGCGCCGAATGCGTTCTCCTGCATACGCCGGAGAGCGTCATCTGCGAACTAACGGGTAACTGCATGCTCGGCAACATTCAAGAGGGCCAGTTTTTAGGGCCGGTACCGTATCGGACTTTGGATAACCAGGTTGACAGGGACGCATATCACGGGATGCTAGCGTGTCTGAAACGGGACATTGTGCGGTATTTGCAGACATGGCCGGACACCACCGTAATCGTGCAGGAAATAGCCCTGGGGGACGGCGTCACCGACACCATCTCGGCCATTATAGATGAAACATTCGGTGAGTGTCTTCCCGTACTGGGGGAGGCCCAAGGCGGGTACGCCCTGGTCTGTAGCATGTATCTGCACGTTATCGTCTCCATCTATTCGACAAAAACGGTGTACAACAGTATGCTATTTAAATGCACAAAGAATAAAAAGTACGACTGCATTGCCAAGCGGGTGCGGACAAAATGGATGCGCATGCTATCAACGAAAGATACGTAGGTCCTCGCTGCCACCGTTTGGCCCACGTGGTGCTGCCTAGGACCTTTCTGCTGCATCACGCCATACCCCTGGAGCCCGAGATCATCTTTTCCACCTACACCCGGTTTAGCCGGTCGCCAGGGTCATCCCGCCGGTTGGTGGTGTGTGGGAAACGTGTCCTGCCAGGGGAGGAAAACCAACTTGCGTCTTCACCTTCTGGCTTGGCGCTTAGCCTGCCTCTGTTTTCCCACGATGGGAACTTTCATCCATTTGACATCTCGGTACTGCGCATTTCCTGCCCTGGTTCTAATCTTAGTCTTACTGTCAGATTTCTCTATCTATCTCTGGTGGTGGCTATGGGGGCGGGACGGAATAATGCGCGGAGTCCGACCGTTGACGGGGTATCGCCGCCAGAGGGCGCCGTAGCCCACCCTTTGGAGGAACTGCAGAGGCTGGCGCGTGCTACGCCGGACCCGGCACTCACCCGTGGACCGTTGCAGGTCCTGACCGGCCTTCTCCGCGCAGGGTCAGACGGAGACCGCGCCACTCACCACATGGCGCTCGAGGCTCCGGGAACCGTGCGTGGAGAAAGCCTAGACCCGCCTGTTTCACAGAAGGGGCCAGCGCGCACACGCCACAGGCCACCCCCCGTGCGACTGAGCTTCAACCCCGTCAATGCCGATGTACCCGCTACCTGGCGAGACGCCACTAACGTGTACTCGGGTGCTCCCTACTATGTGTGTGTTTACGAACGCGGTGGCCGTCAGGAAGACGACTGGCTGCCGATACCACTGAGCTTCCCAGAAGAGCCCGTGCCCCCGCCACCGGGCTTAGTGTTCATGGACGACTTGTTCATTAACACGAAGCAGTGCGACTTTGTGGACACGCTAGAGGCCGCCTGTCGCACGCAAGGCTACACGTTGAGACAGCGCGTGCCTGTCGCCATTCCTCGCGACGCGGAAATCGCAGACGCAGTTAAATCGCACTTTTTAGAGGCGTGCCTAGTGTTACGGGGGCTGGCTTCGGAGGCTAGTGCCTGGATAAGAGCTGCCACGTCCCCGCCCCTTGGCCGCCACGCCTGCTGGATGGACGTGTTAGGATTATGGGAAAGCCGCCCCCACACTCTAGGTTTGGAGTTACGCGGCGTAAACTGTGGCGGCACGGACGGTGACTGGTTAGAGATTTTAAAACAGCCCGATGTGCAAAAGACAGTCAGCGGGAGTCTTGTGGCATGCGTGATCGTCACACCCGCATTGGAAGCCTGGCTTGTGTTACCTGGGGGTTTTGCTATTAAAGGCCGCTATAGGGCGTCGAAGGAGGATCTGGTGTTCATTCGAGGCCGCTATGGCTAGCCGGAGGCGCAAACTTCGGAATTTCCTAAACAAGGAATGCATATGGACTGTTAACCCAATGTCAGGGGACCATATCAAGGTCTTTAACGCCTGCACCTCTATCTCGCCGGTGTATGACCCTGAGCTGGTAACCAGCTACGCACTGAGCGTGCCTGCTTACAATGTGTCTGTGGCTATCTTGCTGCATAAAGTCATGGGACCGTGTGTGGCTGTGGGAATTAACGGAGAAATGATCATGTACGTCGTAAGCCAGTGTGTTTCTGTGCGGCCCGTCCCGGGGCGCGATGGTATGGCGCTCATCTACTTTGGACAGTTTCTGGAGGAAGCATCCGGACTGAGATTTCCCTACATTGCTCCGCCGCCGTCGCGCGAACACGTACCTGACCTGACCAGACAAGAATTAGTTCATACCTCCCAGGTGGTGCGCCGCGGCGACCTGACCAATTGCACTATGGGTCTCGAATTCAGGAATGTGAACCCTTTTGTTTGGCTCGGGGGCGGATCGGTGTGGCTGCTGTTCTTGGGCGTGGACTACATGGCGTTCTGTCCGGGTGTCGACGGAATGCCGTCGTTGGCAAGAGTGGCCGCCCTGCTTACCAGGTGCGACCACCCAGACTGTGTCCACTGCCATGGACTCCGTGGACACGTTAATGTATTTCGTGGGTACTGTTCTGCGCAGTCGCCGGGTCTATCTAACATCTGTCCCTGTATCAAATCATGTGGGACCGGGAATGGAGTGACTAGGGTCACTGGAAACAGAAATTTTCTGGGTCTTCTGTTCGATCCCATTGTCCAGAGCAGGGTAACAGCTCTGAAGATAACTAGCCACCCAACCCCCACGCACGTCGAGAATGTGCTAACAGGAGTGCTCGACGACGGCACCTTGGTGCCGTCGTCCAAGGCACCCTGGGTCCTCTTACGAATGTCTGACTACTTCAGCCGCTTGCTGATATATGAGTGTAAAAAACTTAAGGCCCTGGGCTTACGTTCTTATTGAAGCATGTTGCGCACATCAGCGAGCTGGACCGTCCTCCGGGTCGCGTGTAGATTATGGTTCCGTTCTCCTTCTTGATGTTTAAATTTTTGGGGGGGAACCACCGACAAAGCGTCTTTATGATTTCCGCGAACACGGAGTTGGCTACGTGCTTTTGGTGGGCTACGTACCCAATGTTAATGTTCTCTACGGATGCCAGTAGCATGCTGATGATCGCCACCACTATCCATGTCTTTCCGTGTCTCCTTGGTATTAGGAATACGCTTGCCTTTTGCTTAAACGTCTGTAAAACACTGTTTGGAGTTTCAAATAAACCGAAGTACTGCTTAAACAATCCAAACAACTGGTGCGTCTTTTGTGGGGCCTTGATTGAAACCAAAAAGAAAAAAGTGTGCATTACTAGCTGCTGTTGGAAGGGCTCCAGCCAGTGCACCCCGGGAACGTAACAGCCGTTCAGAAAGGACGAAAGGTTAACCAGAAAAGCCTGAAGTTCGCGGTAGACAGAGCAGGCGTGCAGGGAGTCGTGTGTTTTTCTGGCCGCCTGGTACTCGACCAGTTGATCGGCCGTGGAGACGTGCGCGTCCTCGCGCACACACCGCATCTGCAAGTATGTTGATAGGGACTCCAATAGGCGCGGCTTTGCGGGGACGTTGTCCTCGGACGGTCTGGGGGTTCCCACGTCGGGATTTGCTGACGTGGGCGTGGCGGGATGGTGCCGTGTGCAGTATGTTTCCAGGACCGAACTGTATGAGTTTATTCTGTGCACCACGCCAATAAAAGGGTGCGCCATCCGTGCCGTTTTGGGACAGTGTCGCGTGAATGTCGGGGCACTCAGTTCCCACCTCTCTCCGGCGTCTTTGGCGGTCTCCTGCAGGTTGGCGGCAAGGCGCTCCCTGTGACGGCTGAGCAGCATGTTTGCTTTGAGCTCGCTCGTGTCCGAGGGTGACCCGGAGGTGACCAGTAGGTACGTCAAGGGCGTACAACTTGCCCTGGACCTTAGCGAGAACACACCTGGACAATTTAAGTTGATAGAAACTCCCCTGAACAGCTTCCTCTTGGTTTCCAACGTGATGCCCGAGGTCCAGCCAATCTGCAGTGGCCTGCCGGCCTTGCGGCCAGACTTTAGTAATCTCCACTTGCCTAGACTGGAGAAGCTCCAGAGAGTCCTCGGGCAGGGTTTCGGGGCGGCGGGTGAGGAAATCGCACTGGACCCGTCTCACGTAGAAACACACGAAAAGGGCCAGGTGTTCTACAACCACTATGCTACCGAGGAGTGGACGTGGGCTTTGACTCTGAATAAGGATGCGCTCCTTCGGGAGGCTGTAGATGGCCTGTGTGACCCCGGAACTTGGAAGGGTCTTCTTCCTGACGACCCCCTTCCGTTGCTATGGCTGCTGTTCAACGGACCCGCCTCTTTTTGTCGGGCCGACTGTTGCCTGTACAAGCAGCACTGCGGTTACCCGGGCCCGGTGCTACTTCCAGGTCACATGTACGCTCCCAAACGGGATCTTTTGTCGTTCGTTAATCATGCCCTGAAGTACACCAAGTTTCTATACGGAGATTTTTCCGGGACATGGGCGGCGGCTTGCCGCCCGCCATTCGCTACTTCTCGGATACAAAGGGTAGTGAGTCAGATGAAAATCATAGATGCTTCCGACACTTACATTTCCCACACCTGCCTCTTGTGTCACATATATCAGCAAAATAGCATAATTGCGGGTCAGGGGACCCACGTGGGTGGAATCCTACTGTTGAGTGGAAAAGGGACCCAGTATATAACAGGCAATGTTCAGACCCAAAGGTGTCCAACTACGGGCGACTATCTAATCATCCCATCGTATGACATACCGGCGATCATCACCATGATCAAGGAGAATGGACTCAACCAACTCTAAAAGAGAGTTTATTAAGTCGGCTCTGGAGGCCAACATCAACAGGAGGGCAGCTGTATCGCTATTTGATCGTTTTGGGGGTAGCAGCGCCGTGTTTGAGAAGCAGTTTCAGGACGCACAGCATGCCGTCAGGGCCCACGGTGCACTGAAGCGCGAAGCCGAGCTCGGGACTCTGGTACGCAAGGCGGGCCAGAGGTTTGAGGCGCTGAAAAGGGAACGGTCAATTTTGCGCCAGCCGCGCGACCTCCCACGGGTCGCCGACATTGACGCCCTGGTCGACGCCGTCGCGGACCTCAAAGAAGAGGTGGCCGTGCGCCTAGATGCGCTGGAAGAGAATGGAGAGGAGACCCCCACTCACTCCTCTTCGGAGATCAAGGACACAATCGTCAGGTGGAGGCTTGACGATTTGCCCCCGGTGTGCCCTGAAACTCCCTAAGGCTACCCGGATTTCAGAGAGACCCTGGGCGTCCACATGGCAGCTGAATCAGCATATACAGGTGTCCAAGACTAAAAAGGCCACCGCGTATCTTAAAGCGCCCCGTGAATGGGGGCAGTGCACGCACCAGGATCCAGACTGGTCCAAGCGTCTGGGTCGTGGCGCCTTTGGCATAATCGTCCCTATCTCCGAGGATCTGTGTGTGAAGCAGTTTGATAGCCGCCGGGAGTTTTTCTACGAGGCAATTGCCAACGACCTGATGCAGGCCACCCGAGAGAGGTACCCCATGCATTCTGGTGGATCTAGACTGCTAGGATTCGTGCAGCCTTGCATACCCTGTAGATCGATTGTGTATCCTAGAATGAAGTGCAACCTGCTGCAGCTGGACTGGAGTCAGGTCAACCTGAGTGTCATGGCGGCGGAGTTCACCGGCCTAATGGCGGCGGTGTCCTTTCTAAACAGATACTGTGGCATGGTGCACTGCGACGTTAGTCCAGACAATATTTTGGCCACAGGAGACCTAACGCCCATGAACCCCGGGAGGCTGGTCCTTACCGATTTCGGTTCCGTTGCGCTACACTCTGGGAGCAAGTGGACTAACCTTGTGGTGACCTCTAACCTGGGGTTTAAGCAACACTGCTACGACTTCAGGGTGCCACCCAAACTCATTTGTAAGCATCTCTATAAGCCGTCTTGCGTCCTCTTCCAGTGTTACCTATCCAGTCTCGGTAAGATGCACGCGCAGGTATTGGACCAACCGTACCCTATCAGCCCTAACATGGGACTGACCATCGACATGTCCTCGTTGGGCTACACTCTGCTGACATGCCTGGAACTCTATCTCGATCTGCCGCTAAACAACCCTCTGAAGTTCTTGGGTTCAGCCACCAGAGACGGACGCCCCGAACCCATGTACTACTTGGGCTTCATGATTCCCAGGGTGGTGATGACTCAGATCCTGTCCGCTGTGTGGACCATGACGCTTGACCTGGGACTAGATTGCACCGGCAAAGCCCAGGCGATTCCCATGCGACAGGAGCACCAGCTGGCGTTTCAGAAGCAGTGCTATTTATATAAAGCCAACCAAAAGGCAGAGTCGTTAGCGAACTGCTCCGATAAGCTAAACTGCCCCATGTTAAAGTCTCTCGTTAGAAAGCTACTAGAGCGAGACTTTTTCAACCATGGAGGCCACCCCCACACCCGCGGACTTGTTTTCTGAAGACTATCTGGTTGACACCCTGGATGGGTTAACAGTGGATGACCAACAGGCTGTCCTCGCAAGCTTGAGCTTTTCAAAGTTTCTAAAGCACGCCAAGGTTCGAGACTGGTGCGCACAGGCCAAGATCCAACCCAGCATGCCTGCGCTGCGCATGGCTTACAACTATTTCCTTTTTTCAAAAGTGGGCGAGTTTATTGGTAGTGAGGATGTGTGTAACTTTTTCGTGGACCGTGTGTTTGGTGGTGTCAGGTTACTGGACGTGGCCAGCGTGTACGCCGCCTGTTCGCAAATGAACGCACATCAGCGGCACCACATCTGCTGTCTAGTGGAGAGGGCCACTAGTAGTCAGAGTCTGAACCCCGTGTGGGACGCCCTGCGAGACGGAATTATATCTTCATCCAAGTTTCACTGGGCAGTTAAACAACAGAACACTTCAAAAAAGATATTCAGCCCATGGCCTATAACGAACAACCACTTTGTCGCGGGCCCGCTTGCCTTTGGGCTGCGGTGCGAGGAGGTGGTGAAAACGTTGCTGGCCACCCTTTTGCACCCGGACGAGGCAAATTGTCTCGATTATGGGTTTATGCAGAGTCCGCAAAATGGAATATTTGGCGTGTCGCTGGATTTCGCGGCGAACGTCAAAACTGACACCGAGGGTCGTCTACAGTTTGACCCTAACTGTAAAGTGTATGAAATAAAATGCAGGTTCAAGTACACCTTTGCGAAAATGGAGTGTGACCCCATATACGCCGCGTATCAGCGGCTGTACGAGGCACCCGGAAAGCTGGCACTGAAGGACTTCTTCTATAGCATTTCCAAGCCTGCGGTTGAGTACGTGGGACTTGGAAAACTGCCCAGTGAATCTGATTACTTGGTGGCTTATGATCAGGAATGGGAGGCGTGTCCTCGCAAAAAGAGGAAATTAACGCCCCTTCACAATCTTATTAGGGAGTGTATTTTGCACAACTCGACCACGGAGTCTGACGTCTACGTACTTACTGATCCTCAAGATACTCGGGGTCAAATCAGTATTAAAGCCCGCTTCAAAGCCAACCTCTTCGTGAACGTCCGTCACAGCTACTTTTATCAGGTATTGCTGCAGAGTTCGATCGTCGAGGAGTACATTGGCCTAGATAGCGGCATTCCTCGCCTCGGATCACCGAAATACTACATCGCCACCGGCTTCTTCAGAAAGCGGGGCTATCAGGATCCTGTCAACTGTACCATCGGTGGCGATGCTTTAGACCCGCACGTGGAGATTCCTACGCTGCTAATCGTAACCCCCGTCTACTTTCCCCGAGGCGCAAAGCATCGTCTGCTTCACCAAGCTGCCAACTTTTGGTCAAGAAGTGCGAAGGACACCTTTCCATATATCAAATGGGATTTCTCCTATCTATCTGCAAACGTCCCTCACAGCCCGTAGACGTGGACGGGGAACCGCTCGACGTAGTCGTGGACTATGACCCCATTCGCGTTTCAGAAAAGGGCATGTTGCTTGAGCAATCGCAATCCCCATATCCCGCATTAAAAAAGAAGAAAAAAAATAAAGAAGCAATTTATTAAGCAAACAGTATGGTTTTCTGTACGTATTTTATTCCGTGGTGGGTGAAAAATAACGGGGGATGGAGGAAGAGGGATGGGTTTATAATGCCAATATATCAGCTAAATGAATATCATTTGCGTTTCGTCGATTTCACTGTCACTTTCATGGTCGGACTGGTATTGGGTCCTCGGGGCGGGCGTCGATATGTCCTTCACTTTGGCGCGGGCTCTGGTCTTTGCTGGGAGGGGCGGCGGTTTCTGGTGAACAGTCGGAGTTCTATCGACCGTCGGCGCCGACGTCGCCAGAGGCATGTATGCCGCACTCGGCGTACAGAGTCCCCAGTCGCTCCTTATAACGCGTATAACGATGGCTAGGATGCACAGTATAGGGATACAGGAGATATTGATAGCCACTATGTAGTGGAGATTAGCCTGCACGAACGCGTTTTCATACCTGATGACAGGCAGCAGTAGAATCAGATAACCCACGAATACTCCCACGTAAAAGCCTACCTGCCGTCTCATAAACTTTACCAGGAAAAATTCCGTGTTTATGTACCACACGACCGTCAAGGCTAGGAACATGTTCACCGCACCAAAAATGGCGTCTGACACGAGCACGTAAAAGCTGTTGCCAACGGCCATCATGGTGCTCAATGAAAACAGCAGCATTTCCAAGGCGGTTGTTGATAGGTACAGGTTGACGCAGACCGGTTTCCACCGAGTCAGCAGTGACTCCATCATGGTATTATCAGGTACGTGCTGTTCCAGGAGAGGTATTTCCCACTGGGCGGAGTTACATGTTATCAGTGACTGGATGTGGGCAAAGGATATGCAAAAATGAATGCAGTAGACAAAGGCTGCCATAAGTACGTGTTTATATGACAGAACATGGATAAACAGTTGCATGCTCCACATCCTTAAGATGGCGACATAAAGCACGCTATGTGATCCAAGTAGCGCTATCCAGGATTGCATGCTCATCATGGTAGTGGCGTGAACATGCTTGGCCCGATATACGGCCACCGCCGCGAGACAGTAGTATACTATGGCAATGCCGTCCACGATAAAAGTCCAAAATATGTACACCAGCATCTCTGGTTTCTCTAAAAACAGGGTCGGGGTGAGGTGCTTCGCTGAGTTGCGCACCGTGAGGTTTAGCGCGCTGTAGTTTACCAGATTGTTGAAGTAGCAGGGGAAACCAAGGCCCTCGTACGTGGCGGCCATGGGCACGACTGCAGAGCAAATGTACATAATTACAGCCACAAACAACAGCTTGACCCAGGAGGACATGAGAAAACGGTCGCTCTTTGAAGCGCGCATGTTTCTCGGTCTTTTTAACTTTCGCCAGGCGGCGCTGCGGCGGGAGAGCCAATCTGATGCCACTGCCTATCGCGGTTGACTTTTAAATACGCGCCCCGGGCAGAAGCCAGAGGTAGTCGACTCATTGACTCAATGGCAACGAGCGAAGAAACGGCGGCCGGTTATGTCATCGGTGTCTACTTTCACAGCGTTCACGTCCACTGCCGCATTATTGTCTGGCAGGTTAATTTTCTACCCCTGGACCCAAACGACGGGGAGACTGAATGCTACTTTGTGGTGGACACGCTGACGAAAGAGGCGATGGAGCGCATGCCCGAAATCCAGGAATGCGTCCCGTCTATTACTGAACACGCCCGTGACCTGGCGATCTGGGAGTTGGCGCTGCGACTGCAGAATCAGACGATCGTCAAGGCCGTCCGGACAGCGTCGCTTCCGGTGGTTCTAATTATGACTGTGGGTCGCATAGTGAATGATGTGATTCCCTGCCCCAACGTCAGAACACCCAGACCACTAGCCTGTGCTTACCTACACTGTGAGGCGACGGTGACCTTTGAGGTCCCACTAACCGGGCCCGCGGCGTCCACCGGAACGTGGCACAGCTCTATCTATAGGGAATGTGCGATCTCGGCTATCGAGATATGCTTGAAGACCAGTCGAGGCATATACTCCTGCCAGTCGAACGAGGCCCCTGAGGCCAAGAGGGAAAAGCGAGGTTTAGACATATCAGATGTGTTTGTCTGTCTCACGTATGATATCCCTATCGCAGGGCGGGTCCTTTCTCTGCTGGTGCCCCACGCGCCCGCTTTTCACGTCTTATGGATCAATGAGGACAGCAAGTGGAACGGGGCAGCCGTCGAATTTTTCAGAGCCCTACACCATAAGCTGTTCAGTGAACGCAATGGTATACCCCCTCTGTGGTTGTACGTGTTCCCGGGAGCTGTGGAAGAGGGCACAGCCTTTGCGCCATTACTTCCCGCATTCCCTTGCATACCTTTGCGGTATGGGTCGCCTACCTCTCTGGACAGGGCGTCCGTGCAGTGGGACCTATTTGAACCGCACATCCTGACCCACTTTGACGGGATAAAGCGAACTTCTTTGGCAGATACAGTGTTTGGGTACGACTCCCTGGCCATTTCAAGGGAATGTGAAGATCAGTATGTGTGGCCCACGCCTGTCACTGACATTAATATTAATTTGTGCACGGATAGTGACACTATGGCCATCGTTAGAGAACCATCCGGTCTGGTGGCCGTGAATCTAGAAGCCCTGTTGCGCACCGACTCCGTATTATCGCGGGTCTCGTCCATTGTCTCACTCGATACGCTCTTGGACCTTTCCACCCCGGAGTGCCGTAGGAGCGTGGAGCTTAGATACAACTCACTTTTGTCGACTGTATTATCATGGTCCACCTCTAGGGGTCACAAATGGGCCGCAATCGTGAAGTGGAAGTTATTTTTCCTCGTCCAAGCTTTGGAGCCTGAGGTGAGACCTACTGTCCCTGCTTGAAGCGGAGAGGGGGTGGTGCGAGTTGGCAGTTGACGGGTTTGTGATAGCTGGAGTGCTGACCACGGCACAGGACCCATTAACTTTCCTATGTGTTTATTTTTAGCAATGGTCTCCAGAATTCAAGGATCTCAAAAGGGCCTGCCAGATGGCCGGGTTTACTCTGAAGGGGGGGACTTCGGGGGATCTTGTATTCTCATCGCATGCGAACTTGCTCTTTTCAACCTCGATGGGATATTTCCTCCATGCAGGCAGTCCAAGGTCGACAGCGGGGACGGGGGGTGAGCCTAACCCACGTCACATCACCGGACCAGACACTGAGGGAAATGGGGAACACAGAAACTCCCCCAACCTCTGCGGCTTTGTTACCTGGCTGCAAAGCTTAACCACATGCATTGAACGAGCCCTAAACATGCCTCCCGACACTTCCTGGCTGCAGCTGATAGAGGAAGTGATACCCCTGTATTTTCATAGGCGAAGACAAACATCATTCTGGCTCATCCCCCTATCGCACTGTGAAGGGATCCCAGTATGCCCCCCTTTACCATTTGACTGCCTAGCACCAAGGCTGTTTATAGTAACAAAGTCCGGACCCATGTGTTACCGGGCAGGCTTTTCGCTTCCTGTGGATGTTAATTACCTGTTCTATTTAGAGCAGACTCTGAAAGCTGTCCGGCAAGTTAGCCCACAGGAACACAACCCCCAAGACGCAAAGGAAATGACTCTACAGCTAGAGGCCTGGACCAGGCTTTTATCTTTATTTTGAAAAAAGGGAAACAATGGGGGGTTTGAAAAGGGTGCACATTTTCAGATATTTTAAAACTTCATTGTTCTCCAGGTGCTTGGTAAAGATGGTATCACAATAAAAAATGTTTACTGGGTCCGCGCAGGTTTGTTTGTCATCTTCATTCTCTCCACTAGACTCCAGTTTAAAAGACTCTAGATAAATGGGTTTCATTAGTCCCCCCATGGGGGTTGAAGCGTCGCCTATCGCCTTATGAAGCTTAAACATAACGAGTGGGGTGGCCCTGAAATGATCGTCCACGGACAGCTCGTAAACAAAGGCGGCCGTGGCAGTCAACGTCTCTATACCGTGCATGACGAAGGCCGCGTCCATCCCCGGCGTCCTCTCATGTGTCTTTCTGGCGCGACAAATAATAGATCTCAAAAACGTTGGTGACATGTCTCGACAGTTCTCGAGCATCGATAACAGGCAGCAGAGCTCGGTTATGCCGGGAGATGTAGGTCTAAGGAGGCACACTCGCTCTTGGAACACGTGAGGGTGTAGGTCTATGTGGGTCACCATGTCTTCGTGCTCCACCAGGCACACCACCGTAAATCCCACAAAGTTGGGCGAGGACAGGCGAGATTTCACGTGCTCCCTGAGACACGCTATATCTAAGTGGCCCATCACGGACATTTTGGGGGTATTGCTTCCAACCAGTGCGTTGTTTTTCCTATGCACTTCCAGGACAAGGCGGGGCACCACAGGGTGGGGGTATACGGGACAGGCCTCTTCTGACTCGCGAGTCTTCGGGGCATGAGTACTCATTGGCACTCCAGTCAGTCTCGCCAGGGCCCTTTCCAGGGACATTCTCGAAGGGTGGTGTAACTAGACAGTATTTCTGTCCCACGTCGGTTATATACACAAAGAGTCTGCTAGTCTGATATAAATAGGCCGCGATGTCCTGCAAGCTGGAGGATACGAAGGAGTGACTAATGAGCTCCATCTGAAGCAGGTCCGCGATCACATACGTGAATGGACCAAGCAGGATGGATATGGTGTCCTGAGAATAGGTGACGCTGAGCCGCTGCCCTTGGTTGTCAACAACGGGAGCCAGCTTGTAGGTTTGAAACATCTCGCTTTCCCACAGGTTCGTGAGATCTTTCATGCTTTCTCTCACTGGGGGTATGTAAGAAGAGAAAAAGCTATTTAGCACGGCACTGCCCGATGGGATATGGGAAGACGTTAGCTGCAGAGAGGGGTCCTGTAAACGTCCCAGAGATTGAAATGTGTTGGCGGTCAGCAGATTCACACTCCCGGGACCCTTTGCGTCACCGGGCTGTTGGTGTGACAGCTGTGTCTCAATACATTTTAGCCTCTTCATGCAGAGCTCCCTCTCCTTTTCAAGTTGAGTTATTGTGTCAAATTGTTCGTTTATCTGGTTGGTGAGACACTTGAAAACGCTGTTGGACACCTGGCGCCTGAGCCCCTGAGTGGTCGTCTCTTGGCCTGTGCCGAATAGTTTATTCTTGTCTACTATGTTTTGGGACACGTCGGTGACAAAGTCCTCCACGACGTCGGTGACACCGCTCACTGTCTTGTTTTCTGCCAGTTTCATGAGCAGGTTGAGGAGCTCTCGCTTGGGGTCTGTTCTCTGAGAGGCCTGCTCCAGGTGGGTCATGATGTCTTTGTACACATTGTTACAGGCGCTTCCAACGAGGGCCTTGGTGGGGGCTGTGTTCAGGAGCTGGCAAAGTTTTGCGTGCTCTGCCGTCCGGTGACAGCTCATAATGCTGGTATACATCCTCTGAATGGGGCTGTCAAAGATCACCCGCCCAGCCAAGATGGCGGGCATAGTAATCACCTCCACATGAACCCTTTTCTGCTTATACAATCCCACGAAAGTGTTTTTAACACAGTCATAGTCTATGCTCACCTCTGAGTAGCCCGGAATATAGAGGGCGCTTAAACTAGACACCAGGTTGCTAATCTCCTGAGTCACGCTGGTGAGTATCCGGCCTATGGTTTTTTCACCAGAGGCCAGACGCTGGCAATCTTTCATCAGCTGTTCCTGGATAGAGTTAACCAGCTTGTGGTCGGGTGTGTGCTTGACGACTGGTACCATTCCTACCGTGACCACCCAGTCTACGTATCTCTCATACGAGAGCTGTGTCTTGGCGTAGAGGACCCGGTTGATGGCATTGAGAAGCAGGTGGTCTAATGTCATGCGCATAGTCTGGGCCCAGGAGTCGAAGGTTGACCTTCTGTAAGACCCCCACTGTGCTTCCTTTTCTGGCCACCTGGTTTTTGCTGAGGACTCGTATGTCCTCCAGTCGGACAAGACGTGGTCGTAGCTACAGTTGGCCAATGCATTCTTGTACAGGTGGATAAATAGCTGTCTGAAAAAAACACCCGGGTTTCGCAGGCTGCAGTGTAGAGTCTGACCTCTGACATAAGAATACTTGCCTTGCAGGATCTCAAAGAGGGAGATGGACAGCTCGGAAGGGTGCACTGATATGGACGAGCCCAGCCCCGGGTTCATCCTCAACATGACATCGGATGCCAAAGTCAGGAGCGTAGTGGAACAGATTGACAGGTTGTCAAATATCACTACCTCGCCCCCGGAGATGGGCTGGTATGACCTAGAGTTCGATCCACTGGAAGACGAAGGCCCCTTTCTGCCGTTTTCGGCATACGTAATAACGGGGACTGCAGGAGCGGGGAAAAGCACCAGCGTATCCGCCCTACATCAGAATCTCAACTGCCTAATTACGGGGGCTACAGTGGTAGCGGCACAGAATCTTTCCAGGGCTTTAAAGTCCTACTGTCCCACTATATACCACGCCTTCGGATTCAAGAGCAGACACATTAATATCTGCCAGAGGAAAGTGCCCAAGGTAACTCAGTCCTCCATCGAGCAACTCCAGAGATACGAGCTGGCTAGGTACTGGCCAACTGTCACCGATATTATTCGAGAATTTATGCGCAAGAAACAAAAGGGGCAGTATAGCTCCCTCTCTCAAAGCGCTTTCAGACTCCTTTGCCGTATGGGTGGAGCCAATTTGTGGACGAGTAACATTATCGTGATAGACGAAGCTGGAACCCTCTCGTCCCATATTTTGACGGCCGTGGTGTTCTTCTATTGGTTTTACAACAGTTGGCTGGACACCCCGCTATACAGAAATGGTGCCGTGCCTTGCATAGTCTGCGTGGGGTCTCCCACCCAGACGGACGCCTTTCAGTCGGTCTTCAACCACACGCAGCAGAGAAACGAGATATCTGCCTGTGATAATGTGCTCACCTTCCTATTGGGAAAACGTGAGGTTGCAGATTATATTAGGCTGGACGAGAATTGGGCCCTATTTATAAACAATAAGCGCTGTACGGATCCCCAGTTTGGTCACTTGCTGAAGACCTTAGAATATAATCTAGACATATCACCAGAGTTAATGGACTATATAGATAGGTTTGTGGTTCCGAAGAGTAAGATTCTGGACCCGCTCGAGTATGCAGGGTGGACAAGACTCTTCATCTCACACCAGGAGGTGAAGTCTTTTCTGGCAACGCTGCACACCTGCCTGTCGAGTAATAAGGATGCTGTGTCCACAAAGCTTTTCACCTGCCCAGTGGTCTGTGAGGTGTTTACAGAGCCATTTGAGGAGTACAAACGGGCGGTAGGCCTCACACACATGACTCCCATAGAATGGGTAACAAAAAATCTTTTCAGGCTAAGTAACTACTCGCAGTTTGCTGATCAGGACATGGCTGTGGTTGGGACCTATATCACAGACGCGTCCACACAGATCACCTTCGCCACTAAATTTGTCAAAAACAGCTATGCTACCCTTACTGGAAAGACCAAAAAATGTATATGCGGGTTTCACGGGTCATACCAAAGATTCAAGTCCATCCTAGACGGGGAGCTATTTATCGAAAGTCATTCGCACGATAACCCCGCTTATGTGTACAGTTTCCTTAGTACCCTGCTATATAATGCCATGTACTCATTTTACGCGCACGGGGTGAAGCAGGGGCATGAAGAATTCCTCAGGGACCTCAGGGAACTGCCGGTGTCTCAAGAGCTGATCTCTGAGATGAGCTCCGAGGACGTTCTGGGGCAGGAGGGGGACACAGATGCCTTCTACCTCACCGCCAGCCTCCCACCATCCCCCACCCACGCGGCTCTTCCAACACTGGTGGCCTATTACTCCGGGGCCAAGGAACTATTCTGCAACAGGCTGGCCCTGGCACGCCGACACTTTGGTGACGAGTTCCTCCACTCCGATTTTTCAACGTTTACGGTGAACATCGTGGTGCGAGATGGCGTGGACTTTGTGTCCACTTCCCCCGGGCTCCACGGTCTAGTGGCATACGCATCCACTATAGACACCTATATAATCCAGGGATATACGTTCCTCCCAGTGAGATTCGGCCGTCCAGGAGGACAGCGCCTCAGCGAGGACCTGCGCAGAAAGATGCCCTCCATAGTTGTCCAGGACTCATCGGGGTTCATTGCCTGCCTGGAAAATAACGTCACCAAGATGACAGAGACCCTCGAAGGTGGCGACGTGTTTAACATATGTTGTGCAGGGGACTACGGTATCAGTTCTAATCTGGCTATGACCATAGTGAAGGCACAGGGGGTTTCACTAAGTAGGGTGGCCATATCGTTCGGCAACCACCGCAATATCAGAGCCAGTCTAGTGTATGTGGGTGTATCCAGGGCCATCGACGCTCGTTACCTGGTAATGGACAGTAATCCCCTTAAGCTAATGGACCGCGGCGACGCCCAGTCCCCATCCTCAAAGTACATCATCAAAGCCCTATGCAACCCCAAGACTACTCTGATCTACTGACCCGTACCCCTCTCTTAGGACACTGATGTGTTTGGGAATAAAGCATGAGACTTGACACCTATAATGGTCTGTATTGACACCATTCTTTTATTTATCAGTCCAGCCACGGCCAGTTATATGCACCGTTTCCACACAGGGGTGGCGTGGAGGCCAGGATGCGGGTTGGGTCGCTGCACCTGGACCCCGCGGTAGTTGTGCTTCCTGATGAAATCGAGTGGGCGGAAGTACTGGGAGATTGGGTTGGGAGGTGACCCTTTGTGCTCGACGGAGACACGATCACGCTCACGGCGGACGAGGGCTCCTCGTCTGTGTCACTCCCCGAGGATATAATTATCACAGACGCCACTGCTTTGCGGCTTAAGTTTGGTTGTCTCTGGCAGCGCACCACATCCTCGCTACCAGAGGAGGCGGTAGACTGCCTTTTGCGCTTCTGGCCCACGTCCATGAGCCCGATTCTCTGACTCAATACTTCCCCTTGGTCTTCTCCGTCCTCCTCGGACGAGGGTGGCTGGTGGGAAAAATGGCGCGCGTCGGTAAACGCGGCCTCATTGTTCACGTCCGGAGAGTTGGAACTGTCATCGCTATCAGAGTCCGATGTCAGGTCGACGATCGCGGTGGGTGCGGCGCGCAGGGGGCGCCACGAGGGCCCTTCATCAGGGTCGCTGTATGGTGAACTTTGTGTTCCAGGTACACTATTTCTGGAAGCAGGTGAAAGTCCGTATGCCCCGGTCCCAGTGTATGCCGCCATCGGTTCCAGGATAGCAACCCCCTCGTCGTCTGAAGGTGAGAGCCCAGCAGGGGAAAATCCGTCATCCTGACTAACCCATCCCATGGACGCCTCGGACTCCGCCGTGTCCGTTGAACTGCGCACGCGGCCCGCTACCACTGCTACCGGTTTGGGCGTATGGGCCCGTCTGGCCAGAGGCCTCGGGCGCAAGTGAGATAAAGGTTGAAAAAAGTCTGCAGGGTACCCCTCT

AGATTCGCCGCTCGACTGATCCGGGGATATCTGTAGATCCAGAGGGGTTGCTGGCGGCGATGGCGTGTCCTCGGCGAAGACGTCGTCTGGGGCAGACATATCTATCACCGTGGGTCCAGCATAGCCGCGCGGCCTGCCAAATCCTGGAAGTGATGAAAGAGGTGGAGGTGGGAATATGAACTTCACGGGGGGTCGTCTGCGAGGCGCTCCTTCAATTGGAAGCATTCTCTCTTCATCGTGTGTGCTAGACGAGGTCCTCACAAACATCGCCATGGCCTTGTACGGGGTTGACCGCTAGGGGCGGAAATTTACAAAGCACACGAGTTATTGCCTTTACTGCTCCAACAGGCCCCAGTCCACAGTCTCACGCCGGTGGCGAGTCAAATAGTCGTTGGCTAGGTTAAAGTGATTACAGCCCTGGAACCGAGGCCATCGCGAGTGTCGGCCACCAAGAGAGGCCAGCGGAGATGGATGCTGGGCCGTAAGCACCAGGTGTTTCTGTGCGTTTATGAGCGGAGTTCTGTCAATGGCCTTGCGCCCCCACAGGAGAAAAACGCAATGTTCTAACTTTGAGGATATGCTACTGATGATGAAACTCGTGAACCAATCCCAGCCAAGTCCCTCGTGTGAGCCGGCCCTCCCCTTCTCCACCGTCAAAACTGTGTTTAGTAGCAACACACCCTGGCGAGCCCAGCTGTCGAGGCACCCGTGGGAAGGAGTACTGAAATTGGGGACGGAAGCCTCTAGCTCTCTAAAGATGCTTCTCAAACTGGGTGGAACCTGACATTGCGGATCCACACTAAACGCCAGGCCAGTAGCTTGGCCCTTGTGGTACGGGTCCTGGCCTAAGATCACCACTTTAATATCCTCTGGATCGCAGCAGTGGGACCACCACATCAGCTTGTCCTGTGGGGGATACACTGTGGTGGTTAGCCTAAGTTCCCGAATCTGTCTGAGCAGCGAGAGCAGTTTCTGTTTCAGAAATGATGAGAGGCTCAGAAAGGAAATCCACTTAGGTGCCAGTAACAGATCCCGGTCGTCCACCCCCTGACTGATGGATAGGGTGCCCCTAAAGACCGTCTGTTGCAACCATGCGTCCATGTTGAACTTATTTTCCCTTTTGACCTGCGTGCGCTCTCCGGCTGCTGCTTTTAGCCCGAGTCTGACTTCCGCTAACAGAACCTGTCCGGTTCATGGCCTTTCCCACGCTTATTATAATTATGTTTACGTTGTGAATAGAGCTATCTGCAGTGGTCGCGTTAAAACCTACAGTATAGGCCGTCAAACTTCGTTGTAAATACCACAACAACCTCAGGTTTTCCTGCGACGCCCAGGACCCCAATCTTCGAACGACCGCGACTAAAAATGACCTCAGATTAAACCCATTCACGCATGTTTCCACGGTAATGTCGCCTGTTTTGCTTCGCAGCTTGGCTATACAGACCCCGTTGCAGTGATTCGGATCGGCGAAGTGGATAGAGTGGACCGCAAAGAACAACGGCAGGGTAGAGGCTGCCGATGCCTGAATTGCGCAACATGGTAAGGCGACGTATGCGTGAGATGTGACCAATAGGGTGGTCCACAGGACGGCAAATAGCGCAAAGATCCCCATGGGGCAAATCCGGGTTTCACCCTTGTGTTGCCTGGTTCGGTGCTCCCCAGGGAGCCCCCTTCCGTAATATCTGTTTTATATAGTGAGGGTTCACGCATGCGCGAGTCCCGACTAATGAGGACAATTACTGAAATTGACCTTTTCGCGACACGGGGGTGAGGTCTATTTCCCACGACATACTTCCGCGGAAAAATACCCACGCTCCTTAATTTCCGTGGGAAGACGATGGGGGAAATGTGGCATTACCTGACACGGTTTCAATCATACTCATCGTCGGAGCTGTCACACGTCTGGCTGAGATTTTCTAAAAAGTCATCCAATGAATCATCGGAATCATCAGCACACTCTAGAACTACTCCATATGCCGGGGTGCGCGGGGGTCCCGAGTAGTGCACGTCGCCATCGGGAGACACAGATGATGGGTTTGAAATGTCCATACGGGCCGTGTGCACAAGGGTCACGTCCCCATCCCCAACACAAGGACCTTTAGATACCCTCTCCCGGCATGTGCGCGTATCCGGGCAAGCAAGCTGGTGTTCTGGATTCCAAACGTGCCCAGCGGTACCCAAAATCGCCAGGGCGTGTTTTATTATTTCCACAGGAACCGGTTTCTCTAATTGCATCACCAGGGTATCCAAAAGCCGGGCTTCCACGTTGATCCGGCTTACCGACAGTTCTTTCCAGGGTTTCCTGGTGGGGCGCGGCAGCTGACTCAAAAAGGTCACTGCCTCTGCCCATGGGCGGGTGGGTGACAGTCCGCCATACTCTTCCAGGACACTGGCCATGCATGACTCCAACCGTCTCACGTCCGAGGTAATGTGCTCTATGAAGATGTGGTAGAGCCAGCAGACGTTCAAACACGATGAAATCAAGCTAAGCTCCCGCCGGAACTCCACATCCACAAAGGGGTATTGCTCCGGTGTCTGTATTAGGTCTGGAATAGAAAACTCAGAAAAAGACACTGACCCACCAAGGAGAACCTGGCGTCTTGCAAAGTTGATGAGCCCCGCAGAAAGAATGTGTCTCCCGTGGGACAAAGAGCTTGGGGGGGCAGAGATGGCGCTACAGTGGGTGATTTCTTCTACCACGGTCATACATTGGTGGCACCCACAGGCCTGTTCCAGTATCAGCATAAATCTATCTTTGCAGTCATCCCAGATCAAAGTCATGTCAGATGCTGTTGCCTGGCATTTTGCCCGCATGTACATTTCCTGTCCCACATATTTTAACATCTGTAATACTGGAAGTAGATTCAGTCTGGTGTTGAGCCCCCCCGGGGAAGCCAGCGTATGCTTCAGGACCACCAGGGACGCTAAGAACCCCGGGTGTCCGCGCTCCGGAAACAGACCTCTGAGAATACGCTCGGTCTTGACGAAACCCGATGTGGTACCGAATGCCACAATCTGTGCCCTCCAGCTCTCACAATTTTCATCTCCAATACCCGGAATTGGGATACACACCTCCATGTTCAGTCACATGTACGCTAGGGTCTCCCCACCCAACCCCCATAGGACCCAGCTACAGCTTATCCTCCACTAAATACCAGGCAGCTACCGGCGACTCATTAAGCCCCGCCCAGAAACCAGTAGCTGGGTGGCAATGACACGTCCCCTTTAAAAAGTCAACCTTACTCCGCAAGGGGTAGTCTGTTGTGAGAATACTGTCCAGGCAGCCACAAAAATGGCGCAAGATGACAAGGTAAAGATCGACCTTTTTATTGTATACTGAACAATGCGTGTTTACAATGGTGTAGGTGGGAGCAGAGTTCGCCAAGCTCTACGTCCGAACAGTCGGGTGTCAGGGCTCTTATTAAGTGTTCGGTGTACTTGACCAAAGCCGCGGAACCTAGGTTGGGTCTGTACAGGTCGTACCAGGCAAAAAAGGATCGGGCGGTGCTTTTCAGGAGAGTTAGGGACGTGCTGATTATGTGGACAAGCTTCTGCTCGTAAATGCACCGCTGGTACATCTGAACGACAGCTGTCCAAAAAAAACAAAGGTTCAGCTGCACGTTAAAATCTGTATCCTGAAAGTCCTCGTAAATGACAGTTTCTACCAAGAAAAACTTTTTTACCACGCTGGCCATCCACTGAAAGGAGGGAGCACACGTCCCGTTGTGCGTTGTTAGGATATCCCTAACTTCGGAGCGGAGACGGCCGGACGCTCCCACAAAATGGGAGAGGCACCACTCTGTGCAGTCCGCGGTCGGGGGTTCTGATTCCAGGGGCGCCGTGTGGGGGTATTGGAGAGTCAAAACTCTGGGCAGTCCCTTAATGAGCTCTCTCTCAAAACCTATGCAGCCAGCGTCCACTAGTGGCAGCATGCCGTTAATAACACCCCTTATCTTGTCGTTGCCAAGTTTGTACAACTGCTGCAGGGAATAAGCCAAATTCGCCCTAGCCGCGGGAACCAGGTACGGCTCGCTTTGTCGGTGCTGGACCAATATCTGAATGGTCTTTGCAAGGTATAGGGTCTTCTCAACGTTTAGAGCGGGTACGTGGCAGTCTGGATTGAGGGTGGCGACGGACAGGGTATCTAACTCCTGAAGTATCTGATCCCAGGACGGGTAATGATACCTAAACAGATGGTTGAACAGGTGATCTTTAAGGGGCCTTCTCGATGTCATTGTAAAAACTATGACACGCCACTCTCTCCTTAGGGTAAGAAGCTTCGGCGGTCCTGTGTGGAAAGCTTCGTCGGCCTCTCGGACGAACTGAAGGCCCAACTCTACCAGTGTGTGCTCCTTATAAATGACGCATACGAAACAATCTACGATCCCAGTGACCTAAATAGAGTGGTGGAAGATGTGTGCATTCGGATTATGAAAGAATGTTCCAAGCTTGGTGCGCTATGTGGTCTGTTTACAGACATTAACATGTTTAACCTTTTCTGCTTTTTTCGTGCCTCTCGAATGAGGACCAAAGGCGCGGCCGGGTACAACGTGCCATGCGCAGAGGCATCCCAAGGCATTATTCGGATCCTCACGGAGAGGATCTTATTCTGCACAGAAAAGGCATTTCTGACAGCCGCATGCAGCGGGGTGAGCCTGCCTCCAGCCATATGTAAGCTACTACACGAAATATACACTGAAATGAAGGCCAAATGCCTGGGGGCCTGGAGGCGACTCGTCTGCAATCGGAGGCCCATTATGATATTAACCTCTTCCCTACTGAAGCTCTACAACACGTACGATACCGCCGGGCTGCTCTCTGAGCAGTCCAGGGCCCTCTGCCTTTTGGTTTTCCAACCGGTCTACCTTCCGAGGATTATGGCGCCGCTGGAGATCATGACCAAGGGTCAGCTCGCCCCTGAAAACTTTTACAGCATCACCGGTTCTGCTGAGAAACGCCGGCCAATTACCACCGGCAAGGTCACTGGACTGTCCTATCCAGGAAGCGGTCTCATGCCAGAATCTTTAATTTTGCCAATCCTGGAGCCAGGACTGTTGCCGGCTTCCATGGTAGACCTCAGCGATGTGCTGGCAAAACCCGCCGTTATTCTGAGCGCCCCTGCCCTGAGCCAGTTTGTCATTAGCAAACCCCATCCCAACATGCCGCACACCGTCAGCATCATCCCCTTTAACCCATCGGGTACAGACCCGGCGTTTATTAGTACGTGGCAGGCCGCGTCACAGAATATGGTGTACAACACATCCACCGCGCCCTTAAAACCGGCCACCGGTAGTTCACAGACGGTGTCAGTCAAGGCGGTTGCTCAAGGGGCCGTGATTACTGCGACAACGGTGCCGCAGGCAATGCCAGCGCGGGGTACCGGAGGGGAGTTGCCTGTAATGTCAGCGTCCACTCCTGCAAGAGATCAGGTCGCTGCATGTTTTGTCGCAGAGAACACCGGAGATTCTCCCGACAACCCGAGCTCTTTCCTGACGTCATGTCACCCTTGCGATCCGAACACGGTTATAGTGGCCCAGCAATTTCAACCACCGCAATGCGTTACGTTGTTGCAGGTTACCTGTGCCCCCTCTTCGACACCACCCCCCGATTCAACAGTCCGGGCCCCGGTGGTGCAGTTGCCAACAGTAGTCCCTCTGCCGGCCAGCGCGTTCCTCCCGGCGCTCGCCCAACCAGAAGCCTCGGGCGAAGAGCTTCCGGGCGGTCATGACGGAGACCAAGGTGTGCCGTGTAGAGATTCAACGGCGGCGGCTACGGCGGCAGAGGCGACAACACCCAAACGAAAGCAGAGAAGCAAAGAGAGGAGCTCAAAGAAGCGTAAGGCTTTGACCGTGCCAGAAGCCGACACCACGCCATCGACCACGACACCTGGTACCTCTTTGGGATCAATTACCACCCCCCAGGATGTGCACGCCACGGATGTCGCCACGTCTGAGGGACCATCGGAGGCACAACCCCCGCTACTGTCGTTACCCCCGCCACTGGACGTAGATCAGAGTCTATTCGCCCTGTTAGACGAAGCGGGCCCTGAAACATGGGATGTCGGGTCGCCTCTCTCCCCCACTGACGACGCGCTGTTGTCCAGTATTCTGCAAGGACTGTACCAGCTGGACACGCCACCGCCTCTGCGGTCACCCTCCCCCGCTTCCTTCGGCCCGGAGTCTCCGGCGGATATACCGTCACCTTCTGGTGGAGAGTATACGCAACTGCAACCGGTCAGGGCGACCTCGGCGACGCCCGCTAACGAGGTACAGGAGTCCGGCACACTGTACCAGCTGCACCAATGGCGTAATTACTTCCGAGACTGAAGTGTTCGCAAGGGCGTCTGTGCCTGCGTTAACTTCCCAGGCAGTTTATTTTTAACAGTTTGGTGCAAAGTGGAGTTAACCTACAGATTCTACTTAAAATAGCTCATTTTCTCACGAATCTGGTTGATTGTGACTATTTGTGAAACAATAATGATTAAAGGGGGTGGTATTTCCTCCGTTGTCGACTATAACCTGGCGTGTAAACGTGTAACCCTGCCAAATGCCCAGAATGAAGGACATACCTACTAAGAGTTCCCCGGGAACGGACAATTCTGAGAAAGATGAAGCTGTCATTGAGGAAGATCTAAGCCTCAACGGGCAACCATTTTTTACGGACAATACTGACGGTGGGGAAAACGAAGTCTCTTGGACAAGCTCGCTGTTGTCAACCTACGTAGGTTGCCAGCCCCCGGCCATACCGGTCTGTGAAACGGTCATTGACCTTACAGCGCCTTCCCAAAGTGGCGCGCCCGGTGACGAACATCTGCCATGCTCACTGAATGCAGAAACTAAATTCCACATCCCCGATCCTTCCTGGACGCTCTCTCACACACCACCAAGAGGACCACACATTTCGCAACAGCTTCCAACTCGCAGATCCAAGAGGCGACTACATAGAAAGTTTGAAGAGGAACGCTTATGCACTAAGGCCAAACAGGGCGCAGGTCGCCCCGTGCCTGCGTCTGTAGTTAAGGTAGGGAACATCACCCCCCATTATGGGGAAGAACTGACAAGGGGTGACGCCGTCCCAGCCGCCCCTATAACACCCCCCTACCCGCGCGTTCAACGCCCAGCACAGCCCACACATGTCCTGTTTTCTCCTGTTTTTGTCTCTTTAAAGGCCGAAGTATGTGATCAGTCACATTCTCCCACGCGAAAGCAAGGCAGATACGGCCGCGTGTCATCGAAAGCATACACAAGACAGCTGCAGCAGGTATAGACGGGAAACAGGTGTCTATCTTGGCCGGCTGGTTACTCAAATGGGAACAATGGCGCCACCTTGCTGTCTTTGTAGGCATTAGAAGAAAAGGATGCACAACTATGTTTCCTAGCGGCGAGATTGGAGGCACATAAGGAACAGATTATTTTCCTTCGCGACATGCTGATGCGAATGTGCCAGCAGCCAGCGTCGCCAACGGACGCGCCACTCCCACCATGTTGAAGCTTGGTTGTGCCGTCGTCCGGGAGAACCATGCCAGACTTTGTGTGGTAAGAAGGAATTGTTATCCGGCAGCAATATTAAAGGGACCGAAGTTAATCCCTTAATCCTCTGGGATTAATAACCATGAGTTCCACACAGATTCGCACAGAAATCCCTGTGGCGCTCCTAATCCTATGCCTTTGTCTGGTGGCGTGCCATGCCAATTGTCCCACGTATCGTTCGCATTTGGGATTCTGGCAAGAGGGTTGGAGTGGACAGGTTTATCAGGACTGGCTAGGCAGGATGAACTGTTCCTACGAGAATATGACGGCCCTAGAGGCCGTCTCCCTAAACGGGACCAGACTAGCAGCTGGATCTCCGTCGAGTGAGTATCCAAATGTCTCCGTATCTGTTGAAGATACGTCTGCCTCTGGGTCTGGAGAAGATGCAATAGATGAATCGGGGTCGGGGGAGGAAGAGCGTCCCGTGACCTCCCACGTGACTTTTATGACACAAAGCGTCCAGGCCACCACAGAACTGACCGATGCCTTAATATCAGCCTTTTCAGGTGTATTACACGTTTCAACTGTAATCCCTCGCAATTGGGTAAACCGTCGGTGTGTAGGGATAAAGCGTAACCTTACGTTCTGTCTCATCTACAGGATCATATTCATCTGGGGAACCATCCAGGACCACGCGAATTCGCGTATCACCGGTCGCAGAAAACGGCAGAAATAGTGGTGCTAGTAACCGTGTGCCATTTTCTGCCACCACTACAACGACTAGAGGAAGAGACGCGCACTACAATGCAGAAATACGGACCCATCTTTACATACTATGGGCTGTGGGTTTATTGCTGGGACTTGTCCTTATACTTTACCTGTGCGTTCCACGATGCCGGCGTAAGAAACCCTACATAGTGTAACACAAAACCATAAAAGTAAATAAACGTGTTTATTGTTCACATGATAAAGAGTGGTACTCTTTACTGGTTTGGGGGTTGGGTTGTGGCGTGGTGGCTGGTCCGCGGTTCAGTCATCAACCCCCGCCCGTGTTGTCGAGGCTCCTCTTCGTCGCCTGTTATTGGCACCAGGAGGCGGTTTAGCGGTGCCCCCGTCTGACATGCAGACGTCGATTCTAAGCGAAAGTCCCTTCAGGGCATCGTCCACTTGCTTTTGTGTTACAACCTTGCTGAATATTGTCCTGACCCTGGCTTCGATTTTCTTAGCGGCCGCCGCACTCAGTGCACCCACAGTAGCGGTAAGCTGCGCTTCCTTCTCGGTGGCCGTCAGAGGCCGATCTCTCGGATCGGCAGTGGATCCCAGTGCTTTCCGAAGCTCCCGATTCTCCACAGTCAATTGGCTTATCTTTGCGGTTAGGTCTTCCATCGTAAGGTCCTTTTTGGGTCTGCCCCTGGGCGCGGCCATGTCAGGTACGCGTAGATGTACGTGTTGGTGATGCTCACAACAAAAGCCCAAATCCCTCCTTTATACCCAGCTTTAAATACTTTATTGAAAACCCATAGCTTTCGTCAGCGCTTGTGCGAGTAATCACATGCCAGTCTATGCATGGACCACCTCGTCCACAAACTTGAAAAAACAAAGATATACCAGATAGAAAAATGTGGCCACGACGACTAGTAACGCGTTAATCAAGGCCCAGACGCTAGAAAAGCTAGAAAGGGAGGGGCTAAAACTATCCGCGGAACAAGCAACGTCATAGAATCCTGGGGTAGTGACTGATGTGGGACCGGGCGAAGGCCTGGCGCTGAGCCCAGCCGTACTGGGACTAGAACGCTCTGTAGATGATGCGACACCTGTCGAGTTGGCCGTAACCCAGCAGTGACCTAGTATCGAGGCCACAAATAAAGCCAGGGCCACCGTGGACGCTGTCATTATGAACAACCGCCGAGGCTCCAAGCCGTCTATCCAACGTTCCGCGTTCGCCTCTTATATACACTCTGCAATGCAGTCCGACTCTGCCCCTCTACCCAGGGTGGAATATGTGTTCGAAACAAGCAAATTTAGAATGACGTCGAGAGCAAATGAAGCCAGACTCAGACTGACAAATGAGTGTCCGATACTGGTGAGACCCCACGAGCCGTTCATCATGCCCACCGGAATACACTTCACGCGAACCCCTAGCTGCGCTTTCATCCTGACCGGAGAGACCGACAAGGATGTATTTTGCCACACGGGCCTAATCGACGGAGGCTACCGCGGGGAGATACAGGTTATTTTACTCAACAAGAGGAAGTACCCTGTGACGCTGTATCGCGGGGAGCTCAACATCTGCCTGTCTGCTTTCAATTACGTGCTACCTCCGTTGAGGGACGTATCATTCTTAACCCCCCCTATGTATGCAAACGACGCCGGATTTGACGTGATGGTGATGCACTCTATGGTTATCCCTCCTACTACTGACCAACCGTTCATGATATACCTAGGAGTGGAGACCCCAGGCCCCCCTGAACCCCACGTGGCTCTAGCATTGGGGCGATCCGGTCTAGCATCTAGGGGTATAGTTATAGACGTTAGTGAGTGGGGACCGCGAGGATTGCAGCTGAAGTTTTATAACTACTCGGGGCAGCCGTGGCTGGCGCAGCCCGGTAGCCGCATATGCCAGATTGTGTTTGTGGAACGCAGACACATCCTCAAGGGCTTCAAAAAGTGCTTGCGCCATAGGAAGCTAGCTCCTGGCGTCCGTTTCCGGGAGGCTCGAGTGCATTTTCGCGAGGATACAAATAGCGTCCGAAAACATACCCACGAAGACAACCCCGTCCACGAACCCAACGTAGCCACCGCTTCCGCTGACATTCGTGGAACCAAGGGGCTGGGGTCGTCTGGGTTTTAGAGCCGCCGCCAAATGCGGCCAGTTTATTAGGGCGATTCGATCCCGCAACCCACAGCATCCCCCAAATAAAAAAACGAGTGTACACAGCCAATGTTTTTATTATTGTTCGATTCATTACTGGTACCAGAGAATAAAGCCAACCTATGTCGAACCTATCGCGCTTTCTGTCGTCTCTTCCAGGGTTGACGAAGGCCGGGGAGGGATTGACGAATGCATCGCGGAAACGGACGGGTCTTCGGTGGGTGGCTTGGGTAAAGTTGCCTCCGGCTGGCGCGTAACGGCAGGCGTGAGAGGCAATACAGAAGTGGGTTCCGACAAGGAGTGGCTGATCTCAGAGGCCCATATTACCGAGTCGTCTGACGCCATAGCAGTCGCCAGTTTTTCCATCTCCATGAGCGAAACGCATTCCCCGGCCCTTTTGTTTAAGAGGGACTGGAGCGCACTGTCGTCCACGGTAATCTCGCCGACCGCCAAGGCCAGCATTGTGTTCCACACGACGTTCTGAATAGACTGCAGTTTTTTCACCTGGGTTTTCACGGTCTCCTGGCAGCCCGCCGGAATTTTAGCCACGTCAAAACGCTTCAGGTAGTCTGTGATCTTGTTTGACTGTACAGCCAGAAGGTAGGTCTGGTGCAGCGCCGTCGTGCCAAGGTTCGACTGGACAACGTCACCCAGACACACTCCGGGGGGGAGGCCCAAATCTATCTCTTGCCGCCAGCGCTCTGGACAGCCTTCCAGAGGGTCACCGAGGCGCTTGTAAGCGTGGTTGCCGCGTCCAAAAAGGTTTATACCGCAACACGTCCAGGTGTACCATGGAGACGACATACCGCCGCGAGGCGCTGACAGTAAGGGTTATTTTTTGTACGAGTGGCGACAGCGCCGAGACGATCGCCGACGTCCTTACGGGGGCCCCAACGTCAGCGTCCTTCTTTTCTGTACTCCACGACCTTTTTTATTCCCAGATACTCGCCCCCAGGGTAACCCTAAAATTGTGCCTCCCCGCACGGCGTCCTGGCAACGGCACAAGGTGTTCGCCCGTGTTGGTCCTACGTACTGACGCATCAGTGGCCTCGGGGTTCCTTGGCGGCCGGCCACTGGAGGCGTCCGACATTAAATATATGCTGCTCAGCGACCAGACCGCGGGGTTGTTCAAGCCGCTGTTGGAGATAATCGGTGGCGCGCGCGCACCACCAAATCAGGACGCGTGCACTTTCCAGAGCCAGGTGGCCTGGCTCAGAACGAAATTTGTTACCGCATTGAGAAAACTTTACAAGATGACTCCCTCACCCTACTGGATGCTGTCTGCATTTGGCGCTCAGGAAGCCCAGTTCGTCCTGACCAGCTCATTCTATTTTTTTGAACACACTGTGGTCTGTACCACAGAGACAGTTTCTCACCTGTCTAGACTGTTTTCGCCTCAACAGGGACAGACGCTGGTTTCCGTTACCAGCCACGAGGAGCTGGGGCAGCTATACGGCACTTCCCCTTTCAGGCGGCGCGTCCCCGCGTTCGTCGCTTATGTAAAAGAGAAATTAGCGAGAGACAGTCTGGAGACGGAGGCCATCGACCGCACCATAGACCAGATCAGGGGCAAACTCATGCTGTCTAACCAGGACCTGGTCCATTTCATATATATCTCCTTTTATCAGTGCCTCAACAAACGGGCGTTCCTGCGCTACTCTAGACAGACGTCCTCTTCAAGTGCTCTAAGGGAGCTGGGGGAAGACCCTCAATTGTGTGGCGCCCTACACGGGGAGTTTCGTGACCACGTCCAGTCCTACTACCACAAAAAAACCTACCTATCCACTTACATAGACATTCGGTACGTGGGTGGCGTATTACCAGACGGCTATTTTGGCGGGAGTCTTGTAGGCGAGCGGTGCGTTTATTGGTGCGGGCAGTCAAAGGACACGGCCAGCCTGTTGGCCACCATTAGCCAACAGGTGCCGCACCTGAGGTTGCAAAACGAGTTCGCTGGCATGCTAGACGTGGCCGCACTGCGAGGTTCCGATGACGGTCAGTTTAAAGAGGGCCTTTTCTCCCACAGTCAAGCCCTACCCCTGTACAGGTGCGAGTTTCTGGGCAAGCAGTTTTTCACAATGCTTCAGGAAGACGGCCTAGAGCGATACTGGGAGCAAAGTGTGATATTTCCAGGCGACCAGGACTGGGATATGTTATCTGACAAAGACCTCACCTACCGAATTTTTTACCATGACCTCAGCCTATCGCTGCCAACACTGAAGGAACAGCTCCTTGTTTCAAGACACGAATACTTCAACCCTCGCTTGCCAGTGTATAGATGGGTATTAGACTTTGACCTGCCCGTCTGCCGCGACATTGACAGGACATTCGAGGAGGTGCACTCTCTCTGTTGTTCCCTGCGTGAGGCCATACTCGACATCATTCAACTCCTTGGACCAGTGGATCCTCGAACACACCCAGTATATTTTTTCAAATCAGCCTGTCCACCGGACGAGTGGCGCGGCGAAGACGTCGCCAGCACCAGCTTCTGTCGGTGTCATGACAAACTGGGTATGCGTATTATCGTCCCGTTCCCAGAAGGAGTATGCGTCGTTGGGTCGGAGCCCATGGTGGCACTCACTGGCATTCTAAACAGGACGATAAAGCTTGATCCGGAGCTGGTCCACAGATTCCCGTCAATACAAAAAAAGGGGGGCCCTTTCGACTGTGGCATATACGGCCGAGGACGAAGCGTCCGGCTTCCCCACTGTTACAAGGTGGGCTTAGTGGGGGAACTCTGCCGCCTACTGAAGATACTAGTCTGTCACCCCGCCCCCAACGGCAAGGCGCAGTACGTGCGGCGCGCCTTTACGCTTCGCGAACTGCTCCATCACTCCCCGGGCCACAGCGCCGGTCATGTCGGCCGAATCATCTATAGCATCATGGATCGCAATGAGAATTTTTTAGAAAACAAGACCATTAGCTATCTGCCGGCCAAAATACCTCACATCTTTCAGCGGATAGAGACCCTATCCGGTCGTTCAATAGAGGACTGGCTACACTCGGCCGTTTGGGATAAAGCATACGACACTATATGTAAATTTTTCCCAGATGAAAAAGCACAACAGTTTTCTCACGTTGCATTTACGCAACAAGGGGAAAACATCATCCAGTTAAGACCCCGTCAGGGAAGACACTTCCTCTGCATCAACCATAATCATAAAAACAAGTCAAAAACAGTCCGTGTATTCCTTACCCTTCATTCCATTAGGGTGAGCGAAGTCACGGTAACACTTATGAGTCAGTGTTTTGCCAGCAAGTGTAACAATAATGTTCCCACGGCCCATTTTTCGTTTGTGGTACCAGTGGGACTGGCCAGTTAATCCCACTATATAACCTGGCTGCCAGGTTCCCAAAATAGCCCGCGGCATACGGCTCACTTCCCCCCACATTCCCCCCCGTGCACAATATAAGAACCAAAGGACATGGTACAAGCAATGATAGACATGGACATTATGAAGGGCATCCTAGAGGGTAAGTCCTCGTCTACAACAGACTTTTCCCATTTCTAACGTATCGTGCTATCTTCGTCGCCCGGCGGACCATCCCCCCACCCCTCATTTATCGCGTTTGATATTACAGACTCTGTGTCCTCCTCTGAGTTTGACGAATCGAGGGACGACGAGACGGACGCACCGACACTGGAAGACGAGCAATTGTCCGAACCCGCCGAGCCTCCGGCAGACGAGCGCATCCGTGGTACCCAGTCGGCCCAGGGAATCCCACCCCCCCTGGGCCGCATCCCAAAAAAATCTCAAGGTCGTTCTCAACTGCGCAGTGAGATCCAGTTTTGCTCCCCACTGTCTCGACCCAGGTCCCCCTCACCAGTAAACAGGTACGGTAAAAAAATCAAGTTTGGAACCGCCGGTCAAAACACACGTCCTCCCCCTGAAAAGCGTCCTCGGCGCAGACCACGCGACCGCCTACAATACGGCAGAACAACACGGGGCGGACAGTGTCGCGCTGCACCGAAGCGAGCGACCCGCCGTCCGCAGGTCAATTGCCAGCGGCAGGATGACGACGTCAGACAGGGTGTGTCTGACGCCGTAAAGAAACTCAGACTCCCTGCGAGCATGATAATTGACGGTGAGAGCCCCCGCTTCGACGACTCGATCATCCCCCGCCACCATGGCGCATGTTTCAATGTCTTCATTCCCGCCCCACCATCCCACGTCCCGGAGGTGTTTACGGACAGGGATATCACCGCTCTCATAAGAGCAGGGGGCAAAGACGACGAACTCATAAACAAAAAAATCAGCGCAAAAAAGATTGACCACCTCCACAGACAGATGCTGTCTTTTGTGACCAGCCGCCACAATCAAGCGTACTGGGTGAGTTGCCGTCGAGAAACCGCAGCCGCCGGAGGCCTGCAAACGCTTGGGGCTTTCGTGGAGGAACAAATGACGTGGGCCCAGACGGTTGTGCGCCACGGGGGGTGGTTTGATGAGAAGGACATAGATATAATTTTGGACACCGCAATATTTGTCTGCAATGCGTTTGTTACCAGATTTAGATTACTTCATCTTTCCTGCGTTTTTGACAAGCAGAGCGAGCTAGCACTGATCAAACAGGTGGCATATTTGGTAGCGATGGGAAACCGCTTAGTAGAGGCATGTAACCTTCTTGGCGAGGTCAAGCTTAACTTCAGGGGAGGGCTGCTCTTGGCCTTTGTCCTAACTATCCCAGGCATGCAGAGTCGCAGAAGTATTTCTGCGCGCGGACAGGAGCTGTTTAGAACACTTCTGGAATACTACAGGCCAGGGGATGTGATGGGGCTACTAAACGTGATAGTAATGGAACATCACAGCTTGTGCAGAAACAGTGAATGTGCAGCGGCAACCCGGGCCGCAATGGGGTCGGCCAAATTTAACAAGGGTTTATTCTTTTATCCACTTTCTTAAGGATTGCCAAACCCCATGGCAGAGTGTCTCCCGTATTCCATGTAACTCACGTAGCCTTTCTCTAATAAACAAGCTACCTGCAAACTATACACAAATGAAATGAGTCAGGCGTGGTCTCTTCTCTACCGTGAATCGCACCTTAAACACAACACCAGACCGCCACCAGGTGGCACCCAACATCCATTATGGAAAAACCCCGCGCCACCTTCCGCCACGTGGAGCCAACAAACAAGACACACCCGCCAATGTTTTGGTCTCTTTATTGATATGATATACTCCCTCCCATAACAATACGGTGTAGGCATTTTGTATTATTTATTGCATGGCATCCCATAACGGCTTCGGCATTATTTCGAGTACGACGCAGGCGTCTGAGAAATTACTGCACCTCGCCGCAAAGTCTCGCGGGGACGGGGCGTGGGGCTCTAACTTGCCAACCGCCACCGGTTTCCCCAGCCACAGCTTCACCAAAGGACACGTCACGTGAGAGGGTGCTGGTAACGGTGAATTTGCCAACCCCACCAGAAATGTATTCGGGTTAAATATCCTCGTCGGTTTTCCCTGGGGCAGCAAGAGGGGGCCGGAGTCAGGCGGAACGGTATTTCCAATAAAGTGCACGGGCCCGTTATGATAACATACGCAAAATATGCCATTACAAGAGCTAGTCAGCAGAATGCCTTTTGCACATGCGTCCAGCGTATCGCATAGCTCCCGCTTGGCTATCTCGCAGGCCAGGTTTGGCACATTGGGTAGCCATACCTGGCCCGGAGACCCCACTGCACAGTAATGAACTGCGGGGTCCCTACGCAAGGCCGATGAGATTCGACAGCCCGACTGGCTTGTCGTCAGTAACTCATGAACCTGTTCGCCATTATAATACATCCTGATAAACAACCGACCCCAGTCAATGACGGCCTCCTGACCCTCTGCCGTCGTACAAGATGGCACGGGCGTTACAATCTCGCCTGGCAAGCACTGCCCCGGGGAAAAAAATCCCTCTTGCAAGAGACGTGCCATATTGTTAAAATCGTGGACGGCTCCGGCCACGACTCCACATTCCACGCATTGTTCTTCCTCCGGTTTACGTACTCTAAAGACCAGAAAATGGTGTCCATCCTGAGAAATGCCTTTGCCAATCTCTTGTAAACCCCGCGTCCTGCGTAGCGCGGCAAGCATTCGCCTGCGCCCCCTGGTGCCTTTAAACGAGGCGTCCACGGGCATGTTACCCCTTTCGCGGATATACACAACACCCAATTCCCCGTCTCTGCGCCATTCAAAACAGGGGTCCGCGAGGGGCGTAACTGGTATACGGAAGCGGGTGCGCTCTTCGTCTTCCCACTCTACTCCGGGAAATTTTCCACTGTTGACTTGACATACTATCCAATCCTTGATTGACGCTTTCCCCTCACTGGCACCGGTAGATATTCTTAGTTGTCGTGTCCGGCTCCACTCCGTTATCGCAGCCACCACAGCCTGCCGTGTAATATCGCCTGCGGCTGCAGAACCCCCGGTCCCGGAGGGTCCTTCTCCCGGTGACTCCGACCTGGATGGTTCATCGCAAGGAGCCCCGGAGCCAGATGTTCCCGGTGACCCTTGTGACAAACAAGGTTTTTTGGGTATCGCCCCAGGCGCCCCAAAAGGGTTCGGTCTTTGGCCTGGGTCCATTGTCCCGCAACCAGACTAGCTCGCGCCGCAATGTCCAGTGGTAAGCACAGCTATGCCGGGGAGCCACCGGCCATCAGATATAGAGAGGCGACAGGCTCTCTATATATCACGGCTAGGTGGCTGACATATTAGTGGGCCTAGCCGCAGAATTGCCTGGGTAGTCAAAAACCAGCGTTTCTCAAATTAACCGAAACTACATTTTTCTATTTTAAGTACGGGATACAAAGCAGGGTCTGAGGCAATCTGCCGCCCTCCACCCCCACCCACCATACCCAAAAAAGATATGTCAGAAAGAGCACTCTACCTATTAACTCGTGGAGAAACATCATACAAAATCTGTACATTATTTTTAATACTTTAATTTGTGCAGGTTTCTTCACCCCACACCTGCTTTTTGTCTGGTACAAAAAACCACTGCAGGGTCCCGCCTATAGCCAACTCCTAAGCGGGTTTTTTGCTAAAGCACTTTTTTAGACTGTCCCAGAAACCACATAGCTTCCTTTTCACTCATTTGAAAAACAGCCCCGCCCAACTGCCTGGAGAATTTTCCACCCCCTCTACCATTTCGCGCCTTTACCGCTGGTGCGAAATCTAGCCATCCTATCACCGCGGATCCGCTGGACCAATATACCACGCCCACTTTTCGTAATCAGCAACCCTCTACGCCTACACCCCTATGACTGAATATAACCCCCAACAAGGCTATGAAATCATGAATGGTAACTGTCTGGACACCAATCTTCCGCGGGGTGGCGGCAGTGCGACGCAAGTATCCACAATAAATGGTGCAATAATTGGCGAAATGTCGTGTCTGGTTTATTTGGACTACAAGATTACATCCGGTTTTATAATTCACATATATGATCAATGTAGACTATCCCAAATGGAGCCTATAAAAATTTTAACAGTCAAGGGTACATTTTGGAAATTTTCTGTAGATGCCGGGGATGCGCCGAAAAATACCGTCCCGCACGTCACTGGGTTGACGCTCAGCGGTGTCTGTGGGATTGCGGCTGTGGTTGCCAGGTATCGCGCGGTGTTGAACAGCTGCTGCGGAACTCTGGGGCTAAAGCTTCGGAGGATGCGTTCATAGCGGGAATTTGGATTACCAAACCACCAGCCTTCCACTTGAGTGGCGTTTCTGGAGTATATTCCAGACATCGAGCAAAATATTGGGAATCCGTGGCCAAGGCCTTCAAAAACTCGGTTCAAAATCTCCATTTGCTCGGGTGAGGGGACTGTAAGACGCGGTATGCGAAGCAGTTCTGGTACGAAACTCTGACATAGGTGCCCCAACGTATCCCCAACAGGCCAGCTACATAACATTGCCTCGCCCGCGTCACCTTCGCGTCTCAGAGTTCCACGAAGGTTCCCATACACAAAGATTTCCACAACAAAAGACACCCGCTGACTATCAGGGGGATCAAAAAACATCTTTGAAGGTGGCTTTTCGGGACCGGAGTGGCTAACGGGCGTACGCCGCCCGTGCGGGGACCTGGACCTCGGGCGCCGCCTATCCGTGGCCTGTCTGGTTGAGGAGCTCGGTTCCTCCTGCAGCTCAGACAAAATGTTACCCAACCCTTCTTCCCACGTACATATATCCTCTCCTTGAAGGTTCGAGAGCGTAAGAGGGAGACCCAAAGGCGGCGGCACTAAAGATTGTTCTGGTCCATAACCCCCCACTGCATATCTATCTCCAGCATATGTACTAACAAGTGGAACTCTGGGCCTTTCGCCACTACCCGGGCACACACACTCCCGCCGCTCCAGCTCTGTCGGTAAATGCGAAACCTCGGGGTTCACAGCGGGCTCCGGTGCAGAATAAAGCACCGTAGGTTGGAAAACGCGCGGCCCACTGACAGGTAGGGGCGTGGATGCTACAGTGGTAGATGGGGTATCGGAATCCCCAGTGAGGTCAATAATCTCCACTTCGAGGGCACCAGAACTAGTTGTCACGCGTCTGTATCCAGTCGCCATGTTGTCCCCCTGGCAGACGTACGGTATTCCAGACGAGGATGGCTCCTGTCGCTCTGCCACCTCTGGGGTGGGTGGTGCGCCGGCGGAGGGCGTGGCCGACGCGCCACCCTGCGTGTGGGAAAGACCCTGGTTTGGAGCGCCTCCACTAGACCACGGAATCCAAAGCGGTGTGCGAACTTCCGGCACCACGGCGTGACCAACTGGTGGGTGCCAAACAGGCGCGCGTATGGGTCGCGTAGCTGGCGGTTCTGCCAATGGACTCCAATTGTAACATGATGGTTTCGCATACCCGGGCGCGGGGGCGCTGGGCGGTTGAGGTTCGAAGGGATACACCCGCTCACTCGCAGCACCCTGAGGAGCCCGGCCTTCTGTAGATGCCCCGCAAGCGCCTTCGGCACCGGTTTCCCGGCGGGGAAGCCACGCGCTAGCACATTGGCCGCTTTGGGGGAGCAATCCCTGTGGCGCCAGAGGTGCACCCTGGCTGAACTCACCGACAAATGTTCCCGCTTGGGCGTGCGGCGGAATCCAACTGGGGGCAGCAGGATTCAGCTGGCTGCTAGGAATCCCCGTATATGTCCAACGGGGGGAAAGGGGATCAAATTGGCCCGTGGTTGGCGGATGCACTTTCTCCGGGAGACCAGACGCGCCCTGAGGCCACCATCCCGTGACAGGAAGATCTCCCCATGGAAAACACGCAGGTATCCACGGGGACGTAGATGGCAGCCTAGACCCATCGCGCATGGGAGGGGCTAGTTGCCCCGTATCCCCCGGCGTCTGTGCGACGCCGGAGACCCCTGACACAGTACCGGCAAGCCGTGTTTCGTGCTGCGGCTTGGGCGGCGCCGTGCCCGGTAGGCCTGCACCAGATGAGTGAGGGTCTGAAGGGCCGGTCAGCGTTGATGGAGCAGGCGGATCTCCGGGAACCCGCCACGTAAAGGACGAGGCCTGCGTAACTTGTCGCGTCCCAGAGGACCCCATACCTGAGGTAGATGCGCCCTCATTCACTGGTATCCACACGGAGCAGGCAGCCTTCTGTTCAGTCGTTATATCGCCAACATTGTAATAGCGGTTCGATTTCCGAGGGCGACCCCTCAGCCCCGATGGCGCCTTAGGGGGAGCAGGTGCTGCAGCCCCTGCCTCCTCGTAGCTTTGTTCTCTAAGTAAAAGGCACGAGAGTTAACGTGGTTAGGGTACCTAAAGTATTTCCCGCCGACACCAACGCATCAAACCTCACACCCCCTTCCCCGAGTTACATACCTAGTGTCACTGCGTCGCGTAGCCGTGGTTTGCATTGGGGGGGACAACAGACACTGAATAAATCGCTGCAGTTTTTCAGGACCATACGCGGCCCCATAGCAATACGTACAGTTTTTAAACGGCGTTCGCACCAACTGCCATACTACGTAGCTACCACCAAATGTGTCGCTGTACCGTAAATCGTTCCGCACGACGGCCCTCCTGGTTCCACGCAACAGTCTCCCAAAACGTCCATACACCGTCTGTCCCACGACAGGCGATGGTCCGTAGACTCTATCACACTCCTCATCAAATGCATGGTACACCGAATACCAGCCAGGCGGGATATCGCTGCCGGCAGGCAGGGGCGCGGGGGCTGCAAAAAGAAGGTTGTTCCTATCAAACCAGGAAAAATAGGGAAACTTATTGTTTTCAAGGGCATCAATAATCCATAACGTGGCCCATTCTGAGCCACCGGCTTTAGGCATGGTCCGACACAGAAACCGATCGGCGTTCGTCTTTGAGGCACAGTCCCGACTGAGCCTTATAGTGCCCCCCTTCTTGCTATGAAAAAAACCCACGACCGTTACGCAAATTTGAGGAGCTACTCACCTAAAAGTAGCTCCTTTGACAAATGTCCTGGTTTTATACCAATTGTTCACAATGACATATTGTGCTGGCGGAAACAGGTGTCCCGATGTATCCTCGGCAAGTAAGCACCATTACCATGTGCCATCATATTGTGTGGCACAAAAAAAGCAACTTTTCACGCACGCAGCATAAGACCCGAGCCAGTCGCGCCCTCCATCGCGCCTGCGAATTTTCCCACCACCCAATATTGTGGCAGATCTTTCTTATGTATATGTGGTTACAAACACCACGCCCCTTAAGCTGTCCTCTCTCCCAAGGGGACTAGATTATAACAGTGACATACGAAACCGAGACGCTCTCAAATGCTTTCTATTTTATTTATCGATTCCGGGTTAACATAAGCACAGGTAGCTATAAAATCCCCATCCTCTTGACCTGGTAACCCTGGCTTGAGGTTTCCTCTGTTATCAAACAAACCTGACCACAACTGTACAGAGAAAAGTGGGTGAAATGTAGTGTTTATTTTATCCTCACACTTTCACTTAACCACAGCCCGTCAAACCACAGGGACCCTGTTGGCTGACTATTAGTCATCACATGTAACTGAACGCAATCTGAGCTTGATGACGAGGGGGACCATATCGAACTGTTCTGCCGACGTTGGGTCACCTCCGATGAACACAGTTGTTTTTTTAATGTGCTCATGTCCCTGTATGCGATATTGTGCCACATTAAAAACATCCAGAACAGCCCTAGATGACAGTCCGCAGATCACACCAAACTTCTTTGGAGGATTATTTCCATGATATAATACGGTAGACTTGCACAAATTCTTAACATAAATGCCAGATCGGAGAGAAACTATCACAAGACCCGAAGCAAACGAGCGCAGCACGGCCGCCAGCAGGTTAACGTCTCCTGGCCCTGTGTTATTGTCGTCAGGTTTGGGCAACAAAACTCTTAACCCTTTGCGCGAATGCAAGCAAGAGTGGCTAATGTCTGCCAGTGGGTTCTGGGAACATAGAATAAACACCTTTCGTTCCACTTCCAAAGACATTGCAGGGCGGCCAAAATAAAACACTTCCACACCAAGCCTATCGGTTATCATTACTGGCGGCCGTGCCACTCTATAATATGCGGATCTAAGCTTCCTGTGGCGAATGCGCCTCGTGGTAGGCCTCTCGTGTCTCCGTGGCCCATCATCCCATAAAAATTCGCCAACAACTGGCCGGCGTCTGGACGCCGGCGGCAGTCCAGCACCATCATCGACTTCTTCGTCACTTATCTCCAACACATATTCCCCTGCTACATTCTGGGCCTCGAGTGCCCCAGCTAAGTACACATCCTCTACACCCGCCCCGACAGCCGAGGCGGCGATTGAGCCCTCTGTTACCACGCCGCTTGCATCCGTGTCGCCTCCGGGCTGTGATGTTGCGATAACATCCTCTGGGATGCCAAGCAGATCAAAGAGGTCTTCATCGCACATCGCCCTCATTAGCATGTCCATCTCCTGTCCCACGTGGTACATCAATGCACATGCAGATTCTTTATCAAGCAGTGTGAGGTCATCTTCAACGTTGTCTGTGTGCACCGTTGTTTCATCGGCCGGGGGGGGCTGCGAGTCGCTATGACGCGTCGAGGGTCCTTCGTCTCCAGAGCCAGGAGAGTCGGCATTGGCATCATCAACTGGCTGAACCCCAGACGCACTATGGCGCGTCGATGGTCCCTCGTCTCCAGAGTCCTCAGATTCCGCGCCCGTCTGCGTGACCGGCACATCGCAAAAGGCTGGGTGATCCTCCTCACTGGAATCCGAGTTTTCACCCACAAATGGCCTACAGGAAAAAAAAAACAAATATGTCAACCGGACTAGGGTGGCCAAACCATTTGCCCCACCCCTCCCCACTCTTTCCCCAGGGGACACATCTTACCTTGGTCTTCTCCGATGCTTCTCGAGCCGTACACTGTGTTGATACAAAATTTCCCATAGTGATGACCCACTGTGTAGGTGAGTCCTGGCATGAACGCACCACCAGCATTCCTTTACCTCGGCACACAGGAGGCGCCACCTTCTACAATTAATTCCCTGTACGACCTCGTACTCTTCACCTGGCAAGCGTCTAAGGCGCCGCGACGTGGTACATATTTTCCCAAAAGCCGTAATCGGCGAGCCCAGTAAATCTCTGGGATGCAGGCCCTTCGATAGGCATTCCCTCTTAAAATCAATGAAAAACTGTAGGCTATCCAGAGGAATTACGTCATTACGGGCAGCCGGAGCAAGAAATGTTCCAGTAGATCTATCTAGCCACTTGACCAAAGGATATTTATCAGAGTCCAAAGCACCTACAATAAACTCAGAAATCCAGGTAAGCCTGCGTCCCGCCATGTTGACCTGTCAGAATGGTCTGCCTCCGAGCATTACCCCACCTCAACAGAAGTAATCTACTACGCAAACCACAACATGCTTCCTGCAGCTTTAACCTTCAGTCACGGGTCAAAAAGCATTGCCTGTATTAGACACATGTGTTTCTCACTATGAATCGTGTTCTCCAGCGCTGGCAAGAACATCTGGGGTGATGCTGCCCCGGACCAGCTTTGAAACAGGGTATTGCATGCATAATGAAGCCCACATGTTTGTCTTACTTTACTAACCTCATTACCTTGCATTGCAGGGGACACCCCCTTGCCTTGGCAGCTGAGTGAATCCCAACCGCCTAGGAAAAAAATAACCACTCAGACTTTATTTTGCAGCCACACGGTGGCGCTAACCTTTAATGATGTCCCACTCAGTGAGTTTGGCCACTCCCAAGCCCACATGGGCCTACTATAACAGGAAACATAGAAGTTGCGGATAGAGCCTGGTTTCTAACGGCAATGATATTTATAGTGCAAAACGGAGGGCGGTAAGACAAAGGGAGGTACCCGGACAGAGTGACAAGAAGACTTGTCAAAATTTTAGTCTCTGTGGTAAAATGGGGCAAGGTAAATGTGCAAAATGACTGGATAGTGATCCCAGTCATATTCAGGCGACGGCCGGCGGCCCAGAAACAGGGACGCGTACCGGGACCCTTCAGGTTCTCGATTATGTCGCTCCACGTCAAAAGCTTGTTGGATCTCGTGGCGGTGGGACAGGGGCCTACATTTGCCTATTCTTCTTCGCGATGCATTTCCAACAAAGTATGCTGGGTATTCCAATAATCCCTTCAGAAAAATGCCCATGTTTGTACCGATGGCCACAACTCCCATGGAAAACCTGTCCAGCGTCTGTTCCAAAGTTCGGTTTGCGTCCACACTACAGTGGGCCGTTCTGGGAAGTAAGCATTTATACGGGGGTACCGTCTGACATATGTGTTCAGGGGAGGCCTCTGGGACTTGGGAGCAAATAACGATGCCCCCCGTTAAATCAAAGTGGGTCTTCACCTTTTCTCCGAAATAATACACTTCCACCACTAGGGGCACAAGCTTGTCACCCACTTTGTAAATAGCCTGTTTCTTACTCAGGTATGCTGCCACGGATTGGGTGGCGGTTAAGACCTTGGGCCTCATGTCGCTTCCATACCAGTAAAATGTCTGGTCAGCTTTCTCTTGGTCCTCGACGTCCCGGTCATCACGACACAACGGTGGAATACAATCAATAAAATCATCCACATTGTCGGAAGCTTGGAAAGATGAACCCATGACAGAGGCCCCAGGTGCCGAACTCTCAAGGGGATGCGTGGCGGGAAGTACTGAGACACTCTCCGTGGACCCCTCCTCACCTCCCTCCGACTGCATCGGGCCCTGAGGGCTCGCAGTTTCACACAGAAGTTCACTCAGGTCGCCTAAGTCAGGAAGCTCCTGGCCTGAACCCATGACAGAGGCCCCAGGTGCCGAACTCTCAAGGGGATGCGTGGCGGGAAGTACTGAGACACTCTCCGTGGACCCCTCCTCACCTCCCTCCGACTGCATCGGGCCCTGAGGGCTCGCAGTTTCACACAGAAGTTCACCCAGGTCGCCTAAGTCAGGAAGCTCCTGGCCAACATCTGACAAGAGATCTAACAAACACCCCTCAATGTGATCCACCATCGGTAGGCAATCATCCAGCCCACTGACATGACTGGGGACGGGGCCTTCTGGGGAAAATGGGGTTTGCGACTGTCCAGCAGGCGGCGCTAATAAGCCTTGTGTCTCATGTGGAAAAATAACAGGAGAAGGTAAACCCCCCGTTGGCAAACATAGATCCGTCGGGGTGTGCACGTGTAATGGGCCCTGCACCTGGCTCGTGGAGGGACGCGGGGAATCCGGAGCTAATAAGCTCGATGACTGACCAGATGACCCAAACCCCGACGGTTCTGGCTCTTCAAAAAACAAACTGTGCATATCCCTCCCTACAAAACCCTGAGCCCCCACCCAAAGTTCGTTTTCGCTGTCACTCGATTCCGTATCTTCGCTCTGTGACCGTGATGAAACTTCAGCTGCGGAGGATGTTGTGGGCGTGGCGACTGCCGCCGCCTGTTTCCTGGCGGCCTCCCTAAACAAAAGTTAATTACACAAAGGTAAGTCTGAGTGACATCTCCAATTTCCCGTGATGCCCGCTGCACGTACATCCCGCCGCCCACACAACCCACCGCCCAGTACATCAACCATCCTACCTCTGGGCTTTTTTTCTAAGGCTCCTTCTAAGTGCCTTTTCTCTGTGTTTGTCATCATGGGGATAGATCCCAAACAATGCTTTTAGCATGTTTTTCATGGCTGGTTCCTGCGTCAAGTACACAAGACATCCTTCACATCCCTTGTATGGCCTAGGTGTCATAATCCAGCGGTTGAGTTTCATTTTTCCCTTATAGATGGTAAAGGGCCTCTCCTGTCTGGCTCGATTGGCGGTCCTTAATAGCCGTCCAAAGCAGCCCAGGCCAGTCTCAGTCTCCGGGATTTCTGGCAGCCCGTGCCTACGTCGCTCCTCCAAAAATGCCTCATAGAAGTCATCGAAGCCTTCTGGCATTCTCTCCCGCCGGTTTCGACCCGGCACGGTGAATATTCTCTTTTGTTCATCCAACCACCCTACCCCCCAGAAGCGTCCACTGTCTAAAGCATCTATAATAAAGTCCGTGAGCCATTCCGACTCCGTGTAGCGAGGCATCTTTTTAGGCAAAAGCCACGACACAAAACACCTTTTCCGTGGGCGACTTTCTCGCCACAACTAGCTGGACCCCAACCCCACTGGCACGTAGACTCTGTGCCATCTAACAACAAAACTCAATATATGCAGCTCAACACCGCCCCCCCCCCCCAGCCGGTTGTCGGGCTGCGGAAACTTGTGGTTAGAACTCACTACGGAAAAGGGAACCAATGCAGTTGAACTACTGGCACACACCCATAACCCGGGACAGCACCCAGGCACTGTCCACCCTCTAATACAAGCGGCCTTTGGACGCGAGGGAGGGGTGTCATGGTCAACAAACCAAGAAAAACACATGTATTATTCAATTAGCCAACAACTTTATTTATTACCGACAGGAGACATGAGATACATAAATTTCCAACCGTGCATAGGGCCAATACCATCTGTGGAGCGTTAAGTGCCCTGTGGAGTTTTCGCCTAATTAGCTGAATCTCGACCCCCATTGCGGCCAGCATGCTCACGAGGAATAGGCAGCAGAGGCAGGACCTAACTAGGAGCATATCCGGACCTGATCCAAGTATGTGCACCAAGGTGAGCAACACTGCCGCCAAAGGCAGGAGAACAAATAGCGCTCGTCGGGAGGCGACGGATACGCCCACGCATGACAGTAACCCAACATAAAATAGCGTCATATACTTATCCAGGCCAATCAGGACCGGAGTCAGCAGGCCGATCGAGGCCGTCGATATCAGGGTGGCCAGCAGTAAGGTCACAAACACGACAACCTCGCGCCTACAGTAGGCCCAGGCCTGGAACACTGAATAGGTGATGTACTTCCCGGGCATGATGAATATGGCCCTCCTCCTTTGCATTCCGGCCCTGATGTACACATGCTGTTCCAGGTGCCTAAATGCCAAAAGTCCCCCGACCAAGAAGACAATGAAGGGCAGCCAGAAAACGCCGGACACAAAGACCTTCTTAAACAACAGAAGGTAGTACACCATAAATGCTCCGCAGAAGCCCAGCTCATAGTACCTGTGTACTATTGGCGGCGCCTGATACACCGCCGTTGCGGTGGCTAGCGGATAAGGTAACAGCAGTAAACAGTTAAGTACGCACAGACCCGGTATGAAGGGCACACGAGAAAATGTAAACCCAGAAAAGGCCGCGCAAACTACAGCAGCAAACACTGCTGACGCGCAGATCCATTCCAGCCTCCGGTCCAGCTGTTTTTGCGCCGCAGGGCACAGACACATGCATATCAGGGCCAAGTGCGTGACTGGCAGCGACCAGAAAAACACGGCCGTGATCTCTGTGGTAAAGAGTGTGAACGAGTACAGGGCCTTGAAGATAAAACACCACAGAAAGGGGGTCGCCGCCAACGTCCCGCTCAGATAACTGAAGAGCGACAGAGCGCGCTCACTGTCCAGGCGGCACATGGTGTCAAATCAGGGGGTTAAATGTGGTTTTGGGCACCTTCCCACGATCCCTGGACTGGCTCGAGTCTGAGCGCCTCTTGTGAGGCCTCTTTGTGCTGTCCTTAGTTGGCGCCGCTGGGGGGCAGCTGGTGACAGAGGCAGCGTCCTCAGAGGCGTCCTCCAGCGGCCCAAAGGGACCAACTGGTGTGAGAGGGGGAGAATCCGGAGACTCCAATTCCGGCTGCCTCCTGGAGTCCGGTATAGAATCGGGAACCTTTTGCGAAGACTCGCCTCCCTCGGCAGACACAGATCGGTTTACCTCTAAAAGTAGGACACTTAACTTTACGTCACCTGATTGGCAGCCAGTGGGCACACCTTCCACTTCTAATATTTCGTTGGAGTGCCAAATCAGCCCGGGGGTAAACCAACCCGGGACTTTACACAGTCTCAGGGCGGCGATTAAGGACTCCAGGCTAACCCGGCTCAGGGCGTCGGTGTGCACCACGCCCACATCCACCGACTTCTTCCCCTTCAGACCATCCCAGCCAGAAACGGGTTTGGTTTCTGGCTTGAAATCAATGATCTTGCTCACGCCACCAAGAGAAAATGTCACGATCGACAGCGTCTCGCTGACAGACACAGTCACCGTTTGGTCCTCTTTTGTTTTTTGCTGCCTTAGCCACTTAAGTAGGAATGCACCCGTTTTGCCACAGAGGAGAAGCCTGGTGGTCCTACCACCGGCTTCCATCCGATCGTGGAAAGGTAGGATACCCTTTTGGTCCACCACGCTTTTGTGCACGGTGGAGGTGAGGTTGTCCCCGTAGGAAATGGTGGTCCTGACGAACTGCGGTTGGGCCCCCGTATCGCATGCCTCCCCCTTTCGATAAAAGGCTATGCCAGCGTCGAGTACATTCGCACCGAATAGCTCACGCGTGTGCGTGAAGCCGCTACCGACGGACGTATTCCTGAAGCTGAAGCTAACGTCTCCACTGCCTTCCGTGTGTCCCACCAGGGGCGTAAGGGCATTCTTTATTCTTAACCCCAGAACGCCAGCTGTCCCCACGCTGGACAGCACACTGAGGGTTGGCGTGCAAGCCGATCCGTGCACTTGCACTACTCCGGTTTTAGTGGCACTCTTAATGTGTTCATTGACCCTCCTGATTTTAGACAGGAGGGTCACGTCCACCCTGACCCCATAGTGAAAATCCACAGGCATGATTGCGGCCGTAGACGCACAGAGAAATCACAGGAAAGCTGCGCGCACACTGGGTGTTCTGGAGACGATAGACTGCCTTAAATAGAACTTTTAGGGGAGGTGGAAGTGTGCGACATGGACAGGTTAACCTTCACAAATCGTCAGTCACACACGTGGTGTAATCAGAATTGTCTCGCTCAAAAAAATTCACAGCCTTGAAACTGCCGGTGTATGAGAGGGGGCACGCTTCTGGCGGAGGCGTGCCAAATATGGGAGGAACGAAAATATCACGCAGAATCCTGTCAGCGGTGGCTTCCAGGAACCTCCGGATGTCCACCACGTTAACAAGCGTCACCCCGGCCGCCTTGGCCTGGATAAACCGAATCTCAATATTCACTGCCTCCCTGAACAGCGCCTGGACCTCTGCGTGACTGGGTTTTTCCTGTATCTCCACCATAGTGTTGTACAACATACTGGCGGCCTTGGTGTGCAGCAGCTCGTCCCTGGAAATGTAATCGTTGGCAAGGCACACCCCGGGCATGATGCCTCGCACCCTGCACAAACTGATAGAGTAGAAGGAGCTAATAAAGTATATCCCCTCCACAATCAAAAACATCAGAATCTTCTGAGCTTTGGTGGTCGCCTTACGCACCCTGGAGTGAAGCCACTCCAGCTTCTCGCAAAGGGCGGGGTCCAAAATGATCTTGGCAGCATATGCTAGAAGTTCGCCTCGACTGTTGTTGAAAAATATCTTCAAGATATTGGCATACACGACACCGTGGATATTCTCCATGGCAACCTGTTCGGCATAATAGTGGGCCACGTCGTGGCTGTTAAAATTTGTGACAAGGTCCTCAATGTTAAAGTTAACTAGGCGTTCGGCCATTCCCAAAAACGTAAACAAAAATCTATAAAAGTCCTTGTCGGCATCGCTGAGCTGGTGCACGTGGGAAACATCAAGGTGCAGGGGTATCTGGCTAGGAAACCATCGGTTCTGCCAAGTCTCGCGCGTTAGCGCCAAAAATCCGTCGTGATCGCTTGTATACAGAAATCGATCAACTGAATCCATTGGCCTCACCCGGCTTGCAGAGACCTACCTACTGACAGACCAGGCACTCGGGGTCTGCCGCGCAGGACTCCTCCTCCGGGTTTTTAGGTCCGGGTAACCACGCCCCATCTTGTTTCATCCCAGAGTGAGGCGGTGACCCTGGATCTGCCAGGCACTGAAGAGCCGTCAGACTAGATTGCTTCTGAACCCTACAGTAGTACATGAGGGTTTTTAGACCAAGCCTGTATCCATGTAGCAGCAGGTCCCTAAGATAGCTCGCATTCCTGACTCTGTCCTCCTTGAGGAAGAAGCTCATGGACTGGCTCTGGTCTACAAACGGCGCCCTGGCACGAGCCCTATCCAGTAGCTTAAATGGACAGTAATCAAAGGCTGTTAGGAATACCCTATATCTTTCCCTGTGATGCTTGGGGAACGTGGAAACGTCCCCACCATACTGTCTAACCACCCGAAGGTCGTCGGGGAGAACCTTCTTAAAAAAAGTCACATTGGGCCTCAACACCTCTTCTTTATTGGTGACCTTGGAAGATATATTAGCAAAAAAGGGGTACACGGACTCGGCATAGCCAGTTACTTGCGAGGTCCCAGCCGTCGGCATCACCGCCAGAAACTGAGAATTGAATATGCCATGCTCGGCAATGCTCTTTCCCAACGCGTCCCAGCGATGGCGTGGTACAAACGAAGCATCCTCCCCCTCCCATGTTTGCCAATGAAACCTGCCCTTGGCGAAGTTACTGACCTCCCAGCCATGAAATGGGACACCCTGGCCCTCCAAAACAAGGTTGTGACTAGTCTCCACCGCGGTGTAGTACATAGACTGGAATATATTCTTGTCTAACTCAGCGCTCTCAGCATCGAGGTACCCGTACCCCAATTCCGCAAACACATCCGCCAACCCCTGAACACCAATCCCCATAGACCTCTCCTTTTGACCTCGCTCGACCCCCGGTGTTGGATGGGAACCACCCAGAATGCAGGCGTTGATGACGAGGACTGCCACCCTTACTGCGTCGCCCAAGGCCTCAAAACAAAAAAACGGCCTGTTGGCGTCCGTGGTGCCAACCCTCGCGCTTTCAACAGTTCTCAGACACTTTGGAAGGCAGATATTTGCCAGGTTGCACACCGAAGTGTTTCTTCCTGGCAGTTGGACTATCTCTGCACACAAGTTTGAGCAGTTAATGGCCATGCCCTGAGTGTCGGTCCAGTGGTGTTCATTGAGCGCTTCTTTTAAAAGCACGTACGGTGAGCCTGTCTTTATGATGGTGTGGATAAGAGTGAACATCATAGACTTCAACGGCATGCAACTAACGTACTTTCCAGCCCGCACCAGGCGCTCGTATTCGTTATCGAACGCAGCACCGTATAGCTTAATCAAATTGGGGGCGGTGGCTGGATCGAACAAATACCATAACTTGGATGGGTCCTTTTCATACATCCTGAAAAACAATGTTGGGATGCACACGCCCTGAAAGAGACTGTGACATCTGTCGGGATTCTCCGGTAGTTTGGCGTTCAAAAAATCACAGATTTGACTGTGCCAGAGTTCCATGTATGCGCTCGCGCCAACGGGCCTGATGTTATTGTCATTGAAATAATGAACCTGGGCATCCACCAGTTTGAGGCAACTGGCTATGTTCTTTTGGTGGGAGAATGACGTAACATCCAGACCCACGCCTGACTTACTGGCCAGCAACGGACTCATATCGTGGTACAGGGCGTCCAAAGTACCCGGCTCATTCATCATGGAGGGCTGCAGAATAAAACAGCTGGCGAGTTGTCCGCCTTCGACTCCAGCTGAGCGCAGTATTGGCGTGGCGCAGCACACGTGCTGCGCAGCGAGGTAGCCAAAAACGTACTCCACTATAGCCATCTCAGATACAGACTTAGCGTCCTCAATAAGGTCCCGCGCCAACCAATACAGGCATTCATGCTCTAAGCACTGACAGGCAACAAACACGGAAACCCTCATAAACATTTGCGCCACGCTTTCATAGACAGGCTCTGTCCCCATGGTCCTTAGGACGTAAGTATCATACAACCTCACGGCCGATAGGTAGCCACAGTTAAGTGTGTCCTCGTAAGCTTTGGACCGTCTGTAGGCGCACAACATATCTTCCAAGGCATCAATGTTCTTTTGAATAAACGATTCCACCCGATGTCCCAACACGCCTCGAAAAATCCCAAGATACTGCTTGAGAGTCGCTGGGCACCTAGCCTCCATAATTTGGTGCCACAGCCGCCCCGCCATGGCATTGGCCCGCACGTCCCACCCGACCCTAACCTTTAGAAAGTCTATGAGAGATTGGGCACACATATCAAAATCCGACAATTGTCCCGCAGACACCTGAGACCCGCGTCGCTCTGGTGGGACAGCTCCCAAGTGAACCTGACAAAATGTCCGGACAGACATGACCTTACAGAAACACAGTCCAGGGGCCACACGCGGCCTCAAAGTTCGCAAACACCAGTACAGGCAAGGACGTGCCCTTCACGTTCAGACTTTGGTGCACCGGATGAGAATCAAAGGGAACTGTGCCCAGCGTACAAACCGCCCCAAAAACAAGCCGATTTATATACAGCTCGTGCCTCAGCTGAATATACTTGGTCCGGATTACATCCGTAAAGTGATCCTTTATCATGGCCACAACCTCCGCAAAGCCCTTCCCAGACTGGAAAAACGTCAGCGCCATAGATGGTCTCTGGTTCACACGGAGATAAACCAACGAGGCATAAATAGTAACGTTTAGGCCTGCCGGTTCCCGGCGCTGGACCATGGGACATGACTCATCCAAATCAACTAGCATATCACAAGGGAGGGTCAAGCCTACGTGTGCACGGGGCTCGTCCCGGGCCAACCCAACTCCCTTCATGGCGGAGGTGACCTTGGTCACGAAGGTACTGTGGACACTCTGGACCATTGGACCTACTGGGGTAAGGAGGGTATGAAACTCCCCAGTGTCCATGAGTTCACTCAAGTTAGGGATGAAATCCGCCAGGCCGGATCCACTTCCGTACCACACACCGGCCACTTTGTGAGTCTGTGGCGCTTTTGCCGCTTCCATTCCAGAGAGCATAAACAGGGACGTGGGTGTTAGCAGCATATCCATAGACGAGCCGTTGTCCTCCTGCTTGAATGAAAATAAAAAGGTTCCCAGAGGCTCCTGGGGACTAAAGGTCTGTGAATACACGAGGAAATCTCCATAGGTCGGCTGCCTAAACGGCGCCTGCCGCAAGGCCTCATGCAGCGAGCCAACCGTGGGTCGTGTGGACGCCGCATATTTAGAGAGTAAATCCCGCACCCCCCTGGCAAACTCCGGTCCTCTAGTGAGGGATACCCGGTGAGTTGGTGGAGGTAAAAGACCCAACACTTGCCTACCCAGGCGAGCCGCATTTTCAGCCTGCACCTTCATATCCACGCCGGCAATGGACGGCACAGACGCTCTTGAAAAGCTTACCAAAGGCCTGAGTGGGGGAGGCGGGAGCCTTCACCAGACAAAGCTGTTGATGGAATTTCAACTCCGAGGACTGCCGGTGCCTGCCCTCTTAAACAGCAGCACAACAGAGCAGTTTTTAAATACTGTTGCCCAACTGCCGACGGACCTATCAAAATTTATACGCGACTATCGCGTGTTCGCACTGGTTCGCGCGGCGTATTTTTTAGAACCCCCTTCTAGCATCGACCCCCTTGAGGCAGCGCGCGCTCTTGGACGCCTGGTTGATATATTATCATCACAACCACCGCAGAACACCGCACCGGCGCAGCCACCCACCTCCGACGACACCCTGAATAACTGTACATTGCTCAAACTACTAGCCCACTACGCGGATCAGATAGCAGGTTTCAAAACCCCCGCTCTCCCTCCCGTGCCACCTGGAATCATCGGCCTGTTCACATGCGTGGAACAGATGTACCACGCATGTTTTCAGAAATACTGGGCAGCTGCACTACCCCCAATGTGGATACTGACATACGACCCTCCCACTTCTCCGTTACAGGACTGGCTTATAGTCGCCTATGGTAACAAGGAAGGACTGCTACTCCCCTCTGGCATACCCTCGGAGGAGGTGTTAGCCAAAACATTAGTAACAGAACACCACGAGTTGTTCGTATCGCGGTCGAATTCGACCGAGACCGCCGTCACCATGCCCGTATCCAAAGAACGCGCCCTCGCCATCTACCGGGTGTTCGCCAAGGGTGAGGTGGTGGCGGAAAATACTCCCATTCTTGCCTTCACCGACGTGGAACTATCCACACTCAAACCCCACTATCTGTTCATCTATGATTTTATCATAGAGGCATTATGCAAGAGCTACACATACTCATGCACCCAGGCCCGCCTGGAATCCTTTTTGAGCCGAGGTATAGACTTCATGACTGACCTAGGTCAGTACCTAGATACCGCTACTAGCGGCAAGCAGCAGCTGACGCACAGCCAAATAAAGGAAATCAAATACAGGCTGCTAAGCTGCGGTCTCTCGGCTTCCGCGTGTGATGTTTTCAGAACTGTGATCATGACCCTCCCATATCGACCGACCCCCAACCTCGCTAACCTGTCCACGTTTATGGGGATGGTTCACCAACTGACCATGTTCGGACACTATTTCTACCGGTGCCTGGGCAGCTACAGTCCCACCGGCTTGGCCTTCACAGAATTGCAAAAGATACTGACACGCGCCAGCGCGGAGCAAACGGAACGTAACCCGTGGAGACATCCGGGTATCTCGGACATTCCACTGCGTTGGAAAATATCGCGTGCTCTAGCATTCTTCGTCCCTCCGGCCCCCATAAACACTTTGCAGCGCGTGTACGCCGCGCTGCCCTCGCAACTCATGCGGGCCATCTTCGAGATCTCGGTCAAGACCACATGGGGAGGCGCCGTACCGGCAAACCTGGCGCGCGACATTGACACAGGACCGAACACACAACATATCTCCTCCACACCACCGCCCACCCTCAAGGATGTTGAGACATACTGTCAAGGTCTGCGGGTGGGAGACACGGAGTACGATGAGGACATTGTGAGAAGCCCGCTCTTTGCAGACGCGTTTACCAAGAGTCACTTGTTGCCTATACTGCGCGAGGTTCTGGAAAACCGCCTGCAGAAAAACAGAGCTCTGTTTCAGATAAGATGGCTGATAATATTTGCTGCCGAGGCGGCAACCGGGCTCATCCCTGCCAGGCGCCCGCTAGCCAGAGCCTACTTCCACATCATGGACATTCTGGAGGAGAGACATTCCCAAGACGCCCTATACAACCTTTTGGACTGTATCCAGGAGCTCTTCACCCACATCAGGCAGGCTGTTCCAGACGCACAGTGTCCGCACGCCTTTCTACAGTCCCTGTTCGTCTTTCAATTCCGCCCTTTCGTACTCAAACACCAGCAGGGTGTAACCTTGTTTCTAGATGGCTTGCAGACATCCCTCCCCCCGGTGATAAGTCTGGCCAACCTTGGAGACAAGCTGTGTCGTCTCGAGTTCGAGTACGACAGCGAGGGCGACTTCGTGCGCGTGCCAGTTGCACCGCCAGAACAACCACCGCACGTACATCTGTCGCATTTCAAGAAGACAATACAGACCATCGAACAGGCCACCAGGGAGGCCACCGTAGCCATGACAACAATCGCAAAGCCAATATACCCCGCCTACATCCGGTTACTGCAGCGGCTAGAATATCTTAACAGACTCAACCACCACATTCTCAGGATTCCCTTCCCACAGGACGCCCTTTCTGAACTCCAGGAAACCTACCTGGCGGCGTTTGCACGGTTGACAAAATTGGCAGCGGACGCAGCAAACACTTGTAGCTACTCCCTCACCAAGTACTTTGGAGTTTTATTCCAACACCAGCTGGTCCCCACGGCCATCGTTAAAAAACTGCTACATTTCGACGAGGCTAAAGATACCACAGAAGCCTTTTTACAGAGCCTGGCACAACCCGTAGTGCAGGGACAACGGCAGGGGGCGGCTGGCGGGTCGGGTGTCCTGACGCAGAAAGAACTTGAGCTCTTGAACAAAATAAACCCACAGTTTACAGACGCTCAGGCTAACATTCCTCCATCTATTAAACGTTCATATTCAAATAAATATGACGTCCCTGAGGTCTCAGTCGACTGGGAAACGTACTCCCGGTCTGCCTTCGAGGCACCGGACGACGAACTCCGTTTTGTCCCACTGACGCTGGCAGGCCTCCGGAAACTGTTTGTCGAATAGAGGCCATGGCAGCCCAGCCTCTGTACATGGAGGGAATGGCCTCCACCCACCAAGCTAACTGTATATTCGGAGAACATGCTGGATCCCAGTGCCTCAGCAACTGCGTCATGTACCTGGCGTCCAGCTATTATAACAGCGAAACCCCCCTCGTCGACAGAGCCAGCCTGGACGATGTACTTGAACAGGGCATGAGGCTGGACCTCCTCCTACGAAAATCTGGCATGCTGGGATTTAGACAATATGCCCAACTTCATCACATCCCCGGATTCCTCCGCACAGACGACTGGGCCACCAAGATCTTCCAGTCTCCAGAGTTTTATGGGCTCATCGGACAGGACGCGGCCATCCGCGAGCCATTCATCGAGTCCTTGAGGTCGGTTTTGAGTCGAAACTACGCGGGCACGGTACAGTACCTGATCATTATCTGCCAGTCCAAAGCCGGAGCAATCGTCGTCAAGGACAAAACGTATTACATGTTTGACCCCCACTGCATACCAAACATCCCCAACAGTCCTGCACACGTCATAAAGACTAACGACGTTGGCGTTTTATTACCGTACATAGCCACACATGACACTGAATACACCGGGTGCTTCCTTTACTTTATCCCACATGACTACATCAGCCCAGAGCACTACATCGCAAACCACTACCGCACCATTGTGTTCGAAGAACTCCACGGGCCCAGAATGGATATCTCCCGCGGGGTGGAATCATGCTCCATCACCGAAATCACGTCCCCTTCTGTATCCCCCGCGCCTAGTGAGGCACCATTGCGCAGGGACTCCACCCAATCACAAGACGAAACGCGCCCGCGCAGACCTCGCGTCGTCATTCCTCCTTACGATCCGACAGACCGCCCACGACCGCCTCACCAAGACCGCCCGCCAGAGCAGGCAGCGGGATACGGTGGAAACAAAGGACGCGGCGGTAACAAAGGACGCGGCGGAAAGACGGGACGTGGCGGAAATGAAGGACGCGGTGGCCACCAGCCACCAGACGAGCACCAGCCCCCACACATCACCGCGGAACACATGGACCAGTCCGACGGACAAGGCGCCGATGGAGACATGGATAGTACACCCGCAAATGGTGAGACATCCGTTACGGAAACCCCGGGCCCCGAACCCAATCCCCCAGCACGGCCTGACAGAGAGCCACCGCCCACTCCCCCGGCGACCCCAGGCGCCACAGCGCTGCTCTCTGACCTAACTGCCACAAGAGGGCAGAAACGCAAATTTTCCTCGCTTAAAGAATCTTATCCCATCGACAGCCCACCCTCTGACGACGATGATGTGTCCCAGCCCTCCCAACAAACGGCTCCGGATACTGAAGATATTTGGATTGACGACCCACTCACACCCTTGTACCCACTAACGGATACACCATCTTTCGACATAACGGCGGACGTCACACCCGACAACACCCACCCCGAGAAAGCAGCGGACGGGGACTTTACCAACAAGACCACAAGCACGGATGCGGACAGGTATGCCAGCGCCAGTCAGGAATCGCTGGGCACCCTGGTCTCGCCATACGATTTTACAAACTTGGATACACTGCTGGCAGAGCTGGGCCGGTTGGGAACGGCACAGCCTATCCCTGTAATCGTGGACAGACTGACATCGCGACCTTTTCGAGAAGCCAGCGCTCTACAGGCTATGGATAGGATACTAACACACGTGGTCCTAGAATACGGTCTGGTTTCGGGTTACAGCACAGCTGCCCCATCCAAATGCACCCACGTCCTCCAGTTTTTCATTTTGTGGGGCGAAAAACTCGGCATACCAACGGAGGACGCAAAGACGCTCCTGGAAAGCGCACTGGAGATCCCCGCAATGTGCGAGATCGTCCAACAGGGCCGGTTGAAGGAGCCCACGTTCTCCCGCCACATTATAAGCAAGCTAAACCCCTGCTTGGAATCCCTACACGCCACTAGTCGTCAGGACTTCAAGTCCCTGATACAGGCATTCAACGCCGAAGGGATTAGGATCGCCTCGCGTGAGAGGGAGACGTCCATGGCCGAACTGATAGAAACGATAACCGCCCGCCTTAAACCAAATTTTAACATTGTCTGTGCCCGCCAGGACGCACAAACCATTCAAGACGGCGTCGGTCTCCTCAGGGCCGAGGTTAACAAGAGAAACGCACAGATAGCCCAGGAGGCTGCGTATTTTGAGAATATAATCACGGCCCTCTCCACATTCCAACCACCTCCCCAATCGCAACAGACGTTCGAAGTGCTGCCGGACCTCAAACTGCGCACGCTCGTGGAGCACCTGACCCTGGTTGAGGCGCAGGTGACAACGCAAACGGTGGAAAGTCTACAGGCATACCTACAGAGCGCTGCCACTGCTGAGCATCACCTTACCAACGTGCCCAACGTCCACAGTATACTGTCTAACATATCCAACACTCTAAAAGTTATAGATTATGTAATTCCAAAATTTATAATAAACACCGATACACTGGCCCCATATAAACAGCAGTTTTCATATCTGGGGGGTGAACTGGCATCTATGTTCTCCCTTGACTGGCCTCACGCACCTGCAGAGGCGGTAGAGCCACTACCCGTGCTGACTTCTCTGCGAGGTAAAATCGCAGAGGCGCTGACGCGTCAAGAAAACAAAAACGCTGTAGATCAAATTCTAACCGACGCCGAAGGCCTCCTTAAGAACATTACCGATCCAAACGGCGCACACTTCCACGCCCAGGCCGTATCAATTCCAGTGTTAGAAAACTACGTACATAACGCGGGGGTCCTTCTCAAGGGCGAAAAGAGCGAGAGGTTCTCCCGGCTGAAGACCGCCATCCAAAACCTGGTATCCTCCGAATCATTTATCACCGTGACCCTACACAGTACAAACCTTGGAAACCTAGTTACCAACGTACCAAAACTTGGTGAGGCGTTCACCGGGGGCCCGCACCTCCTGACAAGCCCGTCCGTGAGACAGTCCCTTTCCACCCTGTGCACAACCCTGCTGCGAGATGCCCTGGACGCCCTGGAAAAAAAGGATCCGGCCCTTCTTGGTGAGGGGACCACGTTGGCGCTGGAGACACTCCTAGGATACGGGTCGGTGCAGGACTACAAGGAGACGGTACAGATAATATCCAGCCTTGTGGGCATCCAAAAATTAGTCAGGGACCAGGGCGCGGACAAGTGGGCCACTGCCGTGACAAGGCTAACTGACCTCAAATCAACTCTGGCCACGACCGCCATCGAGACGGCTACGAAACGGAAACTATACAGATTGATCCAAAGGGACCTCAAAGAGGCTCAAAAACACGAGACCAATCGGGCCATGGAGGAATGGAAGCAGAAAGTACTGGCTCTTGACAATGCGTCTCCGGAACGTGTCGCCACCCTCCTGCAACAGGCTCCCACCGCGAAGGCTAGAGAGTTTGCAGAGAAGCACTTCAAAATACTACTCCCCGTACCCGCGGACGCCCCCGTCCAAGCGTCTCCAACGCCGATGGAATACAGCGCCAGCCCCCTCCCGGACCCAAAGGATATAGACAGAGCTACATCCATCCACGGGGAACAGGCGTGGAAGAAGATACAGCAGGCGTTCAAGGATTTCAACTTCGCCGTCCTGCGGCCCGCTGACTGGGATGCCCTGGCAGCGGAGTACCAACGCCGTGGTTCGCCCCTTCCGGCGGCCGTGGGTCCAGCGCTCTCAGGGTTCCTGGAGACGATCCTAGGGACGCTGAACGACATCTACATGGATAAGCTCCGCTCCTTTCTGCCCGACGCGCAGCCTTTTCAGGCGCCGCCCTTCGACTGGCTAACGCCGTATCAGGACCAAGTCAGCTTTTTCTTGCGCACCATAGGGCTGCCGCTGGTGCGAGCGCTGGCCGACAAGATCAGCGTGCAGGCACTGAGGCTTAGCCACGCGCTCCAGTCCGGCGATTTGCAGCAGGCCACGGTGGGCACGCCCCTGGAGCTCCCTGCCACAGAGTACGCGCGCATCGCCTCCAACATGAAGTCCGTGTTCAACGACCACGGACTTCAGGTGCGATCAGAGGTCGCGGATTATGTGGAGGCCCAACGAGCCGACGCACACACGCCACACGTCCCACGTCCAAAGATACAGGCACCAAAGACTCTGATTCCACATCCGGACGCAATCGTCGCGGACGGACTACCCGCCTTTCTTAAGACGTCCCTACTGCAGCAAGAGGCCAAACTTCTGGCGCTACAGCGGGCGGACTTCGAGTCGCTCGAGAGCGACATGCGCGCCGCAGAGGCCCAGAGAAAAGCATCGCGCGAGGAAACCCAGCGCAAAATGGCACACGCCATCACTCAGCTCTTACAGCAGGCACCCAGTGCGATCTCGGGGCGCCCGCTATCCTTACAGGACCCGGTGGGCTTCCTCGAGGGCATCATATACGACAAGGTCCTGGAGCGCGAATCCTACGAGACGGGTCTCGAGGGACTGTCCTGGCTCGAGCAGACCATCAAGTCCATCACCGTATACGCTCCCGTAGAGGAGAAGCAAAGAATGCACGTGCTGCTGGACGAGGTGAAAAAGCAGCGAGCAAACACTGAGACCGCTCTCGAGCTAGAGGCCGCGGCTACGCACGGCGACGACGCTAGACTCCTGCAGCGAGCGGTCGATGAGCTGTCACCGTTGCGCGTTAAGGGGGGGAAGGCCGCGGTGGAATCCTGGCGGCAGAAAATCCAAACCCTGAAATCCCTGGTACAGGAAGCGGAGCAGGCCGGCCTCCTGTTGGCCACCATAGACACGGTGGCCGGCCAGGCCCAGGAGACCATATCACCATCCACACTCCAGGGACTGTACCAACAGGGACAGGAGGCCATGGCGGCCATTAAGCGGTTTAGGGACTCGCCCCAGCTAGCTGGCCTGCAGGAAAAGCTGGCCGAGCTACAGCAGTACGTCAAGTACAAGAAGCAGTATCTGGAACACTTTGAGGCCACCCAAAGCGTAGTGTTTACAGCCTTTCCGCTCACACAGGAGGTTACGATCCCAGCCCTGCATTACGCGGGACCTTTCGACAACTTGGAGCGGCTCTCACGATACCTACACATCGGCCAGACGCAGCCGGCTCCGGGACAGTGGCTCCTGACACTTCCCACATTCGACCCCACGCGCCCGGCCTGCGTCCCAGCCGGCGGCCACGAACCCCCGTTGCACAGACAGGTGGTGTTCTCCAGCTTTTTGGAGGCCCAGATCCGATTAGCGTTGTCCGTAGCGGGCCCCGTGCCTGGACGGGGTCTGCCCGGAACACCGCAGATCCGAAGGGGCGTGGAGGCTGCCGCTTGTTTCCTCCACCAGTGGGACGAGATATCTCGCCTCCTTCCAGAGGTACTGGACACCTTTTTCCACAACGCGCCCCTTCCCGCAGAGTCTTCCTCCAATGCTTTCCTGGCCATGTGCGTATTGACGCACCTTGTCTACCTAGCTGGGCGCGCCGTCTTGGGCCCACGGGAGCCGGAGCACGCCGCCCCGGACGCGTACCCAAGGGAGGTGGCGCTGGCCCCGCGCGACCTGACCTACCTTCTACTGGCCATGTGGCCATCTTGGATCTCGGCAATTTTGAAACAGCCTTCGCACGCGGAGGCGGCGCACGCATGTCTTGTCACGCTGCCAACAATGCTCAAGGCTGTGCCGTACCTCACGCTGGAAGCCTCAGCTGGACCACTGCCGGCGGACATGCGCCACTTCGCCACGCCAGAAGCGCGTCTGTTTTTCCCCGCGCGATGGCACCACGTCAACGTGCAGGAGAAACTGTGGCTGCGTAATGATTTTATGTCGCTGTGTCACCGTTCCCCGGGGCGCGCGCGCATAGCCGTCTTGGTGTGGGCCGTCACTTGCCTAGATCCTGAGGTAATAAGGCAGCTGTGGTCCACCTTGCGGCCCCTTACTGCGGATGAATCCGACACGGCTTCTGGACTGCTGCGGGTGCTAGTAGAAATGGAGTTTGGTCCGCCGCCCAAGACGCCGCGGCGGGAGGCGGTGGCGCCCGGCGCAACACTGCCACCGTACCCCTACGGCCTTGCCACCGGCGAGCGCCTGGTCGGCCAGGCGCAGGAACGCTCTGGCGGCGCTGGCAAGATGCCGGTGTCCGGGTTTGAGATAGTTTTAGGCGCACTGCTGTTCCGCGCCCCCCTACGCATTTTCAGCACCGCATCAACCCACAGGATCTCAGATTTCGAGGGCGGTTTCCAGATACTGACTCCTCTCCTGGACTGTTGCCCAGATCGCGAGCCATTCGCCTCCCTGGCCGCCGCACCACGAAGGACGGTGCCACTGGGAGACCCGTGCGCCAACATTCACACCCCCGAAGAGATACAGATCTTTGCGCGTCAAGCCGCCTGGCTTCAATATACCTTCGCAAATTACCAGATCCCCAGCACCGACAACCCGATACCGATCGTTGTGCTAAACGCTAACAATAACCTTGAAAACAGCTACATCCCTCGCGATCGCAAAGCGGACCCGCTACGACCATTCTATGTAGTCCCTCTGAAGCCGCAGGGTAGATGGCCTGAAATAATGACCACAGCAACAACCCCCTGCCGCCTACCGACATCGCCAGAAGAGGCGGGATCACAGTTCGCCAGACTCCTTCAGAGCCAGGTGAGCGCCACATGGTCTGACATCTTCTCCAGGGTTCCCGAGCGCCTCGCTCCCAATGCGCCTCAGAAGAGTTCCCAGACAATGTCAGAAATCCACGAGGTCGCCGCCACGCCGCCACTCACAATCACCCCAAATAAACCGACCGGAACCCCTCACGTCTCCCCGGAGGCTGATCCAATAACAGAACGCAAACGCGGACAGCAGCCGAAGATTGTCGCGGACAACATGCCTAGTCGTATTCTCCCGTCGCTACCGACCCCGAAACCCAGAGAGCCTAGAATCACGCTACCCCACGCACTGCCCGTTATATCACCCCCAGCACATCGCCCGTCGCCTATACCGCATCTGCCAGCACCGCAGGTAACGGAGCCCAAAGGGGTTCTCCAAAGCAAACGTGGAACTCTCGTGCTGCGGCCCGCCGCGGTCATTGACCCACGGAAGCCCGTCTCGGCACCGATCACGCGATATGAGAGGACGGCGCTCCAGCCCCCCCGGACTGAGGGCGAAGGCCGGCGCCCTCCCGACACGCAACCCGTCACTTTAACCTTTCGTCTCCCACCTACCGCACCCACTCCCGCAACTGCAGCCCTAGAAACCAAAACAACTCCCCCATCCACGCCCCCACACGCCATAGACATTAGCCCACCACAGACACCTCCCATGTCCACCTCACCTCACGCGAGAGACACAAGCCCCCCCGCAGAAAAGCGGGCCGCACCCGTCATTCGAGTAATGGCGCCCACGCAACCGTCGGGAGAGGCAAGAGTCAAGCGAGTGGAGATCGAACAGGGCCTTTCCACACGCAATGAAGCCCCTCCCCTTGAACGCTCGAATCACGCCGTGCCCGCCGTTACCCCAAGGCGCACCGTAGCCCGCGAAATCAGGATCCCGCCGGAGATAAAGGCGGGTTGGGACACTGCACCGGACATTCCTCTGCCCCACAGCTCCCCGGAGTCATCCCCACCGACTTCCCCCCAGCCTATCCGCGTGGATGATAAATCGCCTCTTCCCAACCTCGTAGAGAGATACGCGCGGGGTTTCCTGGACACGCCCTCTGTAGAGGTGATGTCCCTGGAAAATCAGGACATCGCCGTGGACCCCGGACTGCTAACCCGCCGGATTCCATCCGTGGTGCCCATGCCCCATCCAATTATGTGGTCACCCATAGTACCCATCAGTTTACAAAACACAGACATAGACACTGCAAAGATAACACTGATTAGTTTTATTAGACGCATCAAACAAAAAGTGGCCGCCCTATCGGCGTCCCTGGCGGAGACGGTTGACAGAATAAAGAAGTGGTACTTGTGACTCCACGGTTGTCCAATCGTTGCCTATTTCTTTTTGCCAGAGGGGGGTTTCCTCGCGTCGGCCACCGCGGGGGCGGCCGTTTCCGTCGTGGATGAGAGGGTTGTGAGAATGTCTGACGCCGGCGACAATGAATGGGGACCAGAGGACAGGGTGGTTATACTGCTTCCCGAGACCCCCAGTGAGTCCTGGCCCCCGGGCGTGGTGCCGGATGCAGGGCCTGGCCTCGAAGGCACGGTGAACGTCCCCGCGTCGTAAGCCGACGCCGCGGAAACTCGGTCAGCGCGCTCGCGCGGTTTCTGATCCCTAAGGGTCTGCAGATGATCCCGCCTTTGAATTCCACCCATCCTCCTCAGATAGGCCTCATAATAATGATGGGCAATTAAGAACACGAGATAGTGTCTCTTTTGCACGAGGTATTCGGCCTGCGACATATTTCCCTGATCCAGGGTATTCATGCGAGCCACCAGGGGATGGTGAGCGTAGTCATGATCCAGTCGCTCCTGGATCACGGGGTCTCTCACCTTAAAGTTGGACATCTTCCACACAGGCGGGCGAAATAGCCTCAGGAGGAACACTTCCCGCAACAGAACTCCAGCAGCTGTGAGGTGAGCTGAAGCAGTCCGCGCACGTCACGGTGCTTTAATAGGGCAGCCTCGCAGTCGGGCGTCCCAAGGCAAGGCACTACAAAACTGACAGTTTGATCTAGGTCTCGAATGGCAAGGGCCGCGTTGTTAGCTAGAACAGCCCTGATTACGACGCGTGCTAGGGTCCCGCGTCCGGTAATATCGCACAGGGGATACACCCTCATATGTTCGCTGCCACAGTAAGAACAGTAGATCCTCCCCGTGGTCGCACAGATGGTGAACTGCTTCTCTTTCCTGTCCCTGCTGAAAAACACGTTGGTGGGAGGAAAATTGACAGTATGAAACTTGCCCCTGCCAAAGTTAAGACAGTGTCCACACTCCATGCACACAACCGCCCGAGCGCAACGCGCCCGCTTGGCAAGGGCCGCGCGGGCCACGCGAGAACAGATGACGGGTATGGACACGCAGGGGGAGAGAACATTGTATGCCAGAAGCCTCCTGCCAAGGTTCCGCACGAGACCAGGTCCCTCCTGCTCGCAGGCGGGCAGCACTACGTGGCGGGACTTAATAAGGCTCAAAAAACACAGTGACCCAAGCATGGCGTCGAACGGGTTACCGCAGGGAACCGTAGGGGCGACGCGCTCCAAGGCCTCCCGGAGGCCGGTATCTGCCGCCCCTATCCCGAGCCCGTTACCGTCTTCGGTCGCAGCCACACCGCGACGGGTGGGCGAGGGCACCTCCAGGAGGGGACGACGCGGCAACGGCCCATGCCACTTCTTCCTTAGCCAGGGTAGCGACGGTGGGGGCTTCGAACAGCAGGTCACTAACGGAAAGCGAGAGCAAAGCGCCAACAGCTTGCAGAGTTGGGCACAGGCCTTGGAAAATGGAAGCGACAGGTATTTTGCCCATACGTGGCGCGGTATCGCCCTAGCATGGTCGGCGGCCTGGGCACGGGACAGCGTCACCACAACCCATACGTGGGCGCCAAGCAGCTGCTGCGCCGCACAAATCTGCGCCTGTTTGGCGACGGTGTCTGAGCCAGCGCGCAACACGGCGATCGCCTGCGCCAGCGACGGGCGGTCCAACAGGTGCCTGGCCCAGGAGGGCATGTTTCCCTGGAAACCCCGCTCCCCGAATATGACAAAAGCCACATATTCCTCCACTGGCACGCCATTCTCGCCCTCGAACACGCGGTGGGCCGTCAGCTGGGCCTCATCCAAACCAAACCAAGACACAAGAAAGCGATCCCAGCGCTGATCCAGGGCCATGACCTTCTCACCAGCGCGACCGCACGGCCTAAGCTCCACTGAAAGGCGCCCAGAATCCGCACAGTCCTACCCCCCTGGCCCGCCCAATATACCGCTGTGACGTCTGATGTACAGGCCCGCGCGTCGCGGCCGTTGGTGGGAAAACCGGCACCACCCTGTGCGGCCGAATCCGCCACGGGGGCTGCCAGACAGTACACTGTCTCCAGCAGCGACTTCAGTCTCTTGTGACTTTTGGGCGTCACCACCAAAAATTGCAAAACCTGCCTGTAGTCCGTGAAGTAGGTACGGCATATTACCATGGAGTTGTACACGCCCAGGTTCTTTGAGAACACCAGGCTCGCCTTGAACTTTGTAAAGTCATCCTGCCCCAGCACGACAGACGTATTTTTGGCAAGGTATACGTCCGACTCCACGGGAAGGACGTGCCCAAACTGGGACACGGCGTCGCTTGGTCGGCACAGAAAGCACTTCAGGGTTGTGGAAAGGCCATTATTCGATATAACAAAGCAGGGAGAGAACGGGTAGTGCATCTCCTCCAGGAGGTGCGCCCAAAACTTATACACAAACTCTAAGTGGTACACGCAACCGTGCTGCATTCTAACCGTACATATGGCGGTAGCACCGCCCTTAGCATAAACTGGGGCCCCGTCGATGCACCGTTCCAAATCCAGGGACTGACCAGACTGTCCCAAGTATGAGGATACCACCCGACACAGTTCGTCCACTACACGCTTACCAACGACACTCATGGCGACAGCGGGGTGGGGCTGGCAAGGCCCCCAAAGCGCGACACCCGCAGTCAATCAGGGCCGTGCCCGCGCCTCGGAGAATACGGCGTCCGTGCTCACGATCTTGCGCAGGACCTGCCTTACCGTGTCCACCTTGCTCTCCAACACCAGAGTATGATCGCAGGCTGCAGGCTGTGCCCGCTGGACGAGAAAGGTTTTTAAATACTGACAGTAGTTGATGGCGTTCAATCTACAATAGATCGTGGGAAATAAAATTTGCATGTCACGAGGCAGAAGCTGGTCAGACGCGTACTCCATGTTGGGTTCCACGGGGAGGGGAACACACGCCCCAAGACACGACGGCGCACATAGGGAGCGGAGCAAACAATTGATTCAAATATTTGACTCCGCAGCGAGCCGGTTTGCAGAGTGGTCACCTGCCCTGCTCCACACCCACCCCCGCGTCTCTTCCAACTCTCAACTCACGATCCAGGGAAACCACCGTCCAGTGGCCATGTTTGTTCCCTGGCAACTCGGTACAATTACCCGTCACCGAGATGAGCTCCAAAAACTACTGGCAGCCTCCCTGCTCCCGGAGCACCCGGAGGAGAGCCTCGGTAACCCCATAATGACACAGATTCACCAGTCGCTCCAACCATCTTCCCCCTGCAGGGTCTGTCAGCTCCTATTTTCTCTGGTCCGCGATTCGTCCACCCCCATGGGTTTCTTCGAGGACTATGCCTGCCTCTGCTTCTTCTGTCTATACGCCCCACACTGCTGGACCTCGACCATGGCGGCAGCGGCAGACCTGTGCGAGATCATGCATCTGCACTTTCCAGAAGAGGAGGCGACATACGGGCTATTCGGACCGGGTCGCCTTATGGGTATCGACTTGCAGCTGCACTTCTTTGTTCAAAAGTGCTTTAAGACCACCGCCGCCGAAAAAATACTGGGAATATCCAACCTGCAATTTTTAAAATCAGAATTCATCCGGGGCATGCTCACAGGCACCATCACCTGCAACTTCTGCTTCAAAACGTCCTGGCCCAGGACAGACAAGGAGGAGGCCACCGGCCCCACCCCATGCTGCCAGATTACAGACACCACCACCGCACCCGCGAGCGGCATACCGGAACTAGCCCGGGCCACATTCTGCGGCGCAAGTCGCCCCACAAAGCCCAGCCTACTTCCCGCGCTAATAGATATCTGGTCCACGAGCTCAGAGCTCCTTGACGAGCCGCGCCCTCGACTGATCGCAAGCGACATGAGTGAACTCAAATCCGTGGTCGCATCCCACGATCCGTTCTTCTCTCCCCCGCTTCAGGCAGACACCTCACAGGGTCCATGTCTGATGCACCCAACCCTGGGGCTACGATACAAAAACGGGACTGCATCCGTCTGCCTCCTCTGCGAGTGCCTTGCGGCACACCCAGAGGCACCCAAGGCGCTGCAGACCCTTCAGTGCGAGGTAATGGGCCATATAGAAAACAACGTAAAGCTGGTAGACAGAATTGCCTTTGTGTTGGACAACCCATTCGCCATGCCATATGTCTCAGATCCGCTACTTAGAGAGCTGATCCGGGGCTGTACCCCACAGGAAATTCACAAGCACCTGTTCTGCGACCCGCTGTGCGCCCTCAATGCTAAGGTGGTGTCAGAGGACGTACTATTCCGCCTGCCCAGGGAGCAGGAGTATAAAAAGCTCAGGGCATCCGCGGCCGCCGGACAGCTCCTCGATGCCAACACCCTGTTCGACTGCGAGGTCGTGCAGACTTTGGTCTTTCTCTTTAAGGGTCTCCAAAACGCCAGGGTGGGGAAAACCACCTCACTAGACATTATTCGGGAGCTAACCGCACAACTAAAAAGACACCGCCTAGACCTGGCCCACCCCTCACAGACGTCACACTTGTACGCTTGAGCTGGTCCCGGGCCTTCGCACCCCATCCACCGATGCCGAAATCAGTGTCCAGCCACATCAGCTTGGCGACCTCAACCGGTCGCAGTGGACCGCGAGACATCAGAAGATGCTTGTCATCCCGCCTGCGGTCGGTCCCGCCCGGGGCGCGAAGCGCCAGCGTCAGCAGCAAGCACAGAAACGGCCTTCGCAAGTTTATCTCAGACAAGGTATTTTTTAGCATCCTATCGCACAGACACGAGCTAGGAGTGGACTTTCTCCGTGAGATGGAGACCCCGATATGCACCTCCAAAACAGTAATGCTGCCCCTAGACCTGTCTACCGTCGCACCCGGCCGCTGCGTCTCCCTCTCTCCGTTTGGACACTCCTCAAACATGGGGTTCCAGTGCGCTCTGTGCCCATCCACAGAAAATCCCACCGTTGCCCAAGGCTCCCGGCCTCAGACAATGGTGGGCGATGCGCTCAAAAAAAATAACGAGCTATGCTCGGTAGCGCTGGCCTTTTATCACCACGCAGACAAAGTGATCCAACACAAGACGTTTTACCTATCACTCCTCAGTCACTCCATGGATGTGGTTCGGCAGAGCTTCCTGCAGCCTGGTCTACTGTACGCTAACCTGGTCCTAAAAACCTTTGGGCACGATCCCCTACCCATCTTCACTACCAACAACGGCATGCTAACAATGTGCATCCTTTTTAAAACCCGGGCACTACATCTGGGAGAAACTGCGCTTAGGCTGCTTATGGATAACCTCCCCAACTACAAGATATCGGCGGACTGCTGCAGACAGTCCTACGTGGTCAAGTTTGTCCCAACGCACCCGGACACCGCAAGCATTGCAGTGCAGGTACACACCATATGCGAAGCGGTTGCGGCGCTAGACTGCACCGACGAGATGCGGGATGACATTCAAAAGGGAACCGCACTTGTCAACGCCCTATAACCTCACATGTAGCCTGTCACCCCAGCTCCTATTGCAACTGACCATGTTCAGGTGGTAATAAAGTCATTAAACGACAAAGTGATTCTTTTAATCTGTTTATTGTTTTTGAACATGTGGCACACGCTGCAATGTACTGCCATGAAAGGTGGTTCTATATCCACCACTTGGCGTCTGCCGAAGTCAGTGCCACAATTTCATTAACAAACAAGGTCAATACATTCTGAGGGAGTGTTTTTTGCCATGGTACCATTCGTGTGGTTTGGGAGAGCGGACGCCATTTGCGTGCAAAATGTGCTTTGCTGGAGGCCAACTTCCGTCGCGCTGGTTGATGCGCGGCACATTGTGTCAACCAGGGCACCCTCCCCCACCGAGTGCTTTAATGCGGAGAGGAATGGTGGCCTGGTTGACACCGCGTGCCGGCCTTCTGAACTGTGACTGTGTTATGAGCCACGGGTATGCCCTCGATACGCCTGCTCTTCAGCATTGTATGTGTTTAATGTTGTGCTTGGTGCAACCGTGATTGTGCTTTTGTATTTTATTTTACTGACACTCTTTGGGAGGGCACGCTAGCTTCAGTGCGCGCCCGTTGCAACTCGTGTCCTGAATGCTACGGGGCCACGCTGGCCACTCGGGGGGACAACACTAATCGCCAACAGACAAACGAGTGGTGGTATCGCCCCAAGCCTCCAGCGCCACCCATTTAGTAACACATCCGGGACATGAACTGCCACAAACACCGTTAAGCCTCTATCCATGCATTGGGATTGGAGTGAGGAGGGAGGAGGGCACCAGGTTCCCGGGGAGGAGGGCACCAGGTTCCCGGGGAGGAGGGCACCAGGTTCCCGGGGAGGAGGGCACCAGGTTCCCGGGGAGGAGGGCACCAGGTTCCCGGGGAGGAGGGCACCAGGTTCCCGGGGAGGAGGGCACCAGGTTCCCGGGGAGGAGGGCACCAGGTTCCCGGGGAGGAGGGCACCAGGTTCCCGGGGAGGAGGGCACCAGGTTCCCGGGGAGGAGGGCACCAGGTTCCCGGGGAGGAGGCTGGGGTGCGCCGCGCCGGGTTCCCGGGGTGCGCCGCGCCGGGTTCCCGGGGTGCGCCGCGCCGGGTTCCTGGGGTGCGCCGCGCCGGGTTCCTGGGGTGCGCCGCGCCGGGTTCCCGGGGTGCGCCGCGCCGGGTTCCCGGGGTGCGCCGCGCCGGGTTCCCGGGGTGCGCCGCGCCGGGTTCCTGGGGTGCGCCGCGCCGGGTTCCTGGGGTGCGCCGCGCCGGGTTCCTGGGGTGCGCCGCGCCGGGTTCCTGGGGTGCGCCGCGCCGGGTTCCTGGGGTGCGCCGCGCCGGGTTCCTGGGGTGCGCCGCGCCGGGTTCCTGGGGTGCGCCGCGCCGGGTTCCTGGGGTGCGGGGTGCGGGGGGGGGGACCGCGCCGGGGTACTGCAGGGTTCGCAGGGTTCGGGGGTACTACCTGGTTCCTGGGGTGTGCCAGGACGGGTTCCTGGGGTGCCACCGCTCCTCGATACGTGTAAATCCAAGAGATCCGTCCTCCGTGCCGCCGCGCGCGTAATGCGCGAGGGGGGTCGGTCTCCCCTCTTCTTTATAGCGTTTCCTGCGAAGGGGGCGTAACCGTAGGACAAACTGCTTATGTAGGGGTTAGCCACCCATTTCCCGGGGCCGCGCCAGAGGTGAGCGTGGACCTAGCATCCCGCTCCCATTTACCGAAACCACCCAGAGGCGAGATTCCAGGGCCGTGACTCACTAGCTCCCCTCCCATCGAACAACCACGCTTGGCTAACACGGCTGGAGTGGCGGTGGGCGGGGCCCCTATAATCCTGGCCCCCATCTACCGAAACGACCCAGTAGAAAAATCCCAACCCCATGACTCATCAGGCCCTATTATATAGAATATCCCAGTAGAGTGACCCAGCTGGTTTCCATAAATGGATATACTTCCGGAAAACGAAGGAGGGTTGAATACAGTTGGGGGTAGTCCGCTGGTATTCCCAGCTGAGGTTGCCTTATTTGGTAATGCTTCCGGAAATACCACCTGAGTACCCCATTGGTTTATACCTTGTTTAATTGTAGAATTACAGCTGGATTTACCCAGCCGGGTTTACGCAGCTGCGTATACCCAGCTGTGTTTACGCAGCGGGGTTTACGCAGCTGGGTAGACCCAGCTGGGTATACCTACTGGAATAGGGGCTGCGATGACTCAGCTGCGCTAGGATTAAAGGATTATATATATATATATAGGAAAAATCAAAACAAAACTCTAATCGCTGATTGGTTCCCGCTGTGGGCCAATCAGCTTGGGAGTTCTAGGGATAGGGGCCAATGGGAGGCCTCCGAATTTGATTGACGGCTGGGGCGTCCAATGGAATGGCGCGGTCGCCTAGCTCGAACGAGATTGGTCGGCCGAATGGGCCAATGGCGGCTCGGAAAACTTTGATTGACGGGCCGGCGGACCAATGGGAGAGGGGCAGAGGATTACGGGGGATTAGCAAATTCAAGATGGCGGCGCCCATAAAATGGCCAAAAATTATAATTTTTCGAGTCGCTCACGGTCCCACCTAGCGGCGTGACCTGGAGGTGACCCCGTGCACCCGGCGCTCTGAATTTTTCTGCGCATGCGCGACTCCTCATCTACATAATTTATGCACATAAAAGGATTAGCGCATGCAAATTAGTCAGATAGCAGGGCCATCCACACTTTATGTTGGCCGCGTGCCAGGCGCCGGCGTGGGCGCCGCGCGCGTGCTCTCTCAGTCGCGCCTAGCTGCTTCCAACAGACAAAAGCGGGGCGTTAGTGAGGGAGTGCGCGCGCTGCGCTGACTTGGCCGATTTCCAGTGCATGCTTTGTCACCCCAGCGCTAGAATGGAATTTTCATTATTGAGCAATTTGGGCACCCTGGGCACGATAACCATACATGGATACACGGGTTCCAAATATGCAAAGTAGACACTAAGGTACCATTTGGCATATTTGGACGTCCTGGGCAGGTTAGCTACCCACCAGAATATATGGGACTCTGGGCAGGATAGCCACCCACAATTGTTTTGCGCCCCTCTTTGGCCAGGGGACCAAGGTCGTATGGTTCGCGCTACACTAAGCCCGAACGTTCAGCTTTGCGTGCTTTCGACGTCCAGGCGGCTGGCACACGGGCCGTGAGCGCCAGCAACATGGGATCATGGTAGTAAGATACAGCATAAATCCCCGTCCGGTGGCGCTCAACGCCAATATGCGCGGCTGCGTGGTATCTCATCGGTGGGCACGCGTACGGTGGTCTCATGGGTATTGGACTTGTAGGCGAGGGGAGGCGCATACGACAAAAATTGCCGCCGTGAAGGTCGGGAACCCGCCCGCGCTTCCGCAAGGCACGGGGCCGCATCGGACACAGGCTAAGCATTAAGGATCATAACACCGCCCTAGAAATGTTTAAGCTGTGACCAAAGCGAACCTCGCATGAGGCATACGCGAGCGTGGAGGTAGGATTCCCAAGGCTATTGAGAGACGGTGGGTGAAATGATGAAGAACACACAGAACAATAACGGGCGACTAGATAAAAAGACTCGCTCAACAGCCCGAAAACCATCAGCCCGCACCGCCGATGGATTAGGTGCTGCTGGACAAGTCTTTCTAAACCCGCGCAGGGTTTGTGTCGATCCAGACGCTTACGAACGCCCGCTTTAAAAACACTATTCATAATTAACAGAAGTTGACACCAGCCCGCAGTTACCCAACCTTCTATTTTTTTGGAGTGTTGACAAGTTTCCATCGCCCGTTTGGCGTTTCCCGCATGGTGTCAAATTAGTGACGCACCCTCCCCCCGTCACTATGGGTTTACCCTGATTTAGTAAGTAAAACTGCCGCCCCCGCCCACTCATTTTTTTACCCTGTTATTTGCTGTATTTACATCTACGGACCCCCTTTTGGTGAGATTGCCGTGGTTCTAAATAACGTTGTGGTTTTCGGACCCTTTCAGGGACCAAATCTTTTACGTGTTGCCAAGGTAGCATTTGCTGGACCCGCATAGGTTTTTGTGGCACCAGGTTATGGTCTTATGAGCGGGCTTGACCGGCAAGTTCCAGGCATCCTAAGTGCTTGATGTAGACCCTTAGGGCACTAGGGACTACCTAGGTCAAACTCCCCCTTAGTCATGACGCCGTGCCCACGAGGTTTGAGAGGCGTAGACATCCGTGTCGACTGCTGGACGGAGGTAGTATAATCAGCTAGGCCTCAGTATTCTATGTAACAAATGAATGCCCTAGAGTACTGCGGTTTAGCTAGTTATACTGCCCGGTTCCACCAGGCGGCGTTGTGGCCACGGGCGGTTCGTCGCTTGGACCTGGAGGGGTGTCACATTCTGTGACCGCGACGTTGACGTTAGACACACGTCGCTGCCGTCCTCAGAATGTGATAGCCCATTACAGGCATTGTAGCTGTTGCGTTGGTTGGGAGTTTGGGGACCAAATTTCTATAATTGGTGTCACCGCGGCAGCTCTAGCCCTGGAAGATCTGGAAGCTTGCTTCAATGGCTCAGATCGACCCGGACTACAGTTAGCGAAGTAGACCCATTATAATCTTAATCTTAAATCTGGTTGACGGACTTTCGCGCCGGGAACACGCAGGTGGCAGCGGATGTGTTTTGCCCAAACACGAGGGTTGCAGGAAACAGGTGCTGCCGGGGATTATGTACAGCTTACACCCAGTTTCCTGTAATCGCCCGCATCCGGCCGTCCTGGGCAGCACCGCACCCTGCGTAAACAACCGCGTACTTTTTCCTCGTCCCCCCACCCCCACATCCTTCCTCCCACCCTGCCAGTCCAACCCGCTTCCTGTTTTATTCGCCTTCAAACAGAAGCACGCATTCTAATGATTCTTACAAAACTTGTTAGTGTTTATTAAATCAGATACATACATTCTACGGACCAAAAATTAGCAACAGCTTGTTATCTATGGTGTATGGCGATAGTGTTGGGAGTGTGATGGGCCGGAAAGGTGAAGGCCCATTAGGGTTTGCACTTGGCGCTGTAGGTCTACTCTTGACAAAGATCTAAGCATTGACATTAGGGCATCCACGTCAGTGGGACCCAGTAGGTCTAAGTTTTCCATACAGTACACCCAGTGTAAGAATGTCTGTGGTGTGCTGCGAGACCCTATAGTGTCCTTGCTTAAAAATATCAAAGACCTAATATCCCTCGCACACAGCTCCCCGTCTACGTGGAGAACAGTGAGCTGATAAGGGCTGAAATAACTCATTGTGCCCGCTAGGTGGCGCTCTAAAAAACGCGGGTCTAAGTGAAGCAGGTCGCGCAAGAGGTCTCTGCGACCTGCACGAAACAGACATTCCGCTAACAGGGGAAACGTTAACCTGCCCTCCTCCTTTAAAGCTCTAAGAGCTCCAATTAATTGGGCCAGTGTGGGTTGAGGTATGAACACGTTTAGGAGGAACAATACCACTTCCCTGTCATCCGTGCCCAGTTTCCGCGCCACCTCACAGAGAACCTCGTAAGTGGCCATGGTGCCGGCTTGTATATGTGAAGGCACCGATGTGGAAAAACAAAGGAAAATTTATTTTTCCGCCCTAAACAAAATCACAAGCTTAATAGCTGTCCAGAATGCGCAGATCAAAGTCCGAAACAGATGTTAGGATCTGTTCCACTGCCGCCTGTAGAACGGAAACATCGCATCCCAATATGCTTGCCAGCTGAGGAACTACCCCACCCGAGTGGGTATCCTGCGGAATGACGTTGGCAGGAACCAACAGCGCACAGCCTGCAGCGCTGATAATAGAGGCGGGCAATGAGCCAGTCTTTGGGTCAACTAAGGCTTTTGTAATCAGGGTGTTGACCTCGTGGTGCCAAAAGTCCAGGTGTTGGGAGCCCCCCAGCAATTTAAGTAACAAGAAGGAAGTGACGTCCGTCGCTAAGACTGCCTCTGTTCGCCACGCCAACTTCTCAAGGAGTTCTTTCTCCTGGTCTATAAGTTCTTGGCGGGAAAAGGAGTCTGCCGCGGCATAGCAAAGTGAACTGGTAGAAATAGGCGTGAGGCTTCTGAGCTTACTGGCCACTAACAGGCAGGCGCTCCCTGTCTTTTGAAAGTGTTCTTTGGACACCTGCTTTATAAGTAGGAGTCTGTCCAAAAGATTAAGGGCCAACGCGACCACGTTAGGTTCTAGGTTGTATTCCTGGCAAACTGAAAACATCCATGTGCCCAGTAACTTACGCATATGCGAAGTAAGAGATTGTTGAAAGGTCCCAAATACAGAGTCAGAAGTTAAAAAGCGCGGCTCAATTTCAAGAATATTGTAAAAGATCCGATCCTCACATAGCGTGGGATCCAGAAGTCCCGAGGGCGGGTTATTGGCAGTTGCCATATAGAGTGGCGAGCGTATGTGGCCTACCTGTAGAGCCTGGAGTTTCAGGGTGCTCTGTCAGGTTCTCCCATCGACGACGCTGGGCCGCGAGAGTACGCTAGCCGTTGTCCGTGTGTTCAGTTGAGGTAGATGGGTCGTGAGAACACTGCCCCACACACACACCAGCACCCATGGCGCCAAATGCAAGTGCGGAGCGGCGACGGTGGCTTCTAGGGAGGAAAAAGGGGGAGAGGTGTGGCTTTTATGTCATTTCCTGTGGAGAGTCCCCAGGACCTTGGTTTTCCCCTGGCTGGGTTAATGGCAGGGGCTTTTTAAACTTAACTATGGAAGATTGTAGGTTTCCTGCCAGGGGGTGACTAGCTTCCCAGGCTAGGCGGGCCATTTGTACTTTCTTACTTGTGTCTTTGTTCTGACAATACACATATACACAATAAGTTATGGGCGACTGGTCTGGTCCAGGGTGGGGCAAGCAGGACACGGGGCCTGCCTTTACTCCTCCAAACTGGAAGGCCTGAGATAATTTTTTAAGTCCGTATGGGTCATTGCCCCAAAAAATCACTGCAAACTTCCATTGACACTTTGGATCTCGTCTTCCATCCTTTCCCAAAAAGCGTCTATAAAAGATGTGTTGTGGCCTAGCTTTCGCAGGACAATCATCTATCTGTCTGTAAGGGACCGGTGGTTGTTGGTATCTTGGATGTGGCTTTTTTGGGTGGGTAACTGGAACGCGCCTCATACGAACTCCAGGTCTGTGGGGTGGTGATGTTCTGAGTACATAGCGGTATTCGCGAGATGGGCCAGGTTGTGGGTCATCGTCTGGTGTATTATCTCCTGGTGGGCTACTGGCAATTTGTTCATGTGTGCTAACAACAGGGTAATCCACTTCCATTTCGTCCTCGGATGACGACCCGTGCAAGATTATGGGCTCTTCCACCGTCTCCTGCTCCTGCTGTTCCACCCCCTGCTGCTCCTGCTCTTCCACCTCTGCTGTTGTGGCTCCTGCTGTTGTGGCTCCTGCTGTTGTGGCTCCTGCTGTTGTGGCTCCTGCTGTTGTGGCTCCTGCTGCTGTGGCTCCTGCTGCTGTGGCTCCTGCTGCTGTGGCTCCTGCTGCTGTGGCTCCTGCTGCTGTGGCTCCTGCTGCTGTGCTGTGGCTCCCGCTGCTGTGGCTCCCGCTGTTGTGGCTCCTGCTGCTGTGGCTCCTGCTGTTGTGGCTCCTGCTGCTGTGGCTCCTGCTGCTGTGGCTCCTGCTGTTGTGGCTCCTGCTGCTGTGGCTCCTGCTGTTGTGGCTCCTGCTGCTGTTGTGAACTTTGGATGCTCAACGTTTTGTTTCCATCGCCCCCGCCTCATCTGTCTCCTGCTCCTCCTCATCATCCTTATTGTCATTGTCATCCTTGTCAACCTGACTTTCCTTGCTAATCTCGTTGTCCCCATTATCCTCGCCAGCCTGATTATTTTCGGAACATTCTTTTTCATTCTTGGATGCTTCTTCTGCAATCTCCGCAAGGAGCACCAACATGGCTGTGTCATCACCCCAGGATCCCTCAGACGGGGATGATGATCCTATGGAGATGGGAGATGTAGGCGGTTGGCGTGGCGGAGTATCGCCATCGCTGGATGATCCCACGTAGATCGGGGACTCTGTGGCCCATGGGGGGTACACACTACGGTTGGCGAAGTCACATCTAGGGGGAGAGACTGGGGGCGACTGACATATTGGGTTTAGTGTAGAGGGACCTTGGGGGGACGATAGCCTTCTTTTTCTCAGGCTACGCAGGGTAGACGGAGCTAAAGAGTCTGGTGACGACTTGGAGGGAGGCTCGGGTGGAGGAGTCGTGGGTGAGTGTGGAGGTGTAGTCTGCTGCGAGGGTGGCGGACGCATAGGTGTTGAAGAGTCTGGCCTTCCTGTAGGACTTGAAAGCGGTGGCCTTTGAGAAGACTCTGGAGACTGCGTGGGTGGCAATGCAGGAGATGGAGAATGAGTATCCGTGGTCCCCGGAGACACAGGATGGGATGGAGGGATTGGGGAGGAAGACGTGGTTACGGGGGGTAAGAGTGCCGGTGGAGGTAAAGGTGTTGCGGGAGCGGGTGAAGGAATGGGAGCCACCGGTAAAGTAGGACTAGACACAAATGCTGGCAGCCCGGATGTGAACACTGTGGGACTTCCTGGTATAGGCAAGGTGTGGGGTCCACATTCCCGGCCGTCGACGGAGTCGGCGACATGCTTCCTTCGCGGTTGTAGATGTAGGTCATCGCCAAGGTCACATCTTTCCGGAGACCTGTTTCGTTTCCTACAACTTCCTCTCGTTAAGGGCGCGCCGGTGCTCCGTCCCGACCTCAGGCGCATTCCCGGGGGCGCCATCCTCGGGAAATCTGGTCTGACAACCAAAGTAAAATTATGGAGGCGGTGGCAGTATATTCACATTATGCAATACCCGTAGTGACCACAAGGGGGAGCTCTCAGACAATTAAGCGGTTACACACAGTAGCAGGCTGCAGTACCGCCCATGGCCACAGGATGGAGATCGCAGACACTGAAACGCTGAAACACAGCATTAAGCTGCAATACCGCCGATGGCCACCAGATGGCACGCGCCGCCAGCAAATTTAAGTCCTGGTGGCTCACCTGCCAGGTAAACAAGGTTAAAGTGGGTTTGCTGGCCTTGCGTTGCCATGGATGCTACCTAGGCAAGTCCAGATATATAATCCGGGCGTGAGAAACAGAAACGGCCAATAACCCATGTTTTTCGAAAACCACCACACACCTTAACACAAATCATGTACACCTGGTATTACTATTTCCCACACATCTTATAGCATTTCAAAGATAAGGGTGCCTTACGGGCCGCCCGAAACAAGTGGGCGGGCGCTACTCACTGTTTATAAGTCAGCCGGACCAAGCTGCTGCTCTTGGGGACGTGACTGCTTCGTGGCGCAGCTGCCTCCAAATGATACACACATTTTTTGATTGTCCCGGGCGCCGCGTAGTGGAGGGCGGAGTTATATCAAGCTACTTTCTGATTGGTGCCCCAGGCAGGACTGCCATAAAAACTGAAGAAGGCGTGTCTGCTTTGCAGAATTTACCCCCCACTGTGCTCCCGGTTGCTGGCACCGGTTCAGTGGTCCGACCTGTCGTCTGTGCTCCCCCGTGGACGACGCCGAGTGCCTCTCGGGGGTCCATGTCTAGCCTCTTCATTTCATTACCTTGGGTGGCGTTCATCTGGCTAGCCCTCCTTGGCGCGGTTGGGGGTGCCCGCGTTCAGGGGCCCATGCGGGGCTCTGCTGCCCTCACCTGCGCCATCACGCCCCGTGCTGACATAGTTAGCGTTACCTGGCAAAAAAGGCAGCTCCCCGGTCCCGTAAACGTCGCCACGTACAGCCATTCATATGGGGTGGTGGTTCAGACCCAGTACCGCCACAAGGCAAATATAACCTGTCCTGGGCTTTGGAACTCTACCCTTGTTATCCATAACCTTGCAGTGGATGATGAGGGCTGTTACCTGTGTATCTTTAACTCATTTGGTGGCCGGCAGGTGTCATGCACAGCCTGCCTGGAAGTGACATCTCCCCCTACTGGACACGTGCAGGTAAATAGCACAGAAGACGCAGACACCGTCACCTGTTTGGCAACTGGTCGCCCACCCCCCAATGTCACCTGGGCCGCACCCTGGAACAACGCCTCTTCTACCCAGGAGCAGTTCACTGACAGTGATGGTCTTACAGTTGCGTGGAGGACCGTGAGGCTGCCGCGTGGGGATAATACCACCCCAAGTGAGGGAATATGTCTCATCACCTGGGGAAATGAGAGCATATCAATCCCGGCTTCTATTCAAGGCCCCTTGGCCCATGACCTTCCCGCGGCCCAGGGAACTCTTGCCGGGGTTGCCATTACTCTGGTGGGCCTATTTGGGATATTCGCATTACATCATTGCCGCCGCAAGCAGGGCGGTGCATCACCTACTTCAGATGACATGGACCCCCTATCCACCCAGTGACTAGATGGACACCCCGTGAACCGTCGTGCTTACCCACCCCCTTCTGATTCTGACAGACAACACTACTATGTCCCAAAGACTGTTTTTTACAGCCCGATGGCCCTTCAGGCCTCCTTGAGTGTCTAGCTGGTCCCGTGGTCATTGTGTGGTTTGGCAGTCACTTCCCCATTTTGGTGTCGCGTTTTGGGTTTTGCCCTGCCCCCAGCCAACGTGGATCATATTCTTTCCCGTCAGGGGAGTGACAAGCTATAGGACAGAAAGGTCACCTGGCCCAAACGGAGGATCCTAGGTGGGTGTGCATTTATTAGACGTTGGTGTGTTGAAGGACGGATCAGGCGGGGAGGAGGGGTGGGGGAGACTTACTGCAGCACTAGGTTAGGTTGAAAGCCGGGGTAAAAGGCGTGGCTAAACAACACCTATACTACTTGTTATTGTAGGCCATGGCGGCCGAGGATTTCCTAACCATCTTCTTAGATGATGATGAATCCTGGAATGAAACTCTAAATATGAGCGGATATGACTACTCTGGAAACTTCAGCCTAGAAGTGAGCGTGTGTGAGATGACCACCGTGGTGCCTTACACGTGGAACGTTGGAATACTCTCTCTGATTTTCCTCATAAATGTTCTTGGAAATGGATTGGTCACCTACATTTTTTGCAAGCACCGATCGCGGGCAGGAGCGATAGATATACTGCTCCTGGGTATCTGCCTAAACTCGCTGTGTCTTAGCATATCTCTATTGGCAGAAGTGTTGATGTTTTTGTTTCCCAATATCATCTCCACAGGCTTGTGCAGACTTGAAATTTTTTTTTACTATTTATATGTCTACTTGGATATCTTCAGTGTTGTGTGCGTCAGTCTAGTGAGGTACCTCCTGGTGGCATATTCTACGCGTTCCTGGCCCAAGAAGCAGTCCCTCGGATGGGTACTGACATCCGCTGCACTGTTAATTGCATTGGTGCTGTCGGGGGATGCCTGTCGGCACAGGAGCAGGGTGGTCGACCCGGTCAGCAAGCAGGCCATGTGTTATGAGAACGCGGGAAACATGACTGCAGACTGGCGACTGCATGTCAGAACCGTGTCAGTTACTGCAGGTTTCCTGTTACCCCTGGCCCTCCTTATTCTGTTTTATGCTCTCACCTGGTGTGTGGTGAGGAGGACAAAGCTGCAAGCCAGGCGGAAGGTAAGGGGGGTGATTGTTGCTGTGGTGCTGCTGTTTTTTGTGTTTTGCTTCCCTTACCACGTACTAAATCTACTGGACACTCTGCTAAGGCGACGCTGGATCCGGGACAGCTGCTATACGCGGGGGTTGATAAACGTGGGTCTGGCAGTAACCTCGTTACTGCAGGCACTGTACAGCGCCGTTGTTCCCCTGATATACTCCTGCCTGGGATCCCTCTTTAGGCAGAGGATGTACGGTCTCTTCCAAAGCCTCAGGCAGTCTTTCATGTCCGGCGCCACCACGTAGCCCGCGGATGTCTACGTGCCCTTCCCCCTTAATTTAATCTAGCCTCCCGTTCCCATGATGCAGAGAGGCGAATCTGGTTTGTACACAGATGTGACTATGTATTTGTTTTATTATGCGATTAAATGAGGGGTCTGATCCCAAAAGCAATGTTTAGTGGTGGTCGTTGATCTTCTTGACGCTCCATAGGTAGATTGACTGGAACGCCATGGCCCACGGGGACATGGACAGGGGTGTTAGGTCTGGTGGAACATGCTGCCACTGCCACGGATGGAACATCAGGGATGGGTCTATGATCAGGGCAGCGTGTCGCCCGTCACTGGATGTAAGTCCGGCCACCGTGGAGTTGCCTGTGGGGTTTCTGGGATAGTGTCTGGCTGGCAGGGTCTCATCCGCGGCATTTCCATGGTAGGTGAGGGTTATCTCGCCTCGCTGTCTCAGTATGTACTCGAGGGCGTCCTGCTCGTACCGGACCCCCAGGTACTCGCCCTGGGCCCAGCTGGGCAGCACCGTCCCCCGCAACACTCGGAGGAAAACGCTCTTAGTGTTCTGAGGGATCTGTATGTTTAGCCAGTGGCTGTCATACAGCTTGGACACGTTGGTCTCCAGGTTTACCGCCCAGCGCTGGGGTGGTGTGGGTCCGTACGTGTATGGTGAGGATTCCGACCGGCCCACTACACCCAGGGCCACCAGAAGCTGGAAGCCCACTTCGCCACAGCAGATGGAGAATGTGTCGGGCCTGTTTAGAAACTCTGTCAGGGTGGAGGCACAGGTAGGGTCGTTACACAGCGCCAGGACCCATCCCCTGGCGCTGGCGTAGCTGGCCTGGCAGCCTGTTCTGAGACATGTAATCAGACCAGAGAACCCCGACAAGGACTGTCCTCGTTTAAGCTCTTCCACAGTCACCGTGGCCACCTCAAAGCCCGTGTTCTGCAACGCGGCCATGAGCGCGTACGGGGCGCTGCTCCCAGGCAGCACCAACGCGGCCACACGGCGCGGGGAGGTGGGGCATGAAAACAGGCGCAGCTGACTCCCAAGGCACATGGCCCTTAGGCTGCCCAGGTGATGCTCCAGACGTCCCAGGTCCTTCCTGTGCATGTCCTCCAGTGGGTGCAGGGGAGGCGTCACCAGGTTCCACATTTCGTCAGAAAAGGAGGTCCATGAGACTTGCAAGGAAGTCAGGGTCTCTTGAAACACAACTGTCTCGTTCTGCAAAATCGTGACGTTGTTGCCTTGTCCCTCGGGGCCAACGGTGCCCAGTGGGTGTGCCACGCAGCGGTAGTCCCTAGCCGCCCGCAGCACCTCTGACAAGTGTACCTGGGGCACCTCAACCAGTGCCCCAGGGGTCTCTGAAACCATAAGTTCGAGCGGGTTAGGGTGGGCGGGTAGTGAGAGCTGCAGTCCCCTGCAGCCGGCCAGGGCCATCTCGATTGCAGATGGGAGAAGCCCCCCGTCCCCTATGTCGTGCCCCGATACAATGAGCCTCTTGGACATCAGGTACTTAACAAGCATGAACAGGCTGGCGACCGTGGACGGGTTCAGAGGGGGTATTGGGTGCCTGGATGCCAGGAAGTTGTGCTCGAAGGTGGACCCGGCTATGAGACAGCTCTGATTCACGGCCAGGTATACCAGGGCGTTGCCTTCGACCTTTACGTCCGGGGTGACCCTGTATCTGGATCCCTTGACCTCGGCCCAGCTGGTAAACACCACCGAGTTGAAGGGAAGGACCTCCACCGTTTCTTGCTGTTGTGTGATGCGCACATGGCGCTCCGAAAGCGTCGGAGAGCTGGCAGCCGAGGAGATGGACAGTGCCACTCCCAGCTCCCGGCAGAATTCCTTGCAGGCGAAGAGGCACTCCTGTAGGAGGCCGGCTTGGTGGTCCTCCGGACTCCACGCCACGGCGCCAGTTAGCACTACGTCCTGGAGCTTGGACACGGGACTGAACATGAGGTTGGTGAGAGCCTCGGTGATGGCATAGGTGGCCCCGGTGGATACATTAGTAGCCATCTTGTAGGCCTGCTCCCCCATGGCCATGGCCTGACCCCTCCACGCTGGCACTGGAAGCAGCTCCTGGGGCAGGGCCTTCACCCAGGTCTCGAAGTCCTTGTGTAGGAGGTTGGCCATGGACGGAGTGATGGCCTCCACCGTGTCGGGCACTCTGGGCGCCACCCTCTCGGCCAGCATGGACGAGTGCAGCACCAGGTGGTAGTCTGAAACCGGTATGTCCAGGGGTCCCACGCCAGCCTGTTGGGCGATGAGGCCGTTGGAGCATCTGTCCATGTGTCGCGTAAAGAACTCCTTGCTGCCAACCGTCGCGTGGCGAAGTAACTGGTGGATTGTGGAGCCGGTGGCAAAAAGGCCCCAGTCAACATCCTCGGGGTGCCCCGAGACGCGGACACCATCGGACAGCGCCAGCCAGGGGGACGGGGGGGTGGACGAGGGCTGGTCTACAGAGAAGACCCTCGTGGTCTCCCCGGTCAGGTCGTCTACTATTCTGATGCCTGGCTGCTCCGAGGTCCTCCCGAGGACCGTTATCTGGCACGCGCACAGGCGCGCGGCGCGCTGCAGTACCTCCAACGGGGTCTCGCCCAGATCCCCAGGCACCGCGCCCGACTCTGCCACCACCGCAAACACCAGGGAGCAATACACGTTGAGAAAGTGCTCTGCCACCGCCGCCTCCACGGCATCCGGACCGGCAGCGGGATCCGCTGGCAGGTGGGCGCGCACCTCGTCGGGTAGCTTGGAGACAAACAGCTCCAGGCCAGTCCGCGGCGCTAGCGCCTGCAGGTGCCTCACCACCGGGGCCGGGTCATGCGATCTGTTTAGTCCGGAGAAGATAGGGCCCTTGGCAAGCCGCTGAACCAGCTTCAGGGTCTCCAAGATGCGCACCGCGTTGTCGGAGCTGTCGCGATAGAGGTTAGGGTAGGTGTCCGGTCCGTCCGTGGGCTCAAACCTGCCCAGACACACCACTGTCTGCTGGGGGATCATCCTTCTCAGGGAGATGCATTCTTTGGAAGTAGTGGTAGAGATGGAGCAGACTGCCAGGGCGTTGCCAGGAGTGGTGGCGATGGTGCGCACCGTTTTTAAGAAACCCCCCAGGGTGGGGACTCCCGCTCCCTGCAGCATCTCGGCCTGCTGTACGCCCTTGGCGAATATGCGACGAAATCGGCTGTGCGCACGGGGTCCCAGGGCCGGTTCGGTGGCATACAGGCCGGTGAGGGCCCCCTGGGTCTGTCCGCCTGGAAACAGGGTGCTGTGAAACAACAGGTTGCCAAGGCCGCGAATACCCCTCTGCACGCTGCTGTGGACGTGGGTGTATGCTCCGTGGATCCCGAATGCCTGTCTGGCACAGTTCCAGGGCCACCGTTCCATGGTGCATCTTCCCGGTATCACAAAGTACCTGGCCACGTTATAATTGTCCCCGGTTGAAGCCTGCACCGCCAGCGGTAGCAGGTCTGCCCCCAGGGATATCATAACAGCCTGCATAATGACATCATCTTCAATGTGTGGCCTAGCCACGGGCTGGGGACCCTCGGGCACTTCCAACCCCTCGTACGGTACCAGGTCAGTATTTTGTGTAAATGCCCTGATAAACTGAGGTGGGTGTGGTTCTAGCAGGGTCTGTGTGATTTTGGACACCAGGTGCCTGCCCACTTCCACTCTAGCCCACTCCTGCAGTCCTAGCTCTCGCAGCAAAACTCCAAGCTCTGTTGACAGTGTTGTGGGCCTGTGGTTTATGTTTGGCCCGTAGCCAAAGGATACAACACGCTCGCTCCCCCGTGGCGGAGACCGCCTGATGACATGGGGATATCCAAGGAGCGGTGACAGCACAGCGAGCACCGTCTGTATTTCCACATCCCGTCTCTCTCGCTCCTCCCTCGAAGTGGGAGGTCTTCGGAAAGTTATCCATAGCAGATAGTAGCCTCCGGTGCCACCGGGTACGAGAGTGAGTGTGCCCGTACGGCTTGTATAAAAGCTGACAAAAGCTTCCTCATCCGCGGTGAGATCACTCTCCAACCACAGCCCAGTGACGTCGTAGGCCATGCCTAGAGGGTGCCCCGCCCCCGGGGACACCCTCTGTAGTCAGACTTGGGTTAGGGCTTTGATTTCTCTGGGGAGTAGGAAGAAACTGAGAATCCCCAAATATTACGCAGGCACAGGTTGCTCTGCAGAGTTTGTTTTCGCTTTCGTGGAATCCACAGTTACATGTAGCCATGTCACTAACCTCAAATACTCAAAAAAAGGTATCGATGTCAAAAAGTCTGTGGTCGTGGAGGGCTAGTTCCTGGGAAATAAAACCTCCTCATACAGGTCGTCTGTCGGTTGGGTGGCGGCTTGGGCGGTGTCTATACGGAAGGCACTCCCACCATCAACCCTTAAAATTGACATTCCTGATTGGTCTGGCTGTGGTGATGGTTCACGTGATTCTTCCGCACCGGACACTAAAATACTGGCATACCCGAATACATTGGGTGGGTCCCTATTCACTTCCTGTAACTCCACCTCGTTCAGTAGTTGGCTCCCGCGGCCCCTATCGGTGACATGGCTAGGTAACCTAACAGGCTGTCTAAAAAAAGGTGGTAATGGTGGAGGGCTAATTACATTATTGCCAAGGTGGTTGTCATTGTGGTGTAAGTTCTGGTCCCGCGTGTATATGCGCCGCCTTCTCTGTCGGTAACTATTTACAGCCACCACTTTTTTTTCCTTATATAAATAAAATACCAGCAGTCCTTGTAACGTACCTAAATTTTAAAATAGAAAAATACTTTTTTTACATAGTATATGTCATGTGGTGATATAATTTATGGTAATTAGTCTTTAACTTACTGAATAGTGTACTGATGCAGATGATCATTGTTAGGATGCCTCTTGTTAAACCACTAGGGAGAAGCATTACTAATTGTACACAACCTCCAGTGTGGGACCACGCTAAAAAATAGAGGATAGTTATGTTTTTGTGTCATTGTTAAAAAATAAAATCCAACTTTATAATGTGGAAAGTGCTACCTACCTGCAATTAAATGCAGCATTCCAGTAACAAGAGACGCCTTGTTAAAACTTTGCCACACATAAAGGCACCATAGAAAAATGAGGCATACAAGAAGCAATATTTCGTGGCCTGTTTATACATAAAAACACAAATTATGTAGTGTACATTTATTTAGATGCTTGAACCATACCATCAATGCTATACAACAAACCTTTGATATTGCCAGTGGTGCTAGTAATTGCAAGTAGTTCGGTTGTTAAAGTTTGTGTGGTTAGCACTCCCCCCTGAAGAAGCAGGAACGTTAATATTCCACGTTTTGGTGGAGCCCATGACTTATGAAGAGTGCTGATTATGCCAGTGAATCCTACAAAAAATGTCGTTAACAGCAACATGTACTATGCACCAACTAAAACTTGTATTTAAAAAATACTTGTTCTGTACCCAAATAAATGCTGGAAAGACTTAATCCTGCAGCGGTGGCAGTACATGTATGCGTTTTACAGCAGGATACAGGGTAACCCATGACCAAGCCTATAAAAAAAGATAACCGAGTGTTAACTTCAGTTTAGTTGACAGGCCTATCTGTGAATTTCCGGTGTGAAAACAGTGGTTAACTTACCTGCACAAATGAAAGAGACAGATGACACTAATTTAATATTGCCTGTTTTCACGGTGGCAAATGCATACAAGAATGGCAAGCTGAGAGTGAAGCTAATTGGAAAAAATATCCAAGACGATTGCCAGGTGTTTGACATGTGGACCAGCATGTTTGTCATCAACACAGCAAACGTTGATGTGAATAATATTCCATGTTCAAGTTGTAATTTACAGGCGGTGCAGTGTTTTATTAACGTCAAAAAAGCTATAAAATACAAAAAGAATGTTAAGTGCCATAATAAGTATGAGTTTGTTGGATATAACATATAGACCCACCATACTACCTACCTGTTACCAGTGATAAAATGGCCGTGGTGATTAAGCAAGCATAGGTTACAGAGTTGGTAGTTGACAAGTTCCAAAGTTTAAGGACAATGCAAGCTATAAAAACAAATGTATGTGAATATACAAGTGTCAGCTTGCAATGTTTATACTACAATGAAAAAAAGGAAAAGCTACTTACCAGGAATTCCTGGTAGCCAGTACTTACCAATAATTCCTAGGTGCCAGGATGAAGGCCATTTAGGATTGTTGCTAGAGATTGCCTGTGTATACTTATTAATAGCACTGACAAACAAAATGCACATAACAAGCAAAGAAGCCATTGTATCAATTGAGTTGGTAGTTACACAGACAAGAGTGATACACCAAAAACATACCAGTAGGGCCCAAAGCCATAAATTCCAGAAGAATATGAGTGTCTTCATTTTGACTAGGTATCCACAGGGCTTACAATAGAGTTGTTGTAAATGGGGTTTGTGATTTTTAAATACGTTTTAGGATTCACGTGATACTAGAAAGGGCATTTAAGGTTTTGTGTACAATTGTTTTAGGAAGCCACATGGAATTTTGCTGATTTCCTAAGTATTCCCGACACAAGGCTCATAAATATTCCGGATACAAGGCTCGTAAATTGTTACAGCTGCAA

>HPV6_STRIPPED4

ATGGAAAGTGCAAATGCCTCCACGTCTGCAACGACCATAGACCAGTTGTGCAAGACGTTTAATCTATCTATGCATACGTTGCAAATTAATTGTGTGTTTTGCAAGAATGCACTGACCACTGCAGAGATTTATTCATATGCATATAAACACCTAAAGGTCCTGTTTCGAGGCGGCTATCCATATGCAGCCTGCGCGTGCTGCCTAGAATTTCATGGAAAAATAAACCAATATAGACACTTTGATTATGCTGGATATGCAACAACAGTTGAAGAAGAAACTAAACAAGACATCTTAGACGTGCTAATTCGGTGCTACCTGTGTCACAAACCGCTGTGTGAAGTAGAAAAGGTAAAACATATACTAACCAAGGCACGGTTCATAAAGCTAAATTGTACGTGGAAGGGTCGCTGCCTACACTGCTGGACAACATGCATGGAAGACATGTTACCCTAAAGGATATTGTATTAGACCTGCAACCTCCAGACCCTGTAGGGTTACATTGCTATGAGCAATTAGTAGACAGCTCAGAAGATGAGGTGGACGAAGTGGACGGACAAGATTCACAACCTTTAAAACAACATTTCCAAATAGTGACCTGTTGCTGTGGATGTGACAGCAACGTTCGACTGGTTGTGCAGTGTACAGAAACAGACATCAGAGAAGTGCAACAGCTTCTGTTGGGAACACTAAACATAGTGTGTCCCATCTGCGCACCGAAGACCTAACAACGATGGCGGACGATTCAGGTACAGAAAATGAGGGGTCTGGGTGTACAGGATGGTTTATGGTAGAAGCTATAGTGCAACACCCAACAGGTACACAAATATCA

ATTGATGACAGCAATATTACACACAATTCACTGGAAGCACAGGCATTGTTTAACAGGCAGGAGGCGGACGCCCATTATGCGACTGTGCAGGACCTAAAACGAAAGTATTTAGGTAGTCCATATGTTAGTCCTATAAACACTATAGCCGAGGCAGTGGAAAGTGAAATAAGTCCACGATTGGACGCCATTAAACTTACAAGACAGCCAAAAAAGGTAAAGCGACGGCTGTTTCAAACCAGGGAACTAACGGACAGTGGATATGGCTATTCTGAAGTGGAAGCTGGAACGGGAACGCAGGTAGAGAAACATGGCGTACCGGAAAATGGGGGAGATGGTCAGGAAAAGGACACAGGAAGGGACATAGAGGGGGAGGAACATACAGAGGCGGAAGCGCCCACAAACAGTGTACGGGAGCATGCAGGCACAGCAGGAATATTGGAATTGTTAAAATGTAAAGATTTACGGGCAGCATTACTTGGTAAGTTTAAAGAATGCTTTGGGCTGTCTTTTATAGATTTAATTAGGCCATTTAAAAGTGATAAAACAACATGTTTAGATTGGGTGGTAGCAGGATTTGGTATACATCATAGCATATCAGAGGCATTTCAAAAATTAATTGAGCCATTAAGTTTATATGCACATATACAATGGCTAACAAATGCATGGGGAATGGTATTGTTAGTATTATTAAGATTTAAAGTAAATAAAAGTAGAAGTACCGTTGCACGTACACTTGCAACGCTATTAAATATACCTGAAAACCAAATGTTAATAGAGCCACCAAAAATACAAAGTGGTGTTGCAGCCCTGTATTGGTTTCGTACAGGTATATCAAATGCCAGTACAGTTATAGGGGAAGCACCAGAATGGATAACACGCCAAACAGTTATTGAACACGGGTTGGCAGACAGTCAGTTTAAATTAACAGAAATGGTGCAGTGGGCATATGATAATGACATATGCGAGGAGAGTGAAATTGCATTTGAATATGCACAAAGGGGAGATTTTGATTCTAATGCACGAGCATTTTTAAATAGCAATATGCAGGCAAAATATGTGAAAGATTGTGCAACTATGTGTAGACATTATAAACATGCAGAAATGAGGAAGATGTCTATAAAACAATGGATAAAACATAGGGGTTCTAAAATAGAAGGCACAGGAAATTGGAAACCAATTGTACAATTCCTACGACATCAAAATATAGAATTCATTCCTTTTTTAACTAAATTTAAATTATGGCTGCACGGTACGCCAAAAAAAAACTGCATAGCCATAGTAGGCCCTCCAGATACTGGGAAATCGTACTTTTGTATGAGTTTAATAAGCTTTCTAGGAGGTACAGTTATTAGTCATGTAAATTCCAGCAGCCATTTTTGGTTGCAACCGTTAGTAGATGCTAAGGTAGCATTGTTAGATGATGCAACACAGCCATGTTGGATATATATGGATACATATATGAGAAATTTGTTAGATGGTAATCCTATGAGTATTGACAGAAAGCATAAAGCATTGACATTAATTAAATGTCCACCTCTGCTAGTAACGTCCAACATAGATATTACTAAAGAAGATAAATATAAGTATTTACATACTAGAGTAACAACATTTACATTTCCAAATCCATTCCCTTTTGACAGAAATGGGAATGCAGTGTATGAACTGTCAAATGCAAACTGGAAATGTTTTTTTGAAAGACTGTCGTCAAGCCTAGACATTCAGGATTCAGAGGACGAGGAAGATGGAAGCAATAGCCAAGCGTTTAGATGCGTGCCAGGAACAGTTGTTAGAACTTTATGAAGAAAACAGTACTGACCTACACAAACATGTATTGCATTGGAAATGCATGAGACATGAAAGTGTATTATTATATAAAGCAAAACAAATGGGCCTAAGCCACATAGGAATGCAAGTAGTGCCACCATTAAAGGTGTCCGAAGCAAAAGGACATAATGCCATTGAAATGCAAATGCATTTAGAATCATTATTAAGGACTGAGTATAGTATGGAACCGTGGACATTACAAGAAACAAGTTATGAAATGTGGCAAACACCACCTAAACGCTGTTTTAAAAAACGGGGCAAAACTGTAGAAGTTAAATTTGATGGCTGTGCAAACAATACAATGGATTATGTGGTATGGACAGATGTGTATGTGCAGGACAATGACACCTGGGTAAAGGTGCATAGTATGGTAGATGCTAAGGGTATATATTACACATGTGGACAATTTAAAACATATTATGTAAACTTTGTAAAAGAGGCAGAAAAGTATGGGAGCACCAAACATTGGGAAGTATGTTATGGCAGCACAGTTATATGTTCTCCTGCATCTGTATCTAGCACTACACAAGAAGTATCCATTCCTGAATCTACTACATACACCCCCGCACAGACCTCCACCCTTGTGTCCTCAAGCACCAAGGAAGACGCAGTGCAAACGCCGCCTAGGAAACGAGCACGAGGAGTCCAACAGTCCCCTTGCAACGCCTTGTGTGTGGCCCACATTGGACCCGTGGACAGTGGAAACCACAACCTCATCACTAACAATCACGACCAGCACCAAAGACGGAACAACAGTAACAGTTCAGCTACGCCTATAGTGCAATTTCAAGGTGAATCCAATTGTTTAAAGTGTTTTAGATATAGGCTAAATGACAGACACAGACATTTATTTGATTTAATATCATCAACGTGGCACTGGGCCTCCTCAAAGGCACCACATAAACATGCCATTGTAACTGTAACCTATGATAGTGAGGAACAAAGGCAACAGTTTTTAGATGTTGTAAAAATACCCCCTACCATTAGCCACAAACTGGGATTTATGTCACTGCACCTATTGTAATTTGTATATATGTAAATGTGTAAATATATGGTATTGGTGTAATACAACTGTACATGTATGGAAGTGGTGCCTGTACAAATAGCTGCAGGAACAACCAGCACATTCATACTGCCTGTTATAATTGCATTTGTTGTATGTTTTGTTAGCATCATACTTATTGTATGGATATCTGAGTTTATTGTGTACACATCTGTGCTAGTACTAACACTGCTTTTATATTTACTATTGTGGCTGCTATTAACAACCCCCTTGCAATTTTTCCTACTAACTCTACTTGTGTGTTACTGTCCCGCATTGTATATACACTACTATATTGTTACCACACAGCAATGATGCTAACATGTCAATTTAATGATGGAGATACCTGGCTGGGTTTGTGGTTGTTATGTGCCTTTATTGTAGGGATGTTGGGGTTATTATTGATGCACTATAGAGCTGTACAAGGGGATAAACACACCAAATGTAAGAAGTGTAACAAACACAACTGTAATGATGATTATGTAACTATGCATTATACTACTGATGGTGATTATATATATATGAATTAGAGTAAACCGTTTTTTATATTTGTAACAGTGTATGCTTTGTATACCATGGCACATAGTAGGGCCCGACGACGCAAGCGTGCGTCAGCTACACAGCTATATCAAACATGTAAACTCACTGGAACATGCCCCCCAGATGTAATTCCTAAGGTGGAGCACAACACCATTGCAGATCAAATATTAAAATGGGGAAGTTTGGGGGTGTTTTTTGGAGGGTTGGGTATAGGCACGGGTTCCGGCACTGGGGGTCGTACTGGCTATGTTCCCTTACAAACTTCTGCAAAACCTTCTATTACTAGTGGGCCTATGGCTCGTCCTCCTGTGGTGGTGGAGCCTGTGGCCCCTTCGGATCCATCCATTGTGTCTTTAATTGAAGAATCGGCAATCATTAACGCAGGGGCGCCTGAAATTGTGCCCCCTGCACACGGTGGGTTTACAATTACATCCTCTGAAACAACTACCCCTGCAATATTGGATGTATCAGTTACTAGTCACACTACTACTAGTATATTTAGAAATCCTGTCTTTACAGAACCTTCTGTAACACAACCCCAACCACCCGTGGAGGCTAATGGACATATATTAATTTCTGCACCCACTGTAACGTCACACCCTATAGAGGAAATTCCTTTAGATACTTTTGTGGTATCATCTAGTGATAGCGGTCCTACATCCAGTACCCCTGTTCCTGGTACTGCACCTCGGCCTCGTGTGGGCCTATATAGTCGTGCATTGCACCAGGTGCAGGTTACAGACCCTGCATTTCTTTCCACTCCTCAACGCTTAATTACATATGATAACCCTGTATATGAAGGGGAGGATGTTAGTGTACAATTTAGTCATGATTCTATACACAATGCACCTGATGAGGCTTTTATGGACATCATTCGTTTGCACAGACCTGCCATTGCGTCCCGACGTGGCCTTGTGCGGTACAGTCGCATTGGACAACGGGGGTCTATGCACACTCGCAGCGGAAAGCACATAGGGGCCCGCATTCATTATTTTTATGATATTTCACCTATTGCACAAGCTGCAGAAGAAATAGAAATGCACCCTCTTGTGGCTGCACAGGATGATACATTTGATATTTATGCTGAATCTTTTGAACCTGGCATTAACCCTACCCAACACCCTGTTACAAATATATCAGATACATATTTAACTTCCACACCTAATACAGTTACACAACCGTGGGGTAACACCACAGTTCCATTGTCACTTCCTAATGACCTGTTTTTACAATCTGGCCCTGATATAACTTTTCCTACTGCACCTATGGGAACACCCTTTAGTCCTGTAACTCCTGCTTTACCTACAGGCCCTGTTTTCATTACAGGTTCTGGATTTTATTTGCATCCTGCATGGTATTTTGCACGTAAACGCCGTAAACGTATTCCCTTATTTTTTTCAGATGTGGCGGCCTAGCGACAGCACAGTATATGTGCCTCCTCCTAACCCTGTATCCAAAGTTGTTGCCACGGATGCTTATGTTACTCGCACCAACATATTTTATCATGCCAGCAGTTCTAGACTTCTTGCAGTGGGACATCCTTATTTTTCCATAAAACGGGCTAACAAAACTGTTGTGCCAAAGGTGTCAGGATATCAATACAGGGTATTTAAGGTGGTGTTACCAGATCCTAACAAATTTGCATTGCCTGACTCGTCTCTTTTCGATCCCACAACACAACGTTTAGTATGGGCATGCACAGGCCTAGAGGTGGGCAGGGGACAGCCATTAGGTGTGGGTGTAAGTGGACATCCTTTCCTAAATAAATATGATGATGTTGAAAATTCAGGGAGTGGTGGTAACCCTGGACAGGATAACAGGGTTAATGTAGGTATGGATTATAAACAAACACAATTATGCATGGTTGGATGTGCCCCCCCTTTGGGCGAGCATTGGGGTAAAGGTAAACAGTGTACTAATACACCTGTACAGGCTGGTGACTGCCCGCCCTTAGAACTTATTACCAGTGTTATACAGGATGGCGATATGGTTGACACAGGCTTTGGTGCTATGAATTTTGCTGATTTGCAGACCAATAAATCAGATGTTCCTATTGACATATGTGGCACTACATGTAAATATCCAGATTATTTACAAATGGCTGCAGACCCATATGGTGATAGATTATTTTTTTTTCTACGGAAGGAACAAATGTTTGCCAGACATTTTTTTAACAGGGCTGGCGAGGTGGGGGAACCTGTGCCTGATACTCTTATAATTAAGGGTAGTGGAAATCGCACGTCTGTAGGGAGTAGTATATATGTTAACACCCCGAGCGGCTCTTTGGTGTCCTCTGAGGCACAATTGTTTAATAAGCCATATTGGCTACAAAAAGCCCAGGGACATAACAATGGTATTTGTTGGGGTAATCAACTGTTTGTTACTGTGGTAGATACCACACGCAGTACCAACATGACATTATGTGCATCCGTAACTACATCTTCCACATACACCAATTCTGATTATAAAGAGTACATGCGTCATGTGGAAGAGTATGATTTACAATTTATTTTTCAATTATGTAGCATTACATTGTCTGCTGAAGTAATGGCCTATATTCACACAATGAATCCCTCTGTTTTGGAAGACTGGAACTTTGGGTTATCGCCTCCCCCAAATGGTACATTAGAAGATACCTATAGGTATGTGCAGTCACAGGCCATTACCTGTCAAAAGCCCACTCCTGAAAAGGAAAAGCCAGATCCCTATAAAAACCTTAGTTTTTGGGAGGTTAATTTAAAAGAAAAGTTTTCTAGTGAATTGGATCAGTATCCTTTGGGACGCAAGTTTTTGTTACAAAGTGGATATAGGGGACGGTCCTCTATTCGTACAGGTGTTAAGCGCCCTGCTGTTTCCAAAGCCTCTGCTGCCCCTAAACGTAAGCGCGCCAAAACTAAAAGGTAATATAGTGCAATAAACAATTACCTCTTGTTACACCCTGTGACTCAGTGGCTGTTGCACGCGTTTTGGTTTGCACGCGCCTT

CCGTTTTCGGTTGCCCTTAGCATACACTTTCCACCACTTTGTTACAACGTGTTTCCTCTTAATCCTATATATTTTGTGCCAGGTACACATTGCCCTGCCAAGTTGATTGCCAAGTGCATCATATCCTGCCAACCACACACCTGGCGCCAGGGTGCGGTATTGCCTTACTCATAAACCTGTCTTTGTGTTATACTTTTATGCACTGTAGCCAACTCTTAAAAGCATTTTTGGCTTGTAGCAGCACATTTTTTTGCTCTTACTGTTTGGTATACAATAACATAAAAATGAGTAACCTAAGGTCACACACCTGCAACCGGTTTCGGTTATCCACACCCTACATATTTCCTTCTTATAGTTAATAACAATCTTAGTTTAAAAAAGAGGAGGGACCGAAAACGGTTCAACCGAAAACGGTTATATATAAACCAGCCCAAAAAATTAGCAGACGAGGCATT

>HPV16_STRIPPED4

ACTACAATAATTCATGTATAAAACTAAGGGCGTAACCGAAATCGGTTGAACCGAAACCGGTTAGTATAAAAGCAGACATTTTATGCACCAAAAGAGAACTGCAATGTTTCAGGACCCACAGGAGCGACCCAGAAAGTTACCACAGTTATGCACAGAGCTGCAAACAAC

GCAACAGTTACTGCGACGTGAGGTATATGACTTTGCTTTTCGGGATTTATGCATAGTATATAGAGATGGGAATCCATATGCTGTATGTGATAAATGTTTAAAGTTTTATTCTAAAATTAGTGAGTATAGACATTATTGTTATAGTTTGTATGGAACAACATTAGAACAGCAATACAACAAACCGTTGTGTGATTTGTTAATTAGGTGTATTAACTGTCAAAAGCCACTGTGTCCTGAAGAAAAGCAAAGACATCTGGACAAAAAGCAAAGATTCCATAATATAAGGGGTCGGTGGACCGGTCGATGTATGTCTTGTTGCAGATCATCAAGAACACGTAGAGAAACCCAGCTGTAATCATGCATGGAGATACACCTACATTGCATGAATATATGTTAGATTTGCAACCAGAGACAACTGATCTCTACTGTTATGAGCAATTAAATGACAGCTCAGAGGAGGAGGATGAAATAGATGGTCCAGCTGGACAAGCAGAACCGGACAGAGCCCATTACAATATTGTAACCTTTTGTTGCAAGTGTGACTCTACGCTTCGGTTGTGCGTACAAAGCACACACGTAGACATTCGTACTTTGGAAGACCTGTTAATGGGCACACTAGGAATTGTGTGCCCCATCTGTTCTCAGAAACCATAATCTACCATGGCTGATCCTGCAGGTACCAATGGGGAAGAGGGTACGGGATGTAATGGATGGTTTTATGTAGAGGCTGTAGTGGAAAAAAAAACAGGGGATGCTATATCAGATGACGAGAACGAAAATGACAGTGATACAGGTGAAGATTTGGTAGATTTTATAGTAAATGATAATGATTATTTAACACAGGCAGAAACAGAGACAGCACATGCGTTGTTTACTGCACAGGAAGCAAAACAACATAGAGATGCAGTACAGGTTCTAAAACGAAAGTATTTGGTAGTCCACTTAGTGATATTAGTGGATGTGTAGACAATAATATTAGTCCTAGATTAAAAGCTATATGTATAGAAAAACAAAGTAGAGCTGCAAAAAGGAGATTATTTGAAAGCGAAGACAGCGGGTATGGCAATACTGAAGTGGAAACTCAGCAGATGTTACAGGTAGAAGGGCGCCATGAGACTGAAACACCATGTAGTCAGTATAGTGGTGGAAGTGGGGGTGGTTGCAGTCAGTACAGTAGTGGAAGTGGGGGAGAGGGTGTTAGTGAAAGACACACTATATGCCAAACACCACTTACAAATATTTTAAATGTACTAAAAACTAGTAATGCAAAGGCAGCAATGTTAGCAAAATTTAAAGAGTTATACGGGGTGAGTTTTTCAGAATTAGTAAGACCATTTAAAAGTAATAAATCAACGTGTTGCGATTGGTGTATTGCTGCATTTGGACTTACACCCAGTATAGCTGACAGTATAAAAACACTATTACAACAATATTGTTTATATTTACACATTCAAAGTTTAGCATGTTCATGGGGAATGGTTGTGTTACTATTAGTAAGATATAAATGTGGAAAAAATAGAGAAACAATTGAAAAATTGCTGTCTAAACTATTATGTGTGTCTCCAATGTGTATGATGATAGAGCCTCCAAAATTGCGTAGTACAGCAGCAGCATTATATTGGTATAAAACAGGTATATCAAATATTAGTGAAGTGTATGGAGACACGCCAGAATGGATACAAAGACAAACAGTATTACAACATAGTTTTAATGATTGTACATTTGAATTATCACAGATGGTACAATGGGCCTACGATAATGACATAGTAGACGATAGTGAAATTGCATATAAATATGCACAATTGGCAGACACTAATAGTAATGCAAGTGCCTTTCTAAAAAGTAATTCACAGGCAAAAATTGTAAAGGATTGTGCAACAATGTGTAGACATTATAAACGAGCAGAAAAAAAACAAATGAGTATGAGTCAATGGATAAAATATAGATGTGATAGGGTAGATGATGGAGGTGATTGGAAGCAAATTGTTATGTTTTTAAGGTATCAAGGTGTAGAGTTTATGTCATTTTTAACTGCATTAAAAAGATTTTTGCAAGGCATACCTAAAAAAAATTGCATATTACTATATGGTGCAGCTAACACAGGTAAATCATTATTTGGTATGAGTTTAATGAAATTTCTGCAAGGGTCTGT

TGGTTACAACCATTAGCAGATGCCAAAATAGGTATGTTAGATGATGCTACAGTGCCCTGTTGGAACTACATAGATGACAATTTAAGAAATGCATTGGATGGAAATTTAGTTTCTATGGATGTAAAGCATAGACCATTGGTACAACTAAAATGCCCTCCATTATTAATTACATCTAACATTAATGCTGGTACAGATTCTAGGTGGCCTTATTTACATAATAGATTGGTGGTGTTTACATTTCCTAATGAGTTTCCATTTGACGAAAACGGAAATCCAGTGTATGAGCTTAATGATAAGAACTGGAAATCCTTTTTCTCAAGGACGTGGTCCAGATTAAGTTTGCACGAGGACGAGGACAAGGAAAACGATGGAGACTCTTTGCCAACGTTTAAATGTGTGTCAGGACAAAATACTAACACATTATGAAAATGATAGTACAGACCTACGTGACCATATAGACTATTGGAAACACATGCGCCTAGAATGTGCTATTTATTACAAGGCCAGAGAAATGGGATTTAAACATATTAACCACCAAGTGGTGCCAACACTGGCTGTATCAAAGAATAAAGCATTACAAGCAATTGAACTGCAACTAACGTTAGAAACAATATATAACTCACAATATAGTAATGAAAAGTGGACATTACAAGACGTTAGCCTTGAAGTGTATTTAACTGCACCAACAGGATGTATAAAAAAACATGGATATACAGTGGAAGTGCAGTTTGATGGAGACATATGCAATACAATGCATTATACAAACTGGACACATATATATATTTGTGAAGAAGCATCAGTAACTGTGGTAGAGGGTCAAGTTGACTATTATGGTTTATATTATGTTCATGAAGGAATACGAACATATTTTGTGCAGTTTAAAGATGATGCAGAAAAATATAGTAAAAATAAAGTATGGGAAGTTCATGCGGGTGGTCAGGTAATATTATGTCCTACATCTGTGTTTAGCAGCAACGAAGTATCCTCTCCTGAAATTATTAGGCAGCACTTGGCCAACCACCCCGCCGCGACCCATACCAAAGCCGTCGCCTTGGGCACCGAAGAAACACAGACGACTATCCAGCGACCAAGATCAGAGCCAGACACCGGAAACCCCTGCCACACCACTAAGTTGTTGCACAGAGACTCAGTGGACAGTGCTCCAATCCTCACTGCATTTAACAGCTCACACAAAGGACGGATTAACTGTAATAGTAACACTACACCCATAGTACATTTAAAAGGTGATGCTAATACTTTAAAATGTTTAAGATATAGATTTAAAAAGCATTGTACATTGTATACTGCAGTGTCGTCTACATGGCATTGGACAGGACATAATGTAAAACATAAAAGTGCAATTGTTACACTTACATATGATAGTGAATGGCAACGTGACCAATTTTTGTCTCAAGTTAAAATACCAAAAACTATTACAGTGTCTACTGGATTTATGTCTATATGACAAATCTTGATACTGCATCCACAACATTACTGGCGTGCTTTTTGCTTTGCTTTGTGTGCTTTTGTGTGTCTGCCTATTAATACGTCCGCTGCTTTTGTCTGTGTCTACATACACATCATTAATAATATTGGTATTACTATTGTGGATAACAGCAGCCTCTGCGTTTAGGTGTTTTATTGTATATATTATATTTGTTTATATACCATTATTTTTAATACATACACATGCACGCTTTTTAATTACATAATGTATATGTACATAATGTAATTGTTACATATAATTGTTGTATACCATAACTTACTACTGTTATTACTTAACAATGCGACACAAACGTTCTGCAAAACGCACAAAACGTGCATCGGCTACCCAACTTTATAAAACATGCAAACAGGCAGGTACATGTCCACCTGACATTATACCTAAGGTTGAAGGCAAAACTATTGCTGAACAAATATTACAATATGGAAGTATGGGTGTATTTTTTGGTGGGTTAGGAATTGGAACAGGGTCGGGTACAGGCGGACGCACTGGGTATATTCCATTGGGAACAAGGCCTCCCACAGCTACAGATACACTTGCTCCTGTAAGACCCCCTTTAACAGTAGATCCTGTGGGCCCTTCTGATCCTTCTATAGTTTCTTTAGTGGAAGAAACTAGTTTTATTGATGCTGGTGCACCAACATCTGTACCTTCCATTCCCCCAGATGTATCAGGATTTAGTATTACTACTTCAACTGATACCACACCTGCTATATTAGATATTAATAATACTGTTACTACTGTTACTACACATAATAATCCCACTTTCACTGACCCATCTGTATTGCAGCCTCCAACACCTGCAGAAACTGGAGGGCATTTTACACTTTCATCATCCACTATTAGTACACATAATTATGAAGAAATTCCTATGGATACATTTATTGTTAGCACAAACCCTAACACAGTAACTAGTAGCACACCCATACCAGGGTCTCGCCCAGTGGCACGCCTAGGATTATATAGTCGCACAACACAACAGGTTAAAGTTGTAGACCCTGCTTTTGTAACCACTCCCACTAAACTTATTACATATGATAATCCTGCATATGAAGGTATAGATGTGGATAATACATTATATTTTTCTAGTAATGATAATAGTATTAATATAGCTCCAGATCCTGACTTTTTGGATATAGTTGCTTTACATTTAGGTACAGTAGAATTGGTAATAAACAAACACTACGTACTCGTAGTGGAAAATCTATAGGTGCTAAGGTACATTATTATTATGATTTAAGTACTATTGATCCTGCAGAAGAAATAGAATTACAAACTATAACACCTTCTACATATACTACCACTTCACATGCAGCCTCACCTACTTCTATTAATAATGGATTATATGATATTTATGCAGATGACTTTATTACAGATACTTCTACAACCCCGGTACCATCTGTACCCTCTACATCTTTATCAGGTTATATTCCTGCAAATACAACAATTCCTTTTGGTGGTGCATACAATATTCCTTTAGTATCAGGTCCTGATATACCCATTAATATAACTGACCAAGCTCCTTCATTAATTCCTATAGTTCCAGGGTCTCCACAATATACAATTATTGCTGATGCAGGTGACTTTTATTTACATCCTAGTTATTACATGTTACGAAAACGACGTAAACGTTTACCATATTTTTTTTCAGATGTCTCTTTGGCTGCCTAGTGAGGCCACTGTCTACTTGCCTCCTGTCCCAGTATCTAAGGTTGTAAGCACGGATGAATATGTTGCACGCACAAACATATATTATCATGCAGGAACATCCAGACTACTTGCAGTTGGACATCCCTATTTTCCTATTAAAAAACCTAACAATAACAAAATATTAGTTCCTAAAGTATCAGGATTACAATACAGGGTATTTAGAATACATTTACCTGACCCCAATAAGTTTGGTTTTCCTGACACCTCATTTTATAATCCAGATACACAGCGGCTGGTTTGGGCCTGTGTAGGTGTTGAGGTAGGTCGTGGTCAGCCATTAGGTGTGGGCATTAGTGGCCATCCTTTATTAAATAAATTGGATGACACAGAAAATGCTAGTGCTTATGCAGCAAATGCAGGTGTGGATAATAGAGAATGTATATCTATGGATTACAAACAAACACAATTGTGTTTAATTGGTTGCAAACCACCTATAGGGGAACACTGGGGCAAAGGATCCCCATGTACCAATGTTGCAGTAAATCCAGGTGATTGTCCACCATTAGAGTTAATAAACACAGTTATTCAGGATGGTGATATGGTTCATACTGGCTTTGGTGCTATGGACTTTACTACATTACAGGCTAACAAAAGTGAAGTTCCACTGGATATTTGTACATCTATTTGCAAATATCCAGATTATATTAAAATGGTGTCAGAACCATATGGCGACAGCTTATTTTTTTATTTACGAAGGGAACAAATGTTTGTTAGACATTTATTTAATAGGGCTGGTACTGTTGGTGAAAATGTACCAGACGATTTATACATTAAAGGCTCTGGGTCTACTGCAAATTTAGCCAGTTCAAATTATTTTCCTACACCTAGTGGTTCTATGGTTACCTCTGATGCCCAAATATTCAATAAACCTTATTGGTTACAACGAGCACAGGGCCACAATAATGGCATTTGTTGGGGTAACCAACTATTTGTTACTGTTGTTGATACTACACGCAGTACAAATATGTCATTATGTGCTGCCATATCTACTTCAGAAACTACATATAAAAATACTAACTTTAAGGAGTACCTACGACATGGGGAGGAATATGATTTACAGTTTATTTTTCAACTGTGCAAAATAACCTTAACTGCAGACGTTATGACATACATACATTCTATGAATTCCACTATTTTGGAGGACTGGAATTTTGGTCTACAACCTCCCCCAGGAGGCACACTAGAAGATACTTATAGGTTTGTAACCCAGGCAATTGCTTGTCAAAAACATACACCTCCAGCACCTAAAGAAGATGATCCCCTTAAAAAATACACTTTTTGGGAAGTAAATTTAAAGGAAAAGTTTTCTGCAGACCTAGATCAGTTTCCTTTAGGACGCAAATTTTTACTACAAGCAGGATTGAAGGCCAAACCAAAATTTACATTAGGAAAACGAAAAGCTACACCCACCACCTCATCTACCTCTACAACTGCTAAACGCAAAAAACGTAAGCTGTAAGTATTGTATGTATGTTGAATTAGTGTTGTTTGTTGTGTATATGTTTGTATGTGCTTGTATGTGCTTGTAAATATTAAGTTGTATGTGTGTTTGTATGTATGGTATAATAAACACGTGTGTATGTGTTTTTAAATGCTTGTGTAACTATTGTGTCATGCAACATAAATAAACTTATTGTTTCAACACCTACTAATTGTGTTGTGGTTATTCATTGTATATAAACTATATTTGCTACATCCTGTTTTTGTTTTATATATACTATATTTTGTAGCGCCAGGCCCATTTTGTAGCTTCAACCGAATTCGGTTGCATGCTTTTTGGCACAAAATGTGTTTTTTTAAATAGTTCTATGTCAGCAACTATGGTTTAAACTTGTACGTTTCCTGCTTGCCATGCGTGCCAAATCCCTGTTTTCCTGACCTGCACTGCTTGCCAACCATTCCATTGTTTTTTACACTGCACTATGTGCAACTACTGAATCACTATGTACATTGTGTCATATAAAATAAATCACTATGCGCCAACGCCTTACATACCGCTGTTAGGCACATATTTTTGGCTTGTTTTAACTAACCTAATTGCATATTTGGCATAAGGTTTAAACTTCTAAGGCCAACTAAATGTCACCCTAGTTCATACATGAACTGTGTAAAGGTTAGTCATACATTGTTCATTTGTAAAACTGCACATGGGTGTGTGCAAACCGATTTTGGGTTACACATTTACAAGCAACTTATATAATAATACTAA

>TTV_STRIPPED4

ATTTTGTGCAGCCCGCCAATTTCTGTTCAAACAGACCAATCAGGACCTTCTACGTGCACCTCCTGGGGCGTGTCTACGAGGTCTATATAAGCAACAGCGGTGACGAATGGTAGAGTTTCTCTTCGCCCGTCCGCGGCGAGAGCGCGAGCGAAGCGAGCGATCGAGCGTCCCATGGGCGGGTGCCGTAGGTGAGTTTACACACCGAAGTCAAGGGGCAATTCGGGCACGGGACTGGCCGGGCTATGGGCAGGGCTCTTAAAAAATTCCCCCGCTCTGTTCTCCGGCAGGACACAAAGTCATGCCGTGGAGACCGCCGGTCCATAACGTGCCAGGTAGAGAGAATCAATGGTTTGCAGCGTTCTTTCACGGTCATGCTGCTTTCTGCGGGTGTGGTGACCCTGTTGGGCATCTTAACGGCATTGCTCCTCGCTTTCCTAACGCCGGTCCACCGAGACCACCTCCAGGGCTAGACCAGCTTAATCCCGAGGGCCCGGCAGGTCCCGGAGGGCCCCCCCGCCATCTTGCCAGCTCTGCCGGCCCCGGCAGACCCTAAACCGGCACCACGGCGTGGTGGTGGGGCAGATGGAGGCGCCGCCGCTGGGGCCGCCGCCGACGCAGACCATACCGGGTACGAAGAAGGAGACCTAGAAGATCTTTTCGCCGCCGCGGCCGAGGACGATATGTGAGTAGGCGGAGGCGCCGCCGCTACTACAGGCGCAGACTGAGACGGGGCAGACGCAGAGGGCGACGAAAGAGACACAGACAGACTCTAGTAGTGAGGCAGTGGCAACCTGACGTTGTTAAAAAGTGTAAAATAACAGGATGGATGCCTCTTATAATCTGTGGCTCTGGAAGCACACAGATGAACTTTATAACTCACATGGACGATACTCCCCCTATGGGATACACCTACGGGGGCAACTTTGTAAATGTAACTTTCAGTCTAGAGGCCATCTATGAACAATTCCTGTACCACAGAAACAGGTGGTCCAGATCTAACCATGACTTAGACCTGGCCAGATACCAAGGAACCACTCTAAAACTTTACAGACACCAAACGGTAGACTATATAGTTAGCTACAACAGAACAGGCCCCTTTACTATAAGTGAAATGACTTACATGAGCACACACCCGGCTCTCATGCTACTACAAAAACATAGAATAGTTGTACCCAGCTTCAGAACCAAGCCAAAAGGCAAAAGAGCCATAAAAATTAGAATAAGGGCCCCAAAACTAATGCTCACCAAGTGGTACTTTACAAAAGACATTTGCTCCATGGGCCTCTTTCAACTAATGGCAACAGCTGCAGAACTTACAAACCCATGGCTCAGAGACACCACAAAAAGCCCAGTAGTTGGCTTCAGAGTCTTAAAAAACAGCTTATACACATGCCTTTCCAACTTAAAGGACCAAGCAATACAAGGTGAAAGAAAGACTGTACAAAATAGATTACACCCAGAAAACCTACATGGCACAGGACCTAATGCTAAAGGCTGGGAATACACATACACAAAACTAATGGCATCTATATTCTACTCAGCCAACAGAAACAGCACCTACAACTGGCAAAACTATCA

GAACAGCAAACTTAAACTTAATTAAAGCGGAATACCTATATCATTACCCTAACAATGTCACACAATCTGACTTTATATTAGACTACACACTAACACACGACTGGGGCATATACAGCCCCTACTACCTAACACCCACCAGAATTAGCCTAGACTGGGACACACCATGGACATATGTAAGATGCAACCCACTATCAGACAAAGGCATAGGTAACAGAATATATGCACAGTGGTGCTCAGAAAAATCCAGTAAATTAGACACCACAAAGAGCAAGTGCATACTAAGAGACTTCCCACTGTGGGCCATGGCCTATGGCTACTGTGACTGGGTGGTGAAGTGCACAGGAGTGTCCAGTGCATGGACAGACATGAGAATAGCCATTATATGTCCCTACACAGAACCAGCACTTATAGGGTCAACAGAAGATGTAGGCTTCATTCCAGTAAGTGACACCTTTTGCAACGGAGACATGCCGTTTCTTGCACCATACATACCTATTACATGGTGGATTAAGTGGTACCCCATGATTACACACCAAAAGGAAGTTCTTGAGGCAATAGTTAACTGTGGACCGTTTGTACCCCGAGACCAAACTTCCCCAGCTTGGGAAATAACCATGGGTTACAAAATGGATTGGAAATGGGGCGGCTCTCCCCTGCCTTCACAGGCAATCGACGACCCCTGCCAGGAGTCCACCCACGAACTTCCCGACCCCGATAGACACCCTCGCATGTTACAAGTCTCTGACCCGACAAAGCTCGGACCGAAGACAGTTTTTCACAAATGGGACTGGAGACGTGGGATGCTTAGCAAAAGAAGTATTAAAAGAGTCCAAGGAGACTCAACAGATGGTGAATATGTTGCAGGACCTTTACCAAGAAAAAGAAACAAGTTCGATACTCGAGTCCAAGGCCCTCCAACCCCAGAAAAAGAAAGTTACACTTTACTCCAAGCCCTCCAAGAGTCGGGGCAAGAGAGCAGCTCAGAGGACCAAGAACAAGCACCCCAAGAAAAAGAGGACCAGAAGGAAGCGCTCATGGAGCAGCTCCAGCTCCAGAAACACCACCAGCGAGTCCTCAAGCGAGGCCTCAAACTCCTCCTCGGAGACGTGCTCCGACTCCGGAGAGGAGTCCACTGGGACCCCCTCCTGTCCTAATTCAAGGTCCCAGTATCCCAGACCTGCTTTTCCCTAACACACAAAAAAAAAAAACGATTTTCCAACTACGACTGGGTGTGCGAGTACGAGCTGACCAAATGGATGGATCGGCCCTTGCGGCACTACCCATCAGACCCCCCTCACTACCCCTGGCTACCAAAAAACCTCCTACCCCTCCTACATGTAGAGTAAGTTTCAAATTAAAGCTCAATGACTAAAATTCAAGGCCGTGGGTGTTTCACTTCATCGGTGTCTACCTCTAAAAGTCACTAAGCACTCCGAGCGTAAGCGAGGAGTGCGACCCCCCTGCCCGGTAGCAACTTCCTCGGGGTCCGGCGCTACGCCTTCGGCTGCGCCGGACGCCTCGGACCCCCCCTCGACCAGAATCGCTCGCGCGATTCGGACCTGCGGCCTCGGGGGGGTCGGGGGCTTCACTAAACAGACTCCGAGGTGCCATTGGACACTGAGGGGGTGAACAGCAACGAAAGTGAGTGGGGCCAAGCTTCGCCATAAGGCCTTTAACTTTGGGTCGCTTGTCAGCAGCTTCCGGGTCCGCCTGGAGGCCGCCATTTTACATTCGGCCGCCATTTTAGGCCCTCGCGGGCCTCCATAGTCGCACATCAGTGACGTCACGGCAGCCATCTTGGCTGTGACGTCAACGTCACGTGGGGAGGACGGCGTGTAACCCGGAAGTCATCCCCATCACGTGACCTGACGTCACGGCCGCCATTTTGTGCTGTCCGCCATCTTGTGACTTCCTTCCGCTTTTTGTAAAAAAAAGAGGAAGTGTGACGTAGC

>VARIOLAV_STRIPPED4

CTCGAGAGTATATGTTGTTGAACGTTATTGTTTGAGAAATAGTTGATGCATCAGAATGGTTTGCATTTATTGTAGACGTAATAATGCACTACATTGTTTCCTCGTCTATCTATATGTTTTGATAAGTTATAACACGTTTCAATCTCTAGTTTTATTTTTTTGTACGTCACATCTTCATCCAGTAGACGACATAGAATACATGTGCAATCTTTGCCACAATCCATAGCTATTTTGGTGCTAATTATTCCTATATTATATTTCACAAAAAATGATGAAGGCAATCATTCCTCATAAGATAATAAAAAGTGTATTGAGAGTGAGTAAGCATGAAAAAGATTTAGTATTTAGCAGTGCGGATATGATCCAAGAGGGTGAAATAGTCGTTCTCGTTCAGAATCTTTTGCAGCATAAGTAGTATGTCGATATACTTATCGTTGAAGACTCTTCCAGAGACGATAGCTAATTGAGTACAAAGTCCAATGATTGCACAAAGTTCTTTGGCGGTTTTCATGGAGTCATTTCTGATGAAACATTTAATGATCTCCACGCAATTGTCTATATTGTCCCACGGAAGTGAATCCGAGAACTCCTTCAACTCGCTACCAAAGAGCTCCGTTGCATCAGTTCTGAAAGAGATGAGAAGCCTGTAAAGAGACCCTGCGCTTTCTCTATGGGTCCATCTATGAGAAACCCACAGGATGTATTCAGTCAGACAATGTCTGACGTCTGCCACGGTATTCAGGGAGTCCTTAGTAGCATGGCAATGACAGGGACTGAACTGGGCACAAGAAGAGGCCATTGTGAAGGTAGACGAAGGTTAACCCGATGGTTAACCGTAGCCGTCTGTGCTAATAGACGGCATTAATTTCCATTTTTTAATGGGGTTGTGGATGAGGAATGAGAGTGTCTTATCTCGTCCTTGGTTTACATGGATCAGTGTGAGAAAAAATATCTTGTATATTATTAACTAAACTAACTCCTTGGTTTCAATTCATGTTTAAAATGATCTATATGTTTTATTAATTTATTATACTGTTAAATGTACTGTATATAATAGTTTCTAACGTGGAGTACAGAATTTTCGGTTAATGAGTTCATGGTAAAGGGCGAATGAATGCCTGCCTGTATTATAATATACATAAGTTAATAGTTTTTATCATATTTTTCTAATACTATATAAAAATATCATTATGTATAATCATTACTGTCGCTATCATTATTGTGTTTGTGTAGTTCTGTTCTATCATCTACATCACTGTCACTGTCACTCTCCCTATATCTTCTAAAATTACAAACAACTGGATATTCGATAACAGCATTTGTGTAGTTTTTGTCTTTTATAGTATATACGTTATTGTCAAAATCTAAACAAATATTGGCATAATACCTATCTATAAGATTAGGATCCATGTTCGAGCATACTAGCATATTTGTAACTTCGTCGTAAAGCGTTAAATCAATAGAATAAACCATTTTGTGGTGCAACTTTTTACGAACTAGTTGTATGAGTTTATCGTTTACATAAGCCATTAACAGCTTTAACAGATGATCTGAGTAATATCCTCTATTATACATTTAATGTTCACGGTCTTAGTATTTTTAGATATCAATTGTAATTTACACCCATATTCTACTCCCTTGTGTAACGTTAGACATTCTAAATCTATAGTATTATCTATTACAGCGTAAAACACATTCAATATTGTATTGTTATATTATTAGTTTATATTACTGAATTAATAATATAAAATTCCCAATCTTGTTATAAACACACACTGAGAAACAGCATAAACACAAAATCCATCAAAAATGTCGATGAAATATCTGATGTTGTTGTTCGCTGCTATGATAATTAGATCATTCGCCAATAGTGGTAACGCTATCGAAACGACATTGTCAGAAATTACAAACACTACAACAGATATTCCAGCTATCAGATTATGCGGTCCAGAGGGAGATAGGTATTGTTTTCACGGTATCTGTATCCACGCTAGAGATATTGACGGTATGTATTGTAGATGCTCTCATGGTTATACAGGCATTAGATGTCAGCATGTAGTATTAGTAGACTATCAACGTTCAGAAAAACCAAACACTACAACGTCATATATCCCATCTCCCGGTATTGTGCTTGTATTATTAGTAAGCATTATTGTGTGTTGTCTATTATTTGTTTATAGGTTCACTCGAAGAACTAATAAATTACCTTTACAAGATATGGTTGTGCCATAATTTTTATAAATTTTTTTATGAGTATTTTTATGAGTATTTTTCAAAAAATGTATAAAGTGTATGTCTTATGTATATTTATAAAAATGCTAAATGTGATGTATCTATGTTATTTGTATTTATCTAAACAATACCTCTACCTCTAGATATTATACAAAAATTTTTTATTTCAGTATATTAAAGTAAAATCTAGTTACCTTGAAAATAAATACAGTGGGTGGTTCTGTATCACCAGTAAGAACATAATGGTCGAACACAGTATCCGATTGAGATTTTGCATACAATACTAGTCTAGAAAGAAATTTGTAATCATCTTCTGTGACGGGAGTCCATATATCTGTATCATCGTCTAGTTTATCAGTGTCCCATGCTATATTCCTGTTATCATCATTAGTTAATGAAAATAACTCTCGTGCTTCAGAAAAGTCAAATATTGTATCCATACATACATCTCCAAAACTATCACTTATACGTTTATCTTTAACGATACCTATACCTAGATGGTTATTTACTAACAGACATTTTCCAGATCTATTGACTATAACTCCTATAGTTTCCACATTAACCAAGTAATGATCATCTATTGTTATATAACAATAACATAACTCTTTTCCATTTTTATCAGTATGTATATCTATATCAACGTCGCCGTAGTGAATAGTAGTCATTGATCTATTATATGAAACGGATATGTCTAGAACGGCAATTGTCTTACGTCCAGTTAACACTTTCTTTGATTTAAAGTCTAGAGTCTTTGCAAACATAATATCCTTATCCGACTTTATATTTCCTGTAGGGTGGTATAATTTTATTTTGCCTCCACATATCGGTGTTTCCAAATATATTACTAGACAATATTCCATATAGTTATTAGTTAAGGGTACCCAATTAGAACACGTACGCTTATTATCATCATTCGGATCGTATTTCATAAAAGTTATTGTACTATCGATGTCAACACATTCTACATTTTTTAATCGTCTATATAGTATTTTTCTGATATTTTCTATAATATCAGAATTGTCTTCCATCGGAAGTTGTATACTATCGGAATCAGTTACATGTTTAAATAATTCTCTGATGTCATTCCTTATACAATCAAATTCATTATTAAACAGTTTAATAGTCTGTAGACCTTTATCGTTGTAAATATCCATTGTCTTATTAGTTACGCTTATTTTTATGTGTTTTATGTTGATTTATTATATTTATAAGAATGATTGTTTGACAAATCACGAGAACTATTAAGACATATATTATTAGAGGTATATATTATAAAAAAGTTTTTGATTACGATGTTATAAGAGGAAAAAGGACACATTAACATCATACATCAATTAACATTCTTATAACATCGTAATCAAAAGAATTGCAATTTTGATGTATAACAACTGTCAATGAATTATGGAATTGTATATTACACATTATACGGTATGTTGGCAACGACAAATACCGATTGGTAATTGTTTACCGGTGTAATAGAATTTATATATCTATTACACCGGCCAAGTATGCATAATAATAAGTTGTGGGTAGTATCATCCACATATTTATAATTTAGGACTTTGTATTCAGTATTTTTGGAATCATAAAAAAAAGTTTTACCATTTTAAAATTTAAAAAGTATTTACATTTTTTTCACTGTTTAGTCGCGGATATGGAATTTGATCCTACCAAAATCAATATATCATCTATAGATCATGTAACAATATTACAATACATAGATGAACCAAATGATATAAGACTAACAGTATGCATTATCCAAAATATTAATAACATTACATATTATATCAATATCACAAAAATAAATCCACATTTGGCTAATCAATTTCGGGCTTGGAAAAAACGTATCGCTGGAAGGGACTATATGACTAACTTATCTAGAGATACAGGAATACAACAATCAAACCTTACTGAAACTATACGTAACTGTCAAAAAAATAGAAACATATATGGTCTATATATACACTACAATTTAGTTATTAATGTGGTTATTGATTGGATAACCGATGTGATTGTTCAATCAATATTAAGAGGGTTGGTAAATTGGTACATAGATAATAATACCTATACTCCAAATACACCCAATAATACAACAACCATTTCTGAGTTGGATATCATCAAAATACTGGATAAATACGAGGACGTATATAAAGTAAGTAAAGAAAAAGAATGTGGAATTTGCTATGAAGTTGTCTACTCAAAACGATTAGAAAACGATCGATACTTTGGTTTATTGGATTCGTGTAATCATATATTTTGCATAACATGTATCAATATATGGCATAGAACACGAAGAGAAACTGGTGCGTCGGATAATTGTCCTATATGTCGTACCCGTTTTAGAAACATAACAATGAGTAAGTTCTATAAGCTAGTTAACTAATAAATAAAAAGTTTAATTTGTTGACGACATATATCGTTATTTTTCTCGTATGAAAGATTAATTTGATTCTAATATAATCTTTAGTATTAGATGAACTTTAATTCAAATTTCCATTAGATTATATCATAAATAAAAATAGTAGCACGTGCTACTTTAGCCAAATATTCTTTTTTGAAACGCCATTTAGCGTAGTGAGGACACAAGTGAACCTATAATGAGCAAATTTATTAGTATCGGTTACATGAAGGACTTTACGTAGAGTGGTGATTCCACCATCTGTGGTACGAATGGTTTCATCTTCTTTGATGCCATCACCCAGATGTTCTATAAACTTGGCATACTCGTCCGATTTCATATCCTTTGCCAACCAATACATATAGCTAAACTTAGGCATATGTTCCACACATCCTGAACAATAAAATTCTCCAGATGATGTTACAATGTCTAGATTTGGACATTTGGTTTCAACCGCGTTAACATATGAGTGAACACAGCCATACATGAAAGCGATGAGAAATAGGATTCTCATCTCGCCAAAATATCACTAGAAAAAATTTATTTATCAATTTTAAAGGTATAAAATACTTATTGTTGTCCTCGTTTGTCAATCATGTTAATGTCTTTAAGATAAGGCAGGCAAATGTTTATAGTATTAAGAATTGGGCAAGCATAAGACATGACACAAAGACCCTTTTTGTATATATAAGTGTAGAAATTATAACATCCATAGTTGGATTTACATAGGTGTCCAATTGGGATCTCACCATCATCGAGATAATTGATGACATCTCCCCCTTTCTTTTTTAGTAGATATTTCATCGTGTAAGAATCAATATTAATATTTCTAAAGTATCCGTGTATAGCCTCTTTATTTACCACAGCTCCATATGCCAACATGCATTCCACTAGATGGATATCGATATCGTCGAATGTCATATACTCAATTAGTATATGTTGGAGGATATTCGAGTTCATTGTTTTCAATATCAAAGAGATGGTTTCCTTATCATTTCTCCATAGTGGTACAATACTACACATTATTCCGTGTGGCTTTCCATTTTCCAAAAACAATTTGACCAAATCTAAATTCACATCTTCATTGTATCTATAATCACTATTCAGATAATCAGCCATAATTACTCGAGTGCAACGTGTTAGATCGTCTATATATGAATAAGCAGTGTTATCTATTCCTTTCATTAACAATTTAACGATGTCTATATCTATATGAGATGACTTAATATAATATTGAAGAGCTGTACAATAGTTTTTATCTATAGAAGACGGCTTGATTCCGTGATTAATTAGACATTTAACAACTTCCGGACGCACATATGCTCTCGTATCCGACTCTGAATACAGATGAGAGATAATATACAAATGCAATACGGTACCGCAATTTTGTGGTTGATAATCATCATACGTGTATCCATACTCGTCATCCTCATAAAGAACATTGCAGCCATTTTCTATGAACAAATCAATAATTTCAGGAACAGGATCATCTGTCATTACATAATTTTCTATAACTGAACGATGGTTTTCACATTTAACACTCAAGTCAAATCCATGTTCTACCAACACCTTTATTAAGTCAACGTTTACATTTTTGGATTTCATATAGTTGAATATATTAAAGTTATTTATGTTGCTAAATCCAGTGGCTTCTAGTAGAGCCATCGCTATATCCTTATTAACTTTAACATGTCTACTATTTGTGTATTCTTCTAATGGGGTAAAATGTCTCCAATTTTTGTGCAATGGATTAGTGCCACTGTCTAGTAGTAGTTTGACGACCTCGACATTATTACAATGCTCATTAAAAAGGTATGCATGTAAAGCATTATTCTTGAATTGGTTCCTGGTATCATTAGGATCTCTGTCTTTCAACATCTGTTTAAGTTCATCGAGAGCTACCTCCTCATTTTCCAGATAGTTAAACATTTTGACTGAATAGAAGTGAATGAGCTACTGTGAACTCTGTACACCCACACAACTAATGTTATTAAATATCATTTTTTGAATATATTTATACCATGTCAAAACTTGTTCAATTATTAATAAAAATAATTTAGTGTTTAAATTTTACCAGTTCCAGATTTCACACCTCCATTAACCCCACTTTTTACACCACAGGACGATCCTCCCCACATTTCACCGCCACCAGATGTATAAGTTTTAGATCCTTTATTACTACCATCATGTCCATGGATAAAGACACTCCACATGCCGCCACTACCTCCTTTAGAAGACATATTAATAAGACTTAAGACAAGTTTAACAATAAAATTAATCACGAGTACCCTACTACCAACCTACACTATTATATGATTATAGTTTCTATTTTTACAGTACCTTGACTAAAGTCTTTAGTCACACAATACTACCAACCTACACTATTATATGATTATAGTTTCTATTTTTATAGGAACACGTACGAGAAAATCAAATGTCTAAGTTCTAACGGTAGTGTTGATAAACGATTGTTATCCACGGATACCTCATTTATCATGTTGTCTATTTTCTTACTTTGTTCTATTAACTTATTAGCGTTGTATATTATTTGATTATAAAACTTATATTGCTGTTTAGTCCACTCTATAAATATCGGATTATTCACATATCGTTTCTTTGTAGGTTTATTTAACTTGTACATCACTGTAAGCATGTCCGTACCATTTATTTTAATTTGACACATATCAGCAATTTCTTTTTTGCATTCGGTTCTACCAACGTAATAATGAAGTGTGGTATATGATCTATAAGTATAATATAGTATCTCTTTTTTCGTAAGATATTAAACAGTTTATCACATCTAGAGATAATTTATCTCGTGATAATATAATAATAGAGTGCGGTTTTACCTATTTTATCATCTATATAATAATAATATAATACTAATAGTAATCGTTATCATTGTACCCACTTGAGAAGTTAGTAACTTTTTTTCTATTATAATCTTGTATCCGTAAGATACATTACTACACATAGGAATTCCCTGATCGAGCAATAGTTTAAATACATCTACATTTGGATTTGATGTAGTTGCGTATTTCTCTACAATATTAATACCATTTTTGCAACTATTTATTTCTAGACCTTTTGTGATTAGTAATCTCAATAATTCTACGTCAATATTATCAGATTCTATATATTCGAATATATCAAAGTCATTGATATTTTTATAATTGGTAGAAGACAATAATGACACCACAACATCAGTTTTGATATTCTTATTTTTCTTGGTAACGTATACATTTAATGAATTTTCATTACGTTCTACCAATGATTGTGCACTGCAGGCATCAAAAGTTTTACAACTATCATAAAGCATACTATCCTATCCAACCATTATGGTATTGATGCTATAGTATATTTTAATTCATTTCATTTATGTGTATTTGGTACGAGCAGACAGCATGAGATCGTATGATTTCAGTACCGTAGTGACAATACTATTAGTTTCAATAAGATCAATCTCTAGATCTATAGAATCAAAACATGATAGGTTAGAAGATAATGAATATCTGTAGGCTTCTTGTTGCACTGTAACTTCTCGTTTTGTTAGATGTTTGTATCATGCTTTAACATTAATGGTACAAATTTTATCCTCGCTTTGTGTATCATATTCGTCCCTACTATAAAATTGTATATTCAGATTATCATGAGATGTGTTTACACTAACTGTATCAATAAACGGCACACCATTTAGTCATAACCGTAATCCAAAAATTTTTAAAGTATATCTTAACGAAAGAAGTTGTATCATCGTTAGGATTTGGTAAATTATTATCTACAGTGTATGGTACTAAATCCTCATAAGTGTATATATCTAGAGTAATGTTTAATTTATCAAATGGTTCATAATATGGATCCTCATGACAATTTCCGAAGACGAAAATGAGATATAGACATGGTTACTCCTTAAAAAAATACGAATAATCGCCTTGGCTATTTAGTAAGTGTCATTTAACACTATACTTATATTAATCCATGGACTCATAATCTCTATACGGGATTAACGGATGTTCTATATACGGGGATGAGTAGTTCTCTTCTTTAACTTTATACTTTTTACTAATCATATTTAGACTAATGTATGGGTAATAGTGTTTGAAGAGCTCGTTCTCATCATCAGAATAAATCAATATCTCTGTTTTTTTGTTATACAGATGTATTACAGCCTCATATATTACGTAATAGAACGTGTCATCTACCTTATTAACTTTCACCGCATAGTTGTTTGCAAATACGGTTAATCCTTTGACCTCGTCGATTTCCGACCAATCTGGGCGTATAATGAATCTGAACTTTAATTTCTTGTAATCATTCGAAATAATTTTTAGTTTGCATCCGTAGTTATCTCCTCTATGCAACTGTAAATTTCTCAACGCGATATCTCCATTAATAATGATGTCAAATTCGTGTTGTATACCCATACTGAATTGATGAACGAACGAATACCGTCGGCGTTAATAGTAATTTACTTTTTCATCTTTACATACTCGGTACTAGTTTTACTATCATAAGTTTATAAATTCCACAAGCTACTATGGAATATACCAACCATCTTAGTATAGAACACATGTCTTAAAGTTATTAATTAATTACATATTGTTTTATATATCGCTACGAATTTAAACAGAGAAATCAGTTAGGAAGAAAAAATTATCTGTCTTCTTCATCATCGTCTATTGGATAACGTCTCTGTATTCTACGATAGAGTGCTACTTTAAGATGAGACATATCCGTGTTATCAAAAATATACTCCATTAAAATGATTATTCCGGCAGCGAACTTGATATTGGATATATCACAACCTTTGTTAATATCTACGACAATAGACAGCAGTCCCATGGTTCCATAAACAGTGAGTTTATCTTTCTTTGAAGAGATATTTTGTAGAGATCTTATAAAACTGTCAAATGACATCGCATTTATATCTTTAGCTAAATCGTATATGTTACCATCGTAATATCTAACCGCGTCTATCTTAAACGTTTCCATCGCTTTAAAGACGTTTCCGATAGATGGTCTCATTTCATCAGTCATACTAAGCCAACAAATATAATCGTGTATAACATCTTTGATAGAATCAGACTCTAAAGAAAACGAATCGGCTTTATTATACACATTCATGATAAACTTAATGAAAAATGTTTTCGTTGTTTAAGTTGGATGAATAGTATGTCTTAATAATTGTTATTATTTCACTAATTAATATTTAGTAATTAGTGCACTCTATAAAAACGAGAATGACATAACTAATCATAACTAGTTATCAAAGTGTCTAGGATGCGTAATTTTTTATATGGTATAGATCCTGTAAGCGTTGTCTGTATTCTGGAGCTATTTTCTCTATCGCATTAGTGAGTTCAGAATATGTTATAAATTTAAATCGAATAACGAACATAACTTTAGTAAAGTTGTCTATATTAACTCTTTTATTTTCCAGCCATCGTAATACCCATGTTTAAGATAGTATATTCTCTAGTTACACTATGGTCTCATCGTTGTTTAGAATATCACATACCGAATCTACATCAAATTTTAGAAATTGGTCTGTGTTACGTATCTCTTCTATATTATTGTTGATGTATTGTCGTAGAAAACTATTACGTAGACCATTTTCTTTATAAAACGAATATATAGTATACCAATTATCTTTTCCGATAATATATTTACACACATAATCCATTCTCTCAATCACTACATCTTTAAGTTTTTCGTTGTTAAGATATTTGGCTAAATTATATAATTCTATTAGATCATTAACAGAATCAGTATATATTTTTCTAGATCCAAAGACGAACTCTTTGGCGTCCTCTATAATATTCCCAGAAAAGATATTTTCGTGTTTTAGTTTATCGAGATCTAATCTGTTCATATACGTATCCATGATTGCGCGGTACGTTATGATAGCCGCATAAAATAAAATCCATTTTCATTTTTAACCAATACTATCTCCTCTATTCATAATTGAGATTGATGTAATACTTTGTTACTTTGAACGTAAAAACAGTACACGGATCCGTATCTCCAACAAGCATGTAGTAATCAAATTTGGTGTTGTTAAACTTCGCAATATTCATTAATTTAGATAGAAATTTATACTCATCATCTGTTTTAGGAATCCATGTATTATTACCACTTTCCAACTTATCATTATCCCAGGCTATGTTTCGTCCATCATCATTGCGCAGAGTGAATAATTCTTCTGTATCCGGCAGTTCAAATATATGATCCATGCATAGATCGGCAAAGCTATCGTAGATGTGATTTTTCCTAAATCGAGTATAAAACTCGTTTACTAGCAAACACTTTCCTGATTTATTGACCAAGACACATATGGTTTCTAAGTCTATCAAGTGGTGGGGATCCATAGTTATGACGCAGTAACATAGATTATTACCTTCTTGACTGTCGCTAATATCTATATATTTATTGTTATCGTATTGGATTCTGCATATAGATGGCTTGTATGTCAAAGATATAGAACACATAACCAATTTATAGTCGCGCTTTACATTCTCGAATCTAAAGTTAAGAGATTTAGAAAACATTATATCCTCGGATGATGTTATCACTGTTTCTGGAGTAGGATATATTAAAGTCTTTACAGATTTCATCCGATTCAAATAAATCACTAAATAATATCCCACATTGTCATCTGTTAGAGTAGTATCATTAAATCTATTATATTTTATGAAAGATATATTACTGCTCACCTCTATATTTCGTACATTTTTAAACTGTTTGTATAATATCTCTCTGATACAATTAGATATATCTATTGTGTCGGTAGACGATACCGTTACATTTGAATTAATGGTGTTCCATTTTACAACTTTTAACAAGTTGACCAATTTATTTCTAATAGTATCAAACTCTCCATGATTAAATATTTTAATAGTATCCATTTTATATCAACGGACACAAAGTAGCTAACATAAACCATTGTATAATTTTTATGTTTTATGTTTATTAGCGTACACATTTTGGAAGTTCCGGTTGCCATGTATTTCCTGGAGAGCAAGTAGATGATGAGGAACCAGATAGTTTATATCCGTACTTGCACGTAAAGTCTACATTGTCATTATACGAATATGATCTTTTAAACCCGCTAGACAAGTATCCGTTTAATATTGTAGGATGTGGACATTTAACAATCTGACACGTGGGTGGATTAGACCATTCTCCTCCTGAACACAGGACACCAGAGTTACCAATCAACGAATATCCACTATTGCAACTATAAGTTACAACGCTTCCATCGGTATAAAAATCATTATATCCGTTATGTCTTCCGTTGGATATAGATGGAGGTAATTGGCATTTAACAGATTCGCAAATAGGTGCCTTGGGATTCCATACCATAGATCCAGTAGATCCTAATTTACAATACGATTTATATTCACCAATCAAATAATATCCGCTATTACAAGAGTACGTTATACTAGAGCCAAAGTCTACTCCGCCAATATCAAGATGGCCATTATCGATATCTCGAGGCGACGGGCATCTCCGTTTAATACATTGATTAAAGAGTGTCCATCCAGTACCTGTACATTTAGCATATATAGGTCCCATTTTTTGCTTTCTGTATCCAGGTAGACATAGATATTCTATAGTGTCTCCTATGTTGTAATTAGCATTAGCATCAGTCTCCACACTATTCTTAAATGTCATATTAATGGGTCGTGACGGAATAGTACAGCATGATAGAACGCATCCTATTCCCAACAATGTCAGGAACGTCACTCTCTCCACCTTCATATTTATTTATCCGTAAAAATGTTATCCTGGACATTGTGCCAATAATAAAAAGCCCATATATGTTCGCTATTGTAGAAATTGTTTTTCATAGTTGCTCAAAAACGATGGCGGTGACTTATGAGTCACGTTACACTTTGGAGTCTCATCTTTAGTAAACATATCATAATATTCGATATTACGAGTTGACACATTGAACAAATCCAAGTATTTGATTTTGGATAATATTCGTATTTTGCATCTGCTATAATTAAGATATAATCGCCACAAAAACACGCGAACATCTTTCCTACATGGTTAAAGTACATGTACAATTCTATCCATTTGTCTTCCTTAACTATATATTTGTATAGATAATTACGAGTCTCGTGTGTAATTCCAGTAATTGCATAGATGTTACCATCGTACTCTACAGCATAAACTATACTATGATGTCTAGGCATGGGAGACTTTTTTATCCAACAATTTTTAGTGAAACATTCCACATCGTTTAATATTACATATTTTTCATACGTGGTATAAACTCCACCCATTACATATATATCATCATTTACGAATACCGACGCGTCTGAATATCTAGGAGTAATTAAGTTTGGATGTCTTATCCATTTCGAAGTGTAGCGTTTTAAATATTTTGCTACACCTGTTGAAATAGAAAATTTTAATTCTCCTATTACATATAACTTTCCATCATTAACACACGTACTAACTTTTGATTTTAACGACGACATATTAGTAACTGTTTTCCAGATCTACCCACGGATACGAAATAAACATGTCTATTGTTAATCATGCCACCAATAATGTATAGACAATTATATAAAACATTTGCATTATAGAATTGTCTATCTATCTGTATTACCGACGATTGTCCAATATTCTGTTCTAGGAGAGTAATGGGTTATTGTGGATATATAATCAGAGTTTTTAATGACTACTATATTATGTTTTATACCATTTCGTGTCACAGGCTTTGTAGATTTGGATATAGCATTGCACATAGTATTAGTCATAAACTTGGGATGTAAACTGTTGATGATATCTACATCGTTTGGATTTTTATGTATCCACTTTAATAATATCATAGCTGTAACATCCTCGTGATTTACGTTGACGTTTTCGTGGGATAAGATAGTTGTTCATCCTTTGATAATTTTCCAAATTCTGGATTGGATGTCACTGCAGTAATATTGTTAATTATTTCTAACATTGACGCATTATATAGTTTTTACACATAGATCTACAACAGGTGGAATATCAATTAATACGGCAGCAACTAGTATCATCTCTACATTGTTTACGGTGATGTTTATCTTCTTCCAGCGTATATAGTCTAATAGCGATTCAAACGCGTGATAGTTTATACCATTCAATATAATCGCTTCATCATTTATATGGTGTTTCTAAATTCGTTTAAAAAATTATACGGAGACGCCGTAATAATTTCCCCATTGATAGAAAATATCACGCTTTCCATTCTTGAAGTACTATAAGTAATTATAGTATAATGTAACGTTTATATATTCAATATTTTTTATAAAAAAATCATTTCGACATTAATTCCTTTTTAAATTTCCGTCTATCATCTATAGAAACATATTCTATGAATTTATAAAATGCTTTTACGTGTCCTATCGTAGGCGATAGAACCGCTAAAAAGCCTATCGAATTTCTACAAAAGAATCTATTATATGGTATAGGGAGAGTATAAAACATTAAATGTCCGTACTTATTAAAGTATTCAGTAGCCAATCCTAACTCTTTCGAATACTTATTAATGGCTCTTATTCTGTACGAATCTATTTTTTTGAACAACGGACCTAGTGGTATATCTTGTTCTATGTATCTAAAATAATGTCTGACAAGATCCGTTAGTTTAATATCCTTAGTCATCTTGTCTAGAATGGCGTATCTAACTGCGGGTTTAGGCTTTAGTTTAGTTTCTATATCTATATCTACATCTATGTCTTTATCCAACACTAAAAATATAATAGCTAATATTTTATTACAATCATCCGGATATTCTTCTACGATCTCACTAACTAATGTTTCTTTGGTTATACTAGTATAGTCACGATCAGACAAATAAAGAAAATCAGATGATTGATGAATAATACATTTAAATTTATCATCTGTAAGATTTTTGAGATATCTCATTAAAATATTATTAGGGTCAGTACTCATTATCATATATTGGCAGCAGCTATTACTTATTTTATTTTATTTTTCTTTATTTTATTATTTTTCACCATATAGATCAATCATTAGATCATCAAAATATGTTTCAATCATCCTAAAGAGTATGGTAAATGACTCTTCCCATCTAATTTCTGGACGTTCACCAATGTCTCTAGCCACTTTGGCACTAATAGCGATCATTCGCTTAACATCTTCTACATTATTAACTGGTTGATTTAATCTATCTAGCAATGGACCGTCGGACAGTGTCATTCTCATGTTCTTAATCAATGTACATACATCACTGTCATCTACCAATTCATCCAACAACATAAGCTTTTCAAAATCATCATTATAATATGATGGATCGCCATCATTTCTCCAAAGAATATATCTAATAAGTAGAGTCCTCATGCTTAGTAATTTAACTATTTTAGTTAACAACTATTTTTTATGTTAAATCGAGTAGAACCACTATGTTAATACTTATTCATATTTTAGTTTTTAGGATTGATAATCAACATAAAAATTAATACATCATCAATTTTGAAAATACTTAGTTTCCACGTAGTCAATGAAACATTTGAGCTCATCGTACAGAACGTTCTCGTACAGGACGTAACTATAAACTGGTTTATATTTGTTCAAGATAGATACAAATCCGATAACTTTTTTGACAAATTCTACGGGATCCACTTTAAAAGTGTCATACCGGGTTCTTTTTATTTTTTTAAACAGATCAATGGTGTGATGTTGATTAGGTCTTTTACGAATTTGATATAAAATAGCGTTCACATATCCTCCATAATGGTCAATCGCCATTTGTTCGTATGTCATAAATTCTTTAATTATATGGCACTGTGTATTATTTAGTTCATCCTTGTTCATCATTAGGAATCTATCCAATATGGCAATTATACTAGAACTATAAGTGTGTTGTAAACACATATTGATGTGTCTGTTTATACAATTAATGATATTTGGATCCATGCTACTACCTTCGGGTAAAATTGGAGAATCGTATACCATTTCTAGTACTTTAGGTTCATTGTTATCCATTGTAGAGGATGACATGCTCGAATCATACAAAAATATATTATTTTTATGTTATTTTGTTAAAAATAATCATCGAATACTTTGTAAGATACTCCTTCATGAACATAATCAGTTACAAAACGTTTATATGAAGTAAAGTATCTACGATTTTTACAAAAGTCCGGATGCATAAGTACAAAGTACGCGATAAACGGAATAATAATAGATTTATCTAGTTTATCTTTTTCTATCGCTTTCATAGTTAGATACATAGTCTCGGAAGTCGGATTATGTAACATCAACTTCGATAAAATTACTGGGTTATTTAGTCTTACGCATTCACTCATACATGTATTACCGTTAACTACAGAGTCTACACTAAAATGATTAAACAATAGATAGTCTACCATTGTTTCGTATTCAGATAGTACAGCGTAGTACATGGCATCTTCACAAATTATATCATTGTCTAATAGATATTTGATGCATCTTATGGATCCCACTTCGACAGCCATCTTAAAATCGGTAGAATCATATTGCTTTCCTTTATCATTAATAATTTCTAGAATATCATCTCTATCATAAAAGATACAAATATTAACTGTTTGATCCGTAATAACATTACTAGTTGATAACAATTTGTTAATAAGATGTGCTGGGCTCAATGTCTTAATAAGAAGTGTAAGAGGACTATCTCCAAATTTGTTTTGTTTATTAACATCCGTTGATGGAAGTAAAAGATTTATAATGTCTACATTCTTGACTGTTTTAGAGCATACAATATGGAGAGGTGTATTTCCATCATGATCTGGTTTTGAGGGGCTAATTCCTAGTTTCATCATCCATGAGATTGTAGAAGCTTTGGGATTGTCTGACATAAGATGTCTATGAATATGATTTTTGCCAAATTTATCTACTATCCTGGCTTCAAATCCAATGGACATTATTTTTTTAAACACTCTTTCTGAAGGATCGGTAGACGCCAACAACGGACCACATCCTTCTTCATCAACCGAGTTGTTAATCTTGGCTCCATACTGTACCAATAAATTTATTCTCTCTATGACTTCATCATCTGTTCCCGAAAGATAATATAGAGGCGTTTTATGCTGTTTATCGCACGCGTTTGGATCTGCGCCGTGCGTCAATAGCATCGCAACTATTCTATTATTATTAATTTTAGAAGCTATATGTAATGGATAATTTCCATCATCATCCGTCTCATTTGGAGAGTATCCTCTATGAAGAAGTTCTTCGACAAATCGTTCATCTAGTCCTTTAATTCCACAATACGCATGTAGAATGTGATAATTTCCAGAAGGTTCAATAGCTTGTAGCATATTCCTAAATACATCTAAATTTTTACTATTATATTTGGTATAAAGAGATAGATAATACTCGGCCGACATAATGTTGTCCATTGTAGTATAAAAATTAATATTTCTATTTCTATTTCTACTATAATTTACTCTCTATAACAAATGTCATAACTTAGTTCTTTTATGTCAAGAAGGCACTGGTTTAGTTCATCTATGAATGTCACACCATAACTACCACGCATACCATACTTAGAGTTATGATAAAGATATTTATCCTTAGGGTGCAGGTAATGGGGATTAATCTTTGTTGGATCAGTCTCTAAGTTAACACATGTCACACAGGATCCATTTATAGTTATATCACATGATGATTTATGGTGAATTGATTCCGGAAGATCGCTATTGTATTTTGTGGTTCCACAATTCATTTCCATACATGTTATTATCACACTGATATTATGATGAACTTTATCCAGCCGCTGGGTTGTAAACAACATAACAGATAGTTTATTATCTTTACCAACTCCCTCTGCCGCCGCAACGAATCTCTGATCCGTATCCATGATGGTCATGTTTATTTCTAGCCCGTATCCAGTCAACACTATGTTAGCATTTCTATCGATATAGCTTTCACTCATATAACACTCACCAATAATAGTAGAATTAATGTCGTAATTTACACCAATAGTAAGTTCGGCGGCAAAGTACCAATACCTGTAATCTTGTCGAGGAGGACATATAGTATTCTTGTATTCTACCGAATACCCAAGAGATGCAATACAAAAGAGTAAGACTAATTTGTAAACCATCTTACTTAAAATATGTAACAATAGTACGATGAATGAGTAATAAGAAATATGAAATCTATCTTATATACACATAATTATTCTATCAATTTTACCAATTAGTTAGTGTTTAATAAAAATATGGGAATCTAATTAATTTTTCCTTACACAATCGACGTACATGAGTCTGAGTTCCTTGTTTTTGCTAATTATTTCATCCAATTTATTATTTTTGACGATGTCAAGATCTTTTGTATAGGAGTCAGACTTGTATTCAACATGCTTTTCTATAATCATTTTTAGCTATTTCGGCATCATCCAATAGTACATTTTCCAGATTAGCAGAATAGATATTAATGTCGTATTTGAACAGAGCCTGTAACACCTCAATGTCTTTATTATCTATAGCCAATTTAATGTCCGGAATGAAGAGAAGGAAATTATTGGTGTTTGACGGCGTCATATAGTCGAGCAAAAGAATCATCATATCCACGTGTCCATTTTTATAGTGGTGTGAATACAACTAAGGAGAATAGCCAGATCAAAAGTAGATGGTATTTCTGAAAGAAAGTATGATATAATACTTACATCATTAAGCATGACGGTATGATAAAATGAAGTTTTCCATCCAGTTTTCCCATAGAACATAAGTCTCCAATTTTTCTTAAAACAGTTTTACCGTTTGTATGTTACCACTATCAACCACATAATACAATACAGTGTTCCCTTGTCATTAAATTGTGAATCATCCAGTCCACTGAATAGCAAAATTTTTACTATTTTGGTATCTTCCAATGTGGCTGCCTAATGTAATGGAAATTCATTCTCTAGAAGATTTTTTAATGCTCCAGCGTTCAACAACGTACATACTAGACGCATGTTATTATCAGCTATTGCATAATACAAGGCACTATGTCCATGGACATCCGCCTTAAATGTATCTTTACTAGAGAGAAAGCTTTTCAGCTGCTTAGACTTCCGAGTATTAATTCGTGACAGATCCATGTCTGAAACGAGACGCTAATTAGTGTATAATTTTGTCATACTGCTCCAGAATTAATAATATCTCTAATAGATTTGATTAGTAGATATATGGCTATCGCAAAACAACATATACACATTTAATAAAAATAATATTCATTAAGGAGATTCAGATTCCGCCGTATCCATAAACAATATTAAGGAGATTCTACCTTACCCATAAACAATATAAATCCAGTAATATCATGTCTAATGATGAACACAAATGGTGTATTAAATTCCAGTTCTTCAGGAGATGATCTCACCGTAGCTACCATGATAGTAGATGCCTCCGCTACAGTTCCTTGTTCGTTGACATCTATCTTTGCATTCTGAAACATTTTATAAATATATAATGGGTCCCTAGTCATATGTTTAAACGACGCATTATCTGGATTAAACATACTAGGAGCCATCATTTCGGCTATCGACTTAATATCCCTCTTATTTTCGATAGAAAATCTAGGGAGTTTAAGATTGTACACTTTATTCCCTAATTGAGACGACCAATAGTCTAATTTTGCAGCCATGATAGAATCTGTGAAATGGGTCATATTATCACCTATTGCCAGATACATACTAATATTAGCATCCTTATACTGAAGGCGCACCATATCATATTCTTCGTCATCGATTGTGATTGTATTTCCTTGTAATTTAGTAACTACGCTCATCATGGGAACCGTTTTCGTGCCGTACTTATTAGTAAAACTAGTATTGTGTGTTTTAGTGATATCAAATGGATATTGCCACGTACCTTTAAAATATATAGTATTAATGATTGCCCATAGAGTATTATTGTCGAGCATAGTAGAATCTACTACATTAGACATACCTGATCTACGTTCTACTATGGAATTAATTTTATTAACCGCATCTCGTCTAAAGTTTAATCTATATAGGCCGAATCTATGATATTGTTGATAATACGACGGTTTAATACACACAGTATTATCTACGAAACTTTGATAAGTTAGATCAGTGTACGTATATTTAGATGTTTTTGGCTTAGCTAATCCTGATATTAATTCTGTAAATGCTGGACCCAGATCTATTTTTCTCAAATCCATAGTCTTCAATAATTCTACTTTAGTATTACCTGATGCAGGCAATAGTGACATAAACATAGAAAACGAATAGCCAAACGGCGAGAAGACAATATTATCATCCTCATTCCCATCTTGAATATTTTTATACGCTACTATACCGGCATTGGTAAATCCTTGCAGGCGATAGGTGAACGCTGTACACGCTAACGATAGTATCAATAACACAATCATGATTTTATGGTATTAATATTTTACCTTATTTTTTATATTTGGTATAAAAATTATTGATGTCTACACATCCTTTTGTAATTGACATCTATATATCCTTTTGTATAATCAATTCTAATCACTTTAACTTTTACAGTTTTCCCTACCAGTTTATCCCTATATTTAAAATATCTATTCATATGCATCTGAGTACTCTCTACCAAGATAGCTTCAGAGTGAGGATAGTCAAAAAGATCAACATATAGAACATAACCATTCTCGTATACTTTGCCCTTTAATACATCACCCACATTGGGCAACGAATAACAAAATACAAGCATCTTGTTAACGGGCTCGTAAATTGGGATAAAAATTATGTTTTTATTATTTTATATTCATTTAATATTCATTCAATATTTACAATATATGTAATCATTATCCAGATTGAACATACAGTTTGCACTTGCCATGATTCGTTATATAACTATTAATATTAACAGTTCGTTTAACGATCATATTATTTTTATGTTTTATTAATAATTGTAAAACATACAATTAAATCAATATAGAGGAAGGGACGGATACTGTCTTTTGTGAGATATAGTTATGGCGACTAAATCAGATTATGAGGATGCTGTTTTTTACTTTGTGGATGATGATGAAATATGTAGTCGTGACTCCATCATCGATCTAATAGATGAATATATCACGTGGAGAAATCATGTTATAGTGTTTAACAAAGATATTACCAGTTGTGGAAGACTCTACAAGGAATTGATGAAGTTCGATGATGCTGCTATACGATACTATGGTATTGATAAAATTAATGAGATTGTCGAGGCTATGAGTGAAGGAGACCACTACATCAATCTTACAGAAGTTCATGATCAGGAAAGTCTATTCGCTACCATAGGAATATGTGCTAAAATCACTGAACATTGGGGATACAAAAAGATTTCAGAATCTAGATTCCAATCATTGGGAAACATTACAGATCTAATGACCGACGATAATATAAACATCTTGATACTTTTTCTAGAAAAAAAATTGAATTGATGATATAGGGTTCTTTATAATGCATTCGTATTCTATATCCGGTGTTAAAAAAATTATCCTATCATGTATTTGAGAGTCTTATATGTAGCAAACATGATAGCTGTGATACCAATAAGTTTTAGATATTCACGCGTGCTAATGTTGGGGATGGTATTATCTGGCGGTGAAATGTCCGTTATATAATCTATAAAACAATCATCGCATATAGTATGCGATAGTAGAGTAAACATTTTTATAGTTTCTACTGGATTCATACATCGTTTACCCAATTCGGTTATGAATGAAATTGTCGCCAATCTTACACCCAACCCCTTGTTATCCATTAGTATAGTATTAACTTCGTTATTTATGTCATAAACTGTAAATGATTCTGTAGATGCCATATCATACATGATATTCATGTCCCTATTATAATCATTATTAACTTTATCACAATACGTGTTGATAATATCTATATATGATCTAGTTTTTGTGGGCAATTGTCTATACAAGTCGTCTAAACGTTGTTTACTCATATAGTATTGAACAGCCATCATTACATGGTCCCGTTCCGTTGATAGATAATCGAGTATGTTAGTGGACTTGTTAAATCTATATACCATATTTTCTGGAAGTGGATATACATAGTTGTGATCATTATTGCTAGCCTTATCTTCTATATCATCTATATCATCTATATCATCTATATCATCTATATCATCTATATCATCTATATCATCTATATCATCTATATCATCTATATCATCTATATCATCTATATCATCTACGATATTATTACACATAAACATCGGCAACATACTATTGTACATTATCTAAGTCCGGTTGATCCAAACCCTTGATCTCCTCTATCTGTACTATCTAGAGA

GAACTTAGGCATGGACATACTAATATCTGTCTTAATTAATTGTCTTTCTCCTGGAGGGATAGTATAATCATAAGCGCTATATAAATCATATCCGGCGGCGTACGGTGATTGTCTAGTAGGAGATTTAGCTCTGTTAGTTTCCTTAACAAATCTAACTGGTGAGTTAATATTCATGTTGAACATAAAACTAATATTTTATTTCAAAATTATTTACCATCCCATATATTCCATGAATAAGTGCGATGATTGTACACTTCTATAGTATCTATTACGAACCACGATAAAATCCTCCTATCAATAGCAGTTTATTATCCACTATTATCAATTCTGGATTATCCCTTTGATAAATAGTATCATCTATCAGAGACCATGTATTGCTGTATTTGTAATAAAATTTAGCATTTCTACCAACCAAGAATAACCTTCTACCGAACACTAACGCGCATGATTTATAATGTGGATAATAAGTTGACGGTCCAAACTGCCACTGATCATGATTGGGTAGCAAATATTCTGTAGTTGTATCCGTTTCAGAATGTCCTCCTATTACGTACATAACATTGTTTATGGATGCCATTGCTGGATTACATCTAGGTTTCAAAAGACTCGGCATATTAACCCAAGCAGCATTCCCGTGGAACCAACGCTCAACAGATGTGGGATTTGGTAGACCTCCTACTACGTATAATTTATTGTTAGCGGGTATCCCGCTAGCATACAGTTTGGGGCTATTCATCGGAGGAATTGGAATCCAATTGTTTGATATATAATTTACCGCTATAGCATTGTTATGTATTTCATTGTTCATCCATCACCGATGAGATATACTACTTCTCCAACATGTGTACTTGTACACATATGGAATATATCTATAATTTGATCCATGTTCATAGGATACTTTATGAATGGATACCTGTATTATTTGCGTGGTTGTTTATCACAATGAAATATTTTGGTACAGTCTAATATCCATTTTACATTATTTATACCTCTGGGTGAAAGATAATTTGACCTGATTACATTTTTGATAAGGAGTAGCAAATTTCCTAATTTATTTCTTCTCTTCGTATACCACTTAATGACAAAATCAACTACATAATCTTCGTCTGGAACATTTAACTCATTGCTTTCTAGAATAAGTTTCAAGATAAATAATCAAAATTGTCTATGATATCCTCTTCCAGTTCCAAAAAGTGTTTGGCAATAAAGTCTTTAGTATGACATAAGAGATTGGATAGTCCGTATTCTATACCCATTATGTAACACTCGACACAATATTCCTTTCTAAAATCTCGTAGTATAAAGTTTATACAAGTGTAGATGATAAATTCTACAGACGTTAATATAGACACGCGTAATAAATTGATGACGTCATGTCTATTATGTATACCTTTCCAGTATATGAGTAAATAACTATAGAAGTTAAACTGTGAATGTCAAGGTCTAGGCAAACCCTCGTAACTTGATCTTTATTTTTTGTGTATTTTTGACGTAAATGTGTGCGAAAATACGGAGATAACTTTTTCAATATTGTAGAATTGACTATTATATTGCCTCCATAGCATTAATAATTGTTTTGAATTTCTTAGTCATAGACAATGCTAATATATTCTTACAGTACATAGTATTGACAAATATCGGCATTTATGTTTCTAACTAAATTAACAATAGTAGTAATTTTTTTTTCAGTTATCATCTATATGACTGTATTTGGATCTTTTGTACATCGCTATTATCGCAATCACTACAATAATTATAATTATTATTGATAGCATTGTTATTAGCACTATCATAATTAAATTATTGATATTCATGGGTGCTGAATAATCGTTATTATCATCATTATCATTTTGTAATTGTGACATCATACTAGATAAATCGTTTGCGCGATTGTTGTGGGAAACGGGCATGGAGGATGAATTATCGTTATTATTATTTAAAGCCTCCCATTCGGATTCACAAATATGACGCGCGTTCAACATTTTATGGAAACTATAATTTTGTGAAAACAGGTAACAAGAAAACTCGTCATCGTTATTTTTAACGATAGTAAACCGATTAAACGTCGCGCTAATTTCTAACGCTAGCGACTCTATTGGATATGGATTTCCAGATATATATCTTTTCAGCTCCCCTACGTATCTATAATCATCTGTGGGAAATGGAAGGTATTTCCATCTATCTAGTGTTCCTAATATCATATGTAGTGGTGTAATAGAACCATTAAGCGCGAAAGATTTTATTTCACATCGTATTTTAACTTCGCAATAATTTCTGGTTAGATAACACACTCTACCAGTTAAGTCAATAATATTAGCCTTTACAGATATATTCATAGTAGTTGTGACAATGACTCCATCTTTTAGATGCGATACTCCTTTATATGAGCCAGAATCTTCGTACCTCAAACTCGATATATTCAAACAAGTTAATGAGATATTAACGCGTTTTATGAACGATGATATGTATCCAGAAGTTCTATCCTCCGTGGCTAACGCTATAACCTTATTATTATAGTACCAAAAAGTGTGATTAATGTGTGACACATTAGCGCATGTACATATTTGTACATTATTGTCTACGTCGTATTCGACACATCCACATACAGCCAACAAATATAAAATGATAAATGCTCTAATGGTGTTCGTACCCATCTTAATGCGGCTTAATAAATGTTTCGATTTCAATTTATTGTAAAAAAGAATCGGTTTTATACTGTTCGAGATTCTCATTGATTATATTCTCATCTATCATCTCCACACAGTCAAATCCGTGGTTAGCATATACCTCATTAACCGATAATAGACTATCAGACTCTTCTATCGTTATAACTCGAGAATATTTAATTTGGTCATTCTTAATCAAGTCAATTATCTTATTTTTAACAAATGTGAGTATTTTACTCATTTTTTATAAAAAACTTTTAGAAATATACAGACTCTATCGCGTGTCTATATCTTCTTTTTATATCCAATGTATTTATATCTGATTTTTCTTCATTTATTATATATAATGGTCCAAATTCTATACGTGTTGCTTCATCCAGATCATTAAGGTTCTTATAATCGCAATATCCTTCTCCTTCCTCTTCTATATCGTCCTTCTTAAACTTATTCTTAGCGTCACAGAATCGACCACAGCAGGATCCCATGACAAGCGTCATATTAAACTAATTCATTTTCAATAATATACTAATTAGTAACGACTATTAAAATAAATATTCTTCATAACCGGTAAGAATAGTAAAAAGACTAAATCTTCACATTGTAACTATTCAGTAGTGTTTACATAAATGAGATGAGAAGATATTCTCTATTTTGGTGGAGGATTATATGATATAATTCGTGGATAATCATTCTTAAGACACATTTCTTCATTCGTAAATCTTTTCACGTTAAATGAGTGTCCATATTTTGCAATTTCTTCATAGGATGGCGGTGTACGTGGACGGTGCTGTTCCTGTTCTTGTTGTGGTCTCCGACTTTCGTGTTTGCGTTTAGATCCCTCCATTATCGCGATTGCATAGATGAGGTACTATTTTATACCTTGTAATTAAATTTTTTTATTAATTAATTAATTAAACGTATAAAAACGTTCCGTATCTGTATTTAAGAGCCAGATTTCGTCTAATAGAACAAATAGCTACAGTAAAAATAACTAGAATAATCGCTACACCCACTAGAAACCACGGATCGTAATACGGCAATCGGTTTTCGATAATAGGTGGAACGTATATTTTATTTAAGGACTTAAGAATTGTCTGTAAACCACAATTTGCTCCCGCCGATCCTGTATTAACTATCTGTAAAAGCATATGTTGGCCGGGCGGAGCCGAACATTCTCCGATATTCAATTTTTGTATATCTATAATGTTATTAACCTCCGCATACGCATTACAGTTTTTTTCTAGCTTGGATACGGCACTAGGTACATCATTTAGATCTATTCCTATTTCCTCAGCGATAGCTCTTCTATCCTTTTCCGGAAGCAATGAAATCACTTCAATAAATGATTCAACCATGAGTGTGAAACTAAGTCTAGTATTACTCATGCATTTGTTAGTTATTCGGAGCGCGCAATTTTTAAACTGTCCTATAACCTCTCCTATATGAATAGCACAAGTGACATTAGTAGGGATAGAATGTTGAGCTAATTTTTGTAAATAACTATCTATAAAAAGATTATACAAAGTTTTAAACTCTTTAGTTTCTGCCATTTATCCAGTCTGAGAAAATGTCCCTCATAATAAATTTTTCCAAGAAACTAATTGGGTGAAGAATGGAAACCTTTAATCTATATTTATCACAGTCTGTTTTGGTACACATGATGAATTCTTCTAATGCTGTACTAAATTCAATATCTTTTTCGATTTCTGGATATGTTTTTAATAAAGTATGAACAAAGAAATGGAAATCGTAATACCAGTTATGTTCAACTTTGAAATTGTTTTTTATTTTCTTGTTAATGATTCCAGCCACTTGGGAAAAGTCAAAGTCGTTTAATGCCGATTTAATACGTTCATTAAAAACAAACTTTTTATCCTTTAGATGAATTATTATTGGTTCATTGGAATCAAAAAGTAAGATATTATCGGGTTTAAGATCTGCGTGTAAAAAGTTGTCGCAACGGGGTAGTTCGTAGATTTTAATGTATAACAGAGCCATCTGTAAAAAGATAAACTTTATGTATTGTACCAAAGATTTAAATCCTAATTTAATAGCTAACTCGGTATCTACTTTATCTGCCGAATACAGTGCTAGGGGAAAAATTATAATATTTCCTCTTTCGTATTCGTAGTTAGTTCGCTTTTCATGTTCGAAAAAGTGAAACATGCGGTTAAAATAGTTTATAACATTAATATTACTGTTAATAACTGCCGGATAAAAGTGGGATAGTAATTTCACGAATTTGATACTGTCCTTTCTCTCGTTAAACGCCTTTAGAAAAACTTTAGAAGAATATCTCAATGAGAGTTCCTGACCGTCCATAGTTTGTATCAATAATAGTAACATATTAAGAACCCGTTTATACAGTGTATGTAAAAATGTTAATTTATAGTTTAATCCCATGGCCAATGCACACACGATTAATTTTTTTTCGTCTCCCTTTAGATTGTTGTATAGAAATTTTGGTACCGTGAACTCCGCCGTAGTTTCCATGGGACTATATAATTTTGTGGCCTCGAATACAAATTTTACTACATAGTTATCTATCTTAAAGACTATACCATATCCTCCTGTAGATATGTGATAAAAATCGTCGTTTATAGGATAAAATCGTTTATCTTTTTGTTGGAAAAATGATGAATTAATGTAATCATTCTCTTCTATCTTTAGTAGTGTTTCCCTATTAAAATTCTTAAAATAATTTAACAATCTAACTGACGGAGCCCAATTTTGGTGTAAATCTAATTGTGACATTATGTTGTTAAAATATAAACAGTCTCCTAATATAACAGTATCAGATAATCTATGTGGAGACATCCATTGATATTCAGGGGATGAATCATTGGCAACACCCATTTATTGTACAAAAAGCCCCAATTTACAAATAAAAGTCCAGGTTTGATAGAGACAAACTATTAACTATCGTGTCTCTGTTTTTAACACCTCCACAGTTTTTAATTTCTTTGGTAATAAAATTATTCACTATATCAGTATCTTCTTTATCTACCAGAGATTTTACTAACTTGATAACCTTGGCTGTCTCATTCAATAGGGTAGTGATATTTGTATGAGTGATATTGATATCTTTTTGAATTGTTTCTTTTAGAAGTGATTCTTTGATGGTGTTAGCATACAAATTACAATAATGCAGAAACTCAGTTAACATACAGGAATTATAGTAAGCCAATTCCAATTGCTGTCCGTATTGTATTAGAGTATCGATATGAGTAATGGTGTCCTTGCGTTTTTCTGATAGAATGCGAGCAGCGATTTTGGCGTTATCATTTGATGATATGTTTGGAATGACGAATCCTGTTTCTACTAACTTTTTGGTAGGACAAAGTGAAACAATCAAGAAGATAGCTTCTCCTCCTATTTGTAGAAGAAATTGAACTCCTCTAGATGATCTACTGACGATAGTATCTCCTTGACAGATATTGGTCCGAATTACAGAAGTACCTGGAATGTAAAGCCCTGAAACCCCCTCATTTTTTAAGCAGATTGTTGCCGTAAATCCTGCACTATGTCCAAGATAGAGAGCTCCTTTGGTGAATCCATCTCTATGTTTCAGTTTAACCAAGAAACAGTCAGCTGGTCTAAAATTTCCATCTCTATCTAATACAGCATCCAACTTGATGTCAGGAACTATGACCGGTTTAATGTTATATGTAACATTAAGTAAATCCTTAAGTTCATAATCATCACTGTCATCAGTTATGTACGATCCAAACAATGTTTCTACCGACATAGTGGATACGAAGATACTATCCATCAGAATGTTTCCCTGATTAGTATTTTCTATATAGCTATTCTTCTTTAAACGATTTTCCAAATCAGTAACTATGTTCATTTTTTTAGGAGTATGACGCCTAGCCAGCATGGAAGAGAATTTTCTAGATCCTCTCTTCAACATCTTTAATCTCAATGGGATGCAAAACCCCATGGTGTAACAACTAACGATAAAAATAATATTGTTTTTCACTTTTTATAATTTTACCATTTGACTCATGACTTCATTGATATCTTTATACGAGCTACGAATATATAATTCTTTATAACTGAACTGAGATATATACACTGGATCTATGGTTTCCATAATTGAGTAAATAAATGCTCTGCAATAACTAATGGCAAATGTATAAAACAACAAAATTATACTAGAGTTGTTAAAGTTAATATTTTCTATTAGTTGTTCCAATAAATTATTTGTTGTGACTGTGTTCAAGTCATAAATCATTTTGATACTATCCAATAAACAGTCTTTAAGTTCTGGAATATTATCATCCCATTGTAAAGCCCCTAATTCGACTATCGAATACCCTGCTCTGGTAGCCGTTTCAATATCGACGGACGTCAATACTGTAATAAAGGTGGTAGTATTGTCATCATCATGATACACAACAGGAATATGGTCGTTAGTAGGTACGGTGACTTTACACAACGCGATATATAACTTTCCTTTTGTGCCATTTTTAACGTAGTTGGGACGTCCTGCAGGGTATTGTTTTGAAGAAATGATATCGAGAACAGATTTGATACGATATTTGTTGGATTTCTGATTATTCACTATAATATAATCTAGACAGATAGATGATTCGATAAATAAAGAAGATATATCGTTGGTAGGATAATACATCCCCATTCCAGTATTCTTGGATACTCTATTGATGACACTAGTTAAGAACATGTCTTCAATTCTAGAAAACGAAAACATCCTACACGGACTCATTAAAACTTCTAGCGCTCCTGATTGTGTCTCAAATGCCTCATACAATGATTTCAAGGATGTCATAGATTCTTTAACCCACGATTTAATATTGCGTTTAGCATCTATTTTTTTTATTAAATCGAATGGTCGGCTCTCTGGTTTGCTACCCCAATGATAACAATAGTCTTGTAAAGATAAACCGCAAGAAAATTTATACGCATCCATCCAAATAACTCTTGCACCATCAGATGATATCAGTATATTATTATAGATTTTCCATCCACAATTATTGGGCCAGTATACTGTTAGCAACGGTATATCGAATAGATTACTCATGTAACCTACTAGAATGATAGTTCGTGTACTAGTCATAATATCTTTAATCCAATCTAAGAAATTTAAAATTAGATCTTTTACACTGTTAAAGTTAACAAAGGTATTACCCGGGTACGTGGATATCATATATGGCATTGGTCCATTATCAGTAATAGCTCCATAAACTGATACGGTGATGGTTTTTATATGTGTTTGATCTAACGAGGAAGAAATTTGCGCCCATAATTCATCTCTAGATATGTATTTAATATCAAACGGTAACACATCAATTTCGGGACGCGTATATGTTTCTAAATTTTTAATCCAAATATAATGATGACCTATATGCCCTATTATCATACTGTCAACTATAGTACACTTAGAGAACTTACGATACATCTGTTTCCTATAATCGTTAAATTTTACAAATCTATAACATGCTAAACCTTTTGACGACAACCATTCATTAATTTCTGATATGGAATCTGTATTCTCGATACAGTATCGTTTTAAAGCCAGTGCTATATCTCCCTGTTCGTGAGAACGCTTTCGTATAATATCAATCAACGGATAATCTGAGGTTTTTGGAGAATAATATGACTCATGATCTATTTCGTCCATAAACAATCTAGACATAGGAATTGGAGGCGATGATCTTAATTTTGTGCAATGAGTAGTTAATCCTATAACTTCTAATCTTGTAATATTCATCATCGACATAATACTATCTATGTTATCATCGTATATTAGTATACCATGACCTTCTTCATTTCGTGCCAAAATGATATACAGTCTTAAATAGTTACGCAATATCTCAATAGTTTCATAATTGTTAGCTGTTTTCATTAAGATTTGTACCCTGTTTAACATGATGGTGTTCTATACGTCTTTATTTTCTATTTTTAATTTTTTAAATTTTTAACGATTTACTGTGGCTAGATACCCAATCTCTCTCAAATATTTTTTTAGCCTCGCTTACAAGCTGTTTATCTATACTATTAAAACTAACAAATCCGTGATTTTGGTAATGGGTTCCGTCAAAATTTGCCGACGTGATATGAACATATTCATCGTCGACTATCAACAATTTTGTATTATTCTGAATAGTGAAAACCTTCACAGATAGATCATTTTGAACACACAACGCATCTAGACTTTCGGCGGTTGCCATAGAATATACGTCGTTCTTATCCCAATTACCAACTAAAAGTCTGATCTTAACTCCTCTATTAATGGCTGCTTCTATAATGGAGTTGTAAATGTCGGGCCAATAGTAGCTATTACCATCGACACGTGTAGTGGGAACTATTGCCAAATGTTCAATATCTATACTAGTTTTAGCTGACCTAAGTTTATCAATAACTACATCGGTATCTAGATCTCTAGAATATCCCAATAGGTGTTCCGGAGAATCAGTAAAGAACACTCCACCTATAGGATTCTTAATATGATACGCAGTGCTAACTGGCAGACAACAAGCCGAAGAGTATAAATTCAACCATGAATTTTTTACGCTATTAAAGGCTTTAAAAGTATCAAATCTTCTACGAAGATCTGTGGCCAGTGGTGGATAATCAGAATATACACCTAACGTTTTAATTGTATGTATAGATCCTCCAGTAAATGACGCGTTTCCTACATAACATCTTTCATCATCTGATACCCAAAAACATCCAAGTAGTAGTCCTACGTTATTTTTTTTATCAATATTAACGGTTATAAAATTTATATCCGGGCAGTGACTTTGTAGCTCTCCCAGATTTCTTTTCCCTCGTTCATCTAGCAAAACTATTATTTTAATCCCTTTTTCAGATGCCTCTTTTAGTTTATCAAAAATAAGCGCTCCCCTAGTCGTACTCAGAGGATTACAACAAAAAGATGCTATGTATATATATTTCTTAGCTAGAGTGATAATTTCGTTAAAACATTCAAATGTTGTCAAATGATCGGATCTAAAATCCATATTTTCTGGTAGTGTTTCTACCAGTCTACATTTTGCTCCCGCAGGTGCTGATGTAAATGGCCACATTTAGTTAACAAAAAAAATTTACATATCCTGTTCCATCAACGATTCTAGAACGTCATCGGTTATATCGCTAAAATCTTCATCAAAGTCGACATCACAACCTAGCTCAGTCAATATATTAAGAAGTTCCGTGATATTATCTTCATCGATTTCTATATCCATATCGGATGTAGATAATTGACTGATTATTGAGTTAAAATCAGTACTAATTTCCAACCCTTCAGAATATACTTTGTGTTTCATGGTAATATCGGTAGCGTATTTAAGTTGGTAGATTTTTCAATTATGCATCAATATAGCAACAGTAGTTCTTGCTCCTCCTTAATTCTAGCATCTTCTTCATTATTTTCTTCTACATACATAAGCATCTCCAATACGTTAGACAGCACACCGATGATGGCGGCCGCCACAGACACGAATATGACTAGACTGATGACCATTTAAAAAAACACTCTAGCTTTCACTTAAACTGTATCGATCATTCTTTTAGCACATGTATAATATAAAAACATTATTCTATTTCGAATTTAGGCTTCCAAAAATTTTTCATCCGTAAACCGATAATAATATATATAGACTTGTTAATAGTTGGAATAAATAGATTAATGCTTAAACTATCATCATCTCCACGATTAGAGATACAATATTTACATTCTTTTTGCTGTTTCGAAACTTTATCAATACACGTTAATACAAACCCAGGAAGGAGATATTGAAACTGAGGCTGTTGAAAATGAAACGGTGAATACAATAGTTCAGATAATGTAAAATCATGATTCCGTATTCTGATGATATTAGAACTACTAATGGATGTCAATGGTATGTATCTAGGAGTATCTATTTTAACAAAGCATCTATTTGCTAATATACAATTATCCTTTTGATTAATTGTTATTTTATTCATATTCTTAAAAGGTTTCATATTTATCAATTCTTCTACATTAAAAATTTCCATTTTTAATTTATGTAGCCCGCAATACTCCTCATTACGTTTCATTTTTTGTCTATAATATCCATTTTGTTTATCTCAGTACATAGATTATCCAATTGAGAAGCACATTTAGTAGTTTTGTACATTTTAAGTTTATTGACGAATCGTCGAAAACTAGTTATAGTTAACATTTTATTATTTGATACCCTGATATTAATACCCCTACCGTTACTATTATTTATAACTGATGTAACCCACGTAACATTAGAATTAATTATCGATAGTAATGCATTCACGCTTCCAAAATTGTCTATTATAAACTCACCAATAATTTTTTTATTGCATGTTTTCATATTCATTAGGATTATCAAATCTTTAATCTTATTACGATTGTATGCGTTAATATTACAAGACGTCATTCTAAAAGACGGAGGATCTCCATCAAATGCCAGACAATCACGTACAAAGAACATGGAAATAGGTTTTGTTCTATTGCGCATCATAGATTCATATAGAACACCCGTAGAAATACTAATTTGTTTTACTCTATAAAATACTAATGCATCTATTTCATCGTTTTGTATAACGTCTTTCCAAGTGGCGAATTCCAAATTTTTTTCATTGATAGTACCAAATTCTTCTATCTCTTTAACTATTTGCATAGATAGGTAATTACAGTGATGTCTACATGCCGTTTTTTGAAACTGAATAGATGCATCTAAAAGCGATGCTACGCTAGTCACGATCACCACTTTCATATTTAGAATATATGTATGTAAAAATATAGTAGAATTTCATTTTGTTTTTTTCTATGCTATAAATGAATTCTCATTTTGCATCTGCCCATACTCCGTTTTATATCAATACCAAAGAAGGAAGATATCTGGTTCTAAAAGCTGTTAAAGTATGCGATGTTAGAACTGTAGAATTCGAAGGAAGTAAAGCTTCCTGCGTACTCAAAGTAGATAAACCCTCATCGCCCGCGAGCGAGAGAAGACCTTCGTCACCGTCCAGATGCGAGAGAATGAATAACCCAGGAAAACAAGTTCCGTTTATGAGGACGGACATGCTACAAAATATGTTCGCGGCTAATCGCGATAATGTAGCTTCTAGACTTTTGTCCTAAAATACTATTATATCTTTTTCGATATTAATAAATCCGTGTCGCCCATGTTTTTTATCTCTTTCAGTATGTGAATAGATAGGTATTTTATCTCTATTCATCATCGAATTTAAGAGATCCGATAAACATTGTTTGTATGCTCCAGATGTCAGCATCTGATACAACAATATATGTGCACATAAACCTCTGGCACTTATTTCGTGTACCTTCCCCTTATCACTAAGGAGAATAGTATTTGAGAAATATGTATACATAATATTATCATGAATTAGATATACAGAATTTGTAACACTCTCGAAATCACACGATGTGTCTGCGTTAAGATCTAATATATCACTCGATAACACATTTTCATCTAGATACACTAGACATTTTTTAAAGCTAAAATAGTCTTTAGTAGTAACAGTAACTACGCGATTATTTTCATCGATGATGCATTTCATCGGCATATTATTACGCTTACCATCAAAGACTATACCATGTGTATATCTAACGTATTCTAGCATAGTTGCCATACGCGCATTAAACTTTTCAGGATCTTTGGATAGATCTTCCAATCTATCTATTTGAGAAAACATTTTTATCATGTTCAATAGTTGAAACGTCGGGTCCACTATATAGATATTATCTATAAAGATTTTAGGAACTACGTTCATGGTATCCTGGCGAATATTAAAACTATCAATGATATGATTATCGTTTTCATCTTTTATCACCATATAGTTTCTAAGATATGGGATTTTACTTAATATAATATTATTTCCCGTAATAAATTTTATTAGAAATGCCAAATCTATAAGAAAAGTCCTAGAATTAGTCTGAAGAATATCTATATCACCGTACCGTATATTTGGATTAATTAGATATAGAGAATATGATCCGTAACATATACAACTTTTATTATGGCGTCTAAGATATTCTTCCATCAACTTATTAACATTTTTGACTAGGGAAGATACATTATGACGTCCCATCACTTTTGCCTTGTCTATTACTGCAACGTTCATAGAATTTAGCATATCTCTTGCCAATTCTTCCATTGATGTTACATTATAAGAAATTTTAGATGAAATTACATTTGGAGCTTTAATAGTAAGAACTCCTAATATGTTCGTGTATGTGGTCACTAATACAGATTGTAGTTCTATAATCGTAAATAATTTACCTATATTATATGTTTGAGTCTGTTTAGAAAAGTAGCTAAGTATACGATCTTTTATTTCTGATGCAGATGTATCAACATCGGAAAAAAATCTTTTTTTATTCTTTTTTACTAAAGATACAAATATGTCTTTGTTAAAAACAGTTATTTTCTGAATATTTCTAGTTTGTAATTTTAACATATGATATTCATTCACACTAGGTAATCTACCTAAATAGGTTTCTATAATCTTTAATGTAATATTAGGAAAAGTATTATGATCAGGATTCCTATTCATTTTGAGGATTTAAAACTCTGATTATTGTCTAATATGGTCTCAACGCAAACTTTTTCACAGAGCGATAGAGTTTTTGATAACTCGTTTTTCTTAAGAAATATAAAACTACTGTCTCCAGAGCTTGCTCTATCTTTTATTTTATCTAATTCGATACAAACTCCTGATACTGGTTCAGAAAGTAATTCATTAATTTTCAGTCCTTTATAGAAGATATTTAATATAGATAATACAAAATTTTCAGTTTTTGATATCGATCTGATTGATCCTAGAACTAGATATATTAATAACGTGCTCATTAGGCAGTTTATGGCAGCTTGATAATTAGATATAGTATATTCCAGTTCATATTTATTAGATACCGCATTGCCCAGATTTTGATATTCTATGAATTCCTCTGAAAATAAATCCAAAATAACTAGACATTCTATTTTTTGTGGATTAGTGTACTCTCTTCCCTCTATCATGTTCACAACTGGTGTCCACGATGATAAATATCTAGAGGGAATATAATATAGTCCATAGGATGCCAATCTAGCAATGTCGAATAACTGTAATTTTATTCTTCGCTCTTCATTATGAATTGATTCTTGAGGTATAAACCTAACACAAATTATATTATTAGACTTTTCGTATGTAATGTCTTTCATGTTATAAGTTTTTAATCCTGGAATAGAATCTATTTTAATGAGGCTTTTAAACGCAGAGTTCTCCAACAAGTCAAAACATAATACTCTGTTGGTTTTCTTATATACAATATTACGATTTTCTTCTTTGAATGGAATAGGTTTTTGAATTAGTTTATAATTACAACATAATAAATAAGGAAGTGTGCAAATAGTACGCGGAAAAAACATAATAGCTCCCCTGTTTTCATCCATGGTTTTAAGTAAATGATCACTGGCTTCTTTAGTCAATGGATATTCGAACATTAACCGTTTCATCATCATTGGACAAAATCCATATTTCTTAATGTAAAGAGTGATCAAATCATTATGTTTATTGTACCATCTTGTTGTAAATGTGTATTCGGTTATCGGATCTGCTCCTTTTTCTATTAAAGTATCGATGTCGATCTCGTCTAAGAATTCAACTATATCGACATATTTCATTTGTATACACATAACCATTACTAACGTAGAATGTATAGGAAGAGATGTAACGGGAACAGGGTTTGTTGATTCGCAAACTATTCTAATACATAATTCTTCTGTTAATACGTCTTGCACGTAATCTATTATAGATGCCAAGATATCTATATAATTATTTTGTAAGATGATGTTAACTATGTGATCTATATAAGTAGTGTAATAATTTATGTATTTCGATATATGTTCCAACTCTGTCTTTGTGATGTCTAGTTTTGTAATATCTATAGCATCCTCAAAAAATATATTCGCATATATTCCCAAGTCTTCAGTTCTATCTTCTAAAAAATCTTCAACGTATGGAATATAATAATCTATTTTACCTCTTCTGATATCATTAATGATATAGTTTTTGACACTATCTTCTGTCAATTGATTCTTATTCACTATATCTAAGAAACGGATAGCGTCCCTAGGACGAACTACTGCCATTAATATCTCTATTATAGCTTCTGGATATAATTCATCTATTATACCAGAATTAATAGGAACTATTCCGTATTTATCTAACATAGTTTTAAGAAAGTCAGAATCTAAGACCTGATGTTCATATATTGGTTCATACATGAAATGATCTCTATTGATGATAGTGACTATTTCGTTCTCTGAAAATTGGTAACTCATTCTATATATGCTTTCCTTGTTGATGAAGGATAGGATATACTCAATAGAATTTGTACCAACAAACTGTTCTCTTATGAATCGTATATCATCATCTGAAATAATCATGTAAGGCATACATTTAACAATAAGAGACTTGTCTCCTGTTATCAATATACTATTCTTGTGATAATTTATGTGTGAGGCAAATTTGTCCACGTTCTTTAATTTTGTTATAGTAGATATCAAATCCAATGGAGCTACAGTTCTTGGCTTAAACAGATATAGTTTTTCTGGAACGAATTCTACAACATTATTATAAAGGACTTTGGGTAGATAAGTGGGATGAAATCCTATTTTAATTAATGCAATAGCCTTGTCCTCGTGCAGATATCCAAACGCTTTTGTGATAGTATGGCATTCATTGTCTAAAAACGCTCTACGAATATCTGTGACAGATATCATCTTTAGAGAATACTAGTCGCGTTAATAGTACTAAAATTTGTATTTTTAATCTATCTCAATAAAAAATTAATATTTATGATTTAATGTATAACTTTACTAACTGTTATTGATAACTAGAATCAGAATCTAATGATGACGTAACCAAGAAGTTTATCTACAGCCAATTTAGCTGCATTATTTTTAGCATCTCGTTTAGATTTTCCATCGGCCTTATCGAATACTCTCCCGTCGATGTCTACACAGGCATAAAATGTAGGAGAGTTACTAGGCCCCACTGATTCAATACGAAAAGACCAATCTCTCTTAGTTATTTGGCAGTACTCATTAATAATGGTGACAGGGTTAGCATTTTTCCAATCAATAATTTTTTTAGCCGGAATAACATCATCAAAAGACTTATGATCCTCTCTCATTGATTTTTCGCGGGATACATCATCTATTATGACGTCAGCCATAGTCATAGCATCCGGCTTATCCGCCTCAGTTGTCATAAACCAACGAGGAGGAATATCGTCGGAGCTGTACACCATAGCACTACGTTGAAGATCGTACAGAGCTTTATTAACTTCTCGCTTCTCCATATTAAGTTGTCTAGTTAGTTGTACAGCAGTAACTCCTTCAAGTCCAATGTTTTTAATAGCCTCACATACAATCTCTGCGTCAGAACGCTCGTCAATATAGATCTTAGACATTTTTAGAGAGAACTACCGCAACTAGTAATAAAACTAATCTATTTTATCATTTTTTTATTCATCATCTTCTGGTGGTTCATCGTTCCTATCGAATGTGACTCTGATTAACCCATCATCTATAGGTGATGCTGGTTCTGGAGATTCTGGAGGAGATGGATTATTATCTGGAAGAATCTCTGTTATTTCCTTGTTTTTATGTATCGATTGCGTTGTAACATTAAGATTGCGAAATGCTCTAAATTTGGGAGGCTTGAAGTGTTGTTTGCAATCTCTACACGCGTGTCTAACTAATGGAGGTTCATCAGCGGCTCTAGTTTGAATCATCATCGGTGTAGTATTCCTACTTTTACAGTTAGGACACGGTGTATTGTATTTCTCGTTGAGAACGTTAAAGTAATCATTGTAACTCACATCCTTTATTTTATCTATATTGTATTCTACTCCTTTCTTAATGCATTTTATACCGAATAAGAGATAGCGAAGGAATTCTTTTTCGGTACCGCTAGTACCCTTAATCATGTCACATAGTGTTTTATATTCCAAATTTGTGGCAATGAACGGTTTATTTCTATACGATAGTTTGTTTCTAGAATCCTTTGAGTATTCTATACCAATATTATTCTTTGATTCGAATTTAGTTTCTTCGATATTGGATTTTGTATTACCTATATTCTTGATGTAGTACTTTGATGATTTTTCCATGGCCCATTCTATTAAGTCTTCCAAGTTGGCATCATCCACATATTGTGATAGTAATTCTCGGATATTAGTAGCGGCTACCGCCATTGATGTTTGTTCATTGGATGAGTAACTACTAATGTATACATTTTCCATTTATAACACGTATGTATTAACTTTGTTCATTTATATTTTTTCATTATTATGTTGATATTAACAAAAGTGAATATATATATGTTAATAATTGTATTGTGGTTATATGGCTACAATTTCATAATGAGCGGAAGTCAGTGTCCGATGATTAATGACGATAGATTTACTCTGAAAAGAAAGTATCAAATCGATAGTGTAGAGTCGACAATGAAAATGGATAAGAAGAGGACAAAGTTTCAAAATAGAGCCAAAATGGTAAAAGAAATAAATCAGACAATAAGAGCAGCACAAACTCATTACGAGACATTGAAACTAGGATACATAAAATTTAAGAAAATGATTAGGACTACTACTTTAGAAGATATAACAACATCTATTCCAAATATTCAGAAAATTTATAAACTATTCTCGGACATTTCAGCCATTGGCAAAGTATCACAGAATCCGAGTAAAATGGCATATGCTTTACTGCTTTACATGTTCCCCAATTTGTTTGGAGATGACCATAGATTCATTCTTTATAGAATGTTTCCAATGAGTAAAATCAAACACAAGATCTTCTCTCCTTTCAAACTTAATCTTATTAGAATATTAGTGGAAGAAAGATTCTATAATAATGAATGCAGATCTAATAAATGGAGAATAATTGGAACACAAGTTGATAAAATGTTGATAGCTGAATCTGATAAATATACAATAGATGCAAGGTATCGCCTAAGACCTATATATAGAATCAAGGGAAAATCTGAAGAAGATACCCTCTTCATCAAACAGATGGTAGACCAATGTGTGACATCCCAGGAATTGGTGGAAAAAGTGTTGAAGATACTGTTTAGAGATTTGTTCAAGAGTGGAGAATACAAAGCGTACAGATACGATGATGACGTAGAAAATGGATTCATTGGATTGGATAAACTAAAATTAAACATTGTTCATGATATAGTTGAACCATGTATGCCTGTTCGTAGGCCAGTGGCTAAGATACTGTGTAAAGAAATGGTAAATAAATACTTTGAGAATCCGCTGCATATTATTGGTAAGAATCTTCAAGAGTGCATTGACTTTGTTAGTGAATAGGCATTTCATCTTTCTCCAATACTAATTCAAATTGTTAAATTAATAATGGATATAGTATAAATAGTTATTAGAATAAGAGTGTAGTATCATAGATAAGTATTTACTTTCTTCTATAAAATGGATTTTATTCGTAGAAAGTATCTTATATACACAGTAGAAAATAATATAGATTTTTTAAAGGATGATACATTAAGTAAAGTAAACAATTTTACCCTCAATCATGTACTAGCTCTCAAGTATCTAGTTAGCAATTTTCCTCAACACGTTATTACTAAGGATGTATTAGCTAATACCAATTTTTTTGTTTTCATACATATGGTACGATGTTGTAAAGTGTACGAAGCGGTTTTACGACACGCATTTGATGCACCCACGTTGTACGTTAAAGCATTGACTAAGAATTATTTATCGTTTAGTAACGCAATACAATCGTACAAGGAAACAGTGCATAAACTAACACAAGATGAAAAATTTTTAGAGGTAGCCGAATACATGGAAGAATTAGGAGAACTTATAGGGGTAAATTATGACTTAGTTCTTAATCCATTATTTCACGGAGGGGAACCCATCAAAGATATGGAAATCATTTTTTTAAAACTGTTTAAGAAAACAGACTTTAAAGTTGTTAAAAAATTAAGTGTTATAAGATTACTTATTTGGGCTTACCTAAGCAAGAAAGATACAGGTATAGAGTTTGCGGATAATGATAGACAAGATATATATACTCTATTTCAACACACTGGTAGAATAGTCCATAGCAATCTAACAGAAACGTTTAGAGATTATATCTTTCCCGGAGATAAGACTAGCTATTGGGTGTGGTTAAACGAAAGTATAGCTAATGATGCAGATATCGTTCTTAATAGACCCGCCATTACCATGTATGATAAAATTCTTAGTTATATATACTCTGAGATAAAACAGGGACGTGTTAATAAAAACATGCTTAAGTTAGTTTATATCTTTGAGCCTGAAAAAGATATCAGAGAACTTCTGCTAGAAATCATATATGATATTCCTGGAGATATCCTATCTATTATTGATGCAAAAAACGACGATTGGAAAAAATATTTTATTAGTTTTTACAAAGCTAATTTTATTAACGGTAATACATTTATTAGCGATAGAACGTTTAACGAGGATTTATTCAGAGTTGTTGTTCAAATAGATCCTGAATATTTTGATAATGAACGAATTATGTCTTTATTCTCTACGAGTGCTGTGGAAATTAAACGATTTGATGAGTTAGATATTAATAACAGTTATATATCTAATATAATTTATGAGGTGAACGATATCACATTAGATACAATGGATGATATGAAGAAGTGCCAAATCTTTAACGAGGATACATCGTATTATGTTAAGGAATACAATACATACCTGTTTTTGAACGAGACGGATCCCATGGTCATAGAGAACGGAATACTAAAGAAACTGTCATCTATAAAATCCAAGAGTAGACGACTGAACTTGTTTAGCAAAAACATTTTAAAATATTATTTAGACGGACAATTGGCTCGTCTAGGTCTTGTGTTAGATGATTATAAAGGAGACTTGTTAGTTAAAATGATAAACCATCTCAAGTTTGTGGAGGATGTATCAGCATTCGTTAGATTTTCTACAGATAAAAACCCTAGTGTTCTTCCATCGCTAATCAACACTATTTTAGCTAGTTATAATATTTCCATCATTGTCCTATTTCAAAAGTTTTTAAGAGATAATCTATATCATGTAGAAAAATTCTTGGATAAAAGCATCCATCTAACCAAGACGGATAAGAAATATATACTTCAATTGATAAGGCACGGCAGATCATAGAACAAACCAAATATATTATTAATAATTTGTATATACATAGATATAATTATCACATATTAAAATTCACACATTTTTGATAAATGGGAACTACTGCAACAATTTAGACTCCCACCAAATTAATGAATAAAGAGAATGCAGAAATGATTTTGGAAAAATTGTTGATCATATAGTTATGTATATTAGTGACGAATCAAGAGATGAAAATAATCCTGAATATATTGATTTTCGTAACAGATACGGAGACTATAGATCTTTCATTATAAAAAGTGATCACGGGTTTGTAAAGATCATGCAGAGAAAAGTTCTCCAAAAACACAACAAATGATAATCAAACACATCTACGAACAATATCTTATTTCGGTATCTGAAGTACTTATTAAAACCTATGATATCCATGGGTGACATAATTACATATAACGGATGTAAAGACAATAAATGGATGCTAGAACAACTCTCTACCCTAAACTTTAACAATCTCCACGTATGGAACTCATGTAGCATAGGTAATGTAACGCGTATATTTTATACATTTTTTAGTTATCTGATGAAAGATAAACTAGATATATAAATAATCCCATTCTAATACTTTAACATGTGTATTACATGCATCTTATTAGAATATTAACCTAACTAAAAGACATAATATAAAAACTTATTACATAGTTGATAAAAAGCGGTAGGCTATAAATATTATGGCAGCAGTCGTTCCGCGTTTTGATGACGTGTACAAAAATGCACAGCGAAGAATTCTAGATCAAGAAACATTTTTTAGTAGAGGTCTAGGTAGACCGTTAATGAAGAACACATATTTGTTTGATAATTACGCGTATGGATGGATACCAGAAACGGCAATTTGGAGTAGTCGATACGCAAACCTAGATGCAAGTGACTATTATCCCATTTCGTTGGGATTACTTAAAAAGTTTAAGTTTCTCATGTCTCTATATAAAGGTCCTATTCCAGTATACGAAGAAAAAGTAAATACTGAATTCATAGCCAATGGATCGTTCTCTGGTAGATACGTATCATATCTTCGAAAGTTTTCTGCTCTTCCAACAAACGAGTTTATTAGTTTTTTGTTACTGACTTCCATTCCAATCTATAATATCTTGTTCTGGTTTAAAAATACTCAGTTTGATATTACTAAACACACATTATTCAGATACGTCTATACAGATAATGCCAAACACCTGGCATTGGCTAGGTATATGCATCAAACAGGAGACTATAAGCCTTTGTTTAGTCGTCTCAAAGAGAATTATATATTTACCGGTCCCGTTCCAATAGGTATCAAAGATATAGATCACCCTAATCTTAGTAGAGCAAGAAGTCCATCCGACTATGAGACATTAGCTAATATTAGTACTATATTGTACTTTACCAAGTATGATCCGGTATTAATGTTTTTATTGTTTTACGTACCTGGGTATTCAATTACTACAAAAATTACTCCAGCAGTAGAGTATCTAATGGATAAACTGAAACTAACAAAGAACGATGTACAACTGTTGTGAATATTTTATGCTTCGTAAAATGTAGGTCTTGAACCAAACATTCTTTCAAAGAATGAGATACATAAAACTTTATTATCCAATAGATTGACTATTTCGGACGTCAATCGTTTAAAGTAAACTTCGTAAAATATTCTTTGATCACTACCGAGTTTAAAACTTCTATCGATAATTGTTTCATATGTTTTAATATTTACAAGTTTTTTGGTCCATGGTACATTAGCCGGACAAATATATGCAAAATAATATCGTTCTCCAAGTTCTATAGTCTCTGGATTATTTTTATTATATTCAGTAACCAAATACATATTAGGGTTATCTGCGGATTTATAATTTAAGTGATGCATTCGACTCAACATAAATAATTCTAGAGGAGACGATCTACTATCAAATTCGGATCGTAAATCTGTTTCTAAAGAACGGAGAATATCTATACATACCTGATTAGAATTCATCCGTCCTTCAGACAACATCTCAGACAGTCTGGTCTTGTATATCTTAATCATATTCTTATGAAACTTGGAAACATCTCTTCTAGTTTCACTAGTACCTTTATTAATTCTCTCAGGTACAGATTTTGAATTCGATGATGCCGAGTATTTCATCGTTGTATATTTCTTCTTCGATTGCATAATCAGATTCTTATATACCGCCTCAAACTCTATTTTAAAATTATTAAACAATACTCTACTATTAATCAGTCGTTCTAATTCCTTTGCTATTTCTATGGACTTATCTACATCTTGACTGTCTATCTCTGTAAACACGGAGTCGGTATCTCCGTACACGCTACGAAAACGAAATCTGTAATCTATAGGCAACGATGTTTTCACAATCGGATTAATATCTCTATCGTCCATATAAAATGGATTACTTAATGGATTGGCAAACCGTAACATACCGTTAGATAACTCTGCTCCATTTAGTACCGATTCTAGATACAAGATCATTCTACGTCCTATGGATGTGCAACTCTTAGCCGAAGCGTATGAGTATAGAGCACTATTTCTAAATCCCATCAGACCATATACTGAGTTGGCTATGATCTTGTACGTATACTGCATGGAATCATAGATGGCCTTTTCAGTTGAACTGGTAGCCTGTTTTAGCATCTTTTTATATCTGGCTCTCTCTGCCAAAAATGTTCTTAATAGTCTAGGAATGGTTCCTTCTATTGATCTATCGAAAATTGCTATTTCAGAGATGAGGTTAGGTAGTCTAGGTTCACAATGAACCGTAATATATCTAGGCGGTGGATATTTCTGAAGCAAGAGCTGATTATTTATTTCTTCTTCCAATCTATTGCTACTAACAACGACACCGACTAATGTTTCCGGAGATAGATTTCCAAAGATACACACATTAGGATACAGACTGTTATAATCAAAGATTAATACATTATTACTAAACATTTTTTGTTTTGGAGCAAATACCTTACCGCCTTCATAAGGAAACTTTTGTTTTGTTTCTGATCTAACTAAGATAGTTTTAGTTTCCAACAATAGCTTTAACAGTGGACCCTTGATGACTGTACTCGCTTTATACCCGAATACCATGGATTGAGGAAGCACATATGTTGACGCACCCGCGTCTGTTTTTGTTTCTACTCCATAATACTCCCACAAATACTGACACAAGCAAGCATCATGAATACAGTATCTAGCCATATCTAAAGCTATGTTTAGATTATAATCCTTATACATCTGTGCTAGATCAACGTCATCCTTTCCGAACGATAATTTATACGTATCATTTGTTAAAGTGGGACATGATAGTACAACTTTGAATCCATTTTCCCAAATATCTTTATGAATTACTTTACATATAATATCATCAACCGTCACATAATTACCTGTGGTTAAAACCTTTGCAAATACAGCGGCTTTGCCTTTAGCGTCCGTAGTATCGTCACCGATGAACGTCATTTCTCTAACTCCTCTATTTAATACTTTACCCATGCAACTAAACGCGTTCTTGGATATAGAATCCAATTTGTACGAATCCAATTTTTCAGATTTTTGAATGAATGAATACAGATCAAAAAATATAGTTCCATTATTGTTATTAACGTGAAACGTAGTATTGGCCATGCCACCTACCCCCTTGTGACTAGACTGATTTCTCTCATAAATACAGAGATGTACAGCTTCCTTTTTGTCTGGAGATCTAAAGATAATCTTCTCTCCGGTTAATAATTCTAGACGATTAGTAATATATCTCAGATCAAAGTTATGTCCGTTAAAGGTAACAATATAGTCGAACGTTAGTTCCAACAATTGTTTAGCTATTTGTAGCAAAACTATTTCAGAACACAGAACTAGTTCTCTCTCATAATCCATTTCCATCAGTGACTGTATCCTCAAACATCCTCTATCGACGGCTTCTTGTATTTCCTGTTCCGTTAACATCTCTTCATTAATAAGCGTAAACAATAATCGTTTACCACTTAAATCGATATAACAGTAACTTGTATGAGAGATTGGGTTAATAAATACAGAAGGAAACTTCTTATCGAAGTGACACTCTATATCTAGAAATAAGTACGATCTTGGGATATCGAATCTAGGTATTTCTTTAGCGAAACAGTTACGTGGATCGCCACAATGATAACATCCATTGTTAATCTTTGTTAAATATTGATCGTCCAACGAATAACATCCGTCTGGAGATATCCCGTTAGAAATATAAAACCAACTAATATTGAGAAATTCATCCATAGTGGCGTTTTGTATGTTGCGTTTCTTTGGCTCTTCTATTAACCACATATCTGCGACGGAGCATTTTCTATCTTTAATATCTAGATTATAACTTATTGTCTCGTCAATGTCTATAGTTCTCATCTTTCCCATTGGCCTCGCATTAAATGGAGGAGGAGCCAATGACTGATATATTTCGTCTGTCACTACGTAATAAAAGTAATGAGGAAATCGTATAAATACTGTCTCGCCATTTCGACATCTGGATTTCAGATATAAAAATCTGTTTTCACCATGACTTTCGAACCAATTAATACACCGAACATCCATTTCTAGAATTTAGAAATATATTTTCATTTAAATGAATCCCAAACATTGGGGAAGAGCCGCTTGGACCATTATTTTTATAGTACTTTCACAAGCGGGTTTAGACGGCAACATAGAAGCGTGTAAACGAAAACTATATACTATAGTCAGCACTCTTCCATGTCCTGCCTGCAGACGACACGCAACTATCGCTATAGAGGACAATAATATTATGTCTAGCAATGATCTAAATTATATTTATTATTTTTTCATCAGATTATTTAACAATTTGGCATCTGATCCCAAATACGCAATCGATGTGTCAAAGGTTAAACCTTTATAAACTTAACCCATTATAAAACTTATGATTAGTTACGACTGAAATAACCGCATGATTATTTTTTGGTATAATTCTACACGGCATAGTTTCTGTAACTATGAATTCTACCCCCGTTACATTTGTATAATCTTTAACAAACAGTAAGGGCTCGTCAAATACGTAAAACTCATTACTTACGATAGAAATAGATCCCTTATCACACTTAAAATAAAAAATATCCTTATCCTTTACCACCAAATAAAATTCTGATTGGTCAATGTGAATGTATTCACTTAACAATTCCACAAATTTATTTATTAACTCCGAGGCACATACATCGTCGGTATTTTTTATGACAAACTTTACTCTTCCAGCATCCGTTTCTAAAAAAATATTAACGAGTTCCATTTATATCATCCAATATTATTGAAATGACGTTGATGGACAGATGATGCAAATAAGAAGGTACAATACCTTTGTCTACCATCTCCTCCAATTCATGCTCTATTTTGTCATTAACTTTAATGTGTGAAAACAGTACGCCACATGCTTCCATAACAGTGTGTAACACTTTTAATACGAAATGTCTGATGGCGACATAATTGTTCAAAACTGCCAATCTATAATAGATGGTGGCCACAATATATTCTATGATGGTATTGAAGAAGATAACAACCTTGGCATATTGATCATTTGATACGGACATGGTATCAACAGATAGCTTGAATGAAAGAGACTCAGTAATCGGAATAAGTGTTTTCTCAATGGAGTGTCCGTATACCAACATGTCTGATATTTTGATGTACACCATCAAATTGTTTAGTTTTCTCTTTTTATCCTCATTAAACAGCATTTCTGTCAATGGACCCCAACATCTTTGACCGATTAAGTTTTGATTGATTCTTCCATGTAAGGCGTATCTAGTCAGATCGTATAATCTAGCCAACAATCCATCGTCGGTGTTTAGGTCACATCGTATACTTTTTAATTCTCTATAGAAGAGAGACACACACCTGGAACAATTACAGACAGTAATTTCTTTATTATCTACAGATGTAAGATACTTGAAGACATTCCTATGATGATGTAGAATTTTTGATAACACTGTATTGATGGTATCTGTTACCATAATACCTTTTATGGCGGACAGTGTCAGAGCACAGGATTTCCAATCTTTTGCAATTTTCAGTACCATTATCTTTGTTTTGATATCTATATTAGATAGCATGGTGCGTCTGACAACACAGGGATTAAGACGAAAAGATGAAATGATTCTATCTACATCTTCAATAGATACCTTGCTATTTTTTCTGGCATTATCTATATGTGAGAGAATATCCTCTAGAGAATCAGTATCCTTTTTGATAATAGTGGATCTCAATGACATGGGACGTCTAAACCTTCTTATTTTATCACCTGTTTGCATGGTGATTTGTCTGCTTTCTTTTAACATGATGTAATCTCTAAATTCATTGGCAAATTGTCTATATCTAAAATCATAATATGAGATGTTTACCTCTACAAATATCTGTTCGTCCAATGTTAGAGTATCTACATCAGTCTTGTATTCCAAATTAAACATGGCAACGGATTTAATTTTATATTCCTCTATTAGGTCCTTGTCGACAATAACAGAGTGCAGATAATCATTTAATCCGTCGTACATGGTTGGAAGATGCGCATTAACAAAATCTTTAATTGTCTTGATGAAGGTGGGACTATATCTAACATCTTGATTAATAAAATTTATAACATTTTCCATAGGATACTTTGTAACTAGCTTTATACACATCTCTTCATCTGTAAGTTTAGACAGAATATCGTGAACAGGTGGTATATTATATTCATCAGATATACGAAGAATAATGTTCAAATCTAGATTGTTTAATATATTATATAGATGTAGTGTAGCTCCTACAGGAATATCTTTAACTAAGTCAATTATTTCATCAACCGTTAGATCTATTTTAAAGTTAATCATATAGGCATTGATTTTTAAAAGGTATGTAGCCTTGATTACATTCTCATTAATTAACCATTCCAAGTCACTATGTGTAAGAAGATTATATTCTATCATAAGCTTGACTACATTTGGTCCCGATACCATTAAAGAATTCTTATGATATAAGGAAACAGCTTTTAGGTACTCATCTACTCTACAAGAATTTTGGAGAGCCTTAACGATATCAGTGACGTTTATTATTTCAGGAGGAAAAAACCTAACATTGAGAATGTCGGAGTTAATAGCTTCCAGATACAGTGATTTTGGCAATAGTCCGTGTAATCCATAATCCAGTAACACGAGCTGGTGCTTGCTAGACACCTTTTCAATGTTTAATTTTTTTGAAATAAGCTTTGATAAAGCCTTCCTTGCAAATTCCGGATACATGAACATGTCGCCGACATGAATGAGTATTTTTTTTCAGTATTTTATATTTTTCTTAACAAGTTCTCAATACACCAATAGATGATAGAATATCACCCAATGCGTCCATGTTGTCTATTTCCAACAGGTCGCTATATCCACCAATAGAAGTTTTCCCAAAAAATATTCTAGGAACAGTTCTACCACCAGTAATTTGTTCAAAATAGTCGTGCAATTTATTTTCGGGTTTAAATTCTTTAATATCGACAATTTCATACGCTCCTCTTTTGAAACTAAACTTATTTAGAATATCCAGTGCGTTTCTACAAAAAGGACATGTAAACTTGACAAAAATTGTGACTTTGTTATTGGTCAACCTTTGTTGTACAAATTCCTCGGCCATTTTAATATTTAAGTGATATAAAACATTCTCGACTTATTTAACTCTTTAGTCGAGATATATGGACGCAGATAGCTATATGATAACCAACTACAGAAGGCGAACGCTATAAAAAACATAATTACGACGAGCATATTCATAAATATTTTTATTCAGCATTACTTGATATAGTAATATTAGGCACAGTCAAACATTCAACCACTCTCGATACATCAACTCTCTCATTTTCTTCAACAAATTCTGCAATATCTTCGTAAAAAGATTCTTGAAACTTTTCAGAATATCTATCGACTTCAGATGAAATAGCGTTCGTCAACATACTATGTTTTGTATACATAAAGGCGCCCATTTCAACAGTTTCTAGTGACAAAATGCTAGCGATCCTAGGATCCTTCAGAATCACATAGATTGACGATTCGTCTCTCTTAGTAACTCTAGTAAAATAATCATACAATCTAGTACTCGAAATAATATTATCCTTGACTTGAGGAGATCTAAACGATCTAGTTTTGAGAACATCGATAAGTTCATCGGGAATGACATACATACTATCTTTAATAGAACTCTTTTCATCCAGTTGAATGGACTCGTCCTTAACCAACTGATTAATAAGATCTTCTATTTTATCATTTTCCAGATGATATGTATGTCCATTAAAGTTAAATTGTGTAGCGCTTCTTTTTAGTCTAGCAGCCAATACTTTAACATCACTAATATCAATATACAAAGGAGATGATTTATCGACGGTATTAAGAATTCGTTTTTCAACATCCGTCAAAACCAATTCCTTTTTGCCTGTATCATCCAGTTTGCCATTCTTTGTAAAGAAATTATTTTCTACTAGACTATTAATAAGACTGATAAGGATTCCTCCATAATTGCACAATCCAAACTTTTTTACAAAACTAGACTTTACGAGATCTACAGGAATGCGTACTTCAGGTTTCTTAGCTTGTGATTTTTTCTTTTGTGGACATTTTCTAGTGACCAACTCATCTACCATTTCATTGATTTTAGCAGTGAAATAAGCTTTCAATGCACGGGCACTGATACTATTAAAAACGAGTTGATCTTCAAATTCCACCATTTAAGTTCACCAAACAACTTTTAAATACAAATATATCAATAGTAGTAGAATAAGAACTATAAAAAAAATAATAATTAACCAATACCAACCCCAACAACCGGTATTATTAGTTGATGTGACAGTTTTCTCATCACTTAGAACAGATTTAACAATTTCTATAAAGTCTGTCAAATCATCTTCCGGAGAACCCATAAATACACCAAATATAGCGGCGTACAACTTATCCATTTATACATTGAATATTGGCTTTTCTTTATCACTATCTTCATCATATTCATCATCGATATCAACAAGTCCCAAATTACGAGCCAAATCTTCTTCTACATTTTCAGTCATTGATACACGTTCACTATCTCCAGAGAGTCCGATAACGTTAGCCACCACTTCTCTATCAATGATTAGTTTCTTAAGTGCAAATGTAATTTTTGTTTCAGTTCCGGATCTATAGAAAACTACAGGTGTGATAATTGCCTTGGCTAATTGTCTTTCTCTTTTACTGAGTGATTCTAGTTCACCTTCTATAGATCTGAGAATGGATAATTCTCCAGTCGAAACATATTCTACCATGGCTCCGTTTAATTTGTTAATGAAGATGGATTCATCCTTAAATGTTTTCTCTGTAATAGTTTCCACCAAAAGACTATGCAAAGAATTTGGAATGCGTTCCTTGTGCTTAATGTTTCCATAGACAGCTTCTAGAAGTTGATACAACATAGGACTAGCCGCGGTAACTTTTATTTTTAGAAAGTATCCATCACTTCTATCTTGTTTAGATTTATTTTTATAAAGTTTAGTCTCTCCTTCCAACATAATAAAAGTGGAAGTCATTTGACTAGATAAACTATCAGTAAGTTTTATAGAGATAGATGAACAATTAGCGTATTGAGAAGCATTTAGTGTGACGTATTCGATACATTTTGCATTAGATTTACTAATCGATTTTGCATACTCTATAACACCCGCACAAGTCTGTAGAGAATCGCTAGATGCTGTAGGTCTTGGTGAAGTTTCAACTCTCTTTTTGATTACCTTACTCATGATTAAACCTAAATAATTATACTTTGTAATATAATGATATATATTTTCACTTTATCTCATTTGAAAATAAAAATGTTTTTGTTTAACCGCTGCATGATGTACAGATTTCGGAATCACAAACCACCAGTGGXTGACAAACATTTTTGTTGGTGTAATAAAAAAAATTATTTAACTTTTCATTAATAGGGATTTGATGTACGTAGCGTACAAAATTATCGTTCCTGGTATATAGATAAAGAGTCCTATATATTTGAAAATCGTTACGGCTCGATTAAACTTTAATGATTGCATTGTGAATATATCATTAGGATTTAACTCCTTGACTATCATGGCGGTGCCAGAAATTACCATCAAAAGCATTAATACAGTTATACCGATCGCAGTTAGAACGGTTATAGCATCCGCCATTTATATCTAAAAATTAGATCAAAGAATATGTGACAAAGTCCTAGTTGTATACTGAGAATTAACGAAACAATGTTTCTTACATATTTTTTTCTTATTAGTAACTGACTTAATAGTAGGAACTGGAAAGCTAGACTTGATTATTCTATAAGTATAAATACCCTTCCAAATAATATTCTCTTTGATAAAAGTTCCAGAAAATGTAGAATTTTTTAAAAAGTTATCTTTTGCTATTACCAAGATTGTGTTTAGACGCTTATTATTAATATGAGTGATAAAATCCACACCGCCTCTAGATATAGCTTTTATTTCCACATTAGATGGTAAATCCAATAGTGAAACTATCTTTTTAGGAATGTATGGACTCGCGTTTAGAGGAGTGAACGTCTTAGGCGTCGGAAAGGATGATTCATCAAACGAATAAACAATTTCACAAATGGATGTTAATGTATTAGTAGGAAATTTTTTGACGCTAGTGGAATTGAAAATTCTAATGGATGATGTTCTACCTATTTCATCAGATAACATGTTAATTTCCGACATCAACGGTTTTAATATTTCGATAATATACGGTAATCTCTCTTTCGGACTTATATAGCTTATTCCACAATACGAGTCATTATATACTCCAAAAAACAAAATAACTAGTATAAAATCTGTATCGAATGGGAAAAACGAAATTATCGACATAGGTATAGAATCCGGAACATTGAACGTATTAATACTTAATTCTTTTTCAGTGGTAAGTACCGATAGGTTATTGACATTGTATGGTTTTAAATATTCTATAACTTGAGACTTGATAGATATTAATGACGAATTGAAAATTATTTTTATCACCACGTGTGTTTCAGGATCGTCATCGATACCCGTCAACCAACCAAACGGAGTAAAATAAATATCATTAATATATGCTCTAGATATTAGTATTTTTATTAATCCTTTGATTATCATCTTCTCGTACGCGAATGATTCCATGATCAAGAGTGATTTGAGAACATCCTCCGGAGTATTAATGGGCTTAGTAAACAGTCCATCGTTGCAATAATAAAAGTTATCCAAGTTAAAGGATATTATGCATTCGTTTAAAGATATTACCTCATCTGACGGAGACAATTTTTTGGTAGGTTTTAGAGACTTTGAAGCTACTTGTTTAACAAAGTTATTCATCGTCGTCTACTATTCTATTTAATTTTGTAGTTAATTTATCACATATCACATTAATTGACTTTTTGGTCCACTTTTCCATACGTTTATATTCTTTTAATCCTGCGTTATCCGTTTCCGTTATATCCAGGGATAGATCTTGCAAGTTAAATAGAATGCTCTTAAATAATGTCATTTTTTTATCCGCTAAAAATTTAAAGAATGTATAAACCTTTTTTAGAGATTTGAAACTCTTAGGTGGTGTCCTAGTACACAATATCATAAACAAACTAATAAACATTCCACATTCAGATTCCAACAGTTGATTAACTTCCACATTAATACAGCCTATTTTCGCTCCAAATATACATTCGAAAAATCTGAATAAAACATCAATGTCGCAATTTGTATTATCCAATACAGAATGTCTGTGATTCGTGTTAAAACCATCGGAGAAGGAATAGAAATAAAAATTATTATAGTGGTGGAATTCAGTTGGAATATTGCCTCCGGAGTCATAAAAGGATACTAAACATTGTTTTTTATCATAAATTACACATTTCCAATGAGACAAATAACAAAATCCAAACATTACAAATCTAGAGGTAGAACTTTTAATTTTGTCTTTAAGTATATACGATAAGATATGTTTATTCATAAACGCGTCAAATTTTTCATGAATCGCTAAGGAGTTTAAGAATCTCATGTCAAATTGTCCTATATAATCCACTTCGGATCCATAAGCAAACTGAGAGACTAAGTTCTTAATACTTCGATTGCTCATCCAGGCTCCTCTCTCAGGCTCTATTTTCATCTTGACGACCTTTGGATTTTCACCAGTATGTATTCCTTTACGTGATAAATCATCGATTTTCAAATCCATTTGTGAGAAGTCTATTGCCTTAGATACTTTTTCCCGTAGTCGAGGTTTAAAGAAATACGCTAACGGTATACTAGTAGGTAACTCAAAGACATCATATATAGAATGATAACGCGTCTTTAACTCATCGGTTAACTCTTTCTTTTGATCGAGTTCGTCGCTACTATTGGGTCTGCTCAGGTGCCTCGACTCTACTAGTTCCAACATCATACCAATAGGAATACAAGACACTTTGCCAGCGGTTGTAGATTTATCATATTTCTCCACTACATATCCGTTACAATTTGTTAAGAATTTAGATACATCTATATTGCTACATAATCCAGCTAGTGAATATATATGACATAATAAATTGGTAAATCCTAGTTCTGGTATTTTACTAATTACTAAATCTGTATATCTTTCCATTTATCATGGAAAAGAATTTACCAGATATCTTCTTTTTTCCAAACTGCGTTAATGTATTCTCTTACAAATATTCACAAGATGAATTTAGTAATATGAGTAACATGGAACGTGATAGTTTCTCTTTGGCGGTGTTTCCAGTTATAAAACATAGATGGCATAACGCACACGTTGTAAAACATAAAGGAATATACAAAGTTAGTACAGAAGCACATGGAAAAAAAGTATCTCCTCCATCACTAGGAAAACCCTCACACATAAACCTAACCGCGAAGCAATATATATACAGTGAACACACAATAAGCTTTGAATGTTATAGTTTTCTAAAATGTATAACAAATGCAGAAATTAATTCGTTCGATGAGTATATATTAAGAGGACTATTAGAAGCTGGTAATAGTTTACAGATATTTTCCAATTCCGTAGGTAAACGAACAGATACTATAGGTGTACTAGGGAATAAGTATCCATTTAGCAAAATTCCATTGGCCTCATTAACTCCTAAAGCACAACGAGAGATATTTTCAGCATGGATTTCTCATAGACCCGTAGTTTTAACTGGAGGAACCGGAGTGGGTAAGACGTCACAGGTACCCAAGTTATTGCTTTGGTTTAATTATTTATTTGGTGGATTCTCTACTCTAGATAAAATCACTGACTTTCACGAAAGACCAGTCATTCTATCTCTTCCTAGGATAGCTTTAGTTAGATTGCATAGCAATACCATTTTAAAATCATTGGGATTTAAGGTACTAGATGGATCTCCTATTTCTTTACGGTACGGATCTATACCGGAAGAATTAATAAACAAACAACCAAAAAAATATGGAATTGTATTTTCTACCCATAAGTTATCTCTAACAAAACTATTTAGTTATGGCACTCTTATTATAGACGAAGTTCATGAGCATGATCAAATAGGAGATATTATTATAGCAGTAGCGAGAAAGCATCATACGAAAATAGATTCTATGTTTTTAATGACTGCCACGTTAGAGGATGACAGGGAACGGCTAAAAGTATTTTTACCTAATCCCGCATTTATACATATTCCTGGAGATACACTGTTTAAAATTAGCGAGGTATTTATTCATAATAAGATAAATCCATCTTCCAGAATGGCATACATAGAAGAAGAAAAGAGAAATTTAGTTACTGCTATACAGATGTATACTCCTCCTGATGGATCATCTGGTATAGTCTTTGTGGCATCCGTTGCACAGTGTCACGAATATAAATCATATTTAGAAAAAAGATTACCGTATGATATGTATATTATTCATGGTAAGGTCTTAGAAATAGACAAAATATTAGAAAAAGTGTATTCATCGCCTAATGTATCGATAATTATTTCTGCTCCTTATTTGGAATCCAGCGTTACTATACACAATGTTACTCACATTTATGATATGGGTAGAGTTTTTGTTCCCGCTCCTTTTGGAGGATCACAACAATTTATTTCTAAATCTATGAGAGATCAACGAAAAGGAAGAGTAGGAAGAGTTAATCCTGGTACATACGTCTATTTCTATGATCTGTCTTATATGAAGTCTATACAGCGAATAAATTCAGAATTTCTACATAATTATATATTGTACGCTAATAAGTTTAATTTAACACTTCCCGAAGATTTGTTTATAATCCCTACAAATTTGGATATTCTATGGCGTACAAAGGAATATATAGACTCGTTCGATATTAGTACAGAAACATGGAATAAATTATTATCCAATTATTATATGAAGATGATAGAGTATGCTAAACTTTATGTACTAAGTCCTATTCTCGCTGAGGAGTTGGATAACTTTGAGAGGACGGGAGAATTAACTAGTATTGTACAAGAAGCCATTTTATCTCTAAATTTACGAATTAAGATTTTAAATTTTAAACATAAAGATAATGATACGTATATACACTTTTGTAAAATATTATTTGGTGTCTATAACGGAACAAACGCTACTATATATTATCATAGACCTCTAACGGGATATATGAATATGATTTCAGATACTATATTTGTTCCTGTAGATAATAACTAAAAATCAAACTCTAATGACCACATCTTTTTTTAGAGATGAAAAATTTTCCACATCTCCTTTTGTAGATATGACTAAACATTTTGCAGAAAAAAGTTTATTAGTGTTTAGATAATCGTATACTTCATCAGTGTAGATAGTAAATGTGAACAGATAAAAGGTATTCTTGCTCAATAGATTGGTAAATTCCATAGAATATATTAATCCTTTTTTCTTGAGATCCCACATCATTTCAACCAGAGAAGTTTTATCCAATAATTTACCTCGTACTATACCACATACAAAACTAGATTTTGTAGTGACGTCGTACCTGGCATTCCTACCAAACAAAATTTTACTTTTAGTTTTTTTAGAAAATTCTAAGGTAGAATCTCTATTTGTCAATATGTCATCTATGGAATTACCACTAGCAAAAAATGATAGAAATATATATTGATACATCGCAGCTGGTTTTGATCTACTATATTTTAAAAACGAATCAGATTCCATAATTGCCTGTATATCATCAGCTGAAAAACTATGTTTTACACGTATTCCTTCGGCATTTCTTTTTAATGATATATCTTGTTTAGACAATGATAAAGTTATCATGTCCATGAGAGACGCGTCTCCGTATCGTATAAATATTTCATTAGATGTTAGACGCTTCATTAGGGGTATACTTCTATAAGGTTTCTTGATTAGCCCATCATTTGTTGCGTCAAGAACTACTATCGGATGTTGTTGGGTATCTCTAGTGTTACACATGGCCTTACTAAAGTTTGGGTAAATAACTATGATATCTCTATTAATTATAGATGTATATATTTCATTCGTCAAGGATATTAGTATCGACTTGCTATCGTCATTAATACGTGTAATGTAATCATATAAATCATGCGATAGCCAAGGAAAATTCAAATAGATGTTCATCATATAATCATCGCTATAATTCATATTAATACGTTGACATTGACTAATTTGTAATATAGCCTCGCCACGAAGAAAGCTCTCGTATTCAGTTTCATCAATAAAGGATACCGTTAAATATAACTGGTTGCCGATTGTCTCATAGTCTATTAAGTGGTAAGTTTCGTACAAATACAGAATCCCTAAAATATTATCTAATGTTGGATTAATCTTTACCATAACTGTATAAAATGGAGACGGAGTCATAACTATTTTACCGTTTGTACTTACTGGAATAGATGAAGGAATAATCTCCGGACATGCTGGTAAAGACCCAAATGTCTGGTTGAAGAAATCCAATGTTCCGGGTCCTAATCTCTTGACAAAAATTACGATATTCGATCCCGATATCCTTTGCATTCTATTTACCAGCATATCACGAACTATATTAAGATTATCTATCATGTCTATTCTCCCACCGTTATATAAATCGCCTCCGCT

TCATTTCTAAAATAGTATTCGTTTTCTAATTCTTTAATGTGAAATCGTATACTAGAAAGGGAAAAATTATCTTTAAGTTTTCCGTTAGAAAAGAACCACGAAACTAATGTTCTGATTGCGTCTGACTCCGTCGCTGAATTAATGGATTTACACCAAAAACTCATATAACTTCTAGATGTAGAAGCATTAGCTAAAAAAATAGTAGAATCAAAGGATATAAGTAGATGTTCCAACAAGTGAGCAATTCCCAAAATTTCATCTATATCATTCTCAAATCCGAAATTAGAAATTCCCAAGTAGATATCTTTTTTCATCCGATCATTAATAAAAATACGAACTTTATTCGGTAAGACGATCATTTACTAAGGAGTAAAATAGGAAGTAATGTTCGTATATCGTTATCATCGTATAAATTAAAGGTGTGTTTTTTACCATTAAGTGACATTATAATTTTACCAATATTGGAATTATAATATAGGTATATTTGCGCACTTGCGACAGTTGATGCATCGGTAAATATAGCTGTATCTAATGTTCTAGTCGGTATTTCATCATTTCGCTGTATAATAATAGCGTTTTCTCTATCTGTTTCCATTACAGCTGCCTGAAGTTTATTGGTCGGATAATATGTAAAATAATAAGAAATACATACGAATAACAAAAATAAAATAAAATATAATAAAGATGCCATTTAGAGATCTAATTTTGTTCAACTTGTCCAAATTCCTACTTACAGAAGATAAGGAATCGTTGGAGATAGTGTCTTCCTTATGTAGAGGATTTGAAATATCTTATGATGACTTGATAACTTACTTTCCAGATAGAAAATATCATAAATATATTTATAAAGTATTTGAACATGTAGATTTATCGGAGGAATTAAGTATGGAATTCCATGATACAACTCTGCGAGATTTAGTCTATCTTAAATTGTATAAGTATTCCAAGTGTATACGACCGTGTTATAAATTAGGAGATAATCTAAAAGGCATAGTTGTTATAAAGGACAGAAATATATATATTAGAGAAGCAAATGATGACTTGATAGAATATCTCCTCAAGGAATACACTCCTCAGATTTATACATATTCTAATGAGCACGTTCCCATAGCTGGTTCAAAATTAATTCTTTGCGGATTTTCTCAAGTTACATTTATGGCGTATACAACGTCTCATATAACAACAAATAAAAAGGTAGATGTTCTCGTTTCCAAAAAATGTATAGATAAACTAGTCGATCCAATAAATTATCAAATACTTCAAAATTTATTTGATAAAGGAAGCGGAACAATAAACAAAATACTCAGGAAGATATTTTATTCGGTAACCGGTGGCCAAACTCCATAATTTGCTTTTTCTATTTCGGATTTTAGAATTTCCAAATTCACCAGCGATTTATCTGTTTTGGTGAAATCCAAGGATTTATTAATGTCCACAAATGCCATTTGTTTTGTCTGTGGATTGTATTTGAAAATGGAAACGATGTAGTTAGATAGATGCGCTGCAAAGTTTCCTATTAGGGTTCCGCGCTTTACGTCACCTAGCATACTTGAATCACCATCCTTTAAAAAAAATGATAAGATATCAACATGGAGTATATCATACTCTGATTTTAATTCTTCTACTGCCTCACTGACATTTTCACAAATACTACAATACGGTTTACCGAAAATAATCAGTACGTTCTTCATTTATGGGTATCAAAAACTTAAAATCGTTACTGCTGGAAAATAAATCACTGACGATATTAGATGATAATTTATACAAAGTATACAATGGAATATTTGTGGATACAATGAGTATTTATATAGCCGTCGCCAATTGTGTCCGAAACTTAGAAGAGTTAACTACGGTATTCATAAAATACGTAAACGGATGGGTAAAAAAGGGAGGGCATGTAACCCTTTTTATCGATAGAGGAAGTATAAAAATTAAACAAAACGTTAGAGACAAAAGACGTAAATATTCTAAATCAACCAAGGACAGAAAAATGTTAGAATTAGAAAAGTGTACATCCAAAATACAAAATGTTACCGGATTTATGGAAGAAGAAATAAAGGCAGAAATACAATTAAAAATCGATAAACTCACATTTCAAATATATTTATCTGATTCTGATAACATAAAAATATCATTGAATGAGATACTAACACATTTCAACAATAATGAGAATGTTACATTATTTTATTGTGATGAACGAGATGCAGAATTTGTTATGTGTCTAGAGGCTAAAACATATTTCTTTACCACAGGAGAATGGCCGTTGATAATAAGTACCGATCAGGATACTATGCTATTTGCGTCTGTTGATAATCATCCTAAGATGATAAAAAACTTAACTCAACTGTTTAAATTTGTTCCATCTGCAGAGGATAACTATTTAGCAAAATTAACGGCGTTAGTGAATGGATGTGATTTCTTTCCTGGACTCTATGGGGCATCTATAACACCCACCAACTTAAACAAAATACAATTGTTTAGTGATTTTACAATCAATAATATAGTCACTAGTTTGGCAATTAAAAATTATTATAGAAAGACTAACTCTACCGTAGACGTGCGTAATATTGTTACGTTTATAAACGATTACGCTAATTTAGACGATGTCTACTCGTATATTCCTCCTTGTCAATGCACTGTTCAAGAATTTATATTTTCCGCATTAGATGAAAAATGGAACGATTTTAAATCATCTTATTTAGAGACCGTTCCGTTACCCTGTCAATTAATGTACGCGTTAGAACCACGTAAGGAGATTGATGTTTCAGAAGTTAAAACTTTATCATCTTATATAGATTTCGAAAATACTAAATCAGATATCGATGTTATAAAATCTATATCCTCGATCTTTGGATATTCTAACGAAAACTGTAACACGATAGTGTTTGGCATCTATAAGGATAATTTACTACTGAGTATAAATAGTTCATTTTACTTTAACAATAGTCTGTTAATAACCAATACTAAAAGTGATAATATAATAAATATAGGTTACTAGATTAAAAATGGTTTTCCAAGTAGTGTGCTCTACATGCGGTAAAGATATTTCTCACGAACGATATAAATTGATTATACGAAAAAAATCATTAAAGGATGTACTCGTTAGTGTAAAGAACAAATGTTGTAGGTTAAAATTATCTACACAAATAGAACCTCAACGTAACTTAACAGTGCAACCTCTATTGGATATAAACTAATATGGATCCGGTTAATTTTATCAAGACATATGCACCTAGAGGTTCTATTATTTTTATTAATTATACTATGTCATTAACAAGTCATTTAAATCCATCGATAGAAAAACATGTGGGTATTTATTATGGTACGTTATTATCGGAACACTTGGTAGTTGAATCAACATATAGAAAAGGAGTTCAAATAGTCCCATTGGATAGTTTTTTTGAAGGATATCTTAGTGCAAAAGTATACATGTTAGAGAATATTCAAGTTATGAAAATAGCAGCTGATACGTCATTAACTTTACTGGGTATTCCATATGGATTTGGTCATAATAGAATGTATTGTTTTAAATTGGTAGCTGAATGTTATAAAAATGCCGGTATTAATACATCATCTAAACGAATATTGGGCAAAGATATTTTTCTAAGCCAAAACTTCACAGATGATAATAGATGGATAAAGATATATGATTCTAATAATTTAACATTTTGGCAAATTGATTACCTTAAAGGGTGAGTTAATATGCATAACTACTCCTCCGTTGTTTTTTCCCTCGTTCTTTTTCTTAACGTTGTTTGCCATCACTCTCATAATGTAAAGATATTCTAAAATGGTAAACTTTTGCATATCGGACGCAGAAATTGGTATAAATGTTGTAATTGTATTATTTCCCGTCAATGGACTAGTCACAGCTCCATCAGTTTTATATCCTTTAGAGTATTTCTCACTAGTGTCTAGCATTTTAGAGCATTCCATGATCTGTTTATCATTGATATTGGCCGGAAAGATAGATTTTTTATTTTTTATTATATTACTATTGGCAATTGTAGATATAACTTCTGGTAAATATTTTTCTACCTTTTCAATCTCTTCTATTTTCAAGCCGGCTATATATTCTGCTATATTATTGCTAGTATCAATACCTTTTCTGGCTAAGAAGTCATATGTGGTATTAACTATATCAGTTTTAACTGGTAGTTCCATTAGCCTTTCCACTTCTGCAGAATAATTAGAAATTGGTTCTTTACCAGAAAATCCAGCTACTATAATAGGCTCACCGATGATCATTGGCAAAATCCTATATTGTACCAAATTAATGAGAGCATATTTCATTTCCAATAATTCTGCTAGTTCTTGAGACATTGATTTATTTGATGAATCTAGTTGGTTCTCTAGATACTCTACCATTTCTGCCGCATACAATAACTTGTTAGATAAAATCAGGGTTATCAAAGTGTTTAGCGTGGCTAGAATAGTGGGCTTACATGTATTAAAGAATGCGGTAGTATGAGTAAACCGTTTTAACGAATTATATAGTCTCCAGAAATCTGTGGCGTTGCATACATGAGCTGAATGACATCGAAGATTGTCCAATATTTTTAATAGCTGCTCTTTGTCCATTATTTCTATATTTGACTCGCAACAATTGTAAATACCATTAATCACTGATTCCTTTTTTGATGCCGGACAATAGCACAATTGTTTAGCTTTGGACTCTATGTATTCAGAATTAATAGATATATCTCTCAATACAGATTGTACTATACATTTTGAAACTATGTCAAAAATTGTAGAACGACGCTGTTCTGCAGCCATTTAACTTTAAATAATTTACAAAAATTTAAAATGAGTATCCGTATAAAAATCGATAAACTGCGCCAAATTGTGGCATATTTTTCAGAGTTCAGTGAAGAAGTGTCTATAAATGTAGACTCAACGGATGAGTTAATGTATATTTTTGCCGCCTTGGGCGGATCTGTAAACATTTGGGCCATTATACCTCTTAGTGCATCAGTGTTCTACCGCGGAGCCGAAAATATTGTGTTTAATCTTCCGGTGTCCAAGGTAAAATCGTGTTTGTGTAGTTTTCACAATGATGCCATCATAGATATAGAACCTGATCTGGAAAATAATCTAGTAAAACTTTCTAGTTATCATGTAGTAAGTGTCGATTGTAACAAGGAACTGATGCCTATTAGGACAGATACTACTATTTGTCTAAGTATAGATCAAAAGAAATCTTACGTGTTTAATTTTCACAAGTATGAAGAAAAATGTTGTGGTAGAACCGTCATTCATCTAGAATGGTTGTTGGGCTTTATTAAGTGTATTAGTCAGCATCAGCATTTGACTATTATGTTTAAAGATGACAATATTATTATGAAGACTCCTGGTAATACTGATGCTTTTTCCAGGGAATATTCTATGACTGAATGTTCTCAAGAACTACAAAAGTTTTCTTTCAAAATAGCTATCTCGTCTCTCAACAAACTACGAGGATTCAAAAAGAGAGTCAATGTTTTTGAAACCAGAATCGTAATGGATAATGACGATAACATTCTAGGAATGTTGTTTTCGGATAGAGTTCAATCCTTTAAGATCAACATCTTTATGGCGTTTTTAGATTAATACTTTCAATGAGATAAATATGGGTGGCGGAGTAAGTGTTGAGCTCCCTAAACGGGATCCGCCTCCGGGAGTACCCACTGATGAGATGTTATTAAATGTTGATAAAATGCATGACGTGATAGCTCCCGCTAAGCTTTTAGAATATGTGCATATAGGACCACTAGCAAAAGATAAAGAGGATAAAGTAAAGAAAAGATATCCAGAGTTTAGATTAGTTAATACAGGACCCGGTGGTCTTTCGGCATTATTAAGACAATCGTATAATGGAACCGCACCCAACTGCTGTCACACTTTTAATCGTACTCATTATTGGAAGAAGGATGGAAAGATATCAGATAAGTATGAAGAGGGTGCAGTATTAGAATCGTGTTGGCCAGACGTTCACGATACCGGAAAATGCGATGTTAATTTATTTGACTGGTGTCAGGGGGATACGTTCGATAGAAACATATGCCATCAGTGGATCGGTTCAGCATTTAATAGGAGTGATAGAACTGTAGAGGGTCAACAATCGTTAATAAATCTGTATAATAAGATGCAAACATTATGTAGTAAAGATGCTAGTGTACCAATATGCGAATCATTTTTGCATCATTTACGCGCACACAATACAGAAGATAGCAAAGAGATGATCGATTATATTCTAAGACAACAGTCTGCGAACTTTAAACAGAAATATATGAGATGTAGTTATCCCACTAGAGATAAGTTAGAAGAGTCATTAAAATATGCGGAACCTCGAGAATGTTGGGATCCAGAGTGTTCGAATGCCAATGTTAATTTCTTACTAACACGTAATTATAATAATTTAGGACTTTGTAATATTGTACGATGTAATACCAGCGTGAACAACTTACAGATGGATAAAACTTCCTCATTAAGATTGTCATGTGGATTAAGCAATAGTGATAGATTTTCTACTGTTCCCGTCAATAGAGCAAAAGTAGTTCAACATAATATTAAACACTCGTTCGATCTAAAATTGCATTTGATTAGTTTATTATCTCTCTTGGTAATATGGATACTAATTGTAGCTATTTAAATGGGTGCCGCGGCAAGCATACAGACGACGGTGAATACACTCAGCGAACGTATCTCGTCTAAATTAGAACAAGAAGCGAATGCTAGTGCTCAAACAAAATGTGATATAGAAATCGGAAATTTTTATATCCGACAAAACCATGGATGTAACCTCACTGTTAAAAATATGTGCTCTGCAGATGCGGATGCTCAGTTGGATGCTGTGTTATCAGCCGCTACAGAAACATATAGTGGATTAACACCGGAACAAAAAGCATACGTACCAGCTATGTTTACGGCTGCGTTAAACATTCAGACGAGTGTAAACACTGTTGTTAGAGATTTTGAAAATTATGTGAAACAGACTTGTAATTCTAGCGCGGTCGTCGATAACAAATTAAAGATACAAAACGTAATTATAGATGAATGTTACGGAGCCCCAGGATCTCCAACAAATTTGGAATTTATTAATACAGGATCTAGCAAAGGAAATTGTGCCATTAAGGCGTTGATGCAATTGACGACTAAGGCCACTACTCAAATAGCACCTAGACAAGTTGCTGGTACAGGAGTTCAGTTTTATATGATTGTTATCGGTGTTATAATATTGGCAGCGTTGTTTATGTACTATGCCAAGCGTATGCTGTTCACGTCCACCAATGATAAAATCAAACTTATTTTAGCCAATAAGGAAAACGTCCATTGGACTACTTACATGGACACATTCTTTAGAACTTCTCCAATGGTTATTGCTACCACGGATATACAAAACTGAAAATATATTGATAATATTTTAATAGATTAACATGGAAGTTATCGCTGATCGTCTAGATGATATAGTGAAACAAAATATAGCGGATGAAAAATTTGTAGATTTTGTTATACACGGTCTAGAGCATCAATGTCCTGCTATACTTCGGCCATTAATTAGGTTGTTTATTGATATACTATTATTTGTTATAGTAATTTATATTTTTACGGTACGTCTAGTAAGTAGAAATTATCAAATGTTGTTGGCGTTATTGGCGCTAGTCATCTCATTAACTATTTTTTATTACTTTATACTATAATAGTACTAGACTGACTTCTAACAAACATCTCACCTGCCATAAATAAATGCTTGATATTAAAGTCTTCTATTTCTAACACTATTCCATCTGTGGAAAATAATACTCTGACATTATCGCTAATTGACACATCGGTGAGTGATATGCCTATAAAGTAATAATCTTCTTTGGGCACATATACCAGTGTACCAGGTTCTAACAACCTATTTACTGGTGCTCCTGTAGCATACTTTTTCTTTACCTTAAGAATATCCATCGTTTGCTTGGTCAATAGCGACAGGTGATTTTTTATCAACCATTCAAAAAAGTAATTGGAGTGTTCATATCCTCTACAGGCTATTGTCTCATGACCGTGTATGAAATTTAAGTAACACGACTGTGGTAGATTTGTTCTATAGAGCCGGTTGCCGCAAATAGATAGAACTACCAATATGTCTGTACAAATGTTAAACATTAATTGATTAACAGAAAAAACAATGTTCGTTCTGGGAATAGAAACCAAATCAAAACAAAATTCATTAGAATATATGTCACGTTTATACATCGAATATAAAATAACTACAGTTTGAAAAATAACAGTATCATTTAAACATTTAACTTGCGGGGTTAATCTCACAACTTTACTGTTTTTGAACTGTTCAAAATATAGCATAGATCCGTGAGAAATACGTTTAGCCGCCTTTAATAGAGGAAATCCCACCGCCTTTTTGGATCTTACCAACGACGATAGTTCTGACCAGCAACTCATTTCTTCATCATCCACCTGTTTTAACATATAATAGGCAGGAGATAGATATCCGTCATTGCAATATTCCTTCTCGTAGGCACACAATCGAATATTGATAAAATCTCCATTCTCTTCTCTGCATTTATTATCTTGTCTTGGTGGTGGGCTAGGCTGTAGTCTTGGTCTATTATTACCATCGTTGTTGAATCTATTTCTGTCATTAAATCTTTCATTTCTTCCTGGTATATTTTTATTACCTCGCTTGGTTGGATTTTTGTCTATATTATCGTTTGTAACATCGGTACGGGTATTCATTTATCACAAAAAAAACTTCTCTAAATGAGTCTACTGCTAGAAAACCTCATCGAAGAAGATACCATATTTTTTGCAGGAAGTATATCTGAGTATGATGATTTACAAATGGTTATTGCCGGTGCAAAATCCAAATTTCCAAGATCTATGCTTTCTATTTTTAATATAGTACCTAGAACAATGTCAAAATATGAGTTGGAGTTAATTCATAACGAGAATATCACAGGGGCAATGTTTACCACAATGTATAATATAAGAAACAATTTGGGTTTAGGTGATGATAAACTAACTATTGAAGCCATTGAAAACTATTTCTTGGATCCTAACAATGAGGTTATGCCTCTTATTATTAATAATACGGATATGACGGCCGTCATTCCTAAAAAAAGTGGTAGGAGAAAGAATAAGAACATGGTTATCTTCCGTCAAGGATCATCACCTATCTTGTGTATTTTCGAAACTCGTAAAAAGATTAATATTTATAAAGAAAATATGGAATCCGCATCGACTGAGTATACACCTATCGGAGACAACAAGGCTTTAATATCTAAATATGCGGGAATTAATGTCCTGAATGTGTATTCTCCTTCCACATCCATGAGATTGAATGCCATTTACGGATTCACCAATAAAAATAAACTAGAAAAACTTAGTACTAATAAGGAACTAGAATTGTATAGTTCTAGCCCTCTTCAAGAACCCATTAGGTTAAATGATTTTCTGGGACTATTGGAATGTGTTAAAAAGAATATTCCTCTAACAGATATTCCGACAAAGGATTGATTACTATAAATGGAGAATGTTCCTAATGTATACTTTAATCCTGTGTTTATAGAGCCCACGTTTAAACATTCTTTATTAAGTGTTTATAAACACAGATTAATAGTTTTATTTGAAGTATTTGTTGTATTCATTCTAATATATGTATTTTTTAGATCTGAATTAAATATGTTCTTCATGCATAAACGAAAAATACCCGATCCTATTGATAGATTACGACGTGCTAATCTAGCGTGTGAAGACGATAAATTAATGATCTATGGATTACCATGGATAACAACTCAAACATCTGCATTATCAATAAATAGTAAACCGATAGTGTATAAAGATTGTGCAAAGCTTTTGCGATCAATAAATGGATCACAACCAGTATCTCTTAACGATGTTCTTCGCAGATGATGATTCATTTTTTAAGTATTTGGCTAGTCAAGATGATGAATCTTCATTATCTGATATATTGCAAATCACTCAATATCTAGACTTTCTGTTATTATTATTGATCCAATCAAAAAATAAATTAGAAGCTGTTGGTCATTGTTATGAATCTCTTTCAGAGGAATACAGACAATTGACAAAATTCACAGACTCTCAAGATTTTAAAAAACTGTTTAACAAGGTCCCTATTGTTACAGATGGAAGGGTCAAACTTAATAAAGGATATTTGTTCGACTTTGTGATTAGTTTGATGCGATTCAAAAAAGAATCAGCTCTAGCTACCACCGCAATAGATCCTGTTAGATACATAGATCCTCGTCGCGATATCGCATTTTCTAACGTGATGGATATATTAAAGTCAAATAAAGCGAAAAATAATTATTCTTTATTGTCATCATGAACGGCGGGCATATTCAGTTGATAATTGGCCCCATGTTTTCAGGTAAAAGTACAGAATTAATTAGACGAGTTAGACGTTATCAAATAGCTCAATATAAATGCGTGACTATAAAATATTCTAACGATAATAGATACGGAACGGGACTATGGACGCATGATAAGAATAATTTTGAAGCATTGGAAGCAACTAAACTATGCGATGTCTTGGAAGCAATTACAGATTTCTCCGTGATAGGTATTGATGAAGGACAGTTCTTTCCAGACATTGTTGAATTCTGTGAGCGTATGGCAAACGAAGGAAAAATAGTTATAGTAGCCGCACTCGATGGGACATTTCAACGTAAACCGTTTAATAATATCTTGGATCTTATTCCATTATCTGAAATGGTGGTAAAACTAACTGCAGTGTGTATGAAATGCTTTAAGGAGGCGTCCTTTTCTAAACGATTAGGTACAGAAACCAAGATAGAAATAATAGGAGGAATTGATATGTATCAATCGGTGTGTAGAAAGTGTTACATCGACTCATAATATTATATTTTTTATCTAAAAAACTAAAAATAAACATTGATTAAATTTTAATATAATACTTAAAAATGGATGTTGTGTCGTTAGATAAACCGTTTATGTATTTTGAGGAAATTGATAATGAGTTAGATTACGAACCAGAAAGTGCAAATGAGGTAGCAAAAAAACTGCCGTATCAAGGACAGTTAAAACTATTACTAGGAGAATTATTTTTTCTTAGTAAGTTACAGCGACACGGTATATTAGATGGTGCCACCGTAGTGTATATAGGATCTGCTCCCGGTACACATATACGTTATTTGAGAGATCATTTCTATAATTTAGGAGTGATCATCAAATGGATGCTAATTGACGGCCGTCATCATGATCCTATTCTAAATGGATTACGTGATGTGACTCTAGTAACTCGGTTCGTTGATGAGGAATATCTACGATCCATCAAAAAACAACTGCATCCTTCTAAGATTATTTTAATTTCTGATGTGCGATCCAAACGAGGAGGAAATGAACCTAGTACTGAGGATTTACTAAGTAATTACGCTCTACAAAATGTCATGATTAGTATTTTAAACCCCGTAGCGTCTAGTCTTAAATGGAGATGCCCGTTTCCAGATCAATGGATTAAGGACTTTTATATCCCACACGGTAATAAAATGTCACAACCTTTTGCTCCTTCATATTCAGCTGAAATGAGATTATTAAGTATTTATACCGGTGAGAACATGAGACTTACTCGAGTTACCAAATCAGATGCTGTAAATTATGAAAAAAAGATGTATTACCTTAATAAGATAGTTCGCAACAAAGTAGTTGTTAACTTTGATTATCCTAATCAGGAATATGACTATTTTCACATGTACTTTATGTTGAGGACCGTATACTGCAATAAAACATTTCCTACTACTAAAGCAAAGGTACTATTTCTACAACAATCTATATTTCGTTTCTTAAATATTCCAACAACATCAACTGAAAAAGTTAGTCATGAACCAATACAATGTAAAGTATCTAGCAAAGATTCTGTGTATAAAAACAGAAATAGCAAGAGATCCGTACGCGGTAATAAATAGAAATGTGCTACTAAGATATACTACCGATATACAGTATAATGATTTAGTTACTTTAATAACCGTTAGACATAAAATTGATTCTATGAAAACTGTGTTTCAGGTATTTAACGAATCATCCATAAATTATACTCCGGTCGATGATGATTACGGAGAACCAATCATTATAACATCGTATCTTCAAAAAGGTCATAACAAGTTTCCTGTAAATTTTCTATACATAGATGTAGTAATATCTGATTTATTTCCTAGCTTTGTTAGACTAAATACTACAGAAACTAATATAGTTAATAGTGTACTACAAACAGGCGATGGTAAAAAGACTCTTCGTCTTCCCAAAATGTTAGAGACGGAAATAGTTGTCAAGATTCTCTATCGTCCTAATATACCATTAAAAATTGTTAGATTTTTCCGCAATAACATGATAACTGGTGTAGAGATAGCCGATAGATCTGTTATTTCAGTCGCTGATTAATCAATTAGTAGAGATGAGATAAGAACATAATAATATATCTTATATCTCGTTTAGAAAAATGCTAATATTAAAATAGCTAACGCTAGTAATCCAATCGGAAGCCATTTGATATCTATAATAGGGTATCTAATTTCCTGATTCAGATAGCGGACAGCTATATTCTCTGTAGCTACTCGTTTGGAATCACAAACATTATTTACATCTAATTTACTATCTGTAATGGAAACGTTTCCCAATGAAATGGTACAATCAGATACATTACATCTTGATATATTTTTTTTTAAAGAGGCTGGTAACAACGCATCGCTTCGTTTACATGGCTCGTACCAACAGTAATAGGGTAATCTTGTATCTATTCCTATCCGTACTATGCTTTTATCAGGATAAATACATTTACATCGTATATCGTCTTTGTTAGCATCACAGAATGCATAAATTTGTTCGTCTGTCATGATAAAAATTTAAAGTGTAAATATAACTATTATTTTTATAGTTGTAATAAAAAGGGAAATTTAATTGTATACCTTTGGTTCTTTAAAAGAAACTGACTTGATAAAAATGGCTGTAATCTCTAAGGTTACGTATAGTCTATATGATCAAAAAGAGATTAATGCTACAGATATTATCATTAGTCATGTTAAAAATGACGACGATATCGGTACCGTTAAAGATGGTAGACTAGGTGCTATGGATGGGGCATTATGTAAGACTTGTGGGAAAACGGAATTGGAATGTTTCGGTCACTGGGGTAAAGTAAGTATCTATAAAACTCATATAGTTAAGCCTGAATTTATTTCAGAAATTATTCGTTTACTGAATCATATATGTATTCACTGCGGATTATTGCGTTCACGAGAACCGTATTCCAACGATATTAACCTAAAAGAGTTATCGGGACACGCTCTTAGGAGATTAAAGGATAAAATATTATCCAAGAAAAAGTCATGTTGGAATAGCGAATGTATGCAACCGTATCAAAAAATTTCTTTTTCAAAGAAAAAGGTTTGTTTCGTCAACAAGTTGGATGATATTAACGTTCCTAATTCTCTCATTTATCAAAAGTTAATTTCTATTCATGAAAAGTTTTGGCCATTATTAGAAATTTATCAATATCCAGCTAACTTATTTTATACAGACTACTTTCCTATCCCTCCGTTGATTATTAGACCGGCTATTAGTTTTTGGATAGATAGTATACCCAAAGAGACCAATGAATTAACTTACTTATTAGGTATGATCGTTAAGAATTGTAACTTGAATGCTGATGAACAGGTTATCCAAAAGGCGGTAATAGAATACGATGATATTAAAATTATTTCTAATAACACTACCAGTATCAATTTATCATATATCACATCCGGCAAAAATAATATGATTAGAAGTTATATCGTCGCCCGGCGAAAAGATCAGACCGCTAGATCTGTAATTGGTCCCAGTACATCTATCACAGTTAATGAGGTAGGAATGCCCGCATATATTAGAAATACACTTACAGAAAAGATATTTGTTAATGCCTTTACAGTGAATAAAGTTAAACAACTATTAGCGTCAAACCAAGTTAAATTTTACTTTAATAAACGATTAAACCAATTAACAAGAATACGTCAAGGAAAGTTTATCAAAAATAAAATACATTTATTGCCTGGTGATTGGGTAGAAGTAGCTGTTCAAGAATATACAAGTATTATTTTTGGAAGACAGCCGTCGCTACATAGATACAACGTCATCGCTTCATCTATCAGAGCTACCGAAGGAGATACTATCAAAATATCTCCCGGAATTGCCAACTCTCAAAATGCTGATTTCGACGGAGATGAAGAATGGATGATATTGGAGCAAAATCCTAAAGCCGTAGTTGAACAGAGTATTCTTATGTATCCGACAACGTTACTCAAACACGATATTCATGGAGCCCCCGTTTATGGATCTATTCAAGATGAAATCGTAGCAGCATATTCATTGTTTAGGATACAAGATCTTTGTTTAGATGAAGTATTGAACATCTTGGGGAAATATGGAAGAGAGTTCGATCCTAAAGGTAAATGTAAATTCAGTGGTAAAGATATCTATACTTACTTAATAGGTGAAAAGATTAATTATCCGGGTCTCTTAAAGGATGGCGAAATTATTGCAAACGATGTAGATAGTAATTTTGTTGTGGCTATGAGGCATCTGTCATTGGCTGGACTCTTATCCGATCATAAGTCGAACGTGGAAGGTATCAACTTTATTATCAAGTCATCTTATGTTTTTAAGAGATATCTATCTATTTACGGTTTTGGGGTGACATTCAAAGATTTGAGACCAAATTCGACGTTCACTAATAAATTGGAGGCCATCAACGTAGAAAAAATAGAACTTATCAAAGAAGCATACGCCAAATATCTCAAAGATGTAAGAGACGGAAAAATAGTTCCATTATCTAAAGCTTTAGAGGCGGACTATGTGGAATCCATGTTATCCAACTTAACAAATCTTAATATCAGAGAGATAGAAGAACATATGAGACAAACGCTGATAGATAATCCAGATAATAACCTCCTGAAAATGGCCAAAGCGGGTTATAAAGTAAATCCCACAGAACTAATGTATATTTTAGGTACTTATGGACAACAGAGGATAGATGGCGAACCAGCAGAGACTCGAGTATTGGGTAGAGTCTTACCTTACTATCTTCCAGACTCTAAGGATCCAGAAGGAAGAGGTTATATTCTTAATTCTTTAACAAAAGGATTAACGGGTTCTCAATATTACTTTTCAATGCTGGTTGCAAGATCTCAATCTACTGATATTGTCTGTGAAACATCGCGTACCGGAACACTGGCTAGAAAAATCATTAAAAAGATGGAGGATATGGTGGTCGACGGATACGGACAAGTAGTTATAGGTAATACGCTCATCAAGTACGCCGCCAATTATACCAAAATTCTAGGCTCAGTATGTAAACCTGTAGATCTTATCTATCCAGATGAGTCCATGACTTGGTATTTGGAAATTAGTGCTCTGTGGAATAAAATAAAACAAGGATTCGTTTACTCTCAGAAACAGAAACTTGCAAAGAAGACATTGGCGCCGTTTAATTTCCTAGTATTTGTCAAACCCACCACTGAGGATAATGCTATTAAGGTTAAGGATCTGTACGATATGATTCATAACGTCATTGATGATGTGAGAGAGAAATACTTCTTTACGGTATCTAATATAGATTTTATGGAGTATATATTCTTGACGCATCTTAATCCTTCTAGAATTAGAATTACAAAAGAAACGGCTATCACTATCTTTGAAAAGTTCTATGAAAAACTCAATTATACTCTAGGTGGTGGAACTCCTATTGGAATTATTTCTGCACAGGTATTGTCTGAGAAGTTTACACAACAAGCCCTGTCCAGTTTTCACACTACTGAAAAAAGCGGTGCTGTCAAACAAAAACTTGGTTTCAATGAGTTTAATAACTTGACTAATTTGAGTAAGAATAAGACCGAAATTATCACTCTGGTATCCGATGATATCTCTAAACTTCAATCTGTTAAGATTAATTTTGAATTTGTATGTTTGGGAGAATTAAATCCAAACATCACTCTTCGAAAAGAAACAGATAGATATGTAGTAGACATAATAGTCAATAGATTATACATCAAGAGAGCAGAAATAACCGAATTAGTCGTCGAATATATGATTGAACGATTTATCTCCTTTAGCGTCATTGTAAAGGAATGGGGTATGGAGACATTCATTGAGGACGAGGATAATATTAGATTTACTGTTTATCTAAATTTCGTTGAACCAGAGGAATTGAATCTTAGTAAGTTTATGATGGTTCTTCCGGGTGCCGCCAACAAGGGCAAGATTAGTAAATTCAAGATTCCTATCTCTGATTATACAGGTTATGACGATTTCAATCAAACAAAAAAGCTCAATAAGATGACTGTAGAACTCATGAATCTAAAAGAATTGGGTTCTTTCGATTTGGAGAACGTTAACGTGTATCCTGGAGTATGGAATACATACGATATCTTCGGTATCGAGGCCGCTCGTGGATACTTATGCGAAGCCATGTTAAACACCTATGGAGAAGGTTTCGATTATCTGTATCAGCCTTGTGATCTTCTCGCTAGTTTACTATGTGCTAGTTACGAACCAGAATCAGTTAATAAATTCAAGTTCGGCGCAGCTAGTACTCTTAAGAGAGCTACGTTTGGAGACAATAAAGCATTGTTAAACGCGGCTCTTCATAAAAAGTCAGAACCTATTAACGATAATAGTAGCTGCCACTTTTTTAGCAAGGTCCCTAATATAGGAACTGGATATTACAAATACTTTATCGACTTGGGTCTTCTCATGAGAATGGAAAGGAAACTATCTGATAAGATATCTTCTCAAAAGATTAAGGAGATGGAAGAAACAGAAGACTTTTAATTCTTATCAATAACATATTTTTCTATGATCTGTCTTTTAAACGATGGATTTTCCACAAATGCGCCTCTCAAGTCCCTCATAGAATGATACACGTATAAAAAATATAACATAGGTGATGACTCCTTATTTTTAGACATTAGATATGCCAAAATCATAGCCCCGCTTCTATTTACTCCCGCAACACAATGAACCAACACGGGCTCGTTTCGTTGATCACATTTAGATAAGAAGGCGGTTACGTCGTCAAAATATTTACTAATATCGGTAGTTGTATCATCTACCAACGGTATATGAATAATATTAATATTAGAGTTAGGTAATGTATATTTATCCATCGTCAAATTTAAAACATATTTGAACTTAACTTCAGATGATGGTGCATTCATAGCATTTTTATAATTTCCCAAATACACATTATTGGTTACTCTTGTCATTATAGTGGGAGATTTGGCTCTGCGCATATCTCCAGTTGAACGTAGTAGTAAGTATTTATACAAACTTTTCTTATCCATTTATAACGTACAAATGGATAAAACTACTTTATCAGTAAACGCGTGTAATTTAGAATACGTTAGAGAAAAGGCTATAGTAGGCGTACAAGCAGCCAAGACATCAACACTTATATTCTTTGTTATTATATTGGCAATTAGTGCGCTATTACTCTGGTTTCAGACGTCTGATAATCCAGTCTTTAATGAATTAACGAGATATATGCGAATTAAAAATACGGTTAACGATTGGAAATCATTAACGGATAGTAAAACAAAATTAGAAAGTGATAGAGGTAGACTTCTAGCCGCTGGTAAGGATGATATATTCGAATTCAAATGTGTGGATTTTGGTGCCTATTTTATAGCTATGCGATTGGATAAGAAAACATATCTACCGCAAGCTATTAGGCGAGGTACTGGAGACGCGTGGATGGTTAAAAAGGCGGCAAAGGTTGATCCATCTGCTCAACAATTTTGTCAGTATTTGATAAAACACAAGTCTAATAATGTTATTACTTGTGGTAATGAGATGTTAAATGAATTAGGTTATAGCGGTTATTTTATGTCGCCACATTGGTGTTCCGATCTTAGTAATATGGAATAGTGTTAGATAAATGCGGTAACAAATGTTCCTGTAAGGAACCATAACAGCTTAGATTTAACGTTAAAGATGAGCATAAACATAATAAACAAAATTACAATCAAACCTATAACATTAATATCAAACAATCCAAAAAATGAAATCAGTGGAGTAGTAAATGCGTACATAACTCCTGGATAACGTTTAACAGCTGCCGTTCCTATTCTAGACCAAAAATTTGGTTTCATGTTTTCAAAGCGGTGTTCTGCAACAAGACGAGGATCGTGTTCTACGTATTTGGCAGAGTTATCCATTATTTGCCTGTTAATCTTTATTTCGTTTTCGATTCTGGCTATTTCAAAATAAAATCCCGATGATAGACCTCCAGACTTTATAATTTCATCTACAATGTTCAGCGCCGTAGTAACTCTAATAATATAGGCTGACAAGCTAACATCATACCCTCCTGTATATGTGAATATGACATGATTTTTGTCCATTACTAGCTCGGTTTTAACTTTATTGCCTGTAATAATTTCTCTCATCTGTAGGATATCTATTTTCTTGTCATGCATTGCCTTTAAGACTGGACGAAGAAACGTAATATCCTCAATAACGTTATCATTTTCTACAATAACTACATATTCTACCTTTTTATTTTCTAACTCGGTAAAAAATTTAGAATCCCATAGGGCTAAATGTCTAGCGATATTTCTTTTCGTTTCCTCTGTACACATGGTGTTACAAAACCCTGAAAAGAAGTGAGTATACTTGTCATCATTTCTAATGTTTCCTCCAGTCCAGTGTATAAACGCATAATCCTTGTAATGATCTGGATCATCCTTGACTATCACAACATTTCTTTTTTCTGGCATAACTTCGTTGTCCTTCACATCATCGAACTTCTGATCATTAATATGCTCATGAAGATTAGGAAATGTTTCTGATGGAGGTCTATCAATAACTGGCACAACAATAACAGGAGTTTTATTCACAGTCGCCATTTAGTTATTGAAATTAATCATATACAACTCTCTAATACGAGTTATATTTTCGTCTATCCATTGTTTCACATTGACATATTTCGACAAAAAGATATAAAATGCGTATTCCAATGCTTCTCTGTTTAATGAATTACTAAAATATACAAACACGTCACTGTCTGGCAATAAATGATATCTTAGAATATTGTAACAATTTATTTTGTATTGCGCATGTTCGTGATCTATGAGTTCTTCTTCGAATGGCATAGGATCTCCGAATCTGAAAATGTATAAATAGGAGTTAGAATAATAATATTTGAGAGTATTGGTAATGTACAAACTCTTTAGCGGTATAATTAGTTTTTTTCTCTCGATTTCTATTTTTAGATGTGATGGAAAAATGACTAATTTTGTAGCATTAGTATCATGAACTCTAATCAAAATCTTAATATCTTCGTCACACGTTAGCTCTTTGAACTGTTTAAGAGATGCATCAGTTGGTTCCACGGATGGAGTAGGTGCAACAATTTTTTGTTCGACATATGTATGTACTGGAGCCATTGTCTTAACTATAATGGTGCTTGTATCGAAAAACTTTAATGTAGATAGCGGAAGCTCTTCGCCGCGACTTTCTACGTCGTAATTAGGTTCTAATGCCGATCTTTGAATGGATACTAGTTTTCTAAGTTCTAATGTGATTCTCTGAAAATGTAAATCCAATTCCTCCGGCATTATAGATGTGTATACATCGGTAAATAAAACTATAGTATCCAACGATCCCTTCTCGCAAATTCTAGTCTTAACCAAGAAATCGTATATAACTACGGAAATGGCGTATTTAAGAGTGGATTCTTCTACCGTTTTGTTCTTGGATTTCATATAAGAAACTATAAAGTCCGCACTACTGTTAAGAATGATTACTAACGCAACTATATAGTTTAAATTAAGCATCTTGGAAACATAAAATAACTCTGTAGACGATACTTGACTTTCGAATAAGTTTGCAGACAAACGAAGAAAGAACAGACCTCTCTTAATTTCAGAAGAAAACTTTTTTTCGTATTCCTGACGTCTAGAGTTTATATCAATAAAAAAGTTAAGAATTAGTCGGTTAATGTTGTATTTCATTACCCAAGTTTGAGATTTCATAATATTATCAAAAGACATGATAATATTAAAGATAAAGCGCTGACTATGCACGAAATAGCTATATGGTTCGCTCAAGAATATAGTCTTGTTAAACGTGGAAACGATAACTGTATTTTTAATTACGTCAGCTGCATCTAAATTAAATATAGGTATATTTATTCCACACACTCTACAATATGCCACACCATCTTCATAATAAATAAATTCATTAGCAAAGTTATTAATTTTAGTGAAATAGTTAGCGTCAACTTTCATAGCTTCCTTCAATCTAATTTGATGCTCACACGGTGCGAATTCCACTTTAACATCTCTTTTCCATGCCTCAGGTTCATCGATCTCTATAATATCTAGTTTCTTGCGTTTAATAAACACGGGTTCGTCTTTCGCAATGAGGTCTGTATAGTAACTATGTAAATGATAACTAGATAGAAAGATGTAGCTATATAGATGACGATCCTTTAAGAGAGGTATGATAACTTTACCCCAATCAGATAAACTATTGTTATGGTCTTCGGAAAAAGAATTTTTATAAATTTTTCCAGTATTTTCCAAATATACGTACTTGACATCTAAAAAATCCTTAATGATAATAGGAATGGATAATCCGTCTATTTTATAAAGAAATACATATCGCACATTATACTTTTTTTTGGAAATGGGAATACCGATGTGTCTACATAAATATGCAAAGTCTAAATATTTTTTAGAGAATCTTAATTGGTCCAAATTCTTTTCCAAGTACGGTAATAGATTTTTCATATTGAACGGTATCTTCTTGATTTCTGGTTCTAATTCCGCATTAAATGATGAAACTAAGTCGCTATTTTTATAACTAACGATTACATCACCTCTAACATCATCATTTACCAGGATACTGATCTTCTTTTGTCGTAAATACATGTCTAATGTGTTAAAAAAAAGATCATACAAGTTATACGTCATTTCATCTGTAGTATTCTTGTCATTGAGTGATAAACTTGTGCTAATCTCTTCTTTAACAGTCTGTTCAAATTTATATCCTATATACGAAAAAATAGCAACCAATGTTTGATCATTCGCGTCAATATTCTGTTCTATTGTAGTGTATAACAATCTTATATCTTCTTCCGTGATAGCTGATACGTTATAAAGGTTGATAACGAAAATATTTTTATTTCGTGAGATAAAGTCATCGTAGGATTTTGGACTTATATTCGCGTCTAGTAGATATGCTTTTATTTTTGGAATAATCTCAATTAGAATAGTCTCTTTAGAGTCCATTTAAAGTTACAAACAACTAGGAAATTGGTTTATAATGTATAATTTTTTTAGTTTTTATAGATTCTTTATTCTATACTTAAAAAATGAAAATAAATACAAAGGTTCTTGAGGGTTGTGTTAAATTGAAAGCGAGAAATAATCATAAATTATTTCATTATCGCGATATCCGTTAAGTTTGTATTGTAATGGCGTGGTCAATTACGAATAAAGCGGATACTAGTAGTTTCACAAAGATGGCTGAAATTAGAGCTCATCTAAGAAATAGCGCTGAAAATAAAGATAAAAACGATGATATTTTCCCGGAAGATGTAATAATTCCATCTACTAAGCCCAAAACCAAACGAGCCACTACTCCTCGTAAACCAGCGGCTACTAAACGATCAACCAAAAAGGATAAAGAAAAGGAGGAAGTGGAAGAAGAAGAAGTAGTTATAGAGGAATATCATCAAACAACTGAAGAAAATTCTCCACCTCCGTCATCATCTCCTGGAGTCGGCAACATTGTAGAAAGCGTGACCGCTGTAGAGCTTGATGATAGCAACGGGGATGATGATAATGATAATGATAATGACGATAATGAACCTATGGTACAAGTTGAAGCTGGTAAAGTAAATCATAGTGCTAGAAGCGATCTCTCTGACCTAAAGGTGGCTACCGACAATATTGTTAAAGATCTTAAGAAAATTATTACTAGAATCTCTGCAGTATCGACTGTTCTAGAGGATGTTCAAGCAGCTGGTATCTCTAGACAATTTACTTCTATGACTAAATCTATTACAACACTATCTGATCTAGTCACCGAGGGAAAATCTAAAGTTGTTCGTAAAAAAGTTAAAACTTGTAAGAAGTAAATGCGTGCACTTTTTTATAAAGATGGTAAACTGTTTACCGATAATAATTTTTTAAATCCTGTATCAGACAATAATCCAGCGTATGAGGTTTTGCAACATGTTAAAATTCCTACTCATTTAACAGATGTAGTAGTATATGGACAAACGTGGGAGGAGGCGTTAACTAGATTAATTTTTGTGGGAAGTGATTCAAAAGGACGTAGACAATACTTTTACGGAAAAATGCATGTACAGAATCGCAACGCTAAAAGAGATCGTATTTTTGTTAGAGTATATAACGTTATGAAACGAATTAATTGTTTTATAAACAAAAATATAAAGAAATCATCCACAGATTCCAATTATCAGTTGGCAGTTTTTATGTTAATGGAAACTATGTTTTTTATTAGATTTGGTAAAATGAAATATCTTAAGGAGAATGAAACAGTAGGGTTATTAACACTAAAAAATAAACACATAGAAATAAGTCCCGATAAAATAGTTATCAAGTTTGTAGGAAAGGACAAAGTTTCACATGAATTTGTTGTTCATAAGTCTAATAGACTATATAAACCGCTATTGAAACTGACGGATGATTCTAGTCCCGAAGAATTTCTGTTCAACAAACTAAGTGAACGAAAGGTATACGAATGTATCAAACAGTTTGGTATTAGAATCAAGGATCTCCGAACGTATGGAGTCAATTATACGTTTTTATATAATTTTTGGACAAATGTAAAGTCCATATCTCCTCTTCCATCACCAAAAAAGTTAATAGCGTTAACTATCAAACAAACTGCTGAAGTGGTAGGTCATACTCCATCAATTTCAAAAAGAGCTTATATGGCAACGACTATTTTAGAAATGGTAAAGGATAAAAATTTTTTAGATGTAGTATCTAAAACTACGTTCGATGAATTCCTATCTATAGTCGTAGATCACGTTAAATCATCTACGGATGGATGATATAGATCCTTACACAAATAATTACAAAACCGATAAATGGAAATGGATAAGCGTATGAAATCTCTCGCAATGACCGCTTTCTTTGGAGAGCTAACCACATTAGATATTATGGCATTAATAATGTCTATATTTAAACGCCATCCAAACAATACCATTTTTTCAGTGGATAAGGATGGTCAGTTTATGATTGATTTCGAATACGATACTTATAAGGCTTCTCAATATTTGGATTTGCCCCTTACTCCGATATCTGGAGATGAATGCAAGACTCACGCATCGAGTATAGCCAAACAATTGGCGTGTGTGGACATTATTAAAGAGGATATTAGCGAATATATCAAAACTACACCCCGTCTTAAACGATTTATAAAAAAATACCGCAATAGATCAGATACTCGTATCAGTCAAGATACAGAAAAGCTTAAAATAGCTCTAGCTAAAGGCATAGATTACGAATATATAAAAGACGCTTGTTAATAAGTAAATGAAAAAAAACTAGTCGTTTATAATAAAACACGATATGGATGCCAACGTAGTATCATCTTCCACTATCGCGACATATATAGACGCTTTAGCAAAGAATGCTTCGGAATTAGAACAGGGTTCTACCGCATACGAAATAAATAATGAATTGGAACTAGTATTTATTAAGCCGCCATTGATTACTTTGACAAATGTAGTAAATATCTCCACGATTCAGGAATCGTTTATCCGATTTACCGTTACTAATAAGGAAGGCGTCAAAATTAGAACTAAGATTCCATTATCTAAGGTACATGGTCTAGATGTAAAAAATGTACAGTTAGTAGATGCTATAGATAACATAGTTTGGGAAAAGAAATCATTAGTGACGGAAAATCGTCTTCATAAAGCATGCTTGTTGAGACTATCAACAGAGGAACGCCATATATTTTTGGATTACAAGAAATATGGATCCTCTATCCGACTAGAATTAGTTAATCTTATTCAAGCAAAAACAAAAAACTTTACGATAGACTTTAAGCTAAAATATTTTCTAGGATCCGGTGCGCAGTCTAAAAGTTCTTTATTGCACGCTATTAATCATCCAAAGTCAAGGCCTAATACATCTCTGGAAATAGAATTCACACCTAGAGACAATGAAACAGTTCCATATGATGAACTAATAAAGGAATTGACGACTTTCTCGCGTCATATATTTATGGCTTCTCCAGAGAATGTAATTCTTTCTCCACCTATTAACGCACCTATAAAGACTTTTATGTTGCCTAAACAAGATATAGTAGGTATGGATCTGGAAAATCTATATGCCGTAACTAAGACTGACGGCATTCCTATAACTATCAGAGTTACATCAAAGGGGTTGTATTGTTATTTTACACATCTTGGTTATATTATTAGATATCCAGTTAAGAGAATAATAGATTCTGAAGTAGTAGTCTTTGGTGAGGCAGTTAAGGATAAGAACTGGACTGTATATCTCATTAAGCTAATAGAGCCCGTAAATGCAATCAGTGATAGACTAGAAGAAAGTAAGTATGTCGAATCTAAACTAGTGGATATTTGTGATCGGATAGTATTCAAGTCAAAGAAATACGAAGGTCCTTTTACTACAACTAGTGAAGTCGTTGATATGTTATCTACATATTTACCAAAGCAACCAGAAGGTGTTATTTTGTTTTATTCAAAGGGACCTAAATCTAACATTGATTTTAAAATCAAAAAGGAGAATACTATAGACCAAACTGTAAATGTAGTATTTAGGTACATGTCCAGTGAACCAATTATCTTTGGAGAATCATCTATCTTTATAGAGTATAAGAAATTTACCAACGATAAAGGCTTTCCTAAAGAATATGGTTCTGGTAAAATTGTGTTATACAACGGCGTTAATTATCTAAATAATATCTATTGTTTGGAATATATTAATACACATAATGAAGTGGGTATTAAGTCCGTGGTTGTACCTATTAAGTTTATAGCAGAATTCTTAGTCAATGGAGAAATACTTAAACCTAGAATCGATAAAACCATGAAATATATTAACTCAGAAGACTATTATGGAAATCAACATAATGTCATAGTTGAACATTTAAGAGATCAAAGCATCAAAATAGGAGATGTCTTTAACGAGGACAAACTATCTGATGTAGGACATCAATATGCCAATAATGATAAATTTAGATTAAATCCAGAAGTTAGTTATTTTACGAATAAAAGAACTAGAGGACCGTTGGGAATTTTATCGAACTACGTCAAGACTCTTCTTATTTCTATGTATTGTTCCAAAACATTTTTAGACGATTCCAACAAACGAAAGGTATTGGCGATTGATTTTGAAAACGGTGCTGACCTGGAAAAATACTTTTATGGAGAGATTGCGTTATTGGTAGCGACGGATCCAGATGCTGATGCTATAGCTAGAGGAAATGAAAGATACAACAAATTAAATTCTGGAATTAAAACCAAGTACTACAAATTTGACTACATTCAGGAAACTATTCGATCCAATACATTTGTATCTAGTGTCAGAGAAGTATTTTATTTTGGAAAGTTTAATATCATCGACTGGCAGTTTGCTATCCATTATTCTTTCCATCCGAGACATTATGCTACCATCATGAATAACTTATCCGAACTAACTGCTTCTGGAGGCAAGGTATTAATTACTACCATGGACGGAGACAAATTATCAAAATTAACCGATAAAAAGACTTTTATAATTCATAAGAATCTACCTAGTAGCGAAAACTATATGTCTGTAGAAAAAATAGCTGATGATAGAATAGTGGTATATAATCCATCAACAATGTCTACTCCAATGACTGAATACATTATCAAAAAGAACGATATAGTTAGAGTGTTTAACGAATACGGATTTGTTCTTGTAGATAATGTTGATTTCGCTACAATTATAGAACGAAGTAAAAAGTTTATTAATGGTGCATCTACAATGGAAGATAGACCGTCTACAAGAAACTTTTTCGAACTAAATAGAGGAGCCATTAAATGTGAAGGTTTAGATGTCGAAGACTTACTTAGTTACTATGTTGTTTATGTCTTTTCTAAGCGGTAAATAATAATATGGTATGGGTTCTGATATCCCCGTTTTAAATGCATTAAATAATTCCAATAGAGCGATTTTTGTTCCTATAGGACCTTCCAACTGTGGATACTCTGTATTGTTAATAGATATATTAATACTTTTGTCGGGTAACAAAGGTTCTACGTCTTCTAAAAATAAAAGTTTGATAACATCTGGCCTGTTCATAAATAAAAACTTGGCGATTCTATATATACTCTTATTATCAAATCTAGCCATTGTCTTATAGATGTGAGCTACTGTAGGTGTACCATTTGATTTTCTTTCTAATACTATATATTTCTCTCGAAGAAGTTCTTGCACATCATCTGGGAATAAAATACTACTGTTGAGTAAATCAGTTATTTTTTTTATATCGATATTGATGGACATTTTTATAGTTAAGGATAATAAGTATCCCAAAGTAGATAACGACGATAACGAAGTATTTATACTTTTAGGAAATCACAATGACTTTATCAGATCAAAATTAACAAAATTAAAGGAGCATGTATTTTTTTCTAAATATATTGTAACTCCAGATACATATGGATCTTTATGTGTAGAATTAAATGGATCTAGTTTTCAGCACGGTGGTAGATATATAGAGGTGGAGGAATTTATAGATGATGGAAGACAAGTTAGATGGTGTTCTACATCCAATCATATATCTGAAGATATACCCGAAGATATACACACTAATAAATTTATTATTTATGATATTTATACGTTTGATTCGTTCAAAAATAAACGATTGGTATTTGTACAGGTACCTACATCATTAGGAGATGATAGCTATTTAACTAACCCGTTATTGTCACCGTATTATTGTAATTCAGTAGCTAGACAAATGGTCAATGATATGATTTTTAATCAAGATTCATTTTTAAAATATTTATTAGAACATCTGATTAGAAGCCACTATAGAGTTTCTAAACATATAACAATAGTTAGATACAAAGATACCGAAGAATTAAATCTAACGAGAATATGTTATAATAGAGATAAGTTTAAGGCGTTTGCATTCGCTTGGTTTAACGGCGTTTTGGAAAATGAAAAAGTACTAGATACGTATAAAAAGGTATCTGATTTGATATAATGAATTCAGTGACTGTATCACACGCACCATATACTATTACTTATCACGATGATTGGGAACCAGTCATGAATCAATTAGTAGAGTTTTATAACGAAGTAGCCAGTTGGCTGCTACGAGACGAGACGTCGCCTATTCCTGATAAGTTCTTTATACAGTTGAAACAACCGCTTAGAAATAAGCGCGTATGTGTGTGTGGTATAGATCCGTATCCGAAAGATGGAACTGGTGTACCGTTCGAGTCACCAAATTTTACAAAAAAATCAATTAAGGAGATAGCATCTTCTATATCTAGATTAACCGGAGTAATTGATTATAAAGGTTATAACCTTAATATAATAGACGGGGTTATACCCTGGAATTATTACTTAAGTTGTAAATTAGGAGAAACAAAAAGTCACGCGATTTACTGGGATAAGATTTCTAAGTTACTGCTGCATCATATAACTAAACACGTTAGGTTTCTTTATTGTTTGGGTAAAACAGATTTCTCGAATATACGGGCCAAGTTAGAATCTCCAGTAACTACCATAGTGGGATATCATCCAGCAGCTAGAGACCGTCAATTCGAGAAAGATAGATCATTTGAAATTATCAACGTTTTACTGGAATTAGACAACAAGGCACCTATAAATTGGGCTCAAGGGTTTATTTATTAATGCTTTAGTGAAATTTTAACTTGTGTTCTAAATGGATGCGGTTATTAGAGGTAATGATGTTATCTTTGTTCTTAAGACTATAGGTGTCCCGTCAGTATGCAGACAAAATGAAGATCCAAGATTTGTAGAAGCATTTAAATGCGACGAGTTAGAAAGATATATTAAGAATAATCCAGAATGTACACTATTCGAAAGTCTTAGGGATGAGGAAGCATATTCTATAGTCAGAATTTTCATGGATGTAGATTTAGACGCGTGTCTAGACGAAATAGATTATTTAACAGCTATTCAAGATTTTATTATCGAGGTGTCAAACTGTGTAGCTAGATTCGCGTTTACAGAATGCGGTGCCATTCATGAAAATGTAATAAAATCCATGAGATCTAATTTTTCATTAACTAAGTCTACAAATAGAGATAAAACAAGTTTTCATATCATCTTTTTAGATACGTATACCACTATGGATACCTTGATAGCCATGAAACGAACACTATTAGAATTAAGTAGATCATCTGAAAATCCACTAACAAGATCGATAGACACTGCCGTATATAGGAAAAAAACAACTCTTCGGGTTGTAGGTACTAGGAAAAATCCAAATTGCGACACTATTCATGTAATGCAACCACCGCATGATAATATAGAAGATTACCTATTCACTTACGTGGATATGAACAACAATAGTTATTACTTTTCTCTACAACGACGATTGGAGGATTTAGTTCCTGATAAGTTATGGGAACCAGGGTTTATTTCATTCGAAGACGCTATAAAAAGAGTTTCAAAAATATTCATTAATTCTATAATAAACTTTAATGATCTCGATGAAAATAATTTTACGACGGTACCACTGGTCATAGATTACGTAACACCTTGTGCATTATGTAAAAAACGATCGCATAAACATCCGCATCAACTATCGTTGGAAAATGATGCTATTAGAATTTACAAAACTGGTAATCCACATAGTTGTAAAGTTAAAATTGTTCCGTTGGATGGTAATAAACTGTTTAATATCGCACAAAGAATTTTAGACACTAACTCTGTTTTATTAACCGAACGAGGAGACCATATAGTTTGGATTAATAATTCATGGAAATTTAACAGCGAAGAACCCTTAATAACAAAACTAATTCTGTCAATAAGACATCAACTACCTAAGGAATATTCAAGCGAATTACTCTGTCCGAGGAAACGAAAGACTGTAGAAGCTAACATACGAGACATGTTAGTAGATTCAGTAGAGACCGATACCTATCCGGATAAACTTCCGTTTAAAAATGGTGTATTGGACCTGGTAGACGGAATGTTTTACTCTGGAGATGATGCTAAAAAATATACGTGTACTGTATCAACCGGATTTAAATTTGACGATACAAAGTTCGTCGAAGATAGTCCAGAAATGGAAGAGTTAATAAATATCATTAACGATATCCAACCATTAACGGATGAAAATAAGAAAAATAGAGAGTTGTACGAAAAAACTTTATCTAGTTGTTTATGCGGTGCTACCAAAGGATGTTTAACATTCTTTTTTGGAGAAACGGCAACTGGAAAGTCGACAACCAAACGTTTGTTAAAGTCTGCTATCAGTGATCTGTTTGTTGAGACGGGTCAAACAATTTTAACAGATGTATTGGATAAAGGACCTAATCCATTTATCGCTAACATGCATTTGAAAAGATCTGTGTTCTGTAGCGAACTACCTGATTTTGCATGTAGTGGGACAAAGAAAATCAGATCTGATAATATTAAAAAGTTGACAGAACCTTGTGTCATTGGAAGACCGTGTTTCTCCAATAAAATTAATAATAGAAACCATGCTACAATCATTATCGATACTAATTACAAACCTGTCTTTGATAGGATAGATAACGCATTAATGAGAAGAATCGCCGTTGTGCGATTCAGAACACACTTTTCTCAACCTTCTGGTAGAGAGGCTGCTGAAAATAATGACGCGTACGATAAAGTCAAACTATTAGACGAGGGGTTAGATGGTAAAATACAAAATAATAGATATAGATTCGCATTTCTATACTTGTTGGTGAAATGGTACAGAAAATATCATGTTCCTATTATGAAACTATATCCTACACCGGAAGAGATTCCTGACTTTGCATTCTATCTCAAAATAGGTACTCTGTTAGTATCTAGCTCTGTAAAGCATATTCCATTAATGACGGACCTCTCCAAAAAGGGATATATATTGCACGATAATGTGGTTACTCTTCCGTTGACTACTTTCCAACAGAAAATATCCAAGTATTTTAATTCTAGACTATTTGGACACGACATAGAGAGCTTCATCAATAGACATAAGAAATTTGCCAATGTTAGTGATGAATATCTACAATATATATTCATAGAGGATATTTCATCTCCGTAAATATATGCTCATATATTTATAGAAGATATTACATATCTAAATGAATACCGGAATCATAGATTTATTTGATAATCATGTTGATAGTATACCAACTATATTACCTCATCAGTTAGCTACTCTAGATTATCTAGTCAGAACTATCATAGATGAGAACAGAAGCGTGTTATTGTTCCATATTATGGGATCAGGTAAAACAATAATCGCTTTGTTGTTCGCCTTGATAGCTTCCAGATTTAAAAAGGTTCACATTCTAGTGCCGAACATTAACATTTTGAAAATTTTTAATTATAATATGGGTGTAGCTATGAACTTGTTTAATGATGAATTCATAGCTGAGAATATCTTTATTCATTCAACAACAAGTTTTTATTCTCTTAATTATAACGATAACGTCATTAATTATAACGGATTATCTCGCTACAATAACTCTATTTTTATCGTTGATGAGGCACATAATATCTTTGGGAATAATACTGGAGAACTTATGACCGTAATAAAAAATAAAAACAAGATTCCTTTTCTACTATTGTCTGGATCTCCCATTACTAACACACCTAATACTCTGGGTCATATTATAGATTTAATGTCCGAAGAGACGATAGATTTTGGTGAGATTATTAGTCGTGGTAAGAAAGTAATTCAGACACTTCTTAACGAACGCGGTGTGAATGTACTCAAGGATTTGCTTAAAGGAAGAATATCATATTACGAAATGCCTGATAAAGATTTACCAACGATAAGATATCACGGACGTAAGTTTCTAGATACTAGAGTAGTATATTGTCACATGTCTAAACTTCAAGAGAGAGATTATATGATTACTAGACGACAGCTATGTTATCATGAAATGTTTGATAAAAATATGTATAACGTGTCAATGGCAGTATTGGGACAACTTAATCTGATGAATAATTTAGATACTTTATTTCAGGAACAGGATAAGGAATTGTACCCAAATCTGAAAATAAATAATGGTGTGTTATACGGAGAAGAATTGGTAACGTTAAACATTAGTTCCAAATTTAAATACTTTATCAATCGGATACAGACACTCAACGGAAAACATTTTATATACTTTTCTAATTCTACATATGGTGGATTAGTAATTAAATATATCATGCTCAGTAATGGATATTCTGAATATAATGGTTCTCAGGGAACTAATCCACATATGATAAACGGCAAACCAAAAACATTTGCTATCGTTACTAGTAAAATGAAATCGTCTTTAGAGGATCTATTAGATGTGTATAATTCTCCTGAAAACGATGATGGTAGTCAATTGATGTTTTTGTTTTCGTCAAACATTATGTCCGAATCCTATACTCTGAAAGAGGTAAGGCATATTTGGTTTATGACTATCCCGGATACTTTTTCTCAATACAACCAAATTCTTGGACGATCTATTAGAAAATTCTCTTACGTCGATATTTCTGAACCCGTTAATGTATATCTTTTAGCAGCCGTATATTCCGATTTCAATGACGAAGTGACGTCATTAAACGATTACACGCAGGATGAATTGATTAATGTTTTACCCTTTGACATTAAAAAGCTGTTGTATCTAAAATTTAAGACTAAAGAAACGAATAGAATATACTCTATTCTTCAAGAGATGTCTGAAACGTATTCTCTTCCACCACATCCATCAATTGTAAAAGTTTTATTGGGAGAATTGGTCAGACAATTTTTTTATAATAATTCTCGTATTAAGTATAACGATGCCAAGTTACTTAAAATGGTTACATCAGTTATAAAAAATAAAGAAGACGCTAGGAATTACATAGATGATATTGTAAACGGTCACTTCTTTGTATCGAATAAAGTATTTGATAAATCTCTTTTATACAAATACGAAAACGATATTATTACAGTACCGTTTAGACTTTCCTACGAACCGTTTGTTTGGGGAGTTAACTTTCGTAAGGAATATAACGTGGTATCTTCTCCATAAAACTGATAAAATATATAAAGAAATAAATGTCGAGCTTTGTTACCAATGGATACCTTCCAGTTACATTGGAACCACATGAGCTGACGTTAGACATAAAAACTAATATTAGGAATGCCGTATATAAGACGTATCTCCATAAAGAAATTAGTGGTAAAATGGCCAAGAAAATAGAAATTTGTAAAGACGTGGAATTACCTCTCGGCGAAATAGTTAATAATTCTGTAGTTATAAACGTTCCGTGTGTAATAACCTACGCGTATTATCACGTTGGGGATATAGTTAGAGGAACATTAAACATCGAAGATGAATCAAATGTAACTATTCAATGCGGAGATTTAATCTGTAAACTAAGTAGAGATTCGGGTACTGTATCATTTAGCGATTCAAAGTACTGCTTTTTTCGAAATGGTAATGCGTATGATAACGGCAGCGAAGTCTCCGCCGTTCTAATGGAGGCTCAACAAGGTACCGAATCTAGTTTTGTTTTTCTCGCGAATATCGTCGACTCATAAGAAAGAGAATAGCGGTAAGTATATATACGAATACTATGGCAATAATTGCGAATGTTTTATTCCCTTCGATATATTTTTGATAATATGAAAAACATGTCTCTCTCAAATCAGACAACCATCTCATAAAATAGTTCTCGCGTGCTGGAGAGGTAGTTGCTGCTCGTATAATCTCCCCAGAATAATATACTTCTGTGTCATCGTTCAATTTATACGGATTTCTATAGTTCTCTGTTATATAATGCGGTTTGCCCTCATGATTAGACAACGACAATAGTGTTCTGAATTTAGATAGTTGATCAGAATGAATGTTTATTGGCGTTGGAAAAATTATCCATACAGCGTCTGCAGAGTGGTTGATAGTTGTTCCTAGATATTTAAAATAATCCAACTTACTAGGCAGCAAATTGTCTAGATAAAATACTGAATCAAACGGTGCAGACGTATTGGCGGTTCTAATGGAATCCAATTGATTGACTATCTTTTGAAAATATACATTTTTATGATCCGATACTTGTAAGAATATAGAAATAATGATAAGTCCATCATCGTGTTTTTTTGCCTCTTCATAAGAACTATATTTTTTCTTATTCCAATGAACAAGATTAATCTCTCCAGAGTATTTGTACACATCTATCAAGTGATTGGATCCATAATCGTCTTCCTTTCCCCAATATATATGTAGTGATGATAACACATATTCATTGGGAAGAAACCCTCCACTTAAATATCCTCCTTTAAAATTAATCCTTACTAGTTTTCCAGTATTCTGGATAGTGGTTGGTTTCGACTCATTATAATGTATGTTTAACGGCTTTAATCGCGCGTTAGAAATTGCTTTTTTAGTTTCTATATTAATAGGAGATAGTTGTTGTGACATAGTAAAAATGAAATGATAACTGTTTAGAAATAGCTCTTAGTATGGGAATTACAATGGATGAGGAAGTGATATTTGAAACTCCTAGAGAATTAATATCTATTAAACGAATAAAAGATATTCCAAGATCAAAAGACACGCACGTGTTTGCTGCGTGTATAACAAGTGACGGATATCCGCTAATAGGAGCTAGAAGAACTTCATTCGCATTCCAGGCGATATTATCTCAACAAAATTCAGATTCTATCTTTAGAGTATCCACTAAACTATTACGGTTTATGTATTACAATGAACTAAGAGAAATCTTTAGACGGTTGAGAAAAGGTTCTATCAACAATATCGATCCTCACTTCGAAGAGTTAATATTATTGGGTGGTAAACTAGATAAAAAGGAATCTATTAAAGATTGTTTAAGAAGAGAATTAAAAGAGGAAAGTGATGAACGTATAACAGTAAAAGAATTCGGAAATGTAATTCTAAAACTTACAACACAGGATAAATTATTTAATAAAGTGTATATAGGTTATTGCATGTCGTGTTTTATTAATCAATCATTAGAGGATTTATCACATACTAGTATTTACAATGTAGAAATTAGAAAGATTAAATCATTAAATGATTGTATTAACGATGATAAATACGAATATCTGTCTTATATTTATAATATGCTAGTTAATAGTAAATGAACTTTTACAGATCTAGTATAATTAGTCAGATTATTAAGTATAATAGACGACTAGCTAAGTCTATTATTTGCGAGGATGACTCTCAAATTATTACACTTACGGCATTCGTTAACCAATGCCTATGGTGTCATAAACGAGTATCCGTGTCCGCTATTTTATTAACTACGGATAACAAAATATTAGTATGTAACAGACGAGATAGTTTTCTCTATTCTGAAATAATTAGAACTAGAAACATGTCTAGAAAGAAACGATTATTTCTAAATTATTCCAATTATTTGAACAAACAGGAAAGAAGTATACTATCGTCATTTTTTTCTCTAGATCCAGCTACGATTGATAATGATAGAATAGACGCTATTTATCCGGGCGGTATACTCAAAAGGGGTGAGAACGTTCCAGAGTGTCTATCCAGGGAAATCAAAGAAGAAGTTAATATAGACAATTCTTTTGTATTCATAGACACTCGGTTTTTTATTCATGGTATCATAGAAGATACCATTATTAACAAATTTTTTGAAGTAATTTTCTTTGTTGGAAGAATATCTCTAACGAGTGATCAAATTATTGATACCTTTAAAAGTAATCATGAAATTAAGGATCTAATATTTTTAGATCCGAATTCAGGTAATGGACTCCAATACGAAATTGCAAAATATGCTCTAGATACTGCAAAACTTAAATGTTATGGCCATAGAGGATGTTATTATGAATCATTAAAAAAATTAACTGAGGATGATTGATTAGAAAATATAAATTAATTTACCATCGTGTATTTTTATAACGGGATTGTCCGGCATATCATGTAGATAGTTACCATCTACATCGTATACTCGACCATCTACGCCTTTAAATCCTCTATTTATTGACATTAATCTATTAGAATTGGAATACCAAATATTAGTACCCTCAATTAGTTTATTGGTAATATTTTTTTTAGACGATAGATCGATGGCTCTTGAAACCAAGGTTTTCCAACCGGACTCGTTATCGATCGGTGAAAAGTCTTTTTCATTAGCATGAATCCATTCTAATGATGTATGTTTAAACACTCTAAACAATTGGACAAATTCTTTTGATTTGCTTTGAATAATTTCAAATAGGTCTTCGTCTACAGTAGGCATACCATTAGATAATCTAGCCATTATAAAGTGCACGTTTACATATCTACGTTCTGGAGGAGTAAGAACGTGACTATTAAGACGAATGGCTCTTCCTACTATTTGACGAAGAGATGCCTCGTTCCAAGTCATATCTAGAATGAAGATATCATTAATTGAGAAAAAACTAATACCCTCGCCTCCACTAGAAGAGAATACACATGTTTTAATGCATTCTCCGTTAGTGTTTGATTCTTGGTTAAACTCAGCCACCGCCTTGATTCTAGTATCTTTTGTTCTAGATGAGAACTCTATATTAGAGATACCAAAGACTTTGAAATATAGTAATAAAATTTCTATTCCTGACTGATTAACAAATGGTTCAAAGACTAGACATTTACCATGTGATGCTAATATTCCCAAACATACATCTATGAATTTGGCGCTTTTCTCTTTTAATTCAGTAAATAGAGAGATATCAGCCGCACTAGCGTCCCCTCCCAATAGTTCTCCCTTTTTAAAGGTATCTAATGCGGATTTAGAAAACTCTCTATCTCTTAATGAGCGTTTAAAGTCATTATATAGGGTTGCTATCTCTTGCGCGTATTCACCCGGATCACGATTTTGTCTTTCAGGAAAGCTATCGAACGTAAACGTAGTAGCCATACGTCGCAGAATTCTAAATGATGATATACCTGTTTTTATTTCAGTAAGTTTAGCCTTTTGATAAATCTCTTCTTGCTTTTTTGACATATTAACGTATCGCATTAATACTGTTTTCTTAGCGAATGATGCAGACCCTTCTACGTCATCAAAAATAGAAAACTCGTTATTAACTATGTACGAACATAGGCCTCCTAGTTTGGAGACTAATTCTTTTTCATTAACTAGACGTTTATTCTCAAATAGCGATTGGTGTTGTAAGGATCCTGGTCGTAGTAAGTTAACCAACATGGTGAATTCTTGCACACTATTGACGATAGGTGTAGCCGATAAACAAATCATCTTATGGTTTTTTAACGCAATGGTCTTAGATAAAAAATTATATACTGAACGAGTAGGACGGATCTTACCATCTTCTTTGATTAATGATTTAGAAATGAAGTTATGACATTCATCAATGATGACGCATATTCTACTCTTGGAATTAATAGTTTTGATATTAGTAAAAAATTTATTTCTAAAATTTTGATCATCGTAATTAATAAAAATACAATCCTTCGTTATCTCTGGAGCGTATCTGAGTATAGTGTTCATCCAAGGATCTTCTATCAAAGCTTTTTTCACTAATAAGATAATAGCCCAATTCGTATAAATATCCTTAAGATGTTTGAGAATATATACAGTAGTCATTGTTTTACCGACACCTGTTTCATGGAACAATAAAAGAGAATGCATACTGTCTAATCCTAAGAAAACTCTTGCTACAAAATGTTGATAATCCTTGAGGCGTACTACGTCTGTTCCCATCATTTCAACAGGCATATTAGTAGTTCTGCGCAAGGCATAATCGATATAGGCCGCATGTGATTTACTCATTTATAGTGATAAGTAATAACTATGTTTTAAAAATCATAACAGTAGTTTAACTAGTCTTCTCTGATGTTTGTTTTCGATACTTTTCGAATCAGAAGTCATACTAGAATAAAGCAACGAGTGAACGTAATAGAGAGCTTCGTATACTCTATTCGAAAACTCTAAGAACTTATTAATGAATTCCGTATCCACTGGATCGTTTAGAATACTAAATTGAACACTGTTCACATCCTTCCAAGAAGAAGACTTAGTAACGGACTTAACATGAGACATAAATAAATCCAAATTTTTTTTACAAACATTACTAGCCACCATAATGGCGCTATCTTTTAACCAGCTATCGCTTACACATTTCAGCAATCTAACATTTTTAAAGAGACTACAATATATTCTCATGGTATCGATTACACCTCTACCGAATAGAGTAGGAAGTTTAATAATACAATATTTTTCGTTTACAAAATCAAATAATGGTCGAAACACGTCAAAGGTTAACATCTTATAATCGCTAATGTATAGATTGTTTTCAGTAAGATGATTATTAGATTTAATAGCATCTCGTTCGCGTTTAAACAGTTTATTGCGTGCGCTAAGATCGGCAACTACTGCGTCCGCTTTAGTACTTCTCCCATAATACTTTACGCTATTAATCTTTAAAATTTCATAGACTTTATCTAGATCGCTTTCTGGTAACATGATATCATGTGTAAAAAGTTTTAACATGTCGGTTGGCATTCTATTTAGATCATTAACTCTAGAAATCTGAAGAAAGTAATTAGCTCCGTATTCCAAACTAGGTAATGGGCTTTTACCTAGAGACAGATTAAGTTCTGGCAATGTTTCATAAAATGGAAGAAGGACATGCGTCCCCTCCCGGATATTTTTTACAATTTCATCCATTTACAACTCTATAGTTTGTTTTCATTATTATTAGTTATTATCTCCCATTATCTTGGTAATACTTACCCCTTGATCGTAAGATACCTTATACAGGTCATTACATACAACTACCAATTGTTTTTGTACATAATAGATTGGATGGTTGACATCCATGGTGGAATAAACTACTCGAACAGATAGTTTATCTTTCCCCCTAGATACATTGGCCGTAATAGTTGTCGGTCTAAAGAATATCTTTGGTGTAAAGTTAAAAGTTAGGGTTCTTGTTCCATTATTGCTTTTTGTCAGTAGTTCATTATAAATTCTCGAGATGGGCCCGTTCTCTGAATATAGAACATCATTTCCAAATCTAACTTCTAGTCTAGAAATAATATCGGTCTTATTCTTAAAATCTATTCCCTTGATGAAGGGATCGTTAATGAACAAATCCTTGGCCTTTGATTCGGCTGATCTATTATCTCCGTTATAGACGTTACGTTGACTAGTCCAAAGACTTACAGGAATAGATGTATCGATGATGTTGATACTATGTGATATGTGTGCAAAGACTGTTCTCTTGGTGGCGTCGCTATATGTTCCAGTAATGGCGGAAAACTTTTTAGAAATGTTATATATAAAAGAATTTTTTCGGGTTCCAAACATTAACAGATTAGTATGAAGATAAACACTCATATTATCAGGAACATTATCAATTTTTACATACACATCAGCATCTTGAATAGAAACGATACCATCTTCTGGAACCTCAACAATCTCGGCAGACTCCGGATAACCAGTTGGTGGGCCATCACTAACAATAACTAGATCATCCAACAATCTACTCACATATGCATCTATATAATCTTTTTCATCTTGTGAGTACCCTGGATACGAAATAAATTTATTATCCGTATTTCCATAATAAGGTTTAGTATAAACAGAGAGCGATGTTGCCGCATGAACTTCAGTTACAGTCGCCGTTGGTTGGTTTATTTGACCTATTACTCTCCTAGGTTTCTCTATAAACGATGGTTTAATTTGTACATTCTTAACCATATATCCAATAAAGCTCAATTCAGGAACATAAACAAATTCTTTGTTGAACGTTTCAAAGTCGAACGAAGAGTCGCGAATAACGATATCGGATACTGGATTGAAGGTCACCGTTACGGTAATTTTTGAATCGGATAGTTTAAGACTGCTAAATGTATCTTCCACATCAAACGGAGTTTTAATATAAACGTATACTGTAGATGGTTCTTTAATAGTGTCATTAGGAGTTAAGCCAATAGAAATATCATTAAGTTCACTAGAATATCCAGAATGTTTCAAAGCAATTGTATTATTGATACAATTATTATATAATTCTTCGCCCTCAATTTCCCAAATAACACCATTACACGAAGAGACCGATACATGATTAATACATTTATATCCAACATATGGTACGTAACCGAATCTTCCCATACCTTTAACTTCTGGAAGTTCCAAACTTAGAACCAAATGATTAAGCGCAGTAATATACTGATCCCTAATTTCGAAGCTAGCGATAGTCTGATTGTCGGGACCATTGTTTGTCATAACTCCGG

TATCGACTGCGAAGACATTAGACCGTTTAATAGAGTCATCCCCACCAATCAACGAATTAATGATAGTATTATTCATTTTCTATTTAAAATGGAAAAAGCTTACAATAAACTCCGTAGAGAAATATCTATAATTTGTGAGTTTTCCTTAAAGTAACAACTTCCGTAAACGCCGTCTTTATCTCTTAGTAAGTTTATTGTATTTATGACCTTTTCCTTATCTTCATAAAATACTAAAGGCAACAAAGAAATTTTTGGTTCTTCTCTAAGAGCTACGTGAGACTTAACCATAGACGCCAACGAATCCCTACATATTTTAGAACAGAAATACCCAACTTCGCCACCCTTGAATGTCTCAATACTAATAGGTCTAAAAACCAAATCTTGATTACAAAACCAACACTTATCAATTACACTATTTGTCTTAATAGACATATCTGCCATAGATTTATAATACTTTGGTAGTATACAAGCGAGTGCTTCTTCTTTAGCGGGCTTAAAGACTGCTTTAGGTGCTGAAATAACCACATCTGGAAGGCTTACTCGCTTAGCCATTTAATTACGGAACTATTTTTTTATACTTCTAATGAGCAAGTAGAAAACCTCTCATCTACAAAAACATACTCGTGTCCATAATCCTCTACCATAGTAACACGTTTTTTAGATCTCATATGTGCTAAAAAGTTTTCCCATACTAACTGATTACTATTATTTTTCGTATAATTTTTAACTGTTTGAGGTTTTAGATTTTTAGTTACAGAAGTAATATCGAATATTTTATCCAAAAAGAATGAGTAATTAATTGTTTTAGAAGGAGTGTTTTCTTGGCAAAAGAATACCAAGTGCTTAAATATTTCTACTACTTCATTAATCTTTTCGGTACTCAGATTCAGTTTCTCATCTTTTACTTGATTGATTATTTCAAAGACTAACTTATAATCCTTTTTATTTATCCTCTCGTTAGCCTTAAGAAAACTAGATACAAAATTTGCATCTACATCATCCGTGGATATTTGATTTTTTTCCATGATATCCAAAAGTTCCGAGATAATTTCTCCAGAACATTGATGAGACAATAATCTCCGCAATACATTTCTCAAATGAATAAGTTTATTAGACACGTGGAAGTTTGACTTTTTTTGTACCTTTGTACATTTTTGAAATACAGACTCGCAAAAAATACAATATTCATATCCTTGTTCAGATACTATACCGTTGTGTCTACAACCGCTACATAATCGTAGATTCATGTTAACACTCTACGTATCTCGTCGTCCAATATTTTATATAAAAACATTTTATTTCTAGACGTTGCCAAAAAATCCTGTAATACTTTTAGTTTTTGGGGTTGTGAATAAAGTATCGCCCTAATATTATTACCATCTTCCGCCAATATAGTAGTTAGATTATCAGCACATGAAGAAAAACACCTCTTAGGTGGATTCAGTACAATGTTATATTTTTCGTACCAACTCATTTAAATATCATAATCTAAAATAGTTCTGTAATATGTCTAGCGCTAATATATTGATCATAATCCTGTGCATAAATTAAAATACAACAATGTCTCGAAATCATTGACATGGCTTCTTCCATAGTTAGAAGATCGTCGTCAAAGTTAGCAACGTGATTCATCAACATTTGCTGTTTTGAGGCAGCAAATACTGAACCGTCACCATTCAACCATTCATAAAAACCATCGTCTGAATCCATTGATAATTTCTTGTACTGGTTTTTGAGAGCTCGCATCAATCTAGCATTTCTAGCTCCCGGATTGAAAACAGAAAGAGGATCGTACATCCAGGGTCCATTTTCTGTAAATAGAATAGTATAATGTCCCTTTAAGAAGATATCAGACGATCCACAATCAAAAAATTGGTCTCCGAGTTTGTAACAGACTGCGGACTTTAACCTATACATGATACCGTTTAGCATGATTTCTGGTGATACGTCAATTGGAGTATCATCTATTAGAGATCTAAAGCCGGTGTAACATTCTCCACCAAACATATTCTTATTCTGACGTCGTTCTACATAAAACATCATTGCTCCATTAACGATAACAGGGGAATGAACAGCACTACCCATCACATTAGTTCCCAATGAATCAATGTGTGTAACTCCAGAACATCTTCCATAGCCTATGTTAGGAGGAGCGAACACTACTCTTCCACTATTGCCATCGAATGCCATAGAATAAATATCCTTGGAATTGATAGAAATCGGACTGTCGGATGTTGTGATCATCTTCATAGGATTAACAACAATGTATGGTGCCGCCTGAAGTTTCATATCGTAACTGATGCCGTTTATAGGTCTAGCCACAGAAACCAATGTAGGTCTAAATCCAACTATAGACAAAATAGAAGCCAATATCTGTTCCTCATCTGTCATAACTTGAGAGCATCCAGTATGAATAATCTTCATTAGATGGGGATCTACCGCATCATCATCGTTACAATAAAAAATTCCCATTCTAATGTTCATAATTGCTTTTCTAATCATGGTATGCATGTTTGCCCTTTGAATCTCTGTGGAAATTAGATCTGATACACCTGTAATCACTATCGGATTATCCTCCGTAAGACGATTAACCAACAACATATAATTATAAGACTTTACTCTTCTAAATTCATAAAGTTGCTGGATTAGACTATATGTGTCTCCATGTACATACGCGTTCTCAAGCGCGGGAAGTTTAATGCCAAATAGTGCCATCAGAATAGGATGAATGTAGTAATTAGTTTCTGGTTTTCTATAAATAAAAGACAAATCTTGCGAGCTAGACATATCGGTAAAATGCATGGATTGGAATCGTGTAGTCGACAGAAGAATATGATGATTAGACGGAGAGTATATTTTATCTAACTCTTTGAGTTGGTCACCAATTCTAGGACTAGCTCGAGAATGAATAAGTACTAAAGGATGAGTACATTTCACAGAAACACTGGCGTTGTTCAATGTGCTCTTTACATGGGTAAGGAGTTGAAATAGCTCGTTTCTATTTGTTCTGACAATATTTAGTTTATTCATAATGTTAAGCATATCCTGAATAGTAAAATTAGATGTGTCATACTTGTTAGTAGTTAGATATTTAGCAATTGCATTCCCATCATTTCTCAATCTCGTACTCCAATCATGTGTGGATGCTACTTCATCTATAGAAACCATACAATCCTTTTTGGTAGTCTGTTGAGCTTGATCATTTCCTGCACGTTTAGGTTTGGTACGTTGATTTCTAGCCCCTGCGGATATAAAGTCATCGTCTACAATTTGGGACAATGAATTGCATACACTACATGACAAAGATTTATCAGAAGTGTGAATATGATCTTCATCTACCAAAGAAAGAGTTTGATTAGTATAACTAGATTTTAGTCCTGTGTTAGATGTTAAAAAAACATCGCTATTGACCACGGCTTCCATTATTTATATTCGTAGTTTTTACTCGAAAGCGTGATTTTAATATCCAATCTTATTACTTTTGGAATCGTTCAAAACCTTTGACTAGTTGTAGAATTTGATCTATTGCCCTACGCGTATACTCCCTTGCATCATATACGTTCGTCACCAGATCGTTTGTTTCGGCCTGAAGTTGGCGCATATCTTTTTCAACACTCGACATGAGATCCTTAAGGGCCATATTGTCTAGATTTTGTTGAGATGTTGCTCCTGGATTTGGATTTTGTTGTGCTGTTGTACATACTGTACCACCAGTAGGTGTAGGAGTACATACAGTGGCCACAATAGGAGGTTGAGGAGGTGTAACTGTCGGAGTAGTACAAGAAATACTTCCATCCGATTGTTGTGTACATGTAGTTGTTGGTAACGTCTGAGAAGGTTGGGTAGATGGCGTCGTCGTCGTCTTTTGATCTTTATTAAATTTAGAGATAATATCCTGAACAGCATTGATCGGCGTCAACGCTGGAAGGAGTGAACTCGGTTGTGTATTATCATCTGCCGACAACCAATCAAAAAGATTAGACATATCCGATGATGTATTAGTTTGTTGTTGTCGTGGTTTTGGTGTAGGAGCCGGTGTAGGTACCTGCAGTGGAGTTATATGAATAGTTGGTTGTAGCGGTTGGATAGGCTGTCTGCTGGCGGCCATCATATTATCTCTAGCTAGTTGTTCTCGCAACTGTCTTTGATAATACGACTCTTGAGACTTTAGTCCTATTTCAATCGCTTCATCCTTTTTTATATCCAGATCCTTTTCTTCAGAATAATAGATTGACGACTTTGGTGTAGAGGATTCTGCCAACCCCTGTGAGAACTTGTTAAAGAAGTCCATTTAAGGCTTTAAAATTGAATTGCGATTATAAGATTAAATGGCAGACACAGACGATATTATCGACTATGAATCCGATGATCTCACTGAATACGAGGATGATGATGAAGAGGAAGAAGATGGAGAGTCACTAGAAACTAGTGATATAGATCCCAAATCTTCTTATAAGATTGTAGAATCAGCATCCACTCATATAGAAGATGCACATTCCAATCTTAAACATATAGGGAATCATATATCTGCTCTTAAACGACGCTATACTAGACGTATAAGTCTATTTGAAATAGCGGGTATAATAGCAGAAAGCTATAACTTGCTTCAACGAGGAAGATTACCTCTAGTTTCAGAATTTTCTAACGAAACGATGAAGCAAAATATGCTACATGTAATTATACAAGAGATAGAGGAGGGTTCTTGTCCTATAGTCATCGAAAAGAACGGAGAATTGTTGTCGGTAAACGATTTTGACAAAGATGGTCTGAAATTCCATCTAGACTATATTATCAAAATTTGGAAACTTCAAAAACGATATTAGAATTTATACGAATATCGTTCTCTAAATGTCACAATCAAGTCTCTCATATTTAGCAGGTTATTGTCGTACTTTATATCGTGTTCATTAACGATATTTTGCAAAATAGTAATGATTCTATCTTCCTTCGATAGATATTCTTCAGAGATTATTGTCTTATATTCTTTCTTGTTATCCGATATAAATTTAATAAGACTTTGAACATTATTAATACCCGTTTGTTTAAATTTTTCTACAGATATTTTAGTTTTGGTAGATTCTATTGTGTCTGTTAATAGACACCCAACATCGACATTCGACGTTAATTGTCTATAAATTAGAGTATAAATTTTAGAAATAACATTAGCAAATTGTTGTGCGTTGATGTCGTTATTCTGAAACAGTATGATTTTAGGTAGCATTTTCTTAACAAAGAGAACGTATTTATTGTTACTCAGTTGAACAGATGATATATCCAGATTACTAACGCATCTGATTCCATATACCAAACTTTCAGAAGAAATGGTGTACAATTGTTTGTATTCATTCAATGTCTCCTTTTCAGAAATTAGTTTAGAGTCAAATACTGCAATAATTTTCAAAAGATAGTTTTCATCAGATAAGATTTTATTTAGTGTAGATATGATAAAACTATTGTTTTGTTGGAGAACTTGATACGCCGCATTCTCTGTAGTCGACGCTCTCAAATGGGAAACAATCTCCATTATTTTTTTGGAATCGGATACTATATCTTCGGTATCTTGACGCAGTCTAGTATACATAGAGTTAAGAGAAATTAGAGTTTGTACATTAAGCAACATGTCTTTAAATGTGGCTACAAACTTTTCCTTTTCCGCATCATCTAGTTTATTATATACCGATTTTACAACGGCGCCAGATTTAAGAAACCAGAATGAAAAACTCTGATAACTACAATATTTCATCATAGTTACAATTTTATCATCTTCTATAGTTGGTGTGATAACACATACCTTTTTCTCCAAGACTGGAACCAACGTCATAAAAATGTTTAAATCAAAATCCATATCAACATCTGATGCGCTAAGACCAGTCTCGCGTTCAAGATTATCTTTACTAATGGTGACGAACTCATCGTATAGAACTCTAAGTTTGTCCATTATTTATTTACAGATTTAGTTGTTTAATTTATTTGTACTCTTCCAAAGTTGGGATAGTATTTTTCTAACGTTGGTATTATATTATTAGGATCTACGTTCATATGTATCATAATATTAATCATCCACGTTTTGATAAATCTATCTTTAGCTTCTGAAATAACGTATTTAAACAAAGGAGAAAAATATTTAGCTACGGCATCAGACGTAATAACATTTTTTGTAAATGTAACGTATTTAGACGAGAGATCTTCGTTAAAAAGTTTTCCATCTATGTAGAATCCATCGGTTGTTAACACCATTCCCGCGTCAGATTGAATAGGAGTTTGAATAGTTTGTTTTGGAAATAGATCCTTCAATAACTTATAGTTGGGTGGGAAAAAATCGATTTTATCACTAGACTCTTTCTTTTTTACTATCATTACCTCATGAACTATTTCTTGAATGAGTATATGTATTTTCTTTCCTATATCGGACGCGTTCATTGGAAAATATACCATGTCGTTAACTATAAGAATATTTTTATCCTCGTTTACAAACTGAATAATATTAGACGTAGTTCGTAAACGAACTATATCATTACCAGTACAACATCTAACTATATGATATCCACTAGTTTCCTTTAGCCGTTTATTATCTTGTTCCATATTAGCAGTCATTCCATCATTTAAGAAGGCGTCAAAGATAATAGGGAGAAATGACATTTTGGATTCTGTTACTACTTTACCAAAATTAAGGATATACGGACTTACTATCTTTTTCTCAACGTCGATTTGATGAACACACGATGAAAATGTGCTTCTATGAGATTGATCATGTAGAAAACAACAAGGGATACAATATTTCCGCATATCATGAAATATATTAAGAAATCCCACCTTATTATATTTCCCCAAAGGATCCATGCACGTAAACATTATATCGTTATCATTAATAAAGACTTCTTTCTCATCGGATCTGTAAAAGTTGTTACTGATTTTTTTCATTCCAGGATCTAGATAATTAATAATGACGGGTTTTCTATTTTTATTCTTTGTATTTTGGCATATCCTAGACCAGTAAACAGTTTCCACTTTGGTAAAATCAGCAGACTTTTGAACGCTATTAAACATGGCATTAATGGCAATAACTAAAAATGTAAAATATTTTTCTATGTTAGGAATATGGTTTTTCACTTTAATAGATATATGGTTTTTGGCCAAAATGATAGATATTTTTTTATCCGAGGATAGTAAAATATTATTAGTCGCCGTCTCTATAAAAATGAAGCTAGTCTCGATATCCAATTTTATTCTAGAATTGATAGGAGTCGCCAAATGTACCTTATACGTTATATCTCCCTTGATGCGTTCCATTTGTGTATCTATATCTGACACAAGATCGGTAAATAGTTTTACGTTATTAATCATCACGGTATCACCGTCGCTAGATAATGCTAATGTTCCATCCAAGTCCCAAATGGAGAGATTTAACTGTTCATCGTTTAGAATAAAATGATTACCGGTCATATTAATAAAGTGTTCATCGTATCTAGATAACAACGACTTATAATTAATGTCCAAGTCTTGAACTCGCTGAATGATCTTTTTTAACCCAGTTAGTTTTAGATTGGTACGAAATATATTGTTAAACTTTGATTCTACAGTAATGTCCAAATCTAGTTGTGGAAATACGTCCATCAGCATTGTTTCAAACTTGATAATATTATTATCCACATCTTCATATGATCCAAATTCTGGAATAGATGTATCACACGCTCTGGCTACCCAGATAACCAAAAAGTCGCACGCTCCAGGATATACATTGTATAAAAAGCTATCGTTTTTAAGTAGTGTTTTTTTTTGCGTATATACGAAGGGATTGAAAATAGTATTATCGACGTAACTATATTCCAAATTATTCTTATGCGAATAGATAATAATATCGTCCTTAATATCTAACAAATTTCCTAAATATCCCTTTAATTGAGTCATTCGAAGCGTCAATAGAATATGTCTCTTAACTATTTCCGGCTGTTGTATATTTAAATGACTTCGTAAGAAATAATATATGGGCGACTTTTCATCTATGTAATCATATGGAGTGAGATATAGGGCTCGTTCTACCTCCTGCCCTTTACCCACCTGTAATACCAATTGTGGACTCACTATATATCGCATATTTATATCGTGGGGTAAAGTGAAAATCTACTACCGATGATGTAAGTCTTACAATGTTTGAACCAGTACCAGATCTTAATTTGGAGGCCTCCGTAGAACTAGGGGAGGTAAATATAGATCAAACAACACCTATGATAAAGGAGAATATCGGTTTTATATCCCGCAGTAGACGTCTATTCGCCCATAGATCTAAGGATGATGAGAGAAAACTAGCACTACGATTCTTTTTACAAAGACTTTATTTTTTAGATCATAGAGAGATTCATTATTTGTTCAGATGCGTTGACGCTGTAAAAGACGTCACTATTACCAAAAAAAATAACATTATTGTGGCGCCTTATATAGCACTTTTAACTATTGCATCAAAAGGATGCAAACTTACAGAAACAATGATTGAAGCATTCTTTCCAGAACTATATAATGAACATAGTAAGAAATTCAAATTCAACTCTCAAGTATCCATCATCCAAGAAAAACTTGGATATCAGTCTGGAAACTATCACGTTTATGATTTTGAACCGTATTACTCTACAGTAGCTTTGGCTATTCGAGATGAACATTCATCTGGCATTTTTAATATCCGTCAAGAGAGTTATCTGGTAAGTTCATTATCTGAAATAACATATAGATTTTATCTAATTAATCTAAAATCTGATCTTGTTCAATGGAGTGCTAGTACGGGCGCTGTAATTAATCAAATGGTAAATACTGTATTGATTACAGTGTATGAAAAGTTACAACTGGTCATAGAAAATGATTCACAATTTATATGTTCATTGGCTGTGGAATCAGAACTTCCAATAAAATTACTTAAAGATAGAAATGAATTATTTACAAAATTCATCAACGAGTTAAAAAAGACCAGTTCATTCAAGATAAGCAAACGTGATAAGGATACGTTACTAAAATATTTTACTTAGGAGTTAGAATTTATAGACGACTCATTTAGTTTATTACTATTACTAGCATTATTGGTATTCTTCTTGTCATCTTGTTCAGAAATATATAACAATGCTATACCTAATACTAAATACATTATCATGCTTGCAATGGCTCTAACAACGACGAACCAAAATGAATTTGGTCGTAGCTTTTGTTCACAAAAATACATAAAGAAATGTCTACATAAATCTATGGCGCCATTGGCATCTTGAAATAGCGCCAGTCCTCCTACAGATTTTAATATAGCTGTATAACATGACATTTATTCATCATCAAAAGAGACAGAGTCACCATCTGTCATATTTAGATTTTTTTTCATGTGTTCAAAGTATCCTCTACTCATTTCATTATAATAGTTTATCATACTTAGAATTTTAGGACGGATCAATGAGTAAGACTTGACTAAATCGTCAGTAGTAATTTGCGCATCGTCTATTCTACATCCGCTTCGTCGAATAATGTATAGCATTGCTTTGAGATTCTCCATAGCTATCAAGTCTTTATACAATGACATGGAAATATCTGTGAACACTTTATACTTCTCCAACATCGATGCCTTAACATCATCACCTACTTTAGCATTGAAAATACGTTCTATTGTGTAGATGGATGTAGCAAGATTTTTAAACAACAATGCCATCTTACATGATGATTGCCTTAAGTCTCCAATCTTTTGTTTAGAATGATTAGCTACAGAGTCCAACGCTTGGCTGACTAGCATATTATTATCTTTAGAAATTGTATTCTTCAATGAGGCGTTTATCATATCTGTAATTTCGTTAGTCATATTACAGTCTGACTGGGTTGTAATGTTATCCAACATATCACCTATGGATACGGTACACGTACCAGCATTTGTAATAATCCTATCTAAGATGTTGTATGGCATTGCGCAGAAAATATCTTCTCCTGTAATATCTCCACTCTCGATAAATCTACTTAGATTATTCTTAAATGCCTTATTCTCTGGAGAAAAGATATCAGTGTCCATCATTTCATTAATAGTATACGCAGAAAAGATACCACGAGTATCAATTCTATCCAAGATACTTATTGGTTCCGAGTCACAAATAATGGTTTCCTCTCCTTCAGGAGATCCTGCATAGAAATATCTAGGACAATAGTTTCTATACTGTCTGTAACTCTGATAATCTCTAAAGTCACTAACTGATACCATGAAATTGAGAAGATCAAACGCTGAAGTAATCAATTTTTCTGCCTCGTTTTTACTACAACTAGTTTTCATCAATGTAGTGACGATGTATTGTTTAGTTACTCTTGGTCTAATACTGATGATAGAGATATTATTACTTCCCATAATGGATCTTCTAGTAGTCACCTTAAAGCCCATTGATGCGAATAGCAGATAGATAAAGTCTTGGTATGACTCCTTTCTAATATAGTACGGACTACCTTTGTCACCCAACTTTATACCCACATAAGCCATAACAACCTCTTTAATAGCCGTTTCATGAGGTTTATCAGCCATGAGCCTAAGTAGTTGGAAGAATCTCATGAATCCCGTCTCAGAAAGTCCTATATGCATGATAGAAGATTTATCTTTCTTGGGAAACTCTCGTATAGTCATAGATGAAATACTCTTTAAAGTTTCTGAAATAAGATCAGTAACAGTCTTACCTCCGACTACTCTGGGTAACAAACATACTCTAATAGGTGTTTTCTCTGCGGAGATAATATCAGAAAGGATAGAGCAATAAGTAGTATTATTGTGATTATAAAGACCGAATACATAACAGGTAGAATTTATAAACATCATGTCCTGAAGGTTTTTAGACTTGTATTCCTCGTAATCCATACCGTCCCAAAACATGGATTTGGTAACTTTGATAGCCGTAGATCTTTGTTCCTTCGCCAACAGGTTAAAGAAATTAATAAAGAATTTGTTGTTTCTATTTATGTCCACAAATTGCATATTTGGAAGTCCCACCGTTACGTTCACTGCAGCATTTTGAGGATCTCGAGTATGAAGTACGATGTTATTGTTTACTGGTATATCTGGAAAGAATTCTACCAGTCTAGGAATAAGAGATTGATATCGCATAGAAATACAAAAGTTAATAATCTCATCATCGAAGATTACTCGGTTACCATTGTAATAAATTGGTACTCTATCATAATCGTCGACAAAGTACTGTTCATACATGATGAGATGTTTATATGTTGGCATAGTAGTGAGATCGACGTTTGGTAATGGCAATGTATTAAGATTAACTCCATAATGTCTAGCAGCATCCGCGATGGCATAAGTGTTGTCAAAGCGGGGTTGATCTTGTGCTGTTATATATTGTCTAACACCTATAAGATTATCAAAATCTTGTCTGCTTAATACACCGTTAACAATTTTCGCCTTGAATTCTTTTATTGGTGCATTAATAACATCCTTATAGAGGATGTTAAACAAATAAGTATTATCAAAGTTAAGATCTGGGTATTTCTTTTCTGCTAGAACATCCATTGAGTCGGAGCCATCTGGTTTAATATAACCACCGATAAATCTAGCTCTGTATTCTGTATCCGTCAATCTAATATTAAGAAGGTGTTGAGTGAAAGGTGGAAGATCGTAAAAGCTGTGAGTATTAATGATAGGGTTAGTTTCCGAACTAATGTTAATTGGGGTATTAATAATATCGATATTTCCAGCGTTAAGTGTAACATTAAACAGTTTTAATTCACGTGACGTGGTATCAATTAAATAATTAATGCCCAATTTGGATATAGCAGCCTGAAGCTCATCTTGTTTAGTTACGGATCCTAATGAGTTATTAAGAAATACATCAAACGGATGAACGAAGGTTGTTTTAAGTTGGTCACATACTTTGTAATCTAGACATAGATGCGGAAGAACGGTAGAAACTATACGAAATAGATATTCAGAGTCCTCTAATTGATCAAGAGTAACTATTGACTTAATAGGCATCATTTATTTAGTATTAAATGACGACCGTACCAGTGACAGATATACAAAACGACTTAATTACAGAGTTTTCAGAAGATAATTATCCATCTAACAAAAATTATGAAATAACTCTTCGTCAAATGTCTATTCTAACTCACGTTAATAACGTGGTAGATAGAGAACATAATGCCGCCGTAGTGTCATCTCCAGAGGAAATATCATCACAACTTAATGAAGATTTATTTCCAGACGATGATTCTCCGGCCACTATTATTGAAAGAGTACAACAACCGCATACTACTATTATTGACGATACGCCACCTCCTACGTTTCGTAGAGAGTTATTGATATCGGAACAACGTCAGCAACGAGAAAAAAGATTTAATATTACGGTATCAAAAAATGCCGAAGCGATAATGGAATCTAGATCGATGATAACTTCTATGCCAACACAAACACCATCCTTGGGAGTAGTTTATGATAAAGATAAAAGAATTCAGATGCTAGAGGATGAAGTGGTTAATCTTAGAAATCAACAATCTAATACAAAATCATCTAATAATTTGGATAATTTTACCAGAATACTATTTGGTAAGACTCCGTATAAATCAACAGAAGTTAATAAGCGTATAGCCATCGTTAATTATGCAAATTTGAATGGGTCCCCCTTATCAGTAGAGGACTTGGATGTTTGTTCAGAGGATGAAATAGATAGAATCTATAAAACGATTAAACAATATCACGAAAGTAGAAAACGAAAAATTATCGTTACTAACGTGATTATTATTGTCATAAACATTATTGAACAGGCATTGCTAAAACTTGGATTTGACGAAATCAAAGGACTGAGTACCGACATCACTTCAGAAATTATCGATGTGGAGATCGGAGACGACTGTGATGCTGTAGCATCAAAACTAGGAATCGGCAACAGTCCGGTTCTTAATATTGTATTGTTTATACTCAAGATATTTGTTAAACGAATTAAAATTATTTAATTTAATACATTCCCATATCCAGACAACAATCGTTTGGATTAATCTGTTCCTGTCGTCTCATACCGGACGACATATTAATCTTTTTATTAGTGGGCATCTTTTTAGATGGTTTCTTTTTCCCAGCATTAACTGATTCGATACCTAGAAGATCGTGATTGATCTCTCCGACCATTCCACGAACTTCTAATTGACCGTCTCTAACGGTACCATAAACTATTTTACCAGCATTAGTAACAGCTTGGACAATCTGACCATCCATTGCGTTGAATGATGTAGTTGCTGTTGTTCTACGTCTAGGAGCACCAGAAGTATTTTTAGAGCTCTTGGATGTTGATGTAGAAGAAGACGAGGATTTTGATTTACATGTGATACATTTTGAACTCTTTGATTTTGTGTCACATGCGCCGGCAGTCACATCTGTTTGAGAATTAAGATTATTGTTGCCTCCTTTGACGGCTGCATCTCCACCGATCTGTGCTAGTAGATTTTTAAGCTGTGGTGTAATCTTATTAACTGTTTCGATATAATCATCGTAACTGCTTCTAACGGCTAAATTTTTTTTATCCGCCATTTAGAAGCTAAAAATATTTTTATTTATACAGAAGATTTAACTAGATTATACAATGAACTAATATGATCCTTTTCCAGATTATTTACAAACCTGGTATTTCTTGTTTCTGGAGGAGGCAAGTTTACATTCGGACTTGGATTTTGTGAGTTCTTGATCTTATTATACATCGCGTATAGGATGGCGACGGTAACTGCTACACAAATACCAATCAAAAGAAGAATACCAATCATTTATTGATAATAACTTCACTATTGATCAAGTATACAATATATCATCTTTTCACTAAATAAGTAGTAATAATGATTCAACAATGTCGAGATATATGAACGATAATAACTTAGTTTACGGAAATATCGCTATGATTGGTATGAATGACTCCACTAACTCTGTGGGGTGCGCAGTGCTTTCCCCACATAGAATAAATTAGCATTCCGACTGTGATAATAATACCAAGTATAAACGACATAATACTCAATACTTTCCATGTACGAGTGGGACTGGTAGACTTACTAAAGTCAATAAAGGCAAAGATACACGAAAGAATCAAAAGAATGATTCCAGCAATTAACACTCCGGAAAAATAATTTCCAATCATAAGCATCATGTCCATTTAACTAATAAAAATTTTAAATCGCCGAATGAACAAAGTGGAATATAAACCATATAAAAACAATAGTTTGTACTGCAAAAATAATATCTATTTTTGTTTTCGAAGATATGGTAAAATTAAATAGTAGTACACAACATGTTATAACTAACAGCAGCAACGGTTCGTAATTACTTATCATTTACTAGACGAAAAGGTGGCGGGATATTTTCTTGCTCAAATAATACGAATATATTACCCATCCATTTTATGCGATGTTTATATACTCTAATCTTTAATAGATCTATAGACGACGGGTTTACCAACAATATAGATTTTATCGATTCATCTAATTTAAACCCTTCCTTAAACGTGAATGATCTATTATCTGGCATAACGATGACCCTACCTGATGAATCAGACAATGTACTGGGCCATGTAGAATAAATTATCAACGAATCATCGTCTACGAACATTTATATCATTTGTTTTAATTTTAGGACGCGAATAAATAGATATAAAATAGAAAATAACAGATATTACAACCAGTGTTATGGCCGCACCCAACCAGGTAGGCAGTTTTATTTTATCTTTTACTACAGGTTCTCCTGGATGTATGTCACCAACTGTAGACGTAGTTCTAGTACAATTAGACGTAAGTTCCGCTTGGGAATTTTTTAACGCTAAAGAGTTAACGTTAATCGTACACCCAACGTATTTACATCTAGTTCTTTGAACATCTTGATTATAATATAACCATTTTCTATCTCTAGATTCGTCAGTGCACTCATGTAACCAACATACCCTAGGTCCTAAATATTTATCTCCGGAATTAGATTTTGGATAATTCGCACACCAACAATTTCTATTTCCTTTATGATCGTTACAAAAGACGTATAATGCCGTATCCCCAAAAGTAAAATAATCAGGACGAATAATTCTAATAAACTCAGAACAATATCTCGCATCCATATGTTTGGAGCAAATATCGGAATAAGTAGACATAGCCGGTTTCCGTTTTACACGTAACCATTCTAAACAATTGGGGTTTCCAGGATCGTTTCTACAAAATCCAGTCATAAAATCGTCACAATGTTCTGTCTTGTAATTATTATTAAATATTTTTGGACAGTGTTTGGTATTTGTCTTTGAACAACATTTTGCTACGCTATCACTATCTCCCAGGAGATAATCCTTTTTTATAAAATGACATCGTTGCCCGGATGCTATATAATCAGTAGCGTGTTTTAAATCCTTAATATATTCAGGAGTTACCTCGTTCTGATAATAGATTAATGTTCCAGGACGAAATTTGAAAGAACTACATGGTTCTCCATGAATTAATACATATTGTTTAGCAAACTCAGGAACTATAAAACTACTACAATGATCTATCGACATACCATCTATCAAACAAAATTTGGGTTTAATTTCTCCTGGAGACGTTTCATAATAATACATATAACTTTCTTCGGCAAACTTAACAGTTCTATTATATTTAGGATAATTAAAACCTAATTCCATATATTTGTCTCGTATATCTGCTATTCCTGATGCTATGTTGATTCTATTAAGAGTAACGGCTGCCCCCATTCTTAATAATCGTCAGTATTTAAACTGTTAAATGTTGGTATATCAACATCTACCTTATTTCCCGCAGTATAAGGTTTGTTGCAGGTATACTGTTCAGGAATGGTTACATTTATACTTCTTCTATAGTCCTGTCTTTCGATGTTCATCACATATGCAAAGAACAAAATAAACAAAATAATGTAAGAAATAATATTAAATATCTGTGAATTCGTAAATACATTAATTGCCATAATAATTACAGCAGCTACAATACACACAATAGACATTCCCACAGTGTTGCCATTACCTCCACGATACATTTGAGTTACTAAGCAATAGGTAATAACTAAGCTAGTAAGAGGCAATAGAAAAGATGAGATAAATATCATCAATATAGAGATTAGAGGAGGGCTATATAGAGCCAAGACGAACAAAATCAAACCGAGTAACGTTCTAACATCATTATTTTTGAAGATTCCCAAATAATCATTCATTCCTCCATAATCGTTTTGCATCATACCTCCATCTTTAGGCATAAACGATTGCTGCTGTTCCTCTGTAAATAAATCTTTATCAAGCACTCCAGCACCCGCAGAGAAGTCGTCAAGCATATTGTAATATCTTAAATAACTCATTTATATATTAAAAAATGTCACTATTAAAGATGGAGTATAATCTTTATGCCGAACTAAAAAAAATAACTTGTGGTCAATCCCTAAGTCTTTTTAACGAAGACGGGGATTTCGTAGAAGTTGAACCGGGATCATTATTTAAGTTTTTGATACCTAAGGGATTTTACTCCTCTCCTTCCGTAAAGACGAGTTTAGTATTCGAGACATTAACAACGACCGATAATAAAATTACTAGTATCAATCCAACAAATGCACCAAAGTTATATCCTCTTCAACACAAAGTCGTATCTGAAGTAGTTTCTAATATGAGGAAAATGATTAAATTAAAACGTCCTCTATACATCACTCTTCACTTGGCGTGTGGATTTGGTAAGACTATTACCACGTGTTATCTTATGGCTACACACGGAAGAAAAACCGTCATTTGTGTACCCAATAAAATGTTAATACATCAATGGAAGACACAGGTAGAGGCAGTCGGATTGGAACATAAGATATCTATAGATGGAGTAAGTAGTTTATTAAAGGAACTAAAGACTCAAAGTCCGGATGTATTAATAGTAGTTAGTAGACATCTGACAAACGATGCCTTTTGTAAATATATCAATAAGCATTATGATTTGTTCATCTTGGATGAATCACATACGTATAATCTGATGAACAATACAGCAGTTACAAGATTTTTAGCGTATTATCCTCCGATGATGTGTTATTTTTTAACTGCTACACCTAGACCATCTAACAGAATTTATTGTAACAGTATTATTAATATTGCCAAGTTATCCGATCTAAAAAAAACTATCTATGCTGTAGATAGTTTTTTTGAGCCATATTCCACAGATAATATTAGACATATGATAAAACGACTAGATGGACCATCTAATAAATATCATATATATACTGAAAAGTTATTATCTGTAGACGAGCCTAGAAATCAACTTATTCTTAATACCCTGGTAGAAGAATTCAAGTCAGGAACTATTAATCGCGTTTTAGTTATTACTAAACTACGTGAACATATGGTATTATTCTACAAACGATTATTAGATCTTTTCGGACCAGAGGTTGTATTTATAGGAGACGCTCAAAATAGACGTACTCCAGATATGGTTAAATCAATCAAGGAACTAAATAGATTTATATTCGTATCCACCTTATTTTATTCCGGTACTGGTTTAGATATTCCTAGTTTGGATTCGTTGTTCATTTGCTCGGCAGTAATCAACAATATGCAAATAGAGCAATTACTAGGGAGGGTATGTCGAGAAACAGAACTATTAGATAGGACGGTATATGTATTTCCTAACACATCCGTCAAAGAAATAAAGTACATGATAGGAAATTTTGTGCAACGAATTATTAGTCTGTCTGTAGATAAACTAGGATTTAAACAAGAAAGTTATCGGAAACATCAAGAATCCGATCCCACTTCTGTATGTACAGCATCCTCCAGAGAAGAACGTGTATTAAATAGAATATTTAACTCGCAAAATCGTTAAGAAGTTTAAGCGACGATCCACATGCTGTACAGGCCAGTGTATTACCCCTCATAGTATTAATATAATCCAATGATACTTTTGTGATGTCGGAAATCTTAACCAATTTAGACTGACAGACAGAACACGTCATGCAATCATCCTCATCGATAACTGTAGTCTTAGGCTTCTTTTTGCGACTCTTCATTCCGGAACGCGCATTGGTGCTATCCATTTAGGTAGTAAAAAATAAGTCAGAATATGCCCTATAGCACGATCGTGTAAAACCTGGTATATCGTCTCTATCTTTATCACAGTATAGTGTATCGACATCTTTATTATTATTGACCTCGTTTATCTCGGAACATGGAATGGGAACATTTTTGTTATCAACGGCCACCTTTGCCTTAATTCCAGATGTTGTAAAATTATAACTAAACAGTCTATCATCGACACAAATGAAATTTTTGTTTAAACGTTTGTAGTTTACGTATGCGGCTCGTTCTCGTCTCATTTTTTCAGATATTGCAGGTACTATAATATTAAAAATAAGAATGAAATAACATAGGATTAAAAATAAAGTTATCATGACTTCTAGCGCTGATTTAACTAACTTAAAAGAATTACTTAGTCTGTACAAAAGTTTGAGATTTTCAGATTCTGCGGCTATAGAGAAGTATAATTCTTTGGTAGAATGGGGAACATCTACTTACTGGAAAATAGGCGTACAAAAGGTAGCTAATGTCGAGACGTCCATATCTGATTATTATGATGAGGTAAAAAATAAACCGTTTAATATTGATCCAGGGTATTATATTTTCTTACCGGTATATTTTGGAAGCGTCTTTATTTATTCGAAGGGTAAAAATATGGTAGAACTTGGATCTGGAAACTCTTTTCAAATACCAGATGATATGCGAAGTGTGTGTAACAAAGTATTAGACGGCGATAACGGAATAGACTTTCTGAGATTTGTTTTGTTAAACAATAGATGGATAATGGAAGACGCTATATCAAAATATCAGTCACCAGTTAATATATTTAAACTAGCGAGTGAGTACGGATTAAACATACCCAACTATTTAGAAATTGAAATAGAGGAAGACACATTATTTGACGACGAGTTATACTCTATTATAGAACGCTCTTTCGATGATAATTTTCCAAAAATATCCATATCGTATATTAAGTTGGGAGAACTTAGGCGGCAAGTTGTAGACTTTTTCAAATTCTCATTCATGTATATTGAGTCCATCAAGGTAGATCGTATAGGGGATAATATTTTTATTCCTAGCGTTATAACAAAATCAGGAAAAAAGATATTAGTAAAAGATGTAGACCATTTAATACGATCCAAGGTTAGAGAACATACATTTGTAAAAGTAAAAAAGAAAAACACATTTTCCATTTTATACGACTATGATGGGAACGGAACAGAAACTAGAGGAGAAGTAATAAAACGAATTATAGACACTATAGGACGAGACTATTATGTTAACGGAAAGTATTTCTCTAAGGTTGGTAGTGCGGGCTTAAAGCAATTGACTAATAAATTAAATATTAATGAGTGCACAACTGTCGATGAGTTAGTTGATGAGATTAATAAATCCGGAACTGTAAAACGAAAAATAAAAACCCAATCAGCATTTGATTTAAGTAGAGAATGTTTGGGATATCCAGAAGCGGATTTTATAACGTTAGTTAATAACATGCGGTTCAAAATAGAAAATTGTAAGGTTGTAAATTTTAATATTGAAAATACTAATTGTTTAAATAACCCAAGTATTGAAACTATATATGGAAACTTTAACCAGTTCGTCTCAATCTTTAATATCGTCACCGATGTCAAAAAAAGATTATTCGAGTGAAATAATATGTGCCTTTGATATAGGTGCAAAAAATCCTGCCAGAACTGTTTTAGAAGTCAAGGATAACTCCGTTAGGGTATTGGATATATCAAAATTAGACTGGAGTTCTGATTGGGAAAGGCGCATAGCTAAAGATTTGTCACAATATGAATACACTACAGTTCTTCTAGAACGTCAGCCTAGAAGGTCGCCATACGTCAAATTTATCTATTTTATTAAAGGCTTTTTATATCATACATCGGCTACCAAAGTTATTTGTGTCTCGCCTGTCATGTCTGGTAATTCATATAGAGATCGAAAAAAGAGATCTGTCGAAGCATTTTTTGATTGGATGGACATATTCGGATTGCGAGACTCCGTTCCAGATAGACGCAAATTAGACGATGTAGCGGATAGTTTCAATTTGGCTATGAGATACGTATTAGATAAATGGAATACTAATTATACACATTATAATAGGTGTAAATCTAGAAATTACATAAAAAAAATGTAATAACGTTAGTAACGCCATTATGGATAATCTATTTACCTTTCTACATGAAATAGAAGATAGATATACCAGAACTATTTTTAACTTTCATCTAATAAGTTGCGATGAAATAGGAGATATATATGGTCTTATGAAAGAACGCATTTCCTCAGAGGATATGTTTGATAATATAGTATATAATAAAGATATACATCCTGCCATTAAGAAACTAGTTTATTGTGACATTCAACTTACTAAACACATTATTAATCAGAATACGTATCCGGTATTTAACGATTCTTCACAAGTGAAATGTTGTCATTATTTCGATATAAACTCAGATAATAGCAATATTAGCTCTCGTACAGTAGAGATATTTGAGAGGGAAAAGTCATCTCTTGTATCATATATTAAAACTACCAATAAGAAGAGAAAAGTCAATTATGGGGAAATAAAGAAAACTGTACATGGAGGCACTAATGCAAATTACTTTTCCGGTAAAAAATCTGATGAGTATCTGAGCACTACAGTCAGGTCCAATATTAATCAACCTTGGATCAAAACCATCTCTAAGAGGATGAGAGTCAATATCATTAATCACTCTATAGTAACGCGTGGAAAAAGCTCCATATTACAAACTATAGAAATTATTTTTACTAATAGAACATGTGTGAAAATATTCAAGGATTCTACTATGCACATTATTCTATCCAAGGACAAGGATGAAAAGGGGTGTATACACATGATTGACAAATTATTCTATGTCTATTATAATTTATTTCTGTTGTTCGAGGATATCATCCAAAACGAGTACTTTAAAGAAGTAGCTAATGTTGTGAACCATGTACTCACGGCTACGGCATTAGATGAGAAATTATTCCTAATTAAGAAAATGGCTAAACACGATGTTTATGGAGTTAGCAATTTCAAAATAGGGATGTTTAACTTGACATTTATTAAGTCATTGGATCATACCGTTTTCCCCTCTCTGTTAGATGAGGATAGTAAAATAAAGTTTTTTAAGGGGAAAAAGCTCAATATTGTAGCATTACGCTCTCTGGAGGATTGTATAAATTACGTGACTAAATCCGAGAATATGATAGAAATGATGAAGGAAAGATCGACTATTTTAAATAGCATAGATATAGAAACGGAATCGGTAGATCGTCTAAAAGATTTGCTTCTAAAATGAAAAAAAACACTGATTCAGAAATGGATCAACGACTCGGGTATAAGTTTTTGGTACCTGATCCTAAAGCCGGAGTTTTTTATAGACCGTTACATTTCCAATATGTATCGTATTCTAATTTTATATTGCATCGATTGCATGGAATCTTGACCGTCAAGCGGCCACTTTTATCGTTTAAGAATAATACAGAACGAATTATGATAGAAATTAGCAATGTTAAAGTGACTCCTCCAGATTACTCACCTATAATCGCGAGTATTAAAGGTAAGAGTTATGACGCATTAGCCACGTTCACTGTAAATATCTTTAAAGAGGTAATGGCCAAAGAGGGTATATCCATCACTAAAATAAGTAGCTATGAGGGAAAAGATTCTCATTTGATAAAAATTCCGCTACTAATAGGATACGGGAATAAAAATCCACTTGATACAGCCAAGTATCTTGTCCCTAACGTCATAGGTGGGGTATTTATCAATAAACAATCTGTCGAAAAAGTAGGAATTAATCTAGTAGAAAAGATTACAACATGGCCAAAATTTAGGGTTGTTAAGCCAAACTCATTCACGTTCTCGTTTTCCTCCGTATCCCCTCCTAATATATTACCGACAAGATATCGCCATTACAAGATATCTCTGGATATATCACAATTGGAAGCGTCGAATATATCATCGACAAAGACATTTATAACGGTCAATATTGTTTTACTGTCTCAATATTTATCTAGAGTGAGTTTAGGATTCATTAGACGTAGTTTATCATACGATATGCCTCCAGAAGTTGTCTATTTAGTAAACGCGATAATAGATAGTGCTAAACGACTTACCGAATCTATTACTGACTTTAATATTGATACGTATATTAATGACCTGGTGGAAGCTGAACACGTTAAACAAAAATCTCAGTTAACGATTAACGAGTTCAAATATGAAATGCTACATAACTTTTTACCTCATATGAACTATACACCCGATCAACTAAAGGGATTTTATATGATATCTTTACTAAGAAAGTTTCTTTACTGTATCTACTACACTTCTAGATATCCAGATAGAGATTCAATGGTTTGTCATCGCATCCTAACATACGGCAAATATTTTGAGACGTTGGCACATGATGAATTAGAGAATTACATAGGCAACATCAGAAACGACATCATGAACAATCACAAGAATAGAGGCACTTACGCGGTAAACATTCATGTACTAACAACTCCCGGACTTAATCACGCGTTTTCTAGCTTATTGAGTGGAAAGTTCAAAAAGTCAGACGGTAGTTATCGAACACATCCTCACTATTCATGGATGCAGAATATTTCTATTCCTAGAAGTGTTGGGTTTTATCCGGATCAAGTAAAGATTTCAAAGATGTTTTCTGTCAGAAAATACCATCCAAGTCAATATCTTTACTTTTGTTCATCAGATGTTCCGGAAAGAGGTCCTCAGGTAGGTTTAGTATCTCAATTGTCTGTCTTAAGTTCCATTACAAATATACTAACGTCTGAGTATTTGGATTTGGAAAAGAAAATTTGTGAGTATATCAGATCATATTATAAAGATGATATAAGTTACTTTGAAACAGGATTTCCAATCACTATAGAAAATGCTCTAGTCGCATCTCTTAATCCAAATATGATATGTGATTTTGTAACTGACTTTAGACGTAGAAAACGGATGGGATTCTTCGGTAACTTGGAGGTAGGTATTACTTTAGTTAGGGATCACATGAATGAAATTCGTATTAATATTGGAGCAGGAAGATTAGTCAGACCATTCTTGGTGGTGGATAACGGAGAGCTCATGATGGATGTGTGTCCGGAGTTAGAAAGCAGATTAGATGACATGACATTCTCTGACATTCAGAAAGAGTTCCCACATGTCATCGAAATGGTAGATATAGAACAATTTACTTTTAGTAACGTATGTGAATCTGTTCAAAAATTTAGAATGATGTCAAAGGATGAAAGAAAGCAATACGATTTATGTGACTTTCCTGCCGAATTTAGAGATGGATATGTAGCATCTTCACTAGTGGGAATCAATCACAATTCTGGTCCAAGAGCTATTCTTGGATGTGCTCAAGCTAAACAAGCTATCTCTTGTCTGAGTTCGGATATACGAAATAAAATAGACAATGGAATTCATTTGATGTATCCAGAGAGGCCAATTGTCATTAGTAAGGCTTTAGAAACTTCAAAGATTGCGGCTAATTGCTTCGGACAACATGTTACTATAGCATTAATGTCGTACAAAGGTATCAATCAAGAGGATGGAATTATCATCAAAAAACAATTTATTCAGAGAGGCGGTCTTGATATTGTTACAGCCAAGAAACACCAAGTAGAAATTCCATTGGAAAACTTTAATAACAAAGAAAGAGATAGGTCTAACGCCTATTCGAAATTAGAAAGTAATGGATTAGTTAGACTGAATGCTTTCTTGGAATCCGGAGACGCTATGGCACGAAATATCTCATCAAGAACTCTTGAAGATGATTTTGCTAGAGATAATCAGATTAGCTTTGATGTTTCCGAGAAATATACCGATATGTACAAATCTCGCGTTGAACGAGTGCAAGTAGAACTTACTGACAAAGTTAAGGTGCGAGTATTAACCATGAAAGAAAGAAGACCCATTCTAGGAGATAAATTTACCACTAGAACGAGTCAAAAGGGAACAGTCGCGTATATCGCAGATGAAACGGAACTTCCATACGACGAAAATGGTATCACACCAGATGTCATTATTAATTCTACATCCATCTTCTCTAGAAAAACTATATCTATGTTGATAGAAGTTATTTTAACAGCCGCATATTCCGCTAAGCCGTACAACAATAAGGGAGAAAACCGACCTGTCTGTTTTCCTAGTAGTAACGAAACATCCATCGATACATATATGCAATTCGCTAAACAATGTTATGAGCATTTAAATCCGAAATTGACCGAGAAAGAATTATCGGATAAAATCTTTTGTGAAAAGATTCTTTATGATCCTGAAACGGATAAGCCTTATGCATCCAAAGTATTTTTTGGACCAATTTATTACTTGCGTCTGAGACATTTAACTCAGGACAAGGCAACCGTTAGATGTAGAGGTAAAAAGACGAAGCTAATTAGACAAGCGAATGAGGGACGAAAACGTGGAGGAGGTATCAAGTTTGGAGAAATGGAGAGAGACTGTTTAATAGCGCATGGCGCAGCCAATACTATTACAGAAGTTTTAAAAGACTCAGAAGAGGATTATCAAGATGTGTATATTTGTGAAAATTGTGGAGACATAGCAGCACAAATCAAAAGTATTAATACATGTCTTAGATGTTCAAAACTTAATCTCTCTCCTCTCTTAACAAAAATTGATACCACGCACGTATCTAAAGTATTTCTTACTCAAATGAACGCCAGAGGCGTAAAAGTCAAATTAGATTTCGAACGAAGACCTCCTTCGTTTTATAAACCATTAGATAAAGTTGATCTCAAGCCGTCTTTTCTGAAGTAGGTTTCTTATTTAGTAGTTGTTGTAGATAATCGTTAATATTATCAAGATAATCCAAATTTAATTCTTCAAATGAAGTAGGATCTTCTATAACTACTTTATCATAGTCTAAAGATGACTGATATTCAGGTTTTGTTGGCGGTTGAGGTGTAACAGCTACTTGAGATACCAAAGTCTGATATTCAGAAAGCTGTGGATGTTTGGGTTCAACATCCACTGATGGTGTTACACCAATCGGTTCAGTGACGTCTGTGGATGGAGGTGCTACTTTTACAGAACCTGTAGCCTTAGTTGTCGACGGAGATACATCTTCAATGCGAGGAAATGTATAATTTGGTAATGTTTCTTATGTGGATCTGAAGAAGAGGTAAGATATCTACTAGAAAGATACCGATCACGTTCTAGTTCTCTTTTGTAGAACTTAACTTTTTCTTTCTCAGCATCTAGTTGATATTCCAACCTCTTCACGTTACTACGTTCATATTCCAATTCACGTTCGCATGGGTTACCTTCGCAGTTTTTACGAGCGATTTCACGTTCCAGATTACGTTTAGCCTTCATGTGTCTCTCACTCTCTCTATCGAGTTTATCAGAGCAGTCTTTCTAAAGGCGATCGAACTCCATAAATTTCTCCAACGATTTGATTGTTTCCATAGATTTCCGAAGTTCAGCTTCTAGGACGGCGATTCTTTTTCTTTCGAATTCACAGCTGGATGTACAACCGTTTCCTCCGTTTCCATTACCATCTCTAAGTTTCTTTCCTAGATCGGCAATCTTTCTCAACATTTCATCCCCATACATTTTACATTCCTCGAGTCTACCGTCGTCGAAATATCTTTCCAACTCCTTTTCGACCTCAATAACTTTAACACGTTGTCTTTCAAGCTCTCGTTTGTAGCAACTATCATTTTATCTGATTCCCTGGCGCGTTTAAGATCTTCATGCAATTGAGTCAGCTCTTAACGCAATCTCTTGCTTCTTCGTCATAGTACTCACAATCACTGTGAGATCCATTGCTACCACGTCTACACTTGGTGAGCTCGCGTTTAAGAGATTCAATTTCCCGTTTGTATTGGTCCATGTCTCCATTGCCGCAACCATTAGATTTACAGGCTGTTAGCTGTCGTTCAAGATCAGAAATACGGTTTTTCTTGGAATTGATTTCGTCAATGTACTTAACATGATTAACATCGAAACACTTATTAAGTTCTTTTTCCAATTCTACGATTTTATTTCTTTCGCGAGTCAATTCCCTCCTGTAGTAGCTATCGGTTTTGTCAGATTCACGCTCTCTACGTAGACTTTCTTGCAAGTTACTAATTTGTTCCCTGACACGTCCAAGTTCAGTTTTATATGCCGAATAGAGTTCTGATTTATCCTTTAAGCAGATCTCTAGCGATTGTTTAAGATCACTAATTCTAGTCTTTAGCCTATTTACCTCCTCAGAAGATGTTCCGTTACCGTTGCGTTTACACTCGTTAAGCTGTCTATCAAGATCCATGATTCTATCTCTAAGACGTTGTATCTCTTTTTCCATATCAGCATTGCTTTCATTATTACGTCTGTAGTCACTTAACTATCTTTCAAGATTTGAAATTCTATCTCTAAGACGTCTCATTTCTATCTGTTTCGGTATTGGTCTTATTATTATGTCTACAGTCGTTCAACTGTCTTTCAAGATCTAATATTCTAGATTGGAGTCTACTAATCTCTGTAAGATTTCCTCCTCCGCTCTCAATGCAGTCGGTCAACTTATTCTCTAGTTCGCTAATACGCGAACGCAGTGCATCAACTTCTTGCGTGTCTTTCTGGTTGTGTGTACATTCATCGAGTCTAGATTCGAGATCTCTAACGCGTCGTCGTTCTTCCTCAAGTTCTCCGCGTACTGCAGAAAGCGTGTCCCTATCTTGTTGATATTTAGCAATTTCTGATTCTAGAGTACTGATTCTGCTCACGTAGTTACTAATAGTTGTCTTAGCCTTATCAAGTTCCTCCTTGTATTTGTCGCATTCCTTGATATCCCTACGAAGTTTGGACAGTTCCCATTCGACATTACGACGTTTATCGATTTCAGCTCGGAGATCGTCATCGCGTTGTTTTAGCCACATACGACTAAGTTCAAGTCTTCCTTGACAAGATCCATCTACTTTTCCATCCCTAATAGTATCCAGTTCCTTTTCTAGTTCTGAACGCATTTCTCGTTCCCTATCAAGCGATTCTTTCAATTCTCGGATAGTCTTCTTATCAATTTCTGATGAATCTGAACCATCATCTGTCCCATTTTGAATATCCCTAAGTTCTTTGATCTCTTTTGTAAGTCGGTCGATTCTTTCGGTTTTATAAACAGAATCCCTTTCCAAAGTCCTAATCTTACTGAGTTTATCACTAAGTTCTGCATTCAATTCGGTGAGTTTTCTCTTGGCTTCTTCCAACTCTGTTTTAAACTCTCCACCATTTCCGCATTCTTCCTCGCATTTATCTAACCATTCAATTAGTTTATTAATAACTAGTTGGTAATCAGCGATTCCTATAGCCGTTCTTGTAATTGTGGGAACATAATTAGGATCTTCTAATGGATTGTATGACTTAATAGCATCATCTTTATCATTATTAGGGGGATGGACAACCTTAATTGGTTGGTCCTCCTTATCTCCTCCAGTAGCATGTGGTTCTTCAATACCAGTGTTAGTAATAGGCTTAGGCAAATGCTTGTCGTACGCGGGCACTTCCTCATCCATTAAGTATTTATAATCGGGTTCTGCTTCAGAATATTCTTTTCTAAGAGACGCGACTTCAGGAGTTAGTAGAAGAACTCTGTTTCTGTATCTATCAACGCTGGAATCAATACTCAAGTTAAGGATAGCGAATACCTCATCGTCATCATCCGTATCTTCTGAAACACCATCATATGACATTTCATGAAGTCTAACGTATTGATAAATAGAATCAGATTTAGTATTAAACAGATCCTTAACCTTTTTAGTAAATGCATATATATATATTAGATCTCCAGATTTCATAATATGATCACATGCCTTAAATGTCAGTGCTTCCATAATATAGTCTGGAACACTAATGGGTGACGAAAAAGATACCGCACCATATGCTACGTTAATAAATAAATCTGAACCACTAAGTAGATAATGATTAATATTAAGGAAGAGGAAATATTCAGTATATAGATATGCCTTAGCATCATATCTTGTACTAAACACACTAAACAGTTTATTGATGTGATCAATTTCCAACAAAACAATTAGAGCAGCAGGAATACCAACAAACATATTACCACAGCCGTATTTTTTATGAATATCACATATCATGTTAAAAAATCTTGATAGAAGAGCAAATATCTCATCTGACTTAATGAGACGTAGTTCAGCAGCAGCATAAGTCATAACTGTAAATAGAACATACTTTCCTGTAGTATTGATTCTAGACTCCGCATCAACACCATTATTAAAAATAGTTTTATATACATCTTTAATCTGCTCACCGTTAATCGTCGAACGTTCTAGTATACGGAAACACTTTGATTTCTTATCTGTAGTTAATGACTTAGTGATATCACGAAGAATATTACGAATTACATTTCTTGTTTTTCTTGAGAGACATGATTCAGAACTCAACTCATCGTTCCATAGTTTTTCTACCTCAGTGGCGAAATCTTTGGAGTGCTTGGTACATTTTTCAATAAGGTTCGTGACCTCCATTTATTATAAAAAATTTTATTCAAAACTTAACTACAATCGGGTAATTATAAGATCGTAGATCTCCCATGTGGTGGAATACTACCATCTATCGCATGTGGATGGACAGTGGGTAATAGCCATGGGAACAGTAATGATTGCATATTTATCTTTCTTGCTAGTATTACTGCATATTGTCCCAATGTTTCGATGTGGTGTTCTAACCTATCAACTGCCGCTGTATCACAACAATAGTGTCCGATGGAATTAAGATTATGATCCAATGTGTTTAATATATGATTATCAAGTCTTATACGATCCGCGTCTTTTTTGACAGGATCAGGTTCTTCTACATGAAGAAGTTTCGGCCTCTTATAATATTCATGTCTGGGAAACGGTGGTCTAGGGTAAGGCTCCGGTATCGGAGTGGGTTTTGGATTATAATCATCATCGTCTACATCATCATCTTTGATATTTATTTTGCTATCTTGATGATGTCCTGTATCAGTTGCATTTTCAACACTCGACTGAATATTAGCACATTCATTGTCTATTATTACCATATTTCTAAACCCAAAATGTATGTGTTGAACATCAGTACTATTGTTGATGAGTCTTATAGCATGAATTCGCTTATCGTTATCGGGTTTAGTTTCTGTAACCTTAACAATTCCTTTTTTATTAAACTCTACATAATCATAACCATTTCTATTGTTTGTTTTAATATAAACGAGTATAGCATCATTGCTAAATTTTTCAATAGTATCGAAAACAGAATATCCCAAACCATATAATATATATTCAGGGACACTCAAACTAAATGTCCAGGATTCTCCTAAATACGTAAACTTTAATAGTGCGAAATCATTCAAAAATCTATCACTTATAGATAGATAGTACATGAATGCGTATAGTAGTCTACCGATCTCTTTATTATGAAAACCGACATTACCATCATATATTTCGTGATATACATGTGATCCGTTTACGTTAAACCATAAATACATGGGTGACCCTATAAACATGAATTTATTTCTAATTCTCAGAGCCATAGTTAATTGACCGTGTAATATTTGCTTACATGCATACTTGATACGATCATTAATAAGATTTTTATTATTGCTCGTTATTTCAGAATCGTATATATAAGGAGTACCATTGTGATTCTTACCAGATATTATACAAAATACTATATATAAAATATATTGACCCACGTTAGTAATCATGTAAATGTTTAATGTTTTAAATTTTGTATTTAATGATTCATTATCATACGCTAGCATGGTCTTGTAATATTCATTCTTTAAAATATAATATTGTGTTAGCCATTGCATTGGAGCTCCTAATGGAGATTTTCTATTCTCGTCCATTTTAGGATATGCTTTCATAAAGTCCCTAATAACTTCGTGAATAATGTTTCTATGTTTTCTACTGATGCATGTATTTGCTTCGATTTTTTTATCCCATGTTTCATCTATCATAGATTTAAACGCAGTAATGCTTGCAACATTAACATCTTGAACCATTGGTACAATTCCGTTCCATAAATTTATAATGTTCACCATTTATATAACTCATTTTTTGAATATACTTTTAATTGAACAAAAGAGTTAAGTTACTCATATGGGCGCCGACCAGTCTGAACATCAATCTTTTTAGCCAGAGATATCATAGCCGCTCTTAGAGTTTCAGCGTGATTTTCCAACCTAAATAGAACGTCATCATTGCGTTTACAACACTTTTCTATTTGTTCAAACTTTGTTGTTACATTAGTAATTTTTTTTTCCAAATTAGTTAGCCGTTGTTTGAGAGTTTCCTCATTATTGTCTCCATCGGCTTTAACAATTGCTTCGCGTTTAGCCTCTGGCTTTTTAGCAGCCTTTGTAGAGAAAAATTCAGTTGCTGGAATTGCAAGATCATCATCTCCAGGGAAAAGAGTTCCGTCCATTTAAAGTACAGATTTTAAAAACTGACACTCTACGTTATTTATATTTGGTGCAACACATGGATTATAAATATCGATGTTAATAACATCAGAAAATGTAAAGTCTATACATTGCGCCATCGTGTTAAATTTTCTAATGGATCTAGTATTATTGGGTCCAACTTCAGCCTGAAATCCAAATATGGAAGCGGATACAAAACCGTTTCCTGGATAAGCCACACATCTCCACTTTTGCTTTACATCAGAAATTGTGTCATTGACATCTTGAACTCTCCTATCTAATGCTGGTGTACCACCTATAGATTTTGAATATTCGAATGCTGCATGAGTAGCATTAAATTCCTTAATATTGCCATAATTCTCATATATTGAGTAACCCTGGATAAAAAGTAAACACACTGCAGCCGTCGCTACCACAATAAAAAAAATTGATAGAGAGTTCATTTATAATCTATTAGAAGCTAACAAAATTTTTTTACACGCATCAGACAATGCTTTAATAAATAGTTCAACATCTACTTTTGTTATATCGAACCGATGATATGATTCTAACCTAGAATTACATCCGAAAAAGTTGACTATGTTCATTGTCATTAAGTCATTAACGAACAATATGCCAGACTCTGGATTATAAGACGATACTGTTTCATCACAATCACCTACCTTAATCATGTGATTATGAATATTGGCTATTAGAGCACCTTCTAAGAAATCTATAATATCTTTGAAACACGATTTAAAATCAAACCACGAATATACTTCTACGAAGAAAGTTAGTTTACCCATAGGAGAGATAACTATAAATGGAGATCTAGATACAAAATCCGGATTCATGATAGTTTTAACATTATTATATTCTCTATTAAATACCTCCACATCTAAAAATGTTAATTTTGAAACTATGTCTTCGTTTATTACCGTACCTGAACTAAACGCTATAAGCTCTATTGTTTGTGAACTCTTTAAACGATATTCTTGAAATACATGTAACAAAGTTTCCTTTAACTCGGTCGGTTTATCTACCATAGTTACAGAATTTGTATCCTTATCTATAATATAATAATCAAAATCGTATAAAGTTATATAATTATCGCGTTCAGATTGTGATCTTTTCAAATAGACTAAAAACCCCATTTCTCTAGTAAGTATCTTATGTATATGTTTGTAAAATATCTTCATGGTGGGAATATGCTCTACCACAGTTAGCCATTCCTCATTGATAGCGGTAGATGTATTAGACAAAACTATTCCAATGTTTAACAAGGGCCATTTTACAAGATTATTAAATCCTTGTTTGATAAATGTAGCTAATGAGGGTTCGAGTTCAACGACGATTGAATTCTCTTCCCGCGGATGCCGCATGATGAACGACTGGATGTTGTTCGATTGATTTGGAATTCTTTTTCGACTTTTTGTTTATATTAAATATTTTAAAATTTATAGCTGATAGCAATTCATGTACTACGGATAATGTAGACGCGTATTGTGCATCGATATCTTTATTATTAGATAAATTTATCAATAAATGTGAGAAGTTTGCCTCGTTAAGGTCTTCCATTTAAATATTATATAAACATTTGTGTTTGTATCTTATTCGTCTTTTATGGAATAGTTTTTAACTAGTAAAGCTGTAATTACATACTTTGTCCGTAAAACATAAATATAAACACCCGCTTTTATCAAACGTTCCAAAAAGTCGTTAGTAGACATTTTTAACATGGTATCTATTTTAAATACACTTAGGTTTTTAGAAAAAACATCATTTTATAATTGTAACGATTCAATAACTAAAGAAAAGATTAAGATTAAACATAAGGGAATGTCATTTGTATTTTATAAGCCAAAGCATTCTACCGTTGTTAAATACTTATCTGGAGGATGTATATATCATGATGATTTGGTTGTATTGGGGAAGGTAACAATTAATGATCTAAAGATGATGCTATTTTACATGGATTTATCATATCATGGAGTGACAAGTAGTGGAGTAATTTACAAATTGGGATCATCCATAGATAGACTTTCTCTAAATAGGACTATTGTTACAAAAGTTAATAACAATTATAACAATTATAACAATTATAACAATTATTATAATTGTTATAATTATGATGATACATTTTTTGACGATGATGATTGATCGCTATTACACAATTTTGTTTTTGTACTTTCTAATATAGTGTTTAGGTTCTTTTTCATATGAGAATATTGATTTACTAAAATATCTATGTTTAACTTTTGTTCTATAACGTCCTTATCGGCGGTATCGGTACATATACGTAATTCACCTTCACAAAATACGGAGTCTTCGATAATAATAGCCAATCGATTATTGGATCTAACCGTCTGTATCATATTCAACATGTTTAATATATCCTTTCGTTTACCCTTTACAGGCATCGATCGTAGCATATTTTCCGCGTCTGAGATGGAAATGTTAAAACTACAAAAATGCGTAATGTTAGCCCGTCCTAATATTGGTACATGCCTATAAGTTTGGCATAGTAGAATAATAGACGTGTTCAAATGCCTTCCAAAGTTTAAGAATTCTATTAGAGTATTGCATTTTGATAGTTTATCACCTACATCATCAAAAATAAGTAAAAAGTGTGCTGATTTTTTATGATTTTGTGCGACAGTAATACATTTTTCTATGTTACTTTTAGTTCGTATTAGATTATATTCTAGAGATTCCTGACTACTAACGAAATTAATATGATTTGGCCAAATGTACCCATCATAATCTGGGTTATAAACGGGTGTAAACAAGAATATATGTTTATATTTTTTAACTAGTGTAGAAAACAGAGATAGTAAATAGATAGTTTTTCCAGATCCAGATCCTCCCGTTAAAACCATTCTAAACGGCATTTTTAATAAATTTTCTCTTAAAAATTGTTTTTCTTGGAAACAATTCATAATTATATTTACAGTTACTAAATTAATTTGATAATAAATCAAAATATGGAAAACTAAGGTCGTTAGTAGGGAGGAGAACAAAGAAGGCACATCGTGATATAAATAATATTTGTTATCATGATGACACCAGAAAACGACGAAGAGCAAACATCTGTGTTCTCCGCCACTGTTTACGGAGACAAAATTCAGGGAAAAAATAAACGCAAACGCGTAATTGGTATATGTATTAGAATATCTATGGTTATTTCACTACTATCTATGATTACCATGTCCGCGTTTCTCATAGTGCGTCTAAATCAATGCATGTCTGCTAACGAGGCTGCTATTACTGACGCCACTGCAGTTGCTGCTGCATTATCTACTCATAGAAAGGTTGCGTCTAGCACTACACAATATAAACACCAAGAAAGCTGTAATGGTTTATATTACCAGGGTTCTTGTTATATATTCCATTCAGACTACCAGTTATTCTCGGATGCTAAAGCAAATTGCGCCACAGAATCATCAACACTACCCAATAAATCTGATGTCTTGACTACCTGGCTCATTGATTATGTTGAGGATACATGGGGATCTGATGGTAATCCAATTACAAAAACTACAACCGATTATCAAGATTCCGATGTATCACAAGAAGTTAGAAAGTATTTTTGTGTTAAAACAATGAACTAATATTTATTTTTGTACATTAATAAATGAAATCGCTTAATAGACAAACTGTAAGTAGGTTTAAGAAGTTGTCGGTGCCGGCCGCTATAATGATGATACTCTCAACCATTATTAGCGGCATAGGAACATTTCTGCATTACAAAGAAGAACTGATGCCTAGTGCTTGCGCCAATGGATGGATACAATACGATAAACATTGTTATTTAGATACTAACATTAAAATGTCTACAGATAATACGGTTTATCAGTGTCGTAAATTACGAGCCAGATTGCCTAGACCGGATACTAGACATCTAAGAGTATTGTTTAGTATTTTTTATAAAGATTATTGGGTAAGTTTAAAAAAGACCAATAATAAATGGTTAGATATTAATAATGATAAAGATATAGATATTAGTAAATTAACAAATTTTAAACAACTAAACAGTACAACGGATGCTGAAGCGTGTTATATATACAAGTCTGGAAAACTGGTTAAAACAGTATGTAAAAGTACTCAATCTGTACTATGTGTTAAAAGATTCTACAAGTAACAACAAAAAATAAAATAATAATAAGTCCTTAACGAACGTTGATGGACACCACGTTTGTTATTACTCCAATGGGTATGCTGACTATAACAGATACATTATATGATGATCTCGATATCTCAATAATGGACTTTATAGGACCATACATTATAGGTAACATAAAAACTGTCCAAATAGATGTACGGGATATAAAATATTCCGACATGCAAAAATGCTACTTTAGCTAAGGGTAAAATAGTTCCTTAGGATTCTAATGATTTGGTTAGATTCAACATTTATAGCATTTGTACCGCATACAGATCAAAAATACCATCATCATAGCATGCGACTATGATACCATGTTAGATATAGAAGGTAAACATCAGCCATTTTATCTATTTACATCTATTGATGTTTTTAACGCTACAATCATAGAAGCGTATAACCTGTATACAGCTGGAGATTATCATCTGATCATCAATCCTTCAGATAATCTGAAAATGAAATTGTAGTTTAATTCTTCATTCTGCATATCAAACGGCAATGGATGGATCATAATTGATGGGAAATGCAATAGTAATTTTTTATCATAAAAGTTGTAAAGTAAATAATAAAACAATAAATATTGAACTAGTAGTACGTATATTGAGCAATCAGAAATGATACTGGTACCTCTTATCACAGTGACCGTAGTTGCGGGAACAATATTAGTATGTTATATATTATATATTTGTAGGAAAAAGATACGTACTGTCTATAATGACAATAAAATTATTATGACAAAATTAAAAAAGATAAAGAGTTCTAATTCCAGCAAATCTAGTAAATCAACTGATAACGAATCAGACTGGGAGGATCACTGTAGTGCTATGGAACAAAATAATGACGTGGATAATATTTCTAGGAATGAGATATTGGATGATGATAGCTTCGCTGGTAGTTTAATATGGGATAACGAATCCAATGTCATAGCGCCTAGCACAGAACACATTTACGATAGTGTTGCTGGAAGCACGCGGCTAATAAATAATGATTGTAATGAACAAACTATTTATCAGAACACTACAGTAATTAATGAGACAGAGACTATTGAAGTACTTAATGAAGATACCAAACAGAATCCTAGCTATTCGTCCAATCCTTTCGTAAATTATAATAAAACCAGTATTTGTAGCAAGTCAAATCCGTTCATTACAGAACTCAACAATAAATTTAGTGAGAATAATCCGTTTAGGAGAGCACATAGCGATGATTACCTAAATAAGCAAGAACATGATGATATAGAATCATCTGTTGTATCATTAGTCTGATTAGTTTCCTTTTTATAAAATTGAAGTAATATTTAGTATTAATTGCTACCGTTACATTGTACAAATGGAGATATTCCCTGTATTTGGCATTTCTAAAATTAGCAATTTTATTGCTAATAATGACTGTAGATATTATATAGATACAGAACATCATTAAAAAATTATACCTAATGAGATCAATAGACAGATGGATGAAATGGTACTTCTTACCAACATCTTAAGCGTAGAAGTTGTAAATAACAATGAGATGTATCATCTTATTCCCCATAGACTATCGATGATTATACTCTGTATTAGTTCTATTGGAAGATGTGTTATCTCTATAGATAATGACGTTAATAACAAAAATATTCTAACCTTTCCCATTGATCATGCTGTAATCATATCCCACTAAGTAAATGTGTCGTAGTTAGTAAGGGTCCTACAACCATATTGGTTGTTAAAGCGGATATACCCAGCAAACGACATACTATATGTAAATAATCTGTCACTGATTAATTATTTGCCGTTGTCTGTATTCATTATTAGACGAGTTACAAACTATTTGGATAGACACATATGCGATCAGATATTTGCTAATAATAAGTGGTATTCCATTATAACCATCGACGATAAGCAGTTTCCTATTCCATCAAACTGTATAGGTATGTCCTCTACCAAGTACATAAATTCTAGCATCGAGCAAGATACTTTAATCCATGTTTGTAACCTCGAGCATCCATTCGACTCAGTATACAAAAATGCAGTCGTACAAATTCTGTACCTATCAAGGAACAAATATTGTACGGTAGAATTGATAATATAAATATGAGCATTAGTATTTCTGTGGATTAATAGATTTCTAGTATGGGATCATTAATCATTTCTAATCTCTAAATACCTCATAAAACAAAAAAGCTATTATCAAATACTGTACGGAATGGATTCATTCTCTTCTCTTTTTATGAAACTCTGTTGTATATCTACTGATAAAACTGGAAGCAAAAAATCTGATAGAAAGAATAAGAATAAGATCAAGGATTATATGGAACACGATTATTATAAAATAACAATAGTTCCTGGTTCCTCTTCCACGTCTACTAGCTCGTGGTATTATACGCATGCCTAGTAATAGTCTCTTTGCGTTGATGGAAAGCAGACTAGAAATAACAGGCTAAAATGTTCAGACACCATAATAGTTCCCAACCCAGATAATAACAGAGTTCCATCAACACATTCCTTTAGACTCAATCCCAATCCCAAAACCGTTAAAATGTATCCGGCCAATTGATAGTAAATAATGAGGTGTACAGCACATGATAATTTACACAGTAACCAAAATGAAAACACTTTAGTAATTATAAGAAATATAGACGGTAATGTCATCATTAACAATCCAATAATATGCCTGAGAGTAAACATTGACGGATAAAACAAAAATGCCCCGCATAACTCTATCATGGCAATAATACAACCAAACACTTGTAAAATTCCTAAATTAGTAGAAAATACAACTGATATCGATGTATAAGCGATTTCGAGGAATAATAAGAACAAAGTAATGCCTGTAAAAATAAACATCAACATTGTATGATGGTCATTAAACCAATTAGTATGACGTTGAACTAATTTCACAGTAGATTTTATTCCGGTGTTATCCTCGCATGTATAAGTACCGGGTAATATATCTTTATACTTTATAATTAATGAAACATCGTTATCAGATAACGAATGGAGTTTGGCACTAGTATGCCATTTACTTAATATGATCGTCTTGGAAGTTTTATTATAAGTTAAAATATTATGGTTGTCCAATTTCCATCTAATATACTTTGTTGGATTATCTATAGTACACGGAATAATGATGGTATCATTGCACGCCGTATACTCTATAGTCTTTGTAGATGTTATAACCACAAAAGTGCAGAGGTATATCAACAATATTCTAACTCTTAACATTTTTATTTATTTAAAATGATACCTTTGTTATTTATTTTATTCTATTTTGCGAACGGTATCGAATGGCATAAGTTTGAAACGAGTGAAAAAATAATTTCTACTTACTTAATAGATGACGTATTATACACGGGTGTTAATGGGGCAGTATATACATTTTCAAATAATAAACTAAACAAAACTGGTTTAGCTAATACTAATTATATCACAACATCTATAAAAGTAGAGGATGTGATACATTAGTATGCGGAACCAATAACGGAAATCCCAAATGTTGGAAAATAGACGGTTCCAAAGATCCAAAACATAGAGGTAGAGGATACGCTCCTTATCAAAATAGTAAAGTAACGATAATCAGTCATAACAAATGTATACTATCTAACATAAACATATCAAAAGAAGGAATTAAACGATGGAGAAGATTTGACGGACCATGTGGTTATGATTTATTTATACACGGCGGATAACGTAATTCCAAAAGATGGTTTACAAGGAGCATTTGTCGATAAAGACGGTACTTATGACAAAGTTTACATTCTTTTCACTGTTACTATCGGCTCAAAGAGAATTGTTAAAATTCCGTATATAGCACAAATGTGCTTAAACGACGAATGTGGTCCATCATCATTGTCTAGTCATAGATGGTCGACGTTGCTCAAAGTCGAATTAGAATGTGACATCGACGGAAGAAGTTATAGTCAAATTAATCATTCTAAAACTATAAAACAGATAATGATACGATACTATATGTATTCTTTGATAGTCCTTTTCCAAGTCCGCATTATGTACCTATTCTATGAATACCATTAAACAATCTTTTTCTACGTCAAATTGGGAGGATATACAAAGCAATTACTGTCTCCAGCTTTTGGTATATGTCTACCAGCTGGAAAAAGTTGTTCCACATAACACGTTTGACGTTATAGAACAATATAATGTACTAGATAATATTATAAAGCCTTTATTTAACCAACCTATCTTCAAAGGACCGTCTGATGTTAAATGGTTCGATATAAAGGAGAAGGAAAATGAACATCGGAAATATAGAATATACTTCATAAAAGAAAATACTATATATTCGTTCAATACAAAATCTAAACAAACTCGTAGCTCGCAAGTCGATGCGCAACTATTTTCAGTAATGGTAACTTCGAAACCGTTATTTATAGCAGATATAGGGATAGAAGTAGGAATGCCACGAATAAAAAATACTTAAAATGTAATCTTAATCGAGTACGCCATATGACAATGAACAAACCTAAGACAAATTATGCTGGTTATGCTTGCTGCGTAATATGCGGTCTAATTGTTGGAATTATTTTTACAGCGACACTATTAAAAGCTGTAGAACGTAAATTAATTCATACACCATTAATAGATAAAACGATAAAAGATGCATATATTAGAGAAGATTGTCCTACTGACTGAATAAGCTATATAATAAATGTATACATTTATCGACTAATCAAAAAACCTGGGAGGAAGGACGTAATGCATGCAAAGCTCTAAATCCAAATTCGGATCTAATTAATATAGAGACTCTAAACGAGTTAAGTTTTTTAAGAAGCCTTAGAAGAGGCTATTGGGTAGGAGAATTCAAAATATTAAACCAGACAACCACGTATAATTTTATAGCTAAAAATGTCACGAAGAATGGAACTAAAAAACGAAATATATTTGTAGTACAACGAATACTCCCAAACTGCATTCATGTTACACTATATAACAATTACACTACATTTTTATCATAACACTACTTTGGTTAGATGTTTTAGAAAAAAAATATCGCCGTACCGTTCTTGTTTTTATAAAAATAACAATTAACAATTATCAAATTTTTTCTTTAATATTTTACGTGGTTGACCATTCGTGGTGGTAAAATAATCTCTTAGTGTTGGAATGGAATGCTGTTTAATGTTTCCACACTCATCGTATATTTTGACGTATGCAGTTACATCGTTTACGCAATAGTCAGACTGTAGTTCTATTATGCTTCCTACATTAGGAGGAACAGTTTTAAAGTCTCTTGGTTTTAATCTATTACCGTTAGTTTTTATGAAATCCTTTGTTTTATCCACTTCACATTTTAAATAAATGTCCACTATACATTCTTCTGTTAATTTTACTAGATCGTCATATGTCATAGAATTTATAGGTTCCATAGTCCATGGATCCAAACTAGTAAACTTCGCGTATACAGTATCGCGATTAGTGTATACACCAACTGTATGAAAATTAAGAAAACAGTTTAATAGATCAACAGAAATATTTAATCCTCCGTTTGATACAGATGCACCATATTTATGGATTTCGGATTCACACGTTGTTTGTCTGAGGGGTTCGTCTAGCGTTGCTTCTACATAAACTTCTATTCCCATATATTCTTTATTATCAGAATCGCATACCGATTTATCATCATACACTGTTTGAAAACTAAATGGTATACACATCAAAATAACAAATACTAACGAGTACATTCTGCAATATTGTTATCGTAATTGGAAAATTAGTGTTCGGGTGAGTTGGATTATGTGAGTACTGGATTGCATATTATATTTTATATTTTATATTTTATATTTTGTAATAAGAATAGAATGCTAATGTCAAGTTTATTCCAATAAATGACTTATTAAAAAACATATATAATAAATAACAATGGCTGAATGGCATAAAATTATCGAGGATATTTCAAAAAATAATAATTTCGAGGATGCCGCCATCGTTGATTACAAGACTACAAAGAATGTTCTAGCGGCTATTCCTAACAGAACATTTGCAAAGATTAATCCGGGCGAAGTTATTCCGCTCATCACTAATCATAATATTCTAAAACCTCTTATTGGTCAGAAGTTTTGTATTGTATATACTAACTCTCTAATGGATGAGAACACGTATGCTATGGAGTTGCTTACTGGGTACGCCCCTGTATCTCCGATCGTTATAGCGAGAACTCATACCGCACTTATATTTTTGATGGGTAAGCCAACAACATCTAGACGTGATGTGTATAGAACGTGTAGAGATCACGCTACCCGTGTACGTGCAACTGGTAATTAAAATAAAAAGTAATATTCATATGTAGTGTTAATTTTAAATGATGATAAAATGGATAATATCCATATTGACGATGTCAATAATGCCGGTATTGGTATACAGCTCATCGATTTTTAGATTTCGTTCAGAGGATGTGGAATTATGTTATGGGAATTTGTATTTTGATAGGATCTATAATAATGTAGTAAATATAAAATATATTCCTGAGCATATTCCATATAAATATAATTTTATTAATCGTACGTTCTCCGTAGATGAACTAGACAATAATGTCTTTTTTACACATGGTTATTTTTTAAAACACAAATATGGTTCACTTAATCCTAGTTTGATTGTCTCATTATCAGGAAACTTAAAATATAATGATATACAATGCTCAGTAAATGTATCGTGCCTCATTAAAAATTTGGCAACGAGTATATCTACTATATTAACATCTAAACATAAGACGTATTCTCTACATCGGTCCAAGTGTATTACTATAATAGGATATGATTCTATTATATGGTATAAAGATATAAATGACAAGTATAATGATATCTATGATTTTACCGCAATATGTATGCTAATAGCGTCTACATTGATAGTGACCATATACGTGTTTAAAAAAATAAAAATGAACTCTTAATTATGCTATTAGAAATGGATAAAATCAAAATTACGGTTGATTCAAAAATTGGTAATGTTGTTACCATATCGTATAACTTGGAAAAAATAACTATTAACGACACACCAAAAAAGAAAAGGATGTATTATTAGCGCAATCAGTTGCTGTTGAAGAGGCAAAAGATGTCAAGGTGGAAGAAAAAAATATCGATATTGAAAATGACAATGACGATGATATGGATGTAGAAAGCGTGTAATACGATCTATAAAAAATAAGTATATAAATACTTTTTATTTACGGTACTCTTGTAGTGGTAATACCCCTACTCGATTATTTTTTTTAAAAAAAAATACTTATTCTGATTATTCTAGCCATTTCCGTGTTCGTTCAAATGCCACATCAAAGATATGGGAGTAGTTGAAATCTAGTTCTGCATTGTTGGCGCGCCTCAAATGTAGTGTTGGATATCTTCAACGTATAGTTGTTGAGTAGTGATGGTTTTCTAAATAGAATTCTCTTCATATCATTCTTGCACGTGTACATTTTTAGCATCCATCTTGGAATCCTAGATCCTTGTTCTATTCCCAATGGTTTCATCAATAGAAGATTAAACATATCGTACGAACACGATGGAGAGTAACCGTAGCAAAAGTAAGCATTTCCTTTAATCTCAGATCCCGGATACTGGATATATTTTACA

TACCGCGTCATCATCGACTGTACGATACATAATGTTACCGTGTTGCGTACATTGCTCGTAAAAGACTTTCGTTAATTTGTCTCCTCCTCCGTAAATTCCAGTGGGTCTTAGGCAACAAGTATACAATTTTGCACCATTCATAATTACAGAATTATTGGCTTTCGTGACCAGTTGCTCGACCATACGTTTACTTTTTGCGTATACATGTCCGGGTGATATATCGTACAGGGTATGCTCATGACCAATGAATGGATTACCGTGTTTATTTGGTCCTATTGCTTCCATGCTACCTAATATAAATCAAATACTTGATTCCTAGGTCTACAGAAGCTGCCAATATAGTCTGTGTTACATAATAGTTTACTTTCATGATTTCATTATCGGTGTATTTTCCAAATACATCCACTAGAGCAACCGTATGAATAATCAGATTTACCCCATCTAGTGCTTCTTTCACCTTATTAAGTCGTTTATATCACATTGTATATAGTTTATAACTTTAACCTTCGATACGAGAGGTTGTGGATCTTCTACGACATTGATAACTCTGATTTCTTGAACATCATCTGCGCTAATTAAAAGTTTTACTATATACCTGCCTAGAAATTCGGCACCACCAGTAACCGCGTACACGGTCATTGCTGCTGTCACTCATAAATATCGGACTACTTATTCTATTTTACAAATAATGGCTGTTTGTATAATAGACCACGATAATATCAGAGGAGTTATTTACTTTGAACCAGTCCATGGAAAAGATAAAGTTTTAGGATCAGTTATTGGATTAAAATCCGGAACGTATAATTTGATAATTCATCGTTACGGGGATATTAGTCGAGGATGTAATTCCATAGGCAGTCCAGAAATATTTATCGGTAACATCTTTGTAAACAGATATGGTGTAGCATATGTTTATTTAGATACAGATGTAAATATATCTACAATTATTGGAAAGGCGTTATCTATTTCAAAAAATGATCAGAGATTAGCGTGTGGAGTTATTGGTATTTCTTACATAAATGAAAAGATAATACATTTTCTTACAATTAACGAGAATGGCGTTTGATATATCAGTTAATGCATCTAAAACAATAAATGCATTAGTTTACTTTTCTACTCAGCAAAATAAATTAGTCATACGTAATGAAGTTAATGATACACACTATACTGTCGAATTTGATAGGGACAAAGTAGTTGACACGTTTATTTCGTATAATAGACATAATGACTCCATAGAGATAAGAGGGGTGCTTCCAGAGGAAACTAATATCGGTTGCACGGTTAATACGCCGGTTAGTATGACTTACTTGTATAATAAGTATAGTTTTAAACTAATTTTAGCAGAATATATAAGACACAGAAATACTGTATCCGGCAATATTTATTCGGCATTGATGACACTAGATGATTTGGTTATTAAACAGTATGGCGACATTGATCTATTATTTAATGAGAAACTTAAAGTGGACTCCGATTCGGGATTATTTGACTTTGTCAACTTTGTAAAGGATATTATATGTTGTGATTCTAGAATAGTAGTAGCTCTATCTAGTCTAGTATCTAAACATTGGGAATTGACAAATAAAAAGTATAGGTGTATGGCATTAGCCGAACATATAGCTGATAGTATTCCAATATCTGAGCTATCTAGACCACGATACAATCTATGTAAGTATTTACGAGGACACACTGAGAGCATAGAGGATGAATTTGATTATTTTGAAGACGATGATTCGTCGACATGTTCTGTCGTAACCGACAGGGAAACGGATGTATAATTTTTTATAGTGTGCAGGATATGATAAAAATATAATTGTTGTATCCATTCCCATTCTAATCACATTATATGATTCTGTAAAAAATTATACTGTAACACAATGAAGTAGTTGCATAGATGTATATAGGTCAGATACTGGTTTGATAAACTTTTTATTCCACATGAGTATGTTTGACTTTATGGTTAGACCCGCATACTTTAACAAATCACTGAAAATTGGAGTTAGGTATTGACATCTCAGAATCAGTTGCCGTTCTGGAACATTAAATGTATTTTTTATGATATATTCCAACGCATTTATGTGGGTATACAACAAGTCATTACTAATAGAGTATTCCAAGAGTTTTAATTGGCTAGTATTTAACAAGAGAAGAGATTTCAACAGACTGTTTATGAACTCGAATGCCGCCTTATTGTCGCTTATATTGATGATGTCGAATTCTCCCAATATCATCACTGATGAGTAGCTCATCTTGTTATCAGGATCCAAGCTTTCTAAAGACGTCATTAAACCCTCGATCATGAATGGATTTATCATCATCGTTTTTATGTTGGACATAAGCTTAGTCCGTTTGTCCACATCTATAGACGATGATTTCTGAATTATTGCATATATCTCTCTCTTTAACTCCAGGAACTTGTCAGGATGGTCCACTTTAACATGTTCTCGCCTAAGAGATAAAAATCTTTGGATGGTTGCATGTGACTTTTCTCTAAATGATGATGTTGCCCAAGATCCTCTCTTAAATGAATCCATCCTATCCTTGTACAAGATGGACAGTCTATTTTCCTTAGATGGTTTAATATTTTTGTTACCCATGATCTATAAAGGTAGACCTAATCGTCTTGGATGACCATATATTTATTTCCAGTTTTATTATACGCATAAATTGTAAAAATATGTTAGGTTTACTAAAATGTCTCGCGGGGCATTAATCGTTTTTGAAGGATTGGACAAATCTGGAAAAACAACACAATGTATGAACATCATGGAATCTATACCGACAAACACGATAAAATATCTTAACTTTCCGCAGAGATCCACTGTCACTGGAAAGATGATAGATGACTATCTAACTCGTAAAAAAACCTATAATGATCATATAGTTAATCTATTATTTTGTGCAAATAGATGGGAGTTTGCATCTTTTATACAAGAACAATTAGAACAGGGAATTACTTTAATAGTTGATAGATACGCGTTCTCTGGAGTAGCATATGCCACCGCTAAAGGCGCGTCAATGACTCTCAGTAAGAGTTATGAATCTGGATTGCCTAAACCCGACTTAGTTATATTCTTGGAATCTGGTAGCAAAGAAATTAATAGAAACGTCGGCGAGGAAATTTATGAAGATGTAGCATTCCAACAAAAGGTATTACAAGAATATAAAAAAATGATTGAAGAAGGAGAAGATATTCATTGGCAAATTATTTCTTCTGAATTCGAGGAAGATGTAAAGAAGGAGTTGATTAAGAATATAGTTATAGAGGCTATACATACGGTTACTGGACCAGTGGGGCAACTGTGGATGTAATAAAGTGAAATTACATTTTTATAAATAGATGTTAGTGCAGTGTTAAAAAATGGATGAAGGATATTACTCTGGCAACTTGGAATCAGTTCTCGGATACGTATCTGATATGCATACTAAACTCGCATCAATAACTCAATTAGTTATTGCCAAGATAGAAACTATAGATAATGATATATTAAACAACGACATTGTAAATTTCATTATGTGTAGATCAAACTTAAATAATCCATTTATCTCTTTCCTAGATACTGTATATACTATTATAGATCAAGAGATCTATCAGAACGAGTTGATTAATTCATTAGACGACAATAAAATTATCGATTGTATAGTTAATAAGTTTATGAGCTTTTATAAGGATAACCTAGAAAATATAGTAGATGCTATCATTACTCTAAAATATATAATGAATAATCCAGATTTTAAAACTACGTATGCAGAAGTACTCGGTTCCAGAATAGCGGATATAGATATTAAACAAGTAATACGTGAGAATATACTACAATTGTCTAATGATATCCGCGAACGATATTTGTGAAAATATTAAAAAAAAATACTTTTTTTATTAAATGACGTCTCTTCGTGAATTTAGAAAATTATGCTGTGCTATATATCACGCATCAGGATATAAAGAAAAATCTAAATTAATTAGAGACTTTATAACAGATAGGGATGATAAATATTTAATCATTAAGCTATTGCTTCCCGGATTAGACGATAGAATTTATAACATGAACGATAAACAAATTATAAAAATATATAGTATAATATTTAAACAATCTCAGAAAGATATGCTACAAGATTTAGGATACGGATATATAGGAGACACTATTAGTACATTCTTCAAAGAGAACACAGAAATCCGTCCAAGAAATAAAAGCATTTTAACTTTAGAAGACGTGGATAGTTTCTTAACTACATTATCATCCATAACTAAAGAATCGCATCAAATAAAATTATTGACTGACATCGCATCCGTTTGTACATGTAATGATTTAAAATGTGTAGTCATGCTTATTGATAAAGATCTAAAAATTAAAGCGGGTCCTCGGTACGTACTTAACGCTATCAGTCCTCATGCCTATGATGTTTTTAGAAAATCTAATAACTTGAAAGAGATAATAGAAAATGAATCTAAACAAAATCTAGACTCTATATCTGTTTCTGTTATGACTCCAATTAATCCCATGTTAGCGGAATCGTGTGATTCTGTCAATAAGGCATTTAAAAAATTTCCATCAGGAATGTTTGCTGAAGTCAAATACGATGGCGAGAGAGTACAAGTTCATAAAAATAATAACGAGTTTGCCTTTTTTAGTAGAAACATGAAACCAGTACTCTCTTATAAAGTGGATTATCTCAAAGAATACATACCGAAAGCATTTAAAAAAGCTACGTCTATCGTATTGGATTCTGAAATTGTTCTTGTAGACGAACATAATGTACAGCTCCCGTTTGGAAGTTTAGGTATACACAAAAAGAAAGAATATAAAAACTCTAACATGTGTTTGTTTGTGTTTGACTGTTTGTACTTTGATGGATTCGATATGACGGACATTCCATTGTACAAACGAAGATCTTTTCTCAAAGATGTTATGGTCGAGATACCCAATAGAATAGTATTCTCAGAGTTGACGAATATTAGTAACGAGTCTCAGTTAACTGACGTATTGGATGATGCACTAACAAGAAA

AATACGTTAAGGGTTTTGCAAGACCAATTAACGATGGTTAAAATTAACAAGGATCCCAAAAAAATTCCAGAGTGGTTGGTAGTTAATAAAATCTATATTCCCGATTTTGTAGTAGAGGATCCGAAACAATCTCAAATATGGGAAATTTCAGGAGCAGAGTTTACATCTTCCAAGTCACATACAGCGAATGGAATATCGATTAGATTTCCTAGATTTACTAGGATTAGAGAAGATAAAACGTGGAAAGAATCTACTCATCTAAACGATTTAGTAAATTTGACTAAATCTTAATAGTTACATACAAACTGAAAATTAAAATAACACTATTTAGTTGGTGATCGCCATGGATGGTGTTATTGTATACTGTCTAAACGCGTTAGTAAAACATGGTGAGGAAATAAATCATATAAAAAATGATTTCATGATTAAACCATGTTGTGAAAGAGTTTGTGAAAAAGTCAAGAACGTACACATCGACGGACAATCTAAAAACAATACAGTGATTGCAGATTTGCCATATCTGGATAATGCTGTATTGGATGTATGCAAATCAGTATATAAAAAGAATGTATCAAGAATATCCAGATTTGCTAATTTGATAAAGATAGATGATGATGACAAGACTCCTACCGGTGTATATAATTATTTTAAACCTAAAGATGCTATTTCTGTTATTATATCCATAGGAAAGGATAAAGATGTCTGTGAACTATTAATCGCATCCGATAAAGCGTGTGCGTGTATAGAGTTAAATTCATATAAAGTAGCCATTCTTCCCATGAATGTTTCCTTCTTTACCAAAGGAAATGCGTCATTGATTATTCTCCTGTTTGACTTCTCTATCAATGCGGCACCTCTCTTAAGAAGTGTAACCGATAATAATGTTGTTATATCTAGACACAAACGTCTTCATGGCGAGATTCCGAGTTCCAATTGGTTCAAGTTTTATATAAGTATAAAGTCCAACTATTGTTCTATATTATATATGGTAGTTGATGGATCTGTGATGTATGCAATAGCTGATAATAAAACTCACACAATTATTAGCAAAAATATATTAGACAATACTACAATTAACGATGAGTGCAGATGCTGTTATTTTGAACCACAGATTAAGATTCTCGATAGAGATGAGATGCTTAATGGATCATCGTGTGATATGAACAGACATTGTATTATGATGAATTTACCAGATATAGGAGAATTCGGTTCCAGTATATTGGGGAAATATGAACCTGACATGATTAAGATTGCTCTTTCAGTGGCTGGTAATTTAATAAGAAATCAAGACTACATTCCCGGGAGACGCGGCTATAGCTACTACGTTTACGGTATAGCCTCTAGATAATTTTTTAAGCACAAAATAAAAAACATAATTTTAAACTAGTCTATTTCATACTATTTTGTGTGATCACCATGGACATAAAGATAGATATTAGTATTTTTGGTGATAAATTTACGGTGACTACTAGGAGGGAAAATGAAGAAAGAAAAAATATCTACCTCTCCAAAAAGAAAAATTTACTACTGATGTTATCAAACCTAATTATCTTGAGCACGATAACTTATTAGATAGAGATGAGATGTCTACTATTCTAGAGGAATATTTTATGTACAGAGGTCTATTAGGCCTCAGAATAAAATATGGACGACTTTTTAACGAAATTAGAAAATTCGACAATGATGCGGAAGAACAATTCGGTACTATAGAAGAACTCAAACAGAAACTTAGATTAAATGCTGAAGAGGGAGCCGATAACTTTATAGATTATATAAAGGTACAAACAGGATATCTATGATAGGATTGTGTGCGTGTGTGGTAGATGTTTGGAGAAAGGAGAAACTGTTTTCTAGATGGAAATATTGTTTACGAGCTATTAAACTGTTTATTGATGATCCCATACTTGATAAGATAAAATCTATACTGCAGAATAGACTAGTGTATGTGGAAATGTTATAGAAAATTAAAAGTTAATGAGAGCAAAATATAATGTTGTATTCTAATCCCATATTTATTATTTTCACGGAGATATAGGTGTAGTTTTGCAGTGACCGTCCTGATAATATTATCTATATGATGGGTGGATATGATCAGTACCCGTATAGAAGTTCAAAGGTTATAGTGTACAATACATGTACAAATTCTTGGATATATGATATACCAGAGCTAAAATATCCTCGTTCTAATTGTGGAGGAGTTGCTGATGATGAATACATTTATTGCATAGGCGATCAGGATTCATCGTTGATATCTAGTATTGATAGATGGAAGCCATCAAAACCATATTGATAAACGTATGCTAAAATGCGAGAGACAAAATGTGATATTGGTGTAGCGATGTTAAACGGATTAATATATGTCATAGGCGGAGTTGTTAAAGGTGACACATGTACCGACACACTTGAGAGTTTATCAGAAGATGGATGGATGATGCATCAACGTCTTCCAATAAATGTCCAATATGTCGACGATTGTTCATACCGGCAAAATTTATATATCAGGAGGCTACACAATAGTAGTGTAGTTAATGGAATATCAAATCTAGTCCTTAGCTATAATCCGATATATGATGAATGGACCAAATTATCATCATTAAATATTCCTAGAATTAATCCTGCTCTATGGTCAGTGCATAATAAAGTATATGTAGGAGGAATATCTGATGATATTCAAACTAATACATCTGAAACATACAACAAAGAAAAAGATCGTTGGACATTGGATAATAGTCACGTGTTACCACGCAATTATATAATGTATAAATGCGAACCGATTAAACATAAATATCCATTGGAAAAACACAGTACACGAATGATTTTCTAAAGTACTTGGAAAGTTTTATATGTAGTTGATAGAACAAAATACATATTTTTTGTAAAAATAAATCACTTTTTATACTAATATGACACGATTGTCAATACTTTTGTTACTAATATCATTAGTATACTCTACACCTTATCCTCAGACACAGATATCTAAAAAAATAGGTGATGATGCAACTCTATCATGTAGTAGAAATAATATAAATGATTATGTTGTTATGAGTGCTTGGTATAAGGAGCCCAATTCCATTATTCTTTTAGCTGCCAAAAGTGACGTCTTGTATTTTGATAATTATACCAAGGATAAAATATCATACGATTCTCCATACGATGATCTAGTTACAACTATTACAATTAAATCATTGACTGCTAAAGATGCCGGTACTTATGTATGTGCATTCTTTATGACATCAACTACAAATGATACTGATAAAGTAGATTATGAAGAATACTCTACAGAGTTGATTGTAAACACAGATAGTGAATCGACTATAGACATAATACTATCTGGATCTTCACATTCACCGGAAACTAGTTCTGAGAAACCTGATTATATAAATAATTTTAATTGCTCGTTGGTATTTGAAATCGCGACTCCGGGACCAATTACTGATAATGTAGAAAATCATACAGACACTGTCACATACACTAGTGATATCATTAATACAGTAAGTACATCATCTAGAGAATCCACAACAGTCAAGACGTCGGGACCAATTACTAATAAAGAAGATCATACAGTCACAGACACTGTCTCATACACTACAGTAAGTACATCATCTGAAATTGTCACTACTAAATCAACCGCCAATGATGCGCACAATGATAATGAACCATCTACTGTGTCACCAACAACTGTAAAAAACATCACGAAATCTATAGGTAAGTATAGTACTAAAGACTATGTCAAAGTATTTGGTATTGCAGCATTAATTATATTGTCGGCCGTGGCAATTTTCTGTATTACGTATTATATATGTAATAAACGTTCACGTAAATACAAAACAGAAAACAAAGTCTAGATTTTGACTTACATAAATGTCTGGAATAGTAAAATCTATCATTTTGAGCGGGTCATCTGGTTTAGGAAAGACAGCCATAGCCAAAAGACTATGGGAATATATTTGGATTTGTGGTGTCCCATACCACTAGATTTCCTCGTCCTATGGAACGAGAAGGTGTTGATTACCATTACGTTAACAGAGAGGCAATCTGGAAGGGAATAGCCACCGGAAACTTTCTAGAACATACTGAGTTTTTAGGAAATATTTACGGAACTTCTAAAACAGCTGTGAATACAGCGGCTATTAATAATCGTATTTGTGTGATGGATTTAAACATCGACGGTGTTAGAAGTCTTAAAAATACTTACCTAATGCCTTACTCGGTGTATATAAAACCTACCTCTCTTAAAATGGTTGAGACCAAGCTTCGTCGTAGAAACACTGAAGCGGACGATAAGATTCATCGTCGCGCGATGTTGGCAAAAACGGATATGGATGAGGCCAACGAAGCAGGTCTATTCAACACTACTATTATTGAAGATAATGTGAATTTAGCATATAGTAAGTTAATTCAGATACTACAGGACCGTATTAGAATGTATTTTAACACTAATTAAGATATAATACTTGACTGACTTAAAACTTGATAATTAATAATATAATTCGTTTTTATATGTGACTATTTTAACGTCTAAATGTATTAGTTAAATATTAAACTTACCACGTAAAACTTAAAATTTAAAATGGTATTTCATTGACGGATGATCATACATTATGAACTTTCAAGGACTTGTCTTAACTGACAATTGCAAAAATCAATGGGTCGTTGGACCATTAATAGGAAAAGGTGGATTCGGTAGTATTTATACTACTAATGACAATAATTATGTAGTAAAAATAGAGCCCAAAGCTAACGGATCATTATTTACTGAACAGGCATTTTATACTAGAGTACTTAAACCATCCGTTATCGAAGAATGGAAAAAATCTCACCATATAAGCCACGTAGGAGTTATCACATGCAAGGCATTTGGTCTATACAAATCCATTAATACGGAATATAGATTCTTGGTAATTAATAGATTGGGTGTAGATCTAGATGCGGTGATCAGGGCTAACAATAATAGACTACCGAAAAGATCGGTGATGTTAGTAGGAATAGAAATCTTGAATACCATACAATTTATGCACGAGCAAGGATATTCTCATGGAAATATTAAAGCGAGCAATATAGTTTTGGATCAAATGGATAAGAATAAATTATATCTAGTGGATTACGGATTGGTTTCTAAATTCATGTCTAACGGCGAACATGTTCCATTTATAAGAAATCCAAATAAAATGGATAATGGTACTCTAGAATTTACACCTATAGATTCACATAAAGGATACGTTGTATCGAGACGTGGAGATCTAGAAACACTTGGATATTGTATGATTAGATGGTTGGGAGGTATCTTGCCATGGACTAAGATAGCTGAAACAAAGAATTGCGCATTAGTAAGTGCTACAAAACAGAAATATGTGAACAATACTACGACTTTGTTAATGACCAGTTTGCAATATGCGCCTAGAGAATTGCTGCAATATATTACCATGGTAAACTCTTTGACATATTTTGAGGAACCCAATTACGATAAGTTTCGGCACATATTAATGCAGGGTGCATATTATTAAGTGTGAGGTTTTGGTTTGATGTAAAATTTTTGTTGATAAAAATTAAAAAATAACTTAATTTATTATTGATCTCGTGTGTACAACCGAAATCATGGCGATTTTTACACACACGTCTTGGCGGGTACGACGAGAATCTTTATACCTTTCCTGGAATATCATTAACTGTTGCCAATGATGTTAGGAAATATTCTGTTGTGTCAGTTTATAATAACAAGTATGACATTGTAAAAGAAATATATGTGGTGTTACAGTTAGGTGAACAAGAGATATATTGGAGCATTGCTGCATATGTTTGAGTGCAATGAATATCTACAAATTGGAGATCCAATCCATGATCAAGAAGGAAATCAAATCTCTATCGTCACATATCGCTACAAAACTACTATGCTCTAAGCGGAATCGGGTACAAAAGTCTAGACTTGTGTTTGGAAGGAGTAGGGATTCATCATCACGTACTTGAAACAGGAAACGCTGTATATGGAAAAGTTCAACATGATTATTCTACTATCAAAGAGAAGGCCAAAGAAATGAGTGCACTTAGTCCAGGACCTATAATTGATTACCACGTCTGGATAGGAGATTGTATTGGTAAAGTTACTGCTGTAGACGTACATGGAAAGGAAATTATGAGAATGTGATTCAAAAAGGGTGCGGTACTACAGATACCAAATCTGGTAAAAGTTAAACTTGGGGATAATGATACAGAAAATCTTTCCACTACCATATCAGCTCTCCTGAATTCCGGCGGCGGTACCATCAAGGTAACCTCTTAGGAAGATCGCGTTAATCATGTACTCATAAAACGATTGGAATCTATACGCCATATGTGGTCTGTCTATGATCGTTTTAATATTGTGAATGGTAAAGAATGCTGTTATATACATCTTCATTCATCTAATCAGAATCTTATGCCTAGTACTGTAAAAACCAATTTGTATATGTGATCTATGACATCATGCATCCAAATGGATCTCATAACTGCTCTAGATTATCTTAGTGAACTTAAGGAATCAAGTGGATAAAGTCCCAAACCAGAATTGCCCAAATTCGATTACCCAGATGGAGTGCAAGACAATGGATCAATTGAGAGATTTGCAGAAGAGTTCTTCAATAGATCAGAACTTCAGGCGGGTGAATCAGTCAAATTCGGTAATTCCGTTAATATTAAAAATACATCTGTTTCAGCTAAGCAACTAAGAACACGTATACGGCAACAGCTTCCCTCTATACTCTCATCTTTTGCCAACACAGAGGGTGGATATTTGTTCATTGGATTGGATAATAATACACACAAAGTAATTGGATTCATGGCGGGACACGACTGCATTAAACTGATAGAGAATGAGATAGAAAAGTGTATTAGACGACTCATGTTGTACATTTCTGTGAGAAAAAGAGGATATCAAATACGCATATAGATTCATCAAGGTATATAAACCTGGGGAGGAGACTACCTCGACATACGTGTGCGCTATCAAAGTGGAAAGATGTTGCTGTGCTGTGTTTACAGATTGGCCAGAATCATGGTATATGGATACTAGTGGTATCAAGAAGTATTCTCCAGATGAATGGGTGTCATCTATAAAATTTTAATTAAGAACTATAGAGAACAAATAATAGGTTGTAATACATATAGACAATAACTAACAATTAATTAGTAACTGTTATCTCTTTTTAACTAACTCTTATATACCTATTAATACATCGTATTATAGTTCTTAATCATTGATTCACTTATTGTTTTAATAAACTAACATTGTTAATTGAAAAGGGATAACATGTTATAGAATATAAATTATATATGGATTTTTTTAAAAAGGAAATACTTGACTGGAGTATATATTTATCTCTTCATTATATAGCACATGCGTGTTCCAATTCTTCCACATCCCATATAATACAGGAATATAATCTTATTCGAACATACAAAAAAGTGGATAAAACAATAGTTGATTTTTTATCTAGGTGGCCAAATTTATTCCATATTTTAGAATATGGGGAAAATATTCTACATATTTATTCTATGGATGATGCTAATACGAATATTATAATTTTTTTTCTAGATAACGTATTAAATATTAATAAGAACGGGTCATTTATACACAATCTCGGGTTATCATCATCCATTAATATAAAAGAATATGTATATCAATTAGTTAATAATGATCATCTAGATAATGGGATAAGACTAATGCTTGAAAATGGACGTAGAACAAGACA

TAAATCATGGATTTTACATAGATGCTGTGGACAGTTACGGTTGTACATTATTACATAGATGTATATATCACTATAAGAAATCAGAATCAGAATCATACAATGAATTAATTAAAATATTGTTAAATAATGGATCAGATGTAGATAAAAAAGATACGCATGGAAACACGCCATTTATCCTATTATGTAAACACGATATAGACAACGTAGAATTGTTTGAGATATGTTTAGAGAATGCTAATATAGACTCTGTAGACTTTAATGGATATACACCTCTTCATTATGTCTCATGTCGCAATAAATATGATTTTGTAAAGTCATTAATTTCTAAAGGAGCAAATGTTAATACGCGTAATAGATTCGGAACTACTCCATTTTATTGTGGAATTATACACGGTATCTCGCTTATAAAACTATATTTGGAATCAGACACAGAGTTAGAAATAGATAATGAACATATAGTTCGTCATTTAATAATTTTTGATGCCGTTGAATCTTTAGATTATCTATTATTTAGAGGAGTTATTGATATTAACTATCGTACTATATACAACGAAACATCTATTTACGACGCTGTTAGTTATAATGCGTATAATATGTTGGTCTATCTATTAAACAGAAATGGTGATTTTGAGACGATTACTACTAGTGGATGTACATGTATTTCGAAAGCAGTTGCGAACAACAACAAAATAATAATGGAAGTACTATTGTCTAAACAACCATCTTTGAAAATTATGATACTGTCTATAATAGCAATTACTAAACATAAACAACATAATACAAATTTATTAAAAATGTGTATAAAATATACTGCGTGTATGACAGATTATGATACTCTTATAGATGTACAGTCGTTACAGCAATATAAATGGTATATTTTAAAATGTTTCGATGAAATAGATATCATGAAGAGATGTTATATAAAAAATAAAACTGTATTCCAATTAGTTTTTTGTACTAAAGACATTAATACTTTAATGAGATACGGCAGACATCCTTCTTTCGTTAAATATACTAGTCTCGACGTATACGGAAGTCGTGTACGTAATATCATAGCATCTATTAGATATCGTCAGAGATTAATTAGTCTATTATCCAAGAAGCTGGATGTGGGAGATAAATGGGCATGTTTTCCTAACGAAATAAAATATAAAATATTGGAAAACTTTAACGATAACGAACTATCGACATATCTAAAAATCTTATAAACACTATTAAAATATAAAACCTAAGTAGGATAAAACACATTACATCATTGTTTCCTTTTATTGCTTAACAGTGTATACTATTTTTAACACTCATAAATAAAAATGAAAACGATTTCCGTTGTTACGTTGTTATGCGTACTACCTGCGGTTGTTTATTCAACATGTACTGTACCCACTATGAATAACGCTAAATTAACGTCTACCGAAACATCGTTTAATGATAAACAAAAAGTTACATTTACATGTGATTCGGGATATTATTCTTTGGATCCAAATGCTGTCTGTGAAACAGATAAATGGAAATACGAAAATCCATGTAAAAAAATGTGTACAGTTTCTGATTATGTCTCTGAACTATATAATAAACCGCTATACGAAGTAAATGCCATCATAACACTAATTTGTAAAGACGAAACAAAATATTTTCGTTGTGAAGAAAAAAATGGAAATACTTCTTGGAATGATACTGTTACGTGTCCTAATGCGGAATGTCAATCTCTTCAATTAGATCACGGATCGTGTCAACCAGTTAAAGAAAAATACTCATTTGGGGAACATATAACTATCAACTGTGATGTTGGATATGAGGTTATTGGTGCTTCGTACATAACTTGTACAGCTAATTCTTGGAATGTTATTCCATCATGTCAACAAAAATGTGATATACCGTCTCTATCTAATGGATTAATTTCCGGATCTACATTTTCTATCGGTGGCGTTATACATCTTAGTTGTAAAAGTGGTTTTATACTAACGGGATCTCCATCATCCACATGTATCGACGGTAAATGGAATCCCGTACTCCCAATATGTATACGATCTAACGAAGAATTTGATCCAGTGGAGGATGGTCCCGATGATGAGACAGATCTGAGCAAACTCTCAAAAGACGTTGTACAATATGAACAAGAAATAGAATCGTTAGAAGCAACTTATCATATAATCATAGTAGCGTTGACAATTATGGGCGTCATATTTTTAATCTCCGTTATAGTATTAGTTTGTTCCTGTAACAAAAATAATGACCAATATAAGTTCCATAAATTGTTGCTGTGAATATAAATCCGTTAAAATAATAAATAATTAATAACGAACAAGTATCAAAAGATTAAAGAATTATAGCTAGAATTAATTGAGATGTCTTCTTCAGTGGATGTTGATATCTACGATGCTGCCAGAGTATTTTTACTTAGGCACTATTATAACAAGAGATTTATTGTGTATGGAAGAAGTAACCATATTACATAATATATACAGGCTATTTACAAGATGCGCCGTACCGTTCGATGATATAGTACGTACTATGCCAAATGAATCACGTGTTTAACAATGGGTGATAGATACACTTAATGGCATAATGATGAATGAACACAATGTTGCTGTGTAGGTACCGGACTACTGTTCATGGAAATGTTTTTCGATTATAATAAAAATAATTCCAAAAATAGCATCATCAACTAATGTATGACATAATTAATAGCGTATCCATAATTCTAGCTAATGAGAGATATAGAAGCGCGTTTAACGACAATGGAATATACATCCGTAGAACTATGATTGACAAGTTGTACGGATACGCATCTCTAACTACTATTGCACGATCGCTGGAGGTGTTTGTTATTATCTGTTGATGCATCTAGTTAGTTTGTAT

AGGTGCAATAGCATTCTATTCGAATAATGAGTATACTCCGTTTAATAAACTTAGTGTAAAACTATATATAGATGGAGTAGATAATATAGAAAATTCATATACTGATAATAATGAATTAGTGTTAATTTTTAAAGAGTACACAGTTTCTATTATTACAGAGTCATACGACGTCGGATTTGATTCCATAGATATAGATGTTATAAACGACTATAAAATTATTGATATGTATACTATTGACTCGTCTACTATTTAATGCAAAGGACACACATGTAGAATATCTACCAAATTATCATGCCATTATGATAAACACCCTTATATTCACAAATATAAGGGTGATAAGCGACAATATTCTATTACTACAAAGGGAAAATGCTATAAAGGAATAAAATATGAAATAAGTACAATGAACGATAATATTCTATTGAGACAACATACTCTTAAAATTGGATCTACTAATATATTCGATAGTTATGGACATAGTAATACATATTATTCAAAATATGGTCTTTAAAAATTTAAAATATATTATCACTTCAACGACAATAGTCAAATAACAGCCAATATGAGATCTGTAATGCTAACAGTTTTGCTAATTAATAGCATAAATGCTACGATAACTAGCTATAAATTCGAATCCGTCAATTTTGATTCCAAAATTGAATGGACTGGCGATGGTCTATACAATATATCCCTTAAAAATTATGGGATAAAGACGTGGCAAACAATGTATACAAATGTACCAGAAGGAACATACGACATATCCGGATTTCCAAAGAATGATTTCGTATCTTTCTGGGTTAAATTTGAACAAGGTGACTATAAAGTGGAAGAGTATTGTACAGGACTATGCATCGAAGTAAAAATCGGACCACCAACTGTAACATTGACTGAATACGACGGCCATATCAATTTGTACATCGAGCATCCGTATGCCACTAGAGGTAGCAAGAAGATTCCTATTTACAAACGTAATGACATGTGTGATATCTACTTGTTGTATACGGCTAACTTCACATTCGGAGATTCTGAAGAACCAGTAACATATAATATTGATGACTACGATTGTACGTCTACAGGTTGCAGCATAGACTTTGCCACAACAGAAAAAGTGTGCGTGACGGCGCAGGGAGCCACAGAAGGATTTCTAGAAAAAATTACTCCATGGAGTTCGGAAGTATGTCTGACACCTAAGAAGAATGTATTTACATGCGCTATTAGATCCAAGGAAGATGTATCCAATTTCAAGGACAAAATGACCAGAGTTATCAAGAGAAAATTTAATAAACAGTCTCAAAATTATATGACCAAATTTCTCGGTACTACGGCTAATGATGTTACTACCGTGATTAGCATGCTAGATTAAATATTTTGGGCAAATCTAAATATTTTTTATAAAAATTAAAAAATCACTTTCGTTATCGATAACTCGAGAACATGGGTTACATATCAACATATTAATGTTGGTGGCATTGTCTAAAACAGTCACGATATCTCTTAAATGCTACAGAAAGGTAGGATTTTATAATCTTTATGGATCTAATGATAGCGAGCAGGAAAATTATTCTCCTGTATCTGTTAAAACTACGATTATCTATTAAAGACAGAGATACTAAATATAGACAATAACATACCATGGAAAAATATTACAGATTTTATTAAACTCTTTTATAGAGAAATGTAATAATTGCTCAAATAATACTATATACAATATCACTATAAAGTCTTGGACTAATTATACTTTGGTAAATGTTACAACTGACAATAGTGTCTACTTAAATGTATCTAGTGATATCGTGATCAATAAAACAGGAAATATTACTACATTCATTAATAATACAGTTAATGGTAATTTATCGATTAGTCTTAATGATACACTAAGTATATACACAGAATCAGAACCTGTGTTTATAGTGGATCCATTCATTACTCTTGATAAATTGTACAACTGTTCGTTCTCCAAACCAATCAATTATGTAAATATCTTTAATACAGACACCAATGGACATTTCTTATGTTATTAATGACAATATAACATTCATTAGTAATATCACATCAAGTACAAAATATATCAACTCATGTGTAAATTCGTCACTGAGTTTACAGATAGCGGTTGTCAGAATACCAGTATTGTTTAATAAAACAATGGAACAATTAGGTATTACTCTAGGATCTGATTTGGAAGCGTATTTCATGTGTAAGTTACAGGAACTTAATTGTGACGCGAGTATTCTCTTGAACAAGGCATCAAAAAATGTAATGGATAGACAATTATCAAAAACTCCAACGAGATAAAAGAGATCCACTGAGATGACTGGAAATCACTTCTGTCTACATAAGAATCAAGCTAGGCTGGAAACTATTTACGAAACTATCAAAATGCAGGGTACAACAAACAATAATGACGGAAGTAAACGAACGTCTCCAATATATGATAACGTTGGCAATACTAAGGATTCATTGACATATGTAAATATTAATAAAGTATATATGAGATACACGTTTTGATAGTCATCCTAATCGTATCGGAGTACGGAGATCATCAAGTTCATCATCCGATATTATTGTAACCACAATGCATTGGCATCAAAAGAAGATAGATGAACTGACATATAGCGAATATAAGGAGATCCAACACTATTAGGAGAAAGCAGGTCATATGGAAGATGTTCAAGCTGTTAATATCAATCTCGATAAACGTGAACAGGCTGTCGGAGCCACAGTT

AAAAACAAAAGTAGTATAGCAATACAACAGAAAAGAGAATATAAAAAATTTATAGCTACTTTCTTTGAGGACTGTTTTCCGGAAGGAAATAAACCTTTGGAATTAGTATACACGTTAGATTATTCTCAAACTCCTAATTATGAAAGACTACGTAGACTAGACTGTTTATACAAGATTGAAATTATATTCTTTTTTATAGAGCGTGTGATGTTACGGATATCTAATAATATTAGACTATCTCTGACACTACACGACCAAATCGATTACTATGGATATCTTCAGGGAAATCGCATCTTCTACGAAAGGAGAGAATGTATTCATTTCTCCAGCGACAATCTCGTCAGTATTGACAATACTGTATTATGGAGCTAATGGATCCACTGCTGAACAGTTATCAAAATATGTAGAAAAGGAGGAGACCATGGATAAAGTTAGCGCTCAGAATATCTCATTCAAATCCATGAATAAAGTATATGGGCGATATTCTGCCGTGTTTAAAAATTCCTTTTTGGGAAAAATTGGCGATAATTTCCAAACTGTTGACTTCACTGATTGTCGCACTATAGATGCTATCAATAAGTGTGTTGATGTCTTTACTGAGGGGAAAATCAATCCACTATTGACTGAACAATTGTCTCCCAATACCTGTCTCCTAGCAATTAGTGCCGTATACTTTAAAGCAAAATGGTTGATACCATTCAAAAAGGAATTTACCAGTGATTATCCATTTTACGTATCACCAACGGAAATGGTAGACGTAAGTATGATGTCTATGTATGGTGAGTCATTTAATTACGCATCTGTAAAAGAATCATTCGGTAACTTTTCAATCATAGAACTGCCATATGTTGGAAATACTAGTATGATGGTCATTCTTCCAGACAAGATTGATGGATTAGAATCCATAAAACAAAATCTAACAGATACAAATTTTAAGAAATGGTGTAACTCTCTGGAAGCTACGTTTATCGATGTACACATTCCCAAGTTTAAGGTAACAGGTTCGTATAATCTGGTGGATACTCTAGTAAAGTTGGGACTGACAGATGTGTTCTATTCAACTGGCGATTATAGCAATATGTGTAATTCAGATGTGAGTGTTGACGCTATGATTCACAAAACGTATATAGATGTCAATGAAGAGTACACAGAAGCAGCTGCAGCAACTTCTGTACTAGTGGCAGACTGTGCATCAACAGTTACAAATGAGTTCTGTGCAGATCATCCGTTCATCTATGTGATTAGGCATGTCGATGGCAAAATTCTTTTCGTTGGTAGATATTGCTCTCCAACAACTAATTAAGCATATTCTTAATATTAGAATATTATATAGTTAAGATTTTTTTAAAAAATAGAAAAAACATGTGGTATTAGTGCAGGTCGTTATTTCTCCCATTGCAATTGGTAAGATGACGGCCAACTTTAGTACCCACGTCTTTTCACCACAGCACTGTGGATGTGACAGACTGACCAGTATTGATGACGTCAGACAATGTTTGACTGAATATATTTATTGGTCGTCCTATGCATACCGCAACAGGCAATGCGCTGGACAGTTGTATTCCACACTCCTCTCTTTTAGAGATGATGCGGAATCAGTGTTCATCGATGTTCGCGAGCTGGTGAAAAATATGCCGTGGGATGATGTCAAAGATTGTGTAGAAATCATCCGTTGTTATATACCGGATGAGCAAAAAACCATCAGAGAGATTTCGGCCATCATCGGACTTTGTGCATATGCTGCTACTTACTGGGGAGGCGAAGACCATCCCACTAGTAACAGTCTGAACGCATTGTTTGTGATGCTTAAAATGCTCAATTACGTGGATTATAACATCATATTCCGGCGTATGAATTGACATTTCTCCCTTTCCCAGAAATAAACTTTTTTACCCACTATAAAATAAAAGAGTATACTACCTATTATATTTCTTCCTATATTTTTTATTCTTCATTAGTTACGACTTTTAACACACCTAAATGTGTCGACAAAGGGCAATAGTTTGCGTCATTCATGGAGTTAGAAAACGAGCCAGTAATCTTACCATGTCCTCAAATAAATACTCTATCGTTTGGATATAATATATTAGATATTTTATGGGAAAACGAGAAGCGGATAATGATAGAATTATACCAATAGATAATTGTAACAATATGCTAATTCTGAACCCGACACAATCAGACTCTGGTATTTATATGTGCATTACCAAGAACGAGACCTACAGTGATATGATGTCGTTAAATTTGACAATTGTGTCTGTCTCAGAATCAAATATAGATCTTATTTAGTATCCACAAATAGTAAATGAGAGATCTACTGGTGAAATGTGACCCAGTAATGCATTTATTGCTAGTAACATAAACACAGATATTATATGGAGCGGACATCAACGCCTTAGAAATAAGAGACTTAAACAACGGACACCTGGAATTATTACCATAGAAGATGTTAGAAAAATGATGCTGGTTATTACACATGTGTTTTAGGGAGTAAAGCATATAACGTAACCAGAATTGTAAAATTAGAGGTATGGGATAAAATAATACCTTCTACTATGCAATTACCAAAAGGCCTTGTAACTTCACTAGGTAGTAATTTGACTATTGTGTGTAAAGTATCGTTGAGACCTCCCACAACGGATGCAGATGTCTTTTGGATAAGTAATGGTATGTATTACGAAGAAGACAATGAGGACGGAGACGGTAGAATAAGTATAGCAAATAAAATCTATACGACCGATAAGAGACGTGTTATTACATTGTGATTAAACATTAATCCTGTCAAGGAAAAAGATGCTACAGCGTTTACTATTCCTAGCATCAGCAAAACAGTTACTATTAGTATAATGTGATTGTATGTTGTTACATTTCCATGTCAAATAGAGTTTATAAAAATTTTTTATACATTATCTTCCAACAAGCAATTGACGAACGTATTACTATGATTAACTCCCACGATACTATGCATATTATTAATCATTAACTTGCAGACTATACCTAGTGCTATTTTGACATACTTGTGTTCTTGTGTAATTGCGGTATCTATATTATTAAAGTACGTAAATCTAGCTATAGTTTTATTATTTAATTTTAGATAATATACCATCTCCTTATTTTTAAAAATTACCACATCCTTTATTAAATCATGGATGGGAATTTCTATGTCATCGTTAGTATATTGTGAACAACAAGAGCAGATATCTATAGGAAAGGGTGGAATGCGATACATTGATCTATGTAGTTTTAAAACACACGAGAACTTTGAAGAATTTATATAAATTATTCCATCGATACATCCTTCTATGTTAATATGTATATATCCAGGAATTCTTTTATGAATATCAGGAAATGTATAAACTAAAACATTGCCCGGATACGGTGCTTCTATTGGCATTATATCCGTTCTTAACTTGCAAAATGTAACCAATACCTTTGCATGGCTTGTTTTGTTCGGTAACGTTAGTTTAAACTTGACGAATGGATTAATTACAATAGCATGATCTGCGCATCTATTAAGTTTTTTTACTTTAACGCCCTTGTATGTTTTTACAGAGACTTTATCTAAATTTCTAGTGCTTGTATGTGTTATAAATATAACTGGATATAGAACTGAATCACCTACCTTAGATACCCAATTACATTTTATCAGATCCAGATAATAAACAAATTTTGTCGCCCTAACTAATTCTATATTGTTATATATTTTACAATTGGTTATGATATCATGTAATAACTTGGAATCTAACGCGCATCGTCGTACGTTTACACAATTGTGATTTAGTATAGTATATCTACACATATATTTTTCCGCGCTATAGTATTCTGGACTAGCGATAAAACTATCGTTATATCTGTCTTCGATGAACTCATCAAGATATTGCTCTCTGTCATATTCATACACCTGCATAAACTTTCTAGACATTGTACAATATGTGTTATTTTAGGATCATATTTACATATTTACGGTATATCAAAGATGTTAGATATAGTTAATAGGACACATATAATAATGAATATTAAACACTAGTATAGCAGAAGTTTATACTCACAAAACATCATAAAATGAGTCGTCGTCTGATTTATGTTTTAAATATCAACCGCAAATCAACTCATAAAATACAAGAGAATGAAATATATACATATTTTAGTTATTGCAATATAGACCATACTTCTACAGAACTTGATTTTGTAGTTAAAAACTATGATCTAAACAGACGACAGCCTGTAACTGGGTATACCGCACTACACTGCTATTTGTATAATAATTACTTTACAAACGATGTACTGAAGGTATTATTAAATCATGGAGTGGATGTAACGATAAAACCCAGTAGCGGACATATGCCTATTTATATATTGCTTACTAGATGTTGCAATATTTCACACAATGTAGTGATAGATATGATAAACAAAGATAAAACCCACTTATCGCATAAAGACTATTCCAACCTACTACTAGAGTATATAAAATCTCGTTACATGCTATTGAAGGAAGAGGATATCGATGAGAACATAGTATCAACTTTATTAGATAAGGGAATCGATCCTAACTTTAAACAAGACGGATATACAGCATTACATTATTATTATTTGTGTCTTGCACACGTTTATAAACCAGGCGAGTGTAGAAAACCGATAACGATAAAAAAGGCCAAGCGAATTATTTCTTTGTTTATACAACATGGAGCTAATCTAAACGCGTTAGATAATTGTGGTAATACACCATTCCATTTGTATCTTAGTATTGAAATGTGTAATAATATCCATATGACTAAAATGCTGTTGACTTTTAATCCGAATTTCGAAATATGTAATAATCATGGATTAACGCCTATACTATGTTATATAACTTCCGACTACATACAACACGATATTCTTGTCATGTTGATACATCACTATGAAACAAATGTTGGAGAAATGCCTATAGATGAGCGTCGTATGATCGTATTCGAGTTTATCAAAACATATTCTACACGTCCGTTAGATTCGATAACTTATTTGATGAATAGGTTTAAAAATATAGATATTCATACCCGCTATGAAGGAAAGACATTATTACACATAGC

GTATTAACGGAGATATAAATGCGTTAACCGACAATAACAAACACGCTATCCAACTCATTATAGATAACAAAGAAAATTCCCAGTATACCATCGATTGTTTATTGTATATACTTAGATATATTGTAGATAAGAATGTGATAAGATCGTTGGTGGATCAACTTCCATATCTACCTATCTTCGATATAAAATCATTTGAGAAATTCATATCATACTGTATACTTTTAGATGACACATTTTACGATAGACACGTTCAGAATCGCGATTCTAAAACTTATCGATACACGTTTTCAAAATACATATCATTCGATAAATACGATAGTATAATAACTAAATGCTATGAAGAAACAATATTGCTCAAACTATCCACTGTTCTAGACACAACACTATATTCTGTTTTAAGATGTCATAATTCGAGAAAGTTAAAAAGATACCTCAGCGTGTTAAAAAAATATAATAATGATAAGTCCTTCAAAATATATTCTAATATTATGAATGAGAGATACCTTAATGTCTATTATAAAGATATGTATGTGTCAAAAGTATACGATAAACTATTTCCTGTTTTCACAGATAAAAAATGTCTACTAACATTACTACCTTCAGAAATTATATATGAAATATTATACATGTTAACAATTTACGATCTTTATAATATATCGTATCCACCTACCAAAGTATAGTTGTATTTGTAATTTTTCTCATACGATGTGTGTAAAAACTGATATTATAAATTATCTTAGTGTCGTATGATGAAGATGACGATGAAAATGATGGTACATATATATTTTGTATCATTATTGTTATTGCTATTCCACAGTTACGCCATAGACATTGAAAATGAAATCACAGATTTCTTCAATAAAATGAAAGATACTCTACCAGCTAAAGACTCTAAATGGTTGAATCCAACATGTATATTTGGAGGCACAATGAATAATATGGCTGCTATAGGAGAGCCATTCAGTGCAAAGTGTCCTCCTATTGAAGACAGTCTTTTATCGCGTAGATATATAAATAAAGACAACGTAGTTAACTGGGAGAAAATAGGAAAAACTAGACGACCTCTTAATAGACGTGTTAAAAACGGTGACTTATGGATAGCCAACTATACATCTAACGACAGTCATCGTATGTATTTGTGTACAGTGATTACAAAGAATGGCGACTGTATTCAAGGTATAGTTAGATCCCATGTACGGAAACCTTCTTCATGCATTCCAGAAATCTATGAACTAGGTACTCATGATAAGTATGGAATAGACTTATACTGTGGAATTATTTACGCAAAACATTATAATAATATAACTTGGTATAAAGATAATAAGGAAATTAATATCGATGATATTAAGTATTCACAAACGGGAAAGGAATTAATTATTCATAATCCAGCATTAGAAGATAGCGGAAGATATGACTGTTATGTTCATTACGACGACGTTAGAATCAAGAATGATATCGTAGTATCAAGATGTAAAATACTTACGGTTATACCGTCACAAGACCACAGGTTTAAACTAATACTAGATTCGAAAATCAACGTAATTATAGGAGAACCTGCCAATATAACATGCACTGCTGTATCAACGTCATTATTGTTCGATGATGTACTGATTGAATGGGAAAATCCATCCGGATGGCTTATAGGATTCGATTTTGATGTATACTCTGTTTTAACTAGTAGAGGCGGTATTACTGAGGCGACCTTGTATTTTAAAAATGTTACTGAAGAATATATAGGTAATACATATAAATGTCGTGGACACAACTATTATTTTGAAAAAACCCTTACAACTACAGTAGTATTGGAGTAAATACACAATGCATTTTTATATACATTACTGAATTATTATTATTAATTATATCGTATTTGTGCTATAGAATGGATGAAGATACGAAACTATCTAGGTATTTGTATCTCACCGATAGAGAACATATAAATGTAGACTCTATTAAACAGTTGTGTAAAATATCAAATCCTAATTCATGTTATAGATGTGGATGTACGGCTTTACATGAGTACTTTTATAATTATAGATCAGTCAACGGAAAATACAAGTATAGATACAACGGTTACTATCAATATTATTCATTTAGCGATTATGAAAATTATAATGAATATTATTATGATGATTATGATAGAACTGGTATGAATAGCGAGAGTGAGAGTGATAATATATCAATCAAAACAGAATATGAGAATGAATACGAATTCTATGATGAAACACAAGATCAAAGTACACAACTAGTAGGTTACGACATTAAACTCAAAACCAATGAGGATGATTTTGTGGATGAATTCTATGGCTATGATAGATCAGTGGGTGTCCATGATTATATAGATAAATCAATTAATAAAGTAGTATATGGAAGAGAGTCTTACGTAAGATGGCGGGATATATGGCAAGAACATAATGATGGTGTATACAGTATAGGAAAGGAGTGCATAGATAATATATACGAAGACAGACATACAGTAGACGAATTCTACAAGATAGACAGCGTATCAAATGTAAATGACGCGGAACACATATCTCAGATAACTAATGATGTATCTACACAAACATGGGAAAAGAAATCAGAGTTAGATAGATACATGGAAATGTATCCTCGTCATAGATATAGTAAGCATTCTATCTTTAAGGGATTTTCTGACAAAGTTAGAAAAAATGATTTAGACATGAATGTGGTAAAAGAATTACTTTCTAACGGCGCATCTCTAACAATCAAGGATAGCAGTAATAAGGATCCAATTGCTGTTTATTTTAGAAGAACGATAATGAATTTAGAAATGATTGATATCATTAACAAACATACAACTATCTATGAACGCCGGTATATAGTACACTCCTATCTAAAAAATTATAGAAATTTTGATTATCCATTTTTCAGGAAGTTAGTTTTGACTAATAAACATTGTCTCGATAATTATTATAATACAGGTGACCGCAGACATGGAACGCCGCTACATATATTGGCGACTAATAAAAAATTAATAACACCTAATTACATGAAGTTATTAGTGTATAACGGAAATGATATAAACGCACGCGGCGAAAATACACAAATGCAAACTCCATTACACAAATATTTGTGTAAATTTGTATATCATAATATTGAATATGGTATCCGATACTATAATGAAAAGATTATAGACGCATTTATAGAGTTAGGAGCCGATCTAACTATTCCAAATGATGATGAAATGATACCAGTAATTTACTGCATACACGTAAATGCTGAATATGGTTATAACAATATTACTAACATAAGGATAATACGTAAACTACTTAATCTTAGCAGACATGCTCCACATAATCTATTTAGAGATCGAGTCATGCACGATTATATAAGTAATACATATATTGATATAGAGTGTTTAGATATTATTAGATCACTGGATGGGTTCGATATTAATTGTTACTTTGAAGGACGTACACCTCTTCATTGCGCTATACAGTATAACTTCACTCATATTGCGGAATACTTATTAGATCGAGGAGCTGATATATCATTAAAGACAGGCGATGGAAAATCTGTATTCGATTTATCGTTATGTAGCTACATACCTCTTAAATGGACTAGTTTTTTGATTAGTCGTCTACCGCCTAAAAGTGTCATGAGCTCACTGACTAACCATATAATAGATTATGTTCTTACAGACAATAAAAATATTATTTGGCAAAGTCAAATGATTAATAAGTATGTGTTGTTACTTGATCCATCCTTTTATTCTAGATTCAGAAATGCTATTAAAAGCAAATTAAATCAATACGATTATCGTTGCTTCAATTACAATAGATATCGTGTTAATAAAAAGTATCGCAAAGTTTTACATGACATTGATATATATATTAAGGATGTACAAGTATTAAAATCTATTCTCATCGCCAATAATGTAACACTATACGACACTATTGTAAATAATACATCAGAGTTTCCTGTACATCGTCTAAACAACAAACAATTAATTAATACAGTAAATTCCAATATATATCATGATCTTATCGAAAAAGTTATTAAAAAATCATTAGAGAAATGTACTCTAACTAATGCCGTCCTCGAGTATATGATCGCATCTCGATCTCAATCATCTTATTTAAGTCGTATTCCTAATGAGATATTACTCAAAATACTATATAACCTCAGCGTCTACGATTTACGCAAACTATATGCAAGATACATGCTAGAGAATGAGACAGATTATCATATAGAGTCAAGGTCAATTTCTACGCAGACTGAATAATGTTATATACACTACATATTTTTATGTCATTATAACTACTTGTAATATTGAAATCGTAATTTGATATATAGTGAAAAATGGATTTTGATGATATTAAACATAATAGACGAGTAGTAAGTAACATTAGCAATCTTTTAGATGATGATATATATTATGCGATGTAATCATAACTATTGGTGATGGAGAAGAAATTAAAGCACATAAAACTATCTTGGCTGCTGGATCTAAATATTTTAGAACACTGTTTACAACACCTATGATAATAAGAGATCTAGTAACTAGAGTAAAAATCTACAGATGATCAATAAAGATTCCGTCAAAAATATTGTACAGTACTTATACAATAGGTATATAAGTTCTATGAATATAATAGATGTATTAAAATGCGCCAACTATCTTCTAATCGATTATCTAGTCACTGACTGCGAATCCTATATTAAAGATTATACTAATCATGATACCTGCATATGTATGTACCATAGGTTAATGAGATGGTTCATATTCCAATAGTCAAATATATTAAACGTATGCTGATGAGTAATATATCAACATTGATAACTACGGACGCATTTAAAAAAACAGTATTTGAAATATTATTTGATATTCTTTCTACTAACGACAATGTATATTTATACAGAGAAGGCTATAAAGTAACGATACTATTGAAGTGGCTAGAATACAACTACATCACAAAAGAACAGCTTTTGTATATACTGTCCTGTATAGATATACAATCTAGATAGAAGTATAGACTACTACTCTATTCAAATAAAACTATAAATATGCATCCCTCATGTATACAATTTCTATTAGATAATAAACAGAATAGAAACATTATACCCCAACCAGTTATGTTTAGCGTGTCATGATACTAAGTATAATGTATGTAACCCGTGCATATTAGTATATAACATTAATACTATGGAATATAGTGTTATTTCCACCATTCCTAATCACATAATTAATTACACATCCGCGATTGTAGATAATGAAATAATAATAGCTGGAGGATATAACTTTAATAATCCATCGTTAAA

GATGTCGTTTTTCTTTGGCTGTAATCGATAACACAATTTATGCTATAGGTGGTCAAAAACTGAAGTTTAACATTATTTATTTATGATATATATTATATATCGGTATCGTTATTGTTTGGTCTATACCATGGATATCTTTAAAGAACTAATCTTAAAATACCCTGATGAAAATGTTTTGATTTCTCCAGTTTCCATTTTATCTACTTTATCTATTCTGAATCATGGAGCAGCTGGTTCTACAGCTGAACAACTATCAAAATATATAGAGAATGTGAATGAAAATACACCCGATGATAAGAAGGATGACAATAATGACATGGACGTAGATGTTCCGTATTGTGCGACACTAGCTATTGCAAATAAAATATACTGTAGCGATAGTATCGAGTTCCACGCCTCCTTCCTACAAAAAATAAAAGATGATTTTCAAACTGTAAACTTTAATAATGCTAACCAAACAAAGGAACTAATCAATGAATGGGTTAAGACGATGACAAATGGTAAAATTAATTCCTTATTGACTAGTCCACTGCCCATTAATACTCGCATGACAGTTGTTAGCGCCGTCCATTTTAAAGCAATGTGGAAATATCCATTTTCTAAACATCTTACATATACAGACAAGTTTTATATTTCTAAAAATATAGTTACCAGCGTTGATATGATGGTGAGCACTGAAAACGACTTACAATATGTACATATTAATGAATTATTTGGAGGATTCTCTATTATCGATATTCCATACGAGGGAAACTCTAGTATGGTAATTATACTACCAGACGACATAGAAGGTTTATATAACATAGAAAAACACATAACTGAAGAAAATTTTAAAAAATGGTGTGGTAAGTTATACACTAAAAGTATAGACTTGTATATGCCAAAGTTTAAACTGAAAATGACAGAATCGTATAATCTGGTACCTATTCTAGAAAATTTAGGACTTACTAATATATTCGGATATTATGCAGATTTTAGCAAGATGTGTAATGAAACTATCACTGTAGAAAAATTTCTACACAAGACGTTTATAGATGTTAATGAGGAGTATACAGAAGCATCGGCTATTACAGGAGTATTTATGACTAACTTTTCGATGGTATATCGTACGAAGGTCTACATAAACCATCCATTCATATACATGATTAAAGACAACACAGGACGTATACTCTTTATAGGGAAATACTGTTATCCACAATAAATATAAACAAATAGACTTTTTATCTCATGTCTAAGAATTACAAAGTAATATAGTTAGTATAACTAAAGTTAATACTACATTAAAACTTAAAAAAAATAATAATATCACTATTACAATTAATAAGTATAAACTATAAACTAAAAATTAAACAATATCGTTATTATATAAGTAATACCAAAATGATGATATACGATTAATAGCCTGTCTTATATTCGTGACTTTATCCATTGCTAGTCACTTTACATTCCCGTTATTCCTCCCATTACGGAAGATAAATCGTTTAATAGTGTAGAGGTATTAGTTTCCTTGTTTAGAGATGATCAAAAAGACTATACGGTAACTTCTCAGTTCAATAACTACACATCGACACCAACGACTGGACTATAAATGTACTATCCGATGGTCTGGATATACCATTGACTAATATAACTTATTGGTCACGGTTTACTATAGGTCGTGTATTGTTCAAATCAGAGTCTGAGGATATTTTCCAAAAGAACATGAGTATTCTAGGTGTCTCTATTGAATGTAAGAAGCCGTCGGTATTACTTACTTTTTGACCGTGTGTAAAATGACTCGAGTATTTGACTTCCAGATATGGATTATTATCGAGGAGACTGTCTAGAAGTCGTTTATATAACAATGACTTATAAAAATACTAAAACTGAAGAGACTGATTACGCATACCTCTCTAATGTGCGGATTCCTGAATACTATCGATTGATGAGTAATGGGGTTGATGGTTGATTATTGATTAGTTTTCCTTATTCTTTTTATTCACATAAAAGAATATTTTTTATAAACATGAAACCACTGTCTAAATGTAATTATGATCTTGATTTATAGATGAAAATCAGCTGCTTTAGATGATTTTGGTTAGTATGTATAATATAAAAAAATAAATATAACTTATTTTAAGATTAAGTGCTATTGTACTTAATTTTTTGTTCTATAAACTGAATATATAGCTACAATTATTGACGGGTTTATTTATGACCGGCAACCATGAATTTACAGAGATTATCTCTGGCTATATATCTTACTGTGACATGTTCGTGGTGTTATGAAACATGTATGAGAAAAACTGCGTTGTTTCATGACAATCAATTGGGGCATGCGGAAGACAATCAAGATAGTGTAGCGTCGCTACCGTACAAGTATCTACAAGTAGTCAATAAAAGAGAACGTAGTAGATTGCTGGCTACGTTTAATTGGACAAGTATAGCTGAGGGTGTTAAAAATGATTTCATTAGAATATGTGATATCAACGGAACATATCTATATAATTATACTATTGCTGCTAGTATGATTATTGATTCCATGGAAGAACTACCAACAATTACAACATATGAACCGTCTACCTATAATTATACTTTCGATAATAGCACTGTTAGTACTACTGAAGAACTAAAAGTGACTCCATCACCAACTACATACGCGACTGTAACAACTCCTCTTCCAACATCATCAGTTCCATATGATCAACGATCTAATAACAATGTAGGTACTATATCTATTCAGATACTGAGTAAAATATTGGGAGTCAATGAAACAGAATTAACTAATTATCTTATTACGCATAAAAATGCCACGGTTGACAATAACACATTGTATGGTAATATAGGATTTTTGGAAATAAATAATTGCTACAATATTTCTGTATCAAATGCTAGTTTTAGAATAACATTAGTAAACAATACTTCTGAAGAAATTGTTATAATGCTAACAGGGACTAGTTCATCCGACACCTTCATATCTTCCACCAATACCACTGAATGTTTGAAAACATTAATCAATAATACGTCGAATATTAGTGATGTAAGTATAACACAAAATATGAATGTAACATCTAATTGTGATAAATGTTCAATGAATTTGATGACATCCGTTATCCCTGCCGTTAAAGAATTTAACAATACGTTGAAAAAAATTGGTGTAAAAGATGATAAAAACAATACAGTATATAACTATTATAATTGTAAACTAACTACAAATTCTACATGTGACGAGTTAATCAATTTAGATGAAGTCATTAACAATATAACTTTGACAAATATTATAAGTAGTAGTGTTTCTACAACCAACAGCAGAAAAAGACGAGATCTGAATGGTGAGTTTGAATTTTCTACATCCGAGGAATTAGATTGTCTTTACAAATCATATGGTGTAAGTGATGATGTAAGTCATTGTTTTTCATCACCTAGACGTAGACGATCTGACGACAAACAGGAGTACACAGAAATGAAATTACTCGACCACGCGAAAAAAGATTTAAGAATAGACAGTGTTATTCCTAGAGGTACAACTCATTTCCAAGTAGGTGCATCTGGAGCAAGTGGTGGTGTTGTAGGTGATAGTTCCCCATTTCAAAATGTTAAATCACGGGCCAGTCTATTGGCAGAAAAAATAATGCCTAGAGTACCCACTACTGCTACCGAAGAGCAGCTATATGCAACTATAAATAGACAAACAAAGTTGCCTGCAGGTGTTAAAAGTACTCCGTTTACAGAGGCGCTTGTGTCTACGATAAACCAAAAGCTTTCTAGTGTTAAAGAGGTAACTTATGCTTCGTCCAATCTGCCAGGATCAAGTGGTTATGTTCATAGACCATCTGATTCTGTTATTTACAGCACTATAAGACGGACACGTTTACCTAGTGATACCGATAGTGATTTTGAGGATATACAAACTGTTGTTAAGGAATATAATGAAAGATATGGTAGACGAGTTAGTAGAACACAGTCATCAAGTAGTGATTTTGAAGATATAGATGAGGTGGTTGCAGAATATAGACAAAAATATGGTGGAGCTAGCCGTGGTAGAACATCGTCATCAAGTAGTAGTGATTTTGAAGATATAGATGAGGTGGTTGCAGAATATAGACAAAAATATGGCAACGCTATGACAAAAGGACGTGGTTCTTCTAAACCGGATCCGTTATATAGTACTGTTAAGAAAACACCTAAAAGTATAGCATCTGGAGTAGACATAGTTTCAAAACAAACAGACTATTCTTTATTACCCGGCGTTAATACTGGCAGTTCTATCGTAACACCTCTCACCAGAAGAGGAGCTACTAGACGACCTAAACGCCCATCTACACCTCCACGCGAAGATCTACCACCGCTTCCTCTAAATCCTCCTTATCGACAACTTTCTCGTGGTGGTGATCATTCTCTACAACAAGTTCCTCAACGTGATTATTCTCCACCACATAGACCTCCTCCACCACTTCCTCCTAAACCAGTTCCAGCTATTCCACCTAGAGATAGTCAACCAAATAATAAAGGATTTAGTAAGTTTGTATCTCCTAGACGGTGTAGAAGATCAACCTCTGGAGTCGTATGTGGTATGATACAATCAAGACCAAACGATGATACATATTCACTTCTTCAACTACCAAAAATTGAACCAGAATATGCGGAGGTAGGTAATGGCTTACCCAAGAACAATGTTCCTGTGATAGGCAATAAACATAGTAAAAAATATACATCGTCGATGTCAAAAATATCAACAAAATTTGATAAATCTATGGCATTTGGAACAGCAATGTTACTAACTGGTCAACAGGCTATTAACCAACAGGATAGATCAACTGCGTTGATTAAAAAAGATCAAATGAGCAAGGACGAAAAAATATTCGAAGCAGTTACAATGACTCTATCAACTATAGGTTCAACGTTGACAACTGCAGGTATGATAGCTCCACCACTAATGATTGCTGGAATAGGTATATCGCTTATATCAGGTATAATAGATACGGCAAAAGATATATATTACCTATTTTCAGGACAGGAGAAGCCAGTAGATCCAGTTATTAAGTTTTTTAACACATACGCTGGATTAGTATCCGATAGTAGTAAAATGGGTGTAAGGAAATGTTTGACGCCTGGAGAAGACACACTTATTTACATCGCATACAAAAACGATTCCAGTTTTAAACAAAATACGGAGGCAATGGCTTTATATTTCTTAGATGTTATCAACTCAGAGATCCTATATCTAAACACATCAAATTTAGTTTTAGAGTATCATCTAAAGGTGGCTTGTCCCATAGGAACATTAAGATCTGTAGATGTGGACATAACTGCGTATACAATATTATATGATACAGCGGATAATATTAAAAAATACAAGTTTATCAGAATGGCAACGCTACTATCCAAACATCCAGTTATTAGATTGACATGTGGTTTAGCAGCAACATTGGTAATTAAACCGTACGAGGTACCCATCAGTGATATGCAACTACTAAAAATGGCAACGCATGGTGAACCAGAATCCACTAAATCAATACCATCTGATGTCTGTGATAGGTATCCTCTAAAGAAATTCTATCTTTTGGCTGGTGGTTGTCCATATGATACATCTCAAACTTTTATTGTACATACTACTTGCAGTATTTTACTAAGAACAGCTACATGGGATCAGTTTAGAAACAGATGGGTGTTACAAAATCCATTTAGGCAAGAAGGGACATATAAGCAACTGTTTACCTTTAGCAAATACGATTTTAACGACACCATAATAGATCCTAATGGTGTGGCGGGTCATGCTAGCTTTTGTACCAATAGAAGCAGCAACCAATGTTTCTGGTCAGAACCTATGATATTGGAAGATGTATCATCGTGTAGTTCTAGAACTAGAAAAATATACGTAAAACTGGGAATATTTAATACTGAAGGGTTTAATAGTTTTGTACTAAACTGTCCAACTGGGTCTACGCCAACATACATCAAAGATAAAAATACGGACAGTAACAATGTTATCATAGAGCTACCTGTAGGTGATTACGGCACTGCCAAATTGTATTCGGTAACAAAACCATCGAGAATAGCTGTATTCTGCACACATAACTATGATAAACGATTCAAATCAGATATTATAGTTCTAATATTTAATAGTATCAGTGGTATTCCATTTTCGAGCATATACACAGGAAGTGTGAACGGTAGAAATAGACTGTTCACCACATTGTCTAAAGGAATGCCATATAGATCAATGTATTGCGATAACAGACGACCAGGTTGTTATTATGCAGGAATACCATTTAATGAAAATAGTGTAGAATCAGATCTGCATTATGGACCAGAAATAATGCTTAAGGAAACATATGACACAAACAGTATTGATCCACAAGTTATAACAAAGTCAAAAACACACTTTCCTACTCCAATAAGTGTAAAATTTACGGTTGACAATTTAGGAAATGGGTATAACAAACCCGAAAATTTTTGGAAAGACGCTAAAAGTAAGAAAAGGACATATAGCGCAATGACAATAAAAATCCTACCATGTACAGTGAGAAATAAAAATGTAGACTTTGGATATAACTATGGACATATTATTTCCAATATGGTTTATGCACAATCTACTAGTCAGGATTATGGAGATGGTACCAACTATACATTTAAATCTGTAAATAGATCCGATCATGAGTGTGAATCTATTTTAGATTTGAAGGCTAAGGAAGTAACTGTGATGTGTCCCGCGTTTAGTATACCAAGAAATATATCAGCGTATGAAGGTCTATGCTTTAGTGTCACTACATCTAAAGATCATTGTGCCTCAAATAAGGAATGGTTAAAATCTTATGGTTATGGGAATACAGATGCTACTAAACAACGTGTCTGTTTTCATCATTGGAATTATGTCACAACGTCGTTGGATTATTACTGTTCATACGAAGATATTTGGAAAAGCGACTGGCCTGACTATGATCCATGTAAGTCATATATTTATATAGAGTATAGAGACATATGGATAGAATCTAAAGTGTTGCAGCAACCTCCTTATACATTCGAATTCACACATGACGATTCTAATGAATACGTGAATAAAGAAATTAGTAACAAATTGAATGATCTGTACAATGAATACAAGAACATTATGGAATATAGCGACGGATCATTGCCTGCTTCTATAAACAGATTAGCAAAATCATTAACTTCAGAGGGTAGAGAAATAGCAAGTGTTAATATAGATGGTAATCTGTTAGATATTGCATATCAAGCAGATAAGGAAAAGATGGCTGATATACAGAACAAAATAAATGACATCACAAGAGATTTATTCATTCACACTTTATCAAACAAAGATATAAAAGACATTATAGAATCCGAAGAAGGTAAGAGATGTTGTATAATAGATGTTAAGAACAATCGTGTTAAAAAGTACTATCCTATTGATAATTATCTATGTGGTACTTTAGATGATTATATATACACTTCTGTAGAATATAACAAATCCTATGTGTTAATAAACGACACTTACATGAGCTATGACTATCTTGAATCATCAGGTGTAGTTGTTTTATCATGTTATGAAATGACTATAATCTCCTTGGATACAAAAGACGCCAAAGATGCTATAGAAGATGAGATAGTAGCAAGTGCTGTAGCCGAAGCATTGAATGACATGTTTAAGGAATTTGATAAAAACGTAAGTGTTATTATAATAAAAGAAGAAGATAATTATCTAAACAGTTCTCCGAATATATACCATATAATATACATCATAGGCGGCACTATTCTGATACTGTTAGTCATTATTTTAATATTGGTAATTTATATAGCATGCAATAAATACAGAACCAGGAAATATAAAATAATGAAAGATGATACTATGAGCATTAAATCTGAGCATCATAATAGTCTTGAAACAGTATCTATGGAAATTATGGATAATCGGTACTAATAAAAAATAGTTTAACTCTTTTTAGAACCAGTTTGGTACTGGAATTTCAGTTCATTCGTTGAGAATATTGATGATTTTTTTTAAGATGATATTTACTTTTTATATGCTTGCATTACAGAATAATATTCACAGGTATTATTAAAATGAGTATCGGAGCTACATTACCATATCATCATCCATGCTCATGTGGATGGAAAATCCATCCATTATATAATTAATGATACGTGCATGTTTGATATATTCTTTAAATGGAACAGACATCACAGACTGAGATACGCAAAGAATCCTACATTCCTAAACTTTGTATCTGACATAAGAACAGTGAGAGGATCGACTCCATAGTGGACAACATTAATACAAACGATAACTTGATTTCAAAATTATCAATGGAGATACAACGTGAAATACTTTATTACTCCATTAAATAATGTATCACTTACGATAATGTCTCTATAAACGACCGGTTTATCATTAATTAATACAATGTCTAAATTCCATAGCATATTACAAAATCACCGTCTAAGATGATAAAAACACGTTTATTTTATATGTCAACCATTACTAAAAAATATATTGTTCTGTTTTTGTTTTCTTTTTCTTACACACATCGCTAATTATGAAAAAGTAAATGATGAAATGTATGAGATGGTGAGATGGACGAGATTGTGAACATTGTTCGTGATAGTATGTGGTTTATACCTAACGTATTTATGGACAACGGTGAGAATGACGGCCACGTTTCTGTCAACAATGTCTGTCATATGTATTTCGCGTTCTTTGATGTTGATACATCGTCTCATCTGTTTAAGCTAGTTATTAAACACTGCGATCTGAATAAACAACTAAAATGTGGTATGTCTCCATTACATTGCTATGTGATGAATACACGATTTAAGCCATCTGTATTAAAGATATTGTTACACAACGGTGTGAATAACTTTGATAATAAGGATAACAAAGGACATATTCCTCTACACCACTATCTGATTTATTCACTATCAATCGATAACAAGGTCTTTGATATACTAACTGACCCCATTGATGACTTTAGTAAATCATCCGATCTATTGCTGTGTTATCTTAGATATAAATTCAATGGGAGATTAAACTACTACGTTCTGTACAAATTATTGACTAAAGGATCTGACCCTAATTGCGTCGACGAGGATGGACTCACTTCTCTTCATTACTACTGTAAACACATATCCGCATTCCACGAAAGTAATTATTACAAGTCAAAGAGTTACACTAAGATGCGAGCTGAGAAGCGATTCATCTATACGATAATAAATCATGGGGCAAACATTAACGCGGTTACGAAAATCGGAAATACGCCGTTACACACTTACCTTCAACAGTATACCAAACATAGTCCTCGTGTGGTGTATGCTCTTTTATCTCGAGGAGCCGATACGAGAATACGTAATAATTTTGATTGTACACCCATCATGGAATACATAAAGAACGATTGTGTAACAGGTCATATTCTCATAATGTTACTCAATTGGCACGAACAAAAATACGGGAAATTACAAAAGGAAGAAGGACACCATTTACTTTATCTATTCATAAAACATAATCAAGGATACGGAAGTCACGCTTTCAATATATTACGGTATCTACTAGATAGGTTCGACATTCAGAAAGACGAATATTATAATACCATGACTCCTCTTCATACAGCCTTCCAGAATTGTAACAACAATGTTGCCTCATACCTTGTATACATCGGATACGACATCAACCTTCCTACTAAAGACGATAAGACAGTATTCGACTTGGTGTTTGAAAACAGAAACATCTTATTCAATGCGGGTGTTATTCACAACATCATCCACCACAGACTGAAAGTATCTCTACCTATGATTAAATCGTTGTTCTATAAGATGTTAGAATTCTCTCCCTACGACGATTACTACGTAAAGAAGATAATAGCCTACTGCATATTAAGGGACGAGTCATTCACGGAACTACATAGTAAATTCTGTTTAAACGAGGACTATAAAAGTGTATTTATGAAAAATATATCATTCGATAAGATAGATTCCATCATCAAAAAATGCAACTGGGACATAAGTCGCCTCAAAGATATTCAAATCTCTGACACCAACCTGTATACTGTATTAAGAACAGAAGACATCCGGTATCGTACCTATCTTAAAGCCATACATTTAGATAGTCACATTTCATTTCCCATGTACGACGATCTTATAGAACAGTGTCATCTATCGATGGAGCGTAAAAGTAAACTCGTCGACAAAGTACTCAATAAATTAAAGTCTACCATCGATGGTCAATCTAGACTATCGTATTTGCCTCCAGAAATTATACGCAACATCATAACCAAGCTAAGCGACTATCATCTAAACAGTATGTTGTACGGAAAGAACCATTACAAATATTATACATGATAGGAAGAAAATATTTAAAAAATATCTATATGATTGGAGAAGTAGGAAACAGGAACACGGTGATTACTACATTATTAAATCATGAAGTCCGTATTATACTTGTATATATTGTTTCTCTCATGTATAATAATAAACGGAAGAGATGCAGCACCGTATACACCACCCAATGGAAAGTGTAAAGACACCGAATACAAACGCCATAATCTGTGTTGTTTATCGTGTCCTCCGGGAACATACGCTTCCAGATTATGTGATAGCAAGACTAACACACAATGTACACCGTGTGGTTCGGGTACCTTTACATCTCGCAATAATCATTTACCCGCTTGTCTAAGTTGTAACGGAAGATGCAATAGTAATCAGGTAGAGACGCGATCGTGTAACACGACTCACAATAGAATCTGTGAATGCTCTCCCGGATATTATTGTCTTCTTAAAGGATCATCCGGATGCAAGGCATGTGTTTCCCAAACAAAATGTGGAATAGGATACGGAGTATCCGGACACACGTCTGTTGGAGACGTCATCTGTTCTCCGTGTGGTTTCGGAACATATTCTCACACCGTCTCTTCCGCAGATAAATGCGAACCCGTACCCAACAATACATTTAACTATATCGATGTGGAAATTACACTGTATCCAGTTAACGACACATCGTGTACTCGGACGACCACTACCGGTCTCAGCGAATCCATCTTAACGTCGGAACTAACTATTACTATGAATCATACAGATTGCAATCCCGTATTTCGTGAGGAATACTTCTCTGTCCTTAATAAGGTAGCAACTTCAGGATTTTTTACAGGAGAAAATAGATATCAAAATATTTCAAAGGTGTGTACTTTAAATTTTGAGATTAAATGTAATAACAAAGGTTCTTCCTTCAAACAGCTAACGAAAGCAAAGAATGATGACGGTATGATGTCGCATTCGGAGACGGTAACTCTAGCGGGTGACTGTCTATCTAGCGTCGACATCTATATACTATATAGTAATACCAATGCTCAAGACTACGAAACTGATACAATCTCTTATCGTGTGGGTAATGTTCTCGATGATGATAGCCATATGCCCGGTAGTTGCAATATACATAAACCGATCACTAATTCCAAACCCACCCGCTTTTTATAGTAAGTTTTTCACCCATAAATAGTAAATACAATAATTAATTTCTCGTAAAAGTAGAAAATATATTCTAATTTATTGCACGGTAAGGAAGTAGAATCATAAAGAACAGTACTCAATCAATAGCAATCATGAAACAATATATCGTCCTGGCATGCATGTGCCTGGCGGCAGCTGCTATGCCTGCCAGTCTTCAGCAATCCTCATCCTCGTGTACTGAAGAAGAAAACAAACATTATATGGGAATCGATGTTATTATCAAAGTCACAAAGCAAGACCAAACACCGACCAATGATAAGATTTGCCAATCCGTAACGGAAATTACAGAGTCCGAGTCAGATCCCGAGGTGGAATCAGAAGATGATTCCACATCAGTCGAGGATGTAGATCCTCCTACCACTTATTACTCCATTATTGGCGGAGGTCTGAGAATGAACTTTGGATTCACCAAATGTCCTCAGATTAAATCCATCTCAGAATCCGCTAATGGAAACGCAGTGAATGCTAGATTGTCCAGCGTGCCCCTAGGACAAGGTAAGGACTCTCCCGCTATCACTCGTGCAGAAGCTCTGGCTATGATCAAAGACTGTGAGCTGTCTATCGACATTAGATGTAGCGAAGAAGAGAAAGACAGCGACATCCAGACCCATCCAGTACTCGAGTCTAACATCTCTCATAAGAAAGTGAGTTACGAAGATATCATCGGTTCAACGATCGTTGATACAAAATGCGTCAAGAATCTAGAGTTTAGTGTTCGTATCGGAGACATGTGCAAGGAATCGTCTGATCTTGAGGTCAAGGATGGATTTAAGTATGTCGACGGATCGGTATCTGAAGGTGTAACCGATGATACTTCACTCATCGATTCAACAAAACTCAAATCGTGTGTCTGAATAAATAACTCTATTCATCCGAGATTCGGGTAAGTAGGGTTAATCGAACGATTTAGTCACACTACGAATTAAAAAAGTGTACCTGACACTATACTCCGGTTTACAAAACAAAAATGTTCTTAACTACATTAACAAAAAGTTATCTCTCGCGACTTCTTCTTTTTCTGTCTCAGTAGTGTGTACGATATGATCACTGTTCATATTCCTATTCTCTCTTCGATGGTCTCACAAA
